# Single-molecule long-read sequencing of the full-length transcriptome of *Rhododendron lapponicum* L.

**DOI:** 10.1038/s41598-020-63814-x

**Published:** 2020-04-21

**Authors:** Xinping Jia, Ling Tang, Xueying Mei, Huazhou Liu, Hairong Luo, Yanming Deng, Jiale Su

**Affiliations:** Institute of Leisure Agriculture, Jiangsu Academy of Agricultural Sciences, Jiangsu Key Laboratory for Horticultural Crop Genetic Improvement, Nanjing, 210014 China

**Keywords:** RNA sequencing, Transcriptomics

## Abstract

*Rhododendron lapponicum* L. is a familiar ornamental plant worldwide with important ornamental and economic value. However, a full-length *R. lapponicum* transcriptome is still lacking. In the present study, we used the Pacific Biosciences single-molecule real-time sequencing technology to generate the *R. lapponicum* transcriptome. A total of 346,270 full-length non-chimeric reads were generated, from which we obtained 75,002 high-quality full-length transcripts. We identified 55,255 complete open reading frames, 7,140 alternative splicing events and 2,011 long non-coding RNAs. In gene annotation analyses, 71,155, 33,653, 30,359 and 31,749 transcripts were assigned to the Nr, GO, COG and KEGG databases, respectively. Additionally, 3,150 transcription factors were detected. KEGG pathway analysis showed that 96 transcripts were identified coding for the enzymes associated with anthocyanin synthesis. Furthermore, we identified 64,327 simple sequence repeats from 45,319 sequences, and 150 pairs of primers were randomly selected to develop SSR markers. This study provides a large number of full-length transcripts, which will facilitate the further study of the genetics of *R. lapponicum*.

## Introduction

*Rhododendron* is the largest genus in Ericaceae, with more than 1000 species of woody plants, either evergreen or deciduous^1^. *Rhododendron* species are widely cultivated around the world ranging from tropical to polar climates, and serve as a potential genetic resource for the development of new plant cultivars adapted to different environmental conditions^2^. *Rhododendron* is a familiar ornamental plant worldwide, and is decorative shrub with beautiful flowers that are widespread around the world. There are a remarkably broad range of rhododendron flower colours, including red, white, yellow, and green and so on. *Rhododendron lapponicum* L., a *Rhododendron* species found in subarctic regions around the world, is a common ornamental plant worldwide^3^. Recent advances in sequencing technology have facilitated genome and transcriptome studies in many species. However, genome and transcriptome sequencing in *R. lapponicum* has lagged behind that in other species, and information about the sequence and structure of its genes is limited. Therefore, the generation of a transcriptome data may establish a very important molecular biology basis for the research of *R. lapponicum*.

The transcriptome reflects the number and type of genes expressed in different cell types and reveals underlying metabolic pathways and genetic mechanisms^4^. Transcriptome sequencing is an efficient and feasible approach for generating a large amount of sequence data, and a large number of cDNA sequences provides a useful resource for genomic and genetic research^5–9^. Thus, third-generation long-read transcriptome sequencing platforms such as the Pacific Biosciences (PacBio), Nanopore and Moleculo platforms were developed. Recently, PacBio single-molecule real-time (SMRT) sequencing technology has served as a better alternative for obtaining full-length transcripts^10,11^. The major advantage of SMRT is the generation of full-length transcript without the need for fragmentation or post-sequencing assembly^12^. SMRT sequencing technology has been used in the transcriptome analysis of both model and nonmodel species^13–15^. Moreover, transcriptome sequencing is a simple and effective strategy for the development of large-scale SSRs at low cost. In recent years, the development of SSR markers by RNA sequencing and it successfully used in genetic improvement has been reported in many nonmodel plants^16–18^.

In this study, we constructed a full-length cDNA library of *R. lapponicum* and analysed it using SMRT sequencing technology. More than 15 Gb of sequencing data was produced, and 75,002 high-quality transcripts were obtained. Based on the obtained transcripts, alternative splicing (AS) analysis, long non-coding RNAs (lncRNA) prediction, transcription factor (TF) classification, open reading frame (ORF) prediction, transcript functional annotation and SSR analysis were performed. This is the first systematic report to characterize the full-length transcriptome of *R. lapponicum* via SMRT sequencing. The transcriptome data generated from this study provide valuable resources for genome annotation that may establish an important basis for future molecular biology research on *Rhododendron* species.

## Materials and Methods

### Plant materials

*Rhododendron lapponicum* L. (Fuli Jinling) was grown at Jiangsu Academy of Agricultural Sciences (Nanjing, China). Samples of the roots, stems, leaves and flowers from three individual plants were collected and frozen in liquid nitrogen, then stored at −70 °C for RNA extraction.

### RNA extraction

Total RNA was extracted using TRIzol LS reagent (Invitrogen, USA) following the manufacturer’s instructions. RNA degradation and contamination were monitored using 1% agarose gels. The purity, concentration and absorption peak of RNA were measured using a NanoDrop 2000 spectrophotometer (Thermo Scientific, USA). RNA quality was determined with the RNA Nano 6000 Assay Kit of the Agilent Bioanalyzer 2100 system (Agilent, USA). The total RNA samples from four tissues were mixed together for the following experiments.

### Library construction and SMRT sequencing

To construct a full-length transcript sequencing library, 5 μg of mixed total RNA was reverse transcribed into cDNA using the Clontech SMARTer cDNA Synthesis Kit (Takara Clontech Biotech, Dalian, China) following the manufacturer’s protocols. Size fractionation and selection (1–2 kb, 2–3 kb, and 3–6 kb) were performed using the BluePippin Size Selection System (Sage Science, USA). Three SMRT sequencing libraries containing fragments of 1–2, 2–3, and 3–6 kb in length were constructed using the Pacific Biosciences DNA Template Prep Kit 2.0. Finally, 1 (1–2 kb), 1 (2–3 kb) and 1 (3–6 kb) SMRT cells were sequenced on the Pacific Bioscience RS II platform.

### Quality filtering and error correction

Raw reads were processed by removing polymerase reads (<50 bp in length). The obtained clean reads were processed into error-corrected ROIs with the following parameters: full passes ≥0 and predicted consensus accuracy >0.75. By identifying the 5′ and 3′ adapters and the poly (A) tail, full-length and non-full-length reads were determined from the ROIs. A full-length read containing both the 5′ and 3′ primer sequences and a poly (A) tail was considered to be a full-length transcript. ROIs with all three elements that did not contain any additional copies of the adapter sequence within the DNA fragment were referred to as full-length non-chimeric (FLNC) reads. Corrected FLNC reads were clustered into transcripts using the ICE algorithm in the PacBio SMRT Analysis (v2.3.0) software. Full-length transcripts with a post-correction accuracy >99% were used for further analysis.

### Prediction of ORFs, lncRNAs, TFs and AS events

To predict ORFs in transcripts, the TransDecoder v2.0.1 tool was used to find potential coding sequences. Based on the obtained transcripts with redundancy removed, we predicted AS events with the software AStalavista^19^. TFs were predicted from the putative protein sequences using the Plant Transcription Factor Database v4.0 tool^20^. We identified unique transcripts without protein-coding potential as candidate lncRNAs with four analysis tools: the coding-non-coding index (CNCI)^21^, the coding potential assessment tool (CPAT)^22^, the coding potential calculator (CPC)^23^, and Pfam protein structure domain analysis^24^.

### Functional annotation

All transcript sequences were analysed for homology via searches against the non-redundant nucleotide database (Nr)^25^, Swiss-Prot protein^26^, protein family (pfam)^27^, evolutionary genealogy of genes: non-supervised orthologous groups (eggNOG)^28^, clusters of orthologous groups of proteins (COG)^29^, eukaryotic ortholog groups (KOG)^30^, gene ontology (GO)^31^, kyoto encyclopedia of genes and genomes (KEGG)^32^ databases with BLAST alignment (E-value ≤ 10^−5^).

### qRT-PCR analysis

Samples of flowers at four flower developmental stages were collected, and RNA was isolated from them using TRizol regent according to the manufacturer’s instructions. The cDNA was synthesized using AMV reverse transcriptase XL for RT-PCR according to the manufacturer’s instructions^33^. The qRT-PCR was performed under the following conditions: 95 °C for 2 min, followed by 40 cycles of 5 s at 95 °C, 30 s at 55–60 °C, and a final melting curve step. Three biological replicates were performed in a Roche 480 LightCycler. Threshold values (CT) were used to quantify relative gene expression using the comparative 2^−ΔΔCt^ method^34^. The information of primer used for qRT-PCR analyses is shown in Table S7.

### Development of SSR markers

For SSRs analysis, transcripts longer than 500 bp were selected and MISA software was used. The parameters were set for identifcation of mono-, di-, tri-, tetra-, penta-, and hexa-nucleotide motifs with a minimum of ten, six, five, five, five, and five, respectively. SSR primers were designed by Batch Primer 3 tool^6^. PCR amplifications were performed using the DNA template extracted from *R. lapponicum*. The PCR products were separated in 8% polyacrylamide gels.

## Results

### SMRT sequencing data

**Figure 1:**
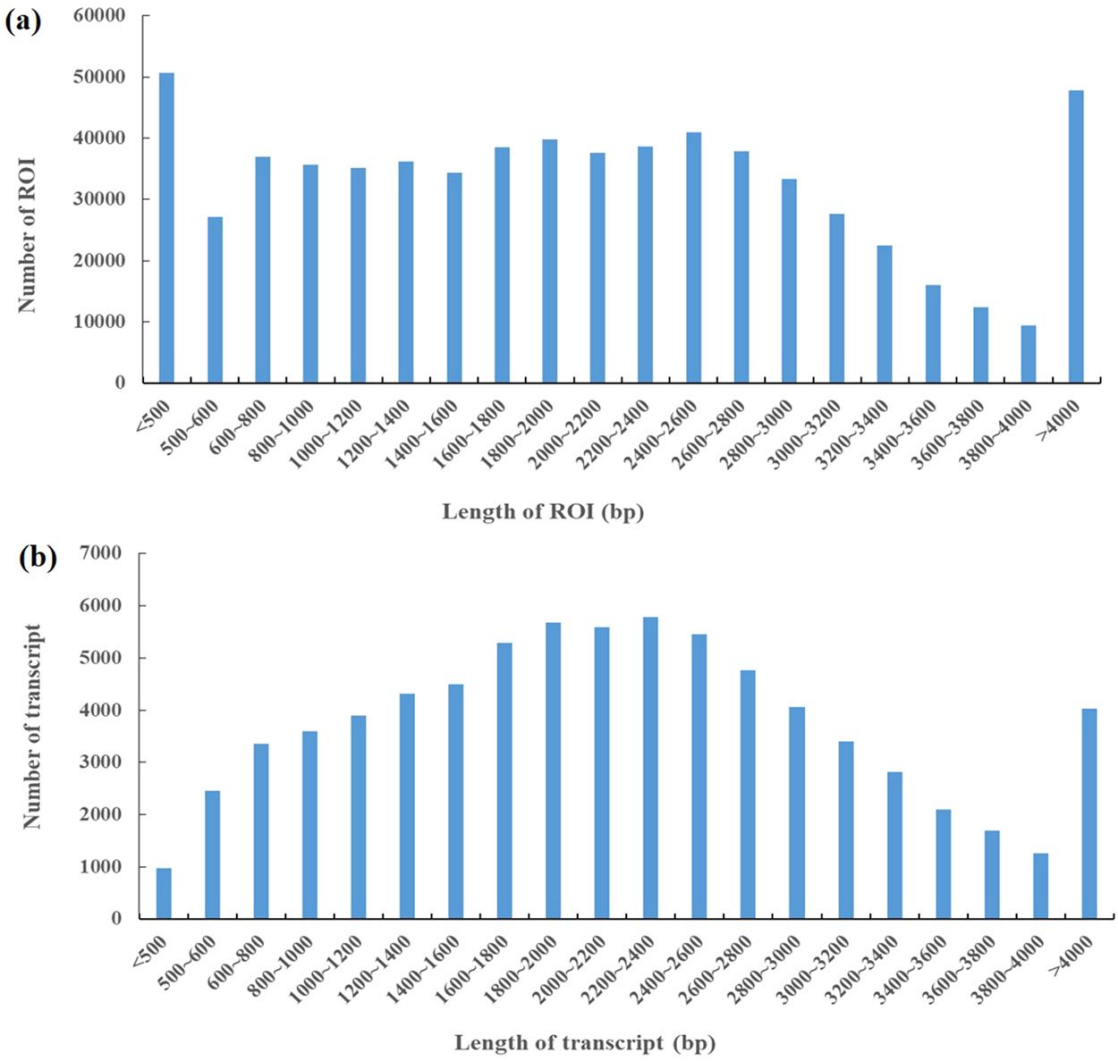
The length distribution of reads of inserts (ROIs) and transcripts. (**a**) The length distribution of 658,338 ROIs. (**b**) The length distribution of 75,002 transcripts.

To obtain a representative full-length transcriptome for *R. lapponicum*, the total RNA from four tissues (root, stem, leaf and flower) were used for the library construction for SMRT sequencing. In this study, we obtained 957,032 polymerase reads from the sequenced library, and a dataset with 15.37 Gb of clean reads was generated (Supplementary Table S1). A total of 658,338 reads of inserts (ROIs) were generated with full passes ≥0 and a consensus accuracy >0.75. Three SMRT cells (1–2 kb, 2–3 kb, and 3–6 kb) were constructed, and the average length of the ROIs in different libraries was between 1,206 bp and 3,519 bp (Table 1). ROIs were classified into 346,270 FLNC and 274,471 non-full-length reads. According to the clustering algorithm of ICE, we obtained 180,047 consensus isoform sequences, including 105,015 high-quality isoforms and 74,963 low-quality isoforms. After the removal of redundant reads, we obtained 75,002 high-quality full-length transcripts. The length distribution of the ROIs and transcripts is shown in Fig. 1.

**Table 1:** Summary of reads of inserts from PacBio single-molecule long-read sequencing.

Size	Reads of insert	Read bases of insert	Mean read length of insert	Mean read quality of insert	Mean number of passes
1–2 K	239,854	289,251,046	1,206	0.97	16
2–3 K	265,435	626,058,946	2,359	0.93	11
3–6 K	153,049	538,326,391	3,519	0.91	8

### Open reading frame and AS event prediction

**Figure 2:**
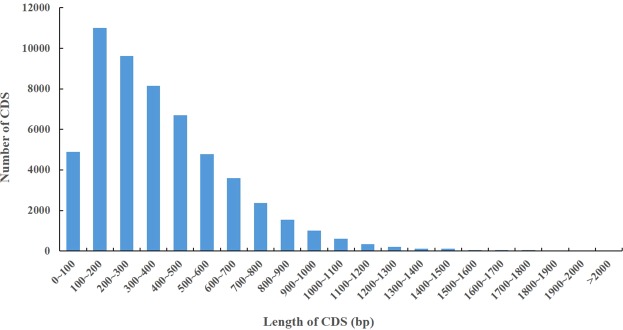
The length distribution of complete open reading frames (ORFs).

Using the software TransDecoder, 72,606 ORFs were predicted. A total of 55,255 complete ORFs were identified, and the length distribution of the complete ORFs was analysed (Fig. 2). Among all transcripts obtained by SMRT sequencing, 7,140 AS events were detected (Supplementary Table S2). Due to the lack of an available *R. lapponicum* reference genome, further characterization of the types of AS events would be warranted in future studies.

### Long non-coding RNA identification

**Figure 3:**
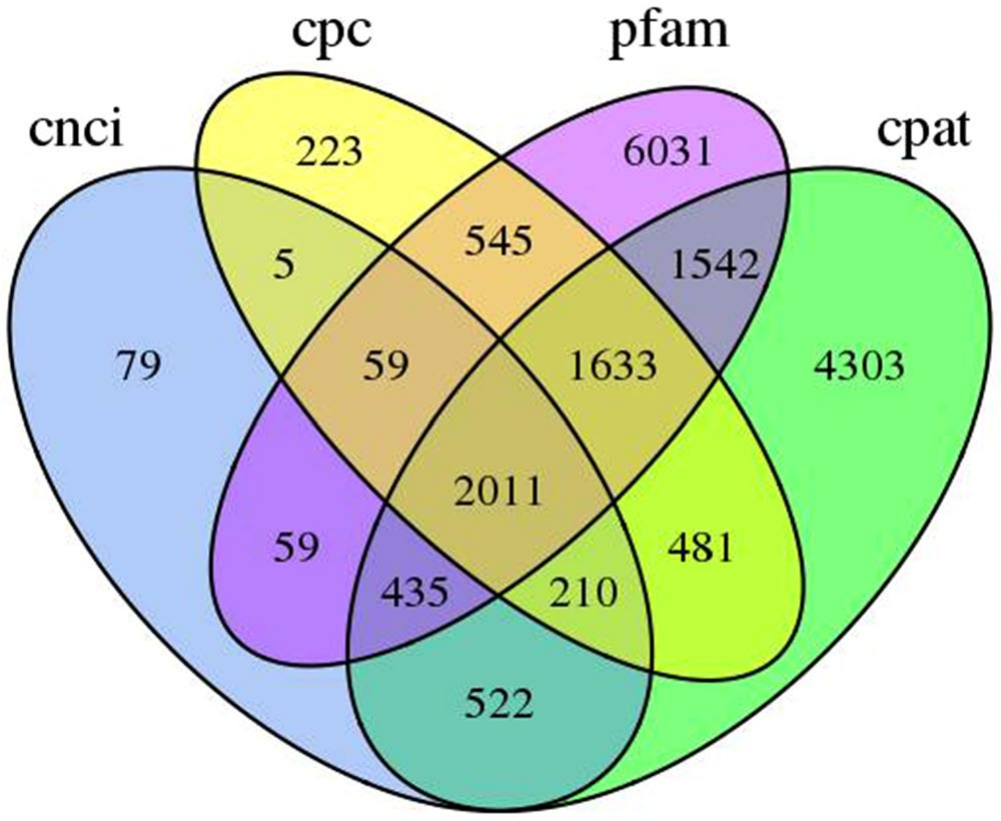
Venn diagram of long non-coding RNAs (lncRNAs) predicted by the Coding-Non-Coding Index (CNCI), Coding Potential Assessment Tool (CPAT), Coding Potential Calculator (CPC) and Pfam protein structure domain analysis methods.

LncRNA are a class of poly-A noncoding RNAs that play roles in the growth and stress responses of plants. In this study, we used four computational approaches to identify lncRNAs, involving the CPC, CNCI, CPAT and Pfam databases. A total of 5,167, 3,380, 11,137 and 12,315 lncRNAs were identified in the CNCI, CPC, CPAT and Pfam databases, respectively (Supplementary Table S3). By filtering transcripts of less than 300 bp, 2,011 transcripts were considered as lncRNAs by all four methods (Fig. 3).

### Transcription factor prediction

**Figure 4:**
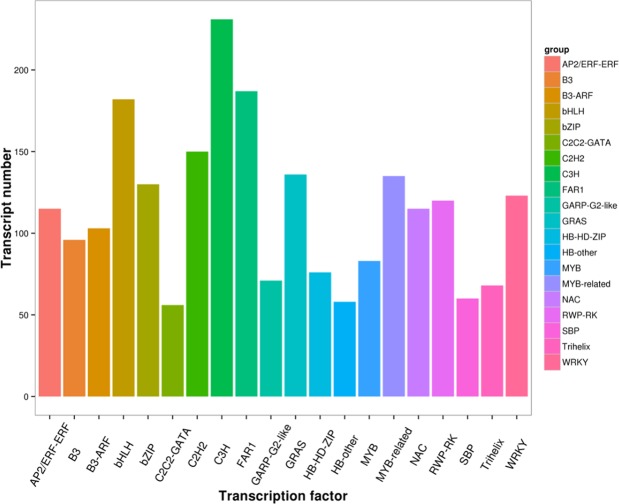
The top 15 transcription factor (TF) families in the *R. lapponicum* transcriptome. The x-axis represents the TFs, and the y-axis indicates the number of transcripts of a specific TF type.

TFs are key regulators of gene expression and play important roles in plant growth and development. In this study, 3,150 putative TFs were identified and divided into 64 TF families (Supplementary Table S4). The TFs in the *R. lapponicum* transcriptome mainly belonged to the C3H (231, 7.33%), FAR1 (187, 5.94%), bHLH (182, 5.78%), C2H2 (150, 4.76%), GRAS (136, 4.32%), MYB-related (135, 4.28%), bZIP (130, 4.13%), WRKY (123, 3.90%), RWP-RK (120, 3.81%), and NAC (115, 3.65%) families (Fig. 4).

### Functional annotation of transcripts

**Figure 5:**
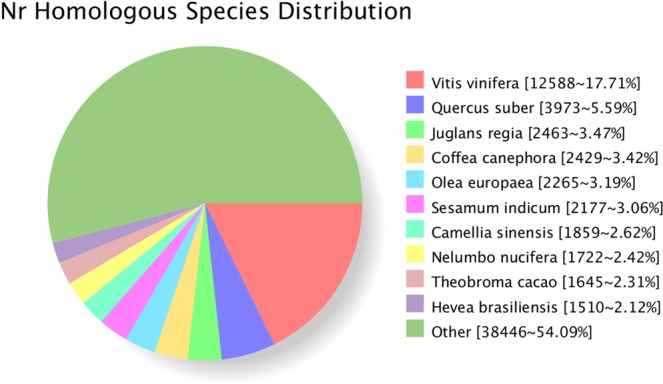
Homologous species distribution of *R. lapponicum* transcripts annotated in the non-redundant (Nr) database. The numbers and frequencies of the main annotated species are shown.

Among the 75,002 transcripts identified, 71,155 (94.87%), 57,837 (77.11%), 33,653 (44.87%), 30,359 (40.48%), 45,925 (61.23%), 69,897 (93.19%), 60,296 (80.39%) and 31,749 (42.33%) transcripts were annotated in the Nr, Swiss-Prot, GO, COG, KOG, eggNOG, Pfam, and KEGG databases (Table 2), respectively. The annotation of the species distribution showed the largest proportion of the transcripts distributed in *Vitis vinifera*, followed by *Quercus suber*, *Juglans regia* and *Coffea canephora* (Fig. 5).

**Table 2:** Summary of the functional annotation of the *R. lapponicum* transcriptome.

Annotated database	Number of transcript hits	Percentage (%)
NR	71,155	94.87
Swiss-Prot	57,837	77.11
GO	33,653	44.87
COG	30,359	40.48
KOG	45,925	61.23
eggNOG	69,897	93.19
Pfam	60,296	80.39
KEGG	31,749	42.33
All annotated	71,386	95.18
All analysed	75,002	100.00

### GO classification

**Figure 6:**
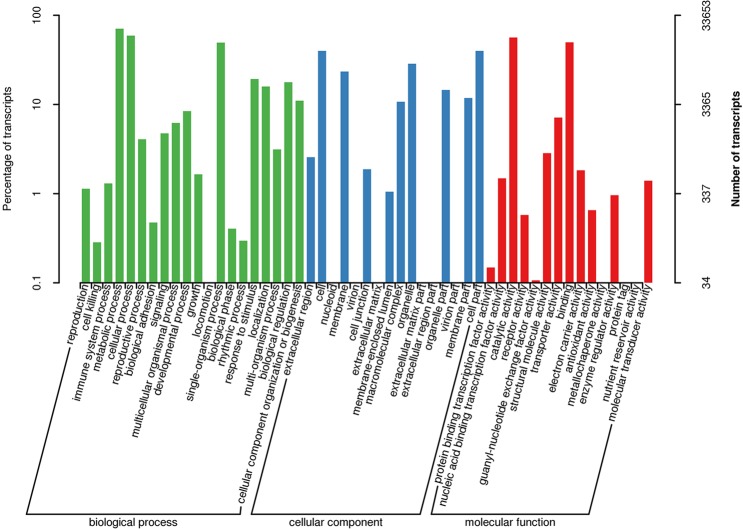
Gene Ontology (GO) classification of *R. lapponicum* transcripts. Green represents biological processes blue represents cellular components, and red represents molecular functions. The x-axis represents GO categories, the right y-axis represents the number of transcripts, and the left y-axis represents the percentage of transcripts.

To classify the gene functions of the transcripts, GO analysis was performed. GO analysis showed the enrichment of 33,653 transcripts categorized into 51 functional groups, which could be divided into three major categories: biological process, cellular component and molecular function (Fig. 6). In the biological process group, catalytic activity and binding were the main categories. In the cellular component group, cell part, cell and organelle were the most frequent categories. In the molecular function group, the genes were involved in catalytic activity, binding and other categories.

### COG classification

**Figure 7:**
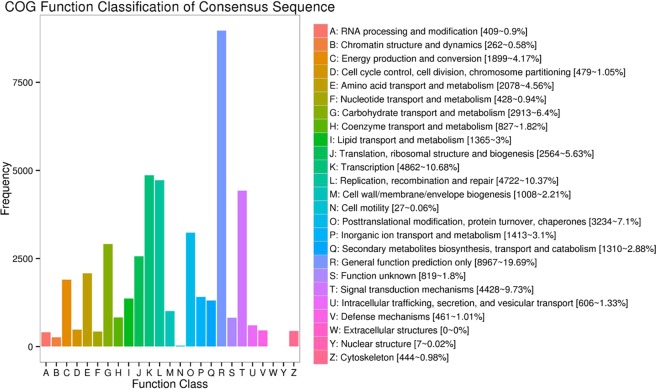
Clusters of Orthologous Groups of protein (COG) classification of *R. lapponicum* transcripts. The x-axis represents the subcategories, and the y-axis indicates the number of transcripts in a specific functional cluster.

To further study the functional annotation and classification of the *R. lapponicum* transcripts, all transcripts were subjected to a search against the Clusters of COG database. COG analysis showed that 30,359 transcripts were assigned to 24 categories (Fig. 7). The largest group was general function prediction only (8,967, 19.69%), followed by transcription (4,862, 10.68%) and then replication, recombination and repair (4,722, 10.37%). The percentages of six groups were less than 1.00%, including RNA processing and modification, nuclear structure, and cell motility.

### KEGG pathway analysis

To identify biological pathways in *R. lapponicum*, transcripts were searched against the KEGG pathway database. A total of 31,749 transcripts were mapped to 128 KEGG functional pathways (Supplementary Table S5). Among these pathways, carbon metabolism (1,378, 4.34%) and protein processing in the endoplasmic reticulum (1290, 4.06%) were the most dominant pathways, followed by the biosynthesis of amino acids (1,196, 3.77%), spliceosomes (946, 2.98%) and ribosomes (909, 2.86%) (Table 3). The KEGG functional classification provided valuable clues for investigating specific processes, functions, and pathways in *R. lapponicum*.

**Table 3:** The top 15 mapped pathways annotated by the KEGG database.

Number	Name of pathway	Pathway ID	Number of transcripts
1	Carbon metabolism	ko01200	1,378
2	Protein processing in endoplasmic reticulum	ko04141	1,290
3	Biosynthesis of amino acids	ko01230	1,196
4	Spliceosome	ko03040	1,049
5	Ribosome	ko03010	946
6	RNA transport	ko03013	909
7	Starch and sucrose metabolism	ko00500	839
8	Plant hormone signal transduction	ko04075	827
9	Oxidative phosphorylation	ko00190	787
10	Glycolysis / Gluconeogenesis	ko00010	740
11	Plant-pathogen interaction	ko04626	701
12	mRNA surveillance pathway	ko03015	664
13	Ubiquitin mediated proteolysis	ko04120	616
14	Amino sugar and nucleotide sugar metabolism	ko00520	581
15	Endocytosis	ko04144	568

### Representative genes in the anthocyanin biosynthesis pathway and expression pattern analysis

**Figure 8:**
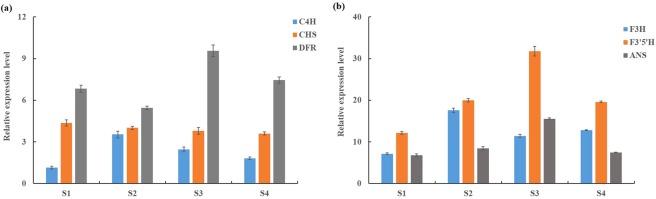
Expression analysis of transcripts involved in flavonoid biosynthesis throughout the flower development in *R. lapponicum*. (**a**) Expression levels of *C4H*, *CHS* and *DFR*. (**b**) Expression levels of *F3H*, *F3*′*5*′*H* and *ANS*. Four flower developmental stages were examined in our study: the budding stage (S1), the initial flowering stage (S3), the full-flowering stage (S3), and the end flowering stage (S4). The qRT-PCR validation of six randomly selected transcripts in the three samples. Columns represent the relative expression levels. Error bars represent the standard deviation from three biological replicates.

Anthocyanins are natural bioactive pigments in plants that play important roles in many physiological functions. Through KEGG analysis, a total of 96 transcripts were identified coding for the enzymes associated with anthocyanin synthesis, which included *trans-cinnamate 4-monooxygenase* (C4H, seven transcripts), *chalcone isomerase* (CHI, 12 transcripts), *chalcone synthase* (CHS, 41 transcripts), *naringenin 3-dioxygenase* (F3H, nine transcripts), *flavonoid 3ʹ-hydroxylase* (F3ʹH, eight transcripts) and *flavonoid 3ʹ*,5*ʹ-hydroxylase* (F3ʹ5ʹH, eight transcripts), *anthocyanidin synthase* (ANS, five transcripts) and *dihydroflavonol 4-reductase* (DFR, six transcripts) (Supplementary Table S6). Six genes related to anthocyanin biosynthesis were randomly selected to perform qRT-PCR analysis. The qRT-PCR analysis showed that the highest expression of *C4H* (F01_cb9736_c8/f1p0/2072) and *F3H* (F01_cb13925_c0/f2p0/1339) was observed at stage S2, while the expression of *F3*′*5*′*H* (F01_cb7576_c7/f2p0/1921), *DFR* (F01_cb3655_c0/f2p0/2991) and *ANS* (F01_cb7563_c31/f1p0/655) was highest at stage S3 (Fig. 8).

### SSR identification

**Figure 9:**
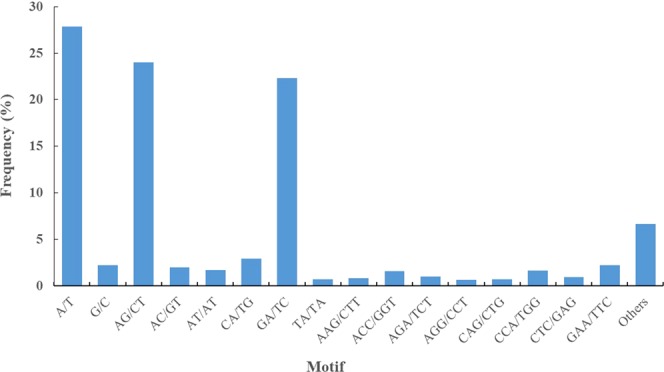
Frequency distribution of SSRs based on motif types. The frequency of the main motif types is shown.

After screening the 74,031 obtained transcripts, 64,327 potential SSRs were identified from 45,319 transcripts. Among these transcripts, 26,312 contained one SSR, and 19,007 contained two loci or more. Furthermore, 26,312 and 19,007 transcripts contained one SSR and at least two SSRs, respectively. In addition, 11,634 SSRs were considered compound formations. As shown in Table 4, the numbers of mono-, di-, tri-, tetra-, penta- and hexa-nucleotide repeats were 18,064, 27,639, 6,494, 228, 114 and 155, respectively. SSRs with 10 repeat units (7,178, 13.62%) were the most abundant, followed by those with 6 (6,496, 12.33%), 11 (4,969, 9.43%) and 7 (4,549, 8.63%) (Table 5). The most frequent motif type was A/T, followed by AG/CT, GA/TC, CA/TG and GAA/TTC (Fig. 9).

**Table 4:** Summary of SSRs identified in the *R. lapponicum* transcriptome.

Searching item	Numbers
Total number of sequences examined	74,031
Total size of examined sequences (bp)	171,584,325
Total number of identified SSRs	64,327
Number of SSR containing sequences	45,319
Number of sequences containing more than one SSR	19,007
Number of SSRs present in compound formation	11,634
Mono-nucleotide	18,064
Di-nucleotide	27,639
Tri-nucleotide	6,494
Tetra-nucleotide	228
Penta-nucleotide	114
Hexa-nucleotide	155

**Table 5:** The distribution of SSRs based on the number of repeat units.

Number of repeat units	Mono	Di	Tri	Tetra	Penta	Hexa	Total	Percentage (%)
5	—	—	3,853	168	83	96	4,200	7.97
6	—	4,917	1,475	46	25	33	6,496	12.33
7	—	3,881	639	12	4	13	4,549	8.63
8	—	3,399	234	1	1	8	3,643	6.91
9	—	3,026	112	1	—	1	3,140	5.96
10	4,635	2,461	81	—	1	—	7,178	13.62
11	2,964	1,968	35	—	—	2	4,969	9.43
12	2,062	1,627	24	—	—	—	3,713	7.05
13	1,359	1,316	26	—	—	—	2,701	5.12
14	1,103	1,104	8	—	—	1	2,216	4.21
≥15	5,941	3,940	7	—	—	1	9,889	18.77

### SSR marker development

Using Primer3.0 software, 40,509 primer pairs were designed, and 150 were randomly selected for PCR (Supplementary Table S8). The PCR products of 127 primer pairs were successfully examined, with an amplification efficiency rate of 84.67%. However, the remaining 23 primer pairs failed to achieve amplification at various annealing temperatures.

## Discussion

SMRT sequencing technology is an efficient and reliable approach for obtaining the full-length transcripts of certain species^35^. Recently, long-read SMRT sequencing has been the most reliable and efficient strategy for whole-transcriptome profiling studies, especially for nonmodel plant species without reference genome sequences. In this study, SMRT sequencing technology was applied to investigate the *R. lapponicum* transcriptome using the PacBio RS II platform. A total of 15.37 Gb of sequencing data were generated, including 658,338 ROIs and 346,270 FLNC reads. The percentage of FLNC reads in all ROIs was 52.59%, and this result was similar findings obtained in alfalfa^36^ and strawberry^37^ by SMRT sequencing. After removing redundant sequences, 75,002 full-length transcripts were obtained. SMRT sequencing can capture the very long nucleotide sequences, where one read usually represents a full-length transcript^13^. The length of the transcripts obtained by SMRT sequencing technology is longer than that of transcripts obtained by next-generation high-throughput sequencing technology. In this study, the average length of the *R. lapponicum* transcripts was 2,509 bp, which was longer than those obtained in seashore paspalum (970 bp)^38^, sweet potato (581 bp)^39^, and sesame (629 bp)^40^ by Illumina sequencing technology. Furthermore, we found that 58.66% of all transcripts were longer than 2,000 bp in this study, and much higher than that in *Rhododendron molle* (7.23%)^41^ and *Neottopteris nidus* (13.63%)^6^ using Illumina sequencing technology. These results indicated that PacBio SMRT sequencing technology is an efficient approach to capture the transcript sequences, especially for long transcript sequences.

Alternative splicing is a major cellular mechanism generating transcriptome diversity and proteome complexity in plants^42^. In this study, 7,140 AS events were detected from the *R. lapponicum* transcripts. In addition, 3,150 TFs that are key components involved in the transcriptional regulatory system were identified. LncRNAs are a novel class of non-coding transcripts with lengths greater than 200 nucleotides that play important roles in many biological processes^43^. LncRNAs are largely involved in regulating plant development and growth, secondary metabolism, and the plant stress response^44^. Recently, an increasing number of studies have focused on the functions of lncRNAs in plants such as in red pineapple^45^ and hot pepper^46^. However, no lncRNAs from *Rhododendron* have been reported. In this study, we identified 2,011 lncRNAs using four methods, and these lncRNAs will be useful for further research in *R. lapponicum*.

A total of 71,386 transcripts were annotated by sequence alignment in eight databases, suggesting that this study generated a very large number of *R. lapponicum* genes. The percentage of annotated transcripts was 95.18%, which was consistent with that in alfalfa^36^ and shrimp^47^. The remaining 3,616 transcripts presented no BLAST matches and might represent *R. lapponicum*-specific genes or unknown genes in *R. lapponicum*. The systematic classification of proteins in the transcriptome is crucial for maximizing the utilization of transcripts for functional and evolutionary studies. The results of GO and COG classification suggested that a large number of transcripts were involved in transcription, replication, recombination and repair, and catalytic activity. There were 31,749 transcripts assigned to specific pathways, such as metabolism, genetic information processing, cellular processes, environmental information processing, and organismal systems pathways. The results of GO, COG and KEGG classification showed that a large number of transcripts had diverse molecular functions and were involved in many biological pathways. Therefore, our data provided abundant genetic information on future molecular survey on the growth and development of *R. lapponicum*.

Flower colour is one of the most important ornamental characteristics of rhododendrons. The biosynthesis of anthocyanin is critical for a wide range of flower colours. Previous studies have shown that C4H, CHS, F3H, F3’H, F3ʹ5ʹH, DFR and ANS are the key enzymes involved in the biosynthesis of anthocyanin for the determination of different flower colours in plants^48^. In the present study, a total of 96 transcripts were identified coding for the enzymes associated with anthocyanin synthesis. Gene expression analysis by qRT-PCR showed that the expression levels of *C4H*, *F3H*, *F3ʹ5ʹH*, *DFR* and *AN*S genes were low at the early flowering developmental stage and increased as the flowers developed. The increases in the expression of these genes were consistent with the changes in anthocyanin content in the flower petals of *R. lapponicum* during flower development. In addition, transcription factors such as those of the MYB, bHLH and WD40 families play a key role by regulating the expression of genes in anthocyanin biosynthesis^49,50^. According to the functional annotation results, 3,150 putative transcription factor genes belonging to 64 TF families were identified. Among these genes, 83 and 182 transcripts belonged to the MYB and bHLH families, respectively (Fig. 4). In conclusion, the identification of key enzymes and related regulatory TF genes involved in anthocyanin biosynthesis and metabolic pathways may contribute to the understanding of colour-regulating mechanisms in rhododendrons.

The rapid development of transcriptome sequencing technology has enabled the massive development of SSR markers^51,52^. In total, 64,327 SSRs were identified from 45,319 SSR-containing sequences, and the average frequency of SSRs was one in 2.67 kb. Among the six types of repeat motifs, dinucleotide repeats were the most abundant. In the present study, the most frequent mono-, di-, and tri-nucleotide motifs were A/T, AG/CT and GAA/TTC, respectively, which was consistent with the results of studies in non-heading Chinese cabbage^53^, rubber tree^54^ and radish^55^. CT/AG/GA/TC were the most abundant motifs, accounting for 92.11% of the total dinucleotide repeats. CT repeats are typically found in transcribed regions that may be involved in antisense transcription and play a role in gene regulation^56,57^. Furthermore, the most abundant mononucleotide motif was A/T, which is thought to be frequent in the genomic sequences of plants^58^. SSR abundance varies among different plant species in different studies. Repeat units of 10, 6, 11, 7, and 5 in SSR sequences accounted for 51.98% of the total SSRs. A total of 150 pairs of PCR primers were designed, and 127 primer pairs successfully amplified PCR products. The failure of 23 primer pairs to achieve amplification might have resulted from the targeting of amplicons with large introns, primers positioned across splice sites or chimeric primers. These results suggested that the development of SSR markers based on *R. lapponicum* transcripts obtained from PacBio SMRT sequencing is an effective and feasible approach. The newly developed SSR markers from our study will provide a valuable genetic tool that be used in studies on genetic diversity, comparative genomics, gene mapping, and population genetics and other types of genetic studies in rhododendron.

In conclusion, we analysed the full-length transcriptome of *R. lapponicum* by using the PacBio SMRT sequencing technology. This study represents the first the third-generation long-read transcriptome sequencing of *R. lapponicum*. Based on the obtained transcriptome data, 7,140 AS events, 2,011 lncRNAs, 55,255 complete ORFs and 3,150 TF members were identified. A total of 96 transcripts were identified coding for the enzymes associated with anthocyanin synthesis. In addition, 64,327 SSRs were detected, and 150 primer pairs were randomly selected to develop SSR markers. The obtained transcriptome data may facilitate further genetic studies on *R. lapponicum*.

## Supplementary information

Supplementary Table S1.

Supplementary Table S2.

Supplementary Table S3.

Supplementary Table S4.

Supplementary Table S5.

Supplementary Table S6.

Supplementary Table S7.

Supplementary Table S8.

## Data Availability

The following information was supplied regarding data availability: Data are available at the Sequence Read Archive (SRA) (https://www.ncbi.nlm.nih.gov/sra) of NCBI, accession number: PRJNA594084.
