# Supplementary material for: Single-molecule long-read sequencing of the full-length transcriptome of Rhododendron lapponicum L
Source: Sci Rep. 2020 Apr 21;10:6755. doi: 10.1038/s41598-020-63814-x (PMC7174332; doi:10.1038/s41598-020-63814-x)
Supplement: Supplementary file 1 — Supplementary Table S1. [file 41598_2020_63814_MOESM1_ESM.pdf]

Single-molecule long-read sequencing of the full-length transcriptome of *Rhododendron lapponicum* L.

Xinping Jia, Ling Tang, Xueying Mei, Huazhou Liu, Hairong Luo, Yanming Deng, Jiale Su

Institute of Leisure Agriculture, Jiangsu Academy of Agricultural Sciences, Jiangsu Key Laboratory for Horticultural Crop Genetic Improvement, Nanjing 210014, China

Table S1 Summary of polymerase reads from PacBio single-molecule long read sequencing

Size	Post-filter polymerase reads	Post-filter total number of subread bases	Post-filter number of subread	Post-filter subreads N50	Post-filter mean subread length
1-2K	387,454	5.27G	2,861,654	1,782	1,659
2-3K	334,062	6.43G	3,573,056	2,531	2,440
3-6K	235,516	3.67G	1,825,478	3,406	3,211
