# Supplementary material for: Single-molecule long-read sequencing of the full-length transcriptome of Rhododendron lapponicum L
Source: Sci Rep. 2020 Apr 21;10:6755. doi: 10.1038/s41598-020-63814-x (PMC7174332; doi:10.1038/s41598-020-63814-x)
Supplement: Supplementary file 3 — Supplementary Table S3. [file 41598_2020_63814_MOESM3_ESM.pdf]

Single-molecule long-read sequencing of the full-length transcriptome of *Rhododendron lapponicum* L.

Xinping Jia, Ling Tang, Xueying Mei, Huazhou Liu, Hairong Luo, Yanming Deng, Jiale Su

Institute of Leisure Agriculture, Jiangsu Academy of Agricultural Sciences, Jiangsu Key Laboratory for Horticultural Crop Genetic Improvement, Nanjing 210014, China

Table S3 Summary of lncRNAs predicted by CPC, CNCI, CPAT, and pfam protein structure domain analysis

ID	cnci	cpat	cpc	pfam
R01_cb8196_c10/f2p0/1238	NA	NA	R01_cb8196_c10/f2p0/1238	R01_cb8196_c10/f2p0/1238
R01_cb10380_c2/f1p0/669	NA	NA	NA	R01_cb10380_c2/f1p0/669
R01_cb8564_c83711/f2p0/3575	NA	R01_cb8564_c83711/f2p0/3575	NA	NA
R01_cb5578_c3/f1p0/2902	NA	R01_cb5578_c3/f1p0/2902	NA	NA
R01_cb6691_c20/f1p0/892	NA	NA	NA	R01_cb6691_c20/f1p0/892
R01_cb8564_c146693/f2p0/2046	R01_cb8564_c146693/f2p0/2046	R01_cb8564_c146693/f2p0/2046	R01_cb8564_c146693/f2p0/2046	R01_cb8564_c146693/f2p0/2046
R01_cb8564_c69331/f3p0/3241	NA	R01_cb8564_c69331/f3p0/3241	NA	NA
R01_cb13003_c5/f1p1/1779	NA	R01_cb13003_c5/f1p1/1779	NA	NA
R01_cb8564_c123957/f1p0/2432	NA	R01_cb8564_c123957/f1p0/2432	NA	NA
R01_cb8030_c5/f1p0/2391	NA	NA	NA	R01_cb8030_c5/f1p0/2391
R01_cb16132_c14/f1p0/806	NA	NA	R01_cb16132_c14/f1p0/806	R01_cb16132_c14/f1p0/806
R01_cb4370_c5/f1p0/2518	NA	NA	NA	R01_cb4370_c5/f1p0/2518
R01_cb9611_c5/f1p0/2100	NA	R01_cb9611_c5/f1p0/2100	NA	R01_cb9611_c5/f1p0/2100
R01_cb18456_c6567/f1p0/933	R01_cb18456_c6567/f1p0/933	NA	NA	NA
R01_cb14239_c16/f1p0/1540	NA	R01_cb14239_c16/f1p0/1540	NA	NA
R01_cb17067_c0/f2p0/1255	NA	NA	R01_cb17067_c0/f2p0/1255	R01_cb17067_c0/f2p0/1255
R01_cb5911_c4/f1p0/3602	NA	NA	NA	R01_cb5911_c4/f1p0/3602
R01_cb3088_c8/f1p0/1078	R01_cb3088_c8/f1p0/1078	R01_cb3088_c8/f1p0/1078	R01_cb3088_c8/f1p0/1078	R01_cb3088_c8/f1p0/1078
R01_cb11665_c0/f1p0/911	R01_cb11665_c0/f1p0/911	R01_cb11665_c0/f1p0/911	R01_cb11665_c0/f1p0/911	R01_cb11665_c0/f1p0/911
R01_cb1074_c3/f1p0/615	R01_cb1074_c3/f1p0/615	R01_cb1074_c3/f1p0/615	R01_cb1074_c3/f1p0/615	R01_cb1074_c3/f1p0/615
R01_cb10391_c5/f1p0/1036	R01_cb10391_c5/f1p0/1036	R01_cb10391_c5/f1p0/1036	NA	R01_cb10391_c5/f1p0/1036
R01_cb4964_c4/f1p0/2572	NA	R01_cb4964_c4/f1p0/2572	NA	NA

R01_cb8564_c113574/f1p0/3901	NA	R01_cb8564_c113574/f1p0/3901	NA	R01_cb8564_c113574/f1p0/3901
R01_cb2749_c17/f1p0/1031	R01_cb2749_c17/f1p0/1031	R01_cb2749_c17/f1p0/1031	R01_cb2749_c17/f1p0/1031	R01_cb2749_c17/f1p0/1031
R01_cb10832_c4/f1p0/412	R01_cb10832_c4/f1p0/412	R01_cb10832_c4/f1p0/412	R01_cb10832_c4/f1p0/412	R01_cb10832_c4/f1p0/412
R01_cb15811_c30/f1p0/1139	NA	R01_cb15811_c30/f1p0/1139	R01_cb15811_c30/f1p0/1139	R01_cb15811_c30/f1p0/1139
R01_cb8564_c4589/f1p0/3384	R01_cb8564_c4589/f1p0/3384	R01_cb8564_c4589/f1p0/3384	NA	R01_cb8564_c4589/f1p0/3384
R01_cb6057_c6/f1p0/3908	NA	R01_cb6057_c6/f1p0/3908	NA	R01_cb6057_c6/f1p0/3908
R01_cb17_c41/f1p0/1777	NA	R01_cb17_c41/f1p0/1777	NA	R01_cb17_c41/f1p0/1777
R01_cb16038_c3/f1p0/452	NA	R01_cb16038_c3/f1p0/452	R01_cb16038_c3/f1p0/452	R01_cb16038_c3/f1p0/452
R01_cb8564_c10048/f2p0/2438	R01_cb8564_c10048/f2p0/2438	R01_cb8564_c10048/f2p0/2438	R01_cb8564_c10048/f2p0/2438	R01_cb8564_c10048/f2p0/2438
R01_cb9107_c7/f1p0/2306	R01_cb9107_c7/f1p0/2306	R01_cb9107_c7/f1p0/2306	NA	R01_cb9107_c7/f1p0/2306
R01_cb8564_c10792/f1p0/3310	NA	R01_cb8564_c10792/f1p0/3310	NA	NA
R01_cb8564_c35088/f1p0/3430	NA	R01_cb8564_c35088/f1p0/3430	NA	NA
R01_cb3370_c2/f1p0/3098	NA	R01_cb3370_c2/f1p0/3098	NA	NA
R01_cb15053_c10/f1p0/816	NA	R01_cb15053_c10/f1p0/816	R01_cb15053_c10/f1p0/816	NA
R01_cb10496_c8/f3p0/1467	NA	NA	NA	R01_cb10496_c8/f3p0/1467
R01_cb8564_c24967/f1p0/1960	NA	R01_cb8564_c24967/f1p0/1960	R01_cb8564_c24967/f1p0/1960	R01_cb8564_c24967/f1p0/1960
R01_cb8564_c88650/f1p0/2939	R01_cb8564_c88650/f1p0/2939	R01_cb8564_c88650/f1p0/2939	R01_cb8564_c88650/f1p0/2939	R01_cb8564_c88650/f1p0/2939
R01_cb9936_c12/f1p0/2901	NA	R01_cb9936_c12/f1p0/2901	R01_cb9936_c12/f1p0/2901	NA
R01_cb8564_c23942/f1p0/3454	NA	NA	NA	R01_cb8564_c23942/f1p0/3454
R01_cb14663_c3/f1p0/1061	NA	R01_cb14663_c3/f1p0/1061	NA	R01_cb14663_c3/f1p0/1061
R01_cb5502_c4/f1p0/2613	NA	R01_cb5502_c4/f1p0/2613	NA	R01_cb5502_c4/f1p0/2613
R01_cb18173_c1/f1p0/645	R01_cb18173_c1/f1p0/645	R01_cb18173_c1/f1p0/645	NA	R01_cb18173_c1/f1p0/645
R01_cb3180_c1/f1p0/3569	NA	NA	NA	R01_cb3180_c1/f1p0/3569

R01_cb279_c33/f5p1/4837	NA	NA	NA	R01_cb279_c33/f5p1/4837
R01_cb4434_c4/f1p1/3289	NA	NA	NA	R01_cb4434_c4/f1p1/3289
R01_cb4657_c9/f1p0/3253	NA	R01_cb4657_c9/f1p0/3253	R01_cb4657_c9/f1p0/3253	NA
R01_cb14751_c3/f1p1/769	NA	R01_cb14751_c3/f1p1/769	NA	NA
R01_cb11142_c2/f1p0/2496	NA	R01_cb11142_c2/f1p0/2496	NA	NA
R01_cb13538_c9/f10p1/776	NA	R01_cb13538_c9/f10p1/776	NA	NA
R01_cb16319_c0/f3p0/836	NA	NA	NA	R01_cb16319_c0/f3p0/836
R01_cb17394_c0/f1p0/1335	NA	NA	NA	R01_cb17394_c0/f1p0/1335
R01_cb14845_c0/f3p1/1699	NA	NA	NA	R01_cb14845_c0/f3p1/1699
R01_cb3025_c7/f1p0/3430	NA	NA	NA	R01_cb3025_c7/f1p0/3430
R01_cb8564_c119069/f1p0/1898	NA	R01_cb8564_c119069/f1p0/1898	R01_cb8564_c119069/f1p0/1898	R01_cb8564_c119069/f1p0/1898
R01_cb8564_c77171/f1p0/3121	NA	R01_cb8564_c77171/f1p0/3121	NA	NA
R01_cb14086_c1/f2p0/704	NA	NA	NA	R01_cb14086_c1/f2p0/704
R01_cb18456_c7514/f1p0/808	NA	R01_cb18456_c7514/f1p0/808	NA	NA
R01_cb8823_c3/f1p2/4298	NA	NA	NA	R01_cb8823_c3/f1p2/4298
R01_cb18383_c0/f1p0/1437	NA	R01_cb18383_c0/f1p0/1437	NA	R01_cb18383_c0/f1p0/1437
R01_cb479_c3/f6p5/4750	NA	NA	NA	R01_cb479_c3/f6p5/4750
R01_cb7695_c0/f1p0/2539	NA	NA	NA	R01_cb7695_c0/f1p0/2539
R01_cb15032_c2/f1p0/1081	NA	NA	NA	R01_cb15032_c2/f1p0/1081
R01_cb8564_c1824/f1p0/2524	NA	R01_cb8564_c1824/f1p0/2524	NA	NA
R01_cb4354_c12/f1p1/3170	NA	NA	NA	R01_cb4354_c12/f1p1/3170
R01_cb1779_c4/f1p0/3691	NA	NA	NA	R01_cb1779_c4/f1p0/3691
R01_cb8564_c124893/f1p0/3487	NA	R01_cb8564_c124893/f1p0/3487	NA	NA
R01_cb8564_c50069/f1p0/2242	R01_cb8564_c50069/f1p0/224	R01_cb8564_c50069/f1p0/2242	NA	R01_cb8564_c50069/f1p0/2242

	2			
R01_cb709_c12/f1p0/2237	NA	R01_cb709_c12/f1p0/2237	NA	NA
R01_cb12452_c10/f1p1/889	NA	R01_cb12452_c10/f1p1/889	NA	NA
R01_cb10186_c4/f1p0/1588	NA	NA	NA	R01_cb10186_c4/f1p0/1588
R01_cb1860_c13/f1p0/2150	NA	NA	NA	R01_cb1860_c13/f1p0/2150
R01_cb3121_c5/f1p0/3594	NA	R01_cb3121_c5/f1p0/3594	R01_cb3121_c5/f1p0/3594	NA
R01_cb14985_c1/f1p0/1395	NA	NA	NA	R01_cb14985_c1/f1p0/1395
R01_cb8564_c11574/f1p0/3962	NA	R01_cb8564_c11574/f1p0/3962	NA	NA
R01_cb1479_c32/f1p0/1175	NA	R01_cb1479_c32/f1p0/1175	NA	NA
R01_cb15828_c0/f1p0/1009	NA	R01_cb15828_c0/f1p0/1009	R01_cb15828_c0/f1p0/1009	R01_cb15828_c0/f1p0/1009
R01_cb684_c2/f1p0/4395	NA	NA	NA	R01_cb684_c2/f1p0/4395
R01_cb8690_c20/f1p0/1538	NA	R01_cb8690_c20/f1p0/1538	NA	R01_cb8690_c20/f1p0/1538
R01_cb8564_c110039/f2p0/4075	NA	R01_cb8564_c110039/f2p0/4075	R01_cb8564_c110039/f2p0/4075	NA
R01_cb8356_c1/f1p0/1927	NA	R01_cb8356_c1/f1p0/1927	NA	NA
R01_cb1567_c1/f1p1/4383	NA	NA	NA	R01_cb1567_c1/f1p1/4383
R01_cb1228_c25/f1p0/2415	NA	NA	NA	R01_cb1228_c25/f1p0/2415
R01_cb8564_c12743/f1p0/2150	NA	NA	NA	R01_cb8564_c12743/f1p0/2150
R01_cb14792_c1/f1p0/1720	NA	R01_cb14792_c1/f1p0/1720	NA	NA
R01_cb2357_c6/f1p0/4143	NA	NA	NA	R01_cb2357_c6/f1p0/4143
R01_cb9009_c6/f1p0/4294	NA	R01_cb9009_c6/f1p0/4294	NA	NA
R01_cb8564_c75993/f1p0/4476	NA	NA	NA	R01_cb8564_c75993/f1p0/4476
R01_cb1030_c18/f1p0/2489	NA	NA	NA	R01_cb1030_c18/f1p0/2489
R01_cb8970_c1/f1p0/2482	NA	R01_cb8970_c1/f1p0/2482	NA	NA
R01_cb1333_c90/f1p0/2033	R01_cb1333_c90/f1p0/2033	R01_cb1333_c90/f1p0/2033	R01_cb1333_c90/f1p0/2033	R01_cb1333_c90/f1p0/2033
R01_cb8564_c91709/f1p1/2474	NA	NA	NA	R01_cb8564_c91709/f1p1/2474

R01_cb17276_c4/f1p0/444	NA	R01_cb17276_c4/f1p0/444	NA	R01_cb17276_c4/f1p0/444
R01_cb12098_c27/f1p0/626	R01_cb12098_c27/f1p0/626	R01_cb12098_c27/f1p0/626	R01_cb12098_c27/f1p0/626	R01_cb12098_c27/f1p0/626
R01_cb16199_c1/f1p0/808	R01_cb16199_c1/f1p0/808	R01_cb16199_c1/f1p0/808	R01_cb16199_c1/f1p0/808	R01_cb16199_c1/f1p0/808
R01_cb9818_c4/f1p0/398	NA	R01_cb9818_c4/f1p0/398	NA	R01_cb9818_c4/f1p0/398
R01_cb11440_c2/f1p0/986	NA	NA	NA	R01_cb11440_c2/f1p0/986
R01_cb12722_c17/f1p0/1712	NA	NA	NA	R01_cb12722_c17/f1p0/1712
R01_cb18178_c1/f1p0/853	NA	NA	NA	R01_cb18178_c1/f1p0/853
R01_cb11190_c2/f1p0/2717	NA	NA	NA	R01_cb11190_c2/f1p0/2717
R01_cb2072_c33/f1p0/3133	NA	NA	NA	R01_cb2072_c33/f1p0/3133
R01_cb6375_c7/f1p0/3856	NA	NA	NA	R01_cb6375_c7/f1p0/3856
R01_cb13545_c37/f1p0/708	NA	NA	NA	R01_cb13545_c37/f1p0/708
R01_cb6792_c8/f1p1/2711	NA	NA	NA	R01_cb6792_c8/f1p1/2711
R01_cb5158_c0/f3p0/1341	NA	NA	NA	R01_cb5158_c0/f3p0/1341
R01_cb10161_c3/f1p0/695	NA	R01_cb10161_c3/f1p0/695	NA	NA
R01_cb18250_c0/f1p0/1093	NA	R01_cb18250_c0/f1p0/1093	NA	NA
R01_cb2970_c3/f2p0/3720	NA	R01_cb2970_c3/f2p0/3720	NA	R01_cb2970_c3/f2p0/3720
R01_cb15701_c1/f1p0/1582	NA	R01_cb15701_c1/f1p0/1582	R01_cb15701_c1/f1p0/1582	R01_cb15701_c1/f1p0/1582
R01_cb13920_c4/f1p0/1358	NA	R01_cb13920_c4/f1p0/1358	NA	NA
R01_cb11828_c0/f1p0/1022	NA	R01_cb11828_c0/f1p0/1022	NA	NA
R01_cb10611_c3/f1p0/2122	NA	NA	NA	R01_cb10611_c3/f1p0/2122
R01_cb8564_c84847/f1p0/2279	NA	R01_cb8564_c84847/f1p0/2279	R01_cb8564_c84847/f1p0/2279	R01_cb8564_c84847/f1p0/2279
R01_cb3079_c5/f1p0/2068	NA	NA	NA	R01_cb3079_c5/f1p0/2068
R01_cb2027_c1/f1p0/4082	NA	NA	R01_cb2027_c1/f1p0/4082	NA
R01_cb10917_c5/f1p0/1172	NA	R01_cb10917_c5/f1p0/1172	NA	NA
R01_cb8564_c37993/f1p0/3281	NA	NA	NA	R01_cb8564_c37993/f1p0/3281
R01_cb101_c1/f1p0/2131	NA	NA	NA	R01_cb101_c1/f1p0/2131

R01_cb7530_c4/f1p1/2539	NA	NA	NA	R01_cb7530_c4/f1p1/2539
R01_cb17363_c10/f1p0/407	R01_cb17363_c10/f1p0/407	R01_cb17363_c10/f1p0/407	R01_cb17363_c10/f1p0/407	NA
R01_cb4943_c0/f1p0/3164	R01_cb4943_c0/f1p0/3164	R01_cb4943_c0/f1p0/3164	NA	NA
R01_cb17973_c53/f1p0/1649	NA	R01_cb17973_c53/f1p0/1649	NA	R01_cb17973_c53/f1p0/1649
R01_cb11344_c3/f1p0/621	R01_cb11344_c3/f1p0/621	R01_cb11344_c3/f1p0/621	NA	R01_cb11344_c3/f1p0/621
R01_cb2161_c12/f1p1/6037	NA	NA	NA	R01_cb2161_c12/f1p1/6037
R01_cb6947_c12/f1p0/2035	NA	NA	NA	R01_cb6947_c12/f1p0/2035
R01_cb1307_c21/f1p0/2796	NA	R01_cb1307_c21/f1p0/2796	R01_cb1307_c21/f1p0/2796	NA
R01_cb11538_c0/f1p0/562	NA	R01_cb11538_c0/f1p0/562	NA	R01_cb11538_c0/f1p0/562
R01_cb18456_c2314/f1p0/497	R01_cb18456_c2314/f1p0/497	R01_cb18456_c2314/f1p0/497	R01_cb18456_c2314/f1p0/497	R01_cb18456_c2314/f1p0/497
R01_cb11212_c2/f1p0/2170	NA	R01_cb11212_c2/f1p0/2170	NA	NA
R01_cb8564_c74859/f1p2/2677	NA	R01_cb8564_c74859/f1p2/2677	NA	NA
R01_cb4268_c2/f1p0/2781	NA	NA	NA	R01_cb4268_c2/f1p0/2781
R01_cb2104_c11/f1p0/1367	NA	NA	R01_cb2104_c11/f1p0/1367	R01_cb2104_c11/f1p0/1367
R01_cb5139_c5/f1p0/3383	NA	NA	NA	R01_cb5139_c5/f1p0/3383
R01_cb12819_c4/f1p0/518	NA	R01_cb12819_c4/f1p0/518	NA	NA
R01_cb8564_c84694/f3p1/2594	NA	NA	NA	R01_cb8564_c84694/f3p1/2594
R01_cb5758_c3/f1p0/3000	NA	R01_cb5758_c3/f1p0/3000	R01_cb5758_c3/f1p0/3000	R01_cb5758_c3/f1p0/3000
R01_cb4338_c5/f1p0/889	NA	NA	NA	R01_cb4338_c5/f1p0/889
R01_cb11677_c1/f1p0/2817	NA	R01_cb11677_c1/f1p0/2817	R01_cb11677_c1/f1p0/2817	R01_cb11677_c1/f1p0/2817
R01_cb17975_c0/f1p0/961	NA	NA	NA	R01_cb17975_c0/f1p0/961
R01_cb6606_c10/f1p0/2105	NA	NA	NA	R01_cb6606_c10/f1p0/2105
R01_cb6715_c1/f1p0/4218	NA	NA	NA	R01_cb6715_c1/f1p0/4218
R01_cb16772_c2/f1p0/1355	NA	NA	NA	R01_cb16772_c2/f1p0/1355
R01_cb18355_c2/f1p0/5068	NA	R01_cb18355_c2/f1p0/5068	R01_cb18355_c2/f1p0/5068	NA
R01_cb10017_c71/f1p0/489	R01_cb10017_c71/f1p0/489	R01_cb10017_c71/f1p0/489	NA	NA

R01_cb2170_c4/f1p0/7876	R01_cb2170_c4/f1p0/7876	NA	NA	NA
R01_cb18456_c3247/f1p0/884	NA	R01_cb18456_c3247/f1p0/884	NA	NA
R01_cb7802_c1/f5p2/1708	NA	NA	NA	R01_cb7802_c1/f5p2/1708
R01_cb17589_c6/f1p0/1450	NA	NA	NA	R01_cb17589_c6/f1p0/1450
R01_cb16269_c1/f1p0/764	NA	NA	NA	R01_cb16269_c1/f1p0/764
R01_cb7441_c6/f1p0/1891	NA	R01_cb7441_c6/f1p0/1891	NA	NA
R01_cb11912_c0/f1p0/1267	NA	NA	NA	R01_cb11912_c0/f1p0/1267
R01_cb13112_c14/f1p0/724	NA	NA	R01_cb13112_c14/f1p0/724	R01_cb13112_c14/f1p0/724
R01_cb13214_c9/f1p0/402	R01_cb13214_c9/f1p0/402	R01_cb13214_c9/f1p0/402	NA	NA
R01_cb1459_c4/f1p1/2579	NA	R01_cb1459_c4/f1p1/2579	NA	NA
R01_cb18409_c72/f1p0/1479	NA	R01_cb18409_c72/f1p0/1479	R01_cb18409_c72/f1p0/1479	R01_cb18409_c72/f1p0/1479
R01_cb18456_c6979/f1p0/492	R01_cb18456_c6979/f1p0/492	R01_cb18456_c6979/f1p0/492	R01_cb18456_c6979/f1p0/492	R01_cb18456_c6979/f1p0/492
R01_cb8909_c10/f1p1/1976	NA	R01_cb8909_c10/f1p1/1976	NA	NA
R01_cb5909_c13/f1p0/782	NA	NA	NA	R01_cb5909_c13/f1p0/782
R01_cb14344_c3/f1p0/1259	NA	R01_cb14344_c3/f1p0/1259	R01_cb14344_c3/f1p0/1259	R01_cb14344_c3/f1p0/1259
R01_cb16755_c1/f1p0/1708	NA	R01_cb16755_c1/f1p0/1708	NA	NA
R01_cb8564_c147548/f5p1/3243	NA	NA	NA	R01_cb8564_c147548/f5p1/3243
R01_cb8131_c4/f1p0/995	NA	NA	NA	R01_cb8131_c4/f1p0/995
R01_cb13943_c6/f1p1/1376	NA	NA	NA	R01_cb13943_c6/f1p1/1376
R01_cb17756_c13/f1p0/1400	R01_cb17756_c13/f1p0/1400	R01_cb17756_c13/f1p0/1400	R01_cb17756_c13/f1p0/1400	R01_cb17756_c13/f1p0/1400
R01_cb2108_c24/f1p0/1390	NA	R01_cb2108_c24/f1p0/1390	NA	NA
R01_cb12432_c1/f22p1/567	NA	NA	R01_cb12432_c1/f22p1/567	R01_cb12432_c1/f22p1/567
R01_cb167_c59/f1p0/2031	NA	NA	NA	R01_cb167_c59/f1p0/2031
R01_cb8564_c88325/f1p0/2456	NA	R01_cb8564_c88325/f1p0/2456	NA	R01_cb8564_c88325/f1p0/2456
R01_cb1178_c10/f1p0/2304	NA	NA	NA	R01_cb1178_c10/f1p0/2304

R01_cb8941_c4/f1p0/1811	NA	NA	NA	R01_cb8941_c4/f1p0/1811
R01_cb8564_c11905/f1p0/3160	NA	R01_cb8564_c11905/f1p0/3160	R01_cb8564_c11905/f1p0/3160	R01_cb8564_c11905/f1p0/3160
R01_cb5234_c10/f1p0/7710	NA	NA	NA	R01_cb5234_c10/f1p0/7710
R01_cb3575_c7/f1p1/3444	NA	NA	NA	R01_cb3575_c7/f1p1/3444
R01_cb10950_c1/f1p0/2520	R01_cb10950_c1/f1p0/2520	R01_cb10950_c1/f1p0/2520	NA	NA
R01_cb2451_c39/f1p0/3469	NA	NA	NA	R01_cb2451_c39/f1p0/3469
R01_cb13801_c3/f4p0/598	NA	R01_cb13801_c3/f4p0/598	NA	NA
R01_cb17973_c47/f1p1/887	NA	NA	R01_cb17973_c47/f1p1/887	R01_cb17973_c47/f1p1/887
R01_cb17973_c38/f1p0/480	NA	R01_cb17973_c38/f1p0/480	R01_cb17973_c38/f1p0/480	NA
R01_cb8564_c84862/f1p2/2064	NA	R01_cb8564_c84862/f1p2/2064	NA	NA
R01_cb8564_c129794/f1p0/2259	R01_cb8564_c129794/f1p0/2259	R01_cb8564_c129794/f1p0/2259	R01_cb8564_c129794/f1p0/2259	R01_cb8564_c129794/f1p0/2259
R01_cb8104_c5/f1p0/1048	NA	NA	NA	R01_cb8104_c5/f1p0/1048
R01_cb16719_c2/f1p0/969	NA	R01_cb16719_c2/f1p0/969	NA	NA
R01_cb5471_c13/f1p1/2689	NA	NA	NA	R01_cb5471_c13/f1p1/2689
R01_cb15777_c2/f1p0/1094	NA	NA	NA	R01_cb15777_c2/f1p0/1094
R01_cb1905_c13/f1p0/3167	NA	R01_cb1905_c13/f1p0/3167	R01_cb1905_c13/f1p0/3167	NA
R01_cb13696_c4/f1p0/1591	NA	NA	NA	R01_cb13696_c4/f1p0/1591
R01_cb9506_c11/f1p0/1083	NA	NA	NA	R01_cb9506_c11/f1p0/1083
R01_cb8564_c91056/f1p2/2552	NA	NA	NA	R01_cb8564_c91056/f1p2/2552
R01_cb6861_c10/f1p0/2927	R01_cb6861_c10/f1p0/2927	R01_cb6861_c10/f1p0/2927	NA	R01_cb6861_c10/f1p0/2927
R01_cb7221_c13/f1p1/2577	NA	NA	NA	R01_cb7221_c13/f1p1/2577
R01_cb9747_c1/f1p1/2201	NA	R01_cb9747_c1/f1p1/2201	NA	NA
R01_cb2164_c1/f1p0/3263	NA	R01_cb2164_c1/f1p0/3263	NA	NA
R01_cb8782_c0/f1p0/2273	NA	NA	NA	R01_cb8782_c0/f1p0/2273
R01_cb8564_c2728/f1p0/2835	NA	R01_cb8564_c2728/f1p0/2835	R01_cb8564_c2728/f1p0/2835	R01_cb8564_c2728/f1p0/2835

R01_cb8564_c111209/f1p0/2907	NA	R01_cb8564_c111209/f1p0/2907	R01_cb8564_c111209/f1p0/2907	R01_cb8564_c111209/f1p0/2907
R01_cb9723_c2/f1p0/1998	NA	NA	NA	R01_cb9723_c2/f1p0/1998
R01_cb6732_c26/f1p0/680	NA	R01_cb6732_c26/f1p0/680	NA	R01_cb6732_c26/f1p0/680
R01_cb17251_c8/f1p0/1878	R01_cb17251_c8/f1p0/1878	R01_cb17251_c8/f1p0/1878	R01_cb17251_c8/f1p0/1878	R01_cb17251_c8/f1p0/1878
R01_cb8564_c109489/f3p0/2914	NA	R01_cb8564_c109489/f3p0/2914	NA	NA
R01_cb18753_c0/f1p1/5035	NA	R01_cb18753_c0/f1p1/5035	R01_cb18753_c0/f1p1/5035	R01_cb18753_c0/f1p1/5035
R01_cb9993_c6/f1p0/1174	NA	NA	NA	R01_cb9993_c6/f1p0/1174
R01_cb17172_c1/f1p0/1363	NA	R01_cb17172_c1/f1p0/1363	NA	R01_cb17172_c1/f1p0/1363
R01_cb4455_c11/f1p1/2840	NA	R01_cb4455_c11/f1p1/2840	NA	NA
R01_cb15998_c2/f1p0/1533	NA	NA	NA	R01_cb15998_c2/f1p0/1533
R01_cb14940_c5/f1p0/1420	NA	R01_cb14940_c5/f1p0/1420	R01_cb14940_c5/f1p0/1420	NA
R01_cb12421_c70/f1p7/721	NA	R01_cb12421_c70/f1p7/721	NA	NA
R01_cb16997_c98/f1p0/2258	NA	R01_cb16997_c98/f1p0/2258	NA	NA
R01_cb13834_c2/f1p0/706	R01_cb13834_c2/f1p0/706	R01_cb13834_c2/f1p0/706	R01_cb13834_c2/f1p0/706	R01_cb13834_c2/f1p0/706
R01_cb8991_c9/f1p0/2306	NA	NA	NA	R01_cb8991_c9/f1p0/2306
R01_cb14761_c2/f2p0/789	R01_cb14761_c2/f2p0/789	R01_cb14761_c2/f2p0/789	R01_cb14761_c2/f2p0/789	R01_cb14761_c2/f2p0/789
R01_cb13249_c2/f1p0/1126	NA	NA	NA	R01_cb13249_c2/f1p0/1126
R01_cb8407_c4/f1p0/918	R01_cb8407_c4/f1p0/918	R01_cb8407_c4/f1p0/918	R01_cb8407_c4/f1p0/918	R01_cb8407_c4/f1p0/918
R01_cb8564_c81705/f1p0/2305	NA	R01_cb8564_c81705/f1p0/2305	NA	NA
R01_cb2845_c4/f2p1/3455	NA	NA	NA	R01_cb2845_c4/f2p1/3455
R01_cb11232_c3/f1p0/2508	NA	R01_cb11232_c3/f1p0/2508	R01_cb11232_c3/f1p0/2508	NA
R01_cb2488_c1/f1p0/3123	NA	R01_cb2488_c1/f1p0/3123	R01_cb2488_c1/f1p0/3123	NA
R01_cb8564_c18112/f1p0/2321	NA	NA	R01_cb8564_c18112/f1p0/2321	R01_cb8564_c18112/f1p0/2321
R01_cb14337_c8/f1p0/709	NA	NA	NA	R01_cb14337_c8/f1p0/709

R01_cb16095_c2/f1p0/1146	NA	R01_cb16095_c2/f1p0/1146	NA	NA
R01_cb10380_c0/f2p1/833	NA	NA	NA	R01_cb10380_c0/f2p1/833
R01_cb15516_c0/f2p0/1385	NA	NA	NA	R01_cb15516_c0/f2p0/1385
R01_cb10611_c5/f1p0/1402	NA	NA	NA	R01_cb10611_c5/f1p0/1402
R01_cb13433_c99/f1p0/650	R01_cb13433_c99/f1p0/650	R01_cb13433_c99/f1p0/650	R01_cb13433_c99/f1p0/650	R01_cb13433_c99/f1p0/650
R01_cb5855_c2/f1p0/2032	NA	R01_cb5855_c2/f1p0/2032	NA	NA
R01_cb8031_c0/f4p0/2437	NA	NA	R01_cb8031_c0/f4p0/2437	R01_cb8031_c0/f4p0/2437
R01_cb9583_c5/f1p0/1887	NA	R01_cb9583_c5/f1p0/1887	NA	NA
R01_cb13283_c10/f1p0/1848	NA	NA	NA	R01_cb13283_c10/f1p0/1848
R01_cb10062_c29/f1p0/688	NA	NA	NA	R01_cb10062_c29/f1p0/688
R01_cb18223_c27/f1p0/739	R01_cb18223_c27/f1p0/739	R01_cb18223_c27/f1p0/739	R01_cb18223_c27/f1p0/739	R01_cb18223_c27/f1p0/739
R01_cb8564_c70645/f1p0/3759	NA	R01_cb8564_c70645/f1p0/3759	R01_cb8564_c70645/f1p0/3759	R01_cb8564_c70645/f1p0/3759
R01_cb2821_c3/f1p0/1030	NA	R01_cb2821_c3/f1p0/1030	NA	NA
R01_cb8564_c2089/f1p0/2900	NA	R01_cb8564_c2089/f1p0/2900	R01_cb8564_c2089/f1p0/2900	R01_cb8564_c2089/f1p0/2900
R01_cb17756_c2/f3p0/314	NA	R01_cb17756_c2/f3p0/314	R01_cb17756_c2/f3p0/314	R01_cb17756_c2/f3p0/314
R01_cb1739_c15/f1p0/5595	NA	NA	NA	R01_cb1739_c15/f1p0/5595
R01_cb10333_c6/f1p0/375	R01_cb10333_c6/f1p0/375	R01_cb10333_c6/f1p0/375	NA	R01_cb10333_c6/f1p0/375
R01_cb3563_c0/f2p0/1482	NA	NA	NA	R01_cb3563_c0/f2p0/1482
R01_cb8107_c0/f2p0/420	R01_cb8107_c0/f2p0/420	R01_cb8107_c0/f2p0/420	R01_cb8107_c0/f2p0/420	R01_cb8107_c0/f2p0/420
R01_cb10353_c2/f1p0/660	NA	R01_cb10353_c2/f1p0/660	R01_cb10353_c2/f1p0/660	R01_cb10353_c2/f1p0/660
R01_cb6037_c1/f2p1/2755	NA	R01_cb6037_c1/f2p1/2755	NA	NA
R01_cb2826_c9/f1p0/2724	NA	R01_cb2826_c9/f1p0/2724	NA	NA
R01_cb7539_c3/f1p0/2258	NA	NA	R01_cb7539_c3/f1p0/2258	R01_cb7539_c3/f1p0/2258
R01_cb8564_c10740/f1p0/2021	NA	R01_cb8564_c10740/f1p0/2021	NA	R01_cb8564_c10740/f1p0/2021
R01_cb8564_c81245/f1p0/2959	NA	R01_cb8564_c81245/f1p0/2959	NA	NA
R01_cb14947_c1/f1p1/567	R01_cb14947_c1/f1p1/567	R01_cb14947_c1/f1p1/567	NA	R01_cb14947_c1/f1p1/567

R01_cb6278_c7/f1p0/2569	NA	NA	NA	R01_cb6278_c7/f1p0/2569
R01_cb1138_c8/f3p0/2824	NA	NA	R01_cb1138_c8/f3p0/2824	NA
R01_cb4529_c13/f1p0/614	NA	R01_cb4529_c13/f1p0/614	NA	NA
R01_cb13021_c0/f4p0/544	R01_cb13021_c0/f4p0/544	R01_cb13021_c0/f4p0/544	NA	NA
R01_cb2804_c141/f1p0/2031	NA	R01_cb2804_c141/f1p0/2031	R01_cb2804_c141/f1p0/2031	R01_cb2804_c141/f1p0/2031
R01_cb5739_c1/f1p0/2437	NA	R01_cb5739_c1/f1p0/2437	R01_cb5739_c1/f1p0/2437	NA
R01_cb16590_c1/f1p0/1306	R01_cb16590_c1/f1p0/1306	R01_cb16590_c1/f1p0/1306	R01_cb16590_c1/f1p0/1306	R01_cb16590_c1/f1p0/1306
R01_cb13715_c0/f1p0/327	R01_cb13715_c0/f1p0/327	R01_cb13715_c0/f1p0/327	R01_cb13715_c0/f1p0/327	R01_cb13715_c0/f1p0/327
R01_cb8564_c73845/f1p0/3547	NA	R01_cb8564_c73845/f1p0/3547	NA	NA
R01_cb8564_c109985/f4p0/3333	NA	R01_cb8564_c109985/f4p0/3333	R01_cb8564_c109985/f4p0/3333	R01_cb8564_c109985/f4p0/3333
R01_cb11624_c2/f1p0/738	R01_cb11624_c2/f1p0/738	R01_cb11624_c2/f1p0/738	R01_cb11624_c2/f1p0/738	R01_cb11624_c2/f1p0/738
R01_cb4469_c12/f1p0/1520	NA	NA	NA	R01_cb4469_c12/f1p0/1520
R01_cb8564_c123532/f1p0/3028	NA	R01_cb8564_c123532/f1p0/3028	R01_cb8564_c123532/f1p0/3028	NA
R01_cb4777_c0/f3p0/733	NA	NA	NA	R01_cb4777_c0/f3p0/733
R01_cb17973_c25/f1p0/888	R01_cb17973_c25/f1p0/888	R01_cb17973_c25/f1p0/888	R01_cb17973_c25/f1p0/888	NA
R01_cb6885_c2/f1p0/2745	NA	NA	NA	R01_cb6885_c2/f1p0/2745
R01_cb13545_c32/f1p0/832	NA	NA	R01_cb13545_c32/f1p0/832	R01_cb13545_c32/f1p0/832
R01_cb10042_c0/f1p0/1693	NA	NA	NA	R01_cb10042_c0/f1p0/1693
R01_cb8564_c111833/f1p0/2574	NA	R01_cb8564_c111833/f1p0/2574	NA	R01_cb8564_c111833/f1p0/2574
R01_cb16125_c21/f1p0/1679	NA	R01_cb16125_c21/f1p0/1679	NA	R01_cb16125_c21/f1p0/1679
R01_cb12165_c16/f1p0/959	NA	R01_cb12165_c16/f1p0/959	NA	NA
R01_cb5647_c0/f4p0/3050	NA	NA	NA	R01_cb5647_c0/f4p0/3050
R01_cb7611_c14/f1p0/2456	NA	NA	NA	R01_cb7611_c14/f1p0/2456

R01_cb8564_c124964/f1p0/2142	NA	NA	NA	R01_cb8564_c124964/f1p0/2142
R01_cb13688_c3/f1p0/1199	NA	R01_cb13688_c3/f1p0/1199	NA	NA
R01_cb8564_c2908/f1p0/4349	NA	R01_cb8564_c2908/f1p0/4349	NA	NA
R01_cb2806_c4/f1p0/3477	NA	NA	NA	R01_cb2806_c4/f1p0/3477
R01_cb3699_c7/f1p0/2126	NA	R01_cb3699_c7/f1p0/2126	NA	NA
R01_cb18591_c1/f1p0/756	NA	R01_cb18591_c1/f1p0/756	R01_cb18591_c1/f1p0/756	R01_cb18591_c1/f1p0/756
R01_cb4627_c1/f1p0/2699	NA	NA	NA	R01_cb4627_c1/f1p0/2699
R01_cb12427_c2/f1p0/1451	NA	R01_cb12427_c2/f1p0/1451	NA	NA
R01_cb16123_c0/f2p0/1577	NA	R01_cb16123_c0/f2p0/1577	NA	NA
R01_cb8564_c14195/f1p0/3596	NA	R01_cb8564_c14195/f1p0/3596	R01_cb8564_c14195/f1p0/3596	R01_cb8564_c14195/f1p0/3596
R01_cb9313_c1/f1p0/2119	NA	R01_cb9313_c1/f1p0/2119	NA	R01_cb9313_c1/f1p0/2119
R01_cb7419_c4/f1p1/2584	NA	NA	NA	R01_cb7419_c4/f1p1/2584
R01_cb8564_c77314/f9p0/3031	NA	R01_cb8564_c77314/f9p0/3031	R01_cb8564_c77314/f9p0/3031	NA
R01_cb8564_c117355/f1p0/2431	NA	R01_cb8564_c117355/f1p0/2431	NA	NA
R01_cb8564_c80964/f1p0/2248	NA	R01_cb8564_c80964/f1p0/2248	R01_cb8564_c80964/f1p0/2248	R01_cb8564_c80964/f1p0/2248
R01_cb18409_c23/f2p0/447	NA	R01_cb18409_c23/f2p0/447	R01_cb18409_c23/f2p0/447	R01_cb18409_c23/f2p0/447
R01_cb7342_c4/f1p1/2046	NA	NA	NA	R01_cb7342_c4/f1p1/2046
R01_cb8706_c2/f1p0/2252	NA	R01_cb8706_c2/f1p0/2252	NA	NA
R01_cb8564_c3039/f1p0/2656	NA	R01_cb8564_c3039/f1p0/2656	NA	NA
R01_cb8564_c39047/f1p0/2845	NA	R01_cb8564_c39047/f1p0/2845	NA	NA
R01_cb13897_c1/f1p1/1422	NA	NA	NA	R01_cb13897_c1/f1p1/1422
R01_cb1124_c16/f1p0/1079	NA	NA	NA	R01_cb1124_c16/f1p0/1079
R01_cb8056_c7/f1p0/4756	NA	R01_cb8056_c7/f1p0/4756	NA	NA
R01_cb16247_c1/f1p0/539	NA	R01_cb16247_c1/f1p0/539	R01_cb16247_c1/f1p0/539	R01_cb16247_c1/f1p0/539

R01_cb9168_c3/f1p0/2165	NA	R01_cb9168_c3/f1p0/2165	NA	NA
R01_cb17098_c0/f1p0/663	R01_cb17098_c0/f1p0/663	R01_cb17098_c0/f1p0/663	NA	NA
R01_cb3655_c0/f2p0/2991	NA	R01_cb3655_c0/f2p0/2991	NA	NA
R01_cb4140_c11/f1p0/3083	NA	NA	NA	R01_cb4140_c11/f1p0/3083
R01_cb12666_c5/f1p0/1889	NA	R01_cb12666_c5/f1p0/1889	NA	NA
R01_cb10261_c2/f1p0/1677	NA	R01_cb10261_c2/f1p0/1677	NA	NA
R01_cb7942_c14/f1p0/1977	NA	R01_cb7942_c14/f1p0/1977	NA	NA
R01_cb17756_c17/f1p0/1768	R01_cb17756_c17/f1p0/1768	R01_cb17756_c17/f1p0/1768	R01_cb17756_c17/f1p0/1768	R01_cb17756_c17/f1p0/1768
R01_cb3941_c87/f1p0/1928	NA	R01_cb3941_c87/f1p0/1928	NA	NA
R01_cb8564_c68740/f3p0/3027	NA	NA	NA	R01_cb8564_c68740/f3p0/3027
R01_cb124_c39/f1p0/2659	NA	R01_cb124_c39/f1p0/2659	NA	R01_cb124_c39/f1p0/2659
R01_cb16950_c0/f1p0/958	NA	R01_cb16950_c0/f1p0/958	NA	NA
R01_cb8564_c85126/f2p0/3277	R01_cb8564_c85126/f2p0/3277	R01_cb8564_c85126/f2p0/3277	NA	NA
R01_cb8564_c14252/f1p0/2308	NA	R01_cb8564_c14252/f1p0/2308	NA	R01_cb8564_c14252/f1p0/2308
R01_cb8564_c91348/f1p0/2713	NA	NA	NA	R01_cb8564_c91348/f1p0/2713
R01_cb11442_c1/f1p0/3117	R01_cb11442_c1/f1p0/3117	R01_cb11442_c1/f1p0/3117	NA	NA
R01_cb9418_c4/f1p1/1988	NA	NA	NA	R01_cb9418_c4/f1p1/1988
R01_cb7296_c1/f4p1/2068	NA	NA	NA	R01_cb7296_c1/f4p1/2068
R01_cb8564_c90285/f1p0/2883	NA	NA	NA	R01_cb8564_c90285/f1p0/2883
R01_cb6802_c66/f1p0/1928	NA	R01_cb6802_c66/f1p0/1928	R01_cb6802_c66/f1p0/1928	R01_cb6802_c66/f1p0/1928
R01_cb1304_c1/f2p0/926	NA	NA	NA	R01_cb1304_c1/f2p0/926
R01_cb8379_c11/f1p0/1372	NA	R01_cb8379_c11/f1p0/1372	NA	NA
R01_cb16256_c1/f1p0/716	R01_cb16256_c1/f1p0/716	R01_cb16256_c1/f1p0/716	R01_cb16256_c1/f1p0/716	R01_cb16256_c1/f1p0/716
R01_cb10365_c14/f1p0/1680	NA	R01_cb10365_c14/f1p0/1680	NA	NA
R01_cb15038_c15/f1p0/489	R01_cb15038_c15/f1p0/489	R01_cb15038_c15/f1p0/489	NA	NA

R01_cb6069_c4/f1p0/2745	NA	NA	NA	R01_cb6069_c4/f1p0/2745
R01_cb8564_c1991/f1p0/2537	NA	R01_cb8564_c1991/f1p0/2537	R01_cb8564_c1991/f1p0/2537	R01_cb8564_c1991/f1p0/2537
R01_cb11838_c1/f1p0/3003	R01_cb11838_c1/f1p0/3003	R01_cb11838_c1/f1p0/3003	R01_cb11838_c1/f1p0/3003	R01_cb11838_c1/f1p0/3003
R01_cb905_c0/f1p0/4576	NA	NA	NA	R01_cb905_c0/f1p0/4576
R01_cb480_c0/f1p1/4778	NA	R01_cb480_c0/f1p1/4778	NA	R01_cb480_c0/f1p1/4778
R01_cb18132_c13/f1p0/1614	NA	R01_cb18132_c13/f1p0/1614	NA	NA
R01_cb10921_c3/f1p0/1562	NA	NA	NA	R01_cb10921_c3/f1p0/1562
R01_cb8564_c79662/f1p0/4430	NA	NA	NA	R01_cb8564_c79662/f1p0/4430
R01_cb12386_c10/f1p0/807	NA	R01_cb12386_c10/f1p0/807	R01_cb12386_c10/f1p0/807	R01_cb12386_c10/f1p0/807
R01_cb3580_c71/f1p1/2713	NA	R01_cb3580_c71/f1p1/2713	NA	NA
R01_cb18526_c12/f1p0/858	R01_cb18526_c12/f1p0/858	R01_cb18526_c12/f1p0/858	R01_cb18526_c12/f1p0/858	R01_cb18526_c12/f1p0/858
R01_cb4211_c1/f6p0/2760	NA	R01_cb4211_c1/f6p0/2760	NA	NA
R01_cb15068_c14/f2p0/1162	NA	R01_cb15068_c14/f2p0/1162	NA	NA
R01_cb124_c43/f1p0/2759	NA	NA	NA	R01_cb124_c43/f1p0/2759
R01_cb4536_c0/f1p1/3324	NA	NA	NA	R01_cb4536_c0/f1p1/3324
R01_cb8564_c79463/f1p0/2118	R01_cb8564_c79463/f1p0/2118	R01_cb8564_c79463/f1p0/2118	NA	R01_cb8564_c79463/f1p0/2118
R01_cb4453_c0/f1p0/3346	NA	NA	NA	R01_cb4453_c0/f1p0/3346
R01_cb8703_c3/f1p0/1515	NA	R01_cb8703_c3/f1p0/1515	NA	NA
R01_cb8564_c84278/f1p0/2530	NA	R01_cb8564_c84278/f1p0/2530	R01_cb8564_c84278/f1p0/2530	NA
R01_cb8564_c115030/f1p0/1946	R01_cb8564_c115030/f1p0/1946	R01_cb8564_c115030/f1p0/1946	R01_cb8564_c115030/f1p0/1946	R01_cb8564_c115030/f1p0/1946
R01_cb10533_c2/f1p0/3054	NA	R01_cb10533_c2/f1p0/3054	NA	R01_cb10533_c2/f1p0/3054
R01_cb2997_c4/f1p0/3859	NA	NA	NA	R01_cb2997_c4/f1p0/3859
R01_cb7114_c7/f1p0/2312	NA	NA	NA	R01_cb7114_c7/f1p0/2312
R01_cb3680_c5/f1p0/1263	NA	NA	NA	R01_cb3680_c5/f1p0/1263

R01_cb392_c12/f1p0/2249	R01_cb392_c12/f1p0/2249	R01_cb392_c12/f1p0/2249	R01_cb392_c12/f1p0/2249	R01_cb392_c12/f1p0/2249
R01_cb14226_c0/f1p0/873	NA	NA	R01_cb14226_c0/f1p0/873	R01_cb14226_c0/f1p0/873
R01_cb8564_c86578/f1p0/2991	NA	R01_cb8564_c86578/f1p0/2991	NA	NA
R01_cb8564_c20384/f1p4/2475	NA	R01_cb8564_c20384/f1p4/2475	NA	R01_cb8564_c20384/f1p4/2475
R01_cb8564_c109543/f3p0/1969	NA	R01_cb8564_c109543/f3p0/1969	NA	R01_cb8564_c109543/f3p0/1969
R01_cb16334_c0/f1p0/939	R01_cb16334_c0/f1p0/939	R01_cb16334_c0/f1p0/939	R01_cb16334_c0/f1p0/939	R01_cb16334_c0/f1p0/939
R01_cb2570_c3/f2p1/2455	NA	NA	NA	R01_cb2570_c3/f2p1/2455
R01_cb9242_c1/f1p0/2019	NA	NA	R01_cb9242_c1/f1p0/2019	NA
R01_cb18055_c0/f1p0/398	NA	NA	R01_cb18055_c0/f1p0/398	R01_cb18055_c0/f1p0/398
R01_cb18456_c2541/f1p0/789	R01_cb18456_c2541/f1p0/789	R01_cb18456_c2541/f1p0/789	R01_cb18456_c2541/f1p0/789	R01_cb18456_c2541/f1p0/789
R01_cb13003_c7/f1p0/1133	NA	R01_cb13003_c7/f1p0/1133	NA	NA
R01_cb18456_c1830/f1p0/1793	NA	R01_cb18456_c1830/f1p0/1793	R01_cb18456_c1830/f1p0/1793	R01_cb18456_c1830/f1p0/1793
R01_cb1892_c3/f1p0/2379	NA	NA	NA	R01_cb1892_c3/f1p0/2379
R01_cb2463_c5/f1p0/2823	NA	NA	NA	R01_cb2463_c5/f1p0/2823
R01_cb2962_c19/f1p0/2430	NA	R01_cb2962_c19/f1p0/2430	R01_cb2962_c19/f1p0/2430	NA
R01_cb8564_c78524/f1p0/3201	NA	R01_cb8564_c78524/f1p0/3201	R01_cb8564_c78524/f1p0/3201	R01_cb8564_c78524/f1p0/3201
R01_cb6285_c2/f1p0/2340	R01_cb6285_c2/f1p0/2340	R01_cb6285_c2/f1p0/2340	R01_cb6285_c2/f1p0/2340	R01_cb6285_c2/f1p0/2340
R01_cb18456_c1853/f1p0/984	NA	NA	NA	R01_cb18456_c1853/f1p0/984
R01_cb3390_c2/f1p0/1813	NA	R01_cb3390_c2/f1p0/1813	NA	NA
R01_cb14968_c16/f1p0/1700	NA	NA	NA	R01_cb14968_c16/f1p0/1700
R01_cb6221_c11/f1p0/2100	NA	R01_cb6221_c11/f1p0/2100	NA	NA
R01_cb1297_c11/f2p0/3854	NA	NA	NA	R01_cb1297_c11/f2p0/3854
R01_cb15034_c3/f1p0/1650	R01_cb15034_c3/f1p0/1650	R01_cb15034_c3/f1p0/1650	NA	R01_cb15034_c3/f1p0/1650
R01_cb8564_c81809/f2p0/2165	NA	NA	NA	R01_cb8564_c81809/f2p0/2165
R01_cb5412_c2/f1p0/3104	NA	NA	NA	R01_cb5412_c2/f1p0/3104

R01_cb8564_c35079/f1p0/4002	NA	R01_cb8564_c35079/f1p0/4002	NA	NA
R01_cb12133_c1/f1p0/1064	NA	NA	NA	R01_cb12133_c1/f1p0/1064
R01_cb18456_c7167/f1p0/604	NA	R01_cb18456_c7167/f1p0/604	NA	NA
R01_cb11988_c9/f1p0/634	NA	R01_cb11988_c9/f1p0/634	NA	NA
R01_cb14344_c4/f1p0/1632	R01_cb14344_c4/f1p0/1632	R01_cb14344_c4/f1p0/1632	R01_cb14344_c4/f1p0/1632	R01_cb14344_c4/f1p0/1632
R01_cb8564_c25480/f2p4/4527	NA	R01_cb8564_c25480/f2p4/4527	R01_cb8564_c25480/f2p4/4527	R01_cb8564_c25480/f2p4/4527
R01_cb699_c2/f1p0/1518	NA	NA	NA	R01_cb699_c2/f1p0/1518
R01_cb18456_c5139/f1p2/634	R01_cb18456_c5139/f1p2/634	NA	NA	NA
R01_cb8433_c13/f1p0/2356	NA	NA	NA	R01_cb8433_c13/f1p0/2356
R01_cb9127_c3/f1p0/2334	NA	R01_cb9127_c3/f1p0/2334	NA	NA
R01_cb13857_c0/f1p0/920	NA	NA	NA	R01_cb13857_c0/f1p0/920
R01_cb4821_c15/f1p0/362	R01_cb4821_c15/f1p0/362	R01_cb4821_c15/f1p0/362	R01_cb4821_c15/f1p0/362	R01_cb4821_c15/f1p0/362
R01_cb11120_c4/f1p0/862	NA	NA	R01_cb11120_c4/f1p0/862	R01_cb11120_c4/f1p0/862
R01_cb16814_c1/f1p0/693	NA	NA	NA	R01_cb16814_c1/f1p0/693
R01_cb12321_c847/f1p0/414	R01_cb12321_c847/f1p0/414	NA	NA	NA
R01_cb8564_c20839/f1p0/1972	NA	NA	NA	R01_cb8564_c20839/f1p0/1972
R01_cb396_c69/f1p0/1086	R01_cb396_c69/f1p0/1086	R01_cb396_c69/f1p0/1086	NA	R01_cb396_c69/f1p0/1086
R01_cb12818_c0/f1p0/940	NA	R01_cb12818_c0/f1p0/940	R01_cb12818_c0/f1p0/940	R01_cb12818_c0/f1p0/940
R01_cb6453_c3/f2p0/2779	NA	R01_cb6453_c3/f2p0/2779	R01_cb6453_c3/f2p0/2779	NA
R01_cb8564_c124865/f1p0/2302	NA	R01_cb8564_c124865/f1p0/2302	NA	R01_cb8564_c124865/f1p0/2302
R01_cb18423_c1/f1p0/602	R01_cb18423_c1/f1p0/602	NA	NA	R01_cb18423_c1/f1p0/602
R01_cb8564_c77683/f1p0/2153	NA	NA	NA	R01_cb8564_c77683/f1p0/2153
R01_cb8564_c9762/f2p1/4234	NA	NA	NA	R01_cb8564_c9762/f2p1/4234
R01_cb11187_c1/f1p0/1994	NA	NA	R01_cb11187_c1/f1p0/1994	R01_cb11187_c1/f1p0/1994
R01_cb5846_c56/f1p0/2248	R01_cb5846_c56/f1p0/2248	R01_cb5846_c56/f1p0/2248	R01_cb5846_c56/f1p0/2248	R01_cb5846_c56/f1p0/2248

R01_cb3368_c1/f1p1/2770	NA	NA	NA	R01_cb3368_c1/f1p1/2770
R01_cb16475_c0/f1p0/625	R01_cb16475_c0/f1p0/625	R01_cb16475_c0/f1p0/625	R01_cb16475_c0/f1p0/625	R01_cb16475_c0/f1p0/625
R01_cb15176_c1/f1p0/948	NA	R01_cb15176_c1/f1p0/948	NA	NA
R01_cb18409_c150/f1p0/498	R01_cb18409_c150/f1p0/498	R01_cb18409_c150/f1p0/498	R01_cb18409_c150/f1p0/498	R01_cb18409_c150/f1p0/498
R01_cb5622_c3/f1p0/1762	NA	NA	NA	R01_cb5622_c3/f1p0/1762
R01_cb8564_c2518/f1p3/2187	NA	R01_cb8564_c2518/f1p3/2187	NA	NA
R01_cb4191_c43/f1p0/3513	NA	R01_cb4191_c43/f1p0/3513	NA	NA
R01_cb13532_c2/f1p3/1066	R01_cb13532_c2/f1p3/1066	NA	R01_cb13532_c2/f1p3/1066	R01_cb13532_c2/f1p3/1066
R01_cb11017_c2/f1p0/823	NA	NA	R01_cb11017_c2/f1p0/823	R01_cb11017_c2/f1p0/823
R01_cb17430_c1/f1p0/1790	R01_cb17430_c1/f1p0/1790	R01_cb17430_c1/f1p0/1790	R01_cb17430_c1/f1p0/1790	R01_cb17430_c1/f1p0/1790
R01_cb9207_c0/f1p0/2153	NA	NA	NA	R01_cb9207_c0/f1p0/2153
R01_cb580_c0/f1p0/4754	NA	R01_cb580_c0/f1p0/4754	NA	NA
R01_cb5533_c18/f2p0/3079	R01_cb5533_c18/f2p0/3079	R01_cb5533_c18/f2p0/3079	R01_cb5533_c18/f2p0/3079	R01_cb5533_c18/f2p0/3079
R01_cb12148_c13/f1p0/797	NA	NA	NA	R01_cb12148_c13/f1p0/797
R01_cb7356_c5/f1p0/2091	NA	NA	NA	R01_cb7356_c5/f1p0/2091
R01_cb9932_c7/f9p0/623	NA	R01_cb9932_c7/f9p0/623	R01_cb9932_c7/f9p0/623	R01_cb9932_c7/f9p0/623
R01_cb8564_c23560/f1p0/2533	NA	R01_cb8564_c23560/f1p0/2533	NA	R01_cb8564_c23560/f1p0/2533
R01_cb8564_c125538/f1p0/2703	R01_cb8564_c125538/f1p0/2703	R01_cb8564_c125538/f1p0/2703	R01_cb8564_c125538/f1p0/2703	R01_cb8564_c125538/f1p0/2703
R01_cb8564_c109164/f5p0/2121	R01_cb8564_c109164/f5p0/2121	R01_cb8564_c109164/f5p0/2121	R01_cb8564_c109164/f5p0/2121	NA
R01_cb8564_c3040/f1p0/2523	NA	R01_cb8564_c3040/f1p0/2523	R01_cb8564_c3040/f1p0/2523	NA
R01_cb14092_c1/f1p0/1038	NA	NA	NA	R01_cb14092_c1/f1p0/1038
R01_cb8031_c2/f2p0/1878	NA	NA	R01_cb8031_c2/f2p0/1878	NA
R01_cb8564_c81895/f1p0/2889	NA	R01_cb8564_c81895/f1p0/2889	R01_cb8564_c81895/f1p0/2889	NA
R01_cb10948_c5/f1p0/3487	NA	NA	NA	R01_cb10948_c5/f1p0/3487

R01_cb6148_c1/f1p0/2678	NA	R01_cb6148_c1/f1p0/2678	NA	NA
R01_cb11213_c2/f1p0/470	R01_cb11213_c2/f1p0/470	R01_cb11213_c2/f1p0/470	NA	NA
R01_cb13178_c2/f5p0/545	NA	R01_cb13178_c2/f5p0/545	NA	NA
R01_cb10458_c7/f1p1/1759	NA	NA	NA	R01_cb10458_c7/f1p1/1759
R01_cb6204_c15/f1p0/2717	NA	NA	NA	R01_cb6204_c15/f1p0/2717
R01_cb4381_c4/f1p0/3153	NA	R01_cb4381_c4/f1p0/3153	NA	NA
R01_cb18456_c5295/f1p2/806	NA	R01_cb18456_c5295/f1p2/806	NA	NA
R01_cb10054_c29/f1p0/1317	NA	R01_cb10054_c29/f1p0/1317	NA	NA
R01_cb13654_c0/f3p1/1096	NA	NA	NA	R01_cb13654_c0/f3p1/1096
R01_cb8564_c4627/f1p0/3317	NA	R01_cb8564_c4627/f1p0/3317	R01_cb8564_c4627/f1p0/3317	R01_cb8564_c4627/f1p0/3317
R01_cb11147_c4/f1p0/624	NA	NA	NA	R01_cb11147_c4/f1p0/624
R01_cb15089_c1/f1p0/444	R01_cb15089_c1/f1p0/444	R01_cb15089_c1/f1p0/444	R01_cb15089_c1/f1p0/444	R01_cb15089_c1/f1p0/444
R01_cb8564_c24150/f1p1/3249	NA	NA	NA	R01_cb8564_c24150/f1p1/3249
R01_cb10795_c2/f1p0/1560	NA	NA	NA	R01_cb10795_c2/f1p0/1560
R01_cb8564_c116875/f2p0/2610	NA	R01_cb8564_c116875/f2p0/2610	R01_cb8564_c116875/f2p0/2610	R01_cb8564_c116875/f2p0/2610
R01_cb1961_c1/f2p0/2344	NA	R01_cb1961_c1/f2p0/2344	NA	NA
R01_cb2836_c14/f1p1/2537	NA	NA	NA	R01_cb2836_c14/f1p1/2537
R01_cb10107_c0/f4p0/1573	NA	NA	NA	R01_cb10107_c0/f4p0/1573
R01_cb9750_c11/f1p0/1846	NA	NA	NA	R01_cb9750_c11/f1p0/1846
R01_cb8564_c35805/f1p0/3489	NA	R01_cb8564_c35805/f1p0/3489	NA	R01_cb8564_c35805/f1p0/3489
R01_cb16452_c13/f1p0/533	R01_cb16452_c13/f1p0/533	R01_cb16452_c13/f1p0/533	R01_cb16452_c13/f1p0/533	R01_cb16452_c13/f1p0/533
R01_cb11017_c4/f1p0/726	NA	NA	R01_cb11017_c4/f1p0/726	R01_cb11017_c4/f1p0/726
R01_cb18456_c5430/f1p3/652	R01_cb18456_c5430/f1p3/652	R01_cb18456_c5430/f1p3/652	R01_cb18456_c5430/f1p3/652	R01_cb18456_c5430/f1p3/652
R01_cb18004_c0/f2p0/574	NA	R01_cb18004_c0/f2p0/574	NA	R01_cb18004_c0/f2p0/574
R01_cb658_c8/f1p0/2734	NA	NA	NA	R01_cb658_c8/f1p0/2734

R01_cb8503_c6/f1p0/330	R01_cb8503_c6/f1p0/330	R01_cb8503_c6/f1p0/330	R01_cb8503_c6/f1p0/330	R01_cb8503_c6/f1p0/330
R01_cb8564_c77553/f2p0/1972	NA	R01_cb8564_c77553/f2p0/1972	R01_cb8564_c77553/f2p0/1972	R01_cb8564_c77553/f2p0/1972
R01_cb5873_c2/f1p0/2402	NA	R01_cb5873_c2/f1p0/2402	NA	NA
R01_cb7023_c7/f1p0/1690	NA	R01_cb7023_c7/f1p0/1690	R01_cb7023_c7/f1p0/1690	R01_cb7023_c7/f1p0/1690
R01_cb13651_c31/f1p0/1464	NA	R01_cb13651_c31/f1p0/1464	NA	NA
R01_cb16564_c5/f1p0/758	R01_cb16564_c5/f1p0/758	R01_cb16564_c5/f1p0/758	R01_cb16564_c5/f1p0/758	R01_cb16564_c5/f1p0/758
R01_cb5736_c11/f1p2/5702	NA	NA	NA	R01_cb5736_c11/f1p2/5702
R01_cb2710_c9/f1p0/2880	NA	NA	NA	R01_cb2710_c9/f1p0/2880
R01_cb18409_c65/f1p0/542	R01_cb18409_c65/f1p0/542	R01_cb18409_c65/f1p0/542	R01_cb18409_c65/f1p0/542	R01_cb18409_c65/f1p0/542
R01_cb14904_c0/f1p0/1239	NA	NA	NA	R01_cb14904_c0/f1p0/1239
R01_cb16558_c0/f1p0/1650	R01_cb16558_c0/f1p0/1650	R01_cb16558_c0/f1p0/1650	R01_cb16558_c0/f1p0/1650	R01_cb16558_c0/f1p0/1650
R01_cb8564_c3744/f3p1/2994	R01_cb8564_c3744/f3p1/2994	R01_cb8564_c3744/f3p1/2994	R01_cb8564_c3744/f3p1/2994	R01_cb8564_c3744/f3p1/2994
R01_cb8564_c77191/f1p0/3234	NA	R01_cb8564_c77191/f1p0/3234	R01_cb8564_c77191/f1p0/3234	NA
R01_cb2826_c3/f1p0/3798	NA	R01_cb2826_c3/f1p0/3798	NA	R01_cb2826_c3/f1p0/3798
R01_cb639_c3/f1p0/3003	NA	NA	NA	R01_cb639_c3/f1p0/3003
R01_cb14278_c9/f1p0/5606	R01_cb14278_c9/f1p0/5606	R01_cb14278_c9/f1p0/5606	NA	NA
R01_cb2082_c21/f1p0/2561	NA	NA	NA	R01_cb2082_c21/f1p0/2561
R01_cb15326_c3/f1p1/1861	NA	R01_cb15326_c3/f1p1/1861	R01_cb15326_c3/f1p1/1861	R01_cb15326_c3/f1p1/1861
R01_cb14720_c2/f1p0/575	NA	NA	NA	R01_cb14720_c2/f1p0/575
R01_cb10873_c7/f5p0/1367	NA	NA	NA	R01_cb10873_c7/f5p0/1367
R01_cb7659_c0/f2p0/2555	NA	R01_cb7659_c0/f2p0/2555	R01_cb7659_c0/f2p0/2555	R01_cb7659_c0/f2p0/2555
R01_cb7528_c5/f1p0/4606	NA	R01_cb7528_c5/f1p0/4606	NA	NA
R01_cb8564_c4495/f1p1/2391	NA	R01_cb8564_c4495/f1p1/2391	R01_cb8564_c4495/f1p1/2391	R01_cb8564_c4495/f1p1/2391
R01_cb13771_c2/f2p0/1265	NA	R01_cb13771_c2/f2p0/1265	R01_cb13771_c2/f2p0/1265	R01_cb13771_c2/f2p0/1265
R01_cb10641_c1/f1p0/1966	NA	NA	NA	R01_cb10641_c1/f1p0/1966
R01_cb6608_c7/f1p0/2631	NA	NA	NA	R01_cb6608_c7/f1p0/2631

R01_cb16645_c31/f1p0/1069	R01_cb16645_c31/f1p0/1069	R01_cb16645_c31/f1p0/1069	R01_cb16645_c31/f1p0/1069	R01_cb16645_c31/f1p0/1069
R01_cb8564_c86878/f1p3/3516	NA	R01_cb8564_c86878/f1p3/3516	NA	R01_cb8564_c86878/f1p3/3516
R01_cb10828_c2/f1p0/2432	NA	R01_cb10828_c2/f1p0/2432	NA	R01_cb10828_c2/f1p0/2432
R01_cb14247_c1/f1p0/1563	NA	R01_cb14247_c1/f1p0/1563	NA	NA
R01_cb3863_c7/f1p1/3404	NA	NA	NA	R01_cb3863_c7/f1p1/3404
R01_cb8564_c21456/f1p0/2826	NA	R01_cb8564_c21456/f1p0/2826	NA	NA
R01_cb289_c18/f1p0/2079	NA	NA	NA	R01_cb289_c18/f1p0/2079
R01_cb3478_c0/f3p0/526	R01_cb3478_c0/f3p0/526	R01_cb3478_c0/f3p0/526	R01_cb3478_c0/f3p0/526	R01_cb3478_c0/f3p0/526
R01_cb18708_c0/f1p0/7035	NA	R01_cb18708_c0/f1p0/7035	NA	NA
R01_cb12932_c2/f1p0/993	NA	R01_cb12932_c2/f1p0/993	NA	NA
R01_cb18611_c1/f1p0/4508	NA	R01_cb18611_c1/f1p0/4508	R01_cb18611_c1/f1p0/4508	NA
R01_cb10015_c159/f1p0/2408	NA	R01_cb10015_c159/f1p0/2408	NA	NA
R01_cb1077_c26/f1p0/2821	NA	NA	NA	R01_cb1077_c26/f1p0/2821
R01_cb3228_c4/f1p0/4440	NA	R01_cb3228_c4/f1p0/4440	NA	NA
R01_cb8564_c9393/f4p0/4187	NA	NA	NA	R01_cb8564_c9393/f4p0/4187
R01_cb12421_c17/f2p0/715	NA	R01_cb12421_c17/f2p0/715	NA	NA
R01_cb18456_c3390/f1p1/1027	NA	R01_cb18456_c3390/f1p1/1027	NA	NA
R01_cb18456_c7595/f1p0/1521	NA	R01_cb18456_c7595/f1p0/1521	NA	R01_cb18456_c7595/f1p0/1521
R01_cb2648_c13/f1p0/2771	NA	NA	NA	R01_cb2648_c13/f1p0/2771
R01_cb5983_c0/f4p1/881	NA	NA	NA	R01_cb5983_c0/f4p1/881
R01_cb3962_c1/f1p0/3111	NA	R01_cb3962_c1/f1p0/3111	R01_cb3962_c1/f1p0/3111	R01_cb3962_c1/f1p0/3111
R01_cb7268_c1/f1p0/2659	NA	NA	R01_cb7268_c1/f1p0/2659	NA
R01_cb7087_c5/f1p0/2262	NA	NA	NA	R01_cb7087_c5/f1p0/2262
R01_cb383_c36/f1p0/1410	NA	R01_cb383_c36/f1p0/1410	NA	R01_cb383_c36/f1p0/1410
R01_cb4340_c2/f1p0/1819	NA	R01_cb4340_c2/f1p0/1819	R01_cb4340_c2/f1p0/1819	R01_cb4340_c2/f1p0/1819
R01_cb17740_c1/f1p0/1364	NA	NA	NA	R01_cb17740_c1/f1p0/1364

R01_cb2445_c5/f2p0/3324	NA	NA	NA	R01_cb2445_c5/f2p0/3324
R01_cb18456_c1380/f1p3/689	NA	R01_cb18456_c1380/f1p3/689	NA	NA
R01_cb2716_c0/f1p0/3829	NA	R01_cb2716_c0/f1p0/3829	NA	NA
R01_cb10920_c10/f1p0/1435	NA	R01_cb10920_c10/f1p0/1435	NA	NA
R01_cb8809_c16/f1p3/2364	NA	NA	NA	R01_cb8809_c16/f1p3/2364
R01_cb10352_c15/f1p1/1494	NA	R01_cb10352_c15/f1p1/1494	NA	NA
R01_cb2431_c3/f1p0/4221	NA	NA	NA	R01_cb2431_c3/f1p0/4221
R01_cb18181_c0/f1p0/1267	NA	NA	NA	R01_cb18181_c0/f1p0/1267
R01_cb16784_c2/f1p0/1591	NA	NA	NA	R01_cb16784_c2/f1p0/1591
R01_cb18480_c2/f1p0/831	NA	R01_cb18480_c2/f1p0/831	NA	R01_cb18480_c2/f1p0/831
R01_cb8564_c124750/f1p0/2176	NA	R01_cb8564_c124750/f1p0/2176	NA	NA
R01_cb15573_c0/f1p0/1344	NA	R01_cb15573_c0/f1p0/1344	NA	NA
R01_cb8417_c23/f1p0/1193	NA	NA	NA	R01_cb8417_c23/f1p0/1193
R01_cb8564_c18910/f1p2/2764	NA	NA	NA	R01_cb8564_c18910/f1p2/2764
R01_cb8564_c39692/f1p0/4120	NA	R01_cb8564_c39692/f1p0/4120	NA	NA
R01_cb9658_c4/f1p0/2058	NA	NA	NA	R01_cb9658_c4/f1p0/2058
R01_cb14835_c10/f1p1/917	NA	R01_cb14835_c10/f1p1/917	NA	R01_cb14835_c10/f1p1/917
R01_cb15233_c1/f3p0/869	NA	NA	R01_cb15233_c1/f3p0/869	R01_cb15233_c1/f3p0/869
R01_cb8564_c41187/f1p0/2814	NA	NA	R01_cb8564_c41187/f1p0/2814	R01_cb8564_c41187/f1p0/2814
R01_cb1813_c44/f9p0/2520	NA	NA	NA	R01_cb1813_c44/f9p0/2520
R01_cb3941_c68/f1p0/3496	NA	R01_cb3941_c68/f1p0/3496	NA	NA
R01_cb8441_c5/f1p0/2025	NA	R01_cb8441_c5/f1p0/2025	NA	R01_cb8441_c5/f1p0/2025
R01_cb11017_c3/f1p0/865	NA	R01_cb11017_c3/f1p0/865	R01_cb11017_c3/f1p0/865	R01_cb11017_c3/f1p0/865
R01_cb8564_c21600/f1p2/3364	NA	R01_cb8564_c21600/f1p2/3364	NA	R01_cb8564_c21600/f1p2/3364
R01_cb9410_c0/f1p1/2063	NA	NA	NA	R01_cb9410_c0/f1p1/2063

R01_cb625_c4/f1p2/3185	NA	NA	NA	R01_cb625_c4/f1p2/3185
R01_cb5774_c2/f1p0/391	NA	R01_cb5774_c2/f1p0/391	NA	R01_cb5774_c2/f1p0/391
R01_cb10767_c1/f1p0/3410	NA	NA	NA	R01_cb10767_c1/f1p0/3410
R01_cb5725_c40/f1p0/2259	NA	NA	NA	R01_cb5725_c40/f1p0/2259
R01_cb11401_c1/f1p1/881	NA	NA	R01_cb11401_c1/f1p1/881	R01_cb11401_c1/f1p1/881
R01_cb9797_c136/f1p0/869	NA	R01_cb9797_c136/f1p0/869	NA	NA
R01_cb5581_c4/f1p0/2101	NA	R01_cb5581_c4/f1p0/2101	NA	NA
R01_cb1508_c3/f1p0/4285	NA	R01_cb1508_c3/f1p0/4285	NA	R01_cb1508_c3/f1p0/4285
R01_cb12827_c0/f2p0/1391	NA	NA	NA	R01_cb12827_c0/f2p0/1391
R01_cb18526_c13/f1p0/1830	NA	R01_cb18526_c13/f1p0/1830	R01_cb18526_c13/f1p0/1830	R01_cb18526_c13/f1p0/1830
R01_cb8564_c880/f2p0/2557	R01_cb8564_c880/f2p0/2557	R01_cb8564_c880/f2p0/2557	R01_cb8564_c880/f2p0/2557	R01_cb8564_c880/f2p0/2557
R01_cb15102_c1/f1p0/890	NA	NA	NA	R01_cb15102_c1/f1p0/890
R01_cb9609_c2/f1p1/1941	NA	NA	NA	R01_cb9609_c2/f1p1/1941
R01_cb8564_c92238/f1p0/2090	NA	NA	NA	R01_cb8564_c92238/f1p0/2090
R01_cb18456_c7437/f1p0/1496	NA	R01_cb18456_c7437/f1p0/1496	R01_cb18456_c7437/f1p0/1496	R01_cb18456_c7437/f1p0/1496
R01_cb16437_c2/f1p0/418	R01_cb16437_c2/f1p0/418	R01_cb16437_c2/f1p0/418	NA	NA
R01_cb4293_c14/f1p0/3805	NA	R01_cb4293_c14/f1p0/3805	NA	NA
R01_cb1077_c7/f3p1/1886	NA	NA	NA	R01_cb1077_c7/f3p1/1886
R01_cb11107_c3/f1p0/6000	NA	R01_cb11107_c3/f1p0/6000	NA	NA
R01_cb8564_c73676/f1p0/2280	NA	NA	NA	R01_cb8564_c73676/f1p0/2280
R01_cb13970_c2/f2p0/998	NA	NA	NA	R01_cb13970_c2/f2p0/998
R01_cb13602_c9/f2p0/1811	NA	NA	NA	R01_cb13602_c9/f2p0/1811
R01_cb9063_c8/f1p0/1064	R01_cb9063_c8/f1p0/1064	R01_cb9063_c8/f1p0/1064	NA	R01_cb9063_c8/f1p0/1064
R01_cb8285_c0/f1p0/2410	NA	NA	NA	R01_cb8285_c0/f1p0/2410
R01_cb8564_c18659/f1p0/2786	NA	R01_cb8564_c18659/f1p0/2786	NA	R01_cb8564_c18659/f1p0/2786
R01_cb4250_c1/f1p0/2776	R01_cb4250_c1/f1p0/2776	R01_cb4250_c1/f1p0/2776	R01_cb4250_c1/f1p0/2776	NA

R01_cb8564_c73895/f1p0/4051	R01_cb8564_c73895/f1p0/4051	R01_cb8564_c73895/f1p0/4051	NA	NA
R01_cb15564_c0/f2p0/817	NA	R01_cb15564_c0/f2p0/817	R01_cb15564_c0/f2p0/817	R01_cb15564_c0/f2p0/817
R01_cb1297_c27/f2p0/4120	NA	NA	NA	R01_cb1297_c27/f2p0/4120
R01_cb7055_c57/f1p1/2206	NA	R01_cb7055_c57/f1p1/2206	NA	NA
R01_cb2691_c5/f1p0/1612	NA	R01_cb2691_c5/f1p0/1612	NA	R01_cb2691_c5/f1p0/1612
R01_cb12897_c1/f2p0/623	R01_cb12897_c1/f2p0/623	R01_cb12897_c1/f2p0/623	NA	NA
R01_cb3046_c12/f1p0/2338	NA	NA	NA	R01_cb3046_c12/f1p0/2338
R01_cb17091_c0/f2p0/1653	R01_cb17091_c0/f2p0/1653	R01_cb17091_c0/f2p0/1653	R01_cb17091_c0/f2p0/1653	R01_cb17091_c0/f2p0/1653
R01_cb4702_c9/f1p0/2908	NA	R01_cb4702_c9/f1p0/2908	R01_cb4702_c9/f1p0/2908	R01_cb4702_c9/f1p0/2908
R01_cb2033_c16/f1p0/2147	NA	NA	NA	R01_cb2033_c16/f1p0/2147
R01_cb8564_c89244/f1p0/2755	NA	R01_cb8564_c89244/f1p0/2755	R01_cb8564_c89244/f1p0/2755	R01_cb8564_c89244/f1p0/2755
R01_cb8564_c70887/f1p0/2052	NA	R01_cb8564_c70887/f1p0/2052	NA	NA
R01_cb14502_c21/f1p0/1607	R01_cb14502_c21/f1p0/1607	R01_cb14502_c21/f1p0/1607	R01_cb14502_c21/f1p0/1607	R01_cb14502_c21/f1p0/1607
R01_cb7976_c1/f2p0/1868	NA	R01_cb7976_c1/f2p0/1868	NA	NA
R01_cb16455_c2/f1p0/533	R01_cb16455_c2/f1p0/533	R01_cb16455_c2/f1p0/533	R01_cb16455_c2/f1p0/533	R01_cb16455_c2/f1p0/533
R01_cb7836_c3/f1p0/1935	NA	NA	NA	R01_cb7836_c3/f1p0/1935
R01_cb285_c18/f1p0/2884	NA	R01_cb285_c18/f1p0/2884	NA	NA
R01_cb18409_c87/f1p0/374	R01_cb18409_c87/f1p0/374	R01_cb18409_c87/f1p0/374	R01_cb18409_c87/f1p0/374	R01_cb18409_c87/f1p0/374
R01_cb2192_c0/f1p0/4018	NA	NA	NA	R01_cb2192_c0/f1p0/4018
R01_cb14125_c55/f1p2/681	NA	R01_cb14125_c55/f1p2/681	R01_cb14125_c55/f1p2/681	NA
R01_cb8564_c76807/f1p0/2283	NA	NA	NA	R01_cb8564_c76807/f1p0/2283
R01_cb14524_c4/f1p0/1330	NA	NA	NA	R01_cb14524_c4/f1p0/1330
R01_cb18386_c8/f1p0/1021	R01_cb18386_c8/f1p0/1021	R01_cb18386_c8/f1p0/1021	NA	R01_cb18386_c8/f1p0/1021
R01_cb7234_c10/f1p1/2606	NA	R01_cb7234_c10/f1p1/2606	NA	NA
R01_cb16875_c3/f1p0/900	NA	NA	NA	R01_cb16875_c3/f1p0/900

R01_cb8564_c89084/f2p0/2474	NA	NA	NA	R01_cb8564_c89084/f2p0/2474
R01_cb18490_c1/f1p0/900	NA	NA	R01_cb18490_c1/f1p0/900	R01_cb18490_c1/f1p0/900
R01_cb11481_c3/f1p0/615	NA	R01_cb11481_c3/f1p0/615	NA	R01_cb11481_c3/f1p0/615
R01_cb12883_c2/f2p0/803	R01_cb12883_c2/f2p0/803	R01_cb12883_c2/f2p0/803	NA	R01_cb12883_c2/f2p0/803
R01_cb18456_c7175/f1p0/506	R01_cb18456_c7175/f1p0/506	R01_cb18456_c7175/f1p0/506	R01_cb18456_c7175/f1p0/506	R01_cb18456_c7175/f1p0/506
R01_cb16061_c2/f1p0/557	NA	R01_cb16061_c2/f1p0/557	NA	NA
R01_cb6456_c0/f2p0/2553	R01_cb6456_c0/f2p0/2553	R01_cb6456_c0/f2p0/2553	NA	R01_cb6456_c0/f2p0/2553
R01_cb1425_c3/f1p0/2584	NA	NA	NA	R01_cb1425_c3/f1p0/2584
R01_cb454_c12/f1p0/4005	NA	R01_cb454_c12/f1p0/4005	NA	R01_cb454_c12/f1p0/4005
R01_cb4532_c4/f1p0/2791	NA	R01_cb4532_c4/f1p0/2791	NA	NA
R01_cb8564_c79596/f1p0/4895	NA	R01_cb8564_c79596/f1p0/4895	NA	NA
R01_cb8564_c51637/f1p7/3093	NA	R01_cb8564_c51637/f1p7/3093	NA	NA
R01_cb8564_c3325/f1p0/2023	NA	R01_cb8564_c3325/f1p0/2023	NA	NA
R01_cb2219_c6/f1p2/1909	NA	R01_cb2219_c6/f1p2/1909	NA	NA
R01_cb7810_c2/f1p0/1116	R01_cb7810_c2/f1p0/1116	R01_cb7810_c2/f1p0/1116	R01_cb7810_c2/f1p0/1116	NA
R01_cb2936_c7/f1p0/2430	NA	R01_cb2936_c7/f1p0/2430	NA	NA
R01_cb10129_c2/f1p0/3753	NA	R01_cb10129_c2/f1p0/3753	NA	NA
R01_cb8564_c89737/f1p0/3593	NA	R01_cb8564_c89737/f1p0/3593	NA	NA
R01_cb8215_c4/f1p0/2103	NA	R01_cb8215_c4/f1p0/2103	NA	NA
R01_cb9540_c0/f1p0/2047	NA	NA	NA	R01_cb9540_c0/f1p0/2047
R01_cb6118_c2/f1p0/2944	NA	R01_cb6118_c2/f1p0/2944	R01_cb6118_c2/f1p0/2944	NA
R01_cb12972_c28/f1p0/932	NA	R01_cb12972_c28/f1p0/932	R01_cb12972_c28/f1p0/932	R01_cb12972_c28/f1p0/932
R01_cb1885_c1/f2p0/2694	NA	R01_cb1885_c1/f2p0/2694	NA	NA
R01_cb8564_c119154/f1p0/2187	NA	NA	R01_cb8564_c119154/f1p0/2187	NA
R01_cb15805_c1/f1p0/628	NA	NA	NA	R01_cb15805_c1/f1p0/628

R01_cb8564_c82516/f1p0/3019	NA	R01_cb8564_c82516/f1p0/3019	NA	NA
R01_cb7645_c5/f1p0/884	R01_cb7645_c5/f1p0/884	NA	R01_cb7645_c5/f1p0/884	R01_cb7645_c5/f1p0/884
R01_cb8564_c85733/f4p1/4182	NA	NA	NA	R01_cb8564_c85733/f4p1/4182
R01_cb11134_c2/f1p0/892	NA	R01_cb11134_c2/f1p0/892	R01_cb11134_c2/f1p0/892	R01_cb11134_c2/f1p0/892
R01_cb2907_c11/f1p0/2418	NA	NA	NA	R01_cb2907_c11/f1p0/2418
R01_cb16177_c2/f1p0/711	NA	NA	NA	R01_cb16177_c2/f1p0/711
R01_cb11397_c4/f1p0/1908	NA	R01_cb11397_c4/f1p0/1908	NA	NA
R01_cb12462_c40/f1p0/769	NA	R01_cb12462_c40/f1p0/769	NA	NA
R01_cb10769_c3/f6p0/1025	NA	NA	NA	R01_cb10769_c3/f6p0/1025
R01_cb9750_c3/f1p0/1926	NA	NA	NA	R01_cb9750_c3/f1p0/1926
R01_cb6704_c2/f1p0/2788	NA	R01_cb6704_c2/f1p0/2788	NA	NA
R01_cb14293_c1/f2p0/903	NA	R01_cb14293_c1/f2p0/903	NA	NA
R01_cb3483_c19/f1p0/2872	NA	NA	NA	R01_cb3483_c19/f1p0/2872
R01_cb17683_c0/f2p0/1527	NA	NA	NA	R01_cb17683_c0/f2p0/1527
R01_cb8564_c87865/f1p0/3752	NA	R01_cb8564_c87865/f1p0/3752	NA	NA
R01_cb4494_c3/f1p0/2950	NA	R01_cb4494_c3/f1p0/2950	NA	NA
R01_cb16242_c12/f1p1/1019	NA	R01_cb16242_c12/f1p1/1019	NA	NA
R01_cb17251_c3/f1p0/767	NA	R01_cb17251_c3/f1p0/767	NA	NA
R01_cb8564_c46392/f1p0/2511	R01_cb8564_c46392/f1p0/2511	R01_cb8564_c46392/f1p0/2511	R01_cb8564_c46392/f1p0/2511	R01_cb8564_c46392/f1p0/2511
R01_cb18456_c7432/f1p0/774	NA	R01_cb18456_c7432/f1p0/774	R01_cb18456_c7432/f1p0/774	R01_cb18456_c7432/f1p0/774
R01_cb8564_c1913/f1p0/2470	R01_cb8564_c1913/f1p0/2470	R01_cb8564_c1913/f1p0/2470	NA	R01_cb8564_c1913/f1p0/2470
R01_cb8564_c115209/f1p2/2422	NA	NA	NA	R01_cb8564_c115209/f1p2/2422
R01_cb18456_c5351/f1p3/831	NA	R01_cb18456_c5351/f1p3/831	NA	NA
R01_cb6931_c0/f3p0/1780	NA	NA	NA	R01_cb6931_c0/f3p0/1780

R01_cb3341_c7/f1p0/2281	NA	R01_cb3341_c7/f1p0/2281	NA	NA
R01_cb119_c29/f1p0/1939	NA	NA	NA	R01_cb119_c29/f1p0/1939
R01_cb8564_c116479/f2p1/3503	R01_cb8564_c116479/f2p1/3503	R01_cb8564_c116479/f2p1/3503	R01_cb8564_c116479/f2p1/3503	R01_cb8564_c116479/f2p1/3503
R01_cb8564_c54312/f1p0/2789	R01_cb8564_c54312/f1p0/2789	R01_cb8564_c54312/f1p0/2789	NA	R01_cb8564_c54312/f1p0/2789
R01_cb2926_c31/f1p0/797	NA	NA	NA	R01_cb2926_c31/f1p0/797
R01_cb14223_c3/f1p0/886	NA	NA	R01_cb14223_c3/f1p0/886	R01_cb14223_c3/f1p0/886
R01_cb2275_c6/f1p0/7298	NA	NA	NA	R01_cb2275_c6/f1p0/7298
R01_cb10623_c2/f1p0/2574	NA	R01_cb10623_c2/f1p0/2574	R01_cb10623_c2/f1p0/2574	NA
R01_cb3888_c10/f1p0/2356	NA	R01_cb3888_c10/f1p0/2356	NA	NA
R01_cb14684_c3/f1p0/1666	NA	R01_cb14684_c3/f1p0/1666	NA	NA
R01_cb10655_c2/f1p0/1974	R01_cb10655_c2/f1p0/1974	R01_cb10655_c2/f1p0/1974	R01_cb10655_c2/f1p0/1974	R01_cb10655_c2/f1p0/1974
R01_cb7057_c3/f1p0/4016	NA	NA	NA	R01_cb7057_c3/f1p0/4016
R01_cb11703_c0/f1p1/861	R01_cb11703_c0/f1p1/861	R01_cb11703_c0/f1p1/861	R01_cb11703_c0/f1p1/861	NA
R01_cb17963_c0/f2p0/680	R01_cb17963_c0/f2p0/680	R01_cb17963_c0/f2p0/680	R01_cb17963_c0/f2p0/680	R01_cb17963_c0/f2p0/680
R01_cb12897_c2/f1p0/623	R01_cb12897_c2/f1p0/623	R01_cb12897_c2/f1p0/623	NA	NA
R01_cb7687_c6/f1p0/2480	NA	NA	NA	R01_cb7687_c6/f1p0/2480
R01_cb8564_c117175/f2p0/2439	R01_cb8564_c117175/f2p0/2439	R01_cb8564_c117175/f2p0/2439	NA	R01_cb8564_c117175/f2p0/2439
R01_cb17475_c3/f1p0/714	NA	R01_cb17475_c3/f1p0/714	R01_cb17475_c3/f1p0/714	R01_cb17475_c3/f1p0/714
R01_cb17103_c3/f1p0/1303	NA	NA	NA	R01_cb17103_c3/f1p0/1303
R01_cb2048_c19/f7p0/1989	NA	NA	NA	R01_cb2048_c19/f7p0/1989
R01_cb10027_c4/f2p4/1653	NA	NA	NA	R01_cb10027_c4/f2p4/1653
R01_cb12480_c5/f1p2/930	NA	R01_cb12480_c5/f1p2/930	R01_cb12480_c5/f1p2/930	R01_cb12480_c5/f1p2/930
R01_cb8564_c1648/f1p0/3092	NA	R01_cb8564_c1648/f1p0/3092	NA	R01_cb8564_c1648/f1p0/3092

R01_cb646_c2/f1p0/4655	NA	NA	NA	R01_cb646_c2/f1p0/4655
R01_cb18456_c7633/f1p0/489	R01_cb18456_c7633/f1p0/489	R01_cb18456_c7633/f1p0/489	R01_cb18456_c7633/f1p0/489	R01_cb18456_c7633/f1p0/489
R01_cb8885_c4/f1p0/2736	NA	NA	NA	R01_cb8885_c4/f1p0/2736
R01_cb9373_c5/f1p0/661	R01_cb9373_c5/f1p0/661	R01_cb9373_c5/f1p0/661	NA	NA
R01_cb6990_c3/f1p0/2720	R01_cb6990_c3/f1p0/2720	R01_cb6990_c3/f1p0/2720	NA	NA
R01_cb8564_c77579/f1p0/2057	NA	NA	NA	R01_cb8564_c77579/f1p0/2057
R01_cb8564_c114759/f1p0/2134	NA	R01_cb8564_c114759/f1p0/2134	R01_cb8564_c114759/f1p0/2134	NA
R01_cb12644_c5/f5p0/555	NA	R01_cb12644_c5/f5p0/555	NA	NA
R01_cb16777_c1/f1p0/633	R01_cb16777_c1/f1p0/633	R01_cb16777_c1/f1p0/633	R01_cb16777_c1/f1p0/633	R01_cb16777_c1/f1p0/633
R01_cb5507_c1/f1p0/2959	NA	R01_cb5507_c1/f1p0/2959	NA	NA
R01_cb6623_c15/f1p0/2688	NA	NA	NA	R01_cb6623_c15/f1p0/2688
R01_cb18352_c0/f1p0/507	R01_cb18352_c0/f1p0/507	R01_cb18352_c0/f1p0/507	R01_cb18352_c0/f1p0/507	R01_cb18352_c0/f1p0/507
R01_cb2826_c2/f2p0/2665	NA	R01_cb2826_c2/f2p0/2665	NA	NA
R01_cb8564_c38562/f1p0/2020	NA	R01_cb8564_c38562/f1p0/2020	R01_cb8564_c38562/f1p0/2020	R01_cb8564_c38562/f1p0/2020
R01_cb4490_c10/f1p0/2154	NA	NA	NA	R01_cb4490_c10/f1p0/2154
R01_cb18456_c7239/f1p0/340	R01_cb18456_c7239/f1p0/340	R01_cb18456_c7239/f1p0/340	R01_cb18456_c7239/f1p0/340	R01_cb18456_c7239/f1p0/340
R01_cb16844_c0/f4p0/1511	NA	NA	NA	R01_cb16844_c0/f4p0/1511
R01_cb14070_c1/f1p0/1408	NA	R01_cb14070_c1/f1p0/1408	R01_cb14070_c1/f1p0/1408	NA
R01_cb17184_c1/f2p0/758	NA	R01_cb17184_c1/f2p0/758	NA	NA
R01_cb2160_c22/f1p0/878	NA	R01_cb2160_c22/f1p0/878	R01_cb2160_c22/f1p0/878	R01_cb2160_c22/f1p0/878
R01_cb8564_c51823/f1p0/3843	R01_cb8564_c51823/f1p0/3843	R01_cb8564_c51823/f1p0/3843	R01_cb8564_c51823/f1p0/3843	R01_cb8564_c51823/f1p0/3843
R01_cb18582_c0/f1p0/1650	NA	R01_cb18582_c0/f1p0/1650	NA	NA
R01_cb6406_c68/f1p0/5111	NA	R01_cb6406_c68/f1p0/5111	NA	R01_cb6406_c68/f1p0/5111
R01_cb3500_c25/f1p0/3053	NA	NA	NA	R01_cb3500_c25/f1p0/3053

R01_cb8975_c4/f1p0/2062	NA	NA	NA	R01_cb8975_c4/f1p0/2062
R01_cb8564_c69340/f2p0/3851	NA	R01_cb8564_c69340/f2p0/3851	NA	NA
R01_cb4923_c1/f5p0/2836	NA	NA	NA	R01_cb4923_c1/f5p0/2836
R01_cb5190_c4/f1p1/2412	NA	NA	NA	R01_cb5190_c4/f1p1/2412
R01_cb11441_c0/f1p0/842	R01_cb11441_c0/f1p0/842	R01_cb11441_c0/f1p0/842	R01_cb11441_c0/f1p0/842	R01_cb11441_c0/f1p0/842
R01_cb6665_c6/f2p0/956	R01_cb6665_c6/f2p0/956	R01_cb6665_c6/f2p0/956	R01_cb6665_c6/f2p0/956	R01_cb6665_c6/f2p0/956
R01_cb2154_c5/f1p1/4034	NA	R01_cb2154_c5/f1p1/4034	NA	NA
R01_cb15214_c0/f7p0/1843	NA	NA	NA	R01_cb15214_c0/f7p0/1843
R01_cb4920_c1/f1p0/1983	R01_cb4920_c1/f1p0/1983	R01_cb4920_c1/f1p0/1983	R01_cb4920_c1/f1p0/1983	R01_cb4920_c1/f1p0/1983
R01_cb11637_c0/f1p0/1510	NA	R01_cb11637_c0/f1p0/1510	NA	NA
R01_cb18456_c4755/f1p0/454	NA	R01_cb18456_c4755/f1p0/454	NA	R01_cb18456_c4755/f1p0/454
R01_cb13289_c9/f1p0/1728	NA	R01_cb13289_c9/f1p0/1728	R01_cb13289_c9/f1p0/1728	NA
R01_cb18456_c6279/f1p0/557	R01_cb18456_c6279/f1p0/557	R01_cb18456_c6279/f1p0/557	NA	R01_cb18456_c6279/f1p0/557
R01_cb8528_c10/f1p0/447	R01_cb8528_c10/f1p0/447	R01_cb8528_c10/f1p0/447	R01_cb8528_c10/f1p0/447	R01_cb8528_c10/f1p0/447
R01_cb709_c3/f1p0/2461	NA	NA	R01_cb709_c3/f1p0/2461	NA
R01_cb2445_c17/f1p0/2819	NA	NA	NA	R01_cb2445_c17/f1p0/2819
R01_cb14796_c5/f2p0/646	NA	R01_cb14796_c5/f2p0/646	NA	NA
R01_cb6004_c5/f1p1/2116	NA	NA	NA	R01_cb6004_c5/f1p1/2116
R01_cb3872_c1/f2p0/3514	NA	R01_cb3872_c1/f2p0/3514	R01_cb3872_c1/f2p0/3514	R01_cb3872_c1/f2p0/3514
R01_cb8564_c76420/f1p0/2783	NA	R01_cb8564_c76420/f1p0/2783	NA	NA
R01_cb8564_c10779/f1p2/2282	NA	NA	NA	R01_cb8564_c10779/f1p2/2282
R01_cb9241_c3/f1p0/1448	R01_cb9241_c3/f1p0/1448	R01_cb9241_c3/f1p0/1448	NA	R01_cb9241_c3/f1p0/1448
R01_cb17241_c1/f1p0/1516	NA	NA	NA	R01_cb17241_c1/f1p0/1516
R01_cb17756_c1/f3p0/381	R01_cb17756_c1/f3p0/381	R01_cb17756_c1/f3p0/381	R01_cb17756_c1/f3p0/381	R01_cb17756_c1/f3p0/381
R01_cb8564_c52937/f1p0/3075	NA	R01_cb8564_c52937/f1p0/3075	NA	R01_cb8564_c52937/f1p0/3075
R01_cb17467_c1/f1p0/1089	R01_cb17467_c1/f1p0/1089	R01_cb17467_c1/f1p0/1089	R01_cb17467_c1/f1p0/1089	R01_cb17467_c1/f1p0/1089

R01_cb13842_c0/f1p0/1450	NA	R01_cb13842_c0/f1p0/1450	NA	NA
R01_cb8564_c68664/f5p0/2301	NA	R01_cb8564_c68664/f5p0/2301	R01_cb8564_c68664/f5p0/2301	R01_cb8564_c68664/f5p0/2301
R01_cb8564_c117101/f1p0/2770	NA	NA	NA	R01_cb8564_c117101/f1p0/2770
R01_cb10641_c4/f1p1/1619	NA	NA	NA	R01_cb10641_c4/f1p1/1619
R01_cb8564_c15731/f1p0/3195	NA	R01_cb8564_c15731/f1p0/3195	NA	R01_cb8564_c15731/f1p0/3195
R01_cb5911_c2/f1p0/1941	NA	NA	NA	R01_cb5911_c2/f1p0/1941
R01_cb12200_c0/f1p0/1686	NA	NA	NA	R01_cb12200_c0/f1p0/1686
R01_cb9052_c1/f1p0/2217	NA	NA	NA	R01_cb9052_c1/f1p0/2217
R01_cb8564_c78144/f1p0/2954	R01_cb8564_c78144/f1p0/2954	R01_cb8564_c78144/f1p0/2954	R01_cb8564_c78144/f1p0/2954	R01_cb8564_c78144/f1p0/2954
R01_cb5159_c4/f2p1/1986	NA	NA	NA	R01_cb5159_c4/f2p1/1986
R01_cb3750_c35/f1p1/2165	NA	NA	NA	R01_cb3750_c35/f1p1/2165
R01_cb3759_c5/f1p0/2655	NA	R01_cb3759_c5/f1p0/2655	NA	NA
R01_cb10145_c2/f1p0/2311	NA	NA	NA	R01_cb10145_c2/f1p0/2311
R01_cb5550_c19/f1p0/3088	NA	R01_cb5550_c19/f1p0/3088	NA	NA
R01_cb3732_c5/f2p0/3545	NA	NA	NA	R01_cb3732_c5/f2p0/3545
R01_cb13024_c2/f1p0/681	NA	NA	NA	R01_cb13024_c2/f1p0/681
R01_cb11953_c3/f1p0/380	R01_cb11953_c3/f1p0/380	R01_cb11953_c3/f1p0/380	R01_cb11953_c3/f1p0/380	R01_cb11953_c3/f1p0/380
R01_cb18456_c7248/f1p0/1461	NA	R01_cb18456_c7248/f1p0/1461	R01_cb18456_c7248/f1p0/1461	NA
R01_cb18456_c6986/f1p0/1316	NA	R01_cb18456_c6986/f1p0/1316	R01_cb18456_c6986/f1p0/1316	R01_cb18456_c6986/f1p0/1316
R01_cb14211_c2/f1p0/1530	NA	NA	NA	R01_cb14211_c2/f1p0/1530
R01_cb8564_c86230/f1p0/2481	NA	R01_cb8564_c86230/f1p0/2481	NA	NA
R01_cb15436_c1/f1p0/799	NA	NA	NA	R01_cb15436_c1/f1p0/799
R01_cb2991_c7/f1p0/2926	NA	NA	NA	R01_cb2991_c7/f1p0/2926
R01_cb9941_c9/f1p0/579	NA	R01_cb9941_c9/f1p0/579	R01_cb9941_c9/f1p0/579	R01_cb9941_c9/f1p0/579

R01_cb9057_c2/f1p0/2081	NA	R01_cb9057_c2/f1p0/2081	NA	NA
R01_cb16660_c1/f2p0/752	NA	R01_cb16660_c1/f2p0/752	NA	R01_cb16660_c1/f2p0/752
R01_cb12057_c14/f1p0/1492	NA	R01_cb12057_c14/f1p0/1492	NA	NA
R01_cb8564_c128333/f1p1/2000	NA	R01_cb8564_c128333/f1p1/2000	NA	NA
R01_cb2334_c7/f1p0/2732	NA	R01_cb2334_c7/f1p0/2732	NA	NA
R01_cb8564_c119927/f1p0/2616	NA	R01_cb8564_c119927/f1p0/2616	NA	NA
R01_cb8564_c85521/f1p0/3423	NA	R01_cb8564_c85521/f1p0/3423	NA	NA
R01_cb10156_c3/f1p0/1158	NA	R01_cb10156_c3/f1p0/1158	NA	NA
R01_cb14627_c6/f1p0/1063	NA	NA	NA	R01_cb14627_c6/f1p0/1063
R01_cb570_c6/f1p0/4743	NA	R01_cb570_c6/f1p0/4743	NA	NA
R01_cb8564_c2546/f1p0/3021	NA	NA	R01_cb8564_c2546/f1p0/3021	R01_cb8564_c2546/f1p0/3021
R01_cb15714_c3/f1p0/705	NA	R01_cb15714_c3/f1p0/705	NA	NA
R01_cb16182_c2/f1p1/1825	NA	NA	NA	R01_cb16182_c2/f1p1/1825
R01_cb17640_c1/f1p0/1318	NA	NA	NA	R01_cb17640_c1/f1p0/1318
R01_cb15145_c2/f1p0/608	NA	R01_cb15145_c2/f1p0/608	R01_cb15145_c2/f1p0/608	NA
R01_cb3359_c26/f1p0/2229	NA	NA	NA	R01_cb3359_c26/f1p0/2229
R01_cb10138_c8/f1p0/780	NA	NA	NA	R01_cb10138_c8/f1p0/780
R01_cb9112_c1/f1p0/2224	NA	R01_cb9112_c1/f1p0/2224	R01_cb9112_c1/f1p0/2224	R01_cb9112_c1/f1p0/2224
R01_cb17201_c0/f1p0/1540	NA	NA	R01_cb17201_c0/f1p0/1540	R01_cb17201_c0/f1p0/1540
R01_cb3053_c3/f1p0/3737	NA	R01_cb3053_c3/f1p0/3737	NA	R01_cb3053_c3/f1p0/3737
R01_cb18409_c41/f1p0/890	NA	NA	NA	R01_cb18409_c41/f1p0/890
R01_cb11685_c0/f1p0/1539	NA	R01_cb11685_c0/f1p0/1539	NA	R01_cb11685_c0/f1p0/1539
R01_cb10118_c1/f1p0/2135	NA	R01_cb10118_c1/f1p0/2135	NA	NA
R01_cb16470_c1/f1p0/701	NA	R01_cb16470_c1/f1p0/701	NA	NA

R01_cb17946_c1/f1p0/657	NA	R01_cb17946_c1/f1p0/657	NA	NA
R01_cb781_c18/f1p0/2084	NA	NA	NA	R01_cb781_c18/f1p0/2084
R01_cb8564_c111062/f1p0/1980	NA	R01_cb8564_c111062/f1p0/1980	NA	NA
R01_cb17736_c0/f1p0/444	NA	NA	R01_cb17736_c0/f1p0/444	R01_cb17736_c0/f1p0/444
R01_cb12178_c3/f2p0/527	R01_cb12178_c3/f2p0/527	R01_cb12178_c3/f2p0/527	NA	NA
R01_cb12603_c7/f1p0/1521	NA	NA	R01_cb12603_c7/f1p0/1521	R01_cb12603_c7/f1p0/1521
R01_cb18456_c7201/f1p0/1409	NA	R01_cb18456_c7201/f1p0/1409	NA	NA
R01_cb16797_c1/f1p0/1311	R01_cb16797_c1/f1p0/1311	R01_cb16797_c1/f1p0/1311	R01_cb16797_c1/f1p0/1311	R01_cb16797_c1/f1p0/1311
R01_cb8564_c69597/f1p0/4104	NA	R01_cb8564_c69597/f1p0/4104	NA	R01_cb8564_c69597/f1p0/4104
R01_cb17990_c0/f1p0/788	R01_cb17990_c0/f1p0/788	R01_cb17990_c0/f1p0/788	R01_cb17990_c0/f1p0/788	R01_cb17990_c0/f1p0/788
R01_cb3563_c8/f1p0/1656	NA	NA	NA	R01_cb3563_c8/f1p0/1656
R01_cb8564_c75856/f1p1/3365	R01_cb8564_c75856/f1p1/3365	R01_cb8564_c75856/f1p1/3365	R01_cb8564_c75856/f1p1/3365	R01_cb8564_c75856/f1p1/3365
R01_cb17236_c1/f2p0/543	NA	R01_cb17236_c1/f2p0/543	R01_cb17236_c1/f2p0/543	R01_cb17236_c1/f2p0/543
R01_cb12946_c3/f1p0/1018	NA	R01_cb12946_c3/f1p0/1018	NA	NA
R01_cb8564_c125018/f1p0/3452	NA	R01_cb8564_c125018/f1p0/3452	NA	NA
R01_cb2027_c3/f1p0/3757	NA	R01_cb2027_c3/f1p0/3757	R01_cb2027_c3/f1p0/3757	NA
R01_cb3145_c2/f1p0/3679	NA	R01_cb3145_c2/f1p0/3679	NA	NA
R01_cb18456_c7953/f6p0/550	NA	R01_cb18456_c7953/f6p0/550	R01_cb18456_c7953/f6p0/550	R01_cb18456_c7953/f6p0/550
R01_cb16403_c1/f1p0/839	NA	R01_cb16403_c1/f1p0/839	R01_cb16403_c1/f1p0/839	R01_cb16403_c1/f1p0/839
R01_cb8564_c17223/f1p0/4169	NA	R01_cb8564_c17223/f1p0/4169	R01_cb8564_c17223/f1p0/4169	NA
R01_cb8564_c117034/f1p1/2624	NA	R01_cb8564_c117034/f1p1/2624	NA	R01_cb8564_c117034/f1p1/2624
R01_cb10708_c1/f1p0/2292	R01_cb10708_c1/f1p0/2292	R01_cb10708_c1/f1p0/2292	NA	NA

R01_cb8564_c80864/f1p0/3876	NA	R01_cb8564_c80864/f1p0/3876	R01_cb8564_c80864/f1p0/3876	R01_cb8564_c80864/f1p0/3876
R01_cb18173_c0/f1p0/614	NA	R01_cb18173_c0/f1p0/614	NA	R01_cb18173_c0/f1p0/614
R01_cb10206_c28/f2p0/1569	NA	R01_cb10206_c28/f2p0/1569	NA	NA
R01_cb7814_c4/f1p0/2751	NA	NA	NA	R01_cb7814_c4/f1p0/2751
R01_cb4785_c7/f1p0/2764	NA	NA	NA	R01_cb4785_c7/f1p0/2764
R01_cb8564_c24387/f1p0/2638	NA	R01_cb8564_c24387/f1p0/2638	NA	NA
R01_cb8324_c10/f1p0/1375	NA	NA	NA	R01_cb8324_c10/f1p0/1375
R01_cb17065_c2/f1p0/1048	NA	R01_cb17065_c2/f1p0/1048	NA	R01_cb17065_c2/f1p0/1048
R01_cb3464_c3/f1p0/3337	NA	NA	NA	R01_cb3464_c3/f1p0/3337
R01_cb15765_c0/f2p0/867	R01_cb15765_c0/f2p0/867	R01_cb15765_c0/f2p0/867	R01_cb15765_c0/f2p0/867	R01_cb15765_c0/f2p0/867
R01_cb18456_c5358/f1p0/875	NA	R01_cb18456_c5358/f1p0/875	NA	NA
R01_cb18287_c5/f1p0/1095	R01_cb18287_c5/f1p0/1095	R01_cb18287_c5/f1p0/1095	R01_cb18287_c5/f1p0/1095	R01_cb18287_c5/f1p0/1095
R01_cb10015_c264/f1p0/568	NA	R01_cb10015_c264/f1p0/568	NA	NA
R01_cb8564_c83310/f1p0/2140	R01_cb8564_c83310/f1p0/2140	R01_cb8564_c83310/f1p0/2140	NA	R01_cb8564_c83310/f1p0/2140
R01_cb9609_c0/f7p1/2034	NA	NA	NA	R01_cb9609_c0/f7p1/2034
R01_cb8564_c112577/f1p0/4024	R01_cb8564_c112577/f1p0/4024	R01_cb8564_c112577/f1p0/4024	R01_cb8564_c112577/f1p0/4024	R01_cb8564_c112577/f1p0/4024
R01_cb4471_c11/f1p0/3111	NA	NA	NA	R01_cb4471_c11/f1p0/3111
R01_cb8564_c23940/f1p0/3025	NA	NA	NA	R01_cb8564_c23940/f1p0/3025
R01_cb15710_c3/f1p0/630	NA	R01_cb15710_c3/f1p0/630	NA	NA
R01_cb16422_c3/f1p0/643	NA	NA	R01_cb16422_c3/f1p0/643	R01_cb16422_c3/f1p0/643
R01_cb5581_c2/f1p0/3067	NA	R01_cb5581_c2/f1p0/3067	NA	NA
R01_cb8564_c80984/f1p0/3227	NA	R01_cb8564_c80984/f1p0/3227	R01_cb8564_c80984/f1p0/3227	NA
R01_cb11230_c1/f1p0/3015	R01_cb11230_c1/f1p0/3015	R01_cb11230_c1/f1p0/3015	R01_cb11230_c1/f1p0/3015	R01_cb11230_c1/f1p0/3015
R01_cb279_c13/f1p0/4553	NA	NA	NA	R01_cb279_c13/f1p0/4553

R01_cb10114_c4/f1p0/1307	NA	R01_cb10114_c4/f1p0/1307	NA	R01_cb10114_c4/f1p0/1307
R01_cb8564_c84266/f1p2/3194	NA	NA	NA	R01_cb8564_c84266/f1p2/3194
R01_cb11248_c0/f2p0/726	NA	R01_cb11248_c0/f2p0/726	NA	NA
R01_cb10732_c2/f1p0/3472	NA	R01_cb10732_c2/f1p0/3472	NA	NA
R01_cb16071_c1/f1p0/813	NA	R01_cb16071_c1/f1p0/813	NA	R01_cb16071_c1/f1p0/813
R01_cb18223_c14/f1p0/1132	R01_cb18223_c14/f1p0/1132	R01_cb18223_c14/f1p0/1132	NA	NA
R01_cb7420_c15/f1p0/499	NA	R01_cb7420_c15/f1p0/499	R01_cb7420_c15/f1p0/499	R01_cb7420_c15/f1p0/499
R01_cb8564_c90640/f1p0/3061	NA	NA	NA	R01_cb8564_c90640/f1p0/3061
R01_cb13703_c4/f1p2/523	R01_cb13703_c4/f1p2/523	NA	NA	NA
R01_cb479_c0/f6p6/4735	NA	NA	NA	R01_cb479_c0/f6p6/4735
R01_cb8564_c16568/f9p0/2643	NA	R01_cb8564_c16568/f9p0/2643	NA	NA
R01_cb7999_c10/f1p0/814	NA	NA	NA	R01_cb7999_c10/f1p0/814
R01_cb7178_c1/f1p0/2668	NA	R01_cb7178_c1/f1p0/2668	NA	NA
R01_cb13438_c0/f3p0/911	NA	NA	NA	R01_cb13438_c0/f3p0/911
R01_cb8127_c4/f1p0/2058	NA	R01_cb8127_c4/f1p0/2058	NA	NA
R01_cb763_c11/f1p0/818	R01_cb763_c11/f1p0/818	R01_cb763_c11/f1p0/818	R01_cb763_c11/f1p0/818	R01_cb763_c11/f1p0/818
R01_cb8564_c79747/f1p0/3160	R01_cb8564_c79747/f1p0/3160	R01_cb8564_c79747/f1p0/3160	R01_cb8564_c79747/f1p0/3160	R01_cb8564_c79747/f1p0/3160
R01_cb714_c13/f1p0/2393	NA	NA	NA	R01_cb714_c13/f1p0/2393
R01_cb931_c2/f1p0/2178	NA	R01_cb931_c2/f1p0/2178	R01_cb931_c2/f1p0/2178	NA
R01_cb11772_c0/f1p0/551	R01_cb11772_c0/f1p0/551	R01_cb11772_c0/f1p0/551	R01_cb11772_c0/f1p0/551	R01_cb11772_c0/f1p0/551
R01_cb977_c1/f1p0/4540	NA	NA	NA	R01_cb977_c1/f1p0/4540
R01_cb18627_c1/f1p0/924	NA	R01_cb18627_c1/f1p0/924	R01_cb18627_c1/f1p0/924	R01_cb18627_c1/f1p0/924
R01_cb16853_c0/f2p0/1229	NA	NA	NA	R01_cb16853_c0/f2p0/1229
R01_cb8324_c1/f3p1/1379	NA	NA	NA	R01_cb8324_c1/f3p1/1379
R01_cb8564_c87724/f1p0/2766	NA	R01_cb8564_c87724/f1p0/2766	NA	NA

R01_cb1938_c8/f1p0/2471	NA	NA	NA	R01_cb1938_c8/f1p0/2471
R01_cb8564_c110276/f2p0/2540	NA	NA	NA	R01_cb8564_c110276/f2p0/2540
R01_cb10154_c0/f1p1/1631	NA	NA	NA	R01_cb10154_c0/f1p1/1631
R01_cb11682_c0/f1p0/1226	NA	NA	NA	R01_cb11682_c0/f1p0/1226
R01_cb3848_c22/f1p1/3080	NA	NA	NA	R01_cb3848_c22/f1p1/3080
R01_cb6608_c13/f1p0/1941	NA	NA	NA	R01_cb6608_c13/f1p0/1941
R01_cb5307_c14/f1p0/3080	NA	NA	NA	R01_cb5307_c14/f1p0/3080
R01_cb4169_c11/f1p0/1444	NA	NA	NA	R01_cb4169_c11/f1p0/1444
R01_cb3287_c4/f1p0/3898	NA	NA	NA	R01_cb3287_c4/f1p0/3898
R01_cb12566_c8/f1p0/495	NA	R01_cb12566_c8/f1p0/495	NA	NA
R01_cb8564_c21087/f1p0/2226	NA	NA	R01_cb8564_c21087/f1p0/2226	R01_cb8564_c21087/f1p0/2226
R01_cb9916_c3/f1p1/1541	NA	NA	NA	R01_cb9916_c3/f1p1/1541
R01_cb18158_c1/f1p0/571	NA	R01_cb18158_c1/f1p0/571	NA	NA
R01_cb16682_c13/f1p0/743	NA	R01_cb16682_c13/f1p0/743	R01_cb16682_c13/f1p0/743	NA
R01_cb8564_c111413/f1p0/2839	NA	NA	NA	R01_cb8564_c111413/f1p0/2839
R01_cb1228_c13/f1p0/2005	NA	R01_cb1228_c13/f1p0/2005	NA	R01_cb1228_c13/f1p0/2005
R01_cb13867_c10/f1p0/1652	R01_cb13867_c10/f1p0/1652	R01_cb13867_c10/f1p0/1652	NA	R01_cb13867_c10/f1p0/1652
R01_cb16303_c9/f1p0/2953	NA	NA	NA	R01_cb16303_c9/f1p0/2953
R01_cb7679_c4/f1p0/3465	NA	R01_cb7679_c4/f1p0/3465	NA	NA
R01_cb2129_c1/f1p0/2099	R01_cb2129_c1/f1p0/2099	R01_cb2129_c1/f1p0/2099	NA	NA
R01_cb4535_c10/f1p0/937	R01_cb4535_c10/f1p0/937	R01_cb4535_c10/f1p0/937	NA	R01_cb4535_c10/f1p0/937
R01_cb17326_c0/f1p0/712	NA	R01_cb17326_c0/f1p0/712	NA	R01_cb17326_c0/f1p0/712
R01_cb13910_c10/f1p0/1230	NA	R01_cb13910_c10/f1p0/1230	R01_cb13910_c10/f1p0/1230	R01_cb13910_c10/f1p0/1230
R01_cb10587_c2/f1p0/2777	R01_cb10587_c2/f1p0/2777	R01_cb10587_c2/f1p0/2777	R01_cb10587_c2/f1p0/2777	NA

R01_cb7224_c14/f1p0/734	NA	R01_cb7224_c14/f1p0/734	NA	R01_cb7224_c14/f1p0/734
R01_cb8407_c1/f2p0/1264	NA	NA	NA	R01_cb8407_c1/f2p0/1264
R01_cb1915_c17/f1p0/2421	NA	R01_cb1915_c17/f1p0/2421	NA	NA
R01_cb13938_c11/f1p0/1528	NA	NA	NA	R01_cb13938_c11/f1p0/1528
R01_cb5736_c2/f1p0/2717	NA	NA	NA	R01_cb5736_c2/f1p0/2717
R01_cb1691_c16/f1p0/3702	NA	R01_cb1691_c16/f1p0/3702	NA	NA
R01_cb1739_c8/f1p0/5910	NA	NA	NA	R01_cb1739_c8/f1p0/5910
R01_cb11870_c0/f1p0/410	R01_cb11870_c0/f1p0/410	R01_cb11870_c0/f1p0/410	R01_cb11870_c0/f1p0/410	NA
R01_cb226_c9/f1p0/5006	NA	NA	NA	R01_cb226_c9/f1p0/5006
R01_cb3575_c4/f1p2/3595	NA	NA	NA	R01_cb3575_c4/f1p2/3595
R01_cb12057_c12/f1p0/580	NA	R01_cb12057_c12/f1p0/580	R01_cb12057_c12/f1p0/580	NA
R01_cb2750_c2/f1p0/2741	NA	R01_cb2750_c2/f1p0/2741	NA	NA
R01_cb6608_c3/f1p0/2812	NA	NA	NA	R01_cb6608_c3/f1p0/2812
R01_cb8564_c76062/f1p0/4468	NA	NA	R01_cb8564_c76062/f1p0/4468	NA
R01_cb3162_c3/f1p0/1716	NA	R01_cb3162_c3/f1p0/1716	NA	NA
R01_cb6636_c1/f1p0/2429	NA	R01_cb6636_c1/f1p0/2429	NA	R01_cb6636_c1/f1p0/2429
R01_cb8564_c49718/f1p0/2132	NA	R01_cb8564_c49718/f1p0/2132	NA	R01_cb8564_c49718/f1p0/2132
R01_cb4900_c10/f1p0/2337	NA	NA	NA	R01_cb4900_c10/f1p0/2337
R01_cb5236_c67/f1p0/1062	NA	NA	NA	R01_cb5236_c67/f1p0/1062
R01_cb16240_c3/f1p0/5417	NA	R01_cb16240_c3/f1p0/5417	NA	NA
R01_cb5000_c5/f1p0/3206	R01_cb5000_c5/f1p0/3206	R01_cb5000_c5/f1p0/3206	R01_cb5000_c5/f1p0/3206	R01_cb5000_c5/f1p0/3206
R01_cb1900_c12/f1p2/3840	NA	NA	NA	R01_cb1900_c12/f1p2/3840
R01_cb8564_c5038/f1p0/2526	NA	NA	NA	R01_cb8564_c5038/f1p0/2526
R01_cb10207_c9/f2p0/645	NA	R01_cb10207_c9/f2p0/645	NA	NA
R01_cb13910_c8/f1p0/1292	NA	R01_cb13910_c8/f1p0/1292	R01_cb13910_c8/f1p0/1292	R01_cb13910_c8/f1p0/1292
R01_cb13545_c57/f1p0/1021	NA	R01_cb13545_c57/f1p0/1021	NA	R01_cb13545_c57/f1p0/1021

R01_cb17269_c2/f3p0/439	NA	NA	R01_cb17269_c2/f3p0/439	R01_cb17269_c2/f3p0/439
R01_cb18456_c1865/f1p0/1641	NA	R01_cb18456_c1865/f1p0/1641	NA	NA
R01_cb1333_c32/f1p0/2647	NA	R01_cb1333_c32/f1p0/2647	R01_cb1333_c32/f1p0/2647	R01_cb1333_c32/f1p0/2647
R01_cb1794_c34/f1p0/863	NA	R01_cb1794_c34/f1p0/863	NA	R01_cb1794_c34/f1p0/863
R01_cb8200_c2/f2p0/934	NA	R01_cb8200_c2/f2p0/934	NA	NA
R01_cb8564_c25125/f1p0/2068	NA	NA	NA	R01_cb8564_c25125/f1p0/2068
R01_cb10310_c3/f1p0/642	R01_cb10310_c3/f1p0/642	R01_cb10310_c3/f1p0/642	R01_cb10310_c3/f1p0/642	R01_cb10310_c3/f1p0/642
R01_cb12422_c9/f1p2/1757	NA	NA	NA	R01_cb12422_c9/f1p2/1757
R01_cb16066_c0/f7p0/484	R01_cb16066_c0/f7p0/484	R01_cb16066_c0/f7p0/484	R01_cb16066_c0/f7p0/484	R01_cb16066_c0/f7p0/484
R01_cb8564_c1028/f2p0/1918	NA	NA	NA	R01_cb8564_c1028/f2p0/1918
R01_cb8564_c79732/f1p0/2999	NA	R01_cb8564_c79732/f1p0/2999	NA	NA
R01_cb18223_c10/f1p0/1469	R01_cb18223_c10/f1p0/1469	NA	NA	NA
R01_cb8564_c112064/f1p0/2786	R01_cb8564_c112064/f1p0/2786	R01_cb8564_c112064/f1p0/2786	R01_cb8564_c112064/f1p0/2786	NA
R01_cb7193_c2/f1p0/3593	NA	R01_cb7193_c2/f1p0/3593	NA	NA
R01_cb13124_c9/f1p0/1633	NA	R01_cb13124_c9/f1p0/1633	NA	NA
R01_cb10332_c1/f1p1/3046	NA	NA	NA	R01_cb10332_c1/f1p1/3046
R01_cb13234_c0/f3p0/592	NA	R01_cb13234_c0/f3p0/592	NA	NA
R01_cb1954_c1/f1p0/4115	NA	R01_cb1954_c1/f1p0/4115	NA	R01_cb1954_c1/f1p0/4115
R01_cb13976_c4/f1p0/466	NA	NA	NA	R01_cb13976_c4/f1p0/466
R01_cb16447_c11/f1p0/918	NA	NA	NA	R01_cb16447_c11/f1p0/918
R01_cb8293_c5/f1p0/416	R01_cb8293_c5/f1p0/416	R01_cb8293_c5/f1p0/416	R01_cb8293_c5/f1p0/416	R01_cb8293_c5/f1p0/416
R01_cb17533_c0/f1p0/677	NA	NA	R01_cb17533_c0/f1p0/677	R01_cb17533_c0/f1p0/677
R01_cb8564_c7774/f3p4/1962	NA	NA	NA	R01_cb8564_c7774/f3p4/1962
R01_cb8564_c69296/f2p0/2798	NA	R01_cb8564_c69296/f2p0/2798	NA	NA
R01_cb18456_c7237/f1p0/982	NA	R01_cb18456_c7237/f1p0/982	R01_cb18456_c7237/f1p0/982	R01_cb18456_c7237/f1p0/982

R01_cb8564_c91808/f1p0/2973	NA	NA	NA	R01_cb8564_c91808/f1p0/2973
R01_cb11477_c1/f1p0/3293	NA	R01_cb11477_c1/f1p0/3293	R01_cb11477_c1/f1p0/3293	NA
R01_cb8564_c80587/f1p0/2798	NA	R01_cb8564_c80587/f1p0/2798	NA	NA
R01_cb11003_c0/f1p0/757	NA	NA	NA	R01_cb11003_c0/f1p0/757
R01_cb10941_c2/f1p1/2105	NA	R01_cb10941_c2/f1p1/2105	NA	NA
R01_cb10676_c4/f2p0/439	NA	NA	NA	R01_cb10676_c4/f2p0/439
R01_cb18456_c5918/f1p0/449	R01_cb18456_c5918/f1p0/449	R01_cb18456_c5918/f1p0/449	R01_cb18456_c5918/f1p0/449	R01_cb18456_c5918/f1p0/449
R01_cb8433_c7/f1p0/2501	R01_cb8433_c7/f1p0/2501	R01_cb8433_c7/f1p0/2501	R01_cb8433_c7/f1p0/2501	R01_cb8433_c7/f1p0/2501
R01_cb13467_c1/f1p0/731	NA	R01_cb13467_c1/f1p0/731	R01_cb13467_c1/f1p0/731	R01_cb13467_c1/f1p0/731
R01_cb4169_c9/f1p0/1562	NA	NA	NA	R01_cb4169_c9/f1p0/1562
R01_cb3848_c17/f1p0/3076	NA	NA	NA	R01_cb3848_c17/f1p0/3076
R01_cb16818_c3/f1p0/745	R01_cb16818_c3/f1p0/745	R01_cb16818_c3/f1p0/745	R01_cb16818_c3/f1p0/745	R01_cb16818_c3/f1p0/745
R01_cb12273_c6/f1p0/1463	NA	NA	NA	R01_cb12273_c6/f1p0/1463
R01_cb8564_c121826/f1p0/2708	NA	R01_cb8564_c121826/f1p0/2708	R01_cb8564_c121826/f1p0/2708	R01_cb8564_c121826/f1p0/2708
R01_cb8564_c120211/f1p0/2363	NA	R01_cb8564_c120211/f1p0/2363	NA	R01_cb8564_c120211/f1p0/2363
R01_cb15207_c124/f5p2/466	R01_cb15207_c124/f5p2/466	R01_cb15207_c124/f5p2/466	R01_cb15207_c124/f5p2/466	R01_cb15207_c124/f5p2/466
R01_cb9993_c5/f1p0/1238	NA	NA	NA	R01_cb9993_c5/f1p0/1238
R01_cb15958_c7/f1p0/1312	NA	R01_cb15958_c7/f1p0/1312	NA	R01_cb15958_c7/f1p0/1312
R01_cb14060_c0/f1p1/1332	NA	NA	NA	R01_cb14060_c0/f1p1/1332
R01_cb8564_c68358/f4p0/2256	NA	NA	R01_cb8564_c68358/f4p0/2256	R01_cb8564_c68358/f4p0/2256
R01_cb17363_c2/f4p1/650	NA	R01_cb17363_c2/f4p1/650	R01_cb17363_c2/f4p1/650	R01_cb17363_c2/f4p1/650
R01_cb14537_c1/f1p0/441	R01_cb14537_c1/f1p0/441	R01_cb14537_c1/f1p0/441	NA	R01_cb14537_c1/f1p0/441
R01_cb14363_c4/f1p0/365	R01_cb14363_c4/f1p0/365	R01_cb14363_c4/f1p0/365	R01_cb14363_c4/f1p0/365	R01_cb14363_c4/f1p0/365
R01_cb16645_c42/f1p0/1259	NA	NA	NA	R01_cb16645_c42/f1p0/1259

R01_cb8564_c117614/f2p1/1909	NA	NA	NA	R01_cb8564_c117614/f2p1/1909
R01_cb8564_c1112/f1p0/1849	NA	NA	NA	R01_cb8564_c1112/f1p0/1849
R01_cb1599_c2/f1p0/3360	NA	NA	NA	R01_cb1599_c2/f1p0/3360
R01_cb1676_c1/f1p0/4216	NA	R01_cb1676_c1/f1p0/4216	NA	NA
R01_cb2314_c8/f1p0/842	R01_cb2314_c8/f1p0/842	R01_cb2314_c8/f1p0/842	NA	NA
R01_cb4576_c167/f1p0/410	R01_cb4576_c167/f1p0/410	R01_cb4576_c167/f1p0/410	R01_cb4576_c167/f1p0/410	R01_cb4576_c167/f1p0/410
R01_cb8288_c5/f1p0/1571	NA	R01_cb8288_c5/f1p0/1571	NA	NA
R01_cb7568_c5/f1p0/476	NA	R01_cb7568_c5/f1p0/476	NA	R01_cb7568_c5/f1p0/476
R01_cb8564_c4133/f1p0/3185	NA	R01_cb8564_c4133/f1p0/3185	NA	NA
R01_cb14524_c12/f1p0/972	NA	NA	NA	R01_cb14524_c12/f1p0/972
R01_cb16937_c1/f1p0/829	NA	NA	NA	R01_cb16937_c1/f1p0/829
R01_cb13068_c4/f1p0/514	NA	R01_cb13068_c4/f1p0/514	NA	NA
R01_cb17184_c2/f1p0/761	NA	R01_cb17184_c2/f1p0/761	NA	NA
R01_cb16725_c4/f4p0/871	R01_cb16725_c4/f4p0/871	NA	NA	NA
R01_cb14720_c7/f11p0/556	NA	NA	NA	R01_cb14720_c7/f11p0/556
R01_cb12600_c2/f1p0/609	NA	R01_cb12600_c2/f1p0/609	NA	R01_cb12600_c2/f1p0/609
R01_cb13778_c3/f1p0/758	R01_cb13778_c3/f1p0/758	R01_cb13778_c3/f1p0/758	R01_cb13778_c3/f1p0/758	R01_cb13778_c3/f1p0/758
R01_cb8564_c123222/f1p0/2361	NA	R01_cb8564_c123222/f1p0/2361	NA	R01_cb8564_c123222/f1p0/2361
R01_cb18404_c2/f1p0/962	NA	R01_cb18404_c2/f1p0/962	NA	NA
R01_cb14481_c7/f1p0/478	R01_cb14481_c7/f1p0/478	R01_cb14481_c7/f1p0/478	NA	R01_cb14481_c7/f1p0/478
R01_cb10350_c6/f1p1/1107	NA	NA	NA	R01_cb10350_c6/f1p1/1107
R01_cb3921_c3/f1p0/1894	NA	NA	NA	R01_cb3921_c3/f1p0/1894
R01_cb8564_c78758/f1p0/3299	NA	R01_cb8564_c78758/f1p0/3299	NA	NA
R01_cb13761_c13/f1p0/1259	R01_cb13761_c13/f1p0/1259	R01_cb13761_c13/f1p0/1259	R01_cb13761_c13/f1p0/1259	R01_cb13761_c13/f1p0/1259

R01_cb10306_c4/f1p0/642	NA	R01_cb10306_c4/f1p0/642	NA	R01_cb10306_c4/f1p0/642
R01_cb15890_c2/f1p0/735	NA	NA	NA	R01_cb15890_c2/f1p0/735
R01_cb8564_c86543/f2p0/2775	NA	R01_cb8564_c86543/f2p0/2775	NA	NA
R01_cb10259_c4/f1p0/2516	NA	R01_cb10259_c4/f1p0/2516	NA	NA
R01_cb16774_c6/f1p1/1292	NA	R01_cb16774_c6/f1p1/1292	NA	NA
R01_cb10693_c1/f2p0/1622	NA	NA	NA	R01_cb10693_c1/f2p0/1622
R01_cb11868_c1/f1p0/597	NA	NA	R01_cb11868_c1/f1p0/597	R01_cb11868_c1/f1p0/597
R01_cb17728_c1/f1p0/1524	NA	R01_cb17728_c1/f1p0/1524	NA	NA
R01_cb7773_c3/f1p0/2091	R01_cb7773_c3/f1p0/2091	R01_cb7773_c3/f1p0/2091	NA	R01_cb7773_c3/f1p0/2091
R01_cb9380_c8/f12p0/1954	NA	NA	NA	R01_cb9380_c8/f12p0/1954
R01_cb8564_c1058/f1p0/2453	NA	R01_cb8564_c1058/f1p0/2453	R01_cb8564_c1058/f1p0/2453	R01_cb8564_c1058/f1p0/2453
R01_cb13802_c1/f1p0/1380	NA	NA	NA	R01_cb13802_c1/f1p0/1380
R01_cb15136_c1/f1p0/567	R01_cb15136_c1/f1p0/567	R01_cb15136_c1/f1p0/567	R01_cb15136_c1/f1p0/567	R01_cb15136_c1/f1p0/567
R01_cb8564_c90076/f1p1/3925	NA	NA	NA	R01_cb8564_c90076/f1p1/3925
R01_cb8121_c4/f1p0/1279	NA	NA	NA	R01_cb8121_c4/f1p0/1279
R01_cb18409_c85/f1p0/1048	R01_cb18409_c85/f1p0/1048	R01_cb18409_c85/f1p0/1048	R01_cb18409_c85/f1p0/1048	R01_cb18409_c85/f1p0/1048
R01_cb17183_c2/f1p2/667	NA	NA	NA	R01_cb17183_c2/f1p2/667
R01_cb8564_c114197/f1p0/2428	R01_cb8564_c114197/f1p0/2428	R01_cb8564_c114197/f1p0/2428	R01_cb8564_c114197/f1p0/2428	NA
R01_cb2243_c2/f1p0/8205	NA	NA	NA	R01_cb2243_c2/f1p0/8205
R01_cb1847_c2/f1p0/4060	NA	NA	NA	R01_cb1847_c2/f1p0/4060
R01_cb18456_c6443/f1p1/629	NA	R01_cb18456_c6443/f1p1/629	NA	NA
R01_cb15526_c0/f2p0/1121	NA	NA	NA	R01_cb15526_c0/f2p0/1121
R01_cb8198_c6/f1p1/1844	NA	NA	NA	R01_cb8198_c6/f1p1/1844
R01_cb8564_c83128/f1p0/3323	NA	NA	NA	R01_cb8564_c83128/f1p0/3323
R01_cb7302_c1/f1p0/2651	NA	NA	NA	R01_cb7302_c1/f1p0/2651

R01_cb7705_c0/f1p0/2553	R01_cb7705_c0/f1p0/2553	R01_cb7705_c0/f1p0/2553	R01_cb7705_c0/f1p0/2553	R01_cb7705_c0/f1p0/2553
R01_cb11936_c24/f1p0/479	R01_cb11936_c24/f1p0/479	R01_cb11936_c24/f1p0/479	R01_cb11936_c24/f1p0/479	R01_cb11936_c24/f1p0/479
R01_cb13467_c0/f2p0/1476	NA	R01_cb13467_c0/f2p0/1476	R01_cb13467_c0/f2p0/1476	R01_cb13467_c0/f2p0/1476
R01_cb13027_c1/f2p0/1126	NA	NA	NA	R01_cb13027_c1/f2p0/1126
R01_cb5659_c65/f1p0/2179	NA	NA	NA	R01_cb5659_c65/f1p0/2179
R01_cb8564_c128731/f1p0/2262	NA	R01_cb8564_c128731/f1p0/2262	R01_cb8564_c128731/f1p0/2262	NA
R01_cb11022_c1/f1p0/2335	NA	NA	R01_cb11022_c1/f1p0/2335	NA
R01_cb18456_c5797/f1p0/856	NA	NA	NA	R01_cb18456_c5797/f1p0/856
R01_cb16915_c2/f1p0/751	R01_cb16915_c2/f1p0/751	R01_cb16915_c2/f1p0/751	NA	NA
R01_cb6758_c7/f1p0/1936	NA	NA	NA	R01_cb6758_c7/f1p0/1936
R01_cb16064_c1/f1p0/1086	NA	NA	NA	R01_cb16064_c1/f1p0/1086
R01_cb8564_c117708/f1p0/1979	R01_cb8564_c117708/f1p0/1979	R01_cb8564_c117708/f1p0/1979	NA	NA
R01_cb14823_c8/f1p0/1380	NA	NA	NA	R01_cb14823_c8/f1p0/1380
R01_cb10782_c1/f1p0/4624	NA	NA	R01_cb10782_c1/f1p0/4624	R01_cb10782_c1/f1p0/4624
R01_cb10260_c1/f1p0/3041	NA	NA	NA	R01_cb10260_c1/f1p0/3041
R01_cb12980_c5/f1p0/300	R01_cb12980_c5/f1p0/300	R01_cb12980_c5/f1p0/300	R01_cb12980_c5/f1p0/300	R01_cb12980_c5/f1p0/300
R01_cb17949_c0/f2p0/553	R01_cb17949_c0/f2p0/553	R01_cb17949_c0/f2p0/553	NA	R01_cb17949_c0/f2p0/553
R01_cb8564_c1646/f1p0/3260	NA	NA	NA	R01_cb8564_c1646/f1p0/3260
R01_cb16277_c3/f1p0/1306	R01_cb16277_c3/f1p0/1306	R01_cb16277_c3/f1p0/1306	NA	NA
R01_cb4373_c9/f1p0/3071	NA	NA	NA	R01_cb4373_c9/f1p0/3071
R01_cb11456_c2/f1p0/3006	NA	R01_cb11456_c2/f1p0/3006	NA	NA
R01_cb10144_c1/f1p1/1897	NA	NA	NA	R01_cb10144_c1/f1p1/1897
R01_cb10694_c5/f1p1/613	R01_cb10694_c5/f1p1/613	NA	NA	R01_cb10694_c5/f1p1/613
R01_cb1610_c6/f1p0/2698	NA	NA	NA	R01_cb1610_c6/f1p0/2698

R01_cb8564_c15369/f2p0/3807	NA	NA	NA	R01_cb8564_c15369/f2p0/3807
R01_cb10952_c1/f1p0/1950	NA	NA	NA	R01_cb10952_c1/f1p0/1950
R01_cb18065_c1/f1p0/1845	R01_cb18065_c1/f1p0/1845	R01_cb18065_c1/f1p0/1845	R01_cb18065_c1/f1p0/1845	R01_cb18065_c1/f1p0/1845
R01_cb4916_c5/f1p0/1981	NA	NA	NA	R01_cb4916_c5/f1p0/1981
R01_cb11717_c2/f1p0/1301	NA	NA	R01_cb11717_c2/f1p0/1301	R01_cb11717_c2/f1p0/1301
R01_cb8136_c5/f1p0/2657	NA	R01_cb8136_c5/f1p0/2657	NA	NA
R01_cb1860_c6/f1p0/3927	NA	NA	NA	R01_cb1860_c6/f1p0/3927
R01_cb14638_c1/f2p0/1355	NA	NA	NA	R01_cb14638_c1/f2p0/1355
R01_cb1856_c0/f1p0/3930	NA	R01_cb1856_c0/f1p0/3930	NA	R01_cb1856_c0/f1p0/3930
R01_cb8564_c45750/f1p0/3236	R01_cb8564_c45750/f1p0/3236	R01_cb8564_c45750/f1p0/3236	R01_cb8564_c45750/f1p0/3236	NA
R01_cb8564_c76909/f2p1/3012	NA	NA	NA	R01_cb8564_c76909/f2p1/3012
R01_cb1938_c4/f1p1/2103	NA	NA	NA	R01_cb1938_c4/f1p1/2103
R01_cb5847_c14/f3p0/2230	NA	NA	NA	R01_cb5847_c14/f3p0/2230
R01_cb8564_c46156/f1p0/2786	NA	R01_cb8564_c46156/f1p0/2786	NA	NA
R01_cb8564_c115624/f1p0/2261	NA	R01_cb8564_c115624/f1p0/2261	NA	NA
R01_cb10270_c1/f1p0/2670	NA	R01_cb10270_c1/f1p0/2670	NA	NA
R01_cb1955_c23/f1p2/2518	NA	NA	NA	R01_cb1955_c23/f1p2/2518
R01_cb17358_c4/f1p0/1871	NA	NA	NA	R01_cb17358_c4/f1p0/1871
R01_cb8529_c7/f1p0/1126	NA	NA	NA	R01_cb8529_c7/f1p0/1126
R01_cb18456_c7379/f1p0/408	R01_cb18456_c7379/f1p0/408	R01_cb18456_c7379/f1p0/408	R01_cb18456_c7379/f1p0/408	R01_cb18456_c7379/f1p0/408
R01_cb2072_c0/f7p0/3079	NA	NA	NA	R01_cb2072_c0/f7p0/3079
R01_cb4180_c8/f1p1/6772	NA	NA	NA	R01_cb4180_c8/f1p1/6772
R01_cb11355_c1/f1p0/2062	NA	R01_cb11355_c1/f1p0/2062	NA	NA
R01_cb15443_c1/f2p0/1068	NA	NA	NA	R01_cb15443_c1/f2p0/1068

R01_cb7821_c3/f1p0/1820	NA	NA	NA	R01_cb7821_c3/f1p0/1820
R01_cb18456_c6847/f1p0/799	NA	R01_cb18456_c6847/f1p0/799	NA	NA
R01_cb18038_c1/f1p1/1813	NA	R01_cb18038_c1/f1p1/1813	NA	NA
R01_cb6332_c6/f1p0/1065	NA	R01_cb6332_c6/f1p0/1065	NA	R01_cb6332_c6/f1p0/1065
R01_cb10664_c0/f1p0/1214	R01_cb10664_c0/f1p0/1214	R01_cb10664_c0/f1p0/1214	R01_cb10664_c0/f1p0/1214	NA
R01_cb8564_c14191/f1p0/4011	NA	R01_cb8564_c14191/f1p0/4011	NA	R01_cb8564_c14191/f1p0/4011
R01_cb5390_c2/f2p0/2906	NA	R01_cb5390_c2/f2p0/2906	NA	NA
R01_cb14238_c1/f4p2/1240	NA	NA	NA	R01_cb14238_c1/f4p2/1240
R01_cb3421_c13/f1p0/4144	NA	NA	NA	R01_cb3421_c13/f1p0/4144
R01_cb15293_c0/f1p0/827	NA	R01_cb15293_c0/f1p0/827	R01_cb15293_c0/f1p0/827	NA
R01_cb12319_c2/f1p0/373	R01_cb12319_c2/f1p0/373	NA	NA	R01_cb12319_c2/f1p0/373
R01_cb18456_c7505/f1p0/720	NA	R01_cb18456_c7505/f1p0/720	NA	NA
R01_cb18628_c1/f1p0/1981	NA	NA	NA	R01_cb18628_c1/f1p0/1981
R01_cb8564_c117154/f1p1/3067	NA	NA	NA	R01_cb8564_c117154/f1p1/3067
R01_cb12743_c4/f1p0/1043	NA	R01_cb12743_c4/f1p0/1043	NA	R01_cb12743_c4/f1p0/1043
R01_cb15279_c0/f1p0/1473	NA	NA	NA	R01_cb15279_c0/f1p0/1473
R01_cb8564_c3668/f1p0/3187	R01_cb8564_c3668/f1p0/3187	R01_cb8564_c3668/f1p0/3187	NA	NA
R01_cb4180_c4/f1p0/3590	NA	NA	NA	R01_cb4180_c4/f1p0/3590
R01_cb10062_c30/f1p0/1248	NA	R01_cb10062_c30/f1p0/1248	NA	NA
R01_cb480_c3/f1p0/3684	NA	NA	NA	R01_cb480_c3/f1p0/3684
R01_cb6071_c8/f1p0/2512	NA	NA	NA	R01_cb6071_c8/f1p0/2512
R01_cb13405_c0/f7p0/835	NA	NA	NA	R01_cb13405_c0/f7p0/835
R01_cb10229_c10/f1p0/1188	R01_cb10229_c10/f1p0/1188	R01_cb10229_c10/f1p0/1188	R01_cb10229_c10/f1p0/1188	R01_cb10229_c10/f1p0/1188
R01_cb17557_c1/f1p0/1756	NA	R01_cb17557_c1/f1p0/1756	NA	R01_cb17557_c1/f1p0/1756
R01_cb18456_c4767/f1p0/370	R01_cb18456_c4767/f1p0/370	R01_cb18456_c4767/f1p0/370	R01_cb18456_c4767/f1p0/370	R01_cb18456_c4767/f1p0/370

R01_cb1178_c22/f1p0/1348	NA	NA	NA	R01_cb1178_c22/f1p0/1348
R01_cb8564_c11904/f1p0/2709	R01_cb8564_c11904/f1p0/2709	R01_cb8564_c11904/f1p0/2709	NA	NA
R01_cb8710_c9/f1p0/3582	NA	NA	R01_cb8710_c9/f1p0/3582	R01_cb8710_c9/f1p0/3582
R01_cb8564_c70727/f1p0/3055	R01_cb8564_c70727/f1p0/3055	R01_cb8564_c70727/f1p0/3055	R01_cb8564_c70727/f1p0/3055	R01_cb8564_c70727/f1p0/3055
R01_cb438_c2/f1p0/4812	NA	R01_cb438_c2/f1p0/4812	NA	R01_cb438_c2/f1p0/4812
R01_cb11453_c1/f1p0/3600	NA	NA	NA	R01_cb11453_c1/f1p0/3600
R01_cb18456_c4754/f1p1/487	R01_cb18456_c4754/f1p1/487	R01_cb18456_c4754/f1p1/487	NA	NA
R01_cb6615_c11/f1p0/2412	NA	R01_cb6615_c11/f1p0/2412	R01_cb6615_c11/f1p0/2412	R01_cb6615_c11/f1p0/2412
R01_cb8564_c121990/f1p0/2391	NA	R01_cb8564_c121990/f1p0/2391	NA	R01_cb8564_c121990/f1p0/2391
R01_cb10080_c13/f1p0/719	NA	R01_cb10080_c13/f1p0/719	NA	NA
R01_cb18223_c25/f1p1/1052	NA	R01_cb18223_c25/f1p1/1052	R01_cb18223_c25/f1p1/1052	R01_cb18223_c25/f1p1/1052
R01_cb8564_c70514/f1p0/2375	NA	NA	NA	R01_cb8564_c70514/f1p0/2375
R01_cb2719_c20/f1p0/2940	NA	NA	R01_cb2719_c20/f1p0/2940	R01_cb2719_c20/f1p0/2940
R01_cb4503_c9/f2p0/3305	NA	R01_cb4503_c9/f2p0/3305	NA	R01_cb4503_c9/f2p0/3305
R01_cb8564_c17855/f1p0/3100	NA	R01_cb8564_c17855/f1p0/3100	NA	NA
R01_cb8564_c1317/f1p0/2253	NA	NA	R01_cb8564_c1317/f1p0/2253	NA
R01_cb1510_c1/f1p0/2691	NA	NA	NA	R01_cb1510_c1/f1p0/2691
R01_cb11613_c0/f1p0/1719	NA	R01_cb11613_c0/f1p0/1719	NA	NA
R01_cb4059_c4/f1p0/2239	NA	NA	NA	R01_cb4059_c4/f1p0/2239
R01_cb6345_c0/f2p1/2864	NA	NA	NA	R01_cb6345_c0/f2p1/2864
R01_cb101_c4/f1p0/2110	NA	NA	NA	R01_cb101_c4/f1p0/2110
R01_cb10244_c1/f1p0/1277	NA	R01_cb10244_c1/f1p0/1277	NA	NA
R01_cb14409_c0/f8p0/636	R01_cb14409_c0/f8p0/636	R01_cb14409_c0/f8p0/636	R01_cb14409_c0/f8p0/636	R01_cb14409_c0/f8p0/636

R01_cb5963_c2/f1p0/3029	NA	R01_cb5963_c2/f1p0/3029	NA	NA
R01_cb6141_c1/f1p0/2928	NA	R01_cb6141_c1/f1p0/2928	NA	NA
R01_cb4444_c6/f1p0/3234	NA	R01_cb4444_c6/f1p0/3234	NA	R01_cb4444_c6/f1p0/3234
R01_cb233_c28/f1p0/2010	NA	NA	NA	R01_cb233_c28/f1p0/2010
R01_cb8564_c1971/f1p0/2751	NA	R01_cb8564_c1971/f1p0/2751	NA	NA
R01_cb8327_c2/f1p0/1824	NA	NA	NA	R01_cb8327_c2/f1p0/1824
R01_cb8925_c16/f1p0/645	NA	R01_cb8925_c16/f1p0/645	NA	NA
R01_cb8564_c74606/f1p0/2240	NA	R01_cb8564_c74606/f1p0/2240	NA	NA
R01_cb5617_c11/f1p0/2501	NA	R01_cb5617_c11/f1p0/2501	NA	NA
R01_cb8478_c8/f2p0/2643	NA	NA	NA	R01_cb8478_c8/f2p0/2643
R01_cb8564_c84947/f1p0/3313	NA	R01_cb8564_c84947/f1p0/3313	NA	R01_cb8564_c84947/f1p0/3313
R01_cb5190_c11/f7p3/2263	NA	NA	NA	R01_cb5190_c11/f7p3/2263
R01_cb18456_c7222/f1p0/1670	NA	R01_cb18456_c7222/f1p0/1670	NA	NA
R01_cb5756_c0/f1p0/2946	R01_cb5756_c0/f1p0/2946	R01_cb5756_c0/f1p0/2946	R01_cb5756_c0/f1p0/2946	R01_cb5756_c0/f1p0/2946
R01_cb10694_c4/f1p0/2489	NA	R01_cb10694_c4/f1p0/2489	R01_cb10694_c4/f1p0/2489	R01_cb10694_c4/f1p0/2489
R01_cb18456_c6458/f1p0/389	R01_cb18456_c6458/f1p0/389	R01_cb18456_c6458/f1p0/389	R01_cb18456_c6458/f1p0/389	R01_cb18456_c6458/f1p0/389
R01_cb12091_c0/f2p0/465	R01_cb12091_c0/f2p0/465	R01_cb12091_c0/f2p0/465	R01_cb12091_c0/f2p0/465	R01_cb12091_c0/f2p0/465
R01_cb13781_c8/f1p0/575	NA	NA	NA	R01_cb13781_c8/f1p0/575
R01_cb15188_c1/f1p0/1754	R01_cb15188_c1/f1p0/1754	R01_cb15188_c1/f1p0/1754	NA	R01_cb15188_c1/f1p0/1754
R01_cb8650_c3/f1p0/2250	R01_cb8650_c3/f1p0/2250	R01_cb8650_c3/f1p0/2250	R01_cb8650_c3/f1p0/2250	NA
R01_cb10076_c3/f1p0/1902	R01_cb10076_c3/f1p0/1902	R01_cb10076_c3/f1p0/1902	NA	R01_cb10076_c3/f1p0/1902
R01_cb16452_c14/f1p0/1698	R01_cb16452_c14/f1p0/1698	R01_cb16452_c14/f1p0/1698	R01_cb16452_c14/f1p0/1698	R01_cb16452_c14/f1p0/1698
R01_cb8564_c2472/f1p0/3325	NA	R01_cb8564_c2472/f1p0/3325	R01_cb8564_c2472/f1p0/3325	R01_cb8564_c2472/f1p0/3325
R01_cb13144_c9/f26p0/480	R01_cb13144_c9/f26p0/480	NA	NA	R01_cb13144_c9/f26p0/480
R01_cb2921_c4/f1p0/1890	NA	NA	NA	R01_cb2921_c4/f1p0/1890
R01_cb11007_c6/f1p0/1412	NA	NA	R01_cb11007_c6/f1p0/1412	NA

R01_cb8564_c115063/f1p0/2302	NA	R01_cb8564_c115063/f1p0/2302	NA	NA
R01_cb8564_c114859/f1p0/2815	NA	R01_cb8564_c114859/f1p0/2815	NA	NA
R01_cb10971_c1/f1p0/2098	NA	R01_cb10971_c1/f1p0/2098	R01_cb10971_c1/f1p0/2098	NA
R01_cb2729_c0/f1p0/3826	R01_cb2729_c0/f1p0/3826	R01_cb2729_c0/f1p0/3826	NA	R01_cb2729_c0/f1p0/3826
R01_cb9877_c1/f1p0/2616	NA	NA	NA	R01_cb9877_c1/f1p0/2616
R01_cb15383_c2/f1p0/578	NA	R01_cb15383_c2/f1p0/578	NA	NA
R01_cb18378_c0/f2p0/646	NA	R01_cb18378_c0/f2p0/646	NA	R01_cb18378_c0/f2p0/646
R01_cb9422_c3/f1p0/2269	R01_cb9422_c3/f1p0/2269	R01_cb9422_c3/f1p0/2269	NA	NA
R01_cb8564_c3876/f1p0/2859	R01_cb8564_c3876/f1p0/2859	R01_cb8564_c3876/f1p0/2859	R01_cb8564_c3876/f1p0/2859	R01_cb8564_c3876/f1p0/2859
R01_cb454_c23/f1p0/2557	NA	NA	NA	R01_cb454_c23/f1p0/2557
R01_cb7184_c7/f1p0/1882	NA	R01_cb7184_c7/f1p0/1882	NA	NA
R01_cb18456_c3582/f1p0/986	NA	R01_cb18456_c3582/f1p0/986	NA	NA
R01_cb6914_c16/f1p0/2349	NA	NA	NA	R01_cb6914_c16/f1p0/2349
R01_cb6284_c4/f3p0/1096	NA	NA	NA	R01_cb6284_c4/f3p0/1096
R01_cb18766_c1/f1p0/1220	NA	R01_cb18766_c1/f1p0/1220	R01_cb18766_c1/f1p0/1220	R01_cb18766_c1/f1p0/1220
R01_cb12641_c27/f1p0/1111	NA	NA	NA	R01_cb12641_c27/f1p0/1111
R01_cb8564_c109799/f2p0/2167	NA	R01_cb8564_c109799/f2p0/2167	NA	NA
R01_cb8564_c146635/f3p0/3607	NA	R01_cb8564_c146635/f3p0/3607	NA	R01_cb8564_c146635/f3p0/3607
R01_cb8564_c19073/f1p0/4292	NA	NA	NA	R01_cb8564_c19073/f1p0/4292
R01_cb11967_c10/f1p0/490	NA	R01_cb11967_c10/f1p0/490	R01_cb11967_c10/f1p0/490	R01_cb11967_c10/f1p0/490
R01_cb8564_c83860/f1p0/2902	NA	R01_cb8564_c83860/f1p0/2902	NA	NA
R01_cb12057_c40/f1p0/752	R01_cb12057_c40/f1p0/752	R01_cb12057_c40/f1p0/752	NA	NA

R01_cb18456_c7234/f1p0/521	R01_cb18456_c7234/f1p0/521	R01_cb18456_c7234/f1p0/521	R01_cb18456_c7234/f1p0/521	R01_cb18456_c7234/f1p0/521
R01_cb16317_c2/f2p0/1287	NA	NA	NA	R01_cb16317_c2/f2p0/1287
R01_cb14920_c3/f1p0/598	NA	R01_cb14920_c3/f1p0/598	NA	NA
R01_cb8564_c18225/f1p0/2693	NA	R01_cb8564_c18225/f1p0/2693	NA	NA
R01_cb14283_c9/f13p1/514	R01_cb14283_c9/f13p1/514	R01_cb14283_c9/f13p1/514	R01_cb14283_c9/f13p1/514	NA
R01_cb14413_c3/f1p0/531	NA	R01_cb14413_c3/f1p0/531	NA	R01_cb14413_c3/f1p0/531
R01_cb14128_c0/f4p0/1013	NA	R01_cb14128_c0/f4p0/1013	NA	NA
R01_cb8564_c4622/f2p2/2848	NA	R01_cb8564_c4622/f2p2/2848	R01_cb8564_c4622/f2p2/2848	R01_cb8564_c4622/f2p2/2848
R01_cb480_c2/f1p0/3018	NA	NA	NA	R01_cb480_c2/f1p0/3018
R01_cb6015_c11/f1p0/2374	R01_cb6015_c11/f1p0/2374	R01_cb6015_c11/f1p0/2374	NA	NA
R01_cb10385_c10/f1p0/1526	NA	R01_cb10385_c10/f1p0/1526	NA	NA
R01_cb11557_c2/f1p1/917	R01_cb11557_c2/f1p1/917	R01_cb11557_c2/f1p1/917	NA	R01_cb11557_c2/f1p1/917
R01_cb1258_c1/f1p0/4428	NA	R01_cb1258_c1/f1p0/4428	R01_cb1258_c1/f1p0/4428	NA
R01_cb14839_c2/f1p0/737	R01_cb14839_c2/f1p0/737	R01_cb14839_c2/f1p0/737	R01_cb14839_c2/f1p0/737	R01_cb14839_c2/f1p0/737
R01_cb8564_c36636/f1p0/2302	NA	R01_cb8564_c36636/f1p0/2302	R01_cb8564_c36636/f1p0/2302	R01_cb8564_c36636/f1p0/2302
R01_cb3907_c4/f1p0/3038	NA	R01_cb3907_c4/f1p0/3038	NA	NA
R01_cb1331_c33/f1p4/1874	NA	R01_cb1331_c33/f1p4/1874	NA	NA
R01_cb15089_c0/f2p0/387	R01_cb15089_c0/f2p0/387	R01_cb15089_c0/f2p0/387	R01_cb15089_c0/f2p0/387	R01_cb15089_c0/f2p0/387
R01_cb16132_c29/f1p0/826	NA	NA	NA	R01_cb16132_c29/f1p0/826
R01_cb639_c9/f1p0/652	NA	NA	NA	R01_cb639_c9/f1p0/652
R01_cb8564_c91180/f1p0/3622	NA	R01_cb8564_c91180/f1p0/3622	R01_cb8564_c91180/f1p0/3622	R01_cb8564_c91180/f1p0/3622
R01_cb110_c18/f1p0/3101	NA	NA	NA	R01_cb110_c18/f1p0/3101
R01_cb8030_c4/f1p0/2295	NA	NA	NA	R01_cb8030_c4/f1p0/2295
R01_cb10875_c1/f1p0/2477	NA	R01_cb10875_c1/f1p0/2477	NA	NA
R01_cb4490_c1/f2p0/2409	NA	NA	NA	R01_cb4490_c1/f2p0/2409
R01_cb10864_c1/f1p0/1920	R01_cb10864_c1/f1p0/1920	R01_cb10864_c1/f1p0/1920	NA	NA

R01_cb16160_c4/f1p1/879	NA	R01_cb16160_c4/f1p1/879	NA	NA
R01_cb11850_c7/f1p0/593	R01_cb11850_c7/f1p0/593	R01_cb11850_c7/f1p0/593	NA	NA
R01_cb2177_c98/f1p1/3415	NA	NA	NA	R01_cb2177_c98/f1p1/3415
R01_cb8669_c10/f1p0/1140	NA	NA	NA	R01_cb8669_c10/f1p0/1140
R01_cb3752_c6/f1p0/1186	NA	NA	NA	R01_cb3752_c6/f1p0/1186
R01_cb18456_c7497/f1p0/562	R01_cb18456_c7497/f1p0/562	R01_cb18456_c7497/f1p0/562	R01_cb18456_c7497/f1p0/562	R01_cb18456_c7497/f1p0/562
R01_cb8564_c16062/f1p0/4315	R01_cb8564_c16062/f1p0/4315	R01_cb8564_c16062/f1p0/4315	NA	R01_cb8564_c16062/f1p0/4315
R01_cb6057_c0/f2p0/3239	NA	R01_cb6057_c0/f2p0/3239	NA	R01_cb6057_c0/f2p0/3239
R01_cb14909_c2/f1p0/797	R01_cb14909_c2/f1p0/797	NA	R01_cb14909_c2/f1p0/797	R01_cb14909_c2/f1p0/797
R01_cb8564_c25451/f1p0/3935	NA	NA	NA	R01_cb8564_c25451/f1p0/3935
R01_cb10063_c7/f2p3/828	NA	R01_cb10063_c7/f2p3/828	NA	NA
R01_cb14980_c0/f5p0/535	NA	R01_cb14980_c0/f5p0/535	NA	NA
R01_cb8687_c4/f1p0/3035	NA	R01_cb8687_c4/f1p0/3035	NA	NA
R01_cb16871_c1/f1p0/1446	NA	NA	NA	R01_cb16871_c1/f1p0/1446
R01_cb709_c4/f1p0/2561	NA	R01_cb709_c4/f1p0/2561	R01_cb709_c4/f1p0/2561	NA
R01_cb3051_c15/f1p0/2400	NA	NA	NA	R01_cb3051_c15/f1p0/2400
R01_cb5953_c4/f1p0/3405	NA	NA	NA	R01_cb5953_c4/f1p0/3405
R01_cb15255_c1/f1p0/760	NA	NA	NA	R01_cb15255_c1/f1p0/760
R01_cb8564_c115297/f1p0/2542	R01_cb8564_c115297/f1p0/2542	R01_cb8564_c115297/f1p0/2542	NA	NA
R01_cb18456_c5804/f1p0/477	R01_cb18456_c5804/f1p0/477	R01_cb18456_c5804/f1p0/477	R01_cb18456_c5804/f1p0/477	R01_cb18456_c5804/f1p0/477
R01_cb18025_c0/f1p0/1087	NA	R01_cb18025_c0/f1p0/1087	NA	NA
R01_cb8564_c19642/f1p0/4457	NA	R01_cb8564_c19642/f1p0/4457	NA	NA
R01_cb8564_c47016/f1p0/2372	R01_cb8564_c47016/f1p0/2372	R01_cb8564_c47016/f1p0/2372	R01_cb8564_c47016/f1p0/2372	R01_cb8564_c47016/f1p0/2372

R01_cb3026_c7/f1p0/2238	NA	R01_cb3026_c7/f1p0/2238	NA	NA
R01_cb13602_c25/f1p0/1663	NA	NA	NA	R01_cb13602_c25/f1p0/1663
R01_cb8564_c1396/f1p0/2152	R01_cb8564_c1396/f1p0/2152	NA	NA	NA
R01_cb1676_c11/f1p0/5120	NA	R01_cb1676_c11/f1p0/5120	NA	NA
R01_cb13322_c1/f1p0/704	NA	R01_cb13322_c1/f1p0/704	NA	NA
R01_cb12152_c3/f1p0/747	NA	NA	R01_cb12152_c3/f1p0/747	R01_cb12152_c3/f1p0/747
R01_cb10961_c1/f1p0/3504	NA	R01_cb10961_c1/f1p0/3504	NA	NA
R01_cb8564_c38225/f1p0/4002	NA	R01_cb8564_c38225/f1p0/4002	NA	NA
R01_cb8564_c49342/f1p0/2182	R01_cb8564_c49342/f1p0/2182	R01_cb8564_c49342/f1p0/2182	R01_cb8564_c49342/f1p0/2182	R01_cb8564_c49342/f1p0/2182
R01_cb3989_c3/f1p1/2020	NA	R01_cb3989_c3/f1p1/2020	NA	R01_cb3989_c3/f1p1/2020
R01_cb15118_c6/f1p1/541	NA	NA	NA	R01_cb15118_c6/f1p1/541
R01_cb16248_c2/f1p0/1067	NA	R01_cb16248_c2/f1p0/1067	NA	NA
R01_cb2803_c20/f1p0/590	NA	R01_cb2803_c20/f1p0/590	R01_cb2803_c20/f1p0/590	R01_cb2803_c20/f1p0/590
R01_cb5659_c101/f45p0/2187	NA	NA	NA	R01_cb5659_c101/f45p0/2187
R01_cb8564_c22153/f1p0/2545	NA	R01_cb8564_c22153/f1p0/2545	R01_cb8564_c22153/f1p0/2545	R01_cb8564_c22153/f1p0/2545
R01_cb12618_c0/f2p0/954	NA	R01_cb12618_c0/f2p0/954	NA	NA
R01_cb2344_c3/f1p0/716	NA	R01_cb2344_c3/f1p0/716	NA	NA
R01_cb1779_c5/f1p0/3168	NA	NA	NA	R01_cb1779_c5/f1p0/3168
R01_cb14522_c1/f1p0/641	NA	R01_cb14522_c1/f1p0/641	R01_cb14522_c1/f1p0/641	R01_cb14522_c1/f1p0/641
R01_cb17553_c5/f1p1/368	NA	R01_cb17553_c5/f1p1/368	R01_cb17553_c5/f1p1/368	R01_cb17553_c5/f1p1/368
R01_cb11124_c2/f1p0/4565	R01_cb11124_c2/f1p0/4565	R01_cb11124_c2/f1p0/4565	NA	NA
R01_cb18214_c2/f1p0/1801	NA	R01_cb18214_c2/f1p0/1801	NA	NA
R01_cb7741_c10/f1p0/515	R01_cb7741_c10/f1p0/515	R01_cb7741_c10/f1p0/515	R01_cb7741_c10/f1p0/515	R01_cb7741_c10/f1p0/515
R01_cb14579_c6/f1p0/686	NA	R01_cb14579_c6/f1p0/686	NA	NA
R01_cb14718_c1/f1p0/1771	NA	R01_cb14718_c1/f1p0/1771	NA	NA

R01_cb18456_c5328/f1p0/695	NA	R01_cb18456_c5328/f1p0/695	NA	NA
R01_cb742_c17/f1p0/2527	NA	R01_cb742_c17/f1p0/2527	NA	NA
R01_cb7828_c1/f2p0/2163	NA	NA	NA	R01_cb7828_c1/f2p0/2163
R01_cb3001_c0/f1p0/3750	NA	R01_cb3001_c0/f1p0/3750	NA	NA
R01_cb8564_c121926/f1p0/1893	NA	R01_cb8564_c121926/f1p0/1893	NA	NA
R01_cb3575_c3/f2p1/3187	NA	NA	NA	R01_cb3575_c3/f2p1/3187
R01_cb3941_c27/f1p0/2126	NA	NA	NA	R01_cb3941_c27/f1p0/2126
R01_cb8564_c72966/f1p0/3209	NA	R01_cb8564_c72966/f1p0/3209	R01_cb8564_c72966/f1p0/3209	R01_cb8564_c72966/f1p0/3209
R01_cb9061_c27/f1p1/2278	NA	NA	NA	R01_cb9061_c27/f1p1/2278
R01_cb326_c10/f1p0/2712	NA	NA	NA	R01_cb326_c10/f1p0/2712
R01_cb2729_c5/f1p0/2525	R01_cb2729_c5/f1p0/2525	R01_cb2729_c5/f1p0/2525	NA	R01_cb2729_c5/f1p0/2525
R01_cb8564_c84760/f1p0/2389	R01_cb8564_c84760/f1p0/2389	R01_cb8564_c84760/f1p0/2389	R01_cb8564_c84760/f1p0/2389	R01_cb8564_c84760/f1p0/2389
R01_cb8564_c87581/f1p0/3082	NA	R01_cb8564_c87581/f1p0/3082	R01_cb8564_c87581/f1p0/3082	R01_cb8564_c87581/f1p0/3082
R01_cb8564_c4910/f1p0/2450	R01_cb8564_c4910/f1p0/2450	R01_cb8564_c4910/f1p0/2450	R01_cb8564_c4910/f1p0/2450	R01_cb8564_c4910/f1p0/2450
R01_cb8564_c4666/f3p0/3234	NA	R01_cb8564_c4666/f3p0/3234	R01_cb8564_c4666/f3p0/3234	R01_cb8564_c4666/f3p0/3234
R01_cb5698_c5/f1p0/2397	R01_cb5698_c5/f1p0/2397	R01_cb5698_c5/f1p0/2397	NA	R01_cb5698_c5/f1p0/2397
R01_cb16937_c0/f1p0/435	R01_cb16937_c0/f1p0/435	R01_cb16937_c0/f1p0/435	NA	R01_cb16937_c0/f1p0/435
R01_cb5554_c5/f1p0/2444	R01_cb5554_c5/f1p0/2444	R01_cb5554_c5/f1p0/2444	R01_cb5554_c5/f1p0/2444	R01_cb5554_c5/f1p0/2444
R01_cb3398_c2/f1p0/3309	R01_cb3398_c2/f1p0/3309	R01_cb3398_c2/f1p0/3309	NA	NA
R01_cb17456_c0/f1p0/1574	NA	R01_cb17456_c0/f1p0/1574	NA	NA
R01_cb7257_c0/f1p0/2663	NA	NA	NA	R01_cb7257_c0/f1p0/2663
R01_cb12620_c17/f3p0/1192	NA	NA	NA	R01_cb12620_c17/f3p0/1192
R01_cb4493_c13/f1p1/4300	NA	NA	NA	R01_cb4493_c13/f1p1/4300
R01_cb11625_c1/f1p0/2559	NA	NA	NA	R01_cb11625_c1/f1p0/2559

R01_cb9797_c36/f1p0/2929	NA	R01_cb9797_c36/f1p0/2929	NA	NA
R01_cb69_c27/f1p1/1310	NA	NA	NA	R01_cb69_c27/f1p1/1310
R01_cb1936_c2/f1p0/2075	R01_cb1936_c2/f1p0/2075	R01_cb1936_c2/f1p0/2075	R01_cb1936_c2/f1p0/2075	R01_cb1936_c2/f1p0/2075
R01_cb1031_c4/f1p0/3637	NA	NA	NA	R01_cb1031_c4/f1p0/3637
R01_cb6802_c72/f1p0/2240	NA	R01_cb6802_c72/f1p0/2240	NA	NA
R01_cb5259_c6/f1p0/3922	NA	NA	R01_cb5259_c6/f1p0/3922	R01_cb5259_c6/f1p0/3922
R01_cb13284_c13/f1p0/1185	NA	NA	NA	R01_cb13284_c13/f1p0/1185
R01_cb13692_c2/f1p0/1508	NA	R01_cb13692_c2/f1p0/1508	NA	NA
R01_cb18456_c6993/f1p2/1172	NA	NA	NA	R01_cb18456_c6993/f1p2/1172
R01_cb18409_c182/f1p0/359	R01_cb18409_c182/f1p0/359	R01_cb18409_c182/f1p0/359	R01_cb18409_c182/f1p0/359	R01_cb18409_c182/f1p0/359
R01_cb8564_c12707/f1p0/3555	NA	R01_cb8564_c12707/f1p0/3555	R01_cb8564_c12707/f1p0/3555	R01_cb8564_c12707/f1p0/3555
R01_cb8564_c17790/f1p0/2283	NA	NA	NA	R01_cb8564_c17790/f1p0/2283
R01_cb5498_c1/f1p0/3651	NA	R01_cb5498_c1/f1p0/3651	R01_cb5498_c1/f1p0/3651	NA
R01_cb6059_c1/f1p0/1894	NA	NA	NA	R01_cb6059_c1/f1p0/1894
R01_cb8868_c1/f1p1/2171	NA	R01_cb8868_c1/f1p1/2171	R01_cb8868_c1/f1p1/2171	R01_cb8868_c1/f1p1/2171
R01_cb8564_c79187/f1p0/2963	NA	R01_cb8564_c79187/f1p0/2963	R01_cb8564_c79187/f1p0/2963	R01_cb8564_c79187/f1p0/2963
R01_cb5168_c2/f1p0/3330	NA	R01_cb5168_c2/f1p0/3330	R01_cb5168_c2/f1p0/3330	R01_cb5168_c2/f1p0/3330
R01_cb8564_c126032/f1p0/3058	NA	NA	NA	R01_cb8564_c126032/f1p0/3058
R01_cb10243_c4/f1p0/829	R01_cb10243_c4/f1p0/829	R01_cb10243_c4/f1p0/829	R01_cb10243_c4/f1p0/829	R01_cb10243_c4/f1p0/829
R01_cb1487_c5/f1p0/1318	NA	NA	NA	R01_cb1487_c5/f1p0/1318
R01_cb6602_c25/f1p0/2937	NA	R01_cb6602_c25/f1p0/2937	NA	R01_cb6602_c25/f1p0/2937
R01_cb8564_c35145/f2p0/3283	NA	R01_cb8564_c35145/f2p0/3283	NA	NA
R01_cb6529_c3/f1p0/2068	NA	NA	NA	R01_cb6529_c3/f1p0/2068
R01_cb2845_c6/f1p0/4170	NA	NA	NA	R01_cb2845_c6/f1p0/4170
R01_cb13726_c0/f2p1/1081	NA	NA	NA	R01_cb13726_c0/f2p1/1081

R01_cb3046_c9/f1p2/2311	NA	R01_cb3046_c9/f1p2/2311	NA	R01_cb3046_c9/f1p2/2311
R01_cb454_c13/f1p0/2437	NA	NA	NA	R01_cb454_c13/f1p0/2437
R01_cb8564_c22011/f1p0/3477	NA	R01_cb8564_c22011/f1p0/3477	R01_cb8564_c22011/f1p0/3477	R01_cb8564_c22011/f1p0/3477
R01_cb15760_c1/f1p0/636	R01_cb15760_c1/f1p0/636	R01_cb15760_c1/f1p0/636	R01_cb15760_c1/f1p0/636	R01_cb15760_c1/f1p0/636
R01_cb12972_c31/f1p0/621	NA	R01_cb12972_c31/f1p0/621	R01_cb12972_c31/f1p0/621	R01_cb12972_c31/f1p0/621
R01_cb17749_c0/f2p0/489	NA	R01_cb17749_c0/f2p0/489	R01_cb17749_c0/f2p0/489	R01_cb17749_c0/f2p0/489
R01_cb16342_c0/f2p0/560	NA	R01_cb16342_c0/f2p0/560	R01_cb16342_c0/f2p0/560	R01_cb16342_c0/f2p0/560
R01_cb5331_c0/f2p0/3049	NA	R01_cb5331_c0/f2p0/3049	NA	NA
R01_cb8564_c76721/f1p0/2245	R01_cb8564_c76721/f1p0/2245	R01_cb8564_c76721/f1p0/2245	NA	NA
R01_cb15714_c2/f1p0/483	NA	R01_cb15714_c2/f1p0/483	NA	NA
R01_cb11870_c1/f1p0/360	R01_cb11870_c1/f1p0/360	R01_cb11870_c1/f1p0/360	R01_cb11870_c1/f1p0/360	R01_cb11870_c1/f1p0/360
R01_cb4047_c18/f14p4/2895	NA	NA	NA	R01_cb4047_c18/f14p4/2895
R01_cb2870_c6/f1p1/3040	NA	NA	NA	R01_cb2870_c6/f1p1/3040
R01_cb7507_c10/f1p0/2506	NA	R01_cb7507_c10/f1p0/2506	NA	NA
R01_cb17973_c37/f1p0/399	R01_cb17973_c37/f1p0/399	R01_cb17973_c37/f1p0/399	R01_cb17973_c37/f1p0/399	NA
R01_cb13990_c1/f1p0/926	R01_cb13990_c1/f1p0/926	R01_cb13990_c1/f1p0/926	NA	R01_cb13990_c1/f1p0/926
R01_cb15186_c22/f1p0/530	R01_cb15186_c22/f1p0/530	R01_cb15186_c22/f1p0/530	R01_cb15186_c22/f1p0/530	NA
R01_cb15578_c3/f1p0/702	NA	R01_cb15578_c3/f1p0/702	NA	R01_cb15578_c3/f1p0/702
R01_cb12603_c9/f1p1/1411	NA	R01_cb12603_c9/f1p1/1411	R01_cb12603_c9/f1p1/1411	R01_cb12603_c9/f1p1/1411
R01_cb2445_c6/f2p0/3518	NA	NA	NA	R01_cb2445_c6/f2p0/3518
R01_cb3352_c2/f1p0/3409	NA	R01_cb3352_c2/f1p0/3409	NA	NA
R01_cb9617_c6/f1p2/1946	NA	NA	NA	R01_cb9617_c6/f1p2/1946
R01_cb16831_c1/f2p0/729	NA	NA	NA	R01_cb16831_c1/f2p0/729
R01_cb454_c2/f4p0/2393	NA	NA	NA	R01_cb454_c2/f4p0/2393
R01_cb17981_c2/f1p0/417	R01_cb17981_c2/f1p0/417	R01_cb17981_c2/f1p0/417	NA	R01_cb17981_c2/f1p0/417

R01_cb10343_c2/f1p0/1518	R01_cb10343_c2/f1p0/1518	R01_cb10343_c2/f1p0/1518	R01_cb10343_c2/f1p0/1518	NA
R01_cb2854_c48/f1p0/2955	NA	R01_cb2854_c48/f1p0/2955	NA	NA
R01_cb8564_c86285/f1p0/2375	NA	NA	NA	R01_cb8564_c86285/f1p0/2375
R01_cb6571_c4/f1p0/1506	NA	R01_cb6571_c4/f1p0/1506	NA	R01_cb6571_c4/f1p0/1506
R01_cb10338_c6/f1p0/1350	NA	R01_cb10338_c6/f1p0/1350	NA	NA
R01_cb7164_c1/f1p0/4448	NA	NA	NA	R01_cb7164_c1/f1p0/4448
R01_cb4169_c10/f1p0/1452	NA	NA	NA	R01_cb4169_c10/f1p0/1452
R01_cb1676_c9/f1p0/594	NA	R01_cb1676_c9/f1p0/594	NA	NA
R01_cb12575_c5/f3p0/968	NA	NA	NA	R01_cb12575_c5/f3p0/968
R01_cb14955_c4/f6p1/1054	NA	NA	NA	R01_cb14955_c4/f6p1/1054
R01_cb10015_c188/f1p0/586	R01_cb10015_c188/f1p0/586	R01_cb10015_c188/f1p0/586	R01_cb10015_c188/f1p0/586	R01_cb10015_c188/f1p0/586
R01_cb8564_c120117/f1p0/2672	R01_cb8564_c120117/f1p0/2672	R01_cb8564_c120117/f1p0/2672	R01_cb8564_c120117/f1p0/2672	NA
R01_cb8564_c21995/f1p0/2135	R01_cb8564_c21995/f1p0/2135	R01_cb8564_c21995/f1p0/2135	NA	NA
R01_cb2104_c9/f1p0/2916	NA	NA	R01_cb2104_c9/f1p0/2916	R01_cb2104_c9/f1p0/2916
R01_cb2676_c7/f1p0/2966	NA	NA	NA	R01_cb2676_c7/f1p0/2966
R01_cb6959_c0/f2p0/2357	NA	NA	NA	R01_cb6959_c0/f2p0/2357
R01_cb8564_c82244/f1p3/3163	NA	R01_cb8564_c82244/f1p3/3163	NA	NA
R01_cb1614_c5/f1p0/2234	NA	NA	NA	R01_cb1614_c5/f1p0/2234
R01_cb8564_c126515/f1p0/2487	R01_cb8564_c126515/f1p0/2487	R01_cb8564_c126515/f1p0/2487	NA	NA
R01_cb8564_c4070/f1p0/2882	NA	R01_cb8564_c4070/f1p0/2882	NA	R01_cb8564_c4070/f1p0/2882
R01_cb17670_c0/f1p0/444	R01_cb17670_c0/f1p0/444	R01_cb17670_c0/f1p0/444	R01_cb17670_c0/f1p0/444	R01_cb17670_c0/f1p0/444
R01_cb385_c8/f1p1/9473	R01_cb385_c8/f1p1/9473	NA	NA	NA
R01_cb18150_c3/f1p0/1737	NA	R01_cb18150_c3/f1p0/1737	R01_cb18150_c3/f1p0/1737	R01_cb18150_c3/f1p0/1737

R01_cb11924_c22/f1p2/514	R01_cb11924_c22/f1p2/514	R01_cb11924_c22/f1p2/514	R01_cb11924_c22/f1p2/514	NA
R01_cb8564_c107873/f2p0/3615	R01_cb8564_c107873/f2p0/3615	R01_cb8564_c107873/f2p0/3615	NA	NA
R01_cb10968_c0/f5p1/605	NA	R01_cb10968_c0/f5p1/605	R01_cb10968_c0/f5p1/605	R01_cb10968_c0/f5p1/605
R01_cb4923_c14/f1p0/1430	NA	R01_cb4923_c14/f1p0/1430	NA	R01_cb4923_c14/f1p0/1430
R01_cb7502_c13/f1p1/2270	NA	NA	R01_cb7502_c13/f1p1/2270	R01_cb7502_c13/f1p1/2270
R01_cb10593_c1/f1p0/3134	NA	R01_cb10593_c1/f1p0/3134	R01_cb10593_c1/f1p0/3134	NA
R01_cb16678_c7/f1p0/1720	NA	R01_cb16678_c7/f1p0/1720	R01_cb16678_c7/f1p0/1720	R01_cb16678_c7/f1p0/1720
R01_cb11522_c2/f1p0/802	NA	R01_cb11522_c2/f1p0/802	NA	R01_cb11522_c2/f1p0/802
R01_cb12750_c3/f1p0/621	NA	R01_cb12750_c3/f1p0/621	NA	R01_cb12750_c3/f1p0/621
R01_cb8809_c1/f10p2/2405	NA	NA	NA	R01_cb8809_c1/f10p2/2405
R01_cb8564_c89534/f1p0/2521	NA	R01_cb8564_c89534/f1p0/2521	NA	NA
R01_cb14004_c14/f1p1/1355	NA	NA	NA	R01_cb14004_c14/f1p1/1355
R01_cb12365_c1/f5p0/651	NA	R01_cb12365_c1/f5p0/651	NA	NA
R01_cb16321_c3/f1p0/1486	R01_cb16321_c3/f1p0/1486	R01_cb16321_c3/f1p0/1486	NA	R01_cb16321_c3/f1p0/1486
R01_cb18373_c0/f1p0/1573	NA	R01_cb18373_c0/f1p0/1573	R01_cb18373_c0/f1p0/1573	R01_cb18373_c0/f1p0/1573
R01_cb4368_c13/f1p0/517	NA	NA	R01_cb4368_c13/f1p0/517	R01_cb4368_c13/f1p0/517
R01_cb8564_c71574/f1p0/3073	NA	R01_cb8564_c71574/f1p0/3073	NA	NA
R01_cb17997_c7/f2p1/919	NA	NA	NA	R01_cb17997_c7/f2p1/919
R01_cb11703_c1/f1p0/2075	NA	R01_cb11703_c1/f1p0/2075	R01_cb11703_c1/f1p0/2075	NA
R01_cb12195_c3/f1p1/1611	NA	R01_cb12195_c3/f1p1/1611	NA	NA
R01_cb4952_c2/f1p0/3420	NA	NA	NA	R01_cb4952_c2/f1p0/3420
R01_cb10383_c2/f1p0/364	NA	NA	R01_cb10383_c2/f1p0/364	R01_cb10383_c2/f1p0/364
R01_cb8564_c122886/f1p1/2748	NA	R01_cb8564_c122886/f1p1/2748	NA	R01_cb8564_c122886/f1p1/2748
R01_cb7757_c10/f1p1/1977	NA	NA	R01_cb7757_c10/f1p1/1977	R01_cb7757_c10/f1p1/1977

R01_cb8564_c23893/f1p0/4543	R01_cb8564_c23893/f1p0/4543	R01_cb8564_c23893/f1p0/4543	R01_cb8564_c23893/f1p0/4543	R01_cb8564_c23893/f1p0/4543
R01_cb10865_c1/f1p0/3004	NA	R01_cb10865_c1/f1p0/3004	NA	R01_cb10865_c1/f1p0/3004
R01_cb18409_c53/f1p0/1144	R01_cb18409_c53/f1p0/1144	R01_cb18409_c53/f1p0/1144	R01_cb18409_c53/f1p0/1144	R01_cb18409_c53/f1p0/1144
R01_cb13875_c5/f1p1/1301	NA	NA	NA	R01_cb13875_c5/f1p1/1301
R01_cb4768_c17/f1p1/2380	NA	R01_cb4768_c17/f1p1/2380	NA	NA
R01_cb14968_c17/f1p0/1774	NA	NA	NA	R01_cb14968_c17/f1p0/1774
R01_cb16828_c1/f1p0/1216	NA	R01_cb16828_c1/f1p0/1216	NA	NA
R01_cb8564_c123310/f1p0/2440	R01_cb8564_c123310/f1p0/2440	R01_cb8564_c123310/f1p0/2440	R01_cb8564_c123310/f1p0/2440	R01_cb8564_c123310/f1p0/2440
R01_cb12722_c4/f3p5/1707	NA	NA	NA	R01_cb12722_c4/f3p5/1707
R01_cb12786_c11/f1p1/726	NA	R01_cb12786_c11/f1p1/726	NA	R01_cb12786_c11/f1p1/726
R01_cb17993_c0/f1p0/421	R01_cb17993_c0/f1p0/421	R01_cb17993_c0/f1p0/421	R01_cb17993_c0/f1p0/421	R01_cb17993_c0/f1p0/421
R01_cb8564_c3077/f1p0/3195	NA	R01_cb8564_c3077/f1p0/3195	R01_cb8564_c3077/f1p0/3195	R01_cb8564_c3077/f1p0/3195
R01_cb8564_c73271/f2p1/2234	R01_cb8564_c73271/f2p1/2234	R01_cb8564_c73271/f2p1/2234	R01_cb8564_c73271/f2p1/2234	R01_cb8564_c73271/f2p1/2234
R01_cb17216_c3/f1p0/443	R01_cb17216_c3/f1p0/443	R01_cb17216_c3/f1p0/443	R01_cb17216_c3/f1p0/443	R01_cb17216_c3/f1p0/443
R01_cb16852_c1/f1p0/1143	NA	R01_cb16852_c1/f1p0/1143	NA	NA
R01_cb14560_c8/f1p0/765	NA	NA	NA	R01_cb14560_c8/f1p0/765
R01_cb10952_c0/f2p0/1453	NA	NA	NA	R01_cb10952_c0/f2p0/1453
R01_cb18427_c1/f1p0/628	NA	NA	NA	R01_cb18427_c1/f1p0/628
R01_cb1333_c71/f1p0/2902	NA	R01_cb1333_c71/f1p0/2902	R01_cb1333_c71/f1p0/2902	NA
R01_cb8564_c86753/f1p0/3161	NA	NA	NA	R01_cb8564_c86753/f1p0/3161
R01_cb9650_c0/f1p0/2021	R01_cb9650_c0/f1p0/2021	R01_cb9650_c0/f1p0/2021	R01_cb9650_c0/f1p0/2021	R01_cb9650_c0/f1p0/2021
R01_cb14131_c5/f1p0/1228	NA	NA	NA	R01_cb14131_c5/f1p0/1228
R01_cb7663_c4/f1p0/2878	NA	NA	NA	R01_cb7663_c4/f1p0/2878

R01_cb11036_c1/f1p0/1951	NA	R01_cb11036_c1/f1p0/1951	NA	NA
R01_cb17973_c52/f1p0/1770	NA	R01_cb17973_c52/f1p0/1770	NA	R01_cb17973_c52/f1p0/1770
R01_cb8564_c149971/f1p13/4827	NA	R01_cb8564_c149971/f1p13/4827	R01_cb8564_c149971/f1p13/4827	R01_cb8564_c149971/f1p13/4827
R01_cb16556_c3/f1p0/1419	NA	NA	NA	R01_cb16556_c3/f1p0/1419
R01_cb10916_c0/f1p0/686	NA	NA	NA	R01_cb10916_c0/f1p0/686
R01_cb7927_c1/f1p0/2046	NA	NA	NA	R01_cb7927_c1/f1p0/2046
R01_cb8564_c107823/f3p0/3389	NA	NA	NA	R01_cb8564_c107823/f3p0/3389
R01_cb17458_c0/f2p0/638	NA	NA	NA	R01_cb17458_c0/f2p0/638
R01_cb13765_c0/f2p0/660	NA	NA	R01_cb13765_c0/f2p0/660	R01_cb13765_c0/f2p0/660
R01_cb381_c5/f1p0/1422	NA	R01_cb381_c5/f1p0/1422	NA	NA
R01_cb8564_c130287/f1p0/2587	R01_cb8564_c130287/f1p0/2587	R01_cb8564_c130287/f1p0/2587	R01_cb8564_c130287/f1p0/2587	R01_cb8564_c130287/f1p0/2587
R01_cb15335_c1/f1p0/896	NA	R01_cb15335_c1/f1p0/896	NA	NA
R01_cb17644_c0/f1p0/711	R01_cb17644_c0/f1p0/711	R01_cb17644_c0/f1p0/711	R01_cb17644_c0/f1p0/711	R01_cb17644_c0/f1p0/711
R01_cb8564_c77607/f1p1/2082	NA	R01_cb8564_c77607/f1p1/2082	NA	NA
R01_cb8564_c17273/f2p0/3222	NA	R01_cb8564_c17273/f2p0/3222	R01_cb8564_c17273/f2p0/3222	R01_cb8564_c17273/f2p0/3222
R01_cb17792_c2/f1p0/1259	NA	NA	NA	R01_cb17792_c2/f1p0/1259
R01_cb1459_c2/f1p1/4317	NA	R01_cb1459_c2/f1p1/4317	NA	NA
R01_cb7986_c17/f1p0/2091	NA	R01_cb7986_c17/f1p0/2091	NA	NA
R01_cb6281_c2/f1p0/1039	NA	NA	NA	R01_cb6281_c2/f1p0/1039
R01_cb18456_c6793/f1p0/649	NA	R01_cb18456_c6793/f1p0/649	R01_cb18456_c6793/f1p0/649	R01_cb18456_c6793/f1p0/649
R01_cb10993_c0/f1p0/1345	NA	NA	NA	R01_cb10993_c0/f1p0/1345
R01_cb2895_c5/f1p0/1683	NA	R01_cb2895_c5/f1p0/1683	NA	NA
R01_cb16646_c0/f1p0/1068	NA	NA	NA	R01_cb16646_c0/f1p0/1068

R01_cb15312_c2/f1p0/728	NA	R01_cb15312_c2/f1p0/728	R01_cb15312_c2/f1p0/728	R01_cb15312_c2/f1p0/728
R01_cb13704_c2/f1p0/1392	NA	R01_cb13704_c2/f1p0/1392	NA	NA
R01_cb18409_c40/f1p0/1884	NA	R01_cb18409_c40/f1p0/1884	R01_cb18409_c40/f1p0/1884	NA
R01_cb506_c8/f1p0/2351	NA	NA	R01_cb506_c8/f1p0/2351	R01_cb506_c8/f1p0/2351
R01_cb8564_c19077/f1p2/4634	NA	R01_cb8564_c19077/f1p2/4634	R01_cb8564_c19077/f1p2/4634	R01_cb8564_c19077/f1p2/4634
R01_cb2183_c1/f3p0/3312	NA	NA	R01_cb2183_c1/f3p0/3312	R01_cb2183_c1/f3p0/3312
R01_cb17697_c1/f1p0/647	NA	NA	R01_cb17697_c1/f1p0/647	R01_cb17697_c1/f1p0/647
R01_cb11624_c1/f1p0/3523	R01_cb11624_c1/f1p0/3523	R01_cb11624_c1/f1p0/3523	R01_cb11624_c1/f1p0/3523	R01_cb11624_c1/f1p0/3523
R01_cb11245_c3/f1p0/1718	NA	R01_cb11245_c3/f1p0/1718	NA	NA
R01_cb8564_c116270/f1p0/2596	NA	R01_cb8564_c116270/f1p0/2596	R01_cb8564_c116270/f1p0/2596	R01_cb8564_c116270/f1p0/2596
R01_cb7611_c4/f1p0/2712	NA	NA	NA	R01_cb7611_c4/f1p0/2712
R01_cb8564_c4475/f1p0/2475	NA	R01_cb8564_c4475/f1p0/2475	NA	NA
R01_cb5929_c7/f1p1/2665	NA	NA	NA	R01_cb5929_c7/f1p1/2665
R01_cb12686_c1/f1p0/447	NA	NA	NA	R01_cb12686_c1/f1p0/447
R01_cb8564_c71789/f1p0/2971	NA	R01_cb8564_c71789/f1p0/2971	NA	R01_cb8564_c71789/f1p0/2971
R01_cb2763_c0/f1p0/3814	NA	R01_cb2763_c0/f1p0/3814	NA	NA
R01_cb8564_c3169/f1p0/3080	NA	R01_cb8564_c3169/f1p0/3080	NA	NA
R01_cb5705_c3/f1p3/2535	NA	R01_cb5705_c3/f1p3/2535	R01_cb5705_c3/f1p3/2535	NA
R01_cb84_c13/f1p0/5191	NA	R01_cb84_c13/f1p0/5191	NA	NA
R01_cb6601_c0/f2p0/959	NA	R01_cb6601_c0/f2p0/959	NA	NA
R01_cb8564_c80479/f1p0/2462	NA	R01_cb8564_c80479/f1p0/2462	R01_cb8564_c80479/f1p0/2462	R01_cb8564_c80479/f1p0/2462
R01_cb11106_c3/f1p0/629	NA	R01_cb11106_c3/f1p0/629	R01_cb11106_c3/f1p0/629	R01_cb11106_c3/f1p0/629
R01_cb2851_c10/f1p0/2281	NA	NA	NA	R01_cb2851_c10/f1p0/2281
R01_cb2536_c13/f1p0/2887	R01_cb2536_c13/f1p0/2887	R01_cb2536_c13/f1p0/2887	NA	NA
R01_cb16413_c1/f1p0/787	R01_cb16413_c1/f1p0/787	R01_cb16413_c1/f1p0/787	R01_cb16413_c1/f1p0/787	R01_cb16413_c1/f1p0/787

R01_cb8564_c9914/f2p1/3768	NA	R01_cb8564_c9914/f2p1/3768	NA	R01_cb8564_c9914/f2p1/3768
R01_cb8173_c15/f83p4/1211	NA	R01_cb8173_c15/f83p4/1211	R01_cb8173_c15/f83p4/1211	R01_cb8173_c15/f83p4/1211
R01_cb5896_c142/f1p0/3587	NA	R01_cb5896_c142/f1p0/3587	NA	NA
R01_cb12554_c2/f1p1/1592	NA	NA	NA	R01_cb12554_c2/f1p1/1592
R01_cb7472_c1/f1p0/2353	NA	NA	NA	R01_cb7472_c1/f1p0/2353
R01_cb5327_c3/f1p0/2049	NA	R01_cb5327_c3/f1p0/2049	R01_cb5327_c3/f1p0/2049	NA
R01_cb510_c7/f1p0/3515	NA	NA	NA	R01_cb510_c7/f1p0/3515
R01_cb11509_c2/f1p0/1979	NA	R01_cb11509_c2/f1p0/1979	NA	R01_cb11509_c2/f1p0/1979
R01_cb8564_c25493/f1p2/2246	NA	R01_cb8564_c25493/f1p2/2246	NA	R01_cb8564_c25493/f1p2/2246
R01_cb1119_c13/f1p0/3370	NA	R01_cb1119_c13/f1p0/3370	NA	NA
R01_cb14067_c5/f1p0/1824	NA	R01_cb14067_c5/f1p0/1824	R01_cb14067_c5/f1p0/1824	R01_cb14067_c5/f1p0/1824
R01_cb16984_c0/f2p0/1439	NA	NA	NA	R01_cb16984_c0/f2p0/1439
R01_cb12402_c2/f1p0/642	NA	R01_cb12402_c2/f1p0/642	R01_cb12402_c2/f1p0/642	R01_cb12402_c2/f1p0/642
R01_cb17973_c22/f1p0/750	NA	R01_cb17973_c22/f1p0/750	R01_cb17973_c22/f1p0/750	R01_cb17973_c22/f1p0/750
R01_cb8564_c91417/f2p0/2105	NA	R01_cb8564_c91417/f2p0/2105	R01_cb8564_c91417/f2p0/2105	R01_cb8564_c91417/f2p0/2105
R01_cb13938_c7/f2p0/1237	NA	NA	NA	R01_cb13938_c7/f2p0/1237
R01_cb8564_c151509/f1p0/2801	NA	R01_cb8564_c151509/f1p0/2801	NA	NA
R01_cb17973_c34/f1p0/741	NA	R01_cb17973_c34/f1p0/741	R01_cb17973_c34/f1p0/741	NA
R01_cb1683_c30/f1p0/2666	NA	R01_cb1683_c30/f1p0/2666	NA	NA
R01_cb8564_c116077/f1p0/2618	NA	R01_cb8564_c116077/f1p0/2618	NA	NA
R01_cb5815_c3/f1p0/2886	NA	NA	NA	R01_cb5815_c3/f1p0/2886
R01_cb8564_c81339/f1p0/2660	NA	NA	NA	R01_cb8564_c81339/f1p0/2660
R01_cb16662_c6/f1p0/5962	NA	NA	R01_cb16662_c6/f1p0/5962	NA
R01_cb5755_c3/f1p0/2933	NA	R01_cb5755_c3/f1p0/2933	NA	NA

R01_cb17901_c1/f1p0/960	R01_cb17901_c1/f1p0/960	R01_cb17901_c1/f1p0/960	R01_cb17901_c1/f1p0/960	R01_cb17901_c1/f1p0/960
R01_cb15125_c1/f1p0/747	R01_cb15125_c1/f1p0/747	R01_cb15125_c1/f1p0/747	R01_cb15125_c1/f1p0/747	R01_cb15125_c1/f1p0/747
R01_cb8564_c115345/f1p0/2353	NA	R01_cb8564_c115345/f1p0/2353	NA	NA
R01_cb8564_c68606/f3p0/2311	NA	NA	R01_cb8564_c68606/f3p0/2311	R01_cb8564_c68606/f3p0/2311
R01_cb8564_c10172/f1p0/2353	NA	NA	NA	R01_cb8564_c10172/f1p0/2353
R01_cb8120_c3/f1p0/2751	NA	NA	NA	R01_cb8120_c3/f1p0/2751
R01_cb17139_c3/f1p1/1263	NA	NA	NA	R01_cb17139_c3/f1p1/1263
R01_cb4293_c25/f1p0/3171	NA	R01_cb4293_c25/f1p0/3171	NA	NA
R01_cb17086_c2/f1p0/1302	NA	R01_cb17086_c2/f1p0/1302	NA	R01_cb17086_c2/f1p0/1302
R01_cb18456_c7212/f1p0/509	R01_cb18456_c7212/f1p0/509	R01_cb18456_c7212/f1p0/509	R01_cb18456_c7212/f1p0/509	R01_cb18456_c7212/f1p0/509
R01_cb1376_c10/f1p0/2448	R01_cb1376_c10/f1p0/2448	R01_cb1376_c10/f1p0/2448	R01_cb1376_c10/f1p0/2448	R01_cb1376_c10/f1p0/2448
R01_cb8564_c128241/f1p0/2723	NA	R01_cb8564_c128241/f1p0/2723	NA	NA
R01_cb11287_c1/f1p0/3609	NA	NA	NA	R01_cb11287_c1/f1p0/3609
R01_cb7748_c2/f2p0/2406	NA	R01_cb7748_c2/f2p0/2406	NA	NA
R01_cb14633_c0/f3p1/917	NA	NA	NA	R01_cb14633_c0/f3p1/917
R01_cb4891_c1/f1p0/3228	NA	NA	NA	R01_cb4891_c1/f1p0/3228
R01_cb2701_c2/f1p0/3840	R01_cb2701_c2/f1p0/3840	R01_cb2701_c2/f1p0/3840	NA	R01_cb2701_c2/f1p0/3840
R01_cb8564_c77521/f1p0/2505	NA	R01_cb8564_c77521/f1p0/2505	NA	NA
R01_cb14351_c0/f13p0/739	NA	NA	NA	R01_cb14351_c0/f13p0/739
R01_cb18456_c2286/f1p0/708	NA	R01_cb18456_c2286/f1p0/708	R01_cb18456_c2286/f1p0/708	R01_cb18456_c2286/f1p0/708
R01_cb1190_c2/f1p0/2447	NA	R01_cb1190_c2/f1p0/2447	NA	NA
R01_cb18456_c342/f1p6/960	NA	R01_cb18456_c342/f1p6/960	NA	NA
R01_cb14352_c0/f2p0/976	NA	NA	R01_cb14352_c0/f2p0/976	R01_cb14352_c0/f2p0/976
R01_cb3363_c17/f1p0/1084	NA	NA	NA	R01_cb3363_c17/f1p0/1084

R01_cb5717_c6/f1p0/3822	NA	NA	NA	R01_cb5717_c6/f1p0/3822
R01_cb9325_c0/f1p0/2118	NA	R01_cb9325_c0/f1p0/2118	R01_cb9325_c0/f1p0/2118	R01_cb9325_c0/f1p0/2118
R01_cb18346_c0/f1p0/1620	NA	R01_cb18346_c0/f1p0/1620	R01_cb18346_c0/f1p0/1620	NA
R01_cb15116_c0/f14p0/995	NA	NA	NA	R01_cb15116_c0/f14p0/995
R01_cb8564_c75018/f1p0/2051	NA	R01_cb8564_c75018/f1p0/2051	NA	R01_cb8564_c75018/f1p0/2051
R01_cb8564_c117577/f1p2/2076	NA	NA	NA	R01_cb8564_c117577/f1p2/2076
R01_cb8564_c138590/f1p1/2653	NA	NA	NA	R01_cb8564_c138590/f1p1/2653
R01_cb14982_c3/f1p0/673	NA	R01_cb14982_c3/f1p0/673	R01_cb14982_c3/f1p0/673	R01_cb14982_c3/f1p0/673
R01_cb8564_c76250/f1p1/3808	NA	NA	NA	R01_cb8564_c76250/f1p1/3808
R01_cb454_c33/f1p0/2337	NA	NA	NA	R01_cb454_c33/f1p0/2337
R01_cb3793_c2/f1p0/3376	NA	NA	NA	R01_cb3793_c2/f1p0/3376
R01_cb10578_c1/f1p0/3966	NA	NA	NA	R01_cb10578_c1/f1p0/3966
R01_cb18762_c1/f1p0/4297	NA	R01_cb18762_c1/f1p0/4297	R01_cb18762_c1/f1p0/4297	R01_cb18762_c1/f1p0/4297
R01_cb12003_c142/f1p2/1168	NA	R01_cb12003_c142/f1p2/1168	NA	NA
R01_cb8564_c33844/f4p0/2678	NA	NA	NA	R01_cb8564_c33844/f4p0/2678
R01_cb9052_c0/f1p0/2202	NA	NA	NA	R01_cb9052_c0/f1p0/2202
R01_cb8564_c71557/f1p1/3111	NA	R01_cb8564_c71557/f1p1/3111	NA	NA
R01_cb16038_c2/f1p0/516	NA	R01_cb16038_c2/f1p0/516	R01_cb16038_c2/f1p0/516	R01_cb16038_c2/f1p0/516
R01_cb8564_c70590/f1p0/3216	NA	R01_cb8564_c70590/f1p0/3216	R01_cb8564_c70590/f1p0/3216	NA
R01_cb13478_c17/f1p0/507	NA	R01_cb13478_c17/f1p0/507	R01_cb13478_c17/f1p0/507	R01_cb13478_c17/f1p0/507
R01_cb8564_c69095/f2p1/4286	NA	R01_cb8564_c69095/f2p1/4286	R01_cb8564_c69095/f2p1/4286	R01_cb8564_c69095/f2p1/4286
R01_cb8564_c1635/f1p0/2390	R01_cb8564_c1635/f1p0/2390	R01_cb8564_c1635/f1p0/2390	R01_cb8564_c1635/f1p0/2390	NA
R01_cb11629_c1/f1p0/3194	NA	R01_cb11629_c1/f1p0/3194	NA	R01_cb11629_c1/f1p0/3194
R01_cb10148_c8/f1p0/1389	NA	NA	NA	R01_cb10148_c8/f1p0/1389

R01_cb10902_c1/f1p0/1552	NA	NA	NA	R01_cb10902_c1/f1p0/1552
R01_cb12383_c14/f1p0/927	R01_cb12383_c14/f1p0/927	NA	NA	NA
R01_cb18451_c3/f1p0/979	NA	NA	NA	R01_cb18451_c3/f1p0/979
R01_cb5756_c1/f1p0/3055	R01_cb5756_c1/f1p0/3055	R01_cb5756_c1/f1p0/3055	R01_cb5756_c1/f1p0/3055	R01_cb5756_c1/f1p0/3055
R01_cb5621_c50/f1p0/693	NA	R01_cb5621_c50/f1p0/693	R01_cb5621_c50/f1p0/693	R01_cb5621_c50/f1p0/693
R01_cb7334_c7/f1p0/2085	NA	NA	NA	R01_cb7334_c7/f1p0/2085
R01_cb8564_c71376/f1p0/3368	NA	NA	NA	R01_cb8564_c71376/f1p0/3368
R01_cb2154_c11/f1p0/2343	NA	R01_cb2154_c11/f1p0/2343	NA	NA
R01_cb5415_c6/f1p0/2542	NA	R01_cb5415_c6/f1p0/2542	NA	R01_cb5415_c6/f1p0/2542
R01_cb6170_c20/f1p0/2823	NA	NA	NA	R01_cb6170_c20/f1p0/2823
R01_cb8564_c51456/f1p0/2928	NA	R01_cb8564_c51456/f1p0/2928	NA	NA
R01_cb16294_c0/f4p0/1001	NA	NA	NA	R01_cb16294_c0/f4p0/1001
R01_cb17550_c1/f1p0/995	NA	R01_cb17550_c1/f1p0/995	NA	R01_cb17550_c1/f1p0/995
R01_cb124_c46/f1p0/3756	NA	NA	NA	R01_cb124_c46/f1p0/3756
R01_cb18132_c26/f1p0/1670	NA	R01_cb18132_c26/f1p0/1670	NA	NA
R01_cb8569_c15/f1p0/5086	NA	R01_cb8569_c15/f1p0/5086	NA	NA
R01_cb7645_c1/f1p0/2570	R01_cb7645_c1/f1p0/2570	R01_cb7645_c1/f1p0/2570	NA	NA
R01_cb13249_c1/f1p1/1112	NA	NA	NA	R01_cb13249_c1/f1p1/1112
R01_cb5790_c3/f1p0/4598	NA	R01_cb5790_c3/f1p0/4598	NA	R01_cb5790_c3/f1p0/4598
R01_cb8564_c90018/f1p0/2229	NA	NA	R01_cb8564_c90018/f1p0/2229	R01_cb8564_c90018/f1p0/2229
R01_cb4824_c7/f1p0/2973	NA	R01_cb4824_c7/f1p0/2973	R01_cb4824_c7/f1p0/2973	R01_cb4824_c7/f1p0/2973
R01_cb8564_c1831/f2p0/4066	NA	R01_cb8564_c1831/f2p0/4066	R01_cb8564_c1831/f2p0/4066	R01_cb8564_c1831/f2p0/4066
R01_cb8564_c107647/f5p1/2575	NA	NA	R01_cb8564_c107647/f5p1/2575	R01_cb8564_c107647/f5p1/2575
R01_cb5304_c23/f1p0/2591	NA	NA	NA	R01_cb5304_c23/f1p0/2591
R01_cb7810_c0/f2p0/2528	R01_cb7810_c0/f2p0/2528	R01_cb7810_c0/f2p0/2528	R01_cb7810_c0/f2p0/2528	NA

R01_cb18386_c7/f1p0/1299	NA	R01_cb18386_c7/f1p0/1299	NA	NA
R01_cb2387_c11/f1p0/3299	NA	R01_cb2387_c11/f1p0/3299	NA	NA
R01_cb2970_c1/f18p0/524	NA	NA	NA	R01_cb2970_c1/f18p0/524
R01_cb13144_c4/f1p0/544	R01_cb13144_c4/f1p0/544	R01_cb13144_c4/f1p0/544	NA	R01_cb13144_c4/f1p0/544
R01_cb11119_c1/f1p0/2722	NA	NA	NA	R01_cb11119_c1/f1p0/2722
R01_cb12623_c3/f1p0/1490	NA	R01_cb12623_c3/f1p0/1490	NA	R01_cb12623_c3/f1p0/1490
R01_cb3301_c8/f1p0/2455	NA	R01_cb3301_c8/f1p0/2455	NA	R01_cb3301_c8/f1p0/2455
R01_cb15214_c1/f1p0/1712	NA	NA	NA	R01_cb15214_c1/f1p0/1712
R01_cb6631_c21/f1p0/2503	NA	NA	NA	R01_cb6631_c21/f1p0/2503
R01_cb18409_c177/f1p0/369	R01_cb18409_c177/f1p0/369	R01_cb18409_c177/f1p0/369	R01_cb18409_c177/f1p0/369	R01_cb18409_c177/f1p0/369
R01_cb16541_c0/f1p0/1140	NA	NA	NA	R01_cb16541_c0/f1p0/1140
R01_cb18301_c51/f1p0/1190	NA	R01_cb18301_c51/f1p0/1190	NA	R01_cb18301_c51/f1p0/1190
R01_cb4676_c2/f1p0/2211	NA	NA	NA	R01_cb4676_c2/f1p0/2211
R01_cb12992_c9/f1p0/977	NA	R01_cb12992_c9/f1p0/977	NA	NA
R01_cb15967_c1/f1p0/557	R01_cb15967_c1/f1p0/557	R01_cb15967_c1/f1p0/557	NA	NA
R01_cb8564_c88698/f1p0/3459	NA	NA	NA	R01_cb8564_c88698/f1p0/3459
R01_cb8564_c21454/f1p0/2405	NA	R01_cb8564_c21454/f1p0/2405	NA	NA
R01_cb8596_c3/f1p0/1801	NA	NA	NA	R01_cb8596_c3/f1p0/1801
R01_cb10014_c15/f1p0/1536	R01_cb10014_c15/f1p0/1536	R01_cb10014_c15/f1p0/1536	NA	NA
R01_cb10556_c2/f1p0/2132	NA	R01_cb10556_c2/f1p0/2132	NA	NA
R01_cb1609_c3/f1p0/2358	NA	R01_cb1609_c3/f1p0/2358	NA	NA
R01_cb8564_c126647/f2p1/2497	NA	R01_cb8564_c126647/f2p1/2497	NA	NA
R01_cb8564_c112825/f1p0/2597	NA	NA	NA	R01_cb8564_c112825/f1p0/2597
R01_cb7946_c5/f1p0/903	R01_cb7946_c5/f1p0/903	R01_cb7946_c5/f1p0/903	R01_cb7946_c5/f1p0/903	R01_cb7946_c5/f1p0/903

R01_cb6281_c1/f1p0/2403	NA	NA	NA	R01_cb6281_c1/f1p0/2403
R01_cb8564_c79804/f1p0/1916	NA	R01_cb8564_c79804/f1p0/1916	NA	NA
R01_cb14125_c92/f1p0/376	R01_cb14125_c92/f1p0/376	R01_cb14125_c92/f1p0/376	R01_cb14125_c92/f1p0/376	NA
R01_cb5900_c41/f1p0/2729	NA	R01_cb5900_c41/f1p0/2729	NA	R01_cb5900_c41/f1p0/2729
R01_cb8564_c77120/f1p0/2159	R01_cb8564_c77120/f1p0/2159	R01_cb8564_c77120/f1p0/2159	NA	R01_cb8564_c77120/f1p0/2159
R01_cb7899_c6/f1p0/1274	NA	NA	NA	R01_cb7899_c6/f1p0/1274
R01_cb8564_c78626/f1p0/2262	NA	R01_cb8564_c78626/f1p0/2262	NA	NA
R01_cb8564_c91190/f1p0/4963	NA	NA	NA	R01_cb8564_c91190/f1p0/4963
R01_cb14316_c3/f1p0/1725	R01_cb14316_c3/f1p0/1725	R01_cb14316_c3/f1p0/1725	R01_cb14316_c3/f1p0/1725	R01_cb14316_c3/f1p0/1725
R01_cb18649_c7/f1p0/5612	NA	NA	NA	R01_cb18649_c7/f1p0/5612
R01_cb8564_c36134/f1p0/3837	NA	R01_cb8564_c36134/f1p0/3837	NA	NA
R01_cb16677_c2/f1p0/1783	NA	R01_cb16677_c2/f1p0/1783	R01_cb16677_c2/f1p0/1783	NA
R01_cb7927_c2/f1p0/2011	NA	NA	R01_cb7927_c2/f1p0/2011	R01_cb7927_c2/f1p0/2011
R01_cb8564_c129049/f1p0/2875	NA	R01_cb8564_c129049/f1p0/2875	NA	NA
R01_cb3774_c78/f1p0/1141	NA	NA	NA	R01_cb3774_c78/f1p0/1141
R01_cb16979_c6/f1p0/1287	R01_cb16979_c6/f1p0/1287	R01_cb16979_c6/f1p0/1287	R01_cb16979_c6/f1p0/1287	NA
R01_cb8564_c42719/f1p0/2476	R01_cb8564_c42719/f1p0/2476	R01_cb8564_c42719/f1p0/2476	R01_cb8564_c42719/f1p0/2476	R01_cb8564_c42719/f1p0/2476
R01_cb13304_c0/f1p0/941	NA	R01_cb13304_c0/f1p0/941	NA	R01_cb13304_c0/f1p0/941
R01_cb18456_c7558/f1p0/1732	NA	R01_cb18456_c7558/f1p0/1732	NA	NA
R01_cb6785_c16/f1p0/2272	NA	R01_cb6785_c16/f1p0/2272	NA	NA
R01_cb8564_c129909/f1p1/2314	NA	R01_cb8564_c129909/f1p1/2314	R01_cb8564_c129909/f1p1/2314	NA
R01_cb3374_c1/f1p0/4903	NA	R01_cb3374_c1/f1p0/4903	R01_cb3374_c1/f1p0/4903	NA

R01_cb8827_c5/f1p0/2191	NA	R01_cb8827_c5/f1p0/2191	NA	NA
R01_cb11271_c2/f1p0/2520	NA	R01_cb11271_c2/f1p0/2520	NA	NA
R01_cb6802_c63/f1p0/2997	R01_cb6802_c63/f1p0/2997	R01_cb6802_c63/f1p0/2997	R01_cb6802_c63/f1p0/2997	R01_cb6802_c63/f1p0/2997
R01_cb13025_c1/f3p0/1232	NA	NA	NA	R01_cb13025_c1/f3p0/1232
R01_cb16132_c35/f1p0/630	R01_cb16132_c35/f1p0/630	R01_cb16132_c35/f1p0/630	R01_cb16132_c35/f1p0/630	R01_cb16132_c35/f1p0/630
R01_cb10993_c1/f1p0/2295	NA	NA	NA	R01_cb10993_c1/f1p0/2295
R01_cb6794_c35/f1p0/2673	NA	R01_cb6794_c35/f1p0/2673	NA	NA
R01_cb2588_c3/f1p0/618	NA	R01_cb2588_c3/f1p0/618	R01_cb2588_c3/f1p0/618	R01_cb2588_c3/f1p0/618
R01_cb454_c34/f1p0/2241	NA	R01_cb454_c34/f1p0/2241	NA	R01_cb454_c34/f1p0/2241
R01_cb16557_c0/f2p0/800	NA	R01_cb16557_c0/f2p0/800	NA	R01_cb16557_c0/f2p0/800
R01_cb10363_c1/f1p0/3898	NA	R01_cb10363_c1/f1p0/3898	R01_cb10363_c1/f1p0/3898	NA
R01_cb4383_c2/f1p0/2404	NA	NA	R01_cb4383_c2/f1p0/2404	NA
R01_cb2177_c76/f1p0/4006	NA	NA	NA	R01_cb2177_c76/f1p0/4006
R01_cb13938_c5/f4p0/1530	NA	NA	NA	R01_cb13938_c5/f4p0/1530
R01_cb3968_c13/f1p0/3540	NA	R01_cb3968_c13/f1p0/3540	NA	NA
R01_cb8564_c85200/f1p0/2237	NA	R01_cb8564_c85200/f1p0/2237	R01_cb8564_c85200/f1p0/2237	R01_cb8564_c85200/f1p0/2237
R01_cb8564_c4867/f1p0/3090	NA	R01_cb8564_c4867/f1p0/3090	NA	NA
R01_cb8564_c11630/f1p0/3365	NA	R01_cb8564_c11630/f1p0/3365	R01_cb8564_c11630/f1p0/3365	R01_cb8564_c11630/f1p0/3365
R01_cb8564_c24669/f1p0/3626	NA	R01_cb8564_c24669/f1p0/3626	R01_cb8564_c24669/f1p0/3626	R01_cb8564_c24669/f1p0/3626
R01_cb7327_c4/f1p0/1677	NA	R01_cb7327_c4/f1p0/1677	NA	NA
R01_cb2378_c26/f1p0/3396	NA	NA	NA	R01_cb2378_c26/f1p0/3396
R01_cb261_c13/f1p0/3047	NA	NA	NA	R01_cb261_c13/f1p0/3047
R01_cb15867_c9/f1p0/1296	NA	R01_cb15867_c9/f1p0/1296	NA	NA
R01_cb1380_c32/f1p0/4415	NA	R01_cb1380_c32/f1p0/4415	NA	NA
R01_cb5900_c52/f1p0/3276	NA	R01_cb5900_c52/f1p0/3276	R01_cb5900_c52/f1p0/3276	R01_cb5900_c52/f1p0/3276
R01_cb8564_c1332/f1p0/2677	NA	NA	NA	R01_cb8564_c1332/f1p0/2677

R01_cb18456_c1987/f1p0/1000	NA	R01_cb18456_c1987/f1p0/1000	NA	NA
R01_cb15396_c1/f1p0/949	NA	NA	NA	R01_cb15396_c1/f1p0/949
R01_cb3854_c2/f1p0/2571	NA	NA	R01_cb3854_c2/f1p0/2571	NA
R01_cb18016_c1/f1p0/416	R01_cb18016_c1/f1p0/416	R01_cb18016_c1/f1p0/416	R01_cb18016_c1/f1p0/416	R01_cb18016_c1/f1p0/416
R01_cb18287_c6/f1p0/783	R01_cb18287_c6/f1p0/783	R01_cb18287_c6/f1p0/783	R01_cb18287_c6/f1p0/783	R01_cb18287_c6/f1p0/783
R01_cb6948_c11/f1p0/2702	NA	NA	NA	R01_cb6948_c11/f1p0/2702
R01_cb4768_c20/f1p0/2201	NA	R01_cb4768_c20/f1p0/2201	NA	NA
R01_cb9352_c9/f1p0/1685	NA	NA	NA	R01_cb9352_c9/f1p0/1685
R01_cb16311_c0/f6p0/1230	NA	NA	NA	R01_cb16311_c0/f6p0/1230
R01_cb8279_c6/f1p1/2612	NA	NA	NA	R01_cb8279_c6/f1p1/2612
R01_cb1021_c6/f1p0/1849	NA	R01_cb1021_c6/f1p0/1849	NA	NA
R01_cb2540_c2/f1p0/3407	NA	R01_cb2540_c2/f1p0/3407	NA	NA
R01_cb8947_c0/f1p0/2228	NA	NA	NA	R01_cb8947_c0/f1p0/2228
R01_cb5190_c2/f3p1/2405	NA	NA	NA	R01_cb5190_c2/f3p1/2405
R01_cb6206_c10/f1p0/609	NA	R01_cb6206_c10/f1p0/609	NA	R01_cb6206_c10/f1p0/609
R01_cb8564_c129502/f1p0/2808	NA	R01_cb8564_c129502/f1p0/2808	NA	NA
R01_cb3149_c67/f1p0/2831	NA	R01_cb3149_c67/f1p0/2831	NA	NA
R01_cb1567_c2/f1p0/4024	NA	NA	NA	R01_cb1567_c2/f1p0/4024
R01_cb1758_c3/f1p0/3123	NA	NA	NA	R01_cb1758_c3/f1p0/3123
R01_cb1666_c9/f1p0/2297	NA	NA	NA	R01_cb1666_c9/f1p0/2297
R01_cb15102_c0/f3p0/776	NA	NA	NA	R01_cb15102_c0/f3p0/776
R01_cb506_c14/f1p0/4569	NA	NA	NA	R01_cb506_c14/f1p0/4569
R01_cb15326_c2/f1p0/1436	NA	R01_cb15326_c2/f1p0/1436	R01_cb15326_c2/f1p0/1436	R01_cb15326_c2/f1p0/1436
R01_cb11278_c1/f3p2/1790	NA	NA	NA	R01_cb11278_c1/f3p2/1790
R01_cb9587_c0/f1p0/2041	R01_cb9587_c0/f1p0/2041	R01_cb9587_c0/f1p0/2041	R01_cb9587_c0/f1p0/2041	R01_cb9587_c0/f1p0/2041

R01_cb9079_c12/f1p0/823	NA	NA	NA	R01_cb9079_c12/f1p0/823
R01_cb2788_c18/f1p0/1890	NA	NA	R01_cb2788_c18/f1p0/1890	R01_cb2788_c18/f1p0/1890
R01_cb1554_c8/f1p0/5253	NA	R01_cb1554_c8/f1p0/5253	R01_cb1554_c8/f1p0/5253	R01_cb1554_c8/f1p0/5253
R01_cb7151_c1/f1p0/2697	R01_cb7151_c1/f1p0/2697	R01_cb7151_c1/f1p0/2697	NA	NA
R01_cb13654_c1/f1p0/1030	NA	NA	NA	R01_cb13654_c1/f1p0/1030
R01_cb8564_c119063/f1p0/2299	NA	R01_cb8564_c119063/f1p0/2299	R01_cb8564_c119063/f1p0/2299	R01_cb8564_c119063/f1p0/2299
R01_cb10024_c516/f1p1/746	NA	NA	R01_cb10024_c516/f1p1/746	NA
R01_cb13667_c0/f6p0/847	R01_cb13667_c0/f6p0/847	R01_cb13667_c0/f6p0/847	R01_cb13667_c0/f6p0/847	R01_cb13667_c0/f6p0/847
R01_cb8564_c69825/f1p0/2606	NA	R01_cb8564_c69825/f1p0/2606	NA	R01_cb8564_c69825/f1p0/2606
R01_cb15682_c2/f1p0/813	NA	NA	R01_cb15682_c2/f1p0/813	R01_cb15682_c2/f1p0/813
R01_cb6365_c14/f1p0/2471	NA	R01_cb6365_c14/f1p0/2471	NA	NA
R01_cb11682_c2/f1p0/1288	NA	NA	NA	R01_cb11682_c2/f1p0/1288
R01_cb15760_c3/f1p0/611	NA	R01_cb15760_c3/f1p0/611	R01_cb15760_c3/f1p0/611	R01_cb15760_c3/f1p0/611
R01_cb16188_c14/f1p0/348	R01_cb16188_c14/f1p0/348	R01_cb16188_c14/f1p0/348	R01_cb16188_c14/f1p0/348	R01_cb16188_c14/f1p0/348
R01_cb16383_c8/f1p0/1866	NA	NA	NA	R01_cb16383_c8/f1p0/1866
R01_cb2073_c0/f1p0/3846	NA	R01_cb2073_c0/f1p0/3846	R01_cb2073_c0/f1p0/3846	R01_cb2073_c0/f1p0/3846
R01_cb8564_c36233/f1p0/3072	NA	NA	R01_cb8564_c36233/f1p0/3072	NA
R01_cb14197_c3/f1p0/1737	NA	NA	NA	R01_cb14197_c3/f1p0/1737
R01_cb4345_c6/f1p1/3369	NA	NA	NA	R01_cb4345_c6/f1p1/3369
R01_cb7214_c8/f1p0/2224	NA	NA	NA	R01_cb7214_c8/f1p0/2224
R01_cb5567_c0/f1p0/3070	NA	NA	R01_cb5567_c0/f1p0/3070	R01_cb5567_c0/f1p0/3070
R01_cb15096_c2/f1p0/662	R01_cb15096_c2/f1p0/662	R01_cb15096_c2/f1p0/662	R01_cb15096_c2/f1p0/662	R01_cb15096_c2/f1p0/662
R01_cb5122_c18/f1p0/2982	NA	NA	NA	R01_cb5122_c18/f1p0/2982
R01_cb14545_c3/f1p1/1803	NA	NA	NA	R01_cb14545_c3/f1p1/1803
R01_cb9587_c1/f1p0/2265	R01_cb9587_c1/f1p0/2265	R01_cb9587_c1/f1p0/2265	R01_cb9587_c1/f1p0/2265	R01_cb9587_c1/f1p0/2265

R01_cb18456_c5435/f1p2/623	R01_cb18456_c5435/f1p2/623	R01_cb18456_c5435/f1p2/623	R01_cb18456_c5435/f1p2/623	R01_cb18456_c5435/f1p2/623
R01_cb8564_c81388/f1p0/3266	R01_cb8564_c81388/f1p0/3266	R01_cb8564_c81388/f1p0/3266	NA	NA
R01_cb18409_c183/f1p0/318	R01_cb18409_c183/f1p0/318	R01_cb18409_c183/f1p0/318	R01_cb18409_c183/f1p0/318	R01_cb18409_c183/f1p0/318
R01_cb14675_c0/f25p2/843	R01_cb14675_c0/f25p2/843	R01_cb14675_c0/f25p2/843	R01_cb14675_c0/f25p2/843	R01_cb14675_c0/f25p2/843
R01_cb3025_c1/f1p0/3471	NA	NA	NA	R01_cb3025_c1/f1p0/3471
R01_cb8564_c129450/f1p0/3060	NA	R01_cb8564_c129450/f1p0/3060	R01_cb8564_c129450/f1p0/3060	R01_cb8564_c129450/f1p0/3060
R01_cb16746_c2/f1p0/1369	NA	NA	NA	R01_cb16746_c2/f1p0/1369
R01_cb8564_c125660/f1p1/2006	NA	NA	NA	R01_cb8564_c125660/f1p1/2006
R01_cb16422_c4/f1p0/1648	NA	R01_cb16422_c4/f1p0/1648	R01_cb16422_c4/f1p0/1648	R01_cb16422_c4/f1p0/1648
R01_cb8564_c110953/f1p0/2771	NA	R01_cb8564_c110953/f1p0/2771	NA	NA
R01_cb4180_c9/f1p0/6243	NA	NA	NA	R01_cb4180_c9/f1p0/6243
R01_cb12397_c2/f1p0/970	R01_cb12397_c2/f1p0/970	R01_cb12397_c2/f1p0/970	R01_cb12397_c2/f1p0/970	R01_cb12397_c2/f1p0/970
R01_cb10889_c0/f1p0/1533	NA	R01_cb10889_c0/f1p0/1533	NA	NA
R01_cb5415_c4/f1p0/3103	NA	NA	NA	R01_cb5415_c4/f1p0/3103
R01_cb606_c27/f1p3/4332	NA	NA	NA	R01_cb606_c27/f1p3/4332
R01_cb15883_c2/f1p0/672	NA	R01_cb15883_c2/f1p0/672	NA	NA
R01_cb7221_c6/f1p0/2447	NA	NA	NA	R01_cb7221_c6/f1p0/2447
R01_cb9763_c9/f1p0/1987	NA	R01_cb9763_c9/f1p0/1987	NA	NA
R01_cb11401_c2/f1p0/2430	NA	R01_cb11401_c2/f1p0/2430	R01_cb11401_c2/f1p0/2430	R01_cb11401_c2/f1p0/2430
R01_cb10398_c4/f1p0/654	R01_cb10398_c4/f1p0/654	R01_cb10398_c4/f1p0/654	NA	R01_cb10398_c4/f1p0/654
R01_cb2571_c7/f3p1/3711	NA	NA	NA	R01_cb2571_c7/f3p1/3711
R01_cb10129_c5/f1p0/550	NA	R01_cb10129_c5/f1p0/550	NA	NA

R01_cb17363_c9/f1p0/400	R01_cb17363_c9/f1p0/400	R01_cb17363_c9/f1p0/400	R01_cb17363_c9/f1p0/400	R01_cb17363_c9/f1p0/400
R01_cb4777_c3/f1p0/717	NA	R01_cb4777_c3/f1p0/717	NA	R01_cb4777_c3/f1p0/717
R01_cb5120_c0/f1p0/3176	NA	NA	NA	R01_cb5120_c0/f1p0/3176
R01_cb18456_c2298/f1p0/329	R01_cb18456_c2298/f1p0/329	R01_cb18456_c2298/f1p0/329	NA	NA
R01_cb8564_c69287/f2p0/1920	NA	R01_cb8564_c69287/f2p0/1920	R01_cb8564_c69287/f2p0/1920	R01_cb8564_c69287/f2p0/1920
R01_cb8564_c1884/f1p0/1965	NA	NA	NA	R01_cb8564_c1884/f1p0/1965
R01_cb5378_c5/f1p0/6519	NA	R01_cb5378_c5/f1p0/6519	R01_cb5378_c5/f1p0/6519	NA
R01_cb16425_c1/f1p0/708	NA	R01_cb16425_c1/f1p0/708	R01_cb16425_c1/f1p0/708	R01_cb16425_c1/f1p0/708
R01_cb18456_c1717/f2p0/1339	NA	R01_cb18456_c1717/f2p0/1339	R01_cb18456_c1717/f2p0/1339	R01_cb18456_c1717/f2p0/1339
R01_cb8564_c2857/f1p0/2224	R01_cb8564_c2857/f1p0/2224	R01_cb8564_c2857/f1p0/2224	R01_cb8564_c2857/f1p0/2224	R01_cb8564_c2857/f1p0/2224
R01_cb8564_c41520/f1p0/2300	R01_cb8564_c41520/f1p0/2300	R01_cb8564_c41520/f1p0/2300	R01_cb8564_c41520/f1p0/2300	R01_cb8564_c41520/f1p0/2300
R01_cb17036_c1/f1p0/936	NA	R01_cb17036_c1/f1p0/936	NA	NA
R01_cb18016_c0/f1p0/445	R01_cb18016_c0/f1p0/445	R01_cb18016_c0/f1p0/445	R01_cb18016_c0/f1p0/445	R01_cb18016_c0/f1p0/445
R01_cb8084_c5/f1p0/1379	NA	NA	NA	R01_cb8084_c5/f1p0/1379
R01_cb8564_c1920/f1p0/2430	NA	R01_cb8564_c1920/f1p0/2430	NA	R01_cb8564_c1920/f1p0/2430
R01_cb12273_c11/f2p0/1517	NA	NA	NA	R01_cb12273_c11/f2p0/1517
R01_cb2701_c4/f1p0/3079	NA	R01_cb2701_c4/f1p0/3079	NA	R01_cb2701_c4/f1p0/3079
R01_cb17809_c0/f3p0/1677	NA	NA	NA	R01_cb17809_c0/f3p0/1677
R01_cb3832_c3/f1p0/3385	NA	R01_cb3832_c3/f1p0/3385	NA	NA
R01_cb8564_c51515/f1p1/2999	NA	R01_cb8564_c51515/f1p1/2999	NA	NA
R01_cb7027_c5/f1p0/1053	R01_cb7027_c5/f1p0/1053	R01_cb7027_c5/f1p0/1053	R01_cb7027_c5/f1p0/1053	R01_cb7027_c5/f1p0/1053
R01_cb5896_c161/f1p0/3233	NA	NA	R01_cb5896_c161/f1p0/3233	R01_cb5896_c161/f1p0/3233
R01_cb647_c7/f1p0/2791	NA	R01_cb647_c7/f1p0/2791	NA	R01_cb647_c7/f1p0/2791
R01_cb11895_c0/f3p0/1393	NA	NA	NA	R01_cb11895_c0/f3p0/1393
R01_cb8609_c13/f1p0/3821	NA	R01_cb8609_c13/f1p0/3821	NA	NA

R01_cb1905_c19/f1p0/2075	NA	NA	R01_cb1905_c19/f1p0/2075	NA
R01_cb8564_c24832/f1p0/4329	NA	NA	NA	R01_cb8564_c24832/f1p0/4329
R01_cb16125_c15/f1p0/1737	NA	R01_cb16125_c15/f1p0/1737	NA	NA
R01_cb14429_c2/f1p0/617	R01_cb14429_c2/f1p0/617	R01_cb14429_c2/f1p0/617	R01_cb14429_c2/f1p0/617	R01_cb14429_c2/f1p0/617
R01_cb8564_c75455/f1p0/3605	NA	R01_cb8564_c75455/f1p0/3605	NA	R01_cb8564_c75455/f1p0/3605
R01_cb7533_c13/f1p0/2022	NA	NA	NA	R01_cb7533_c13/f1p0/2022
R01_cb15682_c0/f5p0/917	NA	NA	NA	R01_cb15682_c0/f5p0/917
R01_cb1813_c39/f1p0/5087	NA	NA	NA	R01_cb1813_c39/f1p0/5087
R01_cb48_c3/f1p2/4932	NA	NA	NA	R01_cb48_c3/f1p2/4932
R01_cb7113_c0/f1p0/2694	NA	NA	NA	R01_cb7113_c0/f1p0/2694
R01_cb14805_c2/f3p0/575	NA	R01_cb14805_c2/f3p0/575	NA	NA
R01_cb8564_c127092/f1p0/2576	NA	R01_cb8564_c127092/f1p0/2576	R01_cb8564_c127092/f1p0/2576	R01_cb8564_c127092/f1p0/2576
R01_cb8564_c124009/f1p0/3402	NA	R01_cb8564_c124009/f1p0/3402	NA	R01_cb8564_c124009/f1p0/3402
R01_cb13127_c4/f1p0/769	NA	R01_cb13127_c4/f1p0/769	NA	NA
R01_cb15255_c0/f3p0/724	NA	NA	NA	R01_cb15255_c0/f3p0/724
R01_cb8379_c15/f1p0/1325	NA	R01_cb8379_c15/f1p0/1325	NA	NA
R01_cb8954_c13/f1p0/2289	NA	R01_cb8954_c13/f1p0/2289	NA	R01_cb8954_c13/f1p0/2289
R01_cb8564_c33591/f5p1/2462	R01_cb8564_c33591/f5p1/2462	R01_cb8564_c33591/f5p1/2462	R01_cb8564_c33591/f5p1/2462	R01_cb8564_c33591/f5p1/2462
R01_cb2845_c10/f1p1/3408	NA	NA	NA	R01_cb2845_c10/f1p1/3408
R01_cb10217_c1/f1p0/1958	NA	NA	NA	R01_cb10217_c1/f1p0/1958
R01_cb119_c14/f1p2/4826	NA	NA	NA	R01_cb119_c14/f1p2/4826
R01_cb9592_c6/f1p1/1652	NA	NA	NA	R01_cb9592_c6/f1p1/1652
R01_cb18409_c127/f1p0/781	R01_cb18409_c127/f1p0/781	R01_cb18409_c127/f1p0/781	R01_cb18409_c127/f1p0/781	R01_cb18409_c127/f1p0/781

R01_cb17562_c2/f5p0/526	NA	R01_cb17562_c2/f5p0/526	NA	NA
R01_cb8564_c70445/f1p0/3004	R01_cb8564_c70445/f1p0/3004	R01_cb8564_c70445/f1p0/3004	NA	NA
R01_cb1346_c9/f1p0/1789	NA	R01_cb1346_c9/f1p0/1789	NA	NA
R01_cb8564_c76406/f1p1/3251	NA	R01_cb8564_c76406/f1p1/3251	NA	NA
R01_cb10539_c0/f13p0/1103	NA	NA	NA	R01_cb10539_c0/f13p0/1103
R01_cb17374_c2/f1p1/790	NA	NA	NA	R01_cb17374_c2/f1p1/790
R01_cb1077_c11/f1p0/2476	NA	R01_cb1077_c11/f1p0/2476	NA	R01_cb1077_c11/f1p0/2476
R01_cb3088_c3/f1p0/3699	NA	R01_cb3088_c3/f1p0/3699	R01_cb3088_c3/f1p0/3699	R01_cb3088_c3/f1p0/3699
R01_cb10787_c1/f1p0/1515	NA	NA	NA	R01_cb10787_c1/f1p0/1515
R01_cb737_c31/f1p0/1563	NA	NA	NA	R01_cb737_c31/f1p0/1563
R01_cb9906_c0/f1p0/1921	NA	R01_cb9906_c0/f1p0/1921	R01_cb9906_c0/f1p0/1921	R01_cb9906_c0/f1p0/1921
R01_cb8564_c85255/f1p1/2783	NA	NA	NA	R01_cb8564_c85255/f1p1/2783
R01_cb7492_c6/f1p1/2833	NA	NA	NA	R01_cb7492_c6/f1p1/2833
R01_cb10948_c6/f1p0/1409	NA	NA	NA	R01_cb10948_c6/f1p0/1409
R01_cb15034_c4/f1p0/1585	NA	NA	NA	R01_cb15034_c4/f1p0/1585
R01_cb8564_c1992/f3p0/2129	R01_cb8564_c1992/f3p0/2129	R01_cb8564_c1992/f3p0/2129	R01_cb8564_c1992/f3p0/2129	R01_cb8564_c1992/f3p0/2129
R01_cb8564_c74830/f1p0/2373	NA	NA	R01_cb8564_c74830/f1p0/2373	NA
R01_cb124_c6/f3p0/2872	NA	NA	NA	R01_cb124_c6/f3p0/2872
R01_cb13033_c2/f2p0/1032	NA	R01_cb13033_c2/f2p0/1032	NA	R01_cb13033_c2/f2p0/1032
R01_cb8564_c23888/f1p0/2702	R01_cb8564_c23888/f1p0/2702	R01_cb8564_c23888/f1p0/2702	R01_cb8564_c23888/f1p0/2702	R01_cb8564_c23888/f1p0/2702
R01_cb11488_c0/f1p0/726	NA	R01_cb11488_c0/f1p0/726	R01_cb11488_c0/f1p0/726	R01_cb11488_c0/f1p0/726
R01_cb18456_c7179/f1p0/640	NA	R01_cb18456_c7179/f1p0/640	R01_cb18456_c7179/f1p0/640	R01_cb18456_c7179/f1p0/640
R01_cb7048_c1/f1p0/2706	NA	R01_cb7048_c1/f1p0/2706	NA	NA
R01_cb16451_c1/f2p0/1240	NA	NA	NA	R01_cb16451_c1/f2p0/1240

R01_cb18456_c5360/f1p4/791	NA	R01_cb18456_c5360/f1p4/791	NA	NA
R01_cb11081_c5/f1p0/1144	NA	R01_cb11081_c5/f1p0/1144	R01_cb11081_c5/f1p0/1144	NA
R01_cb12421_c33/f1p0/658	NA	R01_cb12421_c33/f1p0/658	R01_cb12421_c33/f1p0/658	R01_cb12421_c33/f1p0/658
R01_cb12525_c0/f1p0/1227	NA	R01_cb12525_c0/f1p0/1227	NA	NA
R01_cb17756_c30/f1p0/384	R01_cb17756_c30/f1p0/384	R01_cb17756_c30/f1p0/384	R01_cb17756_c30/f1p0/384	R01_cb17756_c30/f1p0/384
R01_cb8564_c20158/f1p0/3961	NA	NA	NA	R01_cb8564_c20158/f1p0/3961
R01_cb1215_c8/f1p0/3148	NA	R01_cb1215_c8/f1p0/3148	NA	R01_cb1215_c8/f1p0/3148
R01_cb8335_c10/f1p0/2301	NA	NA	NA	R01_cb8335_c10/f1p0/2301
R01_cb14843_c1/f2p0/790	NA	NA	NA	R01_cb14843_c1/f2p0/790
R01_cb8564_c90145/f1p0/2345	NA	R01_cb8564_c90145/f1p0/2345	NA	NA
R01_cb1508_c1/f3p3/1904	NA	NA	NA	R01_cb1508_c1/f3p3/1904
R01_cb3941_c29/f1p0/3410	NA	NA	NA	R01_cb3941_c29/f1p0/3410
R01_cb8564_c85433/f1p0/3121	NA	NA	R01_cb8564_c85433/f1p0/3121	NA
R01_cb4793_c7/f1p1/3859	NA	NA	NA	R01_cb4793_c7/f1p1/3859
R01_cb8564_c24476/f1p0/2808	R01_cb8564_c24476/f1p0/2808	R01_cb8564_c24476/f1p0/2808	R01_cb8564_c24476/f1p0/2808	R01_cb8564_c24476/f1p0/2808
R01_cb1567_c4/f1p0/1922	NA	NA	NA	R01_cb1567_c4/f1p0/1922
R01_cb9354_c3/f1p0/1866	NA	NA	NA	R01_cb9354_c3/f1p0/1866
R01_cb2879_c1/f1p0/3059	NA	R01_cb2879_c1/f1p0/3059	NA	NA
R01_cb13284_c5/f1p0/1429	NA	NA	NA	R01_cb13284_c5/f1p0/1429
R01_cb17448_c5/f1p0/1415	NA	R01_cb17448_c5/f1p0/1415	NA	NA
R01_cb8900_c4/f1p0/2171	NA	NA	NA	R01_cb8900_c4/f1p0/2171
R01_cb13856_c0/f1p0/617	NA	NA	R01_cb13856_c0/f1p0/617	NA
R01_cb12881_c0/f1p0/950	NA	R01_cb12881_c0/f1p0/950	NA	NA
R01_cb16341_c3/f1p0/528	R01_cb16341_c3/f1p0/528	R01_cb16341_c3/f1p0/528	R01_cb16341_c3/f1p0/528	R01_cb16341_c3/f1p0/528
R01_cb16966_c1/f1p0/434	R01_cb16966_c1/f1p0/434	R01_cb16966_c1/f1p0/434	NA	NA

R01_cb7814_c11/f1p0/2626	NA	NA	NA	R01_cb7814_c11/f1p0/2626
R01_cb14030_c4/f4p0/705	NA	R01_cb14030_c4/f4p0/705	NA	NA
R01_cb17_c44/f1p0/1099	NA	R01_cb17_c44/f1p0/1099	NA	NA
R01_cb3445_c1/f2p1/2852	NA	NA	NA	R01_cb3445_c1/f2p1/2852
R01_cb17035_c2/f1p0/403	R01_cb17035_c2/f1p0/403	R01_cb17035_c2/f1p0/403	R01_cb17035_c2/f1p0/403	R01_cb17035_c2/f1p0/403
R01_cb18225_c0/f1p0/389	R01_cb18225_c0/f1p0/389	R01_cb18225_c0/f1p0/389	R01_cb18225_c0/f1p0/389	R01_cb18225_c0/f1p0/389
R01_cb12882_c1/f1p1/1245	NA	NA	NA	R01_cb12882_c1/f1p1/1245
R01_cb10355_c2/f1p0/3063	NA	R01_cb10355_c2/f1p0/3063	NA	NA
R01_cb2875_c20/f1p0/3213	NA	NA	NA	R01_cb2875_c20/f1p0/3213
R01_cb17604_c10/f1p0/520	NA	NA	R01_cb17604_c10/f1p0/520	R01_cb17604_c10/f1p0/520
R01_cb8564_c75293/f1p0/4683	NA	R01_cb8564_c75293/f1p0/4683	NA	NA
R01_cb18456_c7992/f1p0/589	NA	NA	R01_cb18456_c7992/f1p0/589	R01_cb18456_c7992/f1p0/589
R01_cb8564_c3802/f1p0/2813	NA	R01_cb8564_c3802/f1p0/2813	NA	R01_cb8564_c3802/f1p0/2813
R01_cb14067_c3/f1p0/1762	NA	R01_cb14067_c3/f1p0/1762	R01_cb14067_c3/f1p0/1762	R01_cb14067_c3/f1p0/1762
R01_cb13071_c1/f1p0/802	R01_cb13071_c1/f1p0/802	R01_cb13071_c1/f1p0/802	R01_cb13071_c1/f1p0/802	NA
R01_cb8564_c69207/f3p0/2096	NA	R01_cb8564_c69207/f3p0/2096	R01_cb8564_c69207/f3p0/2096	R01_cb8564_c69207/f3p0/2096
R01_cb8564_c10890/f1p0/2612	NA	R01_cb8564_c10890/f1p0/2612	R01_cb8564_c10890/f1p0/2612	NA
R01_cb16929_c3/f1p0/439	NA	R01_cb16929_c3/f1p0/439	NA	R01_cb16929_c3/f1p0/439
R01_cb16342_c2/f1p0/557	R01_cb16342_c2/f1p0/557	R01_cb16342_c2/f1p0/557	R01_cb16342_c2/f1p0/557	R01_cb16342_c2/f1p0/557
R01_cb8564_c7851/f2p0/2741	NA	R01_cb8564_c7851/f2p0/2741	R01_cb8564_c7851/f2p0/2741	NA
R01_cb7221_c15/f2p1/2622	NA	NA	NA	R01_cb7221_c15/f2p1/2622
R01_cb9420_c1/f1p0/2105	NA	NA	NA	R01_cb9420_c1/f1p0/2105
R01_cb13289_c8/f1p2/1749	NA	NA	R01_cb13289_c8/f1p2/1749	NA
R01_cb15058_c1/f1p0/1073	NA	NA	NA	R01_cb15058_c1/f1p0/1073
R01_cb15222_c1/f1p0/684	NA	R01_cb15222_c1/f1p0/684	NA	R01_cb15222_c1/f1p0/684
R01_cb18753_c1/f1p0/1379	NA	R01_cb18753_c1/f1p0/1379	R01_cb18753_c1/f1p0/1379	R01_cb18753_c1/f1p0/1379

R01_cb10051_c1/f1p0/2385	NA	NA	NA	R01_cb10051_c1/f1p0/2385
R01_cb10428_c3/f1p0/2435	NA	R01_cb10428_c3/f1p0/2435	NA	NA
R01_cb8801_c0/f1p0/2266	NA	R01_cb8801_c0/f1p0/2266	NA	NA
R01_cb8564_c52337/f1p0/2050	R01_cb8564_c52337/f1p0/2050	R01_cb8564_c52337/f1p0/2050	R01_cb8564_c52337/f1p0/2050	R01_cb8564_c52337/f1p0/2050
R01_cb6845_c21/f1p1/2394	NA	NA	NA	R01_cb6845_c21/f1p1/2394
R01_cb8529_c3/f1p0/2056	NA	NA	NA	R01_cb8529_c3/f1p0/2056
R01_cb6216_c2/f1p0/2910	NA	NA	NA	R01_cb6216_c2/f1p0/2910
R01_cb5101_c9/f1p0/2315	NA	R01_cb5101_c9/f1p0/2315	NA	NA
R01_cb16410_c0/f2p0/629	NA	NA	NA	R01_cb16410_c0/f2p0/629
R01_cb9724_c5/f1p0/985	NA	R01_cb9724_c5/f1p0/985	NA	NA
R01_cb8564_c120446/f1p0/3511	NA	R01_cb8564_c120446/f1p0/3511	NA	NA
R01_cb12524_c2/f2p4/676	NA	R01_cb12524_c2/f2p4/676	NA	NA
R01_cb11147_c3/f1p0/770	NA	NA	NA	R01_cb11147_c3/f1p0/770
R01_cb5468_c2/f1p0/3107	NA	R01_cb5468_c2/f1p0/3107	NA	NA
R01_cb17483_c0/f2p0/556	R01_cb17483_c0/f2p0/556	R01_cb17483_c0/f2p0/556	R01_cb17483_c0/f2p0/556	R01_cb17483_c0/f2p0/556
R01_cb14672_c3/f1p0/867	NA	NA	NA	R01_cb14672_c3/f1p0/867
R01_cb8564_c123891/f1p0/2434	NA	R01_cb8564_c123891/f1p0/2434	NA	NA
R01_cb18344_c0/f2p0/569	NA	NA	NA	R01_cb18344_c0/f2p0/569
R01_cb11583_c0/f1p0/1384	NA	NA	R01_cb11583_c0/f1p0/1384	R01_cb11583_c0/f1p0/1384
R01_cb8209_c1/f1p0/2427	NA	NA	NA	R01_cb8209_c1/f1p0/2427
R01_cb8564_c107371/f2p3/3175	NA	NA	NA	R01_cb8564_c107371/f2p3/3175
R01_cb3774_c23/f1p0/2036	NA	NA	NA	R01_cb3774_c23/f1p0/2036

R01_cb3667_c6/f1p0/2059	NA	NA	NA	R01_cb3667_c6/f1p0/2059
R01_cb8564_c52008/f1p0/2194	NA	R01_cb8564_c52008/f1p0/2194	NA	NA
R01_cb8564_c112078/f1p0/2630	NA	R01_cb8564_c112078/f1p0/2630	NA	NA
R01_cb8564_c115743/f1p0/2801	NA	R01_cb8564_c115743/f1p0/2801	NA	R01_cb8564_c115743/f1p0/2801
R01_cb8564_c14624/f1p0/1901	NA	R01_cb8564_c14624/f1p0/1901	NA	NA
R01_cb8564_c69034/f2p0/2001	NA	R01_cb8564_c69034/f2p0/2001	R01_cb8564_c69034/f2p0/2001	R01_cb8564_c69034/f2p0/2001
R01_cb8120_c5/f1p0/2294	NA	NA	NA	R01_cb8120_c5/f1p0/2294
R01_cb15811_c14/f1p0/865	NA	R01_cb15811_c14/f1p0/865	R01_cb15811_c14/f1p0/865	R01_cb15811_c14/f1p0/865
R01_cb14533_c2/f1p1/1096	NA	NA	R01_cb14533_c2/f1p1/1096	R01_cb14533_c2/f1p1/1096
R01_cb18456_c1722/f2p0/455	NA	R01_cb18456_c1722/f2p0/455	R01_cb18456_c1722/f2p0/455	R01_cb18456_c1722/f2p0/455
R01_cb540_c2/f1p0/4764	NA	NA	NA	R01_cb540_c2/f1p0/4764
R01_cb8564_c22565/f1p0/3743	NA	NA	R01_cb8564_c22565/f1p0/3743	R01_cb8564_c22565/f1p0/3743
R01_cb8564_c128010/f1p0/3976	NA	R01_cb8564_c128010/f1p0/3976	NA	NA
R01_cb11443_c1/f1p0/3789	NA	R01_cb11443_c1/f1p0/3789	NA	NA
R01_cb18456_c4701/f1p0/349	R01_cb18456_c4701/f1p0/349	R01_cb18456_c4701/f1p0/349	R01_cb18456_c4701/f1p0/349	R01_cb18456_c4701/f1p0/349
R01_cb3941_c93/f1p0/2611	NA	R01_cb3941_c93/f1p0/2611	R01_cb3941_c93/f1p0/2611	R01_cb3941_c93/f1p0/2611
R01_cb392_c14/f1p0/621	NA	R01_cb392_c14/f1p0/621	R01_cb392_c14/f1p0/621	R01_cb392_c14/f1p0/621
R01_cb8564_c78887/f1p0/2186	R01_cb8564_c78887/f1p0/2186	R01_cb8564_c78887/f1p0/2186	R01_cb8564_c78887/f1p0/2186	R01_cb8564_c78887/f1p0/2186
R01_cb10768_c2/f1p0/2062	NA	R01_cb10768_c2/f1p0/2062	NA	NA
R01_cb536_c21/f1p0/498	R01_cb536_c21/f1p0/498	R01_cb536_c21/f1p0/498	R01_cb536_c21/f1p0/498	R01_cb536_c21/f1p0/498
R01_cb10814_c0/f1p0/1169	R01_cb10814_c0/f1p0/1169	R01_cb10814_c0/f1p0/1169	R01_cb10814_c0/f1p0/1169	R01_cb10814_c0/f1p0/1169
R01_cb13490_c2/f2p2/826	NA	R01_cb13490_c2/f2p2/826	R01_cb13490_c2/f2p2/826	R01_cb13490_c2/f2p2/826

R01_cb18456_c7771/f38p0/508	R01_cb18456_c7771/f38p0/508	R01_cb18456_c7771/f38p0/508	R01_cb18456_c7771/f38p0/508	R01_cb18456_c7771/f38p0/508
R01_cb10385_c3/f1p0/3720	NA	R01_cb10385_c3/f1p0/3720	NA	NA
R01_cb5144_c4/f1p0/2056	R01_cb5144_c4/f1p0/2056	R01_cb5144_c4/f1p0/2056	NA	R01_cb5144_c4/f1p0/2056
R01_cb12329_c14/f1p0/1384	NA	NA	NA	R01_cb12329_c14/f1p0/1384
R01_cb8564_c1104/f1p0/2522	NA	R01_cb8564_c1104/f1p0/2522	R01_cb8564_c1104/f1p0/2522	NA
R01_cb16425_c7/f1p0/784	R01_cb16425_c7/f1p0/784	R01_cb16425_c7/f1p0/784	R01_cb16425_c7/f1p0/784	R01_cb16425_c7/f1p0/784
R01_cb14254_c1/f1p0/693	NA	R01_cb14254_c1/f1p0/693	NA	R01_cb14254_c1/f1p0/693
R01_cb8564_c73420/f1p0/2593	NA	R01_cb8564_c73420/f1p0/2593	NA	R01_cb8564_c73420/f1p0/2593
R01_cb12641_c18/f1p0/880	NA	R01_cb12641_c18/f1p0/880	NA	R01_cb12641_c18/f1p0/880
R01_cb3426_c3/f2p1/3451	NA	NA	NA	R01_cb3426_c3/f2p1/3451
R01_cb18349_c1/f1p0/706	NA	R01_cb18349_c1/f1p0/706	NA	NA
R01_cb14334_c2/f2p1/876	NA	R01_cb14334_c2/f2p1/876	NA	NA
R01_cb8564_c124082/f1p0/3008	NA	R01_cb8564_c124082/f1p0/3008	NA	NA
R01_cb8564_c121759/f1p0/2250	NA	NA	R01_cb8564_c121759/f1p0/2250	R01_cb8564_c121759/f1p0/2250
R01_cb15074_c1/f3p0/315	R01_cb15074_c1/f3p0/315	R01_cb15074_c1/f3p0/315	NA	NA
R01_cb2648_c15/f1p0/2639	NA	NA	NA	R01_cb2648_c15/f1p0/2639
R01_cb10620_c9/f1p0/671	R01_cb10620_c9/f1p0/671	R01_cb10620_c9/f1p0/671	NA	NA
R01_cb1230_c8/f1p0/974	NA	R01_cb1230_c8/f1p0/974	R01_cb1230_c8/f1p0/974	R01_cb1230_c8/f1p0/974
R01_cb1228_c28/f1p0/2440	NA	NA	NA	R01_cb1228_c28/f1p0/2440
R01_cb2723_c10/f1p0/2817	NA	R01_cb2723_c10/f1p0/2817	NA	NA
R01_cb3014_c8/f1p0/2250	NA	R01_cb3014_c8/f1p0/2250	NA	NA
R01_cb10166_c3/f1p0/1941	R01_cb10166_c3/f1p0/1941	R01_cb10166_c3/f1p0/1941	NA	NA
R01_cb10587_c0/f2p0/566	NA	R01_cb10587_c0/f2p0/566	R01_cb10587_c0/f2p0/566	R01_cb10587_c0/f2p0/566

R01_cb17897_c0/f2p0/733	NA	NA	NA	R01_cb17897_c0/f2p0/733
R01_cb536_c20/f1p0/745	R01_cb536_c20/f1p0/745	R01_cb536_c20/f1p0/745	R01_cb536_c20/f1p0/745	R01_cb536_c20/f1p0/745
R01_cb16642_c4/f1p1/1366	NA	NA	R01_cb16642_c4/f1p1/1366	R01_cb16642_c4/f1p1/1366
R01_cb781_c2/f3p0/1827	NA	NA	NA	R01_cb781_c2/f3p0/1827
R01_cb1093_c3/f1p0/4314	NA	NA	NA	R01_cb1093_c3/f1p0/4314
R01_cb3243_c3/f1p1/2007	NA	NA	NA	R01_cb3243_c3/f1p1/2007
R01_cb7592_c0/f1p0/2563	NA	R01_cb7592_c0/f1p0/2563	NA	NA
R01_cb11400_c1/f1p0/2487	NA	R01_cb11400_c1/f1p0/2487	NA	NA
R01_cb8564_c24205/f1p0/2604	NA	R01_cb8564_c24205/f1p0/2604	NA	NA
R01_cb17756_c20/f1p0/352	R01_cb17756_c20/f1p0/352	R01_cb17756_c20/f1p0/352	R01_cb17756_c20/f1p0/352	R01_cb17756_c20/f1p0/352
R01_cb5139_c4/f1p0/3171	NA	R01_cb5139_c4/f1p0/3171	R01_cb5139_c4/f1p0/3171	R01_cb5139_c4/f1p0/3171
R01_cb6777_c1/f1p0/2731	R01_cb6777_c1/f1p0/2731	R01_cb6777_c1/f1p0/2731	NA	R01_cb6777_c1/f1p0/2731
R01_cb11662_c1/f1p0/2546	NA	R01_cb11662_c1/f1p0/2546	NA	R01_cb11662_c1/f1p0/2546
R01_cb3807_c3/f1p0/2875	NA	R01_cb3807_c3/f1p0/2875	NA	NA
R01_cb18456_c6442/f1p0/909	R01_cb18456_c6442/f1p0/909	R01_cb18456_c6442/f1p0/909	R01_cb18456_c6442/f1p0/909	NA
R01_cb4223_c1/f1p0/2582	NA	NA	NA	R01_cb4223_c1/f1p0/2582
R01_cb18456_c7502/f1p1/545	NA	NA	NA	R01_cb18456_c7502/f1p1/545
R01_cb7104_c5/f1p0/1869	NA	R01_cb7104_c5/f1p0/1869	NA	NA
R01_cb8564_c116305/f1p0/2020	NA	R01_cb8564_c116305/f1p0/2020	NA	NA
R01_cb8669_c0/f2p0/1888	NA	NA	R01_cb8669_c0/f2p0/1888	NA
R01_cb9803_c2/f1p0/934	R01_cb9803_c2/f1p0/934	R01_cb9803_c2/f1p0/934	R01_cb9803_c2/f1p0/934	R01_cb9803_c2/f1p0/934
R01_cb18456_c4752/f1p1/360	R01_cb18456_c4752/f1p1/360	R01_cb18456_c4752/f1p1/360	R01_cb18456_c4752/f1p1/360	R01_cb18456_c4752/f1p1/360
R01_cb8057_c1/f1p0/3112	R01_cb8057_c1/f1p0/3112	R01_cb8057_c1/f1p0/3112	R01_cb8057_c1/f1p0/3112	R01_cb8057_c1/f1p0/3112
R01_cb17266_c25/f1p0/1381	R01_cb17266_c25/f1p0/1381	R01_cb17266_c25/f1p0/1381	R01_cb17266_c25/f1p0/1381	R01_cb17266_c25/f1p0/1381
R01_cb11501_c1/f1p0/1938	NA	NA	NA	R01_cb11501_c1/f1p0/1938

R01_cb8298_c1/f1p1/2312	R01_cb8298_c1/f1p1/2312	R01_cb8298_c1/f1p1/2312	R01_cb8298_c1/f1p1/2312	R01_cb8298_c1/f1p1/2312
R01_cb8688_c2/f1p0/2404	NA	NA	NA	R01_cb8688_c2/f1p0/2404
R01_cb10386_c0/f2p0/1003	R01_cb10386_c0/f2p0/1003	R01_cb10386_c0/f2p0/1003	R01_cb10386_c0/f2p0/1003	R01_cb10386_c0/f2p0/1003
R01_cb18456_c7678/f1p0/1882	NA	R01_cb18456_c7678/f1p0/1882	NA	NA
R01_cb8564_c1027/f1p0/1955	NA	NA	NA	R01_cb8564_c1027/f1p0/1955
R01_cb17429_c2/f1p0/322	R01_cb17429_c2/f1p0/322	R01_cb17429_c2/f1p0/322	NA	R01_cb17429_c2/f1p0/322
R01_cb17412_c1/f1p0/1450	NA	R01_cb17412_c1/f1p0/1450	R01_cb17412_c1/f1p0/1450	R01_cb17412_c1/f1p0/1450
R01_cb8564_c82859/f1p0/2107	NA	R01_cb8564_c82859/f1p0/2107	NA	R01_cb8564_c82859/f1p0/2107
R01_cb6117_c1/f1p0/2549	R01_cb6117_c1/f1p0/2549	R01_cb6117_c1/f1p0/2549	NA	NA
R01_cb763_c4/f1p0/4192	NA	R01_cb763_c4/f1p0/4192	R01_cb763_c4/f1p0/4192	R01_cb763_c4/f1p0/4192
R01_cb12781_c1/f1p0/558	NA	R01_cb12781_c1/f1p0/558	NA	NA
R01_cb4134_c50/f1p0/2932	NA	NA	NA	R01_cb4134_c50/f1p0/2932
R01_cb8564_c3906/f1p0/2659	NA	R01_cb8564_c3906/f1p0/2659	NA	R01_cb8564_c3906/f1p0/2659
R01_cb8564_c24053/f1p0/2918	NA	R01_cb8564_c24053/f1p0/2918	NA	NA
R01_cb8564_c122281/f1p0/2794	NA	R01_cb8564_c122281/f1p0/2794	NA	NA
R01_cb9986_c3/f1p0/458	NA	NA	NA	R01_cb9986_c3/f1p0/458
R01_cb11905_c8/f1p3/1156	NA	R01_cb11905_c8/f1p3/1156	NA	NA
R01_cb7086_c0/f1p0/2700	NA	NA	NA	R01_cb7086_c0/f1p0/2700
R01_cb5265_c83/f1p1/2645	NA	R01_cb5265_c83/f1p1/2645	NA	NA
R01_cb8564_c86716/f1p0/2030	R01_cb8564_c86716/f1p0/2030	R01_cb8564_c86716/f1p0/2030	R01_cb8564_c86716/f1p0/2030	R01_cb8564_c86716/f1p0/2030
R01_cb10518_c2/f1p0/960	NA	R01_cb10518_c2/f1p0/960	NA	NA
R01_cb17584_c1/f1p0/1478	NA	R01_cb17584_c1/f1p0/1478	NA	NA
R01_cb13438_c3/f1p0/1884	NA	R01_cb13438_c3/f1p0/1884	R01_cb13438_c3/f1p0/1884	NA
R01_cb6742_c12/f1p0/2398	NA	NA	NA	R01_cb6742_c12/f1p0/2398

R01_cb8564_c74006/f1p0/2813	NA	R01_cb8564_c74006/f1p0/2813	NA	NA
R01_cb8062_c1/f2p0/2258	NA	R01_cb8062_c1/f2p0/2258	NA	NA
R01_cb16076_c2/f1p0/669	NA	NA	R01_cb16076_c2/f1p0/669	R01_cb16076_c2/f1p0/669
R01_cb10029_c2815/f2p0/752	NA	R01_cb10029_c2815/f2p0/752	NA	NA
R01_cb8564_c3243/f1p0/1951	R01_cb8564_c3243/f1p0/1951	R01_cb8564_c3243/f1p0/1951	R01_cb8564_c3243/f1p0/1951	R01_cb8564_c3243/f1p0/1951
R01_cb9855_c3/f1p0/3029	NA	R01_cb9855_c3/f1p0/3029	NA	R01_cb9855_c3/f1p0/3029
R01_cb4128_c20/f1p0/3149	NA	NA	NA	R01_cb4128_c20/f1p0/3149
R01_cb18107_c0/f1p0/1163	NA	R01_cb18107_c0/f1p0/1163	NA	R01_cb18107_c0/f1p0/1163
R01_cb5706_c2/f1p0/2577	R01_cb5706_c2/f1p0/2577	R01_cb5706_c2/f1p0/2577	NA	NA
R01_cb12240_c10/f1p0/432	R01_cb12240_c10/f1p0/432	R01_cb12240_c10/f1p0/432	NA	NA
R01_cb10402_c2/f1p0/1390	NA	R01_cb10402_c2/f1p0/1390	NA	NA
R01_cb1077_c8/f2p0/1992	NA	NA	NA	R01_cb1077_c8/f2p0/1992
R01_cb8564_c721/f3p0/3116	R01_cb8564_c721/f3p0/3116	R01_cb8564_c721/f3p0/3116	NA	NA
R01_cb9807_c0/f1p0/1972	NA	R01_cb9807_c0/f1p0/1972	NA	NA
R01_cb13390_c14/f1p0/1686	NA	R01_cb13390_c14/f1p0/1686	NA	R01_cb13390_c14/f1p0/1686
R01_cb13199_c1/f1p0/1603	NA	NA	NA	R01_cb13199_c1/f1p0/1603
R01_cb7303_c6/f1p0/2762	NA	NA	NA	R01_cb7303_c6/f1p0/2762
R01_cb6118_c19/f1p0/430	R01_cb6118_c19/f1p0/430	R01_cb6118_c19/f1p0/430	R01_cb6118_c19/f1p0/430	R01_cb6118_c19/f1p0/430
R01_cb13427_c4/f1p1/891	NA	NA	NA	R01_cb13427_c4/f1p1/891
R01_cb7539_c0/f2p0/2288	NA	NA	R01_cb7539_c0/f2p0/2288	R01_cb7539_c0/f2p0/2288
R01_cb8564_c2729/f1p0/3613	NA	R01_cb8564_c2729/f1p0/3613	R01_cb8564_c2729/f1p0/3613	R01_cb8564_c2729/f1p0/3613
R01_cb17584_c0/f2p0/1649	NA	NA	NA	R01_cb17584_c0/f2p0/1649
R01_cb8564_c10877/f1p0/3585	NA	R01_cb8564_c10877/f1p0/3585	NA	NA
R01_cb4368_c14/f1p0/491	R01_cb4368_c14/f1p0/491	R01_cb4368_c14/f1p0/491	R01_cb4368_c14/f1p0/491	R01_cb4368_c14/f1p0/491
R01_cb2806_c2/f1p0/3415	NA	NA	NA	R01_cb2806_c2/f1p0/3415
R01_cb2313_c7/f1p0/403	NA	R01_cb2313_c7/f1p0/403	R01_cb2313_c7/f1p0/403	R01_cb2313_c7/f1p0/403

R01_cb16078_c2/f3p0/395	R01_cb16078_c2/f3p0/395	R01_cb16078_c2/f3p0/395	R01_cb16078_c2/f3p0/395	NA
R01_cb17797_c0/f1p0/971	NA	R01_cb17797_c0/f1p0/971	NA	NA
R01_cb3228_c3/f1p0/2752	NA	R01_cb3228_c3/f1p0/2752	NA	NA
R01_cb2030_c6/f3p0/581	NA	NA	NA	R01_cb2030_c6/f3p0/581
R01_cb16956_c0/f4p0/544	R01_cb16956_c0/f4p0/544	R01_cb16956_c0/f4p0/544	R01_cb16956_c0/f4p0/544	R01_cb16956_c0/f4p0/544
R01_cb7103_c3/f1p0/1750	NA	R01_cb7103_c3/f1p0/1750	NA	R01_cb7103_c3/f1p0/1750
R01_cb13651_c13/f1p0/1535	NA	R01_cb13651_c13/f1p0/1535	NA	NA
R01_cb10913_c6/f1p0/1886	NA	R01_cb10913_c6/f1p0/1886	NA	R01_cb10913_c6/f1p0/1886
R01_cb14192_c11/f1p0/688	R01_cb14192_c11/f1p0/688	R01_cb14192_c11/f1p0/688	R01_cb14192_c11/f1p0/688	R01_cb14192_c11/f1p0/688
R01_cb8564_c12792/f1p0/3267	NA	R01_cb8564_c12792/f1p0/3267	NA	NA
R01_cb15755_c1/f2p0/815	NA	NA	NA	R01_cb15755_c1/f2p0/815
R01_cb7719_c6/f1p0/5135	NA	R01_cb7719_c6/f1p0/5135	NA	NA
R01_cb2792_c21/f1p0/3291	NA	R01_cb2792_c21/f1p0/3291	NA	NA
R01_cb2682_c5/f1p0/3554	NA	NA	NA	R01_cb2682_c5/f1p0/3554
R01_cb7066_c0/f1p0/2704	NA	R01_cb7066_c0/f1p0/2704	R01_cb7066_c0/f1p0/2704	R01_cb7066_c0/f1p0/2704
R01_cb18634_c5/f1p0/1196	NA	R01_cb18634_c5/f1p0/1196	R01_cb18634_c5/f1p0/1196	R01_cb18634_c5/f1p0/1196
R01_cb3699_c9/f1p0/2741	NA	R01_cb3699_c9/f1p0/2741	NA	NA
R01_cb13054_c1/f1p0/523	NA	NA	R01_cb13054_c1/f1p0/523	R01_cb13054_c1/f1p0/523
R01_cb8564_c121394/f1p0/2533	R01_cb8564_c121394/f1p0/2533	R01_cb8564_c121394/f1p0/2533	R01_cb8564_c121394/f1p0/2533	R01_cb8564_c121394/f1p0/2533
R01_cb14429_c0/f1p0/1028	R01_cb14429_c0/f1p0/1028	R01_cb14429_c0/f1p0/1028	R01_cb14429_c0/f1p0/1028	R01_cb14429_c0/f1p0/1028
R01_cb8564_c80175/f1p0/2488	NA	R01_cb8564_c80175/f1p0/2488	R01_cb8564_c80175/f1p0/2488	NA
R01_cb15173_c1/f1p0/1108	NA	NA	NA	R01_cb15173_c1/f1p0/1108
R01_cb8611_c4/f1p0/2174	NA	NA	NA	R01_cb8611_c4/f1p0/2174
R01_cb15069_c3/f1p1/546	NA	R01_cb15069_c3/f1p1/546	NA	NA
R01_cb781_c38/f1p0/1400	NA	NA	NA	R01_cb781_c38/f1p0/1400

R01_cb2804_c105/f1p0/2811	NA	R01_cb2804_c105/f1p0/2811	R01_cb2804_c105/f1p0/2811	R01_cb2804_c105/f1p0/2811
R01_cb8564_c14097/f1p1/3734	NA	NA	NA	R01_cb8564_c14097/f1p1/3734
R01_cb943_c0/f1p0/4542	NA	NA	NA	R01_cb943_c0/f1p0/4542
R01_cb1247_c0/f1p0/4224	NA	R01_cb1247_c0/f1p0/4224	NA	NA
R01_cb9513_c35/f1p0/2739	NA	R01_cb9513_c35/f1p0/2739	NA	NA
R01_cb18172_c1/f1p0/326	R01_cb18172_c1/f1p0/326	R01_cb18172_c1/f1p0/326	NA	R01_cb18172_c1/f1p0/326
R01_cb950_c45/f1p0/4455	NA	R01_cb950_c45/f1p0/4455	R01_cb950_c45/f1p0/4455	NA
R01_cb8564_c70740/f1p0/3814	NA	R01_cb8564_c70740/f1p0/3814	NA	NA
R01_cb13493_c7/f1p0/406	R01_cb13493_c7/f1p0/406	R01_cb13493_c7/f1p0/406	R01_cb13493_c7/f1p0/406	R01_cb13493_c7/f1p0/406
R01_cb10853_c2/f1p0/2911	NA	R01_cb10853_c2/f1p0/2911	NA	NA
R01_cb8991_c15/f7p1/2056	NA	NA	NA	R01_cb8991_c15/f7p1/2056
R01_cb6251_c1/f3p0/2892	R01_cb6251_c1/f3p0/2892	R01_cb6251_c1/f3p0/2892	NA	R01_cb6251_c1/f3p0/2892
R01_cb16958_c1/f1p0/1052	NA	NA	NA	R01_cb16958_c1/f1p0/1052
R01_cb8020_c1/f1p0/1980	R01_cb8020_c1/f1p0/1980	R01_cb8020_c1/f1p0/1980	R01_cb8020_c1/f1p0/1980	R01_cb8020_c1/f1p0/1980
R01_cb11972_c2/f1p0/1254	NA	NA	NA	R01_cb11972_c2/f1p0/1254
R01_cb17019_c3/f1p0/1166	NA	NA	NA	R01_cb17019_c3/f1p0/1166
R01_cb14264_c0/f3p0/516	NA	NA	R01_cb14264_c0/f3p0/516	R01_cb14264_c0/f3p0/516
R01_cb7825_c1/f2p0/2450	R01_cb7825_c1/f2p0/2450	R01_cb7825_c1/f2p0/2450	NA	R01_cb7825_c1/f2p0/2450
R01_cb10723_c0/f2p0/870	NA	NA	NA	R01_cb10723_c0/f2p0/870
R01_cb17740_c0/f1p0/1594	NA	NA	NA	R01_cb17740_c0/f1p0/1594
R01_cb11063_c0/f2p0/715	NA	NA	NA	R01_cb11063_c0/f2p0/715
R01_cb11321_c2/f1p0/1485	NA	NA	NA	R01_cb11321_c2/f1p0/1485
R01_cb8564_c4581/f1p0/2440	NA	NA	NA	R01_cb8564_c4581/f1p0/2440
R01_cb17154_c2/f1p0/1201	R01_cb17154_c2/f1p0/1201	R01_cb17154_c2/f1p0/1201	R01_cb17154_c2/f1p0/1201	R01_cb17154_c2/f1p0/1201
R01_cb10458_c5/f1p1/2138	NA	NA	NA	R01_cb10458_c5/f1p1/2138
R01_cb8564_c121536/f1p0/279	NA	R01_cb8564_c121536/f1p0/279	NA	R01_cb8564_c121536/f1p0/279

9		9		9
R01_cb4044_c4/f1p0/3942	NA	R01_cb4044_c4/f1p0/3942	NA	NA
R01_cb8564_c124642/f1p0/2345	NA	R01_cb8564_c124642/f1p0/2345	R01_cb8564_c124642/f1p0/2345	R01_cb8564_c124642/f1p0/2345
R01_cb4493_c5/f2p0/1014	NA	NA	NA	R01_cb4493_c5/f2p0/1014
R01_cb8564_c11131/f1p0/4051	NA	R01_cb8564_c11131/f1p0/4051	R01_cb8564_c11131/f1p0/4051	R01_cb8564_c11131/f1p0/4051
R01_cb8564_c3804/f2p0/2944	R01_cb8564_c3804/f2p0/2944	R01_cb8564_c3804/f2p0/2944	R01_cb8564_c3804/f2p0/2944	NA
R01_cb6113_c12/f1p0/844	NA	NA	NA	R01_cb6113_c12/f1p0/844
R01_cb2226_c0/f1p0/4006	NA	NA	NA	R01_cb2226_c0/f1p0/4006
R01_cb5417_c0/f2p0/3113	NA	R01_cb5417_c0/f2p0/3113	R01_cb5417_c0/f2p0/3113	R01_cb5417_c0/f2p0/3113
R01_cb5996_c3/f2p0/2942	NA	R01_cb5996_c3/f2p0/2942	NA	NA
R01_cb13161_c3/f2p0/327	R01_cb13161_c3/f2p0/327	R01_cb13161_c3/f2p0/327	R01_cb13161_c3/f2p0/327	R01_cb13161_c3/f2p0/327
R01_cb16689_c0/f1p0/750	R01_cb16689_c0/f1p0/750	NA	NA	R01_cb16689_c0/f1p0/750
R01_cb11603_c3/f1p0/878	R01_cb11603_c3/f1p0/878	R01_cb11603_c3/f1p0/878	R01_cb11603_c3/f1p0/878	R01_cb11603_c3/f1p0/878
R01_cb1031_c2/f1p0/2730	NA	NA	NA	R01_cb1031_c2/f1p0/2730
R01_cb10641_c3/f1p0/1455	NA	NA	NA	R01_cb10641_c3/f1p0/1455
R01_cb2494_c8/f1p0/3407	R01_cb2494_c8/f1p0/3407	R01_cb2494_c8/f1p0/3407	R01_cb2494_c8/f1p0/3407	R01_cb2494_c8/f1p0/3407
R01_cb1301_c0/f1p0/4386	NA	NA	NA	R01_cb1301_c0/f1p0/4386
R01_cb15277_c1/f2p0/900	NA	R01_cb15277_c1/f2p0/900	NA	NA
R01_cb16752_c4/f1p0/880	NA	R01_cb16752_c4/f1p0/880	NA	R01_cb16752_c4/f1p0/880
R01_cb8102_c24/f1p0/1791	NA	R01_cb8102_c24/f1p0/1791	NA	R01_cb8102_c24/f1p0/1791
R01_cb12452_c5/f1p0/1595	NA	NA	NA	R01_cb12452_c5/f1p0/1595
R01_cb5533_c133/f1p0/2167	NA	NA	NA	R01_cb5533_c133/f1p0/2167
R01_cb12003_c31/f1p0/1799	NA	R01_cb12003_c31/f1p0/1799	NA	NA
R01_cb16721_c6/f1p0/794	NA	R01_cb16721_c6/f1p0/794	R01_cb16721_c6/f1p0/794	R01_cb16721_c6/f1p0/794
R01_cb15804_c1/f2p0/872	NA	NA	NA	R01_cb15804_c1/f2p0/872

R01_cb7810_c3/f1p0/862	R01_cb7810_c3/f1p0/862	R01_cb7810_c3/f1p0/862	R01_cb7810_c3/f1p0/862	NA
R01_cb8564_c80344/f1p0/2421	NA	R01_cb8564_c80344/f1p0/2421	R01_cb8564_c80344/f1p0/2421	R01_cb8564_c80344/f1p0/2421
R01_cb8564_c5186/f1p0/2130	NA	NA	NA	R01_cb8564_c5186/f1p0/2130
R01_cb10014_c22/f7p0/575	NA	R01_cb10014_c22/f7p0/575	NA	NA
R01_cb1896_c66/f1p0/666	R01_cb1896_c66/f1p0/666	R01_cb1896_c66/f1p0/666	R01_cb1896_c66/f1p0/666	R01_cb1896_c66/f1p0/666
R01_cb11918_c19/f5p1/1082	NA	NA	NA	R01_cb11918_c19/f5p1/1082
R01_cb261_c40/f1p0/2567	NA	NA	NA	R01_cb261_c40/f1p0/2567
R01_cb3896_c4/f1p0/2487	R01_cb3896_c4/f1p0/2487	R01_cb3896_c4/f1p0/2487	R01_cb3896_c4/f1p0/2487	NA
R01_cb17183_c3/f1p0/815	NA	NA	NA	R01_cb17183_c3/f1p0/815
R01_cb11742_c0/f2p0/599	NA	R01_cb11742_c0/f2p0/599	NA	NA
R01_cb8564_c78291/f1p0/2076	NA	NA	NA	R01_cb8564_c78291/f1p0/2076
R01_cb8564_c2100/f1p0/2688	NA	NA	NA	R01_cb8564_c2100/f1p0/2688
R01_cb13761_c9/f1p0/1071	R01_cb13761_c9/f1p0/1071	R01_cb13761_c9/f1p0/1071	R01_cb13761_c9/f1p0/1071	R01_cb13761_c9/f1p0/1071
R01_cb8981_c3/f1p0/2033	NA	NA	NA	R01_cb8981_c3/f1p0/2033
R01_cb9380_c5/f1p0/2003	NA	NA	NA	R01_cb9380_c5/f1p0/2003
R01_cb15522_c0/f2p0/986	NA	NA	NA	R01_cb15522_c0/f2p0/986
R01_cb7932_c3/f1p0/464	R01_cb7932_c3/f1p0/464	R01_cb7932_c3/f1p0/464	R01_cb7932_c3/f1p0/464	R01_cb7932_c3/f1p0/464
R01_cb1331_c12/f1p0/2731	NA	R01_cb1331_c12/f1p0/2731	NA	NA
R01_cb16120_c1/f1p0/1022	NA	R01_cb16120_c1/f1p0/1022	R01_cb16120_c1/f1p0/1022	R01_cb16120_c1/f1p0/1022
R01_cb17046_c0/f1p0/1461	NA	NA	NA	R01_cb17046_c0/f1p0/1461
R01_cb10624_c2/f1p0/3472	NA	R01_cb10624_c2/f1p0/3472	NA	NA
R01_cb13398_c3/f1p0/422	NA	R01_cb13398_c3/f1p0/422	NA	NA
R01_cb18760_c0/f1p0/5959	R01_cb18760_c0/f1p0/5959	R01_cb18760_c0/f1p0/5959	R01_cb18760_c0/f1p0/5959	R01_cb18760_c0/f1p0/5959
R01_cb8954_c24/f1p0/731	NA	R01_cb8954_c24/f1p0/731	NA	NA
R01_cb8877_c1/f1p0/2150	NA	NA	NA	R01_cb8877_c1/f1p0/2150
R01_cb18759_c0/f1p0/5269	NA	R01_cb18759_c0/f1p0/5269	NA	NA

R01_cb14078_c2/f1p0/300	R01_cb14078_c2/f1p0/300	R01_cb14078_c2/f1p0/300	R01_cb14078_c2/f1p0/300	R01_cb14078_c2/f1p0/300
R01_cb4471_c2/f2p3/3079	NA	NA	NA	R01_cb4471_c2/f2p3/3079
R01_cb16205_c0/f3p0/1642	NA	NA	NA	R01_cb16205_c0/f3p0/1642
R01_cb8564_c19551/f1p0/3209	R01_cb8564_c19551/f1p0/3209	R01_cb8564_c19551/f1p0/3209	NA	R01_cb8564_c19551/f1p0/3209
R01_cb10127_c2/f1p0/1105	R01_cb10127_c2/f1p0/1105	R01_cb10127_c2/f1p0/1105	NA	R01_cb10127_c2/f1p0/1105
R01_cb8564_c23126/f1p1/4221	NA	NA	NA	R01_cb8564_c23126/f1p1/4221
R01_cb1626_c16/f1p0/2872	NA	NA	NA	R01_cb1626_c16/f1p0/2872
R01_cb9609_c1/f1p1/3401	NA	NA	NA	R01_cb9609_c1/f1p1/3401
R01_cb8564_c80892/f1p1/1944	NA	R01_cb8564_c80892/f1p1/1944	NA	NA
R01_cb4169_c16/f1p0/1095	NA	NA	NA	R01_cb4169_c16/f1p0/1095
R01_cb16631_c1/f1p0/539	NA	R01_cb16631_c1/f1p0/539	NA	R01_cb16631_c1/f1p0/539
R01_cb17412_c0/f1p0/1442	NA	R01_cb17412_c0/f1p0/1442	R01_cb17412_c0/f1p0/1442	R01_cb17412_c0/f1p0/1442
R01_cb10778_c1/f1p0/2691	NA	R01_cb10778_c1/f1p0/2691	NA	NA
R01_cb14131_c4/f2p0/1362	NA	NA	NA	R01_cb14131_c4/f2p0/1362
R01_cb17307_c0/f1p0/1188	R01_cb17307_c0/f1p0/1188	R01_cb17307_c0/f1p0/1188	NA	R01_cb17307_c0/f1p0/1188
R01_cb8564_c52830/f1p0/3091	NA	R01_cb8564_c52830/f1p0/3091	NA	NA
R01_cb2095_c18/f1p1/2010	NA	NA	NA	R01_cb2095_c18/f1p1/2010
R01_cb13766_c7/f1p2/1458	NA	R01_cb13766_c7/f1p2/1458	NA	NA
R01_cb14770_c2/f1p0/1105	NA	NA	NA	R01_cb14770_c2/f1p0/1105
R01_cb6802_c10/f1p0/3714	R01_cb6802_c10/f1p0/3714	R01_cb6802_c10/f1p0/3714	R01_cb6802_c10/f1p0/3714	R01_cb6802_c10/f1p0/3714
R01_cb4233_c81/f1p0/4601	R01_cb4233_c81/f1p0/4601	R01_cb4233_c81/f1p0/4601	R01_cb4233_c81/f1p0/4601	R01_cb4233_c81/f1p0/4601
R01_cb9473_c7/f1p0/1752	NA	R01_cb9473_c7/f1p0/1752	NA	NA
R01_cb9155_c6/f1p0/1036	NA	R01_cb9155_c6/f1p0/1036	NA	NA
R01_cb8564_c90030/f1p0/2483	NA	R01_cb8564_c90030/f1p0/2483	NA	NA
R01_cb8564_c80524/f1p0/3995	NA	R01_cb8564_c80524/f1p0/3995	NA	NA

R01_cb6990_c5/f1p0/2763	NA	R01_cb6990_c5/f1p0/2763	NA	NA
R01_cb18549_c0/f1p0/1077	NA	NA	NA	R01_cb18549_c0/f1p0/1077
R01_cb1297_c15/f1p0/4176	NA	NA	NA	R01_cb1297_c15/f1p0/4176
R01_cb12602_c1/f1p0/1074	NA	R01_cb12602_c1/f1p0/1074	R01_cb12602_c1/f1p0/1074	R01_cb12602_c1/f1p0/1074
R01_cb10272_c3/f5p2/1680	NA	NA	NA	R01_cb10272_c3/f5p2/1680
R01_cb1626_c9/f1p1/2460	NA	NA	NA	R01_cb1626_c9/f1p1/2460
R01_cb13386_c7/f1p0/528	NA	R01_cb13386_c7/f1p0/528	NA	NA
R01_cb18259_c1/f1p0/737	NA	NA	R01_cb18259_c1/f1p0/737	NA
R01_cb2822_c2/f1p0/3718	NA	R01_cb2822_c2/f1p0/3718	NA	NA
R01_cb2602_c13/f1p0/1680	NA	R01_cb2602_c13/f1p0/1680	NA	NA
R01_cb17167_c0/f7p1/583	NA	R01_cb17167_c0/f7p1/583	NA	NA
R01_cb4094_c7/f5p1/2899	NA	R01_cb4094_c7/f5p1/2899	NA	NA
R01_cb8564_c40879/f1p0/2848	R01_cb8564_c40879/f1p0/2848	R01_cb8564_c40879/f1p0/2848	R01_cb8564_c40879/f1p0/2848	R01_cb8564_c40879/f1p0/2848
R01_cb14619_c8/f2p0/1440	NA	R01_cb14619_c8/f2p0/1440	R01_cb14619_c8/f2p0/1440	R01_cb14619_c8/f2p0/1440
R01_cb18456_c5798/f1p0/682	NA	NA	NA	R01_cb18456_c5798/f1p0/682
R01_cb606_c5/f1p1/3393	NA	NA	NA	R01_cb606_c5/f1p1/3393
R01_cb13099_c0/f3p0/653	NA	R01_cb13099_c0/f3p0/653	NA	NA
R01_cb11718_c0/f1p0/893	NA	NA	NA	R01_cb11718_c0/f1p0/893
R01_cb8564_c121217/f1p0/2846	NA	NA	NA	R01_cb8564_c121217/f1p0/2846
R01_cb8564_c4535/f1p1/2090	NA	R01_cb8564_c4535/f1p1/2090	R01_cb8564_c4535/f1p1/2090	R01_cb8564_c4535/f1p1/2090
R01_cb5513_c4/f1p0/2795	NA	NA	NA	R01_cb5513_c4/f1p0/2795
R01_cb5414_c4/f2p0/3076	NA	NA	R01_cb5414_c4/f2p0/3076	NA
R01_cb8779_c14/f1p0/3251	R01_cb8779_c14/f1p0/3251	R01_cb8779_c14/f1p0/3251	NA	NA
R01_cb8564_c51353/f1p0/2964	NA	R01_cb8564_c51353/f1p0/2964	NA	R01_cb8564_c51353/f1p0/2964

R01_cb17199_c5/f1p0/904	NA	R01_cb17199_c5/f1p0/904	NA	NA
R01_cb1124_c7/f1p0/2043	NA	NA	NA	R01_cb1124_c7/f1p0/2043
R01_cb10385_c5/f1p0/1837	NA	R01_cb10385_c5/f1p0/1837	NA	NA
R01_cb11283_c2/f1p0/546	R01_cb11283_c2/f1p0/546	R01_cb11283_c2/f1p0/546	NA	R01_cb11283_c2/f1p0/546
R01_cb11461_c1/f1p0/3729	NA	R01_cb11461_c1/f1p0/3729	NA	R01_cb11461_c1/f1p0/3729
R01_cb11888_c0/f1p0/1138	NA	R01_cb11888_c0/f1p0/1138	R01_cb11888_c0/f1p0/1138	R01_cb11888_c0/f1p0/1138
R01_cb124_c28/f1p0/2797	NA	NA	NA	R01_cb124_c28/f1p0/2797
R01_cb15824_c1/f1p0/1148	NA	NA	NA	R01_cb15824_c1/f1p0/1148
R01_cb15585_c12/f1p0/502	R01_cb15585_c12/f1p0/502	R01_cb15585_c12/f1p0/502	R01_cb15585_c12/f1p0/502	R01_cb15585_c12/f1p0/502
R01_cb7822_c0/f1p0/2475	NA	NA	R01_cb7822_c0/f1p0/2475	R01_cb7822_c0/f1p0/2475
R01_cb3601_c10/f1p0/2500	NA	R01_cb3601_c10/f1p0/2500	NA	NA
R01_cb12001_c4/f1p0/1844	NA	R01_cb12001_c4/f1p0/1844	NA	NA
R01_cb10653_c5/f21p0/526	NA	R01_cb10653_c5/f21p0/526	NA	NA
R01_cb15364_c3/f1p0/1145	NA	NA	NA	R01_cb15364_c3/f1p0/1145
R01_cb4047_c8/f1p0/2511	NA	NA	NA	R01_cb4047_c8/f1p0/2511
R01_cb5042_c6/f1p0/1108	NA	NA	R01_cb5042_c6/f1p0/1108	NA
R01_cb8564_c76570/f1p0/3381	NA	R01_cb8564_c76570/f1p0/3381	NA	NA
R01_cb8198_c4/f1p0/2054	NA	NA	NA	R01_cb8198_c4/f1p0/2054
R01_cb5471_c8/f2p0/2562	NA	NA	NA	R01_cb5471_c8/f2p0/2562
R01_cb6953_c3/f1p0/3317	NA	R01_cb6953_c3/f1p0/3317	NA	R01_cb6953_c3/f1p0/3317
R01_cb8564_c17636/f1p0/2212	R01_cb8564_c17636/f1p0/2212	R01_cb8564_c17636/f1p0/2212	R01_cb8564_c17636/f1p0/2212	R01_cb8564_c17636/f1p0/2212
R01_cb8564_c69530/f1p0/2919	NA	NA	R01_cb8564_c69530/f1p0/2919	NA
R01_cb279_c11/f1p1/4423	NA	NA	NA	R01_cb279_c11/f1p1/4423
R01_cb4932_c4/f1p1/3225	NA	NA	NA	R01_cb4932_c4/f1p1/3225
R01_cb18456_c2305/f1p0/1016	NA	R01_cb18456_c2305/f1p0/1016	NA	NA

R01_cb12286_c7/f1p0/648	R01_cb12286_c7/f1p0/648	R01_cb12286_c7/f1p0/648	NA	NA
R01_cb15345_c26/f1p1/1557	NA	R01_cb15345_c26/f1p1/1557	NA	NA
R01_cb8564_c20932/f1p0/3622	NA	R01_cb8564_c20932/f1p0/3622	NA	R01_cb8564_c20932/f1p0/3622
R01_cb13178_c6/f3p0/494	NA	R01_cb13178_c6/f3p0/494	NA	NA
R01_cb11318_c0/f2p0/1159	NA	NA	NA	R01_cb11318_c0/f2p0/1159
R01_cb8564_c4192/f1p0/2498	NA	R01_cb8564_c4192/f1p0/2498	NA	R01_cb8564_c4192/f1p0/2498
R01_cb4444_c25/f1p0/4660	NA	R01_cb4444_c25/f1p0/4660	NA	NA
R01_cb8506_c3/f1p0/1960	R01_cb8506_c3/f1p0/1960	R01_cb8506_c3/f1p0/1960	NA	NA
R01_cb5251_c2/f1p0/1994	NA	NA	NA	R01_cb5251_c2/f1p0/1994
R01_cb6615_c24/f1p0/874	NA	NA	NA	R01_cb6615_c24/f1p0/874
R01_cb2594_c2/f1p0/622	NA	R01_cb2594_c2/f1p0/622	NA	NA
R01_cb11206_c1/f1p0/2097	R01_cb11206_c1/f1p0/2097	R01_cb11206_c1/f1p0/2097	R01_cb11206_c1/f1p0/2097	R01_cb11206_c1/f1p0/2097
R01_cb10674_c2/f1p1/1881	NA	R01_cb10674_c2/f1p1/1881	NA	NA
R01_cb16182_c1/f1p1/1623	NA	NA	NA	R01_cb16182_c1/f1p1/1623
R01_cb8564_c121789/f1p0/2225	R01_cb8564_c121789/f1p0/2225	R01_cb8564_c121789/f1p0/2225	NA	NA
R01_cb8564_c80737/f1p0/2317	NA	NA	NA	R01_cb8564_c80737/f1p0/2317
R01_cb4128_c23/f1p0/2488	NA	NA	NA	R01_cb4128_c23/f1p0/2488
R01_cb5151_c1/f1p0/2796	NA	NA	NA	R01_cb5151_c1/f1p0/2796
R01_cb6802_c13/f1p0/3135	R01_cb6802_c13/f1p0/3135	R01_cb6802_c13/f1p0/3135	R01_cb6802_c13/f1p0/3135	R01_cb6802_c13/f1p0/3135
R01_cb13761_c11/f1p0/1433	NA	R01_cb13761_c11/f1p0/1433	R01_cb13761_c11/f1p0/1433	R01_cb13761_c11/f1p0/1433
R01_cb3079_c11/f1p0/1658	NA	NA	NA	R01_cb3079_c11/f1p0/1658
R01_cb8564_c70588/f1p0/2189	NA	R01_cb8564_c70588/f1p0/2189	NA	NA
R01_cb18409_c60/f1p0/397	R01_cb18409_c60/f1p0/397	R01_cb18409_c60/f1p0/397	R01_cb18409_c60/f1p0/397	R01_cb18409_c60/f1p0/397
R01_cb8564_c25155/f1p3/4069	NA	R01_cb8564_c25155/f1p3/4069	R01_cb8564_c25155/f1p3/4069	R01_cb8564_c25155/f1p3/4069
R01_cb5647_c4/f1p0/1749	NA	NA	NA	R01_cb5647_c4/f1p0/1749

R01_cb14344_c1/f1p2/1550	NA	R01_cb14344_c1/f1p2/1550	R01_cb14344_c1/f1p2/1550	R01_cb14344_c1/f1p2/1550
R01_cb3445_c6/f1p1/2781	NA	NA	NA	R01_cb3445_c6/f1p1/2781
R01_cb7666_c8/f1p0/2399	NA	R01_cb7666_c8/f1p0/2399	NA	NA
R01_cb8904_c0/f1p0/2239	NA	NA	NA	R01_cb8904_c0/f1p0/2239
R01_cb8564_c22732/f1p0/3568	NA	NA	R01_cb8564_c22732/f1p0/3568	R01_cb8564_c22732/f1p0/3568
R01_cb17110_c2/f1p0/1234	NA	R01_cb17110_c2/f1p0/1234	R01_cb17110_c2/f1p0/1234	R01_cb17110_c2/f1p0/1234
R01_cb18409_c58/f1p0/1658	NA	NA	NA	R01_cb18409_c58/f1p0/1658
R01_cb5190_c5/f1p1/2260	NA	NA	NA	R01_cb5190_c5/f1p1/2260
R01_cb10761_c1/f1p0/2641	R01_cb10761_c1/f1p0/2641	R01_cb10761_c1/f1p0/2641	NA	NA
R01_cb2804_c65/f1p0/3384	NA	R01_cb2804_c65/f1p0/3384	NA	NA
R01_cb8564_c91334/f1p0/3631	NA	R01_cb8564_c91334/f1p0/3631	NA	R01_cb8564_c91334/f1p0/3631
R01_cb13760_c2/f1p1/1807	NA	NA	NA	R01_cb13760_c2/f1p1/1807
R01_cb10054_c55/f2p0/1317	NA	R01_cb10054_c55/f2p0/1317	NA	NA
R01_cb7108_c7/f1p0/2457	NA	NA	NA	R01_cb7108_c7/f1p0/2457
R01_cb6143_c8/f1p0/1742	NA	R01_cb6143_c8/f1p0/1742	R01_cb6143_c8/f1p0/1742	R01_cb6143_c8/f1p0/1742
R01_cb8658_c5/f1p0/3411	NA	NA	NA	R01_cb8658_c5/f1p0/3411
R01_cb1259_c7/f1p1/3256	NA	NA	NA	R01_cb1259_c7/f1p1/3256
R01_cb8564_c118410/f1p1/2510	NA	R01_cb8564_c118410/f1p1/2510	NA	NA
R01_cb15540_c7/f1p1/1414	NA	NA	NA	R01_cb15540_c7/f1p1/1414
R01_cb8400_c2/f2p0/2145	R01_cb8400_c2/f2p0/2145	R01_cb8400_c2/f2p0/2145	R01_cb8400_c2/f2p0/2145	R01_cb8400_c2/f2p0/2145
R01_cb12494_c4/f1p0/1114	NA	R01_cb12494_c4/f1p0/1114	NA	NA
R01_cb12577_c5/f1p1/1133	NA	NA	NA	R01_cb12577_c5/f1p1/1133
R01_cb2807_c6/f1p0/1046	NA	R01_cb2807_c6/f1p0/1046	R01_cb2807_c6/f1p0/1046	R01_cb2807_c6/f1p0/1046
R01_cb8105_c1/f1p0/3855	NA	R01_cb8105_c1/f1p0/3855	NA	NA
R01_cb14839_c1/f1p0/982	NA	NA	R01_cb14839_c1/f1p0/982	R01_cb14839_c1/f1p0/982

R01_cb15364_c0/f1p0/1094	NA	NA	NA	R01_cb15364_c0/f1p0/1094
R01_cb8564_c82568/f7p1/2364	NA	NA	NA	R01_cb8564_c82568/f7p1/2364
R01_cb17756_c31/f1p0/421	R01_cb17756_c31/f1p0/421	R01_cb17756_c31/f1p0/421	R01_cb17756_c31/f1p0/421	R01_cb17756_c31/f1p0/421
R01_cb15864_c1/f1p0/1127	R01_cb15864_c1/f1p0/1127	R01_cb15864_c1/f1p0/1127	R01_cb15864_c1/f1p0/1127	R01_cb15864_c1/f1p0/1127
R01_cb7335_c9/f1p0/3031	NA	R01_cb7335_c9/f1p0/3031	NA	NA
R01_cb18209_c1/f1p0/1129	R01_cb18209_c1/f1p0/1129	R01_cb18209_c1/f1p0/1129	NA	R01_cb18209_c1/f1p0/1129
R01_cb8564_c86288/f1p0/3127	NA	R01_cb8564_c86288/f1p0/3127	R01_cb8564_c86288/f1p0/3127	R01_cb8564_c86288/f1p0/3127
R01_cb10015_c595/f6p1/762	R01_cb10015_c595/f6p1/762	R01_cb10015_c595/f6p1/762	NA	NA
R01_cb17277_c1/f1p0/1589	NA	R01_cb17277_c1/f1p0/1589	NA	R01_cb17277_c1/f1p0/1589
R01_cb18697_c2/f1p0/1535	NA	NA	NA	R01_cb18697_c2/f1p0/1535
R01_cb8564_c1437/f1p0/3322	NA	R01_cb8564_c1437/f1p0/3322	NA	NA
R01_cb3757_c2/f1p0/3524	NA	NA	NA	R01_cb3757_c2/f1p0/3524
R01_cb8564_c127695/f1p0/2536	NA	R01_cb8564_c127695/f1p0/2536	NA	NA
R01_cb18456_c7434/f1p0/848	NA	R01_cb18456_c7434/f1p0/848	R01_cb18456_c7434/f1p0/848	R01_cb18456_c7434/f1p0/848
R01_cb8564_c49600/f2p2/4344	NA	NA	NA	R01_cb8564_c49600/f2p2/4344
R01_cb5896_c155/f1p0/2946	NA	NA	R01_cb5896_c155/f1p0/2946	R01_cb5896_c155/f1p0/2946
R01_cb9533_c10/f1p0/1798	NA	R01_cb9533_c10/f1p0/1798	NA	NA
R01_cb8564_c54023/f1p1/1945	NA	R01_cb8564_c54023/f1p1/1945	NA	R01_cb8564_c54023/f1p1/1945
R01_cb4493_c11/f1p3/4695	NA	NA	NA	R01_cb4493_c11/f1p3/4695
R01_cb438_c8/f1p0/3035	NA	R01_cb438_c8/f1p0/3035	NA	R01_cb438_c8/f1p0/3035
R01_cb3887_c12/f2p0/2396	NA	R01_cb3887_c12/f2p0/2396	NA	NA
R01_cb15373_c2/f1p0/1400	NA	R01_cb15373_c2/f1p0/1400	NA	R01_cb15373_c2/f1p0/1400
R01_cb11110_c3/f1p0/1566	NA	NA	NA	R01_cb11110_c3/f1p0/1566
R01_cb4147_c16/f1p1/4275	NA	R01_cb4147_c16/f1p1/4275	R01_cb4147_c16/f1p1/4275	R01_cb4147_c16/f1p1/4275
R01_cb12972_c0/f4p0/763	NA	R01_cb12972_c0/f4p0/763	R01_cb12972_c0/f4p0/763	R01_cb12972_c0/f4p0/763

R01_cb8564_c91954/f1p0/3366	NA	NA	NA	R01_cb8564_c91954/f1p0/3366
R01_cb14627_c0/f4p0/981	NA	NA	NA	R01_cb14627_c0/f4p0/981
R01_cb14715_c0/f3p0/669	NA	R01_cb14715_c0/f3p0/669	R01_cb14715_c0/f3p0/669	R01_cb14715_c0/f3p0/669
R01_cb8564_c153798/f1p0/2616	NA	R01_cb8564_c153798/f1p0/2616	R01_cb8564_c153798/f1p0/2616	R01_cb8564_c153798/f1p0/2616
R01_cb8564_c92080/f1p0/4203	NA	R01_cb8564_c92080/f1p0/4203	NA	NA
R01_cb8564_c122627/f1p0/2043	NA	R01_cb8564_c122627/f1p0/2043	R01_cb8564_c122627/f1p0/2043	R01_cb8564_c122627/f1p0/2043
R01_cb4785_c2/f3p0/2776	NA	NA	NA	R01_cb4785_c2/f3p0/2776
R01_cb5229_c3/f1p0/3004	NA	NA	NA	R01_cb5229_c3/f1p0/3004
R01_cb16334_c2/f1p0/5402	NA	R01_cb16334_c2/f1p0/5402	R01_cb16334_c2/f1p0/5402	NA
R01_cb3539_c2/f2p0/1181	NA	R01_cb3539_c2/f2p0/1181	NA	NA
R01_cb1388_c31/f1p1/6318	NA	NA	NA	R01_cb1388_c31/f1p1/6318
R01_cb5183_c116/f1p0/5153	NA	NA	NA	R01_cb5183_c116/f1p0/5153
R01_cb8564_c21684/f1p0/2591	NA	NA	R01_cb8564_c21684/f1p0/2591	R01_cb8564_c21684/f1p0/2591
R01_cb8564_c16600/f1p0/4693	NA	R01_cb8564_c16600/f1p0/4693	NA	NA
R01_cb8564_c1116/f1p1/1964	NA	R01_cb8564_c1116/f1p1/1964	NA	NA
R01_cb7304_c2/f1p0/2611	NA	NA	NA	R01_cb7304_c2/f1p0/2611
R01_cb8328_c4/f1p0/1158	R01_cb8328_c4/f1p0/1158	NA	NA	NA
R01_cb9853_c1/f1p0/1907	NA	R01_cb9853_c1/f1p0/1907	R01_cb9853_c1/f1p0/1907	NA
R01_cb1155_c0/f6p2/3977	NA	NA	NA	R01_cb1155_c0/f6p2/3977
R01_cb8564_c69449/f1p0/3123	NA	R01_cb8564_c69449/f1p0/3123	NA	NA
R01_cb13512_c0/f1p0/1232	NA	NA	NA	R01_cb13512_c0/f1p0/1232
R01_cb6547_c3/f1p0/2827	NA	NA	NA	R01_cb6547_c3/f1p0/2827
R01_cb10843_c6/f1p2/1195	NA	NA	NA	R01_cb10843_c6/f1p2/1195
R01_cb4784_c3/f1p1/2082	NA	NA	NA	R01_cb4784_c3/f1p1/2082

R01_cb16447_c4/f1p0/1082	NA	NA	NA	R01_cb16447_c4/f1p0/1082
R01_cb8564_c117842/f1p0/2249	NA	NA	NA	R01_cb8564_c117842/f1p0/2249
R01_cb8564_c53691/f1p0/3380	NA	R01_cb8564_c53691/f1p0/3380	NA	NA
R01_cb3359_c28/f1p0/2729	NA	R01_cb3359_c28/f1p0/2729	NA	NA
R01_cb2991_c16/f1p0/2235	NA	NA	NA	R01_cb2991_c16/f1p0/2235
R01_cb7398_c4/f1p0/1901	NA	NA	NA	R01_cb7398_c4/f1p0/1901
R01_cb2543_c33/f1p0/2215	NA	R01_cb2543_c33/f1p0/2215	R01_cb2543_c33/f1p0/2215	NA
R01_cb6948_c10/f1p0/2541	NA	NA	NA	R01_cb6948_c10/f1p0/2541
R01_cb9522_c3/f1p0/1794	NA	NA	NA	R01_cb9522_c3/f1p0/1794
R01_cb8564_c120555/f1p0/1972	R01_cb8564_c120555/f1p0/1972	R01_cb8564_c120555/f1p0/1972	NA	NA
R01_cb3792_c6/f1p0/5744	NA	NA	NA	R01_cb3792_c6/f1p0/5744
R01_cb8564_c18708/f1p0/3168	NA	R01_cb8564_c18708/f1p0/3168	NA	NA
R01_cb8564_c18027/f1p0/3948	NA	NA	NA	R01_cb8564_c18027/f1p0/3948
R01_cb6742_c9/f1p0/2591	NA	NA	NA	R01_cb6742_c9/f1p0/2591
R01_cb8564_c87465/f2p9/3179	NA	R01_cb8564_c87465/f2p9/3179	NA	R01_cb8564_c87465/f2p9/3179
R01_cb2997_c2/f1p0/3744	NA	NA	NA	R01_cb2997_c2/f1p0/3744
R01_cb5304_c19/f1p0/2807	NA	NA	NA	R01_cb5304_c19/f1p0/2807
R01_cb8564_c117574/f1p0/2644	NA	NA	NA	R01_cb8564_c117574/f1p0/2644
R01_cb8564_c17846/f2p0/3233	NA	NA	NA	R01_cb8564_c17846/f2p0/3233
R01_cb2804_c110/f1p0/2555	NA	R01_cb2804_c110/f1p0/2555	R01_cb2804_c110/f1p0/2555	R01_cb2804_c110/f1p0/2555
R01_cb8564_c116123/f1p0/2206	NA	NA	R01_cb8564_c116123/f1p0/2206	R01_cb8564_c116123/f1p0/2206
R01_cb8564_c123301/f1p0/247	R01_cb8564_c123301/f1p0/24	R01_cb8564_c123301/f1p0/247	R01_cb8564_c123301/f1p0/247	R01_cb8564_c123301/f1p0/247

5	75	5	5	5
R01_cb18456_c5432/f1p0/1276	NA	R01_cb18456_c5432/f1p0/1276	NA	R01_cb18456_c5432/f1p0/1276
R01_cb5042_c2/f1p0/2318	NA	NA	R01_cb5042_c2/f1p0/2318	R01_cb5042_c2/f1p0/2318
R01_cb13057_c4/f1p0/962	NA	NA	NA	R01_cb13057_c4/f1p0/962
R01_cb13026_c5/f1p0/775	NA	R01_cb13026_c5/f1p0/775	NA	R01_cb13026_c5/f1p0/775
R01_cb18456_c6072/f1p0/370	R01_cb18456_c6072/f1p0/370	R01_cb18456_c6072/f1p0/370	R01_cb18456_c6072/f1p0/370	R01_cb18456_c6072/f1p0/370
R01_cb3931_c7/f1p0/694	NA	NA	NA	R01_cb3931_c7/f1p0/694
R01_cb16813_c1/f1p0/372	R01_cb16813_c1/f1p0/372	R01_cb16813_c1/f1p0/372	R01_cb16813_c1/f1p0/372	R01_cb16813_c1/f1p0/372
R01_cb1283_c5/f1p0/3409	NA	NA	NA	R01_cb1283_c5/f1p0/3409
R01_cb8564_c24039/f1p0/1907	NA	R01_cb8564_c24039/f1p0/1907	NA	NA
R01_cb2451_c12/f1p0/3730	NA	NA	NA	R01_cb2451_c12/f1p0/3730
R01_cb6352_c9/f1p0/1956	NA	R01_cb6352_c9/f1p0/1956	NA	R01_cb6352_c9/f1p0/1956
R01_cb6947_c6/f2p0/2118	NA	R01_cb6947_c6/f2p0/2118	NA	R01_cb6947_c6/f2p0/2118
R01_cb15811_c11/f1p0/750	NA	R01_cb15811_c11/f1p0/750	R01_cb15811_c11/f1p0/750	R01_cb15811_c11/f1p0/750
R01_cb10800_c1/f1p0/1522	NA	NA	NA	R01_cb10800_c1/f1p0/1522
R01_cb8564_c18575/f1p0/1939	NA	R01_cb8564_c18575/f1p0/1939	R01_cb8564_c18575/f1p0/1939	R01_cb8564_c18575/f1p0/1939
R01_cb7515_c10/f1p0/2808	NA	NA	NA	R01_cb7515_c10/f1p0/2808
R01_cb18456_c1340/f1p0/507	R01_cb18456_c1340/f1p0/507	R01_cb18456_c1340/f1p0/507	R01_cb18456_c1340/f1p0/507	R01_cb18456_c1340/f1p0/507
R01_cb2048_c13/f1p0/1293	R01_cb2048_c13/f1p0/1293	R01_cb2048_c13/f1p0/1293	NA	NA
R01_cb4676_c3/f1p0/709	R01_cb4676_c3/f1p0/709	R01_cb4676_c3/f1p0/709	R01_cb4676_c3/f1p0/709	R01_cb4676_c3/f1p0/709
R01_cb10865_c3/f1p0/1384	NA	NA	NA	R01_cb10865_c3/f1p0/1384
R01_cb750_c69/f1p0/899	R01_cb750_c69/f1p0/899	R01_cb750_c69/f1p0/899	R01_cb750_c69/f1p0/899	R01_cb750_c69/f1p0/899
R01_cb5552_c0/f2p0/3064	NA	NA	NA	R01_cb5552_c0/f2p0/3064
R01_cb4054_c1/f1p0/3451	NA	R01_cb4054_c1/f1p0/3451	NA	NA
R01_cb15318_c6/f2p3/600	NA	NA	NA	R01_cb15318_c6/f2p3/600
R01_cb8564_c122719/f1p0/257	NA	NA	NA	R01_cb8564_c122719/f1p0/257

9				9
R01_cb14217_c3/f1p0/876	NA	R01_cb14217_c3/f1p0/876	R01_cb14217_c3/f1p0/876	R01_cb14217_c3/f1p0/876
R01_cb1856_c3/f1p0/3033	NA	R01_cb1856_c3/f1p0/3033	NA	R01_cb1856_c3/f1p0/3033
R01_cb6636_c4/f1p0/1118	NA	NA	NA	R01_cb6636_c4/f1p0/1118
R01_cb16182_c4/f1p0/1615	NA	NA	NA	R01_cb16182_c4/f1p0/1615
R01_cb15882_c2/f1p0/1003	NA	R01_cb15882_c2/f1p0/1003	NA	R01_cb15882_c2/f1p0/1003
R01_cb16341_c4/f1p1/545	R01_cb16341_c4/f1p1/545	R01_cb16341_c4/f1p1/545	R01_cb16341_c4/f1p1/545	R01_cb16341_c4/f1p1/545
R01_cb9995_c0/f1p0/1864	NA	R01_cb9995_c0/f1p0/1864	NA	NA
R01_cb14552_c3/f1p0/1689	NA	R01_cb14552_c3/f1p0/1689	NA	NA
R01_cb8564_c119861/f1p0/2226	NA	R01_cb8564_c119861/f1p0/2226	R01_cb8564_c119861/f1p0/2226	R01_cb8564_c119861/f1p0/2226
R01_cb8564_c18488/f1p0/3855	NA	R01_cb8564_c18488/f1p0/3855	NA	NA
R01_cb10808_c0/f1p0/1353	NA	R01_cb10808_c0/f1p0/1353	NA	NA
R01_cb5229_c10/f1p1/603	NA	NA	R01_cb5229_c10/f1p1/603	R01_cb5229_c10/f1p1/603
R01_cb2698_c1/f1p0/3833	NA	NA	NA	R01_cb2698_c1/f1p0/3833
R01_cb10155_c4/f1p0/2424	NA	R01_cb10155_c4/f1p0/2424	NA	NA
R01_cb8706_c6/f1p0/2039	R01_cb8706_c6/f1p0/2039	R01_cb8706_c6/f1p0/2039	NA	R01_cb8706_c6/f1p0/2039
R01_cb17187_c1/f2p0/614	R01_cb17187_c1/f2p0/614	R01_cb17187_c1/f2p0/614	NA	NA
R01_cb13044_c2/f1p0/579	NA	R01_cb13044_c2/f1p0/579	NA	NA
R01_cb8564_c75707/f1p1/4363	NA	NA	NA	R01_cb8564_c75707/f1p1/4363
R01_cb2302_c2/f1p0/3985	NA	R01_cb2302_c2/f1p0/3985	NA	NA
R01_cb12114_c17/f1p0/944	R01_cb12114_c17/f1p0/944	NA	NA	NA
R01_cb8564_c4906/f1p0/2813	NA	NA	NA	R01_cb8564_c4906/f1p0/2813
R01_cb11684_c0/f1p0/1044	NA	R01_cb11684_c0/f1p0/1044	NA	NA
R01_cb8564_c86964/f1p0/2501	R01_cb8564_c86964/f1p0/2501	R01_cb8564_c86964/f1p0/2501	NA	R01_cb8564_c86964/f1p0/2501

R01_cb2055_c71/f1p1/415	R01_cb2055_c71/f1p1/415	R01_cb2055_c71/f1p1/415	R01_cb2055_c71/f1p1/415	R01_cb2055_c71/f1p1/415
R01_cb5900_c132/f1p0/2808	NA	R01_cb5900_c132/f1p0/2808	NA	R01_cb5900_c132/f1p0/2808
R01_cb7777_c1/f1p0/2534	NA	R01_cb7777_c1/f1p0/2534	NA	NA
R01_cb7620_c0/f1p0/2577	NA	R01_cb7620_c0/f1p0/2577	NA	NA
R01_cb8564_c90127/f1p0/3554	NA	R01_cb8564_c90127/f1p0/3554	NA	NA
R01_cb8564_c4630/f1p1/3004	NA	R01_cb8564_c4630/f1p1/3004	R01_cb8564_c4630/f1p1/3004	R01_cb8564_c4630/f1p1/3004
R01_cb16587_c1/f1p0/589	NA	R01_cb16587_c1/f1p0/589	NA	NA
R01_cb10448_c4/f1p0/1242	R01_cb10448_c4/f1p0/1242	R01_cb10448_c4/f1p0/1242	NA	NA
R01_cb8564_c18034/f1p0/4267	NA	R01_cb8564_c18034/f1p0/4267	NA	NA
R01_cb8564_c73937/f2p2/2133	NA	R01_cb8564_c73937/f2p2/2133	R01_cb8564_c73937/f2p2/2133	R01_cb8564_c73937/f2p2/2133
R01_cb8564_c13688/f1p0/2489	NA	R01_cb8564_c13688/f1p0/2489	NA	R01_cb8564_c13688/f1p0/2489
R01_cb4490_c14/f1p0/2307	NA	NA	NA	R01_cb4490_c14/f1p0/2307
R01_cb5032_c3/f1p0/1686	NA	R01_cb5032_c3/f1p0/1686	NA	R01_cb5032_c3/f1p0/1686
R01_cb2445_c21/f1p0/3289	NA	NA	NA	R01_cb2445_c21/f1p0/3289
R01_cb4141_c0/f2p0/3159	NA	R01_cb4141_c0/f2p0/3159	NA	NA
R01_cb8564_c87314/f1p0/2163	NA	R01_cb8564_c87314/f1p0/2163	NA	R01_cb8564_c87314/f1p0/2163
R01_cb1993_c0/f2p1/3166	NA	NA	NA	R01_cb1993_c0/f2p1/3166
R01_cb10615_c0/f1p0/1078	NA	R01_cb10615_c0/f1p0/1078	NA	NA
R01_cb8564_c123948/f1p0/3409	NA	R01_cb8564_c123948/f1p0/3409	NA	NA
R01_cb8131_c1/f1p0/3086	NA	R01_cb8131_c1/f1p0/3086	NA	R01_cb8131_c1/f1p0/3086
R01_cb17721_c1/f1p0/930	NA	R01_cb17721_c1/f1p0/930	R01_cb17721_c1/f1p0/930	R01_cb17721_c1/f1p0/930
R01_cb12552_c0/f1p0/785	NA	NA	NA	R01_cb12552_c0/f1p0/785
R01_cb8564_c122847/f1p0/2777	NA	R01_cb8564_c122847/f1p0/2777	R01_cb8564_c122847/f1p0/2777	NA
R01_cb18581_c1/f1p0/958	NA	NA	NA	R01_cb18581_c1/f1p0/958

R01_cb6546_c2/f2p0/2676	NA	R01_cb6546_c2/f2p0/2676	NA	NA
R01_cb18138_c1/f1p0/1771	NA	NA	NA	R01_cb18138_c1/f1p0/1771
R01_cb8564_c42230/f1p0/4284	NA	R01_cb8564_c42230/f1p0/4284	NA	NA
R01_cb17572_c4/f1p0/802	NA	R01_cb17572_c4/f1p0/802	R01_cb17572_c4/f1p0/802	R01_cb17572_c4/f1p0/802
R01_cb8515_c2/f1p0/2544	NA	NA	NA	R01_cb8515_c2/f1p0/2544
R01_cb17700_c0/f1p0/1131	NA	R01_cb17700_c0/f1p0/1131	R01_cb17700_c0/f1p0/1131	R01_cb17700_c0/f1p0/1131
R01_cb6805_c1/f1p0/2205	NA	NA	NA	R01_cb6805_c1/f1p0/2205
R01_cb3582_c3/f1p0/3545	NA	R01_cb3582_c3/f1p0/3545	NA	R01_cb3582_c3/f1p0/3545
R01_cb9364_c0/f3p0/2104	NA	NA	NA	R01_cb9364_c0/f3p0/2104
R01_cb69_c14/f1p0/3614	NA	NA	NA	R01_cb69_c14/f1p0/3614
R01_cb6004_c7/f1p1/1878	NA	NA	NA	R01_cb6004_c7/f1p1/1878
R01_cb14937_c1/f1p0/807	NA	R01_cb14937_c1/f1p0/807	R01_cb14937_c1/f1p0/807	R01_cb14937_c1/f1p0/807
R01_cb15515_c0/f2p0/1814	R01_cb15515_c0/f2p0/1814	R01_cb15515_c0/f2p0/1814	R01_cb15515_c0/f2p0/1814	R01_cb15515_c0/f2p0/1814
R01_cb14524_c10/f1p0/1106	NA	R01_cb14524_c10/f1p0/1106	NA	NA
R01_cb5258_c3/f1p0/1036	NA	NA	NA	R01_cb5258_c3/f1p0/1036
R01_cb14878_c1/f1p0/744	NA	R01_cb14878_c1/f1p0/744	R01_cb14878_c1/f1p0/744	R01_cb14878_c1/f1p0/744
R01_cb8991_c6/f1p0/2524	NA	NA	NA	R01_cb8991_c6/f1p0/2524
R01_cb8564_c77107/f1p0/2107	R01_cb8564_c77107/f1p0/2107	R01_cb8564_c77107/f1p0/2107	R01_cb8564_c77107/f1p0/2107	R01_cb8564_c77107/f1p0/2107
R01_cb12465_c7/f1p0/516	NA	R01_cb12465_c7/f1p0/516	R01_cb12465_c7/f1p0/516	R01_cb12465_c7/f1p0/516
R01_cb8564_c89153/f1p0/3105	R01_cb8564_c89153/f1p0/3105	R01_cb8564_c89153/f1p0/3105	NA	R01_cb8564_c89153/f1p0/3105
R01_cb4637_c9/f2p0/827	R01_cb4637_c9/f2p0/827	NA	NA	NA
R01_cb7654_c2/f1p0/2492	NA	R01_cb7654_c2/f1p0/2492	NA	R01_cb7654_c2/f1p0/2492
R01_cb8564_c35468/f1p0/4536	NA	R01_cb8564_c35468/f1p0/4536	NA	NA
R01_cb947_c44/f1p0/957	NA	NA	NA	R01_cb947_c44/f1p0/957

R01_cb8564_c1106/f1p0/2055	NA	NA	R01_cb8564_c1106/f1p0/2055	NA
R01_cb6953_c5/f1p0/1068	R01_cb6953_c5/f1p0/1068	R01_cb6953_c5/f1p0/1068	R01_cb6953_c5/f1p0/1068	R01_cb6953_c5/f1p0/1068
R01_cb9630_c36/f1p0/2967	NA	R01_cb9630_c36/f1p0/2967	NA	NA
R01_cb18456_c7608/f1p0/1780	NA	NA	NA	R01_cb18456_c7608/f1p0/1780
R01_cb7741_c13/f7p0/574	R01_cb7741_c13/f7p0/574	R01_cb7741_c13/f7p0/574	R01_cb7741_c13/f7p0/574	R01_cb7741_c13/f7p0/574
R01_cb8564_c74578/f1p0/3393	NA	NA	NA	R01_cb8564_c74578/f1p0/3393
R01_cb9901_c5/f1p0/1372	NA	R01_cb9901_c5/f1p0/1372	NA	NA
R01_cb18429_c0/f1p0/796	NA	R01_cb18429_c0/f1p0/796	NA	NA
R01_cb8564_c45829/f1p0/2882	NA	R01_cb8564_c45829/f1p0/2882	NA	R01_cb8564_c45829/f1p0/2882
R01_cb3017_c4/f1p0/2611	R01_cb3017_c4/f1p0/2611	R01_cb3017_c4/f1p0/2611	R01_cb3017_c4/f1p0/2611	R01_cb3017_c4/f1p0/2611
R01_cb9982_c0/f2p0/1901	NA	R01_cb9982_c0/f2p0/1901	NA	R01_cb9982_c0/f2p0/1901
R01_cb8564_c1998/f1p0/2561	NA	R01_cb8564_c1998/f1p0/2561	NA	NA
R01_cb4166_c2/f1p0/3422	NA	R01_cb4166_c2/f1p0/3422	NA	NA
R01_cb8564_c3271/f1p0/2435	R01_cb8564_c3271/f1p0/2435	R01_cb8564_c3271/f1p0/2435	NA	NA
R01_cb8564_c70449/f1p0/2914	NA	NA	R01_cb8564_c70449/f1p0/2914	NA
R01_cb7064_c11/f1p0/985	R01_cb7064_c11/f1p0/985	R01_cb7064_c11/f1p0/985	R01_cb7064_c11/f1p0/985	R01_cb7064_c11/f1p0/985
R01_cb18335_c0/f1p0/1689	R01_cb18335_c0/f1p0/1689	R01_cb18335_c0/f1p0/1689	R01_cb18335_c0/f1p0/1689	R01_cb18335_c0/f1p0/1689
R01_cb7087_c6/f1p0/2226	NA	R01_cb7087_c6/f1p0/2226	NA	R01_cb7087_c6/f1p0/2226
R01_cb9535_c5/f9p1/1843	NA	R01_cb9535_c5/f9p1/1843	R01_cb9535_c5/f9p1/1843	R01_cb9535_c5/f9p1/1843
R01_cb5428_c0/f1p0/3109	R01_cb5428_c0/f1p0/3109	R01_cb5428_c0/f1p0/3109	NA	NA
R01_cb18456_c5723/f1p8/805	NA	R01_cb18456_c5723/f1p8/805	NA	NA
R01_cb16192_c2/f1p0/1142	NA	R01_cb16192_c2/f1p0/1142	NA	R01_cb16192_c2/f1p0/1142
R01_cb18456_c6507/f1p0/832	NA	R01_cb18456_c6507/f1p0/832	NA	NA
R01_cb10024_c208/f1p0/959	NA	NA	R01_cb10024_c208/f1p0/959	NA
R01_cb8564_c71010/f1p0/3699	NA	R01_cb8564_c71010/f1p0/3699	NA	NA
R01_cb7645_c2/f1p0/2329	R01_cb7645_c2/f1p0/2329	R01_cb7645_c2/f1p0/2329	R01_cb7645_c2/f1p0/2329	R01_cb7645_c2/f1p0/2329

R01_cb6768_c4/f1p0/2585	NA	R01_cb6768_c4/f1p0/2585	NA	NA
R01_cb13452_c0/f1p0/1061	NA	NA	NA	R01_cb13452_c0/f1p0/1061
R01_cb17470_c0/f1p0/1257	NA	R01_cb17470_c0/f1p0/1257	NA	NA
R01_cb18456_c1510/f3p0/589	NA	R01_cb18456_c1510/f3p0/589	R01_cb18456_c1510/f3p0/589	R01_cb18456_c1510/f3p0/589
R01_cb6615_c21/f1p0/1151	NA	NA	NA	R01_cb6615_c21/f1p0/1151
R01_cb15804_c3/f1p0/977	NA	NA	NA	R01_cb15804_c3/f1p0/977
R01_cb17657_c0/f2p0/881	NA	NA	NA	R01_cb17657_c0/f2p0/881
R01_cb8564_c35607/f1p0/2587	NA	R01_cb8564_c35607/f1p0/2587	NA	R01_cb8564_c35607/f1p0/2587
R01_cb18456_c7614/f1p0/956	NA	R01_cb18456_c7614/f1p0/956	NA	NA
R01_cb18456_c6902/f1p0/629	R01_cb18456_c6902/f1p0/629	R01_cb18456_c6902/f1p0/629	NA	R01_cb18456_c6902/f1p0/629
R01_cb14574_c1/f1p0/462	R01_cb14574_c1/f1p0/462	R01_cb14574_c1/f1p0/462	NA	R01_cb14574_c1/f1p0/462
R01_cb8827_c4/f1p0/3061	NA	R01_cb8827_c4/f1p0/3061	NA	NA
R01_cb8564_c84179/f1p0/4163	NA	R01_cb8564_c84179/f1p0/4163	NA	NA
R01_cb2770_c71/f1p0/2441	NA	R01_cb2770_c71/f1p0/2441	R01_cb2770_c71/f1p0/2441	NA
R01_cb18409_c95/f1p0/942	R01_cb18409_c95/f1p0/942	R01_cb18409_c95/f1p0/942	R01_cb18409_c95/f1p0/942	R01_cb18409_c95/f1p0/942
R01_cb679_c13/f1p0/2623	R01_cb679_c13/f1p0/2623	R01_cb679_c13/f1p0/2623	R01_cb679_c13/f1p0/2623	NA
R01_cb8564_c79368/f1p0/2361	NA	R01_cb8564_c79368/f1p0/2361	NA	NA
R01_cb17871_c3/f1p0/857	NA	R01_cb17871_c3/f1p0/857	NA	NA
R01_cb8564_c70508/f1p0/2348	NA	R01_cb8564_c70508/f1p0/2348	NA	NA
R01_cb5808_c4/f1p0/1226	NA	NA	NA	R01_cb5808_c4/f1p0/1226
R01_cb5756_c2/f1p0/1667	R01_cb5756_c2/f1p0/1667	R01_cb5756_c2/f1p0/1667	R01_cb5756_c2/f1p0/1667	R01_cb5756_c2/f1p0/1667
R01_cb391_c5/f1p0/3775	NA	NA	NA	R01_cb391_c5/f1p0/3775
R01_cb17358_c1/f1p0/1623	NA	NA	NA	R01_cb17358_c1/f1p0/1623
R01_cb10436_c4/f1p1/2632	NA	R01_cb10436_c4/f1p1/2632	NA	NA
R01_cb5338_c3/f1p0/1722	NA	R01_cb5338_c3/f1p0/1722	NA	NA
R01_cb16274_c2/f1p0/776	NA	R01_cb16274_c2/f1p0/776	NA	NA

R01_cb8564_c4572/f1p0/2292	NA	R01_cb8564_c4572/f1p0/2292	NA	R01_cb8564_c4572/f1p0/2292
R01_cb17610_c4/f1p0/1359	NA	R01_cb17610_c4/f1p0/1359	NA	R01_cb17610_c4/f1p0/1359
R01_cb8564_c35349/f1p0/2576	R01_cb8564_c35349/f1p0/2576	R01_cb8564_c35349/f1p0/2576	R01_cb8564_c35349/f1p0/2576	R01_cb8564_c35349/f1p0/2576
R01_cb13247_c3/f1p0/450	R01_cb13247_c3/f1p0/450	R01_cb13247_c3/f1p0/450	R01_cb13247_c3/f1p0/450	R01_cb13247_c3/f1p0/450
R01_cb13771_c5/f1p0/1292	NA	R01_cb13771_c5/f1p0/1292	R01_cb13771_c5/f1p0/1292	R01_cb13771_c5/f1p0/1292
R01_cb2367_c0/f1p0/3959	NA	R01_cb2367_c0/f1p0/3959	NA	NA
R01_cb15590_c1/f1p0/679	NA	R01_cb15590_c1/f1p0/679	NA	NA
R01_cb119_c37/f1p0/2258	NA	NA	NA	R01_cb119_c37/f1p0/2258
R01_cb8564_c1799/f2p0/3268	NA	R01_cb8564_c1799/f2p0/3268	NA	NA
R01_cb17973_c7/f2p0/585	R01_cb17973_c7/f2p0/585	R01_cb17973_c7/f2p0/585	R01_cb17973_c7/f2p0/585	R01_cb17973_c7/f2p0/585
R01_cb15772_c2/f1p0/810	NA	NA	R01_cb15772_c2/f1p0/810	R01_cb15772_c2/f1p0/810
R01_cb14924_c8/f1p0/923	NA	R01_cb14924_c8/f1p0/923	R01_cb14924_c8/f1p0/923	R01_cb14924_c8/f1p0/923
R01_cb8803_c1/f1p0/2386	NA	R01_cb8803_c1/f1p0/2386	NA	NA
R01_cb8564_c35539/f1p0/4052	NA	R01_cb8564_c35539/f1p0/4052	NA	R01_cb8564_c35539/f1p0/4052
R01_cb16351_c0/f2p0/569	NA	R01_cb16351_c0/f2p0/569	R01_cb16351_c0/f2p0/569	R01_cb16351_c0/f2p0/569
R01_cb3292_c13/f1p0/3135	NA	R01_cb3292_c13/f1p0/3135	NA	NA
R01_cb380_c15/f1p0/3104	NA	R01_cb380_c15/f1p0/3104	NA	NA
R01_cb18456_c2279/f1p1/622	NA	R01_cb18456_c2279/f1p1/622	NA	NA
R01_cb14341_c2/f1p0/644	R01_cb14341_c2/f1p0/644	R01_cb14341_c2/f1p0/644	R01_cb14341_c2/f1p0/644	R01_cb14341_c2/f1p0/644
R01_cb7497_c21/f9p0/2537	NA	NA	NA	R01_cb7497_c21/f9p0/2537
R01_cb4819_c2/f1p0/3115	NA	R01_cb4819_c2/f1p0/3115	NA	NA
R01_cb2378_c23/f1p0/3951	NA	NA	NA	R01_cb2378_c23/f1p0/3951
R01_cb14882_c8/f1p0/1272	NA	NA	NA	R01_cb14882_c8/f1p0/1272
R01_cb8564_c88607/f1p0/3417	R01_cb8564_c88607/f1p0/3417	R01_cb8564_c88607/f1p0/3417	R01_cb8564_c88607/f1p0/3417	R01_cb8564_c88607/f1p0/3417

R01_cb15796_c3/f1p0/1454	NA	NA	NA	R01_cb15796_c3/f1p0/1454
R01_cb3390_c14/f1p0/1861	NA	R01_cb3390_c14/f1p0/1861	NA	NA
R01_cb10261_c3/f1p0/2105	NA	R01_cb10261_c3/f1p0/2105	NA	NA
R01_cb11448_c2/f1p0/1813	NA	R01_cb11448_c2/f1p0/1813	NA	NA
R01_cb15647_c3/f1p0/1516	NA	NA	NA	R01_cb15647_c3/f1p0/1516
R01_cb11314_c3/f1p0/900	NA	R01_cb11314_c3/f1p0/900	NA	R01_cb11314_c3/f1p0/900
R01_cb8037_c2/f1p0/2480	NA	NA	NA	R01_cb8037_c2/f1p0/2480
R01_cb16662_c2/f1p0/415	R01_cb16662_c2/f1p0/415	R01_cb16662_c2/f1p0/415	R01_cb16662_c2/f1p0/415	R01_cb16662_c2/f1p0/415
R01_cb18456_c7521/f1p0/613	R01_cb18456_c7521/f1p0/613	R01_cb18456_c7521/f1p0/613	R01_cb18456_c7521/f1p0/613	R01_cb18456_c7521/f1p0/613
R01_cb1839_c7/f1p0/1962	NA	R01_cb1839_c7/f1p0/1962	NA	R01_cb1839_c7/f1p0/1962
R01_cb5896_c57/f1p0/1933	NA	NA	NA	R01_cb5896_c57/f1p0/1933
R01_cb293_c4/f1p0/3063	NA	NA	NA	R01_cb293_c4/f1p0/3063
R01_cb2844_c4/f1p0/3076	NA	NA	NA	R01_cb2844_c4/f1p0/3076
R01_cb6399_c12/f1p0/546	R01_cb6399_c12/f1p0/546	R01_cb6399_c12/f1p0/546	NA	NA
R01_cb10587_c5/f1p0/1000	NA	R01_cb10587_c5/f1p0/1000	R01_cb10587_c5/f1p0/1000	R01_cb10587_c5/f1p0/1000
R01_cb14148_c2/f1p0/1258	NA	NA	R01_cb14148_c2/f1p0/1258	R01_cb14148_c2/f1p0/1258
R01_cb9445_c2/f1p0/1972	NA	R01_cb9445_c2/f1p0/1972	R01_cb9445_c2/f1p0/1972	R01_cb9445_c2/f1p0/1972
R01_cb12092_c9/f1p0/1052	NA	R01_cb12092_c9/f1p0/1052	NA	NA
R01_cb18456_c2534/f1p0/1292	NA	R01_cb18456_c2534/f1p0/1292	NA	NA
R01_cb6655_c4/f1p0/443	R01_cb6655_c4/f1p0/443	R01_cb6655_c4/f1p0/443	R01_cb6655_c4/f1p0/443	R01_cb6655_c4/f1p0/443
R01_cb16760_c0/f6p0/388	R01_cb16760_c0/f6p0/388	R01_cb16760_c0/f6p0/388	R01_cb16760_c0/f6p0/388	R01_cb16760_c0/f6p0/388
R01_cb8981_c5/f1p0/1690	NA	R01_cb8981_c5/f1p0/1690	NA	R01_cb8981_c5/f1p0/1690
R01_cb16038_c0/f2p0/505	NA	R01_cb16038_c0/f2p0/505	R01_cb16038_c0/f2p0/505	R01_cb16038_c0/f2p0/505
R01_cb18409_c52/f1p0/594	R01_cb18409_c52/f1p0/594	R01_cb18409_c52/f1p0/594	R01_cb18409_c52/f1p0/594	R01_cb18409_c52/f1p0/594
R01_cb2298_c4/f1p1/3154	NA	NA	NA	R01_cb2298_c4/f1p1/3154
R01_cb8564_c115764/f1p0/262	NA	R01_cb8564_c115764/f1p0/262	NA	NA

6		6		
R01_cb8564_c37220/f1p2/4071	NA	NA	NA	R01_cb8564_c37220/f1p2/4071
R01_cb2445_c11/f1p0/3504	NA	NA	NA	R01_cb2445_c11/f1p0/3504
R01_cb7674_c1/f1p0/2563	NA	R01_cb7674_c1/f1p0/2563	NA	NA
R01_cb14317_c13/f1p0/1454	NA	R01_cb14317_c13/f1p0/1454	NA	NA
R01_cb6042_c1/f1p0/3073	NA	R01_cb6042_c1/f1p0/3073	NA	NA
R01_cb8564_c3333/f1p0/3954	NA	R01_cb8564_c3333/f1p0/3954	NA	NA
R01_cb405_c3/f1p1/3781	NA	R01_cb405_c3/f1p1/3781	NA	NA
R01_cb8104_c2/f1p0/2665	NA	NA	NA	R01_cb8104_c2/f1p0/2665
R01_cb3359_c5/f2p0/3265	NA	R01_cb3359_c5/f2p0/3265	NA	NA
R01_cb4582_c1/f1p0/3310	NA	R01_cb4582_c1/f1p0/3310	NA	NA
R01_cb14005_c1/f1p0/724	NA	R01_cb14005_c1/f1p0/724	R01_cb14005_c1/f1p0/724	NA
R01_cb737_c69/f1p0/1707	NA	R01_cb737_c69/f1p0/1707	NA	NA
R01_cb4123_c100/f1p0/1220	NA	R01_cb4123_c100/f1p0/1220	NA	NA
R01_cb10742_c9/f5p0/879	NA	NA	NA	R01_cb10742_c9/f5p0/879
R01_cb17046_c1/f1p0/1386	NA	NA	NA	R01_cb17046_c1/f1p0/1386
R01_cb7999_c1/f1p0/2485	R01_cb7999_c1/f1p0/2485	NA	NA	R01_cb7999_c1/f1p0/2485
R01_cb1132_c0/f1p0/4461	NA	R01_cb1132_c0/f1p0/4461	NA	NA
R01_cb18108_c0/f2p0/630	NA	NA	NA	R01_cb18108_c0/f2p0/630
R01_cb17118_c7/f1p0/1440	R01_cb17118_c7/f1p0/1440	R01_cb17118_c7/f1p0/1440	R01_cb17118_c7/f1p0/1440	R01_cb17118_c7/f1p0/1440
R01_cb7416_c10/f1p0/1320	NA	NA	NA	R01_cb7416_c10/f1p0/1320
R01_cb8564_c45849/f1p0/2741	NA	R01_cb8564_c45849/f1p0/2741	NA	NA
R01_cb8564_c54478/f1p0/2734	R01_cb8564_c54478/f1p0/2734	R01_cb8564_c54478/f1p0/2734	NA	NA
R01_cb8564_c70110/f1p0/2376	NA	R01_cb8564_c70110/f1p0/2376	NA	NA
R01_cb7089_c2/f1p0/2614	NA	R01_cb7089_c2/f1p0/2614	NA	NA

R01_cb8564_c14159/f1p0/4420	NA	NA	NA	R01_cb8564_c14159/f1p0/4420
R01_cb7303_c15/f1p0/2191	NA	NA	NA	R01_cb7303_c15/f1p0/2191
R01_cb6571_c3/f1p0/1651	NA	NA	NA	R01_cb6571_c3/f1p0/1651
R01_cb3359_c24/f1p0/3501	NA	R01_cb3359_c24/f1p0/3501	R01_cb3359_c24/f1p0/3501	R01_cb3359_c24/f1p0/3501
R01_cb4952_c6/f1p0/1530	NA	NA	NA	R01_cb4952_c6/f1p0/1530
R01_cb3444_c4/f1p0/2189	NA	NA	R01_cb3444_c4/f1p0/2189	R01_cb3444_c4/f1p0/2189
R01_cb8564_c76988/f1p0/3903	NA	R01_cb8564_c76988/f1p0/3903	NA	NA
R01_cb18189_c2/f1p0/302	R01_cb18189_c2/f1p0/302	R01_cb18189_c2/f1p0/302	NA	NA
R01_cb17756_c28/f1p0/632	NA	R01_cb17756_c28/f1p0/632	R01_cb17756_c28/f1p0/632	R01_cb17756_c28/f1p0/632
R01_cb8564_c75254/f1p0/2642	NA	NA	NA	R01_cb8564_c75254/f1p0/2642
R01_cb261_c52/f1p0/1878	NA	NA	NA	R01_cb261_c52/f1p0/1878
R01_cb18301_c41/f1p0/1112	NA	R01_cb18301_c41/f1p0/1112	R01_cb18301_c41/f1p0/1112	R01_cb18301_c41/f1p0/1112
R01_cb8475_c4/f1p0/1264	NA	R01_cb8475_c4/f1p0/1264	NA	NA
R01_cb16415_c1/f1p0/853	NA	R01_cb16415_c1/f1p0/853	NA	NA
R01_cb18456_c7580/f1p0/1737	NA	R01_cb18456_c7580/f1p0/1737	NA	NA
R01_cb5271_c16/f1p0/1936	NA	NA	NA	R01_cb5271_c16/f1p0/1936
R01_cb13185_c0/f1p0/1751	NA	NA	NA	R01_cb13185_c0/f1p0/1751
R01_cb110_c35/f1p0/5257	NA	NA	NA	R01_cb110_c35/f1p0/5257
R01_cb4737_c2/f1p0/3452	NA	NA	NA	R01_cb4737_c2/f1p0/3452
R01_cb1077_c12/f1p0/3339	NA	R01_cb1077_c12/f1p0/3339	NA	NA
R01_cb8564_c1008/f1p0/2295	R01_cb8564_c1008/f1p0/2295	R01_cb8564_c1008/f1p0/2295	NA	NA
R01_cb18456_c1870/f1p0/366	R01_cb18456_c1870/f1p0/366	R01_cb18456_c1870/f1p0/366	R01_cb18456_c1870/f1p0/366	R01_cb18456_c1870/f1p0/366
R01_cb9189_c4/f1p0/2159	NA	R01_cb9189_c4/f1p0/2159	NA	NA
R01_cb15066_c1/f1p1/726	NA	R01_cb15066_c1/f1p1/726	NA	NA
R01_cb12094_c29/f1p0/5160	NA	R01_cb12094_c29/f1p0/5160	NA	NA
R01_cb7484_c2/f1p0/2401	NA	R01_cb7484_c2/f1p0/2401	NA	R01_cb7484_c2/f1p0/2401

R01_cb7362_c10/f1p0/2570	NA	R01_cb7362_c10/f1p0/2570	NA	NA
R01_cb7168_c15/f1p0/2247	NA	NA	NA	R01_cb7168_c15/f1p0/2247
R01_cb1221_c0/f1p0/4421	NA	NA	NA	R01_cb1221_c0/f1p0/4421
R01_cb8564_c1524/f1p0/2360	R01_cb8564_c1524/f1p0/2360	R01_cb8564_c1524/f1p0/2360	R01_cb8564_c1524/f1p0/2360	R01_cb8564_c1524/f1p0/2360
R01_cb11778_c1/f1p0/2667	NA	NA	R01_cb11778_c1/f1p0/2667	NA
R01_cb730_c0/f1p0/4659	NA	R01_cb730_c0/f1p0/4659	NA	R01_cb730_c0/f1p0/4659
R01_cb1340_c2/f1p0/3014	NA	R01_cb1340_c2/f1p0/3014	NA	NA
R01_cb10188_c5/f1p0/2275	R01_cb10188_c5/f1p0/2275	R01_cb10188_c5/f1p0/2275	NA	NA
R01_cb8564_c3042/f1p1/2663	NA	NA	NA	R01_cb8564_c3042/f1p1/2663
R01_cb18181_c1/f1p0/1292	NA	NA	NA	R01_cb18181_c1/f1p0/1292
R01_cb110_c26/f1p1/2308	NA	NA	NA	R01_cb110_c26/f1p1/2308
R01_cb6365_c8/f1p1/2052	NA	R01_cb6365_c8/f1p1/2052	NA	NA
R01_cb17799_c1/f1p0/657	NA	R01_cb17799_c1/f1p0/657	NA	R01_cb17799_c1/f1p0/657
R01_cb10539_c3/f1p0/1144	NA	NA	NA	R01_cb10539_c3/f1p0/1144
R01_cb6974_c1/f1p0/2508	NA	R01_cb6974_c1/f1p0/2508	NA	R01_cb6974_c1/f1p0/2508
R01_cb17973_c45/f1p0/1248	NA	NA	R01_cb17973_c45/f1p0/1248	NA
R01_cb4493_c1/f5p2/4262	NA	NA	NA	R01_cb4493_c1/f5p2/4262
R01_cb9196_c8/f1p0/958	R01_cb9196_c8/f1p0/958	R01_cb9196_c8/f1p0/958	R01_cb9196_c8/f1p0/958	R01_cb9196_c8/f1p0/958
R01_cb5903_c5/f1p0/2158	NA	R01_cb5903_c5/f1p0/2158	NA	R01_cb5903_c5/f1p0/2158
R01_cb1533_c10/f1p0/861	R01_cb1533_c10/f1p0/861	R01_cb1533_c10/f1p0/861	NA	R01_cb1533_c10/f1p0/861
R01_cb17610_c7/f2p0/1728	NA	R01_cb17610_c7/f2p0/1728	NA	R01_cb17610_c7/f2p0/1728
R01_cb11113_c1/f1p0/2246	NA	NA	NA	R01_cb11113_c1/f1p0/2246
R01_cb10015_c288/f1p0/705	R01_cb10015_c288/f1p0/705	R01_cb10015_c288/f1p0/705	R01_cb10015_c288/f1p0/705	NA
R01_cb13064_c1/f1p0/1748	NA	NA	NA	R01_cb13064_c1/f1p0/1748
R01_cb15136_c0/f1p0/1567	NA	NA	NA	R01_cb15136_c0/f1p0/1567
R01_cb8564_c69762/f2p0/4058	NA	R01_cb8564_c69762/f2p0/4058	NA	NA

R01_cb11648_c0/f1p0/1155	NA	NA	NA	R01_cb11648_c0/f1p0/1155
R01_cb15825_c6/f1p0/1524	NA	NA	R01_cb15825_c6/f1p0/1524	R01_cb15825_c6/f1p0/1524
R01_cb4444_c15/f1p0/1913	NA	R01_cb4444_c15/f1p0/1913	NA	NA
R01_cb9222_c17/f1p0/4814	NA	R01_cb9222_c17/f1p0/4814	NA	NA
R01_cb8564_c78005/f1p0/1973	NA	R01_cb8564_c78005/f1p0/1973	NA	NA
R01_cb8564_c4312/f1p0/2961	NA	NA	R01_cb8564_c4312/f1p0/2961	NA
R01_cb18456_c6820/f1p0/532	NA	R01_cb18456_c6820/f1p0/532	R01_cb18456_c6820/f1p0/532	NA
R01_cb11594_c1/f1p0/2093	NA	R01_cb11594_c1/f1p0/2093	NA	R01_cb11594_c1/f1p0/2093
R01_cb438_c11/f1p1/942	NA	R01_cb438_c11/f1p1/942	NA	R01_cb438_c11/f1p1/942
R01_cb8564_c71914/f1p0/2596	NA	R01_cb8564_c71914/f1p0/2596	R01_cb8564_c71914/f1p0/2596	R01_cb8564_c71914/f1p0/2596
R01_cb8260_c15/f1p0/893	R01_cb8260_c15/f1p0/893	R01_cb8260_c15/f1p0/893	R01_cb8260_c15/f1p0/893	R01_cb8260_c15/f1p0/893
R01_cb7592_c2/f1p0/2427	NA	R01_cb7592_c2/f1p0/2427	NA	NA
R01_cb10702_c1/f1p0/2594	NA	R01_cb10702_c1/f1p0/2594	R01_cb10702_c1/f1p0/2594	R01_cb10702_c1/f1p0/2594
R01_cb2104_c8/f1p0/1922	NA	NA	R01_cb2104_c8/f1p0/1922	R01_cb2104_c8/f1p0/1922
R01_cb6645_c0/f1p0/2792	NA	R01_cb6645_c0/f1p0/2792	R01_cb6645_c0/f1p0/2792	R01_cb6645_c0/f1p0/2792
R01_cb10719_c3/f1p0/1171	NA	R01_cb10719_c3/f1p0/1171	NA	NA
R01_cb1594_c15/f1p1/2480	NA	NA	NA	R01_cb1594_c15/f1p1/2480
R01_cb1835_c6/f1p0/1453	NA	R01_cb1835_c6/f1p0/1453	NA	R01_cb1835_c6/f1p0/1453
R01_cb8564_c14030/f1p1/2977	NA	R01_cb8564_c14030/f1p1/2977	R01_cb8564_c14030/f1p1/2977	R01_cb8564_c14030/f1p1/2977
R01_cb7612_c0/f1p1/2574	NA	NA	NA	R01_cb7612_c0/f1p1/2574
R01_cb11708_c1/f1p0/1947	NA	R01_cb11708_c1/f1p0/1947	NA	NA
R01_cb8564_c19611/f1p1/4575	NA	R01_cb8564_c19611/f1p1/4575	NA	R01_cb8564_c19611/f1p1/4575
R01_cb4809_c4/f1p0/3009	NA	NA	NA	R01_cb4809_c4/f1p0/3009
R01_cb3180_c0/f1p0/3698	NA	NA	NA	R01_cb3180_c0/f1p0/3698
R01_cb4964_c6/f1p1/6906	NA	R01_cb4964_c6/f1p1/6906	NA	NA
R01_cb13535_c10/f1p0/1440	NA	R01_cb13535_c10/f1p0/1440	NA	NA

R01_cb14971_c9/f1p0/681	R01_cb14971_c9/f1p0/681	NA	NA	R01_cb14971_c9/f1p0/681
R01_cb3426_c6/f1p0/3094	NA	NA	NA	R01_cb3426_c6/f1p0/3094
R01_cb6758_c8/f1p1/2488	NA	NA	NA	R01_cb6758_c8/f1p1/2488
R01_cb8564_c86664/f1p0/2934	NA	R01_cb8564_c86664/f1p0/2934	NA	NA
R01_cb8564_c34436/f1p2/4197	NA	NA	NA	R01_cb8564_c34436/f1p2/4197
R01_cb261_c20/f1p0/4244	NA	NA	NA	R01_cb261_c20/f1p0/4244
R01_cb8564_c91945/f1p0/4138	NA	R01_cb8564_c91945/f1p0/4138	R01_cb8564_c91945/f1p0/4138	R01_cb8564_c91945/f1p0/4138
R01_cb12152_c4/f1p0/413	NA	R01_cb12152_c4/f1p0/413	R01_cb12152_c4/f1p0/413	R01_cb12152_c4/f1p0/413
R01_cb8901_c6/f1p0/1695	NA	R01_cb8901_c6/f1p0/1695	R01_cb8901_c6/f1p0/1695	NA
R01_cb3750_c3/f4p2/2344	NA	NA	NA	R01_cb3750_c3/f4p2/2344
R01_cb5345_c5/f1p0/3016	R01_cb5345_c5/f1p0/3016	R01_cb5345_c5/f1p0/3016	R01_cb5345_c5/f1p0/3016	R01_cb5345_c5/f1p0/3016
R01_cb9274_c9/f1p0/2424	NA	R01_cb9274_c9/f1p0/2424	NA	NA
R01_cb8564_c1882/f1p2/3030	NA	NA	NA	R01_cb8564_c1882/f1p2/3030
R01_cb4233_c43/f1p0/3399	NA	R01_cb4233_c43/f1p0/3399	NA	R01_cb4233_c43/f1p0/3399
R01_cb6414_c0/f1p1/2869	NA	R01_cb6414_c0/f1p1/2869	R01_cb6414_c0/f1p1/2869	R01_cb6414_c0/f1p1/2869
R01_cb6865_c24/f1p0/1587	NA	R01_cb6865_c24/f1p0/1587	NA	NA
R01_cb4543_c2/f1p0/2903	NA	R01_cb4543_c2/f1p0/2903	R01_cb4543_c2/f1p0/2903	NA
R01_cb3234_c3/f1p0/2215	NA	R01_cb3234_c3/f1p0/2215	NA	NA
R01_cb8564_c126458/f1p0/2314	NA	R01_cb8564_c126458/f1p0/2314	NA	R01_cb8564_c126458/f1p0/2314
R01_cb7822_c5/f1p0/681	NA	NA	NA	R01_cb7822_c5/f1p0/681
R01_cb14708_c4/f1p0/1322	NA	R01_cb14708_c4/f1p0/1322	NA	NA
R01_cb4368_c15/f1p0/473	R01_cb4368_c15/f1p0/473	R01_cb4368_c15/f1p0/473	R01_cb4368_c15/f1p0/473	R01_cb4368_c15/f1p0/473
R01_cb8145_c2/f1p0/1890	NA	NA	NA	R01_cb8145_c2/f1p0/1890
R01_cb11972_c7/f1p0/736	NA	R01_cb11972_c7/f1p0/736	NA	R01_cb11972_c7/f1p0/736
R01_cb6645_c4/f1p0/1775	NA	NA	NA	R01_cb6645_c4/f1p0/1775

R01_cb8120_c4/f1p0/2175	NA	NA	NA	R01_cb8120_c4/f1p0/2175
R01_cb15792_c0/f2p0/1682	NA	R01_cb15792_c0/f2p0/1682	R01_cb15792_c0/f2p0/1682	R01_cb15792_c0/f2p0/1682
R01_cb8564_c111165/f1p0/2201	NA	R01_cb8564_c111165/f1p0/2201	R01_cb8564_c111165/f1p0/2201	R01_cb8564_c111165/f1p0/2201
R01_cb9723_c3/f1p0/1848	NA	NA	NA	R01_cb9723_c3/f1p0/1848
R01_cb16372_c0/f8p0/445	NA	NA	R01_cb16372_c0/f8p0/445	R01_cb16372_c0/f8p0/445
R01_cb8564_c77634/f1p0/4360	R01_cb8564_c77634/f1p0/4360	R01_cb8564_c77634/f1p0/4360	NA	NA
R01_cb1230_c7/f1p0/3618	NA	R01_cb1230_c7/f1p0/3618	R01_cb1230_c7/f1p0/3618	R01_cb1230_c7/f1p0/3618
R01_cb6442_c6/f1p0/2577	R01_cb6442_c6/f1p0/2577	R01_cb6442_c6/f1p0/2577	NA	NA
R01_cb10015_c583/f1p0/479	NA	R01_cb10015_c583/f1p0/479	R01_cb10015_c583/f1p0/479	R01_cb10015_c583/f1p0/479
R01_cb10230_c4/f1p0/435	NA	R01_cb10230_c4/f1p0/435	NA	NA
R01_cb16195_c0/f2p0/1323	NA	R01_cb16195_c0/f2p0/1323	NA	NA
R01_cb16746_c1/f1p0/532	NA	NA	R01_cb16746_c1/f1p0/532	R01_cb16746_c1/f1p0/532
R01_cb10015_c502/f1p0/548	NA	R01_cb10015_c502/f1p0/548	NA	NA
R01_cb11371_c3/f1p0/1425	NA	R01_cb11371_c3/f1p0/1425	NA	NA
R01_cb14532_c1/f1p0/996	NA	NA	NA	R01_cb14532_c1/f1p0/996
R01_cb8564_c110689/f1p0/3321	R01_cb8564_c110689/f1p0/3321	R01_cb8564_c110689/f1p0/3321	R01_cb8564_c110689/f1p0/3321	R01_cb8564_c110689/f1p0/3321
R01_cb9029_c2/f1p0/2126	NA	NA	NA	R01_cb9029_c2/f1p0/2126
R01_cb9433_c0/f1p0/2082	NA	R01_cb9433_c0/f1p0/2082	NA	NA
R01_cb16341_c6/f1p1/517	R01_cb16341_c6/f1p1/517	R01_cb16341_c6/f1p1/517	R01_cb16341_c6/f1p1/517	R01_cb16341_c6/f1p1/517
R01_cb12204_c2/f1p0/1237	NA	R01_cb12204_c2/f1p0/1237	R01_cb12204_c2/f1p0/1237	R01_cb12204_c2/f1p0/1237
R01_cb3613_c0/f1p0/3576	R01_cb3613_c0/f1p0/3576	R01_cb3613_c0/f1p0/3576	R01_cb3613_c0/f1p0/3576	R01_cb3613_c0/f1p0/3576
R01_cb1074_c1/f1p0/4377	R01_cb1074_c1/f1p0/4377	R01_cb1074_c1/f1p0/4377	R01_cb1074_c1/f1p0/4377	R01_cb1074_c1/f1p0/4377
R01_cb6973_c5/f1p0/2729	NA	R01_cb6973_c5/f1p0/2729	NA	NA

R01_cb8564_c75785/f1p0/2612	NA	R01_cb8564_c75785/f1p0/2612	R01_cb8564_c75785/f1p0/2612	R01_cb8564_c75785/f1p0/2612
R01_cb1739_c4/f1p0/3401	NA	NA	NA	R01_cb1739_c4/f1p0/3401
R01_cb8564_c36957/f1p0/3448	NA	R01_cb8564_c36957/f1p0/3448	NA	NA
R01_cb8564_c82013/f1p0/3323	NA	NA	NA	R01_cb8564_c82013/f1p0/3323
R01_cb8564_c15290/f1p0/4550	NA	R01_cb8564_c15290/f1p0/4550	NA	NA
R01_cb8564_c85370/f1p0/3037	NA	R01_cb8564_c85370/f1p0/3037	R01_cb8564_c85370/f1p0/3037	R01_cb8564_c85370/f1p0/3037
R01_cb8865_c2/f1p0/2211	NA	NA	NA	R01_cb8865_c2/f1p0/2211
R01_cb6280_c0/f1p0/2895	R01_cb6280_c0/f1p0/2895	NA	NA	NA
R01_cb4269_c7/f1p0/2973	NA	NA	NA	R01_cb4269_c7/f1p0/2973
R01_cb8564_c113188/f1p0/2625	NA	R01_cb8564_c113188/f1p0/2625	NA	NA
R01_cb17118_c1/f1p0/657	R01_cb17118_c1/f1p0/657	R01_cb17118_c1/f1p0/657	R01_cb17118_c1/f1p0/657	R01_cb17118_c1/f1p0/657
R01_cb2701_c0/f3p0/3296	NA	NA	NA	R01_cb2701_c0/f3p0/3296
R01_cb9577_c4/f1p0/1736	R01_cb9577_c4/f1p0/1736	R01_cb9577_c4/f1p0/1736	R01_cb9577_c4/f1p0/1736	R01_cb9577_c4/f1p0/1736
R01_cb12420_c3/f1p0/1535	NA	NA	NA	R01_cb12420_c3/f1p0/1535
R01_cb9270_c2/f1p0/2756	R01_cb9270_c2/f1p0/2756	R01_cb9270_c2/f1p0/2756	R01_cb9270_c2/f1p0/2756	NA
R01_cb3463_c10/f1p0/2833	NA	R01_cb3463_c10/f1p0/2833	NA	NA
R01_cb8564_c23230/f1p0/3818	NA	R01_cb8564_c23230/f1p0/3818	NA	NA
R01_cb12641_c24/f1p0/1080	NA	NA	NA	R01_cb12641_c24/f1p0/1080
R01_cb12070_c1/f1p0/501	NA	R01_cb12070_c1/f1p0/501	R01_cb12070_c1/f1p0/501	R01_cb12070_c1/f1p0/501
R01_cb2465_c1/f2p0/3700	NA	R01_cb2465_c1/f2p0/3700	NA	R01_cb2465_c1/f2p0/3700
R01_cb14627_c3/f1p0/772	NA	NA	R01_cb14627_c3/f1p0/772	R01_cb14627_c3/f1p0/772
R01_cb8564_c1004/f1p0/2033	R01_cb8564_c1004/f1p0/2033	R01_cb8564_c1004/f1p0/2033	NA	NA
R01_cb18221_c0/f1p0/1090	NA	NA	R01_cb18221_c0/f1p0/1090	R01_cb18221_c0/f1p0/1090
R01_cb2571_c28/f1p0/4193	NA	NA	NA	R01_cb2571_c28/f1p0/4193
R01_cb8356_c0/f1p0/2393	NA	R01_cb8356_c0/f1p0/2393	NA	NA

R01_cb2033_c12/f1p0/3978	NA	NA	NA	R01_cb2033_c12/f1p0/3978
R01_cb18456_c5449/f1p2/597	R01_cb18456_c5449/f1p2/597	R01_cb18456_c5449/f1p2/597	R01_cb18456_c5449/f1p2/597	R01_cb18456_c5449/f1p2/597
R01_cb18192_c3/f1p0/804	R01_cb18192_c3/f1p0/804	R01_cb18192_c3/f1p0/804	R01_cb18192_c3/f1p0/804	NA
R01_cb18119_c0/f1p0/430	NA	R01_cb18119_c0/f1p0/430	NA	R01_cb18119_c0/f1p0/430
R01_cb12593_c4/f1p0/974	NA	R01_cb12593_c4/f1p0/974	NA	NA
R01_cb8564_c90393/f1p0/2948	R01_cb8564_c90393/f1p0/2948	R01_cb8564_c90393/f1p0/2948	R01_cb8564_c90393/f1p0/2948	R01_cb8564_c90393/f1p0/2948
R01_cb17042_c1/f1p0/873	NA	NA	R01_cb17042_c1/f1p0/873	NA
R01_cb8564_c53318/f1p0/3075	NA	R01_cb8564_c53318/f1p0/3075	R01_cb8564_c53318/f1p0/3075	R01_cb8564_c53318/f1p0/3075
R01_cb9271_c0/f4p0/1721	NA	R01_cb9271_c0/f4p0/1721	NA	NA
R01_cb110_c0/f1p0/2226	NA	NA	NA	R01_cb110_c0/f1p0/2226
R01_cb14448_c1/f5p0/768	NA	NA	NA	R01_cb14448_c1/f5p0/768
R01_cb1258_c2/f1p1/3295	R01_cb1258_c2/f1p1/3295	R01_cb1258_c2/f1p1/3295	R01_cb1258_c2/f1p1/3295	R01_cb1258_c2/f1p1/3295
R01_cb1182_c0/f1p0/4439	NA	R01_cb1182_c0/f1p0/4439	NA	NA
R01_cb7841_c3/f1p0/3976	NA	R01_cb7841_c3/f1p0/3976	NA	R01_cb7841_c3/f1p0/3976
R01_cb11396_c1/f1p0/2506	NA	R01_cb11396_c1/f1p0/2506	NA	NA
R01_cb13433_c86/f1p0/2799	NA	R01_cb13433_c86/f1p0/2799	NA	NA
R01_cb11190_c1/f1p0/1730	NA	NA	NA	R01_cb11190_c1/f1p0/1730
R01_cb3791_c12/f1p0/1510	NA	NA	NA	R01_cb3791_c12/f1p0/1510
R01_cb6123_c11/f1p0/2465	NA	R01_cb6123_c11/f1p0/2465	NA	NA
R01_cb8064_c4/f1p0/1357	NA	R01_cb8064_c4/f1p0/1357	NA	NA
R01_cb3732_c24/f1p0/2226	NA	NA	NA	R01_cb3732_c24/f1p0/2226
R01_cb3359_c31/f1p0/2357	NA	R01_cb3359_c31/f1p0/2357	NA	R01_cb3359_c31/f1p0/2357
R01_cb2219_c5/f1p1/1918	NA	NA	NA	R01_cb2219_c5/f1p1/1918
R01_cb11117_c4/f1p1/1055	NA	R01_cb11117_c4/f1p1/1055	NA	NA
R01_cb15721_c0/f1p0/1424	NA	R01_cb15721_c0/f1p0/1424	NA	NA

R01_cb13028_c1/f2p0/809	NA	NA	NA	R01_cb13028_c1/f2p0/809
R01_cb9620_c0/f1p0/2029	R01_cb9620_c0/f1p0/2029	R01_cb9620_c0/f1p0/2029	NA	R01_cb9620_c0/f1p0/2029
R01_cb7498_c0/f1p0/2603	NA	R01_cb7498_c0/f1p0/2603	NA	NA
R01_cb13724_c7/f2p0/439	NA	NA	R01_cb13724_c7/f2p0/439	R01_cb13724_c7/f2p0/439
R01_cb4953_c2/f1p0/3215	R01_cb4953_c2/f1p0/3215	R01_cb4953_c2/f1p0/3215	NA	R01_cb4953_c2/f1p0/3215
R01_cb8564_c53289/f1p0/3265	NA	R01_cb8564_c53289/f1p0/3265	NA	NA
R01_cb12444_c46/f1p0/748	R01_cb12444_c46/f1p0/748	R01_cb12444_c46/f1p0/748	R01_cb12444_c46/f1p0/748	R01_cb12444_c46/f1p0/748
R01_cb8195_c4/f1p0/837	NA	R01_cb8195_c4/f1p0/837	NA	R01_cb8195_c4/f1p0/837
R01_cb1892_c8/f1p0/3052	NA	R01_cb1892_c8/f1p0/3052	NA	NA
R01_cb11002_c0/f2p0/1515	NA	NA	NA	R01_cb11002_c0/f2p0/1515
R01_cb15605_c4/f1p0/849	R01_cb15605_c4/f1p0/849	NA	R01_cb15605_c4/f1p0/849	R01_cb15605_c4/f1p0/849
R01_cb13979_c29/f1p0/440	R01_cb13979_c29/f1p0/440	R01_cb13979_c29/f1p0/440	R01_cb13979_c29/f1p0/440	R01_cb13979_c29/f1p0/440
R01_cb8564_c1049/f1p0/2651	R01_cb8564_c1049/f1p0/2651	R01_cb8564_c1049/f1p0/2651	R01_cb8564_c1049/f1p0/2651	R01_cb8564_c1049/f1p0/2651
R01_cb15804_c2/f1p0/911	NA	NA	NA	R01_cb15804_c2/f1p0/911
R01_cb13507_c1/f1p0/1148	R01_cb13507_c1/f1p0/1148	R01_cb13507_c1/f1p0/1148	R01_cb13507_c1/f1p0/1148	NA
R01_cb8393_c2/f1p0/2003	NA	R01_cb8393_c2/f1p0/2003	NA	NA
R01_cb8564_c3685/f1p0/2452	NA	R01_cb8564_c3685/f1p0/2452	NA	NA
R01_cb2750_c9/f1p0/369	NA	R01_cb2750_c9/f1p0/369	NA	NA
R01_cb6452_c11/f1p0/1407	NA	NA	NA	R01_cb6452_c11/f1p0/1407
R01_cb8564_c129444/f1p0/2374	NA	R01_cb8564_c129444/f1p0/2374	NA	NA
R01_cb6033_c2/f1p0/1294	NA	NA	R01_cb6033_c2/f1p0/1294	R01_cb6033_c2/f1p0/1294
R01_cb8564_c73056/f1p0/4161	NA	R01_cb8564_c73056/f1p0/4161	NA	R01_cb8564_c73056/f1p0/4161
R01_cb11077_c0/f6p1/1624	NA	R01_cb11077_c0/f6p1/1624	R01_cb11077_c0/f6p1/1624	R01_cb11077_c0/f6p1/1624
R01_cb14502_c12/f1p0/746	R01_cb14502_c12/f1p0/746	R01_cb14502_c12/f1p0/746	NA	NA
R01_cb12520_c9/f1p0/873	NA	R01_cb12520_c9/f1p0/873	NA	NA

R01_cb10702_c2/f1p0/1731	NA	NA	NA	R01_cb10702_c2/f1p0/1731
R01_cb4334_c3/f1p0/4377	NA	R01_cb4334_c3/f1p0/4377	R01_cb4334_c3/f1p0/4377	R01_cb4334_c3/f1p0/4377
R01_cb4115_c5/f1p0/2908	NA	NA	NA	R01_cb4115_c5/f1p0/2908
R01_cb8564_c44455/f1p0/2540	NA	R01_cb8564_c44455/f1p0/2540	R01_cb8564_c44455/f1p0/2540	R01_cb8564_c44455/f1p0/2540
R01_cb11767_c0/f1p0/1856	R01_cb11767_c0/f1p0/1856	R01_cb11767_c0/f1p0/1856	R01_cb11767_c0/f1p0/1856	R01_cb11767_c0/f1p0/1856
R01_cb17973_c60/f1p0/1680	R01_cb17973_c60/f1p0/1680	R01_cb17973_c60/f1p0/1680	NA	R01_cb17973_c60/f1p0/1680
R01_cb8354_c11/f1p0/1554	NA	NA	NA	R01_cb8354_c11/f1p0/1554
R01_cb293_c1/f1p0/4190	NA	NA	NA	R01_cb293_c1/f1p0/4190
R01_cb6365_c12/f1p0/2331	NA	R01_cb6365_c12/f1p0/2331	NA	NA
R01_cb8564_c112663/f1p0/2320	NA	R01_cb8564_c112663/f1p0/2320	NA	NA
R01_cb302_c7/f1p0/3991	NA	NA	NA	R01_cb302_c7/f1p0/3991
R01_cb8786_c1/f1p0/2134	NA	NA	NA	R01_cb8786_c1/f1p0/2134
R01_cb15485_c1/f1p0/1209	NA	NA	NA	R01_cb15485_c1/f1p0/1209
R01_cb15849_c0/f1p0/898	NA	NA	NA	R01_cb15849_c0/f1p0/898
R01_cb6354_c1/f1p0/2101	NA	NA	NA	R01_cb6354_c1/f1p0/2101
R01_cb13026_c4/f1p0/679	R01_cb13026_c4/f1p0/679	R01_cb13026_c4/f1p0/679	R01_cb13026_c4/f1p0/679	R01_cb13026_c4/f1p0/679
R01_cb8513_c2/f1p0/1631	NA	NA	NA	R01_cb8513_c2/f1p0/1631
R01_cb13396_c3/f1p0/1287	NA	R01_cb13396_c3/f1p0/1287	R01_cb13396_c3/f1p0/1287	R01_cb13396_c3/f1p0/1287
R01_cb6199_c21/f1p0/3005	NA	NA	NA	R01_cb6199_c21/f1p0/3005
R01_cb8564_c2875/f1p0/3292	NA	R01_cb8564_c2875/f1p0/3292	R01_cb8564_c2875/f1p0/3292	NA
R01_cb13161_c9/f1p0/1486	NA	R01_cb13161_c9/f1p0/1486	NA	R01_cb13161_c9/f1p0/1486
R01_cb10778_c2/f1p0/888	NA	R01_cb10778_c2/f1p0/888	NA	NA
R01_cb4000_c3/f1p0/2269	NA	R01_cb4000_c3/f1p0/2269	NA	NA
R01_cb17973_c14/f1p0/1672	R01_cb17973_c14/f1p0/1672	R01_cb17973_c14/f1p0/1672	R01_cb17973_c14/f1p0/1672	NA
R01_cb18456_c1877/f1p0/496	R01_cb18456_c1877/f1p0/496	R01_cb18456_c1877/f1p0/496	R01_cb18456_c1877/f1p0/496	R01_cb18456_c1877/f1p0/496

R01_cb10742_c1/f1p0/911	NA	NA	NA	R01_cb10742_c1/f1p0/911
R01_cb10017_c146/f1p0/983	NA	R01_cb10017_c146/f1p0/983	NA	NA
R01_cb10145_c7/f2p0/1566	NA	NA	NA	R01_cb10145_c7/f2p0/1566
R01_cb8564_c127412/f1p0/2262	NA	R01_cb8564_c127412/f1p0/2262	NA	R01_cb8564_c127412/f1p0/2262
R01_cb17869_c3/f1p0/1681	NA	NA	NA	R01_cb17869_c3/f1p0/1681
R01_cb7171_c19/f3p1/2330	NA	NA	NA	R01_cb7171_c19/f3p1/2330
R01_cb8564_c34231/f2p1/3144	R01_cb8564_c34231/f2p1/3144	R01_cb8564_c34231/f2p1/3144	R01_cb8564_c34231/f2p1/3144	R01_cb8564_c34231/f2p1/3144
R01_cb3381_c12/f1p0/3573	NA	R01_cb3381_c12/f1p0/3573	NA	R01_cb3381_c12/f1p0/3573
R01_cb6969_c7/f1p0/2591	NA	R01_cb6969_c7/f1p0/2591	NA	NA
R01_cb8564_c22629/f1p0/2616	NA	R01_cb8564_c22629/f1p0/2616	NA	NA
R01_cb8564_c146700/f2p0/2284	NA	R01_cb8564_c146700/f2p0/2284	R01_cb8564_c146700/f2p0/2284	R01_cb8564_c146700/f2p0/2284
R01_cb10775_c0/f1p0/1207	NA	NA	R01_cb10775_c0/f1p0/1207	R01_cb10775_c0/f1p0/1207
R01_cb18456_c7268/f1p4/809	NA	R01_cb18456_c7268/f1p4/809	NA	NA
R01_cb8564_c2801/f1p0/2143	NA	R01_cb8564_c2801/f1p0/2143	NA	NA
R01_cb18624_c1/f1p0/4718	NA	R01_cb18624_c1/f1p0/4718	NA	NA
R01_cb14217_c5/f2p0/775	NA	NA	NA	R01_cb14217_c5/f2p0/775
R01_cb4361_c1/f1p0/2264	NA	R01_cb4361_c1/f1p0/2264	R01_cb4361_c1/f1p0/2264	R01_cb4361_c1/f1p0/2264
R01_cb13428_c6/f1p0/797	NA	R01_cb13428_c6/f1p0/797	NA	NA
R01_cb9856_c4/f1p0/1148	NA	R01_cb9856_c4/f1p0/1148	NA	NA
R01_cb17627_c1/f1p0/474	R01_cb17627_c1/f1p0/474	R01_cb17627_c1/f1p0/474	R01_cb17627_c1/f1p0/474	R01_cb17627_c1/f1p0/474
R01_cb8564_c119075/f1p0/2554	NA	R01_cb8564_c119075/f1p0/2554	NA	R01_cb8564_c119075/f1p0/2554
R01_cb8564_c82675/f1p0/2204	NA	R01_cb8564_c82675/f1p0/2204	R01_cb8564_c82675/f1p0/2204	R01_cb8564_c82675/f1p0/2204

R01_cb8564_c128785/f1p0/2931	NA	R01_cb8564_c128785/f1p0/2931	R01_cb8564_c128785/f1p0/2931	R01_cb8564_c128785/f1p0/2931
R01_cb1758_c0/f3p1/2141	NA	NA	NA	R01_cb1758_c0/f3p1/2141
R01_cb8564_c22247/f1p0/4265	NA	NA	NA	R01_cb8564_c22247/f1p0/4265
R01_cb13862_c3/f1p0/1839	NA	R01_cb13862_c3/f1p0/1839	R01_cb13862_c3/f1p0/1839	R01_cb13862_c3/f1p0/1839
R01_cb8564_c39296/f1p0/2315	NA	NA	R01_cb8564_c39296/f1p0/2315	R01_cb8564_c39296/f1p0/2315
R01_cb8564_c37939/f1p0/4573	NA	R01_cb8564_c37939/f1p0/4573	NA	NA
R01_cb15572_c5/f1p0/1537	NA	R01_cb15572_c5/f1p0/1537	NA	NA
R01_cb8865_c0/f1p0/2207	NA	NA	NA	R01_cb8865_c0/f1p0/2207
R01_cb7019_c0/f1p0/2710	NA	R01_cb7019_c0/f1p0/2710	R01_cb7019_c0/f1p0/2710	R01_cb7019_c0/f1p0/2710
R01_cb18456_c7412/f1p0/350	R01_cb18456_c7412/f1p0/350	R01_cb18456_c7412/f1p0/350	R01_cb18456_c7412/f1p0/350	R01_cb18456_c7412/f1p0/350
R01_cb15768_c5/f1p0/1848	NA	R01_cb15768_c5/f1p0/1848	NA	R01_cb15768_c5/f1p0/1848
R01_cb2616_c3/f1p0/1293	NA	NA	NA	R01_cb2616_c3/f1p0/1293
R01_cb8882_c0/f1p0/2261	NA	R01_cb8882_c0/f1p0/2261	NA	NA
R01_cb15883_c1/f1p0/828	NA	R01_cb15883_c1/f1p0/828	NA	NA
R01_cb4001_c2/f1p0/3202	NA	NA	NA	R01_cb4001_c2/f1p0/3202
R01_cb16383_c0/f2p0/1838	NA	R01_cb16383_c0/f2p0/1838	NA	R01_cb16383_c0/f2p0/1838
R01_cb525_c3/f1p0/4745	NA	NA	NA	R01_cb525_c3/f1p0/4745
R01_cb18014_c1/f1p0/913	NA	NA	NA	R01_cb18014_c1/f1p0/913
R01_cb8564_c79841/f2p0/3174	NA	NA	NA	R01_cb8564_c79841/f2p0/3174
R01_cb7999_c8/f1p0/897	NA	NA	NA	R01_cb7999_c8/f1p0/897
R01_cb17973_c30/f1p0/588	R01_cb17973_c30/f1p0/588	R01_cb17973_c30/f1p0/588	R01_cb17973_c30/f1p0/588	NA
R01_cb5640_c3/f1p1/2302	NA	NA	NA	R01_cb5640_c3/f1p1/2302
R01_cb9354_c2/f1p0/2020	NA	NA	NA	R01_cb9354_c2/f1p0/2020
R01_cb11566_c4/f1p0/420	R01_cb11566_c4/f1p0/420	R01_cb11566_c4/f1p0/420	R01_cb11566_c4/f1p0/420	R01_cb11566_c4/f1p0/420
R01_cb5513_c0/f2p0/2985	NA	NA	NA	R01_cb5513_c0/f2p0/2985

R01_cb5593_c2/f1p0/2301	NA	R01_cb5593_c2/f1p0/2301	NA	NA
R01_cb6845_c17/f1p1/2530	NA	NA	NA	R01_cb6845_c17/f1p1/2530
R01_cb13439_c61/f1p1/1069	R01_cb13439_c61/f1p1/1069	R01_cb13439_c61/f1p1/1069	NA	NA
R01_cb10029_c359/f1p0/1037	NA	R01_cb10029_c359/f1p0/1037	NA	NA
R01_cb18456_c7177/f1p0/556	R01_cb18456_c7177/f1p0/556	R01_cb18456_c7177/f1p0/556	R01_cb18456_c7177/f1p0/556	R01_cb18456_c7177/f1p0/556
R01_cb16552_c1/f1p0/1266	NA	NA	NA	R01_cb16552_c1/f1p0/1266
R01_cb6286_c17/f2p1/2683	NA	NA	NA	R01_cb6286_c17/f2p1/2683
R01_cb16949_c1/f1p0/1799	NA	R01_cb16949_c1/f1p0/1799	NA	NA
R01_cb10681_c2/f1p0/1029	R01_cb10681_c2/f1p0/1029	R01_cb10681_c2/f1p0/1029	NA	R01_cb10681_c2/f1p0/1029
R01_cb4768_c40/f1p1/2478	NA	R01_cb4768_c40/f1p1/2478	NA	NA
R01_cb9201_c7/f1p0/2183	NA	NA	NA	R01_cb9201_c7/f1p0/2183
R01_cb8564_c128014/f1p0/2813	NA	R01_cb8564_c128014/f1p0/2813	NA	NA
R01_cb9704_c2/f1p0/1857	NA	NA	NA	R01_cb9704_c2/f1p0/1857
R01_cb7654_c1/f1p0/2566	NA	R01_cb7654_c1/f1p0/2566	NA	R01_cb7654_c1/f1p0/2566
R01_cb8564_c1324/f1p1/2459	NA	NA	NA	R01_cb8564_c1324/f1p1/2459
R01_cb18632_c3/f1p0/1136	NA	R01_cb18632_c3/f1p0/1136	NA	R01_cb18632_c3/f1p0/1136
R01_cb13938_c23/f1p0/1164	NA	NA	NA	R01_cb13938_c23/f1p0/1164
R01_cb16833_c9/f1p0/6462	NA	R01_cb16833_c9/f1p0/6462	NA	NA
R01_cb10272_c18/f1p2/1699	NA	NA	NA	R01_cb10272_c18/f1p2/1699
R01_cb16248_c0/f2p2/567	NA	R01_cb16248_c0/f2p2/567	NA	NA
R01_cb4368_c1/f9p1/1403	NA	NA	NA	R01_cb4368_c1/f9p1/1403
R01_cb4555_c1/f1p0/3308	NA	R01_cb4555_c1/f1p0/3308	NA	R01_cb4555_c1/f1p0/3308
R01_cb15606_c2/f1p0/993	NA	NA	NA	R01_cb15606_c2/f1p0/993
R01_cb15982_c2/f1p0/869	NA	R01_cb15982_c2/f1p0/869	NA	R01_cb15982_c2/f1p0/869
R01_cb5987_c2/f1p0/1938	NA	NA	NA	R01_cb5987_c2/f1p0/1938

R01_cb13259_c3/f1p0/1611	NA	R01_cb13259_c3/f1p0/1611	NA	NA
R01_cb822_c6/f2p3/4553	NA	NA	NA	R01_cb822_c6/f2p3/4553
R01_cb8564_c161493/f1p2/2096	NA	R01_cb8564_c161493/f1p2/2096	NA	NA
R01_cb16085_c0/f2p0/1547	NA	R01_cb16085_c0/f2p0/1547	R01_cb16085_c0/f2p0/1547	R01_cb16085_c0/f2p0/1547
R01_cb8564_c73731/f2p0/4847	NA	R01_cb8564_c73731/f2p0/4847	NA	R01_cb8564_c73731/f2p0/4847
R01_cb16655_c5/f1p0/1468	NA	R01_cb16655_c5/f1p0/1468	R01_cb16655_c5/f1p0/1468	R01_cb16655_c5/f1p0/1468
R01_cb10392_c3/f1p0/748	NA	R01_cb10392_c3/f1p0/748	NA	NA
R01_cb18456_c2496/f1p0/1862	NA	R01_cb18456_c2496/f1p0/1862	NA	R01_cb18456_c2496/f1p0/1862
R01_cb3337_c3/f1p0/3629	NA	R01_cb3337_c3/f1p0/3629	NA	NA
R01_cb8564_c17286/f1p0/4865	NA	R01_cb8564_c17286/f1p0/4865	NA	NA
R01_cb17475_c0/f1p0/463	R01_cb17475_c0/f1p0/463	R01_cb17475_c0/f1p0/463	R01_cb17475_c0/f1p0/463	R01_cb17475_c0/f1p0/463
R01_cb17043_c1/f1p0/999	NA	NA	NA	R01_cb17043_c1/f1p0/999
R01_cb3001_c3/f1p0/3303	NA	R01_cb3001_c3/f1p0/3303	NA	R01_cb3001_c3/f1p0/3303
R01_cb11352_c0/f3p0/509	NA	R01_cb11352_c0/f3p0/509	NA	NA
R01_cb6553_c4/f1p0/3048	NA	R01_cb6553_c4/f1p0/3048	NA	R01_cb6553_c4/f1p0/3048
R01_cb1569_c2/f2p0/755	R01_cb1569_c2/f2p0/755	R01_cb1569_c2/f2p0/755	NA	NA
R01_cb5853_c2/f1p1/2472	R01_cb5853_c2/f1p1/2472	R01_cb5853_c2/f1p1/2472	R01_cb5853_c2/f1p1/2472	NA
R01_cb8564_c120577/f1p0/2300	R01_cb8564_c120577/f1p0/2300	R01_cb8564_c120577/f1p0/2300	R01_cb8564_c120577/f1p0/2300	NA
R01_cb18456_c7831/f5p0/560	NA	R01_cb18456_c7831/f5p0/560	R01_cb18456_c7831/f5p0/560	R01_cb18456_c7831/f5p0/560
R01_cb684_c0/f1p0/4683	NA	NA	NA	R01_cb684_c0/f1p0/4683
R01_cb8564_c85950/f1p0/3005	NA	R01_cb8564_c85950/f1p0/3005	R01_cb8564_c85950/f1p0/3005	R01_cb8564_c85950/f1p0/3005
R01_cb8564_c118096/f1p0/2820	NA	R01_cb8564_c118096/f1p0/2820	NA	NA
R01_cb8564_c4688/f1p0/4180	NA	R01_cb8564_c4688/f1p0/4180	NA	R01_cb8564_c4688/f1p0/4180

R01_cb8037_c3/f1p0/1939	NA	NA	NA	R01_cb8037_c3/f1p0/1939
R01_cb4180_c2/f1p0/4669	NA	NA	NA	R01_cb4180_c2/f1p0/4669
R01_cb18456_c4740/f1p1/492	R01_cb18456_c4740/f1p1/492	NA	NA	R01_cb18456_c4740/f1p1/492
R01_cb8564_c84603/f1p0/2080	NA	R01_cb8564_c84603/f1p0/2080	NA	NA
R01_cb5725_c26/f1p0/2912	NA	NA	NA	R01_cb5725_c26/f1p0/2912
R01_cb8564_c1447/f1p1/2111	NA	R01_cb8564_c1447/f1p1/2111	NA	NA
R01_cb7197_c8/f1p0/2460	NA	R01_cb7197_c8/f1p0/2460	NA	NA
R01_cb12912_c9/f1p0/1766	NA	R01_cb12912_c9/f1p0/1766	R01_cb12912_c9/f1p0/1766	NA
R01_cb3791_c3/f1p0/2226	NA	NA	NA	R01_cb3791_c3/f1p0/2226
R01_cb8564_c23701/f1p0/3521	NA	R01_cb8564_c23701/f1p0/3521	NA	NA
R01_cb14078_c0/f2p0/583	NA	R01_cb14078_c0/f2p0/583	NA	NA
R01_cb6411_c0/f1p0/2865	NA	NA	NA	R01_cb6411_c0/f1p0/2865
R01_cb12302_c42/f1p0/379	R01_cb12302_c42/f1p0/379	R01_cb12302_c42/f1p0/379	R01_cb12302_c42/f1p0/379	R01_cb12302_c42/f1p0/379
R01_cb8564_c53807/f1p1/2773	R01_cb8564_c53807/f1p1/2773	R01_cb8564_c53807/f1p1/2773	R01_cb8564_c53807/f1p1/2773	R01_cb8564_c53807/f1p1/2773
R01_cb8564_c33756/f5p0/2174	NA	R01_cb8564_c33756/f5p0/2174	R01_cb8564_c33756/f5p0/2174	R01_cb8564_c33756/f5p0/2174
R01_cb7814_c1/f3p1/2550	NA	NA	NA	R01_cb7814_c1/f3p1/2550
R01_cb10458_c2/f2p1/1875	NA	NA	NA	R01_cb10458_c2/f2p1/1875
R01_cb17715_c4/f1p0/1613	NA	NA	NA	R01_cb17715_c4/f1p0/1613
R01_cb16125_c6/f1p0/1476	NA	NA	NA	R01_cb16125_c6/f1p0/1476
R01_cb5595_c20/f1p0/2290	R01_cb5595_c20/f1p0/2290	NA	R01_cb5595_c20/f1p0/2290	R01_cb5595_c20/f1p0/2290
R01_cb4345_c17/f1p0/1868	NA	NA	NA	R01_cb4345_c17/f1p0/1868
R01_cb18529_c0/f1p0/1784	NA	R01_cb18529_c0/f1p0/1784	NA	R01_cb18529_c0/f1p0/1784
R01_cb9612_c1/f1p0/2025	NA	NA	NA	R01_cb9612_c1/f1p0/2025
R01_cb10024_c695/f8p0/663	NA	NA	R01_cb10024_c695/f8p0/663	NA
R01_cb4762_c3/f1p0/1130	NA	R01_cb4762_c3/f1p0/1130	NA	NA

R01_cb8295_c1/f6p0/1642	NA	NA	NA	R01_cb8295_c1/f6p0/1642
R01_cb9916_c2/f1p1/2310	NA	NA	NA	R01_cb9916_c2/f1p1/2310
R01_cb4514_c1/f3p1/1912	NA	NA	NA	R01_cb4514_c1/f3p1/1912
R01_cb8564_c119638/f1p0/2467	NA	R01_cb8564_c119638/f1p0/2467	R01_cb8564_c119638/f1p0/2467	NA
R01_cb13875_c1/f4p1/1304	NA	NA	NA	R01_cb13875_c1/f4p1/1304
R01_cb14502_c16/f1p0/1544	R01_cb14502_c16/f1p0/1544	R01_cb14502_c16/f1p0/1544	NA	R01_cb14502_c16/f1p0/1544
R01_cb7797_c4/f1p0/2493	NA	NA	NA	R01_cb7797_c4/f1p0/2493
R01_cb8564_c69250/f2p0/2678	NA	R01_cb8564_c69250/f2p0/2678	NA	NA
R01_cb8564_c51027/f1p0/3064	NA	NA	R01_cb8564_c51027/f1p0/3064	R01_cb8564_c51027/f1p0/3064
R01_cb7113_c3/f1p0/1224	NA	NA	NA	R01_cb7113_c3/f1p0/1224
R01_cb2471_c20/f1p0/3413	NA	R01_cb2471_c20/f1p0/3413	NA	R01_cb2471_c20/f1p0/3413
R01_cb8564_c3993/f1p0/4381	NA	R01_cb8564_c3993/f1p0/4381	R01_cb8564_c3993/f1p0/4381	R01_cb8564_c3993/f1p0/4381
R01_cb4609_c5/f1p0/743	NA	NA	NA	R01_cb4609_c5/f1p0/743
R01_cb9377_c3/f1p0/1645	NA	NA	NA	R01_cb9377_c3/f1p0/1645
R01_cb8564_c127129/f1p1/2311	NA	NA	R01_cb8564_c127129/f1p1/2311	R01_cb8564_c127129/f1p1/2311
R01_cb5120_c3/f1p0/1716	NA	NA	NA	R01_cb5120_c3/f1p0/1716
R01_cb2925_c1/f1p0/3769	NA	R01_cb2925_c1/f1p0/3769	NA	NA
R01_cb7784_c5/f1p1/2103	NA	R01_cb7784_c5/f1p1/2103	NA	NA
R01_cb8564_c3735/f3p4/2692	NA	NA	NA	R01_cb8564_c3735/f3p4/2692
R01_cb8564_c89585/f1p0/3611	NA	R01_cb8564_c89585/f1p0/3611	NA	NA
R01_cb1279_c72/f1p0/2264	NA	R01_cb1279_c72/f1p0/2264	NA	R01_cb1279_c72/f1p0/2264
R01_cb2378_c31/f1p1/3720	NA	NA	NA	R01_cb2378_c31/f1p1/3720
R01_cb4656_c6/f1p0/1966	NA	R01_cb4656_c6/f1p0/1966	NA	NA
R01_cb2204_c1/f1p0/4012	R01_cb2204_c1/f1p0/4012	R01_cb2204_c1/f1p0/4012	R01_cb2204_c1/f1p0/4012	R01_cb2204_c1/f1p0/4012

R01_cb6480_c3/f1p1/2681	NA	NA	NA	R01_cb6480_c3/f1p1/2681
R01_cb1437_c12/f1p0/2951	NA	NA	NA	R01_cb1437_c12/f1p0/2951
R01_cb9646_c6/f1p0/1128	NA	NA	NA	R01_cb9646_c6/f1p0/1128
R01_cb17756_c16/f1p0/478	R01_cb17756_c16/f1p0/478	R01_cb17756_c16/f1p0/478	R01_cb17756_c16/f1p0/478	R01_cb17756_c16/f1p0/478
R01_cb8564_c69884/f1p1/2650	NA	R01_cb8564_c69884/f1p1/2650	R01_cb8564_c69884/f1p1/2650	R01_cb8564_c69884/f1p1/2650
R01_cb7584_c7/f1p0/2579	R01_cb7584_c7/f1p0/2579	R01_cb7584_c7/f1p0/2579	NA	NA
R01_cb5524_c3/f1p0/2077	NA	R01_cb5524_c3/f1p0/2077	NA	NA
R01_cb18409_c67/f1p0/809	R01_cb18409_c67/f1p0/809	R01_cb18409_c67/f1p0/809	R01_cb18409_c67/f1p0/809	R01_cb18409_c67/f1p0/809
R01_cb9812_c2/f1p0/3208	NA	NA	NA	R01_cb9812_c2/f1p0/3208
R01_cb8564_c68701/f5p0/2447	NA	NA	R01_cb8564_c68701/f5p0/2447	R01_cb8564_c68701/f5p0/2447
R01_cb849_c7/f1p0/2513	NA	R01_cb849_c7/f1p0/2513	NA	NA
R01_cb8564_c23581/f1p0/3057	NA	R01_cb8564_c23581/f1p0/3057	NA	R01_cb8564_c23581/f1p0/3057
R01_cb18456_c207/f1p4/978	NA	R01_cb18456_c207/f1p4/978	NA	NA
R01_cb10075_c41/f1p0/1031	NA	NA	NA	R01_cb10075_c41/f1p0/1031
R01_cb8564_c17408/f3p0/2782	NA	R01_cb8564_c17408/f3p0/2782	NA	R01_cb8564_c17408/f3p0/2782
R01_cb18456_c4759/f1p1/489	R01_cb18456_c4759/f1p1/489	NA	NA	R01_cb18456_c4759/f1p1/489
R01_cb5516_c58/f1p0/3485	NA	R01_cb5516_c58/f1p0/3485	NA	R01_cb5516_c58/f1p0/3485
R01_cb17791_c0/f1p0/1378	NA	NA	NA	R01_cb17791_c0/f1p0/1378
R01_cb9043_c6/f1p0/1371	NA	R01_cb9043_c6/f1p0/1371	NA	R01_cb9043_c6/f1p0/1371
R01_cb416_c32/f1p0/2621	NA	NA	NA	R01_cb416_c32/f1p0/2621
R01_cb7698_c0/f1p0/2542	NA	R01_cb7698_c0/f1p0/2542	NA	NA
R01_cb12421_c0/f99p5/674	NA	R01_cb12421_c0/f99p5/674	NA	NA
R01_cb9143_c23/f4p0/2041	NA	R01_cb9143_c23/f4p0/2041	NA	NA
R01_cb13938_c14/f1p0/1325	NA	NA	NA	R01_cb13938_c14/f1p0/1325
R01_cb17668_c3/f1p0/710	NA	NA	NA	R01_cb17668_c3/f1p0/710
R01_cb8180_c1/f1p0/3811	NA	R01_cb8180_c1/f1p0/3811	NA	NA

R01_cb8564_c125323/f1p0/4191	NA	NA	R01_cb8564_c125323/f1p0/4191	R01_cb8564_c125323/f1p0/4191
R01_cb2156_c24/f1p1/2545	NA	R01_cb2156_c24/f1p1/2545	NA	NA
R01_cb12329_c5/f2p0/1609	NA	NA	NA	R01_cb12329_c5/f2p0/1609
R01_cb8564_c42301/f1p0/3750	NA	R01_cb8564_c42301/f1p0/3750	R01_cb8564_c42301/f1p0/3750	NA
R01_cb18409_c84/f1p0/547	R01_cb18409_c84/f1p0/547	R01_cb18409_c84/f1p0/547	R01_cb18409_c84/f1p0/547	R01_cb18409_c84/f1p0/547
R01_cb9648_c13/f1p0/1753	NA	R01_cb9648_c13/f1p0/1753	NA	NA
R01_cb11147_c2/f1p0/2053	NA	R01_cb11147_c2/f1p0/2053	NA	R01_cb11147_c2/f1p0/2053
R01_cb17314_c1/f1p0/1206	R01_cb17314_c1/f1p0/1206	R01_cb17314_c1/f1p0/1206	R01_cb17314_c1/f1p0/1206	R01_cb17314_c1/f1p0/1206
R01_cb12399_c1/f3p0/435	NA	R01_cb12399_c1/f3p0/435	R01_cb12399_c1/f3p0/435	NA
R01_cb2497_c7/f1p0/2495	NA	NA	NA	R01_cb2497_c7/f1p0/2495
R01_cb15346_c4/f1p1/778	NA	NA	R01_cb15346_c4/f1p1/778	R01_cb15346_c4/f1p1/778
R01_cb2806_c3/f1p0/3653	NA	NA	NA	R01_cb2806_c3/f1p0/3653
R01_cb15448_c1/f1p0/377	R01_cb15448_c1/f1p0/377	R01_cb15448_c1/f1p0/377	NA	R01_cb15448_c1/f1p0/377
R01_cb16248_c1/f1p1/613	NA	R01_cb16248_c1/f1p1/613	NA	NA
R01_cb10483_c0/f1p0/1385	NA	NA	NA	R01_cb10483_c0/f1p0/1385
R01_cb10254_c2/f1p0/498	R01_cb10254_c2/f1p0/498	R01_cb10254_c2/f1p0/498	R01_cb10254_c2/f1p0/498	R01_cb10254_c2/f1p0/498
R01_cb15922_c1/f1p0/1880	NA	R01_cb15922_c1/f1p0/1880	R01_cb15922_c1/f1p0/1880	R01_cb15922_c1/f1p0/1880
R01_cb11354_c5/f1p0/5247	NA	NA	NA	R01_cb11354_c5/f1p0/5247
R01_cb10506_c9/f1p0/1490	NA	R01_cb10506_c9/f1p0/1490	NA	NA
R01_cb11068_c1/f1p0/2816	NA	NA	NA	R01_cb11068_c1/f1p0/2816
R01_cb5449_c19/f1p0/2626	NA	R01_cb5449_c19/f1p0/2626	NA	NA
R01_cb8564_c125927/f1p0/2477	NA	R01_cb8564_c125927/f1p0/2477	R01_cb8564_c125927/f1p0/2477	R01_cb8564_c125927/f1p0/2477
R01_cb9049_c2/f1p0/2201	NA	R01_cb9049_c2/f1p0/2201	NA	R01_cb9049_c2/f1p0/2201
R01_cb8564_c84844/f1p0/1945	R01_cb8564_c84844/f1p0/194	R01_cb8564_c84844/f1p0/1945	NA	R01_cb8564_c84844/f1p0/1945

	5			
R01_cb9225_c1/f1p0/2148	NA	R01_cb9225_c1/f1p0/2148	NA	NA
R01_cb5790_c4/f1p0/1051	NA	NA	NA	R01_cb5790_c4/f1p0/1051
R01_cb8564_c4098/f1p0/3713	NA	R01_cb8564_c4098/f1p0/3713	R01_cb8564_c4098/f1p0/3713	NA
R01_cb3575_c21/f1p0/2927	NA	NA	NA	R01_cb3575_c21/f1p0/2927
R01_cb2367_c2/f1p0/2706	NA	R01_cb2367_c2/f1p0/2706	R01_cb2367_c2/f1p0/2706	NA
R01_cb12093_c3/f1p0/434	NA	R01_cb12093_c3/f1p0/434	NA	NA
R01_cb8564_c2542/f1p0/2705	NA	R01_cb8564_c2542/f1p0/2705	NA	NA
R01_cb12079_c9/f1p0/642	NA	R01_cb12079_c9/f1p0/642	NA	R01_cb12079_c9/f1p0/642
R01_cb8564_c73607/f1p0/2892	NA	R01_cb8564_c73607/f1p0/2892	NA	R01_cb8564_c73607/f1p0/2892
R01_cb1839_c3/f1p0/4333	NA	NA	NA	R01_cb1839_c3/f1p0/4333
R01_cb15396_c0/f1p0/1842	NA	R01_cb15396_c0/f1p0/1842	NA	NA
R01_cb2765_c8/f1p0/2925	NA	R01_cb2765_c8/f1p0/2925	NA	R01_cb2765_c8/f1p0/2925
R01_cb9550_c6/f1p0/1996	NA	R01_cb9550_c6/f1p0/1996	NA	NA
R01_cb13408_c4/f1p0/521	R01_cb13408_c4/f1p0/521	R01_cb13408_c4/f1p0/521	R01_cb13408_c4/f1p0/521	R01_cb13408_c4/f1p0/521
R01_cb3228_c2/f1p0/3680	NA	R01_cb3228_c2/f1p0/3680	NA	NA
R01_cb8564_c1412/f1p0/2246	NA	R01_cb8564_c1412/f1p0/2246	NA	R01_cb8564_c1412/f1p0/2246
R01_cb10310_c1/f1p0/1974	NA	NA	NA	R01_cb10310_c1/f1p0/1974
R01_cb16288_c0/f2p0/702	NA	NA	NA	R01_cb16288_c0/f2p0/702
R01_cb6118_c3/f1p0/2779	NA	R01_cb6118_c3/f1p0/2779	NA	NA
R01_cb4915_c6/f1p0/2189	NA	R01_cb4915_c6/f1p0/2189	NA	NA
R01_cb18260_c0/f1p0/1386	NA	NA	NA	R01_cb18260_c0/f1p0/1386
R01_cb18526_c14/f1p0/1392	R01_cb18526_c14/f1p0/1392	R01_cb18526_c14/f1p0/1392	R01_cb18526_c14/f1p0/1392	R01_cb18526_c14/f1p0/1392
R01_cb4030_c4/f1p0/2419	NA	NA	NA	R01_cb4030_c4/f1p0/2419
R01_cb3031_c3/f1p0/10931	NA	R01_cb3031_c3/f1p0/10931	NA	R01_cb3031_c3/f1p0/10931
R01_cb16376_c4/f1p0/791	NA	R01_cb16376_c4/f1p0/791	NA	NA

R01_cb18456_c7795/f6p0/544	R01_cb18456_c7795/f6p0/544	R01_cb18456_c7795/f6p0/544	R01_cb18456_c7795/f6p0/544	R01_cb18456_c7795/f6p0/544
R01_cb2581_c2/f1p0/2882	NA	R01_cb2581_c2/f1p0/2882	R01_cb2581_c2/f1p0/2882	R01_cb2581_c2/f1p0/2882
R01_cb8564_c73225/f1p0/2221	R01_cb8564_c73225/f1p0/2221	R01_cb8564_c73225/f1p0/2221	R01_cb8564_c73225/f1p0/2221	R01_cb8564_c73225/f1p0/2221
R01_cb18456_c7458/f1p0/619	NA	NA	R01_cb18456_c7458/f1p0/619	R01_cb18456_c7458/f1p0/619
R01_cb8564_c23528/f1p0/4715	NA	R01_cb8564_c23528/f1p0/4715	NA	NA
R01_cb13044_c1/f2p0/629	NA	R01_cb13044_c1/f2p0/629	NA	NA
R01_cb6365_c5/f1p1/2516	NA	R01_cb6365_c5/f1p1/2516	NA	NA
R01_cb4352_c13/f1p0/2355	NA	NA	NA	R01_cb4352_c13/f1p0/2355
R01_cb18182_c1/f1p0/1264	NA	NA	NA	R01_cb18182_c1/f1p0/1264
R01_cb4604_c21/f1p0/3095	R01_cb4604_c21/f1p0/3095	R01_cb4604_c21/f1p0/3095	R01_cb4604_c21/f1p0/3095	R01_cb4604_c21/f1p0/3095
R01_cb9859_c3/f1p0/1042	NA	NA	NA	R01_cb9859_c3/f1p0/1042
R01_cb4490_c5/f1p0/2738	NA	NA	NA	R01_cb4490_c5/f1p0/2738
R01_cb8564_c80017/f1p0/4204	NA	NA	NA	R01_cb8564_c80017/f1p0/4204
R01_cb12896_c3/f1p0/1086	R01_cb12896_c3/f1p0/1086	R01_cb12896_c3/f1p0/1086	R01_cb12896_c3/f1p0/1086	R01_cb12896_c3/f1p0/1086
R01_cb3046_c4/f1p1/3736	NA	R01_cb3046_c4/f1p1/3736	NA	R01_cb3046_c4/f1p1/3736
R01_cb2101_c3/f1p0/4051	NA	R01_cb2101_c3/f1p0/4051	NA	NA
R01_cb14125_c49/f1p0/477	R01_cb14125_c49/f1p0/477	R01_cb14125_c49/f1p0/477	R01_cb14125_c49/f1p0/477	NA
R01_cb1915_c11/f1p0/2308	NA	R01_cb1915_c11/f1p0/2308	NA	NA
R01_cb8564_c4907/f1p0/2588	NA	R01_cb8564_c4907/f1p0/2588	NA	R01_cb8564_c4907/f1p0/2588
R01_cb11184_c2/f1p0/2351	R01_cb11184_c2/f1p0/2351	R01_cb11184_c2/f1p0/2351	NA	NA
R01_cb8564_c36257/f1p0/4640	NA	NA	NA	R01_cb8564_c36257/f1p0/4640
R01_cb16929_c0/f2p0/491	NA	NA	NA	R01_cb16929_c0/f2p0/491
R01_cb6861_c21/f1p0/1681	R01_cb6861_c21/f1p0/1681	R01_cb6861_c21/f1p0/1681	NA	R01_cb6861_c21/f1p0/1681
R01_cb11254_c6/f1p0/1763	NA	NA	NA	R01_cb11254_c6/f1p0/1763
R01_cb8564_c12406/f1p0/3337	NA	R01_cb8564_c12406/f1p0/3337	R01_cb8564_c12406/f1p0/3337	R01_cb8564_c12406/f1p0/3337

R01_cb8564_c4216/f1p0/3677	NA	NA	R01_cb8564_c4216/f1p0/3677	R01_cb8564_c4216/f1p0/3677
R01_cb6163_c18/f1p0/1078	NA	R01_cb6163_c18/f1p0/1078	NA	NA
R01_cb8564_c86398/f1p0/2019	NA	R01_cb8564_c86398/f1p0/2019	R01_cb8564_c86398/f1p0/2019	R01_cb8564_c86398/f1p0/2019
R01_cb10787_c0/f2p0/1172	NA	NA	NA	R01_cb10787_c0/f2p0/1172
R01_cb12714_c37/f1p0/308	R01_cb12714_c37/f1p0/308	R01_cb12714_c37/f1p0/308	R01_cb12714_c37/f1p0/308	R01_cb12714_c37/f1p0/308
R01_cb4715_c2/f1p0/2552	NA	NA	R01_cb4715_c2/f1p0/2552	R01_cb4715_c2/f1p0/2552
R01_cb8564_c121351/f1p0/3350	NA	R01_cb8564_c121351/f1p0/3350	NA	NA
R01_cb1203_c19/f1p0/2824	NA	NA	NA	R01_cb1203_c19/f1p0/2824
R01_cb8564_c14886/f1p1/3641	NA	NA	NA	R01_cb8564_c14886/f1p1/3641
R01_cb13177_c8/f1p0/1612	NA	NA	NA	R01_cb13177_c8/f1p0/1612
R01_cb8564_c89691/f1p0/3554	R01_cb8564_c89691/f1p0/3554	R01_cb8564_c89691/f1p0/3554	R01_cb8564_c89691/f1p0/3554	R01_cb8564_c89691/f1p0/3554
R01_cb1301_c5/f1p0/2947	NA	NA	NA	R01_cb1301_c5/f1p0/2947
R01_cb8564_c10166/f1p0/2336	NA	R01_cb8564_c10166/f1p0/2336	NA	NA
R01_cb18238_c0/f2p0/529	NA	R01_cb18238_c0/f2p0/529	R01_cb18238_c0/f2p0/529	R01_cb18238_c0/f2p0/529
R01_cb5156_c3/f2p2/627	NA	R01_cb5156_c3/f2p2/627	NA	R01_cb5156_c3/f2p2/627
R01_cb4800_c2/f1p0/3257	NA	R01_cb4800_c2/f1p0/3257	NA	NA
R01_cb8187_c1/f1p0/2033	R01_cb8187_c1/f1p0/2033	R01_cb8187_c1/f1p0/2033	R01_cb8187_c1/f1p0/2033	R01_cb8187_c1/f1p0/2033
R01_cb4973_c14/f1p0/444	R01_cb4973_c14/f1p0/444	R01_cb4973_c14/f1p0/444	R01_cb4973_c14/f1p0/444	R01_cb4973_c14/f1p0/444
R01_cb1228_c9/f3p0/2548	NA	NA	NA	R01_cb1228_c9/f3p0/2548
R01_cb5554_c3/f1p0/2720	NA	R01_cb5554_c3/f1p0/2720	NA	NA
R01_cb18604_c1/f1p0/455	NA	R01_cb18604_c1/f1p0/455	NA	R01_cb18604_c1/f1p0/455
R01_cb16984_c2/f1p0/1256	NA	NA	NA	R01_cb16984_c2/f1p0/1256
R01_cb15379_c1/f1p0/1771	NA	NA	R01_cb15379_c1/f1p0/1771	R01_cb15379_c1/f1p0/1771
R01_cb11196_c1/f1p0/3451	NA	R01_cb11196_c1/f1p0/3451	NA	NA

R01_cb8564_c80717/f1p1/2766	NA	R01_cb8564_c80717/f1p1/2766	R01_cb8564_c80717/f1p1/2766	NA
R01_cb14292_c12/f1p0/1656	NA	R01_cb14292_c12/f1p0/1656	NA	NA
R01_cb14613_c0/f1p2/596	NA	NA	NA	R01_cb14613_c0/f1p2/596
R01_cb8564_c14841/f1p0/3290	NA	R01_cb8564_c14841/f1p0/3290	R01_cb8564_c14841/f1p0/3290	R01_cb8564_c14841/f1p0/3290
R01_cb7612_c2/f1p1/2003	NA	NA	NA	R01_cb7612_c2/f1p1/2003
R01_cb11055_c1/f1p0/2751	NA	R01_cb11055_c1/f1p0/2751	NA	NA
R01_cb8564_c24875/f1p0/3485	NA	NA	NA	R01_cb8564_c24875/f1p0/3485
R01_cb10741_c1/f1p0/1891	R01_cb10741_c1/f1p0/1891	R01_cb10741_c1/f1p0/1891	R01_cb10741_c1/f1p0/1891	NA
R01_cb17302_c2/f1p0/711	NA	R01_cb17302_c2/f1p0/711	NA	R01_cb17302_c2/f1p0/711
R01_cb18456_c7093/f1p0/442	R01_cb18456_c7093/f1p0/442	R01_cb18456_c7093/f1p0/442	R01_cb18456_c7093/f1p0/442	R01_cb18456_c7093/f1p0/442
R01_cb5847_c5/f1p0/2404	NA	NA	NA	R01_cb5847_c5/f1p0/2404
R01_cb8023_c6/f1p0/2383	NA	R01_cb8023_c6/f1p0/2383	NA	NA
R01_cb15186_c14/f1p0/562	R01_cb15186_c14/f1p0/562	R01_cb15186_c14/f1p0/562	R01_cb15186_c14/f1p0/562	NA
R01_cb1259_c13/f1p1/3391	NA	NA	NA	R01_cb1259_c13/f1p1/3391
R01_cb8564_c49756/f1p0/4302	NA	NA	NA	R01_cb8564_c49756/f1p0/4302
R01_cb17761_c3/f1p0/778	NA	NA	NA	R01_cb17761_c3/f1p0/778
R01_cb5100_c0/f1p0/3168	NA	R01_cb5100_c0/f1p0/3168	R01_cb5100_c0/f1p0/3168	NA
R01_cb18456_c843/f1p1/795	NA	R01_cb18456_c843/f1p1/795	NA	NA
R01_cb18456_c4055/f1p0/1156	NA	R01_cb18456_c4055/f1p0/1156	NA	NA
R01_cb438_c10/f1p0/1811	NA	R01_cb438_c10/f1p0/1811	NA	R01_cb438_c10/f1p0/1811
R01_cb18044_c0/f1p0/828	R01_cb18044_c0/f1p0/828	R01_cb18044_c0/f1p0/828	R01_cb18044_c0/f1p0/828	R01_cb18044_c0/f1p0/828
R01_cb8564_c20131/f1p1/3881	NA	NA	NA	R01_cb8564_c20131/f1p1/3881
R01_cb781_c16/f1p0/1990	NA	NA	NA	R01_cb781_c16/f1p0/1990
R01_cb18456_c7668/f1p0/470	NA	NA	NA	R01_cb18456_c7668/f1p0/470
R01_cb8564_c4928/f2p0/2999	NA	NA	NA	R01_cb8564_c4928/f2p0/2999
R01_cb3601_c26/f1p0/968	NA	R01_cb3601_c26/f1p0/968	NA	NA

R01_cb18192_c7/f1p0/541	R01_cb18192_c7/f1p0/541	R01_cb18192_c7/f1p0/541	R01_cb18192_c7/f1p0/541	R01_cb18192_c7/f1p0/541
R01_cb8564_c22854/f1p0/3435	NA	R01_cb8564_c22854/f1p0/3435	NA	R01_cb8564_c22854/f1p0/3435
R01_cb18456_c6650/f1p0/664	NA	NA	R01_cb18456_c6650/f1p0/664	R01_cb18456_c6650/f1p0/664
R01_cb8564_c2851/f2p0/2612	NA	NA	NA	R01_cb8564_c2851/f2p0/2612
R01_cb8564_c78756/f1p0/2985	NA	R01_cb8564_c78756/f1p0/2985	NA	NA
R01_cb7168_c17/f1p0/3074	NA	R01_cb7168_c17/f1p0/3074	NA	R01_cb7168_c17/f1p0/3074
R01_cb15796_c2/f2p0/1511	NA	NA	NA	R01_cb15796_c2/f2p0/1511
R01_cb10504_c0/f11p0/521	R01_cb10504_c0/f11p0/521	R01_cb10504_c0/f11p0/521	NA	R01_cb10504_c0/f11p0/521
R01_cb16645_c59/f1p0/1411	NA	R01_cb16645_c59/f1p0/1411	R01_cb16645_c59/f1p0/1411	R01_cb16645_c59/f1p0/1411
R01_cb9116_c7/f1p0/2169	R01_cb9116_c7/f1p0/2169	NA	NA	NA
R01_cb489_c2/f1p0/2009	NA	R01_cb489_c2/f1p0/2009	NA	NA
R01_cb3732_c23/f1p0/2519	NA	NA	NA	R01_cb3732_c23/f1p0/2519
R01_cb17266_c24/f1p0/608	R01_cb17266_c24/f1p0/608	R01_cb17266_c24/f1p0/608	R01_cb17266_c24/f1p0/608	R01_cb17266_c24/f1p0/608
R01_cb13489_c7/f1p0/1342	NA	R01_cb13489_c7/f1p0/1342	NA	NA
R01_cb8564_c123657/f1p0/1999	NA	NA	NA	R01_cb8564_c123657/f1p0/1999
R01_cb8564_c21727/f1p0/3130	NA	NA	NA	R01_cb8564_c21727/f1p0/3130
R01_cb18456_c3441/f1p0/1135	NA	R01_cb18456_c3441/f1p0/1135	NA	NA
R01_cb13252_c4/f1p0/586	R01_cb13252_c4/f1p0/586	R01_cb13252_c4/f1p0/586	NA	NA
R01_cb13169_c1/f1p0/725	R01_cb13169_c1/f1p0/725	R01_cb13169_c1/f1p0/725	R01_cb13169_c1/f1p0/725	R01_cb13169_c1/f1p0/725
R01_cb7502_c8/f1p0/2419	NA	NA	R01_cb7502_c8/f1p0/2419	R01_cb7502_c8/f1p0/2419
R01_cb18054_c3/f1p0/5284	NA	R01_cb18054_c3/f1p0/5284	NA	NA
R01_cb17019_c1/f1p0/1070	NA	NA	NA	R01_cb17019_c1/f1p0/1070
R01_cb11022_c0/f1p0/1052	NA	R01_cb11022_c0/f1p0/1052	R01_cb11022_c0/f1p0/1052	R01_cb11022_c0/f1p0/1052
R01_cb7096_c20/f1p0/2641	NA	NA	NA	R01_cb7096_c20/f1p0/2641
R01_cb16304_c1/f1p0/1546	NA	NA	NA	R01_cb16304_c1/f1p0/1546

R01_cb4768_c24/f1p1/2479	NA	R01_cb4768_c24/f1p1/2479	NA	NA
R01_cb12512_c16/f1p0/1365	NA	R01_cb12512_c16/f1p0/1365	R01_cb12512_c16/f1p0/1365	R01_cb12512_c16/f1p0/1365
R01_cb8985_c7/f1p0/960	NA	R01_cb8985_c7/f1p0/960	NA	NA
R01_cb8564_c9880/f2p0/3052	NA	R01_cb8564_c9880/f2p0/3052	NA	R01_cb8564_c9880/f2p0/3052
R01_cb1686_c12/f1p0/1251	R01_cb1686_c12/f1p0/1251	R01_cb1686_c12/f1p0/1251	R01_cb1686_c12/f1p0/1251	R01_cb1686_c12/f1p0/1251
R01_cb3256_c3/f1p0/3065	NA	R01_cb3256_c3/f1p0/3065	NA	NA
R01_cb18456_c7995/f1p0/560	NA	R01_cb18456_c7995/f1p0/560	R01_cb18456_c7995/f1p0/560	R01_cb18456_c7995/f1p0/560
R01_cb17562_c6/f6p0/579	NA	R01_cb17562_c6/f6p0/579	NA	NA
R01_cb18225_c1/f1p0/395	R01_cb18225_c1/f1p0/395	R01_cb18225_c1/f1p0/395	R01_cb18225_c1/f1p0/395	R01_cb18225_c1/f1p0/395
R01_cb7686_c2/f1p0/2145	NA	NA	NA	R01_cb7686_c2/f1p0/2145
R01_cb4708_c5/f1p0/3255	NA	R01_cb4708_c5/f1p0/3255	NA	NA
R01_cb8564_c12543/f1p0/4114	R01_cb8564_c12543/f1p0/4114	R01_cb8564_c12543/f1p0/4114	R01_cb8564_c12543/f1p0/4114	R01_cb8564_c12543/f1p0/4114
R01_cb4819_c3/f1p1/2594	NA	R01_cb4819_c3/f1p1/2594	NA	NA
R01_cb11429_c4/f1p0/1046	NA	NA	NA	R01_cb11429_c4/f1p0/1046
R01_cb9418_c0/f3p1/1849	NA	NA	NA	R01_cb9418_c0/f3p1/1849
R01_cb18120_c0/f1p0/518	NA	R01_cb18120_c0/f1p0/518	R01_cb18120_c0/f1p0/518	R01_cb18120_c0/f1p0/518
R01_cb18249_c1/f1p0/718	NA	R01_cb18249_c1/f1p0/718	R01_cb18249_c1/f1p0/718	R01_cb18249_c1/f1p0/718
R01_cb14478_c5/f1p0/382	R01_cb14478_c5/f1p0/382	R01_cb14478_c5/f1p0/382	R01_cb14478_c5/f1p0/382	R01_cb14478_c5/f1p0/382
R01_cb16076_c4/f1p0/727	NA	NA	R01_cb16076_c4/f1p0/727	R01_cb16076_c4/f1p0/727
R01_cb8564_c11892/f1p1/3919	NA	NA	NA	R01_cb8564_c11892/f1p1/3919
R01_cb11353_c2/f1p0/2568	NA	R01_cb11353_c2/f1p0/2568	NA	R01_cb11353_c2/f1p0/2568
R01_cb7663_c1/f2p0/1001	NA	NA	NA	R01_cb7663_c1/f2p0/1001
R01_cb16019_c3/f1p0/946	NA	R01_cb16019_c3/f1p0/946	NA	NA
R01_cb8564_c23069/f1p0/2858	NA	NA	NA	R01_cb8564_c23069/f1p0/2858
R01_cb4115_c3/f1p0/3785	NA	R01_cb4115_c3/f1p0/3785	R01_cb4115_c3/f1p0/3785	R01_cb4115_c3/f1p0/3785

R01_cb11692_c1/f1p0/2024	NA	NA	NA	R01_cb11692_c1/f1p0/2024
R01_cb8564_c86404/f2p2/3245	NA	NA	NA	R01_cb8564_c86404/f2p2/3245
R01_cb11938_c9/f1p0/884	NA	R01_cb11938_c9/f1p0/884	R01_cb11938_c9/f1p0/884	R01_cb11938_c9/f1p0/884
R01_cb4603_c2/f1p0/3728	NA	NA	NA	R01_cb4603_c2/f1p0/3728
R01_cb1905_c35/f1p0/2423	NA	R01_cb1905_c35/f1p0/2423	R01_cb1905_c35/f1p0/2423	NA
R01_cb14228_c4/f2p0/714	NA	NA	NA	R01_cb14228_c4/f2p0/714
R01_cb8564_c24830/f1p2/2205	NA	NA	NA	R01_cb8564_c24830/f1p2/2205
R01_cb15695_c0/f1p0/789	R01_cb15695_c0/f1p0/789	R01_cb15695_c0/f1p0/789	R01_cb15695_c0/f1p0/789	R01_cb15695_c0/f1p0/789
R01_cb1432_c2/f1p0/4443	NA	R01_cb1432_c2/f1p0/4443	NA	R01_cb1432_c2/f1p0/4443
R01_cb2018_c0/f1p0/4091	NA	NA	NA	R01_cb2018_c0/f1p0/4091
R01_cb18456_c7107/f1p0/429	NA	R01_cb18456_c7107/f1p0/429	NA	NA
R01_cb69_c35/f1p0/3346	NA	NA	NA	R01_cb69_c35/f1p0/3346
R01_cb8564_c82927/f1p0/2073	NA	R01_cb8564_c82927/f1p0/2073	R01_cb8564_c82927/f1p0/2073	R01_cb8564_c82927/f1p0/2073
R01_cb7538_c14/f1p0/391	R01_cb7538_c14/f1p0/391	R01_cb7538_c14/f1p0/391	NA	NA
R01_cb4911_c1/f1p0/3143	NA	R01_cb4911_c1/f1p0/3143	NA	NA
R01_cb3518_c5/f1p0/3706	NA	R01_cb3518_c5/f1p0/3706	NA	NA
R01_cb8564_c1484/f1p0/3004	NA	R01_cb8564_c1484/f1p0/3004	NA	NA
R01_cb16918_c0/f1p0/843	R01_cb16918_c0/f1p0/843	R01_cb16918_c0/f1p0/843	R01_cb16918_c0/f1p0/843	R01_cb16918_c0/f1p0/843
R01_cb146_c4/f1p0/3484	NA	NA	NA	R01_cb146_c4/f1p0/3484
R01_cb5234_c8/f1p0/3066	NA	NA	NA	R01_cb5234_c8/f1p0/3066
R01_cb5755_c13/f1p0/2471	NA	R01_cb5755_c13/f1p0/2471	NA	R01_cb5755_c13/f1p0/2471
R01_cb15756_c3/f1p0/646	NA	NA	R01_cb15756_c3/f1p0/646	R01_cb15756_c3/f1p0/646
R01_cb4702_c26/f1p0/1026	NA	R01_cb4702_c26/f1p0/1026	NA	R01_cb4702_c26/f1p0/1026
R01_cb11218_c1/f1p0/3101	NA	R01_cb11218_c1/f1p0/3101	NA	NA
R01_cb18569_c0/f1p0/587	R01_cb18569_c0/f1p0/587	R01_cb18569_c0/f1p0/587	NA	R01_cb18569_c0/f1p0/587
R01_cb4074_c7/f1p1/2826	NA	R01_cb4074_c7/f1p1/2826	NA	NA

R01_cb8564_c119359/f1p0/2532	NA	R01_cb8564_c119359/f1p0/2532	NA	NA
R01_cb10232_c2/f2p0/669	NA	R01_cb10232_c2/f2p0/669	NA	NA
R01_cb124_c25/f1p0/3900	NA	NA	NA	R01_cb124_c25/f1p0/3900
R01_cb18456_c5795/f1p0/611	NA	NA	R01_cb18456_c5795/f1p0/611	NA
R01_cb14617_c1/f1p0/1491	NA	R01_cb14617_c1/f1p0/1491	R01_cb14617_c1/f1p0/1491	R01_cb14617_c1/f1p0/1491
R01_cb7128_c9/f1p0/855	NA	NA	NA	R01_cb7128_c9/f1p0/855
R01_cb13771_c9/f4p0/1177	NA	R01_cb13771_c9/f4p0/1177	R01_cb13771_c9/f4p0/1177	R01_cb13771_c9/f4p0/1177
R01_cb5378_c0/f1p0/3125	NA	NA	NA	R01_cb5378_c0/f1p0/3125
R01_cb8564_c124838/f1p1/3152	NA	R01_cb8564_c124838/f1p1/3152	NA	NA
R01_cb6931_c5/f1p0/1796	NA	NA	NA	R01_cb6931_c5/f1p0/1796
R01_cb423_c5/f1p0/2036	NA	NA	NA	R01_cb423_c5/f1p0/2036
R01_cb8564_c71473/f1p0/3250	NA	R01_cb8564_c71473/f1p0/3250	R01_cb8564_c71473/f1p0/3250	R01_cb8564_c71473/f1p0/3250
R01_cb4248_c1/f1p0/3385	NA	R01_cb4248_c1/f1p0/3385	NA	NA
R01_cb8564_c88670/f1p0/2846	NA	R01_cb8564_c88670/f1p0/2846	R01_cb8564_c88670/f1p0/2846	R01_cb8564_c88670/f1p0/2846
R01_cb4614_c1/f1p0/3298	NA	R01_cb4614_c1/f1p0/3298	NA	NA
R01_cb2845_c19/f1p0/3016	NA	NA	NA	R01_cb2845_c19/f1p0/3016
R01_cb7806_c10/f1p0/991	R01_cb7806_c10/f1p0/991	R01_cb7806_c10/f1p0/991	R01_cb7806_c10/f1p0/991	R01_cb7806_c10/f1p0/991
R01_cb13464_c2/f3p0/437	NA	R01_cb13464_c2/f3p0/437	NA	NA
R01_cb17973_c16/f1p0/1313	NA	R01_cb17973_c16/f1p0/1313	NA	R01_cb17973_c16/f1p0/1313
R01_cb12480_c15/f1p0/631	NA	R01_cb12480_c15/f1p0/631	R01_cb12480_c15/f1p0/631	R01_cb12480_c15/f1p0/631
R01_cb12217_c2/f1p0/662	NA	R01_cb12217_c2/f1p0/662	NA	NA
R01_cb8564_c123204/f1p0/2434	NA	NA	NA	R01_cb8564_c123204/f1p0/2434
R01_cb3099_c1/f1p0/3606	R01_cb3099_c1/f1p0/3606	R01_cb3099_c1/f1p0/3606	R01_cb3099_c1/f1p0/3606	R01_cb3099_c1/f1p0/3606

R01_cb10759_c0/f1p0/950	NA	NA	NA	R01_cb10759_c0/f1p0/950
R01_cb15745_c6/f1p0/1124	NA	R01_cb15745_c6/f1p0/1124	R01_cb15745_c6/f1p0/1124	R01_cb15745_c6/f1p0/1124
R01_cb1982_c8/f1p0/863	R01_cb1982_c8/f1p0/863	R01_cb1982_c8/f1p0/863	R01_cb1982_c8/f1p0/863	R01_cb1982_c8/f1p0/863
R01_cb1964_c3/f1p0/3397	NA	R01_cb1964_c3/f1p0/3397	NA	R01_cb1964_c3/f1p0/3397
R01_cb15227_c5/f1p0/1255	NA	NA	NA	R01_cb15227_c5/f1p0/1255
R01_cb8564_c83309/f1p0/2039	NA	R01_cb8564_c83309/f1p0/2039	NA	R01_cb8564_c83309/f1p0/2039
R01_cb13257_c5/f1p0/1109	NA	R01_cb13257_c5/f1p0/1109	NA	NA
R01_cb9179_c0/f1p0/2162	NA	NA	NA	R01_cb9179_c0/f1p0/2162
R01_cb11918_c9/f1p0/766	NA	NA	NA	R01_cb11918_c9/f1p0/766
R01_cb12240_c4/f2p1/605	R01_cb12240_c4/f2p1/605	R01_cb12240_c4/f2p1/605	NA	NA
R01_cb8564_c13387/f1p0/2824	NA	NA	R01_cb8564_c13387/f1p0/2824	R01_cb8564_c13387/f1p0/2824
R01_cb11507_c2/f1p0/1259	R01_cb11507_c2/f1p0/1259	R01_cb11507_c2/f1p0/1259	R01_cb11507_c2/f1p0/1259	R01_cb11507_c2/f1p0/1259
R01_cb15334_c3/f1p0/1167	R01_cb15334_c3/f1p0/1167	R01_cb15334_c3/f1p0/1167	R01_cb15334_c3/f1p0/1167	R01_cb15334_c3/f1p0/1167
R01_cb8564_c116623/f1p0/2525	NA	R01_cb8564_c116623/f1p0/2525	NA	NA
R01_cb11660_c1/f1p0/2817	NA	R01_cb11660_c1/f1p0/2817	NA	NA
R01_cb8564_c112331/f1p4/2084	NA	R01_cb8564_c112331/f1p4/2084	NA	NA
R01_cb18301_c11/f1p0/1709	R01_cb18301_c11/f1p0/1709	R01_cb18301_c11/f1p0/1709	R01_cb18301_c11/f1p0/1709	R01_cb18301_c11/f1p0/1709
R01_cb14968_c18/f1p0/1816	NA	NA	NA	R01_cb14968_c18/f1p0/1816
R01_cb7248_c1/f1p0/3567	NA	R01_cb7248_c1/f1p0/3567	NA	NA
R01_cb4286_c7/f1p0/3033	NA	R01_cb4286_c7/f1p0/3033	NA	NA
R01_cb10844_c2/f1p0/1392	NA	NA	NA	R01_cb10844_c2/f1p0/1392
R01_cb12110_c2/f1p1/658	R01_cb12110_c2/f1p1/658	R01_cb12110_c2/f1p1/658	NA	R01_cb12110_c2/f1p1/658
R01_cb8564_c47502/f1p0/2206	R01_cb8564_c47502/f1p0/2206	R01_cb8564_c47502/f1p0/2206	R01_cb8564_c47502/f1p0/2206	R01_cb8564_c47502/f1p0/2206

R01_cb10702_c0/f1p0/1132	R01_cb10702_c0/f1p0/1132	R01_cb10702_c0/f1p0/1132	R01_cb10702_c0/f1p0/1132	R01_cb10702_c0/f1p0/1132
R01_cb16269_c0/f1p0/1523	R01_cb16269_c0/f1p0/1523	R01_cb16269_c0/f1p0/1523	NA	R01_cb16269_c0/f1p0/1523
R01_cb822_c36/f4p1/2420	NA	NA	NA	R01_cb822_c36/f4p1/2420
R01_cb16875_c1/f1p0/641	R01_cb16875_c1/f1p0/641	R01_cb16875_c1/f1p0/641	R01_cb16875_c1/f1p0/641	R01_cb16875_c1/f1p0/641
R01_cb12740_c14/f1p0/525	R01_cb12740_c14/f1p0/525	R01_cb12740_c14/f1p0/525	NA	R01_cb12740_c14/f1p0/525
R01_cb8564_c554/f4p0/3007	NA	NA	NA	R01_cb8564_c554/f4p0/3007
R01_cb4768_c28/f1p0/2027	NA	R01_cb4768_c28/f1p0/2027	NA	NA
R01_cb18386_c22/f1p0/5179	NA	R01_cb18386_c22/f1p0/5179	NA	NA
R01_cb4198_c0/f2p0/1850	NA	NA	NA	R01_cb4198_c0/f2p0/1850
R01_cb1264_c1/f2p0/4088	NA	NA	NA	R01_cb1264_c1/f2p0/4088
R01_cb8564_c89156/f1p0/3538	NA	R01_cb8564_c89156/f1p0/3538	NA	NA
R01_cb18456_c512/f1p1/991	NA	R01_cb18456_c512/f1p1/991	NA	NA
R01_cb5625_c3/f1p0/2869	NA	R01_cb5625_c3/f1p0/2869	NA	R01_cb5625_c3/f1p0/2869
R01_cb11147_c1/f1p0/1304	NA	R01_cb11147_c1/f1p0/1304	NA	R01_cb11147_c1/f1p0/1304
R01_cb8564_c78670/f1p0/3869	NA	NA	NA	R01_cb8564_c78670/f1p0/3869
R01_cb17314_c0/f1p0/888	R01_cb17314_c0/f1p0/888	R01_cb17314_c0/f1p0/888	R01_cb17314_c0/f1p0/888	R01_cb17314_c0/f1p0/888
R01_cb5659_c76/f1p0/2387	NA	NA	NA	R01_cb5659_c76/f1p0/2387
R01_cb8564_c10936/f1p0/3711	NA	R01_cb8564_c10936/f1p0/3711	NA	R01_cb8564_c10936/f1p0/3711
R01_cb12420_c5/f1p0/1235	NA	NA	NA	R01_cb12420_c5/f1p0/1235
R01_cb8564_c91686/f1p0/3380	NA	NA	NA	R01_cb8564_c91686/f1p0/3380
R01_cb18456_c7187/f1p0/1303	R01_cb18456_c7187/f1p0/1303	R01_cb18456_c7187/f1p0/1303	R01_cb18456_c7187/f1p0/1303	R01_cb18456_c7187/f1p0/1303
R01_cb10175_c0/f2p0/1005	NA	R01_cb10175_c0/f2p0/1005	NA	NA
R01_cb7985_c0/f2p0/2381	NA	R01_cb7985_c0/f2p0/2381	R01_cb7985_c0/f2p0/2381	R01_cb7985_c0/f2p0/2381
R01_cb6645_c1/f1p0/2041	NA	NA	R01_cb6645_c1/f1p0/2041	R01_cb6645_c1/f1p0/2041
R01_cb6802_c6/f1p0/2764	NA	R01_cb6802_c6/f1p0/2764	NA	NA

R01_cb18456_c7803/f6p0/729	NA	R01_cb18456_c7803/f6p0/729	R01_cb18456_c7803/f6p0/729	R01_cb18456_c7803/f6p0/729
R01_cb1377_c4/f1p0/1849	NA	NA	NA	R01_cb1377_c4/f1p0/1849
R01_cb1248_c6/f1p0/6793	NA	NA	NA	R01_cb1248_c6/f1p0/6793
R01_cb18456_c5454/f1p0/1203	NA	R01_cb18456_c5454/f1p0/1203	NA	NA
R01_cb15569_c1/f2p0/1413	NA	NA	R01_cb15569_c1/f2p0/1413	NA
R01_cb15917_c0/f1p0/479	NA	R01_cb15917_c0/f1p0/479	NA	NA
R01_cb8564_c88696/f1p0/3540	NA	NA	NA	R01_cb8564_c88696/f1p0/3540
R01_cb14704_c7/f1p0/1661	NA	R01_cb14704_c7/f1p0/1661	NA	NA
R01_cb9987_c3/f1p0/1686	NA	R01_cb9987_c3/f1p0/1686	NA	R01_cb9987_c3/f1p0/1686
R01_cb10237_c2/f1p0/1256	NA	NA	NA	R01_cb10237_c2/f1p0/1256
R01_cb14727_c0/f2p0/785	NA	NA	R01_cb14727_c0/f2p0/785	R01_cb14727_c0/f2p0/785
R01_cb9422_c4/f1p0/1518	NA	R01_cb9422_c4/f1p0/1518	NA	NA
R01_cb261_c44/f1p0/2433	NA	R01_cb261_c44/f1p0/2433	NA	R01_cb261_c44/f1p0/2433
R01_cb763_c5/f1p0/4241	NA	R01_cb763_c5/f1p0/4241	R01_cb763_c5/f1p0/4241	R01_cb763_c5/f1p0/4241
R01_cb12061_c6/f1p0/796	NA	NA	R01_cb12061_c6/f1p0/796	R01_cb12061_c6/f1p0/796
R01_cb7814_c9/f1p0/2692	NA	NA	NA	R01_cb7814_c9/f1p0/2692
R01_cb8564_c18745/f3p1/4268	R01_cb8564_c18745/f3p1/4268	R01_cb8564_c18745/f3p1/4268	R01_cb8564_c18745/f3p1/4268	R01_cb8564_c18745/f3p1/4268
R01_cb8281_c0/f5p0/2077	NA	NA	NA	R01_cb8281_c0/f5p0/2077
R01_cb2154_c8/f1p0/3166	NA	R01_cb2154_c8/f1p0/3166	NA	NA
R01_cb8564_c124811/f1p0/2286	NA	R01_cb8564_c124811/f1p0/2286	R01_cb8564_c124811/f1p0/2286	R01_cb8564_c124811/f1p0/2286
R01_cb13558_c4/f1p0/696	NA	NA	R01_cb13558_c4/f1p0/696	R01_cb13558_c4/f1p0/696
R01_cb1077_c9/f1p0/4487	NA	R01_cb1077_c9/f1p0/4487	NA	NA
R01_cb11543_c0/f1p0/1881	NA	NA	NA	R01_cb11543_c0/f1p0/1881
R01_cb2571_c22/f1p0/3889	NA	NA	NA	R01_cb2571_c22/f1p0/3889

R01_cb9757_c5/f1p0/2750	NA	R01_cb9757_c5/f1p0/2750	R01_cb9757_c5/f1p0/2750	R01_cb9757_c5/f1p0/2750
R01_cb6752_c1/f1p0/2113	NA	R01_cb6752_c1/f1p0/2113	NA	R01_cb6752_c1/f1p0/2113
R01_cb11743_c0/f1p0/1002	NA	R01_cb11743_c0/f1p0/1002	R01_cb11743_c0/f1p0/1002	R01_cb11743_c0/f1p0/1002
R01_cb18398_c0/f1p0/1444	NA	R01_cb18398_c0/f1p0/1444	R01_cb18398_c0/f1p0/1444	NA
R01_cb9232_c1/f1p0/2771	NA	NA	NA	R01_cb9232_c1/f1p0/2771
R01_cb14502_c17/f1p0/402	R01_cb14502_c17/f1p0/402	R01_cb14502_c17/f1p0/402	R01_cb14502_c17/f1p0/402	R01_cb14502_c17/f1p0/402
R01_cb8564_c3241/f1p0/3032	NA	R01_cb8564_c3241/f1p0/3032	R01_cb8564_c3241/f1p0/3032	R01_cb8564_c3241/f1p0/3032
R01_cb17149_c1/f1p0/1676	R01_cb17149_c1/f1p0/1676	R01_cb17149_c1/f1p0/1676	R01_cb17149_c1/f1p0/1676	R01_cb17149_c1/f1p0/1676
R01_cb4434_c6/f1p0/3018	NA	NA	NA	R01_cb4434_c6/f1p0/3018
R01_cb18513_c0/f1p0/749	R01_cb18513_c0/f1p0/749	R01_cb18513_c0/f1p0/749	R01_cb18513_c0/f1p0/749	R01_cb18513_c0/f1p0/749
R01_cb8564_c112416/f1p0/1908	NA	R01_cb8564_c112416/f1p0/1908	NA	R01_cb8564_c112416/f1p0/1908
R01_cb7653_c10/f1p0/3029	NA	R01_cb7653_c10/f1p0/3029	NA	R01_cb7653_c10/f1p0/3029
R01_cb8564_c69950/f1p0/2760	NA	R01_cb8564_c69950/f1p0/2760	R01_cb8564_c69950/f1p0/2760	NA
R01_cb8564_c121937/f1p0/2803	NA	R01_cb8564_c121937/f1p0/2803	R01_cb8564_c121937/f1p0/2803	R01_cb8564_c121937/f1p0/2803
R01_cb10397_c2/f1p0/816	NA	R01_cb10397_c2/f1p0/816	NA	R01_cb10397_c2/f1p0/816
R01_cb8564_c69529/f1p0/4528	NA	NA	NA	R01_cb8564_c69529/f1p0/4528
R01_cb7552_c1/f1p0/2685	NA	NA	NA	R01_cb7552_c1/f1p0/2685
R01_cb12785_c30/f2p0/1064	NA	NA	R01_cb12785_c30/f2p0/1064	NA
R01_cb4505_c1/f1p0/2787	NA	R01_cb4505_c1/f1p0/2787	R01_cb4505_c1/f1p0/2787	NA
R01_cb8865_c3/f1p0/2075	NA	R01_cb8865_c3/f1p0/2075	NA	R01_cb8865_c3/f1p0/2075
R01_cb18473_c2/f1p1/1761	NA	NA	NA	R01_cb18473_c2/f1p1/1761
R01_cb4094_c19/f1p3/693	NA	R01_cb4094_c19/f1p3/693	NA	NA
R01_cb8809_c13/f1p0/2609	NA	NA	NA	R01_cb8809_c13/f1p0/2609
R01_cb8564_c68762/f3p0/2900	NA	NA	NA	R01_cb8564_c68762/f3p0/2900

R01_cb6454_c11/f1p2/2533	NA	R01_cb6454_c11/f1p2/2533	NA	NA
R01_cb7642_c167/f1p2/2047	R01_cb7642_c167/f1p2/2047	R01_cb7642_c167/f1p2/2047	NA	NA
R01_cb6258_c22/f1p0/2295	NA	NA	NA	R01_cb6258_c22/f1p0/2295
R01_cb10328_c7/f1p1/971	R01_cb10328_c7/f1p1/971	R01_cb10328_c7/f1p1/971	NA	NA
R01_cb8973_c4/f1p0/387	R01_cb8973_c4/f1p0/387	R01_cb8973_c4/f1p0/387	R01_cb8973_c4/f1p0/387	NA
R01_cb17910_c0/f1p0/951	NA	R01_cb17910_c0/f1p0/951	NA	NA
R01_cb8564_c110469/f1p0/2627	NA	R01_cb8564_c110469/f1p0/2627	NA	NA
R01_cb2543_c34/f1p1/2506	R01_cb2543_c34/f1p1/2506	NA	NA	NA
R01_cb947_c12/f1p0/2397	NA	R01_cb947_c12/f1p0/2397	NA	NA
R01_cb5910_c20/f1p0/1822	R01_cb5910_c20/f1p0/1822	R01_cb5910_c20/f1p0/1822	NA	NA
R01_cb12401_c14/f3p1/1451	NA	R01_cb12401_c14/f3p1/1451	NA	NA
R01_cb17044_c16/f1p0/1871	R01_cb17044_c16/f1p0/1871	R01_cb17044_c16/f1p0/1871	R01_cb17044_c16/f1p0/1871	R01_cb17044_c16/f1p0/1871
R01_cb15675_c0/f4p0/503	R01_cb15675_c0/f4p0/503	R01_cb15675_c0/f4p0/503	R01_cb15675_c0/f4p0/503	R01_cb15675_c0/f4p0/503
R01_cb14316_c19/f1p0/399	R01_cb14316_c19/f1p0/399	R01_cb14316_c19/f1p0/399	R01_cb14316_c19/f1p0/399	R01_cb14316_c19/f1p0/399
R01_cb14019_c2/f1p0/1643	NA	R01_cb14019_c2/f1p0/1643	R01_cb14019_c2/f1p0/1643	R01_cb14019_c2/f1p0/1643
R01_cb1459_c7/f1p1/2451	NA	R01_cb1459_c7/f1p1/2451	NA	NA
R01_cb9312_c1/f1p0/2107	NA	R01_cb9312_c1/f1p0/2107	R01_cb9312_c1/f1p0/2107	R01_cb9312_c1/f1p0/2107
R01_cb2686_c5/f1p0/3171	NA	R01_cb2686_c5/f1p0/3171	NA	NA
R01_cb4968_c0/f2p0/764	R01_cb4968_c0/f2p0/764	R01_cb4968_c0/f2p0/764	R01_cb4968_c0/f2p0/764	R01_cb4968_c0/f2p0/764
R01_cb6923_c27/f1p0/2324	NA	R01_cb6923_c27/f1p0/2324	NA	R01_cb6923_c27/f1p0/2324
R01_cb2445_c24/f1p0/3446	NA	NA	NA	R01_cb2445_c24/f1p0/3446
R01_cb11564_c1/f1p0/2645	NA	R01_cb11564_c1/f1p0/2645	NA	NA
R01_cb16312_c2/f1p0/858	NA	R01_cb16312_c2/f1p0/858	NA	NA
R01_cb18456_c4768/f1p0/552	NA	NA	NA	R01_cb18456_c4768/f1p0/552
R01_cb12175_c1/f1p0/351	R01_cb12175_c1/f1p0/351	R01_cb12175_c1/f1p0/351	R01_cb12175_c1/f1p0/351	R01_cb12175_c1/f1p0/351

R01_cb8564_c89955/f1p0/2396	NA	R01_cb8564_c89955/f1p0/2396	R01_cb8564_c89955/f1p0/2396	R01_cb8564_c89955/f1p0/2396
R01_cb18482_c1/f1p0/1852	NA	NA	NA	R01_cb18482_c1/f1p0/1852
R01_cb12059_c11/f1p0/551	NA	R01_cb12059_c11/f1p0/551	NA	NA
R01_cb16387_c14/f1p0/795	R01_cb16387_c14/f1p0/795	R01_cb16387_c14/f1p0/795	R01_cb16387_c14/f1p0/795	R01_cb16387_c14/f1p0/795
R01_cb18456_c2477/f1p0/1616	NA	R01_cb18456_c2477/f1p0/1616	NA	R01_cb18456_c2477/f1p0/1616
R01_cb10413_c3/f1p0/1833	R01_cb10413_c3/f1p0/1833	R01_cb10413_c3/f1p0/1833	R01_cb10413_c3/f1p0/1833	R01_cb10413_c3/f1p0/1833
R01_cb17008_c0/f1p0/898	NA	NA	R01_cb17008_c0/f1p0/898	NA
R01_cb16722_c1/f2p0/500	R01_cb16722_c1/f2p0/500	R01_cb16722_c1/f2p0/500	NA	R01_cb16722_c1/f2p0/500
R01_cb8564_c23105/f3p0/4922	NA	R01_cb8564_c23105/f3p0/4922	NA	NA
R01_cb11376_c2/f1p0/946	NA	R01_cb11376_c2/f1p0/946	NA	R01_cb11376_c2/f1p0/946
R01_cb506_c0/f2p0/4721	NA	NA	NA	R01_cb506_c0/f2p0/4721
R01_cb8213_c1/f4p2/2420	NA	NA	NA	R01_cb8213_c1/f4p2/2420
R01_cb11064_c1/f1p0/2698	NA	R01_cb11064_c1/f1p0/2698	NA	R01_cb11064_c1/f1p0/2698
R01_cb18587_c1/f1p0/968	NA	NA	NA	R01_cb18587_c1/f1p0/968
R01_cb8564_c69463/f1p0/3886	NA	NA	NA	R01_cb8564_c69463/f1p0/3886
R01_cb6113_c5/f1p0/1920	NA	NA	NA	R01_cb6113_c5/f1p0/1920
R01_cb69_c20/f1p0/3744	NA	NA	NA	R01_cb69_c20/f1p0/3744
R01_cb8564_c121948/f1p0/3245	NA	NA	NA	R01_cb8564_c121948/f1p0/3245
R01_cb1085_c16/f1p0/3999	NA	NA	NA	R01_cb1085_c16/f1p0/3999
R01_cb8564_c3064/f2p0/3451	NA	R01_cb8564_c3064/f2p0/3451	NA	R01_cb8564_c3064/f2p0/3451
R01_cb8564_c9972/f2p0/3952	NA	NA	NA	R01_cb8564_c9972/f2p0/3952
R01_cb2701_c5/f1p0/3508	NA	R01_cb2701_c5/f1p0/3508	NA	R01_cb2701_c5/f1p0/3508
R01_cb8564_c72283/f1p0/2502	R01_cb8564_c72283/f1p0/2502	R01_cb8564_c72283/f1p0/2502	R01_cb8564_c72283/f1p0/2502	R01_cb8564_c72283/f1p0/2502
R01_cb8564_c12568/f1p3/3477	NA	R01_cb8564_c12568/f1p3/3477	R01_cb8564_c12568/f1p3/3477	R01_cb8564_c12568/f1p3/3477

R01_cb8564_c3691/f1p0/3023	R01_cb8564_c3691/f1p0/3023	R01_cb8564_c3691/f1p0/3023	R01_cb8564_c3691/f1p0/3023	R01_cb8564_c3691/f1p0/3023
R01_cb10602_c2/f1p0/1509	NA	R01_cb10602_c2/f1p0/1509	NA	NA
R01_cb8564_c127948/f1p0/2638	NA	NA	NA	R01_cb8564_c127948/f1p0/2638
R01_cb7268_c2/f1p0/1955	NA	NA	R01_cb7268_c2/f1p0/1955	NA
R01_cb7484_c8/f1p0/836	NA	R01_cb7484_c8/f1p0/836	NA	R01_cb7484_c8/f1p0/836
R01_cb9359_c5/f1p0/741	NA	R01_cb9359_c5/f1p0/741	R01_cb9359_c5/f1p0/741	R01_cb9359_c5/f1p0/741
R01_cb8564_c108071/f9p6/4539	NA	NA	NA	R01_cb8564_c108071/f9p6/4539
R01_cb14524_c7/f1p1/1689	NA	R01_cb14524_c7/f1p1/1689	NA	NA
R01_cb2619_c5/f1p0/1091	NA	NA	NA	R01_cb2619_c5/f1p0/1091
R01_cb8564_c4957/f1p0/2371	R01_cb8564_c4957/f1p0/2371	R01_cb8564_c4957/f1p0/2371	R01_cb8564_c4957/f1p0/2371	R01_cb8564_c4957/f1p0/2371
R01_cb8265_c1/f1p0/2774	NA	NA	NA	R01_cb8265_c1/f1p0/2774
R01_cb12722_c11/f1p0/539	NA	R01_cb12722_c11/f1p0/539	NA	R01_cb12722_c11/f1p0/539
R01_cb18409_c19/f2p0/522	R01_cb18409_c19/f2p0/522	R01_cb18409_c19/f2p0/522	R01_cb18409_c19/f2p0/522	R01_cb18409_c19/f2p0/522
R01_cb17476_c8/f1p0/779	NA	R01_cb17476_c8/f1p0/779	NA	NA
R01_cb8564_c10008/f3p1/1921	R01_cb8564_c10008/f3p1/1921	R01_cb8564_c10008/f3p1/1921	R01_cb8564_c10008/f3p1/1921	R01_cb8564_c10008/f3p1/1921
R01_cb7618_c8/f1p0/620	R01_cb7618_c8/f1p0/620	NA	NA	NA
R01_cb13626_c1/f1p0/620	NA	R01_cb13626_c1/f1p0/620	NA	R01_cb13626_c1/f1p0/620
R01_cb18539_c0/f1p0/617	NA	R01_cb18539_c0/f1p0/617	R01_cb18539_c0/f1p0/617	R01_cb18539_c0/f1p0/617
R01_cb8564_c13186/f1p0/3566	NA	R01_cb8564_c13186/f1p0/3566	NA	NA
R01_cb8564_c83392/f1p0/2890	NA	NA	NA	R01_cb8564_c83392/f1p0/2890
R01_cb18780_c4/f1p15/7629	NA	R01_cb18780_c4/f1p15/7629	NA	NA
R01_cb9551_c1/f1p0/2283	NA	NA	NA	R01_cb9551_c1/f1p0/2283
R01_cb14831_c3/f1p0/925	NA	R01_cb14831_c3/f1p0/925	NA	NA

R01_cb3295_c0/f3p0/3001	NA	R01_cb3295_c0/f3p0/3001	R01_cb3295_c0/f3p0/3001	R01_cb3295_c0/f3p0/3001
R01_cb6547_c1/f3p1/2576	NA	NA	NA	R01_cb6547_c1/f3p1/2576
R01_cb6141_c2/f1p0/2862	NA	R01_cb6141_c2/f1p0/2862	NA	NA
R01_cb8564_c717/f5p1/2631	NA	R01_cb8564_c717/f5p1/2631	NA	NA
R01_cb1448_c8/f1p0/3617	NA	R01_cb1448_c8/f1p0/3617	NA	NA
R01_cb18159_c0/f1p0/1722	R01_cb18159_c0/f1p0/1722	R01_cb18159_c0/f1p0/1722	R01_cb18159_c0/f1p0/1722	NA
R01_cb15814_c3/f2p0/1207	NA	NA	NA	R01_cb15814_c3/f2p0/1207
R01_cb1673_c1/f1p0/4194	R01_cb1673_c1/f1p0/4194	R01_cb1673_c1/f1p0/4194	NA	R01_cb1673_c1/f1p0/4194
R01_cb8564_c69249/f3p0/2365	NA	R01_cb8564_c69249/f3p0/2365	R01_cb8564_c69249/f3p0/2365	R01_cb8564_c69249/f3p0/2365
R01_cb8564_c91569/f1p0/3035	NA	R01_cb8564_c91569/f1p0/3035	R01_cb8564_c91569/f1p0/3035	NA
R01_cb3478_c4/f1p1/2986	NA	R01_cb3478_c4/f1p1/2986	R01_cb3478_c4/f1p1/2986	R01_cb3478_c4/f1p1/2986
R01_cb8564_c23361/f1p0/3463	NA	R01_cb8564_c23361/f1p0/3463	NA	NA
R01_cb18113_c3/f1p0/913	NA	R01_cb18113_c3/f1p0/913	R01_cb18113_c3/f1p0/913	R01_cb18113_c3/f1p0/913
R01_cb10467_c3/f1p1/2340	NA	R01_cb10467_c3/f1p1/2340	NA	NA
R01_cb6032_c10/f2p0/2950	R01_cb6032_c10/f2p0/2950	R01_cb6032_c10/f2p0/2950	NA	R01_cb6032_c10/f2p0/2950
R01_cb6333_c6/f1p0/2996	NA	R01_cb6333_c6/f1p0/2996	NA	NA
R01_cb4819_c5/f1p0/1224	NA	R01_cb4819_c5/f1p0/1224	R01_cb4819_c5/f1p0/1224	R01_cb4819_c5/f1p0/1224
R01_cb8564_c11209/f1p0/2189	NA	NA	NA	R01_cb8564_c11209/f1p0/2189
R01_cb10556_c3/f1p0/1457	NA	NA	NA	R01_cb10556_c3/f1p0/1457
R01_cb8564_c115621/f1p0/2138	NA	R01_cb8564_c115621/f1p0/2138	R01_cb8564_c115621/f1p0/2138	NA
R01_cb7334_c5/f1p0/1950	NA	NA	NA	R01_cb7334_c5/f1p0/1950
R01_cb6582_c18/f1p0/3412	NA	NA	R01_cb6582_c18/f1p0/3412	R01_cb6582_c18/f1p0/3412
R01_cb16152_c1/f1p0/724	R01_cb16152_c1/f1p0/724	R01_cb16152_c1/f1p0/724	R01_cb16152_c1/f1p0/724	R01_cb16152_c1/f1p0/724
R01_cb15169_c2/f3p0/1079	NA	NA	NA	R01_cb15169_c2/f3p0/1079
R01_cb16465_c3/f1p0/1112	NA	NA	NA	R01_cb16465_c3/f1p0/1112

R01_cb1030_c19/f1p0/4124	NA	NA	NA	R01_cb1030_c19/f1p0/4124
R01_cb3014_c1/f1p0/3259	NA	R01_cb3014_c1/f1p0/3259	NA	NA
R01_cb3295_c1/f1p0/3665	NA	R01_cb3295_c1/f1p0/3665	R01_cb3295_c1/f1p0/3665	R01_cb3295_c1/f1p0/3665
R01_cb10909_c5/f1p0/1742	NA	R01_cb10909_c5/f1p0/1742	NA	R01_cb10909_c5/f1p0/1742
R01_cb8564_c176016/f8p4/4823	NA	R01_cb8564_c176016/f8p4/4823	R01_cb8564_c176016/f8p4/4823	R01_cb8564_c176016/f8p4/4823
R01_cb5800_c2/f1p0/3449	NA	R01_cb5800_c2/f1p0/3449	NA	NA
R01_cb8564_c74065/f1p0/2258	NA	R01_cb8564_c74065/f1p0/2258	NA	NA
R01_cb18409_c50/f1p0/376	R01_cb18409_c50/f1p0/376	R01_cb18409_c50/f1p0/376	R01_cb18409_c50/f1p0/376	R01_cb18409_c50/f1p0/376
R01_cb7257_c2/f1p0/2073	NA	NA	NA	R01_cb7257_c2/f1p0/2073
R01_cb8102_c19/f1p0/1683	NA	NA	NA	R01_cb8102_c19/f1p0/1683
R01_cb2309_c7/f1p0/2411	NA	R01_cb2309_c7/f1p0/2411	NA	NA
R01_cb4030_c1/f1p0/3459	NA	NA	NA	R01_cb4030_c1/f1p0/3459
R01_cb14316_c18/f1p0/485	R01_cb14316_c18/f1p0/485	R01_cb14316_c18/f1p0/485	R01_cb14316_c18/f1p0/485	R01_cb14316_c18/f1p0/485
R01_cb18456_c4709/f1p0/480	NA	NA	NA	R01_cb18456_c4709/f1p0/480
R01_cb16758_c1/f1p0/1652	NA	R01_cb16758_c1/f1p0/1652	NA	NA
R01_cb639_c7/f1p0/748	R01_cb639_c7/f1p0/748	R01_cb639_c7/f1p0/748	NA	R01_cb639_c7/f1p0/748
R01_cb9404_c2/f1p0/2832	R01_cb9404_c2/f1p0/2832	R01_cb9404_c2/f1p0/2832	NA	NA
R01_cb6352_c13/f1p0/5504	R01_cb6352_c13/f1p0/5504	R01_cb6352_c13/f1p0/5504	NA	R01_cb6352_c13/f1p0/5504
R01_cb12740_c12/f1p0/374	R01_cb12740_c12/f1p0/374	R01_cb12740_c12/f1p0/374	R01_cb12740_c12/f1p0/374	R01_cb12740_c12/f1p0/374
R01_cb4715_c0/f1p0/3277	NA	NA	R01_cb4715_c0/f1p0/3277	R01_cb4715_c0/f1p0/3277
R01_cb2771_c13/f1p0/3186	NA	NA	NA	R01_cb2771_c13/f1p0/3186
R01_cb8533_c5/f1p0/2301	NA	R01_cb8533_c5/f1p0/2301	NA	NA
R01_cb8564_c22603/f1p0/2515	NA	R01_cb8564_c22603/f1p0/2515	NA	R01_cb8564_c22603/f1p0/2515
R01_cb11481_c0/f1p0/576	NA	R01_cb11481_c0/f1p0/576	NA	R01_cb11481_c0/f1p0/576
R01_cb18249_c0/f1p0/1819	R01_cb18249_c0/f1p0/1819	R01_cb18249_c0/f1p0/1819	R01_cb18249_c0/f1p0/1819	R01_cb18249_c0/f1p0/1819

R01_cb16097_c0/f1p0/1292	NA	R01_cb16097_c0/f1p0/1292	NA	NA
R01_cb7738_c1/f1p0/2547	NA	NA	NA	R01_cb7738_c1/f1p0/2547
R01_cb7946_c1/f1p0/2399	R01_cb7946_c1/f1p0/2399	R01_cb7946_c1/f1p0/2399	NA	NA
R01_cb8564_c91695/f1p1/2356	NA	R01_cb8564_c91695/f1p1/2356	R01_cb8564_c91695/f1p1/2356	NA
R01_cb8882_c3/f1p0/691	NA	R01_cb8882_c3/f1p0/691	NA	NA
R01_cb3618_c8/f1p0/3557	NA	NA	NA	R01_cb3618_c8/f1p0/3557
R01_cb13318_c0/f2p0/1440	NA	NA	NA	R01_cb13318_c0/f2p0/1440
R01_cb3071_c2/f1p0/1901	NA	NA	NA	R01_cb3071_c2/f1p0/1901
R01_cb5683_c5/f1p0/1541	NA	R01_cb5683_c5/f1p0/1541	NA	NA
R01_cb16246_c2/f1p0/1881	NA	R01_cb16246_c2/f1p0/1881	NA	NA
R01_cb8564_c128354/f1p0/2707	R01_cb8564_c128354/f1p0/2707	R01_cb8564_c128354/f1p0/2707	R01_cb8564_c128354/f1p0/2707	R01_cb8564_c128354/f1p0/2707
R01_cb13146_c12/f72p7/663	R01_cb13146_c12/f72p7/663	R01_cb13146_c12/f72p7/663	R01_cb13146_c12/f72p7/663	R01_cb13146_c12/f72p7/663
R01_cb1398_c13/f1p0/2329	NA	R01_cb1398_c13/f1p0/2329	R01_cb1398_c13/f1p0/2329	NA
R01_cb11465_c0/f1p0/1245	NA	NA	NA	R01_cb11465_c0/f1p0/1245
R01_cb5717_c0/f1p0/3026	NA	NA	NA	R01_cb5717_c0/f1p0/3026
R01_cb2804_c151/f1p0/2463	NA	R01_cb2804_c151/f1p0/2463	R01_cb2804_c151/f1p0/2463	R01_cb2804_c151/f1p0/2463
R01_cb8564_c114729/f1p1/2091	NA	R01_cb8564_c114729/f1p1/2091	R01_cb8564_c114729/f1p1/2091	NA
R01_cb8564_c85227/f1p0/2741	NA	NA	NA	R01_cb8564_c85227/f1p0/2741
R01_cb3281_c2/f2p0/2588	NA	R01_cb3281_c2/f2p0/2588	NA	NA
R01_cb928_c7/f1p0/2634	NA	NA	NA	R01_cb928_c7/f1p0/2634
R01_cb16682_c2/f3p0/954	R01_cb16682_c2/f3p0/954	R01_cb16682_c2/f3p0/954	R01_cb16682_c2/f3p0/954	R01_cb16682_c2/f3p0/954
R01_cb10394_c1/f1p0/1985	NA	NA	NA	R01_cb10394_c1/f1p0/1985
R01_cb8564_c13441/f2p2/3005	NA	NA	NA	R01_cb8564_c13441/f2p2/3005
R01_cb8564_c129572/f1p0/248	NA	R01_cb8564_c129572/f1p0/248	NA	NA

3		3		
R01_cb10640_c6/f1p0/450	R01_cb10640_c6/f1p0/450	R01_cb10640_c6/f1p0/450	R01_cb10640_c6/f1p0/450	R01_cb10640_c6/f1p0/450
R01_cb14267_c2/f1p0/385	R01_cb14267_c2/f1p0/385	R01_cb14267_c2/f1p0/385	R01_cb14267_c2/f1p0/385	R01_cb14267_c2/f1p0/385
R01_cb4410_c4/f1p0/5344	NA	R01_cb4410_c4/f1p0/5344	NA	NA
R01_cb2226_c4/f1p1/4842	NA	NA	NA	R01_cb2226_c4/f1p1/4842
R01_cb18456_c2533/f1p1/1470	NA	R01_cb18456_c2533/f1p1/1470	NA	NA
R01_cb8564_c91933/f1p0/2295	NA	NA	NA	R01_cb8564_c91933/f1p0/2295
R01_cb5017_c3/f1p0/2006	NA	NA	NA	R01_cb5017_c3/f1p0/2006
R01_cb10636_c10/f1p1/1193	NA	R01_cb10636_c10/f1p1/1193	NA	NA
R01_cb17744_c16/f1p0/3043	NA	NA	NA	R01_cb17744_c16/f1p0/3043
R01_cb8564_c81975/f1p2/2386	NA	R01_cb8564_c81975/f1p2/2386	NA	R01_cb8564_c81975/f1p2/2386
R01_cb8564_c4523/f1p0/2561	NA	R01_cb8564_c4523/f1p0/2561	NA	NA
R01_cb18487_c2/f1p0/1532	NA	R01_cb18487_c2/f1p0/1532	R01_cb18487_c2/f1p0/1532	R01_cb18487_c2/f1p0/1532
R01_cb8564_c38665/f1p0/4695	NA	R01_cb8564_c38665/f1p0/4695	NA	NA
R01_cb8564_c146271/f5p0/4276	NA	R01_cb8564_c146271/f5p0/4276	NA	NA
R01_cb4805_c5/f1p0/3640	NA	R01_cb4805_c5/f1p0/3640	NA	NA
R01_cb15537_c4/f1p0/633	NA	R01_cb15537_c4/f1p0/633	R01_cb15537_c4/f1p0/633	R01_cb15537_c4/f1p0/633
R01_cb5331_c5/f1p0/424	R01_cb5331_c5/f1p0/424	R01_cb5331_c5/f1p0/424	R01_cb5331_c5/f1p0/424	R01_cb5331_c5/f1p0/424
R01_cb8564_c91928/f1p0/3426	NA	NA	NA	R01_cb8564_c91928/f1p0/3426
R01_cb1948_c1/f1p0/4109	NA	NA	NA	R01_cb1948_c1/f1p0/4109
R01_cb8564_c111464/f1p0/2032	NA	NA	NA	R01_cb8564_c111464/f1p0/2032
R01_cb8564_c10353/f1p0/3086	NA	R01_cb8564_c10353/f1p0/3086	NA	NA
R01_cb12896_c5/f1p0/545	R01_cb12896_c5/f1p0/545	R01_cb12896_c5/f1p0/545	R01_cb12896_c5/f1p0/545	R01_cb12896_c5/f1p0/545
R01_cb3516_c7/f1p0/2664	NA	NA	NA	R01_cb3516_c7/f1p0/2664

R01_cb3171_c29/f1p0/3071	NA	R01_cb3171_c29/f1p0/3071	NA	R01_cb3171_c29/f1p0/3071
R01_cb8564_c22988/f1p0/2831	NA	R01_cb8564_c22988/f1p0/2831	NA	NA
R01_cb14560_c10/f6p0/844	NA	NA	NA	R01_cb14560_c10/f6p0/844
R01_cb8564_c88915/f1p0/2486	NA	R01_cb8564_c88915/f1p0/2486	NA	NA
R01_cb5815_c6/f1p0/2548	NA	R01_cb5815_c6/f1p0/2548	NA	NA
R01_cb5158_c1/f2p0/1308	NA	NA	R01_cb5158_c1/f2p0/1308	R01_cb5158_c1/f2p0/1308
R01_cb17654_c1/f1p1/382	R01_cb17654_c1/f1p1/382	R01_cb17654_c1/f1p1/382	R01_cb17654_c1/f1p1/382	R01_cb17654_c1/f1p1/382
R01_cb8298_c2/f1p1/2328	R01_cb8298_c2/f1p1/2328	R01_cb8298_c2/f1p1/2328	R01_cb8298_c2/f1p1/2328	R01_cb8298_c2/f1p1/2328
R01_cb11140_c1/f1p0/4513	R01_cb11140_c1/f1p0/4513	R01_cb11140_c1/f1p0/4513	R01_cb11140_c1/f1p0/4513	R01_cb11140_c1/f1p0/4513
R01_cb3972_c1/f1p0/3475	NA	R01_cb3972_c1/f1p0/3475	NA	NA
R01_cb8564_c71065/f1p0/2706	NA	R01_cb8564_c71065/f1p0/2706	NA	NA
R01_cb10628_c1/f1p0/2324	NA	R01_cb10628_c1/f1p0/2324	NA	R01_cb10628_c1/f1p0/2324
R01_cb16191_c5/f1p0/1779	NA	NA	NA	R01_cb16191_c5/f1p0/1779
R01_cb4117_c7/f1p0/1706	NA	NA	NA	R01_cb4117_c7/f1p0/1706
R01_cb260_c3/f1p1/4897	NA	NA	NA	R01_cb260_c3/f1p1/4897
R01_cb8564_c121319/f1p0/2495	NA	R01_cb8564_c121319/f1p0/2495	R01_cb8564_c121319/f1p0/2495	R01_cb8564_c121319/f1p0/2495
R01_cb11429_c1/f1p0/914	NA	NA	NA	R01_cb11429_c1/f1p0/914
R01_cb13283_c3/f1p2/1684	NA	NA	NA	R01_cb13283_c3/f1p2/1684
R01_cb17386_c0/f2p0/609	R01_cb17386_c0/f2p0/609	R01_cb17386_c0/f2p0/609	R01_cb17386_c0/f2p0/609	R01_cb17386_c0/f2p0/609
R01_cb14358_c0/f1p0/643	NA	NA	NA	R01_cb14358_c0/f1p0/643
R01_cb11463_c0/f1p0/572	NA	R01_cb11463_c0/f1p0/572	NA	NA
R01_cb8564_c112582/f1p0/2375	NA	R01_cb8564_c112582/f1p0/2375	NA	R01_cb8564_c112582/f1p0/2375
R01_cb1686_c13/f1p0/975	R01_cb1686_c13/f1p0/975	NA	R01_cb1686_c13/f1p0/975	R01_cb1686_c13/f1p0/975
R01_cb17973_c13/f1p0/676	R01_cb17973_c13/f1p0/676	R01_cb17973_c13/f1p0/676	R01_cb17973_c13/f1p0/676	NA

R01_cb10587_c4/f1p0/802	R01_cb10587_c4/f1p0/802	R01_cb10587_c4/f1p0/802	R01_cb10587_c4/f1p0/802	R01_cb10587_c4/f1p0/802
R01_cb7659_c1/f1p0/2433	NA	R01_cb7659_c1/f1p0/2433	R01_cb7659_c1/f1p0/2433	R01_cb7659_c1/f1p0/2433
R01_cb15299_c2/f1p0/654	R01_cb15299_c2/f1p0/654	R01_cb15299_c2/f1p0/654	R01_cb15299_c2/f1p0/654	NA
R01_cb18195_c0/f2p0/873	R01_cb18195_c0/f2p0/873	R01_cb18195_c0/f2p0/873	R01_cb18195_c0/f2p0/873	R01_cb18195_c0/f2p0/873
R01_cb8564_c5300/f1p0/3199	R01_cb8564_c5300/f1p0/3199	R01_cb8564_c5300/f1p0/3199	NA	NA
R01_cb16192_c0/f3p0/1335	NA	NA	NA	R01_cb16192_c0/f3p0/1335
R01_cb8085_c34/f1p0/436	R01_cb8085_c34/f1p0/436	R01_cb8085_c34/f1p0/436	R01_cb8085_c34/f1p0/436	R01_cb8085_c34/f1p0/436
R01_cb15104_c0/f1p0/863	NA	NA	NA	R01_cb15104_c0/f1p0/863
R01_cb13884_c12/f1p0/774	R01_cb13884_c12/f1p0/774	NA	R01_cb13884_c12/f1p0/774	R01_cb13884_c12/f1p0/774
R01_cb12577_c24/f1p1/929	NA	NA	NA	R01_cb12577_c24/f1p1/929
R01_cb1301_c2/f1p0/2816	NA	NA	NA	R01_cb1301_c2/f1p0/2816
R01_cb17053_c2/f2p0/606	NA	R01_cb17053_c2/f2p0/606	NA	R01_cb17053_c2/f2p0/606
R01_cb13134_c11/f13p0/1303	NA	NA	NA	R01_cb13134_c11/f13p0/1303
R01_cb14179_c1/f2p1/606	NA	NA	NA	R01_cb14179_c1/f2p1/606
R01_cb10918_c0/f2p0/1762	NA	NA	NA	R01_cb10918_c0/f2p0/1762
R01_cb2854_c23/f1p0/2438	NA	R01_cb2854_c23/f1p0/2438	NA	NA
R01_cb8564_c14972/f1p0/2171	NA	NA	NA	R01_cb8564_c14972/f1p0/2171
R01_cb6627_c1/f1p0/2322	NA	R01_cb6627_c1/f1p0/2322	NA	NA
R01_cb8564_c3851/f1p0/3843	NA	NA	NA	R01_cb8564_c3851/f1p0/3843
R01_cb9993_c10/f1p0/1028	NA	NA	NA	R01_cb9993_c10/f1p0/1028
R01_cb8564_c109277/f6p2/2046	NA	NA	NA	R01_cb8564_c109277/f6p2/2046
R01_cb10635_c1/f1p0/2867	R01_cb10635_c1/f1p0/2867	R01_cb10635_c1/f1p0/2867	NA	NA
R01_cb14997_c1/f1p0/789	NA	R01_cb14997_c1/f1p0/789	NA	NA
R01_cb13953_c34/f1p0/904	NA	R01_cb13953_c34/f1p0/904	NA	NA
R01_cb14971_c6/f1p0/1435	R01_cb14971_c6/f1p0/1435	NA	NA	R01_cb14971_c6/f1p0/1435

R01_cb9713_c2/f1p0/1862	R01_cb9713_c2/f1p0/1862	R01_cb9713_c2/f1p0/1862	NA	NA
R01_cb6989_c2/f1p0/2658	NA	R01_cb6989_c2/f1p0/2658	NA	NA
R01_cb8564_c73940/f1p0/2228	NA	R01_cb8564_c73940/f1p0/2228	NA	NA
R01_cb8564_c73163/f1p0/2503	NA	R01_cb8564_c73163/f1p0/2503	NA	NA
R01_cb13217_c2/f2p0/1110	NA	R01_cb13217_c2/f2p0/1110	R01_cb13217_c2/f2p0/1110	R01_cb13217_c2/f2p0/1110
R01_cb422_c6/f1p0/7951	NA	NA	NA	R01_cb422_c6/f1p0/7951
R01_cb14426_c4/f1p0/596	R01_cb14426_c4/f1p0/596	R01_cb14426_c4/f1p0/596	R01_cb14426_c4/f1p0/596	R01_cb14426_c4/f1p0/596
R01_cb8564_c112902/f1p1/4489	NA	R01_cb8564_c112902/f1p1/4489	NA	NA
R01_cb9520_c2/f1p0/1936	NA	R01_cb9520_c2/f1p0/1936	R01_cb9520_c2/f1p0/1936	R01_cb9520_c2/f1p0/1936
R01_cb14524_c8/f1p0/1159	NA	NA	NA	R01_cb14524_c8/f1p0/1159
R01_cb454_c22/f1p0/2434	NA	NA	NA	R01_cb454_c22/f1p0/2434
R01_cb12503_c7/f1p0/1197	R01_cb12503_c7/f1p0/1197	R01_cb12503_c7/f1p0/1197	NA	NA
R01_cb8564_c21230/f2p0/3496	R01_cb8564_c21230/f2p0/3496	R01_cb8564_c21230/f2p0/3496	NA	NA
R01_cb8564_c112596/f1p1/2137	NA	R01_cb8564_c112596/f1p1/2137	NA	NA
R01_cb8564_c113554/f1p0/2949	NA	R01_cb8564_c113554/f1p0/2949	NA	R01_cb8564_c113554/f1p0/2949
R01_cb15537_c1/f1p0/713	NA	R01_cb15537_c1/f1p0/713	R01_cb15537_c1/f1p0/713	R01_cb15537_c1/f1p0/713
R01_cb8564_c3519/f1p0/2826	R01_cb8564_c3519/f1p0/2826	R01_cb8564_c3519/f1p0/2826	NA	NA
R01_cb13033_c3/f1p0/844	NA	R01_cb13033_c3/f1p0/844	R01_cb13033_c3/f1p0/844	R01_cb13033_c3/f1p0/844
R01_cb2926_c14/f1p0/3432	NA	R01_cb2926_c14/f1p0/3432	NA	NA
R01_cb4738_c6/f1p1/1674	NA	R01_cb4738_c6/f1p1/1674	NA	NA
R01_cb15744_c4/f1p0/1789	NA	R01_cb15744_c4/f1p0/1789	NA	R01_cb15744_c4/f1p0/1789
R01_cb11049_c2/f1p0/1962	NA	NA	NA	R01_cb11049_c2/f1p0/1962

R01_cb1822_c3/f1p1/3787	NA	NA	NA	R01_cb1822_c3/f1p1/3787
R01_cb10817_c2/f1p0/1000	NA	NA	NA	R01_cb10817_c2/f1p0/1000
R01_cb15940_c0/f3p0/691	R01_cb15940_c0/f3p0/691	NA	R01_cb15940_c0/f3p0/691	R01_cb15940_c0/f3p0/691
R01_cb12577_c1/f2p2/959	NA	NA	NA	R01_cb12577_c1/f2p2/959
R01_cb16065_c0/f1p0/396	R01_cb16065_c0/f1p0/396	R01_cb16065_c0/f1p0/396	R01_cb16065_c0/f1p0/396	R01_cb16065_c0/f1p0/396
R01_cb12165_c22/f1p0/415	R01_cb12165_c22/f1p0/415	R01_cb12165_c22/f1p0/415	R01_cb12165_c22/f1p0/415	R01_cb12165_c22/f1p0/415
R01_cb12743_c1/f3p0/1017	NA	NA	NA	R01_cb12743_c1/f3p0/1017
R01_cb10843_c3/f2p0/872	NA	NA	NA	R01_cb10843_c3/f2p0/872
R01_cb5540_c6/f1p0/3242	NA	R01_cb5540_c6/f1p0/3242	NA	NA
R01_cb16137_c4/f4p0/1348	NA	NA	NA	R01_cb16137_c4/f4p0/1348
R01_cb4471_c6/f1p1/3051	NA	NA	NA	R01_cb4471_c6/f1p1/3051
R01_cb6121_c1/f2p0/2872	NA	R01_cb6121_c1/f2p0/2872	NA	NA
R01_cb7484_c3/f1p0/2926	NA	R01_cb7484_c3/f1p0/2926	NA	R01_cb7484_c3/f1p0/2926
R01_cb8564_c51420/f1p1/3936	NA	NA	NA	R01_cb8564_c51420/f1p1/3936
R01_cb18045_c1/f1p0/528	NA	R01_cb18045_c1/f1p0/528	NA	R01_cb18045_c1/f1p0/528
R01_cb15060_c3/f1p0/1224	NA	NA	NA	R01_cb15060_c3/f1p0/1224
R01_cb5565_c71/f1p0/3162	R01_cb5565_c71/f1p0/3162	R01_cb5565_c71/f1p0/3162	R01_cb5565_c71/f1p0/3162	R01_cb5565_c71/f1p0/3162
R01_cb8564_c49159/f1p0/2101	R01_cb8564_c49159/f1p0/2101	R01_cb8564_c49159/f1p0/2101	R01_cb8564_c49159/f1p0/2101	R01_cb8564_c49159/f1p0/2101
R01_cb13488_c11/f6p1/814	NA	NA	NA	R01_cb13488_c11/f6p1/814
R01_cb14761_c0/f3p0/704	R01_cb14761_c0/f3p0/704	R01_cb14761_c0/f3p0/704	R01_cb14761_c0/f3p0/704	R01_cb14761_c0/f3p0/704
R01_cb8564_c44941/f1p0/4540	NA	R01_cb8564_c44941/f1p0/4540	NA	NA
R01_cb18456_c7250/f1p0/589	NA	NA	NA	R01_cb18456_c7250/f1p0/589
R01_cb17565_c3/f1p0/1593	R01_cb17565_c3/f1p0/1593	R01_cb17565_c3/f1p0/1593	NA	NA
R01_cb16034_c0/f2p0/411	R01_cb16034_c0/f2p0/411	R01_cb16034_c0/f2p0/411	R01_cb16034_c0/f2p0/411	R01_cb16034_c0/f2p0/411
R01_cb11287_c4/f1p0/762	NA	NA	NA	R01_cb11287_c4/f1p0/762

R01_cb8564_c72011/f1p0/2524	R01_cb8564_c72011/f1p0/2524	R01_cb8564_c72011/f1p0/2524	NA	R01_cb8564_c72011/f1p0/2524
R01_cb5745_c11/f1p0/1408	NA	NA	NA	R01_cb5745_c11/f1p0/1408
R01_cb10024_c510/f1p0/626	R01_cb10024_c510/f1p0/626	NA	R01_cb10024_c510/f1p0/626	R01_cb10024_c510/f1p0/626
R01_cb11630_c1/f1p0/3027	NA	R01_cb11630_c1/f1p0/3027	R01_cb11630_c1/f1p0/3027	R01_cb11630_c1/f1p0/3027
R01_cb8564_c15292/f1p0/2336	NA	R01_cb8564_c15292/f1p0/2336	NA	NA
R01_cb14955_c3/f1p1/1130	NA	NA	NA	R01_cb14955_c3/f1p1/1130
R01_cb4411_c16/f1p0/2254	NA	R01_cb4411_c16/f1p0/2254	R01_cb4411_c16/f1p0/2254	R01_cb4411_c16/f1p0/2254
R01_cb12515_c14/f1p0/640	R01_cb12515_c14/f1p0/640	R01_cb12515_c14/f1p0/640	R01_cb12515_c14/f1p0/640	R01_cb12515_c14/f1p0/640
R01_cb13433_c96/f1p0/971	R01_cb13433_c96/f1p0/971	R01_cb13433_c96/f1p0/971	R01_cb13433_c96/f1p0/971	NA
R01_cb3774_c24/f1p0/2226	NA	NA	NA	R01_cb3774_c24/f1p0/2226
R01_cb6529_c2/f1p0/2832	NA	NA	NA	R01_cb6529_c2/f1p0/2832
R01_cb4524_c18/f1p0/315	R01_cb4524_c18/f1p0/315	R01_cb4524_c18/f1p0/315	R01_cb4524_c18/f1p0/315	R01_cb4524_c18/f1p0/315
R01_cb10423_c5/f1p0/1989	NA	R01_cb10423_c5/f1p0/1989	NA	NA
R01_cb260_c26/f1p0/5382	NA	NA	NA	R01_cb260_c26/f1p0/5382
R01_cb17583_c1/f1p0/1788	NA	NA	NA	R01_cb17583_c1/f1p0/1788
R01_cb16996_c2/f1p0/446	NA	R01_cb16996_c2/f1p0/446	R01_cb16996_c2/f1p0/446	R01_cb16996_c2/f1p0/446
R01_cb8564_c79500/f1p0/4029	NA	NA	NA	R01_cb8564_c79500/f1p0/4029
R01_cb1388_c10/f1p2/4356	NA	NA	NA	R01_cb1388_c10/f1p2/4356
R01_cb13696_c1/f1p0/1166	NA	NA	NA	R01_cb13696_c1/f1p0/1166
R01_cb8102_c17/f1p0/1782	NA	R01_cb8102_c17/f1p0/1782	NA	R01_cb8102_c17/f1p0/1782
R01_cb8785_c6/f1p0/2187	NA	NA	NA	R01_cb8785_c6/f1p0/2187
R01_cb12579_c19/f49p3/975	R01_cb12579_c19/f49p3/975	R01_cb12579_c19/f49p3/975	R01_cb12579_c19/f49p3/975	R01_cb12579_c19/f49p3/975
R01_cb872_c1/f1p0/4457	NA	NA	NA	R01_cb872_c1/f1p0/4457
R01_cb5643_c112/f1p0/1970	NA	R01_cb5643_c112/f1p0/1970	NA	NA
R01_cb18456_c1341/f4p0/621	R01_cb18456_c1341/f4p0/621	R01_cb18456_c1341/f4p0/621	R01_cb18456_c1341/f4p0/621	R01_cb18456_c1341/f4p0/621

R01_cb13287_c2/f6p0/537	NA	R01_cb13287_c2/f6p0/537	NA	R01_cb13287_c2/f6p0/537
R01_cb2352_c10/f1p0/3308	NA	R01_cb2352_c10/f1p0/3308	R01_cb2352_c10/f1p0/3308	R01_cb2352_c10/f1p0/3308
R01_cb18727_c2/f1p0/2155	NA	NA	R01_cb18727_c2/f1p0/2155	R01_cb18727_c2/f1p0/2155
R01_cb302_c3/f2p0/2680	NA	NA	NA	R01_cb302_c3/f2p0/2680
R01_cb2875_c21/f1p1/3307	NA	NA	NA	R01_cb2875_c21/f1p1/3307
R01_cb15465_c0/f2p2/523	R01_cb15465_c0/f2p2/523	NA	R01_cb15465_c0/f2p2/523	R01_cb15465_c0/f2p2/523
R01_cb2715_c3/f1p0/1560	NA	R01_cb2715_c3/f1p0/1560	NA	NA
R01_cb8564_c79363/f1p0/1977	NA	R01_cb8564_c79363/f1p0/1977	NA	NA
R01_cb8564_c15990/f1p0/4062	NA	R01_cb8564_c15990/f1p0/4062	NA	NA
R01_cb17371_c0/f1p0/727	NA	R01_cb17371_c0/f1p0/727	R01_cb17371_c0/f1p0/727	R01_cb17371_c0/f1p0/727
R01_cb8564_c75651/f1p0/1934	R01_cb8564_c75651/f1p0/1934	R01_cb8564_c75651/f1p0/1934	R01_cb8564_c75651/f1p0/1934	R01_cb8564_c75651/f1p0/1934
R01_cb8564_c50128/f1p0/4343	NA	NA	NA	R01_cb8564_c50128/f1p0/4343
R01_cb4338_c4/f1p0/2505	NA	R01_cb4338_c4/f1p0/2505	NA	R01_cb4338_c4/f1p0/2505
R01_cb16613_c1/f1p0/1655	NA	R01_cb16613_c1/f1p0/1655	NA	R01_cb16613_c1/f1p0/1655
R01_cb11263_c1/f1p0/2587	NA	R01_cb11263_c1/f1p0/2587	NA	NA
R01_cb12444_c51/f1p0/758	R01_cb12444_c51/f1p0/758	R01_cb12444_c51/f1p0/758	R01_cb12444_c51/f1p0/758	R01_cb12444_c51/f1p0/758
R01_cb1097_c0/f3p0/3913	NA	NA	NA	R01_cb1097_c0/f3p0/3913
R01_cb1228_c15/f1p0/2516	NA	NA	NA	R01_cb1228_c15/f1p0/2516
R01_cb8564_c12758/f1p0/2368	NA	NA	NA	R01_cb8564_c12758/f1p0/2368
R01_cb13724_c2/f1p2/533	NA	NA	R01_cb13724_c2/f1p2/533	R01_cb13724_c2/f1p2/533
R01_cb15712_c1/f1p0/551	NA	NA	NA	R01_cb15712_c1/f1p0/551
R01_cb3578_c9/f1p0/1757	NA	R01_cb3578_c9/f1p0/1757	NA	R01_cb3578_c9/f1p0/1757
R01_cb18303_c0/f1p0/546	NA	R01_cb18303_c0/f1p0/546	NA	R01_cb18303_c0/f1p0/546
R01_cb8564_c124837/f1p0/2085	NA	R01_cb8564_c124837/f1p0/2085	NA	NA

R01_cb15934_c3/f1p0/777	NA	R01_cb15934_c3/f1p0/777	NA	R01_cb15934_c3/f1p0/777
R01_cb13686_c13/f1p0/1157	NA	R01_cb13686_c13/f1p0/1157	NA	NA
R01_cb8564_c3739/f3p2/2520	NA	NA	NA	R01_cb8564_c3739/f3p2/2520
R01_cb6974_c0/f1p0/2726	NA	R01_cb6974_c0/f1p0/2726	NA	R01_cb6974_c0/f1p0/2726
R01_cb8322_c1/f1p0/2109	NA	NA	NA	R01_cb8322_c1/f1p0/2109
R01_cb6875_c9/f1p0/1845	NA	R01_cb6875_c9/f1p0/1845	NA	NA
R01_cb4649_c0/f1p0/3217	NA	NA	NA	R01_cb4649_c0/f1p0/3217
R01_cb8564_c75742/f1p0/4694	NA	R01_cb8564_c75742/f1p0/4694	NA	NA
R01_cb13746_c0/f6p1/450	R01_cb13746_c0/f6p1/450	R01_cb13746_c0/f6p1/450	NA	NA
R01_cb15326_c1/f1p0/1838	NA	R01_cb15326_c1/f1p0/1838	R01_cb15326_c1/f1p0/1838	R01_cb15326_c1/f1p0/1838
R01_cb9808_c5/f1p0/1886	NA	R01_cb9808_c5/f1p0/1886	NA	NA
R01_cb16606_c6/f1p0/445	R01_cb16606_c6/f1p0/445	R01_cb16606_c6/f1p0/445	NA	R01_cb16606_c6/f1p0/445
R01_cb16532_c0/f4p0/549	NA	R01_cb16532_c0/f4p0/549	NA	NA
R01_cb10015_c314/f1p0/591	NA	R01_cb10015_c314/f1p0/591	NA	NA
R01_cb18456_c2544/f1p0/1197	NA	NA	NA	R01_cb18456_c2544/f1p0/1197
R01_cb2997_c6/f1p0/3552	NA	NA	NA	R01_cb2997_c6/f1p0/3552
R01_cb16736_c2/f1p1/950	NA	R01_cb16736_c2/f1p1/950	NA	NA
R01_cb8279_c1/f2p0/2077	NA	NA	NA	R01_cb8279_c1/f2p0/2077
R01_cb2769_c4/f1p0/2801	NA	R01_cb2769_c4/f1p0/2801	NA	NA
R01_cb684_c1/f1p0/3859	NA	NA	NA	R01_cb684_c1/f1p0/3859
R01_cb2845_c34/f1p0/3557	NA	NA	NA	R01_cb2845_c34/f1p0/3557
R01_cb8779_c9/f1p0/3643	NA	NA	NA	R01_cb8779_c9/f1p0/3643
R01_cb5007_c7/f1p0/2427	NA	R01_cb5007_c7/f1p0/2427	NA	NA
R01_cb8564_c91116/f1p0/3098	NA	R01_cb8564_c91116/f1p0/3098	NA	NA
R01_cb8564_c92144/f1p1/2528	NA	R01_cb8564_c92144/f1p1/2528	R01_cb8564_c92144/f1p1/2528	R01_cb8564_c92144/f1p1/2528
R01_cb709_c6/f1p0/2450	NA	R01_cb709_c6/f1p0/2450	R01_cb709_c6/f1p0/2450	R01_cb709_c6/f1p0/2450

R01_cb8564_c109800/f6p1/3270	NA	NA	NA	R01_cb8564_c109800/f6p1/3270
R01_cb16110_c2/f1p0/910	NA	R01_cb16110_c2/f1p0/910	NA	R01_cb16110_c2/f1p0/910
R01_cb11823_c1/f1p0/2012	R01_cb11823_c1/f1p0/2012	R01_cb11823_c1/f1p0/2012	R01_cb11823_c1/f1p0/2012	R01_cb11823_c1/f1p0/2012
R01_cb6779_c4/f1p1/1903	NA	NA	NA	R01_cb6779_c4/f1p1/1903
R01_cb12443_c8/f1p0/480	R01_cb12443_c8/f1p0/480	R01_cb12443_c8/f1p0/480	R01_cb12443_c8/f1p0/480	R01_cb12443_c8/f1p0/480
R01_cb8564_c13431/f1p0/3551	NA	R01_cb8564_c13431/f1p0/3551	R01_cb8564_c13431/f1p0/3551	R01_cb8564_c13431/f1p0/3551
R01_cb8564_c49958/f1p0/3088	NA	R01_cb8564_c49958/f1p0/3088	NA	NA
R01_cb14634_c58/f1p0/2616	NA	R01_cb14634_c58/f1p0/2616	NA	NA
R01_cb15346_c3/f3p2/764	NA	NA	R01_cb15346_c3/f3p2/764	R01_cb15346_c3/f3p2/764
R01_cb295_c32/f1p2/2147	NA	R01_cb295_c32/f1p2/2147	NA	NA
R01_cb2378_c21/f1p0/3447	NA	NA	NA	R01_cb2378_c21/f1p0/3447
R01_cb15959_c3/f1p0/591	NA	R01_cb15959_c3/f1p0/591	NA	R01_cb15959_c3/f1p0/591
R01_cb1915_c44/f1p0/832	NA	R01_cb1915_c44/f1p0/832	NA	NA
R01_cb8564_c7830/f2p0/2010	R01_cb8564_c7830/f2p0/2010	R01_cb8564_c7830/f2p0/2010	R01_cb8564_c7830/f2p0/2010	R01_cb8564_c7830/f2p0/2010
R01_cb18456_c2538/f1p0/1445	NA	NA	NA	R01_cb18456_c2538/f1p0/1445
R01_cb3848_c18/f1p1/3165	NA	NA	NA	R01_cb3848_c18/f1p1/3165
R01_cb18485_c1/f1p0/476	R01_cb18485_c1/f1p0/476	R01_cb18485_c1/f1p0/476	NA	R01_cb18485_c1/f1p0/476
R01_cb8564_c4497/f1p1/2337	NA	R01_cb8564_c4497/f1p1/2337	R01_cb8564_c4497/f1p1/2337	R01_cb8564_c4497/f1p1/2337
R01_cb9793_c3/f1p1/1975	NA	NA	NA	R01_cb9793_c3/f1p1/1975
R01_cb4762_c2/f1p0/3400	NA	R01_cb4762_c2/f1p0/3400	NA	NA
R01_cb8564_c20845/f1p0/3839	NA	R01_cb8564_c20845/f1p0/3839	NA	NA
R01_cb5896_c139/f1p0/4158	NA	R01_cb5896_c139/f1p0/4158	NA	NA
R01_cb14226_c1/f1p0/757	NA	NA	R01_cb14226_c1/f1p0/757	R01_cb14226_c1/f1p0/757
R01_cb17358_c0/f1p0/1368	NA	NA	NA	R01_cb17358_c0/f1p0/1368
R01_cb8564_c23368/f1p0/2544	NA	R01_cb8564_c23368/f1p0/2544	NA	NA

R01_cb14231_c6/f1p0/1169	NA	R01_cb14231_c6/f1p0/1169	NA	NA
R01_cb11107_c6/f1p0/4998	NA	R01_cb11107_c6/f1p0/4998	NA	NA
R01_cb2451_c23/f4p2/3389	NA	NA	NA	R01_cb2451_c23/f4p2/3389
R01_cb18456_c6904/f1p0/504	R01_cb18456_c6904/f1p0/504	R01_cb18456_c6904/f1p0/504	NA	R01_cb18456_c6904/f1p0/504
R01_cb16070_c0/f1p0/1512	NA	NA	NA	R01_cb16070_c0/f1p0/1512
R01_cb14846_c0/f1p0/1147	NA	R01_cb14846_c0/f1p0/1147	R01_cb14846_c0/f1p0/1147	R01_cb14846_c0/f1p0/1147
R01_cb11248_c2/f1p0/3966	NA	R01_cb11248_c2/f1p0/3966	R01_cb11248_c2/f1p0/3966	NA
R01_cb8564_c51432/f1p0/2855	R01_cb8564_c51432/f1p0/2855	R01_cb8564_c51432/f1p0/2855	R01_cb8564_c51432/f1p0/2855	R01_cb8564_c51432/f1p0/2855
R01_cb16451_c0/f2p0/1367	NA	NA	NA	R01_cb16451_c0/f2p0/1367
R01_cb9797_c61/f1p0/2920	NA	NA	NA	R01_cb9797_c61/f1p0/2920
R01_cb7776_c24/f1p1/1975	NA	NA	NA	R01_cb7776_c24/f1p1/1975
R01_cb16426_c4/f1p0/333	R01_cb16426_c4/f1p0/333	R01_cb16426_c4/f1p0/333	R01_cb16426_c4/f1p0/333	R01_cb16426_c4/f1p0/333
R01_cb16442_c0/f2p0/1405	NA	R01_cb16442_c0/f2p0/1405	R01_cb16442_c0/f2p0/1405	R01_cb16442_c0/f2p0/1405
R01_cb12714_c44/f1p0/666	NA	R01_cb12714_c44/f1p0/666	R01_cb12714_c44/f1p0/666	R01_cb12714_c44/f1p0/666
R01_cb8205_c13/f1p0/2089	NA	R01_cb8205_c13/f1p0/2089	NA	NA
R01_cb1972_c6/f1p1/3771	NA	NA	NA	R01_cb1972_c6/f1p1/3771
R01_cb9105_c4/f1p1/2503	R01_cb9105_c4/f1p1/2503	R01_cb9105_c4/f1p1/2503	R01_cb9105_c4/f1p1/2503	R01_cb9105_c4/f1p1/2503
R01_cb8564_c113842/f1p0/1982	NA	R01_cb8564_c113842/f1p0/1982	NA	NA
R01_cb18456_c4730/f1p2/491	NA	NA	NA	R01_cb18456_c4730/f1p2/491
R01_cb6121_c11/f1p0/2005	NA	R01_cb6121_c11/f1p0/2005	NA	R01_cb6121_c11/f1p0/2005
R01_cb16520_c2/f1p0/1071	NA	NA	NA	R01_cb16520_c2/f1p0/1071
R01_cb18456_c1513/f3p0/459	R01_cb18456_c1513/f3p0/459	R01_cb18456_c1513/f3p0/459	R01_cb18456_c1513/f3p0/459	R01_cb18456_c1513/f3p0/459
R01_cb14647_c3/f2p0/1563	NA	NA	NA	R01_cb14647_c3/f2p0/1563
R01_cb12204_c6/f7p0/1219	R01_cb12204_c6/f7p0/1219	R01_cb12204_c6/f7p0/1219	R01_cb12204_c6/f7p0/1219	R01_cb12204_c6/f7p0/1219

R01_cb17299_c0/f1p0/1077	NA	NA	NA	R01_cb17299_c0/f1p0/1077
R01_cb1779_c8/f1p0/2872	NA	NA	NA	R01_cb1779_c8/f1p0/2872
R01_cb8564_c117051/f1p0/2397	R01_cb8564_c117051/f1p0/2397	R01_cb8564_c117051/f1p0/2397	R01_cb8564_c117051/f1p0/2397	R01_cb8564_c117051/f1p0/2397
R01_cb2729_c4/f1p0/2000	NA	R01_cb2729_c4/f1p0/2000	NA	R01_cb2729_c4/f1p0/2000
R01_cb15845_c1/f1p0/625	NA	R01_cb15845_c1/f1p0/625	NA	NA
R01_cb17266_c27/f1p0/1588	R01_cb17266_c27/f1p0/1588	R01_cb17266_c27/f1p0/1588	R01_cb17266_c27/f1p0/1588	R01_cb17266_c27/f1p0/1588
R01_cb2842_c19/f1p0/2967	NA	R01_cb2842_c19/f1p0/2967	NA	NA
R01_cb8564_c112735/f1p0/2180	NA	R01_cb8564_c112735/f1p0/2180	R01_cb8564_c112735/f1p0/2180	NA
R01_cb8564_c43330/f1p0/3011	NA	R01_cb8564_c43330/f1p0/3011	NA	R01_cb8564_c43330/f1p0/3011
R01_cb11679_c0/f1p0/974	NA	R01_cb11679_c0/f1p0/974	NA	NA
R01_cb17221_c2/f2p0/1091	NA	R01_cb17221_c2/f2p0/1091	NA	R01_cb17221_c2/f2p0/1091
R01_cb8564_c52651/f1p0/3529	NA	R01_cb8564_c52651/f1p0/3529	NA	NA
R01_cb8564_c85766/f1p0/2274	NA	NA	NA	R01_cb8564_c85766/f1p0/2274
R01_cb16982_c0/f2p0/1397	NA	NA	NA	R01_cb16982_c0/f2p0/1397
R01_cb11741_c0/f1p0/1246	NA	R01_cb11741_c0/f1p0/1246	R01_cb11741_c0/f1p0/1246	R01_cb11741_c0/f1p0/1246
R01_cb8564_c81210/f1p0/2478	NA	R01_cb8564_c81210/f1p0/2478	NA	NA
R01_cb9871_c1/f4p2/762	NA	R01_cb9871_c1/f4p2/762	NA	NA
R01_cb16051_c4/f1p0/990	R01_cb16051_c4/f1p0/990	R01_cb16051_c4/f1p0/990	R01_cb16051_c4/f1p0/990	R01_cb16051_c4/f1p0/990
R01_cb15496_c0/f4p0/795	NA	NA	NA	R01_cb15496_c0/f4p0/795
R01_cb14619_c9/f1p0/1471	NA	R01_cb14619_c9/f1p0/1471	R01_cb14619_c9/f1p0/1471	R01_cb14619_c9/f1p0/1471
R01_cb16555_c1/f1p1/733	NA	NA	NA	R01_cb16555_c1/f1p1/733
R01_cb10218_c4/f1p0/976	NA	R01_cb10218_c4/f1p0/976	NA	NA
R01_cb13884_c2/f2p0/528	R01_cb13884_c2/f2p0/528	NA	R01_cb13884_c2/f2p0/528	R01_cb13884_c2/f2p0/528
R01_cb10960_c2/f1p0/1269	NA	NA	NA	R01_cb10960_c2/f1p0/1269

R01_cb8564_c68286/f3p0/2248	NA	R01_cb8564_c68286/f3p0/2248	R01_cb8564_c68286/f3p0/2248	R01_cb8564_c68286/f3p0/2248
R01_cb3596_c3/f1p0/2873	R01_cb3596_c3/f1p0/2873	R01_cb3596_c3/f1p0/2873	NA	R01_cb3596_c3/f1p0/2873
R01_cb9932_c4/f1p0/615	NA	R01_cb9932_c4/f1p0/615	R01_cb9932_c4/f1p0/615	NA
R01_cb8564_c40810/f1p0/3998	NA	NA	NA	R01_cb8564_c40810/f1p0/3998
R01_cb10660_c6/f1p0/392	NA	R01_cb10660_c6/f1p0/392	NA	R01_cb10660_c6/f1p0/392
R01_cb18456_c6564/f1p0/879	R01_cb18456_c6564/f1p0/879	NA	NA	NA
R01_cb3863_c9/f5p0/3156	NA	NA	NA	R01_cb3863_c9/f5p0/3156
R01_cb16656_c0/f1p0/671	NA	R01_cb16656_c0/f1p0/671	NA	NA
R01_cb3840_c3/f1p0/2107	NA	R01_cb3840_c3/f1p0/2107	NA	NA
R01_cb10326_c4/f1p0/2046	R01_cb10326_c4/f1p0/2046	R01_cb10326_c4/f1p0/2046	R01_cb10326_c4/f1p0/2046	R01_cb10326_c4/f1p0/2046
R01_cb11261_c3/f1p0/695	NA	R01_cb11261_c3/f1p0/695	R01_cb11261_c3/f1p0/695	R01_cb11261_c3/f1p0/695
R01_cb6309_c7/f1p0/634	R01_cb6309_c7/f1p0/634	R01_cb6309_c7/f1p0/634	R01_cb6309_c7/f1p0/634	R01_cb6309_c7/f1p0/634
R01_cb13505_c2/f2p0/1492	R01_cb13505_c2/f2p0/1492	R01_cb13505_c2/f2p0/1492	NA	NA
R01_cb4441_c1/f1p0/2698	NA	NA	R01_cb4441_c1/f1p0/2698	NA
R01_cb7392_c1/f1p0/2538	NA	NA	NA	R01_cb7392_c1/f1p0/2538
R01_cb8564_c86250/f1p0/3654	NA	NA	NA	R01_cb8564_c86250/f1p0/3654
R01_cb4657_c27/f1p0/3021	NA	NA	NA	R01_cb4657_c27/f1p0/3021
R01_cb7230_c1/f2p0/1276	NA	R01_cb7230_c1/f2p0/1276	NA	NA
R01_cb17899_c0/f1p0/680	R01_cb17899_c0/f1p0/680	R01_cb17899_c0/f1p0/680	NA	R01_cb17899_c0/f1p0/680
R01_cb11918_c11/f1p1/862	NA	NA	NA	R01_cb11918_c11/f1p1/862
R01_cb17838_c0/f1p0/1140	NA	NA	R01_cb17838_c0/f1p0/1140	NA
R01_cb2817_c3/f1p0/3142	NA	R01_cb2817_c3/f1p0/3142	NA	NA
R01_cb2451_c9/f1p0/3925	NA	NA	NA	R01_cb2451_c9/f1p0/3925
R01_cb5736_c3/f1p2/7025	NA	NA	NA	R01_cb5736_c3/f1p2/7025
R01_cb17013_c2/f1p0/791	NA	NA	NA	R01_cb17013_c2/f1p0/791
R01_cb18203_c0/f1p0/1475	NA	R01_cb18203_c0/f1p0/1475	R01_cb18203_c0/f1p0/1475	R01_cb18203_c0/f1p0/1475

R01_cb16397_c0/f1p0/796	R01_cb16397_c0/f1p0/796	R01_cb16397_c0/f1p0/796	R01_cb16397_c0/f1p0/796	R01_cb16397_c0/f1p0/796
R01_cb4037_c5/f1p0/1879	NA	NA	NA	R01_cb4037_c5/f1p0/1879
R01_cb15735_c1/f1p0/592	R01_cb15735_c1/f1p0/592	R01_cb15735_c1/f1p0/592	NA	NA
R01_cb5808_c1/f2p0/1133	NA	NA	NA	R01_cb5808_c1/f2p0/1133
R01_cb11263_c0/f1p0/638	R01_cb11263_c0/f1p0/638	R01_cb11263_c0/f1p0/638	R01_cb11263_c0/f1p0/638	R01_cb11263_c0/f1p0/638
R01_cb17053_c4/f1p0/722	NA	R01_cb17053_c4/f1p0/722	NA	R01_cb17053_c4/f1p0/722
R01_cb7443_c0/f1p0/2611	NA	NA	NA	R01_cb7443_c0/f1p0/2611
R01_cb18456_c1817/f1p0/1206	R01_cb18456_c1817/f1p0/1206	R01_cb18456_c1817/f1p0/1206	NA	R01_cb18456_c1817/f1p0/1206
R01_cb6845_c36/f11p1/2381	NA	NA	NA	R01_cb6845_c36/f11p1/2381
R01_cb13938_c12/f1p0/1533	NA	NA	NA	R01_cb13938_c12/f1p0/1533
R01_cb1915_c25/f1p0/1838	NA	R01_cb1915_c25/f1p0/1838	NA	NA
R01_cb5896_c75/f1p0/2348	NA	R01_cb5896_c75/f1p0/2348	R01_cb5896_c75/f1p0/2348	R01_cb5896_c75/f1p0/2348
R01_cb8564_c71710/f1p0/1983	NA	R01_cb8564_c71710/f1p0/1983	NA	NA
R01_cb12757_c2/f1p0/1113	NA	NA	NA	R01_cb12757_c2/f1p0/1113
R01_cb6570_c1/f1p0/2821	NA	R01_cb6570_c1/f1p0/2821	NA	R01_cb6570_c1/f1p0/2821
R01_cb18456_c4747/f1p0/430	NA	R01_cb18456_c4747/f1p0/430	R01_cb18456_c4747/f1p0/430	R01_cb18456_c4747/f1p0/430
R01_cb15312_c4/f2p1/655	NA	R01_cb15312_c4/f2p1/655	R01_cb15312_c4/f2p1/655	R01_cb15312_c4/f2p1/655
R01_cb12336_c12/f1p0/1003	NA	R01_cb12336_c12/f1p0/1003	NA	R01_cb12336_c12/f1p0/1003
R01_cb17468_c1/f1p0/816	NA	NA	R01_cb17468_c1/f1p0/816	R01_cb17468_c1/f1p0/816
R01_cb1228_c40/f1p0/2699	NA	NA	NA	R01_cb1228_c40/f1p0/2699
R01_cb8564_c88969/f1p0/2150	NA	R01_cb8564_c88969/f1p0/2150	NA	NA
R01_cb6057_c4/f1p0/1955	NA	NA	NA	R01_cb6057_c4/f1p0/1955
R01_cb4580_c6/f1p0/6353	NA	NA	NA	R01_cb4580_c6/f1p0/6353
R01_cb8564_c75714/f1p0/1945	NA	R01_cb8564_c75714/f1p0/1945	NA	NA
R01_cb14480_c1/f2p0/810	NA	NA	NA	R01_cb14480_c1/f2p0/810

R01_cb2382_c7/f1p0/5459	NA	NA	NA	R01_cb2382_c7/f1p0/5459
R01_cb14547_c15/f2p1/787	NA	R01_cb14547_c15/f2p1/787	NA	NA
R01_cb8564_c23224/f1p0/2195	R01_cb8564_c23224/f1p0/2195	R01_cb8564_c23224/f1p0/2195	NA	NA
R01_cb12696_c13/f1p0/572	NA	R01_cb12696_c13/f1p0/572	NA	NA
R01_cb4094_c24/f1p0/618	NA	R01_cb4094_c24/f1p0/618	NA	NA
R01_cb10913_c0/f5p0/975	NA	NA	NA	R01_cb10913_c0/f5p0/975
R01_cb16867_c1/f1p0/1256	NA	NA	NA	R01_cb16867_c1/f1p0/1256
R01_cb8135_c1/f1p0/2447	NA	R01_cb8135_c1/f1p0/2447	NA	NA
R01_cb3542_c18/f1p0/5167	NA	R01_cb3542_c18/f1p0/5167	NA	NA
R01_cb5554_c8/f1p0/1768	NA	R01_cb5554_c8/f1p0/1768	R01_cb5554_c8/f1p0/1768	NA
R01_cb18456_c7224/f2p0/465	R01_cb18456_c7224/f2p0/465	R01_cb18456_c7224/f2p0/465	R01_cb18456_c7224/f2p0/465	R01_cb18456_c7224/f2p0/465
R01_cb7096_c21/f1p0/2561	NA	NA	NA	R01_cb7096_c21/f1p0/2561
R01_cb8564_c109754/f2p0/1926	NA	NA	NA	R01_cb8564_c109754/f2p0/1926
R01_cb17192_c0/f1p0/969	NA	R01_cb17192_c0/f1p0/969	NA	NA
R01_cb11204_c5/f1p0/1010	NA	NA	NA	R01_cb11204_c5/f1p0/1010
R01_cb454_c19/f1p0/2558	NA	NA	NA	R01_cb454_c19/f1p0/2558
R01_cb13820_c7/f1p0/1282	R01_cb13820_c7/f1p0/1282	R01_cb13820_c7/f1p0/1282	R01_cb13820_c7/f1p0/1282	R01_cb13820_c7/f1p0/1282
R01_cb12641_c34/f1p0/690	NA	R01_cb12641_c34/f1p0/690	R01_cb12641_c34/f1p0/690	R01_cb12641_c34/f1p0/690
R01_cb6888_c3/f1p0/1552	NA	R01_cb6888_c3/f1p0/1552	NA	NA
R01_cb8564_c69069/f2p2/2031	NA	R01_cb8564_c69069/f2p2/2031	R01_cb8564_c69069/f2p2/2031	R01_cb8564_c69069/f2p2/2031
R01_cb8374_c4/f1p0/2094	NA	R01_cb8374_c4/f1p0/2094	NA	NA
R01_cb18456_c6834/f1p0/1557	NA	NA	NA	R01_cb18456_c6834/f1p0/1557
R01_cb2616_c1/f1p0/4840	NA	NA	NA	R01_cb2616_c1/f1p0/4840
R01_cb10133_c7/f1p1/1730	NA	NA	NA	R01_cb10133_c7/f1p1/1730

R01_cb507_c6/f1p0/1656	NA	NA	NA	R01_cb507_c6/f1p0/1656
R01_cb8564_c152980/f1p0/3128	NA	NA	NA	R01_cb8564_c152980/f1p0/3128
R01_cb10337_c6/f1p2/1204	NA	R01_cb10337_c6/f1p2/1204	NA	NA
R01_cb2133_c36/f1p0/2399	NA	R01_cb2133_c36/f1p0/2399	NA	NA
R01_cb11338_c1/f1p1/2802	NA	R01_cb11338_c1/f1p1/2802	NA	R01_cb11338_c1/f1p1/2802
R01_cb12155_c20/f1p1/1710	NA	R01_cb12155_c20/f1p1/1710	NA	NA
R01_cb13042_c4/f1p0/1356	NA	R01_cb13042_c4/f1p0/1356	NA	NA
R01_cb7484_c5/f1p0/3868	NA	R01_cb7484_c5/f1p0/3868	R01_cb7484_c5/f1p0/3868	NA
R01_cb3563_c5/f1p0/2705	NA	NA	NA	R01_cb3563_c5/f1p0/2705
R01_cb10855_c0/f1p0/1369	NA	R01_cb10855_c0/f1p0/1369	NA	NA
R01_cb10543_c1/f1p0/2241	NA	R01_cb10543_c1/f1p0/2241	NA	NA
R01_cb13515_c7/f1p0/1823	NA	R01_cb13515_c7/f1p0/1823	NA	R01_cb13515_c7/f1p0/1823
R01_cb17118_c6/f1p0/1474	R01_cb17118_c6/f1p0/1474	R01_cb17118_c6/f1p0/1474	R01_cb17118_c6/f1p0/1474	R01_cb17118_c6/f1p0/1474
R01_cb365_c1/f2p1/4237	NA	NA	NA	R01_cb365_c1/f2p1/4237
R01_cb10303_c0/f1p0/704	R01_cb10303_c0/f1p0/704	R01_cb10303_c0/f1p0/704	R01_cb10303_c0/f1p0/704	R01_cb10303_c0/f1p0/704
R01_cb4652_c4/f1p0/2883	NA	R01_cb4652_c4/f1p0/2883	NA	NA
R01_cb17274_c3/f1p0/1657	NA	R01_cb17274_c3/f1p0/1657	R01_cb17274_c3/f1p0/1657	R01_cb17274_c3/f1p0/1657
R01_cb191_c15/f1p1/5569	NA	NA	NA	R01_cb191_c15/f1p1/5569
R01_cb18363_c1/f1p0/766	NA	NA	R01_cb18363_c1/f1p0/766	R01_cb18363_c1/f1p0/766
R01_cb11557_c1/f1p0/3488	NA	R01_cb11557_c1/f1p0/3488	NA	R01_cb11557_c1/f1p0/3488
R01_cb11721_c0/f1p0/793	NA	R01_cb11721_c0/f1p0/793	R01_cb11721_c0/f1p0/793	R01_cb11721_c0/f1p0/793
R01_cb8564_c85248/f1p0/3015	NA	R01_cb8564_c85248/f1p0/3015	R01_cb8564_c85248/f1p0/3015	R01_cb8564_c85248/f1p0/3015
R01_cb18456_c2308/f1p0/1537	NA	NA	NA	R01_cb18456_c2308/f1p0/1537
R01_cb13809_c0/f1p0/1776	R01_cb13809_c0/f1p0/1776	R01_cb13809_c0/f1p0/1776	R01_cb13809_c0/f1p0/1776	R01_cb13809_c0/f1p0/1776
R01_cb15145_c1/f1p0/417	NA	R01_cb15145_c1/f1p0/417	R01_cb15145_c1/f1p0/417	NA

R01_cb10024_c318/f1p0/642	NA	NA	R01_cb10024_c318/f1p0/642	NA
R01_cb8564_c88321/f1p0/2683	NA	NA	NA	R01_cb8564_c88321/f1p0/2683
R01_cb14316_c10/f1p0/571	R01_cb14316_c10/f1p0/571	R01_cb14316_c10/f1p0/571	R01_cb14316_c10/f1p0/571	R01_cb14316_c10/f1p0/571
R01_cb4824_c10/f1p0/3091	R01_cb4824_c10/f1p0/3091	R01_cb4824_c10/f1p0/3091	R01_cb4824_c10/f1p0/3091	R01_cb4824_c10/f1p0/3091
R01_cb8564_c69907/f1p0/2519	NA	R01_cb8564_c69907/f1p0/2519	R01_cb8564_c69907/f1p0/2519	R01_cb8564_c69907/f1p0/2519
R01_cb2154_c7/f1p0/3516	R01_cb2154_c7/f1p0/3516	R01_cb2154_c7/f1p0/3516	NA	NA
R01_cb18456_c7425/f1p0/503	R01_cb18456_c7425/f1p0/503	R01_cb18456_c7425/f1p0/503	R01_cb18456_c7425/f1p0/503	R01_cb18456_c7425/f1p0/503
R01_cb2970_c7/f1p0/528	NA	NA	NA	R01_cb2970_c7/f1p0/528
R01_cb9081_c4/f1p0/2358	NA	NA	NA	R01_cb9081_c4/f1p0/2358
R01_cb3582_c2/f1p0/3551	NA	R01_cb3582_c2/f1p0/3551	NA	NA
R01_cb17973_c19/f1p0/1044	NA	R01_cb17973_c19/f1p0/1044	NA	NA
R01_cb8628_c0/f2p0/2305	R01_cb8628_c0/f2p0/2305	R01_cb8628_c0/f2p0/2305	R01_cb8628_c0/f2p0/2305	R01_cb8628_c0/f2p0/2305
R01_cb15286_c6/f1p0/665	NA	R01_cb15286_c6/f1p0/665	NA	NA
R01_cb4273_c13/f1p1/5739	NA	R01_cb4273_c13/f1p1/5739	NA	NA
R01_cb8564_c69195/f2p0/2514	R01_cb8564_c69195/f2p0/2514	R01_cb8564_c69195/f2p0/2514	NA	NA
R01_cb3079_c2/f1p0/3958	NA	NA	NA	R01_cb3079_c2/f1p0/3958
R01_cb17749_c1/f1p0/1516	NA	R01_cb17749_c1/f1p0/1516	R01_cb17749_c1/f1p0/1516	R01_cb17749_c1/f1p0/1516
R01_cb12369_c12/f5p1/561	NA	NA	R01_cb12369_c12/f5p1/561	NA
R01_cb6391_c5/f1p0/2187	NA	R01_cb6391_c5/f1p0/2187	NA	NA
R01_cb9928_c7/f1p0/2076	NA	NA	NA	R01_cb9928_c7/f1p0/2076
R01_cb8564_c22694/f1p0/4624	NA	R01_cb8564_c22694/f1p0/4624	R01_cb8564_c22694/f1p0/4624	R01_cb8564_c22694/f1p0/4624
R01_cb16914_c1/f1p0/1100	NA	R01_cb16914_c1/f1p0/1100	NA	NA
R01_cb10593_c0/f1p0/1416	R01_cb10593_c0/f1p0/1416	R01_cb10593_c0/f1p0/1416	R01_cb10593_c0/f1p0/1416	R01_cb10593_c0/f1p0/1416
R01_cb16861_c2/f1p0/671	NA	R01_cb16861_c2/f1p0/671	NA	NA
R01_cb2133_c43/f1p0/3110	NA	R01_cb2133_c43/f1p0/3110	NA	NA

R01_cb5774_c1/f1p0/3101	NA	R01_cb5774_c1/f1p0/3101	NA	R01_cb5774_c1/f1p0/3101
R01_cb10696_c3/f1p0/1087	R01_cb10696_c3/f1p0/1087	R01_cb10696_c3/f1p0/1087	R01_cb10696_c3/f1p0/1087	NA
R01_cb3426_c14/f1p0/3417	NA	NA	NA	R01_cb3426_c14/f1p0/3417
R01_cb8335_c8/f1p0/2650	NA	NA	NA	R01_cb8335_c8/f1p0/2650
R01_cb6802_c60/f1p0/3428	NA	R01_cb6802_c60/f1p0/3428	R01_cb6802_c60/f1p0/3428	R01_cb6802_c60/f1p0/3428
R01_cb10024_c215/f1p0/586	NA	NA	R01_cb10024_c215/f1p0/586	R01_cb10024_c215/f1p0/586
R01_cb7741_c3/f1p0/2544	NA	NA	R01_cb7741_c3/f1p0/2544	R01_cb7741_c3/f1p0/2544
R01_cb13033_c0/f5p0/948	NA	R01_cb13033_c0/f5p0/948	R01_cb13033_c0/f5p0/948	R01_cb13033_c0/f5p0/948
R01_cb13463_c11/f1p0/556	NA	R01_cb13463_c11/f1p0/556	NA	R01_cb13463_c11/f1p0/556
R01_cb1130_c5/f1p0/2483	NA	R01_cb1130_c5/f1p0/2483	NA	NA
R01_cb14125_c159/f1p0/767	NA	R01_cb14125_c159/f1p0/767	R01_cb14125_c159/f1p0/767	NA
R01_cb2552_c4/f1p0/2025	NA	R01_cb2552_c4/f1p0/2025	NA	NA
R01_cb14832_c0/f2p0/721	NA	NA	NA	R01_cb14832_c0/f2p0/721
R01_cb8564_c116137/f1p0/2429	R01_cb8564_c116137/f1p0/2429	R01_cb8564_c116137/f1p0/2429	NA	R01_cb8564_c116137/f1p0/2429
R01_cb6141_c3/f1p0/2881	NA	R01_cb6141_c3/f1p0/2881	R01_cb6141_c3/f1p0/2881	NA
R01_cb2176_c10/f1p0/797	NA	R01_cb2176_c10/f1p0/797	NA	NA
R01_cb9382_c0/f2p0/2105	NA	NA	NA	R01_cb9382_c0/f2p0/2105
R01_cb10015_c403/f1p0/830	NA	R01_cb10015_c403/f1p0/830	NA	NA
R01_cb2902_c2/f1p0/1960	NA	R01_cb2902_c2/f1p0/1960	NA	NA
R01_cb13602_c23/f1p0/1802	NA	NA	NA	R01_cb13602_c23/f1p0/1802
R01_cb16016_c1/f1p0/1051	NA	NA	NA	R01_cb16016_c1/f1p0/1051
R01_cb1388_c23/f1p1/4341	NA	NA	NA	R01_cb1388_c23/f1p1/4341
R01_cb18456_c7214/f1p0/538	NA	R01_cb18456_c7214/f1p0/538	R01_cb18456_c7214/f1p0/538	R01_cb18456_c7214/f1p0/538
R01_cb8957_c1/f1p0/2225	NA	R01_cb8957_c1/f1p0/2225	NA	NA
R01_cb2415_c12/f1p1/6668	NA	NA	NA	R01_cb2415_c12/f1p1/6668

R01_cb3854_c1/f1p0/2568	NA	NA	R01_cb3854_c1/f1p0/2568	NA
R01_cb2845_c31/f1p0/3444	NA	NA	NA	R01_cb2845_c31/f1p0/3444
R01_cb11230_c3/f1p0/1066	R01_cb11230_c3/f1p0/1066	NA	NA	NA
R01_cb2773_c7/f1p0/3071	NA	R01_cb2773_c7/f1p0/3071	NA	NA
R01_cb17248_c0/f2p0/412	R01_cb17248_c0/f2p0/412	R01_cb17248_c0/f2p0/412	R01_cb17248_c0/f2p0/412	R01_cb17248_c0/f2p0/412
R01_cb5595_c45/f1p0/824	NA	R01_cb5595_c45/f1p0/824	NA	R01_cb5595_c45/f1p0/824
R01_cb10207_c8/f1p0/677	NA	R01_cb10207_c8/f1p0/677	NA	NA
R01_cb8564_c13661/f1p0/2830	NA	R01_cb8564_c13661/f1p0/2830	NA	NA
R01_cb17101_c2/f1p0/1063	NA	NA	NA	R01_cb17101_c2/f1p0/1063
R01_cb13383_c5/f1p0/725	NA	R01_cb13383_c5/f1p0/725	NA	NA
R01_cb8564_c70642/f1p0/2852	NA	R01_cb8564_c70642/f1p0/2852	NA	R01_cb8564_c70642/f1p0/2852
R01_cb13955_c6/f2p0/480	NA	NA	NA	R01_cb13955_c6/f2p0/480
R01_cb13766_c6/f1p0/659	NA	R01_cb13766_c6/f1p0/659	NA	NA
R01_cb7468_c4/f1p0/5986	NA	R01_cb7468_c4/f1p0/5986	NA	NA
R01_cb7296_c9/f1p1/2007	NA	NA	NA	R01_cb7296_c9/f1p1/2007
R01_cb8564_c2572/f1p0/2028	NA	NA	NA	R01_cb8564_c2572/f1p0/2028
R01_cb18456_c4774/f1p1/493	NA	NA	NA	R01_cb18456_c4774/f1p1/493
R01_cb8575_c12/f1p0/362	R01_cb8575_c12/f1p0/362	R01_cb8575_c12/f1p0/362	R01_cb8575_c12/f1p0/362	R01_cb8575_c12/f1p0/362
R01_cb14882_c7/f1p0/1833	NA	R01_cb14882_c7/f1p0/1833	NA	NA
R01_cb18456_c7812/f7p0/505	R01_cb18456_c7812/f7p0/505	R01_cb18456_c7812/f7p0/505	R01_cb18456_c7812/f7p0/505	R01_cb18456_c7812/f7p0/505
R01_cb18223_c22/f1p0/974	R01_cb18223_c22/f1p0/974	R01_cb18223_c22/f1p0/974	R01_cb18223_c22/f1p0/974	R01_cb18223_c22/f1p0/974
R01_cb8564_c88558/f1p0/2939	NA	NA	NA	R01_cb8564_c88558/f1p0/2939
R01_cb1080_c4/f1p0/973	NA	R01_cb1080_c4/f1p0/973	R01_cb1080_c4/f1p0/973	R01_cb1080_c4/f1p0/973
R01_cb9422_c2/f1p0/2085	NA	R01_cb9422_c2/f1p0/2085	NA	NA
R01_cb3156_c15/f1p0/5054	NA	NA	NA	R01_cb3156_c15/f1p0/5054
R01_cb16284_c1/f2p0/583	R01_cb16284_c1/f2p0/583	NA	NA	NA

R01_cb8564_c48449/f1p0/2775	R01_cb8564_c48449/f1p0/2775	R01_cb8564_c48449/f1p0/2775	NA	NA
R01_cb18456_c1497/f1p1/540	NA	NA	NA	R01_cb18456_c1497/f1p1/540
R01_cb11155_c0/f7p0/938	NA	R01_cb11155_c0/f7p0/938	NA	NA
R01_cb8564_c125014/f1p0/2392	NA	NA	NA	R01_cb8564_c125014/f1p0/2392
R01_cb16037_c6/f1p0/913	NA	R01_cb16037_c6/f1p0/913	NA	NA
R01_cb8564_c151174/f1p2/2106	NA	R01_cb8564_c151174/f1p2/2106	NA	NA
R01_cb1905_c11/f1p0/2713	NA	R01_cb1905_c11/f1p0/2713	R01_cb1905_c11/f1p0/2713	NA
R01_cb17885_c11/f1p0/1543	NA	NA	NA	R01_cb17885_c11/f1p0/1543
R01_cb3929_c9/f1p0/3280	R01_cb3929_c9/f1p0/3280	R01_cb3929_c9/f1p0/3280	R01_cb3929_c9/f1p0/3280	R01_cb3929_c9/f1p0/3280
R01_cb2027_c5/f1p0/3571	NA	NA	R01_cb2027_c5/f1p0/3571	NA
R01_cb5100_c1/f1p0/2539	NA	R01_cb5100_c1/f1p0/2539	R01_cb5100_c1/f1p0/2539	NA
R01_cb17895_c0/f1p0/591	NA	NA	NA	R01_cb17895_c0/f1p0/591
R01_cb1658_c14/f1p2/2824	NA	NA	NA	R01_cb1658_c14/f1p2/2824
R01_cb10693_c5/f1p0/1679	NA	R01_cb10693_c5/f1p0/1679	NA	R01_cb10693_c5/f1p0/1679
R01_cb16315_c2/f1p0/1297	NA	R01_cb16315_c2/f1p0/1297	NA	NA
R01_cb4434_c8/f1p1/5298	NA	NA	NA	R01_cb4434_c8/f1p1/5298
R01_cb8564_c19675/f1p0/4124	NA	R01_cb8564_c19675/f1p0/4124	R01_cb8564_c19675/f1p0/4124	R01_cb8564_c19675/f1p0/4124
R01_cb18456_c1554/f3p0/1519	NA	R01_cb18456_c1554/f3p0/1519	R01_cb18456_c1554/f3p0/1519	R01_cb18456_c1554/f3p0/1519
R01_cb7222_c5/f2p0/2309	NA	NA	NA	R01_cb7222_c5/f2p0/2309
R01_cb5896_c64/f1p0/2959	NA	R01_cb5896_c64/f1p0/2959	NA	NA
R01_cb13178_c20/f1p0/679	NA	R01_cb13178_c20/f1p0/679	NA	NA
R01_cb15186_c20/f1p0/527	NA	R01_cb15186_c20/f1p0/527	R01_cb15186_c20/f1p0/527	R01_cb15186_c20/f1p0/527
R01_cb8564_c934/f2p0/2904	NA	R01_cb8564_c934/f2p0/2904	R01_cb8564_c934/f2p0/2904	R01_cb8564_c934/f2p0/2904

R01_cb16729_c1/f1p0/921	NA	NA	NA	R01_cb16729_c1/f1p0/921
R01_cb13857_c2/f1p0/1033	NA	NA	NA	R01_cb13857_c2/f1p0/1033
R01_cb13478_c4/f4p0/593	NA	R01_cb13478_c4/f4p0/593	R01_cb13478_c4/f4p0/593	R01_cb13478_c4/f4p0/593
R01_cb635_c13/f1p0/2491	NA	R01_cb635_c13/f1p0/2491	R01_cb635_c13/f1p0/2491	NA
R01_cb11972_c1/f3p0/1240	NA	NA	NA	R01_cb11972_c1/f3p0/1240
R01_cb2451_c2/f3p2/3379	NA	NA	NA	R01_cb2451_c2/f3p2/3379
R01_cb9201_c6/f1p0/2240	NA	NA	NA	R01_cb9201_c6/f1p0/2240
R01_cb8564_c47145/f1p1/2285	R01_cb8564_c47145/f1p1/2285	R01_cb8564_c47145/f1p1/2285	R01_cb8564_c47145/f1p1/2285	R01_cb8564_c47145/f1p1/2285
R01_cb15631_c0/f3p0/776	NA	NA	R01_cb15631_c0/f3p0/776	R01_cb15631_c0/f3p0/776
R01_cb929_c6/f1p0/1332	NA	NA	NA	R01_cb929_c6/f1p0/1332
R01_cb12575_c2/f1p0/1022	NA	NA	NA	R01_cb12575_c2/f1p0/1022
R01_cb11629_c3/f1p0/2019	NA	NA	NA	R01_cb11629_c3/f1p0/2019
R01_cb16347_c0/f6p0/939	NA	R01_cb16347_c0/f6p0/939	R01_cb16347_c0/f6p0/939	R01_cb16347_c0/f6p0/939
R01_cb8564_c89194/f2p0/4007	NA	NA	NA	R01_cb8564_c89194/f2p0/4007
R01_cb8564_c72321/f1p0/3764	NA	NA	NA	R01_cb8564_c72321/f1p0/3764
R01_cb3637_c6/f1p0/1344	NA	R01_cb3637_c6/f1p0/1344	NA	NA
R01_cb8564_c52563/f2p0/2936	R01_cb8564_c52563/f2p0/2936	R01_cb8564_c52563/f2p0/2936	R01_cb8564_c52563/f2p0/2936	R01_cb8564_c52563/f2p0/2936
R01_cb8564_c3045/f2p0/2896	R01_cb8564_c3045/f2p0/2896	R01_cb8564_c3045/f2p0/2896	R01_cb8564_c3045/f2p0/2896	NA
R01_cb8254_c2/f2p0/2398	NA	NA	NA	R01_cb8254_c2/f2p0/2398
R01_cb10380_c1/f1p0/2987	NA	NA	NA	R01_cb10380_c1/f1p0/2987
R01_cb8336_c5/f1p0/1195	R01_cb8336_c5/f1p0/1195	R01_cb8336_c5/f1p0/1195	R01_cb8336_c5/f1p0/1195	R01_cb8336_c5/f1p0/1195
R01_cb18456_c6858/f1p0/409	NA	R01_cb18456_c6858/f1p0/409	NA	NA
R01_cb8564_c125213/f1p1/2452	NA	NA	NA	R01_cb8564_c125213/f1p1/2452

R01_cb8564_c18096/f1p0/2378	NA	R01_cb8564_c18096/f1p0/2378	R01_cb8564_c18096/f1p0/2378	R01_cb8564_c18096/f1p0/2378
R01_cb8564_c115604/f1p0/2651	NA	R01_cb8564_c115604/f1p0/2651	NA	NA
R01_cb8252_c5/f1p0/2191	NA	NA	NA	R01_cb8252_c5/f1p0/2191
R01_cb8209_c3/f1p0/2130	NA	NA	NA	R01_cb8209_c3/f1p0/2130
R01_cb8564_c17623/f1p0/4985	NA	R01_cb8564_c17623/f1p0/4985	R01_cb8564_c17623/f1p0/4985	NA
R01_cb17250_c0/f2p0/593	NA	R01_cb17250_c0/f2p0/593	NA	R01_cb17250_c0/f2p0/593
R01_cb11590_c1/f1p0/3691	NA	R01_cb11590_c1/f1p0/3691	NA	NA
R01_cb14146_c4/f4p0/1224	NA	NA	NA	R01_cb14146_c4/f4p0/1224
R01_cb1380_c14/f1p0/2371	R01_cb1380_c14/f1p0/2371	R01_cb1380_c14/f1p0/2371	NA	R01_cb1380_c14/f1p0/2371
R01_cb4173_c8/f1p0/3178	NA	R01_cb4173_c8/f1p0/3178	NA	NA
R01_cb8564_c4534/f1p0/2993	NA	R01_cb8564_c4534/f1p0/2993	R01_cb8564_c4534/f1p0/2993	R01_cb8564_c4534/f1p0/2993
R01_cb14316_c17/f1p0/1083	NA	R01_cb14316_c17/f1p0/1083	R01_cb14316_c17/f1p0/1083	R01_cb14316_c17/f1p0/1083
R01_cb13178_c4/f2p0/524	NA	R01_cb13178_c4/f2p0/524	NA	NA
R01_cb1437_c2/f1p0/4325	NA	NA	NA	R01_cb1437_c2/f1p0/4325
R01_cb8564_c51232/f1p0/1905	R01_cb8564_c51232/f1p0/1905	R01_cb8564_c51232/f1p0/1905	R01_cb8564_c51232/f1p0/1905	R01_cb8564_c51232/f1p0/1905
R01_cb17040_c0/f1p0/1080	NA	R01_cb17040_c0/f1p0/1080	NA	R01_cb17040_c0/f1p0/1080
R01_cb12923_c8/f1p1/1062	NA	NA	R01_cb12923_c8/f1p1/1062	R01_cb12923_c8/f1p1/1062
R01_cb8564_c9813/f2p1/2854	NA	R01_cb8564_c9813/f2p1/2854	R01_cb8564_c9813/f2p1/2854	R01_cb8564_c9813/f2p1/2854
R01_cb18409_c7/f4p0/376	R01_cb18409_c7/f4p0/376	R01_cb18409_c7/f4p0/376	R01_cb18409_c7/f4p0/376	R01_cb18409_c7/f4p0/376
R01_cb6636_c3/f1p0/1512	NA	NA	NA	R01_cb6636_c3/f1p0/1512
R01_cb8564_c4491/f1p0/2977	NA	NA	NA	R01_cb8564_c4491/f1p0/2977
R01_cb8564_c469/f3p3/2572	NA	NA	NA	R01_cb8564_c469/f3p3/2572
R01_cb9535_c14/f1p0/2723	NA	R01_cb9535_c14/f1p0/2723	R01_cb9535_c14/f1p0/2723	NA
R01_cb8564_c1860/f1p0/2527	NA	NA	NA	R01_cb8564_c1860/f1p0/2527

R01_cb7293_c2/f1p0/2455	R01_cb7293_c2/f1p0/2455	R01_cb7293_c2/f1p0/2455	NA	NA
R01_cb8564_c115967/f1p0/2355	NA	R01_cb8564_c115967/f1p0/2355	NA	R01_cb8564_c115967/f1p0/2355
R01_cb7954_c2/f1p0/1046	NA	NA	NA	R01_cb7954_c2/f1p0/1046
R01_cb17823_c0/f1p0/1685	NA	R01_cb17823_c0/f1p0/1685	NA	NA
R01_cb8564_c79729/f1p0/2580	NA	NA	NA	R01_cb8564_c79729/f1p0/2580
R01_cb4313_c2/f1p0/3379	NA	R01_cb4313_c2/f1p0/3379	NA	NA
R01_cb8564_c75399/f1p0/3099	NA	NA	NA	R01_cb8564_c75399/f1p0/3099
R01_cb10599_c3/f1p0/1697	NA	R01_cb10599_c3/f1p0/1697	NA	NA
R01_cb5533_c136/f1p0/2939	NA	NA	NA	R01_cb5533_c136/f1p0/2939
R01_cb8564_c115154/f1p0/3120	NA	R01_cb8564_c115154/f1p0/3120	NA	R01_cb8564_c115154/f1p0/3120
R01_cb17973_c40/f1p0/885	R01_cb17973_c40/f1p0/885	R01_cb17973_c40/f1p0/885	R01_cb17973_c40/f1p0/885	NA
R01_cb273_c3/f1p0/2380	NA	R01_cb273_c3/f1p0/2380	NA	NA
R01_cb17266_c11/f1p0/1070	NA	R01_cb17266_c11/f1p0/1070	R01_cb17266_c11/f1p0/1070	R01_cb17266_c11/f1p0/1070
R01_cb10015_c482/f1p0/446	R01_cb10015_c482/f1p0/446	R01_cb10015_c482/f1p0/446	R01_cb10015_c482/f1p0/446	R01_cb10015_c482/f1p0/446
R01_cb16813_c0/f1p0/1207	R01_cb16813_c0/f1p0/1207	R01_cb16813_c0/f1p0/1207	R01_cb16813_c0/f1p0/1207	R01_cb16813_c0/f1p0/1207
R01_cb15053_c9/f1p0/882	NA	R01_cb15053_c9/f1p0/882	R01_cb15053_c9/f1p0/882	NA
R01_cb10912_c8/f1p0/1076	NA	R01_cb10912_c8/f1p0/1076	NA	NA
R01_cb16296_c0/f1p0/1101	NA	NA	NA	R01_cb16296_c0/f1p0/1101
R01_cb3426_c13/f1p0/3035	NA	NA	NA	R01_cb3426_c13/f1p0/3035
R01_cb4198_c3/f1p0/2176	NA	NA	NA	R01_cb4198_c3/f1p0/2176
R01_cb7219_c3/f3p3/2350	NA	NA	NA	R01_cb7219_c3/f3p3/2350
R01_cb14557_c1/f1p0/1434	NA	NA	NA	R01_cb14557_c1/f1p0/1434
R01_cb8564_c43567/f1p0/2412	NA	R01_cb8564_c43567/f1p0/2412	NA	NA
R01_cb12022_c8/f1p0/539	NA	R01_cb12022_c8/f1p0/539	NA	NA

R01_cb12328_c6/f1p1/1569	NA	NA	NA	R01_cb12328_c6/f1p1/1569
R01_cb17538_c0/f2p0/1497	NA	NA	NA	R01_cb17538_c0/f2p0/1497
R01_cb8564_c48640/f1p0/3383	R01_cb8564_c48640/f1p0/3383	R01_cb8564_c48640/f1p0/3383	NA	R01_cb8564_c48640/f1p0/3383
R01_cb14849_c3/f1p0/1248	NA	R01_cb14849_c3/f1p0/1248	NA	NA
R01_cb2822_c3/f1p0/3144	NA	R01_cb2822_c3/f1p0/3144	NA	NA
R01_cb9448_c4/f1p1/2080	NA	NA	NA	R01_cb9448_c4/f1p1/2080
R01_cb10042_c1/f1p0/2066	NA	NA	NA	R01_cb10042_c1/f1p0/2066
R01_cb17236_c3/f1p0/623	R01_cb17236_c3/f1p0/623	R01_cb17236_c3/f1p0/623	R01_cb17236_c3/f1p0/623	R01_cb17236_c3/f1p0/623
R01_cb16645_c60/f1p0/926	R01_cb16645_c60/f1p0/926	R01_cb16645_c60/f1p0/926	R01_cb16645_c60/f1p0/926	R01_cb16645_c60/f1p0/926
R01_cb8564_c1286/f1p0/3383	NA	R01_cb8564_c1286/f1p0/3383	R01_cb8564_c1286/f1p0/3383	R01_cb8564_c1286/f1p0/3383
R01_cb17951_c0/f1p0/1033	NA	NA	NA	R01_cb17951_c0/f1p0/1033
R01_cb1391_c0/f1p0/4343	NA	R01_cb1391_c0/f1p0/4343	NA	NA
R01_cb14523_c0/f2p0/1763	NA	NA	NA	R01_cb14523_c0/f2p0/1763
R01_cb15574_c5/f6p0/548	NA	NA	R01_cb15574_c5/f6p0/548	R01_cb15574_c5/f6p0/548
R01_cb6777_c2/f1p0/328	R01_cb6777_c2/f1p0/328	R01_cb6777_c2/f1p0/328	NA	R01_cb6777_c2/f1p0/328
R01_cb12703_c3/f1p0/1794	NA	NA	NA	R01_cb12703_c3/f1p0/1794
R01_cb16768_c1/f1p0/714	NA	R01_cb16768_c1/f1p0/714	NA	NA
R01_cb2970_c10/f12p0/519	NA	NA	NA	R01_cb2970_c10/f12p0/519
R01_cb17555_c5/f2p0/690	R01_cb17555_c5/f2p0/690	NA	NA	NA
R01_cb8564_c83321/f1p0/2077	NA	R01_cb8564_c83321/f1p0/2077	R01_cb8564_c83321/f1p0/2077	R01_cb8564_c83321/f1p0/2077
R01_cb8564_c68642/f7p2/2593	NA	R01_cb8564_c68642/f7p2/2593	R01_cb8564_c68642/f7p2/2593	R01_cb8564_c68642/f7p2/2593
R01_cb392_c5/f1p0/1982	NA	R01_cb392_c5/f1p0/1982	NA	NA
R01_cb8564_c89795/f1p0/3174	R01_cb8564_c89795/f1p0/3174	R01_cb8564_c89795/f1p0/3174	R01_cb8564_c89795/f1p0/3174	R01_cb8564_c89795/f1p0/3174
R01_cb12554_c1/f1p1/1603	NA	NA	NA	R01_cb12554_c1/f1p1/1603

R01_cb16678_c9/f1p0/680	NA	R01_cb16678_c9/f1p0/680	R01_cb16678_c9/f1p0/680	R01_cb16678_c9/f1p0/680
R01_cb13884_c5/f2p0/931	R01_cb13884_c5/f2p0/931	NA	R01_cb13884_c5/f2p0/931	R01_cb13884_c5/f2p0/931
R01_cb2226_c2/f1p0/3587	NA	NA	NA	R01_cb2226_c2/f1p0/3587
R01_cb16425_c5/f1p0/1201	R01_cb16425_c5/f1p0/1201	R01_cb16425_c5/f1p0/1201	NA	NA
R01_cb11455_c1/f3p0/529	NA	R01_cb11455_c1/f3p0/529	NA	R01_cb11455_c1/f3p0/529
R01_cb16730_c0/f4p0/509	NA	R01_cb16730_c0/f4p0/509	NA	R01_cb16730_c0/f4p0/509
R01_cb8564_c70563/f1p1/2317	NA	R01_cb8564_c70563/f1p1/2317	NA	R01_cb8564_c70563/f1p1/2317
R01_cb10539_c8/f1p0/869	NA	NA	NA	R01_cb10539_c8/f1p0/869
R01_cb15891_c0/f3p0/925	NA	R01_cb15891_c0/f3p0/925	NA	NA
R01_cb5218_c3/f1p0/2697	NA	R01_cb5218_c3/f1p0/2697	NA	NA
R01_cb699_c1/f1p0/2587	NA	NA	NA	R01_cb699_c1/f1p0/2587
R01_cb8564_c14735/f1p0/2354	NA	R01_cb8564_c14735/f1p0/2354	NA	NA
R01_cb14477_c0/f2p1/965	NA	NA	NA	R01_cb14477_c0/f2p1/965
R01_cb16688_c1/f1p1/584	R01_cb16688_c1/f1p1/584	R01_cb16688_c1/f1p1/584	R01_cb16688_c1/f1p1/584	NA
R01_cb15644_c0/f2p0/470	NA	R01_cb15644_c0/f2p0/470	R01_cb15644_c0/f2p0/470	R01_cb15644_c0/f2p0/470
R01_cb3362_c56/f1p0/8236	NA	R01_cb3362_c56/f1p0/8236	NA	NA
R01_cb16297_c2/f1p0/996	NA	R01_cb16297_c2/f1p0/996	NA	NA
R01_cb17973_c49/f1p0/712	NA	NA	R01_cb17973_c49/f1p0/712	R01_cb17973_c49/f1p0/712
R01_cb9274_c8/f1p1/2058	NA	NA	NA	R01_cb9274_c8/f1p1/2058
R01_cb18372_c0/f4p0/2681	NA	NA	NA	R01_cb18372_c0/f4p0/2681
R01_cb8564_c68692/f6p1/3141	R01_cb8564_c68692/f6p1/3141	R01_cb8564_c68692/f6p1/3141	NA	NA
R01_cb15724_c2/f1p0/1445	R01_cb15724_c2/f1p0/1445	R01_cb15724_c2/f1p0/1445	R01_cb15724_c2/f1p0/1445	R01_cb15724_c2/f1p0/1445
R01_cb2756_c14/f1p0/991	NA	NA	R01_cb2756_c14/f1p0/991	R01_cb2756_c14/f1p0/991
R01_cb17166_c2/f1p0/1788	NA	R01_cb17166_c2/f1p0/1788	NA	NA
R01_cb8779_c11/f1p0/3602	NA	R01_cb8779_c11/f1p0/3602	NA	R01_cb8779_c11/f1p0/3602

R01_cb1903_c2/f2p0/2546	NA	R01_cb1903_c2/f2p0/2546	R01_cb1903_c2/f2p0/2546	R01_cb1903_c2/f2p0/2546
R01_cb8779_c4/f1p0/2202	NA	R01_cb8779_c4/f1p0/2202	NA	R01_cb8779_c4/f1p0/2202
R01_cb8564_c52978/f1p0/2089	NA	R01_cb8564_c52978/f1p0/2089	NA	R01_cb8564_c52978/f1p0/2089
R01_cb8564_c3781/f1p0/3919	NA	NA	NA	R01_cb8564_c3781/f1p0/3919
R01_cb10758_c4/f1p0/560	R01_cb10758_c4/f1p0/560	R01_cb10758_c4/f1p0/560	R01_cb10758_c4/f1p0/560	R01_cb10758_c4/f1p0/560
R01_cb15566_c0/f1p0/756	NA	NA	NA	R01_cb15566_c0/f1p0/756
R01_cb1706_c12/f1p0/2418	NA	NA	NA	R01_cb1706_c12/f1p0/2418
R01_cb1706_c16/f1p0/2348	NA	NA	NA	R01_cb1706_c16/f1p0/2348
R01_cb15756_c1/f2p0/628	NA	NA	NA	R01_cb15756_c1/f2p0/628
R01_cb15074_c11/f1p0/907	R01_cb15074_c11/f1p0/907	R01_cb15074_c11/f1p0/907	NA	NA
R01_cb10326_c8/f1p0/1789	R01_cb10326_c8/f1p0/1789	R01_cb10326_c8/f1p0/1789	NA	R01_cb10326_c8/f1p0/1789
R01_cb2845_c25/f1p0/3405	NA	NA	NA	R01_cb2845_c25/f1p0/3405
R01_cb18301_c29/f1p0/1194	NA	R01_cb18301_c29/f1p0/1194	NA	NA
R01_cb8564_c89297/f1p0/2453	NA	NA	NA	R01_cb8564_c89297/f1p0/2453
R01_cb8564_c88606/f1p0/2421	NA	R01_cb8564_c88606/f1p0/2421	R01_cb8564_c88606/f1p0/2421	R01_cb8564_c88606/f1p0/2421
R01_cb16106_c0/f2p1/602	NA	R01_cb16106_c0/f2p1/602	NA	NA
R01_cb16094_c3/f1p0/756	NA	NA	NA	R01_cb16094_c3/f1p0/756
R01_cb18409_c71/f1p0/1795	NA	R01_cb18409_c71/f1p0/1795	NA	NA
R01_cb10086_c2/f4p1/1350	NA	NA	NA	R01_cb10086_c2/f4p1/1350
R01_cb5107_c1/f1p0/1929	NA	R01_cb5107_c1/f1p0/1929	NA	NA
R01_cb17446_c0/f1p0/1723	NA	NA	NA	R01_cb17446_c0/f1p0/1723
R01_cb2807_c5/f1p0/1094	NA	NA	R01_cb2807_c5/f1p0/1094	R01_cb2807_c5/f1p0/1094
R01_cb7463_c9/f1p0/2403	NA	R01_cb7463_c9/f1p0/2403	NA	NA
R01_cb1835_c1/f1p0/4155	NA	NA	NA	R01_cb1835_c1/f1p0/4155
R01_cb4836_c5/f1p1/1805	NA	NA	NA	R01_cb4836_c5/f1p1/1805
R01_cb8564_c119851/f1p0/231	NA	NA	R01_cb8564_c119851/f1p0/231	R01_cb8564_c119851/f1p0/231

0			0	0
R01_cb17103_c4/f1p0/1440	NA	NA	NA	R01_cb17103_c4/f1p0/1440
R01_cb14010_c0/f1p0/1302	NA	NA	NA	R01_cb14010_c0/f1p0/1302
R01_cb8564_c68122/f5p2/2959	NA	NA	NA	R01_cb8564_c68122/f5p2/2959
R01_cb15526_c2/f1p0/1242	NA	NA	NA	R01_cb15526_c2/f1p0/1242
R01_cb10385_c2/f1p0/1609	NA	R01_cb10385_c2/f1p0/1609	NA	NA
R01_cb5720_c5/f1p0/2641	NA	NA	NA	R01_cb5720_c5/f1p0/2641
R01_cb6563_c1/f1p0/3933	NA	R01_cb6563_c1/f1p0/3933	NA	NA
R01_cb16080_c0/f2p0/1212	NA	NA	R01_cb16080_c0/f2p0/1212	NA
R01_cb16396_c0/f1p0/1814	NA	R01_cb16396_c0/f1p0/1814	NA	NA
R01_cb7008_c12/f1p0/3438	NA	R01_cb7008_c12/f1p0/3438	NA	NA
R01_cb2552_c5/f1p0/1147	NA	R01_cb2552_c5/f1p0/1147	R01_cb2552_c5/f1p0/1147	NA
R01_cb13146_c7/f1p0/647	NA	NA	R01_cb13146_c7/f1p0/647	R01_cb13146_c7/f1p0/647
R01_cb17973_c10/f1p0/826	R01_cb17973_c10/f1p0/826	R01_cb17973_c10/f1p0/826	NA	R01_cb17973_c10/f1p0/826
R01_cb17154_c1/f1p0/735	NA	R01_cb17154_c1/f1p0/735	R01_cb17154_c1/f1p0/735	R01_cb17154_c1/f1p0/735
R01_cb9834_c7/f1p0/926	R01_cb9834_c7/f1p0/926	R01_cb9834_c7/f1p0/926	NA	R01_cb9834_c7/f1p0/926
R01_cb2229_c45/f1p0/3297	NA	R01_cb2229_c45/f1p0/3297	NA	NA
R01_cb13761_c0/f1p0/1165	R01_cb13761_c0/f1p0/1165	R01_cb13761_c0/f1p0/1165	R01_cb13761_c0/f1p0/1165	R01_cb13761_c0/f1p0/1165
R01_cb16806_c1/f1p0/691	NA	R01_cb16806_c1/f1p0/691	R01_cb16806_c1/f1p0/691	NA
R01_cb14308_c2/f1p0/550	R01_cb14308_c2/f1p0/550	R01_cb14308_c2/f1p0/550	R01_cb14308_c2/f1p0/550	R01_cb14308_c2/f1p0/550
R01_cb1326_c10/f1p0/3807	NA	R01_cb1326_c10/f1p0/3807	NA	NA
R01_cb18456_c6283/f1p0/883	R01_cb18456_c6283/f1p0/883	R01_cb18456_c6283/f1p0/883	NA	NA
R01_cb16673_c9/f1p1/628	NA	R01_cb16673_c9/f1p1/628	NA	NA
R01_cb12443_c3/f1p0/341	R01_cb12443_c3/f1p0/341	R01_cb12443_c3/f1p0/341	R01_cb12443_c3/f1p0/341	R01_cb12443_c3/f1p0/341
R01_cb4030_c3/f1p0/2235	NA	NA	NA	R01_cb4030_c3/f1p0/2235
R01_cb10846_c3/f1p0/1852	NA	R01_cb10846_c3/f1p0/1852	NA	NA

R01_cb10816_c2/f1p0/2866	NA	R01_cb10816_c2/f1p0/2866	NA	NA
R01_cb8564_c118424/f1p0/2950	NA	NA	NA	R01_cb8564_c118424/f1p0/2950
R01_cb7539_c2/f1p0/2284	NA	NA	R01_cb7539_c2/f1p0/2284	R01_cb7539_c2/f1p0/2284
R01_cb12722_c7/f1p0/1353	NA	NA	NA	R01_cb12722_c7/f1p0/1353
R01_cb1437_c8/f1p1/4051	NA	NA	NA	R01_cb1437_c8/f1p1/4051
R01_cb3228_c1/f2p0/544	NA	R01_cb3228_c1/f2p0/544	NA	NA
R01_cb7254_c6/f1p0/2354	NA	NA	NA	R01_cb7254_c6/f1p0/2354
R01_cb18192_c10/f1p0/737	NA	R01_cb18192_c10/f1p0/737	R01_cb18192_c10/f1p0/737	R01_cb18192_c10/f1p0/737
R01_cb9192_c1/f1p0/2077	NA	NA	NA	R01_cb9192_c1/f1p0/2077
R01_cb15540_c1/f2p2/1575	NA	NA	NA	R01_cb15540_c1/f2p2/1575
R01_cb1203_c25/f1p0/1362	NA	NA	NA	R01_cb1203_c25/f1p0/1362
R01_cb11178_c1/f1p0/2680	NA	R01_cb11178_c1/f1p0/2680	NA	NA
R01_cb8865_c1/f1p0/2492	NA	NA	NA	R01_cb8865_c1/f1p0/2492
R01_cb15931_c0/f3p0/1004	NA	NA	NA	R01_cb15931_c0/f3p0/1004
R01_cb18456_c7681/f1p0/450	R01_cb18456_c7681/f1p0/450	R01_cb18456_c7681/f1p0/450	NA	R01_cb18456_c7681/f1p0/450
R01_cb3498_c20/f1p0/745	R01_cb3498_c20/f1p0/745	R01_cb3498_c20/f1p0/745	NA	NA
R01_cb8564_c659/f1p3/2696	NA	R01_cb8564_c659/f1p3/2696	NA	NA
R01_cb16153_c0/f5p1/1226	NA	NA	R01_cb16153_c0/f5p1/1226	R01_cb16153_c0/f5p1/1226
R01_cb15916_c3/f1p0/1788	NA	NA	NA	R01_cb15916_c3/f1p0/1788
R01_cb13178_c0/f3p0/551	NA	R01_cb13178_c0/f3p0/551	NA	NA
R01_cb15663_c1/f1p0/1537	NA	NA	NA	R01_cb15663_c1/f1p0/1537
R01_cb1964_c2/f1p0/2293	NA	R01_cb1964_c2/f1p0/2293	NA	R01_cb1964_c2/f1p0/2293
R01_cb1581_c6/f1p0/3854	NA	R01_cb1581_c6/f1p0/3854	NA	NA
R01_cb17281_c0/f1p0/1012	NA	NA	NA	R01_cb17281_c0/f1p0/1012
R01_cb10650_c6/f1p0/1828	NA	R01_cb10650_c6/f1p0/1828	NA	NA

R01_cb2602_c3/f1p0/3860	NA	R01_cb2602_c3/f1p0/3860	NA	NA
R01_cb16141_c0/f2p0/581	R01_cb16141_c0/f2p0/581	R01_cb16141_c0/f2p0/581	R01_cb16141_c0/f2p0/581	R01_cb16141_c0/f2p0/581
R01_cb12191_c4/f1p1/838	NA	NA	NA	R01_cb12191_c4/f1p1/838
R01_cb14886_c5/f1p0/848	NA	NA	R01_cb14886_c5/f1p0/848	NA
R01_cb17083_c4/f1p0/963	NA	R01_cb17083_c4/f1p0/963	R01_cb17083_c4/f1p0/963	R01_cb17083_c4/f1p0/963
R01_cb14886_c1/f2p0/335	R01_cb14886_c1/f2p0/335	R01_cb14886_c1/f2p0/335	R01_cb14886_c1/f2p0/335	R01_cb14886_c1/f2p0/335
R01_cb8564_c116212/f1p0/2680	NA	R01_cb8564_c116212/f1p0/2680	R01_cb8564_c116212/f1p0/2680	R01_cb8564_c116212/f1p0/2680
R01_cb8564_c13299/f1p0/3229	NA	R01_cb8564_c13299/f1p0/3229	R01_cb8564_c13299/f1p0/3229	R01_cb8564_c13299/f1p0/3229
R01_cb8814_c12/f1p0/1511	NA	R01_cb8814_c12/f1p0/1511	NA	NA
R01_cb10708_c0/f1p0/1783	NA	R01_cb10708_c0/f1p0/1783	NA	NA
R01_cb4953_c4/f1p0/2996	NA	NA	NA	R01_cb4953_c4/f1p0/2996
R01_cb5480_c1/f1p0/3608	NA	R01_cb5480_c1/f1p0/3608	R01_cb5480_c1/f1p0/3608	NA
R01_cb13273_c0/f2p0/1111	R01_cb13273_c0/f2p0/1111	R01_cb13273_c0/f2p0/1111	R01_cb13273_c0/f2p0/1111	R01_cb13273_c0/f2p0/1111
R01_cb18456_c5911/f1p0/444	R01_cb18456_c5911/f1p0/444	R01_cb18456_c5911/f1p0/444	R01_cb18456_c5911/f1p0/444	R01_cb18456_c5911/f1p0/444
R01_cb17907_c1/f1p0/1120	NA	R01_cb17907_c1/f1p0/1120	NA	NA
R01_cb2022_c17/f1p0/2034	NA	NA	NA	R01_cb2022_c17/f1p0/2034
R01_cb10024_c188/f2p0/742	NA	NA	R01_cb10024_c188/f2p0/742	NA
R01_cb7320_c6/f1p0/4635	NA	NA	NA	R01_cb7320_c6/f1p0/4635
R01_cb18285_c0/f1p0/966	NA	NA	NA	R01_cb18285_c0/f1p0/966
R01_cb13475_c2/f1p0/603	R01_cb13475_c2/f1p0/603	R01_cb13475_c2/f1p0/603	R01_cb13475_c2/f1p0/603	R01_cb13475_c2/f1p0/603
R01_cb15890_c1/f1p0/1180	NA	NA	NA	R01_cb15890_c1/f1p0/1180
R01_cb4785_c8/f2p0/2680	NA	NA	NA	R01_cb4785_c8/f2p0/2680
R01_cb10008_c4/f2p0/799	NA	R01_cb10008_c4/f2p0/799	NA	NA
R01_cb13433_c92/f1p1/1433	NA	R01_cb13433_c92/f1p1/1433	NA	NA
R01_cb8564_c25336/f1p0/1982	R01_cb8564_c25336/f1p0/198	R01_cb8564_c25336/f1p0/1982	R01_cb8564_c25336/f1p0/1982	R01_cb8564_c25336/f1p0/1982

	2			
R01_cb11141_c1/f1p0/1309	NA	NA	NA	R01_cb11141_c1/f1p0/1309
R01_cb8564_c37679/f1p0/4074	NA	NA	NA	R01_cb8564_c37679/f1p0/4074
R01_cb8564_c182504/f1p1/2670	NA	NA	NA	R01_cb8564_c182504/f1p1/2670
R01_cb18456_c1866/f1p0/1138	NA	R01_cb18456_c1866/f1p0/1138	R01_cb18456_c1866/f1p0/1138	R01_cb18456_c1866/f1p0/1138
R01_cb8564_c15756/f1p0/2871	NA	R01_cb8564_c15756/f1p0/2871	NA	NA
R01_cb7625_c9/f1p1/475	NA	NA	NA	R01_cb7625_c9/f1p1/475
R01_cb8564_c85849/f1p0/2262	NA	R01_cb8564_c85849/f1p0/2262	R01_cb8564_c85849/f1p0/2262	R01_cb8564_c85849/f1p0/2262
R01_cb4077_c5/f1p0/2133	NA	R01_cb4077_c5/f1p0/2133	NA	NA
R01_cb8564_c69988/f1p0/2405	NA	R01_cb8564_c69988/f1p0/2405	NA	NA
R01_cb17806_c2/f1p0/827	NA	R01_cb17806_c2/f1p0/827	NA	R01_cb17806_c2/f1p0/827
R01_cb13273_c1/f1p0/1620	R01_cb13273_c1/f1p0/1620	R01_cb13273_c1/f1p0/1620	R01_cb13273_c1/f1p0/1620	R01_cb13273_c1/f1p0/1620
R01_cb8564_c146692/f3p0/2128	NA	NA	NA	R01_cb8564_c146692/f3p0/2128
R01_cb8564_c9368/f3p0/3414	NA	NA	NA	R01_cb8564_c9368/f3p0/3414
R01_cb11189_c0/f1p0/1527	NA	R01_cb11189_c0/f1p0/1527	NA	R01_cb11189_c0/f1p0/1527
R01_cb3426_c16/f1p1/3300	NA	NA	NA	R01_cb3426_c16/f1p1/3300
R01_cb5950_c5/f1p0/2305	NA	NA	NA	R01_cb5950_c5/f1p0/2305
R01_cb17520_c1/f1p0/462	NA	R01_cb17520_c1/f1p0/462	R01_cb17520_c1/f1p0/462	R01_cb17520_c1/f1p0/462
R01_cb5900_c101/f1p0/2044	NA	R01_cb5900_c101/f1p0/2044	NA	R01_cb5900_c101/f1p0/2044
R01_cb8564_c72324/f1p0/2625	R01_cb8564_c72324/f1p0/2625	R01_cb8564_c72324/f1p0/2625	NA	NA
R01_cb9306_c0/f3p1/2058	NA	R01_cb9306_c0/f3p1/2058	NA	NA
R01_cb18456_c1363/f4p0/419	R01_cb18456_c1363/f4p0/419	R01_cb18456_c1363/f4p0/419	R01_cb18456_c1363/f4p0/419	R01_cb18456_c1363/f4p0/419
R01_cb8564_c78003/f1p1/2972	NA	R01_cb8564_c78003/f1p1/2972	R01_cb8564_c78003/f1p1/2972	R01_cb8564_c78003/f1p1/2972

R01_cb8564_c85405/f1p0/3073	NA	R01_cb8564_c85405/f1p0/3073	R01_cb8564_c85405/f1p0/3073	R01_cb8564_c85405/f1p0/3073
R01_cb14120_c0/f1p0/1105	NA	NA	NA	R01_cb14120_c0/f1p0/1105
R01_cb803_c2/f1p0/1849	NA	R01_cb803_c2/f1p0/1849	NA	NA
R01_cb16645_c33/f1p0/1229	NA	R01_cb16645_c33/f1p0/1229	NA	NA
R01_cb11704_c1/f1p0/2570	NA	R01_cb11704_c1/f1p0/2570	NA	NA
R01_cb9617_c13/f1p0/1660	NA	NA	R01_cb9617_c13/f1p0/1660	R01_cb9617_c13/f1p0/1660
R01_cb18556_c1/f1p0/1059	NA	NA	NA	R01_cb18556_c1/f1p0/1059
R01_cb12349_c8/f1p0/1434	NA	R01_cb12349_c8/f1p0/1434	NA	NA
R01_cb6132_c4/f1p0/2723	NA	NA	NA	R01_cb6132_c4/f1p0/2723
R01_cb8564_c70069/f1p0/2273	NA	NA	NA	R01_cb8564_c70069/f1p0/2273
R01_cb16196_c1/f1p0/1726	NA	R01_cb16196_c1/f1p0/1726	R01_cb16196_c1/f1p0/1726	R01_cb16196_c1/f1p0/1726
R01_cb8564_c130103/f1p0/2695	NA	NA	R01_cb8564_c130103/f1p0/2695	R01_cb8564_c130103/f1p0/2695
R01_cb15251_c2/f1p0/1559	NA	NA	NA	R01_cb15251_c2/f1p0/1559
R01_cb11441_c1/f1p0/2643	R01_cb11441_c1/f1p0/2643	R01_cb11441_c1/f1p0/2643	R01_cb11441_c1/f1p0/2643	R01_cb11441_c1/f1p0/2643
R01_cb2822_c0/f2p1/2683	R01_cb2822_c0/f2p1/2683	R01_cb2822_c0/f2p1/2683	NA	NA
R01_cb8543_c2/f1p0/4131	NA	R01_cb8543_c2/f1p0/4131	R01_cb8543_c2/f1p0/4131	NA
R01_cb2423_c29/f1p0/3163	NA	NA	R01_cb2423_c29/f1p0/3163	NA
R01_cb8564_c73565/f1p0/2797	NA	R01_cb8564_c73565/f1p0/2797	NA	NA
R01_cb3637_c0/f1p0/3571	NA	R01_cb3637_c0/f1p0/3571	NA	NA
R01_cb3732_c19/f1p0/2925	NA	NA	NA	R01_cb3732_c19/f1p0/2925
R01_cb6802_c11/f1p0/3639	R01_cb6802_c11/f1p0/3639	R01_cb6802_c11/f1p0/3639	R01_cb6802_c11/f1p0/3639	R01_cb6802_c11/f1p0/3639
R01_cb11446_c1/f1p0/2790	NA	NA	NA	R01_cb11446_c1/f1p0/2790
R01_cb8564_c87490/f1p0/2768	NA	NA	NA	R01_cb8564_c87490/f1p0/2768
R01_cb8564_c112866/f1p0/2579	NA	R01_cb8564_c112866/f1p0/2579	NA	R01_cb8564_c112866/f1p0/2579

R01_cb8564_c118326/f1p0/1878	NA	NA	NA	R01_cb8564_c118326/f1p0/1878
R01_cb15951_c6/f1p0/465	R01_cb15951_c6/f1p0/465	R01_cb15951_c6/f1p0/465	R01_cb15951_c6/f1p0/465	R01_cb15951_c6/f1p0/465
R01_cb4708_c8/f1p0/1739	NA	R01_cb4708_c8/f1p0/1739	NA	NA
R01_cb11160_c1/f1p0/2026	NA	NA	R01_cb11160_c1/f1p0/2026	NA
R01_cb15860_c2/f1p0/609	NA	NA	NA	R01_cb15860_c2/f1p0/609
R01_cb7398_c1/f1p0/3093	NA	NA	NA	R01_cb7398_c1/f1p0/3093
R01_cb2970_c6/f1p0/652	R01_cb2970_c6/f1p0/652	NA	NA	R01_cb2970_c6/f1p0/652
R01_cb4915_c5/f1p0/2344	NA	R01_cb4915_c5/f1p0/2344	NA	NA
R01_cb16078_c7/f1p0/420	R01_cb16078_c7/f1p0/420	R01_cb16078_c7/f1p0/420	R01_cb16078_c7/f1p0/420	NA
R01_cb18516_c1/f1p0/879	R01_cb18516_c1/f1p0/879	R01_cb18516_c1/f1p0/879	R01_cb18516_c1/f1p0/879	R01_cb18516_c1/f1p0/879
R01_cb18456_c7374/f1p0/621	NA	R01_cb18456_c7374/f1p0/621	R01_cb18456_c7374/f1p0/621	R01_cb18456_c7374/f1p0/621
R01_cb649_c35/f1p0/2888	NA	R01_cb649_c35/f1p0/2888	NA	NA
R01_cb4768_c25/f1p0/3150	NA	R01_cb4768_c25/f1p0/3150	NA	NA
R01_cb17676_c1/f1p0/531	NA	R01_cb17676_c1/f1p0/531	NA	R01_cb17676_c1/f1p0/531
R01_cb7403_c6/f1p0/2070	NA	R01_cb7403_c6/f1p0/2070	NA	NA
R01_cb18456_c5452/f1p0/1496	NA	R01_cb18456_c5452/f1p0/1496	NA	R01_cb18456_c5452/f1p0/1496
R01_cb17372_c1/f1p0/546	NA	NA	R01_cb17372_c1/f1p0/546	R01_cb17372_c1/f1p0/546
R01_cb8564_c88724/f1p0/2681	R01_cb8564_c88724/f1p0/2681	R01_cb8564_c88724/f1p0/2681	R01_cb8564_c88724/f1p0/2681	R01_cb8564_c88724/f1p0/2681
R01_cb15168_c1/f1p0/1570	NA	NA	NA	R01_cb15168_c1/f1p0/1570
R01_cb7296_c4/f1p0/3705	NA	NA	NA	R01_cb7296_c4/f1p0/3705
R01_cb11719_c0/f1p0/1370	NA	NA	NA	R01_cb11719_c0/f1p0/1370
R01_cb7899_c1/f1p0/2391	NA	NA	NA	R01_cb7899_c1/f1p0/2391
R01_cb13112_c13/f1p0/909	NA	NA	R01_cb13112_c13/f1p0/909	R01_cb13112_c13/f1p0/909
R01_cb8564_c69636/f1p0/3820	R01_cb8564_c69636/f1p0/382	R01_cb8564_c69636/f1p0/3820	NA	NA

	0			
R01_cb8564_c113069/f1p0/2403	NA	R01_cb8564_c113069/f1p0/2403	R01_cb8564_c113069/f1p0/2403	R01_cb8564_c113069/f1p0/2403
R01_cb1298_c3/f1p1/4105	NA	R01_cb1298_c3/f1p1/4105	R01_cb1298_c3/f1p1/4105	R01_cb1298_c3/f1p1/4105
R01_cb7030_c8/f1p1/2327	NA	NA	NA	R01_cb7030_c8/f1p1/2327
R01_cb15568_c3/f1p0/1032	NA	NA	NA	R01_cb15568_c3/f1p0/1032
R01_cb4141_c5/f1p0/2520	NA	R01_cb4141_c5/f1p0/2520	NA	NA
R01_cb18456_c1539/f3p2/993	NA	R01_cb18456_c1539/f3p2/993	NA	NA
R01_cb8507_c2/f1p1/2344	NA	R01_cb8507_c2/f1p1/2344	NA	NA
R01_cb8564_c69070/f2p0/2710	NA	R01_cb8564_c69070/f2p0/2710	NA	R01_cb8564_c69070/f2p0/2710
R01_cb5480_c2/f1p0/696	R01_cb5480_c2/f1p0/696	R01_cb5480_c2/f1p0/696	R01_cb5480_c2/f1p0/696	R01_cb5480_c2/f1p0/696
R01_cb625_c2/f1p2/4717	NA	NA	NA	R01_cb625_c2/f1p2/4717
R01_cb16871_c2/f1p0/1428	NA	NA	NA	R01_cb16871_c2/f1p0/1428
R01_cb8564_c73920/f1p0/2465	NA	R01_cb8564_c73920/f1p0/2465	NA	R01_cb8564_c73920/f1p0/2465
R01_cb12916_c6/f1p0/671	NA	NA	R01_cb12916_c6/f1p0/671	R01_cb12916_c6/f1p0/671
R01_cb18409_c30/f2p0/442	NA	R01_cb18409_c30/f2p0/442	R01_cb18409_c30/f2p0/442	R01_cb18409_c30/f2p0/442
R01_cb2985_c6/f1p0/3392	NA	NA	NA	R01_cb2985_c6/f1p0/3392
R01_cb18223_c7/f2p2/1729	NA	R01_cb18223_c7/f2p2/1729	NA	R01_cb18223_c7/f2p2/1729
R01_cb8400_c4/f2p0/1858	NA	NA	NA	R01_cb8400_c4/f2p0/1858
R01_cb12534_c3/f1p0/1065	R01_cb12534_c3/f1p0/1065	R01_cb12534_c3/f1p0/1065	R01_cb12534_c3/f1p0/1065	R01_cb12534_c3/f1p0/1065
R01_cb8564_c127487/f2p0/2245	NA	NA	NA	R01_cb8564_c127487/f2p0/2245
R01_cb3517_c6/f1p0/3112	NA	R01_cb3517_c6/f1p0/3112	NA	NA
R01_cb1935_c25/f1p0/581	R01_cb1935_c25/f1p0/581	R01_cb1935_c25/f1p0/581	NA	R01_cb1935_c25/f1p0/581
R01_cb4744_c4/f1p0/1625	NA	R01_cb4744_c4/f1p0/1625	R01_cb4744_c4/f1p0/1625	R01_cb4744_c4/f1p0/1625
R01_cb6278_c11/f1p0/2978	NA	R01_cb6278_c11/f1p0/2978	NA	R01_cb6278_c11/f1p0/2978

R01_cb12369_c13/f1p1/719	NA	NA	R01_cb12369_c13/f1p1/719	NA
R01_cb10106_c6/f1p1/965	NA	R01_cb10106_c6/f1p1/965	NA	NA
R01_cb17708_c2/f1p0/871	R01_cb17708_c2/f1p0/871	R01_cb17708_c2/f1p0/871	R01_cb17708_c2/f1p0/871	R01_cb17708_c2/f1p0/871
R01_cb8564_c77817/f1p0/2256	NA	R01_cb8564_c77817/f1p0/2256	NA	NA
R01_cb10402_c3/f1p0/2605	NA	R01_cb10402_c3/f1p0/2605	NA	NA
R01_cb7720_c6/f1p0/694	R01_cb7720_c6/f1p0/694	NA	NA	R01_cb7720_c6/f1p0/694
R01_cb8283_c8/f1p0/6712	NA	R01_cb8283_c8/f1p0/6712	NA	NA
R01_cb9346_c1/f2p0/995	NA	NA	NA	R01_cb9346_c1/f2p0/995
R01_cb18456_c6623/f1p0/334	R01_cb18456_c6623/f1p0/334	R01_cb18456_c6623/f1p0/334	R01_cb18456_c6623/f1p0/334	R01_cb18456_c6623/f1p0/334
R01_cb6760_c1/f1p0/2583	NA	NA	NA	R01_cb6760_c1/f1p0/2583
R01_cb3601_c8/f1p0/2759	NA	R01_cb3601_c8/f1p0/2759	NA	NA
R01_cb4012_c1/f1p0/3469	NA	R01_cb4012_c1/f1p0/3469	NA	NA
R01_cb15753_c1/f1p0/1641	R01_cb15753_c1/f1p0/1641	R01_cb15753_c1/f1p0/1641	NA	NA
R01_cb8564_c115122/f1p0/2598	NA	NA	NA	R01_cb8564_c115122/f1p0/2598
R01_cb3483_c26/f7p1/2926	NA	NA	NA	R01_cb3483_c26/f7p1/2926
R01_cb12603_c5/f3p0/1448	NA	NA	R01_cb12603_c5/f3p0/1448	R01_cb12603_c5/f3p0/1448
R01_cb15030_c2/f2p0/1420	NA	NA	NA	R01_cb15030_c2/f2p0/1420
R01_cb3294_c1/f1p0/3292	NA	NA	NA	R01_cb3294_c1/f1p0/3292
R01_cb11368_c0/f2p0/604	NA	NA	NA	R01_cb11368_c0/f2p0/604
R01_cb9472_c4/f1p0/1359	NA	NA	NA	R01_cb9472_c4/f1p0/1359
R01_cb8564_c90571/f1p0/3114	NA	R01_cb8564_c90571/f1p0/3114	NA	R01_cb8564_c90571/f1p0/3114
R01_cb16521_c0/f1p0/1199	NA	NA	NA	R01_cb16521_c0/f1p0/1199
R01_cb16447_c2/f1p1/992	NA	NA	NA	R01_cb16447_c2/f1p1/992
R01_cb15811_c28/f1p0/1054	NA	R01_cb15811_c28/f1p0/1054	R01_cb15811_c28/f1p0/1054	R01_cb15811_c28/f1p0/1054
R01_cb260_c10/f1p0/4722	NA	NA	NA	R01_cb260_c10/f1p0/4722

R01_cb8564_c1257/f1p0/4087	NA	R01_cb8564_c1257/f1p0/4087	R01_cb8564_c1257/f1p0/4087	NA
R01_cb8564_c90498/f1p0/3794	NA	R01_cb8564_c90498/f1p0/3794	NA	NA
R01_cb7100_c1/f1p0/2838	R01_cb7100_c1/f1p0/2838	R01_cb7100_c1/f1p0/2838	R01_cb7100_c1/f1p0/2838	R01_cb7100_c1/f1p0/2838
R01_cb15915_c1/f1p0/757	NA	R01_cb15915_c1/f1p0/757	R01_cb15915_c1/f1p0/757	R01_cb15915_c1/f1p0/757
R01_cb3099_c0/f2p1/3378	R01_cb3099_c0/f2p1/3378	R01_cb3099_c0/f2p1/3378	R01_cb3099_c0/f2p1/3378	R01_cb3099_c0/f2p1/3378
R01_cb4047_c12/f1p0/2852	NA	NA	NA	R01_cb4047_c12/f1p0/2852
R01_cb8564_c10722/f1p0/2907	NA	R01_cb8564_c10722/f1p0/2907	NA	R01_cb8564_c10722/f1p0/2907
R01_cb13136_c13/f1p1/857	NA	R01_cb13136_c13/f1p1/857	NA	NA
R01_cb13737_c2/f1p0/1861	R01_cb13737_c2/f1p0/1861	R01_cb13737_c2/f1p0/1861	NA	NA
R01_cb6163_c22/f1p0/953	NA	R01_cb6163_c22/f1p0/953	NA	NA
R01_cb10010_c1/f1p0/2391	NA	R01_cb10010_c1/f1p0/2391	NA	R01_cb10010_c1/f1p0/2391
R01_cb10778_c3/f1p0/858	NA	R01_cb10778_c3/f1p0/858	NA	NA
R01_cb18691_c3/f1p0/748	R01_cb18691_c3/f1p0/748	R01_cb18691_c3/f1p0/748	NA	R01_cb18691_c3/f1p0/748
R01_cb17973_c4/f3p0/755	R01_cb17973_c4/f3p0/755	R01_cb17973_c4/f3p0/755	R01_cb17973_c4/f3p0/755	R01_cb17973_c4/f3p0/755
R01_cb8564_c129513/f1p0/2758	R01_cb8564_c129513/f1p0/2758	R01_cb8564_c129513/f1p0/2758	R01_cb8564_c129513/f1p0/2758	R01_cb8564_c129513/f1p0/2758
R01_cb8625_c2/f1p0/5358	NA	R01_cb8625_c2/f1p0/5358	NA	NA
R01_cb8564_c18629/f1p0/3619	R01_cb8564_c18629/f1p0/3619	R01_cb8564_c18629/f1p0/3619	R01_cb8564_c18629/f1p0/3619	R01_cb8564_c18629/f1p0/3619
R01_cb8564_c34660/f1p0/2579	NA	R01_cb8564_c34660/f1p0/2579	NA	R01_cb8564_c34660/f1p0/2579
R01_cb15934_c2/f1p0/566	NA	R01_cb15934_c2/f1p0/566	NA	R01_cb15934_c2/f1p0/566
R01_cb14141_c0/f1p0/745	R01_cb14141_c0/f1p0/745	R01_cb14141_c0/f1p0/745	R01_cb14141_c0/f1p0/745	R01_cb14141_c0/f1p0/745
R01_cb8564_c9783/f2p3/3332	NA	NA	NA	R01_cb8564_c9783/f2p3/3332
R01_cb5007_c6/f1p0/2296	R01_cb5007_c6/f1p0/2296	R01_cb5007_c6/f1p0/2296	NA	NA
R01_cb18456_c7439/f1p0/658	NA	R01_cb18456_c7439/f1p0/658	R01_cb18456_c7439/f1p0/658	R01_cb18456_c7439/f1p0/658
R01_cb2602_c6/f1p0/2468	NA	NA	NA	R01_cb2602_c6/f1p0/2468

R01_cb4469_c3/f1p0/2816	NA	R01_cb4469_c3/f1p0/2816	NA	R01_cb4469_c3/f1p0/2816
R01_cb8564_c88848/f2p0/2346	NA	NA	R01_cb8564_c88848/f2p0/2346	R01_cb8564_c88848/f2p0/2346
R01_cb12493_c6/f1p0/722	R01_cb12493_c6/f1p0/722	R01_cb12493_c6/f1p0/722	R01_cb12493_c6/f1p0/722	R01_cb12493_c6/f1p0/722
R01_cb5471_c12/f1p0/2589	NA	NA	NA	R01_cb5471_c12/f1p0/2589
R01_cb17723_c1/f1p0/713	NA	NA	R01_cb17723_c1/f1p0/713	NA
R01_cb16275_c0/f3p0/350	R01_cb16275_c0/f3p0/350	R01_cb16275_c0/f3p0/350	NA	NA
R01_cb5204_c19/f1p0/1994	NA	R01_cb5204_c19/f1p0/1994	NA	R01_cb5204_c19/f1p0/1994
R01_cb10917_c4/f1p0/988	NA	NA	NA	R01_cb10917_c4/f1p0/988
R01_cb8564_c72951/f1p0/2113	NA	R01_cb8564_c72951/f1p0/2113	R01_cb8564_c72951/f1p0/2113	R01_cb8564_c72951/f1p0/2113
R01_cb11283_c1/f1p0/2431	NA	R01_cb11283_c1/f1p0/2431	NA	NA
R01_cb17043_c2/f1p0/869	NA	NA	NA	R01_cb17043_c2/f1p0/869
R01_cb8564_c10756/f1p0/3899	NA	R01_cb8564_c10756/f1p0/3899	NA	NA
R01_cb4469_c5/f1p0/2979	NA	NA	NA	R01_cb4469_c5/f1p0/2979
R01_cb12462_c37/f1p0/520	R01_cb12462_c37/f1p0/520	R01_cb12462_c37/f1p0/520	NA	NA
R01_cb8564_c17474/f1p0/2545	NA	NA	NA	R01_cb8564_c17474/f1p0/2545
R01_cb6608_c2/f2p0/3012	NA	NA	NA	R01_cb6608_c2/f2p0/3012
R01_cb16290_c0/f1p0/1541	R01_cb16290_c0/f1p0/1541	R01_cb16290_c0/f1p0/1541	R01_cb16290_c0/f1p0/1541	R01_cb16290_c0/f1p0/1541
R01_cb6631_c34/f4p0/2329	NA	NA	NA	R01_cb6631_c34/f4p0/2329
R01_cb8564_c39348/f1p0/2953	NA	R01_cb8564_c39348/f1p0/2953	NA	NA
R01_cb11294_c3/f1p0/918	R01_cb11294_c3/f1p0/918	R01_cb11294_c3/f1p0/918	NA	R01_cb11294_c3/f1p0/918
R01_cb8564_c126557/f1p0/3867	NA	R01_cb8564_c126557/f1p0/3867	R01_cb8564_c126557/f1p0/3867	NA
R01_cb8564_c121187/f1p0/3158	NA	R01_cb8564_c121187/f1p0/3158	R01_cb8564_c121187/f1p0/3158	R01_cb8564_c121187/f1p0/3158
R01_cb8564_c35375/f1p0/3135	NA	R01_cb8564_c35375/f1p0/3135	NA	NA
R01_cb16627_c0/f2p0/605	R01_cb16627_c0/f2p0/605	R01_cb16627_c0/f2p0/605	R01_cb16627_c0/f2p0/605	R01_cb16627_c0/f2p0/605

R01_cb1658_c45/f1p0/3017	NA	NA	NA	R01_cb1658_c45/f1p0/3017
R01_cb17243_c0/f2p0/1442	NA	NA	NA	R01_cb17243_c0/f2p0/1442
R01_cb18456_c7152/f1p0/470	R01_cb18456_c7152/f1p0/470	R01_cb18456_c7152/f1p0/470	R01_cb18456_c7152/f1p0/470	R01_cb18456_c7152/f1p0/470
R01_cb16612_c1/f1p0/571	NA	R01_cb16612_c1/f1p0/571	NA	NA
R01_cb17843_c0/f1p0/1175	NA	R01_cb17843_c0/f1p0/1175	NA	NA
R01_cb8564_c80680/f1p0/3535	NA	R01_cb8564_c80680/f1p0/3535	R01_cb8564_c80680/f1p0/3535	NA
R01_cb14092_c0/f3p0/1278	NA	NA	NA	R01_cb14092_c0/f3p0/1278
R01_cb3415_c5/f1p1/3345	NA	R01_cb3415_c5/f1p1/3345	NA	NA
R01_cb13772_c7/f1p0/393	R01_cb13772_c7/f1p0/393	R01_cb13772_c7/f1p0/393	R01_cb13772_c7/f1p0/393	R01_cb13772_c7/f1p0/393
R01_cb14610_c1/f2p0/717	NA	R01_cb14610_c1/f2p0/717	R01_cb14610_c1/f2p0/717	R01_cb14610_c1/f2p0/717
R01_cb8823_c6/f1p2/2056	NA	NA	NA	R01_cb8823_c6/f1p2/2056
R01_cb4812_c17/f1p0/3187	NA	R01_cb4812_c17/f1p0/3187	NA	NA
R01_cb8564_c72890/f1p0/3768	NA	R01_cb8564_c72890/f1p0/3768	R01_cb8564_c72890/f1p0/3768	R01_cb8564_c72890/f1p0/3768
R01_cb4625_c0/f1p0/3299	NA	R01_cb4625_c0/f1p0/3299	NA	NA
R01_cb2252_c0/f2p0/1094	NA	NA	NA	R01_cb2252_c0/f2p0/1094
R01_cb8101_c9/f1p0/2203	NA	NA	NA	R01_cb8101_c9/f1p0/2203
R01_cb13462_c0/f2p1/1761	NA	NA	NA	R01_cb13462_c0/f2p1/1761
R01_cb8564_c114038/f1p1/2194	NA	NA	NA	R01_cb8564_c114038/f1p1/2194
R01_cb10970_c8/f1p0/1458	NA	NA	NA	R01_cb10970_c8/f1p0/1458
R01_cb2588_c5/f1p0/471	NA	R01_cb2588_c5/f1p0/471	R01_cb2588_c5/f1p0/471	R01_cb2588_c5/f1p0/471
R01_cb3177_c2/f1p0/2605	NA	NA	NA	R01_cb3177_c2/f1p0/2605
R01_cb7862_c4/f1p0/685	R01_cb7862_c4/f1p0/685	R01_cb7862_c4/f1p0/685	R01_cb7862_c4/f1p0/685	R01_cb7862_c4/f1p0/685
R01_cb8564_c10548/f1p0/2939	NA	R01_cb8564_c10548/f1p0/2939	NA	NA
R01_cb8564_c35315/f1p0/2351	NA	R01_cb8564_c35315/f1p0/2351	NA	NA
R01_cb9935_c1/f1p0/2069	NA	NA	NA	R01_cb9935_c1/f1p0/2069

R01_cb18195_c1/f1p0/788	R01_cb18195_c1/f1p0/788	R01_cb18195_c1/f1p0/788	R01_cb18195_c1/f1p0/788	R01_cb18195_c1/f1p0/788
R01_cb16629_c3/f3p0/890	NA	R01_cb16629_c3/f3p0/890	NA	R01_cb16629_c3/f3p0/890
R01_cb7999_c3/f1p0/1060	NA	NA	NA	R01_cb7999_c3/f1p0/1060
R01_cb8564_c78582/f1p0/3106	NA	R01_cb8564_c78582/f1p0/3106	NA	R01_cb8564_c78582/f1p0/3106
R01_cb14023_c0/f3p0/870	NA	NA	NA	R01_cb14023_c0/f3p0/870
R01_cb18409_c181/f1p0/352	R01_cb18409_c181/f1p0/352	R01_cb18409_c181/f1p0/352	R01_cb18409_c181/f1p0/352	R01_cb18409_c181/f1p0/352
R01_cb15448_c0/f2p0/583	NA	R01_cb15448_c0/f2p0/583	R01_cb15448_c0/f2p0/583	R01_cb15448_c0/f2p0/583
R01_cb11352_c3/f1p0/1019	NA	R01_cb11352_c3/f1p0/1019	NA	NA
R01_cb13590_c1/f3p0/914	NA	R01_cb13590_c1/f3p0/914	R01_cb13590_c1/f3p0/914	R01_cb13590_c1/f3p0/914
R01_cb8564_c83616/f1p0/2570	NA	NA	NA	R01_cb8564_c83616/f1p0/2570
R01_cb8564_c3687/f1p0/1995	NA	R01_cb8564_c3687/f1p0/1995	NA	NA
R01_cb8564_c17515/f1p4/4103	NA	NA	NA	R01_cb8564_c17515/f1p4/4103
R01_cb1190_c12/f1p0/3276	NA	NA	NA	R01_cb1190_c12/f1p0/3276
R01_cb3732_c29/f1p1/2731	NA	NA	NA	R01_cb3732_c29/f1p1/2731
R01_cb5249_c2/f2p0/1894	NA	NA	NA	R01_cb5249_c2/f2p0/1894
R01_cb5482_c5/f1p1/2990	NA	R01_cb5482_c5/f1p1/2990	NA	NA
R01_cb119_c18/f1p1/3694	NA	NA	NA	R01_cb119_c18/f1p1/3694
R01_cb69_c31/f4p0/3533	NA	NA	NA	R01_cb69_c31/f4p0/3533
R01_cb5486_c6/f1p0/1322	R01_cb5486_c6/f1p0/1322	R01_cb5486_c6/f1p0/1322	R01_cb5486_c6/f1p0/1322	R01_cb5486_c6/f1p0/1322
R01_cb16303_c6/f1p0/1732	NA	NA	NA	R01_cb16303_c6/f1p0/1732
R01_cb18456_c7952/f4p2/501	R01_cb18456_c7952/f4p2/501	R01_cb18456_c7952/f4p2/501	NA	NA
R01_cb10024_c261/f1p0/751	NA	NA	R01_cb10024_c261/f1p0/751	NA
R01_cb16278_c0/f1p0/1726	NA	R01_cb16278_c0/f1p0/1726	NA	R01_cb16278_c0/f1p0/1726
R01_cb1240_c26/f1p0/2985	NA	NA	NA	R01_cb1240_c26/f1p0/2985
R01_cb11447_c1/f1p0/2166	NA	R01_cb11447_c1/f1p0/2166	NA	NA
R01_cb12636_c1/f4p0/1521	NA	NA	NA	R01_cb12636_c1/f4p0/1521

R01_cb18024_c1/f1p0/1090	NA	R01_cb18024_c1/f1p0/1090	NA	R01_cb18024_c1/f1p0/1090
R01_cb8564_c22943/f1p0/3151	NA	R01_cb8564_c22943/f1p0/3151	NA	NA
R01_cb8564_c3667/f1p0/2932	NA	NA	NA	R01_cb8564_c3667/f1p0/2932
R01_cb7219_c13/f1p4/2473	NA	NA	NA	R01_cb7219_c13/f1p4/2473
R01_cb10080_c14/f1p0/647	NA	R01_cb10080_c14/f1p0/647	NA	NA
R01_cb13953_c18/f1p1/870	R01_cb13953_c18/f1p1/870	R01_cb13953_c18/f1p1/870	NA	NA
R01_cb10670_c3/f3p0/1012	NA	R01_cb10670_c3/f3p0/1012	NA	NA
R01_cb7632_c25/f1p0/1333	NA	R01_cb7632_c25/f1p0/1333	NA	NA
R01_cb8564_c115387/f1p0/2236	NA	NA	R01_cb8564_c115387/f1p0/2236	R01_cb8564_c115387/f1p0/2236
R01_cb7530_c14/f4p0/2450	NA	NA	NA	R01_cb7530_c14/f4p0/2450
R01_cb10034_c40/f1p0/622	NA	R01_cb10034_c40/f1p0/622	NA	NA
R01_cb17177_c0/f2p0/674	NA	R01_cb17177_c0/f2p0/674	NA	R01_cb17177_c0/f2p0/674
R01_cb8564_c45266/f1p0/2129	NA	R01_cb8564_c45266/f1p0/2129	R01_cb8564_c45266/f1p0/2129	R01_cb8564_c45266/f1p0/2129
R01_cb8564_c76809/f1p0/2176	R01_cb8564_c76809/f1p0/2176	R01_cb8564_c76809/f1p0/2176	NA	NA
R01_cb17052_c8/f1p0/1618	NA	NA	NA	R01_cb17052_c8/f1p0/1618
R01_cb7457_c1/f1p0/2610	R01_cb7457_c1/f1p0/2610	NA	NA	NA
R01_cb6959_c2/f1p0/2602	NA	NA	NA	R01_cb6959_c2/f1p0/2602
R01_cb13809_c5/f1p0/5551	R01_cb13809_c5/f1p0/5551	R01_cb13809_c5/f1p0/5551	R01_cb13809_c5/f1p0/5551	R01_cb13809_c5/f1p0/5551
R01_cb11354_c2/f2p0/1657	NA	NA	NA	R01_cb11354_c2/f2p0/1657
R01_cb3239_c0/f1p0/3675	NA	R01_cb3239_c0/f1p0/3675	NA	NA
R01_cb16068_c0/f2p0/468	NA	R01_cb16068_c0/f2p0/468	NA	R01_cb16068_c0/f2p0/468
R01_cb10286_c1/f1p0/2334	R01_cb10286_c1/f1p0/2334	R01_cb10286_c1/f1p0/2334	R01_cb10286_c1/f1p0/2334	R01_cb10286_c1/f1p0/2334
R01_cb12769_c1/f1p0/1333	NA	NA	NA	R01_cb12769_c1/f1p0/1333
R01_cb16410_c1/f1p0/623	NA	NA	NA	R01_cb16410_c1/f1p0/623

R01_cb8131_c0/f1p0/2456	NA	R01_cb8131_c0/f1p0/2456	NA	R01_cb8131_c0/f1p0/2456
R01_cb1998_c0/f1p0/4047	NA	R01_cb1998_c0/f1p0/4047	R01_cb1998_c0/f1p0/4047	R01_cb1998_c0/f1p0/4047
R01_cb8564_c17591/f1p0/3912	NA	NA	R01_cb8564_c17591/f1p0/3912	R01_cb8564_c17591/f1p0/3912
R01_cb3563_c1/f1p0/3434	NA	R01_cb3563_c1/f1p0/3434	NA	R01_cb3563_c1/f1p0/3434
R01_cb480_c4/f1p0/5717	NA	NA	NA	R01_cb480_c4/f1p0/5717
R01_cb12439_c0/f9p0/551	R01_cb12439_c0/f9p0/551	NA	NA	NA
R01_cb3565_c8/f1p0/1986	NA	R01_cb3565_c8/f1p0/1986	NA	NA
R01_cb3492_c0/f1p0/3604	NA	R01_cb3492_c0/f1p0/3604	NA	NA
R01_cb8564_c84336/f1p0/3480	NA	R01_cb8564_c84336/f1p0/3480	NA	R01_cb8564_c84336/f1p0/3480
R01_cb18456_c1674/f2p0/1614	R01_cb18456_c1674/f2p0/1614	R01_cb18456_c1674/f2p0/1614	NA	NA
R01_cb16124_c2/f1p0/577	NA	NA	R01_cb16124_c2/f1p0/577	R01_cb16124_c2/f1p0/577
R01_cb8564_c125814/f1p0/2108	NA	R01_cb8564_c125814/f1p0/2108	NA	NA
R01_cb17587_c0/f1p0/1830	NA	R01_cb17587_c0/f1p0/1830	R01_cb17587_c0/f1p0/1830	R01_cb17587_c0/f1p0/1830
R01_cb18132_c21/f1p0/1119	NA	R01_cb18132_c21/f1p0/1119	NA	R01_cb18132_c21/f1p0/1119
R01_cb7298_c2/f1p0/3839	R01_cb7298_c2/f1p0/3839	R01_cb7298_c2/f1p0/3839	R01_cb7298_c2/f1p0/3839	R01_cb7298_c2/f1p0/3839
R01_cb12634_c21/f1p0/5456	NA	R01_cb12634_c21/f1p0/5456	NA	NA
R01_cb14426_c5/f1p0/521	R01_cb14426_c5/f1p0/521	R01_cb14426_c5/f1p0/521	R01_cb14426_c5/f1p0/521	R01_cb14426_c5/f1p0/521
R01_cb12273_c9/f2p0/1670	NA	NA	NA	R01_cb12273_c9/f2p0/1670
R01_cb1297_c16/f1p0/3576	NA	NA	NA	R01_cb1297_c16/f1p0/3576
R01_cb8564_c69570/f1p0/2852	R01_cb8564_c69570/f1p0/2852	R01_cb8564_c69570/f1p0/2852	R01_cb8564_c69570/f1p0/2852	R01_cb8564_c69570/f1p0/2852
R01_cb18456_c6452/f1p0/790	NA	R01_cb18456_c6452/f1p0/790	NA	R01_cb18456_c6452/f1p0/790
R01_cb8564_c84364/f1p0/2726	NA	R01_cb8564_c84364/f1p0/2726	NA	NA
R01_cb8564_c88117/f1p0/2677	NA	R01_cb8564_c88117/f1p0/2677	NA	R01_cb8564_c88117/f1p0/2677

R01_cb10015_c202/f1p0/574	NA	R01_cb10015_c202/f1p0/574	NA	NA
R01_cb12376_c38/f1p0/484	R01_cb12376_c38/f1p0/484	R01_cb12376_c38/f1p0/484	R01_cb12376_c38/f1p0/484	R01_cb12376_c38/f1p0/484
R01_cb4523_c2/f1p0/3049	NA	R01_cb4523_c2/f1p0/3049	NA	NA
R01_cb2082_c14/f1p0/3023	NA	NA	NA	R01_cb2082_c14/f1p0/3023
R01_cb9154_c9/f1p1/1598	NA	NA	NA	R01_cb9154_c9/f1p1/1598
R01_cb535_c5/f1p0/4656	NA	NA	NA	R01_cb535_c5/f1p0/4656
R01_cb11561_c1/f1p0/2126	NA	NA	R01_cb11561_c1/f1p0/2126	R01_cb11561_c1/f1p0/2126
R01_cb3138_c2/f1p0/3706	NA	NA	NA	R01_cb3138_c2/f1p0/3706
R01_cb10579_c0/f4p0/993	NA	NA	NA	R01_cb10579_c0/f4p0/993
R01_cb16482_c0/f3p0/1005	NA	R01_cb16482_c0/f3p0/1005	NA	NA
R01_cb9772_c4/f1p0/3466	NA	NA	NA	R01_cb9772_c4/f1p0/3466
R01_cb7533_c5/f1p0/2459	NA	NA	NA	R01_cb7533_c5/f1p0/2459
R01_cb9645_c5/f1p0/1426	NA	R01_cb9645_c5/f1p0/1426	NA	NA
R01_cb261_c16/f1p0/3480	NA	NA	NA	R01_cb261_c16/f1p0/3480
R01_cb8692_c2/f1p0/2298	NA	R01_cb8692_c2/f1p0/2298	NA	NA
R01_cb15423_c1/f1p0/676	NA	R01_cb15423_c1/f1p0/676	R01_cb15423_c1/f1p0/676	R01_cb15423_c1/f1p0/676
R01_cb18456_c1636/f2p0/378	NA	NA	R01_cb18456_c1636/f2p0/378	R01_cb18456_c1636/f2p0/378
R01_cb8568_c11/f1p0/3987	NA	R01_cb8568_c11/f1p0/3987	NA	NA
R01_cb2104_c16/f1p0/5389	NA	R01_cb2104_c16/f1p0/5389	R01_cb2104_c16/f1p0/5389	R01_cb2104_c16/f1p0/5389
R01_cb6206_c4/f1p0/1854	NA	R01_cb6206_c4/f1p0/1854	R01_cb6206_c4/f1p0/1854	NA
R01_cb2033_c22/f1p0/2340	NA	NA	R01_cb2033_c22/f1p0/2340	R01_cb2033_c22/f1p0/2340
R01_cb929_c2/f1p0/3360	NA	NA	NA	R01_cb929_c2/f1p0/3360
R01_cb6644_c0/f1p0/2829	R01_cb6644_c0/f1p0/2829	R01_cb6644_c0/f1p0/2829	NA	NA
R01_cb8564_c148557/f1p5/2802	NA	R01_cb8564_c148557/f1p5/2802	NA	R01_cb8564_c148557/f1p5/2802
R01_cb261_c18/f1p0/2497	NA	NA	NA	R01_cb261_c18/f1p0/2497

R01_cb16921_c1/f1p0/1624	R01_cb16921_c1/f1p0/1624	R01_cb16921_c1/f1p0/1624	R01_cb16921_c1/f1p0/1624	R01_cb16921_c1/f1p0/1624
R01_cb302_c9/f1p2/3522	NA	NA	NA	R01_cb302_c9/f1p2/3522
R01_cb18456_c6853/f1p0/1477	NA	R01_cb18456_c6853/f1p0/1477	NA	R01_cb18456_c6853/f1p0/1477
R01_cb15252_c3/f1p0/1653	NA	NA	NA	R01_cb15252_c3/f1p0/1653
R01_cb1388_c2/f2p0/4190	NA	NA	NA	R01_cb1388_c2/f2p0/4190
R01_cb1116_c1/f1p0/2904	NA	NA	NA	R01_cb1116_c1/f1p0/2904
R01_cb18456_c6124/f1p0/703	NA	NA	NA	R01_cb18456_c6124/f1p0/703
R01_cb10417_c3/f1p0/783	NA	R01_cb10417_c3/f1p0/783	NA	NA
R01_cb16264_c11/f1p0/1258	NA	NA	R01_cb16264_c11/f1p0/1258	R01_cb16264_c11/f1p0/1258
R01_cb16118_c3/f1p0/1328	NA	NA	NA	R01_cb16118_c3/f1p0/1328
R01_cb225_c3/f1p0/4583	NA	NA	NA	R01_cb225_c3/f1p0/4583
R01_cb8388_c6/f1p0/2230	NA	R01_cb8388_c6/f1p0/2230	NA	R01_cb8388_c6/f1p0/2230
R01_cb647_c12/f1p0/4491	NA	R01_cb647_c12/f1p0/4491	NA	R01_cb647_c12/f1p0/4491
R01_cb6188_c1/f1p0/2716	R01_cb6188_c1/f1p0/2716	R01_cb6188_c1/f1p0/2716	R01_cb6188_c1/f1p0/2716	R01_cb6188_c1/f1p0/2716
R01_cb18602_c1/f1p0/833	R01_cb18602_c1/f1p0/833	R01_cb18602_c1/f1p0/833	R01_cb18602_c1/f1p0/833	R01_cb18602_c1/f1p0/833
R01_cb2382_c9/f1p0/5169	NA	NA	NA	R01_cb2382_c9/f1p0/5169
R01_cb13073_c0/f1p0/920	NA	R01_cb13073_c0/f1p0/920	NA	R01_cb13073_c0/f1p0/920
R01_cb1706_c0/f4p0/2938	NA	NA	NA	R01_cb1706_c0/f4p0/2938
R01_cb10074_c7/f3p4/608	R01_cb10074_c7/f3p4/608	R01_cb10074_c7/f3p4/608	R01_cb10074_c7/f3p4/608	R01_cb10074_c7/f3p4/608
R01_cb8250_c1/f1p0/3163	NA	NA	R01_cb8250_c1/f1p0/3163	R01_cb8250_c1/f1p0/3163
R01_cb17623_c1/f1p0/683	NA	NA	NA	R01_cb17623_c1/f1p0/683
R01_cb18456_c6378/f1p0/477	R01_cb18456_c6378/f1p0/477	R01_cb18456_c6378/f1p0/477	NA	R01_cb18456_c6378/f1p0/477
R01_cb3344_c6/f1p0/2126	NA	NA	NA	R01_cb3344_c6/f1p0/2126
R01_cb12577_c14/f1p0/748	NA	NA	R01_cb12577_c14/f1p0/748	R01_cb12577_c14/f1p0/748
R01_cb8564_c48394/f1p0/2949	NA	R01_cb8564_c48394/f1p0/2949	R01_cb8564_c48394/f1p0/2949	R01_cb8564_c48394/f1p0/2949
R01_cb4368_c4/f2p0/1323	NA	NA	NA	R01_cb4368_c4/f2p0/1323

R01_cb15121_c0/f1p0/741	R01_cb15121_c0/f1p0/741	R01_cb15121_c0/f1p0/741	R01_cb15121_c0/f1p0/741	R01_cb15121_c0/f1p0/741
R01_cb3848_c11/f1p0/2441	NA	NA	NA	R01_cb3848_c11/f1p0/2441
R01_cb1228_c50/f1p0/1639	NA	R01_cb1228_c50/f1p0/1639	NA	R01_cb1228_c50/f1p0/1639
R01_cb5824_c8/f1p0/2111	NA	NA	NA	R01_cb5824_c8/f1p0/2111
R01_cb2875_c11/f1p0/3661	NA	NA	NA	R01_cb2875_c11/f1p0/3661
R01_cb8564_c18123/f1p0/2458	NA	NA	NA	R01_cb8564_c18123/f1p0/2458
R01_cb8564_c4689/f1p0/4442	R01_cb8564_c4689/f1p0/4442	R01_cb8564_c4689/f1p0/4442	R01_cb8564_c4689/f1p0/4442	NA
R01_cb9521_c6/f1p0/4198	NA	R01_cb9521_c6/f1p0/4198	NA	NA
R01_cb12368_c5/f1p0/373	R01_cb12368_c5/f1p0/373	R01_cb12368_c5/f1p0/373	R01_cb12368_c5/f1p0/373	R01_cb12368_c5/f1p0/373
R01_cb8564_c23652/f1p0/2627	NA	R01_cb8564_c23652/f1p0/2627	NA	R01_cb8564_c23652/f1p0/2627
R01_cb16416_c1/f1p0/1209	NA	NA	NA	R01_cb16416_c1/f1p0/1209
R01_cb8564_c87040/f1p0/2238	NA	NA	NA	R01_cb8564_c87040/f1p0/2238
R01_cb8564_c77137/f1p0/3949	NA	R01_cb8564_c77137/f1p0/3949	NA	NA
R01_cb17744_c33/f1p0/1153	NA	R01_cb17744_c33/f1p0/1153	NA	R01_cb17744_c33/f1p0/1153
R01_cb10231_c2/f1p0/1191	NA	NA	NA	R01_cb10231_c2/f1p0/1191
R01_cb15273_c8/f4p1/761	NA	NA	R01_cb15273_c8/f4p1/761	R01_cb15273_c8/f4p1/761
R01_cb10314_c8/f1p0/729	NA	R01_cb10314_c8/f1p0/729	NA	NA
R01_cb8564_c113764/f1p0/2478	NA	R01_cb8564_c113764/f1p0/2478	R01_cb8564_c113764/f1p0/2478	R01_cb8564_c113764/f1p0/2478
R01_cb2356_c17/f1p0/4027	NA	R01_cb2356_c17/f1p0/4027	NA	NA
R01_cb12714_c39/f1p0/1127	NA	NA	NA	R01_cb12714_c39/f1p0/1127
R01_cb10873_c8/f1p0/1354	NA	NA	NA	R01_cb10873_c8/f1p0/1354
R01_cb4891_c2/f1p0/3805	NA	R01_cb4891_c2/f1p0/3805	NA	NA
R01_cb8564_c130354/f1p0/2209	NA	NA	NA	R01_cb8564_c130354/f1p0/2209
R01_cb363_c1/f1p0/2190	NA	NA	NA	R01_cb363_c1/f1p0/2190

R01_cb8564_c17119/f1p2/2558	NA	NA	NA	R01_cb8564_c17119/f1p2/2558
R01_cb15168_c0/f1p0/1401	NA	NA	NA	R01_cb15168_c0/f1p0/1401
R01_cb15458_c0/f4p0/1554	NA	NA	NA	R01_cb15458_c0/f4p0/1554
R01_cb18625_c1/f1p0/1464	NA	NA	NA	R01_cb18625_c1/f1p0/1464
R01_cb7342_c0/f1p0/2642	NA	NA	NA	R01_cb7342_c0/f1p0/2642
R01_cb8564_c110356/f1p0/2169	NA	NA	NA	R01_cb8564_c110356/f1p0/2169
R01_cb9154_c2/f2p0/1956	NA	NA	NA	R01_cb9154_c2/f2p0/1956
R01_cb16494_c2/f1p0/1472	NA	NA	NA	R01_cb16494_c2/f1p0/1472
R01_cb8564_c123241/f1p0/2130	NA	R01_cb8564_c123241/f1p0/2130	NA	NA
R01_cb17565_c4/f1p0/1497	NA	R01_cb17565_c4/f1p0/1497	NA	NA
R01_cb18456_c1595/f1p0/981	NA	R01_cb18456_c1595/f1p0/981	NA	NA
R01_cb2875_c9/f1p0/2927	NA	NA	NA	R01_cb2875_c9/f1p0/2927
R01_cb10902_c3/f1p0/1427	NA	NA	NA	R01_cb10902_c3/f1p0/1427
R01_cb1788_c6/f1p0/3148	NA	NA	NA	R01_cb1788_c6/f1p0/3148
R01_cb8564_c112878/f1p0/2103	NA	R01_cb8564_c112878/f1p0/2103	R01_cb8564_c112878/f1p0/2103	R01_cb8564_c112878/f1p0/2103
R01_cb18456_c7340/f1p0/362	R01_cb18456_c7340/f1p0/362	R01_cb18456_c7340/f1p0/362	R01_cb18456_c7340/f1p0/362	NA
R01_cb7506_c5/f1p0/3143	NA	NA	NA	R01_cb7506_c5/f1p0/3143
R01_cb14067_c0/f2p0/1645	NA	NA	R01_cb14067_c0/f2p0/1645	R01_cb14067_c0/f2p0/1645
R01_cb15630_c6/f1p0/813	NA	R01_cb15630_c6/f1p0/813	R01_cb15630_c6/f1p0/813	R01_cb15630_c6/f1p0/813
R01_cb13910_c21/f1p0/1088	NA	R01_cb13910_c21/f1p0/1088	R01_cb13910_c21/f1p0/1088	R01_cb13910_c21/f1p0/1088
R01_cb2540_c1/f1p0/3881	NA	R01_cb2540_c1/f1p0/3881	NA	NA
R01_cb13760_c3/f1p1/1820	NA	NA	NA	R01_cb13760_c3/f1p1/1820
R01_cb11668_c0/f1p0/893	NA	R01_cb11668_c0/f1p0/893	R01_cb11668_c0/f1p0/893	R01_cb11668_c0/f1p0/893

R01_cb10845_c3/f1p0/3316	NA	R01_cb10845_c3/f1p0/3316	NA	NA
R01_cb8064_c6/f1p1/854	NA	R01_cb8064_c6/f1p1/854	NA	R01_cb8064_c6/f1p1/854
R01_cb2875_c7/f1p0/3684	NA	NA	NA	R01_cb2875_c7/f1p0/3684
R01_cb3825_c0/f1p0/3481	NA	R01_cb3825_c0/f1p0/3481	NA	NA
R01_cb1438_c10/f1p5/4153	NA	NA	NA	R01_cb1438_c10/f1p5/4153
R01_cb9812_c1/f1p0/1966	NA	NA	NA	R01_cb9812_c1/f1p0/1966
R01_cb14481_c3/f1p0/716	NA	NA	NA	R01_cb14481_c3/f1p0/716
R01_cb1400_c3/f1p0/1927	NA	R01_cb1400_c3/f1p0/1927	NA	R01_cb1400_c3/f1p0/1927
R01_cb8564_c87548/f1p1/2389	NA	NA	NA	R01_cb8564_c87548/f1p1/2389
R01_cb13118_c4/f1p0/2138	NA	R01_cb13118_c4/f1p0/2138	NA	NA
R01_cb8564_c16185/f1p0/3213	NA	R01_cb8564_c16185/f1p0/3213	NA	NA
R01_cb10723_c2/f1p0/2730	NA	NA	NA	R01_cb10723_c2/f1p0/2730
R01_cb16718_c1/f1p0/418	R01_cb16718_c1/f1p0/418	R01_cb16718_c1/f1p0/418	R01_cb16718_c1/f1p0/418	R01_cb16718_c1/f1p0/418
R01_cb17853_c2/f1p0/927	NA	NA	R01_cb17853_c2/f1p0/927	R01_cb17853_c2/f1p0/927
R01_cb12360_c0/f34p1/793	NA	R01_cb12360_c0/f34p1/793	NA	NA
R01_cb12589_c2/f1p0/875	R01_cb12589_c2/f1p0/875	NA	NA	R01_cb12589_c2/f1p0/875
R01_cb1489_c2/f1p0/3842	NA	NA	R01_cb1489_c2/f1p0/3842	NA
R01_cb15122_c3/f1p0/5153	NA	R01_cb15122_c3/f1p0/5153	NA	NA
R01_cb723_c3/f1p0/2802	NA	R01_cb723_c3/f1p0/2802	NA	NA
R01_cb2061_c11/f1p1/1755	NA	R01_cb2061_c11/f1p1/1755	NA	NA
R01_cb6655_c3/f1p0/967	NA	R01_cb6655_c3/f1p0/967	R01_cb6655_c3/f1p0/967	NA
R01_cb15914_c1/f1p0/1589	R01_cb15914_c1/f1p0/1589	R01_cb15914_c1/f1p0/1589	NA	R01_cb15914_c1/f1p0/1589
R01_cb10843_c5/f1p0/2434	R01_cb10843_c5/f1p0/2434	R01_cb10843_c5/f1p0/2434	R01_cb10843_c5/f1p0/2434	R01_cb10843_c5/f1p0/2434
R01_cb10171_c10/f1p0/1023	NA	NA	NA	R01_cb10171_c10/f1p0/1023
R01_cb8564_c2563/f1p0/3329	NA	R01_cb8564_c2563/f1p0/3329	NA	NA
R01_cb16496_c1/f2p0/1004	NA	R01_cb16496_c1/f2p0/1004	NA	NA

R01_cb8564_c51864/f1p0/2808	NA	R01_cb8564_c51864/f1p0/2808	R01_cb8564_c51864/f1p0/2808	R01_cb8564_c51864/f1p0/2808
R01_cb2676_c3/f2p0/2662	NA	NA	NA	R01_cb2676_c3/f2p0/2662
R01_cb2991_c3/f2p0/2395	NA	NA	NA	R01_cb2991_c3/f2p0/2395
R01_cb5471_c17/f1p0/2727	NA	NA	NA	R01_cb5471_c17/f1p0/2727
R01_cb13755_c0/f2p0/1588	NA	R01_cb13755_c0/f2p0/1588	NA	NA
R01_cb8926_c1/f1p0/2241	R01_cb8926_c1/f1p0/2241	R01_cb8926_c1/f1p0/2241	NA	R01_cb8926_c1/f1p0/2241
R01_cb3192_c6/f1p0/3794	NA	NA	NA	R01_cb3192_c6/f1p0/3794
R01_cb18301_c17/f1p0/1763	R01_cb18301_c17/f1p0/1763	R01_cb18301_c17/f1p0/1763	R01_cb18301_c17/f1p0/1763	R01_cb18301_c17/f1p0/1763
R01_cb3712_c5/f1p1/2972	NA	R01_cb3712_c5/f1p1/2972	NA	NA
R01_cb18456_c7626/f1p0/757	R01_cb18456_c7626/f1p0/757	R01_cb18456_c7626/f1p0/757	R01_cb18456_c7626/f1p0/757	R01_cb18456_c7626/f1p0/757
R01_cb8564_c151660/f1p0/2823	NA	R01_cb8564_c151660/f1p0/2823	R01_cb8564_c151660/f1p0/2823	R01_cb8564_c151660/f1p0/2823
R01_cb8564_c51708/f1p0/2518	NA	R01_cb8564_c51708/f1p0/2518	R01_cb8564_c51708/f1p0/2518	R01_cb8564_c51708/f1p0/2518
R01_cb8564_c81280/f1p0/2846	NA	NA	R01_cb8564_c81280/f1p0/2846	R01_cb8564_c81280/f1p0/2846
R01_cb9759_c2/f1p0/1980	NA	NA	NA	R01_cb9759_c2/f1p0/1980
R01_cb6181_c38/f1p0/2047	NA	R01_cb6181_c38/f1p0/2047	NA	NA
R01_cb10272_c17/f1p1/1759	NA	NA	R01_cb10272_c17/f1p1/1759	R01_cb10272_c17/f1p1/1759
R01_cb10800_c2/f1p0/2620	NA	NA	NA	R01_cb10800_c2/f1p0/2620
R01_cb7962_c8/f1p0/2272	NA	R01_cb7962_c8/f1p0/2272	R01_cb7962_c8/f1p0/2272	R01_cb7962_c8/f1p0/2272
R01_cb3485_c20/f1p0/1001	NA	R01_cb3485_c20/f1p0/1001	R01_cb3485_c20/f1p0/1001	R01_cb3485_c20/f1p0/1001
R01_cb18345_c3/f1p0/799	R01_cb18345_c3/f1p0/799	R01_cb18345_c3/f1p0/799	R01_cb18345_c3/f1p0/799	R01_cb18345_c3/f1p0/799
R01_cb2773_c14/f1p0/2413	R01_cb2773_c14/f1p0/2413	R01_cb2773_c14/f1p0/2413	R01_cb2773_c14/f1p0/2413	NA
R01_cb8564_c3273/f1p0/4325	NA	R01_cb8564_c3273/f1p0/4325	NA	NA
R01_cb8564_c37557/f1p1/3253	NA	R01_cb8564_c37557/f1p1/3253	NA	NA
R01_cb8564_c89816/f1p0/2675	NA	R01_cb8564_c89816/f1p0/2675	NA	NA
R01_cb737_c68/f1p0/2519	NA	R01_cb737_c68/f1p0/2519	NA	NA

R01_cb2782_c0/f1p0/3809	R01_cb2782_c0/f1p0/3809	R01_cb2782_c0/f1p0/3809	R01_cb2782_c0/f1p0/3809	R01_cb2782_c0/f1p0/3809
R01_cb454_c11/f1p0/2332	NA	NA	NA	R01_cb454_c11/f1p0/2332
R01_cb6065_c23/f1p0/1296	NA	NA	NA	R01_cb6065_c23/f1p0/1296
R01_cb16621_c1/f1p0/926	NA	R01_cb16621_c1/f1p0/926	NA	NA
R01_cb17756_c9/f1p0/378	R01_cb17756_c9/f1p0/378	R01_cb17756_c9/f1p0/378	R01_cb17756_c9/f1p0/378	R01_cb17756_c9/f1p0/378
R01_cb5640_c4/f1p0/2159	NA	NA	NA	R01_cb5640_c4/f1p0/2159
R01_cb7367_c8/f1p0/1802	NA	NA	NA	R01_cb7367_c8/f1p0/1802
R01_cb13137_c6/f1p0/1138	NA	R01_cb13137_c6/f1p0/1138	NA	NA
R01_cb8901_c0/f1p0/2237	NA	R01_cb8901_c0/f1p0/2237	NA	R01_cb8901_c0/f1p0/2237
R01_cb8564_c82603/f1p0/3186	NA	R01_cb8564_c82603/f1p0/3186	NA	NA
R01_cb12762_c4/f1p0/865	NA	NA	NA	R01_cb12762_c4/f1p0/865
R01_cb18443_c0/f1p0/428	R01_cb18443_c0/f1p0/428	R01_cb18443_c0/f1p0/428	R01_cb18443_c0/f1p0/428	R01_cb18443_c0/f1p0/428
R01_cb580_c1/f1p0/4871	NA	NA	R01_cb580_c1/f1p0/4871	NA
R01_cb17973_c20/f1p0/638	R01_cb17973_c20/f1p0/638	R01_cb17973_c20/f1p0/638	R01_cb17973_c20/f1p0/638	R01_cb17973_c20/f1p0/638
R01_cb2571_c35/f1p0/3622	NA	NA	NA	R01_cb2571_c35/f1p0/3622
R01_cb10038_c4/f1p0/1141	NA	NA	NA	R01_cb10038_c4/f1p0/1141
R01_cb18456_c2503/f1p0/1724	NA	R01_cb18456_c2503/f1p0/1724	NA	R01_cb18456_c2503/f1p0/1724
R01_cb18456_c6984/f1p1/1282	NA	R01_cb18456_c6984/f1p1/1282	R01_cb18456_c6984/f1p1/1282	R01_cb18456_c6984/f1p1/1282
R01_cb2072_c32/f1p0/3210	NA	NA	NA	R01_cb2072_c32/f1p0/3210
R01_cb167_c43/f1p0/3922	NA	R01_cb167_c43/f1p0/3922	NA	NA
R01_cb5682_c8/f1p0/1039	R01_cb5682_c8/f1p0/1039	R01_cb5682_c8/f1p0/1039	NA	NA
R01_cb15504_c0/f1p0/1239	NA	R01_cb15504_c0/f1p0/1239	NA	NA
R01_cb18456_c6790/f1p0/1522	R01_cb18456_c6790/f1p0/1522	R01_cb18456_c6790/f1p0/1522	R01_cb18456_c6790/f1p0/1522	R01_cb18456_c6790/f1p0/1522
R01_cb8564_c86200/f1p0/2056	R01_cb8564_c86200/f1p0/2056	R01_cb8564_c86200/f1p0/2056	R01_cb8564_c86200/f1p0/2056	R01_cb8564_c86200/f1p0/2056

R01_cb14915_c2/f3p1/1551	NA	NA	NA	R01_cb14915_c2/f3p1/1551
R01_cb11212_c6/f1p0/682	NA	R01_cb11212_c6/f1p0/682	NA	NA
R01_cb18456_c581/f1p0/502	R01_cb18456_c581/f1p0/502	R01_cb18456_c581/f1p0/502	NA	R01_cb18456_c581/f1p0/502
R01_cb5287_c6/f1p0/2126	NA	R01_cb5287_c6/f1p0/2126	NA	NA
R01_cb5533_c135/f1p0/3043	NA	NA	NA	R01_cb5533_c135/f1p0/3043
R01_cb11895_c1/f2p0/1485	NA	NA	NA	R01_cb11895_c1/f2p0/1485
R01_cb13199_c0/f1p0/1627	NA	NA	NA	R01_cb13199_c0/f1p0/1627
R01_cb15540_c5/f1p1/1492	NA	NA	NA	R01_cb15540_c5/f1p1/1492
R01_cb6280_c1/f1p0/2690	NA	R01_cb6280_c1/f1p0/2690	NA	NA
R01_cb8820_c4/f1p0/3519	NA	R01_cb8820_c4/f1p0/3519	NA	NA
R01_cb1302_c2/f1p0/2351	NA	R01_cb1302_c2/f1p0/2351	R01_cb1302_c2/f1p0/2351	R01_cb1302_c2/f1p0/2351
R01_cb5042_c3/f1p0/3257	NA	NA	R01_cb5042_c3/f1p0/3257	R01_cb5042_c3/f1p0/3257
R01_cb8564_c15881/f1p0/3538	R01_cb8564_c15881/f1p0/3538	R01_cb8564_c15881/f1p0/3538	R01_cb8564_c15881/f1p0/3538	R01_cb8564_c15881/f1p0/3538
R01_cb11532_c0/f1p0/925	NA	R01_cb11532_c0/f1p0/925	NA	NA
R01_cb5502_c2/f2p0/1290	NA	NA	NA	R01_cb5502_c2/f2p0/1290
R01_cb16016_c3/f1p0/950	NA	NA	NA	R01_cb16016_c3/f1p0/950
R01_cb2378_c20/f1p0/3349	NA	NA	NA	R01_cb2378_c20/f1p0/3349
R01_cb14573_c29/f1p0/1625	R01_cb14573_c29/f1p0/1625	R01_cb14573_c29/f1p0/1625	R01_cb14573_c29/f1p0/1625	R01_cb14573_c29/f1p0/1625
R01_cb14950_c2/f1p1/1014	NA	R01_cb14950_c2/f1p1/1014	NA	NA
R01_cb8564_c47327/f1p0/4584	NA	R01_cb8564_c47327/f1p0/4584	NA	NA
R01_cb8564_c91176/f1p0/2480	NA	R01_cb8564_c91176/f1p0/2480	NA	NA
R01_cb17721_c0/f1p0/891	NA	R01_cb17721_c0/f1p0/891	R01_cb17721_c0/f1p0/891	R01_cb17721_c0/f1p0/891
R01_cb12821_c1/f2p1/653	NA	R01_cb12821_c1/f2p1/653	NA	NA
R01_cb15550_c0/f1p0/749	NA	NA	NA	R01_cb15550_c0/f1p0/749
R01_cb18456_c4989/f1p0/746	R01_cb18456_c4989/f1p0/746	R01_cb18456_c4989/f1p0/746	R01_cb18456_c4989/f1p0/746	R01_cb18456_c4989/f1p0/746

R01_cb1846_c33/f1p0/3166	NA	R01_cb1846_c33/f1p0/3166	R01_cb1846_c33/f1p0/3166	R01_cb1846_c33/f1p0/3166
R01_cb14286_c3/f1p0/1465	NA	R01_cb14286_c3/f1p0/1465	NA	R01_cb14286_c3/f1p0/1465
R01_cb16210_c0/f3p0/898	NA	NA	NA	R01_cb16210_c0/f3p0/898
R01_cb8564_c4938/f1p0/3004	NA	R01_cb8564_c4938/f1p0/3004	NA	NA
R01_cb119_c41/f1p2/4991	NA	NA	NA	R01_cb119_c41/f1p2/4991
R01_cb2451_c27/f1p1/3408	NA	NA	NA	R01_cb2451_c27/f1p1/3408
R01_cb18456_c7149/f1p0/1356	NA	R01_cb18456_c7149/f1p0/1356	R01_cb18456_c7149/f1p0/1356	R01_cb18456_c7149/f1p0/1356
R01_cb10194_c7/f1p0/921	NA	R01_cb10194_c7/f1p0/921	NA	R01_cb10194_c7/f1p0/921
R01_cb10504_c3/f1p0/636	NA	R01_cb10504_c3/f1p0/636	NA	R01_cb10504_c3/f1p0/636
R01_cb17878_c0/f1p0/1682	NA	NA	NA	R01_cb17878_c0/f1p0/1682
R01_cb8564_c89714/f1p0/3858	NA	R01_cb8564_c89714/f1p0/3858	NA	R01_cb8564_c89714/f1p0/3858
R01_cb5640_c1/f1p0/3049	NA	NA	NA	R01_cb5640_c1/f1p0/3049
R01_cb13010_c3/f1p0/690	NA	R01_cb13010_c3/f1p0/690	NA	NA
R01_cb15154_c4/f1p0/547	NA	NA	R01_cb15154_c4/f1p0/547	R01_cb15154_c4/f1p0/547
R01_cb18109_c0/f1p0/693	R01_cb18109_c0/f1p0/693	R01_cb18109_c0/f1p0/693	NA	NA
R01_cb8564_c78351/f1p0/3803	NA	R01_cb8564_c78351/f1p0/3803	NA	NA
R01_cb16459_c1/f1p0/1167	NA	R01_cb16459_c1/f1p0/1167	NA	NA
R01_cb6720_c4/f1p0/1106	R01_cb6720_c4/f1p0/1106	R01_cb6720_c4/f1p0/1106	R01_cb6720_c4/f1p0/1106	R01_cb6720_c4/f1p0/1106
R01_cb14464_c1/f1p0/1204	NA	NA	NA	R01_cb14464_c1/f1p0/1204
R01_cb150_c5/f1p0/3955	NA	R01_cb150_c5/f1p0/3955	NA	NA
R01_cb3388_c8/f1p0/2986	NA	NA	NA	R01_cb3388_c8/f1p0/2986
R01_cb12587_c12/f1p0/1720	NA	NA	NA	R01_cb12587_c12/f1p0/1720
R01_cb8564_c4113/f2p0/2913	NA	R01_cb8564_c4113/f2p0/2913	R01_cb8564_c4113/f2p0/2913	R01_cb8564_c4113/f2p0/2913
R01_cb13638_c1/f2p0/996	NA	NA	NA	R01_cb13638_c1/f2p0/996
R01_cb2760_c2/f2p0/2091	R01_cb2760_c2/f2p0/2091	R01_cb2760_c2/f2p0/2091	NA	NA
R01_cb18456_c2321/f1p0/608	NA	R01_cb18456_c2321/f1p0/608	NA	R01_cb18456_c2321/f1p0/608

R01_cb18151_c1/f1p0/899	NA	R01_cb18151_c1/f1p0/899	NA	R01_cb18151_c1/f1p0/899
R01_cb8564_c22483/f1p0/3181	R01_cb8564_c22483/f1p0/3181	R01_cb8564_c22483/f1p0/3181	NA	NA
R01_cb8564_c16145/f1p0/3085	NA	R01_cb8564_c16145/f1p0/3085	NA	NA
R01_cb14573_c28/f1p0/1717	NA	R01_cb14573_c28/f1p0/1717	R01_cb14573_c28/f1p0/1717	R01_cb14573_c28/f1p0/1717
R01_cb17442_c1/f1p0/1455	NA	NA	NA	R01_cb17442_c1/f1p0/1455
R01_cb12012_c1/f1p0/1507	NA	NA	NA	R01_cb12012_c1/f1p0/1507
R01_cb2826_c20/f1p0/5103	NA	R01_cb2826_c20/f1p0/5103	NA	NA
R01_cb8564_c50680/f1p0/2269	NA	NA	NA	R01_cb8564_c50680/f1p0/2269
R01_cb6258_c12/f1p0/2772	NA	NA	NA	R01_cb6258_c12/f1p0/2772
R01_cb14639_c3/f1p0/331	R01_cb14639_c3/f1p0/331	R01_cb14639_c3/f1p0/331	R01_cb14639_c3/f1p0/331	R01_cb14639_c3/f1p0/331
R01_cb8564_c72276/f1p0/3795	NA	R01_cb8564_c72276/f1p0/3795	NA	NA
R01_cb11151_c1/f1p0/2174	NA	NA	NA	R01_cb11151_c1/f1p0/2174
R01_cb8564_c68769/f3p0/2103	NA	NA	R01_cb8564_c68769/f3p0/2103	R01_cb8564_c68769/f3p0/2103
R01_cb8564_c19928/f1p0/2100	NA	NA	NA	R01_cb8564_c19928/f1p0/2100
R01_cb7049_c3/f1p0/1587	NA	R01_cb7049_c3/f1p0/1587	NA	NA
R01_cb5622_c7/f1p0/1600	NA	NA	NA	R01_cb5622_c7/f1p0/1600
R01_cb17861_c3/f1p0/1420	NA	NA	NA	R01_cb17861_c3/f1p0/1420
R01_cb2451_c33/f1p1/3069	NA	NA	NA	R01_cb2451_c33/f1p1/3069
R01_cb11301_c1/f1p0/3700	NA	R01_cb11301_c1/f1p0/3700	NA	NA
R01_cb570_c8/f1p0/2010	NA	R01_cb570_c8/f1p0/2010	NA	NA
R01_cb4529_c5/f1p0/3304	NA	R01_cb4529_c5/f1p0/3304	NA	NA
R01_cb8564_c117920/f1p0/2237	NA	R01_cb8564_c117920/f1p0/2237	R01_cb8564_c117920/f1p0/2237	R01_cb8564_c117920/f1p0/2237
R01_cb5900_c104/f1p0/2791	NA	R01_cb5900_c104/f1p0/2791	NA	NA
R01_cb8564_c126148/f1p0/253	R01_cb8564_c126148/f1p0/25	R01_cb8564_c126148/f1p0/253	R01_cb8564_c126148/f1p0/253	R01_cb8564_c126148/f1p0/253

5	35	5	5	5
R01_cb712_c12/f1p0/4255	NA	NA	NA	R01_cb712_c12/f1p0/4255
R01_cb13386_c19/f1p0/1632	NA	R01_cb13386_c19/f1p0/1632	NA	NA
R01_cb8320_c1/f1p1/2333	NA	NA	NA	R01_cb8320_c1/f1p1/2333
R01_cb15116_c4/f1p0/1062	NA	NA	NA	R01_cb15116_c4/f1p0/1062
R01_cb10579_c2/f1p0/3685	NA	R01_cb10579_c2/f1p0/3685	NA	R01_cb10579_c2/f1p0/3685
R01_cb8564_c16521/f3p0/4088	NA	NA	NA	R01_cb8564_c16521/f3p0/4088
R01_cb9319_c6/f1p1/1841	NA	R01_cb9319_c6/f1p1/1841	NA	R01_cb9319_c6/f1p1/1841
R01_cb6319_c17/f1p0/2488	NA	R01_cb6319_c17/f1p0/2488	NA	NA
R01_cb14125_c166/f238p0/682	NA	R01_cb14125_c166/f238p0/682	R01_cb14125_c166/f238p0/682	NA
R01_cb10752_c1/f2p0/1733	NA	R01_cb10752_c1/f2p0/1733	NA	NA
R01_cb3359_c22/f1p0/2040	NA	R01_cb3359_c22/f1p0/2040	NA	NA
R01_cb5698_c4/f1p0/2436	R01_cb5698_c4/f1p0/2436	R01_cb5698_c4/f1p0/2436	NA	R01_cb5698_c4/f1p0/2436
R01_cb8564_c39806/f1p0/2923	NA	NA	NA	R01_cb8564_c39806/f1p0/2923
R01_cb17183_c8/f5p3/776	NA	NA	NA	R01_cb17183_c8/f5p3/776
R01_cb13875_c4/f1p1/1694	NA	NA	NA	R01_cb13875_c4/f1p1/1694
R01_cb7653_c8/f1p0/3624	NA	R01_cb7653_c8/f1p0/3624	NA	R01_cb7653_c8/f1p0/3624
R01_cb11208_c2/f1p0/3597	NA	NA	NA	R01_cb11208_c2/f1p0/3597
R01_cb51_c24/f1p0/2880	NA	R01_cb51_c24/f1p0/2880	NA	NA
R01_cb5208_c10/f1p1/2768	NA	NA	NA	R01_cb5208_c10/f1p1/2768
R01_cb1119_c9/f1p0/2704	NA	R01_cb1119_c9/f1p0/2704	NA	NA
R01_cb1788_c3/f1p0/3440	NA	NA	NA	R01_cb1788_c3/f1p0/3440
R01_cb12739_c8/f1p0/420	R01_cb12739_c8/f1p0/420	R01_cb12739_c8/f1p0/420	NA	R01_cb12739_c8/f1p0/420
R01_cb18456_c4794/f1p0/598	NA	NA	NA	R01_cb18456_c4794/f1p0/598
R01_cb7019_c1/f1p0/1981	NA	R01_cb7019_c1/f1p0/1981	R01_cb7019_c1/f1p0/1981	R01_cb7019_c1/f1p0/1981
R01_cb11358_c0/f1p0/619	NA	R01_cb11358_c0/f1p0/619	R01_cb11358_c0/f1p0/619	R01_cb11358_c0/f1p0/619

R01_cb18456_c6123/f1p0/637	NA	NA	NA	R01_cb18456_c6123/f1p0/637
R01_cb8564_c43748/f1p0/3190	R01_cb8564_c43748/f1p0/3190	R01_cb8564_c43748/f1p0/3190	NA	NA
R01_cb3307_c12/f1p0/2912	NA	R01_cb3307_c12/f1p0/2912	NA	NA
R01_cb3960_c0/f1p0/3436	NA	R01_cb3960_c0/f1p0/3436	R01_cb3960_c0/f1p0/3436	R01_cb3960_c0/f1p0/3436
R01_cb4178_c5/f1p0/2452	NA	NA	NA	R01_cb4178_c5/f1p0/2452
R01_cb17266_c29/f1p0/564	NA	R01_cb17266_c29/f1p0/564	R01_cb17266_c29/f1p0/564	R01_cb17266_c29/f1p0/564
R01_cb8564_c113857/f1p0/3048	NA	NA	NA	R01_cb8564_c113857/f1p0/3048
R01_cb15931_c1/f1p0/977	NA	R01_cb15931_c1/f1p0/977	NA	R01_cb15931_c1/f1p0/977
R01_cb12360_c6/f1p0/539	NA	R01_cb12360_c6/f1p0/539	NA	R01_cb12360_c6/f1p0/539
R01_cb8028_c3/f1p0/4063	NA	R01_cb8028_c3/f1p0/4063	NA	R01_cb8028_c3/f1p0/4063
R01_cb11547_c2/f1p0/3211	R01_cb11547_c2/f1p0/3211	R01_cb11547_c2/f1p0/3211	R01_cb11547_c2/f1p0/3211	R01_cb11547_c2/f1p0/3211
R01_cb10776_c1/f1p0/2288	NA	R01_cb10776_c1/f1p0/2288	NA	NA
R01_cb12221_c0/f1p0/554	R01_cb12221_c0/f1p0/554	R01_cb12221_c0/f1p0/554	R01_cb12221_c0/f1p0/554	NA
R01_cb3051_c10/f1p0/2431	NA	R01_cb3051_c10/f1p0/2431	NA	R01_cb3051_c10/f1p0/2431
R01_cb10080_c4/f1p0/910	NA	R01_cb10080_c4/f1p0/910	NA	NA
R01_cb943_c8/f1p0/5094	NA	NA	NA	R01_cb943_c8/f1p0/5094
R01_cb11248_c4/f1p0/949	NA	R01_cb11248_c4/f1p0/949	NA	NA
R01_cb9847_c17/f1p0/682	NA	R01_cb9847_c17/f1p0/682	R01_cb9847_c17/f1p0/682	NA
R01_cb2812_c4/f2p0/3146	NA	NA	NA	R01_cb2812_c4/f2p0/3146
R01_cb12421_c61/f1p0/653	R01_cb12421_c61/f1p0/653	R01_cb12421_c61/f1p0/653	NA	NA
R01_cb6453_c4/f1p0/2772	NA	R01_cb6453_c4/f1p0/2772	NA	NA
R01_cb11649_c1/f1p0/2149	R01_cb11649_c1/f1p0/2149	R01_cb11649_c1/f1p0/2149	NA	NA
R01_cb13044_c0/f3p0/486	NA	R01_cb13044_c0/f3p0/486	NA	NA
R01_cb6297_c19/f1p0/815	NA	NA	R01_cb6297_c19/f1p0/815	NA

R01_cb17582_c3/f1p0/588	NA	R01_cb17582_c3/f1p0/588	R01_cb17582_c3/f1p0/588	R01_cb17582_c3/f1p0/588
R01_cb8564_c9513/f4p0/2171	R01_cb8564_c9513/f4p0/2171	R01_cb8564_c9513/f4p0/2171	R01_cb8564_c9513/f4p0/2171	R01_cb8564_c9513/f4p0/2171
R01_cb8564_c38151/f1p3/3560	NA	NA	NA	R01_cb8564_c38151/f1p3/3560
R01_cb7646_c8/f1p0/1127	NA	NA	NA	R01_cb7646_c8/f1p0/1127
R01_cb7302_c4/f1p0/955	NA	NA	NA	R01_cb7302_c4/f1p0/955
R01_cb3303_c3/f1p0/1224	R01_cb3303_c3/f1p0/1224	R01_cb3303_c3/f1p0/1224	R01_cb3303_c3/f1p0/1224	R01_cb3303_c3/f1p0/1224
R01_cb8564_c124637/f1p0/2343	NA	R01_cb8564_c124637/f1p0/2343	NA	R01_cb8564_c124637/f1p0/2343
R01_cb1915_c24/f1p0/3278	NA	R01_cb1915_c24/f1p0/3278	NA	NA
R01_cb8564_c79449/f1p0/2980	NA	NA	NA	R01_cb8564_c79449/f1p0/2980
R01_cb8564_c16879/f1p0/3722	NA	NA	NA	R01_cb8564_c16879/f1p0/3722
R01_cb12581_c4/f1p0/1017	NA	R01_cb12581_c4/f1p0/1017	NA	NA
R01_cb11491_c2/f1p0/646	R01_cb11491_c2/f1p0/646	R01_cb11491_c2/f1p0/646	R01_cb11491_c2/f1p0/646	R01_cb11491_c2/f1p0/646
R01_cb18120_c1/f1p0/853	NA	R01_cb18120_c1/f1p0/853	NA	R01_cb18120_c1/f1p0/853
R01_cb10029_c204/f2p0/979	NA	R01_cb10029_c204/f2p0/979	NA	NA
R01_cb8564_c119495/f1p0/1987	R01_cb8564_c119495/f1p0/1987	R01_cb8564_c119495/f1p0/1987	R01_cb8564_c119495/f1p0/1987	R01_cb8564_c119495/f1p0/1987
R01_cb1706_c6/f1p0/2895	NA	NA	NA	R01_cb1706_c6/f1p0/2895
R01_cb8564_c3953/f1p0/2871	NA	R01_cb8564_c3953/f1p0/2871	R01_cb8564_c3953/f1p0/2871	R01_cb8564_c3953/f1p0/2871
R01_cb8564_c69282/f2p0/2953	NA	R01_cb8564_c69282/f2p0/2953	R01_cb8564_c69282/f2p0/2953	NA
R01_cb17939_c1/f1p0/418	NA	R01_cb17939_c1/f1p0/418	NA	R01_cb17939_c1/f1p0/418
R01_cb16075_c3/f1p0/1422	NA	R01_cb16075_c3/f1p0/1422	NA	NA
R01_cb8564_c2031/f1p0/2606	NA	R01_cb8564_c2031/f1p0/2606	R01_cb8564_c2031/f1p0/2606	NA
R01_cb11329_c1/f1p0/1946	R01_cb11329_c1/f1p0/1946	R01_cb11329_c1/f1p0/1946	NA	R01_cb11329_c1/f1p0/1946
R01_cb2543_c45/f1p0/2956	NA	R01_cb2543_c45/f1p0/2956	R01_cb2543_c45/f1p0/2956	NA
R01_cb8564_c17864/f1p3/3853	NA	NA	NA	R01_cb8564_c17864/f1p3/3853

R01_cb17132_c0/f1p0/874	NA	NA	R01_cb17132_c0/f1p0/874	NA
R01_cb7484_c9/f1p0/658	NA	R01_cb7484_c9/f1p0/658	NA	R01_cb7484_c9/f1p0/658
R01_cb9606_c89/f1p0/1399	NA	R01_cb9606_c89/f1p0/1399	NA	NA
R01_cb18456_c7498/f1p0/609	NA	R01_cb18456_c7498/f1p0/609	R01_cb18456_c7498/f1p0/609	NA
R01_cb10277_c4/f1p0/658	NA	R01_cb10277_c4/f1p0/658	NA	R01_cb10277_c4/f1p0/658
R01_cb3992_c4/f1p0/1184	R01_cb3992_c4/f1p0/1184	R01_cb3992_c4/f1p0/1184	R01_cb3992_c4/f1p0/1184	NA
R01_cb5659_c33/f1p0/2120	NA	NA	NA	R01_cb5659_c33/f1p0/2120
R01_cb8564_c185222/f1p3/3772	NA	NA	NA	R01_cb8564_c185222/f1p3/3772
R01_cb723_c0/f1p0/4662	NA	R01_cb723_c0/f1p0/4662	NA	NA
R01_cb11879_c4/f1p0/1353	NA	NA	NA	R01_cb11879_c4/f1p0/1353
R01_cb15460_c0/f12p0/529	NA	R01_cb15460_c0/f12p0/529	NA	R01_cb15460_c0/f12p0/529
R01_cb11106_c1/f1p0/1973	NA	NA	NA	R01_cb11106_c1/f1p0/1973
R01_cb3169_c1/f1p0/3014	NA	NA	NA	R01_cb3169_c1/f1p0/3014
R01_cb6867_c4/f1p0/872	NA	NA	NA	R01_cb6867_c4/f1p0/872
R01_cb3358_c7/f1p0/2790	R01_cb3358_c7/f1p0/2790	R01_cb3358_c7/f1p0/2790	R01_cb3358_c7/f1p0/2790	R01_cb3358_c7/f1p0/2790
R01_cb18409_c91/f1p0/425	R01_cb18409_c91/f1p0/425	R01_cb18409_c91/f1p0/425	R01_cb18409_c91/f1p0/425	R01_cb18409_c91/f1p0/425
R01_cb13428_c7/f1p0/763	NA	R01_cb13428_c7/f1p0/763	NA	NA
R01_cb16154_c0/f1p0/1002	NA	R01_cb16154_c0/f1p0/1002	NA	NA
R01_cb8564_c69217/f2p0/2619	NA	NA	NA	R01_cb8564_c69217/f2p0/2619
R01_cb10236_c2/f1p0/2004	NA	NA	NA	R01_cb10236_c2/f1p0/2004
R01_cb8564_c17422/f1p1/4151	NA	R01_cb8564_c17422/f1p1/4151	NA	R01_cb8564_c17422/f1p1/4151
R01_cb11547_c0/f2p0/649	R01_cb11547_c0/f2p0/649	R01_cb11547_c0/f2p0/649	R01_cb11547_c0/f2p0/649	R01_cb11547_c0/f2p0/649
R01_cb2729_c8/f1p0/1680	NA	R01_cb2729_c8/f1p0/1680	NA	R01_cb2729_c8/f1p0/1680
R01_cb18409_c32/f2p0/371	R01_cb18409_c32/f2p0/371	R01_cb18409_c32/f2p0/371	R01_cb18409_c32/f2p0/371	R01_cb18409_c32/f2p0/371
R01_cb4732_c3/f1p0/6862	NA	NA	NA	R01_cb4732_c3/f1p0/6862

R01_cb15266_c2/f1p0/608	R01_cb15266_c2/f1p0/608	R01_cb15266_c2/f1p0/608	NA	NA
R01_cb7145_c1/f1p0/2686	NA	R01_cb7145_c1/f1p0/2686	NA	NA
R01_cb8107_c2/f1p1/2465	R01_cb8107_c2/f1p1/2465	R01_cb8107_c2/f1p1/2465	R01_cb8107_c2/f1p1/2465	R01_cb8107_c2/f1p1/2465
R01_cb8564_c19208/f1p0/3464	R01_cb8564_c19208/f1p0/3464	R01_cb8564_c19208/f1p0/3464	NA	R01_cb8564_c19208/f1p0/3464
R01_cb13421_c1/f1p0/1230	R01_cb13421_c1/f1p0/1230	R01_cb13421_c1/f1p0/1230	NA	R01_cb13421_c1/f1p0/1230
R01_cb8710_c7/f1p0/3148	NA	R01_cb8710_c7/f1p0/3148	NA	NA
R01_cb8564_c4092/f1p0/3913	NA	R01_cb8564_c4092/f1p0/3913	R01_cb8564_c4092/f1p0/3913	NA
R01_cb8564_c3172/f1p0/2117	NA	NA	NA	R01_cb8564_c3172/f1p0/2117
R01_cb18606_c0/f1p0/488	NA	R01_cb18606_c0/f1p0/488	NA	NA
R01_cb8564_c122477/f1p0/2561	NA	NA	NA	R01_cb8564_c122477/f1p0/2561
R01_cb1894_c4/f1p0/3066	NA	R01_cb1894_c4/f1p0/3066	R01_cb1894_c4/f1p0/3066	R01_cb1894_c4/f1p0/3066
R01_cb8803_c0/f1p0/2266	NA	R01_cb8803_c0/f1p0/2266	NA	NA
R01_cb8564_c126847/f1p0/2680	R01_cb8564_c126847/f1p0/2680	R01_cb8564_c126847/f1p0/2680	R01_cb8564_c126847/f1p0/2680	R01_cb8564_c126847/f1p0/2680
R01_cb7988_c0/f1p1/2527	NA	NA	NA	R01_cb7988_c0/f1p1/2527
R01_cb12779_c0/f2p0/1005	R01_cb12779_c0/f2p0/1005	R01_cb12779_c0/f2p0/1005	R01_cb12779_c0/f2p0/1005	R01_cb12779_c0/f2p0/1005
R01_cb101_c6/f1p0/2290	NA	NA	NA	R01_cb101_c6/f1p0/2290
R01_cb12932_c0/f1p0/779	R01_cb12932_c0/f1p0/779	R01_cb12932_c0/f1p0/779	NA	NA
R01_cb7141_c2/f1p0/2327	NA	R01_cb7141_c2/f1p0/2327	NA	R01_cb7141_c2/f1p0/2327
R01_cb10192_c7/f1p0/1456	NA	NA	NA	R01_cb10192_c7/f1p0/1456
R01_cb8475_c1/f1p0/2078	NA	R01_cb8475_c1/f1p0/2078	NA	R01_cb8475_c1/f1p0/2078
R01_cb14463_c1/f2p0/936	NA	NA	NA	R01_cb14463_c1/f2p0/936
R01_cb124_c18/f1p0/3758	NA	NA	NA	R01_cb124_c18/f1p0/3758
R01_cb9606_c5/f2p1/1916	NA	R01_cb9606_c5/f2p1/1916	NA	NA

R01_cb8564_c25256/f1p0/1962	NA	NA	NA	R01_cb8564_c25256/f1p0/1962
R01_cb6093_c5/f1p0/2717	NA	R01_cb6093_c5/f1p0/2717	NA	NA
R01_cb13279_c2/f1p0/1000	NA	NA	NA	R01_cb13279_c2/f1p0/1000
R01_cb8564_c1468/f1p0/4772	NA	R01_cb8564_c1468/f1p0/4772	NA	NA
R01_cb8400_c0/f6p1/1868	NA	NA	NA	R01_cb8400_c0/f6p1/1868
R01_cb11107_c7/f1p0/2124	NA	R01_cb11107_c7/f1p0/2124	NA	NA
R01_cb18456_c1686/f2p0/1403	NA	R01_cb18456_c1686/f2p0/1403	NA	NA
R01_cb9380_c10/f3p0/1968	NA	NA	NA	R01_cb9380_c10/f3p0/1968
R01_cb6586_c0/f1p0/2816	NA	R01_cb6586_c0/f1p0/2816	NA	NA
R01_cb13982_c2/f1p0/1393	NA	NA	NA	R01_cb13982_c2/f1p0/1393
R01_cb8564_c86688/f1p0/3136	NA	R01_cb8564_c86688/f1p0/3136	NA	NA
R01_cb6947_c8/f2p0/1909	NA	NA	NA	R01_cb6947_c8/f2p0/1909
R01_cb10797_c2/f1p0/742	NA	R01_cb10797_c2/f1p0/742	NA	R01_cb10797_c2/f1p0/742
R01_cb693_c4/f1p0/3062	NA	NA	NA	R01_cb693_c4/f1p0/3062
R01_cb11290_c3/f1p0/3152	NA	R01_cb11290_c3/f1p0/3152	NA	R01_cb11290_c3/f1p0/3152
R01_cb7497_c10/f1p1/3177	NA	NA	NA	R01_cb7497_c10/f1p1/3177
R01_cb6258_c16/f1p1/2765	NA	NA	NA	R01_cb6258_c16/f1p1/2765
R01_cb10936_c5/f1p0/1713	NA	NA	NA	R01_cb10936_c5/f1p0/1713
R01_cb6644_c1/f1p0/3304	NA	R01_cb6644_c1/f1p0/3304	NA	NA
R01_cb8564_c84126/f1p0/3186	NA	R01_cb8564_c84126/f1p0/3186	NA	NA
R01_cb17052_c7/f1p0/577	NA	R01_cb17052_c7/f1p0/577	NA	R01_cb17052_c7/f1p0/577
R01_cb4134_c23/f1p0/2638	NA	NA	NA	R01_cb4134_c23/f1p0/2638
R01_cb3875_c8/f1p0/2382	NA	R01_cb3875_c8/f1p0/2382	NA	NA
R01_cb9359_c1/f1p0/2987	NA	NA	NA	R01_cb9359_c1/f1p0/2987
R01_cb3011_c34/f1p0/3348	NA	R01_cb3011_c34/f1p0/3348	NA	NA
R01_cb17357_c0/f3p0/362	R01_cb17357_c0/f3p0/362	R01_cb17357_c0/f3p0/362	NA	NA

R01_cb7877_c1/f1p0/2502	NA	R01_cb7877_c1/f1p0/2502	R01_cb7877_c1/f1p0/2502	R01_cb7877_c1/f1p0/2502
R01_cb8564_c16296/f1p4/4487	NA	NA	NA	R01_cb8564_c16296/f1p4/4487
R01_cb101_c3/f1p0/2284	NA	NA	NA	R01_cb101_c3/f1p0/2284
R01_cb10651_c1/f1p0/3689	NA	R01_cb10651_c1/f1p0/3689	NA	NA
R01_cb12936_c1/f1p0/501	NA	R01_cb12936_c1/f1p0/501	NA	R01_cb12936_c1/f1p0/501
R01_cb8564_c31883/f5p1/2785	NA	NA	NA	R01_cb8564_c31883/f5p1/2785
R01_cb15449_c2/f1p0/1034	NA	R01_cb15449_c2/f1p0/1034	R01_cb15449_c2/f1p0/1034	R01_cb15449_c2/f1p0/1034
R01_cb17838_c3/f1p0/495	NA	R01_cb17838_c3/f1p0/495	R01_cb17838_c3/f1p0/495	R01_cb17838_c3/f1p0/495
R01_cb11469_c0/f1p0/720	NA	NA	NA	R01_cb11469_c0/f1p0/720
R01_cb14279_c0/f3p0/1689	NA	NA	R01_cb14279_c0/f3p0/1689	R01_cb14279_c0/f3p0/1689
R01_cb9529_c6/f1p0/793	NA	NA	NA	R01_cb9529_c6/f1p0/793
R01_cb10935_c0/f1p0/954	NA	NA	R01_cb10935_c0/f1p0/954	R01_cb10935_c0/f1p0/954
R01_cb9941_c6/f1p0/1628	NA	R01_cb9941_c6/f1p0/1628	NA	R01_cb9941_c6/f1p0/1628
R01_cb17224_c0/f2p0/711	R01_cb17224_c0/f2p0/711	R01_cb17224_c0/f2p0/711	NA	NA
R01_cb4128_c10/f3p1/2897	NA	NA	NA	R01_cb4128_c10/f3p1/2897
R01_cb8564_c81256/f1p0/2508	NA	R01_cb8564_c81256/f1p0/2508	NA	NA
R01_cb18656_c8/f1p0/5500	NA	NA	NA	R01_cb18656_c8/f1p0/5500
R01_cb17755_c1/f1p0/678	NA	NA	NA	R01_cb17755_c1/f1p0/678
R01_cb14758_c3/f1p0/412	NA	R01_cb14758_c3/f1p0/412	R01_cb14758_c3/f1p0/412	R01_cb14758_c3/f1p0/412
R01_cb16364_c1/f1p0/886	NA	R01_cb16364_c1/f1p0/886	NA	NA
R01_cb9606_c88/f1p0/1478	NA	R01_cb9606_c88/f1p0/1478	R01_cb9606_c88/f1p0/1478	R01_cb9606_c88/f1p0/1478
R01_cb4363_c5/f1p0/1115	NA	R01_cb4363_c5/f1p0/1115	NA	NA
R01_cb5114_c2/f1p0/1487	NA	R01_cb5114_c2/f1p0/1487	R01_cb5114_c2/f1p0/1487	R01_cb5114_c2/f1p0/1487
R01_cb1178_c20/f1p0/1387	NA	NA	NA	R01_cb1178_c20/f1p0/1387
R01_cb9129_c2/f1p0/2669	NA	R01_cb9129_c2/f1p0/2669	NA	R01_cb9129_c2/f1p0/2669
R01_cb6758_c4/f1p1/2238	NA	R01_cb6758_c4/f1p1/2238	NA	R01_cb6758_c4/f1p1/2238

R01_cb8564_c78482/f1p0/3189	NA	R01_cb8564_c78482/f1p0/3189	NA	NA
R01_cb18456_c4722/f1p0/486	NA	NA	NA	R01_cb18456_c4722/f1p0/486
R01_cb18456_c7943/f5p0/503	NA	R01_cb18456_c7943/f5p0/503	R01_cb18456_c7943/f5p0/503	R01_cb18456_c7943/f5p0/503
R01_cb14480_c5/f1p1/524	R01_cb14480_c5/f1p1/524	R01_cb14480_c5/f1p1/524	R01_cb14480_c5/f1p1/524	R01_cb14480_c5/f1p1/524
R01_cb12319_c1/f1p0/713	R01_cb12319_c1/f1p0/713	R01_cb12319_c1/f1p0/713	R01_cb12319_c1/f1p0/713	R01_cb12319_c1/f1p0/713
R01_cb2936_c12/f1p0/883	NA	R01_cb2936_c12/f1p0/883	NA	NA
R01_cb8564_c10878/f1p0/4516	NA	R01_cb8564_c10878/f1p0/4516	NA	NA
R01_cb8564_c118137/f1p0/4880	NA	R01_cb8564_c118137/f1p0/4880	NA	NA
R01_cb8160_c1/f1p1/2577	NA	NA	NA	R01_cb8160_c1/f1p1/2577
R01_cb5610_c18/f1p0/2045	NA	R01_cb5610_c18/f1p0/2045	NA	NA
R01_cb1166_c3/f2p0/2498	NA	R01_cb1166_c3/f2p0/2498	NA	NA
R01_cb2875_c1/f2p0/3431	NA	NA	NA	R01_cb2875_c1/f2p0/3431
R01_cb3359_c29/f1p0/2584	NA	R01_cb3359_c29/f1p0/2584	NA	NA
R01_cb4829_c15/f1p0/2914	NA	NA	NA	R01_cb4829_c15/f1p0/2914
R01_cb13884_c15/f1p0/891	R01_cb13884_c15/f1p0/891	R01_cb13884_c15/f1p0/891	R01_cb13884_c15/f1p0/891	R01_cb13884_c15/f1p0/891
R01_cb8564_c115049/f1p0/2307	NA	R01_cb8564_c115049/f1p0/2307	R01_cb8564_c115049/f1p0/2307	R01_cb8564_c115049/f1p0/2307
R01_cb10908_c1/f1p0/2765	NA	R01_cb10908_c1/f1p0/2765	NA	NA
R01_cb1487_c3/f1p0/1560	NA	NA	NA	R01_cb1487_c3/f1p0/1560
R01_cb5676_c12/f1p0/1708	NA	NA	NA	R01_cb5676_c12/f1p0/1708
R01_cb6504_c1/f1p0/2635	R01_cb6504_c1/f1p0/2635	R01_cb6504_c1/f1p0/2635	R01_cb6504_c1/f1p0/2635	NA
R01_cb13765_c1/f1p0/750	NA	NA	NA	R01_cb13765_c1/f1p0/750
R01_cb8564_c117313/f1p0/4753	NA	R01_cb8564_c117313/f1p0/4753	NA	R01_cb8564_c117313/f1p0/4753
R01_cb1982_c6/f1p0/1579	R01_cb1982_c6/f1p0/1579	NA	R01_cb1982_c6/f1p0/1579	R01_cb1982_c6/f1p0/1579

R01_cb8564_c128644/f1p0/2050	NA	NA	NA	R01_cb8564_c128644/f1p0/2050
R01_cb6802_c23/f1p0/4851	NA	R01_cb6802_c23/f1p0/4851	NA	NA
R01_cb5533_c127/f1p0/3774	NA	NA	NA	R01_cb5533_c127/f1p0/3774
R01_cb8564_c120947/f1p0/2719	NA	R01_cb8564_c120947/f1p0/2719	R01_cb8564_c120947/f1p0/2719	R01_cb8564_c120947/f1p0/2719
R01_cb943_c7/f1p0/6060	NA	NA	NA	R01_cb943_c7/f1p0/6060
R01_cb15998_c9/f1p0/1708	NA	NA	NA	R01_cb15998_c9/f1p0/1708
R01_cb18456_c3306/f1p4/1037	NA	R01_cb18456_c3306/f1p4/1037	NA	NA
R01_cb8564_c88101/f1p0/2926	NA	R01_cb8564_c88101/f1p0/2926	NA	NA
R01_cb15407_c8/f1p0/386	R01_cb15407_c8/f1p0/386	R01_cb15407_c8/f1p0/386	R01_cb15407_c8/f1p0/386	R01_cb15407_c8/f1p0/386
R01_cb8564_c118779/f1p0/2652	NA	NA	NA	R01_cb8564_c118779/f1p0/2652
R01_cb8564_c1355/f1p0/2279	NA	R01_cb8564_c1355/f1p0/2279	NA	R01_cb8564_c1355/f1p0/2279
R01_cb5900_c46/f1p0/2044	NA	R01_cb5900_c46/f1p0/2044	NA	R01_cb5900_c46/f1p0/2044
R01_cb18456_c5193/f1p3/628	R01_cb18456_c5193/f1p3/628	R01_cb18456_c5193/f1p3/628	NA	R01_cb18456_c5193/f1p3/628
R01_cb17615_c3/f1p0/503	R01_cb17615_c3/f1p0/503	R01_cb17615_c3/f1p0/503	R01_cb17615_c3/f1p0/503	R01_cb17615_c3/f1p0/503
R01_cb1982_c4/f1p0/1907	NA	NA	R01_cb1982_c4/f1p0/1907	R01_cb1982_c4/f1p0/1907
R01_cb8564_c15226/f1p1/3592	NA	R01_cb8564_c15226/f1p1/3592	NA	NA
R01_cb13895_c3/f1p0/591	NA	NA	NA	R01_cb13895_c3/f1p0/591
R01_cb16464_c3/f1p0/360	R01_cb16464_c3/f1p0/360	R01_cb16464_c3/f1p0/360	R01_cb16464_c3/f1p0/360	R01_cb16464_c3/f1p0/360
R01_cb8564_c91920/f1p0/2089	NA	R01_cb8564_c91920/f1p0/2089	NA	NA
R01_cb8550_c3/f1p0/1983	NA	R01_cb8550_c3/f1p0/1983	NA	NA
R01_cb8564_c86974/f1p0/2846	NA	R01_cb8564_c86974/f1p0/2846	NA	NA
R01_cb10280_c1/f1p0/2387	NA	R01_cb10280_c1/f1p0/2387	NA	NA
R01_cb4583_c12/f1p0/3405	NA	R01_cb4583_c12/f1p0/3405	R01_cb4583_c12/f1p0/3405	NA

R01_cb4001_c1/f1p0/3463	NA	NA	NA	R01_cb4001_c1/f1p0/3463
R01_cb6348_c8/f1p0/2470	NA	NA	NA	R01_cb6348_c8/f1p0/2470
R01_cb9797_c62/f1p0/1923	NA	NA	NA	R01_cb9797_c62/f1p0/1923
R01_cb13481_c9/f1p0/638	R01_cb13481_c9/f1p0/638	R01_cb13481_c9/f1p0/638	R01_cb13481_c9/f1p0/638	R01_cb13481_c9/f1p0/638
R01_cb16449_c1/f1p0/1643	NA	NA	NA	R01_cb16449_c1/f1p0/1643
R01_cb5675_c12/f1p0/2610	NA	R01_cb5675_c12/f1p0/2610	NA	NA
R01_cb14911_c5/f2p0/621	NA	NA	NA	R01_cb14911_c5/f2p0/621
R01_cb6802_c17/f1p0/3640	R01_cb6802_c17/f1p0/3640	R01_cb6802_c17/f1p0/3640	R01_cb6802_c17/f1p0/3640	R01_cb6802_c17/f1p0/3640
R01_cb16898_c0/f1p1/1761	NA	NA	R01_cb16898_c0/f1p1/1761	R01_cb16898_c0/f1p1/1761
R01_cb2367_c6/f1p0/3206	NA	R01_cb2367_c6/f1p0/3206	NA	R01_cb2367_c6/f1p0/3206
R01_cb1331_c44/f1p1/977	NA	R01_cb1331_c44/f1p1/977	NA	NA
R01_cb4443_c8/f1p0/2098	NA	NA	NA	R01_cb4443_c8/f1p0/2098
R01_cb8564_c972/f3p0/2746	NA	NA	NA	R01_cb8564_c972/f3p0/2746
R01_cb2275_c0/f1p0/3981	NA	R01_cb2275_c0/f1p0/3981	R01_cb2275_c0/f1p0/3981	R01_cb2275_c0/f1p0/3981
R01_cb3712_c6/f1p4/1352	NA	R01_cb3712_c6/f1p4/1352	NA	NA
R01_cb8564_c88737/f1p0/3375	NA	R01_cb8564_c88737/f1p0/3375	NA	NA
R01_cb709_c7/f1p0/2038	NA	NA	R01_cb709_c7/f1p0/2038	NA
R01_cb8053_c4/f1p1/2622	NA	NA	NA	R01_cb8053_c4/f1p1/2622
R01_cb2160_c18/f1p1/2853	NA	NA	NA	R01_cb2160_c18/f1p1/2853
R01_cb9745_c0/f1p0/1990	NA	R01_cb9745_c0/f1p0/1990	NA	NA
R01_cb5328_c7/f1p0/1815	NA	R01_cb5328_c7/f1p0/1815	NA	NA
R01_cb18456_c1416/f5p0/1396	NA	R01_cb18456_c1416/f5p0/1396	R01_cb18456_c1416/f5p0/1396	R01_cb18456_c1416/f5p0/1396
R01_cb15361_c1/f1p1/786	NA	NA	R01_cb15361_c1/f1p1/786	R01_cb15361_c1/f1p1/786
R01_cb8564_c87976/f1p0/3738	NA	R01_cb8564_c87976/f1p0/3738	NA	NA
R01_cb7966_c4/f1p0/851	NA	R01_cb7966_c4/f1p0/851	NA	NA
R01_cb17052_c0/f2p0/1426	NA	NA	NA	R01_cb17052_c0/f2p0/1426

R01_cb17106_c1/f1p0/1761	R01_cb17106_c1/f1p0/1761	R01_cb17106_c1/f1p0/1761	R01_cb17106_c1/f1p0/1761	R01_cb17106_c1/f1p0/1761
R01_cb8564_c37658/f1p0/2282	R01_cb8564_c37658/f1p0/2282	R01_cb8564_c37658/f1p0/2282	R01_cb8564_c37658/f1p0/2282	R01_cb8564_c37658/f1p0/2282
R01_cb18456_c7151/f1p0/713	R01_cb18456_c7151/f1p0/713	R01_cb18456_c7151/f1p0/713	R01_cb18456_c7151/f1p0/713	R01_cb18456_c7151/f1p0/713
R01_cb8564_c109761/f2p0/2936	NA	R01_cb8564_c109761/f2p0/2936	R01_cb8564_c109761/f2p0/2936	R01_cb8564_c109761/f2p0/2936
R01_cb4600_c4/f1p0/3249	NA	NA	NA	R01_cb4600_c4/f1p0/3249
R01_cb6456_c1/f2p0/2477	R01_cb6456_c1/f2p0/2477	R01_cb6456_c1/f2p0/2477	NA	R01_cb6456_c1/f2p0/2477
R01_cb18456_c5053/f1p1/655	R01_cb18456_c5053/f1p1/655	NA	R01_cb18456_c5053/f1p1/655	R01_cb18456_c5053/f1p1/655
R01_cb1487_c8/f1p0/1320	NA	NA	NA	R01_cb1487_c8/f1p0/1320
R01_cb13178_c14/f1p0/675	R01_cb13178_c14/f1p0/675	R01_cb13178_c14/f1p0/675	NA	NA
R01_cb793_c0/f1p0/4632	NA	R01_cb793_c0/f1p0/4632	NA	NA
R01_cb5635_c6/f1p0/750	NA	NA	NA	R01_cb5635_c6/f1p0/750
R01_cb18057_c0/f2p1/465	R01_cb18057_c0/f2p1/465	R01_cb18057_c0/f2p1/465	R01_cb18057_c0/f2p1/465	R01_cb18057_c0/f2p1/465
R01_cb5890_c2/f1p0/2643	NA	R01_cb5890_c2/f1p0/2643	NA	NA
R01_cb8564_c79574/f1p0/3664	NA	R01_cb8564_c79574/f1p0/3664	NA	NA
R01_cb4147_c19/f1p0/2317	NA	NA	NA	R01_cb4147_c19/f1p0/2317
R01_cb5471_c11/f1p0/2489	NA	NA	NA	R01_cb5471_c11/f1p0/2489
R01_cb10952_c2/f1p0/1548	NA	NA	NA	R01_cb10952_c2/f1p0/1548
R01_cb8564_c89726/f1p0/2352	R01_cb8564_c89726/f1p0/2352	R01_cb8564_c89726/f1p0/2352	R01_cb8564_c89726/f1p0/2352	R01_cb8564_c89726/f1p0/2352
R01_cb10653_c3/f1p0/1995	R01_cb10653_c3/f1p0/1995	R01_cb10653_c3/f1p0/1995	NA	NA
R01_cb18456_c4974/f1p0/364	R01_cb18456_c4974/f1p0/364	R01_cb18456_c4974/f1p0/364	R01_cb18456_c4974/f1p0/364	R01_cb18456_c4974/f1p0/364
R01_cb8564_c86774/f1p0/3078	NA	NA	NA	R01_cb8564_c86774/f1p0/3078
R01_cb17899_c1/f1p0/413	R01_cb17899_c1/f1p0/413	R01_cb17899_c1/f1p0/413	NA	R01_cb17899_c1/f1p0/413
R01_cb18409_c34/f1p1/966	NA	R01_cb18409_c34/f1p1/966	NA	NA

R01_cb3750_c16/f1p0/3161	NA	NA	NA	R01_cb3750_c16/f1p0/3161
R01_cb17757_c1/f1p0/1269	NA	NA	NA	R01_cb17757_c1/f1p0/1269
R01_cb1964_c6/f1p0/2048	NA	NA	NA	R01_cb1964_c6/f1p0/2048
R01_cb14940_c2/f1p0/732	NA	R01_cb14940_c2/f1p0/732	R01_cb14940_c2/f1p0/732	R01_cb14940_c2/f1p0/732
R01_cb16888_c5/f1p0/774	NA	NA	R01_cb16888_c5/f1p0/774	R01_cb16888_c5/f1p0/774
R01_cb4490_c11/f1p0/2391	NA	NA	NA	R01_cb4490_c11/f1p0/2391
R01_cb15913_c2/f1p0/942	NA	R01_cb15913_c2/f1p0/942	R01_cb15913_c2/f1p0/942	R01_cb15913_c2/f1p0/942
R01_cb13392_c7/f3p0/604	R01_cb13392_c7/f3p0/604	R01_cb13392_c7/f3p0/604	R01_cb13392_c7/f3p0/604	R01_cb13392_c7/f3p0/604
R01_cb17392_c0/f1p0/904	NA	NA	R01_cb17392_c0/f1p0/904	R01_cb17392_c0/f1p0/904
R01_cb16176_c1/f1p0/1202	NA	NA	NA	R01_cb16176_c1/f1p0/1202
R01_cb15681_c1/f1p0/1337	NA	R01_cb15681_c1/f1p0/1337	NA	NA
R01_cb11944_c11/f1p0/1618	NA	NA	R01_cb11944_c11/f1p0/1618	R01_cb11944_c11/f1p0/1618
R01_cb12314_c3/f1p0/545	NA	NA	R01_cb12314_c3/f1p0/545	NA
R01_cb119_c33/f1p0/3141	NA	R01_cb119_c33/f1p0/3141	NA	R01_cb119_c33/f1p0/3141
R01_cb5565_c82/f1p0/1025	R01_cb5565_c82/f1p0/1025	R01_cb5565_c82/f1p0/1025	R01_cb5565_c82/f1p0/1025	R01_cb5565_c82/f1p0/1025
R01_cb7367_c1/f5p1/2481	NA	NA	NA	R01_cb7367_c1/f5p1/2481
R01_cb8564_c2670/f1p0/2170	NA	R01_cb8564_c2670/f1p0/2170	NA	NA
R01_cb10148_c3/f1p0/1907	NA	NA	NA	R01_cb10148_c3/f1p0/1907
R01_cb17656_c0/f7p0/662	NA	R01_cb17656_c0/f7p0/662	R01_cb17656_c0/f7p0/662	R01_cb17656_c0/f7p0/662
R01_cb4555_c5/f1p0/915	NA	NA	NA	R01_cb4555_c5/f1p0/915
R01_cb2033_c9/f1p0/2609	NA	NA	NA	R01_cb2033_c9/f1p0/2609
R01_cb3750_c31/f1p0/2307	NA	NA	NA	R01_cb3750_c31/f1p0/2307
R01_cb8564_c114998/f1p0/3124	NA	NA	NA	R01_cb8564_c114998/f1p0/3124
R01_cb8564_c84495/f1p0/3631	NA	NA	NA	R01_cb8564_c84495/f1p0/3631
R01_cb14715_c2/f2p0/561	R01_cb14715_c2/f2p0/561	R01_cb14715_c2/f2p0/561	R01_cb14715_c2/f2p0/561	R01_cb14715_c2/f2p0/561

R01_cb12931_c18/f1p0/797	R01_cb12931_c18/f1p0/797	R01_cb12931_c18/f1p0/797	R01_cb12931_c18/f1p0/797	R01_cb12931_c18/f1p0/797
R01_cb11141_c2/f1p0/2779	NA	NA	R01_cb11141_c2/f1p0/2779	R01_cb11141_c2/f1p0/2779
R01_cb7171_c12/f1p1/2562	NA	NA	NA	R01_cb7171_c12/f1p1/2562
R01_cb7006_c1/f1p0/1908	NA	R01_cb7006_c1/f1p0/1908	NA	NA
R01_cb9777_c2/f1p0/1970	NA	NA	NA	R01_cb9777_c2/f1p0/1970
R01_cb15428_c1/f1p0/542	R01_cb15428_c1/f1p0/542	R01_cb15428_c1/f1p0/542	R01_cb15428_c1/f1p0/542	R01_cb15428_c1/f1p0/542
R01_cb8564_c38787/f1p0/3773	NA	R01_cb8564_c38787/f1p0/3773	NA	NA
R01_cb8564_c81003/f1p0/4196	NA	NA	NA	R01_cb8564_c81003/f1p0/4196
R01_cb15237_c1/f1p0/1114	NA	R01_cb15237_c1/f1p0/1114	R01_cb15237_c1/f1p0/1114	R01_cb15237_c1/f1p0/1114
R01_cb4105_c13/f1p0/1483	R01_cb4105_c13/f1p0/1483	R01_cb4105_c13/f1p0/1483	R01_cb4105_c13/f1p0/1483	R01_cb4105_c13/f1p0/1483
R01_cb4059_c5/f1p0/1492	NA	NA	NA	R01_cb4059_c5/f1p0/1492
R01_cb8564_c119168/f1p0/2675	R01_cb8564_c119168/f1p0/2675	R01_cb8564_c119168/f1p0/2675	R01_cb8564_c119168/f1p0/2675	R01_cb8564_c119168/f1p0/2675
R01_cb9932_c3/f1p0/802	R01_cb9932_c3/f1p0/802	R01_cb9932_c3/f1p0/802	NA	NA
R01_cb9626_c2/f1p0/3565	NA	R01_cb9626_c2/f1p0/3565	NA	NA
R01_cb13478_c8/f1p0/406	NA	R01_cb13478_c8/f1p0/406	R01_cb13478_c8/f1p0/406	R01_cb13478_c8/f1p0/406
R01_cb1388_c8/f1p0/4718	NA	NA	NA	R01_cb1388_c8/f1p0/4718
R01_cb8032_c1/f1p0/2543	NA	R01_cb8032_c1/f1p0/2543	NA	NA
R01_cb4117_c5/f1p1/3183	NA	NA	NA	R01_cb4117_c5/f1p1/3183
R01_cb12427_c4/f1p0/1352	NA	R01_cb12427_c4/f1p0/1352	NA	R01_cb12427_c4/f1p0/1352
R01_cb1489_c4/f1p0/3013	NA	NA	R01_cb1489_c4/f1p0/3013	NA
R01_cb8110_c1/f1p1/2518	NA	R01_cb8110_c1/f1p1/2518	R01_cb8110_c1/f1p1/2518	NA
R01_cb5075_c3/f1p0/6053	NA	NA	NA	R01_cb5075_c3/f1p0/6053
R01_cb8564_c4909/f1p0/3122	R01_cb8564_c4909/f1p0/3122	R01_cb8564_c4909/f1p0/3122	R01_cb8564_c4909/f1p0/3122	R01_cb8564_c4909/f1p0/3122
R01_cb18456_c6760/f1p0/571	NA	R01_cb18456_c6760/f1p0/571	R01_cb18456_c6760/f1p0/571	NA
R01_cb2528_c1/f1p1/3892	NA	R01_cb2528_c1/f1p1/3892	NA	NA

R01_cb18321_c0/f5p1/332	R01_cb18321_c0/f5p1/332	R01_cb18321_c0/f5p1/332	R01_cb18321_c0/f5p1/332	R01_cb18321_c0/f5p1/332
R01_cb15176_c0/f1p0/813	NA	R01_cb15176_c0/f1p0/813	NA	NA
R01_cb17838_c1/f1p0/1301	R01_cb17838_c1/f1p0/1301	R01_cb17838_c1/f1p0/1301	R01_cb17838_c1/f1p0/1301	R01_cb17838_c1/f1p0/1301
R01_cb17458_c1/f2p0/730	NA	NA	NA	R01_cb17458_c1/f2p0/730
R01_cb2072_c27/f1p0/2074	NA	R01_cb2072_c27/f1p0/2074	NA	NA
R01_cb17668_c2/f2p0/771	NA	NA	NA	R01_cb17668_c2/f2p0/771
R01_cb8823_c4/f1p1/2080	NA	NA	NA	R01_cb8823_c4/f1p1/2080
R01_cb1610_c0/f4p0/2572	NA	NA	NA	R01_cb1610_c0/f4p0/2572
R01_cb17359_c2/f1p0/448	R01_cb17359_c2/f1p0/448	R01_cb17359_c2/f1p0/448	R01_cb17359_c2/f1p0/448	R01_cb17359_c2/f1p0/448
R01_cb8564_c130368/f1p0/3331	R01_cb8564_c130368/f1p0/3331	R01_cb8564_c130368/f1p0/3331	R01_cb8564_c130368/f1p0/3331	R01_cb8564_c130368/f1p0/3331
R01_cb10300_c5/f1p0/1611	NA	NA	NA	R01_cb10300_c5/f1p0/1611
R01_cb6258_c8/f2p1/2472	NA	NA	NA	R01_cb6258_c8/f2p1/2472
R01_cb18456_c7460/f1p0/747	NA	R01_cb18456_c7460/f1p0/747	NA	NA
R01_cb8900_c8/f1p0/1988	NA	NA	NA	R01_cb8900_c8/f1p0/1988
R01_cb5183_c19/f1p0/3158	NA	NA	NA	R01_cb5183_c19/f1p0/3158
R01_cb4824_c0/f3p0/796	NA	R01_cb4824_c0/f3p0/796	NA	R01_cb4824_c0/f3p0/796
R01_cb17398_c2/f1p0/316	R01_cb17398_c2/f1p0/316	R01_cb17398_c2/f1p0/316	R01_cb17398_c2/f1p0/316	R01_cb17398_c2/f1p0/316
R01_cb8564_c34059/f2p0/2420	NA	R01_cb8564_c34059/f2p0/2420	NA	NA
R01_cb8564_c74681/f1p0/2062	NA	R01_cb8564_c74681/f1p0/2062	NA	R01_cb8564_c74681/f1p0/2062
R01_cb1658_c34/f1p0/2745	NA	NA	NA	R01_cb1658_c34/f1p0/2745
R01_cb11278_c4/f1p1/2818	NA	NA	NA	R01_cb11278_c4/f1p1/2818
R01_cb7443_c3/f1p0/751	R01_cb7443_c3/f1p0/751	R01_cb7443_c3/f1p0/751	R01_cb7443_c3/f1p0/751	R01_cb7443_c3/f1p0/751
R01_cb3287_c0/f2p0/1511	NA	NA	NA	R01_cb3287_c0/f2p0/1511
R01_cb6551_c3/f1p0/1692	R01_cb6551_c3/f1p0/1692	R01_cb6551_c3/f1p0/1692	R01_cb6551_c3/f1p0/1692	R01_cb6551_c3/f1p0/1692
R01_cb7096_c45/f1p1/1601	R01_cb7096_c45/f1p1/1601	R01_cb7096_c45/f1p1/1601	R01_cb7096_c45/f1p1/1601	R01_cb7096_c45/f1p1/1601

R01_cb1894_c1/f2p0/2614	NA	R01_cb1894_c1/f2p0/2614	NA	R01_cb1894_c1/f2p0/2614
R01_cb8564_c71555/f2p0/3701	NA	R01_cb8564_c71555/f2p0/3701	NA	NA
R01_cb4563_c9/f1p0/3283	NA	R01_cb4563_c9/f1p0/3283	NA	NA
R01_cb822_c22/f1p0/2751	NA	NA	NA	R01_cb822_c22/f1p0/2751
R01_cb8564_c117666/f1p0/2490	NA	NA	NA	R01_cb8564_c117666/f1p0/2490
R01_cb8126_c3/f1p0/1011	R01_cb8126_c3/f1p0/1011	R01_cb8126_c3/f1p0/1011	NA	NA
R01_cb3774_c57/f1p0/2143	NA	NA	NA	R01_cb3774_c57/f1p0/2143
R01_cb11121_c0/f2p0/1034	NA	NA	NA	R01_cb11121_c0/f2p0/1034
R01_cb11924_c19/f1p0/512	R01_cb11924_c19/f1p0/512	R01_cb11924_c19/f1p0/512	R01_cb11924_c19/f1p0/512	NA
R01_cb8252_c10/f4p0/2328	NA	NA	NA	R01_cb8252_c10/f4p0/2328
R01_cb12037_c6/f1p0/1610	NA	NA	R01_cb12037_c6/f1p0/1610	NA
R01_cb8978_c2/f1p0/3019	R01_cb8978_c2/f1p0/3019	R01_cb8978_c2/f1p0/3019	NA	NA
R01_cb5558_c0/f1p0/3072	NA	R01_cb5558_c0/f1p0/3072	NA	NA
R01_cb2284_c36/f1p0/591	NA	R01_cb2284_c36/f1p0/591	NA	NA
R01_cb17076_c3/f1p0/786	NA	NA	NA	R01_cb17076_c3/f1p0/786
R01_cb16281_c13/f1p0/1150	R01_cb16281_c13/f1p0/1150	NA	NA	NA
R01_cb4370_c14/f1p0/623	R01_cb4370_c14/f1p0/623	R01_cb4370_c14/f1p0/623	NA	R01_cb4370_c14/f1p0/623
R01_cb16414_c0/f2p0/1723	NA	R01_cb16414_c0/f2p0/1723	NA	NA
R01_cb2632_c0/f3p0/669	R01_cb2632_c0/f3p0/669	R01_cb2632_c0/f3p0/669	R01_cb2632_c0/f3p0/669	R01_cb2632_c0/f3p0/669
R01_cb11002_c2/f1p0/1923	NA	R01_cb11002_c2/f1p0/1923	NA	R01_cb11002_c2/f1p0/1923
R01_cb18331_c94/f1p0/4079	NA	R01_cb18331_c94/f1p0/4079	NA	NA
R01_cb8564_c89668/f1p0/2043	NA	R01_cb8564_c89668/f1p0/2043	NA	NA
R01_cb2320_c4/f1p0/4172	NA	NA	NA	R01_cb2320_c4/f1p0/4172
R01_cb4188_c11/f1p0/3048	R01_cb4188_c11/f1p0/3048	R01_cb4188_c11/f1p0/3048	NA	NA
R01_cb8564_c75644/f1p0/2497	NA	R01_cb8564_c75644/f1p0/2497	R01_cb8564_c75644/f1p0/2497	R01_cb8564_c75644/f1p0/2497

R01_cb16100_c6/f1p0/519	R01_cb16100_c6/f1p0/519	R01_cb16100_c6/f1p0/519	R01_cb16100_c6/f1p0/519	R01_cb16100_c6/f1p0/519
R01_cb6802_c73/f1p0/2190	NA	R01_cb6802_c73/f1p0/2190	NA	NA
R01_cb10475_c2/f1p0/2375	NA	R01_cb10475_c2/f1p0/2375	NA	R01_cb10475_c2/f1p0/2375
R01_cb14808_c2/f1p0/579	R01_cb14808_c2/f1p0/579	R01_cb14808_c2/f1p0/579	NA	R01_cb14808_c2/f1p0/579
R01_cb15094_c1/f1p0/1053	NA	NA	NA	R01_cb15094_c1/f1p0/1053
R01_cb1458_c2/f1p0/3204	NA	R01_cb1458_c2/f1p0/3204	NA	NA
R01_cb8564_c15543/f1p1/4073	NA	NA	NA	R01_cb8564_c15543/f1p1/4073
R01_cb16387_c5/f1p0/1527	NA	NA	NA	R01_cb16387_c5/f1p0/1527
R01_cb13996_c6/f1p0/742	NA	R01_cb13996_c6/f1p0/742	NA	NA
R01_cb8564_c148356/f4p0/2099	NA	NA	NA	R01_cb8564_c148356/f4p0/2099
R01_cb5690_c8/f11p0/1087	NA	NA	NA	R01_cb5690_c8/f11p0/1087
R01_cb8564_c13528/f1p0/3013	NA	R01_cb8564_c13528/f1p0/3013	NA	R01_cb8564_c13528/f1p0/3013
R01_cb18654_c5/f1p0/1646	NA	NA	NA	R01_cb18654_c5/f1p0/1646
R01_cb6059_c0/f3p1/2019	NA	NA	NA	R01_cb6059_c0/f3p1/2019
R01_cb18456_c5359/f1p0/910	NA	R01_cb18456_c5359/f1p0/910	NA	NA
R01_cb13663_c1/f1p0/835	NA	NA	NA	R01_cb13663_c1/f1p0/835
R01_cb1915_c19/f1p0/3025	R01_cb1915_c19/f1p0/3025	R01_cb1915_c19/f1p0/3025	NA	NA
R01_cb7143_c13/f1p0/1382	R01_cb7143_c13/f1p0/1382	R01_cb7143_c13/f1p0/1382	NA	R01_cb7143_c13/f1p0/1382
R01_cb14336_c0/f2p1/501	NA	R01_cb14336_c0/f2p1/501	R01_cb14336_c0/f2p1/501	R01_cb14336_c0/f2p1/501
R01_cb8564_c25268/f1p0/3377	NA	R01_cb8564_c25268/f1p0/3377	NA	NA
R01_cb8564_c112333/f1p0/2553	NA	R01_cb8564_c112333/f1p0/2553	NA	NA
R01_cb8564_c11406/f1p0/3425	NA	R01_cb8564_c11406/f1p0/3425	NA	NA
R01_cb11220_c1/f1p0/3263	NA	R01_cb11220_c1/f1p0/3263	NA	NA
R01_cb1626_c5/f1p1/4242	NA	NA	NA	R01_cb1626_c5/f1p1/4242

R01_cb8564_c111149/f1p0/3588	NA	NA	R01_cb8564_c111149/f1p0/3588	R01_cb8564_c111149/f1p0/3588
R01_cb8564_c76727/f1p0/3005	NA	R01_cb8564_c76727/f1p0/3005	NA	R01_cb8564_c76727/f1p0/3005
R01_cb12691_c5/f2p0/1064	NA	R01_cb12691_c5/f2p0/1064	NA	NA
R01_cb11557_c0/f1p1/929	R01_cb11557_c0/f1p1/929	R01_cb11557_c0/f1p1/929	NA	R01_cb11557_c0/f1p1/929
R01_cb8564_c119574/f1p0/1832	NA	NA	NA	R01_cb8564_c119574/f1p0/1832
R01_cb3485_c5/f1p1/2452	NA	R01_cb3485_c5/f1p1/2452	NA	NA
R01_cb13464_c6/f1p0/1344	NA	R01_cb13464_c6/f1p0/1344	NA	NA
R01_cb8564_c72220/f1p1/3491	NA	NA	NA	R01_cb8564_c72220/f1p1/3491
R01_cb10539_c2/f1p0/4443	NA	R01_cb10539_c2/f1p0/4443	NA	R01_cb10539_c2/f1p0/4443
R01_cb15158_c0/f1p0/850	NA	NA	NA	R01_cb15158_c0/f1p0/850
R01_cb4185_c0/f2p0/3264	R01_cb4185_c0/f2p0/3264	R01_cb4185_c0/f2p0/3264	NA	R01_cb4185_c0/f2p0/3264
R01_cb18111_c1/f1p0/1682	NA	R01_cb18111_c1/f1p0/1682	NA	NA
R01_cb11643_c1/f1p0/2151	NA	R01_cb11643_c1/f1p0/2151	NA	NA
R01_cb15811_c34/f1p0/863	NA	R01_cb15811_c34/f1p0/863	R01_cb15811_c34/f1p0/863	R01_cb15811_c34/f1p0/863
R01_cb8564_c19423/f1p0/3401	R01_cb8564_c19423/f1p0/3401	R01_cb8564_c19423/f1p0/3401	NA	NA
R01_cb8564_c115314/f1p0/2940	NA	R01_cb8564_c115314/f1p0/2940	NA	R01_cb8564_c115314/f1p0/2940
R01_cb8564_c109438/f2p1/1976	NA	R01_cb8564_c109438/f2p1/1976	NA	NA
R01_cb14301_c1/f1p0/507	NA	R01_cb14301_c1/f1p0/507	NA	NA
R01_cb9646_c2/f1p0/2155	NA	NA	NA	R01_cb9646_c2/f1p0/2155
R01_cb17853_c3/f1p0/1669	NA	NA	NA	R01_cb17853_c3/f1p0/1669
R01_cb34_c4/f1p0/3669	NA	NA	NA	R01_cb34_c4/f1p0/3669

R01_cb5900_c107/f1p0/2174	NA	R01_cb5900_c107/f1p0/2174	NA	R01_cb5900_c107/f1p0/2174
R01_cb8564_c2795/f1p1/1923	NA	R01_cb8564_c2795/f1p1/1923	NA	NA
R01_cb6658_c2/f1p0/2087	NA	R01_cb6658_c2/f1p0/2087	NA	NA
R01_cb18456_c7150/f1p1/1607	NA	R01_cb18456_c7150/f1p1/1607	R01_cb18456_c7150/f1p1/1607	R01_cb18456_c7150/f1p1/1607
R01_cb14192_c8/f1p0/1817	NA	R01_cb14192_c8/f1p0/1817	R01_cb14192_c8/f1p0/1817	R01_cb14192_c8/f1p0/1817
R01_cb2770_c70/f1p0/2266	NA	R01_cb2770_c70/f1p0/2266	R01_cb2770_c70/f1p0/2266	NA
R01_cb14729_c1/f4p2/649	NA	R01_cb14729_c1/f4p2/649	NA	NA
R01_cb2870_c2/f2p1/2681	NA	NA	NA	R01_cb2870_c2/f2p1/2681
R01_cb9662_c0/f1p0/2008	NA	R01_cb9662_c0/f1p0/2008	NA	R01_cb9662_c0/f1p0/2008
R01_cb16217_c1/f1p0/1058	NA	NA	NA	R01_cb16217_c1/f1p0/1058
R01_cb7398_c7/f1p0/1764	NA	NA	NA	R01_cb7398_c7/f1p0/1764
R01_cb8564_c16834/f1p0/2308	NA	R01_cb8564_c16834/f1p0/2308	R01_cb8564_c16834/f1p0/2308	R01_cb8564_c16834/f1p0/2308
R01_cb2447_c0/f1p0/3927	NA	R01_cb2447_c0/f1p0/3927	NA	NA
R01_cb8564_c69797/f1p0/2711	NA	R01_cb8564_c69797/f1p0/2711	R01_cb8564_c69797/f1p0/2711	R01_cb8564_c69797/f1p0/2711
R01_cb7920_c0/f1p0/2502	NA	NA	R01_cb7920_c0/f1p0/2502	R01_cb7920_c0/f1p0/2502
R01_cb13597_c15/f1p0/504	R01_cb13597_c15/f1p0/504	R01_cb13597_c15/f1p0/504	R01_cb13597_c15/f1p0/504	R01_cb13597_c15/f1p0/504
R01_cb7731_c10/f1p0/468	NA	R01_cb7731_c10/f1p0/468	NA	R01_cb7731_c10/f1p0/468
R01_cb18456_c7236/f1p0/558	R01_cb18456_c7236/f1p0/558	R01_cb18456_c7236/f1p0/558	R01_cb18456_c7236/f1p0/558	R01_cb18456_c7236/f1p0/558
R01_cb11035_c1/f1p0/2518	NA	NA	R01_cb11035_c1/f1p0/2518	R01_cb11035_c1/f1p0/2518
R01_cb1508_c7/f1p0/3465	NA	NA	NA	R01_cb1508_c7/f1p0/3465
R01_cb18456_c1675/f2p0/593	R01_cb18456_c1675/f2p0/593	R01_cb18456_c1675/f2p0/593	R01_cb18456_c1675/f2p0/593	R01_cb18456_c1675/f2p0/593
R01_cb8564_c78224/f1p0/3892	NA	NA	NA	R01_cb8564_c78224/f1p0/3892
R01_cb14221_c0/f1p0/1011	NA	R01_cb14221_c0/f1p0/1011	NA	NA
R01_cb5902_c3/f1p0/1898	NA	R01_cb5902_c3/f1p0/1898	NA	NA
R01_cb8564_c5089/f1p0/2122	NA	NA	NA	R01_cb8564_c5089/f1p0/2122
R01_cb12215_c5/f9p0/973	NA	NA	R01_cb12215_c5/f9p0/973	R01_cb12215_c5/f9p0/973

R01_cb8564_c4723/f1p0/3027	NA	NA	NA	R01_cb8564_c4723/f1p0/3027
R01_cb18619_c1/f1p0/3614	NA	R01_cb18619_c1/f1p0/3614	R01_cb18619_c1/f1p0/3614	NA
R01_cb11078_c2/f1p0/530	NA	R01_cb11078_c2/f1p0/530	R01_cb11078_c2/f1p0/530	R01_cb11078_c2/f1p0/530
R01_cb18659_c1/f1p0/346	NA	R01_cb18659_c1/f1p0/346	R01_cb18659_c1/f1p0/346	NA
R01_cb18456_c6269/f1p0/883	NA	R01_cb18456_c6269/f1p0/883	NA	R01_cb18456_c6269/f1p0/883
R01_cb3308_c12/f1p1/2200	NA	R01_cb3308_c12/f1p1/2200	NA	NA
R01_cb11476_c1/f1p0/2138	NA	R01_cb11476_c1/f1p0/2138	R01_cb11476_c1/f1p0/2138	R01_cb11476_c1/f1p0/2138
R01_cb5667_c27/f1p0/695	NA	R01_cb5667_c27/f1p0/695	NA	R01_cb5667_c27/f1p0/695
R01_cb8564_c50656/f1p0/4642	NA	R01_cb8564_c50656/f1p0/4642	NA	R01_cb8564_c50656/f1p0/4642
R01_cb16093_c1/f1p0/1549	NA	R01_cb16093_c1/f1p0/1549	NA	NA
R01_cb11840_c0/f1p0/969	NA	NA	NA	R01_cb11840_c0/f1p0/969
R01_cb15360_c2/f1p0/1371	R01_cb15360_c2/f1p0/1371	R01_cb15360_c2/f1p0/1371	R01_cb15360_c2/f1p0/1371	R01_cb15360_c2/f1p0/1371
R01_cb7802_c24/f6p1/1743	NA	NA	NA	R01_cb7802_c24/f6p1/1743
R01_cb9985_c0/f1p0/1892	NA	R01_cb9985_c0/f1p0/1892	NA	NA
R01_cb6601_c3/f1p0/973	NA	R01_cb6601_c3/f1p0/973	NA	NA
R01_cb12786_c26/f1p0/613	R01_cb12786_c26/f1p0/613	NA	NA	NA
R01_cb6365_c7/f1p0/3467	NA	R01_cb6365_c7/f1p0/3467	NA	NA
R01_cb9729_c9/f1p0/611	R01_cb9729_c9/f1p0/611	R01_cb9729_c9/f1p0/611	NA	R01_cb9729_c9/f1p0/611
R01_cb18355_c0/f1p0/308	R01_cb18355_c0/f1p0/308	R01_cb18355_c0/f1p0/308	R01_cb18355_c0/f1p0/308	R01_cb18355_c0/f1p0/308
R01_cb7795_c5/f1p0/2209	NA	NA	NA	R01_cb7795_c5/f1p0/2209
R01_cb9535_c22/f1p0/1083	NA	R01_cb9535_c22/f1p0/1083	R01_cb9535_c22/f1p0/1083	R01_cb9535_c22/f1p0/1083
R01_cb13490_c5/f1p0/841	R01_cb13490_c5/f1p0/841	R01_cb13490_c5/f1p0/841	R01_cb13490_c5/f1p0/841	R01_cb13490_c5/f1p0/841
R01_cb9022_c1/f2p0/1726	NA	NA	NA	R01_cb9022_c1/f2p0/1726
R01_cb16997_c58/f1p0/2433	NA	R01_cb16997_c58/f1p0/2433	NA	NA
R01_cb15766_c2/f1p0/1534	NA	NA	NA	R01_cb15766_c2/f1p0/1534
R01_cb16125_c17/f1p0/1780	NA	R01_cb16125_c17/f1p0/1780	NA	NA

R01_cb8564_c4802/f1p0/3192	NA	R01_cb8564_c4802/f1p0/3192	NA	NA
R01_cb18456_c1882/f1p0/956	R01_cb18456_c1882/f1p0/956	R01_cb18456_c1882/f1p0/956	R01_cb18456_c1882/f1p0/956	R01_cb18456_c1882/f1p0/956
R01_cb8564_c84450/f1p0/4007	NA	R01_cb8564_c84450/f1p0/4007	NA	NA
R01_cb8564_c85136/f4p0/2456	NA	R01_cb8564_c85136/f4p0/2456	R01_cb8564_c85136/f4p0/2456	NA
R01_cb17662_c1/f1p0/871	NA	R01_cb17662_c1/f1p0/871	NA	NA
R01_cb7258_c0/f1p0/2661	NA	R01_cb7258_c0/f1p0/2661	R01_cb7258_c0/f1p0/2661	NA
R01_cb14334_c4/f1p0/975	NA	NA	NA	R01_cb14334_c4/f1p0/975
R01_cb7362_c6/f1p1/2256	R01_cb7362_c6/f1p1/2256	R01_cb7362_c6/f1p1/2256	NA	NA
R01_cb13478_c10/f1p0/550	NA	R01_cb13478_c10/f1p0/550	R01_cb13478_c10/f1p0/550	R01_cb13478_c10/f1p0/550
R01_cb8209_c2/f1p0/2365	NA	NA	NA	R01_cb8209_c2/f1p0/2365
R01_cb17044_c17/f1p0/1494	NA	R01_cb17044_c17/f1p0/1494	NA	NA
R01_cb8564_c106631/f6p1/2246	NA	NA	NA	R01_cb8564_c106631/f6p1/2246
R01_cb5686_c11/f1p0/2454	NA	R01_cb5686_c11/f1p0/2454	NA	NA
R01_cb10029_c1336/f1p1/812	NA	R01_cb10029_c1336/f1p1/812	NA	NA
R01_cb16870_c0/f2p0/747	NA	NA	R01_cb16870_c0/f2p0/747	R01_cb16870_c0/f2p0/747
R01_cb17582_c0/f1p0/650	R01_cb17582_c0/f1p0/650	R01_cb17582_c0/f1p0/650	NA	R01_cb17582_c0/f1p0/650
R01_cb9325_c2/f1p0/464	R01_cb9325_c2/f1p0/464	R01_cb9325_c2/f1p0/464	R01_cb9325_c2/f1p0/464	R01_cb9325_c2/f1p0/464
R01_cb3752_c1/f2p0/1494	NA	NA	NA	R01_cb3752_c1/f2p0/1494
R01_cb822_c8/f2p0/4181	NA	NA	NA	R01_cb822_c8/f2p0/4181
R01_cb8564_c118441/f1p0/2931	NA	NA	NA	R01_cb8564_c118441/f1p0/2931
R01_cb2875_c10/f1p0/4041	NA	NA	NA	R01_cb2875_c10/f1p0/4041
R01_cb8564_c23778/f1p0/1942	R01_cb8564_c23778/f1p0/1942	R01_cb8564_c23778/f1p0/1942	R01_cb8564_c23778/f1p0/1942	R01_cb8564_c23778/f1p0/1942
R01_cb3307_c26/f1p2/2430	NA	NA	NA	R01_cb3307_c26/f1p2/2430

R01_cb8564_c4096/f1p0/2104	R01_cb8564_c4096/f1p0/2104	R01_cb8564_c4096/f1p0/2104	R01_cb8564_c4096/f1p0/2104	R01_cb8564_c4096/f1p0/2104
R01_cb3615_c2/f1p0/3065	NA	R01_cb3615_c2/f1p0/3065	NA	NA
R01_cb13985_c7/f1p0/537	NA	R01_cb13985_c7/f1p0/537	NA	NA
R01_cb16515_c1/f1p0/841	NA	NA	NA	R01_cb16515_c1/f1p0/841
R01_cb9003_c6/f1p0/1295	R01_cb9003_c6/f1p0/1295	R01_cb9003_c6/f1p0/1295	R01_cb9003_c6/f1p0/1295	R01_cb9003_c6/f1p0/1295
R01_cb11353_c3/f1p0/890	NA	NA	NA	R01_cb11353_c3/f1p0/890
R01_cb6598_c8/f1p0/1987	NA	R01_cb6598_c8/f1p0/1987	NA	NA
R01_cb676_c6/f1p0/3136	NA	NA	NA	R01_cb676_c6/f1p0/3136
R01_cb8564_c91448/f1p0/2758	NA	R01_cb8564_c91448/f1p0/2758	R01_cb8564_c91448/f1p0/2758	R01_cb8564_c91448/f1p0/2758
R01_cb10015_c225/f1p2/870	R01_cb10015_c225/f1p2/870	R01_cb10015_c225/f1p2/870	NA	R01_cb10015_c225/f1p2/870
R01_cb18456_c5283/f1p0/639	R01_cb18456_c5283/f1p0/639	R01_cb18456_c5283/f1p0/639	NA	R01_cb18456_c5283/f1p0/639
R01_cb15581_c2/f1p0/865	NA	NA	NA	R01_cb15581_c2/f1p0/865
R01_cb9333_c1/f1p0/3393	R01_cb9333_c1/f1p0/3393	R01_cb9333_c1/f1p0/3393	R01_cb9333_c1/f1p0/3393	R01_cb9333_c1/f1p0/3393
R01_cb10337_c3/f1p2/1673	NA	R01_cb10337_c3/f1p2/1673	NA	NA
R01_cb8564_c130109/f1p0/3052	NA	NA	NA	R01_cb8564_c130109/f1p0/3052
R01_cb16180_c0/f2p0/603	R01_cb16180_c0/f2p0/603	R01_cb16180_c0/f2p0/603	R01_cb16180_c0/f2p0/603	R01_cb16180_c0/f2p0/603
R01_cb8564_c40634/f1p0/2728	R01_cb8564_c40634/f1p0/2728	R01_cb8564_c40634/f1p0/2728	R01_cb8564_c40634/f1p0/2728	R01_cb8564_c40634/f1p0/2728
R01_cb9797_c126/f1p0/1106	NA	R01_cb9797_c126/f1p0/1106	NA	NA
R01_cb12024_c12/f1p0/997	NA	NA	NA	R01_cb12024_c12/f1p0/997
R01_cb11900_c2/f2p0/654	NA	R01_cb11900_c2/f2p0/654	NA	R01_cb11900_c2/f2p0/654
R01_cb13284_c7/f1p0/1818	NA	NA	NA	R01_cb13284_c7/f1p0/1818
R01_cb13851_c1/f1p0/1070	NA	R01_cb13851_c1/f1p0/1070	NA	NA
R01_cb13867_c19/f4p3/761	NA	NA	NA	R01_cb13867_c19/f4p3/761
R01_cb16125_c18/f1p0/1753	NA	NA	NA	R01_cb16125_c18/f1p0/1753

R01_cb7797_c8/f1p1/2476	NA	R01_cb7797_c8/f1p1/2476	NA	R01_cb7797_c8/f1p1/2476
R01_cb11925_c14/f1p1/1775	NA	NA	NA	R01_cb11925_c14/f1p1/1775
R01_cb11757_c1/f1p0/3799	NA	R01_cb11757_c1/f1p0/3799	R01_cb11757_c1/f1p0/3799	NA
R01_cb4094_c27/f1p0/537	R01_cb4094_c27/f1p0/537	R01_cb4094_c27/f1p0/537	NA	NA
R01_cb3774_c73/f3p0/1972	NA	NA	NA	R01_cb3774_c73/f3p0/1972
R01_cb2400_c0/f1p0/3947	NA	R01_cb2400_c0/f1p0/3947	NA	NA
R01_cb6615_c9/f1p0/2575	NA	NA	NA	R01_cb6615_c9/f1p0/2575
R01_cb3088_c6/f1p0/2002	R01_cb3088_c6/f1p0/2002	NA	NA	NA
R01_cb3958_c6/f1p0/2932	NA	R01_cb3958_c6/f1p0/2932	NA	NA
R01_cb9529_c0/f3p0/1045	NA	R01_cb9529_c0/f3p0/1045	NA	R01_cb9529_c0/f3p0/1045
R01_cb10725_c0/f1p0/1057	NA	R01_cb10725_c0/f1p0/1057	R01_cb10725_c0/f1p0/1057	R01_cb10725_c0/f1p0/1057
R01_cb8084_c6/f1p0/1314	NA	NA	NA	R01_cb8084_c6/f1p0/1314
R01_cb4642_c8/f1p0/2139	NA	NA	NA	R01_cb4642_c8/f1p0/2139
R01_cb15295_c1/f1p0/1661	NA	NA	NA	R01_cb15295_c1/f1p0/1661
R01_cb8564_c19945/f1p0/3208	NA	R01_cb8564_c19945/f1p0/3208	R01_cb8564_c19945/f1p0/3208	R01_cb8564_c19945/f1p0/3208
R01_cb12943_c3/f1p0/1547	NA	NA	NA	R01_cb12943_c3/f1p0/1547
R01_cb8564_c1044/f1p0/3621	NA	R01_cb8564_c1044/f1p0/3621	NA	R01_cb8564_c1044/f1p0/3621
R01_cb27_c10/f1p0/1363	NA	NA	NA	R01_cb27_c10/f1p0/1363
R01_cb8564_c70156/f1p0/2250	NA	R01_cb8564_c70156/f1p0/2250	NA	NA
R01_cb8564_c117381/f1p0/2327	NA	R01_cb8564_c117381/f1p0/2327	NA	R01_cb8564_c117381/f1p0/2327
R01_cb7329_c4/f1p0/1247	NA	R01_cb7329_c4/f1p0/1247	NA	NA
R01_cb2804_c140/f1p0/2219	NA	R01_cb2804_c140/f1p0/2219	R01_cb2804_c140/f1p0/2219	R01_cb2804_c140/f1p0/2219
R01_cb10764_c5/f1p0/3530	NA	R01_cb10764_c5/f1p0/3530	NA	NA
R01_cb4022_c37/f1p0/1559	NA	NA	NA	R01_cb4022_c37/f1p0/1559
R01_cb8981_c0/f4p1/2043	NA	NA	NA	R01_cb8981_c0/f4p1/2043

R01_cb6093_c2/f2p0/405	R01_cb6093_c2/f2p0/405	R01_cb6093_c2/f2p0/405	R01_cb6093_c2/f2p0/405	R01_cb6093_c2/f2p0/405
R01_cb9485_c2/f1p0/2076	NA	R01_cb9485_c2/f1p0/2076	NA	R01_cb9485_c2/f1p0/2076
R01_cb8279_c14/f1p0/1924	NA	NA	NA	R01_cb8279_c14/f1p0/1924
R01_cb6391_c11/f1p0/2658	NA	R01_cb6391_c11/f1p0/2658	NA	NA
R01_cb2927_c17/f1p0/2828	NA	R01_cb2927_c17/f1p0/2828	NA	NA
R01_cb5846_c42/f1p0/1948	NA	R01_cb5846_c42/f1p0/1948	NA	NA
R01_cb18620_c9/f1p0/1806	NA	NA	NA	R01_cb18620_c9/f1p0/1806
R01_cb17986_c2/f1p0/329	R01_cb17986_c2/f1p0/329	R01_cb17986_c2/f1p0/329	NA	R01_cb17986_c2/f1p0/329
R01_cb16913_c3/f2p0/572	NA	R01_cb16913_c3/f2p0/572	NA	NA
R01_cb10174_c2/f1p1/2740	NA	NA	R01_cb10174_c2/f1p1/2740	NA
R01_cb6456_c4/f1p0/2268	NA	R01_cb6456_c4/f1p0/2268	NA	R01_cb6456_c4/f1p0/2268
R01_cb13161_c7/f1p0/1043	NA	R01_cb13161_c7/f1p0/1043	NA	NA
R01_cb8564_c14928/f1p0/2382	NA	NA	NA	R01_cb8564_c14928/f1p0/2382
R01_cb14246_c0/f1p0/538	NA	R01_cb14246_c0/f1p0/538	R01_cb14246_c0/f1p0/538	NA
R01_cb13724_c8/f1p0/454	NA	NA	R01_cb13724_c8/f1p0/454	R01_cb13724_c8/f1p0/454
R01_cb6213_c11/f1p0/2855	NA	R01_cb6213_c11/f1p0/2855	NA	NA
R01_cb16520_c3/f1p0/1138	NA	NA	NA	R01_cb16520_c3/f1p0/1138
R01_cb18516_c0/f3p0/794	R01_cb18516_c0/f3p0/794	R01_cb18516_c0/f3p0/794	R01_cb18516_c0/f3p0/794	R01_cb18516_c0/f3p0/794
R01_cb13414_c1/f1p0/1601	NA	R01_cb13414_c1/f1p0/1601	NA	NA
R01_cb5911_c5/f1p0/1846	NA	NA	NA	R01_cb5911_c5/f1p0/1846
R01_cb13586_c9/f1p2/913	NA	R01_cb13586_c9/f1p2/913	NA	NA
R01_cb10029_c1416/f1p2/809	NA	R01_cb10029_c1416/f1p2/809	NA	NA
R01_cb8564_c14100/f1p0/4985	NA	R01_cb8564_c14100/f1p0/4985	R01_cb8564_c14100/f1p0/4985	R01_cb8564_c14100/f1p0/4985
R01_cb2959_c7/f1p0/3484	NA	NA	NA	R01_cb2959_c7/f1p0/3484
R01_cb14946_c2/f1p0/886	R01_cb14946_c2/f1p0/886	R01_cb14946_c2/f1p0/886	NA	NA
R01_cb15008_c2/f1p0/1322	NA	R01_cb15008_c2/f1p0/1322	NA	NA

R01_cb8564_c25496/f2p0/2331	NA	R01_cb8564_c25496/f2p0/2331	R01_cb8564_c25496/f2p0/2331	R01_cb8564_c25496/f2p0/2331
R01_cb14556_c0/f6p0/1020	NA	NA	NA	R01_cb14556_c0/f6p0/1020
R01_cb13405_c2/f1p0/668	NA	R01_cb13405_c2/f1p0/668	R01_cb13405_c2/f1p0/668	R01_cb13405_c2/f1p0/668
R01_cb16310_c1/f1p0/1091	NA	R01_cb16310_c1/f1p0/1091	NA	NA
R01_cb17163_c1/f1p0/1082	NA	R01_cb17163_c1/f1p0/1082	R01_cb17163_c1/f1p0/1082	R01_cb17163_c1/f1p0/1082
R01_cb5331_c2/f1p0/694	R01_cb5331_c2/f1p0/694	R01_cb5331_c2/f1p0/694	R01_cb5331_c2/f1p0/694	R01_cb5331_c2/f1p0/694
R01_cb3637_c3/f1p0/1502	NA	R01_cb3637_c3/f1p0/1502	NA	NA
R01_cb8564_c126614/f1p0/4646	NA	R01_cb8564_c126614/f1p0/4646	NA	NA
R01_cb17094_c0/f1p0/422	R01_cb17094_c0/f1p0/422	R01_cb17094_c0/f1p0/422	R01_cb17094_c0/f1p0/422	R01_cb17094_c0/f1p0/422
R01_cb14415_c1/f1p0/989	NA	NA	NA	R01_cb14415_c1/f1p0/989
R01_cb17604_c7/f1p0/566	NA	R01_cb17604_c7/f1p0/566	R01_cb17604_c7/f1p0/566	R01_cb17604_c7/f1p0/566
R01_cb2082_c19/f1p0/2908	NA	NA	NA	R01_cb2082_c19/f1p0/2908
R01_cb3575_c6/f1p1/3530	NA	NA	NA	R01_cb3575_c6/f1p1/3530
R01_cb2219_c9/f1p0/2783	NA	NA	NA	R01_cb2219_c9/f1p0/2783
R01_cb5875_c0/f1p0/2992	R01_cb5875_c0/f1p0/2992	R01_cb5875_c0/f1p0/2992	R01_cb5875_c0/f1p0/2992	R01_cb5875_c0/f1p0/2992
R01_cb17735_c1/f2p0/772	NA	R01_cb17735_c1/f2p0/772	NA	NA
R01_cb4347_c2/f2p0/3350	NA	NA	R01_cb4347_c2/f2p0/3350	NA
R01_cb446_c68/f1p0/780	NA	R01_cb446_c68/f1p0/780	NA	R01_cb446_c68/f1p0/780
R01_cb18037_c0/f2p0/568	NA	R01_cb18037_c0/f2p0/568	R01_cb18037_c0/f2p0/568	R01_cb18037_c0/f2p0/568
R01_cb12767_c3/f4p1/1063	NA	NA	NA	R01_cb12767_c3/f4p1/1063
R01_cb17417_c4/f1p0/344	R01_cb17417_c4/f1p0/344	R01_cb17417_c4/f1p0/344	R01_cb17417_c4/f1p0/344	R01_cb17417_c4/f1p0/344
R01_cb15953_c0/f2p0/968	NA	NA	NA	R01_cb15953_c0/f2p0/968
R01_cb17069_c0/f1p0/1095	NA	R01_cb17069_c0/f1p0/1095	R01_cb17069_c0/f1p0/1095	R01_cb17069_c0/f1p0/1095
R01_cb2761_c8/f1p0/2922	R01_cb2761_c8/f1p0/2922	R01_cb2761_c8/f1p0/2922	NA	R01_cb2761_c8/f1p0/2922
R01_cb18222_c2/f1p0/448	NA	NA	NA	R01_cb18222_c2/f1p0/448

R01_cb8564_c119485/f1p0/2104	NA	R01_cb8564_c119485/f1p0/2104	R01_cb8564_c119485/f1p0/2104	R01_cb8564_c119485/f1p0/2104
R01_cb2844_c0/f1p0/3791	NA	R01_cb2844_c0/f1p0/3791	NA	NA
R01_cb13220_c1/f2p0/1812	NA	NA	NA	R01_cb13220_c1/f2p0/1812
R01_cb8564_c37376/f1p0/2325	R01_cb8564_c37376/f1p0/2325	R01_cb8564_c37376/f1p0/2325	R01_cb8564_c37376/f1p0/2325	R01_cb8564_c37376/f1p0/2325
R01_cb11483_c1/f1p0/2628	NA	NA	NA	R01_cb11483_c1/f1p0/2628
R01_cb8564_c19747/f1p0/2788	NA	R01_cb8564_c19747/f1p0/2788	R01_cb8564_c19747/f1p0/2788	R01_cb8564_c19747/f1p0/2788
R01_cb8564_c34349/f1p0/3514	NA	R01_cb8564_c34349/f1p0/3514	R01_cb8564_c34349/f1p0/3514	R01_cb8564_c34349/f1p0/3514
R01_cb17532_c3/f1p1/5304	NA	NA	NA	R01_cb17532_c3/f1p1/5304
R01_cb8564_c111376/f1p0/3984	NA	NA	NA	R01_cb8564_c111376/f1p0/3984
R01_cb8564_c115478/f1p0/2938	NA	R01_cb8564_c115478/f1p0/2938	R01_cb8564_c115478/f1p0/2938	R01_cb8564_c115478/f1p0/2938
R01_cb17269_c1/f2p0/514	NA	R01_cb17269_c1/f2p0/514	R01_cb17269_c1/f2p0/514	R01_cb17269_c1/f2p0/514
R01_cb3965_c5/f1p1/3316	NA	R01_cb3965_c5/f1p1/3316	NA	NA
R01_cb5896_c164/f1p0/1895	R01_cb5896_c164/f1p0/1895	R01_cb5896_c164/f1p0/1895	R01_cb5896_c164/f1p0/1895	R01_cb5896_c164/f1p0/1895
R01_cb5229_c12/f14p1/1035	NA	NA	NA	R01_cb5229_c12/f14p1/1035
R01_cb1437_c1/f2p0/4008	NA	NA	NA	R01_cb1437_c1/f2p0/4008
R01_cb3500_c20/f1p0/2574	NA	NA	NA	R01_cb3500_c20/f1p0/2574
R01_cb13517_c0/f1p0/961	R01_cb13517_c0/f1p0/961	NA	NA	NA
R01_cb2101_c6/f1p0/2473	R01_cb2101_c6/f1p0/2473	R01_cb2101_c6/f1p0/2473	NA	NA
R01_cb8564_c3800/f1p0/2113	NA	R01_cb8564_c3800/f1p0/2113	R01_cb8564_c3800/f1p0/2113	NA
R01_cb8564_c3171/f1p1/2490	NA	R01_cb8564_c3171/f1p1/2490	NA	NA
R01_cb5208_c3/f1p0/3163	NA	NA	NA	R01_cb5208_c3/f1p0/3163
R01_cb10693_c0/f2p1/1742	NA	NA	NA	R01_cb10693_c0/f2p1/1742

R01_cb15913_c3/f1p0/526	R01_cb15913_c3/f1p0/526	R01_cb15913_c3/f1p0/526	R01_cb15913_c3/f1p0/526	R01_cb15913_c3/f1p0/526
R01_cb8564_c75559/f1p0/2086	NA	R01_cb8564_c75559/f1p0/2086	NA	R01_cb8564_c75559/f1p0/2086
R01_cb11569_c0/f1p0/1142	R01_cb11569_c0/f1p0/1142	R01_cb11569_c0/f1p0/1142	R01_cb11569_c0/f1p0/1142	R01_cb11569_c0/f1p0/1142
R01_cb17052_c1/f1p0/1426	NA	NA	NA	R01_cb17052_c1/f1p0/1426
R01_cb1849_c13/f1p1/1986	NA	NA	NA	R01_cb1849_c13/f1p1/1986
R01_cb1441_c26/f1p0/6400	NA	R01_cb1441_c26/f1p0/6400	NA	NA
R01_cb8564_c48239/f1p0/2761	NA	R01_cb8564_c48239/f1p0/2761	R01_cb8564_c48239/f1p0/2761	R01_cb8564_c48239/f1p0/2761
R01_cb781_c33/f1p0/1887	NA	NA	NA	R01_cb781_c33/f1p0/1887
R01_cb8564_c122775/f1p0/2291	R01_cb8564_c122775/f1p0/2291	NA	R01_cb8564_c122775/f1p0/2291	NA
R01_cb8564_c81433/f1p0/3330	NA	NA	NA	R01_cb8564_c81433/f1p0/3330
R01_cb27_c2/f2p0/1166	NA	NA	NA	R01_cb27_c2/f2p0/1166
R01_cb16145_c2/f1p0/631	NA	R01_cb16145_c2/f1p0/631	R01_cb16145_c2/f1p0/631	NA
R01_cb8564_c85367/f1p0/1928	NA	R01_cb8564_c85367/f1p0/1928	R01_cb8564_c85367/f1p0/1928	R01_cb8564_c85367/f1p0/1928
R01_cb18711_c1/f1p0/988	NA	R01_cb18711_c1/f1p0/988	NA	R01_cb18711_c1/f1p0/988
R01_cb13535_c12/f1p0/1513	NA	R01_cb13535_c12/f1p0/1513	NA	NA
R01_cb8564_c2999/f1p0/2399	NA	NA	NA	R01_cb8564_c2999/f1p0/2399
R01_cb5657_c4/f1p0/2282	NA	NA	NA	R01_cb5657_c4/f1p0/2282
R01_cb18660_c0/f1p0/5189	NA	R01_cb18660_c0/f1p0/5189	R01_cb18660_c0/f1p0/5189	R01_cb18660_c0/f1p0/5189
R01_cb10875_c2/f1p0/1565	NA	R01_cb10875_c2/f1p0/1565	NA	NA
R01_cb10343_c1/f1p0/2337	NA	R01_cb10343_c1/f1p0/2337	NA	NA
R01_cb17090_c1/f1p0/1081	NA	NA	NA	R01_cb17090_c1/f1p0/1081
R01_cb18392_c1/f1p0/993	NA	NA	NA	R01_cb18392_c1/f1p0/993
R01_cb8564_c115131/f1p0/2205	NA	R01_cb8564_c115131/f1p0/2205	NA	R01_cb8564_c115131/f1p0/2205
R01_cb8564_c19993/f1p0/2447	NA	R01_cb8564_c19993/f1p0/2447	NA	NA

R01_cb16191_c2/f1p1/1836	NA	NA	NA	R01_cb16191_c2/f1p1/1836
R01_cb2494_c14/f1p0/921	R01_cb2494_c14/f1p0/921	R01_cb2494_c14/f1p0/921	R01_cb2494_c14/f1p0/921	R01_cb2494_c14/f1p0/921
R01_cb14886_c2/f1p0/664	NA	NA	R01_cb14886_c2/f1p0/664	NA
R01_cb3500_c31/f1p2/3490	NA	NA	NA	R01_cb3500_c31/f1p2/3490
R01_cb2875_c14/f1p1/3543	NA	NA	NA	R01_cb2875_c14/f1p1/3543
R01_cb11250_c2/f1p0/1467	R01_cb11250_c2/f1p0/1467	R01_cb11250_c2/f1p0/1467	R01_cb11250_c2/f1p0/1467	R01_cb11250_c2/f1p0/1467
R01_cb3929_c4/f1p0/3486	NA	R01_cb3929_c4/f1p0/3486	NA	NA
R01_cb15217_c7/f1p0/484	R01_cb15217_c7/f1p0/484	R01_cb15217_c7/f1p0/484	R01_cb15217_c7/f1p0/484	R01_cb15217_c7/f1p0/484
R01_cb1418_c3/f1p0/3102	NA	NA	NA	R01_cb1418_c3/f1p0/3102
R01_cb5369_c4/f1p0/3113	NA	R01_cb5369_c4/f1p0/3113	NA	NA
R01_cb4861_c1/f1p0/2323	NA	R01_cb4861_c1/f1p0/2323	NA	NA
R01_cb8564_c72769/f1p0/4973	NA	NA	NA	R01_cb8564_c72769/f1p0/4973
R01_cb8700_c27/f1p0/1925	NA	R01_cb8700_c27/f1p0/1925	NA	NA
R01_cb11603_c1/f1p0/1913	NA	R01_cb11603_c1/f1p0/1913	NA	NA
R01_cb9653_c10/f1p0/510	R01_cb9653_c10/f1p0/510	R01_cb9653_c10/f1p0/510	R01_cb9653_c10/f1p0/510	R01_cb9653_c10/f1p0/510
R01_cb17084_c0/f2p0/622	R01_cb17084_c0/f2p0/622	R01_cb17084_c0/f2p0/622	NA	NA
R01_cb15312_c3/f1p0/987	NA	NA	R01_cb15312_c3/f1p0/987	R01_cb15312_c3/f1p0/987
R01_cb15814_c6/f2p1/1323	NA	NA	NA	R01_cb15814_c6/f2p1/1323
R01_cb10480_c40/f1p0/383	R01_cb10480_c40/f1p0/383	R01_cb10480_c40/f1p0/383	NA	R01_cb10480_c40/f1p0/383
R01_cb5373_c3/f1p0/3042	NA	NA	NA	R01_cb5373_c3/f1p0/3042
R01_cb14560_c9/f1p0/1215	NA	R01_cb14560_c9/f1p0/1215	NA	R01_cb14560_c9/f1p0/1215
R01_cb11411_c0/f1p0/1838	NA	R01_cb11411_c0/f1p0/1838	R01_cb11411_c0/f1p0/1838	R01_cb11411_c0/f1p0/1838
R01_cb8564_c119824/f1p0/2491	NA	R01_cb8564_c119824/f1p0/2491	R01_cb8564_c119824/f1p0/2491	R01_cb8564_c119824/f1p0/2491
R01_cb15811_c36/f1p1/632	NA	R01_cb15811_c36/f1p1/632	NA	R01_cb15811_c36/f1p1/632
R01_cb8564_c91577/f1p0/2925	NA	R01_cb8564_c91577/f1p0/2925	NA	NA

R01_cb843_c5/f1p0/2329	NA	NA	NA	R01_cb843_c5/f1p0/2329
R01_cb4917_c1/f1p0/2292	NA	NA	NA	R01_cb4917_c1/f1p0/2292
R01_cb15950_c0/f2p1/1402	NA	NA	NA	R01_cb15950_c0/f2p1/1402
R01_cb8564_c78234/f1p0/2871	NA	R01_cb8564_c78234/f1p0/2871	NA	R01_cb8564_c78234/f1p0/2871
R01_cb9272_c1/f2p0/637	NA	R01_cb9272_c1/f2p0/637	R01_cb9272_c1/f2p0/637	R01_cb9272_c1/f2p0/637
R01_cb10653_c4/f1p0/786	R01_cb10653_c4/f1p0/786	R01_cb10653_c4/f1p0/786	NA	NA
R01_cb8564_c1411/f1p0/2310	NA	R01_cb8564_c1411/f1p0/2310	NA	R01_cb8564_c1411/f1p0/2310
R01_cb8564_c86066/f2p0/2719	NA	R01_cb8564_c86066/f2p0/2719	R01_cb8564_c86066/f2p0/2719	NA
R01_cb14533_c3/f1p0/1017	NA	NA	R01_cb14533_c3/f1p0/1017	R01_cb14533_c3/f1p0/1017
R01_cb8030_c2/f1p0/2357	NA	NA	NA	R01_cb8030_c2/f1p0/2357
R01_cb8840_c4/f1p0/802	NA	R01_cb8840_c4/f1p0/802	NA	NA
R01_cb14660_c6/f1p0/565	R01_cb14660_c6/f1p0/565	R01_cb14660_c6/f1p0/565	NA	NA
R01_cb14853_c1/f1p0/668	R01_cb14853_c1/f1p0/668	R01_cb14853_c1/f1p0/668	R01_cb14853_c1/f1p0/668	R01_cb14853_c1/f1p0/668
R01_cb14119_c2/f1p0/654	NA	R01_cb14119_c2/f1p0/654	NA	NA
R01_cb17236_c4/f1p0/791	R01_cb17236_c4/f1p0/791	R01_cb17236_c4/f1p0/791	R01_cb17236_c4/f1p0/791	R01_cb17236_c4/f1p0/791
R01_cb4805_c28/f1p0/752	NA	R01_cb4805_c28/f1p0/752	NA	R01_cb4805_c28/f1p0/752
R01_cb10913_c2/f1p0/2117	NA	R01_cb10913_c2/f1p0/2117	NA	R01_cb10913_c2/f1p0/2117
R01_cb9154_c13/f1p0/1300	NA	NA	NA	R01_cb9154_c13/f1p0/1300
R01_cb8564_c85104/f1p0/2266	NA	R01_cb8564_c85104/f1p0/2266	R01_cb8564_c85104/f1p0/2266	R01_cb8564_c85104/f1p0/2266
R01_cb11744_c0/f1p0/1872	NA	NA	NA	R01_cb11744_c0/f1p0/1872
R01_cb12045_c3/f1p0/1491	NA	NA	NA	R01_cb12045_c3/f1p0/1491
R01_cb15818_c2/f1p0/1426	NA	NA	NA	R01_cb15818_c2/f1p0/1426
R01_cb8564_c123397/f1p0/4138	NA	NA	NA	R01_cb8564_c123397/f1p0/4138
R01_cb13178_c24/f1p0/610	R01_cb13178_c24/f1p0/610	R01_cb13178_c24/f1p0/610	NA	NA
R01_cb8564_c114792/f1p0/280	R01_cb8564_c114792/f1p0/28	R01_cb8564_c114792/f1p0/280	R01_cb8564_c114792/f1p0/280	R01_cb8564_c114792/f1p0/280

9	09	9	9	9
R01_cb12402_c3/f3p1/631	NA	NA	R01_cb12402_c3/f3p1/631	R01_cb12402_c3/f3p1/631
R01_cb13901_c4/f1p0/1146	NA	R01_cb13901_c4/f1p0/1146	NA	NA
R01_cb3888_c1/f2p0/2314	NA	R01_cb3888_c1/f2p0/2314	NA	NA
R01_cb4768_c18/f1p1/2680	NA	R01_cb4768_c18/f1p1/2680	NA	NA
R01_cb8564_c85607/f1p0/2557	NA	NA	NA	R01_cb8564_c85607/f1p0/2557
R01_cb11332_c1/f1p0/2388	NA	R01_cb11332_c1/f1p0/2388	NA	NA
R01_cb7219_c6/f1p3/2633	NA	NA	NA	R01_cb7219_c6/f1p3/2633
R01_cb16899_c1/f1p0/659	NA	R01_cb16899_c1/f1p0/659	NA	NA
R01_cb10024_c691/f4p0/549	NA	R01_cb10024_c691/f4p0/549	NA	NA
R01_cb15428_c2/f1p0/759	NA	NA	R01_cb15428_c2/f1p0/759	R01_cb15428_c2/f1p0/759
R01_cb5297_c1/f1p1/3694	NA	R01_cb5297_c1/f1p1/3694	NA	NA
R01_cb8564_c23847/f1p0/2186	NA	NA	NA	R01_cb8564_c23847/f1p0/2186
R01_cb13026_c3/f1p0/997	R01_cb13026_c3/f1p0/997	R01_cb13026_c3/f1p0/997	R01_cb13026_c3/f1p0/997	R01_cb13026_c3/f1p0/997
R01_cb10799_c1/f1p1/1466	NA	NA	NA	R01_cb10799_c1/f1p1/1466
R01_cb3498_c5/f2p0/1067	NA	R01_cb3498_c5/f2p0/1067	NA	NA
R01_cb15932_c0/f3p0/1233	NA	NA	NA	R01_cb15932_c0/f3p0/1233
R01_cb8786_c3/f1p0/2187	NA	NA	NA	R01_cb8786_c3/f1p0/2187
R01_cb6113_c2/f2p0/1143	NA	NA	NA	R01_cb6113_c2/f2p0/1143
R01_cb2708_c42/f1p2/1291	NA	R01_cb2708_c42/f1p2/1291	NA	NA
R01_cb6845_c10/f1p0/2755	NA	NA	NA	R01_cb6845_c10/f1p0/2755
R01_cb11926_c1/f1p0/622	R01_cb11926_c1/f1p0/622	R01_cb11926_c1/f1p0/622	R01_cb11926_c1/f1p0/622	R01_cb11926_c1/f1p0/622
R01_cb10843_c7/f1p0/627	R01_cb10843_c7/f1p0/627	R01_cb10843_c7/f1p0/627	R01_cb10843_c7/f1p0/627	R01_cb10843_c7/f1p0/627
R01_cb15069_c1/f3p0/552	NA	R01_cb15069_c1/f3p0/552	NA	NA
R01_cb4639_c5/f1p0/2925	NA	NA	NA	R01_cb4639_c5/f1p0/2925
R01_cb2482_c50/f1p0/1645	NA	NA	NA	R01_cb2482_c50/f1p0/1645

R01_cb17090_c0/f2p0/970	NA	NA	NA	R01_cb17090_c0/f2p0/970
R01_cb10205_c1/f1p0/2470	NA	R01_cb10205_c1/f1p0/2470	NA	R01_cb10205_c1/f1p0/2470
R01_cb12620_c7/f1p0/1293	NA	NA	NA	R01_cb12620_c7/f1p0/1293
R01_cb5900_c133/f1p0/2151	R01_cb5900_c133/f1p0/2151	R01_cb5900_c133/f1p0/2151	NA	R01_cb5900_c133/f1p0/2151
R01_cb9388_c0/f1p0/2098	NA	R01_cb9388_c0/f1p0/2098	R01_cb9388_c0/f1p0/2098	R01_cb9388_c0/f1p0/2098
R01_cb5442_c2/f1p0/3163	NA	NA	R01_cb5442_c2/f1p0/3163	R01_cb5442_c2/f1p0/3163
R01_cb8564_c74609/f3p0/2650	NA	NA	R01_cb8564_c74609/f3p0/2650	R01_cb8564_c74609/f3p0/2650
R01_cb3421_c0/f5p1/2836	NA	NA	NA	R01_cb3421_c0/f5p1/2836
R01_cb15465_c5/f1p3/519	R01_cb15465_c5/f1p3/519	NA	R01_cb15465_c5/f1p3/519	R01_cb15465_c5/f1p3/519
R01_cb8564_c125469/f1p0/3715	NA	R01_cb8564_c125469/f1p0/3715	NA	R01_cb8564_c125469/f1p0/3715
R01_cb13134_c8/f1p0/1569	NA	NA	NA	R01_cb13134_c8/f1p0/1569
R01_cb13260_c1/f3p0/811	NA	NA	NA	R01_cb13260_c1/f3p0/811
R01_cb2045_c4/f1p0/3368	NA	NA	NA	R01_cb2045_c4/f1p0/3368
R01_cb8564_c10473/f1p0/2908	R01_cb8564_c10473/f1p0/2908	R01_cb8564_c10473/f1p0/2908	R01_cb8564_c10473/f1p0/2908	R01_cb8564_c10473/f1p0/2908
R01_cb4269_c11/f2p0/2147	NA	NA	NA	R01_cb4269_c11/f2p0/2147
R01_cb8564_c84381/f1p0/2854	NA	R01_cb8564_c84381/f1p0/2854	NA	NA
R01_cb16983_c2/f1p0/1826	NA	R01_cb16983_c2/f1p0/1826	NA	NA
R01_cb17576_c0/f4p1/755	NA	NA	R01_cb17576_c0/f4p1/755	R01_cb17576_c0/f4p1/755
R01_cb1568_c3/f1p0/4264	NA	R01_cb1568_c3/f1p0/4264	NA	R01_cb1568_c3/f1p0/4264
R01_cb6393_c7/f1p0/5586	NA	NA	NA	R01_cb6393_c7/f1p0/5586
R01_cb8564_c19343/f1p0/3358	NA	R01_cb8564_c19343/f1p0/3358	R01_cb8564_c19343/f1p0/3358	R01_cb8564_c19343/f1p0/3358
R01_cb12785_c13/f1p0/912	NA	NA	R01_cb12785_c13/f1p0/912	NA
R01_cb4354_c16/f1p1/2988	NA	NA	NA	R01_cb4354_c16/f1p1/2988
R01_cb18409_c151/f1p0/633	NA	R01_cb18409_c151/f1p0/633	R01_cb18409_c151/f1p0/633	R01_cb18409_c151/f1p0/633

R01_cb14086_c0/f3p0/694	NA	NA	NA	R01_cb14086_c0/f3p0/694
R01_cb16593_c1/f1p0/479	R01_cb16593_c1/f1p0/479	R01_cb16593_c1/f1p0/479	R01_cb16593_c1/f1p0/479	R01_cb16593_c1/f1p0/479
R01_cb18287_c9/f1p0/443	R01_cb18287_c9/f1p0/443	R01_cb18287_c9/f1p0/443	R01_cb18287_c9/f1p0/443	R01_cb18287_c9/f1p0/443
R01_cb6867_c6/f1p0/792	NA	NA	NA	R01_cb6867_c6/f1p0/792
R01_cb8564_c90975/f1p1/2258	R01_cb8564_c90975/f1p1/2258	R01_cb8564_c90975/f1p1/2258	R01_cb8564_c90975/f1p1/2258	R01_cb8564_c90975/f1p1/2258
R01_cb13884_c21/f1p0/889	R01_cb13884_c21/f1p0/889	NA	R01_cb13884_c21/f1p0/889	R01_cb13884_c21/f1p0/889
R01_cb8564_c79338/f1p0/3856	NA	NA	NA	R01_cb8564_c79338/f1p0/3856
R01_cb12827_c1/f2p0/1376	NA	NA	NA	R01_cb12827_c1/f2p0/1376
R01_cb10450_c1/f1p0/2017	R01_cb10450_c1/f1p0/2017	R01_cb10450_c1/f1p0/2017	NA	NA
R01_cb18456_c7206/f1p0/366	R01_cb18456_c7206/f1p0/366	R01_cb18456_c7206/f1p0/366	R01_cb18456_c7206/f1p0/366	R01_cb18456_c7206/f1p0/366
R01_cb6680_c4/f1p0/2326	NA	NA	NA	R01_cb6680_c4/f1p0/2326
R01_cb18649_c2/f1p0/3942	NA	NA	NA	R01_cb18649_c2/f1p0/3942
R01_cb646_c1/f1p0/4677	NA	NA	NA	R01_cb646_c1/f1p0/4677
R01_cb3516_c5/f1p0/2166	NA	NA	NA	R01_cb3516_c5/f1p0/2166
R01_cb13279_c0/f1p0/1113	NA	R01_cb13279_c0/f1p0/1113	R01_cb13279_c0/f1p0/1113	R01_cb13279_c0/f1p0/1113
R01_cb13146_c6/f1p0/1286	NA	R01_cb13146_c6/f1p0/1286	R01_cb13146_c6/f1p0/1286	NA
R01_cb16457_c2/f1p0/511	R01_cb16457_c2/f1p0/511	R01_cb16457_c2/f1p0/511	R01_cb16457_c2/f1p0/511	R01_cb16457_c2/f1p0/511
R01_cb10385_c7/f1p0/1425	NA	R01_cb10385_c7/f1p0/1425	NA	NA
R01_cb17843_c2/f1p0/771	NA	R01_cb17843_c2/f1p0/771	NA	NA
R01_cb11743_c1/f1p0/2887	NA	NA	R01_cb11743_c1/f1p0/2887	R01_cb11743_c1/f1p0/2887
R01_cb5408_c0/f1p0/3110	NA	R01_cb5408_c0/f1p0/3110	NA	NA
R01_cb8564_c83794/f1p0/2318	NA	R01_cb8564_c83794/f1p0/2318	NA	NA
R01_cb8564_c46080/f1p0/2632	NA	R01_cb8564_c46080/f1p0/2632	NA	NA
R01_cb11524_c2/f1p1/1158	NA	NA	NA	R01_cb11524_c2/f1p1/1158
R01_cb10364_c3/f1p0/3091	NA	NA	NA	R01_cb10364_c3/f1p0/3091

R01_cb6008_c0/f1p0/2955	NA	NA	NA	R01_cb6008_c0/f1p0/2955
R01_cb8564_c76969/f1p0/3171	R01_cb8564_c76969/f1p0/3171	R01_cb8564_c76969/f1p0/3171	R01_cb8564_c76969/f1p0/3171	R01_cb8564_c76969/f1p0/3171
R01_cb10668_c11/f2p0/563	NA	R01_cb10668_c11/f2p0/563	NA	NA
R01_cb5455_c2/f1p0/2984	NA	R01_cb5455_c2/f1p0/2984	NA	NA
R01_cb12057_c51/f1p0/977	R01_cb12057_c51/f1p0/977	R01_cb12057_c51/f1p0/977	R01_cb12057_c51/f1p0/977	R01_cb12057_c51/f1p0/977
R01_cb9272_c2/f2p0/658	NA	R01_cb9272_c2/f2p0/658	R01_cb9272_c2/f2p0/658	R01_cb9272_c2/f2p0/658
R01_cb14023_c2/f1p0/466	R01_cb14023_c2/f1p0/466	NA	NA	R01_cb14023_c2/f1p0/466
R01_cb4345_c19/f6p1/3377	NA	NA	NA	R01_cb4345_c19/f6p1/3377
R01_cb9231_c2/f3p0/2135	NA	R01_cb9231_c2/f3p0/2135	R01_cb9231_c2/f3p0/2135	R01_cb9231_c2/f3p0/2135
R01_cb1860_c5/f1p0/4077	NA	NA	NA	R01_cb1860_c5/f1p0/4077
R01_cb12584_c3/f1p0/1577	NA	NA	NA	R01_cb12584_c3/f1p0/1577
R01_cb10434_c2/f1p0/2083	NA	R01_cb10434_c2/f1p0/2083	NA	R01_cb10434_c2/f1p0/2083
R01_cb11190_c3/f1p0/1176	NA	NA	NA	R01_cb11190_c3/f1p0/1176
R01_cb6914_c19/f1p0/2036	NA	R01_cb6914_c19/f1p0/2036	NA	NA
R01_cb16196_c0/f1p0/1689	NA	R01_cb16196_c0/f1p0/1689	R01_cb16196_c0/f1p0/1689	R01_cb16196_c0/f1p0/1689
R01_cb8478_c79/f1p0/2396	NA	NA	NA	R01_cb8478_c79/f1p0/2396
R01_cb6627_c0/f1p0/2804	R01_cb6627_c0/f1p0/2804	R01_cb6627_c0/f1p0/2804	NA	NA
R01_cb10350_c7/f18p1/1046	NA	NA	NA	R01_cb10350_c7/f18p1/1046
R01_cb6802_c20/f1p0/3496	NA	R01_cb6802_c20/f1p0/3496	NA	R01_cb6802_c20/f1p0/3496
R01_cb16645_c50/f1p0/800	NA	R01_cb16645_c50/f1p0/800	NA	NA
R01_cb8564_c78269/f1p0/4689	NA	R01_cb8564_c78269/f1p0/4689	NA	NA
R01_cb16135_c2/f1p0/536	R01_cb16135_c2/f1p0/536	R01_cb16135_c2/f1p0/536	R01_cb16135_c2/f1p0/536	R01_cb16135_c2/f1p0/536
R01_cb8564_c14403/f1p0/2739	NA	NA	NA	R01_cb8564_c14403/f1p0/2739
R01_cb8564_c31974/f8p6/4543	NA	NA	NA	R01_cb8564_c31974/f8p6/4543
R01_cb5177_c4/f1p0/2104	R01_cb5177_c4/f1p0/2104	R01_cb5177_c4/f1p0/2104	R01_cb5177_c4/f1p0/2104	R01_cb5177_c4/f1p0/2104

R01_cb1398_c0/f1p0/4333	NA	R01_cb1398_c0/f1p0/4333	R01_cb1398_c0/f1p0/4333	NA
R01_cb14019_c7/f1p0/1630	NA	R01_cb14019_c7/f1p0/1630	R01_cb14019_c7/f1p0/1630	R01_cb14019_c7/f1p0/1630
R01_cb12133_c0/f1p0/1051	NA	NA	NA	R01_cb12133_c0/f1p0/1051
R01_cb15537_c7/f1p0/658	NA	R01_cb15537_c7/f1p0/658	R01_cb15537_c7/f1p0/658	R01_cb15537_c7/f1p0/658
R01_cb13781_c14/f6p0/563	NA	NA	NA	R01_cb13781_c14/f6p0/563
R01_cb2465_c15/f1p0/346	R01_cb2465_c15/f1p0/346	R01_cb2465_c15/f1p0/346	NA	R01_cb2465_c15/f1p0/346
R01_cb11634_c1/f1p0/2196	NA	R01_cb11634_c1/f1p0/2196	NA	NA
R01_cb7898_c3/f2p0/1492	NA	R01_cb7898_c3/f2p0/1492	NA	R01_cb7898_c3/f2p0/1492
R01_cb9928_c3/f1p0/3979	NA	NA	NA	R01_cb9928_c3/f1p0/3979
R01_cb15910_c2/f1p0/464	R01_cb15910_c2/f1p0/464	R01_cb15910_c2/f1p0/464	NA	NA
R01_cb15227_c6/f2p0/1424	NA	NA	NA	R01_cb15227_c6/f2p0/1424
R01_cb2200_c3/f1p0/3495	R01_cb2200_c3/f1p0/3495	R01_cb2200_c3/f1p0/3495	R01_cb2200_c3/f1p0/3495	R01_cb2200_c3/f1p0/3495
R01_cb12461_c2/f1p0/734	NA	R01_cb12461_c2/f1p0/734	NA	R01_cb12461_c2/f1p0/734
R01_cb2715_c2/f1p0/2264	R01_cb2715_c2/f1p0/2264	R01_cb2715_c2/f1p0/2264	NA	NA
R01_cb18737_c1/f1p0/651	NA	NA	NA	R01_cb18737_c1/f1p0/651
R01_cb2275_c9/f1p0/5025	NA	NA	NA	R01_cb2275_c9/f1p0/5025
R01_cb5251_c5/f1p0/2735	NA	NA	NA	R01_cb5251_c5/f1p0/2735
R01_cb2701_c1/f2p0/1300	NA	NA	NA	R01_cb2701_c1/f2p0/1300
R01_cb18409_c36/f1p0/624	NA	R01_cb18409_c36/f1p0/624	R01_cb18409_c36/f1p0/624	R01_cb18409_c36/f1p0/624
R01_cb7909_c0/f4p0/2280	R01_cb7909_c0/f4p0/2280	R01_cb7909_c0/f4p0/2280	R01_cb7909_c0/f4p0/2280	R01_cb7909_c0/f4p0/2280
R01_cb8564_c3753/f1p0/2799	NA	R01_cb8564_c3753/f1p0/2799	NA	NA
R01_cb8564_c20252/f1p0/4765	NA	R01_cb8564_c20252/f1p0/4765	NA	NA
R01_cb6170_c31/f1p0/2237	NA	NA	NA	R01_cb6170_c31/f1p0/2237
R01_cb18456_c7331/f1p0/1803	NA	R01_cb18456_c7331/f1p0/1803	NA	R01_cb18456_c7331/f1p0/1803
R01_cb8478_c42/f1p0/2103	NA	R01_cb8478_c42/f1p0/2103	R01_cb8478_c42/f1p0/2103	R01_cb8478_c42/f1p0/2103
R01_cb6591_c6/f2p1/728	R01_cb6591_c6/f2p1/728	R01_cb6591_c6/f2p1/728	NA	NA

R01_cb1827_c0/f1p0/4155	NA	R01_cb1827_c0/f1p0/4155	R01_cb1827_c0/f1p0/4155	NA
R01_cb4637_c4/f1p0/2621	R01_cb4637_c4/f1p0/2621	R01_cb4637_c4/f1p0/2621	R01_cb4637_c4/f1p0/2621	R01_cb4637_c4/f1p0/2621
R01_cb17956_c12/f1p0/751	NA	R01_cb17956_c12/f1p0/751	NA	NA
R01_cb11151_c0/f1p0/1503	NA	NA	NA	R01_cb11151_c0/f1p0/1503
R01_cb6170_c24/f1p1/2725	NA	NA	NA	R01_cb6170_c24/f1p1/2725
R01_cb18033_c0/f1p0/500	NA	R01_cb18033_c0/f1p0/500	R01_cb18033_c0/f1p0/500	R01_cb18033_c0/f1p0/500
R01_cb8564_c4091/f1p0/3908	NA	R01_cb8564_c4091/f1p0/3908	R01_cb8564_c4091/f1p0/3908	NA
R01_cb15186_c4/f6p0/461	R01_cb15186_c4/f6p0/461	R01_cb15186_c4/f6p0/461	R01_cb15186_c4/f6p0/461	NA
R01_cb10190_c4/f1p0/1454	R01_cb10190_c4/f1p0/1454	R01_cb10190_c4/f1p0/1454	R01_cb10190_c4/f1p0/1454	R01_cb10190_c4/f1p0/1454
R01_cb10272_c22/f1p0/1736	NA	NA	NA	R01_cb10272_c22/f1p0/1736
R01_cb16823_c1/f1p0/1354	NA	R01_cb16823_c1/f1p0/1354	NA	R01_cb16823_c1/f1p0/1354
R01_cb8564_c41870/f1p0/3436	R01_cb8564_c41870/f1p0/3436	R01_cb8564_c41870/f1p0/3436	NA	R01_cb8564_c41870/f1p0/3436
R01_cb10970_c4/f1p0/1737	NA	NA	NA	R01_cb10970_c4/f1p0/1737
R01_cb473_c0/f1p0/4800	NA	R01_cb473_c0/f1p0/4800	R01_cb473_c0/f1p0/4800	R01_cb473_c0/f1p0/4800
R01_cb9748_c2/f1p0/1968	NA	NA	NA	R01_cb9748_c2/f1p0/1968
R01_cb5690_c4/f1p0/3237	NA	NA	NA	R01_cb5690_c4/f1p0/3237
R01_cb8564_c43373/f1p0/2035	NA	NA	NA	R01_cb8564_c43373/f1p0/2035
R01_cb18456_c4749/f1p0/492	R01_cb18456_c4749/f1p0/492	R01_cb18456_c4749/f1p0/492	R01_cb18456_c4749/f1p0/492	R01_cb18456_c4749/f1p0/492
R01_cb8160_c2/f1p0/2208	NA	NA	NA	R01_cb8160_c2/f1p0/2208
R01_cb7731_c5/f1p0/1555	NA	NA	NA	R01_cb7731_c5/f1p0/1555
R01_cb8564_c122668/f1p0/2438	NA	R01_cb8564_c122668/f1p0/2438	NA	NA
R01_cb2060_c4/f1p1/3578	NA	NA	NA	R01_cb2060_c4/f1p1/3578
R01_cb4352_c11/f1p0/2279	NA	NA	NA	R01_cb4352_c11/f1p0/2279
R01_cb5547_c0/f3p1/3019	NA	NA	NA	R01_cb5547_c0/f3p1/3019

R01_cb6501_c0/f1p1/2842	NA	R01_cb6501_c0/f1p1/2842	NA	NA
R01_cb3072_c7/f1p0/6010	NA	R01_cb3072_c7/f1p0/6010	NA	NA
R01_cb8564_c44420/f1p0/2853	NA	R01_cb8564_c44420/f1p0/2853	NA	NA
R01_cb3281_c3/f1p0/3678	NA	R01_cb3281_c3/f1p0/3678	NA	NA
R01_cb15574_c2/f1p0/503	NA	NA	R01_cb15574_c2/f1p0/503	R01_cb15574_c2/f1p0/503
R01_cb11801_c0/f1p0/1327	NA	NA	NA	R01_cb11801_c0/f1p0/1327
R01_cb4089_c4/f1p0/3057	NA	NA	NA	R01_cb4089_c4/f1p0/3057
R01_cb11825_c1/f1p0/1962	R01_cb11825_c1/f1p0/1962	R01_cb11825_c1/f1p0/1962	R01_cb11825_c1/f1p0/1962	R01_cb11825_c1/f1p0/1962
R01_cb8564_c36823/f1p0/2430	NA	NA	NA	R01_cb8564_c36823/f1p0/2430
R01_cb9664_c0/f1p0/2005	NA	R01_cb9664_c0/f1p0/2005	R01_cb9664_c0/f1p0/2005	R01_cb9664_c0/f1p0/2005
R01_cb6523_c5/f1p0/2346	NA	NA	NA	R01_cb6523_c5/f1p0/2346
R01_cb2497_c8/f1p0/1832	NA	NA	NA	R01_cb2497_c8/f1p0/1832
R01_cb15018_c0/f7p1/531	NA	R01_cb15018_c0/f7p1/531	R01_cb15018_c0/f7p1/531	R01_cb15018_c0/f7p1/531
R01_cb13087_c0/f5p1/508	NA	NA	NA	R01_cb13087_c0/f5p1/508
R01_cb7514_c0/f1p0/2605	R01_cb7514_c0/f1p0/2605	R01_cb7514_c0/f1p0/2605	R01_cb7514_c0/f1p0/2605	R01_cb7514_c0/f1p0/2605
R01_cb8564_c74169/f1p0/3403	R01_cb8564_c74169/f1p0/3403	R01_cb8564_c74169/f1p0/3403	R01_cb8564_c74169/f1p0/3403	NA
R01_cb8564_c3069/f4p0/2737	NA	R01_cb8564_c3069/f4p0/2737	NA	R01_cb8564_c3069/f4p0/2737
R01_cb8564_c116267/f1p5/3275	NA	R01_cb8564_c116267/f1p5/3275	NA	NA
R01_cb5486_c1/f1p1/2323	R01_cb5486_c1/f1p1/2323	R01_cb5486_c1/f1p1/2323	R01_cb5486_c1/f1p1/2323	R01_cb5486_c1/f1p1/2323
R01_cb13441_c0/f2p0/1589	NA	NA	NA	R01_cb13441_c0/f2p0/1589
R01_cb17637_c3/f1p1/923	NA	R01_cb17637_c3/f1p1/923	NA	NA
R01_cb16190_c0/f2p0/552	NA	R01_cb16190_c0/f2p0/552	NA	NA
R01_cb2710_c12/f1p0/2313	NA	R01_cb2710_c12/f1p0/2313	NA	R01_cb2710_c12/f1p0/2313
R01_cb11736_c1/f1p0/2001	R01_cb11736_c1/f1p0/2001	R01_cb11736_c1/f1p0/2001	R01_cb11736_c1/f1p0/2001	NA

R01_cb10811_c4/f2p0/570	NA	R01_cb10811_c4/f2p0/570	NA	NA
R01_cb8564_c115650/f1p1/2216	NA	R01_cb8564_c115650/f1p1/2216	NA	NA
R01_cb6570_c5/f1p0/793	R01_cb6570_c5/f1p0/793	R01_cb6570_c5/f1p0/793	NA	R01_cb6570_c5/f1p0/793
R01_cb8564_c4502/f1p0/1960	NA	NA	NA	R01_cb8564_c4502/f1p0/1960
R01_cb1772_c25/f1p0/3497	NA	R01_cb1772_c25/f1p0/3497	NA	NA
R01_cb9535_c9/f1p0/2200	NA	R01_cb9535_c9/f1p0/2200	R01_cb9535_c9/f1p0/2200	R01_cb9535_c9/f1p0/2200
R01_cb8564_c44016/f2p0/2743	NA	NA	NA	R01_cb8564_c44016/f2p0/2743
R01_cb8564_c23779/f1p0/3567	R01_cb8564_c23779/f1p0/3567	R01_cb8564_c23779/f1p0/3567	R01_cb8564_c23779/f1p0/3567	R01_cb8564_c23779/f1p0/3567
R01_cb5390_c7/f1p0/1946	NA	R01_cb5390_c7/f1p0/1946	NA	NA
R01_cb2771_c8/f1p0/3405	NA	NA	NA	R01_cb2771_c8/f1p0/3405
R01_cb18456_c2515/f1p0/1051	NA	R01_cb18456_c2515/f1p0/1051	NA	R01_cb18456_c2515/f1p0/1051
R01_cb16980_c1/f3p0/1033	NA	NA	NA	R01_cb16980_c1/f3p0/1033
R01_cb11325_c1/f1p0/3215	NA	R01_cb11325_c1/f1p0/3215	NA	R01_cb11325_c1/f1p0/3215
R01_cb17316_c0/f2p0/665	R01_cb17316_c0/f2p0/665	NA	NA	NA
R01_cb10781_c2/f1p0/2241	NA	R01_cb10781_c2/f1p0/2241	R01_cb10781_c2/f1p0/2241	NA
R01_cb1739_c18/f1p0/768	R01_cb1739_c18/f1p0/768	R01_cb1739_c18/f1p0/768	R01_cb1739_c18/f1p0/768	R01_cb1739_c18/f1p0/768
R01_cb8564_c118827/f2p1/2220	R01_cb8564_c118827/f2p1/2220	R01_cb8564_c118827/f2p1/2220	R01_cb8564_c118827/f2p1/2220	R01_cb8564_c118827/f2p1/2220
R01_cb10292_c1/f5p0/1124	NA	NA	NA	R01_cb10292_c1/f5p0/1124
R01_cb10201_c4/f1p0/2090	NA	R01_cb10201_c4/f1p0/2090	NA	NA
R01_cb6845_c9/f2p1/2314	NA	NA	NA	R01_cb6845_c9/f2p1/2314
R01_cb8564_c87792/f1p0/2774	NA	NA	NA	R01_cb8564_c87792/f1p0/2774
R01_cb14472_c6/f1p0/747	NA	R01_cb14472_c6/f1p0/747	NA	R01_cb14472_c6/f1p0/747
R01_cb13608_c6/f1p0/1530	NA	R01_cb13608_c6/f1p0/1530	NA	NA

R01_cb8564_c90429/f1p0/4285	NA	R01_cb8564_c90429/f1p0/4285	NA	NA
R01_cb2895_c3/f1p0/2477	NA	R01_cb2895_c3/f1p0/2477	NA	NA
R01_cb18132_c16/f1p0/1559	NA	R01_cb18132_c16/f1p0/1559	NA	R01_cb18132_c16/f1p0/1559
R01_cb18456_c7428/f1p0/405	R01_cb18456_c7428/f1p0/405	R01_cb18456_c7428/f1p0/405	R01_cb18456_c7428/f1p0/405	R01_cb18456_c7428/f1p0/405
R01_cb8564_c3996/f1p0/2108	NA	NA	R01_cb8564_c3996/f1p0/2108	R01_cb8564_c3996/f1p0/2108
R01_cb18409_c111/f1p0/1749	R01_cb18409_c111/f1p0/1749	R01_cb18409_c111/f1p0/1749	NA	R01_cb18409_c111/f1p0/1749
R01_cb13613_c2/f1p0/1443	NA	NA	NA	R01_cb13613_c2/f1p0/1443
R01_cb3087_c8/f1p0/3738	R01_cb3087_c8/f1p0/3738	R01_cb3087_c8/f1p0/3738	NA	R01_cb3087_c8/f1p0/3738
R01_cb15845_c0/f3p1/658	NA	R01_cb15845_c0/f3p1/658	NA	NA
R01_cb6894_c5/f1p0/2753	NA	R01_cb6894_c5/f1p0/2753	R01_cb6894_c5/f1p0/2753	NA
R01_cb18456_c5374/f1p5/801	NA	R01_cb18456_c5374/f1p5/801	NA	NA
R01_cb10074_c15/f1p4/662	R01_cb10074_c15/f1p4/662	R01_cb10074_c15/f1p4/662	R01_cb10074_c15/f1p4/662	R01_cb10074_c15/f1p4/662
R01_cb4744_c8/f1p0/1289	R01_cb4744_c8/f1p0/1289	R01_cb4744_c8/f1p0/1289	R01_cb4744_c8/f1p0/1289	R01_cb4744_c8/f1p0/1289
R01_cb2399_c12/f1p0/2076	NA	R01_cb2399_c12/f1p0/2076	R01_cb2399_c12/f1p0/2076	R01_cb2399_c12/f1p0/2076
R01_cb261_c17/f1p1/3635	NA	NA	NA	R01_cb261_c17/f1p1/3635
R01_cb13760_c1/f2p0/1489	NA	NA	NA	R01_cb13760_c1/f2p0/1489
R01_cb18456_c6831/f1p0/722	NA	R01_cb18456_c6831/f1p0/722	NA	NA
R01_cb8564_c127279/f1p0/2052	NA	NA	NA	R01_cb8564_c127279/f1p0/2052
R01_cb18456_c7680/f1p0/485	R01_cb18456_c7680/f1p0/485	R01_cb18456_c7680/f1p0/485	NA	R01_cb18456_c7680/f1p0/485
R01_cb5475_c8/f1p1/3007	NA	R01_cb5475_c8/f1p1/3007	NA	NA
R01_cb16527_c1/f1p0/1263	NA	R01_cb16527_c1/f1p0/1263	NA	NA
R01_cb15680_c11/f1p0/493	R01_cb15680_c11/f1p0/493	R01_cb15680_c11/f1p0/493	R01_cb15680_c11/f1p0/493	R01_cb15680_c11/f1p0/493
R01_cb15522_c1/f1p0/1010	NA	NA	NA	R01_cb15522_c1/f1p0/1010
R01_cb18409_c66/f1p0/351	R01_cb18409_c66/f1p0/351	R01_cb18409_c66/f1p0/351	R01_cb18409_c66/f1p0/351	R01_cb18409_c66/f1p0/351
R01_cb4268_c0/f2p0/3087	NA	NA	NA	R01_cb4268_c0/f2p0/3087

R01_cb18065_c0/f1p0/1830	NA	R01_cb18065_c0/f1p0/1830	R01_cb18065_c0/f1p0/1830	NA
R01_cb12450_c0/f2p0/796	R01_cb12450_c0/f2p0/796	R01_cb12450_c0/f2p0/796	R01_cb12450_c0/f2p0/796	R01_cb12450_c0/f2p0/796
R01_cb3518_c7/f1p0/3655	NA	NA	NA	R01_cb3518_c7/f1p0/3655
R01_cb8564_c4732/f3p0/3755	NA	NA	NA	R01_cb8564_c4732/f3p0/3755
R01_cb10453_c2/f2p0/1102	NA	NA	R01_cb10453_c2/f2p0/1102	R01_cb10453_c2/f2p0/1102
R01_cb4945_c4/f1p0/2080	NA	R01_cb4945_c4/f1p0/2080	NA	NA
R01_cb18456_c1294/f9p0/374	NA	R01_cb18456_c1294/f9p0/374	R01_cb18456_c1294/f9p0/374	R01_cb18456_c1294/f9p0/374
R01_cb8564_c52554/f1p3/3826	NA	NA	NA	R01_cb8564_c52554/f1p3/3826
R01_cb8478_c19/f1p0/2531	R01_cb8478_c19/f1p0/2531	R01_cb8478_c19/f1p0/2531	NA	NA
R01_cb18456_c3840/f1p0/935	NA	R01_cb18456_c3840/f1p0/935	NA	NA
R01_cb18443_c1/f1p0/352	NA	R01_cb18443_c1/f1p0/352	NA	R01_cb18443_c1/f1p0/352
R01_cb11787_c1/f1p0/3167	NA	R01_cb11787_c1/f1p0/3167	NA	NA
R01_cb3124_c5/f1p0/968	NA	R01_cb3124_c5/f1p0/968	NA	R01_cb3124_c5/f1p0/968
R01_cb3388_c11/f1p0/3143	NA	NA	NA	R01_cb3388_c11/f1p0/3143
R01_cb4117_c3/f1p0/3377	NA	NA	NA	R01_cb4117_c3/f1p0/3377
R01_cb18409_c89/f1p0/396	R01_cb18409_c89/f1p0/396	R01_cb18409_c89/f1p0/396	R01_cb18409_c89/f1p0/396	R01_cb18409_c89/f1p0/396
R01_cb3132_c5/f1p0/1825	NA	NA	NA	R01_cb3132_c5/f1p0/1825
R01_cb13910_c25/f11p1/787	NA	R01_cb13910_c25/f11p1/787	NA	NA
R01_cb13463_c13/f1p1/1233	NA	NA	NA	R01_cb13463_c13/f1p1/1233
R01_cb9592_c4/f1p1/1927	NA	NA	NA	R01_cb9592_c4/f1p1/1927
R01_cb15724_c1/f2p0/1155	NA	R01_cb15724_c1/f2p0/1155	R01_cb15724_c1/f2p0/1155	R01_cb15724_c1/f2p0/1155
R01_cb3104_c1/f1p0/3078	NA	NA	R01_cb3104_c1/f1p0/3078	R01_cb3104_c1/f1p0/3078
R01_cb11222_c1/f1p0/2234	R01_cb11222_c1/f1p0/2234	R01_cb11222_c1/f1p0/2234	NA	R01_cb11222_c1/f1p0/2234
R01_cb10029_c1141/f1p0/893	NA	R01_cb10029_c1141/f1p0/893	NA	NA
R01_cb5234_c2/f1p0/3147	NA	NA	NA	R01_cb5234_c2/f1p0/3147
R01_cb17329_c0/f2p0/1184	NA	NA	NA	R01_cb17329_c0/f2p0/1184

R01_cb8564_c115669/f1p0/2356	NA	R01_cb8564_c115669/f1p0/2356	NA	NA
R01_cb14576_c0/f1p0/918	NA	NA	R01_cb14576_c0/f1p0/918	R01_cb14576_c0/f1p0/918
R01_cb17398_c1/f1p0/918	NA	R01_cb17398_c1/f1p0/918	R01_cb17398_c1/f1p0/918	R01_cb17398_c1/f1p0/918
R01_cb3997_c1/f1p0/3471	NA	NA	NA	R01_cb3997_c1/f1p0/3471
R01_cb17756_c22/f1p0/1432	R01_cb17756_c22/f1p0/1432	R01_cb17756_c22/f1p0/1432	R01_cb17756_c22/f1p0/1432	NA
R01_cb16310_c0/f1p0/1502	NA	R01_cb16310_c0/f1p0/1502	R01_cb16310_c0/f1p0/1502	NA
R01_cb693_c2/f1p0/4679	NA	NA	NA	R01_cb693_c2/f1p0/4679
R01_cb15574_c3/f1p0/554	NA	NA	R01_cb15574_c3/f1p0/554	R01_cb15574_c3/f1p0/554
R01_cb8564_c37539/f1p0/2105	NA	R01_cb8564_c37539/f1p0/2105	NA	NA
R01_cb3997_c4/f1p0/1734	NA	NA	NA	R01_cb3997_c4/f1p0/1734
R01_cb11081_c8/f3p1/698	NA	R01_cb11081_c8/f3p1/698	R01_cb11081_c8/f3p1/698	R01_cb11081_c8/f3p1/698
R01_cb16070_c1/f1p0/964	NA	R01_cb16070_c1/f1p0/964	R01_cb16070_c1/f1p0/964	R01_cb16070_c1/f1p0/964
R01_cb18132_c2/f1p0/1057	R01_cb18132_c2/f1p0/1057	R01_cb18132_c2/f1p0/1057	R01_cb18132_c2/f1p0/1057	R01_cb18132_c2/f1p0/1057
R01_cb8564_c46895/f1p0/2426	NA	R01_cb8564_c46895/f1p0/2426	NA	NA
R01_cb12932_c1/f1p0/868	NA	R01_cb12932_c1/f1p0/868	NA	NA
R01_cb8564_c81886/f1p0/2814	R01_cb8564_c81886/f1p0/2814	R01_cb8564_c81886/f1p0/2814	R01_cb8564_c81886/f1p0/2814	R01_cb8564_c81886/f1p0/2814
R01_cb2771_c15/f1p0/2825	NA	NA	NA	R01_cb2771_c15/f1p0/2825
R01_cb8222_c0/f3p0/2427	R01_cb8222_c0/f3p0/2427	NA	R01_cb8222_c0/f3p0/2427	R01_cb8222_c0/f3p0/2427
R01_cb1388_c27/f1p0/2564	NA	R01_cb1388_c27/f1p0/2564	R01_cb1388_c27/f1p0/2564	NA
R01_cb8564_c118142/f1p0/2095	NA	R01_cb8564_c118142/f1p0/2095	NA	R01_cb8564_c118142/f1p0/2095
R01_cb5007_c11/f1p0/454	R01_cb5007_c11/f1p0/454	R01_cb5007_c11/f1p0/454	NA	NA
R01_cb8564_c128937/f1p0/2105	NA	NA	R01_cb8564_c128937/f1p0/2105	R01_cb8564_c128937/f1p0/2105

R01_cb13449_c0/f1p1/655	NA	NA	NA	R01_cb13449_c0/f1p1/655
R01_cb17375_c0/f1p0/1561	NA	R01_cb17375_c0/f1p0/1561	NA	NA
R01_cb11234_c2/f1p0/1079	NA	R01_cb11234_c2/f1p0/1079	NA	NA
R01_cb6623_c11/f1p0/4586	NA	R01_cb6623_c11/f1p0/4586	NA	NA
R01_cb2154_c4/f2p1/1756	R01_cb2154_c4/f2p1/1756	R01_cb2154_c4/f2p1/1756	NA	NA
R01_cb11865_c3/f1p0/1184	NA	NA	NA	R01_cb11865_c3/f1p0/1184
R01_cb4254_c2/f1p0/3624	R01_cb4254_c2/f1p0/3624	R01_cb4254_c2/f1p0/3624	NA	NA
R01_cb11291_c1/f1p0/1923	R01_cb11291_c1/f1p0/1923	R01_cb11291_c1/f1p0/1923	R01_cb11291_c1/f1p0/1923	R01_cb11291_c1/f1p0/1923
R01_cb11538_c2/f1p0/463	NA	R01_cb11538_c2/f1p0/463	NA	R01_cb11538_c2/f1p0/463
R01_cb8564_c90015/f1p0/3817	NA	R01_cb8564_c90015/f1p0/3817	NA	NA
R01_cb6602_c46/f1p0/3277	NA	NA	NA	R01_cb6602_c46/f1p0/3277
R01_cb8564_c11196/f5p0/2303	NA	R01_cb8564_c11196/f5p0/2303	NA	NA
R01_cb8564_c87199/f1p0/3286	NA	R01_cb8564_c87199/f1p0/3286	NA	NA
R01_cb15904_c0/f1p0/458	NA	R01_cb15904_c0/f1p0/458	R01_cb15904_c0/f1p0/458	R01_cb15904_c0/f1p0/458
R01_cb10070_c4/f4p0/470	R01_cb10070_c4/f4p0/470	R01_cb10070_c4/f4p0/470	NA	NA
R01_cb9455_c1/f1p0/2626	NA	R01_cb9455_c1/f1p0/2626	NA	NA
R01_cb13583_c4/f1p0/1289	NA	NA	NA	R01_cb13583_c4/f1p0/1289
R01_cb9855_c6/f1p0/2887	NA	R01_cb9855_c6/f1p0/2887	NA	R01_cb9855_c6/f1p0/2887
R01_cb6406_c37/f1p0/3004	NA	R01_cb6406_c37/f1p0/3004	NA	NA
R01_cb16993_c1/f1p0/1410	NA	NA	R01_cb16993_c1/f1p0/1410	NA
R01_cb10782_c3/f1p0/962	NA	R01_cb10782_c3/f1p0/962	R01_cb10782_c3/f1p0/962	R01_cb10782_c3/f1p0/962
R01_cb16520_c0/f4p0/1010	NA	NA	NA	R01_cb16520_c0/f4p0/1010
R01_cb12420_c0/f3p0/1198	NA	NA	NA	R01_cb12420_c0/f3p0/1198
R01_cb4248_c4/f1p0/1483	NA	R01_cb4248_c4/f1p0/1483	NA	NA
R01_cb534_c1/f1p0/1898	NA	R01_cb534_c1/f1p0/1898	NA	NA
R01_cb8564_c35960/f3p0/3902	R01_cb8564_c35960/f3p0/390	R01_cb8564_c35960/f3p0/3902	R01_cb8564_c35960/f3p0/3902	R01_cb8564_c35960/f3p0/3902

	2			
R01_cb8564_c25643/f1p0/2449	NA	R01_cb8564_c25643/f1p0/2449	NA	NA
R01_cb13693_c6/f1p0/655	R01_cb13693_c6/f1p0/655	R01_cb13693_c6/f1p0/655	R01_cb13693_c6/f1p0/655	NA
R01_cb10074_c20/f28p2/568	R01_cb10074_c20/f28p2/568	R01_cb10074_c20/f28p2/568	R01_cb10074_c20/f28p2/568	R01_cb10074_c20/f28p2/568
R01_cb10903_c6/f1p0/624	NA	R01_cb10903_c6/f1p0/624	NA	R01_cb10903_c6/f1p0/624
R01_cb8564_c18174/f1p0/2275	R01_cb8564_c18174/f1p0/2275	R01_cb8564_c18174/f1p0/2275	NA	NA
R01_cb15294_c0/f2p0/1330	NA	R01_cb15294_c0/f2p0/1330	R01_cb15294_c0/f2p0/1330	R01_cb15294_c0/f2p0/1330
R01_cb10873_c2/f1p0/1388	NA	NA	NA	R01_cb10873_c2/f1p0/1388
R01_cb11417_c1/f1p0/2745	NA	R01_cb11417_c1/f1p0/2745	NA	NA
R01_cb4989_c12/f2p0/468	R01_cb4989_c12/f2p0/468	R01_cb4989_c12/f2p0/468	R01_cb4989_c12/f2p0/468	NA
R01_cb8564_c69121/f2p0/1984	NA	R01_cb8564_c69121/f2p0/1984	R01_cb8564_c69121/f2p0/1984	R01_cb8564_c69121/f2p0/1984
R01_cb5220_c3/f1p0/3151	R01_cb5220_c3/f1p0/3151	R01_cb5220_c3/f1p0/3151	R01_cb5220_c3/f1p0/3151	R01_cb5220_c3/f1p0/3151
R01_cb3471_c7/f1p0/1188	NA	NA	NA	R01_cb3471_c7/f1p0/1188
R01_cb8564_c2878/f1p0/2627	NA	NA	R01_cb8564_c2878/f1p0/2627	NA
R01_cb10076_c8/f1p0/1357	NA	R01_cb10076_c8/f1p0/1357	NA	NA
R01_cb8564_c113229/f1p0/2279	NA	R01_cb8564_c113229/f1p0/2279	R01_cb8564_c113229/f1p0/2279	R01_cb8564_c113229/f1p0/2279
R01_cb12223_c0/f2p0/699	R01_cb12223_c0/f2p0/699	R01_cb12223_c0/f2p0/699	R01_cb12223_c0/f2p0/699	R01_cb12223_c0/f2p0/699
R01_cb10648_c5/f1p1/798	NA	R01_cb10648_c5/f1p1/798	NA	NA
R01_cb10851_c4/f1p1/759	R01_cb10851_c4/f1p1/759	NA	R01_cb10851_c4/f1p1/759	R01_cb10851_c4/f1p1/759
R01_cb14627_c2/f2p0/735	NA	NA	R01_cb14627_c2/f2p0/735	R01_cb14627_c2/f2p0/735
R01_cb13422_c1/f1p0/918	NA	R01_cb13422_c1/f1p0/918	R01_cb13422_c1/f1p0/918	R01_cb13422_c1/f1p0/918
R01_cb8564_c14045/f1p0/3389	NA	R01_cb8564_c14045/f1p0/3389	NA	NA
R01_cb11072_c3/f3p0/468	R01_cb11072_c3/f3p0/468	R01_cb11072_c3/f3p0/468	NA	NA
R01_cb14648_c5/f1p0/584	NA	R01_cb14648_c5/f1p0/584	NA	NA

R01_cb10017_c138/f1p0/867	NA	R01_cb10017_c138/f1p0/867	NA	NA
R01_cb5943_c3/f1p0/2903	NA	NA	NA	R01_cb5943_c3/f1p0/2903
R01_cb8564_c1649/f1p0/2395	NA	R01_cb8564_c1649/f1p0/2395	NA	NA
R01_cb10269_c4/f1p0/2253	NA	R01_cb10269_c4/f1p0/2253	NA	NA
R01_cb8294_c1/f1p0/2409	NA	R01_cb8294_c1/f1p0/2409	NA	NA
R01_cb499_c90/f1p2/1505	NA	R01_cb499_c90/f1p2/1505	NA	NA
R01_cb8564_c20527/f1p0/2861	NA	R01_cb8564_c20527/f1p0/2861	R01_cb8564_c20527/f1p0/2861	R01_cb8564_c20527/f1p0/2861
R01_cb4529_c8/f1p0/838	NA	R01_cb4529_c8/f1p0/838	NA	NA
R01_cb8564_c77303/f1p0/1949	NA	NA	NA	R01_cb8564_c77303/f1p0/1949
R01_cb4925_c4/f2p0/3222	NA	NA	NA	R01_cb4925_c4/f2p0/3222
R01_cb8564_c84562/f2p0/4125	NA	NA	NA	R01_cb8564_c84562/f2p0/4125
R01_cb1847_c1/f1p0/4156	NA	NA	NA	R01_cb1847_c1/f1p0/4156
R01_cb18456_c6689/f1p0/903	NA	R01_cb18456_c6689/f1p0/903	NA	NA
R01_cb18409_c63/f1p0/1703	NA	R01_cb18409_c63/f1p0/1703	R01_cb18409_c63/f1p0/1703	R01_cb18409_c63/f1p0/1703
R01_cb13057_c2/f2p0/377	R01_cb13057_c2/f2p0/377	R01_cb13057_c2/f2p0/377	R01_cb13057_c2/f2p0/377	R01_cb13057_c2/f2p0/377
R01_cb1378_c12/f1p0/805	R01_cb1378_c12/f1p0/805	R01_cb1378_c12/f1p0/805	R01_cb1378_c12/f1p0/805	R01_cb1378_c12/f1p0/805
R01_cb11629_c4/f1p0/1480	NA	NA	NA	R01_cb11629_c4/f1p0/1480
R01_cb8084_c9/f1p0/1149	NA	NA	NA	R01_cb8084_c9/f1p0/1149
R01_cb4637_c10/f1p0/736	R01_cb4637_c10/f1p0/736	R01_cb4637_c10/f1p0/736	NA	NA
R01_cb16888_c11/f4p0/886	NA	NA	R01_cb16888_c11/f4p0/886	R01_cb16888_c11/f4p0/886
R01_cb4917_c3/f1p0/3806	NA	NA	NA	R01_cb4917_c3/f1p0/3806
R01_cb18456_c5011/f1p0/862	R01_cb18456_c5011/f1p0/862	R01_cb18456_c5011/f1p0/862	NA	R01_cb18456_c5011/f1p0/862
R01_cb16005_c3/f1p0/765	NA	R01_cb16005_c3/f1p0/765	NA	NA
R01_cb12714_c19/f1p0/1877	NA	R01_cb12714_c19/f1p0/1877	NA	NA
R01_cb8564_c24430/f1p1/2543	NA	R01_cb8564_c24430/f1p1/2543	NA	NA
R01_cb17655_c2/f2p0/595	R01_cb17655_c2/f2p0/595	R01_cb17655_c2/f2p0/595	R01_cb17655_c2/f2p0/595	R01_cb17655_c2/f2p0/595

R01_cb14283_c6/f1p0/741	NA	R01_cb14283_c6/f1p0/741	R01_cb14283_c6/f1p0/741	R01_cb14283_c6/f1p0/741
R01_cb8486_c6/f1p0/1775	NA	R01_cb8486_c6/f1p0/1775	NA	NA
R01_cb18237_c0/f1p0/1098	NA	NA	NA	R01_cb18237_c0/f1p0/1098
R01_cb11704_c0/f1p0/1063	NA	R01_cb11704_c0/f1p0/1063	NA	NA
R01_cb225_c1/f1p0/4907	NA	NA	NA	R01_cb225_c1/f1p0/4907
R01_cb1568_c8/f1p0/1980	NA	NA	NA	R01_cb1568_c8/f1p0/1980
R01_cb6069_c5/f1p0/2802	NA	NA	NA	R01_cb6069_c5/f1p0/2802
R01_cb6211_c55/f1p0/2825	NA	R01_cb6211_c55/f1p0/2825	NA	NA
R01_cb10118_c4/f1p0/693	NA	R01_cb10118_c4/f1p0/693	NA	NA
R01_cb9465_c1/f1p1/2022	NA	R01_cb9465_c1/f1p1/2022	NA	NA
R01_cb11397_c2/f1p1/596	NA	R01_cb11397_c2/f1p1/596	NA	NA
R01_cb18456_c7647/f1p1/861	NA	R01_cb18456_c7647/f1p1/861	NA	NA
R01_cb3838_c17/f1p0/2585	NA	R01_cb3838_c17/f1p0/2585	NA	NA
R01_cb16303_c5/f1p0/308	R01_cb16303_c5/f1p0/308	R01_cb16303_c5/f1p0/308	R01_cb16303_c5/f1p0/308	R01_cb16303_c5/f1p0/308
R01_cb8564_c20604/f1p0/4741	NA	R01_cb8564_c20604/f1p0/4741	R01_cb8564_c20604/f1p0/4741	R01_cb8564_c20604/f1p0/4741
R01_cb8564_c89072/f1p0/1906	NA	NA	NA	R01_cb8564_c89072/f1p0/1906
R01_cb8564_c79156/f1p0/3394	R01_cb8564_c79156/f1p0/3394	R01_cb8564_c79156/f1p0/3394	R01_cb8564_c79156/f1p0/3394	R01_cb8564_c79156/f1p0/3394
R01_cb10293_c12/f1p0/1815	R01_cb10293_c12/f1p0/1815	R01_cb10293_c12/f1p0/1815	R01_cb10293_c12/f1p0/1815	R01_cb10293_c12/f1p0/1815
R01_cb8564_c15535/f1p0/1921	NA	R01_cb8564_c15535/f1p0/1921	NA	NA
R01_cb15911_c0/f1p0/1558	NA	R01_cb15911_c0/f1p0/1558	NA	R01_cb15911_c0/f1p0/1558
R01_cb18370_c0/f1p0/668	NA	NA	R01_cb18370_c0/f1p0/668	R01_cb18370_c0/f1p0/668
R01_cb8234_c13/f1p0/2314	NA	NA	NA	R01_cb8234_c13/f1p0/2314
R01_cb15353_c3/f1p0/1129	NA	NA	NA	R01_cb15353_c3/f1p0/1129
R01_cb8564_c3724/f1p0/2996	R01_cb8564_c3724/f1p0/2996	R01_cb8564_c3724/f1p0/2996	R01_cb8564_c3724/f1p0/2996	NA
R01_cb11204_c3/f1p0/889	NA	R01_cb11204_c3/f1p0/889	NA	R01_cb11204_c3/f1p0/889

R01_cb8564_c3745/f1p0/3841	NA	R01_cb8564_c3745/f1p0/3841	R01_cb8564_c3745/f1p0/3841	R01_cb8564_c3745/f1p0/3841
R01_cb8564_c78656/f1p0/2329	R01_cb8564_c78656/f1p0/2329	R01_cb8564_c78656/f1p0/2329	R01_cb8564_c78656/f1p0/2329	R01_cb8564_c78656/f1p0/2329
R01_cb7875_c1/f1p0/2030	NA	NA	NA	R01_cb7875_c1/f1p0/2030
R01_cb8564_c162316/f1p1/2224	NA	R01_cb8564_c162316/f1p1/2224	NA	NA
R01_cb2985_c4/f1p0/3241	NA	NA	NA	R01_cb2985_c4/f1p0/3241
R01_cb1993_c8/f1p1/3209	NA	NA	NA	R01_cb1993_c8/f1p1/3209
R01_cb6158_c2/f1p0/1006	NA	NA	NA	R01_cb6158_c2/f1p0/1006
R01_cb8478_c1/f6p0/2460	NA	NA	NA	R01_cb8478_c1/f6p0/2460
R01_cb8564_c1110/f1p0/2070	NA	NA	NA	R01_cb8564_c1110/f1p0/2070
R01_cb11868_c0/f3p0/349	NA	R01_cb11868_c0/f3p0/349	R01_cb11868_c0/f3p0/349	R01_cb11868_c0/f3p0/349
R01_cb17973_c54/f1p0/550	R01_cb17973_c54/f1p0/550	R01_cb17973_c54/f1p0/550	R01_cb17973_c54/f1p0/550	R01_cb17973_c54/f1p0/550
R01_cb6570_c2/f1p0/2836	NA	R01_cb6570_c2/f1p0/2836	R01_cb6570_c2/f1p0/2836	R01_cb6570_c2/f1p0/2836
R01_cb10273_c2/f1p1/2244	NA	R01_cb10273_c2/f1p1/2244	NA	NA
R01_cb4504_c0/f8p0/3277	NA	NA	NA	R01_cb4504_c0/f8p0/3277
R01_cb8564_c19092/f1p0/2838	NA	R01_cb8564_c19092/f1p0/2838	NA	NA
R01_cb12301_c133/f1p0/937	NA	R01_cb12301_c133/f1p0/937	NA	NA
R01_cb17131_c0/f3p0/545	NA	R01_cb17131_c0/f3p0/545	NA	NA
R01_cb7103_c2/f2p0/2787	R01_cb7103_c2/f2p0/2787	R01_cb7103_c2/f2p0/2787	NA	R01_cb7103_c2/f2p0/2787
R01_cb15053_c7/f1p0/724	NA	R01_cb15053_c7/f1p0/724	R01_cb15053_c7/f1p0/724	NA
R01_cb11308_c0/f1p0/955	NA	R01_cb11308_c0/f1p0/955	NA	R01_cb11308_c0/f1p0/955
R01_cb17678_c0/f1p0/431	NA	R01_cb17678_c0/f1p0/431	R01_cb17678_c0/f1p0/431	R01_cb17678_c0/f1p0/431
R01_cb4490_c18/f1p0/2440	NA	NA	NA	R01_cb4490_c18/f1p0/2440
R01_cb8564_c99051/f1p7/4137	NA	R01_cb8564_c99051/f1p7/4137	R01_cb8564_c99051/f1p7/4137	R01_cb8564_c99051/f1p7/4137
R01_cb680_c1/f1p0/4121	NA	R01_cb680_c1/f1p0/4121	NA	NA

R01_cb8564_c13973/f1p0/2791	NA	R01_cb8564_c13973/f1p0/2791	R01_cb8564_c13973/f1p0/2791	R01_cb8564_c13973/f1p0/2791
R01_cb11912_c1/f1p0/1837	NA	NA	NA	R01_cb11912_c1/f1p0/1837
R01_cb8564_c89948/f1p0/2252	NA	NA	NA	R01_cb8564_c89948/f1p0/2252
R01_cb8564_c24444/f1p0/4497	NA	NA	NA	R01_cb8564_c24444/f1p0/4497
R01_cb10755_c3/f1p0/2369	R01_cb10755_c3/f1p0/2369	R01_cb10755_c3/f1p0/2369	NA	NA
R01_cb4489_c12/f1p0/1870	NA	R01_cb4489_c12/f1p0/1870	NA	R01_cb4489_c12/f1p0/1870
R01_cb15805_c2/f1p0/632	NA	NA	NA	R01_cb15805_c2/f1p0/632
R01_cb8564_c21412/f1p1/3294	NA	R01_cb8564_c21412/f1p1/3294	NA	NA
R01_cb3438_c28/f1p0/2170	R01_cb3438_c28/f1p0/2170	R01_cb3438_c28/f1p0/2170	R01_cb3438_c28/f1p0/2170	R01_cb3438_c28/f1p0/2170
R01_cb4953_c11/f1p0/444	R01_cb4953_c11/f1p0/444	R01_cb4953_c11/f1p0/444	NA	R01_cb4953_c11/f1p0/444
R01_cb16858_c0/f1p0/762	NA	R01_cb16858_c0/f1p0/762	NA	R01_cb16858_c0/f1p0/762
R01_cb10881_c2/f1p0/2250	NA	R01_cb10881_c2/f1p0/2250	R01_cb10881_c2/f1p0/2250	R01_cb10881_c2/f1p0/2250
R01_cb17493_c0/f1p0/1641	R01_cb17493_c0/f1p0/1641	NA	NA	NA
R01_cb11081_c7/f1p0/550	NA	R01_cb11081_c7/f1p0/550	R01_cb11081_c7/f1p0/550	R01_cb11081_c7/f1p0/550
R01_cb7945_c4/f1p0/1053	NA	R01_cb7945_c4/f1p0/1053	NA	R01_cb7945_c4/f1p0/1053
R01_cb16250_c1/f1p0/430	R01_cb16250_c1/f1p0/430	R01_cb16250_c1/f1p0/430	R01_cb16250_c1/f1p0/430	R01_cb16250_c1/f1p0/430
R01_cb4321_c3/f1p0/2323	NA	R01_cb4321_c3/f1p0/2323	NA	NA
R01_cb18456_c5106/f1p1/856	NA	R01_cb18456_c5106/f1p1/856	NA	NA
R01_cb11065_c4/f1p0/1358	NA	NA	NA	R01_cb11065_c4/f1p0/1358
R01_cb9472_c0/f1p0/2145	NA	NA	NA	R01_cb9472_c0/f1p0/2145
R01_cb2936_c8/f1p0/2509	NA	R01_cb2936_c8/f1p0/2509	NA	NA
R01_cb18287_c7/f1p0/408	R01_cb18287_c7/f1p0/408	R01_cb18287_c7/f1p0/408	R01_cb18287_c7/f1p0/408	R01_cb18287_c7/f1p0/408
R01_cb10646_c0/f1p0/998	NA	NA	R01_cb10646_c0/f1p0/998	R01_cb10646_c0/f1p0/998
R01_cb16552_c3/f1p0/1286	NA	NA	NA	R01_cb16552_c3/f1p0/1286
R01_cb11212_c0/f15p0/720	NA	R01_cb11212_c0/f15p0/720	NA	NA
R01_cb735_c1/f1p1/4165	NA	NA	NA	R01_cb735_c1/f1p1/4165

R01_cb8564_c22083/f1p0/4374	NA	NA	NA	R01_cb8564_c22083/f1p0/4374
R01_cb10064_c3/f1p0/1626	R01_cb10064_c3/f1p0/1626	R01_cb10064_c3/f1p0/1626	R01_cb10064_c3/f1p0/1626	R01_cb10064_c3/f1p0/1626
R01_cb8564_c117585/f1p0/2391	NA	R01_cb8564_c117585/f1p0/2391	NA	R01_cb8564_c117585/f1p0/2391
R01_cb7537_c8/f1p0/2129	NA	NA	NA	R01_cb7537_c8/f1p0/2129
R01_cb5471_c7/f1p0/3091	NA	NA	NA	R01_cb5471_c7/f1p0/3091
R01_cb2027_c8/f1p0/3051	NA	NA	R01_cb2027_c8/f1p0/3051	NA
R01_cb1523_c13/f1p0/3636	R01_cb1523_c13/f1p0/3636	R01_cb1523_c13/f1p0/3636	R01_cb1523_c13/f1p0/3636	R01_cb1523_c13/f1p0/3636
R01_cb538_c7/f1p0/3352	NA	R01_cb538_c7/f1p0/3352	NA	NA
R01_cb17997_c9/f1p0/1018	NA	NA	NA	R01_cb17997_c9/f1p0/1018
R01_cb8564_c77748/f1p0/4086	NA	R01_cb8564_c77748/f1p0/4086	NA	NA
R01_cb17384_c2/f1p0/1560	NA	NA	NA	R01_cb17384_c2/f1p0/1560
R01_cb18456_c5923/f1p0/419	R01_cb18456_c5923/f1p0/419	R01_cb18456_c5923/f1p0/419	R01_cb18456_c5923/f1p0/419	R01_cb18456_c5923/f1p0/419
R01_cb17635_c1/f1p0/526	R01_cb17635_c1/f1p0/526	R01_cb17635_c1/f1p0/526	R01_cb17635_c1/f1p0/526	R01_cb17635_c1/f1p0/526
R01_cb11869_c11/f1p1/840	R01_cb11869_c11/f1p1/840	R01_cb11869_c11/f1p1/840	NA	NA
R01_cb6377_c1/f1p0/2545	NA	NA	NA	R01_cb6377_c1/f1p0/2545
R01_cb3558_c11/f1p0/2820	NA	NA	NA	R01_cb3558_c11/f1p0/2820
R01_cb8564_c3281/f1p0/2701	NA	R01_cb8564_c3281/f1p0/2701	NA	NA
R01_cb18456_c7347/f1p0/605	R01_cb18456_c7347/f1p0/605	R01_cb18456_c7347/f1p0/605	R01_cb18456_c7347/f1p0/605	R01_cb18456_c7347/f1p0/605
R01_cb9485_c1/f1p0/2070	NA	R01_cb9485_c1/f1p0/2070	NA	R01_cb9485_c1/f1p0/2070
R01_cb10029_c1108/f1p3/887	NA	R01_cb10029_c1108/f1p3/887	NA	NA
R01_cb3426_c17/f1p0/3178	NA	NA	NA	R01_cb3426_c17/f1p0/3178
R01_cb8085_c14/f1p0/2012	NA	R01_cb8085_c14/f1p0/2012	NA	NA
R01_cb18587_c0/f1p0/1391	NA	R01_cb18587_c0/f1p0/1391	NA	R01_cb18587_c0/f1p0/1391
R01_cb8564_c20383/f1p1/2928	NA	NA	NA	R01_cb8564_c20383/f1p1/2928
R01_cb5324_c3/f1p0/820	NA	R01_cb5324_c3/f1p0/820	R01_cb5324_c3/f1p0/820	R01_cb5324_c3/f1p0/820

R01_cb15714_c0/f6p0/560	NA	R01_cb15714_c0/f6p0/560	NA	NA
R01_cb17084_c4/f1p0/716	R01_cb17084_c4/f1p0/716	R01_cb17084_c4/f1p0/716	NA	NA
R01_cb7027_c0/f1p0/2718	R01_cb7027_c0/f1p0/2718	R01_cb7027_c0/f1p0/2718	R01_cb7027_c0/f1p0/2718	R01_cb7027_c0/f1p0/2718
R01_cb16981_c12/f1p0/835	NA	NA	NA	R01_cb16981_c12/f1p0/835
R01_cb1437_c0/f2p1/3930	NA	NA	NA	R01_cb1437_c0/f2p1/3930
R01_cb8564_c20780/f1p0/3653	NA	R01_cb8564_c20780/f1p0/3653	NA	NA
R01_cb3939_c20/f1p0/2304	NA	R01_cb3939_c20/f1p0/2304	NA	R01_cb3939_c20/f1p0/2304
R01_cb13318_c3/f1p0/1520	NA	NA	NA	R01_cb13318_c3/f1p0/1520
R01_cb8564_c52855/f1p0/3194	NA	NA	R01_cb8564_c52855/f1p0/3194	NA
R01_cb10024_c418/f1p0/638	NA	R01_cb10024_c418/f1p0/638	R01_cb10024_c418/f1p0/638	NA
R01_cb18433_c0/f1p0/553	R01_cb18433_c0/f1p0/553	R01_cb18433_c0/f1p0/553	NA	R01_cb18433_c0/f1p0/553
R01_cb11816_c0/f1p0/1374	NA	NA	NA	R01_cb11816_c0/f1p0/1374
R01_cb18372_c8/f1p0/2774	NA	R01_cb18372_c8/f1p0/2774	NA	NA
R01_cb4799_c6/f1p2/2506	NA	R01_cb4799_c6/f1p2/2506	NA	NA
R01_cb11723_c4/f1p0/3330	NA	NA	NA	R01_cb11723_c4/f1p0/3330
R01_cb8564_c2130/f1p0/2044	NA	R01_cb8564_c2130/f1p0/2044	NA	NA
R01_cb17579_c0/f2p0/542	NA	R01_cb17579_c0/f2p0/542	R01_cb17579_c0/f2p0/542	R01_cb17579_c0/f2p0/542
R01_cb11815_c0/f1p0/506	NA	R01_cb11815_c0/f1p0/506	NA	R01_cb11815_c0/f1p0/506
R01_cb15951_c8/f1p0/653	NA	R01_cb15951_c8/f1p0/653	R01_cb15951_c8/f1p0/653	R01_cb15951_c8/f1p0/653
R01_cb15287_c1/f1p0/459	NA	R01_cb15287_c1/f1p0/459	NA	R01_cb15287_c1/f1p0/459
R01_cb8970_c0/f1p0/2222	R01_cb8970_c0/f1p0/2222	R01_cb8970_c0/f1p0/2222	R01_cb8970_c0/f1p0/2222	R01_cb8970_c0/f1p0/2222
R01_cb11353_c0/f2p0/814	NA	R01_cb11353_c0/f2p0/814	NA	R01_cb11353_c0/f2p0/814
R01_cb1860_c12/f1p3/2509	NA	NA	NA	R01_cb1860_c12/f1p3/2509
R01_cb7014_c10/f1p0/639	NA	R01_cb7014_c10/f1p0/639	NA	R01_cb7014_c10/f1p0/639
R01_cb10810_c0/f2p1/815	NA	NA	NA	R01_cb10810_c0/f2p1/815
R01_cb8564_c69375/f2p0/2764	NA	R01_cb8564_c69375/f2p0/2764	NA	R01_cb8564_c69375/f2p0/2764

R01_cb4465_c3/f1p0/2359	NA	R01_cb4465_c3/f1p0/2359	NA	R01_cb4465_c3/f1p0/2359
R01_cb2765_c3/f1p0/3628	NA	NA	NA	R01_cb2765_c3/f1p0/3628
R01_cb11141_c7/f1p0/1284	NA	NA	NA	R01_cb11141_c7/f1p0/1284
R01_cb8564_c111231/f1p1/2676	NA	R01_cb8564_c111231/f1p1/2676	NA	NA
R01_cb8564_c19217/f1p0/2062	NA	R01_cb8564_c19217/f1p0/2062	NA	NA
R01_cb3757_c3/f1p0/3313	NA	NA	NA	R01_cb3757_c3/f1p0/3313
R01_cb5659_c43/f1p0/2132	NA	NA	NA	R01_cb5659_c43/f1p0/2132
R01_cb8564_c51248/f1p0/2265	NA	R01_cb8564_c51248/f1p0/2265	NA	R01_cb8564_c51248/f1p0/2265
R01_cb16487_c1/f1p0/546	R01_cb16487_c1/f1p0/546	R01_cb16487_c1/f1p0/546	NA	R01_cb16487_c1/f1p0/546
R01_cb2378_c24/f1p0/3289	NA	NA	NA	R01_cb2378_c24/f1p0/3289
R01_cb7806_c4/f1p0/1821	NA	R01_cb7806_c4/f1p0/1821	R01_cb7806_c4/f1p0/1821	R01_cb7806_c4/f1p0/1821
R01_cb14547_c17/f1p1/777	NA	R01_cb14547_c17/f1p1/777	NA	NA
R01_cb8097_c9/f1p0/3499	NA	R01_cb8097_c9/f1p0/3499	NA	NA
R01_cb17548_c0/f1p0/840	NA	R01_cb17548_c0/f1p0/840	R01_cb17548_c0/f1p0/840	R01_cb17548_c0/f1p0/840
R01_cb12696_c9/f1p0/788	R01_cb12696_c9/f1p0/788	R01_cb12696_c9/f1p0/788	NA	NA
R01_cb5259_c7/f1p0/3967	NA	R01_cb5259_c7/f1p0/3967	R01_cb5259_c7/f1p0/3967	R01_cb5259_c7/f1p0/3967
R01_cb4169_c5/f1p0/3737	NA	R01_cb4169_c5/f1p0/3737	R01_cb4169_c5/f1p0/3737	NA
R01_cb352_c8/f1p0/1740	NA	NA	NA	R01_cb352_c8/f1p0/1740
R01_cb11533_c0/f1p0/809	NA	R01_cb11533_c0/f1p0/809	R01_cb11533_c0/f1p0/809	R01_cb11533_c0/f1p0/809
R01_cb4123_c105/f1p1/1055	NA	R01_cb4123_c105/f1p1/1055	NA	NA
R01_cb8409_c4/f1p0/2044	NA	NA	NA	R01_cb8409_c4/f1p0/2044
R01_cb18456_c6263/f1p0/787	NA	R01_cb18456_c6263/f1p0/787	NA	R01_cb18456_c6263/f1p0/787
R01_cb12156_c2/f1p1/692	R01_cb12156_c2/f1p1/692	R01_cb12156_c2/f1p1/692	NA	NA
R01_cb7632_c28/f1p0/541	R01_cb7632_c28/f1p0/541	R01_cb7632_c28/f1p0/541	R01_cb7632_c28/f1p0/541	R01_cb7632_c28/f1p0/541
R01_cb17792_c1/f1p0/1110	NA	NA	NA	R01_cb17792_c1/f1p0/1110

R01_cb18456_c5455/f1p0/1774	NA	R01_cb18456_c5455/f1p0/1774	NA	NA
R01_cb10206_c17/f1p0/1410	NA	R01_cb10206_c17/f1p0/1410	NA	NA
R01_cb8564_c18289/f3p1/3147	NA	R01_cb8564_c18289/f3p1/3147	R01_cb8564_c18289/f3p1/3147	NA
R01_cb13933_c0/f1p0/1314	NA	R01_cb13933_c0/f1p0/1314	NA	NA
R01_cb8564_c1826/f1p1/4529	NA	R01_cb8564_c1826/f1p1/4529	NA	NA
R01_cb14469_c6/f2p2/577	NA	R01_cb14469_c6/f2p2/577	R01_cb14469_c6/f2p2/577	R01_cb14469_c6/f2p2/577
R01_cb9797_c37/f1p0/2407	NA	R01_cb9797_c37/f1p0/2407	NA	NA
R01_cb8564_c122970/f1p0/3771	NA	R01_cb8564_c122970/f1p0/3771	NA	R01_cb8564_c122970/f1p0/3771
R01_cb8564_c90782/f1p0/2732	NA	NA	NA	R01_cb8564_c90782/f1p0/2732
R01_cb8122_c3/f1p0/3468	NA	NA	NA	R01_cb8122_c3/f1p0/3468
R01_cb658_c3/f1p0/2803	NA	NA	NA	R01_cb658_c3/f1p0/2803
R01_cb7584_c14/f1p0/1402	NA	R01_cb7584_c14/f1p0/1402	NA	NA
R01_cb8564_c23795/f1p0/4903	NA	R01_cb8564_c23795/f1p0/4903	NA	NA
R01_cb2138_c2/f1p1/4037	NA	R01_cb2138_c2/f1p1/4037	NA	NA
R01_cb13867_c14/f3p1/654	NA	NA	NA	R01_cb13867_c14/f3p1/654
R01_cb7714_c6/f1p1/2387	NA	NA	NA	R01_cb7714_c6/f1p1/2387
R01_cb10562_c1/f1p0/2694	NA	NA	R01_cb10562_c1/f1p0/2694	R01_cb10562_c1/f1p0/2694
R01_cb16645_c53/f1p0/926	NA	R01_cb16645_c53/f1p0/926	NA	NA
R01_cb8564_c1548/f1p0/2907	R01_cb8564_c1548/f1p0/2907	R01_cb8564_c1548/f1p0/2907	R01_cb8564_c1548/f1p0/2907	R01_cb8564_c1548/f1p0/2907
R01_cb11379_c0/f1p0/867	NA	R01_cb11379_c0/f1p0/867	NA	R01_cb11379_c0/f1p0/867
R01_cb12328_c7/f1p0/1568	NA	NA	NA	R01_cb12328_c7/f1p0/1568
R01_cb15600_c1/f1p0/877	NA	NA	NA	R01_cb15600_c1/f1p0/877
R01_cb8564_c5054/f1p0/2981	NA	R01_cb8564_c5054/f1p0/2981	NA	NA
R01_cb9960_c2/f1p0/1993	NA	NA	NA	R01_cb9960_c2/f1p0/1993
R01_cb8564_c89241/f1p0/2497	NA	NA	NA	R01_cb8564_c89241/f1p0/2497

R01_cb8564_c69326/f4p0/2439	NA	R01_cb8564_c69326/f4p0/2439	R01_cb8564_c69326/f4p0/2439	R01_cb8564_c69326/f4p0/2439
R01_cb15671_c1/f1p0/1106	NA	R01_cb15671_c1/f1p0/1106	NA	R01_cb15671_c1/f1p0/1106
R01_cb7695_c1/f1p0/2000	NA	NA	NA	R01_cb7695_c1/f1p0/2000
R01_cb8564_c118917/f1p0/2553	NA	R01_cb8564_c118917/f1p0/2553	NA	NA
R01_cb12003_c105/f1p0/926	NA	R01_cb12003_c105/f1p0/926	NA	NA
R01_cb14107_c6/f1p0/1325	NA	NA	R01_cb14107_c6/f1p0/1325	R01_cb14107_c6/f1p0/1325
R01_cb1835_c4/f1p0/3465	NA	NA	NA	R01_cb1835_c4/f1p0/3465
R01_cb10132_c24/f1p1/1035	NA	R01_cb10132_c24/f1p1/1035	NA	NA
R01_cb8564_c18065/f1p0/3238	NA	NA	NA	R01_cb8564_c18065/f1p0/3238
R01_cb18456_c2262/f1p0/595	NA	R01_cb18456_c2262/f1p0/595	R01_cb18456_c2262/f1p0/595	R01_cb18456_c2262/f1p0/595
R01_cb10346_c1/f1p0/3723	NA	R01_cb10346_c1/f1p0/3723	NA	NA
R01_cb17572_c0/f3p0/786	NA	NA	NA	R01_cb17572_c0/f3p0/786
R01_cb6253_c3/f1p0/452	R01_cb6253_c3/f1p0/452	R01_cb6253_c3/f1p0/452	R01_cb6253_c3/f1p0/452	R01_cb6253_c3/f1p0/452
R01_cb4185_c2/f1p0/2697	NA	NA	NA	R01_cb4185_c2/f1p0/2697
R01_cb8564_c107268/f7p1/3924	NA	NA	NA	R01_cb8564_c107268/f7p1/3924
R01_cb13205_c7/f1p0/1529	R01_cb13205_c7/f1p0/1529	R01_cb13205_c7/f1p0/1529	NA	NA
R01_cb8564_c16218/f1p0/3279	NA	R01_cb8564_c16218/f1p0/3279	NA	NA
R01_cb8051_c5/f1p0/2367	NA	NA	NA	R01_cb8051_c5/f1p0/2367
R01_cb6463_c4/f1p1/2826	NA	NA	R01_cb6463_c4/f1p1/2826	NA
R01_cb8055_c5/f1p0/625	NA	NA	R01_cb8055_c5/f1p0/625	R01_cb8055_c5/f1p0/625
R01_cb8790_c10/f1p0/605	NA	R01_cb8790_c10/f1p0/605	NA	R01_cb8790_c10/f1p0/605
R01_cb11768_c0/f1p0/738	R01_cb11768_c0/f1p0/738	R01_cb11768_c0/f1p0/738	R01_cb11768_c0/f1p0/738	R01_cb11768_c0/f1p0/738
R01_cb920_c23/f1p0/1292	NA	NA	NA	R01_cb920_c23/f1p0/1292
R01_cb7329_c3/f1p0/2137	NA	R01_cb7329_c3/f1p0/2137	NA	NA

R01_cb16066_c5/f1p0/649	R01_cb16066_c5/f1p0/649	R01_cb16066_c5/f1p0/649	R01_cb16066_c5/f1p0/649	R01_cb16066_c5/f1p0/649
R01_cb13144_c6/f1p0/489	NA	R01_cb13144_c6/f1p0/489	NA	R01_cb13144_c6/f1p0/489
R01_cb12329_c6/f2p0/910	NA	NA	NA	R01_cb12329_c6/f2p0/910
R01_cb8564_c128713/f1p0/2064	NA	NA	NA	R01_cb8564_c128713/f1p0/2064
R01_cb8564_c16444/f5p10/3053	NA	R01_cb8564_c16444/f5p10/3053	NA	R01_cb8564_c16444/f5p10/3053
R01_cb166_c12/f1p0/2392	NA	NA	NA	R01_cb166_c12/f1p0/2392
R01_cb7994_c11/f1p0/5318	R01_cb7994_c11/f1p0/5318	R01_cb7994_c11/f1p0/5318	NA	NA
R01_cb4490_c4/f1p0/2857	R01_cb4490_c4/f1p0/2857	R01_cb4490_c4/f1p0/2857	R01_cb4490_c4/f1p0/2857	R01_cb4490_c4/f1p0/2857
R01_cb8564_c49292/f1p0/3520	NA	NA	NA	R01_cb8564_c49292/f1p0/3520
R01_cb8564_c54303/f1p0/2267	NA	NA	NA	R01_cb8564_c54303/f1p0/2267
R01_cb16669_c0/f2p0/711	R01_cb16669_c0/f2p0/711	R01_cb16669_c0/f2p0/711	R01_cb16669_c0/f2p0/711	R01_cb16669_c0/f2p0/711
R01_cb4490_c20/f1p0/1884	NA	NA	NA	R01_cb4490_c20/f1p0/1884
R01_cb14535_c0/f2p0/1464	NA	NA	R01_cb14535_c0/f2p0/1464	NA
R01_cb13761_c14/f1p0/516	R01_cb13761_c14/f1p0/516	R01_cb13761_c14/f1p0/516	R01_cb13761_c14/f1p0/516	R01_cb13761_c14/f1p0/516
R01_cb8564_c80761/f1p0/2138	NA	NA	NA	R01_cb8564_c80761/f1p0/2138
R01_cb11262_c2/f1p0/1208	NA	R01_cb11262_c2/f1p0/1208	NA	NA
R01_cb17973_c0/f14p1/909	NA	NA	R01_cb17973_c0/f14p1/909	R01_cb17973_c0/f14p1/909
R01_cb8564_c41755/f1p1/3136	NA	R01_cb8564_c41755/f1p1/3136	R01_cb8564_c41755/f1p1/3136	NA
R01_cb12802_c2/f1p0/1315	R01_cb12802_c2/f1p0/1315	R01_cb12802_c2/f1p0/1315	NA	NA
R01_cb7841_c6/f1p0/606	NA	NA	NA	R01_cb7841_c6/f1p0/606
R01_cb9049_c3/f1p0/3510	NA	R01_cb9049_c3/f1p0/3510	NA	R01_cb9049_c3/f1p0/3510
R01_cb16747_c0/f2p0/1469	NA	NA	NA	R01_cb16747_c0/f2p0/1469
R01_cb16216_c1/f1p0/825	NA	NA	NA	R01_cb16216_c1/f1p0/825
R01_cb8937_c1/f1p0/2278	NA	NA	NA	R01_cb8937_c1/f1p0/2278

R01_cb13795_c1/f2p0/1200	NA	R01_cb13795_c1/f2p0/1200	NA	NA
R01_cb8564_c24034/f1p0/2193	NA	NA	NA	R01_cb8564_c24034/f1p0/2193
R01_cb8564_c15651/f1p0/1993	NA	R01_cb8564_c15651/f1p0/1993	NA	NA
R01_cb12516_c0/f7p0/439	NA	NA	NA	R01_cb12516_c0/f7p0/439
R01_cb8564_c3177/f1p0/2729	NA	R01_cb8564_c3177/f1p0/2729	R01_cb8564_c3177/f1p0/2729	R01_cb8564_c3177/f1p0/2729
R01_cb8040_c14/f1p0/3613	NA	NA	R01_cb8040_c14/f1p0/3613	R01_cb8040_c14/f1p0/3613
R01_cb3575_c10/f1p0/3008	NA	NA	NA	R01_cb3575_c10/f1p0/3008
R01_cb15360_c5/f1p0/1040	NA	R01_cb15360_c5/f1p0/1040	NA	NA
R01_cb8087_c12/f1p0/988	NA	R01_cb8087_c12/f1p0/988	NA	NA
R01_cb15128_c0/f3p0/1626	NA	NA	NA	R01_cb15128_c0/f3p0/1626
R01_cb8564_c70240/f1p0/3561	NA	R01_cb8564_c70240/f1p0/3561	NA	NA
R01_cb3381_c41/f1p0/2116	NA	NA	NA	R01_cb3381_c41/f1p0/2116
R01_cb8564_c117589/f1p0/2950	NA	NA	NA	R01_cb8564_c117589/f1p0/2950
R01_cb18409_c61/f1p0/702	R01_cb18409_c61/f1p0/702	R01_cb18409_c61/f1p0/702	R01_cb18409_c61/f1p0/702	R01_cb18409_c61/f1p0/702
R01_cb9047_c3/f1p1/2053	NA	NA	NA	R01_cb9047_c3/f1p1/2053
R01_cb11287_c0/f1p0/665	NA	NA	NA	R01_cb11287_c0/f1p0/665
R01_cb10024_c266/f1p2/852	NA	NA	R01_cb10024_c266/f1p2/852	NA
R01_cb18456_c7551/f1p0/1750	NA	R01_cb18456_c7551/f1p0/1750	NA	NA
R01_cb15326_c7/f1p0/1319	R01_cb15326_c7/f1p0/1319	R01_cb15326_c7/f1p0/1319	R01_cb15326_c7/f1p0/1319	R01_cb15326_c7/f1p0/1319
R01_cb16558_c1/f1p0/1622	R01_cb16558_c1/f1p0/1622	R01_cb16558_c1/f1p0/1622	R01_cb16558_c1/f1p0/1622	R01_cb16558_c1/f1p0/1622
R01_cb15417_c3/f5p0/618	NA	R01_cb15417_c3/f5p0/618	R01_cb15417_c3/f5p0/618	NA
R01_cb13241_c0/f2p0/401	R01_cb13241_c0/f2p0/401	R01_cb13241_c0/f2p0/401	R01_cb13241_c0/f2p0/401	R01_cb13241_c0/f2p0/401
R01_cb8564_c4104/f1p0/3152	R01_cb8564_c4104/f1p0/3152	R01_cb8564_c4104/f1p0/3152	R01_cb8564_c4104/f1p0/3152	NA
R01_cb6922_c5/f1p0/1222	NA	NA	NA	R01_cb6922_c5/f1p0/1222
R01_cb293_c6/f1p0/3758	NA	NA	NA	R01_cb293_c6/f1p0/3758

R01_cb1178_c24/f1p0/1294	NA	NA	NA	R01_cb1178_c24/f1p0/1294
R01_cb16387_c13/f1p0/1656	NA	NA	NA	R01_cb16387_c13/f1p0/1656
R01_cb5168_c3/f1p0/1497	R01_cb5168_c3/f1p0/1497	R01_cb5168_c3/f1p0/1497	R01_cb5168_c3/f1p0/1497	R01_cb5168_c3/f1p0/1497
R01_cb1297_c6/f1p2/4121	NA	NA	NA	R01_cb1297_c6/f1p2/4121
R01_cb5533_c167/f1p0/872	NA	R01_cb5533_c167/f1p0/872	NA	R01_cb5533_c167/f1p0/872
R01_cb13938_c10/f1p0/1187	NA	NA	NA	R01_cb13938_c10/f1p0/1187
R01_cb12332_c14/f1p0/1053	NA	R01_cb12332_c14/f1p0/1053	NA	NA
R01_cb10231_c4/f1p0/1464	NA	NA	NA	R01_cb10231_c4/f1p0/1464
R01_cb8564_c83382/f1p0/1930	NA	R01_cb8564_c83382/f1p0/1930	NA	R01_cb8564_c83382/f1p0/1930
R01_cb8564_c22573/f1p0/2588	NA	NA	NA	R01_cb8564_c22573/f1p0/2588
R01_cb13181_c14/f1p0/1460	NA	R01_cb13181_c14/f1p0/1460	NA	NA
R01_cb6473_c2/f1p1/2843	NA	R01_cb6473_c2/f1p1/2843	NA	NA
R01_cb17901_c0/f1p0/824	R01_cb17901_c0/f1p0/824	R01_cb17901_c0/f1p0/824	R01_cb17901_c0/f1p0/824	R01_cb17901_c0/f1p0/824
R01_cb7950_c2/f1p0/1051	NA	R01_cb7950_c2/f1p0/1051	R01_cb7950_c2/f1p0/1051	R01_cb7950_c2/f1p0/1051
R01_cb11649_c0/f1p0/1545	R01_cb11649_c0/f1p0/1545	R01_cb11649_c0/f1p0/1545	NA	NA
R01_cb1106_c4/f1p0/2102	NA	NA	NA	R01_cb1106_c4/f1p0/2102
R01_cb14351_c2/f1p0/671	NA	NA	NA	R01_cb14351_c2/f1p0/671
R01_cb897_c39/f1p0/2987	NA	NA	NA	R01_cb897_c39/f1p0/2987
R01_cb11434_c3/f1p0/1539	NA	NA	R01_cb11434_c3/f1p0/1539	R01_cb11434_c3/f1p0/1539
R01_cb13840_c7/f1p0/946	NA	R01_cb13840_c7/f1p0/946	NA	NA
R01_cb13178_c9/f2p0/584	NA	R01_cb13178_c9/f2p0/584	NA	NA
R01_cb6990_c6/f1p0/2668	NA	R01_cb6990_c6/f1p0/2668	NA	NA
R01_cb18456_c6097/f1p0/624	NA	R01_cb18456_c6097/f1p0/624	NA	R01_cb18456_c6097/f1p0/624
R01_cb647_c3/f3p2/1480	NA	NA	NA	R01_cb647_c3/f3p2/1480
R01_cb18730_c2/f1p0/3305	NA	R01_cb18730_c2/f1p0/3305	NA	NA
R01_cb10713_c3/f1p0/937	NA	R01_cb10713_c3/f1p0/937	NA	NA

R01_cb3941_c88/f1p0/1953	NA	R01_cb3941_c88/f1p0/1953	NA	NA
R01_cb5690_c6/f1p0/648	NA	NA	NA	R01_cb5690_c6/f1p0/648
R01_cb18353_c1/f1p0/1301	NA	R01_cb18353_c1/f1p0/1301	R01_cb18353_c1/f1p0/1301	R01_cb18353_c1/f1p0/1301
R01_cb2492_c0/f1p0/3882	NA	NA	NA	R01_cb2492_c0/f1p0/3882
R01_cb10162_c6/f1p1/1718	NA	R01_cb10162_c6/f1p1/1718	NA	NA
R01_cb11447_c2/f1p0/1654	NA	R01_cb11447_c2/f1p0/1654	NA	R01_cb11447_c2/f1p0/1654
R01_cb13938_c21/f1p0/836	NA	NA	NA	R01_cb13938_c21/f1p0/836
R01_cb14675_c11/f1p0/856	R01_cb14675_c11/f1p0/856	R01_cb14675_c11/f1p0/856	R01_cb14675_c11/f1p0/856	R01_cb14675_c11/f1p0/856
R01_cb8564_c7832/f2p1/2061	NA	NA	NA	R01_cb8564_c7832/f2p1/2061
R01_cb11583_c1/f1p0/2174	R01_cb11583_c1/f1p0/2174	R01_cb11583_c1/f1p0/2174	R01_cb11583_c1/f1p0/2174	R01_cb11583_c1/f1p0/2174
R01_cb302_c23/f1p1/1809	NA	NA	NA	R01_cb302_c23/f1p1/1809
R01_cb15293_c1/f1p0/760	NA	NA	R01_cb15293_c1/f1p0/760	NA
R01_cb9057_c4/f1p0/2149	NA	R01_cb9057_c4/f1p0/2149	NA	NA
R01_cb6406_c56/f1p0/3033	NA	R01_cb6406_c56/f1p0/3033	NA	R01_cb6406_c56/f1p0/3033
R01_cb18456_c1861/f1p0/1099	NA	R01_cb18456_c1861/f1p0/1099	NA	NA
R01_cb8478_c68/f1p0/1971	NA	R01_cb8478_c68/f1p0/1971	R01_cb8478_c68/f1p0/1971	NA
R01_cb10133_c4/f1p1/1973	NA	NA	NA	R01_cb10133_c4/f1p1/1973
R01_cb8248_c1/f1p0/2419	NA	NA	R01_cb8248_c1/f1p0/2419	R01_cb8248_c1/f1p0/2419
R01_cb15352_c1/f2p2/909	NA	NA	NA	R01_cb15352_c1/f2p2/909
R01_cb15682_c1/f1p0/955	NA	NA	NA	R01_cb15682_c1/f1p0/955
R01_cb8313_c6/f1p0/2290	NA	R01_cb8313_c6/f1p0/2290	NA	R01_cb8313_c6/f1p0/2290
R01_cb3026_c6/f1p0/1903	NA	R01_cb3026_c6/f1p0/1903	NA	NA
R01_cb8564_c123944/f1p0/2814	NA	NA	NA	R01_cb8564_c123944/f1p0/2814
R01_cb8111_c0/f1p0/2521	NA	NA	NA	R01_cb8111_c0/f1p0/2521
R01_cb1610_c5/f1p0/2559	NA	NA	NA	R01_cb1610_c5/f1p0/2559

R01_cb4923_c3/f1p0/2890	NA	NA	NA	R01_cb4923_c3/f1p0/2890
R01_cb8564_c51035/f2p2/4216	NA	NA	NA	R01_cb8564_c51035/f2p2/4216
R01_cb6947_c13/f1p0/3658	NA	R01_cb6947_c13/f1p0/3658	NA	R01_cb6947_c13/f1p0/3658
R01_cb5943_c2/f1p0/2980	NA	NA	NA	R01_cb5943_c2/f1p0/2980
R01_cb8564_c114927/f1p0/2666	NA	R01_cb8564_c114927/f1p0/2666	R01_cb8564_c114927/f1p0/2666	R01_cb8564_c114927/f1p0/2666
R01_cb8564_c36706/f1p0/2946	NA	NA	NA	R01_cb8564_c36706/f1p0/2946
R01_cb7497_c3/f4p0/2581	NA	NA	NA	R01_cb7497_c3/f4p0/2581
R01_cb8564_c90245/f1p0/2589	NA	R01_cb8564_c90245/f1p0/2589	NA	NA
R01_cb644_c10/f1p0/2355	NA	NA	NA	R01_cb644_c10/f1p0/2355
R01_cb18456_c3558/f1p0/1170	NA	R01_cb18456_c3558/f1p0/1170	NA	NA
R01_cb618_c10/f1p0/1375	NA	NA	NA	R01_cb618_c10/f1p0/1375
R01_cb9343_c1/f1p0/2112	NA	R01_cb9343_c1/f1p0/2112	NA	NA
R01_cb18166_c1/f1p0/342	NA	R01_cb18166_c1/f1p0/342	NA	NA
R01_cb2042_c3/f1p0/2619	NA	R01_cb2042_c3/f1p0/2619	NA	R01_cb2042_c3/f1p0/2619
R01_cb6923_c34/f1p0/1671	R01_cb6923_c34/f1p0/1671	R01_cb6923_c34/f1p0/1671	R01_cb6923_c34/f1p0/1671	R01_cb6923_c34/f1p0/1671
R01_cb8564_c1482/f1p0/1923	NA	R01_cb8564_c1482/f1p0/1923	NA	NA
R01_cb1366_c1/f1p0/1985	R01_cb1366_c1/f1p0/1985	R01_cb1366_c1/f1p0/1985	R01_cb1366_c1/f1p0/1985	R01_cb1366_c1/f1p0/1985
R01_cb8564_c23283/f1p0/2050	NA	R01_cb8564_c23283/f1p0/2050	R01_cb8564_c23283/f1p0/2050	R01_cb8564_c23283/f1p0/2050
R01_cb8564_c20135/f1p0/3340	NA	NA	NA	R01_cb8564_c20135/f1p0/3340
R01_cb8564_c73656/f1p0/2410	NA	R01_cb8564_c73656/f1p0/2410	R01_cb8564_c73656/f1p0/2410	R01_cb8564_c73656/f1p0/2410
R01_cb12465_c8/f1p0/393	NA	R01_cb12465_c8/f1p0/393	NA	NA
R01_cb8564_c35893/f2p0/2773	R01_cb8564_c35893/f2p0/2773	R01_cb8564_c35893/f2p0/2773	R01_cb8564_c35893/f2p0/2773	R01_cb8564_c35893/f2p0/2773
R01_cb8564_c4101/f1p0/3275	NA	R01_cb8564_c4101/f1p0/3275	R01_cb8564_c4101/f1p0/3275	NA
R01_cb3068_c2/f1p0/3739	NA	NA	NA	R01_cb3068_c2/f1p0/3739

R01_cb1135_c1/f1p0/4464	NA	R01_cb1135_c1/f1p0/4464	NA	NA
R01_cb5547_c1/f1p0/3078	NA	NA	NA	R01_cb5547_c1/f1p0/3078
R01_cb14715_c4/f1p0/516	R01_cb14715_c4/f1p0/516	R01_cb14715_c4/f1p0/516	R01_cb14715_c4/f1p0/516	R01_cb14715_c4/f1p0/516
R01_cb4374_c3/f1p0/2557	NA	R01_cb4374_c3/f1p0/2557	NA	NA
R01_cb6642_c0/f3p0/2709	NA	NA	NA	R01_cb6642_c0/f3p0/2709
R01_cb8564_c21270/f1p0/4601	NA	NA	NA	R01_cb8564_c21270/f1p0/4601
R01_cb8564_c86591/f1p1/3640	NA	NA	NA	R01_cb8564_c86591/f1p1/3640
R01_cb2352_c11/f1p0/2274	R01_cb2352_c11/f1p0/2274	R01_cb2352_c11/f1p0/2274	R01_cb2352_c11/f1p0/2274	R01_cb2352_c11/f1p0/2274
R01_cb8564_c70272/f1p0/3476	NA	R01_cb8564_c70272/f1p0/3476	NA	NA
R01_cb3584_c4/f1p0/3441	NA	NA	NA	R01_cb3584_c4/f1p0/3441
R01_cb14064_c0/f1p0/1684	NA	R01_cb14064_c0/f1p0/1684	NA	NA
R01_cb17514_c0/f3p1/383	NA	R01_cb17514_c0/f3p1/383	R01_cb17514_c0/f3p1/383	R01_cb17514_c0/f3p1/383
R01_cb11330_c2/f1p0/719	NA	NA	NA	R01_cb11330_c2/f1p0/719
R01_cb12893_c28/f1p3/1386	NA	R01_cb12893_c28/f1p3/1386	NA	NA
R01_cb8564_c9750/f2p0/3506	NA	R01_cb8564_c9750/f2p0/3506	NA	NA
R01_cb8564_c53685/f1p1/2562	NA	R01_cb8564_c53685/f1p1/2562	NA	NA
R01_cb11204_c0/f2p0/895	NA	NA	NA	R01_cb11204_c0/f2p0/895
R01_cb2761_c9/f1p0/3396	NA	R01_cb2761_c9/f1p0/3396	NA	NA
R01_cb8564_c123889/f1p0/2931	NA	R01_cb8564_c123889/f1p0/2931	NA	NA
R01_cb11361_c2/f1p0/2841	NA	R01_cb11361_c2/f1p0/2841	NA	R01_cb11361_c2/f1p0/2841
R01_cb15595_c0/f2p0/599	NA	NA	NA	R01_cb15595_c0/f2p0/599
R01_cb6286_c15/f1p0/1743	NA	NA	NA	R01_cb6286_c15/f1p0/1743
R01_cb10007_c24/f1p0/518	R01_cb10007_c24/f1p0/518	R01_cb10007_c24/f1p0/518	R01_cb10007_c24/f1p0/518	R01_cb10007_c24/f1p0/518
R01_cb8564_c90725/f1p0/2935	NA	R01_cb8564_c90725/f1p0/2935	NA	NA
R01_cb13041_c8/f1p0/865	NA	R01_cb13041_c8/f1p0/865	NA	R01_cb13041_c8/f1p0/865

R01_cb4657_c7/f1p0/4018	NA	NA	R01_cb4657_c7/f1p0/4018	NA
R01_cb14323_c9/f2p0/770	NA	R01_cb14323_c9/f2p0/770	NA	NA
R01_cb8564_c115200/f1p0/2282	NA	R01_cb8564_c115200/f1p0/2282	NA	NA
R01_cb3949_c8/f1p1/3712	NA	R01_cb3949_c8/f1p1/3712	NA	NA
R01_cb17303_c3/f1p0/881	NA	R01_cb17303_c3/f1p0/881	NA	NA
R01_cb15553_c3/f3p0/1794	NA	NA	NA	R01_cb15553_c3/f3p0/1794
R01_cb12383_c9/f1p0/1820	NA	R01_cb12383_c9/f1p0/1820	NA	NA
R01_cb15938_c1/f1p0/867	NA	NA	NA	R01_cb15938_c1/f1p0/867
R01_cb15582_c0/f1p0/752	NA	NA	NA	R01_cb15582_c0/f1p0/752
R01_cb10742_c10/f1p0/915	NA	NA	R01_cb10742_c10/f1p0/915	R01_cb10742_c10/f1p0/915
R01_cb11722_c1/f1p0/3069	NA	NA	NA	R01_cb11722_c1/f1p0/3069
R01_cb12437_c0/f3p1/776	NA	R01_cb12437_c0/f3p1/776	R01_cb12437_c0/f3p1/776	R01_cb12437_c0/f3p1/776
R01_cb11667_c1/f1p0/4128	NA	R01_cb11667_c1/f1p0/4128	R01_cb11667_c1/f1p0/4128	NA
R01_cb6528_c4/f1p0/2794	NA	R01_cb6528_c4/f1p0/2794	NA	NA
R01_cb12972_c34/f1p0/458	R01_cb12972_c34/f1p0/458	R01_cb12972_c34/f1p0/458	R01_cb12972_c34/f1p0/458	R01_cb12972_c34/f1p0/458
R01_cb3564_c14/f1p0/2619	NA	NA	NA	R01_cb3564_c14/f1p0/2619
R01_cb10166_c2/f1p0/812	NA	R01_cb10166_c2/f1p0/812	NA	NA
R01_cb7806_c1/f1p0/2342	NA	R01_cb7806_c1/f1p0/2342	R01_cb7806_c1/f1p0/2342	R01_cb7806_c1/f1p0/2342
R01_cb10015_c586/f1p0/523	NA	R01_cb10015_c586/f1p0/523	R01_cb10015_c586/f1p0/523	NA
R01_cb8190_c2/f3p0/1707	NA	NA	NA	R01_cb8190_c2/f3p0/1707
R01_cb12443_c1/f1p2/540	R01_cb12443_c1/f1p2/540	R01_cb12443_c1/f1p2/540	R01_cb12443_c1/f1p2/540	R01_cb12443_c1/f1p2/540
R01_cb18456_c7994/f1p2/707	NA	NA	NA	R01_cb18456_c7994/f1p2/707
R01_cb11668_c1/f1p0/2707	NA	R01_cb11668_c1/f1p0/2707	R01_cb11668_c1/f1p0/2707	R01_cb11668_c1/f1p0/2707
R01_cb2985_c15/f1p2/3532	NA	NA	NA	R01_cb2985_c15/f1p2/3532
R01_cb10676_c0/f9p0/481	R01_cb10676_c0/f9p0/481	R01_cb10676_c0/f9p0/481	NA	R01_cb10676_c0/f9p0/481

R01_cb11204_c2/f1p0/922	NA	NA	NA	R01_cb11204_c2/f1p0/922
R01_cb17582_c5/f1p0/649	R01_cb17582_c5/f1p0/649	R01_cb17582_c5/f1p0/649	R01_cb17582_c5/f1p0/649	R01_cb17582_c5/f1p0/649
R01_cb4777_c4/f1p0/696	NA	NA	NA	R01_cb4777_c4/f1p0/696
R01_cb6633_c16/f1p0/2313	NA	R01_cb6633_c16/f1p0/2313	NA	NA
R01_cb18470_c1/f1p0/885	NA	R01_cb18470_c1/f1p0/885	NA	R01_cb18470_c1/f1p0/885
R01_cb8564_c76090/f1p0/3995	NA	R01_cb8564_c76090/f1p0/3995	NA	R01_cb8564_c76090/f1p0/3995
R01_cb8246_c0/f1p0/2432	NA	R01_cb8246_c0/f1p0/2432	NA	NA
R01_cb9008_c3/f1p1/2214	NA	NA	R01_cb9008_c3/f1p1/2214	NA
R01_cb8564_c70950/f1p0/1916	NA	R01_cb8564_c70950/f1p0/1916	R01_cb8564_c70950/f1p0/1916	R01_cb8564_c70950/f1p0/1916
R01_cb6507_c8/f1p0/1130	NA	NA	NA	R01_cb6507_c8/f1p0/1130
R01_cb3941_c95/f1p0/2002	NA	NA	NA	R01_cb3941_c95/f1p0/2002
R01_cb8564_c70960/f1p0/2362	NA	NA	NA	R01_cb8564_c70960/f1p0/2362
R01_cb18456_c1111/f1p3/1088	NA	R01_cb18456_c1111/f1p3/1088	NA	NA
R01_cb4822_c0/f1p0/3252	NA	R01_cb4822_c0/f1p0/3252	R01_cb4822_c0/f1p0/3252	R01_cb4822_c0/f1p0/3252
R01_cb5351_c0/f1p1/3121	NA	NA	NA	R01_cb5351_c0/f1p1/3121
R01_cb14675_c6/f1p0/753	R01_cb14675_c6/f1p0/753	R01_cb14675_c6/f1p0/753	R01_cb14675_c6/f1p0/753	R01_cb14675_c6/f1p0/753
R01_cb16515_c0/f1p0/837	NA	NA	NA	R01_cb16515_c0/f1p0/837
R01_cb8564_c71038/f1p0/3922	NA	R01_cb8564_c71038/f1p0/3922	R01_cb8564_c71038/f1p0/3922	R01_cb8564_c71038/f1p0/3922
R01_cb1729_c25/f2p4/3649	NA	NA	NA	R01_cb1729_c25/f2p4/3649
R01_cb16997_c51/f1p0/3275	NA	R01_cb16997_c51/f1p0/3275	NA	NA
R01_cb11286_c2/f1p0/670	NA	R01_cb11286_c2/f1p0/670	NA	NA
R01_cb8564_c119567/f1p0/3103	NA	R01_cb8564_c119567/f1p0/3103	NA	NA
R01_cb18175_c0/f1p0/1009	NA	R01_cb18175_c0/f1p0/1009	R01_cb18175_c0/f1p0/1009	R01_cb18175_c0/f1p0/1009
R01_cb8564_c21507/f1p0/2810	NA	NA	NA	R01_cb8564_c21507/f1p0/2810
R01_cb2226_c6/f1p0/3264	NA	NA	NA	R01_cb2226_c6/f1p0/3264

R01_cb11283_c4/f1p0/625	NA	R01_cb11283_c4/f1p0/625	NA	R01_cb11283_c4/f1p0/625
R01_cb9352_c4/f1p0/2066	NA	NA	NA	R01_cb9352_c4/f1p0/2066
R01_cb6666_c7/f1p0/2673	NA	NA	NA	R01_cb6666_c7/f1p0/2673
R01_cb1779_c7/f1p0/4115	NA	NA	NA	R01_cb1779_c7/f1p0/4115
R01_cb18456_c7140/f1p0/1822	NA	R01_cb18456_c7140/f1p0/1822	R01_cb18456_c7140/f1p0/1822	R01_cb18456_c7140/f1p0/1822
R01_cb1113_c3/f1p1/4206	R01_cb1113_c3/f1p1/4206	R01_cb1113_c3/f1p1/4206	NA	R01_cb1113_c3/f1p1/4206
R01_cb8564_c34611/f1p0/2586	NA	R01_cb8564_c34611/f1p0/2586	NA	NA
R01_cb2675_c19/f1p0/2756	NA	NA	NA	R01_cb2675_c19/f1p0/2756
R01_cb17307_c1/f1p0/1008	R01_cb17307_c1/f1p0/1008	R01_cb17307_c1/f1p0/1008	NA	R01_cb17307_c1/f1p0/1008
R01_cb2082_c36/f1p0/2660	NA	NA	NA	R01_cb2082_c36/f1p0/2660
R01_cb17443_c0/f3p0/424	NA	NA	NA	R01_cb17443_c0/f3p0/424
R01_cb280_c82/f1p0/3129	NA	R01_cb280_c82/f1p0/3129	NA	NA
R01_cb2779_c6/f1p0/1723	NA	NA	NA	R01_cb2779_c6/f1p0/1723
R01_cb16937_c3/f1p0/568	NA	R01_cb16937_c3/f1p0/568	NA	R01_cb16937_c3/f1p0/568
R01_cb2823_c1/f1p0/3417	NA	NA	R01_cb2823_c1/f1p0/3417	NA
R01_cb9916_c1/f1p0/1925	NA	NA	NA	R01_cb9916_c1/f1p0/1925
R01_cb15883_c3/f1p0/701	NA	R01_cb15883_c3/f1p0/701	NA	NA
R01_cb7593_c0/f1p0/2578	NA	NA	R01_cb7593_c0/f1p0/2578	NA
R01_cb4345_c15/f1p0/2192	NA	NA	NA	R01_cb4345_c15/f1p0/2192
R01_cb8564_c22964/f1p2/1995	NA	R01_cb8564_c22964/f1p2/1995	NA	NA
R01_cb2632_c7/f1p0/5264	NA	R01_cb2632_c7/f1p0/5264	NA	NA
R01_cb16736_c1/f2p1/689	NA	R01_cb16736_c1/f2p1/689	NA	NA
R01_cb8564_c84492/f1p0/2051	NA	NA	NA	R01_cb8564_c84492/f1p0/2051
R01_cb8564_c77086/f1p0/3582	NA	R01_cb8564_c77086/f1p0/3582	NA	R01_cb8564_c77086/f1p0/3582
R01_cb8564_c90314/f1p0/3467	NA	R01_cb8564_c90314/f1p0/3467	NA	NA
R01_cb17760_c1/f1p0/1328	NA	NA	NA	R01_cb17760_c1/f1p0/1328

R01_cb4493_c10/f1p2/4658	NA	NA	NA	R01_cb4493_c10/f1p2/4658
R01_cb15423_c2/f1p1/830	NA	R01_cb15423_c2/f1p1/830	R01_cb15423_c2/f1p1/830	R01_cb15423_c2/f1p1/830
R01_cb7603_c7/f1p0/375	R01_cb7603_c7/f1p0/375	R01_cb7603_c7/f1p0/375	R01_cb7603_c7/f1p0/375	R01_cb7603_c7/f1p0/375
R01_cb17_c16/f1p0/3971	NA	NA	NA	R01_cb17_c16/f1p0/3971
R01_cb8564_c4721/f1p0/4316	NA	NA	NA	R01_cb8564_c4721/f1p0/4316
R01_cb15474_c3/f1p0/1822	NA	NA	NA	R01_cb15474_c3/f1p0/1822
R01_cb17412_c4/f1p0/749	NA	R01_cb17412_c4/f1p0/749	R01_cb17412_c4/f1p0/749	R01_cb17412_c4/f1p0/749
R01_cb8564_c18146/f4p0/2170	R01_cb8564_c18146/f4p0/2170	R01_cb8564_c18146/f4p0/2170	R01_cb8564_c18146/f4p0/2170	R01_cb8564_c18146/f4p0/2170
R01_cb9576_c15/f1p0/1419	NA	NA	NA	R01_cb9576_c15/f1p0/1419
R01_cb8564_c21556/f1p0/3881	NA	NA	NA	R01_cb8564_c21556/f1p0/3881
R01_cb11241_c3/f1p0/1434	NA	R01_cb11241_c3/f1p0/1434	NA	NA
R01_cb8564_c10587/f1p1/3424	NA	R01_cb8564_c10587/f1p1/3424	NA	R01_cb8564_c10587/f1p1/3424
R01_cb7682_c2/f1p0/2436	NA	R01_cb7682_c2/f1p0/2436	NA	NA
R01_cb15755_c0/f3p0/930	NA	NA	NA	R01_cb15755_c0/f3p0/930
R01_cb8564_c89996/f1p1/2847	NA	R01_cb8564_c89996/f1p1/2847	NA	R01_cb8564_c89996/f1p1/2847
R01_cb8564_c119319/f1p0/2305	R01_cb8564_c119319/f1p0/2305	R01_cb8564_c119319/f1p0/2305	R01_cb8564_c119319/f1p0/2305	R01_cb8564_c119319/f1p0/2305
R01_cb8564_c69853/f1p0/2180	NA	NA	R01_cb8564_c69853/f1p0/2180	R01_cb8564_c69853/f1p0/2180
R01_cb17956_c13/f3p0/629	NA	R01_cb17956_c13/f3p0/629	NA	NA
R01_cb8564_c10202/f1p0/3998	NA	R01_cb8564_c10202/f1p0/3998	NA	R01_cb8564_c10202/f1p0/3998
R01_cb8564_c129896/f1p0/4743	NA	R01_cb8564_c129896/f1p0/4743	NA	NA
R01_cb4505_c0/f1p0/3329	NA	NA	R01_cb4505_c0/f1p0/3329	NA
R01_cb10026_c2/f1p0/1082	NA	R01_cb10026_c2/f1p0/1082	NA	NA
R01_cb17179_c2/f1p0/1779	NA	R01_cb17179_c2/f1p0/1779	NA	NA

R01_cb8564_c4001/f1p0/2849	NA	NA	NA	R01_cb8564_c4001/f1p0/2849
R01_cb3388_c1/f1p0/3640	NA	NA	NA	R01_cb3388_c1/f1p0/3640
R01_cb10787_c7/f1p0/1352	NA	NA	NA	R01_cb10787_c7/f1p0/1352
R01_cb18456_c7215/f1p0/447	R01_cb18456_c7215/f1p0/447	R01_cb18456_c7215/f1p0/447	R01_cb18456_c7215/f1p0/447	R01_cb18456_c7215/f1p0/447
R01_cb18156_c1/f1p0/976	NA	NA	R01_cb18156_c1/f1p0/976	R01_cb18156_c1/f1p0/976
R01_cb8409_c8/f1p1/2318	NA	NA	NA	R01_cb8409_c8/f1p1/2318
R01_cb10015_c340/f1p0/544	NA	R01_cb10015_c340/f1p0/544	NA	NA
R01_cb15373_c1/f1p0/381	R01_cb15373_c1/f1p0/381	R01_cb15373_c1/f1p0/381	R01_cb15373_c1/f1p0/381	R01_cb15373_c1/f1p0/381
R01_cb3426_c7/f1p0/2789	NA	NA	NA	R01_cb3426_c7/f1p0/2789
R01_cb11608_c0/f1p0/1104	R01_cb11608_c0/f1p0/1104	R01_cb11608_c0/f1p0/1104	R01_cb11608_c0/f1p0/1104	R01_cb11608_c0/f1p0/1104
R01_cb15541_c1/f2p0/956	NA	NA	NA	R01_cb15541_c1/f2p0/956
R01_cb11063_c1/f1p0/1953	NA	R01_cb11063_c1/f1p0/1953	NA	R01_cb11063_c1/f1p0/1953
R01_cb8564_c24276/f1p0/4454	NA	R01_cb8564_c24276/f1p0/4454	NA	NA
R01_cb11804_c0/f1p0/517	R01_cb11804_c0/f1p0/517	R01_cb11804_c0/f1p0/517	R01_cb11804_c0/f1p0/517	R01_cb11804_c0/f1p0/517
R01_cb9472_c3/f1p0/1395	NA	NA	NA	R01_cb9472_c3/f1p0/1395
R01_cb3424_c2/f1p1/3604	NA	NA	NA	R01_cb3424_c2/f1p1/3604
R01_cb7612_c6/f1p0/2279	NA	NA	NA	R01_cb7612_c6/f1p0/2279
R01_cb9448_c7/f1p1/1843	NA	NA	NA	R01_cb9448_c7/f1p1/1843
R01_cb3376_c1/f1p0/3642	R01_cb3376_c1/f1p0/3642	R01_cb3376_c1/f1p0/3642	NA	R01_cb3376_c1/f1p0/3642
R01_cb9222_c23/f1p0/2509	NA	R01_cb9222_c23/f1p0/2509	R01_cb9222_c23/f1p0/2509	R01_cb9222_c23/f1p0/2509
R01_cb1839_c1/f3p1/4160	NA	NA	NA	R01_cb1839_c1/f3p1/4160
R01_cb8814_c4/f1p0/2257	NA	R01_cb8814_c4/f1p0/2257	NA	NA
R01_cb8564_c22143/f1p1/2486	NA	R01_cb8564_c22143/f1p1/2486	NA	NA
R01_cb1612_c6/f1p5/3499	NA	NA	NA	R01_cb1612_c6/f1p5/3499
R01_cb15186_c7/f5p0/439	R01_cb15186_c7/f5p0/439	R01_cb15186_c7/f5p0/439	R01_cb15186_c7/f5p0/439	NA
R01_cb8811_c2/f1p0/2284	NA	R01_cb8811_c2/f1p0/2284	NA	NA

R01_cb18456_c5288/f1p0/1071	NA	R01_cb18456_c5288/f1p0/1071	NA	R01_cb18456_c5288/f1p0/1071
R01_cb8564_c19645/f1p0/2667	NA	R01_cb8564_c19645/f1p0/2667	NA	NA
R01_cb18456_c888/f1p3/796	NA	R01_cb18456_c888/f1p3/796	NA	NA
R01_cb18039_c0/f1p0/749	NA	NA	R01_cb18039_c0/f1p0/749	R01_cb18039_c0/f1p0/749
R01_cb10337_c9/f1p2/373	R01_cb10337_c9/f1p2/373	R01_cb10337_c9/f1p2/373	NA	R01_cb10337_c9/f1p2/373
R01_cb7838_c3/f1p0/2768	NA	R01_cb7838_c3/f1p0/2768	NA	R01_cb7838_c3/f1p0/2768
R01_cb707_c7/f1p0/497	R01_cb707_c7/f1p0/497	R01_cb707_c7/f1p0/497	R01_cb707_c7/f1p0/497	R01_cb707_c7/f1p0/497
R01_cb8564_c75518/f1p0/3000	R01_cb8564_c75518/f1p0/3000	R01_cb8564_c75518/f1p0/3000	R01_cb8564_c75518/f1p0/3000	R01_cb8564_c75518/f1p0/3000
R01_cb1628_c4/f1p0/2042	NA	NA	NA	R01_cb1628_c4/f1p0/2042
R01_cb16119_c0/f2p0/425	NA	R01_cb16119_c0/f2p0/425	R01_cb16119_c0/f2p0/425	R01_cb16119_c0/f2p0/425
R01_cb16613_c2/f1p0/1093	NA	NA	NA	R01_cb16613_c2/f1p0/1093
R01_cb18456_c1831/f1p0/1418	R01_cb18456_c1831/f1p0/1418	R01_cb18456_c1831/f1p0/1418	R01_cb18456_c1831/f1p0/1418	R01_cb18456_c1831/f1p0/1418
R01_cb4576_c94/f1p0/2970	NA	NA	NA	R01_cb4576_c94/f1p0/2970
R01_cb8564_c4639/f1p0/2649	NA	R01_cb8564_c4639/f1p0/2649	NA	NA
R01_cb18287_c1/f1p0/905	NA	R01_cb18287_c1/f1p0/905	R01_cb18287_c1/f1p0/905	R01_cb18287_c1/f1p0/905
R01_cb8564_c85118/f1p0/4007	NA	R01_cb8564_c85118/f1p0/4007	NA	NA
R01_cb6758_c2/f1p0/2762	NA	NA	NA	R01_cb6758_c2/f1p0/2762
R01_cb9382_c1/f1p0/2149	NA	NA	NA	R01_cb9382_c1/f1p0/2149
R01_cb8564_c84564/f1p0/2312	NA	NA	NA	R01_cb8564_c84564/f1p0/2312
R01_cb8564_c20545/f1p0/4047	NA	R01_cb8564_c20545/f1p0/4047	NA	NA
R01_cb8564_c116543/f1p0/2241	NA	R01_cb8564_c116543/f1p0/2241	R01_cb8564_c116543/f1p0/2241	R01_cb8564_c116543/f1p0/2241
R01_cb13045_c9/f1p0/784	NA	NA	R01_cb13045_c9/f1p0/784	R01_cb13045_c9/f1p0/784
R01_cb3120_c47/f1p1/3369	R01_cb3120_c47/f1p1/3369	R01_cb3120_c47/f1p1/3369	NA	NA

R01_cb10025_c11/f1p0/1638	R01_cb10025_c11/f1p0/1638	R01_cb10025_c11/f1p0/1638	NA	NA
R01_cb2576_c0/f3p0/3843	NA	NA	R01_cb2576_c0/f3p0/3843	NA
R01_cb12641_c12/f1p0/1005	NA	NA	NA	R01_cb12641_c12/f1p0/1005
R01_cb5373_c7/f1p0/2625	NA	NA	NA	R01_cb5373_c7/f1p0/2625
R01_cb13279_c3/f1p0/881	NA	NA	NA	R01_cb13279_c3/f1p0/881
R01_cb693_c5/f1p0/2880	NA	NA	NA	R01_cb693_c5/f1p0/2880
R01_cb18386_c4/f1p1/1388	NA	R01_cb18386_c4/f1p1/1388	NA	NA
R01_cb5830_c0/f2p1/2108	NA	NA	NA	R01_cb5830_c0/f2p1/2108
R01_cb16860_c0/f3p0/830	NA	R01_cb16860_c0/f3p0/830	NA	NA
R01_cb1230_c4/f1p0/3498	NA	R01_cb1230_c4/f1p0/3498	R01_cb1230_c4/f1p0/3498	R01_cb1230_c4/f1p0/3498
R01_cb8564_c128525/f1p0/3961	NA	R01_cb8564_c128525/f1p0/3961	R01_cb8564_c128525/f1p0/3961	NA
R01_cb8564_c34985/f1p0/3209	R01_cb8564_c34985/f1p0/3209	R01_cb8564_c34985/f1p0/3209	R01_cb8564_c34985/f1p0/3209	R01_cb8564_c34985/f1p0/3209
R01_cb4962_c2/f1p0/2493	NA	R01_cb4962_c2/f1p0/2493	NA	NA
R01_cb12505_c9/f1p0/310	R01_cb12505_c9/f1p0/310	R01_cb12505_c9/f1p0/310	R01_cb12505_c9/f1p0/310	R01_cb12505_c9/f1p0/310
R01_cb10024_c663/f1p0/683	NA	NA	R01_cb10024_c663/f1p0/683	R01_cb10024_c663/f1p0/683
R01_cb3445_c5/f1p1/2894	NA	NA	NA	R01_cb3445_c5/f1p1/2894
R01_cb8564_c127999/f1p0/2454	NA	R01_cb8564_c127999/f1p0/2454	NA	NA
R01_cb8564_c114241/f1p0/2219	NA	R01_cb8564_c114241/f1p0/2219	R01_cb8564_c114241/f1p0/2219	R01_cb8564_c114241/f1p0/2219
R01_cb8564_c22989/f1p0/4427	NA	R01_cb8564_c22989/f1p0/4427	NA	NA
R01_cb3359_c20/f1p0/4121	NA	R01_cb3359_c20/f1p0/4121	NA	NA
R01_cb3103_c0/f1p0/3719	R01_cb3103_c0/f1p0/3719	R01_cb3103_c0/f1p0/3719	NA	NA
R01_cb15934_c0/f1p0/798	NA	R01_cb15934_c0/f1p0/798	NA	R01_cb15934_c0/f1p0/798

R01_cb4000_c2/f1p0/2213	NA	R01_cb4000_c2/f1p0/2213	NA	NA
R01_cb8564_c41423/f1p1/2878	NA	NA	R01_cb8564_c41423/f1p1/2878	NA
R01_cb16387_c11/f1p0/1673	NA	NA	NA	R01_cb16387_c11/f1p0/1673
R01_cb8564_c74184/f1p1/3323	NA	R01_cb8564_c74184/f1p1/3323	NA	R01_cb8564_c74184/f1p1/3323
R01_cb4800_c3/f1p0/3705	NA	R01_cb4800_c3/f1p0/3705	NA	NA
R01_cb4434_c5/f1p0/3356	NA	NA	NA	R01_cb4434_c5/f1p0/3356
R01_cb709_c13/f1p0/2493	NA	R01_cb709_c13/f1p0/2493	NA	NA
R01_cb5486_c5/f1p0/2267	R01_cb5486_c5/f1p0/2267	R01_cb5486_c5/f1p0/2267	R01_cb5486_c5/f1p0/2267	R01_cb5486_c5/f1p0/2267
R01_cb6057_c1/f1p0/2947	NA	NA	NA	R01_cb6057_c1/f1p0/2947
R01_cb8564_c88797/f3p1/1986	R01_cb8564_c88797/f3p1/1986	NA	NA	NA
R01_cb1653_c2/f1p0/4075	NA	NA	NA	R01_cb1653_c2/f1p0/4075
R01_cb8324_c6/f1p0/1418	NA	NA	NA	R01_cb8324_c6/f1p0/1418
R01_cb17863_c1/f1p0/1480	NA	NA	NA	R01_cb17863_c1/f1p0/1480
R01_cb6507_c10/f1p0/1082	NA	NA	NA	R01_cb6507_c10/f1p0/1082
R01_cb8564_c53520/f1p0/2997	NA	NA	R01_cb8564_c53520/f1p0/2997	NA
R01_cb13585_c5/f1p0/428	R01_cb13585_c5/f1p0/428	R01_cb13585_c5/f1p0/428	NA	NA
R01_cb8564_c83513/f1p0/2970	NA	NA	NA	R01_cb8564_c83513/f1p0/2970
R01_cb6406_c14/f1p0/2905	NA	R01_cb6406_c14/f1p0/2905	NA	NA
R01_cb8564_c88825/f1p0/3058	R01_cb8564_c88825/f1p0/3058	NA	NA	NA
R01_cb3543_c5/f1p0/3177	NA	R01_cb3543_c5/f1p0/3177	NA	NA
R01_cb6356_c4/f1p0/1535	NA	NA	NA	R01_cb6356_c4/f1p0/1535
R01_cb1333_c36/f1p0/3149	R01_cb1333_c36/f1p0/3149	R01_cb1333_c36/f1p0/3149	R01_cb1333_c36/f1p0/3149	R01_cb1333_c36/f1p0/3149
R01_cb5421_c8/f1p0/1843	NA	R01_cb5421_c8/f1p0/1843	R01_cb5421_c8/f1p0/1843	NA
R01_cb6802_c35/f1p0/1940	NA	R01_cb6802_c35/f1p0/1940	R01_cb6802_c35/f1p0/1940	R01_cb6802_c35/f1p0/1940

R01_cb8564_c77611/f1p0/2668	NA	R01_cb8564_c77611/f1p0/2668	R01_cb8564_c77611/f1p0/2668	R01_cb8564_c77611/f1p0/2668
R01_cb16086_c0/f2p0/386	R01_cb16086_c0/f2p0/386	R01_cb16086_c0/f2p0/386	R01_cb16086_c0/f2p0/386	R01_cb16086_c0/f2p0/386
R01_cb6686_c2/f1p0/2779	NA	R01_cb6686_c2/f1p0/2779	R01_cb6686_c2/f1p0/2779	NA
R01_cb15118_c5/f1p0/604	NA	NA	NA	R01_cb15118_c5/f1p0/604
R01_cb3169_c2/f1p0/1985	NA	NA	NA	R01_cb3169_c2/f1p0/1985
R01_cb8107_c3/f1p0/658	R01_cb8107_c3/f1p0/658	R01_cb8107_c3/f1p0/658	R01_cb8107_c3/f1p0/658	R01_cb8107_c3/f1p0/658
R01_cb545_c2/f1p0/4751	NA	R01_cb545_c2/f1p0/4751	NA	NA
R01_cb14141_c4/f1p0/877	NA	R01_cb14141_c4/f1p0/877	R01_cb14141_c4/f1p0/877	R01_cb14141_c4/f1p0/877
R01_cb18257_c0/f1p0/1739	NA	NA	NA	R01_cb18257_c0/f1p0/1739
R01_cb965_c51/f1p0/2659	NA	R01_cb965_c51/f1p0/2659	NA	NA
R01_cb17756_c19/f1p0/1743	NA	R01_cb17756_c19/f1p0/1743	R01_cb17756_c19/f1p0/1743	NA
R01_cb8564_c110324/f1p0/1912	NA	R01_cb8564_c110324/f1p0/1912	NA	R01_cb8564_c110324/f1p0/1912
R01_cb10444_c2/f1p0/2271	NA	R01_cb10444_c2/f1p0/2271	NA	NA
R01_cb12165_c20/f1p1/970	NA	R01_cb12165_c20/f1p1/970	NA	NA
R01_cb13440_c1/f1p0/483	NA	R01_cb13440_c1/f1p0/483	NA	R01_cb13440_c1/f1p0/483
R01_cb11830_c0/f1p0/855	NA	R01_cb11830_c0/f1p0/855	R01_cb11830_c0/f1p0/855	R01_cb11830_c0/f1p0/855
R01_cb16136_c8/f1p0/1730	NA	NA	NA	R01_cb16136_c8/f1p0/1730
R01_cb11250_c1/f1p0/1955	R01_cb11250_c1/f1p0/1955	R01_cb11250_c1/f1p0/1955	R01_cb11250_c1/f1p0/1955	R01_cb11250_c1/f1p0/1955
R01_cb8564_c24180/f1p0/4190	NA	NA	NA	R01_cb8564_c24180/f1p0/4190
R01_cb8564_c3818/f1p1/3602	NA	R01_cb8564_c3818/f1p1/3602	NA	NA
R01_cb11636_c3/f1p0/2559	R01_cb11636_c3/f1p0/2559	R01_cb11636_c3/f1p0/2559	NA	NA
R01_cb14982_c2/f2p0/511	NA	NA	R01_cb14982_c2/f2p0/511	R01_cb14982_c2/f2p0/511
R01_cb16706_c0/f2p0/895	R01_cb16706_c0/f2p0/895	R01_cb16706_c0/f2p0/895	NA	R01_cb16706_c0/f2p0/895
R01_cb6365_c4/f1p2/2876	NA	R01_cb6365_c4/f1p2/2876	NA	NA
R01_cb5698_c8/f1p0/1782	NA	R01_cb5698_c8/f1p0/1782	NA	NA

R01_cb8564_c119490/f1p0/2228	NA	R01_cb8564_c119490/f1p0/2228	NA	R01_cb8564_c119490/f1p0/2228
R01_cb10034_c46/f1p6/1244	NA	R01_cb10034_c46/f1p6/1244	NA	NA
R01_cb7014_c3/f1p1/2715	NA	R01_cb7014_c3/f1p1/2715	NA	NA
R01_cb2309_c4/f1p0/2747	NA	R01_cb2309_c4/f1p0/2747	NA	NA
R01_cb8564_c49686/f1p0/2254	NA	R01_cb8564_c49686/f1p0/2254	NA	NA
R01_cb8564_c19798/f1p0/4232	R01_cb8564_c19798/f1p0/4232	R01_cb8564_c19798/f1p0/4232	NA	R01_cb8564_c19798/f1p0/4232
R01_cb4514_c0/f4p1/1950	NA	NA	NA	R01_cb4514_c0/f4p1/1950
R01_cb18199_c1/f1p0/629	NA	R01_cb18199_c1/f1p0/629	NA	R01_cb18199_c1/f1p0/629
R01_cb8564_c1880/f1p0/3005	NA	NA	NA	R01_cb8564_c1880/f1p0/3005
R01_cb7654_c4/f1p0/497	NA	R01_cb7654_c4/f1p0/497	NA	R01_cb7654_c4/f1p0/497
R01_cb7946_c4/f1p0/1103	NA	R01_cb7946_c4/f1p0/1103	R01_cb7946_c4/f1p0/1103	NA
R01_cb4653_c1/f1p0/3426	NA	R01_cb4653_c1/f1p0/3426	NA	R01_cb4653_c1/f1p0/3426
R01_cb2042_c1/f1p0/2332	NA	NA	NA	R01_cb2042_c1/f1p0/2332
R01_cb8564_c22258/f1p0/3986	NA	R01_cb8564_c22258/f1p0/3986	NA	R01_cb8564_c22258/f1p0/3986
R01_cb8564_c76928/f1p1/2983	NA	R01_cb8564_c76928/f1p1/2983	R01_cb8564_c76928/f1p1/2983	NA
R01_cb15811_c5/f7p0/809	NA	R01_cb15811_c5/f7p0/809	R01_cb15811_c5/f7p0/809	R01_cb15811_c5/f7p0/809
R01_cb16178_c1/f1p0/1078	R01_cb16178_c1/f1p0/1078	R01_cb16178_c1/f1p0/1078	R01_cb16178_c1/f1p0/1078	R01_cb16178_c1/f1p0/1078
R01_cb8564_c1408/f1p0/3090	NA	R01_cb8564_c1408/f1p0/3090	NA	NA
R01_cb8564_c79171/f1p0/2207	R01_cb8564_c79171/f1p0/2207	R01_cb8564_c79171/f1p0/2207	R01_cb8564_c79171/f1p0/2207	R01_cb8564_c79171/f1p0/2207
R01_cb4414_c3/f1p0/2241	NA	R01_cb4414_c3/f1p0/2241	NA	NA
R01_cb13766_c11/f1p0/634	R01_cb13766_c11/f1p0/634	R01_cb13766_c11/f1p0/634	R01_cb13766_c11/f1p0/634	NA
R01_cb8564_c12071/f1p0/3873	NA	R01_cb8564_c12071/f1p0/3873	NA	NA
R01_cb16564_c1/f1p0/1132	NA	R01_cb16564_c1/f1p0/1132	R01_cb16564_c1/f1p0/1132	R01_cb16564_c1/f1p0/1132

R01_cb7019_c2/f1p0/2573	NA	R01_cb7019_c2/f1p0/2573	R01_cb7019_c2/f1p0/2573	R01_cb7019_c2/f1p0/2573
R01_cb9606_c64/f1p0/1698	NA	R01_cb9606_c64/f1p0/1698	NA	NA
R01_cb4576_c133/f1p0/2323	R01_cb4576_c133/f1p0/2323	R01_cb4576_c133/f1p0/2323	R01_cb4576_c133/f1p0/2323	R01_cb4576_c133/f1p0/2323
R01_cb3368_c0/f1p0/3642	NA	NA	NA	R01_cb3368_c0/f1p0/3642
R01_cb15533_c11/f1p0/1222	NA	R01_cb15533_c11/f1p0/1222	NA	NA
R01_cb14321_c1/f1p0/1621	NA	R01_cb14321_c1/f1p0/1621	NA	NA
R01_cb9506_c7/f1p1/1246	NA	NA	NA	R01_cb9506_c7/f1p1/1246
R01_cb8900_c10/f1p0/2151	NA	NA	NA	R01_cb8900_c10/f1p0/2151
R01_cb8564_c69376/f2p0/2056	NA	R01_cb8564_c69376/f2p0/2056	NA	NA
R01_cb14523_c2/f1p0/1724	NA	NA	NA	R01_cb14523_c2/f1p0/1724
R01_cb18456_c1415/f1p0/449	R01_cb18456_c1415/f1p0/449	R01_cb18456_c1415/f1p0/449	R01_cb18456_c1415/f1p0/449	R01_cb18456_c1415/f1p0/449
R01_cb2528_c2/f1p0/4010	NA	R01_cb2528_c2/f1p0/4010	NA	NA
R01_cb9305_c3/f1p1/2433	NA	R01_cb9305_c3/f1p1/2433	NA	NA
R01_cb4499_c5/f1p0/3094	NA	NA	NA	R01_cb4499_c5/f1p0/3094
R01_cb18456_c1644/f2p0/558	NA	R01_cb18456_c1644/f2p0/558	R01_cb18456_c1644/f2p0/558	NA
R01_cb2577_c11/f1p0/3419	NA	R01_cb2577_c11/f1p0/3419	NA	NA
R01_cb13490_c4/f1p0/961	NA	R01_cb13490_c4/f1p0/961	R01_cb13490_c4/f1p0/961	R01_cb13490_c4/f1p0/961
R01_cb3558_c7/f1p2/2733	NA	NA	NA	R01_cb3558_c7/f1p2/2733
R01_cb16146_c2/f1p0/1257	NA	R01_cb16146_c2/f1p0/1257	NA	NA
R01_cb8564_c120080/f1p0/2166	NA	R01_cb8564_c120080/f1p0/2166	NA	NA
R01_cb12292_c16/f1p0/1881	NA	R01_cb12292_c16/f1p0/1881	NA	NA
R01_cb2870_c5/f1p0/3045	NA	NA	NA	R01_cb2870_c5/f1p0/3045
R01_cb13165_c2/f1p0/559	NA	R01_cb13165_c2/f1p0/559	NA	NA
R01_cb1936_c1/f1p0/3489	R01_cb1936_c1/f1p0/3489	R01_cb1936_c1/f1p0/3489	R01_cb1936_c1/f1p0/3489	R01_cb1936_c1/f1p0/3489
R01_cb8564_c13297/f1p0/2043	NA	R01_cb8564_c13297/f1p0/2043	NA	R01_cb8564_c13297/f1p0/2043

R01_cb8564_c15952/f1p0/4578	NA	R01_cb8564_c15952/f1p0/4578	R01_cb8564_c15952/f1p0/4578	NA
R01_cb8564_c73180/f1p0/3827	NA	NA	NA	R01_cb8564_c73180/f1p0/3827
R01_cb10017_c48/f1p2/979	NA	R01_cb10017_c48/f1p2/979	NA	NA
R01_cb18456_c6666/f1p0/535	NA	R01_cb18456_c6666/f1p0/535	R01_cb18456_c6666/f1p0/535	R01_cb18456_c6666/f1p0/535
R01_cb8564_c939/f2p0/3121	NA	NA	NA	R01_cb8564_c939/f2p0/3121
R01_cb8564_c84431/f1p0/2738	NA	R01_cb8564_c84431/f1p0/2738	NA	NA
R01_cb8564_c89179/f1p0/3763	NA	R01_cb8564_c89179/f1p0/3763	NA	NA
R01_cb1967_c18/f1p1/2406	NA	R01_cb1967_c18/f1p1/2406	R01_cb1967_c18/f1p1/2406	R01_cb1967_c18/f1p1/2406
R01_cb11234_c3/f1p0/1603	NA	R01_cb11234_c3/f1p0/1603	NA	NA
R01_cb17337_c1/f1p0/1348	R01_cb17337_c1/f1p0/1348	R01_cb17337_c1/f1p0/1348	R01_cb17337_c1/f1p0/1348	R01_cb17337_c1/f1p0/1348
R01_cb5676_c3/f1p0/3038	NA	NA	NA	R01_cb5676_c3/f1p0/3038
R01_cb9895_c8/f1p0/1643	NA	R01_cb9895_c8/f1p0/1643	NA	NA
R01_cb8173_c11/f1p3/1095	NA	R01_cb8173_c11/f1p3/1095	R01_cb8173_c11/f1p3/1095	R01_cb8173_c11/f1p3/1095
R01_cb5288_c5/f1p0/2975	NA	R01_cb5288_c5/f1p0/2975	NA	NA
R01_cb8564_c75002/f1p0/2669	R01_cb8564_c75002/f1p0/2669	R01_cb8564_c75002/f1p0/2669	R01_cb8564_c75002/f1p0/2669	NA
R01_cb6258_c10/f1p0/2621	NA	NA	NA	R01_cb6258_c10/f1p0/2621
R01_cb15669_c4/f1p0/409	R01_cb15669_c4/f1p0/409	R01_cb15669_c4/f1p0/409	R01_cb15669_c4/f1p0/409	R01_cb15669_c4/f1p0/409
R01_cb7530_c2/f1p0/2595	NA	NA	NA	R01_cb7530_c2/f1p0/2595
R01_cb8564_c71113/f1p0/2280	NA	NA	NA	R01_cb8564_c71113/f1p0/2280
R01_cb8564_c4481/f2p0/3109	NA	NA	NA	R01_cb8564_c4481/f2p0/3109
R01_cb10665_c2/f1p0/2949	R01_cb10665_c2/f1p0/2949	R01_cb10665_c2/f1p0/2949	NA	R01_cb10665_c2/f1p0/2949
R01_cb10536_c0/f1p0/1572	NA	R01_cb10536_c0/f1p0/1572	R01_cb10536_c0/f1p0/1572	R01_cb10536_c0/f1p0/1572
R01_cb14907_c2/f1p0/1693	NA	R01_cb14907_c2/f1p0/1693	NA	NA
R01_cb2985_c7/f1p0/3138	NA	NA	NA	R01_cb2985_c7/f1p0/3138
R01_cb5471_c20/f3p0/2612	NA	NA	NA	R01_cb5471_c20/f3p0/2612

R01_cb6356_c7/f1p0/438	R01_cb6356_c7/f1p0/438	R01_cb6356_c7/f1p0/438	NA	R01_cb6356_c7/f1p0/438
R01_cb6179_c0/f1p0/2919	NA	R01_cb6179_c0/f1p0/2919	NA	NA
R01_cb3415_c3/f1p0/4126	NA	R01_cb3415_c3/f1p0/4126	NA	NA
R01_cb10586_c2/f1p0/2001	NA	NA	NA	R01_cb10586_c2/f1p0/2001
R01_cb8441_c3/f2p1/1334	NA	NA	NA	R01_cb8441_c3/f2p1/1334
R01_cb18456_c7205/f1p0/917	R01_cb18456_c7205/f1p0/917	R01_cb18456_c7205/f1p0/917	R01_cb18456_c7205/f1p0/917	R01_cb18456_c7205/f1p0/917
R01_cb8564_c18465/f1p0/2920	NA	R01_cb8564_c18465/f1p0/2920	R01_cb8564_c18465/f1p0/2920	R01_cb8564_c18465/f1p0/2920
R01_cb402_c31/f1p0/1999	NA	NA	NA	R01_cb402_c31/f1p0/1999
R01_cb10560_c5/f1p0/1521	NA	R01_cb10560_c5/f1p0/1521	NA	NA
R01_cb13283_c9/f1p0/1806	NA	NA	NA	R01_cb13283_c9/f1p0/1806
R01_cb13204_c2/f1p0/1415	NA	NA	NA	R01_cb13204_c2/f1p0/1415
R01_cb8564_c78637/f1p0/2064	NA	R01_cb8564_c78637/f1p0/2064	R01_cb8564_c78637/f1p0/2064	R01_cb8564_c78637/f1p0/2064
R01_cb5717_c7/f1p0/2086	NA	R01_cb5717_c7/f1p0/2086	NA	R01_cb5717_c7/f1p0/2086
R01_cb4123_c78/f1p1/1364	NA	R01_cb4123_c78/f1p1/1364	NA	NA
R01_cb15443_c3/f1p0/1223	NA	NA	NA	R01_cb15443_c3/f1p0/1223
R01_cb9530_c4/f1p1/1777	NA	NA	NA	R01_cb9530_c4/f1p1/1777
R01_cb15568_c5/f1p0/947	NA	NA	NA	R01_cb15568_c5/f1p0/947
R01_cb8564_c22529/f1p0/2467	NA	R01_cb8564_c22529/f1p0/2467	R01_cb8564_c22529/f1p0/2467	R01_cb8564_c22529/f1p0/2467
R01_cb1113_c2/f2p2/3098	NA	R01_cb1113_c2/f2p2/3098	NA	R01_cb1113_c2/f2p2/3098
R01_cb16935_c0/f1p0/1450	NA	NA	R01_cb16935_c0/f1p0/1450	R01_cb16935_c0/f1p0/1450
R01_cb8564_c70849/f1p0/4109	NA	R01_cb8564_c70849/f1p0/4109	NA	NA
R01_cb5090_c6/f1p0/2471	NA	NA	NA	R01_cb5090_c6/f1p0/2471
R01_cb18456_c6671/f1p0/532	R01_cb18456_c6671/f1p0/532	R01_cb18456_c6671/f1p0/532	R01_cb18456_c6671/f1p0/532	R01_cb18456_c6671/f1p0/532
R01_cb14254_c0/f1p0/871	NA	R01_cb14254_c0/f1p0/871	NA	R01_cb14254_c0/f1p0/871
R01_cb9376_c0/f1p0/2100	R01_cb9376_c0/f1p0/2100	R01_cb9376_c0/f1p0/2100	NA	NA
R01_cb11850_c10/f4p0/485	NA	R01_cb11850_c10/f4p0/485	NA	NA

R01_cb2253_c9/f1p1/1001	NA	R01_cb2253_c9/f1p1/1001	NA	NA
R01_cb15436_c2/f1p1/750	NA	NA	NA	R01_cb15436_c2/f1p1/750
R01_cb3863_c2/f1p0/3505	NA	NA	NA	R01_cb3863_c2/f1p0/3505
R01_cb8564_c10976/f1p0/4679	R01_cb8564_c10976/f1p0/4679	R01_cb8564_c10976/f1p0/4679	NA	NA
R01_cb8564_c79071/f1p0/1947	R01_cb8564_c79071/f1p0/1947	R01_cb8564_c79071/f1p0/1947	R01_cb8564_c79071/f1p0/1947	R01_cb8564_c79071/f1p0/1947
R01_cb17558_c0/f3p0/781	R01_cb17558_c0/f3p0/781	R01_cb17558_c0/f3p0/781	R01_cb17558_c0/f3p0/781	R01_cb17558_c0/f3p0/781
R01_cb1969_c4/f1p0/1750	R01_cb1969_c4/f1p0/1750	R01_cb1969_c4/f1p0/1750	R01_cb1969_c4/f1p0/1750	R01_cb1969_c4/f1p0/1750
R01_cb8564_c159669/f1p1/2190	NA	R01_cb8564_c159669/f1p1/2190	R01_cb8564_c159669/f1p1/2190	R01_cb8564_c159669/f1p1/2190
R01_cb218_c15/f1p0/4605	NA	NA	NA	R01_cb218_c15/f1p0/4605
R01_cb8849_c1/f1p0/2395	NA	NA	NA	R01_cb8849_c1/f1p0/2395
R01_cb18286_c0/f1p0/1126	NA	R01_cb18286_c0/f1p0/1126	R01_cb18286_c0/f1p0/1126	R01_cb18286_c0/f1p0/1126
R01_cb3564_c12/f1p0/3482	NA	NA	NA	R01_cb3564_c12/f1p0/3482
R01_cb8564_c15671/f1p0/3039	NA	R01_cb8564_c15671/f1p0/3039	NA	NA
R01_cb14869_c3/f1p0/896	NA	NA	R01_cb14869_c3/f1p0/896	R01_cb14869_c3/f1p0/896
R01_cb14816_c10/f1p0/640	R01_cb14816_c10/f1p0/640	R01_cb14816_c10/f1p0/640	R01_cb14816_c10/f1p0/640	R01_cb14816_c10/f1p0/640
R01_cb15151_c1/f1p0/967	NA	NA	R01_cb15151_c1/f1p0/967	R01_cb15151_c1/f1p0/967
R01_cb10089_c9/f1p1/1077	NA	R01_cb10089_c9/f1p1/1077	NA	NA
R01_cb15894_c0/f1p0/927	R01_cb15894_c0/f1p0/927	R01_cb15894_c0/f1p0/927	R01_cb15894_c0/f1p0/927	R01_cb15894_c0/f1p0/927
R01_cb8779_c8/f1p0/2841	NA	NA	NA	R01_cb8779_c8/f1p0/2841
R01_cb5633_c11/f5p1/2057	R01_cb5633_c11/f5p1/2057	R01_cb5633_c11/f5p1/2057	NA	R01_cb5633_c11/f5p1/2057
R01_cb7836_c5/f1p0/1885	NA	NA	NA	R01_cb7836_c5/f1p0/1885
R01_cb17127_c4/f1p0/7136	NA	R01_cb17127_c4/f1p0/7136	NA	R01_cb17127_c4/f1p0/7136
R01_cb92_c4/f1p0/2333	NA	NA	NA	R01_cb92_c4/f1p0/2333

R01_cb12743_c3/f1p0/1050	NA	NA	NA	R01_cb12743_c3/f1p0/1050
R01_cb1656_c16/f1p0/3581	R01_cb1656_c16/f1p0/3581	R01_cb1656_c16/f1p0/3581	NA	NA
R01_cb8564_c109387/f4p0/2127	NA	NA	NA	R01_cb8564_c109387/f4p0/2127
R01_cb10583_c1/f1p0/3015	NA	R01_cb10583_c1/f1p0/3015	NA	R01_cb10583_c1/f1p0/3015
R01_cb8475_c6/f1p0/647	NA	R01_cb8475_c6/f1p0/647	NA	R01_cb8475_c6/f1p0/647
R01_cb14805_c6/f1p0/738	R01_cb14805_c6/f1p0/738	R01_cb14805_c6/f1p0/738	NA	NA
R01_cb12973_c22/f1p0/453	NA	R01_cb12973_c22/f1p0/453	NA	NA
R01_cb8564_c17933/f1p0/2278	NA	NA	NA	R01_cb8564_c17933/f1p0/2278
R01_cb14179_c5/f4p1/491	NA	NA	NA	R01_cb14179_c5/f4p1/491
R01_cb6719_c81/f1p0/2048	R01_cb6719_c81/f1p0/2048	R01_cb6719_c81/f1p0/2048	R01_cb6719_c81/f1p0/2048	R01_cb6719_c81/f1p0/2048
R01_cb2367_c3/f1p0/3067	R01_cb2367_c3/f1p0/3067	R01_cb2367_c3/f1p0/3067	R01_cb2367_c3/f1p0/3067	NA
R01_cb9606_c90/f1p0/1361	NA	R01_cb9606_c90/f1p0/1361	NA	R01_cb9606_c90/f1p0/1361
R01_cb6608_c15/f1p0/2369	NA	NA	NA	R01_cb6608_c15/f1p0/2369
R01_cb17814_c1/f1p1/657	NA	R01_cb17814_c1/f1p1/657	R01_cb17814_c1/f1p1/657	R01_cb17814_c1/f1p1/657
R01_cb8564_c11499/f1p1/2333	NA	NA	R01_cb8564_c11499/f1p1/2333	NA
R01_cb8104_c1/f1p0/2460	NA	NA	NA	R01_cb8104_c1/f1p0/2460
R01_cb12505_c8/f1p0/815	NA	R01_cb12505_c8/f1p0/815	NA	NA
R01_cb10505_c2/f1p0/3676	R01_cb10505_c2/f1p0/3676	R01_cb10505_c2/f1p0/3676	NA	NA
R01_cb8548_c2/f1p0/2336	NA	R01_cb8548_c2/f1p0/2336	NA	NA
R01_cb15846_c2/f1p1/1565	NA	R01_cb15846_c2/f1p1/1565	NA	NA
R01_cb8564_c120421/f1p0/3623	NA	R01_cb8564_c120421/f1p0/3623	NA	NA
R01_cb17973_c50/f1p0/842	NA	R01_cb17973_c50/f1p0/842	R01_cb17973_c50/f1p0/842	R01_cb17973_c50/f1p0/842
R01_cb8564_c70829/f1p0/3036	NA	NA	R01_cb8564_c70829/f1p0/3036	NA
R01_cb16861_c0/f2p0/768	NA	R01_cb16861_c0/f2p0/768	NA	NA

R01_cb8564_c23584/f1p4/2247	NA	NA	NA	R01_cb8564_c23584/f1p4/2247
R01_cb9759_c1/f2p1/1826	NA	NA	NA	R01_cb9759_c1/f2p1/1826
R01_cb10880_c8/f1p0/773	NA	R01_cb10880_c8/f1p0/773	NA	R01_cb10880_c8/f1p0/773
R01_cb8564_c2561/f1p0/2609	NA	R01_cb8564_c2561/f1p0/2609	R01_cb8564_c2561/f1p0/2609	NA
R01_cb18456_c2505/f1p0/488	R01_cb18456_c2505/f1p0/488	R01_cb18456_c2505/f1p0/488	R01_cb18456_c2505/f1p0/488	R01_cb18456_c2505/f1p0/488
R01_cb8564_c89631/f1p0/2976	R01_cb8564_c89631/f1p0/2976	R01_cb8564_c89631/f1p0/2976	NA	R01_cb8564_c89631/f1p0/2976
R01_cb16261_c2/f1p0/1266	R01_cb16261_c2/f1p0/1266	R01_cb16261_c2/f1p0/1266	R01_cb16261_c2/f1p0/1266	R01_cb16261_c2/f1p0/1266
R01_cb8564_c123784/f1p0/2528	NA	R01_cb8564_c123784/f1p0/2528	NA	NA
R01_cb9729_c4/f1p0/1602	R01_cb9729_c4/f1p0/1602	R01_cb9729_c4/f1p0/1602	R01_cb9729_c4/f1p0/1602	R01_cb9729_c4/f1p0/1602
R01_cb2045_c15/f4p1/2535	NA	NA	NA	R01_cb2045_c15/f4p1/2535
R01_cb6606_c8/f1p0/2513	NA	NA	NA	R01_cb6606_c8/f1p0/2513
R01_cb8564_c66201/f2p1/2384	NA	R01_cb8564_c66201/f2p1/2384	NA	R01_cb8564_c66201/f2p1/2384
R01_cb10475_c3/f1p0/1463	NA	R01_cb10475_c3/f1p0/1463	NA	R01_cb10475_c3/f1p0/1463
R01_cb7494_c2/f1p0/574	R01_cb7494_c2/f1p0/574	R01_cb7494_c2/f1p0/574	R01_cb7494_c2/f1p0/574	R01_cb7494_c2/f1p0/574
R01_cb7254_c5/f1p0/1881	NA	NA	NA	R01_cb7254_c5/f1p0/1881
R01_cb17354_c0/f2p1/1179	NA	NA	NA	R01_cb17354_c0/f2p1/1179
R01_cb8564_c3008/f1p0/2656	NA	NA	NA	R01_cb8564_c3008/f1p0/2656
R01_cb13289_c13/f2p2/1692	NA	NA	R01_cb13289_c13/f2p2/1692	NA
R01_cb11045_c0/f2p0/1862	NA	R01_cb11045_c0/f2p0/1862	NA	NA
R01_cb8564_c124678/f1p1/2933	NA	R01_cb8564_c124678/f1p1/2933	NA	NA
R01_cb3630_c1/f1p0/3551	NA	NA	R01_cb3630_c1/f1p0/3551	R01_cb3630_c1/f1p0/3551
R01_cb16092_c2/f2p0/508	R01_cb16092_c2/f2p0/508	R01_cb16092_c2/f2p0/508	R01_cb16092_c2/f2p0/508	R01_cb16092_c2/f2p0/508
R01_cb17486_c2/f1p0/569	NA	R01_cb17486_c2/f1p0/569	NA	R01_cb17486_c2/f1p0/569

R01_cb9763_c17/f1p0/1715	NA	R01_cb9763_c17/f1p0/1715	NA	NA
R01_cb10750_c1/f1p0/1977	R01_cb10750_c1/f1p0/1977	R01_cb10750_c1/f1p0/1977	R01_cb10750_c1/f1p0/1977	R01_cb10750_c1/f1p0/1977
R01_cb15188_c2/f1p0/1780	R01_cb15188_c2/f1p0/1780	R01_cb15188_c2/f1p0/1780	NA	R01_cb15188_c2/f1p0/1780
R01_cb10539_c4/f1p2/1124	NA	NA	NA	R01_cb10539_c4/f1p2/1124
R01_cb1487_c2/f1p0/3909	NA	NA	NA	R01_cb1487_c2/f1p0/3909
R01_cb10217_c2/f1p3/1693	NA	NA	NA	R01_cb10217_c2/f1p3/1693
R01_cb4263_c7/f1p0/586	R01_cb4263_c7/f1p0/586	R01_cb4263_c7/f1p0/586	NA	NA
R01_cb12584_c0/f2p0/1141	NA	NA	NA	R01_cb12584_c0/f2p0/1141
R01_cb1215_c15/f1p0/3339	NA	R01_cb1215_c15/f1p0/3339	NA	R01_cb1215_c15/f1p0/3339
R01_cb17550_c0/f1p0/836	NA	NA	R01_cb17550_c0/f1p0/836	R01_cb17550_c0/f1p0/836
R01_cb10915_c1/f1p0/2132	NA	R01_cb10915_c1/f1p0/2132	NA	NA
R01_cb10145_c6/f1p0/1592	NA	NA	NA	R01_cb10145_c6/f1p0/1592
R01_cb4141_c10/f1p0/2489	NA	R01_cb4141_c10/f1p0/2489	NA	NA
R01_cb13290_c2/f2p0/715	NA	R01_cb13290_c2/f2p0/715	NA	NA
R01_cb5223_c4/f1p0/2643	NA	R01_cb5223_c4/f1p0/2643	NA	NA
R01_cb14373_c5/f1p0/1437	NA	R01_cb14373_c5/f1p0/1437	R01_cb14373_c5/f1p0/1437	R01_cb14373_c5/f1p0/1437
R01_cb7544_c3/f1p0/1896	NA	R01_cb7544_c3/f1p0/1896	NA	NA
R01_cb8564_c128749/f1p0/2577	NA	NA	NA	R01_cb8564_c128749/f1p0/2577
R01_cb3344_c8/f1p1/2760	NA	NA	NA	R01_cb3344_c8/f1p1/2760
R01_cb11017_c1/f1p0/2135	NA	R01_cb11017_c1/f1p0/2135	R01_cb11017_c1/f1p0/2135	R01_cb11017_c1/f1p0/2135
R01_cb1333_c84/f1p0/3338	NA	R01_cb1333_c84/f1p0/3338	R01_cb1333_c84/f1p0/3338	R01_cb1333_c84/f1p0/3338
R01_cb16230_c1/f1p0/915	R01_cb16230_c1/f1p0/915	R01_cb16230_c1/f1p0/915	R01_cb16230_c1/f1p0/915	R01_cb16230_c1/f1p0/915
R01_cb7587_c1/f1p0/2581	NA	R01_cb7587_c1/f1p0/2581	NA	NA
R01_cb16172_c4/f1p0/5261	R01_cb16172_c4/f1p0/5261	R01_cb16172_c4/f1p0/5261	R01_cb16172_c4/f1p0/5261	NA
R01_cb11037_c0/f1p0/943	NA	NA	NA	R01_cb11037_c0/f1p0/943

R01_cb10787_c6/f2p0/1136	NA	NA	NA	R01_cb10787_c6/f2p0/1136
R01_cb2952_c2/f1p0/2978	NA	R01_cb2952_c2/f1p0/2978	NA	NA
R01_cb13585_c6/f1p0/1002	NA	R01_cb13585_c6/f1p0/1002	R01_cb13585_c6/f1p0/1002	R01_cb13585_c6/f1p0/1002
R01_cb218_c7/f1p0/4552	NA	NA	NA	R01_cb218_c7/f1p0/4552
R01_cb14068_c2/f1p0/1091	NA	R01_cb14068_c2/f1p0/1091	NA	NA
R01_cb8564_c40333/f1p2/4446	NA	NA	NA	R01_cb8564_c40333/f1p2/4446
R01_cb18456_c6928/f1p0/498	R01_cb18456_c6928/f1p0/498	R01_cb18456_c6928/f1p0/498	R01_cb18456_c6928/f1p0/498	R01_cb18456_c6928/f1p0/498
R01_cb14301_c0/f4p0/508	NA	R01_cb14301_c0/f4p0/508	NA	NA
R01_cb1377_c1/f1p0/2050	NA	NA	NA	R01_cb1377_c1/f1p0/2050
R01_cb8564_c121637/f1p0/3305	NA	R01_cb8564_c121637/f1p0/3305	NA	R01_cb8564_c121637/f1p0/3305
R01_cb15207_c2/f4p3/735	NA	R01_cb15207_c2/f4p3/735	NA	NA
R01_cb8564_c37416/f1p0/2155	R01_cb8564_c37416/f1p0/2155	R01_cb8564_c37416/f1p0/2155	R01_cb8564_c37416/f1p0/2155	R01_cb8564_c37416/f1p0/2155
R01_cb1259_c14/f1p0/3145	NA	NA	NA	R01_cb1259_c14/f1p0/3145
R01_cb10970_c11/f1p0/1730	NA	NA	NA	R01_cb10970_c11/f1p0/1730
R01_cb8564_c42417/f1p0/2894	NA	R01_cb8564_c42417/f1p0/2894	NA	R01_cb8564_c42417/f1p0/2894
R01_cb8564_c44931/f1p0/4411	NA	R01_cb8564_c44931/f1p0/4411	NA	NA
R01_cb9617_c9/f1p0/1935	NA	NA	NA	R01_cb9617_c9/f1p0/1935
R01_cb13392_c12/f1p0/964	NA	R01_cb13392_c12/f1p0/964	NA	NA
R01_cb6507_c12/f1p0/913	NA	NA	NA	R01_cb6507_c12/f1p0/913
R01_cb1998_c3/f1p0/2873	NA	R01_cb1998_c3/f1p0/2873	R01_cb1998_c3/f1p0/2873	R01_cb1998_c3/f1p0/2873
R01_cb15353_c0/f1p0/1386	NA	NA	NA	R01_cb15353_c0/f1p0/1386
R01_cb8020_c0/f1p0/2479	R01_cb8020_c0/f1p0/2479	R01_cb8020_c0/f1p0/2479	R01_cb8020_c0/f1p0/2479	R01_cb8020_c0/f1p0/2479
R01_cb897_c17/f1p0/3323	NA	R01_cb897_c17/f1p0/3323	NA	R01_cb897_c17/f1p0/3323
R01_cb8713_c1/f1p0/2286	NA	R01_cb8713_c1/f1p0/2286	NA	NA

R01_cb1800_c5/f1p1/2166	NA	NA	NA	R01_cb1800_c5/f1p1/2166
R01_cb17196_c2/f1p0/333	R01_cb17196_c2/f1p0/333	R01_cb17196_c2/f1p0/333	R01_cb17196_c2/f1p0/333	R01_cb17196_c2/f1p0/333
R01_cb7533_c12/f1p0/2253	NA	NA	NA	R01_cb7533_c12/f1p0/2253
R01_cb10398_c5/f1p0/1033	NA	R01_cb10398_c5/f1p0/1033	NA	R01_cb10398_c5/f1p0/1033
R01_cb6823_c4/f1p0/1086	NA	NA	NA	R01_cb6823_c4/f1p0/1086
R01_cb8564_c86799/f1p0/2117	NA	R01_cb8564_c86799/f1p0/2117	NA	R01_cb8564_c86799/f1p0/2117
R01_cb8564_c2586/f1p0/3527	NA	R01_cb8564_c2586/f1p0/3527	NA	R01_cb8564_c2586/f1p0/3527
R01_cb9972_c3/f1p0/1160	R01_cb9972_c3/f1p0/1160	R01_cb9972_c3/f1p0/1160	NA	NA
R01_cb12602_c0/f1p0/1745	NA	R01_cb12602_c0/f1p0/1745	NA	R01_cb12602_c0/f1p0/1745
R01_cb8023_c13/f1p0/1364	NA	NA	R01_cb8023_c13/f1p0/1364	R01_cb8023_c13/f1p0/1364
R01_cb18456_c6737/f1p4/765	NA	R01_cb18456_c6737/f1p4/765	NA	NA
R01_cb6945_c2/f1p1/1785	R01_cb6945_c2/f1p1/1785	NA	NA	R01_cb6945_c2/f1p1/1785
R01_cb13386_c14/f1p0/455	R01_cb13386_c14/f1p0/455	R01_cb13386_c14/f1p0/455	NA	NA
R01_cb18456_c1682/f2p0/573	R01_cb18456_c1682/f2p0/573	R01_cb18456_c1682/f2p0/573	R01_cb18456_c1682/f2p0/573	R01_cb18456_c1682/f2p0/573
R01_cb13726_c3/f1p0/861	NA	NA	NA	R01_cb13726_c3/f1p0/861
R01_cb11343_c3/f1p1/1815	NA	NA	NA	R01_cb11343_c3/f1p1/1815
R01_cb18574_c1/f1p0/855	NA	NA	NA	R01_cb18574_c1/f1p0/855
R01_cb15491_c2/f1p0/1656	NA	NA	NA	R01_cb15491_c2/f1p0/1656
R01_cb3483_c14/f1p0/2857	NA	NA	NA	R01_cb3483_c14/f1p0/2857
R01_cb18393_c1/f1p0/1743	R01_cb18393_c1/f1p0/1743	R01_cb18393_c1/f1p0/1743	R01_cb18393_c1/f1p0/1743	R01_cb18393_c1/f1p0/1743
R01_cb8751_c4/f1p0/2162	NA	NA	NA	R01_cb8751_c4/f1p0/2162
R01_cb781_c29/f1p0/2108	NA	NA	NA	R01_cb781_c29/f1p0/2108
R01_cb13662_c3/f1p0/641	R01_cb13662_c3/f1p0/641	R01_cb13662_c3/f1p0/641	NA	R01_cb13662_c3/f1p0/641
R01_cb2061_c4/f1p0/4072	NA	R01_cb2061_c4/f1p0/4072	NA	NA
R01_cb8564_c161243/f1p0/2731	NA	R01_cb8564_c161243/f1p0/2731	NA	NA

R01_cb8564_c115196/f1p0/2341	NA	NA	NA	R01_cb8564_c115196/f1p0/2341
R01_cb14209_c2/f1p0/760	NA	NA	R01_cb14209_c2/f1p0/760	R01_cb14209_c2/f1p0/760
R01_cb16447_c10/f15p3/905	NA	NA	NA	R01_cb16447_c10/f15p3/905
R01_cb8564_c1510/f1p0/2041	NA	R01_cb8564_c1510/f1p0/2041	NA	NA
R01_cb14664_c5/f1p0/1279	NA	R01_cb14664_c5/f1p0/1279	NA	NA
R01_cb2204_c2/f1p0/2049	R01_cb2204_c2/f1p0/2049	R01_cb2204_c2/f1p0/2049	R01_cb2204_c2/f1p0/2049	R01_cb2204_c2/f1p0/2049
R01_cb14770_c3/f3p0/1056	NA	NA	NA	R01_cb14770_c3/f3p0/1056
R01_cb12114_c11/f1p0/1029	NA	NA	NA	R01_cb12114_c11/f1p0/1029
R01_cb18409_c124/f1p0/397	R01_cb18409_c124/f1p0/397	R01_cb18409_c124/f1p0/397	R01_cb18409_c124/f1p0/397	R01_cb18409_c124/f1p0/397
R01_cb9287_c3/f1p0/705	R01_cb9287_c3/f1p0/705	R01_cb9287_c3/f1p0/705	R01_cb9287_c3/f1p0/705	R01_cb9287_c3/f1p0/705
R01_cb18456_c825/f1p7/800	NA	R01_cb18456_c825/f1p7/800	NA	NA
R01_cb9507_c4/f1p0/523	R01_cb9507_c4/f1p0/523	R01_cb9507_c4/f1p0/523	R01_cb9507_c4/f1p0/523	R01_cb9507_c4/f1p0/523
R01_cb18353_c0/f1p0/1355	NA	R01_cb18353_c0/f1p0/1355	R01_cb18353_c0/f1p0/1355	R01_cb18353_c0/f1p0/1355
R01_cb16665_c3/f1p0/1004	NA	NA	R01_cb16665_c3/f1p0/1004	R01_cb16665_c3/f1p0/1004
R01_cb13230_c0/f5p0/640	NA	R01_cb13230_c0/f5p0/640	NA	NA
R01_cb1683_c33/f1p0/2889	NA	R01_cb1683_c33/f1p0/2889	NA	NA
R01_cb8564_c52452/f1p0/3727	NA	R01_cb8564_c52452/f1p0/3727	NA	NA
R01_cb8564_c118043/f1p0/2613	NA	R01_cb8564_c118043/f1p0/2613	NA	NA
R01_cb14125_c120/f1p0/602	NA	NA	R01_cb14125_c120/f1p0/602	NA
R01_cb16071_c0/f1p0/1005	R01_cb16071_c0/f1p0/1005	R01_cb16071_c0/f1p0/1005	NA	R01_cb16071_c0/f1p0/1005
R01_cb302_c5/f1p3/3518	NA	NA	NA	R01_cb302_c5/f1p3/3518
R01_cb1161_c6/f1p0/1574	NA	NA	NA	R01_cb1161_c6/f1p0/1574
R01_cb2022_c19/f1p0/1643	NA	NA	NA	R01_cb2022_c19/f1p0/1643
R01_cb5443_c4/f1p0/2834	R01_cb5443_c4/f1p0/2834	R01_cb5443_c4/f1p0/2834	NA	NA

R01_cb8564_c85517/f1p0/2859	NA	NA	NA	R01_cb8564_c85517/f1p0/2859
R01_cb10238_c4/f1p0/381	R01_cb10238_c4/f1p0/381	R01_cb10238_c4/f1p0/381	NA	NA
R01_cb6181_c55/f1p1/2098	NA	R01_cb6181_c55/f1p1/2098	NA	NA
R01_cb9897_c7/f1p1/1738	NA	R01_cb9897_c7/f1p1/1738	NA	NA
R01_cb381_c1/f1p0/3954	NA	R01_cb381_c1/f1p0/3954	NA	NA
R01_cb14905_c0/f1p0/590	NA	NA	R01_cb14905_c0/f1p0/590	R01_cb14905_c0/f1p0/590
R01_cb8564_c1323/f1p1/2810	NA	NA	NA	R01_cb8564_c1323/f1p1/2810
R01_cb13886_c2/f1p0/510	NA	R01_cb13886_c2/f1p0/510	NA	NA
R01_cb8991_c4/f1p0/2079	NA	NA	NA	R01_cb8991_c4/f1p0/2079
R01_cb4036_c3/f1p1/2063	NA	R01_cb4036_c3/f1p1/2063	NA	NA
R01_cb8564_c85325/f1p0/2780	R01_cb8564_c85325/f1p0/2780	R01_cb8564_c85325/f1p0/2780	R01_cb8564_c85325/f1p0/2780	NA
R01_cb8564_c82130/f1p0/4463	NA	R01_cb8564_c82130/f1p0/4463	NA	R01_cb8564_c82130/f1p0/4463
R01_cb15418_c5/f1p1/1059	NA	NA	NA	R01_cb15418_c5/f1p1/1059
R01_cb8564_c76139/f1p0/2005	NA	R01_cb8564_c76139/f1p0/2005	R01_cb8564_c76139/f1p0/2005	R01_cb8564_c76139/f1p0/2005
R01_cb8564_c86161/f2p0/2876	NA	R01_cb8564_c86161/f2p0/2876	NA	NA
R01_cb18409_c149/f1p0/501	NA	R01_cb18409_c149/f1p0/501	NA	NA
R01_cb8564_c68745/f3p0/2856	NA	R01_cb8564_c68745/f3p0/2856	NA	NA
R01_cb8564_c24818/f1p0/1944	NA	R01_cb8564_c24818/f1p0/1944	NA	NA
R01_cb18456_c7122/f1p0/415	NA	R01_cb18456_c7122/f1p0/415	R01_cb18456_c7122/f1p0/415	R01_cb18456_c7122/f1p0/415
R01_cb9292_c21/f1p0/778	R01_cb9292_c21/f1p0/778	R01_cb9292_c21/f1p0/778	NA	R01_cb9292_c21/f1p0/778
R01_cb15093_c1/f1p0/1775	NA	NA	NA	R01_cb15093_c1/f1p0/1775
R01_cb8564_c69618/f1p0/2659	NA	NA	NA	R01_cb8564_c69618/f1p0/2659
R01_cb6682_c6/f1p1/2237	NA	R01_cb6682_c6/f1p1/2237	NA	NA
R01_cb13430_c0/f1p0/1552	NA	NA	R01_cb13430_c0/f1p0/1552	R01_cb13430_c0/f1p0/1552
R01_cb10230_c6/f3p0/478	NA	R01_cb10230_c6/f3p0/478	NA	NA

R01_cb12397_c0/f1p0/788	R01_cb12397_c0/f1p0/788	R01_cb12397_c0/f1p0/788	R01_cb12397_c0/f1p0/788	NA
R01_cb15155_c0/f1p0/942	NA	R01_cb15155_c0/f1p0/942	NA	NA
R01_cb8564_c69996/f1p0/2880	NA	R01_cb8564_c69996/f1p0/2880	NA	NA
R01_cb14344_c0/f1p0/900	R01_cb14344_c0/f1p0/900	R01_cb14344_c0/f1p0/900	R01_cb14344_c0/f1p0/900	R01_cb14344_c0/f1p0/900
R01_cb6602_c40/f1p0/3304	NA	NA	NA	R01_cb6602_c40/f1p0/3304
R01_cb9154_c16/f1p0/1122	NA	NA	NA	R01_cb9154_c16/f1p0/1122
R01_cb15950_c1/f2p1/1352	NA	NA	NA	R01_cb15950_c1/f2p1/1352
R01_cb4233_c198/f1p0/2372	NA	NA	R01_cb4233_c198/f1p0/2372	R01_cb4233_c198/f1p0/2372
R01_cb8564_c124170/f1p0/1999	R01_cb8564_c124170/f1p0/1999	R01_cb8564_c124170/f1p0/1999	NA	R01_cb8564_c124170/f1p0/1999
R01_cb8564_c15807/f1p1/2210	NA	R01_cb8564_c15807/f1p1/2210	NA	NA
R01_cb7603_c1/f1p0/2574	NA	R01_cb7603_c1/f1p0/2574	NA	NA
R01_cb11430_c2/f2p0/704	NA	R01_cb11430_c2/f2p0/704	NA	R01_cb11430_c2/f2p0/704
R01_cb7412_c19/f1p0/674	NA	NA	NA	R01_cb7412_c19/f1p0/674
R01_cb8564_c91159/f1p0/3159	NA	NA	NA	R01_cb8564_c91159/f1p0/3159
R01_cb8564_c12915/f1p1/4387	NA	NA	NA	R01_cb8564_c12915/f1p1/4387
R01_cb13429_c7/f1p0/349	R01_cb13429_c7/f1p0/349	R01_cb13429_c7/f1p0/349	NA	NA
R01_cb12620_c13/f1p0/1193	NA	NA	NA	R01_cb12620_c13/f1p0/1193
R01_cb18456_c7108/f1p0/504	NA	NA	R01_cb18456_c7108/f1p0/504	R01_cb18456_c7108/f1p0/504
R01_cb12242_c0/f5p1/886	NA	NA	NA	R01_cb12242_c0/f5p1/886
R01_cb18337_c2/f1p0/1750	NA	R01_cb18337_c2/f1p0/1750	R01_cb18337_c2/f1p0/1750	R01_cb18337_c2/f1p0/1750
R01_cb13099_c1/f1p0/599	NA	R01_cb13099_c1/f1p0/599	NA	NA
R01_cb8564_c86686/f1p0/3278	NA	R01_cb8564_c86686/f1p0/3278	NA	R01_cb8564_c86686/f1p0/3278
R01_cb16678_c12/f1p0/1880	NA	R01_cb16678_c12/f1p0/1880	NA	NA
R01_cb5690_c3/f1p0/3019	NA	NA	NA	R01_cb5690_c3/f1p0/3019
R01_cb8564_c10817/f1p0/2936	NA	NA	R01_cb8564_c10817/f1p0/2936	R01_cb8564_c10817/f1p0/2936

R01_cb13517_c1/f1p0/1036	R01_cb13517_c1/f1p0/1036	NA	NA	NA
R01_cb15811_c44/f2p0/929	NA	R01_cb15811_c44/f2p0/929	R01_cb15811_c44/f2p0/929	R01_cb15811_c44/f2p0/929
R01_cb16294_c2/f2p0/1067	NA	R01_cb16294_c2/f2p0/1067	NA	R01_cb16294_c2/f2p0/1067
R01_cb17993_c1/f1p0/603	R01_cb17993_c1/f1p0/603	R01_cb17993_c1/f1p0/603	R01_cb17993_c1/f1p0/603	R01_cb17993_c1/f1p0/603
R01_cb515_c6/f1p0/1914	NA	R01_cb515_c6/f1p0/1914	R01_cb515_c6/f1p0/1914	R01_cb515_c6/f1p0/1914
R01_cb8564_c83342/f1p0/3084	NA	NA	NA	R01_cb8564_c83342/f1p0/3084
R01_cb8564_c112262/f1p0/1971	NA	R01_cb8564_c112262/f1p0/1971	R01_cb8564_c112262/f1p0/1971	NA
R01_cb8564_c121835/f1p0/2351	NA	R01_cb8564_c121835/f1p0/2351	R01_cb8564_c121835/f1p0/2351	R01_cb8564_c121835/f1p0/2351
R01_cb2314_c7/f1p1/856	NA	R01_cb2314_c7/f1p1/856	NA	NA
R01_cb3071_c0/f2p0/1737	NA	NA	NA	R01_cb3071_c0/f2p0/1737
R01_cb18655_c6/f1p0/1607	NA	R01_cb18655_c6/f1p0/1607	NA	NA
R01_cb12785_c19/f1p0/1005	NA	NA	R01_cb12785_c19/f1p0/1005	NA
R01_cb12215_c3/f1p0/559	NA	NA	R01_cb12215_c3/f1p0/559	R01_cb12215_c3/f1p0/559
R01_cb8564_c71985/f1p0/4896	NA	NA	NA	R01_cb8564_c71985/f1p0/4896
R01_cb8564_c43803/f1p0/2712	NA	R01_cb8564_c43803/f1p0/2712	NA	NA
R01_cb8564_c11823/f1p0/4069	R01_cb8564_c11823/f1p0/4069	R01_cb8564_c11823/f1p0/4069	NA	NA
R01_cb15873_c3/f1p0/880	NA	R01_cb15873_c3/f1p0/880	R01_cb15873_c3/f1p0/880	NA
R01_cb7802_c3/f4p0/2215	NA	NA	NA	R01_cb7802_c3/f4p0/2215
R01_cb6591_c8/f1p0/744	R01_cb6591_c8/f1p0/744	R01_cb6591_c8/f1p0/744	NA	NA
R01_cb15622_c0/f2p0/782	NA	NA	NA	R01_cb15622_c0/f2p0/782
R01_cb5635_c0/f2p0/780	NA	NA	NA	R01_cb5635_c0/f2p0/780
R01_cb12421_c26/f1p0/632	R01_cb12421_c26/f1p0/632	R01_cb12421_c26/f1p0/632	NA	NA
R01_cb8564_c73853/f1p0/2443	NA	NA	NA	R01_cb8564_c73853/f1p0/2443

R01_cb18456_c4896/f1p0/1243	R01_cb18456_c4896/f1p0/1243	R01_cb18456_c4896/f1p0/1243	NA	NA
R01_cb16362_c0/f1p0/1532	NA	NA	NA	R01_cb16362_c0/f1p0/1532
R01_cb8564_c91312/f1p0/2845	NA	R01_cb8564_c91312/f1p0/2845	NA	NA
R01_cb8564_c4499/f1p0/2986	NA	NA	NA	R01_cb8564_c4499/f1p0/2986
R01_cb3763_c57/f1p0/3068	NA	NA	NA	R01_cb3763_c57/f1p0/3068
R01_cb2914_c10/f1p0/2828	NA	NA	NA	R01_cb2914_c10/f1p0/2828
R01_cb6348_c7/f1p0/2667	NA	NA	NA	R01_cb6348_c7/f1p0/2667
R01_cb6170_c32/f1p1/2657	NA	NA	NA	R01_cb6170_c32/f1p1/2657
R01_cb15600_c0/f4p1/592	NA	NA	NA	R01_cb15600_c0/f4p1/592
R01_cb12393_c3/f1p0/780	NA	R01_cb12393_c3/f1p0/780	NA	R01_cb12393_c3/f1p0/780
R01_cb8564_c77297/f1p0/3490	NA	R01_cb8564_c77297/f1p0/3490	NA	NA
R01_cb18456_c6084/f1p1/756	NA	NA	NA	R01_cb18456_c6084/f1p1/756
R01_cb8564_c11886/f1p0/2921	NA	NA	NA	R01_cb8564_c11886/f1p0/2921
R01_cb7875_c2/f1p0/1524	NA	NA	NA	R01_cb7875_c2/f1p0/1524
R01_cb10482_c20/f1p0/1368	NA	NA	NA	R01_cb10482_c20/f1p0/1368
R01_cb18456_c4796/f1p0/517	NA	R01_cb18456_c4796/f1p0/517	R01_cb18456_c4796/f1p0/517	R01_cb18456_c4796/f1p0/517
R01_cb4493_c21/f1p4/4506	NA	NA	NA	R01_cb4493_c21/f1p4/4506
R01_cb8564_c69705/f1p0/2450	NA	NA	NA	R01_cb8564_c69705/f1p0/2450
R01_cb12421_c73/f1p0/609	R01_cb12421_c73/f1p0/609	R01_cb12421_c73/f1p0/609	NA	NA
R01_cb416_c76/f1p1/2886	NA	R01_cb416_c76/f1p1/2886	NA	R01_cb416_c76/f1p1/2886
R01_cb1686_c10/f1p0/1341	NA	NA	R01_cb1686_c10/f1p0/1341	R01_cb1686_c10/f1p0/1341
R01_cb4198_c5/f1p0/2116	NA	NA	NA	R01_cb4198_c5/f1p0/2116
R01_cb14924_c4/f2p0/540	NA	R01_cb14924_c4/f2p0/540	NA	R01_cb14924_c4/f2p0/540
R01_cb11251_c1/f1p0/2148	NA	R01_cb11251_c1/f1p0/2148	R01_cb11251_c1/f1p0/2148	R01_cb11251_c1/f1p0/2148
R01_cb2583_c1/f1p1/3596	NA	R01_cb2583_c1/f1p1/3596	NA	NA

R01_cb964_c23/f1p0/3892	NA	NA	NA	R01_cb964_c23/f1p0/3892
R01_cb17756_c0/f3p0/356	R01_cb17756_c0/f3p0/356	R01_cb17756_c0/f3p0/356	R01_cb17756_c0/f3p0/356	R01_cb17756_c0/f3p0/356
R01_cb8564_c53550/f1p0/2563	R01_cb8564_c53550/f1p0/2563	R01_cb8564_c53550/f1p0/2563	R01_cb8564_c53550/f1p0/2563	R01_cb8564_c53550/f1p0/2563
R01_cb8564_c4097/f1p0/2885	R01_cb8564_c4097/f1p0/2885	R01_cb8564_c4097/f1p0/2885	R01_cb8564_c4097/f1p0/2885	NA
R01_cb12577_c21/f1p1/1024	NA	NA	NA	R01_cb12577_c21/f1p1/1024
R01_cb3426_c5/f1p0/3693	NA	NA	NA	R01_cb3426_c5/f1p0/3693
R01_cb18456_c3716/f1p0/1590	NA	R01_cb18456_c3716/f1p0/1590	NA	R01_cb18456_c3716/f1p0/1590
R01_cb8564_c14529/f1p0/2680	NA	R01_cb8564_c14529/f1p0/2680	NA	NA
R01_cb12606_c21/f1p0/1147	NA	R01_cb12606_c21/f1p0/1147	NA	NA
R01_cb3138_c1/f2p0/586	NA	NA	NA	R01_cb3138_c1/f2p0/586
R01_cb16537_c2/f1p0/584	R01_cb16537_c2/f1p0/584	R01_cb16537_c2/f1p0/584	NA	R01_cb16537_c2/f1p0/584
R01_cb15233_c4/f1p0/912	NA	NA	R01_cb15233_c4/f1p0/912	R01_cb15233_c4/f1p0/912
R01_cb6965_c5/f1p1/2751	NA	R01_cb6965_c5/f1p1/2751	NA	NA
R01_cb8564_c129021/f1p0/3021	NA	NA	NA	R01_cb8564_c129021/f1p0/3021
R01_cb8564_c18362/f1p0/1901	NA	NA	NA	R01_cb8564_c18362/f1p0/1901
R01_cb14660_c7/f1p0/1442	NA	R01_cb14660_c7/f1p0/1442	NA	R01_cb14660_c7/f1p0/1442
R01_cb8564_c70265/f2p0/2288	NA	R01_cb8564_c70265/f2p0/2288	R01_cb8564_c70265/f2p0/2288	R01_cb8564_c70265/f2p0/2288
R01_cb16160_c2/f2p0/864	NA	R01_cb16160_c2/f2p0/864	NA	NA
R01_cb8213_c3/f1p0/2342	NA	NA	NA	R01_cb8213_c3/f1p0/2342
R01_cb18456_c6451/f1p0/1258	R01_cb18456_c6451/f1p0/1258	R01_cb18456_c6451/f1p0/1258	R01_cb18456_c6451/f1p0/1258	NA
R01_cb8564_c48200/f1p0/2126	NA	R01_cb8564_c48200/f1p0/2126	NA	NA
R01_cb8564_c78274/f1p0/3327	R01_cb8564_c78274/f1p0/3327	R01_cb8564_c78274/f1p0/3327	R01_cb8564_c78274/f1p0/3327	R01_cb8564_c78274/f1p0/3327

R01_cb7806_c3/f1p0/1914	NA	R01_cb7806_c3/f1p0/1914	R01_cb7806_c3/f1p0/1914	R01_cb7806_c3/f1p0/1914
R01_cb1739_c21/f1p1/5419	NA	NA	NA	R01_cb1739_c21/f1p1/5419
R01_cb5579_c0/f1p0/2937	NA	NA	NA	R01_cb5579_c0/f1p0/2937
R01_cb18456_c5287/f1p0/1305	NA	R01_cb18456_c5287/f1p0/1305	NA	R01_cb18456_c5287/f1p0/1305
R01_cb6615_c4/f1p0/2600	NA	NA	NA	R01_cb6615_c4/f1p0/2600
R01_cb8564_c81743/f1p0/2364	R01_cb8564_c81743/f1p0/2364	R01_cb8564_c81743/f1p0/2364	R01_cb8564_c81743/f1p0/2364	R01_cb8564_c81743/f1p0/2364
R01_cb8905_c5/f1p0/1929	NA	NA	NA	R01_cb8905_c5/f1p0/1929
R01_cb1779_c11/f1p0/5053	NA	NA	NA	R01_cb1779_c11/f1p0/5053
R01_cb7296_c3/f1p1/2653	NA	NA	NA	R01_cb7296_c3/f1p1/2653
R01_cb16784_c3/f1p0/1566	NA	NA	NA	R01_cb16784_c3/f1p0/1566
R01_cb8564_c89731/f1p0/3439	NA	R01_cb8564_c89731/f1p0/3439	R01_cb8564_c89731/f1p0/3439	R01_cb8564_c89731/f1p0/3439
R01_cb2970_c9/f5p0/510	NA	NA	NA	R01_cb2970_c9/f5p0/510
R01_cb3103_c2/f1p0/649	R01_cb3103_c2/f1p0/649	R01_cb3103_c2/f1p0/649	NA	R01_cb3103_c2/f1p0/649
R01_cb2875_c17/f1p0/3371	NA	NA	NA	R01_cb2875_c17/f1p0/3371
R01_cb17891_c0/f1p0/1291	NA	NA	NA	R01_cb17891_c0/f1p0/1291
R01_cb12821_c3/f1p0/613	NA	R01_cb12821_c3/f1p0/613	NA	NA
R01_cb6239_c4/f1p0/2736	NA	R01_cb6239_c4/f1p0/2736	NA	NA
R01_cb4685_c2/f1p0/3259	NA	R01_cb4685_c2/f1p0/3259	NA	R01_cb4685_c2/f1p0/3259
R01_cb8882_c1/f1p0/3027	NA	R01_cb8882_c1/f1p0/3027	NA	NA
R01_cb17172_c0/f1p0/1777	NA	R01_cb17172_c0/f1p0/1777	NA	R01_cb17172_c0/f1p0/1777
R01_cb16794_c1/f1p0/1589	NA	R01_cb16794_c1/f1p0/1589	NA	R01_cb16794_c1/f1p0/1589
R01_cb2873_c8/f1p2/2892	NA	R01_cb2873_c8/f1p2/2892	NA	NA
R01_cb2320_c11/f1p0/3551	NA	NA	NA	R01_cb2320_c11/f1p0/3551
R01_cb16964_c2/f1p0/1068	NA	R01_cb16964_c2/f1p0/1068	R01_cb16964_c2/f1p0/1068	NA
R01_cb8564_c39051/f1p0/2801	NA	NA	NA	R01_cb8564_c39051/f1p0/2801

R01_cb7612_c7/f1p0/2393	NA	NA	NA	R01_cb7612_c7/f1p0/2393
R01_cb13433_c100/f1p0/362	R01_cb13433_c100/f1p0/362	R01_cb13433_c100/f1p0/362	R01_cb13433_c100/f1p0/362	R01_cb13433_c100/f1p0/362
R01_cb17640_c2/f1p0/425	R01_cb17640_c2/f1p0/425	R01_cb17640_c2/f1p0/425	NA	R01_cb17640_c2/f1p0/425
R01_cb17775_c0/f1p0/870	NA	R01_cb17775_c0/f1p0/870	NA	R01_cb17775_c0/f1p0/870
R01_cb16276_c3/f1p0/1333	NA	R01_cb16276_c3/f1p0/1333	NA	NA
R01_cb13577_c0/f3p0/464	R01_cb13577_c0/f3p0/464	R01_cb13577_c0/f3p0/464	R01_cb13577_c0/f3p0/464	NA
R01_cb11751_c1/f1p0/2086	NA	R01_cb11751_c1/f1p0/2086	NA	NA
R01_cb13459_c1/f1p0/970	NA	R01_cb13459_c1/f1p0/970	NA	NA
R01_cb13178_c11/f1p1/593	NA	R01_cb13178_c11/f1p1/593	NA	NA
R01_cb10329_c3/f1p2/4618	NA	R01_cb10329_c3/f1p2/4618	NA	NA
R01_cb6571_c0/f1p0/2817	NA	NA	NA	R01_cb6571_c0/f1p0/2817
R01_cb7819_c1/f2p0/2529	NA	NA	NA	R01_cb7819_c1/f2p0/2529
R01_cb7639_c16/f1p0/5774	NA	R01_cb7639_c16/f1p0/5774	NA	NA
R01_cb454_c35/f1p0/2896	NA	NA	NA	R01_cb454_c35/f1p0/2896
R01_cb4870_c2/f1p0/3004	R01_cb4870_c2/f1p0/3004	R01_cb4870_c2/f1p0/3004	R01_cb4870_c2/f1p0/3004	NA
R01_cb8564_c129342/f1p0/2752	NA	NA	NA	R01_cb8564_c129342/f1p0/2752
R01_cb8564_c107886/f2p1/2151	NA	R01_cb8564_c107886/f2p1/2151	NA	R01_cb8564_c107886/f2p1/2151
R01_cb12451_c7/f1p0/977	NA	NA	NA	R01_cb12451_c7/f1p0/977
R01_cb16851_c0/f2p0/669	NA	R01_cb16851_c0/f2p0/669	NA	NA
R01_cb17839_c1/f1p0/579	NA	R01_cb17839_c1/f1p0/579	NA	R01_cb17839_c1/f1p0/579
R01_cb11319_c2/f1p0/1052	R01_cb11319_c2/f1p0/1052	R01_cb11319_c2/f1p0/1052	R01_cb11319_c2/f1p0/1052	R01_cb11319_c2/f1p0/1052
R01_cb8564_c50797/f1p0/3963	NA	NA	NA	R01_cb8564_c50797/f1p0/3963
R01_cb13856_c2/f1p0/700	NA	NA	R01_cb13856_c2/f1p0/700	NA
R01_cb15418_c7/f1p1/935	NA	R01_cb15418_c7/f1p1/935	NA	R01_cb15418_c7/f1p1/935

R01_cb10872_c2/f1p0/376	R01_cb10872_c2/f1p0/376	R01_cb10872_c2/f1p0/376	R01_cb10872_c2/f1p0/376	R01_cb10872_c2/f1p0/376
R01_cb6990_c9/f1p0/652	NA	R01_cb6990_c9/f1p0/652	NA	NA
R01_cb18838_c0/f1p0/6091	NA	R01_cb18838_c0/f1p0/6091	NA	NA
R01_cb8564_c49041/f1p0/2182	NA	R01_cb8564_c49041/f1p0/2182	R01_cb8564_c49041/f1p0/2182	NA
R01_cb14444_c0/f3p0/1339	NA	R01_cb14444_c0/f3p0/1339	R01_cb14444_c0/f3p0/1339	R01_cb14444_c0/f3p0/1339
R01_cb13890_c0/f1p0/1063	NA	NA	R01_cb13890_c0/f1p0/1063	R01_cb13890_c0/f1p0/1063
R01_cb12240_c12/f51p1/550	R01_cb12240_c12/f51p1/550	R01_cb12240_c12/f51p1/550	NA	NA
R01_cb423_c9/f1p0/1743	NA	R01_cb423_c9/f1p0/1743	NA	R01_cb423_c9/f1p0/1743
R01_cb8564_c83215/f1p0/3558	NA	R01_cb8564_c83215/f1p0/3558	NA	R01_cb8564_c83215/f1p0/3558
R01_cb8356_c4/f1p0/661	NA	R01_cb8356_c4/f1p0/661	R01_cb8356_c4/f1p0/661	R01_cb8356_c4/f1p0/661
R01_cb8564_c39580/f1p0/1920	NA	R01_cb8564_c39580/f1p0/1920	NA	R01_cb8564_c39580/f1p0/1920
R01_cb10304_c3/f1p0/3412	NA	R01_cb10304_c3/f1p0/3412	NA	NA
R01_cb13327_c4/f1p0/745	NA	R01_cb13327_c4/f1p0/745	NA	NA
R01_cb15683_c5/f1p0/1438	NA	NA	NA	R01_cb15683_c5/f1p0/1438
R01_cb8564_c13379/f1p0/4305	NA	NA	NA	R01_cb8564_c13379/f1p0/4305
R01_cb4128_c14/f1p1/2357	NA	NA	NA	R01_cb4128_c14/f1p1/2357
R01_cb14723_c1/f3p0/988	NA	NA	NA	R01_cb14723_c1/f3p0/988
R01_cb2060_c5/f1p1/3821	NA	NA	NA	R01_cb2060_c5/f1p1/3821
R01_cb5682_c10/f1p0/586	NA	R01_cb5682_c10/f1p0/586	NA	R01_cb5682_c10/f1p0/586
R01_cb17037_c1/f1p0/861	R01_cb17037_c1/f1p0/861	R01_cb17037_c1/f1p0/861	NA	NA
R01_cb12399_c0/f4p0/474	NA	R01_cb12399_c0/f4p0/474	NA	NA
R01_cb3846_c4/f1p0/3686	NA	R01_cb3846_c4/f1p0/3686	NA	NA
R01_cb17417_c2/f1p0/553	R01_cb17417_c2/f1p0/553	R01_cb17417_c2/f1p0/553	R01_cb17417_c2/f1p0/553	NA
R01_cb12785_c14/f1p0/1127	NA	NA	R01_cb12785_c14/f1p0/1127	NA
R01_cb13667_c2/f1p0/880	R01_cb13667_c2/f1p0/880	R01_cb13667_c2/f1p0/880	R01_cb13667_c2/f1p0/880	R01_cb13667_c2/f1p0/880
R01_cb13862_c6/f1p0/1233	NA	R01_cb13862_c6/f1p0/1233	R01_cb13862_c6/f1p0/1233	R01_cb13862_c6/f1p0/1233

R01_cb2033_c21/f15p0/2349	NA	NA	NA	R01_cb2033_c21/f15p0/2349
R01_cb18409_c76/f1p0/464	NA	R01_cb18409_c76/f1p0/464	R01_cb18409_c76/f1p0/464	R01_cb18409_c76/f1p0/464
R01_cb11872_c4/f1p0/1415	NA	R01_cb11872_c4/f1p0/1415	NA	NA
R01_cb16345_c2/f1p0/708	R01_cb16345_c2/f1p0/708	R01_cb16345_c2/f1p0/708	NA	NA
R01_cb9993_c4/f1p0/1252	NA	NA	NA	R01_cb9993_c4/f1p0/1252
R01_cb13910_c7/f1p0/1523	R01_cb13910_c7/f1p0/1523	R01_cb13910_c7/f1p0/1523	R01_cb13910_c7/f1p0/1523	R01_cb13910_c7/f1p0/1523
R01_cb11140_c0/f1p0/1842	NA	R01_cb11140_c0/f1p0/1842	R01_cb11140_c0/f1p0/1842	R01_cb11140_c0/f1p0/1842
R01_cb16072_c0/f2p0/1152	NA	NA	NA	R01_cb16072_c0/f2p0/1152
R01_cb4732_c0/f1p0/3272	NA	NA	NA	R01_cb4732_c0/f1p0/3272
R01_cb8564_c74299/f1p0/3003	R01_cb8564_c74299/f1p0/3003	R01_cb8564_c74299/f1p0/3003	R01_cb8564_c74299/f1p0/3003	R01_cb8564_c74299/f1p0/3003
R01_cb48_c8/f1p2/5695	NA	NA	NA	R01_cb48_c8/f1p2/5695
R01_cb506_c10/f1p0/2181	NA	R01_cb506_c10/f1p0/2181	NA	R01_cb506_c10/f1p0/2181
R01_cb8640_c5/f1p0/3356	NA	R01_cb8640_c5/f1p0/3356	NA	NA
R01_cb18456_c1588/f3p1/1236	NA	NA	NA	R01_cb18456_c1588/f3p1/1236
R01_cb8564_c24335/f1p0/3305	NA	NA	NA	R01_cb8564_c24335/f1p0/3305
R01_cb2637_c13/f1p0/2273	NA	R01_cb2637_c13/f1p0/2273	NA	NA
R01_cb15553_c1/f1p0/1633	NA	NA	NA	R01_cb15553_c1/f1p0/1633
R01_cb2407_c9/f1p0/1111	NA	NA	NA	R01_cb2407_c9/f1p0/1111
R01_cb6206_c2/f1p0/3233	NA	R01_cb6206_c2/f1p0/3233	NA	NA
R01_cb17589_c2/f1p0/1728	NA	NA	NA	R01_cb17589_c2/f1p0/1728
R01_cb12373_c0/f1p0/697	R01_cb12373_c0/f1p0/697	R01_cb12373_c0/f1p0/697	R01_cb12373_c0/f1p0/697	R01_cb12373_c0/f1p0/697
R01_cb8564_c21952/f1p0/2843	NA	R01_cb8564_c21952/f1p0/2843	NA	NA
R01_cb16264_c3/f2p1/417	R01_cb16264_c3/f2p1/417	R01_cb16264_c3/f2p1/417	NA	R01_cb16264_c3/f2p1/417
R01_cb16118_c0/f2p0/1380	NA	NA	NA	R01_cb16118_c0/f2p0/1380
R01_cb9775_c1/f1p0/2072	NA	NA	NA	R01_cb9775_c1/f1p0/2072

R01_cb12716_c2/f1p0/625	NA	NA	NA	R01_cb12716_c2/f1p0/625
R01_cb8564_c88809/f1p0/2451	NA	R01_cb8564_c88809/f1p0/2451	NA	NA
R01_cb3464_c7/f1p0/4610	NA	NA	NA	R01_cb3464_c7/f1p0/4610
R01_cb8564_c126029/f1p0/3077	R01_cb8564_c126029/f1p0/3077	R01_cb8564_c126029/f1p0/3077	R01_cb8564_c126029/f1p0/3077	R01_cb8564_c126029/f1p0/3077
R01_cb7114_c3/f5p1/1295	NA	NA	NA	R01_cb7114_c3/f5p1/1295
R01_cb5117_c2/f1p1/3003	NA	NA	NA	R01_cb5117_c2/f1p1/3003
R01_cb17150_c0/f2p0/832	R01_cb17150_c0/f2p0/832	R01_cb17150_c0/f2p0/832	R01_cb17150_c0/f2p0/832	R01_cb17150_c0/f2p0/832
R01_cb1675_c5/f1p0/2009	NA	NA	NA	R01_cb1675_c5/f1p0/2009
R01_cb3483_c22/f1p0/2877	NA	NA	NA	R01_cb3483_c22/f1p0/2877
R01_cb13826_c7/f1p0/929	NA	R01_cb13826_c7/f1p0/929	NA	NA
R01_cb8564_c34491/f1p0/4804	NA	R01_cb8564_c34491/f1p0/4804	NA	NA
R01_cb9875_c6/f1p0/1133	NA	NA	NA	R01_cb9875_c6/f1p0/1133
R01_cb10298_c1/f1p0/2692	NA	R01_cb10298_c1/f1p0/2692	NA	NA
R01_cb11244_c0/f1p0/1196	NA	R01_cb11244_c0/f1p0/1196	NA	NA
R01_cb17942_c0/f1p1/516	NA	R01_cb17942_c0/f1p1/516	NA	NA
R01_cb14271_c0/f1p0/1758	NA	R01_cb14271_c0/f1p0/1758	NA	NA
R01_cb14625_c6/f1p1/1637	NA	R01_cb14625_c6/f1p1/1637	NA	NA
R01_cb8564_c67827/f7p0/3512	NA	R01_cb8564_c67827/f7p0/3512	NA	NA
R01_cb8345_c3/f1p0/1846	NA	NA	NA	R01_cb8345_c3/f1p0/1846
R01_cb17979_c1/f1p0/1255	NA	R01_cb17979_c1/f1p0/1255	NA	R01_cb17979_c1/f1p0/1255
R01_cb12057_c52/f1p0/766	NA	R01_cb12057_c52/f1p0/766	R01_cb12057_c52/f1p0/766	R01_cb12057_c52/f1p0/766
R01_cb8564_c1287/f1p1/2398	NA	R01_cb8564_c1287/f1p1/2398	NA	NA
R01_cb8564_c86374/f1p0/3181	R01_cb8564_c86374/f1p0/3181	R01_cb8564_c86374/f1p0/3181	R01_cb8564_c86374/f1p0/3181	R01_cb8564_c86374/f1p0/3181
R01_cb3254_c2/f1p0/1573	NA	R01_cb3254_c2/f1p0/1573	R01_cb3254_c2/f1p0/1573	R01_cb3254_c2/f1p0/1573

R01_cb10843_c1/f2p0/748	NA	NA	NA	R01_cb10843_c1/f2p0/748
R01_cb4165_c0/f1p0/3415	NA	NA	NA	R01_cb4165_c0/f1p0/3415
R01_cb4352_c12/f1p0/2311	NA	NA	NA	R01_cb4352_c12/f1p0/2311
R01_cb15735_c0/f11p0/402	R01_cb15735_c0/f11p0/402	NA	NA	NA
R01_cb2298_c3/f1p0/3950	NA	R01_cb2298_c3/f1p0/3950	NA	R01_cb2298_c3/f1p0/3950
R01_cb8564_c118109/f1p0/2145	NA	R01_cb8564_c118109/f1p0/2145	R01_cb8564_c118109/f1p0/2145	R01_cb8564_c118109/f1p0/2145
R01_cb18313_c0/f1p0/1037	NA	NA	NA	R01_cb18313_c0/f1p0/1037
R01_cb6204_c9/f1p3/2742	NA	NA	NA	R01_cb6204_c9/f1p3/2742
R01_cb17082_c5/f1p0/1165	R01_cb17082_c5/f1p0/1165	R01_cb17082_c5/f1p0/1165	NA	NA
R01_cb15350_c1/f1p0/803	NA	NA	NA	R01_cb15350_c1/f1p0/803
R01_cb12003_c58/f1p0/1835	NA	R01_cb12003_c58/f1p0/1835	NA	NA
R01_cb13396_c6/f1p0/1379	NA	R01_cb13396_c6/f1p0/1379	R01_cb13396_c6/f1p0/1379	R01_cb13396_c6/f1p0/1379
R01_cb6706_c6/f1p0/1327	NA	NA	NA	R01_cb6706_c6/f1p0/1327
R01_cb3639_c2/f1p0/5253	NA	NA	NA	R01_cb3639_c2/f1p0/5253
R01_cb17973_c29/f1p0/619	NA	R01_cb17973_c29/f1p0/619	R01_cb17973_c29/f1p0/619	NA
R01_cb11010_c1/f1p0/4836	R01_cb11010_c1/f1p0/4836	R01_cb11010_c1/f1p0/4836	NA	R01_cb11010_c1/f1p0/4836
R01_cb15914_c2/f1p0/865	NA	NA	NA	R01_cb15914_c2/f1p0/865
R01_cb8564_c112591/f1p0/2670	NA	R01_cb8564_c112591/f1p0/2670	NA	R01_cb8564_c112591/f1p0/2670
R01_cb7419_c9/f1p1/2574	NA	NA	NA	R01_cb7419_c9/f1p1/2574
R01_cb14741_c1/f1p1/807	NA	NA	NA	R01_cb14741_c1/f1p1/807
R01_cb4141_c11/f1p0/2811	NA	R01_cb4141_c11/f1p0/2811	NA	NA
R01_cb11830_c1/f1p0/1919	NA	R01_cb11830_c1/f1p0/1919	R01_cb11830_c1/f1p0/1919	R01_cb11830_c1/f1p0/1919
R01_cb3265_c18/f1p0/3071	NA	R01_cb3265_c18/f1p0/3071	NA	NA
R01_cb5258_c2/f1p0/3657	NA	NA	NA	R01_cb5258_c2/f1p0/3657

R01_cb12340_c12/f1p0/532	R01_cb12340_c12/f1p0/532	R01_cb12340_c12/f1p0/532	R01_cb12340_c12/f1p0/532	R01_cb12340_c12/f1p0/532
R01_cb6951_c7/f1p0/2994	NA	R01_cb6951_c7/f1p0/2994	NA	R01_cb6951_c7/f1p0/2994
R01_cb8564_c12751/f1p0/3357	NA	R01_cb8564_c12751/f1p0/3357	NA	NA
R01_cb1860_c11/f1p1/2436	NA	NA	NA	R01_cb1860_c11/f1p1/2436
R01_cb9964_c7/f1p0/1113	NA	NA	NA	R01_cb9964_c7/f1p0/1113
R01_cb6253_c2/f1p0/1391	NA	R01_cb6253_c2/f1p0/1391	R01_cb6253_c2/f1p0/1391	R01_cb6253_c2/f1p0/1391
R01_cb8564_c81851/f2p0/3845	NA	NA	NA	R01_cb8564_c81851/f2p0/3845
R01_cb8564_c4117/f2p0/2818	NA	R01_cb8564_c4117/f2p0/2818	R01_cb8564_c4117/f2p0/2818	R01_cb8564_c4117/f2p0/2818
R01_cb8564_c84398/f1p0/2239	NA	R01_cb8564_c84398/f1p0/2239	NA	R01_cb8564_c84398/f1p0/2239
R01_cb18456_c6465/f1p1/661	NA	R01_cb18456_c6465/f1p1/661	NA	NA
R01_cb9455_c0/f1p0/2078	NA	R01_cb9455_c0/f1p0/2078	NA	R01_cb9455_c0/f1p0/2078
R01_cb14684_c0/f4p0/664	NA	R01_cb14684_c0/f4p0/664	NA	NA
R01_cb15045_c8/f1p0/485	R01_cb15045_c8/f1p0/485	R01_cb15045_c8/f1p0/485	R01_cb15045_c8/f1p0/485	R01_cb15045_c8/f1p0/485
R01_cb12399_c4/f1p0/581	NA	R01_cb12399_c4/f1p0/581	R01_cb12399_c4/f1p0/581	NA
R01_cb11249_c2/f1p0/2360	R01_cb11249_c2/f1p0/2360	R01_cb11249_c2/f1p0/2360	NA	NA
R01_cb507_c0/f1p0/4657	NA	NA	NA	R01_cb507_c0/f1p0/4657
R01_cb10527_c1/f1p0/2657	NA	R01_cb10527_c1/f1p0/2657	R01_cb10527_c1/f1p0/2657	NA
R01_cb8564_c109847/f2p0/3757	NA	R01_cb8564_c109847/f2p0/3757	NA	R01_cb8564_c109847/f2p0/3757
R01_cb18409_c96/f2p0/327	R01_cb18409_c96/f2p0/327	R01_cb18409_c96/f2p0/327	R01_cb18409_c96/f2p0/327	R01_cb18409_c96/f2p0/327
R01_cb4708_c2/f1p0/3281	NA	R01_cb4708_c2/f1p0/3281	NA	NA
R01_cb7563_c32/f1p0/597	NA	R01_cb7563_c32/f1p0/597	NA	NA
R01_cb17150_c1/f1p0/1055	NA	R01_cb17150_c1/f1p0/1055	R01_cb17150_c1/f1p0/1055	R01_cb17150_c1/f1p0/1055
R01_cb11019_c0/f1p1/1574	NA	NA	NA	R01_cb11019_c0/f1p1/1574
R01_cb9108_c1/f1p0/2099	NA	NA	NA	R01_cb9108_c1/f1p0/2099
R01_cb7999_c9/f1p0/874	NA	NA	NA	R01_cb7999_c9/f1p0/874

R01_cb2718_c8/f1p1/3776	NA	R01_cb2718_c8/f1p1/3776	NA	NA
R01_cb18456_c2518/f1p0/1789	NA	NA	NA	R01_cb18456_c2518/f1p0/1789
R01_cb7584_c10/f1p0/2518	NA	R01_cb7584_c10/f1p0/2518	NA	NA
R01_cb4784_c7/f1p0/2151	NA	NA	NA	R01_cb4784_c7/f1p0/2151
R01_cb11200_c0/f2p0/339	R01_cb11200_c0/f2p0/339	R01_cb11200_c0/f2p0/339	NA	R01_cb11200_c0/f2p0/339
R01_cb7997_c3/f1p0/2486	NA	R01_cb7997_c3/f1p0/2486	NA	NA
R01_cb12696_c14/f1p1/659	NA	R01_cb12696_c14/f1p1/659	NA	NA
R01_cb8564_c2906/f1p0/1988	NA	R01_cb8564_c2906/f1p0/1988	NA	R01_cb8564_c2906/f1p0/1988
R01_cb10830_c3/f1p0/806	NA	R01_cb10830_c3/f1p0/806	NA	R01_cb10830_c3/f1p0/806
R01_cb13597_c19/f2p1/1046	NA	R01_cb13597_c19/f2p1/1046	NA	NA
R01_cb4490_c8/f1p0/2882	NA	R01_cb4490_c8/f1p0/2882	NA	NA
R01_cb14660_c4/f1p1/1621	NA	R01_cb14660_c4/f1p1/1621	NA	R01_cb14660_c4/f1p1/1621
R01_cb2314_c6/f1p0/1479	R01_cb2314_c6/f1p0/1479	R01_cb2314_c6/f1p0/1479	NA	NA
R01_cb15811_c40/f1p0/607	R01_cb15811_c40/f1p0/607	R01_cb15811_c40/f1p0/607	R01_cb15811_c40/f1p0/607	R01_cb15811_c40/f1p0/607
R01_cb16205_c1/f1p0/1797	NA	NA	NA	R01_cb16205_c1/f1p0/1797
R01_cb11430_c1/f1p0/1927	R01_cb11430_c1/f1p0/1927	R01_cb11430_c1/f1p0/1927	NA	R01_cb11430_c1/f1p0/1927
R01_cb4836_c1/f3p1/2016	NA	NA	NA	R01_cb4836_c1/f3p1/2016
R01_cb11565_c0/f1p0/1531	NA	R01_cb11565_c0/f1p0/1531	NA	R01_cb11565_c0/f1p0/1531
R01_cb11971_c3/f1p0/690	NA	NA	NA	R01_cb11971_c3/f1p0/690
R01_cb1666_c2/f2p0/2331	NA	NA	NA	R01_cb1666_c2/f2p0/2331
R01_cb1016_c9/f1p0/866	R01_cb1016_c9/f1p0/866	R01_cb1016_c9/f1p0/866	NA	R01_cb1016_c9/f1p0/866
R01_cb8564_c127137/f1p1/2655	NA	R01_cb8564_c127137/f1p1/2655	NA	NA
R01_cb16133_c2/f1p0/698	NA	NA	NA	R01_cb16133_c2/f1p0/698
R01_cb7795_c7/f5p1/2561	NA	NA	NA	R01_cb7795_c7/f5p1/2561
R01_cb9286_c2/f1p0/2429	NA	R01_cb9286_c2/f1p0/2429	NA	NA

R01_cb8564_c74842/f1p0/3214	R01_cb8564_c74842/f1p0/3214	R01_cb8564_c74842/f1p0/3214	R01_cb8564_c74842/f1p0/3214	R01_cb8564_c74842/f1p0/3214
R01_cb8564_c855/f2p0/2300	NA	R01_cb8564_c855/f2p0/2300	NA	NA
R01_cb12577_c25/f1p0/1056	NA	NA	NA	R01_cb12577_c25/f1p0/1056
R01_cb8564_c127233/f1p0/2096	NA	R01_cb8564_c127233/f1p0/2096	R01_cb8564_c127233/f1p0/2096	R01_cb8564_c127233/f1p0/2096
R01_cb18456_c4720/f1p0/340	R01_cb18456_c4720/f1p0/340	R01_cb18456_c4720/f1p0/340	R01_cb18456_c4720/f1p0/340	R01_cb18456_c4720/f1p0/340
R01_cb10029_c683/f1p0/1180	NA	R01_cb10029_c683/f1p0/1180	NA	NA
R01_cb868_c44/f1p0/2852	NA	R01_cb868_c44/f1p0/2852	NA	NA
R01_cb2226_c7/f1p0/3551	NA	NA	NA	R01_cb2226_c7/f1p0/3551
R01_cb3988_c3/f1p0/3690	NA	R01_cb3988_c3/f1p0/3690	NA	NA
R01_cb10015_c224/f1p0/579	NA	R01_cb10015_c224/f1p0/579	R01_cb10015_c224/f1p0/579	NA
R01_cb17127_c0/f1p1/1059	NA	R01_cb17127_c0/f1p1/1059	NA	R01_cb17127_c0/f1p1/1059
R01_cb8564_c44734/f1p1/3476	NA	R01_cb8564_c44734/f1p1/3476	NA	NA
R01_cb6760_c2/f1p0/1778	NA	NA	NA	R01_cb6760_c2/f1p0/1778
R01_cb8564_c69562/f1p0/1967	NA	R01_cb8564_c69562/f1p0/1967	NA	NA
R01_cb14125_c165/f1p0/795	NA	R01_cb14125_c165/f1p0/795	R01_cb14125_c165/f1p0/795	NA
R01_cb8564_c71877/f1p0/2253	NA	R01_cb8564_c71877/f1p0/2253	NA	NA
R01_cb8564_c9531/f3p0/2496	R01_cb8564_c9531/f3p0/2496	R01_cb8564_c9531/f3p0/2496	R01_cb8564_c9531/f3p0/2496	R01_cb8564_c9531/f3p0/2496
R01_cb15572_c6/f3p0/1241	NA	R01_cb15572_c6/f3p0/1241	NA	NA
R01_cb8564_c16545/f1p0/3368	R01_cb8564_c16545/f1p0/3368	R01_cb8564_c16545/f1p0/3368	NA	NA
R01_cb13513_c6/f6p1/692	NA	R01_cb13513_c6/f6p1/692	NA	NA
R01_cb8564_c977/f4p0/3093	NA	R01_cb8564_c977/f4p0/3093	NA	R01_cb8564_c977/f4p0/3093
R01_cb8564_c80852/f1p0/2182	R01_cb8564_c80852/f1p0/2182	R01_cb8564_c80852/f1p0/2182	NA	R01_cb8564_c80852/f1p0/2182

R01_cb15621_c0/f3p0/597	R01_cb15621_c0/f3p0/597	R01_cb15621_c0/f3p0/597	R01_cb15621_c0/f3p0/597	NA
R01_cb13532_c0/f24p4/1115	R01_cb13532_c0/f24p4/1115	NA	R01_cb13532_c0/f24p4/1115	R01_cb13532_c0/f24p4/1115
R01_cb5960_c3/f1p0/2695	NA	R01_cb5960_c3/f1p0/2695	NA	NA
R01_cb18103_c7/f1p0/926	NA	R01_cb18103_c7/f1p0/926	R01_cb18103_c7/f1p0/926	NA
R01_cb18456_c7627/f1p0/1639	NA	R01_cb18456_c7627/f1p0/1639	R01_cb18456_c7627/f1p0/1639	R01_cb18456_c7627/f1p0/1639
R01_cb6923_c9/f3p0/2350	NA	NA	NA	R01_cb6923_c9/f3p0/2350
R01_cb402_c26/f1p0/3950	NA	R01_cb402_c26/f1p0/3950	NA	R01_cb402_c26/f1p0/3950
R01_cb2311_c12/f1p0/519	NA	NA	NA	R01_cb2311_c12/f1p0/519
R01_cb2594_c0/f1p0/3843	R01_cb2594_c0/f1p0/3843	R01_cb2594_c0/f1p0/3843	R01_cb2594_c0/f1p0/3843	R01_cb2594_c0/f1p0/3843
R01_cb8564_c51926/f1p1/3365	NA	R01_cb8564_c51926/f1p1/3365	NA	NA
R01_cb218_c21/f1p0/4777	NA	NA	NA	R01_cb218_c21/f1p0/4777
R01_cb11128_c2/f1p0/2839	NA	NA	NA	R01_cb11128_c2/f1p0/2839
R01_cb647_c9/f1p0/2063	NA	R01_cb647_c9/f1p0/2063	NA	R01_cb647_c9/f1p0/2063
R01_cb2048_c16/f1p0/1038	R01_cb2048_c16/f1p0/1038	R01_cb2048_c16/f1p0/1038	NA	NA
R01_cb3359_c3/f3p1/2296	NA	R01_cb3359_c3/f3p1/2296	NA	NA
R01_cb7964_c0/f3p1/2292	NA	NA	NA	R01_cb7964_c0/f3p1/2292
R01_cb11344_c1/f1p0/4741	NA	R01_cb11344_c1/f1p0/4741	NA	NA
R01_cb8564_c14406/f1p0/3781	R01_cb8564_c14406/f1p0/3781	R01_cb8564_c14406/f1p0/3781	R01_cb8564_c14406/f1p0/3781	R01_cb8564_c14406/f1p0/3781
R01_cb8564_c70120/f1p0/3094	NA	R01_cb8564_c70120/f1p0/3094	NA	NA
R01_cb12112_c0/f8p0/555	NA	R01_cb12112_c0/f8p0/555	NA	NA
R01_cb8564_c128029/f1p0/2697	NA	R01_cb8564_c128029/f1p0/2697	NA	NA
R01_cb8441_c10/f1p0/1310	NA	NA	NA	R01_cb8441_c10/f1p0/1310
R01_cb14657_c1/f1p0/1104	R01_cb14657_c1/f1p0/1104	R01_cb14657_c1/f1p0/1104	R01_cb14657_c1/f1p0/1104	R01_cb14657_c1/f1p0/1104
R01_cb9775_c0/f1p0/1981	NA	NA	NA	R01_cb9775_c0/f1p0/1981

R01_cb6877_c1/f1p0/2406	NA	R01_cb6877_c1/f1p0/2406	NA	NA
R01_cb5896_c50/f1p0/2391	NA	R01_cb5896_c50/f1p0/2391	NA	R01_cb5896_c50/f1p0/2391
R01_cb1961_c2/f1p0/4122	NA	R01_cb1961_c2/f1p0/4122	NA	NA
R01_cb6802_c2/f3p0/2295	NA	R01_cb6802_c2/f3p0/2295	NA	NA
R01_cb4131_c10/f1p0/2731	R01_cb4131_c10/f1p0/2731	R01_cb4131_c10/f1p0/2731	NA	NA
R01_cb6758_c3/f1p0/2268	NA	R01_cb6758_c3/f1p0/2268	NA	R01_cb6758_c3/f1p0/2268
R01_cb18233_c0/f1p0/563	R01_cb18233_c0/f1p0/563	R01_cb18233_c0/f1p0/563	NA	R01_cb18233_c0/f1p0/563
R01_cb16431_c0/f2p0/644	NA	R01_cb16431_c0/f2p0/644	NA	NA
R01_cb12064_c5/f1p0/1603	R01_cb12064_c5/f1p0/1603	R01_cb12064_c5/f1p0/1603	NA	R01_cb12064_c5/f1p0/1603
R01_cb16136_c3/f1p0/1189	NA	NA	NA	R01_cb16136_c3/f1p0/1189
R01_cb8564_c85667/f1p0/2158	NA	R01_cb8564_c85667/f1p0/2158	R01_cb8564_c85667/f1p0/2158	NA
R01_cb955_c26/f1p0/2746	NA	R01_cb955_c26/f1p0/2746	NA	R01_cb955_c26/f1p0/2746
R01_cb2536_c24/f1p0/921	NA	R01_cb2536_c24/f1p0/921	NA	NA
R01_cb11128_c3/f2p0/1635	NA	NA	NA	R01_cb11128_c3/f2p0/1635
R01_cb9287_c0/f1p0/2081	NA	R01_cb9287_c0/f1p0/2081	NA	NA
R01_cb8564_c124660/f1p0/2536	NA	NA	NA	R01_cb8564_c124660/f1p0/2536
R01_cb8564_c1576/f1p0/2379	R01_cb8564_c1576/f1p0/2379	R01_cb8564_c1576/f1p0/2379	R01_cb8564_c1576/f1p0/2379	R01_cb8564_c1576/f1p0/2379
R01_cb6606_c9/f1p0/2424	NA	R01_cb6606_c9/f1p0/2424	R01_cb6606_c9/f1p0/2424	R01_cb6606_c9/f1p0/2424
R01_cb8564_c85054/f1p0/2329	NA	R01_cb8564_c85054/f1p0/2329	R01_cb8564_c85054/f1p0/2329	R01_cb8564_c85054/f1p0/2329
R01_cb14125_c130/f1p0/803	NA	R01_cb14125_c130/f1p0/803	R01_cb14125_c130/f1p0/803	NA
R01_cb18524_c0/f2p0/1202	NA	NA	NA	R01_cb18524_c0/f2p0/1202
R01_cb4134_c6/f3p0/2551	NA	NA	NA	R01_cb4134_c6/f3p0/2551
R01_cb8564_c4952/f1p0/2167	R01_cb8564_c4952/f1p0/2167	R01_cb8564_c4952/f1p0/2167	R01_cb8564_c4952/f1p0/2167	R01_cb8564_c4952/f1p0/2167
R01_cb10587_c1/f1p0/698	R01_cb10587_c1/f1p0/698	R01_cb10587_c1/f1p0/698	R01_cb10587_c1/f1p0/698	R01_cb10587_c1/f1p0/698
R01_cb8564_c90040/f1p1/2670	NA	R01_cb8564_c90040/f1p1/2670	NA	NA

R01_cb3793_c0/f2p2/3531	NA	NA	NA	R01_cb3793_c0/f2p2/3531
R01_cb18456_c7501/f1p0/545	NA	NA	NA	R01_cb18456_c7501/f1p0/545
R01_cb8564_c82157/f1p0/2510	R01_cb8564_c82157/f1p0/2510	R01_cb8564_c82157/f1p0/2510	NA	R01_cb8564_c82157/f1p0/2510
R01_cb5725_c15/f1p0/3169	NA	NA	NA	R01_cb5725_c15/f1p0/3169
R01_cb10827_c2/f1p0/842	R01_cb10827_c2/f1p0/842	R01_cb10827_c2/f1p0/842	R01_cb10827_c2/f1p0/842	R01_cb10827_c2/f1p0/842
R01_cb18456_c6438/f1p0/564	NA	R01_cb18456_c6438/f1p0/564	NA	NA
R01_cb2378_c30/f1p0/3817	NA	NA	NA	R01_cb2378_c30/f1p0/3817
R01_cb5830_c1/f1p1/3019	NA	NA	NA	R01_cb5830_c1/f1p1/3019
R01_cb18614_c0/f1p0/5053	NA	NA	NA	R01_cb18614_c0/f1p0/5053
R01_cb13895_c1/f2p0/557	NA	NA	NA	R01_cb13895_c1/f2p0/557
R01_cb7171_c17/f1p0/2352	NA	NA	NA	R01_cb7171_c17/f1p0/2352
R01_cb6258_c4/f2p1/2492	NA	NA	NA	R01_cb6258_c4/f2p1/2492
R01_cb12203_c0/f1p0/1598	R01_cb12203_c0/f1p0/1598	R01_cb12203_c0/f1p0/1598	NA	R01_cb12203_c0/f1p0/1598
R01_cb17302_c3/f1p0/666	R01_cb17302_c3/f1p0/666	R01_cb17302_c3/f1p0/666	NA	R01_cb17302_c3/f1p0/666
R01_cb13384_c5/f5p0/1010	NA	NA	NA	R01_cb13384_c5/f5p0/1010
R01_cb10254_c3/f1p0/544	R01_cb10254_c3/f1p0/544	R01_cb10254_c3/f1p0/544	R01_cb10254_c3/f1p0/544	R01_cb10254_c3/f1p0/544
R01_cb11614_c0/f1p0/1013	R01_cb11614_c0/f1p0/1013	R01_cb11614_c0/f1p0/1013	NA	R01_cb11614_c0/f1p0/1013
R01_cb8131_c2/f1p0/1066	NA	NA	NA	R01_cb8131_c2/f1p0/1066
R01_cb8564_c3736/f1p1/2386	NA	NA	NA	R01_cb8564_c3736/f1p1/2386
R01_cb7765_c23/f1p0/2481	NA	R01_cb7765_c23/f1p0/2481	NA	NA
R01_cb2025_c4/f1p0/3760	NA	R01_cb2025_c4/f1p0/3760	NA	NA
R01_cb3215_c2/f4p0/2455	NA	R01_cb3215_c2/f4p0/2455	NA	R01_cb3215_c2/f4p0/2455
R01_cb6615_c14/f1p0/2490	NA	R01_cb6615_c14/f1p0/2490	R01_cb6615_c14/f1p0/2490	R01_cb6615_c14/f1p0/2490
R01_cb13972_c6/f1p5/775	NA	R01_cb13972_c6/f1p5/775	NA	NA
R01_cb8564_c120255/f1p0/348	NA	R01_cb8564_c120255/f1p0/348	NA	NA

5		5		
R01_cb13261_c0/f1p0/838	NA	NA	NA	R01_cb13261_c0/f1p0/838
R01_cb6608_c19/f1p0/2592	NA	NA	NA	R01_cb6608_c19/f1p0/2592
R01_cb18240_c0/f2p0/629	NA	R01_cb18240_c0/f2p0/629	R01_cb18240_c0/f2p0/629	R01_cb18240_c0/f2p0/629
R01_cb8564_c9691/f2p0/3771	NA	R01_cb8564_c9691/f2p0/3771	NA	NA
R01_cb17763_c1/f1p0/1123	NA	NA	NA	R01_cb17763_c1/f1p0/1123
R01_cb9798_c8/f1p0/2060	NA	R01_cb9798_c8/f1p0/2060	NA	NA
R01_cb5837_c11/f1p0/1991	R01_cb5837_c11/f1p0/1991	R01_cb5837_c11/f1p0/1991	R01_cb5837_c11/f1p0/1991	NA
R01_cb7642_c211/f1p0/1727	NA	R01_cb7642_c211/f1p0/1727	NA	NA
R01_cb8564_c120962/f1p0/2518	NA	NA	NA	R01_cb8564_c120962/f1p0/2518
R01_cb3215_c16/f1p0/3484	NA	R01_cb3215_c16/f1p0/3484	R01_cb3215_c16/f1p0/3484	R01_cb3215_c16/f1p0/3484
R01_cb18692_c1/f1p0/1763	NA	R01_cb18692_c1/f1p0/1763	R01_cb18692_c1/f1p0/1763	R01_cb18692_c1/f1p0/1763
R01_cb18456_c7189/f1p0/531	R01_cb18456_c7189/f1p0/531	R01_cb18456_c7189/f1p0/531	R01_cb18456_c7189/f1p0/531	R01_cb18456_c7189/f1p0/531
R01_cb7128_c8/f1p0/1081	NA	R01_cb7128_c8/f1p0/1081	NA	NA
R01_cb10259_c3/f2p0/340	R01_cb10259_c3/f2p0/340	R01_cb10259_c3/f2p0/340	R01_cb10259_c3/f2p0/340	R01_cb10259_c3/f2p0/340
R01_cb18131_c0/f1p0/435	R01_cb18131_c0/f1p0/435	R01_cb18131_c0/f1p0/435	R01_cb18131_c0/f1p0/435	R01_cb18131_c0/f1p0/435
R01_cb18066_c2/f1p0/792	NA	R01_cb18066_c2/f1p0/792	NA	R01_cb18066_c2/f1p0/792
R01_cb8564_c80835/f1p0/2866	NA	NA	NA	R01_cb8564_c80835/f1p0/2866
R01_cb8564_c4686/f1p0/3506	NA	R01_cb8564_c4686/f1p0/3506	NA	NA
R01_cb8564_c125054/f1p0/1888	R01_cb8564_c125054/f1p0/1888	R01_cb8564_c125054/f1p0/1888	R01_cb8564_c125054/f1p0/1888	R01_cb8564_c125054/f1p0/1888
R01_cb4258_c2/f1p0/3140	NA	R01_cb4258_c2/f1p0/3140	NA	NA
R01_cb5021_c1/f2p0/3182	NA	R01_cb5021_c1/f2p0/3182	NA	R01_cb5021_c1/f2p0/3182
R01_cb11090_c4/f1p0/1434	NA	R01_cb11090_c4/f1p0/1434	NA	NA
R01_cb12449_c2/f1p0/1676	NA	R01_cb12449_c2/f1p0/1676	NA	NA

R01_cb3532_c5/f1p0/1699	NA	R01_cb3532_c5/f1p0/1699	NA	NA
R01_cb8564_c109562/f1p0/1901	NA	NA	R01_cb8564_c109562/f1p0/1901	R01_cb8564_c109562/f1p0/1901
R01_cb11147_c0/f2p0/742	NA	NA	NA	R01_cb11147_c0/f2p0/742
R01_cb8681_c1/f1p0/2273	R01_cb8681_c1/f1p0/2273	R01_cb8681_c1/f1p0/2273	R01_cb8681_c1/f1p0/2273	R01_cb8681_c1/f1p0/2273
R01_cb11900_c1/f9p0/640	NA	R01_cb11900_c1/f9p0/640	NA	R01_cb11900_c1/f9p0/640
R01_cb6666_c12/f1p0/884	NA	NA	NA	R01_cb6666_c12/f1p0/884
R01_cb6625_c13/f1p0/1286	NA	R01_cb6625_c13/f1p0/1286	NA	NA
R01_cb15913_c0/f1p0/1519	NA	R01_cb15913_c0/f1p0/1519	R01_cb15913_c0/f1p0/1519	R01_cb15913_c0/f1p0/1519
R01_cb4916_c0/f6p1/2108	NA	NA	NA	R01_cb4916_c0/f6p1/2108
R01_cb11278_c0/f6p2/1831	NA	NA	NA	R01_cb11278_c0/f6p2/1831
R01_cb10015_c408/f1p0/626	NA	R01_cb10015_c408/f1p0/626	NA	NA
R01_cb17084_c1/f1p0/615	R01_cb17084_c1/f1p0/615	R01_cb17084_c1/f1p0/615	NA	NA
R01_cb9513_c18/f1p0/3291	NA	R01_cb9513_c18/f1p0/3291	NA	NA
R01_cb8564_c8714/f12p0/2566	NA	NA	NA	R01_cb8564_c8714/f12p0/2566
R01_cb8609_c26/f1p0/2139	NA	R01_cb8609_c26/f1p0/2139	NA	NA
R01_cb8564_c2907/f1p0/4168	NA	R01_cb8564_c2907/f1p0/4168	NA	NA
R01_cb1398_c1/f1p0/3187	NA	R01_cb1398_c1/f1p0/3187	R01_cb1398_c1/f1p0/3187	NA
R01_cb16146_c3/f1p0/576	NA	R01_cb16146_c3/f1p0/576	NA	NA
R01_cb8564_c114390/f1p0/2247	R01_cb8564_c114390/f1p0/2247	R01_cb8564_c114390/f1p0/2247	NA	NA
R01_cb12094_c38/f1p0/300	R01_cb12094_c38/f1p0/300	R01_cb12094_c38/f1p0/300	R01_cb12094_c38/f1p0/300	R01_cb12094_c38/f1p0/300
R01_cb15053_c12/f4p0/633	NA	R01_cb15053_c12/f4p0/633	R01_cb15053_c12/f4p0/633	NA
R01_cb12057_c47/f1p1/869	NA	R01_cb12057_c47/f1p1/869	NA	NA
R01_cb3388_c14/f1p0/2251	NA	R01_cb3388_c14/f1p0/2251	NA	NA
R01_cb18015_c0/f1p0/969	NA	NA	NA	R01_cb18015_c0/f1p0/969

R01_cb18553_c1/f1p0/1330	NA	NA	NA	R01_cb18553_c1/f1p0/1330
R01_cb4345_c18/f1p0/1393	NA	NA	NA	R01_cb4345_c18/f1p0/1393
R01_cb9277_c0/f2p0/2114	R01_cb9277_c0/f2p0/2114	R01_cb9277_c0/f2p0/2114	R01_cb9277_c0/f2p0/2114	R01_cb9277_c0/f2p0/2114
R01_cb16005_c0/f2p0/668	NA	R01_cb16005_c0/f2p0/668	NA	NA
R01_cb8441_c6/f1p0/2298	NA	NA	NA	R01_cb8441_c6/f1p0/2298
R01_cb8564_c53082/f1p0/1966	R01_cb8564_c53082/f1p0/1966	R01_cb8564_c53082/f1p0/1966	R01_cb8564_c53082/f1p0/1966	R01_cb8564_c53082/f1p0/1966
R01_cb454_c41/f1p0/5181	NA	NA	NA	R01_cb454_c41/f1p0/5181
R01_cb7441_c11/f1p0/574	NA	R01_cb7441_c11/f1p0/574	R01_cb7441_c11/f1p0/574	R01_cb7441_c11/f1p0/574
R01_cb8564_c12382/f1p0/2497	R01_cb8564_c12382/f1p0/2497	R01_cb8564_c12382/f1p0/2497	R01_cb8564_c12382/f1p0/2497	R01_cb8564_c12382/f1p0/2497
R01_cb17872_c4/f1p0/801	NA	NA	NA	R01_cb17872_c4/f1p0/801
R01_cb18028_c2/f1p1/1409	NA	R01_cb18028_c2/f1p1/1409	NA	NA
R01_cb8564_c1377/f1p0/2527	NA	R01_cb8564_c1377/f1p0/2527	NA	NA
R01_cb8941_c0/f1p0/2230	NA	NA	NA	R01_cb8941_c0/f1p0/2230
R01_cb2737_c16/f1p0/3175	NA	R01_cb2737_c16/f1p0/3175	NA	NA
R01_cb8173_c7/f1p3/1979	NA	R01_cb8173_c7/f1p3/1979	R01_cb8173_c7/f1p3/1979	R01_cb8173_c7/f1p3/1979
R01_cb8310_c4/f1p0/395	R01_cb8310_c4/f1p0/395	R01_cb8310_c4/f1p0/395	R01_cb8310_c4/f1p0/395	R01_cb8310_c4/f1p0/395
R01_cb8564_c14414/f1p0/3224	NA	R01_cb8564_c14414/f1p0/3224	NA	R01_cb8564_c14414/f1p0/3224
R01_cb2072_c36/f1p0/3424	R01_cb2072_c36/f1p0/3424	R01_cb2072_c36/f1p0/3424	R01_cb2072_c36/f1p0/3424	R01_cb2072_c36/f1p0/3424
R01_cb11183_c5/f3p0/1743	NA	NA	NA	R01_cb11183_c5/f3p0/1743
R01_cb17249_c0/f1p0/1449	NA	R01_cb17249_c0/f1p0/1449	NA	NA
R01_cb4248_c2/f1p0/3709	NA	R01_cb4248_c2/f1p0/3709	NA	NA
R01_cb11049_c5/f1p0/1588	NA	NA	NA	R01_cb11049_c5/f1p0/1588
R01_cb1718_c5/f1p1/2111	NA	NA	NA	R01_cb1718_c5/f1p1/2111
R01_cb12213_c4/f1p1/818	NA	R01_cb12213_c4/f1p1/818	NA	NA

R01_cb17165_c0/f2p0/606	NA	R01_cb17165_c0/f2p0/606	NA	NA
R01_cb6529_c1/f2p0/2615	NA	NA	NA	R01_cb6529_c1/f2p0/2615
R01_cb2494_c10/f1p0/979	R01_cb2494_c10/f1p0/979	R01_cb2494_c10/f1p0/979	R01_cb2494_c10/f1p0/979	R01_cb2494_c10/f1p0/979
R01_cb7170_c1/f1p0/3006	NA	NA	NA	R01_cb7170_c1/f1p0/3006
R01_cb5373_c6/f1p0/2748	NA	NA	NA	R01_cb5373_c6/f1p0/2748
R01_cb8564_c74106/f1p0/3040	NA	R01_cb8564_c74106/f1p0/3040	NA	R01_cb8564_c74106/f1p0/3040
R01_cb18409_c70/f3p0/423	R01_cb18409_c70/f3p0/423	R01_cb18409_c70/f3p0/423	R01_cb18409_c70/f3p0/423	R01_cb18409_c70/f3p0/423
R01_cb11514_c0/f1p0/428	R01_cb11514_c0/f1p0/428	R01_cb11514_c0/f1p0/428	NA	R01_cb11514_c0/f1p0/428
R01_cb16583_c1/f1p0/824	NA	R01_cb16583_c1/f1p0/824	R01_cb16583_c1/f1p0/824	R01_cb16583_c1/f1p0/824
R01_cb8360_c2/f2p0/2406	R01_cb8360_c2/f2p0/2406	R01_cb8360_c2/f2p0/2406	NA	NA
R01_cb6631_c20/f1p1/2421	NA	NA	NA	R01_cb6631_c20/f1p1/2421
R01_cb9270_c3/f1p0/2645	NA	R01_cb9270_c3/f1p0/2645	R01_cb9270_c3/f1p0/2645	NA
R01_cb18427_c0/f1p0/744	NA	NA	NA	R01_cb18427_c0/f1p0/744
R01_cb18456_c7242/f1p0/641	R01_cb18456_c7242/f1p0/641	R01_cb18456_c7242/f1p0/641	R01_cb18456_c7242/f1p0/641	R01_cb18456_c7242/f1p0/641
R01_cb8564_c46257/f1p0/3584	NA	R01_cb8564_c46257/f1p0/3584	NA	NA
R01_cb12803_c4/f3p0/1233	NA	NA	NA	R01_cb12803_c4/f3p0/1233
R01_cb14532_c0/f1p0/948	NA	R01_cb14532_c0/f1p0/948	NA	R01_cb14532_c0/f1p0/948
R01_cb8564_c110470/f1p0/2727	NA	R01_cb8564_c110470/f1p0/2727	NA	NA
R01_cb8767_c21/f1p0/308	R01_cb8767_c21/f1p0/308	R01_cb8767_c21/f1p0/308	R01_cb8767_c21/f1p0/308	R01_cb8767_c21/f1p0/308
R01_cb17402_c0/f1p0/452	R01_cb17402_c0/f1p0/452	R01_cb17402_c0/f1p0/452	R01_cb17402_c0/f1p0/452	R01_cb17402_c0/f1p0/452
R01_cb6914_c25/f1p0/1399	NA	NA	NA	R01_cb6914_c25/f1p0/1399
R01_cb7230_c13/f1p0/740	NA	R01_cb7230_c13/f1p0/740	NA	R01_cb7230_c13/f1p0/740
R01_cb8564_c52365/f1p0/2644	NA	NA	NA	R01_cb8564_c52365/f1p0/2644
R01_cb872_c3/f1p0/4371	NA	NA	NA	R01_cb872_c3/f1p0/4371
R01_cb4978_c2/f1p0/3209	NA	R01_cb4978_c2/f1p0/3209	NA	R01_cb4978_c2/f1p0/3209

R01_cb7215_c0/f1p0/2670	NA	NA	NA	R01_cb7215_c0/f1p0/2670
R01_cb8564_c22602/f1p4/3938	NA	R01_cb8564_c22602/f1p4/3938	NA	NA
R01_cb8849_c2/f1p0/2466	NA	NA	NA	R01_cb8849_c2/f1p0/2466
R01_cb17326_c1/f1p0/752	NA	R01_cb17326_c1/f1p0/752	NA	NA
R01_cb4223_c0/f1p0/3399	NA	NA	NA	R01_cb4223_c0/f1p0/3399
R01_cb7544_c2/f1p0/2479	NA	R01_cb7544_c2/f1p0/2479	NA	NA
R01_cb15275_c2/f1p0/607	NA	R01_cb15275_c2/f1p0/607	R01_cb15275_c2/f1p0/607	R01_cb15275_c2/f1p0/607
R01_cb9530_c1/f1p0/2055	NA	NA	NA	R01_cb9530_c1/f1p0/2055
R01_cb12336_c13/f1p0/757	NA	R01_cb12336_c13/f1p0/757	NA	NA
R01_cb4286_c12/f1p0/2641	NA	R01_cb4286_c12/f1p0/2641	NA	NA
R01_cb13033_c8/f1p0/927	NA	R01_cb13033_c8/f1p0/927	R01_cb13033_c8/f1p0/927	R01_cb13033_c8/f1p0/927
R01_cb14004_c10/f1p0/1492	NA	NA	NA	R01_cb14004_c10/f1p0/1492
R01_cb14812_c20/f1p1/426	R01_cb14812_c20/f1p1/426	R01_cb14812_c20/f1p1/426	NA	NA
R01_cb15582_c1/f1p0/1582	NA	NA	NA	R01_cb15582_c1/f1p0/1582
R01_cb4653_c0/f1p0/3288	NA	NA	NA	R01_cb4653_c0/f1p0/3288
R01_cb14751_c0/f1p0/588	NA	R01_cb14751_c0/f1p0/588	NA	NA
R01_cb8564_c11674/f1p0/2675	NA	R01_cb8564_c11674/f1p0/2675	NA	R01_cb8564_c11674/f1p0/2675
R01_cb8564_c90559/f1p0/2150	NA	R01_cb8564_c90559/f1p0/2150	NA	NA
R01_cb4960_c0/f2p0/3162	NA	R01_cb4960_c0/f2p0/3162	R01_cb4960_c0/f2p0/3162	R01_cb4960_c0/f2p0/3162
R01_cb6660_c1/f1p0/2728	NA	R01_cb6660_c1/f1p0/2728	NA	NA
R01_cb5223_c3/f1p0/3048	NA	R01_cb5223_c3/f1p0/3048	NA	NA
R01_cb4649_c3/f1p0/2161	NA	NA	NA	R01_cb4649_c3/f1p0/2161
R01_cb7111_c4/f1p0/2495	NA	R01_cb7111_c4/f1p0/2495	NA	NA
R01_cb15118_c3/f3p0/554	NA	NA	NA	R01_cb15118_c3/f3p0/554
R01_cb7806_c2/f1p0/2497	NA	R01_cb7806_c2/f1p0/2497	R01_cb7806_c2/f1p0/2497	R01_cb7806_c2/f1p0/2497
R01_cb13528_c3/f1p0/1234	NA	NA	NA	R01_cb13528_c3/f1p0/1234

R01_cb11646_c1/f1p0/1708	NA	NA	NA	R01_cb11646_c1/f1p0/1708
R01_cb260_c17/f1p0/2753	NA	R01_cb260_c17/f1p0/2753	NA	R01_cb260_c17/f1p0/2753
R01_cb8564_c12028/f1p0/3854	NA	R01_cb8564_c12028/f1p0/3854	NA	R01_cb8564_c12028/f1p0/3854
R01_cb8564_c5187/f1p1/3167	NA	R01_cb8564_c5187/f1p1/3167	NA	NA
R01_cb18800_c1/f1p1/6954	NA	R01_cb18800_c1/f1p1/6954	NA	NA
R01_cb2686_c2/f1p0/4322	NA	R01_cb2686_c2/f1p0/4322	NA	NA
R01_cb14656_c5/f1p1/1089	NA	R01_cb14656_c5/f1p1/1089	NA	NA
R01_cb14591_c4/f1p0/431	R01_cb14591_c4/f1p0/431	R01_cb14591_c4/f1p0/431	NA	R01_cb14591_c4/f1p0/431
R01_cb13965_c1/f1p0/946	NA	R01_cb13965_c1/f1p0/946	NA	NA
R01_cb8173_c1/f12p3/1196	NA	R01_cb8173_c1/f12p3/1196	R01_cb8173_c1/f12p3/1196	R01_cb8173_c1/f12p3/1196
R01_cb4583_c10/f1p0/3406	NA	R01_cb4583_c10/f1p0/3406	R01_cb4583_c10/f1p0/3406	NA
R01_cb93_c6/f1p0/5054	NA	NA	NA	R01_cb93_c6/f1p0/5054
R01_cb8564_c87303/f1p0/2564	NA	R01_cb8564_c87303/f1p0/2564	NA	NA
R01_cb2640_c10/f1p0/3278	NA	R01_cb2640_c10/f1p0/3278	NA	NA
R01_cb16981_c2/f4p1/712	NA	NA	NA	R01_cb16981_c2/f4p1/712
R01_cb5657_c6/f1p0/2412	NA	NA	NA	R01_cb5657_c6/f1p0/2412
R01_cb16534_c1/f1p0/1414	NA	R01_cb16534_c1/f1p0/1414	NA	NA
R01_cb8564_c17724/f1p0/2921	NA	R01_cb8564_c17724/f1p0/2921	NA	R01_cb8564_c17724/f1p0/2921
R01_cb6752_c0/f1p0/2776	NA	R01_cb6752_c0/f1p0/2776	NA	R01_cb6752_c0/f1p0/2776
R01_cb6281_c0/f1p0/2892	NA	NA	NA	R01_cb6281_c0/f1p0/2892
R01_cb1228_c33/f1p0/2917	NA	NA	NA	R01_cb1228_c33/f1p0/2917
R01_cb4491_c6/f1p0/406	R01_cb4491_c6/f1p0/406	R01_cb4491_c6/f1p0/406	R01_cb4491_c6/f1p0/406	R01_cb4491_c6/f1p0/406
R01_cb2870_c9/f1p0/2871	NA	NA	NA	R01_cb2870_c9/f1p0/2871
R01_cb18655_c2/f1p0/1336	NA	NA	NA	R01_cb18655_c2/f1p0/1336
R01_cb11841_c1/f1p0/3551	NA	R01_cb11841_c1/f1p0/3551	NA	R01_cb11841_c1/f1p0/3551
R01_cb12024_c16/f1p0/997	NA	NA	NA	R01_cb12024_c16/f1p0/997

R01_cb15438_c4/f1p0/698	NA	R01_cb15438_c4/f1p0/698	NA	NA
R01_cb8564_c91941/f1p0/2521	NA	R01_cb8564_c91941/f1p0/2521	R01_cb8564_c91941/f1p0/2521	R01_cb8564_c91941/f1p0/2521
R01_cb10015_c597/f2p0/491	R01_cb10015_c597/f2p0/491	R01_cb10015_c597/f2p0/491	R01_cb10015_c597/f2p0/491	NA
R01_cb3739_c2/f1p2/4063	R01_cb3739_c2/f1p2/4063	R01_cb3739_c2/f1p2/4063	R01_cb3739_c2/f1p2/4063	R01_cb3739_c2/f1p2/4063
R01_cb5831_c2/f1p0/2779	NA	R01_cb5831_c2/f1p0/2779	R01_cb5831_c2/f1p0/2779	R01_cb5831_c2/f1p0/2779
R01_cb12114_c7/f1p0/1209	NA	NA	NA	R01_cb12114_c7/f1p0/1209
R01_cb18256_c1/f1p0/884	NA	R01_cb18256_c1/f1p0/884	NA	R01_cb18256_c1/f1p0/884
R01_cb18456_c6121/f1p0/479	NA	R01_cb18456_c6121/f1p0/479	R01_cb18456_c6121/f1p0/479	R01_cb18456_c6121/f1p0/479
R01_cb8564_c21926/f1p0/3009	NA	R01_cb8564_c21926/f1p0/3009	NA	NA
R01_cb18315_c1/f1p0/1537	NA	R01_cb18315_c1/f1p0/1537	R01_cb18315_c1/f1p0/1537	R01_cb18315_c1/f1p0/1537
R01_cb18456_c7512/f1p0/722	NA	R01_cb18456_c7512/f1p0/722	R01_cb18456_c7512/f1p0/722	R01_cb18456_c7512/f1p0/722
R01_cb8564_c86114/f2p0/3093	R01_cb8564_c86114/f2p0/3093	R01_cb8564_c86114/f2p0/3093	R01_cb8564_c86114/f2p0/3093	R01_cb8564_c86114/f2p0/3093
R01_cb10039_c2/f1p0/2178	NA	R01_cb10039_c2/f1p0/2178	NA	NA
R01_cb13914_c8/f1p0/1455	R01_cb13914_c8/f1p0/1455	R01_cb13914_c8/f1p0/1455	NA	R01_cb13914_c8/f1p0/1455
R01_cb8564_c118261/f1p0/2871	NA	R01_cb8564_c118261/f1p0/2871	NA	R01_cb8564_c118261/f1p0/2871
R01_cb10093_c7/f1p0/1667	NA	R01_cb10093_c7/f1p0/1667	NA	R01_cb10093_c7/f1p0/1667
R01_cb15469_c2/f1p0/1116	NA	R01_cb15469_c2/f1p0/1116	NA	NA
R01_cb8564_c39104/f1p0/2333	R01_cb8564_c39104/f1p0/2333	R01_cb8564_c39104/f1p0/2333	NA	NA
R01_cb10030_c88/f1p0/817	NA	R01_cb10030_c88/f1p0/817	NA	NA
R01_cb12319_c3/f1p0/580	R01_cb12319_c3/f1p0/580	R01_cb12319_c3/f1p0/580	R01_cb12319_c3/f1p0/580	R01_cb12319_c3/f1p0/580
R01_cb7545_c6/f1p0/2482	NA	R01_cb7545_c6/f1p0/2482	NA	NA
R01_cb5847_c2/f2p0/2171	NA	NA	NA	R01_cb5847_c2/f2p0/2171
R01_cb4354_c30/f8p3/3233	NA	NA	NA	R01_cb4354_c30/f8p3/3233

R01_cb5026_c4/f1p0/1579	NA	NA	NA	R01_cb5026_c4/f1p0/1579
R01_cb18456_c4780/f1p0/511	NA	R01_cb18456_c4780/f1p0/511	NA	R01_cb18456_c4780/f1p0/511
R01_cb7945_c0/f1p0/2497	NA	R01_cb7945_c0/f1p0/2497	NA	R01_cb7945_c0/f1p0/2497
R01_cb18100_c1/f1p0/1125	NA	R01_cb18100_c1/f1p0/1125	R01_cb18100_c1/f1p0/1125	R01_cb18100_c1/f1p0/1125
R01_cb17236_c2/f1p0/848	R01_cb17236_c2/f1p0/848	R01_cb17236_c2/f1p0/848	R01_cb17236_c2/f1p0/848	R01_cb17236_c2/f1p0/848
R01_cb18032_c0/f1p0/1122	NA	NA	NA	R01_cb18032_c0/f1p0/1122
R01_cb1116_c4/f1p0/2849	NA	NA	NA	R01_cb1116_c4/f1p0/2849
R01_cb11099_c2/f1p0/1823	NA	NA	NA	R01_cb11099_c2/f1p0/1823
R01_cb14108_c3/f1p1/888	R01_cb14108_c3/f1p1/888	NA	NA	R01_cb14108_c3/f1p1/888
R01_cb1297_c7/f2p0/3854	NA	NA	NA	R01_cb1297_c7/f2p0/3854
R01_cb17579_c3/f1p0/5395	NA	R01_cb17579_c3/f1p0/5395	NA	NA
R01_cb5442_c0/f1p0/2982	NA	NA	R01_cb5442_c0/f1p0/2982	R01_cb5442_c0/f1p0/2982
R01_cb18456_c7344/f1p0/756	NA	R01_cb18456_c7344/f1p0/756	NA	NA
R01_cb4949_c2/f1p0/3213	NA	R01_cb4949_c2/f1p0/3213	NA	NA
R01_cb18409_c39/f1p0/444	R01_cb18409_c39/f1p0/444	R01_cb18409_c39/f1p0/444	R01_cb18409_c39/f1p0/444	R01_cb18409_c39/f1p0/444
R01_cb4916_c8/f1p0/2155	NA	NA	NA	R01_cb4916_c8/f1p0/2155
R01_cb16968_c2/f1p0/1243	NA	NA	NA	R01_cb16968_c2/f1p0/1243
R01_cb10163_c5/f1p0/813	NA	R01_cb10163_c5/f1p0/813	R01_cb10163_c5/f1p0/813	R01_cb10163_c5/f1p0/813
R01_cb15922_c0/f1p0/1840	NA	R01_cb15922_c0/f1p0/1840	R01_cb15922_c0/f1p0/1840	R01_cb15922_c0/f1p0/1840
R01_cb10542_c7/f1p0/979	NA	R01_cb10542_c7/f1p0/979	NA	R01_cb10542_c7/f1p0/979
R01_cb8564_c78723/f1p0/2738	NA	NA	NA	R01_cb8564_c78723/f1p0/2738
R01_cb14307_c3/f1p0/1672	NA	R01_cb14307_c3/f1p0/1672	NA	NA
R01_cb2378_c29/f1p0/3449	NA	NA	NA	R01_cb2378_c29/f1p0/3449
R01_cb10015_c489/f1p0/575	NA	R01_cb10015_c489/f1p0/575	NA	NA
R01_cb8564_c25687/f1p0/3582	NA	NA	NA	R01_cb8564_c25687/f1p0/3582
R01_cb8564_c117293/f1p0/225	NA	R01_cb8564_c117293/f1p0/225	NA	R01_cb8564_c117293/f1p0/225

4		4		4
R01_cb4117_c0/f6p2/1639	NA	NA	NA	R01_cb4117_c0/f6p2/1639
R01_cb8564_c82584/f1p0/3616	NA	R01_cb8564_c82584/f1p0/3616	NA	R01_cb8564_c82584/f1p0/3616
R01_cb4795_c2/f1p0/3264	NA	R01_cb4795_c2/f1p0/3264	NA	R01_cb4795_c2/f1p0/3264
R01_cb5753_c14/f1p1/2728	NA	R01_cb5753_c14/f1p1/2728	NA	NA
R01_cb17584_c2/f1p0/1461	NA	NA	NA	R01_cb17584_c2/f1p0/1461
R01_cb8564_c128060/f1p0/2162	NA	R01_cb8564_c128060/f1p0/2162	NA	R01_cb8564_c128060/f1p0/2162
R01_cb8877_c2/f1p0/2017	NA	NA	NA	R01_cb8877_c2/f1p0/2017
R01_cb4514_c2/f1p0/3337	NA	NA	NA	R01_cb4514_c2/f1p0/3337
R01_cb8564_c125217/f1p0/2307	NA	NA	R01_cb8564_c125217/f1p0/2307	NA
R01_cb4317_c12/f1p0/2105	NA	R01_cb4317_c12/f1p0/2105	R01_cb4317_c12/f1p0/2105	R01_cb4317_c12/f1p0/2105
R01_cb16645_c49/f1p0/1660	NA	R01_cb16645_c49/f1p0/1660	NA	NA
R01_cb11212_c1/f3p0/664	NA	R01_cb11212_c1/f3p0/664	NA	NA
R01_cb8564_c4496/f1p1/2482	NA	R01_cb8564_c4496/f1p1/2482	R01_cb8564_c4496/f1p1/2482	R01_cb8564_c4496/f1p1/2482
R01_cb8564_c50086/f1p0/3752	NA	R01_cb8564_c50086/f1p0/3752	NA	NA
R01_cb6004_c1/f1p1/2953	NA	NA	NA	R01_cb6004_c1/f1p1/2953
R01_cb8564_c53029/f1p0/2512	NA	R01_cb8564_c53029/f1p0/2512	NA	R01_cb8564_c53029/f1p0/2512
R01_cb4453_c2/f1p0/2616	NA	NA	NA	R01_cb4453_c2/f1p0/2616
R01_cb7027_c3/f1p0/1722	NA	R01_cb7027_c3/f1p0/1722	NA	NA
R01_cb10849_c3/f1p0/2946	NA	R01_cb10849_c3/f1p0/2946	NA	NA
R01_cb7401_c9/f1p0/2249	NA	R01_cb7401_c9/f1p0/2249	R01_cb7401_c9/f1p0/2249	R01_cb7401_c9/f1p0/2249
R01_cb18137_c1/f1p0/1363	NA	NA	NA	R01_cb18137_c1/f1p0/1363
R01_cb2581_c1/f1p0/3884	R01_cb2581_c1/f1p0/3884	R01_cb2581_c1/f1p0/3884	R01_cb2581_c1/f1p0/3884	R01_cb2581_c1/f1p0/3884
R01_cb6057_c7/f1p0/1762	NA	NA	NA	R01_cb6057_c7/f1p0/1762

R01_cb11929_c0/f2p0/1689	NA	R01_cb11929_c0/f2p0/1689	R01_cb11929_c0/f2p0/1689	R01_cb11929_c0/f2p0/1689
R01_cb14704_c26/f1p0/1469	NA	R01_cb14704_c26/f1p0/1469	NA	NA
R01_cb15012_c1/f1p0/1699	NA	R01_cb15012_c1/f1p0/1699	NA	R01_cb15012_c1/f1p0/1699
R01_cb16718_c0/f4p0/1155	NA	R01_cb16718_c0/f4p0/1155	NA	R01_cb16718_c0/f4p0/1155
R01_cb14711_c1/f2p0/1357	NA	R01_cb14711_c1/f2p0/1357	NA	NA
R01_cb8564_c3683/f1p0/4183	R01_cb8564_c3683/f1p0/4183	R01_cb8564_c3683/f1p0/4183	R01_cb8564_c3683/f1p0/4183	NA
R01_cb8564_c4582/f1p0/2713	NA	NA	NA	R01_cb8564_c4582/f1p0/2713
R01_cb4861_c2/f1p0/2642	NA	R01_cb4861_c2/f1p0/2642	NA	R01_cb4861_c2/f1p0/2642
R01_cb3485_c8/f1p0/2724	NA	R01_cb3485_c8/f1p0/2724	NA	NA
R01_cb8564_c20587/f1p0/3263	NA	R01_cb8564_c20587/f1p0/3263	NA	NA
R01_cb18456_c5422/f1p0/328	R01_cb18456_c5422/f1p0/328	R01_cb18456_c5422/f1p0/328	NA	NA
R01_cb929_c8/f2p1/1843	NA	NA	NA	R01_cb929_c8/f2p1/1843
R01_cb8564_c10586/f1p1/2704	NA	R01_cb8564_c10586/f1p1/2704	NA	NA
R01_cb3703_c6/f1p0/1503	NA	NA	NA	R01_cb3703_c6/f1p0/1503
R01_cb10024_c550/f1p1/731	NA	NA	R01_cb10024_c550/f1p1/731	NA
R01_cb1392_c8/f1p0/2168	NA	NA	NA	R01_cb1392_c8/f1p0/2168
R01_cb8823_c10/f1p0/1628	R01_cb8823_c10/f1p0/1628	R01_cb8823_c10/f1p0/1628	R01_cb8823_c10/f1p0/1628	R01_cb8823_c10/f1p0/1628
R01_cb3103_c3/f1p0/619	R01_cb3103_c3/f1p0/619	NA	NA	R01_cb3103_c3/f1p0/619
R01_cb2160_c2/f5p1/2874	NA	NA	NA	R01_cb2160_c2/f5p1/2874
R01_cb16583_c0/f1p0/825	R01_cb16583_c0/f1p0/825	R01_cb16583_c0/f1p0/825	R01_cb16583_c0/f1p0/825	R01_cb16583_c0/f1p0/825
R01_cb8564_c126610/f1p0/2193	NA	R01_cb8564_c126610/f1p0/2193	R01_cb8564_c126610/f1p0/2193	R01_cb8564_c126610/f1p0/2193
R01_cb9448_c5/f1p1/3236	NA	NA	NA	R01_cb9448_c5/f1p1/3236
R01_cb302_c10/f1p0/2852	NA	NA	NA	R01_cb302_c10/f1p0/2852
R01_cb4198_c11/f1p0/1410	NA	R01_cb4198_c11/f1p0/1410	NA	R01_cb4198_c11/f1p0/1410
R01_cb2161_c11/f1p0/3342	NA	R01_cb2161_c11/f1p0/3342	NA	R01_cb2161_c11/f1p0/3342

R01_cb17545_c0/f2p0/644	NA	R01_cb17545_c0/f2p0/644	NA	NA
R01_cb8564_c23592/f1p1/1957	R01_cb8564_c23592/f1p1/1957	R01_cb8564_c23592/f1p1/1957	R01_cb8564_c23592/f1p1/1957	R01_cb8564_c23592/f1p1/1957
R01_cb18456_c7431/f1p0/725	NA	NA	R01_cb18456_c7431/f1p0/725	R01_cb18456_c7431/f1p0/725
R01_cb3813_c2/f1p0/4306	NA	R01_cb3813_c2/f1p0/4306	NA	NA
R01_cb6839_c4/f1p0/1242	NA	R01_cb6839_c4/f1p0/1242	NA	NA
R01_cb16341_c0/f10p1/607	R01_cb16341_c0/f10p1/607	R01_cb16341_c0/f10p1/607	R01_cb16341_c0/f10p1/607	R01_cb16341_c0/f10p1/607
R01_cb16624_c3/f1p0/805	NA	R01_cb16624_c3/f1p0/805	NA	NA
R01_cb6824_c6/f1p0/2411	NA	R01_cb6824_c6/f1p0/2411	NA	NA
R01_cb8564_c18046/f1p0/4035	NA	R01_cb8564_c18046/f1p0/4035	NA	NA
R01_cb11693_c2/f1p0/655	NA	R01_cb11693_c2/f1p0/655	NA	NA
R01_cb8564_c90448/f1p0/3951	NA	R01_cb8564_c90448/f1p0/3951	NA	R01_cb8564_c90448/f1p0/3951
R01_cb8564_c33125/f7p0/2843	R01_cb8564_c33125/f7p0/2843	R01_cb8564_c33125/f7p0/2843	R01_cb8564_c33125/f7p0/2843	R01_cb8564_c33125/f7p0/2843
R01_cb8564_c84197/f1p0/2017	R01_cb8564_c84197/f1p0/2017	R01_cb8564_c84197/f1p0/2017	R01_cb8564_c84197/f1p0/2017	R01_cb8564_c84197/f1p0/2017
R01_cb2226_c11/f1p0/410	NA	R01_cb2226_c11/f1p0/410	R01_cb2226_c11/f1p0/410	R01_cb2226_c11/f1p0/410
R01_cb2765_c2/f1p0/3515	NA	NA	NA	R01_cb2765_c2/f1p0/3515
R01_cb3359_c19/f1p0/4140	NA	R01_cb3359_c19/f1p0/4140	NA	NA
R01_cb4134_c47/f2p2/2895	NA	NA	NA	R01_cb4134_c47/f2p2/2895
R01_cb402_c32/f1p0/2175	NA	R01_cb402_c32/f1p0/2175	NA	R01_cb402_c32/f1p0/2175
R01_cb14822_c4/f1p0/1148	R01_cb14822_c4/f1p0/1148	R01_cb14822_c4/f1p0/1148	R01_cb14822_c4/f1p0/1148	R01_cb14822_c4/f1p0/1148
R01_cb69_c8/f1p0/3468	NA	NA	NA	R01_cb69_c8/f1p0/3468
R01_cb8564_c12266/f2p6/4222	NA	R01_cb8564_c12266/f2p6/4222	R01_cb8564_c12266/f2p6/4222	R01_cb8564_c12266/f2p6/4222
R01_cb8453_c3/f1p0/2969	NA	R01_cb8453_c3/f1p0/2969	NA	NA
R01_cb12837_c9/f1p4/694	NA	R01_cb12837_c9/f1p4/694	NA	NA

R01_cb6913_c1/f1p0/2981	NA	NA	NA	R01_cb6913_c1/f1p0/2981
R01_cb8564_c115087/f1p0/2589	NA	R01_cb8564_c115087/f1p0/2589	R01_cb8564_c115087/f1p0/2589	R01_cb8564_c115087/f1p0/2589
R01_cb1961_c4/f1p0/927	NA	R01_cb1961_c4/f1p0/927	NA	NA
R01_cb8564_c850/f2p0/2585	NA	NA	NA	R01_cb8564_c850/f2p0/2585
R01_cb4818_c1/f2p0/3114	NA	R01_cb4818_c1/f2p0/3114	NA	NA
R01_cb3756_c15/f1p0/2679	NA	R01_cb3756_c15/f1p0/2679	NA	R01_cb3756_c15/f1p0/2679
R01_cb10960_c1/f1p0/3420	NA	NA	NA	R01_cb10960_c1/f1p0/3420
R01_cb10190_c0/f2p0/1716	R01_cb10190_c0/f2p0/1716	R01_cb10190_c0/f2p0/1716	R01_cb10190_c0/f2p0/1716	R01_cb10190_c0/f2p0/1716
R01_cb18456_c4735/f1p0/483	NA	NA	NA	R01_cb18456_c4735/f1p0/483
R01_cb13052_c1/f1p0/1774	NA	NA	NA	R01_cb13052_c1/f1p0/1774
R01_cb8564_c945/f5p5/4929	NA	NA	NA	R01_cb8564_c945/f5p5/4929
R01_cb12421_c47/f1p0/708	R01_cb12421_c47/f1p0/708	R01_cb12421_c47/f1p0/708	NA	NA
R01_cb12641_c11/f1p2/1411	NA	NA	NA	R01_cb12641_c11/f1p2/1411
R01_cb18290_c1/f2p0/648	R01_cb18290_c1/f2p0/648	R01_cb18290_c1/f2p0/648	NA	NA
R01_cb8564_c92135/f1p0/3001	NA	R01_cb8564_c92135/f1p0/3001	R01_cb8564_c92135/f1p0/3001	R01_cb8564_c92135/f1p0/3001
R01_cb4536_c3/f1p0/3276	NA	NA	NA	R01_cb4536_c3/f1p0/3276
R01_cb8564_c124728/f1p0/2228	NA	NA	NA	R01_cb8564_c124728/f1p0/2228
R01_cb18456_c6366/f1p0/1735	NA	R01_cb18456_c6366/f1p0/1735	NA	R01_cb18456_c6366/f1p0/1735
R01_cb13158_c1/f1p0/945	NA	NA	NA	R01_cb13158_c1/f1p0/945
R01_cb8564_c53454/f1p0/3141	NA	NA	R01_cb8564_c53454/f1p0/3141	R01_cb8564_c53454/f1p0/3141
R01_cb9910_c1/f1p0/1994	NA	R01_cb9910_c1/f1p0/1994	R01_cb9910_c1/f1p0/1994	R01_cb9910_c1/f1p0/1994
R01_cb2577_c16/f1p0/800	NA	NA	NA	R01_cb2577_c16/f1p0/800
R01_cb2856_c2/f1p0/3498	NA	R01_cb2856_c2/f1p0/3498	NA	NA
R01_cb13545_c36/f1p0/1853	R01_cb13545_c36/f1p0/1853	R01_cb13545_c36/f1p0/1853	NA	NA

R01_cb12914_c14/f1p0/501	NA	R01_cb12914_c14/f1p0/501	R01_cb12914_c14/f1p0/501	NA
R01_cb7257_c5/f1p0/1451	NA	NA	NA	R01_cb7257_c5/f1p0/1451
R01_cb7298_c1/f1p0/2736	R01_cb7298_c1/f1p0/2736	R01_cb7298_c1/f1p0/2736	R01_cb7298_c1/f1p0/2736	R01_cb7298_c1/f1p0/2736
R01_cb12520_c8/f1p0/312	R01_cb12520_c8/f1p0/312	R01_cb12520_c8/f1p0/312	NA	NA
R01_cb13601_c4/f1p0/817	NA	NA	NA	R01_cb13601_c4/f1p0/817
R01_cb8564_c68731/f4p0/2840	R01_cb8564_c68731/f4p0/2840	R01_cb8564_c68731/f4p0/2840	R01_cb8564_c68731/f4p0/2840	R01_cb8564_c68731/f4p0/2840
R01_cb8564_c36812/f1p0/2231	NA	NA	NA	R01_cb8564_c36812/f1p0/2231
R01_cb16555_c4/f1p0/445	NA	NA	NA	R01_cb16555_c4/f1p0/445
R01_cb15100_c1/f1p0/761	NA	NA	NA	R01_cb15100_c1/f1p0/761
R01_cb10713_c0/f1p0/1578	NA	R01_cb10713_c0/f1p0/1578	NA	NA
R01_cb8564_c83795/f2p1/2099	NA	R01_cb8564_c83795/f2p1/2099	R01_cb8564_c83795/f2p1/2099	R01_cb8564_c83795/f2p1/2099
R01_cb17363_c8/f1p0/757	NA	R01_cb17363_c8/f1p0/757	R01_cb17363_c8/f1p0/757	R01_cb17363_c8/f1p0/757
R01_cb2160_c11/f1p1/2859	NA	NA	NA	R01_cb2160_c11/f1p1/2859
R01_cb8285_c1/f1p0/2591	NA	NA	NA	R01_cb8285_c1/f1p0/2591
R01_cb12563_c5/f1p0/1064	NA	R01_cb12563_c5/f1p0/1064	NA	NA
R01_cb8564_c79125/f3p5/2158	NA	R01_cb8564_c79125/f3p5/2158	R01_cb8564_c79125/f3p5/2158	R01_cb8564_c79125/f3p5/2158
R01_cb16452_c3/f1p0/381	R01_cb16452_c3/f1p0/381	R01_cb16452_c3/f1p0/381	R01_cb16452_c3/f1p0/381	R01_cb16452_c3/f1p0/381
R01_cb15680_c4/f1p0/1710	NA	NA	NA	R01_cb15680_c4/f1p0/1710
R01_cb8564_c89842/f1p0/2302	R01_cb8564_c89842/f1p0/2302	R01_cb8564_c89842/f1p0/2302	NA	NA
R01_cb1610_c3/f1p0/3811	NA	NA	NA	R01_cb1610_c3/f1p0/3811
R01_cb10909_c3/f1p0/1894	NA	NA	NA	R01_cb10909_c3/f1p0/1894
R01_cb10070_c2/f1p0/3977	NA	NA	NA	R01_cb10070_c2/f1p0/3977
R01_cb8564_c129323/f1p0/2414	NA	NA	NA	R01_cb8564_c129323/f1p0/2414

R01_cb7623_c28/f1p1/2038	NA	R01_cb7623_c28/f1p1/2038	NA	NA
R01_cb12645_c1/f1p0/1486	NA	R01_cb12645_c1/f1p0/1486	NA	NA
R01_cb8564_c53457/f2p0/2821	R01_cb8564_c53457/f2p0/2821	R01_cb8564_c53457/f2p0/2821	R01_cb8564_c53457/f2p0/2821	R01_cb8564_c53457/f2p0/2821
R01_cb4543_c1/f1p0/3821	NA	R01_cb4543_c1/f1p0/3821	R01_cb4543_c1/f1p0/3821	NA
R01_cb17431_c0/f3p0/1394	NA	NA	NA	R01_cb17431_c0/f3p0/1394
R01_cb17637_c4/f1p0/586	NA	R01_cb17637_c4/f1p0/586	NA	NA
R01_cb17623_c2/f1p0/634	NA	NA	NA	R01_cb17623_c2/f1p0/634
R01_cb4784_c11/f2p1/2086	NA	NA	NA	R01_cb4784_c11/f2p1/2086
R01_cb17305_c3/f2p1/753	NA	R01_cb17305_c3/f2p1/753	NA	R01_cb17305_c3/f2p1/753
R01_cb9771_c0/f1p0/1982	NA	NA	NA	R01_cb9771_c0/f1p0/1982
R01_cb18456_c6669/f1p1/530	R01_cb18456_c6669/f1p1/530	R01_cb18456_c6669/f1p1/530	R01_cb18456_c6669/f1p1/530	R01_cb18456_c6669/f1p1/530
R01_cb18730_c1/f1p0/1824	R01_cb18730_c1/f1p0/1824	R01_cb18730_c1/f1p0/1824	R01_cb18730_c1/f1p0/1824	R01_cb18730_c1/f1p0/1824
R01_cb2698_c3/f1p0/1964	NA	NA	NA	R01_cb2698_c3/f1p0/1964
R01_cb17361_c0/f2p0/380	R01_cb17361_c0/f2p0/380	R01_cb17361_c0/f2p0/380	R01_cb17361_c0/f2p0/380	R01_cb17361_c0/f2p0/380
R01_cb13503_c0/f1p0/972	NA	R01_cb13503_c0/f1p0/972	NA	R01_cb13503_c0/f1p0/972
R01_cb8564_c43362/f1p0/3852	NA	R01_cb8564_c43362/f1p0/3852	NA	R01_cb8564_c43362/f1p0/3852
R01_cb9271_c2/f2p0/1798	NA	R01_cb9271_c2/f2p0/1798	NA	NA
R01_cb3516_c0/f2p0/2634	NA	NA	NA	R01_cb3516_c0/f2p0/2634
R01_cb14267_c1/f1p0/1285	NA	R01_cb14267_c1/f1p0/1285	NA	R01_cb14267_c1/f1p0/1285
R01_cb18456_c7309/f1p0/831	R01_cb18456_c7309/f1p0/831	R01_cb18456_c7309/f1p0/831	R01_cb18456_c7309/f1p0/831	R01_cb18456_c7309/f1p0/831
R01_cb18728_c1/f1p0/3563	NA	NA	NA	R01_cb18728_c1/f1p0/3563
R01_cb6278_c4/f1p0/3465	NA	NA	NA	R01_cb6278_c4/f1p0/3465
R01_cb12246_c5/f1p0/515	R01_cb12246_c5/f1p0/515	R01_cb12246_c5/f1p0/515	R01_cb12246_c5/f1p0/515	R01_cb12246_c5/f1p0/515
R01_cb12837_c1/f1p4/665	NA	R01_cb12837_c1/f1p4/665	NA	NA
R01_cb18201_c1/f1p0/716	R01_cb18201_c1/f1p0/716	R01_cb18201_c1/f1p0/716	R01_cb18201_c1/f1p0/716	R01_cb18201_c1/f1p0/716

R01_cb16426_c2/f1p0/726	NA	R01_cb16426_c2/f1p0/726	NA	NA
R01_cb7490_c0/f2p0/1667	NA	R01_cb7490_c0/f2p0/1667	NA	NA
R01_cb12722_c23/f1p1/1704	NA	NA	NA	R01_cb12722_c23/f1p1/1704
R01_cb188_c2/f1p1/3428	NA	NA	NA	R01_cb188_c2/f1p1/3428
R01_cb14480_c8/f1p0/613	NA	R01_cb14480_c8/f1p0/613	R01_cb14480_c8/f1p0/613	R01_cb14480_c8/f1p0/613
R01_cb13184_c1/f1p0/483	R01_cb13184_c1/f1p0/483	R01_cb13184_c1/f1p0/483	R01_cb13184_c1/f1p0/483	R01_cb13184_c1/f1p0/483
R01_cb1849_c22/f1p0/1764	NA	NA	NA	R01_cb1849_c22/f1p0/1764
R01_cb7096_c26/f1p1/2579	NA	R01_cb7096_c26/f1p1/2579	NA	NA
R01_cb8564_c79050/f1p0/2508	R01_cb8564_c79050/f1p0/2508	R01_cb8564_c79050/f1p0/2508	NA	NA
R01_cb8564_c121529/f1p0/3063	NA	R01_cb8564_c121529/f1p0/3063	NA	NA
R01_cb7278_c4/f1p0/2180	NA	R01_cb7278_c4/f1p0/2180	NA	NA
R01_cb16410_c2/f1p1/529	NA	NA	NA	R01_cb16410_c2/f1p1/529
R01_cb14343_c0/f5p0/585	NA	R01_cb14343_c0/f5p0/585	NA	NA
R01_cb14291_c5/f1p0/1400	NA	NA	NA	R01_cb14291_c5/f1p0/1400
R01_cb9750_c4/f1p1/1951	NA	NA	NA	R01_cb9750_c4/f1p1/1951
R01_cb7238_c3/f1p0/931	NA	R01_cb7238_c3/f1p0/931	NA	NA
R01_cb735_c5/f1p1/2153	NA	NA	NA	R01_cb735_c5/f1p1/2153
R01_cb18456_c1258/f4p4/671	NA	R01_cb18456_c1258/f4p4/671	NA	NA
R01_cb8564_c82723/f1p0/3877	NA	R01_cb8564_c82723/f1p0/3877	NA	R01_cb8564_c82723/f1p0/3877
R01_cb10024_c231/f1p0/748	NA	R01_cb10024_c231/f1p0/748	NA	NA
R01_cb17000_c1/f1p0/381	R01_cb17000_c1/f1p0/381	R01_cb17000_c1/f1p0/381	R01_cb17000_c1/f1p0/381	R01_cb17000_c1/f1p0/381
R01_cb2451_c31/f1p1/3158	NA	NA	NA	R01_cb2451_c31/f1p1/3158
R01_cb18197_c2/f1p0/413	NA	NA	R01_cb18197_c2/f1p0/413	R01_cb18197_c2/f1p0/413
R01_cb8564_c117246/f1p0/234	NA	R01_cb8564_c117246/f1p0/234	NA	NA

7		7		
R01_cb12504_c3/f1p0/817	R01_cb12504_c3/f1p0/817	R01_cb12504_c3/f1p0/817	NA	R01_cb12504_c3/f1p0/817
R01_cb9829_c0/f2p2/1972	NA	NA	NA	R01_cb9829_c0/f2p2/1972
R01_cb18207_c3/f1p0/674	R01_cb18207_c3/f1p0/674	R01_cb18207_c3/f1p0/674	R01_cb18207_c3/f1p0/674	R01_cb18207_c3/f1p0/674
R01_cb13570_c19/f1p1/1629	NA	R01_cb13570_c19/f1p1/1629	NA	NA
R01_cb18409_c27/f2p0/476	R01_cb18409_c27/f2p0/476	R01_cb18409_c27/f2p0/476	R01_cb18409_c27/f2p0/476	R01_cb18409_c27/f2p0/476
R01_cb8564_c22201/f1p0/2016	NA	R01_cb8564_c22201/f1p0/2016	R01_cb8564_c22201/f1p0/2016	R01_cb8564_c22201/f1p0/2016
R01_cb8486_c2/f1p0/2933	NA	R01_cb8486_c2/f1p0/2933	NA	NA
R01_cb6236_c3/f1p1/1832	NA	NA	NA	R01_cb6236_c3/f1p1/1832
R01_cb4657_c19/f1p0/3255	NA	R01_cb4657_c19/f1p0/3255	NA	R01_cb4657_c19/f1p0/3255
R01_cb6606_c36/f1p0/575	R01_cb6606_c36/f1p0/575	R01_cb6606_c36/f1p0/575	R01_cb6606_c36/f1p0/575	R01_cb6606_c36/f1p0/575
R01_cb48_c6/f1p0/4255	NA	NA	NA	R01_cb48_c6/f1p0/4255
R01_cb7177_c16/f1p1/603	NA	R01_cb7177_c16/f1p1/603	NA	R01_cb7177_c16/f1p1/603
R01_cb18286_c1/f1p0/649	NA	R01_cb18286_c1/f1p0/649	R01_cb18286_c1/f1p0/649	R01_cb18286_c1/f1p0/649
R01_cb8450_c0/f1p0/2366	NA	R01_cb8450_c0/f1p0/2366	NA	NA
R01_cb8030_c6/f2p0/2476	NA	NA	NA	R01_cb8030_c6/f2p0/2476
R01_cb16394_c2/f1p0/656	R01_cb16394_c2/f1p0/656	R01_cb16394_c2/f1p0/656	R01_cb16394_c2/f1p0/656	R01_cb16394_c2/f1p0/656
R01_cb10404_c1/f1p0/1471	NA	R01_cb10404_c1/f1p0/1471	NA	NA
R01_cb4233_c22/f4p0/2215	NA	R01_cb4233_c22/f4p0/2215	R01_cb4233_c22/f4p0/2215	R01_cb4233_c22/f4p0/2215
R01_cb8564_c114239/f1p0/2013	NA	R01_cb8564_c114239/f1p0/2013	R01_cb8564_c114239/f1p0/2013	R01_cb8564_c114239/f1p0/2013
R01_cb17756_c18/f1p0/479	R01_cb17756_c18/f1p0/479	R01_cb17756_c18/f1p0/479	R01_cb17756_c18/f1p0/479	R01_cb17756_c18/f1p0/479
R01_cb4805_c26/f1p0/1241	NA	NA	NA	R01_cb4805_c26/f1p0/1241
R01_cb18409_c37/f1p0/705	NA	R01_cb18409_c37/f1p0/705	R01_cb18409_c37/f1p0/705	R01_cb18409_c37/f1p0/705
R01_cb8564_c25056/f1p0/3992	NA	NA	NA	R01_cb8564_c25056/f1p0/3992
R01_cb2154_c6/f1p1/3494	NA	R01_cb2154_c6/f1p1/3494	NA	NA

R01_cb9047_c2/f1p0/2190	NA	NA	NA	R01_cb9047_c2/f1p0/2190
R01_cb12369_c10/f1p0/591	NA	NA	R01_cb12369_c10/f1p0/591	NA
R01_cb8564_c13489/f1p1/3370	NA	R01_cb8564_c13489/f1p1/3370	R01_cb8564_c13489/f1p1/3370	NA
R01_cb323_c4/f1p1/1598	NA	R01_cb323_c4/f1p1/1598	NA	NA
R01_cb15777_c3/f1p0/1011	NA	NA	NA	R01_cb15777_c3/f1p0/1011
R01_cb18456_c1270/f7p0/532	NA	R01_cb18456_c1270/f7p0/532	R01_cb18456_c1270/f7p0/532	R01_cb18456_c1270/f7p0/532
R01_cb12520_c6/f1p0/497	NA	R01_cb12520_c6/f1p0/497	NA	NA
R01_cb11595_c1/f1p0/2065	NA	R01_cb11595_c1/f1p0/2065	NA	NA
R01_cb822_c32/f1p1/5821	NA	NA	NA	R01_cb822_c32/f1p1/5821
R01_cb8564_c69295/f2p0/2365	R01_cb8564_c69295/f2p0/2365	R01_cb8564_c69295/f2p0/2365	R01_cb8564_c69295/f2p0/2365	R01_cb8564_c69295/f2p0/2365
R01_cb5929_c4/f1p0/2726	NA	NA	NA	R01_cb5929_c4/f1p0/2726
R01_cb8564_c54465/f1p10/4380	NA	R01_cb8564_c54465/f1p10/4380	R01_cb8564_c54465/f1p10/4380	R01_cb8564_c54465/f1p10/4380
R01_cb12520_c4/f1p0/593	NA	R01_cb12520_c4/f1p0/593	NA	NA
R01_cb1030_c26/f1p0/3872	NA	R01_cb1030_c26/f1p0/3872	NA	NA
R01_cb15683_c3/f1p0/1701	NA	NA	NA	R01_cb15683_c3/f1p0/1701
R01_cb2804_c93/f1p0/3636	NA	R01_cb2804_c93/f1p0/3636	NA	NA
R01_cb18126_c0/f2p0/1239	NA	NA	NA	R01_cb18126_c0/f2p0/1239
R01_cb16452_c12/f1p0/954	NA	R01_cb16452_c12/f1p0/954	R01_cb16452_c12/f1p0/954	R01_cb16452_c12/f1p0/954
R01_cb1259_c24/f1p0/3670	NA	NA	NA	R01_cb1259_c24/f1p0/3670
R01_cb1388_c6/f1p0/4623	NA	NA	NA	R01_cb1388_c6/f1p0/4623
R01_cb6101_c4/f1p0/2753	NA	R01_cb6101_c4/f1p0/2753	NA	NA
R01_cb13516_c4/f1p0/1066	NA	R01_cb13516_c4/f1p0/1066	NA	NA
R01_cb1138_c54/f74p1/813	NA	NA	R01_cb1138_c54/f74p1/813	R01_cb1138_c54/f74p1/813
R01_cb11451_c0/f1p0/1319	NA	R01_cb11451_c0/f1p0/1319	NA	NA

R01_cb8564_c38676/f2p0/4590	NA	R01_cb8564_c38676/f2p0/4590	R01_cb8564_c38676/f2p0/4590	NA
R01_cb12148_c15/f1p0/613	R01_cb12148_c15/f1p0/613	R01_cb12148_c15/f1p0/613	R01_cb12148_c15/f1p0/613	R01_cb12148_c15/f1p0/613
R01_cb9594_c6/f1p0/2076	R01_cb9594_c6/f1p0/2076	R01_cb9594_c6/f1p0/2076	NA	NA
R01_cb11972_c0/f3p0/1590	NA	NA	NA	R01_cb11972_c0/f3p0/1590
R01_cb8766_c3/f1p1/2108	NA	R01_cb8766_c3/f1p1/2108	NA	NA
R01_cb13230_c1/f8p0/643	NA	R01_cb13230_c1/f8p0/643	NA	NA
R01_cb5641_c10/f1p0/2482	NA	R01_cb5641_c10/f1p0/2482	NA	NA
R01_cb15740_c0/f2p0/626	NA	R01_cb15740_c0/f2p0/626	NA	NA
R01_cb6377_c3/f1p0/2534	NA	NA	NA	R01_cb6377_c3/f1p0/2534
R01_cb2226_c5/f1p0/4574	NA	NA	NA	R01_cb2226_c5/f1p0/4574
R01_cb8564_c24589/f1p0/3831	NA	R01_cb8564_c24589/f1p0/3831	NA	R01_cb8564_c24589/f1p0/3831
R01_cb8564_c113776/f1p0/1903	NA	NA	NA	R01_cb8564_c113776/f1p0/1903
R01_cb18076_c1/f1p1/1784	NA	R01_cb18076_c1/f1p1/1784	R01_cb18076_c1/f1p1/1784	R01_cb18076_c1/f1p1/1784
R01_cb18110_c0/f2p0/412	R01_cb18110_c0/f2p0/412	R01_cb18110_c0/f2p0/412	R01_cb18110_c0/f2p0/412	R01_cb18110_c0/f2p0/412
R01_cb3854_c0/f1p0/3504	NA	R01_cb3854_c0/f1p0/3504	R01_cb3854_c0/f1p0/3504	NA
R01_cb2482_c27/f1p0/2405	NA	NA	NA	R01_cb2482_c27/f1p0/2405
R01_cb16066_c2/f2p0/548	R01_cb16066_c2/f2p0/548	R01_cb16066_c2/f2p0/548	R01_cb16066_c2/f2p0/548	NA
R01_cb2352_c7/f1p0/2975	NA	R01_cb2352_c7/f1p0/2975	R01_cb2352_c7/f1p0/2975	R01_cb2352_c7/f1p0/2975
R01_cb10292_c4/f1p0/1095	NA	R01_cb10292_c4/f1p0/1095	NA	R01_cb10292_c4/f1p0/1095
R01_cb11434_c2/f1p0/1168	NA	R01_cb11434_c2/f1p0/1168	R01_cb11434_c2/f1p0/1168	R01_cb11434_c2/f1p0/1168
R01_cb14239_c20/f1p0/1013	NA	R01_cb14239_c20/f1p0/1013	NA	NA
R01_cb4047_c4/f3p2/2787	NA	NA	NA	R01_cb4047_c4/f3p2/2787
R01_cb8564_c50280/f1p1/3354	NA	NA	NA	R01_cb8564_c50280/f1p1/3354
R01_cb10294_c7/f1p0/1637	NA	R01_cb10294_c7/f1p0/1637	NA	NA
R01_cb8564_c12936/f1p0/2488	NA	R01_cb8564_c12936/f1p0/2488	NA	NA

R01_cb8564_c126992/f1p0/2332	NA	R01_cb8564_c126992/f1p0/2332	R01_cb8564_c126992/f1p0/2332	R01_cb8564_c126992/f1p0/2332
R01_cb18409_c21/f3p0/636	R01_cb18409_c21/f3p0/636	R01_cb18409_c21/f3p0/636	R01_cb18409_c21/f3p0/636	R01_cb18409_c21/f3p0/636
R01_cb14550_c1/f1p0/1088	NA	R01_cb14550_c1/f1p0/1088	NA	NA
R01_cb4824_c14/f1p0/3079	NA	R01_cb4824_c14/f1p0/3079	R01_cb4824_c14/f1p0/3079	R01_cb4824_c14/f1p0/3079
R01_cb18456_c6078/f1p1/662	R01_cb18456_c6078/f1p1/662	R01_cb18456_c6078/f1p1/662	R01_cb18456_c6078/f1p1/662	R01_cb18456_c6078/f1p1/662
R01_cb261_c21/f1p0/2395	NA	NA	NA	R01_cb261_c21/f1p0/2395
R01_cb10587_c3/f1p0/1346	R01_cb10587_c3/f1p0/1346	R01_cb10587_c3/f1p0/1346	R01_cb10587_c3/f1p0/1346	R01_cb10587_c3/f1p0/1346
R01_cb10615_c1/f1p0/2361	NA	R01_cb10615_c1/f1p0/2361	NA	NA
R01_cb13603_c8/f1p0/539	R01_cb13603_c8/f1p0/539	R01_cb13603_c8/f1p0/539	R01_cb13603_c8/f1p0/539	R01_cb13603_c8/f1p0/539
R01_cb9081_c5/f1p0/393	R01_cb9081_c5/f1p0/393	R01_cb9081_c5/f1p0/393	R01_cb9081_c5/f1p0/393	R01_cb9081_c5/f1p0/393
R01_cb12634_c1/f1p0/1616	NA	R01_cb12634_c1/f1p0/1616	R01_cb12634_c1/f1p0/1616	R01_cb12634_c1/f1p0/1616
R01_cb7255_c22/f1p0/2485	NA	R01_cb7255_c22/f1p0/2485	NA	NA
R01_cb15618_c0/f1p0/1084	NA	NA	NA	R01_cb15618_c0/f1p0/1084
R01_cb6845_c11/f1p0/2823	NA	NA	NA	R01_cb6845_c11/f1p0/2823
R01_cb16535_c2/f1p0/398	R01_cb16535_c2/f1p0/398	R01_cb16535_c2/f1p0/398	NA	R01_cb16535_c2/f1p0/398
R01_cb2700_c11/f1p0/2641	NA	R01_cb2700_c11/f1p0/2641	NA	NA
R01_cb7954_c1/f1p0/3299	NA	R01_cb7954_c1/f1p0/3299	NA	R01_cb7954_c1/f1p0/3299
R01_cb8564_c128383/f1p0/2806	NA	R01_cb8564_c128383/f1p0/2806	R01_cb8564_c128383/f1p0/2806	R01_cb8564_c128383/f1p0/2806
R01_cb8564_c89038/f1p0/2799	NA	NA	NA	R01_cb8564_c89038/f1p0/2799
R01_cb8564_c21799/f1p1/2554	NA	R01_cb8564_c21799/f1p1/2554	R01_cb8564_c21799/f1p1/2554	NA
R01_cb7087_c3/f1p0/2513	NA	NA	NA	R01_cb7087_c3/f1p0/2513
R01_cb3699_c15/f1p0/3193	NA	R01_cb3699_c15/f1p0/3193	NA	NA
R01_cb15844_c3/f1p0/743	R01_cb15844_c3/f1p0/743	R01_cb15844_c3/f1p0/743	R01_cb15844_c3/f1p0/743	R01_cb15844_c3/f1p0/743
R01_cb943_c6/f1p0/7019	NA	R01_cb943_c6/f1p0/7019	NA	R01_cb943_c6/f1p0/7019

R01_cb3563_c10/f1p0/1620	NA	NA	NA	R01_cb3563_c10/f1p0/1620
R01_cb17375_c1/f1p0/1899	NA	R01_cb17375_c1/f1p0/1899	R01_cb17375_c1/f1p0/1899	R01_cb17375_c1/f1p0/1899
R01_cb15730_c7/f1p0/727	NA	R01_cb15730_c7/f1p0/727	NA	R01_cb15730_c7/f1p0/727
R01_cb5637_c4/f1p0/3052	NA	R01_cb5637_c4/f1p0/3052	NA	NA
R01_cb10172_c8/f1p0/491	R01_cb10172_c8/f1p0/491	R01_cb10172_c8/f1p0/491	R01_cb10172_c8/f1p0/491	R01_cb10172_c8/f1p0/491
R01_cb4315_c0/f1p0/3374	NA	R01_cb4315_c0/f1p0/3374	NA	R01_cb4315_c0/f1p0/3374
R01_cb12628_c2/f1p0/690	NA	R01_cb12628_c2/f1p0/690	R01_cb12628_c2/f1p0/690	R01_cb12628_c2/f1p0/690
R01_cb18456_c7370/f1p0/1428	NA	R01_cb18456_c7370/f1p0/1428	R01_cb18456_c7370/f1p0/1428	R01_cb18456_c7370/f1p0/1428
R01_cb16475_c1/f1p0/459	R01_cb16475_c1/f1p0/459	R01_cb16475_c1/f1p0/459	R01_cb16475_c1/f1p0/459	R01_cb16475_c1/f1p0/459
R01_cb6715_c2/f1p0/6268	NA	NA	NA	R01_cb6715_c2/f1p0/6268
R01_cb8034_c4/f1p0/2400	NA	R01_cb8034_c4/f1p0/2400	NA	NA
R01_cb17475_c1/f1p0/680	NA	R01_cb17475_c1/f1p0/680	R01_cb17475_c1/f1p0/680	R01_cb17475_c1/f1p0/680
R01_cb8660_c6/f1p0/2305	NA	NA	NA	R01_cb8660_c6/f1p0/2305
R01_cb9791_c2/f1p0/1020	NA	NA	R01_cb9791_c2/f1p0/1020	R01_cb9791_c2/f1p0/1020
R01_cb11287_c2/f1p0/718	NA	NA	NA	R01_cb11287_c2/f1p0/718
R01_cb4352_c8/f1p0/2475	NA	NA	NA	R01_cb4352_c8/f1p0/2475
R01_cb1813_c11/f1p0/2690	NA	NA	NA	R01_cb1813_c11/f1p0/2690
R01_cb3381_c29/f1p0/2827	NA	NA	NA	R01_cb3381_c29/f1p0/2827
R01_cb15537_c0/f2p0/567	NA	R01_cb15537_c0/f2p0/567	R01_cb15537_c0/f2p0/567	R01_cb15537_c0/f2p0/567
R01_cb119_c5/f2p0/2267	NA	NA	NA	R01_cb119_c5/f2p0/2267
R01_cb5000_c6/f1p0/3452	R01_cb5000_c6/f1p0/3452	R01_cb5000_c6/f1p0/3452	R01_cb5000_c6/f1p0/3452	R01_cb5000_c6/f1p0/3452
R01_cb6861_c14/f1p0/2704	NA	R01_cb6861_c14/f1p0/2704	NA	NA
R01_cb17973_c46/f1p0/1810	NA	NA	R01_cb17973_c46/f1p0/1810	R01_cb17973_c46/f1p0/1810
R01_cb11368_c1/f1p0/2504	NA	R01_cb11368_c1/f1p0/2504	NA	R01_cb11368_c1/f1p0/2504
R01_cb124_c20/f1p0/3752	NA	NA	NA	R01_cb124_c20/f1p0/3752
R01_cb15365_c6/f1p0/412	R01_cb15365_c6/f1p0/412	R01_cb15365_c6/f1p0/412	R01_cb15365_c6/f1p0/412	R01_cb15365_c6/f1p0/412

R01_cb3821_c26/f1p0/3122	R01_cb3821_c26/f1p0/3122	R01_cb3821_c26/f1p0/3122	NA	NA
R01_cb8111_c2/f1p0/1357	NA	NA	NA	R01_cb8111_c2/f1p0/1357
R01_cb750_c11/f1p0/4465	NA	R01_cb750_c11/f1p0/4465	NA	NA
R01_cb18456_c1525/f3p0/751	R01_cb18456_c1525/f3p0/751	R01_cb18456_c1525/f3p0/751	R01_cb18456_c1525/f3p0/751	R01_cb18456_c1525/f3p0/751
R01_cb2698_c8/f1p0/1316	NA	NA	NA	R01_cb2698_c8/f1p0/1316
R01_cb4768_c36/f1p0/2589	NA	R01_cb4768_c36/f1p0/2589	NA	NA
R01_cb18456_c7393/f1p0/753	NA	R01_cb18456_c7393/f1p0/753	R01_cb18456_c7393/f1p0/753	R01_cb18456_c7393/f1p0/753
R01_cb18407_c0/f1p0/1353	NA	NA	NA	R01_cb18407_c0/f1p0/1353
R01_cb11832_c0/f1p0/1296	NA	NA	R01_cb11832_c0/f1p0/1296	NA
R01_cb8564_c34110/f2p1/1939	NA	NA	NA	R01_cb8564_c34110/f2p1/1939
R01_cb8820_c5/f1p0/1671	NA	R01_cb8820_c5/f1p0/1671	NA	NA
R01_cb3760_c11/f6p1/2235	NA	NA	NA	R01_cb3760_c11/f6p1/2235
R01_cb8564_c24389/f1p0/2236	R01_cb8564_c24389/f1p0/2236	R01_cb8564_c24389/f1p0/2236	NA	NA
R01_cb8564_c127636/f1p0/2157	NA	NA	NA	R01_cb8564_c127636/f1p0/2157
R01_cb18233_c1/f1p0/1393	NA	NA	NA	R01_cb18233_c1/f1p0/1393
R01_cb5900_c74/f1p0/3322	NA	R01_cb5900_c74/f1p0/3322	R01_cb5900_c74/f1p0/3322	R01_cb5900_c74/f1p0/3322
R01_cb9772_c6/f1p0/1847	NA	R01_cb9772_c6/f1p0/1847	NA	NA
R01_cb12972_c4/f2p0/666	NA	R01_cb12972_c4/f2p0/666	R01_cb12972_c4/f2p0/666	R01_cb12972_c4/f2p0/666
R01_cb18456_c7618/f1p0/1166	NA	R01_cb18456_c7618/f1p0/1166	R01_cb18456_c7618/f1p0/1166	R01_cb18456_c7618/f1p0/1166
R01_cb2095_c9/f1p1/3903	NA	NA	NA	R01_cb2095_c9/f1p1/3903
R01_cb11554_c0/f1p0/934	NA	R01_cb11554_c0/f1p0/934	R01_cb11554_c0/f1p0/934	R01_cb11554_c0/f1p0/934
R01_cb11455_c2/f1p0/2807	NA	R01_cb11455_c2/f1p0/2807	NA	R01_cb11455_c2/f1p0/2807
R01_cb1739_c7/f1p0/5956	NA	NA	NA	R01_cb1739_c7/f1p0/5956
R01_cb17097_c0/f1p0/643	NA	R01_cb17097_c0/f1p0/643	NA	R01_cb17097_c0/f1p0/643

R01_cb8564_c76262/f1p0/3001	NA	R01_cb8564_c76262/f1p0/3001	NA	NA
R01_cb6365_c0/f5p2/1956	NA	R01_cb6365_c0/f5p2/1956	NA	NA
R01_cb6328_c4/f1p0/698	NA	R01_cb6328_c4/f1p0/698	R01_cb6328_c4/f1p0/698	R01_cb6328_c4/f1p0/698
R01_cb8564_c117203/f1p0/2885	NA	R01_cb8564_c117203/f1p0/2885	R01_cb8564_c117203/f1p0/2885	R01_cb8564_c117203/f1p0/2885
R01_cb8049_c20/f1p0/847	NA	R01_cb8049_c20/f1p0/847	NA	NA
R01_cb8564_c69830/f1p0/2648	NA	R01_cb8564_c69830/f1p0/2648	NA	NA
R01_cb3359_c30/f1p0/2249	NA	R01_cb3359_c30/f1p0/2249	NA	R01_cb3359_c30/f1p0/2249
R01_cb8564_c126440/f1p0/2600	NA	R01_cb8564_c126440/f1p0/2600	R01_cb8564_c126440/f1p0/2600	R01_cb8564_c126440/f1p0/2600
R01_cb18556_c0/f1p0/974	NA	NA	NA	R01_cb18556_c0/f1p0/974
R01_cb2719_c70/f1p0/672	R01_cb2719_c70/f1p0/672	R01_cb2719_c70/f1p0/672	R01_cb2719_c70/f1p0/672	R01_cb2719_c70/f1p0/672
R01_cb8564_c42343/f1p2/2933	NA	R01_cb8564_c42343/f1p2/2933	NA	NA
R01_cb16013_c0/f1p0/1384	NA	R01_cb16013_c0/f1p0/1384	NA	NA
R01_cb15188_c0/f1p0/1551	R01_cb15188_c0/f1p0/1551	R01_cb15188_c0/f1p0/1551	NA	R01_cb15188_c0/f1p0/1551
R01_cb11690_c1/f1p0/2286	NA	NA	R01_cb11690_c1/f1p0/2286	NA
R01_cb13392_c26/f1p3/960	NA	R01_cb13392_c26/f1p3/960	NA	NA
R01_cb2344_c1/f1p0/4165	R01_cb2344_c1/f1p0/4165	R01_cb2344_c1/f1p0/4165	NA	NA
R01_cb5255_c6/f1p0/2876	NA	R01_cb5255_c6/f1p0/2876	R01_cb5255_c6/f1p0/2876	R01_cb5255_c6/f1p0/2876
R01_cb3750_c30/f1p0/2586	NA	NA	NA	R01_cb3750_c30/f1p0/2586
R01_cb15443_c2/f1p0/1227	NA	NA	NA	R01_cb15443_c2/f1p0/1227
R01_cb10034_c32/f1p0/595	NA	R01_cb10034_c32/f1p0/595	NA	NA
R01_cb13386_c11/f1p0/1430	NA	R01_cb13386_c11/f1p0/1430	NA	NA
R01_cb16867_c0/f1p0/1340	NA	NA	NA	R01_cb16867_c0/f1p0/1340
R01_cb363_c5/f1p0/2564	NA	R01_cb363_c5/f1p0/2564	NA	R01_cb363_c5/f1p0/2564
R01_cb8564_c12442/f1p0/2779	NA	R01_cb8564_c12442/f1p0/2779	R01_cb8564_c12442/f1p0/2779	R01_cb8564_c12442/f1p0/2779

R01_cb18409_c3/f2p0/428	R01_cb18409_c3/f2p0/428	R01_cb18409_c3/f2p0/428	R01_cb18409_c3/f2p0/428	R01_cb18409_c3/f2p0/428
R01_cb8564_c46990/f1p0/4414	NA	NA	NA	R01_cb8564_c46990/f1p0/4414
R01_cb4795_c3/f1p0/1980	NA	R01_cb4795_c3/f1p0/1980	NA	NA
R01_cb8564_c51855/f1p0/2961	NA	NA	R01_cb8564_c51855/f1p0/2961	NA
R01_cb1522_c0/f1p0/4289	NA	R01_cb1522_c0/f1p0/4289	NA	NA
R01_cb13583_c9/f2p0/1172	NA	NA	NA	R01_cb13583_c9/f2p0/1172
R01_cb3309_c8/f1p0/1954	NA	R01_cb3309_c8/f1p0/1954	NA	NA
R01_cb10970_c7/f1p0/1091	NA	NA	NA	R01_cb10970_c7/f1p0/1091
R01_cb18456_c2292/f1p0/693	NA	R01_cb18456_c2292/f1p0/693	R01_cb18456_c2292/f1p0/693	R01_cb18456_c2292/f1p0/693
R01_cb8564_c49390/f1p0/2842	R01_cb8564_c49390/f1p0/2842	R01_cb8564_c49390/f1p0/2842	R01_cb8564_c49390/f1p0/2842	R01_cb8564_c49390/f1p0/2842
R01_cb8564_c116364/f1p0/1895	NA	NA	NA	R01_cb8564_c116364/f1p0/1895
R01_cb8564_c91973/f1p0/2452	R01_cb8564_c91973/f1p0/2452	R01_cb8564_c91973/f1p0/2452	NA	NA
R01_cb8564_c51888/f1p0/2833	NA	R01_cb8564_c51888/f1p0/2833	NA	R01_cb8564_c51888/f1p0/2833
R01_cb18409_c103/f1p0/442	R01_cb18409_c103/f1p0/442	R01_cb18409_c103/f1p0/442	R01_cb18409_c103/f1p0/442	R01_cb18409_c103/f1p0/442
R01_cb8564_c751/f2p0/2625	NA	R01_cb8564_c751/f2p0/2625	NA	NA
R01_cb8564_c5246/f1p0/2196	NA	R01_cb8564_c5246/f1p0/2196	NA	NA
R01_cb18287_c8/f1p0/449	R01_cb18287_c8/f1p0/449	R01_cb18287_c8/f1p0/449	R01_cb18287_c8/f1p0/449	R01_cb18287_c8/f1p0/449
R01_cb1745_c2/f1p0/3206	NA	R01_cb1745_c2/f1p0/3206	NA	NA
R01_cb9347_c7/f1p0/1792	NA	R01_cb9347_c7/f1p0/1792	NA	NA
R01_cb8564_c148946/f1p1/3399	NA	R01_cb8564_c148946/f1p1/3399	NA	NA
R01_cb4529_c10/f1p0/746	NA	R01_cb4529_c10/f1p0/746	NA	NA
R01_cb1892_c4/f1p0/3473	R01_cb1892_c4/f1p0/3473	R01_cb1892_c4/f1p0/3473	NA	R01_cb1892_c4/f1p0/3473

R01_cb8564_c91980/f1p0/1973	R01_cb8564_c91980/f1p0/1973	NA	NA	NA
R01_cb4455_c19/f1p0/876	R01_cb4455_c19/f1p0/876	R01_cb4455_c19/f1p0/876	NA	R01_cb4455_c19/f1p0/876
R01_cb4533_c1/f1p0/3594	NA	R01_cb4533_c1/f1p0/3594	NA	R01_cb4533_c1/f1p0/3594
R01_cb10812_c1/f1p0/2522	NA	R01_cb10812_c1/f1p0/2522	NA	R01_cb10812_c1/f1p0/2522
R01_cb13877_c3/f1p0/1270	NA	NA	NA	R01_cb13877_c3/f1p0/1270
R01_cb8414_c3/f1p0/1459	NA	NA	NA	R01_cb8414_c3/f1p0/1459
R01_cb16981_c13/f1p1/600	NA	NA	NA	R01_cb16981_c13/f1p1/600
R01_cb13199_c2/f1p0/1799	NA	NA	NA	R01_cb13199_c2/f1p0/1799
R01_cb13802_c2/f1p0/682	NA	NA	R01_cb13802_c2/f1p0/682	R01_cb13802_c2/f1p0/682
R01_cb7687_c8/f8p0/2464	NA	NA	NA	R01_cb7687_c8/f8p0/2464
R01_cb18406_c1/f1p0/703	NA	R01_cb18406_c1/f1p0/703	R01_cb18406_c1/f1p0/703	R01_cb18406_c1/f1p0/703
R01_cb8564_c112307/f1p0/2255	NA	R01_cb8564_c112307/f1p0/2255	R01_cb8564_c112307/f1p0/2255	NA
R01_cb11766_c1/f1p0/2462	NA	NA	R01_cb11766_c1/f1p0/2462	NA
R01_cb10415_c0/f3p0/1058	NA	R01_cb10415_c0/f3p0/1058	NA	NA
R01_cb18191_c2/f1p0/1455	NA	NA	NA	R01_cb18191_c2/f1p0/1455
R01_cb5110_c2/f1p0/1943	NA	R01_cb5110_c2/f1p0/1943	NA	NA
R01_cb13287_c6/f1p1/632	R01_cb13287_c6/f1p1/632	R01_cb13287_c6/f1p1/632	NA	R01_cb13287_c6/f1p1/632
R01_cb17522_c0/f1p0/1119	NA	NA	NA	R01_cb17522_c0/f1p0/1119
R01_cb2382_c0/f4p1/5337	NA	NA	NA	R01_cb2382_c0/f4p1/5337
R01_cb6615_c7/f1p0/2918	NA	NA	R01_cb6615_c7/f1p0/2918	R01_cb6615_c7/f1p0/2918
R01_cb699_c0/f1p0/4676	NA	NA	NA	R01_cb699_c0/f1p0/4676
R01_cb11825_c0/f1p0/615	R01_cb11825_c0/f1p0/615	R01_cb11825_c0/f1p0/615	R01_cb11825_c0/f1p0/615	R01_cb11825_c0/f1p0/615
R01_cb17485_c0/f1p0/927	NA	NA	NA	R01_cb17485_c0/f1p0/927
R01_cb8564_c126966/f1p5/272	R01_cb8564_c126966/f1p5/27	R01_cb8564_c126966/f1p5/272	NA	NA

0	20	0		
R01_cb8564_c74355/f1p1/2682	NA	R01_cb8564_c74355/f1p1/2682	NA	NA
R01_cb12811_c8/f1p0/686	NA	R01_cb12811_c8/f1p0/686	NA	R01_cb12811_c8/f1p0/686
R01_cb15237_c4/f1p0/5009	NA	R01_cb15237_c4/f1p0/5009	NA	NA
R01_cb17760_c3/f1p0/1801	NA	NA	NA	R01_cb17760_c3/f1p0/1801
R01_cb9750_c6/f1p0/1895	NA	NA	NA	R01_cb9750_c6/f1p0/1895
R01_cb16338_c2/f1p0/856	NA	R01_cb16338_c2/f1p0/856	NA	R01_cb16338_c2/f1p0/856
R01_cb2991_c20/f1p0/1382	NA	NA	NA	R01_cb2991_c20/f1p0/1382
R01_cb10913_c7/f1p0/1183	NA	R01_cb10913_c7/f1p0/1183	NA	R01_cb10913_c7/f1p0/1183
R01_cb9236_c0/f1p1/2144	NA	NA	NA	R01_cb9236_c0/f1p1/2144
R01_cb422_c5/f1p0/3346	NA	NA	NA	R01_cb422_c5/f1p0/3346
R01_cb8564_c43401/f1p2/3025	NA	R01_cb8564_c43401/f1p2/3025	NA	NA
R01_cb5287_c2/f1p0/2768	NA	R01_cb5287_c2/f1p0/2768	NA	NA
R01_cb6758_c1/f3p0/1900	NA	NA	NA	R01_cb6758_c1/f3p0/1900
R01_cb8564_c24565/f1p0/3307	NA	R01_cb8564_c24565/f1p0/3307	NA	R01_cb8564_c24565/f1p0/3307
R01_cb16210_c3/f1p0/904	NA	NA	NA	R01_cb16210_c3/f1p0/904
R01_cb17068_c1/f1p0/1570	R01_cb17068_c1/f1p0/1570	R01_cb17068_c1/f1p0/1570	R01_cb17068_c1/f1p0/1570	R01_cb17068_c1/f1p0/1570
R01_cb8564_c112134/f1p0/2518	NA	NA	NA	R01_cb8564_c112134/f1p0/2518
R01_cb17644_c2/f1p0/386	NA	NA	NA	R01_cb17644_c2/f1p0/386
R01_cb12504_c2/f1p0/1579	NA	R01_cb12504_c2/f1p0/1579	NA	R01_cb12504_c2/f1p0/1579
R01_cb5246_c7/f1p0/753	R01_cb5246_c7/f1p0/753	R01_cb5246_c7/f1p0/753	R01_cb5246_c7/f1p0/753	R01_cb5246_c7/f1p0/753
R01_cb13026_c2/f2p0/1558	NA	R01_cb13026_c2/f2p0/1558	R01_cb13026_c2/f2p0/1558	R01_cb13026_c2/f2p0/1558
R01_cb4886_c2/f1p0/1930	NA	R01_cb4886_c2/f1p0/1930	NA	NA
R01_cb7023_c6/f1p0/1630	NA	R01_cb7023_c6/f1p0/1630	R01_cb7023_c6/f1p0/1630	R01_cb7023_c6/f1p0/1630
R01_cb12722_c30/f67p3/1580	NA	NA	NA	R01_cb12722_c30/f67p3/1580

R01_cb12998_c4/f1p0/1068	R01_cb12998_c4/f1p0/1068	R01_cb12998_c4/f1p0/1068	R01_cb12998_c4/f1p0/1068	R01_cb12998_c4/f1p0/1068
R01_cb9704_c3/f1p0/1002	NA	NA	NA	R01_cb9704_c3/f1p0/1002
R01_cb11026_c7/f1p0/698	R01_cb11026_c7/f1p0/698	R01_cb11026_c7/f1p0/698	R01_cb11026_c7/f1p0/698	R01_cb11026_c7/f1p0/698
R01_cb18456_c7557/f1p1/1605	NA	R01_cb18456_c7557/f1p1/1605	NA	NA
R01_cb8736_c5/f1p0/1859	NA	R01_cb8736_c5/f1p0/1859	NA	NA
R01_cb15540_c2/f2p2/1499	NA	NA	NA	R01_cb15540_c2/f2p2/1499
R01_cb3539_c3/f1p0/3596	NA	R01_cb3539_c3/f1p0/3596	NA	NA
R01_cb636_c7/f1p0/1833	NA	NA	NA	R01_cb636_c7/f1p0/1833
R01_cb7358_c3/f1p0/2425	NA	R01_cb7358_c3/f1p0/2425	NA	R01_cb7358_c3/f1p0/2425
R01_cb13435_c12/f1p0/1852	NA	NA	NA	R01_cb13435_c12/f1p0/1852
R01_cb13133_c4/f1p0/1181	R01_cb13133_c4/f1p0/1181	R01_cb13133_c4/f1p0/1181	R01_cb13133_c4/f1p0/1181	R01_cb13133_c4/f1p0/1181
R01_cb3045_c6/f1p0/2444	NA	R01_cb3045_c6/f1p0/2444	NA	NA
R01_cb4294_c24/f1p0/2507	R01_cb4294_c24/f1p0/2507	R01_cb4294_c24/f1p0/2507	NA	NA
R01_cb17_c6/f1p0/5004	NA	NA	NA	R01_cb17_c6/f1p0/5004
R01_cb14125_c54/f1p0/628	NA	R01_cb14125_c54/f1p0/628	R01_cb14125_c54/f1p0/628	NA
R01_cb7836_c0/f1p0/2522	NA	NA	NA	R01_cb7836_c0/f1p0/2522
R01_cb7419_c14/f3p1/2624	NA	NA	NA	R01_cb7419_c14/f3p1/2624
R01_cb15294_c2/f1p0/1595	R01_cb15294_c2/f1p0/1595	R01_cb15294_c2/f1p0/1595	R01_cb15294_c2/f1p0/1595	R01_cb15294_c2/f1p0/1595
R01_cb2334_c2/f1p0/3969	NA	R01_cb2334_c2/f1p0/3969	NA	NA
R01_cb11257_c1/f1p0/786	NA	NA	NA	R01_cb11257_c1/f1p0/786
R01_cb8564_c77387/f1p0/4197	NA	R01_cb8564_c77387/f1p0/4197	NA	NA
R01_cb15379_c2/f1p0/1238	R01_cb15379_c2/f1p0/1238	R01_cb15379_c2/f1p0/1238	R01_cb15379_c2/f1p0/1238	R01_cb15379_c2/f1p0/1238
R01_cb2387_c5/f1p0/2939	NA	R01_cb2387_c5/f1p0/2939	NA	R01_cb2387_c5/f1p0/2939
R01_cb11277_c1/f1p1/1966	NA	R01_cb11277_c1/f1p1/1966	NA	NA
R01_cb16653_c3/f1p0/1725	NA	NA	NA	R01_cb16653_c3/f1p0/1725
R01_cb8564_c88491/f1p0/2512	NA	R01_cb8564_c88491/f1p0/2512	NA	NA

R01_cb2822_c1/f2p0/2694	NA	R01_cb2822_c1/f2p0/2694	NA	NA
R01_cb2573_c1/f1p0/3879	NA	NA	R01_cb2573_c1/f1p0/3879	NA
R01_cb18456_c7621/f1p0/1467	NA	R01_cb18456_c7621/f1p0/1467	NA	R01_cb18456_c7621/f1p0/1467
R01_cb8564_c12365/f1p0/3798	R01_cb8564_c12365/f1p0/3798	NA	NA	NA
R01_cb16682_c9/f1p0/847	R01_cb16682_c9/f1p0/847	R01_cb16682_c9/f1p0/847	R01_cb16682_c9/f1p0/847	R01_cb16682_c9/f1p0/847
R01_cb8564_c129160/f1p0/2693	NA	R01_cb8564_c129160/f1p0/2693	NA	R01_cb8564_c129160/f1p0/2693
R01_cb16321_c1/f1p0/1314	NA	R01_cb16321_c1/f1p0/1314	NA	R01_cb16321_c1/f1p0/1314
R01_cb15873_c1/f2p0/1546	R01_cb15873_c1/f2p0/1546	R01_cb15873_c1/f2p0/1546	NA	NA
R01_cb1012_c18/f1p0/1719	NA	R01_cb1012_c18/f1p0/1719	NA	NA
R01_cb11411_c1/f1p0/1885	NA	R01_cb11411_c1/f1p0/1885	R01_cb11411_c1/f1p0/1885	R01_cb11411_c1/f1p0/1885
R01_cb13857_c1/f1p0/1030	NA	NA	NA	R01_cb13857_c1/f1p0/1030
R01_cb14344_c6/f1p0/1326	NA	R01_cb14344_c6/f1p0/1326	R01_cb14344_c6/f1p0/1326	R01_cb14344_c6/f1p0/1326
R01_cb18456_c7181/f1p0/1377	R01_cb18456_c7181/f1p0/1377	R01_cb18456_c7181/f1p0/1377	R01_cb18456_c7181/f1p0/1377	R01_cb18456_c7181/f1p0/1377
R01_cb9214_c6/f2p0/1064	NA	R01_cb9214_c6/f2p0/1064	NA	NA
R01_cb14599_c0/f2p0/1471	R01_cb14599_c0/f2p0/1471	NA	NA	R01_cb14599_c0/f2p0/1471
R01_cb9154_c3/f1p0/2170	NA	R01_cb9154_c3/f1p0/2170	NA	R01_cb9154_c3/f1p0/2170
R01_cb8564_c125216/f1p0/2375	NA	R01_cb8564_c125216/f1p0/2375	R01_cb8564_c125216/f1p0/2375	R01_cb8564_c125216/f1p0/2375
R01_cb16082_c5/f1p0/691	NA	NA	NA	R01_cb16082_c5/f1p0/691
R01_cb13867_c15/f10p2/797	NA	NA	NA	R01_cb13867_c15/f10p2/797
R01_cb8564_c15085/f1p0/2489	NA	R01_cb8564_c15085/f1p0/2489	NA	R01_cb8564_c15085/f1p0/2489
R01_cb8564_c71769/f1p0/1908	NA	NA	R01_cb8564_c71769/f1p0/1908	R01_cb8564_c71769/f1p0/1908
R01_cb14883_c2/f1p0/1543	NA	R01_cb14883_c2/f1p0/1543	NA	NA

R01_cb8023_c10/f1p0/2224	NA	NA	R01_cb8023_c10/f1p0/2224	R01_cb8023_c10/f1p0/2224
R01_cb3218_c1/f1p0/3683	NA	NA	NA	R01_cb3218_c1/f1p0/3683
R01_cb2383_c12/f1p1/2020	NA	R01_cb2383_c12/f1p1/2020	NA	NA
R01_cb18456_c7209/f1p0/640	R01_cb18456_c7209/f1p0/640	R01_cb18456_c7209/f1p0/640	R01_cb18456_c7209/f1p0/640	R01_cb18456_c7209/f1p0/640
R01_cb6873_c0/f1p0/2745	NA	R01_cb6873_c0/f1p0/2745	NA	NA
R01_cb9359_c4/f1p0/1039	NA	NA	NA	R01_cb9359_c4/f1p0/1039
R01_cb2031_c1/f1p2/4205	NA	NA	NA	R01_cb2031_c1/f1p2/4205
R01_cb18456_c6805/f1p0/438	R01_cb18456_c6805/f1p0/438	R01_cb18456_c6805/f1p0/438	R01_cb18456_c6805/f1p0/438	NA
R01_cb18584_c1/f1p1/1646	NA	NA	NA	R01_cb18584_c1/f1p1/1646
R01_cb11449_c1/f1p0/2586	NA	R01_cb11449_c1/f1p0/2586	NA	NA
R01_cb17126_c0/f2p0/1021	NA	NA	NA	R01_cb17126_c0/f2p0/1021
R01_cb4952_c7/f1p0/1045	NA	NA	NA	R01_cb4952_c7/f1p0/1045
R01_cb12827_c2/f1p0/1364	NA	NA	NA	R01_cb12827_c2/f1p0/1364
R01_cb8564_c86327/f1p1/3488	NA	NA	NA	R01_cb8564_c86327/f1p1/3488
R01_cb3303_c2/f1p0/3381	NA	R01_cb3303_c2/f1p0/3381	R01_cb3303_c2/f1p0/3381	R01_cb3303_c2/f1p0/3381
R01_cb5120_c1/f1p0/2655	NA	NA	NA	R01_cb5120_c1/f1p0/2655
R01_cb7497_c4/f2p0/2558	NA	NA	NA	R01_cb7497_c4/f2p0/2558
R01_cb10610_c1/f1p0/1938	NA	NA	NA	R01_cb10610_c1/f1p0/1938
R01_cb5183_c115/f1p0/1016	NA	NA	NA	R01_cb5183_c115/f1p0/1016
R01_cb18456_c7373/f1p0/1529	NA	R01_cb18456_c7373/f1p0/1529	R01_cb18456_c7373/f1p0/1529	R01_cb18456_c7373/f1p0/1529
R01_cb16946_c1/f1p0/896	NA	NA	NA	R01_cb16946_c1/f1p0/896
R01_cb242_c22/f1p0/3715	NA	NA	NA	R01_cb242_c22/f1p0/3715
R01_cb10192_c8/f1p0/1407	NA	NA	NA	R01_cb10192_c8/f1p0/1407
R01_cb8564_c71951/f1p0/2329	NA	R01_cb8564_c71951/f1p0/2329	NA	NA
R01_cb17973_c58/f1p0/1273	NA	R01_cb17973_c58/f1p0/1273	R01_cb17973_c58/f1p0/1273	R01_cb17973_c58/f1p0/1273
R01_cb16326_c0/f3p1/734	NA	R01_cb16326_c0/f3p1/734	R01_cb16326_c0/f3p1/734	R01_cb16326_c0/f3p1/734

R01_cb14977_c5/f1p0/1742	R01_cb14977_c5/f1p0/1742	R01_cb14977_c5/f1p0/1742	R01_cb14977_c5/f1p0/1742	R01_cb14977_c5/f1p0/1742
R01_cb8564_c118521/f1p0/2064	NA	R01_cb8564_c118521/f1p0/2064	NA	NA
R01_cb1391_c1/f1p0/3632	R01_cb1391_c1/f1p0/3632	R01_cb1391_c1/f1p0/3632	NA	NA
R01_cb155_c12/f1p0/2278	NA	R01_cb155_c12/f1p0/2278	NA	R01_cb155_c12/f1p0/2278
R01_cb7356_c7/f1p0/1722	NA	NA	NA	R01_cb7356_c7/f1p0/1722
R01_cb16164_c0/f1p0/636	NA	R01_cb16164_c0/f1p0/636	NA	NA
R01_cb3645_c45/f1p0/397	R01_cb3645_c45/f1p0/397	R01_cb3645_c45/f1p0/397	R01_cb3645_c45/f1p0/397	R01_cb3645_c45/f1p0/397
R01_cb13089_c1/f1p0/1745	NA	R01_cb13089_c1/f1p0/1745	NA	NA
R01_cb8564_c77498/f1p0/3030	NA	NA	NA	R01_cb8564_c77498/f1p0/3030
R01_cb191_c7/f1p1/4776	NA	NA	NA	R01_cb191_c7/f1p1/4776
R01_cb16594_c0/f2p1/1216	NA	NA	NA	R01_cb16594_c0/f2p1/1216
R01_cb16042_c1/f1p0/1336	NA	R01_cb16042_c1/f1p0/1336	NA	R01_cb16042_c1/f1p0/1336
R01_cb8564_c1325/f1p1/2589	NA	NA	NA	R01_cb8564_c1325/f1p1/2589
R01_cb9871_c4/f1p0/704	NA	R01_cb9871_c4/f1p0/704	NA	NA
R01_cb1203_c2/f1p2/4432	NA	NA	NA	R01_cb1203_c2/f1p2/4432
R01_cb2710_c2/f1p0/2584	NA	NA	NA	R01_cb2710_c2/f1p0/2584
R01_cb2991_c4/f1p0/3750	NA	NA	NA	R01_cb2991_c4/f1p0/3750
R01_cb8564_c84717/f1p0/2742	NA	R01_cb8564_c84717/f1p0/2742	NA	NA
R01_cb4295_c15/f1p0/2702	R01_cb4295_c15/f1p0/2702	R01_cb4295_c15/f1p0/2702	R01_cb4295_c15/f1p0/2702	R01_cb4295_c15/f1p0/2702
R01_cb14873_c4/f1p0/5015	NA	R01_cb14873_c4/f1p0/5015	NA	NA
R01_cb3426_c8/f1p0/1904	NA	NA	NA	R01_cb3426_c8/f1p0/1904
R01_cb12023_c4/f1p0/726	NA	NA	NA	R01_cb12023_c4/f1p0/726
R01_cb2707_c29/f1p1/3207	NA	R01_cb2707_c29/f1p1/3207	NA	NA
R01_cb7741_c8/f1p0/573	R01_cb7741_c8/f1p0/573	R01_cb7741_c8/f1p0/573	R01_cb7741_c8/f1p0/573	R01_cb7741_c8/f1p0/573
R01_cb5858_c6/f1p0/2783	NA	R01_cb5858_c6/f1p0/2783	NA	NA

R01_cb11652_c0/f1p0/852	NA	R01_cb11652_c0/f1p0/852	NA	NA
R01_cb7778_c0/f1p1/2538	R01_cb7778_c0/f1p1/2538	R01_cb7778_c0/f1p1/2538	R01_cb7778_c0/f1p1/2538	R01_cb7778_c0/f1p1/2538
R01_cb15564_c4/f1p0/996	R01_cb15564_c4/f1p0/996	R01_cb15564_c4/f1p0/996	R01_cb15564_c4/f1p0/996	R01_cb15564_c4/f1p0/996
R01_cb2543_c52/f1p0/571	R01_cb2543_c52/f1p0/571	R01_cb2543_c52/f1p0/571	NA	NA
R01_cb1203_c20/f1p0/2906	NA	NA	NA	R01_cb1203_c20/f1p0/2906
R01_cb2131_c2/f1p0/4025	NA	R01_cb2131_c2/f1p0/4025	NA	NA
R01_cb8564_c87105/f1p0/4959	NA	R01_cb8564_c87105/f1p0/4959	NA	NA
R01_cb9451_c0/f1p0/1946	NA	NA	NA	R01_cb9451_c0/f1p0/1946
R01_cb5246_c3/f1p0/1919	NA	R01_cb5246_c3/f1p0/1919	R01_cb5246_c3/f1p0/1919	R01_cb5246_c3/f1p0/1919
R01_cb822_c19/f1p2/4163	NA	NA	NA	R01_cb822_c19/f1p2/4163
R01_cb8564_c16608/f1p0/2865	R01_cb8564_c16608/f1p0/2865	R01_cb8564_c16608/f1p0/2865	R01_cb8564_c16608/f1p0/2865	R01_cb8564_c16608/f1p0/2865
R01_cb1729_c20/f1p4/3827	NA	NA	NA	R01_cb1729_c20/f1p4/3827
R01_cb18456_c7631/f1p0/409	NA	R01_cb18456_c7631/f1p0/409	NA	R01_cb18456_c7631/f1p0/409
R01_cb1377_c3/f1p0/1881	NA	NA	NA	R01_cb1377_c3/f1p0/1881
R01_cb2845_c5/f1p0/3797	NA	NA	NA	R01_cb2845_c5/f1p0/3797
R01_cb16442_c2/f1p0/474	R01_cb16442_c2/f1p0/474	R01_cb16442_c2/f1p0/474	R01_cb16442_c2/f1p0/474	R01_cb16442_c2/f1p0/474
R01_cb8564_c1007/f1p0/1898	R01_cb8564_c1007/f1p0/1898	R01_cb8564_c1007/f1p0/1898	R01_cb8564_c1007/f1p0/1898	R01_cb8564_c1007/f1p0/1898
R01_cb8564_c110727/f1p0/2990	NA	NA	R01_cb8564_c110727/f1p0/2990	R01_cb8564_c110727/f1p0/2990
R01_cb8564_c88002/f2p0/3341	NA	R01_cb8564_c88002/f2p0/3341	R01_cb8564_c88002/f2p0/3341	R01_cb8564_c88002/f2p0/3341
R01_cb8564_c70867/f9p1/2542	NA	NA	NA	R01_cb8564_c70867/f9p1/2542
R01_cb17091_c3/f1p0/671	R01_cb17091_c3/f1p0/671	R01_cb17091_c3/f1p0/671	R01_cb17091_c3/f1p0/671	R01_cb17091_c3/f1p0/671
R01_cb18456_c6434/f1p0/489	R01_cb18456_c6434/f1p0/489	R01_cb18456_c6434/f1p0/489	R01_cb18456_c6434/f1p0/489	R01_cb18456_c6434/f1p0/489
R01_cb8564_c5268/f1p0/3194	NA	R01_cb8564_c5268/f1p0/3194	NA	NA
R01_cb9903_c4/f1p0/1776	NA	R01_cb9903_c4/f1p0/1776	NA	NA

R01_cb14886_c0/f3p0/703	NA	NA	R01_cb14886_c0/f3p0/703	NA
R01_cb4585_c0/f2p0/2163	NA	NA	NA	R01_cb4585_c0/f2p0/2163
R01_cb16095_c1/f1p0/559	R01_cb16095_c1/f1p0/559	R01_cb16095_c1/f1p0/559	R01_cb16095_c1/f1p0/559	R01_cb16095_c1/f1p0/559
R01_cb13545_c64/f1p0/1791	R01_cb13545_c64/f1p0/1791	R01_cb13545_c64/f1p0/1791	NA	R01_cb13545_c64/f1p0/1791
R01_cb8564_c124715/f1p0/2015	NA	NA	NA	R01_cb8564_c124715/f1p0/2015
R01_cb8564_c10630/f1p2/2801	NA	NA	NA	R01_cb8564_c10630/f1p2/2801
R01_cb16396_c1/f1p0/1781	NA	R01_cb16396_c1/f1p0/1781	NA	NA
R01_cb5290_c33/f1p0/3137	NA	NA	NA	R01_cb5290_c33/f1p0/3137
R01_cb8564_c20976/f1p0/3927	NA	R01_cb8564_c20976/f1p0/3927	NA	R01_cb8564_c20976/f1p0/3927
R01_cb17100_c1/f1p0/780	NA	R01_cb17100_c1/f1p0/780	NA	NA
R01_cb8564_c122868/f1p0/2629	NA	R01_cb8564_c122868/f1p0/2629	NA	NA
R01_cb15077_c4/f1p0/1256	NA	NA	NA	R01_cb15077_c4/f1p0/1256
R01_cb4822_c1/f1p0/2484	R01_cb4822_c1/f1p0/2484	R01_cb4822_c1/f1p0/2484	R01_cb4822_c1/f1p0/2484	R01_cb4822_c1/f1p0/2484
R01_cb7754_c2/f1p0/640	R01_cb7754_c2/f1p0/640	R01_cb7754_c2/f1p0/640	NA	R01_cb7754_c2/f1p0/640
R01_cb8785_c1/f3p0/2272	NA	NA	NA	R01_cb8785_c1/f3p0/2272
R01_cb11004_c2/f1p0/1892	NA	R01_cb11004_c2/f1p0/1892	R01_cb11004_c2/f1p0/1892	R01_cb11004_c2/f1p0/1892
R01_cb10815_c7/f1p0/333	R01_cb10815_c7/f1p0/333	R01_cb10815_c7/f1p0/333	R01_cb10815_c7/f1p0/333	R01_cb10815_c7/f1p0/333
R01_cb2893_c0/f3p0/3751	NA	R01_cb2893_c0/f3p0/3751	NA	R01_cb2893_c0/f3p0/3751
R01_cb18798_c1/f1p1/6607	NA	R01_cb18798_c1/f1p1/6607	R01_cb18798_c1/f1p1/6607	NA
R01_cb1860_c31/f1p0/2802	NA	NA	NA	R01_cb1860_c31/f1p0/2802
R01_cb8525_c1/f1p0/686	NA	R01_cb8525_c1/f1p0/686	NA	NA
R01_cb5783_c1/f1p0/2788	NA	R01_cb5783_c1/f1p0/2788	NA	NA
R01_cb8564_c20840/f1p3/2713	NA	R01_cb8564_c20840/f1p3/2713	R01_cb8564_c20840/f1p3/2713	R01_cb8564_c20840/f1p3/2713
R01_cb18456_c7436/f1p0/884	R01_cb18456_c7436/f1p0/884	R01_cb18456_c7436/f1p0/884	R01_cb18456_c7436/f1p0/884	R01_cb18456_c7436/f1p0/884

R01_cb8564_c4900/f4p1/3332	NA	NA	NA	R01_cb8564_c4900/f4p1/3332
R01_cb12326_c2/f1p0/639	NA	NA	NA	R01_cb12326_c2/f1p0/639
R01_cb1146_c10/f1p0/4139	NA	R01_cb1146_c10/f1p0/4139	NA	NA
R01_cb2030_c5/f1p0/577	NA	NA	NA	R01_cb2030_c5/f1p0/577
R01_cb18765_c1/f1p0/1512	R01_cb18765_c1/f1p0/1512	R01_cb18765_c1/f1p0/1512	NA	R01_cb18765_c1/f1p0/1512
R01_cb8564_c14798/f1p0/3645	R01_cb8564_c14798/f1p0/3645	R01_cb8564_c14798/f1p0/3645	NA	R01_cb8564_c14798/f1p0/3645
R01_cb6819_c8/f3p0/5435	NA	NA	NA	R01_cb6819_c8/f3p0/5435
R01_cb4352_c15/f3p0/2438	NA	NA	NA	R01_cb4352_c15/f3p0/2438
R01_cb6272_c3/f3p2/2571	NA	NA	NA	R01_cb6272_c3/f3p2/2571
R01_cb7514_c1/f1p0/2863	R01_cb7514_c1/f1p0/2863	R01_cb7514_c1/f1p0/2863	R01_cb7514_c1/f1p0/2863	R01_cb7514_c1/f1p0/2863
R01_cb4943_c1/f1p0/2633	NA	R01_cb4943_c1/f1p0/2633	R01_cb4943_c1/f1p0/2633	NA
R01_cb11511_c1/f1p0/4636	NA	R01_cb11511_c1/f1p0/4636	NA	NA
R01_cb1001_c3/f1p0/3391	NA	R01_cb1001_c3/f1p0/3391	R01_cb1001_c3/f1p0/3391	R01_cb1001_c3/f1p0/3391
R01_cb12641_c29/f1p0/993	NA	NA	NA	R01_cb12641_c29/f1p0/993
R01_cb11470_c1/f1p0/2083	NA	R01_cb11470_c1/f1p0/2083	NA	R01_cb11470_c1/f1p0/2083
R01_cb17191_c1/f1p0/1511	NA	R01_cb17191_c1/f1p0/1511	NA	NA
R01_cb13774_c0/f2p0/964	NA	NA	NA	R01_cb13774_c0/f2p0/964
R01_cb8564_c68612/f4p1/2746	NA	R01_cb8564_c68612/f4p1/2746	NA	NA
R01_cb14729_c6/f1p2/634	NA	R01_cb14729_c6/f1p2/634	NA	NA
R01_cb1178_c13/f1p0/2397	NA	NA	NA	R01_cb1178_c13/f1p0/2397
R01_cb5122_c14/f1p0/2441	NA	NA	NA	R01_cb5122_c14/f1p0/2441
R01_cb204_c0/f2p1/5152	NA	NA	NA	R01_cb204_c0/f2p1/5152
R01_cb8564_c80419/f1p0/2649	NA	R01_cb8564_c80419/f1p0/2649	NA	NA
R01_cb8564_c50317/f1p0/2438	R01_cb8564_c50317/f1p0/2438	R01_cb8564_c50317/f1p0/2438	R01_cb8564_c50317/f1p0/2438	R01_cb8564_c50317/f1p0/2438

R01_cb14502_c13/f1p0/999	R01_cb14502_c13/f1p0/999	R01_cb14502_c13/f1p0/999	NA	R01_cb14502_c13/f1p0/999
R01_cb8564_c1512/f1p0/2347	NA	R01_cb8564_c1512/f1p0/2347	NA	R01_cb8564_c1512/f1p0/2347
R01_cb2200_c4/f1p1/1423	NA	R01_cb2200_c4/f1p1/1423	R01_cb2200_c4/f1p1/1423	NA
R01_cb9612_c0/f5p0/1063	NA	NA	NA	R01_cb9612_c0/f5p0/1063
R01_cb7725_c5/f1p0/2458	NA	R01_cb7725_c5/f1p0/2458	NA	NA
R01_cb11425_c0/f1p0/1855	R01_cb11425_c0/f1p0/1855	R01_cb11425_c0/f1p0/1855	NA	R01_cb11425_c0/f1p0/1855
R01_cb18456_c7787/f1p0/682	NA	NA	NA	R01_cb18456_c7787/f1p0/682
R01_cb8564_c47398/f1p0/2719	NA	NA	NA	R01_cb8564_c47398/f1p0/2719
R01_cb13161_c14/f1p0/1746	NA	R01_cb13161_c14/f1p0/1746	NA	NA
R01_cb6861_c9/f1p0/3465	R01_cb6861_c9/f1p0/3465	R01_cb6861_c9/f1p0/3465	R01_cb6861_c9/f1p0/3465	R01_cb6861_c9/f1p0/3465
R01_cb2946_c5/f1p0/2333	NA	NA	NA	R01_cb2946_c5/f1p0/2333
R01_cb2506_c3/f1p0/2099	NA	R01_cb2506_c3/f1p0/2099	NA	NA
R01_cb7741_c12/f1p0/457	R01_cb7741_c12/f1p0/457	R01_cb7741_c12/f1p0/457	R01_cb7741_c12/f1p0/457	R01_cb7741_c12/f1p0/457
R01_cb8564_c146882/f3p8/4113	NA	NA	NA	R01_cb8564_c146882/f3p8/4113
R01_cb17786_c0/f1p1/1235	NA	R01_cb17786_c0/f1p1/1235	NA	NA
R01_cb8564_c88081/f1p0/3233	NA	R01_cb8564_c88081/f1p0/3233	R01_cb8564_c88081/f1p0/3233	R01_cb8564_c88081/f1p0/3233
R01_cb6845_c22/f1p0/2629	NA	NA	NA	R01_cb6845_c22/f1p0/2629
R01_cb5900_c135/f1p0/2776	NA	R01_cb5900_c135/f1p0/2776	NA	R01_cb5900_c135/f1p0/2776
R01_cb8564_c177306/f1p0/2309	NA	NA	NA	R01_cb8564_c177306/f1p0/2309
R01_cb8564_c24584/f1p0/3255	NA	R01_cb8564_c24584/f1p0/3255	NA	NA
R01_cb8564_c3507/f1p0/2893	R01_cb8564_c3507/f1p0/2893	R01_cb8564_c3507/f1p0/2893	NA	NA
R01_cb8564_c22600/f1p0/3119	NA	R01_cb8564_c22600/f1p0/3119	R01_cb8564_c22600/f1p0/3119	R01_cb8564_c22600/f1p0/3119
R01_cb17987_c1/f1p0/1160	NA	NA	NA	R01_cb17987_c1/f1p0/1160
R01_cb17052_c4/f1p0/1874	NA	NA	NA	R01_cb17052_c4/f1p0/1874

R01_cb17763_c0/f1p0/1176	NA	NA	NA	R01_cb17763_c0/f1p0/1176
R01_cb2238_c15/f1p0/3318	NA	R01_cb2238_c15/f1p0/3318	NA	NA
R01_cb8564_c112371/f1p0/2779	NA	R01_cb8564_c112371/f1p0/2779	NA	R01_cb8564_c112371/f1p0/2779
R01_cb13872_c5/f1p0/1280	R01_cb13872_c5/f1p0/1280	R01_cb13872_c5/f1p0/1280	NA	R01_cb13872_c5/f1p0/1280
R01_cb11109_c5/f1p0/1353	NA	NA	NA	R01_cb11109_c5/f1p0/1353
R01_cb11810_c1/f1p0/2214	NA	R01_cb11810_c1/f1p0/2214	NA	NA
R01_cb1982_c2/f1p0/2401	NA	NA	R01_cb1982_c2/f1p0/2401	R01_cb1982_c2/f1p0/2401
R01_cb12228_c23/f1p0/786	NA	R01_cb12228_c23/f1p0/786	NA	R01_cb12228_c23/f1p0/786
R01_cb7876_c2/f3p0/2504	NA	R01_cb7876_c2/f3p0/2504	NA	NA
R01_cb16020_c0/f2p0/486	NA	R01_cb16020_c0/f2p0/486	R01_cb16020_c0/f2p0/486	R01_cb16020_c0/f2p0/486
R01_cb8564_c90669/f1p0/2003	NA	NA	NA	R01_cb8564_c90669/f1p0/2003
R01_cb8564_c34518/f1p0/3136	NA	R01_cb8564_c34518/f1p0/3136	NA	NA
R01_cb9916_c0/f2p0/1517	NA	NA	NA	R01_cb9916_c0/f2p0/1517
R01_cb15757_c6/f1p0/1633	NA	NA	NA	R01_cb15757_c6/f1p0/1633
R01_cb4295_c12/f1p0/3169	R01_cb4295_c12/f1p0/3169	R01_cb4295_c12/f1p0/3169	NA	NA
R01_cb6623_c25/f1p0/2847	NA	NA	NA	R01_cb6623_c25/f1p0/2847
R01_cb4652_c8/f1p0/852	R01_cb4652_c8/f1p0/852	R01_cb4652_c8/f1p0/852	R01_cb4652_c8/f1p0/852	R01_cb4652_c8/f1p0/852
R01_cb8991_c7/f1p0/2097	NA	NA	NA	R01_cb8991_c7/f1p0/2097
R01_cb8564_c46350/f1p0/3446	NA	NA	NA	R01_cb8564_c46350/f1p0/3446
R01_cb8564_c4095/f1p0/4108	R01_cb8564_c4095/f1p0/4108	R01_cb8564_c4095/f1p0/4108	R01_cb8564_c4095/f1p0/4108	R01_cb8564_c4095/f1p0/4108
R01_cb8564_c71655/f1p0/3284	NA	R01_cb8564_c71655/f1p0/3284	R01_cb8564_c71655/f1p0/3284	R01_cb8564_c71655/f1p0/3284
R01_cb15531_c1/f1p0/1352	NA	R01_cb15531_c1/f1p0/1352	NA	R01_cb15531_c1/f1p0/1352
R01_cb115_c0/f1p0/4959	NA	NA	NA	R01_cb115_c0/f1p0/4959
R01_cb8295_c4/f1p0/2139	NA	NA	NA	R01_cb8295_c4/f1p0/2139
R01_cb10968_c1/f2p0/673	NA	R01_cb10968_c1/f2p0/673	R01_cb10968_c1/f2p0/673	R01_cb10968_c1/f2p0/673

R01_cb3218_c3/f1p0/4354	NA	NA	NA	R01_cb3218_c3/f1p0/4354
R01_cb7985_c4/f1p0/2097	NA	R01_cb7985_c4/f1p0/2097	R01_cb7985_c4/f1p0/2097	NA
R01_cb13996_c3/f2p1/740	NA	R01_cb13996_c3/f2p1/740	NA	NA
R01_cb6272_c1/f10p2/2582	NA	NA	NA	R01_cb6272_c1/f10p2/2582
R01_cb8564_c79032/f3p0/2300	NA	NA	NA	R01_cb8564_c79032/f3p0/2300
R01_cb18409_c78/f1p0/403	R01_cb18409_c78/f1p0/403	R01_cb18409_c78/f1p0/403	R01_cb18409_c78/f1p0/403	R01_cb18409_c78/f1p0/403
R01_cb352_c2/f1p0/2991	NA	R01_cb352_c2/f1p0/2991	NA	R01_cb352_c2/f1p0/2991
R01_cb8564_c122131/f1p0/4065	NA	R01_cb8564_c122131/f1p0/4065	R01_cb8564_c122131/f1p0/4065	NA
R01_cb3853_c3/f1p0/2530	NA	R01_cb3853_c3/f1p0/2530	NA	NA
R01_cb8564_c3779/f1p0/2570	NA	R01_cb8564_c3779/f1p0/2570	R01_cb8564_c3779/f1p0/2570	R01_cb8564_c3779/f1p0/2570
R01_cb14004_c3/f5p1/1366	NA	NA	NA	R01_cb14004_c3/f5p1/1366
R01_cb647_c13/f1p0/1621	NA	NA	NA	R01_cb647_c13/f1p0/1621
R01_cb4115_c12/f1p0/1357	NA	NA	NA	R01_cb4115_c12/f1p0/1357
R01_cb6059_c4/f1p0/2020	NA	NA	NA	R01_cb6059_c4/f1p0/2020
R01_cb11323_c0/f1p0/1289	NA	NA	NA	R01_cb11323_c0/f1p0/1289
R01_cb8564_c19850/f1p0/1904	NA	NA	NA	R01_cb8564_c19850/f1p0/1904
R01_cb16966_c2/f1p0/647	R01_cb16966_c2/f1p0/647	R01_cb16966_c2/f1p0/647	NA	NA
R01_cb18409_c99/f1p0/415	R01_cb18409_c99/f1p0/415	R01_cb18409_c99/f1p0/415	R01_cb18409_c99/f1p0/415	R01_cb18409_c99/f1p0/415
R01_cb8564_c73348/f1p0/1997	R01_cb8564_c73348/f1p0/1997	R01_cb8564_c73348/f1p0/1997	R01_cb8564_c73348/f1p0/1997	R01_cb8564_c73348/f1p0/1997
R01_cb17103_c0/f2p0/1327	NA	NA	NA	R01_cb17103_c0/f2p0/1327
R01_cb4949_c3/f1p0/3120	NA	NA	NA	R01_cb4949_c3/f1p0/3120
R01_cb15069_c4/f1p0/442	NA	R01_cb15069_c4/f1p0/442	NA	NA
R01_cb11879_c7/f1p0/1721	NA	NA	NA	R01_cb11879_c7/f1p0/1721
R01_cb8564_c21275/f3p4/4047	NA	NA	NA	R01_cb8564_c21275/f3p4/4047

R01_cb5378_c2/f1p0/2616	NA	NA	NA	R01_cb5378_c2/f1p0/2616
R01_cb6513_c2/f1p0/2836	NA	NA	NA	R01_cb6513_c2/f1p0/2836
R01_cb13146_c10/f8p2/607	NA	R01_cb13146_c10/f8p2/607	R01_cb13146_c10/f8p2/607	R01_cb13146_c10/f8p2/607
R01_cb4128_c27/f1p0/1127	NA	NA	NA	R01_cb4128_c27/f1p0/1127
R01_cb12656_c5/f1p0/1803	NA	NA	NA	R01_cb12656_c5/f1p0/1803
R01_cb13667_c1/f1p0/815	NA	R01_cb13667_c1/f1p0/815	R01_cb13667_c1/f1p0/815	R01_cb13667_c1/f1p0/815
R01_cb8279_c0/f5p0/1893	NA	NA	NA	R01_cb8279_c0/f5p0/1893
R01_cb18456_c7743/f132p2/612	R01_cb18456_c7743/f132p2/612	R01_cb18456_c7743/f132p2/612	R01_cb18456_c7743/f132p2/612	R01_cb18456_c7743/f132p2/612
R01_cb2682_c2/f1p0/3291	NA	NA	NA	R01_cb2682_c2/f1p0/3291
R01_cb2298_c2/f1p0/3767	NA	R01_cb2298_c2/f1p0/3767	R01_cb2298_c2/f1p0/3767	R01_cb2298_c2/f1p0/3767
R01_cb9960_c3/f1p0/1881	NA	NA	NA	R01_cb9960_c3/f1p0/1881
R01_cb8564_c89144/f1p0/2639	NA	R01_cb8564_c89144/f1p0/2639	NA	NA
R01_cb8564_c77590/f1p5/2762	NA	NA	NA	R01_cb8564_c77590/f1p5/2762
R01_cb2309_c9/f1p0/1954	NA	R01_cb2309_c9/f1p0/1954	R01_cb2309_c9/f1p0/1954	NA
R01_cb9504_c3/f1p0/492	R01_cb9504_c3/f1p0/492	R01_cb9504_c3/f1p0/492	R01_cb9504_c3/f1p0/492	R01_cb9504_c3/f1p0/492
R01_cb15312_c1/f1p1/711	NA	R01_cb15312_c1/f1p1/711	R01_cb15312_c1/f1p1/711	R01_cb15312_c1/f1p1/711
R01_cb14909_c0/f1p0/664	R01_cb14909_c0/f1p0/664	R01_cb14909_c0/f1p0/664	R01_cb14909_c0/f1p0/664	R01_cb14909_c0/f1p0/664
R01_cb15552_c3/f1p0/1704	NA	NA	R01_cb15552_c3/f1p0/1704	R01_cb15552_c3/f1p0/1704
R01_cb18542_c0/f1p0/752	NA	R01_cb18542_c0/f1p0/752	NA	NA
R01_cb11544_c2/f1p0/1768	NA	R01_cb11544_c2/f1p0/1768	NA	NA
R01_cb10145_c1/f2p0/1612	NA	NA	NA	R01_cb10145_c1/f2p0/1612
R01_cb8564_c88953/f1p0/3767	NA	R01_cb8564_c88953/f1p0/3767	NA	NA
R01_cb4094_c8/f2p0/2920	NA	R01_cb4094_c8/f2p0/2920	NA	NA
R01_cb18335_c2/f1p0/994	R01_cb18335_c2/f1p0/994	R01_cb18335_c2/f1p0/994	R01_cb18335_c2/f1p0/994	R01_cb18335_c2/f1p0/994
R01_cb8564_c72602/f1p0/2743	NA	NA	NA	R01_cb8564_c72602/f1p0/2743

R01_cb1487_c10/f1p0/1153	NA	NA	NA	R01_cb1487_c10/f1p0/1153
R01_cb14054_c12/f1p0/536	R01_cb14054_c12/f1p0/536	R01_cb14054_c12/f1p0/536	R01_cb14054_c12/f1p0/536	R01_cb14054_c12/f1p0/536
R01_cb8434_c2/f1p0/2304	NA	R01_cb8434_c2/f1p0/2304	NA	NA
R01_cb3658_c10/f1p0/2198	NA	NA	NA	R01_cb3658_c10/f1p0/2198
R01_cb1594_c6/f1p1/2130	NA	NA	NA	R01_cb1594_c6/f1p1/2130
R01_cb8564_c130313/f1p0/2329	NA	R01_cb8564_c130313/f1p0/2329	NA	NA
R01_cb13284_c1/f5p0/1845	NA	NA	NA	R01_cb13284_c1/f5p0/1845
R01_cb5452_c2/f1p0/972	NA	NA	NA	R01_cb5452_c2/f1p0/972
R01_cb6608_c10/f1p0/2307	NA	NA	NA	R01_cb6608_c10/f1p0/2307
R01_cb369_c15/f1p0/1175	NA	NA	NA	R01_cb369_c15/f1p0/1175
R01_cb2418_c9/f1p0/3155	NA	R01_cb2418_c9/f1p0/3155	R01_cb2418_c9/f1p0/3155	R01_cb2418_c9/f1p0/3155
R01_cb8564_c4200/f1p0/2947	R01_cb8564_c4200/f1p0/2947	R01_cb8564_c4200/f1p0/2947	NA	NA
R01_cb8485_c9/f1p0/805	NA	R01_cb8485_c9/f1p0/805	R01_cb8485_c9/f1p0/805	R01_cb8485_c9/f1p0/805
R01_cb7010_c17/f1p1/1911	NA	NA	NA	R01_cb7010_c17/f1p1/1911
R01_cb10121_c2/f1p0/2778	NA	R01_cb10121_c2/f1p0/2778	NA	R01_cb10121_c2/f1p0/2778
R01_cb13662_c2/f1p0/492	R01_cb13662_c2/f1p0/492	R01_cb13662_c2/f1p0/492	NA	R01_cb13662_c2/f1p0/492
R01_cb12577_c19/f3p0/365	R01_cb12577_c19/f3p0/365	R01_cb12577_c19/f3p0/365	R01_cb12577_c19/f3p0/365	R01_cb12577_c19/f3p0/365
R01_cb5896_c102/f1p0/2978	NA	NA	R01_cb5896_c102/f1p0/2978	NA
R01_cb16705_c0/f1p0/828	NA	R01_cb16705_c0/f1p0/828	R01_cb16705_c0/f1p0/828	R01_cb16705_c0/f1p0/828
R01_cb10024_c427/f1p0/631	NA	R01_cb10024_c427/f1p0/631	NA	NA
R01_cb13012_c6/f1p1/1128	NA	NA	NA	R01_cb13012_c6/f1p1/1128
R01_cb18456_c1698/f2p1/564	R01_cb18456_c1698/f2p1/564	R01_cb18456_c1698/f2p1/564	R01_cb18456_c1698/f2p1/564	R01_cb18456_c1698/f2p1/564
R01_cb12057_c17/f1p0/1531	NA	R01_cb12057_c17/f1p0/1531	NA	NA
R01_cb3744_c9/f1p0/3017	NA	NA	NA	R01_cb3744_c9/f1p0/3017
R01_cb12785_c21/f1p0/843	NA	NA	R01_cb12785_c21/f1p0/843	NA

R01_cb14909_c3/f1p0/694	R01_cb14909_c3/f1p0/694	NA	NA	R01_cb14909_c3/f1p0/694
R01_cb6947_c11/f1p0/4007	NA	R01_cb6947_c11/f1p0/4007	NA	R01_cb6947_c11/f1p0/4007
R01_cb17734_c1/f1p0/1628	NA	NA	NA	R01_cb17734_c1/f1p0/1628
R01_cb1860_c7/f3p3/2443	NA	NA	NA	R01_cb1860_c7/f3p3/2443
R01_cb12421_c27/f2p0/525	NA	R01_cb12421_c27/f2p0/525	NA	NA
R01_cb11370_c4/f1p0/2621	NA	NA	NA	R01_cb11370_c4/f1p0/2621
R01_cb11318_c1/f1p0/2588	NA	NA	NA	R01_cb11318_c1/f1p0/2588
R01_cb11208_c3/f1p0/1456	NA	NA	NA	R01_cb11208_c3/f1p0/1456
R01_cb7064_c5/f2p0/599	R01_cb7064_c5/f2p0/599	R01_cb7064_c5/f2p0/599	R01_cb7064_c5/f2p0/599	R01_cb7064_c5/f2p0/599
R01_cb14139_c9/f1p0/1662	NA	R01_cb14139_c9/f1p0/1662	NA	NA
R01_cb8942_c61/f1p0/2131	NA	R01_cb8942_c61/f1p0/2131	NA	NA
R01_cb11654_c1/f1p0/2059	NA	NA	NA	R01_cb11654_c1/f1p0/2059
R01_cb9350_c4/f1p0/1655	NA	R01_cb9350_c4/f1p0/1655	NA	NA
R01_cb13938_c18/f4p0/1404	NA	NA	NA	R01_cb13938_c18/f4p0/1404
R01_cb8564_c79829/f1p0/2440	NA	NA	NA	R01_cb8564_c79829/f1p0/2440
R01_cb1336_c2/f1p0/2147	NA	NA	NA	R01_cb1336_c2/f1p0/2147
R01_cb14236_c0/f1p0/617	R01_cb14236_c0/f1p0/617	R01_cb14236_c0/f1p0/617	R01_cb14236_c0/f1p0/617	R01_cb14236_c0/f1p0/617
R01_cb12329_c15/f1p0/1249	NA	NA	NA	R01_cb12329_c15/f1p0/1249
R01_cb7302_c3/f1p0/1867	NA	NA	NA	R01_cb7302_c3/f1p0/1867
R01_cb18301_c55/f1p0/1041	NA	R01_cb18301_c55/f1p0/1041	R01_cb18301_c55/f1p0/1041	R01_cb18301_c55/f1p0/1041
R01_cb8545_c6/f1p0/872	NA	NA	R01_cb8545_c6/f1p0/872	R01_cb8545_c6/f1p0/872
R01_cb18439_c0/f2p0/1428	NA	R01_cb18439_c0/f2p0/1428	R01_cb18439_c0/f2p0/1428	R01_cb18439_c0/f2p0/1428
R01_cb8478_c17/f1p0/3310	NA	R01_cb8478_c17/f1p0/3310	NA	NA
R01_cb8564_c73670/f1p0/1929	NA	NA	NA	R01_cb8564_c73670/f1p0/1929
R01_cb5517_c1/f1p0/2876	NA	NA	R01_cb5517_c1/f1p0/2876	NA
R01_cb6994_c5/f1p0/7066	NA	R01_cb6994_c5/f1p0/7066	NA	NA

R01_cb18456_c6628/f1p0/903	NA	NA	NA	R01_cb18456_c6628/f1p0/903
R01_cb8564_c146682/f3p0/2552	NA	R01_cb8564_c146682/f3p0/2552	R01_cb8564_c146682/f3p0/2552	R01_cb8564_c146682/f3p0/2552
R01_cb8564_c121469/f1p0/2615	NA	R01_cb8564_c121469/f1p0/2615	NA	NA
R01_cb14886_c4/f1p0/773	NA	NA	R01_cb14886_c4/f1p0/773	NA
R01_cb8564_c125619/f1p0/3880	NA	R01_cb8564_c125619/f1p0/3880	NA	NA
R01_cb17756_c27/f1p0/462	R01_cb17756_c27/f1p0/462	R01_cb17756_c27/f1p0/462	R01_cb17756_c27/f1p0/462	R01_cb17756_c27/f1p0/462
R01_cb14322_c1/f2p2/1452	NA	R01_cb14322_c1/f2p2/1452	R01_cb14322_c1/f2p2/1452	R01_cb14322_c1/f2p2/1452
R01_cb8564_c12923/f1p0/2513	NA	R01_cb8564_c12923/f1p0/2513	NA	R01_cb8564_c12923/f1p0/2513
R01_cb261_c2/f3p0/2415	NA	NA	NA	R01_cb261_c2/f3p0/2415
R01_cb7757_c15/f1p0/670	NA	NA	R01_cb7757_c15/f1p0/670	R01_cb7757_c15/f1p0/670
R01_cb8564_c22048/f5p0/4425	NA	NA	NA	R01_cb8564_c22048/f5p0/4425
R01_cb15096_c3/f1p0/1532	NA	NA	NA	R01_cb15096_c3/f1p0/1532
R01_cb16270_c2/f1p0/406	R01_cb16270_c2/f1p0/406	NA	R01_cb16270_c2/f1p0/406	R01_cb16270_c2/f1p0/406
R01_cb15644_c2/f2p0/467	NA	R01_cb15644_c2/f2p0/467	R01_cb15644_c2/f2p0/467	R01_cb15644_c2/f2p0/467
R01_cb13478_c9/f1p0/552	NA	R01_cb13478_c9/f1p0/552	R01_cb13478_c9/f1p0/552	R01_cb13478_c9/f1p0/552
R01_cb8356_c3/f1p0/1027	NA	R01_cb8356_c3/f1p0/1027	R01_cb8356_c3/f1p0/1027	R01_cb8356_c3/f1p0/1027
R01_cb8564_c72659/f1p0/4124	NA	NA	NA	R01_cb8564_c72659/f1p0/4124
R01_cb17392_c1/f1p1/969	NA	R01_cb17392_c1/f1p1/969	R01_cb17392_c1/f1p1/969	R01_cb17392_c1/f1p1/969
R01_cb11918_c23/f1p0/1229	NA	NA	NA	R01_cb11918_c23/f1p0/1229
R01_cb7214_c9/f1p0/2049	NA	NA	NA	R01_cb7214_c9/f1p0/2049
R01_cb14843_c0/f2p0/1417	NA	NA	NA	R01_cb14843_c0/f2p0/1417
R01_cb11124_c3/f1p0/951	R01_cb11124_c3/f1p0/951	R01_cb11124_c3/f1p0/951	R01_cb11124_c3/f1p0/951	R01_cb11124_c3/f1p0/951
R01_cb7334_c3/f1p0/2645	NA	NA	NA	R01_cb7334_c3/f1p0/2645

R01_cb1915_c46/f1p0/614	R01_cb1915_c46/f1p0/614	R01_cb1915_c46/f1p0/614	R01_cb1915_c46/f1p0/614	R01_cb1915_c46/f1p0/614
R01_cb8564_c43626/f1p0/2815	NA	NA	NA	R01_cb8564_c43626/f1p0/2815
R01_cb8564_c92212/f1p0/2726	R01_cb8564_c92212/f1p0/2726	R01_cb8564_c92212/f1p0/2726	R01_cb8564_c92212/f1p0/2726	R01_cb8564_c92212/f1p0/2726
R01_cb4352_c6/f1p0/2422	NA	NA	NA	R01_cb4352_c6/f1p0/2422
R01_cb8564_c110889/f1p1/2516	NA	R01_cb8564_c110889/f1p1/2516	NA	NA
R01_cb14469_c5/f5p2/672	R01_cb14469_c5/f5p2/672	R01_cb14469_c5/f5p2/672	R01_cb14469_c5/f5p2/672	R01_cb14469_c5/f5p2/672
R01_cb11621_c1/f1p0/2911	NA	R01_cb11621_c1/f1p0/2911	NA	NA
R01_cb10443_c1/f1p1/2146	R01_cb10443_c1/f1p1/2146	R01_cb10443_c1/f1p1/2146	R01_cb10443_c1/f1p1/2146	NA
R01_cb11603_c0/f1p0/1217	NA	R01_cb11603_c0/f1p0/1217	NA	NA
R01_cb2619_c3/f1p1/2037	NA	NA	NA	R01_cb2619_c3/f1p1/2037
R01_cb12677_c0/f5p0/936	NA	NA	NA	R01_cb12677_c0/f5p0/936
R01_cb13545_c66/f1p0/1196	NA	R01_cb13545_c66/f1p0/1196	NA	R01_cb13545_c66/f1p0/1196
R01_cb3929_c14/f1p0/3015	NA	NA	NA	R01_cb3929_c14/f1p0/3015
R01_cb9232_c2/f1p0/2604	NA	NA	NA	R01_cb9232_c2/f1p0/2604
R01_cb5394_c1/f1p0/3111	NA	NA	NA	R01_cb5394_c1/f1p0/3111
R01_cb18369_c0/f1p0/562	R01_cb18369_c0/f1p0/562	R01_cb18369_c0/f1p0/562	R01_cb18369_c0/f1p0/562	R01_cb18369_c0/f1p0/562
R01_cb18456_c5206/f1p0/1413	NA	R01_cb18456_c5206/f1p0/1413	NA	NA
R01_cb18456_c3893/f1p1/1629	NA	NA	NA	R01_cb18456_c3893/f1p1/1629
R01_cb2959_c3/f1p0/3317	NA	NA	NA	R01_cb2959_c3/f1p0/3317
R01_cb14481_c5/f1p0/466	NA	R01_cb14481_c5/f1p0/466	NA	R01_cb14481_c5/f1p0/466
R01_cb2698_c5/f1p0/1933	NA	NA	NA	R01_cb2698_c5/f1p0/1933
R01_cb12191_c2/f4p1/769	NA	NA	NA	R01_cb12191_c2/f4p1/769
R01_cb8564_c50051/f1p0/2016	NA	NA	NA	R01_cb8564_c50051/f1p0/2016
R01_cb8819_c4/f2p0/2165	NA	NA	NA	R01_cb8819_c4/f2p0/2165

R01_cb9201_c4/f1p0/2116	NA	NA	NA	R01_cb9201_c4/f1p0/2116
R01_cb8564_c129649/f1p0/3138	R01_cb8564_c129649/f1p0/3138	R01_cb8564_c129649/f1p0/3138	R01_cb8564_c129649/f1p0/3138	R01_cb8564_c129649/f1p0/3138
R01_cb15950_c3/f1p0/1525	R01_cb15950_c3/f1p0/1525	R01_cb15950_c3/f1p0/1525	NA	R01_cb15950_c3/f1p0/1525
R01_cb2482_c51/f1p0/1527	R01_cb2482_c51/f1p0/1527	R01_cb2482_c51/f1p0/1527	NA	R01_cb2482_c51/f1p0/1527
R01_cb110_c24/f1p0/4568	NA	NA	NA	R01_cb110_c24/f1p0/4568
R01_cb4444_c22/f1p0/3073	NA	R01_cb4444_c22/f1p0/3073	NA	NA
R01_cb10364_c1/f2p0/1719	NA	NA	NA	R01_cb10364_c1/f2p0/1719
R01_cb9799_c3/f1p0/2671	R01_cb9799_c3/f1p0/2671	R01_cb9799_c3/f1p0/2671	R01_cb9799_c3/f1p0/2671	R01_cb9799_c3/f1p0/2671
R01_cb9123_c1/f1p0/2566	NA	R01_cb9123_c1/f1p0/2566	NA	NA
R01_cb17405_c0/f1p0/1832	NA	R01_cb17405_c0/f1p0/1832	NA	NA
R01_cb5183_c82/f1p1/1654	NA	NA	NA	R01_cb5183_c82/f1p1/1654
R01_cb18456_c1338/f2p0/461	R01_cb18456_c1338/f2p0/461	R01_cb18456_c1338/f2p0/461	R01_cb18456_c1338/f2p0/461	R01_cb18456_c1338/f2p0/461
R01_cb10905_c5/f1p0/2890	NA	NA	NA	R01_cb10905_c5/f1p0/2890
R01_cb6631_c24/f1p0/2092	NA	NA	NA	R01_cb6631_c24/f1p0/2092
R01_cb8564_c84613/f1p0/3096	NA	R01_cb8564_c84613/f1p0/3096	NA	R01_cb8564_c84613/f1p0/3096
R01_cb18200_c2/f1p0/390	R01_cb18200_c2/f1p0/390	R01_cb18200_c2/f1p0/390	R01_cb18200_c2/f1p0/390	R01_cb18200_c2/f1p0/390
R01_cb11116_c0/f1p0/1869	NA	NA	NA	R01_cb11116_c0/f1p0/1869
R01_cb10326_c9/f1p0/941	NA	R01_cb10326_c9/f1p0/941	NA	NA
R01_cb8939_c6/f1p0/2221	NA	R01_cb8939_c6/f1p0/2221	NA	NA
R01_cb16706_c3/f1p0/774	NA	R01_cb16706_c3/f1p0/774	NA	R01_cb16706_c3/f1p0/774
R01_cb709_c9/f1p0/2033	NA	R01_cb709_c9/f1p0/2033	NA	NA
R01_cb16677_c0/f1p0/1522	R01_cb16677_c0/f1p0/1522	R01_cb16677_c0/f1p0/1522	R01_cb16677_c0/f1p0/1522	R01_cb16677_c0/f1p0/1522
R01_cb2420_c8/f1p0/2228	NA	R01_cb2420_c8/f1p0/2228	NA	R01_cb2420_c8/f1p0/2228
R01_cb10738_c13/f1p0/583	NA	R01_cb10738_c13/f1p0/583	NA	R01_cb10738_c13/f1p0/583
R01_cb5953_c0/f4p0/2900	NA	NA	NA	R01_cb5953_c0/f4p0/2900

R01_cb17357_c1/f1p0/373	R01_cb17357_c1/f1p0/373	R01_cb17357_c1/f1p0/373	NA	NA
R01_cb3432_c1/f3p0/3558	R01_cb3432_c1/f3p0/3558	R01_cb3432_c1/f3p0/3558	NA	NA
R01_cb8564_c53293/f1p0/2959	NA	R01_cb8564_c53293/f1p0/2959	R01_cb8564_c53293/f1p0/2959	R01_cb8564_c53293/f1p0/2959
R01_cb9848_c5/f1p0/773	NA	NA	NA	R01_cb9848_c5/f1p0/773
R01_cb8564_c16265/f1p0/3359	NA	R01_cb8564_c16265/f1p0/3359	NA	NA
R01_cb18456_c7778/f23p0/582	NA	NA	R01_cb18456_c7778/f23p0/582	R01_cb18456_c7778/f23p0/582
R01_cb18745_c3/f1p0/1409	NA	NA	NA	R01_cb18745_c3/f1p0/1409
R01_cb5874_c37/f1p0/973	NA	NA	NA	R01_cb5874_c37/f1p0/973
R01_cb10012_c2/f1p0/3244	NA	R01_cb10012_c2/f1p0/3244	NA	NA
R01_cb13290_c8/f1p0/1002	NA	R01_cb13290_c8/f1p0/1002	NA	NA
R01_cb8564_c38241/f1p0/2298	NA	R01_cb8564_c38241/f1p0/2298	R01_cb8564_c38241/f1p0/2298	R01_cb8564_c38241/f1p0/2298
R01_cb9593_c13/f1p0/418	R01_cb9593_c13/f1p0/418	R01_cb9593_c13/f1p0/418	NA	R01_cb9593_c13/f1p0/418
R01_cb8564_c114193/f1p0/2482	NA	R01_cb8564_c114193/f1p0/2482	NA	NA
R01_cb8564_c21849/f1p3/3972	NA	R01_cb8564_c21849/f1p3/3972	R01_cb8564_c21849/f1p3/3972	NA
R01_cb10970_c3/f1p0/1004	NA	NA	NA	R01_cb10970_c3/f1p0/1004
R01_cb17374_c4/f1p1/826	NA	NA	NA	R01_cb17374_c4/f1p1/826
R01_cb11491_c0/f1p0/1758	R01_cb11491_c0/f1p0/1758	R01_cb11491_c0/f1p0/1758	R01_cb11491_c0/f1p0/1758	R01_cb11491_c0/f1p0/1758
R01_cb12609_c0/f5p0/770	NA	NA	NA	R01_cb12609_c0/f5p0/770
R01_cb2205_c1/f1p0/4012	NA	R01_cb2205_c1/f1p0/4012	NA	R01_cb2205_c1/f1p0/4012
R01_cb12652_c0/f1p0/936	NA	NA	NA	R01_cb12652_c0/f1p0/936
R01_cb8564_c107826/f2p1/3335	NA	R01_cb8564_c107826/f2p1/3335	NA	NA
R01_cb8564_c116710/f1p0/3159	NA	R01_cb8564_c116710/f1p0/3159	NA	NA
R01_cb4529_c16/f2p0/753	NA	R01_cb4529_c16/f2p0/753	NA	NA

R01_cb16387_c2/f1p0/1651	NA	NA	NA	R01_cb16387_c2/f1p0/1651
R01_cb8386_c3/f1p1/1067	NA	R01_cb8386_c3/f1p1/1067	R01_cb8386_c3/f1p1/1067	R01_cb8386_c3/f1p1/1067
R01_cb6740_c8/f1p0/2782	NA	NA	NA	R01_cb6740_c8/f1p0/2782
R01_cb8564_c127693/f1p0/3092	NA	NA	NA	R01_cb8564_c127693/f1p0/3092
R01_cb7877_c0/f2p0/1003	NA	NA	NA	R01_cb7877_c0/f2p0/1003
R01_cb18456_c5734/f1p0/938	NA	R01_cb18456_c5734/f1p0/938	NA	NA
R01_cb17432_c7/f3p0/639	NA	R01_cb17432_c7/f3p0/639	NA	NA
R01_cb12775_c2/f1p2/1418	NA	NA	NA	R01_cb12775_c2/f1p2/1418
R01_cb5815_c13/f1p0/1007	R01_cb5815_c13/f1p0/1007	R01_cb5815_c13/f1p0/1007	NA	R01_cb5815_c13/f1p0/1007
R01_cb9535_c15/f1p1/2071	NA	R01_cb9535_c15/f1p1/2071	R01_cb9535_c15/f1p1/2071	R01_cb9535_c15/f1p1/2071
R01_cb8564_c22413/f1p0/3916	NA	R01_cb8564_c22413/f1p0/3916	NA	NA
R01_cb2918_c14/f1p0/3768	NA	NA	NA	R01_cb2918_c14/f1p0/3768
R01_cb2042_c6/f1p0/2338	NA	NA	NA	R01_cb2042_c6/f1p0/2338
R01_cb11431_c3/f1p0/1165	NA	R01_cb11431_c3/f1p0/1165	NA	R01_cb11431_c3/f1p0/1165
R01_cb7821_c1/f1p0/2414	R01_cb7821_c1/f1p0/2414	R01_cb7821_c1/f1p0/2414	R01_cb7821_c1/f1p0/2414	R01_cb7821_c1/f1p0/2414
R01_cb7168_c18/f1p0/2936	NA	R01_cb7168_c18/f1p0/2936	NA	R01_cb7168_c18/f1p0/2936
R01_cb8564_c52369/f1p0/3232	NA	NA	NA	R01_cb8564_c52369/f1p0/3232
R01_cb4094_c16/f1p4/978	NA	R01_cb4094_c16/f1p4/978	NA	NA
R01_cb8564_c126868/f1p0/3804	NA	R01_cb8564_c126868/f1p0/3804	NA	NA
R01_cb10318_c4/f1p0/1697	NA	R01_cb10318_c4/f1p0/1697	NA	R01_cb10318_c4/f1p0/1697
R01_cb14715_c1/f2p1/572	R01_cb14715_c1/f2p1/572	R01_cb14715_c1/f2p1/572	R01_cb14715_c1/f2p1/572	R01_cb14715_c1/f2p1/572
R01_cb5580_c0/f1p0/3069	NA	R01_cb5580_c0/f1p0/3069	R01_cb5580_c0/f1p0/3069	NA
R01_cb14828_c0/f1p0/1265	NA	R01_cb14828_c0/f1p0/1265	R01_cb14828_c0/f1p0/1265	R01_cb14828_c0/f1p0/1265
R01_cb8564_c10387/f1p0/4385	NA	NA	NA	R01_cb8564_c10387/f1p0/4385

R01_cb17363_c12/f1p0/1314	R01_cb17363_c12/f1p0/1314	R01_cb17363_c12/f1p0/1314	R01_cb17363_c12/f1p0/1314	R01_cb17363_c12/f1p0/1314
R01_cb8564_c13780/f1p0/4204	NA	R01_cb8564_c13780/f1p0/4204	R01_cb8564_c13780/f1p0/4204	R01_cb8564_c13780/f1p0/4204
R01_cb16799_c0/f6p0/728	NA	NA	NA	R01_cb16799_c0/f6p0/728
R01_cb10930_c1/f1p0/2737	R01_cb10930_c1/f1p0/2737	R01_cb10930_c1/f1p0/2737	R01_cb10930_c1/f1p0/2737	R01_cb10930_c1/f1p0/2737
R01_cb8441_c0/f3p0/1280	NA	NA	NA	R01_cb8441_c0/f3p0/1280
R01_cb16494_c3/f4p0/1448	NA	NA	R01_cb16494_c3/f4p0/1448	R01_cb16494_c3/f4p0/1448
R01_cb17033_c3/f1p0/1128	NA	NA	NA	R01_cb17033_c3/f1p0/1128
R01_cb10373_c10/f1p0/487	NA	R01_cb10373_c10/f1p0/487	NA	NA
R01_cb10496_c7/f3p0/1482	NA	NA	NA	R01_cb10496_c7/f3p0/1482
R01_cb16876_c0/f1p0/1250	NA	NA	NA	R01_cb16876_c0/f1p0/1250
R01_cb10360_c15/f1p0/598	R01_cb10360_c15/f1p0/598	R01_cb10360_c15/f1p0/598	R01_cb10360_c15/f1p0/598	R01_cb10360_c15/f1p0/598
R01_cb3610_c0/f1p0/3576	NA	R01_cb3610_c0/f1p0/3576	NA	NA
R01_cb16677_c1/f1p0/464	R01_cb16677_c1/f1p0/464	R01_cb16677_c1/f1p0/464	R01_cb16677_c1/f1p0/464	R01_cb16677_c1/f1p0/464
R01_cb17732_c2/f1p0/1043	NA	R01_cb17732_c2/f1p0/1043	R01_cb17732_c2/f1p0/1043	R01_cb17732_c2/f1p0/1043
R01_cb8564_c112005/f1p0/2719	R01_cb8564_c112005/f1p0/2719	R01_cb8564_c112005/f1p0/2719	R01_cb8564_c112005/f1p0/2719	R01_cb8564_c112005/f1p0/2719
R01_cb11717_c0/f1p0/501	R01_cb11717_c0/f1p0/501	R01_cb11717_c0/f1p0/501	R01_cb11717_c0/f1p0/501	R01_cb11717_c0/f1p0/501
R01_cb13955_c4/f1p0/552	R01_cb13955_c4/f1p0/552	NA	NA	R01_cb13955_c4/f1p0/552
R01_cb14708_c0/f2p0/1091	NA	R01_cb14708_c0/f2p0/1091	NA	NA
R01_cb13490_c10/f1p0/817	R01_cb13490_c10/f1p0/817	R01_cb13490_c10/f1p0/817	R01_cb13490_c10/f1p0/817	R01_cb13490_c10/f1p0/817
R01_cb2804_c101/f1p0/2548	NA	R01_cb2804_c101/f1p0/2548	NA	NA
R01_cb12575_c4/f1p0/1423	NA	NA	NA	R01_cb12575_c4/f1p0/1423
R01_cb9658_c2/f1p0/2335	NA	NA	NA	R01_cb9658_c2/f1p0/2335
R01_cb6147_c9/f1p0/2311	NA	R01_cb6147_c9/f1p0/2311	NA	NA
R01_cb16772_c0/f3p0/1461	NA	NA	NA	R01_cb16772_c0/f3p0/1461
R01_cb8564_c9730/f3p9/4143	NA	R01_cb8564_c9730/f3p9/4143	R01_cb8564_c9730/f3p9/4143	R01_cb8564_c9730/f3p9/4143

R01_cb18764_c0/f1p2/7450	NA	NA	NA	R01_cb18764_c0/f1p2/7450
R01_cb16958_c0/f2p0/985	NA	NA	NA	R01_cb16958_c0/f2p0/985
R01_cb1847_c4/f1p0/4203	NA	NA	NA	R01_cb1847_c4/f1p0/4203
R01_cb15207_c14/f3p6/895	NA	R01_cb15207_c14/f3p6/895	NA	NA
R01_cb17604_c8/f1p0/570	NA	R01_cb17604_c8/f1p0/570	R01_cb17604_c8/f1p0/570	R01_cb17604_c8/f1p0/570
R01_cb12174_c3/f1p1/671	R01_cb12174_c3/f1p1/671	R01_cb12174_c3/f1p1/671	NA	R01_cb12174_c3/f1p1/671
R01_cb1138_c41/f1p0/3215	NA	NA	R01_cb1138_c41/f1p0/3215	NA
R01_cb8844_c0/f1p0/2253	NA	R01_cb8844_c0/f1p0/2253	NA	NA
R01_cb18520_c0/f2p0/1271	NA	NA	NA	R01_cb18520_c0/f2p0/1271
R01_cb1279_c85/f1p0/1794	NA	R01_cb1279_c85/f1p0/1794	NA	NA
R01_cb13318_c1/f1p0/1654	NA	NA	NA	R01_cb13318_c1/f1p0/1654
R01_cb1027_c5/f1p0/4911	NA	R01_cb1027_c5/f1p0/4911	NA	NA
R01_cb1398_c15/f1p0/5057	NA	R01_cb1398_c15/f1p0/5057	R01_cb1398_c15/f1p0/5057	NA
R01_cb8564_c79983/f1p0/1945	NA	R01_cb8564_c79983/f1p0/1945	NA	NA
R01_cb11667_c0/f1p0/1403	NA	NA	R01_cb11667_c0/f1p0/1403	NA
R01_cb15074_c10/f1p0/587	R01_cb15074_c10/f1p0/587	R01_cb15074_c10/f1p0/587	R01_cb15074_c10/f1p0/587	R01_cb15074_c10/f1p0/587
R01_cb10034_c43/f1p2/953	NA	R01_cb10034_c43/f1p2/953	NA	NA
R01_cb18456_c6626/f1p0/723	NA	R01_cb18456_c6626/f1p0/723	NA	NA
R01_cb10015_c231/f1p0/502	R01_cb10015_c231/f1p0/502	R01_cb10015_c231/f1p0/502	NA	NA
R01_cb8564_c38653/f1p1/2281	NA	R01_cb8564_c38653/f1p1/2281	NA	NA
R01_cb7962_c1/f2p1/1860	NA	NA	NA	R01_cb7962_c1/f2p1/1860
R01_cb8564_c74141/f1p0/4167	R01_cb8564_c74141/f1p0/4167	R01_cb8564_c74141/f1p0/4167	R01_cb8564_c74141/f1p0/4167	R01_cb8564_c74141/f1p0/4167
R01_cb242_c19/f1p0/4151	NA	NA	NA	R01_cb242_c19/f1p0/4151
R01_cb345_c31/f1p0/1002	NA	R01_cb345_c31/f1p0/1002	NA	NA
R01_cb3222_c14/f1p0/472	R01_cb3222_c14/f1p0/472	R01_cb3222_c14/f1p0/472	R01_cb3222_c14/f1p0/472	R01_cb3222_c14/f1p0/472

R01_cb17129_c2/f1p0/1790	NA	R01_cb17129_c2/f1p0/1790	NA	NA
R01_cb7051_c1/f1p0/2781	NA	NA	NA	R01_cb7051_c1/f1p0/2781
R01_cb3131_c7/f1p0/7419	NA	R01_cb3131_c7/f1p0/7419	NA	NA
R01_cb14261_c3/f1p0/1245	R01_cb14261_c3/f1p0/1245	R01_cb14261_c3/f1p0/1245	R01_cb14261_c3/f1p0/1245	R01_cb14261_c3/f1p0/1245
R01_cb8564_c88894/f1p0/3482	NA	R01_cb8564_c88894/f1p0/3482	NA	NA
R01_cb1780_c13/f1p0/725	NA	R01_cb1780_c13/f1p0/725	NA	NA
R01_cb1626_c13/f1p0/4596	NA	NA	NA	R01_cb1626_c13/f1p0/4596
R01_cb2161_c9/f1p0/2205	NA	NA	NA	R01_cb2161_c9/f1p0/2205
R01_cb16220_c1/f1p0/708	R01_cb16220_c1/f1p0/708	NA	NA	R01_cb16220_c1/f1p0/708
R01_cb4829_c41/f1p0/3122	NA	NA	NA	R01_cb4829_c41/f1p0/3122
R01_cb14074_c12/f1p0/990	R01_cb14074_c12/f1p0/990	R01_cb14074_c12/f1p0/990	NA	R01_cb14074_c12/f1p0/990
R01_cb14137_c1/f1p0/864	NA	NA	NA	R01_cb14137_c1/f1p0/864
R01_cb14292_c13/f1p0/1808	NA	R01_cb14292_c13/f1p0/1808	NA	NA
R01_cb5635_c2/f1p0/3436	NA	NA	NA	R01_cb5635_c2/f1p0/3436
R01_cb7966_c2/f1p0/2492	R01_cb7966_c2/f1p0/2492	R01_cb7966_c2/f1p0/2492	NA	NA
R01_cb2854_c39/f1p0/3359	NA	R01_cb2854_c39/f1p0/3359	NA	NA
R01_cb13352_c1/f1p0/800	NA	NA	R01_cb13352_c1/f1p0/800	R01_cb13352_c1/f1p0/800
R01_cb14729_c5/f1p0/730	NA	R01_cb14729_c5/f1p0/730	NA	NA
R01_cb6015_c6/f1p0/2740	NA	R01_cb6015_c6/f1p0/2740	NA	NA
R01_cb548_c60/f1p0/2889	NA	R01_cb548_c60/f1p0/2889	NA	NA
R01_cb8564_c18280/f1p0/1947	R01_cb8564_c18280/f1p0/1947	R01_cb8564_c18280/f1p0/1947	NA	NA
R01_cb14156_c1/f1p0/881	NA	R01_cb14156_c1/f1p0/881	R01_cb14156_c1/f1p0/881	R01_cb14156_c1/f1p0/881
R01_cb6278_c18/f1p0/1671	NA	NA	NA	R01_cb6278_c18/f1p0/1671
R01_cb8564_c52284/f1p0/3798	NA	R01_cb8564_c52284/f1p0/3798	R01_cb8564_c52284/f1p0/3798	R01_cb8564_c52284/f1p0/3798
R01_cb4838_c3/f1p0/1177	NA	R01_cb4838_c3/f1p0/1177	NA	NA

R01_cb17052_c5/f1p0/1297	NA	NA	NA	R01_cb17052_c5/f1p0/1297
R01_cb6348_c12/f4p0/1764	NA	NA	NA	R01_cb6348_c12/f4p0/1764
R01_cb316_c21/f1p0/2442	NA	NA	NA	R01_cb316_c21/f1p0/2442
R01_cb18605_c1/f1p0/868	NA	R01_cb18605_c1/f1p0/868	R01_cb18605_c1/f1p0/868	R01_cb18605_c1/f1p0/868
R01_cb4639_c10/f1p0/2558	R01_cb4639_c10/f1p0/2558	R01_cb4639_c10/f1p0/2558	NA	R01_cb4639_c10/f1p0/2558
R01_cb10718_c3/f1p0/4101	NA	R01_cb10718_c3/f1p0/4101	NA	NA
R01_cb8564_c121938/f1p0/2254	NA	NA	NA	R01_cb8564_c121938/f1p0/2254
R01_cb16428_c1/f1p0/607	R01_cb16428_c1/f1p0/607	R01_cb16428_c1/f1p0/607	R01_cb16428_c1/f1p0/607	NA
R01_cb15045_c0/f4p0/1428	NA	NA	NA	R01_cb15045_c0/f4p0/1428
R01_cb6204_c5/f2p3/2918	NA	NA	NA	R01_cb6204_c5/f2p3/2918
R01_cb11891_c3/f1p0/1790	R01_cb11891_c3/f1p0/1790	R01_cb11891_c3/f1p0/1790	R01_cb11891_c3/f1p0/1790	R01_cb11891_c3/f1p0/1790
R01_cb8281_c6/f1p0/2076	NA	NA	NA	R01_cb8281_c6/f1p0/2076
R01_cb8564_c114202/f1p0/2504	R01_cb8564_c114202/f1p0/2504	R01_cb8564_c114202/f1p0/2504	R01_cb8564_c114202/f1p0/2504	NA
R01_cb18132_c12/f1p0/1726	NA	R01_cb18132_c12/f1p0/1726	NA	NA
R01_cb9682_c4/f1p1/1998	NA	NA	NA	R01_cb9682_c4/f1p1/1998
R01_cb9506_c6/f1p0/3026	R01_cb9506_c6/f1p0/3026	R01_cb9506_c6/f1p0/3026	NA	R01_cb9506_c6/f1p0/3026
R01_cb8564_c76021/f1p0/2284	NA	R01_cb8564_c76021/f1p0/2284	R01_cb8564_c76021/f1p0/2284	NA
R01_cb6328_c1/f1p0/2584	NA	NA	NA	R01_cb6328_c1/f1p0/2584
R01_cb7590_c2/f1p0/2581	R01_cb7590_c2/f1p0/2581	R01_cb7590_c2/f1p0/2581	NA	NA
R01_cb11221_c1/f1p0/3463	NA	R01_cb11221_c1/f1p0/3463	NA	NA
R01_cb17033_c4/f1p0/1406	NA	NA	NA	R01_cb17033_c4/f1p0/1406
R01_cb16881_c1/f2p1/1533	NA	NA	NA	R01_cb16881_c1/f2p1/1533
R01_cb9937_c3/f1p0/1348	NA	R01_cb9937_c3/f1p0/1348	R01_cb9937_c3/f1p0/1348	R01_cb9937_c3/f1p0/1348
R01_cb5745_c4/f1p0/2698	NA	R01_cb5745_c4/f1p0/2698	NA	R01_cb5745_c4/f1p0/2698

R01_cb10083_c5/f1p0/2795	NA	R01_cb10083_c5/f1p0/2795	NA	NA
R01_cb8564_c36813/f1p0/3856	NA	R01_cb8564_c36813/f1p0/3856	R01_cb8564_c36813/f1p0/3856	R01_cb8564_c36813/f1p0/3856
R01_cb8564_c91397/f1p0/2896	NA	NA	NA	R01_cb8564_c91397/f1p0/2896
R01_cb14045_c7/f1p0/1601	NA	NA	NA	R01_cb14045_c7/f1p0/1601
R01_cb8564_c16259/f1p0/3399	NA	R01_cb8564_c16259/f1p0/3399	NA	NA
R01_cb1487_c7/f1p0/1224	NA	NA	NA	R01_cb1487_c7/f1p0/1224
R01_cb4799_c14/f1p0/2731	NA	R01_cb4799_c14/f1p0/2731	R01_cb4799_c14/f1p0/2731	R01_cb4799_c14/f1p0/2731
R01_cb16594_c1/f1p1/1222	NA	R01_cb16594_c1/f1p1/1222	NA	R01_cb16594_c1/f1p1/1222
R01_cb11538_c1/f1p0/2006	R01_cb11538_c1/f1p0/2006	R01_cb11538_c1/f1p0/2006	NA	R01_cb11538_c1/f1p0/2006
R01_cb14283_c3/f1p0/810	R01_cb14283_c3/f1p0/810	R01_cb14283_c3/f1p0/810	R01_cb14283_c3/f1p0/810	NA
R01_cb7066_c2/f1p0/2488	NA	R01_cb7066_c2/f1p0/2488	R01_cb7066_c2/f1p0/2488	R01_cb7066_c2/f1p0/2488
R01_cb4368_c5/f1p0/3249	NA	NA	NA	R01_cb4368_c5/f1p0/3249
R01_cb8564_c72400/f1p0/2371	R01_cb8564_c72400/f1p0/2371	R01_cb8564_c72400/f1p0/2371	R01_cb8564_c72400/f1p0/2371	R01_cb8564_c72400/f1p0/2371
R01_cb6278_c3/f1p0/2981	NA	NA	NA	R01_cb6278_c3/f1p0/2981
R01_cb8294_c0/f3p0/2141	NA	R01_cb8294_c0/f3p0/2141	NA	NA
R01_cb13645_c4/f1p0/611	R01_cb13645_c4/f1p0/611	R01_cb13645_c4/f1p0/611	R01_cb13645_c4/f1p0/611	R01_cb13645_c4/f1p0/611
R01_cb10453_c5/f1p0/1216	NA	R01_cb10453_c5/f1p0/1216	R01_cb10453_c5/f1p0/1216	R01_cb10453_c5/f1p0/1216
R01_cb9968_c6/f1p2/1828	NA	R01_cb9968_c6/f1p2/1828	NA	NA
R01_cb1860_c16/f1p1/2491	NA	NA	NA	R01_cb1860_c16/f1p1/2491
R01_cb17635_c0/f1p0/510	R01_cb17635_c0/f1p0/510	R01_cb17635_c0/f1p0/510	R01_cb17635_c0/f1p0/510	R01_cb17635_c0/f1p0/510
R01_cb17812_c0/f2p0/664	R01_cb17812_c0/f2p0/664	R01_cb17812_c0/f2p0/664	R01_cb17812_c0/f2p0/664	R01_cb17812_c0/f2p0/664
R01_cb5114_c3/f1p0/912	R01_cb5114_c3/f1p0/912	R01_cb5114_c3/f1p0/912	R01_cb5114_c3/f1p0/912	R01_cb5114_c3/f1p0/912
R01_cb14611_c2/f1p0/642	NA	NA	NA	R01_cb14611_c2/f1p0/642
R01_cb11234_c1/f1p0/3532	NA	R01_cb11234_c1/f1p0/3532	NA	NA
R01_cb13161_c28/f1p0/490	R01_cb13161_c28/f1p0/490	R01_cb13161_c28/f1p0/490	R01_cb13161_c28/f1p0/490	R01_cb13161_c28/f1p0/490

R01_cb1819_c1/f1p0/2303	NA	R01_cb1819_c1/f1p0/2303	NA	NA
R01_cb6405_c7/f1p0/2728	NA	R01_cb6405_c7/f1p0/2728	NA	NA
R01_cb11653_c0/f1p0/1890	NA	R01_cb11653_c0/f1p0/1890	NA	NA
R01_cb12240_c11/f1p0/551	R01_cb12240_c11/f1p0/551	R01_cb12240_c11/f1p0/551	NA	NA
R01_cb18281_c0/f1p0/1295	NA	NA	NA	R01_cb18281_c0/f1p0/1295
R01_cb8564_c631/f4p0/2066	NA	NA	NA	R01_cb8564_c631/f4p0/2066
R01_cb16613_c0/f1p0/1050	NA	NA	NA	R01_cb16613_c0/f1p0/1050
R01_cb17363_c5/f1p1/661	R01_cb17363_c5/f1p1/661	R01_cb17363_c5/f1p1/661	R01_cb17363_c5/f1p1/661	R01_cb17363_c5/f1p1/661
R01_cb1388_c13/f1p0/4064	NA	NA	NA	R01_cb1388_c13/f1p0/4064
R01_cb8285_c4/f1p0/1361	NA	NA	NA	R01_cb8285_c4/f1p0/1361
R01_cb8564_c81854/f1p0/2013	R01_cb8564_c81854/f1p0/2013	R01_cb8564_c81854/f1p0/2013	R01_cb8564_c81854/f1p0/2013	NA
R01_cb1398_c14/f1p0/1993	NA	R01_cb1398_c14/f1p0/1993	R01_cb1398_c14/f1p0/1993	NA
R01_cb11855_c0/f4p0/575	R01_cb11855_c0/f4p0/575	R01_cb11855_c0/f4p0/575	NA	NA
R01_cb10031_c5/f1p0/3145	NA	R01_cb10031_c5/f1p0/3145	NA	R01_cb10031_c5/f1p0/3145
R01_cb3757_c0/f1p0/3534	NA	NA	NA	R01_cb3757_c0/f1p0/3534
R01_cb8564_c15978/f1p0/3844	NA	R01_cb8564_c15978/f1p0/3844	NA	NA
R01_cb416_c58/f1p0/2505	NA	R01_cb416_c58/f1p0/2505	NA	R01_cb416_c58/f1p0/2505
R01_cb1023_c3/f1p0/1479	NA	R01_cb1023_c3/f1p0/1479	NA	NA
R01_cb8564_c86424/f1p0/2025	R01_cb8564_c86424/f1p0/2025	R01_cb8564_c86424/f1p0/2025	R01_cb8564_c86424/f1p0/2025	R01_cb8564_c86424/f1p0/2025
R01_cb16942_c0/f2p0/603	NA	R01_cb16942_c0/f2p0/603	R01_cb16942_c0/f2p0/603	R01_cb16942_c0/f2p0/603
R01_cb5021_c0/f4p0/1500	NA	NA	NA	R01_cb5021_c0/f4p0/1500
R01_cb10534_c0/f3p0/919	NA	R01_cb10534_c0/f3p0/919	R01_cb10534_c0/f3p0/919	R01_cb10534_c0/f3p0/919
R01_cb8603_c1/f1p0/3454	NA	R01_cb8603_c1/f1p0/3454	NA	NA
R01_cb7954_c3/f1p0/996	NA	NA	NA	R01_cb7954_c3/f1p0/996

R01_cb14070_c0/f1p0/1393	NA	R01_cb14070_c0/f1p0/1393	R01_cb14070_c0/f1p0/1393	R01_cb14070_c0/f1p0/1393
R01_cb18346_c1/f1p0/889	R01_cb18346_c1/f1p0/889	R01_cb18346_c1/f1p0/889	R01_cb18346_c1/f1p0/889	NA
R01_cb14409_c2/f1p0/1807	NA	R01_cb14409_c2/f1p0/1807	R01_cb14409_c2/f1p0/1807	R01_cb14409_c2/f1p0/1807
R01_cb16003_c4/f1p0/1712	NA	NA	NA	R01_cb16003_c4/f1p0/1712
R01_cb15904_c1/f1p0/607	NA	R01_cb15904_c1/f1p0/607	R01_cb15904_c1/f1p0/607	R01_cb15904_c1/f1p0/607
R01_cb5855_c1/f1p0/2909	NA	R01_cb5855_c1/f1p0/2909	NA	NA
R01_cb2049_c6/f1p0/7394	NA	R01_cb2049_c6/f1p0/7394	NA	NA
R01_cb5935_c6/f1p0/937	NA	R01_cb5935_c6/f1p0/937	NA	NA
R01_cb16664_c4/f1p0/575	NA	NA	NA	R01_cb16664_c4/f1p0/575
R01_cb16370_c0/f1p0/478	NA	R01_cb16370_c0/f1p0/478	NA	R01_cb16370_c0/f1p0/478
R01_cb2845_c9/f1p0/3409	NA	NA	NA	R01_cb2845_c9/f1p0/3409
R01_cb12329_c26/f4p2/1461	NA	NA	NA	R01_cb12329_c26/f4p2/1461
R01_cb8564_c50347/f1p0/3158	NA	R01_cb8564_c50347/f1p0/3158	NA	R01_cb8564_c50347/f1p0/3158
R01_cb4524_c5/f1p0/3116	NA	R01_cb4524_c5/f1p0/3116	NA	NA
R01_cb15418_c4/f1p1/780	NA	NA	NA	R01_cb15418_c4/f1p1/780
R01_cb8564_c1543/f1p0/2375	NA	NA	NA	R01_cb8564_c1543/f1p0/2375
R01_cb17052_c2/f1p0/1584	NA	R01_cb17052_c2/f1p0/1584	NA	R01_cb17052_c2/f1p0/1584
R01_cb18456_c7596/f1p0/1340	R01_cb18456_c7596/f1p0/1340	R01_cb18456_c7596/f1p0/1340	NA	R01_cb18456_c7596/f1p0/1340
R01_cb8722_c0/f1p0/2289	NA	R01_cb8722_c0/f1p0/2289	NA	NA
R01_cb1031_c0/f1p0/4508	NA	NA	NA	R01_cb1031_c0/f1p0/4508
R01_cb10558_c5/f1p0/1007	NA	NA	NA	R01_cb10558_c5/f1p0/1007
R01_cb15069_c7/f4p0/505	NA	R01_cb15069_c7/f4p0/505	NA	NA
R01_cb7598_c0/f1p0/2576	NA	R01_cb7598_c0/f1p0/2576	R01_cb7598_c0/f1p0/2576	R01_cb7598_c0/f1p0/2576
R01_cb13027_c2/f1p0/975	NA	NA	NA	R01_cb13027_c2/f1p0/975
R01_cb4000_c1/f1p0/2545	R01_cb4000_c1/f1p0/2545	R01_cb4000_c1/f1p0/2545	NA	NA

R01_cb13059_c3/f1p0/1353	NA	R01_cb13059_c3/f1p0/1353	NA	NA
R01_cb15564_c2/f1p0/928	NA	R01_cb15564_c2/f1p0/928	R01_cb15564_c2/f1p0/928	R01_cb15564_c2/f1p0/928
R01_cb14805_c0/f7p0/623	NA	R01_cb14805_c0/f7p0/623	NA	NA
R01_cb2022_c0/f12p0/2116	NA	NA	NA	R01_cb2022_c0/f12p0/2116
R01_cb9646_c4/f1p0/1827	NA	NA	NA	R01_cb9646_c4/f1p0/1827
R01_cb9922_c0/f1p0/1925	NA	NA	NA	R01_cb9922_c0/f1p0/1925
R01_cb8564_c111155/f1p0/2917	NA	R01_cb8564_c111155/f1p0/2917	NA	R01_cb8564_c111155/f1p0/2917
R01_cb8564_c48905/f1p0/2042	NA	R01_cb8564_c48905/f1p0/2042	NA	R01_cb8564_c48905/f1p0/2042
R01_cb18456_c1508/f3p0/700	NA	NA	NA	R01_cb18456_c1508/f3p0/700
R01_cb7017_c2/f1p0/2742	NA	NA	R01_cb7017_c2/f1p0/2742	NA
R01_cb173_c25/f1p0/2391	NA	NA	NA	R01_cb173_c25/f1p0/2391
R01_cb12666_c2/f1p1/703	NA	R01_cb12666_c2/f1p1/703	NA	NA
R01_cb3575_c12/f1p0/3408	NA	NA	NA	R01_cb3575_c12/f1p0/3408
R01_cb10725_c1/f1p0/2420	NA	R01_cb10725_c1/f1p0/2420	R01_cb10725_c1/f1p0/2420	R01_cb10725_c1/f1p0/2420
R01_cb17_c23/f1p0/6113	NA	NA	NA	R01_cb17_c23/f1p0/6113
R01_cb3426_c18/f1p1/3249	NA	NA	NA	R01_cb3426_c18/f1p1/3249
R01_cb14079_c0/f1p0/486	NA	R01_cb14079_c0/f1p0/486	R01_cb14079_c0/f1p0/486	R01_cb14079_c0/f1p0/486
R01_cb261_c48/f1p1/2183	NA	R01_cb261_c48/f1p1/2183	NA	NA
R01_cb12445_c0/f8p0/988	NA	R01_cb12445_c0/f8p0/988	NA	NA
R01_cb12963_c1/f3p0/1289	NA	NA	NA	R01_cb12963_c1/f3p0/1289
R01_cb7506_c4/f1p0/3483	NA	NA	NA	R01_cb7506_c4/f1p0/3483
R01_cb13681_c4/f1p0/979	NA	NA	R01_cb13681_c4/f1p0/979	R01_cb13681_c4/f1p0/979
R01_cb15383_c0/f2p1/577	NA	R01_cb15383_c0/f2p1/577	NA	NA
R01_cb17335_c1/f1p0/892	NA	R01_cb17335_c1/f1p0/892	R01_cb17335_c1/f1p0/892	R01_cb17335_c1/f1p0/892
R01_cb17268_c1/f1p0/1100	R01_cb17268_c1/f1p0/1100	R01_cb17268_c1/f1p0/1100	NA	NA

R01_cb10217_c5/f1p3/1772	NA	NA	NA	R01_cb10217_c5/f1p3/1772
R01_cb10226_c3/f1p0/732	NA	R01_cb10226_c3/f1p0/732	R01_cb10226_c3/f1p0/732	R01_cb10226_c3/f1p0/732
R01_cb8564_c36482/f1p0/2839	NA	R01_cb8564_c36482/f1p0/2839	NA	R01_cb8564_c36482/f1p0/2839
R01_cb10378_c4/f1p0/564	NA	R01_cb10378_c4/f1p0/564	NA	R01_cb10378_c4/f1p0/564
R01_cb4584_c2/f4p0/3284	NA	NA	NA	R01_cb4584_c2/f4p0/3284
R01_cb280_c73/f1p0/4006	NA	R01_cb280_c73/f1p0/4006	NA	NA
R01_cb13809_c2/f1p0/1672	R01_cb13809_c2/f1p0/1672	R01_cb13809_c2/f1p0/1672	R01_cb13809_c2/f1p0/1672	R01_cb13809_c2/f1p0/1672
R01_cb13602_c27/f1p0/1665	NA	R01_cb13602_c27/f1p0/1665	NA	NA
R01_cb4030_c2/f1p0/2488	NA	NA	NA	R01_cb4030_c2/f1p0/2488
R01_cb9846_c0/f1p0/1944	NA	R01_cb9846_c0/f1p0/1944	R01_cb9846_c0/f1p0/1944	R01_cb9846_c0/f1p0/1944
R01_cb11168_c0/f3p0/644	NA	R01_cb11168_c0/f3p0/644	R01_cb11168_c0/f3p0/644	R01_cb11168_c0/f3p0/644
R01_cb7789_c2/f1p0/2538	NA	R01_cb7789_c2/f1p0/2538	NA	NA
R01_cb8564_c113131/f1p0/2318	NA	R01_cb8564_c113131/f1p0/2318	R01_cb8564_c113131/f1p0/2318	NA
R01_cb8564_c153873/f1p6/4745	NA	R01_cb8564_c153873/f1p6/4745	R01_cb8564_c153873/f1p6/4745	R01_cb8564_c153873/f1p6/4745
R01_cb3518_c3/f1p0/3655	NA	R01_cb3518_c3/f1p0/3655	NA	NA
R01_cb3121_c4/f1p1/3640	NA	R01_cb3121_c4/f1p1/3640	R01_cb3121_c4/f1p1/3640	NA
R01_cb16432_c0/f1p0/493	NA	R01_cb16432_c0/f1p0/493	NA	NA
R01_cb7533_c10/f1p0/2160	NA	NA	NA	R01_cb7533_c10/f1p0/2160
R01_cb10564_c1/f1p0/3075	R01_cb10564_c1/f1p0/3075	R01_cb10564_c1/f1p0/3075	NA	R01_cb10564_c1/f1p0/3075
R01_cb8564_c83630/f1p0/2884	NA	R01_cb8564_c83630/f1p0/2884	NA	R01_cb8564_c83630/f1p0/2884
R01_cb16100_c7/f1p0/711	R01_cb16100_c7/f1p0/711	NA	NA	NA
R01_cb15474_c4/f1p0/1728	NA	NA	NA	R01_cb15474_c4/f1p0/1728
R01_cb6662_c9/f5p0/1818	NA	NA	NA	R01_cb6662_c9/f5p0/1818
R01_cb18077_c1/f1p0/361	R01_cb18077_c1/f1p0/361	R01_cb18077_c1/f1p0/361	R01_cb18077_c1/f1p0/361	R01_cb18077_c1/f1p0/361

R01_cb12243_c0/f1p0/1698	NA	R01_cb12243_c0/f1p0/1698	NA	R01_cb12243_c0/f1p0/1698
R01_cb15825_c4/f1p0/1179	NA	NA	R01_cb15825_c4/f1p0/1179	R01_cb15825_c4/f1p0/1179
R01_cb9692_c1/f1p0/1801	NA	NA	NA	R01_cb9692_c1/f1p0/1801
R01_cb7836_c1/f1p0/1931	NA	R01_cb7836_c1/f1p0/1931	NA	R01_cb7836_c1/f1p0/1931
R01_cb7802_c10/f1p0/1775	NA	NA	NA	R01_cb7802_c10/f1p0/1775
R01_cb8564_c90587/f1p0/3448	NA	R01_cb8564_c90587/f1p0/3448	NA	NA
R01_cb13021_c3/f1p0/417	R01_cb13021_c3/f1p0/417	R01_cb13021_c3/f1p0/417	NA	NA
R01_cb8564_c10238/f1p0/3068	NA	R01_cb8564_c10238/f1p0/3068	NA	R01_cb8564_c10238/f1p0/3068
R01_cb4180_c0/f1p0/3410	NA	R01_cb4180_c0/f1p0/3410	NA	R01_cb4180_c0/f1p0/3410
R01_cb16521_c1/f1p0/926	NA	NA	NA	R01_cb16521_c1/f1p0/926
R01_cb8975_c11/f1p0/340	R01_cb8975_c11/f1p0/340	R01_cb8975_c11/f1p0/340	R01_cb8975_c11/f1p0/340	R01_cb8975_c11/f1p0/340
R01_cb1138_c53/f1p1/1002	NA	NA	R01_cb1138_c53/f1p1/1002	R01_cb1138_c53/f1p1/1002
R01_cb8564_c12898/f1p0/3109	NA	NA	NA	R01_cb8564_c12898/f1p0/3109
R01_cb13287_c11/f8p0/574	NA	R01_cb13287_c11/f8p0/574	NA	R01_cb13287_c11/f8p0/574
R01_cb7345_c8/f1p0/2440	NA	R01_cb7345_c8/f1p0/2440	NA	NA
R01_cb13872_c4/f1p0/1346	NA	R01_cb13872_c4/f1p0/1346	NA	NA
R01_cb4548_c2/f1p0/4653	NA	R01_cb4548_c2/f1p0/4653	NA	NA
R01_cb1203_c14/f1p0/4382	NA	NA	NA	R01_cb1203_c14/f1p0/4382
R01_cb1860_c34/f3p4/2559	NA	NA	NA	R01_cb1860_c34/f3p4/2559
R01_cb4229_c4/f1p0/2456	NA	NA	NA	R01_cb4229_c4/f1p0/2456
R01_cb8564_c89855/f1p0/2077	NA	NA	NA	R01_cb8564_c89855/f1p0/2077
R01_cb17640_c0/f2p0/1240	NA	NA	NA	R01_cb17640_c0/f2p0/1240
R01_cb5983_c7/f1p0/859	NA	NA	NA	R01_cb5983_c7/f1p0/859
R01_cb625_c8/f1p0/3111	NA	NA	NA	R01_cb625_c8/f1p0/3111
R01_cb8564_c4199/f1p0/2665	NA	R01_cb8564_c4199/f1p0/2665	NA	NA
R01_cb8564_c23958/f1p0/2286	NA	NA	NA	R01_cb8564_c23958/f1p0/2286

R01_cb14477_c4/f1p0/1075	NA	NA	NA	R01_cb14477_c4/f1p0/1075
R01_cb8564_c80230/f1p0/3341	NA	R01_cb8564_c80230/f1p0/3341	R01_cb8564_c80230/f1p0/3341	R01_cb8564_c80230/f1p0/3341
R01_cb2960_c4/f1p0/2302	NA	NA	NA	R01_cb2960_c4/f1p0/2302
R01_cb7579_c8/f1p0/2343	NA	NA	NA	R01_cb7579_c8/f1p0/2343
R01_cb11220_c0/f1p0/1669	NA	R01_cb11220_c0/f1p0/1669	NA	NA
R01_cb2382_c8/f1p0/5285	NA	NA	NA	R01_cb2382_c8/f1p0/5285
R01_cb14316_c9/f1p0/1705	R01_cb14316_c9/f1p0/1705	R01_cb14316_c9/f1p0/1705	R01_cb14316_c9/f1p0/1705	R01_cb14316_c9/f1p0/1705
R01_cb11718_c2/f1p0/799	NA	NA	NA	R01_cb11718_c2/f1p0/799
R01_cb4600_c5/f1p2/2237	NA	NA	NA	R01_cb4600_c5/f1p2/2237
R01_cb14316_c4/f1p1/1084	R01_cb14316_c4/f1p1/1084	R01_cb14316_c4/f1p1/1084	R01_cb14316_c4/f1p1/1084	R01_cb14316_c4/f1p1/1084
R01_cb17358_c2/f1p0/1362	NA	NA	NA	R01_cb17358_c2/f1p0/1362
R01_cb2352_c12/f1p0/1756	NA	R01_cb2352_c12/f1p0/1756	R01_cb2352_c12/f1p0/1756	R01_cb2352_c12/f1p0/1756
R01_cb17955_c0/f1p0/1007	NA	R01_cb17955_c0/f1p0/1007	R01_cb17955_c0/f1p0/1007	R01_cb17955_c0/f1p0/1007
R01_cb8564_c24246/f1p0/3791	NA	R01_cb8564_c24246/f1p0/3791	NA	R01_cb8564_c24246/f1p0/3791
R01_cb13037_c1/f1p0/1236	NA	NA	R01_cb13037_c1/f1p0/1236	R01_cb13037_c1/f1p0/1236
R01_cb8073_c4/f1p0/3283	NA	R01_cb8073_c4/f1p0/3283	NA	NA
R01_cb2182_c6/f1p0/2121	NA	R01_cb2182_c6/f1p0/2121	NA	R01_cb2182_c6/f1p0/2121
R01_cb7450_c1/f1p0/2612	NA	R01_cb7450_c1/f1p0/2612	NA	NA
R01_cb8564_c16796/f1p0/3587	NA	NA	R01_cb8564_c16796/f1p0/3587	R01_cb8564_c16796/f1p0/3587
R01_cb5482_c11/f1p0/2785	NA	R01_cb5482_c11/f1p0/2785	NA	NA
R01_cb1378_c5/f1p0/2898	NA	R01_cb1378_c5/f1p0/2898	NA	NA
R01_cb4182_c3/f1p0/3446	NA	R01_cb4182_c3/f1p0/3446	NA	NA
R01_cb7303_c13/f1p0/2572	NA	NA	NA	R01_cb7303_c13/f1p0/2572
R01_cb8564_c10634/f1p0/3176	NA	NA	NA	R01_cb8564_c10634/f1p0/3176
R01_cb3941_c23/f1p0/3486	NA	NA	NA	R01_cb3941_c23/f1p0/3486
R01_cb14576_c6/f1p0/999	NA	NA	R01_cb14576_c6/f1p0/999	R01_cb14576_c6/f1p0/999

R01_cb8564_c4105/f1p0/2220	NA	R01_cb8564_c4105/f1p0/2220	NA	NA
R01_cb6893_c2/f1p0/2744	NA	R01_cb6893_c2/f1p0/2744	NA	R01_cb6893_c2/f1p0/2744
R01_cb5258_c0/f3p0/1031	NA	NA	NA	R01_cb5258_c0/f3p0/1031
R01_cb14444_c2/f1p0/1612	NA	R01_cb14444_c2/f1p0/1612	R01_cb14444_c2/f1p0/1612	R01_cb14444_c2/f1p0/1612
R01_cb5995_c10/f1p0/2599	R01_cb5995_c10/f1p0/2599	R01_cb5995_c10/f1p0/2599	NA	NA
R01_cb12785_c32/f13p0/989	NA	R01_cb12785_c32/f13p0/989	R01_cb12785_c32/f13p0/989	R01_cb12785_c32/f13p0/989
R01_cb2311_c3/f1p0/3980	NA	NA	NA	R01_cb2311_c3/f1p0/3980
R01_cb8564_c75195/f1p0/2825	NA	NA	R01_cb8564_c75195/f1p0/2825	R01_cb8564_c75195/f1p0/2825
R01_cb13565_c68/f1p0/1697	NA	R01_cb13565_c68/f1p0/1697	NA	NA
R01_cb9612_c2/f1p0/2534	NA	R01_cb9612_c2/f1p0/2534	NA	R01_cb9612_c2/f1p0/2534
R01_cb6636_c0/f1p0/2802	NA	R01_cb6636_c0/f1p0/2802	R01_cb6636_c0/f1p0/2802	R01_cb6636_c0/f1p0/2802
R01_cb4972_c25/f1p0/3382	NA	R01_cb4972_c25/f1p0/3382	NA	NA
R01_cb10481_c6/f1p0/430	R01_cb10481_c6/f1p0/430	R01_cb10481_c6/f1p0/430	R01_cb10481_c6/f1p0/430	R01_cb10481_c6/f1p0/430
R01_cb8564_c51860/f1p0/2747	R01_cb8564_c51860/f1p0/2747	R01_cb8564_c51860/f1p0/2747	R01_cb8564_c51860/f1p0/2747	R01_cb8564_c51860/f1p0/2747
R01_cb8564_c86805/f1p0/3202	NA	R01_cb8564_c86805/f1p0/3202	NA	NA
R01_cb9101_c1/f1p0/2193	NA	R01_cb9101_c1/f1p0/2193	NA	NA
R01_cb8564_c19822/f1p0/3534	NA	R01_cb8564_c19822/f1p0/3534	NA	NA
R01_cb12776_c1/f1p0/1435	NA	NA	NA	R01_cb12776_c1/f1p0/1435
R01_cb5287_c0/f2p0/2885	NA	R01_cb5287_c0/f2p0/2885	NA	NA
R01_cb8564_c1990/f1p0/2615	NA	R01_cb8564_c1990/f1p0/2615	R01_cb8564_c1990/f1p0/2615	R01_cb8564_c1990/f1p0/2615
R01_cb8564_c70289/f1p1/2295	NA	R01_cb8564_c70289/f1p1/2295	NA	R01_cb8564_c70289/f1p1/2295
R01_cb18310_c1/f1p0/1530	NA	NA	NA	R01_cb18310_c1/f1p0/1530
R01_cb8564_c45922/f1p0/3553	NA	NA	NA	R01_cb8564_c45922/f1p0/3553
R01_cb13485_c5/f1p0/627	NA	R01_cb13485_c5/f1p0/627	NA	NA
R01_cb18409_c26/f2p0/365	NA	R01_cb18409_c26/f2p0/365	R01_cb18409_c26/f2p0/365	R01_cb18409_c26/f2p0/365

R01_cb16262_c2/f1p0/302	R01_cb16262_c2/f1p0/302	R01_cb16262_c2/f1p0/302	R01_cb16262_c2/f1p0/302	R01_cb16262_c2/f1p0/302
R01_cb8564_c91441/f1p0/3028	NA	NA	NA	R01_cb8564_c91441/f1p0/3028
R01_cb15778_c3/f1p0/1005	NA	NA	NA	R01_cb15778_c3/f1p0/1005
R01_cb2779_c5/f1p0/1790	NA	NA	NA	R01_cb2779_c5/f1p0/1790
R01_cb5297_c6/f1p0/974	NA	R01_cb5297_c6/f1p0/974	NA	NA
R01_cb8751_c5/f1p0/2014	NA	NA	NA	R01_cb8751_c5/f1p0/2014
R01_cb13545_c54/f1p0/961	NA	R01_cb13545_c54/f1p0/961	NA	R01_cb13545_c54/f1p0/961
R01_cb13490_c7/f1p0/867	R01_cb13490_c7/f1p0/867	R01_cb13490_c7/f1p0/867	R01_cb13490_c7/f1p0/867	R01_cb13490_c7/f1p0/867
R01_cb10104_c4/f1p0/1231	NA	NA	NA	R01_cb10104_c4/f1p0/1231
R01_cb7035_c2/f1p0/2020	NA	R01_cb7035_c2/f1p0/2020	NA	NA
R01_cb8564_c157784/f1p1/2430	NA	R01_cb8564_c157784/f1p1/2430	NA	NA
R01_cb4269_c3/f2p0/2105	NA	NA	NA	R01_cb4269_c3/f2p0/2105
R01_cb11202_c1/f1p0/3445	R01_cb11202_c1/f1p0/3445	R01_cb11202_c1/f1p0/3445	NA	NA
R01_cb8564_c121885/f1p0/2853	NA	R01_cb8564_c121885/f1p0/2853	R01_cb8564_c121885/f1p0/2853	R01_cb8564_c121885/f1p0/2853
R01_cb5720_c4/f1p0/2945	NA	NA	NA	R01_cb5720_c4/f1p0/2945
R01_cb6870_c2/f1p0/1399	R01_cb6870_c2/f1p0/1399	NA	NA	NA
R01_cb14882_c5/f1p0/1070	NA	R01_cb14882_c5/f1p0/1070	R01_cb14882_c5/f1p0/1070	NA
R01_cb4233_c31/f3p0/2762	NA	NA	R01_cb4233_c31/f3p0/2762	R01_cb4233_c31/f3p0/2762
R01_cb17053_c5/f1p0/561	R01_cb17053_c5/f1p0/561	R01_cb17053_c5/f1p0/561	R01_cb17053_c5/f1p0/561	R01_cb17053_c5/f1p0/561
R01_cb10054_c35/f1p1/579	NA	R01_cb10054_c35/f1p1/579	NA	R01_cb10054_c35/f1p1/579
R01_cb1095_c4/f1p0/3876	NA	NA	NA	R01_cb1095_c4/f1p0/3876
R01_cb10758_c3/f1p0/1980	NA	NA	NA	R01_cb10758_c3/f1p0/1980
R01_cb3426_c10/f1p0/2226	NA	NA	NA	R01_cb3426_c10/f1p0/2226
R01_cb4657_c13/f1p0/2324	NA	R01_cb4657_c13/f1p0/2324	R01_cb4657_c13/f1p0/2324	NA

R01_cb8564_c77153/f1p0/2697	NA	R01_cb8564_c77153/f1p0/2697	NA	NA
R01_cb8564_c122654/f1p0/3796	NA	R01_cb8564_c122654/f1p0/3796	NA	NA
R01_cb6732_c24/f1p0/979	NA	R01_cb6732_c24/f1p0/979	NA	NA
R01_cb10077_c8/f1p1/1157	NA	R01_cb10077_c8/f1p1/1157	NA	NA
R01_cb8564_c1264/f1p0/2196	NA	R01_cb8564_c1264/f1p0/2196	NA	R01_cb8564_c1264/f1p0/2196
R01_cb3483_c11/f1p0/2887	NA	NA	NA	R01_cb3483_c11/f1p0/2887
R01_cb2223_c4/f1p0/1184	NA	NA	R01_cb2223_c4/f1p0/1184	R01_cb2223_c4/f1p0/1184
R01_cb8250_c0/f1p0/2424	R01_cb8250_c0/f1p0/2424	R01_cb8250_c0/f1p0/2424	R01_cb8250_c0/f1p0/2424	R01_cb8250_c0/f1p0/2424
R01_cb6948_c2/f1p0/2724	NA	R01_cb6948_c2/f1p0/2724	NA	R01_cb6948_c2/f1p0/2724
R01_cb14019_c0/f1p0/1520	NA	R01_cb14019_c0/f1p0/1520	R01_cb14019_c0/f1p0/1520	R01_cb14019_c0/f1p0/1520
R01_cb13463_c19/f10p1/1392	NA	NA	NA	R01_cb13463_c19/f10p1/1392
R01_cb17910_c1/f1p0/1156	NA	R01_cb17910_c1/f1p0/1156	NA	R01_cb17910_c1/f1p0/1156
R01_cb2765_c4/f1p0/2896	NA	NA	NA	R01_cb2765_c4/f1p0/2896
R01_cb10687_c0/f4p0/1528	NA	NA	NA	R01_cb10687_c0/f4p0/1528
R01_cb5911_c0/f3p1/1643	NA	NA	NA	R01_cb5911_c0/f3p1/1643
R01_cb18456_c1820/f1p0/543	NA	NA	NA	R01_cb18456_c1820/f1p0/543
R01_cb8298_c5/f1p0/707	R01_cb8298_c5/f1p0/707	R01_cb8298_c5/f1p0/707	R01_cb8298_c5/f1p0/707	R01_cb8298_c5/f1p0/707
R01_cb8564_c77543/f1p0/2600	NA	R01_cb8564_c77543/f1p0/2600	NA	R01_cb8564_c77543/f1p0/2600
R01_cb8564_c89616/f1p0/2465	NA	NA	NA	R01_cb8564_c89616/f1p0/2465
R01_cb8564_c69453/f1p0/3058	R01_cb8564_c69453/f1p0/3058	R01_cb8564_c69453/f1p0/3058	R01_cb8564_c69453/f1p0/3058	R01_cb8564_c69453/f1p0/3058
R01_cb2378_c13/f1p1/3463	NA	NA	NA	R01_cb2378_c13/f1p1/3463
R01_cb8564_c107827/f2p0/2110	NA	R01_cb8564_c107827/f2p0/2110	R01_cb8564_c107827/f2p0/2110	R01_cb8564_c107827/f2p0/2110
R01_cb5963_c1/f1p0/2966	NA	R01_cb5963_c1/f1p0/2966	NA	NA

R01_cb17904_c2/f1p0/724	R01_cb17904_c2/f1p0/724	R01_cb17904_c2/f1p0/724	R01_cb17904_c2/f1p0/724	R01_cb17904_c2/f1p0/724
R01_cb18456_c3695/f1p0/1345	NA	R01_cb18456_c3695/f1p0/1345	NA	R01_cb18456_c3695/f1p0/1345
R01_cb16617_c2/f2p0/1612	NA	NA	NA	R01_cb16617_c2/f2p0/1612
R01_cb17703_c1/f1p0/713	R01_cb17703_c1/f1p0/713	R01_cb17703_c1/f1p0/713	R01_cb17703_c1/f1p0/713	R01_cb17703_c1/f1p0/713
R01_cb12579_c2/f2p1/987	R01_cb12579_c2/f2p1/987	R01_cb12579_c2/f2p1/987	R01_cb12579_c2/f2p1/987	R01_cb12579_c2/f2p1/987
R01_cb8564_c121924/f1p0/2905	NA	R01_cb8564_c121924/f1p0/2905	NA	NA
R01_cb5900_c73/f1p0/3430	NA	R01_cb5900_c73/f1p0/3430	NA	R01_cb5900_c73/f1p0/3430
R01_cb6523_c9/f1p0/2498	NA	NA	NA	R01_cb6523_c9/f1p0/2498
R01_cb6615_c10/f1p0/2408	NA	NA	NA	R01_cb6615_c10/f1p0/2408
R01_cb15760_c0/f1p0/441	R01_cb15760_c0/f1p0/441	R01_cb15760_c0/f1p0/441	R01_cb15760_c0/f1p0/441	R01_cb15760_c0/f1p0/441
R01_cb14107_c1/f1p0/1327	NA	NA	R01_cb14107_c1/f1p0/1327	R01_cb14107_c1/f1p0/1327
R01_cb6874_c4/f1p0/2343	NA	NA	NA	R01_cb6874_c4/f1p0/2343
R01_cb13914_c7/f1p0/753	NA	R01_cb13914_c7/f1p0/753	NA	R01_cb13914_c7/f1p0/753
R01_cb402_c24/f1p1/3130	NA	R01_cb402_c24/f1p1/3130	NA	R01_cb402_c24/f1p1/3130
R01_cb8279_c2/f1p0/2382	NA	R01_cb8279_c2/f1p0/2382	R01_cb8279_c2/f1p0/2382	R01_cb8279_c2/f1p0/2382
R01_cb11966_c10/f1p0/1567	R01_cb11966_c10/f1p0/1567	R01_cb11966_c10/f1p0/1567	NA	NA
R01_cb7810_c4/f1p0/773	R01_cb7810_c4/f1p0/773	R01_cb7810_c4/f1p0/773	R01_cb7810_c4/f1p0/773	NA
R01_cb14502_c15/f1p0/1205	NA	R01_cb14502_c15/f1p0/1205	NA	R01_cb14502_c15/f1p0/1205
R01_cb8564_c11345/f1p1/2994	NA	R01_cb8564_c11345/f1p1/2994	NA	NA
R01_cb18319_c1/f1p0/1509	NA	NA	NA	R01_cb18319_c1/f1p0/1509
R01_cb2141_c1/f1p0/1910	R01_cb2141_c1/f1p0/1910	R01_cb2141_c1/f1p0/1910	R01_cb2141_c1/f1p0/1910	R01_cb2141_c1/f1p0/1910
R01_cb1488_c4/f1p0/2489	NA	R01_cb1488_c4/f1p0/2489	NA	NA
R01_cb3699_c5/f1p0/2467	NA	R01_cb3699_c5/f1p0/2467	NA	NA
R01_cb17912_c1/f1p0/1460	NA	NA	R01_cb17912_c1/f1p0/1460	NA
R01_cb8564_c79053/f1p0/3219	R01_cb8564_c79053/f1p0/321	R01_cb8564_c79053/f1p0/3219	R01_cb8564_c79053/f1p0/3219	R01_cb8564_c79053/f1p0/3219

	9			
R01_cb8564_c120809/f2p0/2596	R01_cb8564_c120809/f2p0/2596	R01_cb8564_c120809/f2p0/2596	R01_cb8564_c120809/f2p0/2596	R01_cb8564_c120809/f2p0/2596
R01_cb4603_c3/f1p0/2973	NA	R01_cb4603_c3/f1p0/2973	NA	R01_cb4603_c3/f1p0/2973
R01_cb10711_c2/f1p0/1004	NA	R01_cb10711_c2/f1p0/1004	NA	NA
R01_cb10310_c4/f1p1/1855	NA	NA	NA	R01_cb10310_c4/f1p1/1855
R01_cb8564_c45015/f1p0/2577	R01_cb8564_c45015/f1p0/2577	R01_cb8564_c45015/f1p0/2577	NA	NA
R01_cb8564_c48083/f1p0/3187	NA	R01_cb8564_c48083/f1p0/3187	NA	NA
R01_cb18456_c4795/f1p0/515	NA	NA	NA	R01_cb18456_c4795/f1p0/515
R01_cb6028_c3/f1p0/2608	NA	R01_cb6028_c3/f1p0/2608	NA	NA
R01_cb8564_c75436/f1p0/3300	NA	R01_cb8564_c75436/f1p0/3300	NA	NA
R01_cb5165_c5/f1p0/2592	NA	R01_cb5165_c5/f1p0/2592	NA	NA
R01_cb16958_c2/f1p0/1491	NA	NA	NA	R01_cb16958_c2/f1p0/1491
R01_cb8564_c47861/f1p0/2233	R01_cb8564_c47861/f1p0/2233	R01_cb8564_c47861/f1p0/2233	R01_cb8564_c47861/f1p0/2233	R01_cb8564_c47861/f1p0/2233
R01_cb1894_c8/f1p0/1469	NA	NA	NA	R01_cb1894_c8/f1p0/1469
R01_cb8564_c74430/f1p0/2728	NA	R01_cb8564_c74430/f1p0/2728	NA	NA
R01_cb8564_c15127/f1p0/4930	NA	R01_cb8564_c15127/f1p0/4930	NA	R01_cb8564_c15127/f1p0/4930
R01_cb16598_c1/f1p0/737	R01_cb16598_c1/f1p0/737	R01_cb16598_c1/f1p0/737	R01_cb16598_c1/f1p0/737	R01_cb16598_c1/f1p0/737
R01_cb7548_c20/f1p0/2052	NA	R01_cb7548_c20/f1p0/2052	NA	NA
R01_cb10226_c0/f1p0/762	NA	R01_cb10226_c0/f1p0/762	R01_cb10226_c0/f1p0/762	R01_cb10226_c0/f1p0/762
R01_cb3929_c6/f1p0/1893	NA	R01_cb3929_c6/f1p0/1893	NA	NA
R01_cb4627_c2/f1p0/2517	NA	NA	NA	R01_cb4627_c2/f1p0/2517
R01_cb11126_c2/f1p0/664	NA	R01_cb11126_c2/f1p0/664	NA	NA
R01_cb14228_c14/f4p1/682	NA	NA	NA	R01_cb14228_c14/f4p1/682

R01_cb18081_c1/f1p0/807	NA	R01_cb18081_c1/f1p0/807	NA	NA
R01_cb12315_c2/f1p0/902	NA	R01_cb12315_c2/f1p0/902	NA	R01_cb12315_c2/f1p0/902
R01_cb8564_c24523/f1p0/2612	NA	R01_cb8564_c24523/f1p0/2612	NA	R01_cb8564_c24523/f1p0/2612
R01_cb8564_c5259/f1p0/3208	NA	R01_cb8564_c5259/f1p0/3208	NA	R01_cb8564_c5259/f1p0/3208
R01_cb17300_c0/f1p1/941	NA	NA	NA	R01_cb17300_c0/f1p1/941
R01_cb12003_c86/f1p0/1356	NA	R01_cb12003_c86/f1p0/1356	NA	NA
R01_cb2568_c2/f1p0/4726	NA	NA	R01_cb2568_c2/f1p0/4726	R01_cb2568_c2/f1p0/4726
R01_cb8564_c34938/f1p0/4052	R01_cb8564_c34938/f1p0/4052	R01_cb8564_c34938/f1p0/4052	R01_cb8564_c34938/f1p0/4052	R01_cb8564_c34938/f1p0/4052
R01_cb8564_c19247/f1p0/4989	NA	NA	R01_cb8564_c19247/f1p0/4989	NA
R01_cb14415_c0/f2p0/990	NA	NA	NA	R01_cb14415_c0/f2p0/990
R01_cb8564_c3780/f1p0/2807	NA	NA	NA	R01_cb8564_c3780/f1p0/2807
R01_cb8564_c1205/f1p0/2486	NA	R01_cb8564_c1205/f1p0/2486	NA	NA
R01_cb7742_c11/f1p0/2033	NA	R01_cb7742_c11/f1p0/2033	NA	NA
R01_cb7795_c1/f2p1/2486	NA	NA	NA	R01_cb7795_c1/f2p1/2486
R01_cb2229_c78/f1p0/1470	NA	NA	NA	R01_cb2229_c78/f1p0/1470
R01_cb2398_c0/f2p0/2375	NA	NA	NA	R01_cb2398_c0/f2p0/2375
R01_cb12577_c7/f1p0/775	NA	NA	NA	R01_cb12577_c7/f1p0/775
R01_cb7565_c1/f1p0/2723	NA	NA	R01_cb7565_c1/f1p0/2723	R01_cb7565_c1/f1p0/2723
R01_cb8926_c0/f1p0/2232	NA	R01_cb8926_c0/f1p0/2232	NA	R01_cb8926_c0/f1p0/2232
R01_cb14949_c3/f1p0/1027	NA	R01_cb14949_c3/f1p0/1027	NA	NA
R01_cb8564_c89643/f1p0/2101	NA	R01_cb8564_c89643/f1p0/2101	NA	R01_cb8564_c89643/f1p0/2101
R01_cb3874_c1/f1p0/3050	NA	R01_cb3874_c1/f1p0/3050	R01_cb3874_c1/f1p0/3050	R01_cb3874_c1/f1p0/3050
R01_cb8564_c15236/f1p0/3360	R01_cb8564_c15236/f1p0/3360	R01_cb8564_c15236/f1p0/3360	R01_cb8564_c15236/f1p0/3360	R01_cb8564_c15236/f1p0/3360
R01_cb12579_c8/f1p0/539	R01_cb12579_c8/f1p0/539	R01_cb12579_c8/f1p0/539	R01_cb12579_c8/f1p0/539	R01_cb12579_c8/f1p0/539

R01_cb14726_c0/f1p0/1005	NA	NA	NA	R01_cb14726_c0/f1p0/1005
R01_cb5896_c45/f1p0/2541	R01_cb5896_c45/f1p0/2541	R01_cb5896_c45/f1p0/2541	NA	R01_cb5896_c45/f1p0/2541
R01_cb6272_c6/f1p2/2581	NA	NA	NA	R01_cb6272_c6/f1p2/2581
R01_cb11473_c2/f1p0/1478	NA	NA	NA	R01_cb11473_c2/f1p0/1478
R01_cb1458_c1/f1p0/4317	NA	R01_cb1458_c1/f1p0/4317	NA	NA
R01_cb644_c12/f1p0/1004	NA	NA	NA	R01_cb644_c12/f1p0/1004
R01_cb18595_c1/f1p0/1656	NA	NA	NA	R01_cb18595_c1/f1p0/1656
R01_cb1813_c10/f1p0/2610	NA	NA	NA	R01_cb1813_c10/f1p0/2610
R01_cb18456_c7227/f1p0/608	R01_cb18456_c7227/f1p0/608	R01_cb18456_c7227/f1p0/608	R01_cb18456_c7227/f1p0/608	R01_cb18456_c7227/f1p0/608
R01_cb646_c0/f1p0/4686	NA	NA	NA	R01_cb646_c0/f1p0/4686
R01_cb14322_c2/f1p1/1273	NA	R01_cb14322_c2/f1p1/1273	R01_cb14322_c2/f1p1/1273	R01_cb14322_c2/f1p1/1273
R01_cb3320_c6/f1p0/3548	NA	R01_cb3320_c6/f1p0/3548	NA	R01_cb3320_c6/f1p0/3548
R01_cb4836_c3/f1p0/3245	NA	NA	NA	R01_cb4836_c3/f1p0/3245
R01_cb5394_c0/f5p0/2782	NA	NA	NA	R01_cb5394_c0/f5p0/2782
R01_cb1856_c2/f1p0/2572	NA	R01_cb1856_c2/f1p0/2572	NA	R01_cb1856_c2/f1p0/2572
R01_cb18740_c1/f1p1/821	NA	R01_cb18740_c1/f1p1/821	NA	NA
R01_cb16981_c0/f10p1/740	NA	NA	NA	R01_cb16981_c0/f10p1/740
R01_cb8564_c68327/f5p0/4045	NA	NA	NA	R01_cb8564_c68327/f5p0/4045
R01_cb6969_c4/f1p0/3739	NA	R01_cb6969_c4/f1p0/3739	NA	NA
R01_cb13545_c44/f1p0/601	NA	R01_cb13545_c44/f1p0/601	R01_cb13545_c44/f1p0/601	R01_cb13545_c44/f1p0/601
R01_cb2806_c5/f1p0/2104	NA	NA	NA	R01_cb2806_c5/f1p0/2104
R01_cb8564_c86701/f1p0/2895	NA	R01_cb8564_c86701/f1p0/2895	NA	NA
R01_cb4981_c2/f1p0/3208	NA	R01_cb4981_c2/f1p0/3208	NA	R01_cb4981_c2/f1p0/3208
R01_cb17381_c8/f1p0/525	NA	R01_cb17381_c8/f1p0/525	NA	R01_cb17381_c8/f1p0/525
R01_cb8612_c1/f1p0/2818	R01_cb8612_c1/f1p0/2818	R01_cb8612_c1/f1p0/2818	R01_cb8612_c1/f1p0/2818	R01_cb8612_c1/f1p0/2818
R01_cb6874_c9/f2p0/2438	NA	NA	NA	R01_cb6874_c9/f2p0/2438

R01_cb4603_c1/f1p2/3272	NA	R01_cb4603_c1/f1p2/3272	NA	R01_cb4603_c1/f1p2/3272
R01_cb11469_c1/f1p0/2941	NA	NA	NA	R01_cb11469_c1/f1p0/2941
R01_cb7876_c8/f1p1/703	NA	R01_cb7876_c8/f1p1/703	NA	NA
R01_cb18387_c1/f1p0/351	R01_cb18387_c1/f1p0/351	R01_cb18387_c1/f1p0/351	R01_cb18387_c1/f1p0/351	R01_cb18387_c1/f1p0/351
R01_cb17342_c1/f1p0/499	NA	R01_cb17342_c1/f1p0/499	NA	R01_cb17342_c1/f1p0/499
R01_cb11762_c1/f1p0/2990	NA	R01_cb11762_c1/f1p0/2990	NA	NA
R01_cb1228_c54/f4p0/2725	NA	NA	NA	R01_cb1228_c54/f4p0/2725
R01_cb16340_c0/f4p0/514	R01_cb16340_c0/f4p0/514	R01_cb16340_c0/f4p0/514	R01_cb16340_c0/f4p0/514	NA
R01_cb6729_c0/f1p0/2776	NA	NA	NA	R01_cb6729_c0/f1p0/2776
R01_cb10917_c3/f1p0/974	NA	R01_cb10917_c3/f1p0/974	NA	R01_cb10917_c3/f1p0/974
R01_cb8564_c78447/f1p0/3003	NA	R01_cb8564_c78447/f1p0/3003	NA	NA
R01_cb6530_c27/f1p0/2468	NA	R01_cb6530_c27/f1p0/2468	NA	NA
R01_cb6802_c83/f1p0/2222	NA	R01_cb6802_c83/f1p0/2222	NA	NA
R01_cb13045_c8/f1p0/1304	R01_cb13045_c8/f1p0/1304	R01_cb13045_c8/f1p0/1304	R01_cb13045_c8/f1p0/1304	R01_cb13045_c8/f1p0/1304
R01_cb8564_c46605/f1p0/2240	NA	NA	NA	R01_cb8564_c46605/f1p0/2240
R01_cb8564_c122187/f1p0/3216	NA	R01_cb8564_c122187/f1p0/3216	R01_cb8564_c122187/f1p0/3216	R01_cb8564_c122187/f1p0/3216
R01_cb8564_c71605/f1p0/1934	NA	R01_cb8564_c71605/f1p0/1934	R01_cb8564_c71605/f1p0/1934	R01_cb8564_c71605/f1p0/1934
R01_cb8564_c2877/f1p0/3298	NA	NA	R01_cb8564_c2877/f1p0/3298	NA
R01_cb12587_c5/f1p0/1700	NA	NA	NA	R01_cb12587_c5/f1p0/1700
R01_cb2676_c0/f31p2/2795	NA	NA	NA	R01_cb2676_c0/f31p2/2795
R01_cb8564_c115494/f1p0/3548	NA	NA	NA	R01_cb8564_c115494/f1p0/3548
R01_cb8174_c2/f1p0/2443	NA	R01_cb8174_c2/f1p0/2443	NA	NA
R01_cb18456_c5406/f1p2/817	NA	R01_cb18456_c5406/f1p2/817	NA	NA
R01_cb3808_c4/f1p0/1543	NA	NA	NA	R01_cb3808_c4/f1p0/1543

R01_cb16629_c2/f1p0/780	NA	R01_cb16629_c2/f1p0/780	NA	R01_cb16629_c2/f1p0/780
R01_cb11157_c1/f1p0/3224	NA	R01_cb11157_c1/f1p0/3224	NA	NA
R01_cb11401_c0/f3p1/783	NA	NA	R01_cb11401_c0/f3p1/783	R01_cb11401_c0/f3p1/783
R01_cb105_c29/f1p0/887	NA	R01_cb105_c29/f1p0/887	R01_cb105_c29/f1p0/887	R01_cb105_c29/f1p0/887
R01_cb10034_c59/f1p0/571	NA	R01_cb10034_c59/f1p0/571	NA	NA
R01_cb2406_c1/f1p0/3763	NA	R01_cb2406_c1/f1p0/3763	NA	NA
R01_cb8564_c119292/f1p0/4478	NA	R01_cb8564_c119292/f1p0/4478	R01_cb8564_c119292/f1p0/4478	R01_cb8564_c119292/f1p0/4478
R01_cb8946_c1/f1p1/2231	NA	NA	NA	R01_cb8946_c1/f1p1/2231
R01_cb17780_c1/f1p0/484	R01_cb17780_c1/f1p0/484	R01_cb17780_c1/f1p0/484	R01_cb17780_c1/f1p0/484	R01_cb17780_c1/f1p0/484
R01_cb18456_c1714/f2p0/404	R01_cb18456_c1714/f2p0/404	NA	R01_cb18456_c1714/f2p0/404	R01_cb18456_c1714/f2p0/404
R01_cb18456_c3319/f1p0/572	R01_cb18456_c3319/f1p0/572	R01_cb18456_c3319/f1p0/572	R01_cb18456_c3319/f1p0/572	NA
R01_cb13170_c2/f1p0/565	R01_cb13170_c2/f1p0/565	R01_cb13170_c2/f1p0/565	NA	R01_cb13170_c2/f1p0/565
R01_cb8564_c115281/f2p0/2239	NA	R01_cb8564_c115281/f2p0/2239	R01_cb8564_c115281/f2p0/2239	NA
R01_cb11895_c2/f1p0/1425	NA	NA	NA	R01_cb11895_c2/f1p0/1425
R01_cb16616_c2/f1p0/625	NA	NA	R01_cb16616_c2/f1p0/625	NA
R01_cb17390_c0/f1p0/833	R01_cb17390_c0/f1p0/833	R01_cb17390_c0/f1p0/833	R01_cb17390_c0/f1p0/833	R01_cb17390_c0/f1p0/833
R01_cb8564_c72284/f1p1/2943	R01_cb8564_c72284/f1p1/2943	R01_cb8564_c72284/f1p1/2943	R01_cb8564_c72284/f1p1/2943	R01_cb8564_c72284/f1p1/2943
R01_cb8564_c5001/f2p1/3690	NA	R01_cb8564_c5001/f2p1/3690	NA	NA
R01_cb5477_c0/f1p0/3078	NA	NA	NA	R01_cb5477_c0/f1p0/3078
R01_cb4935_c20/f1p0/3089	NA	NA	R01_cb4935_c20/f1p0/3089	R01_cb4935_c20/f1p0/3089
R01_cb2804_c150/f1p0/2890	NA	NA	R01_cb2804_c150/f1p0/2890	R01_cb2804_c150/f1p0/2890
R01_cb10272_c31/f7p1/1650	NA	NA	NA	R01_cb10272_c31/f7p1/1650
R01_cb8564_c24340/f1p0/3304	NA	R01_cb8564_c24340/f1p0/3304	NA	NA

R01_cb10453_c6/f1p0/1181	NA	NA	R01_cb10453_c6/f1p0/1181	R01_cb10453_c6/f1p0/1181
R01_cb8564_c4498/f1p0/2985	NA	R01_cb8564_c4498/f1p0/2985	R01_cb8564_c4498/f1p0/2985	R01_cb8564_c4498/f1p0/2985
R01_cb17430_c2/f1p0/830	R01_cb17430_c2/f1p0/830	R01_cb17430_c2/f1p0/830	R01_cb17430_c2/f1p0/830	R01_cb17430_c2/f1p0/830
R01_cb6802_c21/f1p0/2458	R01_cb6802_c21/f1p0/2458	R01_cb6802_c21/f1p0/2458	NA	NA
R01_cb11258_c0/f1p0/1546	R01_cb11258_c0/f1p0/1546	R01_cb11258_c0/f1p0/1546	R01_cb11258_c0/f1p0/1546	R01_cb11258_c0/f1p0/1546
R01_cb10406_c0/f1p0/575	R01_cb10406_c0/f1p0/575	R01_cb10406_c0/f1p0/575	NA	NA
R01_cb3703_c3/f1p0/2083	NA	NA	NA	R01_cb3703_c3/f1p0/2083
R01_cb6086_c0/f1p0/2939	NA	R01_cb6086_c0/f1p0/2939	NA	R01_cb6086_c0/f1p0/2939
R01_cb8564_c34103/f12p2/3309	NA	NA	NA	R01_cb8564_c34103/f12p2/3309
R01_cb10668_c0/f8p0/647	NA	R01_cb10668_c0/f8p0/647	NA	NA
R01_cb10335_c2/f2p0/1857	NA	NA	NA	R01_cb10335_c2/f2p0/1857
R01_cb6701_c1/f1p0/2672	NA	R01_cb6701_c1/f1p0/2672	NA	R01_cb6701_c1/f1p0/2672
R01_cb3359_c25/f1p0/3112	NA	R01_cb3359_c25/f1p0/3112	NA	NA
R01_cb17065_c1/f1p0/1136	NA	R01_cb17065_c1/f1p0/1136	NA	R01_cb17065_c1/f1p0/1136
R01_cb2594_c1/f1p0/2733	NA	R01_cb2594_c1/f1p0/2733	NA	NA
R01_cb8564_c3689/f1p0/2354	R01_cb8564_c3689/f1p0/2354	R01_cb8564_c3689/f1p0/2354	R01_cb8564_c3689/f1p0/2354	R01_cb8564_c3689/f1p0/2354
R01_cb16443_c1/f1p0/356	R01_cb16443_c1/f1p0/356	R01_cb16443_c1/f1p0/356	R01_cb16443_c1/f1p0/356	R01_cb16443_c1/f1p0/356
R01_cb8564_c112458/f1p0/2415	NA	NA	NA	R01_cb8564_c112458/f1p0/2415
R01_cb10133_c5/f1p0/1576	NA	NA	NA	R01_cb10133_c5/f1p0/1576
R01_cb673_c5/f1p0/815	NA	R01_cb673_c5/f1p0/815	NA	R01_cb673_c5/f1p0/815
R01_cb6199_c22/f1p0/2552	NA	NA	NA	R01_cb6199_c22/f1p0/2552
R01_cb7985_c1/f1p0/2489	NA	R01_cb7985_c1/f1p0/2489	R01_cb7985_c1/f1p0/2489	NA
R01_cb8564_c112668/f1p0/4604	NA	R01_cb8564_c112668/f1p0/4604	NA	NA

R01_cb12680_c14/f1p0/1870	NA	R01_cb12680_c14/f1p0/1870	NA	NA
R01_cb8564_c75611/f1p0/3438	NA	R01_cb8564_c75611/f1p0/3438	NA	NA
R01_cb18409_c54/f1p0/1141	NA	NA	NA	R01_cb18409_c54/f1p0/1141
R01_cb11433_c1/f1p0/3176	NA	NA	NA	R01_cb11433_c1/f1p0/3176
R01_cb12785_c36/f1p0/1093	NA	NA	R01_cb12785_c36/f1p0/1093	NA
R01_cb5229_c7/f2p1/1037	NA	NA	NA	R01_cb5229_c7/f2p1/1037
R01_cb14559_c5/f1p0/1507	NA	R01_cb14559_c5/f1p0/1507	NA	NA
R01_cb10074_c10/f1p0/450	R01_cb10074_c10/f1p0/450	R01_cb10074_c10/f1p0/450	R01_cb10074_c10/f1p0/450	R01_cb10074_c10/f1p0/450
R01_cb14661_c1/f1p0/1501	NA	R01_cb14661_c1/f1p0/1501	NA	NA
R01_cb8564_c21231/f1p0/2328	NA	R01_cb8564_c21231/f1p0/2328	NA	NA
R01_cb2253_c4/f1p0/2680	R01_cb2253_c4/f1p0/2680	R01_cb2253_c4/f1p0/2680	NA	NA
R01_cb18191_c0/f1p0/1475	NA	NA	NA	R01_cb18191_c0/f1p0/1475
R01_cb8564_c118417/f1p1/3305	NA	R01_cb8564_c118417/f1p1/3305	NA	NA
R01_cb6591_c9/f1p0/645	R01_cb6591_c9/f1p0/645	R01_cb6591_c9/f1p0/645	NA	NA
R01_cb8564_c21697/f1p3/3741	NA	NA	NA	R01_cb8564_c21697/f1p3/3741
R01_cb11879_c6/f3p0/1734	NA	NA	NA	R01_cb11879_c6/f3p0/1734
R01_cb8564_c44628/f1p0/2828	NA	R01_cb8564_c44628/f1p0/2828	R01_cb8564_c44628/f1p0/2828	NA
R01_cb4584_c3/f3p0/3070	NA	NA	NA	R01_cb4584_c3/f3p0/3070
R01_cb9318_c0/f1p0/2116	NA	NA	NA	R01_cb9318_c0/f1p0/2116
R01_cb9612_c3/f1p0/1109	NA	NA	NA	R01_cb9612_c3/f1p0/1109
R01_cb18735_c1/f1p0/2075	NA	R01_cb18735_c1/f1p0/2075	NA	NA
R01_cb11645_c1/f1p0/2790	NA	R01_cb11645_c1/f1p0/2790	NA	R01_cb11645_c1/f1p0/2790
R01_cb17779_c1/f1p0/1874	R01_cb17779_c1/f1p0/1874	R01_cb17779_c1/f1p0/1874	NA	NA
R01_cb18393_c0/f1p0/1184	R01_cb18393_c0/f1p0/1184	R01_cb18393_c0/f1p0/1184	R01_cb18393_c0/f1p0/1184	R01_cb18393_c0/f1p0/1184
R01_cb5207_c3/f1p0/3203	R01_cb5207_c3/f1p0/3203	R01_cb5207_c3/f1p0/3203	NA	NA

R01_cb17655_c0/f7p0/538	R01_cb17655_c0/f7p0/538	R01_cb17655_c0/f7p0/538	R01_cb17655_c0/f7p0/538	R01_cb17655_c0/f7p0/538
R01_cb9165_c2/f1p0/1120	NA	R01_cb9165_c2/f1p0/1120	NA	R01_cb9165_c2/f1p0/1120
R01_cb16449_c0/f2p0/1558	NA	NA	NA	R01_cb16449_c0/f2p0/1558
R01_cb2494_c13/f1p0/954	R01_cb2494_c13/f1p0/954	R01_cb2494_c13/f1p0/954	R01_cb2494_c13/f1p0/954	R01_cb2494_c13/f1p0/954
R01_cb12273_c7/f1p0/1595	NA	NA	NA	R01_cb12273_c7/f1p0/1595
R01_cb13982_c1/f2p0/1372	NA	NA	NA	R01_cb13982_c1/f2p0/1372
R01_cb12587_c3/f1p0/1762	NA	NA	NA	R01_cb12587_c3/f1p0/1762
R01_cb16779_c2/f1p0/519	NA	R01_cb16779_c2/f1p0/519	R01_cb16779_c2/f1p0/519	R01_cb16779_c2/f1p0/519
R01_cb12223_c2/f1p0/717	NA	R01_cb12223_c2/f1p0/717	R01_cb12223_c2/f1p0/717	R01_cb12223_c2/f1p0/717
R01_cb18456_c4792/f1p1/490	NA	NA	NA	R01_cb18456_c4792/f1p1/490
R01_cb3989_c2/f1p0/3310	NA	NA	NA	R01_cb3989_c2/f1p0/3310
R01_cb2729_c2/f1p0/4076	NA	R01_cb2729_c2/f1p0/4076	NA	R01_cb2729_c2/f1p0/4076
R01_cb3000_c3/f1p0/1940	NA	R01_cb3000_c3/f1p0/1940	R01_cb3000_c3/f1p0/1940	R01_cb3000_c3/f1p0/1940
R01_cb18456_c7208/f1p0/480	R01_cb18456_c7208/f1p0/480	R01_cb18456_c7208/f1p0/480	R01_cb18456_c7208/f1p0/480	R01_cb18456_c7208/f1p0/480
R01_cb18409_c80/f1p0/437	R01_cb18409_c80/f1p0/437	R01_cb18409_c80/f1p0/437	R01_cb18409_c80/f1p0/437	R01_cb18409_c80/f1p0/437
R01_cb7954_c4/f1p0/838	NA	NA	NA	R01_cb7954_c4/f1p0/838
R01_cb9232_c0/f6p0/2143	NA	NA	NA	R01_cb9232_c0/f6p0/2143
R01_cb8564_c16989/f1p0/4696	NA	R01_cb8564_c16989/f1p0/4696	NA	NA
R01_cb16129_c6/f2p0/533	NA	R01_cb16129_c6/f2p0/533	NA	NA
R01_cb8564_c17664/f1p0/2753	NA	R01_cb8564_c17664/f1p0/2753	NA	R01_cb8564_c17664/f1p0/2753
R01_cb5351_c3/f1p0/1587	NA	NA	NA	R01_cb5351_c3/f1p0/1587
R01_cb1658_c22/f1p0/2855	NA	NA	NA	R01_cb1658_c22/f1p0/2855
R01_cb12169_c3/f1p0/345	R01_cb12169_c3/f1p0/345	R01_cb12169_c3/f1p0/345	R01_cb12169_c3/f1p0/345	R01_cb12169_c3/f1p0/345
R01_cb8564_c124936/f1p0/2356	NA	R01_cb8564_c124936/f1p0/2356	R01_cb8564_c124936/f1p0/2356	R01_cb8564_c124936/f1p0/2356
R01_cb2796_c0/f2p0/3781	R01_cb2796_c0/f2p0/3781	R01_cb2796_c0/f2p0/3781	NA	R01_cb2796_c0/f2p0/3781

R01_cb1340_c1/f1p0/4356	NA	R01_cb1340_c1/f1p0/4356	NA	NA
R01_cb279_c9/f1p0/4717	NA	NA	NA	R01_cb279_c9/f1p0/4717
R01_cb16739_c2/f2p0/666	NA	R01_cb16739_c2/f2p0/666	NA	R01_cb16739_c2/f2p0/666
R01_cb14550_c0/f1p0/851	R01_cb14550_c0/f1p0/851	R01_cb14550_c0/f1p0/851	R01_cb14550_c0/f1p0/851	R01_cb14550_c0/f1p0/851
R01_cb5896_c56/f1p0/1933	NA	NA	NA	R01_cb5896_c56/f1p0/1933
R01_cb18334_c0/f1p0/1680	NA	NA	NA	R01_cb18334_c0/f1p0/1680
R01_cb2027_c9/f1p0/2753	NA	R01_cb2027_c9/f1p0/2753	R01_cb2027_c9/f1p0/2753	R01_cb2027_c9/f1p0/2753
R01_cb13510_c10/f1p0/651	NA	R01_cb13510_c10/f1p0/651	NA	NA
R01_cb8564_c19896/f1p0/2705	R01_cb8564_c19896/f1p0/2705	R01_cb8564_c19896/f1p0/2705	R01_cb8564_c19896/f1p0/2705	R01_cb8564_c19896/f1p0/2705
R01_cb8564_c111745/f1p0/2411	NA	R01_cb8564_c111745/f1p0/2411	NA	R01_cb8564_c111745/f1p0/2411
R01_cb535_c2/f1p0/4696	NA	NA	NA	R01_cb535_c2/f1p0/4696
R01_cb8564_c90005/f1p0/3577	R01_cb8564_c90005/f1p0/3577	R01_cb8564_c90005/f1p0/3577	NA	NA
R01_cb18158_c0/f1p0/838	NA	R01_cb18158_c0/f1p0/838	NA	NA
R01_cb8564_c117576/f1p0/2529	NA	R01_cb8564_c117576/f1p0/2529	R01_cb8564_c117576/f1p0/2529	R01_cb8564_c117576/f1p0/2529
R01_cb17381_c0/f7p1/774	NA	NA	NA	R01_cb17381_c0/f7p1/774
R01_cb4490_c7/f1p0/3020	NA	NA	R01_cb4490_c7/f1p0/3020	R01_cb4490_c7/f1p0/3020
R01_cb14566_c4/f1p0/1308	R01_cb14566_c4/f1p0/1308	R01_cb14566_c4/f1p0/1308	R01_cb14566_c4/f1p0/1308	R01_cb14566_c4/f1p0/1308
R01_cb2421_c0/f1p0/3937	NA	R01_cb2421_c0/f1p0/3937	R01_cb2421_c0/f1p0/3937	NA
R01_cb16662_c5/f1p0/522	R01_cb16662_c5/f1p0/522	R01_cb16662_c5/f1p0/522	R01_cb16662_c5/f1p0/522	R01_cb16662_c5/f1p0/522
R01_cb11212_c3/f1p0/1743	NA	R01_cb11212_c3/f1p0/1743	NA	NA
R01_cb5421_c10/f1p0/1589	NA	NA	R01_cb5421_c10/f1p0/1589	NA
R01_cb18222_c1/f1p0/436	NA	NA	NA	R01_cb18222_c1/f1p0/436

R01_cb10077_c4/f1p0/497	NA	R01_cb10077_c4/f1p0/497	NA	NA
R01_cb363_c3/f1p0/2498	NA	R01_cb363_c3/f1p0/2498	NA	R01_cb363_c3/f1p0/2498
R01_cb7215_c1/f1p0/4177	NA	NA	NA	R01_cb7215_c1/f1p0/4177
R01_cb10786_c1/f1p0/4511	NA	R01_cb10786_c1/f1p0/4511	NA	NA
R01_cb10572_c0/f2p0/1145	NA	NA	NA	R01_cb10572_c0/f2p0/1145
R01_cb3888_c11/f1p0/2128	NA	R01_cb3888_c11/f1p0/2128	NA	NA
R01_cb18456_c5323/f1p3/692	NA	R01_cb18456_c5323/f1p3/692	NA	NA
R01_cb8564_c128807/f1p0/3359	NA	NA	NA	R01_cb8564_c128807/f1p0/3359
R01_cb2822_c5/f1p0/2667	R01_cb2822_c5/f1p0/2667	R01_cb2822_c5/f1p0/2667	NA	NA
R01_cb9119_c5/f1p1/2109	NA	NA	NA	R01_cb9119_c5/f1p1/2109
R01_cb8298_c6/f1p0/575	NA	R01_cb8298_c6/f1p0/575	R01_cb8298_c6/f1p0/575	R01_cb8298_c6/f1p0/575
R01_cb3704_c8/f1p0/2187	NA	R01_cb3704_c8/f1p0/2187	NA	NA
R01_cb14238_c0/f9p1/1255	NA	NA	NA	R01_cb14238_c0/f9p1/1255
R01_cb17266_c6/f1p0/1869	R01_cb17266_c6/f1p0/1869	R01_cb17266_c6/f1p0/1869	NA	R01_cb17266_c6/f1p0/1869
R01_cb8564_c2544/f1p0/2597	NA	NA	R01_cb8564_c2544/f1p0/2597	R01_cb8564_c2544/f1p0/2597
R01_cb4702_c19/f1p0/2664	NA	R01_cb4702_c19/f1p0/2664	NA	R01_cb4702_c19/f1p0/2664
R01_cb10525_c4/f1p0/2020	NA	R01_cb10525_c4/f1p0/2020	NA	NA
R01_cb17244_c0/f2p0/556	R01_cb17244_c0/f2p0/556	NA	R01_cb17244_c0/f2p0/556	NA
R01_cb1623_c10/f1p1/4018	NA	R01_cb1623_c10/f1p1/4018	NA	NA
R01_cb7802_c6/f1p0/2531	NA	NA	NA	R01_cb7802_c6/f1p0/2531
R01_cb8564_c1334/f2p0/2671	NA	NA	NA	R01_cb8564_c1334/f2p0/2671
R01_cb3575_c11/f1p0/3124	NA	NA	NA	R01_cb3575_c11/f1p0/3124
R01_cb17593_c0/f1p0/776	NA	R01_cb17593_c0/f1p0/776	R01_cb17593_c0/f1p0/776	R01_cb17593_c0/f1p0/776
R01_cb3483_c21/f1p0/2978	NA	NA	NA	R01_cb3483_c21/f1p0/2978
R01_cb15811_c38/f1p0/917	R01_cb15811_c38/f1p0/917	R01_cb15811_c38/f1p0/917	R01_cb15811_c38/f1p0/917	R01_cb15811_c38/f1p0/917

R01_cb14997_c0/f1p0/693	NA	R01_cb14997_c0/f1p0/693	NA	NA
R01_cb7182_c28/f1p0/2182	NA	R01_cb7182_c28/f1p0/2182	NA	R01_cb7182_c28/f1p0/2182
R01_cb1119_c24/f1p0/796	R01_cb1119_c24/f1p0/796	NA	R01_cb1119_c24/f1p0/796	R01_cb1119_c24/f1p0/796
R01_cb4490_c3/f1p0/3276	NA	NA	R01_cb4490_c3/f1p0/3276	R01_cb4490_c3/f1p0/3276
R01_cb8564_c14828/f1p0/3026	NA	NA	NA	R01_cb8564_c14828/f1p0/3026
R01_cb4657_c18/f1p0/2982	NA	NA	NA	R01_cb4657_c18/f1p0/2982
R01_cb3307_c20/f1p0/4338	NA	R01_cb3307_c20/f1p0/4338	R01_cb3307_c20/f1p0/4338	NA
R01_cb7171_c13/f1p0/2388	NA	NA	NA	R01_cb7171_c13/f1p0/2388
R01_cb7530_c6/f1p1/2464	NA	R01_cb7530_c6/f1p1/2464	NA	R01_cb7530_c6/f1p1/2464
R01_cb9222_c12/f1p0/2459	NA	NA	NA	R01_cb9222_c12/f1p0/2459
R01_cb8564_c82071/f1p0/3108	R01_cb8564_c82071/f1p0/3108	R01_cb8564_c82071/f1p0/3108	NA	R01_cb8564_c82071/f1p0/3108
R01_cb3215_c20/f1p1/2217	R01_cb3215_c20/f1p1/2217	R01_cb3215_c20/f1p1/2217	NA	NA
R01_cb10567_c0/f1p0/1210	NA	R01_cb10567_c0/f1p0/1210	R01_cb10567_c0/f1p0/1210	R01_cb10567_c0/f1p0/1210
R01_cb3540_c3/f1p0/4041	NA	R01_cb3540_c3/f1p0/4041	NA	NA
R01_cb3732_c4/f2p0/2684	NA	NA	NA	R01_cb3732_c4/f2p0/2684
R01_cb8564_c89686/f1p0/4796	NA	R01_cb8564_c89686/f1p0/4796	NA	R01_cb8564_c89686/f1p0/4796
R01_cb950_c11/f2p1/4346	NA	R01_cb950_c11/f2p1/4346	R01_cb950_c11/f2p1/4346	NA
R01_cb8564_c87060/f1p0/1992	NA	R01_cb8564_c87060/f1p0/1992	R01_cb8564_c87060/f1p0/1992	R01_cb8564_c87060/f1p0/1992
R01_cb14278_c2/f2p0/819	R01_cb14278_c2/f2p0/819	R01_cb14278_c2/f2p0/819	R01_cb14278_c2/f2p0/819	R01_cb14278_c2/f2p0/819
R01_cb16162_c5/f1p1/995	NA	R01_cb16162_c5/f1p1/995	NA	NA
R01_cb4329_c9/f1p2/1405	NA	R01_cb4329_c9/f1p2/1405	NA	NA
R01_cb8564_c12139/f1p4/3380	NA	R01_cb8564_c12139/f1p4/3380	R01_cb8564_c12139/f1p4/3380	R01_cb8564_c12139/f1p4/3380
R01_cb10141_c11/f1p0/974	NA	R01_cb10141_c11/f1p0/974	NA	NA
R01_cb12757_c0/f10p0/1044	NA	NA	NA	R01_cb12757_c0/f10p0/1044
R01_cb13390_c15/f1p0/1688	NA	R01_cb13390_c15/f1p0/1688	NA	R01_cb13390_c15/f1p0/1688

R01_cb10903_c4/f1p0/5094	NA	R01_cb10903_c4/f1p0/5094	NA	R01_cb10903_c4/f1p0/5094
R01_cb859_c5/f1p0/2869	NA	NA	NA	R01_cb859_c5/f1p0/2869
R01_cb4471_c12/f1p0/2778	NA	NA	NA	R01_cb4471_c12/f1p0/2778
R01_cb18456_c6786/f1p0/559	R01_cb18456_c6786/f1p0/559	R01_cb18456_c6786/f1p0/559	NA	R01_cb18456_c6786/f1p0/559
R01_cb13532_c4/f1p0/964	R01_cb13532_c4/f1p0/964	NA	R01_cb13532_c4/f1p0/964	R01_cb13532_c4/f1p0/964
R01_cb8194_c1/f1p0/2434	NA	R01_cb8194_c1/f1p0/2434	NA	R01_cb8194_c1/f1p0/2434
R01_cb17997_c2/f2p0/452	NA	R01_cb17997_c2/f2p0/452	NA	R01_cb17997_c2/f2p0/452
R01_cb12577_c8/f1p3/988	NA	NA	NA	R01_cb12577_c8/f1p3/988
R01_cb10620_c3/f1p0/2818	NA	R01_cb10620_c3/f1p0/2818	NA	NA
R01_cb16394_c1/f1p0/765	R01_cb16394_c1/f1p0/765	NA	R01_cb16394_c1/f1p0/765	R01_cb16394_c1/f1p0/765
R01_cb18086_c0/f1p0/551	NA	R01_cb18086_c0/f1p0/551	NA	R01_cb18086_c0/f1p0/551
R01_cb1846_c32/f1p0/3091	NA	R01_cb1846_c32/f1p0/3091	NA	NA
R01_cb10679_c4/f6p0/491	R01_cb10679_c4/f6p0/491	R01_cb10679_c4/f6p0/491	NA	R01_cb10679_c4/f6p0/491
R01_cb18456_c1864/f1p0/1407	NA	R01_cb18456_c1864/f1p0/1407	NA	NA
R01_cb8564_c69012/f2p0/2088	NA	R01_cb8564_c69012/f2p0/2088	NA	NA
R01_cb18301_c6/f1p0/1748	NA	R01_cb18301_c6/f1p0/1748	R01_cb18301_c6/f1p0/1748	R01_cb18301_c6/f1p0/1748
R01_cb18456_c817/f1p1/487	R01_cb18456_c817/f1p1/487	R01_cb18456_c817/f1p1/487	NA	R01_cb18456_c817/f1p1/487
R01_cb1038_c1/f1p0/4670	NA	R01_cb1038_c1/f1p0/4670	NA	NA
R01_cb13418_c26/f1p0/5325	NA	R01_cb13418_c26/f1p0/5325	NA	NA
R01_cb8564_c114281/f1p0/2328	NA	R01_cb8564_c114281/f1p0/2328	NA	NA
R01_cb2104_c14/f1p0/702	NA	R01_cb2104_c14/f1p0/702	R01_cb2104_c14/f1p0/702	R01_cb2104_c14/f1p0/702
R01_cb8564_c13272/f1p0/3761	NA	NA	NA	R01_cb8564_c13272/f1p0/3761
R01_cb13408_c1/f1p0/1396	NA	NA	NA	R01_cb13408_c1/f1p0/1396
R01_cb302_c11/f1p1/2721	NA	NA	NA	R01_cb302_c11/f1p1/2721
R01_cb17266_c3/f1p0/1412	NA	R01_cb17266_c3/f1p0/1412	NA	NA

R01_cb18409_c62/f1p0/1807	R01_cb18409_c62/f1p0/1807	R01_cb18409_c62/f1p0/1807	R01_cb18409_c62/f1p0/1807	NA
R01_cb8409_c1/f3p1/2260	NA	NA	NA	R01_cb8409_c1/f3p1/2260
R01_cb2219_c1/f1p1/3999	NA	R01_cb2219_c1/f1p1/3999	NA	NA
R01_cb1618_c9/f1p0/2077	NA	R01_cb1618_c9/f1p0/2077	NA	NA
R01_cb8564_c117669/f1p0/3532	NA	NA	NA	R01_cb8564_c117669/f1p0/3532
R01_cb3791_c8/f1p0/1798	NA	NA	NA	R01_cb3791_c8/f1p0/1798
R01_cb2377_c13/f1p0/2163	NA	NA	NA	R01_cb2377_c13/f1p0/2163
R01_cb18409_c179/f1p0/335	R01_cb18409_c179/f1p0/335	R01_cb18409_c179/f1p0/335	R01_cb18409_c179/f1p0/335	R01_cb18409_c179/f1p0/335
R01_cb12620_c12/f1p0/1003	NA	NA	NA	R01_cb12620_c12/f1p0/1003
R01_cb2160_c7/f1p1/2805	NA	NA	NA	R01_cb2160_c7/f1p1/2805
R01_cb7962_c11/f1p1/1802	NA	NA	NA	R01_cb7962_c11/f1p1/1802
R01_cb6352_c8/f1p0/2668	R01_cb6352_c8/f1p0/2668	R01_cb6352_c8/f1p0/2668	NA	R01_cb6352_c8/f1p0/2668
R01_cb14971_c10/f1p0/320	R01_cb14971_c10/f1p0/320	R01_cb14971_c10/f1p0/320	R01_cb14971_c10/f1p0/320	R01_cb14971_c10/f1p0/320
R01_cb12148_c16/f1p0/1860	NA	R01_cb12148_c16/f1p0/1860	NA	R01_cb12148_c16/f1p0/1860
R01_cb18456_c1880/f1p0/696	R01_cb18456_c1880/f1p0/696	R01_cb18456_c1880/f1p0/696	R01_cb18456_c1880/f1p0/696	R01_cb18456_c1880/f1p0/696
R01_cb8823_c7/f1p0/2210	NA	NA	NA	R01_cb8823_c7/f1p0/2210
R01_cb18456_c7118/f1p0/860	NA	R01_cb18456_c7118/f1p0/860	NA	NA
R01_cb5151_c2/f1p0/1636	NA	NA	NA	R01_cb5151_c2/f1p0/1636
R01_cb15011_c5/f1p0/878	NA	R01_cb15011_c5/f1p0/878	NA	NA
R01_cb8564_c13067/f1p0/2126	R01_cb8564_c13067/f1p0/2126	R01_cb8564_c13067/f1p0/2126	R01_cb8564_c13067/f1p0/2126	R01_cb8564_c13067/f1p0/2126
R01_cb8564_c120663/f1p0/2553	R01_cb8564_c120663/f1p0/2553	R01_cb8564_c120663/f1p0/2553	R01_cb8564_c120663/f1p0/2553	R01_cb8564_c120663/f1p0/2553
R01_cb4128_c11/f1p0/3379	NA	NA	NA	R01_cb4128_c11/f1p0/3379
R01_cb5952_c5/f1p0/1361	NA	NA	NA	R01_cb5952_c5/f1p0/1361

R01_cb7806_c9/f1p0/1648	NA	R01_cb7806_c9/f1p0/1648	R01_cb7806_c9/f1p0/1648	R01_cb7806_c9/f1p0/1648
R01_cb14437_c5/f1p0/471	NA	R01_cb14437_c5/f1p0/471	R01_cb14437_c5/f1p0/471	NA
R01_cb7964_c4/f1p2/2820	NA	NA	NA	R01_cb7964_c4/f1p2/2820
R01_cb14573_c3/f4p0/444	R01_cb14573_c3/f4p0/444	R01_cb14573_c3/f4p0/444	NA	NA
R01_cb14409_c3/f1p0/372	NA	R01_cb14409_c3/f1p0/372	R01_cb14409_c3/f1p0/372	R01_cb14409_c3/f1p0/372
R01_cb7087_c8/f1p0/763	NA	NA	R01_cb7087_c8/f1p0/763	R01_cb7087_c8/f1p0/763
R01_cb18539_c1/f1p0/417	R01_cb18539_c1/f1p0/417	R01_cb18539_c1/f1p0/417	R01_cb18539_c1/f1p0/417	R01_cb18539_c1/f1p0/417
R01_cb8564_c48244/f1p0/4543	NA	R01_cb8564_c48244/f1p0/4543	R01_cb8564_c48244/f1p0/4543	R01_cb8564_c48244/f1p0/4543
R01_cb781_c19/f1p1/2021	NA	NA	NA	R01_cb781_c19/f1p1/2021
R01_cb18456_c2525/f1p0/1343	NA	R01_cb18456_c2525/f1p0/1343	NA	R01_cb18456_c2525/f1p0/1343
R01_cb6885_c5/f1p0/3957	NA	R01_cb6885_c5/f1p0/3957	NA	NA
R01_cb8564_c70921/f1p0/2533	NA	R01_cb8564_c70921/f1p0/2533	NA	NA
R01_cb14535_c1/f1p0/1145	NA	NA	R01_cb14535_c1/f1p0/1145	NA
R01_cb4576_c78/f1p0/2184	NA	NA	NA	R01_cb4576_c78/f1p0/2184
R01_cb8384_c2/f1p2/2308	NA	R01_cb8384_c2/f1p2/2308	NA	NA
R01_cb625_c13/f1p0/3947	R01_cb625_c13/f1p0/3947	R01_cb625_c13/f1p0/3947	R01_cb625_c13/f1p0/3947	R01_cb625_c13/f1p0/3947
R01_cb8564_c9903/f2p0/4546	NA	R01_cb8564_c9903/f2p0/4546	NA	R01_cb8564_c9903/f2p0/4546
R01_cb8134_c2/f1p0/787	NA	R01_cb8134_c2/f1p0/787	R01_cb8134_c2/f1p0/787	R01_cb8134_c2/f1p0/787
R01_cb6706_c4/f2p0/2793	NA	R01_cb6706_c4/f2p0/2793	R01_cb6706_c4/f2p0/2793	R01_cb6706_c4/f2p0/2793
R01_cb8564_c15481/f1p1/3541	NA	R01_cb8564_c15481/f1p1/3541	NA	NA
R01_cb506_c19/f1p0/4701	NA	NA	NA	R01_cb506_c19/f1p0/4701
R01_cb3732_c12/f1p1/2738	NA	NA	NA	R01_cb3732_c12/f1p1/2738
R01_cb10314_c9/f2p2/565	R01_cb10314_c9/f2p2/565	R01_cb10314_c9/f2p2/565	NA	NA
R01_cb17527_c0/f1p0/1019	R01_cb17527_c0/f1p0/1019	R01_cb17527_c0/f1p0/1019	R01_cb17527_c0/f1p0/1019	NA
R01_cb15584_c2/f1p0/1708	NA	R01_cb15584_c2/f1p0/1708	NA	NA
R01_cb18456_c1878/f1p0/471	R01_cb18456_c1878/f1p0/471	R01_cb18456_c1878/f1p0/471	R01_cb18456_c1878/f1p0/471	R01_cb18456_c1878/f1p0/471

R01_cb2104_c12/f1p0/1216	NA	NA	R01_cb2104_c12/f1p0/1216	R01_cb2104_c12/f1p0/1216
R01_cb14564_c1/f1p0/561	R01_cb14564_c1/f1p0/561	R01_cb14564_c1/f1p0/561	NA	NA
R01_cb17811_c1/f1p0/742	NA	NA	NA	R01_cb17811_c1/f1p0/742
R01_cb4602_c5/f1p0/2199	R01_cb4602_c5/f1p0/2199	R01_cb4602_c5/f1p0/2199	NA	NA
R01_cb945_c14/f1p0/2568	NA	R01_cb945_c14/f1p0/2568	NA	NA
R01_cb9365_c4/f1p1/1978	NA	NA	NA	R01_cb9365_c4/f1p1/1978
R01_cb8564_c71413/f1p0/2773	R01_cb8564_c71413/f1p0/277 3	R01_cb8564_c71413/f1p0/2773	R01_cb8564_c71413/f1p0/2773	R01_cb8564_c71413/f1p0/2773
R01_cb16672_c0/f2p0/519	R01_cb16672_c0/f2p0/519	R01_cb16672_c0/f2p0/519	R01_cb16672_c0/f2p0/519	NA
R01_cb17693_c0/f1p0/385	R01_cb17693_c0/f1p0/385	R01_cb17693_c0/f1p0/385	R01_cb17693_c0/f1p0/385	R01_cb17693_c0/f1p0/385
R01_cb10780_c1/f1p0/3248	R01_cb10780_c1/f1p0/3248	R01_cb10780_c1/f1p0/3248	R01_cb10780_c1/f1p0/3248	R01_cb10780_c1/f1p0/3248
R01_cb8970_c3/f1p0/527	R01_cb8970_c3/f1p0/527	R01_cb8970_c3/f1p0/527	R01_cb8970_c3/f1p0/527	R01_cb8970_c3/f1p0/527
R01_cb11044_c2/f1p0/1717	NA	R01_cb11044_c2/f1p0/1717	NA	NA
R01_cb10343_c5/f1p0/1879	NA	R01_cb10343_c5/f1p0/1879	NA	NA
R01_cb4489_c10/f1p0/2997	NA	R01_cb4489_c10/f1p0/2997	NA	R01_cb4489_c10/f1p0/2997
R01_cb357_c0/f12p2/905	NA	NA	NA	R01_cb357_c0/f12p2/905
R01_cb18045_c2/f1p0/491	NA	R01_cb18045_c2/f1p0/491	NA	R01_cb18045_c2/f1p0/491
R01_cb8123_c4/f1p0/3382	NA	R01_cb8123_c4/f1p0/3382	NA	NA
R01_cb16997_c68/f1p0/2309	NA	NA	NA	R01_cb16997_c68/f1p0/2309
R01_cb18409_c86/f1p0/983	R01_cb18409_c86/f1p0/983	R01_cb18409_c86/f1p0/983	R01_cb18409_c86/f1p0/983	R01_cb18409_c86/f1p0/983
R01_cb7055_c64/f1p0/1405	NA	R01_cb7055_c64/f1p0/1405	NA	NA
R01_cb4140_c9/f1p0/2412	NA	R01_cb4140_c9/f1p0/2412	NA	NA
R01_cb7409_c2/f1p0/2622	NA	R01_cb7409_c2/f1p0/2622	NA	NA
R01_cb16967_c0/f1p0/1616	NA	NA	NA	R01_cb16967_c0/f1p0/1616
R01_cb7221_c4/f1p1/2600	NA	NA	NA	R01_cb7221_c4/f1p1/2600
R01_cb6258_c15/f1p0/2539	NA	NA	NA	R01_cb6258_c15/f1p0/2539

R01_cb10972_c3/f1p0/960	NA	NA	R01_cb10972_c3/f1p0/960	R01_cb10972_c3/f1p0/960
R01_cb8669_c11/f1p0/1138	NA	NA	NA	R01_cb8669_c11/f1p0/1138
R01_cb18456_c1677/f1p0/872	NA	R01_cb18456_c1677/f1p0/872	NA	NA
R01_cb7611_c3/f1p0/2574	NA	NA	NA	R01_cb7611_c3/f1p0/2574
R01_cb7253_c9/f1p0/2565	NA	R01_cb7253_c9/f1p0/2565	NA	NA
R01_cb1626_c4/f2p1/4537	NA	NA	NA	R01_cb1626_c4/f2p1/4537
R01_cb9668_c0/f1p1/1993	NA	R01_cb9668_c0/f1p1/1993	NA	NA
R01_cb7064_c17/f1p0/3300	NA	R01_cb7064_c17/f1p0/3300	NA	NA
R01_cb1523_c2/f1p0/4204	NA	R01_cb1523_c2/f1p0/4204	NA	NA
R01_cb770_c11/f1p0/432	R01_cb770_c11/f1p0/432	R01_cb770_c11/f1p0/432	R01_cb770_c11/f1p0/432	R01_cb770_c11/f1p0/432
R01_cb9351_c0/f1p0/2102	R01_cb9351_c0/f1p0/2102	R01_cb9351_c0/f1p0/2102	R01_cb9351_c0/f1p0/2102	NA
R01_cb17600_c1/f1p0/1139	NA	R01_cb17600_c1/f1p0/1139	NA	NA
R01_cb9825_c1/f1p0/4342	NA	NA	R01_cb9825_c1/f1p0/4342	R01_cb9825_c1/f1p0/4342
R01_cb6081_c0/f2p0/2855	NA	R01_cb6081_c0/f2p0/2855	R01_cb6081_c0/f2p0/2855	R01_cb6081_c0/f2p0/2855
R01_cb12421_c63/f1p0/637	NA	R01_cb12421_c63/f1p0/637	NA	NA
R01_cb17652_c0/f1p0/1003	NA	NA	R01_cb17652_c0/f1p0/1003	R01_cb17652_c0/f1p0/1003
R01_cb8564_c121135/f1p0/2544	NA	NA	NA	R01_cb8564_c121135/f1p0/2544
R01_cb8027_c6/f1p0/2577	NA	NA	NA	R01_cb8027_c6/f1p0/2577
R01_cb12023_c1/f1p0/742	NA	NA	NA	R01_cb12023_c1/f1p0/742
R01_cb6623_c24/f1p1/2737	NA	R01_cb6623_c24/f1p1/2737	NA	NA
R01_cb14502_c18/f1p0/1655	NA	NA	R01_cb14502_c18/f1p0/1655	NA
R01_cb8564_c11389/f1p0/2269	R01_cb8564_c11389/f1p0/2269	R01_cb8564_c11389/f1p0/2269	R01_cb8564_c11389/f1p0/2269	R01_cb8564_c11389/f1p0/2269
R01_cb8564_c76824/f1p0/4260	NA	R01_cb8564_c76824/f1p0/4260	NA	R01_cb8564_c76824/f1p0/4260
R01_cb8564_c117218/f1p0/221	NA	R01_cb8564_c117218/f1p0/221	R01_cb8564_c117218/f1p0/221	NA

7		7	7	
R01_cb12603_c15/f1p0/1620	NA	NA	R01_cb12603_c15/f1p0/1620	R01_cb12603_c15/f1p0/1620
R01_cb7611_c11/f1p1/2476	NA	NA	NA	R01_cb7611_c11/f1p1/2476
R01_cb11455_c0/f3p0/576	NA	R01_cb11455_c0/f3p0/576	NA	R01_cb11455_c0/f3p0/576
R01_cb11038_c2/f1p0/2589	NA	NA	NA	R01_cb11038_c2/f1p0/2589
R01_cb3491_c4/f1p0/2063	NA	R01_cb3491_c4/f1p0/2063	NA	NA
R01_cb13462_c4/f1p0/1761	NA	R01_cb13462_c4/f1p0/1761	NA	R01_cb13462_c4/f1p0/1761
R01_cb18456_c1457/f3p0/360	R01_cb18456_c1457/f3p0/360	R01_cb18456_c1457/f3p0/360	R01_cb18456_c1457/f3p0/360	R01_cb18456_c1457/f3p0/360
R01_cb8564_c119518/f1p0/2479	NA	R01_cb8564_c119518/f1p0/2479	NA	NA
R01_cb3678_c17/f1p0/2471	NA	R01_cb3678_c17/f1p0/2471	NA	R01_cb3678_c17/f1p0/2471
R01_cb17498_c0/f1p1/1395	NA	NA	NA	R01_cb17498_c0/f1p1/1395
R01_cb16403_c0/f1p0/1864	NA	R01_cb16403_c0/f1p0/1864	R01_cb16403_c0/f1p0/1864	R01_cb16403_c0/f1p0/1864
R01_cb8564_c15434/f4p0/2668	NA	NA	NA	R01_cb8564_c15434/f4p0/2668
R01_cb18456_c4056/f1p0/1520	NA	NA	NA	R01_cb18456_c4056/f1p0/1520
R01_cb8669_c3/f1p0/2234	NA	NA	R01_cb8669_c3/f1p0/2234	R01_cb8669_c3/f1p0/2234
R01_cb1941_c6/f2p1/2058	NA	R01_cb1941_c6/f2p1/2058	NA	NA
R01_cb4923_c2/f1p0/3225	NA	NA	NA	R01_cb4923_c2/f1p0/3225
R01_cb2072_c44/f1p0/2957	NA	R01_cb2072_c44/f1p0/2957	NA	R01_cb2072_c44/f1p0/2957
R01_cb10404_c4/f1p0/1707	NA	R01_cb10404_c4/f1p0/1707	NA	NA
R01_cb6507_c5/f1p0/2184	NA	NA	NA	R01_cb6507_c5/f1p0/2184
R01_cb12641_c20/f1p0/1120	NA	NA	NA	R01_cb12641_c20/f1p0/1120
R01_cb5207_c8/f1p0/566	R01_cb5207_c8/f1p0/566	R01_cb5207_c8/f1p0/566	NA	NA
R01_cb4152_c8/f1p0/3099	NA	R01_cb4152_c8/f1p0/3099	NA	R01_cb4152_c8/f1p0/3099
R01_cb7611_c19/f1p0/3611	NA	R01_cb7611_c19/f1p0/3611	NA	NA
R01_cb17118_c5/f1p0/593	R01_cb17118_c5/f1p0/593	R01_cb17118_c5/f1p0/593	R01_cb17118_c5/f1p0/593	R01_cb17118_c5/f1p0/593

R01_cb8564_c70755/f1p0/2552	NA	NA	NA	R01_cb8564_c70755/f1p0/2552
R01_cb5373_c1/f2p0/2534	NA	NA	NA	R01_cb5373_c1/f2p0/2534
R01_cb6802_c34/f1p0/4811	NA	R01_cb6802_c34/f1p0/4811	R01_cb6802_c34/f1p0/4811	R01_cb6802_c34/f1p0/4811
R01_cb12662_c5/f2p0/1554	NA	R01_cb12662_c5/f2p0/1554	NA	NA
R01_cb18456_c7308/f1p0/920	R01_cb18456_c7308/f1p0/920	R01_cb18456_c7308/f1p0/920	NA	R01_cb18456_c7308/f1p0/920
R01_cb15474_c5/f1p0/1558	R01_cb15474_c5/f1p0/1558	NA	NA	R01_cb15474_c5/f1p0/1558
R01_cb7214_c2/f1p0/2219	NA	NA	NA	R01_cb7214_c2/f1p0/2219
R01_cb4147_c45/f1p0/726	NA	R01_cb4147_c45/f1p0/726	NA	R01_cb4147_c45/f1p0/726
R01_cb2895_c6/f1p0/1707	NA	R01_cb2895_c6/f1p0/1707	NA	NA
R01_cb3213_c32/f1p0/479	R01_cb3213_c32/f1p0/479	R01_cb3213_c32/f1p0/479	R01_cb3213_c32/f1p0/479	R01_cb3213_c32/f1p0/479
R01_cb17468_c0/f2p0/759	NA	NA	R01_cb17468_c0/f2p0/759	R01_cb17468_c0/f2p0/759
R01_cb8295_c5/f5p0/2401	NA	NA	NA	R01_cb8295_c5/f5p0/2401
R01_cb17313_c2/f1p0/6472	R01_cb17313_c2/f1p0/6472	R01_cb17313_c2/f1p0/6472	R01_cb17313_c2/f1p0/6472	NA
R01_cb12767_c5/f1p1/1228	NA	NA	NA	R01_cb12767_c5/f1p1/1228
R01_cb18456_c6818/f1p0/353	R01_cb18456_c6818/f1p0/353	R01_cb18456_c6818/f1p0/353	R01_cb18456_c6818/f1p0/353	NA
R01_cb6839_c2/f1p0/2761	NA	R01_cb6839_c2/f1p0/2761	NA	NA
R01_cb11090_c5/f1p0/1735	NA	R01_cb11090_c5/f1p0/1735	NA	NA
R01_cb18727_c1/f1p0/1217	NA	R01_cb18727_c1/f1p0/1217	R01_cb18727_c1/f1p0/1217	R01_cb18727_c1/f1p0/1217
R01_cb9886_c0/f1p0/1902	NA	R01_cb9886_c0/f1p0/1902	NA	R01_cb9886_c0/f1p0/1902
R01_cb18156_c0/f1p0/977	NA	NA	R01_cb18156_c0/f1p0/977	R01_cb18156_c0/f1p0/977
R01_cb10214_c2/f1p0/1453	R01_cb10214_c2/f1p0/1453	R01_cb10214_c2/f1p0/1453	R01_cb10214_c2/f1p0/1453	R01_cb10214_c2/f1p0/1453
R01_cb8564_c24791/f1p0/4031	NA	R01_cb8564_c24791/f1p0/4031	NA	NA
R01_cb8564_c37095/f1p0/2917	NA	NA	R01_cb8564_c37095/f1p0/2917	R01_cb8564_c37095/f1p0/2917
R01_cb14517_c11/f1p1/1829	NA	NA	NA	R01_cb14517_c11/f1p1/1829
R01_cb11057_c2/f1p0/1759	NA	NA	NA	R01_cb11057_c2/f1p0/1759
R01_cb16178_c2/f1p0/984	R01_cb16178_c2/f1p0/984	R01_cb16178_c2/f1p0/984	R01_cb16178_c2/f1p0/984	R01_cb16178_c2/f1p0/984

R01_cb1938_c6/f1p1/2278	NA	NA	NA	R01_cb1938_c6/f1p1/2278
R01_cb8564_c1040/f1p0/2002	R01_cb8564_c1040/f1p0/2002	R01_cb8564_c1040/f1p0/2002	R01_cb8564_c1040/f1p0/2002	R01_cb8564_c1040/f1p0/2002
R01_cb14286_c6/f1p0/646	R01_cb14286_c6/f1p0/646	R01_cb14286_c6/f1p0/646	R01_cb14286_c6/f1p0/646	R01_cb14286_c6/f1p0/646
R01_cb13490_c3/f2p0/752	R01_cb13490_c3/f2p0/752	R01_cb13490_c3/f2p0/752	R01_cb13490_c3/f2p0/752	R01_cb13490_c3/f2p0/752
R01_cb5659_c90/f1p0/2324	NA	NA	NA	R01_cb5659_c90/f1p0/2324
R01_cb8564_c66340/f2p1/2873	NA	NA	NA	R01_cb8564_c66340/f2p1/2873
R01_cb8564_c109997/f2p0/2937	R01_cb8564_c109997/f2p0/2937	R01_cb8564_c109997/f2p0/2937	R01_cb8564_c109997/f2p0/2937	R01_cb8564_c109997/f2p0/2937
R01_cb13602_c24/f1p0/1606	NA	NA	NA	R01_cb13602_c24/f1p0/1606
R01_cb18409_c109/f1p0/876	R01_cb18409_c109/f1p0/876	R01_cb18409_c109/f1p0/876	R01_cb18409_c109/f1p0/876	R01_cb18409_c109/f1p0/876
R01_cb7822_c6/f1p0/496	NA	NA	NA	R01_cb7822_c6/f1p0/496
R01_cb8564_c120121/f1p0/2816	R01_cb8564_c120121/f1p0/2816	R01_cb8564_c120121/f1p0/2816	NA	NA
R01_cb8564_c21582/f1p0/4177	NA	NA	NA	R01_cb8564_c21582/f1p0/4177
R01_cb7219_c11/f1p3/2453	NA	NA	NA	R01_cb7219_c11/f1p3/2453
R01_cb8564_c10963/f1p0/2691	NA	R01_cb8564_c10963/f1p0/2691	NA	R01_cb8564_c10963/f1p0/2691
R01_cb5586_c8/f1p0/2480	NA	R01_cb5586_c8/f1p0/2480	NA	R01_cb5586_c8/f1p0/2480
R01_cb13867_c13/f1p0/736	NA	NA	NA	R01_cb13867_c13/f1p0/736
R01_cb8564_c147145/f37p3/2018	NA	NA	NA	R01_cb8564_c147145/f37p3/2018
R01_cb14620_c6/f1p0/1780	NA	NA	NA	R01_cb14620_c6/f1p0/1780
R01_cb16342_c1/f1p0/641	R01_cb16342_c1/f1p0/641	R01_cb16342_c1/f1p0/641	R01_cb16342_c1/f1p0/641	R01_cb16342_c1/f1p0/641
R01_cb17319_c0/f1p0/1732	NA	R01_cb17319_c0/f1p0/1732	R01_cb17319_c0/f1p0/1732	NA
R01_cb17137_c6/f1p0/575	NA	R01_cb17137_c6/f1p0/575	NA	R01_cb17137_c6/f1p0/575
R01_cb8564_c74972/f1p0/2743	NA	R01_cb8564_c74972/f1p0/2743	NA	R01_cb8564_c74972/f1p0/2743
R01_cb13812_c0/f3p0/1232	NA	NA	NA	R01_cb13812_c0/f3p0/1232

R01_cb17711_c2/f1p0/1797	NA	R01_cb17711_c2/f1p0/1797	NA	NA
R01_cb10504_c1/f1p0/2631	NA	R01_cb10504_c1/f1p0/2631	NA	R01_cb10504_c1/f1p0/2631
R01_cb8564_c21982/f1p0/3812	NA	R01_cb8564_c21982/f1p0/3812	NA	NA
R01_cb8564_c37799/f1p0/2001	R01_cb8564_c37799/f1p0/2001	R01_cb8564_c37799/f1p0/2001	R01_cb8564_c37799/f1p0/2001	R01_cb8564_c37799/f1p0/2001
R01_cb11967_c16/f1p0/392	R01_cb11967_c16/f1p0/392	R01_cb11967_c16/f1p0/392	R01_cb11967_c16/f1p0/392	R01_cb11967_c16/f1p0/392
R01_cb12688_c2/f1p0/442	R01_cb12688_c2/f1p0/442	R01_cb12688_c2/f1p0/442	NA	R01_cb12688_c2/f1p0/442
R01_cb17706_c6/f1p1/580	R01_cb17706_c6/f1p1/580	R01_cb17706_c6/f1p1/580	R01_cb17706_c6/f1p1/580	R01_cb17706_c6/f1p1/580
R01_cb18529_c1/f1p0/711	R01_cb18529_c1/f1p0/711	R01_cb18529_c1/f1p0/711	R01_cb18529_c1/f1p0/711	R01_cb18529_c1/f1p0/711
R01_cb2698_c7/f1p0/1527	NA	NA	NA	R01_cb2698_c7/f1p0/1527
R01_cb8564_c124363/f1p0/2375	NA	NA	NA	R01_cb8564_c124363/f1p0/2375
R01_cb4342_c4/f1p0/2299	NA	R01_cb4342_c4/f1p0/2299	NA	NA
R01_cb16566_c1/f1p0/1302	NA	R01_cb16566_c1/f1p0/1302	R01_cb16566_c1/f1p0/1302	R01_cb16566_c1/f1p0/1302
R01_cb9242_c0/f1p0/2146	NA	NA	R01_cb9242_c0/f1p0/2146	NA
R01_cb14352_c1/f1p0/481	NA	R01_cb14352_c1/f1p0/481	R01_cb14352_c1/f1p0/481	R01_cb14352_c1/f1p0/481
R01_cb13001_c4/f1p0/892	NA	NA	NA	R01_cb13001_c4/f1p0/892
R01_cb8564_c79896/f1p0/2804	NA	R01_cb8564_c79896/f1p0/2804	NA	NA
R01_cb11524_c1/f1p0/2290	NA	NA	NA	R01_cb11524_c1/f1p0/2290
R01_cb13279_c1/f1p0/894	NA	NA	NA	R01_cb13279_c1/f1p0/894
R01_cb10024_c249/f1p0/691	R01_cb10024_c249/f1p0/691	R01_cb10024_c249/f1p0/691	R01_cb10024_c249/f1p0/691	NA
R01_cb8564_c115046/f1p1/2486	NA	R01_cb8564_c115046/f1p1/2486	NA	NA
R01_cb18760_c1/f1p0/453	R01_cb18760_c1/f1p0/453	R01_cb18760_c1/f1p0/453	R01_cb18760_c1/f1p0/453	R01_cb18760_c1/f1p0/453
R01_cb6141_c5/f1p0/752	NA	R01_cb6141_c5/f1p0/752	NA	NA
R01_cb2880_c3/f1p0/3627	NA	R01_cb2880_c3/f1p0/3627	NA	R01_cb2880_c3/f1p0/3627

R01_cb6354_c3/f1p0/5050	NA	NA	NA	R01_cb6354_c3/f1p0/5050
R01_cb11386_c1/f1p0/2702	NA	R01_cb11386_c1/f1p0/2702	NA	NA
R01_cb13433_c40/f1p0/800	NA	R01_cb13433_c40/f1p0/800	NA	R01_cb13433_c40/f1p0/800
R01_cb8453_c1/f1p0/2265	NA	R01_cb8453_c1/f1p0/2265	NA	NA
R01_cb16425_c6/f1p0/1285	R01_cb16425_c6/f1p0/1285	R01_cb16425_c6/f1p0/1285	R01_cb16425_c6/f1p0/1285	NA
R01_cb2674_c6/f1p0/2252	NA	R01_cb2674_c6/f1p0/2252	R01_cb2674_c6/f1p0/2252	NA
R01_cb9448_c18/f2p0/1769	NA	NA	NA	R01_cb9448_c18/f2p0/1769
R01_cb6802_c81/f1p0/2298	NA	R01_cb6802_c81/f1p0/2298	NA	NA
R01_cb5122_c19/f1p0/2936	NA	NA	NA	R01_cb5122_c19/f1p0/2936
R01_cb18113_c2/f1p0/865	NA	NA	NA	R01_cb18113_c2/f1p0/865
R01_cb12774_c0/f2p0/906	NA	R01_cb12774_c0/f2p0/906	NA	NA
R01_cb8564_c69337/f4p0/2096	NA	R01_cb8564_c69337/f4p0/2096	R01_cb8564_c69337/f4p0/2096	R01_cb8564_c69337/f4p0/2096
R01_cb12641_c9/f2p1/1037	NA	NA	NA	R01_cb12641_c9/f2p1/1037
R01_cb18456_c7592/f1p0/811	NA	R01_cb18456_c7592/f1p0/811	NA	R01_cb18456_c7592/f1p0/811
R01_cb16273_c0/f1p0/585	NA	R01_cb16273_c0/f1p0/585	NA	R01_cb16273_c0/f1p0/585
R01_cb8582_c1/f1p0/2167	NA	R01_cb8582_c1/f1p0/2167	NA	NA
R01_cb4583_c11/f1p0/3476	NA	R01_cb4583_c11/f1p0/3476	R01_cb4583_c11/f1p0/3476	NA
R01_cb8564_c116826/f1p0/3636	NA	R01_cb8564_c116826/f1p0/3636	NA	NA
R01_cb10314_c10/f1p3/565	NA	R01_cb10314_c10/f1p3/565	NA	NA
R01_cb2773_c1/f2p0/2520	NA	R01_cb2773_c1/f2p0/2520	R01_cb2773_c1/f2p0/2520	R01_cb2773_c1/f2p0/2520
R01_cb8564_c33685/f2p0/2837	R01_cb8564_c33685/f2p0/2837	R01_cb8564_c33685/f2p0/2837	R01_cb8564_c33685/f2p0/2837	R01_cb8564_c33685/f2p0/2837
R01_cb3564_c11/f1p0/3379	NA	NA	NA	R01_cb3564_c11/f1p0/3379
R01_cb16476_c0/f5p0/1206	NA	R01_cb16476_c0/f5p0/1206	R01_cb16476_c0/f5p0/1206	R01_cb16476_c0/f5p0/1206
R01_cb15260_c4/f2p0/642	NA	R01_cb15260_c4/f2p0/642	NA	NA

R01_cb11043_c6/f1p0/559	NA	R01_cb11043_c6/f1p0/559	NA	NA
R01_cb14502_c3/f5p0/328	R01_cb14502_c3/f5p0/328	R01_cb14502_c3/f5p0/328	R01_cb14502_c3/f5p0/328	R01_cb14502_c3/f5p0/328
R01_cb18705_c4/f1p0/1376	NA	R01_cb18705_c4/f1p0/1376	R01_cb18705_c4/f1p0/1376	R01_cb18705_c4/f1p0/1376
R01_cb16486_c0/f4p0/813	NA	R01_cb16486_c0/f4p0/813	R01_cb16486_c0/f4p0/813	R01_cb16486_c0/f4p0/813
R01_cb15297_c0/f1p0/1003	R01_cb15297_c0/f1p0/1003	R01_cb15297_c0/f1p0/1003	R01_cb15297_c0/f1p0/1003	R01_cb15297_c0/f1p0/1003
R01_cb8564_c87457/f2p0/2828	NA	R01_cb8564_c87457/f2p0/2828	NA	NA
R01_cb12147_c7/f1p0/801	NA	R01_cb12147_c7/f1p0/801	R01_cb12147_c7/f1p0/801	NA
R01_cb18614_c1/f1p0/1281	NA	NA	NA	R01_cb18614_c1/f1p0/1281
R01_cb8812_c2/f1p0/1987	R01_cb8812_c2/f1p0/1987	R01_cb8812_c2/f1p0/1987	R01_cb8812_c2/f1p0/1987	NA
R01_cb2729_c9/f1p0/4977	NA	R01_cb2729_c9/f1p0/4977	NA	R01_cb2729_c9/f1p0/4977
R01_cb5490_c0/f1p0/3087	NA	R01_cb5490_c0/f1p0/3087	NA	NA
R01_cb6654_c1/f2p0/2765	NA	R01_cb6654_c1/f2p0/2765	NA	NA
R01_cb15925_c2/f1p0/1565	NA	R01_cb15925_c2/f1p0/1565	NA	NA
R01_cb8564_c85863/f1p2/2224	NA	R01_cb8564_c85863/f1p2/2224	NA	NA
R01_cb310_c5/f1p0/4264	NA	R01_cb310_c5/f1p0/4264	NA	NA
R01_cb18456_c7361/f1p0/574	R01_cb18456_c7361/f1p0/574	R01_cb18456_c7361/f1p0/574	R01_cb18456_c7361/f1p0/574	R01_cb18456_c7361/f1p0/574
R01_cb16092_c4/f2p0/455	R01_cb16092_c4/f2p0/455	R01_cb16092_c4/f2p0/455	R01_cb16092_c4/f2p0/455	R01_cb16092_c4/f2p0/455
R01_cb8564_c14452/f1p0/3201	NA	R01_cb8564_c14452/f1p0/3201	NA	NA
R01_cb8564_c116186/f3p1/2503	NA	NA	NA	R01_cb8564_c116186/f3p1/2503
R01_cb5567_c1/f1p0/2620	NA	NA	R01_cb5567_c1/f1p0/2620	R01_cb5567_c1/f1p0/2620
R01_cb10323_c3/f1p0/761	NA	NA	R01_cb10323_c3/f1p0/761	R01_cb10323_c3/f1p0/761
R01_cb14585_c0/f4p0/557	NA	R01_cb14585_c0/f4p0/557	NA	R01_cb14585_c0/f4p0/557
R01_cb18497_c1/f1p0/821	NA	NA	R01_cb18497_c1/f1p0/821	R01_cb18497_c1/f1p0/821
R01_cb15695_c1/f1p0/794	R01_cb15695_c1/f1p0/794	R01_cb15695_c1/f1p0/794	R01_cb15695_c1/f1p0/794	R01_cb15695_c1/f1p0/794
R01_cb352_c6/f1p0/1908	NA	NA	NA	R01_cb352_c6/f1p0/1908

R01_cb1998_c5/f1p0/3344	R01_cb1998_c5/f1p0/3344	R01_cb1998_c5/f1p0/3344	R01_cb1998_c5/f1p0/3344	R01_cb1998_c5/f1p0/3344
R01_cb2866_c0/f2p0/3795	NA	NA	NA	R01_cb2866_c0/f2p0/3795
R01_cb18456_c1868/f1p0/859	NA	R01_cb18456_c1868/f1p0/859	NA	R01_cb18456_c1868/f1p0/859
R01_cb5190_c8/f1p1/2343	NA	NA	NA	R01_cb5190_c8/f1p1/2343
R01_cb8564_c87448/f1p0/2561	NA	R01_cb8564_c87448/f1p0/2561	NA	NA
R01_cb6216_c0/f3p0/2670	NA	NA	NA	R01_cb6216_c0/f3p0/2670
R01_cb16052_c14/f1p0/1756	NA	R01_cb16052_c14/f1p0/1756	R01_cb16052_c14/f1p0/1756	NA
R01_cb17199_c4/f1p0/1138	NA	R01_cb17199_c4/f1p0/1138	NA	NA
R01_cb13982_c3/f1p0/1371	NA	R01_cb13982_c3/f1p0/1371	NA	R01_cb13982_c3/f1p0/1371
R01_cb8564_c48237/f1p0/3233	NA	NA	NA	R01_cb8564_c48237/f1p0/3233
R01_cb8564_c112351/f1p0/2257	NA	R01_cb8564_c112351/f1p0/2257	R01_cb8564_c112351/f1p0/2257	NA
R01_cb146_c3/f1p0/4949	NA	NA	NA	R01_cb146_c3/f1p0/4949
R01_cb2501_c31/f1p0/1967	NA	R01_cb2501_c31/f1p0/1967	NA	NA
R01_cb8564_c3707/f1p0/3217	NA	NA	NA	R01_cb8564_c3707/f1p0/3217
R01_cb10022_c4/f1p0/925	R01_cb10022_c4/f1p0/925	R01_cb10022_c4/f1p0/925	NA	NA
R01_cb3046_c7/f1p0/3074	NA	NA	NA	R01_cb3046_c7/f1p0/3074
R01_cb12240_c9/f1p0/570	R01_cb12240_c9/f1p0/570	R01_cb12240_c9/f1p0/570	NA	NA
R01_cb5640_c2/f1p0/2174	NA	NA	NA	R01_cb5640_c2/f1p0/2174
R01_cb14648_c8/f1p0/855	NA	R01_cb14648_c8/f1p0/855	R01_cb14648_c8/f1p0/855	NA
R01_cb8564_c52388/f1p0/4989	NA	R01_cb8564_c52388/f1p0/4989	NA	NA
R01_cb15811_c31/f1p0/980	NA	R01_cb15811_c31/f1p0/980	R01_cb15811_c31/f1p0/980	R01_cb15811_c31/f1p0/980
R01_cb3929_c15/f1p0/2722	R01_cb3929_c15/f1p0/2722	R01_cb3929_c15/f1p0/2722	R01_cb3929_c15/f1p0/2722	R01_cb3929_c15/f1p0/2722
R01_cb5469_c10/f1p0/2248	NA	R01_cb5469_c10/f1p0/2248	NA	NA
R01_cb10849_c9/f1p0/1005	NA	R01_cb10849_c9/f1p0/1005	NA	R01_cb10849_c9/f1p0/1005
R01_cb6163_c11/f1p0/2243	R01_cb6163_c11/f1p0/2243	R01_cb6163_c11/f1p0/2243	NA	NA

R01_cb3637_c1/f1p0/3433	NA	R01_cb3637_c1/f1p0/3433	NA	NA
R01_cb14223_c4/f1p0/1205	NA	R01_cb14223_c4/f1p0/1205	NA	NA
R01_cb14514_c0/f1p0/1755	NA	R01_cb14514_c0/f1p0/1755	NA	NA
R01_cb15132_c1/f1p0/798	NA	R01_cb15132_c1/f1p0/798	NA	NA
R01_cb18409_c142/f1p1/1127	NA	R01_cb18409_c142/f1p1/1127	NA	NA
R01_cb10797_c3/f1p0/712	NA	R01_cb10797_c3/f1p0/712	NA	R01_cb10797_c3/f1p0/712
R01_cb3142_c0/f2p0/3395	R01_cb3142_c0/f2p0/3395	R01_cb3142_c0/f2p0/3395	NA	NA
R01_cb17266_c4/f1p0/990	NA	R01_cb17266_c4/f1p0/990	R01_cb17266_c4/f1p0/990	R01_cb17266_c4/f1p0/990
R01_cb9038_c6/f1p0/1957	NA	R01_cb9038_c6/f1p0/1957	NA	NA
R01_cb8379_c5/f1p1/2387	NA	R01_cb8379_c5/f1p1/2387	NA	NA
R01_cb18456_c7233/f1p0/575	NA	R01_cb18456_c7233/f1p0/575	R01_cb18456_c7233/f1p0/575	R01_cb18456_c7233/f1p0/575
R01_cb7219_c2/f5p3/2591	NA	NA	NA	R01_cb7219_c2/f5p3/2591
R01_cb10029_c103/f1p6/796	NA	R01_cb10029_c103/f1p6/796	NA	NA
R01_cb14181_c0/f6p0/526	NA	NA	NA	R01_cb14181_c0/f6p0/526
R01_cb13724_c4/f1p0/429	NA	NA	R01_cb13724_c4/f1p0/429	R01_cb13724_c4/f1p0/429
R01_cb4536_c6/f1p0/1015	NA	NA	NA	R01_cb4536_c6/f1p0/1015
R01_cb14521_c0/f2p0/753	NA	NA	NA	R01_cb14521_c0/f2p0/753
R01_cb3176_c2/f1p0/3696	NA	R01_cb3176_c2/f1p0/3696	NA	NA
R01_cb8564_c24824/f1p0/4019	NA	R01_cb8564_c24824/f1p0/4019	NA	NA
R01_cb11742_c1/f1p0/3299	R01_cb11742_c1/f1p0/3299	R01_cb11742_c1/f1p0/3299	NA	NA
R01_cb5351_c2/f1p0/1682	NA	R01_cb5351_c2/f1p0/1682	NA	R01_cb5351_c2/f1p0/1682
R01_cb11439_c0/f1p0/499	R01_cb11439_c0/f1p0/499	R01_cb11439_c0/f1p0/499	R01_cb11439_c0/f1p0/499	R01_cb11439_c0/f1p0/499
R01_cb8564_c48286/f1p0/1988	NA	R01_cb8564_c48286/f1p0/1988	NA	NA
R01_cb9119_c4/f1p0/2214	NA	NA	NA	R01_cb9119_c4/f1p0/2214
R01_cb7877_c3/f1p0/2308	NA	R01_cb7877_c3/f1p0/2308	R01_cb7877_c3/f1p0/2308	R01_cb7877_c3/f1p0/2308
R01_cb2200_c5/f1p1/501	NA	R01_cb2200_c5/f1p1/501	NA	R01_cb2200_c5/f1p1/501

R01_cb5703_c0/f1p0/3031	NA	R01_cb5703_c0/f1p0/3031	R01_cb5703_c0/f1p0/3031	R01_cb5703_c0/f1p0/3031
R01_cb11141_c4/f1p0/1912	NA	NA	NA	R01_cb11141_c4/f1p0/1912
R01_cb16839_c2/f1p0/1628	NA	NA	NA	R01_cb16839_c2/f1p0/1628
R01_cb15701_c3/f1p0/1551	NA	R01_cb15701_c3/f1p0/1551	NA	R01_cb15701_c3/f1p0/1551
R01_cb10229_c3/f1p0/2205	R01_cb10229_c3/f1p0/2205	R01_cb10229_c3/f1p0/2205	NA	R01_cb10229_c3/f1p0/2205
R01_cb4280_c1/f1p0/3387	NA	R01_cb4280_c1/f1p0/3387	R01_cb4280_c1/f1p0/3387	R01_cb4280_c1/f1p0/3387
R01_cb8564_c18306/f1p0/2224	R01_cb8564_c18306/f1p0/2224	R01_cb8564_c18306/f1p0/2224	R01_cb8564_c18306/f1p0/2224	R01_cb8564_c18306/f1p0/2224
R01_cb1473_c4/f1p0/2367	NA	R01_cb1473_c4/f1p0/2367	R01_cb1473_c4/f1p0/2367	R01_cb1473_c4/f1p0/2367
R01_cb18301_c18/f1p0/1613	NA	R01_cb18301_c18/f1p0/1613	R01_cb18301_c18/f1p0/1613	NA
R01_cb13892_c7/f1p0/838	NA	R01_cb13892_c7/f1p0/838	NA	NA
R01_cb10196_c2/f1p0/609	NA	NA	NA	R01_cb10196_c2/f1p0/609
R01_cb1824_c2/f1p1/3327	NA	R01_cb1824_c2/f1p1/3327	NA	NA
R01_cb15462_c1/f1p0/1238	NA	R01_cb15462_c1/f1p0/1238	R01_cb15462_c1/f1p0/1238	NA
R01_cb16504_c4/f1p0/1795	NA	NA	NA	R01_cb16504_c4/f1p0/1795
R01_cb12863_c0/f2p0/451	R01_cb12863_c0/f2p0/451	NA	NA	R01_cb12863_c0/f2p0/451
R01_cb13497_c12/f16p1/856	NA	NA	NA	R01_cb13497_c12/f16p1/856
R01_cb10600_c1/f1p0/3337	NA	R01_cb10600_c1/f1p0/3337	R01_cb10600_c1/f1p0/3337	R01_cb10600_c1/f1p0/3337
R01_cb8564_c31823/f8p0/2321	NA	NA	NA	R01_cb8564_c31823/f8p0/2321
R01_cb13910_c1/f6p0/1533	NA	R01_cb13910_c1/f6p0/1533	R01_cb13910_c1/f6p0/1533	R01_cb13910_c1/f6p0/1533
R01_cb9928_c6/f1p0/2054	NA	NA	NA	R01_cb9928_c6/f1p0/2054
R01_cb12584_c2/f1p0/1188	NA	NA	NA	R01_cb12584_c2/f1p0/1188
R01_cb478_c21/f1p0/2997	NA	NA	NA	R01_cb478_c21/f1p0/2997
R01_cb14153_c1/f1p0/683	R01_cb14153_c1/f1p0/683	R01_cb14153_c1/f1p0/683	R01_cb14153_c1/f1p0/683	R01_cb14153_c1/f1p0/683
R01_cb10668_c10/f1p0/636	NA	R01_cb10668_c10/f1p0/636	NA	NA
R01_cb8564_c115114/f1p0/195	NA	R01_cb8564_c115114/f1p0/195	NA	NA

9		9		
R01_cb13910_c6/f1p0/1579	NA	R01_cb13910_c6/f1p0/1579	R01_cb13910_c6/f1p0/1579	R01_cb13910_c6/f1p0/1579
R01_cb3320_c11/f1p0/2373	NA	R01_cb3320_c11/f1p0/2373	R01_cb3320_c11/f1p0/2373	R01_cb3320_c11/f1p0/2373
R01_cb12221_c1/f1p0/643	R01_cb12221_c1/f1p0/643	R01_cb12221_c1/f1p0/643	R01_cb12221_c1/f1p0/643	NA
R01_cb16341_c10/f1p0/863	NA	R01_cb16341_c10/f1p0/863	R01_cb16341_c10/f1p0/863	R01_cb16341_c10/f1p0/863
R01_cb7506_c1/f1p0/2365	NA	NA	NA	R01_cb7506_c1/f1p0/2365
R01_cb4576_c129/f1p0/2318	R01_cb4576_c129/f1p0/2318	R01_cb4576_c129/f1p0/2318	NA	R01_cb4576_c129/f1p0/2318
R01_cb2838_c6/f1p0/2525	NA	R01_cb2838_c6/f1p0/2525	NA	NA
R01_cb11676_c0/f1p0/1279	NA	NA	NA	R01_cb11676_c0/f1p0/1279
R01_cb7735_c7/f1p0/1232	NA	R01_cb7735_c7/f1p0/1232	NA	R01_cb7735_c7/f1p0/1232
R01_cb13696_c3/f1p0/1110	NA	NA	NA	R01_cb13696_c3/f1p0/1110
R01_cb18084_c0/f1p0/898	NA	R01_cb18084_c0/f1p0/898	NA	NA
R01_cb13485_c4/f1p0/675	NA	R01_cb13485_c4/f1p0/675	NA	NA
R01_cb11430_c0/f2p0/693	NA	R01_cb11430_c0/f2p0/693	NA	R01_cb11430_c0/f2p0/693
R01_cb17731_c1/f1p0/625	NA	R01_cb17731_c1/f1p0/625	NA	NA
R01_cb8710_c11/f1p0/2201	NA	NA	NA	R01_cb8710_c11/f1p0/2201
R01_cb10208_c1/f1p0/2821	NA	R01_cb10208_c1/f1p0/2821	NA	NA
R01_cb13151_c1/f1p0/1812	R01_cb13151_c1/f1p0/1812	R01_cb13151_c1/f1p0/1812	NA	NA
R01_cb18409_c68/f1p0/356	R01_cb18409_c68/f1p0/356	R01_cb18409_c68/f1p0/356	R01_cb18409_c68/f1p0/356	R01_cb18409_c68/f1p0/356
R01_cb17254_c1/f1p0/925	R01_cb17254_c1/f1p0/925	R01_cb17254_c1/f1p0/925	R01_cb17254_c1/f1p0/925	R01_cb17254_c1/f1p0/925
R01_cb1095_c15/f1p1/5852	NA	NA	NA	R01_cb1095_c15/f1p1/5852
R01_cb10111_c3/f1p0/1907	NA	R01_cb10111_c3/f1p0/1907	NA	NA
R01_cb206_c7/f1p0/3217	NA	R01_cb206_c7/f1p0/3217	NA	NA
R01_cb502_c11/f2p0/2142	R01_cb502_c11/f2p0/2142	R01_cb502_c11/f2p0/2142	NA	NA
R01_cb5536_c0/f1p0/3079	NA	R01_cb5536_c0/f1p0/3079	NA	NA
R01_cb12023_c0/f14p0/695	NA	R01_cb12023_c0/f14p0/695	NA	R01_cb12023_c0/f14p0/695

R01_cb10043_c13/f1p0/1036	NA	NA	NA	R01_cb10043_c13/f1p0/1036
R01_cb18456_c6761/f1p0/744	R01_cb18456_c6761/f1p0/744	R01_cb18456_c6761/f1p0/744	NA	NA
R01_cb8786_c5/f1p0/1325	NA	NA	NA	R01_cb8786_c5/f1p0/1325
R01_cb8564_c87747/f1p0/3271	R01_cb8564_c87747/f1p0/3271	R01_cb8564_c87747/f1p0/3271	NA	NA
R01_cb16747_c4/f1p0/1508	NA	NA	NA	R01_cb16747_c4/f1p0/1508
R01_cb4085_c0/f1p0/3440	NA	R01_cb4085_c0/f1p0/3440	NA	NA
R01_cb8564_c17982/f1p0/3250	NA	R01_cb8564_c17982/f1p0/3250	NA	NA
R01_cb12176_c2/f1p0/1520	R01_cb12176_c2/f1p0/1520	R01_cb12176_c2/f1p0/1520	NA	R01_cb12176_c2/f1p0/1520
R01_cb16459_c3/f1p1/619	R01_cb16459_c3/f1p1/619	R01_cb16459_c3/f1p1/619	NA	NA
R01_cb6608_c20/f4p1/2518	NA	NA	NA	R01_cb6608_c20/f4p1/2518
R01_cb14120_c1/f1p0/1017	NA	NA	NA	R01_cb14120_c1/f1p0/1017
R01_cb3074_c3/f1p0/1052	NA	NA	NA	R01_cb3074_c3/f1p0/1052
R01_cb11714_c1/f1p0/3415	R01_cb11714_c1/f1p0/3415	R01_cb11714_c1/f1p0/3415	R01_cb11714_c1/f1p0/3415	R01_cb11714_c1/f1p0/3415
R01_cb5208_c4/f1p0/3236	NA	NA	NA	R01_cb5208_c4/f1p0/3236
R01_cb15745_c5/f3p0/1126	NA	R01_cb15745_c5/f3p0/1126	R01_cb15745_c5/f3p0/1126	R01_cb15745_c5/f3p0/1126
R01_cb18568_c1/f1p0/638	R01_cb18568_c1/f1p0/638	NA	NA	R01_cb18568_c1/f1p0/638
R01_cb13449_c9/f1p0/712	NA	NA	NA	R01_cb13449_c9/f1p0/712
R01_cb14565_c0/f2p0/922	NA	NA	NA	R01_cb14565_c0/f2p0/922
R01_cb17181_c7/f1p0/1391	R01_cb17181_c7/f1p0/1391	R01_cb17181_c7/f1p0/1391	R01_cb17181_c7/f1p0/1391	R01_cb17181_c7/f1p0/1391
R01_cb11017_c5/f1p0/1431	NA	R01_cb11017_c5/f1p0/1431	R01_cb11017_c5/f1p0/1431	R01_cb11017_c5/f1p0/1431
R01_cb18456_c6660/f1p0/506	NA	R01_cb18456_c6660/f1p0/506	NA	NA
R01_cb3138_c7/f1p0/629	NA	NA	NA	R01_cb3138_c7/f1p0/629
R01_cb10768_c4/f1p0/1853	R01_cb10768_c4/f1p0/1853	R01_cb10768_c4/f1p0/1853	NA	R01_cb10768_c4/f1p0/1853
R01_cb14356_c3/f1p0/1612	NA	R01_cb14356_c3/f1p0/1612	NA	NA
R01_cb13449_c10/f1p0/868	NA	NA	NA	R01_cb13449_c10/f1p0/868

R01_cb16206_c1/f1p0/1549	NA	NA	NA	R01_cb16206_c1/f1p0/1549
R01_cb11646_c0/f2p0/1800	NA	NA	NA	R01_cb11646_c0/f2p0/1800
R01_cb737_c48/f1p0/609	R01_cb737_c48/f1p0/609	R01_cb737_c48/f1p0/609	R01_cb737_c48/f1p0/609	R01_cb737_c48/f1p0/609
R01_cb6706_c3/f2p0/1553	NA	NA	NA	R01_cb6706_c3/f2p0/1553
R01_cb1739_c13/f1p1/5711	NA	NA	NA	R01_cb1739_c13/f1p1/5711
R01_cb11048_c0/f1p0/1242	R01_cb11048_c0/f1p0/1242	R01_cb11048_c0/f1p0/1242	R01_cb11048_c0/f1p0/1242	R01_cb11048_c0/f1p0/1242
R01_cb8564_c10684/f1p0/3561	R01_cb8564_c10684/f1p0/3561	R01_cb8564_c10684/f1p0/3561	NA	R01_cb8564_c10684/f1p0/3561
R01_cb16645_c56/f1p0/1670	NA	R01_cb16645_c56/f1p0/1670	NA	NA
R01_cb14750_c0/f4p0/951	NA	NA	NA	R01_cb14750_c0/f4p0/951
R01_cb8080_c2/f1p0/2442	NA	NA	R01_cb8080_c2/f1p0/2442	NA
R01_cb16367_c6/f1p0/523	R01_cb16367_c6/f1p0/523	NA	NA	NA
R01_cb11652_c1/f1p0/3207	NA	R01_cb11652_c1/f1p0/3207	NA	NA
R01_cb12610_c1/f1p0/813	NA	NA	NA	R01_cb12610_c1/f1p0/813
R01_cb8564_c1855/f1p0/2496	R01_cb8564_c1855/f1p0/2496	R01_cb8564_c1855/f1p0/2496	R01_cb8564_c1855/f1p0/2496	R01_cb8564_c1855/f1p0/2496
R01_cb17437_c0/f2p0/867	NA	R01_cb17437_c0/f2p0/867	NA	R01_cb17437_c0/f2p0/867
R01_cb18494_c0/f1p0/1412	NA	R01_cb18494_c0/f1p0/1412	R01_cb18494_c0/f1p0/1412	R01_cb18494_c0/f1p0/1412
R01_cb8564_c21103/f4p0/2248	R01_cb8564_c21103/f4p0/2248	R01_cb8564_c21103/f4p0/2248	NA	R01_cb8564_c21103/f4p0/2248
R01_cb8564_c2610/f1p0/2831	R01_cb8564_c2610/f1p0/2831	R01_cb8564_c2610/f1p0/2831	NA	R01_cb8564_c2610/f1p0/2831
R01_cb15574_c6/f2p0/530	NA	NA	R01_cb15574_c6/f2p0/530	R01_cb15574_c6/f2p0/530
R01_cb6616_c7/f1p0/1644	NA	NA	R01_cb6616_c7/f1p0/1644	R01_cb6616_c7/f1p0/1644
R01_cb3922_c22/f1p1/1626	NA	R01_cb3922_c22/f1p1/1626	NA	NA
R01_cb8564_c119962/f1p0/2258	NA	NA	NA	R01_cb8564_c119962/f1p0/2258
R01_cb2765_c11/f3p0/3044	NA	NA	NA	R01_cb2765_c11/f3p0/3044

R01_cb8865_c4/f1p0/2090	NA	NA	NA	R01_cb8865_c4/f1p0/2090
R01_cb6706_c0/f5p0/2760	NA	R01_cb6706_c0/f5p0/2760	R01_cb6706_c0/f5p0/2760	R01_cb6706_c0/f5p0/2760
R01_cb8564_c86340/f2p0/2725	NA	NA	R01_cb8564_c86340/f2p0/2725	R01_cb8564_c86340/f2p0/2725
R01_cb11106_c0/f2p0/1739	NA	NA	NA	R01_cb11106_c0/f2p0/1739
R01_cb10783_c0/f8p0/461	R01_cb10783_c0/f8p0/461	R01_cb10783_c0/f8p0/461	R01_cb10783_c0/f8p0/461	R01_cb10783_c0/f8p0/461
R01_cb3632_c2/f1p0/2876	NA	R01_cb3632_c2/f1p0/2876	NA	R01_cb3632_c2/f1p0/2876
R01_cb8564_c14260/f1p0/2798	NA	R01_cb8564_c14260/f1p0/2798	NA	NA
R01_cb8564_c17117/f1p0/4269	NA	R01_cb8564_c17117/f1p0/4269	NA	NA
R01_cb507_c3/f1p0/2874	NA	R01_cb507_c3/f1p0/2874	NA	R01_cb507_c3/f1p0/2874
R01_cb10238_c2/f1p0/3142	NA	R01_cb10238_c2/f1p0/3142	NA	NA
R01_cb8564_c129409/f1p0/2483	NA	R01_cb8564_c129409/f1p0/2483	NA	R01_cb8564_c129409/f1p0/2483
R01_cb11458_c1/f1p0/2593	NA	R01_cb11458_c1/f1p0/2593	R01_cb11458_c1/f1p0/2593	R01_cb11458_c1/f1p0/2593
R01_cb9324_c6/f2p0/1934	NA	R01_cb9324_c6/f2p0/1934	NA	NA
R01_cb15851_c0/f1p0/996	NA	R01_cb15851_c0/f1p0/996	NA	NA
R01_cb8310_c3/f1p0/2269	NA	R01_cb8310_c3/f1p0/2269	R01_cb8310_c3/f1p0/2269	R01_cb8310_c3/f1p0/2269
R01_cb10321_c2/f1p0/2281	R01_cb10321_c2/f1p0/2281	R01_cb10321_c2/f1p0/2281	R01_cb10321_c2/f1p0/2281	R01_cb10321_c2/f1p0/2281
R01_cb9662_c1/f1p0/1913	NA	NA	NA	R01_cb9662_c1/f1p0/1913
R01_cb2737_c22/f1p0/3196	NA	R01_cb2737_c22/f1p0/3196	NA	NA
R01_cb17498_c1/f1p0/855	NA	NA	NA	R01_cb17498_c1/f1p0/855
R01_cb8564_c123647/f1p0/1917	R01_cb8564_c123647/f1p0/1917	R01_cb8564_c123647/f1p0/1917	R01_cb8564_c123647/f1p0/1917	R01_cb8564_c123647/f1p0/1917
R01_cb2101_c8/f1p0/1743	NA	R01_cb2101_c8/f1p0/1743	R01_cb2101_c8/f1p0/1743	R01_cb2101_c8/f1p0/1743
R01_cb13836_c0/f1p0/432	R01_cb13836_c0/f1p0/432	R01_cb13836_c0/f1p0/432	NA	NA
R01_cb3391_c6/f1p0/2647	R01_cb3391_c6/f1p0/2647	R01_cb3391_c6/f1p0/2647	R01_cb3391_c6/f1p0/2647	NA
R01_cb8040_c12/f1p0/1866	NA	R01_cb8040_c12/f1p0/1866	R01_cb8040_c12/f1p0/1866	R01_cb8040_c12/f1p0/1866

R01_cb13134_c10/f3p0/1178	NA	NA	NA	R01_cb13134_c10/f3p0/1178
R01_cb13880_c4/f1p0/681	R01_cb13880_c4/f1p0/681	R01_cb13880_c4/f1p0/681	R01_cb13880_c4/f1p0/681	R01_cb13880_c4/f1p0/681
R01_cb7595_c20/f1p0/730	NA	R01_cb7595_c20/f1p0/730	NA	R01_cb7595_c20/f1p0/730
R01_cb17819_c0/f2p0/1164	NA	NA	NA	R01_cb17819_c0/f2p0/1164
R01_cb2588_c2/f1p0/661	NA	R01_cb2588_c2/f1p0/661	R01_cb2588_c2/f1p0/661	R01_cb2588_c2/f1p0/661
R01_cb1119_c5/f1p0/2355	NA	R01_cb1119_c5/f1p0/2355	NA	NA
R01_cb8128_c0/f1p0/2447	R01_cb8128_c0/f1p0/2447	R01_cb8128_c0/f1p0/2447	R01_cb8128_c0/f1p0/2447	R01_cb8128_c0/f1p0/2447
R01_cb10993_c3/f1p0/1477	NA	NA	NA	R01_cb10993_c3/f1p0/1477
R01_cb9841_c3/f1p0/1798	NA	NA	NA	R01_cb9841_c3/f1p0/1798
R01_cb4471_c7/f1p0/2973	NA	NA	NA	R01_cb4471_c7/f1p0/2973
R01_cb16720_c0/f2p0/1444	NA	NA	NA	R01_cb16720_c0/f2p0/1444
R01_cb15659_c0/f1p0/1491	NA	NA	NA	R01_cb15659_c0/f1p0/1491
R01_cb826_c7/f1p0/2574	NA	R01_cb826_c7/f1p0/2574	R01_cb826_c7/f1p0/2574	R01_cb826_c7/f1p0/2574
R01_cb13165_c0/f2p0/487	NA	R01_cb13165_c0/f2p0/487	NA	NA
R01_cb8564_c116311/f1p0/4078	NA	NA	NA	R01_cb8564_c116311/f1p0/4078
R01_cb18456_c6462/f1p0/735	R01_cb18456_c6462/f1p0/735	R01_cb18456_c6462/f1p0/735	NA	NA
R01_cb14523_c1/f1p0/758	NA	NA	NA	R01_cb14523_c1/f1p0/758
R01_cb18456_c7176/f1p0/1692	R01_cb18456_c7176/f1p0/1692	R01_cb18456_c7176/f1p0/1692	R01_cb18456_c7176/f1p0/1692	NA
R01_cb945_c11/f1p0/3380	NA	R01_cb945_c11/f1p0/3380	NA	NA
R01_cb3065_c1/f1p0/3738	R01_cb3065_c1/f1p0/3738	R01_cb3065_c1/f1p0/3738	NA	NA
R01_cb17527_c1/f1p0/389	R01_cb17527_c1/f1p0/389	R01_cb17527_c1/f1p0/389	R01_cb17527_c1/f1p0/389	R01_cb17527_c1/f1p0/389
R01_cb8564_c80459/f1p0/2346	R01_cb8564_c80459/f1p0/2346	R01_cb8564_c80459/f1p0/2346	R01_cb8564_c80459/f1p0/2346	R01_cb8564_c80459/f1p0/2346
R01_cb1938_c7/f1p0/2462	NA	NA	NA	R01_cb1938_c7/f1p0/2462

R01_cb16172_c0/f1p0/1837	NA	R01_cb16172_c0/f1p0/1837	R01_cb16172_c0/f1p0/1837	NA
R01_cb6377_c4/f1p0/2670	NA	NA	NA	R01_cb6377_c4/f1p0/2670
R01_cb9473_c10/f1p0/998	NA	R01_cb9473_c10/f1p0/998	R01_cb9473_c10/f1p0/998	NA
R01_cb18373_c1/f1p0/705	R01_cb18373_c1/f1p0/705	R01_cb18373_c1/f1p0/705	R01_cb18373_c1/f1p0/705	R01_cb18373_c1/f1p0/705
R01_cb12369_c11/f66p0/601	NA	NA	R01_cb12369_c11/f66p0/601	NA
R01_cb17402_c1/f1p0/911	NA	NA	NA	R01_cb17402_c1/f1p0/911
R01_cb1_c15/f3p0/2120	NA	NA	NA	R01_cb1_c15/f3p0/2120
R01_cb8564_c12484/f1p0/2841	NA	NA	NA	R01_cb8564_c12484/f1p0/2841
R01_cb10104_c0/f5p0/1085	NA	NA	NA	R01_cb10104_c0/f5p0/1085
R01_cb15260_c5/f1p0/604	R01_cb15260_c5/f1p0/604	R01_cb15260_c5/f1p0/604	NA	NA
R01_cb8564_c115447/f1p0/2386	NA	NA	NA	R01_cb8564_c115447/f1p0/2386
R01_cb10398_c20/f12p0/721	NA	NA	NA	R01_cb10398_c20/f12p0/721
R01_cb1119_c4/f1p1/4438	NA	R01_cb1119_c4/f1p1/4438	NA	NA
R01_cb8672_c7/f1p0/1868	NA	R01_cb8672_c7/f1p0/1868	R01_cb8672_c7/f1p0/1868	R01_cb8672_c7/f1p0/1868
R01_cb7035_c0/f2p0/2711	NA	R01_cb7035_c0/f2p0/2711	R01_cb7035_c0/f2p0/2711	NA
R01_cb13811_c6/f1p0/1104	NA	R01_cb13811_c6/f1p0/1104	NA	NA
R01_cb416_c23/f1p0/2818	NA	R01_cb416_c23/f1p0/2818	NA	NA
R01_cb3359_c21/f1p0/3156	NA	R01_cb3359_c21/f1p0/3156	NA	NA
R01_cb3568_c4/f1p0/2044	NA	R01_cb3568_c4/f1p0/2044	NA	NA
R01_cb13996_c5/f1p0/819	NA	R01_cb13996_c5/f1p0/819	NA	NA
R01_cb3563_c7/f1p0/2009	NA	NA	NA	R01_cb3563_c7/f1p0/2009
R01_cb6719_c18/f1p0/2795	NA	R01_cb6719_c18/f1p0/2795	R01_cb6719_c18/f1p0/2795	NA
R01_cb15618_c1/f1p0/961	NA	NA	NA	R01_cb15618_c1/f1p0/961
R01_cb14656_c9/f1p0/459	R01_cb14656_c9/f1p0/459	R01_cb14656_c9/f1p0/459	R01_cb14656_c9/f1p0/459	R01_cb14656_c9/f1p0/459
R01_cb11038_c0/f3p0/1047	NA	NA	NA	R01_cb11038_c0/f3p0/1047

R01_cb14576_c7/f1p0/1516	NA	R01_cb14576_c7/f1p0/1516	R01_cb14576_c7/f1p0/1516	R01_cb14576_c7/f1p0/1516
R01_cb18344_c1/f2p0/630	NA	NA	NA	R01_cb18344_c1/f2p0/630
R01_cb7329_c2/f1p0/2646	R01_cb7329_c2/f1p0/2646	R01_cb7329_c2/f1p0/2646	NA	NA
R01_cb2492_c1/f1p0/1945	NA	NA	NA	R01_cb2492_c1/f1p0/1945
R01_cb7962_c5/f1p0/2184	NA	NA	NA	R01_cb7962_c5/f1p0/2184
R01_cb8564_c122202/f1p1/1935	NA	NA	R01_cb8564_c122202/f1p1/1935	R01_cb8564_c122202/f1p1/1935
R01_cb647_c8/f1p0/1962	NA	R01_cb647_c8/f1p0/1962	NA	R01_cb647_c8/f1p0/1962
R01_cb11799_c12/f2p0/1161	NA	R01_cb11799_c12/f2p0/1161	NA	NA
R01_cb4169_c15/f1p0/1203	NA	NA	NA	R01_cb4169_c15/f1p0/1203
R01_cb8564_c71066/f1p0/2382	NA	R01_cb8564_c71066/f1p0/2382	NA	NA
R01_cb8564_c113186/f1p0/2216	NA	R01_cb8564_c113186/f1p0/2216	NA	NA
R01_cb8564_c22746/f1p0/2508	NA	R01_cb8564_c22746/f1p0/2508	NA	NA
R01_cb13337_c4/f1p0/1179	NA	R01_cb13337_c4/f1p0/1179	NA	R01_cb13337_c4/f1p0/1179
R01_cb4824_c12/f1p0/3203	R01_cb4824_c12/f1p0/3203	R01_cb4824_c12/f1p0/3203	R01_cb4824_c12/f1p0/3203	R01_cb4824_c12/f1p0/3203
R01_cb8564_c53036/f1p0/2917	NA	NA	R01_cb8564_c53036/f1p0/2917	R01_cb8564_c53036/f1p0/2917
R01_cb8564_c17716/f1p0/3028	NA	R01_cb8564_c17716/f1p0/3028	NA	NA
R01_cb5900_c48/f1p0/3487	NA	R01_cb5900_c48/f1p0/3487	NA	R01_cb5900_c48/f1p0/3487
R01_cb10591_c6/f1p0/690	R01_cb10591_c6/f1p0/690	R01_cb10591_c6/f1p0/690	NA	R01_cb10591_c6/f1p0/690
R01_cb14706_c4/f1p0/610	R01_cb14706_c4/f1p0/610	NA	R01_cb14706_c4/f1p0/610	R01_cb14706_c4/f1p0/610
R01_cb13096_c22/f1p0/624	NA	R01_cb13096_c22/f1p0/624	NA	NA
R01_cb8564_c38917/f1p0/3160	R01_cb8564_c38917/f1p0/3160	R01_cb8564_c38917/f1p0/3160	R01_cb8564_c38917/f1p0/3160	R01_cb8564_c38917/f1p0/3160
R01_cb120_c10/f1p0/2003	NA	R01_cb120_c10/f1p0/2003	NA	R01_cb120_c10/f1p0/2003
R01_cb2223_c5/f1p0/694	R01_cb2223_c5/f1p0/694	R01_cb2223_c5/f1p0/694	R01_cb2223_c5/f1p0/694	R01_cb2223_c5/f1p0/694

R01_cb8564_c129713/f1p0/2322	NA	R01_cb8564_c129713/f1p0/2322	NA	R01_cb8564_c129713/f1p0/2322
R01_cb15811_c7/f3p0/1029	R01_cb15811_c7/f3p0/1029	R01_cb15811_c7/f3p0/1029	R01_cb15811_c7/f3p0/1029	R01_cb15811_c7/f3p0/1029
R01_cb2960_c1/f1p0/3760	NA	NA	NA	R01_cb2960_c1/f1p0/3760
R01_cb15814_c0/f5p1/1353	NA	NA	NA	R01_cb15814_c0/f5p1/1353
R01_cb2219_c8/f1p1/1916	NA	R01_cb2219_c8/f1p1/1916	NA	R01_cb2219_c8/f1p1/1916
R01_cb5841_c51/f1p0/1108	R01_cb5841_c51/f1p0/1108	R01_cb5841_c51/f1p0/1108	NA	NA
R01_cb8564_c4043/f1p0/2276	NA	NA	NA	R01_cb8564_c4043/f1p0/2276
R01_cb8564_c3242/f1p0/2731	R01_cb8564_c3242/f1p0/2731	R01_cb8564_c3242/f1p0/2731	R01_cb8564_c3242/f1p0/2731	NA
R01_cb16997_c108/f1p0/2138	NA	NA	NA	R01_cb16997_c108/f1p0/2138
R01_cb5905_c3/f1p0/2087	NA	R01_cb5905_c3/f1p0/2087	NA	NA
R01_cb6153_c1/f2p0/3052	NA	NA	NA	R01_cb6153_c1/f2p0/3052
R01_cb15386_c3/f1p2/523	NA	R01_cb15386_c3/f1p2/523	NA	R01_cb15386_c3/f1p2/523
R01_cb5229_c13/f1p1/2074	NA	NA	NA	R01_cb5229_c13/f1p1/2074
R01_cb8922_c1/f1p0/2352	R01_cb8922_c1/f1p0/2352	R01_cb8922_c1/f1p0/2352	R01_cb8922_c1/f1p0/2352	NA
R01_cb8564_c82779/f1p0/2722	NA	R01_cb8564_c82779/f1p0/2722	NA	R01_cb8564_c82779/f1p0/2722
R01_cb10358_c0/f3p0/883	NA	NA	NA	R01_cb10358_c0/f3p0/883
R01_cb4563_c27/f1p0/2598	NA	R01_cb4563_c27/f1p0/2598	NA	NA
R01_cb8564_c82232/f1p1/3574	NA	R01_cb8564_c82232/f1p1/3574	NA	NA
R01_cb416_c54/f1p0/2035	NA	R01_cb416_c54/f1p0/2035	NA	R01_cb416_c54/f1p0/2035
R01_cb8564_c74051/f1p0/2917	NA	R01_cb8564_c74051/f1p0/2917	R01_cb8564_c74051/f1p0/2917	R01_cb8564_c74051/f1p0/2917
R01_cb376_c2/f1p0/2089	NA	R01_cb376_c2/f1p0/2089	NA	NA
R01_cb10106_c5/f1p1/824	NA	R01_cb10106_c5/f1p1/824	NA	NA
R01_cb8564_c118391/f1p0/2115	NA	R01_cb8564_c118391/f1p0/2115	NA	NA
R01_cb16672_c2/f1p0/665	R01_cb16672_c2/f1p0/665	R01_cb16672_c2/f1p0/665	R01_cb16672_c2/f1p0/665	NA

R01_cb8564_c16718/f1p0/3257	R01_cb8564_c16718/f1p0/3257	R01_cb8564_c16718/f1p0/3257	R01_cb8564_c16718/f1p0/3257	R01_cb8564_c16718/f1p0/3257
R01_cb9535_c28/f9p0/481	NA	R01_cb9535_c28/f9p0/481	R01_cb9535_c28/f9p0/481	R01_cb9535_c28/f9p0/481
R01_cb12110_c1/f1p1/622	R01_cb12110_c1/f1p1/622	R01_cb12110_c1/f1p1/622	NA	R01_cb12110_c1/f1p1/622
R01_cb11376_c0/f2p0/817	R01_cb11376_c0/f2p0/817	R01_cb11376_c0/f2p0/817	R01_cb11376_c0/f2p0/817	R01_cb11376_c0/f2p0/817
R01_cb8564_c4653/f2p0/2778	NA	R01_cb8564_c4653/f2p0/2778	NA	R01_cb8564_c4653/f2p0/2778
R01_cb6769_c18/f1p0/401	R01_cb6769_c18/f1p0/401	R01_cb6769_c18/f1p0/401	R01_cb6769_c18/f1p0/401	R01_cb6769_c18/f1p0/401
R01_cb2030_c2/f1p0/4096	NA	R01_cb2030_c2/f1p0/4096	NA	R01_cb2030_c2/f1p0/4096
R01_cb1676_c12/f1p0/5084	NA	R01_cb1676_c12/f1p0/5084	NA	NA
R01_cb654_c13/f1p0/623	NA	R01_cb654_c13/f1p0/623	NA	R01_cb654_c13/f1p0/623
R01_cb12176_c0/f3p0/709	NA	R01_cb12176_c0/f3p0/709	NA	R01_cb12176_c0/f3p0/709
R01_cb8564_c41314/f1p0/3957	NA	NA	NA	R01_cb8564_c41314/f1p0/3957
R01_cb8564_c52940/f1p2/3963	NA	NA	NA	R01_cb8564_c52940/f1p2/3963
R01_cb13743_c3/f1p1/1455	NA	NA	NA	R01_cb13743_c3/f1p1/1455
R01_cb18456_c6668/f1p0/485	R01_cb18456_c6668/f1p0/485	R01_cb18456_c6668/f1p0/485	R01_cb18456_c6668/f1p0/485	R01_cb18456_c6668/f1p0/485
R01_cb1333_c27/f1p0/3280	R01_cb1333_c27/f1p0/3280	R01_cb1333_c27/f1p0/3280	R01_cb1333_c27/f1p0/3280	R01_cb1333_c27/f1p0/3280
R01_cb10585_c0/f1p0/1616	NA	R01_cb10585_c0/f1p0/1616	NA	NA
R01_cb8564_c50208/f1p0/2195	NA	R01_cb8564_c50208/f1p0/2195	NA	NA
R01_cb8107_c1/f1p1/2459	R01_cb8107_c1/f1p1/2459	R01_cb8107_c1/f1p1/2459	R01_cb8107_c1/f1p1/2459	R01_cb8107_c1/f1p1/2459
R01_cb10295_c0/f2p0/1574	NA	R01_cb10295_c0/f2p0/1574	NA	NA
R01_cb6885_c1/f2p0/1890	NA	NA	NA	R01_cb6885_c1/f2p0/1890
R01_cb4323_c0/f1p0/3380	NA	NA	NA	R01_cb4323_c0/f1p0/3380
R01_cb11595_c2/f1p0/707	NA	R01_cb11595_c2/f1p0/707	R01_cb11595_c2/f1p0/707	NA
R01_cb8564_c19531/f1p0/1964	NA	NA	NA	R01_cb8564_c19531/f1p0/1964
R01_cb18409_c57/f1p0/427	R01_cb18409_c57/f1p0/427	R01_cb18409_c57/f1p0/427	R01_cb18409_c57/f1p0/427	R01_cb18409_c57/f1p0/427
R01_cb10866_c5/f1p0/1274	NA	R01_cb10866_c5/f1p0/1274	NA	NA

R01_cb14524_c1/f1p0/1321	NA	NA	NA	R01_cb14524_c1/f1p0/1321
R01_cb1178_c5/f2p0/1389	NA	NA	NA	R01_cb1178_c5/f2p0/1389
R01_cb1993_c2/f1p1/4105	NA	NA	NA	R01_cb1993_c2/f1p1/4105
R01_cb16896_c1/f1p0/1445	NA	R01_cb16896_c1/f1p0/1445	R01_cb16896_c1/f1p0/1445	R01_cb16896_c1/f1p0/1445
R01_cb10378_c0/f2p0/498	R01_cb10378_c0/f2p0/498	R01_cb10378_c0/f2p0/498	NA	R01_cb10378_c0/f2p0/498
R01_cb8539_c7/f1p0/2209	NA	R01_cb8539_c7/f1p0/2209	NA	NA
R01_cb5659_c102/f46p0/2178	NA	NA	NA	R01_cb5659_c102/f46p0/2178
R01_cb18535_c0/f1p0/838	NA	NA	NA	R01_cb18535_c0/f1p0/838
R01_cb12589_c6/f2p0/809	NA	NA	NA	R01_cb12589_c6/f2p0/809
R01_cb8564_c36735/f1p2/2783	NA	R01_cb8564_c36735/f1p2/2783	NA	NA
R01_cb9960_c6/f1p0/1645	NA	NA	NA	R01_cb9960_c6/f1p0/1645
R01_cb9334_c1/f1p0/2106	NA	R01_cb9334_c1/f1p0/2106	NA	NA
R01_cb8564_c89502/f1p0/2946	NA	R01_cb8564_c89502/f1p0/2946	NA	NA
R01_cb2644_c9/f1p0/4119	NA	R01_cb2644_c9/f1p0/4119	NA	R01_cb2644_c9/f1p0/4119
R01_cb18721_c1/f1p0/1646	NA	R01_cb18721_c1/f1p0/1646	NA	NA
R01_cb16338_c0/f5p1/1132	NA	NA	NA	R01_cb16338_c0/f5p1/1132
R01_cb101_c5/f1p0/3045	NA	NA	NA	R01_cb101_c5/f1p0/3045
R01_cb313_c14/f1p0/2248	NA	R01_cb313_c14/f1p0/2248	NA	R01_cb313_c14/f1p0/2248
R01_cb8564_c75318/f1p0/2619	NA	NA	NA	R01_cb8564_c75318/f1p0/2619
R01_cb289_c21/f1p0/1853	NA	R01_cb289_c21/f1p0/1853	NA	NA
R01_cb18456_c3283/f1p3/986	NA	R01_cb18456_c3283/f1p3/986	NA	NA
R01_cb14751_c1/f1p0/748	NA	R01_cb14751_c1/f1p0/748	NA	NA
R01_cb7257_c1/f1p0/2322	NA	NA	NA	R01_cb7257_c1/f1p0/2322
R01_cb15154_c6/f1p0/860	NA	NA	NA	R01_cb15154_c6/f1p0/860
R01_cb12865_c22/f1p0/438	NA	R01_cb12865_c22/f1p0/438	NA	NA
R01_cb8564_c119911/f1p0/210	NA	R01_cb8564_c119911/f1p0/210	R01_cb8564_c119911/f1p0/210	R01_cb8564_c119911/f1p0/210

2		2	2	2
R01_cb14293_c3/f1p0/1096	NA	R01_cb14293_c3/f1p0/1096	NA	NA
R01_cb12566_c10/f1p0/348	R01_cb12566_c10/f1p0/348	R01_cb12566_c10/f1p0/348	R01_cb12566_c10/f1p0/348	R01_cb12566_c10/f1p0/348
R01_cb18456_c5355/f1p0/796	NA	R01_cb18456_c5355/f1p0/796	NA	NA
R01_cb14004_c6/f1p1/1238	NA	NA	NA	R01_cb14004_c6/f1p1/1238
R01_cb13889_c16/f1p0/874	NA	R01_cb13889_c16/f1p0/874	NA	NA
R01_cb13586_c6/f1p0/448	NA	R01_cb13586_c6/f1p0/448	NA	NA
R01_cb16176_c0/f1p0/1117	NA	NA	NA	R01_cb16176_c0/f1p0/1117
R01_cb12743_c6/f5p0/974	NA	NA	NA	R01_cb12743_c6/f5p0/974
R01_cb3797_c9/f1p0/8016	NA	NA	NA	R01_cb3797_c9/f1p0/8016
R01_cb9222_c18/f1p0/2637	NA	R01_cb9222_c18/f1p0/2637	NA	NA
R01_cb7296_c2/f2p1/2070	NA	NA	NA	R01_cb7296_c2/f2p1/2070
R01_cb1240_c8/f1p0/2560	NA	R01_cb1240_c8/f1p0/2560	NA	NA
R01_cb8564_c123884/f1p0/2933	NA	R01_cb8564_c123884/f1p0/2933	R01_cb8564_c123884/f1p0/2933	R01_cb8564_c123884/f1p0/2933
R01_cb10091_c2/f1p0/3144	NA	R01_cb10091_c2/f1p0/3144	NA	NA
R01_cb2445_c4/f2p0/3645	NA	NA	NA	R01_cb2445_c4/f2p0/3645
R01_cb14694_c1/f1p0/1038	NA	NA	NA	R01_cb14694_c1/f1p0/1038
R01_cb16915_c1/f2p0/660	NA	R01_cb16915_c1/f2p0/660	NA	NA
R01_cb5807_c3/f1p0/2811	R01_cb5807_c3/f1p0/2811	R01_cb5807_c3/f1p0/2811	NA	R01_cb5807_c3/f1p0/2811
R01_cb8564_c72227/f2p1/4420	NA	NA	NA	R01_cb8564_c72227/f2p1/4420
R01_cb10221_c0/f3p0/1818	NA	R01_cb10221_c0/f3p0/1818	NA	R01_cb10221_c0/f3p0/1818
R01_cb2389_c12/f1p0/2660	NA	NA	NA	R01_cb2389_c12/f1p0/2660
R01_cb14316_c7/f1p0/1684	NA	R01_cb14316_c7/f1p0/1684	R01_cb14316_c7/f1p0/1684	R01_cb14316_c7/f1p0/1684
R01_cb11205_c0/f1p0/1620	NA	R01_cb11205_c0/f1p0/1620	NA	NA
R01_cb17583_c2/f1p1/1583	NA	NA	NA	R01_cb17583_c2/f1p1/1583

R01_cb18123_c0/f1p0/903	NA	R01_cb18123_c0/f1p0/903	NA	R01_cb18123_c0/f1p0/903
R01_cb3919_c1/f1p0/1252	NA	R01_cb3919_c1/f1p0/1252	NA	NA
R01_cb6586_c4/f1p0/1876	NA	R01_cb6586_c4/f1p0/1876	NA	NA
R01_cb9411_c2/f1p0/2414	R01_cb9411_c2/f1p0/2414	R01_cb9411_c2/f1p0/2414	R01_cb9411_c2/f1p0/2414	R01_cb9411_c2/f1p0/2414
R01_cb7600_c22/f1p0/340	R01_cb7600_c22/f1p0/340	R01_cb7600_c22/f1p0/340	R01_cb7600_c22/f1p0/340	R01_cb7600_c22/f1p0/340
R01_cb3218_c8/f1p0/2827	NA	NA	NA	R01_cb3218_c8/f1p0/2827
R01_cb8564_c34243/f2p0/3193	R01_cb8564_c34243/f2p0/3193	R01_cb8564_c34243/f2p0/3193	R01_cb8564_c34243/f2p0/3193	R01_cb8564_c34243/f2p0/3193
R01_cb8564_c21867/f1p1/3561	NA	R01_cb8564_c21867/f1p1/3561	NA	R01_cb8564_c21867/f1p1/3561
R01_cb17266_c10/f1p0/1080	NA	R01_cb17266_c10/f1p0/1080	R01_cb17266_c10/f1p0/1080	R01_cb17266_c10/f1p0/1080
R01_cb8564_c847/f2p0/2724	R01_cb8564_c847/f2p0/2724	R01_cb8564_c847/f2p0/2724	NA	NA
R01_cb11003_c1/f1p0/1895	NA	R01_cb11003_c1/f1p0/1895	NA	R01_cb11003_c1/f1p0/1895
R01_cb7953_c6/f1p0/3068	NA	R01_cb7953_c6/f1p0/3068	NA	NA
R01_cb8564_c115781/f1p0/2431	NA	R01_cb8564_c115781/f1p0/2431	NA	R01_cb8564_c115781/f1p0/2431
R01_cb8564_c120503/f1p0/1998	NA	NA	NA	R01_cb8564_c120503/f1p0/1998
R01_cb4469_c4/f1p0/4596	NA	NA	NA	R01_cb4469_c4/f1p0/4596
R01_cb11612_c1/f1p0/4652	NA	NA	NA	R01_cb11612_c1/f1p0/4652
R01_cb4501_c4/f1p0/3015	NA	NA	NA	R01_cb4501_c4/f1p0/3015
R01_cb7335_c4/f2p0/3632	NA	R01_cb7335_c4/f2p0/3632	NA	R01_cb7335_c4/f2p0/3632
R01_cb10288_c1/f1p0/2983	R01_cb10288_c1/f1p0/2983	R01_cb10288_c1/f1p0/2983	NA	NA
R01_cb48_c11/f1p2/5127	NA	NA	NA	R01_cb48_c11/f1p2/5127
R01_cb8564_c88253/f1p0/4290	R01_cb8564_c88253/f1p0/4290	R01_cb8564_c88253/f1p0/4290	NA	R01_cb8564_c88253/f1p0/4290
R01_cb5533_c24/f1p0/2861	R01_cb5533_c24/f1p0/2861	R01_cb5533_c24/f1p0/2861	R01_cb5533_c24/f1p0/2861	R01_cb5533_c24/f1p0/2861

R01_cb12360_c5/f1p0/516	R01_cb12360_c5/f1p0/516	R01_cb12360_c5/f1p0/516	NA	NA
R01_cb18409_c92/f1p0/382	R01_cb18409_c92/f1p0/382	R01_cb18409_c92/f1p0/382	R01_cb18409_c92/f1p0/382	R01_cb18409_c92/f1p0/382
R01_cb69_c36/f1p0/868	NA	NA	NA	R01_cb69_c36/f1p0/868
R01_cb17562_c5/f1p0/519	NA	R01_cb17562_c5/f1p0/519	NA	NA
R01_cb18359_c2/f1p0/1790	NA	NA	NA	R01_cb18359_c2/f1p0/1790
R01_cb11697_c1/f1p0/3198	NA	R01_cb11697_c1/f1p0/3198	NA	NA
R01_cb5815_c2/f1p0/3001	NA	NA	NA	R01_cb5815_c2/f1p0/3001
R01_cb12650_c1/f1p0/525	NA	R01_cb12650_c1/f1p0/525	NA	NA
R01_cb18297_c0/f2p0/1609	NA	NA	NA	R01_cb18297_c0/f2p0/1609
R01_cb18002_c1/f1p0/1003	NA	R01_cb18002_c1/f1p0/1003	NA	NA
R01_cb15019_c8/f1p1/916	R01_cb15019_c8/f1p1/916	R01_cb15019_c8/f1p1/916	NA	NA
R01_cb9646_c3/f1p0/1831	NA	NA	NA	R01_cb9646_c3/f1p0/1831
R01_cb18482_c0/f1p0/1690	NA	NA	NA	R01_cb18482_c0/f1p0/1690
R01_cb2744_c5/f1p0/820	NA	NA	NA	R01_cb2744_c5/f1p0/820
R01_cb8564_c2465/f1p0/2936	NA	R01_cb8564_c2465/f1p0/2936	NA	R01_cb8564_c2465/f1p0/2936
R01_cb5156_c1/f1p0/3165	NA	NA	NA	R01_cb5156_c1/f1p0/3165
R01_cb18456_c6102/f1p0/695	NA	NA	NA	R01_cb18456_c6102/f1p0/695
R01_cb11827_c1/f1p0/1893	NA	NA	NA	R01_cb11827_c1/f1p0/1893
R01_cb8564_c84129/f1p0/2206	NA	R01_cb8564_c84129/f1p0/2206	NA	NA
R01_cb18456_c7195/f1p0/695	R01_cb18456_c7195/f1p0/695	R01_cb18456_c7195/f1p0/695	R01_cb18456_c7195/f1p0/695	R01_cb18456_c7195/f1p0/695
R01_cb15287_c0/f1p0/561	NA	NA	NA	R01_cb15287_c0/f1p0/561
R01_cb5412_c0/f2p0/2007	NA	NA	NA	R01_cb5412_c0/f2p0/2007
R01_cb11321_c0/f2p0/1358	NA	NA	NA	R01_cb11321_c0/f2p0/1358
R01_cb18456_c7249/f1p0/450	R01_cb18456_c7249/f1p0/450	R01_cb18456_c7249/f1p0/450	R01_cb18456_c7249/f1p0/450	R01_cb18456_c7249/f1p0/450
R01_cb16645_c14/f1p0/1352	NA	R01_cb16645_c14/f1p0/1352	NA	R01_cb16645_c14/f1p0/1352
R01_cb14239_c17/f1p0/699	R01_cb14239_c17/f1p0/699	R01_cb14239_c17/f1p0/699	NA	R01_cb14239_c17/f1p0/699

R01_cb10196_c1/f1p0/3039	NA	R01_cb10196_c1/f1p0/3039	NA	R01_cb10196_c1/f1p0/3039
R01_cb7171_c5/f1p1/2635	NA	NA	NA	R01_cb7171_c5/f1p1/2635
R01_cb17669_c0/f2p0/582	NA	R01_cb17669_c0/f2p0/582	NA	R01_cb17669_c0/f2p0/582
R01_cb4269_c2/f2p0/807	R01_cb4269_c2/f2p0/807	R01_cb4269_c2/f2p0/807	NA	R01_cb4269_c2/f2p0/807
R01_cb11375_c1/f1p1/3084	NA	R01_cb11375_c1/f1p1/3084	NA	NA
R01_cb10611_c4/f1p0/1378	NA	NA	NA	R01_cb10611_c4/f1p0/1378
R01_cb293_c5/f1p0/4488	NA	NA	NA	R01_cb293_c5/f1p0/4488
R01_cb5506_c4/f1p0/990	NA	NA	NA	R01_cb5506_c4/f1p0/990
R01_cb8941_c5/f1p0/1527	NA	NA	NA	R01_cb8941_c5/f1p0/1527
R01_cb16645_c52/f1p0/1476	NA	R01_cb16645_c52/f1p0/1476	NA	NA
R01_cb18211_c0/f1p0/504	NA	NA	NA	R01_cb18211_c0/f1p0/504
R01_cb2022_c12/f1p1/2286	NA	NA	NA	R01_cb2022_c12/f1p1/2286
R01_cb17329_c1/f1p0/1028	NA	NA	NA	R01_cb17329_c1/f1p0/1028
R01_cb10397_c5/f1p0/1155	NA	R01_cb10397_c5/f1p0/1155	NA	R01_cb10397_c5/f1p0/1155
R01_cb15346_c1/f12p2/660	NA	NA	R01_cb15346_c1/f12p2/660	R01_cb15346_c1/f12p2/660
R01_cb16184_c1/f1p0/418	R01_cb16184_c1/f1p0/418	R01_cb16184_c1/f1p0/418	R01_cb16184_c1/f1p0/418	R01_cb16184_c1/f1p0/418
R01_cb11050_c5/f1p0/1720	NA	NA	NA	R01_cb11050_c5/f1p0/1720
R01_cb2027_c4/f1p0/3050	NA	NA	R01_cb2027_c4/f1p0/3050	NA
R01_cb13884_c4/f3p0/437	R01_cb13884_c4/f3p0/437	NA	R01_cb13884_c4/f3p0/437	R01_cb13884_c4/f3p0/437
R01_cb8564_c14734/f1p0/3360	NA	R01_cb8564_c14734/f1p0/3360	NA	R01_cb8564_c14734/f1p0/3360
R01_cb8564_c127384/f1p0/3185	NA	R01_cb8564_c127384/f1p0/3185	NA	NA
R01_cb13392_c31/f1p0/469	NA	NA	R01_cb13392_c31/f1p0/469	R01_cb13392_c31/f1p0/469
R01_cb17297_c1/f1p0/577	NA	R01_cb17297_c1/f1p0/577	R01_cb17297_c1/f1p0/577	R01_cb17297_c1/f1p0/577
R01_cb8564_c1024/f1p0/3041	NA	R01_cb8564_c1024/f1p0/3041	NA	NA
R01_cb18456_c7196/f1p0/455	R01_cb18456_c7196/f1p0/455	R01_cb18456_c7196/f1p0/455	R01_cb18456_c7196/f1p0/455	R01_cb18456_c7196/f1p0/455

R01_cb18456_c765/f1p11/807	NA	R01_cb18456_c765/f1p11/807	NA	NA
R01_cb5246_c2/f1p0/2737	R01_cb5246_c2/f1p0/2737	R01_cb5246_c2/f1p0/2737	R01_cb5246_c2/f1p0/2737	R01_cb5246_c2/f1p0/2737
R01_cb12636_c2/f2p0/1527	NA	NA	NA	R01_cb12636_c2/f2p0/1527
R01_cb12045_c5/f1p0/6393	NA	R01_cb12045_c5/f1p0/6393	NA	R01_cb12045_c5/f1p0/6393
R01_cb10921_c1/f1p0/2459	NA	NA	NA	R01_cb10921_c1/f1p0/2459
R01_cb8564_c77837/f1p0/4056	R01_cb8564_c77837/f1p0/4056	R01_cb8564_c77837/f1p0/4056	NA	NA
R01_cb6553_c2/f1p0/2826	NA	R01_cb6553_c2/f1p0/2826	NA	R01_cb6553_c2/f1p0/2826
R01_cb18456_c2063/f1p1/1677	NA	R01_cb18456_c2063/f1p1/1677	NA	NA
R01_cb8564_c1993/f1p0/2538	NA	R01_cb8564_c1993/f1p0/2538	NA	NA
R01_cb7502_c2/f1p0/2600	NA	NA	NA	R01_cb7502_c2/f1p0/2600
R01_cb3922_c24/f1p0/615	NA	R01_cb3922_c24/f1p0/615	R01_cb3922_c24/f1p0/615	R01_cb3922_c24/f1p0/615
R01_cb18456_c6618/f1p0/416	R01_cb18456_c6618/f1p0/416	R01_cb18456_c6618/f1p0/416	R01_cb18456_c6618/f1p0/416	R01_cb18456_c6618/f1p0/416
R01_cb8564_c40533/f1p0/2719	NA	R01_cb8564_c40533/f1p0/2719	R01_cb8564_c40533/f1p0/2719	R01_cb8564_c40533/f1p0/2719
R01_cb18409_c101/f1p0/1007	R01_cb18409_c101/f1p0/1007	R01_cb18409_c101/f1p0/1007	R01_cb18409_c101/f1p0/1007	R01_cb18409_c101/f1p0/1007
R01_cb8088_c1/f1p0/2570	NA	R01_cb8088_c1/f1p0/2570	NA	NA
R01_cb8564_c72625/f1p0/2535	R01_cb8564_c72625/f1p0/2535	R01_cb8564_c72625/f1p0/2535	R01_cb8564_c72625/f1p0/2535	R01_cb8564_c72625/f1p0/2535
R01_cb13692_c3/f1p0/1607	NA	R01_cb13692_c3/f1p0/1607	NA	NA
R01_cb17761_c0/f4p0/769	NA	NA	NA	R01_cb17761_c0/f4p0/769
R01_cb15963_c2/f1p0/518	NA	R01_cb15963_c2/f1p0/518	R01_cb15963_c2/f1p0/518	NA
R01_cb18192_c2/f2p0/1180	NA	R01_cb18192_c2/f2p0/1180	R01_cb18192_c2/f2p0/1180	R01_cb18192_c2/f2p0/1180
R01_cb6602_c47/f1p0/3008	NA	NA	NA	R01_cb6602_c47/f1p0/3008
R01_cb16078_c6/f1p1/620	R01_cb16078_c6/f1p1/620	R01_cb16078_c6/f1p1/620	R01_cb16078_c6/f1p1/620	NA
R01_cb1119_c7/f1p0/2404	NA	R01_cb1119_c7/f1p0/2404	R01_cb1119_c7/f1p0/2404	NA
R01_cb12623_c2/f1p0/1115	NA	R01_cb12623_c2/f1p0/1115	R01_cb12623_c2/f1p0/1115	NA

R01_cb8564_c75753/f1p0/3425	NA	R01_cb8564_c75753/f1p0/3425	NA	R01_cb8564_c75753/f1p0/3425
R01_cb2378_c12/f1p0/2671	NA	NA	NA	R01_cb2378_c12/f1p0/2671
R01_cb11025_c1/f1p0/3772	NA	NA	NA	R01_cb11025_c1/f1p0/3772
R01_cb9272_c3/f1p0/4598	NA	NA	NA	R01_cb9272_c3/f1p0/4598
R01_cb5499_c0/f2p0/2164	NA	R01_cb5499_c0/f2p0/2164	R01_cb5499_c0/f2p0/2164	R01_cb5499_c0/f2p0/2164
R01_cb16356_c0/f1p0/1260	NA	R01_cb16356_c0/f1p0/1260	NA	R01_cb16356_c0/f1p0/1260
R01_cb8564_c9887/f2p0/2563	R01_cb8564_c9887/f2p0/2563	R01_cb8564_c9887/f2p0/2563	NA	R01_cb8564_c9887/f2p0/2563
R01_cb2632_c4/f1p0/3384	NA	R01_cb2632_c4/f1p0/3384	NA	NA
R01_cb8564_c32509/f1p1/2880	NA	R01_cb8564_c32509/f1p1/2880	NA	NA
R01_cb8564_c90311/f1p0/3083	NA	R01_cb8564_c90311/f1p0/3083	R01_cb8564_c90311/f1p0/3083	NA
R01_cb8564_c1449/f1p0/2343	NA	NA	NA	R01_cb8564_c1449/f1p0/2343
R01_cb10029_c2822/f9p0/631	NA	R01_cb10029_c2822/f9p0/631	NA	NA
R01_cb1849_c25/f18p1/1918	NA	NA	NA	R01_cb1849_c25/f18p1/1918
R01_cb6615_c5/f1p0/3050	NA	NA	R01_cb6615_c5/f1p0/3050	R01_cb6615_c5/f1p0/3050
R01_cb10948_c10/f4p0/1324	NA	NA	NA	R01_cb10948_c10/f4p0/1324
R01_cb8564_c69742/f1p0/4372	NA	NA	NA	R01_cb8564_c69742/f1p0/4372
R01_cb8564_c70174/f1p0/1958	NA	R01_cb8564_c70174/f1p0/1958	NA	NA
R01_cb11429_c5/f1p0/1866	NA	NA	NA	R01_cb11429_c5/f1p0/1866
R01_cb18725_c0/f1p0/5915	NA	NA	NA	R01_cb18725_c0/f1p0/5915
R01_cb8564_c120462/f1p0/2066	NA	R01_cb8564_c120462/f1p0/2066	NA	NA
R01_cb4076_c4/f1p0/2859	R01_cb4076_c4/f1p0/2859	R01_cb4076_c4/f1p0/2859	R01_cb4076_c4/f1p0/2859	R01_cb4076_c4/f1p0/2859
R01_cb8564_c112100/f1p0/3100	NA	R01_cb8564_c112100/f1p0/3100	R01_cb8564_c112100/f1p0/3100	NA
R01_cb8564_c72494/f1p0/3734	NA	NA	NA	R01_cb8564_c72494/f1p0/3734
R01_cb8564_c146513/f2p1/328	NA	NA	NA	R01_cb8564_c146513/f2p1/328

1				1
R01_cb3303_c1/f1p0/3667	NA	R01_cb3303_c1/f1p0/3667	R01_cb3303_c1/f1p0/3667	R01_cb3303_c1/f1p0/3667
R01_cb18456_c5727/f1p0/849	NA	R01_cb18456_c5727/f1p0/849	NA	NA
R01_cb18709_c0/f1p1/5732	NA	R01_cb18709_c0/f1p1/5732	NA	NA
R01_cb8564_c70179/f1p0/2630	NA	NA	NA	R01_cb8564_c70179/f1p0/2630
R01_cb10071_c4/f1p0/541	NA	R01_cb10071_c4/f1p0/541	NA	NA
R01_cb7757_c12/f1p1/1610	NA	NA	R01_cb7757_c12/f1p1/1610	R01_cb7757_c12/f1p1/1610
R01_cb8564_c2598/f1p0/4915	NA	NA	NA	R01_cb8564_c2598/f1p0/4915
R01_cb14787_c8/f1p0/1267	NA	R01_cb14787_c8/f1p0/1267	NA	NA
R01_cb16462_c1/f1p0/1127	NA	NA	NA	R01_cb16462_c1/f1p0/1127
R01_cb1049_c7/f1p0/4504	NA	R01_cb1049_c7/f1p0/4504	NA	R01_cb1049_c7/f1p0/4504
R01_cb9592_c2/f1p0/2038	NA	NA	NA	R01_cb9592_c2/f1p0/2038
R01_cb8564_c77598/f1p1/2007	R01_cb8564_c77598/f1p1/2007	R01_cb8564_c77598/f1p1/2007	NA	NA
R01_cb14524_c2/f1p0/1611	NA	NA	NA	R01_cb14524_c2/f1p0/1611
R01_cb18456_c1863/f1p0/878	NA	R01_cb18456_c1863/f1p0/878	NA	NA
R01_cb14660_c2/f2p0/728	NA	R01_cb14660_c2/f2p0/728	NA	R01_cb14660_c2/f2p0/728
R01_cb10031_c6/f1p0/757	NA	R01_cb10031_c6/f1p0/757	NA	NA
R01_cb8564_c4911/f1p0/2481	R01_cb8564_c4911/f1p0/2481	R01_cb8564_c4911/f1p0/2481	R01_cb8564_c4911/f1p0/2481	R01_cb8564_c4911/f1p0/2481
R01_cb1905_c26/f1p0/2195	R01_cb1905_c26/f1p0/2195	R01_cb1905_c26/f1p0/2195	R01_cb1905_c26/f1p0/2195	R01_cb1905_c26/f1p0/2195
R01_cb8564_c17893/f3p0/4091	NA	R01_cb8564_c17893/f3p0/4091	NA	NA
R01_cb11135_c4/f1p0/1315	NA	R01_cb11135_c4/f1p0/1315	R01_cb11135_c4/f1p0/1315	R01_cb11135_c4/f1p0/1315
R01_cb16098_c1/f1p0/618	R01_cb16098_c1/f1p0/618	R01_cb16098_c1/f1p0/618	R01_cb16098_c1/f1p0/618	R01_cb16098_c1/f1p0/618
R01_cb6845_c40/f2p0/2333	NA	NA	NA	R01_cb6845_c40/f2p0/2333
R01_cb15081_c6/f8p1/954	NA	R01_cb15081_c6/f8p1/954	NA	NA
R01_cb3269_c11/f1p0/2163	NA	R01_cb3269_c11/f1p0/2163	NA	NA

R01_cb8564_c45235/f1p1/2650	NA	NA	NA	R01_cb8564_c45235/f1p1/2650
R01_cb812_c8/f1p1/3695	NA	R01_cb812_c8/f1p1/3695	NA	NA
R01_cb2960_c2/f1p0/2155	NA	NA	NA	R01_cb2960_c2/f1p0/2155
R01_cb6547_c9/f1p1/1933	NA	NA	NA	R01_cb6547_c9/f1p1/1933
R01_cb18456_c4956/f1p3/1222	NA	R01_cb18456_c4956/f1p3/1222	NA	NA
R01_cb7228_c0/f9p1/2624	NA	NA	NA	R01_cb7228_c0/f9p1/2624
R01_cb10303_c1/f1p0/2876	NA	R01_cb10303_c1/f1p0/2876	R01_cb10303_c1/f1p0/2876	NA
R01_cb8564_c75788/f1p0/2238	NA	NA	R01_cb8564_c75788/f1p0/2238	R01_cb8564_c75788/f1p0/2238
R01_cb2959_c5/f1p0/3349	NA	NA	NA	R01_cb2959_c5/f1p0/3349
R01_cb13433_c128/f1p0/5565	NA	NA	NA	R01_cb13433_c128/f1p0/5565
R01_cb9617_c18/f2p3/1848	NA	NA	NA	R01_cb9617_c18/f2p3/1848
R01_cb8564_c22798/f1p0/2414	NA	NA	R01_cb8564_c22798/f1p0/2414	NA
R01_cb17894_c0/f2p0/871	R01_cb17894_c0/f2p0/871	R01_cb17894_c0/f2p0/871	R01_cb17894_c0/f2p0/871	R01_cb17894_c0/f2p0/871
R01_cb18456_c7617/f1p0/1413	NA	R01_cb18456_c7617/f1p0/1413	NA	R01_cb18456_c7617/f1p0/1413
R01_cb2674_c16/f1p0/3023	NA	R01_cb2674_c16/f1p0/3023	NA	R01_cb2674_c16/f1p0/3023
R01_cb16645_c30/f1p0/380	R01_cb16645_c30/f1p0/380	R01_cb16645_c30/f1p0/380	R01_cb16645_c30/f1p0/380	R01_cb16645_c30/f1p0/380
R01_cb8905_c8/f1p1/1392	NA	NA	NA	R01_cb8905_c8/f1p1/1392
R01_cb8564_c72710/f1p2/3413	NA	NA	NA	R01_cb8564_c72710/f1p2/3413
R01_cb2200_c2/f1p0/4021	NA	NA	R01_cb2200_c2/f1p0/4021	R01_cb2200_c2/f1p0/4021
R01_cb1788_c7/f1p0/2957	NA	R01_cb1788_c7/f1p0/2957	NA	R01_cb1788_c7/f1p0/2957
R01_cb502_c19/f1p0/2650	NA	R01_cb502_c19/f1p0/2650	NA	NA
R01_cb5339_c3/f1p0/2386	NA	NA	NA	R01_cb5339_c3/f1p0/2386
R01_cb18750_c0/f1p0/5164	NA	NA	NA	R01_cb18750_c0/f1p0/5164
R01_cb8564_c115317/f1p0/2597	NA	R01_cb8564_c115317/f1p0/2597	NA	NA
R01_cb6278_c12/f1p0/2879	NA	NA	NA	R01_cb6278_c12/f1p0/2879

R01_cb11774_c1/f1p0/2870	R01_cb11774_c1/f1p0/2870	R01_cb11774_c1/f1p0/2870	NA	NA
R01_cb15114_c0/f4p0/975	NA	NA	NA	R01_cb15114_c0/f4p0/975
R01_cb8564_c53156/f1p0/2866	NA	NA	NA	R01_cb8564_c53156/f1p0/2866
R01_cb11488_c1/f1p0/1908	NA	R01_cb11488_c1/f1p0/1908	R01_cb11488_c1/f1p0/1908	R01_cb11488_c1/f1p0/1908
R01_cb8564_c108411/f1p1/3146	NA	R01_cb8564_c108411/f1p1/3146	NA	NA
R01_cb12045_c4/f1p1/1348	NA	NA	NA	R01_cb12045_c4/f1p1/1348
R01_cb5950_c19/f1p0/2780	NA	R01_cb5950_c19/f1p0/2780	NA	NA
R01_cb17786_c1/f1p0/468	R01_cb17786_c1/f1p0/468	R01_cb17786_c1/f1p0/468	R01_cb17786_c1/f1p0/468	R01_cb17786_c1/f1p0/468
R01_cb3774_c43/f1p1/2434	NA	R01_cb3774_c43/f1p1/2434	NA	NA
R01_cb8564_c43335/f1p0/2669	R01_cb8564_c43335/f1p0/2669	R01_cb8564_c43335/f1p0/2669	R01_cb8564_c43335/f1p0/2669	R01_cb8564_c43335/f1p0/2669
R01_cb15702_c0/f1p0/451	R01_cb15702_c0/f1p0/451	R01_cb15702_c0/f1p0/451	R01_cb15702_c0/f1p0/451	R01_cb15702_c0/f1p0/451
R01_cb16822_c1/f1p0/923	NA	R01_cb16822_c1/f1p0/923	NA	NA
R01_cb16541_c1/f1p0/1504	NA	NA	NA	R01_cb16541_c1/f1p0/1504
R01_cb4370_c7/f1p0/2660	NA	NA	NA	R01_cb4370_c7/f1p0/2660
R01_cb17659_c0/f1p1/1568	NA	NA	NA	R01_cb17659_c0/f1p1/1568
R01_cb4708_c1/f2p0/1581	NA	R01_cb4708_c1/f2p0/1581	NA	NA
R01_cb14004_c21/f15p1/1437	NA	NA	NA	R01_cb14004_c21/f15p1/1437
R01_cb4657_c10/f1p0/4691	NA	R01_cb4657_c10/f1p0/4691	NA	NA
R01_cb1892_c2/f1p0/4133	NA	R01_cb1892_c2/f1p0/4133	NA	R01_cb1892_c2/f1p0/4133
R01_cb8564_c91081/f1p0/2806	R01_cb8564_c91081/f1p0/2806	R01_cb8564_c91081/f1p0/2806	NA	NA
R01_cb10198_c1/f4p1/606	NA	R01_cb10198_c1/f4p1/606	NA	NA
R01_cb15591_c9/f1p0/521	NA	R01_cb15591_c9/f1p0/521	R01_cb15591_c9/f1p0/521	NA
R01_cb9234_c2/f1p0/1510	NA	R01_cb9234_c2/f1p0/1510	NA	R01_cb9234_c2/f1p0/1510

R01_cb4576_c34/f1p0/2188	NA	R01_cb4576_c34/f1p0/2188	NA	NA
R01_cb7484_c4/f1p0/2339	NA	R01_cb7484_c4/f1p0/2339	NA	R01_cb7484_c4/f1p0/2339
R01_cb13733_c3/f1p0/1227	NA	R01_cb13733_c3/f1p0/1227	NA	NA
R01_cb8564_c38656/f1p0/3990	R01_cb8564_c38656/f1p0/3990	R01_cb8564_c38656/f1p0/3990	R01_cb8564_c38656/f1p0/3990	NA
R01_cb8564_c75099/f1p1/2485	NA	NA	NA	R01_cb8564_c75099/f1p1/2485
R01_cb14566_c2/f1p0/1746	NA	R01_cb14566_c2/f1p0/1746	R01_cb14566_c2/f1p0/1746	R01_cb14566_c2/f1p0/1746
R01_cb8564_c3059/f1p0/2839	NA	R01_cb8564_c3059/f1p0/2839	NA	R01_cb8564_c3059/f1p0/2839
R01_cb12223_c3/f1p0/435	R01_cb12223_c3/f1p0/435	R01_cb12223_c3/f1p0/435	R01_cb12223_c3/f1p0/435	R01_cb12223_c3/f1p0/435
R01_cb7032_c4/f1p0/2058	NA	NA	NA	R01_cb7032_c4/f1p0/2058
R01_cb11307_c3/f1p0/819	R01_cb11307_c3/f1p0/819	R01_cb11307_c3/f1p0/819	R01_cb11307_c3/f1p0/819	R01_cb11307_c3/f1p0/819
R01_cb13780_c4/f1p0/1353	NA	R01_cb13780_c4/f1p0/1353	NA	R01_cb13780_c4/f1p0/1353
R01_cb16888_c7/f1p0/1000	NA	NA	R01_cb16888_c7/f1p0/1000	R01_cb16888_c7/f1p0/1000
R01_cb15352_c7/f6p2/757	NA	R01_cb15352_c7/f6p2/757	NA	R01_cb15352_c7/f6p2/757
R01_cb16365_c3/f1p0/1755	NA	R01_cb16365_c3/f1p0/1755	NA	R01_cb16365_c3/f1p0/1755
R01_cb12037_c10/f1p0/643	R01_cb12037_c10/f1p0/643	NA	R01_cb12037_c10/f1p0/643	NA
R01_cb18131_c1/f1p0/1572	NA	NA	NA	R01_cb18131_c1/f1p0/1572
R01_cb15318_c2/f2p1/570	R01_cb15318_c2/f2p1/570	NA	NA	NA
R01_cb6326_c72/f1p0/532	R01_cb6326_c72/f1p0/532	R01_cb6326_c72/f1p0/532	NA	R01_cb6326_c72/f1p0/532
R01_cb6480_c0/f2p1/2702	NA	NA	NA	R01_cb6480_c0/f2p1/2702
R01_cb6570_c3/f1p0/2579	NA	R01_cb6570_c3/f1p0/2579	NA	R01_cb6570_c3/f1p0/2579
R01_cb15207_c51/f1p2/833	NA	R01_cb15207_c51/f1p2/833	R01_cb15207_c51/f1p2/833	NA
R01_cb8564_c46054/f1p0/2739	R01_cb8564_c46054/f1p0/2739	R01_cb8564_c46054/f1p0/2739	NA	R01_cb8564_c46054/f1p0/2739
R01_cb10272_c19/f1p0/1707	NA	R01_cb10272_c19/f1p0/1707	R01_cb10272_c19/f1p0/1707	R01_cb10272_c19/f1p0/1707
R01_cb13184_c3/f1p0/438	R01_cb13184_c3/f1p0/438	R01_cb13184_c3/f1p0/438	R01_cb13184_c3/f1p0/438	R01_cb13184_c3/f1p0/438

R01_cb8564_c82043/f1p0/2070	R01_cb8564_c82043/f1p0/2070	R01_cb8564_c82043/f1p0/2070	R01_cb8564_c82043/f1p0/2070	R01_cb8564_c82043/f1p0/2070
R01_cb8564_c4493/f1p1/3835	NA	R01_cb8564_c4493/f1p1/3835	R01_cb8564_c4493/f1p1/3835	R01_cb8564_c4493/f1p1/3835
R01_cb882_c8/f1p0/1341	NA	R01_cb882_c8/f1p0/1341	NA	R01_cb882_c8/f1p0/1341
R01_cb8564_c1735/f1p0/2133	NA	R01_cb8564_c1735/f1p0/2133	NA	R01_cb8564_c1735/f1p0/2133
R01_cb16704_c4/f1p0/1790	NA	R01_cb16704_c4/f1p0/1790	NA	R01_cb16704_c4/f1p0/1790
R01_cb10888_c2/f1p0/2349	NA	NA	NA	R01_cb10888_c2/f1p0/2349
R01_cb8122_c4/f1p0/2280	NA	NA	NA	R01_cb8122_c4/f1p0/2280
R01_cb5676_c9/f1p0/2906	NA	NA	NA	R01_cb5676_c9/f1p0/2906
R01_cb18456_c3620/f1p0/387	R01_cb18456_c3620/f1p0/387	R01_cb18456_c3620/f1p0/387	R01_cb18456_c3620/f1p0/387	R01_cb18456_c3620/f1p0/387
R01_cb11353_c1/f1p0/892	NA	NA	NA	R01_cb11353_c1/f1p0/892
R01_cb8549_c0/f1p0/2338	NA	NA	NA	R01_cb8549_c0/f1p0/2338
R01_cb8564_c109430/f3p0/2244	NA	NA	R01_cb8564_c109430/f3p0/2244	R01_cb8564_c109430/f3p0/2244
R01_cb18409_c98/f1p0/726	R01_cb18409_c98/f1p0/726	R01_cb18409_c98/f1p0/726	R01_cb18409_c98/f1p0/726	R01_cb18409_c98/f1p0/726
R01_cb13026_c6/f1p0/795	NA	R01_cb13026_c6/f1p0/795	NA	R01_cb13026_c6/f1p0/795
R01_cb507_c5/f1p0/1714	NA	NA	NA	R01_cb507_c5/f1p0/1714
R01_cb12869_c13/f1p0/1587	NA	NA	NA	R01_cb12869_c13/f1p0/1587
R01_cb11645_c0/f1p0/639	NA	R01_cb11645_c0/f1p0/639	NA	R01_cb11645_c0/f1p0/639
R01_cb8354_c7/f1p0/2141	NA	NA	NA	R01_cb8354_c7/f1p0/2141
R01_cb18456_c7251/f1p0/734	NA	NA	NA	R01_cb18456_c7251/f1p0/734
R01_cb8564_c90539/f1p0/3298	NA	NA	NA	R01_cb8564_c90539/f1p0/3298
R01_cb3602_c4/f2p1/618	R01_cb3602_c4/f2p1/618	R01_cb3602_c4/f2p1/618	NA	NA
R01_cb11072_c2/f2p1/509	NA	R01_cb11072_c2/f2p1/509	NA	NA
R01_cb13527_c2/f4p1/766	NA	NA	NA	R01_cb13527_c2/f4p1/766
R01_cb8564_c118507/f1p0/242	NA	R01_cb8564_c118507/f1p0/242	NA	NA

0		0		
R01_cb16248_c3/f1p0/677	NA	R01_cb16248_c3/f1p0/677	NA	NA
R01_cb6032_c41/f1p0/2686	NA	R01_cb6032_c41/f1p0/2686	NA	R01_cb6032_c41/f1p0/2686
R01_cb16261_c0/f1p0/556	NA	R01_cb16261_c0/f1p0/556	NA	R01_cb16261_c0/f1p0/556
R01_cb11924_c9/f4p0/607	R01_cb11924_c9/f4p0/607	R01_cb11924_c9/f4p0/607	R01_cb11924_c9/f4p0/607	NA
R01_cb1733_c0/f1p0/4190	NA	R01_cb1733_c0/f1p0/4190	NA	NA
R01_cb8564_c3143/f1p0/2688	NA	NA	NA	R01_cb8564_c3143/f1p0/2688
R01_cb5041_c1/f1p0/3242	NA	R01_cb5041_c1/f1p0/3242	NA	NA
R01_cb16866_c7/f1p0/8222	NA	R01_cb16866_c7/f1p0/8222	R01_cb16866_c7/f1p0/8222	NA
R01_cb12215_c2/f1p0/1138	NA	NA	NA	R01_cb12215_c2/f1p0/1138
R01_cb11060_c2/f1p0/2069	NA	R01_cb11060_c2/f1p0/2069	NA	NA
R01_cb6719_c19/f1p0/2110	R01_cb6719_c19/f1p0/2110	R01_cb6719_c19/f1p0/2110	R01_cb6719_c19/f1p0/2110	R01_cb6719_c19/f1p0/2110
R01_cb15006_c2/f1p0/1191	NA	R01_cb15006_c2/f1p0/1191	NA	NA
R01_cb2710_c8/f1p0/3470	NA	R01_cb2710_c8/f1p0/3470	NA	R01_cb2710_c8/f1p0/3470
R01_cb3791_c2/f1p0/3527	NA	NA	NA	R01_cb3791_c2/f1p0/3527
R01_cb2160_c15/f1p0/3121	NA	NA	NA	R01_cb2160_c15/f1p0/3121
R01_cb11377_c2/f1p0/2982	NA	R01_cb11377_c2/f1p0/2982	NA	NA
R01_cb8564_c112282/f1p0/2640	NA	R01_cb8564_c112282/f1p0/2640	R01_cb8564_c112282/f1p0/2640	R01_cb8564_c112282/f1p0/2640
R01_cb8564_c81124/f1p1/2509	NA	R01_cb8564_c81124/f1p1/2509	NA	NA
R01_cb454_c39/f1p0/2534	NA	NA	NA	R01_cb454_c39/f1p0/2534
R01_cb13910_c13/f1p0/1490	NA	R01_cb13910_c13/f1p0/1490	R01_cb13910_c13/f1p0/1490	R01_cb13910_c13/f1p0/1490
R01_cb5042_c1/f1p0/3196	NA	NA	R01_cb5042_c1/f1p0/3196	R01_cb5042_c1/f1p0/3196
R01_cb17648_c0/f1p0/1665	NA	R01_cb17648_c0/f1p0/1665	NA	NA
R01_cb16037_c7/f1p0/777	NA	R01_cb16037_c7/f1p0/777	NA	NA
R01_cb5783_c0/f1p0/3011	NA	R01_cb5783_c0/f1p0/3011	NA	R01_cb5783_c0/f1p0/3011

R01_cb6553_c1/f2p0/1270	NA	NA	NA	R01_cb6553_c1/f2p0/1270
R01_cb2779_c4/f1p0/1936	NA	NA	NA	R01_cb2779_c4/f1p0/1936
R01_cb18409_c79/f1p0/1069	R01_cb18409_c79/f1p0/1069	R01_cb18409_c79/f1p0/1069	R01_cb18409_c79/f1p0/1069	R01_cb18409_c79/f1p0/1069
R01_cb8564_c82425/f1p0/2589	R01_cb8564_c82425/f1p0/2589	R01_cb8564_c82425/f1p0/2589	NA	NA
R01_cb8630_c1/f1p0/2821	NA	R01_cb8630_c1/f1p0/2821	NA	NA
R01_cb12916_c4/f2p4/756	NA	NA	R01_cb12916_c4/f2p4/756	R01_cb12916_c4/f2p4/756
R01_cb17898_c0/f2p0/972	R01_cb17898_c0/f2p0/972	R01_cb17898_c0/f2p0/972	NA	R01_cb17898_c0/f2p0/972
R01_cb7394_c0/f3p0/2628	NA	R01_cb7394_c0/f3p0/2628	NA	NA
R01_cb1568_c1/f5p2/2091	NA	NA	NA	R01_cb1568_c1/f5p2/2091
R01_cb17266_c21/f1p0/1508	NA	R01_cb17266_c21/f1p0/1508	R01_cb17266_c21/f1p0/1508	R01_cb17266_c21/f1p0/1508
R01_cb15824_c0/f3p0/1143	NA	NA	NA	R01_cb15824_c0/f3p0/1143
R01_cb17729_c1/f1p0/1499	NA	R01_cb17729_c1/f1p0/1499	NA	NA
R01_cb3716_c17/f1p0/1871	NA	NA	NA	R01_cb3716_c17/f1p0/1871
R01_cb7133_c3/f1p0/2463	R01_cb7133_c3/f1p0/2463	R01_cb7133_c3/f1p0/2463	NA	NA
R01_cb191_c1/f1p2/4439	NA	NA	NA	R01_cb191_c1/f1p2/4439
R01_cb8564_c120568/f1p0/2827	NA	R01_cb8564_c120568/f1p0/2827	NA	R01_cb8564_c120568/f1p0/2827
R01_cb9720_c0/f2p0/1729	NA	NA	NA	R01_cb9720_c0/f2p0/1729
R01_cb18456_c7564/f1p0/402	R01_cb18456_c7564/f1p0/402	R01_cb18456_c7564/f1p0/402	R01_cb18456_c7564/f1p0/402	R01_cb18456_c7564/f1p0/402
R01_cb12336_c16/f1p0/1480	R01_cb12336_c16/f1p0/1480	R01_cb12336_c16/f1p0/1480	NA	R01_cb12336_c16/f1p0/1480
R01_cb13161_c16/f1p0/1616	NA	NA	NA	R01_cb13161_c16/f1p0/1616
R01_cb2970_c4/f1p0/2104	NA	NA	NA	R01_cb2970_c4/f1p0/2104
R01_cb6615_c26/f1p0/739	NA	NA	R01_cb6615_c26/f1p0/739	R01_cb6615_c26/f1p0/739
R01_cb365_c8/f1p0/3146	NA	NA	NA	R01_cb365_c8/f1p0/3146
R01_cb8564_c83677/f1p0/2971	NA	R01_cb8564_c83677/f1p0/2971	R01_cb8564_c83677/f1p0/2971	R01_cb8564_c83677/f1p0/2971

R01_cb13144_c3/f3p0/554	R01_cb13144_c3/f3p0/554	R01_cb13144_c3/f3p0/554	NA	R01_cb13144_c3/f3p0/554
R01_cb4657_c6/f1p0/2955	NA	NA	NA	R01_cb4657_c6/f1p0/2955
R01_cb1686_c11/f1p0/1327	R01_cb1686_c11/f1p0/1327	NA	R01_cb1686_c11/f1p0/1327	R01_cb1686_c11/f1p0/1327
R01_cb10693_c4/f1p1/1797	NA	NA	NA	R01_cb10693_c4/f1p1/1797
R01_cb3307_c19/f1p0/2611	NA	R01_cb3307_c19/f1p0/2611	NA	NA
R01_cb14639_c2/f2p0/514	NA	R01_cb14639_c2/f2p0/514	NA	NA
R01_cb6715_c0/f1p0/2796	NA	NA	NA	R01_cb6715_c0/f1p0/2796
R01_cb5486_c3/f1p1/2478	R01_cb5486_c3/f1p1/2478	R01_cb5486_c3/f1p1/2478	R01_cb5486_c3/f1p1/2478	R01_cb5486_c3/f1p1/2478
R01_cb18456_c7387/f1p0/972	R01_cb18456_c7387/f1p0/972	R01_cb18456_c7387/f1p0/972	R01_cb18456_c7387/f1p0/972	R01_cb18456_c7387/f1p0/972
R01_cb6012_c18/f1p1/1271	NA	NA	NA	R01_cb6012_c18/f1p1/1271
R01_cb1301_c6/f1p0/3235	NA	NA	NA	R01_cb1301_c6/f1p0/3235
R01_cb8564_c72992/f1p0/3120	NA	R01_cb8564_c72992/f1p0/3120	NA	NA
R01_cb18657_c1/f1p0/762	NA	R01_cb18657_c1/f1p0/762	R01_cb18657_c1/f1p0/762	R01_cb18657_c1/f1p0/762
R01_cb15035_c4/f1p0/951	NA	R01_cb15035_c4/f1p0/951	NA	NA
R01_cb5554_c9/f1p0/1602	NA	R01_cb5554_c9/f1p0/1602	R01_cb5554_c9/f1p0/1602	R01_cb5554_c9/f1p0/1602
R01_cb8564_c40405/f1p0/2909	NA	R01_cb8564_c40405/f1p0/2909	NA	R01_cb8564_c40405/f1p0/2909
R01_cb6393_c4/f1p1/2649	NA	NA	NA	R01_cb6393_c4/f1p1/2649
R01_cb7502_c10/f1p0/2512	NA	NA	R01_cb7502_c10/f1p0/2512	R01_cb7502_c10/f1p0/2512
R01_cb17973_c57/f1p0/1315	NA	R01_cb17973_c57/f1p0/1315	R01_cb17973_c57/f1p0/1315	R01_cb17973_c57/f1p0/1315
R01_cb12057_c44/f1p0/487	R01_cb12057_c44/f1p0/487	R01_cb12057_c44/f1p0/487	R01_cb12057_c44/f1p0/487	R01_cb12057_c44/f1p0/487
R01_cb7096_c11/f1p0/2716	NA	R01_cb7096_c11/f1p0/2716	NA	NA
R01_cb1515_c0/f1p0/4284	NA	R01_cb1515_c0/f1p0/4284	R01_cb1515_c0/f1p0/4284	NA
R01_cb15998_c0/f2p0/1642	NA	NA	NA	R01_cb15998_c0/f2p0/1642
R01_cb4580_c4/f1p0/3132	NA	NA	NA	R01_cb4580_c4/f1p0/3132
R01_cb15951_c5/f1p0/590	NA	R01_cb15951_c5/f1p0/590	R01_cb15951_c5/f1p0/590	R01_cb15951_c5/f1p0/590
R01_cb8564_c83110/f1p1/2442	NA	R01_cb8564_c83110/f1p1/2442	NA	NA

R01_cb11352_c2/f1p0/2732	NA	R01_cb11352_c2/f1p0/2732	NA	R01_cb11352_c2/f1p0/2732
R01_cb17177_c1/f2p0/698	NA	R01_cb17177_c1/f2p0/698	NA	R01_cb17177_c1/f2p0/698
R01_cb18456_c7556/f1p0/1139	NA	R01_cb18456_c7556/f1p0/1139	NA	NA
R01_cb2133_c49/f1p0/2352	NA	R01_cb2133_c49/f1p0/2352	NA	NA
R01_cb2834_c16/f1p0/2041	NA	NA	NA	R01_cb2834_c16/f1p0/2041
R01_cb4183_c2/f1p0/1744	NA	R01_cb4183_c2/f1p0/1744	NA	NA
R01_cb10694_c6/f9p0/596	R01_cb10694_c6/f9p0/596	NA	NA	R01_cb10694_c6/f9p0/596
R01_cb8564_c19292/f1p0/2784	R01_cb8564_c19292/f1p0/2784	R01_cb8564_c19292/f1p0/2784	R01_cb8564_c19292/f1p0/2784	R01_cb8564_c19292/f1p0/2784
R01_cb3307_c43/f1p0/1180	NA	NA	NA	R01_cb3307_c43/f1p0/1180
R01_cb16913_c7/f1p0/723	NA	R01_cb16913_c7/f1p0/723	NA	NA
R01_cb10806_c2/f1p0/858	NA	R01_cb10806_c2/f1p0/858	NA	NA
R01_cb4803_c19/f2p0/2151	NA	R01_cb4803_c19/f2p0/2151	NA	NA
R01_cb8564_c84686/f2p0/3781	NA	R01_cb8564_c84686/f2p0/3781	NA	R01_cb8564_c84686/f2p0/3781
R01_cb5062_c7/f1p0/2640	NA	R01_cb5062_c7/f1p0/2640	NA	NA
R01_cb13398_c0/f3p0/657	R01_cb13398_c0/f3p0/657	NA	NA	NA
R01_cb4637_c8/f3p0/844	R01_cb4637_c8/f3p0/844	NA	NA	NA
R01_cb10437_c0/f3p0/1073	NA	NA	NA	R01_cb10437_c0/f3p0/1073
R01_cb11187_c2/f1p0/1444	NA	NA	R01_cb11187_c2/f1p0/1444	R01_cb11187_c2/f1p0/1444
R01_cb11880_c8/f2p0/463	NA	R01_cb11880_c8/f2p0/463	R01_cb11880_c8/f2p0/463	NA
R01_cb5331_c6/f1p0/358	R01_cb5331_c6/f1p0/358	R01_cb5331_c6/f1p0/358	R01_cb5331_c6/f1p0/358	R01_cb5331_c6/f1p0/358
R01_cb1300_c1/f1p0/3303	NA	R01_cb1300_c1/f1p0/3303	R01_cb1300_c1/f1p0/3303	NA
R01_cb2952_c1/f1p0/3769	NA	R01_cb2952_c1/f1p0/3769	NA	NA
R01_cb13748_c50/f1p0/2299	NA	R01_cb13748_c50/f1p0/2299	NA	NA
R01_cb10401_c3/f1p1/625	R01_cb10401_c3/f1p1/625	NA	NA	R01_cb10401_c3/f1p1/625
R01_cb13884_c18/f1p0/765	R01_cb13884_c18/f1p0/765	NA	R01_cb13884_c18/f1p0/765	R01_cb13884_c18/f1p0/765

R01_cb15168_c4/f1p0/1841	NA	NA	NA	R01_cb15168_c4/f1p0/1841
R01_cb2844_c7/f1p0/2064	NA	NA	NA	R01_cb2844_c7/f1p0/2064
R01_cb5471_c0/f7p0/2667	NA	NA	NA	R01_cb5471_c0/f7p0/2667
R01_cb167_c58/f1p0/3304	NA	NA	NA	R01_cb167_c58/f1p0/3304
R01_cb6236_c4/f1p1/1795	NA	NA	NA	R01_cb6236_c4/f1p1/1795
R01_cb10339_c4/f1p0/1086	NA	R01_cb10339_c4/f1p0/1086	NA	NA
R01_cb15624_c3/f2p0/649	NA	R01_cb15624_c3/f2p0/649	R01_cb15624_c3/f2p0/649	R01_cb15624_c3/f2p0/649
R01_cb8564_c40140/f2p0/3120	R01_cb8564_c40140/f2p0/3120	R01_cb8564_c40140/f2p0/3120	R01_cb8564_c40140/f2p0/3120	R01_cb8564_c40140/f2p0/3120
R01_cb3832_c10/f1p0/579	NA	NA	NA	R01_cb3832_c10/f1p0/579
R01_cb8564_c23062/f1p3/3805	NA	R01_cb8564_c23062/f1p3/3805	NA	NA
R01_cb1846_c13/f1p0/3358	NA	R01_cb1846_c13/f1p0/3358	NA	NA
R01_cb18456_c945/f1p1/809	NA	R01_cb18456_c945/f1p1/809	NA	NA
R01_cb8564_c3161/f1p1/2405	NA	R01_cb8564_c3161/f1p1/2405	NA	NA
R01_cb15918_c9/f1p0/553	R01_cb15918_c9/f1p0/553	R01_cb15918_c9/f1p0/553	R01_cb15918_c9/f1p0/553	R01_cb15918_c9/f1p0/553
R01_cb6456_c8/f1p0/913	NA	NA	NA	R01_cb6456_c8/f1p0/913
R01_cb16686_c1/f1p0/578	R01_cb16686_c1/f1p0/578	R01_cb16686_c1/f1p0/578	R01_cb16686_c1/f1p0/578	R01_cb16686_c1/f1p0/578
R01_cb10880_c6/f1p0/499	R01_cb10880_c6/f1p0/499	R01_cb10880_c6/f1p0/499	NA	R01_cb10880_c6/f1p0/499
R01_cb8564_c12740/f1p0/4531	NA	R01_cb8564_c12740/f1p0/4531	NA	R01_cb8564_c12740/f1p0/4531
R01_cb8040_c16/f1p0/2545	NA	R01_cb8040_c16/f1p0/2545	NA	NA
R01_cb1333_c83/f1p0/3385	R01_cb1333_c83/f1p0/3385	R01_cb1333_c83/f1p0/3385	R01_cb1333_c83/f1p0/3385	R01_cb1333_c83/f1p0/3385
R01_cb11278_c3/f1p0/2107	NA	R01_cb11278_c3/f1p0/2107	R01_cb11278_c3/f1p0/2107	R01_cb11278_c3/f1p0/2107
R01_cb7219_c5/f1p0/2701	NA	NA	NA	R01_cb7219_c5/f1p0/2701
R01_cb11918_c17/f1p0/1272	NA	NA	NA	R01_cb11918_c17/f1p0/1272
R01_cb4501_c3/f1p0/3331	NA	NA	NA	R01_cb4501_c3/f1p0/3331
R01_cb3538_c4/f1p0/3597	NA	R01_cb3538_c4/f1p0/3597	NA	NA

R01_cb13761_c4/f1p0/1251	NA	R01_cb13761_c4/f1p0/1251	NA	NA
R01_cb2698_c2/f1p0/1886	NA	NA	NA	R01_cb2698_c2/f1p0/1886
R01_cb18456_c1030/f1p1/992	NA	R01_cb18456_c1030/f1p1/992	NA	NA
R01_cb13867_c18/f1p0/834	NA	NA	NA	R01_cb13867_c18/f1p0/834
R01_cb8564_c68300/f5p1/2665	NA	NA	R01_cb8564_c68300/f5p1/2665	NA
R01_cb2698_c6/f1p0/1752	NA	NA	NA	R01_cb2698_c6/f1p0/1752
R01_cb8564_c78655/f1p0/4933	NA	R01_cb8564_c78655/f1p0/4933	NA	NA
R01_cb8564_c38793/f1p0/3221	NA	R01_cb8564_c38793/f1p0/3221	R01_cb8564_c38793/f1p0/3221	NA
R01_cb16328_c1/f1p0/1725	NA	R01_cb16328_c1/f1p0/1725	NA	NA
R01_cb14532_c2/f1p0/554	R01_cb14532_c2/f1p0/554	NA	R01_cb14532_c2/f1p0/554	R01_cb14532_c2/f1p0/554
R01_cb7231_c21/f1p0/891	R01_cb7231_c21/f1p0/891	R01_cb7231_c21/f1p0/891	R01_cb7231_c21/f1p0/891	R01_cb7231_c21/f1p0/891
R01_cb8564_c5088/f11p4/2131	NA	NA	NA	R01_cb8564_c5088/f11p4/2131
R01_cb11560_c1/f1p0/2849	NA	NA	NA	R01_cb11560_c1/f1p0/2849
R01_cb8564_c48806/f2p0/4863	NA	R01_cb8564_c48806/f2p0/4863	NA	R01_cb8564_c48806/f2p0/4863
R01_cb15676_c3/f1p0/1620	NA	NA	NA	R01_cb15676_c3/f1p0/1620
R01_cb9313_c7/f1p0/1378	NA	R01_cb9313_c7/f1p0/1378	NA	NA
R01_cb14091_c1/f1p0/1634	NA	R01_cb14091_c1/f1p0/1634	NA	NA
R01_cb13131_c24/f1p0/1510	NA	R01_cb13131_c24/f1p0/1510	NA	NA
R01_cb8564_c85386/f1p0/3916	NA	R01_cb8564_c85386/f1p0/3916	NA	NA
R01_cb11808_c1/f1p0/2737	NA	NA	NA	R01_cb11808_c1/f1p0/2737
R01_cb5248_c16/f1p0/2669	NA	R01_cb5248_c16/f1p0/2669	R01_cb5248_c16/f1p0/2669	R01_cb5248_c16/f1p0/2669
R01_cb6935_c1/f1p0/2251	NA	R01_cb6935_c1/f1p0/2251	NA	NA
R01_cb6631_c10/f1p0/2272	NA	NA	NA	R01_cb6631_c10/f1p0/2272
R01_cb8564_c81859/f1p0/2968	R01_cb8564_c81859/f1p0/2968	R01_cb8564_c81859/f1p0/2968	NA	NA
R01_cb11167_c2/f1p0/448	R01_cb11167_c2/f1p0/448	R01_cb11167_c2/f1p0/448	R01_cb11167_c2/f1p0/448	R01_cb11167_c2/f1p0/448

R01_cb10792_c0/f1p0/1754	R01_cb10792_c0/f1p0/1754	R01_cb10792_c0/f1p0/1754	R01_cb10792_c0/f1p0/1754	R01_cb10792_c0/f1p0/1754
R01_cb8564_c120682/f1p0/4126	NA	NA	R01_cb8564_c120682/f1p0/4126	R01_cb8564_c120682/f1p0/4126
R01_cb8564_c37795/f1p0/3540	NA	NA	R01_cb8564_c37795/f1p0/3540	R01_cb8564_c37795/f1p0/3540
R01_cb5929_c8/f1p2/2509	NA	NA	NA	R01_cb5929_c8/f1p2/2509
R01_cb15713_c0/f2p0/349	R01_cb15713_c0/f2p0/349	R01_cb15713_c0/f2p0/349	R01_cb15713_c0/f2p0/349	R01_cb15713_c0/f2p0/349
R01_cb12003_c35/f1p0/1418	NA	R01_cb12003_c35/f1p0/1418	NA	NA
R01_cb5622_c2/f1p0/2496	NA	NA	NA	R01_cb5622_c2/f1p0/2496
R01_cb8564_c3801/f1p0/2058	NA	R01_cb8564_c3801/f1p0/2058	R01_cb8564_c3801/f1p0/2058	NA
R01_cb1905_c2/f3p0/2454	NA	R01_cb1905_c2/f3p0/2454	R01_cb1905_c2/f3p0/2454	NA
R01_cb18409_c102/f1p0/603	R01_cb18409_c102/f1p0/603	R01_cb18409_c102/f1p0/603	R01_cb18409_c102/f1p0/603	R01_cb18409_c102/f1p0/603
R01_cb14574_c0/f3p0/879	NA	NA	NA	R01_cb14574_c0/f3p0/879
R01_cb1860_c35/f18p4/2527	NA	NA	NA	R01_cb1860_c35/f18p4/2527
R01_cb12847_c0/f4p0/1466	NA	NA	NA	R01_cb12847_c0/f4p0/1466
R01_cb15914_c0/f1p0/987	NA	NA	NA	R01_cb15914_c0/f1p0/987
R01_cb8564_c2850/f1p0/3044	NA	R01_cb8564_c2850/f1p0/3044	R01_cb8564_c2850/f1p0/3044	NA
R01_cb13134_c3/f1p0/1383	NA	NA	NA	R01_cb13134_c3/f1p0/1383
R01_cb8564_c21470/f1p0/3642	R01_cb8564_c21470/f1p0/3642	R01_cb8564_c21470/f1p0/3642	R01_cb8564_c21470/f1p0/3642	R01_cb8564_c21470/f1p0/3642
R01_cb6719_c57/f1p0/3012	R01_cb6719_c57/f1p0/3012	NA	NA	R01_cb6719_c57/f1p0/3012
R01_cb14831_c5/f1p0/1090	NA	NA	NA	R01_cb14831_c5/f1p0/1090
R01_cb8564_c84227/f1p0/4645	NA	R01_cb8564_c84227/f1p0/4645	R01_cb8564_c84227/f1p0/4645	R01_cb8564_c84227/f1p0/4645
R01_cb18456_c7577/f1p0/861	NA	R01_cb18456_c7577/f1p0/861	R01_cb18456_c7577/f1p0/861	R01_cb18456_c7577/f1p0/861
R01_cb13254_c5/f1p0/1299	NA	R01_cb13254_c5/f1p0/1299	NA	R01_cb13254_c5/f1p0/1299
R01_cb15588_c2/f1p0/1647	NA	R01_cb15588_c2/f1p0/1647	NA	NA
R01_cb15474_c2/f1p0/1866	NA	NA	NA	R01_cb15474_c2/f1p0/1866

R01_cb10024_c401/f1p0/464	R01_cb10024_c401/f1p0/464	R01_cb10024_c401/f1p0/464	R01_cb10024_c401/f1p0/464	NA
R01_cb12837_c19/f1p0/773	NA	R01_cb12837_c19/f1p0/773	NA	NA
R01_cb12828_c5/f1p1/1544	NA	R01_cb12828_c5/f1p1/1544	NA	NA
R01_cb5828_c1/f1p0/3002	NA	NA	NA	R01_cb5828_c1/f1p0/3002
R01_cb14126_c0/f1p0/926	R01_cb14126_c0/f1p0/926	NA	NA	NA
R01_cb17921_c1/f1p0/786	NA	R01_cb17921_c1/f1p0/786	NA	NA
R01_cb15849_c1/f1p0/852	NA	NA	NA	R01_cb15849_c1/f1p0/852
R01_cb8564_c90138/f1p0/3662	NA	NA	NA	R01_cb8564_c90138/f1p0/3662
R01_cb14228_c15/f2p0/623	NA	NA	NA	R01_cb14228_c15/f2p0/623
R01_cb14487_c0/f2p0/1251	NA	NA	NA	R01_cb14487_c0/f2p0/1251
R01_cb3421_c16/f1p0/2946	NA	NA	NA	R01_cb3421_c16/f1p0/2946
R01_cb10280_c3/f1p0/1292	NA	R01_cb10280_c3/f1p0/1292	NA	NA
R01_cb10712_c2/f1p1/3797	NA	R01_cb10712_c2/f1p1/3797	NA	NA
R01_cb4584_c10/f1p0/3485	NA	NA	NA	R01_cb4584_c10/f1p0/3485
R01_cb13433_c174/f1p0/1039	NA	R01_cb13433_c174/f1p0/1039	NA	NA
R01_cb1297_c8/f2p0/3590	NA	NA	NA	R01_cb1297_c8/f2p0/3590
R01_cb8564_c117838/f1p0/1993	NA	R01_cb8564_c117838/f1p0/1993	NA	NA
R01_cb5745_c10/f3p0/1397	NA	NA	NA	R01_cb5745_c10/f3p0/1397
R01_cb737_c11/f2p0/348	R01_cb737_c11/f2p0/348	R01_cb737_c11/f2p0/348	R01_cb737_c11/f2p0/348	R01_cb737_c11/f2p0/348
R01_cb3570_c10/f1p0/2945	NA	NA	NA	R01_cb3570_c10/f1p0/2945
R01_cb3695_c1/f1p0/3553	NA	NA	NA	R01_cb3695_c1/f1p0/3553
R01_cb4945_c16/f1p0/1836	NA	R01_cb4945_c16/f1p0/1836	NA	NA
R01_cb2104_c5/f1p0/2660	NA	R01_cb2104_c5/f1p0/2660	R01_cb2104_c5/f1p0/2660	R01_cb2104_c5/f1p0/2660
R01_cb8564_c4624/f1p1/1970	NA	R01_cb8564_c4624/f1p1/1970	R01_cb8564_c4624/f1p1/1970	R01_cb8564_c4624/f1p1/1970
R01_cb8564_c84460/f1p0/2544	NA	R01_cb8564_c84460/f1p0/2544	NA	NA

R01_cb4493_c9/f1p0/4495	NA	NA	NA	R01_cb4493_c9/f1p0/4495
R01_cb8564_c4492/f1p0/2634	NA	R01_cb8564_c4492/f1p0/2634	NA	R01_cb8564_c4492/f1p0/2634
R01_cb15093_c2/f1p0/1481	NA	NA	NA	R01_cb15093_c2/f1p0/1481
R01_cb12094_c20/f1p0/1794	NA	R01_cb12094_c20/f1p0/1794	NA	NA
R01_cb8335_c13/f1p0/2125	NA	NA	NA	R01_cb8335_c13/f1p0/2125
R01_cb7611_c6/f1p0/2472	NA	NA	NA	R01_cb7611_c6/f1p0/2472
R01_cb4762_c1/f1p0/3263	NA	R01_cb4762_c1/f1p0/3263	NA	NA
R01_cb8564_c91447/f1p0/2758	NA	NA	R01_cb8564_c91447/f1p0/2758	R01_cb8564_c91447/f1p0/2758
R01_cb7642_c243/f1p0/604	NA	R01_cb7642_c243/f1p0/604	NA	NA
R01_cb6170_c5/f3p0/2859	NA	NA	NA	R01_cb6170_c5/f3p0/2859
R01_cb8564_c77410/f1p0/2021	NA	R01_cb8564_c77410/f1p0/2021	R01_cb8564_c77410/f1p0/2021	R01_cb8564_c77410/f1p0/2021
R01_cb16979_c17/f1p0/1821	R01_cb16979_c17/f1p0/1821	R01_cb16979_c17/f1p0/1821	NA	NA
R01_cb3243_c4/f1p1/1979	NA	NA	NA	R01_cb3243_c4/f1p1/1979
R01_cb17196_c1/f1p0/840	R01_cb17196_c1/f1p0/840	R01_cb17196_c1/f1p0/840	R01_cb17196_c1/f1p0/840	R01_cb17196_c1/f1p0/840
R01_cb8564_c34043/f2p0/3199	R01_cb8564_c34043/f2p0/319 9	R01_cb8564_c34043/f2p0/3199	R01_cb8564_c34043/f2p0/3199	R01_cb8564_c34043/f2p0/3199
R01_cb5258_c4/f2p0/1009	NA	NA	NA	R01_cb5258_c4/f2p0/1009
R01_cb6507_c7/f1p0/1069	NA	NA	NA	R01_cb6507_c7/f1p0/1069
R01_cb13064_c2/f1p0/1598	NA	NA	NA	R01_cb13064_c2/f1p0/1598
R01_cb18689_c1/f1p0/2692	NA	R01_cb18689_c1/f1p0/2692	R01_cb18689_c1/f1p0/2692	NA
R01_cb1812_c58/f1p0/2398	NA	R01_cb1812_c58/f1p0/2398	NA	NA
R01_cb17137_c3/f1p1/1861	NA	R01_cb17137_c3/f1p1/1861	NA	NA
R01_cb16627_c1/f1p0/544	R01_cb16627_c1/f1p0/544	R01_cb16627_c1/f1p0/544	R01_cb16627_c1/f1p0/544	R01_cb16627_c1/f1p0/544
R01_cb6399_c13/f1p0/473	R01_cb6399_c13/f1p0/473	R01_cb6399_c13/f1p0/473	NA	NA
R01_cb18623_c1/f1p0/3283	NA	R01_cb18623_c1/f1p0/3283	R01_cb18623_c1/f1p0/3283	NA
R01_cb12696_c16/f1p4/819	NA	R01_cb12696_c16/f1p4/819	NA	NA

R01_cb11250_c0/f1p0/841	R01_cb11250_c0/f1p0/841	R01_cb11250_c0/f1p0/841	R01_cb11250_c0/f1p0/841	R01_cb11250_c0/f1p0/841
R01_cb18132_c25/f1p0/958	NA	R01_cb18132_c25/f1p0/958	R01_cb18132_c25/f1p0/958	NA
R01_cb2497_c5/f1p0/3897	NA	NA	NA	R01_cb2497_c5/f1p0/3897
R01_cb11081_c4/f1p0/692	NA	R01_cb11081_c4/f1p0/692	R01_cb11081_c4/f1p0/692	R01_cb11081_c4/f1p0/692
R01_cb6345_c3/f1p0/638	NA	NA	NA	R01_cb6345_c3/f1p0/638
R01_cb15811_c19/f1p0/435	NA	R01_cb15811_c19/f1p0/435	R01_cb15811_c19/f1p0/435	R01_cb15811_c19/f1p0/435
R01_cb9119_c3/f3p2/2173	NA	NA	NA	R01_cb9119_c3/f3p2/2173
R01_cb8564_c24550/f1p0/2875	NA	R01_cb8564_c24550/f1p0/2875	NA	NA
R01_cb7027_c6/f1p0/5802	NA	R01_cb7027_c6/f1p0/5802	NA	NA
R01_cb1170_c0/f1p0/4446	R01_cb1170_c0/f1p0/4446	R01_cb1170_c0/f1p0/4446	R01_cb1170_c0/f1p0/4446	NA
R01_cb16655_c3/f1p0/1854	R01_cb16655_c3/f1p0/1854	R01_cb16655_c3/f1p0/1854	NA	NA
R01_cb17383_c2/f1p0/870	NA	NA	NA	R01_cb17383_c2/f1p0/870
R01_cb11165_c8/f1p0/1161	NA	NA	NA	R01_cb11165_c8/f1p0/1161
R01_cb12012_c0/f1p0/1269	NA	NA	NA	R01_cb12012_c0/f1p0/1269
R01_cb15907_c2/f1p0/681	NA	R01_cb15907_c2/f1p0/681	NA	R01_cb15907_c2/f1p0/681
R01_cb8564_c81991/f3p1/3306	NA	NA	NA	R01_cb8564_c81991/f3p1/3306
R01_cb8564_c130246/f1p1/2268	NA	NA	NA	R01_cb8564_c130246/f1p1/2268
R01_cb16238_c1/f1p0/1061	NA	R01_cb16238_c1/f1p0/1061	NA	NA
R01_cb7926_c3/f1p0/2347	R01_cb7926_c3/f1p0/2347	R01_cb7926_c3/f1p0/2347	NA	R01_cb7926_c3/f1p0/2347
R01_cb8027_c5/f1p0/2360	NA	R01_cb8027_c5/f1p0/2360	NA	NA
R01_cb8564_c91691/f1p0/3046	NA	NA	NA	R01_cb8564_c91691/f1p0/3046
R01_cb15756_c0/f2p0/644	NA	NA	NA	R01_cb15756_c0/f2p0/644
R01_cb8564_c83619/f1p0/2534	NA	NA	NA	R01_cb8564_c83619/f1p0/2534
R01_cb7899_c3/f1p0/1819	NA	NA	NA	R01_cb7899_c3/f1p0/1819
R01_cb12401_c4/f1p0/440	R01_cb12401_c4/f1p0/440	R01_cb12401_c4/f1p0/440	R01_cb12401_c4/f1p0/440	NA

R01_cb8564_c1105/f1p0/2127	NA	NA	R01_cb8564_c1105/f1p0/2127	NA
R01_cb8564_c35399/f1p0/4553	NA	NA	NA	R01_cb8564_c35399/f1p0/4553
R01_cb11900_c3/f2p0/419	NA	R01_cb11900_c3/f2p0/419	NA	R01_cb11900_c3/f2p0/419
R01_cb18456_c2519/f1p0/1727	NA	R01_cb18456_c2519/f1p0/1727	NA	R01_cb18456_c2519/f1p0/1727
R01_cb15912_c2/f1p0/1329	NA	NA	NA	R01_cb15912_c2/f1p0/1329
R01_cb9260_c2/f1p1/1973	NA	R01_cb9260_c2/f1p1/1973	NA	R01_cb9260_c2/f1p1/1973
R01_cb8564_c82332/f1p0/2714	NA	R01_cb8564_c82332/f1p0/2714	NA	NA
R01_cb10055_c19/f1p0/759	NA	R01_cb10055_c19/f1p0/759	NA	NA
R01_cb8564_c119335/f1p0/2881	NA	R01_cb8564_c119335/f1p0/2881	NA	NA
R01_cb12496_c11/f1p0/1288	NA	R01_cb12496_c11/f1p0/1288	NA	NA
R01_cb17315_c0/f1p0/484	NA	R01_cb17315_c0/f1p0/484	NA	NA
R01_cb18261_c2/f1p0/792	R01_cb18261_c2/f1p0/792	NA	NA	NA
R01_cb1860_c18/f1p0/2458	NA	NA	NA	R01_cb1860_c18/f1p0/2458
R01_cb18456_c6074/f1p1/640	R01_cb18456_c6074/f1p1/640	R01_cb18456_c6074/f1p1/640	R01_cb18456_c6074/f1p1/640	R01_cb18456_c6074/f1p1/640
R01_cb10437_c2/f1p0/2241	NA	R01_cb10437_c2/f1p0/2241	R01_cb10437_c2/f1p0/2241	R01_cb10437_c2/f1p0/2241
R01_cb17626_c1/f1p0/1152	NA	R01_cb17626_c1/f1p0/1152	NA	NA
R01_cb18005_c6/f1p0/1205	NA	R01_cb18005_c6/f1p0/1205	R01_cb18005_c6/f1p0/1205	R01_cb18005_c6/f1p0/1205
R01_cb18649_c3/f1p0/5011	NA	NA	NA	R01_cb18649_c3/f1p0/5011
R01_cb18456_c7507/f1p0/1050	NA	R01_cb18456_c7507/f1p0/1050	R01_cb18456_c7507/f1p0/1050	R01_cb18456_c7507/f1p0/1050
R01_cb18600_c1/f1p0/1478	NA	R01_cb18600_c1/f1p0/1478	R01_cb18600_c1/f1p0/1478	R01_cb18600_c1/f1p0/1478
R01_cb1236_c1/f1p0/2256	NA	R01_cb1236_c1/f1p0/2256	NA	NA
R01_cb7303_c16/f7p0/2554	NA	NA	NA	R01_cb7303_c16/f7p0/2554
R01_cb4708_c7/f1p0/1963	NA	R01_cb4708_c7/f1p0/1963	NA	NA
R01_cb10029_c658/f1p0/783	NA	R01_cb10029_c658/f1p0/783	NA	NA
R01_cb12037_c9/f1p0/1595	NA	NA	R01_cb12037_c9/f1p0/1595	NA

R01_cb15747_c0/f2p0/579	R01_cb15747_c0/f2p0/579	R01_cb15747_c0/f2p0/579	NA	R01_cb15747_c0/f2p0/579
R01_cb11769_c1/f1p0/2426	NA	R01_cb11769_c1/f1p0/2426	NA	NA
R01_cb18456_c1434/f3p0/444	NA	R01_cb18456_c1434/f3p0/444	R01_cb18456_c1434/f3p0/444	R01_cb18456_c1434/f3p0/444
R01_cb7802_c11/f1p1/1784	NA	NA	NA	R01_cb7802_c11/f1p1/1784
R01_cb8564_c121475/f1p0/2123	NA	R01_cb8564_c121475/f1p0/2123	NA	R01_cb8564_c121475/f1p0/2123
R01_cb18456_c7766/f49p2/805	R01_cb18456_c7766/f49p2/805	NA	NA	NA
R01_cb3482_c34/f1p0/1154	R01_cb3482_c34/f1p0/1154	R01_cb3482_c34/f1p0/1154	NA	NA
R01_cb9513_c37/f1p1/2223	NA	R01_cb9513_c37/f1p1/2223	NA	NA
R01_cb3518_c21/f1p0/1435	NA	NA	NA	R01_cb3518_c21/f1p0/1435
R01_cb5324_c1/f1p0/3819	R01_cb5324_c1/f1p0/3819	R01_cb5324_c1/f1p0/3819	R01_cb5324_c1/f1p0/3819	R01_cb5324_c1/f1p0/3819
R01_cb567_c21/f1p0/2857	NA	NA	NA	R01_cb567_c21/f1p0/2857
R01_cb11251_c2/f1p0/1662	NA	R01_cb11251_c2/f1p0/1662	R01_cb11251_c2/f1p0/1662	R01_cb11251_c2/f1p0/1662
R01_cb13601_c2/f2p0/846	NA	NA	NA	R01_cb13601_c2/f2p0/846
R01_cb8564_c85196/f1p0/2974	NA	R01_cb8564_c85196/f1p0/2974	NA	R01_cb8564_c85196/f1p0/2974
R01_cb8564_c22080/f1p0/4057	NA	R01_cb8564_c22080/f1p0/4057	NA	NA
R01_cb6644_c3/f1p0/447	R01_cb6644_c3/f1p0/447	R01_cb6644_c3/f1p0/447	R01_cb6644_c3/f1p0/447	R01_cb6644_c3/f1p0/447
R01_cb1228_c21/f1p0/2612	NA	NA	NA	R01_cb1228_c21/f1p0/2612
R01_cb4657_c21/f1p0/2134	NA	R01_cb4657_c21/f1p0/2134	R01_cb4657_c21/f1p0/2134	NA
R01_cb6839_c6/f1p0/856	NA	R01_cb6839_c6/f1p0/856	R01_cb6839_c6/f1p0/856	R01_cb6839_c6/f1p0/856
R01_cb11023_c1/f1p0/2469	NA	R01_cb11023_c1/f1p0/2469	NA	NA
R01_cb1301_c4/f1p0/3013	NA	NA	NA	R01_cb1301_c4/f1p0/3013
R01_cb1905_c22/f1p0/2509	NA	NA	R01_cb1905_c22/f1p0/2509	R01_cb1905_c22/f1p0/2509
R01_cb15860_c1/f1p0/613	NA	R01_cb15860_c1/f1p0/613	NA	R01_cb15860_c1/f1p0/613
R01_cb13910_c2/f2p0/1454	R01_cb13910_c2/f2p0/1454	R01_cb13910_c2/f2p0/1454	R01_cb13910_c2/f2p0/1454	R01_cb13910_c2/f2p0/1454

R01_cb11376_c1/f1p0/3720	R01_cb11376_c1/f1p0/3720	R01_cb11376_c1/f1p0/3720	R01_cb11376_c1/f1p0/3720	R01_cb11376_c1/f1p0/3720
R01_cb13033_c7/f1p0/946	NA	R01_cb13033_c7/f1p0/946	R01_cb13033_c7/f1p0/946	R01_cb13033_c7/f1p0/946
R01_cb8564_c21912/f1p0/2701	NA	R01_cb8564_c21912/f1p0/2701	NA	NA
R01_cb17790_c1/f1p0/1110	NA	R01_cb17790_c1/f1p0/1110	NA	NA
R01_cb8564_c69437/f1p0/2197	NA	NA	NA	R01_cb8564_c69437/f1p0/2197
R01_cb8564_c50652/f1p0/4850	NA	R01_cb8564_c50652/f1p0/4850	R01_cb8564_c50652/f1p0/4850	R01_cb8564_c50652/f1p0/4850
R01_cb16069_c3/f1p0/433	R01_cb16069_c3/f1p0/433	R01_cb16069_c3/f1p0/433	R01_cb16069_c3/f1p0/433	R01_cb16069_c3/f1p0/433
R01_cb11465_c1/f1p0/2244	NA	NA	NA	R01_cb11465_c1/f1p0/2244
R01_cb18020_c0/f2p0/417	R01_cb18020_c0/f2p0/417	R01_cb18020_c0/f2p0/417	R01_cb18020_c0/f2p0/417	R01_cb18020_c0/f2p0/417
R01_cb9445_c0/f2p0/2026	R01_cb9445_c0/f2p0/2026	R01_cb9445_c0/f2p0/2026	R01_cb9445_c0/f2p0/2026	R01_cb9445_c0/f2p0/2026
R01_cb18705_c1/f1p0/1197	R01_cb18705_c1/f1p0/1197	R01_cb18705_c1/f1p0/1197	R01_cb18705_c1/f1p0/1197	R01_cb18705_c1/f1p0/1197
R01_cb12785_c23/f1p0/931	NA	R01_cb12785_c23/f1p0/931	R01_cb12785_c23/f1p0/931	R01_cb12785_c23/f1p0/931
R01_cb14522_c0/f2p0/1641	R01_cb14522_c0/f2p0/1641	R01_cb14522_c0/f2p0/1641	R01_cb14522_c0/f2p0/1641	R01_cb14522_c0/f2p0/1641
R01_cb10802_c1/f1p0/2343	NA	R01_cb10802_c1/f1p0/2343	NA	NA
R01_cb13897_c0/f1p0/323	R01_cb13897_c0/f1p0/323	R01_cb13897_c0/f1p0/323	R01_cb13897_c0/f1p0/323	R01_cb13897_c0/f1p0/323
R01_cb15053_c2/f2p0/730	NA	R01_cb15053_c2/f2p0/730	R01_cb15053_c2/f2p0/730	NA
R01_cb6251_c0/f4p0/694	NA	R01_cb6251_c0/f4p0/694	NA	NA
R01_cb8564_c86540/f1p0/3465	NA	NA	NA	R01_cb8564_c86540/f1p0/3465
R01_cb5929_c0/f2p2/2556	NA	NA	NA	R01_cb5929_c0/f2p2/2556
R01_cb3745_c1/f1p0/2535	R01_cb3745_c1/f1p0/2535	R01_cb3745_c1/f1p0/2535	R01_cb3745_c1/f1p0/2535	NA
R01_cb9790_c2/f1p0/1891	NA	R01_cb9790_c2/f1p0/1891	NA	NA
R01_cb8564_c90834/f1p0/3532	R01_cb8564_c90834/f1p0/3532	NA	NA	NA
R01_cb12677_c1/f1p0/883	NA	R01_cb12677_c1/f1p0/883	NA	R01_cb12677_c1/f1p0/883
R01_cb10392_c1/f1p0/2682	NA	R01_cb10392_c1/f1p0/2682	NA	NA
R01_cb5664_c1/f1p0/2358	R01_cb5664_c1/f1p0/2358	R01_cb5664_c1/f1p0/2358	R01_cb5664_c1/f1p0/2358	R01_cb5664_c1/f1p0/2358

R01_cb18456_c6791/f1p0/924	R01_cb18456_c6791/f1p0/924	R01_cb18456_c6791/f1p0/924	R01_cb18456_c6791/f1p0/924	R01_cb18456_c6791/f1p0/924
R01_cb6125_c0/f1p0/2935	R01_cb6125_c0/f1p0/2935	R01_cb6125_c0/f1p0/2935	R01_cb6125_c0/f1p0/2935	R01_cb6125_c0/f1p0/2935
R01_cb10693_c2/f1p1/2329	NA	NA	NA	R01_cb10693_c2/f1p1/2329
R01_cb13781_c11/f2p0/631	NA	NA	NA	R01_cb13781_c11/f2p0/631
R01_cb17967_c1/f1p0/1125	NA	R01_cb17967_c1/f1p0/1125	NA	NA
R01_cb11491_c1/f1p0/3826	R01_cb11491_c1/f1p0/3826	R01_cb11491_c1/f1p0/3826	R01_cb11491_c1/f1p0/3826	R01_cb11491_c1/f1p0/3826
R01_cb11551_c1/f1p0/2866	NA	NA	NA	R01_cb11551_c1/f1p0/2866
R01_cb4824_c8/f1p0/3007	NA	R01_cb4824_c8/f1p0/3007	R01_cb4824_c8/f1p0/3007	R01_cb4824_c8/f1p0/3007
R01_cb11041_c1/f1p0/1938	NA	R01_cb11041_c1/f1p0/1938	NA	NA
R01_cb9108_c3/f1p0/1311	NA	NA	NA	R01_cb9108_c3/f1p0/1311
R01_cb8053_c9/f9p3/2468	NA	NA	NA	R01_cb8053_c9/f9p3/2468
R01_cb18301_c16/f1p0/1538	R01_cb18301_c16/f1p0/1538	R01_cb18301_c16/f1p0/1538	R01_cb18301_c16/f1p0/1538	R01_cb18301_c16/f1p0/1538
R01_cb5635_c1/f1p0/3052	NA	NA	NA	R01_cb5635_c1/f1p0/3052
R01_cb15951_c1/f2p0/655	NA	R01_cb15951_c1/f2p0/655	R01_cb15951_c1/f2p0/655	R01_cb15951_c1/f2p0/655
R01_cb11473_c0/f1p0/960	NA	R01_cb11473_c0/f1p0/960	NA	NA
R01_cb18456_c7544/f1p0/1144	NA	R01_cb18456_c7544/f1p0/1144	NA	NA
R01_cb8564_c73072/f1p0/2431	NA	NA	NA	R01_cb8564_c73072/f1p0/2431
R01_cb16692_c0/f5p0/484	NA	NA	NA	R01_cb16692_c0/f5p0/484
R01_cb18456_c1779/f1p1/1333	NA	R01_cb18456_c1779/f1p1/1333	NA	NA
R01_cb13808_c0/f3p1/624	NA	R01_cb13808_c0/f3p1/624	NA	R01_cb13808_c0/f3p1/624
R01_cb12003_c136/f1p0/1359	NA	R01_cb12003_c136/f1p0/1359	NA	NA
R01_cb8564_c17689/f1p0/3643	NA	R01_cb8564_c17689/f1p0/3643	R01_cb8564_c17689/f1p0/3643	R01_cb8564_c17689/f1p0/3643
R01_cb8564_c110827/f1p0/1850	NA	NA	NA	R01_cb8564_c110827/f1p0/1850
R01_cb6591_c18/f1p1/710	R01_cb6591_c18/f1p1/710	R01_cb6591_c18/f1p1/710	NA	NA
R01_cb18476_c1/f1p0/738	NA	NA	NA	R01_cb18476_c1/f1p0/738

R01_cb2403_c3/f1p0/1688	R01_cb2403_c3/f1p0/1688	R01_cb2403_c3/f1p0/1688	R01_cb2403_c3/f1p0/1688	R01_cb2403_c3/f1p0/1688
R01_cb12363_c7/f1p0/1153	NA	NA	NA	R01_cb12363_c7/f1p0/1153
R01_cb8564_c91896/f1p0/2444	NA	R01_cb8564_c91896/f1p0/2444	NA	NA
R01_cb13545_c55/f1p0/1266	NA	R01_cb13545_c55/f1p0/1266	NA	R01_cb13545_c55/f1p0/1266
R01_cb8564_c80576/f1p0/2840	NA	NA	NA	R01_cb8564_c80576/f1p0/2840
R01_cb8564_c69139/f2p0/3547	NA	R01_cb8564_c69139/f2p0/3547	NA	NA
R01_cb8564_c3046/f1p0/4037	NA	R01_cb8564_c3046/f1p0/4037	NA	NA
R01_cb14261_c2/f1p0/1483	R01_cb14261_c2/f1p0/1483	R01_cb14261_c2/f1p0/1483	R01_cb14261_c2/f1p0/1483	R01_cb14261_c2/f1p0/1483
R01_cb8564_c83126/f1p0/3424	R01_cb8564_c83126/f1p0/3424	R01_cb8564_c83126/f1p0/3424	NA	NA
R01_cb8564_c25173/f1p1/3583	NA	NA	NA	R01_cb8564_c25173/f1p1/3583
R01_cb3791_c17/f4p2/1499	NA	NA	NA	R01_cb3791_c17/f4p2/1499
R01_cb1425_c5/f1p0/3737	NA	NA	NA	R01_cb1425_c5/f1p0/3737
R01_cb18355_c1/f1p0/958	R01_cb18355_c1/f1p0/958	R01_cb18355_c1/f1p0/958	R01_cb18355_c1/f1p0/958	NA
R01_cb2763_c5/f1p0/1506	NA	R01_cb2763_c5/f1p0/1506	NA	NA
R01_cb8564_c42131/f1p0/3182	R01_cb8564_c42131/f1p0/3182	R01_cb8564_c42131/f1p0/3182	R01_cb8564_c42131/f1p0/3182	R01_cb8564_c42131/f1p0/3182
R01_cb10983_c0/f1p0/1170	NA	NA	NA	R01_cb10983_c0/f1p0/1170
R01_cb8564_c87127/f1p0/1943	NA	R01_cb8564_c87127/f1p0/1943	R01_cb8564_c87127/f1p0/1943	R01_cb8564_c87127/f1p0/1943
R01_cb5054_c4/f1p0/1732	NA	R01_cb5054_c4/f1p0/1732	NA	NA
R01_cb1964_c0/f2p0/2285	NA	R01_cb1964_c0/f2p0/2285	NA	R01_cb1964_c0/f2p0/2285
R01_cb6452_c13/f1p0/1330	NA	NA	NA	R01_cb6452_c13/f1p0/1330
R01_cb9523_c3/f1p0/1657	NA	R01_cb9523_c3/f1p0/1657	NA	NA
R01_cb18456_c1854/f1p0/1089	NA	NA	NA	R01_cb18456_c1854/f1p0/1089
R01_cb16981_c6/f1p1/538	NA	NA	NA	R01_cb16981_c6/f1p1/538
R01_cb4180_c7/f1p1/6833	NA	NA	NA	R01_cb4180_c7/f1p1/6833

R01_cb11120_c1/f1p0/667	NA	NA	R01_cb11120_c1/f1p0/667	R01_cb11120_c1/f1p0/667
R01_cb13012_c5/f1p0/1143	NA	NA	NA	R01_cb13012_c5/f1p0/1143
R01_cb15459_c0/f3p0/806	NA	R01_cb15459_c0/f3p0/806	NA	NA
R01_cb8414_c2/f1p0/1509	NA	NA	NA	R01_cb8414_c2/f1p0/1509
R01_cb17838_c2/f1p0/459	NA	NA	R01_cb17838_c2/f1p0/459	R01_cb17838_c2/f1p0/459
R01_cb8564_c122267/f1p0/2906	R01_cb8564_c122267/f1p0/2906	R01_cb8564_c122267/f1p0/2906	R01_cb8564_c122267/f1p0/2906	NA
R01_cb1230_c6/f1p0/4152	NA	R01_cb1230_c6/f1p0/4152	R01_cb1230_c6/f1p0/4152	R01_cb1230_c6/f1p0/4152
R01_cb11178_c2/f1p0/650	R01_cb11178_c2/f1p0/650	R01_cb11178_c2/f1p0/650	R01_cb11178_c2/f1p0/650	R01_cb11178_c2/f1p0/650
R01_cb8564_c120922/f1p0/3037	NA	R01_cb8564_c120922/f1p0/3037	NA	R01_cb8564_c120922/f1p0/3037
R01_cb7193_c1/f1p0/2668	NA	R01_cb7193_c1/f1p0/2668	NA	NA
R01_cb18653_c1/f1p0/1682	NA	R01_cb18653_c1/f1p0/1682	NA	R01_cb18653_c1/f1p0/1682
R01_cb4354_c11/f1p0/3303	NA	NA	NA	R01_cb4354_c11/f1p0/3303
R01_cb3584_c1/f1p0/3586	NA	R01_cb3584_c1/f1p0/3586	NA	R01_cb3584_c1/f1p0/3586
R01_cb6615_c6/f1p0/2350	NA	NA	R01_cb6615_c6/f1p0/2350	R01_cb6615_c6/f1p0/2350
R01_cb11523_c1/f1p0/2651	NA	R01_cb11523_c1/f1p0/2651	NA	NA
R01_cb17973_c35/f1p0/681	R01_cb17973_c35/f1p0/681	R01_cb17973_c35/f1p0/681	R01_cb17973_c35/f1p0/681	R01_cb17973_c35/f1p0/681
R01_cb12421_c25/f1p0/581	R01_cb12421_c25/f1p0/581	R01_cb12421_c25/f1p0/581	NA	NA
R01_cb8564_c124167/f1p0/3100	NA	R01_cb8564_c124167/f1p0/3100	NA	NA
R01_cb14107_c0/f6p0/1343	NA	NA	R01_cb14107_c0/f6p0/1343	R01_cb14107_c0/f6p0/1343
R01_cb5706_c0/f2p1/2686	NA	R01_cb5706_c0/f2p1/2686	NA	NA
R01_cb7926_c2/f1p0/2500	R01_cb7926_c2/f1p0/2500	R01_cb7926_c2/f1p0/2500	NA	R01_cb7926_c2/f1p0/2500
R01_cb8564_c1832/f1p0/2519	R01_cb8564_c1832/f1p0/2519	R01_cb8564_c1832/f1p0/2519	R01_cb8564_c1832/f1p0/2519	R01_cb8564_c1832/f1p0/2519
R01_cb8564_c15411/f1p0/3552	NA	R01_cb8564_c15411/f1p0/3552	NA	NA

R01_cb5297_c0/f1p0/3126	NA	R01_cb5297_c0/f1p0/3126	NA	NA
R01_cb4834_c48/f1p0/984	R01_cb4834_c48/f1p0/984	R01_cb4834_c48/f1p0/984	R01_cb4834_c48/f1p0/984	R01_cb4834_c48/f1p0/984
R01_cb15200_c5/f1p0/954	NA	R01_cb15200_c5/f1p0/954	NA	NA
R01_cb15487_c0/f1p0/842	NA	R01_cb15487_c0/f1p0/842	R01_cb15487_c0/f1p0/842	R01_cb15487_c0/f1p0/842
R01_cb8564_c112816/f1p0/2711	NA	R01_cb8564_c112816/f1p0/2711	NA	NA
R01_cb18456_c5284/f1p0/1261	NA	R01_cb18456_c5284/f1p0/1261	NA	NA
R01_cb8564_c78998/f1p0/3377	NA	R01_cb8564_c78998/f1p0/3377	NA	NA
R01_cb8564_c73321/f1p0/4292	NA	R01_cb8564_c73321/f1p0/4292	NA	NA
R01_cb4825_c13/f1p0/4647	NA	NA	NA	R01_cb4825_c13/f1p0/4647
R01_cb8564_c9925/f2p0/2973	NA	NA	NA	R01_cb8564_c9925/f2p0/2973
R01_cb14660_c9/f1p0/1232	NA	R01_cb14660_c9/f1p0/1232	NA	R01_cb14660_c9/f1p0/1232
R01_cb18456_c7139/f1p0/806	NA	R01_cb18456_c7139/f1p0/806	NA	NA
R01_cb17722_c1/f1p0/343	NA	R01_cb17722_c1/f1p0/343	NA	NA
R01_cb7828_c4/f1p0/2108	NA	NA	NA	R01_cb7828_c4/f1p0/2108
R01_cb10472_c2/f1p0/1568	R01_cb10472_c2/f1p0/1568	R01_cb10472_c2/f1p0/1568	R01_cb10472_c2/f1p0/1568	R01_cb10472_c2/f1p0/1568
R01_cb13884_c23/f1p0/541	R01_cb13884_c23/f1p0/541	NA	R01_cb13884_c23/f1p0/541	R01_cb13884_c23/f1p0/541
R01_cb8564_c39189/f1p1/2975	NA	R01_cb8564_c39189/f1p1/2975	NA	NA
R01_cb6147_c10/f1p0/3193	NA	R01_cb6147_c10/f1p0/3193	NA	NA
R01_cb13686_c5/f2p1/1612	NA	R01_cb13686_c5/f2p1/1612	NA	NA
R01_cb7731_c15/f1p0/2544	NA	NA	NA	R01_cb7731_c15/f1p0/2544
R01_cb10936_c4/f1p0/1784	NA	NA	NA	R01_cb10936_c4/f1p0/1784
R01_cb5448_c2/f1p0/3092	NA	R01_cb5448_c2/f1p0/3092	NA	NA
R01_cb7051_c0/f1p0/2666	NA	NA	NA	R01_cb7051_c0/f1p0/2666
R01_cb15710_c4/f1p0/800	NA	R01_cb15710_c4/f1p0/800	NA	NA
R01_cb3339_c10/f1p0/1322	NA	NA	NA	R01_cb3339_c10/f1p0/1322

R01_cb4128_c19/f1p0/2260	NA	NA	NA	R01_cb4128_c19/f1p0/2260
R01_cb3426_c22/f1p0/3122	NA	NA	NA	R01_cb3426_c22/f1p0/3122
R01_cb17939_c0/f2p0/473	R01_cb17939_c0/f2p0/473	R01_cb17939_c0/f2p0/473	NA	R01_cb17939_c0/f2p0/473
R01_cb5324_c0/f1p0/3110	R01_cb5324_c0/f1p0/3110	R01_cb5324_c0/f1p0/3110	R01_cb5324_c0/f1p0/3110	R01_cb5324_c0/f1p0/3110
R01_cb8564_c83579/f1p0/1981	NA	R01_cb8564_c83579/f1p0/1981	R01_cb8564_c83579/f1p0/1981	R01_cb8564_c83579/f1p0/1981
R01_cb10310_c0/f1p0/2004	NA	NA	NA	R01_cb10310_c0/f1p0/2004
R01_cb2164_c8/f1p0/2033	NA	R01_cb2164_c8/f1p0/2033	NA	R01_cb2164_c8/f1p0/2033
R01_cb8564_c1854/f1p0/3435	NA	R01_cb8564_c1854/f1p0/3435	R01_cb8564_c1854/f1p0/3435	R01_cb8564_c1854/f1p0/3435
R01_cb17756_c3/f2p0/476	R01_cb17756_c3/f2p0/476	R01_cb17756_c3/f2p0/476	R01_cb17756_c3/f2p0/476	R01_cb17756_c3/f2p0/476
R01_cb18456_c1700/f2p0/341	R01_cb18456_c1700/f2p0/341	R01_cb18456_c1700/f2p0/341	R01_cb18456_c1700/f2p0/341	R01_cb18456_c1700/f2p0/341
R01_cb4347_c5/f1p0/607	NA	R01_cb4347_c5/f1p0/607	R01_cb4347_c5/f1p0/607	NA
R01_cb13493_c1/f1p0/1366	NA	R01_cb13493_c1/f1p0/1366	NA	NA
R01_cb12603_c13/f1p0/914	NA	NA	R01_cb12603_c13/f1p0/914	R01_cb12603_c13/f1p0/914
R01_cb16980_c3/f2p0/1124	NA	NA	NA	R01_cb16980_c3/f2p0/1124
R01_cb17869_c2/f1p1/1777	NA	NA	NA	R01_cb17869_c2/f1p1/1777
R01_cb8564_c70122/f1p0/2814	NA	R01_cb8564_c70122/f1p0/2814	NA	NA
R01_cb16774_c7/f1p0/1053	NA	R01_cb16774_c7/f1p0/1053	NA	NA
R01_cb17717_c0/f1p0/959	NA	NA	R01_cb17717_c0/f1p0/959	R01_cb17717_c0/f1p0/959
R01_cb15136_c2/f1p0/612	NA	R01_cb15136_c2/f1p0/612	R01_cb15136_c2/f1p0/612	R01_cb15136_c2/f1p0/612
R01_cb8564_c126285/f1p0/2412	R01_cb8564_c126285/f1p0/2412	R01_cb8564_c126285/f1p0/2412	R01_cb8564_c126285/f1p0/2412	R01_cb8564_c126285/f1p0/2412
R01_cb17336_c0/f1p0/655	NA	R01_cb17336_c0/f1p0/655	R01_cb17336_c0/f1p0/655	R01_cb17336_c0/f1p0/655
R01_cb15796_c6/f1p0/1301	NA	NA	NA	R01_cb15796_c6/f1p0/1301
R01_cb5246_c6/f1p0/799	NA	R01_cb5246_c6/f1p0/799	R01_cb5246_c6/f1p0/799	R01_cb5246_c6/f1p0/799
R01_cb646_c3/f1p0/4014	NA	NA	NA	R01_cb646_c3/f1p0/4014
R01_cb15495_c2/f1p0/466	R01_cb15495_c2/f1p0/466	R01_cb15495_c2/f1p0/466	R01_cb15495_c2/f1p0/466	NA

R01_cb17187_c0/f12p0/601	R01_cb17187_c0/f12p0/601	NA	NA	NA
R01_cb8564_c76556/f1p0/2353	NA	R01_cb8564_c76556/f1p0/2353	NA	NA
R01_cb13118_c0/f1p0/942	NA	NA	NA	R01_cb13118_c0/f1p0/942
R01_cb6719_c80/f1p0/2471	NA	R01_cb6719_c80/f1p0/2471	NA	NA
R01_cb16125_c7/f1p0/1621	NA	NA	NA	R01_cb16125_c7/f1p0/1621
R01_cb4744_c3/f1p0/2848	NA	R01_cb4744_c3/f1p0/2848	R01_cb4744_c3/f1p0/2848	R01_cb4744_c3/f1p0/2848
R01_cb18456_c7435/f1p0/649	R01_cb18456_c7435/f1p0/649	R01_cb18456_c7435/f1p0/649	R01_cb18456_c7435/f1p0/649	R01_cb18456_c7435/f1p0/649
R01_cb1437_c7/f1p0/3821	NA	NA	NA	R01_cb1437_c7/f1p0/3821
R01_cb15100_c0/f6p0/793	NA	NA	NA	R01_cb15100_c0/f6p0/793
R01_cb18166_c0/f1p0/426	NA	R01_cb18166_c0/f1p0/426	NA	NA
R01_cb6424_c3/f1p0/2553	NA	R01_cb6424_c3/f1p0/2553	NA	NA
R01_cb9179_c1/f1p0/2156	NA	R01_cb9179_c1/f1p0/2156	NA	R01_cb9179_c1/f1p0/2156
R01_cb13405_c3/f1p0/732	NA	NA	NA	R01_cb13405_c3/f1p0/732
R01_cb9232_c5/f1p0/371	NA	R01_cb9232_c5/f1p0/371	R01_cb9232_c5/f1p0/371	R01_cb9232_c5/f1p0/371
R01_cb15789_c1/f1p0/284	R01_cb15789_c1/f1p0/284	R01_cb15789_c1/f1p0/284	R01_cb15789_c1/f1p0/284	R01_cb15789_c1/f1p0/284
R01_cb2494_c5/f1p0/3920	NA	R01_cb2494_c5/f1p0/3920	NA	NA
R01_cb16662_c7/f4p0/521	R01_cb16662_c7/f4p0/521	R01_cb16662_c7/f4p0/521	R01_cb16662_c7/f4p0/521	R01_cb16662_c7/f4p0/521
R01_cb7133_c1/f1p0/2694	R01_cb7133_c1/f1p0/2694	R01_cb7133_c1/f1p0/2694	NA	NA
R01_cb11701_c0/f1p0/783	NA	NA	NA	R01_cb11701_c0/f1p0/783
R01_cb6547_c2/f2p1/2686	NA	NA	NA	R01_cb6547_c2/f2p1/2686
R01_cb13445_c2/f1p0/665	R01_cb13445_c2/f1p0/665	R01_cb13445_c2/f1p0/665	NA	NA
R01_cb11075_c0/f1p0/699	NA	R01_cb11075_c0/f1p0/699	NA	NA
R01_cb3359_c15/f1p0/3164	NA	R01_cb3359_c15/f1p0/3164	NA	NA
R01_cb6240_c6/f1p1/2905	NA	R01_cb6240_c6/f1p1/2905	NA	NA
R01_cb6631_c25/f1p0/1946	NA	NA	NA	R01_cb6631_c25/f1p0/1946
R01_cb18456_c5376/f1p0/1429	NA	R01_cb18456_c5376/f1p0/1429	NA	NA

R01_cb8564_c10187/f1p0/2124	R01_cb8564_c10187/f1p0/2124	R01_cb8564_c10187/f1p0/2124	R01_cb8564_c10187/f1p0/2124	R01_cb8564_c10187/f1p0/2124
R01_cb11099_c1/f1p0/2096	NA	NA	NA	R01_cb11099_c1/f1p0/2096
R01_cb4805_c25/f1p0/1488	NA	R01_cb4805_c25/f1p0/1488	NA	R01_cb4805_c25/f1p0/1488
R01_cb10121_c0/f5p0/754	NA	R01_cb10121_c0/f5p0/754	NA	R01_cb10121_c0/f5p0/754
R01_cb11480_c0/f1p0/1304	R01_cb11480_c0/f1p0/1304	R01_cb11480_c0/f1p0/1304	R01_cb11480_c0/f1p0/1304	R01_cb11480_c0/f1p0/1304
R01_cb13880_c2/f1p0/1819	NA	R01_cb13880_c2/f1p0/1819	R01_cb13880_c2/f1p0/1819	NA
R01_cb8564_c70540/f1p0/4401	NA	R01_cb8564_c70540/f1p0/4401	NA	R01_cb8564_c70540/f1p0/4401
R01_cb17268_c2/f1p0/500	NA	R01_cb17268_c2/f1p0/500	NA	NA
R01_cb1487_c1/f1p0/3827	NA	NA	NA	R01_cb1487_c1/f1p0/3827
R01_cb16005_c1/f2p0/630	NA	R01_cb16005_c1/f2p0/630	NA	NA
R01_cb4989_c52/f1p2/1036	R01_cb4989_c52/f1p2/1036	R01_cb4989_c52/f1p2/1036	NA	R01_cb4989_c52/f1p2/1036
R01_cb18456_c5325/f1p4/800	NA	R01_cb18456_c5325/f1p4/800	NA	NA
R01_cb1706_c4/f1p0/3976	NA	NA	NA	R01_cb1706_c4/f1p0/3976
R01_cb6802_c41/f1p0/3431	NA	R01_cb6802_c41/f1p0/3431	NA	NA
R01_cb1554_c6/f1p0/1460	NA	NA	NA	R01_cb1554_c6/f1p0/1460
R01_cb5900_c99/f1p0/3038	NA	R01_cb5900_c99/f1p0/3038	NA	NA
R01_cb18456_c7323/f1p0/752	NA	R01_cb18456_c7323/f1p0/752	NA	NA
R01_cb4233_c203/f1p0/2028	NA	NA	NA	R01_cb4233_c203/f1p0/2028
R01_cb3426_c12/f1p1/3048	NA	NA	NA	R01_cb3426_c12/f1p1/3048
R01_cb1849_c14/f1p1/1916	NA	NA	NA	R01_cb1849_c14/f1p1/1916
R01_cb261_c43/f1p0/2533	NA	NA	NA	R01_cb261_c43/f1p0/2533
R01_cb6443_c1/f1p0/2958	R01_cb6443_c1/f1p0/2958	R01_cb6443_c1/f1p0/2958	R01_cb6443_c1/f1p0/2958	R01_cb6443_c1/f1p0/2958
R01_cb18301_c2/f2p0/1752	NA	NA	NA	R01_cb18301_c2/f2p0/1752
R01_cb16981_c11/f1p2/746	NA	NA	NA	R01_cb16981_c11/f1p2/746
R01_cb8564_c2989/f1p0/2431	NA	R01_cb8564_c2989/f1p0/2431	R01_cb8564_c2989/f1p0/2431	R01_cb8564_c2989/f1p0/2431

R01_cb15085_c4/f1p0/636	NA	R01_cb15085_c4/f1p0/636	NA	NA
R01_cb15844_c0/f1p0/682	R01_cb15844_c0/f1p0/682	R01_cb15844_c0/f1p0/682	R01_cb15844_c0/f1p0/682	R01_cb15844_c0/f1p0/682
R01_cb11453_c0/f5p0/987	NA	NA	NA	R01_cb11453_c0/f5p0/987
R01_cb4582_c2/f1p0/3375	NA	R01_cb4582_c2/f1p0/3375	NA	NA
R01_cb1969_c3/f1p0/3150	NA	R01_cb1969_c3/f1p0/3150	R01_cb1969_c3/f1p0/3150	NA
R01_cb6802_c38/f1p0/2886	NA	R01_cb6802_c38/f1p0/2886	NA	NA
R01_cb12311_c1/f1p0/1218	R01_cb12311_c1/f1p0/1218	R01_cb12311_c1/f1p0/1218	NA	R01_cb12311_c1/f1p0/1218
R01_cb8546_c1/f2p0/1736	NA	NA	NA	R01_cb8546_c1/f2p0/1736
R01_cb882_c7/f1p0/1215	NA	NA	NA	R01_cb882_c7/f1p0/1215
R01_cb10556_c9/f1p0/1619	NA	NA	NA	R01_cb10556_c9/f1p0/1619
R01_cb302_c6/f1p0/3162	NA	NA	NA	R01_cb302_c6/f1p0/3162
R01_cb11808_c0/f1p1/1464	NA	NA	NA	R01_cb11808_c0/f1p1/1464
R01_cb6309_c3/f1p0/2749	NA	R01_cb6309_c3/f1p0/2749	NA	NA
R01_cb6406_c50/f1p0/2717	NA	R01_cb6406_c50/f1p0/2717	NA	R01_cb6406_c50/f1p0/2717
R01_cb8564_c77232/f1p0/2806	NA	NA	NA	R01_cb8564_c77232/f1p0/2806
R01_cb8564_c23387/f1p0/2531	NA	NA	NA	R01_cb8564_c23387/f1p0/2531
R01_cb8564_c34119/f4p0/3135	NA	R01_cb8564_c34119/f4p0/3135	R01_cb8564_c34119/f4p0/3135	R01_cb8564_c34119/f4p0/3135
R01_cb8977_c7/f1p0/1977	NA	R01_cb8977_c7/f1p0/1977	NA	R01_cb8977_c7/f1p0/1977
R01_cb8564_c23605/f1p3/4219	NA	R01_cb8564_c23605/f1p3/4219	R01_cb8564_c23605/f1p3/4219	R01_cb8564_c23605/f1p3/4219
R01_cb10964_c1/f1p0/3951	R01_cb10964_c1/f1p0/3951	R01_cb10964_c1/f1p0/3951	NA	R01_cb10964_c1/f1p0/3951
R01_cb8564_c15505/f1p1/2740	R01_cb8564_c15505/f1p1/2740	NA	R01_cb8564_c15505/f1p1/2740	R01_cb8564_c15505/f1p1/2740
R01_cb9448_c6/f1p1/2077	NA	NA	NA	R01_cb9448_c6/f1p1/2077
R01_cb8564_c13587/f1p1/2298	NA	NA	NA	R01_cb8564_c13587/f1p1/2298
R01_cb1248_c9/f1p0/6348	NA	NA	NA	R01_cb1248_c9/f1p0/6348
R01_cb13118_c3/f1p0/1964	NA	R01_cb13118_c3/f1p0/1964	NA	NA

R01_cb16926_c4/f1p0/1767	NA	R01_cb16926_c4/f1p0/1767	NA	NA
R01_cb14401_c0/f4p0/506	NA	R01_cb14401_c0/f4p0/506	NA	NA
R01_cb13686_c15/f1p1/1835	NA	R01_cb13686_c15/f1p1/1835	NA	NA
R01_cb7230_c7/f1p0/1948	NA	R01_cb7230_c7/f1p0/1948	NA	NA
R01_cb18456_c7593/f1p0/770	R01_cb18456_c7593/f1p0/770	R01_cb18456_c7593/f1p0/770	NA	R01_cb18456_c7593/f1p0/770
R01_cb8564_c1892/f1p0/2987	NA	NA	NA	R01_cb8564_c1892/f1p0/2987
R01_cb10623_c1/f2p0/546	NA	R01_cb10623_c1/f2p0/546	R01_cb10623_c1/f2p0/546	NA
R01_cb2845_c21/f1p0/3681	NA	NA	NA	R01_cb2845_c21/f1p0/3681
R01_cb8564_c129610/f1p0/2386	R01_cb8564_c129610/f1p0/2386	R01_cb8564_c129610/f1p0/2386	R01_cb8564_c129610/f1p0/2386	R01_cb8564_c129610/f1p0/2386
R01_cb18445_c0/f1p0/942	NA	R01_cb18445_c0/f1p0/942	R01_cb18445_c0/f1p0/942	R01_cb18445_c0/f1p0/942
R01_cb8564_c77520/f1p0/3886	NA	R01_cb8564_c77520/f1p0/3886	NA	NA
R01_cb5769_c2/f1p0/3018	NA	R01_cb5769_c2/f1p0/3018	NA	NA
R01_cb3500_c28/f1p0/2679	NA	NA	NA	R01_cb3500_c28/f1p0/2679
R01_cb13910_c24/f1p0/1099	R01_cb13910_c24/f1p0/1099	R01_cb13910_c24/f1p0/1099	R01_cb13910_c24/f1p0/1099	R01_cb13910_c24/f1p0/1099
R01_cb9951_c1/f1p0/2059	NA	R01_cb9951_c1/f1p0/2059	NA	NA
R01_cb18456_c1834/f1p0/359	R01_cb18456_c1834/f1p0/359	R01_cb18456_c1834/f1p0/359	R01_cb18456_c1834/f1p0/359	R01_cb18456_c1834/f1p0/359
R01_cb8564_c25146/f1p0/3529	NA	R01_cb8564_c25146/f1p0/3529	R01_cb8564_c25146/f1p0/3529	R01_cb8564_c25146/f1p0/3529
R01_cb16153_c6/f1p0/1191	R01_cb16153_c6/f1p0/1191	R01_cb16153_c6/f1p0/1191	R01_cb16153_c6/f1p0/1191	R01_cb16153_c6/f1p0/1191
R01_cb14107_c3/f1p0/1338	NA	NA	R01_cb14107_c3/f1p0/1338	R01_cb14107_c3/f1p0/1338
R01_cb8975_c9/f1p0/1783	NA	NA	NA	R01_cb8975_c9/f1p0/1783
R01_cb3756_c23/f1p0/1356	NA	R01_cb3756_c23/f1p0/1356	R01_cb3756_c23/f1p0/1356	R01_cb3756_c23/f1p0/1356
R01_cb7023_c3/f1p1/2449	NA	R01_cb7023_c3/f1p1/2449	R01_cb7023_c3/f1p1/2449	R01_cb7023_c3/f1p1/2449
R01_cb10034_c53/f2p1/549	NA	R01_cb10034_c53/f2p1/549	NA	NA
R01_cb18699_c0/f1p0/6015	R01_cb18699_c0/f1p0/6015	R01_cb18699_c0/f1p0/6015	NA	NA
R01_cb10679_c3/f6p0/486	NA	NA	NA	R01_cb10679_c3/f6p0/486

R01_cb13762_c8/f1p0/1530	NA	R01_cb13762_c8/f1p0/1530	NA	NA
R01_cb8564_c115588/f1p0/2132	NA	R01_cb8564_c115588/f1p0/2132	NA	R01_cb8564_c115588/f1p0/2132
R01_cb18086_c1/f1p0/513	NA	R01_cb18086_c1/f1p0/513	NA	R01_cb18086_c1/f1p0/513
R01_cb8564_c2629/f1p2/2078	NA	R01_cb8564_c2629/f1p2/2078	NA	NA
R01_cb18456_c5168/f1p0/856	R01_cb18456_c5168/f1p0/856	R01_cb18456_c5168/f1p0/856	NA	R01_cb18456_c5168/f1p0/856
R01_cb12785_c9/f1p1/1033	NA	NA	R01_cb12785_c9/f1p1/1033	NA
R01_cb13026_c1/f2p0/452	R01_cb13026_c1/f2p0/452	R01_cb13026_c1/f2p0/452	R01_cb13026_c1/f2p0/452	R01_cb13026_c1/f2p0/452
R01_cb1077_c10/f1p0/3011	NA	R01_cb1077_c10/f1p0/3011	NA	NA
R01_cb18313_c1/f1p0/898	NA	NA	NA	R01_cb18313_c1/f1p0/898
R01_cb18062_c2/f1p0/734	R01_cb18062_c2/f1p0/734	R01_cb18062_c2/f1p0/734	R01_cb18062_c2/f1p0/734	R01_cb18062_c2/f1p0/734
R01_cb8564_c76220/f1p0/2932	NA	R01_cb8564_c76220/f1p0/2932	R01_cb8564_c76220/f1p0/2932	NA
R01_cb7243_c1/f1p0/2653	NA	NA	NA	R01_cb7243_c1/f1p0/2653
R01_cb8564_c988/f1p0/2635	NA	R01_cb8564_c988/f1p0/2635	R01_cb8564_c988/f1p0/2635	R01_cb8564_c988/f1p0/2635
R01_cb8564_c85777/f1p0/2224	R01_cb8564_c85777/f1p0/2224	NA	R01_cb8564_c85777/f1p0/2224	R01_cb8564_c85777/f1p0/2224
R01_cb4657_c29/f1p0/2319	NA	R01_cb4657_c29/f1p0/2319	R01_cb4657_c29/f1p0/2319	NA
R01_cb12024_c6/f1p0/959	NA	NA	NA	R01_cb12024_c6/f1p0/959
R01_cb11075_c1/f1p0/3494	NA	R01_cb11075_c1/f1p0/3494	NA	NA
R01_cb6507_c4/f1p0/2292	NA	NA	R01_cb6507_c4/f1p0/2292	R01_cb6507_c4/f1p0/2292
R01_cb10359_c2/f1p0/2675	NA	NA	NA	R01_cb10359_c2/f1p0/2675
R01_cb5896_c140/f1p0/2367	NA	R01_cb5896_c140/f1p0/2367	NA	R01_cb5896_c140/f1p0/2367
R01_cb15313_c1/f1p0/1841	NA	NA	R01_cb15313_c1/f1p0/1841	R01_cb15313_c1/f1p0/1841
R01_cb3324_c5/f1p0/2017	NA	R01_cb3324_c5/f1p0/2017	NA	R01_cb3324_c5/f1p0/2017
R01_cb13427_c5/f1p0/712	NA	NA	R01_cb13427_c5/f1p0/712	R01_cb13427_c5/f1p0/712
R01_cb4657_c14/f1p0/3546	NA	NA	NA	R01_cb4657_c14/f1p0/3546

R01_cb9552_c2/f1p0/2047	NA	NA	NA	R01_cb9552_c2/f1p0/2047
R01_cb13027_c0/f2p0/1040	NA	NA	NA	R01_cb13027_c0/f2p0/1040
R01_cb16926_c2/f1p0/1100	NA	R01_cb16926_c2/f1p0/1100	NA	NA
R01_cb10927_c1/f1p0/2928	NA	R01_cb10927_c1/f1p0/2928	NA	NA
R01_cb5929_c6/f1p1/2534	NA	NA	NA	R01_cb5929_c6/f1p1/2534
R01_cb5139_c7/f1p1/2751	NA	NA	NA	R01_cb5139_c7/f1p1/2751
R01_cb16426_c1/f1p0/1401	NA	R01_cb16426_c1/f1p0/1401	NA	NA
R01_cb3492_c2/f1p0/3445	NA	R01_cb3492_c2/f1p0/3445	NA	NA
R01_cb8564_c89458/f2p0/3479	NA	R01_cb8564_c89458/f2p0/3479	NA	NA
R01_cb10413_c1/f1p0/2321	R01_cb10413_c1/f1p0/2321	R01_cb10413_c1/f1p0/2321	R01_cb10413_c1/f1p0/2321	R01_cb10413_c1/f1p0/2321
R01_cb8564_c1078/f1p0/2012	NA	NA	NA	R01_cb8564_c1078/f1p0/2012
R01_cb18535_c1/f1p0/807	NA	R01_cb18535_c1/f1p0/807	NA	R01_cb18535_c1/f1p0/807
R01_cb18192_c5/f1p0/1798	R01_cb18192_c5/f1p0/1798	R01_cb18192_c5/f1p0/1798	R01_cb18192_c5/f1p0/1798	R01_cb18192_c5/f1p0/1798
R01_cb1894_c2/f1p0/4133	NA	R01_cb1894_c2/f1p0/4133	NA	R01_cb1894_c2/f1p0/4133
R01_cb12873_c2/f1p0/811	NA	R01_cb12873_c2/f1p0/811	NA	NA
R01_cb16984_c1/f1p0/1248	NA	NA	NA	R01_cb16984_c1/f1p0/1248
R01_cb9699_c0/f2p0/2010	NA	NA	NA	R01_cb9699_c0/f2p0/2010
R01_cb1078_c7/f1p0/3914	NA	NA	NA	R01_cb1078_c7/f1p0/3914
R01_cb8564_c110588/f1p0/1905	NA	NA	NA	R01_cb8564_c110588/f1p0/1905
R01_cb2095_c4/f2p2/2126	NA	NA	NA	R01_cb2095_c4/f2p2/2126
R01_cb8564_c128659/f1p0/1925	NA	R01_cb8564_c128659/f1p0/1925	NA	R01_cb8564_c128659/f1p0/1925
R01_cb1905_c5/f2p0/3069	NA	R01_cb1905_c5/f2p0/3069	R01_cb1905_c5/f2p0/3069	R01_cb1905_c5/f2p0/3069
R01_cb11802_c1/f1p0/2381	R01_cb11802_c1/f1p0/2381	R01_cb11802_c1/f1p0/2381	NA	R01_cb11802_c1/f1p0/2381
R01_cb7141_c1/f1p0/2689	NA	R01_cb7141_c1/f1p0/2689	NA	R01_cb7141_c1/f1p0/2689

R01_cb5208_c9/f1p1/2940	NA	NA	NA	R01_cb5208_c9/f1p1/2940
R01_cb2807_c4/f1p0/1596	NA	NA	R01_cb2807_c4/f1p0/1596	R01_cb2807_c4/f1p0/1596
R01_cb18456_c7783/f3p3/694	NA	R01_cb18456_c7783/f3p3/694	R01_cb18456_c7783/f3p3/694	R01_cb18456_c7783/f3p3/694
R01_cb1134_c4/f1p0/1917	NA	NA	NA	R01_cb1134_c4/f1p0/1917
R01_cb12505_c4/f1p0/1745	NA	R01_cb12505_c4/f1p0/1745	NA	NA
R01_cb8564_c126074/f1p0/2700	NA	NA	NA	R01_cb8564_c126074/f1p0/2700
R01_cb7137_c1/f1p0/2652	NA	R01_cb7137_c1/f1p0/2652	R01_cb7137_c1/f1p0/2652	NA
R01_cb8564_c17390/f1p0/2016	NA	NA	NA	R01_cb8564_c17390/f1p0/2016
R01_cb16133_c4/f1p0/837	NA	R01_cb16133_c4/f1p0/837	NA	R01_cb16133_c4/f1p0/837
R01_cb9882_c8/f1p0/835	R01_cb9882_c8/f1p0/835	R01_cb9882_c8/f1p0/835	R01_cb9882_c8/f1p0/835	NA
R01_cb15208_c7/f1p0/422	R01_cb15208_c7/f1p0/422	R01_cb15208_c7/f1p0/422	NA	R01_cb15208_c7/f1p0/422
R01_cb7570_c1/f1p0/2371	NA	R01_cb7570_c1/f1p0/2371	NA	NA
R01_cb8564_c125535/f1p0/2294	NA	R01_cb8564_c125535/f1p0/2294	NA	R01_cb8564_c125535/f1p0/2294
R01_cb11746_c0/f1p0/1494	NA	NA	NA	R01_cb11746_c0/f1p0/1494
R01_cb8564_c76784/f1p0/2432	NA	R01_cb8564_c76784/f1p0/2432	NA	NA
R01_cb6819_c2/f1p0/3315	NA	NA	NA	R01_cb6819_c2/f1p0/3315
R01_cb8564_c4869/f1p0/3091	NA	NA	NA	R01_cb8564_c4869/f1p0/3091
R01_cb8564_c15921/f1p11/1869	NA	R01_cb8564_c15921/f1p11/1869	NA	NA
R01_cb16888_c8/f1p0/862	NA	NA	R01_cb16888_c8/f1p0/862	R01_cb16888_c8/f1p0/862
R01_cb11007_c4/f2p0/633	NA	R01_cb11007_c4/f2p0/633	NA	R01_cb11007_c4/f2p0/633
R01_cb10830_c2/f1p0/1276	NA	NA	NA	R01_cb10830_c2/f1p0/1276
R01_cb8564_c73982/f1p0/2430	NA	NA	NA	R01_cb8564_c73982/f1p0/2430
R01_cb18622_c2/f1p0/748	NA	R01_cb18622_c2/f1p0/748	NA	R01_cb18622_c2/f1p0/748

R01_cb18690_c1/f1p1/483	R01_cb18690_c1/f1p1/483	R01_cb18690_c1/f1p1/483	R01_cb18690_c1/f1p1/483	R01_cb18690_c1/f1p1/483
R01_cb8266_c4/f1p0/1755	NA	R01_cb8266_c4/f1p0/1755	NA	R01_cb8266_c4/f1p0/1755
R01_cb18676_c5/f1p0/1059	NA	R01_cb18676_c5/f1p0/1059	R01_cb18676_c5/f1p0/1059	R01_cb18676_c5/f1p0/1059
R01_cb8564_c87901/f1p0/3281	NA	R01_cb8564_c87901/f1p0/3281	NA	NA
R01_cb7367_c5/f1p1/2604	NA	NA	NA	R01_cb7367_c5/f1p1/2604
R01_cb4269_c10/f1p0/1931	NA	NA	NA	R01_cb4269_c10/f1p0/1931
R01_cb3929_c8/f1p0/4508	NA	R01_cb3929_c8/f1p0/4508	R01_cb3929_c8/f1p0/4508	NA
R01_cb12039_c1/f2p0/370	NA	R01_cb12039_c1/f2p0/370	R01_cb12039_c1/f2p0/370	R01_cb12039_c1/f2p0/370
R01_cb8564_c77450/f1p0/2895	NA	R01_cb8564_c77450/f1p0/2895	NA	NA
R01_cb14783_c0/f1p0/703	NA	R01_cb14783_c0/f1p0/703	R01_cb14783_c0/f1p0/703	R01_cb14783_c0/f1p0/703
R01_cb10029_c724/f1p0/699	NA	R01_cb10029_c724/f1p0/699	NA	NA
R01_cb10049_c0/f1p0/1662	R01_cb10049_c0/f1p0/1662	R01_cb10049_c0/f1p0/1662	R01_cb10049_c0/f1p0/1662	R01_cb10049_c0/f1p0/1662
R01_cb10726_c1/f1p0/2117	R01_cb10726_c1/f1p0/2117	R01_cb10726_c1/f1p0/2117	NA	NA
R01_cb9723_c0/f3p0/1581	NA	NA	NA	R01_cb9723_c0/f3p0/1581
R01_cb8279_c4/f1p0/4395	NA	NA	NA	R01_cb8279_c4/f1p0/4395
R01_cb1119_c3/f1p1/4475	NA	R01_cb1119_c3/f1p1/4475	R01_cb1119_c3/f1p1/4475	NA
R01_cb3218_c5/f1p0/2663	NA	NA	NA	R01_cb3218_c5/f1p0/2663
R01_cb5758_c2/f1p0/2177	R01_cb5758_c2/f1p0/2177	R01_cb5758_c2/f1p0/2177	R01_cb5758_c2/f1p0/2177	R01_cb5758_c2/f1p0/2177
R01_cb8564_c122060/f1p0/3330	R01_cb8564_c122060/f1p0/3330	NA	NA	R01_cb8564_c122060/f1p0/3330
R01_cb8564_c84907/f1p0/3145	R01_cb8564_c84907/f1p0/3145	R01_cb8564_c84907/f1p0/3145	NA	NA
R01_cb3941_c90/f1p0/4349	NA	R01_cb3941_c90/f1p0/4349	NA	NA
R01_cb3578_c6/f1p0/745	R01_cb3578_c6/f1p0/745	R01_cb3578_c6/f1p0/745	R01_cb3578_c6/f1p0/745	R01_cb3578_c6/f1p0/745
R01_cb4328_c8/f1p0/2449	NA	R01_cb4328_c8/f1p0/2449	NA	NA
R01_cb8564_c20162/f1p0/3449	NA	R01_cb8564_c20162/f1p0/3449	NA	R01_cb8564_c20162/f1p0/3449

R01_cb3025_c6/f1p0/2486	NA	NA	NA	R01_cb3025_c6/f1p0/2486
R01_cb7530_c10/f1p1/2529	NA	NA	NA	R01_cb7530_c10/f1p1/2529
R01_cb18456_c7232/f1p0/593	R01_cb18456_c7232/f1p0/593	R01_cb18456_c7232/f1p0/593	R01_cb18456_c7232/f1p0/593	R01_cb18456_c7232/f1p0/593
R01_cb10015_c353/f1p1/908	NA	R01_cb10015_c353/f1p1/908	NA	NA
R01_cb8564_c80473/f1p0/3519	NA	R01_cb8564_c80473/f1p0/3519	R01_cb8564_c80473/f1p0/3519	R01_cb8564_c80473/f1p0/3519
R01_cb8478_c47/f1p0/2734	NA	NA	NA	R01_cb8478_c47/f1p0/2734
R01_cb15680_c3/f1p0/1859	NA	NA	NA	R01_cb15680_c3/f1p0/1859
R01_cb2069_c2/f1p0/4066	NA	R01_cb2069_c2/f1p0/4066	NA	NA
R01_cb1993_c3/f1p1/3160	NA	NA	NA	R01_cb1993_c3/f1p1/3160
R01_cb1155_c4/f1p0/4388	NA	NA	NA	R01_cb1155_c4/f1p0/4388
R01_cb3732_c9/f1p0/2702	NA	NA	NA	R01_cb3732_c9/f1p0/2702
R01_cb1052_c15/f1p0/4466	NA	R01_cb1052_c15/f1p0/4466	NA	NA
R01_cb8564_c10155/f1p0/2920	NA	R01_cb8564_c10155/f1p0/2920	R01_cb8564_c10155/f1p0/2920	NA
R01_cb8564_c48900/f1p0/2938	NA	NA	NA	R01_cb8564_c48900/f1p0/2938
R01_cb2164_c16/f1p0/2809	R01_cb2164_c16/f1p0/2809	R01_cb2164_c16/f1p0/2809	R01_cb2164_c16/f1p0/2809	R01_cb2164_c16/f1p0/2809
R01_cb7020_c0/f2p0/2584	NA	R01_cb7020_c0/f2p0/2584	NA	NA
R01_cb4824_c11/f1p0/3500	R01_cb4824_c11/f1p0/3500	R01_cb4824_c11/f1p0/3500	R01_cb4824_c11/f1p0/3500	R01_cb4824_c11/f1p0/3500
R01_cb4744_c5/f1p0/1551	NA	R01_cb4744_c5/f1p0/1551	R01_cb4744_c5/f1p0/1551	R01_cb4744_c5/f1p0/1551
R01_cb2344_c0/f1p0/3977	R01_cb2344_c0/f1p0/3977	R01_cb2344_c0/f1p0/3977	NA	NA
R01_cb2052_c1/f2p0/4068	NA	R01_cb2052_c1/f2p0/4068	NA	NA
R01_cb1398_c11/f1p0/2067	NA	R01_cb1398_c11/f1p0/2067	R01_cb1398_c11/f1p0/2067	NA
R01_cb1993_c6/f1p1/3216	NA	NA	NA	R01_cb1993_c6/f1p1/3216
R01_cb9851_c3/f1p0/2725	R01_cb9851_c3/f1p0/2725	R01_cb9851_c3/f1p0/2725	R01_cb9851_c3/f1p0/2725	R01_cb9851_c3/f1p0/2725
R01_cb735_c2/f1p0/2629	NA	NA	NA	R01_cb735_c2/f1p0/2629
R01_cb8564_c20126/f1p0/3488	NA	R01_cb8564_c20126/f1p0/3488	R01_cb8564_c20126/f1p0/3488	R01_cb8564_c20126/f1p0/3488
R01_cb17954_c0/f1p0/839	R01_cb17954_c0/f1p0/839	R01_cb17954_c0/f1p0/839	NA	NA

R01_cb10653_c2/f3p0/439	R01_cb10653_c2/f3p0/439	R01_cb10653_c2/f3p0/439	NA	NA
R01_cb18226_c0/f1p0/414	R01_cb18226_c0/f1p0/414	R01_cb18226_c0/f1p0/414	R01_cb18226_c0/f1p0/414	R01_cb18226_c0/f1p0/414
R01_cb9506_c9/f1p0/1111	NA	NA	NA	R01_cb9506_c9/f1p0/1111
R01_cb7502_c6/f1p0/2067	NA	NA	NA	R01_cb7502_c6/f1p0/2067
R01_cb8475_c5/f1p0/1159	NA	R01_cb8475_c5/f1p0/1159	NA	NA
R01_cb17507_c0/f2p0/604	NA	R01_cb17507_c0/f2p0/604	R01_cb17507_c0/f2p0/604	R01_cb17507_c0/f2p0/604
R01_cb12579_c12/f1p0/1340	NA	R01_cb12579_c12/f1p0/1340	R01_cb12579_c12/f1p0/1340	R01_cb12579_c12/f1p0/1340
R01_cb15190_c0/f1p0/782	NA	R01_cb15190_c0/f1p0/782	NA	NA
R01_cb998_c2/f2p0/505	R01_cb998_c2/f2p0/505	R01_cb998_c2/f2p0/505	NA	NA
R01_cb8564_c89806/f1p0/2503	R01_cb8564_c89806/f1p0/2503	R01_cb8564_c89806/f1p0/2503	NA	NA
R01_cb8564_c116631/f1p0/2583	NA	R01_cb8564_c116631/f1p0/2583	R01_cb8564_c116631/f1p0/2583	NA
R01_cb7588_c3/f1p1/2578	NA	R01_cb7588_c3/f1p1/2578	NA	NA
R01_cb18715_c1/f1p0/721	NA	NA	NA	R01_cb18715_c1/f1p0/721
R01_cb3518_c9/f1p0/3635	NA	R01_cb3518_c9/f1p0/3635	NA	NA
R01_cb7109_c19/f1p0/649	NA	R01_cb7109_c19/f1p0/649	NA	NA
R01_cb8799_c0/f3p0/2216	NA	NA	NA	R01_cb8799_c0/f3p0/2216
R01_cb8564_c92216/f1p0/3345	NA	R01_cb8564_c92216/f1p0/3345	R01_cb8564_c92216/f1p0/3345	R01_cb8564_c92216/f1p0/3345
R01_cb6608_c6/f1p1/2415	NA	NA	NA	R01_cb6608_c6/f1p1/2415
R01_cb17721_c4/f1p0/1623	NA	R01_cb17721_c4/f1p0/1623	NA	R01_cb17721_c4/f1p0/1623
R01_cb14795_c4/f2p0/657	NA	R01_cb14795_c4/f2p0/657	R01_cb14795_c4/f2p0/657	R01_cb14795_c4/f2p0/657
R01_cb17159_c1/f1p0/719	NA	NA	NA	R01_cb17159_c1/f1p0/719
R01_cb13146_c9/f1p1/658	NA	NA	R01_cb13146_c9/f1p1/658	NA
R01_cb17562_c7/f1p0/608	NA	R01_cb17562_c7/f1p0/608	NA	NA
R01_cb17321_c0/f1p0/1816	NA	R01_cb17321_c0/f1p0/1816	R01_cb17321_c0/f1p0/1816	NA

R01_cb5297_c9/f1p0/886	NA	R01_cb5297_c9/f1p0/886	NA	NA
R01_cb17033_c0/f1p0/1184	NA	NA	NA	R01_cb17033_c0/f1p0/1184
R01_cb7027_c4/f1p0/1300	R01_cb7027_c4/f1p0/1300	R01_cb7027_c4/f1p0/1300	R01_cb7027_c4/f1p0/1300	R01_cb7027_c4/f1p0/1300
R01_cb1178_c19/f1p0/1460	NA	NA	NA	R01_cb1178_c19/f1p0/1460
R01_cb8564_c85602/f1p0/4985	NA	R01_cb8564_c85602/f1p0/4985	NA	R01_cb8564_c85602/f1p0/4985
R01_cb16850_c0/f1p0/1708	NA	R01_cb16850_c0/f1p0/1708	R01_cb16850_c0/f1p0/1708	NA
R01_cb822_c16/f1p3/2585	NA	NA	NA	R01_cb822_c16/f1p3/2585
R01_cb6574_c0/f1p0/2834	NA	R01_cb6574_c0/f1p0/2834	NA	NA
R01_cb18456_c8001/f1p0/487	R01_cb18456_c8001/f1p0/487	R01_cb18456_c8001/f1p0/487	NA	NA
R01_cb5658_c4/f1p0/2844	NA	NA	NA	R01_cb5658_c4/f1p0/2844
R01_cb16341_c7/f1p0/460	R01_cb16341_c7/f1p0/460	R01_cb16341_c7/f1p0/460	R01_cb16341_c7/f1p0/460	R01_cb16341_c7/f1p0/460
R01_cb8087_c5/f1p0/3215	NA	R01_cb8087_c5/f1p0/3215	NA	NA
R01_cb8219_c10/f1p1/2990	NA	R01_cb8219_c10/f1p1/2990	NA	NA
R01_cb12443_c5/f1p0/527	R01_cb12443_c5/f1p0/527	R01_cb12443_c5/f1p0/527	R01_cb12443_c5/f1p0/527	R01_cb12443_c5/f1p0/527
R01_cb8564_c90360/f1p0/2334	NA	R01_cb8564_c90360/f1p0/2334	R01_cb8564_c90360/f1p0/2334	NA
R01_cb8564_c1022/f1p0/2466	R01_cb8564_c1022/f1p0/2466	R01_cb8564_c1022/f1p0/2466	R01_cb8564_c1022/f1p0/2466	NA
R01_cb13545_c15/f2p0/675	R01_cb13545_c15/f2p0/675	R01_cb13545_c15/f2p0/675	R01_cb13545_c15/f2p0/675	R01_cb13545_c15/f2p0/675
R01_cb7806_c8/f1p0/1654	R01_cb7806_c8/f1p0/1654	R01_cb7806_c8/f1p0/1654	R01_cb7806_c8/f1p0/1654	R01_cb7806_c8/f1p0/1654
R01_cb8084_c3/f1p1/2128	NA	NA	NA	R01_cb8084_c3/f1p1/2128
R01_cb1388_c1/f3p2/4341	NA	NA	NA	R01_cb1388_c1/f3p2/4341
R01_cb1599_c0/f1p0/4125	NA	NA	NA	R01_cb1599_c0/f1p0/4125
R01_cb4600_c17/f4p1/2134	NA	NA	NA	R01_cb4600_c17/f4p1/2134
R01_cb11019_c2/f1p0/1867	NA	NA	NA	R01_cb11019_c2/f1p0/1867
R01_cb2161_c14/f1p0/3605	NA	NA	NA	R01_cb2161_c14/f1p0/3605
R01_cb13862_c1/f2p0/1481	NA	R01_cb13862_c1/f2p0/1481	R01_cb13862_c1/f2p0/1481	R01_cb13862_c1/f2p0/1481
R01_cb15958_c6/f1p0/974	R01_cb15958_c6/f1p0/974	R01_cb15958_c6/f1p0/974	NA	R01_cb15958_c6/f1p0/974

R01_cb18152_c0/f1p0/525	NA	R01_cb18152_c0/f1p0/525	NA	NA
R01_cb11931_c5/f2p0/422	NA	R01_cb11931_c5/f2p0/422	NA	NA
R01_cb8564_c83469/f1p0/2306	NA	NA	R01_cb8564_c83469/f1p0/2306	R01_cb8564_c83469/f1p0/2306
R01_cb15765_c1/f1p0/808	NA	R01_cb15765_c1/f1p0/808	R01_cb15765_c1/f1p0/808	R01_cb15765_c1/f1p0/808
R01_cb18192_c13/f1p0/1373	NA	R01_cb18192_c13/f1p0/1373	R01_cb18192_c13/f1p0/1373	NA
R01_cb3426_c11/f1p1/3455	NA	NA	NA	R01_cb3426_c11/f1p1/3455
R01_cb12374_c4/f1p1/453	NA	R01_cb12374_c4/f1p1/453	R01_cb12374_c4/f1p1/453	R01_cb12374_c4/f1p1/453
R01_cb15811_c23/f1p0/1267	R01_cb15811_c23/f1p0/1267	R01_cb15811_c23/f1p0/1267	R01_cb15811_c23/f1p0/1267	R01_cb15811_c23/f1p0/1267
R01_cb15440_c3/f1p0/442	NA	R01_cb15440_c3/f1p0/442	R01_cb15440_c3/f1p0/442	R01_cb15440_c3/f1p0/442
R01_cb10327_c9/f1p0/1463	NA	R01_cb10327_c9/f1p0/1463	NA	NA
R01_cb8334_c0/f1p0/2398	NA	R01_cb8334_c0/f1p0/2398	NA	NA
R01_cb8564_c1357/f1p0/3246	NA	NA	NA	R01_cb8564_c1357/f1p0/3246
R01_cb787_c0/f1p0/4629	R01_cb787_c0/f1p0/4629	R01_cb787_c0/f1p0/4629	NA	NA
R01_cb7023_c4/f1p0/2631	NA	R01_cb7023_c4/f1p0/2631	R01_cb7023_c4/f1p0/2631	R01_cb7023_c4/f1p0/2631
R01_cb17013_c0/f2p0/693	NA	NA	NA	R01_cb17013_c0/f2p0/693
R01_cb10008_c3/f1p0/2867	NA	NA	NA	R01_cb10008_c3/f1p0/2867
R01_cb8564_c21988/f1p0/2578	NA	R01_cb8564_c21988/f1p0/2578	R01_cb8564_c21988/f1p0/2578	R01_cb8564_c21988/f1p0/2578
R01_cb14341_c0/f1p0/608	R01_cb14341_c0/f1p0/608	R01_cb14341_c0/f1p0/608	R01_cb14341_c0/f1p0/608	R01_cb14341_c0/f1p0/608
R01_cb18423_c2/f1p0/533	NA	NA	NA	R01_cb18423_c2/f1p0/533
R01_cb8564_c17470/f1p0/2900	NA	R01_cb8564_c17470/f1p0/2900	NA	NA
R01_cb18511_c1/f1p0/1184	R01_cb18511_c1/f1p0/1184	R01_cb18511_c1/f1p0/1184	R01_cb18511_c1/f1p0/1184	NA
R01_cb10198_c2/f2p0/668	NA	R01_cb10198_c2/f2p0/668	NA	NA
R01_cb14058_c1/f1p0/1424	NA	R01_cb14058_c1/f1p0/1424	NA	NA
R01_cb1686_c4/f1p0/2096	NA	NA	R01_cb1686_c4/f1p0/2096	NA
R01_cb15811_c20/f1p0/1153	NA	R01_cb15811_c20/f1p0/1153	R01_cb15811_c20/f1p0/1153	R01_cb15811_c20/f1p0/1153
R01_cb8564_c25152/f1p0/3291	NA	R01_cb8564_c25152/f1p0/3291	NA	NA

R01_cb12443_c6/f1p0/502	NA	R01_cb12443_c6/f1p0/502	R01_cb12443_c6/f1p0/502	R01_cb12443_c6/f1p0/502
R01_cb11754_c1/f1p0/3465	NA	R01_cb11754_c1/f1p0/3465	NA	NA
R01_cb14314_c0/f2p0/327	R01_cb14314_c0/f2p0/327	R01_cb14314_c0/f2p0/327	R01_cb14314_c0/f2p0/327	R01_cb14314_c0/f2p0/327
R01_cb7687_c2/f4p1/2416	NA	NA	NA	R01_cb7687_c2/f4p1/2416
R01_cb2415_c7/f1p0/3359	R01_cb2415_c7/f1p0/3359	R01_cb2415_c7/f1p0/3359	R01_cb2415_c7/f1p0/3359	R01_cb2415_c7/f1p0/3359
R01_cb7687_c3/f1p0/2557	NA	NA	NA	R01_cb7687_c3/f1p0/2557
R01_cb7197_c4/f1p0/3076	NA	R01_cb7197_c4/f1p0/3076	NA	NA
R01_cb13178_c13/f1p0/587	NA	R01_cb13178_c13/f1p0/587	NA	NA
R01_cb17532_c4/f1p0/5275	NA	NA	NA	R01_cb17532_c4/f1p0/5275
R01_cb1097_c9/f1p0/4066	NA	NA	NA	R01_cb1097_c9/f1p0/4066
R01_cb8564_c120522/f1p0/2575	R01_cb8564_c120522/f1p0/2575	R01_cb8564_c120522/f1p0/2575	R01_cb8564_c120522/f1p0/2575	R01_cb8564_c120522/f1p0/2575
R01_cb2072_c34/f1p0/3119	NA	NA	NA	R01_cb2072_c34/f1p0/3119
R01_cb15811_c13/f1p0/750	R01_cb15811_c13/f1p0/750	R01_cb15811_c13/f1p0/750	R01_cb15811_c13/f1p0/750	R01_cb15811_c13/f1p0/750
R01_cb12587_c6/f1p0/1591	NA	NA	NA	R01_cb12587_c6/f1p0/1591
R01_cb13442_c1/f1p0/937	NA	NA	NA	R01_cb13442_c1/f1p0/937
R01_cb9154_c4/f1p0/2109	NA	NA	NA	R01_cb9154_c4/f1p0/2109
R01_cb15724_c3/f1p0/1046	NA	R01_cb15724_c3/f1p0/1046	R01_cb15724_c3/f1p0/1046	R01_cb15724_c3/f1p0/1046
R01_cb10837_c3/f1p0/2634	R01_cb10837_c3/f1p0/2634	R01_cb10837_c3/f1p0/2634	R01_cb10837_c3/f1p0/2634	R01_cb10837_c3/f1p0/2634
R01_cb15092_c9/f1p0/436	NA	R01_cb15092_c9/f1p0/436	NA	R01_cb15092_c9/f1p0/436
R01_cb1092_c0/f1p0/4479	NA	R01_cb1092_c0/f1p0/4479	NA	R01_cb1092_c0/f1p0/4479
R01_cb18385_c0/f1p0/1881	NA	R01_cb18385_c0/f1p0/1881	NA	NA
R01_cb8102_c20/f21p0/1923	NA	NA	NA	R01_cb8102_c20/f21p0/1923
R01_cb3859_c0/f3p1/1826	NA	NA	NA	R01_cb3859_c0/f3p1/1826
R01_cb4223_c2/f1p0/2041	NA	NA	NA	R01_cb4223_c2/f1p0/2041
R01_cb15853_c0/f10p0/495	R01_cb15853_c0/f10p0/495	R01_cb15853_c0/f10p0/495	NA	R01_cb15853_c0/f10p0/495

R01_cb18456_c4928/f1p0/1102	R01_cb18456_c4928/f1p0/1102	R01_cb18456_c4928/f1p0/1102	NA	NA
R01_cb7110_c0/f2p0/1460	NA	NA	NA	R01_cb7110_c0/f2p0/1460
R01_cb6318_c23/f1p1/2399	NA	R01_cb6318_c23/f1p1/2399	NA	NA
R01_cb4484_c0/f1p0/3132	NA	R01_cb4484_c0/f1p0/3132	NA	NA
R01_cb9214_c11/f1p0/2500	NA	R01_cb9214_c11/f1p0/2500	NA	NA
R01_cb17756_c23/f1p0/369	NA	R01_cb17756_c23/f1p0/369	R01_cb17756_c23/f1p0/369	R01_cb17756_c23/f1p0/369
R01_cb17016_c1/f1p0/1317	NA	NA	NA	R01_cb17016_c1/f1p0/1317
R01_cb3732_c8/f1p0/2774	NA	NA	NA	R01_cb3732_c8/f1p0/2774
R01_cb13028_c2/f1p0/893	NA	NA	NA	R01_cb13028_c2/f1p0/893
R01_cb10750_c0/f2p0/782	NA	NA	R01_cb10750_c0/f2p0/782	R01_cb10750_c0/f2p0/782
R01_cb8564_c1410/f1p0/3134	NA	R01_cb8564_c1410/f1p0/3134	NA	NA
R01_cb4532_c2/f1p1/3318	NA	R01_cb4532_c2/f1p1/3318	NA	NA
R01_cb8564_c88936/f1p1/2270	NA	R01_cb8564_c88936/f1p1/2270	R01_cb8564_c88936/f1p1/2270	R01_cb8564_c88936/f1p1/2270
R01_cb6253_c0/f1p0/2902	NA	R01_cb6253_c0/f1p0/2902	R01_cb6253_c0/f1p0/2902	R01_cb6253_c0/f1p0/2902
R01_cb15963_c1/f1p0/669	NA	R01_cb15963_c1/f1p0/669	R01_cb15963_c1/f1p0/669	NA
R01_cb3088_c7/f1p0/1809	NA	R01_cb3088_c7/f1p0/1809	R01_cb3088_c7/f1p0/1809	R01_cb3088_c7/f1p0/1809
R01_cb18457_c0/f1p0/1409	NA	NA	NA	R01_cb18457_c0/f1p0/1409
R01_cb8564_c47497/f1p0/2695	R01_cb8564_c47497/f1p0/2695	R01_cb8564_c47497/f1p0/2695	R01_cb8564_c47497/f1p0/2695	R01_cb8564_c47497/f1p0/2695
R01_cb5641_c6/f1p1/1919	NA	NA	NA	R01_cb5641_c6/f1p1/1919
R01_cb2154_c9/f1p0/2457	NA	R01_cb2154_c9/f1p0/2457	NA	NA
R01_cb16636_c2/f1p1/1850	NA	R01_cb16636_c2/f1p1/1850	NA	NA
R01_cb8564_c53806/f1p3/3974	NA	NA	NA	R01_cb8564_c53806/f1p3/3974
R01_cb10637_c0/f1p0/1826	NA	R01_cb10637_c0/f1p0/1826	R01_cb10637_c0/f1p0/1826	R01_cb10637_c0/f1p0/1826
R01_cb8564_c90042/f1p0/3733	NA	R01_cb8564_c90042/f1p0/3733	NA	NA

R01_cb15998_c1/f1p0/1758	NA	NA	NA	R01_cb15998_c1/f1p0/1758
R01_cb18603_c0/f1p0/498	R01_cb18603_c0/f1p0/498	R01_cb18603_c0/f1p0/498	R01_cb18603_c0/f1p0/498	R01_cb18603_c0/f1p0/498
R01_cb17207_c1/f1p0/715	NA	NA	NA	R01_cb17207_c1/f1p0/715
R01_cb3464_c0/f3p1/3585	NA	NA	NA	R01_cb3464_c0/f3p1/3585
R01_cb2425_c9/f5p0/2281	NA	NA	NA	R01_cb2425_c9/f5p0/2281
R01_cb11395_c3/f3p0/1346	NA	NA	NA	R01_cb11395_c3/f3p0/1346
R01_cb18209_c0/f3p0/1446	NA	R01_cb18209_c0/f3p0/1446	NA	R01_cb18209_c0/f3p0/1446
R01_cb10837_c5/f1p0/705	NA	R01_cb10837_c5/f1p0/705	NA	R01_cb10837_c5/f1p0/705
R01_cb10029_c1275/f1p3/881	NA	R01_cb10029_c1275/f1p3/881	NA	NA
R01_cb11291_c0/f1p0/621	R01_cb11291_c0/f1p0/621	R01_cb11291_c0/f1p0/621	R01_cb11291_c0/f1p0/621	R01_cb11291_c0/f1p0/621
R01_cb10148_c13/f3p0/1791	NA	NA	NA	R01_cb10148_c13/f3p0/1791
R01_cb3757_c1/f1p0/3354	NA	NA	NA	R01_cb3757_c1/f1p0/3354
R01_cb8564_c123568/f1p0/2478	NA	NA	NA	R01_cb8564_c123568/f1p0/2478
R01_cb2629_c30/f1p0/3025	NA	NA	NA	R01_cb2629_c30/f1p0/3025
R01_cb10742_c8/f1p0/973	NA	NA	NA	R01_cb10742_c8/f1p0/973
R01_cb4102_c1/f1p0/3375	NA	R01_cb4102_c1/f1p0/3375	NA	NA
R01_cb8298_c0/f1p0/2384	R01_cb8298_c0/f1p0/2384	R01_cb8298_c0/f1p0/2384	R01_cb8298_c0/f1p0/2384	R01_cb8298_c0/f1p0/2384
R01_cb8564_c71883/f1p0/2864	NA	R01_cb8564_c71883/f1p0/2864	NA	NA
R01_cb5659_c20/f5p0/2269	NA	NA	NA	R01_cb5659_c20/f5p0/2269
R01_cb8564_c16003/f1p0/4482	NA	R01_cb8564_c16003/f1p0/4482	NA	NA
R01_cb10088_c12/f1p0/1263	NA	R01_cb10088_c12/f1p0/1263	NA	NA
R01_cb13396_c2/f2p0/1273	NA	R01_cb13396_c2/f2p0/1273	R01_cb13396_c2/f2p0/1273	R01_cb13396_c2/f2p0/1273
R01_cb3370_c1/f1p0/3646	NA	R01_cb3370_c1/f1p0/3646	NA	NA
R01_cb12373_c1/f1p0/465	R01_cb12373_c1/f1p0/465	R01_cb12373_c1/f1p0/465	R01_cb12373_c1/f1p0/465	R01_cb12373_c1/f1p0/465
R01_cb709_c10/f1p0/2582	NA	R01_cb709_c10/f1p0/2582	NA	NA

R01_cb454_c31/f1p0/2723	NA	NA	NA	R01_cb454_c31/f1p0/2723
R01_cb6819_c6/f1p0/5409	NA	NA	NA	R01_cb6819_c6/f1p0/5409
R01_cb18218_c0/f2p0/1448	NA	NA	NA	R01_cb18218_c0/f2p0/1448
R01_cb2780_c6/f1p1/3788	NA	R01_cb2780_c6/f1p1/3788	NA	NA
R01_cb14134_c2/f1p0/1181	NA	R01_cb14134_c2/f1p0/1181	NA	NA
R01_cb14684_c2/f1p0/604	NA	R01_cb14684_c2/f1p0/604	NA	NA
R01_cb8564_c76073/f1p0/2976	NA	R01_cb8564_c76073/f1p0/2976	R01_cb8564_c76073/f1p0/2976	R01_cb8564_c76073/f1p0/2976
R01_cb16268_c1/f1p0/786	R01_cb16268_c1/f1p0/786	R01_cb16268_c1/f1p0/786	R01_cb16268_c1/f1p0/786	R01_cb16268_c1/f1p0/786
R01_cb12837_c23/f1p0/755	NA	R01_cb12837_c23/f1p0/755	NA	NA
R01_cb4471_c5/f1p1/3026	NA	R01_cb4471_c5/f1p1/3026	NA	R01_cb4471_c5/f1p1/3026
R01_cb14223_c0/f5p0/449	NA	NA	R01_cb14223_c0/f5p0/449	R01_cb14223_c0/f5p0/449
R01_cb18456_c4798/f1p1/481	R01_cb18456_c4798/f1p1/481	NA	NA	R01_cb18456_c4798/f1p1/481
R01_cb1682_c5/f1p1/3916	NA	NA	NA	R01_cb1682_c5/f1p1/3916
R01_cb8564_c112476/f1p0/1961	NA	NA	NA	R01_cb8564_c112476/f1p0/1961
R01_cb4594_c2/f1p0/3067	NA	NA	NA	R01_cb4594_c2/f1p0/3067
R01_cb2991_c5/f1p1/2528	NA	NA	NA	R01_cb2991_c5/f1p1/2528
R01_cb12573_c19/f1p0/798	NA	R01_cb12573_c19/f1p0/798	NA	NA
R01_cb8564_c91354/f1p0/3094	NA	NA	NA	R01_cb8564_c91354/f1p0/3094
R01_cb8564_c4634/f2p2/3151	NA	R01_cb8564_c4634/f2p2/3151	R01_cb8564_c4634/f2p2/3151	R01_cb8564_c4634/f2p2/3151
R01_cb9824_c31/f1p0/495	R01_cb9824_c31/f1p0/495	R01_cb9824_c31/f1p0/495	R01_cb9824_c31/f1p0/495	R01_cb9824_c31/f1p0/495
R01_cb4639_c9/f1p0/2392	NA	NA	NA	R01_cb4639_c9/f1p0/2392
R01_cb8564_c125229/f2p0/2553	NA	NA	NA	R01_cb8564_c125229/f2p0/2553
R01_cb8162_c2/f1p0/2194	NA	NA	NA	R01_cb8162_c2/f1p0/2194
R01_cb8954_c11/f1p0/3820	NA	NA	NA	R01_cb8954_c11/f1p0/3820

R01_cb6922_c4/f1p0/1252	NA	NA	NA	R01_cb6922_c4/f1p0/1252
R01_cb8564_c76538/f1p0/3810	R01_cb8564_c76538/f1p0/3810	R01_cb8564_c76538/f1p0/3810	R01_cb8564_c76538/f1p0/3810	R01_cb8564_c76538/f1p0/3810
R01_cb17050_c0/f3p0/1730	R01_cb17050_c0/f3p0/1730	R01_cb17050_c0/f3p0/1730	R01_cb17050_c0/f3p0/1730	R01_cb17050_c0/f3p0/1730
R01_cb8564_c86657/f1p0/3031	NA	NA	NA	R01_cb8564_c86657/f1p0/3031
R01_cb17711_c1/f1p0/1361	NA	R01_cb17711_c1/f1p0/1361	NA	NA
R01_cb625_c9/f1p2/3104	NA	NA	NA	R01_cb625_c9/f1p2/3104
R01_cb2744_c3/f1p0/1362	NA	R01_cb2744_c3/f1p0/1362	NA	R01_cb2744_c3/f1p0/1362
R01_cb6362_c3/f1p1/2875	NA	R01_cb6362_c3/f1p1/2875	NA	NA
R01_cb2042_c5/f1p0/2493	NA	R01_cb2042_c5/f1p0/2493	NA	R01_cb2042_c5/f1p0/2493
R01_cb8564_c86976/f1p0/2183	NA	R01_cb8564_c86976/f1p0/2183	NA	NA
R01_cb18456_c6347/f1p0/1018	NA	R01_cb18456_c6347/f1p0/1018	NA	NA
R01_cb14153_c4/f1p0/1159	NA	R01_cb14153_c4/f1p0/1159	R01_cb14153_c4/f1p0/1159	NA
R01_cb11027_c0/f1p1/604	R01_cb11027_c0/f1p1/604	R01_cb11027_c0/f1p1/604	NA	R01_cb11027_c0/f1p1/604
R01_cb12524_c12/f2p1/619	NA	R01_cb12524_c12/f2p1/619	NA	NA
R01_cb8564_c86787/f1p0/2176	NA	R01_cb8564_c86787/f1p0/2176	NA	R01_cb8564_c86787/f1p0/2176
R01_cb18577_c1/f1p0/959	NA	NA	NA	R01_cb18577_c1/f1p0/959
R01_cb8564_c2548/f1p1/2656	NA	NA	R01_cb8564_c2548/f1p1/2656	R01_cb8564_c2548/f1p1/2656
R01_cb8564_c44341/f1p0/4569	NA	R01_cb8564_c44341/f1p0/4569	NA	R01_cb8564_c44341/f1p0/4569
R01_cb8564_c90788/f1p0/2216	NA	R01_cb8564_c90788/f1p0/2216	NA	NA
R01_cb18358_c0/f1p0/527	NA	R01_cb18358_c0/f1p0/527	R01_cb18358_c0/f1p0/527	R01_cb18358_c0/f1p0/527
R01_cb8376_c0/f2p0/1873	NA	NA	NA	R01_cb8376_c0/f2p0/1873
R01_cb17703_c0/f1p0/1359	NA	R01_cb17703_c0/f1p0/1359	NA	R01_cb17703_c0/f1p0/1359
R01_cb11331_c3/f1p0/2375	NA	R01_cb11331_c3/f1p0/2375	NA	NA
R01_cb7775_c2/f1p0/2100	NA	NA	NA	R01_cb7775_c2/f1p0/2100
R01_cb9643_c4/f1p0/1253	NA	R01_cb9643_c4/f1p0/1253	R01_cb9643_c4/f1p0/1253	R01_cb9643_c4/f1p0/1253

R01_cb18500_c0/f2p0/660	NA	R01_cb18500_c0/f2p0/660	NA	R01_cb18500_c0/f2p0/660
R01_cb18621_c1/f1p0/477	NA	R01_cb18621_c1/f1p0/477	NA	R01_cb18621_c1/f1p0/477
R01_cb13638_c4/f1p0/1123	NA	NA	NA	R01_cb13638_c4/f1p0/1123
R01_cb18655_c5/f1p1/1401	NA	R01_cb18655_c5/f1p1/1401	NA	R01_cb18655_c5/f1p1/1401
R01_cb8564_c84851/f2p0/2694	NA	R01_cb8564_c84851/f2p0/2694	R01_cb8564_c84851/f2p0/2694	R01_cb8564_c84851/f2p0/2694
R01_cb18186_c0/f1p0/540	NA	R01_cb18186_c0/f1p0/540	R01_cb18186_c0/f1p0/540	R01_cb18186_c0/f1p0/540
R01_cb8538_c0/f2p0/2101	NA	NA	NA	R01_cb8538_c0/f2p0/2101
R01_cb8564_c41184/f1p0/2767	NA	R01_cb8564_c41184/f1p0/2767	R01_cb8564_c41184/f1p0/2767	R01_cb8564_c41184/f1p0/2767
R01_cb8564_c82386/f1p1/2330	NA	NA	NA	R01_cb8564_c82386/f1p1/2330
R01_cb781_c36/f1p0/1809	NA	NA	NA	R01_cb781_c36/f1p0/1809
R01_cb8564_c80423/f1p0/3018	NA	R01_cb8564_c80423/f1p0/3018	NA	NA
R01_cb5952_c4/f1p0/1479	R01_cb5952_c4/f1p0/1479	R01_cb5952_c4/f1p0/1479	NA	R01_cb5952_c4/f1p0/1479
R01_cb18557_c0/f2p0/887	NA	NA	NA	R01_cb18557_c0/f2p0/887
R01_cb18757_c7/f1p0/2486	R01_cb18757_c7/f1p0/2486	R01_cb18757_c7/f1p0/2486	R01_cb18757_c7/f1p0/2486	R01_cb18757_c7/f1p0/2486
R01_cb8564_c88557/f1p3/2757	NA	NA	NA	R01_cb8564_c88557/f1p3/2757
R01_cb18456_c6115/f1p0/821	NA	R01_cb18456_c6115/f1p0/821	R01_cb18456_c6115/f1p0/821	NA
R01_cb14675_c1/f4p1/859	R01_cb14675_c1/f4p1/859	R01_cb14675_c1/f4p1/859	R01_cb14675_c1/f4p1/859	R01_cb14675_c1/f4p1/859
R01_cb8564_c1326/f1p1/3181	NA	NA	NA	R01_cb8564_c1326/f1p1/3181
R01_cb1618_c7/f1p0/4246	NA	R01_cb1618_c7/f1p0/4246	NA	NA
R01_cb8564_c77158/f1p0/4524	NA	NA	NA	R01_cb8564_c77158/f1p0/4524
R01_cb10867_c3/f1p0/4372	NA	NA	NA	R01_cb10867_c3/f1p0/4372
R01_cb5373_c4/f1p0/2557	NA	NA	NA	R01_cb5373_c4/f1p0/2557
R01_cb10292_c3/f1p0/1200	NA	NA	NA	R01_cb10292_c3/f1p0/1200
R01_cb4141_c4/f1p1/2427	NA	R01_cb4141_c4/f1p1/2427	NA	NA
R01_cb1333_c44/f1p0/2307	NA	R01_cb1333_c44/f1p0/2307	R01_cb1333_c44/f1p0/2307	R01_cb1333_c44/f1p0/2307
R01_cb778_c27/f1p0/979	NA	R01_cb778_c27/f1p0/979	NA	R01_cb778_c27/f1p0/979

R01_cb17604_c5/f1p0/1439	NA	R01_cb17604_c5/f1p0/1439	R01_cb17604_c5/f1p0/1439	R01_cb17604_c5/f1p0/1439
R01_cb8564_c88764/f1p0/2888	NA	R01_cb8564_c88764/f1p0/2888	NA	R01_cb8564_c88764/f1p0/2888
R01_cb12_c16/f1p0/3169	NA	NA	NA	R01_cb12_c16/f1p0/3169
R01_cb1230_c2/f1p0/4109	NA	R01_cb1230_c2/f1p0/4109	R01_cb1230_c2/f1p0/4109	R01_cb1230_c2/f1p0/4109
R01_cb12461_c1/f4p0/522	NA	R01_cb12461_c1/f4p0/522	NA	R01_cb12461_c1/f4p0/522
R01_cb17374_c3/f1p0/668	R01_cb17374_c3/f1p0/668	R01_cb17374_c3/f1p0/668	R01_cb17374_c3/f1p0/668	R01_cb17374_c3/f1p0/668
R01_cb8564_c23304/f1p0/2812	NA	R01_cb8564_c23304/f1p0/2812	NA	NA
R01_cb11869_c9/f1p0/421	R01_cb11869_c9/f1p0/421	R01_cb11869_c9/f1p0/421	NA	NA
R01_cb15151_c0/f1p0/913	NA	NA	R01_cb15151_c0/f1p0/913	R01_cb15151_c0/f1p0/913
R01_cb16045_c2/f1p0/765	NA	R01_cb16045_c2/f1p0/765	NA	NA
R01_cb8564_c1800/f2p0/2439	NA	R01_cb8564_c1800/f2p0/2439	NA	NA
R01_cb10522_c7/f1p0/1216	NA	R01_cb10522_c7/f1p0/1216	NA	NA
R01_cb15644_c1/f1p0/721	NA	R01_cb15644_c1/f1p0/721	R01_cb15644_c1/f1p0/721	R01_cb15644_c1/f1p0/721
R01_cb16963_c0/f1p0/553	NA	R01_cb16963_c0/f1p0/553	NA	NA
R01_cb197_c10/f1p0/6515	NA	R01_cb197_c10/f1p0/6515	NA	NA
R01_cb8564_c1887/f1p0/2257	NA	NA	NA	R01_cb8564_c1887/f1p0/2257
R01_cb4490_c2/f1p0/3341	R01_cb4490_c2/f1p0/3341	R01_cb4490_c2/f1p0/3341	R01_cb4490_c2/f1p0/3341	R01_cb4490_c2/f1p0/3341
R01_cb8564_c5266/f1p0/3784	NA	R01_cb8564_c5266/f1p0/3784	NA	NA
R01_cb3478_c2/f1p1/3546	R01_cb3478_c2/f1p1/3546	R01_cb3478_c2/f1p1/3546	R01_cb3478_c2/f1p1/3546	R01_cb3478_c2/f1p1/3546
R01_cb5007_c4/f2p0/760	NA	R01_cb5007_c4/f2p0/760	NA	NA
R01_cb8564_c86075/f1p0/3136	NA	R01_cb8564_c86075/f1p0/3136	R01_cb8564_c86075/f1p0/3136	NA
R01_cb15106_c5/f1p0/884	R01_cb15106_c5/f1p0/884	R01_cb15106_c5/f1p0/884	R01_cb15106_c5/f1p0/884	NA
R01_cb4564_c8/f1p0/3311	NA	R01_cb4564_c8/f1p0/3311	NA	NA
R01_cb8549_c1/f1p0/2974	NA	NA	NA	R01_cb8549_c1/f1p0/2974
R01_cb5499_c3/f1p0/3045	R01_cb5499_c3/f1p0/3045	R01_cb5499_c3/f1p0/3045	R01_cb5499_c3/f1p0/3045	R01_cb5499_c3/f1p0/3045
R01_cb6894_c3/f1p0/6832	NA	R01_cb6894_c3/f1p0/6832	NA	R01_cb6894_c3/f1p0/6832

R01_cb16746_c0/f1p0/1047	NA	NA	NA	R01_cb16746_c0/f1p0/1047
R01_cb3088_c1/f1p0/3724	NA	R01_cb3088_c1/f1p0/3724	R01_cb3088_c1/f1p0/3724	R01_cb3088_c1/f1p0/3724
R01_cb644_c11/f1p0/3471	NA	NA	NA	R01_cb644_c11/f1p0/3471
R01_cb8564_c121515/f1p0/2382	NA	NA	NA	R01_cb8564_c121515/f1p0/2382
R01_cb10180_c2/f1p0/2978	NA	R01_cb10180_c2/f1p0/2978	NA	NA
R01_cb18456_c7372/f1p0/1325	NA	R01_cb18456_c7372/f1p0/1325	R01_cb18456_c7372/f1p0/1325	R01_cb18456_c7372/f1p0/1325
R01_cb2026_c1/f1p0/4080	NA	R01_cb2026_c1/f1p0/4080	R01_cb2026_c1/f1p0/4080	R01_cb2026_c1/f1p0/4080
R01_cb17058_c3/f5p0/502	R01_cb17058_c3/f5p0/502	NA	R01_cb17058_c3/f5p0/502	R01_cb17058_c3/f5p0/502
R01_cb8564_c20762/f1p0/3469	NA	NA	NA	R01_cb8564_c20762/f1p0/3469
R01_cb7347_c0/f2p0/2646	R01_cb7347_c0/f2p0/2646	R01_cb7347_c0/f2p0/2646	R01_cb7347_c0/f2p0/2646	R01_cb7347_c0/f2p0/2646
R01_cb3398_c1/f1p0/3523	NA	R01_cb3398_c1/f1p0/3523	NA	NA
R01_cb16782_c1/f1p0/503	R01_cb16782_c1/f1p0/503	R01_cb16782_c1/f1p0/503	R01_cb16782_c1/f1p0/503	R01_cb16782_c1/f1p0/503
R01_cb13867_c5/f2p1/768	NA	NA	NA	R01_cb13867_c5/f2p1/768
R01_cb15564_c3/f1p0/968	R01_cb15564_c3/f1p0/968	R01_cb15564_c3/f1p0/968	R01_cb15564_c3/f1p0/968	R01_cb15564_c3/f1p0/968
R01_cb506_c9/f1p0/2299	NA	R01_cb506_c9/f1p0/2299	NA	R01_cb506_c9/f1p0/2299
R01_cb8564_c72426/f1p0/3257	R01_cb8564_c72426/f1p0/3257	R01_cb8564_c72426/f1p0/3257	NA	NA
R01_cb11752_c0/f1p0/1739	R01_cb11752_c0/f1p0/1739	R01_cb11752_c0/f1p0/1739	R01_cb11752_c0/f1p0/1739	R01_cb11752_c0/f1p0/1739
R01_cb9841_c2/f1p0/1928	NA	NA	NA	R01_cb9841_c2/f1p0/1928
R01_cb14744_c1/f1p0/1346	NA	NA	NA	R01_cb14744_c1/f1p0/1346
R01_cb4233_c202/f1p0/2119	NA	NA	R01_cb4233_c202/f1p0/2119	R01_cb4233_c202/f1p0/2119
R01_cb2719_c22/f1p0/3504	NA	NA	NA	R01_cb2719_c22/f1p0/3504
R01_cb9427_c8/f1p0/1043	NA	NA	NA	R01_cb9427_c8/f1p0/1043
R01_cb8564_c80557/f1p0/3996	NA	R01_cb8564_c80557/f1p0/3996	NA	R01_cb8564_c80557/f1p0/3996
R01_cb14942_c1/f1p0/855	NA	NA	NA	R01_cb14942_c1/f1p0/855

R01_cb8564_c88554/f1p0/4219	NA	R01_cb8564_c88554/f1p0/4219	NA	NA
R01_cb16341_c1/f2p0/486	R01_cb16341_c1/f2p0/486	R01_cb16341_c1/f2p0/486	R01_cb16341_c1/f2p0/486	R01_cb16341_c1/f2p0/486
R01_cb8564_c25663/f1p0/4167	NA	R01_cb8564_c25663/f1p0/4167	NA	NA
R01_cb8564_c124400/f1p0/2661	NA	R01_cb8564_c124400/f1p0/2661	NA	NA
R01_cb15797_c1/f1p0/1512	NA	NA	NA	R01_cb15797_c1/f1p0/1512
R01_cb261_c19/f1p0/3704	NA	NA	NA	R01_cb261_c19/f1p0/3704
R01_cb18328_c1/f1p0/554	R01_cb18328_c1/f1p0/554	R01_cb18328_c1/f1p0/554	NA	R01_cb18328_c1/f1p0/554
R01_cb12579_c7/f1p3/976	R01_cb12579_c7/f1p3/976	R01_cb12579_c7/f1p3/976	R01_cb12579_c7/f1p3/976	R01_cb12579_c7/f1p3/976
R01_cb18456_c6877/f1p0/848	NA	R01_cb18456_c6877/f1p0/848	NA	NA
R01_cb5498_c0/f1p0/3086	NA	R01_cb5498_c0/f1p0/3086	R01_cb5498_c0/f1p0/3086	NA
R01_cb13289_c12/f5p2/1840	NA	NA	R01_cb13289_c12/f5p2/1840	NA
R01_cb16920_c1/f1p0/642	R01_cb16920_c1/f1p0/642	R01_cb16920_c1/f1p0/642	R01_cb16920_c1/f1p0/642	R01_cb16920_c1/f1p0/642
R01_cb11043_c2/f1p0/2759	NA	R01_cb11043_c2/f1p0/2759	NA	NA
R01_cb1030_c10/f1p0/3270	NA	R01_cb1030_c10/f1p0/3270	NA	NA
R01_cb15733_c1/f3p1/1136	NA	R01_cb15733_c1/f3p1/1136	NA	NA
R01_cb16881_c4/f1p0/1593	NA	NA	NA	R01_cb16881_c4/f1p0/1593
R01_cb5900_c97/f1p0/2618	NA	R01_cb5900_c97/f1p0/2618	NA	NA
R01_cb8564_c74806/f3p0/3981	R01_cb8564_c74806/f3p0/3981	R01_cb8564_c74806/f3p0/3981	R01_cb8564_c74806/f3p0/3981	R01_cb8564_c74806/f3p0/3981
R01_cb18434_c1/f1p0/1092	NA	R01_cb18434_c1/f1p0/1092	NA	NA
R01_cb18365_c0/f1p0/1179	NA	R01_cb18365_c0/f1p0/1179	R01_cb18365_c0/f1p0/1179	R01_cb18365_c0/f1p0/1179
R01_cb2773_c10/f1p0/3070	NA	R01_cb2773_c10/f1p0/3070	NA	NA
R01_cb2536_c34/f1p0/1089	NA	R01_cb2536_c34/f1p0/1089	NA	NA
R01_cb8564_c74546/f1p0/2866	NA	NA	NA	R01_cb8564_c74546/f1p0/2866
R01_cb3218_c10/f1p0/2802	NA	NA	NA	R01_cb3218_c10/f1p0/2802

R01_cb3558_c10/f1p0/3091	NA	NA	NA	R01_cb3558_c10/f1p0/3091
R01_cb13458_c2/f1p0/571	NA	R01_cb13458_c2/f1p0/571	NA	NA
R01_cb5373_c2/f1p0/2829	NA	NA	NA	R01_cb5373_c2/f1p0/2829
R01_cb11513_c1/f1p0/2127	R01_cb11513_c1/f1p0/2127	R01_cb11513_c1/f1p0/2127	NA	R01_cb11513_c1/f1p0/2127
R01_cb14069_c1/f1p0/1465	NA	R01_cb14069_c1/f1p0/1465	NA	NA
R01_cb8564_c84530/f1p0/2483	NA	NA	NA	R01_cb8564_c84530/f1p0/2483
R01_cb18409_c165/f1p0/438	R01_cb18409_c165/f1p0/438	R01_cb18409_c165/f1p0/438	R01_cb18409_c165/f1p0/438	R01_cb18409_c165/f1p0/438
R01_cb8564_c157987/f1p6/2222	NA	R01_cb8564_c157987/f1p6/2222	R01_cb8564_c157987/f1p6/2222	R01_cb8564_c157987/f1p6/2222
R01_cb10944_c0/f2p0/933	NA	NA	NA	R01_cb10944_c0/f2p0/933
R01_cb1666_c13/f1p0/1804	NA	NA	NA	R01_cb1666_c13/f1p0/1804
R01_cb7999_c6/f1p0/981	NA	NA	NA	R01_cb7999_c6/f1p0/981
R01_cb18456_c5292/f1p1/835	NA	R01_cb18456_c5292/f1p1/835	NA	NA
R01_cb18456_c7530/f1p0/616	R01_cb18456_c7530/f1p0/616	R01_cb18456_c7530/f1p0/616	R01_cb18456_c7530/f1p0/616	R01_cb18456_c7530/f1p0/616
R01_cb438_c5/f1p0/1932	NA	R01_cb438_c5/f1p0/1932	NA	R01_cb438_c5/f1p0/1932
R01_cb3791_c16/f1p0/447	NA	NA	NA	R01_cb3791_c16/f1p0/447
R01_cb10233_c2/f1p0/1932	NA	R01_cb10233_c2/f1p0/1932	NA	NA
R01_cb16192_c1/f4p1/1266	NA	NA	NA	R01_cb16192_c1/f4p1/1266
R01_cb952_c0/f1p0/4548	R01_cb952_c0/f1p0/4548	R01_cb952_c0/f1p0/4548	R01_cb952_c0/f1p0/4548	R01_cb952_c0/f1p0/4548
R01_cb5569_c2/f1p0/949	NA	R01_cb5569_c2/f1p0/949	NA	NA
R01_cb5120_c2/f1p0/1874	NA	NA	NA	R01_cb5120_c2/f1p0/1874
R01_cb8564_c13899/f1p0/1939	R01_cb8564_c13899/f1p0/1939	R01_cb8564_c13899/f1p0/1939	NA	NA
R01_cb2367_c4/f1p0/1959	NA	R01_cb2367_c4/f1p0/1959	R01_cb2367_c4/f1p0/1959	NA
R01_cb15432_c2/f1p0/797	NA	R01_cb15432_c2/f1p0/797	NA	NA
R01_cb8564_c84992/f1p0/3087	NA	R01_cb8564_c84992/f1p0/3087	NA	NA

R01_cb9448_c16/f1p0/1145	NA	NA	NA	R01_cb9448_c16/f1p0/1145
R01_cb10238_c3/f1p0/830	NA	R01_cb10238_c3/f1p0/830	R01_cb10238_c3/f1p0/830	R01_cb10238_c3/f1p0/830
R01_cb8564_c15273/f1p0/2696	NA	R01_cb8564_c15273/f1p0/2696	NA	NA
R01_cb14166_c4/f1p0/451	R01_cb14166_c4/f1p0/451	NA	R01_cb14166_c4/f1p0/451	R01_cb14166_c4/f1p0/451
R01_cb8265_c0/f1p0/2422	NA	NA	NA	R01_cb8265_c0/f1p0/2422
R01_cb11410_c3/f1p0/2577	R01_cb11410_c3/f1p0/2577	R01_cb11410_c3/f1p0/2577	NA	NA
R01_cb12039_c2/f7p0/402	NA	R01_cb12039_c2/f7p0/402	R01_cb12039_c2/f7p0/402	R01_cb12039_c2/f7p0/402
R01_cb2674_c33/f1p0/1972	NA	NA	R01_cb2674_c33/f1p0/1972	R01_cb2674_c33/f1p0/1972
R01_cb12154_c3/f1p0/853	NA	R01_cb12154_c3/f1p0/853	NA	R01_cb12154_c3/f1p0/853
R01_cb13933_c7/f1p0/1621	NA	R01_cb13933_c7/f1p0/1621	NA	NA
R01_cb8239_c0/f3p0/2354	NA	NA	NA	R01_cb8239_c0/f3p0/2354
R01_cb18456_c3304/f1p0/975	NA	R01_cb18456_c3304/f1p0/975	NA	NA
R01_cb18590_c0/f1p0/1553	NA	R01_cb18590_c0/f1p0/1553	NA	NA
R01_cb8564_c91202/f1p0/3276	R01_cb8564_c91202/f1p0/3276	R01_cb8564_c91202/f1p0/3276	R01_cb8564_c91202/f1p0/3276	R01_cb8564_c91202/f1p0/3276
R01_cb7334_c4/f1p0/2579	NA	NA	NA	R01_cb7334_c4/f1p0/2579
R01_cb1336_c0/f2p0/1399	NA	NA	NA	R01_cb1336_c0/f2p0/1399
R01_cb10250_c5/f1p0/3153	NA	R01_cb10250_c5/f1p0/3153	NA	NA
R01_cb4861_c3/f1p0/2723	R01_cb4861_c3/f1p0/2723	R01_cb4861_c3/f1p0/2723	NA	R01_cb4861_c3/f1p0/2723
R01_cb10383_c0/f1p0/482	NA	NA	R01_cb10383_c0/f1p0/482	R01_cb10383_c0/f1p0/482
R01_cb17097_c1/f1p0/864	NA	NA	NA	R01_cb17097_c1/f1p0/864
R01_cb7214_c5/f1p0/1888	NA	NA	NA	R01_cb7214_c5/f1p0/1888
R01_cb9378_c18/f1p0/329	R01_cb9378_c18/f1p0/329	R01_cb9378_c18/f1p0/329	R01_cb9378_c18/f1p0/329	R01_cb9378_c18/f1p0/329
R01_cb8122_c9/f1p0/620	R01_cb8122_c9/f1p0/620	R01_cb8122_c9/f1p0/620	R01_cb8122_c9/f1p0/620	R01_cb8122_c9/f1p0/620
R01_cb8564_c110738/f1p0/2222	R01_cb8564_c110738/f1p0/2222	R01_cb8564_c110738/f1p0/2222	R01_cb8564_c110738/f1p0/2222	R01_cb8564_c110738/f1p0/2222

R01_cb2679_c2/f1p0/3844	NA	R01_cb2679_c2/f1p0/3844	NA	NA
R01_cb104_c21/f1p0/1517	NA	NA	NA	R01_cb104_c21/f1p0/1517
R01_cb8564_c109844/f2p0/3619	NA	R01_cb8564_c109844/f2p0/3619	NA	NA
R01_cb5139_c24/f1p1/1064	NA	R01_cb5139_c24/f1p1/1064	R01_cb5139_c24/f1p1/1064	R01_cb5139_c24/f1p1/1064
R01_cb2718_c12/f1p0/3521	NA	R01_cb2718_c12/f1p0/3521	NA	NA
R01_cb8564_c4969/f1p0/2987	R01_cb8564_c4969/f1p0/2987	R01_cb8564_c4969/f1p0/2987	R01_cb8564_c4969/f1p0/2987	R01_cb8564_c4969/f1p0/2987
R01_cb13601_c0/f2p0/803	NA	NA	NA	R01_cb13601_c0/f2p0/803
R01_cb16972_c2/f1p0/1858	NA	NA	NA	R01_cb16972_c2/f1p0/1858
R01_cb18300_c2/f1p0/876	NA	NA	NA	R01_cb18300_c2/f1p0/876
R01_cb7222_c9/f2p0/2183	NA	R01_cb7222_c9/f2p0/2183	NA	R01_cb7222_c9/f2p0/2183
R01_cb8564_c78285/f1p0/3648	NA	R01_cb8564_c78285/f1p0/3648	NA	R01_cb8564_c78285/f1p0/3648
R01_cb6197_c1/f1p0/828	NA	R01_cb6197_c1/f1p0/828	NA	NA
R01_cb8564_c9475/f1p0/2144	NA	R01_cb8564_c9475/f1p0/2144	NA	R01_cb8564_c9475/f1p0/2144
R01_cb17076_c2/f1p0/572	R01_cb17076_c2/f1p0/572	NA	NA	R01_cb17076_c2/f1p0/572
R01_cb10764_c8/f1p0/1323	R01_cb10764_c8/f1p0/1323	R01_cb10764_c8/f1p0/1323	NA	NA
R01_cb17562_c4/f1p0/589	NA	R01_cb17562_c4/f1p0/589	NA	NA
R01_cb11129_c0/f1p0/1587	R01_cb11129_c0/f1p0/1587	R01_cb11129_c0/f1p0/1587	NA	R01_cb11129_c0/f1p0/1587
R01_cb16705_c1/f1p0/629	NA	R01_cb16705_c1/f1p0/629	NA	R01_cb16705_c1/f1p0/629
R01_cb8564_c123192/f1p0/1931	NA	R01_cb8564_c123192/f1p0/1931	R01_cb8564_c123192/f1p0/1931	R01_cb8564_c123192/f1p0/1931
R01_cb8564_c14764/f1p0/4059	NA	R01_cb8564_c14764/f1p0/4059	NA	NA
R01_cb17522_c2/f1p0/6609	NA	NA	NA	R01_cb17522_c2/f1p0/6609
R01_cb5221_c19/f1p0/3839	NA	NA	NA	R01_cb5221_c19/f1p0/3839
R01_cb18456_c2280/f1p0/763	NA	R01_cb18456_c2280/f1p0/763	NA	R01_cb18456_c2280/f1p0/763
R01_cb8564_c127660/f1p1/227	NA	NA	NA	R01_cb8564_c127660/f1p1/227

4				4
R01_cb7356_c4/f1p1/2638	NA	NA	NA	R01_cb7356_c4/f1p1/2638
R01_cb10192_c10/f1p0/1303	NA	NA	NA	R01_cb10192_c10/f1p0/1303
R01_cb12369_c8/f1p1/677	NA	NA	R01_cb12369_c8/f1p1/677	NA
R01_cb8564_c41631/f1p0/2140	NA	R01_cb8564_c41631/f1p0/2140	R01_cb8564_c41631/f1p0/2140	R01_cb8564_c41631/f1p0/2140
R01_cb8564_c48619/f1p0/4132	R01_cb8564_c48619/f1p0/4132	R01_cb8564_c48619/f1p0/4132	NA	R01_cb8564_c48619/f1p0/4132
R01_cb8564_c124765/f1p0/1997	NA	R01_cb8564_c124765/f1p0/1997	R01_cb8564_c124765/f1p0/1997	R01_cb8564_c124765/f1p0/1997
R01_cb3124_c4/f1p0/1921	NA	R01_cb3124_c4/f1p0/1921	R01_cb3124_c4/f1p0/1921	R01_cb3124_c4/f1p0/1921
R01_cb8564_c16122/f1p0/3280	NA	R01_cb8564_c16122/f1p0/3280	R01_cb8564_c16122/f1p0/3280	R01_cb8564_c16122/f1p0/3280
R01_cb4469_c6/f1p0/2503	NA	NA	NA	R01_cb4469_c6/f1p0/2503
R01_cb17973_c26/f1p0/742	NA	R01_cb17973_c26/f1p0/742	R01_cb17973_c26/f1p0/742	NA
R01_cb13289_c6/f2p2/1685	NA	NA	R01_cb13289_c6/f2p2/1685	NA
R01_cb8564_c120674/f1p0/3586	R01_cb8564_c120674/f1p0/3586	R01_cb8564_c120674/f1p0/3586	R01_cb8564_c120674/f1p0/3586	R01_cb8564_c120674/f1p0/3586
R01_cb14657_c3/f1p0/1824	R01_cb14657_c3/f1p0/1824	R01_cb14657_c3/f1p0/1824	R01_cb14657_c3/f1p0/1824	R01_cb14657_c3/f1p0/1824
R01_cb279_c12/f1p1/4491	NA	NA	NA	R01_cb279_c12/f1p1/4491
R01_cb18456_c5105/f1p0/481	R01_cb18456_c5105/f1p0/481	R01_cb18456_c5105/f1p0/481	NA	R01_cb18456_c5105/f1p0/481
R01_cb18386_c9/f1p0/1249	NA	R01_cb18386_c9/f1p0/1249	NA	R01_cb18386_c9/f1p0/1249
R01_cb3213_c31/f1p0/649	R01_cb3213_c31/f1p0/649	R01_cb3213_c31/f1p0/649	R01_cb3213_c31/f1p0/649	R01_cb3213_c31/f1p0/649
R01_cb17345_c1/f1p0/494	R01_cb17345_c1/f1p0/494	R01_cb17345_c1/f1p0/494	R01_cb17345_c1/f1p0/494	R01_cb17345_c1/f1p0/494
R01_cb8564_c20404/f1p3/3974	NA	NA	NA	R01_cb8564_c20404/f1p3/3974
R01_cb8564_c16226/f1p0/3424	NA	R01_cb8564_c16226/f1p0/3424	NA	NA
R01_cb48_c1/f1p3/4991	NA	NA	NA	R01_cb48_c1/f1p3/4991
R01_cb8564_c19108/f1p0/2604	NA	R01_cb8564_c19108/f1p0/2604	NA	R01_cb8564_c19108/f1p0/2604

R01_cb1398_c12/f1p0/2711	NA	R01_cb1398_c12/f1p0/2711	R01_cb1398_c12/f1p0/2711	NA
R01_cb8407_c0/f8p0/1219	NA	NA	NA	R01_cb8407_c0/f8p0/1219
R01_cb12154_c4/f1p0/407	R01_cb12154_c4/f1p0/407	R01_cb12154_c4/f1p0/407	NA	R01_cb12154_c4/f1p0/407
R01_cb12775_c3/f1p2/1319	NA	NA	NA	R01_cb12775_c3/f1p2/1319
R01_cb3074_c1/f1p0/2352	NA	NA	NA	R01_cb3074_c1/f1p0/2352
R01_cb3558_c5/f1p2/2851	NA	NA	NA	R01_cb3558_c5/f1p2/2851
R01_cb18456_c5183/f1p2/640	NA	NA	NA	R01_cb18456_c5183/f1p2/640
R01_cb9359_c0/f1p0/2116	NA	NA	NA	R01_cb9359_c0/f1p0/2116
R01_cb15630_c3/f1p0/1818	NA	R01_cb15630_c3/f1p0/1818	R01_cb15630_c3/f1p0/1818	R01_cb15630_c3/f1p0/1818
R01_cb18456_c5767/f1p0/1871	NA	R01_cb18456_c5767/f1p0/1871	NA	R01_cb18456_c5767/f1p0/1871
R01_cb17183_c7/f8p1/670	NA	NA	NA	R01_cb17183_c7/f8p1/670
R01_cb12620_c8/f1p0/1180	NA	NA	NA	R01_cb12620_c8/f1p0/1180
R01_cb13955_c2/f2p0/572	NA	NA	NA	R01_cb13955_c2/f2p0/572
R01_cb15334_c2/f1p0/371	R01_cb15334_c2/f1p0/371	R01_cb15334_c2/f1p0/371	R01_cb15334_c2/f1p0/371	R01_cb15334_c2/f1p0/371
R01_cb9062_c1/f1p0/2200	NA	R01_cb9062_c1/f1p0/2200	NA	R01_cb9062_c1/f1p0/2200
R01_cb16055_c0/f1p0/385	NA	R01_cb16055_c0/f1p0/385	NA	R01_cb16055_c0/f1p0/385
R01_cb10086_c8/f14p2/1399	NA	NA	NA	R01_cb10086_c8/f14p2/1399
R01_cb8407_c3/f1p0/2387	R01_cb8407_c3/f1p0/2387	R01_cb8407_c3/f1p0/2387	R01_cb8407_c3/f1p0/2387	R01_cb8407_c3/f1p0/2387
R01_cb13025_c4/f2p0/1450	NA	NA	NA	R01_cb13025_c4/f2p0/1450
R01_cb2625_c17/f1p0/1136	NA	R01_cb2625_c17/f1p0/1136	NA	NA
R01_cb16797_c0/f2p0/442	NA	NA	NA	R01_cb16797_c0/f2p0/442
R01_cb5782_c10/f1p0/2925	NA	NA	NA	R01_cb5782_c10/f1p0/2925
R01_cb16465_c1/f1p0/879	NA	NA	NA	R01_cb16465_c1/f1p0/879
R01_cb13234_c1/f1p0/542	NA	R01_cb13234_c1/f1p0/542	R01_cb13234_c1/f1p0/542	NA
R01_cb8564_c73173/f1p0/3891	NA	R01_cb8564_c73173/f1p0/3891	NA	NA
R01_cb8564_c110045/f2p1/220	NA	R01_cb8564_c110045/f2p1/220	NA	NA

1		1		
R01_cb13428_c15/f1p0/619	NA	R01_cb13428_c15/f1p0/619	NA	R01_cb13428_c15/f1p0/619
R01_cb16884_c0/f2p0/335	R01_cb16884_c0/f2p0/335	R01_cb16884_c0/f2p0/335	R01_cb16884_c0/f2p0/335	R01_cb16884_c0/f2p0/335
R01_cb8564_c69132/f2p0/2211	NA	R01_cb8564_c69132/f2p0/2211	R01_cb8564_c69132/f2p0/2211	R01_cb8564_c69132/f2p0/2211
R01_cb15595_c2/f1p0/696	NA	NA	NA	R01_cb15595_c2/f1p0/696
R01_cb13761_c10/f1p0/1253	R01_cb13761_c10/f1p0/1253	R01_cb13761_c10/f1p0/1253	R01_cb13761_c10/f1p0/1253	R01_cb13761_c10/f1p0/1253
R01_cb3787_c3/f1p0/1921	NA	R01_cb3787_c3/f1p0/1921	NA	NA
R01_cb14783_c1/f1p0/505	R01_cb14783_c1/f1p0/505	R01_cb14783_c1/f1p0/505	R01_cb14783_c1/f1p0/505	R01_cb14783_c1/f1p0/505
R01_cb5896_c18/f2p0/1997	NA	NA	NA	R01_cb5896_c18/f2p0/1997
R01_cb8564_c13774/f1p0/2253	R01_cb8564_c13774/f1p0/2253	R01_cb8564_c13774/f1p0/2253	R01_cb8564_c13774/f1p0/2253	R01_cb8564_c13774/f1p0/2253
R01_cb7927_c0/f1p0/2499	NA	R01_cb7927_c0/f1p0/2499	R01_cb7927_c0/f1p0/2499	R01_cb7927_c0/f1p0/2499
R01_cb18426_c1/f1p0/1174	R01_cb18426_c1/f1p0/1174	R01_cb18426_c1/f1p0/1174	R01_cb18426_c1/f1p0/1174	R01_cb18426_c1/f1p0/1174
R01_cb15346_c7/f1p0/874	NA	R01_cb15346_c7/f1p0/874	R01_cb15346_c7/f1p0/874	R01_cb15346_c7/f1p0/874
R01_cb7348_c15/f1p0/1656	NA	R01_cb7348_c15/f1p0/1656	NA	NA
R01_cb3218_c7/f1p0/2559	NA	NA	NA	R01_cb3218_c7/f1p0/2559
R01_cb16076_c1/f2p0/746	NA	NA	R01_cb16076_c1/f2p0/746	R01_cb16076_c1/f2p0/746
R01_cb8564_c21233/f2p0/3428	NA	NA	NA	R01_cb8564_c21233/f2p0/3428
R01_cb3243_c1/f1p0/3684	NA	NA	NA	R01_cb3243_c1/f1p0/3684
R01_cb13743_c4/f1p1/1447	NA	NA	NA	R01_cb13743_c4/f1p1/1447
R01_cb16993_c0/f1p0/1589	NA	NA	R01_cb16993_c0/f1p0/1589	NA
R01_cb18223_c20/f1p1/997	R01_cb18223_c20/f1p1/997	R01_cb18223_c20/f1p1/997	R01_cb18223_c20/f1p1/997	R01_cb18223_c20/f1p1/997
R01_cb3339_c13/f1p0/486	R01_cb3339_c13/f1p0/486	R01_cb3339_c13/f1p0/486	R01_cb3339_c13/f1p0/486	R01_cb3339_c13/f1p0/486
R01_cb10555_c5/f1p0/3929	NA	R01_cb10555_c5/f1p0/3929	NA	NA
R01_cb8564_c73244/f1p0/2654	NA	NA	NA	R01_cb8564_c73244/f1p0/2654
R01_cb8564_c4614/f1p2/2942	NA	R01_cb8564_c4614/f1p2/2942	R01_cb8564_c4614/f1p2/2942	R01_cb8564_c4614/f1p2/2942

R01_cb5117_c1/f1p0/3980	NA	NA	NA	R01_cb5117_c1/f1p0/3980
R01_cb6113_c3/f1p0/2938	NA	NA	NA	R01_cb6113_c3/f1p0/2938
R01_cb8564_c5042/f2p1/3154	NA	NA	NA	R01_cb8564_c5042/f2p1/3154
R01_cb14542_c2/f1p0/1220	NA	NA	NA	R01_cb14542_c2/f1p0/1220
R01_cb4702_c25/f1p0/1095	NA	NA	NA	R01_cb4702_c25/f1p0/1095
R01_cb14540_c7/f1p1/1690	NA	R01_cb14540_c7/f1p1/1690	NA	NA
R01_cb9608_c5/f1p0/2097	NA	NA	NA	R01_cb9608_c5/f1p0/2097
R01_cb6615_c15/f1p0/2361	NA	NA	R01_cb6615_c15/f1p0/2361	R01_cb6615_c15/f1p0/2361
R01_cb11370_c3/f1p0/1229	NA	NA	NA	R01_cb11370_c3/f1p0/1229
R01_cb5832_c0/f2p0/2622	R01_cb5832_c0/f2p0/2622	R01_cb5832_c0/f2p0/2622	R01_cb5832_c0/f2p0/2622	R01_cb5832_c0/f2p0/2622
R01_cb17589_c3/f1p0/1321	NA	NA	NA	R01_cb17589_c3/f1p0/1321
R01_cb18456_c4775/f1p0/481	R01_cb18456_c4775/f1p0/481	NA	NA	R01_cb18456_c4775/f1p0/481
R01_cb18337_c1/f1p0/731	R01_cb18337_c1/f1p0/731	R01_cb18337_c1/f1p0/731	R01_cb18337_c1/f1p0/731	R01_cb18337_c1/f1p0/731
R01_cb1758_c6/f1p0/2223	NA	NA	NA	R01_cb1758_c6/f1p0/2223
R01_cb5657_c5/f1p0/2202	NA	NA	NA	R01_cb5657_c5/f1p0/2202
R01_cb11848_c5/f6p0/837	R01_cb11848_c5/f6p0/837	R01_cb11848_c5/f6p0/837	R01_cb11848_c5/f6p0/837	R01_cb11848_c5/f6p0/837
R01_cb8564_c18668/f1p0/2122	R01_cb8564_c18668/f1p0/2122	R01_cb8564_c18668/f1p0/2122	R01_cb8564_c18668/f1p0/2122	R01_cb8564_c18668/f1p0/2122
R01_cb6158_c0/f1p0/2915	NA	R01_cb6158_c0/f1p0/2915	NA	R01_cb6158_c0/f1p0/2915
R01_cb17604_c2/f3p0/630	NA	R01_cb17604_c2/f3p0/630	R01_cb17604_c2/f3p0/630	R01_cb17604_c2/f3p0/630
R01_cb2253_c1/f2p0/3998	NA	R01_cb2253_c1/f2p0/3998	NA	NA
R01_cb13781_c12/f5p0/592	NA	NA	NA	R01_cb13781_c12/f5p0/592
R01_cb10390_c5/f2p1/1345	NA	R01_cb10390_c5/f2p1/1345	NA	NA
R01_cb8564_c89781/f1p0/2441	NA	R01_cb8564_c89781/f1p0/2441	NA	R01_cb8564_c89781/f1p0/2441
R01_cb8039_c1/f1p0/2579	NA	R01_cb8039_c1/f1p0/2579	NA	NA
R01_cb15823_c0/f8p0/733	R01_cb15823_c0/f8p0/733	NA	NA	NA

R01_cb8564_c1438/f1p0/2329	NA	R01_cb8564_c1438/f1p0/2329	NA	R01_cb8564_c1438/f1p0/2329
R01_cb17977_c1/f1p0/751	NA	R01_cb17977_c1/f1p0/751	R01_cb17977_c1/f1p0/751	NA
R01_cb8564_c12717/f1p0/4583	NA	R01_cb8564_c12717/f1p0/4583	R01_cb8564_c12717/f1p0/4583	NA
R01_cb8564_c19209/f2p0/3017	NA	R01_cb8564_c19209/f2p0/3017	NA	R01_cb8564_c19209/f2p0/3017
R01_cb16645_c29/f1p0/1190	NA	R01_cb16645_c29/f1p0/1190	R01_cb16645_c29/f1p0/1190	R01_cb16645_c29/f1p0/1190
R01_cb12912_c6/f1p0/1393	NA	R01_cb12912_c6/f1p0/1393	NA	R01_cb12912_c6/f1p0/1393
R01_cb3483_c25/f6p1/2943	NA	NA	NA	R01_cb3483_c25/f6p1/2943
R01_cb1626_c21/f5p1/2846	NA	NA	NA	R01_cb1626_c21/f5p1/2846
R01_cb6345_c2/f1p1/2613	NA	R01_cb6345_c2/f1p1/2613	R01_cb6345_c2/f1p1/2613	R01_cb6345_c2/f1p1/2613
R01_cb15045_c2/f2p0/1307	NA	NA	NA	R01_cb15045_c2/f2p0/1307
R01_cb17371_c1/f1p0/688	NA	R01_cb17371_c1/f1p0/688	R01_cb17371_c1/f1p0/688	R01_cb17371_c1/f1p0/688
R01_cb18409_c112/f1p0/411	R01_cb18409_c112/f1p0/411	R01_cb18409_c112/f1p0/411	R01_cb18409_c112/f1p0/411	R01_cb18409_c112/f1p0/411
R01_cb15784_c2/f1p0/1655	NA	NA	NA	R01_cb15784_c2/f1p0/1655
R01_cb13430_c2/f1p0/1246	NA	NA	NA	R01_cb13430_c2/f1p0/1246
R01_cb4491_c2/f1p1/3300	NA	R01_cb4491_c2/f1p1/3300	NA	NA
R01_cb8564_c79549/f1p0/2853	NA	R01_cb8564_c79549/f1p0/2853	R01_cb8564_c79549/f1p0/2853	NA
R01_cb2048_c17/f1p0/1983	NA	NA	NA	R01_cb2048_c17/f1p0/1983
R01_cb8335_c17/f3p0/2378	NA	NA	NA	R01_cb8335_c17/f3p0/2378
R01_cb8564_c17723/f1p0/2448	NA	R01_cb8564_c17723/f1p0/2448	R01_cb8564_c17723/f1p0/2448	NA
R01_cb12024_c14/f1p0/953	NA	R01_cb12024_c14/f1p0/953	NA	R01_cb12024_c14/f1p0/953
R01_cb14307_c5/f1p0/6345	NA	R01_cb14307_c5/f1p0/6345	NA	NA
R01_cb3363_c3/f1p0/2627	NA	R01_cb3363_c3/f1p0/2627	NA	NA
R01_cb5850_c0/f1p0/3001	NA	R01_cb5850_c0/f1p0/3001	R01_cb5850_c0/f1p0/3001	R01_cb5850_c0/f1p0/3001
R01_cb843_c3/f1p0/3210	NA	NA	NA	R01_cb843_c3/f1p0/3210
R01_cb8564_c78970/f1p0/2778	NA	R01_cb8564_c78970/f1p0/2778	R01_cb8564_c78970/f1p0/2778	NA
R01_cb8564_c46566/f1p0/2996	NA	R01_cb8564_c46566/f1p0/2996	NA	R01_cb8564_c46566/f1p0/2996

R01_cb5847_c8/f1p0/2313	NA	NA	NA	R01_cb5847_c8/f1p0/2313
R01_cb8564_c14653/f1p0/4755	NA	R01_cb8564_c14653/f1p0/4755	R01_cb8564_c14653/f1p0/4755	R01_cb8564_c14653/f1p0/4755
R01_cb8564_c70447/f1p0/1903	NA	R01_cb8564_c70447/f1p0/1903	R01_cb8564_c70447/f1p0/1903	NA
R01_cb18121_c0/f2p0/1674	NA	NA	NA	R01_cb18121_c0/f2p0/1674
R01_cb6147_c34/f1p0/2786	NA	R01_cb6147_c34/f1p0/2786	NA	NA
R01_cb11539_c1/f1p0/1903	NA	NA	NA	R01_cb11539_c1/f1p0/1903
R01_cb13396_c5/f1p0/1269	NA	R01_cb13396_c5/f1p0/1269	R01_cb13396_c5/f1p0/1269	R01_cb13396_c5/f1p0/1269
R01_cb1849_c16/f1p1/2012	NA	NA	NA	R01_cb1849_c16/f1p1/2012
R01_cb2570_c15/f2p1/2439	NA	NA	NA	R01_cb2570_c15/f2p1/2439
R01_cb8564_c12583/f1p0/4273	NA	NA	NA	R01_cb8564_c12583/f1p0/4273
R01_cb4960_c2/f1p0/4400	NA	R01_cb4960_c2/f1p0/4400	R01_cb4960_c2/f1p0/4400	R01_cb4960_c2/f1p0/4400
R01_cb16992_c5/f1p0/751	NA	R01_cb16992_c5/f1p0/751	NA	NA
R01_cb14781_c2/f1p0/1665	NA	R01_cb14781_c2/f1p0/1665	NA	NA
R01_cb8564_c10491/f1p0/1993	NA	R01_cb8564_c10491/f1p0/1993	NA	R01_cb8564_c10491/f1p0/1993
R01_cb6272_c7/f1p1/1942	NA	NA	NA	R01_cb6272_c7/f1p1/1942
R01_cb16062_c0/f7p0/697	NA	R01_cb16062_c0/f7p0/697	NA	NA
R01_cb8944_c2/f1p0/1807	R01_cb8944_c2/f1p0/1807	R01_cb8944_c2/f1p0/1807	NA	NA
R01_cb16526_c8/f1p0/1473	NA	R01_cb16526_c8/f1p0/1473	NA	NA
R01_cb8564_c71836/f1p0/2272	R01_cb8564_c71836/f1p0/2272	R01_cb8564_c71836/f1p0/2272	R01_cb8564_c71836/f1p0/2272	NA
R01_cb2894_c13/f1p0/358	R01_cb2894_c13/f1p0/358	R01_cb2894_c13/f1p0/358	R01_cb2894_c13/f1p0/358	NA
R01_cb8564_c117197/f1p0/2207	NA	NA	NA	R01_cb8564_c117197/f1p0/2207
R01_cb7663_c7/f2p0/1035	NA	NA	NA	R01_cb7663_c7/f2p0/1035
R01_cb12639_c0/f2p0/778	NA	R01_cb12639_c0/f2p0/778	NA	NA
R01_cb6495_c30/f1p0/588	NA	R01_cb6495_c30/f1p0/588	NA	R01_cb6495_c30/f1p0/588

R01_cb14186_c1/f1p0/977	NA	R01_cb14186_c1/f1p0/977	NA	NA
R01_cb9368_c5/f1p0/1264	NA	NA	NA	R01_cb9368_c5/f1p0/1264
R01_cb3531_c18/f1p0/2707	NA	R01_cb3531_c18/f1p0/2707	NA	NA
R01_cb18456_c6290/f1p0/631	R01_cb18456_c6290/f1p0/631	R01_cb18456_c6290/f1p0/631	NA	R01_cb18456_c6290/f1p0/631
R01_cb18765_c0/f1p0/5283	NA	R01_cb18765_c0/f1p0/5283	NA	NA
R01_cb3483_c6/f1p0/3047	NA	NA	NA	R01_cb3483_c6/f1p0/3047
R01_cb14709_c1/f1p0/1436	NA	R01_cb14709_c1/f1p0/1436	NA	NA
R01_cb14566_c0/f3p0/627	R01_cb14566_c0/f3p0/627	R01_cb14566_c0/f3p0/627	R01_cb14566_c0/f3p0/627	R01_cb14566_c0/f3p0/627
R01_cb1788_c4/f1p0/3161	NA	NA	NA	R01_cb1788_c4/f1p0/3161
R01_cb2845_c13/f1p1/3510	NA	NA	NA	R01_cb2845_c13/f1p1/3510
R01_cb18745_c1/f1p0/513	R01_cb18745_c1/f1p0/513	NA	R01_cb18745_c1/f1p0/513	R01_cb18745_c1/f1p0/513
R01_cb2026_c0/f2p0/3902	R01_cb2026_c0/f2p0/3902	R01_cb2026_c0/f2p0/3902	R01_cb2026_c0/f2p0/3902	R01_cb2026_c0/f2p0/3902
R01_cb8564_c17747/f1p0/3700	NA	R01_cb8564_c17747/f1p0/3700	NA	NA
R01_cb5331_c1/f1p0/739	R01_cb5331_c1/f1p0/739	R01_cb5331_c1/f1p0/739	R01_cb5331_c1/f1p0/739	R01_cb5331_c1/f1p0/739
R01_cb11141_c8/f1p0/742	NA	NA	NA	R01_cb11141_c8/f1p0/742
R01_cb18456_c6850/f1p0/994	R01_cb18456_c6850/f1p0/994	R01_cb18456_c6850/f1p0/994	NA	NA
R01_cb2844_c1/f1p0/2387	NA	NA	NA	R01_cb2844_c1/f1p0/2387
R01_cb8564_c83840/f1p0/2061	NA	NA	NA	R01_cb8564_c83840/f1p0/2061
R01_cb8564_c131632/f2p0/2188	NA	NA	NA	R01_cb8564_c131632/f2p0/2188
R01_cb10228_c6/f1p1/817	NA	R01_cb10228_c6/f1p1/817	R01_cb10228_c6/f1p1/817	R01_cb10228_c6/f1p1/817
R01_cb2803_c7/f1p0/4315	NA	R01_cb2803_c7/f1p0/4315	NA	NA
R01_cb10024_c195/f1p0/624	NA	NA	R01_cb10024_c195/f1p0/624	NA
R01_cb11354_c0/f2p0/1773	NA	NA	NA	R01_cb11354_c0/f2p0/1773
R01_cb6851_c2/f1p0/2590	NA	R01_cb6851_c2/f1p0/2590	NA	NA
R01_cb14148_c5/f1p0/1278	NA	NA	R01_cb14148_c5/f1p0/1278	R01_cb14148_c5/f1p0/1278

R01_cb18418_c1/f1p0/427	R01_cb18418_c1/f1p0/427	R01_cb18418_c1/f1p0/427	R01_cb18418_c1/f1p0/427	R01_cb18418_c1/f1p0/427
R01_cb9418_c1/f1p1/2092	NA	NA	NA	R01_cb9418_c1/f1p1/2092
R01_cb4233_c79/f1p0/2292	NA	R01_cb4233_c79/f1p0/2292	NA	R01_cb4233_c79/f1p0/2292
R01_cb17415_c5/f1p0/324	R01_cb17415_c5/f1p0/324	R01_cb17415_c5/f1p0/324	R01_cb17415_c5/f1p0/324	R01_cb17415_c5/f1p0/324
R01_cb16763_c1/f1p0/1737	NA	R01_cb16763_c1/f1p0/1737	NA	NA
R01_cb4327_c13/f1p0/1067	R01_cb4327_c13/f1p0/1067	R01_cb4327_c13/f1p0/1067	NA	R01_cb4327_c13/f1p0/1067
R01_cb18780_c7/f1p10/7292	NA	R01_cb18780_c7/f1p10/7292	NA	NA
R01_cb8564_c123805/f1p4/2237	NA	R01_cb8564_c123805/f1p4/2237	NA	NA
R01_cb16476_c3/f1p0/1357	NA	R01_cb16476_c3/f1p0/1357	R01_cb16476_c3/f1p0/1357	R01_cb16476_c3/f1p0/1357
R01_cb16839_c1/f1p0/1616	NA	NA	NA	R01_cb16839_c1/f1p0/1616
R01_cb1438_c19/f1p0/2633	NA	NA	NA	R01_cb1438_c19/f1p0/2633
R01_cb14002_c6/f1p1/718	NA	R01_cb14002_c6/f1p1/718	R01_cb14002_c6/f1p1/718	R01_cb14002_c6/f1p1/718
R01_cb3750_c9/f1p0/2220	NA	NA	NA	R01_cb3750_c9/f1p0/2220
R01_cb10015_c401/f1p0/647	NA	R01_cb10015_c401/f1p0/647	NA	NA
R01_cb8529_c9/f1p0/821	NA	R01_cb8529_c9/f1p0/821	R01_cb8529_c9/f1p0/821	R01_cb8529_c9/f1p0/821
R01_cb12617_c7/f1p0/1281	NA	R01_cb12617_c7/f1p0/1281	R01_cb12617_c7/f1p0/1281	R01_cb12617_c7/f1p0/1281
R01_cb11587_c1/f1p0/3580	NA	R01_cb11587_c1/f1p0/3580	NA	NA
R01_cb8564_c68710/f3p1/3032	NA	NA	NA	R01_cb8564_c68710/f3p1/3032
R01_cb4269_c5/f1p0/2116	NA	NA	NA	R01_cb4269_c5/f1p0/2116
R01_cb7653_c9/f1p0/3045	NA	NA	NA	R01_cb7653_c9/f1p0/3045
R01_cb18074_c0/f3p0/325	R01_cb18074_c0/f3p0/325	R01_cb18074_c0/f3p0/325	R01_cb18074_c0/f3p0/325	R01_cb18074_c0/f3p0/325
R01_cb16425_c4/f1p0/1239	NA	R01_cb16425_c4/f1p0/1239	NA	NA
R01_cb13049_c22/f1p0/469	R01_cb13049_c22/f1p0/469	R01_cb13049_c22/f1p0/469	NA	R01_cb13049_c22/f1p0/469
R01_cb13682_c8/f10p0/760	R01_cb13682_c8/f10p0/760	R01_cb13682_c8/f10p0/760	NA	R01_cb13682_c8/f10p0/760
R01_cb3752_c7/f1p0/3475	NA	NA	NA	R01_cb3752_c7/f1p0/3475

R01_cb16581_c2/f1p0/386	R01_cb16581_c2/f1p0/386	R01_cb16581_c2/f1p0/386	NA	NA
R01_cb872_c2/f1p0/2964	NA	NA	NA	R01_cb872_c2/f1p0/2964
R01_cb3464_c2/f1p0/4818	NA	NA	NA	R01_cb3464_c2/f1p0/4818
R01_cb18487_c0/f3p0/860	NA	R01_cb18487_c0/f3p0/860	NA	NA
R01_cb10384_c5/f1p0/3285	NA	R01_cb10384_c5/f1p0/3285	NA	R01_cb10384_c5/f1p0/3285
R01_cb16678_c10/f1p0/724	NA	R01_cb16678_c10/f1p0/724	R01_cb16678_c10/f1p0/724	R01_cb16678_c10/f1p0/724
R01_cb14720_c5/f1p0/504	NA	NA	NA	R01_cb14720_c5/f1p0/504
R01_cb1077_c21/f1p0/3053	NA	R01_cb1077_c21/f1p0/3053	R01_cb1077_c21/f1p0/3053	R01_cb1077_c21/f1p0/3053
R01_cb9960_c7/f1p0/1570	NA	NA	NA	R01_cb9960_c7/f1p0/1570
R01_cb15932_c1/f2p0/406	NA	NA	R01_cb15932_c1/f2p0/406	R01_cb15932_c1/f2p0/406
R01_cb8564_c118967/f1p0/2070	NA	NA	NA	R01_cb8564_c118967/f1p0/2070
R01_cb14373_c2/f2p0/803	NA	R01_cb14373_c2/f2p0/803	NA	NA
R01_cb11532_c2/f1p0/1057	NA	R01_cb11532_c2/f1p0/1057	NA	NA
R01_cb6802_c67/f1p0/2285	NA	R01_cb6802_c67/f1p0/2285	R01_cb6802_c67/f1p0/2285	R01_cb6802_c67/f1p0/2285
R01_cb15811_c15/f1p0/745	R01_cb15811_c15/f1p0/745	R01_cb15811_c15/f1p0/745	R01_cb15811_c15/f1p0/745	R01_cb15811_c15/f1p0/745
R01_cb15202_c3/f1p0/555	NA	R01_cb15202_c3/f1p0/555	NA	NA
R01_cb8409_c5/f1p0/2657	NA	NA	NA	R01_cb8409_c5/f1p0/2657
R01_cb8564_c122048/f1p0/2289	R01_cb8564_c122048/f1p0/2289	R01_cb8564_c122048/f1p0/2289	NA	NA
R01_cb14481_c0/f2p0/586	NA	NA	NA	R01_cb14481_c0/f2p0/586
R01_cb8660_c4/f3p0/2144	NA	NA	NA	R01_cb8660_c4/f3p0/2144
R01_cb8973_c1/f1p0/2223	R01_cb8973_c1/f1p0/2223	R01_cb8973_c1/f1p0/2223	NA	NA
R01_cb7595_c19/f1p0/737	NA	R01_cb7595_c19/f1p0/737	NA	R01_cb7595_c19/f1p0/737
R01_cb17973_c56/f1p0/1679	NA	R01_cb17973_c56/f1p0/1679	NA	R01_cb17973_c56/f1p0/1679
R01_cb1052_c14/f1p0/4280	NA	R01_cb1052_c14/f1p0/4280	NA	NA

R01_cb15092_c8/f1p0/748	NA	NA	NA	R01_cb15092_c8/f1p0/748
R01_cb10372_c10/f1p0/461	R01_cb10372_c10/f1p0/461	R01_cb10372_c10/f1p0/461	NA	NA
R01_cb16723_c2/f1p0/1296	NA	R01_cb16723_c2/f1p0/1296	NA	NA
R01_cb18285_c1/f1p0/1004	NA	NA	NA	R01_cb18285_c1/f1p0/1004
R01_cb7457_c2/f1p0/2769	R01_cb7457_c2/f1p0/2769	R01_cb7457_c2/f1p0/2769	NA	NA
R01_cb14761_c6/f1p0/852	NA	NA	R01_cb14761_c6/f1p0/852	R01_cb14761_c6/f1p0/852
R01_cb4023_c10/f1p1/3217	NA	R01_cb4023_c10/f1p1/3217	NA	NA
R01_cb14843_c3/f1p0/845	NA	NA	NA	R01_cb14843_c3/f1p0/845
R01_cb1254_c8/f1p1/4309	NA	R01_cb1254_c8/f1p1/4309	NA	NA
R01_cb10413_c2/f1p0/1779	NA	R01_cb10413_c2/f1p0/1779	R01_cb10413_c2/f1p0/1779	R01_cb10413_c2/f1p0/1779
R01_cb2959_c0/f1p0/3757	NA	NA	NA	R01_cb2959_c0/f1p0/3757
R01_cb15107_c1/f1p1/550	NA	R01_cb15107_c1/f1p1/550	NA	R01_cb15107_c1/f1p1/550
R01_cb16504_c0/f5p0/1765	NA	NA	NA	R01_cb16504_c0/f5p0/1765
R01_cb9606_c69/f1p0/1761	NA	R01_cb9606_c69/f1p0/1761	R01_cb9606_c69/f1p0/1761	R01_cb9606_c69/f1p0/1761
R01_cb5659_c29/f2p0/2193	NA	NA	NA	R01_cb5659_c29/f2p0/2193
R01_cb1772_c11/f1p0/3842	NA	R01_cb1772_c11/f1p0/3842	NA	NA
R01_cb14316_c12/f1p0/444	R01_cb14316_c12/f1p0/444	R01_cb14316_c12/f1p0/444	R01_cb14316_c12/f1p0/444	R01_cb14316_c12/f1p0/444
R01_cb2048_c8/f1p0/2138	NA	NA	NA	R01_cb2048_c8/f1p0/2138
R01_cb14179_c4/f4p1/512	NA	NA	R01_cb14179_c4/f4p1/512	R01_cb14179_c4/f4p1/512
R01_cb3421_c17/f1p1/2912	NA	NA	NA	R01_cb3421_c17/f1p1/2912
R01_cb8564_c1403/f1p0/2069	NA	R01_cb8564_c1403/f1p0/2069	R01_cb8564_c1403/f1p0/2069	R01_cb8564_c1403/f1p0/2069
R01_cb8564_c150188/f1p0/3276	NA	NA	NA	R01_cb8564_c150188/f1p0/3276
R01_cb12297_c1/f1p0/651	NA	NA	NA	R01_cb12297_c1/f1p0/651
R01_cb10930_c0/f1p0/831	R01_cb10930_c0/f1p0/831	R01_cb10930_c0/f1p0/831	R01_cb10930_c0/f1p0/831	R01_cb10930_c0/f1p0/831
R01_cb14403_c7/f1p0/546	R01_cb14403_c7/f1p0/546	NA	R01_cb14403_c7/f1p0/546	R01_cb14403_c7/f1p0/546

R01_cb8564_c1322/f1p1/4079	NA	NA	NA	R01_cb8564_c1322/f1p1/4079
R01_cb1138_c58/f1p1/889	NA	NA	R01_cb1138_c58/f1p1/889	R01_cb1138_c58/f1p1/889
R01_cb8564_c124149/f1p0/3342	NA	R01_cb8564_c124149/f1p0/3342	NA	NA
R01_cb7497_c22/f1p0/2606	NA	NA	NA	R01_cb7497_c22/f1p0/2606
R01_cb17973_c18/f1p0/1316	NA	R01_cb17973_c18/f1p0/1316	NA	NA
R01_cb6779_c2/f3p0/1760	NA	NA	NA	R01_cb6779_c2/f3p0/1760
R01_cb8564_c16457/f1p0/2899	NA	R01_cb8564_c16457/f1p0/2899	NA	NA
R01_cb3007_c3/f1p0/3424	NA	R01_cb3007_c3/f1p0/3424	NA	NA
R01_cb10034_c25/f1p0/521	NA	R01_cb10034_c25/f1p0/521	NA	NA
R01_cb3712_c1/f2p0/3548	R01_cb3712_c1/f2p0/3548	R01_cb3712_c1/f2p0/3548	NA	NA
R01_cb8564_c19634/f1p1/3199	NA	R01_cb8564_c19634/f1p1/3199	NA	R01_cb8564_c19634/f1p1/3199
R01_cb11212_c9/f1p0/758	NA	R01_cb11212_c9/f1p0/758	NA	NA
R01_cb3971_c1/f1p0/3443	NA	R01_cb3971_c1/f1p0/3443	NA	NA
R01_cb9713_c0/f1p0/2005	R01_cb9713_c0/f1p0/2005	R01_cb9713_c0/f1p0/2005	NA	NA
R01_cb7945_c3/f1p0/2023	NA	NA	NA	R01_cb7945_c3/f1p0/2023
R01_cb2320_c0/f3p1/3932	NA	NA	NA	R01_cb2320_c0/f3p1/3932
R01_cb8564_c2545/f1p0/3411	R01_cb8564_c2545/f1p0/3411	NA	NA	NA
R01_cb13662_c0/f1p0/639	R01_cb13662_c0/f1p0/639	R01_cb13662_c0/f1p0/639	NA	R01_cb13662_c0/f1p0/639
R01_cb8564_c114298/f1p0/2460	NA	NA	NA	R01_cb8564_c114298/f1p0/2460
R01_cb8564_c89913/f1p1/3572	NA	NA	NA	R01_cb8564_c89913/f1p1/3572
R01_cb3287_c2/f1p0/3389	NA	NA	NA	R01_cb3287_c2/f1p0/3389
R01_cb11025_c0/f4p0/1518	NA	NA	NA	R01_cb11025_c0/f4p0/1518
R01_cb10545_c1/f1p0/1948	R01_cb10545_c1/f1p0/1948	R01_cb10545_c1/f1p0/1948	R01_cb10545_c1/f1p0/1948	R01_cb10545_c1/f1p0/1948
R01_cb8564_c19484/f1p0/3244	R01_cb8564_c19484/f1p0/324	R01_cb8564_c19484/f1p0/3244	NA	R01_cb8564_c19484/f1p0/3244

	4			
R01_cb12319_c0/f1p0/589	R01_cb12319_c0/f1p0/589	NA	NA	R01_cb12319_c0/f1p0/589
R01_cb15551_c1/f1p0/856	NA	R01_cb15551_c1/f1p0/856	NA	NA
R01_cb2224_c2/f1p0/4006	NA	R01_cb2224_c2/f1p0/4006	NA	NA
R01_cb18456_c5436/f1p0/598	R01_cb18456_c5436/f1p0/598	R01_cb18456_c5436/f1p0/598	R01_cb18456_c5436/f1p0/598	R01_cb18456_c5436/f1p0/598
R01_cb11535_c1/f1p0/1910	R01_cb11535_c1/f1p0/1910	R01_cb11535_c1/f1p0/1910	NA	NA
R01_cb6198_c0/f1p0/2916	NA	R01_cb6198_c0/f1p0/2916	NA	R01_cb6198_c0/f1p0/2916
R01_cb11316_c0/f1p0/1092	NA	R01_cb11316_c0/f1p0/1092	NA	R01_cb11316_c0/f1p0/1092
R01_cb16526_c6/f1p0/1280	NA	R01_cb16526_c6/f1p0/1280	NA	NA
R01_cb8564_c42130/f1p0/3239	NA	NA	NA	R01_cb8564_c42130/f1p0/3239
R01_cb7286_c5/f1p1/2594	NA	R01_cb7286_c5/f1p1/2594	NA	NA
R01_cb5983_c2/f2p0/824	NA	NA	NA	R01_cb5983_c2/f2p0/824
R01_cb335_c1/f1p0/4866	NA	NA	NA	R01_cb335_c1/f1p0/4866
R01_cb10024_c395/f1p1/744	NA	NA	R01_cb10024_c395/f1p1/744	R01_cb10024_c395/f1p1/744
R01_cb7565_c2/f1p0/1575	NA	NA	R01_cb7565_c2/f1p0/1575	R01_cb7565_c2/f1p0/1575
R01_cb8564_c18794/f1p0/4376	NA	NA	NA	R01_cb8564_c18794/f1p0/4376
R01_cb2543_c35/f1p0/2891	NA	R01_cb2543_c35/f1p0/2891	NA	NA
R01_cb6143_c12/f1p0/948	NA	R01_cb6143_c12/f1p0/948	NA	NA
R01_cb12421_c19/f2p0/616	R01_cb12421_c19/f2p0/616	R01_cb12421_c19/f2p0/616	NA	NA
R01_cb10229_c9/f1p0/1628	R01_cb10229_c9/f1p0/1628	R01_cb10229_c9/f1p0/1628	NA	R01_cb10229_c9/f1p0/1628
R01_cb8564_c69948/f1p0/2609	NA	NA	R01_cb8564_c69948/f1p0/2609	NA
R01_cb8564_c45501/f1p1/2240	NA	R01_cb8564_c45501/f1p1/2240	NA	NA
R01_cb17756_c15/f1p0/1779	NA	R01_cb17756_c15/f1p0/1779	R01_cb17756_c15/f1p0/1779	R01_cb17756_c15/f1p0/1779
R01_cb8564_c124692/f1p0/2033	NA	NA	NA	R01_cb8564_c124692/f1p0/2033
R01_cb8564_c92247/f2p0/2361	R01_cb8564_c92247/f2p0/236	R01_cb8564_c92247/f2p0/2361	R01_cb8564_c92247/f2p0/2361	R01_cb8564_c92247/f2p0/2361

	1			
R01_cb10237_c0/f8p0/1279	NA	NA	NA	R01_cb10237_c0/f8p0/1279
R01_cb8564_c34590/f3p0/3393	NA	R01_cb8564_c34590/f3p0/3393	NA	R01_cb8564_c34590/f3p0/3393
R01_cb6631_c30/f1p1/2206	NA	NA	NA	R01_cb6631_c30/f1p1/2206
R01_cb16269_c2/f1p0/656	NA	R01_cb16269_c2/f1p0/656	NA	R01_cb16269_c2/f1p0/656
R01_cb18456_c5156/f1p2/593	R01_cb18456_c5156/f1p2/593	R01_cb18456_c5156/f1p2/593	NA	R01_cb18456_c5156/f1p2/593
R01_cb6953_c2/f1p0/2636	NA	R01_cb6953_c2/f1p0/2636	NA	NA
R01_cb13914_c5/f1p0/735	NA	R01_cb13914_c5/f1p0/735	NA	R01_cb13914_c5/f1p0/735
R01_cb6369_c6/f1p0/2455	NA	NA	NA	R01_cb6369_c6/f1p0/2455
R01_cb2159_c3/f1p0/3188	NA	R01_cb2159_c3/f1p0/3188	NA	NA
R01_cb12634_c25/f1p0/5582	NA	R01_cb12634_c25/f1p0/5582	NA	NA
R01_cb412_c8/f1p0/3694	NA	NA	NA	R01_cb412_c8/f1p0/3694
R01_cb10920_c14/f1p0/948	NA	R01_cb10920_c14/f1p0/948	NA	NA
R01_cb950_c26/f1p0/3270	NA	R01_cb950_c26/f1p0/3270	R01_cb950_c26/f1p0/3270	NA
R01_cb8564_c146370/f3p0/2664	NA	NA	R01_cb8564_c146370/f3p0/2664	R01_cb8564_c146370/f3p0/2664
R01_cb2729_c3/f1p0/1910	NA	R01_cb2729_c3/f1p0/1910	NA	R01_cb2729_c3/f1p0/1910
R01_cb8564_c52715/f3p0/2152	R01_cb8564_c52715/f3p0/2152	R01_cb8564_c52715/f3p0/2152	R01_cb8564_c52715/f3p0/2152	R01_cb8564_c52715/f3p0/2152
R01_cb9506_c5/f1p0/2064	NA	NA	NA	R01_cb9506_c5/f1p0/2064
R01_cb18050_c0/f2p0/1027	NA	NA	R01_cb18050_c0/f2p0/1027	R01_cb18050_c0/f2p0/1027
R01_cb11283_c5/f2p0/470	NA	R01_cb11283_c5/f2p0/470	NA	R01_cb11283_c5/f2p0/470
R01_cb8334_c2/f1p0/1913	NA	R01_cb8334_c2/f1p0/1913	NA	NA
R01_cb8564_c51841/f1p0/2363	NA	R01_cb8564_c51841/f1p0/2363	NA	NA
R01_cb18456_c5294/f1p3/810	NA	R01_cb18456_c5294/f1p3/810	NA	NA
R01_cb15533_c16/f1p0/1556	R01_cb15533_c16/f1p0/1556	NA	R01_cb15533_c16/f1p0/1556	R01_cb15533_c16/f1p0/1556

R01_cb6004_c3/f1p1/2038	NA	NA	NA	R01_cb6004_c3/f1p1/2038
R01_cb8564_c46857/f1p0/2753	NA	R01_cb8564_c46857/f1p0/2753	NA	NA
R01_cb18452_c1/f1p0/582	NA	R01_cb18452_c1/f1p0/582	NA	R01_cb18452_c1/f1p0/582
R01_cb11088_c0/f1p0/1268	NA	NA	NA	R01_cb11088_c0/f1p0/1268
R01_cb10014_c24/f2p0/533	NA	R01_cb10014_c24/f2p0/533	NA	NA
R01_cb13901_c2/f1p0/401	R01_cb13901_c2/f1p0/401	R01_cb13901_c2/f1p0/401	NA	R01_cb13901_c2/f1p0/401
R01_cb5517_c0/f1p0/3079	NA	NA	R01_cb5517_c0/f1p0/3079	NA
R01_cb13418_c31/f1p0/388	R01_cb13418_c31/f1p0/388	R01_cb13418_c31/f1p0/388	R01_cb13418_c31/f1p0/388	R01_cb13418_c31/f1p0/388
R01_cb1433_c6/f1p0/2612	NA	R01_cb1433_c6/f1p0/2612	NA	R01_cb1433_c6/f1p0/2612
R01_cb8793_c0/f2p0/2269	R01_cb8793_c0/f2p0/2269	R01_cb8793_c0/f2p0/2269	R01_cb8793_c0/f2p0/2269	R01_cb8793_c0/f2p0/2269
R01_cb16824_c2/f1p1/900	NA	NA	NA	R01_cb16824_c2/f1p1/900
R01_cb11517_c1/f1p0/2922	NA	R01_cb11517_c1/f1p0/2922	NA	NA
R01_cb1378_c14/f1p0/648	R01_cb1378_c14/f1p0/648	R01_cb1378_c14/f1p0/648	R01_cb1378_c14/f1p0/648	R01_cb1378_c14/f1p0/648
R01_cb12774_c1/f1p0/1122	R01_cb12774_c1/f1p0/1122	R01_cb12774_c1/f1p0/1122	R01_cb12774_c1/f1p0/1122	R01_cb12774_c1/f1p0/1122
R01_cb7080_c1/f1p0/2547	NA	R01_cb7080_c1/f1p0/2547	NA	NA
R01_cb8564_c19334/f1p0/2329	NA	R01_cb8564_c19334/f1p0/2329	NA	R01_cb8564_c19334/f1p0/2329
R01_cb8564_c3074/f1p0/3838	R01_cb8564_c3074/f1p0/3838	R01_cb8564_c3074/f1p0/3838	NA	R01_cb8564_c3074/f1p0/3838
R01_cb5552_c3/f1p0/1049	NA	NA	NA	R01_cb5552_c3/f1p0/1049
R01_cb12750_c2/f1p0/652	NA	R01_cb12750_c2/f1p0/652	NA	R01_cb12750_c2/f1p0/652
R01_cb2581_c4/f1p0/884	NA	R01_cb2581_c4/f1p0/884	R01_cb2581_c4/f1p0/884	R01_cb2581_c4/f1p0/884
R01_cb15053_c6/f1p0/929	NA	R01_cb15053_c6/f1p0/929	R01_cb15053_c6/f1p0/929	NA
R01_cb14927_c3/f1p0/690	R01_cb14927_c3/f1p0/690	R01_cb14927_c3/f1p0/690	R01_cb14927_c3/f1p0/690	R01_cb14927_c3/f1p0/690
R01_cb9593_c9/f1p0/2421	R01_cb9593_c9/f1p0/2421	R01_cb9593_c9/f1p0/2421	NA	NA
R01_cb17035_c1/f1p0/440	R01_cb17035_c1/f1p0/440	R01_cb17035_c1/f1p0/440	R01_cb17035_c1/f1p0/440	R01_cb17035_c1/f1p0/440
R01_cb3735_c10/f1p0/368	R01_cb3735_c10/f1p0/368	R01_cb3735_c10/f1p0/368	R01_cb3735_c10/f1p0/368	R01_cb3735_c10/f1p0/368
R01_cb10913_c4/f1p0/1053	NA	NA	NA	R01_cb10913_c4/f1p0/1053

R01_cb8111_c1/f1p0/2250	NA	NA	NA	R01_cb8111_c1/f1p0/2250
R01_cb3303_c4/f1p0/6230	NA	R01_cb3303_c4/f1p0/6230	R01_cb3303_c4/f1p0/6230	R01_cb3303_c4/f1p0/6230
R01_cb6448_c6/f1p0/3003	NA	R01_cb6448_c6/f1p0/3003	NA	NA
R01_cb2497_c9/f4p0/3867	NA	NA	NA	R01_cb2497_c9/f4p0/3867
R01_cb9236_c1/f1p0/2605	NA	NA	NA	R01_cb9236_c1/f1p0/2605
R01_cb4944_c10/f1p0/1431	R01_cb4944_c10/f1p0/1431	R01_cb4944_c10/f1p0/1431	NA	R01_cb4944_c10/f1p0/1431
R01_cb10145_c0/f3p0/1580	NA	NA	NA	R01_cb10145_c0/f3p0/1580
R01_cb6272_c5/f1p2/2588	NA	NA	NA	R01_cb6272_c5/f1p2/2588
R01_cb7982_c1/f1p0/2007	NA	NA	NA	R01_cb7982_c1/f1p0/2007
R01_cb14316_c11/f1p0/707	R01_cb14316_c11/f1p0/707	R01_cb14316_c11/f1p0/707	R01_cb14316_c11/f1p0/707	R01_cb14316_c11/f1p0/707
R01_cb17467_c0/f1p0/802	R01_cb17467_c0/f1p0/802	R01_cb17467_c0/f1p0/802	R01_cb17467_c0/f1p0/802	R01_cb17467_c0/f1p0/802
R01_cb14943_c3/f1p0/1770	NA	R01_cb14943_c3/f1p0/1770	NA	NA
R01_cb8564_c69452/f1p1/2532	NA	R01_cb8564_c69452/f1p1/2532	R01_cb8564_c69452/f1p1/2532	R01_cb8564_c69452/f1p1/2532
R01_cb2357_c3/f1p0/4314	NA	NA	NA	R01_cb2357_c3/f1p0/4314
R01_cb13713_c7/f1p0/1520	NA	R01_cb13713_c7/f1p0/1520	NA	NA
R01_cb18456_c1656/f2p0/510	NA	R01_cb18456_c1656/f2p0/510	R01_cb18456_c1656/f2p0/510	R01_cb18456_c1656/f2p0/510
R01_cb1392_c12/f1p0/2918	NA	NA	NA	R01_cb1392_c12/f1p0/2918
R01_cb11481_c2/f1p0/563	NA	R01_cb11481_c2/f1p0/563	NA	R01_cb11481_c2/f1p0/563
R01_cb16712_c1/f1p1/1173	NA	NA	NA	R01_cb16712_c1/f1p1/1173
R01_cb10930_c2/f1p0/567	R01_cb10930_c2/f1p0/567	R01_cb10930_c2/f1p0/567	R01_cb10930_c2/f1p0/567	R01_cb10930_c2/f1p0/567
R01_cb8376_c6/f1p0/814	NA	R01_cb8376_c6/f1p0/814	R01_cb8376_c6/f1p0/814	R01_cb8376_c6/f1p0/814
R01_cb12574_c1/f1p0/1859	R01_cb12574_c1/f1p0/1859	R01_cb12574_c1/f1p0/1859	R01_cb12574_c1/f1p0/1859	R01_cb12574_c1/f1p0/1859
R01_cb11032_c4/f1p0/833	NA	NA	NA	R01_cb11032_c4/f1p0/833
R01_cb18456_c5429/f1p0/1055	R01_cb18456_c5429/f1p0/1055	R01_cb18456_c5429/f1p0/1055	R01_cb18456_c5429/f1p0/1055	R01_cb18456_c5429/f1p0/1055
R01_cb2494_c7/f1p0/4101	NA	R01_cb2494_c7/f1p0/4101	NA	NA

R01_cb14817_c0/f1p0/1796	NA	R01_cb14817_c0/f1p0/1796	R01_cb14817_c0/f1p0/1796	R01_cb14817_c0/f1p0/1796
R01_cb18270_c0/f3p0/1009	NA	NA	NA	R01_cb18270_c0/f3p0/1009
R01_cb12098_c39/f1p0/422	R01_cb12098_c39/f1p0/422	R01_cb12098_c39/f1p0/422	R01_cb12098_c39/f1p0/422	R01_cb12098_c39/f1p0/422
R01_cb9912_c3/f1p0/1992	NA	R01_cb9912_c3/f1p0/1992	NA	NA
R01_cb16146_c1/f2p0/557	NA	R01_cb16146_c1/f2p0/557	NA	NA
R01_cb8564_c72933/f1p0/2989	NA	R01_cb8564_c72933/f1p0/2989	NA	R01_cb8564_c72933/f1p0/2989
R01_cb8564_c80475/f1p0/2363	R01_cb8564_c80475/f1p0/2363	R01_cb8564_c80475/f1p0/2363	R01_cb8564_c80475/f1p0/2363	R01_cb8564_c80475/f1p0/2363
R01_cb416_c17/f1p0/1988	NA	R01_cb416_c17/f1p0/1988	NA	R01_cb416_c17/f1p0/1988
R01_cb5103_c9/f1p0/1895	NA	R01_cb5103_c9/f1p0/1895	NA	NA
R01_cb3502_c1/f1p0/3612	NA	NA	NA	R01_cb3502_c1/f1p0/3612
R01_cb8564_c25499/f1p0/2032	NA	R01_cb8564_c25499/f1p0/2032	R01_cb8564_c25499/f1p0/2032	NA
R01_cb11664_c3/f1p0/910	NA	R01_cb11664_c3/f1p0/910	NA	NA
R01_cb8849_c0/f2p0/1558	NA	NA	NA	R01_cb8849_c0/f2p0/1558
R01_cb1739_c19/f1p0/1232	NA	R01_cb1739_c19/f1p0/1232	R01_cb1739_c19/f1p0/1232	R01_cb1739_c19/f1p0/1232
R01_cb3667_c3/f1p0/3564	NA	NA	NA	R01_cb3667_c3/f1p0/3564
R01_cb4584_c16/f1p0/3319	NA	NA	NA	R01_cb4584_c16/f1p0/3319
R01_cb2594_c3/f1p0/554	NA	R01_cb2594_c3/f1p0/554	NA	NA
R01_cb8564_c90811/f1p0/3128	NA	R01_cb8564_c90811/f1p0/3128	R01_cb8564_c90811/f1p0/3128	NA
R01_cb17118_c3/f1p0/608	NA	R01_cb17118_c3/f1p0/608	R01_cb17118_c3/f1p0/608	R01_cb17118_c3/f1p0/608
R01_cb11246_c1/f1p0/2206	NA	R01_cb11246_c1/f1p0/2206	NA	NA
R01_cb15701_c9/f1p0/1645	NA	R01_cb15701_c9/f1p0/1645	NA	NA
R01_cb1207_c16/f1p0/3156	NA	NA	NA	R01_cb1207_c16/f1p0/3156
R01_cb7158_c0/f1p0/2685	NA	R01_cb7158_c0/f1p0/2685	R01_cb7158_c0/f1p0/2685	R01_cb7158_c0/f1p0/2685
R01_cb5896_c51/f1p0/2173	NA	R01_cb5896_c51/f1p0/2173	NA	NA
R01_cb14799_c15/f1p0/358	R01_cb14799_c15/f1p0/358	R01_cb14799_c15/f1p0/358	R01_cb14799_c15/f1p0/358	R01_cb14799_c15/f1p0/358

R01_cb17738_c0/f1p0/1658	NA	NA	NA	R01_cb17738_c0/f1p0/1658
R01_cb17456_c1/f1p0/1684	NA	R01_cb17456_c1/f1p0/1684	R01_cb17456_c1/f1p0/1684	R01_cb17456_c1/f1p0/1684
R01_cb8564_c1919/f1p0/2484	NA	NA	R01_cb8564_c1919/f1p0/2484	R01_cb8564_c1919/f1p0/2484
R01_cb17404_c1/f1p0/1469	R01_cb17404_c1/f1p0/1469	R01_cb17404_c1/f1p0/1469	R01_cb17404_c1/f1p0/1469	R01_cb17404_c1/f1p0/1469
R01_cb16678_c6/f1p0/1856	NA	R01_cb16678_c6/f1p0/1856	R01_cb16678_c6/f1p0/1856	R01_cb16678_c6/f1p0/1856
R01_cb14446_c2/f1p0/677	R01_cb14446_c2/f1p0/677	R01_cb14446_c2/f1p0/677	NA	NA
R01_cb8178_c5/f1p0/1295	NA	R01_cb8178_c5/f1p0/1295	NA	NA
R01_cb14565_c1/f1p0/948	NA	R01_cb14565_c1/f1p0/948	NA	R01_cb14565_c1/f1p0/948
R01_cb1813_c6/f3p1/2562	NA	NA	NA	R01_cb1813_c6/f3p1/2562
R01_cb1739_c9/f1p0/6056	NA	NA	NA	R01_cb1739_c9/f1p0/6056
R01_cb8564_c69835/f1p0/2719	NA	R01_cb8564_c69835/f1p0/2719	NA	R01_cb8564_c69835/f1p0/2719
R01_cb4062_c3/f1p0/921	NA	R01_cb4062_c3/f1p0/921	NA	NA
R01_cb10974_c2/f1p0/3806	NA	R01_cb10974_c2/f1p0/3806	NA	NA
R01_cb416_c42/f1p0/2638	NA	NA	NA	R01_cb416_c42/f1p0/2638
R01_cb11303_c2/f1p0/3394	NA	R01_cb11303_c2/f1p0/3394	NA	NA
R01_cb4584_c6/f1p0/3607	NA	NA	NA	R01_cb4584_c6/f1p0/3607
R01_cb18456_c1883/f1p0/1219	R01_cb18456_c1883/f1p0/1219	R01_cb18456_c1883/f1p0/1219	R01_cb18456_c1883/f1p0/1219	R01_cb18456_c1883/f1p0/1219
R01_cb1418_c4/f1p0/3446	NA	NA	NA	R01_cb1418_c4/f1p0/3446
R01_cb8051_c8/f2p0/2289	NA	NA	NA	R01_cb8051_c8/f2p0/2289
R01_cb3582_c12/f1p0/1714	R01_cb3582_c12/f1p0/1714	R01_cb3582_c12/f1p0/1714	R01_cb3582_c12/f1p0/1714	R01_cb3582_c12/f1p0/1714
R01_cb11463_c1/f1p0/2811	R01_cb11463_c1/f1p0/2811	R01_cb11463_c1/f1p0/2811	NA	NA
R01_cb11507_c3/f1p0/1559	NA	NA	NA	R01_cb11507_c3/f1p0/1559
R01_cb11893_c1/f1p0/1204	NA	NA	NA	R01_cb11893_c1/f1p0/1204
R01_cb8564_c112962/f1p0/2169	NA	NA	NA	R01_cb8564_c112962/f1p0/2169

R01_cb8564_c11627/f1p0/3735	NA	R01_cb8564_c11627/f1p0/3735	R01_cb8564_c11627/f1p0/3735	R01_cb8564_c11627/f1p0/3735
R01_cb8564_c45827/f2p2/4435	NA	NA	NA	R01_cb8564_c45827/f2p2/4435
R01_cb18243_c1/f3p1/677	NA	R01_cb18243_c1/f3p1/677	R01_cb18243_c1/f3p1/677	NA
R01_cb15273_c7/f1p0/872	NA	NA	R01_cb15273_c7/f1p0/872	R01_cb15273_c7/f1p0/872
R01_cb3618_c9/f1p0/2646	NA	NA	NA	R01_cb3618_c9/f1p0/2646
R01_cb3426_c9/f1p0/3425	NA	NA	NA	R01_cb3426_c9/f1p0/3425
R01_cb3716_c9/f1p0/2561	NA	NA	NA	R01_cb3716_c9/f1p0/2561
R01_cb4286_c11/f1p0/2813	NA	R01_cb4286_c11/f1p0/2813	NA	NA
R01_cb8564_c85128/f1p0/3287	NA	R01_cb8564_c85128/f1p0/3287	NA	NA
R01_cb2804_c109/f1p0/1890	NA	R01_cb2804_c109/f1p0/1890	R01_cb2804_c109/f1p0/1890	R01_cb2804_c109/f1p0/1890
R01_cb13545_c47/f1p0/918	NA	R01_cb13545_c47/f1p0/918	NA	NA
R01_cb10559_c3/f1p0/346	R01_cb10559_c3/f1p0/346	R01_cb10559_c3/f1p0/346	R01_cb10559_c3/f1p0/346	R01_cb10559_c3/f1p0/346
R01_cb12421_c50/f1p0/597	R01_cb12421_c50/f1p0/597	R01_cb12421_c50/f1p0/597	R01_cb12421_c50/f1p0/597	R01_cb12421_c50/f1p0/597
R01_cb3641_c2/f1p0/1911	NA	R01_cb3641_c2/f1p0/1911	NA	NA
R01_cb8564_c3699/f1p0/3713	R01_cb8564_c3699/f1p0/3713	R01_cb8564_c3699/f1p0/3713	R01_cb8564_c3699/f1p0/3713	R01_cb8564_c3699/f1p0/3713
R01_cb7890_c0/f1p0/2511	R01_cb7890_c0/f1p0/2511	R01_cb7890_c0/f1p0/2511	R01_cb7890_c0/f1p0/2511	R01_cb7890_c0/f1p0/2511
R01_cb5808_c5/f1p0/1115	NA	NA	NA	R01_cb5808_c5/f1p0/1115
R01_cb8564_c82877/f1p0/2948	NA	R01_cb8564_c82877/f1p0/2948	NA	NA
R01_cb10743_c1/f1p0/2920	NA	R01_cb10743_c1/f1p0/2920	R01_cb10743_c1/f1p0/2920	R01_cb10743_c1/f1p0/2920
R01_cb17930_c1/f2p0/1523	NA	R01_cb17930_c1/f2p0/1523	NA	R01_cb17930_c1/f2p0/1523
R01_cb8564_c46212/f3p1/4373	NA	NA	NA	R01_cb8564_c46212/f3p1/4373
R01_cb17052_c3/f1p0/1786	NA	NA	NA	R01_cb17052_c3/f1p0/1786
R01_cb6371_c3/f1p0/1888	NA	NA	NA	R01_cb6371_c3/f1p0/1888
R01_cb2307_c7/f1p0/3667	NA	R01_cb2307_c7/f1p0/3667	NA	NA
R01_cb8051_c2/f1p0/2474	NA	NA	NA	R01_cb8051_c2/f1p0/2474
R01_cb8982_c0/f2p0/2127	NA	NA	NA	R01_cb8982_c0/f2p0/2127

R01_cb2708_c23/f1p0/2261	NA	NA	R01_cb2708_c23/f1p0/2261	R01_cb2708_c23/f1p0/2261
R01_cb9448_c9/f1p1/1806	NA	NA	NA	R01_cb9448_c9/f1p1/1806
R01_cb1860_c23/f1p0/2594	NA	NA	NA	R01_cb1860_c23/f1p0/2594
R01_cb11733_c2/f1p0/1505	NA	NA	NA	R01_cb11733_c2/f1p0/1505
R01_cb891_c14/f4p1/3325	NA	R01_cb891_c14/f4p1/3325	R01_cb891_c14/f4p1/3325	NA
R01_cb17218_c1/f1p0/945	NA	NA	NA	R01_cb17218_c1/f1p0/945
R01_cb11975_c6/f1p0/1473	NA	R01_cb11975_c6/f1p0/1473	R01_cb11975_c6/f1p0/1473	R01_cb11975_c6/f1p0/1473
R01_cb18456_c1822/f1p0/606	NA	NA	NA	R01_cb18456_c1822/f1p0/606
R01_cb10188_c6/f1p0/962	R01_cb10188_c6/f1p0/962	R01_cb10188_c6/f1p0/962	R01_cb10188_c6/f1p0/962	R01_cb10188_c6/f1p0/962
R01_cb10056_c6/f1p0/1438	R01_cb10056_c6/f1p0/1438	R01_cb10056_c6/f1p0/1438	R01_cb10056_c6/f1p0/1438	R01_cb10056_c6/f1p0/1438
R01_cb8564_c84403/f1p0/3160	NA	R01_cb8564_c84403/f1p0/3160	R01_cb8564_c84403/f1p0/3160	NA
R01_cb8564_c48301/f1p2/3934	NA	NA	NA	R01_cb8564_c48301/f1p2/3934
R01_cb7087_c1/f1p0/2331	NA	NA	NA	R01_cb7087_c1/f1p0/2331
R01_cb8564_c18679/f1p1/2628	NA	R01_cb8564_c18679/f1p1/2628	NA	NA
R01_cb18456_c7582/f1p0/1283	NA	R01_cb18456_c7582/f1p0/1283	NA	NA
R01_cb8324_c2/f2p0/1444	NA	NA	NA	R01_cb8324_c2/f2p0/1444
R01_cb15951_c7/f1p0/404	R01_cb15951_c7/f1p0/404	R01_cb15951_c7/f1p0/404	R01_cb15951_c7/f1p0/404	R01_cb15951_c7/f1p0/404
R01_cb18305_c0/f2p0/346	R01_cb18305_c0/f2p0/346	R01_cb18305_c0/f2p0/346	R01_cb18305_c0/f2p0/346	R01_cb18305_c0/f2p0/346
R01_cb10260_c2/f1p0/1126	NA	R01_cb10260_c2/f1p0/1126	NA	NA
R01_cb5054_c3/f1p0/2548	NA	NA	R01_cb5054_c3/f1p0/2548	R01_cb5054_c3/f1p0/2548
R01_cb8564_c110092/f1p3/2724	NA	NA	R01_cb8564_c110092/f1p3/2724	NA
R01_cb11171_c1/f1p1/2450	NA	R01_cb11171_c1/f1p1/2450	R01_cb11171_c1/f1p1/2450	NA
R01_cb13024_c1/f1p0/373	NA	R01_cb13024_c1/f1p0/373	NA	R01_cb13024_c1/f1p0/373
R01_cb12328_c2/f1p1/1478	NA	NA	NA	R01_cb12328_c2/f1p1/1478
R01_cb18409_c97/f1p0/623	NA	R01_cb18409_c97/f1p0/623	R01_cb18409_c97/f1p0/623	R01_cb18409_c97/f1p0/623

R01_cb2031_c3/f1p0/3924	NA	NA	NA	R01_cb2031_c3/f1p0/3924
R01_cb383_c43/f1p0/575	NA	R01_cb383_c43/f1p0/575	NA	R01_cb383_c43/f1p0/575
R01_cb8564_c52736/f1p0/4819	NA	NA	NA	R01_cb8564_c52736/f1p0/4819
R01_cb8941_c3/f1p0/1925	NA	NA	NA	R01_cb8941_c3/f1p0/1925
R01_cb8122_c5/f1p0/2216	NA	R01_cb8122_c5/f1p0/2216	NA	R01_cb8122_c5/f1p0/2216
R01_cb14472_c3/f3p0/860	NA	NA	NA	R01_cb14472_c3/f3p0/860
R01_cb5952_c1/f1p0/2861	NA	NA	NA	R01_cb5952_c1/f1p0/2861
R01_cb17276_c1/f1p0/770	NA	R01_cb17276_c1/f1p0/770	NA	R01_cb17276_c1/f1p0/770
R01_cb7552_c2/f1p0/445	R01_cb7552_c2/f1p0/445	R01_cb7552_c2/f1p0/445	R01_cb7552_c2/f1p0/445	R01_cb7552_c2/f1p0/445
R01_cb8564_c112330/f1p0/2046	R01_cb8564_c112330/f1p0/2046	R01_cb8564_c112330/f1p0/2046	R01_cb8564_c112330/f1p0/2046	NA
R01_cb8564_c122622/f1p0/2900	NA	R01_cb8564_c122622/f1p0/2900	NA	NA
R01_cb5857_c30/f1p0/1721	NA	R01_cb5857_c30/f1p0/1721	NA	NA
R01_cb10830_c0/f1p0/1392	NA	NA	NA	R01_cb10830_c0/f1p0/1392
R01_cb16913_c2/f1p0/505	NA	R01_cb16913_c2/f1p0/505	NA	NA
R01_cb2044_c2/f1p0/2652	NA	NA	NA	R01_cb2044_c2/f1p0/2652
R01_cb5625_c5/f1p0/2328	NA	R01_cb5625_c5/f1p0/2328	NA	R01_cb5625_c5/f1p0/2328
R01_cb12315_c3/f1p1/1414	NA	NA	NA	R01_cb12315_c3/f1p1/1414
R01_cb11414_c1/f1p0/3896	NA	R01_cb11414_c1/f1p0/3896	NA	R01_cb11414_c1/f1p0/3896
R01_cb8564_c123819/f1p0/2118	NA	R01_cb8564_c123819/f1p0/2118	R01_cb8564_c123819/f1p0/2118	NA
R01_cb18780_c10/f1p3/5598	NA	R01_cb18780_c10/f1p3/5598	NA	R01_cb18780_c10/f1p3/5598
R01_cb7380_c0/f1p0/2635	R01_cb7380_c0/f1p0/2635	R01_cb7380_c0/f1p0/2635	NA	R01_cb7380_c0/f1p0/2635
R01_cb13191_c3/f1p0/1446	NA	NA	NA	R01_cb13191_c3/f1p0/1446
R01_cb18456_c7229/f1p1/587	NA	R01_cb18456_c7229/f1p1/587	R01_cb18456_c7229/f1p1/587	R01_cb18456_c7229/f1p1/587

R01_cb8564_c127284/f1p0/2847	NA	R01_cb8564_c127284/f1p0/2847	NA	R01_cb8564_c127284/f1p0/2847
R01_cb5896_c159/f1p0/2030	NA	R01_cb5896_c159/f1p0/2030	R01_cb5896_c159/f1p0/2030	R01_cb5896_c159/f1p0/2030
R01_cb14153_c0/f1p0/1644	NA	R01_cb14153_c0/f1p0/1644	NA	NA
R01_cb14402_c1/f2p0/1540	NA	R01_cb14402_c1/f2p0/1540	NA	R01_cb14402_c1/f2p0/1540
R01_cb3483_c8/f1p0/2848	NA	NA	NA	R01_cb3483_c8/f1p0/2848
R01_cb12003_c145/f1p0/1405	NA	R01_cb12003_c145/f1p0/1405	NA	NA
R01_cb13880_c3/f1p0/1846	NA	R01_cb13880_c3/f1p0/1846	NA	NA
R01_cb18456_c1867/f1p0/1690	NA	R01_cb18456_c1867/f1p0/1690	NA	NA
R01_cb7114_c6/f1p0/2697	NA	NA	NA	R01_cb7114_c6/f1p0/2697
R01_cb17721_c2/f1p0/1819	NA	NA	NA	R01_cb17721_c2/f1p0/1819
R01_cb15828_c5/f1p0/917	NA	R01_cb15828_c5/f1p0/917	R01_cb15828_c5/f1p0/917	R01_cb15828_c5/f1p0/917
R01_cb18435_c1/f1p0/671	R01_cb18435_c1/f1p0/671	R01_cb18435_c1/f1p0/671	R01_cb18435_c1/f1p0/671	R01_cb18435_c1/f1p0/671
R01_cb2771_c1/f5p1/3188	NA	NA	NA	R01_cb2771_c1/f5p1/3188
R01_cb8483_c3/f1p0/2470	NA	NA	NA	R01_cb8483_c3/f1p0/2470
R01_cb8564_c4159/f1p2/3687	NA	NA	NA	R01_cb8564_c4159/f1p2/3687
R01_cb16370_c1/f1p0/795	NA	R01_cb16370_c1/f1p0/795	NA	R01_cb16370_c1/f1p0/795
R01_cb15082_c2/f1p0/1819	NA	R01_cb15082_c2/f1p0/1819	NA	NA
R01_cb13025_c13/f1p0/1286	NA	NA	NA	R01_cb13025_c13/f1p0/1286
R01_cb3500_c12/f1p0/3013	NA	NA	NA	R01_cb3500_c12/f1p0/3013
R01_cb8564_c19058/f1p0/2206	NA	R01_cb8564_c19058/f1p0/2206	R01_cb8564_c19058/f1p0/2206	R01_cb8564_c19058/f1p0/2206
R01_cb8104_c4/f1p0/2404	NA	NA	NA	R01_cb8104_c4/f1p0/2404
R01_cb17973_c55/f1p0/1322	NA	R01_cb17973_c55/f1p0/1322	NA	R01_cb17973_c55/f1p0/1322
R01_cb14108_c6/f1p0/905	R01_cb14108_c6/f1p0/905	NA	NA	R01_cb14108_c6/f1p0/905
R01_cb18456_c4952/f1p0/570	R01_cb18456_c4952/f1p0/570	R01_cb18456_c4952/f1p0/570	NA	R01_cb18456_c4952/f1p0/570
R01_cb12037_c15/f1p0/966	NA	NA	R01_cb12037_c15/f1p0/966	NA

R01_cb11566_c3/f1p0/481	R01_cb11566_c3/f1p0/481	R01_cb11566_c3/f1p0/481	R01_cb11566_c3/f1p0/481	R01_cb11566_c3/f1p0/481
R01_cb15192_c4/f1p0/352	R01_cb15192_c4/f1p0/352	R01_cb15192_c4/f1p0/352	NA	R01_cb15192_c4/f1p0/352
R01_cb9313_c6/f1p0/1413	NA	R01_cb9313_c6/f1p0/1413	NA	NA
R01_cb18456_c3313/f1p0/795	NA	R01_cb18456_c3313/f1p0/795	NA	NA
R01_cb17309_c0/f1p1/1552	NA	NA	R01_cb17309_c0/f1p1/1552	R01_cb17309_c0/f1p1/1552
R01_cb12852_c1/f1p1/1050	NA	NA	R01_cb12852_c1/f1p1/1050	R01_cb12852_c1/f1p1/1050
R01_cb9108_c2/f1p0/1666	NA	NA	NA	R01_cb9108_c2/f1p0/1666
R01_cb14877_c1/f2p0/425	R01_cb14877_c1/f2p0/425	R01_cb14877_c1/f2p0/425	R01_cb14877_c1/f2p0/425	R01_cb14877_c1/f2p0/425
R01_cb3854_c5/f1p0/2584	NA	NA	R01_cb3854_c5/f1p0/2584	NA
R01_cb3699_c8/f1p0/3392	NA	R01_cb3699_c8/f1p0/3392	NA	NA
R01_cb18569_c1/f1p0/619	R01_cb18569_c1/f1p0/619	R01_cb18569_c1/f1p0/619	NA	R01_cb18569_c1/f1p0/619
R01_cb843_c1/f5p1/2346	NA	NA	NA	R01_cb843_c1/f5p1/2346
R01_cb3120_c46/f1p0/1970	NA	NA	R01_cb3120_c46/f1p0/1970	NA
R01_cb8564_c116780/f1p1/1900	NA	R01_cb8564_c116780/f1p1/1900	NA	NA
R01_cb737_c50/f1p0/1437	R01_cb737_c50/f1p0/1437	R01_cb737_c50/f1p0/1437	NA	NA
R01_cb8564_c25092/f1p0/4072	NA	NA	NA	R01_cb8564_c25092/f1p0/4072
R01_cb17306_c0/f1p1/984	R01_cb17306_c0/f1p1/984	R01_cb17306_c0/f1p1/984	R01_cb17306_c0/f1p1/984	R01_cb17306_c0/f1p1/984
R01_cb6983_c1/f1p0/2842	NA	NA	NA	R01_cb6983_c1/f1p0/2842
R01_cb12830_c4/f1p0/441	R01_cb12830_c4/f1p0/441	R01_cb12830_c4/f1p0/441	R01_cb12830_c4/f1p0/441	R01_cb12830_c4/f1p0/441
R01_cb18456_c7449/f1p0/310	R01_cb18456_c7449/f1p0/310	R01_cb18456_c7449/f1p0/310	R01_cb18456_c7449/f1p0/310	R01_cb18456_c7449/f1p0/310
R01_cb4250_c2/f1p0/4608	NA	NA	NA	R01_cb4250_c2/f1p0/4608
R01_cb6480_c4/f1p1/2665	NA	NA	NA	R01_cb6480_c4/f1p1/2665
R01_cb5946_c15/f1p0/1178	NA	R01_cb5946_c15/f1p0/1178	NA	NA
R01_cb17131_c1/f2p0/537	NA	R01_cb17131_c1/f2p0/537	NA	NA
R01_cb8564_c112226/f1p0/248	R01_cb8564_c112226/f1p0/24	R01_cb8564_c112226/f1p0/248	NA	NA

2	82	2		
R01_cb16564_c0/f1p0/801	NA	R01_cb16564_c0/f1p0/801	R01_cb16564_c0/f1p0/801	R01_cb16564_c0/f1p0/801
R01_cb293_c3/f1p0/2953	NA	NA	NA	R01_cb293_c3/f1p0/2953
R01_cb14963_c4/f1p0/602	NA	R01_cb14963_c4/f1p0/602	NA	NA
R01_cb12360_c8/f1p1/758	NA	R01_cb12360_c8/f1p1/758	NA	NA
R01_cb13701_c0/f2p0/841	NA	NA	NA	R01_cb13701_c0/f2p0/841
R01_cb18526_c11/f1p0/1551	NA	R01_cb18526_c11/f1p0/1551	NA	R01_cb18526_c11/f1p0/1551
R01_cb8564_c74541/f1p1/3268	NA	R01_cb8564_c74541/f1p1/3268	NA	NA
R01_cb1077_c4/f6p0/3211	NA	NA	NA	R01_cb1077_c4/f6p0/3211
R01_cb3826_c13/f1p0/488	R01_cb3826_c13/f1p0/488	R01_cb3826_c13/f1p0/488	NA	R01_cb3826_c13/f1p0/488
R01_cb2022_c7/f1p0/3946	NA	NA	NA	R01_cb2022_c7/f1p0/3946
R01_cb12123_c1/f4p0/472	NA	R01_cb12123_c1/f4p0/472	R01_cb12123_c1/f4p0/472	R01_cb12123_c1/f4p0/472
R01_cb3732_c17/f1p0/2194	NA	NA	NA	R01_cb3732_c17/f1p0/2194
R01_cb8612_c0/f1p0/2319	R01_cb8612_c0/f1p0/2319	R01_cb8612_c0/f1p0/2319	R01_cb8612_c0/f1p0/2319	R01_cb8612_c0/f1p0/2319
R01_cb16991_c0/f2p1/604	NA	R01_cb16991_c0/f2p1/604	NA	R01_cb16991_c0/f2p1/604
R01_cb12641_c13/f1p0/926	NA	NA	NA	R01_cb12641_c13/f1p0/926
R01_cb14566_c1/f1p0/573	R01_cb14566_c1/f1p0/573	R01_cb14566_c1/f1p0/573	R01_cb14566_c1/f1p0/573	R01_cb14566_c1/f1p0/573
R01_cb5659_c69/f1p0/2199	NA	NA	NA	R01_cb5659_c69/f1p0/2199
R01_cb12334_c0/f2p0/544	NA	R01_cb12334_c0/f2p0/544	NA	NA
R01_cb8564_c82138/f1p0/4103	NA	NA	NA	R01_cb8564_c82138/f1p0/4103
R01_cb10039_c4/f1p0/1325	NA	R01_cb10039_c4/f1p0/1325	NA	NA
R01_cb8514_c3/f1p0/1154	NA	NA	NA	R01_cb8514_c3/f1p0/1154
R01_cb3026_c2/f2p0/2050	NA	R01_cb3026_c2/f2p0/2050	NA	NA
R01_cb16369_c1/f1p0/382	R01_cb16369_c1/f1p0/382	R01_cb16369_c1/f1p0/382	NA	R01_cb16369_c1/f1p0/382
R01_cb15778_c2/f1p0/1166	NA	NA	NA	R01_cb15778_c2/f1p0/1166
R01_cb9517_c2/f2p1/2083	NA	R01_cb9517_c2/f2p1/2083	NA	NA

R01_cb8564_c16263/f1p0/2381	R01_cb8564_c16263/f1p0/2381	R01_cb8564_c16263/f1p0/2381	R01_cb8564_c16263/f1p0/2381	R01_cb8564_c16263/f1p0/2381
R01_cb8564_c84338/f1p0/2695	NA	R01_cb8564_c84338/f1p0/2695	NA	R01_cb8564_c84338/f1p0/2695
R01_cb8564_c14856/f1p0/3558	NA	R01_cb8564_c14856/f1p0/3558	NA	NA
R01_cb11706_c1/f1p0/2387	NA	R01_cb11706_c1/f1p0/2387	R01_cb11706_c1/f1p0/2387	R01_cb11706_c1/f1p0/2387
R01_cb11121_c1/f1p0/3192	NA	NA	NA	R01_cb11121_c1/f1p0/3192
R01_cb14341_c1/f1p0/682	R01_cb14341_c1/f1p0/682	R01_cb14341_c1/f1p0/682	R01_cb14341_c1/f1p0/682	R01_cb14341_c1/f1p0/682
R01_cb8564_c3055/f1p0/1930	R01_cb8564_c3055/f1p0/1930	R01_cb8564_c3055/f1p0/1930	R01_cb8564_c3055/f1p0/1930	R01_cb8564_c3055/f1p0/1930
R01_cb4691_c3/f1p0/999	NA	R01_cb4691_c3/f1p0/999	NA	NA
R01_cb15958_c2/f1p0/1721	NA	R01_cb15958_c2/f1p0/1721	NA	NA
R01_cb3763_c14/f1p0/3662	NA	NA	NA	R01_cb3763_c14/f1p0/3662
R01_cb12585_c14/f1p0/778	NA	R01_cb12585_c14/f1p0/778	NA	NA
R01_cb3359_c0/f4p1/3589	NA	NA	R01_cb3359_c0/f4p1/3589	R01_cb3359_c0/f4p1/3589
R01_cb17756_c12/f1p0/1766	R01_cb17756_c12/f1p0/1766	R01_cb17756_c12/f1p0/1766	R01_cb17756_c12/f1p0/1766	R01_cb17756_c12/f1p0/1766
R01_cb1981_c18/f1p0/2047	NA	NA	NA	R01_cb1981_c18/f1p0/2047
R01_cb11506_c1/f1p0/3589	R01_cb11506_c1/f1p0/3589	R01_cb11506_c1/f1p0/3589	R01_cb11506_c1/f1p0/3589	NA
R01_cb18724_c0/f1p0/5140	NA	R01_cb18724_c0/f1p0/5140	NA	R01_cb18724_c0/f1p0/5140
R01_cb2602_c5/f1p0/2227	NA	NA	NA	R01_cb2602_c5/f1p0/2227
R01_cb8564_c91930/f1p0/3149	NA	R01_cb8564_c91930/f1p0/3149	NA	NA
R01_cb8513_c0/f1p0/2274	NA	NA	NA	R01_cb8513_c0/f1p0/2274
R01_cb5558_c6/f1p0/1048	NA	R01_cb5558_c6/f1p0/1048	NA	NA
R01_cb16799_c2/f1p0/744	NA	NA	NA	R01_cb16799_c2/f1p0/744
R01_cb8564_c51952/f1p0/4840	NA	R01_cb8564_c51952/f1p0/4840	R01_cb8564_c51952/f1p0/4840	R01_cb8564_c51952/f1p0/4840
R01_cb8564_c33746/f6p0/3485	R01_cb8564_c33746/f6p0/3485	R01_cb8564_c33746/f6p0/3485	R01_cb8564_c33746/f6p0/3485	R01_cb8564_c33746/f6p0/3485
R01_cb16540_c5/f2p0/583	NA	NA	R01_cb16540_c5/f2p0/583	R01_cb16540_c5/f2p0/583

R01_cb8564_c121658/f1p0/2496	NA	R01_cb8564_c121658/f1p0/2496	NA	NA
R01_cb18496_c0/f2p0/470	R01_cb18496_c0/f2p0/470	NA	NA	R01_cb18496_c0/f2p0/470
R01_cb17331_c0/f1p0/565	NA	NA	R01_cb17331_c0/f1p0/565	R01_cb17331_c0/f1p0/565
R01_cb8564_c19120/f1p0/3323	NA	R01_cb8564_c19120/f1p0/3323	NA	NA
R01_cb13453_c14/f1p0/1521	NA	NA	NA	R01_cb13453_c14/f1p0/1521
R01_cb8564_c15799/f1p0/2924	NA	R01_cb8564_c15799/f1p0/2924	NA	R01_cb8564_c15799/f1p0/2924
R01_cb18456_c5635/f1p0/508	NA	R01_cb18456_c5635/f1p0/508	NA	R01_cb18456_c5635/f1p0/508
R01_cb17371_c2/f1p0/591	R01_cb17371_c2/f1p0/591	R01_cb17371_c2/f1p0/591	R01_cb17371_c2/f1p0/591	R01_cb17371_c2/f1p0/591
R01_cb15585_c20/f1p0/756	R01_cb15585_c20/f1p0/756	R01_cb15585_c20/f1p0/756	NA	NA
R01_cb8900_c2/f1p0/2007	NA	NA	NA	R01_cb8900_c2/f1p0/2007
R01_cb4657_c16/f1p0/4283	NA	NA	NA	R01_cb4657_c16/f1p0/4283
R01_cb3832_c4/f1p0/2183	NA	R01_cb3832_c4/f1p0/2183	NA	NA
R01_cb7023_c0/f2p1/2542	NA	R01_cb7023_c0/f2p1/2542	R01_cb7023_c0/f2p1/2542	R01_cb7023_c0/f2p1/2542
R01_cb15114_c1/f1p0/970	NA	R01_cb15114_c1/f1p0/970	NA	R01_cb15114_c1/f1p0/970
R01_cb8564_c4620/f1p2/2929	NA	R01_cb8564_c4620/f1p2/2929	R01_cb8564_c4620/f1p2/2929	R01_cb8564_c4620/f1p2/2929
R01_cb15005_c6/f1p0/336	R01_cb15005_c6/f1p0/336	R01_cb15005_c6/f1p0/336	R01_cb15005_c6/f1p0/336	R01_cb15005_c6/f1p0/336
R01_cb12757_c1/f1p0/944	NA	NA	NA	R01_cb12757_c1/f1p0/944
R01_cb8335_c16/f1p0/2566	NA	NA	NA	R01_cb8335_c16/f1p0/2566
R01_cb9793_c2/f2p2/1955	NA	NA	NA	R01_cb9793_c2/f2p2/1955
R01_cb10082_c4/f1p0/2999	NA	R01_cb10082_c4/f1p0/2999	NA	NA
R01_cb1085_c11/f1p1/4093	NA	NA	NA	R01_cb1085_c11/f1p1/4093
R01_cb6602_c41/f1p1/2258	NA	NA	NA	R01_cb6602_c41/f1p1/2258
R01_cb2804_c308/f1p0/585	R01_cb2804_c308/f1p0/585	R01_cb2804_c308/f1p0/585	R01_cb2804_c308/f1p0/585	R01_cb2804_c308/f1p0/585
R01_cb12114_c8/f1p0/1430	NA	NA	NA	R01_cb12114_c8/f1p0/1430
R01_cb10363_c0/f1p0/609	NA	R01_cb10363_c0/f1p0/609	NA	R01_cb10363_c0/f1p0/609

R01_cb15018_c3/f1p1/600	NA	R01_cb15018_c3/f1p1/600	R01_cb15018_c3/f1p1/600	R01_cb15018_c3/f1p1/600
R01_cb18456_c6114/f1p0/556	NA	NA	R01_cb18456_c6114/f1p0/556	NA
R01_cb18456_c7278/f1p0/932	R01_cb18456_c7278/f1p0/932	R01_cb18456_c7278/f1p0/932	R01_cb18456_c7278/f1p0/932	R01_cb18456_c7278/f1p0/932
R01_cb1056_c0/f1p0/4488	NA	R01_cb1056_c0/f1p0/4488	NA	NA
R01_cb5229_c4/f1p0/2716	NA	R01_cb5229_c4/f1p0/2716	NA	R01_cb5229_c4/f1p0/2716
R01_cb10306_c5/f1p0/3253	R01_cb10306_c5/f1p0/3253	R01_cb10306_c5/f1p0/3253	NA	R01_cb10306_c5/f1p0/3253
R01_cb18456_c5825/f1p0/959	NA	R01_cb18456_c5825/f1p0/959	NA	NA
R01_cb18526_c9/f1p0/701	NA	R01_cb18526_c9/f1p0/701	NA	NA
R01_cb6194_c3/f1p0/2912	NA	R01_cb6194_c3/f1p0/2912	NA	NA
R01_cb18456_c2537/f1p0/1445	NA	R01_cb18456_c2537/f1p0/1445	NA	NA
R01_cb14620_c1/f1p0/1701	NA	NA	R01_cb14620_c1/f1p0/1701	R01_cb14620_c1/f1p0/1701
R01_cb5952_c0/f2p0/1065	NA	NA	NA	R01_cb5952_c0/f2p0/1065
R01_cb18241_c2/f1p0/1042	R01_cb18241_c2/f1p0/1042	R01_cb18241_c2/f1p0/1042	NA	NA
R01_cb8564_c4193/f1p0/3949	NA	R01_cb8564_c4193/f1p0/3949	NA	NA
R01_cb8564_c19221/f2p0/2424	NA	NA	NA	R01_cb8564_c19221/f2p0/2424
R01_cb13601_c1/f2p0/943	NA	NA	NA	R01_cb13601_c1/f2p0/943
R01_cb16910_c1/f1p0/1331	R01_cb16910_c1/f1p0/1331	NA	NA	R01_cb16910_c1/f1p0/1331
R01_cb8564_c74067/f1p0/3829	NA	R01_cb8564_c74067/f1p0/3829	NA	NA
R01_cb10443_c0/f1p0/1365	NA	R01_cb10443_c0/f1p0/1365	NA	NA
R01_cb15963_c3/f1p0/736	NA	NA	R01_cb15963_c3/f1p0/736	NA
R01_cb4952_c3/f1p0/2896	NA	NA	NA	R01_cb4952_c3/f1p0/2896
R01_cb13038_c0/f1p0/1234	NA	R01_cb13038_c0/f1p0/1234	NA	NA
R01_cb8564_c111470/f1p0/2319	NA	R01_cb8564_c111470/f1p0/2319	NA	R01_cb8564_c111470/f1p0/2319
R01_cb15060_c0/f1p0/1608	NA	NA	NA	R01_cb15060_c0/f1p0/1608
R01_cb10454_c16/f1p0/1092	NA	R01_cb10454_c16/f1p0/1092	NA	R01_cb10454_c16/f1p0/1092

R01_cb13610_c2/f1p0/757	NA	R01_cb13610_c2/f1p0/757	NA	NA
R01_cb14896_c1/f1p1/1836	R01_cb14896_c1/f1p1/1836	R01_cb14896_c1/f1p1/1836	R01_cb14896_c1/f1p1/1836	R01_cb14896_c1/f1p1/1836
R01_cb8364_c2/f1p0/2377	R01_cb8364_c2/f1p0/2377	R01_cb8364_c2/f1p0/2377	R01_cb8364_c2/f1p0/2377	R01_cb8364_c2/f1p0/2377
R01_cb8564_c91280/f1p0/1967	NA	NA	NA	R01_cb8564_c91280/f1p0/1967
R01_cb8564_c44954/f1p0/2471	NA	R01_cb8564_c44954/f1p0/2471	NA	R01_cb8564_c44954/f1p0/2471
R01_cb12240_c8/f1p0/579	R01_cb12240_c8/f1p0/579	R01_cb12240_c8/f1p0/579	NA	NA
R01_cb10087_c2/f1p3/1128	NA	R01_cb10087_c2/f1p3/1128	NA	NA
R01_cb12191_c5/f1p0/713	NA	R01_cb12191_c5/f1p0/713	NA	R01_cb12191_c5/f1p0/713
R01_cb10024_c332/f1p0/681	NA	NA	R01_cb10024_c332/f1p0/681	NA
R01_cb18655_c12/f1p0/5964	NA	NA	NA	R01_cb18655_c12/f1p0/5964
R01_cb14286_c4/f1p0/1200	NA	R01_cb14286_c4/f1p0/1200	NA	R01_cb14286_c4/f1p0/1200
R01_cb3120_c52/f1p1/2585	NA	R01_cb3120_c52/f1p1/2585	NA	NA
R01_cb7579_c0/f1p0/2597	NA	NA	NA	R01_cb7579_c0/f1p0/2597
R01_cb5475_c5/f1p0/2918	NA	R01_cb5475_c5/f1p0/2918	NA	NA
R01_cb18456_c2288/f1p0/635	R01_cb18456_c2288/f1p0/635	R01_cb18456_c2288/f1p0/635	R01_cb18456_c2288/f1p0/635	R01_cb18456_c2288/f1p0/635
R01_cb8564_c12586/f1p0/2756	NA	R01_cb8564_c12586/f1p0/2756	NA	R01_cb8564_c12586/f1p0/2756
R01_cb5595_c34/f1p0/3013	NA	R01_cb5595_c34/f1p0/3013	NA	R01_cb5595_c34/f1p0/3013
R01_cb3558_c13/f10p0/2716	NA	NA	NA	R01_cb3558_c13/f10p0/2716
R01_cb10694_c0/f2p1/575	NA	NA	NA	R01_cb10694_c0/f2p1/575
R01_cb480_c5/f1p0/5296	NA	NA	NA	R01_cb480_c5/f1p0/5296
R01_cb3564_c25/f1p1/3034	NA	NA	NA	R01_cb3564_c25/f1p1/3034
R01_cb12373_c3/f1p0/958	R01_cb12373_c3/f1p0/958	R01_cb12373_c3/f1p0/958	R01_cb12373_c3/f1p0/958	R01_cb12373_c3/f1p0/958
R01_cb10723_c1/f2p0/714	NA	NA	NA	R01_cb10723_c1/f2p0/714
R01_cb15574_c4/f1p0/502	NA	NA	R01_cb15574_c4/f1p0/502	R01_cb15574_c4/f1p0/502
R01_cb8564_c21771/f1p0/3179	NA	NA	R01_cb8564_c21771/f1p0/3179	R01_cb8564_c21771/f1p0/3179
R01_cb7303_c12/f1p0/2436	NA	NA	NA	R01_cb7303_c12/f1p0/2436

R01_cb8564_c3799/f1p0/2802	R01_cb8564_c3799/f1p0/2802	R01_cb8564_c3799/f1p0/2802	NA	NA
R01_cb17580_c1/f1p0/1793	NA	NA	NA	R01_cb17580_c1/f1p0/1793
R01_cb1247_c1/f1p0/3154	NA	R01_cb1247_c1/f1p0/3154	NA	NA
R01_cb8564_c156200/f1p3/3087	NA	NA	NA	R01_cb8564_c156200/f1p3/3087
R01_cb4639_c7/f1p0/2470	NA	R01_cb4639_c7/f1p0/2470	NA	R01_cb4639_c7/f1p0/2470
R01_cb8564_c19386/f2p0/3961	NA	NA	NA	R01_cb8564_c19386/f2p0/3961
R01_cb11307_c4/f1p0/704	R01_cb11307_c4/f1p0/704	R01_cb11307_c4/f1p0/704	R01_cb11307_c4/f1p0/704	R01_cb11307_c4/f1p0/704
R01_cb6829_c11/f1p0/360	R01_cb6829_c11/f1p0/360	R01_cb6829_c11/f1p0/360	R01_cb6829_c11/f1p0/360	R01_cb6829_c11/f1p0/360
R01_cb3088_c2/f1p0/3439	NA	R01_cb3088_c2/f1p0/3439	R01_cb3088_c2/f1p0/3439	R01_cb3088_c2/f1p0/3439
R01_cb3445_c2/f1p1/3625	NA	NA	NA	R01_cb3445_c2/f1p1/3625
R01_cb6285_c1/f1p0/2620	NA	R01_cb6285_c1/f1p0/2620	R01_cb6285_c1/f1p0/2620	R01_cb6285_c1/f1p0/2620
R01_cb4478_c0/f1p0/3336	NA	NA	NA	R01_cb4478_c0/f1p0/3336
R01_cb2870_c11/f1p1/2718	NA	NA	NA	R01_cb2870_c11/f1p1/2718
R01_cb2385_c6/f1p0/1818	NA	R01_cb2385_c6/f1p0/1818	NA	NA
R01_cb11246_c0/f1p0/772	R01_cb11246_c0/f1p0/772	R01_cb11246_c0/f1p0/772	NA	NA
R01_cb1203_c12/f1p2/4460	NA	NA	NA	R01_cb1203_c12/f1p2/4460
R01_cb9791_c0/f4p0/1965	NA	R01_cb9791_c0/f4p0/1965	NA	R01_cb9791_c0/f4p0/1965
R01_cb7219_c7/f1p2/2549	NA	NA	NA	R01_cb7219_c7/f1p2/2549
R01_cb13497_c5/f1p0/1729	NA	R01_cb13497_c5/f1p0/1729	NA	R01_cb13497_c5/f1p0/1729
R01_cb15959_c8/f1p0/977	R01_cb15959_c8/f1p0/977	R01_cb15959_c8/f1p0/977	NA	R01_cb15959_c8/f1p0/977
R01_cb2761_c7/f1p0/3531	NA	R01_cb2761_c7/f1p0/3531	NA	NA
R01_cb2839_c6/f1p0/5031	NA	R01_cb2839_c6/f1p0/5031	NA	NA
R01_cb8564_c127692/f1p0/2182	NA	R01_cb8564_c127692/f1p0/2182	R01_cb8564_c127692/f1p0/2182	R01_cb8564_c127692/f1p0/2182
R01_cb10648_c2/f1p0/2342	R01_cb10648_c2/f1p0/2342	R01_cb10648_c2/f1p0/2342	R01_cb10648_c2/f1p0/2342	R01_cb10648_c2/f1p0/2342

R01_cb18629_c1/f1p0/1411	NA	R01_cb18629_c1/f1p0/1411	R01_cb18629_c1/f1p0/1411	R01_cb18629_c1/f1p0/1411
R01_cb9664_c2/f1p0/2566	NA	NA	R01_cb9664_c2/f1p0/2566	R01_cb9664_c2/f1p0/2566
R01_cb8564_c82067/f1p0/2959	NA	NA	NA	R01_cb8564_c82067/f1p0/2959
R01_cb14527_c0/f2p0/796	NA	R01_cb14527_c0/f2p0/796	R01_cb14527_c0/f2p0/796	R01_cb14527_c0/f2p0/796
R01_cb5900_c76/f1p0/3351	R01_cb5900_c76/f1p0/3351	R01_cb5900_c76/f1p0/3351	NA	R01_cb5900_c76/f1p0/3351
R01_cb18456_c6678/f1p0/522	R01_cb18456_c6678/f1p0/522	R01_cb18456_c6678/f1p0/522	R01_cb18456_c6678/f1p0/522	R01_cb18456_c6678/f1p0/522
R01_cb8564_c7790/f7p0/2258	NA	R01_cb8564_c7790/f7p0/2258	R01_cb8564_c7790/f7p0/2258	R01_cb8564_c7790/f7p0/2258
R01_cb8991_c14/f1p0/1263	NA	NA	NA	R01_cb8991_c14/f1p0/1263
R01_cb2421_c1/f1p0/2510	NA	R01_cb2421_c1/f1p0/2510	R01_cb2421_c1/f1p0/2510	NA
R01_cb13064_c6/f1p0/312	R01_cb13064_c6/f1p0/312	R01_cb13064_c6/f1p0/312	R01_cb13064_c6/f1p0/312	R01_cb13064_c6/f1p0/312
R01_cb8564_c85011/f1p0/2932	NA	R01_cb8564_c85011/f1p0/2932	NA	R01_cb8564_c85011/f1p0/2932
R01_cb7030_c28/f1p0/549	NA	R01_cb7030_c28/f1p0/549	NA	NA
R01_cb18456_c6220/f1p0/687	R01_cb18456_c6220/f1p0/687	R01_cb18456_c6220/f1p0/687	NA	R01_cb18456_c6220/f1p0/687
R01_cb2104_c6/f1p0/3853	NA	R01_cb2104_c6/f1p0/3853	R01_cb2104_c6/f1p0/3853	R01_cb2104_c6/f1p0/3853
R01_cb8564_c51150/f1p0/3341	NA	NA	NA	R01_cb8564_c51150/f1p0/3341
R01_cb13178_c5/f2p0/540	NA	R01_cb13178_c5/f2p0/540	NA	NA
R01_cb8564_c1057/f1p0/2347	NA	R01_cb8564_c1057/f1p0/2347	NA	R01_cb8564_c1057/f1p0/2347
R01_cb11733_c0/f1p0/1333	NA	NA	NA	R01_cb11733_c0/f1p0/1333
R01_cb3992_c8/f13p0/883	NA	NA	NA	R01_cb3992_c8/f13p0/883
R01_cb8564_c20099/f1p0/3387	NA	R01_cb8564_c20099/f1p0/3387	R01_cb8564_c20099/f1p0/3387	R01_cb8564_c20099/f1p0/3387
R01_cb10872_c1/f1p0/2992	NA	R01_cb10872_c1/f1p0/2992	NA	NA
R01_cb7334_c13/f1p0/1691	NA	NA	NA	R01_cb7334_c13/f1p0/1691
R01_cb8564_c21367/f1p0/2871	NA	R01_cb8564_c21367/f1p0/2871	NA	NA
R01_cb10272_c11/f1p0/2538	NA	NA	NA	R01_cb10272_c11/f1p0/2538
R01_cb6507_c11/f1p0/1009	NA	NA	NA	R01_cb6507_c11/f1p0/1009
R01_cb2018_c2/f1p0/1820	NA	R01_cb2018_c2/f1p0/1820	NA	NA

R01_cb3500_c18/f1p0/2999	NA	NA	NA	R01_cb3500_c18/f1p0/2999
R01_cb9504_c0/f2p0/1821	NA	R01_cb9504_c0/f2p0/1821	NA	NA
R01_cb3941_c26/f1p0/2346	NA	NA	NA	R01_cb3941_c26/f1p0/2346
R01_cb9530_c3/f1p0/1994	NA	NA	NA	R01_cb9530_c3/f1p0/1994
R01_cb8564_c81762/f1p0/3842	NA	R01_cb8564_c81762/f1p0/3842	NA	NA
R01_cb8564_c75981/f2p0/4046	NA	R01_cb8564_c75981/f2p0/4046	NA	NA
R01_cb8564_c107755/f9p0/2141	NA	R01_cb8564_c107755/f9p0/2141	R01_cb8564_c107755/f9p0/2141	R01_cb8564_c107755/f9p0/2141
R01_cb6802_c12/f1p0/3195	NA	R01_cb6802_c12/f1p0/3195	R01_cb6802_c12/f1p0/3195	NA
R01_cb10641_c2/f1p0/1760	NA	NA	NA	R01_cb10641_c2/f1p0/1760
R01_cb7992_c2/f1p0/2126	NA	NA	NA	R01_cb7992_c2/f1p0/2126
R01_cb8564_c4585/f2p0/3415	R01_cb8564_c4585/f2p0/3415	R01_cb8564_c4585/f2p0/3415	NA	R01_cb8564_c4585/f2p0/3415
R01_cb18456_c2316/f1p2/711	NA	R01_cb18456_c2316/f1p2/711	NA	R01_cb18456_c2316/f1p2/711
R01_cb17248_c2/f1p0/360	NA	R01_cb17248_c2/f1p0/360	R01_cb17248_c2/f1p0/360	R01_cb17248_c2/f1p0/360
R01_cb17712_c1/f1p0/481	NA	R01_cb17712_c1/f1p0/481	NA	NA
R01_cb1178_c8/f1p0/2736	NA	NA	NA	R01_cb1178_c8/f1p0/2736
R01_cb4576_c41/f1p0/3473	NA	NA	NA	R01_cb4576_c41/f1p0/3473
R01_cb8564_c5090/f1p2/2223	NA	NA	NA	R01_cb8564_c5090/f1p2/2223
R01_cb11286_c1/f1p0/1925	NA	R01_cb11286_c1/f1p0/1925	NA	NA
R01_cb7219_c9/f1p0/2265	NA	NA	NA	R01_cb7219_c9/f1p0/2265
R01_cb10764_c4/f1p0/723	R01_cb10764_c4/f1p0/723	R01_cb10764_c4/f1p0/723	NA	NA
R01_cb480_c1/f1p0/3737	NA	NA	NA	R01_cb480_c1/f1p0/3737
R01_cb9833_c1/f1p0/3484	NA	R01_cb9833_c1/f1p0/3484	R01_cb9833_c1/f1p0/3484	NA
R01_cb7615_c0/f1p0/2592	NA	R01_cb7615_c0/f1p0/2592	NA	NA
R01_cb11569_c2/f1p0/1187	R01_cb11569_c2/f1p0/1187	R01_cb11569_c2/f1p0/1187	R01_cb11569_c2/f1p0/1187	R01_cb11569_c2/f1p0/1187
R01_cb454_c4/f3p0/2268	NA	NA	NA	R01_cb454_c4/f3p0/2268

R01_cb18780_c2/f1p26/7582	NA	R01_cb18780_c2/f1p26/7582	NA	NA
R01_cb13178_c28/f1p0/534	NA	R01_cb13178_c28/f1p0/534	NA	NA
R01_cb13598_c0/f1p0/614	NA	R01_cb13598_c0/f1p0/614	NA	R01_cb13598_c0/f1p0/614
R01_cb12286_c1/f17p5/881	NA	R01_cb12286_c1/f17p5/881	NA	NA
R01_cb8564_c1356/f1p0/2384	NA	NA	NA	R01_cb8564_c1356/f1p0/2384
R01_cb12587_c13/f1p0/1569	NA	NA	NA	R01_cb12587_c13/f1p0/1569
R01_cb8660_c13/f1p0/1379	NA	NA	NA	R01_cb8660_c13/f1p0/1379
R01_cb12616_c3/f1p1/1188	NA	NA	NA	R01_cb12616_c3/f1p1/1188
R01_cb12421_c60/f1p0/507	R01_cb12421_c60/f1p0/507	R01_cb12421_c60/f1p0/507	R01_cb12421_c60/f1p0/507	R01_cb12421_c60/f1p0/507
R01_cb16991_c2/f1p0/669	R01_cb16991_c2/f1p0/669	R01_cb16991_c2/f1p0/669	R01_cb16991_c2/f1p0/669	R01_cb16991_c2/f1p0/669
R01_cb12321_c846/f1p0/381	R01_cb12321_c846/f1p0/381	R01_cb12321_c846/f1p0/381	NA	NA
R01_cb12421_c64/f1p0/570	NA	R01_cb12421_c64/f1p0/570	NA	NA
R01_cb18018_c1/f1p0/456	R01_cb18018_c1/f1p0/456	R01_cb18018_c1/f1p0/456	R01_cb18018_c1/f1p0/456	R01_cb18018_c1/f1p0/456
R01_cb8564_c115326/f1p0/2118	NA	NA	NA	R01_cb8564_c115326/f1p0/2118
R01_cb8564_c18936/f1p0/2700	NA	R01_cb8564_c18936/f1p0/2700	R01_cb8564_c18936/f1p0/2700	R01_cb8564_c18936/f1p0/2700
R01_cb6082_c2/f1p2/2709	NA	R01_cb6082_c2/f1p2/2709	NA	NA
R01_cb6802_c8/f1p0/2292	NA	R01_cb6802_c8/f1p0/2292	NA	R01_cb6802_c8/f1p0/2292
R01_cb10689_c1/f1p0/3785	NA	NA	NA	R01_cb10689_c1/f1p0/3785
R01_cb8564_c15803/f3p0/2256	NA	NA	NA	R01_cb8564_c15803/f3p0/2256
R01_cb11721_c1/f1p0/2425	NA	R01_cb11721_c1/f1p0/2425	R01_cb11721_c1/f1p0/2425	R01_cb11721_c1/f1p0/2425
R01_cb18409_c44/f1p0/830	R01_cb18409_c44/f1p0/830	R01_cb18409_c44/f1p0/830	R01_cb18409_c44/f1p0/830	R01_cb18409_c44/f1p0/830
R01_cb11499_c1/f1p0/2061	NA	NA	NA	R01_cb11499_c1/f1p0/2061
R01_cb8328_c5/f1p0/916	R01_cb8328_c5/f1p0/916	R01_cb8328_c5/f1p0/916	NA	NA
R01_cb8564_c4100/f1p0/2890	R01_cb8564_c4100/f1p0/2890	R01_cb8564_c4100/f1p0/2890	R01_cb8564_c4100/f1p0/2890	NA
R01_cb12421_c24/f1p0/708	NA	R01_cb12421_c24/f1p0/708	NA	NA

R01_cb5514_c2/f1p1/2935	NA	R01_cb5514_c2/f1p1/2935	NA	R01_cb5514_c2/f1p1/2935
R01_cb17363_c6/f1p1/692	R01_cb17363_c6/f1p1/692	R01_cb17363_c6/f1p1/692	R01_cb17363_c6/f1p1/692	R01_cb17363_c6/f1p1/692
R01_cb11793_c1/f1p0/3842	NA	R01_cb11793_c1/f1p0/3842	NA	R01_cb11793_c1/f1p0/3842
R01_cb18471_c1/f1p0/1001	NA	NA	NA	R01_cb18471_c1/f1p0/1001
R01_cb8564_c89159/f2p0/2212	NA	R01_cb8564_c89159/f2p0/2212	R01_cb8564_c89159/f2p0/2212	R01_cb8564_c89159/f2p0/2212
R01_cb5536_c1/f1p0/2994	NA	R01_cb5536_c1/f1p0/2994	NA	NA
R01_cb5659_c83/f1p0/2549	NA	NA	NA	R01_cb5659_c83/f1p0/2549
R01_cb8564_c69134/f2p0/1990	NA	R01_cb8564_c69134/f2p0/1990	R01_cb8564_c69134/f2p0/1990	R01_cb8564_c69134/f2p0/1990
R01_cb3997_c5/f1p0/1500	NA	NA	NA	R01_cb3997_c5/f1p0/1500
R01_cb9154_c7/f1p0/1653	NA	NA	NA	R01_cb9154_c7/f1p0/1653
R01_cb2445_c2/f2p0/3403	NA	NA	NA	R01_cb2445_c2/f2p0/3403
R01_cb7345_c24/f1p0/492	NA	NA	NA	R01_cb7345_c24/f1p0/492
R01_cb17636_c2/f1p0/1147	NA	R01_cb17636_c2/f1p0/1147	NA	NA
R01_cb18456_c7485/f1p0/575	NA	R01_cb18456_c7485/f1p0/575	R01_cb18456_c7485/f1p0/575	NA
R01_cb3103_c1/f1p0/2830	R01_cb3103_c1/f1p0/2830	R01_cb3103_c1/f1p0/2830	NA	R01_cb3103_c1/f1p0/2830
R01_cb8564_c81887/f1p0/3974	NA	R01_cb8564_c81887/f1p0/3974	R01_cb8564_c81887/f1p0/3974	R01_cb8564_c81887/f1p0/3974
R01_cb6812_c0/f3p0/377	R01_cb6812_c0/f3p0/377	R01_cb6812_c0/f3p0/377	NA	R01_cb6812_c0/f3p0/377
R01_cb11763_c1/f1p0/3148	NA	NA	NA	R01_cb11763_c1/f1p0/3148
R01_cb15861_c0/f1p0/1249	NA	NA	NA	R01_cb15861_c0/f1p0/1249
R01_cb8564_c942/f2p0/4968	R01_cb8564_c942/f2p0/4968	R01_cb8564_c942/f2p0/4968	NA	NA
R01_cb2022_c14/f1p0/2422	NA	NA	NA	R01_cb2022_c14/f1p0/2422
R01_cb8564_c125480/f1p1/2540	NA	R01_cb8564_c125480/f1p1/2540	NA	NA
R01_cb8564_c82735/f1p0/2122	NA	NA	R01_cb8564_c82735/f1p0/2122	R01_cb8564_c82735/f1p0/2122
R01_cb822_c17/f1p0/2461	NA	R01_cb822_c17/f1p0/2461	NA	R01_cb822_c17/f1p0/2461
R01_cb2701_c3/f1p0/3424	NA	R01_cb2701_c3/f1p0/3424	NA	R01_cb2701_c3/f1p0/3424

R01_cb4185_c4/f1p0/3018	NA	NA	NA	R01_cb4185_c4/f1p0/3018
R01_cb12722_c14/f1p0/1554	NA	NA	NA	R01_cb12722_c14/f1p0/1554
R01_cb8564_c119976/f1p0/1951	NA	NA	NA	R01_cb8564_c119976/f1p0/1951
R01_cb8173_c5/f1p3/2267	NA	R01_cb8173_c5/f1p3/2267	R01_cb8173_c5/f1p3/2267	NA
R01_cb17033_c2/f1p0/1107	NA	NA	NA	R01_cb17033_c2/f1p0/1107
R01_cb18456_c3688/f1p0/1243	NA	R01_cb18456_c3688/f1p0/1243	NA	NA
R01_cb8564_c82480/f1p0/2150	R01_cb8564_c82480/f1p0/2150	R01_cb8564_c82480/f1p0/2150	R01_cb8564_c82480/f1p0/2150	NA
R01_cb10978_c2/f1p0/1299	NA	R01_cb10978_c2/f1p0/1299	NA	NA
R01_cb8187_c0/f1p0/2429	R01_cb8187_c0/f1p0/2429	R01_cb8187_c0/f1p0/2429	R01_cb8187_c0/f1p0/2429	R01_cb8187_c0/f1p0/2429
R01_cb4147_c21/f1p0/2272	NA	NA	NA	R01_cb4147_c21/f1p0/2272
R01_cb8564_c79900/f1p0/4511	R01_cb8564_c79900/f1p0/4511	R01_cb8564_c79900/f1p0/4511	R01_cb8564_c79900/f1p0/4511	R01_cb8564_c79900/f1p0/4511
R01_cb8564_c77385/f1p0/2220	NA	R01_cb8564_c77385/f1p0/2220	NA	NA
R01_cb1228_c14/f1p0/2404	NA	NA	NA	R01_cb1228_c14/f1p0/2404
R01_cb13016_c6/f1p0/334	R01_cb13016_c6/f1p0/334	R01_cb13016_c6/f1p0/334	R01_cb13016_c6/f1p0/334	R01_cb13016_c6/f1p0/334
R01_cb8564_c127124/f1p0/1926	NA	NA	NA	R01_cb8564_c127124/f1p0/1926
R01_cb8102_c8/f1p3/1987	NA	NA	NA	R01_cb8102_c8/f1p3/1987
R01_cb2298_c6/f1p1/2290	NA	NA	NA	R01_cb2298_c6/f1p1/2290
R01_cb17452_c5/f1p1/877	NA	R01_cb17452_c5/f1p1/877	NA	NA
R01_cb8564_c148897/f2p2/3139	NA	NA	NA	R01_cb8564_c148897/f2p2/3139
R01_cb10711_c1/f1p0/2530	NA	R01_cb10711_c1/f1p0/2530	NA	NA
R01_cb18456_c7547/f1p1/980	R01_cb18456_c7547/f1p1/980	R01_cb18456_c7547/f1p1/980	R01_cb18456_c7547/f1p1/980	R01_cb18456_c7547/f1p1/980

R01_cb10453_c4/f1p0/1053	NA	NA	R01_cb10453_c4/f1p0/1053	R01_cb10453_c4/f1p0/1053
R01_cb8564_c114031/f1p0/3559	NA	NA	NA	R01_cb8564_c114031/f1p0/3559
R01_cb17442_c2/f1p1/1710	NA	NA	NA	R01_cb17442_c2/f1p1/1710
R01_cb10448_c2/f1p0/3242	NA	R01_cb10448_c2/f1p0/3242	NA	NA
R01_cb8564_c18740/f1p0/3877	NA	NA	NA	R01_cb8564_c18740/f1p0/3877
R01_cb10909_c6/f1p0/1784	NA	NA	NA	R01_cb10909_c6/f1p0/1784
R01_cb14212_c0/f2p0/972	NA	R01_cb14212_c0/f2p0/972	R01_cb14212_c0/f2p0/972	R01_cb14212_c0/f2p0/972
R01_cb6631_c31/f1p0/1246	NA	NA	NA	R01_cb6631_c31/f1p0/1246
R01_cb18223_c12/f1p0/807	R01_cb18223_c12/f1p0/807	R01_cb18223_c12/f1p0/807	NA	NA
R01_cb8564_c3058/f1p0/4227	NA	R01_cb8564_c3058/f1p0/4227	NA	R01_cb8564_c3058/f1p0/4227
R01_cb8564_c79525/f1p0/2829	NA	NA	NA	R01_cb8564_c79525/f1p0/2829
R01_cb1178_c11/f1p0/2043	NA	NA	NA	R01_cb1178_c11/f1p0/2043
R01_cb1398_c5/f1p0/3236	NA	R01_cb1398_c5/f1p0/3236	R01_cb1398_c5/f1p0/3236	NA
R01_cb12451_c2/f1p0/1013	NA	R01_cb12451_c2/f1p0/1013	NA	NA
R01_cb8564_c112683/f1p0/2927	NA	NA	NA	R01_cb8564_c112683/f1p0/2927
R01_cb13914_c11/f1p0/714	NA	NA	NA	R01_cb13914_c11/f1p0/714
R01_cb1900_c6/f1p1/2700	NA	NA	NA	R01_cb1900_c6/f1p1/2700
R01_cb8564_c83468/f1p0/3918	NA	NA	NA	R01_cb8564_c83468/f1p0/3918
R01_cb18409_c74/f1p0/978	NA	R01_cb18409_c74/f1p0/978	R01_cb18409_c74/f1p0/978	R01_cb18409_c74/f1p0/978
R01_cb7957_c1/f1p0/2607	NA	R01_cb7957_c1/f1p0/2607	R01_cb7957_c1/f1p0/2607	NA
R01_cb17079_c1/f1p0/474	R01_cb17079_c1/f1p0/474	NA	R01_cb17079_c1/f1p0/474	R01_cb17079_c1/f1p0/474
R01_cb6869_c15/f1p0/538	NA	R01_cb6869_c15/f1p0/538	NA	NA
R01_cb11442_c0/f1p1/738	R01_cb11442_c0/f1p1/738	R01_cb11442_c0/f1p1/738	R01_cb11442_c0/f1p1/738	R01_cb11442_c0/f1p1/738
R01_cb1264_c3/f1p0/4126	NA	NA	NA	R01_cb1264_c3/f1p0/4126

R01_cb15035_c5/f1p0/694	NA	R01_cb15035_c5/f1p0/694	R01_cb15035_c5/f1p0/694	R01_cb15035_c5/f1p0/694
R01_cb5513_c2/f1p0/3090	NA	NA	NA	R01_cb5513_c2/f1p0/3090
R01_cb9799_c0/f1p0/1973	NA	R01_cb9799_c0/f1p0/1973	R01_cb9799_c0/f1p0/1973	NA
R01_cb10420_c10/f3p0/1786	NA	NA	NA	R01_cb10420_c10/f3p0/1786
R01_cb10427_c4/f1p0/2507	NA	NA	NA	R01_cb10427_c4/f1p0/2507
R01_cb10015_c604/f2p0/590	NA	R01_cb10015_c604/f2p0/590	NA	NA
R01_cb18456_c7307/f1p0/554	NA	R01_cb18456_c7307/f1p0/554	NA	R01_cb18456_c7307/f1p0/554
R01_cb13025_c15/f1p0/1471	NA	NA	NA	R01_cb13025_c15/f1p0/1471
R01_cb17570_c1/f1p0/726	R01_cb17570_c1/f1p0/726	NA	NA	NA
R01_cb8564_c71952/f1p1/1942	NA	R01_cb8564_c71952/f1p1/1942	R01_cb8564_c71952/f1p1/1942	R01_cb8564_c71952/f1p1/1942
R01_cb18760_c3/f1p0/6396	NA	R01_cb18760_c3/f1p0/6396	R01_cb18760_c3/f1p0/6396	NA
R01_cb8564_c47840/f1p0/2036	NA	NA	NA	R01_cb8564_c47840/f1p0/2036
R01_cb10266_c4/f1p0/2826	NA	R01_cb10266_c4/f1p0/2826	NA	NA
R01_cb796_c9/f1p0/2562	NA	R01_cb796_c9/f1p0/2562	NA	NA
R01_cb15994_c2/f1p0/463	NA	R01_cb15994_c2/f1p0/463	NA	NA
R01_cb6163_c4/f2p0/1141	NA	R01_cb6163_c4/f2p0/1141	NA	NA
R01_cb3872_c3/f1p1/575	NA	R01_cb3872_c3/f1p1/575	NA	R01_cb3872_c3/f1p1/575
R01_cb787_c2/f1p0/1571	R01_cb787_c2/f1p0/1571	R01_cb787_c2/f1p0/1571	NA	NA
R01_cb8564_c3923/f1p0/3032	R01_cb8564_c3923/f1p0/3032	R01_cb8564_c3923/f1p0/3032	R01_cb8564_c3923/f1p0/3032	R01_cb8564_c3923/f1p0/3032
R01_cb11476_c0/f1p0/1726	NA	R01_cb11476_c0/f1p0/1726	R01_cb11476_c0/f1p0/1726	R01_cb11476_c0/f1p0/1726
R01_cb14715_c3/f1p0/446	NA	R01_cb14715_c3/f1p0/446	R01_cb14715_c3/f1p0/446	R01_cb14715_c3/f1p0/446
R01_cb15443_c0/f2p0/1143	NA	NA	NA	R01_cb15443_c0/f2p0/1143
R01_cb1721_c1/f1p0/4139	NA	R01_cb1721_c1/f1p0/4139	R01_cb1721_c1/f1p0/4139	NA
R01_cb7533_c4/f1p0/2219	NA	NA	NA	R01_cb7533_c4/f1p0/2219
R01_cb7116_c7/f1p0/1884	NA	R01_cb7116_c7/f1p0/1884	NA	NA
R01_cb8799_c1/f1p0/2237	NA	NA	NA	R01_cb8799_c1/f1p0/2237

R01_cb18787_c1/f1p0/5167	R01_cb18787_c1/f1p0/5167	R01_cb18787_c1/f1p0/5167	R01_cb18787_c1/f1p0/5167	R01_cb18787_c1/f1p0/5167
R01_cb12882_c0/f1p0/1844	NA	NA	NA	R01_cb12882_c0/f1p0/1844
R01_cb9114_c0/f1p1/2181	NA	R01_cb9114_c0/f1p1/2181	NA	NA
R01_cb8564_c109512/f3p0/1967	NA	NA	NA	R01_cb8564_c109512/f3p0/1967
R01_cb18175_c1/f1p0/830	NA	R01_cb18175_c1/f1p0/830	R01_cb18175_c1/f1p0/830	R01_cb18175_c1/f1p0/830
R01_cb4098_c50/f1p0/3087	NA	R01_cb4098_c50/f1p0/3087	NA	R01_cb4098_c50/f1p0/3087
R01_cb14002_c3/f1p0/809	NA	NA	NA	R01_cb14002_c3/f1p0/809
R01_cb11931_c9/f1p0/367	R01_cb11931_c9/f1p0/367	R01_cb11931_c9/f1p0/367	R01_cb11931_c9/f1p0/367	R01_cb11931_c9/f1p0/367
R01_cb13762_c29/f1p0/1180	NA	R01_cb13762_c29/f1p0/1180	R01_cb13762_c29/f1p0/1180	R01_cb13762_c29/f1p0/1180
R01_cb1333_c85/f1p0/2873	R01_cb1333_c85/f1p0/2873	R01_cb1333_c85/f1p0/2873	R01_cb1333_c85/f1p0/2873	R01_cb1333_c85/f1p0/2873
R01_cb3703_c8/f1p0/1397	NA	NA	NA	R01_cb3703_c8/f1p0/1397
R01_cb17935_c3/f1p0/572	R01_cb17935_c3/f1p0/572	R01_cb17935_c3/f1p0/572	NA	R01_cb17935_c3/f1p0/572
R01_cb13485_c6/f32p0/652	NA	R01_cb13485_c6/f32p0/652	NA	NA
R01_cb1078_c0/f3p0/3821	NA	NA	NA	R01_cb1078_c0/f3p0/3821
R01_cb13864_c0/f2p0/1342	NA	R01_cb13864_c0/f2p0/1342	NA	NA
R01_cb18456_c1908/f1p0/688	NA	R01_cb18456_c1908/f1p0/688	NA	NA
R01_cb14619_c6/f1p0/679	NA	R01_cb14619_c6/f1p0/679	R01_cb14619_c6/f1p0/679	R01_cb14619_c6/f1p0/679
R01_cb8564_c86567/f4p1/2966	NA	R01_cb8564_c86567/f4p1/2966	NA	NA
R01_cb2200_c0/f4p1/733	NA	NA	NA	R01_cb2200_c0/f4p1/733
R01_cb10029_c98/f1p7/798	NA	R01_cb10029_c98/f1p7/798	NA	NA
R01_cb13651_c27/f1p0/1775	R01_cb13651_c27/f1p0/1775	R01_cb13651_c27/f1p0/1775	R01_cb13651_c27/f1p0/1775	R01_cb13651_c27/f1p0/1775
R01_cb7398_c6/f1p0/1846	NA	NA	NA	R01_cb7398_c6/f1p0/1846
R01_cb15864_c2/f1p0/1018	NA	R01_cb15864_c2/f1p0/1018	R01_cb15864_c2/f1p0/1018	R01_cb15864_c2/f1p0/1018
R01_cb8785_c3/f1p0/3260	NA	NA	NA	R01_cb8785_c3/f1p0/3260
R01_cb15012_c0/f1p0/972	NA	R01_cb15012_c0/f1p0/972	NA	R01_cb15012_c0/f1p0/972

R01_cb8564_c87662/f4p1/3732	R01_cb8564_c87662/f4p1/3732	R01_cb8564_c87662/f4p1/3732	NA	NA
R01_cb2033_c2/f2p0/3608	NA	NA	NA	R01_cb2033_c2/f2p0/3608
R01_cb10759_c1/f1p0/3008	R01_cb10759_c1/f1p0/3008	R01_cb10759_c1/f1p0/3008	NA	R01_cb10759_c1/f1p0/3008
R01_cb8564_c72622/f1p1/3614	NA	NA	NA	R01_cb8564_c72622/f1p1/3614
R01_cb8564_c3994/f2p0/3304	NA	NA	R01_cb8564_c3994/f2p0/3304	R01_cb8564_c3994/f2p0/3304
R01_cb8564_c16587/f4p0/3516	NA	NA	NA	R01_cb8564_c16587/f4p0/3516
R01_cb10207_c1/f5p0/743	NA	R01_cb10207_c1/f5p0/743	NA	NA
R01_cb18456_c5748/f1p0/495	NA	NA	NA	R01_cb18456_c5748/f1p0/495
R01_cb11120_c2/f1p1/2560	NA	NA	R01_cb11120_c2/f1p1/2560	R01_cb11120_c2/f1p1/2560
R01_cb18839_c0/f1p0/5272	NA	R01_cb18839_c0/f1p0/5272	NA	NA
R01_cb6608_c14/f1p0/2469	NA	NA	NA	R01_cb6608_c14/f1p0/2469
R01_cb2219_c7/f1p0/2387	NA	NA	NA	R01_cb2219_c7/f1p0/2387
R01_cb8564_c46913/f1p0/2191	NA	R01_cb8564_c46913/f1p0/2191	NA	NA
R01_cb5159_c5/f1p0/3169	NA	R01_cb5159_c5/f1p0/3169	NA	NA
R01_cb3997_c0/f2p0/1326	NA	NA	NA	R01_cb3997_c0/f2p0/1326
R01_cb10924_c3/f1p0/397	R01_cb10924_c3/f1p0/397	R01_cb10924_c3/f1p0/397	NA	NA
R01_cb8286_c10/f1p0/2326	NA	NA	NA	R01_cb8286_c10/f1p0/2326
R01_cb9847_c5/f1p0/1686	NA	R01_cb9847_c5/f1p0/1686	NA	NA
R01_cb8564_c5267/f1p0/3264	NA	R01_cb8564_c5267/f1p0/3264	NA	NA
R01_cb8085_c35/f1p0/337	R01_cb8085_c35/f1p0/337	R01_cb8085_c35/f1p0/337	R01_cb8085_c35/f1p0/337	R01_cb8085_c35/f1p0/337
R01_cb225_c2/f1p0/4622	NA	NA	NA	R01_cb225_c2/f1p0/4622
R01_cb8728_c3/f1p0/2811	NA	R01_cb8728_c3/f1p0/2811	NA	NA
R01_cb18456_c5443/f1p0/608	R01_cb18456_c5443/f1p0/608	R01_cb18456_c5443/f1p0/608	R01_cb18456_c5443/f1p0/608	R01_cb18456_c5443/f1p0/608
R01_cb15807_c0/f5p0/677	NA	NA	NA	R01_cb15807_c0/f5p0/677
R01_cb11235_c0/f1p0/1879	NA	R01_cb11235_c0/f1p0/1879	NA	NA

R01_cb14373_c4/f1p0/856	NA	R01_cb14373_c4/f1p0/856	R01_cb14373_c4/f1p0/856	R01_cb14373_c4/f1p0/856
R01_cb8564_c23163/f1p0/4734	NA	R01_cb8564_c23163/f1p0/4734	R01_cb8564_c23163/f1p0/4734	NA
R01_cb8564_c115077/f1p0/2820	NA	NA	NA	R01_cb8564_c115077/f1p0/2820
R01_cb5378_c4/f1p0/331	NA	R01_cb5378_c4/f1p0/331	R01_cb5378_c4/f1p0/331	R01_cb5378_c4/f1p0/331
R01_cb8564_c17709/f1p0/3279	NA	R01_cb8564_c17709/f1p0/3279	R01_cb8564_c17709/f1p0/3279	R01_cb8564_c17709/f1p0/3279
R01_cb13583_c0/f6p0/1507	NA	NA	NA	R01_cb13583_c0/f6p0/1507
R01_cb13297_c2/f1p0/958	NA	R01_cb13297_c2/f1p0/958	NA	R01_cb13297_c2/f1p0/958
R01_cb17358_c5/f1p0/1420	NA	NA	NA	R01_cb17358_c5/f1p0/1420
R01_cb10970_c5/f1p0/1213	NA	NA	NA	R01_cb10970_c5/f1p0/1213
R01_cb2993_c4/f1p0/2345	NA	R01_cb2993_c4/f1p0/2345	NA	NA
R01_cb17265_c1/f1p0/1088	R01_cb17265_c1/f1p0/1088	R01_cb17265_c1/f1p0/1088	R01_cb17265_c1/f1p0/1088	R01_cb17265_c1/f1p0/1088
R01_cb15218_c0/f1p0/1341	NA	R01_cb15218_c0/f1p0/1341	R01_cb15218_c0/f1p0/1341	R01_cb15218_c0/f1p0/1341
R01_cb13990_c2/f1p0/759	R01_cb13990_c2/f1p0/759	NA	NA	R01_cb13990_c2/f1p0/759
R01_cb15811_c21/f1p0/847	R01_cb15811_c21/f1p0/847	R01_cb15811_c21/f1p0/847	R01_cb15811_c21/f1p0/847	R01_cb15811_c21/f1p0/847
R01_cb4345_c5/f1p1/3251	NA	NA	NA	R01_cb4345_c5/f1p1/3251
R01_cb16532_c1/f1p0/645	NA	R01_cb16532_c1/f1p0/645	NA	NA
R01_cb18456_c5456/f1p0/877	R01_cb18456_c5456/f1p0/877	R01_cb18456_c5456/f1p0/877	NA	R01_cb18456_c5456/f1p0/877
R01_cb8564_c125291/f1p0/3569	NA	R01_cb8564_c125291/f1p0/3569	NA	NA
R01_cb8191_c3/f1p0/2058	NA	R01_cb8191_c3/f1p0/2058	NA	NA
R01_cb8564_c13852/f1p0/2417	NA	R01_cb8564_c13852/f1p0/2417	NA	R01_cb8564_c13852/f1p0/2417
R01_cb16293_c5/f1p0/875	NA	NA	R01_cb16293_c5/f1p0/875	R01_cb16293_c5/f1p0/875
R01_cb15200_c2/f2p0/1439	R01_cb15200_c2/f2p0/1439	NA	NA	NA
R01_cb3555_c3/f1p0/2050	NA	NA	NA	R01_cb3555_c3/f1p0/2050
R01_cb3294_c0/f1p0/3666	NA	NA	NA	R01_cb3294_c0/f1p0/3666

R01_cb8564_c82703/f1p0/3089	NA	R01_cb8564_c82703/f1p0/3089	NA	NA
R01_cb13438_c4/f1p0/791	NA	NA	NA	R01_cb13438_c4/f1p0/791
R01_cb15855_c2/f1p0/1261	NA	NA	NA	R01_cb15855_c2/f1p0/1261
R01_cb8564_c41692/f1p3/2987	NA	R01_cb8564_c41692/f1p3/2987	NA	NA
R01_cb5139_c12/f1p0/3085	NA	NA	NA	R01_cb5139_c12/f1p0/3085
R01_cb8809_c8/f1p1/2899	NA	NA	NA	R01_cb8809_c8/f1p1/2899
R01_cb16645_c22/f1p0/384	R01_cb16645_c22/f1p0/384	R01_cb16645_c22/f1p0/384	R01_cb16645_c22/f1p0/384	R01_cb16645_c22/f1p0/384
R01_cb18132_c11/f1p0/1793	NA	R01_cb18132_c11/f1p0/1793	NA	NA
R01_cb8564_c23203/f1p1/3864	NA	NA	NA	R01_cb8564_c23203/f1p1/3864
R01_cb8564_c50736/f1p0/2717	R01_cb8564_c50736/f1p0/2717	R01_cb8564_c50736/f1p0/2717	R01_cb8564_c50736/f1p0/2717	R01_cb8564_c50736/f1p0/2717
R01_cb10948_c4/f1p0/1242	R01_cb10948_c4/f1p0/1242	R01_cb10948_c4/f1p0/1242	R01_cb10948_c4/f1p0/1242	R01_cb10948_c4/f1p0/1242
R01_cb8564_c91272/f1p0/1917	NA	R01_cb8564_c91272/f1p0/1917	NA	NA
R01_cb2512_c4/f1p0/2433	NA	NA	NA	R01_cb2512_c4/f1p0/2433
R01_cb8564_c3001/f1p0/2451	NA	NA	NA	R01_cb8564_c3001/f1p0/2451
R01_cb18456_c7202/f1p0/621	NA	R01_cb18456_c7202/f1p0/621	R01_cb18456_c7202/f1p0/621	R01_cb18456_c7202/f1p0/621
R01_cb7942_c11/f1p1/4993	NA	R01_cb7942_c11/f1p1/4993	NA	NA
R01_cb6851_c1/f1p0/4156	NA	R01_cb6851_c1/f1p0/4156	NA	NA
R01_cb7611_c7/f1p0/2543	NA	NA	NA	R01_cb7611_c7/f1p0/2543
R01_cb2993_c0/f1p0/3751	NA	R01_cb2993_c0/f1p0/3751	R01_cb2993_c0/f1p0/3751	NA
R01_cb8564_c77129/f1p0/2424	NA	NA	NA	R01_cb8564_c77129/f1p0/2424
R01_cb11717_c1/f1p0/3372	NA	R01_cb11717_c1/f1p0/3372	R01_cb11717_c1/f1p0/3372	R01_cb11717_c1/f1p0/3372
R01_cb8564_c87987/f1p0/2617	NA	NA	NA	R01_cb8564_c87987/f1p0/2617
R01_cb16146_c4/f1p0/310	R01_cb16146_c4/f1p0/310	R01_cb16146_c4/f1p0/310	R01_cb16146_c4/f1p0/310	R01_cb16146_c4/f1p0/310
R01_cb2576_c12/f1p0/2804	R01_cb2576_c12/f1p0/2804	R01_cb2576_c12/f1p0/2804	R01_cb2576_c12/f1p0/2804	R01_cb2576_c12/f1p0/2804
R01_cb18409_c15/f3p0/572	R01_cb18409_c15/f3p0/572	R01_cb18409_c15/f3p0/572	R01_cb18409_c15/f3p0/572	R01_cb18409_c15/f3p0/572

R01_cb12165_c24/f1p1/637	R01_cb12165_c24/f1p1/637	R01_cb12165_c24/f1p1/637	NA	NA
R01_cb8564_c112971/f1p0/2064	NA	R01_cb8564_c112971/f1p0/2064	NA	NA
R01_cb123_c5/f1p0/3222	NA	NA	NA	R01_cb123_c5/f1p0/3222
R01_cb8564_c80293/f2p0/2883	NA	NA	NA	R01_cb8564_c80293/f2p0/2883
R01_cb7362_c11/f1p0/2393	R01_cb7362_c11/f1p0/2393	R01_cb7362_c11/f1p0/2393	R01_cb7362_c11/f1p0/2393	NA
R01_cb8564_c92253/f1p0/2117	NA	R01_cb8564_c92253/f1p0/2117	NA	NA
R01_cb13884_c3/f3p0/581	R01_cb13884_c3/f3p0/581	NA	R01_cb13884_c3/f3p0/581	R01_cb13884_c3/f3p0/581
R01_cb8219_c24/f1p0/2245	NA	R01_cb8219_c24/f1p0/2245	NA	NA
R01_cb4295_c11/f1p0/3015	R01_cb4295_c11/f1p0/3015	R01_cb4295_c11/f1p0/3015	NA	NA
R01_cb2225_c7/f1p0/2823	NA	R01_cb2225_c7/f1p0/2823	NA	NA
R01_cb15334_c4/f1p0/1859	R01_cb15334_c4/f1p0/1859	R01_cb15334_c4/f1p0/1859	R01_cb15334_c4/f1p0/1859	R01_cb15334_c4/f1p0/1859
R01_cb8564_c4477/f1p0/3457	NA	NA	NA	R01_cb8564_c4477/f1p0/3457
R01_cb499_c50/f1p0/2769	NA	R01_cb499_c50/f1p0/2769	NA	NA
R01_cb1554_c5/f1p0/3937	NA	NA	NA	R01_cb1554_c5/f1p0/3937
R01_cb3026_c3/f1p0/3742	NA	R01_cb3026_c3/f1p0/3742	NA	NA
R01_cb4768_c16/f1p0/1948	NA	R01_cb4768_c16/f1p0/1948	NA	NA
R01_cb8564_c21316/f1p2/2982	NA	NA	NA	R01_cb8564_c21316/f1p2/2982
R01_cb4059_c2/f1p0/3449	NA	NA	NA	R01_cb4059_c2/f1p0/3449
R01_cb8564_c121298/f1p0/2247	NA	NA	NA	R01_cb8564_c121298/f1p0/2247
R01_cb9507_c2/f1p0/2136	NA	R01_cb9507_c2/f1p0/2136	NA	NA
R01_cb917_c5/f1p0/4567	NA	NA	NA	R01_cb917_c5/f1p0/4567
R01_cb11924_c12/f1p1/438	R01_cb11924_c12/f1p1/438	R01_cb11924_c12/f1p1/438	R01_cb11924_c12/f1p1/438	NA
R01_cb16645_c15/f1p0/1413	NA	R01_cb16645_c15/f1p0/1413	NA	R01_cb16645_c15/f1p0/1413
R01_cb8564_c41324/f1p0/3027	NA	NA	NA	R01_cb8564_c41324/f1p0/3027

R01_cb8564_c113357/f1p0/2637	NA	NA	NA	R01_cb8564_c113357/f1p0/2637
R01_cb17973_c59/f1p0/1683	NA	NA	R01_cb17973_c59/f1p0/1683	NA
R01_cb8564_c73974/f1p1/2661	R01_cb8564_c73974/f1p1/2661	R01_cb8564_c73974/f1p1/2661	R01_cb8564_c73974/f1p1/2661	R01_cb8564_c73974/f1p1/2661
R01_cb5951_c4/f1p0/2340	NA	R01_cb5951_c4/f1p0/2340	NA	NA
R01_cb1333_c21/f2p0/2545	R01_cb1333_c21/f2p0/2545	R01_cb1333_c21/f2p0/2545	R01_cb1333_c21/f2p0/2545	R01_cb1333_c21/f2p0/2545
R01_cb8881_c19/f1p0/1619	NA	NA	NA	R01_cb8881_c19/f1p0/1619
R01_cb2895_c0/f1p0/3773	NA	R01_cb2895_c0/f1p0/3773	NA	NA
R01_cb15179_c1/f1p1/1415	NA	NA	NA	R01_cb15179_c1/f1p1/1415
R01_cb8564_c86410/f1p0/3195	NA	NA	R01_cb8564_c86410/f1p0/3195	R01_cb8564_c86410/f1p0/3195
R01_cb4009_c3/f1p0/3411	NA	R01_cb4009_c3/f1p0/3411	NA	NA
R01_cb1488_c7/f1p0/1425	NA	NA	NA	R01_cb1488_c7/f1p0/1425
R01_cb8564_c17884/f1p0/2817	NA	R01_cb8564_c17884/f1p0/2817	R01_cb8564_c17884/f1p0/2817	R01_cb8564_c17884/f1p0/2817
R01_cb8239_c1/f1p0/2422	NA	NA	NA	R01_cb8239_c1/f1p0/2422
R01_cb11608_c1/f1p0/2932	NA	R01_cb11608_c1/f1p0/2932	R01_cb11608_c1/f1p0/2932	R01_cb11608_c1/f1p0/2932
R01_cb2018_c1/f1p0/3340	NA	NA	NA	R01_cb2018_c1/f1p0/3340
R01_cb8564_c10791/f1p0/3599	NA	R01_cb8564_c10791/f1p0/3599	R01_cb8564_c10791/f1p0/3599	R01_cb8564_c10791/f1p0/3599
R01_cb18492_c1/f1p0/1349	NA	NA	NA	R01_cb18492_c1/f1p0/1349
R01_cb123_c0/f2p0/4981	NA	NA	NA	R01_cb123_c0/f2p0/4981
R01_cb8564_c111929/f1p0/2254	NA	R01_cb8564_c111929/f1p0/2254	NA	NA
R01_cb8014_c3/f1p0/4117	NA	R01_cb8014_c3/f1p0/4117	NA	NA
R01_cb2779_c3/f1p1/3765	NA	R01_cb2779_c3/f1p1/3765	NA	R01_cb2779_c3/f1p1/3765
R01_cb2206_c8/f1p1/1928	R01_cb2206_c8/f1p1/1928	R01_cb2206_c8/f1p1/1928	NA	NA
R01_cb8564_c47240/f1p0/2783	NA	R01_cb8564_c47240/f1p0/2783	NA	NA

R01_cb5728_c3/f1p0/3034	NA	R01_cb5728_c3/f1p0/3034	NA	NA
R01_cb18261_c1/f1p0/771	R01_cb18261_c1/f1p0/771	NA	NA	NA
R01_cb2378_c39/f37p0/3339	NA	NA	NA	R01_cb2378_c39/f37p0/3339
R01_cb11520_c1/f1p0/1918	NA	R01_cb11520_c1/f1p0/1918	NA	NA
R01_cb5304_c4/f1p0/3674	NA	NA	NA	R01_cb5304_c4/f1p0/3674
R01_cb11065_c1/f1p0/2267	NA	NA	NA	R01_cb11065_c1/f1p0/2267
R01_cb5512_c16/f1p0/2552	NA	NA	R01_cb5512_c16/f1p0/2552	NA
R01_cb8252_c6/f1p0/2382	NA	NA	NA	R01_cb8252_c6/f1p0/2382
R01_cb14353_c1/f1p0/1613	NA	R01_cb14353_c1/f1p0/1613	R01_cb14353_c1/f1p0/1613	R01_cb14353_c1/f1p0/1613
R01_cb18497_c0/f1p0/1820	R01_cb18497_c0/f1p0/1820	R01_cb18497_c0/f1p0/1820	R01_cb18497_c0/f1p0/1820	R01_cb18497_c0/f1p0/1820
R01_cb1905_c30/f1p0/3060	NA	R01_cb1905_c30/f1p0/3060	R01_cb1905_c30/f1p0/3060	R01_cb1905_c30/f1p0/3060
R01_cb7395_c4/f1p0/2685	NA	R01_cb7395_c4/f1p0/2685	NA	NA
R01_cb5778_c4/f1p0/295	R01_cb5778_c4/f1p0/295	R01_cb5778_c4/f1p0/295	R01_cb5778_c4/f1p0/295	R01_cb5778_c4/f1p0/295
R01_cb3270_c30/f1p0/789	R01_cb3270_c30/f1p0/789	R01_cb3270_c30/f1p0/789	NA	R01_cb3270_c30/f1p0/789
R01_cb11279_c3/f1p0/2374	NA	R01_cb11279_c3/f1p0/2374	NA	NA
R01_cb8564_c123127/f1p1/2451	NA	R01_cb8564_c123127/f1p1/2451	R01_cb8564_c123127/f1p1/2451	R01_cb8564_c123127/f1p1/2451
R01_cb1042_c4/f1p0/2464	NA	R01_cb1042_c4/f1p0/2464	NA	R01_cb1042_c4/f1p0/2464
R01_cb10480_c25/f1p0/2414	NA	R01_cb10480_c25/f1p0/2414	NA	NA
R01_cb10379_c1/f1p0/2442	R01_cb10379_c1/f1p0/2442	R01_cb10379_c1/f1p0/2442	NA	NA
R01_cb7519_c6/f1p0/2438	NA	NA	NA	R01_cb7519_c6/f1p0/2438
R01_cb16566_c0/f1p0/737	NA	R01_cb16566_c0/f1p0/737	R01_cb16566_c0/f1p0/737	R01_cb16566_c0/f1p0/737
R01_cb6008_c3/f1p0/2598	NA	NA	NA	R01_cb6008_c3/f1p0/2598
R01_cb8851_c4/f2p0/1421	NA	NA	NA	R01_cb8851_c4/f2p0/1421
R01_cb9682_c1/f1p0/2011	NA	NA	NA	R01_cb9682_c1/f1p0/2011
R01_cb6812_c6/f1p1/2649	NA	R01_cb6812_c6/f1p1/2649	NA	NA

R01_cb8057_c0/f1p0/2473	R01_cb8057_c0/f1p0/2473	R01_cb8057_c0/f1p0/2473	R01_cb8057_c0/f1p0/2473	R01_cb8057_c0/f1p0/2473
R01_cb15154_c8/f1p1/846	NA	NA	NA	R01_cb15154_c8/f1p1/846
R01_cb16929_c1/f1p0/716	NA	R01_cb16929_c1/f1p0/716	NA	NA
R01_cb12577_c23/f1p1/1018	NA	NA	NA	R01_cb12577_c23/f1p1/1018
R01_cb5902_c0/f1p0/2977	NA	R01_cb5902_c0/f1p0/2977	NA	NA
R01_cb3550_c3/f1p0/1739	NA	NA	NA	R01_cb3550_c3/f1p0/1739
R01_cb18113_c1/f1p0/991	NA	NA	NA	R01_cb18113_c1/f1p0/991
R01_cb10751_c2/f1p0/2547	NA	R01_cb10751_c2/f1p0/2547	NA	NA
R01_cb18260_c1/f1p0/1561	NA	NA	NA	R01_cb18260_c1/f1p0/1561
R01_cb13530_c1/f1p0/694	NA	R01_cb13530_c1/f1p0/694	NA	NA
R01_cb6686_c3/f1p0/2629	NA	R01_cb6686_c3/f1p0/2629	R01_cb6686_c3/f1p0/2629	NA
R01_cb8564_c11169/f1p0/3716	R01_cb8564_c11169/f1p0/3716	R01_cb8564_c11169/f1p0/3716	NA	R01_cb8564_c11169/f1p0/3716
R01_cb18192_c6/f1p0/1332	NA	R01_cb18192_c6/f1p0/1332	R01_cb18192_c6/f1p0/1332	R01_cb18192_c6/f1p0/1332
R01_cb14174_c2/f1p2/1445	NA	NA	NA	R01_cb14174_c2/f1p2/1445
R01_cb3192_c11/f1p0/3371	NA	NA	NA	R01_cb3192_c11/f1p0/3371
R01_cb13821_c1/f1p0/490	NA	NA	R01_cb13821_c1/f1p0/490	R01_cb13821_c1/f1p0/490
R01_cb16443_c0/f4p0/528	NA	R01_cb16443_c0/f4p0/528	NA	NA
R01_cb18010_c1/f1p0/791	NA	R01_cb18010_c1/f1p0/791	R01_cb18010_c1/f1p0/791	R01_cb18010_c1/f1p0/791
R01_cb4342_c1/f2p0/3323	NA	R01_cb4342_c1/f2p0/3323	NA	NA
R01_cb10635_c0/f1p0/1625	R01_cb10635_c0/f1p0/1625	R01_cb10635_c0/f1p0/1625	NA	NA
R01_cb18531_c1/f1p0/705	NA	NA	NA	R01_cb18531_c1/f1p0/705
R01_cb16747_c5/f1p0/1473	NA	NA	NA	R01_cb16747_c5/f1p0/1473
R01_cb9236_c2/f1p0/1808	NA	NA	NA	R01_cb9236_c2/f1p0/1808
R01_cb9922_c1/f1p0/2012	NA	NA	NA	R01_cb9922_c1/f1p0/2012
R01_cb5498_c3/f1p0/2270	R01_cb5498_c3/f1p0/2270	R01_cb5498_c3/f1p0/2270	R01_cb5498_c3/f1p0/2270	R01_cb5498_c3/f1p0/2270

R01_cb17284_c0/f1p0/736	NA	NA	NA	R01_cb17284_c0/f1p0/736
R01_cb10217_c4/f1p1/1685	NA	NA	NA	R01_cb10217_c4/f1p1/1685
R01_cb124_c54/f1p0/3344	NA	NA	NA	R01_cb124_c54/f1p0/3344
R01_cb10769_c2/f1p0/4601	NA	R01_cb10769_c2/f1p0/4601	NA	R01_cb10769_c2/f1p0/4601
R01_cb16981_c5/f1p0/820	NA	NA	NA	R01_cb16981_c5/f1p0/820
R01_cb14704_c24/f1p0/1363	NA	R01_cb14704_c24/f1p0/1363	NA	NA
R01_cb8564_c88368/f1p0/2538	NA	R01_cb8564_c88368/f1p0/2538	R01_cb8564_c88368/f1p0/2538	R01_cb8564_c88368/f1p0/2538
R01_cb12677_c3/f1p0/932	NA	NA	NA	R01_cb12677_c3/f1p0/932
R01_cb8564_c86739/f1p0/3142	NA	NA	NA	R01_cb8564_c86739/f1p0/3142
R01_cb4604_c20/f1p0/3971	NA	R01_cb4604_c20/f1p0/3971	R01_cb4604_c20/f1p0/3971	R01_cb4604_c20/f1p0/3971
R01_cb3862_c64/f1p0/349	R01_cb3862_c64/f1p0/349	R01_cb3862_c64/f1p0/349	R01_cb3862_c64/f1p0/349	R01_cb3862_c64/f1p0/349
R01_cb12972_c22/f1p0/1490	R01_cb12972_c22/f1p0/1490	R01_cb12972_c22/f1p0/1490	R01_cb12972_c22/f1p0/1490	R01_cb12972_c22/f1p0/1490
R01_cb17861_c2/f1p0/1492	NA	NA	NA	R01_cb17861_c2/f1p0/1492
R01_cb4838_c2/f1p0/2549	NA	R01_cb4838_c2/f1p0/2549	NA	R01_cb4838_c2/f1p0/2549
R01_cb8564_c119767/f1p0/1892	NA	NA	NA	R01_cb8564_c119767/f1p0/1892
R01_cb9780_c3/f1p0/2086	NA	NA	NA	R01_cb9780_c3/f1p0/2086
R01_cb6570_c4/f1p0/831	R01_cb6570_c4/f1p0/831	R01_cb6570_c4/f1p0/831	NA	R01_cb6570_c4/f1p0/831
R01_cb8564_c16212/f1p0/3078	NA	R01_cb8564_c16212/f1p0/3078	NA	NA
R01_cb16752_c3/f1p0/1348	NA	NA	NA	R01_cb16752_c3/f1p0/1348
R01_cb8100_c18/f1p0/416	R01_cb8100_c18/f1p0/416	NA	R01_cb8100_c18/f1p0/416	R01_cb8100_c18/f1p0/416
R01_cb3341_c6/f1p0/4031	NA	NA	NA	R01_cb3341_c6/f1p0/4031
R01_cb10572_c1/f2p0/1064	NA	NA	NA	R01_cb10572_c1/f2p0/1064
R01_cb10549_c4/f1p0/1005	NA	R01_cb10549_c4/f1p0/1005	NA	NA
R01_cb16552_c0/f1p0/1144	NA	NA	NA	R01_cb16552_c0/f1p0/1144
R01_cb18456_c6765/f1p0/576	NA	NA	NA	R01_cb18456_c6765/f1p0/576

R01_cb3026_c5/f1p0/2242	NA	R01_cb3026_c5/f1p0/2242	NA	NA
R01_cb8564_c17935/f1p0/3432	NA	R01_cb8564_c17935/f1p0/3432	R01_cb8564_c17935/f1p0/3432	R01_cb8564_c17935/f1p0/3432
R01_cb11315_c3/f1p0/2149	NA	R01_cb11315_c3/f1p0/2149	NA	NA
R01_cb8564_c2876/f1p0/2619	NA	R01_cb8564_c2876/f1p0/2619	NA	NA
R01_cb18456_c7280/f1p0/647	NA	R01_cb18456_c7280/f1p0/647	NA	R01_cb18456_c7280/f1p0/647
R01_cb17299_c2/f1p0/801	NA	NA	NA	R01_cb17299_c2/f1p0/801
R01_cb9818_c3/f1p0/680	NA	NA	NA	R01_cb9818_c3/f1p0/680
R01_cb5172_c6/f1p0/3465	NA	NA	NA	R01_cb5172_c6/f1p0/3465
R01_cb8564_c4093/f1p0/2499	R01_cb8564_c4093/f1p0/2499	R01_cb8564_c4093/f1p0/2499	R01_cb8564_c4093/f1p0/2499	NA
R01_cb18409_c18/f3p0/453	NA	R01_cb18409_c18/f3p0/453	R01_cb18409_c18/f3p0/453	R01_cb18409_c18/f3p0/453
R01_cb8564_c10669/f1p0/3821	NA	R01_cb8564_c10669/f1p0/3821	R01_cb8564_c10669/f1p0/3821	R01_cb8564_c10669/f1p0/3821
R01_cb8564_c23042/f2p0/1986	NA	R01_cb8564_c23042/f2p0/1986	R01_cb8564_c23042/f2p0/1986	R01_cb8564_c23042/f2p0/1986
R01_cb12374_c5/f1p0/746	R01_cb12374_c5/f1p0/746	R01_cb12374_c5/f1p0/746	R01_cb12374_c5/f1p0/746	NA
R01_cb17925_c1/f1p0/1082	NA	R01_cb17925_c1/f1p0/1082	R01_cb17925_c1/f1p0/1082	R01_cb17925_c1/f1p0/1082
R01_cb6874_c8/f1p0/2553	NA	NA	NA	R01_cb6874_c8/f1p0/2553
R01_cb7087_c2/f1p0/2263	NA	NA	NA	R01_cb7087_c2/f1p0/2263
R01_cb10272_c24/f1p0/1559	NA	NA	NA	R01_cb10272_c24/f1p0/1559
R01_cb8564_c9751/f2p0/2617	NA	R01_cb8564_c9751/f2p0/2617	R01_cb8564_c9751/f2p0/2617	R01_cb8564_c9751/f2p0/2617
R01_cb18456_c7562/f1p0/331	R01_cb18456_c7562/f1p0/331	R01_cb18456_c7562/f1p0/331	R01_cb18456_c7562/f1p0/331	R01_cb18456_c7562/f1p0/331
R01_cb1626_c6/f1p1/2824	NA	NA	NA	R01_cb1626_c6/f1p1/2824
R01_cb8062_c2/f1p0/2470	NA	R01_cb8062_c2/f1p0/2470	NA	NA
R01_cb13193_c12/f1p0/579	R01_cb13193_c12/f1p0/579	R01_cb13193_c12/f1p0/579	R01_cb13193_c12/f1p0/579	R01_cb13193_c12/f1p0/579
R01_cb10572_c4/f1p0/1063	NA	NA	NA	R01_cb10572_c4/f1p0/1063
R01_cb14619_c0/f4p0/683	R01_cb14619_c0/f4p0/683	R01_cb14619_c0/f4p0/683	R01_cb14619_c0/f4p0/683	R01_cb14619_c0/f4p0/683
R01_cb15959_c10/f1p0/821	NA	R01_cb15959_c10/f1p0/821	NA	R01_cb15959_c10/f1p0/821
R01_cb454_c24/f1p0/2537	NA	NA	NA	R01_cb454_c24/f1p0/2537

R01_cb18456_c1365/f6p0/483	R01_cb18456_c1365/f6p0/483	R01_cb18456_c1365/f6p0/483	R01_cb18456_c1365/f6p0/483	R01_cb18456_c1365/f6p0/483
R01_cb10880_c1/f6p1/750	NA	NA	NA	R01_cb10880_c1/f6p1/750
R01_cb8564_c37360/f1p0/4555	R01_cb8564_c37360/f1p0/4555	R01_cb8564_c37360/f1p0/4555	R01_cb8564_c37360/f1p0/4555	NA
R01_cb8564_c124644/f1p0/2236	NA	R01_cb8564_c124644/f1p0/2236	R01_cb8564_c124644/f1p0/2236	R01_cb8564_c124644/f1p0/2236
R01_cb4357_c0/f1p2/3359	NA	R01_cb4357_c0/f1p2/3359	NA	NA
R01_cb3054_c1/f1p0/3734	NA	NA	NA	R01_cb3054_c1/f1p0/3734
R01_cb12587_c9/f1p0/1679	NA	NA	NA	R01_cb12587_c9/f1p0/1679
R01_cb8564_c17029/f1p0/3716	NA	NA	R01_cb8564_c17029/f1p0/3716	NA
R01_cb8564_c71174/f1p0/3442	NA	R01_cb8564_c71174/f1p0/3442	R01_cb8564_c71174/f1p0/3442	NA
R01_cb14758_c0/f1p0/1850	NA	R01_cb14758_c0/f1p0/1850	R01_cb14758_c0/f1p0/1850	R01_cb14758_c0/f1p0/1850
R01_cb8564_c50945/f1p0/2016	NA	NA	R01_cb8564_c50945/f1p0/2016	R01_cb8564_c50945/f1p0/2016
R01_cb12579_c6/f1p0/887	R01_cb12579_c6/f1p0/887	R01_cb12579_c6/f1p0/887	R01_cb12579_c6/f1p0/887	R01_cb12579_c6/f1p0/887
R01_cb8564_c13599/f1p0/2571	NA	R01_cb8564_c13599/f1p0/2571	NA	NA
R01_cb8564_c4615/f1p3/3089	NA	R01_cb8564_c4615/f1p3/3089	R01_cb8564_c4615/f1p3/3089	R01_cb8564_c4615/f1p3/3089
R01_cb8564_c81694/f1p0/2065	NA	R01_cb8564_c81694/f1p0/2065	NA	NA
R01_cb10425_c1/f2p1/1163	NA	NA	R01_cb10425_c1/f2p1/1163	R01_cb10425_c1/f2p1/1163
R01_cb15768_c2/f1p0/1269	NA	NA	NA	R01_cb15768_c2/f1p0/1269
R01_cb9983_c1/f1p0/4616	R01_cb9983_c1/f1p0/4616	R01_cb9983_c1/f1p0/4616	R01_cb9983_c1/f1p0/4616	R01_cb9983_c1/f1p0/4616
R01_cb12326_c0/f4p0/522	NA	R01_cb12326_c0/f4p0/522	NA	R01_cb12326_c0/f4p0/522
R01_cb10779_c2/f1p0/681	R01_cb10779_c2/f1p0/681	R01_cb10779_c2/f1p0/681	NA	NA
R01_cb1706_c1/f4p0/3014	NA	NA	NA	R01_cb1706_c1/f4p0/3014
R01_cb2205_c4/f1p1/1715	NA	NA	NA	R01_cb2205_c4/f1p1/1715
R01_cb14019_c3/f1p0/1373	NA	R01_cb14019_c3/f1p0/1373	R01_cb14019_c3/f1p0/1373	R01_cb14019_c3/f1p0/1373
R01_cb11542_c0/f1p0/562	NA	NA	NA	R01_cb11542_c0/f1p0/562

R01_cb8449_c1/f2p2/2097	NA	NA	NA	R01_cb8449_c1/f2p2/2097
R01_cb8564_c49888/f1p0/2560	NA	R01_cb8564_c49888/f1p0/2560	NA	R01_cb8564_c49888/f1p0/2560
R01_cb13334_c7/f1p0/340	NA	R01_cb13334_c7/f1p0/340	NA	R01_cb13334_c7/f1p0/340
R01_cb3808_c3/f1p1/2690	NA	NA	NA	R01_cb3808_c3/f1p1/2690
R01_cb9502_c0/f1p0/2066	NA	NA	NA	R01_cb9502_c0/f1p0/2066
R01_cb17633_c0/f1p0/1362	NA	R01_cb17633_c0/f1p0/1362	NA	NA
R01_cb11975_c4/f1p1/1091	NA	R01_cb11975_c4/f1p1/1091	R01_cb11975_c4/f1p1/1091	R01_cb11975_c4/f1p1/1091
R01_cb14739_c1/f1p1/676	NA	R01_cb14739_c1/f1p1/676	NA	R01_cb14739_c1/f1p1/676
R01_cb8564_c125834/f1p0/1917	NA	R01_cb8564_c125834/f1p0/1917	NA	NA
R01_cb5190_c10/f1p0/1874	NA	NA	NA	R01_cb5190_c10/f1p0/1874
R01_cb11479_c1/f1p0/2264	NA	NA	NA	R01_cb11479_c1/f1p0/2264
R01_cb6111_c27/f1p0/1495	NA	R01_cb6111_c27/f1p0/1495	NA	NA
R01_cb7248_c2/f1p0/1330	NA	NA	NA	R01_cb7248_c2/f1p0/1330
R01_cb3695_c2/f1p1/2846	NA	NA	NA	R01_cb3695_c2/f1p1/2846
R01_cb8324_c4/f1p0/2447	NA	NA	R01_cb8324_c4/f1p0/2447	R01_cb8324_c4/f1p0/2447
R01_cb4870_c0/f1p0/3229	NA	R01_cb4870_c0/f1p0/3229	NA	NA
R01_cb7191_c19/f1p0/724	NA	R01_cb7191_c19/f1p0/724	NA	NA
R01_cb13478_c16/f5p1/508	NA	R01_cb13478_c16/f5p1/508	R01_cb13478_c16/f5p1/508	R01_cb13478_c16/f5p1/508
R01_cb9432_c0/f2p0/2062	NA	NA	NA	R01_cb9432_c0/f2p0/2062
R01_cb8564_c53118/f1p0/2515	NA	NA	NA	R01_cb8564_c53118/f1p0/2515
R01_cb8564_c117861/f1p0/2243	NA	R01_cb8564_c117861/f1p0/2243	NA	NA
R01_cb11483_c0/f1p0/1580	NA	NA	NA	R01_cb11483_c0/f1p0/1580
R01_cb8564_c91900/f1p0/3885	NA	NA	R01_cb8564_c91900/f1p0/3885	NA
R01_cb14985_c3/f1p0/1302	NA	NA	NA	R01_cb14985_c3/f1p0/1302

R01_cb8564_c53630/f1p0/2621	NA	R01_cb8564_c53630/f1p0/2621	R01_cb8564_c53630/f1p0/2621	R01_cb8564_c53630/f1p0/2621
R01_cb14702_c4/f1p0/535	NA	R01_cb14702_c4/f1p0/535	NA	NA
R01_cb3526_c9/f1p0/2691	NA	NA	NA	R01_cb3526_c9/f1p0/2691
R01_cb8564_c69796/f1p0/2630	NA	R01_cb8564_c69796/f1p0/2630	NA	NA
R01_cb13217_c4/f1p0/1250	NA	R01_cb13217_c4/f1p0/1250	R01_cb13217_c4/f1p0/1250	R01_cb13217_c4/f1p0/1250
R01_cb8564_c82514/f1p0/2202	NA	R01_cb8564_c82514/f1p0/2202	NA	R01_cb8564_c82514/f1p0/2202
R01_cb18010_c0/f1p0/747	NA	R01_cb18010_c0/f1p0/747	R01_cb18010_c0/f1p0/747	R01_cb18010_c0/f1p0/747
R01_cb16293_c14/f1p0/921	NA	R01_cb16293_c14/f1p0/921	NA	R01_cb16293_c14/f1p0/921
R01_cb6332_c3/f1p0/2116	NA	R01_cb6332_c3/f1p0/2116	NA	R01_cb6332_c3/f1p0/2116
R01_cb11193_c1/f1p0/2732	NA	R01_cb11193_c1/f1p0/2732	NA	NA
R01_cb6081_c2/f1p0/2993	R01_cb6081_c2/f1p0/2993	R01_cb6081_c2/f1p0/2993	R01_cb6081_c2/f1p0/2993	R01_cb6081_c2/f1p0/2993
R01_cb8564_c90226/f1p0/2290	R01_cb8564_c90226/f1p0/2290	R01_cb8564_c90226/f1p0/2290	R01_cb8564_c90226/f1p0/2290	R01_cb8564_c90226/f1p0/2290
R01_cb12785_c16/f1p0/1203	NA	NA	R01_cb12785_c16/f1p0/1203	NA
R01_cb5676_c4/f1p0/2818	NA	NA	NA	R01_cb5676_c4/f1p0/2818
R01_cb11032_c1/f1p0/2619	NA	NA	NA	R01_cb11032_c1/f1p0/2619
R01_cb2302_c3/f1p0/4027	NA	R01_cb2302_c3/f1p0/4027	NA	NA
R01_cb6393_c6/f1p0/2088	NA	NA	NA	R01_cb6393_c6/f1p0/2088
R01_cb14709_c16/f1p0/1225	NA	R01_cb14709_c16/f1p0/1225	NA	NA
R01_cb13652_c0/f2p1/1268	NA	R01_cb13652_c0/f2p1/1268	NA	NA
R01_cb17997_c8/f9p1/1015	NA	R01_cb17997_c8/f9p1/1015	NA	R01_cb17997_c8/f9p1/1015
R01_cb261_c45/f1p0/1957	NA	NA	NA	R01_cb261_c45/f1p0/1957
R01_cb6718_c6/f1p0/2064	NA	R01_cb6718_c6/f1p0/2064	NA	R01_cb6718_c6/f1p0/2064
R01_cb8564_c46752/f1p0/1940	NA	NA	NA	R01_cb8564_c46752/f1p0/1940
R01_cb8037_c6/f1p0/1299	NA	NA	NA	R01_cb8037_c6/f1p0/1299
R01_cb6365_c20/f1p0/777	R01_cb6365_c20/f1p0/777	R01_cb6365_c20/f1p0/777	R01_cb6365_c20/f1p0/777	R01_cb6365_c20/f1p0/777

R01_cb4370_c3/f1p0/3357	NA	NA	NA	R01_cb4370_c3/f1p0/3357
R01_cb9512_c9/f1p0/2068	NA	R01_cb9512_c9/f1p0/2068	NA	NA
R01_cb352_c3/f1p0/2624	NA	NA	NA	R01_cb352_c3/f1p0/2624
R01_cb7962_c3/f2p0/2206	NA	NA	NA	R01_cb7962_c3/f2p0/2206
R01_cb10049_c1/f1p0/2124	R01_cb10049_c1/f1p0/2124	R01_cb10049_c1/f1p0/2124	R01_cb10049_c1/f1p0/2124	R01_cb10049_c1/f1p0/2124
R01_cb5287_c8/f1p0/1706	NA	R01_cb5287_c8/f1p0/1706	NA	NA
R01_cb11081_c3/f1p0/2748	NA	NA	R01_cb11081_c3/f1p0/2748	R01_cb11081_c3/f1p0/2748
R01_cb18287_c2/f1p0/1786	NA	NA	R01_cb18287_c2/f1p0/1786	NA
R01_cb8564_c111232/f1p0/2097	NA	NA	R01_cb8564_c111232/f1p0/2097	R01_cb8564_c111232/f1p0/2097
R01_cb15514_c1/f1p0/1477	NA	R01_cb15514_c1/f1p0/1477	R01_cb15514_c1/f1p0/1477	R01_cb15514_c1/f1p0/1477
R01_cb9993_c1/f1p0/1883	NA	R01_cb9993_c1/f1p0/1883	NA	R01_cb9993_c1/f1p0/1883
R01_cb3079_c4/f1p0/2325	NA	R01_cb3079_c4/f1p0/2325	NA	R01_cb3079_c4/f1p0/2325
R01_cb14316_c20/f1p0/1079	NA	NA	R01_cb14316_c20/f1p0/1079	R01_cb14316_c20/f1p0/1079
R01_cb13910_c12/f1p0/1443	NA	R01_cb13910_c12/f1p0/1443	R01_cb13910_c12/f1p0/1443	R01_cb13910_c12/f1p0/1443
R01_cb9985_c1/f1p0/2529	R01_cb9985_c1/f1p0/2529	R01_cb9985_c1/f1p0/2529	NA	NA
R01_cb11844_c1/f1p0/1879	R01_cb11844_c1/f1p0/1879	R01_cb11844_c1/f1p0/1879	R01_cb11844_c1/f1p0/1879	R01_cb11844_c1/f1p0/1879
R01_cb10873_c4/f1p0/1374	NA	NA	NA	R01_cb10873_c4/f1p0/1374
R01_cb8564_c90205/f1p0/2522	R01_cb8564_c90205/f1p0/2522	R01_cb8564_c90205/f1p0/2522	NA	R01_cb8564_c90205/f1p0/2522
R01_cb6204_c6/f1p1/2162	NA	NA	NA	R01_cb6204_c6/f1p1/2162
R01_cb14337_c6/f1p0/978	R01_cb14337_c6/f1p0/978	R01_cb14337_c6/f1p0/978	R01_cb14337_c6/f1p0/978	R01_cb14337_c6/f1p0/978
R01_cb1016_c2/f1p0/4525	NA	R01_cb1016_c2/f1p0/4525	NA	NA
R01_cb119_c9/f1p0/4967	NA	NA	NA	R01_cb119_c9/f1p0/4967
R01_cb10720_c2/f1p1/1093	NA	NA	NA	R01_cb10720_c2/f1p1/1093
R01_cb8692_c9/f1p0/1475	R01_cb8692_c9/f1p0/1475	R01_cb8692_c9/f1p0/1475	R01_cb8692_c9/f1p0/1475	R01_cb8692_c9/f1p0/1475

R01_cb7964_c1/f2p2/2299	NA	NA	NA	R01_cb7964_c1/f2p2/2299
R01_cb6802_c22/f1p1/2325	NA	R01_cb6802_c22/f1p1/2325	NA	NA
R01_cb12096_c0/f1p0/770	R01_cb12096_c0/f1p0/770	R01_cb12096_c0/f1p0/770	R01_cb12096_c0/f1p0/770	R01_cb12096_c0/f1p0/770
R01_cb3390_c1/f2p0/1899	NA	R01_cb3390_c1/f2p0/1899	NA	NA
R01_cb9351_c1/f1p0/1917	NA	R01_cb9351_c1/f1p0/1917	R01_cb9351_c1/f1p0/1917	NA
R01_cb14067_c4/f1p0/1862	NA	NA	R01_cb14067_c4/f1p0/1862	R01_cb14067_c4/f1p0/1862
R01_cb8564_c3079/f1p0/2671	R01_cb8564_c3079/f1p0/2671	R01_cb8564_c3079/f1p0/2671	NA	NA
R01_cb15168_c3/f1p0/1481	NA	NA	NA	R01_cb15168_c3/f1p0/1481
R01_cb8564_c43594/f1p1/3729	NA	R01_cb8564_c43594/f1p1/3729	R01_cb8564_c43594/f1p1/3729	R01_cb8564_c43594/f1p1/3729
R01_cb8564_c77641/f1p0/3884	NA	R01_cb8564_c77641/f1p0/3884	NA	NA
R01_cb18632_c11/f1p0/5362	NA	NA	NA	R01_cb18632_c11/f1p0/5362
R01_cb8564_c80249/f1p0/3633	NA	R01_cb8564_c80249/f1p0/3633	NA	R01_cb8564_c80249/f1p0/3633
R01_cb12427_c3/f1p0/1826	NA	R01_cb12427_c3/f1p0/1826	R01_cb12427_c3/f1p0/1826	R01_cb12427_c3/f1p0/1826
R01_cb8564_c86656/f1p0/3356	NA	NA	NA	R01_cb8564_c86656/f1p0/3356
R01_cb6_c8/f1p0/3736	NA	R01_cb6_c8/f1p0/3736	NA	NA
R01_cb9610_c1/f1p0/2045	NA	NA	NA	R01_cb9610_c1/f1p0/2045
R01_cb16956_c1/f1p0/696	NA	R01_cb16956_c1/f1p0/696	R01_cb16956_c1/f1p0/696	R01_cb16956_c1/f1p0/696
R01_cb17354_c3/f1p0/1686	NA	NA	NA	R01_cb17354_c3/f1p0/1686
R01_cb5717_c2/f1p0/4037	NA	NA	NA	R01_cb5717_c2/f1p0/4037
R01_cb18456_c1654/f2p0/898	NA	NA	NA	R01_cb18456_c1654/f2p0/898
R01_cb8564_c3743/f1p1/3652	NA	R01_cb8564_c3743/f1p1/3652	R01_cb8564_c3743/f1p1/3652	R01_cb8564_c3743/f1p1/3652
R01_cb8564_c125560/f1p0/2254	R01_cb8564_c125560/f1p0/2254	R01_cb8564_c125560/f1p0/2254	R01_cb8564_c125560/f1p0/2254	R01_cb8564_c125560/f1p0/2254
R01_cb3641_c0/f3p0/623	R01_cb3641_c0/f3p0/623	R01_cb3641_c0/f3p0/623	NA	NA
R01_cb8725_c0/f1p0/2288	NA	R01_cb8725_c0/f1p0/2288	NA	NA
R01_cb8564_c126861/f1p0/203	NA	R01_cb8564_c126861/f1p0/203	R01_cb8564_c126861/f1p0/203	R01_cb8564_c126861/f1p0/203

9		9	9	9
R01_cb124_c29/f1p0/2489	NA	NA	NA	R01_cb124_c29/f1p0/2489
R01_cb12421_c23/f1p0/561	NA	R01_cb12421_c23/f1p0/561	NA	NA
R01_cb438_c14/f11p1/825	NA	NA	NA	R01_cb438_c14/f11p1/825
R01_cb6446_c14/f7p0/600	NA	R01_cb6446_c14/f7p0/600	NA	NA
R01_cb15102_c2/f1p0/764	NA	NA	NA	R01_cb15102_c2/f1p0/764
R01_cb15562_c4/f1p0/1499	NA	R01_cb15562_c4/f1p0/1499	NA	R01_cb15562_c4/f1p0/1499
R01_cb10659_c5/f1p0/766	R01_cb10659_c5/f1p0/766	R01_cb10659_c5/f1p0/766	NA	R01_cb10659_c5/f1p0/766
R01_cb506_c1/f1p0/4782	NA	NA	NA	R01_cb506_c1/f1p0/4782
R01_cb1938_c0/f2p0/2341	NA	NA	NA	R01_cb1938_c0/f2p0/2341
R01_cb18456_c6117/f1p0/696	NA	NA	NA	R01_cb18456_c6117/f1p0/696
R01_cb8281_c7/f1p0/2303	NA	NA	NA	R01_cb8281_c7/f1p0/2303
R01_cb2494_c3/f15p0/975	R01_cb2494_c3/f15p0/975	R01_cb2494_c3/f15p0/975	R01_cb2494_c3/f15p0/975	R01_cb2494_c3/f15p0/975
R01_cb3426_c20/f1p0/3131	NA	NA	NA	R01_cb3426_c20/f1p0/3131
R01_cb6286_c10/f1p1/2556	NA	NA	NA	R01_cb6286_c10/f1p1/2556
R01_cb16769_c0/f1p0/664	NA	R01_cb16769_c0/f1p0/664	R01_cb16769_c0/f1p0/664	R01_cb16769_c0/f1p0/664
R01_cb17458_c2/f1p0/779	NA	NA	NA	R01_cb17458_c2/f1p0/779
R01_cb8564_c82850/f1p0/2031	NA	R01_cb8564_c82850/f1p0/2031	NA	NA
R01_cb8564_c1109/f1p0/2071	NA	R01_cb8564_c1109/f1p0/2071	NA	R01_cb8564_c1109/f1p0/2071
R01_cb3872_c2/f1p1/623	NA	R01_cb3872_c2/f1p1/623	NA	R01_cb3872_c2/f1p1/623
R01_cb2314_c4/f1p0/3457	NA	R01_cb2314_c4/f1p0/3457	NA	NA
R01_cb8564_c16081/f1p0/3057	R01_cb8564_c16081/f1p0/3057	R01_cb8564_c16081/f1p0/3057	NA	R01_cb8564_c16081/f1p0/3057
R01_cb6356_c5/f1p0/1477	NA	R01_cb6356_c5/f1p0/1477	R01_cb6356_c5/f1p0/1477	R01_cb6356_c5/f1p0/1477
R01_cb6823_c1/f1p0/2285	NA	NA	NA	R01_cb6823_c1/f1p0/2285
R01_cb5565_c60/f1p0/2829	NA	R01_cb5565_c60/f1p0/2829	NA	NA

R01_cb6982_c0/f2p0/2725	NA	R01_cb6982_c0/f2p0/2725	R01_cb6982_c0/f2p0/2725	NA
R01_cb18456_c1326/f2p0/510	R01_cb18456_c1326/f2p0/510	R01_cb18456_c1326/f2p0/510	R01_cb18456_c1326/f2p0/510	R01_cb18456_c1326/f2p0/510
R01_cb18456_c6454/f1p0/992	R01_cb18456_c6454/f1p0/992	R01_cb18456_c6454/f1p0/992	NA	R01_cb18456_c6454/f1p0/992
R01_cb8564_c80568/f1p0/3614	NA	NA	NA	R01_cb8564_c80568/f1p0/3614
R01_cb9380_c11/f2p0/1933	NA	NA	NA	R01_cb9380_c11/f2p0/1933
R01_cb15023_c0/f2p0/1549	NA	NA	NA	R01_cb15023_c0/f2p0/1549
R01_cb10420_c7/f1p0/1848	NA	NA	NA	R01_cb10420_c7/f1p0/1848
R01_cb2950_c15/f1p0/606	NA	R01_cb2950_c15/f1p0/606	NA	R01_cb2950_c15/f1p0/606
R01_cb13426_c2/f1p0/924	NA	R01_cb13426_c2/f1p0/924	NA	NA
R01_cb1813_c9/f1p0/2541	NA	NA	NA	R01_cb1813_c9/f1p0/2541
R01_cb10015_c307/f1p3/635	R01_cb10015_c307/f1p3/635	R01_cb10015_c307/f1p3/635	R01_cb10015_c307/f1p3/635	NA
R01_cb7738_c4/f1p0/2536	NA	NA	NA	R01_cb7738_c4/f1p0/2536
R01_cb6906_c5/f1p1/4379	NA	R01_cb6906_c5/f1p1/4379	R01_cb6906_c5/f1p1/4379	R01_cb6906_c5/f1p1/4379
R01_cb763_c9/f1p0/942	R01_cb763_c9/f1p0/942	R01_cb763_c9/f1p0/942	R01_cb763_c9/f1p0/942	R01_cb763_c9/f1p0/942
R01_cb10323_c2/f1p0/2774	NA	NA	R01_cb10323_c2/f1p0/2774	NA
R01_cb15458_c2/f1p0/1574	NA	R01_cb15458_c2/f1p0/1574	NA	R01_cb15458_c2/f1p0/1574
R01_cb8564_c17244/f1p0/3238	NA	NA	NA	R01_cb8564_c17244/f1p0/3238
R01_cb18456_c7238/f1p0/632	NA	R01_cb18456_c7238/f1p0/632	R01_cb18456_c7238/f1p0/632	R01_cb18456_c7238/f1p0/632
R01_cb5900_c50/f1p0/3583	R01_cb5900_c50/f1p0/3583	R01_cb5900_c50/f1p0/3583	NA	NA
R01_cb9423_c1/f1p0/3076	R01_cb9423_c1/f1p0/3076	R01_cb9423_c1/f1p0/3076	NA	R01_cb9423_c1/f1p0/3076
R01_cb11085_c0/f1p0/1465	NA	NA	NA	R01_cb11085_c0/f1p0/1465
R01_cb8564_c122381/f1p0/2410	NA	R01_cb8564_c122381/f1p0/2410	R01_cb8564_c122381/f1p0/2410	R01_cb8564_c122381/f1p0/2410
R01_cb3071_c1/f1p0/3725	NA	R01_cb3071_c1/f1p0/3725	NA	R01_cb3071_c1/f1p0/3725
R01_cb4038_c3/f1p0/2343	NA	R01_cb4038_c3/f1p0/2343	NA	NA
R01_cb18456_c1884/f1p0/561	R01_cb18456_c1884/f1p0/561	R01_cb18456_c1884/f1p0/561	R01_cb18456_c1884/f1p0/561	R01_cb18456_c1884/f1p0/561

R01_cb16098_c4/f1p0/754	NA	R01_cb16098_c4/f1p0/754	R01_cb16098_c4/f1p0/754	R01_cb16098_c4/f1p0/754
R01_cb8564_c122234/f1p1/2786	NA	R01_cb8564_c122234/f1p1/2786	NA	NA
R01_cb8564_c43113/f1p0/4042	NA	R01_cb8564_c43113/f1p0/4042	NA	R01_cb8564_c43113/f1p0/4042
R01_cb14146_c3/f1p0/1425	NA	NA	NA	R01_cb14146_c3/f1p0/1425
R01_cb16739_c4/f2p0/703	NA	NA	NA	R01_cb16739_c4/f2p0/703
R01_cb18208_c0/f2p0/1087	NA	R01_cb18208_c0/f2p0/1087	R01_cb18208_c0/f2p0/1087	R01_cb18208_c0/f2p0/1087
R01_cb2252_c1/f1p0/4037	NA	NA	NA	R01_cb2252_c1/f1p0/4037
R01_cb8564_c122395/f1p0/2229	NA	NA	NA	R01_cb8564_c122395/f1p0/2229
R01_cb16027_c7/f1p0/969	NA	R01_cb16027_c7/f1p0/969	NA	NA
R01_cb15797_c3/f1p0/1081	NA	NA	NA	R01_cb15797_c3/f1p0/1081
R01_cb10291_c3/f1p0/1527	NA	R01_cb10291_c3/f1p0/1527	NA	R01_cb10291_c3/f1p0/1527
R01_cb10246_c4/f1p1/2420	NA	R01_cb10246_c4/f1p1/2420	NA	NA
R01_cb709_c19/f1p0/1584	R01_cb709_c19/f1p0/1584	R01_cb709_c19/f1p0/1584	R01_cb709_c19/f1p0/1584	R01_cb709_c19/f1p0/1584
R01_cb7178_c2/f1p0/2582	NA	R01_cb7178_c2/f1p0/2582	NA	NA
R01_cb17986_c1/f1p0/1442	NA	NA	NA	R01_cb17986_c1/f1p0/1442
R01_cb4708_c6/f1p0/2403	NA	R01_cb4708_c6/f1p0/2403	NA	NA
R01_cb709_c5/f1p0/3139	NA	NA	R01_cb709_c5/f1p0/3139	R01_cb709_c5/f1p0/3139
R01_cb4825_c12/f1p0/4476	NA	NA	NA	R01_cb4825_c12/f1p0/4476
R01_cb12057_c34/f1p1/1327	NA	R01_cb12057_c34/f1p1/1327	NA	NA
R01_cb3602_c7/f1p2/2820	NA	R01_cb3602_c7/f1p2/2820	NA	NA
R01_cb14747_c0/f2p0/1174	NA	R01_cb14747_c0/f2p0/1174	R01_cb14747_c0/f2p0/1174	R01_cb14747_c0/f2p0/1174
R01_cb2770_c64/f1p0/2189	NA	R01_cb2770_c64/f1p0/2189	R01_cb2770_c64/f1p0/2189	R01_cb2770_c64/f1p0/2189
R01_cb5633_c81/f1p0/2727	NA	R01_cb5633_c81/f1p0/2727	NA	NA
R01_cb8564_c109312/f6p1/297	R01_cb8564_c109312/f6p1/29	R01_cb8564_c109312/f6p1/297	R01_cb8564_c109312/f6p1/297	R01_cb8564_c109312/f6p1/297

8	78	8	8	8
R01_cb3000_c1/f1p0/2248	NA	R01_cb3000_c1/f1p0/2248	R01_cb3000_c1/f1p0/2248	R01_cb3000_c1/f1p0/2248
R01_cb3309_c11/f1p0/1951	NA	R01_cb3309_c11/f1p0/1951	NA	NA
R01_cb4368_c11/f1p0/1240	NA	NA	NA	R01_cb4368_c11/f1p0/1240
R01_cb6615_c19/f1p0/1446	NA	NA	NA	R01_cb6615_c19/f1p0/1446
R01_cb8564_c87244/f1p0/4403	NA	NA	NA	R01_cb8564_c87244/f1p0/4403
R01_cb16657_c2/f1p0/854	NA	NA	NA	R01_cb16657_c2/f1p0/854
R01_cb7948_c0/f2p1/2493	NA	NA	NA	R01_cb7948_c0/f2p1/2493
R01_cb8564_c110681/f1p0/2716	R01_cb8564_c110681/f1p0/2716	R01_cb8564_c110681/f1p0/2716	NA	NA
R01_cb5943_c5/f1p0/1383	NA	NA	NA	R01_cb5943_c5/f1p0/1383
R01_cb4242_c1/f1p0/3142	NA	R01_cb4242_c1/f1p0/3142	NA	NA
R01_cb18456_c7489/f1p0/1815	NA	R01_cb18456_c7489/f1p0/1815	R01_cb18456_c7489/f1p0/1815	NA
R01_cb8564_c1210/f1p0/2120	NA	R01_cb8564_c1210/f1p0/2120	NA	NA
R01_cb18409_c13/f5p0/652	NA	R01_cb18409_c13/f5p0/652	R01_cb18409_c13/f5p0/652	R01_cb18409_c13/f5p0/652
R01_cb7693_c2/f1p0/2493	NA	NA	R01_cb7693_c2/f1p0/2493	R01_cb7693_c2/f1p0/2493
R01_cb2095_c14/f1p0/4037	NA	NA	NA	R01_cb2095_c14/f1p0/4037
R01_cb11102_c3/f1p0/1143	NA	NA	NA	R01_cb11102_c3/f1p0/1143
R01_cb12852_c4/f1p0/1014	NA	NA	R01_cb12852_c4/f1p0/1014	R01_cb12852_c4/f1p0/1014
R01_cb12769_c0/f2p0/1303	NA	NA	NA	R01_cb12769_c0/f2p0/1303
R01_cb5896_c158/f1p0/2497	NA	NA	NA	R01_cb5896_c158/f1p0/2497
R01_cb7595_c18/f1p0/771	R01_cb7595_c18/f1p0/771	R01_cb7595_c18/f1p0/771	R01_cb7595_c18/f1p0/771	R01_cb7595_c18/f1p0/771
R01_cb8171_c5/f1p0/395	NA	R01_cb8171_c5/f1p0/395	NA	NA
R01_cb8345_c2/f1p0/2284	NA	NA	NA	R01_cb8345_c2/f1p0/2284
R01_cb8564_c2026/f1p0/3142	NA	R01_cb8564_c2026/f1p0/3142	NA	NA
R01_cb2959_c1/f1p0/3734	NA	NA	NA	R01_cb2959_c1/f1p0/3734

R01_cb13363_c1/f1p0/1680	NA	R01_cb13363_c1/f1p0/1680	NA	NA
R01_cb10392_c2/f1p0/797	NA	R01_cb10392_c2/f1p0/797	NA	NA
R01_cb13334_c5/f1p0/1152	NA	R01_cb13334_c5/f1p0/1152	NA	NA
R01_cb12442_c4/f1p0/1027	NA	R01_cb12442_c4/f1p0/1027	NA	R01_cb12442_c4/f1p0/1027
R01_cb16794_c2/f1p0/868	R01_cb16794_c2/f1p0/868	R01_cb16794_c2/f1p0/868	R01_cb16794_c2/f1p0/868	R01_cb16794_c2/f1p0/868
R01_cb8564_c37627/f1p0/2498	NA	R01_cb8564_c37627/f1p0/2498	NA	NA
R01_cb8564_c71355/f1p0/2540	NA	R01_cb8564_c71355/f1p0/2540	R01_cb8564_c71355/f1p0/2540	R01_cb8564_c71355/f1p0/2540
R01_cb8564_c18383/f1p1/3801	NA	R01_cb8564_c18383/f1p1/3801	NA	NA
R01_cb13203_c3/f1p1/943	NA	R01_cb13203_c3/f1p1/943	NA	NA
R01_cb8793_c1/f1p0/540	R01_cb8793_c1/f1p0/540	R01_cb8793_c1/f1p0/540	R01_cb8793_c1/f1p0/540	R01_cb8793_c1/f1p0/540
R01_cb9846_c1/f1p0/2044	NA	R01_cb9846_c1/f1p0/2044	R01_cb9846_c1/f1p0/2044	R01_cb9846_c1/f1p0/2044
R01_cb4501_c5/f1p0/3632	NA	NA	NA	R01_cb4501_c5/f1p0/3632
R01_cb7257_c4/f1p0/2327	NA	NA	NA	R01_cb7257_c4/f1p0/2327
R01_cb8564_c41939/f1p0/2349	NA	R01_cb8564_c41939/f1p0/2349	NA	NA
R01_cb12785_c12/f1p0/738	NA	R01_cb12785_c12/f1p0/738	R01_cb12785_c12/f1p0/738	R01_cb12785_c12/f1p0/738
R01_cb8564_c80794/f1p0/2427	R01_cb8564_c80794/f1p0/2427	R01_cb8564_c80794/f1p0/2427	R01_cb8564_c80794/f1p0/2427	R01_cb8564_c80794/f1p0/2427
R01_cb15101_c4/f1p0/424	NA	NA	R01_cb15101_c4/f1p0/424	R01_cb15101_c4/f1p0/424
R01_cb6464_c5/f1p0/2806	NA	NA	NA	R01_cb6464_c5/f1p0/2806
R01_cb17582_c1/f1p0/1745	NA	R01_cb17582_c1/f1p0/1745	R01_cb17582_c1/f1p0/1745	R01_cb17582_c1/f1p0/1745
R01_cb8914_c7/f1p0/2021	NA	R01_cb8914_c7/f1p0/2021	NA	NA
R01_cb12352_c11/f1p0/505	R01_cb12352_c11/f1p0/505	R01_cb12352_c11/f1p0/505	R01_cb12352_c11/f1p0/505	R01_cb12352_c11/f1p0/505
R01_cb8564_c87286/f1p0/2748	R01_cb8564_c87286/f1p0/2748	R01_cb8564_c87286/f1p0/2748	R01_cb8564_c87286/f1p0/2748	R01_cb8564_c87286/f1p0/2748
R01_cb16766_c1/f1p0/992	NA	NA	NA	R01_cb16766_c1/f1p0/992
R01_cb12119_c11/f1p0/945	NA	NA	NA	R01_cb12119_c11/f1p0/945

R01_cb16749_c6/f1p0/862	R01_cb16749_c6/f1p0/862	R01_cb16749_c6/f1p0/862	NA	NA
R01_cb8564_c24102/f1p0/2869	NA	R01_cb8564_c24102/f1p0/2869	NA	NA
R01_cb17756_c5/f2p0/334	R01_cb17756_c5/f2p0/334	R01_cb17756_c5/f2p0/334	R01_cb17756_c5/f2p0/334	R01_cb17756_c5/f2p0/334
R01_cb8564_c128307/f1p0/2776	NA	R01_cb8564_c128307/f1p0/2776	R01_cb8564_c128307/f1p0/2776	R01_cb8564_c128307/f1p0/2776
R01_cb13979_c28/f1p0/580	NA	R01_cb13979_c28/f1p0/580	R01_cb13979_c28/f1p0/580	R01_cb13979_c28/f1p0/580
R01_cb4708_c20/f1p0/613	NA	NA	R01_cb4708_c20/f1p0/613	R01_cb4708_c20/f1p0/613
R01_cb11932_c21/f1p0/360	R01_cb11932_c21/f1p0/360	R01_cb11932_c21/f1p0/360	R01_cb11932_c21/f1p0/360	R01_cb11932_c21/f1p0/360
R01_cb1827_c1/f1p0/3991	NA	R01_cb1827_c1/f1p0/3991	R01_cb1827_c1/f1p0/3991	NA
R01_cb2033_c13/f1p0/2928	NA	NA	R01_cb2033_c13/f1p0/2928	R01_cb2033_c13/f1p0/2928
R01_cb2851_c12/f1p0/2159	NA	NA	NA	R01_cb2851_c12/f1p0/2159
R01_cb8564_c125339/f1p1/2645	NA	R01_cb8564_c125339/f1p1/2645	NA	NA
R01_cb18727_c0/f1p0/5051	NA	NA	NA	R01_cb18727_c0/f1p0/5051
R01_cb18456_c7314/f1p0/887	NA	NA	R01_cb18456_c7314/f1p0/887	R01_cb18456_c7314/f1p0/887
R01_cb8564_c71990/f1p0/2407	NA	R01_cb8564_c71990/f1p0/2407	NA	NA
R01_cb2204_c4/f1p0/3794	R01_cb2204_c4/f1p0/3794	R01_cb2204_c4/f1p0/3794	R01_cb2204_c4/f1p0/3794	R01_cb2204_c4/f1p0/3794
R01_cb1178_c29/f1p0/1155	NA	NA	NA	R01_cb1178_c29/f1p0/1155
R01_cb15207_c115/f1p4/1059	NA	R01_cb15207_c115/f1p4/1059	NA	NA
R01_cb12634_c2/f1p0/4959	NA	R01_cb12634_c2/f1p0/4959	NA	NA
R01_cb3219_c0/f2p0/629	NA	NA	NA	R01_cb3219_c0/f2p0/629
R01_cb18739_c1/f1p0/2916	NA	R01_cb18739_c1/f1p0/2916	R01_cb18739_c1/f1p0/2916	R01_cb18739_c1/f1p0/2916
R01_cb8786_c0/f4p0/2072	NA	NA	NA	R01_cb8786_c0/f4p0/2072
R01_cb9552_c3/f1p0/2100	NA	NA	NA	R01_cb9552_c3/f1p0/2100
R01_cb8564_c4737/f1p0/2587	NA	R01_cb8564_c4737/f1p0/2587	NA	R01_cb8564_c4737/f1p0/2587
R01_cb10107_c2/f1p0/1456	NA	NA	NA	R01_cb10107_c2/f1p0/1456

R01_cb8564_c73405/f1p0/3260	NA	R01_cb8564_c73405/f1p0/3260	R01_cb8564_c73405/f1p0/3260	R01_cb8564_c73405/f1p0/3260
R01_cb16888_c2/f1p0/984	NA	NA	R01_cb16888_c2/f1p0/984	R01_cb16888_c2/f1p0/984
R01_cb8564_c83831/f1p0/4086	NA	R01_cb8564_c83831/f1p0/4086	NA	NA
R01_cb11295_c1/f1p0/2991	R01_cb11295_c1/f1p0/2991	R01_cb11295_c1/f1p0/2991	R01_cb11295_c1/f1p0/2991	R01_cb11295_c1/f1p0/2991
R01_cb6682_c11/f1p0/671	NA	R01_cb6682_c11/f1p0/671	NA	R01_cb6682_c11/f1p0/671
R01_cb8975_c2/f3p0/2010	NA	NA	NA	R01_cb8975_c2/f3p0/2010
R01_cb1378_c13/f1p0/682	NA	R01_cb1378_c13/f1p0/682	R01_cb1378_c13/f1p0/682	R01_cb1378_c13/f1p0/682
R01_cb8564_c13232/f1p0/3746	R01_cb8564_c13232/f1p0/3746	R01_cb8564_c13232/f1p0/3746	R01_cb8564_c13232/f1p0/3746	R01_cb8564_c13232/f1p0/3746
R01_cb8380_c1/f1p0/2382	R01_cb8380_c1/f1p0/2382	NA	NA	NA
R01_cb18147_c0/f1p0/412	R01_cb18147_c0/f1p0/412	R01_cb18147_c0/f1p0/412	R01_cb18147_c0/f1p0/412	R01_cb18147_c0/f1p0/412
R01_cb8564_c24544/f3p2/3517	NA	NA	NA	R01_cb8564_c24544/f3p2/3517
R01_cb4944_c3/f1p1/3219	NA	R01_cb4944_c3/f1p1/3219	NA	NA
R01_cb1435_c6/f1p0/1800	NA	NA	NA	R01_cb1435_c6/f1p0/1800
R01_cb5927_c5/f1p0/3187	NA	R01_cb5927_c5/f1p0/3187	NA	NA
R01_cb8564_c794/f2p1/3802	NA	R01_cb8564_c794/f2p1/3802	R01_cb8564_c794/f2p1/3802	R01_cb8564_c794/f2p1/3802
R01_cb6033_c1/f1p0/2556	NA	NA	NA	R01_cb6033_c1/f1p0/2556
R01_cb15485_c0/f1p0/1744	NA	NA	NA	R01_cb15485_c0/f1p0/1744
R01_cb18645_c0/f1p0/5162	NA	R01_cb18645_c0/f1p0/5162	R01_cb18645_c0/f1p0/5162	R01_cb18645_c0/f1p0/5162
R01_cb18456_c7976/f1p0/1559	NA	NA	NA	R01_cb18456_c7976/f1p0/1559
R01_cb17248_c1/f1p0/378	R01_cb17248_c1/f1p0/378	R01_cb17248_c1/f1p0/378	R01_cb17248_c1/f1p0/378	R01_cb17248_c1/f1p0/378
R01_cb13501_c8/f1p0/484	NA	R01_cb13501_c8/f1p0/484	NA	NA
R01_cb7946_c0/f2p0/2481	NA	R01_cb7946_c0/f2p0/2481	R01_cb7946_c0/f2p0/2481	NA
R01_cb5896_c44/f1p0/2069	R01_cb5896_c44/f1p0/2069	R01_cb5896_c44/f1p0/2069	NA	R01_cb5896_c44/f1p0/2069
R01_cb11476_c2/f1p0/1732	NA	R01_cb11476_c2/f1p0/1732	R01_cb11476_c2/f1p0/1732	R01_cb11476_c2/f1p0/1732
R01_cb6004_c2/f1p1/2023	NA	NA	NA	R01_cb6004_c2/f1p1/2023

R01_cb6258_c7/f2p1/2564	NA	NA	NA	R01_cb6258_c7/f2p1/2564
R01_cb6491_c1/f1p0/2843	NA	R01_cb6491_c1/f1p0/2843	NA	R01_cb6491_c1/f1p0/2843
R01_cb16867_c3/f1p0/1296	NA	R01_cb16867_c3/f1p0/1296	NA	R01_cb16867_c3/f1p0/1296
R01_cb3138_c0/f3p0/717	NA	NA	NA	R01_cb3138_c0/f3p0/717
R01_cb8564_c81920/f1p1/3054	NA	R01_cb8564_c81920/f1p1/3054	NA	R01_cb8564_c81920/f1p1/3054
R01_cb17374_c1/f3p1/768	NA	NA	NA	R01_cb17374_c1/f3p1/768
R01_cb8564_c41826/f1p0/2225	NA	R01_cb8564_c41826/f1p0/2225	NA	R01_cb8564_c41826/f1p0/2225
R01_cb4422_c0/f1p0/3347	NA	R01_cb4422_c0/f1p0/3347	NA	R01_cb4422_c0/f1p0/3347
R01_cb8564_c70521/f2p1/2089	NA	R01_cb8564_c70521/f2p1/2089	R01_cb8564_c70521/f2p1/2089	R01_cb8564_c70521/f2p1/2089
R01_cb15345_c8/f1p0/951	NA	R01_cb15345_c8/f1p0/951	NA	R01_cb15345_c8/f1p0/951
R01_cb14542_c3/f1p0/961	NA	NA	NA	R01_cb14542_c3/f1p0/961
R01_cb2352_c9/f1p0/2081	R01_cb2352_c9/f1p0/2081	R01_cb2352_c9/f1p0/2081	R01_cb2352_c9/f1p0/2081	R01_cb2352_c9/f1p0/2081
R01_cb8564_c501/f6p0/2535	R01_cb8564_c501/f6p0/2535	R01_cb8564_c501/f6p0/2535	R01_cb8564_c501/f6p0/2535	R01_cb8564_c501/f6p0/2535
R01_cb1300_c0/f1p0/4386	NA	R01_cb1300_c0/f1p0/4386	NA	NA
R01_cb1279_c84/f1p0/2814	R01_cb1279_c84/f1p0/2814	R01_cb1279_c84/f1p0/2814	R01_cb1279_c84/f1p0/2814	R01_cb1279_c84/f1p0/2814
R01_cb2002_c0/f2p0/561	R01_cb2002_c0/f2p0/561	R01_cb2002_c0/f2p0/561	NA	NA
R01_cb10766_c2/f1p0/2637	NA	R01_cb10766_c2/f1p0/2637	NA	NA
R01_cb6501_c2/f1p1/2316	NA	R01_cb6501_c2/f1p1/2316	NA	NA
R01_cb2710_c7/f1p0/3633	R01_cb2710_c7/f1p0/3633	R01_cb2710_c7/f1p0/3633	R01_cb2710_c7/f1p0/3633	R01_cb2710_c7/f1p0/3633
R01_cb7489_c2/f1p0/2148	NA	R01_cb7489_c2/f1p0/2148	NA	NA
R01_cb14757_c7/f1p0/1772	NA	R01_cb14757_c7/f1p0/1772	NA	NA
R01_cb15105_c4/f1p0/645	NA	R01_cb15105_c4/f1p0/645	NA	NA
R01_cb2156_c45/f1p0/2503	NA	R01_cb2156_c45/f1p0/2503	NA	NA
R01_cb16052_c17/f1p0/869	NA	R01_cb16052_c17/f1p0/869	R01_cb16052_c17/f1p0/869	NA
R01_cb8564_c126699/f1p0/2086	NA	R01_cb8564_c126699/f1p0/2086	NA	NA

R01_cb15094_c0/f1p0/1056	NA	NA	NA	R01_cb15094_c0/f1p0/1056
R01_cb4295_c8/f1p0/3379	R01_cb4295_c8/f1p0/3379	R01_cb4295_c8/f1p0/3379	NA	NA
R01_cb10674_c0/f8p1/391	NA	R01_cb10674_c0/f8p1/391	NA	R01_cb10674_c0/f8p1/391
R01_cb8198_c2/f1p0/2433	NA	NA	NA	R01_cb8198_c2/f1p0/2433
R01_cb8564_c132693/f2p4/3304	NA	R01_cb8564_c132693/f2p4/3304	NA	R01_cb8564_c132693/f2p4/3304
R01_cb18590_c1/f1p0/1096	NA	R01_cb18590_c1/f1p0/1096	NA	R01_cb18590_c1/f1p0/1096
R01_cb8126_c5/f1p0/806	R01_cb8126_c5/f1p0/806	R01_cb8126_c5/f1p0/806	NA	NA
R01_cb8564_c121605/f1p0/2736	R01_cb8564_c121605/f1p0/2736	R01_cb8564_c121605/f1p0/2736	R01_cb8564_c121605/f1p0/2736	R01_cb8564_c121605/f1p0/2736
R01_cb8564_c130347/f1p1/2277	NA	R01_cb8564_c130347/f1p1/2277	NA	R01_cb8564_c130347/f1p1/2277
R01_cb3382_c2/f1p0/3382	NA	R01_cb3382_c2/f1p0/3382	NA	NA
R01_cb14307_c2/f1p0/1253	NA	R01_cb14307_c2/f1p0/1253	NA	NA
R01_cb16118_c5/f1p0/766	NA	R01_cb16118_c5/f1p0/766	R01_cb16118_c5/f1p0/766	R01_cb16118_c5/f1p0/766
R01_cb6024_c5/f1p0/2279	NA	NA	NA	R01_cb6024_c5/f1p0/2279
R01_cb15832_c0/f2p0/1001	NA	R01_cb15832_c0/f2p0/1001	NA	R01_cb15832_c0/f2p0/1001
R01_cb18456_c6112/f1p0/829	NA	NA	NA	R01_cb18456_c6112/f1p0/829
R01_cb18073_c1/f1p0/529	R01_cb18073_c1/f1p0/529	R01_cb18073_c1/f1p0/529	R01_cb18073_c1/f1p0/529	R01_cb18073_c1/f1p0/529
R01_cb17430_c0/f1p0/1151	R01_cb17430_c0/f1p0/1151	R01_cb17430_c0/f1p0/1151	R01_cb17430_c0/f1p0/1151	R01_cb17430_c0/f1p0/1151
R01_cb7265_c4/f1p0/2467	NA	R01_cb7265_c4/f1p0/2467	NA	NA
R01_cb8564_c72331/f1p0/3536	NA	R01_cb8564_c72331/f1p0/3536	NA	R01_cb8564_c72331/f1p0/3536
R01_cb260_c16/f1p0/4789	NA	NA	NA	R01_cb260_c16/f1p0/4789
R01_cb7741_c11/f1p0/465	R01_cb7741_c11/f1p0/465	R01_cb7741_c11/f1p0/465	R01_cb7741_c11/f1p0/465	R01_cb7741_c11/f1p0/465
R01_cb17921_c0/f2p0/694	NA	R01_cb17921_c0/f2p0/694	NA	NA
R01_cb4707_c3/f1p0/2710	NA	NA	NA	R01_cb4707_c3/f1p0/2710

R01_cb18132_c5/f1p0/1551	NA	R01_cb18132_c5/f1p0/1551	NA	NA
R01_cb16376_c3/f1p2/763	NA	R01_cb16376_c3/f1p2/763	NA	NA
R01_cb17982_c0/f1p0/1159	NA	NA	NA	R01_cb17982_c0/f1p0/1159
R01_cb5622_c8/f1p0/1579	NA	NA	NA	R01_cb5622_c8/f1p0/1579
R01_cb8564_c113662/f1p0/2289	NA	NA	NA	R01_cb8564_c113662/f1p0/2289
R01_cb8564_c48321/f1p0/3936	R01_cb8564_c48321/f1p0/3936	R01_cb8564_c48321/f1p0/3936	R01_cb8564_c48321/f1p0/3936	R01_cb8564_c48321/f1p0/3936
R01_cb5114_c0/f1p0/3173	R01_cb5114_c0/f1p0/3173	R01_cb5114_c0/f1p0/3173	R01_cb5114_c0/f1p0/3173	NA
R01_cb14091_c0/f2p0/1721	NA	R01_cb14091_c0/f2p0/1721	NA	R01_cb14091_c0/f2p0/1721
R01_cb17715_c2/f1p0/1433	NA	NA	NA	R01_cb17715_c2/f1p0/1433
R01_cb8564_c113234/f1p0/2194	NA	R01_cb8564_c113234/f1p0/2194	NA	NA
R01_cb4744_c6/f1p0/1496	NA	NA	NA	R01_cb4744_c6/f1p0/1496
R01_cb10488_c1/f1p0/2004	R01_cb10488_c1/f1p0/2004	R01_cb10488_c1/f1p0/2004	R01_cb10488_c1/f1p0/2004	R01_cb10488_c1/f1p0/2004
R01_cb13396_c7/f1p0/1530	NA	R01_cb13396_c7/f1p0/1530	R01_cb13396_c7/f1p0/1530	R01_cb13396_c7/f1p0/1530
R01_cb1847_c0/f3p0/2840	NA	NA	NA	R01_cb1847_c0/f3p0/2840
R01_cb3618_c5/f1p0/2772	NA	NA	NA	R01_cb3618_c5/f1p0/2772
R01_cb1198_c27/f1p1/1495	NA	NA	NA	R01_cb1198_c27/f1p1/1495
R01_cb6236_c1/f1p0/2908	NA	NA	NA	R01_cb6236_c1/f1p0/2908
R01_cb454_c28/f1p0/3850	NA	R01_cb454_c28/f1p0/3850	NA	R01_cb454_c28/f1p0/3850
R01_cb13178_c8/f2p1/584	NA	R01_cb13178_c8/f2p1/584	NA	NA
R01_cb18836_c0/f1p0/5394	NA	NA	NA	R01_cb18836_c0/f1p0/5394
R01_cb8084_c10/f6p1/2469	NA	NA	NA	R01_cb8084_c10/f6p1/2469
R01_cb8863_c4/f1p0/2019	R01_cb8863_c4/f1p0/2019	R01_cb8863_c4/f1p0/2019	NA	NA
R01_cb10314_c3/f1p0/3223	NA	R01_cb10314_c3/f1p0/3223	R01_cb10314_c3/f1p0/3223	R01_cb10314_c3/f1p0/3223

R01_cb9529_c1/f2p0/947	NA	NA	NA	R01_cb9529_c1/f2p0/947
R01_cb16341_c2/f1p0/545	R01_cb16341_c2/f1p0/545	R01_cb16341_c2/f1p0/545	R01_cb16341_c2/f1p0/545	R01_cb16341_c2/f1p0/545
R01_cb10096_c4/f1p0/2125	R01_cb10096_c4/f1p0/2125	R01_cb10096_c4/f1p0/2125	NA	R01_cb10096_c4/f1p0/2125
R01_cb13545_c30/f1p1/553	NA	R01_cb13545_c30/f1p1/553	NA	R01_cb13545_c30/f1p1/553
R01_cb10906_c0/f1p0/1657	NA	NA	NA	R01_cb10906_c0/f1p0/1657
R01_cb69_c23/f1p0/3553	NA	NA	NA	R01_cb69_c23/f1p0/3553
R01_cb10034_c58/f1p3/1621	NA	R01_cb10034_c58/f1p3/1621	NA	NA
R01_cb17811_c0/f3p0/673	R01_cb17811_c0/f3p0/673	NA	NA	R01_cb17811_c0/f3p0/673
R01_cb1497_c6/f1p0/2701	NA	NA	NA	R01_cb1497_c6/f1p0/2701
R01_cb3426_c24/f1p0/2863	NA	NA	NA	R01_cb3426_c24/f1p0/2863
R01_cb5054_c1/f1p0/3564	NA	R01_cb5054_c1/f1p0/3564	NA	NA
R01_cb5417_c3/f1p0/3252	NA	NA	NA	R01_cb5417_c3/f1p0/3252
R01_cb8564_c89748/f3p1/2815	NA	R01_cb8564_c89748/f3p1/2815	NA	R01_cb8564_c89748/f3p1/2815
R01_cb16412_c3/f1p0/1754	NA	R01_cb16412_c3/f1p0/1754	NA	NA
R01_cb261_c27/f1p0/2445	NA	NA	NA	R01_cb261_c27/f1p0/2445
R01_cb18400_c1/f1p0/1781	NA	NA	NA	R01_cb18400_c1/f1p0/1781
R01_cb8564_c22325/f3p0/3387	NA	NA	NA	R01_cb8564_c22325/f3p0/3387
R01_cb4580_c2/f1p0/4527	NA	NA	NA	R01_cb4580_c2/f1p0/4527
R01_cb2161_c2/f2p0/3620	NA	NA	NA	R01_cb2161_c2/f2p0/3620
R01_cb8564_c72401/f1p0/4486	NA	R01_cb8564_c72401/f1p0/4486	NA	NA
R01_cb18456_c2299/f1p0/432	R01_cb18456_c2299/f1p0/432	R01_cb18456_c2299/f1p0/432	R01_cb18456_c2299/f1p0/432	NA
R01_cb14433_c1/f1p0/1516	NA	NA	NA	R01_cb14433_c1/f1p0/1516
R01_cb1568_c4/f1p0/3080	NA	NA	NA	R01_cb1568_c4/f1p0/3080
R01_cb11630_c0/f1p0/1176	NA	R01_cb11630_c0/f1p0/1176	R01_cb11630_c0/f1p0/1176	R01_cb11630_c0/f1p0/1176
R01_cb12830_c1/f6p0/519	R01_cb12830_c1/f6p0/519	NA	NA	NA
R01_cb8564_c19304/f1p0/4013	NA	R01_cb8564_c19304/f1p0/4013	R01_cb8564_c19304/f1p0/4013	R01_cb8564_c19304/f1p0/4013

R01_cb8564_c145739/f4p0/2456	NA	NA	R01_cb8564_c145739/f4p0/2456	R01_cb8564_c145739/f4p0/2456
R01_cb17015_c1/f1p0/987	R01_cb17015_c1/f1p0/987	R01_cb17015_c1/f1p0/987	R01_cb17015_c1/f1p0/987	R01_cb17015_c1/f1p0/987
R01_cb16984_c3/f1p0/1010	NA	R01_cb16984_c3/f1p0/1010	NA	R01_cb16984_c3/f1p0/1010
R01_cb6197_c0/f2p0/2919	NA	R01_cb6197_c0/f2p0/2919	NA	NA
R01_cb9157_c7/f1p0/1802	NA	NA	NA	R01_cb9157_c7/f1p0/1802
R01_cb226_c3/f1p0/2740	NA	NA	NA	R01_cb226_c3/f1p0/2740
R01_cb8564_c89003/f1p0/2479	NA	NA	NA	R01_cb8564_c89003/f1p0/2479
R01_cb12240_c6/f1p1/688	R01_cb12240_c6/f1p1/688	R01_cb12240_c6/f1p1/688	NA	NA
R01_cb15009_c2/f1p0/777	NA	R01_cb15009_c2/f1p0/777	NA	NA
R01_cb8564_c35300/f1p2/3912	NA	NA	NA	R01_cb8564_c35300/f1p2/3912
R01_cb15861_c1/f1p0/1155	NA	NA	NA	R01_cb15861_c1/f1p0/1155
R01_cb13566_c4/f1p0/765	NA	R01_cb13566_c4/f1p0/765	NA	NA
R01_cb8564_c16862/f1p0/2896	NA	R01_cb8564_c16862/f1p0/2896	R01_cb8564_c16862/f1p0/2896	R01_cb8564_c16862/f1p0/2896
R01_cb1135_c3/f1p0/2050	NA	R01_cb1135_c3/f1p0/2050	NA	NA
R01_cb5165_c3/f1p0/1980	NA	R01_cb5165_c3/f1p0/1980	NA	NA
R01_cb17230_c0/f4p0/629	NA	NA	NA	R01_cb17230_c0/f4p0/629
R01_cb14465_c3/f1p0/696	NA	R01_cb14465_c3/f1p0/696	R01_cb14465_c3/f1p0/696	R01_cb14465_c3/f1p0/696
R01_cb15334_c0/f2p0/414	R01_cb15334_c0/f2p0/414	R01_cb15334_c0/f2p0/414	R01_cb15334_c0/f2p0/414	R01_cb15334_c0/f2p0/414
R01_cb8512_c2/f1p0/2321	NA	R01_cb8512_c2/f1p0/2321	NA	NA
R01_cb16118_c4/f1p0/1198	NA	NA	NA	R01_cb16118_c4/f1p0/1198
R01_cb781_c0/f11p1/1956	NA	NA	NA	R01_cb781_c0/f11p1/1956
R01_cb9155_c2/f1p0/2259	NA	R01_cb9155_c2/f1p0/2259	NA	NA
R01_cb5440_c12/f1p0/470	R01_cb5440_c12/f1p0/470	R01_cb5440_c12/f1p0/470	R01_cb5440_c12/f1p0/470	R01_cb5440_c12/f1p0/470
R01_cb10519_c5/f1p0/3036	NA	R01_cb10519_c5/f1p0/3036	NA	NA
R01_cb10532_c3/f1p0/544	R01_cb10532_c3/f1p0/544	R01_cb10532_c3/f1p0/544	R01_cb10532_c3/f1p0/544	R01_cb10532_c3/f1p0/544

R01_cb15745_c2/f1p0/457	R01_cb15745_c2/f1p0/457	R01_cb15745_c2/f1p0/457	R01_cb15745_c2/f1p0/457	R01_cb15745_c2/f1p0/457
R01_cb15701_c2/f1p0/1558	NA	R01_cb15701_c2/f1p0/1558	NA	R01_cb15701_c2/f1p0/1558
R01_cb10262_c9/f1p0/1498	NA	R01_cb10262_c9/f1p0/1498	NA	R01_cb10262_c9/f1p0/1498
R01_cb10596_c8/f1p0/856	NA	R01_cb10596_c8/f1p0/856	NA	NA
R01_cb4229_c1/f1p0/3397	NA	NA	NA	R01_cb4229_c1/f1p0/3397
R01_cb8564_c13869/f1p0/3450	NA	R01_cb8564_c13869/f1p0/3450	NA	NA
R01_cb9704_c1/f1p0/2876	NA	NA	NA	R01_cb9704_c1/f1p0/2876
R01_cb18039_c1/f1p0/771	NA	NA	R01_cb18039_c1/f1p0/771	R01_cb18039_c1/f1p0/771
R01_cb1939_c19/f1p0/1220	R01_cb1939_c19/f1p0/1220	R01_cb1939_c19/f1p0/1220	R01_cb1939_c19/f1p0/1220	R01_cb1939_c19/f1p0/1220
R01_cb8564_c123945/f1p0/1938	NA	R01_cb8564_c123945/f1p0/1938	R01_cb8564_c123945/f1p0/1938	R01_cb8564_c123945/f1p0/1938
R01_cb8564_c89494/f1p0/1933	NA	R01_cb8564_c89494/f1p0/1933	NA	NA
R01_cb8564_c125031/f1p0/2554	NA	NA	NA	R01_cb8564_c125031/f1p0/2554
R01_cb7491_c3/f1p0/1223	NA	NA	NA	R01_cb7491_c3/f1p0/1223
R01_cb4676_c0/f2p1/2636	NA	NA	NA	R01_cb4676_c0/f2p1/2636
R01_cb525_c5/f1p0/2035	NA	NA	NA	R01_cb525_c5/f1p0/2035
R01_cb10022_c3/f1p0/1013	NA	R01_cb10022_c3/f1p0/1013	NA	NA
R01_cb8564_c4474/f1p0/2973	NA	R01_cb8564_c4474/f1p0/2973	NA	NA
R01_cb13289_c1/f6p2/1789	NA	R01_cb13289_c1/f6p2/1789	R01_cb13289_c1/f6p2/1789	NA
R01_cb2451_c22/f1p2/3339	NA	NA	NA	R01_cb2451_c22/f1p2/3339
R01_cb5386_c73/f1p0/1424	NA	R01_cb5386_c73/f1p0/1424	NA	R01_cb5386_c73/f1p0/1424
R01_cb15945_c0/f2p0/462	R01_cb15945_c0/f2p0/462	NA	NA	R01_cb15945_c0/f2p0/462
R01_cb16645_c41/f1p0/1182	NA	R01_cb16645_c41/f1p0/1182	NA	R01_cb16645_c41/f1p0/1182
R01_cb3622_c9/f1p0/2510	NA	R01_cb3622_c9/f1p0/2510	NA	NA
R01_cb18456_c5306/f1p3/796	NA	R01_cb18456_c5306/f1p3/796	NA	NA

R01_cb6586_c1/f1p0/2064	NA	R01_cb6586_c1/f1p0/2064	NA	NA
R01_cb11828_c1/f1p0/2017	NA	R01_cb11828_c1/f1p0/2017	R01_cb11828_c1/f1p0/2017	NA
R01_cb12521_c2/f1p0/1283	NA	NA	NA	R01_cb12521_c2/f1p0/1283
R01_cb6737_c11/f1p0/3297	R01_cb6737_c11/f1p0/3297	R01_cb6737_c11/f1p0/3297	R01_cb6737_c11/f1p0/3297	R01_cb6737_c11/f1p0/3297
R01_cb10639_c1/f1p0/1983	NA	R01_cb10639_c1/f1p0/1983	R01_cb10639_c1/f1p0/1983	R01_cb10639_c1/f1p0/1983
R01_cb8978_c0/f3p0/706	NA	R01_cb8978_c0/f3p0/706	NA	NA
R01_cb13602_c26/f1p0/1802	NA	NA	NA	R01_cb13602_c26/f1p0/1802
R01_cb3618_c11/f1p0/2951	NA	NA	NA	R01_cb3618_c11/f1p0/2951
R01_cb11686_c0/f1p0/1232	NA	NA	NA	R01_cb11686_c0/f1p0/1232
R01_cb720_c4/f1p0/2836	NA	R01_cb720_c4/f1p0/2836	NA	NA
R01_cb9598_c11/f2p0/1690	NA	R01_cb9598_c11/f2p0/1690	NA	NA
R01_cb8564_c150564/f1p2/1934	NA	NA	NA	R01_cb8564_c150564/f1p2/1934
R01_cb17975_c1/f1p0/1057	NA	NA	NA	R01_cb17975_c1/f1p0/1057
R01_cb14610_c0/f3p0/702	NA	R01_cb14610_c0/f3p0/702	R01_cb14610_c0/f3p0/702	R01_cb14610_c0/f3p0/702
R01_cb8564_c40218/f1p0/3146	NA	R01_cb8564_c40218/f1p0/3146	NA	NA
R01_cb16990_c6/f1p0/1760	NA	NA	NA	R01_cb16990_c6/f1p0/1760
R01_cb10877_c1/f1p0/4278	NA	R01_cb10877_c1/f1p0/4278	NA	NA
R01_cb8564_c84897/f1p1/3769	NA	NA	NA	R01_cb8564_c84897/f1p1/3769
R01_cb618_c7/f1p0/4514	NA	NA	NA	R01_cb618_c7/f1p0/4514
R01_cb13613_c0/f2p0/1532	NA	R01_cb13613_c0/f2p0/1532	NA	R01_cb13613_c0/f2p0/1532
R01_cb3596_c1/f1p0/2163	NA	NA	NA	R01_cb3596_c1/f1p0/2163
R01_cb18456_c3358/f1p0/1132	NA	R01_cb18456_c3358/f1p0/1132	NA	NA
R01_cb11429_c2/f1p0/2387	NA	R01_cb11429_c2/f1p0/2387	NA	R01_cb11429_c2/f1p0/2387
R01_cb1819_c3/f1p0/2470	NA	R01_cb1819_c3/f1p0/2470	R01_cb1819_c3/f1p0/2470	NA
R01_cb8564_c17754/f1p0/2925	NA	R01_cb8564_c17754/f1p0/2925	NA	NA

R01_cb18456_c4741/f1p2/497	NA	NA	NA	R01_cb18456_c4741/f1p2/497
R01_cb6499_c16/f1p0/2036	NA	R01_cb6499_c16/f1p0/2036	NA	NA
R01_cb5682_c9/f1p0/940	R01_cb5682_c9/f1p0/940	R01_cb5682_c9/f1p0/940	NA	NA
R01_cb4128_c21/f1p0/3017	NA	NA	NA	R01_cb4128_c21/f1p0/3017
R01_cb10086_c4/f1p0/2322	NA	NA	NA	R01_cb10086_c4/f1p0/2322
R01_cb2161_c10/f1p0/3620	NA	NA	NA	R01_cb2161_c10/f1p0/3620
R01_cb18456_c1798/f1p1/1438	NA	R01_cb18456_c1798/f1p1/1438	NA	NA
R01_cb8564_c128182/f1p0/2570	NA	R01_cb8564_c128182/f1p0/2570	NA	NA
R01_cb14723_c0/f3p0/1104	NA	NA	NA	R01_cb14723_c0/f3p0/1104
R01_cb17918_c4/f1p0/1546	NA	NA	NA	R01_cb17918_c4/f1p0/1546
R01_cb4900_c21/f1p0/2508	NA	NA	NA	R01_cb4900_c21/f1p0/2508
R01_cb2352_c24/f1p0/5226	NA	NA	R01_cb2352_c24/f1p0/5226	R01_cb2352_c24/f1p0/5226
R01_cb16834_c0/f1p0/597	NA	R01_cb16834_c0/f1p0/597	NA	R01_cb16834_c0/f1p0/597
R01_cb8328_c1/f1p0/2492	R01_cb8328_c1/f1p0/2492	NA	NA	NA
R01_cb10594_c0/f6p0/1247	NA	NA	NA	R01_cb10594_c0/f6p0/1247
R01_cb3527_c19/f1p0/2224	NA	R01_cb3527_c19/f1p0/2224	NA	NA
R01_cb8564_c127167/f1p0/2148	NA	NA	NA	R01_cb8564_c127167/f1p0/2148
R01_cb6720_c5/f1p0/505	R01_cb6720_c5/f1p0/505	R01_cb6720_c5/f1p0/505	R01_cb6720_c5/f1p0/505	R01_cb6720_c5/f1p0/505
R01_cb8564_c19566/f1p0/2152	NA	R01_cb8564_c19566/f1p0/2152	R01_cb8564_c19566/f1p0/2152	R01_cb8564_c19566/f1p0/2152
R01_cb15535_c10/f1p5/804	NA	R01_cb15535_c10/f1p5/804	NA	R01_cb15535_c10/f1p5/804
R01_cb390_c9/f1p0/3130	NA	R01_cb390_c9/f1p0/3130	NA	NA
R01_cb8564_c38338/f1p0/2889	R01_cb8564_c38338/f1p0/2889	R01_cb8564_c38338/f1p0/2889	R01_cb8564_c38338/f1p0/2889	R01_cb8564_c38338/f1p0/2889
R01_cb10521_c15/f1p0/460	NA	R01_cb10521_c15/f1p0/460	NA	R01_cb10521_c15/f1p0/460

R01_cb14613_c1/f1p2/715	NA	NA	NA	R01_cb14613_c1/f1p2/715
R01_cb8564_c83678/f1p0/1927	NA	R01_cb8564_c83678/f1p0/1927	R01_cb8564_c83678/f1p0/1927	R01_cb8564_c83678/f1p0/1927
R01_cb2884_c3/f1p0/1955	NA	NA	NA	R01_cb2884_c3/f1p0/1955
R01_cb14987_c0/f3p0/614	R01_cb14987_c0/f3p0/614	R01_cb14987_c0/f3p0/614	R01_cb14987_c0/f3p0/614	R01_cb14987_c0/f3p0/614
R01_cb17453_c1/f1p0/1003	NA	NA	R01_cb17453_c1/f1p0/1003	R01_cb17453_c1/f1p0/1003
R01_cb8564_c17412/f1p0/2278	NA	NA	NA	R01_cb8564_c17412/f1p0/2278
R01_cb17672_c21/f1p0/517	R01_cb17672_c21/f1p0/517	R01_cb17672_c21/f1p0/517	R01_cb17672_c21/f1p0/517	R01_cb17672_c21/f1p0/517
R01_cb3667_c4/f1p1/2784	NA	NA	NA	R01_cb3667_c4/f1p1/2784
R01_cb9305_c5/f1p1/2699	NA	R01_cb9305_c5/f1p1/2699	NA	NA
R01_cb6551_c2/f1p0/2632	NA	R01_cb6551_c2/f1p0/2632	NA	NA
R01_cb9365_c5/f1p0/1795	NA	NA	NA	R01_cb9365_c5/f1p0/1795
R01_cb8564_c79100/f1p0/2453	NA	NA	NA	R01_cb8564_c79100/f1p0/2453
R01_cb8564_c111189/f1p0/3072	NA	R01_cb8564_c111189/f1p0/3072	NA	NA
R01_cb8564_c129815/f1p0/2904	NA	NA	R01_cb8564_c129815/f1p0/2904	R01_cb8564_c129815/f1p0/2904
R01_cb15682_c4/f1p0/775	NA	NA	NA	R01_cb15682_c4/f1p0/775
R01_cb8564_c82681/f1p1/3061	NA	NA	NA	R01_cb8564_c82681/f1p1/3061
R01_cb10648_c1/f1p0/483	R01_cb10648_c1/f1p0/483	R01_cb10648_c1/f1p0/483	NA	R01_cb10648_c1/f1p0/483
R01_cb6655_c0/f1p1/2797	NA	R01_cb6655_c0/f1p1/2797	R01_cb6655_c0/f1p1/2797	NA
R01_cb8564_c114233/f1p0/3713	NA	R01_cb8564_c114233/f1p0/3713	NA	NA
R01_cb9381_c6/f1p0/2060	NA	R01_cb9381_c6/f1p0/2060	NA	NA
R01_cb124_c16/f1p0/2795	NA	NA	NA	R01_cb124_c16/f1p0/2795
R01_cb8564_c84596/f1p0/2235	R01_cb8564_c84596/f1p0/2235	R01_cb8564_c84596/f1p0/2235	R01_cb8564_c84596/f1p0/2235	NA

R01_cb16274_c0/f2p0/615	NA	R01_cb16274_c0/f2p0/615	NA	NA
R01_cb5554_c6/f1p0/2898	R01_cb5554_c6/f1p0/2898	R01_cb5554_c6/f1p0/2898	R01_cb5554_c6/f1p0/2898	R01_cb5554_c6/f1p0/2898
R01_cb8564_c86556/f6p0/2422	NA	R01_cb8564_c86556/f6p0/2422	R01_cb8564_c86556/f6p0/2422	R01_cb8564_c86556/f6p0/2422
R01_cb4997_c19/f1p0/1813	NA	R01_cb4997_c19/f1p0/1813	R01_cb4997_c19/f1p0/1813	R01_cb4997_c19/f1p0/1813
R01_cb13289_c5/f1p0/1757	NA	NA	R01_cb13289_c5/f1p0/1757	NA
R01_cb8564_c2097/f1p0/3145	NA	R01_cb8564_c2097/f1p0/3145	R01_cb8564_c2097/f1p0/3145	NA
R01_cb14353_c3/f1p0/838	R01_cb14353_c3/f1p0/838	R01_cb14353_c3/f1p0/838	R01_cb14353_c3/f1p0/838	R01_cb14353_c3/f1p0/838
R01_cb8564_c87396/f1p0/3094	NA	R01_cb8564_c87396/f1p0/3094	NA	R01_cb8564_c87396/f1p0/3094
R01_cb7155_c11/f1p0/2559	NA	NA	NA	R01_cb7155_c11/f1p0/2559
R01_cb18452_c0/f1p0/1850	R01_cb18452_c0/f1p0/1850	R01_cb18452_c0/f1p0/1850	R01_cb18452_c0/f1p0/1850	R01_cb18452_c0/f1p0/1850
R01_cb8564_c2905/f1p0/3126	NA	R01_cb8564_c2905/f1p0/3126	NA	R01_cb8564_c2905/f1p0/3126
R01_cb8564_c13666/f1p1/1994	R01_cb8564_c13666/f1p1/1994	R01_cb8564_c13666/f1p1/1994	NA	R01_cb8564_c13666/f1p1/1994
R01_cb332_c11/f1p0/5243	NA	R01_cb332_c11/f1p0/5243	NA	NA
R01_cb7957_c0/f1p0/2491	NA	R01_cb7957_c0/f1p0/2491	NA	NA
R01_cb9562_c8/f1p0/1400	R01_cb9562_c8/f1p0/1400	R01_cb9562_c8/f1p0/1400	R01_cb9562_c8/f1p0/1400	R01_cb9562_c8/f1p0/1400
R01_cb15024_c3/f3p0/821	NA	NA	NA	R01_cb15024_c3/f3p0/821
R01_cb10913_c3/f1p0/1028	NA	NA	NA	R01_cb10913_c3/f1p0/1028
R01_cb5632_c20/f1p1/2425	NA	R01_cb5632_c20/f1p1/2425	NA	NA
R01_cb15038_c26/f1p0/365	R01_cb15038_c26/f1p0/365	R01_cb15038_c26/f1p0/365	NA	NA
R01_cb8564_c3684/f1p0/2489	R01_cb8564_c3684/f1p0/2489	R01_cb8564_c3684/f1p0/2489	R01_cb8564_c3684/f1p0/2489	R01_cb8564_c3684/f1p0/2489
R01_cb8564_c109929/f1p0/2520	NA	NA	NA	R01_cb8564_c109929/f1p0/2520
R01_cb17973_c24/f1p0/867	NA	R01_cb17973_c24/f1p0/867	R01_cb17973_c24/f1p0/867	NA
R01_cb14763_c0/f1p0/1292	NA	NA	NA	R01_cb14763_c0/f1p0/1292
R01_cb3224_c3/f1p0/3016	NA	R01_cb3224_c3/f1p0/3016	NA	R01_cb3224_c3/f1p0/3016

R01_cb11471_c0/f1p0/843	NA	NA	R01_cb11471_c0/f1p0/843	R01_cb11471_c0/f1p0/843
R01_cb8564_c119036/f1p0/2844	NA	NA	NA	R01_cb8564_c119036/f1p0/2844
R01_cb8564_c81976/f1p0/2827	NA	R01_cb8564_c81976/f1p0/2827	R01_cb8564_c81976/f1p0/2827	R01_cb8564_c81976/f1p0/2827
R01_cb1437_c6/f1p0/3928	NA	NA	NA	R01_cb1437_c6/f1p0/3928
R01_cb8564_c107814/f3p0/2123	NA	R01_cb8564_c107814/f3p0/2123	R01_cb8564_c107814/f3p0/2123	R01_cb8564_c107814/f3p0/2123
R01_cb15462_c0/f1p0/993	R01_cb15462_c0/f1p0/993	R01_cb15462_c0/f1p0/993	R01_cb15462_c0/f1p0/993	NA
R01_cb8564_c130233/f1p0/2396	NA	R01_cb8564_c130233/f1p0/2396	R01_cb8564_c130233/f1p0/2396	R01_cb8564_c130233/f1p0/2396
R01_cb9535_c13/f1p0/2492	NA	R01_cb9535_c13/f1p0/2492	NA	R01_cb9535_c13/f1p0/2492
R01_cb8564_c19761/f1p0/3250	NA	R01_cb8564_c19761/f1p0/3250	R01_cb8564_c19761/f1p0/3250	NA
R01_cb8564_c121421/f1p1/2345	NA	NA	NA	R01_cb8564_c121421/f1p1/2345
R01_cb2632_c9/f1p0/603	NA	R01_cb2632_c9/f1p0/603	R01_cb2632_c9/f1p0/603	R01_cb2632_c9/f1p0/603
R01_cb11319_c0/f1p0/1318	R01_cb11319_c0/f1p0/1318	R01_cb11319_c0/f1p0/1318	R01_cb11319_c0/f1p0/1318	R01_cb11319_c0/f1p0/1318
R01_cb13118_c5/f1p0/1644	NA	R01_cb13118_c5/f1p0/1644	NA	R01_cb13118_c5/f1p0/1644
R01_cb18456_c6440/f1p0/650	R01_cb18456_c6440/f1p0/650	R01_cb18456_c6440/f1p0/650	R01_cb18456_c6440/f1p0/650	R01_cb18456_c6440/f1p0/650
R01_cb535_c0/f1p0/4763	NA	NA	NA	R01_cb535_c0/f1p0/4763
R01_cb8564_c4607/f1p0/4078	NA	R01_cb8564_c4607/f1p0/4078	NA	NA
R01_cb6169_c8/f1p0/1219	NA	R01_cb6169_c8/f1p0/1219	NA	R01_cb6169_c8/f1p0/1219
R01_cb10258_c4/f1p0/1074	R01_cb10258_c4/f1p0/1074	R01_cb10258_c4/f1p0/1074	NA	R01_cb10258_c4/f1p0/1074
R01_cb18409_c176/f1p0/350	R01_cb18409_c176/f1p0/350	NA	R01_cb18409_c176/f1p0/350	R01_cb18409_c176/f1p0/350
R01_cb119_c10/f1p0/4794	NA	NA	NA	R01_cb119_c10/f1p0/4794
R01_cb11126_c0/f1p0/385	NA	R01_cb11126_c0/f1p0/385	NA	R01_cb11126_c0/f1p0/385
R01_cb8564_c13129/f1p0/3847	R01_cb8564_c13129/f1p0/384	R01_cb8564_c13129/f1p0/3847	NA	NA

	7			
R01_cb8145_c3/f1p0/2978	NA	NA	NA	R01_cb8145_c3/f1p0/2978
R01_cb18222_c0/f2p0/439	NA	NA	NA	R01_cb18222_c0/f2p0/439
R01_cb3618_c2/f8p0/2786	NA	NA	NA	R01_cb3618_c2/f8p0/2786
R01_cb3929_c7/f1p0/3311	NA	R01_cb3929_c7/f1p0/3311	NA	NA
R01_cb2002_c4/f1p0/607	R01_cb2002_c4/f1p0/607	R01_cb2002_c4/f1p0/607	NA	NA
R01_cb16616_c1/f1p0/605	NA	NA	NA	R01_cb16616_c1/f1p0/605
R01_cb10945_c3/f1p0/792	NA	R01_cb10945_c3/f1p0/792	R01_cb10945_c3/f1p0/792	R01_cb10945_c3/f1p0/792
R01_cb16387_c6/f1p0/1877	NA	NA	NA	R01_cb16387_c6/f1p0/1877
R01_cb8779_c13/f1p0/2714	NA	R01_cb8779_c13/f1p0/2714	NA	R01_cb8779_c13/f1p0/2714
R01_cb6069_c0/f4p0/2844	NA	NA	NA	R01_cb6069_c0/f4p0/2844
R01_cb17488_c1/f1p0/1624	NA	NA	NA	R01_cb17488_c1/f1p0/1624
R01_cb5828_c4/f1p0/2616	NA	NA	NA	R01_cb5828_c4/f1p0/2616
R01_cb12521_c5/f1p0/1338	NA	NA	NA	R01_cb12521_c5/f1p0/1338
R01_cb4136_c1/f1p0/3273	R01_cb4136_c1/f1p0/3273	R01_cb4136_c1/f1p0/3273	R01_cb4136_c1/f1p0/3273	NA
R01_cb8564_c147236/f34p1/4140	NA	NA	NA	R01_cb8564_c147236/f34p1/4140
R01_cb2843_c13/f1p2/2666	NA	NA	NA	R01_cb2843_c13/f1p2/2666
R01_cb18456_c6081/f1p0/729	NA	R01_cb18456_c6081/f1p0/729	R01_cb18456_c6081/f1p0/729	R01_cb18456_c6081/f1p0/729
R01_cb17914_c0/f2p0/1416	NA	NA	NA	R01_cb17914_c0/f2p0/1416
R01_cb17284_c2/f1p0/644	NA	NA	NA	R01_cb17284_c2/f1p0/644
R01_cb16180_c1/f1p0/888	R01_cb16180_c1/f1p0/888	R01_cb16180_c1/f1p0/888	R01_cb16180_c1/f1p0/888	R01_cb16180_c1/f1p0/888
R01_cb8564_c72682/f1p0/4419	NA	R01_cb8564_c72682/f1p0/4419	R01_cb8564_c72682/f1p0/4419	R01_cb8564_c72682/f1p0/4419
R01_cb8400_c3/f1p0/2238	R01_cb8400_c3/f1p0/2238	R01_cb8400_c3/f1p0/2238	R01_cb8400_c3/f1p0/2238	R01_cb8400_c3/f1p0/2238
R01_cb8564_c83237/f1p0/3616	NA	R01_cb8564_c83237/f1p0/3616	NA	NA
R01_cb6645_c3/f1p0/1886	NA	NA	NA	R01_cb6645_c3/f1p0/1886

R01_cb8564_c84127/f1p1/2448	NA	R01_cb8564_c84127/f1p1/2448	NA	R01_cb8564_c84127/f1p1/2448
R01_cb14286_c0/f3p0/1237	R01_cb14286_c0/f3p0/1237	R01_cb14286_c0/f3p0/1237	NA	R01_cb14286_c0/f3p0/1237
R01_cb10038_c6/f1p0/1713	NA	NA	NA	R01_cb10038_c6/f1p0/1713
R01_cb8564_c23967/f1p0/4475	NA	NA	NA	R01_cb8564_c23967/f1p0/4475
R01_cb10668_c1/f5p0/617	NA	R01_cb10668_c1/f5p0/617	NA	NA
R01_cb18241_c0/f1p0/983	NA	R01_cb18241_c0/f1p0/983	NA	NA
R01_cb4178_c4/f1p0/2194	NA	NA	NA	R01_cb4178_c4/f1p0/2194
R01_cb4134_c46/f5p0/2542	NA	NA	NA	R01_cb4134_c46/f5p0/2542
R01_cb14547_c5/f6p2/665	NA	R01_cb14547_c5/f6p2/665	NA	NA
R01_cb12114_c2/f15p1/1310	NA	NA	NA	R01_cb12114_c2/f15p1/1310
R01_cb1964_c7/f1p0/1223	NA	NA	NA	R01_cb1964_c7/f1p0/1223
R01_cb10024_c191/f1p1/2942	R01_cb10024_c191/f1p1/2942	R01_cb10024_c191/f1p1/2942	R01_cb10024_c191/f1p1/2942	NA
R01_cb16720_c1/f2p0/1455	NA	NA	NA	R01_cb16720_c1/f2p0/1455
R01_cb5357_c9/f1p0/2509	NA	R01_cb5357_c9/f1p0/2509	NA	NA
R01_cb8564_c1575/f1p0/1934	NA	R01_cb8564_c1575/f1p0/1934	R01_cb8564_c1575/f1p0/1934	R01_cb8564_c1575/f1p0/1934
R01_cb11646_c2/f1p0/1951	NA	NA	NA	R01_cb11646_c2/f1p0/1951
R01_cb8448_c5/f1p0/4066	NA	R01_cb8448_c5/f1p0/4066	NA	NA
R01_cb18456_c4764/f1p0/544	R01_cb18456_c4764/f1p0/544	R01_cb18456_c4764/f1p0/544	NA	R01_cb18456_c4764/f1p0/544
R01_cb10865_c2/f1p0/1428	NA	NA	NA	R01_cb10865_c2/f1p0/1428
R01_cb14474_c0/f2p0/649	NA	NA	NA	R01_cb14474_c0/f2p0/649
R01_cb9874_c12/f1p0/1909	NA	R01_cb9874_c12/f1p0/1909	NA	NA
R01_cb10254_c1/f1p0/2096	NA	R01_cb10254_c1/f1p0/2096	NA	R01_cb10254_c1/f1p0/2096
R01_cb13475_c3/f1p0/843	R01_cb13475_c3/f1p0/843	R01_cb13475_c3/f1p0/843	R01_cb13475_c3/f1p0/843	R01_cb13475_c3/f1p0/843
R01_cb18456_c7616/f1p0/1056	NA	R01_cb18456_c7616/f1p0/1056	R01_cb18456_c7616/f1p0/1056	R01_cb18456_c7616/f1p0/1056
R01_cb8564_c84378/f1p1/2886	NA	R01_cb8564_c84378/f1p1/2886	NA	R01_cb8564_c84378/f1p1/2886
R01_cb8564_c77215/f1p0/3514	NA	R01_cb8564_c77215/f1p0/3514	R01_cb8564_c77215/f1p0/3514	R01_cb8564_c77215/f1p0/3514

R01_cb16762_c0/f2p0/586	NA	R01_cb16762_c0/f2p0/586	NA	NA
R01_cb17101_c0/f1p0/1233	NA	NA	NA	R01_cb17101_c0/f1p0/1233
R01_cb8564_c21721/f1p0/2784	NA	R01_cb8564_c21721/f1p0/2784	NA	R01_cb8564_c21721/f1p0/2784
R01_cb15679_c3/f1p0/1333	NA	NA	NA	R01_cb15679_c3/f1p0/1333
R01_cb9877_c5/f1p0/1492	NA	NA	NA	R01_cb9877_c5/f1p0/1492
R01_cb16090_c0/f1p0/596	NA	R01_cb16090_c0/f1p0/596	R01_cb16090_c0/f1p0/596	R01_cb16090_c0/f1p0/596
R01_cb9519_c2/f1p0/1949	NA	R01_cb9519_c2/f1p0/1949	NA	NA
R01_cb18633_c1/f1p0/493	R01_cb18633_c1/f1p0/493	R01_cb18633_c1/f1p0/493	R01_cb18633_c1/f1p0/493	R01_cb18633_c1/f1p0/493
R01_cb8564_c111186/f1p0/3114	R01_cb8564_c111186/f1p0/3114	R01_cb8564_c111186/f1p0/3114	R01_cb8564_c111186/f1p0/3114	R01_cb8564_c111186/f1p0/3114
R01_cb15605_c3/f1p0/857	R01_cb15605_c3/f1p0/857	NA	R01_cb15605_c3/f1p0/857	R01_cb15605_c3/f1p0/857
R01_cb13970_c0/f8p0/989	NA	NA	NA	R01_cb13970_c0/f8p0/989
R01_cb2638_c4/f1p0/5862	NA	NA	NA	R01_cb2638_c4/f1p0/5862
R01_cb4198_c10/f1p0/1818	NA	NA	NA	R01_cb4198_c10/f1p0/1818
R01_cb4632_c1/f1p0/2685	NA	R01_cb4632_c1/f1p0/2685	NA	NA
R01_cb17251_c6/f1p0/468	NA	R01_cb17251_c6/f1p0/468	NA	NA
R01_cb14316_c21/f1p0/1403	NA	R01_cb14316_c21/f1p0/1403	R01_cb14316_c21/f1p0/1403	R01_cb14316_c21/f1p0/1403
R01_cb18257_c1/f1p0/1729	NA	NA	NA	R01_cb18257_c1/f1p0/1729
R01_cb10198_c3/f1p0/2554	NA	R01_cb10198_c3/f1p0/2554	NA	NA
R01_cb5664_c3/f1p0/764	NA	R01_cb5664_c3/f1p0/764	R01_cb5664_c3/f1p0/764	R01_cb5664_c3/f1p0/764
R01_cb5026_c3/f1p0/3688	NA	R01_cb5026_c3/f1p0/3688	NA	R01_cb5026_c3/f1p0/3688
R01_cb4848_c26/f1p0/1125	R01_cb4848_c26/f1p0/1125	R01_cb4848_c26/f1p0/1125	R01_cb4848_c26/f1p0/1125	R01_cb4848_c26/f1p0/1125
R01_cb8453_c7/f1p0/764	NA	R01_cb8453_c7/f1p0/764	NA	NA
R01_cb7875_c0/f1p0/2502	NA	NA	NA	R01_cb7875_c0/f1p0/2502
R01_cb8564_c82215/f1p0/2881	NA	NA	NA	R01_cb8564_c82215/f1p0/2881
R01_cb18456_c6659/f1p0/906	NA	R01_cb18456_c6659/f1p0/906	NA	NA

R01_cb8564_c11775/f1p2/3080	NA	NA	NA	R01_cb8564_c11775/f1p2/3080
R01_cb15434_c4/f1p0/1772	NA	R01_cb15434_c4/f1p0/1772	NA	R01_cb15434_c4/f1p0/1772
R01_cb16979_c10/f1p0/1003	NA	R01_cb16979_c10/f1p0/1003	NA	NA
R01_cb8564_c74332/f1p1/3294	NA	R01_cb8564_c74332/f1p1/3294	R01_cb8564_c74332/f1p1/3294	R01_cb8564_c74332/f1p1/3294
R01_cb1425_c1/f1p0/4329	NA	NA	NA	R01_cb1425_c1/f1p0/4329
R01_cb10879_c1/f1p1/2306	NA	NA	R01_cb10879_c1/f1p1/2306	R01_cb10879_c1/f1p1/2306
R01_cb10350_c2/f2p2/1101	NA	NA	NA	R01_cb10350_c2/f2p2/1101
R01_cb14019_c8/f1p0/1839	NA	R01_cb14019_c8/f1p0/1839	R01_cb14019_c8/f1p0/1839	R01_cb14019_c8/f1p0/1839
R01_cb17393_c0/f1p0/523	NA	R01_cb17393_c0/f1p0/523	R01_cb17393_c0/f1p0/523	R01_cb17393_c0/f1p0/523
R01_cb17125_c0/f1p1/566	NA	NA	NA	R01_cb17125_c0/f1p1/566
R01_cb9114_c1/f1p1/2578	NA	R01_cb9114_c1/f1p1/2578	NA	NA
R01_cb13254_c2/f1p0/1860	NA	R01_cb13254_c2/f1p0/1860	R01_cb13254_c2/f1p0/1860	R01_cb13254_c2/f1p0/1860
R01_cb3712_c4/f1p1/2242	R01_cb3712_c4/f1p1/2242	R01_cb3712_c4/f1p1/2242	NA	NA
R01_cb13941_c2/f1p0/1788	NA	R01_cb13941_c2/f1p0/1788	NA	NA
R01_cb18456_c1826/f1p2/1166	NA	R01_cb18456_c1826/f1p2/1166	R01_cb18456_c1826/f1p2/1166	R01_cb18456_c1826/f1p2/1166
R01_cb2946_c7/f3p0/3714	NA	NA	NA	R01_cb2946_c7/f3p0/3714
R01_cb3256_c6/f1p0/2095	NA	NA	NA	R01_cb3256_c6/f1p0/2095
R01_cb11254_c7/f1p0/1743	NA	NA	NA	R01_cb11254_c7/f1p0/1743
R01_cb8564_c21503/f1p0/4870	NA	R01_cb8564_c21503/f1p0/4870	NA	NA
R01_cb1523_c7/f1p0/2440	NA	R01_cb1523_c7/f1p0/2440	NA	R01_cb1523_c7/f1p0/2440
R01_cb5900_c158/f1p0/1925	R01_cb5900_c158/f1p0/1925	R01_cb5900_c158/f1p0/1925	R01_cb5900_c158/f1p0/1925	R01_cb5900_c158/f1p0/1925
R01_cb16249_c0/f2p0/1122	NA	NA	NA	R01_cb16249_c0/f2p0/1122
R01_cb13647_c2/f3p0/710	NA	R01_cb13647_c2/f3p0/710	NA	R01_cb13647_c2/f3p0/710
R01_cb4233_c194/f1p0/2645	R01_cb4233_c194/f1p0/2645	R01_cb4233_c194/f1p0/2645	R01_cb4233_c194/f1p0/2645	R01_cb4233_c194/f1p0/2645
R01_cb16263_c0/f3p0/542	R01_cb16263_c0/f3p0/542	R01_cb16263_c0/f3p0/542	R01_cb16263_c0/f3p0/542	R01_cb16263_c0/f3p0/542
R01_cb18456_c6670/f2p0/477	R01_cb18456_c6670/f2p0/477	R01_cb18456_c6670/f2p0/477	R01_cb18456_c6670/f2p0/477	R01_cb18456_c6670/f2p0/477

R01_cb8564_c20382/f1p0/3171	NA	R01_cb8564_c20382/f1p0/3171	NA	NA
R01_cb4368_c6/f1p1/3020	NA	NA	NA	R01_cb4368_c6/f1p1/3020
R01_cb8564_c81520/f1p0/2464	NA	R01_cb8564_c81520/f1p0/2464	NA	NA
R01_cb4953_c8/f1p0/1585	NA	NA	NA	R01_cb4953_c8/f1p0/1585
R01_cb17446_c1/f1p0/1452	NA	NA	NA	R01_cb17446_c1/f1p0/1452
R01_cb15825_c5/f1p0/1163	NA	NA	R01_cb15825_c5/f1p0/1163	R01_cb15825_c5/f1p0/1163
R01_cb10909_c0/f6p0/1815	NA	NA	NA	R01_cb10909_c0/f6p0/1815
R01_cb4639_c4/f1p0/3629	NA	R01_cb4639_c4/f1p0/3629	NA	R01_cb4639_c4/f1p0/3629
R01_cb18456_c7146/f1p0/734	NA	R01_cb18456_c7146/f1p0/734	R01_cb18456_c7146/f1p0/734	R01_cb18456_c7146/f1p0/734
R01_cb13319_c19/f1p0/365	R01_cb13319_c19/f1p0/365	R01_cb13319_c19/f1p0/365	R01_cb13319_c19/f1p0/365	R01_cb13319_c19/f1p0/365
R01_cb8564_c87734/f1p1/2826	NA	NA	NA	R01_cb8564_c87734/f1p1/2826
R01_cb10475_c1/f1p0/1472	NA	R01_cb10475_c1/f1p0/1472	NA	NA
R01_cb2821_c0/f1p0/3647	NA	R01_cb2821_c0/f1p0/3647	NA	NA
R01_cb13146_c3/f1p0/514	R01_cb13146_c3/f1p0/514	R01_cb13146_c3/f1p0/514	R01_cb13146_c3/f1p0/514	R01_cb13146_c3/f1p0/514
R01_cb5316_c0/f1p0/3033	NA	R01_cb5316_c0/f1p0/3033	NA	NA
R01_cb4370_c4/f1p0/2998	NA	NA	NA	R01_cb4370_c4/f1p0/2998
R01_cb7484_c1/f1p0/3557	NA	R01_cb7484_c1/f1p0/3557	NA	NA
R01_cb11695_c2/f1p0/620	NA	NA	NA	R01_cb11695_c2/f1p0/620
R01_cb5217_c5/f1p0/1148	NA	NA	NA	R01_cb5217_c5/f1p0/1148
R01_cb6149_c0/f1p0/2925	NA	R01_cb6149_c0/f1p0/2925	NA	NA
R01_cb10375_c5/f1p2/1608	NA	NA	NA	R01_cb10375_c5/f1p2/1608
R01_cb7497_c19/f1p0/1727	NA	NA	NA	R01_cb7497_c19/f1p0/1727
R01_cb6406_c45/f1p0/2958	NA	R01_cb6406_c45/f1p0/2958	NA	R01_cb6406_c45/f1p0/2958
R01_cb14761_c7/f1p0/1768	R01_cb14761_c7/f1p0/1768	R01_cb14761_c7/f1p0/1768	R01_cb14761_c7/f1p0/1768	R01_cb14761_c7/f1p0/1768
R01_cb8564_c15253/f1p3/4330	NA	R01_cb8564_c15253/f1p3/4330	NA	R01_cb8564_c15253/f1p3/4330
R01_cb8973_c2/f1p0/2001	R01_cb8973_c2/f1p0/2001	R01_cb8973_c2/f1p0/2001	NA	NA

R01_cb12379_c0/f3p1/587	R01_cb12379_c0/f3p1/587	R01_cb12379_c0/f3p1/587	R01_cb12379_c0/f3p1/587	R01_cb12379_c0/f3p1/587
R01_cb10345_c4/f1p0/1777	NA	NA	NA	R01_cb10345_c4/f1p0/1777
R01_cb7877_c2/f1p0/2718	NA	R01_cb7877_c2/f1p0/2718	R01_cb7877_c2/f1p0/2718	R01_cb7877_c2/f1p0/2718
R01_cb6170_c12/f1p1/2612	NA	NA	NA	R01_cb6170_c12/f1p1/2612
R01_cb8564_c76977/f1p0/2097	NA	NA	NA	R01_cb8564_c76977/f1p0/2097
R01_cb920_c6/f1p0/4092	NA	NA	NA	R01_cb920_c6/f1p0/4092
R01_cb16812_c0/f2p0/675	R01_cb16812_c0/f2p0/675	R01_cb16812_c0/f2p0/675	R01_cb16812_c0/f2p0/675	NA
R01_cb4609_c1/f1p0/3297	NA	R01_cb4609_c1/f1p0/3297	NA	R01_cb4609_c1/f1p0/3297
R01_cb8564_c18742/f1p0/4462	NA	R01_cb8564_c18742/f1p0/4462	NA	NA
R01_cb9500_c7/f1p0/2195	NA	NA	NA	R01_cb9500_c7/f1p0/2195
R01_cb1594_c9/f1p0/2443	NA	NA	NA	R01_cb1594_c9/f1p0/2443
R01_cb3017_c10/f1p0/5945	NA	R01_cb3017_c10/f1p0/5945	NA	NA
R01_cb8564_c53969/f1p0/2351	R01_cb8564_c53969/f1p0/2351	R01_cb8564_c53969/f1p0/2351	R01_cb8564_c53969/f1p0/2351	R01_cb8564_c53969/f1p0/2351
R01_cb14708_c2/f1p0/891	NA	R01_cb14708_c2/f1p0/891	NA	NA
R01_cb6732_c27/f1p1/540	R01_cb6732_c27/f1p1/540	R01_cb6732_c27/f1p1/540	NA	R01_cb6732_c27/f1p1/540
R01_cb13433_c38/f1p0/1350	NA	NA	NA	R01_cb13433_c38/f1p0/1350
R01_cb16945_c2/f1p0/1505	NA	R01_cb16945_c2/f1p0/1505	NA	NA
R01_cb8564_c494/f6p1/2566	NA	NA	R01_cb8564_c494/f6p1/2566	R01_cb8564_c494/f6p1/2566
R01_cb18601_c0/f2p0/579	R01_cb18601_c0/f2p0/579	R01_cb18601_c0/f2p0/579	R01_cb18601_c0/f2p0/579	R01_cb18601_c0/f2p0/579
R01_cb10658_c4/f1p0/670	NA	R01_cb10658_c4/f1p0/670	NA	NA
R01_cb8564_c17335/f1p0/2809	NA	R01_cb8564_c17335/f1p0/2809	NA	R01_cb8564_c17335/f1p0/2809
R01_cb8564_c90714/f1p0/3784	NA	R01_cb8564_c90714/f1p0/3784	NA	NA
R01_cb12939_c8/f1p0/1310	NA	R01_cb12939_c8/f1p0/1310	NA	NA
R01_cb2845_c38/f1p0/1808	NA	NA	NA	R01_cb2845_c38/f1p0/1808
R01_cb8564_c120044/f1p0/244	R01_cb8564_c120044/f1p0/24	R01_cb8564_c120044/f1p0/244	R01_cb8564_c120044/f1p0/244	R01_cb8564_c120044/f1p0/244

1	41	1	1	1
R01_cb11888_c1/f1p0/1739	R01_cb11888_c1/f1p0/1739	R01_cb11888_c1/f1p0/1739	R01_cb11888_c1/f1p0/1739	R01_cb11888_c1/f1p0/1739
R01_cb13103_c4/f1p0/567	NA	R01_cb13103_c4/f1p0/567	NA	NA
R01_cb8564_c36067/f1p0/3044	R01_cb8564_c36067/f1p0/3044	R01_cb8564_c36067/f1p0/3044	R01_cb8564_c36067/f1p0/3044	R01_cb8564_c36067/f1p0/3044
R01_cb8564_c119299/f1p0/3248	NA	NA	NA	R01_cb8564_c119299/f1p0/3248
R01_cb10571_c2/f1p0/985	NA	R01_cb10571_c2/f1p0/985	NA	NA
R01_cb3341_c5/f1p0/3652	NA	R01_cb3341_c5/f1p0/3652	NA	R01_cb3341_c5/f1p0/3652
R01_cb11918_c20/f1p0/1139	NA	NA	NA	R01_cb11918_c20/f1p0/1139
R01_cb13502_c2/f1p0/1429	NA	R01_cb13502_c2/f1p0/1429	NA	R01_cb13502_c2/f1p0/1429
R01_cb7027_c2/f1p0/1772	NA	R01_cb7027_c2/f1p0/1772	NA	NA
R01_cb8564_c25599/f1p0/3149	R01_cb8564_c25599/f1p0/3149	R01_cb8564_c25599/f1p0/3149	R01_cb8564_c25599/f1p0/3149	R01_cb8564_c25599/f1p0/3149
R01_cb16662_c4/f1p0/591	R01_cb16662_c4/f1p0/591	R01_cb16662_c4/f1p0/591	R01_cb16662_c4/f1p0/591	R01_cb16662_c4/f1p0/591
R01_cb11242_c2/f1p0/2487	NA	R01_cb11242_c2/f1p0/2487	NA	NA
R01_cb8564_c108825/f1p3/1911	NA	NA	NA	R01_cb8564_c108825/f1p3/1911
R01_cb18523_c0/f1p0/1806	NA	R01_cb18523_c0/f1p0/1806	NA	R01_cb18523_c0/f1p0/1806
R01_cb11102_c2/f1p0/2548	NA	R01_cb11102_c2/f1p0/2548	NA	R01_cb11102_c2/f1p0/2548
R01_cb14816_c6/f1p0/756	NA	R01_cb14816_c6/f1p0/756	NA	NA
R01_cb17413_c4/f1p0/1639	NA	R01_cb17413_c4/f1p0/1639	NA	NA
R01_cb4185_c6/f1p0/1375	NA	NA	NA	R01_cb4185_c6/f1p0/1375
R01_cb12204_c1/f2p0/1289	R01_cb12204_c1/f2p0/1289	R01_cb12204_c1/f2p0/1289	R01_cb12204_c1/f2p0/1289	R01_cb12204_c1/f2p0/1289
R01_cb18456_c1461/f4p0/588	R01_cb18456_c1461/f4p0/588	R01_cb18456_c1461/f4p0/588	R01_cb18456_c1461/f4p0/588	R01_cb18456_c1461/f4p0/588
R01_cb11471_c1/f1p0/3135	NA	R01_cb11471_c1/f1p0/3135	R01_cb11471_c1/f1p0/3135	R01_cb11471_c1/f1p0/3135

R01_cb27_c12/f1p0/1234	NA	NA	NA	R01_cb27_c12/f1p0/1234
R01_cb4066_c10/f1p1/3649	NA	R01_cb4066_c10/f1p1/3649	NA	NA
R01_cb18087_c3/f1p0/1151	R01_cb18087_c3/f1p0/1151	R01_cb18087_c3/f1p0/1151	NA	R01_cb18087_c3/f1p0/1151
R01_cb8564_c127970/f1p0/2751	NA	R01_cb8564_c127970/f1p0/2751	NA	R01_cb8564_c127970/f1p0/2751
R01_cb3618_c12/f1p0/2946	NA	NA	NA	R01_cb3618_c12/f1p0/2946
R01_cb7302_c2/f1p0/3538	NA	R01_cb7302_c2/f1p0/3538	NA	R01_cb7302_c2/f1p0/3538
R01_cb3500_c24/f1p0/3152	NA	NA	NA	R01_cb3500_c24/f1p0/3152
R01_cb123_c6/f1p0/5119	NA	NA	NA	R01_cb123_c6/f1p0/5119
R01_cb4001_c0/f3p0/806	NA	NA	NA	R01_cb4001_c0/f3p0/806
R01_cb8759_c1/f1p0/2690	NA	R01_cb8759_c1/f1p0/2690	NA	R01_cb8759_c1/f1p0/2690
R01_cb5258_c1/f1p0/3137	NA	R01_cb5258_c1/f1p0/3137	NA	R01_cb5258_c1/f1p0/3137
R01_cb781_c40/f1p0/1031	NA	NA	NA	R01_cb781_c40/f1p0/1031
R01_cb8564_c78298/f1p0/3862	R01_cb8564_c78298/f1p0/3862	R01_cb8564_c78298/f1p0/3862	R01_cb8564_c78298/f1p0/3862	R01_cb8564_c78298/f1p0/3862
R01_cb16061_c1/f1p0/497	NA	R01_cb16061_c1/f1p0/497	NA	NA
R01_cb5659_c70/f1p0/2387	NA	NA	NA	R01_cb5659_c70/f1p0/2387
R01_cb105_c21/f1p0/2514	NA	R01_cb105_c21/f1p0/2514	R01_cb105_c21/f1p0/2514	R01_cb105_c21/f1p0/2514
R01_cb943_c5/f1p0/7144	NA	NA	NA	R01_cb943_c5/f1p0/7144
R01_cb8564_c42176/f1p0/3183	NA	NA	NA	R01_cb8564_c42176/f1p0/3183
R01_cb1936_c0/f1p0/4121	NA	R01_cb1936_c0/f1p0/4121	R01_cb1936_c0/f1p0/4121	R01_cb1936_c0/f1p0/4121
R01_cb9487_c4/f1p0/3425	NA	R01_cb9487_c4/f1p0/3425	NA	NA
R01_cb1023_c0/f1p0/4515	NA	R01_cb1023_c0/f1p0/4515	NA	NA
R01_cb11037_c3/f1p0/1642	NA	R01_cb11037_c3/f1p0/1642	NA	R01_cb11037_c3/f1p0/1642
R01_cb7536_c4/f1p0/443	NA	R01_cb7536_c4/f1p0/443	R01_cb7536_c4/f1p0/443	R01_cb7536_c4/f1p0/443
R01_cb5122_c5/f1p0/3166	NA	NA	NA	R01_cb5122_c5/f1p0/3166

R01_cb9529_c3/f1p0/1074	NA	NA	NA	R01_cb9529_c3/f1p0/1074
R01_cb15488_c1/f1p0/1876	NA	R01_cb15488_c1/f1p0/1876	NA	NA
R01_cb454_c6/f1p0/4805	NA	NA	NA	R01_cb454_c6/f1p0/4805
R01_cb12480_c4/f1p0/718	R01_cb12480_c4/f1p0/718	R01_cb12480_c4/f1p0/718	R01_cb12480_c4/f1p0/718	R01_cb12480_c4/f1p0/718
R01_cb8564_c110370/f1p0/3044	NA	R01_cb8564_c110370/f1p0/3044	NA	NA
R01_cb18456_c3397/f1p0/1025	R01_cb18456_c3397/f1p0/1025	NA	NA	NA
R01_cb2771_c4/f1p0/3815	NA	NA	NA	R01_cb2771_c4/f1p0/3815
R01_cb5889_c1/f1p0/3412	NA	NA	NA	R01_cb5889_c1/f1p0/3412
R01_cb16125_c10/f1p0/1414	NA	R01_cb16125_c10/f1p0/1414	NA	R01_cb16125_c10/f1p0/1414
R01_cb1352_c2/f1p0/1936	NA	R01_cb1352_c2/f1p0/1936	NA	NA
R01_cb8639_c0/f1p0/2324	NA	R01_cb8639_c0/f1p0/2324	NA	NA
R01_cb16913_c4/f1p0/588	NA	R01_cb16913_c4/f1p0/588	NA	NA
R01_cb8564_c22596/f1p0/2059	NA	R01_cb8564_c22596/f1p0/2059	R01_cb8564_c22596/f1p0/2059	R01_cb8564_c22596/f1p0/2059
R01_cb8324_c0/f6p0/1480	NA	NA	NA	R01_cb8324_c0/f6p0/1480
R01_cb1594_c8/f2p0/2363	NA	NA	NA	R01_cb1594_c8/f2p0/2363
R01_cb8564_c90273/f1p0/2581	NA	R01_cb8564_c90273/f1p0/2581	NA	NA
R01_cb9222_c14/f1p0/2490	NA	R01_cb9222_c14/f1p0/2490	NA	NA
R01_cb16092_c3/f1p1/533	R01_cb16092_c3/f1p1/533	R01_cb16092_c3/f1p1/533	R01_cb16092_c3/f1p1/533	R01_cb16092_c3/f1p1/533
R01_cb8564_c71841/f1p0/2705	NA	R01_cb8564_c71841/f1p0/2705	R01_cb8564_c71841/f1p0/2705	R01_cb8564_c71841/f1p0/2705
R01_cb1087_c3/f1p0/2382	NA	NA	R01_cb1087_c3/f1p0/2382	R01_cb1087_c3/f1p0/2382
R01_cb16311_c3/f1p0/1175	NA	NA	NA	R01_cb16311_c3/f1p0/1175
R01_cb11850_c3/f4p1/599	NA	R01_cb11850_c3/f4p1/599	NA	NA
R01_cb8564_c80943/f1p0/2583	NA	R01_cb8564_c80943/f1p0/2583	NA	NA
R01_cb5659_c53/f1p0/2198	NA	R01_cb5659_c53/f1p0/2198	NA	NA

R01_cb6894_c2/f1p0/1023	NA	R01_cb6894_c2/f1p0/1023	R01_cb6894_c2/f1p0/1023	R01_cb6894_c2/f1p0/1023
R01_cb15990_c0/f4p0/1141	NA	NA	NA	R01_cb15990_c0/f4p0/1141
R01_cb7296_c10/f1p1/1975	NA	NA	NA	R01_cb7296_c10/f1p1/1975
R01_cb13690_c7/f1p0/314	R01_cb13690_c7/f1p0/314	R01_cb13690_c7/f1p0/314	R01_cb13690_c7/f1p0/314	R01_cb13690_c7/f1p0/314
R01_cb693_c7/f1p0/2901	NA	NA	NA	R01_cb693_c7/f1p0/2901
R01_cb14547_c18/f1p2/650	NA	R01_cb14547_c18/f1p2/650	NA	NA
R01_cb14493_c4/f1p0/1047	NA	R01_cb14493_c4/f1p0/1047	R01_cb14493_c4/f1p0/1047	R01_cb14493_c4/f1p0/1047
R01_cb1626_c8/f1p1/2673	NA	NA	NA	R01_cb1626_c8/f1p1/2673
R01_cb18712_c1/f1p0/2646	R01_cb18712_c1/f1p0/2646	R01_cb18712_c1/f1p0/2646	R01_cb18712_c1/f1p0/2646	R01_cb18712_c1/f1p0/2646
R01_cb9420_c0/f2p0/2092	NA	NA	NA	R01_cb9420_c0/f2p0/2092
R01_cb12192_c0/f9p0/429	NA	R01_cb12192_c0/f9p0/429	NA	NA
R01_cb1533_c9/f1p0/1687	NA	R01_cb1533_c9/f1p0/1687	NA	NA
R01_cb8564_c74334/f1p0/2963	NA	R01_cb8564_c74334/f1p0/2963	NA	NA
R01_cb1095_c18/f1p1/5545	NA	NA	NA	R01_cb1095_c18/f1p1/5545
R01_cb5595_c6/f1p0/2093	NA	R01_cb5595_c6/f1p0/2093	NA	NA
R01_cb14940_c6/f1p0/685	NA	NA	R01_cb14940_c6/f1p0/685	R01_cb14940_c6/f1p0/685
R01_cb18456_c6698/f1p0/652	NA	R01_cb18456_c6698/f1p0/652	NA	NA
R01_cb1881_c3/f1p0/3916	NA	R01_cb1881_c3/f1p0/3916	NA	NA
R01_cb11254_c1/f2p0/1762	NA	NA	NA	R01_cb11254_c1/f2p0/1762
R01_cb15934_c1/f1p0/620	R01_cb15934_c1/f1p0/620	R01_cb15934_c1/f1p0/620	NA	R01_cb15934_c1/f1p0/620
R01_cb6686_c0/f2p0/2564	NA	R01_cb6686_c0/f2p0/2564	R01_cb6686_c0/f2p0/2564	NA
R01_cb1119_c14/f1p0/2501	NA	R01_cb1119_c14/f1p0/2501	NA	NA
R01_cb8564_c20867/f1p0/1948	NA	R01_cb8564_c20867/f1p0/1948	NA	R01_cb8564_c20867/f1p0/1948
R01_cb13563_c3/f1p0/851	NA	R01_cb13563_c3/f1p0/851	NA	NA
R01_cb110_c11/f1p1/3647	NA	NA	NA	R01_cb110_c11/f1p1/3647
R01_cb8345_c0/f1p0/2394	NA	NA	NA	R01_cb8345_c0/f1p0/2394

R01_cb2878_c6/f1p0/1687	NA	R01_cb2878_c6/f1p0/1687	R01_cb2878_c6/f1p0/1687	R01_cb2878_c6/f1p0/1687
R01_cb11695_c0/f1p1/995	NA	NA	NA	R01_cb11695_c0/f1p1/995
R01_cb16665_c0/f1p0/977	NA	NA	R01_cb16665_c0/f1p0/977	R01_cb16665_c0/f1p0/977
R01_cb8414_c1/f1p0/2405	R01_cb8414_c1/f1p0/2405	R01_cb8414_c1/f1p0/2405	NA	R01_cb8414_c1/f1p0/2405
R01_cb8564_c17696/f1p0/3569	NA	R01_cb8564_c17696/f1p0/3569	R01_cb8564_c17696/f1p0/3569	R01_cb8564_c17696/f1p0/3569
R01_cb5533_c168/f1p0/444	R01_cb5533_c168/f1p0/444	R01_cb5533_c168/f1p0/444	R01_cb5533_c168/f1p0/444	R01_cb5533_c168/f1p0/444
R01_cb8564_c115280/f1p0/2295	NA	NA	NA	R01_cb8564_c115280/f1p0/2295
R01_cb8564_c1994/f1p0/2024	NA	NA	NA	R01_cb8564_c1994/f1p0/2024
R01_cb6147_c35/f1p0/2856	NA	R01_cb6147_c35/f1p0/2856	NA	NA
R01_cb8564_c17194/f1p0/4162	NA	R01_cb8564_c17194/f1p0/4162	NA	NA
R01_cb2357_c9/f1p1/3967	NA	NA	NA	R01_cb2357_c9/f1p1/3967
R01_cb12556_c1/f1p0/1614	NA	R01_cb12556_c1/f1p0/1614	NA	R01_cb12556_c1/f1p0/1614
R01_cb13433_c49/f1p0/3824	NA	R01_cb13433_c49/f1p0/3824	NA	NA
R01_cb11145_c1/f1p0/2190	R01_cb11145_c1/f1p0/2190	R01_cb11145_c1/f1p0/2190	R01_cb11145_c1/f1p0/2190	R01_cb11145_c1/f1p0/2190
R01_cb5702_c6/f1p0/1304	NA	R01_cb5702_c6/f1p0/1304	NA	NA
R01_cb11637_c1/f1p0/2015	NA	R01_cb11637_c1/f1p0/2015	NA	NA
R01_cb10214_c1/f1p0/1935	R01_cb10214_c1/f1p0/1935	R01_cb10214_c1/f1p0/1935	R01_cb10214_c1/f1p0/1935	R01_cb10214_c1/f1p0/1935
R01_cb9882_c0/f16p1/1018	R01_cb9882_c0/f16p1/1018	NA	NA	NA
R01_cb8564_c48385/f1p0/3125	NA	R01_cb8564_c48385/f1p0/3125	NA	NA
R01_cb11709_c1/f1p0/3558	NA	NA	NA	R01_cb11709_c1/f1p0/3558
R01_cb5929_c2/f1p0/2974	NA	NA	NA	R01_cb5929_c2/f1p0/2974
R01_cb15555_c2/f1p0/1365	NA	R01_cb15555_c2/f1p0/1365	NA	NA
R01_cb11733_c1/f1p0/2247	NA	NA	NA	R01_cb11733_c1/f1p0/2247
R01_cb15483_c0/f1p0/1039	NA	R01_cb15483_c0/f1p0/1039	NA	NA
R01_cb8564_c85678/f1p0/3912	NA	R01_cb8564_c85678/f1p0/3912	NA	NA

R01_cb8564_c147879/f1p0/2955	NA	R01_cb8564_c147879/f1p0/2955	NA	NA
R01_cb8564_c16877/f1p0/2124	R01_cb8564_c16877/f1p0/2124	R01_cb8564_c16877/f1p0/2124	NA	NA
R01_cb12616_c2/f1p0/1464	NA	NA	NA	R01_cb12616_c2/f1p0/1464
R01_cb14537_c0/f1p0/511	R01_cb14537_c0/f1p0/511	R01_cb14537_c0/f1p0/511	NA	R01_cb14537_c0/f1p0/511
R01_cb8564_c21793/f1p0/2841	NA	R01_cb8564_c21793/f1p0/2841	NA	NA
R01_cb8564_c117016/f1p0/2202	NA	R01_cb8564_c117016/f1p0/2202	NA	NA
R01_cb11804_c1/f1p0/3756	R01_cb11804_c1/f1p0/3756	R01_cb11804_c1/f1p0/3756	R01_cb11804_c1/f1p0/3756	R01_cb11804_c1/f1p0/3756
R01_cb8564_c48585/f1p0/2528	NA	NA	NA	R01_cb8564_c48585/f1p0/2528
R01_cb1519_c31/f1p0/1681	NA	R01_cb1519_c31/f1p0/1681	NA	NA
R01_cb8564_c122688/f1p0/2138	NA	NA	R01_cb8564_c122688/f1p0/2138	NA
R01_cb551_c19/f1p0/2034	NA	R01_cb551_c19/f1p0/2034	NA	NA
R01_cb8564_c114136/f1p0/2409	NA	R01_cb8564_c114136/f1p0/2409	NA	NA
R01_cb9814_c4/f1p0/666	NA	R01_cb9814_c4/f1p0/666	NA	R01_cb9814_c4/f1p0/666
R01_cb17163_c0/f1p0/1035	NA	R01_cb17163_c0/f1p0/1035	R01_cb17163_c0/f1p0/1035	R01_cb17163_c0/f1p0/1035
R01_cb5255_c11/f1p0/1222	NA	R01_cb5255_c11/f1p0/1222	R01_cb5255_c11/f1p0/1222	R01_cb5255_c11/f1p0/1222
R01_cb8564_c78107/f1p1/2897	NA	R01_cb8564_c78107/f1p1/2897	NA	NA
R01_cb16818_c4/f1p0/825	R01_cb16818_c4/f1p0/825	R01_cb16818_c4/f1p0/825	R01_cb16818_c4/f1p0/825	R01_cb16818_c4/f1p0/825
R01_cb8564_c14685/f1p0/2165	NA	R01_cb8564_c14685/f1p0/2165	NA	R01_cb8564_c14685/f1p0/2165
R01_cb17931_c1/f3p0/468	R01_cb17931_c1/f3p0/468	NA	NA	R01_cb17931_c1/f3p0/468
R01_cb16706_c4/f1p0/701	NA	R01_cb16706_c4/f1p0/701	NA	R01_cb16706_c4/f1p0/701
R01_cb1459_c3/f1p1/3193	NA	R01_cb1459_c3/f1p1/3193	NA	NA

R01_cb8564_c179151/f1p1/2045	NA	R01_cb8564_c179151/f1p1/2045	R01_cb8564_c179151/f1p1/2045	R01_cb8564_c179151/f1p1/2045
R01_cb18456_c1819/f1p0/838	NA	NA	NA	R01_cb18456_c1819/f1p0/838
R01_cb8564_c70436/f1p0/3032	NA	NA	NA	R01_cb8564_c70436/f1p0/3032
R01_cb16797_c2/f1p0/454	R01_cb16797_c2/f1p0/454	R01_cb16797_c2/f1p0/454	NA	R01_cb16797_c2/f1p0/454
R01_cb8564_c121844/f1p0/1927	NA	R01_cb8564_c121844/f1p0/1927	NA	NA
R01_cb18456_c6640/f1p0/577	R01_cb18456_c6640/f1p0/577	R01_cb18456_c6640/f1p0/577	R01_cb18456_c6640/f1p0/577	R01_cb18456_c6640/f1p0/577
R01_cb7256_c5/f1p0/1439	NA	R01_cb7256_c5/f1p0/1439	NA	NA
R01_cb3921_c1/f1p0/2047	NA	NA	NA	R01_cb3921_c1/f1p0/2047
R01_cb2845_c36/f1p0/3417	NA	NA	NA	R01_cb2845_c36/f1p0/3417
R01_cb18456_c868/f1p0/484	NA	NA	NA	R01_cb18456_c868/f1p0/484
R01_cb12321_c851/f1p0/546	R01_cb12321_c851/f1p0/546	R01_cb12321_c851/f1p0/546	R01_cb12321_c851/f1p0/546	NA
R01_cb18208_c1/f1p0/1093	NA	R01_cb18208_c1/f1p0/1093	R01_cb18208_c1/f1p0/1093	R01_cb18208_c1/f1p0/1093
R01_cb8564_c82335/f1p0/2498	NA	R01_cb8564_c82335/f1p0/2498	NA	NA
R01_cb8564_c118758/f1p0/2812	NA	R01_cb8564_c118758/f1p0/2812	NA	R01_cb8564_c118758/f1p0/2812
R01_cb15337_c2/f1p0/1634	NA	NA	NA	R01_cb15337_c2/f1p0/1634
R01_cb3121_c1/f1p0/3714	NA	R01_cb3121_c1/f1p0/3714	R01_cb3121_c1/f1p0/3714	NA
R01_cb1096_c9/f1p0/2933	NA	NA	NA	R01_cb1096_c9/f1p0/2933
R01_cb1230_c5/f1p0/2699	NA	R01_cb1230_c5/f1p0/2699	R01_cb1230_c5/f1p0/2699	R01_cb1230_c5/f1p0/2699
R01_cb8414_c0/f1p0/2375	NA	NA	NA	R01_cb8414_c0/f1p0/2375
R01_cb18456_c6070/f1p0/443	NA	R01_cb18456_c6070/f1p0/443	NA	R01_cb18456_c6070/f1p0/443
R01_cb822_c4/f3p2/2466	NA	NA	NA	R01_cb822_c4/f3p2/2466
R01_cb6606_c7/f2p0/2812	NA	NA	NA	R01_cb6606_c7/f2p0/2812
R01_cb11841_c0/f1p0/1393	NA	R01_cb11841_c0/f1p0/1393	NA	R01_cb11841_c0/f1p0/1393

R01_cb8564_c120096/f1p0/3462	NA	R01_cb8564_c120096/f1p0/3462	NA	R01_cb8564_c120096/f1p0/3462
R01_cb17412_c2/f1p0/770	NA	R01_cb17412_c2/f1p0/770	NA	R01_cb17412_c2/f1p0/770
R01_cb18005_c4/f1p0/480	NA	NA	R01_cb18005_c4/f1p0/480	R01_cb18005_c4/f1p0/480
R01_cb893_c12/f1p0/5658	NA	NA	NA	R01_cb893_c12/f1p0/5658
R01_cb12459_c8/f1p0/532	R01_cb12459_c8/f1p0/532	R01_cb12459_c8/f1p0/532	R01_cb12459_c8/f1p0/532	R01_cb12459_c8/f1p0/532
R01_cb1283_c4/f1p0/3882	NA	NA	NA	R01_cb1283_c4/f1p0/3882
R01_cb2160_c12/f1p0/4626	NA	NA	NA	R01_cb2160_c12/f1p0/4626
R01_cb2729_c1/f1p0/1933	R01_cb2729_c1/f1p0/1933	R01_cb2729_c1/f1p0/1933	NA	R01_cb2729_c1/f1p0/1933
R01_cb1178_c26/f1p0/1213	NA	R01_cb1178_c26/f1p0/1213	NA	R01_cb1178_c26/f1p0/1213
R01_cb2045_c3/f1p0/4072	NA	R01_cb2045_c3/f1p0/4072	NA	R01_cb2045_c3/f1p0/4072
R01_cb16979_c5/f1p0/774	R01_cb16979_c5/f1p0/774	R01_cb16979_c5/f1p0/774	R01_cb16979_c5/f1p0/774	R01_cb16979_c5/f1p0/774
R01_cb18456_c1855/f1p1/1135	NA	NA	NA	R01_cb18456_c1855/f1p1/1135
R01_cb11126_c1/f1p0/2685	R01_cb11126_c1/f1p0/2685	R01_cb11126_c1/f1p0/2685	NA	NA
R01_cb8564_c25411/f1p0/2393	NA	NA	NA	R01_cb8564_c25411/f1p0/2393
R01_cb8564_c13615/f1p0/3945	NA	R01_cb8564_c13615/f1p0/3945	NA	NA
R01_cb8513_c1/f1p0/2026	NA	NA	NA	R01_cb8513_c1/f1p0/2026
R01_cb8564_c15171/f1p0/3320	NA	R01_cb8564_c15171/f1p0/3320	NA	NA
R01_cb12023_c2/f1p0/760	NA	NA	NA	R01_cb12023_c2/f1p0/760
R01_cb16076_c5/f1p0/650	NA	NA	R01_cb16076_c5/f1p0/650	R01_cb16076_c5/f1p0/650
R01_cb11485_c1/f1p0/3256	NA	R01_cb11485_c1/f1p0/3256	NA	NA
R01_cb8564_c73047/f1p0/2636	NA	R01_cb8564_c73047/f1p0/2636	NA	R01_cb8564_c73047/f1p0/2636
R01_cb8564_c88399/f1p0/3343	NA	NA	NA	R01_cb8564_c88399/f1p0/3343
R01_cb16106_c2/f1p0/869	NA	R01_cb16106_c2/f1p0/869	NA	R01_cb16106_c2/f1p0/869
R01_cb17577_c0/f1p0/1621	NA	NA	NA	R01_cb17577_c0/f1p0/1621
R01_cb5127_c9/f1p0/2886	NA	R01_cb5127_c9/f1p0/2886	NA	NA

R01_cb8564_c2719/f1p0/3200	R01_cb8564_c2719/f1p0/3200	R01_cb8564_c2719/f1p0/3200	NA	NA
R01_cb8564_c120225/f1p1/2746	NA	R01_cb8564_c120225/f1p1/2746	NA	NA
R01_cb8564_c83233/f1p0/2747	NA	R01_cb8564_c83233/f1p0/2747	NA	NA
R01_cb5297_c5/f1p0/1689	NA	R01_cb5297_c5/f1p0/1689	NA	NA
R01_cb17987_c0/f3p0/1138	NA	NA	NA	R01_cb17987_c0/f3p0/1138
R01_cb10983_c2/f1p0/1018	NA	NA	NA	R01_cb10983_c2/f1p0/1018
R01_cb8564_c116417/f1p0/2093	R01_cb8564_c116417/f1p0/2093	R01_cb8564_c116417/f1p0/2093	R01_cb8564_c116417/f1p0/2093	R01_cb8564_c116417/f1p0/2093
R01_cb6624_c2/f1p0/3013	NA	R01_cb6624_c2/f1p0/3013	NA	R01_cb6624_c2/f1p0/3013
R01_cb10792_c1/f1p0/2174	NA	R01_cb10792_c1/f1p0/2174	NA	R01_cb10792_c1/f1p0/2174
R01_cb14502_c6/f1p0/946	NA	R01_cb14502_c6/f1p0/946	NA	R01_cb14502_c6/f1p0/946
R01_cb8564_c107842/f3p0/2058	R01_cb8564_c107842/f3p0/2058	R01_cb8564_c107842/f3p0/2058	R01_cb8564_c107842/f3p0/2058	R01_cb8564_c107842/f3p0/2058
R01_cb10292_c5/f1p1/1146	NA	NA	NA	R01_cb10292_c5/f1p1/1146
R01_cb5190_c7/f1p2/2292	NA	R01_cb5190_c7/f1p2/2292	NA	R01_cb5190_c7/f1p2/2292
R01_cb18456_c7183/f1p0/513	R01_cb18456_c7183/f1p0/513	R01_cb18456_c7183/f1p0/513	R01_cb18456_c7183/f1p0/513	R01_cb18456_c7183/f1p0/513
R01_cb14078_c1/f1p0/538	NA	R01_cb14078_c1/f1p0/538	NA	NA
R01_cb5900_c42/f1p0/2348	NA	NA	R01_cb5900_c42/f1p0/2348	R01_cb5900_c42/f1p0/2348
R01_cb8564_c71135/f1p0/2328	NA	R01_cb8564_c71135/f1p0/2328	NA	NA
R01_cb15751_c1/f1p0/1256	R01_cb15751_c1/f1p0/1256	R01_cb15751_c1/f1p0/1256	R01_cb15751_c1/f1p0/1256	R01_cb15751_c1/f1p0/1256
R01_cb16524_c0/f5p0/468	R01_cb16524_c0/f5p0/468	NA	NA	R01_cb16524_c0/f5p0/468
R01_cb8564_c4629/f1p0/2950	NA	R01_cb8564_c4629/f1p0/2950	R01_cb8564_c4629/f1p0/2950	R01_cb8564_c4629/f1p0/2950
R01_cb955_c62/f1p0/922	NA	R01_cb955_c62/f1p0/922	NA	NA
R01_cb130_c1/f1p0/4953	NA	R01_cb130_c1/f1p0/4953	NA	NA
R01_cb17054_c0/f2p0/453	R01_cb17054_c0/f2p0/453	R01_cb17054_c0/f2p0/453	R01_cb17054_c0/f2p0/453	R01_cb17054_c0/f2p0/453

R01_cb18456_c7496/f1p0/1356	R01_cb18456_c7496/f1p0/1356	R01_cb18456_c7496/f1p0/1356	R01_cb18456_c7496/f1p0/1356	R01_cb18456_c7496/f1p0/1356
R01_cb10472_c0/f1p0/542	NA	R01_cb10472_c0/f1p0/542	R01_cb10472_c0/f1p0/542	R01_cb10472_c0/f1p0/542
R01_cb5782_c15/f1p0/1333	NA	R01_cb5782_c15/f1p0/1333	NA	NA
R01_cb9376_c1/f1p0/3273	NA	R01_cb9376_c1/f1p0/3273	R01_cb9376_c1/f1p0/3273	R01_cb9376_c1/f1p0/3273
R01_cb5017_c1/f1p0/3198	NA	NA	NA	R01_cb5017_c1/f1p0/3198
R01_cb13512_c1/f1p0/1038	NA	NA	NA	R01_cb13512_c1/f1p0/1038
R01_cb11713_c1/f1p0/1896	NA	R01_cb11713_c1/f1p0/1896	NA	R01_cb11713_c1/f1p0/1896
R01_cb17721_c3/f1p0/1668	NA	R01_cb17721_c3/f1p0/1668	NA	NA
R01_cb9036_c4/f1p0/2432	NA	R01_cb9036_c4/f1p0/2432	NA	NA
R01_cb10229_c4/f1p0/2704	NA	R01_cb10229_c4/f1p0/2704	NA	NA
R01_cb16505_c1/f1p0/573	NA	R01_cb16505_c1/f1p0/573	R01_cb16505_c1/f1p0/573	NA
R01_cb6976_c4/f1p0/2088	NA	R01_cb6976_c4/f1p0/2088	NA	NA
R01_cb34_c2/f1p0/2495	NA	NA	NA	R01_cb34_c2/f1p0/2495
R01_cb13118_c2/f1p0/5011	NA	R01_cb13118_c2/f1p0/5011	NA	NA
R01_cb8564_c109476/f3p1/2122	NA	NA	NA	R01_cb8564_c109476/f3p1/2122
R01_cb18456_c6420/f1p0/461	NA	NA	R01_cb18456_c6420/f1p0/461	R01_cb18456_c6420/f1p0/461
R01_cb16689_c1/f1p0/799	R01_cb16689_c1/f1p0/799	NA	NA	R01_cb16689_c1/f1p0/799
R01_cb8564_c77269/f1p0/3758	NA	NA	NA	R01_cb8564_c77269/f1p0/3758
R01_cb13711_c2/f1p0/1069	NA	R01_cb13711_c2/f1p0/1069	NA	NA
R01_cb10849_c5/f1p0/491	R01_cb10849_c5/f1p0/491	R01_cb10849_c5/f1p0/491	NA	R01_cb10849_c5/f1p0/491
R01_cb15118_c4/f2p1/430	NA	NA	NA	R01_cb15118_c4/f2p1/430
R01_cb11629_c0/f1p0/1315	NA	NA	NA	R01_cb11629_c0/f1p0/1315
R01_cb13876_c2/f5p0/1596	NA	NA	NA	R01_cb13876_c2/f5p0/1596
R01_cb2826_c8/f1p0/3752	NA	R01_cb2826_c8/f1p0/3752	NA	NA

R01_cb4511_c0/f1p0/3341	NA	R01_cb4511_c0/f1p0/3341	NA	NA
R01_cb10448_c3/f1p0/1403	R01_cb10448_c3/f1p0/1403	R01_cb10448_c3/f1p0/1403	NA	NA
R01_cb7110_c2/f1p0/3690	NA	NA	R01_cb7110_c2/f1p0/3690	NA
R01_cb13191_c1/f1p0/1268	NA	NA	NA	R01_cb13191_c1/f1p0/1268
R01_cb8564_c121608/f1p0/3484	NA	R01_cb8564_c121608/f1p0/3484	R01_cb8564_c121608/f1p0/3484	NA
R01_cb14433_c2/f1p0/1612	NA	NA	NA	R01_cb14433_c2/f1p0/1612
R01_cb5875_c1/f1p0/2860	R01_cb5875_c1/f1p0/2860	R01_cb5875_c1/f1p0/2860	R01_cb5875_c1/f1p0/2860	R01_cb5875_c1/f1p0/2860
R01_cb14343_c1/f1p0/570	NA	R01_cb14343_c1/f1p0/570	NA	NA
R01_cb5743_c26/f1p0/1137	NA	R01_cb5743_c26/f1p0/1137	NA	NA
R01_cb17147_c1/f1p0/1466	NA	R01_cb17147_c1/f1p0/1466	NA	NA
R01_cb2161_c6/f1p0/3650	NA	NA	NA	R01_cb2161_c6/f1p0/3650
R01_cb352_c4/f1p0/1968	NA	NA	NA	R01_cb352_c4/f1p0/1968
R01_cb9532_c5/f1p0/893	NA	R01_cb9532_c5/f1p0/893	R01_cb9532_c5/f1p0/893	R01_cb9532_c5/f1p0/893
R01_cb18456_c6532/f1p0/594	NA	R01_cb18456_c6532/f1p0/594	R01_cb18456_c6532/f1p0/594	NA
R01_cb13485_c1/f1p0/669	NA	R01_cb13485_c1/f1p0/669	NA	NA
R01_cb2088_c1/f1p0/4057	NA	NA	NA	R01_cb2088_c1/f1p0/4057
R01_cb16555_c3/f1p0/508	NA	NA	NA	R01_cb16555_c3/f1p0/508
R01_cb2756_c2/f1p0/3820	NA	NA	NA	R01_cb2756_c2/f1p0/3820
R01_cb12803_c1/f1p0/1194	NA	NA	NA	R01_cb12803_c1/f1p0/1194
R01_cb15651_c1/f3p0/782	NA	R01_cb15651_c1/f3p0/782	NA	NA
R01_cb12321_c867/f1p0/343	R01_cb12321_c867/f1p0/343	R01_cb12321_c867/f1p0/343	NA	NA
R01_cb8564_c88373/f1p0/2526	NA	R01_cb8564_c88373/f1p0/2526	R01_cb8564_c88373/f1p0/2526	R01_cb8564_c88373/f1p0/2526
R01_cb10979_c4/f1p0/1409	NA	R01_cb10979_c4/f1p0/1409	NA	NA
R01_cb7221_c3/f3p3/2658	NA	NA	NA	R01_cb7221_c3/f3p3/2658
R01_cb6274_c8/f1p0/2262	NA	NA	NA	R01_cb6274_c8/f1p0/2262

R01_cb2213_c1/f1p0/4372	NA	R01_cb2213_c1/f1p0/4372	NA	R01_cb2213_c1/f1p0/4372
R01_cb1610_c4/f1p0/2665	NA	NA	NA	R01_cb1610_c4/f1p0/2665
R01_cb11257_c0/f2p0/730	NA	NA	NA	R01_cb11257_c0/f2p0/730
R01_cb14937_c6/f1p0/1051	NA	NA	R01_cb14937_c6/f1p0/1051	R01_cb14937_c6/f1p0/1051
R01_cb17756_c32/f1p0/1590	R01_cb17756_c32/f1p0/1590	R01_cb17756_c32/f1p0/1590	R01_cb17756_c32/f1p0/1590	R01_cb17756_c32/f1p0/1590
R01_cb7243_c2/f1p0/2633	NA	NA	NA	R01_cb7243_c2/f1p0/2633
R01_cb18061_c3/f1p0/1047	NA	R01_cb18061_c3/f1p0/1047	NA	NA
R01_cb15251_c3/f1p0/1683	NA	NA	NA	R01_cb15251_c3/f1p0/1683
R01_cb18456_c7147/f1p0/1822	NA	R01_cb18456_c7147/f1p0/1822	R01_cb18456_c7147/f1p0/1822	R01_cb18456_c7147/f1p0/1822
R01_cb6406_c51/f1p0/2760	NA	R01_cb6406_c51/f1p0/2760	NA	NA
R01_cb13097_c3/f1p0/332	R01_cb13097_c3/f1p0/332	R01_cb13097_c3/f1p0/332	R01_cb13097_c3/f1p0/332	R01_cb13097_c3/f1p0/332
R01_cb13230_c3/f1p0/1512	NA	R01_cb13230_c3/f1p0/1512	NA	NA
R01_cb4321_c10/f1p0/504	R01_cb4321_c10/f1p0/504	R01_cb4321_c10/f1p0/504	R01_cb4321_c10/f1p0/504	R01_cb4321_c10/f1p0/504
R01_cb693_c13/f1p0/2877	NA	NA	NA	R01_cb693_c13/f1p0/2877
R01_cb4997_c6/f1p0/2419	NA	NA	NA	R01_cb4997_c6/f1p0/2419
R01_cb14480_c6/f1p0/689	R01_cb14480_c6/f1p0/689	R01_cb14480_c6/f1p0/689	R01_cb14480_c6/f1p0/689	R01_cb14480_c6/f1p0/689
R01_cb3139_c15/f1p0/490	R01_cb3139_c15/f1p0/490	R01_cb3139_c15/f1p0/490	R01_cb3139_c15/f1p0/490	R01_cb3139_c15/f1p0/490
R01_cb8564_c69032/f2p0/3113	R01_cb8564_c69032/f2p0/3113	R01_cb8564_c69032/f2p0/3113	NA	R01_cb8564_c69032/f2p0/3113
R01_cb8564_c107839/f2p1/2411	NA	R01_cb8564_c107839/f2p1/2411	NA	R01_cb8564_c107839/f2p1/2411
R01_cb8564_c122058/f1p1/1944	NA	R01_cb8564_c122058/f1p1/1944	NA	NA
R01_cb9588_c6/f1p1/1687	R01_cb9588_c6/f1p1/1687	R01_cb9588_c6/f1p1/1687	R01_cb9588_c6/f1p1/1687	R01_cb9588_c6/f1p1/1687
R01_cb5183_c33/f1p0/2800	NA	NA	NA	R01_cb5183_c33/f1p0/2800
R01_cb218_c14/f1p0/4667	NA	NA	NA	R01_cb218_c14/f1p0/4667

R01_cb13699_c0/f1p0/1350	NA	NA	NA	R01_cb13699_c0/f1p0/1350
R01_cb13524_c3/f1p0/1102	NA	R01_cb13524_c3/f1p0/1102	NA	NA
R01_cb8564_c109822/f2p1/4639	NA	R01_cb8564_c109822/f2p1/4639	NA	NA
R01_cb8564_c88619/f1p0/4510	R01_cb8564_c88619/f1p0/4510	R01_cb8564_c88619/f1p0/4510	R01_cb8564_c88619/f1p0/4510	NA
R01_cb1163_c77/f1p0/581	R01_cb1163_c77/f1p0/581	R01_cb1163_c77/f1p0/581	NA	R01_cb1163_c77/f1p0/581
R01_cb2104_c4/f1p0/4051	NA	R01_cb2104_c4/f1p0/4051	R01_cb2104_c4/f1p0/4051	R01_cb2104_c4/f1p0/4051
R01_cb9334_c5/f1p0/1137	NA	R01_cb9334_c5/f1p0/1137	NA	NA
R01_cb6127_c4/f1p0/666	NA	R01_cb6127_c4/f1p0/666	NA	NA
R01_cb1581_c4/f2p0/3773	R01_cb1581_c4/f2p0/3773	R01_cb1581_c4/f2p0/3773	NA	NA
R01_cb8564_c83438/f1p1/3175	NA	NA	NA	R01_cb8564_c83438/f1p1/3175
R01_cb18359_c0/f1p0/1599	NA	NA	NA	R01_cb18359_c0/f1p0/1599
R01_cb10880_c10/f9p2/794	NA	NA	NA	R01_cb10880_c10/f9p2/794
R01_cb10567_c3/f1p0/1009	R01_cb10567_c3/f1p0/1009	R01_cb10567_c3/f1p0/1009	R01_cb10567_c3/f1p0/1009	R01_cb10567_c3/f1p0/1009
R01_cb13203_c4/f1p0/2433	NA	R01_cb13203_c4/f1p0/2433	NA	R01_cb13203_c4/f1p0/2433
R01_cb18456_c3758/f1p0/937	R01_cb18456_c3758/f1p0/937	R01_cb18456_c3758/f1p0/937	R01_cb18456_c3758/f1p0/937	R01_cb18456_c3758/f1p0/937
R01_cb12737_c0/f11p0/602	NA	R01_cb12737_c0/f11p0/602	NA	NA
R01_cb7795_c2/f1p0/3536	NA	NA	NA	R01_cb7795_c2/f1p0/3536
R01_cb1376_c7/f1p0/2863	NA	NA	NA	R01_cb1376_c7/f1p0/2863
R01_cb7945_c2/f1p1/4130	NA	R01_cb7945_c2/f1p1/4130	NA	R01_cb7945_c2/f1p1/4130
R01_cb8564_c120860/f1p0/2957	NA	R01_cb8564_c120860/f1p0/2957	NA	NA
R01_cb8564_c184312/f1p0/2279	NA	R01_cb8564_c184312/f1p0/2279	R01_cb8564_c184312/f1p0/2279	R01_cb8564_c184312/f1p0/2279
R01_cb7208_c1/f1p0/2672	NA	R01_cb7208_c1/f1p0/2672	NA	NA

R01_cb2765_c1/f1p0/3808	NA	NA	NA	R01_cb2765_c1/f1p0/3808
R01_cb17690_c1/f1p0/1884	R01_cb17690_c1/f1p0/1884	R01_cb17690_c1/f1p0/1884	R01_cb17690_c1/f1p0/1884	R01_cb17690_c1/f1p0/1884
R01_cb15740_c1/f1p0/663	NA	R01_cb15740_c1/f1p0/663	NA	NA
R01_cb11173_c4/f1p0/595	NA	R01_cb11173_c4/f1p0/595	NA	NA
R01_cb8564_c90196/f1p0/2276	NA	NA	NA	R01_cb8564_c90196/f1p0/2276
R01_cb4147_c14/f1p0/3211	NA	R01_cb4147_c14/f1p0/3211	NA	NA
R01_cb2875_c22/f1p0/3329	NA	NA	NA	R01_cb2875_c22/f1p0/3329
R01_cb10600_c0/f1p0/940	NA	NA	NA	R01_cb10600_c0/f1p0/940
R01_cb2072_c38/f1p0/1966	NA	NA	NA	R01_cb2072_c38/f1p0/1966
R01_cb12332_c7/f1p0/979	NA	R01_cb12332_c7/f1p0/979	NA	NA
R01_cb18116_c0/f1p0/1574	NA	R01_cb18116_c0/f1p0/1574	NA	NA
R01_cb10579_c4/f1p0/1005	NA	NA	NA	R01_cb10579_c4/f1p0/1005
R01_cb16886_c0/f3p0/1312	NA	NA	R01_cb16886_c0/f3p0/1312	R01_cb16886_c0/f3p0/1312
R01_cb3307_c18/f1p0/3518	NA	R01_cb3307_c18/f1p0/3518	NA	NA
R01_cb9759_c4/f1p1/3348	NA	NA	NA	R01_cb9759_c4/f1p1/3348
R01_cb13193_c8/f1p0/605	R01_cb13193_c8/f1p0/605	R01_cb13193_c8/f1p0/605	R01_cb13193_c8/f1p0/605	R01_cb13193_c8/f1p0/605
R01_cb8978_c1/f1p0/2217	R01_cb8978_c1/f1p0/2217	R01_cb8978_c1/f1p0/2217	NA	NA
R01_cb11002_c1/f2p0/1589	NA	NA	NA	R01_cb11002_c1/f2p0/1589
R01_cb12444_c50/f1p0/650	R01_cb12444_c50/f1p0/650	R01_cb12444_c50/f1p0/650	R01_cb12444_c50/f1p0/650	R01_cb12444_c50/f1p0/650
R01_cb8564_c85368/f1p0/2518	R01_cb8564_c85368/f1p0/2518	R01_cb8564_c85368/f1p0/2518	R01_cb8564_c85368/f1p0/2518	R01_cb8564_c85368/f1p0/2518
R01_cb18409_c93/f1p0/756	R01_cb18409_c93/f1p0/756	R01_cb18409_c93/f1p0/756	R01_cb18409_c93/f1p0/756	R01_cb18409_c93/f1p0/756
R01_cb15190_c2/f1p0/982	NA	R01_cb15190_c2/f1p0/982	NA	NA
R01_cb9341_c20/f1p0/2938	NA	R01_cb9341_c20/f1p0/2938	R01_cb9341_c20/f1p0/2938	NA
R01_cb9029_c0/f1p0/2206	NA	NA	NA	R01_cb9029_c0/f1p0/2206
R01_cb12003_c33/f1p0/1360	NA	R01_cb12003_c33/f1p0/1360	NA	NA

R01_cb3169_c3/f1p0/2107	NA	NA	NA	R01_cb3169_c3/f1p0/2107
R01_cb8564_c90923/f1p1/2586	NA	R01_cb8564_c90923/f1p1/2586	R01_cb8564_c90923/f1p1/2586	NA
R01_cb293_c8/f1p0/4316	NA	NA	NA	R01_cb293_c8/f1p0/4316
R01_cb8564_c4978/f2p0/2100	NA	R01_cb8564_c4978/f2p0/2100	NA	NA
R01_cb14619_c7/f1p0/5856	NA	R01_cb14619_c7/f1p0/5856	NA	NA
R01_cb11050_c3/f1p0/2668	NA	R01_cb11050_c3/f1p0/2668	NA	NA
R01_cb12577_c11/f1p2/909	NA	NA	NA	R01_cb12577_c11/f1p2/909
R01_cb14960_c2/f1p0/788	NA	R01_cb14960_c2/f1p0/788	NA	NA
R01_cb8040_c15/f1p0/1895	NA	R01_cb8040_c15/f1p0/1895	NA	R01_cb8040_c15/f1p0/1895
R01_cb10634_c2/f1p1/2846	NA	R01_cb10634_c2/f1p1/2846	NA	NA
R01_cb5339_c4/f1p0/2382	NA	NA	NA	R01_cb5339_c4/f1p0/2382
R01_cb8564_c77472/f1p0/3454	NA	NA	NA	R01_cb8564_c77472/f1p0/3454
R01_cb9643_c2/f1p0/1659	NA	R01_cb9643_c2/f1p0/1659	R01_cb9643_c2/f1p0/1659	NA
R01_cb8173_c4/f1p3/2003	NA	R01_cb8173_c4/f1p3/2003	R01_cb8173_c4/f1p3/2003	NA
R01_cb10118_c2/f1p0/616	NA	R01_cb10118_c2/f1p0/616	NA	R01_cb10118_c2/f1p0/616
R01_cb8564_c1644/f1p0/2393	NA	NA	R01_cb8564_c1644/f1p0/2393	R01_cb8564_c1644/f1p0/2393
R01_cb3432_c7/f1p1/718	NA	R01_cb3432_c7/f1p1/718	NA	NA
R01_cb8564_c25049/f1p0/3150	NA	R01_cb8564_c25049/f1p0/3150	NA	NA
R01_cb13761_c8/f1p0/717	R01_cb13761_c8/f1p0/717	R01_cb13761_c8/f1p0/717	R01_cb13761_c8/f1p0/717	R01_cb13761_c8/f1p0/717
R01_cb6384_c0/f2p0/2878	NA	R01_cb6384_c0/f2p0/2878	R01_cb6384_c0/f2p0/2878	R01_cb6384_c0/f2p0/2878
R01_cb289_c24/f1p0/1250	NA	R01_cb289_c24/f1p0/1250	NA	R01_cb289_c24/f1p0/1250
R01_cb9252_c2/f1p0/2527	NA	R01_cb9252_c2/f1p0/2527	R01_cb9252_c2/f1p0/2527	R01_cb9252_c2/f1p0/2527
R01_cb16396_c2/f1p0/1321	NA	R01_cb16396_c2/f1p0/1321	NA	NA
R01_cb9317_c10/f1p1/1720	NA	R01_cb9317_c10/f1p1/1720	NA	NA
R01_cb10697_c6/f1p0/820	NA	R01_cb10697_c6/f1p0/820	NA	NA
R01_cb8564_c15960/f1p0/3558	NA	NA	R01_cb8564_c15960/f1p0/3558	R01_cb8564_c15960/f1p0/3558

R01_cb27_c0/f3p0/1066	NA	NA	NA	R01_cb27_c0/f3p0/1066
R01_cb17761_c2/f2p0/753	NA	NA	NA	R01_cb17761_c2/f2p0/753
R01_cb13433_c104/f1p0/615	NA	R01_cb13433_c104/f1p0/615	NA	NA
R01_cb17260_c0/f1p0/473	R01_cb17260_c0/f1p0/473	R01_cb17260_c0/f1p0/473	R01_cb17260_c0/f1p0/473	R01_cb17260_c0/f1p0/473
R01_cb88_c25/f1p0/2551	NA	NA	NA	R01_cb88_c25/f1p0/2551
R01_cb16474_c0/f2p0/1518	NA	NA	NA	R01_cb16474_c0/f2p0/1518
R01_cb10024_c345/f1p3/778	NA	NA	R01_cb10024_c345/f1p3/778	NA
R01_cb14223_c2/f3p0/399	R01_cb14223_c2/f3p0/399	R01_cb14223_c2/f3p0/399	R01_cb14223_c2/f3p0/399	R01_cb14223_c2/f3p0/399
R01_cb14196_c0/f1p0/1220	NA	R01_cb14196_c0/f1p0/1220	R01_cb14196_c0/f1p0/1220	R01_cb14196_c0/f1p0/1220
R01_cb11450_c0/f1p0/1165	NA	NA	NA	R01_cb11450_c0/f1p0/1165
R01_cb11771_c1/f1p0/3223	NA	R01_cb11771_c1/f1p0/3223	R01_cb11771_c1/f1p0/3223	R01_cb11771_c1/f1p0/3223
R01_cb6885_c0/f4p1/1919	NA	NA	NA	R01_cb6885_c0/f4p1/1919
R01_cb8564_c42955/f1p0/4148	R01_cb8564_c42955/f1p0/4148	R01_cb8564_c42955/f1p0/4148	R01_cb8564_c42955/f1p0/4148	R01_cb8564_c42955/f1p0/4148
R01_cb13259_c4/f1p0/368	R01_cb13259_c4/f1p0/368	R01_cb13259_c4/f1p0/368	R01_cb13259_c4/f1p0/368	NA
R01_cb2472_c2/f1p0/3916	NA	NA	R01_cb2472_c2/f1p0/3916	NA
R01_cb226_c8/f1p0/5018	NA	NA	NA	R01_cb226_c8/f1p0/5018
R01_cb3516_c4/f1p0/2693	NA	NA	NA	R01_cb3516_c4/f1p0/2693
R01_cb6845_c38/f13p2/2516	NA	NA	NA	R01_cb6845_c38/f13p2/2516
R01_cb12740_c6/f2p0/479	R01_cb12740_c6/f2p0/479	R01_cb12740_c6/f2p0/479	NA	R01_cb12740_c6/f2p0/479
R01_cb6297_c3/f2p0/3335	NA	NA	R01_cb6297_c3/f2p0/3335	NA
R01_cb18456_c7578/f1p0/744	R01_cb18456_c7578/f1p0/744	R01_cb18456_c7578/f1p0/744	R01_cb18456_c7578/f1p0/744	R01_cb18456_c7578/f1p0/744
R01_cb893_c4/f1p0/2859	NA	NA	NA	R01_cb893_c4/f1p0/2859
R01_cb8564_c88984/f1p0/3187	NA	R01_cb8564_c88984/f1p0/3187	NA	R01_cb8564_c88984/f1p0/3187
R01_cb18704_c1/f1p0/720	R01_cb18704_c1/f1p0/720	R01_cb18704_c1/f1p0/720	R01_cb18704_c1/f1p0/720	NA
R01_cb13903_c2/f1p0/943	NA	R01_cb13903_c2/f1p0/943	NA	NA

R01_cb11321_c1/f1p0/2453	NA	NA	NA	R01_cb11321_c1/f1p0/2453
R01_cb5412_c3/f1p0/2987	NA	NA	NA	R01_cb5412_c3/f1p0/2987
R01_cb9237_c11/f1p0/1417	NA	R01_cb9237_c11/f1p0/1417	NA	NA
R01_cb18456_c3611/f1p4/1109	NA	R01_cb18456_c3611/f1p4/1109	NA	NA
R01_cb8564_c87992/f1p0/4208	NA	R01_cb8564_c87992/f1p0/4208	NA	NA
R01_cb8564_c85251/f1p0/2819	NA	R01_cb8564_c85251/f1p0/2819	NA	R01_cb8564_c85251/f1p0/2819
R01_cb9658_c1/f2p0/1942	NA	NA	NA	R01_cb9658_c1/f2p0/1942
R01_cb3563_c2/f1p0/3312	NA	R01_cb3563_c2/f1p0/3312	NA	R01_cb3563_c2/f1p0/3312
R01_cb15469_c7/f1p0/1338	NA	R01_cb15469_c7/f1p0/1338	NA	NA
R01_cb2314_c5/f1p0/3736	NA	R01_cb2314_c5/f1p0/3736	NA	NA
R01_cb1031_c3/f1p0/6376	NA	NA	NA	R01_cb1031_c3/f1p0/6376
R01_cb13761_c1/f1p0/558	R01_cb13761_c1/f1p0/558	R01_cb13761_c1/f1p0/558	R01_cb13761_c1/f1p0/558	R01_cb13761_c1/f1p0/558
R01_cb5158_c5/f1p0/1406	NA	NA	NA	R01_cb5158_c5/f1p0/1406
R01_cb18021_c0/f2p0/643	NA	R01_cb18021_c0/f2p0/643	R01_cb18021_c0/f2p0/643	R01_cb18021_c0/f2p0/643
R01_cb1138_c46/f1p0/716	R01_cb1138_c46/f1p0/716	NA	R01_cb1138_c46/f1p0/716	R01_cb1138_c46/f1p0/716
R01_cb8564_c18520/f2p0/3299	NA	NA	NA	R01_cb8564_c18520/f2p0/3299
R01_cb6571_c2/f1p0/2224	NA	R01_cb6571_c2/f1p0/2224	NA	R01_cb6571_c2/f1p0/2224
R01_cb5234_c6/f1p0/2095	R01_cb5234_c6/f1p0/2095	R01_cb5234_c6/f1p0/2095	NA	R01_cb5234_c6/f1p0/2095
R01_cb11630_c2/f1p0/1652	NA	R01_cb11630_c2/f1p0/1652	R01_cb11630_c2/f1p0/1652	R01_cb11630_c2/f1p0/1652
R01_cb12485_c0/f2p0/1365	NA	NA	NA	R01_cb12485_c0/f2p0/1365
R01_cb16730_c1/f1p0/539	NA	R01_cb16730_c1/f1p0/539	NA	R01_cb16730_c1/f1p0/539
R01_cb8564_c76686/f1p0/3709	NA	NA	NA	R01_cb8564_c76686/f1p0/3709
R01_cb9975_c1/f1p0/2600	NA	NA	NA	R01_cb9975_c1/f1p0/2600
R01_cb8564_c3548/f1p5/2797	NA	R01_cb8564_c3548/f1p5/2797	NA	NA
R01_cb16913_c5/f1p0/800	NA	R01_cb16913_c5/f1p0/800	NA	NA
R01_cb10140_c2/f1p0/2321	NA	R01_cb10140_c2/f1p0/2321	NA	NA

R01_cb14542_c4/f1p0/1085	NA	NA	NA	R01_cb14542_c4/f1p0/1085
R01_cb3059_c1/f1p0/2343	NA	R01_cb3059_c1/f1p0/2343	NA	R01_cb3059_c1/f1p0/2343
R01_cb8564_c10623/f1p0/3768	NA	R01_cb8564_c10623/f1p0/3768	NA	NA
R01_cb10966_c0/f1p0/1793	NA	R01_cb10966_c0/f1p0/1793	R01_cb10966_c0/f1p0/1793	R01_cb10966_c0/f1p0/1793
R01_cb5219_c5/f1p0/3091	NA	R01_cb5219_c5/f1p0/3091	NA	NA
R01_cb8564_c121314/f1p0/2623	R01_cb8564_c121314/f1p0/2623	R01_cb8564_c121314/f1p0/2623	R01_cb8564_c121314/f1p0/2623	NA
R01_cb8789_c1/f1p0/2302	NA	NA	NA	R01_cb8789_c1/f1p0/2302
R01_cb8564_c114604/f3p0/2235	NA	NA	NA	R01_cb8564_c114604/f3p0/2235
R01_cb5062_c8/f1p0/2633	NA	R01_cb5062_c8/f1p0/2633	NA	NA
R01_cb5246_c1/f1p0/2846	R01_cb5246_c1/f1p0/2846	R01_cb5246_c1/f1p0/2846	R01_cb5246_c1/f1p0/2846	R01_cb5246_c1/f1p0/2846
R01_cb8564_c53792/f1p0/4512	R01_cb8564_c53792/f1p0/4512	R01_cb8564_c53792/f1p0/4512	NA	NA
R01_cb18192_c8/f1p0/705	NA	R01_cb18192_c8/f1p0/705	R01_cb18192_c8/f1p0/705	R01_cb18192_c8/f1p0/705
R01_cb6348_c9/f1p0/1853	NA	NA	NA	R01_cb6348_c9/f1p0/1853
R01_cb4715_c1/f1p0/2611	NA	NA	R01_cb4715_c1/f1p0/2611	R01_cb4715_c1/f1p0/2611
R01_cb17744_c7/f2p0/3036	NA	R01_cb17744_c7/f2p0/3036	NA	NA
R01_cb10822_c1/f1p0/2352	NA	R01_cb10822_c1/f1p0/2352	NA	NA
R01_cb8564_c126750/f2p1/2190	NA	NA	NA	R01_cb8564_c126750/f2p1/2190
R01_cb4249_c4/f1p0/4246	NA	R01_cb4249_c4/f1p0/4246	R01_cb4249_c4/f1p0/4246	NA
R01_cb194_c17/f1p0/3170	R01_cb194_c17/f1p0/3170	R01_cb194_c17/f1p0/3170	R01_cb194_c17/f1p0/3170	R01_cb194_c17/f1p0/3170
R01_cb8564_c20159/f1p0/1939	NA	R01_cb8564_c20159/f1p0/1939	NA	NA
R01_cb8564_c87147/f1p0/3283	NA	NA	NA	R01_cb8564_c87147/f1p0/3283
R01_cb18456_c6086/f1p0/1076	NA	NA	R01_cb18456_c6086/f1p0/1076	R01_cb18456_c6086/f1p0/1076

R01_cb13884_c17/f1p0/1021	NA	NA	R01_cb13884_c17/f1p0/1021	R01_cb13884_c17/f1p0/1021
R01_cb3164_c1/f1p0/3697	NA	R01_cb3164_c1/f1p0/3697	NA	NA
R01_cb4233_c204/f1p0/3424	NA	R01_cb4233_c204/f1p0/3424	NA	NA
R01_cb454_c1/f3p0/2382	NA	NA	NA	R01_cb454_c1/f3p0/2382
R01_cb18265_c1/f1p0/407	NA	R01_cb18265_c1/f1p0/407	NA	R01_cb18265_c1/f1p0/407
R01_cb11682_c1/f1p0/2157	NA	NA	NA	R01_cb11682_c1/f1p0/2157
R01_cb2434_c3/f1p0/3369	NA	NA	NA	R01_cb2434_c3/f1p0/3369
R01_cb1224_c17/f1p0/4295	NA	NA	NA	R01_cb1224_c17/f1p0/4295
R01_cb2026_c2/f1p0/3974	R01_cb2026_c2/f1p0/3974	R01_cb2026_c2/f1p0/3974	R01_cb2026_c2/f1p0/3974	R01_cb2026_c2/f1p0/3974
R01_cb8564_c69487/f2p0/2258	NA	R01_cb8564_c69487/f2p0/2258	R01_cb8564_c69487/f2p0/2258	R01_cb8564_c69487/f2p0/2258
R01_cb5896_c157/f1p0/3375	NA	R01_cb5896_c157/f1p0/3375	R01_cb5896_c157/f1p0/3375	NA
R01_cb3563_c6/f1p0/2153	NA	NA	NA	R01_cb3563_c6/f1p0/2153
R01_cb8564_c1404/f1p0/3610	NA	R01_cb8564_c1404/f1p0/3610	R01_cb8564_c1404/f1p0/3610	R01_cb8564_c1404/f1p0/3610
R01_cb2770_c58/f1p0/3220	NA	R01_cb2770_c58/f1p0/3220	NA	NA
R01_cb4911_c4/f1p0/2755	NA	R01_cb4911_c4/f1p0/2755	NA	NA
R01_cb10406_c5/f1p0/1620	R01_cb10406_c5/f1p0/1620	R01_cb10406_c5/f1p0/1620	NA	NA
R01_cb12616_c0/f1p1/1401	NA	NA	NA	R01_cb12616_c0/f1p1/1401
R01_cb59_c4/f1p1/2682	NA	R01_cb59_c4/f1p1/2682	R01_cb59_c4/f1p1/2682	R01_cb59_c4/f1p1/2682
R01_cb18456_c6073/f1p0/468	NA	R01_cb18456_c6073/f1p0/468	NA	NA
R01_cb14639_c4/f19p0/457	R01_cb14639_c4/f19p0/457	R01_cb14639_c4/f19p0/457	NA	NA
R01_cb11685_c1/f1p0/2910	NA	R01_cb11685_c1/f1p0/2910	R01_cb11685_c1/f1p0/2910	R01_cb11685_c1/f1p0/2910
R01_cb12572_c9/f1p0/808	NA	NA	R01_cb12572_c9/f1p0/808	R01_cb12572_c9/f1p0/808
R01_cb8564_c130034/f1p0/2943	NA	R01_cb8564_c130034/f1p0/2943	NA	R01_cb8564_c130034/f1p0/2943
R01_cb8564_c83618/f1p0/2660	NA	R01_cb8564_c83618/f1p0/2660	R01_cb8564_c83618/f1p0/2660	NA
R01_cb10437_c1/f1p0/854	R01_cb10437_c1/f1p0/854	R01_cb10437_c1/f1p0/854	R01_cb10437_c1/f1p0/854	R01_cb10437_c1/f1p0/854

R01_cb4469_c7/f1p0/3119	NA	NA	NA	R01_cb4469_c7/f1p0/3119
R01_cb12288_c0/f4p0/598	R01_cb12288_c0/f4p0/598	R01_cb12288_c0/f4p0/598	R01_cb12288_c0/f4p0/598	R01_cb12288_c0/f4p0/598
R01_cb9274_c27/f1p0/2089	NA	NA	NA	R01_cb9274_c27/f1p0/2089
R01_cb12152_c0/f3p1/867	NA	NA	R01_cb12152_c0/f3p1/867	R01_cb12152_c0/f3p1/867
R01_cb7221_c2/f3p2/2669	NA	NA	NA	R01_cb7221_c2/f3p2/2669
R01_cb15952_c1/f1p0/1351	NA	NA	NA	R01_cb15952_c1/f1p0/1351
R01_cb9285_c0/f1p0/2127	NA	R01_cb9285_c0/f1p0/2127	NA	NA
R01_cb17231_c4/f1p0/734	NA	NA	NA	R01_cb17231_c4/f1p0/734
R01_cb18187_c0/f2p0/830	NA	NA	NA	R01_cb18187_c0/f2p0/830
R01_cb10375_c3/f1p0/1643	NA	NA	NA	R01_cb10375_c3/f1p0/1643
R01_cb17792_c0/f1p0/1223	NA	NA	NA	R01_cb17792_c0/f1p0/1223
R01_cb17271_c0/f2p0/634	NA	R01_cb17271_c0/f2p0/634	R01_cb17271_c0/f2p0/634	R01_cb17271_c0/f2p0/634
R01_cb9882_c3/f1p0/1971	R01_cb9882_c3/f1p0/1971	R01_cb9882_c3/f1p0/1971	NA	NA
R01_cb8564_c86833/f1p0/3511	R01_cb8564_c86833/f1p0/3511	R01_cb8564_c86833/f1p0/3511	R01_cb8564_c86833/f1p0/3511	R01_cb8564_c86833/f1p0/3511
R01_cb5246_c0/f1p0/3012	R01_cb5246_c0/f1p0/3012	R01_cb5246_c0/f1p0/3012	R01_cb5246_c0/f1p0/3012	R01_cb5246_c0/f1p0/3012
R01_cb8564_c78185/f1p0/2766	NA	R01_cb8564_c78185/f1p0/2766	R01_cb8564_c78185/f1p0/2766	R01_cb8564_c78185/f1p0/2766
R01_cb17132_c1/f1p0/685	NA	NA	R01_cb17132_c1/f1p0/685	NA
R01_cb2159_c2/f1p0/3833	NA	R01_cb2159_c2/f1p0/3833	NA	NA
R01_cb13021_c1/f3p0/501	R01_cb13021_c1/f3p0/501	R01_cb13021_c1/f3p0/501	NA	NA
R01_cb8564_c119207/f1p0/2465	NA	R01_cb8564_c119207/f1p0/2465	R01_cb8564_c119207/f1p0/2465	R01_cb8564_c119207/f1p0/2465
R01_cb14019_c4/f1p0/1553	NA	R01_cb14019_c4/f1p0/1553	R01_cb14019_c4/f1p0/1553	R01_cb14019_c4/f1p0/1553
R01_cb10229_c2/f1p0/1728	R01_cb10229_c2/f1p0/1728	R01_cb10229_c2/f1p0/1728	NA	R01_cb10229_c2/f1p0/1728
R01_cb18456_c7581/f1p0/1653	NA	R01_cb18456_c7581/f1p0/1653	NA	NA
R01_cb16856_c3/f1p0/1170	NA	NA	NA	R01_cb16856_c3/f1p0/1170

R01_cb218_c1/f5p1/4661	NA	NA	NA	R01_cb218_c1/f5p1/4661
R01_cb14307_c1/f2p0/1592	NA	R01_cb14307_c1/f2p0/1592	NA	NA
R01_cb8564_c37464/f1p0/2862	NA	NA	NA	R01_cb8564_c37464/f1p0/2862
R01_cb11558_c1/f1p0/4620	R01_cb11558_c1/f1p0/4620	R01_cb11558_c1/f1p0/4620	R01_cb11558_c1/f1p0/4620	R01_cb11558_c1/f1p0/4620
R01_cb1612_c9/f1p4/3376	NA	NA	NA	R01_cb1612_c9/f1p4/3376
R01_cb11632_c1/f1p0/2477	NA	R01_cb11632_c1/f1p0/2477	NA	NA
R01_cb5659_c32/f1p0/2068	NA	NA	NA	R01_cb5659_c32/f1p0/2068
R01_cb15651_c3/f1p0/564	NA	R01_cb15651_c3/f1p0/564	NA	NA
R01_cb6278_c16/f1p0/1750	NA	NA	NA	R01_cb6278_c16/f1p0/1750
R01_cb11206_c0/f1p0/1015	NA	R01_cb11206_c0/f1p0/1015	NA	R01_cb11206_c0/f1p0/1015
R01_cb14004_c5/f1p1/1187	NA	NA	NA	R01_cb14004_c5/f1p1/1187
R01_cb6327_c2/f1p0/2581	NA	R01_cb6327_c2/f1p0/2581	R01_cb6327_c2/f1p0/2581	R01_cb6327_c2/f1p0/2581
R01_cb8564_c123839/f1p0/2304	NA	NA	R01_cb8564_c123839/f1p0/2304	R01_cb8564_c123839/f1p0/2304
R01_cb13386_c16/f1p0/1396	NA	R01_cb13386_c16/f1p0/1396	NA	NA
R01_cb1138_c55/f2p1/913	NA	NA	R01_cb1138_c55/f2p1/913	R01_cb1138_c55/f2p1/913
R01_cb18033_c1/f1p0/479	R01_cb18033_c1/f1p0/479	R01_cb18033_c1/f1p0/479	R01_cb18033_c1/f1p0/479	R01_cb18033_c1/f1p0/479
R01_cb8564_c114342/f1p0/2738	NA	NA	NA	R01_cb8564_c114342/f1p0/2738
R01_cb17773_c1/f1p0/599	R01_cb17773_c1/f1p0/599	R01_cb17773_c1/f1p0/599	R01_cb17773_c1/f1p0/599	R01_cb17773_c1/f1p0/599
R01_cb12924_c0/f1p0/1807	R01_cb12924_c0/f1p0/1807	R01_cb12924_c0/f1p0/1807	NA	R01_cb12924_c0/f1p0/1807
R01_cb4707_c0/f1p0/3293	NA	NA	NA	R01_cb4707_c0/f1p0/3293
R01_cb16383_c7/f1p0/1854	NA	R01_cb16383_c7/f1p0/1854	NA	R01_cb16383_c7/f1p0/1854
R01_cb17398_c7/f1p0/1684	NA	R01_cb17398_c7/f1p0/1684	NA	NA
R01_cb9592_c1/f8p1/1581	NA	NA	NA	R01_cb9592_c1/f8p1/1581
R01_cb17756_c7/f2p0/783	R01_cb17756_c7/f2p0/783	R01_cb17756_c7/f2p0/783	R01_cb17756_c7/f2p0/783	R01_cb17756_c7/f2p0/783

R01_cb11146_c1/f1p0/3190	R01_cb11146_c1/f1p0/3190	R01_cb11146_c1/f1p0/3190	NA	R01_cb11146_c1/f1p0/3190
R01_cb17821_c1/f1p0/1365	NA	R01_cb17821_c1/f1p0/1365	NA	NA
R01_cb15188_c3/f1p0/606	NA	R01_cb15188_c3/f1p0/606	NA	R01_cb15188_c3/f1p0/606
R01_cb4960_c6/f1p0/796	R01_cb4960_c6/f1p0/796	R01_cb4960_c6/f1p0/796	R01_cb4960_c6/f1p0/796	R01_cb4960_c6/f1p0/796
R01_cb8564_c77690/f1p0/3725	NA	R01_cb8564_c77690/f1p0/3725	NA	NA
R01_cb6523_c0/f6p0/2407	NA	NA	NA	R01_cb6523_c0/f6p0/2407
R01_cb8564_c110997/f1p1/2499	NA	R01_cb8564_c110997/f1p1/2499	NA	NA
R01_cb18847_c0/f1p0/5087	NA	R01_cb18847_c0/f1p0/5087	NA	NA
R01_cb8564_c124064/f1p1/2791	NA	R01_cb8564_c124064/f1p1/2791	R01_cb8564_c124064/f1p1/2791	NA
R01_cb16038_c1/f2p1/549	NA	R01_cb16038_c1/f2p1/549	R01_cb16038_c1/f2p1/549	R01_cb16038_c1/f2p1/549
R01_cb8279_c11/f1p0/2005	NA	NA	NA	R01_cb8279_c11/f1p0/2005
R01_cb8564_c25182/f1p0/3606	NA	R01_cb8564_c25182/f1p0/3606	NA	NA
R01_cb8564_c87867/f1p0/3820	NA	R01_cb8564_c87867/f1p0/3820	NA	NA
R01_cb14854_c9/f1p0/408	R01_cb14854_c9/f1p0/408	R01_cb14854_c9/f1p0/408	R01_cb14854_c9/f1p0/408	R01_cb14854_c9/f1p0/408
R01_cb18103_c4/f2p0/1677	NA	R01_cb18103_c4/f2p0/1677	NA	NA
R01_cb1573_c28/f1p0/2024	NA	NA	NA	R01_cb1573_c28/f1p0/2024
R01_cb16264_c17/f1p0/554	R01_cb16264_c17/f1p0/554	R01_cb16264_c17/f1p0/554	R01_cb16264_c17/f1p0/554	R01_cb16264_c17/f1p0/554
R01_cb12767_c6/f1p1/1044	NA	R01_cb12767_c6/f1p1/1044	NA	R01_cb12767_c6/f1p1/1044
R01_cb7214_c10/f1p0/1871	NA	NA	NA	R01_cb7214_c10/f1p0/1871
R01_cb10364_c6/f1p0/1793	NA	NA	NA	R01_cb10364_c6/f1p0/1793
R01_cb12242_c2/f1p0/872	NA	NA	NA	R01_cb12242_c2/f1p0/872
R01_cb2403_c4/f1p0/803	NA	R01_cb2403_c4/f1p0/803	R01_cb2403_c4/f1p0/803	R01_cb2403_c4/f1p0/803
R01_cb16814_c2/f1p0/477	R01_cb16814_c2/f1p0/477	R01_cb16814_c2/f1p0/477	NA	R01_cb16814_c2/f1p0/477
R01_cb8564_c14160/f1p0/3054	NA	NA	NA	R01_cb8564_c14160/f1p0/3054

R01_cb709_c18/f1p0/1642	NA	R01_cb709_c18/f1p0/1642	R01_cb709_c18/f1p0/1642	R01_cb709_c18/f1p0/1642
R01_cb18200_c1/f1p0/1475	R01_cb18200_c1/f1p0/1475	R01_cb18200_c1/f1p0/1475	R01_cb18200_c1/f1p0/1475	R01_cb18200_c1/f1p0/1475
R01_cb18456_c7394/f1p0/1133	NA	NA	R01_cb18456_c7394/f1p0/1133	NA
R01_cb9367_c0/f2p0/2124	NA	NA	NA	R01_cb9367_c0/f2p0/2124
R01_cb13875_c10/f1p1/1513	NA	NA	NA	R01_cb13875_c10/f1p1/1513
R01_cb18290_c5/f1p0/669	R01_cb18290_c5/f1p0/669	R01_cb18290_c5/f1p0/669	NA	NA
R01_cb8564_c122011/f1p0/2947	NA	R01_cb8564_c122011/f1p0/2947	NA	R01_cb8564_c122011/f1p0/2947
R01_cb8564_c42178/f1p0/2207	NA	NA	NA	R01_cb8564_c42178/f1p0/2207
R01_cb8564_c88952/f1p0/2454	NA	NA	NA	R01_cb8564_c88952/f1p0/2454
R01_cb6377_c2/f1p0/2528	NA	NA	NA	R01_cb6377_c2/f1p0/2528
R01_cb8564_c122473/f2p0/1979	NA	NA	R01_cb8564_c122473/f2p0/1979	R01_cb8564_c122473/f2p0/1979
R01_cb9271_c3/f1p0/2135	NA	R01_cb9271_c3/f1p0/2135	NA	NA
R01_cb15672_c1/f1p0/1541	NA	NA	NA	R01_cb15672_c1/f1p0/1541
R01_cb14576_c1/f1p0/1802	NA	NA	R01_cb14576_c1/f1p0/1802	R01_cb14576_c1/f1p0/1802
R01_cb14125_c154/f1p0/1749	NA	R01_cb14125_c154/f1p0/1749	NA	R01_cb14125_c154/f1p0/1749
R01_cb18492_c0/f1p0/905	NA	NA	NA	R01_cb18492_c0/f1p0/905
R01_cb14060_c1/f1p0/624	R01_cb14060_c1/f1p0/624	R01_cb14060_c1/f1p0/624	R01_cb14060_c1/f1p0/624	R01_cb14060_c1/f1p0/624
R01_cb17082_c4/f1p0/634	R01_cb17082_c4/f1p0/634	R01_cb17082_c4/f1p0/634	R01_cb17082_c4/f1p0/634	R01_cb17082_c4/f1p0/634
R01_cb124_c2/f4p0/2458	NA	NA	NA	R01_cb124_c2/f4p0/2458
R01_cb9540_c1/f1p0/2093	R01_cb9540_c1/f1p0/2093	R01_cb9540_c1/f1p0/2093	NA	R01_cb9540_c1/f1p0/2093
R01_cb13513_c5/f1p0/677	NA	R01_cb13513_c5/f1p0/677	NA	NA
R01_cb5421_c6/f1p0/2803	NA	NA	R01_cb5421_c6/f1p0/2803	NA
R01_cb17855_c1/f1p0/767	NA	NA	NA	R01_cb17855_c1/f1p0/767
R01_cb8710_c3/f2p0/2101	NA	R01_cb8710_c3/f2p0/2101	R01_cb8710_c3/f2p0/2101	R01_cb8710_c3/f2p0/2101

R01_cb10771_c3/f10p0/631	NA	NA	R01_cb10771_c3/f10p0/631	R01_cb10771_c3/f10p0/631
R01_cb8564_c124842/f1p0/2463	NA	R01_cb8564_c124842/f1p0/2463	R01_cb8564_c124842/f1p0/2463	NA
R01_cb6106_c26/f1p0/735	R01_cb6106_c26/f1p0/735	R01_cb6106_c26/f1p0/735	NA	R01_cb6106_c26/f1p0/735
R01_cb8797_c1/f1p0/2456	NA	R01_cb8797_c1/f1p0/2456	R01_cb8797_c1/f1p0/2456	R01_cb8797_c1/f1p0/2456
R01_cb7114_c12/f16p1/1407	NA	NA	NA	R01_cb7114_c12/f16p1/1407
R01_cb15364_c2/f1p0/1256	NA	NA	NA	R01_cb15364_c2/f1p0/1256
R01_cb4489_c8/f1p0/2559	NA	R01_cb4489_c8/f1p0/2559	NA	R01_cb4489_c8/f1p0/2559
R01_cb16732_c1/f1p0/546	NA	R01_cb16732_c1/f1p0/546	NA	R01_cb16732_c1/f1p0/546
R01_cb16290_c1/f1p0/1087	NA	R01_cb16290_c1/f1p0/1087	R01_cb16290_c1/f1p0/1087	R01_cb16290_c1/f1p0/1087
R01_cb13651_c22/f1p0/970	NA	R01_cb13651_c22/f1p0/970	NA	NA
R01_cb8564_c82698/f1p0/4117	NA	NA	NA	R01_cb8564_c82698/f1p0/4117
R01_cb8560_c0/f4p2/2329	NA	NA	NA	R01_cb8560_c0/f4p2/2329
R01_cb15449_c0/f1p0/1414	R01_cb15449_c0/f1p0/1414	R01_cb15449_c0/f1p0/1414	R01_cb15449_c0/f1p0/1414	R01_cb15449_c0/f1p0/1414
R01_cb7564_c37/f1p0/3940	NA	R01_cb7564_c37/f1p0/3940	NA	NA
R01_cb3632_c0/f1p0/3572	NA	NA	NA	R01_cb3632_c0/f1p0/3572
R01_cb2632_c8/f1p0/1556	NA	R01_cb2632_c8/f1p0/1556	R01_cb2632_c8/f1p0/1556	R01_cb2632_c8/f1p0/1556
R01_cb977_c4/f1p0/2908	NA	NA	NA	R01_cb977_c4/f1p0/2908
R01_cb11149_c1/f1p0/2502	NA	NA	R01_cb11149_c1/f1p0/2502	NA
R01_cb16706_c6/f1p0/1339	R01_cb16706_c6/f1p0/1339	R01_cb16706_c6/f1p0/1339	R01_cb16706_c6/f1p0/1339	R01_cb16706_c6/f1p0/1339
R01_cb7612_c4/f1p1/3029	NA	NA	NA	R01_cb7612_c4/f1p1/3029
R01_cb7308_c10/f1p0/1431	NA	NA	NA	R01_cb7308_c10/f1p0/1431
R01_cb3921_c2/f1p0/2076	NA	NA	NA	R01_cb3921_c2/f1p0/2076
R01_cb6258_c24/f1p1/2622	NA	NA	NA	R01_cb6258_c24/f1p1/2622
R01_cb6507_c6/f1p2/1136	NA	NA	NA	R01_cb6507_c6/f1p2/1136
R01_cb17973_c8/f2p0/554	R01_cb17973_c8/f2p0/554	R01_cb17973_c8/f2p0/554	R01_cb17973_c8/f2p0/554	NA

R01_cb4573_c0/f2p0/3041	R01_cb4573_c0/f2p0/3041	R01_cb4573_c0/f2p0/3041	R01_cb4573_c0/f2p0/3041	R01_cb4573_c0/f2p0/3041
R01_cb18456_c6361/f1p0/486	R01_cb18456_c6361/f1p0/486	R01_cb18456_c6361/f1p0/486	R01_cb18456_c6361/f1p0/486	R01_cb18456_c6361/f1p0/486
R01_cb15904_c3/f1p0/1213	NA	R01_cb15904_c3/f1p0/1213	R01_cb15904_c3/f1p0/1213	R01_cb15904_c3/f1p0/1213
R01_cb17773_c0/f1p0/1513	NA	R01_cb17773_c0/f1p0/1513	R01_cb17773_c0/f1p0/1513	NA
R01_cb10401_c2/f1p0/4006	NA	NA	NA	R01_cb10401_c2/f1p0/4006
R01_cb11776_c1/f1p0/3563	R01_cb11776_c1/f1p0/3563	R01_cb11776_c1/f1p0/3563	NA	NA
R01_cb6861_c8/f1p0/3931	NA	R01_cb6861_c8/f1p0/3931	NA	R01_cb6861_c8/f1p0/3931
R01_cb8564_c34126/f2p0/3326	NA	R01_cb8564_c34126/f2p0/3326	NA	NA
R01_cb8564_c25561/f1p0/2461	NA	R01_cb8564_c25561/f1p0/2461	R01_cb8564_c25561/f1p0/2461	R01_cb8564_c25561/f1p0/2461
R01_cb12493_c4/f1p0/336	R01_cb12493_c4/f1p0/336	R01_cb12493_c4/f1p0/336	R01_cb12493_c4/f1p0/336	R01_cb12493_c4/f1p0/336
R01_cb4768_c89/f1p0/2920	NA	R01_cb4768_c89/f1p0/2920	R01_cb4768_c89/f1p0/2920	R01_cb4768_c89/f1p0/2920
R01_cb4590_c11/f1p0/2954	NA	NA	NA	R01_cb4590_c11/f1p0/2954
R01_cb8564_c39636/f1p0/2896	NA	R01_cb8564_c39636/f1p0/2896	R01_cb8564_c39636/f1p0/2896	R01_cb8564_c39636/f1p0/2896
R01_cb8564_c39892/f1p0/2908	R01_cb8564_c39892/f1p0/2908	R01_cb8564_c39892/f1p0/2908	NA	NA
R01_cb14328_c2/f1p0/790	NA	NA	R01_cb14328_c2/f1p0/790	R01_cb14328_c2/f1p0/790
R01_cb8564_c75371/f1p0/1913	NA	R01_cb8564_c75371/f1p0/1913	NA	NA
R01_cb507_c4/f1p0/3466	NA	NA	NA	R01_cb507_c4/f1p0/3466
R01_cb17363_c11/f1p0/1824	NA	R01_cb17363_c11/f1p0/1824	R01_cb17363_c11/f1p0/1824	R01_cb17363_c11/f1p0/1824
R01_cb16757_c2/f1p0/1800	NA	R01_cb16757_c2/f1p0/1800	NA	NA
R01_cb15334_c1/f2p0/366	NA	R01_cb15334_c1/f2p0/366	R01_cb15334_c1/f2p0/366	R01_cb15334_c1/f2p0/366
R01_cb9287_c2/f1p0/1231	NA	R01_cb9287_c2/f1p0/1231	NA	NA
R01_cb693_c0/f6p0/2904	NA	NA	NA	R01_cb693_c0/f6p0/2904
R01_cb16290_c4/f1p0/1075	NA	R01_cb16290_c4/f1p0/1075	R01_cb16290_c4/f1p0/1075	R01_cb16290_c4/f1p0/1075
R01_cb3759_c16/f1p0/3176	NA	R01_cb3759_c16/f1p0/3176	NA	NA
R01_cb18456_c7970/f1p0/474	R01_cb18456_c7970/f1p0/474	R01_cb18456_c7970/f1p0/474	R01_cb18456_c7970/f1p0/474	R01_cb18456_c7970/f1p0/474

R01_cb17997_c6/f1p0/1060	NA	NA	NA	R01_cb17997_c6/f1p0/1060
R01_cb8564_c112293/f1p0/2155	NA	R01_cb8564_c112293/f1p0/2155	NA	NA
R01_cb13284_c9/f1p0/1620	NA	NA	NA	R01_cb13284_c9/f1p0/1620
R01_cb8564_c19716/f1p0/2217	NA	NA	NA	R01_cb8564_c19716/f1p0/2217
R01_cb15685_c1/f1p0/717	NA	R01_cb15685_c1/f1p0/717	NA	R01_cb15685_c1/f1p0/717
R01_cb1113_c4/f1p1/2699	NA	R01_cb1113_c4/f1p1/2699	NA	NA
R01_cb14421_c1/f1p0/919	NA	NA	NA	R01_cb14421_c1/f1p0/919
R01_cb12722_c9/f1p4/1835	NA	NA	NA	R01_cb12722_c9/f1p4/1835
R01_cb6406_c52/f1p0/3671	NA	R01_cb6406_c52/f1p0/3671	NA	R01_cb6406_c52/f1p0/3671
R01_cb8564_c70226/f1p0/2215	R01_cb8564_c70226/f1p0/2215	R01_cb8564_c70226/f1p0/2215	R01_cb8564_c70226/f1p0/2215	NA
R01_cb5504_c25/f1p0/2640	NA	R01_cb5504_c25/f1p0/2640	NA	R01_cb5504_c25/f1p0/2640
R01_cb16739_c1/f2p0/750	NA	NA	NA	R01_cb16739_c1/f2p0/750
R01_cb3132_c8/f1p0/1609	NA	NA	NA	R01_cb3132_c8/f1p0/1609
R01_cb7092_c0/f2p1/2704	NA	R01_cb7092_c0/f2p1/2704	NA	NA
R01_cb17925_c0/f1p0/1096	NA	NA	R01_cb17925_c0/f1p0/1096	R01_cb17925_c0/f1p0/1096
R01_cb11051_c7/f1p0/685	NA	R01_cb11051_c7/f1p0/685	NA	R01_cb11051_c7/f1p0/685
R01_cb7507_c11/f1p0/1603	NA	R01_cb7507_c11/f1p0/1603	NA	NA
R01_cb17192_c4/f1p0/1017	NA	R01_cb17192_c4/f1p0/1017	NA	NA
R01_cb10775_c2/f1p0/1221	NA	NA	R01_cb10775_c2/f1p0/1221	R01_cb10775_c2/f1p0/1221
R01_cb11167_c0/f1p0/310	R01_cb11167_c0/f1p0/310	R01_cb11167_c0/f1p0/310	R01_cb11167_c0/f1p0/310	R01_cb11167_c0/f1p0/310
R01_cb8564_c74474/f1p0/3918	NA	R01_cb8564_c74474/f1p0/3918	NA	NA
R01_cb5986_c0/f1p0/2957	NA	R01_cb5986_c0/f1p0/2957	NA	NA
R01_cb18526_c3/f1p0/554	R01_cb18526_c3/f1p0/554	R01_cb18526_c3/f1p0/554	R01_cb18526_c3/f1p0/554	R01_cb18526_c3/f1p0/554
R01_cb15807_c2/f1p0/695	NA	NA	NA	R01_cb15807_c2/f1p0/695

R01_cb2072_c31/f1p0/2869	NA	NA	NA	R01_cb2072_c31/f1p0/2869
R01_cb18409_c110/f1p0/508	R01_cb18409_c110/f1p0/508	R01_cb18409_c110/f1p0/508	R01_cb18409_c110/f1p0/508	R01_cb18409_c110/f1p0/508
R01_cb14839_c0/f1p0/855	NA	NA	R01_cb14839_c0/f1p0/855	R01_cb14839_c0/f1p0/855
R01_cb226_c12/f1p0/5289	NA	NA	NA	R01_cb226_c12/f1p0/5289
R01_cb12292_c13/f1p0/1000	NA	R01_cb12292_c13/f1p0/1000	NA	NA
R01_cb18456_c5803/f1p0/387	R01_cb18456_c5803/f1p0/387	R01_cb18456_c5803/f1p0/387	R01_cb18456_c5803/f1p0/387	R01_cb18456_c5803/f1p0/387
R01_cb5900_c49/f1p0/1960	NA	NA	NA	R01_cb5900_c49/f1p0/1960
R01_cb1625_c1/f1p0/2747	NA	NA	NA	R01_cb1625_c1/f1p0/2747
R01_cb10034_c57/f1p0/608	NA	R01_cb10034_c57/f1p0/608	NA	NA
R01_cb4180_c5/f1p0/3863	NA	R01_cb4180_c5/f1p0/3863	NA	R01_cb4180_c5/f1p0/3863
R01_cb14377_c1/f1p0/1462	NA	NA	NA	R01_cb14377_c1/f1p0/1462
R01_cb3634_c2/f1p0/2100	NA	R01_cb3634_c2/f1p0/2100	NA	NA
R01_cb8564_c82635/f1p0/2823	NA	R01_cb8564_c82635/f1p0/2823	NA	R01_cb8564_c82635/f1p0/2823
R01_cb8564_c77006/f1p0/2229	NA	R01_cb8564_c77006/f1p0/2229	NA	R01_cb8564_c77006/f1p0/2229
R01_cb8564_c5039/f1p0/3047	NA	NA	NA	R01_cb8564_c5039/f1p0/3047
R01_cb17388_c15/f1p0/751	R01_cb17388_c15/f1p0/751	R01_cb17388_c15/f1p0/751	R01_cb17388_c15/f1p0/751	R01_cb17388_c15/f1p0/751
R01_cb10482_c21/f1p3/1026	NA	R01_cb10482_c21/f1p3/1026	NA	NA
R01_cb13602_c4/f2p0/1770	NA	NA	NA	R01_cb13602_c4/f2p0/1770
R01_cb8564_c89017/f1p0/2254	NA	R01_cb8564_c89017/f1p0/2254	NA	R01_cb8564_c89017/f1p0/2254
R01_cb15516_c5/f1p0/1772	NA	R01_cb15516_c5/f1p0/1772	NA	R01_cb15516_c5/f1p0/1772
R01_cb15279_c1/f1p0/1492	NA	NA	NA	R01_cb15279_c1/f1p0/1492
R01_cb8564_c78032/f1p0/1858	NA	NA	NA	R01_cb8564_c78032/f1p0/1858
R01_cb5214_c5/f1p1/2841	NA	R01_cb5214_c5/f1p1/2841	R01_cb5214_c5/f1p1/2841	NA
R01_cb10717_c2/f1p0/2474	NA	R01_cb10717_c2/f1p0/2474	NA	NA
R01_cb15891_c6/f1p0/1371	NA	R01_cb15891_c6/f1p0/1371	NA	NA
R01_cb6672_c1/f1p0/2797	NA	NA	NA	R01_cb6672_c1/f1p0/2797

R01_cb7708_c8/f1p0/743	NA	R01_cb7708_c8/f1p0/743	NA	NA
R01_cb15984_c3/f1p0/1675	NA	R01_cb15984_c3/f1p0/1675	NA	NA
R01_cb3739_c3/f1p1/2887	R01_cb3739_c3/f1p1/2887	R01_cb3739_c3/f1p1/2887	R01_cb3739_c3/f1p1/2887	R01_cb3739_c3/f1p1/2887
R01_cb6113_c4/f1p0/2618	R01_cb6113_c4/f1p0/2618	R01_cb6113_c4/f1p0/2618	NA	R01_cb6113_c4/f1p0/2618
R01_cb2045_c5/f1p0/2453	NA	NA	NA	R01_cb2045_c5/f1p0/2453
R01_cb8564_c18924/f1p0/3930	NA	R01_cb8564_c18924/f1p0/3930	NA	NA
R01_cb8564_c47461/f1p0/4325	NA	NA	NA	R01_cb8564_c47461/f1p0/4325
R01_cb9342_c10/f1p0/1672	NA	R01_cb9342_c10/f1p0/1672	NA	NA
R01_cb8564_c84780/f1p1/2711	NA	R01_cb8564_c84780/f1p1/2711	R01_cb8564_c84780/f1p1/2711	R01_cb8564_c84780/f1p1/2711
R01_cb11796_c1/f1p0/3530	NA	R01_cb11796_c1/f1p0/3530	R01_cb11796_c1/f1p0/3530	R01_cb11796_c1/f1p0/3530
R01_cb12187_c0/f3p0/1404	NA	NA	NA	R01_cb12187_c0/f3p0/1404
R01_cb16706_c5/f1p0/690	NA	R01_cb16706_c5/f1p0/690	NA	R01_cb16706_c5/f1p0/690
R01_cb12421_c62/f1p0/640	NA	R01_cb12421_c62/f1p0/640	NA	NA
R01_cb2022_c8/f1p0/3727	NA	NA	NA	R01_cb2022_c8/f1p0/3727
R01_cb8564_c9427/f3p0/2261	NA	NA	NA	R01_cb8564_c9427/f3p0/2261
R01_cb5158_c2/f1p0/3008	NA	NA	NA	R01_cb5158_c2/f1p0/3008
R01_cb8564_c90033/f1p0/2734	R01_cb8564_c90033/f1p0/2734	R01_cb8564_c90033/f1p0/2734	NA	NA
R01_cb8778_c4/f1p1/823	NA	R01_cb8778_c4/f1p1/823	NA	R01_cb8778_c4/f1p1/823
R01_cb6103_c6/f1p0/2906	NA	R01_cb6103_c6/f1p0/2906	NA	NA
R01_cb8564_c74585/f1p0/3890	NA	R01_cb8564_c74585/f1p0/3890	R01_cb8564_c74585/f1p0/3890	R01_cb8564_c74585/f1p0/3890
R01_cb6431_c29/f1p1/2534	NA	R01_cb6431_c29/f1p1/2534	NA	NA
R01_cb279_c8/f1p0/4893	NA	NA	NA	R01_cb279_c8/f1p0/4893
R01_cb13532_c10/f1p1/1100	R01_cb13532_c10/f1p1/1100	NA	R01_cb13532_c10/f1p1/1100	R01_cb13532_c10/f1p1/1100
R01_cb15465_c3/f1p3/574	R01_cb15465_c3/f1p3/574	NA	R01_cb15465_c3/f1p3/574	R01_cb15465_c3/f1p3/574
R01_cb18132_c19/f1p0/658	NA	R01_cb18132_c19/f1p0/658	R01_cb18132_c19/f1p0/658	R01_cb18132_c19/f1p0/658

R01_cb18456_c7385/f1p0/447	R01_cb18456_c7385/f1p0/447	R01_cb18456_c7385/f1p0/447	R01_cb18456_c7385/f1p0/447	R01_cb18456_c7385/f1p0/447
R01_cb1020_c46/f1p0/440	R01_cb1020_c46/f1p0/440	R01_cb1020_c46/f1p0/440	R01_cb1020_c46/f1p0/440	R01_cb1020_c46/f1p0/440
R01_cb13535_c14/f1p0/838	NA	R01_cb13535_c14/f1p0/838	NA	R01_cb13535_c14/f1p0/838
R01_cb2396_c8/f1p0/2562	NA	R01_cb2396_c8/f1p0/2562	NA	NA
R01_cb8564_c71885/f2p1/2637	NA	NA	NA	R01_cb8564_c71885/f2p1/2637
R01_cb7784_c7/f1p0/2404	NA	R01_cb7784_c7/f1p0/2404	NA	NA
R01_cb4489_c6/f2p1/2678	NA	R01_cb4489_c6/f2p1/2678	NA	R01_cb4489_c6/f2p1/2678
R01_cb8564_c89565/f1p0/2829	NA	R01_cb8564_c89565/f1p0/2829	NA	R01_cb8564_c89565/f1p0/2829
R01_cb8564_c71819/f1p0/4142	NA	NA	NA	R01_cb8564_c71819/f1p0/4142
R01_cb3046_c5/f1p0/3275	NA	NA	NA	R01_cb3046_c5/f1p0/3275
R01_cb8564_c82357/f1p0/2491	NA	NA	R01_cb8564_c82357/f1p0/2491	R01_cb8564_c82357/f1p0/2491
R01_cb4900_c17/f1p1/2753	NA	NA	NA	R01_cb4900_c17/f1p1/2753
R01_cb16422_c1/f3p0/753	NA	NA	NA	R01_cb16422_c1/f3p0/753
R01_cb18196_c1/f1p0/1338	NA	R01_cb18196_c1/f1p0/1338	NA	R01_cb18196_c1/f1p0/1338
R01_cb17984_c2/f3p0/1241	NA	NA	NA	R01_cb17984_c2/f3p0/1241
R01_cb7010_c12/f2p1/2966	NA	R01_cb7010_c12/f2p1/2966	NA	NA
R01_cb9937_c1/f1p0/1919	NA	R01_cb9937_c1/f1p0/1919	NA	NA
R01_cb18109_c1/f1p0/444	R01_cb18109_c1/f1p0/444	R01_cb18109_c1/f1p0/444	NA	NA
R01_cb13353_c2/f1p0/581	R01_cb13353_c2/f1p0/581	NA	NA	R01_cb13353_c2/f1p0/581
R01_cb16383_c2/f2p0/1209	NA	R01_cb16383_c2/f2p0/1209	NA	R01_cb16383_c2/f2p0/1209
R01_cb2767_c2/f1p0/3869	NA	R01_cb2767_c2/f1p0/3869	NA	NA
R01_cb8564_c69111/f2p0/2818	NA	NA	NA	R01_cb8564_c69111/f2p0/2818
R01_cb17602_c2/f1p0/1447	NA	NA	NA	R01_cb17602_c2/f1p0/1447
R01_cb14286_c1/f1p0/1099	NA	R01_cb14286_c1/f1p0/1099	NA	R01_cb14286_c1/f1p0/1099
R01_cb5159_c13/f1p0/1964	NA	NA	NA	R01_cb5159_c13/f1p0/1964
R01_cb8564_c87017/f1p0/2003	R01_cb8564_c87017/f1p0/200	R01_cb8564_c87017/f1p0/2003	NA	R01_cb8564_c87017/f1p0/2003

	3			
R01_cb18456_c7506/f1p0/731	R01_cb18456_c7506/f1p0/731	R01_cb18456_c7506/f1p0/731	NA	NA
R01_cb8564_c11951/f1p0/3240	NA	R01_cb8564_c11951/f1p0/3240	NA	R01_cb8564_c11951/f1p0/3240
R01_cb8564_c4834/f1p0/3516	R01_cb8564_c4834/f1p0/3516	R01_cb8564_c4834/f1p0/3516	R01_cb8564_c4834/f1p0/3516	R01_cb8564_c4834/f1p0/3516
R01_cb8946_c2/f1p0/2040	NA	NA	NA	R01_cb8946_c2/f1p0/2040
R01_cb709_c1/f1p0/2627	NA	NA	R01_cb709_c1/f1p0/2627	NA
R01_cb4719_c20/f1p0/1514	NA	NA	R01_cb4719_c20/f1p0/1514	R01_cb4719_c20/f1p0/1514
R01_cb11732_c1/f1p0/3497	NA	R01_cb11732_c1/f1p0/3497	NA	NA
R01_cb1938_c5/f1p0/2356	NA	NA	NA	R01_cb1938_c5/f1p0/2356
R01_cb14751_c4/f1p0/607	NA	R01_cb14751_c4/f1p0/607	NA	NA
R01_cb8564_c68577/f4p0/4465	NA	R01_cb8564_c68577/f4p0/4465	R01_cb8564_c68577/f4p0/4465	NA
R01_cb4368_c0/f33p1/1481	NA	NA	NA	R01_cb4368_c0/f33p1/1481
R01_cb5900_c44/f1p0/2368	NA	R01_cb5900_c44/f1p0/2368	NA	R01_cb5900_c44/f1p0/2368
R01_cb191_c4/f1p0/3237	NA	NA	NA	R01_cb191_c4/f1p0/3237
R01_cb17466_c0/f2p0/1697	NA	NA	R01_cb17466_c0/f2p0/1697	NA
R01_cb7663_c3/f1p0/2573	NA	R01_cb7663_c3/f1p0/2573	R01_cb7663_c3/f1p0/2573	R01_cb7663_c3/f1p0/2573
R01_cb16439_c0/f1p0/832	NA	NA	NA	R01_cb16439_c0/f1p0/832
R01_cb119_c23/f1p0/3973	NA	NA	NA	R01_cb119_c23/f1p0/3973
R01_cb5378_c3/f1p0/1563	R01_cb5378_c3/f1p0/1563	R01_cb5378_c3/f1p0/1563	R01_cb5378_c3/f1p0/1563	R01_cb5378_c3/f1p0/1563
R01_cb4657_c12/f1p0/2084	NA	R01_cb4657_c12/f1p0/2084	R01_cb4657_c12/f1p0/2084	NA
R01_cb8564_c35910/f1p0/3231	NA	NA	NA	R01_cb8564_c35910/f1p0/3231
R01_cb7862_c1/f2p0/1017	NA	R01_cb7862_c1/f2p0/1017	R01_cb7862_c1/f2p0/1017	R01_cb7862_c1/f2p0/1017
R01_cb12636_c4/f1p0/1468	NA	NA	NA	R01_cb12636_c4/f1p0/1468
R01_cb3500_c22/f1p0/3320	NA	NA	NA	R01_cb3500_c22/f1p0/3320
R01_cb18456_c7603/f1p0/559	NA	R01_cb18456_c7603/f1p0/559	R01_cb18456_c7603/f1p0/559	R01_cb18456_c7603/f1p0/559
R01_cb16590_c0/f2p0/1477	R01_cb16590_c0/f2p0/1477	R01_cb16590_c0/f2p0/1477	R01_cb16590_c0/f2p0/1477	R01_cb16590_c0/f2p0/1477

R01_cb16997_c89/f1p0/2621	NA	R01_cb16997_c89/f1p0/2621	NA	NA
R01_cb8907_c0/f3p0/897	R01_cb8907_c0/f3p0/897	R01_cb8907_c0/f3p0/897	R01_cb8907_c0/f3p0/897	R01_cb8907_c0/f3p0/897
R01_cb8564_c15836/f2p2/2956	NA	R01_cb8564_c15836/f2p2/2956	NA	NA
R01_cb4533_c0/f1p0/3317	NA	R01_cb4533_c0/f1p0/3317	NA	R01_cb4533_c0/f1p0/3317
R01_cb2884_c1/f2p0/2257	NA	NA	NA	R01_cb2884_c1/f2p0/2257
R01_cb10606_c0/f1p0/1733	NA	R01_cb10606_c0/f1p0/1733	NA	NA
R01_cb781_c17/f1p0/1917	NA	NA	NA	R01_cb781_c17/f1p0/1917
R01_cb5218_c2/f1p0/3153	NA	R01_cb5218_c2/f1p0/3153	NA	NA
R01_cb8564_c122421/f1p0/2089	NA	NA	NA	R01_cb8564_c122421/f1p0/2089
R01_cb8564_c35718/f1p0/2911	NA	NA	R01_cb8564_c35718/f1p0/2911	NA
R01_cb10976_c1/f1p0/2738	NA	R01_cb10976_c1/f1p0/2738	NA	NA
R01_cb7747_c5/f1p0/2463	NA	R01_cb7747_c5/f1p0/2463	R01_cb7747_c5/f1p0/2463	R01_cb7747_c5/f1p0/2463
R01_cb6591_c5/f2p0/2613	R01_cb6591_c5/f2p0/2613	R01_cb6591_c5/f2p0/2613	NA	NA
R01_cb18456_c6300/f1p0/559	R01_cb18456_c6300/f1p0/559	R01_cb18456_c6300/f1p0/559	R01_cb18456_c6300/f1p0/559	R01_cb18456_c6300/f1p0/559
R01_cb7950_c0/f3p0/2499	NA	R01_cb7950_c0/f3p0/2499	NA	R01_cb7950_c0/f3p0/2499
R01_cb1905_c24/f1p0/2875	NA	R01_cb1905_c24/f1p0/2875	R01_cb1905_c24/f1p0/2875	R01_cb1905_c24/f1p0/2875
R01_cb8564_c119692/f1p0/2900	R01_cb8564_c119692/f1p0/2900	R01_cb8564_c119692/f1p0/2900	NA	NA
R01_cb11677_c0/f1p0/1016	NA	R01_cb11677_c0/f1p0/1016	R01_cb11677_c0/f1p0/1016	R01_cb11677_c0/f1p0/1016
R01_cb1835_c2/f1p0/4206	NA	NA	NA	R01_cb1835_c2/f1p0/4206
R01_cb10554_c3/f1p0/3885	NA	R01_cb10554_c3/f1p0/3885	NA	NA
R01_cb2198_c1/f1p0/3995	NA	NA	R01_cb2198_c1/f1p0/3995	R01_cb2198_c1/f1p0/3995
R01_cb8564_c3066/f1p1/3917	NA	R01_cb8564_c3066/f1p1/3917	NA	R01_cb8564_c3066/f1p1/3917
R01_cb13012_c2/f2p1/1113	NA	NA	NA	R01_cb13012_c2/f2p1/1113
R01_cb15951_c0/f2p0/476	R01_cb15951_c0/f2p0/476	R01_cb15951_c0/f2p0/476	R01_cb15951_c0/f2p0/476	R01_cb15951_c0/f2p0/476

R01_cb17737_c1/f1p0/1258	R01_cb17737_c1/f1p0/1258	R01_cb17737_c1/f1p0/1258	R01_cb17737_c1/f1p0/1258	NA
R01_cb11774_c0/f1p0/857	NA	NA	NA	R01_cb11774_c0/f1p0/857
R01_cb15361_c0/f8p1/810	NA	NA	R01_cb15361_c0/f8p1/810	R01_cb15361_c0/f8p1/810
R01_cb10506_c6/f1p0/410	R01_cb10506_c6/f1p0/410	R01_cb10506_c6/f1p0/410	R01_cb10506_c6/f1p0/410	R01_cb10506_c6/f1p0/410
R01_cb2087_c5/f1p0/3142	NA	R01_cb2087_c5/f1p0/3142	NA	NA
R01_cb8564_c119077/f1p0/4938	NA	R01_cb8564_c119077/f1p0/4938	NA	NA
R01_cb8564_c14854/f2p0/2226	NA	NA	NA	R01_cb8564_c14854/f2p0/2226
R01_cb17022_c0/f1p0/818	R01_cb17022_c0/f1p0/818	R01_cb17022_c0/f1p0/818	NA	NA
R01_cb2895_c1/f1p0/2164	NA	R01_cb2895_c1/f1p0/2164	NA	NA
R01_cb1686_c5/f1p0/2279	NA	NA	R01_cb1686_c5/f1p0/2279	R01_cb1686_c5/f1p0/2279
R01_cb709_c11/f1p0/2068	R01_cb709_c11/f1p0/2068	R01_cb709_c11/f1p0/2068	R01_cb709_c11/f1p0/2068	R01_cb709_c11/f1p0/2068
R01_cb15017_c5/f1p0/1470	NA	R01_cb15017_c5/f1p0/1470	NA	NA
R01_cb17411_c0/f3p0/1546	NA	R01_cb17411_c0/f3p0/1546	NA	NA
R01_cb8564_c88457/f1p0/2242	NA	R01_cb8564_c88457/f1p0/2242	NA	NA
R01_cb18456_c2339/f1p0/916	NA	R01_cb18456_c2339/f1p0/916	NA	NA
R01_cb11244_c1/f1p0/2673	NA	R01_cb11244_c1/f1p0/2673	NA	NA
R01_cb7537_c1/f2p1/2157	NA	NA	NA	R01_cb7537_c1/f2p1/2157
R01_cb4846_c3/f1p0/2798	NA	R01_cb4846_c3/f1p0/2798	NA	NA
R01_cb8564_c129754/f1p0/2346	NA	R01_cb8564_c129754/f1p0/2346	NA	NA
R01_cb7528_c4/f1p0/4063	NA	R01_cb7528_c4/f1p0/4063	NA	NA
R01_cb16718_c5/f1p0/401	R01_cb16718_c5/f1p0/401	R01_cb16718_c5/f1p0/401	R01_cb16718_c5/f1p0/401	R01_cb16718_c5/f1p0/401
R01_cb6574_c3/f1p0/1915	NA	R01_cb6574_c3/f1p0/1915	R01_cb6574_c3/f1p0/1915	R01_cb6574_c3/f1p0/1915
R01_cb8564_c1020/f1p0/3486	NA	R01_cb8564_c1020/f1p0/3486	NA	R01_cb8564_c1020/f1p0/3486
R01_cb11534_c0/f1p0/1805	NA	R01_cb11534_c0/f1p0/1805	NA	NA

R01_cb16652_c3/f1p0/1316	NA	R01_cb16652_c3/f1p0/1316	NA	NA
R01_cb8564_c85245/f1p0/3173	NA	NA	NA	R01_cb8564_c85245/f1p0/3173
R01_cb8564_c40846/f1p0/1937	NA	R01_cb8564_c40846/f1p0/1937	NA	NA
R01_cb10217_c3/f1p1/1696	NA	NA	NA	R01_cb10217_c3/f1p1/1696
R01_cb10659_c6/f1p0/1478	NA	R01_cb10659_c6/f1p0/1478	NA	NA
R01_cb18758_c1/f1p0/313	R01_cb18758_c1/f1p0/313	R01_cb18758_c1/f1p0/313	R01_cb18758_c1/f1p0/313	R01_cb18758_c1/f1p0/313
R01_cb8564_c24863/f1p0/2842	R01_cb8564_c24863/f1p0/2842	R01_cb8564_c24863/f1p0/2842	R01_cb8564_c24863/f1p0/2842	R01_cb8564_c24863/f1p0/2842
R01_cb9256_c0/f1p0/2138	NA	NA	NA	R01_cb9256_c0/f1p0/2138
R01_cb14547_c13/f3p2/698	NA	R01_cb14547_c13/f3p2/698	NA	NA
R01_cb8279_c10/f1p0/2442	NA	NA	NA	R01_cb8279_c10/f1p0/2442
R01_cb9529_c5/f1p0/908	NA	NA	NA	R01_cb9529_c5/f1p0/908
R01_cb8564_c36533/f1p0/4031	NA	R01_cb8564_c36533/f1p0/4031	NA	R01_cb8564_c36533/f1p0/4031
R01_cb947_c45/f1p0/853	R01_cb947_c45/f1p0/853	R01_cb947_c45/f1p0/853	R01_cb947_c45/f1p0/853	R01_cb947_c45/f1p0/853
R01_cb3745_c0/f1p0/3539	R01_cb3745_c0/f1p0/3539	R01_cb3745_c0/f1p0/3539	R01_cb3745_c0/f1p0/3539	NA
R01_cb11554_c1/f1p0/2411	NA	R01_cb11554_c1/f1p0/2411	NA	NA
R01_cb8564_c16286/f1p0/3853	R01_cb8564_c16286/f1p0/3853	R01_cb8564_c16286/f1p0/3853	R01_cb8564_c16286/f1p0/3853	R01_cb8564_c16286/f1p0/3853
R01_cb11542_c1/f1p0/2639	NA	NA	NA	R01_cb11542_c1/f1p0/2639
R01_cb17331_c1/f1p0/541	NA	NA	R01_cb17331_c1/f1p0/541	R01_cb17331_c1/f1p0/541
R01_cb12156_c1/f3p1/636	R01_cb12156_c1/f3p1/636	R01_cb12156_c1/f3p1/636	NA	NA
R01_cb7032_c0/f1p0/2714	NA	R01_cb7032_c0/f1p0/2714	NA	R01_cb7032_c0/f1p0/2714
R01_cb10968_c2/f1p1/2608	NA	R01_cb10968_c2/f1p1/2608	R01_cb10968_c2/f1p1/2608	R01_cb10968_c2/f1p1/2608
R01_cb18456_c2271/f1p0/1842	NA	R01_cb18456_c2271/f1p0/1842	NA	NA
R01_cb14578_c1/f1p0/1500	NA	R01_cb14578_c1/f1p0/1500	NA	NA
R01_cb8564_c11380/f1p0/2676	NA	R01_cb8564_c11380/f1p0/2676	R01_cb8564_c11380/f1p0/2676	NA

R01_cb12070_c0/f1p0/595	R01_cb12070_c0/f1p0/595	R01_cb12070_c0/f1p0/595	R01_cb12070_c0/f1p0/595	R01_cb12070_c0/f1p0/595
R01_cb18398_c2/f1p0/1588	NA	R01_cb18398_c2/f1p0/1588	NA	NA
R01_cb18080_c0/f2p0/733	R01_cb18080_c0/f2p0/733	R01_cb18080_c0/f2p0/733	R01_cb18080_c0/f2p0/733	R01_cb18080_c0/f2p0/733
R01_cb506_c7/f1p0/3519	NA	NA	NA	R01_cb506_c7/f1p0/3519
R01_cb10010_c2/f1p0/1014	NA	R01_cb10010_c2/f1p0/1014	R01_cb10010_c2/f1p0/1014	NA
R01_cb8564_c13087/f1p1/3138	NA	R01_cb8564_c13087/f1p1/3138	NA	NA
R01_cb8564_c42049/f1p0/4782	NA	R01_cb8564_c42049/f1p0/4782	NA	NA
R01_cb8087_c4/f1p0/2454	NA	R01_cb8087_c4/f1p0/2454	NA	NA
R01_cb16178_c0/f1p0/598	R01_cb16178_c0/f1p0/598	R01_cb16178_c0/f1p0/598	R01_cb16178_c0/f1p0/598	R01_cb16178_c0/f1p0/598
R01_cb4469_c8/f1p0/2281	NA	NA	NA	R01_cb4469_c8/f1p0/2281
R01_cb11824_c0/f1p0/678	R01_cb11824_c0/f1p0/678	R01_cb11824_c0/f1p0/678	R01_cb11824_c0/f1p0/678	R01_cb11824_c0/f1p0/678
R01_cb8564_c24947/f1p0/3141	NA	NA	NA	R01_cb8564_c24947/f1p0/3141
R01_cb11008_c0/f2p0/547	R01_cb11008_c0/f2p0/547	R01_cb11008_c0/f2p0/547	R01_cb11008_c0/f2p0/547	R01_cb11008_c0/f2p0/547
R01_cb8564_c75889/f1p0/4082	R01_cb8564_c75889/f1p0/4082	R01_cb8564_c75889/f1p0/4082	NA	R01_cb8564_c75889/f1p0/4082
R01_cb12686_c2/f1p0/505	NA	NA	NA	R01_cb12686_c2/f1p0/505
R01_cb18766_c0/f1p0/5342	NA	R01_cb18766_c0/f1p0/5342	R01_cb18766_c0/f1p0/5342	R01_cb18766_c0/f1p0/5342
R01_cb10875_c0/f1p0/1519	NA	R01_cb10875_c0/f1p0/1519	NA	NA
R01_cb2451_c19/f1p0/3664	NA	NA	NA	R01_cb2451_c19/f1p0/3664
R01_cb18456_c3750/f1p0/882	NA	R01_cb18456_c3750/f1p0/882	NA	NA
R01_cb15186_c8/f4p1/543	R01_cb15186_c8/f4p1/543	R01_cb15186_c8/f4p1/543	R01_cb15186_c8/f4p1/543	NA
R01_cb14935_c2/f1p0/1520	R01_cb14935_c2/f1p0/1520	R01_cb14935_c2/f1p0/1520	NA	R01_cb14935_c2/f1p0/1520
R01_cb13858_c3/f1p0/345	R01_cb13858_c3/f1p0/345	R01_cb13858_c3/f1p0/345	R01_cb13858_c3/f1p0/345	R01_cb13858_c3/f1p0/345
R01_cb9081_c1/f1p0/2377	NA	NA	NA	R01_cb9081_c1/f1p0/2377
R01_cb6015_c10/f1p0/1949	NA	R01_cb6015_c10/f1p0/1949	NA	NA
R01_cb7551_c16/f1p0/704	R01_cb7551_c16/f1p0/704	R01_cb7551_c16/f1p0/704	R01_cb7551_c16/f1p0/704	R01_cb7551_c16/f1p0/704

R01_cb3341_c12/f1p0/1697	NA	R01_cb3341_c12/f1p0/1697	NA	NA
R01_cb18456_c1824/f1p0/1737	NA	NA	R01_cb18456_c1824/f1p0/1737	NA
R01_cb8564_c47920/f1p0/2894	NA	R01_cb8564_c47920/f1p0/2894	NA	NA
R01_cb6204_c12/f1p3/2884	NA	NA	NA	R01_cb6204_c12/f1p3/2884
R01_cb1915_c29/f1p0/1752	NA	NA	NA	R01_cb1915_c29/f1p0/1752
R01_cb3730_c34/f1p0/472	R01_cb3730_c34/f1p0/472	R01_cb3730_c34/f1p0/472	R01_cb3730_c34/f1p0/472	R01_cb3730_c34/f1p0/472
R01_cb13384_c2/f2p0/1153	NA	NA	NA	R01_cb13384_c2/f2p0/1153
R01_cb2568_c6/f1p0/2781	R01_cb2568_c6/f1p0/2781	R01_cb2568_c6/f1p0/2781	R01_cb2568_c6/f1p0/2781	R01_cb2568_c6/f1p0/2781
R01_cb9582_c5/f1p0/2027	NA	R01_cb9582_c5/f1p0/2027	NA	NA
R01_cb17204_c0/f2p0/648	R01_cb17204_c0/f2p0/648	R01_cb17204_c0/f2p0/648	NA	NA
R01_cb11148_c4/f2p0/550	NA	R01_cb11148_c4/f2p0/550	NA	R01_cb11148_c4/f2p0/550
R01_cb10664_c1/f1p0/2500	R01_cb10664_c1/f1p0/2500	R01_cb10664_c1/f1p0/2500	R01_cb10664_c1/f1p0/2500	NA
R01_cb11310_c1/f1p0/2474	R01_cb11310_c1/f1p0/2474	R01_cb11310_c1/f1p0/2474	R01_cb11310_c1/f1p0/2474	R01_cb11310_c1/f1p0/2474
R01_cb16134_c2/f1p0/1632	NA	R01_cb16134_c2/f1p0/1632	NA	NA
R01_cb5168_c4/f1p0/1155	R01_cb5168_c4/f1p0/1155	R01_cb5168_c4/f1p0/1155	R01_cb5168_c4/f1p0/1155	R01_cb5168_c4/f1p0/1155
R01_cb8564_c3874/f1p0/2929	R01_cb8564_c3874/f1p0/2929	R01_cb8564_c3874/f1p0/2929	R01_cb8564_c3874/f1p0/2929	R01_cb8564_c3874/f1p0/2929
R01_cb12585_c26/f1p0/515	R01_cb12585_c26/f1p0/515	R01_cb12585_c26/f1p0/515	R01_cb12585_c26/f1p0/515	R01_cb12585_c26/f1p0/515
R01_cb8900_c1/f1p0/2444	NA	NA	NA	R01_cb8900_c1/f1p0/2444
R01_cb10681_c5/f1p0/1069	NA	NA	NA	R01_cb10681_c5/f1p0/1069
R01_cb8564_c48531/f1p0/4342	NA	R01_cb8564_c48531/f1p0/4342	NA	NA
R01_cb8564_c120991/f1p0/2687	NA	R01_cb8564_c120991/f1p0/2687	R01_cb8564_c120991/f1p0/2687	R01_cb8564_c120991/f1p0/2687
R01_cb11446_c0/f1p0/1380	NA	R01_cb11446_c0/f1p0/1380	NA	NA
R01_cb8564_c79322/f1p0/3340	NA	NA	NA	R01_cb8564_c79322/f1p0/3340
R01_cb12063_c7/f1p0/1417	NA	NA	NA	R01_cb12063_c7/f1p0/1417
R01_cb8564_c74999/f1p0/2465	NA	R01_cb8564_c74999/f1p0/2465	NA	NA

R01_cb8564_c4583/f5p0/2597	NA	NA	NA	R01_cb8564_c4583/f5p0/2597
R01_cb6459_c3/f1p0/2915	NA	R01_cb6459_c3/f1p0/2915	NA	NA
R01_cb18456_c7311/f1p3/713	R01_cb18456_c7311/f1p3/713	R01_cb18456_c7311/f1p3/713	NA	NA
R01_cb5233_c0/f1p0/3145	NA	R01_cb5233_c0/f1p0/3145	NA	NA
R01_cb8905_c4/f1p1/2111	NA	NA	NA	R01_cb8905_c4/f1p1/2111
R01_cb17167_c2/f1p0/608	NA	R01_cb17167_c2/f1p0/608	NA	NA
R01_cb17266_c18/f1p0/1246	NA	R01_cb17266_c18/f1p0/1246	NA	NA
R01_cb15741_c4/f1p0/5541	R01_cb15741_c4/f1p0/5541	R01_cb15741_c4/f1p0/5541	NA	NA
R01_cb7303_c1/f4p1/2576	NA	NA	NA	R01_cb7303_c1/f4p1/2576
R01_cb3131_c4/f1p0/1505	NA	R01_cb3131_c4/f1p0/1505	NA	NA
R01_cb15003_c0/f4p0/716	R01_cb15003_c0/f4p0/716	R01_cb15003_c0/f4p0/716	R01_cb15003_c0/f4p0/716	R01_cb15003_c0/f4p0/716
R01_cb4354_c5/f3p1/3366	NA	NA	NA	R01_cb4354_c5/f3p1/3366
R01_cb2959_c2/f1p0/3487	NA	NA	NA	R01_cb2959_c2/f1p0/3487
R01_cb16447_c7/f1p1/912	NA	NA	R01_cb16447_c7/f1p1/912	R01_cb16447_c7/f1p1/912
R01_cb8169_c0/f1p0/2438	NA	R01_cb8169_c0/f1p0/2438	NA	NA
R01_cb7693_c1/f1p0/2557	NA	NA	NA	R01_cb7693_c1/f1p0/2557
R01_cb17069_c1/f1p0/735	NA	R01_cb17069_c1/f1p0/735	NA	R01_cb17069_c1/f1p0/735
R01_cb5676_c1/f6p0/2903	NA	NA	NA	R01_cb5676_c1/f6p0/2903
R01_cb6169_c5/f1p0/1654	NA	R01_cb6169_c5/f1p0/1654	NA	NA
R01_cb9613_c6/f1p0/1672	NA	R01_cb9613_c6/f1p0/1672	NA	NA
R01_cb8564_c24172/f1p0/4877	NA	R01_cb8564_c24172/f1p0/4877	R01_cb8564_c24172/f1p0/4877	R01_cb8564_c24172/f1p0/4877
R01_cb8564_c13534/f1p0/3084	NA	R01_cb8564_c13534/f1p0/3084	NA	R01_cb8564_c13534/f1p0/3084
R01_cb12273_c5/f1p0/1561	NA	NA	NA	R01_cb12273_c5/f1p0/1561
R01_cb9841_c1/f1p0/1930	NA	NA	NA	R01_cb9841_c1/f1p0/1930
R01_cb3997_c2/f1p0/3489	NA	NA	NA	R01_cb3997_c2/f1p0/3489
R01_cb17537_c3/f1p0/1507	NA	R01_cb17537_c3/f1p0/1507	NA	NA

R01_cb18255_c0/f2p0/863	R01_cb18255_c0/f2p0/863	R01_cb18255_c0/f2p0/863	R01_cb18255_c0/f2p0/863	R01_cb18255_c0/f2p0/863
R01_cb3381_c6/f2p0/763	R01_cb3381_c6/f2p0/763	R01_cb3381_c6/f2p0/763	R01_cb3381_c6/f2p0/763	R01_cb3381_c6/f2p0/763
R01_cb4469_c11/f1p0/1822	NA	NA	NA	R01_cb4469_c11/f1p0/1822
R01_cb14125_c91/f1p0/675	NA	R01_cb14125_c91/f1p0/675	R01_cb14125_c91/f1p0/675	NA
R01_cb135_c47/f1p2/2136	NA	R01_cb135_c47/f1p2/2136	NA	NA
R01_cb8564_c23949/f1p0/2943	NA	R01_cb8564_c23949/f1p0/2943	NA	R01_cb8564_c23949/f1p0/2943
R01_cb8564_c3877/f1p0/2862	R01_cb8564_c3877/f1p0/2862	R01_cb8564_c3877/f1p0/2862	R01_cb8564_c3877/f1p0/2862	R01_cb8564_c3877/f1p0/2862
R01_cb17604_c6/f1p0/625	NA	R01_cb17604_c6/f1p0/625	R01_cb17604_c6/f1p0/625	R01_cb17604_c6/f1p0/625
R01_cb8564_c1883/f1p0/2869	NA	NA	NA	R01_cb8564_c1883/f1p0/2869
R01_cb2588_c4/f1p0/540	R01_cb2588_c4/f1p0/540	R01_cb2588_c4/f1p0/540	R01_cb2588_c4/f1p0/540	R01_cb2588_c4/f1p0/540
R01_cb8564_c21584/f2p5/3932	NA	R01_cb8564_c21584/f2p5/3932	R01_cb8564_c21584/f2p5/3932	R01_cb8564_c21584/f2p5/3932
R01_cb18132_c7/f1p0/1239	NA	R01_cb18132_c7/f1p0/1239	NA	NA
R01_cb5976_c5/f1p0/1263	NA	R01_cb5976_c5/f1p0/1263	NA	R01_cb5976_c5/f1p0/1263
R01_cb8564_c71150/f1p0/2448	NA	R01_cb8564_c71150/f1p0/2448	NA	NA
R01_cb6740_c10/f1p0/4763	NA	R01_cb6740_c10/f1p0/4763	NA	NA
R01_cb8564_c71119/f1p0/3003	R01_cb8564_c71119/f1p0/3003	R01_cb8564_c71119/f1p0/3003	R01_cb8564_c71119/f1p0/3003	R01_cb8564_c71119/f1p0/3003
R01_cb8564_c4479/f1p0/3012	NA	NA	NA	R01_cb8564_c4479/f1p0/3012
R01_cb15175_c2/f1p0/660	NA	NA	NA	R01_cb15175_c2/f1p0/660
R01_cb8564_c91619/f1p0/3725	NA	R01_cb8564_c91619/f1p0/3725	R01_cb8564_c91619/f1p0/3725	R01_cb8564_c91619/f1p0/3725
R01_cb8564_c123717/f1p0/2918	NA	NA	NA	R01_cb8564_c123717/f1p0/2918
R01_cb16945_c3/f1p0/1647	R01_cb16945_c3/f1p0/1647	R01_cb16945_c3/f1p0/1647	R01_cb16945_c3/f1p0/1647	R01_cb16945_c3/f1p0/1647
R01_cb8564_c74104/f1p0/2277	NA	NA	NA	R01_cb8564_c74104/f1p0/2277
R01_cb8564_c3997/f1p0/2183	NA	NA	R01_cb8564_c3997/f1p0/2183	R01_cb8564_c3997/f1p0/2183
R01_cb412_c4/f1p0/3252	NA	NA	NA	R01_cb412_c4/f1p0/3252

R01_cb14882_c4/f1p1/1019	NA	R01_cb14882_c4/f1p1/1019	NA	NA
R01_cb9805_c0/f1p0/1972	NA	R01_cb9805_c0/f1p0/1972	R01_cb9805_c0/f1p0/1972	NA
R01_cb16470_c0/f6p0/659	NA	R01_cb16470_c0/f6p0/659	NA	NA
R01_cb7714_c15/f1p0/2031	NA	NA	NA	R01_cb7714_c15/f1p0/2031
R01_cb9143_c123/f1p0/2290	NA	R01_cb9143_c123/f1p0/2290	NA	NA
R01_cb1610_c1/f2p0/2723	NA	NA	NA	R01_cb1610_c1/f2p0/2723
R01_cb261_c15/f1p0/3068	NA	NA	NA	R01_cb261_c15/f1p0/3068
R01_cb2989_c9/f1p0/2038	NA	NA	NA	R01_cb2989_c9/f1p0/2038
R01_cb12767_c13/f1p0/1024	NA	NA	NA	R01_cb12767_c13/f1p0/1024
R01_cb16991_c3/f1p0/413	R01_cb16991_c3/f1p0/413	R01_cb16991_c3/f1p0/413	R01_cb16991_c3/f1p0/413	R01_cb16991_c3/f1p0/413
R01_cb2160_c6/f1p1/3786	NA	NA	NA	R01_cb2160_c6/f1p1/3786
R01_cb18456_c6120/f1p0/777	NA	NA	NA	R01_cb18456_c6120/f1p0/777
R01_cb9409_c2/f1p0/2056	NA	R01_cb9409_c2/f1p0/2056	NA	NA
R01_cb8582_c0/f1p0/2321	NA	R01_cb8582_c0/f1p0/2321	NA	R01_cb8582_c0/f1p0/2321
R01_cb11261_c2/f1p0/2163	R01_cb11261_c2/f1p0/2163	R01_cb11261_c2/f1p0/2163	NA	NA
R01_cb18409_c81/f1p0/1272	R01_cb18409_c81/f1p0/1272	R01_cb18409_c81/f1p0/1272	R01_cb18409_c81/f1p0/1272	R01_cb18409_c81/f1p0/1272
R01_cb10262_c10/f1p0/488	NA	R01_cb10262_c10/f1p0/488	R01_cb10262_c10/f1p0/488	R01_cb10262_c10/f1p0/488
R01_cb13558_c1/f3p0/631	NA	NA	R01_cb13558_c1/f3p0/631	R01_cb13558_c1/f3p0/631
R01_cb8564_c4109/f1p0/2778	R01_cb8564_c4109/f1p0/2778	R01_cb8564_c4109/f1p0/2778	R01_cb8564_c4109/f1p0/2778	R01_cb8564_c4109/f1p0/2778
R01_cb5682_c11/f1p0/488	R01_cb5682_c11/f1p0/488	R01_cb5682_c11/f1p0/488	R01_cb5682_c11/f1p0/488	R01_cb5682_c11/f1p0/488
R01_cb1259_c21/f1p0/3481	NA	NA	NA	R01_cb1259_c21/f1p0/3481
R01_cb17484_c0/f2p0/1133	NA	NA	NA	R01_cb17484_c0/f2p0/1133
R01_cb8969_c1/f1p0/1893	NA	R01_cb8969_c1/f1p0/1893	R01_cb8969_c1/f1p0/1893	R01_cb8969_c1/f1p0/1893
R01_cb8564_c89944/f1p0/3176	NA	NA	NA	R01_cb8564_c89944/f1p0/3176
R01_cb4226_c4/f1p0/2756	NA	R01_cb4226_c4/f1p0/2756	NA	NA
R01_cb16174_c2/f1p0/1199	NA	R01_cb16174_c2/f1p0/1199	NA	NA

R01_cb14316_c6/f1p0/1839	R01_cb14316_c6/f1p0/1839	R01_cb14316_c6/f1p0/1839	R01_cb14316_c6/f1p0/1839	R01_cb14316_c6/f1p0/1839
R01_cb8564_c130214/f1p1/2920	NA	R01_cb8564_c130214/f1p1/2920	R01_cb8564_c130214/f1p1/2920	NA
R01_cb18456_c7655/f1p0/465	R01_cb18456_c7655/f1p0/465	R01_cb18456_c7655/f1p0/465	R01_cb18456_c7655/f1p0/465	R01_cb18456_c7655/f1p0/465
R01_cb8564_c85134/f2p0/2014	NA	R01_cb8564_c85134/f2p0/2014	R01_cb8564_c85134/f2p0/2014	R01_cb8564_c85134/f2p0/2014
R01_cb8564_c92362/f1p0/3943	NA	R01_cb8564_c92362/f1p0/3943	NA	NA
R01_cb416_c29/f1p0/2757	NA	NA	NA	R01_cb416_c29/f1p0/2757
R01_cb12240_c5/f1p1/622	R01_cb12240_c5/f1p1/622	R01_cb12240_c5/f1p1/622	NA	NA
R01_cb8564_c4902/f1p0/3303	NA	R01_cb8564_c4902/f1p0/3303	NA	R01_cb8564_c4902/f1p0/3303
R01_cb7333_c0/f3p0/2980	NA	R01_cb7333_c0/f3p0/2980	NA	R01_cb7333_c0/f3p0/2980
R01_cb1905_c37/f1p0/2427	NA	R01_cb1905_c37/f1p0/2427	R01_cb1905_c37/f1p0/2427	NA
R01_cb8564_c16144/f1p0/3618	NA	NA	NA	R01_cb8564_c16144/f1p0/3618
R01_cb14856_c1/f1p0/1755	NA	R01_cb14856_c1/f1p0/1755	NA	R01_cb14856_c1/f1p0/1755
R01_cb16125_c20/f1p0/1652	NA	R01_cb16125_c20/f1p0/1652	R01_cb16125_c20/f1p0/1652	R01_cb16125_c20/f1p0/1652
R01_cb17904_c0/f1p0/610	R01_cb17904_c0/f1p0/610	R01_cb17904_c0/f1p0/610	R01_cb17904_c0/f1p0/610	R01_cb17904_c0/f1p0/610
R01_cb2804_c99/f1p0/2179	NA	R01_cb2804_c99/f1p0/2179	NA	NA
R01_cb18456_c7473/f1p0/1338	NA	NA	NA	R01_cb18456_c7473/f1p0/1338
R01_cb8160_c0/f1p1/2444	NA	NA	NA	R01_cb8160_c0/f1p1/2444
R01_cb18508_c1/f1p0/314	R01_cb18508_c1/f1p0/314	R01_cb18508_c1/f1p0/314	R01_cb18508_c1/f1p0/314	R01_cb18508_c1/f1p0/314
R01_cb12473_c3/f1p0/659	NA	R01_cb12473_c3/f1p0/659	R01_cb12473_c3/f1p0/659	R01_cb12473_c3/f1p0/659
R01_cb16022_c1/f1p0/1359	NA	R01_cb16022_c1/f1p0/1359	NA	NA
R01_cb10083_c11/f1p0/1261	NA	R01_cb10083_c11/f1p0/1261	NA	NA
R01_cb6779_c1/f3p0/1914	NA	NA	NA	R01_cb6779_c1/f3p0/1914
R01_cb2807_c3/f1p0/1894	R01_cb2807_c3/f1p0/1894	R01_cb2807_c3/f1p0/1894	R01_cb2807_c3/f1p0/1894	R01_cb2807_c3/f1p0/1894
R01_cb7441_c9/f1p0/1153	NA	NA	NA	R01_cb7441_c9/f1p0/1153
R01_cb5807_c9/f1p0/464	NA	R01_cb5807_c9/f1p0/464	NA	NA

R01_cb8564_c145443/f2p0/2287	NA	NA	NA	R01_cb8564_c145443/f2p0/2287
R01_cb124_c19/f1p0/3959	NA	NA	NA	R01_cb124_c19/f1p0/3959
R01_cb18456_c7368/f1p0/1505	NA	R01_cb18456_c7368/f1p0/1505	R01_cb18456_c7368/f1p0/1505	R01_cb18456_c7368/f1p0/1505
R01_cb8564_c36226/f1p0/3283	R01_cb8564_c36226/f1p0/3283	R01_cb8564_c36226/f1p0/3283	R01_cb8564_c36226/f1p0/3283	R01_cb8564_c36226/f1p0/3283
R01_cb3848_c15/f1p0/2348	NA	NA	NA	R01_cb3848_c15/f1p0/2348
R01_cb5502_c3/f1p1/3086	NA	NA	NA	R01_cb5502_c3/f1p1/3086
R01_cb380_c8/f1p1/4390	NA	R01_cb380_c8/f1p1/4390	NA	NA
R01_cb15207_c80/f1p0/981	NA	R01_cb15207_c80/f1p0/981	NA	NA
R01_cb9606_c65/f1p0/1786	NA	R01_cb9606_c65/f1p0/1786	NA	R01_cb9606_c65/f1p0/1786
R01_cb8564_c81222/f1p0/2638	NA	NA	NA	R01_cb8564_c81222/f1p0/2638
R01_cb8564_c4652/f3p0/3053	R01_cb8564_c4652/f3p0/3053	R01_cb8564_c4652/f3p0/3053	NA	R01_cb8564_c4652/f3p0/3053
R01_cb8564_c23979/f1p0/4568	NA	R01_cb8564_c23979/f1p0/4568	NA	R01_cb8564_c23979/f1p0/4568
R01_cb17973_c1/f7p0/508	R01_cb17973_c1/f7p0/508	R01_cb17973_c1/f7p0/508	R01_cb17973_c1/f7p0/508	R01_cb17973_c1/f7p0/508
R01_cb17363_c7/f1p1/571	R01_cb17363_c7/f1p1/571	R01_cb17363_c7/f1p1/571	R01_cb17363_c7/f1p1/571	R01_cb17363_c7/f1p1/571
R01_cb11709_c2/f1p0/1396	NA	NA	NA	R01_cb11709_c2/f1p0/1396
R01_cb5755_c12/f1p0/2708	NA	NA	NA	R01_cb5755_c12/f1p0/2708
R01_cb6534_c3/f1p0/723	NA	NA	NA	R01_cb6534_c3/f1p0/723
R01_cb8564_c50670/f1p0/3680	NA	R01_cb8564_c50670/f1p0/3680	NA	R01_cb8564_c50670/f1p0/3680
R01_cb6285_c0/f1p0/2892	R01_cb6285_c0/f1p0/2892	R01_cb6285_c0/f1p0/2892	R01_cb6285_c0/f1p0/2892	R01_cb6285_c0/f1p0/2892
R01_cb8564_c4217/f1p0/4767	NA	NA	R01_cb8564_c4217/f1p0/4767	R01_cb8564_c4217/f1p0/4767
R01_cb16945_c0/f1p0/1426	R01_cb16945_c0/f1p0/1426	R01_cb16945_c0/f1p0/1426	R01_cb16945_c0/f1p0/1426	R01_cb16945_c0/f1p0/1426
R01_cb17354_c1/f1p0/1440	NA	NA	NA	R01_cb17354_c1/f1p0/1440
R01_cb3026_c0/f3p0/578	NA	R01_cb3026_c0/f3p0/578	NA	NA
R01_cb8564_c109812/f2p1/368	NA	NA	NA	R01_cb8564_c109812/f2p1/368

7				7
R01_cb8198_c5/f1p1/1990	NA	NA	NA	R01_cb8198_c5/f1p1/1990
R01_cb5943_c9/f4p0/1361	NA	NA	NA	R01_cb5943_c9/f4p0/1361
R01_cb4249_c9/f1p0/631	NA	NA	NA	R01_cb4249_c9/f1p0/631
R01_cb5632_c9/f1p0/3493	NA	R01_cb5632_c9/f1p0/3493	NA	NA
R01_cb13786_c0/f2p0/596	NA	NA	NA	R01_cb13786_c0/f2p0/596
R01_cb3848_c1/f4p0/2367	NA	NA	NA	R01_cb3848_c1/f4p0/2367
R01_cb8564_c77662/f1p0/3894	NA	R01_cb8564_c77662/f1p0/3894	NA	NA
R01_cb8564_c14552/f1p0/2951	R01_cb8564_c14552/f1p0/295 1	R01_cb8564_c14552/f1p0/2951	R01_cb8564_c14552/f1p0/2951	R01_cb8564_c14552/f1p0/2951
R01_cb12696_c18/f1p3/711	NA	R01_cb12696_c18/f1p3/711	NA	NA
R01_cb15676_c2/f1p0/1197	NA	NA	NA	R01_cb15676_c2/f1p0/1197
R01_cb12008_c2/f1p0/1640	NA	NA	NA	R01_cb12008_c2/f1p0/1640
R01_cb2845_c17/f1p0/3838	NA	NA	NA	R01_cb2845_c17/f1p0/3838
R01_cb17196_c0/f1p0/735	NA	R01_cb17196_c0/f1p0/735	R01_cb17196_c0/f1p0/735	R01_cb17196_c0/f1p0/735
R01_cb8564_c51796/f1p0/2631	R01_cb8564_c51796/f1p0/263 1	R01_cb8564_c51796/f1p0/2631	R01_cb8564_c51796/f1p0/2631	R01_cb8564_c51796/f1p0/2631
R01_cb13768_c23/f1p0/439	NA	R01_cb13768_c23/f1p0/439	NA	NA
R01_cb3582_c6/f1p1/2497	NA	NA	NA	R01_cb3582_c6/f1p1/2497
R01_cb18836_c1/f1p0/5315	NA	NA	NA	R01_cb18836_c1/f1p0/5315
R01_cb4380_c9/f1p0/962	NA	NA	NA	R01_cb4380_c9/f1p0/962
R01_cb11361_c1/f3p0/872	NA	NA	NA	R01_cb11361_c1/f3p0/872
R01_cb11117_c2/f1p1/3308	NA	R01_cb11117_c2/f1p1/3308	NA	NA
R01_cb10720_c1/f1p0/2161	NA	R01_cb10720_c1/f1p0/2161	NA	R01_cb10720_c1/f1p0/2161
R01_cb10630_c1/f1p0/3182	R01_cb10630_c1/f1p0/3182	NA	NA	NA
R01_cb14329_c0/f3p0/837	R01_cb14329_c0/f3p0/837	NA	NA	NA

R01_cb11924_c10/f3p0/486	NA	R01_cb11924_c10/f3p0/486	R01_cb11924_c10/f3p0/486	NA
R01_cb17347_c3/f1p0/873	NA	NA	NA	R01_cb17347_c3/f1p0/873
R01_cb14761_c4/f1p0/799	NA	R01_cb14761_c4/f1p0/799	R01_cb14761_c4/f1p0/799	NA
R01_cb14329_c5/f1p0/872	R01_cb14329_c5/f1p0/872	NA	NA	NA
R01_cb8564_c78456/f1p0/2589	NA	R01_cb8564_c78456/f1p0/2589	NA	NA
R01_cb8564_c69240/f4p0/4134	NA	NA	NA	R01_cb8564_c69240/f4p0/4134
R01_cb1095_c11/f1p0/3070	NA	NA	NA	R01_cb1095_c11/f1p0/3070
R01_cb7642_c212/f1p4/1760	NA	R01_cb7642_c212/f1p4/1760	NA	NA
R01_cb11975_c2/f1p1/962	NA	R01_cb11975_c2/f1p1/962	R01_cb11975_c2/f1p1/962	R01_cb11975_c2/f1p1/962
R01_cb10182_c0/f1p0/1487	NA	R01_cb10182_c0/f1p0/1487	NA	NA
R01_cb8682_c2/f1p0/2252	NA	R01_cb8682_c2/f1p0/2252	NA	NA
R01_cb15252_c12/f1p0/3614	NA	NA	NA	R01_cb15252_c12/f1p0/3614
R01_cb9_c3/f1p0/2562	NA	R01_cb9_c3/f1p0/2562	NA	NA
R01_cb18372_c16/f1p0/2987	R01_cb18372_c16/f1p0/2987	R01_cb18372_c16/f1p0/2987	NA	NA
R01_cb2538_c3/f1p0/1585	NA	NA	NA	R01_cb2538_c3/f1p0/1585
R01_cb18666_c1/f1p0/1263	NA	R01_cb18666_c1/f1p0/1263	R01_cb18666_c1/f1p0/1263	R01_cb18666_c1/f1p0/1263
R01_cb5904_c11/f1p0/1421	NA	R01_cb5904_c11/f1p0/1421	NA	NA
R01_cb10071_c2/f1p0/1614	R01_cb10071_c2/f1p0/1614	R01_cb10071_c2/f1p0/1614	R01_cb10071_c2/f1p0/1614	R01_cb10071_c2/f1p0/1614
R01_cb115_c1/f1p0/4742	NA	NA	NA	R01_cb115_c1/f1p0/4742
R01_cb7268_c3/f1p3/2423	NA	NA	R01_cb7268_c3/f1p3/2423	NA
R01_cb9192_c2/f1p0/2582	NA	NA	NA	R01_cb9192_c2/f1p0/2582
R01_cb1935_c24/f1p0/881	NA	R01_cb1935_c24/f1p0/881	NA	R01_cb1935_c24/f1p0/881
R01_cb17079_c0/f1p0/1488	NA	R01_cb17079_c0/f1p0/1488	NA	NA
R01_cb11715_c1/f1p0/2823	NA	R01_cb11715_c1/f1p0/2823	NA	NA
R01_cb7392_c0/f2p0/2457	NA	NA	NA	R01_cb7392_c0/f2p0/2457
R01_cb13938_c20/f1p0/1134	NA	NA	NA	R01_cb13938_c20/f1p0/1134

R01_cb10873_c1/f1p0/3029	NA	NA	NA	R01_cb10873_c1/f1p0/3029
R01_cb7419_c5/f1p1/2191	NA	NA	NA	R01_cb7419_c5/f1p1/2191
R01_cb1248_c5/f1p0/6631	NA	NA	NA	R01_cb1248_c5/f1p0/6631
R01_cb14455_c18/f1p0/421	R01_cb14455_c18/f1p0/421	R01_cb14455_c18/f1p0/421	R01_cb14455_c18/f1p0/421	R01_cb14455_c18/f1p0/421
R01_cb1849_c15/f1p1/2085	NA	NA	NA	R01_cb1849_c15/f1p1/2085
R01_cb1508_c10/f1p0/1976	NA	NA	NA	R01_cb1508_c10/f1p0/1976
R01_cb10800_c4/f1p0/1517	NA	NA	NA	R01_cb10800_c4/f1p0/1517
R01_cb666_c2/f1p0/2897	NA	NA	NA	R01_cb666_c2/f1p0/2897
R01_cb7595_c17/f1p0/834	NA	R01_cb7595_c17/f1p0/834	NA	NA
R01_cb15834_c1/f1p0/681	NA	NA	NA	R01_cb15834_c1/f1p0/681
R01_cb8564_c54093/f1p1/2870	NA	R01_cb8564_c54093/f1p1/2870	NA	NA
R01_cb11790_c0/f1p0/1881	NA	R01_cb11790_c0/f1p0/1881	NA	NA
R01_cb6865_c15/f1p0/2248	NA	R01_cb6865_c15/f1p0/2248	NA	NA
R01_cb16397_c1/f1p0/607	R01_cb16397_c1/f1p0/607	R01_cb16397_c1/f1p0/607	R01_cb16397_c1/f1p0/607	R01_cb16397_c1/f1p0/607
R01_cb8564_c123053/f1p1/2096	NA	NA	NA	R01_cb8564_c123053/f1p1/2096
R01_cb13979_c7/f1p0/1231	R01_cb13979_c7/f1p0/1231	NA	NA	NA
R01_cb12273_c2/f1p0/1623	NA	NA	NA	R01_cb12273_c2/f1p0/1623
R01_cb8564_c789/f3p0/2586	NA	R01_cb8564_c789/f3p0/2586	R01_cb8564_c789/f3p0/2586	NA
R01_cb4117_c1/f2p0/2944	NA	NA	NA	R01_cb4117_c1/f2p0/2944
R01_cb8564_c11613/f1p0/2758	NA	R01_cb8564_c11613/f1p0/2758	NA	NA
R01_cb16452_c4/f1p0/684	NA	R01_cb16452_c4/f1p0/684	R01_cb16452_c4/f1p0/684	R01_cb16452_c4/f1p0/684
R01_cb16098_c3/f3p0/754	R01_cb16098_c3/f3p0/754	R01_cb16098_c3/f3p0/754	R01_cb16098_c3/f3p0/754	R01_cb16098_c3/f3p0/754
R01_cb8564_c9884/f2p0/2565	NA	R01_cb8564_c9884/f2p0/2565	R01_cb8564_c9884/f2p0/2565	R01_cb8564_c9884/f2p0/2565
R01_cb13103_c2/f1p0/464	R01_cb13103_c2/f1p0/464	R01_cb13103_c2/f1p0/464	R01_cb13103_c2/f1p0/464	R01_cb13103_c2/f1p0/464
R01_cb1093_c0/f1p0/4471	NA	NA	NA	R01_cb1093_c0/f1p0/4471

R01_cb2451_c18/f1p0/2265	NA	NA	NA	R01_cb2451_c18/f1p0/2265
R01_cb8564_c23602/f1p0/2921	NA	R01_cb8564_c23602/f1p0/2921	R01_cb8564_c23602/f1p0/2921	R01_cb8564_c23602/f1p0/2921
R01_cb8564_c23111/f1p0/4221	NA	NA	NA	R01_cb8564_c23111/f1p0/4221
R01_cb8564_c34188/f2p3/4546	NA	NA	NA	R01_cb8564_c34188/f2p3/4546
R01_cb8564_c89386/f1p0/2244	NA	R01_cb8564_c89386/f1p0/2244	NA	NA
R01_cb12785_c20/f1p0/359	R01_cb12785_c20/f1p0/359	R01_cb12785_c20/f1p0/359	R01_cb12785_c20/f1p0/359	R01_cb12785_c20/f1p0/359
R01_cb8564_c78565/f1p1/2318	NA	R01_cb8564_c78565/f1p1/2318	NA	NA
R01_cb12498_c3/f1p0/739	NA	R01_cb12498_c3/f1p0/739	NA	NA
R01_cb13141_c0/f2p0/950	NA	R01_cb13141_c0/f2p0/950	NA	NA
R01_cb391_c4/f1p0/4291	NA	NA	NA	R01_cb391_c4/f1p0/4291
R01_cb4600_c14/f1p0/2035	NA	NA	NA	R01_cb4600_c14/f1p0/2035
R01_cb5811_c6/f1p0/1837	NA	R01_cb5811_c6/f1p0/1837	NA	NA
R01_cb13284_c8/f1p0/1100	NA	NA	NA	R01_cb13284_c8/f1p0/1100
R01_cb8564_c109382/f1p1/2939	R01_cb8564_c109382/f1p1/2939	R01_cb8564_c109382/f1p1/2939	R01_cb8564_c109382/f1p1/2939	R01_cb8564_c109382/f1p1/2939
R01_cb1905_c23/f1p0/2032	NA	R01_cb1905_c23/f1p0/2032	R01_cb1905_c23/f1p0/2032	NA
R01_cb8564_c16240/f1p3/2671	NA	NA	NA	R01_cb8564_c16240/f1p3/2671
R01_cb10101_c4/f1p0/420	NA	R01_cb10101_c4/f1p0/420	NA	R01_cb10101_c4/f1p0/420
R01_cb1307_c22/f1p0/4955	R01_cb1307_c22/f1p0/4955	R01_cb1307_c22/f1p0/4955	R01_cb1307_c22/f1p0/4955	R01_cb1307_c22/f1p0/4955
R01_cb8564_c119262/f1p0/2228	NA	R01_cb8564_c119262/f1p0/2228	R01_cb8564_c119262/f1p0/2228	R01_cb8564_c119262/f1p0/2228
R01_cb18409_c114/f1p0/474	R01_cb18409_c114/f1p0/474	R01_cb18409_c114/f1p0/474	R01_cb18409_c114/f1p0/474	R01_cb18409_c114/f1p0/474
R01_cb18236_c1/f1p0/752	NA	R01_cb18236_c1/f1p0/752	NA	R01_cb18236_c1/f1p0/752
R01_cb17394_c1/f1p0/1029	NA	NA	NA	R01_cb17394_c1/f1p0/1029
R01_cb9178_c0/f1p0/2147	NA	R01_cb9178_c0/f1p0/2147	R01_cb9178_c0/f1p0/2147	NA
R01_cb1779_c6/f1p0/3786	NA	NA	NA	R01_cb1779_c6/f1p0/3786

R01_cb4233_c73/f1p0/3408	NA	R01_cb4233_c73/f1p0/3408	NA	NA
R01_cb6377_c0/f1p0/2879	NA	NA	NA	R01_cb6377_c0/f1p0/2879
R01_cb8564_c72601/f1p0/2783	R01_cb8564_c72601/f1p0/2783	R01_cb8564_c72601/f1p0/2783	R01_cb8564_c72601/f1p0/2783	R01_cb8564_c72601/f1p0/2783
R01_cb15425_c2/f1p0/527	NA	R01_cb15425_c2/f1p0/527	NA	NA
R01_cb8564_c115403/f1p0/2492	NA	R01_cb8564_c115403/f1p0/2492	R01_cb8564_c115403/f1p0/2492	R01_cb8564_c115403/f1p0/2492
R01_cb18456_c6817/f1p0/528	NA	R01_cb18456_c6817/f1p0/528	R01_cb18456_c6817/f1p0/528	NA
R01_cb14922_c2/f1p0/900	NA	NA	NA	R01_cb14922_c2/f1p0/900
R01_cb3922_c8/f1p0/4175	NA	R01_cb3922_c8/f1p0/4175	NA	NA
R01_cb16612_c0/f2p0/562	NA	R01_cb16612_c0/f2p0/562	NA	NA
R01_cb18224_c2/f1p0/1671	NA	NA	NA	R01_cb18224_c2/f1p0/1671
R01_cb8564_c70172/f1p0/2498	R01_cb8564_c70172/f1p0/2498	R01_cb8564_c70172/f1p0/2498	R01_cb8564_c70172/f1p0/2498	R01_cb8564_c70172/f1p0/2498
R01_cb4230_c2/f1p0/2518	R01_cb4230_c2/f1p0/2518	R01_cb4230_c2/f1p0/2518	R01_cb4230_c2/f1p0/2518	R01_cb4230_c2/f1p0/2518
R01_cb7693_c7/f1p0/5458	NA	R01_cb7693_c7/f1p0/5458	R01_cb7693_c7/f1p0/5458	R01_cb7693_c7/f1p0/5458
R01_cb8564_c70200/f1p0/3569	NA	R01_cb8564_c70200/f1p0/3569	R01_cb8564_c70200/f1p0/3569	R01_cb8564_c70200/f1p0/3569
R01_cb8007_c1/f1p0/2733	R01_cb8007_c1/f1p0/2733	R01_cb8007_c1/f1p0/2733	NA	NA
R01_cb3464_c5/f1p0/2790	NA	NA	NA	R01_cb3464_c5/f1p0/2790
R01_cb12722_c19/f1p2/1665	NA	NA	NA	R01_cb12722_c19/f1p2/1665
R01_cb16387_c9/f1p0/1504	NA	NA	NA	R01_cb16387_c9/f1p0/1504
R01_cb7304_c0/f2p0/2239	NA	NA	NA	R01_cb7304_c0/f2p0/2239
R01_cb18395_c1/f1p0/706	NA	R01_cb18395_c1/f1p0/706	R01_cb18395_c1/f1p0/706	R01_cb18395_c1/f1p0/706
R01_cb10133_c6/f1p1/1304	NA	NA	NA	R01_cb10133_c6/f1p1/1304
R01_cb6802_c61/f1p0/2216	R01_cb6802_c61/f1p0/2216	R01_cb6802_c61/f1p0/2216	R01_cb6802_c61/f1p0/2216	R01_cb6802_c61/f1p0/2216
R01_cb5775_c4/f1p0/2914	NA	R01_cb5775_c4/f1p0/2914	NA	R01_cb5775_c4/f1p0/2914

R01_cb8388_c4/f1p0/1967	NA	R01_cb8388_c4/f1p0/1967	NA	NA
R01_cb11698_c0/f1p0/1039	NA	NA	NA	R01_cb11698_c0/f1p0/1039
R01_cb8564_c49791/f1p0/4590	NA	NA	NA	R01_cb8564_c49791/f1p0/4590
R01_cb16125_c11/f1p0/1851	NA	R01_cb16125_c11/f1p0/1851	NA	R01_cb16125_c11/f1p0/1851
R01_cb10024_c300/f1p3/1162	NA	NA	R01_cb10024_c300/f1p3/1162	NA
R01_cb3483_c24/f1p0/2925	NA	NA	NA	R01_cb3483_c24/f1p0/2925
R01_cb1238_c18/f1p0/1027	R01_cb1238_c18/f1p0/1027	R01_cb1238_c18/f1p0/1027	R01_cb1238_c18/f1p0/1027	R01_cb1238_c18/f1p0/1027
R01_cb17106_c2/f1p0/1644	NA	R01_cb17106_c2/f1p0/1644	R01_cb17106_c2/f1p0/1644	R01_cb17106_c2/f1p0/1644
R01_cb69_c24/f1p0/3522	NA	NA	NA	R01_cb69_c24/f1p0/3522
R01_cb10254_c0/f1p0/657	NA	R01_cb10254_c0/f1p0/657	R01_cb10254_c0/f1p0/657	R01_cb10254_c0/f1p0/657
R01_cb15655_c1/f1p0/1850	NA	R01_cb15655_c1/f1p0/1850	NA	NA
R01_cb8564_c119177/f1p0/2132	NA	R01_cb8564_c119177/f1p0/2132	NA	NA
R01_cb8564_c23677/f1p0/2303	R01_cb8564_c23677/f1p0/2303	R01_cb8564_c23677/f1p0/2303	R01_cb8564_c23677/f1p0/2303	R01_cb8564_c23677/f1p0/2303
R01_cb8564_c3951/f1p0/3719	NA	R01_cb8564_c3951/f1p0/3719	NA	R01_cb8564_c3951/f1p0/3719
R01_cb3046_c10/f1p0/2958	NA	NA	NA	R01_cb3046_c10/f1p0/2958
R01_cb10292_c2/f1p0/3157	NA	R01_cb10292_c2/f1p0/3157	NA	R01_cb10292_c2/f1p0/3157
R01_cb3745_c2/f1p0/3392	NA	R01_cb3745_c2/f1p0/3392	R01_cb3745_c2/f1p0/3392	NA
R01_cb12294_c4/f4p0/614	NA	R01_cb12294_c4/f4p0/614	NA	NA
R01_cb506_c11/f1p1/4629	NA	NA	NA	R01_cb506_c11/f1p1/4629
R01_cb8564_c72148/f3p0/3260	NA	R01_cb8564_c72148/f3p0/3260	R01_cb8564_c72148/f3p0/3260	NA
R01_cb12151_c12/f1p0/723	NA	R01_cb12151_c12/f1p0/723	NA	NA
R01_cb6069_c2/f1p0/2944	NA	NA	NA	R01_cb6069_c2/f1p0/2944
R01_cb14480_c7/f1p0/1514	R01_cb14480_c7/f1p0/1514	R01_cb14480_c7/f1p0/1514	NA	R01_cb14480_c7/f1p0/1514
R01_cb9552_c4/f1p0/2192	NA	NA	NA	R01_cb9552_c4/f1p0/2192

R01_cb5174_c23/f1p0/1396	NA	R01_cb5174_c23/f1p0/1396	NA	R01_cb5174_c23/f1p0/1396
R01_cb9079_c11/f1p0/1031	NA	NA	R01_cb9079_c11/f1p0/1031	NA
R01_cb11455_c4/f2p0/498	NA	R01_cb11455_c4/f2p0/498	NA	R01_cb11455_c4/f2p0/498
R01_cb3760_c5/f1p0/3536	NA	NA	NA	R01_cb3760_c5/f1p0/3536
R01_cb18433_c1/f1p0/877	R01_cb18433_c1/f1p0/877	R01_cb18433_c1/f1p0/877	NA	R01_cb18433_c1/f1p0/877
R01_cb17706_c1/f3p0/569	R01_cb17706_c1/f3p0/569	R01_cb17706_c1/f3p0/569	R01_cb17706_c1/f3p0/569	R01_cb17706_c1/f3p0/569
R01_cb8564_c85845/f1p0/2281	NA	NA	R01_cb8564_c85845/f1p0/2281	NA
R01_cb15337_c3/f1p0/1784	NA	NA	NA	R01_cb15337_c3/f1p0/1784
R01_cb10337_c1/f1p2/1924	R01_cb10337_c1/f1p2/1924	R01_cb10337_c1/f1p2/1924	NA	NA
R01_cb18456_c7213/f1p0/693	R01_cb18456_c7213/f1p0/693	R01_cb18456_c7213/f1p0/693	R01_cb18456_c7213/f1p0/693	R01_cb18456_c7213/f1p0/693
R01_cb3750_c5/f2p2/2925	NA	NA	NA	R01_cb3750_c5/f2p2/2925
R01_cb17483_c1/f1p0/537	R01_cb17483_c1/f1p0/537	R01_cb17483_c1/f1p0/537	R01_cb17483_c1/f1p0/537	R01_cb17483_c1/f1p0/537
R01_cb1533_c5/f1p0/2746	NA	R01_cb1533_c5/f1p0/2746	NA	NA
R01_cb18223_c21/f1p0/481	R01_cb18223_c21/f1p0/481	R01_cb18223_c21/f1p0/481	R01_cb18223_c21/f1p0/481	R01_cb18223_c21/f1p0/481
R01_cb16125_c23/f1p0/585	R01_cb16125_c23/f1p0/585	R01_cb16125_c23/f1p0/585	R01_cb16125_c23/f1p0/585	R01_cb16125_c23/f1p0/585
R01_cb15751_c0/f1p0/1852	R01_cb15751_c0/f1p0/1852	R01_cb15751_c0/f1p0/1852	R01_cb15751_c0/f1p0/1852	R01_cb15751_c0/f1p0/1852
R01_cb8564_c1409/f1p0/2948	R01_cb8564_c1409/f1p0/2948	R01_cb8564_c1409/f1p0/2948	NA	NA
R01_cb15034_c1/f1p0/1224	R01_cb15034_c1/f1p0/1224	R01_cb15034_c1/f1p0/1224	NA	R01_cb15034_c1/f1p0/1224
R01_cb8564_c36768/f1p0/4007	NA	R01_cb8564_c36768/f1p0/4007	NA	NA
R01_cb1788_c1/f1p0/3299	NA	NA	NA	R01_cb1788_c1/f1p0/3299
R01_cb3703_c0/f3p1/3550	NA	NA	NA	R01_cb3703_c0/f3p1/3550
R01_cb12059_c9/f1p4/1010	NA	R01_cb12059_c9/f1p4/1010	NA	NA
R01_cb10058_c6/f2p1/1685	NA	R01_cb10058_c6/f2p1/1685	NA	NA
R01_cb10353_c0/f1p0/636	NA	R01_cb10353_c0/f1p0/636	R01_cb10353_c0/f1p0/636	NA
R01_cb7612_c3/f1p1/2038	NA	NA	NA	R01_cb7612_c3/f1p1/2038
R01_cb8564_c52680/f1p0/2870	NA	R01_cb8564_c52680/f1p0/2870	R01_cb8564_c52680/f1p0/2870	NA

R01_cb15252_c6/f1p0/1728	NA	R01_cb15252_c6/f1p0/1728	NA	NA
R01_cb5659_c42/f1p0/3218	NA	NA	NA	R01_cb5659_c42/f1p0/3218
R01_cb2170_c0/f1p0/4029	NA	NA	NA	R01_cb2170_c0/f1p0/4029
R01_cb573_c16/f1p0/2689	NA	R01_cb573_c16/f1p0/2689	NA	R01_cb573_c16/f1p0/2689
R01_cb15844_c1/f1p0/741	R01_cb15844_c1/f1p0/741	R01_cb15844_c1/f1p0/741	R01_cb15844_c1/f1p0/741	R01_cb15844_c1/f1p0/741
R01_cb6628_c2/f1p0/2578	NA	R01_cb6628_c2/f1p0/2578	NA	NA
R01_cb8975_c3/f1p0/2222	NA	NA	NA	R01_cb8975_c3/f1p0/2222
R01_cb18107_c1/f1p0/1204	NA	NA	NA	R01_cb18107_c1/f1p0/1204
R01_cb4529_c6/f1p2/4180	NA	R01_cb4529_c6/f1p2/4180	NA	NA
R01_cb14004_c15/f1p1/1445	NA	NA	NA	R01_cb14004_c15/f1p1/1445
R01_cb15864_c0/f1p0/1348	NA	R01_cb15864_c0/f1p0/1348	R01_cb15864_c0/f1p0/1348	R01_cb15864_c0/f1p0/1348
R01_cb6802_c74/f1p0/2372	NA	R01_cb6802_c74/f1p0/2372	NA	NA
R01_cb15222_c6/f1p0/691	R01_cb15222_c6/f1p0/691	R01_cb15222_c6/f1p0/691	NA	R01_cb15222_c6/f1p0/691
R01_cb7836_c4/f1p0/1915	NA	R01_cb7836_c4/f1p0/1915	NA	R01_cb7836_c4/f1p0/1915
R01_cb8564_c91271/f1p0/3105	NA	R01_cb8564_c91271/f1p0/3105	NA	R01_cb8564_c91271/f1p0/3105
R01_cb4352_c7/f1p0/2277	NA	NA	NA	R01_cb4352_c7/f1p0/2277
R01_cb17333_c3/f1p0/1654	NA	R01_cb17333_c3/f1p0/1654	NA	R01_cb17333_c3/f1p0/1654
R01_cb11506_c0/f1p0/687	R01_cb11506_c0/f1p0/687	R01_cb11506_c0/f1p0/687	R01_cb11506_c0/f1p0/687	R01_cb11506_c0/f1p0/687
R01_cb8564_c53400/f1p2/3991	R01_cb8564_c53400/f1p2/3991	R01_cb8564_c53400/f1p2/3991	R01_cb8564_c53400/f1p2/3991	R01_cb8564_c53400/f1p2/3991
R01_cb3492_c1/f1p0/3348	NA	R01_cb3492_c1/f1p0/3348	NA	NA
R01_cb8564_c126630/f1p0/2236	NA	R01_cb8564_c126630/f1p0/2236	NA	NA
R01_cb14620_c5/f1p0/1877	NA	NA	R01_cb14620_c5/f1p0/1877	NA
R01_cb141_c2/f1p0/4942	NA	NA	NA	R01_cb141_c2/f1p0/4942
R01_cb8327_c0/f1p0/2370	NA	NA	NA	R01_cb8327_c0/f1p0/2370

R01_cb2170_c5/f1p0/6505	NA	NA	NA	R01_cb2170_c5/f1p0/6505
R01_cb13228_c4/f1p0/883	NA	R01_cb13228_c4/f1p0/883	R01_cb13228_c4/f1p0/883	R01_cb13228_c4/f1p0/883
R01_cb18035_c6/f1p0/351	R01_cb18035_c6/f1p0/351	R01_cb18035_c6/f1p0/351	R01_cb18035_c6/f1p0/351	R01_cb18035_c6/f1p0/351
R01_cb8564_c74642/f1p0/2006	NA	R01_cb8564_c74642/f1p0/2006	NA	R01_cb8564_c74642/f1p0/2006
R01_cb8564_c3688/f1p0/2353	NA	R01_cb8564_c3688/f1p0/2353	R01_cb8564_c3688/f1p0/2353	R01_cb8564_c3688/f1p0/2353
R01_cb3757_c4/f1p0/2679	NA	NA	NA	R01_cb3757_c4/f1p0/2679
R01_cb13578_c3/f1p0/1078	NA	R01_cb13578_c3/f1p0/1078	NA	NA
R01_cb453_c10/f1p1/2563	NA	R01_cb453_c10/f1p1/2563	NA	NA
R01_cb5635_c3/f1p0/2927	NA	NA	NA	R01_cb5635_c3/f1p0/2927
R01_cb10029_c255/f1p0/777	NA	R01_cb10029_c255/f1p0/777	NA	NA
R01_cb15709_c5/f1p0/551	R01_cb15709_c5/f1p0/551	R01_cb15709_c5/f1p0/551	NA	R01_cb15709_c5/f1p0/551
R01_cb14353_c0/f1p0/1201	NA	NA	NA	R01_cb14353_c0/f1p0/1201
R01_cb5847_c6/f1p0/2573	NA	NA	NA	R01_cb5847_c6/f1p0/2573
R01_cb3971_c4/f1p0/2436	NA	R01_cb3971_c4/f1p0/2436	NA	NA
R01_cb8564_c3065/f1p0/3414	NA	R01_cb8564_c3065/f1p0/3414	NA	R01_cb8564_c3065/f1p0/3414
R01_cb11918_c15/f1p0/1747	NA	NA	NA	R01_cb11918_c15/f1p0/1747
R01_cb4147_c17/f1p0/2280	NA	R01_cb4147_c17/f1p0/2280	NA	NA
R01_cb12837_c3/f1p2/709	NA	R01_cb12837_c3/f1p2/709	NA	NA
R01_cb8564_c91819/f1p0/2197	NA	R01_cb8564_c91819/f1p0/2197	NA	NA
R01_cb15659_c2/f1p0/1527	NA	NA	NA	R01_cb15659_c2/f1p0/1527
R01_cb5412_c6/f1p0/2031	NA	NA	NA	R01_cb5412_c6/f1p0/2031
R01_cb18417_c0/f1p0/604	NA	R01_cb18417_c0/f1p0/604	R01_cb18417_c0/f1p0/604	R01_cb18417_c0/f1p0/604
R01_cb18132_c14/f1p0/1820	NA	R01_cb18132_c14/f1p0/1820	NA	NA
R01_cb8564_c25660/f1p0/2099	R01_cb8564_c25660/f1p0/2099	R01_cb8564_c25660/f1p0/2099	NA	R01_cb8564_c25660/f1p0/2099
R01_cb1894_c6/f1p0/2061	NA	R01_cb1894_c6/f1p0/2061	NA	R01_cb1894_c6/f1p0/2061

R01_cb8564_c123817/f1p0/2095	NA	R01_cb8564_c123817/f1p0/2095	NA	NA
R01_cb5862_c0/f1p0/2994	NA	R01_cb5862_c0/f1p0/2994	R01_cb5862_c0/f1p0/2994	R01_cb5862_c0/f1p0/2994
R01_cb12329_c3/f3p0/939	NA	NA	NA	R01_cb12329_c3/f3p0/939
R01_cb8564_c80343/f1p0/2617	R01_cb8564_c80343/f1p0/2617	R01_cb8564_c80343/f1p0/2617	R01_cb8564_c80343/f1p0/2617	R01_cb8564_c80343/f1p0/2617
R01_cb6411_c1/f1p0/3261	NA	NA	NA	R01_cb6411_c1/f1p0/3261
R01_cb10943_c3/f1p0/2689	NA	NA	NA	R01_cb10943_c3/f1p0/2689
R01_cb8564_c126890/f1p0/3020	NA	R01_cb8564_c126890/f1p0/3020	NA	NA
R01_cb15388_c11/f1p0/1743	NA	R01_cb15388_c11/f1p0/1743	NA	NA
R01_cb8564_c82746/f1p0/2594	NA	R01_cb8564_c82746/f1p0/2594	NA	NA
R01_cb4147_c22/f1p0/2769	NA	NA	NA	R01_cb4147_c22/f1p0/2769
R01_cb3556_c6/f1p0/3509	NA	R01_cb3556_c6/f1p0/3509	NA	NA
R01_cb11271_c1/f1p0/565	NA	R01_cb11271_c1/f1p0/565	NA	NA
R01_cb8564_c70550/f1p0/2913	NA	NA	R01_cb8564_c70550/f1p0/2913	R01_cb8564_c70550/f1p0/2913
R01_cb124_c7/f2p0/3544	NA	NA	NA	R01_cb124_c7/f2p0/3544
R01_cb9320_c1/f1p0/2028	NA	R01_cb9320_c1/f1p0/2028	R01_cb9320_c1/f1p0/2028	NA
R01_cb13561_c1/f1p0/1209	NA	R01_cb13561_c1/f1p0/1209	NA	NA
R01_cb8564_c49341/f1p0/2817	NA	R01_cb8564_c49341/f1p0/2817	NA	NA
R01_cb8564_c80228/f1p0/3046	NA	R01_cb8564_c80228/f1p0/3046	NA	NA
R01_cb15326_c8/f1p0/1693	R01_cb15326_c8/f1p0/1693	R01_cb15326_c8/f1p0/1693	R01_cb15326_c8/f1p0/1693	R01_cb15326_c8/f1p0/1693
R01_cb16422_c5/f1p0/1868	NA	R01_cb16422_c5/f1p0/1868	R01_cb16422_c5/f1p0/1868	R01_cb16422_c5/f1p0/1868
R01_cb8198_c3/f1p1/1936	NA	NA	NA	R01_cb8198_c3/f1p1/1936
R01_cb1594_c14/f5p0/2426	NA	NA	NA	R01_cb1594_c14/f5p0/2426
R01_cb11563_c0/f1p0/1260	NA	NA	NA	R01_cb11563_c0/f1p0/1260

R01_cb17775_c1/f1p0/706	NA	R01_cb17775_c1/f1p0/706	R01_cb17775_c1/f1p0/706	R01_cb17775_c1/f1p0/706
R01_cb10810_c2/f2p1/796	NA	NA	NA	R01_cb10810_c2/f2p1/796
R01_cb8564_c127007/f1p0/2166	NA	NA	R01_cb8564_c127007/f1p0/2166	R01_cb8564_c127007/f1p0/2166
R01_cb10724_c2/f1p0/1098	NA	NA	NA	R01_cb10724_c2/f1p0/1098
R01_cb11318_c2/f1p0/616	NA	NA	NA	R01_cb11318_c2/f1p0/616
R01_cb7563_c31/f1p0/655	NA	R01_cb7563_c31/f1p0/655	NA	NA
R01_cb1228_c5/f5p0/2625	NA	NA	NA	R01_cb1228_c5/f5p0/2625
R01_cb2843_c17/f12p4/2666	NA	NA	NA	R01_cb2843_c17/f12p4/2666
R01_cb10820_c1/f1p0/4353	NA	R01_cb10820_c1/f1p0/4353	NA	R01_cb10820_c1/f1p0/4353
R01_cb1905_c32/f1p0/2036	NA	NA	R01_cb1905_c32/f1p0/2036	NA
R01_cb5724_c6/f1p0/2264	NA	R01_cb5724_c6/f1p0/2264	NA	NA
R01_cb3320_c12/f1p0/1908	NA	R01_cb3320_c12/f1p0/1908	R01_cb3320_c12/f1p0/1908	R01_cb3320_c12/f1p0/1908
R01_cb2907_c1/f1p0/3762	NA	NA	NA	R01_cb2907_c1/f1p0/3762
R01_cb13478_c15/f7p1/531	NA	R01_cb13478_c15/f7p1/531	R01_cb13478_c15/f7p1/531	R01_cb13478_c15/f7p1/531
R01_cb9272_c6/f1p0/748	NA	NA	R01_cb9272_c6/f1p0/748	R01_cb9272_c6/f1p0/748
R01_cb6553_c0/f3p0/1174	NA	NA	NA	R01_cb6553_c0/f3p0/1174
R01_cb8417_c5/f2p0/2381	NA	NA	NA	R01_cb8417_c5/f2p0/2381
R01_cb15581_c0/f4p0/910	NA	NA	NA	R01_cb15581_c0/f4p0/910
R01_cb10979_c5/f1p0/506	R01_cb10979_c5/f1p0/506	R01_cb10979_c5/f1p0/506	NA	NA
R01_cb14982_c0/f2p0/612	NA	NA	R01_cb14982_c0/f2p0/612	R01_cb14982_c0/f2p0/612
R01_cb16217_c0/f1p0/1013	NA	NA	NA	R01_cb16217_c0/f1p0/1013
R01_cb1095_c8/f1p0/2141	NA	NA	NA	R01_cb1095_c8/f1p0/2141
R01_cb5429_c2/f1p0/752	NA	R01_cb5429_c2/f1p0/752	R01_cb5429_c2/f1p0/752	R01_cb5429_c2/f1p0/752
R01_cb17216_c1/f1p0/538	NA	R01_cb17216_c1/f1p0/538	R01_cb17216_c1/f1p0/538	R01_cb17216_c1/f1p0/538
R01_cb8564_c44681/f1p0/2675	NA	R01_cb8564_c44681/f1p0/2675	R01_cb8564_c44681/f1p0/2675	R01_cb8564_c44681/f1p0/2675

R01_cb17790_c0/f1p0/571	NA	R01_cb17790_c0/f1p0/571	NA	NA
R01_cb3388_c6/f1p0/2642	NA	NA	NA	R01_cb3388_c6/f1p0/2642
R01_cb8564_c9710/f2p15/4287	NA	R01_cb8564_c9710/f2p15/4287	R01_cb8564_c9710/f2p15/4287	R01_cb8564_c9710/f2p15/4287
R01_cb15879_c1/f2p0/1091	NA	R01_cb15879_c1/f2p0/1091	NA	NA
R01_cb17855_c0/f2p1/796	NA	R01_cb17855_c0/f2p1/796	NA	R01_cb17855_c0/f2p1/796
R01_cb9797_c60/f1p0/2904	NA	NA	NA	R01_cb9797_c60/f1p0/2904
R01_cb8564_c13734/f1p0/3266	NA	R01_cb8564_c13734/f1p0/3266	NA	NA
R01_cb6574_c1/f1p0/2496	NA	R01_cb6574_c1/f1p0/2496	R01_cb6574_c1/f1p0/2496	R01_cb6574_c1/f1p0/2496
R01_cb6655_c2/f1p0/1676	NA	R01_cb6655_c2/f1p0/1676	R01_cb6655_c2/f1p0/1676	NA
R01_cb18456_c7463/f1p0/1807	NA	R01_cb18456_c7463/f1p0/1807	R01_cb18456_c7463/f1p0/1807	R01_cb18456_c7463/f1p0/1807
R01_cb13914_c12/f36p1/729	NA	NA	NA	R01_cb13914_c12/f36p1/729
R01_cb8564_c70142/f1p0/2485	R01_cb8564_c70142/f1p0/2485	R01_cb8564_c70142/f1p0/2485	R01_cb8564_c70142/f1p0/2485	R01_cb8564_c70142/f1p0/2485
R01_cb1993_c4/f1p1/3171	NA	NA	NA	R01_cb1993_c4/f1p1/3171
R01_cb15096_c0/f1p0/1393	NA	NA	NA	R01_cb15096_c0/f1p0/1393
R01_cb10138_c9/f28p4/982	NA	NA	NA	R01_cb10138_c9/f28p4/982
R01_cb15427_c1/f1p0/1149	NA	R01_cb15427_c1/f1p0/1149	NA	NA
R01_cb1964_c8/f1p0/764	NA	R01_cb1964_c8/f1p0/764	NA	R01_cb1964_c8/f1p0/764
R01_cb8564_c126971/f1p0/2316	R01_cb8564_c126971/f1p0/2316	R01_cb8564_c126971/f1p0/2316	R01_cb8564_c126971/f1p0/2316	R01_cb8564_c126971/f1p0/2316
R01_cb5471_c21/f2p0/2530	NA	NA	NA	R01_cb5471_c21/f2p0/2530
R01_cb2357_c11/f1p0/5825	NA	NA	NA	R01_cb2357_c11/f1p0/5825
R01_cb10970_c9/f1p0/1293	NA	NA	NA	R01_cb10970_c9/f1p0/1293
R01_cb9154_c11/f1p0/1346	NA	NA	NA	R01_cb9154_c11/f1p0/1346
R01_cb8564_c47737/f7p5/4351	NA	NA	NA	R01_cb8564_c47737/f7p5/4351
R01_cb16664_c6/f8p0/544	NA	NA	NA	R01_cb16664_c6/f8p0/544

R01_cb8564_c82906/f1p1/2331	NA	NA	NA	R01_cb8564_c82906/f1p1/2331
R01_cb8564_c81378/f1p0/3909	NA	NA	NA	R01_cb8564_c81378/f1p0/3909
R01_cb8564_c78770/f1p0/3662	NA	NA	NA	R01_cb8564_c78770/f1p0/3662
R01_cb8564_c34173/f4p0/2374	NA	R01_cb8564_c34173/f4p0/2374	NA	NA
R01_cb4953_c5/f1p1/1763	NA	NA	NA	R01_cb4953_c5/f1p1/1763
R01_cb8564_c86163/f1p1/3120	NA	NA	R01_cb8564_c86163/f1p1/3120	R01_cb8564_c86163/f1p1/3120
R01_cb10837_c8/f13p1/653	NA	R01_cb10837_c8/f13p1/653	NA	R01_cb10837_c8/f13p1/653
R01_cb1228_c11/f1p0/2648	NA	NA	NA	R01_cb1228_c11/f1p0/2648
R01_cb10226_c4/f1p0/1358	NA	NA	R01_cb10226_c4/f1p0/1358	R01_cb10226_c4/f1p0/1358
R01_cb8564_c20497/f1p0/3369	NA	R01_cb8564_c20497/f1p0/3369	NA	NA
R01_cb8564_c75238/f1p0/1942	NA	R01_cb8564_c75238/f1p0/1942	R01_cb8564_c75238/f1p0/1942	NA
R01_cb2378_c19/f1p2/3428	NA	NA	NA	R01_cb2378_c19/f1p2/3428
R01_cb8564_c19698/f1p2/3079	NA	NA	NA	R01_cb8564_c19698/f1p2/3079
R01_cb16311_c2/f1p0/784	NA	NA	NA	R01_cb16311_c2/f1p0/784
R01_cb9682_c6/f1p1/1955	NA	NA	NA	R01_cb9682_c6/f1p1/1955
R01_cb11148_c2/f1p0/542	NA	R01_cb11148_c2/f1p0/542	NA	R01_cb11148_c2/f1p0/542
R01_cb15107_c0/f1p1/891	NA	R01_cb15107_c0/f1p1/891	NA	NA
R01_cb11303_c6/f1p0/477	NA	R01_cb11303_c6/f1p0/477	NA	NA
R01_cb8376_c5/f1p0/2409	R01_cb8376_c5/f1p0/2409	R01_cb8376_c5/f1p0/2409	R01_cb8376_c5/f1p0/2409	R01_cb8376_c5/f1p0/2409
R01_cb7472_c0/f1p0/2617	NA	R01_cb7472_c0/f1p0/2617	NA	R01_cb7472_c0/f1p0/2617
R01_cb2494_c6/f1p0/2872	NA	R01_cb2494_c6/f1p0/2872	NA	NA
R01_cb8564_c17508/f1p0/4193	NA	NA	R01_cb8564_c17508/f1p0/4193	NA
R01_cb5847_c11/f1p0/2217	NA	NA	NA	R01_cb5847_c11/f1p0/2217
R01_cb2352_c8/f1p0/2989	NA	NA	R01_cb2352_c8/f1p0/2989	R01_cb2352_c8/f1p0/2989
R01_cb15290_c0/f1p0/526	R01_cb15290_c0/f1p0/526	R01_cb15290_c0/f1p0/526	R01_cb15290_c0/f1p0/526	R01_cb15290_c0/f1p0/526
R01_cb17451_c2/f1p0/846	NA	NA	NA	R01_cb17451_c2/f1p0/846

R01_cb18132_c15/f1p0/1348	NA	R01_cb18132_c15/f1p0/1348	NA	NA
R01_cb11295_c0/f1p0/637	R01_cb11295_c0/f1p0/637	NA	R01_cb11295_c0/f1p0/637	R01_cb11295_c0/f1p0/637
R01_cb3582_c9/f1p0/2457	NA	NA	NA	R01_cb3582_c9/f1p0/2457
R01_cb18199_c2/f1p0/400	R01_cb18199_c2/f1p0/400	R01_cb18199_c2/f1p0/400	R01_cb18199_c2/f1p0/400	R01_cb18199_c2/f1p0/400
R01_cb8564_c24016/f1p1/3082	NA	NA	NA	R01_cb8564_c24016/f1p1/3082
R01_cb11905_c15/f1p0/857	NA	R01_cb11905_c15/f1p0/857	NA	NA
R01_cb18132_c24/f1p0/589	NA	R01_cb18132_c24/f1p0/589	R01_cb18132_c24/f1p0/589	R01_cb18132_c24/f1p0/589
R01_cb14169_c1/f1p0/1149	NA	R01_cb14169_c1/f1p0/1149	NA	NA
R01_cb13783_c4/f1p0/338	R01_cb13783_c4/f1p0/338	R01_cb13783_c4/f1p0/338	R01_cb13783_c4/f1p0/338	R01_cb13783_c4/f1p0/338
R01_cb10648_c9/f1p0/1176	NA	R01_cb10648_c9/f1p0/1176	NA	NA
R01_cb11072_c4/f1p0/2463	R01_cb11072_c4/f1p0/2463	R01_cb11072_c4/f1p0/2463	NA	NA
R01_cb12579_c13/f1p1/1099	R01_cb12579_c13/f1p1/1099	R01_cb12579_c13/f1p1/1099	R01_cb12579_c13/f1p1/1099	R01_cb12579_c13/f1p1/1099
R01_cb8564_c89247/f1p0/3102	NA	R01_cb8564_c89247/f1p0/3102	NA	R01_cb8564_c89247/f1p0/3102
R01_cb873_c9/f4p0/2933	NA	NA	NA	R01_cb873_c9/f4p0/2933
R01_cb9373_c1/f2p0/2064	NA	R01_cb9373_c1/f2p0/2064	NA	NA
R01_cb13778_c2/f1p0/1426	NA	R01_cb13778_c2/f1p0/1426	NA	NA
R01_cb5950_c6/f1p0/2341	NA	NA	NA	R01_cb5950_c6/f1p0/2341
R01_cb8564_c124605/f1p0/2372	NA	NA	NA	R01_cb8564_c124605/f1p0/2372
R01_cb2277_c30/f1p0/346	R01_cb2277_c30/f1p0/346	R01_cb2277_c30/f1p0/346	R01_cb2277_c30/f1p0/346	R01_cb2277_c30/f1p0/346
R01_cb10921_c6/f1p0/1495	NA	NA	NA	R01_cb10921_c6/f1p0/1495
R01_cb11274_c2/f1p0/2546	NA	R01_cb11274_c2/f1p0/2546	NA	NA
R01_cb13914_c4/f1p0/677	NA	R01_cb13914_c4/f1p0/677	NA	R01_cb13914_c4/f1p0/677
R01_cb135_c16/f1p0/4235	NA	R01_cb135_c16/f1p0/4235	NA	NA
R01_cb8564_c81714/f3p1/2660	NA	NA	NA	R01_cb8564_c81714/f3p1/2660
R01_cb13776_c6/f1p0/724	NA	R01_cb13776_c6/f1p0/724	NA	R01_cb13776_c6/f1p0/724

R01_cb10203_c0/f1p0/726	R01_cb10203_c0/f1p0/726	NA	NA	R01_cb10203_c0/f1p0/726
R01_cb9459_c12/f10p0/1844	NA	NA	NA	R01_cb9459_c12/f10p0/1844
R01_cb5132_c3/f1p0/3552	NA	R01_cb5132_c3/f1p0/3552	NA	NA
R01_cb10359_c1/f1p0/1450	NA	NA	NA	R01_cb10359_c1/f1p0/1450
R01_cb14915_c1/f3p1/1477	NA	NA	NA	R01_cb14915_c1/f3p1/1477
R01_cb7096_c8/f2p1/2560	NA	NA	NA	R01_cb7096_c8/f2p1/2560
R01_cb9993_c2/f1p0/2237	R01_cb9993_c2/f1p0/2237	R01_cb9993_c2/f1p0/2237	NA	R01_cb9993_c2/f1p0/2237
R01_cb9864_c0/f1p0/1948	NA	R01_cb9864_c0/f1p0/1948	NA	NA
R01_cb8564_c18927/f1p0/3935	NA	R01_cb8564_c18927/f1p0/3935	NA	NA
R01_cb9423_c3/f1p0/1436	NA	R01_cb9423_c3/f1p0/1436	NA	R01_cb9423_c3/f1p0/1436
R01_cb2445_c22/f1p0/3331	NA	NA	NA	R01_cb2445_c22/f1p0/3331
R01_cb17886_c0/f1p0/1458	NA	NA	NA	R01_cb17886_c0/f1p0/1458
R01_cb4489_c17/f1p0/670	NA	R01_cb4489_c17/f1p0/670	NA	NA
R01_cb1698_c7/f1p0/3196	NA	NA	NA	R01_cb1698_c7/f1p0/3196
R01_cb2125_c10/f2p0/1566	R01_cb2125_c10/f2p0/1566	R01_cb2125_c10/f2p0/1566	R01_cb2125_c10/f2p0/1566	R01_cb2125_c10/f2p0/1566
R01_cb13582_c4/f1p0/1391	NA	R01_cb13582_c4/f1p0/1391	NA	NA
R01_cb18456_c1879/f1p0/754	NA	R01_cb18456_c1879/f1p0/754	R01_cb18456_c1879/f1p0/754	R01_cb18456_c1879/f1p0/754
R01_cb17950_c1/f1p0/977	NA	R01_cb17950_c1/f1p0/977	R01_cb17950_c1/f1p0/977	NA
R01_cb17220_c0/f2p0/1200	NA	R01_cb17220_c0/f2p0/1200	NA	NA
R01_cb8564_c80086/f1p0/3379	NA	NA	R01_cb8564_c80086/f1p0/3379	R01_cb8564_c80086/f1p0/3379
R01_cb10764_c6/f1p0/742	R01_cb10764_c6/f1p0/742	R01_cb10764_c6/f1p0/742	NA	NA
R01_cb8564_c70273/f1p0/2002	NA	R01_cb8564_c70273/f1p0/2002	R01_cb8564_c70273/f1p0/2002	R01_cb8564_c70273/f1p0/2002
R01_cb3575_c15/f1p0/2164	NA	NA	NA	R01_cb3575_c15/f1p0/2164
R01_cb8564_c38316/f1p0/4521	R01_cb8564_c38316/f1p0/4521	R01_cb8564_c38316/f1p0/4521	R01_cb8564_c38316/f1p0/4521	R01_cb8564_c38316/f1p0/4521
R01_cb17973_c32/f1p0/554	NA	R01_cb17973_c32/f1p0/554	R01_cb17973_c32/f1p0/554	NA

R01_cb1706_c5/f1p0/2389	NA	NA	NA	R01_cb1706_c5/f1p0/2389
R01_cb14883_c4/f1p0/1220	NA	R01_cb14883_c4/f1p0/1220	NA	R01_cb14883_c4/f1p0/1220
R01_cb6456_c7/f1p0/975	NA	NA	NA	R01_cb6456_c7/f1p0/975
R01_cb658_c2/f2p0/2863	NA	NA	NA	R01_cb658_c2/f2p0/2863
R01_cb17817_c1/f1p0/414	R01_cb17817_c1/f1p0/414	R01_cb17817_c1/f1p0/414	NA	R01_cb17817_c1/f1p0/414
R01_cb17589_c5/f1p0/343	NA	R01_cb17589_c5/f1p0/343	R01_cb17589_c5/f1p0/343	R01_cb17589_c5/f1p0/343
R01_cb13513_c4/f1p2/701	NA	R01_cb13513_c4/f1p2/701	NA	NA
R01_cb11049_c1/f1p0/2139	NA	NA	NA	R01_cb11049_c1/f1p0/2139
R01_cb3017_c0/f1p0/3746	NA	R01_cb3017_c0/f1p0/3746	NA	NA
R01_cb3138_c5/f1p0/3627	NA	R01_cb3138_c5/f1p0/3627	NA	R01_cb3138_c5/f1p0/3627
R01_cb8564_c91585/f1p0/3131	NA	R01_cb8564_c91585/f1p0/3131	NA	NA
R01_cb8564_c4616/f1p1/3023	NA	R01_cb8564_c4616/f1p1/3023	R01_cb8564_c4616/f1p1/3023	R01_cb8564_c4616/f1p1/3023
R01_cb5950_c20/f1p0/1817	NA	NA	NA	R01_cb5950_c20/f1p0/1817
R01_cb9352_c3/f1p0/2124	NA	NA	NA	R01_cb9352_c3/f1p0/2124
R01_cb506_c15/f1p0/2848	NA	NA	NA	R01_cb506_c15/f1p0/2848
R01_cb12045_c0/f1p0/1607	NA	NA	NA	R01_cb12045_c0/f1p0/1607
R01_cb15533_c17/f1p0/743	NA	NA	NA	R01_cb15533_c17/f1p0/743
R01_cb6802_c37/f1p0/3820	NA	R01_cb6802_c37/f1p0/3820	R01_cb6802_c37/f1p0/3820	R01_cb6802_c37/f1p0/3820
R01_cb18103_c6/f1p0/1356	NA	R01_cb18103_c6/f1p0/1356	NA	NA
R01_cb8564_c15997/f1p0/4783	NA	R01_cb8564_c15997/f1p0/4783	NA	NA
R01_cb8564_c3666/f1p0/2796	NA	NA	NA	R01_cb8564_c3666/f1p0/2796
R01_cb4229_c5/f1p0/2565	NA	NA	NA	R01_cb4229_c5/f1p0/2565
R01_cb18470_c0/f1p0/723	NA	R01_cb18470_c0/f1p0/723	NA	R01_cb18470_c0/f1p0/723
R01_cb3763_c27/f1p0/2368	NA	NA	NA	R01_cb3763_c27/f1p0/2368
R01_cb8564_c70656/f1p0/1915	NA	R01_cb8564_c70656/f1p0/1915	NA	R01_cb8564_c70656/f1p0/1915
R01_cb13304_c1/f1p0/1119	NA	NA	NA	R01_cb13304_c1/f1p0/1119

R01_cb1228_c35/f1p0/3000	NA	NA	NA	R01_cb1228_c35/f1p0/3000
R01_cb4243_c0/f2p0/2997	NA	R01_cb4243_c0/f2p0/2997	R01_cb4243_c0/f2p0/2997	NA
R01_cb7766_c4/f1p0/2455	NA	R01_cb7766_c4/f1p0/2455	NA	NA
R01_cb6779_c6/f1p0/1810	NA	NA	NA	R01_cb6779_c6/f1p0/1810
R01_cb3613_c1/f1p0/3196	R01_cb3613_c1/f1p0/3196	R01_cb3613_c1/f1p0/3196	NA	R01_cb3613_c1/f1p0/3196
R01_cb1767_c37/f1p0/1947	NA	R01_cb1767_c37/f1p0/1947	R01_cb1767_c37/f1p0/1947	NA
R01_cb6204_c10/f1p1/2537	NA	NA	NA	R01_cb6204_c10/f1p1/2537
R01_cb15929_c0/f1p0/933	NA	R01_cb15929_c0/f1p0/933	R01_cb15929_c0/f1p0/933	R01_cb15929_c0/f1p0/933
R01_cb8564_c109666/f1p0/1989	NA	NA	NA	R01_cb8564_c109666/f1p0/1989
R01_cb12945_c2/f1p0/1406	NA	NA	NA	R01_cb12945_c2/f1p0/1406
R01_cb6008_c1/f1p0/3191	NA	NA	NA	R01_cb6008_c1/f1p0/3191
R01_cb10043_c9/f1p1/1230	NA	R01_cb10043_c9/f1p1/1230	NA	NA
R01_cb8564_c88385/f1p0/2356	NA	R01_cb8564_c88385/f1p0/2356	NA	R01_cb8564_c88385/f1p0/2356
R01_cb16043_c0/f2p0/841	R01_cb16043_c0/f2p0/841	R01_cb16043_c0/f2p0/841	R01_cb16043_c0/f2p0/841	NA
R01_cb7819_c0/f2p0/1374	NA	NA	NA	R01_cb7819_c0/f2p0/1374
R01_cb535_c1/f1p0/4438	NA	NA	NA	R01_cb535_c1/f1p0/4438
R01_cb14292_c17/f1p0/446	R01_cb14292_c17/f1p0/446	NA	NA	NA
R01_cb7214_c1/f1p0/2667	NA	NA	NA	R01_cb7214_c1/f1p0/2667
R01_cb8501_c34/f1p0/544	R01_cb8501_c34/f1p0/544	R01_cb8501_c34/f1p0/544	R01_cb8501_c34/f1p0/544	R01_cb8501_c34/f1p0/544
R01_cb4094_c12/f1p0/3199	R01_cb4094_c12/f1p0/3199	R01_cb4094_c12/f1p0/3199	NA	NA
R01_cb7458_c4/f1p0/409	R01_cb7458_c4/f1p0/409	R01_cb7458_c4/f1p0/409	R01_cb7458_c4/f1p0/409	R01_cb7458_c4/f1p0/409
R01_cb7962_c12/f1p0/1652	NA	NA	NA	R01_cb7962_c12/f1p0/1652
R01_cb8564_c17975/f1p0/3360	NA	R01_cb8564_c17975/f1p0/3360	R01_cb8564_c17975/f1p0/3360	R01_cb8564_c17975/f1p0/3360
R01_cb8123_c3/f1p0/2320	NA	R01_cb8123_c3/f1p0/2320	NA	NA
R01_cb9364_c1/f1p0/2425	NA	NA	NA	R01_cb9364_c1/f1p0/2425

R01_cb9513_c33/f1p0/2509	NA	R01_cb9513_c33/f1p0/2509	NA	NA
R01_cb10520_c8/f1p1/1678	NA	NA	NA	R01_cb10520_c8/f1p1/1678
R01_cb8564_c86127/f1p0/2979	NA	R01_cb8564_c86127/f1p0/2979	NA	NA
R01_cb8564_c76659/f1p0/1894	NA	R01_cb8564_c76659/f1p0/1894	R01_cb8564_c76659/f1p0/1894	NA
R01_cb11795_c1/f1p0/2869	NA	R01_cb11795_c1/f1p0/2869	NA	NA
R01_cb7303_c8/f1p0/2578	NA	NA	NA	R01_cb7303_c8/f1p0/2578
R01_cb12883_c7/f1p2/949	NA	R01_cb12883_c7/f1p2/949	NA	NA
R01_cb8428_c7/f1p0/497	R01_cb8428_c7/f1p0/497	R01_cb8428_c7/f1p0/497	R01_cb8428_c7/f1p0/497	R01_cb8428_c7/f1p0/497
R01_cb8564_c72997/f1p0/2150	R01_cb8564_c72997/f1p0/2150	R01_cb8564_c72997/f1p0/2150	R01_cb8564_c72997/f1p0/2150	R01_cb8564_c72997/f1p0/2150
R01_cb8564_c125231/f1p0/2379	NA	R01_cb8564_c125231/f1p0/2379	NA	NA
R01_cb8564_c72734/f1p0/3132	NA	R01_cb8564_c72734/f1p0/3132	NA	NA
R01_cb16452_c2/f1p0/917	R01_cb16452_c2/f1p0/917	R01_cb16452_c2/f1p0/917	R01_cb16452_c2/f1p0/917	R01_cb16452_c2/f1p0/917
R01_cb16092_c1/f1p0/552	R01_cb16092_c1/f1p0/552	R01_cb16092_c1/f1p0/552	R01_cb16092_c1/f1p0/552	R01_cb16092_c1/f1p0/552
R01_cb6513_c3/f1p0/2187	NA	NA	NA	R01_cb6513_c3/f1p0/2187
R01_cb14353_c4/f1p0/1127	NA	NA	NA	R01_cb14353_c4/f1p0/1127
R01_cb6945_c3/f1p0/1227	R01_cb6945_c3/f1p0/1227	NA	NA	R01_cb6945_c3/f1p0/1227
R01_cb8564_c13723/f1p4/3217	NA	R01_cb8564_c13723/f1p4/3217	R01_cb8564_c13723/f1p4/3217	R01_cb8564_c13723/f1p4/3217
R01_cb7611_c12/f1p0/2555	NA	NA	NA	R01_cb7611_c12/f1p0/2555
R01_cb8564_c83742/f1p0/2904	NA	NA	NA	R01_cb8564_c83742/f1p0/2904
R01_cb16061_c0/f1p0/416	NA	R01_cb16061_c0/f1p0/416	NA	NA
R01_cb15605_c5/f2p0/635	R01_cb15605_c5/f2p0/635	NA	R01_cb15605_c5/f2p0/635	R01_cb15605_c5/f2p0/635
R01_cb17973_c21/f1p0/744	R01_cb17973_c21/f1p0/744	R01_cb17973_c21/f1p0/744	R01_cb17973_c21/f1p0/744	R01_cb17973_c21/f1p0/744
R01_cb15045_c6/f1p0/1489	NA	NA	NA	R01_cb15045_c6/f1p0/1489
R01_cb17194_c0/f2p0/588	NA	R01_cb17194_c0/f2p0/588	NA	NA

R01_cb18456_c6641/f1p0/468	R01_cb18456_c6641/f1p0/468	R01_cb18456_c6641/f1p0/468	R01_cb18456_c6641/f1p0/468	R01_cb18456_c6641/f1p0/468
R01_cb5635_c5/f1p0/766	NA	NA	NA	R01_cb5635_c5/f1p0/766
R01_cb18409_c158/f1p0/407	R01_cb18409_c158/f1p0/407	R01_cb18409_c158/f1p0/407	R01_cb18409_c158/f1p0/407	R01_cb18409_c158/f1p0/407
R01_cb14897_c12/f1p0/1814	NA	R01_cb14897_c12/f1p0/1814	R01_cb14897_c12/f1p0/1814	R01_cb14897_c12/f1p0/1814
R01_cb18207_c2/f1p0/743	R01_cb18207_c2/f1p0/743	R01_cb18207_c2/f1p0/743	R01_cb18207_c2/f1p0/743	R01_cb18207_c2/f1p0/743
R01_cb17639_c4/f1p0/784	NA	R01_cb17639_c4/f1p0/784	NA	NA
R01_cb18456_c6071/f1p0/644	NA	R01_cb18456_c6071/f1p0/644	R01_cb18456_c6071/f1p0/644	R01_cb18456_c6071/f1p0/644
R01_cb1758_c2/f1p0/3336	NA	NA	NA	R01_cb1758_c2/f1p0/3336
R01_cb14854_c3/f2p0/866	NA	R01_cb14854_c3/f2p0/866	NA	NA
R01_cb6352_c3/f1p0/2877	NA	R01_cb6352_c3/f1p0/2877	NA	NA
R01_cb12701_c2/f2p0/941	R01_cb12701_c2/f2p0/941	R01_cb12701_c2/f2p0/941	NA	NA
R01_cb9201_c2/f1p0/3381	NA	NA	NA	R01_cb9201_c2/f1p0/3381
R01_cb981_c5/f2p0/2058	R01_cb981_c5/f2p0/2058	R01_cb981_c5/f2p0/2058	R01_cb981_c5/f2p0/2058	R01_cb981_c5/f2p0/2058
R01_cb7368_c2/f1p0/1989	R01_cb7368_c2/f1p0/1989	R01_cb7368_c2/f1p0/1989	NA	R01_cb7368_c2/f1p0/1989
R01_cb7836_c2/f1p0/2354	NA	R01_cb7836_c2/f1p0/2354	NA	R01_cb7836_c2/f1p0/2354
R01_cb8564_c1256/f1p0/2155	NA	R01_cb8564_c1256/f1p0/2155	R01_cb8564_c1256/f1p0/2155	NA
R01_cb18456_c764/f1p0/526	R01_cb18456_c764/f1p0/526	R01_cb18456_c764/f1p0/526	NA	R01_cb18456_c764/f1p0/526
R01_cb18445_c1/f1p0/845	NA	R01_cb18445_c1/f1p0/845	R01_cb18445_c1/f1p0/845	R01_cb18445_c1/f1p0/845
R01_cb15772_c0/f2p0/759	NA	NA	R01_cb15772_c0/f2p0/759	R01_cb15772_c0/f2p0/759
R01_cb13647_c0/f6p0/665	NA	R01_cb13647_c0/f6p0/665	NA	R01_cb13647_c0/f6p0/665
R01_cb4674_c74/f1p0/3043	NA	R01_cb4674_c74/f1p0/3043	NA	R01_cb4674_c74/f1p0/3043
R01_cb9852_c20/f1p0/1870	NA	R01_cb9852_c20/f1p0/1870	NA	NA
R01_cb8564_c47779/f1p0/2789	R01_cb8564_c47779/f1p0/278 9	R01_cb8564_c47779/f1p0/2789	R01_cb8564_c47779/f1p0/2789	R01_cb8564_c47779/f1p0/2789
R01_cb8564_c17441/f3p0/4144	NA	NA	NA	R01_cb8564_c17441/f3p0/4144
R01_cb2344_c2/f1p0/932	R01_cb2344_c2/f1p0/932	R01_cb2344_c2/f1p0/932	NA	NA

R01_cb8564_c114653/f1p0/4035	NA	R01_cb8564_c114653/f1p0/4035	NA	NA
R01_cb16832_c1/f1p0/564	R01_cb16832_c1/f1p0/564	NA	NA	R01_cb16832_c1/f1p0/564
R01_cb10944_c1/f1p0/2431	NA	R01_cb10944_c1/f1p0/2431	R01_cb10944_c1/f1p0/2431	R01_cb10944_c1/f1p0/2431
R01_cb11558_c0/f1p0/746	R01_cb11558_c0/f1p0/746	R01_cb11558_c0/f1p0/746	R01_cb11558_c0/f1p0/746	R01_cb11558_c0/f1p0/746
R01_cb13580_c0/f2p0/983	NA	NA	NA	R01_cb13580_c0/f2p0/983
R01_cb18577_c0/f1p0/463	R01_cb18577_c0/f1p0/463	R01_cb18577_c0/f1p0/463	NA	R01_cb18577_c0/f1p0/463
R01_cb124_c13/f1p0/3616	NA	NA	NA	R01_cb124_c13/f1p0/3616
R01_cb8564_c91317/f1p0/2833	NA	NA	NA	R01_cb8564_c91317/f1p0/2833
R01_cb11283_c3/f1p0/464	R01_cb11283_c3/f1p0/464	R01_cb11283_c3/f1p0/464	R01_cb11283_c3/f1p0/464	R01_cb11283_c3/f1p0/464
R01_cb2030_c4/f1p0/544	R01_cb2030_c4/f1p0/544	R01_cb2030_c4/f1p0/544	NA	R01_cb2030_c4/f1p0/544
R01_cb9418_c10/f1p1/1829	NA	NA	NA	R01_cb9418_c10/f1p1/1829
R01_cb11455_c3/f1p0/530	NA	R01_cb11455_c3/f1p0/530	NA	R01_cb11455_c3/f1p0/530
R01_cb15739_c0/f2p0/1397	R01_cb15739_c0/f2p0/1397	R01_cb15739_c0/f2p0/1397	NA	NA
R01_cb17823_c1/f1p0/1495	NA	R01_cb17823_c1/f1p0/1495	NA	NA
R01_cb14863_c2/f1p0/1105	NA	R01_cb14863_c2/f1p0/1105	R01_cb14863_c2/f1p0/1105	R01_cb14863_c2/f1p0/1105
R01_cb15578_c5/f1p0/520	R01_cb15578_c5/f1p0/520	R01_cb15578_c5/f1p0/520	R01_cb15578_c5/f1p0/520	R01_cb15578_c5/f1p0/520
R01_cb242_c26/f1p0/3976	NA	R01_cb242_c26/f1p0/3976	NA	R01_cb242_c26/f1p0/3976
R01_cb14971_c8/f1p0/399	R01_cb14971_c8/f1p0/399	NA	NA	R01_cb14971_c8/f1p0/399
R01_cb18456_c7240/f1p0/521	R01_cb18456_c7240/f1p0/521	R01_cb18456_c7240/f1p0/521	R01_cb18456_c7240/f1p0/521	R01_cb18456_c7240/f1p0/521
R01_cb18456_c7252/f20p3/715	NA	R01_cb18456_c7252/f20p3/715	R01_cb18456_c7252/f20p3/715	R01_cb18456_c7252/f20p3/715
R01_cb8564_c115392/f1p0/3012	NA	R01_cb8564_c115392/f1p0/3012	NA	R01_cb8564_c115392/f1p0/3012
R01_cb10038_c2/f1p0/2659	NA	R01_cb10038_c2/f1p0/2659	NA	R01_cb10038_c2/f1p0/2659
R01_cb11072_c0/f7p0/405	R01_cb11072_c0/f7p0/405	R01_cb11072_c0/f7p0/405	NA	NA
R01_cb6737_c47/f1p0/1101	NA	NA	NA	R01_cb6737_c47/f1p0/1101

R01_cb15811_c17/f1p0/1051	NA	R01_cb15811_c17/f1p0/1051	R01_cb15811_c17/f1p0/1051	R01_cb15811_c17/f1p0/1051
R01_cb8564_c75531/f1p0/3115	R01_cb8564_c75531/f1p0/3115	R01_cb8564_c75531/f1p0/3115	R01_cb8564_c75531/f1p0/3115	R01_cb8564_c75531/f1p0/3115
R01_cb12418_c1/f1p0/474	R01_cb12418_c1/f1p0/474	R01_cb12418_c1/f1p0/474	NA	R01_cb12418_c1/f1p0/474
R01_cb10205_c3/f1p0/402	R01_cb10205_c3/f1p0/402	R01_cb10205_c3/f1p0/402	R01_cb10205_c3/f1p0/402	R01_cb10205_c3/f1p0/402
R01_cb17543_c0/f2p0/502	NA	NA	R01_cb17543_c0/f2p0/502	R01_cb17543_c0/f2p0/502
R01_cb9729_c7/f1p0/804	NA	R01_cb9729_c7/f1p0/804	R01_cb9729_c7/f1p0/804	NA
R01_cb8447_c9/f1p1/2420	NA	NA	NA	R01_cb8447_c9/f1p1/2420
R01_cb4679_c2/f1p0/3286	NA	R01_cb4679_c2/f1p0/3286	NA	NA
R01_cb16125_c24/f1p0/1428	NA	R01_cb16125_c24/f1p0/1428	NA	NA
R01_cb5144_c6/f1p0/955	NA	R01_cb5144_c6/f1p0/955	NA	R01_cb5144_c6/f1p0/955
R01_cb8564_c73505/f1p0/2565	R01_cb8564_c73505/f1p0/2565	R01_cb8564_c73505/f1p0/2565	R01_cb8564_c73505/f1p0/2565	R01_cb8564_c73505/f1p0/2565
R01_cb10549_c5/f1p0/806	NA	R01_cb10549_c5/f1p0/806	NA	NA
R01_cb12897_c0/f2p0/717	R01_cb12897_c0/f2p0/717	R01_cb12897_c0/f2p0/717	NA	NA
R01_cb6170_c10/f2p0/2672	NA	NA	NA	R01_cb6170_c10/f2p0/2672
R01_cb1207_c14/f1p0/1998	NA	NA	NA	R01_cb1207_c14/f1p0/1998
R01_cb16274_c1/f1p0/649	NA	R01_cb16274_c1/f1p0/649	NA	NA
R01_cb8564_c1019/f1p0/2974	NA	R01_cb8564_c1019/f1p0/2974	NA	NA
R01_cb8564_c82912/f1p2/2538	NA	R01_cb8564_c82912/f1p2/2538	NA	NA
R01_cb3022_c8/f1p0/2347	NA	R01_cb3022_c8/f1p0/2347	NA	NA
R01_cb8564_c82687/f1p0/3370	NA	R01_cb8564_c82687/f1p0/3370	NA	NA
R01_cb763_c1/f1p0/4636	NA	R01_cb763_c1/f1p0/4636	R01_cb763_c1/f1p0/4636	R01_cb763_c1/f1p0/4636
R01_cb11328_c2/f1p0/2836	NA	R01_cb11328_c2/f1p0/2836	NA	NA
R01_cb13663_c0/f2p0/786	NA	R01_cb13663_c0/f2p0/786	NA	R01_cb13663_c0/f2p0/786
R01_cb18456_c4751/f1p1/559	R01_cb18456_c4751/f1p1/559	R01_cb18456_c4751/f1p1/559	R01_cb18456_c4751/f1p1/559	R01_cb18456_c4751/f1p1/559

R01_cb13462_c2/f1p0/1771	NA	NA	NA	R01_cb13462_c2/f1p0/1771
R01_cb8564_c82591/f1p0/4158	NA	R01_cb8564_c82591/f1p0/4158	NA	NA
R01_cb8564_c2613/f1p0/3842	NA	R01_cb8564_c2613/f1p0/3842	NA	R01_cb8564_c2613/f1p0/3842
R01_cb8564_c85339/f1p0/2450	NA	R01_cb8564_c85339/f1p0/2450	NA	R01_cb8564_c85339/f1p0/2450
R01_cb8564_c75802/f1p0/4636	NA	R01_cb8564_c75802/f1p0/4636	NA	NA
R01_cb7873_c1/f1p0/2332	NA	R01_cb7873_c1/f1p0/2332	NA	R01_cb7873_c1/f1p0/2332
R01_cb15883_c0/f1p0/673	R01_cb15883_c0/f1p0/673	R01_cb15883_c0/f1p0/673	NA	NA
R01_cb12402_c4/f3p1/595	NA	NA	R01_cb12402_c4/f3p1/595	R01_cb12402_c4/f3p1/595
R01_cb8564_c81984/f1p0/2454	NA	R01_cb8564_c81984/f1p0/2454	NA	NA
R01_cb18136_c1/f1p0/445	NA	R01_cb18136_c1/f1p0/445	NA	NA
R01_cb16476_c4/f1p0/1265	R01_cb16476_c4/f1p0/1265	R01_cb16476_c4/f1p0/1265	R01_cb16476_c4/f1p0/1265	R01_cb16476_c4/f1p0/1265
R01_cb2223_c1/f1p0/2395	NA	NA	NA	R01_cb2223_c1/f1p0/2395
R01_cb2072_c5/f2p0/3034	NA	NA	NA	R01_cb2072_c5/f2p0/3034
R01_cb15911_c1/f1p0/1420	NA	R01_cb15911_c1/f1p0/1420	NA	R01_cb15911_c1/f1p0/1420
R01_cb13980_c2/f1p0/1412	NA	NA	NA	R01_cb13980_c2/f1p0/1412
R01_cb9606_c41/f1p0/1956	NA	NA	R01_cb9606_c41/f1p0/1956	R01_cb9606_c41/f1p0/1956
R01_cb1438_c12/f1p0/3268	NA	NA	NA	R01_cb1438_c12/f1p0/3268
R01_cb8564_c83628/f1p0/3221	NA	R01_cb8564_c83628/f1p0/3221	NA	NA
R01_cb7010_c7/f4p1/2320	NA	R01_cb7010_c7/f4p1/2320	NA	NA
R01_cb4809_c3/f1p1/2534	NA	R01_cb4809_c3/f1p1/2534	NA	NA
R01_cb2445_c7/f2p0/3470	NA	NA	NA	R01_cb2445_c7/f2p0/3470
R01_cb8564_c19874/f1p0/3596	NA	R01_cb8564_c19874/f1p0/3596	NA	NA
R01_cb18704_c0/f1p0/5707	NA	R01_cb18704_c0/f1p0/5707	R01_cb18704_c0/f1p0/5707	NA
R01_cb18456_c7961/f1p0/519	R01_cb18456_c7961/f1p0/519	R01_cb18456_c7961/f1p0/519	R01_cb18456_c7961/f1p0/519	R01_cb18456_c7961/f1p0/519
R01_cb14905_c1/f1p0/630	NA	NA	R01_cb14905_c1/f1p0/630	R01_cb14905_c1/f1p0/630
R01_cb8564_c37245/f1p0/2951	NA	R01_cb8564_c37245/f1p0/2951	NA	NA

R01_cb8564_c129371/f1p0/2096	NA	R01_cb8564_c129371/f1p0/2096	R01_cb8564_c129371/f1p0/2096	R01_cb8564_c129371/f1p0/2096
R01_cb7033_c3/f1p0/2985	NA	R01_cb7033_c3/f1p0/2985	NA	NA
R01_cb14044_c19/f1p0/1646	NA	R01_cb14044_c19/f1p0/1646	NA	NA
R01_cb6719_c47/f1p0/2096	R01_cb6719_c47/f1p0/2096	R01_cb6719_c47/f1p0/2096	NA	R01_cb6719_c47/f1p0/2096
R01_cb18456_c1871/f1p0/893	NA	R01_cb18456_c1871/f1p0/893	NA	NA
R01_cb6885_c6/f1p0/2457	R01_cb6885_c6/f1p0/2457	R01_cb6885_c6/f1p0/2457	R01_cb6885_c6/f1p0/2457	R01_cb6885_c6/f1p0/2457
R01_cb7194_c4/f1p0/3202	NA	R01_cb7194_c4/f1p0/3202	NA	NA
R01_cb8564_c21207/f1p0/4093	NA	NA	NA	R01_cb8564_c21207/f1p0/4093
R01_cb11047_c2/f1p0/5269	NA	NA	NA	R01_cb11047_c2/f1p0/5269
R01_cb10376_c3/f1p0/3263	R01_cb10376_c3/f1p0/3263	R01_cb10376_c3/f1p0/3263	R01_cb10376_c3/f1p0/3263	R01_cb10376_c3/f1p0/3263
R01_cb723_c1/f1p0/2894	NA	R01_cb723_c1/f1p0/2894	NA	NA
R01_cb8564_c89776/f1p0/2655	NA	R01_cb8564_c89776/f1p0/2655	NA	NA
R01_cb8564_c3144/f3p2/2153	NA	NA	NA	R01_cb8564_c3144/f3p2/2153
R01_cb17085_c0/f1p0/1413	NA	NA	NA	R01_cb17085_c0/f1p0/1413
R01_cb12534_c0/f9p0/989	NA	R01_cb12534_c0/f9p0/989	R01_cb12534_c0/f9p0/989	R01_cb12534_c0/f9p0/989
R01_cb6247_c5/f1p0/1307	NA	R01_cb6247_c5/f1p0/1307	NA	R01_cb6247_c5/f1p0/1307
R01_cb293_c7/f1p0/4528	NA	NA	NA	R01_cb293_c7/f1p0/4528
R01_cb9906_c2/f1p0/1839	NA	R01_cb9906_c2/f1p0/1839	R01_cb9906_c2/f1p0/1839	R01_cb9906_c2/f1p0/1839
R01_cb11642_c1/f1p0/3328	NA	NA	R01_cb11642_c1/f1p0/3328	R01_cb11642_c1/f1p0/3328
R01_cb8564_c10402/f1p1/2617	NA	R01_cb8564_c10402/f1p1/2617	NA	NA
R01_cb10844_c4/f1p0/1329	NA	R01_cb10844_c4/f1p0/1329	NA	R01_cb10844_c4/f1p0/1329
R01_cb8564_c1751/f1p0/2432	NA	R01_cb8564_c1751/f1p0/2432	NA	R01_cb8564_c1751/f1p0/2432
R01_cb13033_c1/f4p0/1205	NA	R01_cb13033_c1/f4p0/1205	NA	R01_cb13033_c1/f4p0/1205
R01_cb13776_c5/f1p0/343	R01_cb13776_c5/f1p0/343	R01_cb13776_c5/f1p0/343	R01_cb13776_c5/f1p0/343	R01_cb13776_c5/f1p0/343
R01_cb8564_c1402/f1p0/2306	NA	R01_cb8564_c1402/f1p0/2306	R01_cb8564_c1402/f1p0/2306	R01_cb8564_c1402/f1p0/2306

R01_cb10673_c1/f1p0/3365	NA	R01_cb10673_c1/f1p0/3365	NA	NA
R01_cb7506_c2/f1p0/2311	NA	NA	NA	R01_cb7506_c2/f1p0/2311
R01_cb14052_c0/f1p0/436	NA	R01_cb14052_c0/f1p0/436	NA	R01_cb14052_c0/f1p0/436
R01_cb10323_c1/f1p0/860	NA	NA	R01_cb10323_c1/f1p0/860	R01_cb10323_c1/f1p0/860
R01_cb8564_c4220/f2p0/2906	R01_cb8564_c4220/f2p0/2906	R01_cb8564_c4220/f2p0/2906	R01_cb8564_c4220/f2p0/2906	R01_cb8564_c4220/f2p0/2906
R01_cb17495_c4/f1p0/765	R01_cb17495_c4/f1p0/765	R01_cb17495_c4/f1p0/765	R01_cb17495_c4/f1p0/765	R01_cb17495_c4/f1p0/765
R01_cb16710_c0/f1p0/541	NA	R01_cb16710_c0/f1p0/541	R01_cb16710_c0/f1p0/541	NA
R01_cb5900_c152/f1p0/2667	NA	R01_cb5900_c152/f1p0/2667	NA	R01_cb5900_c152/f1p0/2667
R01_cb8564_c118788/f2p0/2730	NA	R01_cb8564_c118788/f2p0/2730	NA	NA
R01_cb13073_c2/f1p0/582	NA	R01_cb13073_c2/f1p0/582	NA	R01_cb13073_c2/f1p0/582
R01_cb7219_c10/f1p4/2444	NA	NA	NA	R01_cb7219_c10/f1p4/2444
R01_cb11744_c1/f1p0/2053	NA	NA	NA	R01_cb11744_c1/f1p0/2053
R01_cb18310_c0/f1p0/1110	NA	NA	NA	R01_cb18310_c0/f1p0/1110
R01_cb11431_c0/f1p0/1200	NA	NA	NA	R01_cb11431_c0/f1p0/1200
R01_cb11642_c0/f1p0/1873	NA	NA	NA	R01_cb11642_c0/f1p0/1873
R01_cb8564_c53263/f1p0/4074	NA	R01_cb8564_c53263/f1p0/4074	NA	R01_cb8564_c53263/f1p0/4074
R01_cb8564_c3147/f1p0/2665	NA	R01_cb8564_c3147/f1p0/2665	NA	R01_cb8564_c3147/f1p0/2665
R01_cb6017_c4/f1p0/2952	NA	R01_cb6017_c4/f1p0/2952	NA	R01_cb6017_c4/f1p0/2952
R01_cb1686_c1/f1p0/4237	NA	R01_cb1686_c1/f1p0/4237	R01_cb1686_c1/f1p0/4237	R01_cb1686_c1/f1p0/4237
R01_cb9464_c4/f1p0/822	NA	R01_cb9464_c4/f1p0/822	NA	R01_cb9464_c4/f1p0/822
R01_cb17723_c0/f1p0/938	NA	NA	R01_cb17723_c0/f1p0/938	NA
R01_cb2049_c7/f1p0/417	R01_cb2049_c7/f1p0/417	R01_cb2049_c7/f1p0/417	R01_cb2049_c7/f1p0/417	R01_cb2049_c7/f1p0/417
R01_cb7137_c0/f1p0/2680	NA	R01_cb7137_c0/f1p0/2680	R01_cb7137_c0/f1p0/2680	R01_cb7137_c0/f1p0/2680
R01_cb6874_c5/f1p0/2567	NA	NA	NA	R01_cb6874_c5/f1p0/2567
R01_cb13497_c8/f1p1/888	NA	NA	NA	R01_cb13497_c8/f1p1/888

R01_cb6893_c3/f1p0/3849	NA	R01_cb6893_c3/f1p0/3849	NA	R01_cb6893_c3/f1p0/3849
R01_cb16213_c0/f1p0/1773	NA	NA	NA	R01_cb16213_c0/f1p0/1773
R01_cb2884_c0/f2p0/3781	NA	NA	NA	R01_cb2884_c0/f2p0/3781
R01_cb8564_c3934/f1p0/4228	NA	R01_cb8564_c3934/f1p0/4228	R01_cb8564_c3934/f1p0/4228	NA
R01_cb8564_c19159/f1p0/2904	NA	NA	NA	R01_cb8564_c19159/f1p0/2904
R01_cb8564_c20510/f1p0/2983	R01_cb8564_c20510/f1p0/2983	R01_cb8564_c20510/f1p0/2983	NA	R01_cb8564_c20510/f1p0/2983
R01_cb3971_c2/f1p0/3505	NA	R01_cb3971_c2/f1p0/3505	NA	NA
R01_cb10457_c2/f1p0/2021	NA	R01_cb10457_c2/f1p0/2021	NA	R01_cb10457_c2/f1p0/2021
R01_cb10070_c3/f1p0/833	NA	NA	NA	R01_cb10070_c3/f1p0/833
R01_cb18456_c6265/f1p0/814	NA	NA	NA	R01_cb18456_c6265/f1p0/814
R01_cb4893_c18/f1p0/573	R01_cb4893_c18/f1p0/573	R01_cb4893_c18/f1p0/573	R01_cb4893_c18/f1p0/573	R01_cb4893_c18/f1p0/573
R01_cb11278_c2/f1p0/1699	NA	R01_cb11278_c2/f1p0/1699	R01_cb11278_c2/f1p0/1699	R01_cb11278_c2/f1p0/1699
R01_cb1849_c21/f1p1/1806	NA	NA	NA	R01_cb1849_c21/f1p1/1806
R01_cb17582_c4/f1p0/710	NA	R01_cb17582_c4/f1p0/710	R01_cb17582_c4/f1p0/710	R01_cb17582_c4/f1p0/710
R01_cb16996_c1/f1p0/824	R01_cb16996_c1/f1p0/824	R01_cb16996_c1/f1p0/824	R01_cb16996_c1/f1p0/824	R01_cb16996_c1/f1p0/824
R01_cb7345_c7/f1p0/2464	NA	R01_cb7345_c7/f1p0/2464	NA	NA
R01_cb8564_c77201/f1p0/2102	NA	R01_cb8564_c77201/f1p0/2102	R01_cb8564_c77201/f1p0/2102	R01_cb8564_c77201/f1p0/2102
R01_cb8037_c5/f1p0/1371	NA	NA	NA	R01_cb8037_c5/f1p0/1371
R01_cb8564_c51296/f1p0/2027	NA	R01_cb8564_c51296/f1p0/2027	R01_cb8564_c51296/f1p0/2027	R01_cb8564_c51296/f1p0/2027
R01_cb4471_c4/f1p0/3540	NA	NA	NA	R01_cb4471_c4/f1p0/3540
R01_cb17333_c2/f1p0/1535	NA	R01_cb17333_c2/f1p0/1535	NA	R01_cb17333_c2/f1p0/1535
R01_cb4543_c0/f1p0/3310	NA	R01_cb4543_c0/f1p0/3310	R01_cb4543_c0/f1p0/3310	NA
R01_cb13392_c9/f2p0/484	NA	NA	R01_cb13392_c9/f2p0/484	R01_cb13392_c9/f2p0/484
R01_cb3483_c9/f1p0/2825	NA	NA	NA	R01_cb3483_c9/f1p0/2825
R01_cb13762_c33/f1p0/1048	NA	R01_cb13762_c33/f1p0/1048	R01_cb13762_c33/f1p0/1048	R01_cb13762_c33/f1p0/1048

R01_cb1905_c8/f1p0/2229	NA	R01_cb1905_c8/f1p0/2229	R01_cb1905_c8/f1p0/2229	NA
R01_cb18817_c0/f2p0/5685	NA	NA	NA	R01_cb18817_c0/f2p0/5685
R01_cb13941_c4/f1p0/1008	NA	R01_cb13941_c4/f1p0/1008	NA	R01_cb13941_c4/f1p0/1008
R01_cb7416_c12/f1p0/1304	NA	NA	NA	R01_cb7416_c12/f1p0/1304
R01_cb8564_c3067/f1p0/2488	NA	R01_cb8564_c3067/f1p0/2488	NA	R01_cb8564_c3067/f1p0/2488
R01_cb3703_c5/f1p1/2985	NA	R01_cb3703_c5/f1p1/2985	R01_cb3703_c5/f1p1/2985	R01_cb3703_c5/f1p1/2985
R01_cb10691_c5/f1p0/1270	NA	R01_cb10691_c5/f1p0/1270	NA	NA
R01_cb12321_c654/f1p0/537	NA	R01_cb12321_c654/f1p0/537	NA	NA
R01_cb8660_c14/f1p0/946	R01_cb8660_c14/f1p0/946	R01_cb8660_c14/f1p0/946	NA	R01_cb8660_c14/f1p0/946
R01_cb5046_c5/f1p0/1852	NA	R01_cb5046_c5/f1p0/1852	NA	NA
R01_cb16369_c0/f1p0/541	NA	R01_cb16369_c0/f1p0/541	NA	R01_cb16369_c0/f1p0/541
R01_cb1488_c5/f1p0/2442	NA	R01_cb1488_c5/f1p0/2442	NA	NA
R01_cb2588_c1/f1p0/3874	NA	R01_cb2588_c1/f1p0/3874	R01_cb2588_c1/f1p0/3874	R01_cb2588_c1/f1p0/3874
R01_cb14542_c6/f1p0/1272	NA	NA	NA	R01_cb14542_c6/f1p0/1272
R01_cb842_c2/f1p0/4603	NA	R01_cb842_c2/f1p0/4603	NA	R01_cb842_c2/f1p0/4603
R01_cb8564_c69823/f1p0/2349	NA	NA	NA	R01_cb8564_c69823/f1p0/2349
R01_cb5811_c14/f1p0/1001	NA	R01_cb5811_c14/f1p0/1001	NA	R01_cb5811_c14/f1p0/1001
R01_cb15663_c0/f2p0/1628	NA	NA	NA	R01_cb15663_c0/f2p0/1628
R01_cb11974_c3/f1p0/374	NA	R01_cb11974_c3/f1p0/374	NA	NA
R01_cb9617_c14/f1p0/1639	NA	NA	R01_cb9617_c14/f1p0/1639	R01_cb9617_c14/f1p0/1639
R01_cb13135_c2/f1p0/1002	NA	R01_cb13135_c2/f1p0/1002	NA	NA
R01_cb8564_c5258/f1p0/2514	NA	R01_cb8564_c5258/f1p0/2514	NA	NA
R01_cb11376_c3/f1p1/614	NA	R01_cb11376_c3/f1p1/614	NA	R01_cb11376_c3/f1p1/614
R01_cb13953_c17/f1p2/877	NA	R01_cb13953_c17/f1p2/877	NA	NA
R01_cb8789_c0/f2p0/2281	NA	R01_cb8789_c0/f2p0/2281	NA	NA
R01_cb8564_c83855/f1p0/3065	NA	R01_cb8564_c83855/f1p0/3065	R01_cb8564_c83855/f1p0/3065	R01_cb8564_c83855/f1p0/3065

R01_cb6686_c1/f1p0/2801	NA	R01_cb6686_c1/f1p0/2801	R01_cb6686_c1/f1p0/2801	NA
R01_cb8564_c72631/f1p0/2252	NA	NA	NA	R01_cb8564_c72631/f1p0/2252
R01_cb10190_c6/f1p0/1605	NA	R01_cb10190_c6/f1p0/1605	R01_cb10190_c6/f1p0/1605	R01_cb10190_c6/f1p0/1605
R01_cb8564_c2015/f1p0/2862	NA	R01_cb8564_c2015/f1p0/2862	R01_cb8564_c2015/f1p0/2862	NA
R01_cb4652_c1/f1p0/3202	NA	R01_cb4652_c1/f1p0/3202	NA	NA
R01_cb8823_c1/f1p0/2258	NA	R01_cb8823_c1/f1p0/2258	R01_cb8823_c1/f1p0/2258	R01_cb8823_c1/f1p0/2258
R01_cb8564_c74705/f1p0/4795	NA	R01_cb8564_c74705/f1p0/4795	NA	R01_cb8564_c74705/f1p0/4795
R01_cb2894_c6/f1p0/3214	NA	R01_cb2894_c6/f1p0/3214	NA	NA
R01_cb18409_c56/f1p0/836	R01_cb18409_c56/f1p0/836	R01_cb18409_c56/f1p0/836	R01_cb18409_c56/f1p0/836	R01_cb18409_c56/f1p0/836
R01_cb18456_c1616/f2p2/728	R01_cb18456_c1616/f2p2/728	R01_cb18456_c1616/f2p2/728	R01_cb18456_c1616/f2p2/728	R01_cb18456_c1616/f2p2/728
R01_cb16784_c1/f1p0/1525	NA	NA	NA	R01_cb16784_c1/f1p0/1525
R01_cb7911_c1/f1p0/2380	NA	NA	NA	R01_cb7911_c1/f1p0/2380
R01_cb1096_c3/f2p0/2251	NA	NA	NA	R01_cb1096_c3/f2p0/2251
R01_cb3757_c5/f1p0/2581	NA	NA	NA	R01_cb3757_c5/f1p0/2581
R01_cb4625_c1/f1p0/3002	NA	R01_cb4625_c1/f1p0/3002	NA	NA
R01_cb8564_c118310/f1p0/1939	NA	NA	NA	R01_cb8564_c118310/f1p0/1939
R01_cb8564_c75756/f2p0/1973	NA	R01_cb8564_c75756/f2p0/1973	R01_cb8564_c75756/f2p0/1973	R01_cb8564_c75756/f2p0/1973
R01_cb18409_c163/f1p0/428	R01_cb18409_c163/f1p0/428	R01_cb18409_c163/f1p0/428	R01_cb18409_c163/f1p0/428	R01_cb18409_c163/f1p0/428
R01_cb14635_c5/f1p0/692	R01_cb14635_c5/f1p0/692	R01_cb14635_c5/f1p0/692	R01_cb14635_c5/f1p0/692	R01_cb14635_c5/f1p0/692
R01_cb13184_c2/f1p0/499	R01_cb13184_c2/f1p0/499	R01_cb13184_c2/f1p0/499	R01_cb13184_c2/f1p0/499	R01_cb13184_c2/f1p0/499
R01_cb10483_c2/f1p1/1808	NA	NA	NA	R01_cb10483_c2/f1p1/1808
R01_cb16672_c1/f2p0/705	R01_cb16672_c1/f2p0/705	R01_cb16672_c1/f2p0/705	R01_cb16672_c1/f2p0/705	NA
R01_cb5331_c3/f1p0/587	R01_cb5331_c3/f1p0/587	R01_cb5331_c3/f1p0/587	R01_cb5331_c3/f1p0/587	R01_cb5331_c3/f1p0/587
R01_cb48_c7/f1p0/4085	NA	NA	NA	R01_cb48_c7/f1p0/4085
R01_cb11473_c1/f1p0/2543	NA	NA	NA	R01_cb11473_c1/f1p0/2543

R01_cb8564_c15007/f1p0/3414	NA	R01_cb8564_c15007/f1p0/3414	NA	NA
R01_cb14164_c0/f1p0/1705	NA	NA	R01_cb14164_c0/f1p0/1705	R01_cb14164_c0/f1p0/1705
R01_cb1706_c11/f1p0/2510	NA	NA	NA	R01_cb1706_c11/f1p0/2510
R01_cb8564_c108686/f8p0/2394	NA	NA	NA	R01_cb8564_c108686/f8p0/2394
R01_cb16135_c0/f1p0/615	NA	NA	NA	R01_cb16135_c0/f1p0/615
R01_cb7443_c1/f1p0/2613	NA	NA	NA	R01_cb7443_c1/f1p0/2613
R01_cb5090_c14/f1p0/523	NA	R01_cb5090_c14/f1p0/523	R01_cb5090_c14/f1p0/523	R01_cb5090_c14/f1p0/523
R01_cb8564_c79197/f2p0/3089	R01_cb8564_c79197/f2p0/3089	R01_cb8564_c79197/f2p0/3089	NA	NA
R01_cb9772_c5/f1p0/2170	R01_cb9772_c5/f1p0/2170	R01_cb9772_c5/f1p0/2170	R01_cb9772_c5/f1p0/2170	R01_cb9772_c5/f1p0/2170
R01_cb18409_c31/f2p0/833	R01_cb18409_c31/f2p0/833	R01_cb18409_c31/f2p0/833	R01_cb18409_c31/f2p0/833	R01_cb18409_c31/f2p0/833
R01_cb12496_c7/f1p0/1620	NA	R01_cb12496_c7/f1p0/1620	NA	NA
R01_cb3320_c13/f1p0/2406	NA	R01_cb3320_c13/f1p0/2406	R01_cb3320_c13/f1p0/2406	R01_cb3320_c13/f1p0/2406
R01_cb8037_c1/f2p0/1508	NA	NA	NA	R01_cb8037_c1/f2p0/1508
R01_cb10549_c2/f1p0/973	NA	R01_cb10549_c2/f1p0/973	NA	NA
R01_cb18151_c0/f1p0/800	NA	NA	NA	R01_cb18151_c0/f1p0/800
R01_cb8564_c127591/f1p0/2476	NA	R01_cb8564_c127591/f1p0/2476	NA	NA
R01_cb16303_c3/f1p0/904	NA	R01_cb16303_c3/f1p0/904	R01_cb16303_c3/f1p0/904	R01_cb16303_c3/f1p0/904
R01_cb8564_c21611/f1p1/3531	NA	R01_cb8564_c21611/f1p1/3531	NA	NA
R01_cb1085_c8/f1p1/4090	NA	NA	NA	R01_cb1085_c8/f1p1/4090
R01_cb10594_c1/f1p0/1672	NA	NA	NA	R01_cb10594_c1/f1p0/1672
R01_cb13532_c8/f23p2/1128	R01_cb13532_c8/f23p2/1128	NA	R01_cb13532_c8/f23p2/1128	R01_cb13532_c8/f23p2/1128
R01_cb18220_c0/f1p0/794	NA	R01_cb18220_c0/f1p0/794	NA	NA
R01_cb9628_c18/f1p0/776	R01_cb9628_c18/f1p0/776	R01_cb9628_c18/f1p0/776	NA	NA

R01_cb8084_c0/f7p0/1210	NA	NA	NA	R01_cb8084_c0/f7p0/1210
R01_cb18456_c1479/f3p0/426	R01_cb18456_c1479/f3p0/426	R01_cb18456_c1479/f3p0/426	R01_cb18456_c1479/f3p0/426	R01_cb18456_c1479/f3p0/426
R01_cb5808_c2/f1p0/3006	NA	NA	NA	R01_cb5808_c2/f1p0/3006
R01_cb11194_c0/f2p0/1719	NA	NA	NA	R01_cb11194_c0/f2p0/1719
R01_cb5554_c2/f1p0/2738	R01_cb5554_c2/f1p0/2738	R01_cb5554_c2/f1p0/2738	R01_cb5554_c2/f1p0/2738	NA
R01_cb3120_c50/f1p0/3165	R01_cb3120_c50/f1p0/3165	R01_cb3120_c50/f1p0/3165	NA	NA
R01_cb3246_c5/f1p0/568	R01_cb3246_c5/f1p0/568	R01_cb3246_c5/f1p0/568	NA	R01_cb3246_c5/f1p0/568
R01_cb11780_c0/f1p0/1100	NA	NA	NA	R01_cb11780_c0/f1p0/1100
R01_cb18456_c6106/f1p0/381	R01_cb18456_c6106/f1p0/381	NA	R01_cb18456_c6106/f1p0/381	R01_cb18456_c6106/f1p0/381
R01_cb3637_c5/f1p0/1392	NA	R01_cb3637_c5/f1p0/1392	NA	NA
R01_cb8564_c71403/f1p0/3069	NA	R01_cb8564_c71403/f1p0/3069	R01_cb8564_c71403/f1p0/3069	R01_cb8564_c71403/f1p0/3069
R01_cb14106_c2/f1p0/1040	R01_cb14106_c2/f1p0/1040	R01_cb14106_c2/f1p0/1040	NA	NA
R01_cb2027_c6/f1p0/2718	NA	R01_cb2027_c6/f1p0/2718	R01_cb2027_c6/f1p0/2718	R01_cb2027_c6/f1p0/2718
R01_cb12399_c5/f1p0/588	NA	R01_cb12399_c5/f1p0/588	R01_cb12399_c5/f1p0/588	NA
R01_cb13024_c0/f4p0/689	NA	NA	NA	R01_cb13024_c0/f4p0/689
R01_cb18471_c0/f1p0/998	NA	NA	NA	R01_cb18471_c0/f1p0/998
R01_cb10165_c3/f1p0/987	NA	R01_cb10165_c3/f1p0/987	R01_cb10165_c3/f1p0/987	NA
R01_cb16141_c2/f1p0/1885	R01_cb16141_c2/f1p0/1885	R01_cb16141_c2/f1p0/1885	R01_cb16141_c2/f1p0/1885	R01_cb16141_c2/f1p0/1885
R01_cb8827_c2/f2p0/2199	NA	R01_cb8827_c2/f2p0/2199	NA	NA
R01_cb12421_c45/f1p0/644	R01_cb12421_c45/f1p0/644	R01_cb12421_c45/f1p0/644	NA	NA
R01_cb8564_c828/f1p0/1978	NA	R01_cb8564_c828/f1p0/1978	NA	NA
R01_cb5051_c1/f1p0/2919	NA	R01_cb5051_c1/f1p0/2919	R01_cb5051_c1/f1p0/2919	R01_cb5051_c1/f1p0/2919
R01_cb8564_c23476/f1p0/3764	NA	NA	NA	R01_cb8564_c23476/f1p0/3764
R01_cb8564_c89753/f1p0/1969	NA	R01_cb8564_c89753/f1p0/1969	NA	NA
R01_cb9606_c85/f1p0/1596	NA	NA	R01_cb9606_c85/f1p0/1596	R01_cb9606_c85/f1p0/1596
R01_cb8564_c71668/f1p0/2070	NA	NA	NA	R01_cb8564_c71668/f1p0/2070

R01_cb8564_c33846/f3p0/2319	NA	R01_cb8564_c33846/f3p0/2319	R01_cb8564_c33846/f3p0/2319	R01_cb8564_c33846/f3p0/2319
R01_cb12824_c15/f1p0/757	NA	NA	R01_cb12824_c15/f1p0/757	R01_cb12824_c15/f1p0/757
R01_cb3431_c3/f1p0/3516	NA	NA	NA	R01_cb3431_c3/f1p0/3516
R01_cb17214_c1/f1p0/1158	NA	NA	NA	R01_cb17214_c1/f1p0/1158
R01_cb1981_c13/f1p0/2717	NA	R01_cb1981_c13/f1p0/2717	NA	NA
R01_cb14557_c2/f1p0/1802	NA	NA	NA	R01_cb14557_c2/f1p0/1802
R01_cb8564_c117000/f1p0/2259	NA	NA	NA	R01_cb8564_c117000/f1p0/2259
R01_cb16556_c2/f1p0/494	NA	R01_cb16556_c2/f1p0/494	NA	R01_cb16556_c2/f1p0/494
R01_cb10375_c7/f1p0/1131	NA	NA	NA	R01_cb10375_c7/f1p0/1131
R01_cb11046_c2/f1p1/2451	NA	R01_cb11046_c2/f1p1/2451	R01_cb11046_c2/f1p1/2451	R01_cb11046_c2/f1p1/2451
R01_cb11447_c0/f1p0/1847	NA	R01_cb11447_c0/f1p0/1847	R01_cb11447_c0/f1p0/1847	R01_cb11447_c0/f1p0/1847
R01_cb6000_c22/f1p0/2286	NA	R01_cb6000_c22/f1p0/2286	NA	NA
R01_cb14409_c1/f3p0/576	NA	R01_cb14409_c1/f3p0/576	R01_cb14409_c1/f3p0/576	R01_cb14409_c1/f3p0/576
R01_cb16153_c2/f2p0/850	NA	NA	R01_cb16153_c2/f2p0/850	R01_cb16153_c2/f2p0/850
R01_cb16019_c4/f1p0/1610	R01_cb16019_c4/f1p0/1610	R01_cb16019_c4/f1p0/1610	R01_cb16019_c4/f1p0/1610	R01_cb16019_c4/f1p0/1610
R01_cb16678_c1/f1p0/1341	NA	NA	R01_cb16678_c1/f1p0/1341	R01_cb16678_c1/f1p0/1341
R01_cb9993_c3/f1p0/2514	NA	R01_cb9993_c3/f1p0/2514	NA	R01_cb9993_c3/f1p0/2514
R01_cb8102_c5/f1p0/2455	NA	NA	NA	R01_cb8102_c5/f1p0/2455
R01_cb8564_c123359/f1p1/1989	NA	NA	NA	R01_cb8564_c123359/f1p1/1989
R01_cb10968_c3/f1p0/639	NA	R01_cb10968_c3/f1p0/639	R01_cb10968_c3/f1p0/639	R01_cb10968_c3/f1p0/639
R01_cb5902_c4/f1p0/527	NA	R01_cb5902_c4/f1p0/527	NA	NA
R01_cb104_c13/f1p0/2645	NA	NA	NA	R01_cb104_c13/f1p0/2645
R01_cb18409_c180/f1p0/373	R01_cb18409_c180/f1p0/373	R01_cb18409_c180/f1p0/373	R01_cb18409_c180/f1p0/373	R01_cb18409_c180/f1p0/373
R01_cb14946_c1/f1p0/1288	R01_cb14946_c1/f1p0/1288	R01_cb14946_c1/f1p0/1288	NA	NA

R01_cb14576_c4/f1p0/936	NA	NA	R01_cb14576_c4/f1p0/936	R01_cb14576_c4/f1p0/936
R01_cb16304_c3/f1p0/1715	NA	NA	NA	R01_cb16304_c3/f1p0/1715
R01_cb9443_c0/f1p0/2114	NA	R01_cb9443_c0/f1p0/2114	NA	NA
R01_cb9932_c2/f1p0/2662	NA	R01_cb9932_c2/f1p0/2662	R01_cb9932_c2/f1p0/2662	R01_cb9932_c2/f1p0/2662
R01_cb917_c6/f1p0/4659	NA	NA	NA	R01_cb917_c6/f1p0/4659
R01_cb12119_c5/f1p0/1146	NA	R01_cb12119_c5/f1p0/1146	R01_cb12119_c5/f1p0/1146	NA
R01_cb2376_c18/f10p1/3642	NA	NA	NA	R01_cb2376_c18/f10p1/3642
R01_cb17126_c1/f1p0/1260	NA	NA	NA	R01_cb17126_c1/f1p0/1260
R01_cb8564_c20190/f1p2/4575	NA	NA	NA	R01_cb8564_c20190/f1p2/4575
R01_cb4134_c28/f1p1/2717	NA	NA	NA	R01_cb4134_c28/f1p1/2717
R01_cb16723_c3/f1p0/499	R01_cb16723_c3/f1p0/499	R01_cb16723_c3/f1p0/499	R01_cb16723_c3/f1p0/499	R01_cb16723_c3/f1p0/499
R01_cb18456_c7148/f1p0/1595	NA	R01_cb18456_c7148/f1p0/1595	R01_cb18456_c7148/f1p0/1595	R01_cb18456_c7148/f1p0/1595
R01_cb2571_c37/f1p1/3764	NA	NA	NA	R01_cb2571_c37/f1p1/3764
R01_cb12603_c18/f1p0/1827	NA	NA	R01_cb12603_c18/f1p0/1827	R01_cb12603_c18/f1p0/1827
R01_cb10024_c526/f1p2/754	NA	R01_cb10024_c526/f1p2/754	NA	NA
R01_cb12432_c3/f3p1/657	NA	NA	R01_cb12432_c3/f3p1/657	R01_cb12432_c3/f3p1/657
R01_cb8564_c116843/f1p0/2789	NA	NA	R01_cb8564_c116843/f1p0/2789	R01_cb8564_c116843/f1p0/2789
R01_cb7329_c6/f1p0/950	NA	R01_cb7329_c6/f1p0/950	NA	NA
R01_cb3371_c4/f1p0/3647	NA	R01_cb3371_c4/f1p0/3647	NA	NA
R01_cb17003_c0/f1p0/1199	NA	R01_cb17003_c0/f1p0/1199	NA	R01_cb17003_c0/f1p0/1199
R01_cb8564_c157677/f1p0/2142	NA	R01_cb8564_c157677/f1p0/2142	R01_cb8564_c157677/f1p0/2142	R01_cb8564_c157677/f1p0/2142
R01_cb15428_c0/f3p0/780	NA	NA	R01_cb15428_c0/f3p0/780	R01_cb15428_c0/f3p0/780
R01_cb1203_c21/f1p0/2229	NA	NA	NA	R01_cb1203_c21/f1p0/2229
R01_cb8564_c71513/f1p0/2076	NA	NA	NA	R01_cb8564_c71513/f1p0/2076

R01_cb8564_c73343/f1p0/2005	NA	R01_cb8564_c73343/f1p0/2005	NA	NA
R01_cb952_c1/f1p0/4472	R01_cb952_c1/f1p0/4472	R01_cb952_c1/f1p0/4472	R01_cb952_c1/f1p0/4472	NA
R01_cb18456_c7455/f1p0/1446	NA	R01_cb18456_c7455/f1p0/1446	NA	R01_cb18456_c7455/f1p0/1446
R01_cb416_c7/f4p0/3143	NA	R01_cb416_c7/f4p0/3143	NA	NA
R01_cb16645_c51/f1p0/997	NA	R01_cb16645_c51/f1p0/997	NA	NA
R01_cb15755_c2/f1p0/765	NA	R01_cb15755_c2/f1p0/765	NA	R01_cb15755_c2/f1p0/765
R01_cb8564_c146594/f5p1/2739	NA	R01_cb8564_c146594/f5p1/2739	R01_cb8564_c146594/f5p1/2739	R01_cb8564_c146594/f5p1/2739
R01_cb3359_c60/f1p0/490	NA	R01_cb3359_c60/f1p0/490	NA	NA
R01_cb1224_c19/f1p1/4162	NA	NA	NA	R01_cb1224_c19/f1p1/4162
R01_cb14125_c67/f1p0/559	NA	R01_cb14125_c67/f1p0/559	R01_cb14125_c67/f1p0/559	NA
R01_cb5108_c28/f1p0/2142	NA	R01_cb5108_c28/f1p0/2142	NA	NA
R01_cb8564_c84445/f1p0/2154	NA	R01_cb8564_c84445/f1p0/2154	R01_cb8564_c84445/f1p0/2154	R01_cb8564_c84445/f1p0/2154
R01_cb763_c10/f1p0/860	R01_cb763_c10/f1p0/860	R01_cb763_c10/f1p0/860	R01_cb763_c10/f1p0/860	R01_cb763_c10/f1p0/860
R01_cb11343_c1/f1p1/1893	NA	NA	NA	R01_cb11343_c1/f1p1/1893
R01_cb10024_c295/f1p0/564	NA	NA	R01_cb10024_c295/f1p0/564	NA
R01_cb8564_c82297/f1p0/3607	NA	NA	NA	R01_cb8564_c82297/f1p0/3607
R01_cb8564_c81595/f1p0/2803	NA	R01_cb8564_c81595/f1p0/2803	NA	NA
R01_cb7946_c2/f1p0/2121	NA	R01_cb7946_c2/f1p0/2121	R01_cb7946_c2/f1p0/2121	NA
R01_cb18575_c0/f2p0/1063	R01_cb18575_c0/f2p0/1063	R01_cb18575_c0/f2p0/1063	R01_cb18575_c0/f2p0/1063	R01_cb18575_c0/f2p0/1063
R01_cb14863_c1/f1p1/941	NA	R01_cb14863_c1/f1p1/941	R01_cb14863_c1/f1p1/941	R01_cb14863_c1/f1p1/941
R01_cb12972_c29/f1p0/1456	NA	R01_cb12972_c29/f1p0/1456	NA	R01_cb12972_c29/f1p0/1456
R01_cb8564_c127806/f1p0/2484	NA	R01_cb8564_c127806/f1p0/2484	NA	R01_cb8564_c127806/f1p0/2484
R01_cb3826_c11/f1p0/1554	R01_cb3826_c11/f1p0/1554	R01_cb3826_c11/f1p0/1554	NA	R01_cb3826_c11/f1p0/1554
R01_cb8564_c51485/f1p0/4405	NA	R01_cb8564_c51485/f1p0/4405	NA	NA

R01_cb15990_c1/f2p0/1153	NA	NA	NA	R01_cb15990_c1/f2p0/1153
R01_cb10347_c7/f1p0/966	NA	R01_cb10347_c7/f1p0/966	NA	NA
R01_cb8564_c68180/f8p0/3575	NA	NA	NA	R01_cb8564_c68180/f8p0/3575
R01_cb13083_c1/f1p0/503	R01_cb13083_c1/f1p0/503	NA	R01_cb13083_c1/f1p0/503	R01_cb13083_c1/f1p0/503
R01_cb17731_c2/f1p0/612	NA	R01_cb17731_c2/f1p0/612	NA	NA
R01_cb13910_c22/f1p1/1851	NA	R01_cb13910_c22/f1p1/1851	R01_cb13910_c22/f1p1/1851	R01_cb13910_c22/f1p1/1851
R01_cb1263_c1/f1p0/4218	NA	R01_cb1263_c1/f1p0/4218	NA	NA
R01_cb6884_c10/f1p0/2549	NA	R01_cb6884_c10/f1p0/2549	NA	NA
R01_cb7890_c1/f1p0/2072	R01_cb7890_c1/f1p0/2072	R01_cb7890_c1/f1p0/2072	R01_cb7890_c1/f1p0/2072	R01_cb7890_c1/f1p0/2072
R01_cb4233_c205/f1p0/3584	R01_cb4233_c205/f1p0/3584	R01_cb4233_c205/f1p0/3584	R01_cb4233_c205/f1p0/3584	NA
R01_cb7497_c5/f1p1/3232	NA	NA	NA	R01_cb7497_c5/f1p1/3232
R01_cb8564_c76648/f1p0/2870	NA	NA	NA	R01_cb8564_c76648/f1p0/2870
R01_cb10088_c30/f1p0/1003	NA	R01_cb10088_c30/f1p0/1003	NA	NA
R01_cb737_c57/f1p0/1427	NA	R01_cb737_c57/f1p0/1427	NA	NA
R01_cb13761_c15/f1p0/1137	R01_cb13761_c15/f1p0/1137	R01_cb13761_c15/f1p0/1137	R01_cb13761_c15/f1p0/1137	R01_cb13761_c15/f1p0/1137
R01_cb18481_c0/f1p0/374	R01_cb18481_c0/f1p0/374	R01_cb18481_c0/f1p0/374	NA	R01_cb18481_c0/f1p0/374
R01_cb9410_c2/f2p1/2008	NA	NA	NA	R01_cb9410_c2/f2p1/2008
R01_cb8710_c8/f1p0/3734	NA	R01_cb8710_c8/f1p0/3734	NA	NA
R01_cb2843_c6/f1p4/2841	NA	NA	NA	R01_cb2843_c6/f1p4/2841
R01_cb8564_c1825/f1p0/4282	NA	R01_cb8564_c1825/f1p0/4282	R01_cb8564_c1825/f1p0/4282	NA
R01_cb13169_c0/f1p0/547	R01_cb13169_c0/f1p0/547	R01_cb13169_c0/f1p0/547	R01_cb13169_c0/f1p0/547	R01_cb13169_c0/f1p0/547
R01_cb8564_c1972/f1p0/2732	R01_cb8564_c1972/f1p0/2732	R01_cb8564_c1972/f1p0/2732	NA	NA
R01_cb18324_c1/f1p0/1717	NA	R01_cb18324_c1/f1p0/1717	NA	NA
R01_cb18845_c0/f2p0/5160	NA	R01_cb18845_c0/f2p0/5160	NA	NA
R01_cb17533_c1/f1p0/702	NA	NA	R01_cb17533_c1/f1p0/702	R01_cb17533_c1/f1p0/702
R01_cb14125_c58/f1p0/921	R01_cb14125_c58/f1p0/921	R01_cb14125_c58/f1p0/921	R01_cb14125_c58/f1p0/921	NA

R01_cb8564_c16927/f1p0/4700	NA	R01_cb8564_c16927/f1p0/4700	NA	R01_cb8564_c16927/f1p0/4700
R01_cb18132_c6/f1p0/1723	NA	R01_cb18132_c6/f1p0/1723	NA	NA
R01_cb8564_c117091/f1p0/2493	NA	R01_cb8564_c117091/f1p0/2493	NA	NA
R01_cb6615_c20/f1p0/1263	NA	NA	NA	R01_cb6615_c20/f1p0/1263
R01_cb2765_c5/f1p0/3605	NA	NA	NA	R01_cb2765_c5/f1p0/3605
R01_cb8564_c122549/f1p0/2541	NA	R01_cb8564_c122549/f1p0/2541	NA	NA
R01_cb4094_c29/f1p0/407	R01_cb4094_c29/f1p0/407	R01_cb4094_c29/f1p0/407	R01_cb4094_c29/f1p0/407	R01_cb4094_c29/f1p0/407
R01_cb10192_c3/f3p0/1329	NA	NA	NA	R01_cb10192_c3/f3p0/1329
R01_cb18386_c6/f1p0/1008	NA	NA	NA	R01_cb18386_c6/f1p0/1008
R01_cb15418_c3/f1p0/1155	NA	NA	NA	R01_cb15418_c3/f1p0/1155
R01_cb17973_c51/f1p0/1205	NA	NA	R01_cb17973_c51/f1p0/1205	NA
R01_cb12955_c14/f1p0/777	NA	R01_cb12955_c14/f1p0/777	NA	NA
R01_cb3081_c35/f1p0/1192	NA	NA	NA	R01_cb3081_c35/f1p0/1192
R01_cb17648_c1/f1p0/815	NA	R01_cb17648_c1/f1p0/815	NA	R01_cb17648_c1/f1p0/815
R01_cb4477_c0/f1p0/3331	NA	NA	NA	R01_cb4477_c0/f1p0/3331
R01_cb11226_c1/f1p0/2696	NA	R01_cb11226_c1/f1p0/2696	NA	NA
R01_cb14611_c1/f1p0/570	NA	NA	NA	R01_cb14611_c1/f1p0/570
R01_cb13910_c23/f1p0/1150	R01_cb13910_c23/f1p0/1150	R01_cb13910_c23/f1p0/1150	R01_cb13910_c23/f1p0/1150	R01_cb13910_c23/f1p0/1150
R01_cb10281_c8/f1p0/774	R01_cb10281_c8/f1p0/774	R01_cb10281_c8/f1p0/774	R01_cb10281_c8/f1p0/774	R01_cb10281_c8/f1p0/774
R01_cb12577_c15/f1p1/1098	NA	NA	NA	R01_cb12577_c15/f1p1/1098
R01_cb6216_c5/f1p1/2842	NA	NA	NA	R01_cb6216_c5/f1p1/2842
R01_cb124_c45/f1p0/3856	NA	NA	NA	R01_cb124_c45/f1p0/3856
R01_cb12061_c3/f1p0/909	NA	NA	R01_cb12061_c3/f1p0/909	R01_cb12061_c3/f1p0/909
R01_cb8564_c19106/f1p1/3385	NA	NA	NA	R01_cb8564_c19106/f1p1/3385

R01_cb302_c22/f1p1/3121	NA	NA	NA	R01_cb302_c22/f1p1/3121
R01_cb12061_c4/f1p0/868	NA	NA	R01_cb12061_c4/f1p0/868	R01_cb12061_c4/f1p0/868
R01_cb8564_c15054/f1p0/2411	NA	NA	NA	R01_cb8564_c15054/f1p0/2411
R01_cb449_c9/f1p0/1986	NA	NA	NA	R01_cb449_c9/f1p0/1986
R01_cb12521_c3/f1p0/1002	NA	NA	NA	R01_cb12521_c3/f1p0/1002
R01_cb3079_c0/f10p0/1714	NA	NA	NA	R01_cb3079_c0/f10p0/1714
R01_cb18314_c0/f2p0/525	R01_cb18314_c0/f2p0/525	R01_cb18314_c0/f2p0/525	R01_cb18314_c0/f2p0/525	R01_cb18314_c0/f2p0/525
R01_cb7828_c3/f1p0/2524	NA	NA	NA	R01_cb7828_c3/f1p0/2524
R01_cb3556_c4/f1p0/3583	NA	R01_cb3556_c4/f1p0/3583	NA	NA
R01_cb177_c0/f2p0/2287	NA	NA	NA	R01_cb177_c0/f2p0/2287
R01_cb4136_c0/f1p0/3416	R01_cb4136_c0/f1p0/3416	R01_cb4136_c0/f1p0/3416	R01_cb4136_c0/f1p0/3416	NA
R01_cb11576_c1/f1p0/2492	NA	NA	R01_cb11576_c1/f1p0/2492	NA
R01_cb8564_c111865/f1p0/3154	NA	R01_cb8564_c111865/f1p0/3154	R01_cb8564_c111865/f1p0/3154	NA
R01_cb8564_c4518/f1p0/2908	NA	NA	R01_cb8564_c4518/f1p0/2908	NA
R01_cb8564_c110028/f2p0/2413	NA	R01_cb8564_c110028/f2p0/2413	R01_cb8564_c110028/f2p0/2413	R01_cb8564_c110028/f2p0/2413
R01_cb8564_c121871/f1p0/2404	NA	R01_cb8564_c121871/f1p0/2404	R01_cb8564_c121871/f1p0/2404	NA
R01_cb8564_c71207/f1p0/4053	NA	R01_cb8564_c71207/f1p0/4053	NA	NA
R01_cb8564_c129510/f1p0/2174	NA	R01_cb8564_c129510/f1p0/2174	R01_cb8564_c129510/f1p0/2174	R01_cb8564_c129510/f1p0/2174
R01_cb18540_c1/f1p0/1000	R01_cb18540_c1/f1p0/1000	R01_cb18540_c1/f1p0/1000	NA	NA
R01_cb15624_c2/f1p0/540	NA	R01_cb15624_c2/f1p0/540	R01_cb15624_c2/f1p0/540	R01_cb15624_c2/f1p0/540
R01_cb18456_c6982/f1p1/577	R01_cb18456_c6982/f1p1/577	R01_cb18456_c6982/f1p1/577	R01_cb18456_c6982/f1p1/577	R01_cb18456_c6982/f1p1/577
R01_cb9704_c0/f1p0/2009	NA	NA	NA	R01_cb9704_c0/f1p0/2009

R01_cb3615_c4/f1p0/3002	NA	R01_cb3615_c4/f1p0/3002	NA	NA
R01_cb8564_c43829/f1p0/4379	NA	NA	NA	R01_cb8564_c43829/f1p0/4379
R01_cb8564_c1833/f1p0/2496	R01_cb8564_c1833/f1p0/2496	R01_cb8564_c1833/f1p0/2496	R01_cb8564_c1833/f1p0/2496	R01_cb8564_c1833/f1p0/2496
R01_cb3794_c2/f1p0/3320	NA	R01_cb3794_c2/f1p0/3320	NA	NA
R01_cb5502_c1/f4p1/2543	NA	NA	NA	R01_cb5502_c1/f4p1/2543
R01_cb8564_c13270/f1p0/4132	NA	R01_cb8564_c13270/f1p0/4132	R01_cb8564_c13270/f1p0/4132	NA
R01_cb5168_c5/f1p0/1092	NA	R01_cb5168_c5/f1p0/1092	R01_cb5168_c5/f1p0/1092	R01_cb5168_c5/f1p0/1092
R01_cb8564_c23172/f1p0/2885	R01_cb8564_c23172/f1p0/2885	R01_cb8564_c23172/f1p0/2885	R01_cb8564_c23172/f1p0/2885	R01_cb8564_c23172/f1p0/2885
R01_cb16978_c1/f1p0/886	R01_cb16978_c1/f1p0/886	NA	R01_cb16978_c1/f1p0/886	R01_cb16978_c1/f1p0/886
R01_cb8564_c34222/f2p0/2540	R01_cb8564_c34222/f2p0/2540	R01_cb8564_c34222/f2p0/2540	R01_cb8564_c34222/f2p0/2540	R01_cb8564_c34222/f2p0/2540
R01_cb13894_c4/f1p0/670	R01_cb13894_c4/f1p0/670	R01_cb13894_c4/f1p0/670	NA	NA
R01_cb8501_c28/f1p0/1957	NA	R01_cb8501_c28/f1p0/1957	NA	NA
R01_cb5713_c11/f1p0/937	R01_cb5713_c11/f1p0/937	R01_cb5713_c11/f1p0/937	R01_cb5713_c11/f1p0/937	R01_cb5713_c11/f1p0/937
R01_cb8564_c120089/f1p0/3325	NA	R01_cb8564_c120089/f1p0/3325	R01_cb8564_c120089/f1p0/3325	NA
R01_cb3949_c4/f1p0/3484	NA	NA	NA	R01_cb3949_c4/f1p0/3484
R01_cb14284_c1/f1p0/743	NA	R01_cb14284_c1/f1p0/743	NA	NA
R01_cb15582_c2/f1p0/1436	NA	NA	NA	R01_cb15582_c2/f1p0/1436
R01_cb13544_c0/f4p1/1570	NA	NA	NA	R01_cb13544_c0/f4p1/1570
R01_cb8564_c119651/f1p1/2856	NA	NA	NA	R01_cb8564_c119651/f1p1/2856
R01_cb4345_c14/f1p1/3316	NA	NA	NA	R01_cb4345_c14/f1p1/3316
R01_cb12837_c15/f1p3/562	NA	R01_cb12837_c15/f1p3/562	NA	NA
R01_cb9592_c9/f2p2/1628	NA	NA	NA	R01_cb9592_c9/f2p2/1628

R01_cb8564_c50708/f2p0/3011	R01_cb8564_c50708/f2p0/3011	R01_cb8564_c50708/f2p0/3011	R01_cb8564_c50708/f2p0/3011	R01_cb8564_c50708/f2p0/3011
R01_cb10292_c0/f4p0/1111	NA	NA	NA	R01_cb10292_c0/f4p0/1111
R01_cb8564_c53424/f1p0/2985	NA	NA	R01_cb8564_c53424/f1p0/2985	R01_cb8564_c53424/f1p0/2985
R01_cb18456_c7560/f1p0/543	NA	R01_cb18456_c7560/f1p0/543	NA	R01_cb18456_c7560/f1p0/543
R01_cb8564_c109814/f2p0/1887	R01_cb8564_c109814/f2p0/1887	R01_cb8564_c109814/f2p0/1887	NA	R01_cb8564_c109814/f2p0/1887
R01_cb10775_c1/f1p0/2754	R01_cb10775_c1/f1p0/2754	R01_cb10775_c1/f1p0/2754	R01_cb10775_c1/f1p0/2754	R01_cb10775_c1/f1p0/2754
R01_cb13545_c59/f1p0/586	R01_cb13545_c59/f1p0/586	R01_cb13545_c59/f1p0/586	R01_cb13545_c59/f1p0/586	R01_cb13545_c59/f1p0/586
R01_cb16439_c1/f1p0/834	NA	NA	NA	R01_cb16439_c1/f1p0/834
R01_cb8564_c111500/f1p0/3065	NA	NA	NA	R01_cb8564_c111500/f1p0/3065
R01_cb17973_c6/f2p0/918	R01_cb17973_c6/f2p0/918	R01_cb17973_c6/f2p0/918	R01_cb17973_c6/f2p0/918	R01_cb17973_c6/f2p0/918
R01_cb8478_c44/f1p0/3234	NA	R01_cb8478_c44/f1p0/3234	NA	R01_cb8478_c44/f1p0/3234
R01_cb4147_c18/f1p0/2386	NA	NA	NA	R01_cb4147_c18/f1p0/2386
R01_cb18087_c0/f1p0/799	NA	R01_cb18087_c0/f1p0/799	NA	R01_cb18087_c0/f1p0/799
R01_cb13889_c18/f1p0/539	R01_cb13889_c18/f1p0/539	R01_cb13889_c18/f1p0/539	NA	NA
R01_cb18301_c7/f1p0/1719	R01_cb18301_c7/f1p0/1719	R01_cb18301_c7/f1p0/1719	R01_cb18301_c7/f1p0/1719	R01_cb18301_c7/f1p0/1719
R01_cb18756_c1/f1p0/2022	NA	R01_cb18756_c1/f1p0/2022	R01_cb18756_c1/f1p0/2022	R01_cb18756_c1/f1p0/2022
R01_cb12149_c17/f1p0/1518	R01_cb12149_c17/f1p0/1518	R01_cb12149_c17/f1p0/1518	NA	NA
R01_cb17094_c1/f1p0/979	R01_cb17094_c1/f1p0/979	R01_cb17094_c1/f1p0/979	R01_cb17094_c1/f1p0/979	R01_cb17094_c1/f1p0/979
R01_cb6839_c3/f1p0/2558	NA	R01_cb6839_c3/f1p0/2558	NA	NA
R01_cb12329_c20/f1p0/934	NA	NA	NA	R01_cb12329_c20/f1p0/934
R01_cb1998_c6/f1p0/2457	NA	R01_cb1998_c6/f1p0/2457	R01_cb1998_c6/f1p0/2457	NA
R01_cb12842_c3/f1p0/383	R01_cb12842_c3/f1p0/383	R01_cb12842_c3/f1p0/383	R01_cb12842_c3/f1p0/383	NA
R01_cb9614_c2/f1p0/1819	NA	NA	NA	R01_cb9614_c2/f1p0/1819

R01_cb17885_c4/f1p0/970	NA	R01_cb17885_c4/f1p0/970	NA	NA
R01_cb8564_c74038/f1p0/4350	NA	R01_cb8564_c74038/f1p0/4350	R01_cb8564_c74038/f1p0/4350	R01_cb8564_c74038/f1p0/4350
R01_cb8564_c76384/f1p0/2231	NA	NA	NA	R01_cb8564_c76384/f1p0/2231
R01_cb8564_c4718/f1p0/3695	NA	NA	NA	R01_cb8564_c4718/f1p0/3695
R01_cb6258_c17/f1p0/2464	NA	NA	R01_cb6258_c17/f1p0/2464	R01_cb6258_c17/f1p0/2464
R01_cb4490_c13/f1p0/2545	NA	NA	NA	R01_cb4490_c13/f1p0/2545
R01_cb12516_c3/f1p0/555	R01_cb12516_c3/f1p0/555	NA	NA	R01_cb12516_c3/f1p0/555
R01_cb12775_c4/f1p0/1459	NA	NA	NA	R01_cb12775_c4/f1p0/1459
R01_cb12443_c2/f1p0/419	R01_cb12443_c2/f1p0/419	R01_cb12443_c2/f1p0/419	R01_cb12443_c2/f1p0/419	R01_cb12443_c2/f1p0/419
R01_cb8564_c1435/f1p0/1939	NA	R01_cb8564_c1435/f1p0/1939	R01_cb8564_c1435/f1p0/1939	NA
R01_cb1333_c20/f2p1/4359	NA	R01_cb1333_c20/f2p1/4359	R01_cb1333_c20/f2p1/4359	R01_cb1333_c20/f2p1/4359
R01_cb14758_c1/f1p0/1383	NA	R01_cb14758_c1/f1p0/1383	R01_cb14758_c1/f1p0/1383	R01_cb14758_c1/f1p0/1383
R01_cb14624_c4/f1p0/534	R01_cb14624_c4/f1p0/534	R01_cb14624_c4/f1p0/534	R01_cb14624_c4/f1p0/534	R01_cb14624_c4/f1p0/534
R01_cb1706_c2/f2p0/2994	NA	NA	NA	R01_cb1706_c2/f2p0/2994
R01_cb8564_c73594/f1p0/2698	NA	NA	NA	R01_cb8564_c73594/f1p0/2698
R01_cb15149_c0/f3p0/424	R01_cb15149_c0/f3p0/424	R01_cb15149_c0/f3p0/424	NA	NA
R01_cb8564_c110432/f1p0/4489	NA	R01_cb8564_c110432/f1p0/4489	NA	NA
R01_cb8564_c3049/f1p0/3118	NA	R01_cb8564_c3049/f1p0/3118	R01_cb8564_c3049/f1p0/3118	R01_cb8564_c3049/f1p0/3118
R01_cb18489_c1/f1p0/1306	NA	R01_cb18489_c1/f1p0/1306	R01_cb18489_c1/f1p0/1306	R01_cb18489_c1/f1p0/1306
R01_cb7368_c1/f1p0/2387	NA	R01_cb7368_c1/f1p0/2387	NA	R01_cb7368_c1/f1p0/2387
R01_cb8564_c76115/f1p0/2356	NA	R01_cb8564_c76115/f1p0/2356	NA	NA
R01_cb10034_c28/f1p5/2081	NA	R01_cb10034_c28/f1p5/2081	NA	NA
R01_cb18456_c7244/f1p0/1336	NA	R01_cb18456_c7244/f1p0/1336	R01_cb18456_c7244/f1p0/1336	R01_cb18456_c7244/f1p0/1336
R01_cb8650_c4/f1p1/2111	R01_cb8650_c4/f1p1/2111	R01_cb8650_c4/f1p1/2111	R01_cb8650_c4/f1p1/2111	NA
R01_cb18764_c1/f1p0/1518	R01_cb18764_c1/f1p0/1518	R01_cb18764_c1/f1p0/1518	R01_cb18764_c1/f1p0/1518	R01_cb18764_c1/f1p0/1518

R01_cb8564_c126035/f2p0/2239	NA	R01_cb8564_c126035/f2p0/2239	NA	R01_cb8564_c126035/f2p0/2239
R01_cb16426_c3/f1p0/678	NA	R01_cb16426_c3/f1p0/678	NA	NA
R01_cb18456_c7977/f1p0/395	R01_cb18456_c7977/f1p0/395	R01_cb18456_c7977/f1p0/395	R01_cb18456_c7977/f1p0/395	R01_cb18456_c7977/f1p0/395
R01_cb6508_c13/f1p0/2792	NA	R01_cb6508_c13/f1p0/2792	NA	NA
R01_cb13726_c4/f1p1/1007	NA	NA	NA	R01_cb13726_c4/f1p1/1007
R01_cb11112_c1/f1p0/3116	NA	R01_cb11112_c1/f1p0/3116	NA	NA
R01_cb14502_c5/f2p0/1300	NA	R01_cb14502_c5/f2p0/1300	R01_cb14502_c5/f2p0/1300	R01_cb14502_c5/f2p0/1300
R01_cb10637_c1/f1p0/3698	NA	R01_cb10637_c1/f1p0/3698	R01_cb10637_c1/f1p0/3698	R01_cb10637_c1/f1p0/3698
R01_cb9418_c3/f1p1/1920	NA	R01_cb9418_c3/f1p1/1920	NA	R01_cb9418_c3/f1p1/1920
R01_cb8007_c0/f1p0/2483	NA	R01_cb8007_c0/f1p0/2483	NA	NA
R01_cb8564_c1754/f1p0/4035	NA	NA	NA	R01_cb8564_c1754/f1p0/4035
R01_cb13502_c1/f1p0/1346	NA	R01_cb13502_c1/f1p0/1346	NA	R01_cb13502_c1/f1p0/1346
R01_cb15570_c2/f1p0/1661	R01_cb15570_c2/f1p0/1661	R01_cb15570_c2/f1p0/1661	R01_cb15570_c2/f1p0/1661	R01_cb15570_c2/f1p0/1661
R01_cb8216_c3/f1p0/2346	NA	R01_cb8216_c3/f1p0/2346	NA	NA
R01_cb17956_c5/f1p0/705	R01_cb17956_c5/f1p0/705	R01_cb17956_c5/f1p0/705	NA	NA
R01_cb18427_c2/f1p0/652	NA	NA	NA	R01_cb18427_c2/f1p0/652
R01_cb17402_c2/f1p0/1034	NA	NA	NA	R01_cb17402_c2/f1p0/1034
R01_cb8564_c37949/f1p0/4680	NA	R01_cb8564_c37949/f1p0/4680	NA	NA
R01_cb13845_c1/f1p0/652	R01_cb13845_c1/f1p0/652	R01_cb13845_c1/f1p0/652	NA	NA
R01_cb5183_c110/f1p0/1341	NA	NA	NA	R01_cb5183_c110/f1p0/1341
R01_cb10797_c1/f1p0/3720	NA	R01_cb10797_c1/f1p0/3720	NA	R01_cb10797_c1/f1p0/3720
R01_cb8564_c43079/f1p0/1963	R01_cb8564_c43079/f1p0/1963	R01_cb8564_c43079/f1p0/1963	R01_cb8564_c43079/f1p0/1963	R01_cb8564_c43079/f1p0/1963
R01_cb3699_c13/f1p0/2720	NA	R01_cb3699_c13/f1p0/2720	NA	NA
R01_cb14753_c1/f2p0/1551	NA	R01_cb14753_c1/f2p0/1551	NA	NA

R01_cb9029_c3/f1p0/1834	NA	NA	NA	R01_cb9029_c3/f1p0/1834
R01_cb18456_c7423/f1p0/309	R01_cb18456_c7423/f1p0/309	R01_cb18456_c7423/f1p0/309	R01_cb18456_c7423/f1p0/309	R01_cb18456_c7423/f1p0/309
R01_cb5139_c9/f1p0/3187	NA	NA	NA	R01_cb5139_c9/f1p0/3187
R01_cb8126_c1/f2p0/2416	NA	R01_cb8126_c1/f2p0/2416	NA	NA
R01_cb4744_c2/f1p0/3502	NA	R01_cb4744_c2/f1p0/3502	R01_cb4744_c2/f1p0/3502	R01_cb4744_c2/f1p0/3502
R01_cb981_c12/f1p0/3088	NA	R01_cb981_c12/f1p0/3088	NA	NA
R01_cb2061_c9/f1p1/1858	NA	R01_cb2061_c9/f1p1/1858	NA	NA
R01_cb1418_c0/f2p0/3458	NA	NA	NA	R01_cb1418_c0/f2p0/3458
R01_cb16138_c4/f1p0/842	R01_cb16138_c4/f1p0/842	R01_cb16138_c4/f1p0/842	R01_cb16138_c4/f1p0/842	R01_cb16138_c4/f1p0/842
R01_cb8564_c4122/f1p0/2429	NA	R01_cb8564_c4122/f1p0/2429	NA	NA
R01_cb2629_c15/f1p0/2175	NA	R01_cb2629_c15/f1p0/2175	NA	NA
R01_cb8564_c80781/f1p0/2804	NA	R01_cb8564_c80781/f1p0/2804	NA	R01_cb8564_c80781/f1p0/2804
R01_cb10842_c2/f1p0/2349	NA	R01_cb10842_c2/f1p0/2349	NA	R01_cb10842_c2/f1p0/2349
R01_cb18456_c6118/f1p0/763	NA	NA	NA	R01_cb18456_c6118/f1p0/763
R01_cb11105_c3/f1p0/1347	NA	R01_cb11105_c3/f1p0/1347	NA	NA
R01_cb18409_c120/f1p0/1060	R01_cb18409_c120/f1p0/1060	R01_cb18409_c120/f1p0/1060	R01_cb18409_c120/f1p0/1060	R01_cb18409_c120/f1p0/1060
R01_cb2072_c20/f1p0/2470	NA	NA	NA	R01_cb2072_c20/f1p0/2470
R01_cb4678_c2/f1p0/2481	NA	R01_cb4678_c2/f1p0/2481	NA	NA
R01_cb13191_c0/f2p0/1238	NA	NA	NA	R01_cb13191_c0/f2p0/1238
R01_cb14109_c2/f1p0/316	R01_cb14109_c2/f1p0/316	R01_cb14109_c2/f1p0/316	R01_cb14109_c2/f1p0/316	R01_cb14109_c2/f1p0/316
R01_cb8939_c5/f1p0/2247	NA	R01_cb8939_c5/f1p0/2247	NA	NA
R01_cb5351_c1/f1p0/2013	NA	NA	NA	R01_cb5351_c1/f1p0/2013
R01_cb8564_c33786/f3p0/2615	NA	R01_cb8564_c33786/f3p0/2615	NA	NA
R01_cb12702_c2/f1p0/629	R01_cb12702_c2/f1p0/629	R01_cb12702_c2/f1p0/629	R01_cb12702_c2/f1p0/629	R01_cb12702_c2/f1p0/629
R01_cb17826_c0/f1p0/1328	NA	NA	NA	R01_cb17826_c0/f1p0/1328
R01_cb8564_c2035/f1p0/2544	NA	R01_cb8564_c2035/f1p0/2544	NA	NA

R01_cb16205_c2/f1p0/1681	NA	NA	NA	R01_cb16205_c2/f1p0/1681
R01_cb11524_c0/f2p1/1126	NA	NA	NA	R01_cb11524_c0/f2p1/1126
R01_cb17269_c3/f2p0/579	NA	NA	R01_cb17269_c3/f2p0/579	R01_cb17269_c3/f2p0/579
R01_cb16172_c2/f1p0/1370	NA	R01_cb16172_c2/f1p0/1370	R01_cb16172_c2/f1p0/1370	NA
R01_cb10197_c2/f1p0/879	R01_cb10197_c2/f1p0/879	R01_cb10197_c2/f1p0/879	R01_cb10197_c2/f1p0/879	R01_cb10197_c2/f1p0/879
R01_cb5625_c6/f1p0/1376	NA	NA	NA	R01_cb5625_c6/f1p0/1376
R01_cb13287_c1/f7p0/571	NA	R01_cb13287_c1/f7p0/571	NA	R01_cb13287_c1/f7p0/571
R01_cb12297_c0/f2p0/609	NA	NA	NA	R01_cb12297_c0/f2p0/609
R01_cb10117_c22/f12p0/353	R01_cb10117_c22/f12p0/353	R01_cb10117_c22/f12p0/353	R01_cb10117_c22/f12p0/353	R01_cb10117_c22/f12p0/353
R01_cb17084_c2/f1p0/480	NA	R01_cb17084_c2/f1p0/480	NA	NA
R01_cb8669_c2/f1p0/2229	NA	NA	R01_cb8669_c2/f1p0/2229	R01_cb8669_c2/f1p0/2229
R01_cb8564_c76526/f1p0/3221	NA	R01_cb8564_c76526/f1p0/3221	NA	NA
R01_cb18360_c0/f2p0/675	NA	R01_cb18360_c0/f2p0/675	R01_cb18360_c0/f2p0/675	R01_cb18360_c0/f2p0/675
R01_cb261_c47/f1p0/2204	NA	R01_cb261_c47/f1p0/2204	NA	NA
R01_cb7087_c4/f1p0/2209	NA	NA	NA	R01_cb7087_c4/f1p0/2209
R01_cb9347_c2/f1p0/2109	NA	R01_cb9347_c2/f1p0/2109	NA	NA
R01_cb12946_c1/f1p0/678	R01_cb12946_c1/f1p0/678	R01_cb12946_c1/f1p0/678	R01_cb12946_c1/f1p0/678	NA
R01_cb4313_c1/f2p0/2541	NA	R01_cb4313_c1/f2p0/2541	NA	NA
R01_cb3791_c1/f3p0/1750	NA	NA	NA	R01_cb3791_c1/f3p0/1750
R01_cb8564_c4708/f1p0/3069	NA	R01_cb8564_c4708/f1p0/3069	R01_cb8564_c4708/f1p0/3069	R01_cb8564_c4708/f1p0/3069
R01_cb8564_c126584/f1p0/2460	NA	NA	NA	R01_cb8564_c126584/f1p0/2460
R01_cb226_c7/f1p0/5055	NA	NA	NA	R01_cb226_c7/f1p0/5055
R01_cb14004_c22/f1p0/550	NA	NA	NA	R01_cb14004_c22/f1p0/550
R01_cb8564_c22636/f1p0/2704	NA	R01_cb8564_c22636/f1p0/2704	NA	NA
R01_cb11453_c2/f1p0/959	NA	NA	NA	R01_cb11453_c2/f1p0/959

R01_cb8564_c68757/f3p0/3422	R01_cb8564_c68757/f3p0/3422	R01_cb8564_c68757/f3p0/3422	R01_cb8564_c68757/f3p0/3422	R01_cb8564_c68757/f3p0/3422
R01_cb8564_c36635/f1p0/4030	NA	NA	NA	R01_cb8564_c36635/f1p0/4030
R01_cb17207_c0/f3p0/724	NA	NA	NA	R01_cb17207_c0/f3p0/724
R01_cb9990_c0/f1p0/1892	NA	NA	NA	R01_cb9990_c0/f1p0/1892
R01_cb14019_c1/f1p0/1824	NA	R01_cb14019_c1/f1p0/1824	R01_cb14019_c1/f1p0/1824	R01_cb14019_c1/f1p0/1824
R01_cb8564_c22500/f1p0/3275	NA	R01_cb8564_c22500/f1p0/3275	R01_cb8564_c22500/f1p0/3275	NA
R01_cb18456_c6425/f1p0/502	R01_cb18456_c6425/f1p0/502	R01_cb18456_c6425/f1p0/502	R01_cb18456_c6425/f1p0/502	NA
R01_cb13101_c1/f1p0/1314	NA	NA	NA	R01_cb13101_c1/f1p0/1314
R01_cb1093_c2/f1p0/4429	NA	NA	NA	R01_cb1093_c2/f1p0/4429
R01_cb18348_c2/f1p0/1259	NA	R01_cb18348_c2/f1p0/1259	NA	NA
R01_cb6327_c0/f1p0/2887	NA	R01_cb6327_c0/f1p0/2887	R01_cb6327_c0/f1p0/2887	R01_cb6327_c0/f1p0/2887
R01_cb8564_c40367/f1p0/2463	NA	NA	R01_cb8564_c40367/f1p0/2463	R01_cb8564_c40367/f1p0/2463
R01_cb13781_c10/f7p0/614	NA	NA	NA	R01_cb13781_c10/f7p0/614
R01_cb1682_c7/f1p2/3680	NA	NA	NA	R01_cb1682_c7/f1p2/3680
R01_cb6608_c9/f1p1/2617	NA	NA	NA	R01_cb6608_c9/f1p1/2617
R01_cb4368_c12/f1p0/675	NA	NA	NA	R01_cb4368_c12/f1p0/675
R01_cb10024_c500/f1p0/692	NA	NA	R01_cb10024_c500/f1p0/692	NA
R01_cb2763_c6/f1p0/1445	NA	R01_cb2763_c6/f1p0/1445	NA	NA
R01_cb17744_c8/f1p0/447	R01_cb17744_c8/f1p0/447	R01_cb17744_c8/f1p0/447	NA	NA
R01_cb3359_c23/f1p0/3467	NA	R01_cb3359_c23/f1p0/3467	R01_cb3359_c23/f1p0/3467	R01_cb3359_c23/f1p0/3467
R01_cb8564_c78644/f1p0/2442	R01_cb8564_c78644/f1p0/2442	R01_cb8564_c78644/f1p0/2442	NA	R01_cb8564_c78644/f1p0/2442
R01_cb18301_c42/f1p0/1749	NA	NA	NA	R01_cb18301_c42/f1p0/1749
R01_cb10027_c9/f1p1/1643	NA	NA	NA	R01_cb10027_c9/f1p1/1643
R01_cb18124_c2/f1p0/1422	NA	R01_cb18124_c2/f1p0/1422	NA	NA

R01_cb12003_c74/f1p0/1823	NA	R01_cb12003_c74/f1p0/1823	NA	NA
R01_cb10615_c2/f1p0/1333	NA	R01_cb10615_c2/f1p0/1333	NA	NA
R01_cb119_c44/f2p0/3502	NA	NA	NA	R01_cb119_c44/f2p0/3502
R01_cb8564_c18784/f1p0/2016	NA	R01_cb8564_c18784/f1p0/2016	NA	NA
R01_cb16580_c2/f4p0/1145	NA	NA	NA	R01_cb16580_c2/f4p0/1145
R01_cb1837_c12/f1p0/1586	NA	R01_cb1837_c12/f1p0/1586	NA	R01_cb1837_c12/f1p0/1586
R01_cb15540_c3/f1p1/1396	NA	R01_cb15540_c3/f1p1/1396	NA	R01_cb15540_c3/f1p1/1396
R01_cb8180_c2/f1p0/1605	NA	R01_cb8180_c2/f1p0/1605	NA	NA
R01_cb8564_c122291/f1p0/2986	NA	NA	NA	R01_cb8564_c122291/f1p0/2986
R01_cb18456_c7266/f1p0/615	NA	R01_cb18456_c7266/f1p0/615	NA	NA
R01_cb5929_c3/f1p2/2584	NA	NA	NA	R01_cb5929_c3/f1p2/2584
R01_cb11120_c3/f1p0/558	NA	NA	R01_cb11120_c3/f1p0/558	R01_cb11120_c3/f1p0/558
R01_cb8564_c76035/f1p0/2086	R01_cb8564_c76035/f1p0/2086	R01_cb8564_c76035/f1p0/2086	R01_cb8564_c76035/f1p0/2086	R01_cb8564_c76035/f1p0/2086
R01_cb7167_c48/f1p0/2824	R01_cb7167_c48/f1p0/2824	R01_cb7167_c48/f1p0/2824	R01_cb7167_c48/f1p0/2824	R01_cb7167_c48/f1p0/2824
R01_cb13433_c87/f1p0/1925	NA	R01_cb13433_c87/f1p0/1925	NA	NA
R01_cb3088_c5/f1p0/2366	NA	R01_cb3088_c5/f1p0/2366	R01_cb3088_c5/f1p0/2366	R01_cb3088_c5/f1p0/2366
R01_cb10291_c2/f1p0/2325	NA	NA	NA	R01_cb10291_c2/f1p0/2325
R01_cb2037_c13/f1p0/5035	NA	R01_cb2037_c13/f1p0/5035	NA	NA
R01_cb15326_c6/f1p0/1229	R01_cb15326_c6/f1p0/1229	R01_cb15326_c6/f1p0/1229	R01_cb15326_c6/f1p0/1229	R01_cb15326_c6/f1p0/1229
R01_cb8564_c18631/f1p0/2064	NA	NA	NA	R01_cb8564_c18631/f1p0/2064
R01_cb5847_c7/f1p0/2904	NA	NA	NA	R01_cb5847_c7/f1p0/2904
R01_cb17102_c0/f3p0/423	R01_cb17102_c0/f3p0/423	R01_cb17102_c0/f3p0/423	R01_cb17102_c0/f3p0/423	R01_cb17102_c0/f3p0/423
R01_cb438_c7/f1p0/2770	NA	R01_cb438_c7/f1p0/2770	NA	R01_cb438_c7/f1p0/2770
R01_cb15116_c3/f1p0/1614	NA	NA	R01_cb15116_c3/f1p0/1614	R01_cb15116_c3/f1p0/1614

R01_cb10742_c5/f1p0/949	NA	NA	NA	R01_cb10742_c5/f1p0/949
R01_cb8907_c2/f1p0/3003	R01_cb8907_c2/f1p0/3003	R01_cb8907_c2/f1p0/3003	R01_cb8907_c2/f1p0/3003	R01_cb8907_c2/f1p0/3003
R01_cb5007_c5/f1p0/3201	NA	R01_cb5007_c5/f1p0/3201	NA	NA
R01_cb11572_c1/f1p0/2221	NA	NA	NA	R01_cb11572_c1/f1p0/2221
R01_cb16136_c0/f2p0/1338	NA	NA	NA	R01_cb16136_c0/f2p0/1338
R01_cb17238_c1/f1p0/888	NA	NA	R01_cb17238_c1/f1p0/888	R01_cb17238_c1/f1p0/888
R01_cb8564_c20155/f3p1/4475	NA	R01_cb8564_c20155/f3p1/4475	R01_cb8564_c20155/f3p1/4475	R01_cb8564_c20155/f3p1/4475
R01_cb16459_c2/f1p0/1204	NA	R01_cb16459_c2/f1p0/1204	NA	NA
R01_cb18047_c0/f2p0/1152	NA	R01_cb18047_c0/f2p0/1152	R01_cb18047_c0/f2p0/1152	R01_cb18047_c0/f2p0/1152
R01_cb8564_c3669/f1p0/1927	NA	R01_cb8564_c3669/f1p0/1927	NA	NA
R01_cb1170_c1/f1p0/2088	NA	R01_cb1170_c1/f1p0/2088	R01_cb1170_c1/f1p0/2088	NA
R01_cb8564_c68974/f3p1/3540	NA	NA	NA	R01_cb8564_c68974/f3p1/3540
R01_cb11261_c0/f3p0/700	NA	R01_cb11261_c0/f3p0/700	R01_cb11261_c0/f3p0/700	R01_cb11261_c0/f3p0/700
R01_cb7356_c6/f1p0/1732	NA	NA	NA	R01_cb7356_c6/f1p0/1732
R01_cb8564_c43449/f2p0/4408	NA	R01_cb8564_c43449/f2p0/4408	NA	NA
R01_cb16913_c0/f1p0/566	NA	R01_cb16913_c0/f1p0/566	NA	NA
R01_cb6671_c2/f1p0/1376	NA	R01_cb6671_c2/f1p0/1376	R01_cb6671_c2/f1p0/1376	R01_cb6671_c2/f1p0/1376
R01_cb3388_c4/f1p0/2798	NA	R01_cb3388_c4/f1p0/2798	NA	R01_cb3388_c4/f1p0/2798
R01_cb18541_c0/f1p0/867	R01_cb18541_c0/f1p0/867	R01_cb18541_c0/f1p0/867	NA	NA
R01_cb16799_c3/f1p0/758	NA	NA	NA	R01_cb16799_c3/f1p0/758
R01_cb9723_c1/f1p0/1995	NA	NA	NA	R01_cb9723_c1/f1p0/1995
R01_cb2445_c25/f1p0/2518	NA	R01_cb2445_c25/f1p0/2518	NA	R01_cb2445_c25/f1p0/2518
R01_cb13910_c20/f1p0/1107	R01_cb13910_c20/f1p0/1107	R01_cb13910_c20/f1p0/1107	R01_cb13910_c20/f1p0/1107	R01_cb13910_c20/f1p0/1107
R01_cb10594_c2/f1p0/2401	NA	NA	NA	R01_cb10594_c2/f1p0/2401
R01_cb8564_c2570/f1p0/2598	NA	R01_cb8564_c2570/f1p0/2598	NA	NA
R01_cb6170_c14/f1p0/2773	NA	NA	NA	R01_cb6170_c14/f1p0/2773

R01_cb850_c20/f1p0/1927	NA	NA	NA	R01_cb850_c20/f1p0/1927
R01_cb12315_c1/f1p1/1143	NA	R01_cb12315_c1/f1p1/1143	NA	R01_cb12315_c1/f1p1/1143
R01_cb15460_c2/f1p0/1111	NA	R01_cb15460_c2/f1p0/1111	NA	R01_cb15460_c2/f1p0/1111
R01_cb1952_c9/f1p0/4112	R01_cb1952_c9/f1p0/4112	R01_cb1952_c9/f1p0/4112	NA	NA
R01_cb12226_c2/f1p0/599	NA	R01_cb12226_c2/f1p0/599	NA	NA
R01_cb8564_c89499/f1p0/1910	NA	R01_cb8564_c89499/f1p0/1910	R01_cb8564_c89499/f1p0/1910	R01_cb8564_c89499/f1p0/1910
R01_cb1097_c8/f1p0/2417	NA	R01_cb1097_c8/f1p0/2417	NA	R01_cb1097_c8/f1p0/2417
R01_cb5443_c11/f1p0/2439	NA	R01_cb5443_c11/f1p0/2439	NA	NA
R01_cb12_c49/f1p0/3000	NA	R01_cb12_c49/f1p0/3000	NA	NA
R01_cb1230_c0/f2p0/4428	NA	R01_cb1230_c0/f2p0/4428	R01_cb1230_c0/f2p0/4428	R01_cb1230_c0/f2p0/4428
R01_cb10024_c326/f1p0/755	NA	R01_cb10024_c326/f1p0/755	NA	NA
R01_cb2654_c0/f1p0/3834	NA	NA	NA	R01_cb2654_c0/f1p0/3834
R01_cb18409_c28/f2p0/418	R01_cb18409_c28/f2p0/418	R01_cb18409_c28/f2p0/418	R01_cb18409_c28/f2p0/418	R01_cb18409_c28/f2p0/418
R01_cb9591_c3/f1p0/1917	NA	R01_cb9591_c3/f1p0/1917	NA	NA
R01_cb10211_c9/f1p0/1107	NA	NA	NA	R01_cb10211_c9/f1p0/1107
R01_cb13598_c2/f1p0/495	NA	R01_cb13598_c2/f1p0/495	NA	R01_cb13598_c2/f1p0/495
R01_cb8564_c35078/f2p0/2657	NA	NA	NA	R01_cb8564_c35078/f2p0/2657
R01_cb10359_c6/f1p0/1432	NA	NA	NA	R01_cb10359_c6/f1p0/1432
R01_cb12316_c5/f1p0/639	NA	R01_cb12316_c5/f1p0/639	NA	NA
R01_cb7035_c1/f1p0/4129	NA	R01_cb7035_c1/f1p0/4129	R01_cb7035_c1/f1p0/4129	NA
R01_cb16948_c1/f1p0/1422	NA	NA	NA	R01_cb16948_c1/f1p0/1422
R01_cb8564_c156631/f1p1/3967	NA	NA	NA	R01_cb8564_c156631/f1p1/3967
R01_cb18456_c7563/f1p0/579	R01_cb18456_c7563/f1p0/579	R01_cb18456_c7563/f1p0/579	R01_cb18456_c7563/f1p0/579	R01_cb18456_c7563/f1p0/579
R01_cb11046_c1/f3p1/496	NA	R01_cb11046_c1/f3p1/496	NA	R01_cb11046_c1/f3p1/496
R01_cb8564_c83212/f1p0/2606	NA	R01_cb8564_c83212/f1p0/2606	NA	R01_cb8564_c83212/f1p0/2606

R01_cb16806_c0/f1p0/715	NA	R01_cb16806_c0/f1p0/715	R01_cb16806_c0/f1p0/715	NA
R01_cb7223_c1/f1p0/2500	R01_cb7223_c1/f1p0/2500	R01_cb7223_c1/f1p0/2500	R01_cb7223_c1/f1p0/2500	R01_cb7223_c1/f1p0/2500
R01_cb18456_c6093/f1p0/302	R01_cb18456_c6093/f1p0/302	R01_cb18456_c6093/f1p0/302	R01_cb18456_c6093/f1p0/302	R01_cb18456_c6093/f1p0/302
R01_cb10778_c0/f1p0/798	NA	R01_cb10778_c0/f1p0/798	NA	NA
R01_cb13289_c11/f1p2/1730	NA	R01_cb13289_c11/f1p2/1730	R01_cb13289_c11/f1p2/1730	NA
R01_cb6959_c3/f1p0/2664	NA	NA	NA	R01_cb6959_c3/f1p0/2664
R01_cb14399_c0/f1p0/695	NA	R01_cb14399_c0/f1p0/695	NA	R01_cb14399_c0/f1p0/695
R01_cb8564_c122271/f1p0/2171	NA	R01_cb8564_c122271/f1p0/2171	NA	NA
R01_cb16833_c35/f1p1/1073	R01_cb16833_c35/f1p1/1073	R01_cb16833_c35/f1p1/1073	NA	R01_cb16833_c35/f1p1/1073
R01_cb12024_c3/f3p0/909	NA	NA	NA	R01_cb12024_c3/f3p0/909
R01_cb15473_c3/f1p0/1657	NA	R01_cb15473_c3/f1p0/1657	NA	NA
R01_cb3618_c25/f1p0/2107	NA	NA	NA	R01_cb3618_c25/f1p0/2107
R01_cb6399_c8/f1p0/1599	R01_cb6399_c8/f1p0/1599	R01_cb6399_c8/f1p0/1599	NA	NA
R01_cb12421_c44/f1p0/727	R01_cb12421_c44/f1p0/727	R01_cb12421_c44/f1p0/727	NA	NA
R01_cb12294_c3/f1p0/669	NA	R01_cb12294_c3/f1p0/669	NA	NA
R01_cb9402_c0/f1p0/2094	R01_cb9402_c0/f1p0/2094	R01_cb9402_c0/f1p0/2094	R01_cb9402_c0/f1p0/2094	R01_cb9402_c0/f1p0/2094
R01_cb3848_c7/f1p0/3003	NA	NA	NA	R01_cb3848_c7/f1p0/3003
R01_cb15930_c0/f2p0/418	NA	R01_cb15930_c0/f2p0/418	NA	R01_cb15930_c0/f2p0/418
R01_cb17371_c3/f1p0/636	NA	R01_cb17371_c3/f1p0/636	R01_cb17371_c3/f1p0/636	R01_cb17371_c3/f1p0/636
R01_cb14940_c3/f1p0/796	NA	R01_cb14940_c3/f1p0/796	R01_cb14940_c3/f1p0/796	R01_cb14940_c3/f1p0/796
R01_cb8564_c49593/f1p0/2995	NA	R01_cb8564_c49593/f1p0/2995	NA	NA
R01_cb16425_c8/f1p0/974	NA	R01_cb16425_c8/f1p0/974	R01_cb16425_c8/f1p0/974	R01_cb16425_c8/f1p0/974
R01_cb8148_c9/f1p0/1061	R01_cb8148_c9/f1p0/1061	R01_cb8148_c9/f1p0/1061	R01_cb8148_c9/f1p0/1061	R01_cb8148_c9/f1p0/1061
R01_cb14344_c7/f1p0/1144	NA	R01_cb14344_c7/f1p0/1144	R01_cb14344_c7/f1p0/1144	R01_cb14344_c7/f1p0/1144
R01_cb18456_c1862/f1p0/1739	R01_cb18456_c1862/f1p0/173	R01_cb18456_c1862/f1p0/1739	R01_cb18456_c1862/f1p0/1739	R01_cb18456_c1862/f1p0/1739

	9			
R01_cb6948_c5/f1p0/2544	NA	NA	NA	R01_cb6948_c5/f1p0/2544
R01_cb8564_c81685/f1p0/2236	NA	R01_cb8564_c81685/f1p0/2236	R01_cb8564_c81685/f1p0/2236	R01_cb8564_c81685/f1p0/2236
R01_cb17678_c1/f1p0/335	R01_cb17678_c1/f1p0/335	R01_cb17678_c1/f1p0/335	R01_cb17678_c1/f1p0/335	R01_cb17678_c1/f1p0/335
R01_cb8564_c4864/f1p0/3201	R01_cb8564_c4864/f1p0/3201	R01_cb8564_c4864/f1p0/3201	NA	R01_cb8564_c4864/f1p0/3201
R01_cb8564_c13085/f1p0/2474	NA	NA	NA	R01_cb8564_c13085/f1p0/2474
R01_cb11128_c7/f1p0/1535	NA	NA	NA	R01_cb11128_c7/f1p0/1535
R01_cb8746_c1/f1p0/2389	NA	NA	NA	R01_cb8746_c1/f1p0/2389
R01_cb8564_c121285/f1p0/2852	NA	R01_cb8564_c121285/f1p0/2852	NA	R01_cb8564_c121285/f1p0/2852
R01_cb5000_c25/f1p0/5093	NA	R01_cb5000_c25/f1p0/5093	R01_cb5000_c25/f1p0/5093	NA
R01_cb13161_c20/f1p0/560	R01_cb13161_c20/f1p0/560	R01_cb13161_c20/f1p0/560	R01_cb13161_c20/f1p0/560	R01_cb13161_c20/f1p0/560
R01_cb17356_c0/f1p0/1272	NA	NA	NA	R01_cb17356_c0/f1p0/1272
R01_cb18526_c10/f1p0/647	NA	R01_cb18526_c10/f1p0/647	NA	NA
R01_cb8564_c130175/f1p0/1893	NA	R01_cb8564_c130175/f1p0/1893	NA	NA
R01_cb10496_c2/f1p0/2309	NA	NA	NA	R01_cb10496_c2/f1p0/2309
R01_cb9858_c2/f1p0/2061	NA	R01_cb9858_c2/f1p0/2061	R01_cb9858_c2/f1p0/2061	R01_cb9858_c2/f1p0/2061
R01_cb14148_c0/f1p0/1292	NA	NA	R01_cb14148_c0/f1p0/1292	R01_cb14148_c0/f1p0/1292
R01_cb8564_c17126/f1p0/3574	NA	R01_cb8564_c17126/f1p0/3574	NA	NA
R01_cb4347_c1/f2p0/894	NA	R01_cb4347_c1/f2p0/894	R01_cb4347_c1/f2p0/894	NA
R01_cb10237_c1/f1p0/2822	NA	NA	NA	R01_cb10237_c1/f1p0/2822
R01_cb16370_c3/f1p0/633	NA	R01_cb16370_c3/f1p0/633	NA	R01_cb16370_c3/f1p0/633
R01_cb17154_c0/f2p0/1071	R01_cb17154_c0/f2p0/1071	R01_cb17154_c0/f2p0/1071	R01_cb17154_c0/f2p0/1071	R01_cb17154_c0/f2p0/1071
R01_cb4062_c1/f1p0/3007	R01_cb4062_c1/f1p0/3007	R01_cb4062_c1/f1p0/3007	R01_cb4062_c1/f1p0/3007	R01_cb4062_c1/f1p0/3007
R01_cb15473_c5/f1p0/1662	R01_cb15473_c5/f1p0/1662	R01_cb15473_c5/f1p0/1662	NA	R01_cb15473_c5/f1p0/1662

R01_cb18456_c4760/f1p3/486	R01_cb18456_c4760/f1p3/486	R01_cb18456_c4760/f1p3/486	NA	R01_cb18456_c4760/f1p3/486
R01_cb6456_c3/f1p0/2341	R01_cb6456_c3/f1p0/2341	R01_cb6456_c3/f1p0/2341	R01_cb6456_c3/f1p0/2341	R01_cb6456_c3/f1p0/2341
R01_cb15950_c5/f1p0/1531	NA	NA	NA	R01_cb15950_c5/f1p0/1531
R01_cb12811_c9/f1p0/925	R01_cb12811_c9/f1p0/925	R01_cb12811_c9/f1p0/925	R01_cb12811_c9/f1p0/925	R01_cb12811_c9/f1p0/925
R01_cb18409_c73/f1p0/406	R01_cb18409_c73/f1p0/406	R01_cb18409_c73/f1p0/406	R01_cb18409_c73/f1p0/406	R01_cb18409_c73/f1p0/406
R01_cb9448_c11/f2p0/1755	NA	NA	NA	R01_cb9448_c11/f2p0/1755
R01_cb8564_c69061/f2p0/2215	NA	NA	R01_cb8564_c69061/f2p0/2215	R01_cb8564_c69061/f2p0/2215
R01_cb4054_c2/f1p0/2025	NA	R01_cb4054_c2/f1p0/2025	NA	NA
R01_cb8831_c12/f1p1/2130	NA	NA	NA	R01_cb8831_c12/f1p1/2130
R01_cb8564_c90750/f1p0/2475	NA	NA	NA	R01_cb8564_c90750/f1p0/2475
R01_cb13178_c21/f1p0/503	NA	R01_cb13178_c21/f1p0/503	NA	NA
R01_cb17807_c0/f1p0/822	NA	NA	NA	R01_cb17807_c0/f1p0/822
R01_cb17381_c6/f1p0/1452	NA	R01_cb17381_c6/f1p0/1452	NA	R01_cb17381_c6/f1p0/1452
R01_cb17197_c2/f1p0/403	R01_cb17197_c2/f1p0/403	R01_cb17197_c2/f1p0/403	R01_cb17197_c2/f1p0/403	R01_cb17197_c2/f1p0/403
R01_cb16460_c2/f1p0/604	NA	R01_cb16460_c2/f1p0/604	R01_cb16460_c2/f1p0/604	R01_cb16460_c2/f1p0/604
R01_cb8564_c88874/f1p0/3390	NA	R01_cb8564_c88874/f1p0/3390	R01_cb8564_c88874/f1p0/3390	NA
R01_cb12228_c10/f1p0/1781	NA	R01_cb12228_c10/f1p0/1781	NA	NA
R01_cb16450_c0/f5p0/1015	NA	R01_cb16450_c0/f5p0/1015	NA	NA
R01_cb8569_c13/f1p0/825	R01_cb8569_c13/f1p0/825	R01_cb8569_c13/f1p0/825	NA	R01_cb8569_c13/f1p0/825
R01_cb8564_c120546/f1p0/2467	R01_cb8564_c120546/f1p0/2467	R01_cb8564_c120546/f1p0/2467	NA	NA
R01_cb392_c0/f2p1/1752	R01_cb392_c0/f2p1/1752	R01_cb392_c0/f2p1/1752	R01_cb392_c0/f2p1/1752	R01_cb392_c0/f2p1/1752
R01_cb17239_c1/f1p0/566	R01_cb17239_c1/f1p0/566	R01_cb17239_c1/f1p0/566	NA	R01_cb17239_c1/f1p0/566
R01_cb7390_c12/f1p0/1196	NA	R01_cb7390_c12/f1p0/1196	NA	NA
R01_cb3502_c6/f1p0/2135	NA	NA	NA	R01_cb3502_c6/f1p0/2135
R01_cb18523_c2/f1p0/5611	NA	R01_cb18523_c2/f1p0/5611	NA	NA

R01_cb15591_c3/f1p1/907	NA	R01_cb15591_c3/f1p1/907	NA	NA
R01_cb13178_c16/f1p0/584	NA	R01_cb13178_c16/f1p0/584	NA	NA
R01_cb14023_c1/f1p0/818	NA	NA	NA	R01_cb14023_c1/f1p0/818
R01_cb8564_c128844/f1p0/2505	NA	R01_cb8564_c128844/f1p0/2505	NA	R01_cb8564_c128844/f1p0/2505
R01_cb8564_c117557/f1p0/2730	NA	R01_cb8564_c117557/f1p0/2730	NA	NA
R01_cb7982_c4/f1p0/1981	NA	NA	NA	R01_cb7982_c4/f1p0/1981
R01_cb17813_c0/f1p0/1627	NA	R01_cb17813_c0/f1p0/1627	NA	NA
R01_cb2804_c73/f1p0/3721	NA	R01_cb2804_c73/f1p0/3721	NA	NA
R01_cb3861_c0/f1p0/3506	NA	R01_cb3861_c0/f1p0/3506	NA	NA
R01_cb7806_c5/f1p0/1767	NA	R01_cb7806_c5/f1p0/1767	R01_cb7806_c5/f1p0/1767	R01_cb7806_c5/f1p0/1767
R01_cb6950_c35/f1p0/1271	NA	NA	NA	R01_cb6950_c35/f1p0/1271
R01_cb10536_c1/f1p0/3926	NA	R01_cb10536_c1/f1p0/3926	R01_cb10536_c1/f1p0/3926	R01_cb10536_c1/f1p0/3926
R01_cb17737_c0/f1p0/1106	NA	R01_cb17737_c0/f1p0/1106	R01_cb17737_c0/f1p0/1106	NA
R01_cb3702_c2/f1p0/3550	NA	R01_cb3702_c2/f1p0/3550	NA	NA
R01_cb3716_c10/f1p0/3127	NA	NA	NA	R01_cb3716_c10/f1p0/3127
R01_cb8564_c4698/f1p0/3199	NA	R01_cb8564_c4698/f1p0/3199	NA	NA
R01_cb11450_c1/f1p0/2051	NA	NA	R01_cb11450_c1/f1p0/2051	R01_cb11450_c1/f1p0/2051
R01_cb14263_c0/f2p0/1061	R01_cb14263_c0/f2p0/1061	R01_cb14263_c0/f2p0/1061	R01_cb14263_c0/f2p0/1061	R01_cb14263_c0/f2p0/1061
R01_cb8564_c11142/f1p0/2111	NA	NA	NA	R01_cb8564_c11142/f1p0/2111
R01_cb16765_c1/f1p0/782	NA	NA	R01_cb16765_c1/f1p0/782	R01_cb16765_c1/f1p0/782
R01_cb8564_c626/f4p1/2238	NA	NA	NA	R01_cb8564_c626/f4p1/2238
R01_cb10818_c2/f1p0/3390	NA	R01_cb10818_c2/f1p0/3390	NA	NA
R01_cb16856_c2/f2p0/1185	NA	NA	NA	R01_cb16856_c2/f2p0/1185
R01_cb10627_c1/f2p0/763	NA	R01_cb10627_c1/f2p0/763	NA	NA

R01_cb6802_c62/f1p0/2495	R01_cb6802_c62/f1p0/2495	R01_cb6802_c62/f1p0/2495	R01_cb6802_c62/f1p0/2495	NA
R01_cb625_c14/f1p0/2067	R01_cb625_c14/f1p0/2067	R01_cb625_c14/f1p0/2067	NA	R01_cb625_c14/f1p0/2067
R01_cb16722_c0/f2p0/511	R01_cb16722_c0/f2p0/511	R01_cb16722_c0/f2p0/511	NA	R01_cb16722_c0/f2p0/511
R01_cb14878_c0/f1p0/762	NA	R01_cb14878_c0/f1p0/762	R01_cb14878_c0/f1p0/762	R01_cb14878_c0/f1p0/762
R01_cb7171_c18/f1p0/2147	NA	NA	NA	R01_cb7171_c18/f1p0/2147
R01_cb8564_c116398/f1p1/2024	NA	R01_cb8564_c116398/f1p1/2024	R01_cb8564_c116398/f1p1/2024	NA
R01_cb9987_c1/f1p1/1892	NA	R01_cb9987_c1/f1p1/1892	NA	R01_cb9987_c1/f1p1/1892
R01_cb8564_c1804/f1p0/3212	NA	R01_cb8564_c1804/f1p0/3212	R01_cb8564_c1804/f1p0/3212	R01_cb8564_c1804/f1p0/3212
R01_cb2243_c0/f1p0/3999	NA	NA	NA	R01_cb2243_c0/f1p0/3999
R01_cb8564_c123066/f1p0/2932	NA	NA	R01_cb8564_c123066/f1p0/2932	R01_cb8564_c123066/f1p0/2932
R01_cb8564_c41379/f1p0/3270	R01_cb8564_c41379/f1p0/3270	R01_cb8564_c41379/f1p0/3270	R01_cb8564_c41379/f1p0/3270	R01_cb8564_c41379/f1p0/3270
R01_cb3992_c1/f1p0/3457	NA	NA	NA	R01_cb3992_c1/f1p0/3457
R01_cb1119_c15/f1p0/2159	NA	R01_cb1119_c15/f1p0/2159	NA	NA
R01_cb6499_c15/f1p1/1972	NA	R01_cb6499_c15/f1p1/1972	NA	NA
R01_cb14751_c2/f1p0/688	NA	R01_cb14751_c2/f1p0/688	NA	NA
R01_cb640_c6/f1p0/3220	NA	NA	R01_cb640_c6/f1p0/3220	NA
R01_cb5378_c1/f1p0/2801	NA	NA	NA	R01_cb5378_c1/f1p0/2801
R01_cb9988_c3/f1p0/1707	R01_cb9988_c3/f1p0/1707	NA	NA	NA
R01_cb1863_c29/f1p0/1662	NA	NA	NA	R01_cb1863_c29/f1p0/1662
R01_cb6615_c29/f1p0/857	NA	R01_cb6615_c29/f1p0/857	R01_cb6615_c29/f1p0/857	R01_cb6615_c29/f1p0/857
R01_cb18456_c6432/f1p0/607	R01_cb18456_c6432/f1p0/607	R01_cb18456_c6432/f1p0/607	NA	NA
R01_cb1839_c5/f1p0/4173	NA	NA	NA	R01_cb1839_c5/f1p0/4173
R01_cb9409_c3/f1p0/1367	NA	NA	NA	R01_cb9409_c3/f1p0/1367

R01_cb8564_c72986/f1p0/2988	NA	NA	NA	R01_cb8564_c72986/f1p0/2988
R01_cb3863_c8/f3p0/3240	NA	NA	NA	R01_cb3863_c8/f3p0/3240
R01_cb17315_c1/f1p0/604	NA	R01_cb17315_c1/f1p0/604	NA	NA
R01_cb8564_c114001/f1p0/2053	NA	R01_cb8564_c114001/f1p0/2053	NA	NA
R01_cb4085_c2/f1p0/1476	NA	R01_cb4085_c2/f1p0/1476	NA	NA
R01_cb14328_c3/f1p0/802	NA	NA	R01_cb14328_c3/f1p0/802	R01_cb14328_c3/f1p0/802
R01_cb8564_c38417/f1p0/2173	NA	NA	NA	R01_cb8564_c38417/f1p0/2173
R01_cb6419_c21/f1p0/871	NA	R01_cb6419_c21/f1p0/871	NA	NA
R01_cb14907_c0/f1p0/1589	NA	R01_cb14907_c0/f1p0/1589	NA	NA
R01_cb8564_c15719/f1p0/1926	NA	NA	NA	R01_cb8564_c15719/f1p0/1926
R01_cb13463_c16/f1p0/418	R01_cb13463_c16/f1p0/418	R01_cb13463_c16/f1p0/418	R01_cb13463_c16/f1p0/418	R01_cb13463_c16/f1p0/418
R01_cb16819_c2/f7p1/661	NA	NA	NA	R01_cb16819_c2/f7p1/661
R01_cb10540_c4/f1p0/2579	NA	R01_cb10540_c4/f1p0/2579	NA	NA
R01_cb18752_c1/f1p0/1296	NA	R01_cb18752_c1/f1p0/1296	NA	NA
R01_cb15094_c2/f1p0/1108	NA	NA	NA	R01_cb15094_c2/f1p0/1108
R01_cb2970_c2/f2p0/415	NA	R01_cb2970_c2/f2p0/415	NA	R01_cb2970_c2/f2p0/415
R01_cb1905_c25/f1p0/2081	NA	R01_cb1905_c25/f1p0/2081	R01_cb1905_c25/f1p0/2081	NA
R01_cb8120_c6/f1p0/2284	NA	NA	NA	R01_cb8120_c6/f1p0/2284
R01_cb17610_c6/f1p0/1790	NA	NA	NA	R01_cb17610_c6/f1p0/1790
R01_cb5452_c0/f1p0/3097	NA	NA	NA	R01_cb5452_c0/f1p0/3097
R01_cb17000_c0/f2p0/491	R01_cb17000_c0/f2p0/491	R01_cb17000_c0/f2p0/491	R01_cb17000_c0/f2p0/491	R01_cb17000_c0/f2p0/491
R01_cb1155_c18/f1p1/4197	NA	NA	NA	R01_cb1155_c18/f1p1/4197
R01_cb13884_c1/f9p0/539	R01_cb13884_c1/f9p0/539	NA	R01_cb13884_c1/f9p0/539	R01_cb13884_c1/f9p0/539
R01_cb10795_c0/f1p0/1581	NA	NA	NA	R01_cb10795_c0/f1p0/1581
R01_cb10993_c2/f1p0/1320	NA	NA	NA	R01_cb10993_c2/f1p0/1320

R01_cb3808_c2/f1p0/3519	NA	NA	NA	R01_cb3808_c2/f1p0/3519
R01_cb8564_c68676/f6p1/2219	NA	R01_cb8564_c68676/f6p1/2219	R01_cb8564_c68676/f6p1/2219	R01_cb8564_c68676/f6p1/2219
R01_cb7533_c1/f4p0/2243	NA	NA	NA	R01_cb7533_c1/f4p0/2243
R01_cb8564_c78463/f1p0/2428	NA	R01_cb8564_c78463/f1p0/2428	NA	R01_cb8564_c78463/f1p0/2428
R01_cb18456_c5763/f1p0/1775	NA	R01_cb18456_c5763/f1p0/1775	NA	R01_cb18456_c5763/f1p0/1775
R01_cb3320_c16/f1p0/2305	NA	R01_cb3320_c16/f1p0/2305	R01_cb3320_c16/f1p0/2305	R01_cb3320_c16/f1p0/2305
R01_cb13165_c1/f1p0/533	R01_cb13165_c1/f1p0/533	R01_cb13165_c1/f1p0/533	NA	R01_cb13165_c1/f1p0/533
R01_cb8970_c2/f1p0/1764	NA	R01_cb8970_c2/f1p0/1764	NA	NA
R01_cb8564_c23681/f1p0/2579	NA	R01_cb8564_c23681/f1p0/2579	NA	R01_cb8564_c23681/f1p0/2579
R01_cb15853_c1/f6p1/539	NA	R01_cb15853_c1/f6p1/539	NA	R01_cb15853_c1/f6p1/539
R01_cb5674_c0/f1p0/3039	R01_cb5674_c0/f1p0/3039	R01_cb5674_c0/f1p0/3039	R01_cb5674_c0/f1p0/3039	NA
R01_cb14656_c2/f1p0/589	R01_cb14656_c2/f1p0/589	R01_cb14656_c2/f1p0/589	R01_cb14656_c2/f1p0/589	R01_cb14656_c2/f1p0/589
R01_cb13488_c1/f2p0/745	NA	NA	NA	R01_cb13488_c1/f2p0/745
R01_cb12893_c14/f2p0/815	NA	R01_cb12893_c14/f2p0/815	R01_cb12893_c14/f2p0/815	NA
R01_cb7254_c9/f1p0/2112	NA	NA	NA	R01_cb7254_c9/f1p0/2112
R01_cb4609_c4/f1p0/756	NA	R01_cb4609_c4/f1p0/756	NA	R01_cb4609_c4/f1p0/756
R01_cb8564_c91034/f1p1/3457	R01_cb8564_c91034/f1p1/3457	R01_cb8564_c91034/f1p1/3457	NA	NA
R01_cb18830_c1/f1p0/6537	NA	R01_cb18830_c1/f1p0/6537	NA	NA
R01_cb12174_c0/f3p0/655	NA	R01_cb12174_c0/f3p0/655	NA	R01_cb12174_c0/f3p0/655
R01_cb11204_c1/f1p0/2933	NA	R01_cb11204_c1/f1p0/2933	NA	R01_cb11204_c1/f1p0/2933
R01_cb8122_c8/f10p0/2218	NA	NA	NA	R01_cb8122_c8/f10p0/2218
R01_cb2806_c1/f1p0/3022	NA	NA	NA	R01_cb2806_c1/f1p0/3022
R01_cb18456_c7565/f1p0/1269	R01_cb18456_c7565/f1p0/1269	R01_cb18456_c7565/f1p0/1269	R01_cb18456_c7565/f1p0/1269	R01_cb18456_c7565/f1p0/1269
R01_cb16979_c11/f1p0/914	NA	R01_cb16979_c11/f1p0/914	NA	NA

R01_cb8564_c77752/f1p1/3809	NA	R01_cb8564_c77752/f1p1/3809	NA	NA
R01_cb15588_c0/f1p0/577	NA	R01_cb15588_c0/f1p0/577	NA	R01_cb15588_c0/f1p0/577
R01_cb13184_c0/f2p0/607	NA	R01_cb13184_c0/f2p0/607	R01_cb13184_c0/f2p0/607	R01_cb13184_c0/f2p0/607
R01_cb3536_c2/f1p0/3608	NA	R01_cb3536_c2/f1p0/3608	NA	R01_cb3536_c2/f1p0/3608
R01_cb8564_c99491/f1p0/2703	NA	R01_cb8564_c99491/f1p0/2703	NA	R01_cb8564_c99491/f1p0/2703
R01_cb8564_c46039/f1p0/2420	NA	NA	R01_cb8564_c46039/f1p0/2420	R01_cb8564_c46039/f1p0/2420
R01_cb12496_c12/f1p0/774	NA	R01_cb12496_c12/f1p0/774	NA	NA
R01_cb8564_c11099/f1p1/4643	NA	R01_cb8564_c11099/f1p1/4643	NA	NA
R01_cb5943_c6/f1p0/1364	NA	NA	NA	R01_cb5943_c6/f1p0/1364
R01_cb12552_c3/f1p0/779	NA	NA	NA	R01_cb12552_c3/f1p0/779
R01_cb14019_c6/f1p0/1358	R01_cb14019_c6/f1p0/1358	R01_cb14019_c6/f1p0/1358	R01_cb14019_c6/f1p0/1358	R01_cb14019_c6/f1p0/1358
R01_cb8564_c85207/f1p0/2727	NA	R01_cb8564_c85207/f1p0/2727	NA	NA
R01_cb5249_c3/f1p0/3143	NA	R01_cb5249_c3/f1p0/3143	NA	R01_cb5249_c3/f1p0/3143
R01_cb11535_c0/f1p0/725	R01_cb11535_c0/f1p0/725	R01_cb11535_c0/f1p0/725	R01_cb11535_c0/f1p0/725	R01_cb11535_c0/f1p0/725
R01_cb17869_c0/f2p0/1665	NA	NA	NA	R01_cb17869_c0/f2p0/1665
R01_cb16993_c2/f1p0/1461	NA	NA	R01_cb16993_c2/f1p0/1461	NA
R01_cb8564_c24445/f1p0/4124	NA	NA	NA	R01_cb8564_c24445/f1p0/4124
R01_cb435_c5/f1p0/1428	NA	NA	NA	R01_cb435_c5/f1p0/1428
R01_cb8564_c69065/f2p0/3980	NA	R01_cb8564_c69065/f2p0/3980	R01_cb8564_c69065/f2p0/3980	R01_cb8564_c69065/f2p0/3980
R01_cb5862_c1/f1p0/1944	NA	R01_cb5862_c1/f1p0/1944	R01_cb5862_c1/f1p0/1944	R01_cb5862_c1/f1p0/1944
R01_cb2845_c11/f1p0/3300	NA	NA	NA	R01_cb2845_c11/f1p0/3300
R01_cb8564_c121611/f1p0/2323	NA	R01_cb8564_c121611/f1p0/2323	R01_cb8564_c121611/f1p0/2323	R01_cb8564_c121611/f1p0/2323
R01_cb8564_c81815/f1p0/2165	NA	R01_cb8564_c81815/f1p0/2165	R01_cb8564_c81815/f1p0/2165	R01_cb8564_c81815/f1p0/2165
R01_cb2929_c8/f1p0/1889	NA	NA	R01_cb2929_c8/f1p0/1889	NA
R01_cb14634_c28/f1p1/2221	NA	NA	NA	R01_cb14634_c28/f1p1/2221

R01_cb7947_c5/f1p1/2305	NA	NA	NA	R01_cb7947_c5/f1p1/2305
R01_cb4370_c6/f1p0/2015	NA	NA	NA	R01_cb4370_c6/f1p0/2015
R01_cb9154_c6/f1p0/1781	NA	NA	NA	R01_cb9154_c6/f1p0/1781
R01_cb2826_c10/f1p0/2973	R01_cb2826_c10/f1p0/2973	R01_cb2826_c10/f1p0/2973	NA	NA
R01_cb15778_c4/f1p0/1256	NA	NA	NA	R01_cb15778_c4/f1p0/1256
R01_cb5026_c2/f1p0/3198	NA	R01_cb5026_c2/f1p0/3198	NA	R01_cb5026_c2/f1p0/3198
R01_cb4166_c5/f1p0/2886	NA	R01_cb4166_c5/f1p0/2886	NA	NA
R01_cb644_c13/f1p0/1014	NA	NA	NA	R01_cb644_c13/f1p0/1014
R01_cb8441_c7/f1p0/1376	NA	NA	NA	R01_cb8441_c7/f1p0/1376
R01_cb8564_c935/f2p0/2617	NA	R01_cb8564_c935/f2p0/2617	NA	NA
R01_cb10638_c1/f1p0/3205	NA	R01_cb10638_c1/f1p0/3205	NA	NA
R01_cb10801_c4/f1p0/551	NA	R01_cb10801_c4/f1p0/551	NA	NA
R01_cb3618_c23/f1p0/2587	NA	NA	NA	R01_cb3618_c23/f1p0/2587
R01_cb11924_c23/f157p2/558	R01_cb11924_c23/f157p2/558	R01_cb11924_c23/f157p2/558	R01_cb11924_c23/f157p2/558	NA
R01_cb12329_c13/f1p0/1468	NA	NA	NA	R01_cb12329_c13/f1p0/1468
R01_cb10272_c30/f1p2/1750	NA	NA	NA	R01_cb10272_c30/f1p2/1750
R01_cb17119_c2/f4p0/717	NA	NA	NA	R01_cb17119_c2/f4p0/717
R01_cb5976_c2/f1p0/2282	NA	R01_cb5976_c2/f1p0/2282	NA	R01_cb5976_c2/f1p0/2282
R01_cb13803_c6/f1p0/645	NA	R01_cb13803_c6/f1p0/645	NA	NA
R01_cb3000_c2/f1p0/2342	NA	R01_cb3000_c2/f1p0/2342	R01_cb3000_c2/f1p0/2342	R01_cb3000_c2/f1p0/2342
R01_cb6935_c0/f1p0/2736	NA	R01_cb6935_c0/f1p0/2736	NA	NA
R01_cb8564_c1547/f1p0/2233	R01_cb8564_c1547/f1p0/2233	NA	NA	NA
R01_cb708_c5/f1p0/2959	R01_cb708_c5/f1p0/2959	R01_cb708_c5/f1p0/2959	R01_cb708_c5/f1p0/2959	R01_cb708_c5/f1p0/2959
R01_cb18456_c3894/f1p0/1063	R01_cb18456_c3894/f1p0/1063	R01_cb18456_c3894/f1p0/1063	NA	R01_cb18456_c3894/f1p0/1063
R01_cb9928_c5/f1p0/2028	NA	NA	NA	R01_cb9928_c5/f1p0/2028

R01_cb12641_c17/f1p0/888	NA	NA	NA	R01_cb12641_c17/f1p0/888
R01_cb380_c7/f1p0/4788	NA	R01_cb380_c7/f1p0/4788	NA	NA
R01_cb9112_c0/f1p0/2186	NA	R01_cb9112_c0/f1p0/2186	R01_cb9112_c0/f1p0/2186	R01_cb9112_c0/f1p0/2186
R01_cb6737_c12/f1p0/4635	NA	R01_cb6737_c12/f1p0/4635	NA	R01_cb6737_c12/f1p0/4635
R01_cb10893_c1/f1p0/2844	NA	R01_cb10893_c1/f1p0/2844	NA	NA
R01_cb13856_c1/f1p0/573	NA	NA	R01_cb13856_c1/f1p0/573	NA
R01_cb6446_c54/f1p0/1098	NA	R01_cb6446_c54/f1p0/1098	NA	NA
R01_cb18001_c1/f1p1/909	NA	NA	NA	R01_cb18001_c1/f1p1/909
R01_cb13178_c25/f10p0/546	NA	R01_cb13178_c25/f10p0/546	NA	NA
R01_cb9532_c4/f1p0/1876	NA	R01_cb9532_c4/f1p0/1876	NA	NA
R01_cb8564_c78182/f1p0/2217	NA	R01_cb8564_c78182/f1p0/2217	R01_cb8564_c78182/f1p0/2217	R01_cb8564_c78182/f1p0/2217
R01_cb12329_c22/f1p0/1574	NA	NA	NA	R01_cb12329_c22/f1p0/1574
R01_cb12837_c22/f1p3/718	NA	R01_cb12837_c22/f1p3/718	NA	NA
R01_cb10015_c418/f1p1/722	NA	R01_cb10015_c418/f1p1/722	NA	NA
R01_cb2022_c16/f1p0/1807	NA	NA	NA	R01_cb2022_c16/f1p0/1807
R01_cb16633_c1/f2p0/1768	R01_cb16633_c1/f2p0/1768	R01_cb16633_c1/f2p0/1768	R01_cb16633_c1/f2p0/1768	R01_cb16633_c1/f2p0/1768
R01_cb15739_c2/f1p0/1108	NA	R01_cb15739_c2/f1p0/1108	NA	NA
R01_cb3632_c1/f1p0/2963	NA	NA	NA	R01_cb3632_c1/f1p0/2963
R01_cb2192_c1/f1p0/3640	NA	NA	NA	R01_cb2192_c1/f1p0/3640
R01_cb5987_c0/f1p0/2971	NA	NA	NA	R01_cb5987_c0/f1p0/2971
R01_cb13764_c1/f1p0/821	NA	NA	R01_cb13764_c1/f1p0/821	R01_cb13764_c1/f1p0/821
R01_cb13202_c20/f1p0/706	NA	R01_cb13202_c20/f1p0/706	NA	NA
R01_cb8751_c6/f1p0/2115	NA	NA	NA	R01_cb8751_c6/f1p0/2115
R01_cb17077_c0/f1p0/1093	NA	NA	NA	R01_cb17077_c0/f1p0/1093
R01_cb8400_c1/f1p1/1838	NA	NA	NA	R01_cb8400_c1/f1p1/1838
R01_cb8564_c12343/f1p0/3935	NA	R01_cb8564_c12343/f1p0/3935	NA	NA

R01_cb3580_c61/f1p1/2489	NA	R01_cb3580_c61/f1p1/2489	NA	NA
R01_cb9859_c4/f1p0/522	NA	NA	NA	R01_cb9859_c4/f1p0/522
R01_cb10936_c3/f1p0/1966	NA	NA	NA	R01_cb10936_c3/f1p0/1966
R01_cb18456_c7990/f4p1/640	NA	R01_cb18456_c7990/f4p1/640	R01_cb18456_c7990/f4p1/640	R01_cb18456_c7990/f4p1/640
R01_cb18456_c4573/f1p1/644	NA	NA	NA	R01_cb18456_c4573/f1p1/644
R01_cb15945_c1/f1p0/616	NA	NA	NA	R01_cb15945_c1/f1p0/616
R01_cb16946_c0/f2p0/888	NA	NA	NA	R01_cb16946_c0/f2p0/888
R01_cb14786_c1/f1p0/356	R01_cb14786_c1/f1p0/356	R01_cb14786_c1/f1p0/356	R01_cb14786_c1/f1p0/356	R01_cb14786_c1/f1p0/356
R01_cb8564_c121996/f1p0/2142	NA	R01_cb8564_c121996/f1p0/2142	NA	NA
R01_cb15730_c4/f1p0/669	NA	R01_cb15730_c4/f1p0/669	NA	R01_cb15730_c4/f1p0/669
R01_cb8660_c15/f1p0/5408	NA	NA	NA	R01_cb8660_c15/f1p0/5408
R01_cb18456_c7490/f1p0/1391	NA	NA	NA	R01_cb18456_c7490/f1p0/1391
R01_cb3582_c10/f1p1/2415	NA	NA	NA	R01_cb3582_c10/f1p1/2415
R01_cb6278_c9/f1p0/2648	NA	NA	NA	R01_cb6278_c9/f1p0/2648
R01_cb2570_c12/f1p0/2654	NA	NA	NA	R01_cb2570_c12/f1p0/2654
R01_cb8564_c79763/f1p0/4065	NA	R01_cb8564_c79763/f1p0/4065	R01_cb8564_c79763/f1p0/4065	NA
R01_cb8564_c139516/f1p0/1964	NA	R01_cb8564_c139516/f1p0/1964	NA	NA
R01_cb7757_c13/f1p0/609	NA	NA	R01_cb7757_c13/f1p0/609	R01_cb7757_c13/f1p0/609
R01_cb6147_c36/f1p0/2635	NA	R01_cb6147_c36/f1p0/2635	NA	NA
R01_cb8564_c85066/f1p0/1989	NA	R01_cb8564_c85066/f1p0/1989	NA	R01_cb8564_c85066/f1p0/1989
R01_cb8564_c69138/f4p0/2133	R01_cb8564_c69138/f4p0/2133	R01_cb8564_c69138/f4p0/2133	NA	NA
R01_cb8564_c69666/f1p0/4138	NA	R01_cb8564_c69666/f1p0/4138	R01_cb8564_c69666/f1p0/4138	R01_cb8564_c69666/f1p0/4138
R01_cb4354_c14/f1p0/3085	NA	NA	NA	R01_cb4354_c14/f1p0/3085

R01_cb5641_c3/f1p0/2521	NA	NA	NA	R01_cb5641_c3/f1p0/2521
R01_cb9061_c17/f1p0/2115	NA	NA	NA	R01_cb9061_c17/f1p0/2115
R01_cb14316_c14/f1p0/471	R01_cb14316_c14/f1p0/471	R01_cb14316_c14/f1p0/471	R01_cb14316_c14/f1p0/471	R01_cb14316_c14/f1p0/471
R01_cb9506_c4/f2p0/1046	NA	NA	NA	R01_cb9506_c4/f2p0/1046
R01_cb8564_c47695/f1p0/2559	R01_cb8564_c47695/f1p0/2559	R01_cb8564_c47695/f1p0/2559	R01_cb8564_c47695/f1p0/2559	R01_cb8564_c47695/f1p0/2559
R01_cb2804_c111/f1p0/2745	NA	R01_cb2804_c111/f1p0/2745	R01_cb2804_c111/f1p0/2745	R01_cb2804_c111/f1p0/2745
R01_cb1894_c3/f1p0/3285	R01_cb1894_c3/f1p0/3285	R01_cb1894_c3/f1p0/3285	NA	R01_cb1894_c3/f1p0/3285
R01_cb18409_c90/f1p0/1029	R01_cb18409_c90/f1p0/1029	R01_cb18409_c90/f1p0/1029	R01_cb18409_c90/f1p0/1029	R01_cb18409_c90/f1p0/1029
R01_cb18100_c0/f1p0/1804	NA	NA	NA	R01_cb18100_c0/f1p0/1804
R01_cb8136_c4/f1p0/2437	NA	R01_cb8136_c4/f1p0/2437	NA	NA
R01_cb8564_c17217/f1p0/4378	NA	NA	NA	R01_cb8564_c17217/f1p0/4378
R01_cb5040_c17/f1p1/3129	NA	NA	NA	R01_cb5040_c17/f1p1/3129
R01_cb16752_c0/f4p2/1249	NA	NA	NA	R01_cb16752_c0/f4p2/1249
R01_cb18319_c0/f1p0/1238	R01_cb18319_c0/f1p0/1238	R01_cb18319_c0/f1p0/1238	R01_cb18319_c0/f1p0/1238	R01_cb18319_c0/f1p0/1238
R01_cb11625_c0/f1p0/943	NA	NA	NA	R01_cb11625_c0/f1p0/943
R01_cb4584_c1/f2p0/2926	NA	NA	NA	R01_cb4584_c1/f2p0/2926
R01_cb6538_c3/f1p0/3815	NA	R01_cb6538_c3/f1p0/3815	NA	NA
R01_cb3307_c6/f2p0/2644	NA	R01_cb3307_c6/f2p0/2644	NA	NA
R01_cb16383_c1/f2p0/1715	NA	R01_cb16383_c1/f2p0/1715	R01_cb16383_c1/f2p0/1715	NA
R01_cb13501_c7/f1p0/472	NA	R01_cb13501_c7/f1p0/472	NA	NA
R01_cb1459_c5/f1p2/3360	NA	NA	NA	R01_cb1459_c5/f1p2/3360
R01_cb18456_c4716/f1p0/503	R01_cb18456_c4716/f1p0/503	R01_cb18456_c4716/f1p0/503	NA	R01_cb18456_c4716/f1p0/503
R01_cb8564_c35197/f1p0/3278	R01_cb8564_c35197/f1p0/3278	R01_cb8564_c35197/f1p0/3278	R01_cb8564_c35197/f1p0/3278	R01_cb8564_c35197/f1p0/3278
R01_cb18456_c4057/f1p0/1655	NA	R01_cb18456_c4057/f1p0/1655	NA	NA

R01_cb16135_c1/f1p0/747	NA	NA	NA	R01_cb16135_c1/f1p0/747
R01_cb3703_c7/f1p0/1455	NA	NA	NA	R01_cb3703_c7/f1p0/1455
R01_cb2320_c7/f1p0/4028	NA	NA	NA	R01_cb2320_c7/f1p0/4028
R01_cb2640_c15/f1p0/2346	NA	R01_cb2640_c15/f1p0/2346	NA	NA
R01_cb13502_c0/f2p0/1503	NA	R01_cb13502_c0/f2p0/1503	NA	R01_cb13502_c0/f2p0/1503
R01_cb10453_c0/f5p0/1132	NA	NA	R01_cb10453_c0/f5p0/1132	R01_cb10453_c0/f5p0/1132
R01_cb14977_c4/f1p0/1489	R01_cb14977_c4/f1p0/1489	R01_cb14977_c4/f1p0/1489	R01_cb14977_c4/f1p0/1489	R01_cb14977_c4/f1p0/1489
R01_cb3585_c25/f1p2/2717	NA	R01_cb3585_c25/f1p2/2717	NA	NA
R01_cb9882_c6/f1p0/1046	R01_cb9882_c6/f1p0/1046	NA	R01_cb9882_c6/f1p0/1046	NA
R01_cb8564_c1362/f1p0/3486	NA	R01_cb8564_c1362/f1p0/3486	NA	NA
R01_cb7485_c2/f1p0/3048	R01_cb7485_c2/f1p0/3048	R01_cb7485_c2/f1p0/3048	R01_cb7485_c2/f1p0/3048	R01_cb7485_c2/f1p0/3048
R01_cb3794_c4/f1p0/2414	R01_cb3794_c4/f1p0/2414	R01_cb3794_c4/f1p0/2414	NA	NA
R01_cb2851_c6/f1p0/2221	NA	NA	NA	R01_cb2851_c6/f1p0/2221
R01_cb15882_c1/f1p1/1493	NA	NA	NA	R01_cb15882_c1/f1p1/1493
R01_cb11109_c3/f1p0/1475	NA	NA	NA	R01_cb11109_c3/f1p0/1475
R01_cb7401_c3/f1p0/2319	NA	NA	NA	R01_cb7401_c3/f1p0/2319
R01_cb18136_c0/f1p0/569	NA	R01_cb18136_c0/f1p0/569	NA	NA
R01_cb11931_c15/f1p0/323	R01_cb11931_c15/f1p0/323	R01_cb11931_c15/f1p0/323	R01_cb11931_c15/f1p0/323	R01_cb11931_c15/f1p0/323
R01_cb763_c7/f1p0/3711	NA	R01_cb763_c7/f1p0/3711	R01_cb763_c7/f1p0/3711	R01_cb763_c7/f1p0/3711
R01_cb11076_c0/f1p0/1361	NA	R01_cb11076_c0/f1p0/1361	R01_cb11076_c0/f1p0/1361	R01_cb11076_c0/f1p0/1361
R01_cb7096_c16/f1p0/2638	NA	NA	NA	R01_cb7096_c16/f1p0/2638
R01_cb13680_c7/f1p0/428	R01_cb13680_c7/f1p0/428	R01_cb13680_c7/f1p0/428	NA	R01_cb13680_c7/f1p0/428
R01_cb9798_c5/f1p0/2665	NA	R01_cb9798_c5/f1p0/2665	NA	NA
R01_cb3774_c35/f1p0/2110	NA	R01_cb3774_c35/f1p0/2110	NA	NA
R01_cb18303_c1/f1p0/1171	NA	R01_cb18303_c1/f1p0/1171	NA	R01_cb18303_c1/f1p0/1171
R01_cb3079_c6/f1p0/2497	NA	NA	NA	R01_cb3079_c6/f1p0/2497

R01_cb9614_c1/f1p0/2097	NA	NA	NA	R01_cb9614_c1/f1p0/2097
R01_cb8564_c22926/f1p0/2247	NA	R01_cb8564_c22926/f1p0/2247	NA	NA
R01_cb4614_c3/f1p0/3038	NA	R01_cb4614_c3/f1p0/3038	NA	NA
R01_cb14832_c1/f1p0/797	NA	NA	NA	R01_cb14832_c1/f1p0/797
R01_cb1178_c27/f1p0/1197	NA	NA	NA	R01_cb1178_c27/f1p0/1197
R01_cb5983_c6/f1p0/923	NA	NA	NA	R01_cb5983_c6/f1p0/923
R01_cb5902_c2/f1p0/2787	NA	R01_cb5902_c2/f1p0/2787	NA	NA
R01_cb11301_c2/f1p0/1249	NA	R01_cb11301_c2/f1p0/1249	NA	NA
R01_cb17604_c11/f36p0/533	NA	NA	NA	R01_cb17604_c11/f36p0/533
R01_cb8564_c18777/f1p0/2490	NA	R01_cb8564_c18777/f1p0/2490	NA	NA
R01_cb8564_c123394/f1p0/2341	R01_cb8564_c123394/f1p0/2341	R01_cb8564_c123394/f1p0/2341	R01_cb8564_c123394/f1p0/2341	R01_cb8564_c123394/f1p0/2341
R01_cb2445_c19/f1p0/2598	NA	NA	NA	R01_cb2445_c19/f1p0/2598
R01_cb8564_c80370/f1p0/3493	NA	R01_cb8564_c80370/f1p0/3493	R01_cb8564_c80370/f1p0/3493	NA
R01_cb1093_c1/f1p0/4261	NA	NA	NA	R01_cb1093_c1/f1p0/4261
R01_cb16177_c0/f6p0/860	NA	NA	NA	R01_cb16177_c0/f6p0/860
R01_cb8564_c73785/f1p0/2184	NA	R01_cb8564_c73785/f1p0/2184	R01_cb8564_c73785/f1p0/2184	R01_cb8564_c73785/f1p0/2184
R01_cb13433_c97/f1p0/830	R01_cb13433_c97/f1p0/830	R01_cb13433_c97/f1p0/830	R01_cb13433_c97/f1p0/830	R01_cb13433_c97/f1p0/830
R01_cb625_c15/f1p0/3549	R01_cb625_c15/f1p0/3549	R01_cb625_c15/f1p0/3549	R01_cb625_c15/f1p0/3549	R01_cb625_c15/f1p0/3549
R01_cb17432_c5/f1p0/1768	NA	R01_cb17432_c5/f1p0/1768	NA	NA
R01_cb9129_c1/f1p0/2112	NA	NA	NA	R01_cb9129_c1/f1p0/2112
R01_cb5339_c0/f3p0/2454	NA	NA	NA	R01_cb5339_c0/f3p0/2454
R01_cb2082_c8/f2p0/2508	NA	NA	NA	R01_cb2082_c8/f2p0/2508
R01_cb6286_c19/f1p0/2614	NA	NA	NA	R01_cb6286_c19/f1p0/2614
R01_cb16172_c1/f1p0/1268	R01_cb16172_c1/f1p0/1268	R01_cb16172_c1/f1p0/1268	R01_cb16172_c1/f1p0/1268	NA
R01_cb15352_c6/f7p2/759	NA	NA	NA	R01_cb15352_c6/f7p2/759

R01_cb18456_c4705/f1p0/522	NA	NA	NA	R01_cb18456_c4705/f1p0/522
R01_cb8564_c121942/f1p0/2724	NA	R01_cb8564_c121942/f1p0/2724	NA	R01_cb8564_c121942/f1p0/2724
R01_cb9960_c4/f1p0/1841	NA	NA	NA	R01_cb9960_c4/f1p0/1841
R01_cb10015_c330/f1p0/590	NA	R01_cb10015_c330/f1p0/590	R01_cb10015_c330/f1p0/590	NA
R01_cb7033_c2/f1p0/2717	NA	R01_cb7033_c2/f1p0/2717	NA	NA
R01_cb18456_c5889/f1p0/446	R01_cb18456_c5889/f1p0/446	R01_cb18456_c5889/f1p0/446	NA	R01_cb18456_c5889/f1p0/446
R01_cb17739_c1/f1p0/430	R01_cb17739_c1/f1p0/430	R01_cb17739_c1/f1p0/430	R01_cb17739_c1/f1p0/430	R01_cb17739_c1/f1p0/430
R01_cb1854_c5/f1p0/4153	NA	R01_cb1854_c5/f1p0/4153	NA	NA
R01_cb10948_c8/f1p0/1369	NA	NA	NA	R01_cb10948_c8/f1p0/1369
R01_cb6399_c3/f3p0/535	R01_cb6399_c3/f3p0/535	R01_cb6399_c3/f3p0/535	NA	NA
R01_cb18409_c51/f1p0/384	R01_cb18409_c51/f1p0/384	R01_cb18409_c51/f1p0/384	R01_cb18409_c51/f1p0/384	R01_cb18409_c51/f1p0/384
R01_cb15186_c12/f1p0/507	NA	R01_cb15186_c12/f1p0/507	R01_cb15186_c12/f1p0/507	NA
R01_cb15118_c8/f1p0/548	R01_cb15118_c8/f1p0/548	NA	NA	R01_cb15118_c8/f1p0/548
R01_cb4824_c1/f2p0/2790	NA	R01_cb4824_c1/f2p0/2790	NA	R01_cb4824_c1/f2p0/2790
R01_cb18456_c7445/f1p0/968	NA	R01_cb18456_c7445/f1p0/968	R01_cb18456_c7445/f1p0/968	R01_cb18456_c7445/f1p0/968
R01_cb5664_c2/f1p0/2292	NA	R01_cb5664_c2/f1p0/2292	R01_cb5664_c2/f1p0/2292	R01_cb5664_c2/f1p0/2292
R01_cb8501_c40/f1p0/407	R01_cb8501_c40/f1p0/407	R01_cb8501_c40/f1p0/407	R01_cb8501_c40/f1p0/407	R01_cb8501_c40/f1p0/407
R01_cb8564_c69028/f2p0/2446	NA	R01_cb8564_c69028/f2p0/2446	NA	NA
R01_cb4952_c4/f1p0/1716	NA	NA	NA	R01_cb4952_c4/f1p0/1716
R01_cb10230_c5/f1p1/714	NA	R01_cb10230_c5/f1p1/714	NA	NA
R01_cb13783_c5/f1p0/309	R01_cb13783_c5/f1p0/309	R01_cb13783_c5/f1p0/309	R01_cb13783_c5/f1p0/309	R01_cb13783_c5/f1p0/309
R01_cb8564_c48909/f1p0/2687	NA	NA	NA	R01_cb8564_c48909/f1p0/2687
R01_cb11929_c2/f1p0/1841	NA	R01_cb11929_c2/f1p0/1841	R01_cb11929_c2/f1p0/1841	R01_cb11929_c2/f1p0/1841
R01_cb8564_c1413/f1p0/2505	NA	R01_cb8564_c1413/f1p0/2505	NA	NA
R01_cb10771_c1/f1p0/3003	NA	NA	R01_cb10771_c1/f1p0/3003	R01_cb10771_c1/f1p0/3003

R01_cb7008_c32/f1p0/2486	NA	R01_cb7008_c32/f1p0/2486	NA	NA
R01_cb8564_c80900/f1p1/2361	NA	NA	NA	R01_cb8564_c80900/f1p1/2361
R01_cb13193_c2/f2p0/1555	R01_cb13193_c2/f2p0/1555	R01_cb13193_c2/f2p0/1555	R01_cb13193_c2/f2p0/1555	R01_cb13193_c2/f2p0/1555
R01_cb11177_c1/f1p0/3117	NA	R01_cb11177_c1/f1p0/3117	NA	NA
R01_cb2104_c18/f17p1/1424	NA	NA	R01_cb2104_c18/f17p1/1424	R01_cb2104_c18/f17p1/1424
R01_cb9779_c1/f1p0/2179	NA	NA	NA	R01_cb9779_c1/f1p0/2179
R01_cb416_c13/f1p0/4820	NA	R01_cb416_c13/f1p0/4820	NA	R01_cb416_c13/f1p0/4820
R01_cb7564_c35/f1p0/2473	NA	R01_cb7564_c35/f1p0/2473	NA	NA
R01_cb12579_c5/f1p0/936	NA	R01_cb12579_c5/f1p0/936	R01_cb12579_c5/f1p0/936	R01_cb12579_c5/f1p0/936
R01_cb8564_c113524/f1p0/1967	NA	R01_cb8564_c113524/f1p0/1967	NA	R01_cb8564_c113524/f1p0/1967
R01_cb17266_c28/f1p0/721	R01_cb17266_c28/f1p0/721	R01_cb17266_c28/f1p0/721	R01_cb17266_c28/f1p0/721	R01_cb17266_c28/f1p0/721
R01_cb8809_c7/f1p0/4039	NA	R01_cb8809_c7/f1p0/4039	NA	R01_cb8809_c7/f1p0/4039
R01_cb1119_c6/f1p0/2308	NA	R01_cb1119_c6/f1p0/2308	NA	NA
R01_cb8564_c52637/f1p0/2340	NA	R01_cb8564_c52637/f1p0/2340	NA	R01_cb8564_c52637/f1p0/2340
R01_cb8564_c92323/f1p0/3640	NA	R01_cb8564_c92323/f1p0/3640	NA	NA
R01_cb18301_c33/f1p0/304	R01_cb18301_c33/f1p0/304	R01_cb18301_c33/f1p0/304	R01_cb18301_c33/f1p0/304	R01_cb18301_c33/f1p0/304
R01_cb13317_c3/f1p0/1730	R01_cb13317_c3/f1p0/1730	R01_cb13317_c3/f1p0/1730	NA	R01_cb13317_c3/f1p0/1730
R01_cb5902_c1/f1p0/2259	NA	R01_cb5902_c1/f1p0/2259	NA	NA
R01_cb11895_c3/f1p0/5507	NA	NA	NA	R01_cb11895_c3/f1p0/5507
R01_cb11633_c0/f1p0/1899	NA	R01_cb11633_c0/f1p0/1899	NA	NA
R01_cb8564_c77699/f1p0/2085	R01_cb8564_c77699/f1p0/2085	R01_cb8564_c77699/f1p0/2085	NA	R01_cb8564_c77699/f1p0/2085
R01_cb5952_c7/f1p0/950	NA	NA	NA	R01_cb5952_c7/f1p0/950
R01_cb18456_c4726/f1p0/487	NA	NA	NA	R01_cb18456_c4726/f1p0/487
R01_cb8564_c4556/f1p0/3011	NA	R01_cb8564_c4556/f1p0/3011	NA	NA

R01_cb8564_c86179/f1p0/2927	NA	R01_cb8564_c86179/f1p0/2927	NA	NA
R01_cb6513_c4/f1p0/1892	NA	NA	NA	R01_cb6513_c4/f1p0/1892
R01_cb8564_c74639/f1p0/2310	NA	R01_cb8564_c74639/f1p0/2310	R01_cb8564_c74639/f1p0/2310	R01_cb8564_c74639/f1p0/2310
R01_cb18197_c3/f1p0/1143	NA	R01_cb18197_c3/f1p0/1143	R01_cb18197_c3/f1p0/1143	R01_cb18197_c3/f1p0/1143
R01_cb3861_c2/f1p0/2983	NA	R01_cb3861_c2/f1p0/2983	NA	NA
R01_cb18135_c0/f1p0/900	NA	R01_cb18135_c0/f1p0/900	R01_cb18135_c0/f1p0/900	R01_cb18135_c0/f1p0/900
R01_cb8564_c126545/f1p0/2581	NA	NA	NA	R01_cb8564_c126545/f1p0/2581
R01_cb14575_c6/f1p1/431	R01_cb14575_c6/f1p1/431	R01_cb14575_c6/f1p1/431	NA	R01_cb14575_c6/f1p1/431
R01_cb4923_c11/f1p0/2754	NA	NA	NA	R01_cb4923_c11/f1p0/2754
R01_cb8564_c21834/f1p0/3062	R01_cb8564_c21834/f1p0/3062	R01_cb8564_c21834/f1p0/3062	R01_cb8564_c21834/f1p0/3062	R01_cb8564_c21834/f1p0/3062
R01_cb2710_c5/f1p0/2884	NA	NA	NA	R01_cb2710_c5/f1p0/2884
R01_cb1626_c17/f1p0/3006	NA	NA	NA	R01_cb1626_c17/f1p0/3006
R01_cb16335_c0/f1p2/785	NA	NA	NA	R01_cb16335_c0/f1p2/785
R01_cb8564_c40511/f1p0/2548	NA	NA	NA	R01_cb8564_c40511/f1p0/2548
R01_cb7017_c4/f1p0/1330	NA	R01_cb7017_c4/f1p0/1330	R01_cb7017_c4/f1p0/1330	R01_cb7017_c4/f1p0/1330
R01_cb13463_c8/f1p0/1370	NA	R01_cb13463_c8/f1p0/1370	NA	R01_cb13463_c8/f1p0/1370
R01_cb5775_c3/f1p0/3003	NA	R01_cb5775_c3/f1p0/3003	NA	NA
R01_cb5390_c1/f2p0/2938	NA	R01_cb5390_c1/f2p0/2938	NA	NA
R01_cb18172_c0/f1p0/694	NA	NA	NA	R01_cb18172_c0/f1p0/694
R01_cb17829_c0/f2p0/423	R01_cb17829_c0/f2p0/423	R01_cb17829_c0/f2p0/423	R01_cb17829_c0/f2p0/423	R01_cb17829_c0/f2p0/423
R01_cb8078_c2/f1p0/2364	NA	R01_cb8078_c2/f1p0/2364	NA	R01_cb8078_c2/f1p0/2364
R01_cb10159_c3/f1p0/1119	NA	R01_cb10159_c3/f1p0/1119	NA	NA
R01_cb13439_c21/f1p0/551	NA	R01_cb13439_c21/f1p0/551	NA	NA
R01_cb454_c3/f4p0/2389	NA	NA	NA	R01_cb454_c3/f4p0/2389

R01_cb14947_c0/f2p1/522	R01_cb14947_c0/f2p1/522	R01_cb14947_c0/f2p1/522	NA	R01_cb14947_c0/f2p1/522
R01_cb18456_c7203/f1p0/731	NA	R01_cb18456_c7203/f1p0/731	R01_cb18456_c7203/f1p0/731	R01_cb18456_c7203/f1p0/731
R01_cb15911_c3/f1p0/1589	NA	R01_cb15911_c3/f1p0/1589	NA	R01_cb15911_c3/f1p0/1589
R01_cb10029_c240/f1p0/433	NA	R01_cb10029_c240/f1p0/433	R01_cb10029_c240/f1p0/433	R01_cb10029_c240/f1p0/433
R01_cb13478_c5/f3p0/424	NA	R01_cb13478_c5/f3p0/424	R01_cb13478_c5/f3p0/424	R01_cb13478_c5/f3p0/424
R01_cb10958_c2/f1p0/3151	R01_cb10958_c2/f1p0/3151	R01_cb10958_c2/f1p0/3151	NA	NA
R01_cb416_c12/f2p0/2821	NA	R01_cb416_c12/f2p0/2821	NA	NA
R01_cb8564_c84065/f1p0/3380	NA	R01_cb8564_c84065/f1p0/3380	NA	R01_cb8564_c84065/f1p0/3380
R01_cb10818_c4/f1p0/596	NA	R01_cb10818_c4/f1p0/596	NA	R01_cb10818_c4/f1p0/596
R01_cb10314_c5/f1p0/667	NA	R01_cb10314_c5/f1p0/667	NA	NA
R01_cb10378_c3/f1p0/442	NA	R01_cb10378_c3/f1p0/442	NA	R01_cb10378_c3/f1p0/442
R01_cb18553_c0/f1p0/1296	NA	NA	NA	R01_cb18553_c0/f1p0/1296
R01_cb18511_c0/f1p0/478	R01_cb18511_c0/f1p0/478	R01_cb18511_c0/f1p0/478	R01_cb18511_c0/f1p0/478	R01_cb18511_c0/f1p0/478
R01_cb2946_c6/f1p0/2232	NA	NA	NA	R01_cb2946_c6/f1p0/2232
R01_cb10010_c3/f1p0/1790	NA	R01_cb10010_c3/f1p0/1790	NA	R01_cb10010_c3/f1p0/1790
R01_cb8564_c16650/f1p0/2552	NA	R01_cb8564_c16650/f1p0/2552	NA	NA
R01_cb11924_c24/f1p0/475	R01_cb11924_c24/f1p0/475	R01_cb11924_c24/f1p0/475	R01_cb11924_c24/f1p0/475	NA
R01_cb14477_c2/f1p0/980	NA	NA	NA	R01_cb14477_c2/f1p0/980
R01_cb2463_c4/f1p0/2906	NA	NA	NA	R01_cb2463_c4/f1p0/2906
R01_cb8973_c3/f1p0/526	NA	R01_cb8973_c3/f1p0/526	NA	NA
R01_cb7761_c26/f1p0/801	R01_cb7761_c26/f1p0/801	R01_cb7761_c26/f1p0/801	R01_cb7761_c26/f1p0/801	R01_cb7761_c26/f1p0/801
R01_cb9853_c3/f1p0/741	NA	NA	R01_cb9853_c3/f1p0/741	R01_cb9853_c3/f1p0/741
R01_cb12815_c2/f1p0/1648	R01_cb12815_c2/f1p0/1648	R01_cb12815_c2/f1p0/1648	NA	NA
R01_cb3359_c35/f1p0/1632	NA	R01_cb3359_c35/f1p0/1632	NA	NA
R01_cb14993_c1/f1p0/929	NA	R01_cb14993_c1/f1p0/929	NA	R01_cb14993_c1/f1p0/929
R01_cb16295_c2/f1p0/1524	NA	NA	NA	R01_cb16295_c2/f1p0/1524

R01_cb5946_c17/f1p1/890	NA	NA	NA	R01_cb5946_c17/f1p1/890
R01_cb8564_c146532/f8p0/2639	NA	NA	NA	R01_cb8564_c146532/f8p0/2639
R01_cb15150_c0/f4p0/566	NA	R01_cb15150_c0/f4p0/566	NA	R01_cb15150_c0/f4p0/566
R01_cb8564_c81199/f1p0/3038	NA	R01_cb8564_c81199/f1p0/3038	NA	NA
R01_cb5513_c6/f1p0/2890	NA	NA	NA	R01_cb5513_c6/f1p0/2890
R01_cb8564_c129756/f1p0/2608	NA	R01_cb8564_c129756/f1p0/2608	R01_cb8564_c129756/f1p0/2608	R01_cb8564_c129756/f1p0/2608
R01_cb795_c2/f1p0/3065	R01_cb795_c2/f1p0/3065	R01_cb795_c2/f1p0/3065	R01_cb795_c2/f1p0/3065	R01_cb795_c2/f1p0/3065
R01_cb15766_c1/f1p0/1574	NA	NA	NA	R01_cb15766_c1/f1p0/1574
R01_cb12498_c0/f2p0/1415	NA	R01_cb12498_c0/f2p0/1415	NA	NA
R01_cb12898_c2/f1p1/758	NA	R01_cb12898_c2/f1p1/758	NA	NA
R01_cb18044_c3/f1p0/966	R01_cb18044_c3/f1p0/966	R01_cb18044_c3/f1p0/966	R01_cb18044_c3/f1p0/966	R01_cb18044_c3/f1p0/966
R01_cb16334_c1/f1p0/1310	NA	R01_cb16334_c1/f1p0/1310	R01_cb16334_c1/f1p0/1310	R01_cb16334_c1/f1p0/1310
R01_cb1788_c2/f1p0/3011	NA	NA	NA	R01_cb1788_c2/f1p0/3011
R01_cb13558_c2/f1p0/707	NA	NA	R01_cb13558_c2/f1p0/707	R01_cb13558_c2/f1p0/707
R01_cb8564_c36799/f1p0/4885	NA	NA	NA	R01_cb8564_c36799/f1p0/4885
R01_cb17885_c20/f1p0/1166	R01_cb17885_c20/f1p0/1166	NA	NA	NA
R01_cb8281_c8/f1p0/2274	NA	NA	NA	R01_cb8281_c8/f1p0/2274
R01_cb8564_c115260/f1p1/1895	NA	R01_cb8564_c115260/f1p1/1895	NA	R01_cb8564_c115260/f1p1/1895
R01_cb15535_c11/f1p1/392	R01_cb15535_c11/f1p1/392	R01_cb15535_c11/f1p1/392	R01_cb15535_c11/f1p1/392	R01_cb15535_c11/f1p1/392
R01_cb124_c23/f1p0/3623	NA	NA	NA	R01_cb124_c23/f1p0/3623
R01_cb8564_c89541/f1p0/2001	NA	R01_cb8564_c89541/f1p0/2001	R01_cb8564_c89541/f1p0/2001	R01_cb8564_c89541/f1p0/2001
R01_cb6547_c4/f2p1/2736	NA	NA	NA	R01_cb6547_c4/f2p1/2736
R01_cb9663_c4/f1p0/1762	NA	R01_cb9663_c4/f1p0/1762	NA	NA

R01_cb3888_c4/f1p0/3311	NA	R01_cb3888_c4/f1p0/3311	NA	NA
R01_cb18456_c1860/f1p0/1860	NA	R01_cb18456_c1860/f1p0/1860	NA	R01_cb18456_c1860/f1p0/1860
R01_cb693_c3/f1p0/3671	NA	NA	NA	R01_cb693_c3/f1p0/3671
R01_cb5625_c2/f1p0/3617	NA	NA	NA	R01_cb5625_c2/f1p0/3617
R01_cb12421_c74/f9p5/702	NA	R01_cb12421_c74/f9p5/702	NA	NA
R01_cb9256_c1/f1p0/2700	NA	NA	NA	R01_cb9256_c1/f1p0/2700
R01_cb18422_c1/f1p0/553	NA	R01_cb18422_c1/f1p0/553	NA	NA
R01_cb10978_c1/f1p0/2956	NA	R01_cb10978_c1/f1p0/2956	NA	NA
R01_cb15932_c3/f1p0/770	NA	NA	NA	R01_cb15932_c3/f1p0/770
R01_cb647_c4/f3p0/3277	NA	R01_cb647_c4/f3p0/3277	R01_cb647_c4/f3p0/3277	NA
R01_cb16127_c0/f2p0/540	R01_cb16127_c0/f2p0/540	R01_cb16127_c0/f2p0/540	NA	R01_cb16127_c0/f2p0/540
R01_cb3813_c4/f1p0/1550	NA	R01_cb3813_c4/f1p0/1550	NA	NA
R01_cb17577_c1/f1p0/1564	NA	NA	NA	R01_cb17577_c1/f1p0/1564
R01_cb12785_c29/f9p1/969	NA	NA	R01_cb12785_c29/f9p1/969	NA
R01_cb7693_c4/f1p0/2666	NA	R01_cb7693_c4/f1p0/2666	R01_cb7693_c4/f1p0/2666	R01_cb7693_c4/f1p0/2666
R01_cb16782_c3/f1p0/611	R01_cb16782_c3/f1p0/611	R01_cb16782_c3/f1p0/611	R01_cb16782_c3/f1p0/611	R01_cb16782_c3/f1p0/611
R01_cb11767_c1/f1p0/2007	R01_cb11767_c1/f1p0/2007	R01_cb11767_c1/f1p0/2007	R01_cb11767_c1/f1p0/2007	R01_cb11767_c1/f1p0/2007
R01_cb12480_c6/f1p0/553	R01_cb12480_c6/f1p0/553	R01_cb12480_c6/f1p0/553	R01_cb12480_c6/f1p0/553	R01_cb12480_c6/f1p0/553
R01_cb6615_c3/f1p0/2809	NA	NA	NA	R01_cb6615_c3/f1p0/2809
R01_cb2674_c7/f1p0/2708	NA	NA	NA	R01_cb2674_c7/f1p0/2708
R01_cb18456_c7625/f1p1/798	NA	R01_cb18456_c7625/f1p1/798	NA	R01_cb18456_c7625/f1p1/798
R01_cb8564_c85403/f1p0/2182	R01_cb8564_c85403/f1p0/2182	R01_cb8564_c85403/f1p0/2182	NA	NA
R01_cb15487_c1/f1p0/389	R01_cb15487_c1/f1p0/389	R01_cb15487_c1/f1p0/389	R01_cb15487_c1/f1p0/389	R01_cb15487_c1/f1p0/389
R01_cb8564_c68979/f2p1/2038	NA	NA	R01_cb8564_c68979/f2p1/2038	R01_cb8564_c68979/f2p1/2038
R01_cb18192_c12/f1p0/768	R01_cb18192_c12/f1p0/768	R01_cb18192_c12/f1p0/768	R01_cb18192_c12/f1p0/768	NA

R01_cb17052_c6/f1p0/1486	NA	NA	NA	R01_cb17052_c6/f1p0/1486
R01_cb10019_c2/f1p1/3311	NA	R01_cb10019_c2/f1p1/3311	NA	NA
R01_cb3307_c44/f1p0/1073	NA	NA	NA	R01_cb3307_c44/f1p0/1073
R01_cb8564_c23850/f1p0/3925	NA	R01_cb8564_c23850/f1p0/3925	NA	NA
R01_cb13655_c1/f1p0/924	NA	R01_cb13655_c1/f1p0/924	NA	NA
R01_cb14308_c3/f1p0/1223	NA	R01_cb14308_c3/f1p0/1223	NA	NA
R01_cb14763_c1/f1p0/1791	NA	NA	NA	R01_cb14763_c1/f1p0/1791
R01_cb2970_c5/f1p1/1749	NA	R01_cb2970_c5/f1p1/1749	NA	NA
R01_cb1259_c4/f1p0/4412	NA	NA	NA	R01_cb1259_c4/f1p0/4412
R01_cb7368_c0/f1p0/2617	NA	R01_cb7368_c0/f1p0/2617	NA	R01_cb7368_c0/f1p0/2617
R01_cb5000_c14/f1p0/2767	NA	NA	NA	R01_cb5000_c14/f1p0/2767
R01_cb18409_c38/f1p0/416	R01_cb18409_c38/f1p0/416	R01_cb18409_c38/f1p0/416	R01_cb18409_c38/f1p0/416	R01_cb18409_c38/f1p0/416
R01_cb8564_c90445/f1p0/2792	R01_cb8564_c90445/f1p0/2792	R01_cb8564_c90445/f1p0/2792	NA	R01_cb8564_c90445/f1p0/2792
R01_cb16769_c1/f1p0/1432	R01_cb16769_c1/f1p0/1432	R01_cb16769_c1/f1p0/1432	R01_cb16769_c1/f1p0/1432	R01_cb16769_c1/f1p0/1432
R01_cb2238_c40/f1p0/2390	NA	NA	NA	R01_cb2238_c40/f1p0/2390
R01_cb8564_c71261/f1p2/2275	NA	R01_cb8564_c71261/f1p2/2275	R01_cb8564_c71261/f1p2/2275	R01_cb8564_c71261/f1p2/2275
R01_cb15435_c0/f1p0/1831	NA	R01_cb15435_c0/f1p0/1831	NA	NA
R01_cb18456_c1859/f1p0/1467	NA	R01_cb18456_c1859/f1p0/1467	NA	NA
R01_cb13820_c5/f1p0/999	NA	R01_cb13820_c5/f1p0/999	NA	NA
R01_cb4531_c5/f1p0/2282	NA	NA	NA	R01_cb4531_c5/f1p0/2282
R01_cb15273_c4/f1p0/974	NA	NA	R01_cb15273_c4/f1p0/974	NA
R01_cb4679_c0/f2p0/3167	NA	R01_cb4679_c0/f2p0/3167	NA	NA
R01_cb10644_c3/f1p0/5384	NA	R01_cb10644_c3/f1p0/5384	NA	NA
R01_cb8564_c124784/f1p0/1996	NA	R01_cb8564_c124784/f1p0/1996	NA	NA

R01_cb18456_c6568/f1p0/548	NA	NA	NA	R01_cb18456_c6568/f1p0/548
R01_cb9771_c1/f1p1/2599	NA	NA	NA	R01_cb9771_c1/f1p1/2599
R01_cb13545_c19/f1p0/743	R01_cb13545_c19/f1p0/743	R01_cb13545_c19/f1p0/743	R01_cb13545_c19/f1p0/743	R01_cb13545_c19/f1p0/743
R01_cb17560_c1/f1p0/618	R01_cb17560_c1/f1p0/618	NA	NA	R01_cb17560_c1/f1p0/618
R01_cb1894_c9/f1p0/1465	NA	NA	NA	R01_cb1894_c9/f1p0/1465
R01_cb17850_c0/f2p0/1308	NA	R01_cb17850_c0/f2p0/1308	R01_cb17850_c0/f2p0/1308	R01_cb17850_c0/f2p0/1308
R01_cb5159_c15/f1p0/1954	NA	NA	NA	R01_cb5159_c15/f1p0/1954
R01_cb293_c2/f1p1/4354	NA	NA	NA	R01_cb293_c2/f1p1/4354
R01_cb8564_c21068/f1p0/3700	NA	R01_cb8564_c21068/f1p0/3700	NA	NA
R01_cb1612_c15/f2p3/3007	NA	NA	NA	R01_cb1612_c15/f2p3/3007
R01_cb10687_c2/f2p1/1715	NA	NA	NA	R01_cb10687_c2/f2p1/1715
R01_cb10015_c593/f4p0/567	R01_cb10015_c593/f4p0/567	R01_cb10015_c593/f4p0/567	R01_cb10015_c593/f4p0/567	NA
R01_cb9472_c1/f1p0/2121	NA	NA	NA	R01_cb9472_c1/f1p0/2121
R01_cb8564_c118304/f1p0/2654	NA	R01_cb8564_c118304/f1p0/2654	NA	R01_cb8564_c118304/f1p0/2654
R01_cb9915_c2/f1p0/1798	NA	NA	NA	R01_cb9915_c2/f1p0/1798
R01_cb6879_c5/f1p0/2258	NA	NA	NA	R01_cb6879_c5/f1p0/2258
R01_cb5911_c3/f1p0/3105	NA	NA	NA	R01_cb5911_c3/f1p0/3105
R01_cb8887_c3/f1p0/2242	NA	R01_cb8887_c3/f1p0/2242	NA	R01_cb8887_c3/f1p0/2242
R01_cb9594_c5/f1p0/2037	R01_cb9594_c5/f1p0/2037	R01_cb9594_c5/f1p0/2037	NA	NA
R01_cb3145_c1/f1p0/3709	NA	R01_cb3145_c1/f1p0/3709	NA	NA
R01_cb1225_c1/f1p0/2000	NA	R01_cb1225_c1/f1p0/2000	NA	NA
R01_cb8564_c2627/f1p0/2600	R01_cb8564_c2627/f1p0/2600	R01_cb8564_c2627/f1p0/2600	NA	R01_cb8564_c2627/f1p0/2600
R01_cb10080_c5/f1p0/2509	R01_cb10080_c5/f1p0/2509	R01_cb10080_c5/f1p0/2509	NA	NA
R01_cb8564_c115744/f1p0/2647	R01_cb8564_c115744/f1p0/2647	R01_cb8564_c115744/f1p0/2647	R01_cb8564_c115744/f1p0/2647	R01_cb8564_c115744/f1p0/2647

R01_cb18456_c2511/f1p0/347	R01_cb18456_c2511/f1p0/347	R01_cb18456_c2511/f1p0/347	R01_cb18456_c2511/f1p0/347	R01_cb18456_c2511/f1p0/347
R01_cb8564_c19624/f1p0/2697	NA	R01_cb8564_c19624/f1p0/2697	NA	NA
R01_cb3716_c16/f1p0/2300	NA	NA	NA	R01_cb3716_c16/f1p0/2300
R01_cb1626_c7/f1p1/2840	NA	NA	NA	R01_cb1626_c7/f1p1/2840
R01_cb15176_c3/f1p0/1043	R01_cb15176_c3/f1p0/1043	R01_cb15176_c3/f1p0/1043	NA	NA
R01_cb7342_c3/f1p0/2044	NA	NA	NA	R01_cb7342_c3/f1p0/2044
R01_cb8305_c6/f1p0/2221	NA	R01_cb8305_c6/f1p0/2221	NA	NA
R01_cb7219_c16/f3p2/2487	NA	NA	NA	R01_cb7219_c16/f3p2/2487
R01_cb5547_c2/f1p0/2129	NA	NA	NA	R01_cb5547_c2/f1p0/2129
R01_cb12152_c1/f3p0/709	NA	NA	R01_cb12152_c1/f3p0/709	R01_cb12152_c1/f3p0/709
R01_cb5683_c4/f1p0/2487	NA	R01_cb5683_c4/f1p0/2487	NA	NA
R01_cb17973_c36/f1p0/1061	NA	R01_cb17973_c36/f1p0/1061	R01_cb17973_c36/f1p0/1061	R01_cb17973_c36/f1p0/1061
R01_cb8564_c76611/f1p0/2650	NA	NA	NA	R01_cb8564_c76611/f1p0/2650
R01_cb18335_c1/f1p0/1654	R01_cb18335_c1/f1p0/1654	R01_cb18335_c1/f1p0/1654	R01_cb18335_c1/f1p0/1654	R01_cb18335_c1/f1p0/1654
R01_cb18409_c113/f1p0/664	NA	R01_cb18409_c113/f1p0/664	R01_cb18409_c113/f1p0/664	R01_cb18409_c113/f1p0/664
R01_cb18265_c0/f1p0/682	NA	NA	NA	R01_cb18265_c0/f1p0/682
R01_cb10029_c426/f1p0/614	NA	R01_cb10029_c426/f1p0/614	R01_cb10029_c426/f1p0/614	R01_cb10029_c426/f1p0/614
R01_cb625_c17/f1p0/1040	R01_cb625_c17/f1p0/1040	R01_cb625_c17/f1p0/1040	R01_cb625_c17/f1p0/1040	R01_cb625_c17/f1p0/1040
R01_cb17575_c1/f1p0/777	NA	NA	NA	R01_cb17575_c1/f1p0/777
R01_cb12003_c32/f1p0/1862	NA	R01_cb12003_c32/f1p0/1862	NA	NA
R01_cb12120_c1/f1p0/1642	NA	R01_cb12120_c1/f1p0/1642	NA	NA
R01_cb16066_c4/f1p0/509	R01_cb16066_c4/f1p0/509	R01_cb16066_c4/f1p0/509	R01_cb16066_c4/f1p0/509	R01_cb16066_c4/f1p0/509
R01_cb3859_c2/f1p0/1982	NA	NA	NA	R01_cb3859_c2/f1p0/1982
R01_cb7506_c3/f1p0/2275	NA	NA	NA	R01_cb7506_c3/f1p0/2275
R01_cb18637_c1/f1p0/731	R01_cb18637_c1/f1p0/731	R01_cb18637_c1/f1p0/731	R01_cb18637_c1/f1p0/731	R01_cb18637_c1/f1p0/731
R01_cb4555_c2/f1p0/3510	NA	R01_cb4555_c2/f1p0/3510	NA	R01_cb4555_c2/f1p0/3510

R01_cb12641_c8/f3p0/979	NA	NA	NA	R01_cb12641_c8/f3p0/979
R01_cb1438_c3/f3p3/4154	NA	NA	NA	R01_cb1438_c3/f3p3/4154
R01_cb12469_c11/f1p0/527	NA	R01_cb12469_c11/f1p0/527	NA	NA
R01_cb6069_c3/f1p0/2911	NA	NA	NA	R01_cb6069_c3/f1p0/2911
R01_cb3564_c8/f1p0/3644	NA	NA	NA	R01_cb3564_c8/f1p0/3644
R01_cb5090_c11/f1p0/2102	NA	R01_cb5090_c11/f1p0/2102	NA	NA
R01_cb6204_c0/f24p2/2501	NA	NA	NA	R01_cb6204_c0/f24p2/2501
R01_cb3247_c1/f1p0/2062	NA	NA	NA	R01_cb3247_c1/f1p0/2062
R01_cb8564_c10041/f6p0/3368	NA	R01_cb8564_c10041/f6p0/3368	NA	NA
R01_cb8564_c91906/f1p0/3084	R01_cb8564_c91906/f1p0/3084	R01_cb8564_c91906/f1p0/3084	R01_cb8564_c91906/f1p0/3084	R01_cb8564_c91906/f1p0/3084
R01_cb6867_c5/f1p0/817	NA	R01_cb6867_c5/f1p0/817	NA	R01_cb6867_c5/f1p0/817
R01_cb12600_c1/f1p0/780	R01_cb12600_c1/f1p0/780	NA	NA	R01_cb12600_c1/f1p0/780
R01_cb16935_c1/f1p0/1650	NA	NA	R01_cb16935_c1/f1p0/1650	R01_cb16935_c1/f1p0/1650
R01_cb18365_c1/f1p0/1154	NA	R01_cb18365_c1/f1p0/1154	R01_cb18365_c1/f1p0/1154	R01_cb18365_c1/f1p0/1154
R01_cb3074_c0/f1p0/3702	NA	NA	NA	R01_cb3074_c0/f1p0/3702
R01_cb8785_c2/f1p0/2198	NA	NA	NA	R01_cb8785_c2/f1p0/2198
R01_cb8564_c116490/f1p0/2832	NA	R01_cb8564_c116490/f1p0/2832	NA	NA
R01_cb13490_c9/f1p0/804	R01_cb13490_c9/f1p0/804	R01_cb13490_c9/f1p0/804	R01_cb13490_c9/f1p0/804	R01_cb13490_c9/f1p0/804
R01_cb1333_c39/f1p0/3450	R01_cb1333_c39/f1p0/3450	R01_cb1333_c39/f1p0/3450	R01_cb1333_c39/f1p0/3450	R01_cb1333_c39/f1p0/3450
R01_cb8564_c83177/f1p0/2084	NA	R01_cb8564_c83177/f1p0/2084	NA	R01_cb8564_c83177/f1p0/2084
R01_cb8564_c146112/f14p3/2729	NA	NA	NA	R01_cb8564_c146112/f14p3/2729
R01_cb5758_c4/f1p0/1141	NA	R01_cb5758_c4/f1p0/1141	R01_cb5758_c4/f1p0/1141	R01_cb5758_c4/f1p0/1141
R01_cb13497_c4/f2p1/944	NA	NA	NA	R01_cb13497_c4/f2p1/944

R01_cb15116_c2/f6p0/976	NA	NA	NA	R01_cb15116_c2/f6p0/976
R01_cb5697_c4/f1p1/3696	NA	R01_cb5697_c4/f1p1/3696	NA	R01_cb5697_c4/f1p1/3696
R01_cb8564_c10712/f1p0/2394	NA	R01_cb8564_c10712/f1p0/2394	R01_cb8564_c10712/f1p0/2394	R01_cb8564_c10712/f1p0/2394
R01_cb8564_c110438/f1p0/2116	NA	R01_cb8564_c110438/f1p0/2116	R01_cb8564_c110438/f1p0/2116	R01_cb8564_c110438/f1p0/2116
R01_cb4375_c5/f1p0/3262	NA	R01_cb4375_c5/f1p0/3262	NA	NA
R01_cb8564_c79130/f1p0/1904	NA	R01_cb8564_c79130/f1p0/1904	NA	NA
R01_cb8564_c88383/f1p0/1935	NA	NA	NA	R01_cb8564_c88383/f1p0/1935
R01_cb14977_c6/f1p0/1715	R01_cb14977_c6/f1p0/1715	R01_cb14977_c6/f1p0/1715	R01_cb14977_c6/f1p0/1715	R01_cb14977_c6/f1p0/1715
R01_cb18409_c43/f1p0/1577	NA	R01_cb18409_c43/f1p0/1577	R01_cb18409_c43/f1p0/1577	R01_cb18409_c43/f1p0/1577
R01_cb16535_c0/f1p0/770	NA	R01_cb16535_c0/f1p0/770	NA	R01_cb16535_c0/f1p0/770
R01_cb9898_c1/f1p0/1925	NA	R01_cb9898_c1/f1p0/1925	NA	NA
R01_cb10468_c3/f1p2/1245	R01_cb10468_c3/f1p2/1245	R01_cb10468_c3/f1p2/1245	NA	R01_cb10468_c3/f1p2/1245
R01_cb14982_c1/f2p0/633	NA	R01_cb14982_c1/f2p0/633	R01_cb14982_c1/f2p0/633	R01_cb14982_c1/f2p0/633
R01_cb960_c1/f1p0/4546	NA	R01_cb960_c1/f1p0/4546	NA	NA
R01_cb14301_c2/f1p0/558	NA	R01_cb14301_c2/f1p0/558	NA	NA
R01_cb6141_c6/f1p0/512	R01_cb6141_c6/f1p0/512	R01_cb6141_c6/f1p0/512	R01_cb6141_c6/f1p0/512	R01_cb6141_c6/f1p0/512
R01_cb8564_c130081/f1p0/3189	NA	R01_cb8564_c130081/f1p0/3189	R01_cb8564_c130081/f1p0/3189	NA
R01_cb8564_c122639/f1p0/2418	NA	R01_cb8564_c122639/f1p0/2418	NA	R01_cb8564_c122639/f1p0/2418
R01_cb10015_c589/f1p0/554	NA	R01_cb10015_c589/f1p0/554	NA	NA
R01_cb4233_c179/f1p0/3264	NA	R01_cb4233_c179/f1p0/3264	NA	NA
R01_cb2844_c6/f1p0/2159	NA	NA	NA	R01_cb2844_c6/f1p0/2159
R01_cb18456_c7491/f1p0/1798	NA	R01_cb18456_c7491/f1p0/1798	NA	R01_cb18456_c7491/f1p0/1798
R01_cb6976_c2/f1p0/1874	NA	R01_cb6976_c2/f1p0/1874	NA	NA

R01_cb13392_c11/f1p0/699	NA	R01_cb13392_c11/f1p0/699	NA	NA
R01_cb8564_c110692/f1p0/1943	NA	R01_cb8564_c110692/f1p0/1943	NA	NA
R01_cb2959_c8/f1p0/3480	NA	NA	NA	R01_cb2959_c8/f1p0/3480
R01_cb7254_c3/f2p0/2654	NA	NA	NA	R01_cb7254_c3/f2p0/2654
R01_cb8564_c39718/f1p0/2899	NA	NA	R01_cb8564_c39718/f1p0/2899	NA
R01_cb18456_c7538/f1p0/604	R01_cb18456_c7538/f1p0/604	R01_cb18456_c7538/f1p0/604	R01_cb18456_c7538/f1p0/604	R01_cb18456_c7538/f1p0/604
R01_cb2568_c5/f1p0/3253	NA	R01_cb2568_c5/f1p0/3253	R01_cb2568_c5/f1p0/3253	R01_cb2568_c5/f1p0/3253
R01_cb5042_c8/f1p0/1108	NA	NA	R01_cb5042_c8/f1p0/1108	NA
R01_cb16125_c19/f1p0/1653	NA	R01_cb16125_c19/f1p0/1653	R01_cb16125_c19/f1p0/1653	R01_cb16125_c19/f1p0/1653
R01_cb500_c82/f1p0/1399	NA	R01_cb500_c82/f1p0/1399	NA	NA
R01_cb2946_c1/f1p0/3592	NA	NA	NA	R01_cb2946_c1/f1p0/3592
R01_cb2160_c13/f1p0/3048	NA	NA	NA	R01_cb2160_c13/f1p0/3048
R01_cb16104_c0/f4p1/645	NA	R01_cb16104_c0/f4p1/645	R01_cb16104_c0/f4p1/645	R01_cb16104_c0/f4p1/645
R01_cb18192_c14/f1p0/573	NA	NA	R01_cb18192_c14/f1p0/573	NA
R01_cb666_c1/f1p0/4314	NA	R01_cb666_c1/f1p0/4314	NA	NA
R01_cb7087_c0/f1p0/2707	NA	NA	NA	R01_cb7087_c0/f1p0/2707
R01_cb17255_c2/f1p0/791	NA	R01_cb17255_c2/f1p0/791	NA	NA
R01_cb15034_c2/f1p0/1493	NA	R01_cb15034_c2/f1p0/1493	NA	R01_cb15034_c2/f1p0/1493
R01_cb18526_c2/f1p0/532	R01_cb18526_c2/f1p0/532	R01_cb18526_c2/f1p0/532	R01_cb18526_c2/f1p0/532	R01_cb18526_c2/f1p0/532
R01_cb8564_c110436/f1p0/2513	NA	R01_cb8564_c110436/f1p0/2513	NA	NA
R01_cb8564_c146231/f12p0/2444	NA	R01_cb8564_c146231/f12p0/2444	R01_cb8564_c146231/f12p0/2444	R01_cb8564_c146231/f12p0/2444
R01_cb12739_c3/f1p0/489	R01_cb12739_c3/f1p0/489	R01_cb12739_c3/f1p0/489	R01_cb12739_c3/f1p0/489	R01_cb12739_c3/f1p0/489
R01_cb14223_c1/f4p0/469	R01_cb14223_c1/f4p0/469	R01_cb14223_c1/f4p0/469	R01_cb14223_c1/f4p0/469	R01_cb14223_c1/f4p0/469

R01_cb10300_c1/f1p0/1859	NA	R01_cb10300_c1/f1p0/1859	NA	R01_cb10300_c1/f1p0/1859
R01_cb6982_c1/f1p0/2694	NA	R01_cb6982_c1/f1p0/2694	NA	R01_cb6982_c1/f1p0/2694
R01_cb8376_c1/f1p0/2386	R01_cb8376_c1/f1p0/2386	R01_cb8376_c1/f1p0/2386	R01_cb8376_c1/f1p0/2386	R01_cb8376_c1/f1p0/2386
R01_cb17607_c1/f1p0/1249	NA	NA	NA	R01_cb17607_c1/f1p0/1249
R01_cb8564_c35586/f1p1/1997	NA	R01_cb8564_c35586/f1p1/1997	NA	NA
R01_cb13834_c3/f1p0/1663	R01_cb13834_c3/f1p0/1663	R01_cb13834_c3/f1p0/1663	NA	NA
R01_cb15245_c0/f1p0/1270	NA	R01_cb15245_c0/f1p0/1270	NA	NA
R01_cb8564_c89898/f1p0/3616	NA	R01_cb8564_c89898/f1p0/3616	NA	NA
R01_cb7649_c10/f1p0/2271	NA	NA	NA	R01_cb7649_c10/f1p0/2271
R01_cb8564_c114048/f1p0/4574	NA	R01_cb8564_c114048/f1p0/4574	NA	NA
R01_cb8564_c11339/f4p6/4947	NA	R01_cb8564_c11339/f4p6/4947	R01_cb8564_c11339/f4p6/4947	R01_cb8564_c11339/f4p6/4947
R01_cb10689_c2/f1p0/1433	NA	NA	NA	R01_cb10689_c2/f1p0/1433
R01_cb10728_c2/f1p1/1257	NA	R01_cb10728_c2/f1p1/1257	R01_cb10728_c2/f1p1/1257	NA
R01_cb438_c6/f1p0/3240	NA	R01_cb438_c6/f1p0/3240	NA	R01_cb438_c6/f1p0/3240
R01_cb18372_c11/f1p0/4185	NA	R01_cb18372_c11/f1p0/4185	NA	NA
R01_cb16678_c5/f1p0/935	NA	R01_cb16678_c5/f1p0/935	R01_cb16678_c5/f1p0/935	R01_cb16678_c5/f1p0/935
R01_cb3563_c4/f1p0/2024	NA	NA	NA	R01_cb3563_c4/f1p0/2024
R01_cb5850_c2/f1p0/668	R01_cb5850_c2/f1p0/668	R01_cb5850_c2/f1p0/668	R01_cb5850_c2/f1p0/668	R01_cb5850_c2/f1p0/668
R01_cb17756_c6/f2p0/360	R01_cb17756_c6/f2p0/360	R01_cb17756_c6/f2p0/360	R01_cb17756_c6/f2p0/360	R01_cb17756_c6/f2p0/360
R01_cb10024_c251/f1p7/878	NA	R01_cb10024_c251/f1p7/878	NA	NA
R01_cb8023_c3/f1p0/2442	NA	NA	R01_cb8023_c3/f1p0/2442	R01_cb8023_c3/f1p0/2442
R01_cb15060_c1/f1p0/1073	NA	NA	NA	R01_cb15060_c1/f1p0/1073
R01_cb18407_c1/f1p0/1186	NA	NA	NA	R01_cb18407_c1/f1p0/1186
R01_cb15929_c1/f1p0/1088	NA	R01_cb15929_c1/f1p0/1088	NA	R01_cb15929_c1/f1p0/1088
R01_cb18456_c7306/f1p1/649	NA	R01_cb18456_c7306/f1p1/649	NA	NA

R01_cb8564_c10056/f2p0/2254	NA	NA	NA	R01_cb8564_c10056/f2p0/2254
R01_cb16166_c6/f1p0/532	R01_cb16166_c6/f1p0/532	R01_cb16166_c6/f1p0/532	R01_cb16166_c6/f1p0/532	R01_cb16166_c6/f1p0/532
R01_cb4637_c6/f1p0/747	R01_cb4637_c6/f1p0/747	NA	NA	NA
R01_cb8611_c3/f1p0/2091	NA	NA	NA	R01_cb8611_c3/f1p0/2091
R01_cb14444_c3/f1p0/1119	NA	R01_cb14444_c3/f1p0/1119	R01_cb14444_c3/f1p0/1119	R01_cb14444_c3/f1p0/1119
R01_cb6863_c0/f1p0/2750	R01_cb6863_c0/f1p0/2750	R01_cb6863_c0/f1p0/2750	R01_cb6863_c0/f1p0/2750	R01_cb6863_c0/f1p0/2750
R01_cb1905_c34/f1p0/2216	NA	R01_cb1905_c34/f1p0/2216	R01_cb1905_c34/f1p0/2216	NA
R01_cb8779_c10/f1p0/4827	NA	NA	NA	R01_cb8779_c10/f1p0/4827
R01_cb13954_c1/f1p0/673	NA	R01_cb13954_c1/f1p0/673	NA	NA
R01_cb9359_c2/f1p0/1540	NA	NA	NA	R01_cb9359_c2/f1p0/1540
R01_cb13832_c5/f1p0/980	NA	R01_cb13832_c5/f1p0/980	NA	NA
R01_cb12556_c4/f1p0/421	NA	R01_cb12556_c4/f1p0/421	NA	R01_cb12556_c4/f1p0/421
R01_cb3415_c1/f1p2/3524	NA	R01_cb3415_c1/f1p2/3524	NA	NA
R01_cb8380_c3/f1p0/2054	NA	R01_cb8380_c3/f1p0/2054	NA	NA
R01_cb16669_c1/f1p0/677	R01_cb16669_c1/f1p0/677	R01_cb16669_c1/f1p0/677	R01_cb16669_c1/f1p0/677	R01_cb16669_c1/f1p0/677
R01_cb8019_c7/f1p1/1708	NA	NA	NA	R01_cb8019_c7/f1p1/1708
R01_cb15701_c4/f1p0/1743	R01_cb15701_c4/f1p0/1743	R01_cb15701_c4/f1p0/1743	NA	NA
R01_cb13505_c7/f1p0/603	R01_cb13505_c7/f1p0/603	R01_cb13505_c7/f1p0/603	NA	NA
R01_cb16960_c2/f1p0/673	NA	R01_cb16960_c2/f1p0/673	NA	R01_cb16960_c2/f1p0/673
R01_cb3750_c17/f1p0/2207	NA	NA	NA	R01_cb3750_c17/f1p0/2207
R01_cb18455_c0/f1p0/803	R01_cb18455_c0/f1p0/803	R01_cb18455_c0/f1p0/803	R01_cb18455_c0/f1p0/803	R01_cb18455_c0/f1p0/803
R01_cb18321_c1/f1p1/334	R01_cb18321_c1/f1p1/334	R01_cb18321_c1/f1p1/334	R01_cb18321_c1/f1p1/334	R01_cb18321_c1/f1p1/334
R01_cb507_c2/f1p0/2884	NA	R01_cb507_c2/f1p0/2884	NA	R01_cb507_c2/f1p0/2884
R01_cb2851_c0/f1p0/3790	NA	R01_cb2851_c0/f1p0/3790	NA	NA
R01_cb13384_c3/f2p0/1063	NA	NA	NA	R01_cb13384_c3/f2p0/1063
R01_cb17_c21/f1p0/6823	NA	NA	NA	R01_cb17_c21/f1p0/6823

R01_cb3492_c5/f1p0/725	NA	NA	NA	R01_cb3492_c5/f1p0/725
R01_cb11150_c2/f1p0/2464	NA	R01_cb11150_c2/f1p0/2464	NA	NA
R01_cb17637_c1/f1p0/617	NA	R01_cb17637_c1/f1p0/617	NA	NA
R01_cb18545_c1/f1p0/522	R01_cb18545_c1/f1p0/522	R01_cb18545_c1/f1p0/522	R01_cb18545_c1/f1p0/522	R01_cb18545_c1/f1p0/522
R01_cb8249_c3/f1p0/2375	NA	R01_cb8249_c3/f1p0/2375	NA	NA
R01_cb10701_c4/f1p0/413	R01_cb10701_c4/f1p0/413	R01_cb10701_c4/f1p0/413	NA	NA
R01_cb18456_c6266/f1p0/1395	NA	NA	NA	R01_cb18456_c6266/f1p0/1395
R01_cb13980_c1/f1p0/1511	NA	NA	NA	R01_cb13980_c1/f1p0/1511
R01_cb16831_c0/f3p0/678	NA	NA	NA	R01_cb16831_c0/f3p0/678
R01_cb3162_c1/f1p0/3706	NA	R01_cb3162_c1/f1p0/3706	NA	R01_cb3162_c1/f1p0/3706
R01_cb17637_c2/f1p0/568	NA	R01_cb17637_c2/f1p0/568	NA	NA
R01_cb13710_c3/f1p0/362	R01_cb13710_c3/f1p0/362	R01_cb13710_c3/f1p0/362	R01_cb13710_c3/f1p0/362	R01_cb13710_c3/f1p0/362
R01_cb8564_c121564/f1p0/2376	NA	R01_cb8564_c121564/f1p0/2376	NA	NA
R01_cb9906_c1/f1p0/3344	R01_cb9906_c1/f1p0/3344	R01_cb9906_c1/f1p0/3344	R01_cb9906_c1/f1p0/3344	R01_cb9906_c1/f1p0/3344
R01_cb10068_c23/f1p0/1377	NA	R01_cb10068_c23/f1p0/1377	NA	NA
R01_cb1378_c15/f1p0/603	NA	R01_cb1378_c15/f1p0/603	R01_cb1378_c15/f1p0/603	R01_cb1378_c15/f1p0/603
R01_cb6090_c1/f1p0/2937	NA	NA	NA	R01_cb6090_c1/f1p0/2937
R01_cb5000_c7/f1p0/4027	R01_cb5000_c7/f1p0/4027	R01_cb5000_c7/f1p0/4027	R01_cb5000_c7/f1p0/4027	R01_cb5000_c7/f1p0/4027
R01_cb17886_c1/f1p0/1421	NA	NA	NA	R01_cb17886_c1/f1p0/1421
R01_cb18456_c3354/f1p10/1022	NA	R01_cb18456_c3354/f1p10/1022	NA	NA
R01_cb2959_c4/f1p0/3532	NA	NA	NA	R01_cb2959_c4/f1p0/3532
R01_cb11072_c1/f4p1/549	NA	R01_cb11072_c1/f4p1/549	NA	NA
R01_cb4357_c1/f1p0/3408	NA	R01_cb4357_c1/f1p0/3408	NA	NA
R01_cb470_c13/f1p0/2876	NA	R01_cb470_c13/f1p0/2876	NA	NA

R01_cb17873_c1/f1p0/1385	NA	R01_cb17873_c1/f1p0/1385	NA	NA
R01_cb17300_c1/f1p0/818	NA	NA	NA	R01_cb17300_c1/f1p0/818
R01_cb13910_c17/f1p0/1426	R01_cb13910_c17/f1p0/1426	R01_cb13910_c17/f1p0/1426	R01_cb13910_c17/f1p0/1426	R01_cb13910_c17/f1p0/1426
R01_cb6163_c8/f1p0/2271	NA	R01_cb6163_c8/f1p0/2271	NA	NA
R01_cb10314_c2/f2p2/703	NA	R01_cb10314_c2/f2p2/703	NA	NA
R01_cb9648_c15/f1p0/1532	NA	NA	NA	R01_cb9648_c15/f1p0/1532
R01_cb11796_c0/f1p0/1501	NA	R01_cb11796_c0/f1p0/1501	NA	NA
R01_cb18456_c6441/f1p0/843	NA	NA	R01_cb18456_c6441/f1p0/843	R01_cb18456_c6441/f1p0/843
R01_cb17474_c0/f2p0/423	NA	R01_cb17474_c0/f2p0/423	NA	NA
R01_cb822_c20/f1p1/2333	NA	NA	NA	R01_cb822_c20/f1p1/2333
R01_cb2959_c9/f1p0/2767	NA	NA	NA	R01_cb2959_c9/f1p0/2767
R01_cb8564_c40675/f1p0/2856	NA	R01_cb8564_c40675/f1p0/2856	NA	R01_cb8564_c40675/f1p0/2856
R01_cb8564_c145727/f5p1/2610	NA	R01_cb8564_c145727/f5p1/2610	NA	NA
R01_cb14937_c0/f6p0/869	NA	NA	R01_cb14937_c0/f6p0/869	R01_cb14937_c0/f6p0/869
R01_cb14125_c125/f1p0/628	NA	R01_cb14125_c125/f1p0/628	R01_cb14125_c125/f1p0/628	NA
R01_cb1178_c16/f1p0/1671	NA	NA	NA	R01_cb1178_c16/f1p0/1671
R01_cb11178_c0/f1p0/891	R01_cb11178_c0/f1p0/891	R01_cb11178_c0/f1p0/891	R01_cb11178_c0/f1p0/891	R01_cb11178_c0/f1p0/891
R01_cb2708_c13/f1p0/3388	R01_cb2708_c13/f1p0/3388	R01_cb2708_c13/f1p0/3388	NA	R01_cb2708_c13/f1p0/3388
R01_cb7584_c9/f1p0/2139	NA	R01_cb7584_c9/f1p0/2139	NA	NA
R01_cb929_c4/f1p1/2480	NA	NA	NA	R01_cb929_c4/f1p1/2480
R01_cb16465_c0/f1p1/675	R01_cb16465_c0/f1p1/675	R01_cb16465_c0/f1p1/675	R01_cb16465_c0/f1p1/675	R01_cb16465_c0/f1p1/675
R01_cb18387_c0/f3p0/336	NA	R01_cb18387_c0/f3p0/336	R01_cb18387_c0/f3p0/336	R01_cb18387_c0/f3p0/336
R01_cb1739_c12/f1p0/551	NA	NA	NA	R01_cb1739_c12/f1p0/551
R01_cb4681_c0/f1p1/3285	NA	NA	R01_cb4681_c0/f1p1/3285	NA
R01_cb5373_c0/f5p0/2920	NA	NA	NA	R01_cb5373_c0/f5p0/2920

R01_cb18196_c0/f1p0/615	R01_cb18196_c0/f1p0/615	R01_cb18196_c0/f1p0/615	NA	R01_cb18196_c0/f1p0/615
R01_cb15308_c3/f1p0/911	NA	NA	R01_cb15308_c3/f1p0/911	R01_cb15308_c3/f1p0/911
R01_cb14726_c1/f1p0/958	NA	NA	NA	R01_cb14726_c1/f1p0/958
R01_cb8564_c4488/f1p0/2817	NA	NA	NA	R01_cb8564_c4488/f1p0/2817
R01_cb18456_c7522/f1p0/775	R01_cb18456_c7522/f1p0/775	R01_cb18456_c7522/f1p0/775	R01_cb18456_c7522/f1p0/775	R01_cb18456_c7522/f1p0/775
R01_cb8564_c73473/f1p1/2503	NA	R01_cb8564_c73473/f1p1/2503	R01_cb8564_c73473/f1p1/2503	NA
R01_cb15665_c0/f1p0/1425	NA	R01_cb15665_c0/f1p0/1425	NA	NA
R01_cb4147_c3/f2p0/2421	NA	NA	NA	R01_cb4147_c3/f2p0/2421
R01_cb15999_c0/f3p0/477	NA	R01_cb15999_c0/f3p0/477	NA	NA
R01_cb10246_c3/f1p0/341	R01_cb10246_c3/f1p0/341	R01_cb10246_c3/f1p0/341	R01_cb10246_c3/f1p0/341	R01_cb10246_c3/f1p0/341
R01_cb17612_c2/f1p1/1031	NA	R01_cb17612_c2/f1p1/1031	NA	NA
R01_cb1887_c3/f1p0/2571	NA	NA	NA	R01_cb1887_c3/f1p0/2571
R01_cb4981_c3/f1p0/3014	NA	R01_cb4981_c3/f1p0/3014	NA	R01_cb4981_c3/f1p0/3014
R01_cb882_c6/f1p0/5018	NA	NA	NA	R01_cb882_c6/f1p0/5018
R01_cb18273_c1/f1p0/1345	NA	R01_cb18273_c1/f1p0/1345	NA	NA
R01_cb10414_c2/f1p1/3224	NA	R01_cb10414_c2/f1p1/3224	NA	NA
R01_cb3287_c5/f1p0/3334	NA	NA	NA	R01_cb3287_c5/f1p0/3334
R01_cb7108_c6/f1p0/2585	NA	NA	NA	R01_cb7108_c6/f1p0/2585
R01_cb10809_c1/f1p0/2163	NA	R01_cb10809_c1/f1p0/2163	NA	NA
R01_cb9088_c0/f2p0/2120	NA	NA	NA	R01_cb9088_c0/f2p0/2120
R01_cb5815_c5/f1p0/2550	NA	R01_cb5815_c5/f1p0/2550	NA	NA
R01_cb18456_c3241/f1p0/941	NA	R01_cb18456_c3241/f1p0/941	NA	NA
R01_cb15725_c7/f5p0/1434	NA	NA	NA	R01_cb15725_c7/f5p0/1434
R01_cb8564_c71381/f1p0/3321	NA	R01_cb8564_c71381/f1p0/3321	NA	NA
R01_cb6666_c9/f1p0/2543	NA	NA	NA	R01_cb6666_c9/f1p0/2543
R01_cb14898_c0/f1p0/1836	NA	NA	NA	R01_cb14898_c0/f1p0/1836

R01_cb8564_c120273/f1p0/2137	NA	R01_cb8564_c120273/f1p0/2137	NA	NA
R01_cb8564_c130068/f1p1/3908	R01_cb8564_c130068/f1p1/3908	R01_cb8564_c130068/f1p1/3908	R01_cb8564_c130068/f1p1/3908	R01_cb8564_c130068/f1p1/3908
R01_cb11802_c0/f1p0/864	R01_cb11802_c0/f1p0/864	R01_cb11802_c0/f1p0/864	NA	R01_cb11802_c0/f1p0/864
R01_cb9281_c3/f1p0/2611	NA	NA	NA	R01_cb9281_c3/f1p0/2611
R01_cb12972_c27/f1p0/525	NA	R01_cb12972_c27/f1p0/525	R01_cb12972_c27/f1p0/525	R01_cb12972_c27/f1p0/525
R01_cb1982_c1/f1p0/3354	R01_cb1982_c1/f1p0/3354	R01_cb1982_c1/f1p0/3354	R01_cb1982_c1/f1p0/3354	R01_cb1982_c1/f1p0/3354
R01_cb3774_c3/f8p0/1883	NA	NA	NA	R01_cb3774_c3/f8p0/1883
R01_cb3074_c2/f1p0/1125	NA	NA	NA	R01_cb3074_c2/f1p0/1125
R01_cb7803_c0/f1p0/2531	NA	R01_cb7803_c0/f1p0/2531	NA	NA
R01_cb8564_c25121/f1p0/1830	NA	R01_cb8564_c25121/f1p0/1830	R01_cb8564_c25121/f1p0/1830	R01_cb8564_c25121/f1p0/1830
R01_cb18287_c4/f1p0/521	NA	R01_cb18287_c4/f1p0/521	R01_cb18287_c4/f1p0/521	R01_cb18287_c4/f1p0/521
R01_cb11257_c2/f1p0/3533	NA	NA	NA	R01_cb11257_c2/f1p0/3533
R01_cb7999_c2/f1p0/2565	R01_cb7999_c2/f1p0/2565	R01_cb7999_c2/f1p0/2565	NA	R01_cb7999_c2/f1p0/2565
R01_cb4198_c7/f1p0/2272	NA	NA	NA	R01_cb4198_c7/f1p0/2272
R01_cb1668_c11/f1p0/2234	NA	R01_cb1668_c11/f1p0/2234	NA	NA
R01_cb12972_c18/f1p0/1604	NA	R01_cb12972_c18/f1p0/1604	NA	NA
R01_cb2870_c18/f2p1/2618	NA	NA	NA	R01_cb2870_c18/f2p1/2618
R01_cb8564_c9731/f2p0/2421	NA	R01_cb8564_c9731/f2p0/2421	NA	NA
R01_cb2688_c3/f1p0/2921	R01_cb2688_c3/f1p0/2921	R01_cb2688_c3/f1p0/2921	R01_cb2688_c3/f1p0/2921	R01_cb2688_c3/f1p0/2921
R01_cb5533_c143/f1p0/2760	NA	R01_cb5533_c143/f1p0/2760	NA	NA
R01_cb8564_c4719/f1p0/2639	NA	R01_cb8564_c4719/f1p0/2639	NA	R01_cb8564_c4719/f1p0/2639
R01_cb2676_c22/f1p0/2029	NA	NA	NA	R01_cb2676_c22/f1p0/2029
R01_cb3219_c2/f1p0/3884	NA	R01_cb3219_c2/f1p0/3884	NA	R01_cb3219_c2/f1p0/3884
R01_cb18417_c1/f1p0/1805	NA	R01_cb18417_c1/f1p0/1805	R01_cb18417_c1/f1p0/1805	R01_cb18417_c1/f1p0/1805

R01_cb11201_c4/f1p0/579	R01_cb11201_c4/f1p0/579	R01_cb11201_c4/f1p0/579	NA	NA
R01_cb16185_c0/f1p0/468	R01_cb16185_c0/f1p0/468	R01_cb16185_c0/f1p0/468	R01_cb16185_c0/f1p0/468	R01_cb16185_c0/f1p0/468
R01_cb8564_c52738/f1p0/3595	NA	NA	NA	R01_cb8564_c52738/f1p0/3595
R01_cb18370_c1/f1p0/692	R01_cb18370_c1/f1p0/692	R01_cb18370_c1/f1p0/692	R01_cb18370_c1/f1p0/692	R01_cb18370_c1/f1p0/692
R01_cb16772_c1/f1p0/1441	NA	NA	NA	R01_cb16772_c1/f1p0/1441
R01_cb14639_c5/f1p0/409	NA	R01_cb14639_c5/f1p0/409	NA	NA
R01_cb11574_c1/f1p0/1977	NA	NA	NA	R01_cb11574_c1/f1p0/1977
R01_cb6424_c2/f1p0/2856	NA	R01_cb6424_c2/f1p0/2856	NA	NA
R01_cb14469_c2/f1p2/688	NA	R01_cb14469_c2/f1p2/688	R01_cb14469_c2/f1p2/688	R01_cb14469_c2/f1p2/688
R01_cb17042_c0/f1p1/1015	NA	R01_cb17042_c0/f1p1/1015	R01_cb17042_c0/f1p1/1015	R01_cb17042_c0/f1p1/1015
R01_cb15019_c1/f4p0/564	R01_cb15019_c1/f4p0/564	R01_cb15019_c1/f4p0/564	R01_cb15019_c1/f4p0/564	R01_cb15019_c1/f4p0/564
R01_cb5782_c4/f2p0/2916	NA	NA	NA	R01_cb5782_c4/f2p0/2916
R01_cb8568_c21/f1p0/2336	R01_cb8568_c21/f1p0/2336	R01_cb8568_c21/f1p0/2336	R01_cb8568_c21/f1p0/2336	R01_cb8568_c21/f1p0/2336
R01_cb16092_c5/f1p0/420	R01_cb16092_c5/f1p0/420	R01_cb16092_c5/f1p0/420	R01_cb16092_c5/f1p0/420	R01_cb16092_c5/f1p0/420
R01_cb13464_c10/f30p2/598	NA	R01_cb13464_c10/f30p2/598	NA	NA
R01_cb8564_c115053/f1p0/3109	NA	R01_cb8564_c115053/f1p0/3109	NA	NA
R01_cb8564_c106041/f2p2/2940	NA	NA	NA	R01_cb8564_c106041/f2p2/2940
R01_cb16653_c0/f2p0/1555	NA	NA	NA	R01_cb16653_c0/f2p0/1555
R01_cb7450_c7/f1p0/5653	NA	R01_cb7450_c7/f1p0/5653	NA	NA
R01_cb7029_c1/f1p0/2712	NA	R01_cb7029_c1/f1p0/2712	R01_cb7029_c1/f1p0/2712	R01_cb7029_c1/f1p0/2712
R01_cb110_c9/f1p0/3058	NA	NA	NA	R01_cb110_c9/f1p0/3058
R01_cb3791_c4/f1p0/2359	NA	NA	NA	R01_cb3791_c4/f1p0/2359
R01_cb17765_c0/f3p0/654	NA	R01_cb17765_c0/f3p0/654	R01_cb17765_c0/f3p0/654	R01_cb17765_c0/f3p0/654
R01_cb18456_c3308/f1p3/1087	NA	R01_cb18456_c3308/f1p3/1087	NA	NA

R01_cb7595_c16/f1p0/1434	NA	R01_cb7595_c16/f1p0/1434	NA	NA
R01_cb6328_c2/f1p0/2742	NA	NA	NA	R01_cb6328_c2/f1p0/2742
R01_cb8564_c2592/f1p0/4172	NA	R01_cb8564_c2592/f1p0/4172	NA	NA
R01_cb8863_c5/f1p0/2026	NA	R01_cb8863_c5/f1p0/2026	NA	NA
R01_cb1813_c13/f1p0/2526	NA	NA	NA	R01_cb1813_c13/f1p0/2526
R01_cb12012_c2/f1p0/1624	NA	NA	NA	R01_cb12012_c2/f1p0/1624
R01_cb8365_c2/f1p0/2001	NA	R01_cb8365_c2/f1p0/2001	NA	NA
R01_cb5040_c69/f1p1/3154	NA	NA	NA	R01_cb5040_c69/f1p1/3154
R01_cb8564_c121005/f1p1/1998	NA	NA	NA	R01_cb8564_c121005/f1p1/1998
R01_cb5131_c3/f1p0/2671	NA	R01_cb5131_c3/f1p0/2671	NA	NA
R01_cb4169_c7/f1p0/1995	NA	NA	NA	R01_cb4169_c7/f1p0/1995
R01_cb17184_c3/f1p0/572	R01_cb17184_c3/f1p0/572	R01_cb17184_c3/f1p0/572	NA	NA
R01_cb8564_c3819/f1p1/2846	NA	NA	NA	R01_cb8564_c3819/f1p1/2846
R01_cb10604_c1/f1p0/2230	NA	R01_cb10604_c1/f1p0/2230	R01_cb10604_c1/f1p0/2230	NA
R01_cb422_c0/f1p0/4825	NA	NA	NA	R01_cb422_c0/f1p0/4825
R01_cb17652_c1/f1p0/1020	NA	NA	R01_cb17652_c1/f1p0/1020	R01_cb17652_c1/f1p0/1020
R01_cb5745_c5/f1p0/1695	NA	NA	NA	R01_cb5745_c5/f1p0/1695
R01_cb2400_c1/f1p0/2223	NA	R01_cb2400_c1/f1p0/2223	R01_cb2400_c1/f1p0/2223	R01_cb2400_c1/f1p0/2223
R01_cb9270_c4/f1p0/2740	R01_cb9270_c4/f1p0/2740	R01_cb9270_c4/f1p0/2740	R01_cb9270_c4/f1p0/2740	R01_cb9270_c4/f1p0/2740
R01_cb11793_c0/f1p0/1497	NA	R01_cb11793_c0/f1p0/1497	NA	NA
R01_cb8564_c80211/f1p0/4044	R01_cb8564_c80211/f1p0/4044	R01_cb8564_c80211/f1p0/4044	R01_cb8564_c80211/f1p0/4044	R01_cb8564_c80211/f1p0/4044
R01_cb6982_c2/f1p0/1326	R01_cb6982_c2/f1p0/1326	R01_cb6982_c2/f1p0/1326	R01_cb6982_c2/f1p0/1326	R01_cb6982_c2/f1p0/1326
R01_cb8564_c86065/f1p0/3002	R01_cb8564_c86065/f1p0/3002	R01_cb8564_c86065/f1p0/3002	R01_cb8564_c86065/f1p0/3002	R01_cb8564_c86065/f1p0/3002

R01_cb11451_c1/f1p0/2542	NA	R01_cb11451_c1/f1p0/2542	NA	R01_cb11451_c1/f1p0/2542
R01_cb5533_c166/f1p0/684	R01_cb5533_c166/f1p0/684	R01_cb5533_c166/f1p0/684	R01_cb5533_c166/f1p0/684	R01_cb5533_c166/f1p0/684
R01_cb313_c17/f1p0/3434	NA	R01_cb313_c17/f1p0/3434	NA	NA
R01_cb9656_c0/f1p1/2020	NA	NA	NA	R01_cb9656_c0/f1p1/2020
R01_cb17_c19/f1p0/2966	NA	NA	NA	R01_cb17_c19/f1p0/2966
R01_cb11413_c3/f1p0/1901	NA	R01_cb11413_c3/f1p0/1901	R01_cb11413_c3/f1p0/1901	R01_cb11413_c3/f1p0/1901
R01_cb10029_c107/f1p3/795	NA	R01_cb10029_c107/f1p3/795	NA	NA
R01_cb8564_c80030/f1p0/3424	NA	R01_cb8564_c80030/f1p0/3424	NA	R01_cb8564_c80030/f1p0/3424
R01_cb18456_c6962/f1p0/640	R01_cb18456_c6962/f1p0/640	R01_cb18456_c6962/f1p0/640	NA	NA
R01_cb16896_c0/f1p0/1117	NA	R01_cb16896_c0/f1p0/1117	NA	R01_cb16896_c0/f1p0/1117
R01_cb3896_c3/f1p0/3491	R01_cb3896_c3/f1p0/3491	NA	NA	R01_cb3896_c3/f1p0/3491
R01_cb14157_c2/f1p0/1488	NA	R01_cb14157_c2/f1p0/1488	NA	NA
R01_cb8628_c2/f1p0/1062	NA	NA	NA	R01_cb8628_c2/f1p0/1062
R01_cb16341_c9/f1p0/440	R01_cb16341_c9/f1p0/440	R01_cb16341_c9/f1p0/440	R01_cb16341_c9/f1p0/440	R01_cb16341_c9/f1p0/440
R01_cb1228_c51/f1p0/1417	NA	NA	NA	R01_cb1228_c51/f1p0/1417
R01_cb8564_c34638/f1p0/2817	NA	R01_cb8564_c34638/f1p0/2817	NA	NA
R01_cb8564_c87713/f1p0/2432	NA	R01_cb8564_c87713/f1p0/2432	NA	NA
R01_cb10015_c246/f1p0/690	R01_cb10015_c246/f1p0/690	R01_cb10015_c246/f1p0/690	NA	NA
R01_cb13261_c3/f1p0/883	NA	NA	NA	R01_cb13261_c3/f1p0/883
R01_cb8564_c73593/f1p0/3554	NA	R01_cb8564_c73593/f1p0/3554	NA	NA
R01_cb2352_c4/f1p0/3973	NA	R01_cb2352_c4/f1p0/3973	R01_cb2352_c4/f1p0/3973	R01_cb2352_c4/f1p0/3973
R01_cb4041_c0/f2p0/3129	NA	R01_cb4041_c0/f2p0/3129	NA	R01_cb4041_c0/f2p0/3129
R01_cb8564_c127788/f1p0/2800	NA	R01_cb8564_c127788/f1p0/2800	NA	NA
R01_cb13675_c2/f1p0/792	NA	NA	R01_cb13675_c2/f1p0/792	NA
R01_cb8564_c133426/f1p2/279	NA	R01_cb8564_c133426/f1p2/279	NA	NA

7		7		
R01_cb10649_c2/f1p0/826	NA	R01_cb10649_c2/f1p0/826	NA	NA
R01_cb8564_c123147/f1p0/2657	NA	NA	NA	R01_cb8564_c123147/f1p0/2657
R01_cb14945_c6/f1p0/1110	NA	R01_cb14945_c6/f1p0/1110	NA	NA
R01_cb8564_c127658/f1p0/3567	NA	NA	NA	R01_cb8564_c127658/f1p0/3567
R01_cb10190_c2/f1p0/2194	R01_cb10190_c2/f1p0/2194	R01_cb10190_c2/f1p0/2194	R01_cb10190_c2/f1p0/2194	R01_cb10190_c2/f1p0/2194
R01_cb17744_c17/f1p0/3235	NA	NA	NA	R01_cb17744_c17/f1p0/3235
R01_cb8868_c0/f2p0/2247	NA	R01_cb8868_c0/f2p0/2247	R01_cb8868_c0/f2p0/2247	R01_cb8868_c0/f2p0/2247
R01_cb10849_c7/f1p0/671	NA	R01_cb10849_c7/f1p0/671	NA	R01_cb10849_c7/f1p0/671
R01_cb2007_c2/f2p1/2738	NA	R01_cb2007_c2/f2p1/2738	NA	NA
R01_cb17453_c0/f1p0/763	NA	NA	R01_cb17453_c0/f1p0/763	R01_cb17453_c0/f1p0/763
R01_cb8345_c1/f1p0/2379	NA	NA	NA	R01_cb8345_c1/f1p0/2379
R01_cb15550_c1/f1p0/622	R01_cb15550_c1/f1p0/622	R01_cb15550_c1/f1p0/622	NA	R01_cb15550_c1/f1p0/622
R01_cb8564_c50449/f1p0/3504	NA	R01_cb8564_c50449/f1p0/3504	NA	NA
R01_cb9467_c28/f1p0/2130	NA	R01_cb9467_c28/f1p0/2130	NA	NA
R01_cb18456_c7319/f1p0/480	R01_cb18456_c7319/f1p0/480	R01_cb18456_c7319/f1p0/480	R01_cb18456_c7319/f1p0/480	R01_cb18456_c7319/f1p0/480
R01_cb8564_c118694/f1p0/2303	NA	R01_cb8564_c118694/f1p0/2303	NA	NA
R01_cb12775_c0/f3p2/1307	NA	NA	NA	R01_cb12775_c0/f3p2/1307
R01_cb124_c26/f1p0/3756	NA	NA	NA	R01_cb124_c26/f1p0/3756
R01_cb17049_c1/f1p0/700	NA	R01_cb17049_c1/f1p0/700	NA	R01_cb17049_c1/f1p0/700
R01_cb6513_c1/f3p0/2254	NA	NA	NA	R01_cb6513_c1/f3p0/2254
R01_cb17266_c5/f1p0/943	NA	R01_cb17266_c5/f1p0/943	R01_cb17266_c5/f1p0/943	R01_cb17266_c5/f1p0/943
R01_cb1468_c8/f1p0/2956	NA	R01_cb1468_c8/f1p0/2956	NA	NA

R01_cb16814_c0/f1p0/719	NA	NA	NA	R01_cb16814_c0/f1p0/719
R01_cb8564_c118539/f1p0/2391	R01_cb8564_c118539/f1p0/2391	R01_cb8564_c118539/f1p0/2391	R01_cb8564_c118539/f1p0/2391	R01_cb8564_c118539/f1p0/2391
R01_cb14083_c1/f1p0/900	NA	R01_cb14083_c1/f1p0/900	NA	NA
R01_cb18456_c6975/f1p0/362	R01_cb18456_c6975/f1p0/362	R01_cb18456_c6975/f1p0/362	R01_cb18456_c6975/f1p0/362	R01_cb18456_c6975/f1p0/362
R01_cb2779_c1/f1p1/3812	NA	NA	NA	R01_cb2779_c1/f1p1/3812
R01_cb15911_c2/f1p0/1696	NA	R01_cb15911_c2/f1p0/1696	NA	NA
R01_cb18456_c5603/f1p0/1203	NA	R01_cb18456_c5603/f1p0/1203	NA	NA
R01_cb8564_c120968/f2p0/2695	NA	R01_cb8564_c120968/f2p0/2695	NA	R01_cb8564_c120968/f2p0/2695
R01_cb8681_c2/f1p0/2210	NA	R01_cb8681_c2/f1p0/2210	R01_cb8681_c2/f1p0/2210	R01_cb8681_c2/f1p0/2210
R01_cb18014_c0/f1p0/1261	NA	NA	NA	R01_cb18014_c0/f1p0/1261
R01_cb3813_c3/f1p0/1831	NA	R01_cb3813_c3/f1p0/1831	NA	NA
R01_cb8564_c69617/f4p0/3013	NA	R01_cb8564_c69617/f4p0/3013	NA	R01_cb8564_c69617/f4p0/3013
R01_cb6597_c15/f1p0/2081	NA	NA	NA	R01_cb6597_c15/f1p0/2081
R01_cb8564_c3047/f1p0/2490	NA	R01_cb8564_c3047/f1p0/2490	R01_cb8564_c3047/f1p0/2490	R01_cb8564_c3047/f1p0/2490
R01_cb8564_c75247/f1p2/2626	R01_cb8564_c75247/f1p2/2626	R01_cb8564_c75247/f1p2/2626	NA	NA
R01_cb10799_c2/f1p0/3682	NA	R01_cb10799_c2/f1p0/3682	NA	R01_cb10799_c2/f1p0/3682
R01_cb10648_c3/f1p1/797	NA	R01_cb10648_c3/f1p1/797	NA	NA
R01_cb8564_c3324/f1p0/2768	R01_cb8564_c3324/f1p0/2768	R01_cb8564_c3324/f1p0/2768	R01_cb8564_c3324/f1p0/2768	R01_cb8564_c3324/f1p0/2768
R01_cb18456_c5636/f1p0/490	NA	NA	NA	R01_cb18456_c5636/f1p0/490
R01_cb16665_c1/f1p0/996	NA	NA	R01_cb16665_c1/f1p0/996	R01_cb16665_c1/f1p0/996
R01_cb4410_c0/f2p0/3318	NA	R01_cb4410_c0/f2p0/3318	NA	R01_cb4410_c0/f2p0/3318
R01_cb10015_c611/f15p0/580	R01_cb10015_c611/f15p0/580	R01_cb10015_c611/f15p0/580	R01_cb10015_c611/f15p0/580	NA
R01_cb13761_c12/f1p0/655	R01_cb13761_c12/f1p0/655	R01_cb13761_c12/f1p0/655	R01_cb13761_c12/f1p0/655	R01_cb13761_c12/f1p0/655

R01_cb6328_c0/f4p0/2707	NA	NA	NA	R01_cb6328_c0/f4p0/2707
R01_cb3859_c1/f1p0/3510	NA	R01_cb3859_c1/f1p0/3510	NA	R01_cb3859_c1/f1p0/3510
R01_cb5042_c4/f1p0/3767	NA	NA	R01_cb5042_c4/f1p0/3767	NA
R01_cb13545_c53/f1p0/416	R01_cb13545_c53/f1p0/416	R01_cb13545_c53/f1p0/416	R01_cb13545_c53/f1p0/416	R01_cb13545_c53/f1p0/416
R01_cb8564_c14679/f1p0/3095	R01_cb8564_c14679/f1p0/3095	R01_cb8564_c14679/f1p0/3095	R01_cb8564_c14679/f1p0/3095	R01_cb8564_c14679/f1p0/3095
R01_cb4631_c12/f1p0/2111	NA	NA	NA	R01_cb4631_c12/f1p0/2111
R01_cb647_c14/f1p2/1446	NA	NA	NA	R01_cb647_c14/f1p2/1446
R01_cb11771_c0/f1p0/1449	NA	NA	R01_cb11771_c0/f1p0/1449	R01_cb11771_c0/f1p0/1449
R01_cb8564_c70659/f1p0/4083	NA	NA	NA	R01_cb8564_c70659/f1p0/4083
R01_cb9777_c3/f1p0/1950	NA	NA	NA	R01_cb9777_c3/f1p0/1950
R01_cb6706_c1/f3p0/1450	NA	NA	NA	R01_cb6706_c1/f3p0/1450
R01_cb8023_c5/f1p0/2277	NA	NA	R01_cb8023_c5/f1p0/2277	R01_cb8023_c5/f1p0/2277
R01_cb6170_c17/f1p1/2649	NA	R01_cb6170_c17/f1p1/2649	NA	R01_cb6170_c17/f1p1/2649
R01_cb438_c9/f1p0/2672	NA	NA	NA	R01_cb438_c9/f1p0/2672
R01_cb8564_c25521/f1p0/2483	NA	NA	NA	R01_cb8564_c25521/f1p0/2483
R01_cb15255_c2/f1p0/849	NA	NA	NA	R01_cb15255_c2/f1p0/849
R01_cb2045_c6/f1p0/2314	NA	NA	NA	R01_cb2045_c6/f1p0/2314
R01_cb8564_c19603/f1p0/2850	NA	NA	NA	R01_cb8564_c19603/f1p0/2850
R01_cb8564_c122642/f1p0/3006	R01_cb8564_c122642/f1p0/3006	R01_cb8564_c122642/f1p0/3006	R01_cb8564_c122642/f1p0/3006	R01_cb8564_c122642/f1p0/3006
R01_cb8564_c90576/f2p0/2673	NA	NA	R01_cb8564_c90576/f2p0/2673	R01_cb8564_c90576/f2p0/2673
R01_cb8564_c1189/f1p0/2710	NA	R01_cb8564_c1189/f1p0/2710	NA	NA
R01_cb5533_c132/f1p0/2007	NA	NA	NA	R01_cb5533_c132/f1p0/2007
R01_cb6113_c6/f1p0/2008	NA	NA	NA	R01_cb6113_c6/f1p0/2008
R01_cb8564_c38068/f1p0/3466	R01_cb8564_c38068/f1p0/346	R01_cb8564_c38068/f1p0/3466	R01_cb8564_c38068/f1p0/3466	R01_cb8564_c38068/f1p0/3466

	6			
R01_cb4334_c2/f1p0/3372	NA	R01_cb4334_c2/f1p0/3372	NA	NA
R01_cb525_c10/f6p0/2020	NA	NA	NA	R01_cb525_c10/f6p0/2020
R01_cb17791_c1/f1p0/1721	NA	NA	NA	R01_cb17791_c1/f1p0/1721
R01_cb1813_c7/f2p1/2805	NA	NA	NA	R01_cb1813_c7/f2p1/2805
R01_cb14615_c3/f1p0/1131	NA	NA	NA	R01_cb14615_c3/f1p0/1131
R01_cb2682_c1/f1p0/3843	NA	NA	NA	R01_cb2682_c1/f1p0/3843
R01_cb10409_c10/f1p0/924	NA	R01_cb10409_c10/f1p0/924	NA	NA
R01_cb8564_c17839/f2p0/4068	R01_cb8564_c17839/f2p0/406<br>8	R01_cb8564_c17839/f2p0/4068	R01_cb8564_c17839/f2p0/4068	R01_cb8564_c17839/f2p0/4068
R01_cb8564_c3602/f1p0/3523	NA	R01_cb8564_c3602/f1p0/3523	NA	NA
R01_cb16383_c3/f1p0/1718	NA	R01_cb16383_c3/f1p0/1718	NA	R01_cb16383_c3/f1p0/1718
R01_cb8564_c52810/f1p0/3803	NA	R01_cb8564_c52810/f1p0/3803	NA	R01_cb8564_c52810/f1p0/3803
R01_cb11662_c2/f1p0/1096	NA	NA	NA	R01_cb11662_c2/f1p0/1096
R01_cb8564_c80050/f1p0/2530	NA	NA	NA	R01_cb8564_c80050/f1p0/2530
R01_cb14940_c4/f1p0/663	NA	R01_cb14940_c4/f1p0/663	R01_cb14940_c4/f1p0/663	R01_cb14940_c4/f1p0/663
R01_cb10600_c2/f1p0/838	NA	NA	NA	R01_cb10600_c2/f1p0/838
R01_cb2674_c25/f1p0/2966	NA	R01_cb2674_c25/f1p0/2966	NA	R01_cb2674_c25/f1p0/2966
R01_cb14125_c124/f1p0/597	NA	R01_cb14125_c124/f1p0/597	R01_cb14125_c124/f1p0/597	NA
R01_cb2520_c11/f1p0/3186	NA	R01_cb2520_c11/f1p0/3186	NA	NA
R01_cb8564_c14666/f1p0/2420	NA	R01_cb8564_c14666/f1p0/2420	NA	NA
R01_cb176_c2/f1p0/2483	NA	NA	NA	R01_cb176_c2/f1p0/2483
R01_cb3929_c5/f1p0/2160	R01_cb3929_c5/f1p0/2160	R01_cb3929_c5/f1p0/2160	NA	NA
R01_cb8279_c17/f1p0/5208	NA	NA	NA	R01_cb8279_c17/f1p0/5208
R01_cb11614_c1/f1p0/2308	NA	NA	NA	R01_cb11614_c1/f1p0/2308
R01_cb10541_c3/f1p0/617	R01_cb10541_c3/f1p0/617	R01_cb10541_c3/f1p0/617	R01_cb10541_c3/f1p0/617	R01_cb10541_c3/f1p0/617

R01_cb1905_c3/f2p0/2341	NA	NA	R01_cb1905_c3/f2p0/2341	NA
R01_cb5658_c3/f1p0/2974	NA	R01_cb5658_c3/f1p0/2974	NA	R01_cb5658_c3/f1p0/2974
R01_cb7708_c3/f1p0/2328	NA	R01_cb7708_c3/f1p0/2328	NA	NA
R01_cb5117_c4/f1p0/837	R01_cb5117_c4/f1p0/837	R01_cb5117_c4/f1p0/837	R01_cb5117_c4/f1p0/837	R01_cb5117_c4/f1p0/837
R01_cb4679_c4/f1p0/1131	NA	R01_cb4679_c4/f1p0/1131	NA	NA
R01_cb12775_c1/f2p1/1261	NA	NA	NA	R01_cb12775_c1/f2p1/1261
R01_cb4178_c1/f1p0/3412	NA	R01_cb4178_c1/f1p0/3412	NA	R01_cb4178_c1/f1p0/3412
R01_cb3426_c4/f1p0/3628	NA	NA	NA	R01_cb3426_c4/f1p0/3628
R01_cb1077_c42/f1p0/2003	NA	NA	NA	R01_cb1077_c42/f1p0/2003
R01_cb1248_c3/f1p0/3470	NA	NA	NA	R01_cb1248_c3/f1p0/3470
R01_cb2771_c12/f1p1/2633	NA	NA	NA	R01_cb2771_c12/f1p1/2633
R01_cb17266_c19/f1p0/1545	R01_cb17266_c19/f1p0/1545	R01_cb17266_c19/f1p0/1545	NA	R01_cb17266_c19/f1p0/1545
R01_cb7441_c10/f1p0/587	NA	R01_cb7441_c10/f1p0/587	R01_cb7441_c10/f1p0/587	R01_cb7441_c10/f1p0/587
R01_cb9784_c1/f1p0/2033	R01_cb9784_c1/f1p0/2033	R01_cb9784_c1/f1p0/2033	R01_cb9784_c1/f1p0/2033	R01_cb9784_c1/f1p0/2033
R01_cb8564_c33818/f3p0/2136	R01_cb8564_c33818/f3p0/2136	R01_cb8564_c33818/f3p0/2136	R01_cb8564_c33818/f3p0/2136	R01_cb8564_c33818/f3p0/2136
R01_cb17756_c24/f1p0/770	R01_cb17756_c24/f1p0/770	R01_cb17756_c24/f1p0/770	R01_cb17756_c24/f1p0/770	R01_cb17756_c24/f1p0/770
R01_cb6680_c3/f1p0/2510	NA	NA	NA	R01_cb6680_c3/f1p0/2510
R01_cb7010_c16/f1p1/1971	NA	NA	NA	R01_cb7010_c16/f1p1/1971
R01_cb12589_c1/f2p0/1003	NA	NA	NA	R01_cb12589_c1/f2p0/1003
R01_cb8564_c54057/f1p0/3595	NA	R01_cb8564_c54057/f1p0/3595	NA	R01_cb8564_c54057/f1p0/3595
R01_cb17162_c0/f2p0/565	NA	NA	NA	R01_cb17162_c0/f2p0/565
R01_cb2851_c2/f1p0/2309	NA	NA	NA	R01_cb2851_c2/f1p0/2309
R01_cb1388_c37/f1p0/5272	NA	NA	NA	R01_cb1388_c37/f1p0/5272
R01_cb8564_c1021/f1p0/2810	NA	R01_cb8564_c1021/f1p0/2810	NA	NA
R01_cb11009_c0/f2p0/525	R01_cb11009_c0/f2p0/525	R01_cb11009_c0/f2p0/525	R01_cb11009_c0/f2p0/525	R01_cb11009_c0/f2p0/525

R01_cb843_c4/f1p1/2300	NA	NA	NA	R01_cb843_c4/f1p1/2300
R01_cb737_c56/f1p0/2325	R01_cb737_c56/f1p0/2325	R01_cb737_c56/f1p0/2325	R01_cb737_c56/f1p0/2325	NA
R01_cb3680_c3/f1p0/3095	NA	NA	NA	R01_cb3680_c3/f1p0/3095
R01_cb8564_c71559/f1p0/4717	R01_cb8564_c71559/f1p0/4717	R01_cb8564_c71559/f1p0/4717	R01_cb8564_c71559/f1p0/4717	NA
R01_cb14166_c3/f1p0/583	R01_cb14166_c3/f1p0/583	R01_cb14166_c3/f1p0/583	R01_cb14166_c3/f1p0/583	R01_cb14166_c3/f1p0/583
R01_cb18350_c1/f1p0/1195	NA	NA	NA	R01_cb18350_c1/f1p0/1195
R01_cb1228_c39/f1p0/2553	NA	NA	NA	R01_cb1228_c39/f1p0/2553
R01_cb8564_c15388/f1p0/3616	NA	R01_cb8564_c15388/f1p0/3616	NA	NA
R01_cb17951_c1/f1p0/1268	NA	NA	NA	R01_cb17951_c1/f1p0/1268
R01_cb7814_c13/f4p1/2570	NA	NA	NA	R01_cb7814_c13/f4p1/2570
R01_cb6238_c15/f1p0/2699	NA	R01_cb6238_c15/f1p0/2699	NA	NA
R01_cb12577_c4/f1p0/447	R01_cb12577_c4/f1p0/447	R01_cb12577_c4/f1p0/447	R01_cb12577_c4/f1p0/447	R01_cb12577_c4/f1p0/447
R01_cb7087_c7/f1p0/1773	NA	NA	NA	R01_cb7087_c7/f1p0/1773
R01_cb6845_c19/f1p0/2408	NA	NA	NA	R01_cb6845_c19/f1p0/2408
R01_cb12328_c3/f1p1/1591	NA	NA	NA	R01_cb12328_c3/f1p1/1591
R01_cb14596_c1/f1p0/1482	NA	R01_cb14596_c1/f1p0/1482	R01_cb14596_c1/f1p0/1482	R01_cb14596_c1/f1p0/1482
R01_cb15157_c4/f1p1/895	NA	R01_cb15157_c4/f1p1/895	NA	NA
R01_cb13767_c8/f1p0/429	R01_cb13767_c8/f1p0/429	R01_cb13767_c8/f1p0/429	NA	R01_cb13767_c8/f1p0/429
R01_cb10029_c1010/f1p0/871	NA	R01_cb10029_c1010/f1p0/871	NA	NA
R01_cb8981_c1/f1p1/2166	NA	NA	NA	R01_cb8981_c1/f1p1/2166
R01_cb5297_c3/f1p0/2341	R01_cb5297_c3/f1p0/2341	R01_cb5297_c3/f1p0/2341	NA	NA
R01_cb13834_c4/f1p0/1110	NA	R01_cb13834_c4/f1p0/1110	NA	NA
R01_cb14425_c0/f6p0/630	NA	NA	NA	R01_cb14425_c0/f6p0/630
R01_cb6534_c2/f1p0/2807	NA	R01_cb6534_c2/f1p0/2807	NA	NA
R01_cb10263_c2/f1p0/3546	NA	R01_cb10263_c2/f1p0/3546	NA	NA

R01_cb12871_c5/f1p0/463	R01_cb12871_c5/f1p0/463	R01_cb12871_c5/f1p0/463	NA	R01_cb12871_c5/f1p0/463
R01_cb10880_c9/f2p1/777	NA	NA	NA	R01_cb10880_c9/f2p1/777
R01_cb15773_c2/f1p0/524	R01_cb15773_c2/f1p0/524	R01_cb15773_c2/f1p0/524	NA	NA
R01_cb6170_c4/f2p2/2886	NA	NA	NA	R01_cb6170_c4/f2p2/2886
R01_cb10815_c6/f1p0/607	R01_cb10815_c6/f1p0/607	R01_cb10815_c6/f1p0/607	R01_cb10815_c6/f1p0/607	R01_cb10815_c6/f1p0/607
R01_cb18456_c7386/f1p0/866	R01_cb18456_c7386/f1p0/866	R01_cb18456_c7386/f1p0/866	R01_cb18456_c7386/f1p0/866	R01_cb18456_c7386/f1p0/866
R01_cb3774_c17/f1p1/3530	NA	NA	NA	R01_cb3774_c17/f1p1/3530
R01_cb9154_c8/f1p0/1641	NA	NA	NA	R01_cb9154_c8/f1p0/1641
R01_cb8975_c6/f1p0/2082	NA	NA	NA	R01_cb8975_c6/f1p0/2082
R01_cb3017_c6/f1p0/2424	R01_cb3017_c6/f1p0/2424	R01_cb3017_c6/f1p0/2424	R01_cb3017_c6/f1p0/2424	R01_cb3017_c6/f1p0/2424
R01_cb12480_c10/f1p0/514	R01_cb12480_c10/f1p0/514	R01_cb12480_c10/f1p0/514	R01_cb12480_c10/f1p0/514	NA
R01_cb15371_c13/f46p0/657	NA	R01_cb15371_c13/f46p0/657	NA	NA
R01_cb18456_c7377/f1p1/599	NA	NA	NA	R01_cb18456_c7377/f1p1/599
R01_cb15789_c0/f1p0/1295	NA	R01_cb15789_c0/f1p0/1295	R01_cb15789_c0/f1p0/1295	NA
R01_cb8564_c70600/f1p2/3373	NA	NA	NA	R01_cb8564_c70600/f1p2/3373
R01_cb15869_c0/f1p2/968	R01_cb15869_c0/f1p2/968	NA	NA	NA
R01_cb13696_c2/f1p0/1495	NA	NA	NA	R01_cb13696_c2/f1p0/1495
R01_cb10970_c10/f1p0/1276	NA	NA	NA	R01_cb10970_c10/f1p0/1276
R01_cb11744_c2/f1p0/977	NA	NA	NA	R01_cb11744_c2/f1p0/977
R01_cb12575_c3/f1p0/781	NA	NA	NA	R01_cb12575_c3/f1p0/781
R01_cb1228_c29/f1p0/2586	NA	NA	NA	R01_cb1228_c29/f1p0/2586
R01_cb10060_c2/f1p0/2699	NA	R01_cb10060_c2/f1p0/2699	NA	NA
R01_cb18456_c7433/f1p0/585	NA	R01_cb18456_c7433/f1p0/585	R01_cb18456_c7433/f1p0/585	R01_cb18456_c7433/f1p0/585
R01_cb8089_c1/f1p0/2464	NA	R01_cb8089_c1/f1p0/2464	R01_cb8089_c1/f1p0/2464	NA
R01_cb11507_c1/f1p0/3442	NA	NA	NA	R01_cb11507_c1/f1p0/3442
R01_cb5183_c36/f1p0/2369	NA	NA	NA	R01_cb5183_c36/f1p0/2369

R01_cb17690_c0/f2p0/446	R01_cb17690_c0/f2p0/446	R01_cb17690_c0/f2p0/446	R01_cb17690_c0/f2p0/446	R01_cb17690_c0/f2p0/446
R01_cb8080_c0/f2p0/2374	NA	NA	R01_cb8080_c0/f2p0/2374	NA
R01_cb2161_c13/f1p0/5935	NA	NA	NA	R01_cb2161_c13/f1p0/5935
R01_cb593_c9/f1p0/4075	NA	R01_cb593_c9/f1p0/4075	NA	NA
R01_cb15149_c3/f1p0/886	R01_cb15149_c3/f1p0/886	R01_cb15149_c3/f1p0/886	NA	NA
R01_cb8279_c12/f1p0/2210	NA	NA	NA	R01_cb8279_c12/f1p0/2210
R01_cb18409_c94/f1p0/521	R01_cb18409_c94/f1p0/521	R01_cb18409_c94/f1p0/521	R01_cb18409_c94/f1p0/521	R01_cb18409_c94/f1p0/521
R01_cb18610_c5/f1p0/1491	NA	NA	NA	R01_cb18610_c5/f1p0/1491
R01_cb4916_c7/f1p0/2248	NA	NA	NA	R01_cb4916_c7/f1p0/2248
R01_cb15018_c2/f1p1/645	NA	R01_cb15018_c2/f1p1/645	R01_cb15018_c2/f1p1/645	R01_cb15018_c2/f1p1/645
R01_cb15251_c0/f2p0/1736	NA	NA	NA	R01_cb15251_c0/f2p0/1736
R01_cb5952_c2/f1p0/2152	NA	NA	NA	R01_cb5952_c2/f1p0/2152
R01_cb13137_c3/f2p0/1214	NA	R01_cb13137_c3/f2p0/1214	NA	NA
R01_cb2644_c43/f1p0/492	R01_cb2644_c43/f1p0/492	R01_cb2644_c43/f1p0/492	R01_cb2644_c43/f1p0/492	R01_cb2644_c43/f1p0/492
R01_cb8564_c44645/f2p0/2348	R01_cb8564_c44645/f2p0/2348	R01_cb8564_c44645/f2p0/2348	R01_cb8564_c44645/f2p0/2348	R01_cb8564_c44645/f2p0/2348
R01_cb15891_c4/f1p0/1791	NA	R01_cb15891_c4/f1p0/1791	NA	NA
R01_cb3639_c0/f1p0/3568	NA	R01_cb3639_c0/f1p0/3568	NA	R01_cb3639_c0/f1p0/3568
R01_cb16966_c0/f2p0/549	NA	R01_cb16966_c0/f2p0/549	NA	NA
R01_cb1675_c0/f4p0/3209	NA	NA	NA	R01_cb1675_c0/f4p0/3209
R01_cb8564_c74023/f1p0/2120	NA	R01_cb8564_c74023/f1p0/2120	R01_cb8564_c74023/f1p0/2120	R01_cb8564_c74023/f1p0/2120
R01_cb8564_c1414/f1p0/2727	NA	R01_cb8564_c1414/f1p0/2727	NA	NA
R01_cb8564_c90385/f1p0/3230	NA	R01_cb8564_c90385/f1p0/3230	NA	R01_cb8564_c90385/f1p0/3230
R01_cb18192_c9/f1p0/987	NA	R01_cb18192_c9/f1p0/987	R01_cb18192_c9/f1p0/987	R01_cb18192_c9/f1p0/987
R01_cb17421_c0/f1p0/552	NA	R01_cb17421_c0/f1p0/552	NA	NA
R01_cb10402_c6/f1p0/686	NA	R01_cb10402_c6/f1p0/686	NA	NA

R01_cb8564_c80418/f1p0/3809	R01_cb8564_c80418/f1p0/3809	R01_cb8564_c80418/f1p0/3809	R01_cb8564_c80418/f1p0/3809	R01_cb8564_c80418/f1p0/3809
R01_cb8564_c16861/f1p0/2967	NA	R01_cb8564_c16861/f1p0/2967	NA	R01_cb8564_c16861/f1p0/2967
R01_cb8564_c4621/f1p0/3187	NA	R01_cb8564_c4621/f1p0/3187	R01_cb8564_c4621/f1p0/3187	R01_cb8564_c4621/f1p0/3187
R01_cb405_c17/f1p0/2482	NA	R01_cb405_c17/f1p0/2482	NA	NA
R01_cb8564_c88600/f1p0/3686	NA	R01_cb8564_c88600/f1p0/3686	NA	NA
R01_cb10021_c3/f1p0/2850	R01_cb10021_c3/f1p0/2850	R01_cb10021_c3/f1p0/2850	NA	NA
R01_cb18733_c1/f1p0/521	NA	R01_cb18733_c1/f1p0/521	R01_cb18733_c1/f1p0/521	R01_cb18733_c1/f1p0/521
R01_cb18456_c7828/f2p1/635	NA	NA	R01_cb18456_c7828/f2p1/635	R01_cb18456_c7828/f2p1/635
R01_cb1068_c3/f1p0/2777	NA	R01_cb1068_c3/f1p0/2777	NA	NA
R01_cb2378_c11/f1p1/4562	NA	NA	NA	R01_cb2378_c11/f1p1/4562
R01_cb4363_c1/f2p0/2715	R01_cb4363_c1/f2p0/2715	R01_cb4363_c1/f2p0/2715	NA	R01_cb4363_c1/f2p0/2715
R01_cb11673_c1/f1p0/2191	NA	R01_cb11673_c1/f1p0/2191	NA	NA
R01_cb17582_c2/f1p0/544	R01_cb17582_c2/f1p0/544	R01_cb17582_c2/f1p0/544	R01_cb17582_c2/f1p0/544	R01_cb17582_c2/f1p0/544
R01_cb14209_c1/f1p0/864	NA	R01_cb14209_c1/f1p0/864	R01_cb14209_c1/f1p0/864	R01_cb14209_c1/f1p0/864
R01_cb6582_c20/f1p0/1979	R01_cb6582_c20/f1p0/1979	R01_cb6582_c20/f1p0/1979	R01_cb6582_c20/f1p0/1979	R01_cb6582_c20/f1p0/1979
R01_cb7687_c7/f1p0/2442	NA	NA	NA	R01_cb7687_c7/f1p0/2442
R01_cb8564_c82173/f1p0/3046	NA	R01_cb8564_c82173/f1p0/3046	NA	NA
R01_cb14656_c4/f1p0/551	R01_cb14656_c4/f1p0/551	R01_cb14656_c4/f1p0/551	NA	NA
R01_cb14077_c2/f1p0/876	NA	R01_cb14077_c2/f1p0/876	NA	NA
R01_cb2073_c2/f1p0/5178	NA	NA	NA	R01_cb2073_c2/f1p0/5178
R01_cb4169_c3/f1p0/3414	NA	NA	NA	R01_cb4169_c3/f1p0/3414
R01_cb16960_c1/f2p0/553	NA	R01_cb16960_c1/f2p0/553	NA	R01_cb16960_c1/f2p0/553
R01_cb16646_c4/f1p0/920	NA	NA	NA	R01_cb16646_c4/f1p0/920
R01_cb14797_c0/f2p0/735	NA	R01_cb14797_c0/f2p0/735	NA	NA
R01_cb17937_c0/f2p0/1087	NA	NA	R01_cb17937_c0/f2p0/1087	R01_cb17937_c0/f2p0/1087

R01_cb11885_c4/f1p0/1695	NA	R01_cb11885_c4/f1p0/1695	NA	NA
R01_cb527_c2/f1p0/2024	NA	R01_cb527_c2/f1p0/2024	NA	NA
R01_cb8564_c20280/f1p0/2622	NA	R01_cb8564_c20280/f1p0/2622	NA	NA
R01_cb18759_c1/f1p0/3589	R01_cb18759_c1/f1p0/3589	R01_cb18759_c1/f1p0/3589	NA	NA
R01_cb16850_c1/f1p0/781	NA	R01_cb16850_c1/f1p0/781	R01_cb16850_c1/f1p0/781	NA
R01_cb14971_c11/f1p0/594	R01_cb14971_c11/f1p0/594	R01_cb14971_c11/f1p0/594	R01_cb14971_c11/f1p0/594	R01_cb14971_c11/f1p0/594
R01_cb8564_c9931/f2p0/2258	NA	NA	NA	R01_cb8564_c9931/f2p0/2258
R01_cb12504_c4/f1p0/1675	NA	R01_cb12504_c4/f1p0/1675	NA	R01_cb12504_c4/f1p0/1675
R01_cb3412_c1/f1p0/2908	NA	R01_cb3412_c1/f1p0/2908	NA	NA
R01_cb5159_c11/f1p0/2222	NA	NA	NA	R01_cb5159_c11/f1p0/2222
R01_cb6081_c4/f1p0/2311	NA	R01_cb6081_c4/f1p0/2311	R01_cb6081_c4/f1p0/2311	NA
R01_cb4113_c2/f1p1/3393	NA	R01_cb4113_c2/f1p1/3393	NA	NA
R01_cb4603_c0/f2p1/1242	NA	NA	NA	R01_cb4603_c0/f2p1/1242
R01_cb8564_c86444/f1p0/2782	NA	R01_cb8564_c86444/f1p0/2782	NA	R01_cb8564_c86444/f1p0/2782
R01_cb7079_c5/f1p0/2517	NA	NA	NA	R01_cb7079_c5/f1p0/2517
R01_cb11671_c1/f1p0/4005	NA	R01_cb11671_c1/f1p0/4005	NA	NA
R01_cb18456_c7247/f1p0/534	R01_cb18456_c7247/f1p0/534	R01_cb18456_c7247/f1p0/534	R01_cb18456_c7247/f1p0/534	R01_cb18456_c7247/f1p0/534
R01_cb8564_c128819/f1p0/2461	NA	NA	NA	R01_cb8564_c128819/f1p0/2461
R01_cb11439_c1/f1p0/2628	NA	R01_cb11439_c1/f1p0/2628	R01_cb11439_c1/f1p0/2628	NA
R01_cb18456_c6881/f1p0/854	NA	R01_cb18456_c6881/f1p0/854	NA	NA
R01_cb15175_c1/f1p0/868	NA	NA	NA	R01_cb15175_c1/f1p0/868
R01_cb2095_c10/f1p1/2150	NA	NA	NA	R01_cb2095_c10/f1p1/2150
R01_cb10015_c543/f1p0/867	NA	R01_cb10015_c543/f1p0/867	NA	NA
R01_cb15045_c5/f1p1/794	NA	R01_cb15045_c5/f1p1/794	NA	R01_cb15045_c5/f1p1/794
R01_cb18473_c1/f1p0/1627	NA	NA	NA	R01_cb18473_c1/f1p0/1627

R01_cb960_c3/f1p0/4686	NA	R01_cb960_c3/f1p0/4686	NA	R01_cb960_c3/f1p0/4686
R01_cb15514_c0/f1p0/1028	R01_cb15514_c0/f1p0/1028	R01_cb15514_c0/f1p0/1028	NA	R01_cb15514_c0/f1p0/1028
R01_cb8564_c4473/f2p0/4098	NA	NA	R01_cb8564_c4473/f2p0/4098	R01_cb8564_c4473/f2p0/4098
R01_cb12722_c22/f1p2/1519	NA	NA	NA	R01_cb12722_c22/f1p2/1519
R01_cb5850_c1/f1p0/3457	NA	R01_cb5850_c1/f1p0/3457	R01_cb5850_c1/f1p0/3457	R01_cb5850_c1/f1p0/3457
R01_cb6427_c0/f1p0/2858	NA	NA	NA	R01_cb6427_c0/f1p0/2858
R01_cb12612_c1/f1p0/1781	NA	R01_cb12612_c1/f1p0/1781	NA	NA
R01_cb11743_c3/f1p0/2319	NA	R01_cb11743_c3/f1p0/2319	NA	NA
R01_cb4953_c3/f1p0/3107	NA	NA	NA	R01_cb4953_c3/f1p0/3107
R01_cb8564_c69317/f2p0/2565	NA	R01_cb8564_c69317/f2p0/2565	NA	NA
R01_cb6286_c14/f1p0/1844	NA	NA	NA	R01_cb6286_c14/f1p0/1844
R01_cb8537_c15/f1p0/985	NA	NA	NA	R01_cb8537_c15/f1p0/985
R01_cb12784_c3/f1p0/1147	NA	R01_cb12784_c3/f1p0/1147	NA	R01_cb12784_c3/f1p0/1147
R01_cb5852_c1/f1p0/2994	NA	R01_cb5852_c1/f1p0/2994	NA	NA
R01_cb8564_c10432/f1p0/2146	NA	R01_cb8564_c10432/f1p0/2146	NA	R01_cb8564_c10432/f1p0/2146
R01_cb8564_c122122/f1p0/2495	NA	R01_cb8564_c122122/f1p0/2495	R01_cb8564_c122122/f1p0/2495	R01_cb8564_c122122/f1p0/2495
R01_cb5595_c14/f1p0/2659	NA	NA	NA	R01_cb5595_c14/f1p0/2659
R01_cb8779_c6/f1p0/3403	NA	R01_cb8779_c6/f1p0/3403	NA	R01_cb8779_c6/f1p0/3403
R01_cb2107_c7/f1p0/2748	NA	R01_cb2107_c7/f1p0/2748	NA	NA
R01_cb4147_c23/f1p0/2431	NA	R01_cb4147_c23/f1p0/2431	NA	NA
R01_cb14283_c5/f1p0/795	NA	R01_cb14283_c5/f1p0/795	R01_cb14283_c5/f1p0/795	R01_cb14283_c5/f1p0/795
R01_cb8660_c8/f1p0/2113	NA	NA	NA	R01_cb8660_c8/f1p0/2113
R01_cb9888_c5/f1p0/1422	NA	R01_cb9888_c5/f1p0/1422	NA	NA
R01_cb9700_c6/f1p0/1641	NA	R01_cb9700_c6/f1p0/1641	NA	NA
R01_cb9367_c3/f1p0/1075	NA	R01_cb9367_c3/f1p0/1075	NA	R01_cb9367_c3/f1p0/1075

R01_cb18197_c1/f1p0/559	NA	R01_cb18197_c1/f1p0/559	R01_cb18197_c1/f1p0/559	R01_cb18197_c1/f1p0/559
R01_cb12418_c0/f1p0/554	R01_cb12418_c0/f1p0/554	R01_cb12418_c0/f1p0/554	R01_cb12418_c0/f1p0/554	R01_cb12418_c0/f1p0/554
R01_cb8564_c74520/f1p0/3076	NA	NA	NA	R01_cb8564_c74520/f1p0/3076
R01_cb8564_c92111/f1p0/2633	NA	R01_cb8564_c92111/f1p0/2633	R01_cb8564_c92111/f1p0/2633	NA
R01_cb4536_c1/f1p1/3272	NA	NA	NA	R01_cb4536_c1/f1p1/3272
R01_cb8569_c11/f1p0/1064	NA	R01_cb8569_c11/f1p0/1064	NA	NA
R01_cb17576_c1/f2p0/840	NA	NA	R01_cb17576_c1/f2p0/840	R01_cb17576_c1/f2p0/840
R01_cb8564_c122105/f1p0/2845	NA	R01_cb8564_c122105/f1p0/2845	NA	NA
R01_cb2845_c30/f1p3/3567	NA	NA	NA	R01_cb2845_c30/f1p3/3567
R01_cb8564_c34291/f1p0/4647	NA	NA	NA	R01_cb8564_c34291/f1p0/4647
R01_cb17734_c0/f1p0/892	NA	NA	NA	R01_cb17734_c0/f1p0/892
R01_cb8564_c10819/f1p1/3887	NA	R01_cb8564_c10819/f1p1/3887	R01_cb8564_c10819/f1p1/3887	NA
R01_cb8564_c83183/f1p0/4016	NA	R01_cb8564_c83183/f1p0/4016	NA	NA
R01_cb4821_c6/f1p0/3180	NA	R01_cb4821_c6/f1p0/3180	NA	NA
R01_cb8669_c5/f1p0/1843	NA	R01_cb8669_c5/f1p0/1843	R01_cb8669_c5/f1p0/1843	NA
R01_cb17274_c1/f1p0/880	NA	NA	R01_cb17274_c1/f1p0/880	R01_cb17274_c1/f1p0/880
R01_cb8564_c2686/f1p1/2051	NA	R01_cb8564_c2686/f1p1/2051	NA	NA
R01_cb8900_c0/f1p0/2223	NA	NA	NA	R01_cb8900_c0/f1p0/2223
R01_cb5736_c5/f1p0/2180	NA	NA	NA	R01_cb5736_c5/f1p0/2180
R01_cb2997_c0/f3p0/3489	NA	NA	NA	R01_cb2997_c0/f3p0/3489
R01_cb8254_c3/f1p0/2422	NA	NA	NA	R01_cb8254_c3/f1p0/2422
R01_cb16999_c0/f1p0/933	R01_cb16999_c0/f1p0/933	R01_cb16999_c0/f1p0/933	NA	NA
R01_cb15907_c1/f1p0/696	R01_cb15907_c1/f1p0/696	R01_cb15907_c1/f1p0/696	NA	R01_cb15907_c1/f1p0/696
R01_cb8564_c2470/f1p0/3910	NA	R01_cb8564_c2470/f1p0/3910	NA	NA
R01_cb18456_c6431/f1p0/500	R01_cb18456_c6431/f1p0/500	R01_cb18456_c6431/f1p0/500	NA	NA

R01_cb10024_c285/f1p0/656	NA	NA	R01_cb10024_c285/f1p0/656	NA
R01_cb4371_c4/f1p0/2305	NA	R01_cb4371_c4/f1p0/2305	NA	NA
R01_cb8942_c23/f1p2/3072	NA	R01_cb8942_c23/f1p2/3072	NA	NA
R01_cb15907_c0/f1p0/713	NA	R01_cb15907_c0/f1p0/713	NA	R01_cb15907_c0/f1p0/713
R01_cb9448_c8/f1p1/1865	NA	NA	NA	R01_cb9448_c8/f1p1/1865
R01_cb16034_c2/f1p0/390	R01_cb16034_c2/f1p0/390	R01_cb16034_c2/f1p0/390	R01_cb16034_c2/f1p0/390	R01_cb16034_c2/f1p0/390
R01_cb11030_c0/f1p0/1209	NA	R01_cb11030_c0/f1p0/1209	NA	NA
R01_cb13686_c11/f1p1/1157	NA	R01_cb13686_c11/f1p1/1157	NA	NA
R01_cb18723_c1/f1p0/341	R01_cb18723_c1/f1p0/341	R01_cb18723_c1/f1p0/341	R01_cb18723_c1/f1p0/341	R01_cb18723_c1/f1p0/341
R01_cb4469_c9/f1p0/2235	NA	NA	NA	R01_cb4469_c9/f1p0/2235
R01_cb9410_c5/f2p1/2049	NA	NA	NA	R01_cb9410_c5/f2p1/2049
R01_cb2878_c5/f1p0/3337	NA	R01_cb2878_c5/f1p0/3337	NA	NA
R01_cb16293_c10/f1p0/865	NA	NA	R01_cb16293_c10/f1p0/865	R01_cb16293_c10/f1p0/865
R01_cb723_c2/f1p0/3494	R01_cb723_c2/f1p0/3494	R01_cb723_c2/f1p0/3494	NA	NA
R01_cb18456_c1509/f3p0/524	R01_cb18456_c1509/f3p0/524	R01_cb18456_c1509/f3p0/524	R01_cb18456_c1509/f3p0/524	R01_cb18456_c1509/f3p0/524
R01_cb17620_c0/f1p0/1645	R01_cb17620_c0/f1p0/1645	R01_cb17620_c0/f1p0/1645	NA	NA
R01_cb8564_c73046/f1p0/2679	NA	NA	NA	R01_cb8564_c73046/f1p0/2679
R01_cb16153_c9/f2p0/690	NA	NA	R01_cb16153_c9/f2p0/690	R01_cb16153_c9/f2p0/690
R01_cb2667_c4/f1p0/2890	NA	R01_cb2667_c4/f1p0/2890	NA	NA
R01_cb8564_c38564/f2p0/2549	NA	R01_cb8564_c38564/f2p0/2549	R01_cb8564_c38564/f2p0/2549	R01_cb8564_c38564/f2p0/2549
R01_cb8564_c90099/f1p0/3063	NA	R01_cb8564_c90099/f1p0/3063	NA	NA
R01_cb14675_c5/f1p2/939	R01_cb14675_c5/f1p2/939	R01_cb14675_c5/f1p2/939	R01_cb14675_c5/f1p2/939	R01_cb14675_c5/f1p2/939
R01_cb17221_c3/f1p0/1610	NA	R01_cb17221_c3/f1p0/1610	NA	R01_cb17221_c3/f1p0/1610
R01_cb10390_c12/f1p0/832	NA	R01_cb10390_c12/f1p0/832	NA	NA
R01_cb15908_c2/f1p0/1420	NA	NA	NA	R01_cb15908_c2/f1p0/1420
R01_cb17381_c5/f1p0/768	NA	NA	NA	R01_cb17381_c5/f1p0/768

R01_cb11843_c0/f1p0/995	NA	R01_cb11843_c0/f1p0/995	NA	NA
R01_cb4117_c6/f1p1/1742	NA	R01_cb4117_c6/f1p1/1742	NA	R01_cb4117_c6/f1p1/1742
R01_cb8564_c13302/f2p0/1920	NA	R01_cb8564_c13302/f2p0/1920	R01_cb8564_c13302/f2p0/1920	R01_cb8564_c13302/f2p0/1920
R01_cb6473_c13/f1p0/1888	NA	R01_cb6473_c13/f1p0/1888	NA	NA
R01_cb12698_c18/f1p1/1108	NA	R01_cb12698_c18/f1p1/1108	NA	NA
R01_cb12334_c1/f1p0/444	R01_cb12334_c1/f1p0/444	R01_cb12334_c1/f1p0/444	NA	NA
R01_cb2382_c6/f1p1/5561	NA	NA	NA	R01_cb2382_c6/f1p1/5561
R01_cb18456_c7207/f1p0/606	NA	R01_cb18456_c7207/f1p0/606	NA	NA
R01_cb6571_c1/f1p0/2221	NA	R01_cb6571_c1/f1p0/2221	NA	R01_cb6571_c1/f1p0/2221
R01_cb11064_c0/f1p0/497	NA	NA	NA	R01_cb11064_c0/f1p0/497
R01_cb10658_c6/f1p0/3185	R01_cb10658_c6/f1p0/3185	R01_cb10658_c6/f1p0/3185	NA	NA
R01_cb5893_c0/f3p0/507	R01_cb5893_c0/f3p0/507	R01_cb5893_c0/f3p0/507	R01_cb5893_c0/f3p0/507	R01_cb5893_c0/f3p0/507
R01_cb2357_c8/f1p0/2643	NA	NA	NA	R01_cb2357_c8/f1p0/2643
R01_cb6602_c26/f1p0/2955	NA	R01_cb6602_c26/f1p0/2955	NA	R01_cb6602_c26/f1p0/2955
R01_cb11332_c2/f1p0/1012	NA	NA	NA	R01_cb11332_c2/f1p0/1012
R01_cb16762_c1/f1p0/630	NA	R01_cb16762_c1/f1p0/630	NA	NA
R01_cb13396_c4/f1p0/1537	NA	R01_cb13396_c4/f1p0/1537	R01_cb13396_c4/f1p0/1537	R01_cb13396_c4/f1p0/1537
R01_cb4469_c1/f2p0/1533	NA	NA	NA	R01_cb4469_c1/f2p0/1533
R01_cb18580_c0/f1p0/1375	NA	NA	R01_cb18580_c0/f1p0/1375	NA
R01_cb16424_c0/f2p0/830	NA	R01_cb16424_c0/f2p0/830	NA	R01_cb16424_c0/f2p0/830
R01_cb10029_c92/f1p7/800	NA	R01_cb10029_c92/f1p7/800	NA	NA
R01_cb8564_c14881/f1p1/2242	NA	NA	NA	R01_cb8564_c14881/f1p1/2242
R01_cb8564_c89576/f1p0/3861	NA	NA	NA	R01_cb8564_c89576/f1p0/3861
R01_cb124_c24/f1p0/3642	NA	NA	NA	R01_cb124_c24/f1p0/3642
R01_cb14817_c1/f1p0/1251	NA	NA	R01_cb14817_c1/f1p0/1251	R01_cb14817_c1/f1p0/1251
R01_cb12176_c1/f1p0/575	NA	R01_cb12176_c1/f1p0/575	NA	R01_cb12176_c1/f1p0/575

R01_cb11363_c1/f1p0/4033	NA	R01_cb11363_c1/f1p0/4033	NA	NA
R01_cb8564_c978/f1p2/2552	NA	R01_cb8564_c978/f1p2/2552	NA	NA
R01_cb17542_c1/f1p0/655	NA	R01_cb17542_c1/f1p0/655	R01_cb17542_c1/f1p0/655	R01_cb17542_c1/f1p0/655
R01_cb3201_c81/f1p0/6719	NA	R01_cb3201_c81/f1p0/6719	NA	NA
R01_cb1097_c3/f1p0/4198	NA	NA	NA	R01_cb1097_c3/f1p0/4198
R01_cb4953_c0/f12p0/1594	NA	NA	NA	R01_cb4953_c0/f12p0/1594
R01_cb17556_c0/f1p0/1248	NA	R01_cb17556_c0/f1p0/1248	NA	NA
R01_cb763_c0/f2p0/4159	NA	R01_cb763_c0/f2p0/4159	R01_cb763_c0/f2p0/4159	R01_cb763_c0/f2p0/4159
R01_cb15552_c1/f2p0/942	NA	NA	NA	R01_cb15552_c1/f2p0/942
R01_cb822_c11/f1p1/2602	NA	NA	NA	R01_cb822_c11/f1p1/2602
R01_cb13186_c5/f1p0/637	R01_cb13186_c5/f1p0/637	R01_cb13186_c5/f1p0/637	R01_cb13186_c5/f1p0/637	NA
R01_cb18581_c0/f1p0/1050	NA	NA	NA	R01_cb18581_c0/f1p0/1050
R01_cb1333_c28/f1p0/2464	R01_cb1333_c28/f1p0/2464	R01_cb1333_c28/f1p0/2464	R01_cb1333_c28/f1p0/2464	R01_cb1333_c28/f1p0/2464
R01_cb5659_c31/f1p1/2500	NA	NA	NA	R01_cb5659_c31/f1p1/2500
R01_cb15732_c0/f1p0/884	NA	R01_cb15732_c0/f1p0/884	NA	NA
R01_cb9552_c1/f4p0/1921	NA	NA	NA	R01_cb9552_c1/f4p0/1921
R01_cb16834_c1/f1p0/802	NA	NA	NA	R01_cb16834_c1/f1p0/802
R01_cb17347_c4/f1p0/498	R01_cb17347_c4/f1p0/498	R01_cb17347_c4/f1p0/498	NA	R01_cb17347_c4/f1p0/498
R01_cb8564_c83266/f1p0/2067	NA	R01_cb8564_c83266/f1p0/2067	NA	R01_cb8564_c83266/f1p0/2067
R01_cb15432_c4/f1p0/628	NA	R01_cb15432_c4/f1p0/628	NA	NA
R01_cb12803_c2/f1p0/1341	NA	NA	NA	R01_cb12803_c2/f1p0/1341
R01_cb3752_c0/f3p1/1540	NA	NA	NA	R01_cb3752_c0/f3p1/1540
R01_cb14757_c4/f1p0/1874	NA	R01_cb14757_c4/f1p0/1874	NA	NA
R01_cb2324_c10/f1p0/3917	NA	R01_cb2324_c10/f1p0/3917	NA	NA
R01_cb6989_c1/f1p0/2721	NA	R01_cb6989_c1/f1p0/2721	NA	NA
R01_cb8564_c3178/f1p0/2670	R01_cb8564_c3178/f1p0/2670	R01_cb8564_c3178/f1p0/2670	R01_cb8564_c3178/f1p0/2670	NA

R01_cb15712_c0/f2p0/541	NA	NA	NA	R01_cb15712_c0/f2p0/541
R01_cb18409_c75/f1p0/1035	R01_cb18409_c75/f1p0/1035	R01_cb18409_c75/f1p0/1035	R01_cb18409_c75/f1p0/1035	R01_cb18409_c75/f1p0/1035
R01_cb11019_c1/f1p0/1947	NA	NA	NA	R01_cb11019_c1/f1p0/1947
R01_cb18456_c1487/f1p0/540	R01_cb18456_c1487/f1p0/540	R01_cb18456_c1487/f1p0/540	NA	NA
R01_cb4634_c4/f1p0/3075	NA	R01_cb4634_c4/f1p0/3075	NA	NA
R01_cb11924_c21/f1p1/526	R01_cb11924_c21/f1p1/526	R01_cb11924_c21/f1p1/526	R01_cb11924_c21/f1p1/526	NA
R01_cb17151_c1/f1p0/1007	R01_cb17151_c1/f1p0/1007	R01_cb17151_c1/f1p0/1007	R01_cb17151_c1/f1p0/1007	R01_cb17151_c1/f1p0/1007
R01_cb9106_c7/f1p0/3165	R01_cb9106_c7/f1p0/3165	R01_cb9106_c7/f1p0/3165	R01_cb9106_c7/f1p0/3165	R01_cb9106_c7/f1p0/3165
R01_cb10207_c6/f1p0/658	NA	R01_cb10207_c6/f1p0/658	NA	NA
R01_cb7999_c11/f1p0/793	NA	NA	NA	R01_cb7999_c11/f1p0/793
R01_cb8564_c15343/f1p0/3588	NA	R01_cb8564_c15343/f1p0/3588	NA	R01_cb8564_c15343/f1p0/3588
R01_cb4223_c3/f1p0/2029	NA	NA	NA	R01_cb4223_c3/f1p0/2029
R01_cb1458_c6/f1p0/3084	NA	R01_cb1458_c6/f1p0/3084	NA	NA
R01_cb6517_c55/f1p0/1589	NA	R01_cb6517_c55/f1p0/1589	NA	R01_cb6517_c55/f1p0/1589
R01_cb8564_c3245/f1p0/2141	NA	R01_cb8564_c3245/f1p0/2141	NA	NA
R01_cb1380_c45/f1p0/3005	NA	R01_cb1380_c45/f1p0/3005	NA	NA
R01_cb12420_c1/f2p0/1344	NA	NA	NA	R01_cb12420_c1/f2p0/1344
R01_cb3449_c51/f1p0/339	NA	R01_cb3449_c51/f1p0/339	NA	NA
R01_cb14500_c2/f1p0/508	R01_cb14500_c2/f1p0/508	R01_cb14500_c2/f1p0/508	NA	R01_cb14500_c2/f1p0/508
R01_cb8564_c10814/f1p0/3217	NA	NA	NA	R01_cb8564_c10814/f1p0/3217
R01_cb10215_c6/f1p0/678	NA	R01_cb10215_c6/f1p0/678	NA	NA
R01_cb10567_c2/f1p0/872	R01_cb10567_c2/f1p0/872	R01_cb10567_c2/f1p0/872	R01_cb10567_c2/f1p0/872	R01_cb10567_c2/f1p0/872
R01_cb17345_c0/f2p0/696	R01_cb17345_c0/f2p0/696	R01_cb17345_c0/f2p0/696	R01_cb17345_c0/f2p0/696	R01_cb17345_c0/f2p0/696
R01_cb17405_c1/f1p0/1209	NA	R01_cb17405_c1/f1p0/1209	NA	NA
R01_cb12286_c6/f1p0/774	NA	R01_cb12286_c6/f1p0/774	NA	NA
R01_cb10609_c1/f1p0/2785	NA	R01_cb10609_c1/f1p0/2785	NA	NA

R01_cb8564_c36064/f1p0/2046	NA	R01_cb8564_c36064/f1p0/2046	R01_cb8564_c36064/f1p0/2046	R01_cb8564_c36064/f1p0/2046
R01_cb11123_c0/f2p0/845	NA	R01_cb11123_c0/f2p0/845	NA	R01_cb11123_c0/f2p0/845
R01_cb15959_c4/f1p0/1507	NA	NA	NA	R01_cb15959_c4/f1p0/1507
R01_cb12972_c30/f1p0/1457	R01_cb12972_c30/f1p0/1457	R01_cb12972_c30/f1p0/1457	R01_cb12972_c30/f1p0/1457	R01_cb12972_c30/f1p0/1457
R01_cb1827_c2/f1p0/4139	NA	R01_cb1827_c2/f1p0/4139	R01_cb1827_c2/f1p0/4139	NA
R01_cb1138_c50/f1p0/813	R01_cb1138_c50/f1p0/813	NA	NA	R01_cb1138_c50/f1p0/813
R01_cb10236_c6/f1p0/1618	NA	R01_cb10236_c6/f1p0/1618	R01_cb10236_c6/f1p0/1618	NA
R01_cb929_c7/f3p1/1831	NA	NA	NA	R01_cb929_c7/f3p1/1831
R01_cb8564_c73443/f1p0/4382	NA	R01_cb8564_c73443/f1p0/4382	R01_cb8564_c73443/f1p0/4382	R01_cb8564_c73443/f1p0/4382
R01_cb8564_c1895/f1p0/2189	NA	NA	NA	R01_cb8564_c1895/f1p0/2189
R01_cb8126_c2/f1p0/2450	NA	R01_cb8126_c2/f1p0/2450	NA	NA
R01_cb5815_c9/f1p0/1387	NA	NA	NA	R01_cb5815_c9/f1p0/1387
R01_cb8564_c119671/f1p0/2360	NA	R01_cb8564_c119671/f1p0/2360	R01_cb8564_c119671/f1p0/2360	R01_cb8564_c119671/f1p0/2360
R01_cb4287_c1/f1p0/2677	NA	R01_cb4287_c1/f1p0/2677	R01_cb4287_c1/f1p0/2677	R01_cb4287_c1/f1p0/2677
R01_cb12444_c53/f1p0/784	R01_cb12444_c53/f1p0/784	R01_cb12444_c53/f1p0/784	R01_cb12444_c53/f1p0/784	R01_cb12444_c53/f1p0/784
R01_cb8564_c3176/f1p0/2324	NA	R01_cb8564_c3176/f1p0/2324	NA	NA
R01_cb13603_c2/f1p0/814	NA	R01_cb13603_c2/f1p0/814	NA	NA
R01_cb8564_c79521/f2p0/2343	NA	R01_cb8564_c79521/f2p0/2343	R01_cb8564_c79521/f2p0/2343	R01_cb8564_c79521/f2p0/2343
R01_cb18456_c1382/f6p0/701	NA	NA	NA	R01_cb18456_c1382/f6p0/701
R01_cb5942_c1/f1p0/2920	NA	NA	NA	R01_cb5942_c1/f1p0/2920
R01_cb8564_c20829/f1p0/2257	NA	R01_cb8564_c20829/f1p0/2257	NA	NA
R01_cb10189_c4/f1p0/2649	NA	R01_cb10189_c4/f1p0/2649	NA	NA
R01_cb2177_c99/f1p0/3339	NA	R01_cb2177_c99/f1p0/3339	NA	R01_cb2177_c99/f1p0/3339
R01_cb14678_c0/f3p0/537	R01_cb14678_c0/f3p0/537	NA	R01_cb14678_c0/f3p0/537	R01_cb14678_c0/f3p0/537
R01_cb5167_c22/f1p0/324	R01_cb5167_c22/f1p0/324	R01_cb5167_c22/f1p0/324	R01_cb5167_c22/f1p0/324	R01_cb5167_c22/f1p0/324

R01_cb18456_c7542/f1p0/583	NA	NA	NA	R01_cb18456_c7542/f1p0/583
R01_cb7603_c2/f1p0/2773	NA	R01_cb7603_c2/f1p0/2773	NA	NA
R01_cb99_c19/f1p0/967	NA	NA	NA	R01_cb99_c19/f1p0/967
R01_cb8564_c38431/f1p0/3078	NA	R01_cb8564_c38431/f1p0/3078	NA	R01_cb8564_c38431/f1p0/3078
R01_cb261_c6/f2p0/2276	NA	R01_cb261_c6/f2p0/2276	R01_cb261_c6/f2p0/2276	NA
R01_cb7010_c19/f1p1/1926	NA	NA	NA	R01_cb7010_c19/f1p1/1926
R01_cb7741_c1/f3p0/538	R01_cb7741_c1/f3p0/538	R01_cb7741_c1/f3p0/538	R01_cb7741_c1/f3p0/538	R01_cb7741_c1/f3p0/538
R01_cb8564_c109380/f4p0/2067	R01_cb8564_c109380/f4p0/2067	R01_cb8564_c109380/f4p0/2067	R01_cb8564_c109380/f4p0/2067	R01_cb8564_c109380/f4p0/2067
R01_cb7749_c5/f1p0/1533	NA	NA	NA	R01_cb7749_c5/f1p0/1533
R01_cb2875_c0/f7p0/3313	NA	NA	NA	R01_cb2875_c0/f7p0/3313
R01_cb8564_c11555/f1p0/3755	NA	NA	NA	R01_cb8564_c11555/f1p0/3755
R01_cb12620_c9/f1p0/1192	NA	NA	NA	R01_cb12620_c9/f1p0/1192
R01_cb827_c1/f1p0/3960	NA	NA	NA	R01_cb827_c1/f1p0/3960
R01_cb423_c7/f1p0/2240	NA	NA	NA	R01_cb423_c7/f1p0/2240
R01_cb8564_c80425/f1p0/2938	NA	NA	NA	R01_cb8564_c80425/f1p0/2938
R01_cb14286_c2/f1p0/1146	NA	R01_cb14286_c2/f1p0/1146	NA	R01_cb14286_c2/f1p0/1146
R01_cb8564_c1296/f1p1/2175	NA	NA	NA	R01_cb8564_c1296/f1p1/2175
R01_cb8564_c52308/f1p0/4637	NA	R01_cb8564_c52308/f1p0/4637	NA	NA
R01_cb3797_c8/f1p0/1773	NA	NA	NA	R01_cb3797_c8/f1p0/1773
R01_cb8564_c124934/f1p0/2464	R01_cb8564_c124934/f1p0/2464	R01_cb8564_c124934/f1p0/2464	NA	R01_cb8564_c124934/f1p0/2464
R01_cb4185_c3/f1p0/3063	R01_cb4185_c3/f1p0/3063	R01_cb4185_c3/f1p0/3063	R01_cb4185_c3/f1p0/3063	R01_cb4185_c3/f1p0/3063
R01_cb13900_c4/f1p0/452	NA	R01_cb13900_c4/f1p0/452	NA	R01_cb13900_c4/f1p0/452
R01_cb363_c0/f1p0/4854	R01_cb363_c0/f1p0/4854	R01_cb363_c0/f1p0/4854	NA	R01_cb363_c0/f1p0/4854
R01_cb10893_c2/f1p0/518	R01_cb10893_c2/f1p0/518	R01_cb10893_c2/f1p0/518	NA	NA

R01_cb8564_c12865/f1p0/4006	NA	NA	NA	R01_cb8564_c12865/f1p0/4006
R01_cb6608_c11/f1p0/2405	NA	NA	NA	R01_cb6608_c11/f1p0/2405
R01_cb6352_c5/f1p0/1928	R01_cb6352_c5/f1p0/1928	R01_cb6352_c5/f1p0/1928	NA	R01_cb6352_c5/f1p0/1928
R01_cb13449_c6/f1p1/1318	NA	NA	NA	R01_cb13449_c6/f1p1/1318
R01_cb8564_c126160/f1p0/2517	NA	R01_cb8564_c126160/f1p0/2517	R01_cb8564_c126160/f1p0/2517	R01_cb8564_c126160/f1p0/2517
R01_cb8564_c51697/f1p0/2941	R01_cb8564_c51697/f1p0/2941	R01_cb8564_c51697/f1p0/2941	R01_cb8564_c51697/f1p0/2941	R01_cb8564_c51697/f1p0/2941
R01_cb11025_c2/f1p0/1387	NA	NA	NA	R01_cb11025_c2/f1p0/1387
R01_cb10572_c2/f2p0/1073	NA	NA	NA	R01_cb10572_c2/f2p0/1073
R01_cb6615_c28/f2p0/876	NA	NA	NA	R01_cb6615_c28/f2p0/876
R01_cb8564_c81628/f1p1/2479	NA	NA	NA	R01_cb8564_c81628/f1p1/2479
R01_cb12574_c0/f1p0/1178	NA	R01_cb12574_c0/f1p0/1178	R01_cb12574_c0/f1p0/1178	R01_cb12574_c0/f1p0/1178
R01_cb8564_c9458/f4p16/4874	NA	R01_cb8564_c9458/f4p16/4874	R01_cb8564_c9458/f4p16/4874	R01_cb8564_c9458/f4p16/4874
R01_cb10453_c7/f1p0/408	R01_cb10453_c7/f1p0/408	R01_cb10453_c7/f1p0/408	R01_cb10453_c7/f1p0/408	R01_cb10453_c7/f1p0/408
R01_cb13895_c6/f12p0/525	NA	NA	NA	R01_cb13895_c6/f12p0/525
R01_cb8391_c1/f1p0/2380	NA	R01_cb8391_c1/f1p0/2380	NA	NA
R01_cb18456_c1468/f3p0/1754	NA	R01_cb18456_c1468/f3p0/1754	R01_cb18456_c1468/f3p0/1754	R01_cb18456_c1468/f3p0/1754
R01_cb16440_c1/f1p0/430	R01_cb16440_c1/f1p0/430	R01_cb16440_c1/f1p0/430	R01_cb16440_c1/f1p0/430	R01_cb16440_c1/f1p0/430
R01_cb18456_c1260/f11p0/580	NA	R01_cb18456_c1260/f11p0/580	R01_cb18456_c1260/f11p0/580	R01_cb18456_c1260/f11p0/580
R01_cb11984_c26/f1p0/566	NA	R01_cb11984_c26/f1p0/566	NA	NA
R01_cb13386_c12/f1p0/1226	NA	R01_cb13386_c12/f1p0/1226	NA	NA
R01_cb15512_c1/f1p0/1831	NA	R01_cb15512_c1/f1p0/1831	NA	NA
R01_cb3907_c6/f1p0/2579	NA	R01_cb3907_c6/f1p0/2579	NA	NA
R01_cb11522_c1/f1p0/4183	R01_cb11522_c1/f1p0/4183	R01_cb11522_c1/f1p0/4183	NA	R01_cb11522_c1/f1p0/4183
R01_cb18694_c1/f1p0/867	NA	NA	NA	R01_cb18694_c1/f1p0/867

R01_cb8564_c41693/f1p0/2038	NA	NA	R01_cb8564_c41693/f1p0/2038	NA
R01_cb1887_c4/f1p0/2632	NA	NA	NA	R01_cb1887_c4/f1p0/2632
R01_cb8564_c89056/f1p0/1956	NA	R01_cb8564_c89056/f1p0/1956	R01_cb8564_c89056/f1p0/1956	R01_cb8564_c89056/f1p0/1956
R01_cb10616_c3/f1p0/2697	NA	NA	NA	R01_cb10616_c3/f1p0/2697
R01_cb17199_c2/f1p0/1081	NA	R01_cb17199_c2/f1p0/1081	NA	NA
R01_cb2298_c8/f13p1/2289	NA	NA	NA	R01_cb2298_c8/f13p1/2289
R01_cb9951_c3/f1p0/1285	R01_cb9951_c3/f1p0/1285	R01_cb9951_c3/f1p0/1285	NA	R01_cb9951_c3/f1p0/1285
R01_cb5324_c2/f1p0/881	R01_cb5324_c2/f1p0/881	R01_cb5324_c2/f1p0/881	R01_cb5324_c2/f1p0/881	R01_cb5324_c2/f1p0/881
R01_cb17717_c1/f1p0/951	NA	NA	R01_cb17717_c1/f1p0/951	R01_cb17717_c1/f1p0/951
R01_cb18456_c6792/f1p0/746	NA	R01_cb18456_c6792/f1p0/746	R01_cb18456_c6792/f1p0/746	R01_cb18456_c6792/f1p0/746
R01_cb1830_c1/f1p0/4665	NA	R01_cb1830_c1/f1p0/4665	NA	NA
R01_cb13012_c4/f1p0/1167	NA	NA	NA	R01_cb13012_c4/f1p0/1167
R01_cb5208_c5/f1p0/2628	NA	NA	NA	R01_cb5208_c5/f1p0/2628
R01_cb1097_c2/f1p0/3917	NA	NA	NA	R01_cb1097_c2/f1p0/3917
R01_cb8564_c109285/f4p0/2584	R01_cb8564_c109285/f4p0/2584	R01_cb8564_c109285/f4p0/2584	R01_cb8564_c109285/f4p0/2584	R01_cb8564_c109285/f4p0/2584
R01_cb4321_c7/f1p0/1381	NA	R01_cb4321_c7/f1p0/1381	NA	NA
R01_cb8564_c75166/f1p1/2653	NA	NA	NA	R01_cb8564_c75166/f1p1/2653
R01_cb18757_c1/f1p0/3326	NA	NA	NA	R01_cb18757_c1/f1p0/3326
R01_cb8564_c110982/f1p0/2561	NA	R01_cb8564_c110982/f1p0/2561	NA	R01_cb8564_c110982/f1p0/2561
R01_cb7029_c0/f2p0/2473	NA	R01_cb7029_c0/f2p0/2473	R01_cb7029_c0/f2p0/2473	R01_cb7029_c0/f2p0/2473
R01_cb4692_c1/f1p0/3283	NA	R01_cb4692_c1/f1p0/3283	NA	NA
R01_cb5569_c0/f1p0/3070	NA	R01_cb5569_c0/f1p0/3070	NA	NA
R01_cb3639_c1/f1p0/2018	NA	NA	NA	R01_cb3639_c1/f1p0/2018
R01_cb13590_c0/f3p0/787	NA	R01_cb13590_c0/f3p0/787	R01_cb13590_c0/f3p0/787	R01_cb13590_c0/f3p0/787

R01_cb3243_c2/f1p0/3677	NA	NA	NA	R01_cb3243_c2/f1p0/3677
R01_cb11134_c0/f1p0/982	NA	R01_cb11134_c0/f1p0/982	R01_cb11134_c0/f1p0/982	R01_cb11134_c0/f1p0/982
R01_cb8564_c5087/f1p0/3205	NA	NA	NA	R01_cb8564_c5087/f1p0/3205
R01_cb14706_c2/f2p1/593	R01_cb14706_c2/f2p1/593	R01_cb14706_c2/f2p1/593	R01_cb14706_c2/f2p1/593	R01_cb14706_c2/f2p1/593
R01_cb5987_c3/f1p0/1602	NA	NA	NA	R01_cb5987_c3/f1p0/1602
R01_cb9866_c2/f1p0/2072	NA	NA	NA	R01_cb9866_c2/f1p0/2072
R01_cb14729_c4/f1p2/652	NA	R01_cb14729_c4/f1p2/652	NA	NA
R01_cb2002_c3/f1p0/724	NA	R01_cb2002_c3/f1p0/724	NA	NA
R01_cb8545_c5/f1p0/875	NA	NA	R01_cb8545_c5/f1p0/875	R01_cb8545_c5/f1p0/875
R01_cb14610_c3/f1p0/513	R01_cb14610_c3/f1p0/513	R01_cb14610_c3/f1p0/513	R01_cb14610_c3/f1p0/513	R01_cb14610_c3/f1p0/513
R01_cb7088_c10/f1p0/2704	NA	R01_cb7088_c10/f1p0/2704	NA	NA
R01_cb10725_c4/f1p0/1028	NA	R01_cb10725_c4/f1p0/1028	R01_cb10725_c4/f1p0/1028	R01_cb10725_c4/f1p0/1028
R01_cb10878_c1/f1p0/3270	NA	R01_cb10878_c1/f1p0/3270	NA	NA
R01_cb10038_c0/f8p0/899	NA	NA	NA	R01_cb10038_c0/f8p0/899
R01_cb18456_c3612/f1p0/1082	NA	R01_cb18456_c3612/f1p0/1082	NA	NA
R01_cb12234_c2/f1p0/403	R01_cb12234_c2/f1p0/403	R01_cb12234_c2/f1p0/403	R01_cb12234_c2/f1p0/403	R01_cb12234_c2/f1p0/403
R01_cb2738_c1/f1p0/3829	NA	NA	NA	R01_cb2738_c1/f1p0/3829
R01_cb12945_c1/f1p0/1386	NA	NA	NA	R01_cb12945_c1/f1p0/1386
R01_cb10272_c27/f1p0/1290	NA	NA	R01_cb10272_c27/f1p0/1290	R01_cb10272_c27/f1p0/1290
R01_cb12286_c18/f1p0/911	NA	R01_cb12286_c18/f1p0/911	NA	NA
R01_cb16785_c1/f1p0/1773	NA	R01_cb16785_c1/f1p0/1773	NA	NA
R01_cb18413_c0/f1p0/1007	NA	R01_cb18413_c0/f1p0/1007	NA	NA
R01_cb513_c11/f1p0/1899	R01_cb513_c11/f1p0/1899	R01_cb513_c11/f1p0/1899	NA	NA
R01_cb1228_c47/f1p0/2325	NA	NA	NA	R01_cb1228_c47/f1p0/2325
R01_cb8459_c0/f2p0/2367	NA	R01_cb8459_c0/f2p0/2367	R01_cb8459_c0/f2p0/2367	NA
R01_cb13178_c27/f10p0/541	NA	R01_cb13178_c27/f10p0/541	NA	NA

R01_cb17765_c3/f1p0/1599	NA	R01_cb17765_c3/f1p0/1599	R01_cb17765_c3/f1p0/1599	R01_cb17765_c3/f1p0/1599
R01_cb11918_c2/f3p1/1220	NA	NA	NA	R01_cb11918_c2/f3p1/1220
R01_cb18409_c55/f1p0/411	R01_cb18409_c55/f1p0/411	R01_cb18409_c55/f1p0/411	R01_cb18409_c55/f1p0/411	R01_cb18409_c55/f1p0/411
R01_cb5208_c14/f1p0/2726	NA	NA	NA	R01_cb5208_c14/f1p0/2726
R01_cb10681_c4/f3p0/933	NA	NA	NA	R01_cb10681_c4/f3p0/933
R01_cb9320_c0/f1p0/2123	NA	R01_cb9320_c0/f1p0/2123	R01_cb9320_c0/f1p0/2123	NA
R01_cb8564_c73461/f1p0/3149	NA	R01_cb8564_c73461/f1p0/3149	NA	NA
R01_cb7940_c36/f1p1/1937	NA	R01_cb7940_c36/f1p1/1937	NA	NA
R01_cb9530_c2/f1p0/2426	NA	NA	NA	R01_cb9530_c2/f1p0/2426
R01_cb1346_c6/f1p0/4350	NA	R01_cb1346_c6/f1p0/4350	NA	NA
R01_cb18377_c0/f1p0/756	R01_cb18377_c0/f1p0/756	R01_cb18377_c0/f1p0/756	R01_cb18377_c0/f1p0/756	R01_cb18377_c0/f1p0/756
R01_cb17008_c2/f1p0/819	NA	NA	R01_cb17008_c2/f1p0/819	NA
R01_cb10003_c0/f1p0/1886	NA	R01_cb10003_c0/f1p0/1886	NA	R01_cb10003_c0/f1p0/1886
R01_cb17805_c2/f1p0/618	NA	R01_cb17805_c2/f1p0/618	NA	R01_cb17805_c2/f1p0/618
R01_cb18118_c0/f1p0/1543	NA	NA	NA	R01_cb18118_c0/f1p0/1543
R01_cb8213_c8/f4p3/2432	NA	NA	NA	R01_cb8213_c8/f4p3/2432
R01_cb2602_c14/f1p0/1520	R01_cb2602_c14/f1p0/1520	R01_cb2602_c14/f1p0/1520	NA	R01_cb2602_c14/f1p0/1520
R01_cb8564_c20052/f1p0/2339	NA	R01_cb8564_c20052/f1p0/2339	NA	R01_cb8564_c20052/f1p0/2339
R01_cb16829_c0/f1p0/1387	NA	R01_cb16829_c0/f1p0/1387	NA	NA
R01_cb8564_c145851/f5p1/2705	NA	NA	NA	R01_cb8564_c145851/f5p1/2705
R01_cb15853_c2/f1p1/524	NA	R01_cb15853_c2/f1p1/524	NA	R01_cb15853_c2/f1p1/524
R01_cb9630_c39/f1p0/2941	NA	R01_cb9630_c39/f1p0/2941	NA	NA
R01_cb507_c1/f1p0/4600	NA	NA	NA	R01_cb507_c1/f1p0/4600
R01_cb18456_c4765/f1p0/489	NA	NA	NA	R01_cb18456_c4765/f1p0/489
R01_cb7738_c2/f1p0/2225	NA	NA	NA	R01_cb7738_c2/f1p0/2225

R01_cb18456_c7204/f1p0/598	R01_cb18456_c7204/f1p0/598	R01_cb18456_c7204/f1p0/598	R01_cb18456_c7204/f1p0/598	R01_cb18456_c7204/f1p0/598
R01_cb8564_c111463/f2p0/4294	NA	R01_cb8564_c111463/f2p0/4294	NA	NA
R01_cb8564_c110236/f1p0/2530	NA	R01_cb8564_c110236/f1p0/2530	R01_cb8564_c110236/f1p0/2530	R01_cb8564_c110236/f1p0/2530
R01_cb10228_c4/f1p0/746	R01_cb10228_c4/f1p0/746	R01_cb10228_c4/f1p0/746	R01_cb10228_c4/f1p0/746	R01_cb10228_c4/f1p0/746
R01_cb17335_c0/f1p0/480	NA	R01_cb17335_c0/f1p0/480	R01_cb17335_c0/f1p0/480	NA
R01_cb10192_c0/f5p0/1335	NA	NA	NA	R01_cb10192_c0/f5p0/1335
R01_cb412_c12/f3p1/4582	NA	NA	NA	R01_cb412_c12/f3p1/4582
R01_cb10402_c4/f1p0/1399	NA	R01_cb10402_c4/f1p0/1399	NA	NA
R01_cb9272_c0/f3p0/2072	NA	R01_cb9272_c0/f3p0/2072	R01_cb9272_c0/f3p0/2072	R01_cb9272_c0/f3p0/2072
R01_cb14708_c1/f1p0/934	NA	R01_cb14708_c1/f1p0/934	NA	NA
R01_cb1628_c1/f2p0/1924	NA	NA	NA	R01_cb1628_c1/f2p0/1924
R01_cb11603_c2/f1p0/1065	NA	R01_cb11603_c2/f1p0/1065	NA	NA
R01_cb17324_c0/f2p0/817	NA	R01_cb17324_c0/f2p0/817	R01_cb17324_c0/f2p0/817	R01_cb17324_c0/f2p0/817
R01_cb16596_c20/f1p0/721	NA	NA	NA	R01_cb16596_c20/f1p0/721
R01_cb8564_c80765/f1p0/2029	NA	NA	NA	R01_cb8564_c80765/f1p0/2029
R01_cb10034_c64/f1p0/1617	NA	R01_cb10034_c64/f1p0/1617	NA	NA
R01_cb5593_c0/f1p0/3063	NA	R01_cb5593_c0/f1p0/3063	R01_cb5593_c0/f1p0/3063	R01_cb5593_c0/f1p0/3063
R01_cb2274_c9/f1p1/2153	NA	R01_cb2274_c9/f1p1/2153	NA	NA
R01_cb10207_c3/f1p0/4283	NA	R01_cb10207_c3/f1p0/4283	R01_cb10207_c3/f1p0/4283	R01_cb10207_c3/f1p0/4283
R01_cb9937_c0/f9p0/1283	NA	R01_cb9937_c0/f9p0/1283	R01_cb9937_c0/f9p0/1283	R01_cb9937_c0/f9p0/1283
R01_cb9037_c1/f2p0/3528	NA	R01_cb9037_c1/f2p0/3528	NA	NA
R01_cb15353_c1/f1p0/1329	NA	NA	NA	R01_cb15353_c1/f1p0/1329
R01_cb11141_c9/f1p0/1231	NA	NA	NA	R01_cb11141_c9/f1p0/1231
R01_cb8564_c33830/f4p1/1979	NA	NA	NA	R01_cb8564_c33830/f4p1/1979

R01_cb11569_c1/f1p0/4589	R01_cb11569_c1/f1p0/4589	R01_cb11569_c1/f1p0/4589	R01_cb11569_c1/f1p0/4589	R01_cb11569_c1/f1p0/4589
R01_cb14251_c5/f1p2/1275	NA	R01_cb14251_c5/f1p2/1275	NA	NA
R01_cb6586_c3/f1p0/2782	NA	R01_cb6586_c3/f1p0/2782	NA	NA
R01_cb8564_c40975/f1p1/2422	NA	NA	NA	R01_cb8564_c40975/f1p1/2422
R01_cb2571_c21/f1p1/3851	NA	NA	NA	R01_cb2571_c21/f1p1/3851
R01_cb8564_c4134/f1p0/2932	NA	R01_cb8564_c4134/f1p0/2932	NA	R01_cb8564_c4134/f1p0/2932
R01_cb644_c5/f1p0/4671	NA	NA	NA	R01_cb644_c5/f1p0/4671
R01_cb8564_c112417/f1p0/2056	R01_cb8564_c112417/f1p0/2056	R01_cb8564_c112417/f1p0/2056	R01_cb8564_c112417/f1p0/2056	R01_cb8564_c112417/f1p0/2056
R01_cb8564_c72849/f1p0/2326	NA	NA	NA	R01_cb8564_c72849/f1p0/2326
R01_cb4478_c1/f1p0/3008	NA	R01_cb4478_c1/f1p0/3008	NA	NA
R01_cb188_c8/f1p0/1931	NA	NA	NA	R01_cb188_c8/f1p0/1931
R01_cb8564_c117483/f1p0/2158	R01_cb8564_c117483/f1p0/2158	R01_cb8564_c117483/f1p0/2158	NA	NA
R01_cb4744_c7/f1p0/1486	NA	R01_cb4744_c7/f1p0/1486	R01_cb4744_c7/f1p0/1486	R01_cb4744_c7/f1p0/1486
R01_cb8564_c35473/f1p0/2841	NA	NA	NA	R01_cb8564_c35473/f1p0/2841
R01_cb7693_c3/f1p0/2570	NA	R01_cb7693_c3/f1p0/2570	R01_cb7693_c3/f1p0/2570	R01_cb7693_c3/f1p0/2570
R01_cb11046_c0/f9p0/487	NA	R01_cb11046_c0/f9p0/487	NA	R01_cb11046_c0/f9p0/487
R01_cb14316_c13/f1p0/609	R01_cb14316_c13/f1p0/609	R01_cb14316_c13/f1p0/609	R01_cb14316_c13/f1p0/609	R01_cb14316_c13/f1p0/609
R01_cb8564_c35514/f1p0/4199	NA	R01_cb8564_c35514/f1p0/4199	R01_cb8564_c35514/f1p0/4199	NA
R01_cb13884_c14/f1p0/565	R01_cb13884_c14/f1p0/565	NA	R01_cb13884_c14/f1p0/565	R01_cb13884_c14/f1p0/565
R01_cb16968_c0/f1p0/1336	NA	NA	NA	R01_cb16968_c0/f1p0/1336
R01_cb5214_c10/f9p1/2990	NA	R01_cb5214_c10/f9p1/2990	R01_cb5214_c10/f9p1/2990	NA
R01_cb6802_c36/f1p0/3647	R01_cb6802_c36/f1p0/3647	R01_cb6802_c36/f1p0/3647	R01_cb6802_c36/f1p0/3647	R01_cb6802_c36/f1p0/3647
R01_cb2357_c0/f3p1/3958	NA	NA	NA	R01_cb2357_c0/f3p1/3958
R01_cb3926_c3/f1p0/1949	NA	R01_cb3926_c3/f1p0/1949	NA	NA

R01_cb7999_c4/f1p0/989	NA	NA	NA	R01_cb7999_c4/f1p0/989
R01_cb8564_c81496/f1p0/4041	NA	R01_cb8564_c81496/f1p0/4041	NA	NA
R01_cb18610_c2/f1p0/5235	NA	R01_cb18610_c2/f1p0/5235	NA	NA
R01_cb3426_c25/f1p0/3366	NA	NA	NA	R01_cb3426_c25/f1p0/3366
R01_cb8564_c80489/f1p0/1988	R01_cb8564_c80489/f1p0/1988	R01_cb8564_c80489/f1p0/1988	NA	NA
R01_cb8564_c50869/f1p0/3578	NA	R01_cb8564_c50869/f1p0/3578	NA	NA
R01_cb13203_c1/f1p0/830	R01_cb13203_c1/f1p0/830	R01_cb13203_c1/f1p0/830	R01_cb13203_c1/f1p0/830	R01_cb13203_c1/f1p0/830
R01_cb17839_c0/f1p0/757	NA	R01_cb17839_c0/f1p0/757	NA	R01_cb17839_c0/f1p0/757
R01_cb11535_c2/f1p0/792	R01_cb11535_c2/f1p0/792	R01_cb11535_c2/f1p0/792	R01_cb11535_c2/f1p0/792	R01_cb11535_c2/f1p0/792
R01_cb12445_c3/f3p0/993	NA	R01_cb12445_c3/f3p0/993	NA	NA
R01_cb10662_c4/f1p0/1487	NA	R01_cb10662_c4/f1p0/1487	NA	R01_cb10662_c4/f1p0/1487
R01_cb8564_c3741/f1p0/2794	NA	R01_cb8564_c3741/f1p0/2794	R01_cb8564_c3741/f1p0/2794	R01_cb8564_c3741/f1p0/2794
R01_cb18374_c1/f1p0/1256	NA	R01_cb18374_c1/f1p0/1256	NA	NA
R01_cb8564_c15644/f1p0/3192	NA	NA	NA	R01_cb8564_c15644/f1p0/3192
R01_cb6532_c6/f1p0/1540	NA	NA	NA	R01_cb6532_c6/f1p0/1540
R01_cb7741_c4/f1p0/2284	R01_cb7741_c4/f1p0/2284	R01_cb7741_c4/f1p0/2284	R01_cb7741_c4/f1p0/2284	R01_cb7741_c4/f1p0/2284
R01_cb18456_c1716/f2p0/1857	NA	R01_cb18456_c1716/f2p0/1857	R01_cb18456_c1716/f2p0/1857	R01_cb18456_c1716/f2p0/1857
R01_cb14125_c138/f1p0/1025	NA	R01_cb14125_c138/f1p0/1025	NA	R01_cb14125_c138/f1p0/1025
R01_cb4501_c13/f1p0/2489	NA	NA	NA	R01_cb4501_c13/f1p0/2489
R01_cb15207_c120/f1p0/650	NA	R01_cb15207_c120/f1p0/650	R01_cb15207_c120/f1p0/650	NA
R01_cb18392_c0/f1p1/973	NA	NA	NA	R01_cb18392_c0/f1p1/973
R01_cb7258_c4/f1p0/1670	NA	R01_cb7258_c4/f1p0/1670	R01_cb7258_c4/f1p0/1670	NA
R01_cb11745_c2/f1p0/999	R01_cb11745_c2/f1p0/999	R01_cb11745_c2/f1p0/999	R01_cb11745_c2/f1p0/999	R01_cb11745_c2/f1p0/999
R01_cb6983_c0/f1p0/2724	NA	R01_cb6983_c0/f1p0/2724	R01_cb6983_c0/f1p0/2724	NA
R01_cb5631_c26/f1p1/2402	NA	R01_cb5631_c26/f1p1/2402	NA	R01_cb5631_c26/f1p1/2402

R01_cb4300_c4/f1p0/1885	R01_cb4300_c4/f1p0/1885	R01_cb4300_c4/f1p0/1885	R01_cb4300_c4/f1p0/1885	NA
R01_cb8564_c72586/f1p0/3056	NA	NA	R01_cb8564_c72586/f1p0/3056	R01_cb8564_c72586/f1p0/3056
R01_cb9196_c6/f1p0/1977	NA	R01_cb9196_c6/f1p0/1977	NA	NA
R01_cb6982_c4/f1p0/5150	NA	R01_cb6982_c4/f1p0/5150	NA	NA
R01_cb8982_c1/f1p0/2213	NA	NA	NA	R01_cb8982_c1/f1p0/2213
R01_cb8564_c3076/f1p0/2807	NA	R01_cb8564_c3076/f1p0/2807	NA	R01_cb8564_c3076/f1p0/2807
R01_cb13169_c2/f1p0/649	R01_cb13169_c2/f1p0/649	R01_cb13169_c2/f1p0/649	R01_cb13169_c2/f1p0/649	R01_cb13169_c2/f1p0/649
R01_cb14761_c8/f1p0/949	NA	R01_cb14761_c8/f1p0/949	R01_cb14761_c8/f1p0/949	R01_cb14761_c8/f1p0/949
R01_cb15480_c0/f2p0/573	NA	R01_cb15480_c0/f2p0/573	NA	NA
R01_cb2445_c23/f1p0/3339	NA	NA	NA	R01_cb2445_c23/f1p0/3339
R01_cb8564_c121875/f1p0/1960	NA	NA	NA	R01_cb8564_c121875/f1p0/1960
R01_cb11073_c1/f1p0/3410	NA	R01_cb11073_c1/f1p0/3410	NA	NA
R01_cb18456_c1527/f3p0/631	R01_cb18456_c1527/f3p0/631	R01_cb18456_c1527/f3p0/631	R01_cb18456_c1527/f3p0/631	NA
R01_cb8564_c126874/f1p0/2195	NA	NA	NA	R01_cb8564_c126874/f1p0/2195
R01_cb14375_c1/f1p0/1220	NA	R01_cb14375_c1/f1p0/1220	NA	NA
R01_cb4824_c4/f1p0/3555	R01_cb4824_c4/f1p0/3555	R01_cb4824_c4/f1p0/3555	R01_cb4824_c4/f1p0/3555	R01_cb4824_c4/f1p0/3555
R01_cb8564_c115222/f1p0/2238	NA	NA	NA	R01_cb8564_c115222/f1p0/2238
R01_cb10679_c1/f1p0/2332	R01_cb10679_c1/f1p0/2332	R01_cb10679_c1/f1p0/2332	R01_cb10679_c1/f1p0/2332	R01_cb10679_c1/f1p0/2332
R01_cb8564_c91216/f1p0/2554	NA	NA	NA	R01_cb8564_c91216/f1p0/2554
R01_cb14125_c123/f1p0/617	NA	R01_cb14125_c123/f1p0/617	R01_cb14125_c123/f1p0/617	NA
R01_cb8116_c2/f2p0/2414	R01_cb8116_c2/f2p0/2414	R01_cb8116_c2/f2p0/2414	R01_cb8116_c2/f2p0/2414	NA
R01_cb9119_c6/f1p2/2148	NA	NA	NA	R01_cb9119_c6/f1p2/2148
R01_cb14195_c5/f1p0/475	R01_cb14195_c5/f1p0/475	R01_cb14195_c5/f1p0/475	R01_cb14195_c5/f1p0/475	R01_cb14195_c5/f1p0/475

R01_cb8564_c40193/f1p0/3545	NA	R01_cb8564_c40193/f1p0/3545	R01_cb8564_c40193/f1p0/3545	R01_cb8564_c40193/f1p0/3545
R01_cb1998_c4/f1p0/3483	NA	R01_cb1998_c4/f1p0/3483	R01_cb1998_c4/f1p0/3483	NA
R01_cb14421_c2/f1p0/937	NA	NA	NA	R01_cb14421_c2/f1p0/937
R01_cb805_c9/f1p0/1709	NA	NA	NA	R01_cb805_c9/f1p0/1709
R01_cb15890_c0/f1p0/759	NA	NA	NA	R01_cb15890_c0/f1p0/759
R01_cb12314_c8/f7p1/552	NA	NA	R01_cb12314_c8/f7p1/552	NA
R01_cb1398_c10/f1p0/1985	NA	R01_cb1398_c10/f1p0/1985	R01_cb1398_c10/f1p0/1985	NA
R01_cb7825_c5/f1p0/1011	NA	R01_cb7825_c5/f1p0/1011	NA	NA
R01_cb12192_c2/f1p0/1204	R01_cb12192_c2/f1p0/1204	R01_cb12192_c2/f1p0/1204	R01_cb12192_c2/f1p0/1204	R01_cb12192_c2/f1p0/1204
R01_cb1398_c3/f1p0/4846	NA	R01_cb1398_c3/f1p0/4846	R01_cb1398_c3/f1p0/4846	R01_cb1398_c3/f1p0/4846
R01_cb8564_c15894/f1p0/2875	NA	NA	NA	R01_cb8564_c15894/f1p0/2875
R01_cb822_c26/f1p0/2757	NA	NA	NA	R01_cb822_c26/f1p0/2757
R01_cb8564_c114711/f1p0/2060	NA	NA	NA	R01_cb8564_c114711/f1p0/2060
R01_cb8779_c3/f1p0/2326	NA	NA	NA	R01_cb8779_c3/f1p0/2326
R01_cb12936_c0/f1p0/803	NA	R01_cb12936_c0/f1p0/803	NA	NA
R01_cb11035_c0/f1p0/1363	NA	NA	R01_cb11035_c0/f1p0/1363	R01_cb11035_c0/f1p0/1363
R01_cb8564_c82617/f1p1/2534	NA	NA	NA	R01_cb8564_c82617/f1p1/2534
R01_cb15880_c0/f1p0/558	R01_cb15880_c0/f1p0/558	NA	R01_cb15880_c0/f1p0/558	R01_cb15880_c0/f1p0/558
R01_cb16442_c1/f1p0/1061	NA	R01_cb16442_c1/f1p0/1061	R01_cb16442_c1/f1p0/1061	R01_cb16442_c1/f1p0/1061
R01_cb4602_c8/f1p0/2365	NA	R01_cb4602_c8/f1p0/2365	NA	R01_cb4602_c8/f1p0/2365
R01_cb14720_c1/f11p0/540	NA	NA	NA	R01_cb14720_c1/f11p0/540
R01_cb8115_c2/f1p0/379	R01_cb8115_c2/f1p0/379	R01_cb8115_c2/f1p0/379	R01_cb8115_c2/f1p0/379	R01_cb8115_c2/f1p0/379
R01_cb234_c7/f1p1/3110	NA	NA	NA	R01_cb234_c7/f1p1/3110
R01_cb3022_c27/f1p0/555	NA	R01_cb3022_c27/f1p0/555	NA	NA
R01_cb8564_c77005/f1p1/2490	NA	R01_cb8564_c77005/f1p1/2490	NA	NA

R01_cb16815_c0/f1p0/711	NA	R01_cb16815_c0/f1p0/711	NA	NA
R01_cb3578_c8/f1p0/761	NA	R01_cb3578_c8/f1p0/761	NA	R01_cb3578_c8/f1p0/761
R01_cb8897_c0/f1p0/2243	NA	R01_cb8897_c0/f1p0/2243	NA	NA
R01_cb6615_c13/f1p0/2451	NA	NA	R01_cb6615_c13/f1p0/2451	R01_cb6615_c13/f1p0/2451
R01_cb8564_c9748/f3p2/4681	NA	R01_cb8564_c9748/f3p2/4681	NA	NA
R01_cb8564_c4626/f1p0/3211	NA	R01_cb8564_c4626/f1p0/3211	R01_cb8564_c4626/f1p0/3211	R01_cb8564_c4626/f1p0/3211
R01_cb8564_c17637/f1p0/3299	R01_cb8564_c17637/f1p0/3299	R01_cb8564_c17637/f1p0/3299	R01_cb8564_c17637/f1p0/3299	R01_cb8564_c17637/f1p0/3299
R01_cb18280_c0/f1p0/1564	R01_cb18280_c0/f1p0/1564	R01_cb18280_c0/f1p0/1564	NA	NA
R01_cb2275_c1/f1p0/3022	NA	R01_cb2275_c1/f1p0/3022	R01_cb2275_c1/f1p0/3022	R01_cb2275_c1/f1p0/3022
R01_cb18456_c7601/f1p0/1376	NA	R01_cb18456_c7601/f1p0/1376	R01_cb18456_c7601/f1p0/1376	R01_cb18456_c7601/f1p0/1376
R01_cb8564_c118186/f1p0/2178	NA	NA	R01_cb8564_c118186/f1p0/2178	R01_cb8564_c118186/f1p0/2178
R01_cb625_c3/f1p0/3983	NA	NA	NA	R01_cb625_c3/f1p0/3983
R01_cb15053_c8/f1p0/373	R01_cb15053_c8/f1p0/373	R01_cb15053_c8/f1p0/373	R01_cb15053_c8/f1p0/373	NA
R01_cb4669_c17/f1p0/2363	R01_cb4669_c17/f1p0/2363	R01_cb4669_c17/f1p0/2363	NA	NA
R01_cb18045_c0/f1p0/475	R01_cb18045_c0/f1p0/475	R01_cb18045_c0/f1p0/475	R01_cb18045_c0/f1p0/475	R01_cb18045_c0/f1p0/475
R01_cb8564_c113684/f1p0/3476	NA	R01_cb8564_c113684/f1p0/3476	NA	NA
R01_cb781_c28/f1p0/1999	NA	NA	NA	R01_cb781_c28/f1p0/1999
R01_cb3637_c4/f1p0/1412	NA	R01_cb3637_c4/f1p0/1412	NA	NA
R01_cb18359_c1/f1p0/1680	NA	NA	NA	R01_cb18359_c1/f1p0/1680
R01_cb8564_c25695/f1p1/2271	NA	R01_cb8564_c25695/f1p1/2271	NA	R01_cb8564_c25695/f1p1/2271
R01_cb4134_c19/f1p1/2903	NA	NA	NA	R01_cb4134_c19/f1p1/2903
R01_cb15607_c0/f1p0/617	NA	NA	R01_cb15607_c0/f1p0/617	NA
R01_cb4295_c39/f1p0/1929	NA	R01_cb4295_c39/f1p0/1929	NA	NA

R01_cb8564_c107887/f1p0/2716	NA	R01_cb8564_c107887/f1p0/2716	NA	NA
R01_cb10024_c565/f1p1/620	NA	NA	R01_cb10024_c565/f1p1/620	NA
R01_cb8564_c35464/f1p1/3983	NA	R01_cb8564_c35464/f1p1/3983	R01_cb8564_c35464/f1p1/3983	NA
R01_cb7334_c1/f3p0/1895	NA	NA	NA	R01_cb7334_c1/f3p0/1895
R01_cb12620_c15/f42p0/1044	NA	NA	NA	R01_cb12620_c15/f42p0/1044
R01_cb18528_c1/f1p0/678	NA	R01_cb18528_c1/f1p0/678	NA	NA
R01_cb16841_c1/f1p0/441	NA	R01_cb16841_c1/f1p0/441	NA	NA
R01_cb8564_c126368/f1p0/2603	NA	NA	NA	R01_cb8564_c126368/f1p0/2603
R01_cb8564_c16926/f1p0/2964	R01_cb8564_c16926/f1p0/2964	R01_cb8564_c16926/f1p0/2964	R01_cb8564_c16926/f1p0/2964	R01_cb8564_c16926/f1p0/2964
R01_cb7731_c2/f3p0/1452	NA	NA	NA	R01_cb7731_c2/f3p0/1452
R01_cb10197_c1/f1p0/2608	R01_cb10197_c1/f1p0/2608	R01_cb10197_c1/f1p0/2608	R01_cb10197_c1/f1p0/2608	R01_cb10197_c1/f1p0/2608
R01_cb8564_c41853/f1p0/3754	NA	NA	NA	R01_cb8564_c41853/f1p0/3754
R01_cb11271_c0/f2p0/582	NA	R01_cb11271_c0/f2p0/582	NA	NA
R01_cb16125_c22/f1p0/1760	NA	NA	R01_cb16125_c22/f1p0/1760	R01_cb16125_c22/f1p0/1760
R01_cb8564_c112444/f1p0/3012	NA	R01_cb8564_c112444/f1p0/3012	NA	NA
R01_cb14675_c8/f1p0/840	R01_cb14675_c8/f1p0/840	R01_cb14675_c8/f1p0/840	R01_cb14675_c8/f1p0/840	R01_cb14675_c8/f1p0/840
R01_cb18456_c7273/f1p0/538	R01_cb18456_c7273/f1p0/538	R01_cb18456_c7273/f1p0/538	NA	NA
R01_cb16341_c12/f1p0/647	R01_cb16341_c12/f1p0/647	R01_cb16341_c12/f1p0/647	R01_cb16341_c12/f1p0/647	R01_cb16341_c12/f1p0/647
R01_cb8564_c4687/f1p0/2736	NA	R01_cb8564_c4687/f1p0/2736	NA	NA
R01_cb9610_c0/f1p0/2033	NA	NA	NA	R01_cb9610_c0/f1p0/2033
R01_cb16631_c0/f1p0/565	NA	R01_cb16631_c0/f1p0/565	NA	R01_cb16631_c0/f1p0/565
R01_cb7280_c3/f2p0/2696	R01_cb7280_c3/f2p0/2696	R01_cb7280_c3/f2p0/2696	NA	NA

R01_cb16645_c54/f1p0/709	NA	R01_cb16645_c54/f1p0/709	NA	NA
R01_cb8564_c115836/f1p0/2206	NA	R01_cb8564_c115836/f1p0/2206	NA	R01_cb8564_c115836/f1p0/2206
R01_cb11734_c1/f1p0/4290	NA	R01_cb11734_c1/f1p0/4290	NA	NA
R01_cb16872_c9/f1p0/753	NA	R01_cb16872_c9/f1p0/753	NA	NA
R01_cb16098_c2/f1p0/621	R01_cb16098_c2/f1p0/621	R01_cb16098_c2/f1p0/621	R01_cb16098_c2/f1p0/621	R01_cb16098_c2/f1p0/621
R01_cb18645_c1/f1p0/1684	R01_cb18645_c1/f1p0/1684	R01_cb18645_c1/f1p0/1684	R01_cb18645_c1/f1p0/1684	R01_cb18645_c1/f1p0/1684
R01_cb14166_c0/f4p0/640	R01_cb14166_c0/f4p0/640	NA	R01_cb14166_c0/f4p0/640	R01_cb14166_c0/f4p0/640
R01_cb3536_c0/f2p0/1378	NA	NA	NA	R01_cb3536_c0/f2p0/1378
R01_cb8564_c22806/f1p0/3041	NA	R01_cb8564_c22806/f1p0/3041	NA	NA
R01_cb18409_c82/f1p0/452	NA	R01_cb18409_c82/f1p0/452	R01_cb18409_c82/f1p0/452	R01_cb18409_c82/f1p0/452
R01_cb8232_c0/f1p0/2423	NA	R01_cb8232_c0/f1p0/2423	R01_cb8232_c0/f1p0/2423	NA
R01_cb15371_c4/f1p0/624	NA	R01_cb15371_c4/f1p0/624	NA	NA
R01_cb18290_c4/f1p0/617	R01_cb18290_c4/f1p0/617	R01_cb18290_c4/f1p0/617	NA	NA
R01_cb5709_c2/f1p0/2876	NA	NA	NA	R01_cb5709_c2/f1p0/2876
R01_cb10045_c32/f1p0/1369	NA	R01_cb10045_c32/f1p0/1369	NA	NA
R01_cb8564_c43296/f1p0/2175	R01_cb8564_c43296/f1p0/2175	R01_cb8564_c43296/f1p0/2175	R01_cb8564_c43296/f1p0/2175	R01_cb8564_c43296/f1p0/2175
R01_cb16250_c0/f1p0/1742	NA	R01_cb16250_c0/f1p0/1742	R01_cb16250_c0/f1p0/1742	R01_cb16250_c0/f1p0/1742
R01_cb18301_c9/f1p0/645	R01_cb18301_c9/f1p0/645	R01_cb18301_c9/f1p0/645	R01_cb18301_c9/f1p0/645	R01_cb18301_c9/f1p0/645
R01_cb8349_c56/f1p0/446	R01_cb8349_c56/f1p0/446	R01_cb8349_c56/f1p0/446	R01_cb8349_c56/f1p0/446	R01_cb8349_c56/f1p0/446
R01_cb6307_c27/f1p0/3542	NA	NA	NA	R01_cb6307_c27/f1p0/3542
R01_cb14968_c15/f1p0/1687	NA	NA	NA	R01_cb14968_c15/f1p0/1687
R01_cb13488_c3/f1p0/575	NA	R01_cb13488_c3/f1p0/575	NA	R01_cb13488_c3/f1p0/575
R01_cb8564_c107831/f1p0/2250	NA	R01_cb8564_c107831/f1p0/2250	R01_cb8564_c107831/f1p0/2250	R01_cb8564_c107831/f1p0/2250

R01_cb8564_c88286/f1p0/2450	NA	R01_cb8564_c88286/f1p0/2450	R01_cb8564_c88286/f1p0/2450	NA
R01_cb1228_c24/f1p1/2664	NA	NA	NA	R01_cb1228_c24/f1p1/2664
R01_cb16540_c0/f11p0/827	NA	NA	R01_cb16540_c0/f11p0/827	R01_cb16540_c0/f11p0/827
R01_cb10114_c0/f1p0/1117	NA	R01_cb10114_c0/f1p0/1117	NA	NA
R01_cb2988_c9/f1p0/2293	NA	NA	NA	R01_cb2988_c9/f1p0/2293
R01_cb8688_c4/f1p0/2314	NA	NA	NA	R01_cb8688_c4/f1p0/2314
R01_cb13449_c7/f5p1/705	NA	NA	NA	R01_cb13449_c7/f5p1/705
R01_cb11553_c1/f1p0/2733	R01_cb11553_c1/f1p0/2733	R01_cb11553_c1/f1p0/2733	R01_cb11553_c1/f1p0/2733	NA
R01_cb5153_c7/f1p0/3152	NA	R01_cb5153_c7/f1p0/3152	NA	NA
R01_cb1473_c3/f1p0/2023	NA	R01_cb1473_c3/f1p0/2023	R01_cb1473_c3/f1p0/2023	R01_cb1473_c3/f1p0/2023
R01_cb13884_c11/f1p0/555	R01_cb13884_c11/f1p0/555	R01_cb13884_c11/f1p0/555	R01_cb13884_c11/f1p0/555	R01_cb13884_c11/f1p0/555
R01_cb8564_c75327/f1p0/2046	NA	R01_cb8564_c75327/f1p0/2046	R01_cb8564_c75327/f1p0/2046	R01_cb8564_c75327/f1p0/2046
R01_cb18456_c7113/f1p0/485	R01_cb18456_c7113/f1p0/485	R01_cb18456_c7113/f1p0/485	R01_cb18456_c7113/f1p0/485	R01_cb18456_c7113/f1p0/485
R01_cb7654_c3/f1p0/510	NA	R01_cb7654_c3/f1p0/510	NA	R01_cb7654_c3/f1p0/510
R01_cb1599_c1/f1p0/2663	NA	NA	NA	R01_cb1599_c1/f1p0/2663
R01_cb8564_c2582/f1p0/2831	NA	R01_cb8564_c2582/f1p0/2831	NA	NA
R01_cb6398_c19/f1p0/1160	NA	R01_cb6398_c19/f1p0/1160	NA	NA
R01_cb8564_c71049/f2p0/2966	R01_cb8564_c71049/f2p0/2966	R01_cb8564_c71049/f2p0/2966	R01_cb8564_c71049/f2p0/2966	R01_cb8564_c71049/f2p0/2966
R01_cb6666_c3/f2p0/2457	NA	NA	NA	R01_cb6666_c3/f2p0/2457
R01_cb11041_c6/f1p0/792	NA	R01_cb11041_c6/f1p0/792	NA	NA
R01_cb12496_c8/f1p0/338	R01_cb12496_c8/f1p0/338	R01_cb12496_c8/f1p0/338	R01_cb12496_c8/f1p0/338	R01_cb12496_c8/f1p0/338
R01_cb18568_c0/f1p0/589	R01_cb18568_c0/f1p0/589	NA	NA	R01_cb18568_c0/f1p0/589
R01_cb1259_c27/f1p0/2795	NA	NA	NA	R01_cb1259_c27/f1p0/2795
R01_cb5096_c1/f1p0/3169	NA	R01_cb5096_c1/f1p0/3169	NA	R01_cb5096_c1/f1p0/3169
R01_cb15093_c4/f1p0/842	NA	NA	NA	R01_cb15093_c4/f1p0/842

R01_cb18456_c4772/f1p0/481	R01_cb18456_c4772/f1p0/481	NA	NA	R01_cb18456_c4772/f1p0/481
R01_cb8564_c38983/f1p0/3279	R01_cb8564_c38983/f1p0/3279	R01_cb8564_c38983/f1p0/3279	NA	R01_cb8564_c38983/f1p0/3279
R01_cb10698_c1/f1p0/4008	NA	R01_cb10698_c1/f1p0/4008	NA	NA
R01_cb402_c23/f1p1/2136	NA	R01_cb402_c23/f1p1/2136	NA	R01_cb402_c23/f1p1/2136
R01_cb8564_c3607/f1p0/2889	NA	R01_cb8564_c3607/f1p0/2889	NA	NA
R01_cb1905_c15/f1p0/3162	NA	R01_cb1905_c15/f1p0/3162	R01_cb1905_c15/f1p0/3162	NA
R01_cb11101_c2/f1p0/2288	NA	R01_cb11101_c2/f1p0/2288	NA	NA
R01_cb7133_c2/f1p0/2452	NA	R01_cb7133_c2/f1p0/2452	NA	NA
R01_cb8564_c81521/f2p1/4466	NA	R01_cb8564_c81521/f2p1/4466	NA	NA
R01_cb4469_c0/f4p0/1536	NA	NA	NA	R01_cb4469_c0/f4p0/1536
R01_cb7842_c13/f1p0/2319	NA	R01_cb7842_c13/f1p0/2319	R01_cb7842_c13/f1p0/2319	NA
R01_cb10200_c5/f1p0/767	NA	R01_cb10200_c5/f1p0/767	NA	NA
R01_cb8564_c20866/f2p0/2614	NA	R01_cb8564_c20866/f2p0/2614	NA	R01_cb8564_c20866/f2p0/2614
R01_cb8564_c85131/f1p3/1987	NA	R01_cb8564_c85131/f1p3/1987	NA	NA
R01_cb4614_c2/f1p0/2134	NA	R01_cb4614_c2/f1p0/2134	NA	NA
R01_cb12603_c10/f1p0/1345	NA	NA	R01_cb12603_c10/f1p0/1345	R01_cb12603_c10/f1p0/1345
R01_cb14186_c0/f1p0/1114	NA	R01_cb14186_c0/f1p0/1114	NA	NA
R01_cb12983_c18/f1p0/577	NA	R01_cb12983_c18/f1p0/577	R01_cb12983_c18/f1p0/577	R01_cb12983_c18/f1p0/577
R01_cb12512_c2/f2p3/1432	NA	R01_cb12512_c2/f2p3/1432	R01_cb12512_c2/f2p3/1432	R01_cb12512_c2/f2p3/1432
R01_cb12609_c1/f1p0/776	NA	NA	NA	R01_cb12609_c1/f1p0/776
R01_cb12722_c12/f1p0/380	R01_cb12722_c12/f1p0/380	R01_cb12722_c12/f1p0/380	NA	R01_cb12722_c12/f1p0/380
R01_cb2022_c10/f1p0/2385	NA	NA	NA	R01_cb2022_c10/f1p0/2385
R01_cb15807_c1/f2p0/754	NA	NA	NA	R01_cb15807_c1/f2p0/754
R01_cb10065_c2/f1p0/2654	NA	R01_cb10065_c2/f1p0/2654	NA	NA
R01_cb9682_c3/f1p1/1949	NA	NA	NA	R01_cb9682_c3/f1p1/1949

R01_cb16185_c1/f1p0/1366	R01_cb16185_c1/f1p0/1366	R01_cb16185_c1/f1p0/1366	R01_cb16185_c1/f1p0/1366	R01_cb16185_c1/f1p0/1366
R01_cb8564_c113160/f1p0/2115	NA	NA	NA	R01_cb8564_c113160/f1p0/2115
R01_cb18456_c1031/f1p0/628	NA	R01_cb18456_c1031/f1p0/628	NA	NA
R01_cb929_c3/f1p0/2496	NA	NA	NA	R01_cb929_c3/f1p0/2496
R01_cb2737_c43/f1p0/3713	NA	R01_cb2737_c43/f1p0/3713	NA	NA
R01_cb18456_c5921/f1p0/708	NA	R01_cb18456_c5921/f1p0/708	R01_cb18456_c5921/f1p0/708	R01_cb18456_c5921/f1p0/708
R01_cb3774_c22/f1p0/3380	NA	NA	NA	R01_cb3774_c22/f1p0/3380
R01_cb15207_c85/f1p0/824	NA	R01_cb15207_c85/f1p0/824	NA	NA
R01_cb7519_c15/f1p1/728	NA	NA	NA	R01_cb7519_c15/f1p1/728
R01_cb2773_c2/f1p0/3812	R01_cb2773_c2/f1p0/3812	R01_cb2773_c2/f1p0/3812	R01_cb2773_c2/f1p0/3812	R01_cb2773_c2/f1p0/3812
R01_cb15534_c10/f1p0/568	R01_cb15534_c10/f1p0/568	R01_cb15534_c10/f1p0/568	NA	NA
R01_cb17805_c0/f7p0/514	R01_cb17805_c0/f7p0/514	R01_cb17805_c0/f7p0/514	NA	R01_cb17805_c0/f7p0/514
R01_cb8564_c42802/f1p0/4924	NA	R01_cb8564_c42802/f1p0/4924	R01_cb8564_c42802/f1p0/4924	R01_cb8564_c42802/f1p0/4924
R01_cb8564_c24309/f1p0/4229	NA	R01_cb8564_c24309/f1p0/4229	NA	NA
R01_cb14152_c3/f1p0/868	NA	NA	NA	R01_cb14152_c3/f1p0/868
R01_cb9784_c2/f1p0/1445	R01_cb9784_c2/f1p0/1445	R01_cb9784_c2/f1p0/1445	R01_cb9784_c2/f1p0/1445	R01_cb9784_c2/f1p0/1445
R01_cb2936_c6/f1p0/3561	NA	R01_cb2936_c6/f1p0/3561	NA	NA
R01_cb12039_c0/f14p0/423	NA	R01_cb12039_c0/f14p0/423	R01_cb12039_c0/f14p0/423	R01_cb12039_c0/f14p0/423
R01_cb13760_c0/f9p1/1821	NA	NA	NA	R01_cb13760_c0/f9p1/1821
R01_cb8564_c85502/f1p0/3131	NA	R01_cb8564_c85502/f1p0/3131	R01_cb8564_c85502/f1p0/3131	R01_cb8564_c85502/f1p0/3131
R01_cb1342_c2/f1p0/4368	NA	R01_cb1342_c2/f1p0/4368	NA	NA
R01_cb8564_c34076/f3p0/2476	R01_cb8564_c34076/f3p0/2476	R01_cb8564_c34076/f3p0/2476	R01_cb8564_c34076/f3p0/2476	R01_cb8564_c34076/f3p0/2476
R01_cb13603_c3/f1p0/579	R01_cb13603_c3/f1p0/579	R01_cb13603_c3/f1p0/579	R01_cb13603_c3/f1p0/579	R01_cb13603_c3/f1p0/579
R01_cb7802_c20/f1p0/1296	NA	NA	NA	R01_cb7802_c20/f1p0/1296

R01_cb15615_c3/f1p0/693	NA	R01_cb15615_c3/f1p0/693	NA	NA
R01_cb9813_c27/f1p0/1927	NA	R01_cb9813_c27/f1p0/1927	NA	R01_cb9813_c27/f1p0/1927
R01_cb14448_c0/f5p0/594	NA	NA	NA	R01_cb14448_c0/f5p0/594
R01_cb12875_c1/f1p0/642	NA	R01_cb12875_c1/f1p0/642	R01_cb12875_c1/f1p0/642	R01_cb12875_c1/f1p0/642
R01_cb124_c36/f1p0/2750	NA	NA	NA	R01_cb124_c36/f1p0/2750
R01_cb3229_c19/f1p0/2319	NA	NA	NA	R01_cb3229_c19/f1p0/2319
R01_cb18192_c0/f3p0/468	NA	R01_cb18192_c0/f3p0/468	R01_cb18192_c0/f3p0/468	NA
R01_cb12192_c1/f2p0/419	R01_cb12192_c1/f2p0/419	R01_cb12192_c1/f2p0/419	NA	NA
R01_cb8564_c71665/f3p0/2489	NA	R01_cb8564_c71665/f3p0/2489	R01_cb8564_c71665/f3p0/2489	R01_cb8564_c71665/f3p0/2489
R01_cb9492_c1/f1p0/1958	NA	NA	R01_cb9492_c1/f1p0/1958	NA
R01_cb18476_c2/f1p0/1221	NA	NA	NA	R01_cb18476_c2/f1p0/1221
R01_cb7379_c5/f1p0/2417	NA	R01_cb7379_c5/f1p0/2417	NA	NA
R01_cb13146_c2/f1p1/738	NA	NA	R01_cb13146_c2/f1p1/738	R01_cb13146_c2/f1p1/738
R01_cb16078_c5/f1p0/458	R01_cb16078_c5/f1p0/458	R01_cb16078_c5/f1p0/458	R01_cb16078_c5/f1p0/458	NA
R01_cb18200_c0/f1p0/535	R01_cb18200_c0/f1p0/535	R01_cb18200_c0/f1p0/535	R01_cb18200_c0/f1p0/535	R01_cb18200_c0/f1p0/535
R01_cb13010_c2/f1p0/1387	NA	R01_cb13010_c2/f1p0/1387	NA	NA
R01_cb14558_c1/f1p0/557	NA	R01_cb14558_c1/f1p0/557	NA	NA
R01_cb4960_c5/f1p0/2432	NA	R01_cb4960_c5/f1p0/2432	R01_cb4960_c5/f1p0/2432	R01_cb4960_c5/f1p0/2432
R01_cb18630_c0/f1p0/5566	R01_cb18630_c0/f1p0/5566	R01_cb18630_c0/f1p0/5566	R01_cb18630_c0/f1p0/5566	R01_cb18630_c0/f1p0/5566
R01_cb13478_c2/f7p1/518	NA	R01_cb13478_c2/f7p1/518	R01_cb13478_c2/f7p1/518	R01_cb13478_c2/f7p1/518
R01_cb11686_c1/f1p0/3017	NA	NA	NA	R01_cb11686_c1/f1p0/3017
R01_cb8564_c73783/f1p0/2163	NA	R01_cb8564_c73783/f1p0/2163	NA	NA
R01_cb8564_c78748/f1p0/3419	NA	R01_cb8564_c78748/f1p0/3419	NA	NA
R01_cb8564_c19812/f1p0/4646	NA	R01_cb8564_c19812/f1p0/4646	R01_cb8564_c19812/f1p0/4646	R01_cb8564_c19812/f1p0/4646
R01_cb1969_c1/f1p0/4106	R01_cb1969_c1/f1p0/4106	R01_cb1969_c1/f1p0/4106	R01_cb1969_c1/f1p0/4106	NA
R01_cb10074_c8/f1p1/3367	NA	NA	NA	R01_cb10074_c8/f1p1/3367

R01_cb8564_c69172/f2p0/2078	NA	R01_cb8564_c69172/f2p0/2078	R01_cb8564_c69172/f2p0/2078	R01_cb8564_c69172/f2p0/2078
R01_cb8564_c114168/f1p0/2849	NA	R01_cb8564_c114168/f1p0/2849	R01_cb8564_c114168/f1p0/2849	R01_cb8564_c114168/f1p0/2849
R01_cb5042_c0/f2p0/2155	NA	NA	R01_cb5042_c0/f2p0/2155	NA
R01_cb13289_c4/f2p2/1680	NA	R01_cb13289_c4/f2p2/1680	R01_cb13289_c4/f2p2/1680	NA
R01_cb17244_c1/f2p0/511	R01_cb17244_c1/f2p0/511	NA	NA	NA
R01_cb6534_c1/f1p0/2836	NA	R01_cb6534_c1/f1p0/2836	NA	NA
R01_cb11867_c3/f1p0/1755	NA	R01_cb11867_c3/f1p0/1755	NA	NA
R01_cb920_c22/f8p1/4290	NA	NA	NA	R01_cb920_c22/f8p1/4290
R01_cb8564_c778/f2p0/2111	NA	R01_cb8564_c778/f2p0/2111	R01_cb8564_c778/f2p0/2111	R01_cb8564_c778/f2p0/2111
R01_cb13433_c46/f1p0/2902	NA	NA	NA	R01_cb13433_c46/f1p0/2902
R01_cb10198_c0/f8p0/547	NA	R01_cb10198_c0/f8p0/547	NA	NA
R01_cb18462_c0/f2p0/965	R01_cb18462_c0/f2p0/965	R01_cb18462_c0/f2p0/965	R01_cb18462_c0/f2p0/965	R01_cb18462_c0/f2p0/965
R01_cb11029_c0/f4p1/1072	NA	NA	NA	R01_cb11029_c0/f4p1/1072
R01_cb18456_c6878/f1p1/793	NA	R01_cb18456_c6878/f1p1/793	NA	NA
R01_cb7596_c2/f1p0/2917	NA	R01_cb7596_c2/f1p0/2917	R01_cb7596_c2/f1p0/2917	R01_cb7596_c2/f1p0/2917
R01_cb48_c12/f1p1/5066	NA	NA	NA	R01_cb48_c12/f1p1/5066
R01_cb5554_c1/f1p0/2350	NA	R01_cb5554_c1/f1p0/2350	R01_cb5554_c1/f1p0/2350	NA
R01_cb8564_c20959/f1p0/3066	NA	R01_cb8564_c20959/f1p0/3066	NA	NA
R01_cb13037_c2/f1p0/1345	NA	NA	R01_cb13037_c2/f1p0/1345	R01_cb13037_c2/f1p0/1345
R01_cb9643_c1/f1p0/2189	NA	R01_cb9643_c1/f1p0/2189	NA	NA
R01_cb17317_c1/f1p0/934	R01_cb17317_c1/f1p0/934	R01_cb17317_c1/f1p0/934	NA	NA
R01_cb16125_c12/f1p0/823	R01_cb16125_c12/f1p0/823	R01_cb16125_c12/f1p0/823	R01_cb16125_c12/f1p0/823	R01_cb16125_c12/f1p0/823
R01_cb18456_c5450/f1p2/667	R01_cb18456_c5450/f1p2/667	R01_cb18456_c5450/f1p2/667	R01_cb18456_c5450/f1p2/667	R01_cb18456_c5450/f1p2/667
R01_cb8564_c121161/f1p0/4754	NA	R01_cb8564_c121161/f1p0/4754	NA	NA

R01_cb8650_c0/f3p0/2134	R01_cb8650_c0/f3p0/2134	R01_cb8650_c0/f3p0/2134	R01_cb8650_c0/f3p0/2134	NA
R01_cb11228_c2/f1p1/2322	R01_cb11228_c2/f1p1/2322	NA	NA	NA
R01_cb3046_c0/f6p0/2414	NA	NA	NA	R01_cb3046_c0/f6p0/2414
R01_cb4269_c6/f1p0/3009	NA	NA	NA	R01_cb4269_c6/f1p0/3009
R01_cb10375_c6/f1p1/1556	NA	NA	NA	R01_cb10375_c6/f1p1/1556
R01_cb2803_c5/f1p0/2932	NA	R01_cb2803_c5/f1p0/2932	NA	NA
R01_cb17756_c8/f1p0/818	R01_cb17756_c8/f1p0/818	R01_cb17756_c8/f1p0/818	R01_cb17756_c8/f1p0/818	R01_cb17756_c8/f1p0/818
R01_cb5122_c11/f1p0/3059	NA	NA	NA	R01_cb5122_c11/f1p0/3059
R01_cb8564_c66851/f4p0/2051	NA	NA	NA	R01_cb8564_c66851/f4p0/2051
R01_cb11029_c2/f1p0/2559	NA	R01_cb11029_c2/f1p0/2559	NA	NA
R01_cb2033_c17/f1p0/2200	NA	NA	NA	R01_cb2033_c17/f1p0/2200
R01_cb8564_c85279/f1p0/3482	NA	NA	NA	R01_cb8564_c85279/f1p0/3482
R01_cb10138_c7/f1p1/1451	NA	R01_cb10138_c7/f1p1/1451	R01_cb10138_c7/f1p1/1451	R01_cb10138_c7/f1p1/1451
R01_cb10970_c0/f2p0/1446	NA	NA	NA	R01_cb10970_c0/f2p0/1446
R01_cb8564_c46882/f1p0/2937	NA	R01_cb8564_c46882/f1p0/2937	R01_cb8564_c46882/f1p0/2937	NA
R01_cb8564_c83850/f1p0/2010	NA	R01_cb8564_c83850/f1p0/2010	NA	NA
R01_cb8564_c1090/f1p0/2642	NA	R01_cb8564_c1090/f1p0/2642	NA	NA
R01_cb8823_c9/f1p0/1972	NA	NA	NA	R01_cb8823_c9/f1p0/1972
R01_cb11606_c0/f1p0/1753	NA	R01_cb11606_c0/f1p0/1753	NA	NA
R01_cb10383_c1/f1p0/2490	NA	R01_cb10383_c1/f1p0/2490	R01_cb10383_c1/f1p0/2490	R01_cb10383_c1/f1p0/2490
R01_cb8564_c122781/f1p0/2486	NA	R01_cb8564_c122781/f1p0/2486	R01_cb8564_c122781/f1p0/2486	R01_cb8564_c122781/f1p0/2486
R01_cb4037_c1/f1p0/3449	R01_cb4037_c1/f1p0/3449	R01_cb4037_c1/f1p0/3449	NA	R01_cb4037_c1/f1p0/3449
R01_cb15811_c27/f1p0/1080	NA	R01_cb15811_c27/f1p0/1080	R01_cb15811_c27/f1p0/1080	R01_cb15811_c27/f1p0/1080
R01_cb8564_c42354/f1p0/3356	NA	NA	NA	R01_cb8564_c42354/f1p0/3356
R01_cb8564_c112394/f1p0/233	NA	R01_cb8564_c112394/f1p0/233	NA	NA

6		6		
R01_cb8951_c6/f1p1/1756	NA	R01_cb8951_c6/f1p1/1756	NA	NA
R01_cb14656_c3/f1p3/575	R01_cb14656_c3/f1p3/575	R01_cb14656_c3/f1p3/575	NA	NA
R01_cb2843_c7/f1p0/4056	NA	NA	NA	R01_cb2843_c7/f1p0/4056
R01_cb2875_c13/f1p1/2551	NA	NA	NA	R01_cb2875_c13/f1p1/2551
R01_cb8564_c114122/f1p0/2470	NA	R01_cb8564_c114122/f1p0/2470	R01_cb8564_c114122/f1p0/2470	R01_cb8564_c114122/f1p0/2470
R01_cb16881_c2/f1p0/696	NA	NA	NA	R01_cb16881_c2/f1p0/696
R01_cb7334_c12/f6p0/1866	NA	NA	NA	R01_cb7334_c12/f6p0/1866
R01_cb5758_c0/f1p0/3020	NA	R01_cb5758_c0/f1p0/3020	R01_cb5758_c0/f1p0/3020	R01_cb5758_c0/f1p0/3020
R01_cb5900_c106/f1p0/2045	NA	R01_cb5900_c106/f1p0/2045	NA	R01_cb5900_c106/f1p0/2045
R01_cb8564_c44173/f1p0/2802	R01_cb8564_c44173/f1p0/2802	R01_cb8564_c44173/f1p0/2802	R01_cb8564_c44173/f1p0/2802	R01_cb8564_c44173/f1p0/2802
R01_cb8564_c76105/f1p0/3001	NA	R01_cb8564_c76105/f1p0/3001	NA	NA
R01_cb8660_c12/f1p0/1891	NA	NA	NA	R01_cb8660_c12/f1p0/1891
R01_cb3516_c1/f1p0/3607	NA	NA	NA	R01_cb3516_c1/f1p0/3607
R01_cb6547_c5/f1p0/2728	NA	NA	NA	R01_cb6547_c5/f1p0/2728
R01_cb15951_c2/f1p0/1885	NA	R01_cb15951_c2/f1p0/1885	NA	NA
R01_cb2870_c3/f1p1/3794	NA	NA	NA	R01_cb2870_c3/f1p1/3794
R01_cb4443_c1/f1p0/3343	NA	R01_cb4443_c1/f1p0/3343	NA	NA
R01_cb15093_c0/f4p2/1881	NA	NA	NA	R01_cb15093_c0/f4p2/1881
R01_cb7184_c9/f1p0/410	R01_cb7184_c9/f1p0/410	R01_cb7184_c9/f1p0/410	NA	R01_cb7184_c9/f1p0/410
R01_cb6198_c1/f1p0/2650	NA	R01_cb6198_c1/f1p0/2650	NA	R01_cb6198_c1/f1p0/2650
R01_cb10923_c3/f1p0/626	NA	NA	NA	R01_cb10923_c3/f1p0/626
R01_cb4639_c2/f1p0/3298	NA	NA	NA	R01_cb4639_c2/f1p0/3298
R01_cb11431_c2/f1p0/1126	NA	NA	NA	R01_cb11431_c2/f1p0/1126

R01_cb8564_c20664/f3p4/3119	NA	NA	NA	R01_cb8564_c20664/f3p4/3119
R01_cb11676_c1/f1p0/2624	NA	NA	NA	R01_cb11676_c1/f1p0/2624
R01_cb2243_c3/f1p0/5429	NA	NA	NA	R01_cb2243_c3/f1p0/5429
R01_cb5558_c2/f1p0/2901	NA	R01_cb5558_c2/f1p0/2901	NA	R01_cb5558_c2/f1p0/2901
R01_cb822_c10/f2p0/2473	NA	NA	NA	R01_cb822_c10/f2p0/2473
R01_cb8564_c78923/f1p0/3273	NA	NA	NA	R01_cb8564_c78923/f1p0/3273
R01_cb17053_c3/f2p0/756	NA	R01_cb17053_c3/f2p0/756	NA	R01_cb17053_c3/f2p0/756
R01_cb18456_c3707/f1p0/926	NA	NA	NA	R01_cb18456_c3707/f1p0/926
R01_cb6452_c12/f1p0/1361	NA	NA	NA	R01_cb6452_c12/f1p0/1361
R01_cb8564_c54019/f1p1/2940	NA	R01_cb8564_c54019/f1p1/2940	NA	NA
R01_cb10441_c2/f1p0/2219	NA	R01_cb10441_c2/f1p0/2219	NA	NA
R01_cb16120_c0/f3p0/1061	NA	R01_cb16120_c0/f3p0/1061	R01_cb16120_c0/f3p0/1061	R01_cb16120_c0/f3p0/1061
R01_cb12786_c13/f1p0/571	NA	NA	NA	R01_cb12786_c13/f1p0/571
R01_cb1207_c6/f1p0/2380	NA	NA	NA	R01_cb1207_c6/f1p0/2380
R01_cb5921_c53/f1p0/1605	NA	NA	NA	R01_cb5921_c53/f1p0/1605
R01_cb9788_c53/f1p0/1645	NA	R01_cb9788_c53/f1p0/1645	NA	NA
R01_cb6238_c8/f1p0/2406	NA	R01_cb6238_c8/f1p0/2406	NA	NA
R01_cb10917_c2/f1p0/2295	NA	R01_cb10917_c2/f1p0/2295	NA	NA
R01_cb2048_c12/f1p0/1302	NA	R01_cb2048_c12/f1p0/1302	NA	NA
R01_cb8564_c148506/f1p1/2161	NA	R01_cb8564_c148506/f1p1/2161	NA	NA
R01_cb13563_c1/f1p0/985	NA	R01_cb13563_c1/f1p0/985	NA	NA
R01_cb2692_c9/f1p0/687	R01_cb2692_c9/f1p0/687	NA	NA	NA
R01_cb12740_c13/f1p0/467	R01_cb12740_c13/f1p0/467	R01_cb12740_c13/f1p0/467	NA	NA
R01_cb8564_c92286/f1p0/2973	R01_cb8564_c92286/f1p0/2973	NA	NA	NA

R01_cb17239_c0/f1p0/1574	NA	R01_cb17239_c0/f1p0/1574	NA	NA
R01_cb6591_c16/f14p1/664	R01_cb6591_c16/f14p1/664	R01_cb6591_c16/f14p1/664	NA	NA
R01_cb17887_c1/f1p0/1240	NA	NA	NA	R01_cb17887_c1/f1p0/1240
R01_cb3307_c4/f2p0/2996	NA	NA	NA	R01_cb3307_c4/f2p0/2996
R01_cb10252_c2/f1p0/802	R01_cb10252_c2/f1p0/802	R01_cb10252_c2/f1p0/802	NA	NA
R01_cb13427_c2/f1p0/657	NA	NA	R01_cb13427_c2/f1p0/657	R01_cb13427_c2/f1p0/657
R01_cb3483_c3/f6p0/2856	NA	NA	NA	R01_cb3483_c3/f6p0/2856
R01_cb14576_c2/f1p0/948	NA	NA	NA	R01_cb14576_c2/f1p0/948
R01_cb12837_c14/f1p0/669	NA	R01_cb12837_c14/f1p0/669	NA	NA
R01_cb8102_c6/f1p2/2413	NA	NA	NA	R01_cb8102_c6/f1p2/2413
R01_cb14792_c0/f1p0/576	NA	R01_cb14792_c0/f1p0/576	NA	NA
R01_cb15672_c0/f1p0/1373	NA	NA	NA	R01_cb15672_c0/f1p0/1373
R01_cb6530_c81/f1p0/895	NA	NA	NA	R01_cb6530_c81/f1p0/895
R01_cb8564_c86518/f1p0/2868	NA	R01_cb8564_c86518/f1p0/2868	NA	NA
R01_cb8564_c21204/f1p5/3369	NA	R01_cb8564_c21204/f1p5/3369	NA	R01_cb8564_c21204/f1p5/3369
R01_cb16428_c0/f1p0/954	R01_cb16428_c0/f1p0/954	R01_cb16428_c0/f1p0/954	R01_cb16428_c0/f1p0/954	NA
R01_cb17266_c17/f1p0/1336	NA	R01_cb17266_c17/f1p0/1336	NA	NA
R01_cb4732_c1/f1p0/3141	NA	NA	NA	R01_cb4732_c1/f1p0/3141
R01_cb8564_c80052/f1p0/2362	NA	R01_cb8564_c80052/f1p0/2362	R01_cb8564_c80052/f1p0/2362	R01_cb8564_c80052/f1p0/2362
R01_cb17016_c2/f1p0/1330	NA	NA	NA	R01_cb17016_c2/f1p0/1330
R01_cb15714_c1/f6p0/677	NA	R01_cb15714_c1/f6p0/677	NA	NA
R01_cb5533_c106/f1p0/3274	NA	R01_cb5533_c106/f1p0/3274	NA	NA
R01_cb17989_c0/f2p0/1560	NA	R01_cb17989_c0/f2p0/1560	R01_cb17989_c0/f2p0/1560	NA
R01_cb17102_c1/f1p1/502	NA	R01_cb17102_c1/f1p1/502	R01_cb17102_c1/f1p1/502	R01_cb17102_c1/f1p1/502
R01_cb17035_c0/f2p1/829	NA	NA	NA	R01_cb17035_c0/f2p1/829
R01_cb12487_c8/f1p0/1681	NA	R01_cb12487_c8/f1p0/1681	NA	NA

R01_cb8564_c78035/f1p0/2846	NA	NA	NA	R01_cb8564_c78035/f1p0/2846
R01_cb8564_c80007/f1p0/4408	NA	R01_cb8564_c80007/f1p0/4408	R01_cb8564_c80007/f1p0/4408	R01_cb8564_c80007/f1p0/4408
R01_cb8564_c88788/f1p0/1896	NA	R01_cb8564_c88788/f1p0/1896	NA	NA
R01_cb5681_c1/f1p0/3077	NA	NA	NA	R01_cb5681_c1/f1p0/3077
R01_cb8564_c85912/f1p1/2858	NA	R01_cb8564_c85912/f1p1/2858	NA	NA
R01_cb3750_c6/f2p0/2746	NA	NA	NA	R01_cb3750_c6/f2p0/2746
R01_cb13101_c2/f1p0/1390	R01_cb13101_c2/f1p0/1390	NA	NA	R01_cb13101_c2/f1p0/1390
R01_cb14286_c5/f1p0/1067	R01_cb14286_c5/f1p0/1067	R01_cb14286_c5/f1p0/1067	NA	R01_cb14286_c5/f1p0/1067
R01_cb14283_c4/f1p2/713	R01_cb14283_c4/f1p2/713	R01_cb14283_c4/f1p2/713	R01_cb14283_c4/f1p2/713	NA
R01_cb11614_c2/f1p0/930	NA	NA	NA	R01_cb11614_c2/f1p0/930
R01_cb17221_c1/f2p0/1003	NA	R01_cb17221_c1/f2p0/1003	NA	R01_cb17221_c1/f2p0/1003
R01_cb8564_c69101/f2p0/2723	NA	NA	NA	R01_cb8564_c69101/f2p0/2723
R01_cb18261_c0/f1p0/788	R01_cb18261_c0/f1p0/788	NA	NA	NA
R01_cb11722_c0/f2p0/840	NA	NA	NA	R01_cb11722_c0/f2p0/840
R01_cb12003_c146/f1p0/831	NA	R01_cb12003_c146/f1p0/831	R01_cb12003_c146/f1p0/831	NA
R01_cb9611_c6/f1p0/2498	NA	R01_cb9611_c6/f1p0/2498	NA	NA
R01_cb16098_c0/f1p0/779	R01_cb16098_c0/f1p0/779	R01_cb16098_c0/f1p0/779	R01_cb16098_c0/f1p0/779	R01_cb16098_c0/f1p0/779
R01_cb8564_c12085/f1p0/2304	R01_cb8564_c12085/f1p0/2304	R01_cb8564_c12085/f1p0/2304	NA	R01_cb8564_c12085/f1p0/2304
R01_cb16555_c2/f1p1/514	NA	NA	NA	R01_cb16555_c2/f1p1/514
R01_cb14502_c20/f1p0/451	R01_cb14502_c20/f1p0/451	R01_cb14502_c20/f1p0/451	R01_cb14502_c20/f1p0/451	NA
R01_cb17996_c2/f2p1/694	NA	NA	NA	R01_cb17996_c2/f2p1/694
R01_cb5790_c0/f4p0/1266	NA	NA	NA	R01_cb5790_c0/f4p0/1266
R01_cb18385_c1/f1p0/1882	NA	R01_cb18385_c1/f1p0/1882	NA	NA
R01_cb15730_c9/f2p0/617	NA	NA	NA	R01_cb15730_c9/f2p0/617
R01_cb8564_c112543/f1p0/217	NA	R01_cb8564_c112543/f1p0/217	NA	NA

3		3		
R01_cb8564_c25562/f4p3/4181	NA	NA	NA	R01_cb8564_c25562/f4p3/4181
R01_cb15797_c2/f1p0/1502	NA	NA	NA	R01_cb15797_c2/f1p0/1502
R01_cb8564_c123295/f1p0/2263	R01_cb8564_c123295/f1p0/2263	R01_cb8564_c123295/f1p0/2263	R01_cb8564_c123295/f1p0/2263	R01_cb8564_c123295/f1p0/2263
R01_cb977_c0/f2p0/3854	NA	NA	NA	R01_cb977_c0/f2p0/3854
R01_cb12946_c10/f1p0/616	NA	R01_cb12946_c10/f1p0/616	NA	NA
R01_cb8946_c3/f2p0/2196	NA	NA	NA	R01_cb8946_c3/f2p0/2196
R01_cb18456_c7641/f1p0/395	R01_cb18456_c7641/f1p0/395	R01_cb18456_c7641/f1p0/395	R01_cb18456_c7641/f1p0/395	R01_cb18456_c7641/f1p0/395
R01_cb11131_c2/f1p0/2518	NA	NA	NA	R01_cb11131_c2/f1p0/2518
R01_cb8564_c81461/f1p1/2199	NA	R01_cb8564_c81461/f1p1/2199	R01_cb8564_c81461/f1p1/2199	R01_cb8564_c81461/f1p1/2199
R01_cb8564_c115526/f1p0/2377	NA	R01_cb8564_c115526/f1p0/2377	R01_cb8564_c115526/f1p0/2377	R01_cb8564_c115526/f1p0/2377
R01_cb3584_c3/f1p0/2151	NA	R01_cb3584_c3/f1p0/2151	NA	R01_cb3584_c3/f1p0/2151
R01_cb8324_c7/f1p0/1415	NA	NA	NA	R01_cb8324_c7/f1p0/1415
R01_cb13938_c17/f1p0/1419	NA	NA	NA	R01_cb13938_c17/f1p0/1419
R01_cb1178_c4/f2p0/1928	NA	NA	NA	R01_cb1178_c4/f2p0/1928
R01_cb8564_c18198/f1p0/2953	NA	R01_cb8564_c18198/f1p0/2953	NA	NA
R01_cb16839_c0/f1p0/1757	NA	NA	NA	R01_cb16839_c0/f1p0/1757
R01_cb8564_c49143/f1p0/4983	NA	R01_cb8564_c49143/f1p0/4983	NA	R01_cb8564_c49143/f1p0/4983
R01_cb6427_c1/f1p0/3055	NA	NA	NA	R01_cb6427_c1/f1p0/3055
R01_cb9448_c12/f1p1/1731	NA	NA	NA	R01_cb9448_c12/f1p1/1731
R01_cb18456_c1378/f3p0/559	R01_cb18456_c1378/f3p0/559	R01_cb18456_c1378/f3p0/559	R01_cb18456_c1378/f3p0/559	R01_cb18456_c1378/f3p0/559
R01_cb2682_c6/f5p1/3358	NA	NA	NA	R01_cb2682_c6/f5p1/3358
R01_cb8564_c37875/f1p1/2576	NA	R01_cb8564_c37875/f1p1/2576	NA	R01_cb8564_c37875/f1p1/2576
R01_cb10971_c0/f1p1/1867	NA	NA	R01_cb10971_c0/f1p1/1867	NA

R01_cb3359_c27/f1p0/2765	NA	R01_cb3359_c27/f1p0/2765	NA	NA
R01_cb7194_c3/f1p0/2677	NA	R01_cb7194_c3/f1p0/2677	NA	NA
R01_cb3545_c19/f1p0/1377	NA	NA	NA	R01_cb3545_c19/f1p0/1377
R01_cb12421_c58/f1p0/574	NA	R01_cb12421_c58/f1p0/574	NA	NA
R01_cb124_c41/f1p0/3234	NA	NA	NA	R01_cb124_c41/f1p0/3234
R01_cb7114_c1/f4p1/1447	NA	NA	NA	R01_cb7114_c1/f4p1/1447
R01_cb9750_c5/f1p0/1899	NA	NA	NA	R01_cb9750_c5/f1p0/1899
R01_cb10032_c2/f1p0/3742	NA	R01_cb10032_c2/f1p0/3742	NA	R01_cb10032_c2/f1p0/3742
R01_cb6931_c2/f2p0/2369	NA	NA	NA	R01_cb6931_c2/f2p0/2369
R01_cb17383_c1/f1p0/1097	NA	NA	NA	R01_cb17383_c1/f1p0/1097
R01_cb6706_c2/f3p0/1609	NA	NA	NA	R01_cb6706_c2/f3p0/1609
R01_cb3744_c16/f1p0/2234	NA	NA	NA	R01_cb3744_c16/f1p0/2234
R01_cb3046_c8/f1p0/2508	NA	NA	NA	R01_cb3046_c8/f1p0/2508
R01_cb8564_c42583/f1p0/1936	NA	R01_cb8564_c42583/f1p0/1936	NA	R01_cb8564_c42583/f1p0/1936
R01_cb610_c2/f1p0/3227	NA	NA	NA	R01_cb610_c2/f1p0/3227
R01_cb14596_c0/f1p0/375	R01_cb14596_c0/f1p0/375	R01_cb14596_c0/f1p0/375	R01_cb14596_c0/f1p0/375	R01_cb14596_c0/f1p0/375
R01_cb8564_c127989/f1p0/2263	R01_cb8564_c127989/f1p0/2263	R01_cb8564_c127989/f1p0/2263	R01_cb8564_c127989/f1p0/2263	R01_cb8564_c127989/f1p0/2263
R01_cb8564_c17183/f1p0/2176	R01_cb8564_c17183/f1p0/2176	R01_cb8564_c17183/f1p0/2176	R01_cb8564_c17183/f1p0/2176	R01_cb8564_c17183/f1p0/2176
R01_cb3426_c23/f1p0/3049	NA	NA	NA	R01_cb3426_c23/f1p0/3049
R01_cb14166_c2/f1p0/684	NA	NA	R01_cb14166_c2/f1p0/684	R01_cb14166_c2/f1p0/684
R01_cb6654_c0/f2p0/777	NA	R01_cb6654_c0/f2p0/777	NA	NA
R01_cb8564_c74522/f2p3/2461	NA	R01_cb8564_c74522/f2p3/2461	R01_cb8564_c74522/f2p3/2461	R01_cb8564_c74522/f2p3/2461
R01_cb15460_c1/f2p0/474	R01_cb15460_c1/f2p0/474	R01_cb15460_c1/f2p0/474	R01_cb15460_c1/f2p0/474	R01_cb15460_c1/f2p0/474
R01_cb13137_c10/f1p0/601	R01_cb13137_c10/f1p0/601	R01_cb13137_c10/f1p0/601	R01_cb13137_c10/f1p0/601	R01_cb13137_c10/f1p0/601

R01_cb13766_c9/f1p0/363	R01_cb13766_c9/f1p0/363	R01_cb13766_c9/f1p0/363	NA	NA
R01_cb12785_c26/f1p0/1428	NA	NA	R01_cb12785_c26/f1p0/1428	R01_cb12785_c26/f1p0/1428
R01_cb8564_c4480/f1p0/2899	NA	NA	NA	R01_cb8564_c4480/f1p0/2899
R01_cb9643_c3/f1p0/1558	R01_cb9643_c3/f1p0/1558	R01_cb9643_c3/f1p0/1558	R01_cb9643_c3/f1p0/1558	R01_cb9643_c3/f1p0/1558
R01_cb11150_c1/f1p0/1860	NA	R01_cb11150_c1/f1p0/1860	NA	NA
R01_cb4951_c4/f1p0/1904	NA	R01_cb4951_c4/f1p0/1904	NA	NA
R01_cb18660_c1/f1p0/357	R01_cb18660_c1/f1p0/357	R01_cb18660_c1/f1p0/357	R01_cb18660_c1/f1p0/357	R01_cb18660_c1/f1p0/357
R01_cb8564_c71907/f1p0/3048	NA	R01_cb8564_c71907/f1p0/3048	NA	NA
R01_cb2367_c7/f1p0/767	R01_cb2367_c7/f1p0/767	R01_cb2367_c7/f1p0/767	R01_cb2367_c7/f1p0/767	R01_cb2367_c7/f1p0/767
R01_cb2082_c12/f1p0/2788	NA	NA	NA	R01_cb2082_c12/f1p0/2788
R01_cb14660_c3/f1p0/1416	R01_cb14660_c3/f1p0/1416	R01_cb14660_c3/f1p0/1416	NA	R01_cb14660_c3/f1p0/1416
R01_cb5948_c9/f1p1/2969	NA	NA	NA	R01_cb5948_c9/f1p1/2969
R01_cb10980_c2/f1p0/681	R01_cb10980_c2/f1p0/681	NA	R01_cb10980_c2/f1p0/681	R01_cb10980_c2/f1p0/681
R01_cb2351_c1/f1p0/3788	NA	R01_cb2351_c1/f1p0/3788	NA	NA
R01_cb567_c8/f1p0/3388	NA	R01_cb567_c8/f1p0/3388	NA	NA
R01_cb15417_c1/f1p0/772	NA	R01_cb15417_c1/f1p0/772	R01_cb15417_c1/f1p0/772	NA
R01_cb8564_c44027/f1p0/2617	NA	R01_cb8564_c44027/f1p0/2617	R01_cb8564_c44027/f1p0/2617	R01_cb8564_c44027/f1p0/2617
R01_cb9648_c2/f2p0/686	NA	NA	NA	R01_cb9648_c2/f2p0/686
R01_cb5358_c1/f1p0/3589	NA	R01_cb5358_c1/f1p0/3589	NA	NA
R01_cb8564_c130375/f1p0/1911	NA	R01_cb8564_c130375/f1p0/1911	R01_cb8564_c130375/f1p0/1911	NA
R01_cb2088_c3/f1p0/3560	NA	NA	NA	R01_cb2088_c3/f1p0/3560
R01_cb7419_c11/f1p1/2569	NA	NA	NA	R01_cb7419_c11/f1p1/2569
R01_cb438_c3/f1p0/3438	NA	R01_cb438_c3/f1p0/3438	NA	R01_cb438_c3/f1p0/3438
R01_cb2807_c1/f1p0/3693	NA	R01_cb2807_c1/f1p0/3693	R01_cb2807_c1/f1p0/3693	R01_cb2807_c1/f1p0/3693
R01_cb18456_c7550/f1p0/1776	NA	NA	R01_cb18456_c7550/f1p0/1776	R01_cb18456_c7550/f1p0/1776

R01_cb17700_c1/f1p0/1131	NA	R01_cb17700_c1/f1p0/1131	R01_cb17700_c1/f1p0/1131	R01_cb17700_c1/f1p0/1131
R01_cb12480_c9/f1p0/843	NA	NA	R01_cb12480_c9/f1p0/843	NA
R01_cb1155_c5/f1p0/2631	NA	NA	NA	R01_cb1155_c5/f1p0/2631
R01_cb11219_c2/f1p0/2877	NA	R01_cb11219_c2/f1p0/2877	NA	NA
R01_cb7535_c8/f1p0/2595	R01_cb7535_c8/f1p0/2595	R01_cb7535_c8/f1p0/2595	NA	R01_cb7535_c8/f1p0/2595
R01_cb2445_c8/f2p0/3318	NA	NA	NA	R01_cb2445_c8/f2p0/3318
R01_cb15725_c10/f2p0/1431	NA	NA	NA	R01_cb15725_c10/f2p0/1431
R01_cb18135_c1/f1p0/864	NA	R01_cb18135_c1/f1p0/864	R01_cb18135_c1/f1p0/864	R01_cb18135_c1/f1p0/864
R01_cb8564_c110854/f1p0/3149	NA	R01_cb8564_c110854/f1p0/3149	NA	NA
R01_cb13981_c1/f1p0/905	NA	R01_cb13981_c1/f1p0/905	NA	NA
R01_cb3787_c2/f1p0/3093	NA	R01_cb3787_c2/f1p0/3093	NA	NA
R01_cb8449_c4/f1p1/2010	NA	NA	NA	R01_cb8449_c4/f1p1/2010
R01_cb10611_c1/f2p0/1452	NA	NA	NA	R01_cb10611_c1/f2p0/1452
R01_cb9998_c1/f1p0/3512	NA	R01_cb9998_c1/f1p0/3512	NA	NA
R01_cb8564_c71575/f1p1/3050	NA	R01_cb8564_c71575/f1p1/3050	NA	NA
R01_cb10530_c1/f1p0/2633	NA	NA	NA	R01_cb10530_c1/f1p0/2633
R01_cb8227_c10/f1p0/2310	NA	R01_cb8227_c10/f1p0/2310	NA	NA
R01_cb2738_c0/f2p0/2124	NA	NA	NA	R01_cb2738_c0/f2p0/2124
R01_cb1739_c11/f1p0/483	R01_cb1739_c11/f1p0/483	NA	NA	R01_cb1739_c11/f1p0/483
R01_cb7114_c8/f1p0/3569	NA	NA	NA	R01_cb7114_c8/f1p0/3569
R01_cb15676_c0/f1p0/1601	NA	NA	NA	R01_cb15676_c0/f1p0/1601
R01_cb12444_c49/f1p0/1060	NA	R01_cb12444_c49/f1p0/1060	NA	NA
R01_cb10344_c4/f1p1/1136	NA	NA	NA	R01_cb10344_c4/f1p1/1136
R01_cb5482_c10/f1p0/3244	NA	R01_cb5482_c10/f1p0/3244	R01_cb5482_c10/f1p0/3244	NA
R01_cb12617_c4/f1p0/620	NA	R01_cb12617_c4/f1p0/620	NA	R01_cb12617_c4/f1p0/620

R01_cb12321_c181/f6p0/671	NA	R01_cb12321_c181/f6p0/671	NA	NA
R01_cb7736_c8/f1p0/3317	NA	R01_cb7736_c8/f1p0/3317	NA	NA
R01_cb12645_c0/f1p0/1679	NA	R01_cb12645_c0/f1p0/1679	NA	R01_cb12645_c0/f1p0/1679
R01_cb8564_c119694/f4p0/2330	NA	NA	NA	R01_cb8564_c119694/f4p0/2330
R01_cb7362_c3/f3p1/2642	R01_cb7362_c3/f3p1/2642	R01_cb7362_c3/f3p1/2642	NA	NA
R01_cb7845_c1/f1p0/2303	NA	R01_cb7845_c1/f1p0/2303	NA	NA
R01_cb16960_c0/f2p0/657	NA	R01_cb16960_c0/f2p0/657	NA	R01_cb16960_c0/f2p0/657
R01_cb18409_c10/f5p0/916	NA	NA	NA	R01_cb18409_c10/f5p0/916
R01_cb8564_c41242/f1p0/3140	NA	NA	NA	R01_cb8564_c41242/f1p0/3140
R01_cb363_c4/f1p0/2373	NA	NA	NA	R01_cb363_c4/f1p0/2373
R01_cb10228_c8/f37p2/746	NA	R01_cb10228_c8/f37p2/746	R01_cb10228_c8/f37p2/746	R01_cb10228_c8/f37p2/746
R01_cb15811_c39/f1p0/972	NA	R01_cb15811_c39/f1p0/972	R01_cb15811_c39/f1p0/972	R01_cb15811_c39/f1p0/972
R01_cb9305_c12/f1p1/1823	NA	NA	NA	R01_cb9305_c12/f1p1/1823
R01_cb2715_c4/f1p0/811	NA	R01_cb2715_c4/f1p0/811	NA	NA
R01_cb14646_c0/f4p0/717	R01_cb14646_c0/f4p0/717	NA	NA	NA
R01_cb3281_c14/f1p0/2440	NA	R01_cb3281_c14/f1p0/2440	NA	NA
R01_cb15379_c0/f1p0/1725	NA	R01_cb15379_c0/f1p0/1725	R01_cb15379_c0/f1p0/1725	R01_cb15379_c0/f1p0/1725
R01_cb13980_c0/f1p0/1631	NA	NA	NA	R01_cb13980_c0/f1p0/1631
R01_cb15998_c8/f1p0/1636	NA	NA	NA	R01_cb15998_c8/f1p0/1636
R01_cb17824_c0/f1p0/1802	R01_cb17824_c0/f1p0/1802	R01_cb17824_c0/f1p0/1802	R01_cb17824_c0/f1p0/1802	R01_cb17824_c0/f1p0/1802
R01_cb8564_c129254/f1p0/2303	NA	R01_cb8564_c129254/f1p0/2303	R01_cb8564_c129254/f1p0/2303	R01_cb8564_c129254/f1p0/2303
R01_cb13545_c17/f1p0/737	NA	R01_cb13545_c17/f1p0/737	R01_cb13545_c17/f1p0/737	NA
R01_cb3601_c22/f1p0/1018	NA	R01_cb3601_c22/f1p0/1018	NA	NA
R01_cb8564_c77640/f1p0/2583	NA	R01_cb8564_c77640/f1p0/2583	R01_cb8564_c77640/f1p0/2583	R01_cb8564_c77640/f1p0/2583

R01_cb3539_c4/f1p0/3571	NA	R01_cb3539_c4/f1p0/3571	NA	NA
R01_cb8564_c152349/f1p3/3967	NA	NA	NA	R01_cb8564_c152349/f1p3/3967
R01_cb5983_c5/f1p1/1039	NA	NA	NA	R01_cb5983_c5/f1p1/1039
R01_cb12512_c4/f1p2/1452	NA	R01_cb12512_c4/f1p2/1452	R01_cb12512_c4/f1p2/1452	R01_cb12512_c4/f1p2/1452
R01_cb15901_c0/f2p0/1670	NA	R01_cb15901_c0/f2p0/1670	NA	NA
R01_cb3586_c14/f1p0/2399	NA	NA	NA	R01_cb3586_c14/f1p0/2399
R01_cb15346_c6/f1p0/618	NA	NA	R01_cb15346_c6/f1p0/618	R01_cb15346_c6/f1p0/618
R01_cb18358_c1/f1p0/1464	R01_cb18358_c1/f1p0/1464	R01_cb18358_c1/f1p0/1464	R01_cb18358_c1/f1p0/1464	R01_cb18358_c1/f1p0/1464
R01_cb18005_c2/f1p0/619	NA	NA	R01_cb18005_c2/f1p0/619	NA
R01_cb3963_c2/f1p0/2208	NA	R01_cb3963_c2/f1p0/2208	NA	NA
R01_cb8564_c22650/f1p0/3988	NA	R01_cb8564_c22650/f1p0/3988	NA	NA
R01_cb11434_c0/f2p0/1777	NA	NA	R01_cb11434_c0/f2p0/1777	R01_cb11434_c0/f2p0/1777
R01_cb8285_c2/f1p0/1948	NA	NA	NA	R01_cb8285_c2/f1p0/1948
R01_cb10740_c2/f1p0/2758	NA	R01_cb10740_c2/f1p0/2758	NA	NA
R01_cb8937_c0/f1p0/2230	NA	NA	NA	R01_cb8937_c0/f1p0/2230
R01_cb16153_c5/f1p0/692	NA	NA	R01_cb16153_c5/f1p0/692	R01_cb16153_c5/f1p0/692
R01_cb8564_c4734/f1p0/2654	NA	NA	R01_cb8564_c4734/f1p0/2654	R01_cb8564_c4734/f1p0/2654
R01_cb13488_c8/f1p1/915	NA	NA	NA	R01_cb13488_c8/f1p1/915
R01_cb12148_c14/f1p0/667	NA	R01_cb12148_c14/f1p0/667	NA	R01_cb12148_c14/f1p0/667
R01_cb14228_c9/f1p0/606	NA	NA	NA	R01_cb14228_c9/f1p0/606
R01_cb12515_c8/f1p1/1782	NA	R01_cb12515_c8/f1p1/1782	NA	NA
R01_cb11123_c2/f1p0/689	NA	R01_cb11123_c2/f1p0/689	NA	R01_cb11123_c2/f1p0/689
R01_cb18476_c0/f1p0/814	NA	NA	NA	R01_cb18476_c0/f1p0/814
R01_cb6534_c0/f2p0/701	NA	NA	NA	R01_cb6534_c0/f2p0/701
R01_cb18224_c1/f1p0/1554	NA	NA	NA	R01_cb18224_c1/f1p0/1554

R01_cb18456_c7632/f1p0/550	NA	NA	NA	R01_cb18456_c7632/f1p0/550
R01_cb18853_c1/f1p0/5106	NA	R01_cb18853_c1/f1p0/5106	NA	R01_cb18853_c1/f1p0/5106
R01_cb6059_c2/f1p1/2947	NA	NA	NA	R01_cb6059_c2/f1p1/2947
R01_cb1594_c10/f1p0/2394	NA	NA	NA	R01_cb1594_c10/f1p0/2394
R01_cb8564_c111266/f1p0/3561	NA	NA	NA	R01_cb8564_c111266/f1p0/3561
R01_cb2104_c13/f1p0/1024	NA	NA	R01_cb2104_c13/f1p0/1024	R01_cb2104_c13/f1p0/1024
R01_cb8564_c116757/f8p2/2119	NA	NA	NA	R01_cb8564_c116757/f8p2/2119
R01_cb9359_c3/f1p0/1679	NA	NA	NA	R01_cb9359_c3/f1p0/1679
R01_cb16930_c1/f1p0/785	R01_cb16930_c1/f1p0/785	R01_cb16930_c1/f1p0/785	R01_cb16930_c1/f1p0/785	R01_cb16930_c1/f1p0/785
R01_cb8335_c0/f4p1/2388	NA	NA	NA	R01_cb8335_c0/f4p1/2388
R01_cb3848_c8/f1p1/3035	NA	NA	NA	R01_cb3848_c8/f1p1/3035
R01_cb8564_c114092/f1p0/3222	R01_cb8564_c114092/f1p0/3222	R01_cb8564_c114092/f1p0/3222	R01_cb8564_c114092/f1p0/3222	R01_cb8564_c114092/f1p0/3222
R01_cb16782_c2/f1p0/636	R01_cb16782_c2/f1p0/636	R01_cb16782_c2/f1p0/636	R01_cb16782_c2/f1p0/636	R01_cb16782_c2/f1p0/636
R01_cb18694_c0/f1p0/5743	R01_cb18694_c0/f1p0/5743	R01_cb18694_c0/f1p0/5743	NA	NA
R01_cb8564_c45133/f1p0/1926	R01_cb8564_c45133/f1p0/1926	R01_cb8564_c45133/f1p0/1926	R01_cb8564_c45133/f1p0/1926	R01_cb8564_c45133/f1p0/1926
R01_cb110_c33/f1p0/6698	NA	NA	NA	R01_cb110_c33/f1p0/6698
R01_cb4656_c5/f1p0/2544	NA	R01_cb4656_c5/f1p0/2544	NA	NA
R01_cb15173_c0/f1p0/1153	NA	NA	NA	R01_cb15173_c0/f1p0/1153
R01_cb8324_c12/f1p0/1325	NA	NA	NA	R01_cb8324_c12/f1p0/1325
R01_cb9529_c4/f1p0/949	NA	NA	NA	R01_cb9529_c4/f1p0/949
R01_cb3797_c12/f1p1/6141	NA	NA	NA	R01_cb3797_c12/f1p1/6141
R01_cb17756_c26/f1p0/1649	R01_cb17756_c26/f1p0/1649	R01_cb17756_c26/f1p0/1649	R01_cb17756_c26/f1p0/1649	R01_cb17756_c26/f1p0/1649

R01_cb13586_c4/f1p0/1157	NA	R01_cb13586_c4/f1p0/1157	NA	NA
R01_cb8564_c129816/f1p0/2078	R01_cb8564_c129816/f1p0/2078	R01_cb8564_c129816/f1p0/2078	R01_cb8564_c129816/f1p0/2078	R01_cb8564_c129816/f1p0/2078
R01_cb9760_c0/f1p0/1985	NA	R01_cb9760_c0/f1p0/1985	R01_cb9760_c0/f1p0/1985	R01_cb9760_c0/f1p0/1985
R01_cb8564_c381/f4p3/1955	NA	NA	NA	R01_cb8564_c381/f4p3/1955
R01_cb18456_c1689/f2p0/482	R01_cb18456_c1689/f2p0/482	R01_cb18456_c1689/f2p0/482	R01_cb18456_c1689/f2p0/482	R01_cb18456_c1689/f2p0/482
R01_cb10010_c0/f1p0/1371	NA	R01_cb10010_c0/f1p0/1371	NA	R01_cb10010_c0/f1p0/1371
R01_cb12023_c8/f1p0/786	NA	NA	NA	R01_cb12023_c8/f1p0/786
R01_cb15878_c8/f1p0/611	NA	R01_cb15878_c8/f1p0/611	NA	NA
R01_cb4146_c6/f1p0/2808	NA	R01_cb4146_c6/f1p0/2808	NA	NA
R01_cb6294_c2/f1p0/2891	NA	R01_cb6294_c2/f1p0/2891	NA	NA
R01_cb8564_c14728/f1p0/2835	NA	R01_cb8564_c14728/f1p0/2835	NA	NA
R01_cb10386_c3/f1p2/1775	R01_cb10386_c3/f1p2/1775	R01_cb10386_c3/f1p2/1775	NA	R01_cb10386_c3/f1p2/1775
R01_cb10241_c3/f1p1/2051	NA	R01_cb10241_c3/f1p1/2051	R01_cb10241_c3/f1p1/2051	NA
R01_cb1124_c15/f1p0/1216	NA	NA	NA	R01_cb1124_c15/f1p0/1216
R01_cb12824_c1/f6p0/674	NA	NA	R01_cb12824_c1/f6p0/674	R01_cb12824_c1/f6p0/674
R01_cb12912_c2/f1p0/1874	NA	R01_cb12912_c2/f1p0/1874	NA	R01_cb12912_c2/f1p0/1874
R01_cb11674_c1/f1p0/2815	NA	R01_cb11674_c1/f1p0/2815	R01_cb11674_c1/f1p0/2815	R01_cb11674_c1/f1p0/2815
R01_cb8564_c82192/f1p0/2137	NA	R01_cb8564_c82192/f1p0/2137	NA	NA
R01_cb18456_c4846/f1p1/1274	NA	R01_cb18456_c4846/f1p1/1274	NA	NA
R01_cb11972_c4/f1p0/1207	NA	NA	NA	R01_cb11972_c4/f1p0/1207
R01_cb1438_c8/f1p0/4349	NA	NA	NA	R01_cb1438_c8/f1p0/4349
R01_cb8564_c3875/f1p0/2853	R01_cb8564_c3875/f1p0/2853	R01_cb8564_c3875/f1p0/2853	R01_cb8564_c3875/f1p0/2853	R01_cb8564_c3875/f1p0/2853
R01_cb3550_c0/f5p0/2847	NA	NA	NA	R01_cb3550_c0/f5p0/2847
R01_cb11372_c1/f1p0/3352	NA	R01_cb11372_c1/f1p0/3352	NA	NA
R01_cb14805_c5/f1p0/619	R01_cb14805_c5/f1p0/619	R01_cb14805_c5/f1p0/619	NA	NA

R01_cb9511_c2/f1p0/2059	NA	R01_cb9511_c2/f1p0/2059	NA	NA
R01_cb16646_c3/f1p0/1856	NA	NA	NA	R01_cb16646_c3/f1p0/1856
R01_cb8546_c2/f2p0/1759	NA	NA	NA	R01_cb8546_c2/f2p0/1759
R01_cb10628_c0/f1p0/793	NA	R01_cb10628_c0/f1p0/793	NA	R01_cb10628_c0/f1p0/793
R01_cb10226_c1/f1p0/2354	R01_cb10226_c1/f1p0/2354	R01_cb10226_c1/f1p0/2354	R01_cb10226_c1/f1p0/2354	R01_cb10226_c1/f1p0/2354
R01_cb8564_c44301/f1p0/3723	NA	NA	R01_cb8564_c44301/f1p0/3723	R01_cb8564_c44301/f1p0/3723
R01_cb15552_c2/f1p0/845	NA	R01_cb15552_c2/f1p0/845	NA	R01_cb15552_c2/f1p0/845
R01_cb16416_c2/f1p0/1199	NA	NA	NA	R01_cb16416_c2/f1p0/1199
R01_cb6162_c2/f1p0/1338	R01_cb6162_c2/f1p0/1338	R01_cb6162_c2/f1p0/1338	R01_cb6162_c2/f1p0/1338	R01_cb6162_c2/f1p0/1338
R01_cb4836_c6/f1p0/5743	NA	R01_cb4836_c6/f1p0/5743	NA	R01_cb4836_c6/f1p0/5743
R01_cb8564_c12935/f1p0/2562	NA	R01_cb8564_c12935/f1p0/2562	R01_cb8564_c12935/f1p0/2562	R01_cb8564_c12935/f1p0/2562
R01_cb7828_c2/f2p0/2025	NA	NA	NA	R01_cb7828_c2/f2p0/2025
R01_cb280_c86/f1p0/2559	NA	R01_cb280_c86/f1p0/2559	NA	NA
R01_cb14472_c0/f20p1/755	NA	NA	NA	R01_cb14472_c0/f20p1/755
R01_cb11150_c3/f1p0/628	NA	R01_cb11150_c3/f1p0/628	NA	NA
R01_cb12847_c1/f1p0/1438	NA	NA	NA	R01_cb12847_c1/f1p0/1438
R01_cb7821_c0/f1p0/2526	R01_cb7821_c0/f1p0/2526	R01_cb7821_c0/f1p0/2526	R01_cb7821_c0/f1p0/2526	R01_cb7821_c0/f1p0/2526
R01_cb14526_c7/f1p0/458	NA	R01_cb14526_c7/f1p0/458	R01_cb14526_c7/f1p0/458	R01_cb14526_c7/f1p0/458
R01_cb14152_c2/f1p0/775	NA	NA	NA	R01_cb14152_c2/f1p0/775
R01_cb14085_c0/f1p0/598	NA	R01_cb14085_c0/f1p0/598	NA	NA
R01_cb11208_c1/f2p0/1334	NA	NA	NA	R01_cb11208_c1/f2p0/1334
R01_cb8564_c71667/f1p0/3239	R01_cb8564_c71667/f1p0/3239	R01_cb8564_c71667/f1p0/3239	NA	NA
R01_cb15912_c1/f1p0/1764	NA	NA	NA	R01_cb15912_c1/f1p0/1764
R01_cb16782_c0/f1p0/407	R01_cb16782_c0/f1p0/407	R01_cb16782_c0/f1p0/407	R01_cb16782_c0/f1p0/407	R01_cb16782_c0/f1p0/407
R01_cb8564_c1636/f1p0/2152	R01_cb8564_c1636/f1p0/2152	R01_cb8564_c1636/f1p0/2152	NA	NA

R01_cb12839_c6/f1p0/516	NA	R01_cb12839_c6/f1p0/516	NA	NA
R01_cb12314_c2/f1p1/624	NA	NA	R01_cb12314_c2/f1p1/624	NA
R01_cb8564_c5185/f1p6/1948	NA	NA	NA	R01_cb8564_c5185/f1p6/1948
R01_cb7730_c18/f1p0/2091	NA	NA	NA	R01_cb7730_c18/f1p0/2091
R01_cb8564_c25428/f1p0/2720	NA	R01_cb8564_c25428/f1p0/2720	NA	NA
R01_cb6631_c14/f1p0/2754	NA	NA	NA	R01_cb6631_c14/f1p0/2754
R01_cb12054_c33/f1p0/362	R01_cb12054_c33/f1p0/362	R01_cb12054_c33/f1p0/362	R01_cb12054_c33/f1p0/362	R01_cb12054_c33/f1p0/362
R01_cb8564_c38921/f1p0/2056	NA	R01_cb8564_c38921/f1p0/2056	NA	NA
R01_cb14971_c0/f3p0/701	R01_cb14971_c0/f3p0/701	NA	NA	R01_cb14971_c0/f3p0/701
R01_cb11150_c0/f2p0/823	NA	R01_cb11150_c0/f2p0/823	NA	NA
R01_cb10359_c3/f1p0/1247	NA	NA	NA	R01_cb10359_c3/f1p0/1247
R01_cb2177_c49/f1p2/3375	NA	R01_cb2177_c49/f1p2/3375	NA	NA
R01_cb18301_c15/f1p0/972	R01_cb18301_c15/f1p0/972	R01_cb18301_c15/f1p0/972	R01_cb18301_c15/f1p0/972	R01_cb18301_c15/f1p0/972
R01_cb6802_c24/f1p0/4072	NA	R01_cb6802_c24/f1p0/4072	NA	NA
R01_cb5756_c3/f1p0/1507	R01_cb5756_c3/f1p0/1507	R01_cb5756_c3/f1p0/1507	R01_cb5756_c3/f1p0/1507	R01_cb5756_c3/f1p0/1507
R01_cb18409_c48/f1p0/401	R01_cb18409_c48/f1p0/401	R01_cb18409_c48/f1p0/401	R01_cb18409_c48/f1p0/401	R01_cb18409_c48/f1p0/401
R01_cb15724_c5/f1p0/1249	R01_cb15724_c5/f1p0/1249	R01_cb15724_c5/f1p0/1249	R01_cb15724_c5/f1p0/1249	R01_cb15724_c5/f1p0/1249
R01_cb8564_c1046/f1p0/4229	NA	R01_cb8564_c1046/f1p0/4229	NA	NA
R01_cb1077_c23/f1p1/2042	NA	NA	NA	R01_cb1077_c23/f1p1/2042
R01_cb1887_c1/f1p0/1966	NA	NA	NA	R01_cb1887_c1/f1p0/1966
R01_cb11693_c1/f1p0/1972	NA	R01_cb11693_c1/f1p0/1972	NA	NA
R01_cb10493_c2/f1p0/2221	NA	NA	NA	R01_cb10493_c2/f1p0/2221
R01_cb10034_c34/f1p4/1711	NA	R01_cb10034_c34/f1p4/1711	NA	NA
R01_cb8564_c91234/f1p0/4224	NA	NA	NA	R01_cb8564_c91234/f1p0/4224
R01_cb4839_c4/f1p0/3248	NA	R01_cb4839_c4/f1p0/3248	NA	NA
R01_cb8564_c50439/f1p0/2188	NA	R01_cb8564_c50439/f1p0/2188	R01_cb8564_c50439/f1p0/2188	R01_cb8564_c50439/f1p0/2188

R01_cb8564_c9977/f2p1/2598	NA	NA	NA	R01_cb8564_c9977/f2p1/2598
R01_cb4074_c6/f1p0/3445	NA	R01_cb4074_c6/f1p0/3445	NA	NA
R01_cb8564_c3048/f1p0/2616	NA	R01_cb8564_c3048/f1p0/2616	R01_cb8564_c3048/f1p0/2616	R01_cb8564_c3048/f1p0/2616
R01_cb18423_c0/f1p0/497	NA	NA	NA	R01_cb18423_c0/f1p0/497
R01_cb16145_c0/f1p0/1000	NA	R01_cb16145_c0/f1p0/1000	R01_cb16145_c0/f1p0/1000	NA
R01_cb7504_c9/f1p0/2486	NA	R01_cb7504_c9/f1p0/2486	NA	NA
R01_cb8564_c68423/f4p0/2640	NA	NA	NA	R01_cb8564_c68423/f4p0/2640
R01_cb14463_c4/f1p0/489	NA	NA	NA	R01_cb14463_c4/f1p0/489
R01_cb11183_c3/f1p0/2053	NA	NA	NA	R01_cb11183_c3/f1p0/2053
R01_cb15342_c2/f1p0/785	NA	R01_cb15342_c2/f1p0/785	NA	NA
R01_cb3760_c3/f3p1/2548	NA	NA	NA	R01_cb3760_c3/f3p1/2548
R01_cb8564_c1857/f1p0/2458	NA	R01_cb8564_c1857/f1p0/2458	R01_cb8564_c1857/f1p0/2458	R01_cb8564_c1857/f1p0/2458
R01_cb4315_c2/f1p0/786	NA	R01_cb4315_c2/f1p0/786	NA	R01_cb4315_c2/f1p0/786
R01_cb8564_c75745/f1p1/2647	NA	R01_cb8564_c75745/f1p1/2647	NA	NA
R01_cb14696_c4/f1p0/378	R01_cb14696_c4/f1p0/378	R01_cb14696_c4/f1p0/378	R01_cb14696_c4/f1p0/378	R01_cb14696_c4/f1p0/378
R01_cb15468_c8/f1p0/1883	NA	R01_cb15468_c8/f1p0/1883	NA	NA
R01_cb7584_c13/f1p2/1804	NA	R01_cb7584_c13/f1p2/1804	NA	NA
R01_cb8564_c4219/f1p0/2959	R01_cb8564_c4219/f1p0/2959	R01_cb8564_c4219/f1p0/2959	R01_cb8564_c4219/f1p0/2959	R01_cb8564_c4219/f1p0/2959
R01_cb7276_c17/f1p0/533	NA	R01_cb7276_c17/f1p0/533	R01_cb7276_c17/f1p0/533	R01_cb7276_c17/f1p0/533
R01_cb8564_c81741/f1p0/2084	NA	R01_cb8564_c81741/f1p0/2084	NA	R01_cb8564_c81741/f1p0/2084
R01_cb5565_c74/f1p0/1859	NA	R01_cb5565_c74/f1p0/1859	NA	NA
R01_cb4674_c89/f1p0/1671	NA	NA	NA	R01_cb4674_c89/f1p0/1671
R01_cb8564_c4899/f1p0/3376	NA	R01_cb8564_c4899/f1p0/3376	NA	R01_cb8564_c4899/f1p0/3376
R01_cb8564_c10643/f1p0/3397	NA	R01_cb8564_c10643/f1p0/3397	NA	NA
R01_cb13638_c0/f5p0/1098	NA	NA	NA	R01_cb13638_c0/f5p0/1098
R01_cb18361_c0/f2p0/483	NA	R01_cb18361_c0/f2p0/483	R01_cb18361_c0/f2p0/483	R01_cb18361_c0/f2p0/483

R01_cb5725_c17/f1p0/2282	NA	R01_cb5725_c17/f1p0/2282	NA	R01_cb5725_c17/f1p0/2282
R01_cb18456_c7628/f1p0/458	R01_cb18456_c7628/f1p0/458	R01_cb18456_c7628/f1p0/458	R01_cb18456_c7628/f1p0/458	R01_cb18456_c7628/f1p0/458
R01_cb2571_c24/f1p0/3923	NA	NA	NA	R01_cb2571_c24/f1p0/3923
R01_cb10077_c2/f2p0/1145	NA	R01_cb10077_c2/f2p0/1145	NA	NA
R01_cb17110_c0/f2p0/822	R01_cb17110_c0/f2p0/822	R01_cb17110_c0/f2p0/822	R01_cb17110_c0/f2p0/822	R01_cb17110_c0/f2p0/822
R01_cb7254_c11/f1p0/1680	NA	NA	NA	R01_cb7254_c11/f1p0/1680
R01_cb14682_c1/f1p0/911	NA	R01_cb14682_c1/f1p0/911	NA	NA
R01_cb1392_c5/f1p0/2403	NA	NA	NA	R01_cb1392_c5/f1p0/2403
R01_cb10713_c4/f1p0/1797	NA	R01_cb10713_c4/f1p0/1797	NA	R01_cb10713_c4/f1p0/1797
R01_cb18132_c18/f1p0/1884	NA	R01_cb18132_c18/f1p0/1884	NA	NA
R01_cb12495_c2/f1p0/1842	NA	R01_cb12495_c2/f1p0/1842	NA	NA
R01_cb14037_c4/f1p0/680	NA	NA	R01_cb14037_c4/f1p0/680	NA
R01_cb10017_c82/f1p0/917	NA	R01_cb10017_c82/f1p0/917	NA	NA
R01_cb1437_c3/f1p0/4973	NA	NA	NA	R01_cb1437_c3/f1p0/4973
R01_cb8564_c123882/f1p0/2943	NA	R01_cb8564_c123882/f1p0/2943	R01_cb8564_c123882/f1p0/2943	R01_cb8564_c123882/f1p0/2943
R01_cb2674_c29/f1p0/2727	NA	NA	NA	R01_cb2674_c29/f1p0/2727
R01_cb8564_c76712/f1p0/2263	R01_cb8564_c76712/f1p0/2263	R01_cb8564_c76712/f1p0/2263	R01_cb8564_c76712/f1p0/2263	NA
R01_cb6032_c18/f1p0/2989	NA	R01_cb6032_c18/f1p0/2989	R01_cb6032_c18/f1p0/2989	R01_cb6032_c18/f1p0/2989
R01_cb560_c16/f1p0/1285	NA	NA	NA	R01_cb560_c16/f1p0/1285
R01_cb15714_c4/f1p0/604	NA	R01_cb15714_c4/f1p0/604	NA	NA
R01_cb15418_c2/f3p1/836	NA	NA	NA	R01_cb15418_c2/f3p1/836
R01_cb2333_c0/f1p0/3971	R01_cb2333_c0/f1p0/3971	R01_cb2333_c0/f1p0/3971	R01_cb2333_c0/f1p0/3971	NA
R01_cb2701_c7/f1p0/2595	NA	NA	NA	R01_cb2701_c7/f1p0/2595
R01_cb13408_c0/f3p0/1179	NA	NA	NA	R01_cb13408_c0/f3p0/1179

R01_cb8564_c83551/f1p0/3123	NA	R01_cb8564_c83551/f1p0/3123	NA	R01_cb8564_c83551/f1p0/3123
R01_cb8564_c38568/f1p0/2321	NA	NA	NA	R01_cb8564_c38568/f1p0/2321
R01_cb2177_c153/f1p0/1742	NA	R01_cb2177_c153/f1p0/1742	NA	NA
R01_cb14256_c2/f1p0/1569	NA	R01_cb14256_c2/f1p0/1569	NA	NA
R01_cb16120_c2/f1p0/982	NA	R01_cb16120_c2/f1p0/982	R01_cb16120_c2/f1p0/982	R01_cb16120_c2/f1p0/982
R01_cb9793_c5/f1p0/1162	NA	NA	R01_cb9793_c5/f1p0/1162	R01_cb9793_c5/f1p0/1162
R01_cb868_c27/f1p0/3308	NA	R01_cb868_c27/f1p0/3308	NA	NA
R01_cb17805_c1/f1p0/522	NA	R01_cb17805_c1/f1p0/522	NA	R01_cb17805_c1/f1p0/522
R01_cb10717_c5/f1p0/1797	NA	R01_cb10717_c5/f1p0/1797	NA	NA
R01_cb10432_c6/f1p0/4529	NA	R01_cb10432_c6/f1p0/4529	NA	NA
R01_cb6508_c16/f1p1/2368	NA	R01_cb6508_c16/f1p1/2368	NA	NA
R01_cb6039_c56/f1p1/3085	NA	R01_cb6039_c56/f1p1/3085	R01_cb6039_c56/f1p1/3085	NA
R01_cb3381_c5/f2p0/2469	NA	NA	NA	R01_cb3381_c5/f2p0/2469
R01_cb3848_c28/f4p0/2408	NA	NA	NA	R01_cb3848_c28/f4p0/2408
R01_cb11718_c1/f1p0/3893	NA	NA	NA	R01_cb11718_c1/f1p0/3893
R01_cb8564_c23189/f1p0/2373	NA	R01_cb8564_c23189/f1p0/2373	R01_cb8564_c23189/f1p0/2373	R01_cb8564_c23189/f1p0/2373
R01_cb11706_c0/f1p0/1158	NA	R01_cb11706_c0/f1p0/1158	R01_cb11706_c0/f1p0/1158	R01_cb11706_c0/f1p0/1158
R01_cb8564_c123469/f1p0/3334	NA	R01_cb8564_c123469/f1p0/3334	NA	NA
R01_cb8831_c5/f1p0/2282	NA	NA	R01_cb8831_c5/f1p0/2282	R01_cb8831_c5/f1p0/2282
R01_cb8564_c74209/f1p0/2540	NA	R01_cb8564_c74209/f1p0/2540	NA	NA
R01_cb2854_c5/f2p0/3548	NA	R01_cb2854_c5/f2p0/3548	NA	NA
R01_cb18456_c5125/f1p0/653	R01_cb18456_c5125/f1p0/653	R01_cb18456_c5125/f1p0/653	R01_cb18456_c5125/f1p0/653	R01_cb18456_c5125/f1p0/653
R01_cb15959_c7/f1p0/644	NA	R01_cb15959_c7/f1p0/644	NA	R01_cb15959_c7/f1p0/644
R01_cb8560_c1/f1p0/2563	NA	NA	NA	R01_cb8560_c1/f1p0/2563
R01_cb4734_c0/f1p0/3205	NA	R01_cb4734_c0/f1p0/3205	R01_cb4734_c0/f1p0/3205	R01_cb4734_c0/f1p0/3205

R01_cb9797_c51/f2p0/2505	NA	NA	NA	R01_cb9797_c51/f2p0/2505
R01_cb18456_c6672/f1p0/620	NA	R01_cb18456_c6672/f1p0/620	R01_cb18456_c6672/f1p0/620	R01_cb18456_c6672/f1p0/620
R01_cb2581_c0/f2p0/1099	NA	R01_cb2581_c0/f2p0/1099	R01_cb2581_c0/f2p0/1099	R01_cb2581_c0/f2p0/1099
R01_cb17118_c4/f1p0/1886	NA	R01_cb17118_c4/f1p0/1886	R01_cb17118_c4/f1p0/1886	R01_cb17118_c4/f1p0/1886
R01_cb18456_c3340/f1p1/984	NA	R01_cb18456_c3340/f1p1/984	NA	NA
R01_cb12967_c7/f1p0/975	NA	R01_cb12967_c7/f1p0/975	NA	NA
R01_cb8564_c85965/f1p0/2336	R01_cb8564_c85965/f1p0/2336	R01_cb8564_c85965/f1p0/2336	R01_cb8564_c85965/f1p0/2336	R01_cb8564_c85965/f1p0/2336
R01_cb1301_c3/f1p0/2903	NA	NA	NA	R01_cb1301_c3/f1p0/2903
R01_cb584_c20/f1p0/4710	NA	R01_cb584_c20/f1p0/4710	NA	NA
R01_cb16332_c1/f1p0/1369	NA	R01_cb16332_c1/f1p0/1369	R01_cb16332_c1/f1p0/1369	R01_cb16332_c1/f1p0/1369
R01_cb10074_c19/f3p2/555	R01_cb10074_c19/f3p2/555	R01_cb10074_c19/f3p2/555	R01_cb10074_c19/f3p2/555	R01_cb10074_c19/f3p2/555
R01_cb8564_c78250/f1p0/1901	R01_cb8564_c78250/f1p0/1901	R01_cb8564_c78250/f1p0/1901	R01_cb8564_c78250/f1p0/1901	R01_cb8564_c78250/f1p0/1901
R01_cb16038_c4/f1p1/477	NA	R01_cb16038_c4/f1p1/477	R01_cb16038_c4/f1p1/477	R01_cb16038_c4/f1p1/477
R01_cb11589_c0/f1p0/1162	NA	R01_cb11589_c0/f1p0/1162	NA	NA
R01_cb9850_c4/f1p0/756	NA	R01_cb9850_c4/f1p0/756	NA	NA
R01_cb5042_c9/f1p0/983	NA	NA	R01_cb5042_c9/f1p0/983	NA
R01_cb1488_c3/f1p0/3463	NA	R01_cb1488_c3/f1p0/3463	NA	NA
R01_cb6954_c3/f1p0/497	R01_cb6954_c3/f1p0/497	R01_cb6954_c3/f1p0/497	R01_cb6954_c3/f1p0/497	R01_cb6954_c3/f1p0/497
R01_cb18343_c0/f1p0/1791	NA	R01_cb18343_c0/f1p0/1791	NA	NA
R01_cb8564_c83657/f1p0/2627	NA	R01_cb8564_c83657/f1p0/2627	NA	NA
R01_cb15425_c0/f3p0/1033	NA	R01_cb15425_c0/f3p0/1033	NA	NA
R01_cb18456_c7086/f1p0/1800	NA	R01_cb18456_c7086/f1p0/1800	NA	NA
R01_cb8564_c3053/f2p2/4010	NA	R01_cb8564_c3053/f2p2/4010	R01_cb8564_c3053/f2p2/4010	R01_cb8564_c3053/f2p2/4010
R01_cb12680_c15/f1p0/1755	R01_cb12680_c15/f1p0/1755	R01_cb12680_c15/f1p0/1755	NA	NA

R01_cb18211_c1/f1p0/461	NA	R01_cb18211_c1/f1p0/461	NA	R01_cb18211_c1/f1p0/461
R01_cb8564_c83948/f1p0/3284	NA	R01_cb8564_c83948/f1p0/3284	R01_cb8564_c83948/f1p0/3284	R01_cb8564_c83948/f1p0/3284
R01_cb392_c13/f1p0/1064	R01_cb392_c13/f1p0/1064	R01_cb392_c13/f1p0/1064	NA	R01_cb392_c13/f1p0/1064
R01_cb124_c52/f1p0/2890	NA	NA	NA	R01_cb124_c52/f1p0/2890
R01_cb9088_c1/f1p0/2195	NA	NA	NA	R01_cb9088_c1/f1p0/2195
R01_cb938_c50/f1p0/700	R01_cb938_c50/f1p0/700	R01_cb938_c50/f1p0/700	NA	R01_cb938_c50/f1p0/700
R01_cb8564_c80143/f2p0/4971	NA	R01_cb8564_c80143/f2p0/4971	R01_cb8564_c80143/f2p0/4971	R01_cb8564_c80143/f2p0/4971
R01_cb4503_c10/f1p0/3330	NA	R01_cb4503_c10/f1p0/3330	NA	R01_cb4503_c10/f1p0/3330
R01_cb8564_c20519/f1p0/3425	R01_cb8564_c20519/f1p0/3425	R01_cb8564_c20519/f1p0/3425	R01_cb8564_c20519/f1p0/3425	R01_cb8564_c20519/f1p0/3425
R01_cb18714_c1/f1p0/1400	NA	NA	NA	R01_cb18714_c1/f1p0/1400
R01_cb7368_c3/f1p0/1498	NA	R01_cb7368_c3/f1p0/1498	NA	R01_cb7368_c3/f1p0/1498
R01_cb8084_c2/f3p1/2279	NA	NA	NA	R01_cb8084_c2/f3p1/2279
R01_cb12587_c4/f1p0/1767	NA	NA	NA	R01_cb12587_c4/f1p0/1767
R01_cb10228_c3/f1p0/1532	NA	NA	R01_cb10228_c3/f1p0/1532	NA
R01_cb1998_c1/f1p0/2460	NA	R01_cb1998_c1/f1p0/2460	R01_cb1998_c1/f1p0/2460	R01_cb1998_c1/f1p0/2460
R01_cb14694_c2/f1p0/1209	NA	NA	NA	R01_cb14694_c2/f1p0/1209
R01_cb10830_c1/f1p0/2271	NA	NA	NA	R01_cb10830_c1/f1p0/2271
R01_cb4458_c1/f1p0/2901	NA	NA	NA	R01_cb4458_c1/f1p0/2901
R01_cb8253_c4/f1p0/2419	NA	R01_cb8253_c4/f1p0/2419	NA	R01_cb8253_c4/f1p0/2419
R01_cb8564_c117138/f1p0/4110	NA	R01_cb8564_c117138/f1p0/4110	NA	NA
R01_cb13445_c1/f1p0/590	NA	R01_cb13445_c1/f1p0/590	NA	NA
R01_cb2677_c32/f1p0/3741	NA	R01_cb2677_c32/f1p0/3741	NA	NA
R01_cb11205_c1/f1p0/2021	NA	R01_cb11205_c1/f1p0/2021	NA	NA
R01_cb8564_c91068/f1p0/3080	R01_cb8564_c91068/f1p0/308	R01_cb8564_c91068/f1p0/3080	R01_cb8564_c91068/f1p0/3080	R01_cb8564_c91068/f1p0/3080

	0			
R01_cb18456_c7199/f1p0/1869	NA	R01_cb18456_c7199/f1p0/1869	NA	NA
R01_cb8564_c129149/f1p0/2439	NA	NA	NA	R01_cb8564_c129149/f1p0/2439
R01_cb18456_c7462/f1p0/1282	R01_cb18456_c7462/f1p0/1282	R01_cb18456_c7462/f1p0/1282	R01_cb18456_c7462/f1p0/1282	R01_cb18456_c7462/f1p0/1282
R01_cb18456_c3577/f1p0/809	NA	R01_cb18456_c3577/f1p0/809	NA	NA
R01_cb8335_c3/f2p0/2388	NA	NA	NA	R01_cb8335_c3/f2p0/2388
R01_cb16718_c2/f1p0/334	R01_cb16718_c2/f1p0/334	R01_cb16718_c2/f1p0/334	R01_cb16718_c2/f1p0/334	R01_cb16718_c2/f1p0/334
R01_cb8564_c52119/f1p0/2979	NA	NA	NA	R01_cb8564_c52119/f1p0/2979
R01_cb3132_c0/f2p0/1470	R01_cb3132_c0/f2p0/1470	R01_cb3132_c0/f2p0/1470	NA	R01_cb3132_c0/f2p0/1470
R01_cb8564_c121591/f1p0/2169	NA	R01_cb8564_c121591/f1p0/2169	NA	R01_cb8564_c121591/f1p0/2169
R01_cb16422_c2/f1p0/1761	NA	R01_cb16422_c2/f1p0/1761	R01_cb16422_c2/f1p0/1761	R01_cb16422_c2/f1p0/1761
R01_cb8900_c7/f1p0/1920	NA	NA	NA	R01_cb8900_c7/f1p0/1920
R01_cb8564_c10270/f1p0/3438	NA	R01_cb8564_c10270/f1p0/3438	NA	NA
R01_cb15949_c1/f1p0/992	NA	NA	NA	R01_cb15949_c1/f1p0/992
R01_cb15712_c3/f1p0/6308	NA	R01_cb15712_c3/f1p0/6308	NA	R01_cb15712_c3/f1p0/6308
R01_cb631_c14/f1p0/2844	NA	R01_cb631_c14/f1p0/2844	NA	NA
R01_cb402_c27/f1p0/3012	NA	NA	NA	R01_cb402_c27/f1p0/3012
R01_cb5246_c4/f1p1/1828	R01_cb5246_c4/f1p1/1828	R01_cb5246_c4/f1p1/1828	R01_cb5246_c4/f1p1/1828	R01_cb5246_c4/f1p1/1828
R01_cb13801_c2/f1p0/620	NA	R01_cb13801_c2/f1p0/620	NA	NA
R01_cb14472_c7/f1p0/354	R01_cb14472_c7/f1p0/354	R01_cb14472_c7/f1p0/354	NA	R01_cb14472_c7/f1p0/354
R01_cb4249_c3/f1p0/3392	NA	R01_cb4249_c3/f1p0/3392	R01_cb4249_c3/f1p0/3392	NA
R01_cb4378_c6/f1p0/1725	NA	NA	NA	R01_cb4378_c6/f1p0/1725
R01_cb2870_c14/f1p0/2569	NA	NA	NA	R01_cb2870_c14/f1p0/2569

R01_cb4861_c0/f1p0/3233	NA	R01_cb4861_c0/f1p0/3233	NA	R01_cb4861_c0/f1p0/3233
R01_cb5622_c4/f1p0/1701	NA	NA	NA	R01_cb5622_c4/f1p0/1701
R01_cb8564_c53816/f1p0/2916	NA	R01_cb8564_c53816/f1p0/2916	NA	R01_cb8564_c53816/f1p0/2916
R01_cb10364_c7/f1p0/1783	NA	NA	NA	R01_cb10364_c7/f1p0/1783
R01_cb8564_c3998/f1p0/2346	NA	NA	R01_cb8564_c3998/f1p0/2346	R01_cb8564_c3998/f1p0/2346
R01_cb8564_c72169/f1p0/3201	NA	R01_cb8564_c72169/f1p0/3201	NA	NA
R01_cb1259_c26/f1p2/3479	NA	NA	NA	R01_cb1259_c26/f1p2/3479
R01_cb4941_c1/f1p0/3363	NA	NA	NA	R01_cb4941_c1/f1p0/3363
R01_cb8564_c11163/f1p0/2029	NA	NA	NA	R01_cb8564_c11163/f1p0/2029
R01_cb15308_c0/f4p0/759	NA	NA	R01_cb15308_c0/f4p0/759	R01_cb15308_c0/f4p0/759
R01_cb10609_c2/f1p0/731	NA	R01_cb10609_c2/f1p0/731	NA	NA
R01_cb8564_c1358/f1p0/2279	NA	NA	NA	R01_cb8564_c1358/f1p0/2279
R01_cb8564_c123670/f1p0/2039	NA	R01_cb8564_c123670/f1p0/2039	R01_cb8564_c123670/f1p0/2039	R01_cb8564_c123670/f1p0/2039
R01_cb12931_c19/f1p0/374	R01_cb12931_c19/f1p0/374	R01_cb12931_c19/f1p0/374	R01_cb12931_c19/f1p0/374	R01_cb12931_c19/f1p0/374
R01_cb13776_c3/f1p0/1861	NA	NA	NA	R01_cb13776_c3/f1p0/1861
R01_cb14174_c4/f1p0/1348	NA	NA	NA	R01_cb14174_c4/f1p0/1348
R01_cb16499_c0/f7p1/1114	NA	R01_cb16499_c0/f7p1/1114	NA	NA
R01_cb17653_c2/f1p0/1656	NA	NA	NA	R01_cb17653_c2/f1p0/1656
R01_cb18456_c5010/f1p0/1704	NA	R01_cb18456_c5010/f1p0/1704	NA	NA
R01_cb5705_c2/f1p4/2396	NA	NA	R01_cb5705_c2/f1p4/2396	NA
R01_cb9535_c21/f1p0/1132	NA	R01_cb9535_c21/f1p0/1132	R01_cb9535_c21/f1p0/1132	R01_cb9535_c21/f1p0/1132
R01_cb8564_c117896/f1p0/2328	R01_cb8564_c117896/f1p0/2328	R01_cb8564_c117896/f1p0/2328	R01_cb8564_c117896/f1p0/2328	R01_cb8564_c117896/f1p0/2328
R01_cb12767_c14/f11p1/933	NA	NA	NA	R01_cb12767_c14/f11p1/933
R01_cb4532_c5/f1p1/2905	NA	R01_cb4532_c5/f1p1/2905	NA	NA

R01_cb8232_c2/f1p0/1283	R01_cb8232_c2/f1p0/1283	R01_cb8232_c2/f1p0/1283	R01_cb8232_c2/f1p0/1283	NA
R01_cb17363_c3/f2p0/748	NA	R01_cb17363_c3/f2p0/748	R01_cb17363_c3/f2p0/748	NA
R01_cb11815_c1/f1p0/3535	NA	R01_cb11815_c1/f1p0/3535	NA	NA
R01_cb8564_c17175/f1p0/2669	NA	R01_cb8564_c17175/f1p0/2669	R01_cb8564_c17175/f1p0/2669	R01_cb8564_c17175/f1p0/2669
R01_cb945_c10/f1p0/3512	NA	R01_cb945_c10/f1p0/3512	NA	NA
R01_cb10236_c8/f1p0/1225	NA	R01_cb10236_c8/f1p0/1225	NA	R01_cb10236_c8/f1p0/1225
R01_cb8564_c36663/f1p0/2776	NA	NA	NA	R01_cb8564_c36663/f1p0/2776
R01_cb5156_c0/f69p3/807	NA	NA	NA	R01_cb5156_c0/f69p3/807
R01_cb14595_c6/f1p0/597	R01_cb14595_c6/f1p0/597	R01_cb14595_c6/f1p0/597	R01_cb14595_c6/f1p0/597	R01_cb14595_c6/f1p0/597
R01_cb14564_c0/f6p0/818	NA	R01_cb14564_c0/f6p0/818	R01_cb14564_c0/f6p0/818	NA
R01_cb1594_c4/f1p0/2740	NA	NA	NA	R01_cb1594_c4/f1p0/2740
R01_cb10688_c4/f1p0/1048	NA	R01_cb10688_c4/f1p0/1048	NA	R01_cb10688_c4/f1p0/1048
R01_cb7506_c0/f1p0/2600	NA	NA	NA	R01_cb7506_c0/f1p0/2600
R01_cb8564_c126834/f1p1/2899	NA	R01_cb8564_c126834/f1p1/2899	R01_cb8564_c126834/f1p1/2899	R01_cb8564_c126834/f1p1/2899
R01_cb18456_c2306/f1p0/445	NA	R01_cb18456_c2306/f1p0/445	R01_cb18456_c2306/f1p0/445	R01_cb18456_c2306/f1p0/445
R01_cb18055_c1/f1p0/473	NA	NA	NA	R01_cb18055_c1/f1p0/473
R01_cb261_c54/f1p0/943	R01_cb261_c54/f1p0/943	R01_cb261_c54/f1p0/943	R01_cb261_c54/f1p0/943	R01_cb261_c54/f1p0/943
R01_cb15590_c0/f1p0/709	NA	R01_cb15590_c0/f1p0/709	NA	NA
R01_cb1388_c21/f1p0/2483	NA	R01_cb1388_c21/f1p0/2483	R01_cb1388_c21/f1p0/2483	R01_cb1388_c21/f1p0/2483
R01_cb18456_c7584/f1p0/998	R01_cb18456_c7584/f1p0/998	R01_cb18456_c7584/f1p0/998	R01_cb18456_c7584/f1p0/998	R01_cb18456_c7584/f1p0/998
R01_cb14332_c0/f2p0/1153	R01_cb14332_c0/f2p0/1153	R01_cb14332_c0/f2p0/1153	NA	R01_cb14332_c0/f2p0/1153
R01_cb5425_c10/f1p0/3099	NA	R01_cb5425_c10/f1p0/3099	NA	NA
R01_cb6179_c1/f1p0/2170	NA	R01_cb6179_c1/f1p0/2170	NA	NA
R01_cb8564_c4472/f1p0/2965	NA	NA	R01_cb8564_c4472/f1p0/2965	R01_cb8564_c4472/f1p0/2965
R01_cb7219_c21/f2p1/2451	NA	NA	NA	R01_cb7219_c21/f2p1/2451

R01_cb8564_c23809/f1p0/2906	NA	R01_cb8564_c23809/f1p0/2906	NA	NA
R01_cb7519_c12/f1p1/1370	NA	R01_cb7519_c12/f1p1/1370	NA	R01_cb7519_c12/f1p1/1370
R01_cb5847_c3/f1p0/2997	NA	R01_cb5847_c3/f1p0/2997	NA	R01_cb5847_c3/f1p0/2997
R01_cb14675_c9/f1p0/726	NA	R01_cb14675_c9/f1p0/726	R01_cb14675_c9/f1p0/726	R01_cb14675_c9/f1p0/726
R01_cb13026_c7/f1p0/990	R01_cb13026_c7/f1p0/990	R01_cb13026_c7/f1p0/990	NA	R01_cb13026_c7/f1p0/990
R01_cb6615_c8/f1p0/4079	NA	NA	NA	R01_cb6615_c8/f1p0/4079
R01_cb16425_c9/f1p0/803	R01_cb16425_c9/f1p0/803	R01_cb16425_c9/f1p0/803	R01_cb16425_c9/f1p0/803	R01_cb16425_c9/f1p0/803
R01_cb422_c7/f1p1/6992	NA	NA	NA	R01_cb422_c7/f1p1/6992
R01_cb17128_c2/f1p0/1039	NA	R01_cb17128_c2/f1p0/1039	NA	NA
R01_cb6356_c6/f1p0/1021	NA	R01_cb6356_c6/f1p0/1021	R01_cb6356_c6/f1p0/1021	R01_cb6356_c6/f1p0/1021
R01_cb16653_c1/f1p0/1631	NA	NA	NA	R01_cb16653_c1/f1p0/1631
R01_cb8564_c111997/f1p0/2548	R01_cb8564_c111997/f1p0/2548	R01_cb8564_c111997/f1p0/2548	NA	R01_cb8564_c111997/f1p0/2548
R01_cb3518_c14/f1p2/1777	NA	NA	NA	R01_cb3518_c14/f1p2/1777
R01_cb8564_c10300/f1p0/2657	NA	NA	NA	R01_cb8564_c10300/f1p0/2657
R01_cb1938_c12/f1p1/2274	NA	NA	NA	R01_cb1938_c12/f1p1/2274
R01_cb15462_c2/f1p0/527	NA	R01_cb15462_c2/f1p0/527	R01_cb15462_c2/f1p0/527	NA
R01_cb12242_c1/f1p0/825	NA	NA	NA	R01_cb12242_c1/f1p0/825
R01_cb5775_c9/f1p0/1798	NA	R01_cb5775_c9/f1p0/1798	NA	NA
R01_cb14752_c1/f1p0/866	NA	NA	NA	R01_cb14752_c1/f1p0/866
R01_cb17068_c0/f1p0/1743	NA	R01_cb17068_c0/f1p0/1743	R01_cb17068_c0/f1p0/1743	R01_cb17068_c0/f1p0/1743
R01_cb17216_c4/f1p0/488	NA	R01_cb17216_c4/f1p0/488	R01_cb17216_c4/f1p0/488	R01_cb17216_c4/f1p0/488
R01_cb18456_c3711/f1p0/1750	NA	R01_cb18456_c3711/f1p0/1750	NA	R01_cb18456_c3711/f1p0/1750
R01_cb16304_c2/f1p0/1698	NA	NA	NA	R01_cb16304_c2/f1p0/1698
R01_cb2082_c32/f1p0/2897	NA	NA	NA	R01_cb2082_c32/f1p0/2897
R01_cb5373_c5/f1p0/2899	NA	NA	NA	R01_cb5373_c5/f1p0/2899

R01_cb11358_c1/f1p0/2709	NA	R01_cb11358_c1/f1p0/2709	R01_cb11358_c1/f1p0/2709	R01_cb11358_c1/f1p0/2709
R01_cb11267_c0/f1p0/1468	NA	R01_cb11267_c0/f1p0/1468	NA	NA
R01_cb3954_c3/f1p0/395	R01_cb3954_c3/f1p0/395	R01_cb3954_c3/f1p0/395	R01_cb3954_c3/f1p0/395	R01_cb3954_c3/f1p0/395
R01_cb17956_c4/f1p0/945	NA	R01_cb17956_c4/f1p0/945	NA	NA
R01_cb8564_c81089/f1p1/2976	NA	R01_cb8564_c81089/f1p1/2976	NA	NA
R01_cb14761_c1/f2p0/780	NA	R01_cb14761_c1/f2p0/780	R01_cb14761_c1/f2p0/780	R01_cb14761_c1/f2p0/780
R01_cb17145_c0/f2p0/1001	NA	R01_cb17145_c0/f2p0/1001	NA	NA
R01_cb4185_c5/f1p0/1429	NA	R01_cb4185_c5/f1p0/1429	NA	R01_cb4185_c5/f1p0/1429
R01_cb18201_c0/f1p0/916	NA	R01_cb18201_c0/f1p0/916	R01_cb18201_c0/f1p0/916	R01_cb18201_c0/f1p0/916
R01_cb13433_c98/f1p0/975	NA	NA	NA	R01_cb13433_c98/f1p0/975
R01_cb11060_c1/f1p0/1246	NA	R01_cb11060_c1/f1p0/1246	NA	NA
R01_cb16642_c3/f1p0/1200	NA	NA	R01_cb16642_c3/f1p0/1200	R01_cb16642_c3/f1p0/1200
R01_cb8031_c3/f1p0/1528	NA	NA	R01_cb8031_c3/f1p0/1528	R01_cb8031_c3/f1p0/1528
R01_cb8564_c79060/f1p0/2839	NA	R01_cb8564_c79060/f1p0/2839	R01_cb8564_c79060/f1p0/2839	R01_cb8564_c79060/f1p0/2839
R01_cb1333_c38/f1p0/2096	R01_cb1333_c38/f1p0/2096	R01_cb1333_c38/f1p0/2096	R01_cb1333_c38/f1p0/2096	R01_cb1333_c38/f1p0/2096
R01_cb10806_c0/f1p0/1055	NA	R01_cb10806_c0/f1p0/1055	NA	NA
R01_cb6181_c26/f1p0/2938	NA	R01_cb6181_c26/f1p0/2938	NA	NA
R01_cb8564_c20564/f1p0/3303	R01_cb8564_c20564/f1p0/3303	R01_cb8564_c20564/f1p0/3303	NA	R01_cb8564_c20564/f1p0/3303
R01_cb11844_c0/f1p0/1732	R01_cb11844_c0/f1p0/1732	R01_cb11844_c0/f1p0/1732	R01_cb11844_c0/f1p0/1732	R01_cb11844_c0/f1p0/1732
R01_cb8564_c42299/f1p0/3200	NA	R01_cb8564_c42299/f1p0/3200	NA	NA
R01_cb18456_c7191/f1p0/429	R01_cb18456_c7191/f1p0/429	R01_cb18456_c7191/f1p0/429	R01_cb18456_c7191/f1p0/429	R01_cb18456_c7191/f1p0/429
R01_cb5139_c25/f1p0/1029	NA	R01_cb5139_c25/f1p0/1029	R01_cb5139_c25/f1p0/1029	R01_cb5139_c25/f1p0/1029
R01_cb6508_c6/f1p0/2824	NA	R01_cb6508_c6/f1p0/2824	NA	NA
R01_cb8991_c2/f2p0/2016	NA	NA	NA	R01_cb8991_c2/f2p0/2016
R01_cb1967_c12/f1p0/3184	R01_cb1967_c12/f1p0/3184	R01_cb1967_c12/f1p0/3184	R01_cb1967_c12/f1p0/3184	R01_cb1967_c12/f1p0/3184

R01_cb16617_c0/f2p0/1486	NA	NA	NA	R01_cb16617_c0/f2p0/1486
R01_cb8564_c7779/f10p2/4939	NA	NA	NA	R01_cb8564_c7779/f10p2/4939
R01_cb13178_c1/f7p0/580	NA	R01_cb13178_c1/f7p0/580	NA	NA
R01_cb16979_c9/f1p0/694	NA	R01_cb16979_c9/f1p0/694	NA	NA
R01_cb13875_c7/f1p0/1471	NA	NA	NA	R01_cb13875_c7/f1p0/1471
R01_cb8139_c10/f1p0/3065	NA	R01_cb8139_c10/f1p0/3065	NA	NA
R01_cb6334_c3/f1p0/2879	NA	R01_cb6334_c3/f1p0/2879	NA	NA
R01_cb8107_c4/f1p0/622	R01_cb8107_c4/f1p0/622	R01_cb8107_c4/f1p0/622	R01_cb8107_c4/f1p0/622	R01_cb8107_c4/f1p0/622
R01_cb10676_c5/f5p0/468	NA	NA	NA	R01_cb10676_c5/f5p0/468
R01_cb18447_c0/f2p0/1014	NA	R01_cb18447_c0/f2p0/1014	R01_cb18447_c0/f2p0/1014	R01_cb18447_c0/f2p0/1014
R01_cb4300_c2/f1p0/2636	NA	R01_cb4300_c2/f1p0/2636	NA	NA
R01_cb18456_c3722/f1p0/1251	R01_cb18456_c3722/f1p0/1251	R01_cb18456_c3722/f1p0/1251	NA	R01_cb18456_c3722/f1p0/1251
R01_cb2536_c31/f2p0/2648	R01_cb2536_c31/f2p0/2648	R01_cb2536_c31/f2p0/2648	NA	NA
R01_cb18720_c1/f1p1/2800	R01_cb18720_c1/f1p1/2800	R01_cb18720_c1/f1p1/2800	R01_cb18720_c1/f1p1/2800	NA
R01_cb18485_c0/f1p0/570	R01_cb18485_c0/f1p0/570	R01_cb18485_c0/f1p0/570	R01_cb18485_c0/f1p0/570	R01_cb18485_c0/f1p0/570
R01_cb14005_c4/f1p0/773	R01_cb14005_c4/f1p0/773	R01_cb14005_c4/f1p0/773	R01_cb14005_c4/f1p0/773	R01_cb14005_c4/f1p0/773
R01_cb4722_c3/f1p0/852	NA	R01_cb4722_c3/f1p0/852	NA	R01_cb4722_c3/f1p0/852
R01_cb8564_c4632/f1p0/3266	NA	R01_cb8564_c4632/f1p0/3266	R01_cb8564_c4632/f1p0/3266	R01_cb8564_c4632/f1p0/3266
R01_cb8564_c90609/f1p0/4125	NA	R01_cb8564_c90609/f1p0/4125	NA	NA
R01_cb17309_c2/f1p1/1188	NA	NA	R01_cb17309_c2/f1p1/1188	R01_cb17309_c2/f1p1/1188
R01_cb11572_c0/f1p0/378	R01_cb11572_c0/f1p0/378	R01_cb11572_c0/f1p0/378	R01_cb11572_c0/f1p0/378	R01_cb11572_c0/f1p0/378
R01_cb8564_c14962/f1p0/2093	NA	NA	NA	R01_cb8564_c14962/f1p0/2093
R01_cb5622_c5/f1p0/1617	NA	NA	NA	R01_cb5622_c5/f1p0/1617
R01_cb9037_c3/f1p0/2159	NA	R01_cb9037_c3/f1p0/2159	NA	NA
R01_cb15615_c1/f1p0/634	NA	R01_cb15615_c1/f1p0/634	NA	NA

R01_cb506_c18/f1p0/2271	NA	NA	R01_cb506_c18/f1p0/2271	R01_cb506_c18/f1p0/2271
R01_cb7151_c3/f1p0/2229	R01_cb7151_c3/f1p0/2229	R01_cb7151_c3/f1p0/2229	R01_cb7151_c3/f1p0/2229	R01_cb7151_c3/f1p0/2229
R01_cb6845_c8/f2p1/2728	NA	NA	NA	R01_cb6845_c8/f2p1/2728
R01_cb1178_c21/f1p0/1378	NA	NA	NA	R01_cb1178_c21/f1p0/1378
R01_cb2738_c5/f1p0/2058	NA	NA	NA	R01_cb2738_c5/f1p0/2058
R01_cb8564_c1030/f1p0/1954	NA	R01_cb8564_c1030/f1p0/1954	NA	NA
R01_cb15768_c0/f6p0/1168	NA	NA	NA	R01_cb15768_c0/f6p0/1168
R01_cb2082_c37/f7p0/2806	NA	NA	NA	R01_cb2082_c37/f7p0/2806
R01_cb8564_c69007/f2p0/2967	NA	R01_cb8564_c69007/f2p0/2967	R01_cb8564_c69007/f2p0/2967	NA
R01_cb10906_c1/f1p0/2092	NA	NA	NA	R01_cb10906_c1/f1p0/2092
R01_cb11770_c1/f1p0/2393	NA	R01_cb11770_c1/f1p0/2393	NA	NA
R01_cb5893_c3/f1p0/2183	R01_cb5893_c3/f1p0/2183	R01_cb5893_c3/f1p0/2183	R01_cb5893_c3/f1p0/2183	R01_cb5893_c3/f1p0/2183
R01_cb12422_c18/f1p0/1709	NA	NA	NA	R01_cb12422_c18/f1p0/1709
R01_cb8564_c77719/f1p0/3011	NA	R01_cb8564_c77719/f1p0/3011	R01_cb8564_c77719/f1p0/3011	R01_cb8564_c77719/f1p0/3011
R01_cb8564_c127023/f1p0/2228	NA	R01_cb8564_c127023/f1p0/2228	R01_cb8564_c127023/f1p0/2228	NA
R01_cb2033_c8/f1p0/2427	NA	NA	R01_cb2033_c8/f1p0/2427	R01_cb2033_c8/f1p0/2427
R01_cb13885_c5/f1p0/671	NA	R01_cb13885_c5/f1p0/671	NA	R01_cb13885_c5/f1p0/671
R01_cb15186_c26/f15p0/475	R01_cb15186_c26/f15p0/475	R01_cb15186_c26/f15p0/475	R01_cb15186_c26/f15p0/475	NA
R01_cb17973_c27/f1p0/1103	NA	R01_cb17973_c27/f1p0/1103	R01_cb17973_c27/f1p0/1103	R01_cb17973_c27/f1p0/1103
R01_cb2710_c1/f1p0/3833	NA	NA	NA	R01_cb2710_c1/f1p0/3833
R01_cb8564_c21735/f1p0/4287	NA	R01_cb8564_c21735/f1p0/4287	NA	NA
R01_cb13025_c9/f1p0/1535	NA	NA	NA	R01_cb13025_c9/f1p0/1535
R01_cb10606_c2/f1p0/1156	R01_cb10606_c2/f1p0/1156	R01_cb10606_c2/f1p0/1156	NA	NA
R01_cb18409_c77/f1p0/1058	R01_cb18409_c77/f1p0/1058	R01_cb18409_c77/f1p0/1058	R01_cb18409_c77/f1p0/1058	NA
R01_cb8564_c23727/f1p0/2755	NA	R01_cb8564_c23727/f1p0/2755	NA	NA

R01_cb15418_c0/f4p1/876	NA	NA	NA	R01_cb15418_c0/f4p1/876
R01_cb8564_c112163/f1p0/2459	NA	R01_cb8564_c112163/f1p0/2459	NA	NA
R01_cb17756_c14/f1p0/1565	R01_cb17756_c14/f1p0/1565	R01_cb17756_c14/f1p0/1565	R01_cb17756_c14/f1p0/1565	R01_cb17756_c14/f1p0/1565
R01_cb2095_c11/f1p3/2401	NA	NA	NA	R01_cb2095_c11/f1p3/2401
R01_cb13766_c10/f1p0/888	NA	R01_cb13766_c10/f1p0/888	NA	NA
R01_cb4702_c28/f1p0/957	NA	NA	NA	R01_cb4702_c28/f1p0/957
R01_cb4128_c17/f1p0/2338	NA	NA	NA	R01_cb4128_c17/f1p0/2338
R01_cb18348_c0/f1p0/1144	NA	R01_cb18348_c0/f1p0/1144	NA	NA
R01_cb16465_c4/f1p0/1058	NA	NA	NA	R01_cb16465_c4/f1p0/1058
R01_cb9882_c7/f1p0/984	R01_cb9882_c7/f1p0/984	NA	NA	NA
R01_cb8564_c77311/f1p0/2386	NA	NA	NA	R01_cb8564_c77311/f1p0/2386
R01_cb2543_c36/f1p1/2737	NA	R01_cb2543_c36/f1p1/2737	NA	NA
R01_cb1155_c22/f1p0/4835	NA	NA	NA	R01_cb1155_c22/f1p0/4835
R01_cb7519_c21/f1p0/2280	NA	NA	NA	R01_cb7519_c21/f1p0/2280
R01_cb17106_c0/f3p0/1466	NA	NA	R01_cb17106_c0/f3p0/1466	R01_cb17106_c0/f3p0/1466
R01_cb5608_c49/f1p0/3694	NA	NA	NA	R01_cb5608_c49/f1p0/3694
R01_cb8564_c46968/f1p0/2274	NA	NA	NA	R01_cb8564_c46968/f1p0/2274
R01_cb17195_c0/f1p0/322	R01_cb17195_c0/f1p0/322	R01_cb17195_c0/f1p0/322	NA	NA
R01_cb8573_c4/f1p0/1868	NA	NA	NA	R01_cb8573_c4/f1p0/1868
R01_cb17442_c0/f2p0/1363	NA	NA	NA	R01_cb17442_c0/f2p0/1363
R01_cb14680_c2/f1p0/440	R01_cb14680_c2/f1p0/440	R01_cb14680_c2/f1p0/440	R01_cb14680_c2/f1p0/440	R01_cb14680_c2/f1p0/440
R01_cb6700_c9/f1p0/2495	NA	NA	NA	R01_cb6700_c9/f1p0/2495
R01_cb3818_c18/f1p0/2496	NA	R01_cb3818_c18/f1p0/2496	NA	NA
R01_cb12226_c3/f1p0/373	R01_cb12226_c3/f1p0/373	NA	R01_cb12226_c3/f1p0/373	NA
R01_cb9984_c1/f1p0/2507	R01_cb9984_c1/f1p0/2507	R01_cb9984_c1/f1p0/2507	R01_cb9984_c1/f1p0/2507	R01_cb9984_c1/f1p0/2507

R01_cb8564_c15158/f1p5/4376	NA	R01_cb8564_c15158/f1p5/4376	R01_cb8564_c15158/f1p5/4376	R01_cb8564_c15158/f1p5/4376
R01_cb8564_c68563/f3p0/3011	NA	R01_cb8564_c68563/f3p0/3011	NA	NA
R01_cb5595_c12/f1p0/2082	NA	R01_cb5595_c12/f1p0/2082	NA	NA
R01_cb11212_c5/f1p0/696	NA	R01_cb11212_c5/f1p0/696	NA	NA
R01_cb16888_c3/f1p0/1008	NA	NA	R01_cb16888_c3/f1p0/1008	R01_cb16888_c3/f1p0/1008
R01_cb260_c19/f1p0/2307	NA	NA	NA	R01_cb260_c19/f1p0/2307
R01_cb9487_c14/f1p0/1063	NA	R01_cb9487_c14/f1p0/1063	NA	R01_cb9487_c14/f1p0/1063
R01_cb9841_c0/f8p0/1829	NA	NA	NA	R01_cb9841_c0/f8p0/1829
R01_cb544_c6/f1p0/4746	NA	NA	NA	R01_cb544_c6/f1p0/4746
R01_cb13910_c14/f1p0/1630	NA	R01_cb13910_c14/f1p0/1630	R01_cb13910_c14/f1p0/1630	R01_cb13910_c14/f1p0/1630
R01_cb17676_c0/f2p0/523	NA	R01_cb17676_c0/f2p0/523	NA	R01_cb17676_c0/f2p0/523
R01_cb2367_c5/f1p0/2867	NA	R01_cb2367_c5/f1p0/2867	R01_cb2367_c5/f1p0/2867	NA
R01_cb4030_c0/f2p0/2589	NA	NA	NA	R01_cb4030_c0/f2p0/2589
R01_cb8564_c80921/f1p1/2309	NA	NA	NA	R01_cb8564_c80921/f1p1/2309
R01_cb8564_c85478/f1p0/2221	NA	R01_cb8564_c85478/f1p0/2221	R01_cb8564_c85478/f1p0/2221	R01_cb8564_c85478/f1p0/2221
R01_cb11210_c2/f1p0/1268	NA	R01_cb11210_c2/f1p0/1268	R01_cb11210_c2/f1p0/1268	R01_cb11210_c2/f1p0/1268
R01_cb8564_c40788/f1p0/2101	NA	R01_cb8564_c40788/f1p0/2101	NA	R01_cb8564_c40788/f1p0/2101
R01_cb416_c33/f1p1/2908	NA	NA	NA	R01_cb416_c33/f1p1/2908
R01_cb8564_c78312/f1p0/3944	NA	R01_cb8564_c78312/f1p0/3944	NA	NA
R01_cb8209_c0/f2p0/2294	NA	NA	NA	R01_cb8209_c0/f2p0/2294
R01_cb8564_c1351/f5p1/2265	NA	NA	NA	R01_cb8564_c1351/f5p1/2265
R01_cb2314_c2/f1p0/3840	R01_cb2314_c2/f1p0/3840	R01_cb2314_c2/f1p0/3840	NA	NA
R01_cb8564_c1353/f1p0/3452	NA	R01_cb8564_c1353/f1p0/3452	NA	NA
R01_cb7439_c0/f1p0/2616	NA	R01_cb7439_c0/f1p0/2616	NA	NA
R01_cb8564_c4071/f1p0/2728	NA	R01_cb8564_c4071/f1p0/2728	NA	R01_cb8564_c4071/f1p0/2728
R01_cb17003_c1/f1p0/1176	NA	NA	NA	R01_cb17003_c1/f1p0/1176

R01_cb4117_c2/f1p0/3430	NA	NA	NA	R01_cb4117_c2/f1p0/3430
R01_cb4702_c8/f1p0/3284	NA	NA	NA	R01_cb4702_c8/f1p0/3284
R01_cb8564_c111632/f1p0/2604	NA	R01_cb8564_c111632/f1p0/2604	NA	NA
R01_cb8023_c12/f1p0/1479	NA	NA	R01_cb8023_c12/f1p0/1479	R01_cb8023_c12/f1p0/1479
R01_cb14657_c2/f1p0/1784	R01_cb14657_c2/f1p0/1784	R01_cb14657_c2/f1p0/1784	R01_cb14657_c2/f1p0/1784	R01_cb14657_c2/f1p0/1784
R01_cb8564_c52843/f2p0/2733	NA	R01_cb8564_c52843/f2p0/2733	NA	R01_cb8564_c52843/f2p0/2733
R01_cb18456_c4374/f1p0/644	NA	NA	NA	R01_cb18456_c4374/f1p0/644
R01_cb8564_c130195/f1p0/2809	R01_cb8564_c130195/f1p0/2809	R01_cb8564_c130195/f1p0/2809	NA	NA
R01_cb13488_c12/f1p0/818	NA	NA	NA	R01_cb13488_c12/f1p0/818
R01_cb7528_c2/f1p0/3135	NA	R01_cb7528_c2/f1p0/3135	NA	NA
R01_cb8564_c11512/f1p0/1974	NA	R01_cb8564_c11512/f1p0/1974	NA	NA
R01_cb18022_c1/f1p0/1596	R01_cb18022_c1/f1p0/1596	R01_cb18022_c1/f1p0/1596	R01_cb18022_c1/f1p0/1596	R01_cb18022_c1/f1p0/1596
R01_cb12824_c20/f1p0/1592	NA	R01_cb12824_c20/f1p0/1592	NA	NA
R01_cb16652_c4/f1p0/362	R01_cb16652_c4/f1p0/362	R01_cb16652_c4/f1p0/362	R01_cb16652_c4/f1p0/362	R01_cb16652_c4/f1p0/362
R01_cb5051_c0/f3p0/3220	R01_cb5051_c0/f3p0/3220	R01_cb5051_c0/f3p0/3220	R01_cb5051_c0/f3p0/3220	R01_cb5051_c0/f3p0/3220
R01_cb5739_c0/f1p0/2876	NA	R01_cb5739_c0/f1p0/2876	R01_cb5739_c0/f1p0/2876	R01_cb5739_c0/f1p0/2876
R01_cb15466_c4/f1p0/537	R01_cb15466_c4/f1p0/537	R01_cb15466_c4/f1p0/537	NA	NA
R01_cb15449_c1/f1p0/1317	NA	R01_cb15449_c1/f1p0/1317	R01_cb15449_c1/f1p0/1317	R01_cb15449_c1/f1p0/1317
R01_cb16662_c3/f1p0/360	R01_cb16662_c3/f1p0/360	R01_cb16662_c3/f1p0/360	R01_cb16662_c3/f1p0/360	R01_cb16662_c3/f1p0/360
R01_cb10693_c3/f1p1/1536	NA	NA	NA	R01_cb10693_c3/f1p1/1536
R01_cb13421_c0/f1p0/1070	NA	R01_cb13421_c0/f1p0/1070	NA	NA
R01_cb8564_c22279/f1p0/2983	NA	R01_cb8564_c22279/f1p0/2983	NA	NA
R01_cb16648_c0/f1p0/1224	NA	R01_cb16648_c0/f1p0/1224	R01_cb16648_c0/f1p0/1224	R01_cb16648_c0/f1p0/1224
R01_cb18456_c6833/f1p0/736	R01_cb18456_c6833/f1p0/736	R01_cb18456_c6833/f1p0/736	NA	R01_cb18456_c6833/f1p0/736

R01_cb18187_c1/f1p0/743	NA	NA	NA	R01_cb18187_c1/f1p0/743
R01_cb9897_c4/f1p0/1935	NA	R01_cb9897_c4/f1p0/1935	NA	NA
R01_cb18456_c2501/f1p0/704	NA	R01_cb18456_c2501/f1p0/704	NA	NA
R01_cb8564_c14244/f1p1/2174	NA	R01_cb8564_c14244/f1p1/2174	NA	NA
R01_cb8002_c2/f1p0/304	R01_cb8002_c2/f1p0/304	NA	R01_cb8002_c2/f1p0/304	R01_cb8002_c2/f1p0/304
R01_cb9614_c0/f1p0/2025	NA	NA	NA	R01_cb9614_c0/f1p0/2025
R01_cb454_c10/f1p0/2459	NA	NA	NA	R01_cb454_c10/f1p0/2459
R01_cb8564_c1827/f1p1/4356	NA	R01_cb8564_c1827/f1p1/4356	R01_cb8564_c1827/f1p1/4356	NA
R01_cb10074_c13/f1p0/474	NA	R01_cb10074_c13/f1p0/474	R01_cb10074_c13/f1p0/474	R01_cb10074_c13/f1p0/474
R01_cb18494_c1/f1p0/776	NA	R01_cb18494_c1/f1p0/776	R01_cb18494_c1/f1p0/776	R01_cb18494_c1/f1p0/776
R01_cb2845_c29/f1p0/3315	NA	NA	NA	R01_cb2845_c29/f1p0/3315
R01_cb10583_c4/f1p0/5305	NA	R01_cb10583_c4/f1p0/5305	NA	NA
R01_cb18386_c5/f1p0/1465	NA	R01_cb18386_c5/f1p0/1465	NA	NA
R01_cb12912_c7/f1p0/480	NA	R01_cb12912_c7/f1p0/480	NA	R01_cb12912_c7/f1p0/480
R01_cb18537_c1/f1p0/1709	NA	R01_cb18537_c1/f1p0/1709	NA	NA
R01_cb5582_c4/f1p0/1289	NA	NA	NA	R01_cb5582_c4/f1p0/1289
R01_cb10042_c3/f1p0/1672	NA	NA	NA	R01_cb10042_c3/f1p0/1672
R01_cb10107_c1/f1p0/2150	NA	NA	NA	R01_cb10107_c1/f1p0/2150
R01_cb535_c4/f1p0/4795	NA	NA	NA	R01_cb535_c4/f1p0/4795
R01_cb10145_c4/f1p0/1575	NA	NA	NA	R01_cb10145_c4/f1p0/1575
R01_cb13112_c3/f2p0/761	NA	R01_cb13112_c3/f2p0/761	R01_cb13112_c3/f2p0/761	R01_cb13112_c3/f2p0/761
R01_cb6485_c2/f1p0/2384	NA	R01_cb6485_c2/f1p0/2384	NA	NA
R01_cb11148_c1/f1p0/2728	R01_cb11148_c1/f1p0/2728	R01_cb11148_c1/f1p0/2728	R01_cb11148_c1/f1p0/2728	NA
R01_cb11329_c2/f1p0/837	NA	R01_cb11329_c2/f1p0/837	NA	NA
R01_cb8831_c9/f1p1/2228	NA	R01_cb8831_c9/f1p1/2228	NA	R01_cb8831_c9/f1p1/2228
R01_cb2204_c5/f1p0/5403	R01_cb2204_c5/f1p0/5403	R01_cb2204_c5/f1p0/5403	R01_cb2204_c5/f1p0/5403	R01_cb2204_c5/f1p0/5403

R01_cb8564_c3879/f1p0/2868	NA	NA	NA	R01_cb8564_c3879/f1p0/2868
R01_cb3618_c27/f1p0/2821	NA	NA	NA	R01_cb3618_c27/f1p0/2821
R01_cb10943_c10/f1p0/1666	NA	NA	NA	R01_cb10943_c10/f1p0/1666
R01_cb8400_c5/f1p0/1347	NA	NA	NA	R01_cb8400_c5/f1p0/1347
R01_cb18456_c3225/f1p0/875	NA	R01_cb18456_c3225/f1p0/875	NA	NA
R01_cb3650_c15/f1p0/1409	NA	R01_cb3650_c15/f1p0/1409	R01_cb3650_c15/f1p0/1409	R01_cb3650_c15/f1p0/1409
R01_cb18456_c7461/f1p1/491	R01_cb18456_c7461/f1p1/491	R01_cb18456_c7461/f1p1/491	NA	R01_cb18456_c7461/f1p1/491
R01_cb9707_c0/f1p0/2002	NA	NA	NA	R01_cb9707_c0/f1p0/2002
R01_cb9993_c8/f1p0/1104	NA	NA	NA	R01_cb9993_c8/f1p0/1104
R01_cb6529_c4/f1p0/2774	NA	NA	NA	R01_cb6529_c4/f1p0/2774
R01_cb8564_c129970/f1p0/2025	NA	NA	R01_cb8564_c129970/f1p0/2025	R01_cb8564_c129970/f1p0/2025
R01_cb9160_c2/f2p0/2150	NA	R01_cb9160_c2/f2p0/2150	R01_cb9160_c2/f2p0/2150	R01_cb9160_c2/f2p0/2150
R01_cb8094_c8/f1p0/1118	R01_cb8094_c8/f1p0/1118	R01_cb8094_c8/f1p0/1118	NA	R01_cb8094_c8/f1p0/1118
R01_cb10272_c9/f3p2/1688	NA	NA	NA	R01_cb10272_c9/f3p2/1688
R01_cb12912_c0/f3p0/1732	NA	R01_cb12912_c0/f3p0/1732	NA	R01_cb12912_c0/f3p0/1732
R01_cb6258_c28/f3p1/2727	NA	NA	NA	R01_cb6258_c28/f3p1/2727
R01_cb13463_c4/f2p1/1348	NA	NA	NA	R01_cb13463_c4/f2p1/1348
R01_cb6069_c7/f1p0/2320	NA	NA	NA	R01_cb6069_c7/f1p0/2320
R01_cb15937_c3/f1p0/537	R01_cb15937_c3/f1p0/537	R01_cb15937_c3/f1p0/537	NA	R01_cb15937_c3/f1p0/537
R01_cb3426_c28/f2p1/3133	NA	NA	NA	R01_cb3426_c28/f2p1/3133
R01_cb9492_c0/f1p0/2032	NA	NA	R01_cb9492_c0/f1p0/2032	NA
R01_cb13445_c0/f1p0/599	NA	R01_cb13445_c0/f1p0/599	NA	NA
R01_cb8298_c3/f1p1/2277	R01_cb8298_c3/f1p1/2277	R01_cb8298_c3/f1p1/2277	R01_cb8298_c3/f1p1/2277	R01_cb8298_c3/f1p1/2277
R01_cb10847_c2/f1p0/1850	NA	R01_cb10847_c2/f1p0/1850	NA	NA
R01_cb10191_c2/f1p0/1632	NA	NA	NA	R01_cb10191_c2/f1p0/1632

R01_cb1628_c3/f1p0/2275	NA	NA	NA	R01_cb1628_c3/f1p0/2275
R01_cb5297_c7/f1p0/914	NA	R01_cb5297_c7/f1p0/914	NA	NA
R01_cb8564_c124360/f1p0/2240	NA	R01_cb8564_c124360/f1p0/2240	R01_cb8564_c124360/f1p0/2240	R01_cb8564_c124360/f1p0/2240
R01_cb10049_c2/f1p0/565	R01_cb10049_c2/f1p0/565	R01_cb10049_c2/f1p0/565	R01_cb10049_c2/f1p0/565	R01_cb10049_c2/f1p0/565
R01_cb6348_c4/f1p0/2643	NA	NA	NA	R01_cb6348_c4/f1p0/2643
R01_cb15461_c4/f1p0/990	R01_cb15461_c4/f1p0/990	R01_cb15461_c4/f1p0/990	NA	R01_cb15461_c4/f1p0/990
R01_cb16645_c27/f1p0/1774	NA	R01_cb16645_c27/f1p0/1774	R01_cb16645_c27/f1p0/1774	R01_cb16645_c27/f1p0/1774
R01_cb9949_c1/f1p0/4100	NA	R01_cb9949_c1/f1p0/4100	R01_cb9949_c1/f1p0/4100	NA
R01_cb8564_c89368/f1p0/3386	NA	NA	NA	R01_cb8564_c89368/f1p0/3386
R01_cb2700_c3/f1p0/2436	NA	R01_cb2700_c3/f1p0/2436	NA	NA
R01_cb7334_c9/f2p0/1757	NA	NA	NA	R01_cb7334_c9/f2p0/1757
R01_cb16808_c1/f1p0/693	NA	R01_cb16808_c1/f1p0/693	NA	NA
R01_cb6048_c5/f1p0/2730	NA	R01_cb6048_c5/f1p0/2730	NA	NA
R01_cb6014_c2/f1p0/2966	NA	NA	NA	R01_cb6014_c2/f1p0/2966
R01_cb8564_c44139/f1p0/3415	NA	R01_cb8564_c44139/f1p0/3415	NA	R01_cb8564_c44139/f1p0/3415
R01_cb8564_c15336/f2p1/3893	NA	NA	NA	R01_cb8564_c15336/f2p1/3893
R01_cb3344_c5/f1p0/4842	NA	NA	NA	R01_cb3344_c5/f1p0/4842
R01_cb454_c7/f1p0/2374	NA	NA	NA	R01_cb454_c7/f1p0/2374
R01_cb4501_c11/f1p0/2194	NA	NA	NA	R01_cb4501_c11/f1p0/2194
R01_cb4198_c4/f1p0/2477	NA	NA	NA	R01_cb4198_c4/f1p0/2477
R01_cb8564_c81836/f1p0/1971	R01_cb8564_c81836/f1p0/1971	R01_cb8564_c81836/f1p0/1971	NA	R01_cb8564_c81836/f1p0/1971
R01_cb15828_c1/f1p0/958	NA	R01_cb15828_c1/f1p0/958	R01_cb15828_c1/f1p0/958	R01_cb15828_c1/f1p0/958
R01_cb3750_c20/f1p3/3049	NA	NA	NA	R01_cb3750_c20/f1p3/3049
R01_cb10337_c7/f1p2/785	NA	R01_cb10337_c7/f1p2/785	NA	NA

R01_cb5386_c56/f1p1/2155	NA	R01_cb5386_c56/f1p1/2155	NA	NA
R01_cb5468_c3/f1p0/2739	NA	R01_cb5468_c3/f1p0/2739	NA	NA
R01_cb5054_c0/f1p0/3185	NA	R01_cb5054_c0/f1p0/3185	NA	NA
R01_cb16273_c1/f1p0/592	NA	R01_cb16273_c1/f1p0/592	NA	R01_cb16273_c1/f1p0/592
R01_cb11148_c5/f1p0/549	NA	R01_cb11148_c5/f1p0/549	NA	R01_cb11148_c5/f1p0/549
R01_cb10340_c3/f1p0/2798	NA	R01_cb10340_c3/f1p0/2798	NA	NA
R01_cb5297_c2/f1p0/2708	NA	R01_cb5297_c2/f1p0/2708	NA	NA
R01_cb8564_c15044/f1p0/3492	NA	R01_cb8564_c15044/f1p0/3492	NA	NA
R01_cb1505_c65/f4p0/1424	NA	R01_cb1505_c65/f4p0/1424	R01_cb1505_c65/f4p0/1424	R01_cb1505_c65/f4p0/1424
R01_cb8600_c1/f1p0/2322	NA	R01_cb8600_c1/f1p0/2322	NA	NA
R01_cb8564_c79863/f1p0/2280	NA	NA	NA	R01_cb8564_c79863/f1p0/2280
R01_cb14660_c8/f1p0/831	NA	R01_cb14660_c8/f1p0/831	NA	R01_cb14660_c8/f1p0/831
R01_cb18090_c1/f1p0/746	NA	R01_cb18090_c1/f1p0/746	NA	NA
R01_cb8564_c106737/f2p0/2097	NA	NA	NA	R01_cb8564_c106737/f2p0/2097
R01_cb12003_c147/f1p0/1313	NA	R01_cb12003_c147/f1p0/1313	R01_cb12003_c147/f1p0/1313	NA
R01_cb6629_c1/f1p0/2809	NA	R01_cb6629_c1/f1p0/2809	NA	NA
R01_cb4657_c8/f1p0/3762	NA	NA	R01_cb4657_c8/f1p0/3762	NA
R01_cb13572_c0/f2p0/558	NA	NA	NA	R01_cb13572_c0/f2p0/558
R01_cb2875_c8/f1p0/3946	NA	NA	NA	R01_cb2875_c8/f1p0/3946
R01_cb3941_c39/f1p0/4473	NA	R01_cb3941_c39/f1p0/4473	NA	NA
R01_cb1388_c0/f3p0/4208	NA	NA	NA	R01_cb1388_c0/f3p0/4208
R01_cb5017_c2/f1p0/2301	NA	NA	NA	R01_cb5017_c2/f1p0/2301
R01_cb8564_c77049/f1p0/2003	R01_cb8564_c77049/f1p0/2003	R01_cb8564_c77049/f1p0/2003	R01_cb8564_c77049/f1p0/2003	R01_cb8564_c77049/f1p0/2003
R01_cb17452_c6/f1p0/937	NA	R01_cb17452_c6/f1p0/937	NA	NA

R01_cb3550_c1/f1p0/3606	NA	R01_cb3550_c1/f1p0/3606	NA	R01_cb3550_c1/f1p0/3606
R01_cb8564_c21550/f1p0/2219	NA	R01_cb8564_c21550/f1p0/2219	NA	NA
R01_cb3068_c3/f1p0/2722	NA	NA	NA	R01_cb3068_c3/f1p0/2722
R01_cb10200_c4/f1p0/3731	NA	R01_cb10200_c4/f1p0/3731	NA	NA
R01_cb13565_c20/f1p0/866	NA	R01_cb13565_c20/f1p0/866	NA	NA
R01_cb4321_c9/f1p0/999	NA	NA	NA	R01_cb4321_c9/f1p0/999
R01_cb18365_c2/f1p0/881	R01_cb18365_c2/f1p0/881	R01_cb18365_c2/f1p0/881	R01_cb18365_c2/f1p0/881	R01_cb18365_c2/f1p0/881
R01_cb8564_c10832/f3p0/2415	NA	R01_cb8564_c10832/f3p0/2415	R01_cb8564_c10832/f3p0/2415	R01_cb8564_c10832/f3p0/2415
R01_cb449_c13/f1p0/2593	NA	NA	NA	R01_cb449_c13/f1p0/2593
R01_cb18232_c0/f2p0/497	R01_cb18232_c0/f2p0/497	R01_cb18232_c0/f2p0/497	R01_cb18232_c0/f2p0/497	R01_cb18232_c0/f2p0/497
R01_cb18456_c7602/f1p0/414	R01_cb18456_c7602/f1p0/414	R01_cb18456_c7602/f1p0/414	R01_cb18456_c7602/f1p0/414	R01_cb18456_c7602/f1p0/414
R01_cb13907_c7/f1p0/870	NA	NA	NA	R01_cb13907_c7/f1p0/870
R01_cb625_c11/f1p0/1768	NA	R01_cb625_c11/f1p0/1768	NA	NA
R01_cb17744_c15/f1p0/4007	NA	NA	NA	R01_cb17744_c15/f1p0/4007
R01_cb10417_c2/f1p0/837	NA	R01_cb10417_c2/f1p0/837	NA	NA
R01_cb15891_c2/f1p0/820	NA	R01_cb15891_c2/f1p0/820	NA	NA
R01_cb13894_c3/f1p0/546	NA	R01_cb13894_c3/f1p0/546	NA	NA
R01_cb822_c9/f1p3/4612	NA	NA	NA	R01_cb822_c9/f1p3/4612
R01_cb17520_c0/f2p0/1050	NA	NA	NA	R01_cb17520_c0/f2p0/1050
R01_cb8120_c2/f1p1/2430	NA	NA	NA	R01_cb8120_c2/f1p1/2430
R01_cb18409_c128/f1p0/964	NA	R01_cb18409_c128/f1p0/964	NA	NA
R01_cb15796_c7/f1p0/938	NA	NA	NA	R01_cb15796_c7/f1p0/938
R01_cb4825_c11/f1p0/4859	NA	NA	NA	R01_cb4825_c11/f1p0/4859
R01_cb8564_c15536/f1p0/2715	R01_cb8564_c15536/f1p0/2715	R01_cb8564_c15536/f1p0/2715	R01_cb8564_c15536/f1p0/2715	R01_cb8564_c15536/f1p0/2715
R01_cb11938_c3/f2p0/360	NA	R01_cb11938_c3/f2p0/360	NA	R01_cb11938_c3/f2p0/360

R01_cb3938_c0/f1p0/3479	NA	R01_cb3938_c0/f1p0/3479	NA	NA
R01_cb6861_c3/f1p0/3622	NA	R01_cb6861_c3/f1p0/3622	R01_cb6861_c3/f1p0/3622	R01_cb6861_c3/f1p0/3622
R01_cb11676_c2/f1p0/1089	NA	NA	NA	R01_cb11676_c2/f1p0/1089
R01_cb4519_c0/f1p0/3323	NA	R01_cb4519_c0/f1p0/3323	NA	NA
R01_cb16997_c70/f1p0/2346	NA	R01_cb16997_c70/f1p0/2346	NA	R01_cb16997_c70/f1p0/2346
R01_cb8564_c112837/f1p0/2319	NA	NA	NA	R01_cb8564_c112837/f1p0/2319
R01_cb9365_c2/f1p1/2005	NA	NA	NA	R01_cb9365_c2/f1p1/2005
R01_cb1860_c15/f1p2/2457	NA	NA	NA	R01_cb1860_c15/f1p2/2457
R01_cb8564_c185851/f1p0/2077	NA	R01_cb8564_c185851/f1p0/2077	R01_cb8564_c185851/f1p0/2077	R01_cb8564_c185851/f1p0/2077
R01_cb12314_c4/f1p0/422	NA	NA	R01_cb12314_c4/f1p0/422	NA
R01_cb7254_c7/f1p0/2281	NA	NA	NA	R01_cb7254_c7/f1p0/2281
R01_cb3307_c7/f2p0/3452	NA	NA	NA	R01_cb3307_c7/f2p0/3452
R01_cb12686_c0/f2p0/436	NA	NA	NA	R01_cb12686_c0/f2p0/436
R01_cb8564_c118029/f1p0/2642	NA	NA	NA	R01_cb8564_c118029/f1p0/2642
R01_cb11395_c4/f1p0/1250	NA	NA	NA	R01_cb11395_c4/f1p0/1250
R01_cb8975_c5/f1p0/2225	NA	R01_cb8975_c5/f1p0/2225	NA	R01_cb8975_c5/f1p0/2225
R01_cb18409_c88/f1p0/760	R01_cb18409_c88/f1p0/760	R01_cb18409_c88/f1p0/760	R01_cb18409_c88/f1p0/760	R01_cb18409_c88/f1p0/760
R01_cb16312_c1/f1p0/962	NA	R01_cb16312_c1/f1p0/962	NA	NA
R01_cb920_c17/f1p0/4399	NA	NA	NA	R01_cb920_c17/f1p0/4399
R01_cb13433_c34/f2p0/2517	R01_cb13433_c34/f2p0/2517	R01_cb13433_c34/f2p0/2517	NA	R01_cb13433_c34/f2p0/2517
R01_cb18456_c4734/f1p0/513	NA	NA	NA	R01_cb18456_c4734/f1p0/513
R01_cb10029_c1463/f1p0/790	NA	R01_cb10029_c1463/f1p0/790	NA	NA
R01_cb11243_c0/f1p0/1580	NA	R01_cb11243_c0/f1p0/1580	NA	NA

R01_cb12554_c4/f1p0/1603	NA	NA	NA	R01_cb12554_c4/f1p0/1603
R01_cb10306_c1/f2p0/850	R01_cb10306_c1/f2p0/850	R01_cb10306_c1/f2p0/850	NA	R01_cb10306_c1/f2p0/850
R01_cb17363_c4/f1p0/1060	R01_cb17363_c4/f1p0/1060	R01_cb17363_c4/f1p0/1060	R01_cb17363_c4/f1p0/1060	NA
R01_cb12444_c57/f1p0/497	NA	R01_cb12444_c57/f1p0/497	R01_cb12444_c57/f1p0/497	R01_cb12444_c57/f1p0/497
R01_cb13488_c7/f1p0/1010	NA	R01_cb13488_c7/f1p0/1010	NA	R01_cb13488_c7/f1p0/1010
R01_cb8088_c0/f1p0/2457	NA	R01_cb8088_c0/f1p0/2457	NA	NA
R01_cb639_c1/f1p0/4709	NA	R01_cb639_c1/f1p0/4709	NA	R01_cb639_c1/f1p0/4709
R01_cb18591_c0/f1p0/692	R01_cb18591_c0/f1p0/692	R01_cb18591_c0/f1p0/692	R01_cb18591_c0/f1p0/692	R01_cb18591_c0/f1p0/692
R01_cb18456_c4786/f1p0/485	R01_cb18456_c4786/f1p0/485	R01_cb18456_c4786/f1p0/485	NA	R01_cb18456_c4786/f1p0/485
R01_cb2272_c4/f1p0/2783	NA	NA	NA	R01_cb2272_c4/f1p0/2783
R01_cb9882_c4/f1p0/1622	R01_cb9882_c4/f1p0/1622	R01_cb9882_c4/f1p0/1622	R01_cb9882_c4/f1p0/1622	R01_cb9882_c4/f1p0/1622
R01_cb1982_c5/f1p0/1626	R01_cb1982_c5/f1p0/1626	R01_cb1982_c5/f1p0/1626	R01_cb1982_c5/f1p0/1626	R01_cb1982_c5/f1p0/1626
R01_cb2788_c5/f1p0/4276	NA	R01_cb2788_c5/f1p0/4276	NA	NA
R01_cb16319_c1/f1p0/807	NA	NA	NA	R01_cb16319_c1/f1p0/807
R01_cb8564_c51299/f1p0/3097	NA	R01_cb8564_c51299/f1p0/3097	NA	NA
R01_cb8564_c20204/f1p0/3519	NA	R01_cb8564_c20204/f1p0/3519	NA	NA
R01_cb17878_c1/f1p0/1825	NA	NA	R01_cb17878_c1/f1p0/1825	R01_cb17878_c1/f1p0/1825
R01_cb8564_c43143/f1p0/4280	NA	R01_cb8564_c43143/f1p0/4280	NA	NA
R01_cb17374_c0/f5p1/712	NA	NA	NA	R01_cb17374_c0/f5p1/712
R01_cb6608_c5/f1p1/2467	NA	NA	NA	R01_cb6608_c5/f1p1/2467
R01_cb17082_c1/f1p0/585	R01_cb17082_c1/f1p0/585	R01_cb17082_c1/f1p0/585	R01_cb17082_c1/f1p0/585	R01_cb17082_c1/f1p0/585
R01_cb742_c24/f1p0/1369	NA	NA	NA	R01_cb742_c24/f1p0/1369
R01_cb10251_c2/f1p0/4086	NA	R01_cb10251_c2/f1p0/4086	NA	NA
R01_cb14226_c3/f1p0/688	NA	NA	R01_cb14226_c3/f1p0/688	R01_cb14226_c3/f1p0/688
R01_cb5144_c5/f1p0/1716	NA	R01_cb5144_c5/f1p0/1716	NA	R01_cb5144_c5/f1p0/1716
R01_cb4639_c8/f1p0/2579	NA	NA	NA	R01_cb4639_c8/f1p0/2579

R01_cb8564_c125207/f1p0/2587	NA	R01_cb8564_c125207/f1p0/2587	NA	NA
R01_cb6802_c39/f1p0/3232	R01_cb6802_c39/f1p0/3232	R01_cb6802_c39/f1p0/3232	NA	NA
R01_cb8564_c51335/f1p0/2219	NA	R01_cb8564_c51335/f1p0/2219	R01_cb8564_c51335/f1p0/2219	R01_cb8564_c51335/f1p0/2219
R01_cb8037_c4/f1p0/1609	NA	NA	NA	R01_cb8037_c4/f1p0/1609
R01_cb2160_c20/f1p0/3175	NA	NA	NA	R01_cb2160_c20/f1p0/3175
R01_cb12634_c0/f1p0/602	R01_cb12634_c0/f1p0/602	R01_cb12634_c0/f1p0/602	R01_cb12634_c0/f1p0/602	R01_cb12634_c0/f1p0/602
R01_cb12641_c15/f1p0/689	NA	R01_cb12641_c15/f1p0/689	R01_cb12641_c15/f1p0/689	R01_cb12641_c15/f1p0/689
R01_cb11547_c1/f1p0/1452	R01_cb11547_c1/f1p0/1452	R01_cb11547_c1/f1p0/1452	R01_cb11547_c1/f1p0/1452	R01_cb11547_c1/f1p0/1452
R01_cb15032_c1/f1p0/1100	NA	NA	NA	R01_cb15032_c1/f1p0/1100
R01_cb5720_c1/f1p0/3027	NA	NA	NA	R01_cb5720_c1/f1p0/3027
R01_cb2637_c10/f1p0/2610	NA	R01_cb2637_c10/f1p0/2610	NA	NA
R01_cb8564_c69701/f1p0/3032	R01_cb8564_c69701/f1p0/3032	R01_cb8564_c69701/f1p0/3032	R01_cb8564_c69701/f1p0/3032	R01_cb8564_c69701/f1p0/3032
R01_cb9606_c84/f1p0/1598	NA	NA	NA	R01_cb9606_c84/f1p0/1598
R01_cb8564_c86196/f1p0/2588	NA	NA	NA	R01_cb8564_c86196/f1p0/2588
R01_cb4744_c0/f2p0/2942	NA	R01_cb4744_c0/f2p0/2942	R01_cb4744_c0/f2p0/2942	R01_cb4744_c0/f2p0/2942
R01_cb6170_c23/f1p1/2670	NA	NA	NA	R01_cb6170_c23/f1p1/2670
R01_cb6501_c3/f1p1/2434	NA	R01_cb6501_c3/f1p1/2434	NA	NA
R01_cb18081_c0/f1p0/1114	NA	R01_cb18081_c0/f1p0/1114	NA	NA
R01_cb5190_c3/f1p0/3169	NA	NA	NA	R01_cb5190_c3/f1p0/3169
R01_cb2533_c10/f1p0/2206	NA	R01_cb2533_c10/f1p0/2206	NA	NA
R01_cb8564_c24026/f1p0/4553	NA	R01_cb8564_c24026/f1p0/4553	NA	NA
R01_cb2154_c12/f1p0/2249	NA	R01_cb2154_c12/f1p0/2249	NA	NA
R01_cb8564_c124040/f1p0/1994	NA	R01_cb8564_c124040/f1p0/1994	NA	R01_cb8564_c124040/f1p0/1994

R01_cb13726_c2/f1p0/834	NA	NA	NA	R01_cb13726_c2/f1p0/834
R01_cb15855_c1/f1p1/1233	NA	NA	NA	R01_cb15855_c1/f1p1/1233
R01_cb18456_c1825/f1p0/1371	NA	R01_cb18456_c1825/f1p0/1371	NA	NA
R01_cb4229_c3/f1p0/3383	NA	NA	NA	R01_cb4229_c3/f1p0/3383
R01_cb11109_c1/f1p0/2040	NA	NA	NA	R01_cb11109_c1/f1p0/2040
R01_cb9646_c1/f1p0/2024	NA	NA	NA	R01_cb9646_c1/f1p0/2024
R01_cb10230_c10/f7p0/530	NA	R01_cb10230_c10/f7p0/530	NA	NA
R01_cb12422_c7/f1p0/1802	NA	NA	NA	R01_cb12422_c7/f1p0/1802
R01_cb2902_c0/f1p0/3769	NA	R01_cb2902_c0/f1p0/3769	NA	NA
R01_cb6794_c33/f1p0/2230	NA	R01_cb6794_c33/f1p0/2230	NA	R01_cb6794_c33/f1p0/2230
R01_cb1437_c4/f1p0/3935	NA	NA	NA	R01_cb1437_c4/f1p0/3935
R01_cb15647_c1/f1p0/1131	NA	NA	NA	R01_cb15647_c1/f1p0/1131
R01_cb14956_c2/f7p0/768	NA	R01_cb14956_c2/f7p0/768	NA	R01_cb14956_c2/f7p0/768
R01_cb8564_c68819/f5p0/2092	NA	R01_cb8564_c68819/f5p0/2092	R01_cb8564_c68819/f5p0/2092	R01_cb8564_c68819/f5p0/2092
R01_cb5331_c4/f1p0/581	R01_cb5331_c4/f1p0/581	R01_cb5331_c4/f1p0/581	R01_cb5331_c4/f1p0/581	R01_cb5331_c4/f1p0/581
R01_cb16266_c0/f2p0/596	R01_cb16266_c0/f2p0/596	R01_cb16266_c0/f2p0/596	R01_cb16266_c0/f2p0/596	R01_cb16266_c0/f2p0/596
R01_cb7214_c12/f1p1/1806	NA	NA	NA	R01_cb7214_c12/f1p1/1806
R01_cb9987_c2/f1p0/2237	NA	R01_cb9987_c2/f1p0/2237	NA	R01_cb9987_c2/f1p0/2237
R01_cb18456_c4663/f1p2/481	R01_cb18456_c4663/f1p2/481	R01_cb18456_c4663/f1p2/481	NA	R01_cb18456_c4663/f1p2/481
R01_cb12785_c35/f1p0/980	NA	NA	R01_cb12785_c35/f1p0/980	NA
R01_cb18153_c2/f1p0/423	R01_cb18153_c2/f1p0/423	R01_cb18153_c2/f1p0/423	NA	R01_cb18153_c2/f1p0/423
R01_cb8564_c81876/f1p0/2906	NA	R01_cb8564_c81876/f1p0/2906	NA	NA
R01_cb226_c4/f1p0/2584	NA	NA	NA	R01_cb226_c4/f1p0/2584
R01_cb13025_c3/f2p1/1451	NA	NA	NA	R01_cb13025_c3/f2p1/1451
R01_cb7731_c4/f1p0/1810	R01_cb7731_c4/f1p0/1810	R01_cb7731_c4/f1p0/1810	NA	R01_cb7731_c4/f1p0/1810
R01_cb15710_c1/f1p0/472	NA	R01_cb15710_c1/f1p0/472	NA	R01_cb15710_c1/f1p0/472

R01_cb8991_c12/f1p0/1805	NA	NA	NA	R01_cb8991_c12/f1p0/1805
R01_cb12603_c4/f3p0/1706	NA	NA	R01_cb12603_c4/f3p0/1706	R01_cb12603_c4/f3p0/1706
R01_cb17023_c3/f1p0/1027	NA	NA	R01_cb17023_c3/f1p0/1027	R01_cb17023_c3/f1p0/1027
R01_cb9373_c2/f1p0/2856	R01_cb9373_c2/f1p0/2856	R01_cb9373_c2/f1p0/2856	NA	NA
R01_cb8564_c15674/f1p1/3939	NA	NA	R01_cb8564_c15674/f1p1/3939	NA
R01_cb18456_c3244/f1p2/984	NA	R01_cb18456_c3244/f1p2/984	NA	NA
R01_cb7214_c11/f1p0/1870	NA	NA	NA	R01_cb7214_c11/f1p0/1870
R01_cb17715_c1/f1p0/1776	NA	NA	NA	R01_cb17715_c1/f1p0/1776
R01_cb8564_c35935/f1p0/4149	NA	NA	NA	R01_cb8564_c35935/f1p0/4149
R01_cb10368_c3/f1p0/764	NA	R01_cb10368_c3/f1p0/764	R01_cb10368_c3/f1p0/764	R01_cb10368_c3/f1p0/764
R01_cb10398_c19/f1p0/607	NA	R01_cb10398_c19/f1p0/607	R01_cb10398_c19/f1p0/607	R01_cb10398_c19/f1p0/607
R01_cb15413_c1/f1p0/696	NA	R01_cb15413_c1/f1p0/696	NA	NA
R01_cb8564_c124906/f1p0/2560	NA	NA	NA	R01_cb8564_c124906/f1p0/2560
R01_cb3667_c10/f1p0/1178	NA	NA	NA	R01_cb3667_c10/f1p0/1178
R01_cb2851_c9/f1p0/2585	NA	NA	NA	R01_cb2851_c9/f1p0/2585
R01_cb9846_c2/f1p0/1844	NA	R01_cb9846_c2/f1p0/1844	R01_cb9846_c2/f1p0/1844	R01_cb9846_c2/f1p0/1844
R01_cb16341_c8/f1p0/526	R01_cb16341_c8/f1p0/526	R01_cb16341_c8/f1p0/526	R01_cb16341_c8/f1p0/526	R01_cb16341_c8/f1p0/526
R01_cb7367_c0/f7p1/2403	NA	NA	NA	R01_cb7367_c0/f7p1/2403
R01_cb8564_c81742/f1p0/3716	NA	NA	NA	R01_cb8564_c81742/f1p0/3716
R01_cb2991_c13/f1p0/2718	NA	NA	NA	R01_cb2991_c13/f1p0/2718
R01_cb4570_c3/f1p0/3808	NA	R01_cb4570_c3/f1p0/3808	NA	NA
R01_cb5468_c4/f1p0/3066	NA	R01_cb5468_c4/f1p0/3066	NA	NA
R01_cb14882_c0/f1p0/1874	NA	NA	NA	R01_cb14882_c0/f1p0/1874
R01_cb12361_c5/f1p0/1778	NA	NA	R01_cb12361_c5/f1p0/1778	R01_cb12361_c5/f1p0/1778
R01_cb3219_c1/f1p1/3698	NA	R01_cb3219_c1/f1p1/3698	NA	R01_cb3219_c1/f1p1/3698

R01_cb8564_c87681/f1p0/3343	NA	R01_cb8564_c87681/f1p0/3343	R01_cb8564_c87681/f1p0/3343	R01_cb8564_c87681/f1p0/3343
R01_cb14882_c6/f1p0/997	R01_cb14882_c6/f1p0/997	R01_cb14882_c6/f1p0/997	NA	NA
R01_cb7100_c0/f1p0/2696	R01_cb7100_c0/f1p0/2696	R01_cb7100_c0/f1p0/2696	R01_cb7100_c0/f1p0/2696	R01_cb7100_c0/f1p0/2696
R01_cb5669_c9/f1p0/1210	NA	R01_cb5669_c9/f1p0/1210	NA	NA
R01_cb14660_c10/f1p0/549	NA	R01_cb14660_c10/f1p0/549	R01_cb14660_c10/f1p0/549	R01_cb14660_c10/f1p0/549
R01_cb12003_c65/f1p0/1149	NA	R01_cb12003_c65/f1p0/1149	NA	NA
R01_cb15951_c4/f1p0/698	NA	R01_cb15951_c4/f1p0/698	R01_cb15951_c4/f1p0/698	R01_cb15951_c4/f1p0/698
R01_cb13545_c18/f1p0/776	R01_cb13545_c18/f1p0/776	R01_cb13545_c18/f1p0/776	NA	R01_cb13545_c18/f1p0/776
R01_cb8564_c72788/f1p0/2855	NA	R01_cb8564_c72788/f1p0/2855	NA	R01_cb8564_c72788/f1p0/2855
R01_cb8564_c34238/f4p1/4317	NA	NA	NA	R01_cb8564_c34238/f4p1/4317
R01_cb10307_c4/f1p0/2845	NA	R01_cb10307_c4/f1p0/2845	NA	NA
R01_cb8564_c113568/f1p0/2455	R01_cb8564_c113568/f1p0/2455	R01_cb8564_c113568/f1p0/2455	R01_cb8564_c113568/f1p0/2455	R01_cb8564_c113568/f1p0/2455
R01_cb16137_c2/f1p0/1718	NA	NA	NA	R01_cb16137_c2/f1p0/1718
R01_cb14156_c0/f1p0/545	R01_cb14156_c0/f1p0/545	R01_cb14156_c0/f1p0/545	R01_cb14156_c0/f1p0/545	R01_cb14156_c0/f1p0/545
R01_cb18456_c7559/f1p0/818	R01_cb18456_c7559/f1p0/818	R01_cb18456_c7559/f1p0/818	R01_cb18456_c7559/f1p0/818	R01_cb18456_c7559/f1p0/818
R01_cb8564_c24460/f1p0/4466	NA	R01_cb8564_c24460/f1p0/4466	NA	NA
R01_cb14758_c4/f1p0/575	NA	R01_cb14758_c4/f1p0/575	R01_cb14758_c4/f1p0/575	R01_cb14758_c4/f1p0/575
R01_cb8564_c110624/f1p0/2725	NA	R01_cb8564_c110624/f1p0/2725	R01_cb8564_c110624/f1p0/2725	R01_cb8564_c110624/f1p0/2725
R01_cb16728_c0/f2p0/1120	NA	NA	NA	R01_cb16728_c0/f2p0/1120
R01_cb18506_c1/f1p0/724	NA	R01_cb18506_c1/f1p0/724	R01_cb18506_c1/f1p0/724	R01_cb18506_c1/f1p0/724
R01_cb14080_c6/f1p0/1818	NA	R01_cb14080_c6/f1p0/1818	NA	NA
R01_cb1510_c0/f1p0/4164	NA	NA	NA	R01_cb1510_c0/f1p0/4164
R01_cb8564_c91083/f1p0/2192	NA	R01_cb8564_c91083/f1p0/2192	NA	R01_cb8564_c91083/f1p0/2192
R01_cb9720_c6/f1p0/1821	NA	NA	NA	R01_cb9720_c6/f1p0/1821

R01_cb10609_c3/f1p0/695	NA	R01_cb10609_c3/f1p0/695	NA	NA
R01_cb7806_c6/f1p0/1731	NA	R01_cb7806_c6/f1p0/1731	R01_cb7806_c6/f1p0/1731	R01_cb7806_c6/f1p0/1731
R01_cb8564_c14987/f1p0/2611	NA	NA	NA	R01_cb8564_c14987/f1p0/2611
R01_cb17956_c8/f1p0/1065	NA	R01_cb17956_c8/f1p0/1065	NA	NA
R01_cb12504_c0/f2p0/938	NA	NA	NA	R01_cb12504_c0/f2p0/938
R01_cb14235_c2/f1p0/478	R01_cb14235_c2/f1p0/478	R01_cb14235_c2/f1p0/478	NA	R01_cb14235_c2/f1p0/478
R01_cb5533_c138/f1p0/2944	NA	R01_cb5533_c138/f1p0/2944	NA	R01_cb5533_c138/f1p0/2944
R01_cb10743_c0/f1p0/792	NA	R01_cb10743_c0/f1p0/792	R01_cb10743_c0/f1p0/792	NA
R01_cb545_c4/f1p0/2126	NA	R01_cb545_c4/f1p0/2126	NA	NA
R01_cb1779_c1/f1p0/4179	NA	NA	NA	R01_cb1779_c1/f1p0/4179
R01_cb17744_c32/f1p0/1223	NA	NA	NA	R01_cb17744_c32/f1p0/1223
R01_cb6153_c4/f1p0/3039	NA	NA	NA	R01_cb6153_c4/f1p0/3039
R01_cb8564_c4695/f1p0/2526	R01_cb8564_c4695/f1p0/2526	R01_cb8564_c4695/f1p0/2526	NA	NA
R01_cb14675_c2/f3p1/856	NA	R01_cb14675_c2/f3p1/856	R01_cb14675_c2/f3p1/856	R01_cb14675_c2/f3p1/856
R01_cb17400_c0/f1p0/725	NA	R01_cb17400_c0/f1p0/725	R01_cb17400_c0/f1p0/725	NA
R01_cb8564_c91308/f1p0/3701	NA	R01_cb8564_c91308/f1p0/3701	NA	NA
R01_cb12321_c644/f1p0/708	R01_cb12321_c644/f1p0/708	R01_cb12321_c644/f1p0/708	R01_cb12321_c644/f1p0/708	R01_cb12321_c644/f1p0/708
R01_cb8564_c25012/f1p1/2631	NA	NA	NA	R01_cb8564_c25012/f1p1/2631
R01_cb6090_c10/f1p0/2595	NA	NA	NA	R01_cb6090_c10/f1p0/2595
R01_cb9353_c8/f1p0/557	R01_cb9353_c8/f1p0/557	R01_cb9353_c8/f1p0/557	NA	NA
R01_cb18456_c7103/f1p1/1164	NA	R01_cb18456_c7103/f1p1/1164	R01_cb18456_c7103/f1p1/1164	R01_cb18456_c7103/f1p1/1164
R01_cb14721_c1/f1p0/395	R01_cb14721_c1/f1p0/395	R01_cb14721_c1/f1p0/395	R01_cb14721_c1/f1p0/395	R01_cb14721_c1/f1p0/395
R01_cb2293_c23/f1p0/354	R01_cb2293_c23/f1p0/354	R01_cb2293_c23/f1p0/354	R01_cb2293_c23/f1p0/354	R01_cb2293_c23/f1p0/354
R01_cb7032_c1/f1p0/1901	NA	R01_cb7032_c1/f1p0/1901	NA	NA
R01_cb4637_c3/f1p0/3297	R01_cb4637_c3/f1p0/3297	NA	NA	NA
R01_cb18467_c0/f2p0/1134	NA	R01_cb18467_c0/f2p0/1134	NA	R01_cb18467_c0/f2p0/1134

R01_cb1224_c13/f1p1/3758	NA	NA	NA	R01_cb1224_c13/f1p1/3758
R01_cb8564_c77903/f1p1/4534	NA	R01_cb8564_c77903/f1p1/4534	NA	NA
R01_cb357_c1/f1p2/4848	NA	NA	NA	R01_cb357_c1/f1p2/4848
R01_cb18456_c2499/f1p0/1363	NA	R01_cb18456_c2499/f1p0/1363	NA	R01_cb18456_c2499/f1p0/1363
R01_cb1993_c1/f2p0/3051	NA	NA	NA	R01_cb1993_c1/f2p0/3051
R01_cb13910_c5/f1p0/1513	NA	R01_cb13910_c5/f1p0/1513	R01_cb13910_c5/f1p0/1513	R01_cb13910_c5/f1p0/1513
R01_cb5893_c2/f1p0/2002	NA	R01_cb5893_c2/f1p0/2002	R01_cb5893_c2/f1p0/2002	R01_cb5893_c2/f1p0/2002
R01_cb8216_c2/f1p0/2427	NA	R01_cb8216_c2/f1p0/2427	NA	NA
R01_cb4208_c7/f1p0/2028	NA	R01_cb4208_c7/f1p0/2028	NA	NA
R01_cb18456_c3392/f1p6/758	NA	R01_cb18456_c3392/f1p6/758	NA	NA
R01_cb8564_c151357/f1p3/2495	NA	R01_cb8564_c151357/f1p3/2495	NA	NA
R01_cb10457_c4/f1p0/1087	R01_cb10457_c4/f1p0/1087	R01_cb10457_c4/f1p0/1087	R01_cb10457_c4/f1p0/1087	R01_cb10457_c4/f1p0/1087
R01_cb16125_c4/f1p0/1477	NA	R01_cb16125_c4/f1p0/1477	NA	NA
R01_cb8564_c75384/f1p1/3644	NA	NA	NA	R01_cb8564_c75384/f1p1/3644
R01_cb763_c3/f1p0/3898	NA	R01_cb763_c3/f1p0/3898	R01_cb763_c3/f1p0/3898	R01_cb763_c3/f1p0/3898
R01_cb18334_c1/f1p0/1740	NA	NA	NA	R01_cb18334_c1/f1p0/1740
R01_cb15928_c0/f4p0/873	NA	NA	NA	R01_cb15928_c0/f4p0/873
R01_cb9351_c2/f1p0/1990	NA	R01_cb9351_c2/f1p0/1990	R01_cb9351_c2/f1p0/1990	NA
R01_cb8564_c12541/f1p0/3791	R01_cb8564_c12541/f1p0/3791	R01_cb8564_c12541/f1p0/3791	R01_cb8564_c12541/f1p0/3791	R01_cb8564_c12541/f1p0/3791
R01_cb16867_c2/f1p0/1415	NA	NA	NA	R01_cb16867_c2/f1p0/1415
R01_cb11530_c3/f1p0/938	NA	R01_cb11530_c3/f1p0/938	NA	NA
R01_cb8564_c3057/f1p0/2651	NA	R01_cb8564_c3057/f1p0/2651	R01_cb8564_c3057/f1p0/2651	R01_cb8564_c3057/f1p0/2651
R01_cb17861_c1/f1p1/1418	NA	NA	NA	R01_cb17861_c1/f1p1/1418
R01_cb13696_c0/f2p0/1445	NA	NA	NA	R01_cb13696_c0/f2p0/1445

R01_cb124_c47/f1p0/3771	NA	NA	NA	R01_cb124_c47/f1p0/3771
R01_cb14334_c6/f3p0/859	NA	NA	NA	R01_cb14334_c6/f3p0/859
R01_cb8564_c17427/f1p0/2705	NA	NA	NA	R01_cb8564_c17427/f1p0/2705
R01_cb2843_c12/f1p0/2967	NA	NA	NA	R01_cb2843_c12/f1p0/2967
R01_cb18669_c1/f1p0/1087	R01_cb18669_c1/f1p0/1087	R01_cb18669_c1/f1p0/1087	R01_cb18669_c1/f1p0/1087	R01_cb18669_c1/f1p0/1087
R01_cb13778_c0/f1p0/1159	NA	NA	R01_cb13778_c0/f1p0/1159	NA
R01_cb17231_c2/f1p0/743	NA	NA	NA	R01_cb17231_c2/f1p0/743
R01_cb8564_c3270/f1p0/4948	NA	NA	NA	R01_cb8564_c3270/f1p0/4948
R01_cb18692_c0/f1p0/5018	NA	R01_cb18692_c0/f1p0/5018	NA	NA
R01_cb10819_c2/f1p0/1448	NA	R01_cb10819_c2/f1p0/1448	NA	R01_cb10819_c2/f1p0/1448
R01_cb7740_c0/f1p0/2542	NA	R01_cb7740_c0/f1p0/2542	R01_cb7740_c0/f1p0/2542	R01_cb7740_c0/f1p0/2542
R01_cb11698_c1/f1p0/3856	NA	NA	NA	R01_cb11698_c1/f1p0/3856
R01_cb17418_c0/f2p0/1610	NA	NA	NA	R01_cb17418_c0/f2p0/1610
R01_cb3610_c1/f1p0/2101	NA	R01_cb3610_c1/f1p0/2101	NA	NA
R01_cb14620_c0/f2p0/1771	NA	NA	R01_cb14620_c0/f2p0/1771	R01_cb14620_c0/f2p0/1771
R01_cb12636_c0/f8p0/1482	NA	NA	NA	R01_cb12636_c0/f8p0/1482
R01_cb3310_c9/f1p0/679	R01_cb3310_c9/f1p0/679	R01_cb3310_c9/f1p0/679	R01_cb3310_c9/f1p0/679	R01_cb3310_c9/f1p0/679
R01_cb5900_c47/f1p0/1949	NA	R01_cb5900_c47/f1p0/1949	NA	R01_cb5900_c47/f1p0/1949
R01_cb3797_c3/f1p0/3023	NA	NA	NA	R01_cb3797_c3/f1p0/3023
R01_cb12174_c1/f1p0/615	NA	R01_cb12174_c1/f1p0/615	NA	R01_cb12174_c1/f1p0/615
R01_cb17037_c2/f1p0/1366	R01_cb17037_c2/f1p0/1366	R01_cb17037_c2/f1p0/1366	NA	NA
R01_cb15753_c0/f1p0/1582	NA	R01_cb15753_c0/f1p0/1582	NA	NA
R01_cb14942_c2/f1p0/860	NA	NA	NA	R01_cb14942_c2/f1p0/860
R01_cb9402_c1/f1p0/2289	R01_cb9402_c1/f1p0/2289	R01_cb9402_c1/f1p0/2289	R01_cb9402_c1/f1p0/2289	R01_cb9402_c1/f1p0/2289
R01_cb119_c32/f1p0/2471	NA	NA	NA	R01_cb119_c32/f1p0/2471
R01_cb10517_c2/f1p0/434	NA	R01_cb10517_c2/f1p0/434	NA	NA

R01_cb4846_c11/f1p0/1842	NA	R01_cb4846_c11/f1p0/1842	NA	NA
R01_cb13260_c2/f1p0/730	NA	NA	NA	R01_cb13260_c2/f1p0/730
R01_cb8564_c70908/f1p0/2481	NA	NA	NA	R01_cb8564_c70908/f1p0/2481
R01_cb8564_c50883/f1p0/3589	R01_cb8564_c50883/f1p0/3589	R01_cb8564_c50883/f1p0/3589	NA	R01_cb8564_c50883/f1p0/3589
R01_cb5647_c3/f1p0/3223	NA	NA	NA	R01_cb5647_c3/f1p0/3223
R01_cb8564_c84119/f1p0/4188	NA	NA	NA	R01_cb8564_c84119/f1p0/4188
R01_cb18456_c7352/f1p0/586	R01_cb18456_c7352/f1p0/586	R01_cb18456_c7352/f1p0/586	R01_cb18456_c7352/f1p0/586	R01_cb18456_c7352/f1p0/586
R01_cb8564_c82142/f1p0/3467	R01_cb8564_c82142/f1p0/3467	R01_cb8564_c82142/f1p0/3467	NA	NA
R01_cb8564_c91504/f1p0/2024	R01_cb8564_c91504/f1p0/2024	R01_cb8564_c91504/f1p0/2024	R01_cb8564_c91504/f1p0/2024	R01_cb8564_c91504/f1p0/2024
R01_cb7608_c1/f1p0/2572	NA	R01_cb7608_c1/f1p0/2572	NA	NA
R01_cb12782_c3/f1p0/908	NA	NA	NA	R01_cb12782_c3/f1p0/908
R01_cb17195_c1/f1p0/519	NA	R01_cb17195_c1/f1p0/519	NA	NA
R01_cb3997_c3/f1p0/2106	NA	NA	NA	R01_cb3997_c3/f1p0/2106
R01_cb3180_c2/f1p0/3347	NA	NA	NA	R01_cb3180_c2/f1p0/3347
R01_cb5705_c1/f1p0/3027	NA	R01_cb5705_c1/f1p0/3027	R01_cb5705_c1/f1p0/3027	NA
R01_cb18034_c1/f1p0/1550	NA	R01_cb18034_c1/f1p0/1550	NA	R01_cb18034_c1/f1p0/1550
R01_cb18409_c117/f1p0/649	R01_cb18409_c117/f1p0/649	R01_cb18409_c117/f1p0/649	R01_cb18409_c117/f1p0/649	R01_cb18409_c117/f1p0/649
R01_cb15630_c2/f1p0/1683	NA	R01_cb15630_c2/f1p0/1683	NA	NA
R01_cb18161_c0/f1p0/511	R01_cb18161_c0/f1p0/511	R01_cb18161_c0/f1p0/511	R01_cb18161_c0/f1p0/511	R01_cb18161_c0/f1p0/511
R01_cb13731_c9/f1p0/759	NA	R01_cb13731_c9/f1p0/759	NA	NA
R01_cb1331_c43/f1p4/1872	NA	R01_cb1331_c43/f1p4/1872	NA	NA
R01_cb10779_c0/f2p0/625	R01_cb10779_c0/f2p0/625	R01_cb10779_c0/f2p0/625	NA	NA
R01_cb18748_c1/f1p0/1451	R01_cb18748_c1/f1p0/1451	R01_cb18748_c1/f1p0/1451	R01_cb18748_c1/f1p0/1451	R01_cb18748_c1/f1p0/1451

R01_cb8564_c129959/f1p0/3247	NA	R01_cb8564_c129959/f1p0/3247	NA	NA
R01_cb13093_c7/f1p0/583	NA	R01_cb13093_c7/f1p0/583	NA	NA
R01_cb16153_c8/f1p0/677	NA	NA	R01_cb16153_c8/f1p0/677	R01_cb16153_c8/f1p0/677
R01_cb10884_c1/f1p0/3843	NA	R01_cb10884_c1/f1p0/3843	NA	NA
R01_cb18456_c5290/f1p0/886	NA	R01_cb18456_c5290/f1p0/886	NA	R01_cb18456_c5290/f1p0/886
R01_cb15844_c2/f1p0/842	R01_cb15844_c2/f1p0/842	R01_cb15844_c2/f1p0/842	R01_cb15844_c2/f1p0/842	R01_cb15844_c2/f1p0/842
R01_cb16303_c8/f1p0/2780	NA	R01_cb16303_c8/f1p0/2780	NA	NA
R01_cb9856_c1/f1p0/1949	NA	R01_cb9856_c1/f1p0/1949	NA	NA
R01_cb16981_c14/f1p1/692	NA	NA	NA	R01_cb16981_c14/f1p1/692
R01_cb3026_c8/f1p0/1664	NA	R01_cb3026_c8/f1p0/1664	R01_cb3026_c8/f1p0/1664	R01_cb3026_c8/f1p0/1664
R01_cb14307_c12/f1p0/308	R01_cb14307_c12/f1p0/308	R01_cb14307_c12/f1p0/308	R01_cb14307_c12/f1p0/308	R01_cb14307_c12/f1p0/308
R01_cb8564_c14540/f1p0/3382	R01_cb8564_c14540/f1p0/3382	R01_cb8564_c14540/f1p0/3382	R01_cb8564_c14540/f1p0/3382	NA
R01_cb6758_c6/f1p0/2435	NA	NA	NA	R01_cb6758_c6/f1p0/2435
R01_cb699_c4/f1p0/1340	NA	NA	NA	R01_cb699_c4/f1p0/1340
R01_cb16673_c8/f1p0/394	R01_cb16673_c8/f1p0/394	R01_cb16673_c8/f1p0/394	R01_cb16673_c8/f1p0/394	R01_cb16673_c8/f1p0/394
R01_cb10272_c10/f1p0/2065	NA	NA	NA	R01_cb10272_c10/f1p0/2065
R01_cb15580_c5/f1p0/1731	NA	R01_cb15580_c5/f1p0/1731	NA	NA
R01_cb8564_c42401/f1p0/3662	NA	R01_cb8564_c42401/f1p0/3662	NA	NA
R01_cb4453_c1/f1p0/2779	NA	NA	NA	R01_cb4453_c1/f1p0/2779
R01_cb16732_c2/f1p0/563	NA	NA	NA	R01_cb16732_c2/f1p0/563
R01_cb10155_c6/f1p0/521	NA	NA	NA	R01_cb10155_c6/f1p0/521
R01_cb1849_c12/f1p0/2290	NA	NA	NA	R01_cb1849_c12/f1p0/2290
R01_cb8564_c37851/f1p0/2980	NA	R01_cb8564_c37851/f1p0/2980	R01_cb8564_c37851/f1p0/2980	NA
R01_cb2875_c16/f1p0/3132	NA	NA	NA	R01_cb2875_c16/f1p0/3132

R01_cb9222_c21/f1p0/2318	NA	R01_cb9222_c21/f1p0/2318	R01_cb9222_c21/f1p0/2318	NA
R01_cb10962_c1/f1p0/1058	NA	R01_cb10962_c1/f1p0/1058	NA	NA
R01_cb8564_c129693/f1p0/2912	NA	R01_cb8564_c129693/f1p0/2912	NA	R01_cb8564_c129693/f1p0/2912
R01_cb3320_c4/f1p1/3654	NA	R01_cb3320_c4/f1p1/3654	NA	NA
R01_cb6658_c7/f1p1/1192	NA	NA	NA	R01_cb6658_c7/f1p1/1192
R01_cb18456_c6264/f1p0/628	NA	R01_cb18456_c6264/f1p0/628	NA	NA
R01_cb454_c9/f1p0/2795	NA	NA	NA	R01_cb454_c9/f1p0/2795
R01_cb6152_c1/f2p0/1940	NA	R01_cb6152_c1/f2p0/1940	NA	NA
R01_cb2788_c4/f1p0/3827	NA	NA	NA	R01_cb2788_c4/f1p0/3827
R01_cb7303_c14/f1p0/2482	NA	NA	NA	R01_cb7303_c14/f1p0/2482
R01_cb454_c8/f1p0/2277	NA	NA	NA	R01_cb454_c8/f1p0/2277
R01_cb16426_c0/f2p0/656	NA	R01_cb16426_c0/f2p0/656	NA	NA
R01_cb8564_c4055/f1p0/3722	R01_cb8564_c4055/f1p0/3722	R01_cb8564_c4055/f1p0/3722	NA	NA
R01_cb8564_c69349/f2p0/2368	NA	NA	R01_cb8564_c69349/f2p0/2368	R01_cb8564_c69349/f2p0/2368
R01_cb1666_c3/f1p1/4226	NA	NA	NA	R01_cb1666_c3/f1p1/4226
R01_cb4830_c21/f1p0/2564	NA	R01_cb4830_c21/f1p0/2564	NA	NA
R01_cb16638_c5/f1p0/529	NA	R01_cb16638_c5/f1p0/529	NA	NA
R01_cb2884_c5/f1p0/3541	NA	NA	NA	R01_cb2884_c5/f1p0/3541
R01_cb3307_c25/f1p0/2537	NA	NA	NA	R01_cb3307_c25/f1p0/2537
R01_cb10192_c1/f3p0/1351	NA	NA	NA	R01_cb10192_c1/f3p0/1351
R01_cb5197_c6/f1p0/3323	NA	R01_cb5197_c6/f1p0/3323	NA	NA
R01_cb3307_c27/f1p0/3461	NA	NA	NA	R01_cb3307_c27/f1p0/3461
R01_cb13249_c0/f8p1/1086	NA	NA	NA	R01_cb13249_c0/f8p1/1086
R01_cb8957_c4/f1p0/1084	NA	NA	NA	R01_cb8957_c4/f1p0/1084
R01_cb9260_c1/f1p0/2138	NA	NA	NA	R01_cb9260_c1/f1p0/2138

R01_cb8564_c71657/f1p0/2664	NA	NA	NA	R01_cb8564_c71657/f1p0/2664
R01_cb13626_c0/f1p0/477	NA	R01_cb13626_c0/f1p0/477	NA	R01_cb13626_c0/f1p0/477
R01_cb13161_c19/f1p0/534	R01_cb13161_c19/f1p0/534	R01_cb13161_c19/f1p0/534	NA	R01_cb13161_c19/f1p0/534
R01_cb8564_c84711/f1p0/2095	NA	NA	R01_cb8564_c84711/f1p0/2095	R01_cb8564_c84711/f1p0/2095
R01_cb10116_c1/f1p0/1916	NA	R01_cb10116_c1/f1p0/1916	NA	NA
R01_cb8564_c119258/f1p0/2481	NA	NA	NA	R01_cb8564_c119258/f1p0/2481
R01_cb11017_c0/f1p0/1566	NA	R01_cb11017_c0/f1p0/1566	NA	R01_cb11017_c0/f1p0/1566
R01_cb15009_c0/f2p0/776	NA	R01_cb15009_c0/f2p0/776	NA	NA
R01_cb12480_c11/f1p0/535	R01_cb12480_c11/f1p0/535	R01_cb12480_c11/f1p0/535	R01_cb12480_c11/f1p0/535	NA
R01_cb8564_c14387/f1p0/2648	NA	R01_cb8564_c14387/f1p0/2648	R01_cb8564_c14387/f1p0/2648	R01_cb8564_c14387/f1p0/2648
R01_cb8564_c90942/f1p0/3161	R01_cb8564_c90942/f1p0/3161	R01_cb8564_c90942/f1p0/3161	R01_cb8564_c90942/f1p0/3161	R01_cb8564_c90942/f1p0/3161
R01_cb8796_c5/f1p0/1571	NA	R01_cb8796_c5/f1p0/1571	NA	NA
R01_cb8564_c34749/f1p0/4828	NA	NA	NA	R01_cb8564_c34749/f1p0/4828
R01_cb9769_c2/f1p0/458	NA	R01_cb9769_c2/f1p0/458	NA	R01_cb9769_c2/f1p0/458
R01_cb11520_c0/f1p0/1560	NA	R01_cb11520_c0/f1p0/1560	NA	NA
R01_cb17973_c42/f1p0/551	NA	R01_cb17973_c42/f1p0/551	R01_cb17973_c42/f1p0/551	NA
R01_cb7502_c5/f1p1/2335	NA	NA	NA	R01_cb7502_c5/f1p1/2335
R01_cb15640_c5/f1p0/829	NA	R01_cb15640_c5/f1p0/829	NA	NA
R01_cb5896_c152/f1p0/1996	R01_cb5896_c152/f1p0/1996	R01_cb5896_c152/f1p0/1996	R01_cb5896_c152/f1p0/1996	R01_cb5896_c152/f1p0/1996
R01_cb1982_c9/f1p0/637	R01_cb1982_c9/f1p0/637	R01_cb1982_c9/f1p0/637	R01_cb1982_c9/f1p0/637	R01_cb1982_c9/f1p0/637
R01_cb8564_c70535/f1p0/3076	NA	R01_cb8564_c70535/f1p0/3076	NA	NA
R01_cb3131_c2/f1p0/2835	NA	R01_cb3131_c2/f1p0/2835	NA	NA
R01_cb3120_c48/f1p1/2486	NA	R01_cb3120_c48/f1p1/2486	NA	NA
R01_cb6631_c35/f13p1/2152	NA	NA	NA	R01_cb6631_c35/f13p1/2152

R01_cb8564_c71532/f1p0/3010	NA	R01_cb8564_c71532/f1p0/3010	NA	NA
R01_cb13260_c0/f4p0/857	NA	NA	NA	R01_cb13260_c0/f4p0/857
R01_cb8564_c81333/f1p0/2975	NA	NA	NA	R01_cb8564_c81333/f1p0/2975
R01_cb1264_c4/f1p0/4092	NA	NA	NA	R01_cb1264_c4/f1p0/4092
R01_cb9472_c2/f1p0/1675	NA	NA	NA	R01_cb9472_c2/f1p0/1675
R01_cb18051_c5/f1p0/1040	R01_cb18051_c5/f1p0/1040	R01_cb18051_c5/f1p0/1040	R01_cb18051_c5/f1p0/1040	R01_cb18051_c5/f1p0/1040
R01_cb625_c0/f3p1/3209	NA	NA	NA	R01_cb625_c0/f3p1/3209
R01_cb17765_c1/f1p0/1649	NA	R01_cb17765_c1/f1p0/1649	R01_cb17765_c1/f1p0/1649	R01_cb17765_c1/f1p0/1649
R01_cb15936_c0/f1p0/777	R01_cb15936_c0/f1p0/777	NA	R01_cb15936_c0/f1p0/777	R01_cb15936_c0/f1p0/777
R01_cb9759_c3/f1p1/1890	NA	NA	NA	R01_cb9759_c3/f1p1/1890
R01_cb18456_c6448/f1p0/707	R01_cb18456_c6448/f1p0/707	NA	NA	NA
R01_cb3472_c4/f1p0/1815	NA	R01_cb3472_c4/f1p0/1815	NA	NA
R01_cb18059_c0/f1p0/1045	R01_cb18059_c0/f1p0/1045	R01_cb18059_c0/f1p0/1045	R01_cb18059_c0/f1p0/1045	R01_cb18059_c0/f1p0/1045
R01_cb635_c11/f1p0/3042	NA	R01_cb635_c11/f1p0/3042	NA	R01_cb635_c11/f1p0/3042
R01_cb17644_c1/f1p0/372	NA	NA	NA	R01_cb17644_c1/f1p0/372
R01_cb8222_c1/f1p0/550	R01_cb8222_c1/f1p0/550	R01_cb8222_c1/f1p0/550	R01_cb8222_c1/f1p0/550	R01_cb8222_c1/f1p0/550
R01_cb12837_c17/f1p7/648	NA	R01_cb12837_c17/f1p7/648	NA	NA
R01_cb9189_c2/f2p0/2107	NA	R01_cb9189_c2/f2p0/2107	NA	NA
R01_cb2357_c1/f3p1/3937	NA	NA	NA	R01_cb2357_c1/f3p1/3937
R01_cb18456_c5442/f1p0/379	R01_cb18456_c5442/f1p0/379	R01_cb18456_c5442/f1p0/379	R01_cb18456_c5442/f1p0/379	R01_cb18456_c5442/f1p0/379
R01_cb8564_c41306/f1p0/3967	NA	R01_cb8564_c41306/f1p0/3967	R01_cb8564_c41306/f1p0/3967	R01_cb8564_c41306/f1p0/3967
R01_cb8825_c3/f1p0/2143	NA	R01_cb8825_c3/f1p0/2143	NA	NA
R01_cb8564_c78049/f1p0/2173	R01_cb8564_c78049/f1p0/2173	R01_cb8564_c78049/f1p0/2173	R01_cb8564_c78049/f1p0/2173	R01_cb8564_c78049/f1p0/2173
R01_cb8564_c74917/f1p1/2870	NA	R01_cb8564_c74917/f1p1/2870	NA	R01_cb8564_c74917/f1p1/2870
R01_cb8564_c91256/f1p0/3296	R01_cb8564_c91256/f1p0/329	R01_cb8564_c91256/f1p0/3296	R01_cb8564_c91256/f1p0/3296	R01_cb8564_c91256/f1p0/3296

	6			
R01_cb12314_c9/f4p1/532	NA	NA	R01_cb12314_c9/f4p1/532	NA
R01_cb4342_c2/f1p0/3243	NA	R01_cb4342_c2/f1p0/3243	NA	NA
R01_cb15457_c7/f1p0/1214	NA	R01_cb15457_c7/f1p0/1214	NA	R01_cb15457_c7/f1p0/1214
R01_cb5151_c0/f1p0/3168	NA	NA	NA	R01_cb5151_c0/f1p0/3168
R01_cb1138_c5/f5p3/860	R01_cb1138_c5/f5p3/860	NA	NA	R01_cb1138_c5/f5p3/860
R01_cb6990_c4/f1p0/2354	R01_cb6990_c4/f1p0/2354	R01_cb6990_c4/f1p0/2354	NA	NA
R01_cb4469_c2/f1p0/3340	NA	NA	NA	R01_cb4469_c2/f1p0/3340
R01_cb9974_c3/f1p0/1393	NA	R01_cb9974_c3/f1p0/1393	NA	R01_cb9974_c3/f1p0/1393
R01_cb8564_c111723/f1p0/2006	NA	R01_cb8564_c111723/f1p0/2006	R01_cb8564_c111723/f1p0/2006	R01_cb8564_c111723/f1p0/2006
R01_cb14153_c3/f1p0/1306	R01_cb14153_c3/f1p0/1306	R01_cb14153_c3/f1p0/1306	NA	NA
R01_cb16486_c1/f2p0/1075	NA	R01_cb16486_c1/f2p0/1075	R01_cb16486_c1/f2p0/1075	R01_cb16486_c1/f2p0/1075
R01_cb416_c25/f1p0/3907	NA	NA	NA	R01_cb416_c25/f1p0/3907
R01_cb10051_c3/f1p0/946	NA	NA	NA	R01_cb10051_c3/f1p0/946
R01_cb8564_c14346/f1p4/3686	NA	R01_cb8564_c14346/f1p4/3686	R01_cb8564_c14346/f1p4/3686	R01_cb8564_c14346/f1p4/3686
R01_cb1905_c14/f1p0/2728	NA	R01_cb1905_c14/f1p0/2728	R01_cb1905_c14/f1p0/2728	NA
R01_cb2987_c1/f1p0/3748	NA	R01_cb2987_c1/f1p0/3748	NA	NA
R01_cb16921_c3/f1p0/1790	NA	R01_cb16921_c3/f1p0/1790	R01_cb16921_c3/f1p0/1790	R01_cb16921_c3/f1p0/1790
R01_cb13583_c8/f1p0/1422	NA	NA	NA	R01_cb13583_c8/f1p0/1422
R01_cb4765_c3/f1p0/3221	R01_cb4765_c3/f1p0/3221	R01_cb4765_c3/f1p0/3221	R01_cb4765_c3/f1p0/3221	R01_cb4765_c3/f1p0/3221
R01_cb4340_c1/f1p0/3432	NA	NA	R01_cb4340_c1/f1p0/3432	R01_cb4340_c1/f1p0/3432
R01_cb8564_c121914/f1p0/2671	NA	R01_cb8564_c121914/f1p0/2671	NA	NA
R01_cb12827_c4/f1p1/1489	NA	NA	NA	R01_cb12827_c4/f1p1/1489
R01_cb13315_c0/f2p0/904	NA	R01_cb13315_c0/f2p0/904	NA	NA

R01_cb13808_c1/f1p0/743	NA	R01_cb13808_c1/f1p0/743	NA	R01_cb13808_c1/f1p0/743
R01_cb191_c13/f1p0/525	NA	R01_cb191_c13/f1p0/525	R01_cb191_c13/f1p0/525	R01_cb191_c13/f1p0/525
R01_cb10920_c11/f1p0/1457	NA	R01_cb10920_c11/f1p0/1457	NA	NA
R01_cb15995_c1/f2p0/760	NA	NA	NA	R01_cb15995_c1/f2p0/760
R01_cb5304_c3/f1p0/2687	NA	NA	NA	R01_cb5304_c3/f1p0/2687
R01_cb8564_c25142/f1p1/4429	NA	R01_cb8564_c25142/f1p1/4429	R01_cb8564_c25142/f1p1/4429	R01_cb8564_c25142/f1p1/4429
R01_cb5513_c1/f1p0/3071	NA	NA	NA	R01_cb5513_c1/f1p0/3071
R01_cb11869_c7/f1p0/580	NA	R01_cb11869_c7/f1p0/580	NA	NA
R01_cb8564_c78882/f1p0/2391	NA	NA	NA	R01_cb8564_c78882/f1p0/2391
R01_cb8564_c78578/f1p0/3318	NA	NA	NA	R01_cb8564_c78578/f1p0/3318
R01_cb8159_c1/f1p0/2391	NA	R01_cb8159_c1/f1p0/2391	NA	NA
R01_cb3516_c3/f1p0/2828	NA	R01_cb3516_c3/f1p0/2828	NA	R01_cb3516_c3/f1p0/2828
R01_cb8564_c39130/f3p0/2526	NA	R01_cb8564_c39130/f3p0/2526	NA	R01_cb8564_c39130/f3p0/2526
R01_cb8564_c22901/f1p0/3801	NA	R01_cb8564_c22901/f1p0/3801	NA	NA
R01_cb763_c8/f1p0/1321	NA	R01_cb763_c8/f1p0/1321	R01_cb763_c8/f1p0/1321	R01_cb763_c8/f1p0/1321
R01_cb3464_c8/f1p0/1553	NA	NA	NA	R01_cb3464_c8/f1p0/1553
R01_cb8564_c38512/f1p0/3619	NA	NA	R01_cb8564_c38512/f1p0/3619	R01_cb8564_c38512/f1p0/3619
R01_cb288_c11/f1p0/1164	NA	NA	NA	R01_cb288_c11/f1p0/1164
R01_cb5608_c48/f1p0/2689	NA	R01_cb5608_c48/f1p0/2689	R01_cb5608_c48/f1p0/2689	R01_cb5608_c48/f1p0/2689
R01_cb8564_c4631/f1p0/3294	NA	R01_cb8564_c4631/f1p0/3294	R01_cb8564_c4631/f1p0/3294	R01_cb8564_c4631/f1p0/3294
R01_cb10793_c1/f1p0/2837	R01_cb10793_c1/f1p0/2837	R01_cb10793_c1/f1p0/2837	NA	NA
R01_cb8564_c3038/f1p0/2664	NA	R01_cb8564_c3038/f1p0/2664	NA	NA
R01_cb9252_c4/f1p0/682	R01_cb9252_c4/f1p0/682	R01_cb9252_c4/f1p0/682	R01_cb9252_c4/f1p0/682	R01_cb9252_c4/f1p0/682
R01_cb8564_c117283/f1p0/2941	NA	NA	R01_cb8564_c117283/f1p0/2941	NA
R01_cb17659_c1/f1p1/1464	NA	NA	NA	R01_cb17659_c1/f1p1/1464

R01_cb8276_c6/f1p1/3242	NA	R01_cb8276_c6/f1p1/3242	NA	NA
R01_cb13771_c7/f1p0/1009	NA	R01_cb13771_c7/f1p0/1009	R01_cb13771_c7/f1p0/1009	R01_cb13771_c7/f1p0/1009
R01_cb4536_c5/f1p0/1031	NA	NA	NA	R01_cb4536_c5/f1p0/1031
R01_cb14261_c0/f1p0/662	R01_cb14261_c0/f1p0/662	R01_cb14261_c0/f1p0/662	R01_cb14261_c0/f1p0/662	R01_cb14261_c0/f1p0/662
R01_cb11408_c2/f1p1/2605	NA	R01_cb11408_c2/f1p1/2605	NA	NA
R01_cb2445_c3/f3p0/3409	NA	NA	NA	R01_cb2445_c3/f3p0/3409
R01_cb8564_c116509/f1p0/3009	NA	NA	NA	R01_cb8564_c116509/f1p0/3009
R01_cb8564_c15922/f1p0/2749	NA	NA	NA	R01_cb8564_c15922/f1p0/2749
R01_cb17202_c0/f1p0/346	R01_cb17202_c0/f1p0/346	R01_cb17202_c0/f1p0/346	R01_cb17202_c0/f1p0/346	R01_cb17202_c0/f1p0/346
R01_cb14911_c2/f1p0/770	NA	NA	NA	R01_cb14911_c2/f1p0/770
R01_cb8564_c113904/f1p0/2973	R01_cb8564_c113904/f1p0/2973	R01_cb8564_c113904/f1p0/2973	R01_cb8564_c113904/f1p0/2973	R01_cb8564_c113904/f1p0/2973
R01_cb10425_c2/f1p1/2683	NA	NA	R01_cb10425_c2/f1p1/2683	R01_cb10425_c2/f1p1/2683
R01_cb13012_c0/f3p0/1080	NA	NA	NA	R01_cb13012_c0/f3p0/1080
R01_cb7182_c18/f1p0/3116	NA	R01_cb7182_c18/f1p0/3116	NA	NA
R01_cb16125_c8/f1p0/1802	NA	R01_cb16125_c8/f1p0/1802	NA	NA
R01_cb8564_c76302/f1p0/4652	NA	R01_cb8564_c76302/f1p0/4652	NA	NA
R01_cb8564_c1722/f1p0/2580	NA	R01_cb8564_c1722/f1p0/2580	NA	NA
R01_cb665_c6/f1p0/2146	NA	NA	NA	R01_cb665_c6/f1p0/2146
R01_cb8162_c0/f1p0/2440	NA	R01_cb8162_c0/f1p0/2440	R01_cb8162_c0/f1p0/2440	R01_cb8162_c0/f1p0/2440
R01_cb2568_c7/f1p0/1765	R01_cb2568_c7/f1p0/1765	R01_cb2568_c7/f1p0/1765	R01_cb2568_c7/f1p0/1765	NA
R01_cb10566_c1/f2p0/1407	NA	NA	NA	R01_cb10566_c1/f2p0/1407
R01_cb2925_c3/f1p0/3108	NA	R01_cb2925_c3/f1p0/3108	NA	NA
R01_cb8564_c75872/f1p0/2250	NA	R01_cb8564_c75872/f1p0/2250	R01_cb8564_c75872/f1p0/2250	R01_cb8564_c75872/f1p0/2250
R01_cb18426_c0/f1p0/1802	R01_cb18426_c0/f1p0/1802	R01_cb18426_c0/f1p0/1802	R01_cb18426_c0/f1p0/1802	R01_cb18426_c0/f1p0/1802

R01_cb6591_c4/f3p0/632	R01_cb6591_c4/f3p0/632	R01_cb6591_c4/f3p0/632	NA	NA
R01_cb11533_c1/f1p0/3865	R01_cb11533_c1/f1p0/3865	R01_cb11533_c1/f1p0/3865	R01_cb11533_c1/f1p0/3865	R01_cb11533_c1/f1p0/3865
R01_cb10865_c0/f1p0/1516	NA	NA	NA	R01_cb10865_c0/f1p0/1516
R01_cb5681_c0/f1p0/3039	NA	R01_cb5681_c0/f1p0/3039	NA	R01_cb5681_c0/f1p0/3039
R01_cb10905_c12/f1p0/996	NA	R01_cb10905_c12/f1p0/996	NA	R01_cb10905_c12/f1p0/996
R01_cb12038_c29/f1p2/1808	NA	R01_cb12038_c29/f1p2/1808	NA	NA
R01_cb18456_c5739/f1p0/853	NA	R01_cb18456_c5739/f1p0/853	NA	NA
R01_cb18456_c7197/f1p0/561	R01_cb18456_c7197/f1p0/561	R01_cb18456_c7197/f1p0/561	R01_cb18456_c7197/f1p0/561	R01_cb18456_c7197/f1p0/561
R01_cb8564_c129337/f1p0/2511	NA	NA	NA	R01_cb8564_c129337/f1p0/2511
R01_cb18059_c1/f1p0/534	R01_cb18059_c1/f1p0/534	R01_cb18059_c1/f1p0/534	R01_cb18059_c1/f1p0/534	R01_cb18059_c1/f1p0/534
R01_cb2198_c4/f1p0/1321	NA	NA	R01_cb2198_c4/f1p0/1321	R01_cb2198_c4/f1p0/1321
R01_cb8564_c1985/f1p0/3118	NA	R01_cb8564_c1985/f1p0/3118	R01_cb8564_c1985/f1p0/3118	R01_cb8564_c1985/f1p0/3118
R01_cb1666_c5/f1p3/2503	NA	NA	NA	R01_cb1666_c5/f1p3/2503
R01_cb8475_c3/f1p0/3491	NA	R01_cb8475_c3/f1p0/3491	NA	NA
R01_cb13888_c0/f5p0/619	R01_cb13888_c0/f5p0/619	R01_cb13888_c0/f5p0/619	R01_cb13888_c0/f5p0/619	R01_cb13888_c0/f5p0/619
R01_cb2042_c2/f1p0/2780	NA	NA	NA	R01_cb2042_c2/f1p0/2780
R01_cb5659_c68/f1p0/2200	NA	NA	NA	R01_cb5659_c68/f1p0/2200
R01_cb9448_c15/f1p0/1430	NA	NA	NA	R01_cb9448_c15/f1p0/1430
R01_cb2985_c2/f4p2/3365	NA	NA	NA	R01_cb2985_c2/f4p2/3365
R01_cb15683_c6/f1p0/1620	NA	NA	NA	R01_cb15683_c6/f1p0/1620
R01_cb5635_c4/f1p0/793	NA	NA	NA	R01_cb5635_c4/f1p0/793
R01_cb11102_c0/f2p0/905	NA	NA	NA	R01_cb11102_c0/f2p0/905
R01_cb12437_c1/f1p0/459	R01_cb12437_c1/f1p0/459	R01_cb12437_c1/f1p0/459	R01_cb12437_c1/f1p0/459	R01_cb12437_c1/f1p0/459
R01_cb12098_c38/f1p0/833	R01_cb12098_c38/f1p0/833	R01_cb12098_c38/f1p0/833	R01_cb12098_c38/f1p0/833	R01_cb12098_c38/f1p0/833
R01_cb16459_c0/f1p0/1377	R01_cb16459_c0/f1p0/1377	R01_cb16459_c0/f1p0/1377	R01_cb16459_c0/f1p0/1377	NA

R01_cb14085_c1/f1p0/652	NA	R01_cb14085_c1/f1p0/652	NA	NA
R01_cb13275_c1/f1p0/418	R01_cb13275_c1/f1p0/418	R01_cb13275_c1/f1p0/418	NA	NA
R01_cb4169_c4/f1p0/3340	NA	NA	NA	R01_cb4169_c4/f1p0/3340
R01_cb11631_c3/f1p1/1766	NA	R01_cb11631_c3/f1p1/1766	NA	NA
R01_cb4368_c8/f1p1/1542	NA	NA	NA	R01_cb4368_c8/f1p1/1542
R01_cb16024_c5/f1p0/577	R01_cb16024_c5/f1p0/577	NA	NA	NA
R01_cb12213_c3/f1p1/829	NA	R01_cb12213_c3/f1p1/829	NA	NA
R01_cb1905_c12/f1p0/3147	NA	R01_cb1905_c12/f1p0/3147	R01_cb1905_c12/f1p0/3147	NA
R01_cb7690_c0/f1p0/2559	NA	R01_cb7690_c0/f1p0/2559	NA	NA
R01_cb15122_c0/f3p0/881	NA	R01_cb15122_c0/f3p0/881	NA	NA
R01_cb13019_c0/f11p2/632	NA	NA	R01_cb13019_c0/f11p2/632	NA
R01_cb8564_c3686/f1p0/3543	NA	R01_cb8564_c3686/f1p0/3543	NA	NA
R01_cb10015_c162/f1p0/575	NA	R01_cb10015_c162/f1p0/575	R01_cb10015_c162/f1p0/575	NA
R01_cb12743_c5/f1p0/703	NA	R01_cb12743_c5/f1p0/703	NA	R01_cb12743_c5/f1p0/703
R01_cb5808_c3/f1p0/2793	NA	NA	NA	R01_cb5808_c3/f1p0/2793
R01_cb2836_c9/f1p0/2465	NA	NA	NA	R01_cb2836_c9/f1p0/2465
R01_cb12938_c7/f1p0/483	R01_cb12938_c7/f1p0/483	R01_cb12938_c7/f1p0/483	R01_cb12938_c7/f1p0/483	R01_cb12938_c7/f1p0/483
R01_cb15537_c5/f1p0/1802	R01_cb15537_c5/f1p0/1802	R01_cb15537_c5/f1p0/1802	R01_cb15537_c5/f1p0/1802	NA
R01_cb8564_c17323/f1p0/2493	NA	R01_cb8564_c17323/f1p0/2493	R01_cb8564_c17323/f1p0/2493	R01_cb8564_c17323/f1p0/2493
R01_cb10386_c1/f1p0/2111	R01_cb10386_c1/f1p0/2111	R01_cb10386_c1/f1p0/2111	R01_cb10386_c1/f1p0/2111	R01_cb10386_c1/f1p0/2111
R01_cb6615_c23/f1p0/1004	NA	NA	NA	R01_cb6615_c23/f1p0/1004
R01_cb136_c2/f1p0/1762	NA	R01_cb136_c2/f1p0/1762	NA	NA
R01_cb2637_c11/f1p0/2585	NA	R01_cb2637_c11/f1p0/2585	NA	NA
R01_cb8564_c74050/f1p1/3406	NA	NA	NA	R01_cb8564_c74050/f1p1/3406
R01_cb6153_c2/f1p0/2933	NA	NA	NA	R01_cb6153_c2/f1p0/2933
R01_cb3874_c0/f1p0/3504	R01_cb3874_c0/f1p0/3504	R01_cb3874_c0/f1p0/3504	R01_cb3874_c0/f1p0/3504	R01_cb3874_c0/f1p0/3504

R01_cb8040_c22/f1p0/1484	NA	R01_cb8040_c22/f1p0/1484	R01_cb8040_c22/f1p0/1484	NA
R01_cb17266_c23/f1p0/1582	R01_cb17266_c23/f1p0/1582	R01_cb17266_c23/f1p0/1582	NA	R01_cb17266_c23/f1p0/1582
R01_cb8564_c47229/f1p0/2561	NA	R01_cb8564_c47229/f1p0/2561	NA	R01_cb8564_c47229/f1p0/2561
R01_cb9130_c3/f1p0/827	NA	R01_cb9130_c3/f1p0/827	NA	NA
R01_cb4490_c16/f1p0/2383	NA	NA	NA	R01_cb4490_c16/f1p0/2383
R01_cb7686_c1/f1p0/2418	NA	NA	NA	R01_cb7686_c1/f1p0/2418
R01_cb5579_c3/f1p0/2378	NA	NA	NA	R01_cb5579_c3/f1p0/2378
R01_cb8564_c88855/f1p0/2600	NA	NA	NA	R01_cb8564_c88855/f1p0/2600
R01_cb7960_c26/f1p0/1559	R01_cb7960_c26/f1p0/1559	R01_cb7960_c26/f1p0/1559	R01_cb7960_c26/f1p0/1559	NA
R01_cb16504_c2/f1p0/1676	NA	NA	NA	R01_cb16504_c2/f1p0/1676
R01_cb8441_c4/f1p0/2369	NA	NA	NA	R01_cb8441_c4/f1p0/2369
R01_cb2847_c1/f1p0/2017	NA	R01_cb2847_c1/f1p0/2017	NA	NA
R01_cb10586_c3/f1p0/1736	NA	NA	NA	R01_cb10586_c3/f1p0/1736
R01_cb14125_c97/f2p0/690	NA	R01_cb14125_c97/f2p0/690	R01_cb14125_c97/f2p0/690	NA
R01_cb8564_c128965/f1p0/2370	NA	R01_cb8564_c128965/f1p0/2370	NA	NA
R01_cb8564_c3946/f1p0/3184	R01_cb8564_c3946/f1p0/3184	R01_cb8564_c3946/f1p0/3184	R01_cb8564_c3946/f1p0/3184	R01_cb8564_c3946/f1p0/3184
R01_cb7114_c9/f1p0/1792	NA	NA	NA	R01_cb7114_c9/f1p0/1792
R01_cb18456_c3327/f1p0/1071	NA	R01_cb18456_c3327/f1p0/1071	NA	NA
R01_cb8564_c115544/f1p0/2007	R01_cb8564_c115544/f1p0/2007	R01_cb8564_c115544/f1p0/2007	NA	NA
R01_cb8162_c1/f1p0/2330	NA	R01_cb8162_c1/f1p0/2330	NA	R01_cb8162_c1/f1p0/2330
R01_cb3680_c2/f1p0/3549	NA	NA	NA	R01_cb3680_c2/f1p0/3549
R01_cb2425_c6/f1p0/2303	NA	NA	NA	R01_cb2425_c6/f1p0/2303
R01_cb14787_c7/f1p0/1107	NA	R01_cb14787_c7/f1p0/1107	NA	NA
R01_cb18005_c0/f6p0/707	NA	NA	NA	R01_cb18005_c0/f6p0/707

R01_cb3154_c2/f1p0/3704	NA	R01_cb3154_c2/f1p0/3704	NA	NA
R01_cb16809_c2/f1p0/501	NA	R01_cb16809_c2/f1p0/501	NA	NA
R01_cb15578_c6/f1p0/621	NA	R01_cb15578_c6/f1p0/621	NA	R01_cb15578_c6/f1p0/621
R01_cb11477_c0/f1p0/1212	NA	R01_cb11477_c0/f1p0/1212	NA	NA
R01_cb15273_c3/f2p0/1239	NA	NA	NA	R01_cb15273_c3/f2p0/1239
R01_cb15949_c0/f2p1/1029	NA	NA	NA	R01_cb15949_c0/f2p1/1029
R01_cb2773_c9/f1p0/2545	R01_cb2773_c9/f1p0/2545	R01_cb2773_c9/f1p0/2545	NA	NA
R01_cb14493_c6/f1p0/1013	NA	R01_cb14493_c6/f1p0/1013	R01_cb14493_c6/f1p0/1013	R01_cb14493_c6/f1p0/1013
R01_cb18456_c5441/f1p2/604	R01_cb18456_c5441/f1p2/604	R01_cb18456_c5441/f1p2/604	R01_cb18456_c5441/f1p2/604	R01_cb18456_c5441/f1p2/604
R01_cb17345_c2/f1p0/435	R01_cb17345_c2/f1p0/435	R01_cb17345_c2/f1p0/435	R01_cb17345_c2/f1p0/435	R01_cb17345_c2/f1p0/435
R01_cb7653_c3/f1p0/2544	NA	R01_cb7653_c3/f1p0/2544	NA	NA
R01_cb17098_c1/f1p0/697	R01_cb17098_c1/f1p0/697	R01_cb17098_c1/f1p0/697	NA	NA
R01_cb8564_c90451/f1p0/2698	R01_cb8564_c90451/f1p0/2698	R01_cb8564_c90451/f1p0/2698	R01_cb8564_c90451/f1p0/2698	R01_cb8564_c90451/f1p0/2698
R01_cb8564_c125504/f1p0/2676	NA	R01_cb8564_c125504/f1p0/2676	NA	NA
R01_cb639_c5/f1p0/886	NA	NA	NA	R01_cb639_c5/f1p0/886
R01_cb8564_c1853/f2p0/2478	NA	R01_cb8564_c1853/f2p0/2478	R01_cb8564_c1853/f2p0/2478	R01_cb8564_c1853/f2p0/2478
R01_cb13131_c34/f1p1/1713	NA	R01_cb13131_c34/f1p1/1713	NA	NA
R01_cb18372_c22/f1p0/2571	NA	R01_cb18372_c22/f1p0/2571	R01_cb18372_c22/f1p0/2571	R01_cb18372_c22/f1p0/2571
R01_cb8564_c15409/f1p1/4804	NA	R01_cb8564_c15409/f1p1/4804	NA	NA
R01_cb17423_c0/f3p0/789	NA	NA	NA	R01_cb17423_c0/f3p0/789
R01_cb11087_c2/f1p0/4793	NA	R01_cb11087_c2/f1p0/4793	NA	NA
R01_cb6202_c2/f1p0/2915	NA	R01_cb6202_c2/f1p0/2915	NA	NA
R01_cb6101_c1/f1p0/2933	NA	R01_cb6101_c1/f1p0/2933	NA	NA
R01_cb14481_c6/f1p1/1053	NA	NA	NA	R01_cb14481_c6/f1p1/1053

R01_cb8564_c81953/f1p0/3828	R01_cb8564_c81953/f1p0/3828	R01_cb8564_c81953/f1p0/3828	R01_cb8564_c81953/f1p0/3828	R01_cb8564_c81953/f1p0/3828
R01_cb8564_c16518/f1p0/3639	NA	NA	NA	R01_cb8564_c16518/f1p0/3639
R01_cb8564_c129933/f1p0/2634	NA	R01_cb8564_c129933/f1p0/2634	NA	NA
R01_cb8564_c91025/f1p1/2344	NA	NA	NA	R01_cb8564_c91025/f1p1/2344
R01_cb14573_c24/f1p0/1876	NA	R01_cb14573_c24/f1p0/1876	NA	NA
R01_cb3593_c8/f1p0/1266	NA	R01_cb3593_c8/f1p0/1266	NA	R01_cb3593_c8/f1p0/1266
R01_cb8564_c41329/f3p0/2272	R01_cb8564_c41329/f3p0/2272	R01_cb8564_c41329/f3p0/2272	R01_cb8564_c41329/f3p0/2272	R01_cb8564_c41329/f3p0/2272
R01_cb3857_c20/f1p0/1834	NA	R01_cb3857_c20/f1p0/1834	NA	R01_cb3857_c20/f1p0/1834
R01_cb15725_c4/f1p0/1432	NA	NA	NA	R01_cb15725_c4/f1p0/1432
R01_cb8564_c2547/f1p0/2521	NA	R01_cb8564_c2547/f1p0/2521	R01_cb8564_c2547/f1p0/2521	R01_cb8564_c2547/f1p0/2521
R01_cb8564_c50301/f1p0/2398	NA	R01_cb8564_c50301/f1p0/2398	NA	NA
R01_cb3483_c7/f1p0/2730	NA	NA	NA	R01_cb3483_c7/f1p0/2730
R01_cb2320_c10/f1p0/3766	NA	R01_cb2320_c10/f1p0/3766	NA	R01_cb2320_c10/f1p0/3766
R01_cb8564_c53693/f2p0/3706	NA	R01_cb8564_c53693/f2p0/3706	NA	NA
R01_cb5595_c39/f1p0/2521	NA	NA	NA	R01_cb5595_c39/f1p0/2521
R01_cb13982_c6/f1p0/1111	NA	NA	NA	R01_cb13982_c6/f1p0/1111
R01_cb11190_c0/f3p0/1684	NA	NA	NA	R01_cb11190_c0/f3p0/1684
R01_cb10923_c1/f1p0/2249	NA	R01_cb10923_c1/f1p0/2249	R01_cb10923_c1/f1p0/2249	R01_cb10923_c1/f1p0/2249
R01_cb10145_c5/f1p0/1761	NA	NA	NA	R01_cb10145_c5/f1p0/1761
R01_cb18456_c3724/f1p0/1121	R01_cb18456_c3724/f1p0/1121	R01_cb18456_c3724/f1p0/1121	NA	R01_cb18456_c3724/f1p0/1121
R01_cb13638_c2/f1p0/1162	NA	NA	NA	R01_cb13638_c2/f1p0/1162
R01_cb8564_c78493/f1p0/3078	NA	R01_cb8564_c78493/f1p0/3078	NA	NA

R01_cb13112_c9/f3p1/909	NA	NA	R01_cb13112_c9/f3p1/909	R01_cb13112_c9/f3p1/909
R01_cb10040_c1/f1p0/2331	NA	R01_cb10040_c1/f1p0/2331	NA	NA
R01_cb8131_c3/f1p0/1050	NA	NA	NA	R01_cb8131_c3/f1p0/1050
R01_cb8564_c35234/f1p0/2655	NA	NA	NA	R01_cb8564_c35234/f1p0/2655
R01_cb5339_c6/f1p0/2507	NA	NA	NA	R01_cb5339_c6/f1p0/2507
R01_cb12365_c2/f3p0/604	NA	R01_cb12365_c2/f3p0/604	NA	NA
R01_cb15457_c9/f1p0/1732	NA	R01_cb15457_c9/f1p0/1732	NA	R01_cb15457_c9/f1p0/1732
R01_cb8564_c69283/f2p2/1886	NA	R01_cb8564_c69283/f2p2/1886	R01_cb8564_c69283/f2p2/1886	R01_cb8564_c69283/f2p2/1886
R01_cb18073_c0/f1p0/985	NA	R01_cb18073_c0/f1p0/985	R01_cb18073_c0/f1p0/985	R01_cb18073_c0/f1p0/985
R01_cb5659_c75/f2p0/2112	NA	NA	NA	R01_cb5659_c75/f2p0/2112
R01_cb6580_c0/f1p0/2812	R01_cb6580_c0/f1p0/2812	NA	NA	NA
R01_cb8564_c114253/f1p0/2644	R01_cb8564_c114253/f1p0/2644	R01_cb8564_c114253/f1p0/2644	R01_cb8564_c114253/f1p0/2644	R01_cb8564_c114253/f1p0/2644
R01_cb502_c29/f1p0/2029	NA	R01_cb502_c29/f1p0/2029	NA	NA
R01_cb5428_c2/f1p0/1624	NA	R01_cb5428_c2/f1p0/1624	NA	NA
R01_cb18287_c3/f1p0/1559	NA	NA	NA	R01_cb18287_c3/f1p0/1559
R01_cb13270_c3/f4p0/1393	NA	NA	NA	R01_cb13270_c3/f4p0/1393
R01_cb572_c4/f1p0/3571	R01_cb572_c4/f1p0/3571	R01_cb572_c4/f1p0/3571	NA	NA
R01_cb12023_c5/f1p0/888	NA	NA	NA	R01_cb12023_c5/f1p0/888
R01_cb3281_c4/f1p0/3248	NA	R01_cb3281_c4/f1p0/3248	NA	NA
R01_cb988_c2/f1p0/917	NA	R01_cb988_c2/f1p0/917	R01_cb988_c2/f1p0/917	R01_cb988_c2/f1p0/917
R01_cb68_c8/f1p0/3018	NA	NA	NA	R01_cb68_c8/f1p0/3018
R01_cb8564_c88023/f1p0/3490	NA	R01_cb8564_c88023/f1p0/3490	R01_cb8564_c88023/f1p0/3490	R01_cb8564_c88023/f1p0/3490
R01_cb4538_c1/f2p0/3318	NA	R01_cb4538_c1/f2p0/3318	NA	NA
R01_cb8564_c81525/f1p0/3901	NA	R01_cb8564_c81525/f1p0/3901	NA	NA
R01_cb10152_c4/f1p0/660	R01_cb10152_c4/f1p0/660	NA	NA	NA

R01_cb15941_c2/f1p0/1296	NA	NA	NA	R01_cb15941_c2/f1p0/1296
R01_cb8564_c77551/f1p0/2749	NA	NA	R01_cb8564_c77551/f1p0/2749	R01_cb8564_c77551/f1p0/2749
R01_cb15952_c0/f1p0/1341	NA	NA	NA	R01_cb15952_c0/f1p0/1341
R01_cb14221_c4/f1p0/1002	NA	R01_cb14221_c4/f1p0/1002	NA	NA
R01_cb14898_c1/f1p0/1681	NA	R01_cb14898_c1/f1p0/1681	NA	R01_cb14898_c1/f1p0/1681
R01_cb16952_c2/f1p0/1168	NA	R01_cb16952_c2/f1p0/1168	NA	NA
R01_cb16648_c1/f1p0/1583	NA	R01_cb16648_c1/f1p0/1583	NA	R01_cb16648_c1/f1p0/1583
R01_cb920_c20/f1p1/4332	NA	NA	NA	R01_cb920_c20/f1p1/4332
R01_cb8809_c9/f1p0/2312	NA	NA	NA	R01_cb8809_c9/f1p0/2312
R01_cb8564_c16642/f3p1/3588	NA	R01_cb8564_c16642/f3p1/3588	NA	R01_cb8564_c16642/f3p1/3588
R01_cb14955_c2/f2p1/1088	NA	NA	NA	R01_cb14955_c2/f2p1/1088
R01_cb1996_c0/f1p0/4100	NA	NA	NA	R01_cb1996_c0/f1p0/4100
R01_cb18456_c7579/f1p0/1474	NA	R01_cb18456_c7579/f1p0/1474	NA	R01_cb18456_c7579/f1p0/1474
R01_cb18456_c7180/f2p0/512	R01_cb18456_c7180/f2p0/512	R01_cb18456_c7180/f2p0/512	R01_cb18456_c7180/f2p0/512	R01_cb18456_c7180/f2p0/512
R01_cb14153_c2/f1p0/1014	NA	R01_cb14153_c2/f1p0/1014	NA	NA
R01_cb18733_c0/f1p0/5403	NA	R01_cb18733_c0/f1p0/5403	NA	R01_cb18733_c0/f1p0/5403
R01_cb1739_c14/f1p0/5622	NA	NA	NA	R01_cb1739_c14/f1p0/5622
R01_cb8564_c70300/f2p0/2933	R01_cb8564_c70300/f2p0/2933	R01_cb8564_c70300/f2p0/2933	R01_cb8564_c70300/f2p0/2933	R01_cb8564_c70300/f2p0/2933
R01_cb17865_c3/f1p5/5005	NA	NA	NA	R01_cb17865_c3/f1p5/5005
R01_cb8564_c19769/f1p0/3665	NA	R01_cb8564_c19769/f1p0/3665	NA	R01_cb8564_c19769/f1p0/3665
R01_cb3177_c0/f1p0/3696	NA	NA	NA	R01_cb3177_c0/f1p0/3696
R01_cb16577_c1/f1p0/654	NA	R01_cb16577_c1/f1p0/654	NA	R01_cb16577_c1/f1p0/654
R01_cb10724_c0/f1p0/1019	NA	NA	NA	R01_cb10724_c0/f1p0/1019
R01_cb868_c34/f1p0/3812	NA	R01_cb868_c34/f1p0/3812	NA	NA
R01_cb6147_c38/f1p0/2414	NA	R01_cb6147_c38/f1p0/2414	NA	NA

R01_cb14971_c4/f1p0/1647	NA	R01_cb14971_c4/f1p0/1647	NA	NA
R01_cb1124_c4/f1p0/4458	NA	NA	NA	R01_cb1124_c4/f1p0/4458
R01_cb12057_c24/f1p1/995	NA	R01_cb12057_c24/f1p1/995	NA	NA
R01_cb13772_c6/f1p0/535	NA	R01_cb13772_c6/f1p0/535	NA	NA
R01_cb4257_c11/f1p0/3120	NA	R01_cb4257_c11/f1p0/3120	NA	R01_cb4257_c11/f1p0/3120
R01_cb14704_c8/f1p0/1779	R01_cb14704_c8/f1p0/1779	R01_cb14704_c8/f1p0/1779	NA	R01_cb14704_c8/f1p0/1779
R01_cb7214_c4/f1p0/2395	NA	NA	NA	R01_cb7214_c4/f1p0/2395
R01_cb8564_c43733/f1p0/3701	NA	NA	R01_cb8564_c43733/f1p0/3701	NA
R01_cb15491_c0/f2p0/1598	NA	NA	NA	R01_cb15491_c0/f2p0/1598
R01_cb17933_c3/f1p1/1170	NA	R01_cb17933_c3/f1p1/1170	NA	NA
R01_cb8564_c70220/f1p0/2788	NA	R01_cb8564_c70220/f1p0/2788	NA	NA
R01_cb1438_c18/f1p0/2810	NA	NA	NA	R01_cb1438_c18/f1p0/2810
R01_cb2309_c6/f1p0/2551	NA	R01_cb2309_c6/f1p0/2551	NA	NA
R01_cb3972_c2/f1p0/2502	NA	R01_cb3972_c2/f1p0/2502	NA	R01_cb3972_c2/f1p0/2502
R01_cb14960_c4/f1p0/1099	NA	R01_cb14960_c4/f1p0/1099	NA	NA
R01_cb8564_c123277/f1p0/2905	NA	R01_cb8564_c123277/f1p0/2905	NA	NA
R01_cb6136_c1/f1p0/2462	NA	R01_cb6136_c1/f1p0/2462	NA	NA
R01_cb14853_c0/f1p0/717	R01_cb14853_c0/f1p0/717	R01_cb14853_c0/f1p0/717	R01_cb14853_c0/f1p0/717	R01_cb14853_c0/f1p0/717
R01_cb16022_c2/f1p0/1335	NA	R01_cb16022_c2/f1p0/1335	NA	NA
R01_cb6680_c1/f1p0/2375	NA	NA	NA	R01_cb6680_c1/f1p0/2375
R01_cb11057_c0/f1p0/1828	NA	NA	NA	R01_cb11057_c0/f1p0/1828
R01_cb16809_c0/f2p0/546	NA	R01_cb16809_c0/f2p0/546	NA	NA
R01_cb18456_c6887/f1p0/1212	NA	R01_cb18456_c6887/f1p0/1212	NA	NA
R01_cb14816_c2/f1p0/949	NA	R01_cb14816_c2/f1p0/949	NA	NA
R01_cb18456_c7524/f1p0/943	NA	R01_cb18456_c7524/f1p0/943	R01_cb18456_c7524/f1p0/943	R01_cb18456_c7524/f1p0/943

R01_cb18409_c104/f1p0/1443	R01_cb18409_c104/f1p0/1443	R01_cb18409_c104/f1p0/1443	R01_cb18409_c104/f1p0/1443	R01_cb18409_c104/f1p0/1443
R01_cb8905_c6/f1p1/2018	NA	NA	NA	R01_cb8905_c6/f1p1/2018
R01_cb3365_c2/f1p0/3647	NA	R01_cb3365_c2/f1p0/3647	NA	NA
R01_cb12589_c3/f1p0/705	R01_cb12589_c3/f1p0/705	NA	NA	R01_cb12589_c3/f1p0/705
R01_cb13178_c22/f1p0/646	NA	R01_cb13178_c22/f1p0/646	NA	NA
R01_cb16706_c2/f1p0/677	NA	R01_cb16706_c2/f1p0/677	NA	R01_cb16706_c2/f1p0/677
R01_cb16425_c3/f1p0/659	NA	R01_cb16425_c3/f1p0/659	R01_cb16425_c3/f1p0/659	R01_cb16425_c3/f1p0/659
R01_cb12680_c13/f1p0/1839	NA	R01_cb12680_c13/f1p0/1839	NA	NA
R01_cb6258_c9/f1p1/2903	NA	NA	NA	R01_cb6258_c9/f1p1/2903
R01_cb10034_c52/f1p0/971	NA	R01_cb10034_c52/f1p0/971	NA	NA
R01_cb12963_c3/f1p0/1441	NA	NA	NA	R01_cb12963_c3/f1p0/1441
R01_cb7457_c0/f7p0/431	R01_cb7457_c0/f7p0/431	R01_cb7457_c0/f7p0/431	NA	NA
R01_cb4134_c40/f1p0/2766	NA	NA	NA	R01_cb4134_c40/f1p0/2766
R01_cb5943_c4/f1p0/1485	NA	NA	NA	R01_cb5943_c4/f1p0/1485
R01_cb6393_c2/f1p0/3385	NA	NA	NA	R01_cb6393_c2/f1p0/3385
R01_cb8564_c3170/f1p0/2981	NA	R01_cb8564_c3170/f1p0/2981	NA	NA
R01_cb14723_c4/f1p1/1125	NA	NA	NA	R01_cb14723_c4/f1p1/1125
R01_cb15811_c25/f1p0/1262	R01_cb15811_c25/f1p0/1262	R01_cb15811_c25/f1p0/1262	R01_cb15811_c25/f1p0/1262	R01_cb15811_c25/f1p0/1262
R01_cb13910_c15/f1p0/1125	NA	R01_cb13910_c15/f1p0/1125	R01_cb13910_c15/f1p0/1125	R01_cb13910_c15/f1p0/1125
R01_cb5533_c128/f1p0/3072	NA	NA	NA	R01_cb5533_c128/f1p0/3072
R01_cb8135_c3/f1p1/1229	R01_cb8135_c3/f1p1/1229	R01_cb8135_c3/f1p1/1229	NA	NA
R01_cb17231_c3/f2p0/701	NA	NA	NA	R01_cb17231_c3/f2p0/701
R01_cb2463_c8/f1p0/2182	NA	NA	NA	R01_cb2463_c8/f1p0/2182
R01_cb9928_c4/f1p0/2967	NA	NA	NA	R01_cb9928_c4/f1p0/2967
R01_cb1515_c1/f1p0/2859	NA	R01_cb1515_c1/f1p0/2859	R01_cb1515_c1/f1p0/2859	NA
R01_cb8564_c45851/f1p0/4516	R01_cb8564_c45851/f1p0/451	R01_cb8564_c45851/f1p0/4516	NA	R01_cb8564_c45851/f1p0/4516

	6			
R01_cb8564_c75360/f1p0/3610	NA	R01_cb8564_c75360/f1p0/3610	NA	NA
R01_cb8564_c113809/f1p0/3795	NA	NA	NA	R01_cb8564_c113809/f1p0/3795
R01_cb17833_c1/f1p0/517	R01_cb17833_c1/f1p0/517	R01_cb17833_c1/f1p0/517	R01_cb17833_c1/f1p0/517	R01_cb17833_c1/f1p0/517
R01_cb13862_c8/f1p1/1551	NA	R01_cb13862_c8/f1p1/1551	R01_cb13862_c8/f1p1/1551	R01_cb13862_c8/f1p1/1551
R01_cb10402_c5/f1p0/1553	R01_cb10402_c5/f1p0/1553	R01_cb10402_c5/f1p0/1553	NA	NA
R01_cb18192_c4/f1p0/808	R01_cb18192_c4/f1p0/808	R01_cb18192_c4/f1p0/808	R01_cb18192_c4/f1p0/808	NA
R01_cb8564_c1093/f1p0/3160	NA	NA	NA	R01_cb8564_c1093/f1p0/3160
R01_cb2540_c5/f1p0/500	R01_cb2540_c5/f1p0/500	R01_cb2540_c5/f1p0/500	R01_cb2540_c5/f1p0/500	NA
R01_cb14761_c5/f1p0/1481	NA	R01_cb14761_c5/f1p0/1481	NA	NA
R01_cb12195_c5/f1p1/1744	NA	R01_cb12195_c5/f1p1/1744	NA	NA
R01_cb12750_c6/f1p0/586	NA	R01_cb12750_c6/f1p0/586	NA	R01_cb12750_c6/f1p0/586
R01_cb18456_c6764/f1p0/1294	NA	R01_cb18456_c6764/f1p0/1294	NA	NA
R01_cb10063_c16/f1p0/598	R01_cb10063_c16/f1p0/598	R01_cb10063_c16/f1p0/598	NA	NA
R01_cb18456_c7380/f1p0/453	R01_cb18456_c7380/f1p0/453	R01_cb18456_c7380/f1p0/453	R01_cb18456_c7380/f1p0/453	R01_cb18456_c7380/f1p0/453
R01_cb363_c7/f1p0/1464	NA	NA	NA	R01_cb363_c7/f1p0/1464
R01_cb8564_c42137/f1p0/2446	NA	NA	NA	R01_cb8564_c42137/f1p0/2446
R01_cb11245_c2/f1p0/3396	NA	R01_cb11245_c2/f1p0/3396	NA	NA
R01_cb10114_c1/f1p0/2230	NA	R01_cb10114_c1/f1p0/2230	NA	NA
R01_cb3962_c0/f1p0/3461	R01_cb3962_c0/f1p0/3461	R01_cb3962_c0/f1p0/3461	R01_cb3962_c0/f1p0/3461	R01_cb3962_c0/f1p0/3461
R01_cb8564_c22697/f1p0/2203	NA	R01_cb8564_c22697/f1p0/2203	NA	NA
R01_cb18243_c0/f1p1/685	NA	R01_cb18243_c0/f1p1/685	R01_cb18243_c0/f1p1/685	NA
R01_cb8564_c88064/f1p0/4623	NA	R01_cb8564_c88064/f1p0/4623	NA	NA
R01_cb8564_c71101/f1p0/3411	NA	R01_cb8564_c71101/f1p0/3411	NA	NA
R01_cb8564_c76942/f1p1/2861	NA	NA	NA	R01_cb8564_c76942/f1p1/2861

R01_cb8771_c1/f1p0/2261	R01_cb8771_c1/f1p0/2261	R01_cb8771_c1/f1p0/2261	R01_cb8771_c1/f1p0/2261	R01_cb8771_c1/f1p0/2261
R01_cb14239_c21/f1p0/430	R01_cb14239_c21/f1p0/430	R01_cb14239_c21/f1p0/430	NA	R01_cb14239_c21/f1p0/430
R01_cb6365_c2/f3p0/2541	R01_cb6365_c2/f3p0/2541	R01_cb6365_c2/f3p0/2541	NA	NA
R01_cb13879_c7/f1p1/1466	NA	R01_cb13879_c7/f1p1/1466	NA	NA
R01_cb18088_c0/f3p0/485	NA	NA	NA	R01_cb18088_c0/f3p0/485
R01_cb18301_c50/f1p0/1432	NA	R01_cb18301_c50/f1p0/1432	NA	NA
R01_cb8564_c51372/f1p0/4218	NA	R01_cb8564_c51372/f1p0/4218	R01_cb8564_c51372/f1p0/4218	R01_cb8564_c51372/f1p0/4218
R01_cb8692_c5/f1p0/1761	NA	R01_cb8692_c5/f1p0/1761	NA	NA
R01_cb2870_c8/f1p0/2862	NA	NA	NA	R01_cb2870_c8/f1p0/2862
R01_cb6198_c2/f1p0/2107	NA	R01_cb6198_c2/f1p0/2107	NA	R01_cb6198_c2/f1p0/2107
R01_cb16016_c5/f1p0/984	NA	NA	NA	R01_cb16016_c5/f1p0/984
R01_cb9628_c7/f2p0/2000	NA	R01_cb9628_c7/f2p0/2000	NA	NA
R01_cb8564_c4494/f1p0/2545	NA	R01_cb8564_c4494/f1p0/2545	R01_cb8564_c4494/f1p0/2545	R01_cb8564_c4494/f1p0/2545
R01_cb8991_c10/f1p1/2179	NA	NA	NA	R01_cb8991_c10/f1p1/2179
R01_cb9720_c4/f1p0/1846	NA	NA	NA	R01_cb9720_c4/f1p0/1846
R01_cb8564_c113604/f1p0/2257	NA	R01_cb8564_c113604/f1p0/2257	NA	NA
R01_cb6701_c2/f1p0/2251	R01_cb6701_c2/f1p0/2251	R01_cb6701_c2/f1p0/2251	R01_cb6701_c2/f1p0/2251	R01_cb6701_c2/f1p0/2251
R01_cb16455_c1/f1p0/1119	R01_cb16455_c1/f1p0/1119	R01_cb16455_c1/f1p0/1119	R01_cb16455_c1/f1p0/1119	R01_cb16455_c1/f1p0/1119
R01_cb16645_c24/f1p0/1196	R01_cb16645_c24/f1p0/1196	R01_cb16645_c24/f1p0/1196	R01_cb16645_c24/f1p0/1196	R01_cb16645_c24/f1p0/1196
R01_cb5499_c4/f1p1/2908	R01_cb5499_c4/f1p1/2908	R01_cb5499_c4/f1p1/2908	R01_cb5499_c4/f1p1/2908	R01_cb5499_c4/f1p1/2908
R01_cb8564_c111810/f1p0/1947	NA	R01_cb8564_c111810/f1p0/1947	NA	NA
R01_cb10157_c4/f1p0/1127	NA	R01_cb10157_c4/f1p0/1127	NA	R01_cb10157_c4/f1p0/1127
R01_cb8564_c48497/f1p0/3125	NA	NA	NA	R01_cb8564_c48497/f1p0/3125
R01_cb10663_c2/f1p0/2293	NA	R01_cb10663_c2/f1p0/2293	NA	NA

R01_cb14592_c5/f1p0/604	R01_cb14592_c5/f1p0/604	R01_cb14592_c5/f1p0/604	NA	R01_cb14592_c5/f1p0/604
R01_cb13133_c3/f1p0/1095	R01_cb13133_c3/f1p0/1095	R01_cb13133_c3/f1p0/1095	R01_cb13133_c3/f1p0/1095	R01_cb13133_c3/f1p0/1095
R01_cb15811_c32/f1p0/908	R01_cb15811_c32/f1p0/908	R01_cb15811_c32/f1p0/908	R01_cb15811_c32/f1p0/908	R01_cb15811_c32/f1p0/908
R01_cb1487_c6/f1p0/1268	NA	NA	NA	R01_cb1487_c6/f1p0/1268
R01_cb8564_c4736/f1p0/3076	R01_cb8564_c4736/f1p0/3076	R01_cb8564_c4736/f1p0/3076	NA	NA
R01_cb17587_c1/f1p0/849	R01_cb17587_c1/f1p0/849	R01_cb17587_c1/f1p0/849	R01_cb17587_c1/f1p0/849	R01_cb17587_c1/f1p0/849
R01_cb8564_c3734/f1p0/2289	NA	NA	NA	R01_cb8564_c3734/f1p0/2289
R01_cb1856_c4/f1p0/2934	NA	R01_cb1856_c4/f1p0/2934	NA	R01_cb1856_c4/f1p0/2934
R01_cb8564_c76370/f1p0/1925	NA	R01_cb8564_c76370/f1p0/1925	NA	NA
R01_cb1614_c4/f1p0/4251	NA	R01_cb1614_c4/f1p0/4251	NA	R01_cb1614_c4/f1p0/4251
R01_cb12401_c3/f2p1/1137	NA	R01_cb12401_c3/f2p1/1137	NA	NA
R01_cb12577_c26/f1p0/937	NA	NA	NA	R01_cb12577_c26/f1p0/937
R01_cb572_c3/f1p0/4739	R01_cb572_c3/f1p0/4739	R01_cb572_c3/f1p0/4739	R01_cb572_c3/f1p0/4739	NA
R01_cb8564_c4102/f1p0/2734	NA	R01_cb8564_c4102/f1p0/2734	R01_cb8564_c4102/f1p0/2734	NA
R01_cb5581_c3/f1p0/2033	NA	R01_cb5581_c3/f1p0/2033	NA	NA
R01_cb7980_c1/f1p0/2188	NA	R01_cb7980_c1/f1p0/2188	NA	NA
R01_cb8564_c50277/f1p0/2782	NA	R01_cb8564_c50277/f1p0/2782	NA	R01_cb8564_c50277/f1p0/2782
R01_cb10272_c12/f1p0/4713	NA	NA	R01_cb10272_c12/f1p0/4713	R01_cb10272_c12/f1p0/4713
R01_cb14125_c113/f1p0/1276	NA	R01_cb14125_c113/f1p0/1276	R01_cb14125_c113/f1p0/1276	NA
R01_cb141_c6/f1p0/3332	NA	NA	NA	R01_cb141_c6/f1p0/3332
R01_cb8564_c112533/f1p0/2383	NA	NA	NA	R01_cb8564_c112533/f1p0/2383
R01_cb1915_c23/f1p0/3434	NA	R01_cb1915_c23/f1p0/3434	NA	R01_cb1915_c23/f1p0/3434
R01_cb822_c15/f1p0/2423	NA	NA	NA	R01_cb822_c15/f1p0/2423
R01_cb18147_c1/f1p0/387	R01_cb18147_c1/f1p0/387	R01_cb18147_c1/f1p0/387	R01_cb18147_c1/f1p0/387	R01_cb18147_c1/f1p0/387
R01_cb6086_c1/f1p0/1945	NA	NA	NA	R01_cb6086_c1/f1p0/1945

R01_cb1433_c9/f1p0/3065	NA	R01_cb1433_c9/f1p0/3065	NA	NA
R01_cb15581_c1/f1p0/772	NA	NA	NA	R01_cb15581_c1/f1p0/772
R01_cb8564_c128509/f1p0/2552	NA	NA	NA	R01_cb8564_c128509/f1p0/2552
R01_cb893_c11/f1p0/6190	NA	NA	NA	R01_cb893_c11/f1p0/6190
R01_cb16913_c1/f2p0/519	R01_cb16913_c1/f2p0/519	R01_cb16913_c1/f2p0/519	R01_cb16913_c1/f2p0/519	R01_cb16913_c1/f2p0/519
R01_cb8564_c14116/f1p0/2968	NA	NA	NA	R01_cb8564_c14116/f1p0/2968
R01_cb7182_c11/f1p0/2681	NA	R01_cb7182_c11/f1p0/2681	NA	NA
R01_cb17945_c1/f1p0/727	NA	R01_cb17945_c1/f1p0/727	NA	NA
R01_cb6819_c7/f1p0/5270	NA	NA	NA	R01_cb6819_c7/f1p0/5270
R01_cb15776_c8/f1p1/1537	NA	R01_cb15776_c8/f1p1/1537	NA	NA
R01_cb8564_c2712/f1p1/2572	NA	R01_cb8564_c2712/f1p1/2572	NA	NA
R01_cb8564_c52788/f1p0/3297	NA	NA	NA	R01_cb8564_c52788/f1p0/3297
R01_cb7731_c13/f8p0/1386	NA	NA	NA	R01_cb7731_c13/f8p0/1386
R01_cb15860_c0/f2p0/510	NA	NA	NA	R01_cb15860_c0/f2p0/510
R01_cb8564_c121284/f1p0/2852	NA	R01_cb8564_c121284/f1p0/2852	NA	NA
R01_cb14560_c1/f6p0/752	NA	NA	NA	R01_cb14560_c1/f6p0/752
R01_cb8564_c72074/f1p0/2654	NA	R01_cb8564_c72074/f1p0/2654	R01_cb8564_c72074/f1p0/2654	R01_cb8564_c72074/f1p0/2654
R01_cb18456_c1818/f1p0/1365	NA	NA	NA	R01_cb18456_c1818/f1p0/1365
R01_cb104_c20/f1p0/1879	NA	NA	NA	R01_cb104_c20/f1p0/1879
R01_cb5881_c1/f1p0/2992	NA	R01_cb5881_c1/f1p0/2992	NA	NA
R01_cb5846_c57/f1p0/2797	NA	NA	NA	R01_cb5846_c57/f1p0/2797
R01_cb15298_c0/f3p0/526	R01_cb15298_c0/f3p0/526	R01_cb15298_c0/f3p0/526	R01_cb15298_c0/f3p0/526	R01_cb15298_c0/f3p0/526
R01_cb12319_c4/f1p0/850	NA	NA	NA	R01_cb12319_c4/f1p0/850
R01_cb8564_c89787/f1p0/1945	NA	R01_cb8564_c89787/f1p0/1945	R01_cb8564_c89787/f1p0/1945	R01_cb8564_c89787/f1p0/1945

R01_cb18456_c4790/f1p0/513	NA	NA	NA	R01_cb18456_c4790/f1p0/513
R01_cb3194_c1/f1p0/3738	NA	R01_cb3194_c1/f1p0/3738	NA	NA
R01_cb18386_c15/f1p0/988	NA	R01_cb18386_c15/f1p0/988	R01_cb18386_c15/f1p0/988	R01_cb18386_c15/f1p0/988
R01_cb6680_c5/f1p0/2053	NA	NA	NA	R01_cb6680_c5/f1p0/2053
R01_cb18456_c3719/f1p0/1373	R01_cb18456_c3719/f1p0/1373	R01_cb18456_c3719/f1p0/1373	NA	R01_cb18456_c3719/f1p0/1373
R01_cb8564_c3322/f1p0/2919	NA	R01_cb8564_c3322/f1p0/2919	NA	NA
R01_cb13990_c0/f2p0/889	NA	NA	NA	R01_cb13990_c0/f2p0/889
R01_cb8564_c19197/f1p0/4795	R01_cb8564_c19197/f1p0/4795	R01_cb8564_c19197/f1p0/4795	R01_cb8564_c19197/f1p0/4795	R01_cb8564_c19197/f1p0/4795
R01_cb3445_c3/f1p1/2831	NA	NA	NA	R01_cb3445_c3/f1p1/2831
R01_cb11433_c2/f1p0/1646	NA	NA	NA	R01_cb11433_c2/f1p0/1646
R01_cb18456_c2330/f1p0/644	NA	R01_cb18456_c2330/f1p0/644	R01_cb18456_c2330/f1p0/644	R01_cb18456_c2330/f1p0/644
R01_cb10945_c4/f1p0/702	R01_cb10945_c4/f1p0/702	R01_cb10945_c4/f1p0/702	R01_cb10945_c4/f1p0/702	R01_cb10945_c4/f1p0/702
R01_cb8564_c34605/f2p0/2504	NA	NA	R01_cb8564_c34605/f2p0/2504	R01_cb8564_c34605/f2p0/2504
R01_cb920_c13/f1p2/4504	NA	NA	NA	R01_cb920_c13/f1p2/4504
R01_cb7493_c5/f1p0/621	NA	R01_cb7493_c5/f1p0/621	NA	NA
R01_cb13161_c29/f1p0/356	R01_cb13161_c29/f1p0/356	R01_cb13161_c29/f1p0/356	R01_cb13161_c29/f1p0/356	R01_cb13161_c29/f1p0/356
R01_cb8564_c79290/f1p0/2163	NA	NA	R01_cb8564_c79290/f1p0/2163	R01_cb8564_c79290/f1p0/2163
R01_cb13778_c1/f1p0/599	R01_cb13778_c1/f1p0/599	R01_cb13778_c1/f1p0/599	R01_cb13778_c1/f1p0/599	R01_cb13778_c1/f1p0/599
R01_cb10148_c2/f5p0/1744	NA	NA	NA	R01_cb10148_c2/f5p0/1744
R01_cb18456_c6925/f1p0/480	NA	R01_cb18456_c6925/f1p0/480	NA	R01_cb18456_c6925/f1p0/480
R01_cb18218_c2/f1p0/1412	NA	NA	NA	R01_cb18218_c2/f1p0/1412
R01_cb8564_c76144/f1p0/3302	NA	R01_cb8564_c76144/f1p0/3302	NA	R01_cb8564_c76144/f1p0/3302
R01_cb10293_c6/f1p1/2320	NA	R01_cb10293_c6/f1p1/2320	NA	NA
R01_cb9008_c4/f1p1/2426	NA	NA	R01_cb9008_c4/f1p1/2426	NA

R01_cb8564_c129284/f1p0/2118	NA	R01_cb8564_c129284/f1p0/2118	NA	NA
R01_cb17636_c3/f1p0/1270	R01_cb17636_c3/f1p0/1270	R01_cb17636_c3/f1p0/1270	NA	NA
R01_cb14676_c0/f2p0/1396	NA	R01_cb14676_c0/f2p0/1396	NA	NA
R01_cb13545_c46/f1p0/566	NA	R01_cb13545_c46/f1p0/566	R01_cb13545_c46/f1p0/566	R01_cb13545_c46/f1p0/566
R01_cb8564_c88449/f1p0/2455	NA	R01_cb8564_c88449/f1p0/2455	NA	NA
R01_cb18456_c1133/f3p1/635	NA	R01_cb18456_c1133/f3p1/635	NA	NA
R01_cb8564_c23068/f2p0/3892	NA	R01_cb8564_c23068/f2p0/3892	R01_cb8564_c23068/f2p0/3892	R01_cb8564_c23068/f2p0/3892
R01_cb2445_c27/f1p0/2957	NA	NA	NA	R01_cb2445_c27/f1p0/2957
R01_cb8564_c90701/f1p0/2158	R01_cb8564_c90701/f1p0/2158	R01_cb8564_c90701/f1p0/2158	R01_cb8564_c90701/f1p0/2158	R01_cb8564_c90701/f1p0/2158
R01_cb18599_c0/f1p0/1056	NA	R01_cb18599_c0/f1p0/1056	NA	NA
R01_cb18456_c3723/f1p0/1453	NA	R01_cb18456_c3723/f1p0/1453	NA	R01_cb18456_c3723/f1p0/1453
R01_cb15701_c5/f1p0/1466	NA	R01_cb15701_c5/f1p0/1466	NA	NA
R01_cb8564_c10484/f1p0/2516	NA	R01_cb8564_c10484/f1p0/2516	NA	R01_cb8564_c10484/f1p0/2516
R01_cb16394_c0/f2p0/989	NA	R01_cb16394_c0/f2p0/989	R01_cb16394_c0/f2p0/989	R01_cb16394_c0/f2p0/989
R01_cb639_c8/f1p0/668	NA	NA	NA	R01_cb639_c8/f1p0/668
R01_cb9106_c1/f1p0/2183	NA	NA	R01_cb9106_c1/f1p0/2183	NA
R01_cb6337_c1/f1p0/2134	NA	NA	NA	R01_cb6337_c1/f1p0/2134
R01_cb3358_c0/f2p0/3345	R01_cb3358_c0/f2p0/3345	R01_cb3358_c0/f2p0/3345	R01_cb3358_c0/f2p0/3345	R01_cb3358_c0/f2p0/3345
R01_cb13938_c29/f5p0/1498	NA	NA	NA	R01_cb13938_c29/f5p0/1498
R01_cb8278_c3/f1p0/964	NA	R01_cb8278_c3/f1p0/964	NA	NA
R01_cb7976_c13/f1p0/2220	NA	R01_cb7976_c13/f1p0/2220	NA	NA
R01_cb1039_c47/f1p0/1829	NA	R01_cb1039_c47/f1p0/1829	R01_cb1039_c47/f1p0/1829	R01_cb1039_c47/f1p0/1829
R01_cb10879_c0/f1p0/1650	NA	R01_cb10879_c0/f1p0/1650	R01_cb10879_c0/f1p0/1650	R01_cb10879_c0/f1p0/1650
R01_cb8564_c129858/f1p0/242	NA	NA	NA	R01_cb8564_c129858/f1p0/242

9				9
R01_cb10970_c2/f1p0/2904	NA	NA	NA	R01_cb10970_c2/f1p0/2904
R01_cb1300_c2/f1p0/4092	NA	R01_cb1300_c2/f1p0/4092	NA	NA
R01_cb13938_c1/f5p2/1410	NA	NA	NA	R01_cb13938_c1/f5p2/1410
R01_cb3224_c4/f1p0/3455	NA	R01_cb3224_c4/f1p0/3455	NA	NA
R01_cb8564_c40891/f1p0/4271	NA	R01_cb8564_c40891/f1p0/4271	NA	NA
R01_cb18456_c7193/f1p0/1592	NA	R01_cb18456_c7193/f1p0/1592	R01_cb18456_c7193/f1p0/1592	R01_cb18456_c7193/f1p0/1592
R01_cb525_c4/f1p0/1986	NA	NA	NA	R01_cb525_c4/f1p0/1986
R01_cb14627_c8/f2p0/1061	NA	NA	NA	R01_cb14627_c8/f2p0/1061
R01_cb8564_c24176/f2p0/4094	NA	R01_cb8564_c24176/f2p0/4094	NA	NA
R01_cb1016_c5/f1p0/2657	NA	R01_cb1016_c5/f1p0/2657	NA	NA
R01_cb2738_c8/f1p0/8696	NA	NA	NA	R01_cb2738_c8/f1p0/8696
R01_cb6802_c71/f1p0/2208	NA	R01_cb6802_c71/f1p0/2208	NA	NA
R01_cb261_c23/f1p1/3640	NA	NA	NA	R01_cb261_c23/f1p1/3640
R01_cb11051_c5/f1p0/5432	NA	R01_cb11051_c5/f1p0/5432	NA	R01_cb11051_c5/f1p0/5432
R01_cb16163_c0/f1p0/1863	NA	NA	NA	R01_cb16163_c0/f1p0/1863
R01_cb7268_c4/f1p2/2443	NA	NA	R01_cb7268_c4/f1p2/2443	NA
R01_cb7286_c8/f1p0/990	NA	R01_cb7286_c8/f1p0/990	NA	R01_cb7286_c8/f1p0/990
R01_cb8564_c4691/f1p0/3446	R01_cb8564_c4691/f1p0/3446	R01_cb8564_c4691/f1p0/3446	NA	R01_cb8564_c4691/f1p0/3446
R01_cb3563_c3/f1p0/2975	NA	R01_cb3563_c3/f1p0/2975	NA	R01_cb3563_c3/f1p0/2975
R01_cb8564_c4260/f1p0/2927	NA	R01_cb8564_c4260/f1p0/2927	NA	NA
R01_cb16107_c0/f1p0/1045	NA	NA	R01_cb16107_c0/f1p0/1045	R01_cb16107_c0/f1p0/1045
R01_cb7114_c10/f1p1/1289	NA	NA	NA	R01_cb7114_c10/f1p1/1289
R01_cb9077_c0/f1p0/2197	NA	R01_cb9077_c0/f1p0/2197	NA	NA
R01_cb8564_c53534/f1p0/2382	NA	NA	R01_cb8564_c53534/f1p0/2382	NA
R01_cb15630_c1/f1p1/1848	R01_cb15630_c1/f1p1/1848	R01_cb15630_c1/f1p1/1848	R01_cb15630_c1/f1p1/1848	R01_cb15630_c1/f1p1/1848

R01_cb18456_c4788/f1p0/466	NA	NA	NA	R01_cb18456_c4788/f1p0/466
R01_cb10355_c3/f1p0/638	NA	R01_cb10355_c3/f1p0/638	NA	NA
R01_cb822_c13/f1p0/2650	NA	NA	NA	R01_cb822_c13/f1p0/2650
R01_cb8564_c125450/f1p1/2354	R01_cb8564_c125450/f1p1/2354	R01_cb8564_c125450/f1p1/2354	NA	R01_cb8564_c125450/f1p1/2354
R01_cb6199_c20/f1p0/3192	NA	NA	NA	R01_cb6199_c20/f1p0/3192
R01_cb17084_c3/f1p0/707	R01_cb17084_c3/f1p0/707	R01_cb17084_c3/f1p0/707	NA	R01_cb17084_c3/f1p0/707
R01_cb18456_c6646/f1p0/707	NA	NA	NA	R01_cb18456_c6646/f1p0/707
R01_cb10983_c3/f1p0/839	NA	R01_cb10983_c3/f1p0/839	R01_cb10983_c3/f1p0/839	R01_cb10983_c3/f1p0/839
R01_cb8564_c128065/f1p0/2159	NA	R01_cb8564_c128065/f1p0/2159	NA	NA
R01_cb15811_c2/f11p0/1405	NA	NA	R01_cb15811_c2/f11p0/1405	NA
R01_cb3921_c4/f1p0/1981	NA	NA	NA	R01_cb3921_c4/f1p0/1981
R01_cb2927_c12/f1p1/2996	NA	R01_cb2927_c12/f1p1/2996	NA	NA
R01_cb8564_c75298/f1p0/2148	NA	R01_cb8564_c75298/f1p0/2148	NA	NA
R01_cb8200_c8/f1p0/1425	NA	R01_cb8200_c8/f1p0/1425	R01_cb8200_c8/f1p0/1425	R01_cb8200_c8/f1p0/1425
R01_cb1822_c9/f1p0/2216	NA	NA	NA	R01_cb1822_c9/f1p0/2216
R01_cb8564_c4690/f1p0/3065	R01_cb8564_c4690/f1p0/3065	R01_cb8564_c4690/f1p0/3065	R01_cb8564_c4690/f1p0/3065	R01_cb8564_c4690/f1p0/3065
R01_cb12851_c3/f1p0/1240	NA	NA	NA	R01_cb12851_c3/f1p0/1240
R01_cb8564_c45289/f1p0/2306	NA	R01_cb8564_c45289/f1p0/2306	NA	NA
R01_cb254_c18/f1p0/3813	NA	R01_cb254_c18/f1p0/3813	NA	R01_cb254_c18/f1p0/3813
R01_cb8475_c0/f1p0/2353	NA	R01_cb8475_c0/f1p0/2353	NA	NA
R01_cb8564_c79147/f1p0/3741	NA	R01_cb8564_c79147/f1p0/3741	NA	NA
R01_cb88_c24/f1p0/2478	NA	NA	NA	R01_cb88_c24/f1p0/2478
R01_cb3169_c0/f1p0/3707	NA	NA	NA	R01_cb3169_c0/f1p0/3707
R01_cb10596_c5/f1p0/3599	NA	R01_cb10596_c5/f1p0/3599	NA	NA

R01_cb17346_c1/f1p0/1168	R01_cb17346_c1/f1p0/1168	R01_cb17346_c1/f1p0/1168	R01_cb17346_c1/f1p0/1168	R01_cb17346_c1/f1p0/1168
R01_cb1653_c1/f1p0/4225	NA	NA	NA	R01_cb1653_c1/f1p0/4225
R01_cb625_c5/f1p0/3261	NA	NA	NA	R01_cb625_c5/f1p0/3261
R01_cb124_c8/f2p0/3745	NA	NA	NA	R01_cb124_c8/f2p0/3745
R01_cb8713_c0/f1p0/2285	NA	R01_cb8713_c0/f1p0/2285	NA	NA
R01_cb11988_c7/f1p0/1578	NA	R01_cb11988_c7/f1p0/1578	NA	NA
R01_cb18456_c7936/f3p1/386	R01_cb18456_c7936/f3p1/386	R01_cb18456_c7936/f3p1/386	NA	R01_cb18456_c7936/f3p1/386
R01_cb17531_c4/f1p0/1403	NA	R01_cb17531_c4/f1p0/1403	R01_cb17531_c4/f1p0/1403	R01_cb17531_c4/f1p0/1403
R01_cb10464_c1/f1p0/3172	NA	R01_cb10464_c1/f1p0/3172	NA	NA
R01_cb6616_c10/f1p1/437	R01_cb6616_c10/f1p1/437	R01_cb6616_c10/f1p1/437	R01_cb6616_c10/f1p1/437	R01_cb6616_c10/f1p1/437
R01_cb8564_c4103/f1p0/2941	NA	R01_cb8564_c4103/f1p0/2941	R01_cb8564_c4103/f1p0/2941	NA
R01_cb17114_c0/f2p0/952	NA	NA	NA	R01_cb17114_c0/f2p0/952
R01_cb1203_c4/f1p0/4549	NA	NA	NA	R01_cb1203_c4/f1p0/4549
R01_cb6886_c3/f1p0/2584	NA	R01_cb6886_c3/f1p0/2584	NA	NA
R01_cb8564_c89561/f1p0/2394	NA	R01_cb8564_c89561/f1p0/2394	R01_cb8564_c89561/f1p0/2394	R01_cb8564_c89561/f1p0/2394
R01_cb10190_c3/f1p0/1585	R01_cb10190_c3/f1p0/1585	R01_cb10190_c3/f1p0/1585	R01_cb10190_c3/f1p0/1585	R01_cb10190_c3/f1p0/1585
R01_cb506_c2/f1p0/4681	NA	NA	NA	R01_cb506_c2/f1p0/4681
R01_cb18590_c2/f1p0/703	NA	NA	R01_cb18590_c2/f1p0/703	NA
R01_cb17709_c0/f2p0/547	R01_cb17709_c0/f2p0/547	R01_cb17709_c0/f2p0/547	R01_cb17709_c0/f2p0/547	NA
R01_cb7064_c9/f1p0/1480	NA	R01_cb7064_c9/f1p0/1480	NA	NA
R01_cb14086_c2/f1p0/867	NA	R01_cb14086_c2/f1p0/867	NA	R01_cb14086_c2/f1p0/867
R01_cb8564_c78490/f1p1/3430	NA	NA	NA	R01_cb8564_c78490/f1p1/3430
R01_cb5448_c5/f1p0/1537	NA	R01_cb5448_c5/f1p0/1537	NA	NA
R01_cb7594_c10/f1p0/2210	NA	R01_cb7594_c10/f1p0/2210	NA	NA
R01_cb13875_c2/f3p1/1096	NA	NA	NA	R01_cb13875_c2/f3p1/1096
R01_cb8564_c2983/f1p0/2648	NA	R01_cb8564_c2983/f1p0/2648	R01_cb8564_c2983/f1p0/2648	R01_cb8564_c2983/f1p0/2648

R01_cb2182_c7/f1p0/331	R01_cb2182_c7/f1p0/331	R01_cb2182_c7/f1p0/331	R01_cb2182_c7/f1p0/331	R01_cb2182_c7/f1p0/331
R01_cb1982_c3/f1p0/2118	NA	R01_cb1982_c3/f1p0/2118	R01_cb1982_c3/f1p0/2118	R01_cb1982_c3/f1p0/2118
R01_cb6253_c1/f1p0/2817	R01_cb6253_c1/f1p0/2817	R01_cb6253_c1/f1p0/2817	R01_cb6253_c1/f1p0/2817	R01_cb6253_c1/f1p0/2817
R01_cb10024_c213/f1p0/716	NA	NA	R01_cb10024_c213/f1p0/716	NA
R01_cb8433_c3/f1p0/2627	NA	NA	NA	R01_cb8433_c3/f1p0/2627
R01_cb15784_c1/f2p0/1460	NA	NA	NA	R01_cb15784_c1/f2p0/1460
R01_cb1626_c15/f1p0/2204	NA	NA	NA	R01_cb1626_c15/f1p0/2204
R01_cb8564_c128557/f2p0/2916	R01_cb8564_c128557/f2p0/2916	R01_cb8564_c128557/f2p0/2916	R01_cb8564_c128557/f2p0/2916	R01_cb8564_c128557/f2p0/2916
R01_cb17542_c0/f1p0/427	NA	R01_cb17542_c0/f1p0/427	R01_cb17542_c0/f1p0/427	R01_cb17542_c0/f1p0/427
R01_cb10385_c11/f1p0/1423	R01_cb10385_c11/f1p0/1423	R01_cb10385_c11/f1p0/1423	R01_cb10385_c11/f1p0/1423	R01_cb10385_c11/f1p0/1423
R01_cb17221_c6/f1p0/1522	NA	R01_cb17221_c6/f1p0/1522	NA	NA
R01_cb13999_c9/f1p0/481	R01_cb13999_c9/f1p0/481	R01_cb13999_c9/f1p0/481	R01_cb13999_c9/f1p0/481	R01_cb13999_c9/f1p0/481
R01_cb17780_c0/f1p0/641	R01_cb17780_c0/f1p0/641	R01_cb17780_c0/f1p0/641	R01_cb17780_c0/f1p0/641	R01_cb17780_c0/f1p0/641
R01_cb16370_c2/f1p0/966	NA	R01_cb16370_c2/f1p0/966	NA	R01_cb16370_c2/f1p0/966
R01_cb5640_c7/f2p0/2229	NA	NA	NA	R01_cb5640_c7/f2p0/2229
R01_cb16460_c1/f2p0/544	NA	R01_cb16460_c1/f2p0/544	R01_cb16460_c1/f2p0/544	R01_cb16460_c1/f2p0/544
R01_cb13463_c7/f1p0/368	R01_cb13463_c7/f1p0/368	R01_cb13463_c7/f1p0/368	R01_cb13463_c7/f1p0/368	R01_cb13463_c7/f1p0/368
R01_cb7999_c5/f1p0/996	R01_cb7999_c5/f1p0/996	R01_cb7999_c5/f1p0/996	NA	R01_cb7999_c5/f1p0/996
R01_cb2452_c1/f1p0/3807	NA	R01_cb2452_c1/f1p0/3807	NA	NA
R01_cb9354_c0/f1p0/2101	NA	NA	NA	R01_cb9354_c0/f1p0/2101
R01_cb14399_c1/f1p0/1170	NA	NA	NA	R01_cb14399_c1/f1p0/1170
R01_cb6442_c14/f1p0/1684	R01_cb6442_c14/f1p0/1684	R01_cb6442_c14/f1p0/1684	NA	R01_cb6442_c14/f1p0/1684
R01_cb4634_c17/f1p0/2349	NA	R01_cb4634_c17/f1p0/2349	NA	NA
R01_cb1610_c2/f1p0/4245	NA	NA	NA	R01_cb1610_c2/f1p0/4245
R01_cb1398_c7/f1p0/2599	NA	R01_cb1398_c7/f1p0/2599	R01_cb1398_c7/f1p0/2599	NA

R01_cb8564_c24206/f1p0/2126	NA	R01_cb8564_c24206/f1p0/2126	R01_cb8564_c24206/f1p0/2126	R01_cb8564_c24206/f1p0/2126
R01_cb3759_c6/f1p0/2298	NA	R01_cb3759_c6/f1p0/2298	NA	NA
R01_cb1388_c17/f1p0/4640	NA	NA	NA	R01_cb1388_c17/f1p0/4640
R01_cb15980_c6/f1p0/309	R01_cb15980_c6/f1p0/309	R01_cb15980_c6/f1p0/309	R01_cb15980_c6/f1p0/309	R01_cb15980_c6/f1p0/309
R01_cb8564_c16763/f1p0/4168	NA	NA	NA	R01_cb8564_c16763/f1p0/4168
R01_cb8564_c74352/f1p0/3696	NA	R01_cb8564_c74352/f1p0/3696	NA	R01_cb8564_c74352/f1p0/3696
R01_cb5208_c2/f2p1/2653	NA	NA	NA	R01_cb5208_c2/f2p1/2653
R01_cb18456_c5731/f2p2/812	NA	R01_cb18456_c5731/f2p2/812	NA	NA
R01_cb16341_c11/f1p0/442	R01_cb16341_c11/f1p0/442	R01_cb16341_c11/f1p0/442	R01_cb16341_c11/f1p0/442	R01_cb16341_c11/f1p0/442
R01_cb7822_c3/f1p0/3275	NA	R01_cb7822_c3/f1p0/3275	R01_cb7822_c3/f1p0/3275	R01_cb7822_c3/f1p0/3275
R01_cb8905_c7/f1p0/2108	NA	NA	NA	R01_cb8905_c7/f1p0/2108
R01_cb16422_c0/f4p0/825	NA	NA	NA	R01_cb16422_c0/f4p0/825
R01_cb5853_c3/f1p1/1763	NA	R01_cb5853_c3/f1p1/1763	R01_cb5853_c3/f1p1/1763	NA
R01_cb18456_c1828/f1p0/1498	R01_cb18456_c1828/f1p0/1498	R01_cb18456_c1828/f1p0/1498	R01_cb18456_c1828/f1p0/1498	R01_cb18456_c1828/f1p0/1498
R01_cb4639_c6/f1p0/2609	NA	NA	NA	R01_cb4639_c6/f1p0/2609
R01_cb1788_c0/f1p0/4170	NA	NA	NA	R01_cb1788_c0/f1p0/4170
R01_cb8564_c41059/f1p0/2332	R01_cb8564_c41059/f1p0/2332	R01_cb8564_c41059/f1p0/2332	R01_cb8564_c41059/f1p0/2332	R01_cb8564_c41059/f1p0/2332
R01_cb13501_c9/f1p0/591	NA	R01_cb13501_c9/f1p0/591	NA	NA
R01_cb7695_c2/f1p0/3798	NA	NA	NA	R01_cb7695_c2/f1p0/3798
R01_cb14239_c15/f1p0/814	R01_cb14239_c15/f1p0/814	R01_cb14239_c15/f1p0/814	NA	R01_cb14239_c15/f1p0/814
R01_cb13106_c16/f1p0/585	R01_cb13106_c16/f1p0/585	R01_cb13106_c16/f1p0/585	R01_cb13106_c16/f1p0/585	R01_cb13106_c16/f1p0/585
R01_cb11460_c2/f1p0/4570	NA	R01_cb11460_c2/f1p0/4570	NA	NA
R01_cb8564_c17007/f1p0/3657	NA	NA	NA	R01_cb8564_c17007/f1p0/3657
R01_cb8564_c92112/f1p0/1974	NA	R01_cb8564_c92112/f1p0/1974	R01_cb8564_c92112/f1p0/1974	R01_cb8564_c92112/f1p0/1974

R01_cb2463_c6/f1p0/2503	NA	NA	NA	R01_cb2463_c6/f1p0/2503
R01_cb18456_c1829/f1p0/651	R01_cb18456_c1829/f1p0/651	R01_cb18456_c1829/f1p0/651	R01_cb18456_c1829/f1p0/651	R01_cb18456_c1829/f1p0/651
R01_cb551_c6/f1p0/4749	NA	R01_cb551_c6/f1p0/4749	NA	NA
R01_cb8564_c73825/f1p0/4350	NA	R01_cb8564_c73825/f1p0/4350	NA	NA
R01_cb8564_c16195/f1p1/4149	NA	R01_cb8564_c16195/f1p1/4149	R01_cb8564_c16195/f1p1/4149	R01_cb8564_c16195/f1p1/4149
R01_cb5340_c0/f1p0/3122	R01_cb5340_c0/f1p0/3122	R01_cb5340_c0/f1p0/3122	R01_cb5340_c0/f1p0/3122	NA
R01_cb16383_c9/f1p0/1463	NA	R01_cb16383_c9/f1p0/1463	R01_cb16383_c9/f1p0/1463	NA
R01_cb18519_c0/f2p0/752	NA	NA	NA	R01_cb18519_c0/f2p0/752
R01_cb9430_c0/f1p0/2087	R01_cb9430_c0/f1p0/2087	R01_cb9430_c0/f1p0/2087	R01_cb9430_c0/f1p0/2087	R01_cb9430_c0/f1p0/2087
R01_cb2822_c4/f1p0/3300	NA	R01_cb2822_c4/f1p0/3300	NA	R01_cb2822_c4/f1p0/3300
R01_cb8564_c50698/f1p0/2433	NA	R01_cb8564_c50698/f1p0/2433	R01_cb8564_c50698/f1p0/2433	R01_cb8564_c50698/f1p0/2433
R01_cb9847_c4/f1p0/2763	NA	R01_cb9847_c4/f1p0/2763	NA	NA
R01_cb2960_c3/f1p0/2973	NA	NA	NA	R01_cb2960_c3/f1p0/2973
R01_cb8564_c78516/f1p0/3292	NA	R01_cb8564_c78516/f1p0/3292	NA	NA
R01_cb8068_c4/f1p0/2466	NA	R01_cb8068_c4/f1p0/2466	NA	NA
R01_cb2834_c7/f1p1/2050	NA	NA	NA	R01_cb2834_c7/f1p1/2050
R01_cb8564_c45869/f1p0/3574	NA	NA	NA	R01_cb8564_c45869/f1p0/3574
R01_cb6680_c2/f1p0/2670	NA	NA	NA	R01_cb6680_c2/f1p0/2670
R01_cb8324_c3/f1p0/2404	NA	NA	NA	R01_cb8324_c3/f1p0/2404
R01_cb2180_c1/f1p0/4023	NA	R01_cb2180_c1/f1p0/4023	NA	NA
R01_cb505_c3/f1p0/2298	NA	R01_cb505_c3/f1p0/2298	NA	NA
R01_cb11526_c1/f1p0/2353	NA	R01_cb11526_c1/f1p0/2353	NA	NA
R01_cb12443_c9/f24p2/498	NA	R01_cb12443_c9/f24p2/498	R01_cb12443_c9/f24p2/498	R01_cb12443_c9/f24p2/498
R01_cb373_c40/f1p0/4542	NA	R01_cb373_c40/f1p0/4542	NA	NA
R01_cb6350_c13/f1p1/1393	NA	R01_cb6350_c13/f1p1/1393	R01_cb6350_c13/f1p1/1393	R01_cb6350_c13/f1p1/1393
R01_cb15725_c8/f2p0/1464	NA	NA	NA	R01_cb15725_c8/f2p0/1464

R01_cb1675_c4/f1p0/3406	NA	NA	NA	R01_cb1675_c4/f1p0/3406
R01_cb8014_c2/f1p0/2482	NA	R01_cb8014_c2/f1p0/2482	NA	R01_cb8014_c2/f1p0/2482
R01_cb17200_c0/f1p0/923	NA	R01_cb17200_c0/f1p0/923	R01_cb17200_c0/f1p0/923	R01_cb17200_c0/f1p0/923
R01_cb6348_c5/f1p0/2756	NA	NA	NA	R01_cb6348_c5/f1p0/2756
R01_cb12883_c4/f1p0/506	R01_cb12883_c4/f1p0/506	R01_cb12883_c4/f1p0/506	NA	NA
R01_cb3650_c17/f1p0/477	R01_cb3650_c17/f1p0/477	R01_cb3650_c17/f1p0/477	R01_cb3650_c17/f1p0/477	R01_cb3650_c17/f1p0/477
R01_cb8650_c2/f1p0/2263	NA	R01_cb8650_c2/f1p0/2263	R01_cb8650_c2/f1p0/2263	NA
R01_cb8564_c71121/f1p0/2073	NA	R01_cb8564_c71121/f1p0/2073	NA	NA
R01_cb6452_c6/f1p0/2905	NA	R01_cb6452_c6/f1p0/2905	NA	R01_cb6452_c6/f1p0/2905
R01_cb16997_c20/f1p0/1834	NA	R01_cb16997_c20/f1p0/1834	R01_cb16997_c20/f1p0/1834	R01_cb16997_c20/f1p0/1834
R01_cb6548_c22/f1p0/2266	R01_cb6548_c22/f1p0/2266	R01_cb6548_c22/f1p0/2266	R01_cb6548_c22/f1p0/2266	R01_cb6548_c22/f1p0/2266
R01_cb10496_c1/f4p0/1487	NA	NA	NA	R01_cb10496_c1/f4p0/1487
R01_cb8564_c18554/f1p0/3189	NA	R01_cb8564_c18554/f1p0/3189	R01_cb8564_c18554/f1p0/3189	R01_cb8564_c18554/f1p0/3189
R01_cb4410_c1/f1p0/3728	NA	R01_cb4410_c1/f1p0/3728	NA	R01_cb4410_c1/f1p0/3728
R01_cb10917_c1/f1p0/1091	NA	NA	NA	R01_cb10917_c1/f1p0/1091
R01_cb16192_c5/f1p0/1057	NA	NA	NA	R01_cb16192_c5/f1p0/1057
R01_cb8582_c3/f1p0/1194	NA	R01_cb8582_c3/f1p0/1194	R01_cb8582_c3/f1p0/1194	R01_cb8582_c3/f1p0/1194
R01_cb8564_c4722/f1p0/2354	NA	R01_cb8564_c4722/f1p0/2354	R01_cb8564_c4722/f1p0/2354	R01_cb8564_c4722/f1p0/2354
R01_cb17649_c1/f1p0/663	NA	NA	NA	R01_cb17649_c1/f1p0/663
R01_cb12955_c8/f1p0/1019	NA	R01_cb12955_c8/f1p0/1019	NA	NA
R01_cb8386_c1/f2p0/2386	R01_cb8386_c1/f2p0/2386	R01_cb8386_c1/f2p0/2386	R01_cb8386_c1/f2p0/2386	R01_cb8386_c1/f2p0/2386
R01_cb2804_c55/f2p0/3404	NA	R01_cb2804_c55/f2p0/3404	NA	NA
R01_cb6906_c4/f1p1/4786	NA	NA	R01_cb6906_c4/f1p1/4786	R01_cb6906_c4/f1p1/4786
R01_cb4117_c4/f1p0/3360	NA	NA	NA	R01_cb4117_c4/f1p0/3360
R01_cb18409_c42/f1p0/968	NA	NA	NA	R01_cb18409_c42/f1p0/968
R01_cb14799_c2/f2p1/1119	R01_cb14799_c2/f2p1/1119	R01_cb14799_c2/f2p1/1119	NA	NA

R01_cb5753_c16/f1p1/2807	NA	R01_cb5753_c16/f1p1/2807	NA	NA
R01_cb8710_c14/f1p0/3389	NA	NA	NA	R01_cb8710_c14/f1p0/3389
R01_cb16545_c2/f1p0/349	R01_cb16545_c2/f1p0/349	R01_cb16545_c2/f1p0/349	R01_cb16545_c2/f1p0/349	R01_cb16545_c2/f1p0/349
R01_cb7334_c6/f1p0/2961	NA	NA	NA	R01_cb7334_c6/f1p0/2961
R01_cb18409_c20/f3p0/496	R01_cb18409_c20/f3p0/496	R01_cb18409_c20/f3p0/496	R01_cb18409_c20/f3p0/496	R01_cb18409_c20/f3p0/496
R01_cb17900_c0/f2p0/693	NA	NA	NA	R01_cb17900_c0/f2p0/693
R01_cb352_c0/f3p0/1755	NA	NA	NA	R01_cb352_c0/f3p0/1755
R01_cb16913_c6/f1p0/1025	NA	R01_cb16913_c6/f1p0/1025	NA	NA
R01_cb8564_c73010/f2p0/3305	NA	R01_cb8564_c73010/f2p0/3305	R01_cb8564_c73010/f2p0/3305	R01_cb8564_c73010/f2p0/3305
R01_cb14921_c2/f1p0/1206	NA	NA	NA	R01_cb14921_c2/f1p0/1206
R01_cb15134_c18/f1p0/1662	NA	NA	NA	R01_cb15134_c18/f1p0/1662
R01_cb6601_c1/f1p0/2811	NA	R01_cb6601_c1/f1p0/2811	NA	NA
R01_cb4529_c9/f1p0/780	NA	R01_cb4529_c9/f1p0/780	NA	NA
R01_cb8116_c0/f3p0/685	NA	R01_cb8116_c0/f3p0/685	R01_cb8116_c0/f3p0/685	R01_cb8116_c0/f3p0/685
R01_cb7108_c4/f1p0/2695	NA	NA	NA	R01_cb7108_c4/f1p0/2695
R01_cb2037_c10/f1p0/3646	NA	R01_cb2037_c10/f1p0/3646	NA	NA
R01_cb8564_c3700/f1p0/2899	NA	NA	R01_cb8564_c3700/f1p0/2899	R01_cb8564_c3700/f1p0/2899
R01_cb17905_c0/f2p0/302	R01_cb17905_c0/f2p0/302	R01_cb17905_c0/f2p0/302	R01_cb17905_c0/f2p0/302	R01_cb17905_c0/f2p0/302
R01_cb8564_c76328/f1p0/2005	NA	R01_cb8564_c76328/f1p0/2005	NA	NA
R01_cb8564_c125315/f1p0/2085	R01_cb8564_c125315/f1p0/2085	R01_cb8564_c125315/f1p0/2085	NA	NA
R01_cb16387_c12/f2p0/1709	NA	NA	NA	R01_cb16387_c12/f2p0/1709
R01_cb18695_c0/f1p0/5329	NA	R01_cb18695_c0/f1p0/5329	NA	NA
R01_cb9533_c3/f1p0/3715	NA	R01_cb9533_c3/f1p0/3715	NA	NA
R01_cb12321_c823/f1p0/631	NA	R01_cb12321_c823/f1p0/631	NA	NA
R01_cb17_c15/f1p0/2470	NA	NA	NA	R01_cb17_c15/f1p0/2470

R01_cb13442_c0/f5p0/785	NA	NA	NA	R01_cb13442_c0/f5p0/785
R01_cb4734_c1/f1p0/2986	NA	R01_cb4734_c1/f1p0/2986	R01_cb4734_c1/f1p0/2986	R01_cb4734_c1/f1p0/2986
R01_cb18456_c5726/f1p7/798	NA	R01_cb18456_c5726/f1p7/798	NA	NA
R01_cb7380_c1/f1p0/2467	R01_cb7380_c1/f1p0/2467	R01_cb7380_c1/f1p0/2467	NA	R01_cb7380_c1/f1p0/2467
R01_cb18132_c1/f1p0/1345	R01_cb18132_c1/f1p0/1345	R01_cb18132_c1/f1p0/1345	R01_cb18132_c1/f1p0/1345	NA
R01_cb1594_c11/f1p0/2163	NA	NA	NA	R01_cb1594_c11/f1p0/2163
R01_cb13545_c43/f1p0/755	NA	R01_cb13545_c43/f1p0/755	R01_cb13545_c43/f1p0/755	R01_cb13545_c43/f1p0/755
R01_cb10696_c2/f1p0/3298	R01_cb10696_c2/f1p0/3298	R01_cb10696_c2/f1p0/3298	R01_cb10696_c2/f1p0/3298	R01_cb10696_c2/f1p0/3298
R01_cb12603_c19/f1p0/643	R01_cb12603_c19/f1p0/643	R01_cb12603_c19/f1p0/643	R01_cb12603_c19/f1p0/643	R01_cb12603_c19/f1p0/643
R01_cb8564_c25380/f1p0/3874	NA	R01_cb8564_c25380/f1p0/3874	NA	NA
R01_cb8564_c24078/f1p1/3014	NA	R01_cb8564_c24078/f1p1/3014	R01_cb8564_c24078/f1p1/3014	R01_cb8564_c24078/f1p1/3014
R01_cb6802_c15/f1p0/2731	NA	R01_cb6802_c15/f1p0/2731	NA	NA
R01_cb5364_c4/f1p0/3115	NA	R01_cb5364_c4/f1p0/3115	NA	NA
R01_cb7596_c4/f1p0/2132	NA	R01_cb7596_c4/f1p0/2132	R01_cb7596_c4/f1p0/2132	R01_cb7596_c4/f1p0/2132
R01_cb8564_c4202/f1p0/2116	NA	R01_cb8564_c4202/f1p0/2116	R01_cb8564_c4202/f1p0/2116	R01_cb8564_c4202/f1p0/2116
R01_cb8564_c902/f2p0/2353	R01_cb8564_c902/f2p0/2353	R01_cb8564_c902/f2p0/2353	R01_cb8564_c902/f2p0/2353	R01_cb8564_c902/f2p0/2353
R01_cb16823_c0/f2p0/1305	NA	NA	NA	R01_cb16823_c0/f2p0/1305
R01_cb18502_c0/f2p0/470	NA	R01_cb18502_c0/f2p0/470	R01_cb18502_c0/f2p0/470	R01_cb18502_c0/f2p0/470
R01_cb14920_c1/f1p0/675	NA	R01_cb14920_c1/f1p0/675	NA	NA
R01_cb8564_c112921/f1p0/1978	NA	R01_cb8564_c112921/f1p0/1978	NA	R01_cb8564_c112921/f1p0/1978
R01_cb8564_c13713/f1p1/3756	NA	R01_cb8564_c13713/f1p1/3756	R01_cb8564_c13713/f1p1/3756	NA
R01_cb4469_c10/f1p0/2911	NA	NA	NA	R01_cb4469_c10/f1p0/2911
R01_cb3500_c17/f1p0/2620	NA	NA	NA	R01_cb3500_c17/f1p0/2620
R01_cb15337_c4/f1p0/1562	NA	NA	NA	R01_cb15337_c4/f1p0/1562
R01_cb1658_c11/f1p0/3913	NA	NA	NA	R01_cb1658_c11/f1p0/3913

R01_cb6024_c8/f1p0/1159	NA	R01_cb6024_c8/f1p0/1159	NA	NA
R01_cb2870_c12/f1p0/2612	NA	NA	NA	R01_cb2870_c12/f1p0/2612
R01_cb15537_c8/f1p0/701	NA	R01_cb15537_c8/f1p0/701	NA	R01_cb15537_c8/f1p0/701
R01_cb4131_c13/f1p0/849	NA	NA	NA	R01_cb4131_c13/f1p0/849
R01_cb6158_c3/f1p0/798	NA	NA	NA	R01_cb6158_c3/f1p0/798
R01_cb14664_c3/f1p0/1551	NA	R01_cb14664_c3/f1p0/1551	NA	NA
R01_cb12628_c0/f2p0/1203	NA	NA	NA	R01_cb12628_c0/f2p0/1203
R01_cb16578_c0/f1p0/607	R01_cb16578_c0/f1p0/607	R01_cb16578_c0/f1p0/607	R01_cb16578_c0/f1p0/607	R01_cb16578_c0/f1p0/607
R01_cb15904_c2/f1p0/571	NA	R01_cb15904_c2/f1p0/571	R01_cb15904_c2/f1p0/571	R01_cb15904_c2/f1p0/571
R01_cb8564_c4628/f1p0/3073	NA	R01_cb8564_c4628/f1p0/3073	R01_cb8564_c4628/f1p0/3073	R01_cb8564_c4628/f1p0/3073
R01_cb4352_c0/f4p0/2496	NA	NA	NA	R01_cb4352_c0/f4p0/2496
R01_cb6348_c0/f5p0/1740	NA	NA	NA	R01_cb6348_c0/f5p0/1740
R01_cb14253_c17/f1p0/896	R01_cb14253_c17/f1p0/896	R01_cb14253_c17/f1p0/896	NA	NA
R01_cb6393_c5/f1p0/2217	NA	NA	NA	R01_cb6393_c5/f1p0/2217
R01_cb18456_c7594/f1p0/1061	NA	R01_cb18456_c7594/f1p0/1061	NA	NA
R01_cb11174_c0/f5p0/1491	NA	R01_cb11174_c0/f5p0/1491	NA	NA
R01_cb10339_c3/f1p0/2245	NA	NA	NA	R01_cb10339_c3/f1p0/2245
R01_cb8824_c3/f1p0/1749	NA	R01_cb8824_c3/f1p0/1749	NA	NA
R01_cb9448_c0/f7p1/1767	NA	NA	NA	R01_cb9448_c0/f7p1/1767
R01_cb3430_c0/f1p0/3618	NA	R01_cb3430_c0/f1p0/3618	R01_cb3430_c0/f1p0/3618	R01_cb3430_c0/f1p0/3618
R01_cb6216_c3/f1p0/2363	NA	NA	NA	R01_cb6216_c3/f1p0/2363
R01_cb8232_c1/f1p1/2570	NA	R01_cb8232_c1/f1p1/2570	R01_cb8232_c1/f1p1/2570	NA
R01_cb14228_c10/f1p0/625	NA	NA	NA	R01_cb14228_c10/f1p0/625
R01_cb6602_c44/f1p2/3121	NA	NA	NA	R01_cb6602_c44/f1p2/3121
R01_cb16368_c1/f1p0/914	R01_cb16368_c1/f1p0/914	R01_cb16368_c1/f1p0/914	R01_cb16368_c1/f1p0/914	R01_cb16368_c1/f1p0/914
R01_cb8564_c14664/f1p0/3045	R01_cb8564_c14664/f1p0/304	R01_cb8564_c14664/f1p0/3045	NA	R01_cb8564_c14664/f1p0/3045

	5			
R01_cb11757_c0/f1p0/1007	R01_cb11757_c0/f1p0/1007	R01_cb11757_c0/f1p0/1007	R01_cb11757_c0/f1p0/1007	R01_cb11757_c0/f1p0/1007
R01_cb3573_c1/f1p0/2640	NA	NA	NA	R01_cb3573_c1/f1p0/2640
R01_cb17336_c1/f1p0/501	NA	R01_cb17336_c1/f1p0/501	R01_cb17336_c1/f1p0/501	R01_cb17336_c1/f1p0/501
R01_cb8564_c44706/f1p0/2375	NA	R01_cb8564_c44706/f1p0/2375	NA	R01_cb8564_c44706/f1p0/2375
R01_cb5807_c1/f2p0/427	NA	R01_cb5807_c1/f2p0/427	R01_cb5807_c1/f2p0/427	R01_cb5807_c1/f2p0/427
R01_cb3564_c13/f1p0/1992	NA	NA	NA	R01_cb3564_c13/f1p0/1992
R01_cb454_c29/f1p0/3267	NA	NA	NA	R01_cb454_c29/f1p0/3267
R01_cb4917_c0/f1p0/3125	NA	NA	NA	R01_cb4917_c0/f1p0/3125
R01_cb917_c8/f1p0/2629	NA	R01_cb917_c8/f1p0/2629	NA	R01_cb917_c8/f1p0/2629
R01_cb13390_c22/f1p0/419	R01_cb13390_c22/f1p0/419	R01_cb13390_c22/f1p0/419	R01_cb13390_c22/f1p0/419	R01_cb13390_c22/f1p0/419
R01_cb7945_c1/f1p0/2283	NA	R01_cb7945_c1/f1p0/2283	NA	R01_cb7945_c1/f1p0/2283
R01_cb6602_c50/f1p0/3164	NA	NA	NA	R01_cb6602_c50/f1p0/3164
R01_cb4784_c4/f1p0/3232	NA	NA	NA	R01_cb4784_c4/f1p0/3232
R01_cb10571_c1/f1p0/2782	NA	R01_cb10571_c1/f1p0/2782	NA	NA
R01_cb18456_c5799/f1p0/561	NA	NA	R01_cb18456_c5799/f1p0/561	R01_cb18456_c5799/f1p0/561
R01_cb13488_c2/f1p0/744	NA	NA	NA	R01_cb13488_c2/f1p0/744
R01_cb4117_c8/f1p0/1672	NA	NA	NA	R01_cb4117_c8/f1p0/1672
R01_cb1864_c8/f1p0/1010	NA	R01_cb1864_c8/f1p0/1010	NA	R01_cb1864_c8/f1p0/1010
R01_cb1333_c62/f1p0/3275	NA	R01_cb1333_c62/f1p0/3275	NA	NA
R01_cb11560_c0/f1p0/1218	NA	NA	NA	R01_cb11560_c0/f1p0/1218
R01_cb8564_c47787/f1p0/4152	NA	NA	NA	R01_cb8564_c47787/f1p0/4152
R01_cb5394_c2/f1p0/2758	NA	NA	NA	R01_cb5394_c2/f1p0/2758
R01_cb1283_c0/f1p0/4343	NA	NA	NA	R01_cb1283_c0/f1p0/4343
R01_cb2601_c7/f1p0/423	R01_cb2601_c7/f1p0/423	R01_cb2601_c7/f1p0/423	R01_cb2601_c7/f1p0/423	R01_cb2601_c7/f1p0/423
R01_cb14152_c0/f4p0/790	NA	NA	NA	R01_cb14152_c0/f4p0/790

R01_cb8728_c2/f1p0/2263	NA	R01_cb8728_c2/f1p0/2263	NA	NA
R01_cb822_c31/f1p0/6069	NA	NA	NA	R01_cb822_c31/f1p0/6069
R01_cb5580_c1/f1p0/3717	NA	R01_cb5580_c1/f1p0/3717	R01_cb5580_c1/f1p0/3717	NA
R01_cb12644_c3/f1p0/521	NA	R01_cb12644_c3/f1p0/521	NA	NA
R01_cb13050_c0/f1p0/1337	NA	R01_cb13050_c0/f1p0/1337	NA	NA
R01_cb4243_c1/f1p0/3383	NA	R01_cb4243_c1/f1p0/3383	NA	NA
R01_cb13161_c22/f1p0/1465	NA	R01_cb13161_c22/f1p0/1465	R01_cb13161_c22/f1p0/1465	R01_cb13161_c22/f1p0/1465
R01_cb16564_c3/f1p0/768	R01_cb16564_c3/f1p0/768	R01_cb16564_c3/f1p0/768	R01_cb16564_c3/f1p0/768	R01_cb16564_c3/f1p0/768
R01_cb5234_c9/f1p0/2515	NA	NA	NA	R01_cb5234_c9/f1p0/2515
R01_cb8564_c87726/f1p0/2795	NA	R01_cb8564_c87726/f1p0/2795	NA	NA
R01_cb8564_c33774/f3p0/2387	R01_cb8564_c33774/f3p0/2387	R01_cb8564_c33774/f3p0/2387	R01_cb8564_c33774/f3p0/2387	R01_cb8564_c33774/f3p0/2387
R01_cb4822_c2/f1p0/495	R01_cb4822_c2/f1p0/495	R01_cb4822_c2/f1p0/495	R01_cb4822_c2/f1p0/495	R01_cb4822_c2/f1p0/495
R01_cb8564_c21500/f1p0/4129	NA	R01_cb8564_c21500/f1p0/4129	NA	NA
R01_cb16296_c1/f1p0/870	NA	NA	R01_cb16296_c1/f1p0/870	R01_cb16296_c1/f1p0/870
R01_cb11128_c1/f1p0/1599	NA	R01_cb11128_c1/f1p0/1599	NA	R01_cb11128_c1/f1p0/1599
R01_cb3114_c2/f1p1/3969	NA	NA	NA	R01_cb3114_c2/f1p1/3969
R01_cb8564_c52791/f1p0/4346	NA	R01_cb8564_c52791/f1p0/4346	NA	NA
R01_cb2804_c144/f1p0/2003	NA	R01_cb2804_c144/f1p0/2003	R01_cb2804_c144/f1p0/2003	R01_cb2804_c144/f1p0/2003
R01_cb16018_c4/f1p0/1417	R01_cb16018_c4/f1p0/1417	R01_cb16018_c4/f1p0/1417	R01_cb16018_c4/f1p0/1417	R01_cb16018_c4/f1p0/1417
R01_cb7845_c0/f1p0/2521	NA	R01_cb7845_c0/f1p0/2521	NA	NA
R01_cb11429_c3/f1p0/1331	NA	NA	NA	R01_cb11429_c3/f1p0/1331
R01_cb11833_c1/f1p0/1962	R01_cb11833_c1/f1p0/1962	R01_cb11833_c1/f1p0/1962	R01_cb11833_c1/f1p0/1962	R01_cb11833_c1/f1p0/1962
R01_cb18456_c6444/f1p0/646	R01_cb18456_c6444/f1p0/646	R01_cb18456_c6444/f1p0/646	R01_cb18456_c6444/f1p0/646	R01_cb18456_c6444/f1p0/646
R01_cb11171_c0/f1p0/1251	NA	R01_cb11171_c0/f1p0/1251	NA	R01_cb11171_c0/f1p0/1251
R01_cb18456_c827/f1p1/495	R01_cb18456_c827/f1p1/495	R01_cb18456_c827/f1p1/495	R01_cb18456_c827/f1p1/495	R01_cb18456_c827/f1p1/495

R01_cb13525_c1/f1p0/898	NA	R01_cb13525_c1/f1p0/898	NA	NA
R01_cb494_c17/f1p0/423	R01_cb494_c17/f1p0/423	R01_cb494_c17/f1p0/423	R01_cb494_c17/f1p0/423	R01_cb494_c17/f1p0/423
R01_cb14058_c0/f1p0/686	NA	R01_cb14058_c0/f1p0/686	NA	NA
R01_cb454_c40/f1p0/2415	NA	NA	NA	R01_cb454_c40/f1p0/2415
R01_cb8564_c51500/f1p0/2801	NA	R01_cb8564_c51500/f1p0/2801	NA	R01_cb8564_c51500/f1p0/2801
R01_cb8564_c68292/f4p0/2395	NA	R01_cb8564_c68292/f4p0/2395	R01_cb8564_c68292/f4p0/2395	R01_cb8564_c68292/f4p0/2395
R01_cb391_c0/f1p0/4835	NA	NA	NA	R01_cb391_c0/f1p0/4835
R01_cb7228_c8/f1p0/3020	NA	NA	NA	R01_cb7228_c8/f1p0/3020
R01_cb18456_c1386/f6p0/556	NA	R01_cb18456_c1386/f6p0/556	R01_cb18456_c1386/f6p0/556	R01_cb18456_c1386/f6p0/556
R01_cb10843_c4/f1p0/1752	R01_cb10843_c4/f1p0/1752	R01_cb10843_c4/f1p0/1752	R01_cb10843_c4/f1p0/1752	R01_cb10843_c4/f1p0/1752
R01_cb7064_c7/f1p0/3393	NA	R01_cb7064_c7/f1p0/3393	NA	NA
R01_cb3388_c13/f1p0/2298	NA	R01_cb3388_c13/f1p0/2298	NA	NA
R01_cb8564_c3280/f1p0/3250	NA	R01_cb8564_c3280/f1p0/3250	NA	R01_cb8564_c3280/f1p0/3250
R01_cb8564_c13068/f2p0/4131	NA	R01_cb8564_c13068/f2p0/4131	NA	NA
R01_cb15128_c4/f1p0/1673	NA	NA	NA	R01_cb15128_c4/f1p0/1673
R01_cb10366_c5/f1p0/1573	NA	NA	NA	R01_cb10366_c5/f1p0/1573
R01_cb13345_c0/f9p0/1156	NA	R01_cb13345_c0/f9p0/1156	R01_cb13345_c0/f9p0/1156	R01_cb13345_c0/f9p0/1156
R01_cb18456_c7896/f2p0/344	R01_cb18456_c7896/f2p0/344	R01_cb18456_c7896/f2p0/344	R01_cb18456_c7896/f2p0/344	NA
R01_cb8564_c77197/f1p0/2222	NA	NA	NA	R01_cb8564_c77197/f1p0/2222
R01_cb10034_c77/f1p3/649	NA	R01_cb10034_c77/f1p3/649	NA	NA
R01_cb16574_c1/f1p0/1694	NA	NA	NA	R01_cb16574_c1/f1p0/1694
R01_cb9791_c1/f1p0/1861	NA	R01_cb9791_c1/f1p0/1861	NA	R01_cb9791_c1/f1p0/1861
R01_cb8564_c119356/f1p0/2280	R01_cb8564_c119356/f1p0/2280	R01_cb8564_c119356/f1p0/2280	R01_cb8564_c119356/f1p0/2280	R01_cb8564_c119356/f1p0/2280
R01_cb18456_c7430/f1p0/637	NA	NA	R01_cb18456_c7430/f1p0/637	R01_cb18456_c7430/f1p0/637
R01_cb1945_c19/f1p1/4836	NA	R01_cb1945_c19/f1p1/4836	NA	NA

R01_cb16251_c0/f1p0/1418	NA	R01_cb16251_c0/f1p0/1418	NA	NA
R01_cb2698_c4/f1p0/1944	NA	NA	NA	R01_cb2698_c4/f1p0/1944
R01_cb17861_c0/f3p1/1422	NA	NA	NA	R01_cb17861_c0/f3p1/1422
R01_cb11499_c0/f1p0/1694	NA	NA	NA	R01_cb11499_c0/f1p0/1694
R01_cb8944_c0/f1p0/2225	R01_cb8944_c0/f1p0/2225	R01_cb8944_c0/f1p0/2225	NA	NA
R01_cb8564_c145767/f2p0/2282	NA	R01_cb8564_c145767/f2p0/2282	R01_cb8564_c145767/f2p0/2282	R01_cb8564_c145767/f2p0/2282
R01_cb191_c14/f1p0/5848	NA	NA	NA	R01_cb191_c14/f1p0/5848
R01_cb5904_c25/f2p1/1382	NA	R01_cb5904_c25/f2p1/1382	NA	NA
R01_cb5950_c4/f1p0/2590	NA	NA	NA	R01_cb5950_c4/f1p0/2590
R01_cb16321_c2/f1p0/1730	R01_cb16321_c2/f1p0/1730	R01_cb16321_c2/f1p0/1730	NA	R01_cb16321_c2/f1p0/1730
R01_cb8564_c74524/f1p0/3137	NA	R01_cb8564_c74524/f1p0/3137	NA	NA
R01_cb8564_c21788/f1p0/2347	R01_cb8564_c21788/f1p0/2347	R01_cb8564_c21788/f1p0/2347	R01_cb8564_c21788/f1p0/2347	R01_cb8564_c21788/f1p0/2347
R01_cb5896_c20/f1p0/2762	NA	R01_cb5896_c20/f1p0/2762	NA	NA
R01_cb18542_c1/f1p0/505	NA	R01_cb18542_c1/f1p0/505	NA	NA
R01_cb5896_c133/f1p0/2433	NA	R01_cb5896_c133/f1p0/2433	NA	NA
R01_cb15782_c2/f2p0/1464	NA	NA	NA	R01_cb15782_c2/f2p0/1464
R01_cb2022_c9/f1p1/2351	NA	NA	NA	R01_cb2022_c9/f1p1/2351
R01_cb8261_c0/f1p0/2416	NA	R01_cb8261_c0/f1p0/2416	NA	NA
R01_cb6480_c2/f1p1/2681	NA	NA	NA	R01_cb6480_c2/f1p1/2681
R01_cb7773_c2/f1p0/2538	NA	R01_cb7773_c2/f1p0/2538	NA	R01_cb7773_c2/f1p0/2538
R01_cb16295_c0/f1p0/1333	NA	NA	NA	R01_cb16295_c0/f1p0/1333
R01_cb17744_c18/f1p0/2301	NA	NA	NA	R01_cb17744_c18/f1p0/2301
R01_cb18226_c1/f1p0/1254	R01_cb18226_c1/f1p0/1254	R01_cb18226_c1/f1p0/1254	R01_cb18226_c1/f1p0/1254	R01_cb18226_c1/f1p0/1254
R01_cb16094_c2/f1p0/945	NA	R01_cb16094_c2/f1p0/945	NA	R01_cb16094_c2/f1p0/945

R01_cb8564_c75478/f1p0/3890	R01_cb8564_c75478/f1p0/3890	R01_cb8564_c75478/f1p0/3890	R01_cb8564_c75478/f1p0/3890	R01_cb8564_c75478/f1p0/3890
R01_cb8564_c113838/f1p0/2481	NA	NA	R01_cb8564_c113838/f1p0/2481	R01_cb8564_c113838/f1p0/2481
R01_cb8564_c116622/f1p0/2948	NA	R01_cb8564_c116622/f1p0/2948	NA	NA
R01_cb17266_c20/f1p1/1365	NA	R01_cb17266_c20/f1p1/1365	R01_cb17266_c20/f1p1/1365	R01_cb17266_c20/f1p1/1365
R01_cb2206_c3/f1p1/3943	NA	R01_cb2206_c3/f1p1/3943	NA	NA
R01_cb1333_c50/f1p0/4810	NA	R01_cb1333_c50/f1p0/4810	NA	NA
R01_cb16227_c2/f1p0/821	R01_cb16227_c2/f1p0/821	R01_cb16227_c2/f1p0/821	NA	NA
R01_cb18138_c0/f1p0/397	R01_cb18138_c0/f1p0/397	R01_cb18138_c0/f1p0/397	R01_cb18138_c0/f1p0/397	R01_cb18138_c0/f1p0/397
R01_cb10515_c5/f1p0/570	NA	R01_cb10515_c5/f1p0/570	NA	NA
R01_cb18128_c1/f1p0/1328	NA	NA	NA	R01_cb18128_c1/f1p0/1328
R01_cb3921_c5/f1p0/1879	NA	NA	NA	R01_cb3921_c5/f1p0/1879
R01_cb3582_c4/f1p0/2583	NA	R01_cb3582_c4/f1p0/2583	NA	R01_cb3582_c4/f1p0/2583
R01_cb10704_c8/f1p0/671	R01_cb10704_c8/f1p0/671	R01_cb10704_c8/f1p0/671	R01_cb10704_c8/f1p0/671	R01_cb10704_c8/f1p0/671
R01_cb8564_c83190/f1p1/3641	NA	R01_cb8564_c83190/f1p1/3641	NA	NA
R01_cb5139_c15/f1p0/3114	NA	NA	NA	R01_cb5139_c15/f1p0/3114
R01_cb8564_c39850/f1p0/1894	NA	R01_cb8564_c39850/f1p0/1894	R01_cb8564_c39850/f1p0/1894	R01_cb8564_c39850/f1p0/1894
R01_cb8564_c4637/f1p0/2556	NA	R01_cb8564_c4637/f1p0/2556	NA	NA
R01_cb13682_c3/f1p0/914	R01_cb13682_c3/f1p0/914	R01_cb13682_c3/f1p0/914	NA	R01_cb13682_c3/f1p0/914
R01_cb8456_c157/f1p0/587	NA	R01_cb8456_c157/f1p0/587	NA	NA
R01_cb12314_c5/f1p0/578	NA	NA	R01_cb12314_c5/f1p0/578	NA
R01_cb2425_c7/f1p0/1904	NA	NA	NA	R01_cb2425_c7/f1p0/1904
R01_cb10388_c4/f1p0/1270	NA	NA	R01_cb10388_c4/f1p0/1270	NA
R01_cb454_c27/f1p0/2557	NA	NA	NA	R01_cb454_c27/f1p0/2557

R01_cb4809_c12/f1p0/5024	NA	R01_cb4809_c12/f1p0/5024	NA	R01_cb4809_c12/f1p0/5024
R01_cb7219_c19/f2p3/2439	NA	NA	NA	R01_cb7219_c19/f2p3/2439
R01_cb12896_c2/f1p0/1485	R01_cb12896_c2/f1p0/1485	R01_cb12896_c2/f1p0/1485	R01_cb12896_c2/f1p0/1485	R01_cb12896_c2/f1p0/1485
R01_cb11321_c3/f1p0/1327	NA	NA	NA	R01_cb11321_c3/f1p0/1327
R01_cb6299_c1/f1p0/2896	NA	R01_cb6299_c1/f1p0/2896	NA	NA
R01_cb16125_c14/f1p0/1582	NA	R01_cb16125_c14/f1p0/1582	NA	R01_cb16125_c14/f1p0/1582
R01_cb6938_c1/f1p0/2232	NA	R01_cb6938_c1/f1p0/2232	NA	R01_cb6938_c1/f1p0/2232
R01_cb12480_c16/f1p0/835	NA	R01_cb12480_c16/f1p0/835	R01_cb12480_c16/f1p0/835	R01_cb12480_c16/f1p0/835
R01_cb17130_c0/f1p0/733	R01_cb17130_c0/f1p0/733	R01_cb17130_c0/f1p0/733	R01_cb17130_c0/f1p0/733	R01_cb17130_c0/f1p0/733
R01_cb6057_c2/f1p0/2077	NA	NA	NA	R01_cb6057_c2/f1p0/2077
R01_cb14587_c0/f1p0/780	NA	R01_cb14587_c0/f1p0/780	NA	R01_cb14587_c0/f1p0/780
R01_cb8564_c43422/f1p0/2948	NA	R01_cb8564_c43422/f1p0/2948	R01_cb8564_c43422/f1p0/2948	R01_cb8564_c43422/f1p0/2948
R01_cb4581_c12/f1p0/2085	NA	R01_cb4581_c12/f1p0/2085	R01_cb4581_c12/f1p0/2085	NA
R01_cb14937_c3/f1p0/916	NA	NA	R01_cb14937_c3/f1p0/916	R01_cb14937_c3/f1p0/916
R01_cb12024_c7/f1p0/1003	NA	NA	NA	R01_cb12024_c7/f1p0/1003
R01_cb1626_c14/f1p0/2173	NA	NA	NA	R01_cb1626_c14/f1p0/2173
R01_cb14_c28/f1p0/3251	NA	R01_cb14_c28/f1p0/3251	NA	NA
R01_cb402_c19/f1p0/2579	NA	NA	NA	R01_cb402_c19/f1p0/2579
R01_cb12785_c37/f2p1/929	NA	NA	R01_cb12785_c37/f2p1/929	NA
R01_cb326_c5/f1p0/2325	NA	NA	NA	R01_cb326_c5/f1p0/2325
R01_cb12096_c1/f1p0/1130	NA	R01_cb12096_c1/f1p0/1130	R01_cb12096_c1/f1p0/1130	R01_cb12096_c1/f1p0/1130
R01_cb7528_c3/f1p0/1980	R01_cb7528_c3/f1p0/1980	R01_cb7528_c3/f1p0/1980	R01_cb7528_c3/f1p0/1980	R01_cb7528_c3/f1p0/1980
R01_cb10293_c10/f1p0/1244	NA	R01_cb10293_c10/f1p0/1244	NA	NA
R01_cb4489_c19/f2p0/1037	NA	R01_cb4489_c19/f2p0/1037	NA	NA
R01_cb14816_c9/f1p0/575	R01_cb14816_c9/f1p0/575	R01_cb14816_c9/f1p0/575	R01_cb14816_c9/f1p0/575	R01_cb14816_c9/f1p0/575
R01_cb4866_c8/f1p1/2481	NA	NA	NA	R01_cb4866_c8/f1p1/2481

R01_cb3716_c4/f1p0/3546	NA	NA	NA	R01_cb3716_c4/f1p0/3546
R01_cb6158_c1/f1p0/2514	R01_cb6158_c1/f1p0/2514	R01_cb6158_c1/f1p0/2514	NA	R01_cb6158_c1/f1p0/2514
R01_cb18525_c1/f1p0/522	R01_cb18525_c1/f1p0/522	R01_cb18525_c1/f1p0/522	R01_cb18525_c1/f1p0/522	R01_cb18525_c1/f1p0/522
R01_cb3500_c23/f1p0/3788	NA	NA	NA	R01_cb3500_c23/f1p0/3788
R01_cb8564_c3957/f1p0/2652	NA	NA	NA	R01_cb8564_c3957/f1p0/2652
R01_cb15537_c3/f1p0/608	R01_cb15537_c3/f1p0/608	R01_cb15537_c3/f1p0/608	R01_cb15537_c3/f1p0/608	R01_cb15537_c3/f1p0/608
R01_cb1614_c24/f1p0/452	R01_cb1614_c24/f1p0/452	R01_cb1614_c24/f1p0/452	NA	R01_cb1614_c24/f1p0/452
R01_cb875_c4/f1p0/2945	NA	NA	NA	R01_cb875_c4/f1p0/2945
R01_cb4016_c47/f1p0/1965	R01_cb4016_c47/f1p0/1965	R01_cb4016_c47/f1p0/1965	R01_cb4016_c47/f1p0/1965	R01_cb4016_c47/f1p0/1965
R01_cb7258_c5/f1p0/1369	NA	R01_cb7258_c5/f1p0/1369	R01_cb7258_c5/f1p0/1369	NA
R01_cb5807_c2/f1p0/3003	NA	R01_cb5807_c2/f1p0/3003	NA	R01_cb5807_c2/f1p0/3003
R01_cb11530_c1/f1p0/3901	NA	R01_cb11530_c1/f1p0/3901	NA	NA
R01_cb12301_c74/f1p0/750	NA	R01_cb12301_c74/f1p0/750	NA	NA
R01_cb16256_c0/f1p0/1266	NA	R01_cb16256_c0/f1p0/1266	NA	NA
R01_cb5672_c29/f1p0/1142	NA	R01_cb5672_c29/f1p0/1142	R01_cb5672_c29/f1p0/1142	R01_cb5672_c29/f1p0/1142
R01_cb1138_c51/f1p1/1003	NA	NA	R01_cb1138_c51/f1p1/1003	R01_cb1138_c51/f1p1/1003
R01_cb7499_c2/f1p0/2333	R01_cb7499_c2/f1p0/2333	NA	NA	NA
R01_cb1353_c2/f1p1/1576	NA	R01_cb1353_c2/f1p1/1576	NA	NA
R01_cb8564_c124097/f1p0/2107	R01_cb8564_c124097/f1p0/2107	R01_cb8564_c124097/f1p0/2107	R01_cb8564_c124097/f1p0/2107	R01_cb8564_c124097/f1p0/2107
R01_cb8564_c86997/f1p0/2960	NA	R01_cb8564_c86997/f1p0/2960	NA	NA
R01_cb8564_c10051/f3p1/3001	NA	NA	NA	R01_cb8564_c10051/f3p1/3001
R01_cb13364_c1/f1p2/1348	NA	R01_cb13364_c1/f1p2/1348	NA	NA
R01_cb3006_c6/f1p1/1782	NA	NA	NA	R01_cb3006_c6/f1p1/1782
R01_cb12490_c12/f1p0/1008	NA	R01_cb12490_c12/f1p0/1008	NA	NA
R01_cb16657_c1/f1p0/747	NA	R01_cb16657_c1/f1p0/747	NA	R01_cb16657_c1/f1p0/747

R01_cb9116_c8/f1p1/3762	NA	R01_cb9116_c8/f1p1/3762	NA	NA
R01_cb8349_c36/f1p0/2219	NA	R01_cb8349_c36/f1p0/2219	NA	NA
R01_cb6735_c7/f1p0/1986	NA	R01_cb6735_c7/f1p0/1986	NA	NA
R01_cb8564_c88021/f1p0/3390	NA	NA	NA	R01_cb8564_c88021/f1p0/3390
R01_cb8564_c128337/f1p0/2102	NA	R01_cb8564_c128337/f1p0/2102	NA	R01_cb8564_c128337/f1p0/2102
R01_cb5248_c15/f1p1/3624	NA	R01_cb5248_c15/f1p1/3624	NA	R01_cb5248_c15/f1p1/3624
R01_cb4923_c4/f1p0/2860	NA	NA	NA	R01_cb4923_c4/f1p0/2860
R01_cb18455_c1/f1p0/1452	NA	R01_cb18455_c1/f1p0/1452	NA	R01_cb18455_c1/f1p0/1452
R01_cb8564_c117310/f1p0/2444	NA	R01_cb8564_c117310/f1p0/2444	R01_cb8564_c117310/f1p0/2444	NA
R01_cb8564_c121879/f1p0/2935	R01_cb8564_c121879/f1p0/2935	R01_cb8564_c121879/f1p0/2935	NA	R01_cb8564_c121879/f1p0/2935
R01_cb15701_c8/f1p0/1532	NA	R01_cb15701_c8/f1p0/1532	NA	NA
R01_cb13427_c1/f1p0/875	NA	NA	NA	R01_cb13427_c1/f1p0/875
R01_cb16141_c1/f1p0/1546	R01_cb16141_c1/f1p0/1546	R01_cb16141_c1/f1p0/1546	R01_cb16141_c1/f1p0/1546	R01_cb16141_c1/f1p0/1546
R01_cb17887_c0/f1p0/1156	NA	NA	NA	R01_cb17887_c0/f1p0/1156
R01_cb9380_c4/f1p0/2049	NA	NA	NA	R01_cb9380_c4/f1p0/2049
R01_cb17704_c2/f1p0/1116	NA	R01_cb17704_c2/f1p0/1116	NA	NA
R01_cb8564_c92063/f1p0/2144	R01_cb8564_c92063/f1p0/2144	R01_cb8564_c92063/f1p0/2144	R01_cb8564_c92063/f1p0/2144	R01_cb8564_c92063/f1p0/2144
R01_cb18132_c23/f1p0/1186	NA	R01_cb18132_c23/f1p0/1186	NA	NA
R01_cb2104_c7/f1p0/2209	NA	R01_cb2104_c7/f1p0/2209	R01_cb2104_c7/f1p0/2209	R01_cb2104_c7/f1p0/2209
R01_cb8564_c83927/f1p0/3742	NA	R01_cb8564_c83927/f1p0/3742	NA	NA
R01_cb18705_c0/f1p0/6297	NA	R01_cb18705_c0/f1p0/6297	NA	R01_cb18705_c0/f1p0/6297
R01_cb17918_c3/f1p0/1349	NA	NA	NA	R01_cb17918_c3/f1p0/1349

R01_cb18527_c0/f1p0/1762	NA	NA	NA	R01_cb18527_c0/f1p0/1762
R01_cb5807_c8/f1p0/1053	NA	R01_cb5807_c8/f1p0/1053	R01_cb5807_c8/f1p0/1053	R01_cb5807_c8/f1p0/1053
R01_cb17266_c16/f1p0/1079	R01_cb17266_c16/f1p0/1079	R01_cb17266_c16/f1p0/1079	NA	NA
R01_cb8564_c128459/f1p0/2112	NA	R01_cb8564_c128459/f1p0/2112	NA	R01_cb8564_c128459/f1p0/2112
R01_cb17302_c1/f1p0/1490	NA	NA	NA	R01_cb17302_c1/f1p0/1490
R01_cb8564_c46177/f1p0/1967	NA	R01_cb8564_c46177/f1p0/1967	R01_cb8564_c46177/f1p0/1967	R01_cb8564_c46177/f1p0/1967
R01_cb7564_c36/f1p0/1940	NA	R01_cb7564_c36/f1p0/1940	NA	NA
R01_cb920_c3/f1p1/4571	NA	NA	NA	R01_cb920_c3/f1p1/4571
R01_cb15679_c1/f1p0/1332	NA	NA	NA	R01_cb15679_c1/f1p0/1332
R01_cb18650_c6/f1p2/5458	NA	NA	NA	R01_cb18650_c6/f1p2/5458
R01_cb8582_c2/f1p0/1247	NA	R01_cb8582_c2/f1p0/1247	NA	R01_cb8582_c2/f1p0/1247
R01_cb7333_c2/f1p0/1087	NA	R01_cb7333_c2/f1p0/1087	NA	R01_cb7333_c2/f1p0/1087
R01_cb6141_c0/f4p0/709	NA	R01_cb6141_c0/f4p0/709	NA	NA
R01_cb14205_c1/f3p0/1864	NA	R01_cb14205_c1/f3p0/1864	NA	NA
R01_cb12504_c5/f1p0/984	NA	R01_cb12504_c5/f1p0/984	NA	R01_cb12504_c5/f1p0/984
R01_cb5900_c136/f1p0/2285	NA	R01_cb5900_c136/f1p0/2285	NA	R01_cb5900_c136/f1p0/2285
R01_cb17786_c2/f1p1/514	NA	R01_cb17786_c2/f1p1/514	NA	R01_cb17786_c2/f1p1/514
R01_cb6615_c1/f4p0/1383	NA	NA	NA	R01_cb6615_c1/f4p0/1383
R01_cb12722_c10/f1p0/1359	NA	NA	NA	R01_cb12722_c10/f1p0/1359
R01_cb3699_c20/f1p0/2136	NA	R01_cb3699_c20/f1p0/2136	NA	NA
R01_cb17122_c1/f1p0/843	NA	R01_cb17122_c1/f1p0/843	NA	NA
R01_cb16199_c0/f2p0/837	NA	R01_cb16199_c0/f2p0/837	R01_cb16199_c0/f2p0/837	R01_cb16199_c0/f2p0/837
R01_cb18456_c4643/f1p0/453	NA	R01_cb18456_c4643/f1p0/453	NA	R01_cb18456_c4643/f1p0/453
R01_cb10817_c1/f1p0/4352	NA	NA	NA	R01_cb10817_c1/f1p0/4352
R01_cb15514_c2/f1p0/5584	NA	R01_cb15514_c2/f1p0/5584	R01_cb15514_c2/f1p0/5584	R01_cb15514_c2/f1p0/5584

R01_cb8564_c70941/f1p1/2445	NA	R01_cb8564_c70941/f1p1/2445	R01_cb8564_c70941/f1p1/2445	R01_cb8564_c70941/f1p1/2445
R01_cb14353_c2/f1p0/1293	NA	R01_cb14353_c2/f1p0/1293	R01_cb14353_c2/f1p0/1293	R01_cb14353_c2/f1p0/1293
R01_cb16148_c0/f1p0/1697	NA	R01_cb16148_c0/f1p0/1697	NA	NA
R01_cb2082_c10/f1p0/3869	NA	NA	NA	R01_cb2082_c10/f1p0/3869
R01_cb16953_c0/f1p0/695	NA	NA	R01_cb16953_c0/f1p0/695	R01_cb16953_c0/f1p0/695
R01_cb8564_c127488/f1p0/2913	NA	R01_cb8564_c127488/f1p0/2913	R01_cb8564_c127488/f1p0/2913	R01_cb8564_c127488/f1p0/2913
R01_cb8564_c71606/f1p0/2007	NA	R01_cb8564_c71606/f1p0/2007	NA	R01_cb8564_c71606/f1p0/2007
R01_cb15712_c2/f1p0/687	NA	R01_cb15712_c2/f1p0/687	NA	R01_cb15712_c2/f1p0/687
R01_cb8564_c89497/f1p0/3017	R01_cb8564_c89497/f1p0/3017	R01_cb8564_c89497/f1p0/3017	R01_cb8564_c89497/f1p0/3017	R01_cb8564_c89497/f1p0/3017
R01_cb7766_c6/f1p0/3745	R01_cb7766_c6/f1p0/3745	R01_cb7766_c6/f1p0/3745	NA	NA
R01_cb3359_c18/f1p0/3573	NA	R01_cb3359_c18/f1p0/3573	NA	NA
R01_cb12573_c16/f1p0/747	NA	R01_cb12573_c16/f1p0/747	R01_cb12573_c16/f1p0/747	NA
R01_cb13877_c2/f1p0/1479	NA	NA	NA	R01_cb13877_c2/f1p0/1479
R01_cb8515_c1/f1p0/2347	NA	R01_cb8515_c1/f1p0/2347	NA	NA
R01_cb17708_c0/f1p0/1789	NA	R01_cb17708_c0/f1p0/1789	R01_cb17708_c0/f1p0/1789	NA
R01_cb11099_c0/f3p0/1823	NA	NA	NA	R01_cb11099_c0/f3p0/1823
R01_cb16526_c3/f1p0/847	NA	NA	R01_cb16526_c3/f1p0/847	R01_cb16526_c3/f1p0/847
R01_cb16206_c0/f2p0/1413	NA	NA	NA	R01_cb16206_c0/f2p0/1413
R01_cb3087_c9/f1p0/4385	NA	NA	NA	R01_cb3087_c9/f1p0/4385
R01_cb218_c2/f2p0/4554	NA	NA	NA	R01_cb218_c2/f2p0/4554
R01_cb8982_c2/f3p0/2160	NA	NA	NA	R01_cb8982_c2/f3p0/2160
R01_cb13386_c22/f1p0/1071	NA	R01_cb13386_c22/f1p0/1071	NA	NA
R01_cb8564_c118550/f1p0/3959	NA	R01_cb8564_c118550/f1p0/3959	NA	NA

R01_cb12573_c10/f1p0/646	NA	NA	NA	R01_cb12573_c10/f1p0/646
R01_cb11176_c2/f1p0/2534	R01_cb11176_c2/f1p0/2534	R01_cb11176_c2/f1p0/2534	R01_cb11176_c2/f1p0/2534	R01_cb11176_c2/f1p0/2534
R01_cb8030_c7/f4p0/2422	NA	NA	NA	R01_cb8030_c7/f4p0/2422
R01_cb18456_c7296/f1p0/410	R01_cb18456_c7296/f1p0/410	R01_cb18456_c7296/f1p0/410	R01_cb18456_c7296/f1p0/410	R01_cb18456_c7296/f1p0/410
R01_cb8564_c126964/f1p0/2909	NA	NA	NA	R01_cb8564_c126964/f1p0/2909
R01_cb8564_c87268/f1p0/3745	NA	R01_cb8564_c87268/f1p0/3745	NA	NA
R01_cb8564_c13752/f1p0/4357	NA	NA	NA	R01_cb8564_c13752/f1p0/4357
R01_cb2643_c11/f1p1/3425	NA	R01_cb2643_c11/f1p1/3425	NA	NA
R01_cb9357_c5/f1p0/1681	R01_cb9357_c5/f1p0/1681	R01_cb9357_c5/f1p0/1681	R01_cb9357_c5/f1p0/1681	R01_cb9357_c5/f1p0/1681
R01_cb8564_c37837/f2p0/2810	NA	NA	R01_cb8564_c37837/f2p0/2810	R01_cb8564_c37837/f2p0/2810
R01_cb8564_c88494/f1p1/2983	NA	R01_cb8564_c88494/f1p1/2983	NA	NA
R01_cb8354_c14/f1p0/5298	NA	NA	NA	R01_cb8354_c14/f1p0/5298
R01_cb8564_c2619/f1p0/2635	R01_cb8564_c2619/f1p0/2635	R01_cb8564_c2619/f1p0/2635	NA	R01_cb8564_c2619/f1p0/2635
R01_cb9035_c3/f1p1/2199	NA	NA	NA	R01_cb9035_c3/f1p1/2199
R01_cb11603_c5/f1p0/919	NA	R01_cb11603_c5/f1p0/919	NA	NA
R01_cb16540_c1/f10p0/721	NA	NA	R01_cb16540_c1/f10p0/721	R01_cb16540_c1/f10p0/721
R01_cb12716_c0/f2p0/1491	NA	NA	NA	R01_cb12716_c0/f2p0/1491
R01_cb1425_c7/f1p0/2088	NA	NA	NA	R01_cb1425_c7/f1p0/2088
R01_cb14587_c1/f1p0/865	NA	R01_cb14587_c1/f1p0/865	NA	R01_cb14587_c1/f1p0/865
R01_cb6742_c8/f1p0/2804	NA	NA	NA	R01_cb6742_c8/f1p0/2804
R01_cb8564_c39123/f1p1/3996	R01_cb8564_c39123/f1p1/3996	R01_cb8564_c39123/f1p1/3996	R01_cb8564_c39123/f1p1/3996	R01_cb8564_c39123/f1p1/3996
R01_cb8564_c90564/f1p0/2758	NA	NA	NA	R01_cb8564_c90564/f1p0/2758
R01_cb10226_c2/f1p0/816	NA	R01_cb10226_c2/f1p0/816	R01_cb10226_c2/f1p0/816	R01_cb10226_c2/f1p0/816
R01_cb17266_c33/f1p1/1372	NA	R01_cb17266_c33/f1p1/1372	R01_cb17266_c33/f1p1/1372	R01_cb17266_c33/f1p1/1372

R01_cb17049_c0/f2p0/845	NA	NA	NA	R01_cb17049_c0/f2p0/845
R01_cb4352_c10/f1p0/2137	NA	NA	NA	R01_cb4352_c10/f1p0/2137
R01_cb15569_c0/f5p0/1464	NA	NA	R01_cb15569_c0/f5p0/1464	NA
R01_cb7501_c2/f1p0/2602	NA	NA	NA	R01_cb7501_c2/f1p0/2602
R01_cb8564_c89725/f1p1/3641	NA	NA	NA	R01_cb8564_c89725/f1p1/3641
R01_cb16920_c0/f1p0/523	R01_cb16920_c0/f1p0/523	R01_cb16920_c0/f1p0/523	R01_cb16920_c0/f1p0/523	R01_cb16920_c0/f1p0/523
R01_cb11529_c1/f1p0/1940	NA	R01_cb11529_c1/f1p0/1940	NA	NA
R01_cb6456_c6/f1p0/979	NA	NA	NA	R01_cb6456_c6/f1p0/979
R01_cb8564_c116216/f1p0/4369	R01_cb8564_c116216/f1p0/4369	R01_cb8564_c116216/f1p0/4369	NA	NA
R01_cb9970_c1/f1p0/3500	NA	R01_cb9970_c1/f1p0/3500	NA	NA
R01_cb3131_c0/f1p0/3713	NA	R01_cb3131_c0/f1p0/3713	NA	NA
R01_cb2213_c0/f1p0/3937	NA	NA	NA	R01_cb2213_c0/f1p0/3937
R01_cb4536_c4/f1p0/2190	NA	R01_cb4536_c4/f1p0/2190	NA	NA
R01_cb10376_c2/f1p0/1963	NA	R01_cb10376_c2/f1p0/1963	NA	NA
R01_cb15394_c2/f1p0/386	R01_cb15394_c2/f1p0/386	R01_cb15394_c2/f1p0/386	R01_cb15394_c2/f1p0/386	R01_cb15394_c2/f1p0/386
R01_cb8564_c73456/f2p0/3436	NA	R01_cb8564_c73456/f2p0/3436	R01_cb8564_c73456/f2p0/3436	R01_cb8564_c73456/f2p0/3436
R01_cb18456_c1081/f2p3/622	R01_cb18456_c1081/f2p3/622	R01_cb18456_c1081/f2p3/622	NA	NA
R01_cb8564_c111239/f1p0/3118	NA	NA	NA	R01_cb8564_c111239/f1p0/3118
R01_cb2423_c30/f1p0/2753	NA	R01_cb2423_c30/f1p0/2753	NA	NA
R01_cb11145_c0/f1p0/1407	NA	R01_cb11145_c0/f1p0/1407	NA	R01_cb11145_c0/f1p0/1407
R01_cb8564_c84993/f1p0/2265	NA	R01_cb8564_c84993/f1p0/2265	NA	NA
R01_cb8564_c2587/f1p0/1997	NA	R01_cb8564_c2587/f1p0/1997	R01_cb8564_c2587/f1p0/1997	NA
R01_cb17572_c3/f1p0/780	NA	NA	NA	R01_cb17572_c3/f1p0/780
R01_cb18456_c7190/f1p0/795	R01_cb18456_c7190/f1p0/795	R01_cb18456_c7190/f1p0/795	R01_cb18456_c7190/f1p0/795	R01_cb18456_c7190/f1p0/795

R01_cb3580_c24/f1p0/3582	NA	R01_cb3580_c24/f1p0/3582	NA	NA
R01_cb4352_c3/f2p0/2420	NA	NA	NA	R01_cb4352_c3/f2p0/2420
R01_cb16678_c8/f1p0/1124	NA	R01_cb16678_c8/f1p0/1124	R01_cb16678_c8/f1p0/1124	R01_cb16678_c8/f1p0/1124
R01_cb3376_c0/f3p0/1201	NA	NA	NA	R01_cb3376_c0/f3p0/1201
R01_cb12946_c6/f1p0/402	R01_cb12946_c6/f1p0/402	R01_cb12946_c6/f1p0/402	NA	R01_cb12946_c6/f1p0/402
R01_cb8564_c50202/f1p0/2941	NA	NA	NA	R01_cb8564_c50202/f1p0/2941
R01_cb8564_c2842/f1p0/3509	NA	R01_cb8564_c2842/f1p0/3509	NA	R01_cb8564_c2842/f1p0/3509
R01_cb18409_c49/f1p0/379	R01_cb18409_c49/f1p0/379	R01_cb18409_c49/f1p0/379	R01_cb18409_c49/f1p0/379	R01_cb18409_c49/f1p0/379
R01_cb12773_c1/f1p0/1012	NA	NA	NA	R01_cb12773_c1/f1p0/1012
R01_cb16678_c2/f1p0/1364	NA	R01_cb16678_c2/f1p0/1364	R01_cb16678_c2/f1p0/1364	R01_cb16678_c2/f1p0/1364
R01_cb8564_c92313/f1p0/2936	NA	R01_cb8564_c92313/f1p0/2936	NA	NA
R01_cb7151_c4/f1p0/4007	R01_cb7151_c4/f1p0/4007	R01_cb7151_c4/f1p0/4007	NA	R01_cb7151_c4/f1p0/4007
R01_cb11972_c6/f1p0/665	R01_cb11972_c6/f1p0/665	R01_cb11972_c6/f1p0/665	NA	R01_cb11972_c6/f1p0/665
R01_cb14044_c12/f1p0/1698	NA	R01_cb14044_c12/f1p0/1698	NA	NA
R01_cb2568_c3/f1p0/2129	NA	NA	R01_cb2568_c3/f1p0/2129	R01_cb2568_c3/f1p0/2129
R01_cb4501_c14/f1p0/2920	NA	NA	NA	R01_cb4501_c14/f1p0/2920
R01_cb8564_c11400/f1p0/2922	NA	R01_cb8564_c11400/f1p0/2922	NA	NA
R01_cb16959_c2/f1p0/682	NA	NA	NA	R01_cb16959_c2/f1p0/682
R01_cb18456_c7367/f1p0/1228	NA	NA	R01_cb18456_c7367/f1p0/1228	R01_cb18456_c7367/f1p0/1228
R01_cb8564_c74161/f1p0/2239	NA	R01_cb8564_c74161/f1p0/2239	NA	NA
R01_cb10787_c9/f1p0/1232	NA	R01_cb10787_c9/f1p0/1232	NA	R01_cb10787_c9/f1p0/1232
R01_cb17756_c25/f1p0/509	R01_cb17756_c25/f1p0/509	R01_cb17756_c25/f1p0/509	R01_cb17756_c25/f1p0/509	R01_cb17756_c25/f1p0/509
R01_cb18456_c7081/f1p0/450	R01_cb18456_c7081/f1p0/450	R01_cb18456_c7081/f1p0/450	R01_cb18456_c7081/f1p0/450	NA
R01_cb8476_c2/f1p0/507	R01_cb8476_c2/f1p0/507	R01_cb8476_c2/f1p0/507	R01_cb8476_c2/f1p0/507	R01_cb8476_c2/f1p0/507
R01_cb4293_c26/f1p0/2872	NA	R01_cb4293_c26/f1p0/2872	NA	NA
R01_cb8564_c82059/f1p0/2438	NA	R01_cb8564_c82059/f1p0/2438	R01_cb8564_c82059/f1p0/2438	NA

R01_cb2959_c6/f1p0/3470	NA	NA	NA	R01_cb2959_c6/f1p0/3470
R01_cb8564_c45894/f1p0/2258	NA	NA	NA	R01_cb8564_c45894/f1p0/2258
R01_cb10215_c4/f1p0/609	NA	R01_cb10215_c4/f1p0/609	NA	NA
R01_cb11128_c0/f3p0/1559	NA	NA	NA	R01_cb11128_c0/f3p0/1559
R01_cb10506_c5/f1p0/1571	NA	R01_cb10506_c5/f1p0/1571	NA	NA
R01_cb13449_c8/f1p0/686	NA	NA	NA	R01_cb13449_c8/f1p0/686
R01_cb9262_c3/f1p0/2414	NA	R01_cb9262_c3/f1p0/2414	NA	NA
R01_cb4768_c26/f1p1/2668	NA	R01_cb4768_c26/f1p1/2668	NA	NA
R01_cb2223_c6/f1p0/622	R01_cb2223_c6/f1p0/622	R01_cb2223_c6/f1p0/622	R01_cb2223_c6/f1p0/622	R01_cb2223_c6/f1p0/622
R01_cb12682_c1/f1p1/1667	NA	NA	NA	R01_cb12682_c1/f1p1/1667
R01_cb18456_c1620/f2p0/1175	NA	NA	R01_cb18456_c1620/f2p0/1175	R01_cb18456_c1620/f2p0/1175
R01_cb8564_c119720/f1p0/2418	NA	NA	NA	R01_cb8564_c119720/f1p0/2418
R01_cb15585_c16/f1p0/531	R01_cb15585_c16/f1p0/531	R01_cb15585_c16/f1p0/531	NA	R01_cb15585_c16/f1p0/531
R01_cb2773_c17/f1p0/983	NA	R01_cb2773_c17/f1p0/983	NA	NA
R01_cb15913_c4/f1p0/945	R01_cb15913_c4/f1p0/945	R01_cb15913_c4/f1p0/945	R01_cb15913_c4/f1p0/945	R01_cb15913_c4/f1p0/945
R01_cb8564_c84255/f1p0/2733	NA	NA	NA	R01_cb8564_c84255/f1p0/2733
R01_cb8564_c36548/f1p0/3104	NA	R01_cb8564_c36548/f1p0/3104	NA	R01_cb8564_c36548/f1p0/3104
R01_cb9357_c2/f1p1/2109	NA	NA	NA	R01_cb9357_c2/f1p1/2109
R01_cb3848_c13/f1p0/2535	NA	NA	NA	R01_cb3848_c13/f1p0/2535
R01_cb17297_c0/f1p0/475	R01_cb17297_c0/f1p0/475	R01_cb17297_c0/f1p0/475	R01_cb17297_c0/f1p0/475	R01_cb17297_c0/f1p0/475
R01_cb13910_c4/f1p0/1395	NA	R01_cb13910_c4/f1p0/1395	R01_cb13910_c4/f1p0/1395	R01_cb13910_c4/f1p0/1395
R01_cb17738_c2/f1p0/1769	NA	NA	NA	R01_cb17738_c2/f1p0/1769
