# Supplementary material for: Single-molecule long-read sequencing of the full-length transcriptome of Rhododendron lapponicum L
Source: Sci Rep. 2020 Apr 21;10:6755. doi: 10.1038/s41598-020-63814-x (PMC7174332; doi:10.1038/s41598-020-63814-x)
Supplement: Supplementary file 4 — Supplementary Table S4. [file 41598_2020_63814_MOESM4_ESM.pdf]

Single-molecule long-read sequencing of the full-length transcriptome of *Rhododendron lapponicum* L.

Xinping Jia, Ling Tang, Xueying Mei, Huazhou Liu, Hairong Luo, Yanming Deng, Jiale Su

Institute of Leisure Agriculture, Jiangsu Academy of Agricultural Sciences, Jiangsu Key Laboratory for Horticultural Crop Genetic Improvement, Nanjing 210014, China

Table S4 Summary of TFs identified

#ID	Family	Type
F01_cb10016_c0/f2p0/1728-0F	bZIP	TF
F01_cb10016_c1/f2p0/1827-0F	bZIP	TF
F01_cb10016_c2/f1p0/1840-0F	bZIP	TF
F01_cb1001_c0/f1p0/4503-1F	SET	TR
F01_cb1001_c1/f1p0/4288-0F	SET	TR
F01_cb1001_c10/f1p0/2566-0F	SET	TR
F01_cb1001_c11/f1p0/5852-2F	SET	TR
F01_cb1001_c4/f1p0/4389-2F	SET	TR
F01_cb1001_c8/f1p0/2108-1F	SET	TR
F01_cb10034_c28/f1p5/2081-2R	Others	TR
F01_cb10067_c1/f5p0/1837-0F	Trihelix	TF
F01_cb10067_c2/f1p0/1963-2F	Trihelix	TF
F01_cb10076_c10/f1p0/806-1F	HMG	TR
F01_cb10076_c13/f1p0/1019-0F	HMG	TR
F01_cb10076_c14/f5p0/933-0F	HMG	TR
F01_cb10076_c2/f1p0/1025-1F	HMG	TR
F01_cb10076_c5/f1p0/878-2F	HMG	TR
F01_cb10076_c6/f1p0/949-1F	HMG	TR
F01_cb10076_c7/f1p0/940-0F	HMG	TR
F01_cb10076_c8/f1p0/1357-2F	HMG	TR
F01_cb10076_c9/f10p0/893-0F	HMG	TR
F01_cb10085_c0/f2p0/1767-2F	C2C2-GATA	TF
F01_cb10085_c1/f2p0/1754-2F	C2C2-GATA	TF
F01_cb10085_c2/f1p0/1629-2F	C2C2-GATA	TF

F01_cb10109_c6/f8p2/1221-1F	PLATZ	TF
F01_cb10109_c9/f5p3/1322-0F	PLATZ	TF
F01_cb10117_c10/f1p0/2724-0F	HB-HD-ZIP	TF
F01_cb10117_c15/f1p0/1431-0F	HB-HD-ZIP	TF
F01_cb10117_c21/f38p2/1632-0F	HB-HD-ZIP	TF
F01_cb10117_c5/f2p2/1657-0F	HB-other	TF
F01_cb10117_c8/f1p2/1684-2F	HB-HD-ZIP	TF
F01_cb10117_c9/f1p0/1947-2F	HB-HD-ZIP	TF
F01_cb1011_c10/f1p0/4389-2F	SET	TR
F01_cb1011_c3/f1p0/4520-0F	SET	TR
F01_cb1011_c7/f1p0/4494-2F	SET	TR
F01_cb10146_c3/f1p0/2688-0F	AUX/IAA	TR
F01_cb10146_c3/f1p0/2688-2R	AUX/IAA	TR
F01_cb10146_c5/f1p8/1206-2F	AUX/IAA	TR
F01_cb10149_c0/f1p0/1722-1F	Others	TR
F01_cb10157_c0/f4p0/1568-2F	GeBP	TF
F01_cb10157_c1/f2p0/1630-0F	GeBP	TF
F01_cb10157_c2/f2p0/1545-1F	GeBP	TF
F01_cb10194_c0/f8p0/1318-2F	MYB	TF
F01_cb10194_c2/f1p0/2011-2F	MYB	TF
F01_cb10194_c4/f1p0/1404-0F	MYB	TF
F01_cb10194_c6/f1p1/1456-2F	MYB	TF
F01_cb10212_c0/f3p0/1000-2F	MADS-MIKC	TF
F01_cb10212_c1/f1p0/1976-0F	MADS-M-type	TF
F01_cb10216_c2/f1p0/1904-1F	bHLH	TF
F01_cb10216_c3/f1p0/1404-0F	bHLH	TF

F01_cb10216_c4/f4p0/1536-1F	bHLH	TF
F01_cb10225_c0/f5p0/981-2F	bHLH	TF
F01_cb10225_c1/f2p0/1025-1F	bHLH	TF
F01_cb10225_c2/f1p0/1923-2F	bHLH	TF
F01_cb1024_c0/f1p0/4515-1F	BES1	TF
F01_cb1024_c0/f1p0/4515-2F	BES1	TF
F01_cb1024_c11/f1p0/3144-1F	BES1	TF
F01_cb1024_c14/f1p0/3152-1F	BES1	TF
F01_cb1024_c14/f1p0/3152-2F	BES1	TF
F01_cb1024_c17/f1p0/2936-1F	BES1	TF
F01_cb1024_c3/f1p0/3567-2F	BES1	TF
F01_cb1024_c4/f1p0/2950-2F	BES1	TF
F01_cb1024_c6/f1p0/2936-1F	BES1	TF
F01_cb1024_c9/f1p0/4102-0F	BES1	TF
F01_cb1024_c9/f1p0/4102-2F	BES1	TF
F01_cb10277_c1/f1p0/1796-1F	AP2/ERF-ERF	TF
F01_cb10277_c3/f1p1/1749-2F	AP2/ERF-ERF	TF
F01_cb10287_c1/f1p0/1904-0F	AP2/ERF-ERF	TF
F01_cb10287_c2/f1p0/987-2F	AP2/ERF-ERF	TF
F01_cb10293_c10/f1p0/1244-0F	MYB-related	TF
F01_cb10293_c10/f1p0/1244-0R	MYB-related	TF
F01_cb10293_c11/f1p0/760-0F	MYB-related	TF
F01_cb10293_c14/f6p4/949-2F	MYB	TF
F01_cb10293_c6/f1p1/2320-0F	MYB	TF
F01_cb10293_c7/f1p0/746-2F	MYB-related	TF
F01_cb10297_c1/f1p0/1718-0F	TRAF	TR

F01_cb10297_c10/f1p0/1778-2F	TRAF	TR
F01_cb10297_c2/f1p0/1900-2F	TRAF	TR
F01_cb10297_c3/f1p0/1887-0F	TRAF	TR
F01_cb10297_c4/f1p0/2035-0F	TRAF	TR
F01_cb10297_c5/f1p0/1838-0F	TRAF	TR
F01_cb10297_c7/f1p0/1757-0F	TRAF	TR
F01_cb10297_c8/f2p0/1646-2F	TRAF	TR
F01_cb10300_c2/f1p0/2334-2F	bHLH	TF
F01_cb10300_c4/f1p0/1430-2F	bHLH	TF
F01_cb10300_c6/f1p0/1813-2F	bHLH	TF
F01_cb10300_c7/f1p0/1744-1F	bHLH	TF
F01_cb10301_c1/f1p0/2686-2F	TRAF	TR
F01_cb10310_c0/f1p0/2004-1F	C2H2	TF
F01_cb10310_c1/f1p0/1974-0F	C2H2	TF
F01_cb10310_c4/f1p1/1855-1F	C2H2	TF
F01_cb10314_c10/f1p3/565-1F	zf-HD	TF
F01_cb10314_c2/f2p2/703-2F	zf-HD	TF
F01_cb10314_c5/f1p0/667-2F	zf-HD	TF
F01_cb10314_c8/f1p0/729-2F	zf-HD	TF
F01_cb10314_c9/f2p2/565-1F	zf-HD	TF
F01_cb10330_c1/f1p0/1746-2F	GARP-G2-like	TF
F01_cb10330_c4/f1p0/1465-0F	GARP-G2-like	TF
F01_cb10330_c5/f1p0/1234-2F	GARP-G2-like	TF
F01_cb10330_c6/f1p0/1244-1F	GARP-G2-like	TF
F01_cb10331_c1/f1p0/1922-1F	C2H2	TF
F01_cb10331_c3/f1p2/1317-0F	C2H2	TF

F01_cb10343_c0/f2p0/1206-1F	SOH1	TR
F01_cb10343_c1/f1p0/2337-1F	SOH1	TR
F01_cb10343_c5/f1p0/1879-0F	SOH1	TR
F01_cb10367_c0/f6p2/1078-0F	MADS-MIKC	TF
F01_cb10367_c3/f2p1/1104-0F	MADS-MIKC	TF
F01_cb10367_c4/f1p0/2732-1F	MADS-MIKC	TF
F01_cb10367_c5/f1p0/875-0F	MADS-MIKC	TF
F01_cb10367_c9/f7p2/1046-0F	MADS-MIKC	TF
F01_cb10394_c1/f1p0/1985-0F	C2H2	TF
F01_cb10417_c0/f1p0/962-0F	B3	TF
F01_cb10417_c1/f1p0/3467-0F	B3	TF
F01_cb10417_c2/f1p0/837-1F	B3	TF
F01_cb10417_c3/f1p0/783-1F	B3	TF
F01_cb10417_c4/f1p0/889-0F	B3	TF
F01_cb10417_c5/f1p0/1424-1F	B3	TF
F01_cb10440_c0/f2p0/1764-1F	mTERF	TR
F01_cb10440_c1/f1p0/1690-0F	mTERF	TR
F01_cb10440_c2/f1p0/2030-0F	mTERF	TR
F01_cb10440_c2/f1p0/2030-1F	mTERF	TR
F01_cb10440_c3/f1p0/2001-0F	mTERF	TR
F01_cb10440_c4/f1p0/1748-2F	mTERF	TR
F01_cb10440_c6/f1p0/1468-1F	mTERF	TR
F01_cb10440_c8/f1p0/1840-2F	mTERF	TR
F01_cb10471_c2/f3p1/1771-1F	BBR-BPC	TF
F01_cb10471_c3/f1p0/1767-2F	BBR-BPC	TF
F01_cb10471_c4/f1p1/2111-0F	BBR-BPC	TF

F01_cb10471_c5/f1p1/1667-1F	BBR-BPC	TF
F01_cb10471_c6/f1p0/1683-1F	BBR-BPC	TF
F01_cb1047_c8/f1p4/6596-1R	MYB-related	TF
F01_cb10496_c1/f4p0/1487-1F	C3H	TF
F01_cb10496_c2/f1p0/2309-2F	C3H	TF
F01_cb10496_c7/f3p0/1482-1F	C3H	TF
F01_cb10496_c8/f3p0/1467-2F	C3H	TF
F01_cb104_c11/f1p0/4029-0F	SBP	TF
F01_cb104_c12/f1p0/3686-1F	SBP	TF
F01_cb104_c17/f1p0/3640-1F	SBP	TF
F01_cb104_c18/f1p0/4363-0F	SBP	TF
F01_cb104_c22/f8p2/3884-1F	SBP	TF
F01_cb104_c3/f4p2/3886-2F	SBP	TF
F01_cb104_c5/f1p0/4986-1F	SBP	TF
F01_cb104_c6/f1p0/3759-0F	SBP	TF
F01_cb10522_c10/f8p2/1454-1F	bZIP	TF
F01_cb10522_c2/f1p0/2435-1F	bZIP	TF
F01_cb10522_c3/f1p0/1671-0F	bZIP	TF
F01_cb10522_c6/f1p0/1705-0F	bZIP	TF
F01_cb10522_c9/f12p0/1530-0F	bZIP	TF
F01_cb10527_c1/f1p0/2657-1R	MYB-related	TF
F01_cb10534_c2/f2p1/1206-1F	C2C2-GATA	TF
F01_cb10534_c3/f1p0/3489-2F	C2C2-GATA	TF
F01_cb10534_c4/f1p1/1333-2F	C2C2-GATA	TF
F01_cb10534_c5/f1p0/1161-1F	C2C2-GATA	TF
F01_cb10545_c0/f2p0/1242-2F	AP2/ERF-ERF	TF

F01_cb10552_c0/f4p1/743-1F	SWI/SNF-BAF60b	TR
F01_cb10552_c2/f1p0/2254-0F	SWI/SNF-BAF60b	TR
F01_cb10552_c2/f1p0/2254-2F	SWI/SNF-BAF60b	TR
F01_cb10552_c3/f1p0/763-2F	SWI/SNF-BAF60b	TR
F01_cb10569_c3/f3p1/1359-2F	AP2/ERF-ERF	TF
F01_cb10580_c0/f1p0/1704-2F	C2C2-Dof	TF
F01_cb10580_c1/f1p0/2371-2F	C2C2-Dof	TF
F01_cb10580_c2/f1p0/1796-2F	C2C2-Dof	TF
F01_cb10631_c0/f1p0/1110-2F	Alfin-like	TF
F01_cb10631_c2/f1p0/1074-0F	Alfin-like	TF
F01_cb10651_c0/f1p0/1807-0F	bZIP	TF
F01_cb10655_c1/f1p0/1032-0F	NF-YC	TF
F01_cb10675_c3/f1p0/2177-0F	MYB	TF
F01_cb10675_c4/f1p0/1822-1F	MYB	TF
F01_cb10675_c5/f1p0/1538-2F	MYB-related	TF
F01_cb10675_c6/f1p0/1511-2F	MYB	TF
F01_cb10685_c2/f1p0/1016-1F	C2C2-YABBY	TF
F01_cb10685_c4/f1p0/897-0F	C2C2-YABBY	TF
F01_cb10686_c0/f1p0/1630-2F	BBR-BPC	TF
F01_cb10686_c2/f1p0/1570-2F	BBR-BPC	TF
F01_cb10687_c0/f4p0/1528-1F	C3H	TF
F01_cb10687_c2/f2p1/1715-0F	C3H	TF
F01_cb10697_c3/f1p0/3232-0F	SBP	TF
F01_cb10697_c3/f1p0/3232-2F	SBP	TF
F01_cb10697_c6/f1p0/820-1F	SBP	TF
F01_cb10697_c7/f6p0/887-0F	SBP	TF

F01_cb10707_c0/f1p0/1395-2F	AUX/IAA	TR
F01_cb10707_c2/f1p0/1665-2F	AUX/IAA	TR
F01_cb10707_c3/f1p0/1620-0F	AUX/IAA	TR
F01_cb10708_c0/f1p0/1783-0F	Rcd1-like	TR
F01_cb10708_c1/f1p0/2292-0F	Rcd1-like	TR
F01_cb10708_c1/f1p0/2292-2F	Rcd1-like	TR
F01_cb10710_c0/f1p0/1357-1F	BBR-BPC	TF
F01_cb10710_c1/f1p0/2345-1F	BBR-BPC	TF
F01_cb10710_c2/f1p0/1395-2F	BBR-BPC	TF
F01_cb10712_c2/f1p1/3797-0F	C3H	TF
F01_cb10712_c2/f1p1/3797-1F	C3H	TF
F01_cb10712_c2/f1p1/3797-2F	C3H	TF
F01_cb10712_c3/f1p0/942-1F	C3H	TF
F01_cb10712_c5/f3p0/1597-2F	C3H	TF
F01_cb10715_c1/f1p0/3184-1F	C3H	TF
F01_cb10728_c1/f1p0/1935-0R	Tify	TF
F01_cb10728_c2/f1p1/1257-1R	Tify	TF
F01_cb10734_c1/f1p0/1914-0F	PHD	TR
F01_cb10734_c2/f1p0/1126-0F	PHD	TR
F01_cb10736_c0/f1p0/1267-0F	ARID	TR
F01_cb10736_c0/f1p0/1267-2F	HMG	TR
F01_cb10736_c1/f1p0/3095-2F	ARID	TR
F01_cb10736_c2/f1p0/1061-1F	ARID	TR
F01_cb10737_c0/f4p0/1679-0F	C2C2-GATA	TF
F01_cb10737_c1/f1p0/1858-1F	C2C2-GATA	TF
F01_cb10747_c0/f1p0/1409-0F	B3	TF

F01_cb10755_c2/f2p2/1076-0F	LIM	TF
F01_cb10755_c3/f1p0/2369-1F	Others	TR
F01_cb10779_c0/f2p0/625-1F	bHLH	TF
F01_cb10779_c2/f1p0/681-1F	bHLH	TF
F01_cb10794_c0/f1p0/1847-0F	GRAS	TF
F01_cb10794_c0/f1p0/1847-2F	GRAS	TF
F01_cb10794_c1/f1p0/1941-1F	GRAS	TF
F01_cb10794_c1/f1p0/1941-2F	GRAS	TF
F01_cb10796_c2/f1p0/1474-2F	NAC	TF
F01_cb10796_c6/f1p0/1325-2F	NAC	TF
F01_cb10796_c7/f2p0/1424-1F	NAC	TF
F01_cb10796_c9/f1p0/1462-0F	NAC	TF
F01_cb10819_c0/f1p0/1532-1F	bHLH	TF
F01_cb10819_c1/f1p0/2192-2F	bHLH	TF
F01_cb10819_c3/f1p0/1514-0F	bHLH	TF
F01_cb10835_c0/f3p0/1697-2F	AP2/ERF-AP2	TF
F01_cb10835_c1/f1p0/2503-0F	AP2/ERF-ERF	TF
F01_cb10835_c1/f1p0/2503-2F	AP2/ERF-ERF	TF
F01_cb10839_c1/f2p0/1210-2F	WRKY	TF
F01_cb10839_c2/f1p0/2420-1F	WRKY	TF
F01_cb10839_c3/f1p0/1112-2F	WRKY	TF
F01_cb10839_c5/f2p0/1168-2F	WRKY	TF
F01_cb10842_c1/f1p0/1781-1F	Others	TR
F01_cb10842_c6/f1p0/1044-2F	Others	TR
F01_cb10842_c7/f1p0/1028-1F	Others	TR
F01_cb10842_c8/f6p0/1301-2F	Others	TR

F01_cb10854_c1/f2p1/1540-1F	TAZ	TR
F01_cb10854_c3/f1p0/2817-0F	TAZ	TR
F01_cb10854_c7/f3p1/1572-2F	TAZ	TR
F01_cb10855_c0/f1p0/1369-0F	Tify	TF
F01_cb10855_c0/f1p0/1369-1F	Others	TR
F01_cb10855_c1/f1p0/1950-2F	Tify	TF
F01_cb10855_c2/f1p0/1343-2F	Tify	TF
F01_cb10855_c4/f1p0/1827-0F	Tify	TF
F01_cb1086_c1/f1p0/4509-2F	HSF	TF
F01_cb1086_c2/f1p0/2114-1F	HSF	TF
F01_cb1086_c6/f1p0/2686-2F	HSF	TF
F01_cb1086_c8/f1p2/2297-1F	HSF	TF
F01_cb10882_c0/f1p0/1582-2F	GNAT	TR
F01_cb10882_c1/f1p0/2758-1F	GNAT	TR
F01_cb10883_c1/f1p1/1342-0F	MADS-M-type	TF
F01_cb10883_c2/f1p0/2114-2F	MADS-M-type	TF
F01_cb10901_c0/f1p0/1542-1F	mTERF	TR
F01_cb10901_c0/f1p0/1542-2F	mTERF	TR
F01_cb10901_c1/f1p0/1939-0F	mTERF	TR
F01_cb10901_c2/f1p0/1770-0F	mTERF	TR
F01_cb10901_c3/f1p0/1596-0F	mTERF	TR
F01_cb10905_c3/f1p0/6047-0F	RWP-RK	TF
F01_cb10905_c3/f1p0/6047-2F	bHLH	TF
F01_cb10906_c1/f1p0/2092-0F	SET	TR
F01_cb10928_c0/f2p0/1619-0F	bZIP	TF
F01_cb10928_c1/f1p0/1850-2F	bZIP	TF

F01_cb10928_c3/f1p0/1689-1F	bZIP	TF
F01_cb10932_c5/f1p0/1663-2F	TCP	TF
F01_cb10932_c7/f1p0/1672-1F	TCP	TF
F01_cb10934_c0/f7p0/1199-1F	bZIP	TF
F01_cb10934_c3/f1p0/2605-1F	bZIP	TF
F01_cb10934_c5/f1p0/1190-1F	bZIP	TF
F01_cb10934_c6/f1p0/1185-0F	bZIP	TF
F01_cb10934_c7/f2p0/1121-2F	bZIP	TF
F01_cb10949_c0/f2p0/1197-0F	WRKY	TF
F01_cb10949_c3/f1p0/2940-0F	WRKY	TF
F01_cb10949_c4/f1p0/1390-2F	WRKY	TF
F01_cb10949_c6/f1p0/1080-1F	WRKY	TF
F01_cb10951_c0/f1p0/1115-1F	MYB-related	TF
F01_cb10951_c1/f1p0/2075-2F	MYB-related	TF
F01_cb10951_c2/f1p0/1119-2F	MYB-related	TF
F01_cb10954_c0/f13p0/1830-1F	TUB	TF
F01_cb10954_c1/f1p0/1887-1F	TUB	TF
F01_cb10954_c2/f1p0/1767-0F	TUB	TF
F01_cb10954_c2/f1p0/1767-1F	TUB	TF
F01_cb10984_c0/f4p0/1068-2F	AUX/IAA	TR
F01_cb10984_c1/f1p0/1952-1F	AUX/IAA	TR
F01_cb10984_c1/f1p0/1952-2F	AUX/IAA	TR
F01_cb11010_c0/f3p0/1732-0F	AP2/ERF-ERF	TF
F01_cb11010_c4/f1p0/1728-0F	AP2/ERF-ERF	TF
F01_cb11010_c5/f1p0/1834-2F	AP2/ERF-ERF	TF
F01_cb11028_c0/f1p0/1229-0F	MYB	TF

F01_cb11028_c1/f1p0/2918-2F	MYB	TF
F01_cb11058_c0/f1p0/1748-1F	TUB	TF
F01_cb11058_c1/f1p0/2419-0R	TUB	TF
F01_cb11058_c1/f1p0/2419-1F	TUB	TF
F01_cb11113_c0/f1p0/1450-0F	MADS-M-type	TF
F01_cb11117_c1/f1p1/951-1F	C2C2-LSD	TF
F01_cb11117_c2/f1p1/3308-0F	C2C2-LSD	TF
F01_cb11117_c3/f1p2/1078-1F	C2C2-LSD	TF
F01_cb11117_c4/f1p1/1055-1F	C2C2-LSD	TF
F01_cb11118_c0/f1p0/1597-1F	WRKY	TF
F01_cb11118_c1/f1p0/2955-2F	WRKY	TF
F01_cb11133_c1/f1p0/2603-1F	HSF	TF
F01_cb11133_c2/f1p0/1209-2F	HSF	TF
F01_cb11151_c0/f1p0/1503-1F	C2H2	TF
F01_cb11151_c1/f1p0/2174-2F	C2H2	TF
F01_cb11168_c1/f1p0/1970-0F	C2C2-Dof	TF
F01_cb11189_c0/f1p0/1527-0F	GARP-G2-like	TF
F01_cb11189_c1/f1p0/1926-0F	GARP-G2-like	TF
F01_cb11189_c3/f1p0/1904-2F	GARP-G2-like	TF
F01_cb11199_c3/f1p0/1946-2F	MYB-related	TF
F01_cb11199_c4/f1p0/2106-2F	MYB-related	TF
F01_cb11213_c0/f1p0/1532-0F	C2C2-CO-like	TF
F01_cb11213_c1/f1p0/2821-0F	Others	TR
F01_cb11213_c1/f1p0/2821-1F	Others	TR
F01_cb11213_c2/f1p0/470-0F	Others	TR
F01_cb11215_c2/f1p1/3800-0R	B3	TF

F01_cb11217_c0/f3p0/1673-0F	bZIP	TF
F01_cb11217_c1/f2p0/1583-2F	bZIP	TF
F01_cb11217_c2/f1p0/2080-0F	bZIP	TF
F01_cb11233_c1/f1p0/2442-0F	HB-HD-ZIP	TF
F01_cb11233_c3/f1p1/1474-2F	HB-HD-ZIP	TF
F01_cb11240_c2/f1p0/1856-1F	bHLH	TF
F01_cb11240_c3/f1p0/1825-1F	bHLH	TF
F01_cb11240_c4/f1p0/1711-2F	bHLH	TF
F01_cb11245_c0/f2p0/1092-1F	Tify	TF
F01_cb11245_c1/f2p0/1231-0F	Tify	TF
F01_cb11245_c2/f1p0/3396-0F	Tify	TF
F01_cb11245_c2/f1p0/3396-2F	Others	TR
F01_cb11245_c3/f1p0/1718-1F	Others	TR
F01_cb11245_c3/f1p0/1718-2F	Tify	TF
F01_cb11276_c2/f1p0/1391-1F	B3	TF
F01_cb11277_c0/f2p1/1609-1F	GRAS	TF
F01_cb11277_c1/f1p1/1966-0F	GRAS	TF
F01_cb11277_c1/f1p1/1966-2F	GRAS	TF
F01_cb11290_c4/f1p0/1024-1F	C2H2	TF
F01_cb11290_c6/f1p0/1349-2F	C2H2	TF
F01_cb11330_c1/f1p0/2773-0F	HSF	TF
F01_cb11330_c3/f1p0/1877-2F	HSF	TF
F01_cb11351_c0/f1p0/944-2F	NF-YB	TF
F01_cb11351_c1/f1p0/3360-0F	NF-YB	TF
F01_cb11351_c2/f1p0/974-1F	NF-YB	TF
F01_cb11362_c0/f1p0/975-0F	FAR1	TF

F01_cb11362_c1/f1p0/2434-0F	FAR1	TF
F01_cb11362_c2/f1p0/981-1F	FAR1	TF
F01_cb11380_c0/f1p0/1605-0F	AP2/ERF-ERF	TF
F01_cb11380_c1/f1p0/2062-2F	AP2/ERF-ERF	TF
F01_cb11380_c2/f1p0/1784-0F	AP2/ERF-ERF	TF
F01_cb11381_c0/f1p0/1547-2F	NF-YA	TF
F01_cb11381_c1/f1p0/2416-1F	NF-YA	TF
F01_cb11384_c0/f1p0/1833-1F	FAR1	TF
F01_cb11384_c1/f1p0/1921-1F	FAR1	TF
F01_cb11389_c1/f1p0/1954-0F	SBP	TF
F01_cb11389_c2/f1p0/1676-0F	SBP	TF
F01_cb11389_c3/f1p0/1754-0F	SBP	TF
F01_cb11389_c4/f1p0/1701-0F	SBP	TF
F01_cb11403_c1/f1p0/1722-0F	C2C2-GATA	TF
F01_cb11403_c1/f1p0/1722-2F	Tify	TF
F01_cb11403_c4/f2p0/1632-2F	C2C2-GATA	TF
F01_cb11403_c5/f2p0/1683-1F	C2C2-GATA	TF
F01_cb11408_c0/f2p2/728-2F	bZIP	TF
F01_cb11408_c2/f1p1/2605-2F	bZIP	TF
F01_cb11449_c0/f1p0/974-1F	SBP	TF
F01_cb11449_c0/f1p0/974-2F	SBP	TF
F01_cb11449_c1/f1p0/2586-0F	SBP	TF
F01_cb11449_c1/f1p0/2586-2F	SBP	TF
F01_cb11452_c1/f2p0/1866-2F	bHLH	TF
F01_cb11452_c10/f1p0/1962-0F	bHLH	TF
F01_cb11493_c1/f1p0/1127-0F	C3H	TF

F01_cb11493_c1/f1p0/1127-2F	C3H	TF
F01_cb11493_c2/f1p0/3557-1F	C3H	TF
F01_cb11493_c3/f1p0/1433-2F	C3H	TF
F01_cb11495_c0/f1p0/1331-0F	MYB	TF
F01_cb11495_c1/f1p0/2019-1F	MYB-related	TF
F01_cb11495_c1/f1p0/2019-2F	MYB-related	TF
F01_cb11497_c0/f1p0/1736-1F	WRKY	TF
F01_cb11497_c1/f1p0/2006-0F	WRKY	TF
F01_cb11498_c0/f2p0/1015-1F	MED7	TR
F01_cb11501_c0/f1p0/1463-2F	Others	TR
F01_cb11537_c0/f1p0/1374-1F	Others	TR
F01_cb11537_c1/f1p0/2859-2F	Others	TR
F01_cb11537_c2/f1p0/1364-1F	Others	TR
F01_cb11541_c0/f1p0/1099-0F	B3	TF
F01_cb11555_c0/f1p0/1365-0F	GARP-G2-like	TF
F01_cb11555_c1/f1p0/2126-1F	GARP-G2-like	TF
F01_cb11565_c0/f1p0/1531-0F	mTERF	TR
F01_cb11565_c1/f1p0/2202-0F	mTERF	TR
F01_cb11586_c0/f2p0/1432-0F	SWI/SNF-BAF60b	TR
F01_cb11586_c1/f2p0/1321-1F	SWI/SNF-BAF60b	TR
F01_cb11586_c2/f1p0/3270-0F	SWI/SNF-BAF60b	TR
F01_cb11589_c0/f1p0/1162-1F	MYB-related	TF
F01_cb11589_c1/f1p0/2770-1F	MYB-related	TF
F01_cb11595_c0/f1p0/1020-1F	LOB	TF
F01_cb11595_c1/f1p0/2065-0F	LOB	TF
F01_cb11595_c1/f1p0/2065-2F	LOB	TF

F01_cb11596_c1/f1p0/3195-2F	mTERF	TR
F01_cb11596_c1/f1p0/3195-2R	mTERF	TR
F01_cb11597_c0/f2p0/1427-2F	BBR-BPC	TF
F01_cb11615_c0/f1p0/1362-0F	MYB-related	TF
F01_cb11615_c1/f1p0/2012-1F	MYB-related	TF
F01_cb11615_c2/f1p0/1391-2F	MYB-related	TF
F01_cb11618_c0/f2p0/908-0F	FAR1	TF
F01_cb11632_c1/f1p0/2477-0F	MYB-related	TF
F01_cb11632_c1/f1p0/2477-2F	MYB-related	TF
F01_cb11636_c0/f2p0/1844-0F	bZIP	TF
F01_cb11636_c10/f1p0/1825-1F	bZIP	TF
F01_cb11636_c12/f1p0/1823-1F	bZIP	TF
F01_cb11636_c13/f1p0/1820-0F	bZIP	TF
F01_cb11636_c3/f1p0/2559-0F	bZIP	TF
F01_cb11636_c4/f1p0/1786-0F	bZIP	TF
F01_cb11636_c7/f1p0/1863-1F	bZIP	TF
F01_cb11636_c8/f1p0/2436-0F	bZIP	TF
F01_cb11637_c0/f1p0/1510-1F	SBP	TF
F01_cb11637_c1/f1p0/2015-0F	SBP	TF
F01_cb11637_c1/f1p0/2015-1F	SBP	TF
F01_cb11638_c0/f1p0/1765-0F	mTERF	TR
F01_cb11648_c1/f1p0/3520-1F	MADS-M-type	TF
F01_cb11648_c2/f1p0/1870-0F	MADS-M-type	TF
F01_cb11661_c0/f1p0/1667-1F	GARP-G2-like	TF
F01_cb11661_c1/f1p0/1926-1F	GARP-G2-like	TF
F01_cb11672_c1/f1p0/1988-0F	C2C2-Dof	TF

F01_cb11672_c2/f1p0/1570-1F	C2C2-Dof	TF
F01_cb11687_c0/f1p0/1089-1F	MYB	TF
F01_cb11687_c1/f1p0/2258-0F	MYB-related	TF
F01_cb11687_c1/f1p0/2258-1F	MYB-related	TF
F01_cb11704_c2/f1p0/3738-1R	B3-ARF	TF
F01_cb11728_c0/f1p0/1316-0F	NF-YA	TF
F01_cb11728_c1/f1p0/2287-2F	NF-YA	TF
F01_cb11730_c0/f1p0/1803-1F	AP2/ERF-AP2	TF
F01_cb11730_c1/f1p0/2299-1F	AP2/ERF-ERF	TF
F01_cb11760_c0/f1p0/1120-1F	C2C2-GATA	TF
F01_cb11760_c1/f1p0/3328-2F	C2C2-GATA	TF
F01_cb11766_c0/f2p0/870-0F	SWI/SNF-BAF60b	TR
F01_cb11766_c1/f1p0/2462-1F	SWI/SNF-BAF60b	TR
F01_cb11772_c1/f1p0/2298-2F	SET	TR
F01_cb11778_c0/f1p0/1206-0F	C2H2	TF
F01_cb11778_c1/f1p0/2667-1F	C2H2	TF
F01_cb11784_c0/f1p0/1508-0F	TCP	TF
F01_cb1178_c17/f1p0/1559-2F	SRS	TF
F01_cb11803_c0/f1p0/1137-0F	FAR1	TF
F01_cb11803_c1/f1p0/2877-0F	FAR1	TF
F01_cb11803_c2/f1p0/1104-2F	FAR1	TF
F01_cb11824_c1/f1p0/2561-1F	GARP-G2-like	TF
F01_cb11826_c0/f1p0/1708-1F	bHLH	TF
F01_cb11828_c1/f1p0/2017-0R	MYB	TF
F01_cb11836_c0/f1p0/1194-1F	NAC	TF
F01_cb11836_c1/f1p0/1906-1F	NAC	TF

F01_cb11853_c0/f15p0/1059-2F	C2H2	TF
F01_cb11853_c2/f1p1/1142-1F	C2H2	TF
F01_cb11853_c3/f1p2/1103-2F	C2H2	TF
F01_cb11853_c5/f1p0/1092-0F	C2H2	TF
F01_cb11891_c0/f2p0/703-0F	HMG	TR
F01_cb11891_c1/f1p0/972-1F	HMG	TR
F01_cb11891_c1/f1p0/972-1R	HMG	TR
F01_cb11891_c2/f1p0/814-0F	HMG	TR
F01_cb11893_c0/f1p0/1318-0F	HB-HD-ZIP	TF
F01_cb12021_c2/f1p1/1311-2F	AP2/ERF-ERF	TF
F01_cb12021_c5/f1p0/1024-2F	AP2/ERF-ERF	TF
F01_cb12021_c7/f5p1/1295-0F	AP2/ERF-ERF	TF
F01_cb12021_c8/f13p1/1264-2F	AP2/ERF-ERF	TF
F01_cb12067_c0/f5p0/843-2F	SWI/SNF-BAF60b	TR
F01_cb12067_c1/f1p0/1279-1F	SWI/SNF-BAF60b	TR
F01_cb12067_c1/f1p0/1279-1R	SWI/SNF-BAF60b	TR
F01_cb12067_c2/f1p1/868-1F	SWI/SNF-BAF60b	TR
F01_cb12110_c0/f2p0/1171-1F	Others	TR
F01_cb12113_c0/f8p0/1020-0F	AP2/ERF-ERF	TF
F01_cb12113_c1/f1p0/997-2F	AP2/ERF-ERF	TF
F01_cb12113_c4/f1p0/1121-2F	AP2/ERF-ERF	TF
F01_cb12149_c11/f1p5/1234-0F	AUX/IAA	TR
F01_cb12149_c11/f1p5/1234-1F	AUX/IAA	TR
F01_cb12149_c17/f1p0/1518-0F	AUX/IAA	TR
F01_cb12149_c17/f1p0/1518-2F	AUX/IAA	TR
F01_cb12149_c18/f1p1/1166-0F	AUX/IAA	TR

F01_cb12149_c19/f1p0/1285-1F	AUX/IAA	TR
F01_cb12149_c20/f1p1/1039-2F	AUX/IAA	TR
F01_cb12149_c23/f1p1/1192-2F	AUX/IAA	TR
F01_cb12149_c24/f1p0/1452-1F	AUX/IAA	TR
F01_cb12149_c25/f1p2/1155-0F	AUX/IAA	TR
F01_cb12149_c26/f1p1/1021-0F	AUX/IAA	TR
F01_cb12149_c28/f1p0/1090-0F	AUX/IAA	TR
F01_cb12149_c28/f1p0/1090-1F	AUX/IAA	TR
F01_cb12149_c29/f1p0/1255-0F	AUX/IAA	TR
F01_cb12149_c31/f1p0/1050-2F	AUX/IAA	TR
F01_cb12149_c36/f1p0/1338-0F	AUX/IAA	TR
F01_cb12149_c37/f2p3/1146-1F	AUX/IAA	TR
F01_cb12149_c8/f3p5/1188-0F	AUX/IAA	TR
F01_cb12150_c0/f1p0/1763-1F	AP2/ERF-ERF	TF
F01_cb12150_c2/f1p0/1017-2F	AP2/ERF-ERF	TF
F01_cb12150_c3/f1p0/1758-2F	AP2/ERF-ERF	TF
F01_cb12181_c2/f1p0/1479-1F	NAC	TF
F01_cb12181_c3/f1p0/1159-2F	NAC	TF
F01_cb12181_c4/f1p0/1570-1F	NAC	TF
F01_cb12214_c10/f1p0/1297-0F	MYB	TF
F01_cb12214_c5/f1p0/1243-2F	MYB	TF
F01_cb12230_c1/f1p1/1499-2F	BES1	TF
F01_cb12230_c11/f3p0/1406-1F	BES1	TF
F01_cb12230_c7/f1p0/1675-0F	BES1	TF
F01_cb12323_c1/f25p0/788-0F	MBF1	TR
F01_cb12323_c11/f1p0/758-0F	MBF1	TR

F01_cb12323_c14/f7p0/808-2F	MBF1	TR
F01_cb12323_c17/f8p0/705-2F	MBF1	TR
F01_cb12323_c9/f1p0/877-1F	MBF1	TR
F01_cb12377_c0/f2p0/1601-0F	TRAF	TR
F01_cb12377_c1/f1p0/1751-1F	TRAF	TR
F01_cb1240_c10/f1p1/3810-0F	Others	TR
F01_cb1240_c10/f1p1/3810-2F	Others	TR
F01_cb1240_c16/f1p0/3942-0F	Others	TR
F01_cb1240_c17/f1p0/4879-0F	Others	TR
F01_cb1240_c2/f3p1/4343-1F	Others	TR
F01_cb1240_c22/f1p0/4522-1F	Others	TR
F01_cb1240_c3/f1p1/4420-1F	Others	TR
F01_cb1240_c4/f1p1/4081-2F	Others	TR
F01_cb1240_c6/f1p0/4543-1F	Others	TR
F01_cb1240_c7/f1p0/3554-2F	Others	TR
F01_cb12485_c0/f2p0/1365-1F	Trihelix	TF
F01_cb12497_c0/f3p0/1607-2F	HSF	TF
F01_cb12505_c1/f1p1/853-1F	HMG	TR
F01_cb12505_c10/f7p0/816-2F	HMG	TR
F01_cb12505_c4/f1p0/1745-2F	HMG	TR
F01_cb12505_c6/f1p0/868-0F	HMG	TR
F01_cb12505_c7/f1p0/1718-2F	HMG	TR
F01_cb12545_c2/f1p0/1662-1F	bZIP	TF
F01_cb12545_c3/f1p0/1749-2F	bZIP	TF
F01_cb1254_c13/f1p2/3019-1F	Pseudo ARR-B	TR
F01_cb1254_c17/f1p1/2619-0F	Others	TR

F01_cb1254_c17/f1p1/2619-2F	Others	TR
F01_cb1254_c20/f1p0/2893-0F	Pseudo ARR-B	TR
F01_cb1254_c27/f3p3/2944-2F	Pseudo ARR-B	TR
F01_cb1254_c5/f2p3/2995-0F	Pseudo ARR-B	TR
F01_cb1254_c7/f1p0/4411-0F	Others	TR
F01_cb1254_c7/f1p0/4411-2F	Others	TR
F01_cb1254_c8/f1p1/4309-2F	Others	TR
F01_cb12578_c11/f1p0/1792-0F	AUX/IAA	TR
F01_cb12578_c11/f1p0/1792-1F	AUX/IAA	TR
F01_cb12578_c12/f1p0/1877-1F	AUX/IAA	TR
F01_cb12578_c12/f1p0/1877-2F	AUX/IAA	TR
F01_cb12578_c13/f1p0/1790-1F	AUX/IAA	TR
F01_cb12578_c18/f1p0/1805-0F	AUX/IAA	TR
F01_cb12578_c18/f1p0/1805-1F	AUX/IAA	TR
F01_cb12578_c20/f1p1/1703-2F	AUX/IAA	TR
F01_cb12578_c21/f1p1/1749-2F	AUX/IAA	TR
F01_cb12578_c24/f3p3/1752-1F	AUX/IAA	TR
F01_cb12578_c28/f18p0/1731-0F	AUX/IAA	TR
F01_cb12578_c31/f1p2/1777-1F	AUX/IAA	TR
F01_cb12578_c31/f1p2/1777-2F	AUX/IAA	TR
F01_cb12586_c0/f2p0/1428-0F	PHD	TR
F01_cb12586_c1/f1p0/1542-1F	PHD	TR
F01_cb12586_c2/f1p0/1542-0F	PHD	TR
F01_cb12586_c4/f1p0/1346-0F	PHD	TR
F01_cb12586_c5/f1p0/1583-2F	PHD	TR
F01_cb12588_c2/f1p0/1199-1F	WRKY	TF

F01_cb12633_c1/f2p0/1594-0F	WRKY	TF
F01_cb12633_c5/f1p0/1544-0F	WRKY	TF
F01_cb12633_c6/f1p0/1839-0F	WRKY	TF
F01_cb12724_c0/f1p0/762-2F	GNAT	TR
F01_cb12724_c1/f1p0/858-0F	GNAT	TR
F01_cb12731_c1/f1p0/689-0F	SWI/SNF-BAF60b	TR
F01_cb12768_c1/f2p2/1148-1F	AP2/ERF-ERF	TF
F01_cb12768_c2/f1p2/1248-2F	AP2/ERF-ERF	TF
F01_cb12768_c7/f1p1/1430-0F	AP2/ERF-ERF	TF
F01_cb12768_c8/f1p2/1260-2F	AP2/ERF-ERF	TF
F01_cb12785_c13/f1p0/912-0F	C3H	TF
F01_cb12785_c14/f1p0/1127-1F	C3H	TF
F01_cb12785_c16/f1p0/1203-0F	C3H	TF
F01_cb12785_c19/f1p0/1005-0F	C3H	TF
F01_cb12785_c21/f1p0/843-1F	C3H	TF
F01_cb12785_c26/f1p0/1428-1F	C3H	TF
F01_cb12785_c29/f9p1/969-2F	C3H	TF
F01_cb12785_c32/f13p0/989-0F	C3H	TF
F01_cb12785_c35/f1p0/980-2F	C3H	TF
F01_cb12785_c36/f1p0/1093-1F	C3H	TF
F01_cb12785_c37/f2p1/929-1F	C3H	TF
F01_cb12785_c9/f1p1/1033-2F	C3H	TF
F01_cb12802_c0/f1p0/1125-0F	GARP-G2-like	TF
F01_cb12802_c1/f1p0/1587-1F	GARP-G2-like	TF
F01_cb12828_c1/f1p1/1807-0F	Trihelix	TF
F01_cb12828_c5/f1p1/1544-0F	Trihelix	TF

F01_cb12869_c14/f4p0/1664-1F	bHLH	TF
F01_cb12869_c15/f12p0/1600-1F	bHLH	TF
F01_cb12869_c3/f1p0/1671-2F	bHLH	TF
F01_cb12869_c7/f1p0/1799-1F	bHLH	TF
F01_cb12869_c9/f1p0/1617-0F	bHLH	TF
F01_cb12883_c3/f3p2/871-2F	GNAT	TR
F01_cb12883_c4/f1p0/506-0F	GNAT	TR
F01_cb12883_c5/f1p0/771-2F	GNAT	TR
F01_cb12883_c6/f1p2/761-1F	GNAT	TR
F01_cb12883_c7/f1p2/949-0F	GNAT	TR
F01_cb12883_c8/f1p2/753-2F	GNAT	TR
F01_cb1291_c14/f1p0/2892-1F	TRAF	TR
F01_cb1291_c19/f1p0/2699-2F	TRAF	TR
F01_cb1291_c2/f6p1/2729-2F	TRAF	TR
F01_cb1291_c4/f1p1/4391-0F	TRAF	TR
F01_cb1291_c5/f1p0/4325-2F	TRAF	TR
F01_cb1291_c6/f1p0/2480-2F	TRAF	TR
F01_cb1291_c7/f1p1/2985-0F	TRAF	TR
F01_cb1291_c9/f1p1/2168-1F	TRAF	TR
F01_cb12928_c0/f4p1/1334-0F	C2C2-Dof	TF
F01_cb12928_c1/f1p1/1524-1F	C2C2-Dof	TF
F01_cb12928_c2/f1p1/1511-0F	C2C2-Dof	TF
F01_cb12928_c4/f1p0/1350-2F	C2C2-Dof	TF
F01_cb12936_c0/f1p0/803-1F	MADS-M-type	TF
F01_cb12948_c11/f2p2/1337-2F	MADS-MIKC	TF
F01_cb12948_c2/f1p0/1497-1F	MADS-MIKC	TF

F01_cb12948_c4/f1p2/1293-1F	MADS-MIKC	TF
F01_cb12948_c5/f1p0/1460-1F	MADS-MIKC	TF
F01_cb12948_c6/f1p0/1788-0R	MADS-MIKC	TF
F01_cb12948_c6/f1p0/1788-2F	MADS-MIKC	TF
F01_cb12948_c7/f1p0/1698-1F	MADS-MIKC	TF
F01_cb12983_c7/f1p2/1542-0F	HB-HD-ZIP	TF
F01_cb13002_c0/f3p0/1416-1F	Trihelix	TF
F01_cb13002_c2/f1p0/1196-2F	Trihelix	TF
F01_cb13002_c3/f1p0/1329-2F	Trihelix	TF
F01_cb13038_c0/f1p0/1234-1F	NF-YB	TF
F01_cb13038_c1/f1p0/834-0F	NF-YB	TF
F01_cb13050_c0/f1p0/1337-2F	SBP	TF
F01_cb13050_c1/f1p0/1389-0F	SBP	TF
F01_cb13050_c2/f1p0/1389-0F	SBP	TF
F01_cb13055_c0/f2p1/682-1F	OFP	TF
F01_cb13055_c2/f1p0/583-1F	OFP	TF
F01_cb13080_c0/f2p0/1164-2F	bZIP	TF
F01_cb13082_c10/f1p0/1535-2F	AP2/ERF-ERF	TF
F01_cb13082_c2/f3p0/1615-0F	AP2/ERF-ERF	TF
F01_cb13082_c3/f2p0/1388-2F	AP2/ERF-ERF	TF
F01_cb13082_c9/f1p0/1512-0F	AP2/ERF-ERF	TF
F01_cb13095_c1/f1p0/1486-0F	MYB	TF
F01_cb13118_c1/f1p0/1111-0F	GNAT	TR
F01_cb13152_c0/f2p0/1574-0F	NAC	TF
F01_cb13152_c1/f1p0/1388-0F	NAC	TF
F01_cb13152_c1/f1p0/1388-1F	NAC	TF

F01_cb13152_c2/f1p0/1560-1F	NAC	TF
F01_cb13164_c0/f4p2/1616-0F	C2C2-GATA	TF
F01_cb13164_c2/f1p1/1514-0F	C2C2-GATA	TF
F01_cb13164_c5/f1p0/1648-2F	C2C2-GATA	TF
F01_cb13215_c0/f2p0/1270-0F	HB-HD-ZIP	TF
F01_cb13243_c2/f1p0/1717-1F	C3H	TF
F01_cb13243_c4/f1p0/1340-2F	C3H	TF
F01_cb13243_c8/f1p0/1759-2F	C3H	TF
F01_cb13243_c9/f7p0/1516-2F	C3H	TF
F01_cb13278_c5/f1p1/1155-1F	MYB-related	TF
F01_cb132_c0/f14p0/3618-2F	RB	TR
F01_cb132_c2/f1p0/4955-2F	RB	TR
F01_cb132_c3/f1p0/3919-0F	RB	TR
F01_cb132_c4/f1p0/3541-1F	RB	TR
F01_cb13338_c1/f3p0/1279-0F	BBR-BPC	TF
F01_cb13338_c6/f1p0/1420-1F	BBR-BPC	TF
F01_cb13338_c7/f4p0/1359-1F	BBR-BPC	TF
F01_cb13390_c4/f1p0/1705-1F	TRAF	TR
F01_cb13390_c4/f1p0/1705-2F	TRAF	TR
F01_cb1339_c0/f2p1/4378-1F	AUX/IAA	TR
F01_cb1339_c0/f2p1/4378-2F	B3-ARF	TF
F01_cb1339_c2/f1p0/4262-1F	B3	TF
F01_cb1339_c2/f1p0/4262-2F	AUX/IAA	TR
F01_cb1339_c4/f1p0/4235-2F	B3-ARF	TF
F01_cb1339_c8/f1p1/3971-2F	B3	TF
F01_cb1339_c9/f1p0/3837-1F	B3	TF

F01_cb133_c2/f1p0/3610-2F	DDT	TR
F01_cb133_c4/f1p0/5829-0F	HB-other	TF
F01_cb133_c5/f1p2/5631-2F	HB-other	TF
F01_cb133_c6/f1p2/5625-0F	DDT	TR
F01_cb133_c6/f1p2/5625-1F	HB-other	TF
F01_cb133_c7/f1p1/5392-1F	DDT	TR
F01_cb133_c7/f1p1/5392-2F	HB-other	TF
F01_cb13402_c0/f5p1/1791-1F	bZIP	TF
F01_cb13402_c2/f1p1/1835-1F	bZIP	TF
F01_cb13402_c3/f1p0/1665-1F	bZIP	TF
F01_cb13402_c4/f1p0/1547-0F	bZIP	TF
F01_cb13402_c5/f1p0/1841-0F	bZIP	TF
F01_cb1340_c0/f3p0/1559-2F	mTERF	TR
F01_cb1340_c1/f1p0/4356-1F	mTERF	TR
F01_cb1340_c2/f1p0/3014-0F	mTERF	TR
F01_cb1340_c2/f1p0/3014-2F	mTERF	TR
F01_cb1340_c3/f1p0/2373-0F	mTERF	TR
F01_cb1340_c4/f1p0/1977-0F	mTERF	TR
F01_cb1340_c5/f1p0/1572-1F	mTERF	TR
F01_cb1340_c6/f1p0/1564-0F	mTERF	TR
F01_cb13412_c2/f1p2/1715-1F	NAC	TF
F01_cb1341_c2/f1p1/2875-1F	HB-BELL	TF
F01_cb1341_c3/f1p1/2271-0F	HB-other	TF
F01_cb1341_c4/f1p0/2383-1F	HB-BELL	TF
F01_cb1341_c5/f1p1/2310-2F	HB-BELL	TF
F01_cb13423_c1/f1p0/1833-1F	NAC	TF

F01_cb13433_c123/f1p0/5867-2F	MYB-related	TF
F01_cb13433_c126/f1p0/5680-1F	MYB-related	TF
F01_cb13433_c127/f1p0/5721-1F	MYB-related	TF
F01_cb13433_c41/f1p0/1701-0F	SRS	TF
F01_cb13433_c53/f1p0/4827-1F	MYB-related	TF
F01_cb13433_c54/f1p0/6911-2F	MYB-related	TF
F01_cb13433_c57/f1p0/6389-1F	MYB-related	TF
F01_cb13456_c11/f1p0/1489-1F	PLATZ	TF
F01_cb13456_c11/f1p0/1489-2F	PLATZ	TF
F01_cb13456_c12/f3p1/1486-2F	PLATZ	TF
F01_cb13456_c13/f7p1/1403-2F	PLATZ	TF
F01_cb13456_c2/f2p1/1279-0F	PLATZ	TF
F01_cb13456_c4/f1p1/1442-0F	PLATZ	TF
F01_cb13499_c0/f5p1/1128-2F	AUX/IAA	TR
F01_cb13499_c1/f4p1/1133-1F	AUX/IAA	TR
F01_cb13499_c3/f1p0/1450-0F	AUX/IAA	TR
F01_cb13499_c3/f1p0/1450-1F	AUX/IAA	TR
F01_cb13499_c6/f1p0/1088-2F	AUX/IAA	TR
F01_cb13516_c0/f10p1/1137-2F	AUX/IAA	TR
F01_cb13516_c1/f4p1/1207-0F	AUX/IAA	TR
F01_cb13516_c4/f1p0/1066-0F	AUX/IAA	TR
F01_cb13516_c6/f1p0/1306-0F	AUX/IAA	TR
F01_cb13516_c7/f1p0/1058-1F	AUX/IAA	TR
F01_cb13516_c8/f1p0/1141-1F	AUX/IAA	TR
F01_cb13587_c0/f1p0/1563-0F	DBP	TF
F01_cb13587_c1/f1p0/1516-0F	DBP	TF

F01_cb13600_c0/f3p0/1089-0F	Whirly	TF
F01_cb13600_c1/f1p0/1023-2F	Whirly	TF
F01_cb13608_c1/f3p0/1005-2F	AUX/IAA	TR
F01_cb13608_c5/f1p0/1088-0F	AUX/IAA	TR
F01_cb13608_c6/f1p0/1530-0F	AUX/IAA	TR
F01_cb13608_c6/f1p0/1530-2F	AUX/IAA	TR
F01_cb13622_c1/f1p1/1313-2F	AP2/ERF-ERF	TF
F01_cb13622_c3/f1p0/1028-0F	AP2/ERF-ERF	TF
F01_cb13622_c5/f1p0/1218-0F	AP2/ERF-ERF	TF
F01_cb13675_c1/f1p0/417-1F	C2H2	TF
F01_cb13675_c2/f1p0/792-0F	C2H2	TF
F01_cb13675_c2/f1p0/792-2R	C2H2	TF
F01_cb13675_c3/f1p0/996-2F	C2H2	TF
F01_cb13679_c10/f1p0/1618-1F	AUX/IAA	TR
F01_cb13679_c13/f5p0/1060-2F	AUX/IAA	TR
F01_cb13679_c6/f1p0/875-0F	AUX/IAA	TR
F01_cb13681_c2/f2p0/1375-1F	MYB-related	TF
F01_cb13681_c5/f1p3/1426-2F	MYB-related	TF
F01_cb13707_c1/f1p0/1697-1F	TAZ	TR
F01_cb13707_c2/f1p0/1046-0F	TRAF	TR
F01_cb13707_c4/f4p0/1730-0F	TAZ	TR
F01_cb13720_c13/f1p1/1127-2F	AUX/IAA	TR
F01_cb13720_c16/f1p1/1060-0F	AUX/IAA	TR
F01_cb13720_c16/f1p1/1060-1F	AUX/IAA	TR
F01_cb13720_c16/f1p1/1060-2F	AUX/IAA	TR
F01_cb13720_c2/f4p1/1182-2F	AUX/IAA	TR

F01_cb13720_c3/f1p1/1053-2F	AUX/IAA	TR
F01_cb13720_c5/f2p1/1064-0F	AUX/IAA	TR
F01_cb13720_c5/f2p1/1064-2F	AUX/IAA	TR
F01_cb13720_c7/f1p1/1247-2F	AUX/IAA	TR
F01_cb13720_c9/f1p0/1300-1F	AUX/IAA	TR
F01_cb13768_c11/f1p0/1372-0F	mTERF	TR
F01_cb13768_c12/f1p0/1501-1F	mTERF	TR
F01_cb13768_c13/f1p0/1599-2F	mTERF	TR
F01_cb13768_c15/f1p0/1469-2F	mTERF	TR
F01_cb13768_c17/f1p0/1647-1F	mTERF	TR
F01_cb13768_c18/f1p0/1662-2F	mTERF	TR
F01_cb13768_c19/f1p0/1537-1F	mTERF	TR
F01_cb13768_c20/f1p0/1285-0F	mTERF	TR
F01_cb13768_c20/f1p0/1285-1F	mTERF	TR
F01_cb13768_c23/f1p0/439-1F	mTERF	TR
F01_cb13768_c3/f2p0/1777-0F	mTERF	TR
F01_cb13768_c9/f1p0/1801-1F	mTERF	TR
F01_cb13788_c0/f3p0/1433-0F	TAZ	TR
F01_cb13788_c0/f3p0/1433-2F	TRAF	TR
F01_cb13813_c1/f1p0/1364-1F	C2C2-LSD	TF
F01_cb13817_c1/f1p0/1446-0F	MYB	TF
F01_cb13817_c2/f1p0/1832-1F	MYB	TF
F01_cb13858_c1/f1p0/1067-1F	MADS-MIKC	TF
F01_cb13858_c2/f1p0/1173-2F	MADS-MIKC	TF
F01_cb13877_c1/f1p0/1565-0F	HB-other	TF
F01_cb13877_c4/f1p0/1685-2F	HB-other	TF

F01_cb13882_c0/f9p0/1554-2F	bZIP	TF
F01_cb13882_c10/f1p0/1690-2F	bZIP	TF
F01_cb13882_c13/f2p1/1651-2F	bZIP	TF
F01_cb13882_c2/f4p0/1574-1F	bZIP	TF
F01_cb13882_c8/f1p0/1505-1F	bZIP	TF
F01_cb13892_c10/f1p0/753-1F	MADS-M-type	TF
F01_cb13892_c11/f1p0/1220-1F	MADS-MIKC	TF
F01_cb13892_c3/f1p0/1261-2F	MADS-MIKC	TF
F01_cb13892_c4/f1p0/993-2F	MADS-M-type	TF
F01_cb13892_c5/f1p0/1181-0F	MADS-MIKC	TF
F01_cb13892_c6/f1p0/1107-1F	MADS-MIKC	TF
F01_cb13892_c7/f1p0/838-0F	MADS-MIKC	TF
F01_cb13892_c9/f1p0/765-1F	MADS-MIKC	TF
F01_cb13900_c0/f2p0/1222-0F	GARP-G2-like	TF
F01_cb13900_c2/f1p0/1186-0F	GARP-G2-like	TF
F01_cb13900_c5/f1p0/1230-1F	GARP-G2-like	TF
F01_cb13900_c6/f1p0/1337-2F	GARP-G2-like	TF
F01_cb13913_c2/f7p1/1581-0F	BES1	TF
F01_cb13913_c5/f1p0/1472-1F	BES1	TF
F01_cb13913_c6/f1p1/1684-2F	BES1	TF
F01_cb13928_c0/f1p0/1540-1F	NAC	TF
F01_cb13928_c2/f1p0/1648-0F	NAC	TF
F01_cb13940_c1/f1p0/1605-1F	bHLH	TF
F01_cb13940_c2/f1p0/1527-0F	bHLH	TF
F01_cb13960_c0/f1p0/1079-2F	WRKY	TF
F01_cb13960_c1/f1p0/1634-1F	WRKY	TF

F01_cb13960_c3/f1p0/1618-0F	WRKY	TF
F01_cb13960_c5/f1p0/834-1F	WRKY	TF
F01_cb13960_c6/f1p0/1219-2F	WRKY	TF
F01_cb14000_c0/f2p0/1578-0F	BES1	TF
F01_cb14000_c2/f1p0/1474-2F	BES1	TF
F01_cb14000_c3/f1p0/1464-1F	BES1	TF
F01_cb14009_c2/f1p0/1253-0F	NF-YC	TF
F01_cb14011_c2/f1p0/1295-2F	HB-WOX	TF
F01_cb14035_c3/f1p1/1261-2F	MYB	TF
F01_cb14035_c8/f10p1/1244-2F	MYB	TF
F01_cb14038_c1/f3p0/1022-2F	MYB	TF
F01_cb14045_c6/f1p1/1820-2F	GARP-G2-like	TF
F01_cb14045_c7/f1p0/1601-0F	GARP-G2-like	TF
F01_cb14045_c9/f4p0/1537-1F	GARP-G2-like	TF
F01_cb14057_c0/f3p0/1252-0F	TRAF	TR
F01_cb14073_c0/f1p0/1836-2F	MYB	TF
F01_cb14073_c1/f1p0/1318-1F	MYB	TF
F01_cb14073_c2/f1p0/1147-1F	MYB	TF
F01_cb14085_c2/f1p0/5412-0F	SNF2	TR
F01_cb14085_c2/f1p0/5412-1F	SNF2	TR
F01_cb14085_c2/f1p0/5412-2F	SNF2	TR
F01_cb14151_c0/f1p0/1608-1F	bZIP	TF
F01_cb14151_c1/f1p0/1629-1F	bZIP	TF
F01_cb14151_c2/f1p0/1544-1F	bZIP	TF
F01_cb14184_c0/f3p0/1551-2F	NAC	TF
F01_cb14184_c1/f1p0/1708-0F	NAC	TF

F01_cb14194_c0/f3p0/1736-1F	AP2/ERF-ERF	TF
F01_cb14194_c4/f1p0/1704-2F	AP2/ERF-ERF	TF
F01_cb14242_c0/f1p0/1182-1F	MYB-related	TF
F01_cb14242_c0/f1p0/1182-2F	MYB-related	TF
F01_cb14242_c2/f1p0/1278-0F	MYB	TF
F01_cb14244_c0/f5p1/1104-2F	AP2/ERF-ERF	TF
F01_cb14244_c2/f2p1/1102-1F	AP2/ERF-ERF	TF
F01_cb14244_c7/f1p0/1153-1F	AP2/ERF-ERF	TF
F01_cb14257_c1/f1p0/1851-2F	AUX/IAA	TR
F01_cb14270_c1/f1p0/820-0F	GNAT	TR
F01_cb14270_c2/f1p0/850-0F	GNAT	TR
F01_cb14271_c0/f1p0/1758-0F	BES1	TF
F01_cb14271_c1/f1p0/1674-0F	BES1	TF
F01_cb14271_c2/f1p0/1768-1F	BES1	TF
F01_cb14271_c3/f1p0/1762-1F	BES1	TF
F01_cb14298_c3/f2p0/1513-2F	zf-HD	TF
F01_cb14298_c4/f1p0/1447-0F	zf-HD	TF
F01_cb14323_c0/f7p0/1360-2F	HB-HD-ZIP	TF
F01_cb14323_c3/f1p0/1827-1F	HB-HD-ZIP	TF
F01_cb14323_c5/f1p0/1411-2F	HB-other	TF
F01_cb14323_c6/f1p0/1451-1F	HB-HD-ZIP	TF
F01_cb14323_c9/f2p0/770-1F	HB-HD-ZIP	TF
F01_cb14340_c2/f2p0/987-1F	HB-HD-ZIP	TF
F01_cb14340_c3/f1p0/1111-0F	HB-HD-ZIP	TF
F01_cb14356_c3/f1p0/1612-1F	AP2/ERF-ERF	TF
F01_cb14356_c4/f1p0/1432-2F	AP2/ERF-ERF	TF

F01_cb14356_c5/f1p0/1737-0F	AP2/ERF-ERF	TF
F01_cb14369_c2/f1p0/1361-2F	C2C2-GATA	TF
F01_cb14375_c0/f2p0/1155-1F	GNAT	TR
F01_cb14375_c1/f1p0/1220-1F	GNAT	TR
F01_cb14375_c2/f1p0/1291-1F	GNAT	TR
F01_cb14375_c5/f1p0/1254-1F	GNAT	TR
F01_cb14375_c7/f1p0/1208-1F	GNAT	TR
F01_cb14390_c2/f1p0/1596-1F	GeBP	TF
F01_cb14390_c4/f1p0/1475-0F	GeBP	TF
F01_cb14390_c6/f1p0/1864-1F	GeBP	TF
F01_cb14390_c7/f1p0/1392-1F	GeBP	TF
F01_cb14390_c8/f1p0/1844-0F	GeBP	TF
F01_cb14397_c0/f3p0/1111-2F	HB-HD-ZIP	TF
F01_cb14397_c2/f1p0/1187-2F	HB-HD-ZIP	TF
F01_cb14418_c0/f4p0/1437-0F	AUX/IAA	TR
F01_cb14419_c0/f1p0/1531-0F	NAC	TF
F01_cb14419_c1/f1p0/1390-1F	NAC	TF
F01_cb14426_c0/f2p0/1445-2F	GARP-G2-like	TF
F01_cb14426_c1/f2p0/1310-0F	GARP-G2-like	TF
F01_cb14426_c2/f1p0/1330-0F	GARP-G2-like	TF
F01_cb14458_c1/f1p0/1697-1F	WRKY	TF
F01_cb14458_c1/f1p0/1697-2F	WRKY	TF
F01_cb14480_c2/f1p0/1076-2F	AP2/ERF-ERF	TF
F01_cb14480_c3/f1p0/1151-0F	AP2/ERF-ERF	TF
F01_cb14480_c4/f1p0/1346-1F	AP2/ERF-ERF	TF
F01_cb14488_c2/f1p0/1783-0F	GRAS	TF

F01_cb14500_c0/f1p0/1133-1F	bHLH	TF
F01_cb14500_c1/f1p0/968-1F	bHLH	TF
F01_cb1454_c1/f1p0/4317-2F	B3-ARF	TF
F01_cb1454_c2/f1p0/2893-2F	AUX/IAA	TR
F01_cb1455_c1/f1p1/4291-2F	MYB-related	TF
F01_cb1455_c2/f1p1/3990-2F	MYB-related	TF
F01_cb14582_c0/f1p0/1794-1F	HB-KNOX	TF
F01_cb14582_c3/f1p0/1627-0F	HB-KNOX	TF
F01_cb14624_c2/f1p0/1232-0F	NF-YC	TF
F01_cb14624_c3/f1p0/1110-2F	NF-YC	TF
F01_cb14624_c5/f1p0/1323-1F	NF-YC	TF
F01_cb14624_c6/f6p0/1171-0F	NF-YC	TF
F01_cb14624_c7/f1p0/1180-0F	NF-YC	TF
F01_cb14663_c3/f1p0/1061-1F	Others	TR
F01_cb14663_c4/f1p0/1635-1F	Others	TR
F01_cb14691_c0/f1p0/1259-0F	bZIP	TF
F01_cb14691_c1/f1p0/1313-0F	bZIP	TF
F01_cb14691_c2/f1p0/967-2F	bZIP	TF
F01_cb14698_c0/f2p1/1106-0F	MED6	TR
F01_cb14698_c2/f1p0/880-0F	MED6	TR
F01_cb14698_c4/f1p0/1691-1F	MED6	TR
F01_cb14735_c0/f1p0/1876-1F	HSF	TF
F01_cb14735_c1/f1p0/1839-1F	HSF	TF
F01_cb14735_c3/f1p1/5303-0F	HSF	TF
F01_cb14745_c2/f1p0/1084-1F	NF-YB	TF
F01_cb14745_c3/f1p0/1052-1F	NF-YB	TF

F01_cb1474_c3/f1p0/6921-0F	SNF2	TR
F01_cb14753_c1/f2p0/1551-2F	LOB	TF
F01_cb14753_c3/f1p0/1231-1F	LOB	TF
F01_cb14753_c3/f1p0/1231-2F	LOB	TF
F01_cb14768_c1/f1p0/1282-1F	HSF	TF
F01_cb14811_c0/f2p0/1144-2F	Alfin-like	TF
F01_cb14811_c2/f1p0/1208-0F	Alfin-like	TF
F01_cb14829_c4/f1p1/1036-1F	MYB	TF
F01_cb14829_c6/f1p0/1576-1F	MYB-related	TF
F01_cb14852_c0/f4p0/1444-1F	bZIP	TF
F01_cb14852_c1/f1p0/1470-2F	bZIP	TF
F01_cb14852_c2/f1p0/1434-2F	bZIP	TF
F01_cb14852_c3/f1p0/1424-0F	bZIP	TF
F01_cb14873_c2/f1p1/1184-0F	AP2/ERF-ERF	TF
F01_cb14873_c3/f2p1/1131-2F	AP2/ERF-ERF	TF
F01_cb14873_c5/f2p1/1061-0F	AP2/ERF-ERF	TF
F01_cb14883_c0/f1p0/1402-1F	MYB-related	TF
F01_cb14883_c1/f1p0/1394-2F	MYB-related	TF
F01_cb14883_c2/f1p0/1543-0F	MYB-related	TF
F01_cb14883_c3/f1p0/1454-0F	MYB-related	TF
F01_cb14893_c1/f1p0/1319-1F	GNAT	TR
F01_cb14893_c3/f1p0/1598-1F	GNAT	TR
F01_cb14893_c5/f1p0/1307-2F	GNAT	TR
F01_cb14912_c0/f1p0/1518-1F	Others	TR
F01_cb14912_c1/f1p0/1480-2F	Others	TR
F01_cb14912_c2/f1p0/1499-2F	Others	TR

F01_cb14921_c0/f10p3/1047-1F	bHLH	TF
F01_cb14921_c1/f1p0/950-1F	bHLH	TF
F01_cb14931_c2/f1p1/1300-0F	DBB	TF
F01_cb14931_c3/f1p0/1247-0F	Others	TR
F01_cb14931_c4/f1p1/1296-0F	Others	TR
F01_cb14969_c1/f1p1/1576-1F	SWI/SNF-BAF60b	TR
F01_cb1497_c0/f1p1/4300-2F	PHD	TR
F01_cb1497_c1/f1p0/4043-1F	PHD	TR
F01_cb1497_c3/f1p0/4345-2F	PHD	TR
F01_cb14991_c3/f2p0/929-0F	AUX/IAA	TR
F01_cb14991_c5/f4p0/922-0F	AUX/IAA	TR
F01_cb14991_c6/f3p0/897-0F	AUX/IAA	TR
F01_cb15025_c1/f1p0/1819-0F	C3H	TF
F01_cb15025_c1/f1p0/1819-1F	C3H	TF
F01_cb15028_c0/f1p0/1268-0F	TRAF	TR
F01_cb15028_c2/f1p0/1135-1F	TRAF	TR
F01_cb15048_c3/f1p2/1121-2F	LIM	TF
F01_cb15052_c0/f1p0/1582-1F	C2H2	TF
F01_cb15052_c1/f1p0/1709-0F	C2H2	TF
F01_cb15052_c2/f1p0/1331-1F	C2H2	TF
F01_cb15106_c0/f1p0/1779-1F	Others	TR
F01_cb15106_c5/f1p0/884-0F	Others	TR
F01_cb15127_c1/f1p1/1498-0F	MYB	TF
F01_cb15127_c2/f1p1/5940-1F	MYB-related	TF
F01_cb15127_c2/f1p1/5940-2F	MYB-related	TF
F01_cb15145_c1/f1p0/417-2F	S1Fa-like	TF

F01_cb15145_c2/f1p0/608-1F	S1Fa-like	TF
F01_cb15158_c1/f1p0/1266-2F	NAC	TF
F01_cb15172_c3/f3p1/868-1F	MBF1	TR
F01_cb15172_c6/f1p0/759-0F	MBF1	TR
F01_cb15185_c0/f4p0/720-0F	HMG	TR
F01_cb15185_c1/f1p0/653-0F	HMG	TR
F01_cb1519_c5/f3p0/1913-0F	C3H	TF
F01_cb1523_c0/f1p0/4282-0F	Jumonji	TR
F01_cb1523_c1/f1p0/4350-1F	Jumonji	TR
F01_cb1523_c10/f1p0/2714-2F	Jumonji	TR
F01_cb1523_c12/f1p0/2619-0F	Jumonji	TR
F01_cb1523_c14/f1p0/1782-2F	Jumonji	TR
F01_cb1523_c2/f1p0/4204-0F	Jumonji	TR
F01_cb1523_c2/f1p0/4204-1F	Jumonji	TR
F01_cb1523_c5/f1p0/2853-2F	Jumonji	TR
F01_cb1523_c8/f1p0/4424-1F	Jumonji	TR
F01_cb1523_c9/f1p0/2695-2F	Jumonji	TR
F01_cb15293_c0/f1p0/827-2R	AP2/ERF-ERF	TF
F01_cb15293_c1/f1p0/760-0R	AP2/ERF-ERF	TF
F01_cb15297_c1/f1p0/1662-2F	AP2/ERF-ERF	TF
F01_cb15297_c3/f1p0/1138-0F	AP2/ERF-ERF	TF
F01_cb15301_c0/f1p0/945-2F	PHD	TR
F01_cb15301_c2/f1p0/849-1F	PHD	TR
F01_cb15330_c0/f14p3/1401-1F	PLATZ	TF
F01_cb15330_c3/f1p3/1415-2F	PLATZ	TF
F01_cb15330_c6/f1p1/1450-2F	PLATZ	TF

F01_cb15330_c7/f1p1/1448-0F	PLATZ	TF
F01_cb15357_c2/f1p1/1271-1F	TRAF	TR
F01_cb15357_c4/f7p1/1265-1F	TRAF	TR
F01_cb15393_c4/f1p0/916-0F	HMG	TR
F01_cb15393_c5/f1p1/836-0F	HMG	TR
F01_cb15393_c7/f11p1/856-1F	HMG	TR
F01_cb1540_c11/f1p0/3904-1F	AUX/IAA	TR
F01_cb1540_c11/f1p0/3904-2F	B3-ARF	TF
F01_cb1540_c14/f1p1/3846-1F	B3-ARF	TF
F01_cb1540_c17/f1p0/3387-2F	B3-ARF	TF
F01_cb1540_c20/f1p0/4038-1F	B3-ARF	TF
F01_cb1540_c23/f1p0/1897-2F	AUX/IAA	TR
F01_cb1540_c27/f1p0/3741-0F	B3-ARF	TF
F01_cb1540_c3/f2p0/3962-0F	B3	TF
F01_cb1540_c31/f1p0/3473-1F	B3-ARF	TF
F01_cb1540_c32/f1p0/3950-1F	B3-ARF	TF
F01_cb1540_c34/f1p0/3880-1F	B3-ARF	TF
F01_cb1540_c35/f1p0/3229-1F	B3-ARF	TF
F01_cb1540_c4/f2p0/3874-2F	B3-ARF	TF
F01_cb1540_c7/f1p0/4274-2F	B3	TF
F01_cb15427_c1/f1p0/1149-1F	B3	TF
F01_cb15430_c0/f4p0/1307-0F	zf-HD	TF
F01_cb15435_c0/f1p0/1831-0F	NAC	TF
F01_cb15435_c1/f1p0/1301-2F	NAC	TF
F01_cb15435_c2/f1p0/1732-0F	NAC	TF
F01_cb15435_c3/f1p0/1675-0F	NAC	TF

F01_cb15495_c1/f2p0/1322-0F	C2H2	TF
F01_cb15495_c10/f1p0/1473-2F	C2H2	TF
F01_cb15495_c2/f1p0/466-2F	C2H2	TF
F01_cb15495_c3/f1p0/1339-0F	C2H2	TF
F01_cb15495_c4/f1p0/1107-1F	C2H2	TF
F01_cb15495_c6/f1p0/1348-2F	C2H2	TF
F01_cb15498_c0/f2p0/1291-1F	MYB-related	TF
F01_cb15501_c1/f1p0/1232-0F	AP2/ERF-ERF	TF
F01_cb15501_c5/f6p0/1093-1F	AP2/ERF-ERF	TF
F01_cb15521_c0/f2p0/1238-2F	HB-HD-ZIP	TF
F01_cb15521_c1/f1p0/1283-0F	HB-HD-ZIP	TF
F01_cb15527_c3/f2p0/1695-2F	C3H	TF
F01_cb15527_c4/f1p0/1687-1F	C3H	TF
F01_cb15546_c0/f1p0/1801-0F	TRAF	TR
F01_cb15546_c1/f1p0/1865-2F	TRAF	TR
F01_cb15566_c1/f1p0/1589-1F	SET	TR
F01_cb15572_c2/f1p0/1175-1F	MYB	TF
F01_cb15572_c3/f1p0/1236-1F	MYB-related	TF
F01_cb15572_c4/f1p0/1278-1F	MYB	TF
F01_cb15572_c5/f1p0/1537-1F	MYB	TF
F01_cb15572_c6/f3p0/1241-1F	MYB	TF
F01_cb15575_c11/f6p2/1520-2F	C2C2-GATA	TF
F01_cb15575_c2/f1p1/1401-1F	C2C2-GATA	TF
F01_cb15584_c0/f3p0/1259-1F	LOB	TF
F01_cb15584_c1/f1p0/1269-2F	LOB	TF
F01_cb15584_c2/f1p0/1708-0F	LOB	TF

F01_cb15584_c2/f1p0/1708-2F	LOB	TF
F01_cb15584_c4/f1p0/1780-1F	LOB	TF
F01_cb15587_c0/f2p0/1090-0F	Others	TR
F01_cb15591_c1/f1p1/1413-2F	AUX/IAA	TR
F01_cb15591_c3/f1p1/907-1F	AUX/IAA	TR
F01_cb15591_c7/f1p0/1750-1F	AUX/IAA	TR
F01_cb15591_c9/f1p0/521-0F	AUX/IAA	TR
F01_cb155_c16/f1p0/6222-2F	Others	TR
F01_cb155_c20/f1p1/5063-1F	Others	TR
F01_cb155_c21/f1p0/5298-0F	Others	TR
F01_cb155_c6/f1p0/4973-1F	Others	TR
F01_cb15609_c2/f1p0/1578-1F	NF-YA	TF
F01_cb15609_c3/f4p0/1369-1F	NF-YA	TF
F01_cb15617_c0/f2p0/1727-1F	bZIP	TF
F01_cb15628_c0/f7p0/1214-2F	Others	TR
F01_cb15628_c1/f1p0/1207-2F	Others	TR
F01_cb15648_c0/f2p0/1418-1F	NAC	TF
F01_cb15648_c1/f1p0/1430-0F	NAC	TF
F01_cb15649_c0/f5p0/1619-2F	bZIP	TF
F01_cb15649_c1/f1p0/1748-1F	bZIP	TF
F01_cb15652_c0/f1p0/1590-2F	C2C2-Dof	TF
F01_cb15652_c1/f1p0/1797-2F	C2C2-Dof	TF
F01_cb15666_c0/f1p0/1396-1F	GeBP	TF
F01_cb15666_c1/f1p0/1388-1F	GeBP	TF
F01_cb15686_c0/f2p0/1208-1F	Alfin-like	TF
F01_cb15686_c1/f1p0/1149-2F	Alfin-like	TF

F01_cb15705_c3/f1p0/1369-2F	NF-YC	TF
F01_cb15705_c4/f1p0/1428-1F	NF-YC	TF
F01_cb15705_c5/f1p0/1357-1F	NF-YC	TF
F01_cb15711_c0/f2p0/1393-2F	C2C2-GATA	TF
F01_cb15733_c0/f2p1/1091-2F	Others	TR
F01_cb15733_c1/f3p1/1136-0F	Others	TR
F01_cb15733_c1/f3p1/1136-2F	Others	TR
F01_cb15739_c0/f2p0/1397-2F	MYB-related	TF
F01_cb15739_c1/f1p0/740-1F	MYB	TF
F01_cb15739_c2/f1p0/1108-0F	MYB-related	TF
F01_cb15780_c0/f3p0/1029-0F	C2H2	TF
F01_cb15784_c1/f2p0/1460-2F	C3H	TF
F01_cb15784_c2/f1p0/1655-2F	C3H	TF
F01_cb1579_c0/f2p0/3667-0F	SNF2	TR
F01_cb1579_c1/f1p0/4261-1F	SNF2	TR
F01_cb1579_c11/f1p2/3898-0F	SNF2	TR
F01_cb15817_c0/f1p0/957-1F	Others	TR
F01_cb15817_c2/f1p0/727-1F	Others	TR
F01_cb15817_c3/f1p0/811-2F	Others	TR
F01_cb15851_c0/f1p0/996-0F	NAC	TF
F01_cb15851_c0/f1p0/996-1F	NAC	TF
F01_cb15851_c1/f1p0/1052-1F	NAC	TF
F01_cb15857_c2/f3p0/1419-2F	WRKY	TF
F01_cb15857_c3/f2p0/1434-1F	WRKY	TF
F01_cb15861_c0/f1p0/1249-2F	C2H2	TF
F01_cb15861_c1/f1p0/1155-2F	C2H2	TF

F01_cb15875_c0/f2p0/1127-2F	AP2/ERF-ERF	TF
F01_cb15875_c1/f1p0/1299-0F	AP2/ERF-ERF	TF
F01_cb15892_c2/f1p0/1522-1F	MYB-related	TF
F01_cb15892_c3/f1p1/1541-0F	MYB-related	TF
F01_cb15905_c4/f1p0/1806-0R	MADS-MIKC	TF
F01_cb15919_c0/f3p0/1851-0F	bHLH	TF
F01_cb15938_c0/f1p0/1193-1F	MYB	TF
F01_cb1594_c10/f1p0/2394-1F	C2H2	TF
F01_cb1594_c11/f1p0/2163-2F	C2H2	TF
F01_cb1594_c13/f1p0/2343-0F	C2H2	TF
F01_cb1594_c13/f1p0/2343-2F	C2H2	TF
F01_cb1594_c14/f5p0/2426-1F	C2H2	TF
F01_cb1594_c15/f1p1/2480-2F	C2H2	TF
F01_cb1594_c3/f1p0/4277-2F	C2H2	TF
F01_cb1594_c4/f1p0/2740-1F	C2H2	TF
F01_cb1594_c6/f1p1/2130-1F	C2H2	TF
F01_cb1594_c8/f2p0/2363-1F	C2H2	TF
F01_cb1594_c9/f1p0/2443-0F	C2H2	TF
F01_cb15980_c0/f3p0/1436-0F	bZIP	TF
F01_cb15980_c2/f1p0/1454-2F	bZIP	TF
F01_cb15980_c4/f1p0/1687-2F	bZIP	TF
F01_cb16015_c0/f1p0/1029-2F	B3	TF
F01_cb16015_c1/f1p0/1025-0F	B3	TF
F01_cb16027_c2/f1p0/1556-0F	IWS1	TR
F01_cb16027_c4/f1p0/1226-1F	IWS1	TR
F01_cb16027_c5/f1p0/1555-0F	IWS1	TR

F01_cb16031_c0/f3p0/1653-2F	Trihelix	TF
F01_cb16031_c1/f1p0/1689-0F	Trihelix	TF
F01_cb16033_c0/f3p0/1401-2F	Alfin-like	TF
F01_cb16033_c10/f1p0/1489-2F	Alfin-like	TF
F01_cb16033_c3/f1p0/1307-1F	Alfin-like	TF
F01_cb16033_c4/f1p0/1025-1F	Alfin-like	TF
F01_cb16033_c5/f1p0/1421-1F	Alfin-like	TF
F01_cb16033_c7/f1p0/1696-0F	Alfin-like	TF
F01_cb16033_c8/f1p0/1432-1F	Alfin-like	TF
F01_cb16033_c9/f1p0/1428-2F	Alfin-like	TF
F01_cb16039_c2/f1p0/1094-2F	MYB	TF
F01_cb16039_c3/f1p0/1218-0F	MYB	TF
F01_cb16049_c0/f2p0/1336-2F	C3H	TF
F01_cb16052_c13/f1p0/1376-1F	C3H	TF
F01_cb16052_c19/f1p0/1802-1F	C3H	TF
F01_cb16065_c1/f1p0/1639-1F	HB-HD-ZIP	TF
F01_cb16085_c1/f2p1/1445-2F	bHLH	TF
F01_cb16085_c2/f1p1/1448-2F	bHLH	TF
F01_cb16123_c0/f2p0/1577-0F	mTERF	TR
F01_cb16123_c0/f2p0/1577-2F	mTERF	TR
F01_cb16123_c1/f1p0/1678-2F	mTERF	TR
F01_cb16123_c2/f1p0/1564-1F	mTERF	TR
F01_cb16125_c4/f1p0/1477-1F	B3	TF
F01_cb16125_c5/f1p0/1587-2F	B3	TF
F01_cb16157_c0/f2p0/1546-2F	TAZ	TR
F01_cb16157_c1/f1p1/1613-1F	TAZ	TR

F01_cb16164_c0/f1p0/636-0F	AUX/IAA	TR
F01_cb16164_c1/f1p0/1231-2F	AUX/IAA	TR
F01_cb16183_c0/f1p0/953-2F	MYB	TF
F01_cb16183_c1/f1p0/1385-1F	MYB-related	TF
F01_cb16183_c2/f1p0/1040-0F	MYB-related	TF
F01_cb16183_c2/f1p0/1040-1F	MYB-related	TF
F01_cb16183_c3/f1p0/1375-2F	MYB	TF
F01_cb16183_c4/f1p0/1116-2F	MYB	TF
F01_cb16197_c1/f1p0/1654-1F	MYB	TF
F01_cb16212_c0/f2p0/1337-1F	CSD	TF
F01_cb16262_c0/f3p0/880-2F	AP2/ERF-ERF	TF
F01_cb16262_c1/f3p0/926-1F	AP2/ERF-ERF	TF
F01_cb16262_c3/f1p0/781-0F	AP2/ERF-ERF	TF
F01_cb16262_c4/f1p0/790-2F	AP2/ERF-ERF	TF
F01_cb162_c0/f1p0/4942-0F	Jumonji	TR
F01_cb162_c1/f1p0/3584-0F	Jumonji	TR
F01_cb162_c3/f1p0/4680-1F	Jumonji	TR
F01_cb16348_c0/f3p0/1109-2F	LIM	TF
F01_cb16348_c1/f1p0/1024-1F	LIM	TF
F01_cb16348_c2/f1p0/1084-2F	LIM	TF
F01_cb16375_c0/f1p0/1473-0F	TCP	TF
F01_cb16375_c1/f1p0/1380-0F	TCP	TF
F01_cb16375_c3/f1p0/1461-2F	TCP	TF
F01_cb16375_c4/f1p0/1413-1F	TCP	TF
F01_cb16391_c1/f1p0/1230-2F	C2C2-Dof	TF
F01_cb16391_c2/f1p0/1496-0F	C2C2-Dof	TF

F01_cb16393_c0/f1p0/1117-1F	FAR1	TF
F01_cb16393_c1/f1p0/1053-0F	FAR1	TF
F01_cb16395_c1/f1p0/1529-2F	C3H	TF
F01_cb16418_c1/f1p0/1698-0F	HB-KNOX	TF
F01_cb16444_c2/f1p0/1853-0F	OFP	TF
F01_cb16454_c0/f1p0/1347-2F	PLATZ	TF
F01_cb16454_c4/f1p1/1168-0F	PLATZ	TF
F01_cb16479_c1/f2p0/1043-1F	C2C2-Dof	TF
F01_cb16479_c3/f1p0/1233-0F	C2C2-Dof	TF
F01_cb16479_c4/f2p0/976-2F	C2C2-Dof	TF
F01_cb16505_c0/f5p0/660-1F	S1Fa-like	TF
F01_cb16505_c1/f1p0/573-1F	S1Fa-like	TF
F01_cb16546_c0/f2p0/1656-1F	bHLH	TF
F01_cb16551_c0/f1p0/746-0F	AP2/ERF-ERF	TF
F01_cb16551_c1/f1p0/1140-1F	AP2/ERF-ERF	TF
F01_cb16578_c3/f1p0/5077-2F	RWP-RK	TF
F01_cb16584_c1/f1p0/1103-0F	AP2/ERF-ERF	TF
F01_cb16584_c3/f1p0/1132-2F	AP2/ERF-ERF	TF
F01_cb16589_c0/f1p0/1660-1F	C2C2-GATA	TF
F01_cb16599_c0/f5p0/886-0F	GNAT	TR
F01_cb16599_c2/f1p0/1788-2F	GNAT	TR
F01_cb16599_c3/f1p0/985-1F	GNAT	TR
F01_cb16605_c0/f1p0/1282-2F	SBP	TF
F01_cb16605_c1/f1p0/1630-2F	SBP	TF
F01_cb16655_c2/f2p0/1747-2F	NAC	TF
F01_cb16655_c6/f1p0/1804-0F	NAC	TF

F01_cb16655_c7/f1p0/1290-1F	NAC	TF
F01_cb16661_c1/f1p0/1630-1F	HB-other	TF
F01_cb16661_c2/f1p0/1762-2F	HB-KNOX	TF
F01_cb16661_c3/f1p0/1575-0F	HB-KNOX	TF
F01_cb16661_c4/f1p0/1762-2F	HB-KNOX	TF
F01_cb16661_c5/f1p0/1652-1F	HB-KNOX	TF
F01_cb16661_c6/f1p0/1728-2F	HB-KNOX	TF
F01_cb1668_c11/f1p0/2234-0F	C3H	TF
F01_cb1668_c11/f1p0/2234-1F	C3H	TF
F01_cb1668_c11/f1p0/2234-2F	C3H	TF
F01_cb1668_c15/f1p0/2586-1F	C3H	TF
F01_cb1668_c17/f1p0/1399-0F	C3H	TF
F01_cb1668_c3/f1p1/4216-0F	C3H	TF
F01_cb1668_c3/f1p1/4216-2F	C3H	TF
F01_cb1668_c4/f1p1/2924-0F	C3H	TF
F01_cb1668_c4/f1p1/2924-1F	C3H	TF
F01_cb1668_c5/f1p0/2954-2F	C3H	TF
F01_cb1668_c6/f1p0/1907-0F	C3H	TF
F01_cb1668_c7/f1p1/2154-2F	C3H	TF
F01_cb1668_c8/f1p2/2340-0F	C3H	TF
F01_cb1668_c8/f1p2/2340-2F	C3H	TF
F01_cb16695_c1/f1p0/834-0F	MYB-related	TF
F01_cb16695_c2/f1p0/872-0F	MYB-related	TF
F01_cb166_c10/f1p0/4308-1F	SBP	TF
F01_cb166_c11/f1p0/4188-2F	SBP	TF
F01_cb166_c16/f1p0/4452-2F	SBP	TF

F01_cb166_c21/f4p1/4388-2F	SBP	TF
F01_cb166_c3/f1p1/4936-1F	SBP	TF
F01_cb166_c6/f1p0/4230-2F	SBP	TF
F01_cb166_c9/f1p1/4497-1F	SBP	TF
F01_cb16741_c0/f1p0/1326-2F	MYB	TF
F01_cb16741_c2/f1p0/1262-1F	MYB	TF
F01_cb16747_c1/f2p1/965-1F	bHLH	TF
F01_cb16749_c0/f9p0/1410-0F	NF-YC	TF
F01_cb16749_c4/f1p0/1491-1F	NF-YC	TF
F01_cb16749_c5/f1p0/1823-1F	NF-YC	TF
F01_cb16749_c7/f1p0/1497-2F	NF-YC	TF
F01_cb16753_c0/f9p3/1466-2F	TUB	TF
F01_cb16802_c2/f1p1/1451-2F	MYB-related	TF
F01_cb16802_c3/f1p0/1096-1F	MYB-related	TF
F01_cb16802_c6/f1p0/1368-2F	MYB-related	TF
F01_cb16802_c8/f1p1/1398-2F	MYB-related	TF
F01_cb1680_c10/f1p0/4212-1F	NF-X1	TF
F01_cb1680_c16/f1p0/4127-0F	NF-X1	TF
F01_cb1680_c19/f1p0/3975-2F	NF-X1	TF
F01_cb16820_c0/f2p0/1011-2F	MADS-MIKC	TF
F01_cb16820_c1/f1p0/947-0F	MADS-MIKC	TF
F01_cb1682_c0/f5p2/3786-0F	PHD	TR
F01_cb1682_c6/f1p0/3872-2F	PHD	TR
F01_cb16864_c2/f1p0/1028-2F	FAR1	TF
F01_cb16864_c4/f3p0/921-0F	FAR1	TF
F01_cb16869_c0/f4p1/1687-1F	bZIP	TF

F01_cb1688_c10/f1p0/3802-1F	SNF2	TR
F01_cb1688_c10/f1p0/3802-2F	SNF2	TR
F01_cb1688_c13/f1p0/2298-0F	SNF2	TR
F01_cb1688_c3/f1p1/3708-0F	SNF2	TR
F01_cb1688_c4/f1p1/3873-1F	SNF2	TR
F01_cb1688_c5/f1p0/3706-1F	SNF2	TR
F01_cb1688_c8/f1p1/3849-0F	SNF2	TR
F01_cb1688_c8/f1p1/3849-2F	SNF2	TR
F01_cb1688_c9/f1p0/3870-0F	SNF2	TR
F01_cb16902_c0/f6p0/1202-1F	C2H2	TF
F01_cb16936_c0/f3p0/932-1F	MADS-MIKC	TF
F01_cb16936_c1/f2p0/986-2F	MADS-MIKC	TF
F01_cb16936_c2/f1p0/1507-0F	MADS-MIKC	TF
F01_cb16997_c100/f1p1/3034-0F	RWP-RK	TF
F01_cb16997_c103/f1p0/2729-1F	RWP-RK	TF
F01_cb16997_c105/f1p0/2376-0F	B3-ARF	TF
F01_cb16997_c109/f1p0/3326-0F	RWP-RK	TF
F01_cb16997_c112/f1p0/2249-0F	B3-ARF	TF
F01_cb16997_c115/f1p0/2279-1F	B3-ARF	TF
F01_cb16997_c122/f1p0/2691-2F	RWP-RK	TF
F01_cb16997_c129/f14p1/2502-0F	B3-ARF	TF
F01_cb16997_c132/f1p1/2143-0F	B3	TF
F01_cb16997_c132/f1p1/2143-2F	AUX/IAA	TR
F01_cb16997_c15/f3p0/2493-2F	RWP-RK	TF
F01_cb16997_c17/f2p0/2331-1F	RWP-RK	TF
F01_cb16997_c19/f1p0/1620-1F	RWP-RK	TF

F01_cb16997_c23/f1p0/6285-1F	RWP-RK	TF
F01_cb16997_c49/f1p0/3604-1F	RWP-RK	TF
F01_cb16997_c51/f1p0/3275-2F	RWP-RK	TF
F01_cb16997_c53/f1p2/2222-1F	B3-ARF	TF
F01_cb16997_c53/f1p2/2222-2F	AUX/IAA	TR
F01_cb16997_c56/f1p0/2394-1F	B3	TF
F01_cb16997_c57/f1p0/2337-0F	B3-ARF	TF
F01_cb16997_c58/f1p0/2433-1F	B3-ARF	TF
F01_cb16997_c59/f1p0/4526-1F	RWP-RK	TF
F01_cb16997_c60/f1p0/2509-1F	RWP-RK	TF
F01_cb16997_c62/f1p1/2596-2F	RWP-RK	TF
F01_cb16997_c64/f1p0/2490-2F	RWP-RK	TF
F01_cb16997_c67/f1p0/2441-0F	RWP-RK	TF
F01_cb16997_c69/f1p0/2518-2F	RWP-RK	TF
F01_cb16997_c71/f1p0/2349-1F	RWP-RK	TF
F01_cb16997_c72/f1p0/2574-1F	RWP-RK	TF
F01_cb16997_c77/f1p0/2371-2F	RWP-RK	TF
F01_cb16997_c79/f1p0/2350-2F	RWP-RK	TF
F01_cb16997_c81/f1p0/2411-1F	RWP-RK	TF
F01_cb16997_c84/f1p0/2527-2F	RWP-RK	TF
F01_cb16997_c85/f1p0/2068-2F	RWP-RK	TF
F01_cb16997_c88/f1p0/2985-1F	RWP-RK	TF
F01_cb16997_c89/f1p0/2621-2F	RWP-RK	TF
F01_cb16997_c90/f1p0/2628-2F	RWP-RK	TF
F01_cb16997_c91/f1p0/2673-1F	RWP-RK	TF
F01_cb16997_c92/f1p0/2602-2F	RWP-RK	TF

F01_cb16997_c94/f1p0/3198-2F	RWP-RK	TF
F01_cb16997_c96/f1p0/2831-2F	RWP-RK	TF
F01_cb16997_c98/f1p0/2258-2F	RWP-RK	TF
F01_cb16_c0/f1p0/4992-2F	SNF2	TR
F01_cb16_c1/f1p0/4199-0F	SNF2	TR
F01_cb16_c6/f1p0/5456-0F	SNF2	TR
F01_cb16_c7/f1p0/5159-1F	SNF2	TR
F01_cb16_c8/f1p0/5091-1F	SNF2	TR
F01_cb17006_c2/f1p0/616-1F	GNAT	TR
F01_cb17014_c0/f2p0/1137-2F	GNAT	TR
F01_cb17014_c1/f1p0/1161-1F	GNAT	TR
F01_cb17056_c0/f1p0/1854-0F	bHLH	TF
F01_cb17056_c1/f1p0/1827-0F	bHLH	TF
F01_cb17080_c0/f2p0/1417-2F	NAC	TF
F01_cb17082_c2/f1p0/1712-2F	FAR1	TF
F01_cb17082_c3/f1p0/1761-0F	FAR1	TF
F01_cb17082_c5/f1p0/1165-1F	FAR1	TF
F01_cb17091_c1/f1p0/1592-2F	FAR1	TF
F01_cb17091_c2/f1p0/776-1F	FAR1	TF
F01_cb17091_c4/f1p0/688-2F	FAR1	TF
F01_cb17091_c5/f1p0/702-1F	FAR1	TF
F01_cb17099_c0/f2p0/1509-0F	MYB-related	TF
F01_cb17099_c0/f2p0/1509-1F	MYB-related	TF
F01_cb17099_c1/f1p0/1496-2F	MYB	TF
F01_cb17116_c0/f2p0/1302-1F	mTERF	TR
F01_cb17116_c2/f1p0/1413-1F	mTERF	TR

F01_cb17144_c0/f3p0/1198-1F	MYB-related	TF
F01_cb17144_c1/f1p0/1059-2F	MYB	TF
F01_cb17156_c0/f2p0/1200-0F	MYB-related	TF
F01_cb17157_c0/f2p0/1116-0F	OFP	TF
F01_cb17192_c0/f1p0/969-2F	SWI/SNF-BAF60b	TR
F01_cb17192_c3/f1p0/959-0F	SWI/SNF-BAF60b	TR
F01_cb17192_c4/f1p0/1017-1F	SWI/SNF-BAF60b	TR
F01_cb17213_c1/f1p0/1217-1F	WRKY	TF
F01_cb17213_c2/f1p0/1291-1F	WRKY	TF
F01_cb17233_c0/f1p0/1715-0F	WRKY	TF
F01_cb17233_c1/f1p0/1415-0F	WRKY	TF
F01_cb17246_c0/f2p0/969-2F	NF-YC	TF
F01_cb17255_c2/f1p0/791-1F	HB-HD-ZIP	TF
F01_cb17255_c4/f1p0/1566-0F	HB-HD-ZIP	TF
F01_cb17255_c5/f1p0/684-1F	HB-HD-ZIP	TF
F01_cb17261_c0/f1p0/1182-0F	AP2/ERF-ERF	TF
F01_cb17261_c1/f1p0/1202-0F	AP2/ERF-ERF	TF
F01_cb1726_c0/f1p0/4212-1F	FAR1	TF
F01_cb1726_c2/f1p0/2898-2F	FAR1	TF
F01_cb1726_c3/f1p0/2976-1F	FAR1	TF
F01_cb1726_c4/f1p0/2596-0F	FAR1	TF
F01_cb17285_c0/f1p0/1339-2F	MYB	TF
F01_cb17289_c0/f1p0/1053-0F	GNAT	TR
F01_cb17289_c1/f1p0/1215-0F	GNAT	TR
F01_cb17310_c0/f2p0/1673-2F	MYB	TF
F01_cb17310_c1/f1p0/1679-1F	MYB	TF

F01_cb17312_c1/f1p0/1797-2F	MYB	TF
F01_cb17312_c2/f1p0/1752-1F	MYB	TF
F01_cb17312_c3/f1p0/1636-2F	MYB	TF
F01_cb1733_c0/f1p0/4190-0F	MYB	TF
F01_cb1733_c1/f1p0/3527-2F	MYB	TF
F01_cb17355_c0/f1p0/1830-2F	GeBP	TF
F01_cb173_c12/f1p1/2635-0F	C3H	TF
F01_cb173_c13/f1p0/2536-2F	C3H	TF
F01_cb173_c16/f1p0/2914-0F	C3H	TF
F01_cb173_c17/f1p0/2758-2F	C3H	TF
F01_cb173_c23/f1p0/2812-1F	C3H	TF
F01_cb173_c34/f1p1/2634-0F	C3H	TF
F01_cb173_c9/f1p0/4950-1F	C3H	TF
F01_cb17433_c0/f1p0/1675-0F	NAC	TF
F01_cb17433_c1/f1p0/1713-0F	NAC	TF
F01_cb17433_c2/f1p0/1600-2F	NAC	TF
F01_cb17473_c0/f2p0/1522-1F	MYB-related	TF
F01_cb17473_c0/f2p0/1522-2F	MYB-related	TF
F01_cb17473_c1/f1p0/1511-2F	MYB	TF
F01_cb17473_c2/f1p0/1312-2F	MYB	TF
F01_cb17476_c3/f1p0/1241-0F	NF-YA	TF
F01_cb17476_c5/f1p0/1359-1F	NF-YA	TF
F01_cb17476_c7/f1p0/1500-0F	NF-YA	TF
F01_cb17476_c8/f1p0/779-0F	NF-YA	TF
F01_cb17482_c0/f1p0/1390-0F	MYB-related	TF
F01_cb17482_c1/f1p0/1210-0F	MYB	TF

F01_cb17482_c2/f1p0/1228-2F	MYB	TF
F01_cb17496_c1/f1p0/971-0F	GNAT	TR
F01_cb17496_c2/f1p0/853-0F	GNAT	TR
F01_cb17496_c3/f1p0/877-2F	GNAT	TR
F01_cb17515_c1/f1p1/1273-1F	MADS-MIKC	TF
F01_cb17521_c1/f1p1/1478-0F	HB-other	TF
F01_cb17521_c2/f1p0/1637-1F	HB-HD-ZIP	TF
F01_cb17607_c1/f1p0/1249-2R	C3H	TF
F01_cb17624_c0/f1p1/1178-2F	AP2/ERF-ERF	TF
F01_cb17634_c1/f1p0/1420-0F	MADS-MIKC	TF
F01_cb1763_c1/f1p0/3004-1F	RWP-RK	TF
F01_cb1763_c3/f1p0/2029-1F	RWP-RK	TF
F01_cb17641_c2/f1p0/1589-0F	mTERF	TR
F01_cb17645_c0/f5p0/1152-0F	CSD	TF
F01_cb17666_c1/f1p0/1114-2F	LOB	TF
F01_cb17711_c0/f1p0/1292-2F	bZIP	TF
F01_cb17711_c1/f1p0/1361-1F	bZIP	TF
F01_cb17711_c2/f1p0/1797-0F	bZIP	TF
F01_cb17714_c0/f1p0/1519-1F	C2H2	TF
F01_cb17744_c14/f1p0/4249-1F	C3H	TF
F01_cb17744_c14/f1p0/4249-2F	C3H	TF
F01_cb17744_c15/f1p0/4007-2F	C3H	TF
F01_cb17744_c16/f1p0/3043-2F	C3H	TF
F01_cb17744_c17/f1p0/3235-0F	C3H	TF
F01_cb17744_c18/f1p0/2301-0F	C3H	TF
F01_cb17744_c19/f1p1/1451-0F	DBB	TF

F01_cb17744_c21/f1p1/1357-1F	DBB	TF
F01_cb17744_c27/f1p0/1267-1F	DBB	TF
F01_cb17744_c35/f1p0/1190-1F	Others	TR
F01_cb17744_c36/f1p0/1185-0F	Others	TR
F01_cb17744_c37/f1p0/1184-1F	Others	TR
F01_cb17744_c38/f1p0/1113-1F	DBB	TF
F01_cb17744_c39/f1p0/1107-1F	DBB	TF
F01_cb17744_c40/f1p0/1078-2F	DBB	TF
F01_cb17744_c41/f1p0/1040-1F	DBB	TF
F01_cb17744_c47/f59p1/1275-1F	DBB	TF
F01_cb17744_c5/f3p0/1270-0F	DBB	TF
F01_cb17744_c8/f1p0/447-2F	Others	TR
F01_cb17753_c0/f2p0/1256-2F	C2H2	TF
F01_cb17753_c1/f1p0/1295-1F	C2H2	TF
F01_cb17756_c19/f1p0/1743-2R	bHLH	TF
F01_cb17770_c0/f1p0/1374-1F	HSF	TF
F01_cb17770_c1/f1p0/1278-2F	HSF	TF
F01_cb17774_c0/f1p0/1663-1F	B3	TF
F01_cb17774_c1/f1p0/1619-0F	B3	TF
F01_cb17807_c1/f1p0/941-0F	AP2/ERF-ERF	TF
F01_cb17823_c0/f1p0/1685-2R	C3H	TF
F01_cb17823_c1/f1p0/1495-1R	C3H	TF
F01_cb17835_c1/f1p0/970-0F	bHLH	TF
F01_cb17840_c0/f1p0/1111-0F	HB-KNOX	TF
F01_cb17858_c1/f1p0/1806-1F	GRAS	TF
F01_cb17862_c0/f1p0/1730-2F	NF-YA	TF

F01_cb17862_c2/f1p0/1771-2F	NF-YA	TF
F01_cb17862_c4/f1p0/1707-0F	NF-YA	TF
F01_cb17862_c5/f1p0/1255-1F	NF-YA	TF
F01_cb17924_c0/f1p0/1345-1F	bHLH	TF
F01_cb17965_c1/f1p0/976-0F	Others	TR
F01_cb17977_c0/f2p0/812-0F	C2C2-GATA	TF
F01_cb17977_c1/f1p0/751-2F	C2C2-GATA	TF
F01_cb17977_c2/f1p0/949-0F	C2C2-GATA	TF
F01_cb18006_c0/f1p0/1045-1F	SBP	TF
F01_cb18015_c1/f1p0/941-2F	MYB-related	TF
F01_cb18048_c0/f3p0/1120-1F	AP2/ERF-ERF	TF
F01_cb18048_c3/f1p0/1350-0F	AP2/ERF-ERF	TF
F01_cb18052_c0/f2p0/1572-1F	WRKY	TF
F01_cb18052_c1/f1p0/1626-2F	WRKY	TF
F01_cb18065_c0/f1p0/1830-1R	MYB-related	TF
F01_cb18068_c0/f1p0/1290-1F	MADS-MIKC	TF
F01_cb18068_c1/f1p0/1274-0F	MADS-MIKC	TF
F01_cb18070_c0/f4p0/1143-2F	C2H2	TF
F01_cb18070_c1/f2p0/1056-1F	C2H2	TF
F01_cb18070_c3/f1p0/1199-0F	C2H2	TF
F01_cb18075_c0/f1p0/1355-0F	GARP-G2-like	TF
F01_cb18075_c1/f1p0/1218-1F	GARP-G2-like	TF
F01_cb18099_c0/f1p0/1413-1F	C2C2-Dof	TF
F01_cb18124_c0/f1p0/1449-2F	bHLH	TF
F01_cb18124_c1/f1p0/1506-0F	bHLH	TF
F01_cb18124_c2/f1p0/1422-0F	bHLH	TF

F01_cb18125_c1/f1p0/1242-1F	AP2/ERF-ERF	TF
F01_cb18129_c0/f1p0/1391-1F	MYB-related	TF
F01_cb18129_c1/f1p0/1562-2F	MYB-related	TF
F01_cb18133_c0/f1p0/1317-2F	bZIP	TF
F01_cb18143_c0/f1p0/1740-2F	HSF	TF
F01_cb18143_c1/f1p0/1677-1F	HSF	TF
F01_cb18158_c0/f1p0/838-1F	C2H2	TF
F01_cb18158_c1/f1p0/571-1F	C2H2	TF
F01_cb18180_c1/f1p0/1225-0F	AP2/ERF-ERF	TF
F01_cb18210_c0/f1p0/1365-1F	MYB	TF
F01_cb1822_c15/f1p0/1826-0F	IWS1	TR
F01_cb1822_c16/f1p0/5988-2F	IWS1	TR
F01_cb1822_c17/f1p0/5987-2F	IWS1	TR
F01_cb1822_c8/f1p2/4358-1F	IWS1	TR
F01_cb18239_c0/f1p0/1709-0F	NF-YA	TF
F01_cb18239_c0/f1p0/1709-2F	NF-YA	TF
F01_cb18239_c1/f1p0/1475-2F	NF-YA	TF
F01_cb18239_c2/f1p0/1454-2F	NF-YA	TF
F01_cb18239_c3/f1p0/1627-0F	NF-YA	TF
F01_cb18250_c0/f1p0/1093-1F	NAC	TF
F01_cb18250_c1/f1p0/1493-0F	NAC	TF
F01_cb18253_c0/f1p0/1854-1F	bZIP	TF
F01_cb18253_c1/f1p0/1853-0F	bZIP	TF
F01_cb18258_c0/f1p0/1135-0F	AUX/IAA	TR
F01_cb18258_c1/f1p0/1153-0F	AUX/IAA	TR
F01_cb18277_c0/f1p0/1483-0F	C3H	TF

F01_cb18277_c1/f1p0/1534-0F	C3H	TF
F01_cb18316_c0/f1p0/1427-0F	Trihelix	TF
F01_cb18316_c1/f1p0/1406-2F	Trihelix	TF
F01_cb18330_c1/f1p0/1774-1F	Others	TR
F01_cb18330_c2/f1p0/1336-0F	C2C2-GATA	TF
F01_cb18330_c4/f2p0/1338-0F	C2C2-GATA	TF
F01_cb18339_c2/f1p0/5412-1F	HMG	TR
F01_cb18366_c1/f1p0/898-0F	NF-YB	TF
F01_cb18366_c2/f1p0/818-2F	NF-YB	TF
F01_cb18366_c3/f1p0/845-2F	NF-YB	TF
F01_cb18367_c0/f2p0/1072-0F	WRKY	TF
F01_cb18371_c1/f1p0/1758-0F	TRAF	TR
F01_cb18371_c2/f1p0/1680-0F	TRAF	TR
F01_cb18375_c0/f1p0/1205-2F	MADS-MIKC	TF
F01_cb18396_c0/f2p0/1347-0F	bHLH	TF
F01_cb18403_c0/f1p0/1110-1F	AP2/ERF-ERF	TF
F01_cb18403_c1/f1p0/997-1F	AP2/ERF-ERF	TF
F01_cb18422_c1/f1p0/553-0F	AUX/IAA	TR
F01_cb18422_c1/f1p0/553-2F	AUX/IAA	TR
F01_cb18422_c2/f1p0/1525-2F	AUX/IAA	TR
F01_cb18429_c0/f1p0/796-0F	bHLH	TF
F01_cb18429_c1/f1p0/1155-0F	bHLH	TF
F01_cb18431_c0/f1p0/766-1F	NF-YB	TF
F01_cb18442_c2/f1p0/1878-0F	TRAF	TR
F01_cb18442_c3/f1p0/1737-2F	TRAF	TR
F01_cb18456_c1147/f6p5/994-1F	MADS-MIKC	TF

F01_cb18456_c1202/f1p0/946-2F	MADS-M-type	TF
F01_cb18456_c1577/f3p0/769-0F	MADS-M-type	TF
F01_cb18456_c2271/f1p0/1842-1F	MADS-M-type	TF
F01_cb18456_c2272/f1p0/1581-0F	MADS-MIKC	TF
F01_cb18456_c2278/f1p0/807-2F	MADS-M-type	TF
F01_cb18456_c2279/f1p1/622-1F	MADS-M-type	TF
F01_cb18456_c2282/f1p1/942-1F	MADS-MIKC	TF
F01_cb18456_c2284/f1p0/1131-1F	MADS-MIKC	TF
F01_cb18456_c2330/f1p0/644-2F	C2H2	TF
F01_cb18456_c2341/f1p0/1173-0F	bHLH	TF
F01_cb18456_c6642/f1p0/1248-0F	bHLH	TF
F01_cb18456_c6672/f1p0/620-1F	C2H2	TF
F01_cb18456_c7283/f1p0/1049-1F	bHLH	TF
F01_cb18456_c7394/f1p0/1133-1R	MADS-M-type	TF
F01_cb18456_c7395/f1p1/1610-2R	MADS-MIKC	TF
F01_cb18456_c7528/f1p0/1331-1F	bHLH	TF
F01_cb18465_c0/f2p0/1430-1F	MADS-MIKC	TF
F01_cb18466_c0/f1p0/1231-2F	C2H2	TF
F01_cb18488_c0/f2p0/832-0F	NF-YB	TF
F01_cb18499_c0/f1p0/1716-1F	AP2/ERF-ERF	TF
F01_cb18505_c0/f2p0/1046-1F	NAC	TF
F01_cb18514_c1/f1p0/1476-0F	GNAT	TR
F01_cb18525_c0/f1p0/1320-2F	MADS-MIKC	TF
F01_cb18534_c0/f1p0/1158-2F	NAC	TF
F01_cb18543_c1/f1p0/6028-0F	PHD	TR
F01_cb18543_c2/f1p2/5729-1F	PHD	TR

F01_cb18543_c5/f1p1/5435-2F	PHD	TR
F01_cb1854_c14/f1p0/3082-0F	B3-ARF	TF
F01_cb1854_c18/f1p0/3004-1F	B3-ARF	TF
F01_cb1854_c25/f1p0/3077-1F	B3-ARF	TF
F01_cb1854_c26/f1p0/3151-1F	B3-ARF	TF
F01_cb1854_c31/f1p0/3117-0F	B3-ARF	TF
F01_cb1854_c34/f1p0/3128-2F	B3-ARF	TF
F01_cb1854_c42/f1p0/3192-0F	B3-ARF	TF
F01_cb18550_c1/f1p0/1534-2F	Trihelix	TF
F01_cb18562_c0/f2p0/1202-2F	AP2/ERF-ERF	TF
F01_cb18577_c2/f1p0/1326-2F	TRAF	TR
F01_cb18607_c0/f1p0/5858-2F	Others	TR
F01_cb18609_c2/f1p0/5334-2F	Others	TR
F01_cb18625_c3/f1p1/6280-2F	SNF2	TR
F01_cb18625_c4/f1p1/6078-2F	SNF2	TR
F01_cb18631_c0/f2p0/5921-2F	PHD	TR
F01_cb18634_c6/f1p0/5260-1F	bHLH	TF
F01_cb18637_c0/f1p0/8301-2F	Others	TR
F01_cb18639_c0/f2p0/5077-2F	SWI/SNF-BAF60b	TR
F01_cb18657_c2/f1p0/5718-1F	C2C2-Dof	TF
F01_cb18667_c0/f1p2/5116-1F	Others	TR
F01_cb18667_c2/f1p2/5204-2F	Others	TR
F01_cb18685_c0/f1p1/7263-2F	SNF2	TR
F01_cb18685_c2/f1p1/7183-2F	SNF2	TR
F01_cb18685_c3/f1p0/7890-2F	SNF2	TR
F01_cb18685_c6/f1p0/7141-0F	SNF2	TR

F01_cb18692_c0/f1p0/5018-2F	SET	TR
F01_cb18729_c1/f1p1/1344-2F	DBB	TF
F01_cb18732_c0/f1p0/7623-1F	PHD	TR
F01_cb18734_c0/f1p0/5102-2F	PHD	TR
F01_cb18744_c1/f1p0/3188-2F	GRAS	TF
F01_cb18746_c0/f1p0/4937-1F	Jumonji	TR
F01_cb18746_c1/f1p0/2767-1F	Jumonji	TR
F01_cb18769_c11/f1p0/5395-1F	WRKY	TF
F01_cb18788_c0/f1p0/5832-2F	C2C2-Dof	TF
F01_cb18788_c1/f1p0/5403-1F	C2C2-Dof	TF
F01_cb18793_c1/f1p1/6347-0F	ARID	TR
F01_cb18793_c1/f1p1/6347-2F	PHD	TR
F01_cb18793_c2/f1p2/5878-2F	ARID	TR
F01_cb18795_c0/f1p0/5147-1F	RWP-RK	TF
F01_cb18795_c2/f1p1/7526-2F	RWP-RK	TF
F01_cb18808_c0/f1p0/6564-1F	WRKY	TF
F01_cb18844_c1/f1p0/5518-0F	SWI/SNF-BAF60b	TR
F01_cb18848_c0/f1p0/5897-1F	IWS1	TR
F01_cb18848_c1/f1p2/5728-1F	IWS1	TR
F01_cb1892_c0/f3p1/1308-1F	GeBP	TF
F01_cb1892_c11/f1p0/1691-2F	GeBP	TF
F01_cb1892_c12/f1p0/1676-0F	GeBP	TF
F01_cb1892_c14/f1p0/1636-2F	GeBP	TF
F01_cb1892_c15/f1p1/1628-2F	GeBP	TF
F01_cb1892_c16/f1p0/1617-2F	GeBP	TF
F01_cb1892_c17/f1p0/1593-0F	GeBP	TF

F01_cb1892_c18/f1p0/1578-1F	GeBP	TF
F01_cb1892_c19/f1p1/1547-0F	GeBP	TF
F01_cb1892_c20/f1p0/1622-0F	GeBP	TF
F01_cb1892_c22/f1p0/1429-2F	GeBP	TF
F01_cb1892_c23/f1p0/1423-1F	GeBP	TF
F01_cb1892_c26/f1p0/1317-0F	GeBP	TF
F01_cb1892_c6/f1p0/1890-1F	GeBP	TF
F01_cb1892_c7/f1p2/3204-1F	GeBP	TF
F01_cb1892_c8/f1p0/3052-2F	GeBP	TF
F01_cb1892_c9/f1p1/3106-2F	GeBP	TF
F01_cb1905_c11/f1p0/2713-0F	C3H	TF
F01_cb1905_c12/f1p0/3147-1F	C3H	TF
F01_cb1905_c13/f1p0/3167-0F	C3H	TF
F01_cb1905_c15/f1p0/3162-1F	C3H	TF
F01_cb1905_c19/f1p0/2075-0F	C3H	TF
F01_cb1905_c2/f3p0/2454-0F	C3H	TF
F01_cb1905_c22/f1p0/2509-0F	C3H	TF
F01_cb1905_c23/f1p0/2032-2F	C3H	TF
F01_cb1905_c24/f1p0/2875-0F	C3H	TF
F01_cb1905_c25/f1p0/2081-0F	C3H	TF
F01_cb1905_c3/f2p0/2341-0F	C3H	TF
F01_cb1905_c31/f1p0/3100-1F	C3H	TF
F01_cb1905_c34/f1p0/2216-0F	C3H	TF
F01_cb1905_c35/f1p0/2423-1F	C3H	TF
F01_cb1905_c36/f1p0/2252-2F	C3H	TF
F01_cb1905_c37/f1p0/2427-1F	C3H	TF

F01_cb1905_c5/f2p0/3069-0F	C3H	TF
F01_cb1905_c6/f1p0/4127-2F	C3H	TF
F01_cb1905_c7/f1p0/3406-1F	C3H	TF
F01_cb1905_c8/f1p0/2229-1F	C3H	TF
F01_cb1905_c9/f1p0/3045-1F	C3H	TF
F01_cb1909_c0/f2p0/3866-2F	RWP-RK	TF
F01_cb1909_c10/f1p0/3896-2F	RWP-RK	TF
F01_cb1909_c12/f1p0/3811-0F	RWP-RK	TF
F01_cb1909_c2/f1p0/3883-2F	RWP-RK	TF
F01_cb1909_c3/f1p0/3823-1F	RWP-RK	TF
F01_cb1909_c4/f1p0/3818-0F	RWP-RK	TF
F01_cb1909_c5/f1p0/3837-0F	RWP-RK	TF
F01_cb1909_c6/f1p0/3557-0F	RWP-RK	TF
F01_cb1913_c32/f1p0/3800-2F	Trihelix	TF
F01_cb1913_c34/f1p1/2444-1F	Trihelix	TF
F01_cb1913_c37/f1p0/2644-0F	Trihelix	TF
F01_cb1913_c37/f1p0/2644-2F	Trihelix	TF
F01_cb1913_c39/f1p0/2723-2F	Trihelix	TF
F01_cb1913_c44/f1p0/2236-0F	Trihelix	TF
F01_cb1913_c46/f1p1/2592-2F	Trihelix	TF
F01_cb1913_c50/f1p1/2689-0F	Trihelix	TF
F01_cb1913_c51/f1p0/2531-2F	Trihelix	TF
F01_cb1913_c57/f1p0/2520-0F	Trihelix	TF
F01_cb1913_c57/f1p0/2520-1F	Trihelix	TF
F01_cb1913_c65/f5p2/2595-1F	Trihelix	TF
F01_cb1931_c0/f1p0/4121-1F	B3-ARF	TF

F01_cb1931_c1/f1p0/2305-1F	B3-ARF	TF
F01_cb1952_c22/f1p1/2726-0F	SET	TR
F01_cb1952_c23/f1p1/2727-2F	SET	TR
F01_cb1952_c26/f1p1/2800-2F	SET	TR
F01_cb1952_c28/f1p0/2974-0F	SET	TR
F01_cb1952_c29/f1p0/3038-0F	SET	TR
F01_cb1952_c30/f1p1/2397-2F	SET	TR
F01_cb1952_c32/f1p1/2394-2F	SET	TR
F01_cb1952_c7/f2p0/2740-2F	SET	TR
F01_cb1960_c10/f2p0/3006-1F	Pseudo ARR-B	TR
F01_cb1960_c12/f2p0/3366-1F	Others	TR
F01_cb1960_c14/f1p0/4111-0F	Pseudo ARR-B	TR
F01_cb1960_c15/f1p1/3038-0F	Others	TR
F01_cb1960_c15/f1p1/3038-2F	Others	TR
F01_cb1960_c19/f1p1/3084-0F	Others	TR
F01_cb1960_c19/f1p1/3084-2F	Others	TR
F01_cb1960_c20/f1p0/2337-1F	Pseudo ARR-B	TR
F01_cb1960_c24/f1p1/2785-0F	Others	TR
F01_cb1960_c24/f1p1/2785-2F	Others	TR
F01_cb1960_c28/f1p0/3094-0F	Others	TR
F01_cb1960_c28/f1p0/3094-1F	Pseudo ARR-B	TR
F01_cb1960_c31/f1p0/2904-0F	Others	TR
F01_cb1960_c31/f1p0/2904-2F	Others	TR
F01_cb1960_c37/f1p0/2611-2F	Pseudo ARR-B	TR
F01_cb1960_c41/f1p1/2517-0F	Others	TR
F01_cb1960_c41/f1p1/2517-1F	Others	TR

F01_cb1960_c42/f1p0/3273-2F	Others	TR
F01_cb1960_c43/f1p0/3174-1F	Pseudo ARR-B	TR
F01_cb1960_c45/f1p0/3143-1F	Pseudo ARR-B	TR
F01_cb1960_c52/f1p0/3141-2F	Pseudo ARR-B	TR
F01_cb1960_c58/f1p0/2977-2F	Pseudo ARR-B	TR
F01_cb1960_c63/f1p4/2956-0F	Others	TR
F01_cb1960_c63/f1p4/2956-1F	Others	TR
F01_cb1967_c3/f1p0/4111-2F	C2H2	TF
F01_cb1983_c2/f1p0/3834-1F	DDT	TR
F01_cb1983_c3/f1p0/3888-2F	DDT	TR
F01_cb1_c4/f1p2/4866-1F	SNF2	TR
F01_cb1_c5/f1p0/4921-0F	SNF2	TR
F01_cb1_c5/f1p0/4921-1F	SNF2	TR
F01_cb1_c7/f1p1/2558-2F	SNF2	TR
F01_cb1_c8/f1p0/4614-0F	SNF2	TR
F01_cb1_c9/f1p1/4998-2F	SNF2	TR
F01_cb2075_c1/f1p0/4053-1F	EIL	TF
F01_cb2075_c4/f1p0/2548-0F	EIL	TF
F01_cb2075_c7/f2p1/2280-2F	EIL	TF
F01_cb2084_c11/f1p0/2330-2F	bHLH	TF
F01_cb2084_c12/f1p0/3016-1F	bHLH	TF
F01_cb2084_c13/f1p0/1992-2F	bHLH	TF
F01_cb2084_c14/f1p0/2295-2F	bHLH	TF
F01_cb2084_c20/f4p0/2186-1F	bHLH	TF
F01_cb2084_c3/f2p0/2121-2F	bHLH	TF
F01_cb2084_c5/f1p0/2818-0F	bHLH	TF

F01_cb2084_c7/f1p0/2088-0F	bHLH	TF
F01_cb2084_c9/f1p0/2478-2F	bHLH	TF
F01_cb2122_c10/f2p0/3794-0F	Jumonji	TR
F01_cb2122_c5/f1p0/3100-0F	Jumonji	TR
F01_cb2122_c6/f1p0/2400-1F	Jumonji	TR
F01_cb2122_c9/f1p0/3797-1F	Jumonji	TR
F01_cb2156_c20/f2p3/2707-2F	HB-BELL	TF
F01_cb2156_c31/f1p1/2575-0F	HB-BELL	TF
F01_cb2156_c32/f1p0/2880-0F	HB-BELL	TF
F01_cb2156_c34/f1p0/2846-0F	HB-BELL	TF
F01_cb2156_c46/f1p0/2751-2F	HB-BELL	TF
F01_cb2156_c55/f1p0/2868-2F	HB-BELL	TF
F01_cb2156_c56/f1p0/2935-2F	HB-BELL	TF
F01_cb2156_c57/f1p0/2040-2F	HB-BELL	TF
F01_cb2156_c58/f1p0/2830-2F	HB-other	TF
F01_cb2156_c70/f1p0/3008-0F	HB-BELL	TF
F01_cb2156_c71/f1p0/2653-0F	HB-BELL	TF
F01_cb2172_c11/f1p0/2849-1F	Others	TR
F01_cb2172_c11/f1p0/2849-2F	Others	TR
F01_cb2172_c12/f1p0/3182-1F	Pseudo ARR-B	TR
F01_cb2172_c12/f1p0/3182-2F	Others	TR
F01_cb2172_c2/f3p0/2851-1F	Pseudo ARR-B	TR
F01_cb2172_c21/f1p1/2738-2F	Pseudo ARR-B	TR
F01_cb2172_c22/f7p1/2799-2F	Pseudo ARR-B	TR
F01_cb2172_c5/f2p0/3992-1F	Pseudo ARR-B	TR
F01_cb2172_c8/f1p0/2384-0F	Pseudo ARR-B	TR

F01_cb2172_c9/f1p0/2750-2F	Others	TR
F01_cb2217_c12/f1p0/2420-2F	Jumonji	TR
F01_cb2217_c13/f1p5/3312-0F	Jumonji	TR
F01_cb2217_c15/f5p5/3297-0F	Jumonji	TR
F01_cb2217_c4/f1p4/3161-1F	Jumonji	TR
F01_cb2250_c1/f1p1/3997-2F	PHD	TR
F01_cb2250_c3/f1p1/2308-0F	PHD	TR
F01_cb2250_c8/f3p1/2123-1F	PHD	TR
F01_cb2285_c10/f1p0/2288-1F	C3H	TF
F01_cb2285_c10/f1p0/2288-2F	C3H	TF
F01_cb2285_c12/f1p0/2111-2F	C3H	TF
F01_cb2285_c14/f1p0/2217-2F	C3H	TF
F01_cb2285_c15/f1p0/2154-0F	C3H	TF
F01_cb2285_c15/f1p0/2154-1F	C3H	TF
F01_cb2285_c15/f1p0/2154-2F	C3H	TF
F01_cb2285_c6/f1p0/3984-1F	C3H	TF
F01_cb2285_c6/f1p0/3984-2F	C3H	TF
F01_cb2285_c7/f1p0/1969-2F	C3H	TF
F01_cb2285_c8/f1p0/3929-0F	C3H	TF
F01_cb2285_c8/f1p0/3929-2F	C3H	TF
F01_cb2285_c9/f1p0/2005-0F	C3H	TF
F01_cb2285_c9/f1p0/2005-2F	C3H	TF
F01_cb2295_c2/f1p0/2821-1F	SET	TR
F01_cb2295_c4/f1p0/2810-0F	SET	TR
F01_cb2295_c6/f1p0/8328-0F	SET	TR
F01_cb229_c11/f1p1/4779-2F	SNF2	TR

F01_cb229_c3/f1p1/4847-0F	SNF2	TR
F01_cb229_c8/f1p1/4650-0F	SNF2	TR
F01_cb229_c9/f1p0/4601-2F	SNF2	TR
F01_cb2304_c1/f2p0/2952-0F	SNF2	TR
F01_cb2304_c10/f1p0/6051-1F	SNF2	TR
F01_cb2304_c9/f1p0/6807-0F	SNF2	TR
F01_cb2326_c10/f1p0/1746-0F	Tify	TF
F01_cb2326_c19/f7p0/1550-2F	Tify	TF
F01_cb2326_c21/f5p0/1596-0F	Tify	TF
F01_cb2326_c4/f3p0/1682-0F	Tify	TF
F01_cb2326_c5/f1p0/3979-1F	Others	TR
F01_cb2326_c5/f1p0/3979-2F	Tify	TF
F01_cb2326_c6/f1p0/3266-0F	Tify	TF
F01_cb2326_c6/f1p0/3266-1F	Others	TR
F01_cb2326_c7/f1p0/2619-1F	Tify	TF
F01_cb2326_c7/f1p0/2619-2F	Others	TR
F01_cb2326_c8/f1p0/1998-0F	Tify	TF
F01_cb2326_c9/f1p0/2051-1F	Tify	TF
F01_cb2334_c2/f1p0/3969-1F	C3H	TF
F01_cb2334_c3/f1p0/2839-0F	C3H	TF
F01_cb2334_c4/f1p0/2425-0F	C3H	TF
F01_cb2334_c4/f1p0/2425-1F	C3H	TF
F01_cb2334_c5/f1p0/3343-1F	C3H	TF
F01_cb2334_c6/f1p0/3333-0F	C3H	TF
F01_cb2334_c6/f1p0/3333-1F	C3H	TF
F01_cb2334_c6/f1p0/3333-2F	C3H	TF

F01_cb2334_c8/f1p0/1697-1F	C3H	TF
F01_cb233_c12/f1p0/3006-1F	SET	TR
F01_cb233_c13/f1p0/2651-0F	SET	TR
F01_cb233_c15/f1p0/4550-0F	SET	TR
F01_cb233_c16/f1p0/3723-0F	SET	TR
F01_cb233_c17/f1p0/3370-2F	SET	TR
F01_cb233_c18/f1p0/4196-2F	SET	TR
F01_cb233_c19/f1p0/2802-1F	SET	TR
F01_cb233_c20/f1p0/3028-1F	SET	TR
F01_cb233_c22/f1p0/2838-0F	SET	TR
F01_cb233_c23/f1p0/2935-2F	SET	TR
F01_cb233_c24/f1p0/3435-2F	SET	TR
F01_cb233_c27/f16p1/2985-1F	SET	TR
F01_cb233_c5/f2p0/2943-0F	SET	TR
F01_cb233_c6/f2p1/3007-0F	SET	TR
F01_cb233_c7/f1p0/4912-1F	SET	TR
F01_cb2345_c15/f1p1/2999-0F	HB-HD-ZIP	TF
F01_cb2345_c16/f1p0/2882-1F	HB-HD-ZIP	TF
F01_cb2345_c2/f2p1/3492-1F	HB-HD-ZIP	TF
F01_cb2345_c3/f1p0/3969-1F	HB-HD-ZIP	TF
F01_cb2345_c4/f1p1/3441-0F	HB-other	TF
F01_cb2365_c0/f2p0/2949-0F	B3-ARF	TF
F01_cb2365_c1/f1p0/3963-2F	B3-ARF	TF
F01_cb2365_c2/f1p0/3184-2F	B3-ARF	TF
F01_cb2365_c3/f1p0/2982-2F	B3-ARF	TF
F01_cb2372_c0/f1p0/3991-2F	Jumonji	TR

F01_cb2372_c2/f1p0/4859-0F	Jumonji	TR
F01_cb2372_c4/f1p0/5505-0F	Jumonji	TR
F01_cb2376_c10/f1p1/3620-2F	IWS1	TR
F01_cb2376_c18/f10p1/3642-1F	IWS1	TR
F01_cb2376_c20/f2p1/3661-0F	IWS1	TR
F01_cb237_c1/f3p1/4388-0F	SNF2	TR
F01_cb237_c10/f1p0/4733-1F	SNF2	TR
F01_cb237_c11/f1p0/2052-1F	SNF2	TR
F01_cb237_c11/f1p0/2052-2F	SNF2	TR
F01_cb237_c12/f1p0/4420-1F	SNF2	TR
F01_cb237_c12/f1p0/4420-2F	SNF2	TR
F01_cb237_c13/f1p0/4633-0F	SNF2	TR
F01_cb237_c14/f1p0/4353-1F	SNF2	TR
F01_cb237_c17/f2p0/4311-1F	SNF2	TR
F01_cb237_c2/f1p0/4913-1F	SNF2	TR
F01_cb237_c3/f1p1/4828-0F	SNF2	TR
F01_cb237_c3/f1p1/4828-1F	SNF2	TR
F01_cb237_c3/f1p1/4828-2F	SNF2	TR
F01_cb237_c5/f1p0/2911-0F	SNF2	TR
F01_cb237_c5/f1p0/2911-1F	SNF2	TR
F01_cb237_c6/f1p0/2903-0F	SNF2	TR
F01_cb237_c6/f1p0/2903-1F	SNF2	TR
F01_cb237_c9/f1p0/3796-0F	SNF2	TR
F01_cb2385_c0/f2p0/3557-1F	Coactivator p15	TR
F01_cb2385_c1/f1p0/4132-1F	Coactivator p15	TR
F01_cb2385_c2/f1p0/3690-2F	Coactivator p15	TR

F01_cb2406_c0/f2p0/1703-0F	C2C2-GATA	TF
F01_cb2406_c0/f2p0/1703-2F	Tify	TF
F01_cb2406_c1/f1p0/3763-1F	C2C2-GATA	TF
F01_cb2406_c2/f1p0/2884-0F	Others	TR
F01_cb2406_c3/f1p1/1625-2F	C2C2-GATA	TF
F01_cb2431_c13/f1p1/5944-0F	IWS1	TR
F01_cb2444_c10/f1p0/3260-1F	C3H	TF
F01_cb2444_c8/f1p0/3369-1F	C3H	TF
F01_cb2446_c2/f3p0/3727-2F	SNF2	TR
F01_cb2446_c3/f1p0/3927-0F	SNF2	TR
F01_cb2446_c4/f1p0/3579-0F	SNF2	TR
F01_cb2446_c4/f1p0/3579-1F	SNF2	TR
F01_cb2446_c5/f1p0/3490-1F	SNF2	TR
F01_cb2446_c8/f1p0/3717-1F	SNF2	TR
F01_cb2459_c0/f1p0/3919-0F	B3	TF
F01_cb2459_c0/f1p0/3919-1F	B3	TF
F01_cb2459_c2/f1p0/1843-0F	B3	TF
F01_cb2459_c3/f1p0/3534-0F	B3	TF
F01_cb2459_c4/f1p0/1726-2F	B3	TF
F01_cb2459_c5/f1p0/1705-1F	B3	TF
F01_cb2459_c6/f1p0/1744-0F	B3	TF
F01_cb245_c0/f1p0/4906-1R	C2H2	TF
F01_cb245_c2/f1p0/4025-1R	C2H2	TF
F01_cb2474_c0/f1p0/3915-2F	FAR1	TF
F01_cb2474_c1/f1p0/3818-1F	FAR1	TF
F01_cb2474_c2/f1p0/3949-1F	FAR1	TF

F01_cb2474_c3/f1p0/4162-2F	FAR1	TF
F01_cb2474_c6/f1p0/3752-0F	FAR1	TF
F01_cb2474_c6/f1p0/3752-2F	FAR1	TF
F01_cb2483_c0/f2p0/2861-1F	C2C2-CO-like	TF
F01_cb2483_c1/f1p0/3910-0F	Others	TR
F01_cb2483_c1/f1p0/3910-1F	Others	TR
F01_cb2483_c2/f1p0/1912-0F	Others	TR
F01_cb2483_c2/f1p0/1912-2F	Others	TR
F01_cb2483_c3/f1p0/3161-1F	Others	TR
F01_cb2483_c4/f1p0/1785-0F	C2C2-CO-like	TF
F01_cb2488_c1/f1p0/3123-2R	HB-other	TF
F01_cb2505_c0/f1p0/3821-0F	Jumonji	TR
F01_cb2505_c0/f1p0/3821-1F	Jumonji	TR
F01_cb2505_c2/f1p0/3400-1F	Jumonji	TR
F01_cb2505_c3/f1p0/3388-2F	Jumonji	TR
F01_cb2505_c4/f1p0/3283-1F	Jumonji	TR
F01_cb2529_c0/f2p0/3703-2F	SNF2	TR
F01_cb2529_c5/f1p0/3561-1F	SNF2	TR
F01_cb2529_c6/f1p0/2581-1F	SNF2	TR
F01_cb2529_c7/f1p0/5178-2F	SNF2	TR
F01_cb2533_c16/f1p0/4039-1F	HB-other	TF
F01_cb2533_c17/f1p0/3689-2F	HB-HD-ZIP	TF
F01_cb2533_c20/f1p0/3689-1F	HB-HD-ZIP	TF
F01_cb2533_c26/f1p1/3568-2F	HB-HD-ZIP	TF
F01_cb2533_c28/f1p0/3624-1F	HB-HD-ZIP	TF
F01_cb2533_c30/f1p0/3420-2F	HB-HD-ZIP	TF

F01_cb2533_c52/f1p0/3172-2F	HB-HD-ZIP	TF
F01_cb2533_c53/f1p0/3490-1F	HB-HD-ZIP	TF
F01_cb2533_c57/f1p3/3153-0F	HB-HD-ZIP	TF
F01_cb2533_c6/f2p1/3490-1F	HB-HD-ZIP	TF
F01_cb2533_c65/f1p2/2992-1F	HB-HD-ZIP	TF
F01_cb2572_c0/f2p1/3513-1F	HB-HD-ZIP	TF
F01_cb2572_c6/f1p0/3742-0F	HB-HD-ZIP	TF
F01_cb2599_c18/f1p0/3075-1F	HB-BELL	TF
F01_cb2599_c19/f1p0/3461-0F	HB-BELL	TF
F01_cb2599_c20/f1p0/1890-2F	HB-other	TF
F01_cb2599_c23/f1p0/3316-2F	HB-BELL	TF
F01_cb2599_c28/f10p0/2946-1F	HB-BELL	TF
F01_cb2599_c4/f4p0/2721-1F	HB-BELL	TF
F01_cb2599_c5/f2p1/2802-2F	HB-BELL	TF
F01_cb2599_c7/f1p0/3524-0F	HB-BELL	TF
F01_cb260_c13/f1p2/3725-2F	C2C2-GATA	TF
F01_cb260_c15/f1p0/4067-0F	C2C2-GATA	TF
F01_cb260_c20/f1p0/4102-0F	C2C2-GATA	TF
F01_cb260_c21/f1p3/3927-1F	C2C2-GATA	TF
F01_cb260_c23/f1p0/4082-2F	C2C2-GATA	TF
F01_cb260_c28/f8p1/3718-2F	C2C2-GATA	TF
F01_cb260_c7/f1p1/3641-0F	C2C2-GATA	TF
F01_cb260_c7/f1p1/3641-2F	C2C2-GATA	TF
F01_cb260_c8/f1p0/3825-0F	C2C2-GATA	TF
F01_cb260_c9/f1p1/3879-1F	C2C2-GATA	TF
F01_cb2618_c4/f1p0/4255-1F	C2H2	TF

F01_cb2618_c5/f1p1/1408-2F	C2H2	TF
F01_cb2618_c7/f1p1/1379-2F	C2H2	TF
F01_cb2618_c8/f6p1/1387-2F	C2H2	TF
F01_cb2619_c3/f1p1/2037-2F	C2H2	TF
F01_cb2619_c5/f1p0/1091-0F	C2H2	TF
F01_cb2629_c0/f3p3/4660-1F	C3H	TF
F01_cb2629_c0/f3p3/4660-2F	SWI/SNF-BAF60b	TR
F01_cb2629_c14/f1p0/3350-0F	SWI/SNF-BAF60b	TR
F01_cb2629_c16/f1p0/2938-0F	SWI/SNF-BAF60b	TR
F01_cb2629_c27/f1p2/2884-1F	C3H	TF
F01_cb2629_c3/f1p0/3859-1F	SWI/SNF-BAF60b	TR
F01_cb2638_c1/f1p0/3310-2F	SET	TR
F01_cb2638_c2/f1p0/3093-0F	SET	TR
F01_cb2638_c3/f1p0/3063-0F	SET	TR
F01_cb2640_c10/f1p0/3278-1F	Jumonji	TR
F01_cb2640_c11/f1p0/3319-1F	Jumonji	TR
F01_cb2640_c12/f1p0/3046-2F	Jumonji	TR
F01_cb2640_c13/f1p0/3176-1F	Jumonji	TR
F01_cb2640_c14/f1p1/3398-2F	Jumonji	TR
F01_cb2640_c18/f1p0/3205-0F	Jumonji	TR
F01_cb2640_c18/f1p0/3205-1F	Jumonji	TR
F01_cb2640_c22/f1p1/3609-2F	Jumonji	TR
F01_cb2640_c23/f1p0/2402-1F	Jumonji	TR
F01_cb2640_c24/f1p0/3186-0F	Jumonji	TR
F01_cb2640_c25/f1p0/3308-0F	Jumonji	TR
F01_cb2640_c28/f1p0/3675-1F	Jumonji	TR

F01_cb2640_c28/f1p0/3675-2F	Jumonji	TR
F01_cb2640_c29/f1p0/3441-2F	Jumonji	TR
F01_cb2640_c3/f2p1/3206-2F	Jumonji	TR
F01_cb2640_c31/f3p0/3266-0F	Jumonji	TR
F01_cb2640_c6/f1p0/3857-0F	Jumonji	TR
F01_cb2640_c6/f1p0/3857-1F	Jumonji	TR
F01_cb2640_c8/f1p0/3279-0F	Jumonji	TR
F01_cb2640_c9/f1p0/3551-2F	Jumonji	TR
F01_cb2649_c2/f1p0/2059-1F	GRAS	TF
F01_cb2720_c0/f1p0/3827-0F	PHD	TR
F01_cb2720_c1/f1p0/3716-1F	PHD	TR
F01_cb2737_c1/f4p0/2862-1F	TRAF	TR
F01_cb2737_c12/f1p0/3972-1F	TRAF	TR
F01_cb2737_c13/f1p0/3105-2F	TRAF	TR
F01_cb2737_c14/f1p0/2854-2F	TRAF	TR
F01_cb2737_c15/f1p0/3303-0F	TRAF	TR
F01_cb2737_c19/f1p0/3583-2F	TRAF	TR
F01_cb2737_c20/f1p0/3644-0F	TRAF	TR
F01_cb2737_c21/f1p2/3479-1F	TRAF	TR
F01_cb2737_c22/f1p0/3196-0F	TRAF	TR
F01_cb2737_c22/f1p0/3196-1F	TRAF	TR
F01_cb2737_c23/f1p0/2933-0F	TRAF	TR
F01_cb2737_c27/f1p0/2325-1F	TRAF	TR
F01_cb2737_c43/f1p0/3713-0F	TRAF	TR
F01_cb2737_c43/f1p0/3713-2F	TRAF	TR
F01_cb2737_c50/f1p0/3706-2F	TRAF	TR

F01_cb2737_c54/f1p0/3029-1F	TRAF	TR
F01_cb2737_c63/f1p2/2799-1F	TRAF	TR
F01_cb2737_c64/f1p1/2838-2F	TRAF	TR
F01_cb2737_c9/f2p0/2910-2F	TRAF	TR
F01_cb2747_c16/f1p1/3380-0F	SWI/SNF-SWI3	TR
F01_cb2747_c16/f1p1/3380-1F	MYB-related	TF
F01_cb2760_c0/f3p0/3301-1F	B3	TF
F01_cb2760_c4/f1p0/3330-0F	B3	TF
F01_cb2760_c9/f1p0/3316-2F	B3	TF
F01_cb2792_c13/f1p0/3807-2F	B3-ARF	TF
F01_cb2792_c14/f1p0/3588-0F	B3-ARF	TF
F01_cb2792_c17/f1p0/3095-0F	B3-ARF	TF
F01_cb2792_c18/f1p0/2374-1F	B3-ARF	TF
F01_cb2792_c21/f1p0/3291-0F	B3	TF
F01_cb2792_c24/f1p0/3416-0F	B3-ARF	TF
F01_cb2792_c27/f1p0/3552-1F	B3-ARF	TF
F01_cb2792_c27/f1p0/3552-2F	AUX/IAA	TR
F01_cb2792_c28/f1p0/3225-0F	B3-ARF	TF
F01_cb2792_c29/f1p1/3324-1F	B3-ARF	TF
F01_cb2792_c5/f1p0/3809-1F	AUX/IAA	TR
F01_cb2792_c5/f1p0/3809-2F	B3-ARF	TF
F01_cb2792_c6/f1p0/3198-0F	B3-ARF	TF
F01_cb2792_c7/f1p0/2929-0F	B3-ARF	TF
F01_cb2792_c7/f1p0/2929-2F	AUX/IAA	TR
F01_cb2792_c8/f1p1/3110-0F	AUX/IAA	TR
F01_cb2792_c8/f1p1/3110-1F	B3-ARF	TF

F01_cb2806_c6/f1p0/6088-0F	DDT	TR
F01_cb280_c67/f1p0/2419-2F	NF-YA	TF
F01_cb2812_c4/f2p0/3146-0F	DDT	TR
F01_cb2812_c6/f1p0/3033-2F	DDT	TR
F01_cb2812_c8/f3p0/3209-0F	DDT	TR
F01_cb2899_c0/f4p0/1604-2F	C2C2-GATA	TF
F01_cb2899_c1/f2p0/1740-2F	C2C2-GATA	TF
F01_cb2899_c11/f1p0/1619-0F	C2C2-GATA	TF
F01_cb2899_c12/f1p0/1615-2F	C2C2-GATA	TF
F01_cb2899_c13/f1p0/1590-0F	C2C2-GATA	TF
F01_cb2899_c13/f1p0/1590-1F	Tify	TF
F01_cb2899_c14/f1p0/1484-0F	C2C2-GATA	TF
F01_cb2899_c15/f1p0/1306-0F	C2C2-GATA	TF
F01_cb2899_c2/f1p0/3774-1F	C2C2-GATA	TF
F01_cb2899_c3/f1p0/2878-0F	C2C2-GATA	TF
F01_cb2899_c8/f1p0/2712-2F	C2C2-GATA	TF
F01_cb2899_c9/f1p0/1705-0F	C2C2-GATA	TF
F01_cb2903_c0/f8p1/2229-0F	WRKY	TF
F01_cb2903_c10/f1p0/2263-2F	WRKY	TF
F01_cb2903_c13/f1p0/2228-0F	WRKY	TF
F01_cb2903_c13/f1p0/2228-1F	WRKY	TF
F01_cb2903_c14/f1p0/2117-0F	WRKY	TF
F01_cb2903_c5/f1p0/3769-1F	WRKY	TF
F01_cb2937_c0/f1p0/3765-2F	Others	TR
F01_cb2937_c3/f1p0/3537-2F	Others	TR
F01_cb2937_c4/f1p0/3451-1F	Others	TR

F01_cb2962_c12/f1p0/3385-2F	FAR1	TF
F01_cb2962_c14/f1p0/3047-0F	FAR1	TF
F01_cb2962_c17/f1p0/3231-2F	FAR1	TF
F01_cb2962_c7/f1p0/3759-0F	FAR1	TF
F01_cb2963_c0/f1p0/3758-2F	PHD	TR
F01_cb2963_c1/f1p0/3071-2F	PHD	TR
F01_cb2963_c2/f1p1/3308-0F	PHD	TR
F01_cb2963_c3/f1p0/3590-1F	PHD	TR
F01_cb2966_c0/f3p0/2527-0F	FAR1	TF
F01_cb2966_c1/f2p0/2466-2F	FAR1	TF
F01_cb2966_c2/f1p0/3755-1F	FAR1	TF
F01_cb2966_c4/f1p0/2868-2F	FAR1	TF
F01_cb2966_c5/f1p0/2974-1F	FAR1	TF
F01_cb2966_c6/f1p0/5561-0F	FAR1	TF
F01_cb2966_c6/f1p0/5561-0R	FAR1	TF
F01_cb2966_c6/f1p0/5561-1F	FAR1	TF
F01_cb2977_c0/f1p0/3755-2F	Jumonji	TR
F01_cb3008_c0/f11p3/2616-0F	FAR1	TF
F01_cb3008_c1/f2p2/3740-2F	FAR1	TF
F01_cb3008_c2/f1p1/2485-0F	FAR1	TF
F01_cb3008_c5/f1p1/1912-0F	FAR1	TF
F01_cb3031_c0/f1p0/3742-2F	Jumonji	TR
F01_cb3031_c1/f1p0/3822-2F	Jumonji	TR
F01_cb3031_c2/f1p0/2783-0F	Jumonji	TR
F01_cb3067_c1/f1p0/4064-1F	SNF2	TR
F01_cb3086_c10/f2p1/4066-1F	PHD	TR

F01_cb3086_c14/f1p0/3734-1F	CAMTA	TF
F01_cb3086_c17/f1p0/3322-0F	CAMTA	TF
F01_cb3086_c29/f1p2/3439-0F	CAMTA	TF
F01_cb3086_c3/f3p1/3388-2F	CAMTA	TF
F01_cb3086_c36/f1p1/3238-1F	CAMTA	TF
F01_cb3086_c4/f3p1/3409-0F	CAMTA	TF
F01_cb3086_c40/f1p0/3247-0F	CAMTA	TF
F01_cb3086_c44/f1p0/3558-1F	CAMTA	TF
F01_cb3086_c45/f1p0/3498-2F	CAMTA	TF
F01_cb3086_c53/f1p1/3280-2F	CAMTA	TF
F01_cb3086_c57/f1p0/3323-2F	CAMTA	TF
F01_cb3086_c62/f1p0/6330-0F	CAMTA	TF
F01_cb3121_c1/f1p0/3714-0R	AUX/IAA	TR
F01_cb3121_c1/f1p0/3714-2R	B3-ARF	TF
F01_cb3121_c4/f1p1/3640-1R	B3-ARF	TF
F01_cb3121_c5/f1p0/3594-2R	B3-ARF	TF
F01_cb3149_c15/f1p0/4008-0F	B3-ARF	TF
F01_cb3149_c16/f1p1/4212-0F	AUX/IAA	TR
F01_cb3149_c16/f1p1/4212-2F	B3	TF
F01_cb3149_c17/f1p0/4493-0F	B3-ARF	TF
F01_cb3149_c18/f1p1/3326-1F	B3-ARF	TF
F01_cb3149_c20/f1p0/4424-0F	B3-ARF	TF
F01_cb3149_c24/f1p0/4095-1F	B3-ARF	TF
F01_cb3149_c25/f1p0/3152-2F	B3-ARF	TF
F01_cb3149_c26/f1p0/3554-1F	B3-ARF	TF
F01_cb3149_c29/f1p0/2871-0F	B3	TF

F01_cb3149_c31/f1p0/3457-0F	B3	TF
F01_cb3149_c35/f1p0/3213-2F	B3-ARF	TF
F01_cb3149_c40/f1p0/3643-0F	B3-ARF	TF
F01_cb3149_c42/f1p0/3602-1F	B3-ARF	TF
F01_cb3149_c44/f1p0/3465-2F	B3	TF
F01_cb3149_c47/f1p0/3833-0F	B3	TF
F01_cb3149_c54/f1p0/3496-0F	B3-ARF	TF
F01_cb3149_c56/f1p0/3368-0F	B3-ARF	TF
F01_cb3149_c57/f1p0/2868-0F	FAR1	TF
F01_cb3149_c58/f1p0/3200-1F	B3-ARF	TF
F01_cb3149_c58/f1p0/3200-2F	AUX/IAA	TR
F01_cb3164_c0/f2p0/3680-0F	SET	TR
F01_cb3164_c1/f1p0/3697-0F	SET	TR
F01_cb3203_c12/f1p1/2066-1F	GRAS	TF
F01_cb3203_c22/f1p0/2976-0F	GRAS	TF
F01_cb3203_c22/f1p0/2976-1F	GRAS	TF
F01_cb3203_c23/f4p1/2176-0F	GRAS	TF
F01_cb3203_c5/f2p0/3221-1F	GRAS	TF
F01_cb3203_c6/f1p0/3678-2F	GRAS	TF
F01_cb3203_c7/f1p2/3226-2F	GRAS	TF
F01_cb3211_c1/f1p0/3687-2F	B3	TF
F01_cb3211_c2/f1p1/3277-2F	B3	TF
F01_cb3211_c3/f1p1/3175-1F	B3	TF
F01_cb3211_c4/f1p0/3640-1F	B3	TF
F01_cb3211_c6/f1p0/3543-2F	B3	TF
F01_cb3214_c1/f1p0/3686-2F	CAMTA	TF

F01_cb3214_c3/f1p0/3521-1F	CAMTA	TF
F01_cb3214_c5/f1p0/3377-1F	CAMTA	TF
F01_cb3214_c6/f3p1/3424-2F	CAMTA	TF
F01_cb3226_c0/f4p0/2730-0F	WRKY	TF
F01_cb3226_c0/f4p0/2730-1F	WRKY	TF
F01_cb3226_c10/f1p0/3292-1F	WRKY	TF
F01_cb3226_c12/f1p0/3216-0F	WRKY	TF
F01_cb3226_c16/f1p0/2457-1F	WRKY	TF
F01_cb3226_c20/f1p1/2573-1F	WRKY	TF
F01_cb3226_c22/f2p0/2434-0F	WRKY	TF
F01_cb3226_c5/f2p0/2559-1F	WRKY	TF
F01_cb3226_c6/f1p0/3708-0F	WRKY	TF
F01_cb3226_c6/f1p0/3708-2F	WRKY	TF
F01_cb3226_c7/f1p0/3210-0F	WRKY	TF
F01_cb3226_c8/f1p0/2993-0F	WRKY	TF
F01_cb3226_c8/f1p0/2993-2F	WRKY	TF
F01_cb3226_c9/f1p0/2473-0F	WRKY	TF
F01_cb3227_c10/f2p0/3206-1F	LUG	TR
F01_cb3227_c13/f1p0/3841-2F	LUG	TR
F01_cb3227_c15/f1p1/3305-1F	LUG	TR
F01_cb3227_c25/f1p0/3220-1F	LUG	TR
F01_cb3227_c31/f1p0/3481-2F	LUG	TR
F01_cb3227_c37/f1p0/3185-2F	LUG	TR
F01_cb3227_c41/f9p1/3288-0F	LUG	TR
F01_cb3227_c8/f2p1/3397-0F	LUG	TR
F01_cb3253_c0/f2p0/2494-2F	MYB-related	TF

F01_cb3253_c1/f1p0/3675-2F	MYB-related	TF
F01_cb3253_c2/f1p1/2645-2F	MYB-related	TF
F01_cb3253_c3/f1p0/2624-2F	MYB-related	TF
F01_cb3269_c1/f2p1/3036-0F	SNF2	TR
F01_cb3269_c10/f1p0/2536-0F	SNF2	TR
F01_cb3269_c11/f1p0/2163-2F	SNF2	TR
F01_cb3269_c13/f1p0/2224-0F	SNF2	TR
F01_cb3269_c13/f1p0/2224-2F	SNF2	TR
F01_cb3269_c8/f1p0/3501-0F	SNF2	TR
F01_cb3278_c12/f1p0/3210-0F	FAR1	TF
F01_cb3278_c16/f1p0/3632-1F	FAR1	TF
F01_cb3278_c18/f1p0/3282-0F	FAR1	TF
F01_cb3278_c18/f1p0/3282-1F	FAR1	TF
F01_cb3278_c2/f3p0/3188-1F	FAR1	TF
F01_cb3278_c3/f1p0/3594-0F	FAR1	TF
F01_cb3278_c5/f1p0/3664-1F	FAR1	TF
F01_cb3278_c5/f1p0/3664-2F	FAR1	TF
F01_cb3278_c9/f1p0/3082-0F	FAR1	TF
F01_cb3280_c1/f1p0/2723-2F	bZIP	TF
F01_cb3280_c2/f1p0/2122-0F	bZIP	TF
F01_cb3280_c3/f1p0/1537-2F	bZIP	TF
F01_cb3306_c0/f4p0/3336-1F	Trihelix	TF
F01_cb3306_c11/f1p0/3005-1F	Trihelix	TF
F01_cb3306_c12/f1p0/3175-2F	Trihelix	TF
F01_cb3306_c13/f1p0/2058-0F	Trihelix	TF
F01_cb3306_c20/f1p0/3929-0F	Trihelix	TF

F01_cb3306_c4/f1p0/4279-1F	Trihelix	TF
F01_cb3306_c5/f1p0/3913-1F	Trihelix	TF
F01_cb3306_c9/f1p0/3416-1F	Trihelix	TF
F01_cb3312_c0/f9p2/1172-2F	bZIP	TF
F01_cb3312_c12/f1p0/3200-2F	bZIP	TF
F01_cb3312_c14/f1p1/3482-0F	bZIP	TF
F01_cb3312_c16/f1p0/1452-1F	bZIP	TF
F01_cb3312_c17/f1p0/1262-2F	bZIP	TF
F01_cb3312_c19/f1p0/1144-0F	bZIP	TF
F01_cb3312_c22/f3p2/1210-2F	bZIP	TF
F01_cb3312_c3/f3p0/2369-0F	bZIP	TF
F01_cb3312_c6/f2p0/3040-1F	bZIP	TF
F01_cb3318_c0/f1p0/3663-0F	PHD	TR
F01_cb3318_c1/f1p0/3625-2F	PHD	TR
F01_cb3318_c2/f1p0/3688-0F	PHD	TR
F01_cb3318_c3/f1p0/3536-1F	PHD	TR
F01_cb3318_c4/f1p1/3064-0F	PHD	TR
F01_cb3318_c6/f1p0/2556-2F	PHD	TR
F01_cb3318_c7/f1p0/3560-2F	PHD	TR
F01_cb3339_c12/f13p1/3205-2F	RWP-RK	TF
F01_cb3339_c2/f1p1/3653-2F	RWP-RK	TF
F01_cb3339_c5/f1p1/3094-2F	RWP-RK	TF
F01_cb3339_c8/f1p0/3249-2F	RWP-RK	TF
F01_cb3339_c9/f1p0/2219-2F	RWP-RK	TF
F01_cb3344_c11/f1p0/3514-0F	Jumonji	TR
F01_cb3344_c12/f1p0/2543-1F	Jumonji	TR

F01_cb3344_c13/f1p0/2922-0F	Jumonji	TR
F01_cb3344_c14/f1p0/3327-2F	Jumonji	TR
F01_cb3344_c2/f4p1/3653-0F	Jumonji	TR
F01_cb3344_c4/f2p0/2908-0F	Jumonji	TR
F01_cb3344_c9/f1p0/3064-1F	Jumonji	TR
F01_cb3355_c13/f1p1/2541-2F	C2H2	TF
F01_cb3355_c15/f1p3/2337-2F	C2H2	TF
F01_cb3367_c13/f1p0/1942-0F	GRAS	TF
F01_cb3367_c13/f1p0/1942-1F	GRAS	TF
F01_cb3367_c15/f1p0/3106-1F	GRAS	TF
F01_cb3367_c20/f3p0/3095-1F	GRAS	TF
F01_cb3367_c21/f1p0/3212-1F	GRAS	TF
F01_cb3367_c21/f1p0/3212-2F	GRAS	TF
F01_cb3367_c9/f1p0/3072-2F	GRAS	TF
F01_cb3383_c0/f2p0/3072-1F	SWI/SNF-SWI3	TR
F01_cb3383_c2/f1p0/3641-0F	SWI/SNF-SWI3	TR
F01_cb3383_c3/f1p0/3067-0F	SWI/SNF-SWI3	TR
F01_cb3390_c0/f2p0/3636-0F	mTERF	TR
F01_cb3390_c0/f2p0/3636-2F	mTERF	TR
F01_cb3390_c1/f2p0/1899-0F	mTERF	TR
F01_cb3390_c10/f1p0/2708-1F	mTERF	TR
F01_cb3390_c11/f1p0/2011-0F	mTERF	TR
F01_cb3390_c13/f1p0/2133-0F	mTERF	TR
F01_cb3390_c13/f1p0/2133-2F	mTERF	TR
F01_cb3390_c14/f1p0/1861-0F	mTERF	TR
F01_cb3390_c14/f1p0/1861-2F	mTERF	TR

F01_cb3390_c16/f1p0/1774-2F	mTERF	TR
F01_cb3390_c2/f1p0/1813-0F	mTERF	TR
F01_cb3390_c2/f1p0/1813-1F	mTERF	TR
F01_cb3390_c3/f2p0/2186-2F	mTERF	TR
F01_cb3390_c6/f1p0/2069-0F	mTERF	TR
F01_cb3390_c6/f1p0/2069-2F	mTERF	TR
F01_cb3390_c7/f1p0/2203-0F	mTERF	TR
F01_cb3390_c8/f1p0/2138-0F	mTERF	TR
F01_cb3390_c8/f1p0/2138-2F	mTERF	TR
F01_cb3390_c9/f1p0/2379-0F	mTERF	TR
F01_cb3390_c9/f1p0/2379-1F	mTERF	TR
F01_cb3411_c17/f1p0/3564-0F	C3H	TF
F01_cb3431_c0/f3p2/1268-0F	C2C2-LSD	TF
F01_cb3431_c2/f1p0/2266-1F	C2C2-LSD	TF
F01_cb3431_c2/f1p0/2266-2F	C2C2-LSD	TF
F01_cb3431_c4/f1p0/2338-1F	C2C2-LSD	TF
F01_cb3431_c5/f1p0/2189-0F	C2C2-LSD	TF
F01_cb3449_c10/f1p0/4190-1R	GRAS	TF
F01_cb3451_c0/f1p0/2080-1F	mTERF	TR
F01_cb3451_c0/f1p0/2080-2F	mTERF	TR
F01_cb3451_c1/f2p0/3277-2F	mTERF	TR
F01_cb3451_c2/f1p0/2070-1F	mTERF	TR
F01_cb3451_c3/f1p0/2428-0F	mTERF	TR
F01_cb3451_c3/f1p0/2428-1F	mTERF	TR
F01_cb3451_c4/f1p1/2393-0F	mTERF	TR
F01_cb3451_c4/f1p1/2393-2F	mTERF	TR

F01_cb3451_c6/f1p1/2200-1F	mTERF	TR
F01_cb3451_c7/f2p0/2047-2F	mTERF	TR
F01_cb3451_c8/f1p0/1800-0F	mTERF	TR
F01_cb3457_c0/f2p0/2030-1F	bZIP	TF
F01_cb3457_c10/f1p0/2348-1F	bZIP	TF
F01_cb3457_c11/f1p0/1940-1F	bZIP	TF
F01_cb3457_c12/f1p0/2646-0F	bZIP	TF
F01_cb3457_c13/f1p0/2100-2F	bZIP	TF
F01_cb3457_c14/f1p0/2004-2F	bZIP	TF
F01_cb3457_c2/f1p0/2243-2F	bZIP	TF
F01_cb3457_c3/f1p0/2251-2F	bZIP	TF
F01_cb3457_c8/f1p0/2552-1F	bZIP	TF
F01_cb3471_c0/f1p0/3611-2F	bHLH	TF
F01_cb3471_c1/f1p0/3483-0F	bHLH	TF
F01_cb3471_c3/f1p1/1814-0F	bHLH	TF
F01_cb3471_c4/f1p0/1799-2F	bHLH	TF
F01_cb3471_c5/f1p1/1643-0F	bHLH	TF
F01_cb3520_c5/f1p0/3051-1F	TRAF	TR
F01_cb353_c3/f1p0/4866-1F	Others	TR
F01_cb353_c3/f1p0/4866-2F	Others	TR
F01_cb353_c4/f1p1/3068-0F	Others	TR
F01_cb353_c5/f1p1/2645-2F	C2C2-CO-like	TF
F01_cb353_c6/f1p0/1879-1F	Others	TR
F01_cb353_c8/f2p2/1647-0F	C2C2-CO-like	TF
F01_cb3549_c0/f3p0/1735-0F	HB-KNOX	TF
F01_cb3549_c1/f3p1/1745-2F	HB-KNOX	TF

F01_cb3549_c2/f2p0/1762-1F	HB-KNOX	TF
F01_cb3549_c20/f1p0/851-2F	HB-other	TF
F01_cb3549_c21/f1p0/5108-1F	HB-other	TF
F01_cb3549_c3/f1p0/3595-1F	HB-KNOX	TF
F01_cb3549_c6/f1p2/1934-0F	HB-KNOX	TF
F01_cb3549_c8/f1p0/3448-0F	HB-other	TF
F01_cb3551_c0/f4p0/3532-2F	FAR1	TF
F01_cb3551_c10/f1p0/3333-0F	FAR1	TF
F01_cb3551_c11/f1p0/3439-2F	FAR1	TF
F01_cb3551_c13/f1p0/3934-0F	FAR1	TF
F01_cb3551_c14/f1p1/3279-0F	FAR1	TF
F01_cb3551_c15/f1p0/3307-1F	FAR1	TF
F01_cb3551_c17/f1p1/3045-0F	FAR1	TF
F01_cb3551_c19/f1p0/3455-1F	FAR1	TF
F01_cb3551_c2/f2p2/3461-2F	FAR1	TF
F01_cb3551_c3/f2p0/3540-2F	FAR1	TF
F01_cb3551_c5/f1p2/3595-0F	FAR1	TF
F01_cb3551_c9/f1p0/3631-0F	FAR1	TF
F01_cb3566_c0/f2p1/1967-0F	C2H2	TF
F01_cb3566_c1/f1p1/3588-0F	C2H2	TF
F01_cb3583_c1/f1p0/3595-2F	Jumonji	TR
F01_cb3583_c4/f1p0/3408-0F	Jumonji	TR
F01_cb3586_c0/f4p0/3057-0F	MYB-related	TF
F01_cb3586_c10/f1p0/2722-0F	MYB-related	TF
F01_cb3586_c11/f1p0/2822-1F	MYB-related	TF
F01_cb3586_c13/f1p0/3140-1F	MYB-related	TF

F01_cb3586_c2/f1p0/3545-2F	MYB-related	TF
F01_cb3586_c5/f1p0/2816-0F	MYB-related	TF
F01_cb3586_c6/f1p0/2809-1F	MYB-related	TF
F01_cb3586_c7/f1p0/2846-2F	MYB-related	TF
F01_cb3586_c9/f1p0/3463-2F	MYB-related	TF
F01_cb3597_c11/f1p0/3188-1F	FAR1	TF
F01_cb3597_c13/f1p0/3124-2F	FAR1	TF
F01_cb3597_c30/f5p0/3147-0F	FAR1	TF
F01_cb3597_c30/f5p0/3147-2F	FAR1	TF
F01_cb3597_c5/f1p0/3587-2F	FAR1	TF
F01_cb3597_c6/f1p1/3143-0F	FAR1	TF
F01_cb3597_c9/f1p1/3159-0F	FAR1	TF
F01_cb3603_c26/f1p1/4747-2F	SET	TR
F01_cb3603_c30/f1p0/3545-1F	SET	TR
F01_cb3603_c31/f1p0/4927-0F	SET	TR
F01_cb3603_c36/f1p0/5599-2F	SET	TR
F01_cb3603_c38/f1p0/4973-2F	SET	TR
F01_cb3603_c4/f2p3/5068-1F	SET	TR
F01_cb3603_c40/f1p1/5143-0F	SET	TR
F01_cb3603_c40/f1p1/5143-1F	SET	TR
F01_cb3603_c41/f1p0/5154-1F	SET	TR
F01_cb3603_c42/f1p0/5113-1F	SET	TR
F01_cb3603_c43/f1p2/5123-1F	SET	TR
F01_cb3603_c46/f1p0/5012-0F	SET	TR
F01_cb3628_c2/f1p0/3299-2F	HB-HD-ZIP	TF
F01_cb3628_c3/f1p0/3849-2F	HB-HD-ZIP	TF

F01_cb3628_c7/f1p0/3843-1F	HB-HD-ZIP	TF
F01_cb3628_c8/f1p0/3355-2F	HB-other	TF
F01_cb3644_c15/f1p0/3548-0F	B3	TF
F01_cb3644_c18/f1p1/3159-1F	B3	TF
F01_cb3644_c20/f1p0/3151-0F	B3	TF
F01_cb3644_c22/f1p0/3675-1F	B3	TF
F01_cb3644_c5/f3p0/3297-1F	B3	TF
F01_cb3644_c6/f1p0/3570-0F	B3	TF
F01_cb3644_c7/f1p0/3638-0F	B3	TF
F01_cb3644_c9/f1p0/3316-1F	B3	TF
F01_cb3650_c11/f1p1/1811-0F	B3	TF
F01_cb3650_c11/f1p1/1811-1F	B3	TF
F01_cb3650_c14/f1p1/1651-0F	B3	TF
F01_cb3650_c14/f1p1/1651-1F	B3	TF
F01_cb3650_c18/f7p1/1695-0F	B3	TF
F01_cb3650_c5/f1p1/2879-0F	B3	TF
F01_cb3650_c6/f1p1/2430-1F	B3	TF
F01_cb3650_c6/f1p1/2430-2F	B3	TF
F01_cb3650_c7/f1p0/2118-0F	B3	TF
F01_cb3650_c7/f1p0/2118-2F	B3	TF
F01_cb3650_c8/f1p2/2335-1F	B3	TF
F01_cb3699_c10/f1p0/3357-2F	HSF	TF
F01_cb3699_c11/f1p0/2983-1F	HSF	TF
F01_cb3699_c12/f1p0/3310-0F	HSF	TF
F01_cb3699_c17/f1p0/3321-1F	HSF	TF
F01_cb3699_c2/f1p0/3554-2F	HSF	TF

F01_cb3699_c6/f1p0/3729-1F	HSF	TF
F01_cb369_c1/f2p0/2357-0F	SNF2	TR
F01_cb369_c4/f1p0/4486-0F	SNF2	TR
F01_cb369_c4/f1p0/4486-2F	SNF2	TR
F01_cb369_c5/f1p0/4455-0F	SNF2	TR
F01_cb369_c6/f1p0/4410-2F	SNF2	TR
F01_cb369_c8/f1p0/3502-0F	SNF2	TR
F01_cb369_c8/f1p0/3502-1F	SNF2	TR
F01_cb3750_c16/f1p0/3161-1F	VOZ	TF
F01_cb3750_c17/f1p0/2207-0F	VOZ	TF
F01_cb3750_c20/f1p3/3049-1F	VOZ	TF
F01_cb3750_c3/f4p2/2344-2F	VOZ	TF
F01_cb3750_c30/f1p0/2586-2F	VOZ	TF
F01_cb3750_c31/f1p0/2307-0F	VOZ	TF
F01_cb3750_c35/f1p1/2165-2F	VOZ	TF
F01_cb3750_c5/f2p2/2925-0F	VOZ	TF
F01_cb3750_c5/f2p2/2925-1F	VOZ	TF
F01_cb3750_c6/f2p0/2746-1F	VOZ	TF
F01_cb3750_c9/f1p0/2220-0F	VOZ	TF
F01_cb3755_c10/f2p1/3165-1F	SET	TR
F01_cb3755_c14/f1p1/3790-0F	SET	TR
F01_cb3755_c19/f1p0/3624-0F	SET	TR
F01_cb3755_c30/f1p1/3108-0F	SET	TR
F01_cb3755_c31/f1p0/3146-2F	SET	TR
F01_cb3755_c35/f1p1/2918-0F	SET	TR
F01_cb3755_c41/f1p0/2802-0F	SET	TR

F01_cb3755_c45/f1p0/2895-2F	SET	TR
F01_cb3755_c47/f1p0/3281-2F	SET	TR
F01_cb3755_c50/f1p1/2934-2F	SET	TR
F01_cb3755_c51/f1p1/3015-0F	SET	TR
F01_cb3755_c52/f1p0/2922-1F	SET	TR
F01_cb3755_c57/f1p0/3053-1F	SET	TR
F01_cb3755_c6/f8p1/3083-2F	SET	TR
F01_cb3755_c60/f11p1/2825-2F	SET	TR
F01_cb3755_c62/f9p1/2884-2F	SET	TR
F01_cb3755_c63/f9p2/2822-1F	SET	TR
F01_cb3755_c8/f4p0/2721-1F	SET	TR
F01_cb3764_c0/f3p0/3263-1F	HB-other	TF
F01_cb3764_c1/f1p0/2578-1F	HB-BELL	TF
F01_cb3791_c1/f3p0/1750-2F	HRT	TF
F01_cb3791_c12/f1p0/1510-1F	HRT	TF
F01_cb3791_c17/f4p2/1499-0F	HRT	TF
F01_cb3791_c2/f1p0/3527-1F	HRT	TF
F01_cb3791_c3/f1p0/2226-1F	HRT	TF
F01_cb3791_c4/f1p0/2359-2F	HRT	TF
F01_cb3791_c8/f1p0/1798-0F	HRT	TF
F01_cb3799_c0/f1p0/3527-0F	HB-other	TF
F01_cb3799_c1/f1p0/2994-0F	HB-other	TF
F01_cb3839_c10/f1p0/3225-1F	B3-ARF	TF
F01_cb3839_c12/f2p0/3178-2F	B3-ARF	TF
F01_cb3839_c16/f1p0/2928-1F	B3-ARF	TF
F01_cb3839_c16/f1p0/2928-2F	AUX/IAA	TR

F01_cb3839_c19/f1p0/2723-1F	B3-ARF	TF
F01_cb3839_c22/f7p1/3028-2F	B3-ARF	TF
F01_cb3839_c8/f1p0/2927-1F	B3	TF
F01_cb3842_c0/f2p0/2475-1F	mTERF	TR
F01_cb3842_c1/f1p0/3511-1F	mTERF	TR
F01_cb3842_c4/f1p0/3346-1F	mTERF	TR
F01_cb3842_c5/f1p0/2814-0F	mTERF	TR
F01_cb3842_c6/f1p0/3329-0F	mTERF	TR
F01_cb3842_c6/f1p0/3329-1F	mTERF	TR
F01_cb3842_c6/f1p0/3329-2F	mTERF	TR
F01_cb3886_c1/f3p1/2318-2F	B3	TF
F01_cb3886_c12/f1p1/1735-1F	B3	TF
F01_cb3886_c3/f1p2/2629-1F	B3	TF
F01_cb3886_c4/f1p1/2982-2F	B3	TF
F01_cb3886_c9/f1p2/2477-1F	B3	TF
F01_cb392_c2/f1p0/3348-0F	SET	TR
F01_cb392_c4/f1p1/1991-1F	SET	TR
F01_cb392_c5/f1p0/1982-0F	SET	TR
F01_cb392_c6/f1p0/1997-2F	SET	TR
F01_cb392_c7/f1p0/2120-2F	SET	TR
F01_cb392_c9/f1p0/2892-1F	SET	TR
F01_cb3941_c35/f1p0/3281-0F	FAR1	TF
F01_cb3941_c35/f1p0/3281-2F	FAR1	TF
F01_cb3941_c36/f1p0/3156-0F	FAR1	TF
F01_cb3941_c36/f1p0/3156-1F	FAR1	TF
F01_cb3941_c48/f1p0/3414-0F	FAR1	TF

F01_cb3941_c48/f1p0/3414-2F	FAR1	TF
F01_cb3941_c94/f1p0/2327-1F	FAR1	TF
F01_cb3941_c94/f1p0/2327-2F	FAR1	TF
F01_cb3956_c10/f1p0/2749-0F	DDT	TR
F01_cb3956_c11/f1p0/2270-2F	DDT	TR
F01_cb3956_c2/f1p0/3482-0F	DDT	TR
F01_cb3956_c5/f1p0/2970-0F	DDT	TR
F01_cb3956_c8/f1p0/2721-2F	DDT	TR
F01_cb3956_c9/f1p0/2666-1F	DDT	TR
F01_cb3962_c2/f1p0/2633-0F	FAR1	TF
F01_cb3965_c1/f1p0/3487-1F	TRAF	TR
F01_cb3965_c3/f1p0/2377-0F	TRAF	TR
F01_cb3965_c5/f1p1/3316-1F	TRAF	TR
F01_cb3995_c6/f1p0/3190-1F	Others	TR
F01_cb3995_c8/f1p0/1625-0F	Others	TR
F01_cb4009_c0/f1p0/3464-2F	FAR1	TF
F01_cb4009_c2/f1p0/2929-0F	FAR1	TF
F01_cb4009_c3/f1p0/3411-2F	FAR1	TF
F01_cb4013_c0/f1p0/3469-0F	PHD	TR
F01_cb4013_c2/f1p0/5086-1F	PHD	TR
F01_cb4013_c2/f1p0/5086-2F	DDT	TR
F01_cb401_c79/f1p0/2391-0F	TRAF	TR
F01_cb4025_c2/f1p0/3461-0F	GRAS	TF
F01_cb4025_c5/f2p0/3060-2F	GRAS	TF
F01_cb4025_c6/f1p0/3220-1F	GRAS	TF
F01_cb402_c36/f1p0/5510-1R	MADS-MIKC	TF

F01_cb4042_c11/f1p0/3362-1F	FAR1	TF
F01_cb4042_c12/f1p0/3156-1F	FAR1	TF
F01_cb4042_c14/f1p0/3138-0F	FAR1	TF
F01_cb4042_c14/f1p0/3138-1F	FAR1	TF
F01_cb4042_c3/f1p0/3470-2F	FAR1	TF
F01_cb4042_c5/f1p1/3481-1F	FAR1	TF
F01_cb4042_c8/f1p0/3354-2F	FAR1	TF
F01_cb4042_c9/f1p0/3437-0F	FAR1	TF
F01_cb4074_c11/f1p0/2602-1F	C2H2	TF
F01_cb4074_c13/f1p0/2925-1F	C2H2	TF
F01_cb4074_c19/f3p1/2467-1F	C2H2	TF
F01_cb4074_c3/f2p1/2503-0F	C2H2	TF
F01_cb4074_c4/f2p0/2329-0F	C2H2	TF
F01_cb4074_c6/f1p0/3445-0F	C2H2	TF
F01_cb4074_c7/f1p1/2826-0F	C2H2	TF
F01_cb4074_c7/f1p1/2826-2F	C2H2	TF
F01_cb4074_c9/f1p1/2413-0F	C2H2	TF
F01_cb4088_c2/f1p0/2761-2F	FAR1	TF
F01_cb4088_c4/f1p0/2792-0F	FAR1	TF
F01_cb4098_c63/f1p0/2704-0F	SBP	TF
F01_cb4101_c10/f1p0/3199-0F	RWP-RK	TF
F01_cb4101_c11/f3p1/3262-1F	RWP-RK	TF
F01_cb4101_c12/f1p1/3212-0F	RWP-RK	TF
F01_cb4101_c12/f1p1/3212-2F	RWP-RK	TF
F01_cb4101_c2/f1p0/3435-0F	RWP-RK	TF
F01_cb4101_c6/f1p0/3298-1F	RWP-RK	TF

F01_cb4101_c8/f1p0/3287-0F	RWP-RK	TF
F01_cb4131_c12/f1p1/1212-2F	MYB-related	TF
F01_cb4131_c15/f7p3/1294-0F	MYB-related	TF
F01_cb4131_c5/f15p2/1269-0F	MYB-related	TF
F01_cb4131_c6/f1p0/3311-1F	MYB-related	TF
F01_cb4131_c8/f1p0/3080-1F	MYB-related	TF
F01_cb4131_c9/f1p0/2847-0F	MYB-related	TF
F01_cb4147_c12/f1p0/6657-1F	TAZ	TR
F01_cb4147_c12/f1p0/6657-2F	TAZ	TR
F01_cb4147_c14/f1p0/3211-0F	TAZ	TR
F01_cb4147_c2/f2p1/6129-1F	TAZ	TR
F01_cb4147_c28/f1p0/6755-0F	TAZ	TR
F01_cb4147_c31/f1p0/6049-1F	TAZ	TR
F01_cb4147_c32/f1p1/5785-1F	TAZ	TR
F01_cb4147_c32/f1p1/5785-2F	TAZ	TR
F01_cb4147_c33/f1p0/5961-0F	TAZ	TR
F01_cb4147_c33/f1p0/5961-1F	PHD	TR
F01_cb4147_c33/f1p0/5961-2F	TAZ	TR
F01_cb4147_c38/f1p2/5596-0F	TAZ	TR
F01_cb4147_c38/f1p2/5596-1F	PHD	TR
F01_cb4147_c38/f1p2/5596-2F	TAZ	TR
F01_cb4147_c40/f1p0/5399-2F	TAZ	TR
F01_cb4147_c42/f4p4/5844-0F	TAZ	TR
F01_cb4147_c5/f1p0/2445-1F	TAZ	TR
F01_cb4147_c8/f1p0/3276-2F	TAZ	TR
F01_cb4186_c1/f2p0/1783-0F	Trihelix	TF

F01_cb4186_c10/f2p0/1882-1F	Trihelix	TF
F01_cb4186_c2/f2p1/1773-2F	Trihelix	TF
F01_cb4186_c3/f1p0/3432-1F	Trihelix	TF
F01_cb4186_c6/f1p1/1800-1F	Trihelix	TF
F01_cb4186_c9/f3p1/1894-2F	Trihelix	TF
F01_cb418_c19/f1p0/2326-1F	bZIP	TF
F01_cb418_c2/f2p2/2674-2F	bZIP	TF
F01_cb418_c5/f1p1/2711-2F	bZIP	TF
F01_cb418_c8/f1p1/2632-2F	bZIP	TF
F01_cb418_c9/f1p0/2782-0F	bZIP	TF
F01_cb4209_c12/f1p0/2915-1F	C3H	TF
F01_cb4209_c5/f1p0/3403-0F	C3H	TF
F01_cb4209_c6/f1p0/3222-0F	C3H	TF
F01_cb4209_c6/f1p0/3222-2F	C3H	TF
F01_cb4209_c8/f1p0/3250-0F	C3H	TF
F01_cb4234_c15/f1p2/3200-0F	C3H	TF
F01_cb4234_c18/f1p0/2918-1F	C3H	TF
F01_cb4234_c19/f1p0/3319-1F	C3H	TF
F01_cb4234_c5/f3p2/3150-1F	C3H	TF
F01_cb4235_c11/f1p2/3350-1F	SNF2	TR
F01_cb4235_c12/f1p0/3273-2F	SNF2	TR
F01_cb4235_c4/f1p2/3482-0F	SNF2	TR
F01_cb4235_c5/f1p2/3333-0F	SNF2	TR
F01_cb4235_c6/f1p0/2639-2F	SNF2	TR
F01_cb423_c1/f1p0/4817-1F	E2F-DP	TF
F01_cb4247_c4/f1p0/3145-1F	FAR1	TF

F01_cb4247_c7/f1p1/3200-2F	FAR1	TF
F01_cb4262_c1/f1p0/2252-1F	mTERF	TR
F01_cb4262_c1/f1p0/2252-2F	mTERF	TR
F01_cb4262_c2/f1p0/2277-2F	mTERF	TR
F01_cb4262_c3/f1p0/1156-2F	mTERF	TR
F01_cb4283_c4/f1p0/3896-2F	FAR1	TF
F01_cb4283_c5/f1p1/3520-1F	FAR1	TF
F01_cb4283_c8/f1p0/3419-0F	FAR1	TF
F01_cb4314_c2/f3p0/2111-0F	C3H	TF
F01_cb4314_c4/f1p0/3432-0F	C3H	TF
F01_cb4314_c5/f1p0/3121-0F	C3H	TF
F01_cb4314_c6/f1p0/2072-0F	C3H	TF
F01_cb4314_c7/f1p0/2208-0F	C3H	TF
F01_cb4317_c0/f5p1/3336-0F	SNF2	TR
F01_cb4317_c11/f1p1/3350-2F	SNF2	TR
F01_cb4317_c13/f1p0/3009-1F	SNF2	TR
F01_cb4317_c14/f1p0/2386-0F	SNF2	TR
F01_cb4317_c14/f1p0/2386-1F	SNF2	TR
F01_cb4317_c15/f1p0/3096-1F	SNF2	TR
F01_cb4317_c5/f1p0/4037-0F	SNF2	TR
F01_cb4317_c6/f1p0/3297-0F	SNF2	TR
F01_cb4327_c1/f2p0/2125-0F	IWS1	TR
F01_cb4327_c10/f1p0/1776-1F	IWS1	TR
F01_cb4327_c11/f1p0/1700-0F	IWS1	TR
F01_cb4327_c12/f1p0/1448-1F	IWS1	TR
F01_cb4327_c4/f1p0/2236-0F	IWS1	TR

F01_cb4327_c6/f1p0/2076-0F	IWS1	TR
F01_cb4327_c9/f1p0/1848-0F	IWS1	TR
F01_cb432_c10/f1p1/5326-2F	C3H	TF
F01_cb432_c8/f1p0/5484-2F	C3H	TF
F01_cb432_c9/f1p1/5358-2F	C3H	TF
F01_cb4337_c30/f1p0/2725-0F	C3H	TF
F01_cb4375_c0/f5p0/1767-2F	mTERF	TR
F01_cb4375_c1/f2p1/3160-0F	mTERF	TR
F01_cb4375_c6/f1p0/2971-0F	mTERF	TR
F01_cb4375_c7/f1p0/3057-2F	mTERF	TR
F01_cb4387_c0/f2p0/6053-1F	HB-other	TF
F01_cb4388_c3/f1p0/3270-2F	FAR1	TF
F01_cb4389_c11/f1p1/2597-2F	TCP	TF
F01_cb4389_c12/f1p0/2103-2F	TCP	TF
F01_cb4389_c15/f1p1/2762-2F	TCP	TF
F01_cb4389_c17/f1p0/2434-2F	TCP	TF
F01_cb4389_c22/f1p0/2826-1F	TCP	TF
F01_cb4389_c8/f1p0/3361-2F	TCP	TF
F01_cb4389_c9/f1p1/2744-1F	TCP	TF
F01_cb4427_c0/f1p0/3349-1F	SBP	TF
F01_cb4427_c0/f1p0/3349-2F	SBP	TF
F01_cb4427_c1/f1p0/3309-2F	SBP	TF
F01_cb4427_c3/f1p0/2034-2F	SBP	TF
F01_cb4444_c17/f1p0/3051-1F	GRAS	TF
F01_cb4444_c2/f4p0/2597-2F	GRAS	TF
F01_cb4444_c25/f1p0/4660-1F	GRAS	TF

F01_cb4444_c26/f1p0/2544-1F	GRAS	TF
F01_cb4444_c30/f1p0/2548-1F	GRAS	TF
F01_cb4444_c31/f1p0/2518-0F	GRAS	TF
F01_cb4444_c33/f1p0/3036-1F	GRAS	TF
F01_cb4444_c33/f1p0/3036-2F	GRAS	TF
F01_cb4449_c1/f2p0/3029-2F	BES1	TF
F01_cb4449_c10/f1p0/3005-0F	BES1	TF
F01_cb4449_c11/f1p1/3044-1F	BES1	TF
F01_cb4449_c14/f4p1/3073-1F	BES1	TF
F01_cb4449_c4/f1p0/3414-1F	BES1	TF
F01_cb4449_c6/f1p0/3358-0F	BES1	TF
F01_cb4449_c7/f1p0/2856-0F	BES1	TF
F01_cb4449_c8/f1p0/2993-1F	BES1	TF
F01_cb4449_c9/f1p0/2969-0F	BES1	TF
F01_cb4450_c0/f1p0/3343-0F	CSD	TF
F01_cb4450_c1/f1p0/3275-0F	CSD	TF
F01_cb4465_c0/f2p0/3328-2F	mTERF	TR
F01_cb4465_c1/f1p0/2776-1F	mTERF	TR
F01_cb4465_c2/f1p0/2714-0F	mTERF	TR
F01_cb446_c14/f1p1/2839-0F	EIL	TF
F01_cb446_c16/f1p0/2478-0F	EIL	TF
F01_cb446_c16/f1p0/2478-2F	EIL	TF
F01_cb446_c17/f1p0/2292-1F	EIL	TF
F01_cb446_c18/f1p0/2198-0F	EIL	TF
F01_cb446_c24/f1p3/3787-0F	EIL	TF
F01_cb446_c27/f1p0/2708-0F	EIL	TF

F01_cb446_c28/f1p0/2688-1F	EIL	TF
F01_cb446_c32/f1p0/2721-0F	EIL	TF
F01_cb446_c32/f1p0/2721-1F	EIL	TF
F01_cb446_c4/f1p1/2601-0F	EIL	TF
F01_cb446_c4/f1p1/2601-1F	EIL	TF
F01_cb446_c4/f1p1/2601-2F	EIL	TF
F01_cb446_c45/f1p0/2393-0F	EIL	TF
F01_cb446_c45/f1p0/2393-2F	EIL	TF
F01_cb446_c48/f1p0/2961-0F	EIL	TF
F01_cb446_c49/f1p0/2765-0F	EIL	TF
F01_cb446_c49/f1p0/2765-1F	EIL	TF
F01_cb446_c51/f1p0/2635-1F	EIL	TF
F01_cb446_c53/f1p0/2944-2F	EIL	TF
F01_cb446_c54/f1p0/2863-2F	EIL	TF
F01_cb446_c55/f1p1/2586-0F	EIL	TF
F01_cb446_c57/f1p2/2685-1F	EIL	TF
F01_cb446_c65/f1p0/1483-2F	EIL	TF
F01_cb4478_c1/f1p0/3008-2F	GNAT	TR
F01_cb4480_c0/f4p0/2037-1F	NAC	TF
F01_cb4480_c10/f1p0/2307-0F	NAC	TF
F01_cb4480_c13/f1p0/1871-1F	NAC	TF
F01_cb4480_c16/f3p0/2035-2F	NAC	TF
F01_cb4480_c3/f1p0/3336-2F	NAC	TF
F01_cb4480_c4/f1p0/3113-1F	NAC	TF
F01_cb4480_c7/f1p0/1963-0F	NAC	TF
F01_cb4480_c7/f1p0/1963-1F	NAC	TF

F01_cb4494_c1/f1p0/3279-0F	ARID	TR
F01_cb4494_c2/f1p0/2761-0F	ARID	TR
F01_cb4494_c3/f1p0/2950-1F	ARID	TR
F01_cb4494_c7/f1p0/2870-1F	ARID	TR
F01_cb4520_c1/f4p0/2353-1F	C3H	TF
F01_cb4520_c2/f1p0/3324-0F	C3H	TF
F01_cb4520_c3/f1p0/2384-1F	C3H	TF
F01_cb4520_c5/f1p0/2430-1F	C3H	TF
F01_cb4520_c7/f1p0/2404-0F	C3H	TF
F01_cb4520_c9/f5p0/2406-2F	C3H	TF
F01_cb4529_c10/f1p0/746-2F	zf-HD	TF
F01_cb4529_c13/f1p0/614-0F	zf-HD	TF
F01_cb4529_c16/f2p0/753-2F	zf-HD	TF
F01_cb4529_c5/f1p0/3304-1F	zf-HD	TF
F01_cb4529_c6/f1p2/4180-1F	zf-HD	TF
F01_cb4529_c8/f1p0/838-2F	zf-HD	TF
F01_cb4529_c9/f1p0/780-2F	zf-HD	TF
F01_cb4541_c0/f2p0/3319-0F	bHLH	TF
F01_cb4541_c2/f1p0/1238-0F	bHLH	TF
F01_cb458_c13/f1p0/5055-2F	PHD	TR
F01_cb4594_c3/f1p0/6274-2F	DDT	TR
F01_cb4594_c4/f1p0/6003-0F	DDT	TR
F01_cb4594_c4/f1p0/6003-2F	PHD	TR
F01_cb4604_c18/f1p0/3255-2F	TRAF	TR
F01_cb4604_c19/f1p0/2042-1F	TRAF	TR
F01_cb4634_c9/f1p0/2221-1R	MADS-M-type	TF

F01_cb4635_c2/f1p0/2072-0F	C2C2-CO-like	TF
F01_cb4635_c3/f1p0/1855-1F	C2C2-CO-like	TF
F01_cb4635_c4/f1p0/1745-2F	C2C2-CO-like	TF
F01_cb4662_c0/f2p0/3283-1F	FAR1	TF
F01_cb4662_c1/f2p0/1138-2F	FAR1	TF
F01_cb4667_c4/f1p2/3118-1F	FAR1	TF
F01_cb4667_c7/f1p0/3276-2F	FAR1	TF
F01_cb4670_c1/f3p0/1598-0F	bHLH	TF
F01_cb4670_c10/f1p0/1465-2F	bHLH	TF
F01_cb4670_c2/f2p0/1545-2F	bHLH	TF
F01_cb4670_c4/f1p0/2273-2F	bHLH	TF
F01_cb4670_c7/f1p0/1514-0F	bHLH	TF
F01_cb4670_c8/f1p0/1376-0F	bHLH	TF
F01_cb4670_c9/f2p0/1564-1F	bHLH	TF
F01_cb4684_c13/f1p0/2905-0F	C2H2	TF
F01_cb4684_c3/f1p0/3284-0F	C2H2	TF
F01_cb4707_c4/f1p0/6741-0F	PHD	TR
F01_cb4710_c0/f1p0/3208-0F	SET	TR
F01_cb4710_c4/f1p0/6822-1F	SET	TR
F01_cb4737_c0/f3p0/2432-1F	C2H2	TF
F01_cb4737_c3/f1p0/2429-0F	C2H2	TF
F01_cb4745_c0/f12p0/3046-1F	GRAS	TF
F01_cb4745_c12/f1p0/2017-0F	GRAS	TF
F01_cb4745_c17/f1p0/2837-2F	GRAS	TF
F01_cb4745_c18/f1p0/3074-0F	GRAS	TF
F01_cb4745_c19/f1p0/3044-0F	GRAS	TF

F01_cb4745_c19/f1p0/3044-2F	GRAS	TF
F01_cb4745_c2/f4p1/3063-1F	GRAS	TF
F01_cb4745_c25/f7p0/2733-0F	GRAS	TF
F01_cb4745_c7/f1p0/3655-1F	GRAS	TF
F01_cb4745_c8/f1p0/2875-0F	GRAS	TF
F01_cb4752_c2/f1p0/3014-0F	B3	TF
F01_cb4752_c3/f1p0/2513-1F	B3	TF
F01_cb4752_c3/f1p0/2513-2F	B3	TF
F01_cb4755_c12/f2p0/2459-0F	C3H	TF
F01_cb4755_c3/f2p0/2543-1F	C3H	TF
F01_cb4755_c5/f1p0/2534-1F	C3H	TF
F01_cb4755_c6/f1p0/2908-2F	C3H	TF
F01_cb4755_c7/f1p0/3105-2F	C3H	TF
F01_cb4790_c15/f1p1/2582-1F	GRAS	TF
F01_cb4790_c3/f2p1/2340-2F	GRAS	TF
F01_cb4790_c4/f1p0/3257-2F	GRAS	TF
F01_cb4790_c8/f1p1/2558-0F	GRAS	TF
F01_cb4790_c8/f1p1/2558-1F	GRAS	TF
F01_cb4818_c0/f3p0/1481-0F	B3	TF
F01_cb4818_c0/f3p0/1481-2F	B3	TF
F01_cb4818_c1/f2p0/3114-0F	B3	TF
F01_cb4818_c1/f2p0/3114-2F	B3	TF
F01_cb4818_c2/f1p0/3199-0F	B3	TF
F01_cb4818_c2/f1p0/3199-2F	B3	TF
F01_cb4818_c4/f1p0/2037-0F	B3	TF
F01_cb4818_c5/f1p0/1549-0F	B3	TF

F01_cb4835_c1/f3p1/2340-1F	bHLH	TF
F01_cb4835_c11/f1p0/2717-0F	bHLH	TF
F01_cb4835_c12/f1p0/2189-1F	bHLH	TF
F01_cb4835_c2/f3p0/2243-0F	bHLH	TF
F01_cb4835_c7/f1p0/2458-0F	bHLH	TF
F01_cb4835_c8/f1p0/2582-0F	bHLH	TF
F01_cb4835_c9/f1p0/2177-1F	bHLH	TF
F01_cb4842_c0/f1p0/3243-2F	HB-HD-ZIP	TF
F01_cb4842_c1/f1p0/3389-1F	HB-HD-ZIP	TF
F01_cb4889_c64/f1p0/3070-1F	mTERF	TR
F01_cb4889_c66/f1p1/3206-0F	mTERF	TR
F01_cb4889_c66/f1p1/3206-1F	mTERF	TR
F01_cb4889_c66/f1p1/3206-2F	mTERF	TR
F01_cb4889_c72/f1p0/2961-0F	mTERF	TR
F01_cb4889_c80/f1p0/3020-1F	mTERF	TR
F01_cb4889_c86/f6p0/2960-1F	mTERF	TR
F01_cb4925_c11/f1p2/3304-2F	FAR1	TF
F01_cb4925_c12/f1p1/3169-2F	FAR1	TF
F01_cb4925_c3/f2p2/3330-2F	FAR1	TF
F01_cb4934_c0/f1p0/3222-1F	GARP-G2-like	TF
F01_cb4934_c0/f1p0/3222-2F	Others	TR
F01_cb4934_c1/f1p0/2571-1F	Others	TR
F01_cb4934_c2/f1p0/2274-1F	Others	TR
F01_cb4934_c7/f1p0/2934-0F	GARP-G2-like	TF
F01_cb4942_c11/f1p1/1345-0F	AP2/ERF-ERF	TF
F01_cb4942_c12/f1p1/1339-0F	AP2/ERF-ERF	TF

F01_cb4942_c2/f3p0/1309-2F	AP2/ERF-ERF	TF
F01_cb4942_c5/f2p1/2283-1F	AP2/ERF-ERF	TF
F01_cb4942_c8/f1p0/2187-2F	AP2/ERF-ERF	TF
F01_cb4957_c0/f1p0/3218-0F	HB-other	TF
F01_cb4957_c1/f1p0/3205-1F	HB-HD-ZIP	TF
F01_cb4957_c12/f1p0/1876-0F	HB-HD-ZIP	TF
F01_cb4957_c3/f1p0/3177-2F	HB-HD-ZIP	TF
F01_cb4957_c4/f1p0/3205-1F	HB-HD-ZIP	TF
F01_cb4957_c9/f1p0/3457-2F	HB-HD-ZIP	TF
F01_cb4959_c3/f1p0/3215-0F	SET	TR
F01_cb4959_c4/f1p0/2670-1F	SET	TR
F01_cb4959_c6/f1p1/2644-0F	SET	TR
F01_cb4959_c9/f3p1/2722-2F	SET	TR
F01_cb4968_c1/f1p0/3209-2F	FAR1	TF
F01_cb4968_c2/f1p0/2962-2F	FAR1	TF
F01_cb4968_c3/f1p0/2859-0F	FAR1	TF
F01_cb4968_c4/f1p0/2927-2F	FAR1	TF
F01_cb4968_c5/f1p0/2916-2F	FAR1	TF
F01_cb4968_c7/f1p0/2910-1F	FAR1	TF
F01_cb4973_c1/f1p0/3211-1F	SWI/SNF-SWI3	TR
F01_cb4973_c2/f1p1/2923-0F	MYB-related	TF
F01_cb4973_c2/f1p1/2923-1F	SWI/SNF-SWI3	TR
F01_cb4999_c14/f1p1/3238-1F	SET	TR
F01_cb4999_c17/f1p1/3105-1F	SET	TR
F01_cb4999_c18/f1p0/3066-1F	SET	TR
F01_cb4999_c20/f1p0/5049-2F	SET	TR

F01_cb4999_c22/f5p1/2954-2F	SET	TR
F01_cb4999_c23/f5p0/2983-2F	SET	TR
F01_cb4999_c3/f4p0/3051-2F	SET	TR
F01_cb4999_c7/f1p0/3156-1F	SET	TR
F01_cb500_c15/f1p0/4958-2R	ARID	TR
F01_cb501_c0/f1p0/4783-1F	mTERF	TR
F01_cb501_c1/f1p0/1961-0F	mTERF	TR
F01_cb501_c1/f1p0/1961-2F	mTERF	TR
F01_cb501_c11/f1p0/1842-0F	mTERF	TR
F01_cb501_c11/f1p0/1842-2F	mTERF	TR
F01_cb501_c12/f1p0/1810-1F	mTERF	TR
F01_cb501_c12/f1p0/1810-2F	mTERF	TR
F01_cb501_c2/f1p0/2192-2F	mTERF	TR
F01_cb501_c3/f1p0/2471-2F	mTERF	TR
F01_cb501_c4/f1p0/2008-2F	mTERF	TR
F01_cb501_c5/f1p0/2505-1F	mTERF	TR
F01_cb501_c6/f1p1/1929-2F	mTERF	TR
F01_cb501_c8/f1p0/1917-1F	mTERF	TR
F01_cb501_c9/f1p0/1876-1F	mTERF	TR
F01_cb5020_c0/f1p1/3058-1F	SNF2	TR
F01_cb5020_c2/f1p1/3434-2F	SNF2	TR
F01_cb5020_c3/f1p1/3328-2F	SNF2	TR
F01_cb5020_c4/f1p0/3090-1F	SNF2	TR
F01_cb5082_c0/f3p0/2540-2F	MYB-related	TF
F01_cb5082_c10/f1p0/2806-0F	MYB-related	TF
F01_cb5082_c11/f1p0/2718-2F	MYB-related	TF

F01_cb5082_c12/f1p0/2668-2F	MYB-related	TF
F01_cb5082_c13/f1p0/2435-2F	MYB-related	TF
F01_cb5082_c2/f2p0/2585-0F	MYB-related	TF
F01_cb5082_c5/f1p0/3051-1F	MYB-related	TF
F01_cb5082_c7/f1p0/2984-0F	MYB-related	TF
F01_cb5082_c8/f1p0/2811-0F	MYB-related	TF
F01_cb5082_c9/f1p0/2437-2F	MYB-related	TF
F01_cb5106_c11/f1p1/2803-2F	bHLH	TF
F01_cb5106_c12/f1p0/2863-0F	bHLH	TF
F01_cb5106_c6/f1p0/2987-1F	bHLH	TF
F01_cb5106_c7/f1p0/2281-1F	bHLH	TF
F01_cb5106_c8/f1p0/2333-0F	bHLH	TF
F01_cb5121_c1/f15p0/1009-0F	MADS-MIKC	TF
F01_cb5121_c3/f1p0/3174-2F	MADS-M-type	TF
F01_cb5121_c4/f1p0/2356-2F	MADS-MIKC	TF
F01_cb5121_c5/f1p0/1094-1F	MADS-MIKC	TF
F01_cb5173_c10/f1p0/2708-2F	FAR1	TF
F01_cb5173_c11/f1p0/2729-2F	FAR1	TF
F01_cb5173_c12/f1p0/2675-1F	FAR1	TF
F01_cb5173_c12/f1p0/2675-2F	FAR1	TF
F01_cb5173_c16/f6p0/2587-0F	FAR1	TF
F01_cb5173_c17/f8p0/2561-1F	FAR1	TF
F01_cb5173_c3/f5p0/2764-0F	FAR1	TF
F01_cb5173_c4/f2p0/2756-2F	FAR1	TF
F01_cb5173_c5/f1p0/3165-0F	FAR1	TF
F01_cb5173_c6/f1p0/3163-2F	FAR1	TF

F01_cb5173_c7/f1p0/3308-2F	FAR1	TF
F01_cb5181_c1/f2p1/2850-2F	C3H	TF
F01_cb5181_c2/f2p2/3159-0F	C3H	TF
F01_cb5181_c7/f1p0/3122-1F	C3H	TF
F01_cb5197_c4/f1p0/3085-1F	FAR1	TF
F01_cb5197_c7/f1p0/2919-1F	FAR1	TF
F01_cb5260_c2/f1p0/1431-1F	GeBP	TF
F01_cb5265_c61/f1p3/3584-1F	MYB-related	TF
F01_cb5265_c61/f1p3/3584-2F	MYB-related	TF
F01_cb5265_c62/f1p0/4128-0F	MYB	TF
F01_cb5265_c64/f1p0/3314-1F	MYB-related	TF
F01_cb5265_c66/f1p0/3373-1F	MYB-related	TF
F01_cb5265_c66/f1p0/3373-2F	MYB-related	TF
F01_cb5265_c70/f1p0/3665-2F	MYB	TF
F01_cb5265_c78/f1p0/3290-0F	MYB	TF
F01_cb5265_c79/f1p0/3747-1F	MYB	TF
F01_cb5265_c82/f1p0/3653-1F	MYB	TF
F01_cb5266_c2/f2p2/2704-2F	MYB	TF
F01_cb5266_c4/f2p3/2756-1F	MYB	TF
F01_cb5266_c5/f2p0/2731-2F	MYB-related	TF
F01_cb5290_c11/f4p1/2778-0F	GARP-ARR-B	TF
F01_cb5290_c18/f1p0/3122-1F	GARP-ARR-B	TF
F01_cb5290_c19/f1p1/4140-1F	Others	TR
F01_cb5290_c21/f1p0/2764-2F	GARP-ARR-B	TF
F01_cb5290_c22/f1p1/3256-2F	Others	TR
F01_cb5290_c32/f1p1/2945-2F	GARP-ARR-B	TF

F01_cb5290_c35/f1p1/2948-2F	GARP-ARR-B	TF
F01_cb5290_c38/f1p0/3048-2F	GARP-ARR-B	TF
F01_cb5290_c40/f1p0/2998-1F	GARP-ARR-B	TF
F01_cb5290_c41/f1p1/3038-0F	GARP-ARR-B	TF
F01_cb5290_c43/f1p0/2774-1F	GARP-ARR-B	TF
F01_cb5290_c45/f1p0/2555-2F	GARP-G2-like	TF
F01_cb5290_c46/f1p0/2814-0F	GARP-ARR-B	TF
F01_cb5290_c50/f1p0/2718-1F	GARP-G2-like	TF
F01_cb5290_c50/f1p0/2718-2F	Others	TR
F01_cb5290_c54/f1p0/3428-2F	GARP-ARR-B	TF
F01_cb5290_c64/f1p0/3128-1F	GARP-ARR-B	TF
F01_cb5290_c65/f1p1/2785-2F	GARP-ARR-B	TF
F01_cb5290_c66/f1p1/2753-0F	GARP-ARR-B	TF
F01_cb5290_c76/f8p1/2767-2F	GARP-ARR-B	TF
F01_cb5290_c79/f1p0/2804-0F	GARP-ARR-B	TF
F01_cb5306_c0/f5p0/2026-2F	AP2/ERF-AP2	TF
F01_cb5306_c10/f1p0/2569-1F	AP2/ERF-AP2	TF
F01_cb5306_c13/f1p0/2299-2F	AP2/ERF-AP2	TF
F01_cb5306_c20/f1p0/1640-2F	AP2/ERF-AP2	TF
F01_cb5306_c4/f1p0/3133-1F	AP2/ERF-ERF	TF
F01_cb5306_c5/f1p0/2072-0F	AP2/ERF-ERF	TF
F01_cb5306_c6/f1p0/2511-2F	AP2/ERF-AP2	TF
F01_cb5306_c8/f1p0/1982-1F	AP2/ERF-AP2	TF
F01_cb5307_c0/f9p0/3058-1F	TRAF	TR
F01_cb5307_c16/f1p0/3281-0F	TRAF	TR
F01_cb5307_c19/f2p1/3110-0F	TRAF	TR

F01_cb5307_c20/f1p0/3073-0F	TRAF	TR
F01_cb5307_c22/f2p0/3012-0F	TRAF	TR
F01_cb5307_c23/f1p0/2078-0F	TRAF	TR
F01_cb5307_c3/f1p0/3130-0F	TRAF	TR
F01_cb5307_c4/f1p0/3484-1F	TRAF	TR
F01_cb5307_c7/f1p0/3894-2F	TRAF	TR
F01_cb5307_c9/f1p0/2902-2F	TRAF	TR
F01_cb5322_c10/f1p0/2639-2F	SWI/SNF-BAF60b	TR
F01_cb5322_c13/f1p0/2386-2F	SWI/SNF-BAF60b	TR
F01_cb5322_c16/f9p1/2635-0F	SWI/SNF-BAF60b	TR
F01_cb5322_c3/f1p0/3125-1F	SWI/SNF-BAF60b	TR
F01_cb5322_c4/f1p1/2817-1F	SWI/SNF-BAF60b	TR
F01_cb5322_c5/f1p0/2395-2F	SWI/SNF-BAF60b	TR
F01_cb5326_c0/f5p2/2201-2F	TCP	TF
F01_cb5326_c1/f1p0/3125-1F	TCP	TF
F01_cb5326_c3/f1p2/2172-1F	TCP	TF
F01_cb5335_c1/f4p1/1501-1F	WRKY	TF
F01_cb5335_c11/f5p1/1426-0F	WRKY	TF
F01_cb5335_c5/f1p0/2305-1F	WRKY	TF
F01_cb5335_c5/f1p0/2305-2F	WRKY	TF
F01_cb5335_c6/f1p0/2177-0F	WRKY	TF
F01_cb5335_c6/f1p0/2177-1F	WRKY	TF
F01_cb5335_c7/f1p1/1615-0F	WRKY	TF
F01_cb5335_c8/f1p1/1527-2F	WRKY	TF
F01_cb5335_c9/f1p1/1411-1F	WRKY	TF
F01_cb5340_c0/f1p0/3122-1R	LUG	TR

F01_cb5340_c1/f1p0/3858-1R	LUG	TR
F01_cb5376_c3/f1p0/1886-0F	SET	TR
F01_cb5376_c5/f1p0/2140-1F	SET	TR
F01_cb5382_c1/f2p0/3096-1F	GRAS	TF
F01_cb5382_c2/f1p0/2750-1F	GRAS	TF
F01_cb5382_c3/f1p0/2584-0F	GRAS	TF
F01_cb5382_c3/f1p0/2584-2F	GRAS	TF
F01_cb5388_c0/f3p0/2888-0F	FAR1	TF
F01_cb5388_c1/f2p0/2996-1F	FAR1	TF
F01_cb5388_c3/f1p0/2658-0F	FAR1	TF
F01_cb5388_c4/f1p0/2912-0F	FAR1	TF
F01_cb5419_c1/f1p0/3099-0F	bZIP	TF
F01_cb5419_c2/f1p1/3151-0F	bZIP	TF
F01_cb5419_c5/f1p1/1827-0F	bZIP	TF
F01_cb5419_c6/f1p0/1768-2F	bZIP	TF
F01_cb5419_c8/f1p0/1620-0F	bZIP	TF
F01_cb5438_c3/f1p0/3077-2F	MYB-related	TF
F01_cb5438_c6/f1p0/2257-1F	SWI/SNF-SWI3	TR
F01_cb5438_c6/f1p0/2257-2F	MYB-related	TF
F01_cb5438_c8/f1p0/1725-0F	SWI/SNF-SWI3	TR
F01_cb5438_c8/f1p0/1725-1F	MYB-related	TF
F01_cb5440_c8/f1p1/3070-2F	C3H	TF
F01_cb5443_c0/f4p0/2482-1F	Tify	TF
F01_cb5443_c10/f2p0/2611-2F	Tify	TF
F01_cb5443_c11/f1p0/2439-2F	Tify	TF
F01_cb5443_c16/f1p0/772-1F	Tify	TF

F01_cb5443_c4/f1p0/2834-0F	Others	TR
F01_cb5443_c4/f1p0/2834-2F	Tify	TF
F01_cb5443_c9/f1p0/2456-1F	Tify	TF
F01_cb5448_c2/f1p0/3092-2F	HB-other	TF
F01_cb5448_c3/f1p0/2987-1F	HB-other	TF
F01_cb5448_c4/f1p0/2656-0F	HB-other	TF
F01_cb5448_c5/f1p0/1537-0F	HB-other	TF
F01_cb5448_c6/f1p0/1531-1F	HB-HD-ZIP	TF
F01_cb544_c11/f1p0/3876-2F	ARID	TR
F01_cb544_c12/f1p0/3783-1F	ARID	TR
F01_cb544_c15/f1p1/3794-1F	ARID	TR
F01_cb544_c17/f1p0/3934-2F	ARID	TR
F01_cb544_c22/f1p0/3760-2F	ARID	TR
F01_cb544_c23/f1p4/4136-2F	ARID	TR
F01_cb544_c26/f1p0/3748-2F	ARID	TR
F01_cb544_c6/f1p0/4746-0F	ARID	TR
F01_cb544_c7/f1p0/2959-0F	ARID	TR
F01_cb544_c8/f1p0/3991-1F	ARID	TR
F01_cb544_c9/f1p0/2694-0F	ARID	TR
F01_cb5450_c1/f1p0/2475-1F	Jumonji	TR
F01_cb5450_c2/f1p0/1563-0F	Jumonji	TR
F01_cb5533_c134/f1p0/2453-2F	SET	TR
F01_cb5572_c0/f1p0/3104-1F	B3-ARF	TF
F01_cb5572_c1/f1p0/2760-1F	B3	TF
F01_cb5572_c2/f1p0/2959-2F	B3-ARF	TF
F01_cb5572_c4/f1p0/2711-2F	B3-ARF	TF

F01_cb5609_c0/f4p0/2338-1F	BSD	TF
F01_cb5609_c2/f2p0/3059-2F	BSD	TF
F01_cb5643_c122/f3p0/2775-0F	C3H	TF
F01_cb5643_c128/f1p0/1747-0F	C3H	TF
F01_cb5643_c17/f3p1/2843-2F	C3H	TF
F01_cb5643_c25/f1p0/3322-2F	C3H	TF
F01_cb5643_c27/f1p0/2675-2F	C3H	TF
F01_cb5643_c35/f1p0/2675-1F	C3H	TF
F01_cb5643_c42/f1p0/3067-2F	C3H	TF
F01_cb5643_c44/f1p0/2460-0F	C3H	TF
F01_cb5643_c82/f1p0/2858-2F	C3H	TF
F01_cb5643_c83/f1p0/2526-0F	C3H	TF
F01_cb5643_c92/f1p0/2723-0F	C3H	TF
F01_cb5643_c99/f1p2/2746-2F	C3H	TF
F01_cb5655_c5/f1p1/3035-1F	C3H	TF
F01_cb5655_c6/f1p0/2784-1F	C3H	TF
F01_cb5659_c105/f4p0/2776-1F	bHLH	TF
F01_cb5659_c106/f1p1/2740-2F	bHLH	TF
F01_cb5659_c107/f46p4/2736-1F	bHLH	TF
F01_cb5659_c24/f3p0/3605-2F	bHLH	TF
F01_cb5659_c44/f1p3/2486-2F	bHLH	TF
F01_cb5659_c47/f1p0/2555-2F	bHLH	TF
F01_cb5659_c58/f1p0/3341-2F	bHLH	TF
F01_cb5659_c59/f1p6/2922-1F	bHLH	TF
F01_cb5659_c60/f1p1/2659-2F	bHLH	TF
F01_cb5659_c61/f1p0/2713-1F	bHLH	TF

F01_cb5659_c71/f1p0/3069-1F	bHLH	TF
F01_cb5659_c74/f1p3/2885-2F	bHLH	TF
F01_cb5661_c2/f1p0/2927-0F	GRAS	TF
F01_cb5682_c13/f10p1/1353-1F	bHLH	TF
F01_cb56_c2/f1p0/5001-2F	SNF2	TR
F01_cb56_c3/f1p0/4918-0F	SNF2	TR
F01_cb56_c3/f1p0/4918-1F	SNF2	TR
F01_cb56_c5/f1p0/4943-0F	SNF2	TR
F01_cb56_c5/f1p0/4943-1F	SNF2	TR
F01_cb5714_c1/f1p0/3032-2F	NF-YC	TF
F01_cb5714_c11/f1p0/1261-1F	NF-YC	TF
F01_cb5714_c12/f1p0/1223-0F	NF-YC	TF
F01_cb5714_c13/f1p0/1198-0F	NF-YC	TF
F01_cb5714_c15/f8p0/1309-2F	NF-YC	TF
F01_cb5714_c2/f1p0/2670-2F	NF-YC	TF
F01_cb5714_c3/f1p0/2759-0F	NF-YC	TF
F01_cb5714_c5/f1p0/3572-0F	NF-YC	TF
F01_cb5714_c6/f1p0/1426-1F	NF-YC	TF
F01_cb5714_c7/f1p0/1389-0F	NF-YC	TF
F01_cb5714_c8/f1p0/1350-2F	NF-YC	TF
F01_cb5752_c0/f1p0/3026-0F	HB-BELL	TF
F01_cb5752_c1/f1p0/3028-0F	HB-BELL	TF
F01_cb5781_c3/f1p1/3037-0F	C3H	TF
F01_cb5781_c4/f1p1/2033-1F	C3H	TF
F01_cb5781_c6/f1p0/2146-2F	C3H	TF
F01_cb5782_c1/f2p0/1642-1F	Others	TR

F01_cb5782_c11/f1p1/1705-1F	Others	TR
F01_cb5782_c13/f2p0/1497-0F	Others	TR
F01_cb5782_c14/f1p0/1477-1F	Others	TR
F01_cb5786_c0/f1p0/3012-0F	GRAS	TF
F01_cb5786_c0/f1p0/3012-1F	GRAS	TF
F01_cb5786_c1/f1p0/2920-2F	GRAS	TF
F01_cb5819_c5/f1p0/2298-0F	C3H	TF
F01_cb5836_c1/f2p1/2232-1F	GRAS	TF
F01_cb5836_c2/f2p1/2315-0F	GRAS	TF
F01_cb5836_c2/f2p1/2315-2F	GRAS	TF
F01_cb5836_c6/f1p1/2301-2F	GRAS	TF
F01_cb5836_c7/f1p0/2197-2F	GRAS	TF
F01_cb584_c11/f1p0/3581-2F	CAMTA	TF
F01_cb584_c17/f1p1/3696-2F	CAMTA	TF
F01_cb584_c18/f1p0/3716-2F	CAMTA	TF
F01_cb584_c19/f1p0/3377-0F	CAMTA	TF
F01_cb584_c20/f1p0/4710-0F	CAMTA	TF
F01_cb584_c21/f1p0/3705-0F	CAMTA	TF
F01_cb584_c29/f1p0/4045-1F	CAMTA	TF
F01_cb584_c30/f1p0/3643-0F	CAMTA	TF
F01_cb584_c31/f1p0/4041-0F	CAMTA	TF
F01_cb584_c34/f1p0/3933-0F	CAMTA	TF
F01_cb584_c36/f1p0/3790-1F	CAMTA	TF
F01_cb584_c37/f1p0/3420-1F	CAMTA	TF
F01_cb584_c8/f1p0/4733-1F	CAMTA	TF
F01_cb5861_c0/f2p0/2964-2F	FAR1	TF

F01_cb5861_c1/f1p0/2826-0F	FAR1	TF
F01_cb5890_c1/f1p0/2713-1F	MYB-related	TF
F01_cb5890_c4/f1p0/1073-2F	MYB	TF
F01_cb5891_c10/f1p0/2609-2F	FAR1	TF
F01_cb5891_c11/f1p0/2563-2F	FAR1	TF
F01_cb5891_c2/f1p0/3660-1F	FAR1	TF
F01_cb5891_c3/f1p0/3307-0F	FAR1	TF
F01_cb5891_c8/f1p0/2721-0F	FAR1	TF
F01_cb5891_c9/f1p0/2810-2F	FAR1	TF
F01_cb5896_c116/f1p1/3615-1F	CAMTA	TF
F01_cb5896_c119/f1p3/3813-0F	CAMTA	TF
F01_cb5896_c119/f1p3/3813-2F	CAMTA	TF
F01_cb5896_c123/f1p1/3585-0F	CAMTA	TF
F01_cb5896_c134/f1p0/4910-0F	PHD	TR
F01_cb5896_c135/f1p0/4855-1F	PHD	TR
F01_cb5896_c15/f2p1/3602-0F	CAMTA	TF
F01_cb5896_c157/f1p0/3375-0F	HMG	TR
F01_cb5896_c172/f9p0/3714-1F	CAMTA	TF
F01_cb5931_c10/f1p1/2616-1F	GARP-G2-like	TF
F01_cb5931_c11/f1p0/2398-2F	GARP-G2-like	TF
F01_cb5931_c12/f1p0/2152-0F	GARP-G2-like	TF
F01_cb5931_c14/f1p0/2172-0F	GARP-G2-like	TF
F01_cb5931_c17/f1p0/2381-0F	GARP-G2-like	TF
F01_cb5931_c18/f1p0/2066-0F	GARP-G2-like	TF
F01_cb5931_c19/f2p1/1950-1F	GARP-G2-like	TF
F01_cb5931_c2/f3p0/1968-2F	GARP-G2-like	TF

F01_cb5931_c3/f1p0/2877-1F	GARP-G2-like	TF
F01_cb5931_c4/f1p0/2378-1F	GARP-G2-like	TF
F01_cb5931_c5/f1p0/3756-0F	GARP-G2-like	TF
F01_cb5931_c6/f1p0/2315-0F	GARP-G2-like	TF
F01_cb5931_c8/f1p0/3091-2F	GARP-G2-like	TF
F01_cb5931_c9/f1p0/2483-2F	GARP-G2-like	TF
F01_cb5944_c13/f1p0/2333-0F	PHD	TR
F01_cb5944_c15/f1p0/2797-2F	PHD	TR
F01_cb5944_c17/f1p0/2892-2F	PHD	TR
F01_cb5944_c7/f2p0/2904-0F	PHD	TR
F01_cb5959_c1/f1p0/2966-0F	SBP	TF
F01_cb5959_c2/f1p0/2748-1F	SBP	TF
F01_cb5959_c3/f1p0/2121-2F	SBP	TF
F01_cb5959_c6/f1p0/2817-0F	SBP	TF
F01_cb5996_c0/f6p0/1399-0F	NAC	TF
F01_cb5996_c3/f2p0/2942-1F	NAC	TF
F01_cb5996_c3/f2p0/2942-2F	NAC	TF
F01_cb5996_c4/f1p0/1602-0F	NAC	TF
F01_cb603_c12/f1p0/2110-2F	B3	TF
F01_cb603_c13/f1p0/1717-1F	B3	TF
F01_cb603_c16/f3p0/2747-0F	B3-ARF	TF
F01_cb603_c3/f1p0/4729-2F	B3	TF
F01_cb603_c4/f1p0/2531-1F	B3-ARF	TF
F01_cb603_c5/f1p0/4497-2F	B3	TF
F01_cb603_c7/f1p0/3143-0F	B3-ARF	TF
F01_cb603_c8/f1p0/2924-1F	B3-ARF	TF

F01_cb6054_c15/f1p8/2510-0F	GRAS	TF
F01_cb6054_c15/f1p8/2510-2F	GRAS	TF
F01_cb6054_c19/f1p0/2621-0F	GRAS	TF
F01_cb6054_c20/f1p0/2610-1F	GRAS	TF
F01_cb6054_c23/f1p2/2316-0F	GRAS	TF
F01_cb6054_c29/f1p0/1952-1F	GRAS	TF
F01_cb6054_c29/f1p0/1952-2F	GRAS	TF
F01_cb6054_c31/f1p0/2404-0F	GRAS	TF
F01_cb6054_c33/f1p0/1690-1F	GRAS	TF
F01_cb6066_c0/f1p1/2945-1F	GNAT	TR
F01_cb6066_c2/f1p0/2806-1F	GNAT	TR
F01_cb6066_c3/f1p0/2479-2F	GNAT	TR
F01_cb6066_c4/f1p0/2044-0F	GNAT	TR
F01_cb6066_c6/f1p0/2056-2F	GNAT	TR
F01_cb6066_c7/f1p0/1915-0F	GNAT	TR
F01_cb6091_c1/f1p0/3658-0F	C3H	TF
F01_cb6091_c1/f1p0/3658-1F	C3H	TF
F01_cb6101_c1/f1p0/2933-1F	SET	TR
F01_cb6101_c2/f1p0/3506-0F	SET	TR
F01_cb6101_c3/f1p0/2953-0F	SET	TR
F01_cb6101_c4/f1p0/2753-1F	SET	TR
F01_cb6101_c5/f1p0/2887-0F	SET	TR
F01_cb611_c0/f1p0/4704-1R	LOB	TF
F01_cb611_c4/f1p0/3672-1R	LOB	TF
F01_cb611_c4/f1p0/3672-2R	LOB	TF
F01_cb611_c6/f1p0/5294-1R	LOB	TF

F01_cb611_c7/f1p0/5234-2R	LOB	TF
F01_cb611_c8/f1p0/5147-1R	LOB	TF
F01_cb6134_c12/f1p0/1381-0F	AUX/IAA	TR
F01_cb6134_c14/f1p0/1378-0F	AUX/IAA	TR
F01_cb6134_c15/f1p0/1342-1F	AUX/IAA	TR
F01_cb6134_c18/f9p0/1403-0F	AUX/IAA	TR
F01_cb6134_c18/f9p0/1403-2F	AUX/IAA	TR
F01_cb6134_c5/f2p0/1354-2F	AUX/IAA	TR
F01_cb6134_c6/f1p0/2931-1F	AUX/IAA	TR
F01_cb6134_c6/f1p0/2931-2F	AUX/IAA	TR
F01_cb6134_c7/f1p0/3186-0F	AUX/IAA	TR
F01_cb6134_c7/f1p0/3186-2F	AUX/IAA	TR
F01_cb6134_c8/f1p1/1608-0F	AUX/IAA	TR
F01_cb6134_c9/f1p1/1566-1F	AUX/IAA	TR
F01_cb6147_c0/f6p0/2154-0F	HB-other	TF
F01_cb6147_c26/f1p0/2002-0F	HB-other	TF
F01_cb6147_c29/f1p0/2193-0F	HB-other	TF
F01_cb6147_c38/f1p0/2414-1F	HB-other	TF
F01_cb6176_c0/f2p0/2824-2F	SNF2	TR
F01_cb6176_c1/f1p0/2916-2F	SNF2	TR
F01_cb6176_c2/f1p1/3003-0F	SNF2	TR
F01_cb6185_c11/f1p0/2334-2F	GARP-ARR-B	TF
F01_cb6185_c13/f1p0/2793-0F	Others	TR
F01_cb6185_c13/f1p0/2793-2F	GARP-G2-like	TF
F01_cb6185_c14/f1p0/2545-1F	GARP-G2-like	TF
F01_cb6185_c18/f1p0/2593-2F	GARP-ARR-B	TF

F01_cb6185_c5/f1p0/2923-2F	GARP-ARR-B	TF
F01_cb6185_c6/f1p0/2824-0F	GARP-ARR-B	TF
F01_cb6185_c8/f1p0/2436-1F	GARP-ARR-B	TF
F01_cb61_c1/f2p1/4638-2F	Jumonji	TR
F01_cb61_c2/f1p0/4986-2F	Jumonji	TR
F01_cb61_c3/f1p0/4610-0F	Jumonji	TR
F01_cb61_c3/f1p0/4610-2F	Jumonji	TR
F01_cb6203_c3/f1p0/2225-0F	SET	TR
F01_cb6239_c10/f1p0/2664-0F	SNF2	TR
F01_cb6239_c11/f1p0/2522-0F	SNF2	TR
F01_cb6239_c12/f1p0/2697-1F	SNF2	TR
F01_cb6239_c12/f1p0/2697-2F	SNF2	TR
F01_cb6239_c2/f1p0/3034-2F	SNF2	TR
F01_cb6239_c3/f1p0/2984-0F	SNF2	TR
F01_cb6239_c3/f1p0/2984-1F	SNF2	TR
F01_cb6239_c4/f1p0/2736-0F	SNF2	TR
F01_cb6239_c6/f1p1/2671-1F	SNF2	TR
F01_cb6239_c7/f1p0/2012-2F	SNF2	TR
F01_cb6239_c8/f1p0/2559-1F	SNF2	TR
F01_cb6239_c9/f1p0/2413-2F	SNF2	TR
F01_cb6259_c14/f1p0/2936-1F	HB-BELL	TF
F01_cb6259_c16/f1p0/2866-0F	HB-BELL	TF
F01_cb6259_c17/f1p0/2839-2F	HB-other	TF
F01_cb6259_c20/f3p0/2786-1F	HB-BELL	TF
F01_cb6259_c21/f1p0/1419-2F	HB-other	TF
F01_cb6259_c3/f1p1/2898-1F	HB-other	TF

F01_cb6259_c4/f1p0/2869-2F	HB-other	TF
F01_cb6259_c6/f1p0/3911-1F	HB-BELL	TF
F01_cb6259_c7/f1p0/2932-2F	HB-other	TF
F01_cb6259_c8/f1p0/2869-1F	HB-BELL	TF
F01_cb626_c14/f1p0/2874-1F	Others	TR
F01_cb626_c15/f1p0/3353-2F	Others	TR
F01_cb626_c16/f1p0/3290-2F	Others	TR
F01_cb626_c17/f1p0/4935-0F	B3-ARF	TF
F01_cb626_c39/f1p0/2478-1F	AUX/IAA	TR
F01_cb626_c40/f1p3/4315-2F	B3-ARF	TF
F01_cb626_c41/f1p1/4495-1F	B3-ARF	TF
F01_cb626_c52/f1p0/4764-2F	B3-ARF	TF
F01_cb626_c57/f1p0/4032-0F	B3-ARF	TF
F01_cb626_c58/f1p1/3731-1F	B3	TF
F01_cb626_c59/f1p0/4159-0F	AUX/IAA	TR
F01_cb626_c60/f1p1/4254-0F	B3-ARF	TF
F01_cb626_c61/f1p0/3800-0F	B3-ARF	TF
F01_cb626_c62/f1p1/4231-1F	AUX/IAA	TR
F01_cb626_c62/f1p1/4231-2F	B3-ARF	TF
F01_cb626_c66/f1p0/2695-2F	AUX/IAA	TR
F01_cb626_c67/f1p1/4388-2F	B3-ARF	TF
F01_cb626_c68/f1p1/4195-0F	B3-ARF	TF
F01_cb626_c68/f1p1/4195-2F	AUX/IAA	TR
F01_cb626_c77/f1p0/3018-1F	Others	TR
F01_cb626_c78/f1p0/5392-0F	B3-ARF	TF
F01_cb626_c79/f1p0/5202-0F	AUX/IAA	TR

F01_cb626_c79/f1p0/5202-1F	B3-ARF	TF
F01_cb626_c8/f4p1/4379-2F	B3-ARF	TF
F01_cb626_c89/f1p0/4168-1F	B3	TF
F01_cb626_c91/f1p0/2043-2F	AUX/IAA	TR
F01_cb6313_c0/f2p0/1113-1F	C2C2-YABBY	TF
F01_cb6313_c2/f1p0/2578-1F	C2C2-YABBY	TF
F01_cb6313_c4/f1p0/773-1F	C2C2-YABBY	TF
F01_cb6320_c0/f6p0/1902-0F	WRKY	TF
F01_cb6320_c1/f2p0/1818-0F	WRKY	TF
F01_cb6320_c2/f1p0/2874-0F	WRKY	TF
F01_cb6320_c2/f1p0/2874-1F	WRKY	TF
F01_cb6354_c0/f1p0/2876-1F	MYB-related	TF
F01_cb6354_c1/f1p0/2101-0F	MYB-related	TF
F01_cb6362_c0/f4p0/1243-0F	Rcd1-like	TR
F01_cb6362_c1/f2p1/1377-0F	Rcd1-like	TR
F01_cb6362_c1/f2p1/1377-2F	Rcd1-like	TR
F01_cb6362_c2/f2p1/1459-1F	Rcd1-like	TR
F01_cb6362_c3/f1p1/2875-0F	Rcd1-like	TR
F01_cb6362_c3/f1p1/2875-1F	Rcd1-like	TR
F01_cb6362_c3/f1p1/2875-2F	Rcd1-like	TR
F01_cb6362_c4/f1p1/2389-2F	Rcd1-like	TR
F01_cb6362_c6/f1p0/1222-0F	Rcd1-like	TR
F01_cb6364_c1/f1p0/3064-1F	SET	TR
F01_cb6364_c2/f1p0/1346-1F	SET	TR
F01_cb6381_c0/f5p1/2779-2F	MYB	TF
F01_cb6381_c1/f1p0/2872-2F	MYB	TF

F01_cb6381_c3/f1p0/2832-2F	MYB	TF
F01_cb6381_c4/f1p0/2208-2F	MYB	TF
F01_cb6390_c7/f1p0/2169-0F	TRAF	TR
F01_cb6391_c11/f1p0/2658-1F	WRKY	TF
F01_cb6391_c12/f1p0/2288-1F	WRKY	TF
F01_cb6391_c13/f1p0/2480-0F	WRKY	TF
F01_cb6391_c14/f1p1/2258-0F	WRKY	TF
F01_cb6391_c15/f1p0/2088-2F	WRKY	TF
F01_cb6391_c2/f2p0/2236-1F	WRKY	TF
F01_cb6391_c3/f2p0/2237-2F	WRKY	TF
F01_cb6391_c4/f1p0/2869-0F	WRKY	TF
F01_cb6391_c4/f1p0/2869-1F	WRKY	TF
F01_cb6391_c5/f1p0/2187-0F	WRKY	TF
F01_cb6391_c5/f1p0/2187-1F	WRKY	TF
F01_cb6391_c5/f1p0/2187-2F	WRKY	TF
F01_cb6391_c7/f1p0/2310-2F	WRKY	TF
F01_cb6391_c9/f1p0/2400-1F	WRKY	TF
F01_cb6392_c2/f1p1/2869-0F	HB-HD-ZIP	TF
F01_cb6419_c10/f1p4/1748-1F	C2C2-CO-like	TF
F01_cb6419_c17/f1p2/1539-2F	Others	TR
F01_cb6419_c21/f1p0/871-1F	Others	TR
F01_cb6419_c22/f1p0/859-1F	Others	TR
F01_cb6419_c25/f35p3/1510-0F	C2C2-CO-like	TF
F01_cb6419_c3/f2p0/2540-1F	Others	TR
F01_cb6419_c3/f2p0/2540-2F	DBB	TF
F01_cb6461_c0/f1p0/2849-0F	HSF	TF

F01_cb6463_c10/f1p0/1943-1F	C2H2	TF
F01_cb6463_c17/f1p0/1732-0F	C2H2	TF
F01_cb6463_c2/f3p0/2023-0F	C2H2	TF
F01_cb6463_c5/f1p0/2822-1F	C2H2	TF
F01_cb6463_c6/f1p0/2024-2F	C2H2	TF
F01_cb6463_c7/f1p0/2061-2F	C2H2	TF
F01_cb6473_c2/f1p1/2843-0R	C3H	TF
F01_cb6482_c0/f1p0/2844-1F	EIL	TF
F01_cb6482_c4/f1p0/2701-1F	EIL	TF
F01_cb6482_c5/f1p0/2610-1F	EIL	TF
F01_cb6482_c5/f1p0/2610-2F	EIL	TF
F01_cb6504_c0/f1p0/2844-0F	C2C2-YABBY	TF
F01_cb6504_c0/f1p0/2844-1F	C2C2-YABBY	TF
F01_cb6504_c1/f1p0/2635-2F	C2C2-YABBY	TF
F01_cb6504_c3/f1p0/1353-1F	C2C2-YABBY	TF
F01_cb6504_c4/f1p0/1247-0F	C2C2-YABBY	TF
F01_cb6530_c13/f5p0/2532-0F	Pseudo ARR-B	TR
F01_cb6530_c18/f1p1/2394-0F	Pseudo ARR-B	TR
F01_cb6530_c20/f1p0/2957-1F	Pseudo ARR-B	TR
F01_cb6530_c21/f1p0/2410-1F	Pseudo ARR-B	TR
F01_cb6530_c23/f1p0/2764-0F	Others	TR
F01_cb6530_c23/f1p0/2764-2F	Others	TR
F01_cb6530_c24/f1p0/2522-0F	Others	TR
F01_cb6530_c24/f1p0/2522-2F	Others	TR
F01_cb6530_c25/f1p0/2502-1F	Others	TR
F01_cb6530_c25/f1p0/2502-2F	Others	TR

F01_cb6530_c26/f1p0/2387-1F	Pseudo ARR-B	TR
F01_cb6530_c27/f1p0/2468-1F	Others	TR
F01_cb6530_c35/f1p0/2668-2F	Pseudo ARR-B	TR
F01_cb6530_c36/f1p0/2516-2F	Pseudo ARR-B	TR
F01_cb6530_c37/f1p1/2528-1F	Others	TR
F01_cb6530_c37/f1p1/2528-2F	Others	TR
F01_cb6530_c44/f1p0/2497-2F	Pseudo ARR-B	TR
F01_cb6530_c47/f1p0/2752-1F	Pseudo ARR-B	TR
F01_cb6530_c5/f10p0/2318-1F	Pseudo ARR-B	TR
F01_cb6530_c52/f1p0/2215-0F	Pseudo ARR-B	TR
F01_cb6530_c57/f1p1/2343-0F	Others	TR
F01_cb6530_c57/f1p1/2343-2F	Others	TR
F01_cb6530_c59/f1p0/2656-1F	Others	TR
F01_cb6530_c59/f1p0/2656-2F	Others	TR
F01_cb6530_c6/f10p2/2387-1F	Pseudo ARR-B	TR
F01_cb6530_c60/f1p1/2213-0F	Pseudo ARR-B	TR
F01_cb6530_c62/f1p0/2626-0F	Others	TR
F01_cb6530_c62/f1p0/2626-1F	Others	TR
F01_cb6530_c63/f1p1/2489-2F	Pseudo ARR-B	TR
F01_cb6530_c65/f1p0/1913-0F	Others	TR
F01_cb6530_c65/f1p0/1913-1F	Others	TR
F01_cb6530_c75/f4p0/2693-2F	Pseudo ARR-B	TR
F01_cb6530_c80/f1p0/2511-0F	Others	TR
F01_cb6530_c80/f1p0/2511-1F	Others	TR
F01_cb6533_c0/f5p1/2832-2F	FAR1	TF
F01_cb6548_c10/f1p1/2515-2F	bZIP	TF

F01_cb6548_c12/f1p1/2464-1F	bZIP	TF
F01_cb6548_c13/f1p1/2372-1F	bZIP	TF
F01_cb6548_c16/f1p0/2550-1F	bZIP	TF
F01_cb6548_c17/f1p1/2526-2F	bZIP	TF
F01_cb6548_c2/f6p1/2451-1F	bZIP	TF
F01_cb6548_c7/f1p3/4317-2F	bZIP	TF
F01_cb6548_c8/f1p0/2532-0F	bZIP	TF
F01_cb6580_c2/f1p0/1593-2F	SET	TR
F01_cb6588_c0/f3p0/1488-0F	C2H2	TF
F01_cb6588_c3/f1p0/2893-0F	C2H2	TF
F01_cb6588_c4/f1p0/2564-2F	C2H2	TF
F01_cb6588_c6/f1p0/1563-2F	C2H2	TF
F01_cb6588_c7/f1p0/1500-2F	C2H2	TF
F01_cb6596_c0/f2p0/2822-2F	BBR-BPC	TF
F01_cb6596_c1/f1p0/2804-0F	BBR-BPC	TF
F01_cb6596_c2/f1p0/1246-2F	BBR-BPC	TF
F01_cb6596_c3/f1p0/1178-0F	BBR-BPC	TF
F01_cb6596_c4/f1p0/1084-1F	BBR-BPC	TF
F01_cb6605_c11/f1p1/2320-2F	TUB	TF
F01_cb6605_c12/f1p0/1976-0F	TUB	TF
F01_cb6605_c15/f1p0/2422-0F	TUB	TF
F01_cb6605_c16/f1p0/2227-1F	TUB	TF
F01_cb6605_c3/f2p2/2277-0F	TUB	TF
F01_cb6605_c3/f2p2/2277-1F	TUB	TF
F01_cb6605_c3/f2p2/2277-2F	TUB	TF
F01_cb6605_c6/f1p0/2783-1F	TUB	TF

F01_cb6605_c7/f1p0/3797-1F	TUB	TF
F01_cb6626_c4/f1p0/3001-2F	C3H	TF
F01_cb6626_c5/f1p0/2902-2F	C3H	TF
F01_cb6649_c1/f4p0/1675-1F	GARP-G2-like	TF
F01_cb6649_c5/f1p0/2845-0F	GARP-G2-like	TF
F01_cb6649_c6/f1p0/2300-0F	GARP-G2-like	TF
F01_cb6649_c7/f1p0/1804-2F	GARP-G2-like	TF
F01_cb6649_c8/f1p0/1753-1F	GARP-G2-like	TF
F01_cb666_c0/f1p0/4630-1F	HSF	TF
F01_cb666_c1/f1p0/4314-2F	HSF	TF
F01_cb666_c10/f1p0/2176-0F	HSF	TF
F01_cb666_c11/f1p0/2689-0F	HSF	TF
F01_cb666_c12/f1p0/3434-0F	HSF	TF
F01_cb666_c14/f1p0/2626-0F	HSF	TF
F01_cb666_c3/f1p0/2372-0F	HSF	TF
F01_cb666_c4/f1p0/2217-1F	HSF	TF
F01_cb666_c7/f1p0/2038-0F	HSF	TF
F01_cb666_c9/f1p0/2356-2F	HSF	TF
F01_cb6713_c0/f3p0/2459-2F	TCP	TF
F01_cb6713_c12/f1p0/2639-1F	TCP	TF
F01_cb6713_c14/f1p0/2405-1F	TCP	TF
F01_cb6713_c2/f2p0/2403-0F	TCP	TF
F01_cb6713_c3/f1p0/2796-2F	TCP	TF
F01_cb6713_c5/f1p0/2620-0F	TCP	TF
F01_cb6713_c8/f1p0/2659-2F	TCP	TF
F01_cb6715_c1/f1p0/4218-0F	C3H	TF

F01_cb6715_c2/f1p0/6268-0F	C3H	TF
F01_cb6716_c0/f2p2/2584-1F	C2H2	TF
F01_cb6716_c1/f1p1/2898-0F	C2H2	TF
F01_cb6716_c2/f1p1/2319-0F	C2H2	TF
F01_cb6716_c2/f1p1/2319-2F	C2H2	TF
F01_cb6716_c3/f1p0/2637-1F	C2H2	TF
F01_cb6716_c4/f1p0/2291-2F	C2H2	TF
F01_cb6716_c6/f1p0/2262-2F	C2H2	TF
F01_cb6737_c10/f1p0/4156-0F	MYB	TF
F01_cb6758_c1/f3p0/1900-1F	C3H	TF
F01_cb6758_c2/f1p0/2762-2F	C3H	TF
F01_cb6758_c3/f1p0/2268-2F	C3H	TF
F01_cb6758_c7/f1p0/1936-1F	C3H	TF
F01_cb6767_c31/f13p0/2175-2F	TRAF	TR
F01_cb6767_c7/f1p0/2776-0F	TRAF	TR
F01_cb6770_c4/f1p1/2671-1F	FAR1	TF
F01_cb6783_c0/f2p0/2589-2F	AP2/ERF-ERF	TF
F01_cb6789_c5/f1p0/2546-0F	C3H	TF
F01_cb6789_c7/f1p0/2433-2F	C3H	TF
F01_cb6789_c9/f1p0/2524-1F	C3H	TF
F01_cb6794_c0/f9p3/2687-1F	TRAF	TR
F01_cb6794_c12/f1p1/2526-1F	TRAF	TR
F01_cb6794_c13/f1p0/3113-2F	TRAF	TR
F01_cb6794_c14/f1p3/2709-1F	TRAF	TR
F01_cb6794_c16/f1p0/2530-1F	TRAF	TR
F01_cb6794_c16/f1p0/2530-2F	TRAF	TR

F01_cb6794_c18/f1p1/2705-2F	TRAF	TR
F01_cb6794_c19/f1p1/2702-2F	TRAF	TR
F01_cb6794_c23/f1p1/2708-0F	TRAF	TR
F01_cb6794_c24/f1p0/2733-2F	TRAF	TR
F01_cb6794_c42/f1p0/2644-0F	TRAF	TR
F01_cb6794_c42/f1p0/2644-1F	TRAF	TR
F01_cb6794_c49/f6p1/2503-1F	TRAF	TR
F01_cb6794_c53/f7p2/2471-0F	TRAF	TR
F01_cb6794_c9/f2p0/2356-2F	TRAF	TR
F01_cb6802_c23/f1p0/4851-1F	NF-YA	TF
F01_cb6802_c23/f1p0/4851-2F	NF-YA	TF
F01_cb6802_c24/f1p0/4072-0F	NF-YA	TF
F01_cb6802_c24/f1p0/4072-2F	NF-YA	TF
F01_cb6802_c77/f1p0/2420-2F	NF-YA	TF
F01_cb6808_c10/f1p1/2304-0F	HB-BELL	TF
F01_cb6808_c2/f1p1/2723-1F	HB-BELL	TF
F01_cb6808_c4/f1p0/3737-1F	HB-other	TF
F01_cb6808_c9/f1p1/2175-1F	HB-BELL	TF
F01_cb6842_c0/f3p0/2759-2F	SWI/SNF-SWI3	TR
F01_cb6842_c1/f1p0/2588-1F	SWI/SNF-SWI3	TR
F01_cb6844_c1/f4p0/2603-2F	CPP	TF
F01_cb6844_c13/f9p0/2702-2F	CPP	TF
F01_cb6844_c4/f2p0/2631-1F	CPP	TF
F01_cb6844_c4/f2p0/2631-2F	CPP	TF
F01_cb6844_c7/f1p0/2755-1F	CPP	TF
F01_cb6844_c9/f1p0/2576-1F	CPP	TF

F01_cb6847_c0/f2p0/1252-0F	BBR-BPC	TF
F01_cb6847_c1/f2p0/2774-1F	BBR-BPC	TF
F01_cb6851_c0/f1p0/2755-2F	Alfin-like	TF
F01_cb6851_c1/f1p0/4156-2F	Alfin-like	TF
F01_cb6851_c2/f1p0/2590-0F	Alfin-like	TF
F01_cb6851_c3/f1p0/1276-1F	Alfin-like	TF
F01_cb6851_c4/f1p0/1250-2F	Alfin-like	TF
F01_cb6860_c0/f1p1/2753-1F	Others	TR
F01_cb6860_c1/f1p0/2277-0F	Others	TR
F01_cb6860_c2/f1p0/2407-0F	Others	TR
F01_cb6860_c3/f1p0/1781-0F	Others	TR
F01_cb6860_c4/f1p0/1717-2F	Others	TR
F01_cb6867_c2/f1p0/2663-2F	HB-PHD	TF
F01_cb6867_c3/f1p0/2798-0F	HB-PHD	TF
F01_cb6870_c1/f1p0/2924-0F	FAR1	TF
F01_cb6870_c2/f1p0/1399-1F	FAR1	TF
F01_cb6914_c10/f1p0/1914-1F	C2H2	TF
F01_cb6914_c11/f1p1/2301-0F	C2H2	TF
F01_cb6914_c12/f1p0/1983-2F	C2H2	TF
F01_cb6914_c13/f1p1/2216-0F	C2H2	TF
F01_cb6914_c14/f1p0/1958-0F	C2H2	TF
F01_cb6914_c15/f1p0/2278-2F	C2H2	TF
F01_cb6914_c16/f1p0/2349-2F	C2H2	TF
F01_cb6914_c19/f1p0/2036-0F	C2H2	TF
F01_cb6914_c19/f1p0/2036-2F	C2H2	TF
F01_cb6914_c20/f1p0/1923-1F	C2H2	TF

F01_cb6914_c21/f1p1/2085-1F	C2H2	TF
F01_cb6914_c23/f1p0/2011-0F	C2H2	TF
F01_cb6914_c4/f1p0/2740-2F	C2H2	TF
F01_cb6914_c8/f1p0/2191-0F	C2H2	TF
F01_cb6914_c9/f1p0/2255-0F	C2H2	TF
F01_cb6924_c12/f1p0/2411-2F	GRAS	TF
F01_cb6924_c14/f1p1/2407-0F	GRAS	TF
F01_cb6924_c23/f1p1/2456-0F	GRAS	TF
F01_cb6924_c23/f1p1/2456-1F	GRAS	TF
F01_cb6924_c26/f1p1/2295-2F	GRAS	TF
F01_cb6924_c3/f2p1/2334-0F	GRAS	TF
F01_cb6924_c3/f2p1/2334-1F	GRAS	TF
F01_cb6924_c3/f2p1/2334-2F	GRAS	TF
F01_cb6924_c34/f25p2/2370-0F	GRAS	TF
F01_cb6924_c6/f1p2/2739-0F	GRAS	TF
F01_cb6957_c10/f1p0/1611-1F	GeBP	TF
F01_cb6957_c13/f6p0/2480-2F	GeBP	TF
F01_cb6957_c4/f2p0/1635-2F	GeBP	TF
F01_cb6957_c5/f1p0/2730-2F	GeBP	TF
F01_cb6957_c7/f1p0/2483-2F	GeBP	TF
F01_cb6957_c8/f3p0/2496-0F	GeBP	TF
F01_cb6957_c9/f1p0/1685-0F	GeBP	TF
F01_cb6961_c13/f13p1/2660-1F	C3H	TF
F01_cb6961_c15/f3p2/2652-1F	C3H	TF
F01_cb6961_c7/f1p0/2784-0F	C3H	TF
F01_cb6961_c8/f1p0/2906-2F	C3H	TF

F01_cb6968_c18/f2p0/2681-0F	GRAS	TF
F01_cb6968_c19/f6p0/2660-1F	GRAS	TF
F01_cb6968_c3/f2p0/2613-0F	GRAS	TF
F01_cb6968_c3/f2p0/2613-1F	GRAS	TF
F01_cb6968_c3/f2p0/2613-2F	GRAS	TF
F01_cb6968_c6/f1p0/2273-0F	GRAS	TF
F01_cb6968_c6/f1p0/2273-2F	GRAS	TF
F01_cb6968_c9/f1p0/2204-0F	GRAS	TF
F01_cb6968_c9/f1p0/2204-2F	GRAS	TF
F01_cb7008_c12/f1p0/3438-1R	GRAS	TF
F01_cb7012_c0/f5p0/2486-0F	ARID	TR
F01_cb7012_c1/f1p0/2706-1F	ARID	TR
F01_cb7012_c2/f1p0/2476-0F	ARID	TR
F01_cb7012_c4/f1p0/2516-1F	ARID	TR
F01_cb7015_c1/f2p1/1388-2F	Trihelix	TF
F01_cb7015_c11/f5p0/2542-1F	Trihelix	TF
F01_cb7015_c3/f1p0/2659-0F	Trihelix	TF
F01_cb7015_c4/f1p0/4378-2F	Trihelix	TF
F01_cb7015_c7/f1p0/1458-1F	Trihelix	TF
F01_cb7015_c8/f1p0/1346-2F	Trihelix	TF
F01_cb7024_c0/f2p0/2727-0F	C2C2-Dof	TF
F01_cb7024_c1/f1p0/1496-1F	C2C2-Dof	TF
F01_cb7027_c6/f1p0/5802-2F	GRAS	TF
F01_cb7034_c0/f3p1/2572-0F	B3-ARF	TF
F01_cb7035_c0/f2p0/2711-0R	WRKY	TF
F01_cb7035_c1/f1p0/4129-0R	WRKY	TF

F01_cb7036_c10/f1p0/3316-0F	Others	TR
F01_cb7036_c10/f1p0/3316-1F	GARP-G2-like	TF
F01_cb7036_c11/f1p0/3157-2F	GARP-ARR-B	TF
F01_cb7036_c12/f1p0/2716-0F	GARP-ARR-B	TF
F01_cb7036_c15/f1p0/1899-0F	GARP-ARR-B	TF
F01_cb7036_c19/f1p1/2831-0F	GARP-ARR-B	TF
F01_cb7036_c3/f2p1/2778-0F	Others	TR
F01_cb7036_c3/f2p1/2778-2F	GARP-G2-like	TF
F01_cb7036_c6/f1p0/3409-0F	GARP-ARR-B	TF
F01_cb7036_c8/f1p0/2651-1F	GARP-ARR-B	TF
F01_cb7036_c9/f1p0/2936-2F	Others	TR
F01_cb705_c1/f1p0/4658-1F	C2H2	TF
F01_cb705_c3/f1p0/4558-0F	Jumonji	TR
F01_cb705_c3/f1p0/4558-1F	C2H2	TF
F01_cb7101_c2/f1p0/5603-2F	C3H	TF
F01_cb711_c0/f1p0/4668-0F	B3	TF
F01_cb711_c0/f1p0/4668-1F	B3	TF
F01_cb711_c0/f1p0/4668-2F	B3	TF
F01_cb711_c1/f1p0/2532-2F	B3	TF
F01_cb711_c2/f1p1/4372-0F	B3	TF
F01_cb711_c2/f1p1/4372-1F	B3	TF
F01_cb711_c3/f1p0/4600-0F	B3	TF
F01_cb711_c3/f1p0/4600-1F	B3	TF
F01_cb711_c4/f1p0/2827-0F	B3	TF
F01_cb711_c4/f1p0/2827-1F	B3	TF
F01_cb711_c4/f1p0/2827-2F	B3	TF

F01_cb711_c6/f1p0/1837-0F	B3	TF
F01_cb711_c6/f1p0/1837-1F	B3	TF
F01_cb712_c7/f1p0/2729-0F	PHD	TR
F01_cb7146_c0/f2p0/2641-2F	Tify	TF
F01_cb7146_c1/f1p0/2423-0F	Tify	TF
F01_cb7146_c2/f1p0/2035-1F	Tify	TF
F01_cb7221_c13/f1p1/2577-1F	C3H	TF
F01_cb7221_c15/f2p1/2622-1F	C3H	TF
F01_cb7221_c2/f3p2/2669-1F	C3H	TF
F01_cb7221_c3/f3p3/2658-2F	C3H	TF
F01_cb7221_c4/f1p1/2600-0F	C3H	TF
F01_cb7221_c6/f1p0/2447-0F	C3H	TF
F01_cb7227_c15/f1p0/2366-1F	bHLH	TF
F01_cb7227_c21/f2p1/2381-1F	bHLH	TF
F01_cb7227_c4/f2p1/2393-2F	bHLH	TF
F01_cb7227_c6/f1p1/2555-1F	bHLH	TF
F01_cb7227_c8/f1p0/2289-0F	bHLH	TF
F01_cb7227_c9/f1p1/2479-0F	bHLH	TF
F01_cb7250_c10/f1p0/2442-1F	TUB	TF
F01_cb7250_c10/f1p0/2442-2F	TUB	TF
F01_cb7250_c11/f1p0/2344-1F	TUB	TF
F01_cb7250_c14/f1p0/1978-0F	TUB	TF
F01_cb7250_c14/f1p0/1978-2F	TUB	TF
F01_cb7250_c15/f1p0/1986-1F	TUB	TF
F01_cb7250_c17/f1p0/2045-1F	TUB	TF
F01_cb7250_c5/f1p0/2662-1F	TUB	TF

F01_cb7250_c7/f1p0/2322-2F	TUB	TF
F01_cb7250_c8/f1p0/2123-2F	TUB	TF
F01_cb7268_c1/f1p0/2659-1R	bHLH	TF
F01_cb7268_c2/f1p0/1955-2R	bHLH	TF
F01_cb7268_c3/f1p3/2423-2R	bHLH	TF
F01_cb7268_c4/f1p2/2443-2R	bHLH	TF
F01_cb7277_c0/f1p0/2657-2F	GRAS	TF
F01_cb7277_c1/f1p0/2066-0F	GRAS	TF
F01_cb7277_c1/f1p0/2066-2F	GRAS	TF
F01_cb7326_c0/f3p0/2586-1F	Others	TR
F01_cb7326_c2/f1p0/2642-0F	GARP-G2-like	TF
F01_cb7326_c3/f1p0/2521-0F	Others	TR
F01_cb7326_c5/f1p0/2716-1F	GARP-ARR-B	TF
F01_cb7326_c6/f1p0/2307-1F	Others	TR
F01_cb7329_c10/f2p0/821-0F	C2C2-LSD	TF
F01_cb7329_c11/f15p0/837-1F	C2C2-LSD	TF
F01_cb7329_c2/f1p0/2646-1F	C2C2-LSD	TF
F01_cb7329_c2/f1p0/2646-2F	C2C2-LSD	TF
F01_cb7329_c3/f1p0/2137-0F	C2C2-LSD	TF
F01_cb7329_c3/f1p0/2137-2F	C2C2-LSD	TF
F01_cb7329_c4/f1p0/1247-0F	C2C2-LSD	TF
F01_cb7329_c5/f1p0/1191-1F	C2C2-LSD	TF
F01_cb7329_c6/f1p0/950-0F	C2C2-LSD	TF
F01_cb7329_c7/f1p0/950-2F	C2C2-LSD	TF
F01_cb7329_c8/f2p0/896-0F	C2C2-LSD	TF
F01_cb7337_c1/f1p0/2641-0F	AP2/ERF-ERF	TF

F01_cb7337_c3/f1p0/2569-0F	AP2/ERF-ERF	TF
F01_cb7337_c4/f1p0/2070-0F	AP2/ERF-ERF	TF
F01_cb7337_c6/f1p0/1841-1F	AP2/ERF-ERF	TF
F01_cb7351_c0/f3p1/2134-0F	bHLH	TF
F01_cb7351_c1/f1p0/2638-1F	bHLH	TF
F01_cb7377_c0/f1p0/2631-0F	GRAS	TF
F01_cb7377_c10/f1p0/2010-1F	GRAS	TF
F01_cb7377_c12/f1p0/2428-0F	GRAS	TF
F01_cb7377_c2/f1p0/2583-0F	GRAS	TF
F01_cb7377_c9/f1p0/2341-2F	GRAS	TF
F01_cb7394_c0/f3p0/2628-2R	MYB-related	TF
F01_cb7401_c0/f1p0/2470-1F	bZIP	TF
F01_cb7401_c2/f1p0/2349-1F	bZIP	TF
F01_cb7401_c5/f1p0/2406-2F	bZIP	TF
F01_cb7401_c6/f1p0/2499-1F	bZIP	TF
F01_cb7416_c10/f1p0/1320-2F	C3H	TF
F01_cb7416_c12/f1p0/1304-1F	C3H	TF
F01_cb7416_c3/f1p0/2846-0F	C3H	TF
F01_cb7416_c4/f1p1/3436-2F	C3H	TF
F01_cb7416_c8/f1p0/2805-0F	C3H	TF
F01_cb7420_c0/f7p1/2477-1F	bHLH	TF
F01_cb7420_c1/f2p0/2574-0F	bHLH	TF
F01_cb7420_c13/f1p0/2504-2F	bHLH	TF
F01_cb7420_c3/f1p0/2583-1F	bHLH	TF
F01_cb7420_c5/f1p0/4429-0F	bHLH	TF
F01_cb7420_c9/f1p0/3428-1F	bHLH	TF

F01_cb7424_c0/f2p0/5795-1F	SET	TR
F01_cb7424_c4/f1p0/5945-1F	SET	TR
F01_cb7424_c5/f1p0/5425-0F	SET	TR
F01_cb7424_c6/f1p0/5597-0F	SET	TR
F01_cb7438_c0/f3p0/2229-1F	C3H	TF
F01_cb7438_c1/f1p0/2616-0F	C3H	TF
F01_cb7438_c3/f1p0/2563-0F	C3H	TF
F01_cb7438_c4/f1p0/2337-0F	C3H	TF
F01_cb7438_c6/f1p0/2534-0F	C3H	TF
F01_cb7464_c1/f2p0/2286-0F	ARID	TR
F01_cb7464_c2/f1p0/2609-1F	ARID	TR
F01_cb7464_c3/f1p0/2370-1F	ARID	TR
F01_cb7464_c5/f1p0/2090-1F	ARID	TR
F01_cb7464_c7/f7p0/1963-0F	ARID	TR
F01_cb7473_c15/f3p0/2177-2F	GNAT	TR
F01_cb7473_c22/f1p1/2690-2F	GNAT	TR
F01_cb7473_c24/f1p0/2303-0F	GNAT	TR
F01_cb7473_c26/f1p1/2292-2F	GNAT	TR
F01_cb7473_c27/f1p0/2196-0F	GNAT	TR
F01_cb7477_c3/f1p0/2604-2F	Trihelix	TF
F01_cb7477_c6/f5p0/2322-0F	Trihelix	TF
F01_cb7477_c7/f6p0/2229-1F	Trihelix	TF
F01_cb7491_c2/f1p0/1509-1F	HB-other	TF
F01_cb7505_c0/f6p0/2552-0F	bHLH	TF
F01_cb7505_c1/f1p0/2589-2F	bHLH	TF
F01_cb7505_c2/f1p0/2591-1F	bHLH	TF

F01_cb7505_c3/f1p0/3471-0F	bHLH	TF
F01_cb7505_c5/f1p0/2003-2F	bHLH	TF
F01_cb7515_c12/f1p0/2851-1F	bHLH	TF
F01_cb7515_c14/f3p1/2599-2F	bHLH	TF
F01_cb7515_c2/f1p0/2717-2F	bHLH	TF
F01_cb7515_c3/f1p0/3235-0F	bHLH	TF
F01_cb7515_c8/f1p0/3092-2F	bHLH	TF
F01_cb7515_c9/f1p1/2473-1F	bHLH	TF
F01_cb7536_c0/f4p0/2573-0F	SWI/SNF-BAF60b	TR
F01_cb7536_c1/f1p0/2495-1F	SWI/SNF-BAF60b	TR
F01_cb7546_c0/f2p0/2538-0F	FAR1	TF
F01_cb7546_c1/f1p0/2593-1F	FAR1	TF
F01_cb7578_c0/f2p0/2517-2F	FAR1	TF
F01_cb7578_c5/f1p0/2401-0F	FAR1	TF
F01_cb7609_c1/f1p0/2494-0F	FAR1	TF
F01_cb7609_c2/f1p0/2180-0F	FAR1	TF
F01_cb760_c1/f1p0/4642-1F	SNF2	TR
F01_cb760_c10/f1p0/5334-0F	SNF2	TR
F01_cb760_c7/f1p0/5742-0F	SNF2	TR
F01_cb760_c7/f1p0/5742-1F	SNF2	TR
F01_cb7622_c2/f1p0/2568-1F	Trihelix	TF
F01_cb7622_c3/f1p0/2230-1F	Trihelix	TF
F01_cb7647_c0/f3p1/2342-2F	FAR1	TF
F01_cb7647_c2/f2p0/2361-2F	FAR1	TF
F01_cb7647_c3/f2p0/2256-1F	FAR1	TF
F01_cb7647_c4/f1p1/2573-1F	FAR1	TF

F01_cb7647_c7/f1p0/2390-2F	FAR1	TF
F01_cb7655_c0/f1p0/2566-2F	MYB-related	TF
F01_cb7655_c1/f1p0/1912-0F	MYB-related	TF
F01_cb765_c15/f1p0/3098-0F	SNF2	TR
F01_cb765_c2/f1p1/4647-0F	PHD	TR
F01_cb765_c3/f1p0/4678-2F	SNF2	TR
F01_cb765_c4/f1p0/4269-0F	PHD	TR
F01_cb765_c4/f1p0/4269-2F	SNF2	TR
F01_cb765_c6/f1p0/4514-2F	PHD	TR
F01_cb7661_c2/f1p1/2394-1F	Jumonji	TR
F01_cb7661_c3/f1p1/2127-2F	Jumonji	TR
F01_cb7671_c0/f4p0/2439-1F	C2H2	TF
F01_cb7671_c6/f1p0/2457-0F	C2H2	TF
F01_cb7671_c7/f1p0/2584-2F	C2H2	TF
F01_cb770_c0/f2p0/1445-0F	GeBP	TF
F01_cb770_c1/f2p0/2088-2F	GeBP	TF
F01_cb770_c2/f2p0/2154-1F	GeBP	TF
F01_cb770_c3/f1p1/4637-0F	GeBP	TF
F01_cb770_c4/f1p0/2173-2F	GeBP	TF
F01_cb770_c5/f1p0/2208-0F	GeBP	TF
F01_cb770_c7/f1p0/1765-1F	GeBP	TF
F01_cb770_c9/f1p0/1434-0F	GeBP	TF
F01_cb7727_c0/f1p0/2473-2F	ARID	TR
F01_cb7727_c1/f1p0/2654-2F	ARID	TR
F01_cb7744_c2/f1p0/3029-0F	FAR1	TF
F01_cb7744_c3/f13p1/2516-2F	FAR1	TF

F01_cb7744_c4/f2p0/2472-0F	FAR1	TF
F01_cb7775_c0/f8p0/2245-1F	MYB-related	TF
F01_cb7775_c1/f1p0/2497-2F	MYB-related	TF
F01_cb7775_c3/f1p0/2253-0F	MYB-related	TF
F01_cb7777_c1/f1p0/2534-2F	MADS-M-type	TF
F01_cb7783_c10/f1p0/3451-0F	NAC	TF
F01_cb7783_c10/f1p0/3451-2F	NAC	TF
F01_cb7783_c12/f1p2/2182-0F	NAC	TF
F01_cb7783_c14/f1p1/2060-2F	NAC	TF
F01_cb7783_c17/f1p0/2403-0F	NAC	TF
F01_cb7783_c18/f1p0/2190-0F	NAC	TF
F01_cb7783_c22/f1p1/2195-2F	NAC	TF
F01_cb7783_c26/f1p0/2174-0F	NAC	TF
F01_cb7783_c31/f7p3/2265-1F	NAC	TF
F01_cb7783_c7/f1p2/2533-0F	NAC	TF
F01_cb7794_c1/f3p0/2178-0F	NAC	TF
F01_cb7794_c12/f1p0/1921-2F	NAC	TF
F01_cb7794_c15/f3p0/2130-2F	NAC	TF
F01_cb7794_c16/f5p0/2066-2F	NAC	TF
F01_cb7794_c4/f1p0/2532-2F	NAC	TF
F01_cb7794_c7/f1p0/2104-1F	NAC	TF
F01_cb7794_c9/f1p0/2090-0F	NAC	TF
F01_cb7802_c1/f5p2/1708-0F	C3H	TF
F01_cb7802_c10/f1p0/1775-2F	C3H	TF
F01_cb7802_c11/f1p1/1784-0F	C3H	TF
F01_cb7802_c15/f1p0/1722-2F	C3H	TF

F01_cb7802_c20/f1p0/1296-1F	C3H	TF
F01_cb7802_c21/f1p0/1065-0F	C3H	TF
F01_cb7802_c24/f6p1/1743-0F	C3H	TF
F01_cb7802_c3/f4p0/2215-2F	C3H	TF
F01_cb7802_c6/f1p0/2531-0F	C3H	TF
F01_cb7802_c8/f1p0/1992-0F	C3H	TF
F01_cb7802_c9/f1p0/1789-1F	C3H	TF
F01_cb7826_c1/f2p0/1694-1F	NAC	TF
F01_cb7826_c2/f2p0/2579-2F	NAC	TF
F01_cb7826_c3/f1p0/1930-1F	NAC	TF
F01_cb7826_c4/f1p0/1812-2F	NAC	TF
F01_cb7826_c5/f1p0/1774-1F	NAC	TF
F01_cb7826_c6/f1p0/1693-2F	NAC	TF
F01_cb7839_c0/f1p0/2522-2F	Trihelix	TF
F01_cb7839_c1/f1p0/2164-1F	Trihelix	TF
F01_cb7839_c2/f1p0/1709-1F	Trihelix	TF
F01_cb7839_c3/f1p0/1582-0F	Trihelix	TF
F01_cb7839_c4/f1p0/1475-0F	Trihelix	TF
F01_cb7845_c1/f1p0/2303-0F	HB-other	TF
F01_cb7845_c2/f1p0/2265-1F	HB-BELL	TF
F01_cb7845_c4/f1p0/2414-2F	HB-BELL	TF
F01_cb7851_c0/f3p0/2191-1F	MYB-related	TF
F01_cb7851_c1/f1p0/2518-1F	MYB-related	TF
F01_cb7851_c2/f1p0/2210-2F	MYB-related	TF
F01_cb7851_c3/f1p0/2493-1F	MYB-related	TF
F01_cb7851_c4/f1p0/2193-0F	MYB-related	TF

F01_cb7851_c6/f1p0/2187-2F	MYB-related	TF
F01_cb7862_c0/f2p0/2198-2F	FAR1	TF
F01_cb7892_c1/f1p0/2464-0F	bHLH	TF
F01_cb7892_c2/f1p0/2437-1F	bHLH	TF
F01_cb7900_c1/f1p0/2613-2F	NAC	TF
F01_cb7900_c2/f1p0/1125-1F	NAC	TF
F01_cb7919_c0/f5p0/2485-0F	CPP	TF
F01_cb7919_c2/f1p0/2497-1F	CPP	TF
F01_cb7921_c0/f4p0/2286-0F	MYB	TF
F01_cb7921_c1/f1p0/2500-1F	MYB	TF
F01_cb7921_c4/f1p1/2319-0F	MYB	TF
F01_cb7965_c0/f10p0/2364-0F	IWS1	TR
F01_cb7965_c12/f3p0/2492-0F	IWS1	TR
F01_cb7965_c3/f1p0/3001-2F	IWS1	TR
F01_cb7965_c4/f1p0/2512-0F	IWS1	TR
F01_cb7992_c0/f3p0/1018-1F	bHLH	TF
F01_cb7994_c10/f1p0/1797-1F	HB-other	TF
F01_cb7994_c12/f21p4/2000-2F	HB-KNOX	TF
F01_cb7994_c15/f2p2/1880-1F	HB-KNOX	TF
F01_cb7994_c4/f1p1/2484-2F	HB-KNOX	TF
F01_cb8008_c0/f3p0/1481-1F	B3	TF
F01_cb8008_c3/f1p0/2803-2F	B3	TF
F01_cb8008_c4/f1p0/1536-1F	B3	TF
F01_cb8012_c2/f1p0/2209-0F	AP2/ERF-AP2	TF
F01_cb8012_c3/f1p0/2011-2F	AP2/ERF-AP2	TF
F01_cb8012_c4/f1p0/2147-0F	AP2/ERF-ERF	TF

F01_cb8012_c4/f1p0/2147-2F	AP2/ERF-ERF	TF
F01_cb801_c1/f1p0/3812-1F	SWI/SNF-SWI3	TR
F01_cb801_c2/f1p0/4334-2F	SWI/SNF-SWI3	TR
F01_cb801_c5/f1p0/4078-2F	SWI/SNF-SWI3	TR
F01_cb8021_c4/f2p0/2179-1F	TUB	TF
F01_cb8021_c5/f1p0/2473-2F	TUB	TF
F01_cb8021_c6/f1p1/2110-0F	TUB	TF
F01_cb8021_c6/f1p1/2110-1F	TUB	TF
F01_cb8039_c0/f1p0/2478-0F	HSF	TF
F01_cb8039_c1/f1p0/2579-1F	HSF	TF
F01_cb8039_c1/f1p0/2579-2F	HSF	TF
F01_cb8041_c10/f1p2/2193-1F	NAC	TF
F01_cb8041_c12/f1p0/2361-0F	NAC	TF
F01_cb8041_c13/f1p0/2390-1F	NAC	TF
F01_cb8041_c14/f1p0/2381-0F	NAC	TF
F01_cb8041_c15/f1p1/2453-2F	NAC	TF
F01_cb8041_c16/f1p0/2433-1F	NAC	TF
F01_cb8041_c17/f1p0/2556-2F	NAC	TF
F01_cb8041_c22/f1p0/2444-1F	NAC	TF
F01_cb8041_c22/f1p0/2444-2F	NAC	TF
F01_cb8041_c25/f1p0/2398-1F	NAC	TF
F01_cb8041_c27/f1p2/2321-1F	NAC	TF
F01_cb8041_c6/f1p0/2474-0F	NAC	TF
F01_cb8044_c0/f3p0/2437-2F	MYB-related	TF
F01_cb8044_c1/f1p0/2465-1F	MYB-related	TF
F01_cb8090_c0/f3p0/2304-1F	Others	TR

F01_cb8090_c2/f2p0/2462-2F	Others	TR
F01_cb8103_c10/f1p0/2354-2F	HMG	TR
F01_cb8103_c12/f1p2/2304-1F	HMG	TR
F01_cb8103_c13/f1p0/2001-2F	HMG	TR
F01_cb8103_c18/f31p2/2112-1F	HMG	TR
F01_cb8103_c5/f1p2/2342-0F	HMG	TR
F01_cb8113_c0/f5p1/2291-1F	HMG	TR
F01_cb8113_c2/f1p1/2633-0F	HMG	TR
F01_cb8113_c4/f1p1/3608-1F	HMG	TR
F01_cb8113_c5/f1p1/2305-1F	HMG	TR
F01_cb8113_c6/f1p0/2348-0F	HMG	TR
F01_cb8133_c11/f1p0/2365-1F	GRAS	TF
F01_cb8133_c12/f1p2/2384-1F	GRAS	TF
F01_cb8133_c15/f1p1/2322-0F	GRAS	TF
F01_cb8133_c22/f4p2/2309-2F	GRAS	TF
F01_cb8133_c24/f2p1/2289-2F	GRAS	TF
F01_cb8133_c4/f1p1/2448-2F	GRAS	TF
F01_cb8133_c6/f1p1/2183-2F	GRAS	TF
F01_cb8148_c11/f6p0/2039-1F	bHLH	TF
F01_cb8148_c2/f2p0/2226-1F	bHLH	TF
F01_cb8148_c3/f2p0/1759-2F	bHLH	TF
F01_cb8148_c7/f1p0/2147-1F	bHLH	TF
F01_cb8148_c8/f1p0/1885-2F	bHLH	TF
F01_cb8156_c0/f4p0/1790-0F	GARP-G2-like	TF
F01_cb8156_c2/f1p0/2444-1F	GARP-G2-like	TF
F01_cb8156_c3/f1p1/1819-0F	GARP-G2-like	TF

F01_cb8156_c4/f1p0/2088-1F	GARP-G2-like	TF
F01_cb8156_c6/f1p0/1778-2F	GARP-G2-like	TF
F01_cb8156_c8/f1p0/1578-1F	GARP-G2-like	TF
F01_cb8158_c0/f2p0/2240-2F	NF-YB	TF
F01_cb816_c10/f1p0/3174-0F	IWS1	TR
F01_cb816_c12/f1p0/3158-0F	IWS1	TR
F01_cb816_c16/f1p0/2521-0F	IWS1	TR
F01_cb816_c19/f7p1/2220-1F	IWS1	TR
F01_cb816_c20/f5p2/2306-2F	IWS1	TR
F01_cb816_c3/f3p0/2302-0F	IWS1	TR
F01_cb816_c7/f1p0/2363-2F	IWS1	TR
F01_cb8176_c0/f1p0/2434-2F	TCP	TF
F01_cb8176_c1/f1p0/1853-1F	TCP	TF
F01_cb8176_c2/f1p1/1576-2F	TCP	TF
F01_cb8182_c1/f1p0/2323-2F	FAR1	TF
F01_cb8192_c11/f6p0/2345-1F	C3H	TF
F01_cb8192_c3/f1p0/2430-0F	C3H	TF
F01_cb8192_c3/f1p0/2430-1F	C3H	TF
F01_cb8192_c4/f1p0/2486-0F	C3H	TF
F01_cb8192_c4/f1p0/2486-2F	C3H	TF
F01_cb8208_c0/f1p0/2428-2F	bHLH	TF
F01_cb8208_c1/f1p0/1991-1F	bHLH	TF
F01_cb8215_c8/f1p0/2308-0R	TAZ	TR
F01_cb8229_c1/f1p0/2156-0F	HB-HD-ZIP	TF
F01_cb8229_c2/f1p0/1882-1F	HB-HD-ZIP	TF
F01_cb8229_c3/f1p0/1714-1F	HB-HD-ZIP	TF

F01_cb8230_c1/f1p0/2424-0F	NF-YC	TF
F01_cb8230_c3/f1p0/2044-0F	NF-YC	TF
F01_cb8234_c5/f4p2/3010-0F	C2H2	TF
F01_cb8257_c0/f1p0/2419-2F	C2H2	TF
F01_cb8257_c1/f1p0/2452-2F	C2H2	TF
F01_cb8258_c12/f1p0/2154-0F	C2C2-Dof	TF
F01_cb8258_c13/f1p0/2409-0F	C2C2-Dof	TF
F01_cb8258_c18/f1p0/2040-0F	C2C2-Dof	TF
F01_cb8258_c6/f1p0/2414-1F	C2C2-Dof	TF
F01_cb8258_c6/f1p0/2414-2F	C2C2-Dof	TF
F01_cb8258_c8/f1p0/2039-2F	C2C2-Dof	TF
F01_cb8258_c9/f1p0/2131-0F	C2C2-Dof	TF
F01_cb8268_c0/f5p2/2432-0F	GARP-ARR-B	TF
F01_cb8268_c2/f3p2/2423-0F	GARP-ARR-B	TF
F01_cb8268_c4/f1p2/2376-0F	GARP-ARR-B	TF
F01_cb8277_c2/f1p0/3699-1F	GARP-G2-like	TF
F01_cb8277_c2/f1p0/3699-2F	Others	TR
F01_cb8287_c2/f1p0/2422-0F	mTERF	TR
F01_cb8295_c1/f6p0/1642-1F	C2H2	TF
F01_cb8295_c4/f1p0/2139-0F	C2H2	TF
F01_cb8295_c5/f5p0/2401-2F	C2H2	TF
F01_cb8301_c0/f1p0/2363-0F	mTERF	TR
F01_cb8301_c2/f1p0/2755-1F	mTERF	TR
F01_cb8301_c3/f1p0/2474-2F	mTERF	TR
F01_cb8301_c4/f1p0/1209-1F	mTERF	TR
F01_cb8347_c1/f2p0/2190-0F	mTERF	TR

F01_cb8391_c2/f1p0/1888-2F	GARP-G2-like	TF
F01_cb8391_c3/f1p0/1610-2F	GARP-G2-like	TF
F01_cb8391_c4/f1p0/1467-1F	GARP-G2-like	TF
F01_cb8391_c5/f1p0/1456-0F	GARP-G2-like	TF
F01_cb8391_c7/f1p0/1432-2F	GARP-G2-like	TF
F01_cb8399_c0/f1p1/2379-1F	AP2/ERF-ERF	TF
F01_cb8399_c1/f1p0/1901-1F	AP2/ERF-ERF	TF
F01_cb8399_c4/f1p0/1181-2F	AP2/ERF-ERF	TF
F01_cb8400_c0/f6p1/1868-1F	C3H	TF
F01_cb8400_c1/f1p1/1838-1F	C3H	TF
F01_cb8400_c4/f2p0/1858-2F	C3H	TF
F01_cb8400_c5/f1p0/1347-2F	C3H	TF
F01_cb8424_c0/f1p0/2374-1F	MYB-related	TF
F01_cb8424_c1/f1p0/2377-0F	MYB-related	TF
F01_cb8433_c12/f1p0/2395-1F	FAR1	TF
F01_cb8433_c20/f1p0/1045-1F	FAR1	TF
F01_cb8433_c22/f1p0/5526-0F	FAR1	TF
F01_cb8433_c23/f3p0/1059-1F	FAR1	TF
F01_cb8447_c10/f1p0/2414-2F	MYB-related	TF
F01_cb8447_c13/f3p1/2336-1F	MYB-related	TF
F01_cb8447_c3/f2p1/2371-1F	MYB-related	TF
F01_cb8447_c6/f1p0/2232-2F	MYB-related	TF
F01_cb8458_c1/f1p0/3115-1F	bZIP	TF
F01_cb8463_c0/f1p0/2363-0F	AP2/ERF-ERF	TF
F01_cb8463_c0/f1p0/2363-2F	AP2/ERF-ERF	TF
F01_cb8463_c1/f1p0/2256-2F	AP2/ERF-AP2	TF

F01_cb8469_c0/f4p0/2201-1F	MYB-related	TF
F01_cb8469_c2/f1p0/2360-2F	MYB-related	TF
F01_cb8469_c4/f1p0/3531-2F	MYB-related	TF
F01_cb8469_c6/f1p0/3442-1F	MYB-related	TF
F01_cb8469_c8/f1p0/2270-2F	MYB-related	TF
F01_cb8478_c10/f2p0/3099-1F	FAR1	TF
F01_cb8478_c13/f2p0/3348-2F	FAR1	TF
F01_cb8478_c22/f1p0/3564-0F	FAR1	TF
F01_cb8478_c24/f1p0/3383-0F	FAR1	TF
F01_cb8478_c24/f1p0/3383-2F	FAR1	TF
F01_cb8478_c26/f1p0/3535-0F	FAR1	TF
F01_cb8478_c32/f1p0/3207-0F	FAR1	TF
F01_cb8478_c34/f1p0/3403-2F	FAR1	TF
F01_cb8478_c37/f1p0/3192-0F	FAR1	TF
F01_cb8478_c38/f1p0/3197-0F	FAR1	TF
F01_cb8478_c39/f1p0/3267-0F	FAR1	TF
F01_cb8478_c7/f2p0/3290-1F	FAR1	TF
F01_cb8478_c9/f2p1/3524-2F	FAR1	TF
F01_cb8516_c0/f3p1/2129-2F	TCP	TF
F01_cb8516_c2/f1p0/2182-0F	TCP	TF
F01_cb8516_c3/f1p0/2479-0F	TCP	TF
F01_cb8526_c0/f4p0/2241-0F	HSF	TF
F01_cb8526_c1/f2p0/2143-2F	HSF	TF
F01_cb8526_c3/f1p0/2285-2F	HSF	TF
F01_cb8526_c6/f1p0/2343-1F	HSF	TF
F01_cb8526_c7/f1p0/2227-2F	HSF	TF

F01_cb8526_c8/f1p0/1787-1F	HSF	TF
F01_cb854_c0/f1p0/4601-2F	FAR1	TF
F01_cb854_c1/f1p1/2719-0F	FAR1	TF
F01_cb854_c3/f1p1/2814-1F	FAR1	TF
F01_cb854_c5/f1p0/2064-1F	FAR1	TF
F01_cb854_c6/f1p1/2657-0F	FAR1	TF
F01_cb8553_c18/f12p1/2234-0F	GRAS	TF
F01_cb8553_c19/f11p0/2278-1F	GRAS	TF
F01_cb8553_c3/f8p0/2196-2F	GRAS	TF
F01_cb8553_c4/f4p1/2240-2F	GRAS	TF
F01_cb8553_c8/f1p0/2286-2F	GRAS	TF
F01_cb8553_c9/f1p0/2435-1F	GRAS	TF
F01_cb8564_c10206/f1p0/3499-1F	WRKY	TF
F01_cb8564_c1027/f1p0/1955-0F	C2H2	TF
F01_cb8564_c1028/f2p0/1918-1F	C2H2	TF
F01_cb8564_c103295/f1p0/2626-2F	HB-BELL	TF
F01_cb8564_c10426/f1p0/3142-0F	GRAS	TF
F01_cb8564_c10426/f1p0/3142-2F	GRAS	TF
F01_cb8564_c106647/f6p1/2113-2F	WRKY	TF
F01_cb8564_c10668/f1p0/2523-0F	bHLH	TF
F01_cb8564_c106852/f5p2/2630-2F	GRAS	TF
F01_cb8564_c106911/f7p3/3690-0F	SBP	TF
F01_cb8564_c106917/f6p1/3331-2F	SWI/SNF-BAF60b	TR
F01_cb8564_c107051/f2p1/3797-1F	SBP	TF
F01_cb8564_c107237/f5p1/2872-0F	RWP-RK	TF
F01_cb8564_c107332/f9p1/3308-1F	SWI/SNF-BAF60b	TR

F01_cb8564_c1076/f1p0/2896-0F	HB-BELL	TF
F01_cb8564_c107632/f8p2/2201-1F	C2H2	TF
F01_cb8564_c107657/f8p0/3003-0F	GRAS	TF
F01_cb8564_c107870/f4p1/2211-2F	Trihelix	TF
F01_cb8564_c108232/f2p5/4746-0F	Others	TR
F01_cb8564_c1086/f1p0/3040-1F	C3H	TF
F01_cb8564_c1087/f3p0/2638-0F	C3H	TF
F01_cb8564_c109507/f4p6/2607-1F	NAC	TF
F01_cb8564_c109928/f2p1/3116-1F	B3-ARF	TF
F01_cb8564_c109971/f3p4/2739-2F	HB-HD-ZIP	TF
F01_cb8564_c1100/f2p1/2055-1F	TRAF	TR
F01_cb8564_c110015/f3p0/4441-0F	PHD	TR
F01_cb8564_c110015/f3p0/4441-1F	SET	TR
F01_cb8564_c110086/f2p0/3836-2F	Others	TR
F01_cb8564_c1101/f1p1/1925-0F	TRAF	TR
F01_cb8564_c1102/f1p0/2215-1F	TRAF	TR
F01_cb8564_c11055/f1p2/1904-0F	HB-HD-ZIP	TF
F01_cb8564_c110637/f1p2/2491-0F	FAR1	TF
F01_cb8564_c111594/f1p0/3002-0F	TAZ	TR
F01_cb8564_c11215/f1p0/3486-2F	WRKY	TF
F01_cb8564_c112473/f1p0/3272-0F	WRKY	TF
F01_cb8564_c112632/f1p0/2851-1R	MADS-MIKC	TF
F01_cb8564_c112707/f1p0/2546-0F	WRKY	TF
F01_cb8564_c112719/f1p0/2721-2F	RWP-RK	TF
F01_cb8564_c11301/f2p0/3190-0F	WRKY	TF
F01_cb8564_c113200/f1p0/2705-2F	FAR1	TF

F01_cb8564_c113323/f1p0/1928-1F	mTERF	TR
F01_cb8564_c113379/f1p1/2337-2F	WRKY	TF
F01_cb8564_c113484/f1p0/2649-2F	FAR1	TF
F01_cb8564_c113749/f1p1/2029-2F	AP2/ERF-ERF	TF
F01_cb8564_c113907/f1p2/1941-2F	AP2/ERF-ERF	TF
F01_cb8564_c113961/f1p0/3218-1F	TAZ	TR
F01_cb8564_c114548/f1p0/2001-2F	bHLH	TF
F01_cb8564_c115092/f1p0/2887-0R	TUB	TF
F01_cb8564_c115579/f1p0/2540-1F	bHLH	TF
F01_cb8564_c115771/f1p0/2894-0F	NAC	TF
F01_cb8564_c115945/f1p0/2388-0F	TRAF	TR
F01_cb8564_c115983/f1p0/2126-2F	RWP-RK	TF
F01_cb8564_c11628/f1p0/3577-2F	Jumonji	TR
F01_cb8564_c116305/f1p0/2020-2F	C3H	TF
F01_cb8564_c116852/f1p0/2560-1F	C2C2-GATA	TF
F01_cb8564_c117515/f1p0/2510-2F	RWP-RK	TF
F01_cb8564_c117538/f1p1/2205-2F	WRKY	TF
F01_cb8564_c117852/f1p0/1926-2F	Others	TR
F01_cb8564_c118234/f1p0/2657-0F	AP2/ERF-ERF	TF
F01_cb8564_c118234/f1p0/2657-1F	RWP-RK	TF
F01_cb8564_c11824/f2p0/2433-2F	mTERF	TR
F01_cb8564_c119112/f1p0/3859-2F	Others	TR
F01_cb8564_c119459/f1p0/2673-1F	TUB	TF
F01_cb8564_c119489/f1p2/2132-2F	HB-HD-ZIP	TF
F01_cb8564_c11955/f1p0/3106-0F	B3-ARF	TF
F01_cb8564_c119629/f1p3/2129-0F	AP2/ERF-ERF	TF

F01_cb8564_c119729/f1p1/1931-0F	AUX/IAA	TR
F01_cb8564_c119935/f1p0/3487-0F	TAZ	TR
F01_cb8564_c120024/f1p0/2514-1F	TRAF	TR
F01_cb8564_c120089/f1p0/3325-2R	Others	TR
F01_cb8564_c120246/f1p1/1940-1F	AUX/IAA	TR
F01_cb8564_c120610/f1p0/2686-0F	SNF2	TR
F01_cb8564_c120610/f1p0/2686-1F	SNF2	TR
F01_cb8564_c121017/f1p0/4917-0F	bHLH	TF
F01_cb8564_c121214/f1p0/2308-0F	RWP-RK	TF
F01_cb8564_c121422/f1p3/2066-1F	HB-HD-ZIP	TF
F01_cb8564_c121579/f1p0/2710-0F	GRAS	TF
F01_cb8564_c121579/f1p0/2710-2F	GRAS	TF
F01_cb8564_c122355/f1p0/2994-0F	RWP-RK	TF
F01_cb8564_c122499/f4p1/2386-0F	WRKY	TF
F01_cb8564_c122690/f2p1/2644-2F	RWP-RK	TF
F01_cb8564_c123008/f1p0/2517-2F	FAR1	TF
F01_cb8564_c12334/f3p1/4316-2F	mTERF	TR
F01_cb8564_c123469/f1p0/3334-1F	NAC	TF
F01_cb8564_c123817/f1p0/2095-2F	C3H	TF
F01_cb8564_c123973/f1p0/3276-2F	FAR1	TF
F01_cb8564_c124012/f1p0/2451-2F	WRKY	TF
F01_cb8564_c12433/f1p0/3729-0F	HB-HD-ZIP	TF
F01_cb8564_c124519/f1p0/2115-1F	AP2/ERF-ERF	TF
F01_cb8564_c124567/f1p0/2268-2F	Trihelix	TF
F01_cb8564_c124659/f1p0/2129-2F	AP2/ERF-ERF	TF
F01_cb8564_c124689/f1p0/2316-1F	Others	TR

F01_cb8564_c124703/f1p1/2204-0F	AUX/IAA	TR
F01_cb8564_c124703/f1p1/2204-1F	AUX/IAA	TR
F01_cb8564_c124955/f1p0/2372-0F	GRAS	TF
F01_cb8564_c124955/f1p0/2372-1F	GRAS	TF
F01_cb8564_c124955/f1p0/2372-2F	GRAS	TF
F01_cb8564_c125111/f1p0/2638-0F	SNF2	TR
F01_cb8564_c125111/f1p0/2638-1F	SNF2	TR
F01_cb8564_c125116/f2p1/2358-1F	GRAS	TF
F01_cb8564_c12556/f1p0/4524-0F	MYB	TF
F01_cb8564_c1256/f1p0/2155-0R	TUB	TF
F01_cb8564_c1257/f1p0/4087-0R	TUB	TF
F01_cb8564_c125850/f1p1/2243-2F	mTERF	TR
F01_cb8564_c126419/f1p0/1910-2F	Trihelix	TF
F01_cb8564_c126457/f1p0/4429-0F	PHD	TR
F01_cb8564_c126498/f1p0/2408-1F	WRKY	TF
F01_cb8564_c12677/f2p1/3263-2F	B3-ARF	TF
F01_cb8564_c126796/f1p0/2168-1F	C2C2-CO-like	TF
F01_cb8564_c127138/f1p0/4403-1F	PHD	TR
F01_cb8564_c127138/f1p0/4403-2F	SET	TR
F01_cb8564_c127809/f1p0/2005-1F	AP2/ERF-ERF	TF
F01_cb8564_c127882/f1p0/2154-0F	Others	TR
F01_cb8564_c128164/f1p0/2243-0F	AP2/ERF-ERF	TF
F01_cb8564_c128215/f1p0/2462-2F	FAR1	TF
F01_cb8564_c128322/f1p0/2488-1F	Trihelix	TF
F01_cb8564_c128705/f1p0/2363-0F	Others	TR
F01_cb8564_c128749/f1p0/2577-2F	STAT	TF

F01_cb8564_c128999/f1p2/2626-2F	AUX/IAA	TR
F01_cb8564_c129342/f1p0/2752-0F	STAT	TF
F01_cb8564_c1298/f14p5/2084-1F	WRKY	TF
F01_cb8564_c129967/f2p0/4204-2F	PHD	TR
F01_cb8564_c13000/f2p0/4359-0F	SBP	TF
F01_cb8564_c130327/f1p7/1940-2F	HB-HD-ZIP	TF
F01_cb8564_c130334/f1p0/3467-1F	bHLH	TF
F01_cb8564_c13150/f4p0/2764-0F	GRAS	TF
F01_cb8564_c13280/f3p0/4088-0F	PHD	TR
F01_cb8564_c134008/f1p6/2127-1F	HB-HD-ZIP	TF
F01_cb8564_c13451/f1p2/3193-1F	GRAS	TF
F01_cb8564_c137927/f1p7/1905-1F	HB-HD-ZIP	TF
F01_cb8564_c139145/f1p8/2101-0F	HB-HD-ZIP	TF
F01_cb8564_c14001/f1p0/3475-2F	GRAS	TF
F01_cb8564_c14024/f1p0/4640-0F	GRAS	TF
F01_cb8564_c14024/f1p0/4640-2F	GRAS	TF
F01_cb8564_c14103/f1p0/2351-0F	WRKY	TF
F01_cb8564_c141478/f2p1/3738-2F	GRAS	TF
F01_cb8564_c14157/f1p1/4319-2F	mTERF	TR
F01_cb8564_c14216/f1p0/3375-2F	B3-ARF	TF
F01_cb8564_c14355/f1p0/3726-0F	HB-PHD	TF
F01_cb8564_c14361/f1p0/2209-2F	TRAF	TR
F01_cb8564_c145324/f8p2/2614-0F	SNF2	TR
F01_cb8564_c145331/f4p1/2049-0F	Trihelix	TF
F01_cb8564_c145342/f2p0/2771-0F	bHLH	TF
F01_cb8564_c145452/f2p1/2632-1F	GRAS	TF

F01_cb8564_c14546/f1p5/3300-2F	HB-BELL	TF
F01_cb8564_c145803/f3p4/2658-0F	HB-HD-ZIP	TF
F01_cb8564_c145851/f5p1/2705-2F	STAT	TF
F01_cb8564_c145906/f6p0/3616-1F	HB-PHD	TF
F01_cb8564_c145947/f8p2/2415-0F	C2C2-CO-like	TF
F01_cb8564_c146107/f6p1/2403-0F	C2C2-GATA	TF
F01_cb8564_c146223/f12p1/2980-0F	LUG	TR
F01_cb8564_c146257/f6p6/2574-0F	HB-BELL	TF
F01_cb8564_c146313/f4p3/3697-0F	SBP	TF
F01_cb8564_c146386/f13p5/2801-0F	GRAS	TF
F01_cb8564_c146488/f2p1/2737-2F	HB-other	TF
F01_cb8564_c146864/f4p3/2617-0F	GRAS	TF
F01_cb8564_c147135/f5p2/2714-0F	TUB	TF
F01_cb8564_c147929/f1p0/2042-2F	C2C2-CO-like	TF
F01_cb8564_c148927/f5p0/2405-1F	C2C2-GATA	TF
F01_cb8564_c149594/f1p1/2449-2F	C2C2-CO-like	TF
F01_cb8564_c15031/f1p2/3179-2F	B3-ARF	TF
F01_cb8564_c15033/f1p1/3280-2F	MADS-M-type	TF
F01_cb8564_c150346/f1p2/2814-1F	GRAS	TF
F01_cb8564_c15292/f1p0/2336-2F	SBP	TF
F01_cb8564_c153372/f2p2/3657-2F	SNF2	TR
F01_cb8564_c15372/f1p0/3532-0F	SNF2	TR
F01_cb8564_c15372/f1p0/3532-1F	SNF2	TR
F01_cb8564_c15372/f1p0/3532-2F	SNF2	TR
F01_cb8564_c155769/f1p3/2649-2F	HB-BELL	TF
F01_cb8564_c158006/f2p1/3461-0F	AUX/IAA	TR

F01_cb8564_c158006/f2p1/3461-2F	B3-ARF	TF
F01_cb8564_c158498/f1p2/2482-1F	FAR1	TF
F01_cb8564_c159074/f1p3/2275-0F	Trihelix	TF
F01_cb8564_c159074/f1p3/2275-1F	Trihelix	TF
F01_cb8564_c162316/f1p1/2224-0F	WRKY	TF
F01_cb8564_c162316/f1p1/2224-2F	WRKY	TF
F01_cb8564_c16410/f1p0/2002-2F	TRAF	TR
F01_cb8564_c16421/f1p0/2249-2F	mTERF	TR
F01_cb8564_c16498/f2p2/2818-1F	HB-HD-ZIP	TF
F01_cb8564_c16669/f1p0/2894-1F	mTERF	TR
F01_cb8564_c166913/f2p2/2105-0F	Trihelix	TF
F01_cb8564_c166913/f2p2/2105-1F	Trihelix	TF
F01_cb8564_c16731/f1p0/3593-0F	SNF2	TR
F01_cb8564_c16731/f1p0/3593-2F	SNF2	TR
F01_cb8564_c168338/f2p0/3398-2F	bHLH	TF
F01_cb8564_c17442/f1p1/2792-2F	B3	TF
F01_cb8564_c1759/f1p0/4103-0F	Others	TR
F01_cb8564_c176158/f6p0/2068-2F	C2C2-CO-like	TF
F01_cb8564_c1764/f1p0/2367-0F	Others	TR
F01_cb8564_c1771/f3p1/3767-0F	Others	TR
F01_cb8564_c1772/f1p1/2420-0F	C2C2-CO-like	TF
F01_cb8564_c1773/f2p1/2473-1F	Others	TR
F01_cb8564_c1775/f1p0/2782-1F	Others	TR
F01_cb8564_c1777/f1p0/2673-2F	C2C2-CO-like	TF
F01_cb8564_c1780/f1p1/3143-2F	Others	TR
F01_cb8564_c17806/f1p0/3709-2F	SNF2	TR

F01_cb8564_c1781/f1p1/2101-2F	C2C2-CO-like	TF
F01_cb8564_c1783/f1p0/2556-0F	C2C2-CO-like	TF
F01_cb8564_c1784/f1p0/2603-0F	C2C2-CO-like	TF
F01_cb8564_c181097/f1p0/2142-0F	NAC	TF
F01_cb8564_c18118/f3p4/3114-1F	HB-HD-ZIP	TF
F01_cb8564_c18359/f1p0/3917-1F	SNF2	TR
F01_cb8564_c1861/f1p0/2943-1F	WRKY	TF
F01_cb8564_c1861/f1p0/2943-2F	WRKY	TF
F01_cb8564_c186144/f1p0/3163-1F	B3-ARF	TF
F01_cb8564_c186144/f1p0/3163-2F	AUX/IAA	TR
F01_cb8564_c1864/f1p0/2035-1F	WRKY	TF
F01_cb8564_c1864/f1p0/2035-2F	WRKY	TF
F01_cb8564_c1865/f1p0/2458-1F	WRKY	TF
F01_cb8564_c18654/f1p0/3773-1F	SBP	TF
F01_cb8564_c1868/f1p1/2359-2F	WRKY	TF
F01_cb8564_c18715/f15p2/3609-0F	Jumonji	TR
F01_cb8564_c18743/f1p0/3923-2F	bHLH	TF
F01_cb8564_c18992/f1p0/3876-0F	mTERF	TR
F01_cb8564_c19056/f1p2/2030-2F	AP2/ERF-ERF	TF
F01_cb8564_c1916/f2p0/2923-1F	NF-X1	TF
F01_cb8564_c19742/f1p4/2984-0F	HB-HD-ZIP	TF
F01_cb8564_c19805/f1p0/4483-0F	SNF2	TR
F01_cb8564_c19831/f2p0/3515-1F	mTERF	TR
F01_cb8564_c19910/f1p0/4399-2F	B3	TF
F01_cb8564_c20069/f1p1/3817-0F	MADS-M-type	TF
F01_cb8564_c2041/f2p1/3039-1F	RWP-RK	TF

F01_cb8564_c2042/f2p1/2865-1F	RWP-RK	TF
F01_cb8564_c2059/f3p1/2245-1F	NAC	TF
F01_cb8564_c20602/f1p0/2495-2F	FAR1	TF
F01_cb8564_c2068/f1p0/2547-2F	RWP-RK	TF
F01_cb8564_c20700/f1p0/4463-0F	PHD	TR
F01_cb8564_c20962/f1p0/3220-0F	RWP-RK	TF
F01_cb8564_c21211/f1p0/2788-1F	GRAS	TF
F01_cb8564_c21527/f7p0/3631-1F	HB-PHD	TF
F01_cb8564_c21622/f1p1/2491-2F	AUX/IAA	TR
F01_cb8564_c22308/f1p0/3539-1F	RWP-RK	TF
F01_cb8564_c22388/f10p2/3754-2F	GRAS	TF
F01_cb8564_c22414/f6p0/4311-1F	Jumonji	TR
F01_cb8564_c22414/f6p0/4311-2F	C2H2	TF
F01_cb8564_c22766/f1p1/3652-2F	HB-HD-ZIP	TF
F01_cb8564_c23069/f1p0/2858-2F	C2H2	TF
F01_cb8564_c23436/f1p0/3497-0F	PHD	TR
F01_cb8564_c23436/f1p0/3497-2F	HB-other	TF
F01_cb8564_c23492/f1p0/2909-2F	bHLH	TF
F01_cb8564_c23497/f1p0/4803-1F	C2H2	TF
F01_cb8564_c24511/f1p1/2143-0F	WRKY	TF
F01_cb8564_c24511/f1p1/2143-2F	WRKY	TF
F01_cb8564_c24616/f1p2/2013-0F	TUB	TF
F01_cb8564_c24732/f1p0/3220-0F	TRAF	TR
F01_cb8564_c25256/f1p0/1962-2F	C2H2	TF
F01_cb8564_c25258/f1p0/2537-0F	bHLH	TF
F01_cb8564_c25313/f1p0/3934-0F	TRAF	TR

F01_cb8564_c25473/f1p0/3549-1F	GRAS	TF
F01_cb8564_c25473/f1p0/3549-2F	GRAS	TF
F01_cb8564_c3050/f1p0/3051-1F	FAR1	TF
F01_cb8564_c3051/f1p0/2396-2F	FAR1	TF
F01_cb8564_c3078/f1p0/1893-2F	FAR1	TF
F01_cb8564_c31858/f2p1/3149-1F	B3-ARF	TF
F01_cb8564_c3246/f1p0/3869-2F	GNAT	TR
F01_cb8564_c3275/f1p0/2647-1F	GNAT	TR
F01_cb8564_c3276/f1p0/2732-0F	GNAT	TR
F01_cb8564_c3281/f1p0/2701-2F	GNAT	TR
F01_cb8564_c32899/f13p0/3234-1F	RWP-RK	TF
F01_cb8564_c3309/f1p0/2757-0F	RWP-RK	TF
F01_cb8564_c34027/f4p0/3345-2F	RWP-RK	TF
F01_cb8564_c34066/f4p0/3241-0F	RWP-RK	TF
F01_cb8564_c34233/f3p0/3141-0F	SBP	TF
F01_cb8564_c34553/f1p0/3407-0F	SNF2	TR
F01_cb8564_c36086/f1p0/2874-1F	SNF2	TR
F01_cb8564_c3614/f5p1/2784-0F	HB-HD-ZIP	TF
F01_cb8564_c36410/f1p0/4676-1F	HB-other	TF
F01_cb8564_c36410/f1p0/4676-2F	PHD	TR
F01_cb8564_c3674/f1p0/3030-2F	bHLH	TF
F01_cb8564_c3675/f1p0/2696-0F	bHLH	TF
F01_cb8564_c36758/f1p0/4378-0F	PHD	TR
F01_cb8564_c36758/f1p0/4378-2F	HB-other	TF
F01_cb8564_c3676/f1p0/3441-0F	bHLH	TF
F01_cb8564_c3679/f1p0/2936-0F	bHLH	TF

F01_cb8564_c3680/f1p0/2812-0F	bHLH	TF
F01_cb8564_c3702/f2p1/2964-2F	bHLH	TF
F01_cb8564_c3709/f1p0/3392-0F	bHLH	TF
F01_cb8564_c3710/f1p0/3427-0F	bHLH	TF
F01_cb8564_c3714/f1p0/2897-2F	bHLH	TF
F01_cb8564_c3716/f2p0/3418-1F	bHLH	TF
F01_cb8564_c3717/f1p0/3029-1F	bHLH	TF
F01_cb8564_c3718/f1p0/2760-1F	bHLH	TF
F01_cb8564_c37419/f1p0/3185-0F	RWP-RK	TF
F01_cb8564_c37468/f1p0/3466-2F	SWI/SNF-BAF60b	TR
F01_cb8564_c37594/f3p1/3734-1F	SBP	TF
F01_cb8564_c37737/f1p0/4956-1F	RWP-RK	TF
F01_cb8564_c3811/f1p0/2840-1F	RWP-RK	TF
F01_cb8564_c3812/f2p0/2257-2F	RWP-RK	TF
F01_cb8564_c38940/f1p0/4032-0F	SBP	TF
F01_cb8564_c39144/f1p1/3779-1F	SBP	TF
F01_cb8564_c39181/f2p0/3796-0F	SNF2	TR
F01_cb8564_c39224/f1p0/4176-1F	SNF2	TR
F01_cb8564_c393/f4p2/2731-0F	HB-BELL	TF
F01_cb8564_c39355/f1p0/3531-2F	SWI/SNF-BAF60b	TR
F01_cb8564_c39667/f1p0/2433-2F	C2C2-Dof	TF
F01_cb8564_c39692/f1p0/4120-0R	IWS1	TR
F01_cb8564_c39720/f1p0/4003-1F	SNF2	TR
F01_cb8564_c3985/f1p0/3415-2F	RWP-RK	TF
F01_cb8564_c3987/f1p1/3626-0F	RWP-RK	TF
F01_cb8564_c3987/f1p1/3626-2F	RWP-RK	TF

F01_cb8564_c3988/f1p0/2802-2F	RWP-RK	TF
F01_cb8564_c3990/f1p0/2667-2F	RWP-RK	TF
F01_cb8564_c3992/f1p0/1902-1F	RWP-RK	TF
F01_cb8564_c3999/f1p0/2898-0F	RWP-RK	TF
F01_cb8564_c3999/f1p0/2898-2F	RWP-RK	TF
F01_cb8564_c4002/f1p0/3162-2F	RWP-RK	TF
F01_cb8564_c4003/f1p2/3053-0F	RWP-RK	TF
F01_cb8564_c4004/f1p1/2537-2F	RWP-RK	TF
F01_cb8564_c4005/f1p0/3728-0F	RWP-RK	TF
F01_cb8564_c4005/f1p0/3728-1F	RWP-RK	TF
F01_cb8564_c4006/f1p0/3349-0F	RWP-RK	TF
F01_cb8564_c4006/f1p0/3349-2F	RWP-RK	TF
F01_cb8564_c4008/f1p0/2754-0F	RWP-RK	TF
F01_cb8564_c4010/f1p3/2976-1F	RWP-RK	TF
F01_cb8564_c40127/f1p0/3255-1F	Jumonji	TR
F01_cb8564_c4014/f1p0/2787-1F	RWP-RK	TF
F01_cb8564_c4014/f1p0/2787-2F	RWP-RK	TF
F01_cb8564_c4015/f1p0/3731-0F	RWP-RK	TF
F01_cb8564_c4019/f1p0/4254-0F	RWP-RK	TF
F01_cb8564_c4019/f1p0/4254-1F	RWP-RK	TF
F01_cb8564_c40203/f1p0/3161-0F	SWI/SNF-BAF60b	TR
F01_cb8564_c4022/f1p0/2899-1F	RWP-RK	TF
F01_cb8564_c4023/f1p0/2972-1F	RWP-RK	TF
F01_cb8564_c4025/f1p2/3853-0F	RWP-RK	TF
F01_cb8564_c4025/f1p2/3853-1F	RWP-RK	TF
F01_cb8564_c4027/f1p0/2658-0F	RWP-RK	TF

F01_cb8564_c4030/f1p0/4123-2F	RWP-RK	TF
F01_cb8564_c4033/f1p0/3146-0F	RWP-RK	TF
F01_cb8564_c4037/f1p0/3225-0F	RWP-RK	TF
F01_cb8564_c4037/f1p0/3225-2F	RWP-RK	TF
F01_cb8564_c4038/f1p2/2737-0F	RWP-RK	TF
F01_cb8564_c40403/f2p0/2465-1F	NAC	TF
F01_cb8564_c4045/f1p0/2658-1F	RWP-RK	TF
F01_cb8564_c4045/f1p0/2658-2F	RWP-RK	TF
F01_cb8564_c40701/f1p0/3863-2F	SNF2	TR
F01_cb8564_c4081/f1p0/2462-1F	SNF2	TR
F01_cb8564_c40999/f1p1/3690-0F	SBP	TF
F01_cb8564_c4135/f1p1/4201-1F	FAR1	TF
F01_cb8564_c4136/f2p1/2697-1F	FAR1	TF
F01_cb8564_c4138/f5p2/2729-2F	FAR1	TF
F01_cb8564_c41919/f1p0/2814-2F	RWP-RK	TF
F01_cb8564_c4196/f1p0/2945-1F	TCP	TF
F01_cb8564_c41964/f1p0/3664-0F	NAC	TF
F01_cb8564_c4197/f3p0/2921-1F	TCP	TF
F01_cb8564_c42118/f1p0/3109-2F	GRAS	TF
F01_cb8564_c42230/f1p0/4284-1F	B3	TF
F01_cb8564_c423/f7p1/3074-1F	GRAS	TF
F01_cb8564_c42636/f1p0/3216-0F	HMG	TR
F01_cb8564_c42753/f2p0/2720-0F	NAC	TF
F01_cb8564_c42859/f1p0/3413-0F	SNF2	TR
F01_cb8564_c42859/f1p0/3413-2F	SNF2	TR
F01_cb8564_c42896/f1p1/2299-1F	NAC	TF

F01_cb8564_c43663/f1p0/2765-1F	AP2/ERF-AP2	TF
F01_cb8564_c44585/f1p0/3901-1F	SBP	TF
F01_cb8564_c44682/f1p0/3745-1F	NAC	TF
F01_cb8564_c44720/f1p0/4093-0F	SBP	TF
F01_cb8564_c44792/f1p0/3463-1F	TUB	TF
F01_cb8564_c4499/f1p0/2986-0F	C2H2	TF
F01_cb8564_c4501/f1p0/2987-0F	C2C2-GATA	TF
F01_cb8564_c4504/f1p0/2793-0F	C2C2-GATA	TF
F01_cb8564_c4519/f1p0/2992-2F	TUB	TF
F01_cb8564_c4522/f1p0/2198-2F	TUB	TF
F01_cb8564_c4523/f1p0/2561-1F	TUB	TF
F01_cb8564_c4523/f1p0/2561-2F	TUB	TF
F01_cb8564_c4526/f1p0/2249-0F	TUB	TF
F01_cb8564_c4526/f1p0/2249-2F	TUB	TF
F01_cb8564_c4527/f2p0/2499-2F	TUB	TF
F01_cb8564_c4529/f1p0/2635-1F	TUB	TF
F01_cb8564_c4530/f3p0/2769-1F	TUB	TF
F01_cb8564_c4530/f3p0/2769-2F	TUB	TF
F01_cb8564_c45410/f1p0/4404-2F	PHD	TR
F01_cb8564_c45426/f1p0/2266-1F	NAC	TF
F01_cb8564_c45467/f1p0/2463-2F	LUG	TR
F01_cb8564_c46491/f1p0/3510-1F	B3-ARF	TF
F01_cb8564_c4710/f1p0/3063-0F	WRKY	TF
F01_cb8564_c4715/f6p1/2185-1F	WRKY	TF
F01_cb8564_c47562/f1p0/4869-2F	NAC	TF
F01_cb8564_c47567/f1p0/3302-0F	RWP-RK	TF

F01_cb8564_c48079/f5p1/3512-0F	SWI/SNF-BAF60b	TR
F01_cb8564_c48117/f1p0/2507-2F	RWP-RK	TF
F01_cb8564_c48460/f1p0/2462-2F	TCP	TF
F01_cb8564_c4869/f1p0/3091-1F	C2H2	TF
F01_cb8564_c4884/f1p0/3387-1F	GRAS	TF
F01_cb8564_c4885/f2p1/3179-2F	GRAS	TF
F01_cb8564_c4888/f1p0/3367-0F	GRAS	TF
F01_cb8564_c4890/f2p1/2906-1F	GRAS	TF
F01_cb8564_c4891/f1p0/3367-1F	GRAS	TF
F01_cb8564_c4894/f1p1/2708-0F	GRAS	TF
F01_cb8564_c48947/f1p0/3034-1F	LUG	TR
F01_cb8564_c4895/f1p0/2700-0F	GRAS	TF
F01_cb8564_c49348/f15p0/3022-2F	LUG	TR
F01_cb8564_c4938/f1p0/3004-0F	Others	TR
F01_cb8564_c4939/f1p0/3100-1F	Others	TR
F01_cb8564_c4940/f1p0/2731-2F	Others	TR
F01_cb8564_c4941/f1p0/3015-2F	C2C2-CO-like	TF
F01_cb8564_c4944/f1p0/2151-0F	C2C2-CO-like	TF
F01_cb8564_c4945/f1p0/1950-0F	C2C2-CO-like	TF
F01_cb8564_c4948/f1p0/3126-0F	Others	TR
F01_cb8564_c4948/f1p0/3126-1R	Others	TR
F01_cb8564_c4950/f1p0/2564-0F	Others	TR
F01_cb8564_c4961/f1p0/2082-1F	C2C2-CO-like	TF
F01_cb8564_c4966/f1p0/2245-1F	C2C2-CO-like	TF
F01_cb8564_c4968/f1p0/2194-0F	C2C2-CO-like	TF
F01_cb8564_c49758/f1p0/2412-2F	RWP-RK	TF

F01_cb8564_c50035/f1p0/4246-0F	RWP-RK	TF
F01_cb8564_c50035/f1p0/4246-2F	RWP-RK	TF
F01_cb8564_c50054/f1p0/2877-0F	NAC	TF
F01_cb8564_c50250/f2p0/4315-1F	C2C2-Dof	TF
F01_cb8564_c5046/f1p0/4363-1F	DBP	TF
F01_cb8564_c50713/f2p0/3735-1F	RWP-RK	TF
F01_cb8564_c50800/f1p0/3416-2F	NAC	TF
F01_cb8564_c50920/f1p0/4402-0F	RWP-RK	TF
F01_cb8564_c51208/f1p0/3009-0F	RWP-RK	TF
F01_cb8564_c52182/f1p0/3379-2F	SWI/SNF-BAF60b	TR
F01_cb8564_c52451/f1p0/3796-2F	SNF2	TR
F01_cb8564_c5251/f1p0/3184-0F	TRAF	TR
F01_cb8564_c5251/f1p0/3184-2F	TRAF	TR
F01_cb8564_c5252/f1p0/2216-0F	TRAF	TR
F01_cb8564_c5252/f1p0/2216-2F	TRAF	TR
F01_cb8564_c52554/f1p3/3826-1F	C2H2	TF
F01_cb8564_c5257/f1p0/2539-1F	PHD	TR
F01_cb8564_c52998/f1p1/2110-1F	NAC	TF
F01_cb8564_c52998/f1p1/2110-2F	NAC	TF
F01_cb8564_c53868/f1p0/4757-2F	NAC	TF
F01_cb8564_c54045/f1p0/1961-2F	NAC	TF
F01_cb8564_c54122/f1p1/3367-1F	SWI/SNF-BAF60b	TR
F01_cb8564_c54187/f1p0/3412-1F	SWI/SNF-BAF60b	TR
F01_cb8564_c54212/f1p0/2443-0F	NAC	TF
F01_cb8564_c57586/f4p1/3438-0F	SWI/SNF-BAF60b	TR
F01_cb8564_c608/f6p2/2608-1F	HB-BELL	TF

F01_cb8564_c66437/f7p9/2105-1F	HB-HD-ZIP	TF
F01_cb8564_c675/f4p4/2437-1F	C2C2-CO-like	TF
F01_cb8564_c68331/f3p0/2030-1F	SET	TR
F01_cb8564_c68344/f4p0/2522-1F	mTERF	TR
F01_cb8564_c68846/f10p2/2582-0F	HB-BELL	TF
F01_cb8564_c68939/f2p0/3169-1F	SBP	TF
F01_cb8564_c69115/f2p0/4131-0F	Others	TR
F01_cb8564_c69115/f2p0/4131-1F	Others	TR
F01_cb8564_c69336/f3p0/3731-2F	HB-PHD	TF
F01_cb8564_c69408/f10p0/2152-0F	Others	TR
F01_cb8564_c70010/f1p0/3088-0F	SBP	TF
F01_cb8564_c701/f3p0/3014-1F	Others	TR
F01_cb8564_c70365/f1p1/3583-0F	HB-other	TF
F01_cb8564_c70365/f1p1/3583-2F	PHD	TR
F01_cb8564_c70436/f1p0/3032-0F	C2H2	TF
F01_cb8564_c70911/f1p0/3414-2F	SBP	TF
F01_cb8564_c70960/f1p0/2362-2F	C2H2	TF
F01_cb8564_c71551/f1p0/3242-0F	RWP-RK	TF
F01_cb8564_c71552/f1p0/3651-1F	bHLH	TF
F01_cb8564_c71574/f1p0/3073-1F	MYB-related	TF
F01_cb8564_c71574/f1p0/3073-2F	MYB-related	TF
F01_cb8564_c717/f5p1/2631-0F	NAC	TF
F01_cb8564_c717/f5p1/2631-1F	NAC	TF
F01_cb8564_c71826/f1p0/2193-0F	WRKY	TF
F01_cb8564_c71979/f1p1/2062-0F	HB-HD-ZIP	TF
F01_cb8564_c72081/f1p0/3245-2F	HB-PHD	TF

F01_cb8564_c72135/f1p0/2733-0F	GRAS	TF
F01_cb8564_c72135/f1p0/2733-1F	GRAS	TF
F01_cb8564_c72529/f2p6/2886-0R	MADS-M-type	TF
F01_cb8564_c72552/f1p1/3557-1F	HB-PHD	TF
F01_cb8564_c72622/f1p1/3614-1F	C2H2	TF
F01_cb8564_c72701/f1p0/2931-1F	HB-BELL	TF
F01_cb8564_c72755/f6p0/3094-1F	GRAS	TF
F01_cb8564_c72884/f1p3/3343-0F	B3	TF
F01_cb8564_c73123/f1p0/2143-0F	mTERF	TR
F01_cb8564_c73163/f1p0/2503-2F	WRKY	TF
F01_cb8564_c73301/f1p6/2428-1F	MADS-MIKC	TF
F01_cb8564_c73359/f1p1/3245-2F	HB-HD-ZIP	TF
F01_cb8564_c73422/f1p0/3612-2F	SNF2	TR
F01_cb8564_c73924/f1p0/1935-2F	WRKY	TF
F01_cb8564_c74123/f1p1/4911-1F	Others	TR
F01_cb8564_c74150/f1p4/3524-1F	MADS-MIKC	TF
F01_cb8564_c74150/f1p4/3524-2R	HB-BELL	TF
F01_cb8564_c74345/f1p0/4390-2R	ARID	TR
F01_cb8564_c74520/f1p0/3076-1F	C2H2	TF
F01_cb8564_c74621/f1p0/2635-1F	MADS-M-type	TF
F01_cb8564_c74719/f1p0/2385-0F	C2H2	TF
F01_cb8564_c75099/f1p1/2485-2F	C2H2	TF
F01_cb8564_c75166/f1p1/2653-2F	C2H2	TF
F01_cb8564_c75382/f1p4/4637-0F	Others	TR
F01_cb8564_c75382/f1p4/4637-2F	Others	TR
F01_cb8564_c75436/f1p0/3300-2F	WRKY	TF

F01_cb8564_c75453/f1p2/2270-1F	GRAS	TF
F01_cb8564_c75479/f1p1/2639-2F	MADS-MIKC	TF
F01_cb8564_c75491/f1p0/3002-2F	GRAS	TF
F01_cb8564_c75742/f1p0/4694-1F	SET	TR
F01_cb8564_c75900/f1p3/2677-2F	HB-BELL	TF
F01_cb8564_c76081/f1p0/3554-0F	C2H2	TF
F01_cb8564_c76091/f2p0/3755-2F	MYB	TF
F01_cb8564_c76115/f1p0/2356-1F	WRKY	TF
F01_cb8564_c76250/f1p1/3808-0F	C2H2	TF
F01_cb8564_c76380/f1p1/3217-0R	HB-BELL	TF
F01_cb8564_c76434/f1p0/3111-0F	GRAS	TF
F01_cb8564_c76434/f1p0/3111-1F	GRAS	TF
F01_cb8564_c76434/f1p0/3111-2F	GRAS	TF
F01_cb8564_c76463/f1p1/4672-1F	Jumonji	TR
F01_cb8564_c76495/f1p0/3949-1F	bHLH	TF
F01_cb8564_c7674/f6p3/2437-0F	C2C2-CO-like	TF
F01_cb8564_c77044/f1p0/2776-1F	GRAS	TF
F01_cb8564_c77044/f1p0/2776-2F	GRAS	TF
F01_cb8564_c77055/f1p0/2184-2F	WRKY	TF
F01_cb8564_c77521/f1p0/2505-1F	HB-other	TF
F01_cb8564_c77529/f1p1/3590-0F	MYB	TF
F01_cb8564_c77529/f1p1/3590-2F	MYB-related	TF
F01_cb8564_c77748/f1p0/4086-0F	MADS-M-type	TF
F01_cb8564_c77748/f1p0/4086-1F	WRKY	TF
F01_cb8564_c77804/f4p0/2215-1F	WRKY	TF
F01_cb8564_c77886/f1p0/3549-2F	SBP	TF

F01_cb8564_c77970/f1p0/3545-1F	HB-HD-ZIP	TF
F01_cb8564_c77990/f1p0/2338-2F	HB-other	TF
F01_cb8564_c7800/f4p1/3109-1F	GRAS	TF
F01_cb8564_c78037/f1p1/3201-0R	MADS-MIKC	TF
F01_cb8564_c7835/f2p0/2745-0F	RWP-RK	TF
F01_cb8564_c7849/f2p0/2035-2F	C3H	TF
F01_cb8564_c78507/f1p0/4935-1F	mTERF	TR
F01_cb8564_c78894/f1p0/3527-2F	HB-PHD	TF
F01_cb8564_c78900/f1p3/4750-2F	Others	TR
F01_cb8564_c78972/f1p0/2676-2F	mTERF	TR
F01_cb8564_c79218/f4p0/4174-2F	SET	TR
F01_cb8564_c79543/f1p0/2014-1F	Trihelix	TF
F01_cb8564_c79566/f1p0/3645-1F	RWP-RK	TF
F01_cb8564_c79790/f1p0/3452-0F	Jumonji	TR
F01_cb8564_c79969/f2p0/4362-1F	Others	TR
F01_cb8564_c80291/f2p0/3316-2F	mTERF	TR
F01_cb8564_c80412/f1p0/2903-2F	Others	TR
F01_cb8564_c80882/f1p0/2524-1F	HB-BELL	TF
F01_cb8564_c81031/f1p0/3466-2F	RWP-RK	TF
F01_cb8564_c81286/f1p2/3636-2F	GRAS	TF
F01_cb8564_c81339/f1p0/2660-2F	STAT	TF
F01_cb8564_c81496/f1p0/4041-1F	WRKY	TF
F01_cb8564_c81654/f1p4/4945-1F	Others	TR
F01_cb8564_c81714/f3p1/2660-2F	C2H2	TF
F01_cb8564_c81913/f1p0/1929-2F	WRKY	TF
F01_cb8564_c81984/f1p0/2454-2F	WRKY	TF

F01_cb8564_c82013/f1p0/3323-2F	STAT	TF
F01_cb8564_c82382/f1p0/3622-1F	mTERF	TR
F01_cb8564_c82480/f1p0/2150-2F	MADS-M-type	TF
F01_cb8564_c82558/f1p0/3707-2F	HB-PHD	TF
F01_cb8564_c83008/f1p0/3371-0F	LUG	TR
F01_cb8564_c83336/f1p0/2732-0F	HB-BELL	TF
F01_cb8564_c83474/f1p1/3380-0F	Jumonji	TR
F01_cb8564_c83547/f1p5/3039-2F	HB-BELL	TF
F01_cb8564_c83566/f1p3/4576-2F	Others	TR
F01_cb8564_c83619/f1p0/2534-1F	STAT	TF
F01_cb8564_c83750/f1p0/3418-1F	RWP-RK	TF
F01_cb8564_c83974/f2p0/4733-2F	mTERF	TR
F01_cb8564_c84092/f1p0/2571-0F	Trihelix	TF
F01_cb8564_c84092/f1p0/2571-2F	Trihelix	TF
F01_cb8564_c84130/f1p2/3574-2F	SBP	TF
F01_cb8564_c844/f2p0/2586-0F	bHLH	TF
F01_cb8564_c84436/f1p0/2677-0F	PHD	TR
F01_cb8564_c84436/f1p0/2677-1F	HB-other	TF
F01_cb8564_c84457/f1p2/4101-2F	Others	TR
F01_cb8564_c84910/f1p0/3224-0F	mTERF	TR
F01_cb8564_c85001/f1p0/3853-2F	HB-PHD	TF
F01_cb8564_c85084/f1p0/1955-1F	TRAF	TR
F01_cb8564_c85255/f1p1/2783-1F	C2H2	TF
F01_cb8564_c86003/f1p0/2279-1F	Others	TR
F01_cb8564_c86076/f1p0/4428-2F	Others	TR
F01_cb8564_c86299/f1p1/3123-1F	Others	TR

F01_cb8564_c86299/f1p1/3123-2F	Others	TR
F01_cb8564_c86312/f1p1/2565-0F	Jumonji	TR
F01_cb8564_c86352/f2p0/2565-2F	HB-BELL	TF
F01_cb8564_c86441/f1p0/2175-0F	C2H2	TF
F01_cb8564_c86457/f1p0/4530-0F	MADS-M-type	TF
F01_cb8564_c86457/f1p0/4530-2F	MADS-M-type	TF
F01_cb8564_c86479/f1p0/3266-0F	Jumonji	TR
F01_cb8564_c865/f4p0/2761-0F	bHLH	TF
F01_cb8564_c86688/f1p0/3136-2F	Jumonji	TR
F01_cb8564_c86788/f1p0/2078-2F	WRKY	TF
F01_cb8564_c86873/f1p1/4866-2F	Others	TR
F01_cb8564_c86900/f1p0/3283-1F	SWI/SNF-BAF60b	TR
F01_cb8564_c87010/f1p2/2598-0F	HB-BELL	TF
F01_cb8564_c87256/f1p0/2285-2F	HB-BELL	TF
F01_cb8564_c87817/f1p0/3148-0F	LUG	TR
F01_cb8564_c88377/f1p1/4091-0F	RWP-RK	TF
F01_cb8564_c88377/f1p1/4091-1F	RWP-RK	TF
F01_cb8564_c88512/f1p0/4140-0F	Others	TR
F01_cb8564_c88512/f1p0/4140-1F	Others	TR
F01_cb8564_c88543/f1p0/3439-0F	PHD	TR
F01_cb8564_c88543/f1p0/3439-2F	HB-other	TF
F01_cb8564_c88616/f1p1/2497-0F	HB-other	TF
F01_cb8564_c88686/f1p0/3352-1F	SBP	TF
F01_cb8564_c88743/f1p0/1985-0F	mTERF	TR
F01_cb8564_c88743/f1p0/1985-2F	mTERF	TR
F01_cb8564_c88774/f1p2/3565-1F	SNF2	TR

F01_cb8564_c89502/f1p0/2946-0F	WRKY	TF
F01_cb8564_c89525/f1p0/3645-1F	HB-PHD	TF
F01_cb8564_c89737/f1p0/3593-2F	MADS-M-type	TF
F01_cb8564_c89871/f1p2/3359-2F	B3-ARF	TF
F01_cb8564_c90047/f1p0/3038-0F	Others	TR
F01_cb8564_c90047/f1p0/3038-1F	Others	TR
F01_cb8564_c90076/f1p1/3925-2F	STAT	TF
F01_cb8564_c90128/f1p0/3205-1F	Others	TR
F01_cb8564_c90316/f1p1/3596-2F	SBP	TF
F01_cb8564_c90424/f1p0/2623-2F	HB-BELL	TF
F01_cb8564_c90495/f1p1/4661-2F	Jumonji	TR
F01_cb8564_c90606/f1p0/2204-0F	C2H2	TF
F01_cb8564_c90653/f1p0/3252-0F	WRKY	TF
F01_cb8564_c90653/f1p0/3252-1F	WRKY	TF
F01_cb8564_c90653/f1p0/3252-2F	WRKY	TF
F01_cb8564_c90906/f1p0/3670-2F	Jumonji	TR
F01_cb8564_c910/f6p2/2725-1F	HB-BELL	TF
F01_cb8564_c91136/f1p0/3087-2F	B3-ARF	TF
F01_cb8564_c91169/f1p0/3575-2F	SNF2	TR
F01_cb8564_c91176/f1p0/2480-0F	MADS-M-type	TF
F01_cb8564_c91176/f1p0/2480-2F	MADS-M-type	TF
F01_cb8564_c91216/f1p0/2554-0F	C2H2	TF
F01_cb8564_c9126/f4p0/2131-2F	WRKY	TF
F01_cb8564_c91354/f1p0/3094-0F	C2H2	TF
F01_cb8564_c91515/f1p0/3340-0R	MADS-MIKC	TF
F01_cb8564_c91515/f1p0/3340-2F	HB-other	TF

F01_cb8564_c91515/f1p0/3340-2R	MADS-M-type	TF
F01_cb8564_c91942/f1p0/3111-2F	mTERF	TR
F01_cb8564_c92050/f1p0/1905-1F	mTERF	TR
F01_cb8564_c92050/f1p0/1905-2F	mTERF	TR
F01_cb8564_c92199/f1p0/2079-0F	mTERF	TR
F01_cb8564_c9224/f5p1/2112-0F	WRKY	TF
F01_cb8564_c92362/f1p0/3943-1R	FAR1	TF
F01_cb8564_c9265/f3p1/3275-2F	B3-ARF	TF
F01_cb8564_c927/f7p2/2538-1F	FAR1	TF
F01_cb8564_c93615/f1p2/1871-0F	HB-HD-ZIP	TF
F01_cb8564_c9461/f3p0/2447-0F	WRKY	TF
F01_cb8564_c9657/f4p0/1983-0F	WRKY	TF
F01_cb8564_c9705/f9p0/2288-2F	TRAF	TR
F01_cb8564_c971/f3p0/2749-0F	GRAS	TF
F01_cb8564_c971/f3p0/2749-1F	GRAS	TF
F01_cb8564_c9798/f7p1/3585-1F	MYB	TF
F01_cb8564_c98278/f5p6/2387-1F	NAC	TF
F01_cb8564_c98300/f2p1/2186-1F	WRKY	TF
F01_cb8564_c9837/f2p0/4515-2F	bHLH	TF
F01_cb8564_c9866/f2p0/3472-2F	GRAS	TF
F01_cb8564_c987/f3p1/1950-0F	RWP-RK	TF
F01_cb8576_c1/f2p0/2240-1F	GARP-ARR-B	TF
F01_cb8576_c3/f1p0/2311-2F	Others	TR
F01_cb8576_c4/f1p0/2300-2F	GARP-ARR-B	TF
F01_cb8576_c6/f1p0/2313-0F	GARP-ARR-B	TF
F01_cb8586_c3/f1p1/2345-0F	C3H	TF

F01_cb8586_c5/f1p1/2288-1F	C3H	TF
F01_cb8586_c5/f1p1/2288-2F	C3H	TF
F01_cb8586_c9/f1p3/1411-2F	C3H	TF
F01_cb8590_c1/f1p0/2319-2F	C2C2-CO-like	TF
F01_cb8590_c2/f1p0/2063-2F	C2C2-CO-like	TF
F01_cb8590_c3/f1p0/2298-1F	C2C2-CO-like	TF
F01_cb8590_c5/f1p0/2246-0F	Others	TR
F01_cb8590_c8/f3p0/1964-2F	C2C2-CO-like	TF
F01_cb8606_c2/f1p0/2322-0F	C2C2-Dof	TF
F01_cb8606_c5/f2p0/2106-2F	C2C2-Dof	TF
F01_cb8620_c0/f1p0/2318-0F	mTERF	TR
F01_cb8620_c1/f1p0/2443-1F	mTERF	TR
F01_cb8643_c1/f1p0/2309-1F	C2C2-CO-like	TF
F01_cb8643_c2/f1p0/2364-2F	Others	TR
F01_cb8643_c3/f1p0/1354-1F	Others	TR
F01_cb8643_c5/f1p0/1313-1F	C2C2-CO-like	TF
F01_cb8650_c0/f3p0/2134-1R	B3-ARF	TF
F01_cb8650_c2/f1p0/2263-1R	B3	TF
F01_cb8650_c3/f1p0/2250-0R	AUX/IAA	TR
F01_cb8650_c3/f1p0/2250-2R	B3-ARF	TF
F01_cb8650_c4/f1p1/2111-1R	AUX/IAA	TR
F01_cb8650_c4/f1p1/2111-2R	B3-ARF	TF
F01_cb8662_c0/f4p0/2191-0F	Others	TR
F01_cb8662_c12/f1p0/2017-0F	Others	TR
F01_cb8662_c13/f1p0/2219-1F	Others	TR
F01_cb8662_c2/f1p0/2305-1F	Others	TR

F01_cb8662_c3/f1p0/2862-0F	Others	TR
F01_cb8662_c4/f1p0/1962-1F	Others	TR
F01_cb8662_c5/f1p0/2097-1F	Others	TR
F01_cb8662_c6/f1p0/2072-1F	Others	TR
F01_cb8662_c8/f1p0/2813-1F	Others	TR
F01_cb8662_c9/f1p1/2326-0F	Others	TR
F01_cb8735_c0/f1p0/2263-2F	SET	TR
F01_cb8736_c0/f1p0/1765-1F	C2C2-Dof	TF
F01_cb8736_c1/f1p0/2285-1F	C2C2-Dof	TF
F01_cb8736_c3/f1p0/2166-0F	C2C2-Dof	TF
F01_cb8736_c4/f1p0/1975-2F	C2C2-Dof	TF
F01_cb8736_c5/f1p0/1859-1F	C2C2-Dof	TF
F01_cb8736_c6/f1p0/1826-1F	C2C2-Dof	TF
F01_cb8736_c7/f1p0/1795-2F	C2C2-Dof	TF
F01_cb8742_c0/f1p0/2279-2F	AP2/ERF-ERF	TF
F01_cb8742_c2/f1p0/2097-0F	AP2/ERF-AP2	TF
F01_cb8742_c3/f1p0/2145-0F	AP2/ERF-ERF	TF
F01_cb8742_c4/f1p0/2053-2F	AP2/ERF-AP2	TF
F01_cb8743_c0/f3p0/2049-0F	MYB-related	TF
F01_cb8743_c1/f2p0/1977-0F	MYB-related	TF
F01_cb8743_c2/f1p0/2279-1F	MYB-related	TF
F01_cb8743_c3/f1p0/2167-1F	MYB-related	TF
F01_cb8748_c1/f2p0/1899-2F	AP2/ERF-ERF	TF
F01_cb8748_c3/f1p0/1911-0F	AP2/ERF-ERF	TF
F01_cb8748_c5/f1p1/1962-0F	AP2/ERF-ERF	TF
F01_cb8754_c0/f3p0/1561-0F	C2C2-CO-like	TF

F01_cb8754_c3/f1p0/1687-2F	C2C2-CO-like	TF
F01_cb8754_c4/f1p0/1667-1F	Others	TR
F01_cb8829_c0/f2p0/2146-1F	C2C2-Dof	TF
F01_cb8829_c1/f1p0/2259-2F	C2C2-Dof	TF
F01_cb8829_c3/f1p0/2284-0F	C2C2-Dof	TF
F01_cb8845_c0/f2p0/2260-2F	C2H2	TF
F01_cb8857_c2/f1p0/2216-2F	Trihelix	TF
F01_cb8857_c3/f1p1/2039-0F	Trihelix	TF
F01_cb8857_c4/f1p0/2204-2F	Trihelix	TF
F01_cb8857_c9/f5p1/2109-0F	Trihelix	TF
F01_cb8888_c0/f1p0/2243-0F	mTERF	TR
F01_cb8888_c1/f1p0/2099-1F	mTERF	TR
F01_cb8888_c1/f1p0/2099-2F	mTERF	TR
F01_cb8888_c2/f1p0/2242-2F	mTERF	TR
F01_cb8923_c0/f1p0/2224-0F	WRKY	TF
F01_cb8923_c1/f1p0/2166-2F	WRKY	TF
F01_cb8930_c0/f1p0/2228-2F	Tify	TF
F01_cb8930_c1/f1p0/2241-0F	Tify	TF
F01_cb8930_c2/f1p0/1839-2F	Tify	TF
F01_cb8930_c3/f1p0/1794-0F	Tify	TF
F01_cb8930_c4/f1p0/1315-0F	Tify	TF
F01_cb8939_c1/f4p1/1236-2F	Tify	TF
F01_cb8939_c10/f1p0/1284-2F	Tify	TF
F01_cb8939_c14/f1p0/8770-2F	Tify	TF
F01_cb8939_c16/f3p1/1296-1F	Tify	TF
F01_cb8939_c18/f1p1/1256-2F	Tify	TF

F01_cb8939_c4/f2p0/2227-2F	Tify	TF
F01_cb8939_c5/f1p0/2247-0F	Tify	TF
F01_cb8939_c6/f1p0/2221-1F	Tify	TF
F01_cb8939_c7/f1p2/1298-0F	Tify	TF
F01_cb893_c0/f2p0/10966-2F	SNF2	TR
F01_cb8954_c17/f1p0/1263-2F	GNAT	TR
F01_cb8970_c1/f1p0/2482-0F	MYB-related	TF
F01_cb8970_c2/f1p0/1764-0F	MYB-related	TF
F01_cb8995_c10/f1p0/2440-0F	SWI/SNF-BAF60b	TR
F01_cb8995_c11/f1p0/2278-2F	SWI/SNF-BAF60b	TR
F01_cb8995_c4/f1p0/2502-2F	SWI/SNF-BAF60b	TR
F01_cb8995_c5/f1p0/2524-2F	SWI/SNF-BAF60b	TR
F01_cb8995_c6/f1p0/2230-2F	SWI/SNF-BAF60b	TR
F01_cb8995_c8/f1p0/2000-1F	SWI/SNF-BAF60b	TR
F01_cb9016_c0/f5p0/2211-2F	RWP-RK	TF
F01_cb9016_c5/f1p0/1921-0F	RWP-RK	TF
F01_cb9016_c9/f1p0/2025-0F	RWP-RK	TF
F01_cb9034_c0/f1p0/2207-0F	C2C2-Dof	TF
F01_cb9034_c2/f1p0/2069-1F	C2C2-Dof	TF
F01_cb9049_c4/f1p1/1367-0F	HB-other	TF
F01_cb9049_c6/f1p0/1253-2F	HB-other	TF
F01_cb9079_c11/f1p0/1031-2F	C3H	TF
F01_cb9103_c0/f2p0/1625-0F	bZIP	TF
F01_cb9103_c2/f1p0/2165-0F	bZIP	TF
F01_cb9125_c11/f1p0/1951-0F	bHLH	TF
F01_cb9125_c20/f1p1/1894-1F	bHLH	TF

F01_cb9125_c7/f1p0/2617-1F	bHLH	TF
F01_cb9125_c8/f1p0/1957-1F	bHLH	TF
F01_cb9125_c9/f1p0/2006-1F	bHLH	TF
F01_cb9141_c0/f2p0/1528-0F	bZIP	TF
F01_cb9141_c2/f1p0/1896-2F	bZIP	TF
F01_cb9141_c3/f1p0/1793-2F	bZIP	TF
F01_cb9141_c4/f1p0/1781-1F	bZIP	TF
F01_cb9141_c5/f1p0/1620-0F	bZIP	TF
F01_cb9165_c0/f1p0/2184-2F	AP2/ERF-ERF	TF
F01_cb9165_c1/f1p0/1910-0F	AP2/ERF-ERF	TF
F01_cb9165_c1/f1p0/1910-2F	AP2/ERF-ERF	TF
F01_cb9189_c2/f2p0/2107-0F	Whirly	TF
F01_cb9189_c3/f2p0/1312-1F	Whirly	TF
F01_cb9189_c4/f1p0/2159-2F	Whirly	TF
F01_cb9189_c5/f1p0/1254-1F	Whirly	TF
F01_cb9189_c8/f2p0/1131-0F	Whirly	TF
F01_cb9191_c0/f1p0/2159-0F	MADS-M-type	TF
F01_cb9192_c3/f1p0/1755-0F	GNAT	TR
F01_cb9192_c4/f1p0/1696-0F	GNAT	TR
F01_cb9228_c0/f4p1/2141-1F	bZIP	TF
F01_cb9228_c2/f1p0/3917-0F	bZIP	TF
F01_cb9228_c5/f1p1/2025-0F	bZIP	TF
F01_cb9237_c15/f1p0/2147-1F	AP2/ERF-ERF	TF
F01_cb9237_c16/f1p2/2049-2F	AP2/ERF-ERF	TF
F01_cb9237_c5/f1p1/1918-2F	AP2/ERF-ERF	TF
F01_cb9237_c6/f1p0/2527-0F	AP2/ERF-ERF	TF

F01_cb9237_c7/f1p0/1965-0F	AP2/ERF-ERF	TF
F01_cb9294_c0/f1p0/1892-1F	C3H	TF
F01_cb9294_c1/f2p0/1458-0F	C3H	TF
F01_cb9294_c2/f1p0/2126-2F	C3H	TF
F01_cb9297_c3/f1p1/1908-0F	SET	TR
F01_cb92_c10/f1p1/5011-1F	Others	TR
F01_cb92_c11/f1p0/4947-1F	Others	TR
F01_cb92_c11/f1p0/4947-2F	Others	TR
F01_cb92_c2/f1p0/4936-1F	Others	TR
F01_cb92_c2/f1p0/4936-2F	Others	TR
F01_cb92_c8/f1p0/5075-0F	Others	TR
F01_cb9317_c10/f1p1/1720-2F	AUX/IAA	TR
F01_cb9317_c11/f1p1/1656-2F	AUX/IAA	TR
F01_cb9317_c12/f1p0/1624-0F	AUX/IAA	TR
F01_cb9317_c12/f1p0/1624-1F	AUX/IAA	TR
F01_cb9317_c5/f2p3/1769-0F	AUX/IAA	TR
F01_cb9317_c6/f1p0/2118-0F	AUX/IAA	TR
F01_cb9317_c7/f1p0/4812-0F	AUX/IAA	TR
F01_cb9317_c7/f1p0/4812-2F	AUX/IAA	TR
F01_cb9317_c8/f1p0/1919-1F	AUX/IAA	TR
F01_cb9317_c9/f1p1/1734-0F	AUX/IAA	TR
F01_cb9325_c0/f1p0/2118-0R	C2H2	TF
F01_cb9325_c1/f1p0/2784-1F	HMG	TR
F01_cb9325_c1/f1p0/2784-2R	C2H2	TF
F01_cb9326_c0/f1p0/2116-2F	Trihelix	TF
F01_cb9377_c0/f1p0/2020-0F	TRAF	TR

F01_cb9377_c1/f1p0/1909-2F	TRAF	TR
F01_cb9377_c2/f1p0/1657-0F	TRAF	TR
F01_cb9377_c4/f1p0/1554-2F	TRAF	TR
F01_cb9393_c0/f2p0/1980-1F	C3H	TF
F01_cb9393_c2/f1p0/2047-1F	C3H	TF
F01_cb9393_c5/f1p0/1658-0F	C3H	TF
F01_cb9393_c5/f1p0/1658-2F	C3H	TF
F01_cb9416_c0/f5p0/2093-1F	WRKY	TF
F01_cb9416_c2/f1p0/2094-0F	WRKY	TF
F01_cb9416_c5/f1p0/2153-0F	WRKY	TF
F01_cb9416_c7/f1p0/1853-1F	WRKY	TF
F01_cb9416_c8/f1p0/1831-2F	WRKY	TF
F01_cb9422_c2/f1p0/2085-0F	FAR1	TF
F01_cb9422_c2/f1p0/2085-2F	FAR1	TF
F01_cb9436_c10/f1p0/2077-2F	WRKY	TF
F01_cb9436_c13/f1p0/2211-1F	WRKY	TF
F01_cb9436_c13/f1p0/2211-2F	WRKY	TF
F01_cb9436_c14/f3p0/2049-0F	WRKY	TF
F01_cb9436_c14/f3p0/2049-2F	WRKY	TF
F01_cb9436_c4/f1p0/2516-0F	WRKY	TF
F01_cb9437_c0/f1p0/2082-2F	TRAF	TR
F01_cb9437_c1/f1p0/2267-2F	TRAF	TR
F01_cb947_c12/f1p0/2397-2F	SET	TR
F01_cb9482_c1/f1p0/2038-1F	MYB-related	TF
F01_cb9482_c1/f1p0/2038-2F	MYB-related	TF
F01_cb9482_c4/f1p0/1339-1F	MYB	TF

F01_cb9504_c0/f2p0/1821-2F	GARP-G2-like	TF
F01_cb9504_c1/f1p0/1994-0F	GARP-G2-like	TF
F01_cb9504_c2/f1p0/1693-0F	GARP-G2-like	TF
F01_cb9505_c0/f1p0/2059-0F	SET	TR
F01_cb9505_c1/f1p0/2153-0F	SET	TR
F01_cb9505_c2/f1p0/1943-0F	SET	TR
F01_cb9523_c3/f1p0/1657-1F	C2C2-GATA	TF
F01_cb9549_c0/f3p0/1849-2F	NAC	TF
F01_cb9549_c1/f2p0/1736-1F	NAC	TF
F01_cb9549_c2/f1p0/2047-2F	NAC	TF
F01_cb9549_c3/f1p0/1890-2F	NAC	TF
F01_cb9549_c4/f1p0/1874-0F	NAC	TF
F01_cb9549_c6/f1p0/1758-0F	NAC	TF
F01_cb9549_c7/f1p0/1719-2F	NAC	TF
F01_cb9549_c8/f1p0/1542-2F	NAC	TF
F01_cb9570_c0/f1p0/2043-1F	GRF	TF
F01_cb9570_c1/f1p0/2120-1F	GRF	TF
F01_cb9573_c0/f3p0/2033-0F	E2F-DP	TF
F01_cb9584_c2/f3p0/1964-0F	GRAS	TF
F01_cb9584_c3/f5p0/1956-0F	GRAS	TF
F01_cb9584_c3/f5p0/1956-1F	GRAS	TF
F01_cb9585_c4/f5p2/1968-0F	TCP	TF
F01_cb9596_c0/f3p0/1935-0F	GRAS	TF
F01_cb9596_c0/f3p0/1935-1F	GRAS	TF
F01_cb9596_c3/f1p0/1889-0F	GRAS	TF
F01_cb9596_c4/f1p0/1930-1F	GRAS	TF

F01_cb9597_c0/f3p0/1932-2F	HB-KNOX	TF
F01_cb9597_c1/f3p0/1890-1F	HB-KNOX	TF
F01_cb9597_c3/f2p0/1924-0F	HB-KNOX	TF
F01_cb9597_c5/f1p0/1754-1F	HB-other	TF
F01_cb9597_c7/f1p0/1069-1F	HB-other	TF
F01_cb9621_c12/f1p0/899-2F	MYB	TF
F01_cb9621_c14/f4p0/2004-2F	MYB	TF
F01_cb9621_c4/f2p1/1113-0F	MYB	TF
F01_cb9621_c7/f1p1/1114-0F	MYB	TF
F01_cb9621_c8/f1p0/1100-0F	MYB	TF
F01_cb9632_c0/f1p0/2027-1F	SET	TR
F01_cb9632_c1/f1p0/1923-2F	SET	TR
F01_cb9632_c2/f1p0/1999-1F	SET	TR
F01_cb9644_c10/f1p0/1952-0F	GNAT	TR
F01_cb9644_c3/f1p1/3224-0F	GNAT	TR
F01_cb9644_c4/f1p1/2067-1F	GNAT	TR
F01_cb9644_c7/f1p0/2639-1F	GNAT	TR
F01_cb9653_c4/f1p1/1931-2F	TRAF	TR
F01_cb9673_c1/f1p2/2014-1F	DBP	TF
F01_cb9673_c3/f1p0/1890-1F	DBP	TF
F01_cb9692_c0/f3p0/1992-0F	bHLH	TF
F01_cb9692_c1/f1p0/1801-1F	bHLH	TF
F01_cb9693_c0/f1p0/2046-0F	SET	TR
F01_cb9693_c1/f1p0/1933-1F	SET	TR
F01_cb9693_c2/f1p0/1900-0F	SET	TR
F01_cb9714_c2/f1p0/2093-2F	C2H2	TF

F01_cb9714_c3/f1p0/2243-0F	C2H2	TF
F01_cb9727_c1/f1p0/1995-1F	B3	TF
F01_cb9727_c2/f1p0/2172-2F	B3	TF
F01_cb9727_c4/f1p1/1862-0F	B3	TF
F01_cb9735_c3/f1p0/2913-0F	Trihelix	TF
F01_cb9738_c2/f1p0/1750-2F	mTERF	TR
F01_cb9738_c3/f2p0/1998-0F	mTERF	TR
F01_cb9738_c4/f1p0/1937-2F	mTERF	TR
F01_cb9745_c0/f1p0/1990-2F	MADS-M-type	TF
F01_cb9745_c1/f1p0/2229-0F	MADS-MIKC	TF
F01_cb9745_c2/f1p0/1545-0F	MADS-M-type	TF
F01_cb9745_c3/f1p0/1260-0F	MADS-MIKC	TF
F01_cb9749_c2/f1p0/1908-2F	TCP	TF
F01_cb9749_c3/f1p0/1799-0F	TCP	TF
F01_cb9749_c6/f1p0/1269-0F	TCP	TF
F01_cb9751_c2/f1p0/2163-0F	HSF	TF
F01_cb9751_c4/f1p0/1614-2F	HSF	TF
F01_cb9771_c0/f1p0/1982-0F	SET	TR
F01_cb9771_c1/f1p1/2599-0F	SET	TR
F01_cb9772_c1/f1p0/1982-1F	GARP-G2-like	TF
F01_cb9772_c7/f1p0/1217-2F	GARP-G2-like	TF
F01_cb9777_c0/f2p0/1880-1F	bHLH	TF
F01_cb9777_c1/f2p0/1937-1F	bHLH	TF
F01_cb9777_c2/f1p0/1970-0F	bHLH	TF
F01_cb9777_c3/f1p0/1950-2F	bHLH	TF
F01_cb9777_c5/f1p0/799-0F	bHLH	TF

F01_cb9815_c2/f1p0/2812-0F	C3H	TF
F01_cb9815_c3/f1p0/1942-0F	C3H	TF
F01_cb9815_c4/f1p0/1909-1F	C3H	TF
F01_cb9816_c2/f2p1/1967-0F	bHLH	TF
F01_cb9816_c3/f1p1/1924-2F	bHLH	TF
F01_cb9821_c10/f3p1/1861-1F	TUB	TF
F01_cb9821_c4/f1p1/2016-0F	TUB	TF
F01_cb9821_c7/f1p1/1778-2F	TUB	TF
F01_cb9833_c0/f1p0/1962-0R	AUX/IAA	TR
F01_cb9833_c1/f1p0/3484-0R	AUX/IAA	TR
F01_cb9833_c1/f1p0/3484-1R	AUX/IAA	TR
F01_cb9833_c1/f1p0/3484-2R	AUX/IAA	TR
F01_cb9838_c0/f2p0/1881-0F	HB-other	TF
F01_cb9838_c2/f1p0/1913-2F	HB-KNOX	TF
F01_cb9839_c0/f3p0/1910-1F	C3H	TF
F01_cb9842_c0/f1p0/1957-2F	DBP	TF
F01_cb9853_c0/f1p0/1951-1F	NAC	TF
F01_cb9853_c2/f1p0/1484-0F	NAC	TF
F01_cb9864_c0/f1p0/1948-2F	CPP	TF
F01_cb9864_c1/f1p0/2873-2F	CPP	TF
F01_cb9931_c0/f2p0/1899-1F	mTERF	TR
F01_cb9952_c0/f4p1/1799-2F	C3H	TF
F01_cb9952_c11/f3p1/1866-0F	C3H	TF
F01_cb9952_c2/f3p1/1779-2F	C3H	TF
F01_cb9952_c6/f1p0/1699-1F	C3H	TF
F01_cb9952_c7/f1p0/1663-0F	C3H	TF

F01_cb9952_c7/f1p0/1663-2F	C3H	TF
F01_cb9981_c10/f1p0/1696-1F	bZIP	TF
F01_cb9981_c12/f1p1/5105-0F	bZIP	TF
F01_cb9981_c12/f1p1/5105-2F	bZIP	TF
F01_cb9981_c2/f2p1/1754-0F	bZIP	TF
F01_cb9981_c3/f1p0/1893-2F	bZIP	TF
F01_cb9981_c4/f1p0/2004-2F	bZIP	TF
F01_cb9981_c5/f1p0/1871-2F	bZIP	TF
F01_cb9981_c6/f1p1/1869-0F	bZIP	TF
F01_cb9981_c7/f1p2/1848-2F	bZIP	TF
F01_cb9981_c8/f1p0/1812-2F	bZIP	TF
F01_cb9981_c9/f1p1/1748-2F	bZIP	TF
F01_cb9_c0/f2p0/2178-2F	PHD	TR
F01_cb9_c2/f1p0/2212-1F	PHD	TR
F01_cb9_c3/f1p0/2562-0F	PHD	TR
F01_cb9_c4/f1p0/2454-1F	PHD	TR
F01_cb9_c5/f1p0/2249-0F	PHD	TR
F01_cb10030_c23/f1p1/3564-0R	CMGC_CDK-CRK7-CDK9	PK
F01_cb10096_c0/f4p0/1788-0F	RLK-Pelle_RLCK-VIII	PK
F01_cb10096_c7/f1p0/1710-2F	RLK-Pelle_RLCK-VIII	PK
F01_cb10096_c8/f1p0/1775-0F	RLK-Pelle_RLCK-VIII	PK
F01_cb10096_c8/f1p0/1775-1F	RLK-Pelle_RLCK-VIII	PK
F01_cb10135_c1/f2p0/1634-1F	CMGC_CDK-CCRK	PK
F01_cb10135_c3/f1p0/1799-2F	CMGC_CDK-CCRK	PK
F01_cb10266_c4/f1p0/2826-0F	RLK-Pelle_RLCK-VIIa-2	PK
F01_cb10266_c5/f1p0/1940-0F	RLK-Pelle_RLCK-VIIa-2	PK

F01_cb10266_c6/f1p0/1904-1F	RLK-Pelle_RLCK-VIIa-2	PK
F01_cb10266_c7/f1p0/1776-1F	RLK-Pelle_RLCK-VIIa-2	PK
F01_cb10266_c8/f2p0/1770-1F	RLK-Pelle_RLCK-VIIa-2	PK
F01_cb1026_c0/f1p0/4508-2F	RLK-Pelle_LRR-XIV	PK
F01_cb1026_c3/f1p0/3846-2F	RLK-Pelle_LRR-XIV	PK
F01_cb10280_c0/f19p0/1256-1F	CMGC_CDK-Pl	PK
F01_cb10280_c1/f1p0/2387-0F	CMGC_CDK-Pl	PK
F01_cb10280_c1/f1p0/2387-1F	CMGC_CDK-Pl	PK
F01_cb10280_c3/f1p0/1292-0F	CMGC_CDK-Pl	PK
F01_cb10280_c4/f1p0/1445-0F	CMGC_CDK-Pl	PK
F01_cb10280_c5/f1p0/1799-1F	CMGC_CDK-Pl	PK
F01_cb10323_c2/f1p0/2774-1R	RLK-Pelle_RLCK-XI	PK
F01_cb10334_c0/f11p0/1199-1F	CAMK_CDPK	PK
F01_cb10334_c2/f2p0/966-2F	CAMK_CDPK	PK
F01_cb10334_c4/f1p0/2353-0F	CAMK_CDPK	PK
F01_cb10334_c6/f1p0/999-0F	CAMK_CDPK	PK
F01_cb10334_c7/f1p0/1022-0F	CAMK_CDPK	PK
F01_cb10452_c10/f1p0/1588-2F	CAMK_OST1L	PK
F01_cb10452_c12/f13p0/1712-1F	CAMK_OST1L	PK
F01_cb10452_c3/f1p0/2040-1R	CAMK_OST1L	PK
F01_cb10452_c3/f1p0/2040-2F	CAMK_OST1L	PK
F01_cb10452_c7/f1p0/1508-1F	CAMK_OST1L	PK
F01_cb10452_c8/f1p0/1461-2F	CAMK_OST1L	PK
F01_cb10573_c0/f2p0/1782-0F	Group-Pl-4	PK
F01_cb10688_c1/f6p2/1552-1F	TKL-Pl-4	PK
F01_cb10688_c2/f1p2/1627-2F	TKL-Pl-4	PK

F01_cb10688_c3/f1p1/2522-0F	TKL-Pl-4	PK
F01_cb10688_c5/f1p1/1076-2F	TKL-Pl-4	PK
F01_cb10688_c6/f1p1/1588-1F	TKL-Pl-4	PK
F01_cb1069_c0/f1p0/4487-0F	ULK_ULK4	PK
F01_cb1069_c0/f1p0/4487-1F	ULK_ULK4	PK
F01_cb10705_c12/f1p0/1220-2F	CAMK_CAMKL-CHK1	PK
F01_cb10705_c14/f1p0/1879-1F	CAMK_CAMKL-CHK1	PK
F01_cb10705_c14/f1p0/1879-2F	CAMK_CAMKL-CHK1	PK
F01_cb10705_c17/f3p0/1800-2F	CAMK_CAMKL-CHK1	PK
F01_cb1077_c13/f1p0/2261-1F	STE_STE11	PK
F01_cb1077_c14/f1p0/3474-0F	STE_STE11	PK
F01_cb1077_c27/f1p0/3149-1F	STE_STE11	PK
F01_cb1077_c30/f1p0/2226-0F	STE_STE11	PK
F01_cb1077_c32/f1p0/2415-1F	STE_STE11	PK
F01_cb1077_c39/f1p0/2520-2F	STE_STE11	PK
F01_cb1077_c44/f1p1/2498-1F	STE_STE11	PK
F01_cb1077_c45/f6p0/2505-1F	STE_STE11	PK
F01_cb10821_c0/f2p1/1740-0F	CMGC_MAPK	PK
F01_cb10821_c2/f1p0/1561-0F	CMGC_MAPK	PK
F01_cb10821_c3/f1p0/1928-1F	CMGC_MAPK	PK
F01_cb10821_c6/f1p1/1816-0F	CMGC_MAPK	PK
F01_cb10821_c6/f1p1/1816-2F	CMGC_MAPK	PK
F01_cb10847_c1/f1p0/2847-1F	RLK-Pelle_CR4L	PK
F01_cb10847_c2/f1p0/1850-2F	RLK-Pelle_CR4L	PK
F01_cb10925_c2/f1p0/2405-2R	RLK-Pelle_LRR-XIIIa	PK
F01_cb10939_c0/f3p0/1637-2F	RLK-Pelle_DLSV	PK

F01_cb10939_c1/f3p0/1721-1F	RLK-Pelle_DLSV	PK
F01_cb10939_c2/f1p0/2519-0F	RLK-Pelle_DLSV	PK
F01_cb10939_c2/f1p0/2519-1F	RLK-Pelle_DLSV	PK
F01_cb10939_c2/f1p0/2519-2F	RLK-Pelle_DLSV	PK
F01_cb10939_c3/f1p0/1565-2F	RLK-Pelle_DLSV	PK
F01_cb10939_c4/f1p0/1593-1F	RLK-Pelle_DLSV	PK
F01_cb10939_c5/f1p0/1684-2F	RLK-Pelle_DLSV	PK
F01_cb10939_c6/f1p0/5099-1F	RLK-Pelle_DLSV	PK
F01_cb10939_c6/f1p0/5099-2F	RLK-Pelle_DLSV	PK
F01_cb10965_c0/f3p0/1720-0F	CMGC_GSK	PK
F01_cb10965_c4/f1p1/1818-2F	CMGC_GSK	PK
F01_cb10965_c5/f1p0/1651-0F	CMGC_GSK	PK
F01_cb10965_c5/f1p0/1651-1F	CMGC_GSK	PK
F01_cb10989_c1/f9p1/2579-1F	WNK_NRBP	PK
F01_cb10989_c10/f1p0/3595-0F	WNK_NRBP	PK
F01_cb10989_c11/f1p0/3363-2F	WNK_NRBP	PK
F01_cb10989_c12/f1p0/3180-0F	WNK_NRBP	PK
F01_cb10989_c12/f1p0/3180-1F	WNK_NRBP	PK
F01_cb10989_c16/f1p1/2700-2F	WNK_NRBP	PK
F01_cb10989_c17/f1p1/2555-0F	WNK_NRBP	PK
F01_cb10989_c8/f1p0/3471-0F	WNK_NRBP	PK
F01_cb11079_c4/f1p0/1706-2F	STE_STE7	PK
F01_cb1107_c11/f1p0/6048-0F	TKL-Pl-2	PK
F01_cb1107_c3/f1p0/4155-2F	TKL-Pl-2	PK
F01_cb1107_c6/f1p0/4264-1F	TKL-Pl-2	PK
F01_cb1110_c0/f5p2/1816-2F	RLK-Pelle_RLCK-VIII	PK

F01_cb1110_c11/f1p0/1830-1F	RLK-Pelle_RLCK-VIII	PK
F01_cb1110_c13/f1p0/1784-0F	RLK-Pelle_RLCK-VIII	PK
F01_cb1110_c13/f1p0/1784-1F	RLK-Pelle_RLCK-VIII	PK
F01_cb1110_c21/f4p0/1704-2F	RLK-Pelle_RLCK-VIII	PK
F01_cb1110_c23/f3p0/1698-0F	RLK-Pelle_RLCK-VIII	PK
F01_cb1110_c7/f1p1/4474-0F	RLK-Pelle_RLCK-VIII	PK
F01_cb1110_c8/f1p0/2290-2F	RLK-Pelle_RLCK-VIII	PK
F01_cb1110_c9/f1p1/1847-1F	RLK-Pelle_RLCK-VIII	PK
F01_cb11232_c3/f1p0/2508-0R	CAMK_CDPK	PK
F01_cb11247_c0/f2p0/1571-2F	RLK-Pelle_DLSV	PK
F01_cb11247_c1/f1p0/2876-0F	RLK-Pelle_DLSV	PK
F01_cb11247_c1/f1p0/2876-2F	RLK-Pelle_DLSV	PK
F01_cb11272_c0/f1p0/1684-1F	RLK-Pelle_RLCK-VIIa-2	PK
F01_cb11309_c1/f1p0/2641-0F	WNK_NRBP	PK
F01_cb11309_c1/f1p0/2641-1F	WNK_NRBP	PK
F01_cb11309_c2/f1p0/1295-0F	WNK_NRBP	PK
F01_cb11309_c2/f1p0/1295-2F	WNK_NRBP	PK
F01_cb11309_c4/f1p1/1476-2F	WNK_NRBP	PK
F01_cb11355_c0/f2p0/1740-1F	RLK-Pelle_DLSV	PK
F01_cb11355_c1/f1p0/2062-0F	RLK-Pelle_DLSV	PK
F01_cb11355_c1/f1p0/2062-1F	RLK-Pelle_DLSV	PK
F01_cb11355_c2/f1p0/1643-1F	RLK-Pelle_DLSV	PK
F01_cb11388_c0/f1p0/1626-1F	RLK-Pelle_RLCK-VIIa-2	PK
F01_cb1140_c11/f1p0/3479-0F	TKL_CTR1-DRK-2	PK
F01_cb1140_c19/f1p0/2766-1F	TKL_CTR1-DRK-2	PK
F01_cb1140_c2/f5p0/3323-1F	TKL_CTR1-DRK-2	PK

F01_cb1140_c24/f1p0/3830-0F	TKL_CTR1-DRK-2	PK
F01_cb1140_c24/f1p0/3830-1F	TKL_CTR1-DRK-2	PK
F01_cb1140_c28/f1p0/3350-2F	TKL_CTR1-DRK-2	PK
F01_cb1140_c32/f8p2/3463-0F	TKL_CTR1-DRK-2	PK
F01_cb11577_c1/f1p0/2827-0F	RLK-Pelle_SD-2b	PK
F01_cb11623_c0/f1p0/1017-1F	RLK-Pelle_RLCK-IV	PK
F01_cb11623_c1/f1p0/4995-0F	RLK-Pelle_RLCK-IV	PK
F01_cb11726_c1/f1p0/2400-1R	RLK-Pelle_RLCK-VIIa-1	PK
F01_cb11726_c1/f1p0/2400-2R	RLK-Pelle_RLCK-VIIa-1	PK
F01_cb11735_c1/f1p0/3700-1F	RLK-Pelle_LRR-VII-3	PK
F01_cb11735_c1/f1p0/3700-2F	RLK-Pelle_LRR-VII-3	PK
F01_cb11783_c0/f1p0/1744-1F	RLK-Pelle_LRR-I-2	PK
F01_cb11783_c1/f1p0/2369-0F	RLK-Pelle_LRR-I-2	PK
F01_cb11783_c1/f1p0/2369-2F	RLK-Pelle_LRR-I-2	PK
F01_cb11785_c0/f1p0/1852-0F	RLK-Pelle_C-LEC	PK
F01_cb11785_c1/f1p0/1968-0F	RLK-Pelle_C-LEC	PK
F01_cb11813_c0/f1p0/1690-0F	RLK-Pelle_RLCK-VIIa-2	PK
F01_cb11813_c1/f1p0/2518-1F	RLK-Pelle_RLCK-VIIa-2	PK
F01_cb11813_c1/f1p0/2518-2F	RLK-Pelle_RLCK-VIIa-2	PK
F01_cb12007_c0/f4p1/1721-2F	RLK-Pelle_RLCK-IXb	PK
F01_cb12007_c2/f1p1/1781-1F	RLK-Pelle_RLCK-IXb	PK
F01_cb12007_c3/f1p0/1215-0F	RLK-Pelle_RLCK-IXb	PK
F01_cb12007_c6/f1p0/5755-0F	RLK-Pelle_RLCK-IXb	PK
F01_cb12076_c10/f6p0/1661-2F	CMGC_CK2	PK
F01_cb12076_c2/f2p0/1546-0F	CMGC_CK2	PK
F01_cb12076_c3/f1p0/1417-2F	CMGC_CK2	PK

F01_cb12076_c4/f1p0/1481-0F	CMGC_CK2	PK
F01_cb12076_c4/f1p0/1481-2F	CMGC_CK2	PK
F01_cb12076_c8/f1p0/1627-0F	CMGC_CK2	PK
F01_cb12076_c9/f4p0/1681-1F	CMGC_CK2	PK
F01_cb12094_c14/f1p1/1757-0F	CMGC_GSK	PK
F01_cb12094_c17/f1p0/1812-2F	CMGC_GSK	PK
F01_cb12094_c19/f1p0/1865-0F	CMGC_GSK	PK
F01_cb12094_c2/f19p2/1816-1F	CMGC_GSK	PK
F01_cb12094_c20/f1p0/1794-0F	CMGC_GSK	PK
F01_cb12094_c22/f1p1/1861-2F	CMGC_GSK	PK
F01_cb12094_c23/f1p0/1833-0F	CMGC_GSK	PK
F01_cb12094_c34/f1p1/1801-1F	CMGC_GSK	PK
F01_cb12094_c35/f2p1/1660-1F	CMGC_GSK	PK
F01_cb12239_c11/f1p0/1168-2F	CAMK_OST1L	PK
F01_cb12239_c12/f1p1/1603-1F	CAMK_OST1L	PK
F01_cb12239_c13/f1p0/1266-0F	CAMK_OST1L	PK
F01_cb12239_c15/f1p0/1389-1F	CAMK_OST1L	PK
F01_cb12239_c15/f1p0/1389-2F	CAMK_OST1L	PK
F01_cb12239_c16/f1p0/1735-1F	CAMK_OST1L	PK
F01_cb12239_c2/f2p1/1388-0F	CAMK_OST1L	PK
F01_cb12239_c3/f1p1/1477-0F	CAMK_OST1L	PK
F01_cb12239_c4/f1p0/1681-0F	CAMK_OST1L	PK
F01_cb12239_c4/f1p0/1681-1F	CAMK_OST1L	PK
F01_cb12239_c5/f1p0/1318-0F	CAMK_OST1L	PK
F01_cb12239_c8/f1p0/1565-0F	CAMK_OST1L	PK
F01_cb12397_c0/f1p0/788-1R	RLK-Pelle_LRR-XI-1	PK

F01_cb12529_c10/f2p0/1762-2F	TKL-Pl-4	PK
F01_cb12529_c35/f1p0/1800-2F	TKL-Pl-4	PK
F01_cb12529_c5/f3p0/1846-0F	TKL-Pl-4	PK
F01_cb12533_c0/f2p0/1634-2F	TKL-Pl-4	PK
F01_cb12533_c1/f1p0/1665-1F	TKL-Pl-4	PK
F01_cb12866_c3/f1p0/1872-0F	RLK-Pelle_CR4L	PK
F01_cb12866_c3/f1p0/1872-1F	RLK-Pelle_CrRLK1L-1	PK
F01_cb12866_c3/f1p0/1872-2F	RLK-Pelle_CrRLK1L-1	PK
F01_cb12_c10/f1p0/4331-1F	STE_STE-Pl	PK
F01_cb12_c11/f1p2/4713-1F	STE_STE-Pl	PK
F01_cb12_c15/f1p1/4774-1F	STE_STE-Pl	PK
F01_cb12_c17/f1p0/4826-0F	STE_STE-Pl	PK
F01_cb12_c21/f1p0/4378-0F	STE_STE-Pl	PK
F01_cb12_c22/f1p0/4618-2F	STE_STE-Pl	PK
F01_cb12_c24/f1p0/4291-2F	STE_STE-Pl	PK
F01_cb12_c28/f1p0/4906-1F	STE_STE-Pl	PK
F01_cb12_c29/f1p0/4185-2F	STE_STE-Pl	PK
F01_cb12_c30/f1p2/4443-1F	STE_STE-Pl	PK
F01_cb12_c35/f1p0/4696-1F	STE_STE-Pl	PK
F01_cb12_c35/f1p0/4696-2F	STE_STE-Pl	PK
F01_cb12_c40/f1p0/4680-1F	STE_STE-Pl	PK
F01_cb12_c41/f1p0/4748-1F	STE_STE-Pl	PK
F01_cb12_c45/f1p0/4319-2F	STE_STE-Pl	PK
F01_cb12_c50/f1p0/4439-0F	STE_STE-Pl	PK
F01_cb12_c54/f1p0/8058-1F	STE_STE-Pl	PK
F01_cb12_c56/f1p0/6478-2F	STE_STE-Pl	PK

F01_cb12_c58/f1p0/5377-0F	STE_STE-Pl	PK
F01_cb12_c60/f1p0/5175-2F	STE_STE-Pl	PK
F01_cb12_c64/f1p0/5051-2F	STE_STE-Pl	PK
F01_cb1306_c0/f11p2/3283-0F	TKL_CTR1-DRK-2	PK
F01_cb1306_c1/f7p1/3196-0F	TKL_CTR1-DRK-2	PK
F01_cb1306_c10/f1p1/3298-0F	TKL_CTR1-DRK-2	PK
F01_cb1306_c11/f1p1/3048-2F	TKL_CTR1-DRK-2	PK
F01_cb1306_c16/f1p0/3008-1F	TKL_CTR1-DRK-2	PK
F01_cb1306_c16/f1p0/3008-2F	TKL_CTR1-DRK-2	PK
F01_cb1306_c21/f1p0/2970-1F	TKL_CTR1-DRK-2	PK
F01_cb1306_c25/f1p1/3130-0F	TKL_CTR1-DRK-2	PK
F01_cb1306_c25/f1p1/3130-1F	TKL_CTR1-DRK-2	PK
F01_cb1306_c26/f1p0/3118-1F	TKL_CTR1-DRK-2	PK
F01_cb1306_c27/f1p0/2865-1F	TKL_CTR1-DRK-2	PK
F01_cb1306_c27/f1p0/2865-2F	TKL_CTR1-DRK-2	PK
F01_cb1306_c28/f1p0/5943-1F	TKL_CTR1-DRK-2	PK
F01_cb1306_c35/f1p0/3110-1F	TKL_CTR1-DRK-2	PK
F01_cb1306_c8/f2p2/3284-0F	TKL_CTR1-DRK-2	PK
F01_cb1306_c9/f1p1/4365-2F	TKL_CTR1-DRK-2	PK
F01_cb1309_c0/f9p1/2646-0F	STE_STE11	PK
F01_cb1309_c1/f1p0/2563-0F	STE_STE11	PK
F01_cb1309_c1/f1p0/2563-1F	STE_STE11	PK
F01_cb13131_c0/f16p0/1744-2F	CAMK_CAMKL-CHK1	PK
F01_cb13131_c14/f1p0/1395-2F	CAMK_CAMKL-CHK1	PK
F01_cb13131_c17/f1p0/1657-0F	CAMK_CAMKL-CHK1	PK
F01_cb13131_c17/f1p0/1657-1F	CAMK_CAMKL-CHK1	PK

F01_cb13131_c20/f1p0/1870-2F	CAMK_CAMKL-CHK1	PK
F01_cb13131_c21/f1p0/1874-1F	CAMK_CAMKL-CHK1	PK
F01_cb13131_c23/f1p0/1651-2F	CAMK_CAMKL-CHK1	PK
F01_cb13131_c26/f1p0/1819-1F	CAMK_CAMKL-CHK1	PK
F01_cb13131_c28/f1p0/1654-1F	CAMK_CAMKL-CHK1	PK
F01_cb13131_c34/f1p1/1713-0F	CAMK_CAMKL-CHK1	PK
F01_cb13131_c34/f1p1/1713-1F	CAMK_CAMKL-CHK1	PK
F01_cb1326_c10/f1p0/3807-0F	RLK-Pelle_LRR-Xb-1	PK
F01_cb1326_c10/f1p0/3807-1F	RLK-Pelle_LRR-Xb-1	PK
F01_cb1326_c13/f1p1/4140-0F	RLK-Pelle_LRR-Xb-1	PK
F01_cb1326_c13/f1p1/4140-2F	RLK-Pelle_LRR-Xb-1	PK
F01_cb1326_c20/f1p0/2140-2F	RLK-Pelle_LRR-Xb-1	PK
F01_cb1326_c23/f1p0/3133-2F	RLK-Pelle_LRR-Xb-1	PK
F01_cb1326_c24/f1p0/4269-0F	RLK-Pelle_LRR-Xb-1	PK
F01_cb1326_c34/f5p1/4137-2F	RLK-Pelle_LRR-Xb-1	PK
F01_cb1326_c5/f1p0/4374-0F	RLK-Pelle_LRR-Xb-1	PK
F01_cb1326_c9/f1p0/3549-1F	RLK-Pelle_LRR-Xb-1	PK
F01_cb13288_c1/f1p0/1622-2F	CMGC_GSK	PK
F01_cb13288_c2/f1p0/1761-0F	CMGC_GSK	PK
F01_cb13288_c3/f1p0/1730-1F	CMGC_GSK	PK
F01_cb13288_c5/f1p0/1812-1F	CMGC_GSK	PK
F01_cb13288_c6/f1p0/727-1F	CMGC_GSK	PK
F01_cb13311_c0/f2p0/1319-2F	STE_STE7	PK
F01_cb13311_c4/f1p0/1310-1F	STE_STE7	PK
F01_cb13311_c5/f1p0/1585-0F	STE_STE7	PK
F01_cb13437_c4/f1p0/1747-0F	RLK-Pelle_DLSV	PK

F01_cb1346_c6/f1p0/4350-0R	RLK-Pelle_CR4L	PK
F01_cb13495_c0/f1p0/1252-0F	RLK-Pelle_LRR-XI-1	PK
F01_cb13495_c1/f1p1/1287-2F	RLK-Pelle_LRR-XI-1	PK
F01_cb13496_c0/f4p0/1760-1F	RLK-Pelle_RLCK-VIIa-2	PK
F01_cb13496_c2/f1p0/1741-1F	RLK-Pelle_RLCK-VIIa-2	PK
F01_cb13496_c4/f1p0/1831-0F	RLK-Pelle_RLCK-VIIa-2	PK
F01_cb13496_c5/f1p0/1776-2F	RLK-Pelle_RLCK-VIIa-2	PK
F01_cb13624_c0/f2p0/1436-0F	RLK-Pelle_DLSV	PK
F01_cb13624_c1/f1p0/893-2F	RLK-Pelle_DLSV	PK
F01_cb13624_c2/f1p0/1538-1F	RLK-Pelle_LRR-Xb-1	PK
F01_cb13624_c3/f1p0/1519-0F	RLK-Pelle_DLSV	PK
F01_cb13624_c4/f1p0/1613-0F	RLK-Pelle_DLSV	PK
F01_cb13661_c3/f1p0/1743-0F	TKL-Pl-5	PK
F01_cb13661_c3/f1p0/1743-1F	TKL-Pl-5	PK
F01_cb13661_c3/f1p0/1743-2F	TKL-Pl-5	PK
F01_cb140_c1/f2p0/5012-2F	CMGC_Pl-Tthe	PK
F01_cb140_c2/f2p0/4887-1F	CMGC_Pl-Tthe	PK
F01_cb140_c3/f1p0/4949-1F	CMGC_Pl-Tthe	PK
F01_cb14168_c0/f1p0/1816-1F	RLK-Pelle_LysM	PK
F01_cb14536_c0/f4p0/1758-2F	RLK-Pelle_RLCK-VIIa-2	PK
F01_cb14536_c1/f1p0/1824-1F	RLK-Pelle_RLCK-VIIa-2	PK
F01_cb14536_c2/f1p0/1447-2F	RLK-Pelle_RLCK-VIIa-2	PK
F01_cb1453_c10/f1p0/3066-2F	RLK-Pelle_LRR-I-1	PK
F01_cb1453_c13/f1p1/2563-0F	RLK-Pelle_LRR-I-1	PK
F01_cb1453_c14/f1p0/2699-2F	RLK-Pelle_LRR-I-1	PK
F01_cb1453_c2/f2p0/3081-0F	RLK-Pelle_LRR-I-1	PK

F01_cb1453_c2/f2p0/3081-1F	RLK-Pelle_LRR-I-1	PK
F01_cb1453_c4/f1p0/2990-2F	RLK-Pelle_LRR-I-1	PK
F01_cb1453_c5/f1p0/3040-1F	RLK-Pelle_LRR-I-1	PK
F01_cb1453_c6/f1p0/4435-1F	RLK-Pelle_LRR-I-1	PK
F01_cb1453_c8/f1p0/3087-0F	RLK-Pelle_LRR-I-1	PK
F01_cb1453_c8/f1p0/3087-1F	RLK-Pelle_LRR-I-1	PK
F01_cb1453_c9/f1p0/3235-2F	RLK-Pelle_LRR-I-1	PK
F01_cb14552_c1/f1p0/1806-0F	RLK-Pelle_WAK	PK
F01_cb14552_c3/f1p0/1689-1F	RLK-Pelle_WAK	PK
F01_cb14600_c3/f1p1/1346-2F	WNK_NRBP	PK
F01_cb14672_c1/f1p0/1126-2F	TKL-Pl-4	PK
F01_cb14672_c2/f1p0/1303-1F	TKL-Pl-4	PK
F01_cb14683_c0/f2p0/1748-1F	RLK-Pelle_RLCK-VI	PK
F01_cb14683_c1/f1p0/1744-0F	RLK-Pelle_RLCK-VI	PK
F01_cb1468_c0/f7p1/2932-0F	STE_STE11	PK
F01_cb1468_c1/f4p2/2905-2F	STE_STE11	PK
F01_cb1468_c12/f1p1/3132-0F	STE_STE11	PK
F01_cb1468_c3/f1p0/4312-0F	STE_STE11	PK
F01_cb1468_c4/f1p2/2897-1F	STE_STE11	PK
F01_cb1468_c7/f1p1/3360-0F	STE_STE11	PK
F01_cb1468_c8/f1p0/2956-2F	STE_STE11	PK
F01_cb1468_c9/f1p1/2963-0F	STE_STE11	PK
F01_cb1469_c18/f1p0/3487-2F	RLK-Pelle_LRR-VII-1	PK
F01_cb1469_c21/f1p0/3647-1F	RLK-Pelle_LRR-VII-1	PK
F01_cb1469_c31/f8p2/3549-1F	RLK-Pelle_LRR-VII-1	PK
F01_cb1469_c36/f1p0/1214-0F	RLK-Pelle_LRR-VII-1	PK

F01_cb1469_c6/f2p2/3648-0F	RLK-Pelle_LRR-VII-1	PK
F01_cb14704_c12/f1p0/2420-0F	RLK-Pelle_RLCK-II	PK
F01_cb14704_c13/f1p0/2410-1F	RLK-Pelle_RLCK-II	PK
F01_cb14704_c15/f1p1/2401-1F	RLK-Pelle_RLCK-II	PK
F01_cb14704_c18/f1p0/2184-0F	RLK-Pelle_RLCK-II	PK
F01_cb14704_c19/f1p1/5793-1F	RLK-Pelle_RLCK-II	PK
F01_cb14704_c29/f1p0/5601-1F	RLK-Pelle_RLCK-II	PK
F01_cb14806_c1/f1p0/1602-1F	AGC_RSK-2	PK
F01_cb14806_c2/f1p0/1357-1F	AGC_RSK-2	PK
F01_cb1482_c0/f3p1/4124-0F	TKL_CTR1-DRK-2	PK
F01_cb1482_c1/f1p0/4095-0F	TKL_CTR1-DRK-2	PK
F01_cb1482_c4/f1p1/4863-2F	TKL_CTR1-DRK-2	PK
F01_cb1482_c5/f1p1/4141-0F	TKL_CTR1-DRK-2	PK
F01_cb1482_c5/f1p1/4141-2F	TKL_CTR1-DRK-2	PK
F01_cb1482_c6/f1p1/3871-1F	TKL_CTR1-DRK-2	PK
F01_cb1482_c7/f1p1/3492-1F	TKL_CTR1-DRK-2	PK
F01_cb15071_c0/f6p0/1717-1F	RLK-Pelle_RLCK-VIIa-2	PK
F01_cb15071_c2/f2p0/1680-0F	RLK-Pelle_RLCK-VIIa-2	PK
F01_cb15071_c3/f1p0/1820-2F	RLK-Pelle_RLCK-VIIa-2	PK
F01_cb15071_c5/f1p0/1763-1F	RLK-Pelle_RLCK-VIIa-2	PK
F01_cb15077_c0/f1p0/1511-1F	RLK-Pelle_RLCK-VIIa-2	PK
F01_cb15077_c1/f1p0/1683-0F	RLK-Pelle_RLCK-VIIa-2	PK
F01_cb151_c2/f1p0/4262-0F	TKL-Pl-6	PK
F01_cb151_c2/f1p0/4262-2F	TKL-Pl-6	PK
F01_cb151_c3/f1p1/4867-0F	TKL-Pl-6	PK
F01_cb151_c3/f1p1/4867-1F	TKL-Pl-6	PK

F01_cb151_c4/f1p0/4653-0F	TKL-Pl-6	PK
F01_cb151_c6/f1p0/4591-0F	TKL-Pl-6	PK
F01_cb151_c6/f1p0/4591-1F	TKL-Pl-6	PK
F01_cb151_c6/f1p0/4591-2F	TKL-Pl-6	PK
F01_cb151_c7/f1p0/4348-1F	TKL-Pl-6	PK
F01_cb15250_c0/f2p0/1673-1F	CMGC_MAPK	PK
F01_cb15250_c1/f1p0/1709-0F	CMGC_MAPK	PK
F01_cb15276_c1/f1p0/1292-0F	RLK-Pelle_URK-1	PK
F01_cb15276_c2/f1p0/1105-2F	RLK-Pelle_URK-1	PK
F01_cb15276_c3/f1p0/1318-0F	RLK-Pelle_URK-1	PK
F01_cb15285_c1/f1p1/1724-0F	STE_STE7	PK
F01_cb15462_c2/f1p0/527-0R	RLK-Pelle_WAK_LRK10L-1	PK
F01_cb15588_c2/f1p0/1647-0F	RLK-Pelle_PERK-1	PK
F01_cb15588_c3/f1p0/1471-1F	RLK-Pelle_PERK-1	PK
F01_cb1573_c15/f1p0/3869-0F	CMGC_DYRK-YAK	PK
F01_cb1573_c16/f1p0/3454-0F	CMGC_DYRK-YAK	PK
F01_cb1573_c17/f1p1/3566-1F	CMGC_DYRK-YAK	PK
F01_cb1573_c18/f1p0/3817-1F	CMGC_DYRK-YAK	PK
F01_cb1573_c18/f1p0/3817-2F	CMGC_DYRK-YAK	PK
F01_cb1573_c19/f1p1/3913-0F	CMGC_DYRK-YAK	PK
F01_cb1573_c20/f1p0/3450-0F	CMGC_DYRK-YAK	PK
F01_cb1573_c21/f1p2/3728-2F	CMGC_DYRK-YAK	PK
F01_cb1573_c23/f1p1/3875-1F	CMGC_DYRK-YAK	PK
F01_cb1573_c24/f1p1/3680-0F	CMGC_DYRK-YAK	PK
F01_cb1573_c24/f1p1/3680-2F	CMGC_DYRK-YAK	PK
F01_cb1573_c3/f4p1/3618-2F	CMGC_DYRK-YAK	PK

F01_cb1573_c32/f1p0/2936-1F	CMGC_DYRK-YAK	PK
F01_cb1573_c35/f1p0/2689-0F	CMGC_DYRK-YAK	PK
F01_cb1573_c41/f1p0/1724-0F	CMGC_DYRK-YAK	PK
F01_cb1573_c7/f1p2/3841-1F	CMGC_DYRK-YAK	PK
F01_cb1573_c9/f2p0/3382-1F	CMGC_DYRK-YAK	PK
F01_cb15873_c3/f1p0/880-1F	CAMK_CDPK	PK
F01_cb15877_c0/f1p0/1393-2F	RLK-Pelle_RLCK-VIIa-2	PK
F01_cb16046_c0/f2p0/1662-0F	RLK-Pelle_RLCK-VIIa-2	PK
F01_cb16046_c1/f1p0/1813-0F	RLK-Pelle_RLCK-VIIa-2	PK
F01_cb16046_c1/f1p0/1813-2F	RLK-Pelle_RLCK-VIIa-2	PK
F01_cb16081_c1/f4p1/1595-1F	RLK-Pelle_RLCK-XV	PK
F01_cb16081_c2/f3p1/1402-2F	RLK-Pelle_RLCK-XV	PK
F01_cb16081_c3/f2p0/1740-2F	RLK-Pelle_RLCK-XV	PK
F01_cb16081_c9/f5p1/1786-0F	RLK-Pelle_RLCK-XV	PK
F01_cb1608_c0/f2p1/3657-2F	RLK-Pelle_LRR-III	PK
F01_cb1608_c2/f1p1/3631-0F	RLK-Pelle_LRR-III	PK
F01_cb1608_c4/f1p0/3531-0F	RLK-Pelle_LRR-III	PK
F01_cb16090_c1/f1p0/1134-0F	RLK-Pelle_L-LEC	PK
F01_cb16132_c18/f1p1/1756-0F	CAMK_CAMKL-CHK1	PK
F01_cb16132_c18/f1p1/1756-1F	CAMK_CAMKL-CHK1	PK
F01_cb16132_c23/f1p2/1726-0F	CAMK_CAMKL-CHK1	PK
F01_cb16132_c27/f1p1/1796-2F	CAMK_CAMKL-CHK1	PK
F01_cb16132_c31/f1p0/1829-2F	CAMK_CAMKL-CHK1	PK
F01_cb16132_c36/f1p1/1828-1F	CAMK_CAMKL-CHK1	PK
F01_cb16134_c2/f1p0/1632-0F	RLK-Pelle_DLSV	PK
F01_cb16299_c1/f1p0/1885-2F	RLK-Pelle_RLCK-VIIa-2	PK

F01_cb16299_c3/f1p0/1697-2F	RLK-Pelle_RLCK-VIIa-2	PK
F01_cb16299_c4/f1p0/1771-2F	RLK-Pelle_RLCK-VIIa-2	PK
F01_cb16299_c6/f1p1/1757-0F	RLK-Pelle_RLCK-VIIa-2	PK
F01_cb16299_c6/f1p1/1757-2F	RLK-Pelle_RLCK-VIIa-2	PK
F01_cb16299_c8/f3p1/5415-1F	RLK-Pelle_RLCK-VIIa-2	PK
F01_cb16344_c0/f3p0/1511-1F	RLK-Pelle_DLSV	PK
F01_cb16344_c2/f1p0/1585-1F	RLK-Pelle_DLSV	PK
F01_cb16361_c1/f2p0/1778-2F	RLK-Pelle_RLCK-VIIa-2	PK
F01_cb16430_c1/f1p0/1766-0F	CK1_CK1	PK
F01_cb16488_c2/f2p0/1570-1F	RLK-Pelle_RLCK-VIII	PK
F01_cb16488_c8/f1p0/1732-1F	RLK-Pelle_RLCK-VIII	PK
F01_cb16488_c9/f1p0/1566-2F	RLK-Pelle_RLCK-VIII	PK
F01_cb16508_c0/f1p0/1682-0F	RLK-Pelle_RLCK-XV	PK
F01_cb16508_c1/f1p0/1732-0F	RLK-Pelle_RLCK-XV	PK
F01_cb16508_c2/f1p0/1556-1F	RLK-Pelle_RLCK-XV	PK
F01_cb1654_c0/f2p0/4227-0F	RLK-Pelle_LRR-XII-1	PK
F01_cb1654_c2/f1p0/3835-0F	RLK-Pelle_LRR-XII-1	PK
F01_cb16744_c2/f1p0/1530-0F	RLK-Pelle_RLCK-VIIa-2	PK
F01_cb16744_c3/f1p0/1625-2F	RLK-Pelle_RLCK-VIIa-2	PK
F01_cb16817_c2/f1p0/1493-1F	CAMK_OST1L	PK
F01_cb16817_c3/f1p0/1693-1F	CAMK_OST1L	PK
F01_cb16817_c4/f1p0/1658-1F	CAMK_OST1L	PK
F01_cb16830_c0/f3p0/1253-0F	STE_STE11	PK
F01_cb16907_c0/f2p1/1856-1F	CMGC_MAPK	PK
F01_cb16907_c0/f2p1/1856-2F	CMGC_MAPK	PK
F01_cb16907_c3/f1p0/1849-0F	CMGC_MAPK	PK

F01_cb16907_c3/f1p0/1849-2F	CMGC_MAPK	PK
F01_cb16907_c5/f1p0/1573-0F	CMGC_MAPK	PK
F01_cb16907_c5/f1p0/1573-2F	CMGC_MAPK	PK
F01_cb16970_c5/f1p0/1759-2F	STE_STE7	PK
F01_cb16970_c7/f1p0/5657-0F	STE_STE7	PK
F01_cb16970_c7/f1p0/5657-2F	STE_STE7	PK
F01_cb16974_c1/f1p2/1758-2F	RLK-Pelle_RLCK-V	PK
F01_cb16974_c2/f1p1/1739-1F	RLK-Pelle_RLCK-V	PK
F01_cb16974_c3/f1p0/1764-1F	RLK-Pelle_RLCK-V	PK
F01_cb17001_c0/f3p1/1878-1F	RLK-Pelle_RLCK-VIIa-2	PK
F01_cb1701_c1/f2p1/3968-2F	AGC_MAST	PK
F01_cb1701_c2/f1p0/4012-2F	AGC_MAST	PK
F01_cb1701_c5/f1p0/3219-0F	AGC_MAST	PK
F01_cb1701_c6/f1p0/3989-0F	AGC_MAST	PK
F01_cb1701_c7/f1p0/4121-1F	AGC_MAST	PK
F01_cb17086_c0/f1p0/1379-1F	RLK-Pelle_CR4L	PK
F01_cb17086_c1/f1p0/1418-0F	RLK-Pelle_RLCK-VIIa-2	PK
F01_cb1714_c0/f2p0/4139-2F	RLK-Pelle_LRR-XI-1	PK
F01_cb1714_c1/f2p1/4137-2F	RLK-Pelle_LRR-XI-1	PK
F01_cb1714_c2/f1p0/4405-1F	RLK-Pelle_LRR-XI-1	PK
F01_cb1714_c8/f1p0/1554-1F	RLK-Pelle_LRR-XI-1	PK
F01_cb17293_c0/f2p0/1765-0F	RLK-Pelle_DLSV	PK
F01_cb17293_c1/f1p0/1774-0F	RLK-Pelle_DLSV	PK
F01_cb17293_c3/f1p0/1593-0F	RLK-Pelle_DLSV	PK
F01_cb17378_c1/f1p1/1845-1F	STE_STE7	PK
F01_cb17378_c2/f1p0/1207-2F	STE_STE7	PK

F01_cb17397_c0/f1p0/1631-2F	CMGC_MAPK	PK
F01_cb17397_c1/f1p0/1744-0F	CMGC_MAPK	PK
F01_cb17397_c2/f1p0/5709-2F	CMGC_MAPK	PK
F01_cb17397_c3/f1p0/2619-0F	CMGC_MAPK	PK
F01_cb17397_c4/f1p0/2269-1F	CMGC_MAPK	PK
F01_cb17397_c4/f1p0/2269-2F	CMGC_MAPK	PK
F01_cb17397_c5/f1p1/1634-1F	CMGC_MAPK	PK
F01_cb17397_c7/f1p1/1566-2F	CMGC_MAPK	PK
F01_cb17397_c8/f1p0/1505-0F	CMGC_MAPK	PK
F01_cb17397_c9/f1p1/1416-2F	CMGC_MAPK	PK
F01_cb17428_c1/f1p0/1642-1F	RLK-Pelle_LRR-II	PK
F01_cb1778_c11/f1p1/2906-2F	RLK-Pelle_LRR-IX	PK
F01_cb1778_c14/f1p0/3718-2F	RLK-Pelle_LRR-IX	PK
F01_cb1778_c17/f1p0/3447-2F	RLK-Pelle_LRR-IX	PK
F01_cb1778_c3/f1p0/4178-0F	RLK-Pelle_LRR-IX	PK
F01_cb1778_c7/f1p1/3671-1F	RLK-Pelle_LRR-IX	PK
F01_cb1778_c8/f1p1/3002-1F	RLK-Pelle_LRR-IX	PK
F01_cb1780_c13/f1p0/725-0F	RLK-Pelle_LRR-XIIIa	PK
F01_cb1780_c2/f1p0/4547-0F	RLK-Pelle_LRR-XV	PK
F01_cb1780_c4/f1p1/4362-2F	RLK-Pelle_LRR-XV	PK
F01_cb1780_c7/f1p1/2237-2F	RLK-Pelle_LRR-XV	PK
F01_cb1782_c1/f2p0/2023-1F	CMGC_RCK	PK
F01_cb1782_c2/f1p0/4177-1F	CMGC_RCK	PK
F01_cb1782_c3/f1p0/3452-0F	CMGC_RCK	PK
F01_cb1782_c4/f1p0/4087-0F	CMGC_RCK	PK
F01_cb1782_c4/f1p0/4087-2F	CMGC_RCK	PK

F01_cb1782_c6/f1p0/2143-0F	CMGC_RCK	PK
F01_cb1782_c7/f1p0/1946-2F	CMGC_RCK	PK
F01_cb1782_c8/f1p0/2255-0F	CMGC_RCK	PK
F01_cb17859_c0/f1p0/1025-1F	RLK-Pelle_RLCK-VIIa-2	PK
F01_cb17859_c2/f1p0/1454-1F	RLK-Pelle_RLCK-VIIa-2	PK
F01_cb17896_c2/f1p0/5746-0F	RLK-Pelle_DLSV	PK
F01_cb17896_c2/f1p0/5746-2F	RLK-Pelle_DLSV	PK
F01_cb17896_c3/f1p1/5521-2F	RLK-Pelle_DLSV	PK
F01_cb17896_c4/f1p1/5406-0F	RLK-Pelle_DLSV	PK
F01_cb17973_c17/f1p0/1407-1F	Group-Pl-3	PK
F01_cb17973_c17/f1p0/1407-2F	Group-Pl-3	PK
F01_cb17973_c33/f1p0/1278-1F	Group-Pl-3	PK
F01_cb17973_c33/f1p0/1278-2F	Group-Pl-3	PK
F01_cb18079_c0/f2p0/1626-2F	RLK-Pelle_Extensin	PK
F01_cb18144_c0/f2p0/1467-0F	RLK-Pelle_RLCK-VIIa-2	PK
F01_cb18144_c2/f1p0/1453-1F	RLK-Pelle_RLCK-VIIa-2	PK
F01_cb18288_c1/f1p0/1746-1F	RLK-Pelle_LRR-Xa	PK
F01_cb18288_c2/f1p0/1450-1F	RLK-Pelle_LRR-Xa	PK
F01_cb18332_c2/f1p1/1832-1F	RLK-Pelle_CrRLK1L-1	PK
F01_cb18332_c3/f1p2/1665-0F	RLK-Pelle_RLCK-VIII	PK
F01_cb18332_c6/f1p0/1841-0F	RLK-Pelle_RLCK-VIII	PK
F01_cb18332_c7/f1p2/1609-0F	RLK-Pelle_RLCK-VIII	PK
F01_cb18332_c8/f1p1/1670-1F	RLK-Pelle_RLCK-VIII	PK
F01_cb18332_c9/f1p0/1604-0F	RLK-Pelle_RLCK-VIII	PK
F01_cb18372_c1/f2p0/2056-1F	RLK-Pelle_LRK10L-2	PK
F01_cb18372_c11/f1p0/4185-0F	RLK-Pelle_LRK10L-2	PK

F01_cb18372_c13/f1p0/2046-1F	RLK-Pelle_LRK10L-2	PK
F01_cb18372_c14/f1p0/2197-0F	RLK-Pelle_LRK10L-2	PK
F01_cb18372_c14/f1p0/2197-1F	RLK-Pelle_LRK10L-2	PK
F01_cb18372_c14/f1p0/2197-2F	RLK-Pelle_LRK10L-2	PK
F01_cb18372_c17/f1p0/2038-0F	RLK-Pelle_LRK10L-2	PK
F01_cb18372_c18/f1p0/2238-2F	RLK-Pelle_LRK10L-2	PK
F01_cb18372_c19/f1p0/2121-2F	RLK-Pelle_LRK10L-2	PK
F01_cb18372_c2/f2p0/2027-2F	RLK-Pelle_LRK10L-2	PK
F01_cb18372_c20/f1p0/2079-0F	RLK-Pelle_LRK10L-2	PK
F01_cb18372_c21/f1p0/2218-2F	RLK-Pelle_LRK10L-2	PK
F01_cb18372_c24/f1p0/2094-1F	RLK-Pelle_LRK10L-2	PK
F01_cb18372_c6/f1p0/1942-0F	RLK-Pelle_LRK10L-2	PK
F01_cb18372_c7/f1p0/5609-2F	RLK-Pelle_LRK10L-2	PK
F01_cb18372_c8/f1p0/2774-2F	RLK-Pelle_LRK10L-2	PK
F01_cb18386_c4/f1p1/1388-1F	TKL-Pl-4	PK
F01_cb18386_c5/f1p0/1465-0F	TKL-Pl-4	PK
F01_cb18386_c7/f1p0/1299-2F	TKL-Pl-4	PK
F01_cb18456_c7551/f1p0/1750-0F	CAMK_CDPK	PK
F01_cb18456_c7552/f1p0/1784-1F	CAMK_CDPK	PK
F01_cb18456_c7580/f1p0/1737-0F	CAMK_AMPK	PK
F01_cb1857_c1/f2p0/3686-1F	AGC_RSK-2	PK
F01_cb1857_c1/f2p0/3686-2F	AGC_RSK-2	PK
F01_cb1857_c13/f1p0/3399-0F	AGC_RSK-2	PK
F01_cb1857_c13/f1p0/3399-2F	AGC_RSK-2	PK
F01_cb1857_c15/f1p0/3661-0F	AGC_RSK-2	PK
F01_cb1857_c18/f1p0/3189-2F	AGC_RSK-2	PK

F01_cb1857_c25/f1p0/3413-1F	AGC_RSK-2	PK
F01_cb1857_c4/f2p0/3275-0F	AGC_RSK-2	PK
F01_cb1857_c7/f1p0/3508-2F	AGC_RSK-2	PK
F01_cb1857_c8/f1p1/3635-0F	AGC_RSK-2	PK
F01_cb1857_c8/f1p1/3635-1F	AGC_RSK-2	PK
F01_cb1857_c9/f1p0/3585-0F	AGC_RSK-2	PK
F01_cb1857_c9/f1p0/3585-2F	AGC_RSK-2	PK
F01_cb18616_c3/f1p1/5034-0F	RLK-Pelle_LRR-XI-1	PK
F01_cb18616_c3/f1p1/5034-2F	RLK-Pelle_LRR-XI-1	PK
F01_cb18621_c0/f1p0/6004-0F	RLK-Pelle_DLSV	PK
F01_cb18621_c0/f1p0/6004-1F	RLK-Pelle_DLSV	PK
F01_cb18621_c0/f1p0/6004-2F	RLK-Pelle_DLSV	PK
F01_cb18634_c2/f1p0/5092-2F	RLK-Pelle_CrRLK1L-1	PK
F01_cb18647_c0/f1p0/4880-0F	RLK-Pelle_CrRLK1L-1	PK
F01_cb18647_c0/f1p0/4880-1F	RLK-Pelle_RLCK-VIIa-2	PK
F01_cb18663_c0/f1p0/8486-0F	CMGC_CDK-PITSLRE	PK
F01_cb18663_c2/f1p1/7161-2F	CMGC_CDK-PITSLRE	PK
F01_cb18688_c0/f1p0/5088-1F	CMGC_MAPK	PK
F01_cb18773_c0/f2p3/5493-0F	CMGC_CDKL-Cr	PK
F01_cb18773_c10/f1p3/5620-2F	CMGC_Pl-Tthe	PK
F01_cb18773_c6/f1p1/5702-0F	CMGC_CDKL-Cr	PK
F01_cb18773_c9/f1p0/6228-1F	CMGC_CDKL-Cr	PK
F01_cb18783_c0/f1p0/5398-2F	CAMK_CAMKL-CHK1	PK
F01_cb187_c10/f1p0/2688-1F	AGC_PKA-PKG	PK
F01_cb187_c11/f1p1/3861-2F	AGC_PKA-PKG	PK
F01_cb187_c14/f1p0/2733-0F	AGC_PKA-PKG	PK

F01_cb187_c15/f1p0/4178-0F	AGC_PKA-PKG	PK
F01_cb187_c17/f1p0/4037-1F	AGC_PKA-PKG	PK
F01_cb187_c18/f1p0/4376-0F	AGC_PKA-PKG	PK
F01_cb187_c21/f1p0/4068-1F	AGC_PKA-PKG	PK
F01_cb187_c5/f1p0/3727-0F	AGC_PKA-PKG	PK
F01_cb187_c6/f9p1/4066-1F	AGC_PKA-PKG	PK
F01_cb187_c7/f1p0/4521-0F	AGC_PKA-PKG	PK
F01_cb187_c9/f1p0/4217-0F	AGC_PKA-PKG	PK
F01_cb18802_c1/f1p0/5282-1F	RLK-Pelle_LRR-XI-1	PK
F01_cb18802_c2/f1p0/5074-0F	RLK-Pelle_LRR-XI-1	PK
F01_cb18802_c3/f1p0/5057-0F	RLK-Pelle_LRR-XI-1	PK
F01_cb18808_c1/f1p0/5276-0F	STE_STE11	PK
F01_cb18808_c1/f1p0/5276-1F	STE_STE11	PK
F01_cb18808_c2/f1p0/5842-0F	STE_STE11	PK
F01_cb18808_c2/f1p0/5842-2F	STE_STE11	PK
F01_cb18810_c0/f1p1/6058-0F	RLK-Pelle_RLCK-VIIa-2	PK
F01_cb18810_c0/f1p1/6058-1F	RLK-Pelle_CrRLK1L-1	PK
F01_cb18810_c1/f1p0/5070-0F	RLK-Pelle_RLCK-VIIa-2	PK
F01_cb18810_c1/f1p0/5070-0R	RLK-Pelle_CrRLK1L-1	PK
F01_cb18810_c1/f1p0/5070-1F	RLK-Pelle_CrRLK1L-1	PK
F01_cb18810_c1/f1p0/5070-2R	RLK-Pelle_RLCK-VIIa-2	PK
F01_cb18819_c0/f1p0/5048-0F	TKL-Pl-6	PK
F01_cb18819_c2/f1p0/5197-0F	TKL-Pl-6	PK
F01_cb18819_c2/f1p0/5197-1F	TKL-Pl-6	PK
F01_cb18839_c0/f1p0/5272-0F	RLK-Pelle_DLSV	PK
F01_cb1897_c3/f1p0/4130-2F	RLK-Pelle_LRR-XI-1	PK

F01_cb1897_c5/f1p0/3283-2F	RLK-Pelle_LRR-XI-1	PK
F01_cb1962_c0/f6p3/2505-0F	CMGC_MAPK	PK
F01_cb1962_c1/f4p2/2528-0F	CMGC_MAPK	PK
F01_cb1962_c1/f4p2/2528-1F	CMGC_MAPK	PK
F01_cb1962_c2/f1p2/4105-0F	CMGC_MAPK	PK
F01_cb1962_c2/f1p2/4105-1F	CMGC_MAPK	PK
F01_cb1962_c4/f1p2/2403-0F	CMGC_MAPK	PK
F01_cb1962_c5/f1p4/2574-2F	CMGC_MAPK	PK
F01_cb1962_c7/f1p2/2447-1F	CMGC_MAPK	PK
F01_cb1962_c8/f26p4/2502-1F	CMGC_MAPK	PK
F01_cb2052_c0/f4p0/4081-0F	RLK-Pelle_LRR-III	PK
F01_cb2052_c0/f4p0/4081-1F	RLK-Pelle_LRR-III	PK
F01_cb2052_c1/f2p0/4068-0F	RLK-Pelle_LRR-III	PK
F01_cb2052_c2/f3p0/4040-1F	RLK-Pelle_LRR-III	PK
F01_cb2107_c11/f1p0/2039-1F	CAMK_CAMKL-CHK1	PK
F01_cb2107_c13/f1p0/2033-1F	CAMK_CAMKL-CHK1	PK
F01_cb2107_c14/f1p0/2127-0F	CAMK_CAMKL-CHK1	PK
F01_cb2107_c17/f1p0/1838-0F	CAMK_CAMKL-CHK1	PK
F01_cb2107_c2/f1p0/4062-0F	CAMK_CAMKL-CHK1	PK
F01_cb2107_c2/f1p0/4062-1F	CAMK_CAMKL-CHK1	PK
F01_cb2107_c3/f1p0/2815-1F	CAMK_CAMKL-CHK1	PK
F01_cb2107_c4/f1p0/4645-1F	CAMK_CAMKL-CHK1	PK
F01_cb2107_c6/f1p0/1856-0F	CAMK_CAMKL-CHK1	PK
F01_cb2107_c7/f1p0/2748-1F	CAMK_CAMKL-CHK1	PK
F01_cb2107_c7/f1p0/2748-2F	CAMK_CAMKL-CHK1	PK
F01_cb2107_c8/f1p1/1953-2F	CAMK_CAMKL-CHK1	PK

F01_cb2135_c0/f1p0/4045-1F	RLK-Pelle_Extensin	PK
F01_cb2155_c10/f1p0/3697-0F	STE_STE20-Fray	PK
F01_cb2155_c10/f1p0/3697-1F	STE_STE20-Fray	PK
F01_cb2155_c11/f1p0/2668-2F	STE_STE20-Fray	PK
F01_cb2155_c12/f1p0/2784-1F	STE_STE20-Fray	PK
F01_cb2155_c18/f1p0/3029-0F	STE_STE20-Fray	PK
F01_cb2155_c20/f1p0/3317-0F	STE_STE20-Fray	PK
F01_cb2155_c21/f1p0/3343-1F	STE_STE20-Fray	PK
F01_cb2155_c25/f2p1/2883-1F	STE_STE20-Fray	PK
F01_cb2155_c26/f5p0/2830-2F	STE_STE20-Fray	PK
F01_cb2155_c3/f7p0/2772-1F	STE_STE20-Fray	PK
F01_cb2155_c6/f2p0/3314-0F	STE_STE20-Fray	PK
F01_cb2155_c8/f1p0/4028-1F	STE_STE20-Fray	PK
F01_cb2155_c9/f1p0/3407-2F	STE_STE20-Fray	PK
F01_cb2164_c1/f1p0/3263-0F	STE_STE20-Fray	PK
F01_cb2164_c2/f1p0/3126-2F	STE_STE20-Fray	PK
F01_cb2164_c5/f1p0/2608-0F	STE_STE20-Fray	PK
F01_cb2164_c7/f1p0/2957-0F	STE_STE20-Fray	PK
F01_cb2177_c159/f4p0/3437-1F	RLK-Pelle_LRR-IX	PK
F01_cb2177_c69/f1p0/4458-1F	RLK-Pelle_LRR-IX	PK
F01_cb2177_c72/f1p0/4010-1F	RLK-Pelle_LRR-IX	PK
F01_cb2177_c80/f1p0/3411-0F	RLK-Pelle_LRR-IX	PK
F01_cb2177_c90/f1p0/3677-2F	RLK-Pelle_LRR-IX	PK
F01_cb2177_c91/f1p0/3417-2F	RLK-Pelle_LRR-IX	PK
F01_cb2177_c97/f1p0/2557-2F	RLK-Pelle_LRR-IX	PK
F01_cb2187_c10/f1p1/3921-0F	RLK-Pelle_LRR-XI-1	PK

F01_cb2187_c10/f1p1/3921-2F	RLK-Pelle_LRR-XI-1	PK
F01_cb2187_c12/f1p0/3730-1F	RLK-Pelle_LRR-XI-1	PK
F01_cb2187_c14/f1p0/3388-2F	RLK-Pelle_LRR-XI-1	PK
F01_cb2187_c19/f1p0/3843-0F	RLK-Pelle_LRR-XI-1	PK
F01_cb2187_c20/f1p2/3187-2F	RLK-Pelle_LRR-XI-1	PK
F01_cb2187_c26/f1p2/3431-0F	RLK-Pelle_LRR-XI-1	PK
F01_cb2187_c30/f1p0/3087-0F	RLK-Pelle_LRR-XI-1	PK
F01_cb2187_c30/f1p0/3087-2F	RLK-Pelle_LRR-XI-1	PK
F01_cb2187_c33/f1p0/3495-1F	RLK-Pelle_LRR-XI-1	PK
F01_cb2187_c9/f1p0/3345-1F	RLK-Pelle_LRR-XI-1	PK
F01_cb2195_c19/f1p0/1931-0F	RLK-Pelle_DLSV	PK
F01_cb2195_c19/f1p0/1931-1F	RLK-Pelle_DLSV	PK
F01_cb2195_c2/f3p0/2787-2F	RLK-Pelle_DLSV	PK
F01_cb2195_c20/f1p0/2740-2F	RLK-Pelle_DLSV	PK
F01_cb2195_c22/f1p0/2827-0F	RLK-Pelle_DLSV	PK
F01_cb2195_c23/f1p0/3248-1F	RLK-Pelle_DLSV	PK
F01_cb2195_c26/f1p0/2900-1F	RLK-Pelle_DLSV	PK
F01_cb2195_c27/f1p0/2613-1F	RLK-Pelle_DLSV	PK
F01_cb2195_c3/f3p0/2817-2F	RLK-Pelle_DLSV	PK
F01_cb2208_c10/f1p0/3064-1F	RLK-Pelle_LRR-VIII-1	PK
F01_cb2208_c10/f1p0/3064-2F	RLK-Pelle_LRR-VIII-1	PK
F01_cb2208_c13/f1p0/3223-0F	RLK-Pelle_LRR-VIII-1	PK
F01_cb2208_c13/f1p0/3223-2F	RLK-Pelle_LRR-VIII-1	PK
F01_cb2208_c15/f1p0/2981-1F	RLK-Pelle_LRR-VIII-1	PK
F01_cb2208_c15/f1p0/2981-2F	RLK-Pelle_LRR-VIII-1	PK
F01_cb2208_c16/f1p0/2388-0F	RLK-Pelle_LRR-VIII-1	PK

F01_cb2208_c16/f1p0/2388-2F	RLK-Pelle_LRR-VIII-1	PK
F01_cb2208_c18/f1p0/3335-2F	RLK-Pelle_LRR-VIII-1	PK
F01_cb2208_c2/f4p0/3275-1F	RLK-Pelle_LRR-VIII-1	PK
F01_cb2208_c21/f1p0/2436-1F	RLK-Pelle_LRR-VIII-1	PK
F01_cb2208_c21/f1p0/2436-2F	RLK-Pelle_LRR-VIII-1	PK
F01_cb2208_c22/f1p0/2562-0F	RLK-Pelle_LRR-VIII-1	PK
F01_cb2208_c23/f1p0/3237-2F	RLK-Pelle_LRR-VIII-1	PK
F01_cb2208_c25/f1p0/2989-1F	RLK-Pelle_LRR-VIII-1	PK
F01_cb2208_c27/f1p0/2987-1F	RLK-Pelle_LRR-VIII-1	PK
F01_cb2208_c27/f1p0/2987-2F	RLK-Pelle_LRR-VIII-1	PK
F01_cb2208_c28/f1p1/2703-1F	RLK-Pelle_LRR-VIII-1	PK
F01_cb2208_c30/f1p0/3607-0F	RLK-Pelle_LRR-VIII-1	PK
F01_cb2208_c33/f1p0/3174-0F	RLK-Pelle_LRR-VIII-1	PK
F01_cb2208_c33/f1p0/3174-2F	RLK-Pelle_LRR-VIII-1	PK
F01_cb2208_c34/f1p0/3429-0F	RLK-Pelle_LRR-VIII-1	PK
F01_cb2208_c35/f1p0/3221-1F	RLK-Pelle_LRR-VIII-1	PK
F01_cb2208_c37/f1p0/3146-0F	RLK-Pelle_LRR-VIII-1	PK
F01_cb2208_c38/f1p0/2591-0F	RLK-Pelle_LRR-VIII-1	PK
F01_cb2208_c39/f1p0/2505-2F	RLK-Pelle_LRR-VIII-1	PK
F01_cb2208_c40/f1p0/3281-0F	RLK-Pelle_LRR-VIII-1	PK
F01_cb2208_c42/f1p0/2791-0F	RLK-Pelle_LRR-VIII-1	PK
F01_cb2208_c42/f1p0/2791-2F	RLK-Pelle_LRR-VIII-1	PK
F01_cb2208_c45/f1p0/1853-2F	RLK-Pelle_LRR-VIII-1	PK
F01_cb2208_c46/f1p0/1714-0F	RLK-Pelle_LRR-VIII-1	PK
F01_cb2208_c5/f3p0/3196-1F	RLK-Pelle_LRR-VIII-1	PK
F01_cb2208_c52/f1p1/3627-0F	RLK-Pelle_LRR-VIII-1	PK

F01_cb2208_c7/f2p0/2647-1F	RLK-Pelle_LRR-VIII-1	PK
F01_cb2208_c9/f1p0/3012-1F	RLK-Pelle_LRR-VIII-1	PK
F01_cb2222_c0/f1p0/4007-1F	RLK-Pelle_LRR-VI-2	PK
F01_cb2222_c0/f1p0/4007-2F	RLK-Pelle_LRR-VI-2	PK
F01_cb2222_c1/f1p0/2851-2F	RLK-Pelle_LRR-VI-2	PK
F01_cb2222_c2/f1p0/2410-2F	RLK-Pelle_LRR-VI-2	PK
F01_cb2222_c3/f1p0/2396-1F	RLK-Pelle_LRR-VI-2	PK
F01_cb2242_c1/f4p0/1990-2F	TKL-Pl-5	PK
F01_cb2242_c10/f1p1/2221-1F	TKL-Pl-5	PK
F01_cb2242_c12/f1p0/1844-0F	TKL-Pl-5	PK
F01_cb2242_c14/f1p0/1775-1F	TKL-Pl-5	PK
F01_cb2242_c14/f1p0/1775-2F	TKL-Pl-5	PK
F01_cb2242_c3/f2p1/1866-1F	TKL-Pl-5	PK
F01_cb2242_c5/f2p0/2543-0F	TKL-Pl-5	PK
F01_cb2242_c5/f2p0/2543-1F	TKL-Pl-5	PK
F01_cb2242_c7/f1p0/2048-0F	TKL-Pl-5	PK
F01_cb2242_c7/f1p0/2048-2F	TKL-Pl-5	PK
F01_cb2242_c8/f1p0/2306-1F	TKL-Pl-5	PK
F01_cb2242_c8/f1p0/2306-2F	TKL-Pl-5	PK
F01_cb2263_c0/f4p0/2143-2F	CAMK_CAMKL-LKB	PK
F01_cb2263_c1/f3p1/2288-1F	CAMK_CAMKL-LKB	PK
F01_cb2263_c11/f1p0/1956-1F	CAMK_CAMKL-LKB	PK
F01_cb2263_c12/f1p0/2668-2F	CAMK_CAMKL-LKB	PK
F01_cb2263_c13/f1p0/3861-1F	CAMK_CAMKL-LKB	PK
F01_cb2263_c13/f1p0/3861-2F	CAMK_CAMKL-LKB	PK
F01_cb2263_c15/f1p0/3195-0F	CAMK_CAMKL-LKB	PK

F01_cb2263_c18/f1p0/2268-1F	CAMK_CAMKL-LKB	PK
F01_cb2263_c19/f1p0/2822-0F	CAMK_CAMKL-LKB	PK
F01_cb2263_c2/f3p1/2293-0F	CAMK_CAMKL-LKB	PK
F01_cb2263_c20/f1p0/2614-1F	CAMK_CAMKL-LKB	PK
F01_cb2263_c21/f1p0/2103-1F	CAMK_CAMKL-LKB	PK
F01_cb2263_c22/f1p0/2315-1F	CAMK_CAMKL-LKB	PK
F01_cb2263_c3/f2p0/2490-2F	CAMK_CAMKL-LKB	PK
F01_cb2263_c4/f2p0/2476-1F	CAMK_CAMKL-LKB	PK
F01_cb2263_c4/f2p0/2476-2F	CAMK_CAMKL-LKB	PK
F01_cb2263_c5/f1p0/3994-2F	CAMK_CAMKL-LKB	PK
F01_cb2263_c8/f1p0/2391-1F	CAMK_CAMKL-LKB	PK
F01_cb2263_c8/f1p0/2391-2F	CAMK_CAMKL-LKB	PK
F01_cb2263_c9/f1p0/2491-0F	CAMK_CAMKL-LKB	PK
F01_cb2305_c10/f1p0/2184-0F	NEK	PK
F01_cb2305_c11/f1p0/3032-0F	NEK	PK
F01_cb2305_c11/f1p0/3032-2F	NEK	PK
F01_cb2305_c14/f1p0/2296-1F	NEK	PK
F01_cb2305_c14/f1p0/2296-2F	NEK	PK
F01_cb2305_c15/f1p0/2436-0F	NEK	PK
F01_cb2305_c15/f1p0/2436-2F	NEK	PK
F01_cb2305_c3/f2p0/2548-2F	NEK	PK
F01_cb2305_c4/f2p0/2538-1F	NEK	PK
F01_cb2305_c7/f1p0/2918-1F	NEK	PK
F01_cb2305_c7/f1p0/2918-2F	NEK	PK
F01_cb2305_c8/f1p0/2515-2F	NEK	PK
F01_cb2305_c9/f1p0/3279-0F	NEK	PK

F01_cb2333_c0/f1p0/3971-1R	RLK-Pelle_DLSV	PK
F01_cb2333_c0/f1p0/3971-2R	RLK-Pelle_CR4L	PK
F01_cb2338_c0/f1p0/3970-1F	PEK_GCN2	PK
F01_cb2338_c0/f1p0/3970-2F	PEK_GCN2	PK
F01_cb2338_c1/f1p0/4453-1F	PEK_GCN2	PK
F01_cb2389_c10/f1p0/3549-0F	NEK	PK
F01_cb2389_c15/f1p0/2930-2F	NEK	PK
F01_cb2389_c4/f1p0/3334-0F	NEK	PK
F01_cb2389_c4/f1p0/3334-2F	NEK	PK
F01_cb2389_c5/f1p0/3424-1F	NEK	PK
F01_cb2389_c5/f1p0/3424-2F	NEK	PK
F01_cb2389_c8/f1p0/3551-2F	NEK	PK
F01_cb2389_c9/f1p0/3504-2F	NEK	PK
F01_cb2405_c0/f1p0/3942-2F	TKL-Pl-6	PK
F01_cb2405_c1/f1p0/3572-0F	TKL-Pl-6	PK
F01_cb2405_c1/f1p0/3572-2F	TKL-Pl-6	PK
F01_cb2405_c2/f1p0/2041-0F	TKL-Pl-6	PK
F01_cb2448_c1/f2p0/3376-2F	RLK-Pelle_LRR-XI-1	PK
F01_cb2448_c14/f6p0/3347-1F	RLK-Pelle_LRR-XI-1	PK
F01_cb2448_c2/f1p0/3926-0F	RLK-Pelle_LRR-XI-1	PK
F01_cb2448_c2/f1p0/3926-1F	RLK-Pelle_LRR-XI-1	PK
F01_cb2448_c3/f1p0/3231-2F	RLK-Pelle_LRR-XI-1	PK
F01_cb2448_c4/f1p0/3324-1F	RLK-Pelle_LRR-XI-1	PK
F01_cb2448_c4/f1p0/3324-2F	RLK-Pelle_LRR-XI-1	PK
F01_cb2448_c6/f1p0/3197-0F	RLK-Pelle_LRR-XI-1	PK
F01_cb2482_c30/f13p1/3051-1F	PEK_GCN2	PK

F01_cb2482_c31/f1p1/2865-1F	PEK_GCN2	PK
F01_cb2482_c45/f1p0/3357-2F	PEK_GCN2	PK
F01_cb2482_c57/f13p2/3062-1F	PEK_GCN2	PK
F01_cb2482_c6/f2p1/3099-2F	PEK_GCN2	PK
F01_cb2501_c10/f1p0/2573-0F	STE_STE20-Fray	PK
F01_cb2501_c11/f1p0/2612-1F	STE_STE20-Fray	PK
F01_cb2501_c13/f1p0/2572-2F	STE_STE20-Fray	PK
F01_cb2501_c14/f1p1/2816-2F	STE_STE20-Fray	PK
F01_cb2501_c17/f1p0/3044-0F	STE_STE20-Fray	PK
F01_cb2501_c20/f1p0/3134-1F	STE_STE20-Fray	PK
F01_cb2501_c21/f1p0/3101-0F	STE_STE20-Fray	PK
F01_cb2501_c23/f1p0/3165-2F	STE_STE20-Fray	PK
F01_cb2501_c24/f1p0/2915-0F	STE_STE20-Fray	PK
F01_cb2501_c31/f1p0/1967-1F	STE_STE20-Fray	PK
F01_cb2501_c4/f4p0/2994-1F	STE_STE20-Fray	PK
F01_cb2501_c6/f2p0/2804-0F	STE_STE20-Fray	PK
F01_cb2501_c6/f2p0/2804-2F	STE_STE20-Fray	PK
F01_cb2501_c7/f2p0/2631-2F	STE_STE20-Fray	PK
F01_cb2501_c8/f1p0/3903-1F	STE_STE20-Fray	PK
F01_cb2501_c8/f1p0/3903-2F	STE_STE20-Fray	PK
F01_cb2501_c9/f1p0/2890-1F	STE_STE20-Fray	PK
F01_cb2516_c5/f1p0/3824-2F	RLK-Pelle_LRR-XIIIb	PK
F01_cb2517_c1/f3p0/2265-1F	TKL-Pl-4	PK
F01_cb2517_c11/f1p0/2274-0F	TKL-Pl-4	PK
F01_cb2517_c12/f1p0/2406-0F	TKL-Pl-4	PK
F01_cb2517_c3/f2p0/3772-0F	TKL-Pl-4	PK

F01_cb2517_c3/f2p0/3772-2F	TKL-Pl-4	PK
F01_cb2517_c5/f1p0/2210-0F	TKL-Pl-4	PK
F01_cb2517_c7/f1p0/2289-2F	TKL-Pl-4	PK
F01_cb2517_c8/f1p0/3354-0F	TKL-Pl-4	PK
F01_cb2519_c1/f1p0/3804-1F	RLK-Pelle_LRR-Xb-1	PK
F01_cb2520_c0/f1p0/3145-2F	RLK-Pelle_LRR-VI-1	PK
F01_cb2520_c14/f1p1/2870-2F	RLK-Pelle_LRR-VI-1	PK
F01_cb2520_c17/f1p0/3137-2F	RLK-Pelle_LRR-VI-1	PK
F01_cb2520_c20/f1p1/2903-0F	RLK-Pelle_LRR-VI-1	PK
F01_cb2520_c3/f2p0/2952-0F	RLK-Pelle_LRR-VI-1	PK
F01_cb2520_c4/f2p0/3911-1F	RLK-Pelle_LRR-VI-1	PK
F01_cb2520_c5/f1p0/3341-0F	RLK-Pelle_LRR-VI-1	PK
F01_cb2520_c6/f1p0/4295-0F	RLK-Pelle_LRR-VI-1	PK
F01_cb2520_c7/f1p1/3561-0F	RLK-Pelle_LRR-VI-1	PK
F01_cb2532_c14/f1p0/2373-0F	RLK-Pelle_CrRLK1L-1	PK
F01_cb2532_c18/f3p3/3329-0F	RLK-Pelle_CrRLK1L-1	PK
F01_cb2532_c2/f3p3/3381-0F	RLK-Pelle_CrRLK1L-1	PK
F01_cb2532_c5/f1p3/3858-0F	RLK-Pelle_CrRLK1L-1	PK
F01_cb2579_c0/f2p0/1501-0F	RLK-Pelle_RLCK-VIIa-2	PK
F01_cb2579_c0/f2p0/1501-2F	RLK-Pelle_RLCK-VIIa-2	PK
F01_cb2579_c2/f1p0/5000-0F	RLK-Pelle_RLCK-VIIa-2	PK
F01_cb2579_c2/f1p0/5000-1F	RLK-Pelle_RLCK-VIIa-2	PK
F01_cb2579_c3/f1p0/3543-1F	RLK-Pelle_RLCK-VIIa-2	PK
F01_cb2579_c4/f1p0/1818-1F	RLK-Pelle_RLCK-VIIa-2	PK
F01_cb2579_c4/f1p0/1818-2F	RLK-Pelle_RLCK-VIIa-2	PK
F01_cb2579_c5/f1p0/1601-1F	RLK-Pelle_RLCK-VIIa-2	PK

F01_cb2583_c0/f1p2/3852-0F	CMGC_GSKL	PK
F01_cb2610_c16/f3p0/3772-1F	RLK-Pelle_LRR-XI-1	PK
F01_cb2610_c8/f1p0/4067-0F	RLK-Pelle_LRR-XI-1	PK
F01_cb2643_c10/f1p0/2833-2F	RLK-Pelle_LRR-I-1	PK
F01_cb2643_c11/f1p1/3425-1F	RLK-Pelle_LRR-I-1	PK
F01_cb2643_c11/f1p1/3425-2F	RLK-Pelle_LRR-I-1	PK
F01_cb2643_c12/f1p0/2916-0F	RLK-Pelle_LRR-I-1	PK
F01_cb2643_c13/f1p0/3261-1F	RLK-Pelle_LRR-I-1	PK
F01_cb2643_c20/f1p0/3313-2F	RLK-Pelle_LRR-I-1	PK
F01_cb2643_c4/f2p0/3433-1F	RLK-Pelle_LRR-I-1	PK
F01_cb2643_c6/f1p0/3857-2F	RLK-Pelle_LRR-I-1	PK
F01_cb2643_c9/f1p0/3249-1F	RLK-Pelle_LRR-I-1	PK
F01_cb2655_c0/f1p0/3850-1F	RLK-Pelle_LRR-Xb-1	PK
F01_cb2655_c1/f1p0/4332-2F	RLK-Pelle_LRR-Xb-1	PK
F01_cb2655_c2/f1p0/3073-2F	RLK-Pelle_LRR-Xb-1	PK
F01_cb2663_c0/f7p0/3459-2F	RLK-Pelle_RLCK-IXb	PK
F01_cb2663_c1/f3p0/3484-0F	RLK-Pelle_RLCK-IXb	PK
F01_cb2663_c1/f3p0/3484-1F	RLK-Pelle_RLCK-IXb	PK
F01_cb2663_c10/f1p0/2836-0F	RLK-Pelle_RLCK-IXb	PK
F01_cb2663_c4/f1p0/3376-1F	RLK-Pelle_RLCK-IXb	PK
F01_cb2663_c5/f1p0/3025-0F	RLK-Pelle_RLCK-IXb	PK
F01_cb2663_c6/f1p0/3293-1F	RLK-Pelle_RLCK-IXb	PK
F01_cb2663_c7/f1p0/3242-1F	RLK-Pelle_RLCK-IXb	PK
F01_cb2688_c0/f1p0/3748-2F	CMGC_DYRK-PRP4	PK
F01_cb2688_c1/f1p0/2450-2F	CMGC_DYRK-PRP4	PK
F01_cb2688_c2/f1p0/3661-0F	CMGC_DYRK-PRP4	PK

F01_cb2735_c14/f1p3/3537-0F	RLK-Pelle_LRR-XI-1	PK
F01_cb2735_c15/f1p0/2847-2F	RLK-Pelle_LRR-XI-1	PK
F01_cb2735_c7/f1p3/3636-2F	RLK-Pelle_LRR-XI-1	PK
F01_cb2735_c8/f1p3/4461-1F	RLK-Pelle_LRR-XI-1	PK
F01_cb2770_c12/f1p1/3809-0F	RLK-Pelle_SD-2b	PK
F01_cb2770_c12/f1p1/3809-1F	RLK-Pelle_DLSV	PK
F01_cb2770_c12/f1p1/3809-2F	RLK-Pelle_DLSV	PK
F01_cb2770_c13/f1p1/2635-1F	RLK-Pelle_LRR-III	PK
F01_cb2770_c13/f1p1/2635-2F	RLK-Pelle_DLSV	PK
F01_cb2770_c14/f1p1/3101-1F	RLK-Pelle_DLSV	PK
F01_cb2770_c15/f1p1/3337-0F	RLK-Pelle_DLSV	PK
F01_cb2770_c15/f1p1/3337-1F	RLK-Pelle_DLSV	PK
F01_cb2770_c15/f1p1/3337-2F	RLK-Pelle_DLSV	PK
F01_cb2770_c16/f1p0/3092-1F	RLK-Pelle_DLSV	PK
F01_cb2770_c17/f1p0/3038-1F	RLK-Pelle_DLSV	PK
F01_cb2770_c17/f1p0/3038-2F	RLK-Pelle_DLSV	PK
F01_cb2770_c19/f1p0/3145-1F	RLK-Pelle_DLSV	PK
F01_cb2770_c19/f1p0/3145-2F	RLK-Pelle_DLSV	PK
F01_cb2770_c2/f3p0/2565-1F	RLK-Pelle_DLSV	PK
F01_cb2770_c21/f1p0/3235-1F	RLK-Pelle_DLSV	PK
F01_cb2770_c22/f1p0/3195-1F	RLK-Pelle_DLSV	PK
F01_cb2770_c22/f1p0/3195-2F	RLK-Pelle_CR4L	PK
F01_cb2770_c24/f1p1/2794-1F	RLK-Pelle_DLSV	PK
F01_cb2770_c24/f1p1/2794-2F	RLK-Pelle_DLSV	PK
F01_cb2770_c26/f1p0/4911-0F	RLK-Pelle_DLSV	PK
F01_cb2770_c27/f1p1/2537-1F	RLK-Pelle_DLSV	PK

F01_cb2770_c28/f1p0/3699-0F	RLK-Pelle_DLSV	PK
F01_cb2770_c28/f1p0/3699-1F	RLK-Pelle_DLSV	PK
F01_cb2770_c28/f1p0/3699-2F	RLK-Pelle_DLSV	PK
F01_cb2770_c29/f1p0/2418-0F	RLK-Pelle_DLSV	PK
F01_cb2770_c29/f1p0/2418-2F	RLK-Pelle_DLSV	PK
F01_cb2770_c30/f1p2/2621-0F	RLK-Pelle_DLSV	PK
F01_cb2770_c32/f1p1/2588-0F	RLK-Pelle_DLSV	PK
F01_cb2770_c35/f1p0/2093-1F	RLK-Pelle_DLSV	PK
F01_cb2770_c35/f1p0/2093-2F	RLK-Pelle_DLSV	PK
F01_cb2770_c36/f1p0/3443-1F	RLK-Pelle_DLSV	PK
F01_cb2770_c36/f1p0/3443-2F	RLK-Pelle_DLSV	PK
F01_cb2770_c37/f1p0/2426-1F	RLK-Pelle_DLSV	PK
F01_cb2770_c38/f1p0/3465-0F	RLK-Pelle_DLSV	PK
F01_cb2770_c38/f1p0/3465-1F	RLK-Pelle_DLSV	PK
F01_cb2770_c38/f1p0/3465-2F	RLK-Pelle_DLSV	PK
F01_cb2770_c39/f1p0/2594-2F	RLK-Pelle_DLSV	PK
F01_cb2770_c41/f1p1/2574-2F	RLK-Pelle_DLSV	PK
F01_cb2770_c42/f1p1/2535-2F	RLK-Pelle_DLSV	PK
F01_cb2770_c43/f1p1/2757-1F	RLK-Pelle_DLSV	PK
F01_cb2770_c44/f1p0/3320-0F	RLK-Pelle_DLSV	PK
F01_cb2770_c45/f1p1/2767-2F	RLK-Pelle_DLSV	PK
F01_cb2770_c46/f1p1/3457-1F	RLK-Pelle_DLSV	PK
F01_cb2770_c46/f1p1/3457-2F	RLK-Pelle_DLSV	PK
F01_cb2770_c47/f1p0/3578-1F	RLK-Pelle_DLSV	PK
F01_cb2770_c48/f2p1/2540-0F	RLK-Pelle_DLSV	PK
F01_cb2770_c48/f2p1/2540-1F	RLK-Pelle_DLSV	PK

F01_cb2770_c48/f2p1/2540-2F	RLK-Pelle_DLSV	PK
F01_cb2770_c49/f1p0/2592-1F	RLK-Pelle_DLSV	PK
F01_cb2770_c50/f1p0/2615-1F	RLK-Pelle_DLSV	PK
F01_cb2770_c52/f1p0/2648-2F	RLK-Pelle_DLSV	PK
F01_cb2770_c55/f1p0/2896-1F	RLK-Pelle_DLSV	PK
F01_cb2770_c57/f1p1/3444-1F	RLK-Pelle_DLSV	PK
F01_cb2770_c57/f1p1/3444-2F	RLK-Pelle_DLSV	PK
F01_cb2770_c58/f1p0/3220-0F	RLK-Pelle_DLSV	PK
F01_cb2770_c59/f1p0/2758-2F	RLK-Pelle_DLSV	PK
F01_cb2770_c68/f1p0/2804-2F	RLK-Pelle_DLSV	PK
F01_cb2770_c73/f1p0/3196-0F	RLK-Pelle_DLSV	PK
F01_cb2770_c73/f1p0/3196-2F	RLK-Pelle_DLSV	PK
F01_cb2770_c75/f1p0/2342-1F	RLK-Pelle_DLSV	PK
F01_cb2770_c78/f1p0/1820-0F	RLK-Pelle_DLSV	PK
F01_cb2802_c11/f1p0/2727-1F	CMGC_CDK-CDK8	PK
F01_cb2802_c14/f1p0/2534-1F	CMGC_CDK-CDK8	PK
F01_cb2802_c17/f5p0/2060-1F	CMGC_CDK-CDK8	PK
F01_cb2802_c3/f2p0/1941-2F	CMGC_CDK-CDK8	PK
F01_cb2802_c4/f1p0/3732-2F	CMGC_CDK-CDK8	PK
F01_cb2802_c6/f1p0/1916-1F	CMGC_CDK-CDK8	PK
F01_cb2802_c7/f1p0/1966-1F	CMGC_CDK-CDK8	PK
F01_cb2810_c1/f1p0/3610-1F	RLK-Pelle_LRR-XII-1	PK
F01_cb2810_c2/f1p0/3579-1F	RLK-Pelle_LRR-XII-1	PK
F01_cb2810_c4/f1p0/3486-0F	RLK-Pelle_LRR-XII-1	PK
F01_cb2821_c0/f1p0/3647-1F	CMGC_CLK	PK
F01_cb2821_c1/f1p0/2302-1F	CMGC_CLK	PK

F01_cb2821_c2/f1p0/2624-1F	CMGC_CLK	PK
F01_cb2821_c3/f1p0/1030-0F	CMGC_CLK	PK
F01_cb2821_c3/f1p0/1030-2F	CMGC_CLK	PK
F01_cb2823_c1/f1p0/3417-1R	RLK-Pelle_LRR-XI-1	PK
F01_cb2840_c0/f1p0/3793-1F	RLK-Pelle_LRR-XII-1	PK
F01_cb2840_c2/f1p0/1528-1F	RLK-Pelle_LRR-XII-1	PK
F01_cb2840_c2/f1p0/1528-2F	RLK-Pelle_LRR-XII-1	PK
F01_cb2849_c10/f1p0/3378-1F	RLK-Pelle_LysM	PK
F01_cb2849_c11/f1p0/2657-1F	RLK-Pelle_LysM	PK
F01_cb2849_c13/f1p0/2942-2F	RLK-Pelle_LysM	PK
F01_cb2849_c16/f3p0/2734-0F	RLK-Pelle_LysM	PK
F01_cb2849_c2/f1p0/3791-0F	RLK-Pelle_LysM	PK
F01_cb2849_c2/f1p0/3791-1F	RLK-Pelle_LysM	PK
F01_cb2849_c2/f1p0/3791-2F	RLK-Pelle_LysM	PK
F01_cb2854_c10/f1p0/3788-1F	RLK-Pelle_LRR-XI-1	PK
F01_cb2854_c11/f1p0/3334-0F	RLK-Pelle_LRR-XI-1	PK
F01_cb2854_c12/f1p0/3908-0F	RLK-Pelle_LRR-XI-1	PK
F01_cb2854_c14/f1p0/3567-1F	RLK-Pelle_LRR-XI-1	PK
F01_cb2854_c15/f1p0/3776-2F	RLK-Pelle_LRR-XI-1	PK
F01_cb2854_c5/f2p0/3548-1F	RLK-Pelle_LRR-XI-1	PK
F01_cb2900_c2/f1p0/3756-0F	SCY1_SCYL2	PK
F01_cb2900_c3/f1p0/3545-1F	SCY1_SCYL2	PK
F01_cb2900_c5/f1p0/2187-2F	SCY1_SCYL2	PK
F01_cb2938_c0/f2p0/3750-1F	RLK-Pelle_LRR-XI-1	PK
F01_cb2938_c1/f1p0/3758-2F	RLK-Pelle_LRR-XI-1	PK
F01_cb2965_c0/f1p0/3440-0F	RLK-Pelle_LRR-XII-1	PK

F01_cb2965_c1/f1p0/3306-1F	RLK-Pelle_LRR-XII-1	PK
F01_cb2965_c1/f1p0/3306-2F	RLK-Pelle_LRR-XII-1	PK
F01_cb2965_c3/f1p0/3423-1F	RLK-Pelle_LRR-XII-1	PK
F01_cb2965_c4/f1p0/3375-1F	RLK-Pelle_LRR-XII-1	PK
F01_cb3115_c0/f1p0/3717-0F	RLK-Pelle_LRR-XI-1	PK
F01_cb3115_c0/f1p0/3717-2F	RLK-Pelle_LRR-XI-1	PK
F01_cb3115_c1/f1p0/3824-0F	RLK-Pelle_LRR-XI-1	PK
F01_cb3115_c1/f1p0/3824-1F	RLK-Pelle_LRR-XI-1	PK
F01_cb3115_c3/f1p0/3418-0F	RLK-Pelle_LRR-XI-1	PK
F01_cb3115_c5/f1p0/3669-0F	RLK-Pelle_LRR-XI-1	PK
F01_cb3130_c0/f2p0/2363-0F	RLK-Pelle_LRR-II	PK
F01_cb3130_c1/f1p0/3713-2F	RLK-Pelle_LRR-II	PK
F01_cb3130_c2/f1p0/2365-0F	RLK-Pelle_LRR-II	PK
F01_cb3130_c4/f1p0/2408-1F	RLK-Pelle_LRR-II	PK
F01_cb3130_c5/f1p0/2742-0F	RLK-Pelle_LRR-II	PK
F01_cb3130_c7/f1p0/2534-2F	RLK-Pelle_LRR-II	PK
F01_cb3134_c0/f3p0/3714-1F	RLK-Pelle_LRR-Xb-1	PK
F01_cb3134_c1/f1p0/3871-2F	RLK-Pelle_LRR-Xb-1	PK
F01_cb3134_c3/f1p0/5077-0F	RLK-Pelle_LRR-Xb-1	PK
F01_cb3170_c0/f2p1/2670-0F	TKL-Pl-4	PK
F01_cb3170_c3/f1p1/2075-1F	TKL-Pl-4	PK
F01_cb3170_c4/f1p1/2150-2F	TKL-Pl-4	PK
F01_cb3170_c5/f1p2/2463-0F	TKL-Pl-4	PK
F01_cb3170_c6/f1p0/2104-0F	TKL-Pl-4	PK
F01_cb3183_c13/f1p3/2674-2F	RLK-Pelle_CrRLK1L-1	PK
F01_cb3183_c3/f2p4/3655-2F	RLK-Pelle_CrRLK1L-1	PK

F01_cb3183_c6/f2p4/3666-1F	RLK-Pelle_CrRLK1L-1	PK
F01_cb3183_c8/f1p3/3030-0F	RLK-Pelle_CrRLK1L-1	PK
F01_cb3230_c1/f1p0/3098-0F	RLK-Pelle_RLCK-IXb	PK
F01_cb3263_c1/f1p0/3442-1F	RLK-Pelle_LRR-XII-1	PK
F01_cb3263_c10/f1p0/1731-1F	RLK-Pelle_LRR-XII-1	PK
F01_cb3263_c3/f1p0/3378-0F	RLK-Pelle_LRR-XII-1	PK
F01_cb3263_c5/f1p0/3305-2F	RLK-Pelle_LRR-XII-1	PK
F01_cb3309_c10/f1p1/3166-1F	CMGC_CLK	PK
F01_cb3309_c11/f1p0/1951-1F	CMGC_CLK	PK
F01_cb3309_c11/f1p0/1951-2F	CMGC_CLK	PK
F01_cb3309_c13/f1p0/2443-2F	CMGC_CLK	PK
F01_cb3309_c14/f1p0/2567-2F	CMGC_CLK	PK
F01_cb3309_c15/f1p0/1803-0F	CMGC_CLK	PK
F01_cb3309_c16/f1p0/1711-0F	CMGC_CLK	PK
F01_cb3309_c2/f2p0/2095-1F	CMGC_CLK	PK
F01_cb3309_c3/f2p0/1731-0F	CMGC_CLK	PK
F01_cb3309_c3/f2p0/1731-1F	CMGC_CLK	PK
F01_cb3309_c4/f1p1/3664-2F	CMGC_CLK	PK
F01_cb3309_c5/f1p0/3239-2F	CMGC_CLK	PK
F01_cb3309_c6/f1p0/2086-2F	CMGC_CLK	PK
F01_cb3309_c7/f1p0/1997-0F	CMGC_CLK	PK
F01_cb3309_c8/f1p0/1954-1F	CMGC_CLK	PK
F01_cb3309_c9/f1p0/2090-1F	CMGC_CLK	PK
F01_cb3326_c2/f1p0/4564-0F	RLK-Pelle_LRR-Xb-2	PK
F01_cb3326_c4/f1p0/3861-1F	RLK-Pelle_LRR-Xb-2	PK
F01_cb3326_c6/f1p0/3863-1F	RLK-Pelle_LRR-Xb-2	PK

F01_cb3337_c0/f2p0/2594-0F	RLK-Pelle_WAK_LRK10L-1	PK
F01_cb3337_c1/f2p0/2494-2F	RLK-Pelle_WAK_LRK10L-1	PK
F01_cb3337_c2/f1p0/3655-1F	RLK-Pelle_WAK_LRK10L-1	PK
F01_cb3337_c3/f1p0/3629-0F	RLK-Pelle_WAK_LRK10L-1	PK
F01_cb3337_c6/f1p0/2762-0F	RLK-Pelle_WAK_LRK10L-1	PK
F01_cb3371_c0/f5p1/2506-2F	CAMK_CDPK	PK
F01_cb3371_c1/f2p1/2479-0F	CAMK_CDPK	PK
F01_cb3371_c1/f2p1/2479-1F	CAMK_CDPK	PK
F01_cb3371_c1/f2p1/2479-2F	CAMK_CDPK	PK
F01_cb3371_c3/f2p1/2565-2F	CAMK_CDPK	PK
F01_cb3371_c4/f1p0/3647-0F	CAMK_CDPK	PK
F01_cb3371_c4/f1p0/3647-2F	CAMK_CDPK	PK
F01_cb3371_c5/f1p0/2283-1F	CAMK_CDPK	PK
F01_cb3371_c9/f1p0/2372-0F	CAMK_CDPK	PK
F01_cb3372_c1/f1p0/3385-0F	RLK-Pelle_LRR-XI-1	PK
F01_cb33_c0/f5p0/1993-2F	CMGC_GSK	PK
F01_cb33_c1/f2p0/2095-1F	CMGC_GSK	PK
F01_cb33_c2/f3p1/1966-2F	CMGC_GSK	PK
F01_cb33_c5/f1p1/2191-0F	CMGC_GSK	PK
F01_cb33_c5/f1p1/2191-2F	CMGC_GSK	PK
F01_cb33_c7/f1p0/1963-2F	CMGC_GSK	PK
F01_cb33_c8/f1p0/1900-0F	CMGC_GSK	PK
F01_cb3435_c0/f3p0/3501-0F	RLK-Pelle_LRR-IX	PK
F01_cb3435_c0/f3p0/3501-2F	RLK-Pelle_LRR-IX	PK
F01_cb3435_c1/f1p0/3625-2F	RLK-Pelle_LRR-IX	PK
F01_cb3435_c4/f1p0/3640-1F	RLK-Pelle_LRR-IX	PK

F01_cb3435_c6/f1p1/3576-0F	RLK-Pelle_LRR-IX	PK
F01_cb3439_c0/f1p0/3625-1F	RLK-Pelle_LRR-XII-1	PK
F01_cb3439_c0/f1p0/3625-2F	RLK-Pelle_LRR-XII-1	PK
F01_cb3439_c1/f1p0/4663-1F	RLK-Pelle_LRR-XII-1	PK
F01_cb3439_c2/f1p0/3280-0F	RLK-Pelle_LRR-XII-1	PK
F01_cb3439_c2/f1p0/3280-1F	RLK-Pelle_LRR-XII-1	PK
F01_cb3439_c3/f1p0/2926-0F	RLK-Pelle_LRR-XII-1	PK
F01_cb3439_c3/f1p0/2926-1F	RLK-Pelle_LRR-XII-1	PK
F01_cb3439_c5/f1p0/2543-2F	RLK-Pelle_LRR-XII-1	PK
F01_cb3470_c1/f1p0/3617-1F	RLK-Pelle_LRR-XIV	PK
F01_cb3470_c2/f1p0/2829-1F	RLK-Pelle_LRR-XIV	PK
F01_cb3470_c3/f1p0/3191-0F	RLK-Pelle_LRR-XIV	PK
F01_cb3470_c5/f1p0/3472-0F	RLK-Pelle_LRR-XIV	PK
F01_cb3470_c6/f1p0/3057-1F	RLK-Pelle_LRR-XIV	PK
F01_cb3470_c7/f1p0/2987-0F	RLK-Pelle_LRR-XIV	PK
F01_cb3474_c0/f1p0/3617-2F	RLK-Pelle_RLCK-VIIa-1	PK
F01_cb3474_c1/f1p0/3693-1F	RLK-Pelle_RLCK-VIIa-1	PK
F01_cb3474_c1/f1p0/3693-2F	RLK-Pelle_RLCK-VIIa-1	PK
F01_cb3527_c1/f1p0/3601-1F	RLK-Pelle_LRK10L-2	PK
F01_cb3527_c10/f1p1/3423-0F	RLK-Pelle_LRK10L-2	PK
F01_cb3527_c12/f1p1/2296-2F	RLK-Pelle_LRK10L-2	PK
F01_cb3527_c14/f1p1/2566-1F	RLK-Pelle_LRK10L-2	PK
F01_cb3527_c15/f1p1/1912-1F	RLK-Pelle_LRK10L-2	PK
F01_cb3527_c16/f1p1/2234-2F	RLK-Pelle_LRK10L-2	PK
F01_cb3527_c19/f1p0/2224-0F	RLK-Pelle_LRK10L-2	PK
F01_cb3527_c19/f1p0/2224-1F	RLK-Pelle_LRK10L-2	PK

F01_cb3527_c21/f1p1/2165-0F	RLK-Pelle_LRK10L-2	PK
F01_cb3527_c22/f1p0/1864-2F	RLK-Pelle_LRK10L-2	PK
F01_cb3527_c5/f1p1/2206-1F	RLK-Pelle_LRK10L-2	PK
F01_cb3527_c7/f1p0/2341-0F	RLK-Pelle_LRK10L-2	PK
F01_cb3531_c12/f1p0/2627-2F	RLK-Pelle_LRR-VI-2	PK
F01_cb3531_c14/f1p0/2881-1F	RLK-Pelle_LRR-VI-2	PK
F01_cb3531_c15/f1p0/2480-2F	RLK-Pelle_LRR-VI-2	PK
F01_cb3531_c17/f1p0/2938-1F	RLK-Pelle_LRR-VI-2	PK
F01_cb3531_c17/f1p0/2938-2F	RLK-Pelle_LRR-VI-2	PK
F01_cb3531_c18/f1p0/2707-0F	RLK-Pelle_LRR-VI-2	PK
F01_cb3531_c20/f1p0/2825-1F	RLK-Pelle_LRR-VI-2	PK
F01_cb3531_c22/f1p0/2608-0F	RLK-Pelle_LRR-VI-2	PK
F01_cb3531_c24/f9p1/3281-2F	RLK-Pelle_LRR-VI-2	PK
F01_cb3531_c4/f2p1/3294-0F	RLK-Pelle_LRR-VI-2	PK
F01_cb3531_c4/f2p1/3294-2F	RLK-Pelle_LRR-VI-2	PK
F01_cb3555_c0/f2p0/5456-0F	TKL-Pl-1	PK
F01_cb3555_c6/f1p0/5501-2F	TKL-Pl-1	PK
F01_cb3555_c7/f1p0/5403-1F	TKL-Pl-1	PK
F01_cb3562_c15/f1p1/2983-1F	RLK-Pelle_RLCK-VI	PK
F01_cb3562_c19/f1p0/3192-0F	RLK-Pelle_RLCK-VI	PK
F01_cb3562_c19/f1p0/3192-1F	RLK-Pelle_RLCK-VI	PK
F01_cb3562_c22/f1p0/2867-0F	RLK-Pelle_RLCK-VI	PK
F01_cb3562_c25/f1p0/3146-2F	RLK-Pelle_RLCK-VI	PK
F01_cb3562_c27/f1p0/3043-1F	RLK-Pelle_RLCK-VI	PK
F01_cb3562_c28/f1p0/2970-0F	RLK-Pelle_RLCK-VI	PK
F01_cb3562_c29/f2p0/2954-2F	RLK-Pelle_RLCK-VI	PK

F01_cb3562_c30/f1p0/2949-0F	RLK-Pelle_RLCK-VI	PK
F01_cb3562_c6/f1p2/3245-2F	RLK-Pelle_RLCK-VI	PK
F01_cb3593_c0/f3p0/2940-1F	RLK-Pelle_RKF3	PK
F01_cb3593_c0/f3p0/2940-2F	RLK-Pelle_RKF3	PK
F01_cb3593_c1/f1p0/3600-1F	RLK-Pelle_RKF3	PK
F01_cb3593_c2/f1p0/2887-1F	RLK-Pelle_RKF3	PK
F01_cb3593_c3/f1p0/2862-0F	RLK-Pelle_RKF3	PK
F01_cb3593_c3/f1p0/2862-1F	RLK-Pelle_RKF3	PK
F01_cb3593_c5/f1p0/2399-2F	RLK-Pelle_RKF3	PK
F01_cb3593_c7/f1p0/2064-1F	RLK-Pelle_RKF3	PK
F01_cb3657_c10/f2p0/3290-2F	RLK-Pelle_LRR-XI-1	PK
F01_cb3657_c4/f1p0/3466-2F	RLK-Pelle_LRR-XI-1	PK
F01_cb3657_c8/f1p0/3296-0F	RLK-Pelle_LRR-XI-1	PK
F01_cb3660_c0/f2p1/2307-0F	RLK-Pelle_LRR-III	PK
F01_cb3660_c1/f2p0/2693-0F	RLK-Pelle_LRR-III	PK
F01_cb3660_c2/f1p1/3569-0F	RLK-Pelle_LRR-III	PK
F01_cb3660_c3/f1p0/2654-2F	RLK-Pelle_LRR-III	PK
F01_cb3660_c5/f1p0/2230-0F	RLK-Pelle_LRR-III	PK
F01_cb3660_c7/f1p0/2652-1F	RLK-Pelle_LRR-III	PK
F01_cb3664_c1/f3p0/2420-1F	CMGC_CDK-PITSLRE	PK
F01_cb3664_c10/f1p0/2454-1F	CMGC_CDK-PITSLRE	PK
F01_cb3664_c4/f1p2/3015-2F	CMGC_CDK-PITSLRE	PK
F01_cb3664_c6/f1p0/3060-2F	CMGC_CDK-PITSLRE	PK
F01_cb3696_c14/f1p0/3559-1F	RLK-Pelle_CrRLK1L-1	PK
F01_cb3696_c18/f1p0/3409-1F	RLK-Pelle_CrRLK1L-1	PK
F01_cb3696_c21/f1p1/3238-2F	RLK-Pelle_CrRLK1L-1	PK

F01_cb3696_c22/f1p0/3426-0F	RLK-Pelle_CrRLK1L-1	PK
F01_cb3696_c29/f1p0/2871-0F	RLK-Pelle_CrRLK1L-1	PK
F01_cb3696_c30/f1p1/3332-0F	RLK-Pelle_CrRLK1L-1	PK
F01_cb3696_c33/f1p0/3129-0F	RLK-Pelle_CrRLK1L-1	PK
F01_cb3696_c33/f1p0/3129-1F	RLK-Pelle_CrRLK1L-1	PK
F01_cb3696_c45/f1p0/2437-1F	RLK-Pelle_CrRLK1L-1	PK
F01_cb3696_c8/f3p0/3274-1F	RLK-Pelle_CrRLK1L-1	PK
F01_cb373_c11/f1p0/3937-0F	AGC_RSK-2	PK
F01_cb373_c11/f1p0/3937-1F	AGC_RSK-2	PK
F01_cb373_c12/f1p1/3932-1F	AGC_RSK-2	PK
F01_cb373_c13/f1p0/3706-0F	AGC_RSK-2	PK
F01_cb373_c16/f1p0/3997-1F	AGC_RSK-2	PK
F01_cb373_c17/f1p0/3689-2F	AGC_RSK-2	PK
F01_cb373_c29/f1p0/4336-0F	AGC_RSK-2	PK
F01_cb373_c32/f1p0/2443-2F	AGC_RSK-2	PK
F01_cb373_c34/f1p0/4280-2F	AGC_RSK-2	PK
F01_cb373_c36/f1p0/4183-2F	AGC_RSK-2	PK
F01_cb373_c37/f1p0/4690-2F	AGC_RSK-2	PK
F01_cb373_c39/f1p0/4415-2F	AGC_RSK-2	PK
F01_cb373_c4/f2p0/3694-2F	AGC_RSK-2	PK
F01_cb373_c40/f1p0/4542-1F	AGC_RSK-2	PK
F01_cb373_c40/f1p0/4542-2F	AGC_RSK-2	PK
F01_cb373_c46/f1p0/6511-1F	AGC_RSK-2	PK
F01_cb373_c48/f1p0/5064-1F	AGC_RSK-2	PK
F01_cb373_c6/f1p0/4615-2F	AGC_RSK-2	PK
F01_cb373_c7/f1p0/3964-2F	AGC_RSK-2	PK

F01_cb373_c8/f1p0/3698-1F	AGC_RSK-2	PK
F01_cb373_c9/f1p0/3771-0F	AGC_RSK-2	PK
F01_cb3779_c1/f1p0/2575-0F	RLK-Pelle_RLCK-VI	PK
F01_cb3779_c1/f1p0/2575-1F	RLK-Pelle_RLCK-VI	PK
F01_cb3854_c0/f1p0/3504-0R	TKL_Gdt	PK
F01_cb3854_c1/f1p0/2568-0R	TKL_Gdt	PK
F01_cb3854_c2/f1p0/2571-2R	TKL_Gdt	PK
F01_cb3854_c4/f1p0/3075-1R	TKL_Gdt	PK
F01_cb3854_c5/f1p0/2584-2R	TKL_Gdt	PK
F01_cb3855_c1/f1p1/3561-2F	RLK-Pelle_SD-2b	PK
F01_cb3855_c5/f1p0/2771-0F	RLK-Pelle_SD-2b	PK
F01_cb3875_c0/f5p0/2930-2F	RLK-Pelle_LRR-I-1	PK
F01_cb3875_c1/f3p1/2386-1F	RLK-Pelle_LRR-I-1	PK
F01_cb3875_c2/f2p1/2402-1F	RLK-Pelle_LRR-I-1	PK
F01_cb3875_c3/f2p0/2412-2F	RLK-Pelle_LRR-I-1	PK
F01_cb3875_c6/f1p0/2825-0F	RLK-Pelle_LRR-I-1	PK
F01_cb3875_c6/f1p0/2825-1F	RLK-Pelle_LRR-I-1	PK
F01_cb3875_c7/f1p0/2347-2F	RLK-Pelle_LRR-I-1	PK
F01_cb3875_c8/f1p0/2382-2F	RLK-Pelle_LRR-I-1	PK
F01_cb3875_c9/f1p0/2412-1F	RLK-Pelle_LRR-I-1	PK
F01_cb3897_c2/f1p0/3326-2F	RLK-Pelle_LRR-XI-1	PK
F01_cb3897_c3/f1p0/3495-1F	RLK-Pelle_LRR-XI-1	PK
F01_cb3903_c0/f2p0/2622-1F	CMGC_CDK-CRK7-CDK9	PK
F01_cb3903_c1/f1p0/3496-0F	CMGC_CDK-CRK7-CDK9	PK
F01_cb3903_c2/f1p0/3465-0F	CMGC_CDK-CRK7-CDK9	PK
F01_cb3926_c1/f1p0/1927-1F	RLK-Pelle_RLCK-IXa	PK

F01_cb3926_c2/f1p0/3089-0F	RLK-Pelle_RLCK-IXa	PK
F01_cb3926_c3/f1p0/1949-2F	RLK-Pelle_RLCK-IXa	PK
F01_cb3939_c10/f3p0/3353-0F	AGC_RSK-2	PK
F01_cb3939_c13/f1p0/3298-0F	AGC_RSK-2	PK
F01_cb3939_c15/f1p0/3290-2F	AGC_RSK-2	PK
F01_cb3939_c16/f1p0/2356-0F	AGC_RSK-2	PK
F01_cb3939_c17/f1p0/2467-0F	AGC_RSK-2	PK
F01_cb3939_c17/f1p0/2467-1F	AGC_RSK-2	PK
F01_cb3939_c21/f1p0/2607-1F	AGC_RSK-2	PK
F01_cb3939_c27/f1p0/2215-1F	AGC_RSK-2	PK
F01_cb3939_c27/f1p0/2215-2F	AGC_RSK-2	PK
F01_cb3939_c29/f1p0/2382-2F	AGC_RSK-2	PK
F01_cb3939_c32/f1p0/2860-1F	AGC_RSK-2	PK
F01_cb3939_c39/f6p0/2469-0F	AGC_RSK-2	PK
F01_cb3939_c42/f13p1/2456-0F	AGC_RSK-2	PK
F01_cb396_c10/f3p0/2297-2F	CAMK_CDPK	PK
F01_cb396_c16/f1p0/2356-1F	CAMK_CDPK	PK
F01_cb396_c18/f1p0/2281-0F	CAMK_CDPK	PK
F01_cb396_c19/f1p0/2179-0F	CAMK_CDPK	PK
F01_cb396_c19/f1p0/2179-1F	CAMK_CDPK	PK
F01_cb396_c25/f1p0/3318-0F	CAMK_CDPK	PK
F01_cb396_c29/f1p0/2646-2F	CAMK_CDPK	PK
F01_cb396_c30/f1p0/2423-0F	CAMK_CDPK	PK
F01_cb396_c30/f1p0/2423-2F	CAMK_CDPK	PK
F01_cb396_c4/f9p0/2621-0F	CAMK_CDPK	PK
F01_cb396_c41/f1p0/2356-1F	CAMK_CDPK	PK

F01_cb396_c42/f1p0/2343-0F	CAMK_CDPK	PK
F01_cb396_c45/f1p0/2634-2F	CAMK_CDPK	PK
F01_cb396_c48/f1p0/2172-2F	CAMK_CDPK	PK
F01_cb396_c49/f1p0/3077-2F	CAMK_CDPK	PK
F01_cb396_c50/f1p0/2950-0F	CAMK_CDPK	PK
F01_cb396_c51/f1p1/2465-2F	CAMK_CDPK	PK
F01_cb396_c57/f1p0/2029-0F	CAMK_CDPK	PK
F01_cb396_c67/f28p0/2542-0F	CAMK_CDPK	PK
F01_cb3988_c0/f2p0/3006-0F	RLK-Pelle_PERK-1	PK
F01_cb3988_c1/f1p1/3470-1F	RLK-Pelle_PERK-1	PK
F01_cb3988_c15/f1p0/2853-0F	RLK-Pelle_PERK-1	PK
F01_cb3988_c5/f1p0/3981-0F	RLK-Pelle_PERK-1	PK
F01_cb3988_c6/f1p0/2823-0F	RLK-Pelle_PERK-1	PK
F01_cb3988_c7/f1p1/3083-0F	RLK-Pelle_PERK-1	PK
F01_cb3988_c9/f1p0/2730-1F	RLK-Pelle_PERK-1	PK
F01_cb3988_c9/f1p0/2730-2F	RLK-Pelle_PERK-1	PK
F01_cb3993_c0/f1p0/3469-1F	RLK-Pelle_RLCK-V	PK
F01_cb3993_c2/f1p0/1723-0F	RLK-Pelle_RLCK-V	PK
F01_cb4016_c10/f1p0/2636-0F	RLK-Pelle_DLSV	PK
F01_cb4016_c10/f1p0/2636-2F	RLK-Pelle_DLSV	PK
F01_cb4016_c11/f1p0/2688-2F	RLK-Pelle_DLSV	PK
F01_cb4016_c12/f1p0/2681-2F	RLK-Pelle_DLSV	PK
F01_cb4016_c14/f1p0/2928-0F	RLK-Pelle_DLSV	PK
F01_cb4016_c14/f1p0/2928-1F	RLK-Pelle_DLSV	PK
F01_cb4016_c14/f1p0/2928-2F	RLK-Pelle_DLSV	PK
F01_cb4016_c15/f1p1/2488-1F	RLK-Pelle_DLSV	PK

F01_cb4016_c16/f3p2/2553-1F	RLK-Pelle_DLSV	PK
F01_cb4016_c17/f1p0/2709-0F	RLK-Pelle_DLSV	PK
F01_cb4016_c17/f1p0/2709-2F	RLK-Pelle_DLSV	PK
F01_cb4016_c18/f1p0/2605-1F	RLK-Pelle_DLSV	PK
F01_cb4016_c18/f1p0/2605-2F	RLK-Pelle_DLSV	PK
F01_cb4016_c19/f1p0/2644-1F	RLK-Pelle_DLSV	PK
F01_cb4016_c19/f1p0/2644-2F	RLK-Pelle_DLSV	PK
F01_cb4016_c2/f3p1/2863-1F	RLK-Pelle_DLSV	PK
F01_cb4016_c20/f1p1/2959-1F	RLK-Pelle_DLSV	PK
F01_cb4016_c24/f1p0/2450-1F	RLK-Pelle_DLSV	PK
F01_cb4016_c24/f1p0/2450-2F	RLK-Pelle_DLSV	PK
F01_cb4016_c27/f1p1/3204-0F	RLK-Pelle_DLSV	PK
F01_cb4016_c27/f1p1/3204-1F	RLK-Pelle_DLSV	PK
F01_cb4016_c29/f1p0/2693-1F	RLK-Pelle_DLSV	PK
F01_cb4016_c3/f2p0/2850-0F	RLK-Pelle_DLSV	PK
F01_cb4016_c30/f1p1/3132-1F	RLK-Pelle_DLSV	PK
F01_cb4016_c31/f1p0/2568-2F	RLK-Pelle_DLSV	PK
F01_cb4016_c32/f1p0/1913-1F	RLK-Pelle_DLSV	PK
F01_cb4016_c33/f1p0/2507-1F	RLK-Pelle_DLSV	PK
F01_cb4016_c34/f1p0/2560-0F	RLK-Pelle_DLSV	PK
F01_cb4016_c34/f1p0/2560-2F	RLK-Pelle_DLSV	PK
F01_cb4016_c35/f1p0/2920-0F	RLK-Pelle_DLSV	PK
F01_cb4016_c38/f1p0/2815-1F	RLK-Pelle_DLSV	PK
F01_cb4016_c41/f1p0/3118-0F	RLK-Pelle_LRR-Xb-1	PK
F01_cb4016_c42/f1p1/2580-2F	RLK-Pelle_DLSV	PK
F01_cb4016_c43/f1p0/2750-2F	RLK-Pelle_DLSV	PK

F01_cb4016_c45/f1p0/6266-0F	RLK-Pelle_DLSV	PK
F01_cb4016_c5/f1p0/3485-1F	RLK-Pelle_DLSV	PK
F01_cb4016_c6/f1p1/2956-1F	RLK-Pelle_DLSV	PK
F01_cb4016_c7/f1p0/2879-0F	RLK-Pelle_DLSV	PK
F01_cb4016_c8/f1p0/2818-0F	RLK-Pelle_RKF3	PK
F01_cb4016_c8/f1p0/2818-1F	RLK-Pelle_DLSV	PK
F01_cb4016_c9/f1p1/2685-0F	RLK-Pelle_DLSV	PK
F01_cb4019_c0/f2p0/3413-1F	RLK-Pelle_Singleton	PK
F01_cb4048_c1/f1p1/3452-1F	RLK-Pelle_LRR-XI-1	PK
F01_cb4048_c1/f1p1/3452-2F	RLK-Pelle_LRR-XI-1	PK
F01_cb4065_c0/f1p0/3449-1F	RLK-Pelle_LRR-I-1	PK
F01_cb4065_c0/f1p0/3449-2F	RLK-Pelle_LRR-I-1	PK
F01_cb4065_c1/f1p0/3012-0F	RLK-Pelle_LRR-I-1	PK
F01_cb4067_c11/f1p0/2801-2F	AGC_RSK-2	PK
F01_cb4067_c2/f1p1/3451-0F	AGC_RSK-2	PK
F01_cb4067_c7/f1p0/3749-1F	AGC_RSK-2	PK
F01_cb4067_c8/f1p0/3375-2F	AGC_RSK-2	PK
F01_cb4113_c14/f1p0/3280-1F	RLK-Pelle_LRR-V	PK
F01_cb4113_c2/f1p1/3393-0F	RLK-Pelle_LRR-V	PK
F01_cb4113_c2/f1p1/3393-1F	RLK-Pelle_LRR-V	PK
F01_cb4113_c2/f1p1/3393-2F	RLK-Pelle_LRR-V	PK
F01_cb4113_c21/f1p0/2899-1F	RLK-Pelle_LRR-V	PK
F01_cb4113_c4/f1p0/3418-1F	RLK-Pelle_LRR-V	PK
F01_cb4113_c7/f1p0/3435-2F	RLK-Pelle_LRR-V	PK
F01_cb4113_c8/f1p0/3143-2F	RLK-Pelle_LRR-V	PK
F01_cb4113_c9/f1p0/3342-1F	RLK-Pelle_LRR-V	PK

F01_cb4162_c0/f1p0/3247-2F	CMGC_CDK-CRK7-CDK9	PK
F01_cb4162_c2/f1p1/3150-1F	CMGC_CDK-CRK7-CDK9	PK
F01_cb4162_c3/f1p0/3138-1F	CMGC_CDK-CRK7-CDK9	PK
F01_cb416_c14/f1p0/2799-2F	SCY1_SCYL1	PK
F01_cb416_c19/f1p0/2939-2F	SCY1_SCYL1	PK
F01_cb416_c21/f1p0/3162-0F	SCY1_SCYL1	PK
F01_cb416_c26/f1p0/3038-1F	SCY1_SCYL1	PK
F01_cb416_c28/f1p0/2988-1F	SCY1_SCYL1	PK
F01_cb416_c31/f1p0/3143-2F	SCY1_SCYL1	PK
F01_cb416_c36/f1p0/3277-2F	SCY1_SCYL1	PK
F01_cb416_c40/f1p0/3116-1F	SCY1_SCYL1	PK
F01_cb416_c41/f1p0/3183-0F	SCY1_SCYL1	PK
F01_cb416_c49/f1p0/3164-1F	SCY1_SCYL1	PK
F01_cb416_c51/f1p0/2774-0F	SCY1_SCYL1	PK
F01_cb416_c53/f1p0/2998-0F	SCY1_SCYL1	PK
F01_cb416_c55/f1p1/2871-1F	SCY1_SCYL1	PK
F01_cb416_c62/f1p0/2875-1F	SCY1_SCYL1	PK
F01_cb416_c68/f2p0/3419-0F	SCY1_SCYL1	PK
F01_cb416_c73/f3p0/3007-1F	SCY1_SCYL1	PK
F01_cb4201_c10/f1p0/2976-0F	RLK-Pelle_RLCK-IXb	PK
F01_cb4201_c19/f1p0/2906-1F	RLK-Pelle_RLCK-IXb	PK
F01_cb4201_c19/f1p0/2906-2F	RLK-Pelle_RLCK-IXb	PK
F01_cb4201_c2/f16p0/3072-0F	RLK-Pelle_RLCK-IXb	PK
F01_cb4201_c4/f5p0/2972-0F	RLK-Pelle_RLCK-IXb	PK
F01_cb4208_c10/f1p0/1764-1F	CAMK_OST1L	PK
F01_cb4208_c11/f1p0/1696-0F	CAMK_OST1L	PK

F01_cb4208_c11/f1p0/1696-1F	CAMK_OST1L	PK
F01_cb4208_c16/f1p0/1531-1F	CAMK_OST1L	PK
F01_cb4208_c16/f1p0/1531-2F	CAMK_OST1L	PK
F01_cb4208_c2/f1p0/3403-1F	CAMK_OST1L	PK
F01_cb4208_c2/f1p0/3403-2F	CAMK_OST1L	PK
F01_cb4208_c3/f1p0/2074-0F	CAMK_OST1L	PK
F01_cb4208_c3/f1p0/2074-2F	CAMK_OST1L	PK
F01_cb4208_c5/f1p0/1950-2F	CAMK_OST1L	PK
F01_cb4208_c7/f1p0/2028-0F	CAMK_OST1L	PK
F01_cb4208_c8/f1p0/1816-1F	CAMK_OST1L	PK
F01_cb4219_c1/f1p0/3397-1F	RLK-Pelle_PERK-2	PK
F01_cb4221_c0/f4p0/3358-1F	RLK-Pelle_LRR-XI-1	PK
F01_cb4221_c3/f1p0/3924-0F	RLK-Pelle_LRR-XI-1	PK
F01_cb4221_c3/f1p0/3924-1F	RLK-Pelle_LRR-XI-1	PK
F01_cb4221_c4/f1p1/3385-0F	RLK-Pelle_LRR-XI-1	PK
F01_cb4221_c4/f1p1/3385-2F	RLK-Pelle_LRR-XI-1	PK
F01_cb4233_c106/f1p2/3225-0F	RLK-Pelle_L-LEC	PK
F01_cb4233_c107/f1p0/2385-0F	RLK-Pelle_L-LEC	PK
F01_cb4233_c107/f1p0/2385-1F	RLK-Pelle_L-LEC	PK
F01_cb4233_c109/f1p3/2092-0F	RLK-Pelle_L-LEC	PK
F01_cb4233_c110/f1p1/2847-1F	RLK-Pelle_L-LEC	PK
F01_cb4233_c111/f1p0/2330-0F	RLK-Pelle_L-LEC	PK
F01_cb4233_c112/f1p0/2252-1F	RLK-Pelle_L-LEC	PK
F01_cb4233_c164/f1p1/2453-0F	RLK-Pelle_L-LEC	PK
F01_cb4233_c168/f1p1/2096-1F	RLK-Pelle_L-LEC	PK
F01_cb4233_c168/f1p1/2096-1R	RLK-Pelle_L-LEC	PK

F01_cb4233_c168/f1p1/2096-2F	RLK-Pelle_L-LEC	PK
F01_cb4233_c17/f3p0/2228-2F	RLK-Pelle_L-LEC	PK
F01_cb4233_c230/f1p2/2315-2F	RLK-Pelle_L-LEC	PK
F01_cb4233_c232/f1p0/2301-2F	RLK-Pelle_L-LEC	PK
F01_cb4233_c239/f16p1/2275-0F	RLK-Pelle_L-LEC	PK
F01_cb4233_c240/f11p1/2257-0F	RLK-Pelle_L-LEC	PK
F01_cb4233_c248/f2p0/2257-1F	RLK-Pelle_L-LEC	PK
F01_cb4233_c248/f2p0/2257-2F	RLK-Pelle_L-LEC	PK
F01_cb4233_c38/f2p0/2432-0F	RLK-Pelle_L-LEC	PK
F01_cb4233_c89/f1p1/3782-2F	RLK-Pelle_L-LEC	PK
F01_cb4233_c90/f1p2/2913-1F	RLK-Pelle_L-LEC	PK
F01_cb4233_c92/f1p1/2467-0F	RLK-Pelle_L-LEC	PK
F01_cb4233_c92/f1p1/2467-2F	RLK-Pelle_L-LEC	PK
F01_cb4233_c94/f1p0/2460-0F	RLK-Pelle_L-LEC	PK
F01_cb4233_c94/f1p0/2460-1F	RLK-Pelle_L-LEC	PK
F01_cb4233_c97/f1p1/2296-1F	RLK-Pelle_L-LEC	PK
F01_cb425_c1/f1p1/4818-2F	AGC_MAST	PK
F01_cb425_c3/f1p2/4514-0F	AGC_MAST	PK
F01_cb4260_c1/f1p0/3391-2F	RLK-Pelle_LRR-III	PK
F01_cb4260_c2/f2p2/2188-1F	RLK-Pelle_LRR-III	PK
F01_cb4260_c4/f2p2/2217-1F	RLK-Pelle_LRR-III	PK
F01_cb4260_c5/f1p1/2120-2F	RLK-Pelle_LRR-III	PK
F01_cb4265_c0/f2p1/3418-1F	RLK-Pelle_LRR-XI-1	PK
F01_cb4265_c1/f1p0/2567-2F	RLK-Pelle_LRR-XI-1	PK
F01_cb4272_c10/f1p0/2431-1F	AGC_NDR	PK
F01_cb4272_c11/f1p0/2448-2F	AGC_NDR	PK

F01_cb4272_c13/f1p0/2415-1F	AGC_NDR	PK
F01_cb4272_c15/f1p0/2056-0F	AGC_NDR	PK
F01_cb4272_c17/f3p0/2418-1F	AGC_NDR	PK
F01_cb4272_c5/f1p0/3380-1F	AGC_NDR	PK
F01_cb4272_c7/f1p0/2177-1F	AGC_NDR	PK
F01_cb4272_c9/f1p0/2343-0F	AGC_NDR	PK
F01_cb4275_c0/f2p0/3202-0F	RLK-Pelle_LRR-VII-1	PK
F01_cb4275_c2/f1p0/2002-0F	RLK-Pelle_LRR-VII-1	PK
F01_cb4349_c0/f2p0/2456-0F	RLK-Pelle_LRR-Xb-1	PK
F01_cb4349_c0/f2p0/2456-1F	RLK-Pelle_LRR-Xb-1	PK
F01_cb4349_c1/f1p0/3370-1F	RLK-Pelle_LRR-Xb-1	PK
F01_cb4349_c2/f1p0/2461-0F	RLK-Pelle_LRR-Xb-1	PK
F01_cb4375_c1/f2p1/3160-1F	RLK-Pelle_LRR-XI-1	PK
F01_cb4375_c3/f1p0/3653-2F	RLK-Pelle_LRR-XI-1	PK
F01_cb4375_c4/f1p0/3606-2F	RLK-Pelle_LRR-XI-1	PK
F01_cb4375_c5/f1p0/3262-0F	RLK-Pelle_LRR-XI-1	PK
F01_cb4375_c5/f1p0/3262-1F	RLK-Pelle_LRR-XI-1	PK
F01_cb4375_c6/f1p0/2971-0F	RLK-Pelle_LRR-XI-1	PK
F01_cb4375_c7/f1p0/3057-1F	RLK-Pelle_LRR-XI-1	PK
F01_cb4461_c1/f2p1/2671-0F	RLK-Pelle_RLCK-IXb	PK
F01_cb4461_c10/f2p2/2767-0F	RLK-Pelle_RLCK-IXb	PK
F01_cb4461_c2/f1p2/3341-1F	RLK-Pelle_RLCK-IXb	PK
F01_cb4461_c3/f1p2/2896-2F	RLK-Pelle_RLCK-IXb	PK
F01_cb4461_c4/f1p2/2844-0F	RLK-Pelle_RLCK-IXb	PK
F01_cb4461_c7/f1p2/2804-2F	RLK-Pelle_RLCK-IXb	PK
F01_cb4461_c9/f1p1/1818-0F	RLK-Pelle_RLCK-IXb	PK

F01_cb4461_c9/f1p1/1818-1F	RLK-Pelle_RLCK-IXb	PK
F01_cb4482_c0/f1p0/3335-1F	RLK-Pelle_SD-2b	PK
F01_cb4482_c1/f1p0/2752-2F	RLK-Pelle_SD-2b	PK
F01_cb4482_c2/f1p0/2586-1F	RLK-Pelle_SD-2b	PK
F01_cb4482_c4/f1p0/2722-0F	RLK-Pelle_SD-2b	PK
F01_cb4483_c0/f2p0/2583-0F	RLK-Pelle_SD-2b	PK
F01_cb4483_c1/f1p0/3291-0F	RLK-Pelle_SD-2b	PK
F01_cb4483_c1/f1p0/3291-1F	RLK-Pelle_SD-2b	PK
F01_cb4483_c2/f1p0/3162-1F	RLK-Pelle_SD-2b	PK
F01_cb4483_c3/f1p0/2625-2F	RLK-Pelle_SD-2b	PK
F01_cb4486_c0/f1p0/3330-2F	RLK-Pelle_DLSV	PK
F01_cb4486_c1/f1p0/3190-2F	RLK-Pelle_DLSV	PK
F01_cb450_c1/f2p2/4660-2F	RLK-Pelle_CrRLK1L-1	PK
F01_cb450_c4/f1p1/4687-0F	RLK-Pelle_CrRLK1L-1	PK
F01_cb450_c5/f1p1/3647-0F	RLK-Pelle_CrRLK1L-1	PK
F01_cb4543_c0/f1p0/3310-1R	RLK-Pelle_DLSV	PK
F01_cb4543_c0/f1p0/3310-2R	RLK-Pelle_DLSV	PK
F01_cb4543_c1/f1p0/3821-0R	RLK-Pelle_DLSV	PK
F01_cb4543_c1/f1p0/3821-1R	RLK-Pelle_DLSV	PK
F01_cb4543_c1/f1p0/3821-2R	RLK-Pelle_DLSV	PK
F01_cb4543_c2/f1p0/2903-2R	RLK-Pelle_DLSV	PK
F01_cb4559_c3/f1p0/3310-0F	CMGC_CK2	PK
F01_cb4559_c3/f1p0/3310-1F	CMGC_CK2	PK
F01_cb4559_c4/f1p0/3029-0F	CMGC_CK2	PK
F01_cb4559_c4/f1p0/3029-1F	CMGC_CK2	PK
F01_cb4559_c5/f1p0/2844-0F	CMGC_CK2	PK

F01_cb4559_c6/f1p0/1326-1F	CMGC_CK2	PK
F01_cb4559_c7/f1p0/1283-0F	CMGC_CK2	PK
F01_cb4559_c9/f1p0/1461-0F	CMGC_CK2	PK
F01_cb4563_c10/f1p1/4024-1F	RLK-Pelle_RLCK-IXb	PK
F01_cb4563_c10/f1p1/4024-2F	RLK-Pelle_RLCK-IXb	PK
F01_cb4563_c12/f1p0/3843-2F	RLK-Pelle_RLCK-IXb	PK
F01_cb4563_c16/f1p2/3314-1F	RLK-Pelle_RLCK-IXb	PK
F01_cb4563_c16/f1p2/3314-2F	RLK-Pelle_RLCK-IXb	PK
F01_cb4563_c17/f1p0/2816-0F	RLK-Pelle_RLCK-IXb	PK
F01_cb4563_c17/f1p0/2816-1F	RLK-Pelle_RLCK-IXb	PK
F01_cb4563_c22/f1p0/3155-1F	RLK-Pelle_RLCK-IXb	PK
F01_cb4563_c27/f1p0/2598-1F	RLK-Pelle_RLCK-IXb	PK
F01_cb4563_c29/f1p0/2820-0F	RLK-Pelle_RLCK-IXb	PK
F01_cb4563_c32/f1p0/2938-1F	RLK-Pelle_RLCK-IXb	PK
F01_cb4563_c32/f1p0/2938-2F	RLK-Pelle_RLCK-IXb	PK
F01_cb4563_c33/f1p0/2825-2F	RLK-Pelle_RLCK-IXb	PK
F01_cb4563_c36/f7p0/2932-0F	RLK-Pelle_RLCK-IXb	PK
F01_cb4563_c37/f5p0/2841-2F	RLK-Pelle_RLCK-IXb	PK
F01_cb4563_c6/f6p1/3275-0F	RLK-Pelle_RLCK-IXb	PK
F01_cb4563_c8/f2p2/2875-2F	RLK-Pelle_RLCK-IXb	PK
F01_cb4563_c9/f1p0/3283-1F	RLK-Pelle_RLCK-IXb	PK
F01_cb4576_c125/f1p0/2012-2F	RLK-Pelle_RLCK-VIIa-2	PK
F01_cb4576_c128/f1p0/2398-1F	RLK-Pelle_RLCK-VIIa-2	PK
F01_cb4576_c134/f1p0/2248-1F	RLK-Pelle_RLCK-VIIa-2	PK
F01_cb4576_c16/f2p1/1879-0F	RLK-Pelle_RLCK-VIIa-2	PK
F01_cb4576_c18/f2p0/1935-2F	RLK-Pelle_RLCK-VIIa-2	PK

F01_cb4576_c69/f1p0/4561-0F	STE_STE7	PK
F01_cb4576_c73/f1p1/3381-0F	RLK-Pelle_RLCK-VIIa-2	PK
F01_cb4576_c73/f1p1/3381-2F	RLK-Pelle_RLCK-VIIa-2	PK
F01_cb4576_c95/f1p0/2097-2F	RLK-Pelle_RLCK-VIIa-2	PK
F01_cb4634_c14/f1p0/2408-1F	CK1_CK1	PK
F01_cb4634_c15/f1p0/3263-2F	CK1_CK1	PK
F01_cb4634_c17/f1p0/2349-1F	CK1_CK1	PK
F01_cb4634_c18/f1p0/3191-2F	CK1_CK1	PK
F01_cb4634_c3/f1p0/3290-0F	CK1_CK1	PK
F01_cb4634_c4/f1p0/3075-1F	CK1_CK1	PK
F01_cb4634_c5/f1p0/3037-2F	CK1_CK1	PK
F01_cb4634_c6/f1p0/2211-2F	CK1_CK1	PK
F01_cb4634_c7/f2p0/2230-0F	CK1_CK1	PK
F01_cb4634_c8/f1p0/2660-1F	CK1_CK1	PK
F01_cb4634_c9/f1p0/2221-1F	CK1_CK1	PK
F01_cb4641_c10/f1p0/2354-2F	RLK-Pelle_LRK10L-2	PK
F01_cb4641_c12/f1p1/2986-0F	RLK-Pelle_LRK10L-2	PK
F01_cb4641_c12/f1p1/2986-2F	RLK-Pelle_LRK10L-2	PK
F01_cb4641_c17/f1p0/2432-0F	RLK-Pelle_LRK10L-2	PK
F01_cb4641_c18/f1p0/2337-0F	RLK-Pelle_LRK10L-2	PK
F01_cb4641_c2/f2p1/2256-2F	RLK-Pelle_LRK10L-2	PK
F01_cb4641_c3/f2p1/2387-0F	RLK-Pelle_LRK10L-2	PK
F01_cb4641_c4/f2p1/2180-2F	RLK-Pelle_LRK10L-2	PK
F01_cb4641_c6/f1p0/3294-0F	RLK-Pelle_LRK10L-2	PK
F01_cb4641_c7/f1p1/2278-1F	RLK-Pelle_LRK10L-2	PK
F01_cb4641_c8/f1p1/2416-1F	RLK-Pelle_LRK10L-2	PK

F01_cb4641_c9/f1p0/3152-0F	RLK-Pelle_LRK10L-2	PK
F01_cb4644_c1/f1p0/3405-0F	RLK-Pelle_LRR-XI-1	PK
F01_cb4657_c7/f1p0/4018-0R	RLK-Pelle_SD-2b	PK
F01_cb4657_c8/f1p0/3762-0R	RLK-Pelle_SD-2b	PK
F01_cb4657_c8/f1p0/3762-2R	RLK-Pelle_SD-2b	PK
F01_cb4657_c9/f1p0/3253-0R	RLK-Pelle_SD-2b	PK
F01_cb4658_c0/f1p0/3289-1F	RLK-Pelle_CrRLK1L-1	PK
F01_cb4658_c1/f1p0/2992-1F	RLK-Pelle_CrRLK1L-1	PK
F01_cb4680_c0/f1p0/3288-2F	RLK-Pelle_SD-2b	PK
F01_cb4680_c1/f1p0/3024-2F	RLK-Pelle_SD-2b	PK
F01_cb4680_c2/f1p0/2712-1F	RLK-Pelle_SD-2b	PK
F01_cb4692_c0/f5p1/1918-1F	RLK-Pelle_RLCK-VIIa-2	PK
F01_cb4692_c1/f1p0/3283-2F	RLK-Pelle_RLCK-VIIa-2	PK
F01_cb4692_c2/f1p1/1855-0F	RLK-Pelle_RLCK-VIIa-2	PK
F01_cb4692_c4/f1p0/1054-0F	RLK-Pelle_RLCK-VIIa-2	PK
F01_cb4758_c0/f1p0/3264-1F	RLK-Pelle_LRR-XII-1	PK
F01_cb4758_c1/f1p0/3083-1F	RLK-Pelle_LRR-XII-1	PK
F01_cb4758_c1/f1p0/3083-2F	RLK-Pelle_LRR-XII-1	PK
F01_cb4758_c3/f1p0/3403-1F	RLK-Pelle_LRR-XII-1	PK
F01_cb4758_c3/f1p0/3403-2F	RLK-Pelle_LRR-XII-1	PK
F01_cb4758_c4/f1p0/2881-1F	RLK-Pelle_LRR-XII-1	PK
F01_cb4758_c5/f1p0/3624-1F	RLK-Pelle_LRR-XII-1	PK
F01_cb4771_c1/f3p0/3144-1F	RLK-Pelle_LRR-IX	PK
F01_cb4771_c2/f1p0/3261-0F	RLK-Pelle_LRR-IX	PK
F01_cb4771_c2/f1p0/3261-2F	RLK-Pelle_LRR-IX	PK
F01_cb4771_c3/f1p0/3275-1F	RLK-Pelle_LRR-IX	PK

F01_cb4771_c5/f1p0/2633-1F	RLK-Pelle_LRR-IX	PK
F01_cb4771_c6/f1p0/1174-2F	RLK-Pelle_LRR-IX	PK
F01_cb4774_c11/f1p0/3257-0F	CK1_CK1-Pl	PK
F01_cb4774_c13/f1p0/2724-1F	CK1_CK1-Pl	PK
F01_cb4774_c16/f1p0/2562-1F	CK1_CK1-Pl	PK
F01_cb4774_c20/f1p1/2938-2F	CK1_CK1-Pl	PK
F01_cb4774_c29/f1p0/3161-2F	CK1_CK1-Pl	PK
F01_cb4774_c30/f1p0/2823-1F	CK1_CK1-Pl	PK
F01_cb4774_c33/f1p2/2849-1F	CK1_CK1-Pl	PK
F01_cb4774_c34/f1p0/3232-0F	CK1_CK1-Pl	PK
F01_cb4774_c37/f1p0/3038-1F	CK1_CK1-Pl	PK
F01_cb4774_c40/f1p0/3021-2F	CK1_CK1-Pl	PK
F01_cb4774_c43/f1p0/3179-1F	CK1_CK1-Pl	PK
F01_cb4789_c2/f1p0/2371-1F	RLK-Pelle_WAK_LRK10L-1	PK
F01_cb4789_c4/f1p0/2272-1F	RLK-Pelle_WAK_LRK10L-1	PK
F01_cb4805_c0/f4p0/2407-0F	STE_STE7	PK
F01_cb4805_c1/f3p0/2556-0F	STE_STE7	PK
F01_cb4805_c11/f1p0/2332-1F	STE_STE7	PK
F01_cb4805_c12/f1p0/3191-0F	STE_STE7	PK
F01_cb4805_c13/f1p0/3131-0F	STE_STE7	PK
F01_cb4805_c14/f1p0/2808-0F	STE_STE7	PK
F01_cb4805_c15/f1p0/2174-2F	STE_STE7	PK
F01_cb4805_c16/f1p0/2879-0F	STE_STE7	PK
F01_cb4805_c17/f1p0/2451-1F	STE_STE7	PK
F01_cb4805_c18/f1p0/2325-0F	STE_STE7	PK
F01_cb4805_c19/f1p0/2494-1F	STE_STE7	PK

F01_cb4805_c24/f1p0/1798-2F	STE_STE7	PK
F01_cb4805_c27/f1p0/1186-2F	STE_STE7	PK
F01_cb4805_c5/f1p0/3640-0F	STE_STE7	PK
F01_cb4805_c5/f1p0/3640-2F	STE_STE7	PK
F01_cb4805_c7/f1p0/2117-0F	STE_STE7	PK
F01_cb4805_c9/f1p0/3065-0F	STE_STE7	PK
F01_cb4805_c9/f1p0/3065-2F	STE_STE7	PK
F01_cb4834_c10/f1p0/3243-0F	CK1_CK1-Pl	PK
F01_cb4834_c13/f1p2/2756-2F	CK1_CK1-Pl	PK
F01_cb4834_c14/f1p1/2790-1F	CK1_CK1-Pl	PK
F01_cb4834_c31/f1p0/2857-0F	CK1_CK1-Pl	PK
F01_cb4834_c36/f1p0/2620-2F	CK1_CK1-Pl	PK
F01_cb4834_c37/f1p0/3014-2F	CK1_CK1-Pl	PK
F01_cb4834_c38/f1p0/2881-1F	CK1_CK1-Pl	PK
F01_cb4834_c39/f1p0/2850-1F	CK1_CK1-Pl	PK
F01_cb4834_c44/f1p1/2977-1F	CK1_CK1-Pl	PK
F01_cb4834_c5/f3p0/2725-2F	CK1_CK1-Pl	PK
F01_cb4834_c6/f3p0/2838-2F	CK1_CK1-Pl	PK
F01_cb4834_c8/f2p0/2788-2F	CK1_CK1-Pl	PK
F01_cb4834_c9/f2p4/2896-2F	CK1_CK1-Pl	PK
F01_cb4871_c0/f2p0/3053-1F	RLK-Pelle_LRR-VI-1	PK
F01_cb4871_c1/f2p0/3169-0F	RLK-Pelle_LRR-VI-1	PK
F01_cb4871_c3/f1p0/3099-2F	RLK-Pelle_LRR-VI-1	PK
F01_cb4871_c7/f1p0/1266-1F	RLK-Pelle_LRR-VI-1	PK
F01_cb4877_c1/f2p1/2369-2F	RLK-Pelle_L-LEC	PK
F01_cb4877_c2/f1p0/3363-0F	RLK-Pelle_L-LEC	PK

F01_cb4877_c2/f1p0/3363-2F	RLK-Pelle_L-LEC	PK
F01_cb4877_c3/f1p0/2888-2F	RLK-Pelle_L-LEC	PK
F01_cb4877_c4/f1p1/2530-2F	RLK-Pelle_L-LEC	PK
F01_cb4877_c5/f1p0/1889-0F	RLK-Pelle_L-LEC	PK
F01_cb4877_c5/f1p0/1889-1F	RLK-Pelle_L-LEC	PK
F01_cb4880_c0/f5p1/3228-2F	RLK-Pelle_LRR-XI-1	PK
F01_cb4880_c1/f1p0/2414-2F	RLK-Pelle_LRR-XI-1	PK
F01_cb4880_c2/f1p0/2038-0F	RLK-Pelle_LRR-XI-1	PK
F01_cb4893_c0/f7p1/2413-1F	RLK-Pelle_RLCK-VIIa-1	PK
F01_cb4893_c1/f5p0/2291-2F	RLK-Pelle_RLCK-VIIa-1	PK
F01_cb4893_c13/f1p0/2332-1F	RLK-Pelle_RLCK-VIIa-1	PK
F01_cb4893_c3/f1p0/3233-1F	RLK-Pelle_RLCK-VIIa-1	PK
F01_cb4893_c5/f1p0/2367-0F	RLK-Pelle_RLCK-VIIa-1	PK
F01_cb4893_c7/f1p1/2506-1F	RLK-Pelle_RLCK-VIIa-1	PK
F01_cb4893_c7/f1p1/2506-2F	RLK-Pelle_RLCK-VIIa-1	PK
F01_cb4975_c0/f3p0/3181-0F	RLK-Pelle_LRR-XI-1	PK
F01_cb4975_c1/f2p1/3221-1F	RLK-Pelle_LRR-XI-1	PK
F01_cb4975_c2/f1p0/3183-0F	RLK-Pelle_LRR-XI-1	PK
F01_cb4975_c5/f1p0/3193-2F	RLK-Pelle_LRR-XI-1	PK
F01_cb497_c0/f1p0/4788-0F	RLK-Pelle_PERK-1	PK
F01_cb497_c1/f1p0/2979-1F	RLK-Pelle_PERK-1	PK
F01_cb499_c10/f2p0/3400-0F	RLK-Pelle_DLSV	PK
F01_cb499_c13/f1p0/4098-0F	RLK-Pelle_DLSV	PK
F01_cb499_c13/f1p0/4098-1F	RLK-Pelle_DLSV	PK
F01_cb499_c13/f1p0/4098-2F	RLK-Pelle_DLSV	PK
F01_cb499_c14/f1p0/3097-0F	RLK-Pelle_DLSV	PK

F01_cb499_c15/f1p0/4187-2F	RLK-Pelle_DLSV	PK
F01_cb499_c16/f1p0/3614-2F	RLK-Pelle_DLSV	PK
F01_cb499_c17/f1p1/3623-2F	RLK-Pelle_DLSV	PK
F01_cb499_c19/f1p1/3405-0F	RLK-Pelle_DLSV	PK
F01_cb499_c19/f1p1/3405-1F	RLK-Pelle_PERK-2	PK
F01_cb499_c20/f1p1/3689-0F	RLK-Pelle_DLSV	PK
F01_cb499_c20/f1p1/3689-1F	RLK-Pelle_DLSV	PK
F01_cb499_c21/f1p0/3092-2F	RLK-Pelle_DLSV	PK
F01_cb499_c24/f1p0/3755-0F	RLK-Pelle_DLSV	PK
F01_cb499_c25/f1p0/3238-2F	RLK-Pelle_DLSV	PK
F01_cb499_c26/f1p0/3193-1F	RLK-Pelle_DLSV	PK
F01_cb499_c28/f1p0/3948-1F	RLK-Pelle_DLSV	PK
F01_cb499_c29/f1p0/3667-0F	RLK-Pelle_DLSV	PK
F01_cb499_c30/f1p1/2918-1F	RLK-Pelle_DLSV	PK
F01_cb499_c33/f1p0/3719-1F	RLK-Pelle_DLSV	PK
F01_cb499_c34/f1p0/3463-0F	RLK-Pelle_DLSV	PK
F01_cb499_c37/f1p0/4542-1F	RLK-Pelle_DLSV	PK
F01_cb499_c38/f1p0/3714-2F	RLK-Pelle_DLSV	PK
F01_cb499_c40/f1p0/3109-0F	RLK-Pelle_DLSV	PK
F01_cb499_c40/f1p0/3109-1F	RLK-Pelle_DLSV	PK
F01_cb499_c41/f1p0/3022-2F	RLK-Pelle_DLSV	PK
F01_cb499_c42/f1p0/1931-1F	RLK-Pelle_DLSV	PK
F01_cb499_c42/f1p0/1931-2F	RLK-Pelle_DLSV	PK
F01_cb499_c44/f1p0/2881-1F	RLK-Pelle_DLSV	PK
F01_cb499_c46/f1p0/3594-0F	RLK-Pelle_DLSV	PK
F01_cb499_c48/f1p0/4241-1F	RLK-Pelle_DLSV	PK

F01_cb499_c50/f1p0/2769-0F	RLK-Pelle_DLSV	PK
F01_cb499_c50/f1p0/2769-2F	RLK-Pelle_DLSV	PK
F01_cb5013_c1/f4p0/2651-1F	STE_STE20-YSK	PK
F01_cb5013_c10/f1p0/2544-0F	STE_STE20-YSK	PK
F01_cb5013_c13/f1p0/2846-0F	STE_STE20-YSK	PK
F01_cb5013_c13/f1p0/2846-2F	STE_STE20-YSK	PK
F01_cb5013_c14/f1p0/2747-1F	STE_STE20-YSK	PK
F01_cb5013_c15/f1p0/2652-1F	STE_STE20-YSK	PK
F01_cb5013_c16/f1p0/2722-2F	STE_STE20-YSK	PK
F01_cb5013_c21/f1p0/2775-0F	STE_STE20-YSK	PK
F01_cb5013_c22/f1p0/2641-0F	STE_STE20-YSK	PK
F01_cb5013_c22/f1p0/2641-1F	STE_STE20-YSK	PK
F01_cb5013_c25/f5p0/2757-2F	STE_STE20-YSK	PK
F01_cb5013_c6/f1p0/3199-0F	STE_STE20-YSK	PK
F01_cb5013_c8/f1p0/2599-2F	STE_STE20-YSK	PK
F01_cb5013_c9/f1p0/2494-0F	STE_STE20-YSK	PK
F01_cb5109_c0/f2p0/2754-1F	RLK-Pelle_CR4L	PK
F01_cb5109_c1/f1p0/3177-0F	RLK-Pelle_CR4L	PK
F01_cb5109_c1/f1p0/3177-2F	RLK-Pelle_CR4L	PK
F01_cb5109_c4/f1p0/2758-1F	RLK-Pelle_CR4L	PK
F01_cb5109_c4/f1p0/2758-2F	RLK-Pelle_CR4L	PK
F01_cb5111_c10/f1p0/2552-1F	CMGC_CDK-CRK7-CDK9	PK
F01_cb5111_c12/f1p0/2418-1F	CMGC_CDK-CRK7-CDK9	PK
F01_cb5111_c13/f10p0/2484-1F	CMGC_CDK-CRK7-CDK9	PK
F01_cb5111_c2/f2p0/2492-2F	CMGC_CDK-CRK7-CDK9	PK
F01_cb5111_c4/f1p0/3176-0F	CMGC_CDK-CRK7-CDK9	PK

F01_cb5111_c4/f1p0/3176-1F	CMGC_CDK-CRK7-CDK9	PK
F01_cb5111_c6/f1p0/3148-1F	CMGC_CDK-CRK7-CDK9	PK
F01_cb5111_c8/f1p0/2226-1F	CMGC_CDK-CRK7-CDK9	PK
F01_cb5112_c0/f4p1/2507-1F	RLK-Pelle_LRR-IV	PK
F01_cb5112_c2/f1p1/2437-0F	RLK-Pelle_LRR-IV	PK
F01_cb5112_c3/f1p1/2498-2F	RLK-Pelle_LRR-IV	PK
F01_cb5142_c0/f1p0/3181-2F	RLK-Pelle_LRR-XI-1	PK
F01_cb5142_c1/f1p0/3108-2F	RLK-Pelle_LRR-XI-1	PK
F01_cb5144_c3/f1p0/2512-0F	CAMK_CDPK	PK
F01_cb5191_c0/f2p1/2708-1F	RLK-Pelle_LRR-IV	PK
F01_cb5191_c1/f1p0/3157-1F	RLK-Pelle_LRR-IV	PK
F01_cb5191_c1/f1p0/3157-2F	RLK-Pelle_LRR-IV	PK
F01_cb5191_c2/f1p0/2543-2F	RLK-Pelle_LRR-IV	PK
F01_cb5191_c3/f1p1/2520-2F	RLK-Pelle_LRR-IV	PK
F01_cb5191_c4/f1p1/2579-2F	RLK-Pelle_LRR-IV	PK
F01_cb5215_c3/f1p0/2863-2F	RLK-Pelle_LRR-VI-1	PK
F01_cb5233_c0/f1p0/3145-0F	RLK-Pelle_LRR-VII-2	PK
F01_cb5233_c0/f1p0/3145-1F	RLK-Pelle_LRR-VII-2	PK
F01_cb5233_c0/f1p0/3145-2F	RLK-Pelle_LRR-VII-2	PK
F01_cb5233_c1/f1p0/3273-0F	RLK-Pelle_LRR-VII-2	PK
F01_cb5271_c13/f1p0/2572-1F	WNK_NRBP	PK
F01_cb5271_c13/f1p0/2572-2F	WNK_NRBP	PK
F01_cb5271_c15/f1p0/2907-1F	WNK_NRBP	PK
F01_cb5271_c17/f1p2/2902-0F	WNK_NRBP	PK
F01_cb5271_c17/f1p2/2902-2F	WNK_NRBP	PK
F01_cb5271_c18/f1p2/2761-1F	WNK_NRBP	PK

F01_cb5271_c21/f1p0/2659-1F	WNK_NRBP	PK
F01_cb5271_c4/f1p2/3138-2F	WNK_NRBP	PK
F01_cb5271_c5/f1p2/2317-2F	WNK_NRBP	PK
F01_cb5271_c8/f1p2/2641-0F	WNK_NRBP	PK
F01_cb5271_c8/f1p2/2641-1F	WNK_NRBP	PK
F01_cb5271_c9/f1p0/2773-0F	WNK_NRBP	PK
F01_cb5286_c1/f1p0/3138-1F	RLK-Pelle_LRR-V	PK
F01_cb5286_c3/f1p0/2982-0F	RLK-Pelle_LRR-V	PK
F01_cb5286_c3/f1p0/2982-1F	RLK-Pelle_LRR-V	PK
F01_cb5286_c6/f1p0/2911-2F	RLK-Pelle_LRR-V	PK
F01_cb5286_c7/f1p0/2974-1F	RLK-Pelle_LRR-V	PK
F01_cb5286_c8/f1p0/2815-2F	RLK-Pelle_LRR-V	PK
F01_cb5290_c58/f1p0/2344-0F	AGC_NDR	PK
F01_cb5290_c61/f1p0/2229-0F	AGC_NDR	PK
F01_cb5290_c73/f5p0/2307-1F	AGC_NDR	PK
F01_cb5320_c0/f3p0/3002-1F	RLK-Pelle_CrRLK1L-1	PK
F01_cb5320_c3/f1p0/3096-1F	RLK-Pelle_CrRLK1L-1	PK
F01_cb5320_c5/f1p0/2967-0F	RLK-Pelle_CrRLK1L-1	PK
F01_cb5321_c3/f1p0/2327-2F	TKL-Pl-1	PK
F01_cb5321_c4/f1p0/2599-2F	TKL-Pl-1	PK
F01_cb5321_c6/f1p0/2549-1F	TKL-Pl-1	PK
F01_cb5342_c2/f1p1/2625-1F	RLK-Pelle_LRR-III	PK
F01_cb5357_c1/f3p0/2536-2F	CMGC_CDK-CRK7-CDK9	PK
F01_cb5357_c11/f1p0/2716-1F	CMGC_CDK-CRK7-CDK9	PK
F01_cb5357_c12/f1p0/2313-2F	CMGC_CDK-CRK7-CDK9	PK
F01_cb5357_c2/f1p0/3122-0F	CMGC_CDK-CRK7-CDK9	PK

F01_cb5357_c3/f1p1/3064-1F	CMGC_CDK-CRK7-CDK9	PK
F01_cb5357_c6/f1p1/3394-1F	CMGC_CDK-CRK7-CDK9	PK
F01_cb5357_c8/f1p0/2504-2F	CMGC_CDK-CRK7-CDK9	PK
F01_cb5357_c9/f1p0/2509-1F	CMGC_CDK-CRK7-CDK9	PK
F01_cb5357_c9/f1p0/2509-2F	CMGC_CDK-CRK7-CDK9	PK
F01_cb5367_c1/f1p0/3116-1F	RLK-Pelle_RLCK-VIIa-2	PK
F01_cb5367_c2/f1p0/1997-0F	RLK-Pelle_RLCK-VIIa-2	PK
F01_cb5367_c2/f1p0/1997-2F	RLK-Pelle_RLCK-VIIa-2	PK
F01_cb5367_c3/f1p0/2118-1F	RLK-Pelle_RLCK-VIIa-2	PK
F01_cb5367_c4/f1p0/1945-0F	RLK-Pelle_RLCK-VIIa-2	PK
F01_cb5367_c4/f1p0/1945-0R	RLK-Pelle_RLCK-VIIa-2	PK
F01_cb5367_c4/f1p0/1945-1R	RLK-Pelle_RLCK-VIIa-2	PK
F01_cb5367_c5/f1p0/1827-0F	RLK-Pelle_RLCK-VIIa-2	PK
F01_cb5369_c1/f15p0/1896-2F	RLK-Pelle_SD-2b	PK
F01_cb5369_c12/f1p0/1798-0F	RLK-Pelle_SD-2b	PK
F01_cb5369_c13/f1p0/1756-2F	RLK-Pelle_SD-2b	PK
F01_cb5369_c14/f1p0/1960-1F	RLK-Pelle_SD-2b	PK
F01_cb5369_c3/f3p0/2592-2F	RLK-Pelle_SD-2b	PK
F01_cb5369_c7/f1p0/2712-1F	RLK-Pelle_SD-2b	PK
F01_cb5369_c8/f1p0/2051-2F	RLK-Pelle_SD-2b	PK
F01_cb5436_c2/f1p0/2851-2F	RLK-Pelle_LRR-III	PK
F01_cb5436_c6/f1p1/1613-2F	RLK-Pelle_LRR-III	PK
F01_cb5436_c7/f1p1/2914-1F	RLK-Pelle_LRR-III	PK
F01_cb5441_c2/f1p0/3099-0F	RLK-Pelle_URK-1	PK
F01_cb5441_c2/f1p0/3099-2F	RLK-Pelle_URK-1	PK
F01_cb5441_c4/f1p0/2567-1F	RLK-Pelle_URK-1	PK

F01_cb5441_c5/f1p0/2520-0F	RLK-Pelle_URK-1	PK
F01_cb5441_c6/f1p0/2471-1F	RLK-Pelle_URK-1	PK
F01_cb5441_c8/f4p0/2512-2F	RLK-Pelle_URK-1	PK
F01_cb5447_c3/f5p2/3155-0F	RLK-Pelle_Extensin	PK
F01_cb5447_c33/f1p0/4199-1F	RLK-Pelle_Extensin	PK
F01_cb5447_c33/f1p0/4199-2F	RLK-Pelle_Extensin	PK
F01_cb5447_c34/f1p0/3194-1F	RLK-Pelle_Extensin	PK
F01_cb5447_c38/f1p1/3528-1F	RLK-Pelle_Extensin	PK
F01_cb5447_c39/f1p0/4097-1F	RLK-Pelle_Extensin	PK
F01_cb5447_c39/f1p0/4097-2F	RLK-Pelle_Extensin	PK
F01_cb5447_c40/f1p0/3143-1F	RLK-Pelle_Extensin	PK
F01_cb5447_c41/f1p0/3095-0F	RLK-Pelle_Extensin	PK
F01_cb5447_c41/f1p0/3095-2F	RLK-Pelle_Extensin	PK
F01_cb5459_c3/f1p0/2194-1F	TKL-Pl-4	PK
F01_cb5459_c3/f1p0/2194-2F	TKL-Pl-4	PK
F01_cb5459_c4/f1p0/2364-2F	TKL-Pl-4	PK
F01_cb5459_c5/f1p0/1863-1F	TKL-Pl-4	PK
F01_cb5483_c0/f2p0/2674-0F	STE_STE20-Pl	PK
F01_cb5483_c2/f1p0/2756-0F	STE_STE20-Pl	PK
F01_cb5483_c2/f1p0/2756-2F	STE_STE20-Pl	PK
F01_cb5483_c3/f1p0/2270-1F	STE_STE20-Pl	PK
F01_cb5483_c4/f1p0/2765-2F	STE_STE20-Pl	PK
F01_cb5483_c6/f1p0/2710-1F	STE_STE20-Pl	PK
F01_cb5483_c7/f1p0/2690-1F	STE_STE20-Pl	PK
F01_cb5491_c13/f1p0/3025-1F	TKL_CTR1-DRK-2	PK
F01_cb5491_c4/f1p1/2927-0F	TKL_CTR1-DRK-2	PK

F01_cb5491_c5/f1p0/3302-1F	TKL_CTR1-DRK-2	PK
F01_cb5491_c6/f1p0/3581-0F	TKL_CTR1-DRK-2	PK
F01_cb5491_c6/f1p0/3581-1F	TKL_CTR1-DRK-2	PK
F01_cb5491_c7/f1p0/2954-2F	TKL_CTR1-DRK-2	PK
F01_cb5491_c9/f1p0/3235-0F	TKL_CTR1-DRK-2	PK
F01_cb5504_c13/f1p0/2497-0F	RLK-Pelle_RLCK-IV	PK
F01_cb5504_c13/f1p0/2497-1F	RLK-Pelle_RLCK-IV	PK
F01_cb5504_c14/f1p0/2964-2F	RLK-Pelle_RLCK-IV	PK
F01_cb5504_c15/f1p0/2760-2F	RLK-Pelle_RLCK-IV	PK
F01_cb5504_c16/f1p0/2220-0F	RLK-Pelle_RLCK-IV	PK
F01_cb5504_c17/f1p0/2609-0F	RLK-Pelle_RLCK-IV	PK
F01_cb5504_c17/f1p0/2609-1F	RLK-Pelle_RLCK-IV	PK
F01_cb5504_c17/f1p0/2609-2F	RLK-Pelle_RLCK-IV	PK
F01_cb5504_c18/f1p0/2890-0F	RLK-Pelle_RLCK-IV	PK
F01_cb5504_c18/f1p0/2890-1F	RLK-Pelle_RLCK-IV	PK
F01_cb5504_c18/f1p0/2890-2F	RLK-Pelle_RLCK-IV	PK
F01_cb5504_c20/f1p0/2911-0F	RLK-Pelle_RLCK-IV	PK
F01_cb5504_c27/f1p0/2492-2F	RLK-Pelle_RLCK-IV	PK
F01_cb5504_c37/f1p0/2776-2F	RLK-Pelle_RLCK-IV	PK
F01_cb5504_c38/f1p0/2797-2F	RLK-Pelle_RLCK-IV	PK
F01_cb5504_c6/f6p0/2380-0F	RLK-Pelle_RLCK-IV	PK
F01_cb5511_c0/f1p0/3045-0F	TKL-Pl-5	PK
F01_cb5511_c0/f1p0/3045-1F	TKL-Pl-5	PK
F01_cb5511_c1/f1p0/1933-0F	TKL-Pl-5	PK
F01_cb5511_c2/f1p0/1970-0F	TKL-Pl-5	PK
F01_cb5511_c2/f1p0/1970-1F	TKL-Pl-5	PK

F01_cb5522_c7/f1p0/1984-1F	RLK-Pelle_RLCK-VIIa-2	PK
F01_cb5522_c7/f1p0/1984-2F	RLK-Pelle_RLCK-VIIa-2	PK
F01_cb5531_c0/f1p0/3078-1F	RLK-Pelle_RLCK-IXb	PK
F01_cb5533_c100/f1p0/3079-2F	IRE1	PK
F01_cb5533_c102/f1p0/3223-1F	IRE1	PK
F01_cb5533_c14/f3p0/3182-0F	IRE1	PK
F01_cb5533_c157/f30p1/3193-0F	IRE1	PK
F01_cb5533_c23/f2p0/2999-2F	IRE1	PK
F01_cb5533_c87/f1p0/4042-1F	IRE1	PK
F01_cb5533_c91/f1p0/2376-1F	IRE1	PK
F01_cb5533_c94/f1p0/3604-1F	IRE1	PK
F01_cb5533_c97/f1p0/3240-0F	IRE1	PK
F01_cb5544_c0/f1p0/3075-0F	WNK_NRBP	PK
F01_cb5544_c1/f1p0/2871-1F	WNK_NRBP	PK
F01_cb5544_c7/f1p0/2702-1F	WNK_NRBP	PK
F01_cb5550_c10/f1p0/4832-0F	RLK-Pelle_SD-2b	PK
F01_cb5550_c11/f1p0/2859-0F	RLK-Pelle_SD-2b	PK
F01_cb5550_c11/f1p0/2859-1F	RLK-Pelle_SD-2b	PK
F01_cb5550_c15/f1p0/3028-0F	RLK-Pelle_SD-2b	PK
F01_cb5550_c17/f1p0/3017-2F	RLK-Pelle_SD-2b	PK
F01_cb5550_c19/f1p0/3088-2F	RLK-Pelle_SD-2b	PK
F01_cb5550_c2/f6p2/2945-2F	RLK-Pelle_SD-2b	PK
F01_cb5550_c20/f1p0/3027-0F	RLK-Pelle_SD-2b	PK
F01_cb5550_c21/f1p0/2777-2F	RLK-Pelle_SD-2b	PK
F01_cb5550_c24/f1p0/3585-2F	RLK-Pelle_SD-2b	PK
F01_cb5550_c25/f1p0/3243-1F	RLK-Pelle_SD-2b	PK

F01_cb5550_c26/f1p0/3074-0F	RLK-Pelle_SD-2b	PK
F01_cb5550_c33/f1p0/2639-1F	RLK-Pelle_SD-2b	PK
F01_cb5550_c36/f1p0/2814-1F	RLK-Pelle_SD-2b	PK
F01_cb5550_c36/f1p0/2814-2F	RLK-Pelle_SD-2b	PK
F01_cb5550_c37/f1p1/2659-0F	RLK-Pelle_SD-2b	PK
F01_cb5550_c41/f1p0/2806-2F	RLK-Pelle_SD-2b	PK
F01_cb5550_c7/f2p1/2855-2F	RLK-Pelle_SD-2b	PK
F01_cb5550_c8/f1p0/3082-2F	RLK-Pelle_SD-2b	PK
F01_cb5564_c13/f1p0/1988-1F	CAMK_CDPK	PK
F01_cb5564_c14/f14p1/2858-1F	CAMK_CDPK	PK
F01_cb5564_c17/f1p0/2403-2F	CAMK_CDPK	PK
F01_cb5564_c18/f1p1/2823-2F	CAMK_CDPK	PK
F01_cb5564_c2/f3p1/2892-2F	CAMK_CDPK	PK
F01_cb5564_c21/f1p0/1889-2F	CAMK_CDPK	PK
F01_cb5564_c6/f3p0/2246-1F	CAMK_CDPK	PK
F01_cb5564_c9/f1p0/2154-0F	CAMK_CDPK	PK
F01_cb5578_c0/f1p0/3067-2F	RLK-Pelle_SD-2b	PK
F01_cb5578_c2/f1p0/2748-2F	RLK-Pelle_SD-2b	PK
F01_cb5578_c3/f1p0/2902-0F	RLK-Pelle_SD-2b	PK
F01_cb5578_c3/f1p0/2902-2F	RLK-Pelle_SD-2b	PK
F01_cb5596_c0/f36p1/2642-0F	RLK-Pelle_PERK-2	PK
F01_cb5596_c10/f1p2/2553-1F	RLK-Pelle_PERK-2	PK
F01_cb5596_c12/f2p1/2644-2F	RLK-Pelle_PERK-2	PK
F01_cb5596_c14/f1p0/2891-0F	RLK-Pelle_PERK-2	PK
F01_cb5596_c16/f1p0/3416-2F	RLK-Pelle_PERK-2	PK
F01_cb5596_c18/f1p0/2340-1F	RLK-Pelle_PERK-2	PK

F01_cb5596_c18/f1p0/2340-2F	RLK-Pelle_PERK-2	PK
F01_cb5596_c21/f1p0/2666-0F	RLK-Pelle_PERK-2	PK
F01_cb5596_c22/f1p0/3148-0F	RLK-Pelle_PERK-2	PK
F01_cb5596_c23/f1p0/2671-0F	RLK-Pelle_PERK-2	PK
F01_cb5596_c24/f1p0/2921-0F	RLK-Pelle_PERK-2	PK
F01_cb5596_c24/f1p0/2921-2F	RLK-Pelle_PERK-2	PK
F01_cb5596_c26/f1p1/2546-1F	RLK-Pelle_PERK-2	PK
F01_cb5596_c27/f1p0/2677-1F	RLK-Pelle_PERK-2	PK
F01_cb5596_c28/f1p0/2520-2F	RLK-Pelle_PERK-2	PK
F01_cb5596_c33/f1p0/2807-2F	RLK-Pelle_PERK-2	PK
F01_cb5596_c37/f1p0/2917-2F	RLK-Pelle_PERK-2	PK
F01_cb5596_c38/f1p0/2685-1F	RLK-Pelle_PERK-2	PK
F01_cb5596_c39/f1p0/2882-1F	RLK-Pelle_PERK-2	PK
F01_cb5596_c44/f1p0/2655-1F	RLK-Pelle_PERK-2	PK
F01_cb5596_c6/f10p0/2530-1F	RLK-Pelle_PERK-2	PK
F01_cb5612_c10/f1p0/2551-2F	NEK	PK
F01_cb5612_c13/f3p0/2458-0F	NEK	PK
F01_cb5612_c14/f3p0/2416-1F	NEK	PK
F01_cb5612_c2/f2p0/2528-2F	NEK	PK
F01_cb5612_c3/f1p0/3057-1F	NEK	PK
F01_cb5612_c4/f1p0/2446-2F	NEK	PK
F01_cb5612_c7/f1p0/2803-1F	NEK	PK
F01_cb5612_c8/f1p0/2542-0F	NEK	PK
F01_cb5632_c10/f1p0/2740-0F	RLK-Pelle_L-LEC	PK
F01_cb5632_c10/f1p0/2740-1F	RLK-Pelle_L-LEC	PK
F01_cb5632_c13/f1p0/2425-2F	RLK-Pelle_L-LEC	PK

F01_cb5632_c14/f1p1/2688-0F	RLK-Pelle_L-LEC	PK
F01_cb5632_c16/f1p1/2698-2F	RLK-Pelle_L-LEC	PK
F01_cb5632_c17/f1p0/1897-0F	RLK-Pelle_L-LEC	PK
F01_cb5632_c18/f1p1/3084-1F	RLK-Pelle_L-LEC	PK
F01_cb5632_c20/f1p1/2425-1F	RLK-Pelle_L-LEC	PK
F01_cb5632_c26/f1p1/2618-1F	RLK-Pelle_L-LEC	PK
F01_cb5632_c31/f1p1/2684-1F	RLK-Pelle_L-LEC	PK
F01_cb5632_c34/f1p0/2494-1F	RLK-Pelle_L-LEC	PK
F01_cb5632_c38/f1p0/2556-1F	RLK-Pelle_L-LEC	PK
F01_cb5632_c41/f1p0/1646-1F	RLK-Pelle_L-LEC	PK
F01_cb5632_c42/f2p0/2465-1F	RLK-Pelle_L-LEC	PK
F01_cb5632_c45/f2p1/3154-0F	RLK-Pelle_L-LEC	PK
F01_cb5632_c5/f2p0/2554-1F	RLK-Pelle_L-LEC	PK
F01_cb5632_c9/f1p0/3493-0F	RLK-Pelle_L-LEC	PK
F01_cb5632_c9/f1p0/3493-2F	RLK-Pelle_L-LEC	PK
F01_cb5633_c76/f1p0/2047-2F	CAMK_CAMKL-CHK1	PK
F01_cb5643_c111/f1p1/2459-0F	RLK-Pelle_LRR-II	PK
F01_cb5643_c111/f1p1/2459-2F	RLK-Pelle_LRR-II	PK
F01_cb5643_c113/f1p0/2786-0F	RLK-Pelle_LRR-II	PK
F01_cb5643_c120/f3p1/2448-2F	RLK-Pelle_LRR-II	PK
F01_cb5643_c124/f5p1/2491-1F	RLK-Pelle_LRR-II	PK
F01_cb5643_c45/f1p0/3996-0F	RLK-Pelle_LRR-II	PK
F01_cb5643_c45/f1p0/3996-2F	RLK-Pelle_LRR-II	PK
F01_cb5643_c46/f1p0/3297-2F	RLK-Pelle_LRR-II	PK
F01_cb5643_c47/f1p0/2358-0F	RLK-Pelle_LRR-II	PK
F01_cb5643_c47/f1p0/2358-1F	RLK-Pelle_LRR-II	PK

F01_cb5643_c49/f1p0/2351-0F	RLK-Pelle_LRR-II	PK
F01_cb5643_c52/f1p1/2668-0F	RLK-Pelle_LRR-II	PK
F01_cb5643_c54/f1p0/2813-1F	RLK-Pelle_LRR-II	PK
F01_cb5643_c54/f1p0/2813-2F	RLK-Pelle_LRR-II	PK
F01_cb5643_c56/f1p0/2251-2F	RLK-Pelle_LRR-II	PK
F01_cb5643_c60/f1p1/2497-1F	RLK-Pelle_LRR-II	PK
F01_cb5643_c62/f1p1/3868-2F	RLK-Pelle_LRR-II	PK
F01_cb5643_c64/f1p0/2089-0F	RLK-Pelle_LRR-II	PK
F01_cb5643_c66/f1p0/2234-0F	RLK-Pelle_LRR-II	PK
F01_cb5643_c68/f1p0/3247-2F	RLK-Pelle_LRR-II	PK
F01_cb5643_c69/f1p0/2630-2F	RLK-Pelle_LRR-II	PK
F01_cb5643_c70/f1p1/2237-0F	RLK-Pelle_LRR-II	PK
F01_cb5643_c71/f1p0/2363-2F	RLK-Pelle_LRR-II	PK
F01_cb5643_c72/f1p1/2912-1F	RLK-Pelle_LRR-II	PK
F01_cb5643_c74/f1p0/2864-1F	RLK-Pelle_LRR-II	PK
F01_cb5643_c75/f1p0/2482-1F	RLK-Pelle_LRR-II	PK
F01_cb5643_c76/f1p0/2697-2F	RLK-Pelle_LRR-II	PK
F01_cb5672_c13/f1p0/3266-1F	WNK_NRBP	PK
F01_cb5672_c15/f1p0/3570-1F	WNK_NRBP	PK
F01_cb5672_c16/f1p0/2940-0F	WNK_NRBP	PK
F01_cb5672_c17/f1p0/2844-2F	WNK_NRBP	PK
F01_cb5672_c6/f1p0/3840-1F	WNK_NRBP	PK
F01_cb5672_c6/f1p0/3840-2F	WNK_NRBP	PK
F01_cb5672_c7/f1p0/3125-1F	WNK_NRBP	PK
F01_cb5672_c9/f1p0/2989-2F	WNK_NRBP	PK
F01_cb5687_c10/f1p0/2094-0F	WNK_NRBP	PK

F01_cb5687_c11/f1p0/2528-1F	WNK_NRBP	PK
F01_cb5687_c13/f1p0/2518-2F	WNK_NRBP	PK
F01_cb5687_c16/f1p0/2296-0F	WNK_NRBP	PK
F01_cb5687_c6/f1p1/2721-1F	WNK_NRBP	PK
F01_cb5748_c0/f8p0/1723-1F	CMGC_CK2	PK
F01_cb5748_c2/f1p0/3016-2F	CMGC_CK2	PK
F01_cb5748_c4/f1p0/2096-0F	CMGC_CK2	PK
F01_cb5748_c4/f1p0/2096-1F	CMGC_CK2	PK
F01_cb5748_c6/f1p0/1754-1F	CMGC_CK2	PK
F01_cb5748_c7/f1p0/1637-1F	CMGC_CK2	PK
F01_cb5753_c0/f7p1/2864-0F	CMGC_MAPK	PK
F01_cb5753_c0/f7p1/2864-1F	CMGC_MAPK	PK
F01_cb5753_c11/f2p0/2606-1F	CMGC_MAPK	PK
F01_cb5753_c12/f1p0/3021-2F	CMGC_MAPK	PK
F01_cb5753_c13/f1p0/3054-0F	CMGC_MAPK	PK
F01_cb5753_c14/f1p1/2728-0F	CMGC_MAPK	PK
F01_cb5753_c15/f1p0/2255-0F	CMGC_MAPK	PK
F01_cb5753_c15/f1p0/2255-1F	CMGC_MAPK	PK
F01_cb5753_c15/f1p0/2255-2F	CMGC_MAPK	PK
F01_cb5753_c16/f1p1/2807-1F	CMGC_MAPK	PK
F01_cb5753_c18/f1p0/2096-0F	CMGC_MAPK	PK
F01_cb5753_c19/f1p0/2260-2F	CMGC_MAPK	PK
F01_cb5753_c20/f1p0/2971-1F	CMGC_MAPK	PK
F01_cb5753_c21/f1p0/2865-0F	CMGC_MAPK	PK
F01_cb5753_c21/f1p0/2865-2F	CMGC_MAPK	PK
F01_cb5753_c23/f1p0/2880-0F	CMGC_MAPK	PK

F01_cb5753_c28/f1p0/1949-1F	CMGC_MAPK	PK
F01_cb5753_c29/f1p0/2753-0F	CMGC_MAPK	PK
F01_cb5753_c29/f1p0/2753-1F	CMGC_MAPK	PK
F01_cb5753_c30/f1p0/2911-1F	CMGC_MAPK	PK
F01_cb5753_c31/f1p0/2748-0F	CMGC_MAPK	PK
F01_cb5753_c31/f1p0/2748-2F	CMGC_MAPK	PK
F01_cb5753_c33/f1p0/2714-0F	CMGC_MAPK	PK
F01_cb5753_c33/f1p0/2714-2F	CMGC_MAPK	PK
F01_cb5753_c34/f1p0/2333-1F	CMGC_MAPK	PK
F01_cb5753_c36/f1p0/2398-0F	CMGC_MAPK	PK
F01_cb5753_c39/f1p0/2241-1F	CMGC_MAPK	PK
F01_cb5753_c41/f1p0/5369-0F	CMGC_MAPK	PK
F01_cb5753_c41/f1p0/5369-1F	CMGC_MAPK	PK
F01_cb5753_c5/f3p2/2848-0F	CMGC_MAPK	PK
F01_cb5753_c6/f3p0/2151-2F	CMGC_MAPK	PK
F01_cb5753_c8/f2p1/2741-1F	CMGC_MAPK	PK
F01_cb5753_c8/f2p1/2741-2F	CMGC_MAPK	PK
F01_cb5776_c11/f1p0/2892-1F	RLK-Pelle_DLSV	PK
F01_cb5776_c12/f1p0/2807-1F	RLK-Pelle_DLSV	PK
F01_cb5776_c14/f1p0/1675-0F	RLK-Pelle_DLSV	PK
F01_cb5776_c16/f1p0/1547-1F	RLK-Pelle_DLSV	PK
F01_cb5776_c4/f2p0/3004-1F	RLK-Pelle_DLSV	PK
F01_cb5776_c6/f1p0/3012-0F	RLK-Pelle_DLSV	PK
F01_cb5800_c0/f3p1/2948-0F	RLK-Pelle_LRR-XI-1	PK
F01_cb5800_c4/f1p1/2928-0F	RLK-Pelle_LRR-XI-1	PK
F01_cb5806_c1/f2p1/2477-2F	CMGC_DYRK-PRP4	PK

F01_cb5806_c10/f1p0/2919-0F	CMGC_DYRK-PRP4	PK
F01_cb5806_c2/f1p0/2888-1F	CMGC_DYRK-PRP4	PK
F01_cb5806_c3/f1p1/2760-2F	CMGC_DYRK-PRP4	PK
F01_cb5806_c4/f1p2/2764-0F	CMGC_DYRK-PRP4	PK
F01_cb5806_c7/f1p0/2887-2F	CMGC_DYRK-PRP4	PK
F01_cb5806_c8/f1p1/2966-0F	CMGC_DYRK-PRP4	PK
F01_cb5806_c8/f1p1/2966-2R	CMGC_DYRK-PRP4	PK
F01_cb5806_c9/f1p0/2701-2F	CMGC_DYRK-PRP4	PK
F01_cb5846_c11/f2p0/2656-2F	RLK-Pelle_WAK	PK
F01_cb5846_c13/f1p0/2820-0F	RLK-Pelle_WAK	PK
F01_cb5846_c15/f1p1/2610-0F	RLK-Pelle_WAK	PK
F01_cb5846_c26/f1p0/3075-1F	RLK-Pelle_WAK	PK
F01_cb5846_c27/f1p0/2652-2F	RLK-Pelle_WAK	PK
F01_cb5846_c28/f1p1/2713-0F	RLK-Pelle_WAK	PK
F01_cb5846_c28/f1p1/2713-2F	RLK-Pelle_WAK	PK
F01_cb5846_c29/f1p0/2902-0F	RLK-Pelle_WAK	PK
F01_cb5846_c29/f1p0/2902-1F	RLK-Pelle_WAK_LRK10L-1	PK
F01_cb5846_c3/f2p0/2710-0F	RLK-Pelle_WAK	PK
F01_cb5846_c3/f2p0/2710-1F	RLK-Pelle_WAK	PK
F01_cb5846_c33/f1p0/2324-0F	RLK-Pelle_WAK	PK
F01_cb5846_c34/f1p0/3222-1F	RLK-Pelle_WAK	PK
F01_cb5846_c35/f1p0/3317-1F	RLK-Pelle_WAK	PK
F01_cb5846_c37/f1p0/2702-1F	RLK-Pelle_WAK	PK
F01_cb5846_c38/f1p0/2537-2F	RLK-Pelle_WAK	PK
F01_cb5846_c4/f3p0/2791-1F	RLK-Pelle_WAK	PK
F01_cb5846_c40/f1p0/2790-0F	RLK-Pelle_WAK	PK

F01_cb5846_c43/f1p0/2784-0F	RLK-Pelle_WAK	PK
F01_cb5846_c43/f1p0/2784-1F	RLK-Pelle_WAK	PK
F01_cb5846_c9/f2p0/2777-1F	RLK-Pelle_WAK	PK
F01_cb5869_c0/f1p0/2991-1F	AGC_RSK-2	PK
F01_cb5874_c11/f2p1/2975-0F	WNK_NRBP	PK
F01_cb5874_c13/f1p0/3016-0F	WNK_NRBP	PK
F01_cb5874_c15/f1p0/2254-2F	WNK_NRBP	PK
F01_cb5874_c16/f1p0/3263-1F	WNK_NRBP	PK
F01_cb5874_c22/f1p0/3282-0F	WNK_NRBP	PK
F01_cb5874_c27/f1p0/2672-2F	WNK_NRBP	PK
F01_cb5874_c30/f1p0/2210-0F	WNK_NRBP	PK
F01_cb5874_c33/f1p0/2945-1F	WNK_NRBP	PK
F01_cb5874_c6/f8p0/2864-2F	WNK_NRBP	PK
F01_cb5896_c10/f2p1/2427-0F	RLK-Pelle_DLSV	PK
F01_cb5896_c108/f1p0/2749-0F	RLK-Pelle_DLSV	PK
F01_cb5896_c11/f2p1/2808-0F	RLK-Pelle_DLSV	PK
F01_cb5896_c144/f1p0/2661-2F	RLK-Pelle_LRR-XII-1	PK
F01_cb5896_c146/f1p0/2739-1F	RLK-Pelle_DLSV	PK
F01_cb5896_c148/f1p0/2924-1F	RLK-Pelle_DLSV	PK
F01_cb5896_c2/f6p2/2670-0F	RLK-Pelle_DLSV	PK
F01_cb5896_c5/f4p2/2581-1F	RLK-Pelle_DLSV	PK
F01_cb5896_c60/f1p2/2803-1F	RLK-Pelle_DLSV	PK
F01_cb5896_c61/f1p1/2595-2F	RLK-Pelle_DLSV	PK
F01_cb5896_c64/f1p0/2959-1F	RLK-Pelle_DLSV	PK
F01_cb5896_c64/f1p0/2959-2F	RLK-Pelle_DLSV	PK
F01_cb5896_c65/f1p0/2600-0F	RLK-Pelle_DLSV	PK

F01_cb5896_c67/f1p0/3378-2F	RLK-Pelle_DLSV	PK
F01_cb5896_c68/f1p0/2575-2F	RLK-Pelle_DLSV	PK
F01_cb5896_c97/f1p1/2626-2F	RLK-Pelle_DLSV	PK
F01_cb5910_c10/f1p0/2649-0F	RLK-Pelle_LRR-V	PK
F01_cb5910_c10/f1p0/2649-2F	RLK-Pelle_LRR-V	PK
F01_cb5910_c12/f1p0/2660-1F	RLK-Pelle_LRR-V	PK
F01_cb5910_c12/f1p0/2660-2F	RLK-Pelle_LRR-V	PK
F01_cb5910_c14/f1p0/2772-2F	RLK-Pelle_LRR-V	PK
F01_cb5910_c19/f1p0/2736-0F	RLK-Pelle_LRR-V	PK
F01_cb5910_c8/f2p0/2812-2F	RLK-Pelle_LRR-V	PK
F01_cb5910_c9/f1p0/2993-2F	RLK-Pelle_LRR-V	PK
F01_cb5914_c0/f1p0/2979-2F	RLK-Pelle_RLCK-IXb	PK
F01_cb5954_c0/f1p0/2959-1F	RLK-Pelle_SD-2b	PK
F01_cb5954_c1/f1p0/3443-0F	RLK-Pelle_SD-2b	PK
F01_cb5964_c1/f1p0/2966-1F	RLK-Pelle_Extensin	PK
F01_cb5964_c2/f1p0/2720-2F	RLK-Pelle_Extensin	PK
F01_cb5976_c0/f1p0/2947-1F	RLK-Pelle_RLCK-VIIa-2	PK
F01_cb5976_c0/f1p0/2947-2F	RLK-Pelle_LysM	PK
F01_cb5976_c1/f1p0/3373-0F	RLK-Pelle_LRR-XI-1	PK
F01_cb5976_c1/f1p0/3373-1F	RLK-Pelle_RLCK-VIIa-2	PK
F01_cb5976_c3/f1p0/2633-1F	RLK-Pelle_RLCK-VIIa-2	PK
F01_cb5976_c3/f1p0/2633-2F	RLK-Pelle_LRR-XI-1	PK
F01_cb5976_c4/f1p0/2501-0F	RLK-Pelle_WAK	PK
F01_cb5976_c4/f1p0/2501-2F	RLK-Pelle_L-LEC	PK
F01_cb5990_c0/f2p0/2303-2F	RLK-Pelle_L-LEC	PK
F01_cb5990_c1/f1p0/2959-1F	RLK-Pelle_L-LEC	PK

F01_cb5990_c2/f1p0/2341-0F	RLK-Pelle_L-LEC	PK
F01_cb5990_c3/f1p0/2715-1F	RLK-Pelle_L-LEC	PK
F01_cb6005_c1/f2p1/2623-2F	RLK-Pelle_LRR-III	PK
F01_cb6005_c3/f1p1/2710-0F	RLK-Pelle_LRR-III	PK
F01_cb6005_c4/f1p0/2486-2F	RLK-Pelle_LRR-III	PK
F01_cb6032_c27/f1p0/3675-0F	RLK-Pelle_LRR-XII-1	PK
F01_cb6032_c28/f1p0/3437-1F	RLK-Pelle_LRR-XII-1	PK
F01_cb6032_c29/f1p0/3404-1F	RLK-Pelle_LRR-XII-1	PK
F01_cb6032_c29/f1p0/3404-2F	RLK-Pelle_LRR-XII-1	PK
F01_cb6032_c30/f1p0/2901-0F	RLK-Pelle_LRR-XII-1	PK
F01_cb6032_c33/f1p0/3723-1F	RLK-Pelle_LRR-XII-1	PK
F01_cb6032_c46/f4p0/3527-2F	RLK-Pelle_LRR-XII-1	PK
F01_cb6032_c8/f3p0/3620-0F	RLK-Pelle_LRR-XII-1	PK
F01_cb6039_c19/f2p1/2346-1F	CAMK_CDPK	PK
F01_cb6039_c22/f1p0/2768-0F	CAMK_CDPK	PK
F01_cb6039_c27/f1p0/3037-1F	CAMK_CDPK	PK
F01_cb6039_c28/f1p0/1963-1F	CAMK_CDPK	PK
F01_cb6039_c30/f1p1/2549-1F	CAMK_CDPK	PK
F01_cb6039_c31/f1p0/2292-0F	CAMK_CDPK	PK
F01_cb6039_c31/f1p0/2292-1F	CAMK_CDPK	PK
F01_cb6039_c33/f1p0/2697-0F	CAMK_CDPK	PK
F01_cb6039_c35/f1p1/2387-2F	CAMK_CDPK	PK
F01_cb6039_c38/f1p0/2744-1F	CAMK_CDPK	PK
F01_cb6039_c42/f1p0/2375-0F	CAMK_CDPK	PK
F01_cb6039_c42/f1p0/2375-2F	CAMK_CDPK	PK
F01_cb6039_c51/f1p1/2182-0F	CAMK_CDPK	PK

F01_cb6039_c53/f1p1/2533-1F	CAMK_CDPK	PK
F01_cb6039_c56/f1p1/3085-1R	CAMK_CDPK	PK
F01_cb6039_c56/f1p1/3085-2R	CAMK_CDPK	PK
F01_cb6039_c58/f1p1/2741-1F	CAMK_CDPK	PK
F01_cb6039_c59/f1p0/2684-2F	CAMK_CDPK	PK
F01_cb6039_c63/f1p0/2475-0F	CAMK_CDPK	PK
F01_cb6039_c65/f1p0/2531-1F	CAMK_CDPK	PK
F01_cb6039_c66/f1p0/2569-2F	CAMK_CDPK	PK
F01_cb6039_c68/f1p1/2451-2F	CAMK_CDPK	PK
F01_cb6039_c75/f1p0/2369-2F	CAMK_CDPK	PK
F01_cb6039_c8/f1p0/2609-0F	CAMK_CDPK	PK
F01_cb6039_c81/f1p0/1608-1F	CAMK_CDPK	PK
F01_cb6039_c82/f1p0/1413-2F	CAMK_CDPK	PK
F01_cb6039_c85/f59p1/2520-2F	CAMK_CDPK	PK
F01_cb6039_c86/f5p2/2402-1F	CAMK_CDPK	PK
F01_cb6039_c91/f1p0/1362-0F	CAMK_CDPK	PK
F01_cb6039_c91/f1p0/1362-1F	CAMK_CDPK	PK
F01_cb6103_c0/f5p0/1708-0F	CMGC_CLK	PK
F01_cb6103_c1/f3p0/1732-2F	CMGC_CLK	PK
F01_cb6103_c10/f1p0/1635-1F	CMGC_CLK	PK
F01_cb6103_c10/f1p0/1635-2F	CMGC_CLK	PK
F01_cb6103_c3/f1p0/2950-0F	CMGC_CLK	PK
F01_cb6103_c3/f1p0/2950-1F	CMGC_CLK	PK
F01_cb6103_c4/f1p0/4044-2F	CMGC_CLK	PK
F01_cb6103_c5/f1p0/2571-1F	CMGC_CLK	PK
F01_cb6103_c6/f1p0/2906-0F	CMGC_CLK	PK

F01_cb6103_c7/f1p0/1725-1F	CMGC_CLK	PK
F01_cb6103_c8/f1p0/1672-2F	CMGC_CLK	PK
F01_cb6106_c14/f1p0/2093-0F	CMGC_GSK	PK
F01_cb6106_c14/f1p0/2093-2F	CMGC_GSK	PK
F01_cb6106_c15/f1p0/2059-0F	CMGC_GSK	PK
F01_cb6106_c15/f1p0/2059-1F	CMGC_GSK	PK
F01_cb6106_c16/f1p0/2075-1F	CMGC_GSK	PK
F01_cb6106_c17/f1p0/2044-0F	CMGC_GSK	PK
F01_cb6106_c19/f1p0/2246-0F	CMGC_GSK	PK
F01_cb6106_c23/f14p0/1983-0F	CMGC_GSK	PK
F01_cb6106_c23/f14p0/1983-2F	CMGC_GSK	PK
F01_cb6106_c25/f1p0/2002-1F	CMGC_GSK	PK
F01_cb6106_c3/f2p0/2554-2F	CMGC_GSK	PK
F01_cb6106_c4/f1p0/2932-0F	CMGC_GSK	PK
F01_cb6106_c4/f1p0/2932-1F	CMGC_GSK	PK
F01_cb6106_c5/f1p0/2598-2F	CMGC_GSK	PK
F01_cb6106_c6/f1p0/4071-0F	CMGC_GSK	PK
F01_cb6106_c7/f4p0/1960-0F	CMGC_GSK	PK
F01_cb6106_c8/f1p0/2021-0F	CMGC_GSK	PK
F01_cb6106_c9/f1p0/2392-0F	CMGC_GSK	PK
F01_cb6106_c9/f1p0/2392-1F	CMGC_GSK	PK
F01_cb6142_c0/f1p1/2941-2F	RLK-Pelle_DLSV	PK
F01_cb6142_c1/f1p0/2631-2F	RLK-Pelle_DLSV	PK
F01_cb6142_c2/f1p0/2705-2F	RLK-Pelle_DLSV	PK
F01_cb6142_c4/f1p0/2645-1F	RLK-Pelle_DLSV	PK
F01_cb6152_c1/f2p0/1940-0F	RLK-Pelle_LRR-I-2	PK

F01_cb6152_c1/f2p0/1940-2F	RLK-Pelle_LRR-I-2	PK
F01_cb6152_c2/f1p0/2925-0F	RLK-Pelle_LRR-I-2	PK
F01_cb6152_c2/f1p0/2925-1F	RLK-Pelle_LRR-I-2	PK
F01_cb6152_c3/f1p0/2053-1F	RLK-Pelle_LRR-I-2	PK
F01_cb6154_c11/f1p0/1844-0F	CAMK_CAMKL-CHK1	PK
F01_cb6154_c12/f1p0/1775-1F	CAMK_CAMKL-CHK1	PK
F01_cb6154_c13/f1p0/1708-1F	CAMK_CAMKL-CHK1	PK
F01_cb6154_c16/f1p0/1636-0F	CAMK_CAMKL-CHK1	PK
F01_cb6154_c17/f1p0/5308-1F	CAMK_CAMKL-CHK1	PK
F01_cb6154_c3/f2p0/2864-1F	CAMK_CAMKL-CHK1	PK
F01_cb6154_c4/f2p1/1876-0F	CAMK_CAMKL-CHK1	PK
F01_cb6154_c5/f1p0/2695-0F	CAMK_CAMKL-CHK1	PK
F01_cb6154_c6/f1p0/1975-0F	CAMK_CAMKL-CHK1	PK
F01_cb6154_c7/f1p0/1877-1F	CAMK_CAMKL-CHK1	PK
F01_cb6154_c7/f1p0/1877-2F	CAMK_CAMKL-CHK1	PK
F01_cb6154_c8/f1p1/1841-1F	CAMK_CAMKL-CHK1	PK
F01_cb6154_c9/f1p0/1854-0F	CAMK_CAMKL-CHK1	PK
F01_cb6154_c9/f1p0/1854-1F	CAMK_CAMKL-CHK1	PK
F01_cb6164_c2/f2p0/2370-1F	RLK-Pelle_CrRLK1L-1	PK
F01_cb6164_c5/f1p1/2560-0F	RLK-Pelle_CrRLK1L-1	PK
F01_cb6164_c5/f1p1/2560-1F	RLK-Pelle_CrRLK1L-1	PK
F01_cb6164_c6/f1p0/2802-1F	RLK-Pelle_CrRLK1L-1	PK
F01_cb6164_c7/f1p0/2587-1F	RLK-Pelle_CrRLK1L-1	PK
F01_cb6164_c7/f1p0/2587-2F	RLK-Pelle_CrRLK1L-1	PK
F01_cb6164_c9/f1p0/2594-0F	RLK-Pelle_CrRLK1L-1	PK
F01_cb6164_c9/f1p0/2594-2F	RLK-Pelle_CrRLK1L-1	PK

F01_cb6168_c0/f3p0/2896-0F	RLK-Pelle_WAK_LRK10L-1	PK
F01_cb6168_c5/f1p0/2385-0F	RLK-Pelle_WAK_LRK10L-1	PK
F01_cb6168_c5/f1p0/2385-1F	RLK-Pelle_WAK_LRK10L-1	PK
F01_cb6168_c5/f1p0/2385-2F	RLK-Pelle_WAK_LRK10L-1	PK
F01_cb6172_c0/f3p0/2431-1F	RLK-Pelle_LysM	PK
F01_cb6172_c2/f1p0/2920-1F	RLK-Pelle_LysM	PK
F01_cb6172_c3/f1p0/2797-1F	RLK-Pelle_LysM	PK
F01_cb6172_c6/f1p0/2744-0F	RLK-Pelle_LysM	PK
F01_cb6172_c6/f1p0/2744-1F	RLK-Pelle_LysM	PK
F01_cb6172_c6/f1p0/2744-2F	RLK-Pelle_LysM	PK
F01_cb6180_c2/f1p1/2707-0F	TLK	PK
F01_cb6180_c4/f1p0/2938-0F	TLK	PK
F01_cb6192_c0/f2p0/2889-1F	RLK-Pelle_DLSV	PK
F01_cb6217_c22/f1p0/2893-2F	CMGC_SRPK	PK
F01_cb6217_c3/f5p0/2029-2F	CMGC_SRPK	PK
F01_cb6217_c8/f1p0/2090-2F	CMGC_SRPK	PK
F01_cb6217_c9/f1p0/1956-0F	CMGC_SRPK	PK
F01_cb6257_c0/f3p0/2850-0F	RLK-Pelle_LRR-VI-1	PK
F01_cb6257_c0/f3p0/2850-1F	RLK-Pelle_LRR-VI-1	PK
F01_cb6257_c3/f1p0/2912-0F	RLK-Pelle_LRR-VI-1	PK
F01_cb6257_c4/f1p0/2896-2F	RLK-Pelle_LRR-VI-1	PK
F01_cb6277_c11/f1p1/1926-1F	RLK-Pelle_RLCK-XII-1	PK
F01_cb6277_c13/f1p0/2679-1F	RLK-Pelle_RLCK-XII-1	PK
F01_cb6277_c14/f1p1/2588-1F	RLK-Pelle_RLCK-XII-1	PK
F01_cb6277_c14/f1p1/2588-2F	RLK-Pelle_RLCK-XII-1	PK
F01_cb6277_c15/f1p1/2535-1F	RLK-Pelle_RLCK-XII-1	PK

F01_cb6277_c18/f1p0/2697-1F	RLK-Pelle_RLCK-XII-1	PK
F01_cb6277_c19/f1p1/2357-0F	RLK-Pelle_RLCK-XII-1	PK
F01_cb6277_c20/f1p1/2469-2F	RLK-Pelle_RLCK-XII-1	PK
F01_cb6277_c23/f1p0/2504-1F	RLK-Pelle_RLCK-XII-1	PK
F01_cb6277_c24/f1p0/2733-0F	RLK-Pelle_RLCK-XII-1	PK
F01_cb6277_c26/f1p0/2524-2F	RLK-Pelle_RLCK-XII-1	PK
F01_cb6277_c30/f4p0/2856-2F	RLK-Pelle_RLCK-XII-1	PK
F01_cb6277_c4/f3p1/2489-1F	RLK-Pelle_RLCK-XII-1	PK
F01_cb6277_c5/f2p1/2240-0F	RLK-Pelle_RLCK-XII-1	PK
F01_cb6277_c7/f1p0/2614-1F	RLK-Pelle_RLCK-XII-1	PK
F01_cb6277_c8/f1p0/2793-2F	RLK-Pelle_RLCK-XII-1	PK
F01_cb6277_c9/f1p1/3706-0F	RLK-Pelle_RLCK-XII-1	PK
F01_cb6277_c9/f1p1/3706-2F	RLK-Pelle_RLCK-XII-1	PK
F01_cb6291_c0/f2p0/2911-0F	IRE1	PK
F01_cb6291_c1/f2p0/2894-0F	IRE1	PK
F01_cb6291_c2/f1p0/3168-0F	IRE1	PK
F01_cb6291_c3/f1p0/2781-2F	IRE1	PK
F01_cb6291_c4/f1p0/2908-2F	IRE1	PK
F01_cb6297_c10/f1p0/2453-0F	CAMK_CAMKL-CHK1	PK
F01_cb6297_c11/f1p0/2459-1F	CAMK_CAMKL-CHK1	PK
F01_cb6297_c12/f1p0/2314-2F	CAMK_CAMKL-CHK1	PK
F01_cb6297_c18/f1p0/5398-0F	CAMK_CAMKL-CHK1	PK
F01_cb6297_c20/f14p0/2230-0F	CAMK_CAMKL-CHK1	PK
F01_cb6297_c4/f1p0/2892-0F	CAMK_CAMKL-CHK1	PK
F01_cb6297_c4/f1p0/2892-1F	CAMK_CAMKL-CHK1	PK
F01_cb6297_c6/f1p1/2256-0F	CAMK_CAMKL-CHK1	PK

F01_cb6297_c7/f1p0/2439-0F	CAMK_CAMKL-CHK1	PK
F01_cb6297_c7/f1p0/2439-1F	CAMK_CAMKL-CHK1	PK
F01_cb6304_c1/f1p0/1993-0F	RLK-Pelle_LRR-XIIIa	PK
F01_cb6340_c10/f1p0/2833-1F	CAMK_CDPK	PK
F01_cb6340_c11/f1p1/2433-0F	CAMK_CDPK	PK
F01_cb6340_c11/f1p1/2433-1F	CAMK_CDPK	PK
F01_cb6340_c11/f1p1/2433-2F	CAMK_CDPK	PK
F01_cb6340_c14/f1p0/2054-2F	CAMK_CDPK	PK
F01_cb6340_c15/f1p0/2223-0F	CAMK_CDPK	PK
F01_cb6340_c17/f1p0/2148-1F	CAMK_CDPK	PK
F01_cb6340_c17/f1p0/2148-2F	CAMK_CDPK	PK
F01_cb6340_c22/f1p0/2535-2F	CAMK_CDPK	PK
F01_cb6340_c30/f1p0/2439-0F	CAMK_CDPK	PK
F01_cb6340_c30/f1p0/2439-1F	CAMK_CDPK	PK
F01_cb6340_c37/f1p2/2383-1F	CAMK_CDPK	PK
F01_cb6357_c14/f1p0/2611-0F	AGC_PDK1	PK
F01_cb6357_c3/f1p0/2878-0F	AGC_PDK1	PK
F01_cb6357_c3/f1p0/2878-1F	AGC_PDK1	PK
F01_cb6357_c5/f1p0/2208-1F	AGC_PDK1	PK
F01_cb6357_c5/f1p0/2208-2F	AGC_PDK1	PK
F01_cb6357_c6/f1p0/2668-2F	AGC_PDK1	PK
F01_cb6357_c8/f1p0/2626-0F	AGC_PDK1	PK
F01_cb6357_c9/f1p0/2483-0F	AGC_PDK1	PK
F01_cb6385_c1/f1p1/2871-1F	CAMK_CDPK	PK
F01_cb6385_c2/f1p0/1994-2F	CAMK_CDPK	PK
F01_cb6399_c5/f1p0/2869-0R	CK1_CK1	PK

F01_cb6399_c6/f1p0/2830-2R	CK1_CK1	PK
F01_cb6406_c10/f1p0/2739-0F	RLK-Pelle_CrRLK1L-1	PK
F01_cb6406_c11/f1p0/2536-0F	RLK-Pelle_CrRLK1L-1	PK
F01_cb6406_c11/f1p0/2536-1F	RLK-Pelle_CrRLK1L-1	PK
F01_cb6406_c11/f1p0/2536-2F	RLK-Pelle_CrRLK1L-1	PK
F01_cb6406_c12/f1p0/2317-0F	RLK-Pelle_CrRLK1L-1	PK
F01_cb6406_c13/f1p0/2482-0F	RLK-Pelle_CrRLK1L-1	PK
F01_cb6406_c13/f1p0/2482-2F	RLK-Pelle_CrRLK1L-1	PK
F01_cb6406_c27/f2p1/3235-1F	CMGC_CDK-CRK7-CDK9	PK
F01_cb6406_c37/f1p0/3004-0F	CMGC_CDK-CRK7-CDK9	PK
F01_cb6406_c37/f1p0/3004-1F	CMGC_CDK-CRK7-CDK9	PK
F01_cb6406_c38/f1p0/3157-2F	CMGC_CDK-CRK7-CDK9	PK
F01_cb6406_c39/f1p0/3171-0F	CMGC_CDK-CRK7-CDK9	PK
F01_cb6406_c6/f2p0/3266-2F	CMGC_CDK-CRK7-CDK9	PK
F01_cb6406_c60/f1p0/2062-0F	RLK-Pelle_CrRLK1L-1	PK
F01_cb6406_c63/f1p0/2891-1F	CMGC_CDK-CRK7-CDK9	PK
F01_cb6406_c66/f1p0/1774-0F	RLK-Pelle_CrRLK1L-1	PK
F01_cb6406_c7/f2p1/3045-2F	CMGC_CDK-CRK7-CDK9	PK
F01_cb6406_c9/f1p0/2859-2F	RLK-Pelle_CrRLK1L-1	PK
F01_cb6408_c1/f1p0/3208-0F	TTK	PK
F01_cb6408_c1/f1p0/3208-2F	TTK	PK
F01_cb6433_c2/f1p0/2665-2F	RLK-Pelle_RLCK-XVI	PK
F01_cb6433_c3/f1p0/2643-2F	RLK-Pelle_RLCK-XVI	PK
F01_cb6433_c4/f1p0/2420-0F	RLK-Pelle_RLCK-XVI	PK
F01_cb6433_c5/f1p0/2672-1F	RLK-Pelle_RLCK-XVI	PK
F01_cb6440_c0/f6p0/2480-2F	RLK-Pelle_LRR-III	PK

F01_cb6440_c2/f3p0/2455-1F	RLK-Pelle_LRR-III	PK
F01_cb6440_c2/f3p0/2455-2F	RLK-Pelle_LRR-III	PK
F01_cb6440_c3/f2p0/2488-1F	RLK-Pelle_LRR-III	PK
F01_cb6440_c7/f1p0/2264-2F	RLK-Pelle_LRR-III	PK
F01_cb6445_c2/f3p0/1815-2F	RLK-Pelle_RLCK-VIIa-2	PK
F01_cb6445_c3/f2p0/2196-1F	RLK-Pelle_RLCK-VIIa-2	PK
F01_cb6445_c4/f2p0/1559-2F	RLK-Pelle_RLCK-VIIa-2	PK
F01_cb6445_c6/f1p0/2014-2F	RLK-Pelle_RLCK-VIIa-2	PK
F01_cb6445_c7/f1p0/1629-0F	RLK-Pelle_RLCK-VIIa-2	PK
F01_cb6463_c4/f1p1/2826-1R	CMGC_RCK	PK
F01_cb6483_c0/f4p0/2606-2F	RLK-Pelle_LRR-III	PK
F01_cb6483_c1/f4p0/2645-2F	RLK-Pelle_LRR-III	PK
F01_cb6483_c4/f1p0/2780-1F	RLK-Pelle_LRR-III	PK
F01_cb6483_c5/f1p0/2297-2F	RLK-Pelle_LRR-III	PK
F01_cb6483_c6/f1p0/2684-1F	RLK-Pelle_LRR-III	PK
F01_cb6483_c7/f1p0/2790-0F	RLK-Pelle_LRR-III	PK
F01_cb6483_c7/f1p0/2790-1F	RLK-Pelle_LRR-III	PK
F01_cb6483_c9/f1p0/2727-0F	RLK-Pelle_LRR-III	PK
F01_cb6483_c9/f1p0/2727-2F	RLK-Pelle_LRR-III	PK
F01_cb648_c3/f1p1/3872-0F	RLK-Pelle_LRR-VII-1	PK
F01_cb648_c4/f1p0/3842-2F	RLK-Pelle_LRR-VII-1	PK
F01_cb648_c9/f1p0/3724-1F	RLK-Pelle_LRR-VII-1	PK
F01_cb6491_c2/f1p0/4290-0F	RLK-Pelle_LRR-XII-1	PK
F01_cb6491_c2/f1p0/4290-2F	RLK-Pelle_LRR-XII-1	PK
F01_cb6491_c3/f1p0/3487-0F	RLK-Pelle_LRR-XII-1	PK
F01_cb6491_c5/f1p0/3634-1F	RLK-Pelle_LRR-XII-1	PK

F01_cb6491_c6/f1p1/3274-2F	RLK-Pelle_LRR-XII-1	PK
F01_cb6491_c7/f1p0/3118-1F	RLK-Pelle_LRR-XII-1	PK
F01_cb6491_c8/f1p1/3298-2F	RLK-Pelle_LRR-XII-1	PK
F01_cb6491_c9/f1p0/2466-0F	RLK-Pelle_LRR-XII-1	PK
F01_cb6505_c12/f1p3/2615-1F	RLK-Pelle_LRR-XIIIa	PK
F01_cb6505_c15/f1p0/2339-2F	RLK-Pelle_LRR-XIIIa	PK
F01_cb6505_c19/f1p1/2616-2F	RLK-Pelle_LRR-XIIIa	PK
F01_cb6505_c21/f7p3/2574-0F	RLK-Pelle_LRR-XIIIa	PK
F01_cb6505_c22/f3p2/2613-2F	RLK-Pelle_LRR-XIIIa	PK
F01_cb6505_c5/f1p0/2839-0F	RLK-Pelle_LRR-XIIIa	PK
F01_cb6505_c9/f1p0/2473-2F	RLK-Pelle_LRR-XIIIa	PK
F01_cb6512_c1/f1p0/2849-0F	STE_STE11	PK
F01_cb6512_c1/f1p0/2849-1F	STE_STE11	PK
F01_cb6512_c2/f1p0/2743-0F	STE_STE11	PK
F01_cb6512_c2/f1p0/2743-2F	TKL-Cr-3	PK
F01_cb6549_c2/f1p0/2346-2F	TKL-Pl-7	PK
F01_cb6549_c3/f1p0/2119-1F	TKL-Pl-7	PK
F01_cb6549_c7/f1p0/1775-0F	TKL-Pl-7	PK
F01_cb654_c11/f1p0/1637-1F	RLK-Pelle_PERK-2	PK
F01_cb654_c2/f1p0/4697-0F	RLK-Pelle_PERK-2	PK
F01_cb654_c2/f1p0/4697-1F	RLK-Pelle_PERK-2	PK
F01_cb654_c3/f1p0/4488-0F	RLK-Pelle_PERK-2	PK
F01_cb654_c3/f1p0/4488-1F	RLK-Pelle_PERK-2	PK
F01_cb654_c7/f1p0/2369-0F	RLK-Pelle_PERK-2	PK
F01_cb654_c7/f1p0/2369-1F	RLK-Pelle_PERK-2	PK
F01_cb654_c8/f1p0/2465-0F	RLK-Pelle_PERK-2	PK

F01_cb654_c9/f1p0/2672-1F	RLK-Pelle_PERK-2	PK
F01_cb6554_c0/f4p0/2759-2F	RLK-Pelle_CR4L	PK
F01_cb6554_c2/f2p0/2726-0F	RLK-Pelle_CR4L	PK
F01_cb6564_c0/f1p0/2770-0F	RLK-Pelle_SD-2b	PK
F01_cb6564_c0/f1p0/2770-1F	RLK-Pelle_SD-2b	PK
F01_cb6564_c1/f1p0/2552-1F	RLK-Pelle_SD-2b	PK
F01_cb6564_c1/f1p0/2552-2F	RLK-Pelle_SD-2b	PK
F01_cb6564_c3/f1p0/2730-0F	RLK-Pelle_SD-2b	PK
F01_cb6564_c3/f1p0/2730-1F	RLK-Pelle_SD-2b	PK
F01_cb6575_c0/f3p0/2822-2F	RLK-Pelle_L-LEC	PK
F01_cb6575_c2/f1p0/2916-0F	RLK-Pelle_L-LEC	PK
F01_cb6575_c2/f1p0/2916-2F	RLK-Pelle_L-LEC	PK
F01_cb6575_c4/f1p0/2749-1F	RLK-Pelle_L-LEC	PK
F01_cb6632_c0/f1p0/2800-0F	CMGC_CDK-CRK7-CDK9	PK
F01_cb6632_c1/f1p0/2571-0F	CMGC_CDK-CRK7-CDK9	PK
F01_cb6670_c3/f1p0/3029-0F	RLK-Pelle_SD-2b	PK
F01_cb6670_c6/f1p0/2428-1F	RLK-Pelle_SD-2b	PK
F01_cb6670_c7/f1p1/2713-1F	RLK-Pelle_SD-2b	PK
F01_cb6670_c8/f1p1/2713-2F	RLK-Pelle_SD-2b	PK
F01_cb6689_c16/f1p0/2383-0F	CAMK_CAMKL-CHK1	PK
F01_cb6689_c16/f1p0/2383-1F	CAMK_CAMKL-CHK1	PK
F01_cb6689_c3/f3p0/2447-1F	CAMK_CAMKL-CHK1	PK
F01_cb6689_c30/f2p1/2359-1F	CAMK_CAMKL-CHK1	PK
F01_cb6689_c31/f3p2/2467-2F	CAMK_CAMKL-CHK1	PK
F01_cb6689_c5/f1p0/2769-0F	CAMK_CAMKL-CHK1	PK
F01_cb6689_c8/f1p2/2473-1F	CAMK_CAMKL-CHK1	PK

F01_cb6691_c10/f1p0/2598-2F	RLK-Pelle_LRK10L-2	PK
F01_cb6691_c13/f1p0/2308-2F	RLK-Pelle_LRK10L-2	PK
F01_cb6691_c18/f1p0/1446-1F	RLK-Pelle_LRK10L-2	PK
F01_cb6691_c21/f1p0/2327-0F	RLK-Pelle_LRK10L-2	PK
F01_cb6691_c22/f13p0/2328-1F	RLK-Pelle_LRK10L-2	PK
F01_cb6691_c3/f3p0/2333-1F	RLK-Pelle_LRK10L-2	PK
F01_cb6691_c6/f2p0/2298-2F	RLK-Pelle_LRK10L-2	PK
F01_cb6691_c7/f1p0/2792-2F	RLK-Pelle_LRK10L-2	PK
F01_cb6691_c9/f1p0/2602-0F	RLK-Pelle_LRK10L-2	PK
F01_cb6694_c0/f1p0/2797-1F	RLK-Pelle_DLSV	PK
F01_cb6694_c0/f1p0/2797-2F	RLK-Pelle_DLSV	PK
F01_cb6694_c1/f1p0/2607-0F	RLK-Pelle_DLSV	PK
F01_cb6694_c3/f1p0/1269-0F	RLK-Pelle_DLSV	PK
F01_cb6762_c2/f2p1/2774-2F	RLK-Pelle_LRR-V	PK
F01_cb6762_c6/f1p0/2680-0F	RLK-Pelle_LRR-V	PK
F01_cb6785_c11/f1p0/2230-2F	AGC_NDR	PK
F01_cb6785_c14/f1p0/2376-1F	AGC_NDR	PK
F01_cb6785_c16/f1p0/2272-0F	AGC_NDR	PK
F01_cb6785_c17/f1p0/2283-2F	AGC_NDR	PK
F01_cb6785_c2/f2p0/2387-0F	AGC_NDR	PK
F01_cb6785_c4/f1p0/2768-2F	AGC_NDR	PK
F01_cb6785_c5/f1p0/2019-0F	AGC_NDR	PK
F01_cb6785_c7/f1p0/2303-0F	AGC_NDR	PK
F01_cb6790_c12/f1p0/2413-2F	RLK-Pelle_LRR-III	PK
F01_cb6790_c16/f1p0/2426-0F	RLK-Pelle_LRR-III	PK
F01_cb6790_c16/f1p0/2426-2F	RLK-Pelle_LRR-III	PK

F01_cb6790_c17/f1p0/2515-0F	RLK-Pelle_LRR-III	PK
F01_cb6790_c19/f1p0/2397-2F	RLK-Pelle_LRR-III	PK
F01_cb6790_c2/f2p0/2405-0F	RLK-Pelle_LRR-III	PK
F01_cb6790_c21/f1p0/2623-0F	RLK-Pelle_LRR-III	PK
F01_cb6790_c21/f1p0/2623-1F	RLK-Pelle_LRR-III	PK
F01_cb6790_c23/f1p0/2273-2F	RLK-Pelle_LRR-III	PK
F01_cb6790_c24/f1p0/2508-2F	RLK-Pelle_LRR-III	PK
F01_cb6790_c7/f2p0/2723-1F	RLK-Pelle_LRR-III	PK
F01_cb6790_c9/f1p0/2450-1F	RLK-Pelle_LRR-III	PK
F01_cb6887_c2/f1p2/2927-1F	RLK-Pelle_RLCK-IXb	PK
F01_cb6887_c3/f1p1/2908-0F	RLK-Pelle_RLCK-IXb	PK
F01_cb6887_c4/f1p0/2608-2F	RLK-Pelle_RLCK-IXb	PK
F01_cb6894_c5/f1p0/2753-0R	CAMK_CDPK	PK
F01_cb6903_c10/f1p0/2429-0F	TKL-Pl-1	PK
F01_cb6903_c11/f1p0/2427-0F	TKL-Pl-1	PK
F01_cb6903_c11/f1p0/2427-1F	TKL-Pl-1	PK
F01_cb6903_c13/f1p0/2361-2F	TKL-Pl-1	PK
F01_cb6903_c14/f1p1/2425-1F	TKL-Pl-1	PK
F01_cb6903_c15/f1p0/2342-2F	TKL-Pl-1	PK
F01_cb6903_c16/f1p1/2369-0F	TKL-Pl-1	PK
F01_cb6903_c17/f1p0/2350-0F	TKL-Pl-1	PK
F01_cb6903_c21/f1p0/2350-1F	TKL-Pl-1	PK
F01_cb6903_c24/f1p0/2542-0F	TKL-Pl-1	PK
F01_cb6903_c26/f1p0/2659-0F	TKL-Pl-1	PK
F01_cb6903_c27/f1p0/2646-1F	TKL-Pl-1	PK
F01_cb6903_c29/f1p0/2343-0F	TKL-Pl-1	PK

F01_cb6903_c8/f1p0/2744-1F	TKL-Pl-1	PK
F01_cb690_c11/f1p0/2668-1F	CMGC_DYRK-PRP4	PK
F01_cb690_c5/f1p0/4466-0F	CMGC_DYRK-PRP4	PK
F01_cb690_c7/f1p0/4210-1F	CMGC_DYRK-PRP4	PK
F01_cb690_c8/f1p0/3878-0F	CMGC_DYRK-PRP4	PK
F01_cb6915_c0/f1p0/2741-2F	STE_STE11	PK
F01_cb6915_c2/f1p0/1684-2F	STE_STE11	PK
F01_cb6929_c0/f6p0/2681-1F	RLK-Pelle_L-LEC	PK
F01_cb6929_c11/f3p0/2427-2F	RLK-Pelle_L-LEC	PK
F01_cb6929_c3/f1p0/2665-2F	RLK-Pelle_L-LEC	PK
F01_cb6929_c8/f1p0/2638-0F	RLK-Pelle_L-LEC	PK
F01_cb6951_c5/f1p0/2490-0F	TKL-Pl-4	PK
F01_cb6951_c5/f1p0/2490-2F	TKL-Pl-4	PK
F01_cb6951_c6/f1p0/2600-0F	TKL-Pl-4	PK
F01_cb696_c12/f1p0/4499-0F	TKL-Pl-6	PK
F01_cb696_c15/f1p1/4487-1F	TKL-Pl-6	PK
F01_cb696_c3/f2p2/4704-2F	TKL-Pl-6	PK
F01_cb696_c9/f1p0/4548-2F	TKL-Pl-6	PK
F01_cb6977_c7/f3p0/2504-0F	CAMK_CDPK	PK
F01_cb6993_c10/f1p1/2358-0F	AGC_NDR	PK
F01_cb6993_c11/f1p1/2588-1F	AGC_NDR	PK
F01_cb6993_c15/f1p1/1940-1F	AGC_NDR	PK
F01_cb6993_c17/f1p0/2283-0F	AGC_NDR	PK
F01_cb6993_c18/f3p1/2383-1F	AGC_NDR	PK
F01_cb6993_c19/f3p1/2522-0F	AGC_NDR	PK
F01_cb6993_c5/f1p0/2362-2F	AGC_NDR	PK

F01_cb6993_c6/f1p0/2793-0F	AGC_NDR	PK
F01_cb6993_c7/f1p1/2590-0F	AGC_NDR	PK
F01_cb6998_c1/f1p0/2718-1F	RLK-Pelle_LRR-VI-2	PK
F01_cb6998_c2/f1p0/2676-1F	RLK-Pelle_LRR-VI-2	PK
F01_cb7016_c10/f1p0/3046-2F	TKL_CTR1-DRK-2	PK
F01_cb7016_c11/f1p0/3457-2F	TKL_CTR1-DRK-2	PK
F01_cb7016_c13/f1p0/3328-2F	TKL_CTR1-DRK-2	PK
F01_cb7016_c16/f1p0/3082-0F	TKL_CTR1-DRK-2	PK
F01_cb7016_c17/f1p0/3309-0F	TKL_CTR1-DRK-2	PK
F01_cb7016_c23/f1p0/3517-0F	TKL_CTR1-DRK-2	PK
F01_cb7016_c24/f1p0/3513-2F	TKL_CTR1-DRK-2	PK
F01_cb7016_c27/f1p0/3427-1F	TKL_CTR1-DRK-2	PK
F01_cb7016_c28/f1p0/3512-1F	TKL_CTR1-DRK-2	PK
F01_cb7016_c30/f1p1/3494-0F	TKL_CTR1-DRK-2	PK
F01_cb7016_c8/f1p0/3581-0F	TKL_CTR1-DRK-2	PK
F01_cb7016_c8/f1p0/3581-2F	TKL_CTR1-DRK-2	PK
F01_cb7016_c9/f1p0/3305-2F	TKL_CTR1-DRK-2	PK
F01_cb7074_c0/f1p0/2607-2F	RLK-Pelle_RLCK-XI	PK
F01_cb7074_c2/f1p0/2389-1F	RLK-Pelle_RLCK-XI	PK
F01_cb7074_c3/f1p0/1703-2F	RLK-Pelle_RLCK-XI	PK
F01_cb7088_c10/f1p0/2704-0F	CMGC_GSK	PK
F01_cb7088_c10/f1p0/2704-1F	CMGC_GSK	PK
F01_cb7088_c11/f1p0/2918-2F	CMGC_GSK	PK
F01_cb7088_c12/f1p0/2354-1F	CMGC_GSK	PK
F01_cb7088_c13/f1p0/2088-0F	CMGC_GSK	PK
F01_cb7088_c14/f1p0/2041-0F	CMGC_GSK	PK

F01_cb7088_c15/f1p0/1821-2F	CMGC_GSK	PK
F01_cb7088_c21/f15p0/1737-2F	CMGC_GSK	PK
F01_cb7088_c25/f1p0/1664-0F	CMGC_GSK	PK
F01_cb7088_c4/f5p0/1585-1F	CMGC_GSK	PK
F01_cb7088_c6/f1p0/2700-1F	CMGC_GSK	PK
F01_cb7088_c7/f1p0/1907-1F	CMGC_GSK	PK
F01_cb7088_c9/f1p0/2325-2F	CMGC_GSK	PK
F01_cb7138_c0/f3p0/2364-0F	RLK-Pelle_LRR-III	PK
F01_cb7138_c0/f3p0/2364-1F	RLK-Pelle_LRR-III	PK
F01_cb7138_c2/f1p0/2379-0F	RLK-Pelle_LRR-III	PK
F01_cb7150_c0/f2p0/2634-0F	CAMK_CDPK	PK
F01_cb7150_c1/f1p0/2682-2F	CAMK_CDPK	PK
F01_cb7150_c3/f1p0/3108-2F	CAMK_CDPK	PK
F01_cb7195_c2/f12p0/2637-1F	RLK-Pelle_L-LEC	PK
F01_cb7195_c21/f9p0/2661-1F	RLK-Pelle_L-LEC	PK
F01_cb7195_c22/f3p0/2696-2F	RLK-Pelle_L-LEC	PK
F01_cb7195_c9/f1p0/2681-2F	RLK-Pelle_L-LEC	PK
F01_cb7196_c0/f4p0/2676-0F	RLK-Pelle_SD-2b	PK
F01_cb7196_c1/f1p0/2724-1F	RLK-Pelle_SD-2b	PK
F01_cb7201_c12/f1p0/2678-0F	CAMK_CDPK	PK
F01_cb7201_c15/f1p2/2408-0F	CAMK_CDPK	PK
F01_cb7201_c25/f1p0/2368-0F	CAMK_CDPK	PK
F01_cb7201_c25/f1p0/2368-2F	CAMK_CDPK	PK
F01_cb7201_c28/f1p1/2384-0F	CAMK_CDPK	PK
F01_cb7201_c29/f1p2/2668-0F	CAMK_CDPK	PK
F01_cb7201_c31/f1p1/2612-1F	CAMK_CDPK	PK

F01_cb7201_c34/f1p0/2568-1F	CAMK_CDPK	PK
F01_cb7201_c36/f1p1/2177-2F	CAMK_CDPK	PK
F01_cb7201_c38/f1p0/2544-1F	CAMK_CDPK	PK
F01_cb7201_c44/f1p0/2593-1F	CAMK_CDPK	PK
F01_cb7201_c54/f18p2/2387-2F	CAMK_CDPK	PK
F01_cb7201_c9/f2p2/2605-2F	CAMK_CDPK	PK
F01_cb7231_c17/f39p2/2330-2F	CMGC_MAPK	PK
F01_cb7231_c2/f1p0/2285-1F	CMGC_MAPK	PK
F01_cb7231_c2/f1p0/2285-2F	CMGC_MAPK	PK
F01_cb7231_c6/f1p0/2665-2F	CMGC_MAPK	PK
F01_cb7238_c2/f1p0/2602-0F	TKL-Pl-4	PK
F01_cb7246_c4/f1p2/2674-0F	RLK-Pelle_LRR-III	PK
F01_cb7246_c5/f1p2/2841-1F	RLK-Pelle_LRR-III	PK
F01_cb7246_c6/f1p0/2850-2F	RLK-Pelle_LRR-III	PK
F01_cb7289_c1/f4p0/2280-0F	RLK-Pelle_RKF3	PK
F01_cb7289_c1/f4p0/2280-1F	RLK-Pelle_RKF3	PK
F01_cb7289_c2/f1p1/2655-1F	RLK-Pelle_RKF3	PK
F01_cb7289_c2/f1p1/2655-2F	RLK-Pelle_RKF3	PK
F01_cb7289_c3/f1p0/2299-0F	RLK-Pelle_RKF3	PK
F01_cb7289_c3/f1p0/2299-1F	RLK-Pelle_RKF3	PK
F01_cb7289_c5/f1p0/2601-2F	RLK-Pelle_RKF3	PK
F01_cb7289_c6/f1p0/2261-0F	RLK-Pelle_RKF3	PK
F01_cb7289_c7/f1p0/1719-2F	RLK-Pelle_RKF3	PK
F01_cb7300_c1/f2p1/2552-0F	CMGC_CDK-CRK7-CDK9	PK
F01_cb7300_c3/f1p0/2340-0F	CMGC_CDK-CRK7-CDK9	PK
F01_cb7300_c3/f1p0/2340-2F	CMGC_CDK-CRK7-CDK9	PK

F01_cb7313_c0/f1p0/2641-0F	RLK-Pelle_LRR-XI-1	PK
F01_cb7313_c1/f1p0/3338-2F	RLK-Pelle_LRR-XI-1	PK
F01_cb7321_c14/f2p0/2550-2F	CAMK_CDPK	PK
F01_cb7321_c3/f2p0/2605-2F	CAMK_CDPK	PK
F01_cb7321_c5/f2p0/2199-0F	CAMK_CDPK	PK
F01_cb7321_c6/f1p0/2620-1F	CAMK_CDPK	PK
F01_cb7321_c6/f1p0/2620-2F	CAMK_CDPK	PK
F01_cb7328_c10/f4p0/2211-1F	RLK-Pelle_LRR-VI-2	PK
F01_cb7328_c11/f2p0/2266-1F	RLK-Pelle_LRR-VI-2	PK
F01_cb7328_c2/f1p0/2693-0F	RLK-Pelle_LRR-VI-2	PK
F01_cb7328_c4/f1p0/2098-2F	RLK-Pelle_LRR-VI-2	PK
F01_cb7328_c5/f1p0/3547-0F	RLK-Pelle_LRR-VI-2	PK
F01_cb7328_c5/f1p0/3547-2F	RLK-Pelle_LRR-VI-2	PK
F01_cb7328_c7/f1p0/2466-0F	RLK-Pelle_LRR-VI-2	PK
F01_cb7328_c7/f1p0/2466-2F	RLK-Pelle_LRR-VI-2	PK
F01_cb7328_c9/f2p0/2268-0F	RLK-Pelle_LRR-VI-2	PK
F01_cb7332_c3/f2p0/2489-2F	RLK-Pelle_RLCK-V	PK
F01_cb7332_c4/f1p0/2646-0F	RLK-Pelle_RLCK-V	PK
F01_cb7332_c6/f1p0/2808-2F	RLK-Pelle_RLCK-V	PK
F01_cb7381_c2/f1p0/2630-1F	RLK-Pelle_LRR-II	PK
F01_cb7381_c3/f1p0/2562-2F	RLK-Pelle_LRR-II	PK
F01_cb7381_c4/f1p0/2122-2F	RLK-Pelle_LRR-II	PK
F01_cb7381_c5/f1p0/2233-1F	RLK-Pelle_LRR-II	PK
F01_cb7394_c1/f2p0/1619-1F	RLK-Pelle_RLCK-X	PK
F01_cb7394_c1/f2p0/1619-2F	RLK-Pelle_RLCK-X	PK
F01_cb7394_c4/f1p0/2441-0F	RLK-Pelle_RLCK-X	PK

F01_cb7394_c6/f1p0/1751-2F	RLK-Pelle_RLCK-X	PK
F01_cb7394_c7/f1p0/1507-1F	RLK-Pelle_RLCK-X	PK
F01_cb7394_c7/f1p0/1507-2F	RLK-Pelle_RLCK-X	PK
F01_cb7397_c0/f1p0/2627-0F	RLK-Pelle_CR4L	PK
F01_cb7397_c1/f1p0/2180-0F	RLK-Pelle_CR4L	PK
F01_cb7397_c1/f1p0/2180-1F	RLK-Pelle_CR4L	PK
F01_cb7397_c2/f1p0/1887-0F	RLK-Pelle_CR4L	PK
F01_cb7397_c4/f1p0/1787-1F	RLK-Pelle_CR4L	PK
F01_cb7455_c1/f1p0/2475-1F	RLK-Pelle_LRK10L-2	PK
F01_cb7455_c2/f1p0/2955-2F	RLK-Pelle_LRK10L-2	PK
F01_cb7465_c0/f17p2/2428-1F	CAMK_CDPK	PK
F01_cb7465_c0/f17p2/2428-2F	CAMK_CDPK	PK
F01_cb7465_c11/f1p1/2467-2F	CAMK_CDPK	PK
F01_cb7465_c14/f1p0/2565-0F	CAMK_CDPK	PK
F01_cb7473_c101/f1p0/5112-1F	TKL-Pl-6	PK
F01_cb7473_c101/f1p0/5112-2F	TKL-Pl-6	PK
F01_cb7473_c102/f1p2/4847-1F	TKL-Pl-6	PK
F01_cb7473_c104/f104p3/4707-2F	TKL-Pl-6	PK
F01_cb7473_c32/f1p2/4521-0F	TKL-Pl-6	PK
F01_cb7473_c35/f1p1/4531-1F	TKL-Pl-6	PK
F01_cb7473_c39/f1p3/4702-2F	TKL-Pl-6	PK
F01_cb7473_c69/f1p5/4717-0F	TKL-Pl-6	PK
F01_cb7473_c70/f1p2/3838-2F	TKL-Pl-6	PK
F01_cb7473_c73/f1p0/4374-1F	TKL-Pl-6	PK
F01_cb7473_c75/f1p2/3492-2F	TKL-Pl-6	PK
F01_cb7473_c79/f1p2/4350-1F	TKL-Pl-6	PK

F01_cb7473_c79/f1p2/4350-2F	TKL-Pl-6	PK
F01_cb7473_c81/f1p0/4667-0F	TKL-Pl-6	PK
F01_cb7473_c82/f1p1/4302-0F	TKL-Pl-6	PK
F01_cb7473_c85/f1p0/2404-1F	TKL-Pl-6	PK
F01_cb7473_c88/f1p0/4354-1F	TKL-Pl-6	PK
F01_cb7473_c94/f1p0/5910-0F	TKL-Pl-6	PK
F01_cb7473_c97/f1p0/5726-2F	TKL-Pl-6	PK
F01_cb7473_c98/f1p0/5241-1F	TKL-Pl-6	PK
F01_cb7519_c1/f3p0/1555-0F	CAMK_OST1L	PK
F01_cb7519_c10/f1p0/1531-1F	CAMK_OST1L	PK
F01_cb7519_c20/f2p0/1611-1F	CAMK_OST1L	PK
F01_cb7519_c8/f1p0/1575-1F	CAMK_OST1L	PK
F01_cb7519_c8/f1p0/1575-2F	CAMK_OST1L	PK
F01_cb7519_c9/f1p0/1565-1F	CAMK_OST1L	PK
F01_cb7544_c0/f1p0/2593-1F	RLK-Pelle_LysM	PK
F01_cb7614_c2/f1p0/2572-0F	RLK-Pelle_L-LEC	PK
F01_cb7614_c2/f1p0/2572-1F	RLK-Pelle_L-LEC	PK
F01_cb7614_c4/f1p0/2346-0F	RLK-Pelle_L-LEC	PK
F01_cb7614_c7/f1p0/2088-2F	RLK-Pelle_L-LEC	PK
F01_cb7614_c8/f3p0/2186-1F	RLK-Pelle_L-LEC	PK
F01_cb7614_c9/f1p0/2099-0F	RLK-Pelle_L-LEC	PK
F01_cb7623_c10/f1p1/3196-1F	CAMK_CDPK	PK
F01_cb7623_c10/f1p1/3196-2F	CAMK_CDPK	PK
F01_cb7623_c11/f1p1/2298-0F	CAMK_CDPK	PK
F01_cb7623_c11/f1p1/2298-1F	CAMK_CDPK	PK
F01_cb7623_c13/f1p0/2310-0F	CAMK_CDPK	PK

F01_cb7623_c14/f1p0/2422-0F	CAMK_CDPK	PK
F01_cb7623_c16/f1p0/2535-0F	CAMK_CDPK	PK
F01_cb7623_c16/f1p0/2535-2F	CAMK_CDPK	PK
F01_cb7623_c23/f1p0/2362-0F	CAMK_CDPK	PK
F01_cb7623_c23/f1p0/2362-2F	CAMK_CDPK	PK
F01_cb7623_c25/f1p0/2346-0F	CAMK_CDPK	PK
F01_cb7623_c27/f1p0/2082-1F	CAMK_CDPK	PK
F01_cb7623_c28/f1p1/2038-0F	CAMK_CDPK	PK
F01_cb7623_c34/f2p0/2410-1F	CAMK_CDPK	PK
F01_cb7623_c34/f2p0/2410-2F	CAMK_CDPK	PK
F01_cb7623_c8/f1p0/2569-1F	CAMK_CDPK	PK
F01_cb7636_c1/f1p1/2570-1F	TKL-Pl-4	PK
F01_cb7636_c2/f1p0/2241-2F	TKL-Pl-4	PK
F01_cb7636_c3/f1p0/2438-2F	TKL-Pl-4	PK
F01_cb7636_c5/f1p0/1712-0F	TKL-Pl-4	PK
F01_cb7640_c0/f1p0/2570-1F	RLK-Pelle_DLSV	PK
F01_cb7681_c0/f2p0/2467-0F	RLK-Pelle_DLSV	PK
F01_cb7681_c1/f1p0/2561-1F	RLK-Pelle_DLSV	PK
F01_cb7690_c0/f1p0/2559-0F	RLK-Pelle_LRR-III	PK
F01_cb7690_c0/f1p0/2559-2F	RLK-Pelle_LRR-III	PK
F01_cb7690_c1/f1p0/2545-1F	RLK-Pelle_LRR-III	PK
F01_cb7690_c1/f1p0/2545-2F	RLK-Pelle_LRR-III	PK
F01_cb7718_c0/f1p0/2575-1F	RLK-Pelle_SD-2b	PK
F01_cb7718_c4/f1p0/2261-0F	RLK-Pelle_SD-2b	PK
F01_cb7718_c4/f1p0/2261-1F	RLK-Pelle_SD-2b	PK
F01_cb7719_c0/f3p0/2334-2F	RLK-Pelle_RLCK-VIIa-2	PK

F01_cb7719_c3/f1p0/2551-1F	RLK-Pelle_RLCK-VIIa-2	PK
F01_cb7719_c4/f1p0/2330-0F	RLK-Pelle_RLCK-VIIa-2	PK
F01_cb7719_c5/f1p0/2379-0F	RLK-Pelle_RLCK-VIIa-2	PK
F01_cb7719_c6/f1p0/5135-1F	RLK-Pelle_RLCK-VIIa-2	PK
F01_cb7719_c6/f1p0/5135-2F	RLK-Pelle_RLCK-VIIa-2	PK
F01_cb7748_c0/f3p0/2463-2F	RLK-Pelle_LRR-XI-2	PK
F01_cb7748_c1/f3p0/2333-1F	RLK-Pelle_LRR-XI-2	PK
F01_cb7748_c2/f2p0/2406-0F	RLK-Pelle_LRR-XI-2	PK
F01_cb7748_c2/f2p0/2406-1F	RLK-Pelle_LRR-XI-2	PK
F01_cb7748_c4/f1p0/4792-2F	RLK-Pelle_LRR-XI-2	PK
F01_cb7770_c1/f1p0/2271-0F	NEK	PK
F01_cb7812_c0/f1p0/2576-1F	RLK-Pelle_SD-2b	PK
F01_cb7812_c11/f1p0/2558-2F	RLK-Pelle_SD-2b	PK
F01_cb7812_c12/f1p0/2425-1F	RLK-Pelle_SD-2b	PK
F01_cb7812_c3/f2p0/2427-0F	RLK-Pelle_SD-2b	PK
F01_cb7812_c3/f2p0/2427-1F	RLK-Pelle_SD-2b	PK
F01_cb7812_c9/f1p0/2503-1F	RLK-Pelle_SD-2b	PK
F01_cb7815_c1/f8p1/2084-0F	AGC_NDR	PK
F01_cb7815_c11/f1p1/2093-1F	AGC_NDR	PK
F01_cb7815_c11/f1p1/2093-2F	AGC_NDR	PK
F01_cb7815_c12/f1p0/3859-1F	AGC_NDR	PK
F01_cb7815_c15/f1p1/2179-0F	AGC_NDR	PK
F01_cb7815_c5/f1p0/2383-1F	AGC_NDR	PK
F01_cb7815_c7/f1p0/3828-0F	AGC_NDR	PK
F01_cb7815_c8/f1p0/3813-1F	AGC_NDR	PK
F01_cb7833_c0/f1p1/2523-0F	RLK-Pelle_RLCK-VI	PK

F01_cb7833_c4/f1p1/2093-0F	RLK-Pelle_RLCK-VI	PK
F01_cb7833_c5/f1p0/2433-1F	RLK-Pelle_RLCK-VI	PK
F01_cb7833_c7/f1p0/1964-0F	RLK-Pelle_RLCK-VI	PK
F01_cb7833_c7/f1p0/1964-2F	RLK-Pelle_RLCK-VI	PK
F01_cb7833_c9/f1p0/1865-2F	RLK-Pelle_RLCK-VI	PK
F01_cb7857_c13/f1p0/2356-0F	RLK-Pelle_LRR-Xa	PK
F01_cb7857_c14/f1p1/2180-0F	RLK-Pelle_LRR-Xa	PK
F01_cb7857_c16/f1p0/2520-1F	RLK-Pelle_LRR-Xa	PK
F01_cb7857_c17/f1p1/2293-2F	RLK-Pelle_LRR-Xa	PK
F01_cb7857_c18/f1p0/2248-1F	RLK-Pelle_LRR-Xa	PK
F01_cb7857_c22/f1p1/2443-1F	RLK-Pelle_LRR-Xa	PK
F01_cb7857_c23/f1p1/2477-0F	RLK-Pelle_LRR-Xa	PK
F01_cb7857_c33/f1p1/2317-2F	RLK-Pelle_LRR-Xa	PK
F01_cb7857_c35/f11p2/2269-1F	RLK-Pelle_LRR-Xa	PK
F01_cb7857_c8/f1p0/2518-2F	RLK-Pelle_LRR-Xa	PK
F01_cb7857_c9/f1p0/2620-1F	RLK-Pelle_LRR-Xa	PK
F01_cb7877_c4/f1p0/1633-1F	Aur	PK
F01_cb7877_c5/f1p0/1554-0F	Aur	PK
F01_cb7880_c0/f1p0/2513-1F	RLK-Pelle_RLCK-IXa	PK
F01_cb7880_c0/f1p0/2513-2F	RLK-Pelle_RLCK-IXa	PK
F01_cb7880_c1/f1p0/2383-1F	RLK-Pelle_RLCK-IXa	PK
F01_cb7880_c2/f1p0/2386-0F	RLK-Pelle_RLCK-IXa	PK
F01_cb7880_c2/f1p0/2386-2F	RLK-Pelle_RLCK-IXa	PK
F01_cb7880_c3/f1p0/2527-2F	RLK-Pelle_RLCK-IXa	PK
F01_cb7880_c5/f1p0/2230-0F	RLK-Pelle_RLCK-IXa	PK
F01_cb7884_c0/f6p0/2160-1F	RLK-Pelle_RLCK-VIIa-1	PK

F01_cb7884_c1/f4p0/2102-0F	RLK-Pelle_RLCK-VIIa-1	PK
F01_cb7884_c10/f1p0/2173-0F	RLK-Pelle_RLCK-VIIa-1	PK
F01_cb7884_c13/f1p0/2131-0F	RLK-Pelle_RLCK-VIIa-1	PK
F01_cb7884_c14/f1p0/1994-0F	RLK-Pelle_RLCK-VIIa-1	PK
F01_cb7884_c19/f1p0/1680-1F	RLK-Pelle_RLCK-VIIa-1	PK
F01_cb7884_c20/f1p0/1563-1F	RLK-Pelle_RLCK-VIIa-1	PK
F01_cb7884_c4/f1p0/2511-0F	RLK-Pelle_RLCK-VIIa-1	PK
F01_cb7884_c5/f1p0/2380-2F	RLK-Pelle_RLCK-VIIa-1	PK
F01_cb7884_c8/f1p0/2095-2F	RLK-Pelle_RLCK-VIIa-1	PK
F01_cb7884_c9/f1p0/2372-0F	RLK-Pelle_RLCK-VIIa-1	PK
F01_cb7887_c0/f1p0/2522-1F	RLK-Pelle_PERK-1	PK
F01_cb7887_c0/f1p0/2522-2F	RLK-Pelle_PERK-1	PK
F01_cb7887_c3/f1p0/1941-1F	RLK-Pelle_PERK-1	PK
F01_cb792_c1/f1p1/3799-0F	AGC_RSK-2	PK
F01_cb792_c1/f1p1/3799-1F	AGC_RSK-2	PK
F01_cb792_c11/f1p0/4630-1F	AGC_RSK-2	PK
F01_cb792_c15/f1p0/3428-0F	AGC_RSK-2	PK
F01_cb792_c15/f1p0/3428-2F	AGC_RSK-2	PK
F01_cb792_c16/f1p0/3622-1F	AGC_RSK-2	PK
F01_cb792_c27/f1p3/4257-0F	AGC_RSK-2	PK
F01_cb792_c27/f1p3/4257-2F	AGC_RSK-2	PK
F01_cb792_c30/f1p1/3745-1F	AGC_RSK-2	PK
F01_cb792_c31/f1p0/3278-0F	AGC_RSK-2	PK
F01_cb792_c31/f1p0/3278-2F	AGC_RSK-2	PK
F01_cb792_c32/f1p1/4065-2F	AGC_RSK-2	PK
F01_cb792_c35/f1p1/3973-1F	AGC_RSK-2	PK

F01_cb792_c38/f1p0/3385-0F	AGC_RSK-2	PK
F01_cb792_c38/f1p0/3385-1F	AGC_RSK-2	PK
F01_cb792_c42/f1p0/4087-0F	AGC_RSK-2	PK
F01_cb792_c44/f1p2/4203-2F	AGC_RSK-2	PK
F01_cb792_c47/f1p0/3981-0F	AGC_RSK-2	PK
F01_cb792_c48/f1p0/2773-1F	AGC_RSK-2	PK
F01_cb792_c5/f1p3/3973-2F	AGC_RSK-2	PK
F01_cb792_c53/f1p0/3930-2F	AGC_RSK-2	PK
F01_cb792_c8/f3p0/3700-1F	AGC_RSK-2	PK
F01_cb792_c9/f3p0/3428-2F	AGC_RSK-2	PK
F01_cb7938_c0/f1p0/2496-0F	RLK-Pelle_Extensin	PK
F01_cb7938_c1/f1p0/2905-1F	RLK-Pelle_Extensin	PK
F01_cb7938_c1/f1p0/2905-2F	RLK-Pelle_Extensin	PK
F01_cb7938_c2/f1p0/1721-0F	RLK-Pelle_Extensin	PK
F01_cb7938_c2/f1p0/1721-2F	RLK-Pelle_Extensin	PK
F01_cb793_c2/f1p0/2758-0F	TKL_Gdt	PK
F01_cb7942_c28/f1p0/5231-0F	STE_STE11	PK
F01_cb7956_c0/f1p0/2494-2F	RLK-Pelle_LRR-II	PK
F01_cb7956_c1/f1p1/2308-0F	RLK-Pelle_LRR-II	PK
F01_cb7956_c2/f1p1/2417-0F	RLK-Pelle_LRR-II	PK
F01_cb7956_c4/f1p0/1716-2F	RLK-Pelle_LRR-II	PK
F01_cb7958_c1/f1p0/2525-0F	RLK-Pelle_LRR-VII-2	PK
F01_cb7958_c2/f1p0/2402-0F	RLK-Pelle_LRR-VII-2	PK
F01_cb7958_c3/f1p0/2396-2F	RLK-Pelle_LRR-VII-2	PK
F01_cb7959_c0/f3p1/2489-1F	RLK-Pelle_LRR-III	PK
F01_cb7986_c10/f1p0/2589-0F	CK1_CK1	PK

F01_cb7986_c11/f1p0/2013-1F	CK1_CK1	PK
F01_cb7986_c12/f1p0/2135-0F	CK1_CK1	PK
F01_cb7986_c14/f1p0/2289-0F	CK1_CK1	PK
F01_cb7986_c20/f1p0/2471-2F	CK1_CK1	PK
F01_cb7986_c25/f1p0/2385-1F	CK1_CK1	PK
F01_cb7986_c27/f1p2/2043-0F	CK1_CK1	PK
F01_cb7986_c28/f1p1/2038-1F	CK1_CK1	PK
F01_cb7986_c29/f1p0/2406-2F	CK1_CK1	PK
F01_cb7986_c30/f1p0/2096-2F	CK1_CK1	PK
F01_cb7986_c32/f1p0/2203-1F	CK1_CK1	PK
F01_cb7986_c33/f1p0/2206-1F	CK1_CK1	PK
F01_cb7986_c34/f1p2/2046-0F	CK1_CK1	PK
F01_cb7986_c38/f1p0/2277-1F	CK1_CK1	PK
F01_cb7986_c39/f1p0/2138-1F	CK1_CK1	PK
F01_cb7986_c42/f1p0/2132-1F	CK1_CK1	PK
F01_cb7986_c43/f1p0/1870-2F	CK1_CK1	PK
F01_cb7986_c46/f1p0/1612-0F	CK1_CK1	PK
F01_cb7986_c50/f13p0/2156-0F	CK1_CK1	PK
F01_cb7986_c8/f1p0/2489-0F	CK1_CK1	PK
F01_cb7986_c9/f1p0/2279-1F	CK1_CK1	PK
F01_cb804_c11/f1p0/3984-0F	RLK-Pelle_Extensin	PK
F01_cb804_c12/f1p0/3580-1F	RLK-Pelle_Extensin	PK
F01_cb804_c3/f1p2/3980-1F	RLK-Pelle_Extensin	PK
F01_cb804_c4/f1p0/4404-1F	RLK-Pelle_Extensin	PK
F01_cb804_c4/f1p0/4404-2F	RLK-Pelle_Extensin	PK
F01_cb804_c7/f1p0/3407-0F	RLK-Pelle_Extensin	PK

F01_cb804_c9/f1p2/3622-2F	RLK-Pelle_Extensin	PK
F01_cb8052_c0/f2p0/1908-0F	RLK-Pelle_RLCK-IV	PK
F01_cb8052_c1/f1p0/2472-0F	RLK-Pelle_RLCK-IV	PK
F01_cb8052_c1/f1p0/2472-2F	RLK-Pelle_RLCK-IV	PK
F01_cb8052_c2/f1p0/2271-2F	RLK-Pelle_RLCK-IV	PK
F01_cb808_c5/f1p0/2943-0F	RLK-Pelle_LRR-I-2	PK
F01_cb808_c5/f1p0/2943-1F	RLK-Pelle_LRR-I-2	PK
F01_cb8219_c10/f1p1/2990-2F	RLK-Pelle_LRR-II	PK
F01_cb8219_c12/f1p1/2448-2F	RLK-Pelle_LRR-II	PK
F01_cb8219_c13/f1p0/2684-2F	RLK-Pelle_LRR-II	PK
F01_cb8219_c15/f1p2/2313-1F	RLK-Pelle_LRR-II	PK
F01_cb8219_c21/f1p0/2209-2F	RLK-Pelle_LRR-II	PK
F01_cb8219_c23/f1p0/2536-0F	RLK-Pelle_LRR-II	PK
F01_cb8219_c23/f1p0/2536-1F	RLK-Pelle_LRR-II	PK
F01_cb8219_c24/f1p0/2245-0F	RLK-Pelle_LRR-II	PK
F01_cb8219_c7/f1p0/2426-1F	RLK-Pelle_LRR-II	PK
F01_cb8219_c8/f1p0/2332-2F	RLK-Pelle_LRR-II	PK
F01_cb8219_c9/f1p0/2465-1F	RLK-Pelle_LRR-II	PK
F01_cb8232_c0/f1p0/2423-2R	RLK-Pelle_CrRLK1L-1	PK
F01_cb8232_c1/f1p1/2570-1R	RLK-Pelle_CrRLK1L-1	PK
F01_cb8232_c2/f1p0/1283-2R	RLK-Pelle_CrRLK1L-1	PK
F01_cb8243_c11/f1p0/2473-1F	CAMK_CAMKL-CHK1	PK
F01_cb8243_c13/f1p0/1996-1F	CAMK_CAMKL-CHK1	PK
F01_cb8243_c13/f1p0/1996-2F	CAMK_CAMKL-CHK1	PK
F01_cb8243_c2/f3p0/2267-2F	CAMK_CAMKL-CHK1	PK
F01_cb8243_c23/f1p0/2340-1F	CAMK_CAMKL-CHK1	PK

F01_cb8243_c8/f1p1/2514-0F	CAMK_CAMKL-CHK1	PK
F01_cb8243_c8/f1p1/2514-1F	CAMK_CAMKL-CHK1	PK
F01_cb8243_c9/f1p1/2375-1F	CAMK_CAMKL-CHK1	PK
F01_cb8243_c9/f1p1/2375-2F	CAMK_CAMKL-CHK1	PK
F01_cb8261_c0/f1p0/2416-0F	RLK-Pelle_L-LEC	PK
F01_cb8261_c0/f1p0/2416-2F	RLK-Pelle_L-LEC	PK
F01_cb8261_c1/f1p0/2427-0F	RLK-Pelle_L-LEC	PK
F01_cb8282_c0/f1p0/2410-0F	RLK-Pelle_L-LEC	PK
F01_cb8282_c1/f1p0/2267-1F	RLK-Pelle_L-LEC	PK
F01_cb8304_c0/f1p0/2405-0F	RLK-Pelle_RLCK-XI	PK
F01_cb8313_c10/f1p2/1641-1F	RLK-Pelle_L-LEC	PK
F01_cb8313_c2/f2p2/1637-0F	RLK-Pelle_L-LEC	PK
F01_cb8313_c3/f2p4/1747-0F	RLK-Pelle_L-LEC	PK
F01_cb8313_c4/f2p0/1668-2F	RLK-Pelle_L-LEC	PK
F01_cb8313_c5/f1p0/2403-0F	RLK-Pelle_L-LEC	PK
F01_cb8313_c7/f1p0/1924-0F	RLK-Pelle_L-LEC	PK
F01_cb8313_c8/f1p0/1620-2F	RLK-Pelle_L-LEC	PK
F01_cb8313_c9/f1p0/1605-0F	RLK-Pelle_WAK	PK
F01_cb8316_c1/f1p0/2402-0F	CAMK_CDPK	PK
F01_cb8316_c5/f4p0/2159-1F	CAMK_CDPK	PK
F01_cb8326_c1/f1p0/3568-2F	RLK-Pelle_DLSV	PK
F01_cb8353_c0/f1p0/2372-2F	RLK-Pelle_DLSV	PK
F01_cb8353_c2/f1p0/1626-0F	RLK-Pelle_DLSV	PK
F01_cb8353_c3/f1p0/1612-1F	RLK-Pelle_DLSV	PK
F01_cb8362_c7/f1p0/2209-1F	RLK-Pelle_LRR-XI-2	PK
F01_cb8370_c3/f1p0/2389-0F	RLK-Pelle_RLCK-XII-1	PK

F01_cb8415_c11/f1p0/2377-1F	TKL-Pl-3	PK
F01_cb8415_c12/f3p1/2518-0F	TKL-Pl-3	PK
F01_cb8415_c4/f1p0/3003-2F	TKL-Pl-3	PK
F01_cb8415_c5/f1p0/2267-0F	TKL-Pl-3	PK
F01_cb8415_c5/f1p0/2267-1F	TKL-Pl-3	PK
F01_cb8415_c8/f1p0/2154-0F	TKL-Pl-3	PK
F01_cb8415_c9/f1p0/2373-1F	TKL-Pl-3	PK
F01_cb8430_c12/f1p0/2260-1F	CMGC_CDK-CRK7-CDK9	PK
F01_cb8430_c13/f1p0/2161-1F	CMGC_CDK-CRK7-CDK9	PK
F01_cb8430_c13/f1p0/2161-2F	CMGC_CDK-CRK7-CDK9	PK
F01_cb8430_c19/f6p0/2064-2F	CMGC_CDK-CRK7-CDK9	PK
F01_cb8430_c9/f1p0/2284-2F	CMGC_CDK-CRK7-CDK9	PK
F01_cb8435_c0/f1p0/2370-0F	RLK-Pelle_LRR-I-2	PK
F01_cb8435_c1/f1p0/1902-2F	RLK-Pelle_LRR-I-2	PK
F01_cb8435_c2/f1p0/1682-1F	RLK-Pelle_LRR-I-2	PK
F01_cb8435_c2/f1p0/1682-2F	RLK-Pelle_LRR-I-2	PK
F01_cb847_c0/f1p0/4607-0F	RLK-Pelle_LRR-VIII-1	PK
F01_cb847_c1/f1p0/2020-2F	RLK-Pelle_LRR-VIII-1	PK
F01_cb847_c3/f1p0/3395-2F	RLK-Pelle_LRR-VIII-1	PK
F01_cb8498_c0/f1p0/2351-0F	RLK-Pelle_L-LEC	PK
F01_cb8498_c2/f1p0/2306-2F	RLK-Pelle_L-LEC	PK
F01_cb8518_c0/f5p1/2336-0F	RLK-Pelle_LRR-III	PK
F01_cb8547_c1/f2p0/2162-1F	TKL-Pl-4	PK
F01_cb8547_c1/f2p0/2162-2F	TKL-Pl-4	PK
F01_cb8547_c14/f3p0/2216-0F	TKL-Pl-4	PK
F01_cb8547_c15/f1p0/2174-0F	TKL-Pl-4	PK

F01_cb8547_c15/f1p0/2174-1F	TKL-Pl-4	PK
F01_cb8547_c2/f1p0/2338-0F	TKL-Pl-4	PK
F01_cb8547_c3/f1p0/2081-2F	TKL-Pl-4	PK
F01_cb8547_c4/f1p0/2164-1F	TKL-Pl-4	PK
F01_cb8547_c6/f1p0/2234-1F	TKL-Pl-4	PK
F01_cb8547_c6/f1p0/2234-2F	TKL-Pl-4	PK
F01_cb8547_c7/f1p0/2217-2F	TKL-Pl-4	PK
F01_cb8547_c8/f1p0/2636-2F	TKL-Pl-4	PK
F01_cb8547_c9/f1p0/2136-2F	TKL-Pl-4	PK
F01_cb8564_c10047/f3p1/3273-0F	CMGC_CDK-PITSLRE	PK
F01_cb8564_c10328/f1p1/3525-2F	CMGC_CDK-PITSLRE	PK
F01_cb8564_c10425/f1p0/4814-0F	CMGC_MAPK	PK
F01_cb8564_c10461/f1p0/1967-0F	AGC-Pl	PK
F01_cb8564_c10461/f1p0/1967-1F	AGC-Pl	PK
F01_cb8564_c104640/f4p5/2313-2F	RLK-Pelle_LysM	PK
F01_cb8564_c10551/f1p0/2536-0F	RLK-Pelle_DLSV	PK
F01_cb8564_c105673/f1p1/2557-2F	RLK-Pelle_LysM	PK
F01_cb8564_c106799/f2p0/3168-0F	RLK-Pelle_LRR-IX	PK
F01_cb8564_c106809/f12p4/3320-1F	RLK-Pelle_LRR-VIII-1	PK
F01_cb8564_c106830/f3p0/3099-0F	RLK-Pelle_CrRLK1L-1	PK
F01_cb8564_c106830/f3p0/3099-1F	RLK-Pelle_CrRLK1L-1	PK
F01_cb8564_c106956/f4p6/2390-2F	RLK-Pelle_DLSV	PK
F01_cb8564_c107010/f7p2/3198-1F	RLK-Pelle_DLSV	PK
F01_cb8564_c107325/f11p0/2469-2F	RLK-Pelle_DLSV	PK
F01_cb8564_c107607/f7p0/2484-1F	RLK-Pelle_LRR-II	PK
F01_cb8564_c107625/f7p0/2800-1F	RLK-Pelle_LRR-II	PK

F01_cb8564_c107650/f6p0/2145-2F	RLK-Pelle_LysM	PK
F01_cb8564_c107702/f7p2/2922-1F	RLK-Pelle_RLCK-VI	PK
F01_cb8564_c107754/f5p0/3585-0F	RLK-Pelle_LRR-XIIIb	PK
F01_cb8564_c107794/f3p2/1953-1F	CAMK_CAMKL-CHK1	PK
F01_cb8564_c107859/f5p0/2352-1F	RLK-Pelle_LRR-II	PK
F01_cb8564_c10786/f1p0/2509-1F	CMGC_MAPK	PK
F01_cb8564_c108739/f7p0/2789-2F	CMGC_RCK	PK
F01_cb8564_c108806/f9p0/2182-1F	RLK-Pelle_LysM	PK
F01_cb8564_c108809/f8p2/2590-2F	RLK-Pelle_WAK_LRK10L-1	PK
F01_cb8564_c109293/f3p0/2904-0F	CMGC_RCK	PK
F01_cb8564_c109436/f3p0/3336-2F	RLK-Pelle_RLCK-IXb	PK
F01_cb8564_c109567/f3p0/2271-2F	RLK-Pelle_LysM	PK
F01_cb8564_c109614/f3p0/3516-0F	STE_STE11	PK
F01_cb8564_c109614/f3p0/3516-1F	STE_STE11	PK
F01_cb8564_c109636/f4p0/2195-1F	RLK-Pelle_PERK-1	PK
F01_cb8564_c109730/f3p0/2853-1F	RLK-Pelle_DLSV	PK
F01_cb8564_c109763/f6p0/2213-0F	RLK-Pelle_LRK10L-2	PK
F01_cb8564_c109805/f2p0/2993-2F	RLK-Pelle_RLCK-IXb	PK
F01_cb8564_c109935/f2p1/2100-2F	RLK-Pelle_RLCK-VIIa-2	PK
F01_cb8564_c109983/f2p0/2051-0F	RLK-Pelle_RLCK-IXb	PK
F01_cb8564_c110001/f2p0/2066-0F	RLK-Pelle_RLCK-VIIa-2	PK
F01_cb8564_c110030/f3p0/2151-1F	CAMK_AMPK	PK
F01_cb8564_c110031/f4p0/2010-0F	CAMK_CAMKL-CHK1	PK
F01_cb8564_c110039/f2p0/4075-2R	RLK-Pelle_LRR-XI-2	PK
F01_cb8564_c110170/f1p0/2703-0F	CMGC_RCK	PK
F01_cb8564_c110170/f1p0/2703-2F	CMGC_RCK	PK

F01_cb8564_c110249/f1p5/2441-2F	RLK-Pelle_L-LEC	PK
F01_cb8564_c110331/f1p0/2891-0F	CMGC_RCK	PK
F01_cb8564_c110331/f1p0/2891-1F	CMGC_RCK	PK
F01_cb8564_c110354/f1p0/2697-0F	RLK-Pelle_RLCK-IXb	PK
F01_cb8564_c110369/f2p2/3432-0F	TKL_CTR1-DRK-1	PK
F01_cb8564_c110469/f1p0/2627-2F	CMGC_RCK	PK
F01_cb8564_c110511/f1p0/3279-0F	TKL_CTR1-DRK-1	PK
F01_cb8564_c110511/f1p0/3279-2F	TKL_CTR1-DRK-1	PK
F01_cb8564_c110657/f1p0/3023-0F	CMGC_RCK	PK
F01_cb8564_c110700/f1p0/3062-1F	RLK-Pelle_PERK-1	PK
F01_cb8564_c110700/f1p0/3062-2F	RLK-Pelle_PERK-1	PK
F01_cb8564_c110704/f1p0/3841-0F	STE_STE11	PK
F01_cb8564_c110704/f1p0/3841-1F	STE_STE11	PK
F01_cb8564_c110760/f1p0/2242-1F	CAMK_CAMKL-CHK1	PK
F01_cb8564_c110775/f1p1/2362-1F	RLK-Pelle_L-LEC	PK
F01_cb8564_c110799/f1p1/2389-2F	RLK-Pelle_WAK_LRK10L-1	PK
F01_cb8564_c110818/f1p0/2549-2F	CK1_CK1	PK
F01_cb8564_c110872/f1p0/4089-1F	RLK-Pelle_PERK-1	PK
F01_cb8564_c110897/f1p0/3122-1F	RLK-Pelle_RLCK-IXb	PK
F01_cb8564_c110953/f1p0/2771-0F	CMGC_RCK	PK
F01_cb8564_c110953/f1p0/2771-1F	CMGC_RCK	PK
F01_cb8564_c110954/f1p1/2392-0F	CMGC_RCK	PK
F01_cb8564_c110954/f1p1/2392-1F	CMGC_RCK	PK
F01_cb8564_c111220/f1p0/2588-1F	AGC_RSK-2	PK
F01_cb8564_c111240/f1p1/3895-1F	STE_STE11	PK
F01_cb8564_c111257/f1p0/2726-2F	RLK-Pelle_WAK_LRK10L-1	PK

F01_cb8564_c111279/f1p0/2793-1F	RLK-Pelle_RLCK-IXb	PK
F01_cb8564_c111279/f1p0/2793-2F	RLK-Pelle_RLCK-IXb	PK
F01_cb8564_c111300/f1p1/2327-2F	RLK-Pelle_LRR-II	PK
F01_cb8564_c111346/f1p0/2978-0F	CMGC_RCK	PK
F01_cb8564_c111363/f1p0/2737-0F	CMGC_RCK	PK
F01_cb8564_c111363/f1p0/2737-1F	CMGC_RCK	PK
F01_cb8564_c111370/f1p0/2892-1F	RLK-Pelle_PERK-1	PK
F01_cb8564_c111370/f1p0/2892-2F	RLK-Pelle_PERK-1	PK
F01_cb8564_c111419/f2p1/2850-1F	CAMK_CDPK	PK
F01_cb8564_c111566/f1p0/2381-1F	TKL-Pl-4	PK
F01_cb8564_c111597/f1p0/3403-1F	RLK-Pelle_RLCK-IXb	PK
F01_cb8564_c111765/f1p0/2336-0F	RLK-Pelle_DLSV	PK
F01_cb8564_c11204/f1p1/2554-1F	CMGC_MAPK	PK
F01_cb8564_c112044/f1p4/2411-2F	RLK-Pelle_DLSV	PK
F01_cb8564_c112175/f2p0/2623-1F	CMGC_RCK	PK
F01_cb8564_c112175/f2p0/2623-2F	CMGC_RCK	PK
F01_cb8564_c112185/f1p1/2186-0F	RLK-Pelle_PERK-1	PK
F01_cb8564_c112185/f1p1/2186-2F	RLK-Pelle_PERK-1	PK
F01_cb8564_c112214/f1p0/2132-1F	RLK-Pelle_DLSV	PK
F01_cb8564_c112253/f1p0/2816-2F	CMGC_RCK	PK
F01_cb8564_c112272/f1p0/3791-2F	STE_STE11	PK
F01_cb8564_c11237/f1p0/3521-2F	RLK-Pelle_DLSV	PK
F01_cb8564_c112401/f1p0/2533-2F	RLK-Pelle_LRK10L-2	PK
F01_cb8564_c112503/f1p0/2983-0F	TKL_CTR1-DRK-1	PK
F01_cb8564_c112503/f1p0/2983-2F	TKL_CTR1-DRK-1	PK
F01_cb8564_c11255/f1p0/2899-2F	RLK-Pelle_LRR-I-1	PK

F01_cb8564_c112660/f1p0/2777-1F	STE_STE11	PK
F01_cb8564_c112735/f1p0/2180-0R	RLK-Pelle_RLCK-XII-1	PK
F01_cb8564_c11283/f1p0/3248-0F	RLK-Pelle_LRR-I-1	PK
F01_cb8564_c11283/f1p0/3248-2F	RLK-Pelle_LRR-I-1	PK
F01_cb8564_c112951/f1p1/2676-1F	STE_STE11	PK
F01_cb8564_c112951/f1p1/2676-2F	STE_STE11	PK
F01_cb8564_c113014/f1p0/2963-0F	CMGC_RCK	PK
F01_cb8564_c113014/f1p0/2963-1F	CMGC_RCK	PK
F01_cb8564_c113063/f1p0/2238-0F	AGC-Pl	PK
F01_cb8564_c113063/f1p0/2238-1F	AGC-Pl	PK
F01_cb8564_c113082/f1p0/2984-1F	RLK-Pelle_RLCK-IXb	PK
F01_cb8564_c113090/f2p1/2586-2F	RLK-Pelle_WAK_LRK10L-1	PK
F01_cb8564_c113205/f1p0/3318-1F	RLK-Pelle_RLCK-VIIa-2	PK
F01_cb8564_c11335/f2p0/2147-2F	AGC-Pl	PK
F01_cb8564_c113378/f2p3/2387-0F	RLK-Pelle_L-LEC	PK
F01_cb8564_c113391/f1p0/2206-1F	CAMK_CAMKL-CHK1	PK
F01_cb8564_c113442/f1p1/4271-1F	RLK-Pelle_RLCK-IXb	PK
F01_cb8564_c113452/f1p0/2024-1F	RLK-Pelle_LRK10L-2	PK
F01_cb8564_c113536/f4p0/2375-1F	CAMK_CAMKL-CHK1	PK
F01_cb8564_c113585/f1p0/1949-0F	CAMK_CAMKL-CHK1	PK
F01_cb8564_c113585/f1p0/1949-1F	CAMK_CAMKL-CHK1	PK
F01_cb8564_c113585/f1p0/1949-2F	CAMK_CAMKL-CHK1	PK
F01_cb8564_c113599/f1p0/2144-0F	CMGC_SRPK	PK
F01_cb8564_c113824/f1p0/2966-0F	CMGC_RCK	PK
F01_cb8564_c113878/f1p0/2151-2F	RLK-Pelle_RLCK-XII-1	PK
F01_cb8564_c114061/f1p0/3218-1F	TKL_CTR1-DRK-1	PK

F01_cb8564_c114061/f1p0/3218-2F	TKL_CTR1-DRK-1	PK
F01_cb8564_c114173/f1p0/2891-1F	RLK-Pelle_DLSV	PK
F01_cb8564_c114181/f1p0/3066-0F	TKL_CTR1-DRK-1	PK
F01_cb8564_c114181/f1p0/3066-2F	TKL_CTR1-DRK-1	PK
F01_cb8564_c114210/f5p0/2641-1F	RLK-Pelle_DLSV	PK
F01_cb8564_c114259/f1p0/2848-0F	RLK-Pelle_WAK_LRK10L-1	PK
F01_cb8564_c114307/f1p0/3739-0F	CMGC_RCK	PK
F01_cb8564_c114383/f1p0/2453-1F	CMGC_RCK	PK
F01_cb8564_c114409/f1p0/2716-1F	STE_STE11	PK
F01_cb8564_c114466/f1p0/3000-0F	CMGC_RCK	PK
F01_cb8564_c114521/f1p0/3515-1F	RLK-Pelle_RLCK-VIIa-2	PK
F01_cb8564_c114592/f1p0/2403-0F	RLK-Pelle_DLSV	PK
F01_cb8564_c114592/f1p0/2403-1F	RLK-Pelle_DLSV	PK
F01_cb8564_c114609/f1p0/3442-1F	STE_STE11	PK
F01_cb8564_c114655/f1p0/2695-2F	RLK-Pelle_RLCK-IXb	PK
F01_cb8564_c114894/f1p0/1910-0F	RLK-Pelle_WAK_LRK10L-1	PK
F01_cb8564_c115113/f1p0/2592-0F	RLK-Pelle_RLCK-IXb	PK
F01_cb8564_c115113/f1p0/2592-2F	RLK-Pelle_RLCK-IXb	PK
F01_cb8564_c115114/f1p0/1959-1F	TKL-Pl-4	PK
F01_cb8564_c115114/f1p0/1959-2F	TKL-Pl-4	PK
F01_cb8564_c11519/f1p0/2109-0F	CMGC_CDK-PITSLRE	PK
F01_cb8564_c11519/f1p0/2109-2F	CMGC_CDK-PITSLRE	PK
F01_cb8564_c115329/f1p0/2622-0F	RLK-Pelle_WAK_LRK10L-1	PK
F01_cb8564_c115399/f2p0/2879-1F	TKL_CTR1-DRK-1	PK
F01_cb8564_c115405/f1p0/3948-0F	TKL_CTR1-DRK-1	PK
F01_cb8564_c115486/f1p1/3203-1F	STE_STE11	PK

F01_cb8564_c115557/f1p0/2665-1F	CK1_CK1	PK
F01_cb8564_c115642/f1p0/3794-1F	RLK-Pelle_RLCK-IXb	PK
F01_cb8564_c115775/f1p0/4708-2F	TKL_CTR1-DRK-1	PK
F01_cb8564_c115829/f1p0/2856-0F	RLK-Pelle_RLCK-VIIa-2	PK
F01_cb8564_c115829/f1p0/2856-1F	RLK-Pelle_RLCK-VIIa-2	PK
F01_cb8564_c115862/f1p0/2728-2F	RLK-Pelle_RLCK-XII-1	PK
F01_cb8564_c116001/f1p0/3225-1F	RLK-Pelle_RLCK-IXb	PK
F01_cb8564_c116001/f1p0/3225-2F	RLK-Pelle_RLCK-IXb	PK
F01_cb8564_c116029/f1p0/2889-2F	RLK-Pelle_DLSV	PK
F01_cb8564_c116037/f1p0/3379-1F	TKL_CTR1-DRK-1	PK
F01_cb8564_c116112/f1p0/2411-2F	RLK-Pelle_LRR-IV	PK
F01_cb8564_c116115/f1p0/2124-0F	TKL-Pl-4	PK
F01_cb8564_c116144/f1p0/3080-1F	RLK-Pelle_DLSV	PK
F01_cb8564_c116249/f1p0/2661-0F	CMGC_RCK	PK
F01_cb8564_c116249/f1p0/2661-2F	CMGC_RCK	PK
F01_cb8564_c116449/f1p0/2445-0F	RLK-Pelle_LRK10L-2	PK
F01_cb8564_c116449/f1p0/2445-1F	RLK-Pelle_LRK10L-2	PK
F01_cb8564_c116534/f1p1/2348-0F	RLK-Pelle_PERK-1	PK
F01_cb8564_c116534/f1p1/2348-1F	RLK-Pelle_PERK-1	PK
F01_cb8564_c116548/f1p0/2006-0F	RLK-Pelle_LysM	PK
F01_cb8564_c116603/f1p1/2342-2F	RLK-Pelle_PERK-1	PK
F01_cb8564_c116615/f1p0/3195-1F	CMGC_RCK	PK
F01_cb8564_c116615/f1p0/3195-2F	CMGC_RCK	PK
F01_cb8564_c116627/f1p0/2108-2F	RLK-Pelle_RLCK-IXb	PK
F01_cb8564_c116648/f1p0/2630-2F	RLK-Pelle_DLSV	PK
F01_cb8564_c116701/f1p0/2471-0F	CMGC_RCK	PK

F01_cb8564_c116701/f1p0/2471-2F	CMGC_RCK	PK
F01_cb8564_c116822/f1p0/2723-2F	RLK-Pelle_DLSV	PK
F01_cb8564_c11697/f1p0/4709-1F	RLK-Pelle_CrRLK1L-1	PK
F01_cb8564_c116976/f1p0/2943-1F	CMGC_RCK	PK
F01_cb8564_c117060/f1p0/2233-0F	CAMK_CAMKL-CHK1	PK
F01_cb8564_c117126/f2p0/2610-2F	RLK-Pelle_DLSV	PK
F01_cb8564_c11716/f1p1/3711-1F	RLK-Pelle_LRR-XII-1	PK
F01_cb8564_c117192/f1p0/2991-1F	RLK-Pelle_RLCK-IXb	PK
F01_cb8564_c117256/f1p1/2878-2F	CK1_CK1	PK
F01_cb8564_c117464/f1p0/2604-0F	RLK-Pelle_LRR-II	PK
F01_cb8564_c11759/f10p0/4062-0F	STE_STE11	PK
F01_cb8564_c117641/f1p0/2859-0F	RLK-Pelle_LRR-Xb-1	PK
F01_cb8564_c117641/f1p0/2859-1F	RLK-Pelle_LysM	PK
F01_cb8564_c117663/f1p0/2828-0F	RLK-Pelle_WAK_LRK10L-1	PK
F01_cb8564_c117774/f1p1/2201-0F	RLK-Pelle_L-LEC	PK
F01_cb8564_c117787/f1p1/4351-2F	TKL-Pl-4	PK
F01_cb8564_c117861/f1p0/2243-1F	RLK-Pelle_LRK10L-2	PK
F01_cb8564_c117861/f1p0/2243-2F	RLK-Pelle_LRK10L-2	PK
F01_cb8564_c117889/f1p0/2484-0F	RLK-Pelle_DLSV	PK
F01_cb8564_c118086/f1p0/2187-1F	RLK-Pelle_LysM	PK
F01_cb8564_c118096/f1p0/2820-1F	CMGC_RCK	PK
F01_cb8564_c118096/f1p0/2820-2F	CMGC_RCK	PK
F01_cb8564_c118242/f1p2/3724-2F	STE_STE11	PK
F01_cb8564_c118301/f1p0/2259-0F	RLK-Pelle_RLCK-VI	PK
F01_cb8564_c118313/f1p0/2343-1F	RLK-Pelle_L-LEC	PK
F01_cb8564_c11857/f1p0/3989-2F	RLK-Pelle_RLCK-VIIa-2	PK

F01_cb8564_c118597/f1p0/2325-2F	RLK-Pelle_L-LEC	PK
F01_cb8564_c118611/f1p3/2971-1F	RLK-Pelle_CrRLK1L-1	PK
F01_cb8564_c118901/f1p0/3630-1F	NAK	PK
F01_cb8564_c11895/f2p0/2161-2F	RLK-Pelle_LRK10L-2	PK
F01_cb8564_c118956/f1p0/2861-2F	TKL_CTR1-DRK-1	PK
F01_cb8564_c11907/f1p0/3937-1F	RLK-Pelle_LRR-XI-1	PK
F01_cb8564_c119140/f1p0/3034-1F	RLK-Pelle_RLCK-IXb	PK
F01_cb8564_c119266/f1p0/2858-2F	CK1_CK1-Pl	PK
F01_cb8564_c119337/f1p0/2896-2R	RLK-Pelle_LRR-XI-2	PK
F01_cb8564_c119410/f1p0/2746-1F	CK1_CK1	PK
F01_cb8564_c119434/f1p0/2937-1F	CMGC_RCK	PK
F01_cb8564_c119646/f1p0/2336-2F	RLK-Pelle_DLSV	PK
F01_cb8564_c11969/f1p1/2914-2F	RLK-Pelle_SD-2b	PK
F01_cb8564_c119780/f1p0/2632-0F	RLK-Pelle_LysM	PK
F01_cb8564_c119826/f1p0/2124-2F	CMGC_GSK	PK
F01_cb8564_c1199/f1p0/2887-2F	RLK-Pelle_DLSV	PK
F01_cb8564_c119947/f1p0/2488-1F	RLK-Pelle_WAK_LRK10L-1	PK
F01_cb8564_c119992/f1p1/2694-0F	CMGC_RCK	PK
F01_cb8564_c12002/f1p0/4465-0F	STE_STE11	PK
F01_cb8564_c120113/f1p0/2030-2F	CAMK_AMPK	PK
F01_cb8564_c120178/f1p0/2851-1F	RLK-Pelle_RLCK-IXb	PK
F01_cb8564_c1203/f1p0/2185-0F	RLK-Pelle_DLSV	PK
F01_cb8564_c1203/f1p0/2185-1F	RLK-Pelle_DLSV	PK
F01_cb8564_c1205/f1p0/2486-0F	RLK-Pelle_DLSV	PK
F01_cb8564_c1206/f1p0/2104-0F	RLK-Pelle_DLSV	PK
F01_cb8564_c120613/f1p0/2079-0F	RLK-Pelle_RLCK-IXb	PK

F01_cb8564_c120621/f1p0/2147-1F	RLK-Pelle_PERK-1	PK
F01_cb8564_c120694/f1p0/2801-0F	RLK-Pelle_DLSV	PK
F01_cb8564_c1207/f1p0/2117-0F	TKL-Pl-4	PK
F01_cb8564_c120731/f4p0/3162-2F	RLK-Pelle_LRR-IX	PK
F01_cb8564_c120736/f1p1/3227-0F	TKL_CTR1-DRK-1	PK
F01_cb8564_c120736/f1p1/3227-2F	TKL_CTR1-DRK-1	PK
F01_cb8564_c1208/f1p0/1938-2F	TKL-Pl-4	PK
F01_cb8564_c120913/f1p1/2797-1F	RLK-Pelle_DLSV	PK
F01_cb8564_c120960/f1p0/3590-0F	RLK-Pelle_RLCK-IXb	PK
F01_cb8564_c120960/f1p0/3590-1F	RLK-Pelle_RLCK-IXb	PK
F01_cb8564_c121040/f1p0/3601-0F	STE_STE11	PK
F01_cb8564_c1211/f1p1/4992-2F	RLK-Pelle_LRR-XI-1	PK
F01_cb8564_c121149/f1p0/3251-0F	CMGC_RCK	PK
F01_cb8564_c1212/f1p0/4151-0F	RLK-Pelle_LRR-XI-1	PK
F01_cb8564_c1212/f1p0/4151-2F	RLK-Pelle_LRR-XI-1	PK
F01_cb8564_c121236/f1p4/3795-1F	TKL-Pl-4	PK
F01_cb8564_c121315/f1p0/2356-0F	RLK-Pelle_DLSV	PK
F01_cb8564_c1214/f1p0/3091-0F	RLK-Pelle_LRR-XI-1	PK
F01_cb8564_c121404/f1p6/2450-1F	RLK-Pelle_LysM	PK
F01_cb8564_c121433/f4p1/2731-1F	CMGC_RCK	PK
F01_cb8564_c121448/f1p0/3452-1F	RLK-Pelle_RLCK-IXb	PK
F01_cb8564_c121469/f1p0/2615-0F	CAMK_CDPK	PK
F01_cb8564_c121469/f1p0/2615-2F	CAMK_CDPK	PK
F01_cb8564_c1215/f1p0/3984-1F	RLK-Pelle_LRR-XI-1	PK
F01_cb8564_c121535/f1p0/1945-0F	RLK-Pelle_RLCK-VIII	PK
F01_cb8564_c121572/f1p0/2082-0F	CMGC_SRPK	PK

F01_cb8564_c121572/f1p0/2082-2F	CMGC_SRPK	PK
F01_cb8564_c121652/f1p0/2604-0F	NAK	PK
F01_cb8564_c121718/f1p0/2735-1F	AGC_RSK-2	PK
F01_cb8564_c121915/f1p0/2807-0F	RLK-Pelle_RKF3	PK
F01_cb8564_c12192/f1p0/1925-2F	RLK-Pelle_LRK10L-2	PK
F01_cb8564_c121940/f1p0/2673-0F	RLK-Pelle_RLCK-IXb	PK
F01_cb8564_c121959/f1p0/2723-0F	RLK-Pelle_RLCK-VI	PK
F01_cb8564_c121959/f1p0/2723-2F	RLK-Pelle_RLCK-VI	PK
F01_cb8564_c122242/f1p0/2340-2F	CMGC_MAPK	PK
F01_cb8564_c122273/f1p0/2363-2F	RLK-Pelle_RLCK-IXb	PK
F01_cb8564_c122475/f1p0/2873-0F	CMGC_RCK	PK
F01_cb8564_c122487/f1p0/3064-0F	CMGC_RCK	PK
F01_cb8564_c122615/f2p0/2073-1F	RLK-Pelle_RLCK-XII-1	PK
F01_cb8564_c122671/f1p0/3119-0F	TKL_CTR1-DRK-1	PK
F01_cb8564_c122847/f1p0/2777-0R	CAMK_AMPK	PK
F01_cb8564_c122889/f1p0/3295-0F	CMGC_RCK	PK
F01_cb8564_c122963/f1p0/3278-2F	STE_STE11	PK
F01_cb8564_c123038/f1p0/2682-1F	RLK-Pelle_DLSV	PK
F01_cb8564_c123079/f1p0/1966-1F	RLK-Pelle_RLCK-IXb	PK
F01_cb8564_c123083/f1p0/2536-0F	RLK-Pelle_LRK10L-2	PK
F01_cb8564_c123151/f1p1/3168-2F	RLK-Pelle_RLCK-IXb	PK
F01_cb8564_c123164/f1p0/2333-0F	RLK-Pelle_WAK_LRK10L-1	PK
F01_cb8564_c123171/f1p3/2923-1F	CK1_CK1-Pl	PK
F01_cb8564_c123202/f1p0/2686-2F	RLK-Pelle_LRK10L-2	PK
F01_cb8564_c123207/f1p0/2075-0F	CAMK_CAMKL-CHK1	PK
F01_cb8564_c123403/f1p0/2808-0R	RLK-Pelle_LRR-XI-2	PK

F01_cb8564_c123436/f1p0/3559-2F	RLK-Pelle_RLCK-IXb	PK
F01_cb8564_c123534/f1p0/2994-2F	RLK-Pelle_RLCK-IXb	PK
F01_cb8564_c123711/f1p0/2282-2F	RLK-Pelle_WAK_LRK10L-1	PK
F01_cb8564_c123757/f1p0/2888-0F	RLK-Pelle_CrRLK1L-1	PK
F01_cb8564_c123784/f1p0/2528-1F	CMGC_RCK	PK
F01_cb8564_c123889/f1p0/2931-1F	CMGC_RCK	PK
F01_cb8564_c123889/f1p0/2931-2F	CMGC_RCK	PK
F01_cb8564_c123910/f1p0/1933-2F	RLK-Pelle_DLSV	PK
F01_cb8564_c124059/f1p0/2078-2F	CAMK_AMPK	PK
F01_cb8564_c124114/f4p0/2478-2F	RLK-Pelle_WAK_LRK10L-1	PK
F01_cb8564_c124189/f1p0/2801-1F	CMGC_RCK	PK
F01_cb8564_c124205/f3p0/2236-1F	CMGC_SRPK	PK
F01_cb8564_c124220/f1p0/3115-1F	RLK-Pelle_PERK-1	PK
F01_cb8564_c124226/f1p0/2958-1F	RLK-Pelle_RLCK-IXb	PK
F01_cb8564_c124253/f1p1/3110-1F	TKL_CTR1-DRK-1	PK
F01_cb8564_c124255/f1p0/3874-1F	STE_STE11	PK
F01_cb8564_c124294/f1p0/2474-0F	RLK-Pelle_DLSV	PK
F01_cb8564_c124298/f1p0/2114-1F	CMGC_RCK	PK
F01_cb8564_c124298/f1p0/2114-2F	CMGC_RCK	PK
F01_cb8564_c124354/f1p0/2793-1F	RLK-Pelle_WAK_LRK10L-1	PK
F01_cb8564_c124569/f1p2/2454-2F	RLK-Pelle_DLSV	PK
F01_cb8564_c124590/f2p3/3324-0F	TKL_CTR1-DRK-1	PK
F01_cb8564_c124693/f1p1/2321-0F	TKL_CTR1-DRK-1	PK
F01_cb8564_c124810/f1p0/2494-2F	RLK-Pelle_RKF3	PK
F01_cb8564_c124824/f1p0/3173-0F	CMGC_RCK	PK
F01_cb8564_c124866/f1p1/2690-0F	RLK-Pelle_PERK-1	PK

F01_cb8564_c124866/f1p1/2690-1F	RLK-Pelle_PERK-1	PK
F01_cb8564_c124887/f1p0/1953-0F	CMGC_GSK	PK
F01_cb8564_c124949/f1p0/2727-0F	RLK-Pelle_DLSV	PK
F01_cb8564_c124949/f1p0/2727-1F	RLK-Pelle_DLSV	PK
F01_cb8564_c125018/f1p0/3452-0F	STE_STE11	PK
F01_cb8564_c125018/f1p0/3452-1F	STE_STE11	PK
F01_cb8564_c125018/f1p0/3452-2F	STE_STE11	PK
F01_cb8564_c125048/f1p0/2689-2F	STE_STE11	PK
F01_cb8564_c125152/f1p0/2135-2F	CAMK_CDPK	PK
F01_cb8564_c125211/f1p0/2958-0F	STE_STE11	PK
F01_cb8564_c125384/f1p0/2816-1F	CK1_CK1	PK
F01_cb8564_c125412/f1p0/2422-0F	CMGC_RCK	PK
F01_cb8564_c125421/f1p0/2128-0F	RLK-Pelle_WAK_LRK10L-1	PK
F01_cb8564_c12553/f1p0/3439-1F	RLK-Pelle_RLCK-VIIa-2	PK
F01_cb8564_c125605/f2p0/2787-1F	STE_STE11	PK
F01_cb8564_c125690/f1p1/2868-2F	RLK-Pelle_DLSV	PK
F01_cb8564_c125829/f1p0/3356-0F	RLK-Pelle_RLCK-IXb	PK
F01_cb8564_c125829/f1p0/3356-1F	RLK-Pelle_RLCK-IXb	PK
F01_cb8564_c12594/f1p0/2738-2F	CMGC_MAPK	PK
F01_cb8564_c126020/f1p1/2281-2F	RLK-Pelle_PERK-1	PK
F01_cb8564_c126219/f1p1/2912-0F	TKL-Pl-4	PK
F01_cb8564_c126219/f1p1/2912-1F	TKL-Pl-4	PK
F01_cb8564_c126283/f1p2/3253-0F	TKL_CTR1-DRK-1	PK
F01_cb8564_c126348/f1p0/3601-2F	CMGC_RCK	PK
F01_cb8564_c12635/f1p0/2906-1F	Group-Pl-3	PK
F01_cb8564_c126429/f1p0/2339-0F	CAMK_CAMKL-CHK1	PK

F01_cb8564_c126429/f1p0/2339-2F	CAMK_CAMKL-CHK1	PK
F01_cb8564_c126556/f1p1/2614-2F	CMGC_RCK	PK
F01_cb8564_c126691/f1p1/3817-2F	STE_STE11	PK
F01_cb8564_c126707/f1p0/2918-0F	RLK-Pelle_DLSV	PK
F01_cb8564_c126819/f1p0/3149-0F	TKL_CTR1-DRK-1	PK
F01_cb8564_c126819/f1p0/3149-1F	TKL_CTR1-DRK-1	PK
F01_cb8564_c126961/f1p0/2708-1F	RLK-Pelle_WAK_LRK10L-1	PK
F01_cb8564_c126979/f1p0/2455-1F	RLK-Pelle_WAK_LRK10L-1	PK
F01_cb8564_c1270/f1p0/2203-1F	CAMK_AMPK	PK
F01_cb8564_c127064/f1p0/2665-0F	RLK-Pelle_WAK_LRK10L-1	PK
F01_cb8564_c127064/f1p0/2665-2F	RLK-Pelle_WAK_LRK10L-1	PK
F01_cb8564_c127210/f1p0/2308-0F	RLK-Pelle_LRK10L-2	PK
F01_cb8564_c127211/f1p0/2256-0F	RLK-Pelle_PERK-1	PK
F01_cb8564_c127257/f1p0/3239-0F	TKL_CTR1-DRK-1	PK
F01_cb8564_c127306/f1p0/3040-0F	CMGC_RCK	PK
F01_cb8564_c127335/f1p0/2345-1F	CAMK_CAMKL-CHK1	PK
F01_cb8564_c127485/f1p0/2563-0F	RLK-Pelle_WAK_LRK10L-1	PK
F01_cb8564_c127495/f1p1/2539-1F	RLK-Pelle_RLCK-VIIa-2	PK
F01_cb8564_c12751/f1p0/3357-0F	RLK-Pelle_LRR-VIII-1	PK
F01_cb8564_c12751/f1p0/3357-2F	RLK-Pelle_LRR-VIII-1	PK
F01_cb8564_c127519/f1p0/2902-1F	RLK-Pelle_RLCK-IXb	PK
F01_cb8564_c127591/f1p0/2476-0F	CAMK_CDPK	PK
F01_cb8564_c127591/f1p0/2476-1F	CAMK_CDPK	PK
F01_cb8564_c127715/f2p0/2769-0F	RLK-Pelle_RLCK-IXb	PK
F01_cb8564_c12781/f1p0/3625-0F	RLK-Pelle_LRR-XI-1	PK
F01_cb8564_c127839/f1p0/2470-0F	RLK-Pelle_WAK_LRK10L-1	PK

F01_cb8564_c127879/f1p1/3681-2F	TKL_CTR1-DRK-1	PK
F01_cb8564_c127940/f1p0/2066-1F	RLK-Pelle_LRK10L-2	PK
F01_cb8564_c127962/f1p0/2651-0F	STE_STE11	PK
F01_cb8564_c128052/f1p0/2064-0F	RLK-Pelle_RLCK-VIIa-2	PK
F01_cb8564_c128268/f1p0/2378-0F	RLK-Pelle_L-LEC	PK
F01_cb8564_c128385/f1p0/2575-0F	RLK-Pelle_WAK_LRK10L-1	PK
F01_cb8564_c128396/f1p0/2977-1F	RLK-Pelle_LysM	PK
F01_cb8564_c128453/f1p0/2467-1F	TKL-Pl-4	PK
F01_cb8564_c128479/f1p0/2340-0F	CMGC_RCK	PK
F01_cb8564_c128479/f1p0/2340-1F	CMGC_RCK	PK
F01_cb8564_c128783/f1p0/2185-2F	RLK-Pelle_WAK_LRK10L-1	PK
F01_cb8564_c1288/f1p0/2349-1F	CMGC_MAPK	PK
F01_cb8564_c1288/f1p0/2349-2F	CMGC_MAPK	PK
F01_cb8564_c12886/f1p0/2334-1F	AGC-Pl	PK
F01_cb8564_c128872/f1p0/2506-0F	CMGC_RCK	PK
F01_cb8564_c1289/f1p1/2238-0F	CMGC_MAPK	PK
F01_cb8564_c1290/f1p0/1928-1F	CMGC_MAPK	PK
F01_cb8564_c129038/f1p0/2416-2F	RLK-Pelle_PERK-1	PK
F01_cb8564_c129118/f1p0/2569-0F	CMGC_RCK	PK
F01_cb8564_c129120/f1p0/2596-0F	RLK-Pelle_DLSV	PK
F01_cb8564_c129136/f1p0/2515-0F	RLK-Pelle_WAK_LRK10L-1	PK
F01_cb8564_c129146/f1p0/2383-2F	RLK-Pelle_WAK_LRK10L-1	PK
F01_cb8564_c129154/f1p0/2632-0F	CMGC_RCK	PK
F01_cb8564_c129215/f1p0/2844-2F	STE_STE11	PK
F01_cb8564_c129236/f1p0/2711-0F	RLK-Pelle_WAK_LRK10L-1	PK
F01_cb8564_c129260/f1p0/2454-0F	AGC-Pl	PK

F01_cb8564_c129260/f1p0/2454-2F	AGC-Pl	PK
F01_cb8564_c129286/f1p0/2001-1F	RLK-Pelle_CrRLK1L-1	PK
F01_cb8564_c129352/f1p0/2700-0F	RLK-Pelle_DLSV	PK
F01_cb8564_c129352/f1p0/2700-2F	RLK-Pelle_DLSV	PK
F01_cb8564_c129379/f1p0/3821-1F	RLK-Pelle_RLCK-IXb	PK
F01_cb8564_c129472/f1p0/2679-2F	RLK-Pelle_DLSV	PK
F01_cb8564_c129695/f1p0/2575-2F	RLK-Pelle_RKF3	PK
F01_cb8564_c129919/f1p0/2904-2F	RLK-Pelle_RLCK-IXb	PK
F01_cb8564_c130071/f1p0/4218-2F	STE_STE11	PK
F01_cb8564_c130150/f1p1/2667-2F	CMGC_RCK	PK
F01_cb8564_c130207/f1p0/2575-1F	RLK-Pelle_DLSV	PK
F01_cb8564_c13026/f1p0/4872-0F	RLK-Pelle_CrRLK1L-1	PK
F01_cb8564_c13026/f1p0/4872-2F	RLK-Pelle_RLCK-VIIa-2	PK
F01_cb8564_c130264/f1p0/2624-0F	CMGC_RCK	PK
F01_cb8564_c13031/f1p0/2119-0F	RLK-Pelle_LRR-IV	PK
F01_cb8564_c13031/f1p0/2119-2F	RLK-Pelle_LRR-IV	PK
F01_cb8564_c13077/f1p0/3535-1F	RLK-Pelle_LRR-XII-1	PK
F01_cb8564_c131213/f1p2/2634-0F	RLK-Pelle_RLCK-VI	PK
F01_cb8564_c131213/f1p2/2634-2F	RLK-Pelle_RLCK-VI	PK
F01_cb8564_c132218/f1p5/2319-1F	RLK-Pelle_LysM	PK
F01_cb8564_c13234/f1p0/3522-1F	CMGC_CDK-PITSLRE	PK
F01_cb8564_c13254/f1p0/3855-1F	RLK-Pelle_RLCK-VIIa-2	PK
F01_cb8564_c13467/f1p0/3088-1F	CMGC_CDK-PITSLRE	PK
F01_cb8564_c13502/f1p1/4106-0F	RLK-Pelle_RLCK-VIIa-2	PK
F01_cb8564_c13502/f1p1/4106-1F	RLK-Pelle_CrRLK1L-1	PK
F01_cb8564_c13502/f1p1/4106-2F	RLK-Pelle_RLCK-VIIa-2	PK

F01_cb8564_c13527/f1p0/3096-1F	STE_STE11	PK
F01_cb8564_c13574/f1p0/2342-0F	RLK-Pelle_DLSV	PK
F01_cb8564_c1361/f1p0/3334-1F	CMGC_MAPK	PK
F01_cb8564_c1362/f1p0/3486-1F	CMGC_MAPK	PK
F01_cb8564_c1363/f1p0/2245-1F	CMGC_MAPK	PK
F01_cb8564_c1363/f1p0/2245-2F	CMGC_MAPK	PK
F01_cb8564_c1364/f1p0/2214-1F	CMGC_MAPK	PK
F01_cb8564_c136802/f2p3/2749-2F	RLK-Pelle_RLCK-VI	PK
F01_cb8564_c1370/f1p0/2891-0F	CMGC_MAPK	PK
F01_cb8564_c1371/f2p1/3040-2F	CMGC_MAPK	PK
F01_cb8564_c1372/f1p0/2553-2F	CMGC_MAPK	PK
F01_cb8564_c1374/f1p0/3856-0F	CMGC_MAPK	PK
F01_cb8564_c1374/f1p0/3856-1F	CMGC_MAPK	PK
F01_cb8564_c1374/f1p0/3856-2F	CMGC_MAPK	PK
F01_cb8564_c1375/f1p0/2963-0F	CMGC_MAPK	PK
F01_cb8564_c137667/f2p2/2686-2F	CK1_CK1-Pl	PK
F01_cb8564_c1377/f1p0/2527-1F	CMGC_MAPK	PK
F01_cb8564_c1377/f1p0/2527-2F	CMGC_MAPK	PK
F01_cb8564_c1378/f1p0/2739-0F	CMGC_MAPK	PK
F01_cb8564_c13789/f1p1/3323-1F	CMGC_CDK-PITSLRE	PK
F01_cb8564_c1380/f2p0/2966-1F	CMGC_MAPK	PK
F01_cb8564_c1385/f3p0/2409-0F	CMGC_MAPK	PK
F01_cb8564_c1386/f1p0/2304-1F	CMGC_MAPK	PK
F01_cb8564_c13876/f1p2/3265-1F	CMGC_CDK-PITSLRE	PK
F01_cb8564_c14035/f1p0/3566-0F	RLK-Pelle_RLCK-VIIa-2	PK
F01_cb8564_c14035/f1p0/3566-1F	RLK-Pelle_CrRLK1L-1	PK

F01_cb8564_c14125/f1p1/3319-2F	RLK-Pelle_LRR-XII-1	PK
F01_cb8564_c141549/f3p0/2691-1F	CK1_CK1-Pl	PK
F01_cb8564_c14392/f1p0/3747-0F	CMGC_MAPK	PK
F01_cb8564_c14392/f1p0/3747-1F	CMGC_MAPK	PK
F01_cb8564_c14392/f1p0/3747-2F	CMGC_MAPK	PK
F01_cb8564_c14418/f1p0/3613-1F	RLK-Pelle_RLCK-VIIa-2	PK
F01_cb8564_c14458/f1p0/2514-0F	AGC-Pl	PK
F01_cb8564_c1450/f1p0/3000-1F	RLK-Pelle_LRR-Xa	PK
F01_cb8564_c1451/f4p0/2452-1F	RLK-Pelle_LRR-Xa	PK
F01_cb8564_c1455/f2p1/2542-2F	RLK-Pelle_LRR-Xa	PK
F01_cb8564_c145553/f1p3/3270-0F	WNK_NRBP	PK
F01_cb8564_c145553/f1p3/3270-2F	WNK_NRBP	PK
F01_cb8564_c1456/f2p1/2629-2F	RLK-Pelle_LRR-Xa	PK
F01_cb8564_c145669/f3p0/2544-1F	RLK-Pelle_DLSV	PK
F01_cb8564_c1457/f1p0/2947-2F	RLK-Pelle_LRR-Xa	PK
F01_cb8564_c145775/f16p1/2617-0F	AGC_RSK-2	PK
F01_cb8564_c145775/f16p1/2617-1F	AGC_RSK-2	PK
F01_cb8564_c145991/f10p2/2099-0F	CAMK_AMPK	PK
F01_cb8564_c145998/f5p0/2918-1F	Group-Pl-3	PK
F01_cb8564_c146016/f5p1/3267-0F	RLK-Pelle_LRR-IX	PK
F01_cb8564_c146056/f3p0/3230-2F	RLK-Pelle_RLCK-VIIa-2	PK
F01_cb8564_c146148/f2p5/3291-0F	RLK-Pelle_LRR-VIII-1	PK
F01_cb8564_c14618/f5p1/3581-0F	RLK-Pelle_LRR-XI-1	PK
F01_cb8564_c146191/f1p0/2060-0F	RLK-Pelle_LRR-XI-1	PK
F01_cb8564_c146191/f1p0/2060-1F	RLK-Pelle_LRR-XI-1	PK
F01_cb8564_c1462/f3p1/2646-0F	RLK-Pelle_LRR-Xa	PK

F01_cb8564_c146236/f7p0/3381-1F	RLK-Pelle_SD-2b	PK
F01_cb8564_c146340/f7p1/2494-1F	RLK-Pelle_LRR-II	PK
F01_cb8564_c146477/f9p0/3025-1F	TKL_CTR1-DRK-1	PK
F01_cb8564_c146485/f4p0/2047-2F	RLK-Pelle_RLCK-VIIa-2	PK
F01_cb8564_c146492/f2p2/2580-2F	RLK-Pelle_L-LEC	PK
F01_cb8564_c146651/f7p1/2500-1F	RLK-Pelle_PERK-1	PK
F01_cb8564_c146666/f3p0/3094-2F	RLK-Pelle_RLCK-IXb	PK
F01_cb8564_c146680/f3p1/2465-1F	RLK-Pelle_WAK_LRK10L-1	PK
F01_cb8564_c146687/f3p2/2506-2F	RLK-Pelle_PERK-1	PK
F01_cb8564_c146690/f2p0/2014-2F	RLK-Pelle_RLCK-VIIa-2	PK
F01_cb8564_c146791/f2p0/2690-1F	CK1_CK1-Pl	PK
F01_cb8564_c1468/f1p0/4772-2F	RLK-Pelle_LRR-XI-1	PK
F01_cb8564_c147030/f5p3/2289-2F	RLK-Pelle_L-LEC	PK
F01_cb8564_c147075/f38p6/2315-0F	RLK-Pelle_DLSV	PK
F01_cb8564_c147078/f1p1/2160-1F	RLK-Pelle_PERK-1	PK
F01_cb8564_c147106/f4p0/2896-1F	RLK-Pelle_RLCK-IXb	PK
F01_cb8564_c1473/f1p0/4329-0F	RLK-Pelle_LRR-XI-1	PK
F01_cb8564_c147440/f3p2/2538-0F	RLK-Pelle_L-LEC	PK
F01_cb8564_c1475/f1p0/2478-2F	RLK-Pelle_LRR-XI-1	PK
F01_cb8564_c1476/f1p0/2955-2F	RLK-Pelle_LRR-XI-1	PK
F01_cb8564_c14764/f1p0/4059-2F	STE_STE11	PK
F01_cb8564_c1477/f1p0/3841-1F	RLK-Pelle_LRR-XI-1	PK
F01_cb8564_c1478/f1p1/3616-1F	RLK-Pelle_LRR-XI-1	PK
F01_cb8564_c1480/f3p0/3284-0F	RLK-Pelle_LRR-XI-1	PK
F01_cb8564_c1481/f1p0/3196-0F	RLK-Pelle_LRR-XI-1	PK
F01_cb8564_c14816/f1p0/3943-2F	STE_STE11	PK

F01_cb8564_c1483/f1p0/3257-0F	RLK-Pelle_LRR-XI-1	PK
F01_cb8564_c1484/f1p0/3004-0F	RLK-Pelle_LRR-XI-1	PK
F01_cb8564_c1484/f1p0/3004-1F	RLK-Pelle_LRR-XI-1	PK
F01_cb8564_c1488/f1p0/3089-0F	RLK-Pelle_LRR-XI-1	PK
F01_cb8564_c149007/f1p1/3589-1F	RLK-Pelle_RLCK-VIII	PK
F01_cb8564_c150256/f1p0/2131-1F	RLK-Pelle_RLCK-VIIa-2	PK
F01_cb8564_c15042/f2p0/2695-1F	RLK-Pelle_LRR-I-1	PK
F01_cb8564_c15087/f1p1/3594-2F	CMGC_CDK-PITSLRE	PK
F01_cb8564_c15143/f7p3/4165-1F	RLK-Pelle_LRR-XI-1	PK
F01_cb8564_c152120/f1p3/2607-2F	RLK-Pelle_L-LEC	PK
F01_cb8564_c15227/f1p0/3770-1F	RLK-Pelle_CrRLK1L-1	PK
F01_cb8564_c15227/f1p0/3770-2F	RLK-Pelle_CrRLK1L-1	PK
F01_cb8564_c153439/f1p1/3724-0F	RLK-Pelle_DLSV	PK
F01_cb8564_c153439/f1p1/3724-1F	RLK-Pelle_DLSV	PK
F01_cb8564_c154026/f1p0/2823-0F	CMGC_MAPK	PK
F01_cb8564_c154638/f1p1/3274-2F	RLK-Pelle_LRR-IX	PK
F01_cb8564_c154750/f1p1/2417-0F	RLK-Pelle_CrRLK1L-1	PK
F01_cb8564_c15491/f2p1/2821-0F	CMGC_MAPK	PK
F01_cb8564_c15491/f2p1/2821-1F	CMGC_MAPK	PK
F01_cb8564_c155311/f1p0/3003-0F	RLK-Pelle_RLCK-IXb	PK
F01_cb8564_c15568/f1p0/2322-0F	RLK-Pelle_DLSV	PK
F01_cb8564_c157100/f1p0/2517-0F	RLK-Pelle_LRR-IV	PK
F01_cb8564_c157733/f1p1/3325-0F	RLK-Pelle_DLSV	PK
F01_cb8564_c158886/f1p0/2691-2F	CK1_CK1	PK
F01_cb8564_c159301/f2p0/2930-1F	Group-Pl-3	PK
F01_cb8564_c160054/f1p0/2479-2F	RLK-Pelle_WAK_LRK10L-1	PK

F01_cb8564_c16064/f1p0/3198-2F	RLK-Pelle_LRK10L-2	PK
F01_cb8564_c16070/f1p1/3687-0F	RLK-Pelle_LRR-XII-1	PK
F01_cb8564_c16159/f1p0/3072-0F	RLK-Pelle_CrRLK1L-1	PK
F01_cb8564_c16159/f1p0/3072-2F	RLK-Pelle_RLCK-VIIa-2	PK
F01_cb8564_c16162/f1p1/4558-0F	RLK-Pelle_RLCK-VIIa-2	PK
F01_cb8564_c16162/f1p1/4558-2F	RLK-Pelle_CrRLK1L-1	PK
F01_cb8564_c16220/f12p0/3116-0F	RLK-Pelle_CrRLK1L-1	PK
F01_cb8564_c16231/f1p0/3606-2F	RLK-Pelle_CrRLK1L-1	PK
F01_cb8564_c16349/f1p0/4828-2F	CMGC_CDK-PITSLRE	PK
F01_cb8564_c16468/f1p1/2237-0F	RLK-Pelle_DLSV	PK
F01_cb8564_c16468/f1p1/2237-2F	RLK-Pelle_DLSV	PK
F01_cb8564_c16516/f1p0/4473-1F	RLK-Pelle_LRR-XI-1	PK
F01_cb8564_c16649/f1p0/3722-0F	RLK-Pelle_RLCK-VIII	PK
F01_cb8564_c16650/f1p0/2552-0F	STE_STE11	PK
F01_cb8564_c16650/f1p0/2552-2F	STE_STE11	PK
F01_cb8564_c16688/f1p1/3526-1F	RLK-Pelle_LRR-XII-1	PK
F01_cb8564_c167108/f1p0/2919-1F	WNK_NRBP	PK
F01_cb8564_c16897/f1p0/3118-0F	RLK-Pelle_CrRLK1L-1	PK
F01_cb8564_c16897/f1p0/3118-2F	RLK-Pelle_CrRLK1L-1	PK
F01_cb8564_c16954/f1p1/3233-0F	RLK-Pelle_WAK_LRK10L-1	PK
F01_cb8564_c16954/f1p1/3233-1F	RLK-Pelle_WAK_LRK10L-1	PK
F01_cb8564_c17058/f1p0/3724-1F	RLK-Pelle_LRR-XII-1	PK
F01_cb8564_c171109/f1p1/2188-0F	RLK-Pelle_LysM	PK
F01_cb8564_c17117/f1p0/4269-1F	TKL-Pl-4	PK
F01_cb8564_c17225/f1p1/3107-1F	STE_STE11	PK
F01_cb8564_c17225/f1p1/3107-2F	STE_STE11	PK

F01_cb8564_c17291/f1p0/2775-0F	RLK-Pelle_LRR-XII-1	PK
F01_cb8564_c17291/f1p0/2775-1F	RLK-Pelle_LRR-XII-1	PK
F01_cb8564_c17396/f1p1/3193-0F	RLK-Pelle_LRR-VIII-1	PK
F01_cb8564_c174230/f1p1/2759-0F	CMGC_RCK	PK
F01_cb8564_c17503/f1p0/4953-1F	CMGC_MAPK	PK
F01_cb8564_c175946/f10p1/3002-2F	RLK-Pelle_CrRLK1L-1	PK
F01_cb8564_c17887/f1p0/2275-0F	RLK-Pelle_CrRLK1L-1	PK
F01_cb8564_c17887/f1p0/2275-2F	RLK-Pelle_CrRLK1L-1	PK
F01_cb8564_c18017/f1p0/4120-0F	RLK-Pelle_CrRLK1L-1	PK
F01_cb8564_c18017/f1p0/4120-2F	RLK-Pelle_RLCK-VIIa-2	PK
F01_cb8564_c18173/f1p0/4165-1F	RLK-Pelle_LRR-XI-1	PK
F01_cb8564_c182592/f1p0/2600-2F	CMGC_RCK	PK
F01_cb8564_c18267/f1p0/4385-2F	STE_STE11	PK
F01_cb8564_c18494/f1p0/4108-0F	RLK-Pelle_SD-2b	PK
F01_cb8564_c18681/f1p0/3658-0F	RLK-Pelle_SD-2b	PK
F01_cb8564_c18690/f1p0/2323-2F	RLK-Pelle_DLSV	PK
F01_cb8564_c18753/f1p0/2990-0F	RLK-Pelle_DLSV	PK
F01_cb8564_c18753/f1p0/2990-1F	RLK-Pelle_DLSV	PK
F01_cb8564_c18757/f1p0/2729-1F	RLK-Pelle_DLSV	PK
F01_cb8564_c18757/f1p0/2729-2F	RLK-Pelle_DLSV	PK
F01_cb8564_c18824/f2p0/3618-1F	RLK-Pelle_DLSV	PK
F01_cb8564_c19339/f1p1/3362-2F	CMGC_CDK-PITSLRE	PK
F01_cb8564_c19433/f1p0/3193-1F	RLK-Pelle_LRR-XII-1	PK
F01_cb8564_c19617/f1p0/4907-1F	RLK-Pelle_CrRLK1L-1	PK
F01_cb8564_c19646/f1p0/4614-1F	RLK-Pelle_RLCK-VIIa-2	PK
F01_cb8564_c19646/f1p0/4614-2F	RLK-Pelle_CrRLK1L-1	PK

F01_cb8564_c1970/f1p0/3046-0F	CAMK_CAMKL-CHK1	PK
F01_cb8564_c1971/f1p0/2751-0F	CAMK_CAMKL-CHK1	PK
F01_cb8564_c1971/f1p0/2751-2F	CAMK_CAMKL-CHK1	PK
F01_cb8564_c1972/f1p0/2732-0F	CAMK_CAMKL-CHK1	PK
F01_cb8564_c1974/f1p0/3059-0F	CAMK_CAMKL-CHK1	PK
F01_cb8564_c1974/f1p0/3059-1F	CAMK_CAMKL-CHK1	PK
F01_cb8564_c1974/f1p0/3059-2F	CAMK_CAMKL-CHK1	PK
F01_cb8564_c1976/f1p0/2327-0F	CAMK_CAMKL-CHK1	PK
F01_cb8564_c1976/f1p0/2327-1F	CAMK_CAMKL-CHK1	PK
F01_cb8564_c1977/f1p0/2532-1F	CAMK_CAMKL-CHK1	PK
F01_cb8564_c1978/f1p1/1997-1F	CAMK_CAMKL-CHK1	PK
F01_cb8564_c1981/f1p0/1910-0F	CAMK_CAMKL-CHK1	PK
F01_cb8564_c1983/f1p0/2537-2F	RLK-Pelle_LRK10L-2	PK
F01_cb8564_c1984/f1p0/2181-0F	RLK-Pelle_LRK10L-2	PK
F01_cb8564_c1984/f1p0/2181-1F	RLK-Pelle_LRK10L-2	PK
F01_cb8564_c1984/f1p0/2181-2F	RLK-Pelle_LRK10L-2	PK
F01_cb8564_c19894/f1p0/2891-1F	RLK-Pelle_RLCK-VIIa-2	PK
F01_cb8564_c19894/f1p0/2891-2F	RLK-Pelle_CrRLK1L-1	PK
F01_cb8564_c19999/f1p0/2804-1F	CMGC_MAPK	PK
F01_cb8564_c20025/f1p0/3787-1F	RLK-Pelle_LRR-IX	PK
F01_cb8564_c20112/f1p0/3162-1F	RLK-Pelle_DLSV	PK
F01_cb8564_c20112/f1p0/3162-2F	RLK-Pelle_DLSV	PK
F01_cb8564_c20113/f1p0/2614-1F	RLK-Pelle_DLSV	PK
F01_cb8564_c20119/f1p0/3346-0F	RLK-Pelle_DLSV	PK
F01_cb8564_c20204/f1p0/3519-0F	CMGC_MAPK	PK
F01_cb8564_c20204/f1p0/3519-2F	CMGC_MAPK	PK

F01_cb8564_c20337/f1p1/3326-1F	CMGC_CDK-PITSLRE	PK
F01_cb8564_c2041/f2p1/3039-1F	RLK-Pelle_RLCK-VIIa-2	PK
F01_cb8564_c2041/f2p1/3039-2F	RLK-Pelle_RLCK-VIIa-2	PK
F01_cb8564_c2042/f2p1/2865-0F	RLK-Pelle_RLCK-VIIa-2	PK
F01_cb8564_c2042/f2p1/2865-2F	RLK-Pelle_RLCK-VIIa-2	PK
F01_cb8564_c2045/f1p0/1948-1F	RLK-Pelle_RLCK-VIIa-2	PK
F01_cb8564_c20573/f1p0/3664-1F	RLK-Pelle_LRK10L-2	PK
F01_cb8564_c2068/f1p0/2547-2F	RLK-Pelle_RLCK-VIIa-2	PK
F01_cb8564_c20845/f1p0/3839-1F	TKL-Pl-4	PK
F01_cb8564_c21151/f1p0/3128-0F	RLK-Pelle_DLSV	PK
F01_cb8564_c21334/f1p0/4233-2F	CMGC_CDK-PITSLRE	PK
F01_cb8564_c21471/f1p0/3237-0F	RLK-Pelle_LRK10L-2	PK
F01_cb8564_c21471/f1p0/3237-2F	RLK-Pelle_LRK10L-2	PK
F01_cb8564_c21541/f1p0/2864-0F	Group-Pl-3	PK
F01_cb8564_c21635/f1p0/3294-0F	RLK-Pelle_LRR-I-1	PK
F01_cb8564_c21778/f1p0/3229-1F	RLK-Pelle_DLSV	PK
F01_cb8564_c22298/f1p0/2771-2F	RLK-Pelle_DLSV	PK
F01_cb8564_c22447/f1p0/4272-0F	RLK-Pelle_LRR-XI-1	PK
F01_cb8564_c22452/f1p0/3321-0F	RLK-Pelle_DLSV	PK
F01_cb8564_c22475/f1p0/1967-0F	RLK-Pelle_RLCK-VIIa-2	PK
F01_cb8564_c22475/f1p0/1967-2F	RLK-Pelle_RLCK-VIIa-2	PK
F01_cb8564_c22570/f1p0/3631-1F	RLK-Pelle_LRR-IX	PK
F01_cb8564_c22623/f1p0/3058-0F	RLK-Pelle_LRR-XII-1	PK
F01_cb8564_c22630/f1p0/2676-1F	RLK-Pelle_LRR-XII-1	PK
F01_cb8564_c22662/f1p0/3838-1F	STE_STE11	PK
F01_cb8564_c22662/f1p0/3838-2F	STE_STE11	PK

F01_cb8564_c22900/f1p1/3151-1F	RLK-Pelle_LRR-VIII-1	PK
F01_cb8564_c22900/f1p1/3151-2F	RLK-Pelle_LRR-VIII-1	PK
F01_cb8564_c23001/f1p0/2423-1F	CMGC_CDK-PITSLRE	PK
F01_cb8564_c23009/f1p0/3941-2F	RLK-Pelle_RLCK-VIII	PK
F01_cb8564_c23076/f1p0/3542-1F	RLK-Pelle_LRR-VI-2	PK
F01_cb8564_c23090/f2p0/3109-2F	STE_STE7	PK
F01_cb8564_c23276/f1p0/3993-2F	RLK-Pelle_CrRLK1L-1	PK
F01_cb8564_c23407/f1p0/2923-0F	Group-Pl-3	PK
F01_cb8564_c23426/f1p0/4026-1F	RLK-Pelle_CrRLK1L-1	PK
F01_cb8564_c23426/f1p0/4026-2F	RLK-Pelle_RLCK-VIIa-2	PK
F01_cb8564_c23446/f1p0/2833-0F	RLK-Pelle_CrRLK1L-1	PK
F01_cb8564_c23546/f1p0/4263-2F	CMGC_CDK-PITSLRE	PK
F01_cb8564_c23631/f1p0/4582-0F	RLK-Pelle_DLSV	PK
F01_cb8564_c24281/f1p0/3435-2F	CMGC_CDK-PITSLRE	PK
F01_cb8564_c24283/f2p0/3438-0F	RLK-Pelle_LRR-XII-1	PK
F01_cb8564_c2433/f1p4/3038-1F	CAMK_CDPK	PK
F01_cb8564_c24450/f1p1/2884-1F	RLK-Pelle_LRR-XII-1	PK
F01_cb8564_c24757/f1p0/4390-1F	RLK-Pelle_CrRLK1L-1	PK
F01_cb8564_c24799/f1p2/4162-1F	RLK-Pelle_CrRLK1L-1	PK
F01_cb8564_c24907/f2p1/2371-2F	RLK-Pelle_WAK_LRK10L-1	PK
F01_cb8564_c24976/f1p1/3685-0F	CMGC_CDK-PITSLRE	PK
F01_cb8564_c25024/f1p0/3748-1F	STE_STE11	PK
F01_cb8564_c25105/f3p1/4062-1F	RLK-Pelle_RLCK-VIIa-2	PK
F01_cb8564_c25347/f4p1/3671-2F	RLK-Pelle_LRR-VIII-1	PK
F01_cb8564_c25433/f2p0/2642-1F	RLK-Pelle_DLSV	PK
F01_cb8564_c25442/f1p0/2908-0F	RLK-Pelle_CrRLK1L-1	PK

F01_cb8564_c25444/f2p0/3991-0F	RLK-Pelle_RLCK-VIIa-2	PK
F01_cb8564_c25444/f2p0/3991-1F	RLK-Pelle_CrRLK1L-1	PK
F01_cb8564_c2556/f1p0/2194-0F	RLK-Pelle_LRK10L-2	PK
F01_cb8564_c2556/f1p0/2194-2F	RLK-Pelle_LRK10L-2	PK
F01_cb8564_c2557/f2p0/2375-2F	RLK-Pelle_LRK10L-2	PK
F01_cb8564_c2559/f1p1/2250-0F	RLK-Pelle_LRK10L-2	PK
F01_cb8564_c2560/f3p0/2832-2F	RLK-Pelle_LRK10L-2	PK
F01_cb8564_c25609/f1p0/3062-0F	RLK-Pelle_LRR-XII-1	PK
F01_cb8564_c26895/f3p0/2222-1F	RLK-Pelle_CrRLK1L-1	PK
F01_cb8564_c26915/f3p0/3758-0F	RLK-Pelle_LRR-XII-1	PK
F01_cb8564_c3013/f1p1/2638-2F	RLK-Pelle_WAK_LRK10L-1	PK
F01_cb8564_c31691/f6p2/3305-1F	CMGC_CDK-PITSLRE	PK
F01_cb8564_c31696/f14p0/2701-0F	RLK-Pelle_SD-2b	PK
F01_cb8564_c31960/f12p2/4203-2F	RLK-Pelle_RLCK-VIIa-2	PK
F01_cb8564_c31975/f2p1/2253-2F	STE_STE11	PK
F01_cb8564_c32018/f2p0/4095-0F	RLK-Pelle_RLCK-VIIa-2	PK
F01_cb8564_c32018/f2p0/4095-2F	RLK-Pelle_CrRLK1L-1	PK
F01_cb8564_c32023/f3p0/3253-1F	RLK-Pelle_CrRLK1L-1	PK
F01_cb8564_c32024/f4p1/4197-2F	RLK-Pelle_RLCK-VIIa-2	PK
F01_cb8564_c32899/f13p0/3234-1F	RLK-Pelle_CrRLK1L-1	PK
F01_cb8564_c3319/f1p0/2765-2F	RLK-Pelle_LRR-I-1	PK
F01_cb8564_c3320/f1p0/2486-1F	RLK-Pelle_LRR-I-1	PK
F01_cb8564_c33393/f7p2/3346-2F	RLK-Pelle_DLSV	PK
F01_cb8564_c334/f9p1/2646-0F	RLK-Pelle_LRR-Xa	PK
F01_cb8564_c33672/f2p0/2453-0F	RLK-Pelle_DLSV	PK
F01_cb8564_c33720/f9p0/2495-1F	RLK-Pelle_DLSV	PK

F01_cb8564_c33787/f6p0/2712-0F	RLK-Pelle_CrRLK1L-1	PK
F01_cb8564_c33936/f7p0/2581-0F	RLK-Pelle_DLSV	PK
F01_cb8564_c33936/f7p0/2581-2F	RLK-Pelle_DLSV	PK
F01_cb8564_c34046/f3p0/2667-2F	RLK-Pelle_DLSV	PK
F01_cb8564_c34047/f2p0/3175-0F	RLK-Pelle_DLSV	PK
F01_cb8564_c34050/f2p1/3258-1F	RLK-Pelle_DLSV	PK
F01_cb8564_c34131/f3p0/2847-2F	RLK-Pelle_SD-2b	PK
F01_cb8564_c34141/f2p2/3036-0F	RLK-Pelle_CrRLK1L-1	PK
F01_cb8564_c34141/f2p2/3036-1F	RLK-Pelle_CrRLK1L-1	PK
F01_cb8564_c34141/f2p2/3036-2F	RLK-Pelle_CrRLK1L-1	PK
F01_cb8564_c34176/f3p0/3161-0F	TKL-Pl-4	PK
F01_cb8564_c34189/f2p0/2891-0F	RLK-Pelle_CrRLK1L-1	PK
F01_cb8564_c34195/f2p1/2830-0F	WNK_NRBP	PK
F01_cb8564_c34195/f2p1/2830-2F	WNK_NRBP	PK
F01_cb8564_c34397/f1p1/3138-0F	RLK-Pelle_CrRLK1L-1	PK
F01_cb8564_c34437/f2p1/2950-1F	RLK-Pelle_CrRLK1L-1	PK
F01_cb8564_c34906/f1p0/3905-0F	CMGC_GSK	PK
F01_cb8564_c34920/f1p1/3239-2F	RLK-Pelle_DLSV	PK
F01_cb8564_c34943/f1p0/2034-0F	RLK-Pelle_DLSV	PK
F01_cb8564_c35196/f1p0/3115-1F	RLK-Pelle_DLSV	PK
F01_cb8564_c35196/f1p0/3115-2F	RLK-Pelle_DLSV	PK
F01_cb8564_c35208/f1p0/2151-1F	RLK-Pelle_PERK-1	PK
F01_cb8564_c35259/f2p2/2450-2F	RLK-Pelle_PERK-1	PK
F01_cb8564_c35284/f1p0/4390-2F	RLK-Pelle_LRR-XI-1	PK
F01_cb8564_c35419/f2p4/3123-0F	RLK-Pelle_CrRLK1L-1	PK
F01_cb8564_c35567/f2p1/2669-1F	RLK-Pelle_DLSV	PK

F01_cb8564_c35913/f1p0/2772-2F	RLK-Pelle_DLSV	PK
F01_cb8564_c35970/f1p0/2874-0F	Group-Pl-3	PK
F01_cb8564_c35970/f1p0/2874-1F	Group-Pl-3	PK
F01_cb8564_c35970/f1p0/2874-2F	Group-Pl-3	PK
F01_cb8564_c35971/f1p0/3519-0F	RLK-Pelle_DLSV	PK
F01_cb8564_c36018/f1p0/1971-1F	RLK-Pelle_LRR-XI-1	PK
F01_cb8564_c36029/f1p1/2932-0F	RLK-Pelle_DLSV	PK
F01_cb8564_c36029/f1p1/2932-2F	RLK-Pelle_DLSV	PK
F01_cb8564_c36140/f1p1/4637-2F	RLK-Pelle_DLSV	PK
F01_cb8564_c362/f8p0/2792-0F	CMGC_CDK-CRK7-CDK9	PK
F01_cb8564_c36204/f3p0/3065-1F	RLK-Pelle_CrRLK1L-1	PK
F01_cb8564_c3624/f3p0/2790-1F	CMGC_CDK-CRK7-CDK9	PK
F01_cb8564_c3625/f1p0/2594-2F	CMGC_CDK-CRK7-CDK9	PK
F01_cb8564_c36449/f1p0/3556-0F	RLK-Pelle_LRR-XIIIb	PK
F01_cb8564_c36596/f2p0/2350-1F	RLK-Pelle_DLSV	PK
F01_cb8564_c36775/f1p1/4614-0F	RLK-Pelle_DLSV	PK
F01_cb8564_c36775/f1p1/4614-2F	RLK-Pelle_DLSV	PK
F01_cb8564_c36940/f2p3/3069-2F	RLK-Pelle_CrRLK1L-1	PK
F01_cb8564_c37008/f1p0/2404-2F	RLK-Pelle_DLSV	PK
F01_cb8564_c37049/f1p0/3385-2F	RLK-Pelle_DLSV	PK
F01_cb8564_c37079/f1p0/2659-0F	WNK_NRBP	PK
F01_cb8564_c37163/f1p0/3147-0F	RLK-Pelle_CrRLK1L-1	PK
F01_cb8564_c37163/f1p0/3147-2F	RLK-Pelle_CrRLK1L-1	PK
F01_cb8564_c37274/f1p0/3183-0F	WNK_NRBP	PK
F01_cb8564_c37325/f1p1/2512-0F	CMGC_GSK	PK
F01_cb8564_c37492/f1p0/2985-0F	WNK_NRBP	PK

F01_cb8564_c37571/f1p4/2881-1F	CMGC_GSK	PK
F01_cb8564_c37604/f2p0/3150-0F	RLK-Pelle_CrRLK1L-1	PK
F01_cb8564_c37604/f2p0/3150-1F	RLK-Pelle_CrRLK1L-1	PK
F01_cb8564_c37666/f1p0/2149-0F	RLK-Pelle_DLSV	PK
F01_cb8564_c37674/f1p0/2185-0F	RLK-Pelle_DLSV	PK
F01_cb8564_c37674/f1p0/2185-2F	RLK-Pelle_DLSV	PK
F01_cb8564_c37759/f1p2/4302-1F	RLK-Pelle_LysM	PK
F01_cb8564_c38100/f3p0/3175-0F	RLK-Pelle_CrRLK1L-1	PK
F01_cb8564_c38110/f1p0/4638-1F	RLK-Pelle_DLSV	PK
F01_cb8564_c38178/f1p2/3019-0F	RLK-Pelle_CrRLK1L-1	PK
F01_cb8564_c38178/f1p2/3019-1F	RLK-Pelle_CrRLK1L-1	PK
F01_cb8564_c3837/f2p1/3505-1F	RLK-Pelle_LRR-I-1	PK
F01_cb8564_c3839/f1p0/3128-0F	RLK-Pelle_LRR-I-1	PK
F01_cb8564_c3839/f1p0/3128-2F	RLK-Pelle_LRR-I-1	PK
F01_cb8564_c3841/f1p0/3339-2F	RLK-Pelle_LRR-I-1	PK
F01_cb8564_c38491/f2p0/4386-1F	RLK-Pelle_DLSV	PK
F01_cb8564_c38520/f1p0/3288-0F	RLK-Pelle_DLSV	PK
F01_cb8564_c38653/f1p1/2281-2F	RLK-Pelle_DLSV	PK
F01_cb8564_c38751/f1p0/3392-0F	RLK-Pelle_DLSV	PK
F01_cb8564_c38815/f1p3/3473-1F	RLK-Pelle_LRR-XII-1	PK
F01_cb8564_c38815/f1p3/3473-2F	RLK-Pelle_LRR-XII-1	PK
F01_cb8564_c38880/f1p1/2687-1F	RLK-Pelle_CR4L	PK
F01_cb8564_c38880/f1p1/2687-2F	RLK-Pelle_CrRLK1L-1	PK
F01_cb8564_c39023/f1p0/2641-0F	RLK-Pelle_CrRLK1L-1	PK
F01_cb8564_c39023/f1p0/2641-1F	RLK-Pelle_CrRLK1L-1	PK
F01_cb8564_c39075/f1p0/2655-2F	RLK-Pelle_SD-2b	PK

F01_cb8564_c39637/f1p1/3015-2F	Group-Pl-3	PK
F01_cb8564_c39796/f1p0/3998-0F	RLK-Pelle_CrRLK1L-1	PK
F01_cb8564_c39796/f1p0/3998-1F	RLK-Pelle_RLCK-VIIa-2	PK
F01_cb8564_c39852/f1p0/3673-0F	RLK-Pelle_RLCK-VIIa-2	PK
F01_cb8564_c39852/f1p0/3673-1F	RLK-Pelle_CrRLK1L-1	PK
F01_cb8564_c40026/f1p0/4751-1F	RLK-Pelle_LRR-XI-1	PK
F01_cb8564_c40090/f1p0/3400-1F	RLK-Pelle_DLSV	PK
F01_cb8564_c40090/f1p0/3400-2F	RLK-Pelle_DLSV	PK
F01_cb8564_c40246/f1p1/2822-2F	RLK-Pelle_CrRLK1L-1	PK
F01_cb8564_c40359/f1p0/2646-0F	RLK-Pelle_CrRLK1L-1	PK
F01_cb8564_c40359/f1p0/2646-1F	RLK-Pelle_CrRLK1L-1	PK
F01_cb8564_c40359/f1p0/2646-2F	RLK-Pelle_CrRLK1L-1	PK
F01_cb8564_c40630/f1p0/2696-0F	RLK-Pelle_DLSV	PK
F01_cb8564_c40630/f1p0/2696-2F	RLK-Pelle_DLSV	PK
F01_cb8564_c40648/f1p0/3538-0F	RLK-Pelle_LRR-XI-1	PK
F01_cb8564_c40820/f1p0/3230-0F	RLK-Pelle_DLSV	PK
F01_cb8564_c40820/f1p0/3230-1F	RLK-Pelle_DLSV	PK
F01_cb8564_c40874/f1p7/2355-2F	RLK-Pelle_LysM	PK
F01_cb8564_c40930/f2p0/2663-2F	RLK-Pelle_SD-2b	PK
F01_cb8564_c40939/f1p0/4508-1F	RLK-Pelle_CrRLK1L-1	PK
F01_cb8564_c40939/f1p0/4508-2F	RLK-Pelle_CrRLK1L-1	PK
F01_cb8564_c41128/f1p4/3076-1F	RLK-Pelle_CrRLK1L-1	PK
F01_cb8564_c41169/f1p0/2809-0F	STE_STE11	PK
F01_cb8564_c41286/f1p0/3500-0F	RLK-Pelle_DLSV	PK
F01_cb8564_c41286/f1p0/3500-1F	RLK-Pelle_DLSV	PK
F01_cb8564_c41286/f1p0/3500-2F	RLK-Pelle_DLSV	PK

F01_cb8564_c41565/f2p0/3288-0F	RLK-Pelle_DLSV	PK
F01_cb8564_c41628/f1p0/1940-2F	TKL-Pl-4	PK
F01_cb8564_c41734/f1p0/2770-0F	RLK-Pelle_DLSV	PK
F01_cb8564_c41734/f1p0/2770-2F	RLK-Pelle_DLSV	PK
F01_cb8564_c41739/f1p0/3232-0F	RLK-Pelle_RLCK-VIIa-2	PK
F01_cb8564_c4185/f1p1/2489-0F	RLK-Pelle_SD-2b	PK
F01_cb8564_c4186/f4p1/2803-1F	RLK-Pelle_SD-2b	PK
F01_cb8564_c4188/f1p0/2834-2F	RLK-Pelle_SD-2b	PK
F01_cb8564_c41895/f1p0/4142-1F	PEK_GCN2	PK
F01_cb8564_c4190/f1p2/2940-1F	CK1_CK1	PK
F01_cb8564_c4193/f1p0/3949-2F	CK1_CK1	PK
F01_cb8564_c41939/f1p0/2349-0F	RLK-Pelle_DLSV	PK
F01_cb8564_c4195/f1p1/2403-0F	CK1_CK1	PK
F01_cb8564_c42049/f1p0/4782-0F	RLK-Pelle_CrRLK1L-1	PK
F01_cb8564_c42049/f1p0/4782-1F	RLK-Pelle_CrRLK1L-1	PK
F01_cb8564_c42140/f1p1/3025-1F	RLK-Pelle_CrRLK1L-1	PK
F01_cb8564_c42231/f6p7/2491-0F	RLK-Pelle_LysM	PK
F01_cb8564_c42425/f1p1/3397-0F	RLK-Pelle_DLSV	PK
F01_cb8564_c42602/f1p0/2704-2F	Group-Pl-3	PK
F01_cb8564_c42619/f1p0/3414-0F	RLK-Pelle_DLSV	PK
F01_cb8564_c42619/f1p0/3414-1F	RLK-Pelle_DLSV	PK
F01_cb8564_c42658/f1p0/2401-0F	Group-Pl-3	PK
F01_cb8564_c42658/f1p0/2401-2F	Group-Pl-3	PK
F01_cb8564_c42776/f1p0/2186-1F	WNK_NRBP	PK
F01_cb8564_c42776/f1p0/2186-2F	WNK_NRBP	PK
F01_cb8564_c42832/f1p1/3519-0F	RLK-Pelle_CrRLK1L-1	PK

F01_cb8564_c42889/f3p0/2860-0F	RLK-Pelle_CrRLK1L-1	PK
F01_cb8564_c42889/f3p0/2860-1F	RLK-Pelle_CrRLK1L-1	PK
F01_cb8564_c42897/f4p0/2406-2F	RLK-Pelle_DLSV	PK
F01_cb8564_c42961/f1p0/2560-1F	Group-Pl-3	PK
F01_cb8564_c43017/f1p0/3517-1F	RLK-Pelle_DLSV	PK
F01_cb8564_c43137/f1p0/2726-0F	WNK_NRBP	PK
F01_cb8564_c43137/f1p0/2726-1F	WNK_NRBP	PK
F01_cb8564_c43158/f1p0/4727-0F	RLK-Pelle_LRR-XI-1	PK
F01_cb8564_c43158/f1p0/4727-1F	RLK-Pelle_LRR-XI-1	PK
F01_cb8564_c43158/f1p0/4727-2F	RLK-Pelle_LRR-XI-1	PK
F01_cb8564_c43180/f2p0/2641-0F	RLK-Pelle_SD-2b	PK
F01_cb8564_c43212/f2p1/2417-1F	RLK-Pelle_DLSV	PK
F01_cb8564_c43414/f1p0/3444-0F	RLK-Pelle_CrRLK1L-1	PK
F01_cb8564_c43414/f1p0/3444-1F	RLK-Pelle_DLSV	PK
F01_cb8564_c43414/f1p0/3444-2F	RLK-Pelle_DLSV	PK
F01_cb8564_c43552/f1p0/3829-2F	RLK-Pelle_LRR-XI-1	PK
F01_cb8564_c43730/f1p0/3179-0F	RLK-Pelle_DLSV	PK
F01_cb8564_c43989/f1p0/2902-0F	RLK-Pelle_DLSV	PK
F01_cb8564_c44004/f1p0/2796-0F	RLK-Pelle_CrRLK1L-1	PK
F01_cb8564_c44004/f1p0/2796-1F	RLK-Pelle_CrRLK1L-1	PK
F01_cb8564_c44035/f1p0/3142-1F	WNK_NRBP	PK
F01_cb8564_c44035/f1p0/3142-2F	WNK_NRBP	PK
F01_cb8564_c44206/f1p0/2835-1F	RLK-Pelle_DLSV	PK
F01_cb8564_c44206/f1p0/2835-2F	RLK-Pelle_DLSV	PK
F01_cb8564_c44323/f8p0/2622-0F	WNK_NRBP	PK
F01_cb8564_c44514/f1p0/2728-0F	RLK-Pelle_DLSV	PK

F01_cb8564_c44514/f1p0/2728-2F	RLK-Pelle_DLSV	PK
F01_cb8564_c4489/f1p0/2975-2F	Group-Pl-3	PK
F01_cb8564_c44912/f1p1/2347-0F	RLK-Pelle_RLCK-XII-1	PK
F01_cb8564_c44920/f1p0/3934-1F	WNK_NRBP	PK
F01_cb8564_c45116/f1p0/2875-1F	RLK-Pelle_CrRLK1L-1	PK
F01_cb8564_c45116/f1p0/2875-2F	RLK-Pelle_CrRLK1L-1	PK
F01_cb8564_c45169/f1p0/3762-2F	RLK-Pelle_LRR-XI-1	PK
F01_cb8564_c45229/f4p1/3393-0F	RLK-Pelle_DLSV	PK
F01_cb8564_c45583/f1p0/3371-2F	RLK-Pelle_DLSV	PK
F01_cb8564_c45651/f1p0/3090-0F	Aur	PK
F01_cb8564_c46081/f1p0/3421-1F	RLK-Pelle_DLSV	PK
F01_cb8564_c46200/f1p0/3185-1F	RLK-Pelle_DLSV	PK
F01_cb8564_c46301/f1p3/3891-0F	RLK-Pelle_LysM	PK
F01_cb8564_c46333/f1p1/2412-1F	RLK-Pelle_DLSV	PK
F01_cb8564_c46446/f1p0/2736-0F	RLK-Pelle_CrRLK1L-1	PK
F01_cb8564_c46749/f1p0/4497-2F	CMGC_GSK	PK
F01_cb8564_c46860/f1p0/4047-2F	RLK-Pelle_DLSV	PK
F01_cb8564_c46876/f1p0/4486-1F	RLK-Pelle_DLSV	PK
F01_cb8564_c46993/f1p0/4177-1F	RLK-Pelle_DLSV	PK
F01_cb8564_c47124/f1p0/4568-2F	RLK-Pelle_CrRLK1L-1	PK
F01_cb8564_c47139/f1p2/4516-0F	RLK-Pelle_CrRLK1L-1	PK
F01_cb8564_c47149/f1p1/2601-0F	RLK-Pelle_RLCK-VIIa-2	PK
F01_cb8564_c47149/f1p1/2601-1F	RLK-Pelle_CrRLK1L-1	PK
F01_cb8564_c47331/f1p0/2588-1F	WNK_NRBP	PK
F01_cb8564_c47558/f1p0/1918-2F	RLK-Pelle_RLCK-VIII	PK
F01_cb8564_c47567/f1p0/3302-0F	RLK-Pelle_CrRLK1L-1	PK

F01_cb8564_c47634/f2p1/3095-0F	RLK-Pelle_CrRLK1L-1	PK
F01_cb8564_c47634/f2p1/3095-2F	RLK-Pelle_CrRLK1L-1	PK
F01_cb8564_c47662/f1p0/2984-0F	RLK-Pelle_SD-2b	PK
F01_cb8564_c47713/f1p0/4233-1F	RLK-Pelle_CrRLK1L-1	PK
F01_cb8564_c47741/f2p0/2569-0F	RLK-Pelle_DLSV	PK
F01_cb8564_c47741/f2p0/2569-1F	RLK-Pelle_DLSV	PK
F01_cb8564_c47783/f1p0/2418-2F	RLK-Pelle_DLSV	PK
F01_cb8564_c47929/f1p0/3443-1F	RLK-Pelle_DLSV	PK
F01_cb8564_c47929/f1p0/3443-2F	RLK-Pelle_DLSV	PK
F01_cb8564_c47930/f1p0/3019-2F	NAK	PK
F01_cb8564_c47960/f1p1/4330-0F	RLK-Pelle_LRR-XI-1	PK
F01_cb8564_c47960/f1p1/4330-2F	RLK-Pelle_LRR-XI-1	PK
F01_cb8564_c48054/f1p0/2834-2F	RLK-Pelle_DLSV	PK
F01_cb8564_c48056/f1p1/3301-0F	WNK_NRBP	PK
F01_cb8564_c48056/f1p1/3301-1F	WNK_NRBP	PK
F01_cb8564_c48168/f1p0/4168-0F	RLK-Pelle_LRR-XI-1	PK
F01_cb8564_c48168/f1p0/4168-1F	RLK-Pelle_LRR-XI-1	PK
F01_cb8564_c48282/f1p0/2709-0F	WNK_NRBP	PK
F01_cb8564_c48293/f1p0/3428-0F	CMGC_GSK	PK
F01_cb8564_c48445/f1p2/3004-2F	RLK-Pelle_CrRLK1L-1	PK
F01_cb8564_c48449/f1p0/2775-0F	CMGC_CDK-CRK7-CDK9	PK
F01_cb8564_c48449/f1p0/2775-2F	CMGC_CDK-CRK7-CDK9	PK
F01_cb8564_c48538/f1p0/2599-2F	WNK_NRBP	PK
F01_cb8564_c48551/f1p0/2454-0F	WNK_NRBP	PK
F01_cb8564_c48551/f1p0/2454-2F	WNK_NRBP	PK
F01_cb8564_c48563/f1p0/3640-1F	WNK_NRBP	PK

F01_cb8564_c48607/f1p0/4177-0F	RLK-Pelle_DLSV	PK
F01_cb8564_c48634/f1p0/3498-2F	RLK-Pelle_DLSV	PK
F01_cb8564_c48742/f1p1/3198-1F	RLK-Pelle_RLCK-VIII	PK
F01_cb8564_c48990/f1p2/2740-0F	RLK-Pelle_LysM	PK
F01_cb8564_c4912/f1p0/3121-1F	TKL_CTR1-DRK-2	PK
F01_cb8564_c49125/f1p0/2935-2F	RLK-Pelle_DLSV	PK
F01_cb8564_c4914/f4p0/3012-0F	TKL_CTR1-DRK-2	PK
F01_cb8564_c4915/f1p0/3082-1F	TKL_CTR1-DRK-2	PK
F01_cb8564_c4916/f1p0/2816-0F	TKL_CTR1-DRK-2	PK
F01_cb8564_c4916/f1p0/2816-1F	TKL_CTR1-DRK-2	PK
F01_cb8564_c4917/f1p0/2766-0F	TKL_CTR1-DRK-2	PK
F01_cb8564_c4917/f1p0/2766-2F	TKL_CTR1-DRK-2	PK
F01_cb8564_c49236/f1p1/2666-1F	RLK-Pelle_PERK-1	PK
F01_cb8564_c49291/f1p0/2164-0F	WNK_NRBP	PK
F01_cb8564_c49294/f1p0/4356-0F	RLK-Pelle_DLSV	PK
F01_cb8564_c49294/f1p0/4356-2F	RLK-Pelle_DLSV	PK
F01_cb8564_c49592/f1p0/2266-2F	RLK-Pelle_DLSV	PK
F01_cb8564_c49617/f1p0/3794-2F	RLK-Pelle_LRR-XI-1	PK
F01_cb8564_c49729/f1p0/2870-2F	RLK-Pelle_RLCK-VIIa-2	PK
F01_cb8564_c49738/f1p0/3667-2F	RLK-Pelle_DLSV	PK
F01_cb8564_c50086/f1p0/3752-1F	RLK-Pelle_CrRLK1L-1	PK
F01_cb8564_c50086/f1p0/3752-2F	RLK-Pelle_CrRLK1L-1	PK
F01_cb8564_c50118/f1p0/2385-2F	CAMK_CAMKL-CHK1	PK
F01_cb8564_c50226/f1p0/3953-2F	CMGC_GSK	PK
F01_cb8564_c50450/f9p1/4363-1F	RLK-Pelle_RLCK-VIIa-2	PK
F01_cb8564_c50706/f1p0/4300-0F	Group-Pl-3	PK

F01_cb8564_c50706/f1p0/4300-1F	Group-Pl-3	PK
F01_cb8564_c50777/f1p0/2798-0F	RLK-Pelle_DLSV	PK
F01_cb8564_c50777/f1p0/2798-1F	RLK-Pelle_DLSV	PK
F01_cb8564_c51098/f1p0/2821-1F	RLK-Pelle_DLSV	PK
F01_cb8564_c51098/f1p0/2821-2F	RLK-Pelle_DLSV	PK
F01_cb8564_c511/f5p0/1943-0F	CAMK_CAMKL-CHK1	PK
F01_cb8564_c51208/f1p0/3009-1F	RLK-Pelle_CrRLK1L-1	PK
F01_cb8564_c51208/f1p0/3009-2F	RLK-Pelle_CrRLK1L-1	PK
F01_cb8564_c51244/f1p0/2997-2F	RLK-Pelle_DLSV	PK
F01_cb8564_c51399/f1p0/1945-2F	TKL-Pl-4	PK
F01_cb8564_c51492/f1p0/3591-0F	RLK-Pelle_CrRLK1L-1	PK
F01_cb8564_c51492/f1p0/3591-1F	RLK-Pelle_DLSV	PK
F01_cb8564_c51593/f1p0/4707-1F	RLK-Pelle_DLSV	PK
F01_cb8564_c51647/f1p0/2214-1F	RLK-Pelle_DLSV	PK
F01_cb8564_c51648/f1p0/4091-0F	RLK-Pelle_CrRLK1L-1	PK
F01_cb8564_c51718/f1p0/3732-0F	RLK-Pelle_DLSV	PK
F01_cb8564_c5192/f1p0/2669-0F	RLK-Pelle_DLSV	PK
F01_cb8564_c5192/f1p0/2669-2F	RLK-Pelle_DLSV	PK
F01_cb8564_c5193/f1p0/2677-2F	RLK-Pelle_DLSV	PK
F01_cb8564_c5195/f1p0/2262-0F	RLK-Pelle_DLSV	PK
F01_cb8564_c5195/f1p0/2262-1F	RLK-Pelle_DLSV	PK
F01_cb8564_c5195/f1p0/2262-2F	RLK-Pelle_DLSV	PK
F01_cb8564_c5196/f1p1/2290-0F	RLK-Pelle_DLSV	PK
F01_cb8564_c51979/f1p0/2022-2F	RLK-Pelle_DLSV	PK
F01_cb8564_c52018/f1p0/2577-2F	RLK-Pelle_DLSV	PK
F01_cb8564_c52041/f1p0/2522-0R	RLK-Pelle_PERK-1	PK

F01_cb8564_c52175/f1p3/3008-1F	RLK-Pelle_CrRLK1L-1	PK
F01_cb8564_c52175/f1p3/3008-2F	RLK-Pelle_CrRLK1L-1	PK
F01_cb8564_c52273/f1p0/2352-1F	RLK-Pelle_DLSV	PK
F01_cb8564_c52273/f1p0/2352-2F	RLK-Pelle_DLSV	PK
F01_cb8564_c52297/f1p0/1962-1F	Group-Pl-4	PK
F01_cb8564_c52388/f1p0/4989-0F	RLK-Pelle_DLSV	PK
F01_cb8564_c52388/f1p0/4989-1F	RLK-Pelle_DLSV	PK
F01_cb8564_c52388/f1p0/4989-2F	RLK-Pelle_DLSV	PK
F01_cb8564_c52477/f1p0/3241-2F	RLK-Pelle_DLSV	PK
F01_cb8564_c52539/f1p1/3441-0F	RLK-Pelle_CrRLK1L-1	PK
F01_cb8564_c52539/f1p1/3441-1F	RLK-Pelle_CrRLK1L-1	PK
F01_cb8564_c5271/f1p0/3205-0F	RLK-Pelle_DLSV	PK
F01_cb8564_c5272/f2p0/3163-1F	RLK-Pelle_DLSV	PK
F01_cb8564_c5272/f2p0/3163-2F	RLK-Pelle_DLSV	PK
F01_cb8564_c52735/f1p0/3300-0F	RLK-Pelle_DLSV	PK
F01_cb8564_c52735/f1p0/3300-2F	RLK-Pelle_DLSV	PK
F01_cb8564_c52784/f1p1/2879-0F	RLK-Pelle_CrRLK1L-1	PK
F01_cb8564_c52784/f1p1/2879-2F	RLK-Pelle_CrRLK1L-1	PK
F01_cb8564_c52794/f1p0/1938-0F	TKL-Pl-4	PK
F01_cb8564_c52863/f1p0/2866-1F	RLK-Pelle_DLSV	PK
F01_cb8564_c53087/f1p0/2677-0F	RLK-Pelle_RLCK-VIIa-2	PK
F01_cb8564_c53087/f1p0/2677-2F	RLK-Pelle_CrRLK1L-1	PK
F01_cb8564_c53152/f1p0/2729-0F	RLK-Pelle_DLSV	PK
F01_cb8564_c53152/f1p0/2729-2F	RLK-Pelle_DLSV	PK
F01_cb8564_c53289/f1p0/3265-2F	WNK_NRBP	PK
F01_cb8564_c53446/f1p1/3081-0F	RLK-Pelle_DLSV	PK

F01_cb8564_c53561/f1p0/2359-1F	WNK_NRBP	PK
F01_cb8564_c53561/f1p0/2359-2F	WNK_NRBP	PK
F01_cb8564_c53758/f1p0/2784-1F	RLK-Pelle_SD-2b	PK
F01_cb8564_c53916/f1p0/2590-0F	Group-Pl-3	PK
F01_cb8564_c53916/f1p0/2590-2F	Group-Pl-3	PK
F01_cb8564_c53960/f1p0/2993-0F	RLK-Pelle_CrRLK1L-1	PK
F01_cb8564_c53960/f1p0/2993-1F	RLK-Pelle_CrRLK1L-1	PK
F01_cb8564_c53960/f1p0/2993-2F	RLK-Pelle_CrRLK1L-1	PK
F01_cb8564_c54171/f1p0/3438-0F	RLK-Pelle_RLCK-VI	PK
F01_cb8564_c54206/f2p0/3081-2F	RLK-Pelle_DLSV	PK
F01_cb8564_c543/f5p1/3002-2F	RLK-Pelle_LRR-XI-1	PK
F01_cb8564_c54369/f1p0/3902-1F	RLK-Pelle_DLSV	PK
F01_cb8564_c54486/f1p0/2881-0F	RLK-Pelle_DLSV	PK
F01_cb8564_c54518/f1p0/2781-0F	RLK-Pelle_CrRLK1L-1	PK
F01_cb8564_c54518/f1p0/2781-2F	RLK-Pelle_CrRLK1L-1	PK
F01_cb8564_c58034/f2p2/3989-1F	CMGC_CDK-PITSLRE	PK
F01_cb8564_c620/f4p0/2539-1F	RLK-Pelle_LRR-Xa	PK
F01_cb8564_c65564/f1p0/2521-1F	RLK-Pelle_DLSV	PK
F01_cb8564_c66088/f4p1/4083-1F	RLK-Pelle_RLCK-VIIa-2	PK
F01_cb8564_c66088/f4p1/4083-2F	RLK-Pelle_CrRLK1L-1	PK
F01_cb8564_c66757/f6p0/2263-2F	Group-Pl-4	PK
F01_cb8564_c66813/f6p0/2579-0F	RLK-Pelle_DLSV	PK
F01_cb8564_c66877/f3p0/2938-1F	WNK_NRBP	PK
F01_cb8564_c67321/f1p0/2299-0F	RLK-Pelle_PERK-1	PK
F01_cb8564_c67635/f10p0/2139-2F	RLK-Pelle_LysM	PK
F01_cb8564_c68144/f9p2/2568-0F	RLK-Pelle_LRR-II	PK

F01_cb8564_c68304/f3p0/3488-0F	RLK-Pelle_DLSV	PK
F01_cb8564_c68314/f3p1/1908-0F	CAMK_CDPK	PK
F01_cb8564_c68442/f4p2/1923-2F	CAMK_CDPK	PK
F01_cb8564_c68572/f7p0/3021-2F	RLK-Pelle_LRR-II	PK
F01_cb8564_c68629/f9p0/2229-0F	CMGC_SRPK	PK
F01_cb8564_c68638/f4p1/2882-2F	RLK-Pelle_RLCK-VI	PK
F01_cb8564_c68654/f5p1/2121-2F	CAMK_AMPK	PK
F01_cb8564_c68715/f3p0/3175-0F	RLK-Pelle_LRR-XIIIb	PK
F01_cb8564_c68749/f3p0/3289-2F	RLK-Pelle_DLSV	PK
F01_cb8564_c68921/f2p2/2973-2F	RLK-Pelle_SD-2b	PK
F01_cb8564_c68990/f2p1/2891-2F	RLK-Pelle_CrRLK1L-1	PK
F01_cb8564_c69015/f2p0/2135-0F	RLK-Pelle_SD-2b	PK
F01_cb8564_c69017/f3p0/3411-0F	RLK-Pelle_DLSV	PK
F01_cb8564_c69017/f3p0/3411-1F	RLK-Pelle_DLSV	PK
F01_cb8564_c69097/f2p0/2364-1F	TKL_CTR1-DRK-2	PK
F01_cb8564_c69213/f1p2/1856-0F	CAMK_CAMKL-CHK1	PK
F01_cb8564_c69227/f2p3/3025-1F	RLK-Pelle_CrRLK1L-1	PK
F01_cb8564_c69227/f2p3/3025-2F	RLK-Pelle_CrRLK1L-1	PK
F01_cb8564_c69241/f3p0/2222-1F	RLK-Pelle_LRK10L-2	PK
F01_cb8564_c69266/f2p0/3325-0F	RLK-Pelle_DLSV	PK
F01_cb8564_c69277/f3p1/2291-0F	RLK-Pelle_LRK10L-2	PK
F01_cb8564_c69289/f1p1/3510-0F	RLK-Pelle_DLSV	PK
F01_cb8564_c69289/f1p1/3510-1F	RLK-Pelle_DLSV	PK
F01_cb8564_c69358/f4p1/2369-0F	RLK-Pelle_WAK_LRK10L-1	PK
F01_cb8564_c69366/f5p4/2938-1F	CK1_CK1-Pl	PK
F01_cb8564_c69550/f1p0/2630-2F	RLK-Pelle_DLSV	PK

F01_cb8564_c69852/f1p0/2513-0F	RLK-Pelle_DLSV	PK
F01_cb8564_c69874/f1p0/3374-0R	RLK-Pelle_SD-2b	PK
F01_cb8564_c69874/f1p0/3374-1F	RLK-Pelle_SD-2b	PK
F01_cb8564_c69913/f2p1/3530-1F	RLK-Pelle_DLSV	PK
F01_cb8564_c69925/f1p1/2234-0F	RLK-Pelle_LysM	PK
F01_cb8564_c69946/f1p0/3776-2F	RLK-Pelle_DLSV	PK
F01_cb8564_c70015/f1p0/2936-1F	WNK_NRBP	PK
F01_cb8564_c70068/f1p0/3324-0F	WNK_NRBP	PK
F01_cb8564_c70068/f1p0/3324-2F	WNK_NRBP	PK
F01_cb8564_c70073/f1p0/3142-1F	RLK-Pelle_CrRLK1L-1	PK
F01_cb8564_c70101/f1p0/2241-0F	CK1_CK1-Pl	PK
F01_cb8564_c70118/f1p0/2047-1F	RLK-Pelle_LysM	PK
F01_cb8564_c70118/f1p0/2047-2F	RLK-Pelle_LysM	PK
F01_cb8564_c70307/f2p0/3222-2F	RLK-Pelle_DLSV	PK
F01_cb8564_c70533/f1p0/2295-0F	RLK-Pelle_RLCK-VI	PK
F01_cb8564_c70533/f1p0/2295-2F	RLK-Pelle_RLCK-VI	PK
F01_cb8564_c70578/f1p1/3120-1F	RLK-Pelle_DLSV	PK
F01_cb8564_c70704/f1p0/3537-0F	CAMK_AMPK	PK
F01_cb8564_c70704/f1p0/3537-1F	CAMK_AMPK	PK
F01_cb8564_c70801/f1p0/2684-0F	RLK-Pelle_LRR-II	PK
F01_cb8564_c70819/f1p1/4129-2F	RLK-Pelle_DLSV	PK
F01_cb8564_c70831/f1p0/3329-0F	RLK-Pelle_DLSV	PK
F01_cb8564_c70831/f1p0/3329-2F	RLK-Pelle_DLSV	PK
F01_cb8564_c70927/f1p0/4372-0F	RLK-Pelle_DLSV	PK
F01_cb8564_c71054/f1p0/3048-1F	Group-Pl-3	PK
F01_cb8564_c71076/f1p2/2391-0F	RLK-Pelle_RLCK-VI	PK

F01_cb8564_c71216/f1p0/2088-2F	CK1_CK1-Pl	PK
F01_cb8564_c71239/f1p0/3090-0F	WNK_NRBP	PK
F01_cb8564_c71331/f1p0/1900-0F	RLK-Pelle_LysM	PK
F01_cb8564_c71369/f1p0/2592-0F	NAK	PK
F01_cb8564_c71369/f1p0/2592-2F	NAK	PK
F01_cb8564_c71390/f1p0/3703-1F	RLK-Pelle_RLCK-VIII	PK
F01_cb8564_c71404/f1p0/3980-0F	RLK-Pelle_DLSV	PK
F01_cb8564_c71404/f1p0/3980-2F	RLK-Pelle_DLSV	PK
F01_cb8564_c71546/f1p2/1919-2F	CAMK_CAMKL-CHK1	PK
F01_cb8564_c71564/f1p0/2695-2F	NAK	PK
F01_cb8564_c71618/f1p1/3399-0F	RLK-Pelle_DLSV	PK
F01_cb8564_c71716/f1p0/3226-1F	CMGC_CDK-PITSLRE	PK
F01_cb8564_c71717/f1p0/1993-0F	RLK-Pelle_RLCK-XII-1	PK
F01_cb8564_c71742/f1p0/2675-2F	WNK_NRBP	PK
F01_cb8564_c71747/f1p1/2679-0F	RLK-Pelle_CrRLK1L-1	PK
F01_cb8564_c71747/f1p1/2679-2F	RLK-Pelle_CrRLK1L-1	PK
F01_cb8564_c71888/f1p1/4007-1F	RLK-Pelle_LysM	PK
F01_cb8564_c71897/f1p1/2487-0F	RLK-Pelle_RLCK-VIIa-2	PK
F01_cb8564_c71983/f1p0/2471-2F	NAK	PK
F01_cb8564_c72004/f2p0/2684-0F	RLK-Pelle_CrRLK1L-1	PK
F01_cb8564_c72004/f2p0/2684-1F	RLK-Pelle_CrRLK1L-1	PK
F01_cb8564_c72104/f1p0/2102-1F	RLK-Pelle_RLCK-XII-1	PK
F01_cb8564_c72110/f1p0/3310-2F	RLK-Pelle_DLSV	PK
F01_cb8564_c72121/f1p0/3290-0F	RLK-Pelle_DLSV	PK
F01_cb8564_c72121/f1p0/3290-1F	RLK-Pelle_DLSV	PK
F01_cb8564_c72294/f1p1/3519-0F	RLK-Pelle_DLSV	PK

F01_cb8564_c72303/f1p0/2684-0F	RLK-Pelle_WAK_LRK10L-1	PK
F01_cb8564_c72497/f1p0/2108-2F	RLK-Pelle_LRK10L-2	PK
F01_cb8564_c72545/f1p0/3448-2F	RLK-Pelle_LRR-XI-1	PK
F01_cb8564_c72575/f1p0/1900-1F	RLK-Pelle_RLCK-VIII	PK
F01_cb8564_c72575/f1p0/1900-2F	RLK-Pelle_RLCK-VIII	PK
F01_cb8564_c72702/f1p0/4583-0F	CAMK_AMPK	PK
F01_cb8564_c72702/f1p0/4583-1F	CAMK_AMPK	PK
F01_cb8564_c72739/f1p0/3265-0F	WNK_NRBP	PK
F01_cb8564_c72739/f1p0/3265-1F	WNK_NRBP	PK
F01_cb8564_c72782/f1p0/2689-1F	RLK-Pelle_LRR-II	PK
F01_cb8564_c72832/f1p1/2918-1F	RLK-Pelle_WAK_LRK10L-1	PK
F01_cb8564_c72832/f1p1/2918-2F	RLK-Pelle_WAK_LRK10L-1	PK
F01_cb8564_c73009/f1p1/4319-2F	RLK-Pelle_DLSV	PK
F01_cb8564_c73112/f1p0/3584-0F	RLK-Pelle_SD-2b	PK
F01_cb8564_c73167/f1p0/3510-2F	RLK-Pelle_DLSV	PK
F01_cb8564_c73260/f1p0/3860-2F	RLK-Pelle_RLCK-XII-1	PK
F01_cb8564_c73284/f1p0/3111-0F	RLK-Pelle_DLSV	PK
F01_cb8564_c73328/f1p0/3197-0F	RLK-Pelle_DLSV	PK
F01_cb8564_c73385/f1p0/1981-0F	CAMK_CAMKL-CHK1	PK
F01_cb8564_c73565/f1p0/2797-0F	RLK-Pelle_DLSV	PK
F01_cb8564_c73773/f1p1/3465-0F	RLK-Pelle_DLSV	PK
F01_cb8564_c73794/f1p1/3512-1F	RLK-Pelle_RLCK-VI	PK
F01_cb8564_c73794/f1p1/3512-2F	RLK-Pelle_RLCK-VI	PK
F01_cb8564_c73801/f1p0/2858-2F	CK1_CK1	PK
F01_cb8564_c73802/f1p0/2498-0F	RLK-Pelle_DLSV	PK
F01_cb8564_c73802/f1p0/2498-1F	RLK-Pelle_DLSV	PK

F01_cb8564_c73811/f1p1/3283-1F	RLK-Pelle_LRR-II	PK
F01_cb8564_c73912/f1p0/3538-0F	RLK-Pelle_DLSV	PK
F01_cb8564_c73912/f1p0/3538-1F	RLK-Pelle_DLSV	PK
F01_cb8564_c74067/f1p0/3829-0F	CAMK_AMPK	PK
F01_cb8564_c74067/f1p0/3829-1F	CAMK_AMPK	PK
F01_cb8564_c74097/f1p1/3703-0F	RLK-Pelle_DLSV	PK
F01_cb8564_c74286/f1p0/2484-1F	RLK-Pelle_DLSV	PK
F01_cb8564_c74523/f1p0/2103-2F	RLK-Pelle_LRR-XI-1	PK
F01_cb8564_c74576/f1p0/4386-0F	RLK-Pelle_DLSV	PK
F01_cb8564_c74576/f1p0/4386-2F	RLK-Pelle_DLSV	PK
F01_cb8564_c74652/f1p0/2680-2F	RLK-Pelle_LRR-II	PK
F01_cb8564_c74798/f1p1/2116-0F	RLK-Pelle_WAK_LRK10L-1	PK
F01_cb8564_c74926/f1p0/2151-1F	RLK-Pelle_CrRLK1L-1	PK
F01_cb8564_c75095/f1p1/2355-2F	RLK-Pelle_LysM	PK
F01_cb8564_c75190/f6p0/3003-2F	RLK-Pelle_CrRLK1L-1	PK
F01_cb8564_c75256/f1p0/2082-1F	RLK-Pelle_RLCK-XII-1	PK
F01_cb8564_c75276/f1p0/3372-2F	RLK-Pelle_LRR-IX	PK
F01_cb8564_c75291/f1p0/3859-1F	RLK-Pelle_LysM	PK
F01_cb8564_c75291/f1p0/3859-2F	RLK-Pelle_LysM	PK
F01_cb8564_c75378/f1p0/2685-1F	RLK-Pelle_LRR-XI-1	PK
F01_cb8564_c75378/f1p0/2685-2F	RLK-Pelle_LRR-XI-1	PK
F01_cb8564_c755/f2p0/3389-0F	RLK-Pelle_LRR-XI-1	PK
F01_cb8564_c75505/f1p0/2250-0F	RLK-Pelle_WAK_LRK10L-1	PK
F01_cb8564_c75607/f1p0/2841-0F	CK1_CK1-Pl	PK
F01_cb8564_c75684/f1p0/2133-0F	RLK-Pelle_SD-2b	PK
F01_cb8564_c75765/f1p0/3629-0F	RLK-Pelle_LRR-XII-1	PK

F01_cb8564_c75852/f1p0/3326-0F	RLK-Pelle_DLSV	PK
F01_cb8564_c75852/f1p0/3326-2F	RLK-Pelle_DLSV	PK
F01_cb8564_c75864/f1p0/1902-1F	RLK-Pelle_LysM	PK
F01_cb8564_c75876/f2p0/2376-0F	RLK-Pelle_LysM	PK
F01_cb8564_c75933/f1p0/2952-0F	WNK_NRBP	PK
F01_cb8564_c75933/f1p0/2952-2F	WNK_NRBP	PK
F01_cb8564_c75961/f4p0/3591-0F	RLK-Pelle_LRR-XIIIb	PK
F01_cb8564_c76001/f1p0/3083-1F	RLK-Pelle_LysM	PK
F01_cb8564_c76043/f2p0/2731-2F	CAMK_CDPK	PK
F01_cb8564_c76051/f1p0/2091-0F	RLK-Pelle_SD-2b	PK
F01_cb8564_c76051/f1p0/2091-1F	RLK-Pelle_SD-2b	PK
F01_cb8564_c76143/f1p0/3097-0F	RLK-Pelle_DLSV	PK
F01_cb8564_c76149/f2p8/2118-1F	CAMK_CAMKL-CHK1	PK
F01_cb8564_c76305/f1p0/2353-1F	CMGC_SRPK	PK
F01_cb8564_c76390/f1p1/2940-2F	RLK-Pelle_RLCK-VIIa-2	PK
F01_cb8564_c76490/f3p1/3602-2F	RLK-Pelle_DLSV	PK
F01_cb8564_c76505/f1p1/2102-0F	RLK-Pelle_WAK_LRK10L-1	PK
F01_cb8564_c76505/f1p1/2102-1F	RLK-Pelle_WAK_LRK10L-1	PK
F01_cb8564_c76505/f1p1/2102-2F	RLK-Pelle_WAK_LRK10L-1	PK
F01_cb8564_c76562/f1p1/4711-0F	RLK-Pelle_DLSV	PK
F01_cb8564_c76756/f1p1/3429-2F	RLK-Pelle_RLCK-VI	PK
F01_cb8564_c76929/f1p0/2420-0F	RLK-Pelle_DLSV	PK
F01_cb8564_c76946/f4p0/2069-0F	RLK-Pelle_LRR-XI-1	PK
F01_cb8564_c7697/f20p4/2318-1F	CK1_CK1	PK
F01_cb8564_c770/f2p0/2459-1F	RLK-Pelle_LRR-XI-1	PK
F01_cb8564_c77097/f1p0/3052-0F	RLK-Pelle_DLSV	PK

F01_cb8564_c77176/f1p0/3311-2F	RLK-Pelle_DLSV	PK
F01_cb8564_c77182/f1p0/2215-2F	CAMK_CAMKL-CHK1	PK
F01_cb8564_c77203/f2p0/2820-1F	RLK-Pelle_CrRLK1L-1	PK
F01_cb8564_c77211/f1p0/2910-0F	RLK-Pelle_RLCK-XII-1	PK
F01_cb8564_c77380/f2p0/3010-1F	WNK_NRBP	PK
F01_cb8564_c77380/f2p0/3010-2F	WNK_NRBP	PK
F01_cb8564_c77412/f1p0/4476-2F	RLK-Pelle_LRR-II	PK
F01_cb8564_c7742/f7p1/2556-1F	RLK-Pelle_LRR-Xa	PK
F01_cb8564_c7761/f6p0/2830-2F	CMGC_MAPK	PK
F01_cb8564_c7763/f11p0/3077-1F	TKL_CTR1-DRK-2	PK
F01_cb8564_c77679/f1p0/2688-2F	WNK_NRBP	PK
F01_cb8564_c77775/f1p0/3843-1F	RLK-Pelle_DLSV	PK
F01_cb8564_c7780/f7p0/3197-0F	CMGC_MAPK	PK
F01_cb8564_c779/f2p0/2583-1F	RLK-Pelle_LRR-IV	PK
F01_cb8564_c77901/f1p0/3053-1F	RLK-Pelle_CrRLK1L-1	PK
F01_cb8564_c77903/f1p1/4534-1F	RLK-Pelle_DLSV	PK
F01_cb8564_c77903/f1p1/4534-2F	RLK-Pelle_WAK	PK
F01_cb8564_c78054/f1p0/2071-1F	RLK-Pelle_LRK10L-2	PK
F01_cb8564_c78131/f1p0/3256-0F	RLK-Pelle_DLSV	PK
F01_cb8564_c78131/f1p0/3256-2F	RLK-Pelle_DLSV	PK
F01_cb8564_c78195/f6p2/2734-1F	RLK-Pelle_LRR-VI-2	PK
F01_cb8564_c78426/f1p0/3753-2F	RLK-Pelle_DLSV	PK
F01_cb8564_c78429/f1p0/3306-2F	RLK-Pelle_DLSV	PK
F01_cb8564_c78459/f1p0/1902-1F	CAMK_AMPK	PK
F01_cb8564_c7851/f2p0/2741-0R	RLK-Pelle_RLCK-VIIa-1	PK
F01_cb8564_c7851/f2p0/2741-2R	RLK-Pelle_RLCK-VIIa-1	PK

F01_cb8564_c78527/f1p0/3048-0F	RLK-Pelle_LRR-II	PK
F01_cb8564_c78606/f1p0/3454-2F	RLK-Pelle_DLSV	PK
F01_cb8564_c78701/f2p2/2927-0F	RLK-Pelle_CrRLK1L-1	PK
F01_cb8564_c78848/f1p0/2925-1F	RLK-Pelle_SD-2b	PK
F01_cb8564_c78992/f1p0/2856-1F	CK1_CK1-Pl	PK
F01_cb8564_c79249/f1p0/2653-2F	RLK-Pelle_DLSV	PK
F01_cb8564_c79253/f2p0/4281-0F	RLK-Pelle_LRR-II	PK
F01_cb8564_c79264/f1p3/3503-0F	CAMK_CAMKL-CHK1	PK
F01_cb8564_c79264/f1p3/3503-0R	CAMK_CAMKL-CHK1	PK
F01_cb8564_c79285/f1p0/4747-0F	CAMK_AMPK	PK
F01_cb8564_c79285/f1p0/4747-2F	CAMK_AMPK	PK
F01_cb8564_c79291/f12p0/2841-0F	CAMK_CDPK	PK
F01_cb8564_c79528/f2p1/3397-0F	RLK-Pelle_DLSV	PK
F01_cb8564_c79693/f1p1/2678-2F	RLK-Pelle_DLSV	PK
F01_cb8564_c79730/f1p0/2988-1F	RLK-Pelle_LRR-II	PK
F01_cb8564_c79905/f1p0/2920-2F	RLK-Pelle_DLSV	PK
F01_cb8564_c79924/f1p0/2967-2F	RLK-Pelle_CrRLK1L-1	PK
F01_cb8564_c79945/f2p1/1901-1F	CAMK_CDPK	PK
F01_cb8564_c79983/f1p0/1945-0F	RLK-Pelle_LRR-XI-1	PK
F01_cb8564_c79983/f1p0/1945-1F	RLK-Pelle_LRR-XI-1	PK
F01_cb8564_c79989/f1p1/3569-2F	RLK-Pelle_DLSV	PK
F01_cb8564_c80076/f1p0/3414-2F	RLK-Pelle_LRR-I-1	PK
F01_cb8564_c80108/f1p2/2992-0F	RLK-Pelle_CrRLK1L-1	PK
F01_cb8564_c80108/f1p2/2992-1F	RLK-Pelle_CrRLK1L-1	PK
F01_cb8564_c80108/f1p2/2992-2F	RLK-Pelle_CrRLK1L-1	PK
F01_cb8564_c80123/f1p1/3310-1F	RLK-Pelle_DLSV	PK

F01_cb8564_c80142/f1p0/3421-1F	RLK-Pelle_CrRLK1L-1	PK
F01_cb8564_c80213/f1p0/3027-1F	CK1_CK1	PK
F01_cb8564_c80397/f1p0/2098-1F	CAMK_AMPK	PK
F01_cb8564_c80502/f1p1/2346-1F	RLK-Pelle_RLCK-VI	PK
F01_cb8564_c80838/f1p0/2264-0F	Aur	PK
F01_cb8564_c80883/f1p0/3185-0F	RLK-Pelle_LRR-XI-1	PK
F01_cb8564_c80883/f1p0/3185-1F	RLK-Pelle_LRR-XI-1	PK
F01_cb8564_c80922/f1p0/3708-0F	RLK-Pelle_DLSV	PK
F01_cb8564_c80943/f1p0/2583-1F	RLK-Pelle_DLSV	PK
F01_cb8564_c80959/f1p0/2936-0F	RLK-Pelle_CrRLK1L-1	PK
F01_cb8564_c80971/f1p0/4679-1F	RLK-Pelle_DLSV	PK
F01_cb8564_c81227/f1p0/3713-0F	RLK-Pelle_SD-2b	PK
F01_cb8564_c81246/f1p0/2901-0F	RLK-Pelle_CrRLK1L-1	PK
F01_cb8564_c81281/f1p1/3440-1F	RLK-Pelle_CrRLK1L-1	PK
F01_cb8564_c81424/f1p1/1904-0F	CAMK_CAMKL-CHK1	PK
F01_cb8564_c81525/f1p0/3901-1F	CAMK_CDPK	PK
F01_cb8564_c81658/f1p0/3480-0F	RLK-Pelle_LRR-XIIIb	PK
F01_cb8564_c81677/f1p0/3033-2F	RLK-Pelle_CrRLK1L-1	PK
F01_cb8564_c81690/f1p0/3035-1F	RLK-Pelle_DLSV	PK
F01_cb8564_c81766/f1p1/3467-0F	RLK-Pelle_DLSV	PK
F01_cb8564_c81772/f1p0/3125-0F	RLK-Pelle_LRR-II	PK
F01_cb8564_c81797/f1p0/3382-0F	RLK-Pelle_DLSV	PK
F01_cb8564_c81801/f1p0/2224-0F	RLK-Pelle_DLSV	PK
F01_cb8564_c81827/f1p0/3217-0F	RLK-Pelle_DLSV	PK
F01_cb8564_c81827/f1p0/3217-1F	RLK-Pelle_DLSV	PK
F01_cb8564_c81894/f1p1/3481-2F	RLK-Pelle_CrRLK1L-1	PK

F01_cb8564_c81938/f1p0/2945-0F	RLK-Pelle_RLCK-VI	PK
F01_cb8564_c81974/f1p0/4207-0F	RLK-Pelle_RLCK-VIIb	PK
F01_cb8564_c81978/f1p0/4223-0F	CAMK_AMPK	PK
F01_cb8564_c81978/f1p0/4223-1F	CAMK_AMPK	PK
F01_cb8564_c82081/f1p0/3279-0F	RLK-Pelle_DLSV	PK
F01_cb8564_c82194/f1p1/3394-0F	RLK-Pelle_WAK_LRK10L-1	PK
F01_cb8564_c82280/f2p0/2262-0F	RLK-Pelle_WAK_LRK10L-1	PK
F01_cb8564_c82328/f1p0/3253-0F	RLK-Pelle_DLSV	PK
F01_cb8564_c82328/f1p0/3253-2F	RLK-Pelle_DLSV	PK
F01_cb8564_c82425/f1p0/2589-1F	STE_STE11	PK
F01_cb8564_c82499/f1p1/4897-0F	RLK-Pelle_DLSV	PK
F01_cb8564_c825/f4p0/2287-1F	RLK-Pelle_DLSV	PK
F01_cb8564_c82505/f1p0/2151-1F	RLK-Pelle_LysM	PK
F01_cb8564_c82521/f1p0/2413-1F	RLK-Pelle_LRR-XI-1	PK
F01_cb8564_c82521/f1p0/2413-2F	RLK-Pelle_LRR-XI-1	PK
F01_cb8564_c82687/f1p0/3370-1F	RLK-Pelle_DLSV	PK
F01_cb8564_c82687/f1p0/3370-2F	RLK-Pelle_DLSV	PK
F01_cb8564_c82795/f1p0/2546-0F	RLK-Pelle_LRR-II	PK
F01_cb8564_c82866/f1p0/3058-2F	CK1_CK1-Pl	PK
F01_cb8564_c82948/f1p0/2074-1F	CAMK_AMPK	PK
F01_cb8564_c83023/f1p0/4116-1F	RLK-Pelle_DLSV	PK
F01_cb8564_c83113/f1p0/2883-1F	RLK-Pelle_SD-2b	PK
F01_cb8564_c83159/f1p0/2624-1F	CAMK_CDPK	PK
F01_cb8564_c83173/f1p0/3670-1F	RLK-Pelle_DLSV	PK
F01_cb8564_c83173/f1p0/3670-2F	RLK-Pelle_DLSV	PK
F01_cb8564_c83196/f1p0/2505-0F	RLK-Pelle_DLSV	PK

F01_cb8564_c83196/f1p0/2505-1F	RLK-Pelle_DLSV	PK
F01_cb8564_c83198/f1p0/3397-0F	RLK-Pelle_LRR-XI-1	PK
F01_cb8564_c83198/f1p0/3397-2F	RLK-Pelle_LRR-XI-1	PK
F01_cb8564_c83251/f1p1/3372-0F	RLK-Pelle_DLSV	PK
F01_cb8564_c83694/f1p0/2192-0F	CAMK_AMPK	PK
F01_cb8564_c83711/f2p0/3575-2F	CAMK_AMPK	PK
F01_cb8564_c83750/f1p0/3418-1F	RLK-Pelle_CrRLK1L-1	PK
F01_cb8564_c83803/f1p1/2961-1F	RLK-Pelle_SD-2b	PK
F01_cb8564_c83803/f1p1/2961-2F	RLK-Pelle_SD-2b	PK
F01_cb8564_c83859/f1p1/3286-2F	RLK-Pelle_DLSV	PK
F01_cb8564_c83882/f1p0/2530-0F	RLK-Pelle_CrRLK1L-1	PK
F01_cb8564_c83882/f1p0/2530-1F	RLK-Pelle_CrRLK1L-1	PK
F01_cb8564_c84021/f3p1/2788-2F	RLK-Pelle_RLCK-VI	PK
F01_cb8564_c84068/f1p0/3483-1F	RLK-Pelle_DLSV	PK
F01_cb8564_c84068/f1p0/3483-2F	RLK-Pelle_CrRLK1L-1	PK
F01_cb8564_c84103/f1p0/2993-1F	CK1_CK1-Pl	PK
F01_cb8564_c84155/f1p0/3503-1F	RLK-Pelle_CrRLK1L-1	PK
F01_cb8564_c84421/f1p0/1902-0F	RLK-Pelle_RLCK-XII-1	PK
F01_cb8564_c84435/f1p0/3218-1F	RLK-Pelle_CrRLK1L-1	PK
F01_cb8564_c84676/f2p0/2807-0F	RLK-Pelle_SD-2b	PK
F01_cb8564_c84705/f1p0/1914-1F	RLK-Pelle_DLSV	PK
F01_cb8564_c84750/f1p0/3347-2F	RLK-Pelle_CrRLK1L-1	PK
F01_cb8564_c84828/f1p3/3008-0F	RLK-Pelle_CrRLK1L-1	PK
F01_cb8564_c84828/f1p3/3008-2F	RLK-Pelle_CrRLK1L-1	PK
F01_cb8564_c84898/f1p0/3878-0F	RLK-Pelle_LysM	PK
F01_cb8564_c84922/f2p0/2579-2F	CK1_CK1-Pl	PK

F01_cb8564_c85038/f1p0/3407-1F	RLK-Pelle_SD-2b	PK
F01_cb8564_c85062/f1p1/3553-2F	RLK-Pelle_DLSV	PK
F01_cb8564_c85408/f1p0/2335-1F	RLK-Pelle_DLSV	PK
F01_cb8564_c85451/f1p0/3023-0F	RLK-Pelle_RLCK-VI	PK
F01_cb8564_c85451/f1p0/3023-2F	RLK-Pelle_RLCK-VI	PK
F01_cb8564_c85599/f1p0/3132-2F	RLK-Pelle_DLSV	PK
F01_cb8564_c85725/f1p0/2171-1F	RLK-Pelle_LysM	PK
F01_cb8564_c85969/f1p3/2183-0F	WNK_NRBP	PK
F01_cb8564_c85969/f1p3/2183-1F	WNK_NRBP	PK
F01_cb8564_c85978/f1p1/2839-0F	RLK-Pelle_CrRLK1L-1	PK
F01_cb8564_c85978/f1p1/2839-1F	RLK-Pelle_CrRLK1L-1	PK
F01_cb8564_c86191/f1p0/3642-0F	CAMK_AMPK	PK
F01_cb8564_c86193/f1p0/2558-1F	RLK-Pelle_LRR-I-1	PK
F01_cb8564_c86193/f1p0/2558-2F	RLK-Pelle_LRR-I-1	PK
F01_cb8564_c86324/f1p0/2805-1F	RLK-Pelle_DLSV	PK
F01_cb8564_c86396/f1p0/2097-2F	CAMK_CAMKL-CHK1	PK
F01_cb8564_c86497/f1p0/2897-0F	RLK-Pelle_LysM	PK
F01_cb8564_c86499/f1p1/3322-1F	RLK-Pelle_CrRLK1L-1	PK
F01_cb8564_c86499/f1p1/3322-2F	RLK-Pelle_CrRLK1L-1	PK
F01_cb8564_c86549/f3p0/2562-1F	RLK-Pelle_LRR-II	PK
F01_cb8564_c86669/f1p0/3571-0F	RLK-Pelle_CrRLK1L-1	PK
F01_cb8564_c86829/f1p0/2430-1F	RLK-Pelle_DLSV	PK
F01_cb8564_c86975/f1p0/3253-0F	RLK-Pelle_DLSV	PK
F01_cb8564_c86986/f1p0/3627-2F	RLK-Pelle_DLSV	PK
F01_cb8564_c87000/f1p0/2635-1F	NAK	PK
F01_cb8564_c87035/f1p0/2284-0F	RLK-Pelle_DLSV	PK

F01_cb8564_c87192/f1p0/2491-1F	RLK-Pelle_LRK10L-2	PK
F01_cb8564_c87344/f1p0/2474-0F	CMGC_SRPK	PK
F01_cb8564_c87345/f1p0/2963-1F	RLK-Pelle_CrRLK1L-1	PK
F01_cb8564_c87536/f6p0/2505-0F	RLK-Pelle_LRR-II	PK
F01_cb8564_c87595/f1p0/4323-0F	RLK-Pelle_DLSV	PK
F01_cb8564_c87595/f1p0/4323-2F	RLK-Pelle_DLSV	PK
F01_cb8564_c87635/f1p0/2805-0F	RLK-Pelle_CrRLK1L-1	PK
F01_cb8564_c87635/f1p0/2805-2F	RLK-Pelle_CrRLK1L-1	PK
F01_cb8564_c8768/f7p0/3451-1F	RLK-Pelle_DLSV	PK
F01_cb8564_c87747/f1p0/3271-2F	RLK-Pelle_RLCK-VIIa-2	PK
F01_cb8564_c87786/f1p1/2285-0F	AGC_RSK-2	PK
F01_cb8564_c87786/f1p1/2285-2F	AGC_RSK-2	PK
F01_cb8564_c87855/f1p1/2779-0F	RLK-Pelle_CrRLK1L-1	PK
F01_cb8564_c87859/f1p0/3852-1F	RLK-Pelle_LRR-XI-1	PK
F01_cb8564_c88064/f1p0/4623-0F	CAMK_AMPK	PK
F01_cb8564_c88155/f2p0/4001-2F	RLK-Pelle_SD-2b	PK
F01_cb8564_c88582/f1p0/2884-1F	RLK-Pelle_CrRLK1L-1	PK
F01_cb8564_c88722/f1p0/4185-1F	RLK-Pelle_DLSV	PK
F01_cb8564_c88757/f1p0/2347-2F	RLK-Pelle_DLSV	PK
F01_cb8564_c88819/f1p0/3550-0F	RLK-Pelle_DLSV	PK
F01_cb8564_c88853/f1p0/2988-0F	RLK-Pelle_CrRLK1L-1	PK
F01_cb8564_c88886/f1p0/3995-0F	RLK-Pelle_DLSV	PK
F01_cb8564_c88906/f1p0/3156-1F	RLK-Pelle_RLCK-VI	PK
F01_cb8564_c89596/f1p0/3266-0F	RLK-Pelle_DLSV	PK
F01_cb8564_c89619/f1p0/4818-0F	RLK-Pelle_LRR-I-1	PK
F01_cb8564_c89827/f3p0/3331-2F	RLK-Pelle_DLSV	PK

F01_cb8564_c90031/f1p0/4423-0F	RLK-Pelle_LysM	PK
F01_cb8564_c90046/f1p0/2504-2F	CK1_CK1-Pl	PK
F01_cb8564_c90089/f1p0/3202-2F	RLK-Pelle_RLCK-VIIa-2	PK
F01_cb8564_c90171/f1p0/2007-0F	RLK-Pelle_CrRLK1L-1	PK
F01_cb8564_c90361/f2p0/2743-0F	Group-Pl-3	PK
F01_cb8564_c90462/f1p1/3321-1F	RLK-Pelle_DLSV	PK
F01_cb8564_c90472/f2p0/2171-0F	RLK-Pelle_SD-2b	PK
F01_cb8564_c90587/f1p0/3448-1F	RLK-Pelle_LysM	PK
F01_cb8564_c90675/f1p0/3207-1F	RLK-Pelle_DLSV	PK
F01_cb8564_c90723/f1p0/2496-0F	RLK-Pelle_LRR-II	PK
F01_cb8564_c90831/f2p1/2565-0F	RLK-Pelle_RLCK-VI	PK
F01_cb8564_c90837/f1p0/2097-2F	Aur	PK
F01_cb8564_c90863/f1p0/3539-0F	RLK-Pelle_DLSV	PK
F01_cb8564_c91017/f1p0/2537-1F	RLK-Pelle_RLCK-VI	PK
F01_cb8564_c91017/f1p0/2537-2F	RLK-Pelle_RLCK-VI	PK
F01_cb8564_c91137/f1p0/3102-2F	RLK-Pelle_LRR-II	PK
F01_cb8564_c9117/f2p0/2201-0F	AGC-Pl	PK
F01_cb8564_c9117/f2p0/2201-1F	AGC-Pl	PK
F01_cb8564_c91252/f1p0/3201-0F	RLK-Pelle_LRR-II	PK
F01_cb8564_c91267/f1p0/2862-0F	RLK-Pelle_SD-2b	PK
F01_cb8564_c91276/f1p0/2372-1F	CAMK_CDPK	PK
F01_cb8564_c91405/f1p1/2994-0F	RLK-Pelle_CrRLK1L-1	PK
F01_cb8564_c91405/f1p1/2994-1F	RLK-Pelle_CrRLK1L-1	PK
F01_cb8564_c91454/f1p2/3641-2F	RLK-Pelle_DLSV	PK
F01_cb8564_c91775/f1p1/3299-1F	STE_STE11	PK
F01_cb8564_c91775/f1p1/3299-2F	STE_STE11	PK

F01_cb8564_c91839/f1p0/2218-0F	RLK-Pelle_LRK10L-2	PK
F01_cb8564_c91965/f1p0/4169-2F	CK1_CK1-Pl	PK
F01_cb8564_c92004/f1p0/3165-2F	RLK-Pelle_SD-2b	PK
F01_cb8564_c92057/f1p0/3432-1F	RLK-Pelle_CrRLK1L-1	PK
F01_cb8564_c92122/f1p0/4505-1F	RLK-Pelle_DLSV	PK
F01_cb8564_c92122/f1p0/4505-2F	RLK-Pelle_DLSV	PK
F01_cb8564_c92254/f1p0/3559-2F	RLK-Pelle_DLSV	PK
F01_cb8564_c93761/f8p4/3293-2F	RLK-Pelle_LRR-VIII-1	PK
F01_cb8564_c9410/f3p1/3347-1F	RLK-Pelle_LRR-XII-1	PK
F01_cb8564_c943/f2p0/2919-0F	RLK-Pelle_LRR-XI-1	PK
F01_cb8564_c9440/f6p0/2195-2F	RLK-Pelle_LRK10L-2	PK
F01_cb8564_c9481/f3p0/2666-1F	RLK-Pelle_LRR-VI-2	PK
F01_cb8564_c96400/f1p0/2636-0F	RLK-Pelle_LRR-XI-1	PK
F01_cb8564_c96404/f1p3/3164-0F	RLK-Pelle_CrRLK1L-1	PK
F01_cb8564_c96404/f1p3/3164-2F	RLK-Pelle_CrRLK1L-1	PK
F01_cb8564_c9744/f2p0/4141-0F	RLK-Pelle_CrRLK1L-1	PK
F01_cb8564_c9744/f2p0/4141-2F	RLK-Pelle_RLCK-VIIa-2	PK
F01_cb8564_c9746/f3p3/3969-2F	RLK-Pelle_LRR-XII-1	PK
F01_cb8564_c9771/f3p1/2625-2F	RLK-Pelle_LRR-VI-2	PK
F01_cb8564_c9893/f7p1/3111-0F	RLK-Pelle_LRR-XI-1	PK
F01_cb8564_c9950/f3p1/2453-2F	STE_STE11	PK
F01_cb8564_c9954/f2p0/3282-2F	RLK-Pelle_LRR-XI-1	PK
F01_cb8570_c0/f1p0/2329-0F	RLK-Pelle_WAK_LRK10L-1	PK
F01_cb8570_c1/f1p0/2487-2F	RLK-Pelle_WAK_LRK10L-1	PK
F01_cb8613_c1/f2p0/2410-2F	RLK-Pelle_LRR-III	PK
F01_cb8613_c2/f1p0/2424-0F	RLK-Pelle_LRR-III	PK

F01_cb862_c1/f2p0/3252-0F	RLK-Pelle_LRR-VIII-1	PK
F01_cb862_c10/f1p0/3428-1F	RLK-Pelle_LRR-VIII-1	PK
F01_cb862_c11/f1p0/3220-0F	RLK-Pelle_LRR-VIII-1	PK
F01_cb862_c12/f1p0/3756-2F	RLK-Pelle_LRR-VIII-1	PK
F01_cb862_c15/f1p0/3207-2F	RLK-Pelle_LRR-VIII-1	PK
F01_cb862_c16/f1p0/2631-1F	RLK-Pelle_LRR-VIII-1	PK
F01_cb862_c17/f1p0/2770-1F	RLK-Pelle_LRR-VIII-1	PK
F01_cb862_c18/f1p0/3367-2F	RLK-Pelle_LRR-VIII-1	PK
F01_cb862_c2/f1p0/4599-1F	RLK-Pelle_LRR-VIII-1	PK
F01_cb862_c21/f1p0/3616-2F	RLK-Pelle_LRR-VIII-1	PK
F01_cb862_c22/f1p0/3314-0F	RLK-Pelle_LRR-VIII-1	PK
F01_cb862_c23/f1p0/3466-0F	RLK-Pelle_LRR-VIII-1	PK
F01_cb862_c24/f1p0/3226-0F	RLK-Pelle_LRR-VIII-1	PK
F01_cb862_c25/f1p0/2839-1F	RLK-Pelle_LRR-VIII-1	PK
F01_cb862_c26/f1p0/2964-0F	RLK-Pelle_LRR-VIII-1	PK
F01_cb862_c27/f1p0/3141-0F	RLK-Pelle_LRR-VIII-1	PK
F01_cb862_c31/f1p0/5935-0F	RLK-Pelle_LRR-VIII-1	PK
F01_cb862_c5/f1p0/3238-0F	RLK-Pelle_LRR-VIII-1	PK
F01_cb862_c7/f1p0/2681-0F	RLK-Pelle_LRR-VIII-1	PK
F01_cb862_c8/f1p0/3325-2F	RLK-Pelle_LRR-VIII-1	PK
F01_cb8649_c0/f2p0/2325-2F	RLK-Pelle_LRR-VIII-1	PK
F01_cb8649_c1/f1p0/2228-0F	RLK-Pelle_LRR-VIII-1	PK
F01_cb8649_c1/f1p0/2228-2F	RLK-Pelle_LRR-VIII-1	PK
F01_cb8655_c0/f1p0/2306-1F	RLK-Pelle_L-LEC	PK
F01_cb8658_c0/f1p0/2305-2F	TKL-Pl-4	PK
F01_cb8658_c2/f1p0/2311-0F	TKL-Pl-4	PK

F01_cb8658_c3/f1p0/2265-0F	TKL_Gdt	PK
F01_cb8658_c3/f1p0/2265-2F	TKL-Pl-4	PK
F01_cb8658_c4/f1p0/3502-0F	TKL-Pl-4	PK
F01_cb8658_c6/f1p0/3454-1F	TKL_Gdt	PK
F01_cb8658_c6/f1p0/3454-2F	TKL-Pl-4	PK
F01_cb8659_c0/f2p0/2306-1F	RLK-Pelle_RLCK-XI	PK
F01_cb8659_c0/f2p0/2306-2F	RLK-Pelle_RLCK-XI	PK
F01_cb8659_c1/f1p0/2273-0F	RLK-Pelle_RLCK-XI	PK
F01_cb8659_c3/f1p0/1416-2F	RLK-Pelle_RLCK-XI	PK
F01_cb8675_c0/f1p0/2308-1F	RLK-Pelle_RLCK-XI	PK
F01_cb8675_c1/f1p0/2167-0F	RLK-Pelle_RLCK-XI	PK
F01_cb8687_c10/f2p0/1657-1F	RLK-Pelle_RLCK-VIIa-1	PK
F01_cb8687_c13/f1p0/1583-2F	RLK-Pelle_RLCK-VIIa-1	PK
F01_cb8687_c2/f2p0/1575-0F	RLK-Pelle_RLCK-VIIa-1	PK
F01_cb8687_c3/f1p0/2296-0F	RLK-Pelle_RLCK-VIIa-1	PK
F01_cb8687_c3/f1p0/2296-1F	RLK-Pelle_RLCK-VIIa-1	PK
F01_cb8687_c4/f1p0/3035-1F	RLK-Pelle_RLCK-VIIa-1	PK
F01_cb8687_c5/f1p0/1932-0F	RLK-Pelle_RLCK-VIIa-1	PK
F01_cb8687_c5/f1p0/1932-1F	RLK-Pelle_RLCK-VIIa-1	PK
F01_cb8687_c8/f1p0/1570-1F	RLK-Pelle_RLCK-VIIa-1	PK
F01_cb8698_c0/f12p0/2111-0F	AGC-Pl	PK
F01_cb8698_c7/f1p0/2165-1F	AGC-Pl	PK
F01_cb8698_c8/f1p0/2067-1F	AGC-Pl	PK
F01_cb8764_c1/f6p1/2165-2F	CAMK_CAMKL-CHK1	PK
F01_cb8764_c11/f1p1/2234-2F	CAMK_CAMKL-CHK1	PK
F01_cb8764_c13/f8p0/2189-1F	CAMK_CAMKL-CHK1	PK

F01_cb8764_c4/f1p0/2406-1F	CAMK_CAMKL-CHK1	PK
F01_cb8764_c5/f1p0/3284-2F	CAMK_CAMKL-CHK1	PK
F01_cb8764_c7/f1p0/3184-0F	CAMK_CAMKL-CHK1	PK
F01_cb8764_c7/f1p0/3184-1F	CAMK_CAMKL-CHK1	PK
F01_cb8764_c8/f1p0/2430-0F	CAMK_CAMKL-CHK1	PK
F01_cb8779_c12/f1p0/3324-1R	RLK-Pelle_SD-2b	PK
F01_cb8779_c14/f1p0/3251-1R	RLK-Pelle_SD-2b	PK
F01_cb8779_c14/f1p0/3251-2R	RLK-Pelle_SD-2b	PK
F01_cb8787_c0/f1p0/2276-1F	RLK-Pelle_WAK_LRK10L-1	PK
F01_cb8843_c0/f1p0/2265-2F	TKL-Pl-4	PK
F01_cb8843_c1/f1p0/1986-1F	TKL-Pl-4	PK
F01_cb8843_c2/f1p0/1903-1F	TKL-Pl-4	PK
F01_cb8871_c0/f1p0/2247-0F	AGC_RSK-2	PK
F01_cb8871_c0/f1p0/2247-2F	AGC_RSK-2	PK
F01_cb8871_c1/f1p0/2182-1F	AGC_RSK-2	PK
F01_cb8871_c3/f1p0/1531-2F	AGC_RSK-2	PK
F01_cb8924_c2/f1p1/2141-1F	RLK-Pelle_RLCK-V	PK
F01_cb8924_c3/f1p0/2164-2F	RLK-Pelle_RLCK-V	PK
F01_cb8924_c4/f1p0/2227-2F	RLK-Pelle_RLCK-V	PK
F01_cb8931_c15/f1p0/1567-0F	CAMK_CAMKL-CHK1	PK
F01_cb8931_c15/f1p0/1567-2F	CAMK_CAMKL-CHK1	PK
F01_cb8931_c17/f1p1/2168-1F	CAMK_CAMKL-CHK1	PK
F01_cb8931_c2/f4p1/2097-1F	CAMK_CAMKL-CHK1	PK
F01_cb8931_c20/f1p2/2173-2F	CAMK_CAMKL-CHK1	PK
F01_cb8931_c22/f1p0/814-1F	CAMK_CAMKL-CHK1	PK
F01_cb8931_c5/f4p1/2097-2F	CAMK_CAMKL-CHK1	PK

F01_cb8931_c7/f1p0/2326-0F	CAMK_OST1L	PK
F01_cb8931_c7/f1p0/2326-2F	CAMK_CAMKL-CHK1	PK
F01_cb8972_c1/f1p0/2282-1F	CAMK_CDPK	PK
F01_cb8972_c1/f1p0/2282-2F	CAMK_CDPK	PK
F01_cb8972_c15/f12p0/2217-0F	CAMK_CDPK	PK
F01_cb8972_c7/f1p0/2254-2F	CAMK_CDPK	PK
F01_cb8972_c8/f1p0/2251-1F	CAMK_CDPK	PK
F01_cb8972_c8/f1p0/2251-2F	CAMK_CDPK	PK
F01_cb8972_c9/f1p0/2155-2F	CAMK_CDPK	PK
F01_cb897_c10/f1p0/3008-1F	CK1_CK1-Pl	PK
F01_cb897_c11/f1p1/3229-2F	CK1_CK1-Pl	PK
F01_cb897_c13/f1p1/3220-1F	CK1_CK1-Pl	PK
F01_cb897_c18/f1p0/3399-2F	CK1_CK1-Pl	PK
F01_cb897_c21/f1p0/3157-2F	CK1_CK1-Pl	PK
F01_cb897_c24/f1p0/3809-1F	CK1_CK1-Pl	PK
F01_cb897_c25/f1p0/3279-0F	CK1_CK1-Pl	PK
F01_cb897_c26/f1p0/3536-0F	CK1_CK1-Pl	PK
F01_cb897_c28/f1p0/3140-2F	CK1_CK1-Pl	PK
F01_cb897_c30/f1p0/2403-0F	CK1_CK1-Pl	PK
F01_cb897_c33/f1p0/3077-1F	CK1_CK1-Pl	PK
F01_cb897_c34/f1p0/2834-2F	CK1_CK1-Pl	PK
F01_cb897_c8/f1p0/4517-2F	CK1_CK1-Pl	PK
F01_cb897_c9/f1p1/3651-0F	CK1_CK1-Pl	PK
F01_cb8998_c0/f1p0/2213-0F	TKL-Pl-5	PK
F01_cb8998_c0/f1p0/2213-1F	TKL-Pl-5	PK
F01_cb8998_c0/f1p0/2213-2F	TKL-Pl-5	PK

F01_cb8998_c1/f1p0/2414-1F	TKL-Pl-5	PK
F01_cb9018_c11/f5p0/1856-1F	CMGC_GSK	PK
F01_cb9018_c11/f5p0/1856-2F	CMGC_GSK	PK
F01_cb9018_c12/f19p0/1819-2F	CMGC_GSK	PK
F01_cb9018_c2/f2p1/1808-1F	CMGC_GSK	PK
F01_cb9018_c3/f1p0/2198-1F	CMGC_GSK	PK
F01_cb9018_c6/f1p0/1972-2F	CMGC_GSK	PK
F01_cb9018_c7/f1p0/1749-2F	CMGC_GSK	PK
F01_cb9025_c0/f1p0/2209-0F	RLK-Pelle_RLCK-VIIa-1	PK
F01_cb9025_c1/f1p0/2032-2F	RLK-Pelle_RLCK-VIIa-1	PK
F01_cb9025_c2/f1p0/2097-1F	RLK-Pelle_RLCK-VIIa-1	PK
F01_cb9053_c10/f1p1/1983-2F	CAMK_CAMKL-CHK1	PK
F01_cb9053_c3/f4p1/2186-1F	CAMK_CAMKL-CHK1	PK
F01_cb9053_c6/f1p0/2093-0F	CAMK_CAMKL-CHK1	PK
F01_cb9053_c6/f1p0/2093-2F	CAMK_CAMKL-CHK1	PK
F01_cb9053_c7/f1p0/1807-1F	CAMK_CAMKL-CHK1	PK
F01_cb9053_c7/f1p0/1807-2F	CAMK_CAMKL-CHK1	PK
F01_cb9053_c9/f6p1/2030-1F	CAMK_CAMKL-CHK1	PK
F01_cb9057_c0/f1p0/2199-0F	RLK-Pelle_DLSV	PK
F01_cb9057_c0/f1p0/2199-2F	RLK-Pelle_DLSV	PK
F01_cb9057_c1/f1p0/1949-0F	RLK-Pelle_DLSV	PK
F01_cb9057_c2/f1p0/2081-1F	RLK-Pelle_DLSV	PK
F01_cb9057_c4/f1p0/2149-1F	RLK-Pelle_DLSV	PK
F01_cb9057_c5/f1p0/2010-1F	RLK-Pelle_DLSV	PK
F01_cb9057_c7/f1p0/1426-0F	RLK-Pelle_DLSV	PK
F01_cb9057_c7/f1p0/1426-1F	RLK-Pelle_DLSV	PK

F01_cb9061_c14/f3p0/2326-0F	RLK-Pelle_LRR-III	PK
F01_cb9061_c36/f1p0/2363-1F	RLK-Pelle_LRR-III	PK
F01_cb9061_c36/f1p0/2363-2F	RLK-Pelle_LRR-III	PK
F01_cb9061_c37/f1p0/2495-2F	RLK-Pelle_LRR-III	PK
F01_cb9061_c38/f1p0/2450-2F	RLK-Pelle_LRR-III	PK
F01_cb9061_c40/f1p0/2523-0F	RLK-Pelle_LRR-III	PK
F01_cb9061_c41/f1p0/2654-0F	RLK-Pelle_LRR-III	PK
F01_cb9061_c45/f1p1/2398-1F	RLK-Pelle_LRR-III	PK
F01_cb9061_c45/f1p1/2398-2F	RLK-Pelle_LRR-III	PK
F01_cb9061_c49/f1p0/2410-0F	RLK-Pelle_LRR-III	PK
F01_cb9061_c50/f1p0/2140-0F	RLK-Pelle_LRR-III	PK
F01_cb9061_c50/f1p0/2140-1F	RLK-Pelle_LRR-III	PK
F01_cb9061_c8/f6p0/2350-1F	RLK-Pelle_LRR-III	PK
F01_cb9061_c9/f3p1/2536-0F	RLK-Pelle_LRR-III	PK
F01_cb9064_c12/f1p2/1954-1F	CAMK_CAMKL-CHK1	PK
F01_cb9064_c17/f1p2/2047-2F	CAMK_CAMKL-CHK1	PK
F01_cb9064_c30/f1p0/1539-2F	CAMK_CAMKL-CHK1	PK
F01_cb9064_c39/f1p0/649-0F	CAMK_CAMKL-CHK1	PK
F01_cb9064_c9/f1p0/2197-0F	CAMK_CAMKL-CHK1	PK
F01_cb9075_c0/f2p0/2171-1F	NEK	PK
F01_cb9075_c1/f1p0/2135-2F	NEK	PK
F01_cb9076_c0/f1p0/2192-0F	RLK-Pelle_RLCK-VI	PK
F01_cb9076_c1/f1p0/2158-0F	RLK-Pelle_RLCK-VI	PK
F01_cb9076_c2/f1p0/2130-0F	RLK-Pelle_RLCK-VI	PK
F01_cb9105_c2/f1p1/2185-1F	CMGC_CLK	PK
F01_cb9105_c3/f1p0/1933-2F	CMGC_CLK	PK

F01_cb9105_c8/f2p0/1770-1F	CMGC_CLK	PK
F01_cb9115_c0/f2p0/2025-1F	AGC_RSK-2	PK
F01_cb9115_c2/f1p0/2131-1F	AGC_RSK-2	PK
F01_cb9143_c137/f1p0/3665-0F	CAMK_CDPK	PK
F01_cb9194_c1/f1p0/2285-1F	CAMK_CAMKL-CHK1	PK
F01_cb9250_c1/f1p0/2385-0F	RLK-Pelle_L-LEC	PK
F01_cb9264_c2/f1p0/1981-0F	RLK-Pelle_RLCK-II	PK
F01_cb9264_c4/f2p0/2028-0F	RLK-Pelle_RLCK-II	PK
F01_cb9264_c5/f1p0/2108-1F	RLK-Pelle_RLCK-II	PK
F01_cb9292_c13/f1p0/1702-0F	RLK-Pelle_PERK-2	PK
F01_cb9327_c0/f2p1/2017-1F	TKL-Pl-4	PK
F01_cb9327_c0/f2p1/2017-2F	TKL-Pl-4	PK
F01_cb9327_c1/f2p1/1981-0F	TKL-Pl-4	PK
F01_cb9327_c3/f1p0/2087-0F	TKL-Pl-4	PK
F01_cb9340_c0/f1p0/2116-0F	RLK-Pelle_RLCK-VIIa-1	PK
F01_cb9340_c1/f1p0/2378-0F	RLK-Pelle_RLCK-VIIa-1	PK
F01_cb9340_c1/f1p0/2378-1F	RLK-Pelle_RLCK-VIIa-1	PK
F01_cb9349_c13/f1p1/1813-1F	NAK	PK
F01_cb9349_c16/f1p0/1742-0F	NAK	PK
F01_cb9349_c16/f1p0/1742-2F	NAK	PK
F01_cb9349_c17/f1p0/1743-0F	NAK	PK
F01_cb9349_c21/f5p1/1481-1F	NAK	PK
F01_cb9349_c8/f2p0/1693-1F	NAK	PK
F01_cb9400_c0/f2p0/2063-2F	Group-Pl-3	PK
F01_cb9400_c1/f1p0/2099-1F	Group-Pl-3	PK
F01_cb9400_c2/f1p0/3693-1F	Group-Pl-3	PK

F01_cb9400_c3/f1p0/2055-0F	Group-Pl-3	PK
F01_cb9412_c1/f4p0/2105-1F	RLK-Pelle_RLCK-VIIa-2	PK
F01_cb9412_c2/f1p0/2904-2F	RLK-Pelle_RLCK-VIIa-2	PK
F01_cb9412_c3/f1p0/1975-2F	RLK-Pelle_RLCK-VIIa-2	PK
F01_cb9417_c0/f18p2/1832-2F	TKL-Pl-4	PK
F01_cb9417_c2/f3p2/1912-1F	TKL-Pl-4	PK
F01_cb9417_c3/f1p0/2093-0F	TKL-Pl-4	PK
F01_cb9417_c7/f1p2/1820-0F	TKL-Pl-4	PK
F01_cb944_c61/f1p0/2278-0F	RLK-Pelle_RLCK-V	PK
F01_cb944_c63/f1p1/2372-2F	RLK-Pelle_RLCK-V	PK
F01_cb944_c67/f1p0/1598-1F	RLK-Pelle_RLCK-V	PK
F01_cb944_c96/f9p2/2331-0F	RLK-Pelle_RLCK-V	PK
F01_cb944_c97/f7p2/2379-1F	RLK-Pelle_RLCK-V	PK
F01_cb9465_c0/f6p0/2000-0F	CAMK_CAMKL-CHK1	PK
F01_cb9465_c1/f1p1/2022-1F	CAMK_CAMKL-CHK1	PK
F01_cb9465_c1/f1p1/2022-2F	CAMK_CAMKL-CHK1	PK
F01_cb9465_c2/f1p0/1816-1F	CAMK_CAMKL-CHK1	PK
F01_cb9481_c1/f1p0/2070-1F	CAMK_CDPK	PK
F01_cb9483_c0/f3p0/2156-1F	CAMK_CAMKL-CHK1	PK
F01_cb9483_c12/f1p0/2249-2F	CAMK_CAMKL-CHK1	PK
F01_cb9483_c16/f1p0/1966-0F	CAMK_CAMKL-CHK1	PK
F01_cb9483_c18/f1p0/2201-0F	CAMK_CAMKL-CHK1	PK
F01_cb9483_c4/f2p1/2250-0F	CAMK_CAMKL-CHK1	PK
F01_cb9483_c5/f1p0/3199-0F	CAMK_CAMKL-CHK1	PK
F01_cb9483_c8/f1p0/2183-0F	CAMK_CAMKL-CHK1	PK
F01_cb9483_c8/f1p0/2183-1F	CAMK_CAMKL-CHK1	PK

F01_cb9483_c9/f1p0/2094-0F	CAMK_CAMKL-CHK1	PK
F01_cb9491_c1/f2p0/1929-1F	RLK-Pelle_RLCK-V	PK
F01_cb9491_c10/f3p0/1919-2F	RLK-Pelle_RLCK-V	PK
F01_cb9491_c3/f1p0/1965-0F	RLK-Pelle_RLCK-V	PK
F01_cb9491_c3/f1p0/1965-2F	RLK-Pelle_RLCK-V	PK
F01_cb9491_c4/f1p0/1989-0F	RLK-Pelle_RLCK-V	PK
F01_cb9491_c7/f1p0/1809-1F	RLK-Pelle_RLCK-V	PK
F01_cb9499_c3/f1p0/2215-0F	TKL-Pl-4	PK
F01_cb9499_c6/f1p0/1800-2F	TKL-Pl-4	PK
F01_cb9513_c14/f2p2/1964-0F	CAMK_CAMKL-CHK1	PK
F01_cb9513_c31/f1p0/2513-0F	CAMK_CAMKL-CHK1	PK
F01_cb9513_c31/f1p0/2513-1F	CAMK_CAMKL-CHK1	PK
F01_cb9513_c33/f1p0/2509-0F	CAMK_CAMKL-CHK1	PK
F01_cb9513_c33/f1p0/2509-1F	CAMK_OST1L	PK
F01_cb9513_c35/f1p0/2739-1F	CAMK_CAMKL-CHK1	PK
F01_cb9513_c37/f1p1/2223-0F	CAMK_CAMKL-CHK1	PK
F01_cb9513_c37/f1p1/2223-1F	CAMK_CAMKL-CHK1	PK
F01_cb9513_c38/f1p0/2842-0F	CAMK_CAMKL-CHK1	PK
F01_cb9513_c38/f1p0/2842-1F	CAMK_CAMKL-CHK1	PK
F01_cb9513_c42/f1p0/2488-0F	CAMK_CAMKL-CHK1	PK
F01_cb9513_c42/f1p0/2488-2F	CAMK_CAMKL-CHK1	PK
F01_cb9513_c43/f1p0/2384-0F	CAMK_CAMKL-CHK1	PK
F01_cb9513_c43/f1p0/2384-1F	CAMK_CAMKL-CHK1	PK
F01_cb9513_c46/f1p2/3668-1F	CAMK_CAMKL-CHK1	PK
F01_cb9513_c51/f1p0/2168-0F	CAMK_CAMKL-CHK1	PK
F01_cb9513_c52/f1p0/2397-0F	CAMK_CAMKL-CHK1	PK

F01_cb9513_c52/f1p0/2397-2F	CAMK_CAMKL-CHK1	PK
F01_cb9513_c53/f1p0/2546-0F	RLK-Pelle_L-LEC	PK
F01_cb9513_c77/f1p0/2335-1F	CAMK_CAMKL-CHK1	PK
F01_cb9513_c78/f1p1/2057-1F	CAMK_CAMKL-CHK1	PK
F01_cb9513_c80/f1p0/2294-2F	CAMK_CAMKL-CHK1	PK
F01_cb9513_c81/f1p0/1984-1F	CAMK_CAMKL-CHK1	PK
F01_cb9513_c87/f5p1/1971-1F	CAMK_CAMKL-CHK1	PK
F01_cb9598_c11/f2p0/1690-1F	CAMK_CAMKL-CHK1	PK
F01_cb9598_c11/f2p0/1690-2F	CAMK_CAMKL-CHK1	PK
F01_cb9598_c2/f1p1/2036-0F	CAMK_CAMKL-CHK1	PK
F01_cb9598_c3/f1p0/2266-2F	CAMK_CAMKL-CHK1	PK
F01_cb9598_c5/f1p0/1480-2F	CAMK_CAMKL-CHK1	PK
F01_cb9598_c6/f1p0/1428-1F	CAMK_CAMKL-CHK1	PK
F01_cb961_c0/f1p0/4551-2F	RLK-Pelle_LRR-Xb-1	PK
F01_cb961_c2/f1p0/4748-0F	RLK-Pelle_LRR-Xb-1	PK
F01_cb961_c3/f1p0/4441-0F	RLK-Pelle_LRR-Xb-1	PK
F01_cb961_c3/f1p0/4441-1F	RLK-Pelle_LRR-Xb-1	PK
F01_cb961_c4/f1p0/4404-0F	RLK-Pelle_LRR-Xb-1	PK
F01_cb961_c4/f1p0/4404-1F	RLK-Pelle_LRR-Xb-1	PK
F01_cb9667_c0/f3p0/1941-0F	Group-Pl-4	PK
F01_cb9667_c2/f1p0/2017-2F	Group-Pl-4	PK
F01_cb9667_c4/f1p0/1542-0F	Group-Pl-4	PK
F01_cb9667_c5/f7p0/1958-1F	Group-Pl-4	PK
F01_cb9701_c1/f1p0/2067-1F	CAMK_CDPK	PK
F01_cb9701_c3/f1p0/1897-0F	CAMK_CDPK	PK
F01_cb9701_c3/f1p0/1897-2F	CAMK_CDPK	PK

F01_cb9709_c3/f1p0/2084-1F	RLK-Pelle_RLCK-V	PK
F01_cb9709_c5/f1p0/1785-0F	RLK-Pelle_RLCK-V	PK
F01_cb9755_c0/f1p0/1983-0F	CMGC_CDK-CRK7-CDK9	PK
F01_cb9755_c2/f1p0/1869-0F	CMGC_CDK-CRK7-CDK9	PK
F01_cb9764_c1/f1p0/2030-0F	AGC_RSK-2	PK
F01_cb9764_c2/f1p0/1862-1F	AGC_RSK-2	PK
F01_cb9804_c2/f3p1/1971-0F	CMGC_MAPK	PK
F01_cb9804_c3/f1p1/3028-1F	CMGC_MAPK	PK
F01_cb9804_c5/f1p0/1884-0F	CMGC_MAPK	PK
F01_cb9804_c6/f1p1/1792-0F	CMGC_MAPK	PK
F01_cb9804_c7/f1p1/1683-0F	CMGC_MAPK	PK
F01_cb9809_c0/f1p0/1980-1F	TKL-Pl-5	PK
F01_cb9809_c1/f1p0/2020-2F	TKL-Pl-5	PK
F01_cb9809_c3/f1p1/1875-0F	TKL-Pl-5	PK
F01_cb9809_c3/f1p1/1875-1F	TKL-Pl-5	PK
F01_cb9809_c4/f1p1/1875-0F	TKL-Pl-5	PK
F01_cb9809_c4/f1p1/1875-1F	TKL-Pl-5	PK
F01_cb9809_c4/f1p1/1875-2F	TKL-Pl-5	PK
F01_cb9809_c5/f1p1/1862-0F	TKL-Pl-5	PK
F01_cb9809_c7/f1p0/1782-1F	TKL-Pl-5	PK
F01_cb9831_c3/f1p0/1857-1F	RLK-Pelle_RLCK-VI	PK
F01_cb9831_c5/f4p1/1950-1F	RLK-Pelle_RLCK-VI	PK
F01_cb9884_c0/f1p0/1938-2F	RLK-Pelle_RLCK-VI	PK
F01_cb9884_c1/f1p0/2114-0F	RLK-Pelle_RLCK-VI	PK
F01_cb9905_c0/f2p1/1924-2F	RLK-Pelle_LysM	PK
F01_cb9905_c3/f1p0/1859-2F	RLK-Pelle_LysM	PK

F01_cb9905_c4/f1p0/1859-2F	RLK-Pelle_LysM	PK
F01_cb9920_c0/f1p0/1922-0F	RLK-Pelle_RLCK-Os	PK
F01_cb9980_c2/f1p0/2014-0F	CMGC_CDK-CDK7	PK
F01_cb9980_c3/f1p0/1858-1F	CMGC_CDK-CDK7	PK
F01_cb9980_c6/f1p0/1501-1F	CMGC_CDK-CDK7	PK
F01_cb9994_c16/f2p2/1976-0F	RLK-Pelle_RLCK-XII-1	PK
F01_cb9994_c17/f1p0/1879-0F	RLK-Pelle_RLCK-XII-1	PK
F01_cb9994_c18/f1p0/1919-0F	RLK-Pelle_RLCK-XII-1	PK
F01_cb9994_c18/f1p0/1919-1F	RLK-Pelle_RLCK-XII-1	PK
F01_cb9994_c20/f1p1/2821-0F	RLK-Pelle_RLCK-XII-1	PK
F01_cb9994_c20/f1p1/2821-2F	RLK-Pelle_RLCK-XII-1	PK
F01_cb9994_c24/f1p0/4896-1F	RLK-Pelle_RLCK-XII-1	PK
F01_cb9994_c34/f1p0/2587-0F	RLK-Pelle_RLCK-XII-1	PK
F01_cb9994_c34/f1p0/2587-1F	RLK-Pelle_RLCK-XII-1	PK
F01_cb9994_c37/f1p1/1933-2F	RLK-Pelle_RLCK-XII-1	PK
F01_cb9994_c44/f1p0/2263-0F	RLK-Pelle_RLCK-XII-1	PK
F01_cb9994_c45/f1p0/2003-1F	RLK-Pelle_RLCK-XII-1	PK
F01_cb9994_c47/f1p1/2004-2F	RLK-Pelle_RLCK-XII-1	PK
F01_cb9994_c49/f1p0/2031-2F	RLK-Pelle_RLCK-XII-1	PK
F01_cb9994_c53/f1p0/1698-1F	RLK-Pelle_RLCK-XII-1	PK
F01_cb9994_c54/f1p0/1505-1F	RLK-Pelle_RLCK-XII-1	PK
F01_cb9994_c56/f41p2/2058-0F	RLK-Pelle_RLCK-XII-1	PK
F01_cb9994_c57/f4p0/1785-0F	RLK-Pelle_RLCK-XII-1	PK
F01_cb9994_c60/f1p1/1928-0F	RLK-Pelle_RLCK-XII-1	PK
F01_cb99_c10/f1p0/4693-1F	TKL-Pl-6	PK
F01_cb99_c11/f1p1/3412-1F	TKL-Pl-6	PK

F01_cb99_c12/f1p0/3017-0F	TKL-Pl-6	PK
F01_cb99_c12/f1p0/3017-2F	TKL-Pl-6	PK
F01_cb99_c13/f1p0/2256-1F	TKL-Pl-6	PK
F01_cb99_c3/f1p1/4977-1F	TKL-Pl-6	PK
F01_cb99_c4/f1p1/4938-1F	TKL-Pl-6	PK
F01_cb99_c6/f1p0/4955-1F	TKL-Pl-6	PK
F01_cb99_c7/f1p0/3479-0F	TKL-Pl-6	PK
F01_cb99_c7/f1p0/3479-1F	TKL-Pl-6	PK
F01_cb99_c8/f1p1/4787-2F	TKL-Pl-6	PK
