# Supplementary material for: Single-molecule long-read sequencing of the full-length transcriptome of Rhododendron lapponicum L
Source: Sci Rep. 2020 Apr 21;10:6755. doi: 10.1038/s41598-020-63814-x (PMC7174332; doi:10.1038/s41598-020-63814-x)
Supplement: Supplementary file 5 — Supplementary Table S5. [file 41598_2020_63814_MOESM5_ESM.pdf]

Single-molecule long-read sequencing of the full-length transcriptome of *Rhododendron lapponicum* L.

Xinping Jia, Ling Tang, Xueying Mei, Huazhou Liu, Hairong Luo, Yanming Deng, Jiale Su

Institute of Leisure Agriculture, Jiangsu Academy of Agricultural Sciences, Jiangsu Key Laboratory for Horticultural Crop Genetic Improvement, Nanjing 210014, China

Table S5 Summary of the transcripts annotated to the reference canonical pathways in the KEGG database

Pathway	Pathway_ID	Gene_Number	KOs
Glycolysis / Gluconeogenesis	ko00010	740	K00161+K00161+K00161+K00161+K00927+K00927+K00927+K01623+K01623+K01623+K01623+K01623+K01623+K01623+K01623+K01792+K01792+K01792+K00121+K00121+K00121+K00121+K00121+K00121+K00121+K01803+K01803+K01803+K01803+K01803+K01803+K01803+K01803+K00121+K00121+K01792+K15634+K15634+K01689+K01623+K01623+K01623+K01623+K01623+K01623+K01623+K01623+K01623+K01623+K01623+K01623+K01623+K01623+K01623+K01835+K00128+K00927+K00927+K00927+K00927+K00927+K00927+K00927+K00162+K00162+K00162+K00162+K00162+K00162+K00162+K00162+K00162+K01623+K01623+K01623+K01623+K01623+K01623+K01623+K01623+K01623+K01623+K01623+K01623+K01623+K01792+K01792+K01792+K01792+K01792+K00161+K00873+K01803+K01803+K01803+K00121+K00121+K00131+K00131+K00131+K18857+K03841+K03841+K14085+K14085+K14085+K14085+K14085+K14085+K14085+K14085+K14085+K14085+K14085+K14085+K14085+K14085+K14085+K14085+K01568+K01568+K01568+K01568+K01568+K01568+K00161+K00161+K01834+K01834+K01834+K01834+K00001+K00001+K00001+K01785+K01785+K01785+K01785+K01785+K01785+K00850+K01810+K01810+K00121+K00121+K00121+K00121+K00121+K01623+K01623+K01568+K01568+K01568+K01568+K01568+K01568+K00134+K01689+K01689+K01689+K01689+K01689+K01689+K00134+K18857+K18857+K18857+K00134+K00134+K01689+K00134+K00134+K00134+K01792+K01792+K00627+K00627+K01803+K01803+K01803+K15634+K01803+K01803+K01803+K01803+K01803+K01803+K01803+K01803+K01803+K01803+K01803+K01803+K01803+K00121+K00121+K00121+K00121+K00121+K00121+K00121+K00121+K00121+K00121+K00121+K00121+K00121+K00128+K00128+K00128+K00128+K00128+K00128+K00128+K00128+K00128+K00128+K00128+K00134+K00134+K00134+K00134+K00134+K00134+K00134+K00134+K00134+K00134+K01835+K01835+K01835+K01835+K01835+K01835+K01835+K01835+K01623+K01623+K01623+K01623+K00873+K00873+K00873+K01785+K01785+K01785+K01785+K01785+K01785+K01785+K00128+K00128+K00128+K00128+K00128+K00128+K00128+K00128+K00128+K00128+K00128+K00128+K00128+K00128+K00128+K00873+K00873+K00873+K00873+K01792+K01792+K01792+K01792+K01792+K01792+K00873+K00873+K00873+K00873+K00873+K00873+K00873+K00873+K00873+K00873+K01689+K01689+K01689+K01689+K01689+K00001+K00001+K00001+K00001+K00001+K00001+K00873+K00873+K00873+K00873+K01568+K01568+K01568+K01568+K01568+K01568+K01568+K01568+K01568+K01568+K01568+K00382+K00382+K00382+K00382+K00382+K00382+K00382+K00382+K00382+K00382+K00927+K00927+

	K00927+K00927+K15633+K15633+K15633+K15633+K15633+K15633+K15633+K15633+K15633+K15633+K15633+K15633+K15633+K15633+K15633+K15633+K15633+K15633+K15633+K00121+K00121+K00121+K00121+K00121+K03841+K03841+K03841+K03841+K03841+K03841+K03841+K00850+K00850+K00850+K00850+K00850+K00850+K00850+K00850+K00850+K00850+K03103+K03103+K03103+K03103+K03103+K03103+K03103+K03103+K03103+K03103+K03103+K03103+K03103+K03103+K03103+K03103+K03103+K03103+K00128+K00128+K00128+K00128+K00128+K00128+K00128+K00128+K00128+K00128+K00128+K00128+K00128+K00128+K00128+K00382+K00382+K00382+K00382+K01810+K00627+K00873+K00873+K00873+K01835+K00134+K01623+K00128+K00873+K01810+K00627+K00001+K00134+K01810+K00134+K01835+K00844+K00873+K01610+K01610+K00134+K00844+K00134+K00128+K01610+K00850+K01610+K01610+K01623+K01610+K00850+K01610+K01610+K00873+K01610+K01610+K00627+K00873+K01810+K01623+K00873+K01835+K00134+K01810+K01623+K00134+K00873+K00134+K18857+K00134+K00134+K00873+K00873+K00844+K01835+K00134+K00850+K00873+K00134+K00844+K01810+K01810+K01810+K01810+K01835+K01810+K01810+K01810+K00873+K00873+K00844+K01610+K00844+K00873+K01810+K00873+K01568+K01835+K01895+K00873+K01810+K00627+K01895+K01810+K01835+K00128+K00873+K00128+K01835+K01835+K01835+K01835+K01835+K01835+K01835+K01835+K01835+K01835+K01835+K01835+K01835+K01835+K01835+K01835+K00128+K00850+K00627+K00627+K01810+K01568+K00627+K01568+K00844+K00844+K00844+K00844+K00844+K00844+K00844+K00844+K00844+K00844+K00844+K00844+K00844+K00844+K00134+K00627+K00627+K00627+K01623+K01623+K01568+K00850+K00001+K01610+K00128+K01568+K00850+K01568+K01835+K01835+K01610+K01835+K01810+K01895+K00873+K18857+K00627+K01895+K00844+K00627+K00627+K01810+K00873+K01810+K01810+K00844+K01895+K01895+K01810+K00873+K18857+K01895+K00627+K00627+K01810+K01895+K01810+K01810+K00134+K01803+K18857+K00627+K00844+K00873+K18857+K01610+K18857+K00627+K18857+K18857+K01810+K00873+K00844+K00627+K00873+K01568+K00873+K01689+K01689+K01689+K01689+K00873+K00873+K00850+K00850+K00850+K00850+K00850+K00850+K00850+K00850+K00850+K00850+K00850+K00850+K00873+K00873+K00873+K00873+K00873+K00873+K00873+K00128+K00128+K00128+K03841+K03841+K03841+K03841+K03841+K03841+K03841+K00850+K01689+K01689+K01689+K01689+K00121+K00121+K00121+K00121+K00121+K00121+K00121+K00121+K00121+K00627+K00627+K00627+K00627+K00627+K00627+K00627+K00627+K00627+K00627+K00627+K00850+K00850+K00850+K18857+K18857+K18857+K18857+K18857+K18857+K18857+K18857+K18857+K18857+K00001+K18857+K18857+K18857+K00927+K00927+K00927+K00927+K00162+K00162+K00162

Citrate cycle (TCA cycle)	ko00020	318	K00161+K00161+K00161+K00161+K00236+K00235+K00235+K00235+K00235+K00235+K00235+K00030+K00030+K00030+K00030+K00030+K00030+K00030+K00030+K00030+K00025+K00025+K00025+K00025+K00025+K00025+K00025+K00025+K00025+K00025+K00025+K00031+K00031+K00031+K00031+K00031+K00031+K00031+K00031+K01648+K01648+K01648+K01648+K01648+K01648+K01648+K01648+K01648+K00026+K00026+K00026+K00026+K00026+K00026+K00026+K00026+K00026+K00162+K00162+K00162+K00162+K00162+K00162+K00162+K00162+K00162+K00161+K00025+K00026+K00026+K00026+K01679+K00161+K00161+K00025+K00025+K00026+K00026+K00026+K00026+K00026+K01648+K00030+K00030+K01899+K01899+K01899+K01899+K00627+K00627+K01647+K01648+K01648+K00026+K00026+K00026+K00382+K00382+K00382+K00382+K00382+K00382+K00382+K00382+K00382+K00382+K01647+K01647+K01647+K01647+K01647+K01647+K01647+K00382+K00382+K00382+K00382+K00030+K00030+K00030+K00627+K01648+K01648+K01648+K00234+K01648+K00627+K00031+K01648+K01610+K01610+K01648+K01648+K00030+K01610+K00031+K01610+K01610+K01610+K01610+K01610+K01610+K01610+K00627+K01681+K01648+K01648+K00234+K01648+K01679+K01648+K01648+K01648+K01681+K00164+K01610+K01681+K00164+K00234+K00164+K01648+K00234+K00234+K01681+K00030+K00030+K00627+K01679+K01679+K01679+K01679+K01679+K01679+K01679+K01679+K01679+K01679+K00234+K00627+K00234+K00234+K00627+K00627+K00627+K01648+K00627+K00627+K01648+K01679+K01899+K01610+K00031+K01610+K00234+K00164+K01681+K00164+K00164+K00164+K00164+K01681+K01648+K00627+K01681+K00164+K01648+K01681+K00164+K01681+K00627+K00627+K01648+K00164+K00164+K00164+K01681+K00164+K01681+K01681+K01681+K00164+K00164+K01681+K00164+K01681+K00627+K01681+K01681+K00627+K01681+K01681+K00164+K01681+K00164+K01648+K00031+K01681+K01681+K00164+K01648+K01681+K01679+K00164+K01681+K01681+K01648+K00164+K00627+K01679+K00164+K01681+K01681+K01610+K01681+K00164+K01681+K00627+K01681+K00164+K00164+K01681+K01681+K01681+K01681+K01681+K00627+K01681+K00031+K00164+K01648+K01648+K01648+K01648+K01648+K01648+K01648+K01648+K01648+K01648+K00658+K00658+K00658+K00658+K00658+K00658+K00658+K00658+K01900+K01900+K01900+K01900+K01900+K01900+K00627+K00627+K00627+K00627+K00627+K00627+K00627+K00627+K00627+K00627+K00627+K00031+K00031+K00162+K00162+K00162+K01647+K01647+K01647+K01647+K01647+K01647+K01647+K01647+K01647
Pentose phosphate pathway	ko00030	298	K01623+K01623+K01623+K01623+K01623+K01623+K01623+K01623+K00616+K00616+K00616+K00616+K00616+K00616+K00616+K00616+K00033+K01057+K01057+K01807+K01807+K01807+K01623+K01623+K01623+K01623+K01623+K01623+K01623+K01623+K01623+K01623+K01623+K01623+K01623+K01623+K01623+K01835+K01057+K01623+K01623+K01623+K01623+K01623+K01623+K01623+K01623+K01623+K01623+K01623+K01623+K01623+K00616+K00616+K01057+K01057+K00033+K00033+K00033+

			K00033+K00033+K00033+K00033+K01057+K01057+K01057+K01057+K01057+K01783+K01783+K01783+K00131+K00131+K00131+K00851+K00851+K00852+K03841+K03841+K01807+K01807+K01807+K00036+K00850+K01057+K01057+K01057+K01810+K01810+K00616+K01623+K01623+K01057+K01057+K01807+K01783+K01783+K01783+K01783+K00948+K00948+K00948+K00948+K00948+K00036+K01835+K01835+K01835+K01835+K01835+K01835+K01835+K01835+K01623+K01623+K01623+K01623+K01057+K01057+K01057+K01057+K01057+K00615+K00615+K00615+K00615+K00615+K00615+K00615+K00615+K00615+K00615+K00615+K00615+K00615+K00615+K00615+K00615+K00615+K00615+K00615+K00036+K00036+K00036+K00036+K00036+K00036+K00036+K00036+K00036+K01783+K01783+K01783+K03841+K03841+K03841+K03841+K03841+K03841+K03841+K00850+K00850+K00850+K00850+K00850+K00850+K00850+K00850+K00850+K00850+K00948+K00948+K00036+K00036+K00036+K00036+K00036+K00036+K01810+K01835+K01623+K00036+K00036+K01810+K01810+K01835+K00850+K01623+K00850+K01810+K01623+K00036+K01835+K01810+K01623+K01835+K00850+K01810+K01810+K01810+K01810+K01835+K01810+K01810+K01810+K01810+K01835+K01810+K00036+K01810+K00036+K01835+K01835+K01835+K01835+K01835+K01835+K01835+K01835+K01835+K01835+K01835+K01835+K01835+K01835+K01835+K01835+K01835+K00850+K01810+K00036+K00036+K00036+K00036+K01623+K01623+K00850+K00850+K01835+K01835+K01835+K01810+K01810+K01810+K01810+K01810+K01810+K01810+K01810+K01810+K00036+K00036+K00850+K00850+K00850+K00850+K00850+K00850+K00850+K00850+K00850+K00850+K00850+K00850+K03841+K03841+K03841+K03841+K03841+K03841+K03841+K00850+K00033+K00033+K00033+K00850+K00850+K00850
Pentose and glucuronate interconversions	ko00040	182	K01051+K01051+K01051+K00008+K00008+K00008+K00008+K00963+K00963+K00963+K00963+K01805+K00128+K00008+K00008+K00008+K00011+K00011+K00011+K01783+K01783+K01783+K16190+K16190+K01728+K01728+K01728+K01728+K01728+K00008+K01051+K01051+K01783+K01783+K01783+K01783+K00128+K00128+K00128+K00128+K00128+K00128+K00128+K00128+K00128+K00128+K00128+K12447+K12447+K12447+K12447+K00012+K00012+K00012+K00012+K00012+K00012+K00012+K00128+K00128+K00128+K00128+K00128+K00128+K00128+K00128+K00128+K00128+K00128+K00128+K00128+K00128+K00128+K01728+K01728+K01728+K01728+K01728+K01728+K01728+K01728+K00963+K00963+K00963+K00963+K00963+K01051+K01051+K00854+K00854+K00854+K00854+K01051+K01051+K01051+K01051+K01728+K01728+K01728+K01728+K01728+K00012+K01783+K01783+K01783+K01728+K01728+K01728+K01728+K01728+K01728+K01728+K01728+K00128+K00128+K00128+K00128+K00128+K00128+K00128+K00128+K00128+K00128+K00128+K00128+K00128+K00128+K00128+K00128+K00128+K00128+K00128+K01051+K01051+K01051+K01051+K01051+K01051+K01051+K01051+K01051+K01051+K01051+K01051+K01051+K00128+K00128+K00128+K00128+K00128+K00128+K01051+K01051+

			K01051+K01051+K00128+K00128+K00128+K01051+K01051+K00012+K00012+K00012+K00012+K00012+K00012+K00012+K01051+K01051+K01051+K01051+K01051+K01805+K01805+K01805+K01805+K01805+K01805+K01805+K01805+K01051+K00963+K00963+K00963+K00963
Fructose and mannose metabolism	ko00051	278	K01809+K01809+K01623+K01623+K01623+K01623+K01623+K01623+K01623+K01623+K05305+K05305+K05305+K05305+K05305+K05305+K05305+K05305+K05305+K05305+K05305+K05305+K00847+K00847+K00847+K00847+K01803+K01803+K01803+K01803+K01803+K01803+K01803+K01803+K00008+K00008+K00008+K00008+K01809+K01809+K00966+K01805+K01623+K01623+K01623+K01623+K01623+K01623+K01623+K01623+K01623+K01623+K01623+K01623+K01623+K01623+K01623+K02377+K02377+K02377+K00008+K00008+K00008+K01623+K01623+K01623+K01623+K01623+K01623+K01623+K01623+K01623+K01623+K01623+K01623+K01623+K00011+K00011+K00011+K00847+K00847+K00847+K00847+K00847+K01803+K01803+K01803+K00966+K00966+K00966+K00966+K03841+K03841+K00966+K00966+K00966+K00966+K00966+K00966+K18447+K18447+K00850+K01809+K01809+K17497+K17497+K17497+K17497+K01623+K01623+K01809+K00008+K00847+K00847+K00847+K00847+K00847+K01803+K01803+K01803+K01803+K01803+K01803+K01803+K01803+K01803+K01803+K01803+K01803+K01803+K01803+K01803+K01803+K00966+K00966+K00966+K00966+K00966+K00966+K00966+K01623+K01623+K01623+K01623+K00847+K00847+K00847+K00847+K00847+K00847+K00847+K00847+K00895+K00895+K00895+K00895+K00895+K00895+K00895+K00895+K00895+K01103+K01103+K01103+K01103+K01103+K01103+K01103+K00966+K00966+K00966+K03841+K03841+K03841+K03841+K03841+K03841+K03841+K00850+K00850+K00850+K00850+K00850+K00850+K00850+K00850+K00850+K00850+K01623+K00844+K00844+K00850+K01623+K00850+K01623+K01623+K00844+K00850+K00844+K00844+K00844+K00850+K01809+K00844+K00844+K00844+K00844+K00844+K00844+K00844+K00844+K00844+K00844+K00844+K00844+K00844+K00844+K01623+K01623+K00850+K00850+K00844+K00844+K01803+K00844+K00844+K00850+K00850+K00850+K00850+K00850+K00850+K00850+K00850+K00850+K00850+K00850+K00850+K03841+K03841+K03841+K03841+K03841+K03841+K03841+K00850+K00895+K00895+K00895+K00895+K00895+K00895+K00895+K00895+K00895+K00895+K00895+K00895+K00847+K00847+K00847+K17497+K17497+K17497+K17497+K00966+K00966+K00850+K00850+K00850+K01805+K01805+K01805+K01805+K01805+K01805+K01805+K01805
Galactose metabolism	ko00052	393	K01784+K01784+K01784+K01784+K07407+K07407+K00963+K00963+K00963+K00963+K07407+K01835+K00965+K18819+K18819+K18819+K18819+K18819+K18819+K18819+K18819+K18819+K06617+K06617+K06617+K06617+K06617+K06617+K06617+K00011+K00011+K00011+K01190+K01190+K01190+K01190+K01190+K01190+K01190+K01190+K12309+K07407+K07407+K07407+K01784+K01784+K01187+K01785+K01785+K01785+K01785+K01785+K01785+K00850+K18819+K12447+K12447+K12447+K12447+K06617+

			K06617+K06617+K06617+K06617+K06617+K01835+K01835+K01835+K01835+K01835+K01835+K01835+K01835+K01193+K01193+<br>K01193+K01193+K01193+K01193+K01193+K01193+K01193+K01193+K01193+K01785+K01785+K01785+K01785+K01785+K01785+<br>K01785+K06617+K06617+K06617+K01187+K01187+K06617+K00963+K00963+K00963+K00963+K00963+K06617+K06617+K06617+<br>K06617+K12309+K12309+K12309+K12309+K12309+K12309+K12309+K12309+K12309+K12309+K12309+K12309+K12309+K12309+<br>K06617+K06617+K01193+K01193+K01193+K01193+K01193+K01193+K01193+K01193+K01193+K00850+K00850+K00850+K00850+<br>K00850+K00850+K00850+K00850+K00850+K00850+K07407+K07407+K01835+K06617+K06617+K01187+K01835+K00844+K06617+<br>K00844+K00850+K00850+K01835+K00844+K01835+K00850+K06617+K00844+K01835+K01187+K07407+K00844+K00844+K01835+<br>K01187+K06617+K06617+K06617+K06617+K01835+K06617+K06617+K06617+K06617+K06617+K01187+K01835+K01835+K01835+<br>K01835+K01835+K01835+K01835+K01835+K01835+K01835+K01835+K01835+K01835+K01835+K01835+K01835+K01187+K00850+<br>K01187+K00844+K00844+K00844+K00844+K00844+K00844+K00844+K00844+K00844+K00844+K00844+K00844+K00844+K00844+<br>K01187+K06617+K06617+K06617+K06617+K06617+K06617+K06617+K06617+K06617+K06617+K06617+K06617+K06617+K06617+<br>K06617+K06617+K06617+K06617+K06617+K06617+K06617+K06617+K06617+K06617+K06617+K06617+K06617+K06617+K06617+<br>K06617+K06617+K06617+K06617+K06617+K06617+K06617+K06617+K00850+K06617+K06617+K06617+K06617+K06617+K06617+<br>K06617+K06617+K06617+K06617+K06617+K06617+K06617+K06617+K06617+K06617+K06617+K06617+K06617+K06617+K06617+<br>K06617+K06617+K06617+K06617+K06617+K06617+K06617+K06617+K06617+K06617+K06617+K06617+K06617+K06617+K06617+<br>K06617+K06617+K06617+K06617+K06617+K06617+K06617+K06617+K06617+K06617+K06617+K06617+K06617+K06617+K06617+<br>K06617+K06617+K06617+K06617+K06617+K06617+K06617+K06617+K06617+K06617+K06617+K06617+K06617+K06617+K06617+<br>K06617+K06617+K06617+K06617+K06617+K06617+K06617+K06617+K06617+K06617+K06617+K06617+K06617+K06617+K06617+<br>K06617+K00850+K07407+K06617+K06617+K06617+K06617+K06617+K06617+K01835+K06617+K01835+K01835+K06617+K06617+<br>K06617+K00844+K06617+K06617+K00844+K06617+K06617+K06617+K06617+K06617+K06617+K06617+K06617+K00844+K06617+<br>K06617+K01187+K00844+K06617+K01187+K00850+K00850+K00850+K00850+K00850+K00850+K00850+K00850+K00850+K00850+<br>K00850+K00850+K00850+K01193+K01193+K01193+K01193+K07407+K07407+K07407+K07407+K00850+K00850+K00850+K00963+<br>K00963+K00963+K00963
Ascorbate and aldarate metabolism	ko00053	221	K00434+K00434+K17744+K17744+K10046+K10046+K10046+K10046+K10046+K08232+K08232+K08232+K08232+K08232+K00128+<br>K00434+K00434+K00434+K00434+K00434+K00434+K00434+K00434+K00434+K00434+K00434+K00434+K00434+K00434+K00434+<br>K00434+K00434+K00434+K00434+K00434+K00434+K00434+K00434+K00434+K00434+K00434+K00434+K00434+K00434+K00434+

			K00434+K00434+K00434+K00469+K00469+K00469+K00469+K00469+K00469+K14190+K14190+K16190+K16190+K00434+K17744+K17744+K14085+K14085+K14085+K14085+K14085+K14085+K14085+K14085+K14085+K14085+K14085+K14085+K14085+K14085+K14085+K00434+K00434+K00128+K00128+K00128+K00128+K00128+K00128+K00128+K00128+K00128+K00128+K00128+K00434+K00434+K00434+K00434+K00434+K00434+K12447+K12447+K12447+K12447+K00012+K00012+K00012+K00012+K00012+K00012+K00012+K00012+K00128+K00128+K00128+K00128+K00128+K00128+K00128+K00128+K00128+K00128+K00128+K00128+K00128+K00128+K00128+K00128+K14190+K14190+K14190+K14190+K14190+K00434+K00434+K00434+K08232+K08232+K08232+K08232+K08232+K08232+K08232+K08232+K08232+K08232+K00012+K00128+K00128+K00128+K00128+K00128+K00128+K00128+K00128+K00128+K00128+K08232+K08232+K08232+K08232+K08232+K08232+K08232+K08232+K08232+K08232+K00128+K00128+K00128+K00128+K00128+K00469+K00128+K14190+K00434+K00434+K00128+K08232+K14190+K14190+K14190+K14190+K14190+K14190+K14190+K00469+K00469+K14190+K00128+K00225+K00128+K00128+K00225+K00225+K14190+K14190+K00128+K00225+K00434+K00434+K00423+K00423+K00423+K00128+K00128+K00128+K00434+K00434+K00434+K00434+K00434+K10046+K10046+K10046+K10046+K10046+K10046+K10046+K00012+K00012+K00012+K00012+K00012+K00012+K00012
Fatty acid biosynthesis	ko00061	212	K01897+K00059+K00059+K00059+K00059+K00059+K02160+K02160+K02160+K02160+K09458+K00208+K00208+K00208+K00208+K03921+K03921+K03921+K03921+K03921+K03921+K03921+K10781+K10781+K11262+K11262+K11262+K11262+K11262+K00059+K00059+K02372+K02372+K00208+K00208+K00208+K01963+K10782+K10782+K03921+K01962+K01962+K01962+K01962+K01962+K01962+K01962+K01962+K01962+K01962+K01962+K01962+K00059+K00059+K00059+K00059+K01897+K01897+K01897+K01897+K01897+K01897+K01897+K01897+K01897+K01897+K01897+K01897+K01897+K01962+K01962+K01962+K01962+K01962+K01897+K10782+K10782+K10782+K10782+K00645+K00645+K00645+K01897+K01897+K01897+K01897+K01897+K01897+K01897+K01897+K01897+K01897+K01897+K01897+K01897+K01963+K01897+K01897+K01897+K01897+K01897+K01897+K01897+K01897+K01897+K01897+K01897+K01897+K01897+K01897+K01897+K01897+K01897+K01897+K01897+K01963+K01963+K01963+K01963+K01963+K01963+K01897+K01897+K01897+K09458+K09458+K09458+K09458+K09458+K09458+K01961+K01961+K01961+K10781+K09458+K09458+K09458+K00648+K00648+

			K00648+K00648
Fatty acid elongation	ko00062	78	K10258+K10258+K01074+K01074+K01074+K01074+K01074+K07512+K07512+K07512+K07512+K15397+K10703+K15397+K15397+K15397+K15397+K15397+K15397+K15397+K15397+K15397+K15397+K15397+K15397+K10251+K10251+K10251+K10703+K10703+K15397+K15397+K15397+K15397+K10703+K10703+K01074+K15397+K15397+K15397+K15397+K15397+K15397+K15397+K15397+K15397+K15397+K01068+K01068+K01068+K01068+K01068+K15397+K01074+K01074+K01074+K01074
Fatty acid degradation	ko00071	354	K01897+K00121+K00121+K00121+K00121+K00121+K00121+K00121+K00121+K00121+K00128+K07513+K07513+K00232+K07513+K00121+K00121+K18857+K00626+K00626+K00626+K00626+K00626+K00626+K14085+K14085+K14085+K14085+K14085+K14085+K14085+K14085+K14085+K14085+K14085+K14085+K14085+K14085+K14085+K14085+K00001+K00001+K00001+K00121+K00121+K00121+K00121+K00121+K18857+K18857+K18857+K00232+K00249+K00249+K00249+K00249+K00249+K00249+K00249+K00249+K00249+K00249+K00121+K00121+K00121+K00121+K00121+K00121+K00121+K00121+K00121+K00121+K00121+K00121+K00121+K01897+K01897+K01897+K01897+K01897+K01897+K01897+K01897+K01897+K01897+K01897+K01897+K01897+K00128+K10527+K10527+K10527+K10527+K10527+K10527+K10527+K10527+K01897+K01897+K01897+K01897+K01897+K01897+K01897+K01897+K00001+K00001+K00001+K00001+K00001+K00001+K00232+K00232+K00232+K00232+K00232+K01897+K00232+K00232+K00232+K01897+K01897+K01897+K01897+K01897+K01897+K01897+K00121+K00121+K00121+K00121+K00121+K01897+K01897+K01897+K01897+K01897+K01897+K01897+K01897+K01897+K01897+K01897+K01897+K00128+K00128+K00128+K00128+K00128+K00128+K00128+K00128+K00128+K00128+K00128+K00128+K00128+K00128+K00128+K07513+K07513+K07513+K07513+K07513+K07513+K07513+K01897+K01897+K01897+K00232+K00128+K01897+K01897+K01897+K00001+K01897+K01897+K00128+K01897+K01897+K01897+K01897+K01897+K01897+K01897+K01897+K01897+K18857+K01897+K01897+K01897+K01897+K00249+K01897+K10527+K10527+K10527+K10527+K10527+K10527+K01897+K01897+K01897+K01897+K01897+K10527+

			K10527+K01897+K01897+K01897+K01897+K01897+K00128+K00128+K00128+K10527+K10527+K10527+K10527+K10527+K10527+K10527+K10527+K10527+K10527+K10527+K10527+K07513+K00001+K00128+K18857+K10527+K10527+K18857+K01897+K10527+K18857+K18857+K18857+K18857+K18857+K01897+K01897+K00128+K00128+K00128+K00121+K00121+K00121+K00121+K00121+K00121+K00121+K00121+K00121+K00626+K00626+K00626+K00626+K00626+K00626+K18857+K18857+K18857+K18857+K18857+K18857+K18857+K18857+K18857+K18857+K00001+K18857+K18857+K18857
Synthesis and degradation of ketone bodies	ko00072	28	K01640+K01640+K01640+K01640+K00626+K00626+K00626+K00626+K00626+K00626+K01641+K01641+K01641+K01641+K01641+K01641+K01640+K01640+K01640+K01640+K01640+K01640+K00626+K00626+K00626+K00626+K00626+K00626
Cutin, suberine and wax biosynthesis	ko00073	76	K13356+K13356+K13356+K13356+K13356+K13356+K13356+K13356+K13356+K13356+K13356+K13356+K13356+K13356+K15400+K15400+K17991+K17991+K17991+K15400+K13356+K15404+K15404+K15404+K15404+K15404+K15404+K15404+K15404+K15398+K15398+K15398+K15398+K15398+K15398+K15398+K13356+K15403+K15403+K15403+K15403+K15403+K15403+K15403+K15403+K15403+K15404+K15404+K15404+K15404+K15404+K15404+K15404+K15404+K13356+K13356+K13356+K13356+K13356+K13356+K13356+K13356+K13356+K13356+K13356+K13356+K13356+K13356+K13356+K13407+K13407+K13407+K13407+K15401+K15401+K15402
Steroid biosynthesis	ko00100	125	K00213+K00213+K00213+K00213+K00213+K00227+K00227+K00227+K00227+K00801+K00559+K00559+K00559+K00559+K00559+K00227+K08242+K08242+K08242+K08242+K14423+K14423+K14423+K00213+K00511+K00511+K05917+K05917+K05917+K00801+K00801+K00801+K00801+K00227+K00222+K00559+K01824+K01824+K01824+K01824+K01824+K00222+K00222+K00511+K00511+K00511+K00511+K00511+K00511+K00511+K00511+K00511+K00511+K00511+K00511+K00511+K00511+K00511+K08246+K08246+K01853+K01853+K14424+K14424+K14424+K14424+K09828+K09828+K09828+K09828+K09828+K09828+K09828+K09828+K09828+K09828+K09828+K09828+K09828+K09828+K09828+K00511+K00511+K00511+K00511+K00511+K00511+K00511+K00511+K00511+K01853+K01853+K01853+K01853+K01853+K01853+K01853+K01853+K01853+K01853+K01853+K07748+K07748+K07748+K07748+K14674+K14674+K14674+K14674+K14674+K14674+K14674+K14674+K14674+K14674+K14674+K05917+K05917+K05917+K08242+K05917+K07748+K07748+K07748+K07748
Ubiquinone and other terpenoid-quinone	ko00130	166	K00457+K00591+K00591+K00591+K00487+K00487+K06125+K12502+K12502+K09833+K09833+K09833+K12502+K12502+K12502+K12502+K12502+K12502+K00815+K05928+K05928+K06127+K06127+K06127+K01661+K01661+K00815+K00815+K00815+K00815+K00815+K00815+K02548+K02548+K02548+K02548+K02548+K02548+K02548+K02548+K02548+K02548+K02548+K02548+K02548+

biosynthesis			K02548+K02548+K02548+K02548+K02548+K02548+K01904+K01904+K01904+K01904+K06127+K06127+K06127+K00457+K09833+K09833+K09833+K09833+K09833+K14760+K14760+K14760+K14760+K14760+K02552+K02552+K02552+K01904+K01904+K01904+K01904+K01904+K01904+K01904+K01904+K01904+K01904+K01904+K12501+K12501+K00815+K00815+K00815+K00815+K00815+K06125+K00815+K00815+K00815+K00815+K01661+K00815+K00815+K00815+K01661+K00815+K00815+K00815+K01661+K01661+K01661+K01661+K01661+K06125+K06125+K06125+K06125+K06125+K06125+K03183+K03183+K09834+K09834+K09834+K01904+K01904+K01904+K01904+K01904+K06126+K06126+K06126+K06126+K06126+K01904+K01904+K01904+K01904+K01904+K00487+K00487+K00487+K00487+K00487+K14759+K14759
Oxidative phosphorylation	ko00190	787	K01507+K01507+K01507+K01507+K01507+K01507+K00236+K02115+K02115+K02115+K02115+K03879+K03879+K03965+K03965+K03965+K03965+K03965+K03965+K03965+K03966+K03966+K03966+K03966+K03966+K00235+K00235+K00235+K00235+K00235+K00235+K02135+K02135+K02135+K02135+K11352+K11352+K11352+K11352+K03946+K03946+K03946+K03946+K03946+K02148+K02148+K02148+K02148+K02134+K02134+K02134+K02134+K02134+K02134+K02134+K02134+K03952+K03952+K01507+K01507+K01507+K01507+K02109+K02262+K02109+K02262+K03937+K03937+K03937+K02155+K02155+K02155+K02153+K02153+K02153+K02153+K02257+K02257+K02147+K05575+K02113+K02140+K02140+K02140+K01507+K01507+K01507+K01507+K03939+K03939+K03939+K03939+K05582+K00416+K00416+K02155+K02155+K02266+K02266+K02266+K03934+K03934+K03934+K03934+K03934+K03934+K03934+K03934+K03934+K03934+K03934+K03934+K03934+K03934+K02109+K02109+K02109+K02109+K02109+K02109+K02109+K02109+K02146+K02146+K02146+K02146+K02146+K02155+K02155+K02155+K02155+K02155+K02155+K03943+K03943+K03943+K03943+K03955+K03955+K03955+K03955+K01507+K01507+K01507+K01507+K01507+K01507+K01507+K01507+K03940+K03940+K03940+K03940+K01507+K01507+K01507+K02154+K02154+K02154+K02154+K02154+K02154+K02154+K02154+K02154+K02154+K02154+K02138+K02138+K02138+K02138+K02138+K02138+K02138+K02138+K02138+K02138+K03661+K02112+K05580+K02267+K03941+K03941+K02152+K02152+K03955+K03955+K03952+K03952+K03953+K03953+K03953+K03953+K02265+K02265+K02265+K02265+K02265+K02265+K02111+K02111+K02111+K02110+K02108+K02111+K03938+K03938+K03938+K03938+K02151+K02151+K02151+K02151+K02151+K02151+K03949+K03949+K03949+K05579+K05579+K02267+K02267+K02267+K02152+K02152+K02152+K02152+K02152+K02155+K02155+K02267+K02267+K02112+K00419+K00419+K02140+K02140+K02140+K00417+K00417+K00417+K00417+K00417+K00417+K00417+K00417+K00417+K00417+K11352+K02258+K02258+K02258+K02258+K02258+K02150+

	K02150+K02150+K02150+K02150+K02150+K02150+K03952+K03952+K03952+K11353+K11353+K11353+K11353+K01535+K03949+<br>K03661+K03661+K02154+K00413+K00413+K00413+K00413+K02153+K02153+K02153+K02153+K02153+K02257+K01507+K02115+<br>K02115+K01535+K03963+K03963+K03963+K03963+K03963+K01535+K03950+K03950+K02259+K02259+K01507+K01507+K01507+<br>K01507+K01507+K01507+K01507+K01507+K01507+K01507+K01507+K03955+K02149+K00411+K00411+K00411+K00411+K00411+<br>K01535+K01535+K02154+K01507+K01535+K00412+K00412+K00412+K00412+K01507+K05581+K05581+K05581+K05582+K05574+<br>K01535+K01535+K03935+K03935+K03935+K03945+K03945+K03945+K03945+K03945+K03945+K03945+K03945+K00411+K00411+<br>K00411+K00411+K02144+K02144+K02144+K02144+K02144+K02144+K03950+K03950+K03950+K03950+K03879+K02148+K02148+<br>K02133+K02133+K02133+K02133+K02133+K02133+K02133+K02133+K02133+K02133+K02133+K02133+K02133+K02133+K03942+<br>K03942+K03942+K03942+K03942+K03942+K03942+K03942+K03942+K03942+K03942+K03942+K02155+K00419+K00419+K00419+<br>K05573+K05573+K02145+K02145+K02145+K02145+K02145+K02145+K02145+K02145+K02145+K02145+K02137+K02137+K02137+<br>K02137+K02137+K01535+K01535+K01535+K01535+K02149+K02147+K02147+K02147+K02147+K02147+K02147+K02147+K02147+<br>K02147+K02147+K02147+K02147+K02147+K02147+K03883+K03883+K02128+K01535+K02136+K02136+K02136+K02136+K02136+<br>K02136+K02136+K02136+K01507+K01535+K01507+K01535+K01507+K01535+K01535+K02111+K01535+K01507+K01535+K01535+<br>K01507+K01507+K00234+K01535+K02112+K02112+K02154+K02154+K01535+K02154+K02154+K01507+K01507+K01507+K02112+<br>K02111+K02154+K01507+K01507+K01507+K01507+K01507+K02111+K01507+K01507+K01507+K01507+K02111+K02112+K05572+<br>K01507+K02138+K01507+K02154+K01507+K01507+K01507+K01535+K02138+K02154+K00234+K05575+K01507+K02154+K02111+<br>K01507+K01535+K01507+K01507+K01507+K05579+K01535+K01507+K01535+K01507+K01507+K01507+K01535+K01507+K01535+<br>K01535+K02154+K01507+K01507+K01507+K01507+K00234+K01507+K01507+K01507+K01507+K01535+K01535+K01507+K01535+<br>K01507+K01507+K01507+K01507+K05579+K00234+K02112+K02112+K01535+K00234+K01507+K01507+K01507+K01535+K01507+<br>K01535+K01507+K02111+K02132+K01507+K01535+K01535+K02111+K01507+K01507+K01507+K01507+K02154+K01507+K02112+<br>K01507+K01507+K01535+K01507+K01507+K01535+K01535+K01507+K01507+K01535+K02112+K01507+K01507+K01507+K01507+<br>K02154+K01507+K01507+K01507+K01507+K01507+K01507+K01507+K02111+K01507+K01535+K01507+K01507+K01507+K01507+<br>K02132+K01535+K00234+K02110+K00234+K02111+K01507+K00234+K01507+K01507+K01507+K01507+K01535+K01507+K01507+<br>K01535+K01535+K01535+K01507+K02112+K01507+K01507+K01507+K01535+K01535+K01507+K01507+K01507+K01507+K02110+<br>K01507+K01535+K05579+K05579+K01535+K01507+K01507+K01507+K01507+K01507+K01535+K01507+K01507+K01507+K01507+<br>K01507+K01507+K02112+K01507+K01507+K01535+K01535+K01507+K01535+K01535+K01535+K01535+K01535+K01507+K01507+

			K01507+K01507+K01507+K02132+K01507+K01507+K01507+K01535+K01507+K01507+K01507+K02112+K02112+K02112+K01507+K01507+K02112+K02112+K02112+K02112+K01535+K01507+K01507+K01535+K01535+K01535+K01535+K01507+K01507+K01535+K01507+K01507+K01507+K01507+K01507+K01507+K01535+K01507+K01535+K01535+K01535+K01535+K01535+K01507+K01507+K01507+K01535+K01507+K01507+K01535+K01507+K01535+K01507+K01507+K01507+K01507+K01507+K01507+K01535+K01507+K01507+K01535+K01507+K01507+K01507+K01507+K01507+K01507+K01507+K01507+K01507+K01507+K01507+K01507+K01507+K01535+K02132+K01535+K01507+K01507+K01507+K01535+K00234+K01507+K01535+K01507+K01535+K01535+K02112+K01535+K01507+K01507+K01535+K01507+K01507+K01507+K01535+K01507+K02132+K02112+K01507+K01507+K02132+K01507+K01535+K01507+K01535+K01535+K02111+K01535+K01507+K01507+K02112+K01507+K01507+K01535+K01535+K01507+K02112+K01507+K02112+K01535+K01507+K01535+K01535+K01507+K01507+K01507+K01507+K01507+K01535+K01507+K01535+K03881+K03881+K03881+K03881+K03881+K03881+K01507+K01507+K02133
Photosynthesis	ko00195	274	K03541+K03541+K03541+K03541+K03541+K03541+K03541+K03541+K02639+K02639+K02639+K02115+K02115+K02115+K02115+K02109+K02109+K02695+K02641+K02641+K02641+K02641+K02641+K02113+K02716+K02716+K02716+K02716+K02716+K02716+K02716+K02716+K02716+K02695+K02695+K02695+K02695+K02695+K02695+K02638+K02638+K02638+K02638+K02638+K02638+K02638+K02638+K02109+K02109+K02109+K02109+K02109+K02109+K02109+K02109+K02636+K02636+K02698+K02698+K02698+K02698+K02698+K02698+K02717+K02717+K02717+K02723+K02723+K02723+K02723+K02723+K02701+K02701+K02701+K02701+K02638+K02638+K02638+K02638+K02699+K02699+K02699+K02699+K02699+K02699+K02689+K02689+K02721+K02721+K02721+K02639+K02639+K02639+K03541+K03541+K03541+K03541+K03541+K03541+K03541+K03541+K03541+K03541+K03541+K03541+K02694+K02694+K02694+K02694+K02694+K02694+K02694+K02112+K14332+K14332+K14332+K14332+K14332+K14332+K02111+K02111+K02111+K02110+K02108+K02111+K02705+K02705+K08902+K08902+K08902+K02710+K02710+K02639+K02639+K02112+K08903+K02693+K02693+K02693+K02693+K02693+K02693+K02723+K02723+K02639+K02639+K02639+K02639+K08905+K08905+K08905+K08905+K08905+K08905+K02693+K02693+K02693+K02693+K02693+K02693+K02693+K02693+K02115+K02115+K08906+K08906+K02703+K02703+K02703+K03541+K02692+K02692+K02692+K02692+K02692+K02692+K02693+K02641+K02641+K02641+K02641+K02641+K02641+K02641+K02641+K02641+K02641+K02641+K02641+K02641+K02641+K02712+K02712+K02712+K02637+K02637+K02704+K02704+K02707+K02707+K08901+K08901+K08901+K08901+K08901+K02704+K02111+K02112+K02112+K02690+K02690+K02112+K02111+K02690+K02111+K02111+K02112+K02639+K02111+K02635+K02704+K02704+K02704+K02716+K02112+K02112+K02111+K02111+K02112+K02112+K02111+K02704+K02110+K02111+K02704+K02112+K02635+K02110+K02634+K02634+K

			02704+K02716+K02112+K02112+K02112+K02112+K02112+K02112+K02112+K02112+K02704+K02112+K02705+K02706+K02112+K02705+K02707+K02111+K02112+K02639+K02112+K02112+K02704+K03542+K03542+K03542+K03542+K02717+K02717+K02717+K02717+K02717+K02717+K02717
Photosynthesis - antenna proteins	ko00196	131	K08916+K08916+K08916+K08916+K08916+K08916+K08916+K08916+K08916+K08916+K08916+K08917+K08917+K08917+K08917+K08917+K08917+K08917+K08910+K08910+K08910+K08910+K08910+K08910+K08910+K08913+K08913+K08913+K08913+K08913+K08908+K08908+K08908+K08908+K08908+K08908+K08908+K08908+K08908+K08914+K08914+K08914+K08908+K08908+K08917+K08917+K08917+K08912+K08912+K08912+K08912+K08915+K08915+K08915+K08915+K08915+K08915+K08915+K08915+K08915+K08912+K08909+K08909+K08909+K08909+K08912+K08912+K08912+K08912+K08915+K08915+K08912+K08912+K08915+K08909+K08912+K08909+K08912+K08908+K08908+K08908+K08915+K08913+K14172+K14172+K14172+K08907+K08907+K08907+K08907+K08907+K08907+K08907+K08907+K08907+K08907+K08907+K08907+K08907
Purine metabolism	ko00230	525	K03005+K03005+K03005+K03005+K03787+K03787+K03787+K03787+K03787+K03787+K03787+K03787+K00759+K00759+K03016+K03016+K03016+K03016+K00939+K00939+K00939+K00939+K00939+K00939+K00939+K03008+K03008+K03008+K03015+K03015+K03015+K01519+K01519+K03009+K03009+K03009+K03009+K03009+K13811+K00759+K00759+K03011+K03011+K03011+K03011+K01517+K01517+K00759+K01835+K14641+K14641+K00856+K00856+K00856+K03024+K00759+K03025+K03025+K18532+K18532+K02320+K02320+K02320+K00940+K00940+K00940+K00940+K00940+K00940+K00940+K00940+K00940+K00940+K00940+K00940+K14977+K14977+K00940+K03040+K03020+K03020+K00365+K00873+K03013+K03013+K13484+K13484+K13484+K13484+K02328+K03025+K03025+K03025+K03505+K03505+K03505+K00759+K00759+K00759+K00759+K03013+K18447+K18447+K03014+K03014+K03019+K03007+K03016+K03016+K01522+K01522+K03013+K03013+K00940+K00940+K03012+K03012+K03012+K02685+K00939+K00939+K00939+K03506+K03002+K03002+K03002+K03002+K03013+K03013+K03013+K06928+K02684+K02684+K03000+K03000+K10808+K03021+K03021+K03021+K02325+K02325+K01466+K00940+K00940+K02326+K02999+K02999+K02999+K02999+K02999+K18453+K18453+K01081+K01081+K03021+K03021+K03021+K03021+K03021+K03021+K03021+K01490+K01490+K01490+K01490+K01490+K01490+K01490+K00939+K00939+K00939+K00939+K00939+K00939+K00939+K02327+K02327+K02327+K02327+K13811+K13811+K13811+K13811+K13811+K13811+K00106+K00106+K00106+K00106+K00106+K00106+K03010+K03010+K03010+K03010+K03010+K03010+K03010+K03010+K03010+K03010+K03010+K00948+K00948+K00948+K00948+K00948+K14721+K14721+K14721+

			K14721+K14721+K14721+K14721+K14721+K14721+K14721+K14721+K14721+K00962+K00962+K00962+K00962+K00962+K03014+K03014+K03014+K03014+K03014+K03014+K00088+K00088+K00088+K00088+K00088+K00088+K00088+K00088+K00088+K00088+K01835+K01835+K01835+K01835+K01835+K01835+K01835+K01835+K00873+K00873+K00873+K02083+K02083+K02083+K02083+K02083+K02083+K03006+K03006+K03006+K03006+K03006+K01490+K01490+K01490+K01490+K01490+K00939+K01427+K01427+K01427+K01427+K01427+K01427+K01427+K01427+K01427+K10807+K10807+K00873+K00873+K00873+K00873+K11808+K11808+K11808+K11808+K11808+K11808+K11808+K11808+K11808+K11808+K11808+K11808+K11808+K11808+K11808+K11808+K03043+K03043+K01952+K01952+K01952+K01952+K01952+K01952+K01952+K01952+K01952+K01952+K01951+K01951+K00873+K00873+K00873+K00873+K00873+K00873+K00873+K00873+K00873+K00873+K00873+K00873+K00873+K00873+K01466+K01466+K01466+K01466+K01466+K01945+K01945+K01945+K01945+K02335+K02335+K02335+K00948+K00948+K03013+K03504+K03504+K03504+K03504+K00602+K00602+K00602+K00602+K00602+K00602+K00602+K03787+K03787+K03787+K03787+K00962+K14641+K00873+K00873+K00873+K01835+K14641+K18151+K00873+K01835+K00873+K00873+K00873+K00873+K01835+K00873+K00873+K00873+K01835+K00873+K18151+K01835+K00873+K00873+K14641+K00873+K14641+K14641+K00873+K01835+K14641+K00873+K18151+K18151+K00962+K18151+K02327+K01835+K18151+K18151+K00873+K01835+K01835+K01835+K01835+K01835+K01835+K01835+K01835+K01835+K01835+K01835+K01835+K01835+K01835+K01835+K01835+K18151+K00962+K00962+K03040+K03040+K03040+K03040+K03040+K03040+K00962+K01835+K01835+K01835+K00873+K03018+K00873+K00873+K18453+K03018+K03040+K00962+K14641+K14641+K03043+K00873+K00873+K00873+K03046+K00873+K00939+K00939+K00939+K00939+K00939+K00873+K00873+K00764+K00764+K00873+K00873+K00873+K00873+K00873+K00873+K00873+K03007+K03007+K03007+K03007+K01923+K01923+K01923+K01923+K01923+K01923+K00942+K00942+K00942+K01939+K01939+K01939+K01939+K01756+K01756+K03012+K03012+K03012+K03012+K03023+K00939+K00939+K02325+K02325+K02325+K02325+K02325+K02325+K03787+K03787+K03787+K03787+K03787+K13811+K13811+K13811
Caffeine metabolism	ko00232	7	K00365+K00106+K00106+K00106+K00106+K00106+K00106
Pyrimidine metabolism	ko00240	373	K13800+K13800+K13800+K13800+K13800+K03005+K03005+K03005+K03005+K03787+K03787+K03787+K03787+K03787+K03787+K03787+K03787+K00384+K00384+K00384+K03016+K03016+K03016+K03016+K03008+K03008+K03008+K03015+K03015+K03015+K01465+K01465+K01465+K01465+K01465+K16904+K16904+K01519+K01519+K03009+K03009+K03009+K03009+K03009+K16329+

			K16329+K16329+K16329+K16329+K16329+K03011+K03011+K03011+K03011+K14641+K14641+K00876+K01464+K01464+K03024+K03025+K03025+K02320+K02320+K02320+K00940+K00940+K00940+K00940+K00940+K00940+K00940+K00940+K00940+K00940+K00940+K00940+K01431+K01431+K01431+K01431+K01431+K00940+K03040+K03020+K03020+K13800+K13800+K03013+K03013+K02328+K03025+K03025+K03025+K03505+K03505+K03505+K00761+K00761+K01489+K01489+K03013+K03014+K03014+K03019+K03007+K03016+K03016+K03013+K03013+K00940+K00940+K03012+K03012+K03012+K02685+K03506+K03002+K03002+K03002+K03002+K03013+K03013+K03013+K02684+K02684+K00609+K00609+K03000+K03000+K10808+K03021+K03021+K03021+K02325+K02325+K00940+K00940+K02326+K02999+K02999+K02999+K02999+K02999+K01081+K01081+K03021+K03021+K03021+K03021+K03021+K03021+K03021+K01240+K01240+K01240+K01240+K01240+K01240+K02327+K02327+K02327+K02327+K03010+K03010+K03010+K03010+K03010+K03010+K03010+K03010+K03010+K03010+K03010+K09903+K00857+K00857+K00857+K14721+K14721+K14721+K14721+K14721+K14721+K14721+K14721+K14721+K14721+K14721+K14721+K14721+K14721+K00962+K00962+K00962+K00962+K00962+K03014+K03014+K03014+K03014+K03014+K03014+K01955+K01955+K01955+K01955+K01955+K01955+K01955+K01955+K01955+K01955+K01955+K01955+K03006+K03006+K03006+K03006+K03006+K00384+K00384+K00384+K00384+K10807+K10807+K03043+K03043+K13421+K13421+K13421+K13421+K13421+K00876+K13998+K13998+K13998+K13998+K13998+K13998+K13998+K13998+K13998+K13998+K13998+K01937+K01937+K01937+K01937+K01937+K01937+K01937+K01937+K00207+K00207+K00207+K00207+K00207+K00207+K00207+K00207+K00207+K02335+K02335+K02335+K03013+K03504+K03504+K03504+K03504+K03787+K03787+K03787+K03787+K00962+K14641+K14641+K00761+K01464+K01464+K01464+K01489+K01464+K01464+K01464+K01464+K01464+K01464+K01464+K01464+K01464+K14641+K14641+K01464+K14641+K01464+K14641+K01464+K00962+K02327+K01464+K00962+K00962+K03040+K03040+K03040+K03040+K03040+K03040+K00962+K00761+K03018+K01937+K03018+K03040+K00962+K14641+K14641+K03043+K03046+K13800+K13800+K13800+K13800+K13800+K13800+K13800+K03007+K03007+K03007+K03007+K03012+K03012+K03012+K03012+K03023+K01937+K01937+K00254+K00254+K00254+K02325+K02325+K02325+K02325+K02325+K02325+K01956+K01956+K01956+K03787+K03787+K03787+K03787+K03787
Alanine, aspartate and glutamate metabolism	ko00250	211	K00261+K00261+K14454+K14454+K14454+K14454+K14454+K14455+K14455+K14455+K01915+K13566+K13566+K13566+K00811+K00811+K00811+K16871+K00827+K00827+K01940+K01940+K01940+K01940+K00814+K00814+K01580+K01580+K01580+K00609+K00609+K01755+K01755+K01755+K01755+K01755+K01755+K01755+K01755+K01755+K01755+K01940+K01940+K01940+K01940+K01940+K01940+K01940+K01940+K00261+K00261+K00261+K01915+K01915+K01915+K01915+K01915+K01915+K01915+

			K01915+K01915+K01915+K01915+K01915+K01915+K01915+K01915+K01915+K01915+K01915+K01915+K01915+K01915+K01915+<br>K01915+K01915+K01915+K01915+K01915+K01915+K01915+K01955+K01955+K01955+K01955+K01955+K01955+K01955+K01955+<br>K01955+K01955+K01955+K01955+K00262+K00262+K00262+K00262+K00262+K14454+K14454+K14272+K14272+K14272+K14272+<br>K14272+K14272+K14272+K14272+K00820+K00820+K00820+K00820+K01915+K01915+K00278+K00278+K00278+K00278+K00278+<br>K00278+K00264+K00264+K00264+K00264+K00264+K00264+K00264+K00264+K00264+K00264+K00264+K00264+K00264+K00264+<br>K00830+K00830+K00830+K00830+K00830+K00830+K00830+K00830+K17761+K17761+K17761+K17761+K17761+K17761+K17761+<br>K00294+K00294+K00294+K00294+K00294+K00294+K00294+K01953+K01953+K01953+K01953+K01953+K01953+K00814+K01953+<br>K00814+K00814+K00814+K01953+K01915+K01953+K01953+K13566+K13566+K13566+K00814+K13566+K00814+K13566+K01953+<br>K01580+K01580+K01580+K01580+K01580+K00764+K00764+K00827+K00827+K00827+K00827+K00827+K00827+K00827+K01939+<br>K01939+K01939+K01939+K01756+K01756+K01956+K01956+K01956+K16871+K16871+K16871+K16871+K16871+K16871+K16871+<br>K16871
Glycine, serine and threonine metabolism	ko00260	329	K01696+K01696+K01696+K00276+K01696+K01696+K15893+K15893+K15893+K15893+K15893+K15893+K15893+K15893+K15893+<br>K00133+K00133+K15634+K15634+K12235+K12235+K12235+K12235+K12235+K12235+K12235+K12235+K12235+K12235+K12235+<br>K12235+K12235+K12235+K12235+K12235+K12235+K12235+K12235+K12235+K12235+K12235+K12235+K12235+K12235+K12235+<br>K15919+K01079+K01695+K01695+K01695+K01695+K01695+K00827+K00827+K00605+K00605+K00831+K00831+K00831+K14085+<br>K14085+K14085+K14085+K14085+K14085+K14085+K14085+K14085+K14085+K14085+K14085+K14085+K14085+K14085+K14085+<br>K01834+K01834+K01834+K01834+K01620+K01620+K01620+K00058+K02437+K02437+K02437+K02437+K00058+K00058+K00058+<br>K00058+K00058+K00058+K00058+K00058+K00058+K02437+K06001+K06001+K06001+K06001+K00276+K00276+K00276+K00276+<br>K00276+K00276+K00928+K00928+K00928+K00928+K00928+K00928+K00928+K00928+K15634+K15919+K15919+K15919+K15919+<br>K15919+K15919+K00276+K00276+K00276+K00276+K00276+K00276+K00276+K00276+K00276+K00276+K00276+K00276+K12524+<br>K12524+K12524+K12524+K12524+K12524+K12524+K12524+K12524+K00281+K15918+K15918+K15918+K15918+K15918+K15918+<br>K15918+K15918+K15918+K00058+K00058+K00058+K00058+K00058+K12235+K12235+K12235+K12235+K12235+K12235+K12235+<br>K12235+K12235+K12235+K12235+K12235+K12235+K12235+K12235+K12235+K12235+K12235+K14272+K14272+K14272+K14272+<br>K14272+K14272+K14272+K14272+K00872+K00872+K00130+K00130+K00130+K00130+K00130+K00130+K00130+K00130+K00130+<br>K00130+K00130+K00130+K00130+K00130+K00382+K00382+K00382+K00382+K00382+K00382+K00382+K00382+K00382+K00382+<br>K00600+K00600+K00600+K00600+K00600+K15633+K15633+K15633+K15633+K15633+K15633+K15633+K15633+K15633+K15633+

			K15633+K15633+K15633+K15633+K15633+K15633+K15633+K15633+K15633+K00830+K00830+K00830+K00830+K00830+K00830+K00830+K00830+K00600+K00600+K00600+K00600+K00600+K00600+K00600+K01754+K01754+K01754+K00382+K00382+K00382+K00382+K00281+K00281+K00928+K00276+K15919+K15919+K00605+K00928+K01620+K00928+K00281+K00605+K02437+K02437+K01620+K01620+K00281+K00281+K01620+K00281+K00281+K00281+K00281+K00281+K00281+K00281+K00281+K00281+K00281+K00281+K00281+K00281+K15919+K15919+K00281+K00281+K00281+K00600+K00600+K00600+K00600+K00600+K00600+K00600+K00600+K00600+K00600+K00600+K00600+K00600+K00600+K00600+K00600+K00600+K00827+K00827+K00827+K00827+K00827+K00827+K00827+K01733+K01733+K01733+K01733+K00058+K01733+K01733+K00600+K00600+K00600+K00306+K00306
Cysteine and methionine metabolism	ko00270	395	K00797+K00797+K00797+K00797+K00797+K00797+K00797+K05933+K05933+K05933+K05933+K08963+K08963+K08963+K08963+K00789+K13034+K13034+K13034+K01244+K01244+K01244+K01244+K00640+K00640+K00640+K08967+K08967+K08967+K08967+K14454+K14454+K14454+K14454+K14454+K01738+K01738+K01738+K01738+K14455+K14455+K14455+K00133+K00133+K00558+K01251+K01251+K01251+K01251+K01251+K00025+K00025+K00025+K00025+K00025+K00025+K00025+K00025+K00025+K00025+K00025+K00797+K00797+K00797+K00797+K00797+K00797+K00797+K00797+K13034+K13034+K05933+K05933+K05933+K05933+K05933+K00789+K00789+K00789+K00789+K00789+K00789+K00789+K00789+K00789+K00789+K00789+K00789+K00789+K00789+K01761+K01761+K01761+K01761+K01761+K01761+K01761+K01761+K01761+K01761+K01761+K01761+K00026+K00026+K00026+K00026+K00026+K00026+K00026+K00026+K00026+K08967+K01738+K01738+K01738+K01738+K01738+K01738+K01738+K01738+K01738+K01738+K00789+K00789+K00789+K00789+K00547+K00547+K00547+K17398+K17398+K17398+K17398+K17398+K17398+K17398+K17398+K00811+K00811+K00811+K00025+K00026+K00026+K00026+K00640+K00640+K01738+K01738+K01738+K00640+K00640+K00549+K00549+K17398+K17398+K01244+K01244+K01244+K00558+K00558+K00025+K00025+K05933+K16054+K16054+K00815+K00026+K00026+K00026+K00026+K00026+K01738+K00547+K00547+K01738+K01738+K00815+K00815+K00815+K00815+K00815+K00815+K00547+K00558+K00558+K00928+K00928+K00928+K00928+K00928+K00928+K00928+K00928+K01611+K01611+K01611+K01611+K01611+K01611+K01611+K01760+K01760+K01760+K01760+K01739+K01739+K01739+K01739+K01739+K01739+K01739+K01739+K01739+K01739+K01739+K01739+K00640+K12524+K12524+K12524+K12524+K12524+K12524+K12524+K12524+K12524+K01611+K01611+K01611+K01611+K01611+K00899+K00899+K00899+K00899+K00899+K00899+K00899+K00899+K00899+K00899+K00899+K00899+K14454+K14454+K00789+K00789+K00789+K00789+K00789+K00789+K00789+K00789+K00558+K01244+K01244+K00558+K00558+K00558+K00558+K00558+K00558+K00789+K00789+K00789+K00789+K08967+K08967+K08967+K08967+K08967+K08967+K16054+K16054+K16054+K16054+K16054+K16054+K16054+K16054+K00026+K00026+K00026+K00640+

			K00789+K00789+K05933+K00549+K01251+K00549+K00549+K00549+K00815+K00928+K00815+K00815+K00549+K00815+K00815+K00549+K00815+K00549+K01251+K01738+K00815+K01738+K00815+K01738+K01251+K01251+K00815+K00928+K00789+K00797+K01251+K01251+K00815+K00815+K00928+K00815+K00549+K00549+K00549+K01251+K00549+K01251+K00815+K00549+K00815+K00549+K00549+K00549+K00549+K01738+K00549+K00549+K00815+K00549+K00549+K00549+K00549+K00549+K00549+K01738+K01251+K00549+K00549+K01251+K01251+K01251+K00797+K00797+K00797+K01251+K01251+K01251+K00549+K00549+K00549+K00549+K00549+K00797+K00549+K01251+K00549+K00549+K00549+K01251+K00549+K00549+K00549+K01011+K01011+K01011+K01011+K01011+K01011+K01011+K01011+K00547+K00547+K00547+K00547+K00547+K00547+K00547+K00547+K00547+K00547+K00547+K01761+K01761+K01761+K01761
Valine, leucine and isoleucine degradation	ko00280	285	K00253+K00253+K00253+K00253+K00253+K00253+K00253+K00253+K05605+K05605+K05605+K05605+K05605+K05605+K05605+K00128+K07513+K07513+K01640+K01640+K01640+K01640+K05607+K05607+K05607+K05607+K05607+K07513+K05605+K05605+K05605+K05605+K05605+K05605+K00826+K00826+K00826+K00826+K00826+K00826+K00826+K00826+K00826+K00826+K00826+K00626+K00626+K00626+K00626+K00626+K00626+K14085+K14085+K14085+K14085+K14085+K14085+K14085+K14085+K14085+K14085+K14085+K14085+K14085+K14085+K14085+K14085+K05605+K05605+K05605+K05605+K05605+K05605+K05605+K05605+K05605+K05605+K05605+K05605+K05605+K05607+K05605+K05605+K00249+K00249+K00249+K00249+K00249+K00249+K00249+K00249+K00249+K00249+K05605+K05605+K05605+K05605+K05605+K05605+K05605+K01968+K01968+K01968+K01968+K01968+K01968+K01968+K01968+K01968+K01968+K01968+K01968+K01968+K00128+K00128+K00128+K00128+K00128+K00128+K00128+K00128+K00128+K00128+K00128+K00166+K00166+K00166+K00166+K00166+K00166+K00166+K05605+K05605+K05605+K05605+K05605+K00167+K00167+K00167+K00167+K05605+K05605+K00166+K00166+K00166+K00166+K00166+K00166+K00166+K00128+K00128+K00128+K00128+K00128+K00128+K00128+K00128+K00128+K00128+K00128+K00128+K00128+K00128+K00128+K00140+K00140+K00140+K00140+K00140+K00140+K00140+K00166+K00166+K00140+K00140+K00140+K00140+K00140+K00140+K00382+K00382+K00382+K00382+K00382+K00382+K00382+K00382+K00382+K00382+K01641+K01641+K01641+K01641+K01641+K01641+K00128+K00128+K00128+K00128+K00128+K00128+K00128+K00128+K00128+K00128+K18660+K18660+K18660+K18660+K18660+K18660+K18660+K18660+K18660+K18660+K18660+K00128+K00128+K00128+K00128+K00128+K00382+K00382+K00382+K00382+K07513+K07513+K07513+K07513+K07513+K07513+K07513+K09699+K09699+K09699+K00128+K09699+K09699+K00128+K09699+K09699+K00249+K00140+K09699+K00140+K09699+K09699+K00140+K00128+K00128+K00826+K00128+K00140+K00140+K00140+K07513+K00128+K05607+K05607+K00140+K00140+K00826+K09699+K01969+K01969+K01969+K01969+K01969+K01640+K01640+

			K01640+K01640+K01640+K01640+K00020+K00020+K00128+K00128+K00128+K00626+K00626+K00626+K00626+K00626+K00626
Valine, leucine and isoleucine biosynthesis	ko00290	78	K01703+K01703+K01703+K01703+K00053+K00826+K00826+K00826+K00826+K00826+K00826+K00826+K00826+K00826+K00826+K00826+K01704+K01704+K01704+K01704+K00052+K00052+K01653+K01653+K01653+K01653+K01653+K01653+K01653+K01653+K01703+K01703+K01703+K01703+K01703+K01703+K01703+K01703+K01703+K01703+K01649+K01649+K01649+K01649+K01649+K01649+K01649+K01649+K01649+K01649+K01649+K01649+K00052+K00052+K01687+K01687+K01754+K01754+K01754+K00053+K00053+K00053+K00053+K00053+K00053+K00053+K00053+K00053+K00053+K00826+K00826+K01652+K01652+K01652+K01652+K01652+K01653+K01653
Lysine biosynthesis	ko00300	62	K01714+K01714+K00215+K00215+K00215+K00215+K00133+K00133+K01778+K01778+K01778+K10206+K10206+K10206+K10206+K10206+K10206+K10206+K10206+K14085+K14085+K14085+K14085+K14085+K14085+K14085+K14085+K14085+K14085+K14085+K14085+K14085+K14085+K14085+K14085+K01586+K01586+K01586+K01586+K01586+K01586+K01586+K00928+K00928+K00928+K00928+K00928+K00928+K00928+K00928+K12524+K12524+K12524+K12524+K12524+K12524+K12524+K12524+K12524+K00928+K00928+K00928
Lysine degradation	ko00310	182	K11420+K11420+K11420+K00128+K00626+K00626+K00626+K00626+K00626+K00626+K14085+K14085+K14085+K14085+K14085+K14085+K14085+K14085+K14085+K14085+K14085+K14085+K14085+K14085+K14085+K14085+K11423+K11420+K11420+K11420+K11420+K11420+K11420+K11420+K11420+K11420+K00128+K00128+K00128+K00128+K00128+K00128+K00128+K00128+K00128+K00128+K00128+K11420+K00128+K00128+K00128+K00128+K00128+K00128+K00128+K00128+K00128+K00128+K00128+K00128+K00128+K00128+K00128+K11433+K11433+K11423+K14157+K00128+K00128+K00128+K00128+K00128+K00128+K00128+K00128+K00128+K00128+K00128+K00128+K00128+K00128+K00128+K00128+K00128+K00164+K00164+K00164+K00128+K00128+K00128+K00128+K00164+K11420+K00658+K00658+K00658+K00658+K00658+K00658+K00658+K00658+K00128+K00128+K00128+K11423+K00626+K00626+K00626+K00626+K00626+K00626+K00306+K00306
Arginine and	ko00330	318	K00797+K00797+K00797+K00797+K00797+K00797+K00797+K01259+K01259+K01259+K01259+K12259+K12259+K01438+K01438+

proline metabolism			K00472+K00472+K00472+K00472+K00261+K00261+K01476+K01476+K01476+K01476+K00472+K00472+K00472+K00472+K00472+K14454+K14454+K14454+K14454+K14454+K14455+K14455+K14455+K00620+K00611+K01915+K00797+K00797+K00797+K00797+K00797+K00797+K00797+K00797+K00128+K00818+K00818+K00818+K00818+K00818+K00811+K00811+K00811+K01438+K01438+K01438+K01581+K01581+K01581+K00472+K00472+K00472+K00472+K00472+K00472+K00472+K00472+K00472+K00472+K12251+K12251+K12251+K00472+K00472+K01940+K01940+K01940+K01940+K14085+K00930+K00930+K00472+K00472+K01581+K00818+K00818+K00818+K00818+K01755+K01755+K01755+K01755+K01755+K01755+K01755+K01755+K01755+K01755+K01755+K01611+K01611+K01611+K01611+K01611+K01611+K01611+K13366+K13366+K13366+K13366+K00128+K00128+K00128+K00128+K00128+K00128+K00128+K00128+K00128+K00128+K00128+K01940+K01940+K01940+K01940+K01940+K01940+K01940+K01940+K00261+K00261+K00261+K01611+K01611+K01611+K01611+K01611+K01583+K01583+K01583+K01583+K01915+K17839+K17839+K17839+K17839+K17839+K17839+K17839+K17839+K00262+K00262+K00262+K00262+K00262+K14454+K14454+K00128+K00128+K00128+K00128+K00128+K00128+K00128+K00128+K00128+K00128+K00128+K00128+K00128+K00128+K00128+K01427+K01427+K01427+K01427+K01427+K01427+K01427+K01427+K01427+K01915+K01915+K13427+K13427+K13427+K13427+K13427+K13427+K12657+K12657+K00819+K00819+K00819+K00819+K12657+K12657+K14682+K14682+K14682+K14682+K14682+K00294+K00294+K00294+K00294+K00294+K00294+K00294+K00128+K00128+K00128+K00128+K00128+K00128+K00128+K00128+K00128+K00128+K17839+K17839+K17839+K17839+K17839+K17839+K00128+K00128+K00128+K00128+K00128+K00472+K00128+K00128+K01915+K00797+K00472+K00128+K01438+K00128+K00128+K00472+K00472+K00128+K00797+K00797+K00797+K00797+K01438+K00145+K00145+K00145+K10536+K10536+K10536+K10536+K10536+K00128+K00128+K00128+K14677+K14677+K14677+K00318+K00318+K00318+K00318+K00318+K00318+K00318+K00318
Histidine metabolism	ko00340	116	K00128+K01814+K01814+K01814+K01814+K11755+K11755+K11755+K14085+K14085+K14085+K14085+K14085+K14085+K14085+K14085+K14085+K14085+K14085+K14085+K14085+K14085+K14085+K14085+K01693+K00765+K00765+K00765+K00128+K00128+K00128+K00128+K00128+K00128+K00128+K00128+K00128+K00128+K00128+K01693+K01693+K01693+K01693+K01693+K01693+K00128+K00128+K00128+K00128+K00128+K00128+K00128+K00128+K00128+K00128+K00128+K00128+K00128+K00128+K00128+K11755+K11755+K00128+K00128+K00128+K00128+K00128+K00128+K00128+K00128+K00128+K00128+K00128+K00128+K00128+

			K00128+K00128+K00128+K00128+K01663+K01663+K01663+K00128+K01663+K00128+K00128+K01663+K01663+K00128+K01663+K01663+K01663+K01663+K00013+K00013+K00013+K00013+K00013+K00817+K00817+K00817+K00817+K00817+K00817+K00817+K00817+K00128+K00128+K00128+K00765+K00765+K00765+K00765+K01590+K01590+K01590
Tyrosine metabolism	ko00350	231	K00276+K00121+K00121+K00121+K00121+K00121+K00121+K00121+K00457+K00121+K00121+K14454+K14454+K14454+K14454+K14454+K14455+K14455+K14455+K00451+K00451+K00451+K00451+K00451+K00451+K01800+K01800+K01800+K00811+K00811+K00811+K01555+K01555+K01555+K01557+K01557+K01557+K01557+K01557+K00121+K00121+K18857+K07253+K00001+K00001+K00001+K01592+K01592+K00815+K07253+K00121+K00121+K00121+K00121+K00121+K00815+K00815+K00815+K00815+K00815+K00815+K18857+K18857+K18857+K00276+K00276+K00276+K00276+K00276+K00276+K00121+K00121+K00121+K00121+K00121+K00121+K00121+K00121+K00121+K00121+K00121+K00121+K00121+K00276+K00276+K00276+K00276+K00276+K00276+K00276+K00276+K00276+K00276+K00276+K00276+K00276+K01800+K01800+K01800+K14454+K14454+K00457+K01800+K01800+K01800+K01800+K01800+K01800+K01800+K01800+K01800+K01800+K01800+K01800+K01800+K01800+K01800+K01800+K00001+K00001+K00001+K00001+K00001+K00001+K01592+K01592+K01592+K01592+K00121+K00121+K00121+K00121+K00121+K00815+K00815+K00815+K00276+K00001+K00815+K00815+K01555+K00451+K00815+K00815+K01555+K00815+K00815+K18857+K00815+K00815+K00815+K00815+K00815+K00815+K00001+K00451+K00451+K18857+K00451+K00451+K00451+K00451+K18857+K01555+K00451+K00451+K18857+K18857+K18857+K18857+K18857+K00451+K00817+K00817+K00817+K00817+K00817+K00817+K00817+K00817+K15849+K15849+K15849+K15849+K00121+K00121+K00121+K00121+K00121+K00121+K00121+K00121+K00121+K18857+K18857+K18857+K18857+K18857+K18857+K18857+K18857+K18857+K18857+K00001+K18857+K18857+K18857
Phenylalanine metabolism	ko00360	214	K00430+K00430+K00430+K00430+K00430+K00430+K00430+K00430+K00430+K00430+K00430+K00430+K00430+K00430+K00276+K00457+K14454+K14454+K14454+K14454+K14454+K14455+K14455+K14455+K00430+K00811+K00811+K00811+K00487+K00487+K00430+K00430+K00430+K00430+K07253+K00430+K00815+K07253+K00430+K00430+K00430+K00815+K00815+K00815+K00815+K00815+K00815+K00276+K00276+K00276+K00276+K00276+K00276+K00276+K00276+K00276+K00276+K00276+K00276+K00276+K00276+K00276+K00276+K00276+K00276+K01904+K01904+K01904+K01904+K14454+K14454+K10775+K10775+K10775+K10775+K10775+K10775+K10775+

			K10775+K10775+K00457+K10775+K10775+K00430+K00430+K00430+K00430+K00430+K00430+K00430+K00430+K00588+K00588+K00588+K00588+K00588+K00074+K00074+K00074+K00074+K00430+K00430+K00430+K00430+K00430+K00430+K01904+K01904+K01904+K01904+K01904+K01904+K01904+K01904+K01904+K01904+K01904+K00430+K00815+K00815+K00815+K00276+K00815+K00815+K00815+K00815+K00815+K00815+K00815+K00815+K00815+K00815+K00815+K00815+K00817+K00817+K00817+K00817+K00817+K00817+K00817+K00817+K01904+K01904+K01904+K01904+K01904+K01904+K01904+K01904+K01904+K01904+K15849+K15849+K15849+K15849+K00430+K00487+K00487+K00487+K00487+K00487
Tryptophan metabolism	ko00380	137	K00128+K03781+K03781+K03781+K03781+K03781+K03781+K03781+K03781+K03781+K03781+K11816+K11816+K11816+K11816+K11816+K11816+K00626+K00626+K00626+K00626+K00626+K00626+K14085+K14085+K14085+K14085+K14085+K14085+K14085+K14085+K14085+K14085+K14085+K14085+K14085+K14085+K14085+K14085+K11820+K11820+K00128+K00128+K00128+K00128+K00128+K00128+K00128+K00128+K00128+K00128+K00128+K16903+K16903+K00128+K11816+K00128+K00164+K00164+K00164+K00128+K00128+K00128+K00128+K00164+K11816+K11816+K00128+K00128+K00128+K03781+K03781+K03781+K03781+K03781+K03781+K03781+K03781+K00626+K00626+K00626+K00626+K00626+K00626
Phenylalanine, tyrosine and tryptophan biosynthesis	ko00400	233	K01696+K01696+K01696+K01735+K01735+K01735+K01735+K01735+K05359+K05359+K05359+K01696+K01696+K14454+K14454+K14454+K14454+K14454+K05359+K05359+K05359+K05359+K14455+K14455+K14455+K15227+K15227+K05359+K05359+K00891+K01626+K01626+K01626+K01626+K01626+K01626+K01626+K01626+K01626+K01626+K01626+K01626+K01626+K01695+K01695+K01695+K01695+K01695+K01850+K01850+K01850+K01850+K01850+K01850+K01850+K00811+K00811+K00811+K13832+K13832+K13832+K13832+K13832+K13832+K13832+K13832+K13832+K13832+K13832+K01609+K00815+K01658+K01658+K05359+K00815+K00815+K00815+K00815+K00815+K00815+K01657+K01657+K00891+K00891+K06001+K06001+K06001+K06001+K00891+K00891+K01626+K01626+K01626+K01626+K01626+K01626+K01626+K15227+K15227+K15227+K15227+K15227+K15227+K15227+K13832+K01850+K01850+K01850+K01850+K14454+K14454+K00766+K00766+K00766+K00766+K00766+K13832+K13832+K13832+K13832+

			K13832+K01817+K00815+K00815+K00815+K13832+K00815+K00815+K01657+K00815+K01657+K01657+K00815+K13832+K00815+K01657+K00815+K01657+K00815+K00815+K00815+K01657+K01657+K01657+K01657+K00815+K00815+K00815+K01657+K01657+K01657+K01657+K01657+K01657+K01657+K01657+K01657+K01657+K01657+K01657+K01657+K01657+K01657+K01657+K01657+K01626+K05359+K00800+K00800+K00800+K00800+K00800+K00800+K00800+K00800+K00817+K00817+K00817+K00817+K00817+K00817+K00817+K00817+K05359+K05359+K05359+K05359+K05359+K05359+K15849+K15849+K15849+K15849+K13832+K01736+K01736+K01736+K01736+K01736+K01736+K01736+K01736+K01736+K01626+K01626+K01626+K01626+K01626+K01626
beta-Alanine metabolism	ko00410	245	K00797+K00797+K00797+K00797+K00797+K00797+K00797+K12259+K12259+K00276+K05605+K05605+K05605+K05605+K05605+K05605+K05605+K00797+K00797+K00797+K00797+K00797+K00797+K00797+K00797+K00128+K01464+K01464+K01431+K01431+K01431+K01431+K01431+K05605+K05605+K05605+K05605+K05605+K05605+K14085+K14085+K14085+K14085+K14085+K14085+K14085+K14085+K14085+K14085+K14085+K14085+K14085+K14085+K14085+K14085+K05605+K05605+K05605+K05605+K05605+K05605+K05605+K05605+K05605+K05605+K05605+K05605+K05605+K05605+K05605+K01580+K01580+K01580+K00249+K00249+K00249+K00249+K00249+K00249+K00249+K00249+K00249+K00249+K00276+K00276+K00276+K00276+K00276+K00276+K05605+K05605+K05605+K05605+K05605+K05605+K05605+K13366+K13366+K13366+K13366+K00128+K00128+K00128+K00128+K00128+K00128+K00128+K00128+K00128+K00128+K00128+K00276+K00276+K00276+K00276+K00276+K00276+K00276+K00276+K00276+K00276+K00276+K00276+K05605+K05605+K05605+K05605+K05605+K05605+K05605+K17839+K17839+K17839+K17839+K17839+K17839+K17839+K17839+K00128+K00128+K00128+K00128+K00128+K00128+K00128+K00128+K00128+K00128+K00128+K00128+K00128+K00128+K00128+K00140+K00140+K00140+K00140+K00140+K00140+K00140+K00140+K00140+K00140+K00140+K00140+K00140+K00207+K00207+K00207+K00207+K00207+K00207+K00207+K00207+K00128+K00128+K00128+K00128+K00128+K00128+K00128+K00128+K00128+K00128+K17839+K17839+K17839+K17839+K17839+K17839+K00128+K00128+K00128+K00128+K00128+K00128+K00276+K01464+K01464+K01464+K00128+K01464+K01464+K01464+K01464+K00797+K01464+K01464+K01464+K01464+K00249+K01464+K00140+K01464+K00140+K01464+K00140+K01464+K01464+K00128+K00128+K00128+K00140+K00140+K00140+K00128+K00797+K00797+K00797+K00140+K00797+K00140+K01580+K01580+K01580+K01580+K01580+K00128+K00128+K00128+K01578+K01578+K01578+K01578
Taurine and	ko00430	26	K01580+K01580+K01580+K18592+K18592+K18592+K18592+K18592+K18592+K18592+K18592+K18592+K10712+K10712+K10712+

hypotaurine metabolism			K10712+K10712+K01580+K01580+K01580+K01580+K01580+K10712+K10712+K10712+K10712
Selenocompound metabolism	ko00450	122	K00384+K00384+K00384+K13811+K01761+K01761+K01761+K01761+K01761+K01761+K01761+K01761+K01761+K01761+K01761+K01761+K00549+K00549+K08247+K08247+K08247+K08247+K08247+K08247+K08247+K13811+K13811+K13811+K13811+K13811+K13811+K01760+K01760+K01760+K01760+K01739+K01739+K01739+K01739+K01739+K01739+K01739+K01739+K01739+K01739+K01739+K01739+K00384+K00384+K00384+K00384+K11717+K11717+K11717+K11717+K11717+K11717+K00549+K01874+K01761+K01761+K01761+K01761+K13811+K13811+K13811
Cyanoamino acid metabolism	ko00460	224	K01455+K01455+K01455+K01455+K01455+K01455+K13034+K13034+K13034+K01188+K01188+K01188+K01188+K01188+K01188+K13035+K13035+K13035+K13035+K13035+K13034+K13034+K01188+K05350+K05350+K05350+K05350+K01188+K01188+K01188+K01188+K05349+K05350+K05350+K01188+K05349+K05349+K05350+K05350+K05350+K05350+K05350+K05350+K05350+K05349+K05349+K05349+K05349+K01188+K01188+K01188+K01188+K01188+K01188+K01188+K01188+K01188+K01188+K01188+K01188+K01188+K01188+K00600+K00600+K00600+K00600+K00600+K18592+K18592+K18592+K18592+K18592+K18592+K18592+K18592+K18592+K01188+K01188+K01188+K01188+K01188+K01188+K01188+K01188+K00600+K00600+K00600+K00600+K00600+K00600+K00600+K05349+K05349+K05349+K05350+K05349+K05349+K05349+K01188+K01188+K01188+K01188+K01188+K01188+K01188+K01188+K05349+K01188+K05349+K05349+K01188+K05349+K01188+K01188+K01188+K01188+K01188+K05349+K05349+K05350+K01188+K01188+K01188+K05350+K05349+K01188+K05349+K01188+K05349+K01188+K01188+K05349+K01188+K01188+K01188+K01188+K01188+K05349+K01188+K05349+K05349+K05349+K01188+K05349+K05349+K05349+K05349+K05350+K05349+K05349+K05349+K05349+K05349+K05349+K01188+K05349+K05349+K05349+K05349+K01188+K05350+K05349+K05349+K05349+K05349+K05349+K05349+K05349+K05349+K05349+K05349+K05349+K05349+K05349+K05350+K05349+K05349+K05349+K05349+K13035+K13035+K05350+K05349+K05349+K05349+K05349+K00600+K00600+K00600+K00600+K00600+K00600+K00600+K00600+K00600+K00600+K00600+K00600+K00600+K00600+K00600+K00600+K00600+K01188+K01188+K01188+K01188+K01188+K01188+K01188+K01188+K01188+K01188+K01188+K01188+K01188+K01188+K01188+K00600+K00600+K00600+K01188+K01188+K01188

Glutathione metabolism	ko00480	321	K00797+K00797+K00797+K00797+K00797+K00797+K00797+K00799+K00799+K00799+K00799+K00434+K00434+K00432+K00432+K00432+K00432+K00432+K00432+K00033+K00799+K00799+K00799+K00799+K00797+K00797+K00797+K00797+K00797+K00797+K00797+K00797+K00434+K00434+K00434+K00434+K00434+K00434+K00434+K00434+K00434+K00434+K00434+K00434+K00434+K00434+K00434+K00799+K00031+K00031+K00031+K00031+K00031+K00031+K00031+K00031+K00432+K00432+K00432+K00434+K00434+K00434+K00434+K00434+K00434+K00434+K00434+K00434+K00434+K00434+K00434+K00434+K00434+K00434+K00434+K00434+K00434+K00033+K00033+K00033+K00033+K00033+K00033+K00033+K00799+K00799+K00799+K00799+K00799+K00799+K00799+K00799+K01581+K01581+K01581+K00434+K00799+K00799+K00799+K00799+K00799+K00799+K00799+K00799+K00799+K00799+K00036+K00799+K00799+K00799+K00799+K00432+K00432+K00799+K00799+K00799+K00434+K00434+K00799+K00799+K00799+K00799+K00799+K00799+K00799+K00799+K00799+K10808+K00799+K00799+K01581+K01255+K00799+K00799+K00799+K00799+K00799+K00799+K01919+K00434+K00434+K00434+K00434+K00434+K00434+K01469+K01469+K01469+K01469+K01469+K00036+K00432+K00432+K00432+K00432+K00799+K00799+K00799+K00799+K00799+K00799+K00799+K00799+K00799+K00799+K10807+K10807+K00799+K00036+K00036+K00036+K00036+K00036+K00036+K00036+K00036+K01920+K01920+K01920+K01920+K00434+K00434+K00434+K00799+K18592+K18592+K18592+K18592+K18592+K18592+K18592+K18592+K18592+K01919+K01919+K01919+K01919+K01919+K01919+K01919+K00383+K00383+K00383+K00383+K00383+K00383+K00383+K00383+K00432+K00432+K00432+K00432+K00432+K00432+K00432+K00432+K00036+K00036+K00036+K00036+K00036+K00036+K00799+K00799+K01255+K00036+K00036+K00434+K00434+K00031+K00031+K01255+K01255+K00036+K01255+K01255+K00797+K01255+K01255+K01255+K01256+K00383+K01256+K01256+K01256+K00036+K00383+K00036+K00799+K00036+K00799+K01256+K00036+K01256+K00036+K00036+K01256+K01255+K01256+K01256+K01256+K01256+K00797+K00797+K00797+K01255+K01256+K01256+K01256+K00031+K01255+K01256+K01256+K00797+K01256+K01256+K01256+K01256+K00031+K01256+K00383+K00434+K01256+K01256+K00434+K00036+K00036+K00031+K00799+K00799+K00799+K00434+K00434+K00434+K00434+K00434+K00031+K00031+K00033+K00033+K00033
Starch and sucrose metabolism	ko00500	839	K08679+K08679+K08679+K08679+K08679+K01176+K01176+K01176+K01176+K01177+K00847+K00847+K00847+K00847+K01051+K01051+K01051+K00975+K01188+K01188+K01188+K01188+K01179+K00963+K00963+K00963+K00963+K08679+K08679+K01188+K01188+K01835+K16055+K16055+K16055+K16055+K16055+K16055+K16055+K16055+K16055+K16055+K01177+K01177+K01177+K01177+K01177+K01177+K01177+K01177+K01188+K13648+K13648+K13648+K13648+K13648+K13648+K13648+K05350+K05350+

	K05350+K05350+K08679+K08679+K01188+K01188+K01188+K01188+K08679+K08679+K00847+K00847+K00847+K00847+K00847+<br>K00696+K00696+K00696+K00696+K00696+K00696+K05349+K00695+K00695+K00695+K00705+K01187+K18447+K18447+K13648+<br>K13648+K13648+K13648+K13648+K13648+K13648+K13648+K13648+K13648+K13648+K13648+K13648+K13648+K01810+K01810+<br>K01177+K01177+K01087+K01087+K01087+K01087+K05350+K05350+K00696+K00696+K01177+K01188+K01051+K01051+K05349+<br>K05349+K00847+K00696+K00847+K00847+K00847+K00847+K08679+K08679+K08679+K08679+K08679+K08679+K08679+K08679+<br>K08679+K08679+K08679+K00695+K00695+K00695+K00695+K00695+K00695+K00695+K13648+K13648+K13648+K13648+K13648+<br>K13648+K13648+K13648+K13648+K13648+K13648+K00688+K00688+K00688+K00688+K00688+K00688+K00688+K00688+K00688+<br>K00688+K00688+K13648+K13648+K13648+K13648+K13648+K13648+K13648+K13648+K16055+K16055+K16055+K16055+K16055+<br>K16055+K16055+K16055+K16055+K16055+K16055+K16055+K16055+K00696+K00696+K00695+K00695+K00695+K00695+K00695+<br>K00695+K00695+K00695+K00695+K00695+K00695+K00705+K00705+K00705+K00705+K00696+K01835+K01835+K01835+K01835+<br>K01835+K01835+K01835+K01835+K00975+K00975+K00975+K00975+K00696+K00696+K01177+K01177+K01177+K01177+K01177+<br>K01177+K01177+K01177+K01177+K13648+K13648+K13648+K13648+K13648+K13648+K13648+K13648+K13648+K01193+K01193+<br>K01193+K01193+K01193+K01193+K01193+K01193+K01193+K01193+K01193+K00847+K00847+K00847+K00847+K00847+K00847+<br>K00847+K00847+K00012+K00012+K00012+K00012+K00012+K00012+K00012+K00012+K00703+K00703+K00703+K00703+K00703+<br>K00703+K00703+K00703+K13648+K13648+K13648+K13648+K13648+K13648+K13648+K13648+K13648+K13648+K13648+K05350+<br>K13648+K13648+K13648+K13648+K13648+K13648+K13648+K00703+K00703+K00703+K00770+K00975+K00975+K00975+K00975+<br>K00975+K00975+K00975+K00975+K00975+K00975+K00975+K00975+K00975+K16055+K16055+K16055+K01187+K01187+K13648+<br>K13648+K13648+K13648+K13648+K13648+K13648+K13648+K00703+K00703+K00695+K00695+K00695+K00695+K00695+K00963+<br>K00963+K00963+K00963+K00963+K05350+K05350+K05350+K05350+K05350+K05350+K05349+K05349+K05349+K05349+K13648+<br>K13648+K13648+K13648+K01051+K01051+K15920+K15920+K15920+K15920+K01188+K01188+K01188+K01188+K01188+K01188+<br>K01188+K01188+K01188+K01188+K01188+K01188+K01188+K01188+K13648+K13648+K13648+K13648+K01051+K01051+K01051+<br>K01051+K01193+K01193+K01193+K01193+K01193+K01193+K01193+K01193+K01193+K13648+K13648+K13648+K13648+K13648+<br>K13648+K13648+K13648+K13648+K13648+K13648+K13648+K00012+K01188+K01188+K01188+K01188+K01188+K01188+K01188+<br>K01188+K16055+K16055+K16055+K16055+K16055+K16055+K16055+K16055+K16055+K16055+K16055+K01177+K01177+K01177+<br>K01177+K01177+K00688+K00688+K00688+K00975+K00975+K00975+K00975+K05349+K05349+K05349+K01051+K01051+K01051+<br>K01051+K01051+K01051+K01051+K01051+K01051+K01051+K01051+K01051+K01051+K05350+K00700+K00975+K00700+K00975+

	K05349+K00705+K00700+K00700+K00705+K01810+K05349+K00688+K05349+K01188+K00695+K01188+K01188+K01188+K01835+K00700+K01188+K01188+K01188+K01188+K01810+K05349+K00705+K01188+K05349+K01810+K05349+K00695+K01188+K01187+K01835+K00844+K00705+K05349+K01188+K01188+K00844+K01188+K01188+K01188+K05349+K01810+K05349+K05350+K01188+K01188+K01188+K05350+K05349+K01188+K05349+K01188+K01835+K05349+K01810+K01188+K01188+K05349+K00700+K01188+K01188+K00844+K01835+K01188+K00975+K00700+K01188+K01188+K05349+K00705+K01188+K00844+K05349+K01810+K01810+K01810+K01810+K00700+K01835+K01810+K00696+K01187+K01810+K05349+K05349+K01188+K01810+K00695+K00700+K00844+K05349+K00844+K00696+K01810+K05349+K05349+K05349+K00700+K00700+K00700+K00700+K00700+K00975+K01835+K01187+K01810+K00705+K00705+K00705+K00705+K00695+K05350+K05349+K01810+K00700+K01835+K05349+K05349+K00695+K00695+K05349+K00695+K05349+K05349+K01187+K01188+K01835+K01835+K01835+K01835+K01835+K01835+K01835+K01835+K01835+K00695+K01835+K05349+K01835+K01835+K01835+K01835+K01835+K01835+K01187+K00700+K00975+K05349+K00705+K05349+K05349+K01810+K00700+K00700+K00695+K01188+K00695+K00695+K05350+K00705+K00700+K01187+K00975+K00844+K00844+K00844+K00844+K00844+K00844+K00844+K00844+K00844+K00700+K00844+K00695+K00844+K00844+K00844+K00844+K05349+K05349+K05349+K05349+K05349+K05349+K05349+K05349+K05349+K05349+K05349+K05349+K05349+K00695+K00700+K01187+K00695+K00700+K00700+K05350+K00695+K00695+K00695+K00700+K00695+K00695+K00700+K00695+K00695+K00700+K00696+K05349+K00696+K00700+K01835+K01835+K00696+K05349+K01835+K00705+K00695+K01810+K16055+K16055+K05349+K00688+K00695+K00695+K00700+K16055+K00695+K16055+K00844+K00695+K00688+K00688+K00700+K01810+K01810+K00705+K00700+K01810+K16055+K16055+K00688+K16055+K00844+K00688+K00700+K05349+K01810+K16055+K16055+K00700+K01176+K16055+K00688+K00700+K00695+K00688+K00688+K00695+K16055+K01810+K01176+K00695+K00695+K01810+K00695+K01810+K00695+K00700+K00705+K05350+K00700+K16055+K00688+K00688+K00844+K16055+K05349+K00700+K05349+K13648+K01187+K01810+K00688+K00695+K00700+K00844+K00688+K00695+K00695+K00705+K01187+K05349+K00975+K00700+K00700+K05349+K00696+K01051+K01051+K01051+K01051+K01188+K01188+K16055+K16055+K16055+K16055+K16055+K16055+K16055+K16055+K01188+K01188+K01188+K01188+K01188+K01188+K01188+K01188+K01194+K01194+K01194+K01194+K01194+K01194+K01194+K01194+K01188+K01188+K01051+K01051+K01193+K01193+K01193+K01193+K08679+K08679+K08679+K00847+K00847+K00847+K01188+K01188+K01188+K00012+K00012+K00012+K00012+K00012+K00012+K00012+K01051+K01051+K01051+K01051+K01051+K01051+K00963+K00963+K00963+K00963+K01188+K01188+K01188

N-Glycan biosynthesis	ko00510	240	K12666+K12670+K12670+K12670+K12670+K12670+K12670+K01231+K01231+K01231+K12668+K12668+K12668+K12668+K12668+K12668+K12668+K12668+K00729+K00729+K12666+K12666+K12666+K12666+K12666+K00737+K00737+K00737+K00721+K00721+K00721+K00721+K00721+K00721+K00721+K05546+K00737+K00737+K00737+K03844+K03844+K07432+K07432+K07432+K07432+K07252+K07252+K07252+K03846+K12668+K03847+K03847+K12669+K12669+K09659+K09659+K09659+K09659+K09659+K09659+K12666+K12666+K12666+K12666+K07252+K07441+K07441+K07441+K07441+K07151+K07151+K07151+K07151+K07151+K07151+K07151+K07151+K00721+K00721+K00721+K00721+K00721+K07252+K00737+K00737+K00737+K00737+K00737+K00737+K05546+K03843+K03843+K03843+K12667+K12667+K12667+K12667+K12667+K12667+K12667+K12667+K12667+K12667+K12667+K12667+K12667+K12667+K12667+K12667+K12667+K12667+K01230+K01230+K01230+K01230+K01230+K01230+K01230+K01230+K07151+K07151+K07151+K07151+K07151+K07151+K01230+K01230+K01230+K01230+K01230+K03843+K03843+K03843+K03850+K03850+K03850+K03850+K03850+K03850+K00902+K00902+K00902+K00902+K00902+K00902+K03845+K03845+K03845+K03845+K03845+K03845+K03845+K03845+K00737+K00737+K00737+K00737+K00737+K00736+K00736+K00736+K00736+K00736+K00736+K00736+K00736+K00736+K05546+K12666+K03847+K03847+K03848+K03847+K12666+K12666+K12666+K03846+K03846+K07432+K12666+K12666+K07432+K12666+K03846+K12666+K03846+K03846+K12666+K12666+K03846+K01228+K03846+K03848+K05546+K05546+K01228+K01228+K01228+K01228+K01228+K01228+K01228+K01228+K01228+K03848+K03848+K03848+K03848+K03848+K03848+K03848+K03848+K05546+K05546+K05546+K05546+K03846+K03848+K05546+K03848+K03848+K03849+K03849+K01001+K01001+K01001+K01001+K01001+K01001+K01001+K03842+K03842+K03842+K03842+K03842+K00726+K00726+K00726+K00726+K00726
Other glycan degradation	ko00511	176	K01206+K01190+K01190+K01190+K01190+K01190+K01190+K01190+K01190+K12309+K12373+K01191+K01191+K01444+K01444+K01191+K01191+K01191+K01191+K01191+K01191+K01191+K01191+K01191+K01191+K01191+K12373+K12373+K12373+K12373+K12373+K12373+K12373+K12373+K12373+K12373+K12373+K12373+K12373+K12373+K12373+K12373+K12309+K12309+K12309+K12309+K12309+K12309+K12309+K12309+K12309+K12309+K12309+K12309+K12309+K12309+K01227+K01227+K01227+K01227+K01227+K15923+K01191+K15923+K15923+K15923+K01191+K01191+K17108+K17108+K01191+K17108+K01191+K17108+K15923+K15923+K15923+K01191+K01191+K01191+K17108+K01191+K17108+K17108+K01191+K01191+K01191+K01191+K01191+K01191+K01191+K01191+K01191+K17108+K01191+K01191+K01191+K01191+K01191+K17108+K01191+K01191+K17108+K17108+K01191+K01191+K01191+K01191+K17108+K01191+K17108+K01191+K01191+K01191+K17108+K17108+K01191+K01191+K01191+K01191+K01191+K01191+K01191+K01191+K01191+K15923+K01191+K17108+K01191+K01191+K01191+K01191+K01191+K01191+K01191+K01191+K01191+K15923+K15923+K15923+K17108+K17108+K17108+K01191+K01191+K01191+K01191+K15923+K15923+K01191+K0

			1191+K01191+K01191+K01191+K01191+K15923+K01191+K17108+K17108+K15923+K17108+K01191+K01191+K17108+K15923+K17108+K01191+K17108+K17108+K17108+K17108+K17108+K01191+K01191+K01191+K12373
Other types of O-glycan biosynthesis	ko00514	29	K18134+K18134+K09667+K09667+K09667+K09667+K09667+K09667+K09667+K09667+K09667+K09667+K09667+K09667+K09667+K09667+K09667+K09667+K09667+K09667+K13667+K03691+K03691+K03691+K03691+K03691+K03691+K03691+K03691
Amino sugar and nucleotide sugar metabolism	ko00520	581	K08679+K08679+K08679+K08679+K08679+K01809+K01809+K05305+K05305+K05305+K05305+K05305+K05305+K05305+K05305+K05305+K05305+K05305+K05305+K00847+K00847+K00847+K00847+K01784+K01784+K01784+K01784+K01183+K01183+K01183+K01183+K00975+K10046+K10046+K10046+K10046+K10046+K01183+K01183+K01183+K01183+K01183+K01809+K01809+K00966+K00963+K00963+K00963+K00963+K01183+K08679+K08679+K01835+K01183+K00965+K02377+K02377+K02377+K13648+K13648+K13648+K13648+K13648+K13648+K13648+K01183+K01183+K01183+K01183+K01183+K01183+K01183+K01209+K12451+K12451+K12451+K08679+K08679+K16190+K16190+K08679+K08679+K00847+K00847+K00847+K00847+K00847+K01183+K01183+K01183+K01183+K00972+K00966+K00966+K00966+K00966+K00966+K00966+K00966+K00966+K00966+K00966+K01784+K01784+K00326+K00326+K00326+K01183+K01183+K12373+K13648+K13648+K13648+K13648+K13648+K13648+K13648+K13648+K13648+K13648+K13648+K13648+K13648+K13648+K01809+K01809+K01810+K01810+K17497+K17497+K17497+K17497+K06118+K01183+K01183+K01183+K01183+K01809+K00847+K01209+K01209+K01209+K00847+K00847+K00847+K00847+K08679+K08679+K08679+K08679+K08679+K08679+K08679+K08679+K08679+K08679+K08679+K12446+K12446+K12446+K12446+K12446+K12446+K12446+K12446+K12446+K13648+K13648+K13648+K13648+K13648+K13648+K13648+K13648+K13648+K13648+K13648+K01183+K01183+K01183+K01183+K01183+K01183+K01183+K01183+K01183+K01183+K01183+K01183+K01183+K12449+K12449+K12449+K12449+K12449+K12449+K12449+K12449+K12449+K12449+K12449+K12449+K12449+K13648+K13648+K12373+K12373+K12373+K12373+K12373+K12373+K12373+K12373+K12373+K12373+K12373+K12373+K13648+K13648+K13648+K13648+K13648+K13648+K12450+K12450+K12450+K12450+K12450+K12450+K12450+K12450+K12450+K12450+K12450+K12450+K12450+K12450+K12450+K12450+K12450+K12450+K12447+K12447+K12447+K12447+K01835+K01835+K01835+K01835+K01835+K01835+K01835+K01835+K00975+K00975+K00975+K00975+K00966+K00966+K00966+K00966+K00966+K00966+K00966+K12373+K12373+K12373+K12373+K13648+K13648+K13648+K13648+K13648+K13648+K13648+K13648+K13648+K00847+K00847+K00847+K00847+K00847+K008

			47+K00847+K00847+K00012+K00012+K00012+K00012+K00012+K00012+K00012+K00012+K00820+K00820+K00820+K00820+K13648+K13648+K13648+K13648+K13648+K13648+K13648+K13648+K13648+K13648+K13648+K13648+K13648+K13648+K13648+K13648+K13648+K13648+K00770+K00975+K00975+K00975+K00975+K00975+K00975+K00975+K00975+K00975+K00975+K00975+K00975+K00975+K13648+K13648+K13648+K13648+K13648+K13648+K13648+K13648+K12448+K12448+K12448+K12448+K12448+K12448+K12448+K12448+K12448+K12448+K12448+K12448+K12448+K12448+K12448+K00966+K00966+K00966+K00963+K00963+K00963+K00963+K00963+K13648+K13648+K13648+K13648+K01836+K01836+K01836+K01836+K01836+K01836+K01836+K01836+K01836+K01836+K01836+K15920+K15920+K15920+K15920+K18677+K18677+K18677+K18677+K18677+K13648+K13648+K13648+K13648+K13648+K13648+K13648+K13648+K13648+K13648+K13648+K13648+K13648+K13648+K13648+K00012+K00975+K00975+K00975+K00975+K00975+K00975+K00972+K00972+K01810+K01835+K01209+K01810+K01209+K01810+K01183+K01835+K00844+K00844+K01209+K01183+K01810+K01835+K01810+K01209+K01209+K01209+K00844+K01835+K00975+K00844+K01810+K01810+K01810+K01810+K01835+K01810+K01209+K01810+K01209+K01810+K01209+K01209+K00844+K06118+K01209+K01209+K00844+K01810+K00975+K01835+K01810+K01209+K06118+K01209+K01810+K01209+K01835+K01835+K01835+K01835+K01835+K01835+K01835+K01835+K01835+K01835+K01835+K01835+K01835+K01835+K01835+K01835+K01835+K00975+K01810+K01809+K01209+K00975+K00844+K00844+K00844+K00844+K00844+K00844+K00844+K00844+K00844+K00844+K00844+K00844+K00844+K00844+K06118+K06118+K01835+K01835+K01835+K01810+K00844+K01810+K01810+K01810+K00844+K01810+K01209+K01810+K01810+K01810+K00844+K13648+K01810+K01209+K00844+K01209+K00975+K12373+K08679+K08679+K08679+K00847+K00847+K00847+K10046+K10046+K10046+K10046+K10046+K10046+K10046+K00012+K00012+K00012+K00012+K00012+K00012+K17497+K17497+K17497+K17497+K00966+K00966+K00963+K00963+K00963+K00963+K01183+K01183
Glycosaminoglycan degradation	ko00531	66	K10532+K12309+K07964+K07964+K12373+K10532+K10532+K07964+K07964+K07964+K07964+K07964+K07964+K07964+K07964+K07964+K07964+K07964+K07964+K07964+K12373+K12373+K12373+K12373+K12373+K12373+K12373+K12373+K12373+K12373+K12373+K12373+K12373+K12373+K12373+K12373+K01205+K01205+K12309+K12309+K12309+K12309+K12309+K12309+K12309+K12309+K12309+K12309+K12309+K12309+K12309+K12309+K07964+K07964+K07964+K07964+K07964+K07964+K07964+K07964+K07964+K10532+K10532+K10532+K10532+K12373
Glycerolipid metabolism	ko00561	309	K13519+K13519+K13519+K13519+K13519+K13513+K07407+K07407+K06119+K07407+K13508+K13508+K13508+K13508+K13508+K13508+K00128+K13506+K13506+K13506+K13506+K13506+K13506+K03715+K00011+K00011+K00011+K13508+K13508+K13508+K13508+K13508+K13508+K13508+K13508+K13508+K13508+K13508+K13508+K14457+K14457+K00630+K00630+K00630+K07407+

			K07407+K07407+K14085+K14085+K14085+K14085+K14085+K14085+K14085+K14085+K14085+K14085+K14085+K14085+K14085+K14085+K14085+K14085+K06118+K00679+K00679+K00901+K13523+K13523+K13523+K13523+K13523+K13523+K00901+K00901+K00864+K00864+K00864+K00864+K00864+K06119+K06119+K06119+K06119+K13508+K13508+K13508+K13508+K00128+K00128+K00128+K00128+K00128+K00128+K00128+K00128+K00128+K00128+K00128+K15728+K15728+K15728+K15728+K15728+K15728+K15728+K15918+K15918+K15918+K15918+K15918+K15918+K15918+K15918+K15918+K09480+K09480+K09480+K09480+K09480+K09480+K09480+K09480+K09480+K09480+K13513+K13513+K13513+K00128+K00128+K00128+K00128+K00128+K00128+K00128+K00128+K00128+K00128+K00128+K00128+K00128+K00128+K00128+K15728+K15728+K15728+K15728+K15728+K15728+K15728+K15728+K00901+K00901+K00901+K00901+K13506+K13506+K13506+K13506+K13506+K13506+K13506+K13506+K13506+K13506+K00679+K00679+K00679+K00679+K00679+K00679+K00679+K00679+K00679+K00679+K00679+K00679+K00679+K00679+K00679+K00679+K00679+K00679+K11155+K11155+K11155+K00128+K00128+K00128+K00128+K00128+K00128+K00128+K00128+K00128+K00128+K07407+K07407+K00128+K00128+K00128+K00128+K00128+K09480+K09480+K09480+K09480+K09480+K09480+K09480+K09480+K09480+K09480+K14674+K14674+K14674+K14674+K14674+K14674+K14674+K14674+K14674+K14674+K14674+K00901+K00901+K03715+K00901+K00901+K00901+K00128+K00901+K06119+K00901+K00901+K00901+K00128+K06119+K00863+K03715+K00901+K03715+K00901+K00901+K00901+K00901+K03715+K00901+K06119+K00901+K07407+K06118+K00901+K00901+K00901+K00863+K06118+K00128+K00128+K00863+K00901+K00128+K00901+K00901+K00901+K00901+K06118+K06118+K00863+K00128+K07407+K00863+K00901+K00863+K00901+K00901+K00901+K13523+K00863+K00901+K00901+K00901+K00901+K00901+K00901+K00863+K00901+K00901+K00901+K00901+K00901+K00901+K00128+K00128+K00128+K13508+K13508+K03715+K03715+K03715+K03715+K07407+K07407+K07407+K07407+K13508+K13508+K13508+K13508
Inositol phosphate metabolism	ko00562	242	K00913+K00913+K00913+K00913+K00913+K01803+K01803+K01803+K01803+K01803+K01803+K01803+K01803+K00915+K00915+K00915+K00913+K00913+K00469+K00469+K00469+K00469+K00469+K00469+K00921+K00921+K00921+K00921+K00921+K00921+K00921+K00921+K00921+K00921+K00921+K00921+K01114+K10572+K10572+K10572+K10572+K10572+K00921+K00921+K00921+K00921+K00921+K00921+K00921+K00921+K00921+K00921+K00921+K15422+K15422+K01803+K01803+K01803+K01114+K01114+K01092+K01092+K00999+K00999+K00999+K00999+K00999+K00999+K00913+K01110+K01110+K01110+K01110+K01110+K01110+K01110+K01110+K01110+K01110+K01110+K01110+K01110+K01803+K01803+K01803+K01803+K01803+K01803+K01803+K01803+K01803+K01803+K01803+K01803+K01803+K01803+K01803+K00889+K00889+K00889+K00889+K00889+K00888+K00888+K01106+K01106+K00889+K00889+K00889+K00889+K00140+K00140+K00140+K00140+K00140+K00140+K00140+K00914+K00914+K

			00914+K00914+K00914+K00889+K00889+K00889+K00889+K00889+K00889+K00889+K00889+K00889+K01110+K01110+K05857+K05857+K05857+K05857+K05857+K05857+K05857+K00888+K00888+K00888+K00888+K00888+K00140+K00140+K00140+K00140+K00140+K00140+K10572+K10572+K10572+K10572+K01114+K01114+K01114+K00915+K00915+K00915+K03103+K03103+K03103+K03103+K03103+K03103+K03103+K03103+K03103+K03103+K03103+K03103+K03103+K03103+K03103+K03103+K03103+K03103+K01092+K00469+K05857+K10572+K01114+K10572+K01114+K01110+K10572+K01858+K10572+K01858+K10572+K01858+K01114+K10572+K10572+K01858+K10572+K10572+K01114+K10572+K10572+K01092+K00140+K00140+K00140+K01114+K01114+K01114+K00469+K00469+K01114+K05857+K01858+K00140+K00140+K00140+K01114+K01858+K05857+K05857+K05857+K05857+K01858+K01110+K01858+K01858+K05857+K00140+K15422+K01110+K00140+K05857+K01858+K01803+K15422+K01110+K05857+K05857+K00913+K00913
Glycosylphosphatidylinositol(GPI)-anchor biosynthesis	ko00563	87	K05290+K05290+K05290+K05290+K05290+K07542+K07542+K07542+K07542+K07542+K07542+K05310+K05310+K05310+K05310+K05283+K05283+K05283+K05283+K05283+K05283+K05283+K03434+K03434+K05283+K05283+K05283+K05283+K05285+K05285+K05285+K05285+K03857+K03857+K03857+K03857+K03857+K03860+K03860+K03860+K03860+K05289+K05289+K03861+K05291+K05291+K05291+K05291+K05291+K05291+K05291+K05291+K05291+K05284+K05284+K05284+K05284+K05288+K05288+K05288+K05288+K05288+K05292+K05292+K05287+K05287+K05293+K05293+K05293+K05293+K03434+K05286+K05293+K05293+K07541+K07541+K05286+K05286+K05286+K05286+K05286+K05293+K05293+K05293+K07541+K05283+K05283
Glycerophospholipid metabolism	ko00564	399	K00968+K00968+K00968+K00968+K13510+K13510+K13510+K13510+K13510+K13510+K13510+K13510+K06130+K06130+K06130+K06130+K06130+K06130+K06130+K06130+K00967+K00967+K00967+K00967+K13519+K13519+K13519+K13519+K13519+K13513+K14156+K14156+K14156+K14156+K00993+K00993+K00993+K16860+K16860+K16860+K01115+K01517+K01517+K13508+K13508+K13508+K13508+K13508+K13508+K13506+K13506+K13506+K13506+K13506+K13506+K00894+K00894+K00894+K00894+K01114+K13508+K13508+K13508+K13508+K13508+K13508+K13508+K13508+K13508+K13508+K13508+K13508+K06130+K00967+K00967+K00630+K00630+K00630+K00006+K01126+K01126+K01114+K01114+K01115+K01094+K01115+K01115+K00999+K00999+K00999+K00999+K00999+K00999+K00993+K06129+K00995+K00550+K00550+K06130+K06130+K00901+K13523+K13523+K13523+K13523+K13523+K13523+K01115+K00901+K00901+K01115+K01115+K01115+K01115+K01115+K01115+K13508+K13508+K13508+K13508+K01115+K01115+K01115+K01115+K01115+K01115+K01115+K00967+K01613+K01613+K01613+K15728+K15728+K15728+K15728+K15728+K15728+K15728+K13513+K13513+K13513+K01115+K01115+K01115+K01115+K01115+K01115+K01115+K01115+K01115+K01115+K01115+K05929+K05929+K05929+K05929+K05929+K05929+K05929+K05929+K05929+K05929+K05929+K01115+K01115+K011

			15+K01115+K01115+K01115+K01115+K01115+K01115+K01115+K01115+K01115+K01115+K01115+K01115+K01115+K01115+K01115+K01115+K01115+K01115+K01115+K15728+K15728+K15728+K15728+K15728+K15728+K15728+K15728+K01115+K01115+K01115+K01115+K01115+K01115+K01115+K01115+K01115+K00901+K00901+K00901+K00901+K13506+K13506+K13506+K13506+K13506+K13506+K13506+K13506+K13506+K13506+K01115+K01115+K01115+K01115+K01115+K01115+K01115+K01115+K00993+K00993+K00993+K00993+K00993+K00993+K00993+K00993+K00993+K01126+K01126+K01126+K01126+K01126+K01126+K01126+K01126+K01126+K01126+K01126+K01126+K01126+K01126+K01126+K01126+K01126+K01613+K01613+K01613+K01613+K00006+K00006+K00006+K00006+K00006+K00006+K13510+K13510+K13510+K13510+K13510+K13510+K00981+K00111+K00111+K00111+K00111+K01114+K01114+K01114+K00968+K00968+K00968+K00968+K00968+K00968+K00968+K00968+K14156+K14156+K14156+K14156+K14674+K14674+K14674+K14674+K14674+K14674+K14674+K14674+K14674+K14674+K14674+K00901+K00981+K00981+K00901+K00901+K00901+K00901+K01114+K00901+K00901+K01114+K00901+K00901+K01115+K01115+K01115+K00901+K00901+K00901+K00901+K00901+K01114+K00901+K01114+K00901+K00901+K00901+K01115+K01115+K00901+K01114+K01114+K01115+K01114+K01114+K00901+K00901+K00901+K01115+K00901+K00901+K01115+K01115+K01115+K01115+K01114+K00981+K01115+K01115+K01115+K01115+K01115+K01115+K01115+K01115+K00901+K00981+K00967+K00901+K00006+K00006+K00967+K00901+K01115+K00901+K00967+K13523+K00981+K00901+K00901+K00901+K00967+K00901+K00967+K00006+K00901+K00901+K00901+K00901+K00901+K00967+K00901+K00901+K00901+K13511+K13511+K13511+K13511+K13511+K13511+K08730+K08730+K08730+K08730+K08730+K08730+K13508+K13508+K00981+K00981+K00981+K13508+K13508+K13508+K13508
Ether lipid metabolism	ko00565	149	K13510+K13510+K13510+K13510+K13510+K13510+K13510+K13510+K13519+K13519+K13519+K13519+K13519+K00993+K00993+K00993+K16860+K16860+K16860+K01115+K01114+K01114+K01114+K01115+K01115+K01115+K00993+K01115+K00993+K00993+K00993+K00993+K00993+K00993+K00993+K00993+K00993+K13510+K13510+K13510+K13510+K13510+K01114+K01114+K01114+K14674+K14674+K14674+K14674+K14674+K14674+K14674+K14674+K14674+K14674+K14674+K01114+K01114+K01115+K01115+K01115+K01114+K01114+K01115+K01115+K01114+K01114+K01115+K01114+K01114+K01115+K01115+K01115+K01115+K01115+K01114+K01115+K01115+K01115+K01115+K01115+K01115+K01115+K01115+K01115

Arachidonic acid metabolism	ko00590	49	K00432+K00432+K00432+K00432+K00432+K00432+K00079+K00432+K00432+K00432+K15730+K15730+K15730+K05309+K05309+K00432+K00432+K00432+K00432+K00432+K00432+K18592+K18592+K18592+K18592+K18592+K18592+K18592+K18592+K18592+K00432+K00432+K00432+K00432+K00432+K00432+K00432+K00432+K14674+K14674+K14674+K14674+K14674+K14674+K14674+K14674+K14674+K14674+K14674
Linoleic acid metabolism	ko00591	110	K00454+K00454+K00454+K00454+K00454+K15718+K00454+K00454+K00454+K00454+K00454+K00454+K00454+K00454+K00454+K00454+K00454+K00454+K00454+K14674+K14674+K14674+K14674+K14674+K14674+K14674+K14674+K14674+K14674+K14674+K15718+K00454
alpha-Linolenic acid metabolism	ko00592	266	K05894+K05894+K05894+K05894+K05894+K10525+K01723+K05894+K05894+K08241+K08241+K08241+K08241+K08241+K08241+K08241+K08241+K08241+K08241+K08241+K08241+K08241+K08241+K05894+K05894+K05894+K07513+K07513+K00232+K07513+K18857+K10526+K10525+K10525+K10525+K10525+K10525+K10525+K05894+K05894+K05894+K18857+K18857+K18857+K00232+K08241+K00454+K00454+K00454+K00454+K00454+K00454+K00454+K00454+K00454+K00454+K00454+K00454+K00454+K00454+K00454+K00454+K00454+K00454+K10527+K10527+K10527+K10527+K10527+K10527+K10527+K10527+K00232+K00232+K00232+K00232+K00232+K10528+K10528+K10528+K10528+K10528+K10528+K10528+K10528+K10528+K00232+K00232+K00232+K01723+K01723+K01723+K01723+K01723+K01723+K01723+K01723+K01723+K01723+K07513+K07513+K07513+K07513+K07513+K07513+K07513+K14674+K14674+K14674+K14674+K14674+K14674+K14674+K14674+K14674+K14674+K14674+K00232+K01723+K10526+K10526+K18857+K10526+K10526+K01723+K08241+K10527+K07513+K05894+

			K18857+K10527+K01723+K01723+K10527+K10526+K18857+K10527+K18857+K18857+K18857+K01723+K18857+K18857+K10529+K00454+K08241+K18857+K18857+K18857+K18857+K18857+K18857+K18857+K18857+K18857+K18857+K18857+K18857+K18857
Sphingolipid metabolism	ko00600	142	K07407+K07407+K04718+K04718+K04718+K04718+K04710+K04710+K04711+K04711+K00654+K07407+K04713+K04713+K04713+K04713+K04713+K01190+K01190+K01190+K01190+K01190+K01190+K01190+K01190+K12309+K07407+K07407+K07407+K04716+K04716+K04716+K04716+K01634+K01634+K01634+K01634+K04710+K04710+K04710+K04710+K00654+K00654+K00654+K00654+K00654+K00654+K00654+K00654+K00654+K00654+K00654+K00654+K00654+K00654+K00654+K00654+K00654+K00654+K04711+K04711+K04711+K04711+K12349+K12349+K12349+K12349+K12349+K12349+K12349+K12349+K12349+K12349+K12349+K12349+K12349+K12349+K12349+K12349+K12349+K12309+K12309+K12309+K12309+K12309+K12309+K12309+K12309+K12309+K12309+K12309+K12309+K12309+K12309+K12309+K12309+K12309+K12309+K12309+K12349+K04715+K04715+K04715+K04715+K07407+K07407+K04708+K04708+K04708+K00654+K04716+K00654+K07407+K17108+K17108+K00654+K17108+K17108+K17108+K17108+K17108+K17108+K17108+K17108+K17108+K17108+K17108+K17108+K17108+K17108+K07407+K17108+K17108+K17108+K00654+K00654+K17108+K00654+K17108+K17108+K17108+K17108+K00654+K17108+K17108+K17108+K17108+K17108+K00654+K00654+K07407+K07407+K07407+K07407
Glycosphingolipid biosynthesis - globo series	ko00603	32	K07407+K07407+K07407+K07407+K07407+K07407+K12373+K12373+K12373+K12373+K12373+K12373+K12373+K12373+K12373+K12373+K12373+K12373+K12373+K12373+K12373+K12373+K12373+K07407+K07407+K07407+K07407+K12373+K07407+K07407+K07407+K07407
Glycosphingolipid biosynthesis - ganglio series	ko00604	33	K12309+K12373+K12373+K12373+K12373+K12373+K12373+K12373+K12373+K12373+K12373+K12373+K12373+K12373+K12373+K12373+K12373+K12373+K12309+K12309+K12309+K12309+K12309+K12309+K12309+K12309+K12309+K12309+K12309+K12309+K12309+K12309+K12373
Pyruvate metabolism	ko00620	527	K00161+K00161+K00161+K00161+K02160+K02160+K02160+K02160+K01759+K01759+K00025+K00025+K00025+K00025+K00025+K00025+K00025+K00025+K00025+K00025+K00025+K01069+K01069+K01069+K01069+K00128+K01759+K01759+K00029+K01759+K01759+K01759+K01759+K01759+K01759+K01759+K01759+K00026+K00026+K00026+K00026+K00026+K00026+K00026+K00026+K00026+K00162+K00162+K00162+K00162+K00162+K00162+K00162+K00162+K00162+K11262+K11262+K11262+K11262+K11262+K00161+K00025+K00026+K00026+K00026+K01595+K01595+K00873+K18881+K18881+K18881+K18881+K18881+K01679+K00626+K00626+K00626+K00626+K00626+K00626+K14085+K14085+K14085+K14085+K14085+K14085+K14085+K14085+K14085+K14085+

	K14085+K14085+K14085+K14085+K14085+K14085+K00051+K00051+K00051+K00051+K00161+K00161+K01963+K00025+K00025+K00026+K00026+K00026+K00026+K00026+K01759+K01759+K01512+K01512+K01759+K01759+K00627+K00627+K01962+K01962+K01962+K01962+K01962+K01962+K01962+K01962+K01962+K01962+K01962+K01962+K01595+K01595+K01595+K01595+K01595+K01595+K00128+K00128+K00128+K00128+K00128+K00128+K00128+K00128+K00128+K00128+K00128+K01006+K01006+K01069+K01069+K01069+K01069+K01069+K01069+K01649+K01649+K01649+K01649+K01649+K01649+K01649+K01649+K01649+K01649+K01649+K01649+K00028+K00028+K00028+K00028+K00028+K00028+K00028+K00028+K00028+K00028+K00028+K00028+K00028+K00028+K00028+K00873+K00873+K00873+K00128+K00128+K00128+K00128+K00128+K00128+K00128+K00128+K00128+K00128+K00128+K00128+K00128+K00128+K00128+K01962+K01962+K01962+K01962+K01962+K00873+K00873+K00873+K00873+K00873+K00873+K00873+K00873+K00873+K00873+K00873+K00873+K00873+K00873+K00873+K00873+K00873+K00873+K00026+K00026+K00026+K00382+K00382+K00382+K00382+K00382+K00382+K00382+K00382+K00382+K00382+K00029+K00029+K00029+K00029+K00029+K00029+K00029+K00029+K00029+K00028+K00028+K00028+K00028+K00028+K00028+K00028+K00028+K00028+K00051+K00128+K00128+K00128+K00128+K00128+K00128+K00128+K00128+K00128+K00128+K00128+K00128+K00128+K00128+K00128+K00382+K00382+K00382+K00382+K00102+K00029+K00627+K00873+K00873+K00873+K01595+K01595+K00051+K00029+K01595+K01595+K00128+K00873+K01595+K01595+K00627+K00029+K01595+K01595+K00051+K01595+K00873+K01610+K01610+K00128+K01610+K01610+K01610+K01595+K01610+K01595+K01610+K01610+K00873+K01610+K01610+K01595+K00627+K00873+K00029+K00873+K00029+K01963+K00102+K01595+K00029+K01595+K00873+K01595+K01595+K00873+K00873+K01595+K01595+K01595+K01679+K00873+K01595+K01595+K00051+K01595+K00029+K00029+K00873+K00029+K00873+K00051+K00029+K01610+K00029+K00029+K00029+K01595+K00051+K01595+K00873+K00029+K00029+K00029+K00029+K00102+K00102+K00102+K00873+K00102+K00029+K01895+K00873+K00051+K00029+K00029+K00029+K00627+K01895+K00029+K00029+K00029+K00029+K00029+K00029+K00029+K00029+K00029+K00029+K00029+K00128+K00029+K01679+K01679+K00873+K01679+K01679+K01679+K01679+K01679+K01679+K01679+K01679+K00128+K00029+K00029+K00128+K00627+K00627+K00029+K00029+K00029+K00627+K00029+K00029+K00029+K00029+K00627+K00029+K01963+K01963+K01963+K01963+K01963+K01963+K00627+K00627+K00029+K01679+K00029+K00029+K00029+K00029+K00029+K01610+K00128+K00051+K00029+K00029+K00029+K00029+K00029+K00029+K00102+K01610+K01895+K00873+K00627+K01895+K00627+K00627+K00873+K01895+K01895+K00873+K01895+K00627+K00627+K01895+K01679+K00627+K01679+K00873+K01610+K00627+K00873+K00627+K00873+

			K00873+K00873+K00873+K00873+K00873+K00873+K00873+K00873+K00873+K00873+K00873+K00873+K00873+K00873+K01961+K01961+K01961+K00128+K00128+K00128+K00627+K00627+K00627+K00627+K00627+K00627+K00627+K00627+K00627+K00627+K00627+K00626+K00626+K00626+K00626+K00626+K00626+K01759+K01759+K01759+K01759+K01759+K01759+K01759+K00162+K00162+K00162
Glyoxylate and dicarboxylate metabolism	ko00630	314	K01455+K01455+K01455+K01455+K01455+K01455+K00122+K00122+K15893+K15893+K15893+K15893+K15893+K15893+K 15893+K15893+K15893+K00025+K00025+K00025+K00025+K00025+K00025+K00025+K00025+K00025+K00025+K00025+K01915+K15919+K03781+K03781+K03781+K03781+K03781+K03781+K03781+K03781+K03781+K03781+K00026+K00026+K00026+K00026+K00026+K00026+K00026+K00026+K00026+K11517+K01602+K01602+K01602+K01602+K01602+K01602+K01602+K01602+K01602+K01602+K01602+K00025+K01602+K01602+K01602+K01602+K01602+K01602+K01602+K01602+K01602+K01602+K01602+K01602+K01602+K00026+K00026+K00026+K01091+K00626+K00626+K00626+K00626+K00626+K00626+K00025+K00025+K02437+K02437+K02437+K02437+K11517+K00026+K00026+K00026+K00026+K00026+K02437+K01601+K01601+K01601+K15919+K15919+K15919+K15919+K15919+K15919+K15918+K15918+K15918+K15918+K15918+K15918+K15918+K15918+K15918+K01915+K14272+K14272+K14272+K14272+K14272+K14272+K14272+K14272+K01647+K00284+K00284+K00284+K00284+K00284+K00284+K00284+K00284+K00284+K01915+K01915+K00026+K00026+K00026+K00600+K00600+K00600+K00600+K00600+K11517+K11517+K11517+K11517+K11517+K00830+K00830+K00830+K00830+K00830+K00830+K00830+K00830+K01647+K01647+K01647+K01647+K01647+K01647+K01647+K00600+K00600+K00600+K00600+K00600+K00600+K00600+K15919+K15919+K01915+K01681+K01601+K01681+K01681+K01681+K02437+K02437+K01681+K01602+K01091+K01602+K01602+K01681+K01681+K01681+K01681+K01681+K01681+K01681+K01681+K01091+K01681+K01681+K01681+K01681+K01091+K01091+K01681+K01681+K01681+K01681+K01091+K01681+K01681+K01681+K01681+K01681+K01681+K01091+K01681+K01091+K01681+K15919+K01681+K01681+K15919+K01681+K01681+K01681+K01681+K01602+K01681+K00600+K00600+K00600+K00600+K00600+K00600+K00600+K00600+K00600+K00600+K00600+K00600+K00600+K00600+K00600+K00600+K00600+K00122+K00122+K00122+K00122+K00122+K00122+K00122+K00122+K11517+K11517+K11517+K11517+K00600+K00600+K00600+K03781+K03781+K03781+K03781+K03781+K03781+K03781+K00626+K00626+K00626+K00626+K00626+K00626+K01433+K01433+K01433+K01647+K01647+K01647+K01647+K01647+K01647+K01647+K01647
Propanoate	ko00640	146	K02160+K02160+K02160+K02160+K05605+K05605+K05605+K05605+K05605+K05605+K05605+K11262+K11262+K11262+K11262+

metabolism			K11262+K05605+K05605+K05605+K05605+K05605+K05605+K00626+K00626+K00626+K00626+K00626+K00626+K01963+K05605+K05605+K05605+K05605+K05605+K05605+K05605+K05605+K05605+K05605+K05605+K05605+K05605+K01899+K01899+K01899+K01899+K05605+K05605+K01962+K01962+K01962+K01962+K01962+K01962+K01962+K01962+K01962+K01962+K01962+K01962+K00249+K00249+K00249+K00249+K00249+K00249+K00249+K00249+K00249+K00249+K05605+K05605+K05605+K05605+K05605+K05605+K05605+K05605+K05605+K05605+K05605+K05605+K05605+K05605+K00140+K00140+K00140+K00140+K00140+K00140+K00140+K01962+K01962+K01962+K01962+K01962+K00140+K00140+K00140+K00140+K00140+K00140+K01963+K00249+K00140+K00140+K01895+K00140+K01895+K00140+K00140+K00140+K01963+K01963+K01963+K01963+K01963+K01963+K01899+K01895+K00140+K01895+K01895+K01895+K00140+K01895+K01895+K01961+K01961+K01961+K01900+K01900+K01900+K01900+K01900+K01900+K01578+K01578+K01578+K01578+K00626+K00626+K00626+K00626+K00626+K00626
Butanoate metabolism	ko00650	82	K01640+K01640+K01640+K01640+K16871+K00626+K00626+K00626+K00626+K00626+K00626+K18121+K18121+K18121+K01580+K01580+K01580+K18121+K18121+K01653+K01653+K01653+K01653+K01653+K01653+K01653+K01641+K01641+K01641+K01641+K01641+K01641+K18121+K18121+K18121+K18121+K17761+K17761+K17761+K17761+K17761+K17761+K17761+K00074+K00074+K00074+K00074+K18121+K18121+K01652+K01652+K01652+K01652+K01652+K01580+K01580+K01580+K01580+K01580+K01640+K01640+K01640+K01640+K01640+K01640+K16871+K16871+K16871+K16871+K16871+K16871+K16871+K16871+K01653+K01653+K00626+K00626+K00626+K00626+K00626+K00626
C5-Branched dibasic acid metabolism	ko00660	37	K01703+K01703+K01703+K01703+K01704+K01704+K01704+K01704+K00052+K00052+K01653+K01653+K01653+K01653+K01653+K01653+K01653+K01653+K01703+K01703+K01703+K01703+K01703+K01703+K01703+K01703+K01703+K01703+K00052+K00052+K01652+K01652+K01652+K01652+K01652+K01653+K01653
One carbon pool by folate	ko00670	80	K00605+K00605+K00604+K13998+K13998+K13998+K13998+K13998+K13998+K13998+K13998+K13998+K13998+K13998+K00600+K00600+K00600+K00600+K00600+K01934+K01934+K01934+K01934+K01934+K01938+K01938+K01938+K01938+K01938+K01938+K01938+K01938+K01938+K00297+K00297+K00297+K00297+K00297+K00600+K00600+K00600+K00600+K00600+K00600+K00600+K00602+K00602+K00602+K00602+K00602+K00602+K00602+K00605+K00604+K00604+K00604+K00605+K00600+K01433+K01433+K01433
Carbon fixation in photosynthetic	ko00710	487	K00855+K00855+K00855+K00855+K00855+K00927+K00927+K00927+K01623+K01623+K01623+K01623+K01623+K01623+K01623+K01803+K01803+K01803+K01803+K01803+K01803+K01803+K01803+K14454+K14454+K14454+K14454+K14454+K14455+

organisms	K14455+K14455+K01807+K01807+K01807+K00025+K00025+K00025+K00025+K00025+K00025+K00025+K00025+K00025+K00025+K00025+K01623+K01623+K01623+K01623+K01623+K01623+K01623+K01623+K01623+K01623+K01623+K01623+K01623+K01623+K01623+K00029+K00927+K00927+K00927+K00927+K00927+K00927+K00927+K00026+K00026+K00026+K00026+K00026+K00026+K00026+K00026+K00026+K01623+K01623+K01623+K01623+K01623+K01623+K01623+K01623+K01623+K01623+K01623+K01623+K01623+K01602+K01602+K01602+K01602+K01602+K01602+K01602+K01602+K01602+K01602+K01602+K00025+K01602+K01602+K01602+K01602+K01602+K01602+K01602+K01602+K01602+K01602+K01602+K01602+K01602+K01783+K01783+K01783+K00026+K00026+K00026+K01595+K01595+K01803+K01803+K01803+K03841+K03841+K01807+K01807+K01807+K05298+K05298+K05298+K05298+K05298+K05298+K05298+K00051+K00051+K00051+K00051+K00025+K00025+K00814+K00814+K00026+K00026+K00026+K00026+K00026+K01623+K01623+K00134+K00134+K00134+K00134+K01807+K00134+K05298+K00134+K00134+K00134+K00134+K00134+K00134+K00134+K00134+K00134+K00134+K00134+K00134+K00134+K00134+K00134+K00134+K00134+K00134+K01803+K01803+K01803+K01595+K01595+K01803+K01803+K01803+K01803+K01803+K01803+K01803+K01803+K01803+K01803+K01803+K01803+K01803+K01601+K01601+K01601+K01595+K01595+K01595+K01595+K01783+K01783+K01783+K01783+K01006+K01006+K00134+K00134+K00134+K00134+K00134+K00134+K00134+K00134+K00134+K00134+K05298+K05298+K05298+K05298+K05298+K05298+K05298+K05298+K05298+K00028+K00028+K00028+K00028+K00028+K00028+K00028+K00028+K00028+K00028+K00028+K00028+K00028+K00028+K00028+K01623+K01623+K01623+K01623+K14454+K14454+K14272+K14272+K14272+K14272+K14272+K14272+K14272+K14272+K00615+K00615+K00615+K00615+K00615+K00615+K00615+K00615+K00615+K00615+K00615+K00615+K00615+K00615+K00615+K00615+K00615+K00615+K00615+K00026+K00026+K00026+K00029+K00029+K00029+K00029+K00029+K00029+K00029+K00029+K00029+K00927+K00927+K00927+K00927+K00028+K00028+K00028+K00028+K00028+K00028+K00028+K00028+K00028+K01783+K01783+K01783+K03841+K03841+K03841+K03841+K03841+K03841+K03841+K00051+K00814+K00029+K00814+K00814+K00814+K01595+K01595+K00051+K00134+K01623+K00029+K01595+K01595+K01595+K01595+K00029+K01595+K00134+K00134+K01595+K00051+K01595+K01610+K01610+K00134+K00134+K01610+K01610+K01610+K01623+K01595+K01610+K01595+K01610+K01610+K01610+K01610+K01595+K01623+K00029+K00029+K05298+K00134+K01595+K00029+K01623+K01595+K00134+K01595+K00134+K00134+K00134+K01595+K05298+K01595+K01595+K01595+K01601+K00134+K01595+K01595+K00134+K00051+K01595+K00029+K00029+K00029+K00051+K00029+K01610+K00029+K00029+K00029+K01595+K00051+K01595+K00029+K00029+K00029+K00029+K00029+K00051+K00029+K00029+K00029+K00029+

			K00029+K00134+K00029+K00029+K01623+K00029+K01623+K00029+K00029+K00029+K00029+K01610+K00051+K00029+K00029+K00029+K00029+K00029+K00029+K01610+K01602+K01602+K01602+K00814+K05298+K00814+K05298+K05298+K05298+K05298+K00134+K01803+K01610+K01602+K01100+K01100+K01100+K03841+K03841+K03841+K03841+K03841+K03841+K03841+K00927+K00927+K00927+K00927
Thiamine metabolism	ko00730	44	K03146+K03146+K03146+K03146+K03146+K03146+K03146+K03146+K03146+K03146+K03146+K04487+K04487+K04487+K00949+K00949+K03146+K03146+K03146+K06928+K01662+K01662+K01662+K01662+K01662+K01662+K01662+K01662+K01662+K01662+K01662+K03147+K03147+K01662+K03147+K03147+K01662+K01662+K01662+K01662+K01662+K14153+K14153+K14153
Riboflavin metabolism	ko00740	32	K00794+K00794+K00794+K00794+K00793+K00793+K14652+K18453+K18453+K14652+K14652+K14652+K14652+K00861+K14652+K00861+K00861+K00861+K00861+K00861+K00861+K00861+K00861+K11752+K11752+K11752+K11752+K11752+K18453+K14652+K14652+K14652
Vitamin B6 metabolism	ko00750	33	K08681+K08681+K08681+K13248+K13248+K13248+K13248+K13248+K06215+K06215+K06215+K06215+K06215+K06215+K00831+K00831+K00831+K13248+K13248+K00868+K00868+K13248+K01733+K01733+K01733+K01733+K17759+K17759+K17759+K17759+K17759+K01733+K01733
Nicotinate and nicotinamide metabolism	ko00760	67	K03787+K03787+K03787+K03787+K03787+K03787+K03787+K03787+K00767+K00767+K00767+K03426+K03426+K00858+K00858+K01081+K01081+K06210+K06210+K01240+K01240+K01240+K01240+K01240+K01240+K01950+K01950+K01950+K01950+K01950+K01950+K01950+K00278+K00278+K00278+K00278+K00278+K00278+K00763+K00763+K00763+K00763+K03787+K03787+K03787+K03787+K00858+K00858+K00858+K03517+K00858+K00858+K00858+K00858+K00858+K00858+K00858+K00858+K00858+K03517+K03517+K06210+K03787+K03787+K03787+K03787+K03787
Pantothenate and CoA biosynthesis	ko00770	110	K01922+K01922+K01922+K01922+K01922+K06133+K06133+K06133+K06133+K06133+K06133+K01464+K01464+K00859+K00859+K00859+K00859+K00859+K01431+K01431+K01431+K01431+K01431+K00606+K00606+K00606+K00053+K01598+K01598+K00826+K00826+K00826+K00826+K00826+K00826+K00826+K00826+K00826+K00826+K00826+K02201+K02201+K09680+K01653+K01653+K01653+K01653+K01653+K01653+K01653+K01653+K00606+K00606+K09680+K09680+K09680+K09680+K09680+K00207+K00207+K00207+K00207+K00207+K00207+K00207+K00207+K00207+K01687+K01687+K09680+K09680+K09680+K09680+K09680+K09680+K00053+K00053+K00053+K00053+K01464+K00053+K01464+K01464+K00053+K00053+K01464+K01464+K01464+K00053+K00053+K01464+K00053+K01464+K01464+K01464+K01464+K01464+K01464+K01464+K01464+K01464+K00826+K00826+K01652+K01652+

			K01652+K01652+K01652+K01653+K01653
Biotin metabolism	ko00780	40	K00059+K00059+K00059+K00059+K00059+K09458+K00208+K00208+K00208+K00208+K01942+K01942+K01942+K00059+K00059+K02372+K02372+K00208+K00208+K00208+K01012+K00059+K00059+K00059+K00059+K00833+K00833+K01942+K01942+K09458+K09458+K09458+K09458+K09458+K09458+K00652+K00652+K09458+K09458+K09458
Lipoic acid metabolism	ko00785	12	K03801+K03801+K03801+K03801+K03644+K03644+K03644+K03644+K03644+K03644+K03644+K03644
Folate biosynthesis	ko00790	67	K01307+K01307+K01307+K01307+K01307+K01307+K18482+K18482+K18482+K13950+K13950+K13950+K13950+K01495+K01113+K01113+K01113+K01307+K01307+K03635+K03635+K01633+K01633+K01633+K01633+K01633+K01633+K01633+K03635+K03635+K03635+K03635+K01930+K01930+K01930+K01930+K01930+K01930+K01930+K01930+K01930+K13998+K13998+K13998+K13998+K13998+K13998+K13998+K13998+K13998+K13998+K13998+K13941+K13941+K13941+K01930+K01930+K01930+K01930+K01930+K01930+K01930+K01495+K01495+K01495+K01495+K01495
Porphyrin and chlorophyll metabolism	ko00860	167	K04040+K04040+K04040+K04040+K04040+K02257+K02257+K00510+K00510+K00510+K00510+K00510+K00228+K00228+K00228+K00228+K01599+K01599+K01599+K01599+K01599+K01698+K10960+K10960+K10960+K10960+K00218+K00218+K00218+K00218+K13606+K01749+K01749+K04035+K00218+K01599+K01599+K08099+K02257+K01885+K00218+K00218+K02259+K02259+K13545+K03404+K03404+K03404+K03404+K03404+K03404+K03404+K03403+K03403+K03403+K03403+K03403+K03403+K03403+K18010+K18010+K18010+K18010+K13600+K13600+K13600+K13600+K01885+K01885+K01885+K01885+K01885+K01885+K01885+K01885+K01885+K01885+K01885+K01885+K01885+K01885+K01885+K10960+K10960+K13545+K13545+K08099+K08099+K08099+K08099+K08099+K00231+K00231+K00231+K00231+K00231+K00231+K00231+K00231+K00218+K00218+K00218+K00218+K00218+K00218+K00218+K00218+K00218+K00218+K00218+K04035+K04035+K04035+K04035+K04035+K02492+K02492+K02492+K02492+K02492+K02492+K13606+K13606+K03405+K03405+K03405+K00231+K00231+K00231+K01845+K01845+K01845+K01772+K01772+K01772+K01772+K01772+K01772+K01772+K13606+K13071+K13071+K13606+K01885+K13071+K01885+K13071+K13071+K01772+K01885+K13071+K13606+K13606+K01885+K01885+K01772+K01885+K01885+K01772+K01772+K02492+K02492+K01719+K01719+K01719+K01719+K01719
Terpenoid backbone biosynthesis	ko00900	210	K05356+K03527+K03527+K13789+K13789+K13789+K13789+K13789+K13789+K13789+K00991+K00991+K01597+K01597+K01597+K00869+K00869+K00869+K00869+K00869+K00869+K00919+K00919+K05906+K05906+K15891+K00021+K14066+K01823+K01823+K01823+K01823+K01823+K10960+K10960+K10960+K10960+K00099+K13789+K13789+K13789+K05955+K05955+K05955+K01770+

			K01770+K01770+K01770+K01597+K01597+K13789+K13789+K13789+K13789+K00626+K00626+K00626+K00626+K00626+K00626+K13789+K00787+K00787+K00787+K15889+K03527+K03527+K03526+K03526+K03526+K03526+K03526+K03526+K03526+K03526+K03526+K03526+K03526+K03526+K03526+K03526+K03526+K03526+K03526+K03526+K10960+K10960+K01662+K01662+K01662+K01662+K01662+K01662+K01662+K01662+K01662+K00869+K00869+K00869+K00869+K00869+K00869+K15892+K15892+K15892+K15892+K15892+K15892+K00587+K14066+K14066+K00099+K00099+K00099+K00099+K00021+K00021+K00021+K00021+K11778+K11778+K01641+K01641+K01641+K01641+K01641+K01641+K00938+K00938+K00938+K00938+K00938+K00938+K00938+K00938+K00938+K05954+K05954+K05954+K05954+K01823+K01662+K00021+K00021+K14066+K03527+K01662+K01662+K03527+K03527+K01662+K05955+K03527+K08658+K01823+K08658+K01823+K03527+K01823+K00021+K14066+K03527+K14066+K00021+K00021+K14066+K14066+K00021+K00021+K00021+K00021+K00021+K00021+K00021+K00021+K00021+K00021+K00021+K00021+K00021+K00021+K00021+K00021+K00021+K01662+K01662+K00021+K00021+K01662+K01662+K00021+K14066+K03527+K00021+K00021+K00021+K06013+K06013+K06013+K06013+K11778+K11778+K11778+K00626+K00626+K00626+K00626+K00626+K00626
Monoterpenoid biosynthesis	ko00902	39	K15095+K15086+K15086+K15086+K15086+K15095+K15095+K15095+K15095+K15095+K15095+K15095+K15095+K15095+K15095+K15095+K15095
Limonene and pinene degradation	ko00903	51	K00517+K00517+K00517+K00128+K00128+K00128+K00128+K00128+K00128+K00128+K00128+K00128+K00128+K00128+K00128+K00128+K00128+K00128+K00128+K00128+K00128+K00128+K00517+K00517+K00128+K00128+K00128+K00128+K00128+K00128+K00128+K00128+K00128+K00128+K00517+K00517+K00517+K00517+K00517+K00128+K00128+K00128+K00128+K00128+K00128+K00128+K00128+K00128+K00128+K00128+K00128
Diterpenoid biosynthesis	ko00904	32	K05282+K05282+K05282+K17961+K17961+K17961+K04124+K04121+K04121+K04121+K04121+K17961+K17961+K17961+K17961+K17961+K04120+K04121+K04121+K04121+K04121+K04121+K04121+K04121+K04121+K17982+K17982+K04122+K04122+K04122+K04122+K04122
Brassinosteroid biosynthesis	ko00905	31	K12640+K12640+K15639+K09588+K15639+K15639+K15639+K15639+K15639+K15639+K15639+K15639+K15639+K15639+K15639+K09587+K09587+K12637+K09588+K09588+K09588+K09588+K09588+K09588+K09588+K09588+K12638+K12638+K12638+K12638+K12638
Carotenoid biosynthesis	ko00906	90	K15744+K15746+K15746+K15746+K09839+K09839+K09838+K09838+K09843+K15746+K15746+K15747+K15747+K15747+K15747+K15747+K15747+K02291+K09843+K09843+K09843+K09840+K09840+K09840+K02291+K02291+K02291+K02291+K02291+K02291+

			K02291+K02291+K02291+K02291+K02291+K02291+K02291+K02291+K02291+K02291+K14595+K14595+K06443+K06443+K09841+K09841+K09841+K09841+K09841+K09840+K09840+K09840+K09840+K09840+K09840+K09840+K09840+K09840+K09840+K02293+K02293+K02293+K02293+K02293+K02293+K02293+K09838+K09838+K09838+K09838+K09838+K09838+K09838+K09842+K09842+K06444+K06444+K06444+K00514+K00514+K00514+K00514+K00514+K09835+K09835+K09835+K09837+K09837+K09837+K09837
Zeatin biosynthesis	ko00908	26	K13495+K13495+K00791+K13495+K13495+K13495+K10760+K13495+K13495+K13495+K13495+K13495+K13495+K13495+K13495+K13495+K00279+K00791+K00791+K00791+K00791+K00791+K00791+K00791+K00791+K10717
Sesquiterpenoid and triterpenoid biosynthesis	ko00909	84	K00801+K15891+K15472+K00511+K00511+K00801+K00801+K00801+K00801+K15472+K00511+K00511+K00511+K00511+K00511+K00511+K00511+K00511+K00511+K00511+K00511+K00511+K00511+K00511+K00511+K15813+K15813+K15813+K15813+K15813+K15813+K15813+K15813+K15813+K15813+K15813+K15813+K15813+K15813+K15813+K15813+K15813+K15813+K15813+K15816+K15813+K00511+K00511+K00511+K00511+K00511+K00511+K00511+K00511+K00511+K15803+K15803+K15803+K15803+K15803+K15803
Nitrogen metabolism	ko00910	119	K01455+K01455+K01455+K01455+K01455+K01455+K00261+K00261+K01674+K01673+K01673+K01673+K01673+K01673+K01673+K01673+K01673+K01673+K01673+K01673+K01673+K01673+K01673+K01673+K01673+K01673+K01673+K01915+K01673+K01673+K01725+K01725+K01674+K01673+K01673+K01673+K01673+K01673+K00261+K00261+K00261+K01915+K00262+K00262+K00262+K00262+K00262+K00284+K00284+K00284+K00284+K00284+K00284+K00284+K00284+K00284+K01915+K01915+K01725+K10534+K10534+K00264+K00264+K00264+K00264+K00264+K00264+K00264+K00264+K00264+K00264+K00264+K00264+K00264+K00264+K02575+K02575+K01915+K01673+K01673+K01673+K00366+K00366+K02575+K02575+K02575+K02575+K02575+K02575+K02575+K02575
Sulfur metabolism	ko00920	131	K13034+K13034+K13034+K00640+K00640+K00640+K01738+K01738+K01738+K01738+K13811+K08738+K00387+K13034+K13034+K05907+K05907+K05907+K01738+K01738+K01738+K01738+K01738+K01738+K01738+K01738+K01738+K01738+K08738+K00640+K00640+K01738+K01738+K01738+K00640+K00640+K17725+K17725+K15422+K15422+K17725+K17725+K17725+K17725+K00387+K01082+K01738+K01738+K01738+K13811+K13811+K13811+K13811+K13811+K13811+K01739+K01739+K01739+K01739+K01739+K01739+K01739+K01739+K01739+K01739+K01739+K01739+K00640+K05907+K05907+K00640+K01082+K01082+K01082+K01082+K01082+K01082+K01082+K01082+K01082+K08738+K17725+K00392+K00392+K00392+K00392+K00392+K00392+K17725+K00392+

			K17725+K01738+K01738+K00392+K01738+K17725+K00392+K17725+K00392+K17725+K01738+K17725+K17725+K17725+K01738+K17725+K17725+K17725+K15422+K17725+K17725+K17725+K17725+K17725+K17725+K15422+K17725+K17725+K17725+K17725+K01011+K01011+K01011+K01011+K01011+K01011+K01011+K01011+K13811+K13811+K13811
Phenylpropanoid biosynthesis	ko00940	478	K00430+K00430+K00430+K00430+K00430+K00430+K00430+K00430+K00430+K00430+K00430+K00430+K00430+K00430+K00083+K00083+K00083+K01188+K01188+K01188+K01188+K13065+K13065+K01188+K01188+K00430+K00430+K00430+K00430+K00430+K00430+K00430+K00430+K00430+K00083+K00430+K00430+K00083+K00083+K00083+K00083+K00083+K00083+K00083+K00083+K00083+K00083+K00083+K00083+K00083+K00083+K00083+K00083+K00083+K01188+K00430+K00430+K00430+K13065+K13065+K13065+K13065+K13065+K13065+K13065+K00430+K00430+K00430+K00430+K00430+K00430+K05350+K05350+K05350+K05350+K00430+K00430+K00430+K00430+K00430+K00430+K00430+K00430+K18368+K18368+K18368+K13066+K13066+K00083+K00487+K00487+K01188+K01188+K01188+K01188+K05349+K12355+K00083+K00083+K00083+K00083+K00083+K00083+K00083+K00083+K00083+K00083+K00083+K00430+K00430+K00430+K00430+K00083+K00083+K00430+K00430+K05350+K05350+K01188+K00430+K00430+K05349+K05349+K13066+K09753+K09753+K09753+K09753+K09753+K09753+K09753+K09753+K09753+K09753+K09753+K09753+K09753+K09753+K09753+K09753+K09753+K09753+K09753+K09755+K09755+K09755+K09755+K09755+K09755+K09755+K09755+K09755+K09755+K09755+K01904+K01904+K01904+K01904+K00083+K00083+K00083+K00083+K00083+K10775+K10775+K10775+K10775+K10775+K10775+K10775+K10775+K10775+K05350+K10775+K10775+K13066+K13066+K13066+K13066+K13066+K13066+K13066+K13066+K13066+K13066+K13066+K13066+K05350+K05350+K05350+K05350+K05350+K05350+K05349+K05349+K05349+K05349+K01188+K01188+K01188+K01188+K01188+K01188+K01188+K01188+K01188+K01188+K01188+K01188+K01188+K01188+K13066+K13066+K13066+K13066+K13066+K13066+K13066+K13066+K13066+K13066+K13066+K13066+K13066+K00430+K00430+K00430+K00430+K00430+K00430+K00430+K00430+K01188+K01188+K01188+K01188+K01188+K01188+K01188+K01188+K00588+K00588+K00588+K00588+K00588+K13066+K13066+K13066+K13066+K13066+K09754+K09754+K12355+K12355+K12355+K12355+K12355+K00430+K00430+K00430+K00430+K00430+K00430+K01904+K01904+K01904+K01904+K01904+K01904+K01904+K01904+K05349+K05349+K05349+K01904+K01904+K01904+K05350+K05349+K05349+K05349+K00430+K01188+K01188+K01188+K13066+K01188+K13066+K01188+K01188+K01188+

			K01188+K05349+K01188+K05349+K05349+K01188+K13066+K13066+K05349+K01188+K01188+K01188+K01188+K01188+K13066+K05349+K05349+K05350+K01188+K01188+K01188+K05350+K13066+K05349+K01188+K05349+K01188+K05349+K13066+K01188+K01188+K05349+K01188+K01188+K01188+K01188+K01188+K05349+K01188+K13066+K05349+K13066+K05349+K05349+K01188+K05349+K05349+K05349+K05349+K05350+K05349+K05349+K05349+K05349+K05349+K05349+K01188+K05349+K05349+K05349+K05349+K01188+K05350+K05349+K05349+K05349+K05349+K05349+K05349+K05349+K05349+K05349+K05349+K05349+K05349+K05349+K05350+K00083+K00083+K00083+K00083+K00083+K05349+K05349+K05349+K05349+K05350+K05349+K05349+K05349+K05349+K09755+K09755+K01188+K01188+K01904+K01904+K01904+K01904+K01904+K01904+K01904+K01904+K01904+K01904+K01188+K01188+K01188+K01188+K01188+K01188+K01188+K01188+K12355+K01188+K01188+K00430+K01188+K01188+K01188+K00487+K00487+K00487+K00487+K00487+K13065+K13065+K13065+K13065+K12356+K01188+K01188+K01188
Flavonoid biosynthesis	ko00941	164	K13082+K13082+K13082+K13082+K13082+K00660+K00660+K00660+K13065+K13065+K13082+K13082+K13082+K13065+K13065+K13065+K13065+K13065+K13065+K13065+K05278+K05278+K05278+K05278+K05278+K00475+K00475+K00475+K00475+K00475+K00487+K00487+K05278+K05278+K05278+K05278+K05278+K05278+K13081+K13081+K13081+K00660+K05278+K00660+K05278+K00660+K00660+K01859+K01859+K01859+K01859+K01859+K01859+K01859+K01859+K01859+K01859+K01859+K05278+K13083+K08695+K08695+K08695+K08695+K08695+K08695+K08695+K08695+K08695+K08695+K08695+K08695+K08695+K13081+K13081+K13081+K13081+K13081+K13081+K13081+K13081+K13081+K05280+K05280+K05280+K05280+K05280+K05280+K05280+K05280+K00588+K00588+K00588+K00588+K00588+K05277+K05277+K05277+K05277+K05277+K05277+K05277+K05277+K05277+K05277+K05277+K13083+K13083+K13083+K13083+K13083+K13083+K13083+K00475+K00475+K00475+K00475+K09754+K09754+K00660+K00660+K00660+K00660+K00660+K00660+K00660+K00487+K00487+K00487+K00487+K00487+K13065+K13065+K13065+K13065
Anthocyanin biosynthesis	ko00942	27	K12338+K12338+K12338+K12338+K12338+K12338+K12338+K12338+K12338+K12338+K12338+K12930+K12930+K12338+K12338+K12338+K12338+K12338+K12338+K12338+K12338+K12338+K12338+K12338+K12930+K12930+K12930
Isoflavonoid biosynthesis	ko00943	6	K13260+K13260+K13260+K13260+K13260+K13260
Flavone and flavonol	ko00944	24	K05279+K05279+K05279+K05279+K05279+K05279+K05279+K13083+K05280+K05280+K05280+K05280+K05280+K05280+K05280+K05280+K13083+K13083+K13083+K13083+K13083+K13083+K13083+K05279

biosynthesis			
Stilbenoid, diarylheptanoid and gingerol biosynthesis	ko00945	37	K13065+K13065+K13065+K13065+K13065+K13065+K13065+K13065+K13065+K00487+K00487+K00517+K00517+K00517+K00517+K00517+K00588+K00588+K00588+K00588+K00588+K09754+K09754+K00517+K00517+K00517+K00517+K00517+K00487+K00487+K00487+K00487+K00487+K13065+K13065+K13065+K13065
Isoquinoline alkaloid biosynthesis	ko00950	65	K00276+K14454+K14454+K14454+K14454+K14454+K14455+K14455+K14455+K00811+K00811+K00811+K01592+K01592+K00815+K00815+K00815+K00815+K00815+K00815+K00815+K00276+K00276+K00276+K00276+K00276+K00276+K00276+K00276+K00276+K00276+K00276+K00276+K00276+K00276+K00276+K00276+K00276+K00276+K14454+K14454+K01592+K01592+K01592+K01592+K00815+K00815+K00815+K00276+K00815+K00815+K00815+K00815+K00815+K00815+K00815+K00815+K00815+K00815+K00815+K00815+K15849+K15849+K15849+K15849
Tropane, piperidine and pyridine alkaloid biosynthesis	ko00960	70	K00276+K14454+K14454+K14454+K14454+K14454+K08081+K08081+K08081+K14455+K14455+K14455+K00811+K00811+K00811+K00815+K00815+K00815+K00815+K00815+K00815+K00815+K00276+K00276+K00276+K00276+K00276+K00276+K00276+K00276+K00276+K00276+K00276+K00276+K00276+K00276+K00276+K00276+K00276+K14454+K14454+K00815+K00815+K00815+K00276+K00815+K00815+K00815+K00815+K00815+K00815+K00815+K00815+K00815+K00815+K00815+K00815+K00817+K00817+K00817+K00817+K00817+K00817+K00817+K00817+K15849+K15849+K15849+K15849
Betalain biosynthesis	ko00965	7	K15777+K15777+K15777+K15777+K15777+K15777+K15777
Glucosinolate biosynthesis	ko00966	2	K11820+K11820
Aminoacyl-tRNA biosynthesis	ko00970	386	K01889+K01889+K01889+K01889+K02435+K02435+K01867+K01867+K01889+K01880+K01880+K01880+K01867+K01867+K01889+K01889+K01889+K01889+K01889+K01886+K01886+K01875+K04567+K01881+K01881+K01881+K01881+K01881+K01881+K01881+K01881+K01881+K01881+K01881+K01881+K01881+K01881+K01881+K01881+K01881+K01876+K01876+K01885+K01872+K01872+K01872+K01872+K01872+K01872+K01872+K01872+K01872+K01872+K01872+K01866+K01866+K01866+K01866+K01873+K01876+K01873+K00604+K01870+K01870+K01870+K01870+K01870+K01870+K01870+K01870+K01870+K01870+K14164+K14164+K14164+K14164+K14164+K14164+K01866+K01866+K01866+K01866+K01869+K01869+K01869+K01869+K01869+K01869+K01869+K01869+K01869+K01869+K01869+K01869+K01869+K01869+K01873+K01873+K01873+K01873+K01873+K01873+K01873+K01893+K01893+

			K01893+K01893+K01893+K01872+K01872+K01872+K01872+K01872+K01872+K01872+K01872+K01872+K01872+K01872+K01872+<br>K01872+K01872+K01872+K01872+K01872+K01872+K01872+K01885+K01885+K01885+K01885+K01885+K01885+K01885+K01885+<br>K01885+K01885+K01885+K01885+K01885+K01885+K01885+K01890+K01890+K01890+K01890+K01890+K01890+K01890+K01890+<br>K01890+K01890+K01890+K01890+K01890+K01890+K01890+K01890+K01890+K01890+K01890+K02433+K02433+K02433+K02433+<br>K02433+K02433+K02433+K02433+K02433+K02433+K02433+K01869+K01869+K01869+K01869+K01869+K01869+K01869+K01869+<br>K01869+K01883+K01883+K01883+K01883+K01883+K01883+K01883+K01883+K01883+K01883+K01892+K01892+K01892+K01892+<br>K01887+K01887+K01887+K01887+K01887+K01887+K01887+K01887+K01887+K01883+K01883+K01883+K01883+K01866+K01866+<br>K01866+K01893+K01893+K01893+K01893+K01893+K01868+K01868+K01868+K01868+K04567+K04567+K04567+K04567+K04567+<br>K04567+K01893+K01893+K01868+K01868+K01868+K01868+K01868+K01868+K01868+K01868+K01868+K01876+K01889+K04567+<br>K04567+K01876+K01886+K01876+K01889+K04567+K01889+K01876+K01880+K01876+K01880+K01885+K01889+K01873+K01876+<br>K00604+K00604+K01885+K01880+K01886+K01873+K00604+K04567+K01885+K01889+K01876+K04567+K01873+K01886+K01873+<br>K01876+K04567+K01886+K01880+K01880+K01880+K01873+K04567+K01873+K04567+K01875+K01875+K01875+K01875+K01892+<br>K01886+K04567+K04567+K01886+K01886+K01892+K01873+K01886+K01876+K01876+K01876+K01873+K01873+K01886+K01873+<br>K01873+K01873+K01873+K01892+K04567+K01892+K01876+K04567+K01886+K01885+K01876+K01876+K01886+K01885+K01870+<br>K01870+K01870+K01892+K02433+K04567+K01870+K01876+K01870+K01885+K01870+K01870+K01885+K04567+K01876+K01870+<br>K01876+K04567+K04567+K04567+K04567+K01876+K01886+K04567+K04567+K01870+K01870+K04567+K04567+K01886+K01892+<br>K01870+K01873+K01886+K01889+K01889+K01889+K01889+K01889+K01874+K01874+K01874+K01874+K01874+K01874+K01874+<br>K01874+K01874+K01874+K01874+K01874+K01874+K01874+K02434+K02434+K01875+K01875+K01875+K01875+K01875+K01875+<br>K01875+K01874+K01874+K01874+K01874+K01874+K01874+K01881+K01881+K01881+K01881
Biosynthesis of unsaturated fatty acids	ko01040	132	K10258+K10258+K10256+K10257+K10257+K10257+K00059+K00059+K00059+K00059+K00059+K00507+K00507+K10703+K03921+<br>K03921+K03921+K03921+K03921+K03921+K03921+K07513+K07513+K00232+K07513+K10256+K10256+K10256+K00059+K00059+<br>K10251+K10251+K10256+K10256+K10251+K10257+K10703+K10703+K03921+K10703+K10703+K00232+K00232+K00232+K00232+<br>K00232+K00232+K00232+K00232+K00232+K00232+K00232+K00232+K00232+K00232+K00232+K00232+K00232+K00232+K00232+<br>K00232+K00232+K00232+K00232+K00232+K00232+K00232+K00232+K00232+K00232+K00232+K00232+K00059+K00059+K00059+<br>K00059+K10255+K10255+K10255+K10255+K01068+K01068+K01068+K01068+K01068+K10256+K10256+K10256+K10256+K10256+<br>K10256+K10256+K10256+K10256+K10256+K10256+K10256+K10256+K10256+K10256+K10256+K10256+K10256+K10256+K10256+

			K10256+K10256+K10256+K10256+K00232+K00232+K00232+K00232+K00232+K00232+K00232+K00232+K07513+K07513+K07513+K07513+K07513+K07513+K07513+K00232+K07513+K10257+K10257+K10257+K10257+K10257+K10257
Carbon metabolism	ko01200	1378	K00161+K00161+K00161+K00161+K01455+K01455+K01455+K01455+K01455+K01455+K00236+K00855+K00855+K00855+K00855+K00855+K00927+K00927+K00927+K00235+K00235+K00235+K00235+K00235+K00235+K13034+K13034+K13034+K01623+K01623+K01623+K01623+K01623+K01623+K01623+K01623+K00121+K00121+K00121+K00121+K00121+K00121+K00121+K01803+K01803+K01803+K01803+K01803+K01803+K01803+K01803+K00616+K00616+K00616+K00616+K00616+K00616+K00616+K00616+K00616+K00640+K00640+K00640+K00121+K00121+K02160+K02160+K02160+K02160+K00033+K05605+K05605+K05605+K14454+K14454+K14454+K14454+K14454+K01738+K01738+K01738+K01738+K00122+K00122+K01057+K01057+K00030+K00030+K14455+K14455+K14455+K01807+K01807+K01807+K15634+K15634+K05605+K05605+K05605+K05605+K01689+K00030+K00030+K00030+K00030+K00030+K00030+K00030+K00025+K00025+K00025+K00025+K00025+K00025+K00025+K00025+K00025+K00025+K00025+K01623+K01623+K01623+K01623+K01623+K01623+K01623+K01623+K01623+K01623+K01623+K01623+K01623+K01623+K01623+K13034+K13034+K00031+K00031+K00031+K00031+K00031+K00031+K00031+K00031+K00029+K00927+K00927+K00927+K00927+K00927+K00927+K00927+K00026+K00026+K00026+K00026+K00026+K00026+K00026+K00026+K00026+K01057+K00162+K00162+K00162+K00162+K00162+K00162+K00162+K00162+K00162+K01738+K01738+K01738+K01738+K01738+K01738+K01738+K01738+K01738+K01738+K01079+K01070+K01070+K01070+K01623+K01623+K01623+K01623+K01623+K01623+K01623+K01623+K01623+K01623+K01623+K01623+K01623+K00616+K00616+K01602+K01602+K01602+K01602+K01602+K01602+K01602+K01602+K01602+K01602+K01602+K01057+K01057+K00033+K00033+K00033+K00033+K00033+K00033+K00033+K00161+K00025+K01602+K01602+K01602+K01602+K01602+K01602+K01602+K01602+K01602+K01602+K01602+K01602+K01602+K01057+K01057+K01057+K01057+K01057+K01783+K01783+K01783+K05605+K05605+K05605+K00026+K00026+K00026+K01595+K01595+K00640+K00640+K01738+K01738+K01738+K00640+K00640+K05605+K05605+K05605+K00873+K01803+K01803+K01803+K00121+K00121+K00131+K00131+K00131+K00851+K00851+K01679+K00605+K00605+K00626+K00626+K00626+K00626+K00626+K00626+K00831+K00831+K00831+K03841+K03841+K01807+K01807+K01807+K05298+K05298+K05298+K05298+K05298+K05298+K05298+K00051+K00051+K00051+K00051+K00161+K00161+K00036+K01834+K01834+K01834+K01834+K01963+K00025+K00025+K00058+K00850+K05605+K05605+K05605+K05605+K05605+K05605+K05605+K05605+K05605+K05605+K05605+K05605+K05605+K00058+K00058+K00058+K00058+K00058+K00058+K00058+K00058+K00058+K01057+K01057+K01057+K01810+K01810+K00121+K00121+K00814+K00814+K00026+K00026+K00026+K00026+K00026+K00616+K01738+K00121+K00121+K00121+K01623+K01623+K00030+K00030+K01899+K01899+K01899+

	K01899+K18121+K18121+K18121+K05605+K05605+K01738+K01738+K01057+K01057+K18121+K18121+K00134+K00134+K00134+K00134+K01807+K00134+K05298+K00134+K00134+K00134+K00134+K00134+K00134+K00134+K00134+K00134+K00134+K00134+K01689+K01689+K01689+K01689+K01689+K01689+K00134+K00134+K00134+K01689+K00134+K00134+K00134+K00627+K00627+K01962+K01962+K01962+K01962+K01962+K01962+K01962+K01962+K01962+K01962+K01962+K01962+K01962+K01962+K01962+K01962+K00249+K00249+K00249+K00249+K00249+K00249+K00249+K00249+K00249+K00249+K01803+K01803+K01803+K01803+K01595+K01595+K15634+K01803+K01803+K01803+K01803+K01803+K01803+K01803+K01803+K01803+K01803+K01803+K01803+K01803+K01601+K01601+K01601+K01595+K01595+K01595+K01595+K05605+K05605+K05605+K05605+K05605+K05605+K05605+K01783+K01783+K01783+K01783+K00121+K00121+K00121+K00121+K00121+K00121+K00121+K00121+K00121+K00121+K00121+K00121+K00121+K00948+K00948+K00948+K00948+K00948+K01006+K01006+K00036+K00640+K00281+K00134+K00134+K00134+K00134+K00134+K00134+K00134+K00134+K00134+K00134+K05605+K05605+K05605+K05605+K05605+K05298+K05298+K05298+K05298+K05298+K05298+K05298+K05298+K05298+K05605+K05605+K00028+K00028+K00028+K00028+K00028+K00028+K00028+K00028+K00028+K00028+K00028+K00028+K00028+K00028+K00028+K00058+K00058+K00058+K00058+K00058+K01623+K01623+K01623+K01623+K00873+K00873+K00873+K14454+K14454+K14272+K14272+K14272+K14272+K14272+K14272+K14272+K14272+K01057+K01057+K01057+K01057+K01057+K00140+K00140+K00140+K00140+K00140+K00140+K00140+K01962+K01962+K01962+K01962+K01962+K01647+K00615+K00615+K00615+K00615+K00615+K00615+K00615+K00615+K00615+K00615+K00615+K00615+K00615+K00615+K00615+K00615+K00615+K00615+K00615+K00873+K00873+K00873+K00873+K00036+K00036+K00036+K00036+K00036+K00036+K00036+K00036+K00036+K00873+K00873+K00873+K00873+K00873+K00873+K00873+K00873+K00873+K00873+K01689+K01689+K01689+K01689+K01689+K00873+K00873+K00873+K00873+K00026+K00026+K00026+K00140+K00140+K00140+K00140+K00140+K00140+K00382+K00382+K00382+K00382+K00382+K00382+K00382+K00382+K00382+K00382+K00029+K00029+K00029+K00029+K00029+K00029+K00029+K00029+K00029+K00927+K00927+K00927+K00927+K00600+K00600+K00600+K00600+K00600+K00640+K15633+K18121+K18121+K18121+K18121+K00830+K00830+K00830+K00830+K00830+K00830+K00830+K00830+K00121+K00121+K00121+K00121+K00121+K00028+K00028+K00028+K00028+K00028+K00028+K00028+K00028+K00028+K01783+K01783+K01783+K01647+K01647+K01647+K01647+

	K01647+K01647+K01647+K03841+K03841+K03841+K03841+K03841+K03841+K03841+K00850+K00850+K00850+K00850+K00850+K00850+K00850+K00850+K00850+K00850+K01938+K01938+K01938+K01938+K01938+K01938+K01938+K01938+K00948+K00948+K00297+K00297+K00297+K00297+K00297+K00600+K00600+K00600+K00600+K00600+K00600+K00600+K00051+K01754+K01754+K01754+K00382+K00382+K00382+K00382+K18121+K18121+K00030+K00030+K00030+K00814+K00036+K00036+K00036+K00036+K00036+K00036+K00029+K01810+K00627+K00281+K00281+K00873+K00873+K00873+K00814+K00814+K00814+K01595+K01595+K00051+K00134+K01623+K00029+K00036+K00234+K01595+K00036+K01595+K00873+K01595+K01595+K01810+K00627+K00029+K01595+K00134+K01810+K00134+K01595+K00051+K00844+K01595+K00031+K00873+K01610+K01610+K00134+K00844+K00134+K00030+K01610+K00850+K00605+K00031+K01610+K01610+K01623+K01595+K01610+K00850+K01595+K01610+K01610+K00873+K01610+K01610+K01595+K00627+K01738+K00873+K01810+K01623+K01681+K00029+K00873+K00029+K01738+K01963+K00036+K00863+K05298+K00134+K01595+K01738+K00029+K01810+K01623+K01595+K00134+K00873+K01595+K00134+K00234+K00134+K00134+K01595+K05298+K00873+K00873+K01595+K01595+K00844+K01595+K01601+K01679+K00134+K00850+K00873+K01595+K01595+K00134+K00844+K00051+K01595+K00249+K01810+K01810+K01810+K01810+K01810+K00029+K01681+K01810+K00029+K01810+K00873+K00029+K00873+K00051+K00844+K00164+K00029+K01610+K00140+K01681+K00844+K00164+K00029+K00029+K00029+K01595+K00051+K01595+K00873+K00029+K01810+K00029+K00029+K00234+K00164+K00029+K00140+K00863+K00873+K00234+K00029+K00234+K01895+K00873+K01681+K01810+K00140+K01738+K00051+K00036+K00029+K00030+K00030+K00029+K00029+K00627+K01895+K01810+K00036+K00029+K00029+K00029+K00029+K00029+K00029+K00029+K00029+K00281+K00029+K00029+K00029+K00029+K01679+K01679+K00873+K01679+K01679+K01679+K01679+K01679+K01679+K01679+K01679+K00863+K00029+K00029+K00234+K00850+K00627+K00234+K00234+K00627+K01810+K00036+K00029+K01738+K00029+K00029+K00036+K00036+K00627+K00029+K00029+K00029+K00036+K00844+K00844+K00844+K00844+K00844+K00844+K00844+K00844+K00844+K00844+K00844+K00844+K00844+K00844+K00029+K00134+K00627+K00029+K00140+K00140+K00140+K01963+K01963+K01963+K01963+K01963+K01963+K00627+K00627+K00029+K01679+K01623+K00029+K01623+K01899+K00029+K00029+K00850+K00029+K00029+K01610+K00863+K00605+K00051+K00029+K00029+K00029+K00029+K00029+K00029+K00850+K00031+K01610+K00863+K00234+K01810+K00164+K01681+K01602+K00164+K01895+K00281+K00164+K00140+K00164+K01602+K01602+K00873+K00863+K00164+K00814+K00281+K01681+K05298+K00281+K00627+K01895+K01681+K00164+K00844+K01681+K00164+K00281+K01681+K00627+K00627+K01810+K00873+K00814+K00164+K01810+K01810+K00164+K00164+K01681+K00164+K01681+K00844+K01681+K01895+K01681+K01895+K00164+K01810+K05298+K00140+K00873+K00281+K00164+K01681+K00164+K01681+K00281+

			K01895+K00281+K00627+K01681+K00281+K01681+K00281+K00627+K01681+K01681+K00281+K00164+K01681+K00281+K00164+K00031+K01810+K01895+K00281+K05298+K01810+K01681+K00863+K01681+K01810+K00164+K05298+K01681+K01679+K00281+K00164+K05298+K01681+K00134+K01681+K01803+K00164+K00627+K01679+K00164+K00844+K01681+K00873+K01681+K01610+K00281+K01681+K00281+K00164+K01681+K00627+K01681+K00164+K00164+K00863+K01681+K01810+K00281+K00281+K00873+K00844+K01681+K01681+K01681+K01681+K00627+K01602+K00873+K01681+K00281+K00036+K00036+K00031+K00164+K00600+K00600+K00600+K00600+K00600+K00600+K00600+K00600+K00600+K00600+K00600+K00600+K00600+K00600+K00600+K00600+K00600+K00873+K00122+K00122+K00122+K00122+K00122+K00122+K00122+K00122+K00122+K01689+K01689+K01689+K01689+K00873+K00873+K00850+K00850+K00850+K00850+K00850+K00850+K00850+K00850+K00850+K00850+K00850+K00850+K00873+K00873+K00873+K00873+K00873+K00873+K00873+K01961+K01961+K01961+K00658+K00658+K00658+K00658+K00658+K00658+K00658+K00658+K01100+K01100+K01100+K03841+K03841+K03841+K03841+K03841+K03841+K03841+K01900+K01900+K01900+K01900+K01900+K01900+K00850+K01689+K01689+K01689+K01689+K00121+K00121+K00121+K00121+K00121+K00121+K00121+K00121+K00121+K00627+K00627+K00627+K00627+K00627+K00627+K00627+K00058+K00627+K00627+K00627+K00627+K00600+K00600+K00600+K00031+K00031+K00033+K00033+K00033+K00626+K00626+K00626+K00626+K00626+K00626+K00850+K00850+K00850+K00927+K00927+K00927+K00927+K00162+K00162+K00162+K01647+K01647+K01647+K01647+K01647+K01647+K01647+K01647+K01647
2-Oxocarboxylic acid metabolism	ko01210	234	K01438+K01438+K14454+K14454+K14454+K14454+K14454+K00030+K00030+K14455+K14455+K14455+K00133+K00133+K00620+K00030+K00030+K00030+K00030+K00030+K00030+K00030+K00031+K00031+K00031+K00031+K00031+K00031+K00031+K00031+K01703+K01703+K01703+K01703+K00818+K00818+K00818+K00818+K00818+K00811+K00811+K00811+K01438+K01438+K01438+K00053+K00826+K00826+K00826+K00826+K00826+K00826+K00826+K00826+K00826+K00826+K00826+K01704+K01704+K01704+K01704+K00930+K00930+K00814+K00814+K00052+K00052+K00030+K00030+K11820+K11820+K00818+K00818+K00818+K00818+K00928+K00928+K00928+K00928+K00928+K00928+K00928+K00928+K01653+K01653+K01653+K01653+K01653+K01653+K01653+K01653+K01703+K01703+K01703+K01703+K01703+K01703+K01703+K01703+K01703+K01703+K01649+K01649+K01649+K01649+K01649+K01649+K01649+K01649+K01649+K01649+K01649+K01649+K14454+K14454+K14272+K14272+K14272+K14272+K14272+K14272+K14272+K14272+K01647+K00052+K00052+K01687+K01687+K14682+K14682+K14682+K14682+K14682+K01647+K01647+K01647+K01647+K01647+K01647+K01647+K00030+K00030+K00030+K00814+K00053+K00814+K00053+K00814+K00814+K00928+

			K00053+K00053+K00053+K00031+K00030+K00031+K00053+K00053+K01681+K00053+K00053+K00928+K00053+K00928+K01681+K01681+K01681+K00030+K00030+K01438+K00826+K00031+K01681+K00814+K01681+K01681+K01681+K01681+K00814+K01681+K01681+K01681+K01681+K01681+K01681+K01438+K01681+K01681+K01681+K01681+K01681+K00031+K01681+K01681+K01681+K01681+K01681+K01681+K01681+K01681+K01681+K01681+K00826+K01681+K01681+K01681+K01681+K01681+K01681+K00031+K01652+K01652+K01652+K01652+K01652+K00145+K00145+K00145+K14677+K14677+K14677+K00031+K00031+K01653+K01653+K01647+K01647+K01647+K01647+K01647+K01647+K01647+K01647+K01647
Fatty acid metabolism	ko01212	382	K01897+K10258+K10258+K10256+K01074+K01074+K01074+K01074+K01074+K10257+K10257+K10257+K00059+K00059+K00059+K00059+K00059+K07512+K07512+K07512+K07512+K02160+K02160+K02160+K02160+K09458+K00507+K00507+K10703+K00208+K00208+K00208+K00208+K03921+K03921+K03921+K03921+K03921+K03921+K03921+K10781+K10781+K11262+K11262+K11262+K11262+K11262+K07513+K07513+K00232+K07513+K10256+K10256+K10256+K00626+K00626+K00626+K00626+K00626+K00626+K00059+K00059+K10251+K10251+K02372+K02372+K10256+K10256+K10251+K00208+K00208+K00208+K01963+K10257+K10703+K10703+K03921+K10703+K10703+K01074+K00232+K01962+K01962+K01962+K01962+K01962+K01962+K01962+K01962+K01962+K01962+K01962+K01962+K00232+K00232+K00232+K00232+K00232+K00249+K00249+K00249+K00249+K00249+K00249+K00249+K00249+K00249+K00249+K00059+K00059+K00059+K00059+K01897+K01897+K01897+K01897+K01897+K01897+K01897+K01897+K01897+K01897+K01897+K01897+K01897+K10255+K10255+K10255+K10255+K01962+K01962+K01962+K01962+K01962+K10527+K10527+K10527+K10527+K10527+K10527+K10527+K10527+K01897+K01897+K01897+K01897+K01897+K01897+K01897+K01897+K10256+K00232+K00232+K00232+K00232+K00232+K01897+K00232+K00232+K00232+K01897+K01897+K01897+K01897+K01897+K01897+K01897+K01897+K01897+K01897+K01897+K01897+K01897+K01897+K01897+K01897+K01897+K01897+K01897+K00645+K00645+K00645+K07513+K07513+K07513+K07513+K07513+K07513+K07513+K01897+K01897+K01897+K00232+K01897+K01897+K01897+K01897+K01897+K01897+K01897+K01897+K01897+K01897+K01963+K01897+K01897+K01897+K01897+K01897+K01897+K01897+K01897+K00249+K01897+K10527+K10527+K10527+K10527+K10527+K10527+K01897+K01897+K01897+K01897+K01897+K10527+K10527+K01897+

			K01897+K01897+K01897+K01897+K01963+K01963+K01963+K01963+K01963+K01963+K10527+K10527+K10527+K10527+K10527+K10527+K10527+K10527+K10527+K10527+K10527+K10527+K07513+K10527+K10527+K01897+K10527+K01897+K01897+K09458+K09458+K09458+K09458+K09458+K09458+K01961+K01961+K01961+K10781+K00626+K00626+K00626+K00626+K00626+K00626+K10257+K10257+K10257+K10257+K10257+K10257+K09458+K09458+K09458+K00648+K00648+K00648+K00648+K01074+K01074+K01074+K01074
Degradation of aromatic compounds	ko01220	62	K00121+K00121+K00121+K00121+K00121+K00121+K00121+K00121+K00121+K00487+K00487+K00121+K00121+K00001+K00001+K00001+K00121+K00121+K00121+K00121+K00121+K00121+K00121+K00121+K00121+K00121+K00121+K00121+K00121+K00121+K00121+K00121+K00121+K00121+K00001+K00001+K00001+K00001+K00001+K00001+K00121+K00121+K00121+K00121+K00121+K00001+K00001+K00121+K00121+K00121+K00121+K00121+K00121+K00121+K00121+K00121+K00487+K00487+K00487+K00487+K00487+K00001
Biosynthesis of amino acids	ko01230	1196	K01714+K01714+K00927+K00927+K00927+K01696+K01696+K01696+K00789+K13034+K13034+K13034+K01623+K01623+K01623+K01623+K01623+K01623+K01623+K01623+K01438+K01438+K01803+K01803+K01803+K01803+K01803+K01803+K01803+K01803+K00616+K00616+K00616+K00616+K00616+K00616+K00616+K00616+K00616+K00640+K00640+K00640+K01735+K01735+K01735+K01735+K01735+K00215+K00215+K00215+K00215+K01476+K01476+K01476+K01476+K05359+K05359+K05359+K01696+K01696+K14454+K14454+K14454+K14454+K14454+K01738+K01738+K01738+K01738+K05359+K05359+K05359+K05359+K00030+K00030+K14455+K14455+K14455+K01807+K01807+K01807+K15227+K15227+K00133+K00133+K15634+K15634+K05359+K05359+K00620+K00611+K01689+K00030+K00030+K00030+K00030+K00030+K00030+K00030+K01623+K00891+K01915+K01623+K01623+K01623+K01623+K01623+K01623+K01623+K01623+K01623+K01623+K01623+K01623+K01623+K01623+K13034+K13034+K00789+K00789+K00789+K00789+K00789+K00789+K00789+K00789+K00789+K00789+K00789+K00789+K00789+K00789+K01778+K01778+K01778+K00031+K00031+K00031+K00031+K00031+K00031+K00031+K00031+K01703+K01703+K01703+K01703+K00927+K00927+K00927+K00927+K00927+K00927+K00927+K01814+K01814+K01814+K01814+K01738+K01738+K01738+K01738+K01738+K01738+K01738+K01738+K01738+K01738+K01079+K00789+K00789+K00789+K00789+K01626+K01626+K01626+K01626+K01626+K01626+K01626+K01626+K01626+K01626+K01626+K01626+K01626+K01695+K01695+K01695+K01695+K01695+K01623+K01623+K01623+K01623+K01623+K01623+K01623+K01623+K01623+K01623+K01623+K01623+K01623+K10206+K10206+K10206+K10206+K10206+K10206+K10206+K10206+K01850+K01850+K01850+K01850+K01850+K01850+K01850+K00616+K00616+K00818+K00818+K00818+K00818+

K00818+K00811+K00811+K00811+K01438+K01438+K01438+K00053+K11755+K11755+K11755+K01783+K01783+K01783+K00640+
K00640+K01738+K01738+K01738+K00640+K00640+K13832+K13832+K13832+K13832+K13832+K13832+K13832+K13832+K13832+
K13832+K13832+K00873+K00549+K00549+K01803+K01803+K01803+K01609+K00826+K00826+K00826+K00826+K00826+K00826+
K00826+K00826+K00826+K00826+K00826+K01704+K01704+K01704+K01704+K00831+K00831+K00831+K01940+K01940+K01940+
K01940+K14085+K14085+K14085+K14085+K14085+K14085+K14085+K14085+K14085+K14085+K14085+K14085+K14085+K14085+
K14085+K14085+K01807+K01807+K01807+K01834+K01834+K01834+K01834+K01620+K01620+K01620+K00815+K00058+K00850+
K00058+K00058+K00058+K00058+K00058+K00058+K00058+K00058+K00058+K00930+K00930+K00814+K00814+K00052+K00052+
K00616+K01738+K01623+K01623+K00030+K00030+K01738+K01738+K01658+K01658+K05359+K00815+K00815+K00815+K00815+
K00815+K00815+K00134+K00134+K00134+K00134+K01807+K00134+K00134+K00134+K00134+K00134+K00134+K00134+K00134+
K00134+K00134+K00134+K00134+K00134+K00134+K00134+K00134+K00134+K00134+K00134+K00134+K00134+K00134+K00134+
K00134+K00134+K00134+K00134+K00134+K00134+K00134+K00134+K00134+K00134+K00134+K01689+K01689+K01689+K01689+
K01689+K01689+K00134+K01657+K01657+K00134+K00134+K01689+K00134+K00134+K00134+K00818+K00818+K00818+K00818+
K01693+K01586+K01586+K01586+K01586+K01586+K01586+K01586+K00891+K00891+K01803+K01803+K01803+K06001+K06001+
K06001+K06001+K00928+K00928+K00928+K00928+K00928+K00928+K00928+K00928+K15634+K01803+K01803+K01803+K01803+
K01803+K01803+K01803+K01803+K01803+K01803+K01803+K01803+K01803+K01783+K01783+K01783+K01783+K01755+K01755+
K01755+K01755+K01755+K01755+K01755+K01755+K01755+K01755+K01755+K01653+K01653+K01653+K01653+K01653+K01653+
K01653+K01653+K00891+K00891+K00765+K00765+K00765+K01703+K01703+K01703+K01703+K01703+K01703+K01703+K01703+
K01703+K01703+K00948+K00948+K00948+K00948+K00948+K01940+K01940+K01940+K01940+K01940+K01940+K01940+K01940+
K01693+K01693+K01693+K01693+K01693+K01693+K01760+K01760+K01760+K01760+K01739+K01739+K01739+K01739+K01739+
K01739+K01739+K01739+K01739+K01739+K01739+K01739+K00640+K01626+K01626+K01626+K01626+K01626+K01626+K01626+
K12524+K12524+K12524+K12524+K12524+K12524+K12524+K12524+K12524+K15227+K15227+K15227+K15227+K15227+K15227+
K15227+K00134+K00134+K00134+K00134+K00134+K00134+K00134+K00134+K00134+K00134+K01649+K01649+K01649+K01649+
K01649+K01649+K01649+K01649+K01649+K01649+K01649+K01649+K01915+K01915+K01915+K01915+K01915+K01915+K01915+
K01915+K01915+K01915+K01915+K01915+K01915+K01915+K01915+K01915+K01915+K01915+K01915+K01915+K01915+K01915+
K01915+K01915+K01915+K01915+K01915+K01915+K01915+K13832+K00058+K00058+K00058+K00058+K00058+K01623+K01623+
K01623+K01623+K01850+K01850+K01850+K01850+K00873+K00873+K00873+K14454+K14454+K14272+K14272+K14272+K14272+

	K14272+K14272+K14272+K14272+K00872+K00872+K00789+K00789+K00789+K00789+K00789+K00789+K00789+K00789+K01647+K00052+K00052+K00615+K00615+K01915+K01915+K00615+K00615+K00615+K00615+K00615+K00615+K00615+K00615+K00615+K00615+K00615+K00615+K00615+K00615+K00615+K00615+K00615+K00873+K00873+K00873+K00873+K00264+K00264+K00264+K00264+K00264+K00264+K00264+K00264+K00264+K00264+K00264+K00264+K00264+K00264+K00873+K00873+K00873+K00873+K00873+K00873+K00873+K00873+K00873+K00873+K01689+K01689+K01689+K01689+K01689+K00873+K00873+K00873+K00873+K00766+K00766+K00766+K00766+K00766+K00789+K00789+K00789+K00789+K12657+K12657+K01687+K01687+K00927+K00927+K00927+K00927+K00600+K00600+K00600+K00600+K00600+K00640+K15633+K00789+K00789+K12657+K12657+K13832+K13832+K13832+K13832+K13832+K14682+K14682+K14682+K14682+K14682+K01783+K01783+K01783+K01647+K01647+K01647+K01647+K01647+K01647+K01647+K00850+K00850+K00850+K00850+K00850+K00850+K00850+K00850+K00850+K00850+K00948+K00948+K00600+K00600+K00600+K00600+K00600+K00600+K00600+K01817+K11755+K11755+K01754+K01754+K01754+K00030+K00030+K00030+K00814+K00549+K00549+K00549+K00549+K00873+K00873+K00873+K00053+K00814+K00053+K00814+K00815+K00814+K00928+K00815+K00134+K01623+K00815+K00053+K00549+K00053+K00873+K00053+K13832+K00134+K00815+K00815+K00134+K00031+K00873+K00134+K00134+K00030+K01657+K00850+K00031+K00549+K00815+K00053+K01623+K00053+K00850+K00549+K00873+K01657+K01657+K01738+K00815+K00873+K13832+K01623+K01915+K01681+K00873+K01738+K00053+K00815+K01657+K00053+K00134+K01738+K01623+K00134+K00873+K00815+K00928+K00134+K00134+K00134+K01657+K00053+K00789+K00873+K00873+K00815+K00134+K00850+K01620+K00873+K00815+K00928+K00134+K00815+K00549+K00549+K00549+K01681+K01657+K01657+K00873+K00873+K01657+K00549+K01657+K01681+K00873+K00815+K00549+K00815+K00549+K00549+K00873+K00549+K00549+K00873+K01681+K01738+K00549+K00030+K00030+K00549+K01663+K01663+K00815+K00549+K00549+K01663+K00549+K01438+K00873+K01663+K00549+K00549+K00549+K00826+K00850+K01738+K01663+K01663+K00549+K00549+K00134+K01623+K01623+K01657+K01657+K01657+K01657+K01657+K01657+K01657+K01657+K01657+K01657+K01657+K01657+K01657+K01657+K01657+K01657+K01657+K00850+K01620+K01620+K00850+K00031+K01681+K00873+K00814+K01681+K01663+K01620+K00549+K00549+K01681+K01681+K00549+K01681+K00549+K00873+K00814+K00549+K01681+K01681+K01681+K01681+K00549+K00873+K01681+K01681+K01438+K01681+K01681+K00549+K01681+K01681+K01681+K00031+K00549+K01681+K01681+K01681+K01681+K00134+K01681+K00549+K01803+K01626+K01663+K01681+K00873+K01681+K01663+K05359+K01681+K01663+K01681+K01681+K00826+K01681+K00873+K01681+K01681+K01681+K01681+K00873+K00549+K01681+K00549+K00549+

			K00031+K01652+K01652+K01652+K01652+K01652+K00600+K00600+K00600+K00600+K00600+K00600+K00600+K00600+K00600+K00600+K00600+K00600+K00600+K00600+K00600+K00600+K00600+K00873+K00800+K00800+K00800+K00800+K00800+K00800+K00800+K00800+K00145+K00145+K00145+K01689+K01689+K01689+K01689+K00873+K00873+K00013+K00013+K00013+K00013+K00013+K00850+K00850+K00850+K00850+K00850+K00850+K00850+K00850+K00850+K00850+K00850+K00850+K00817+K00817+K00817+K00817+K00817+K00817+K00817+K00817+K00873+K00873+K00873+K00873+K00873+K00873+K00873+K05359+K05359+K05359+K05359+K05359+K05359+K15849+K15849+K15849+K15849+K00850+K14677+K14677+K14677+K01689+K01689+K01689+K01689+K01733+K01733+K01733+K01733+K13832+K13832+K00765+K00765+K00765+K00765+K00058+K13832+K01736+K01736+K01736+K01736+K01736+K01736+K01736+K01736+K01736+K01733+K01733+K00600+K00600+K00600+K00031+K00031+K01626+K01626+K01626+K01626+K01626+K01626+K01653+K01653+K00850+K00850+K00850+K00927+K00927+K00927+K00927+K01647+K01647+K01647+K01647+K01647+K01647+K01647+K01647+K01647
Vancomycin resistance	ko01502	3	K01000+K01000+K01000
ABC transporters	ko02010	72	K05658+K05658+K05658+K05658+K05643+K05643+K05658+K05658+K05658+K05658+K05674+K05658+K05658+K05658+K05658+K05658+K05658+K05658+K05658+K05658+K05658+K05658+K05658+K05658+K05677+K05674+K05656+K05656+K05656+K05674+K05643+K05643+K05658+K05658+K05658+K05663+K05663+K05663+K05663+K05658+K05674+K05674+K05674+K05674+K05674+K05674+K05658+K05658+K05681+K05681
Ribosome biogenesis in eukaryotes	ko03008	376	K14569+K14569+K14569+K14569+K14569+K14290+K14290+K14290+K14290+K14290+K14290+K14290+K14545+K14545+K07936+K07936+K07936+K07936+K07936+K07936+K07936+K07936+K07936+K14565+K14574+K14574+K12845+K14550+K11130+K11130+K03115+K03115+K03115+K03115+K03115+K03115+K03097+K03097+K03097+K03097+K03097+K03097+K07936+K07936+K07936+K11130+K11130+K11130+K14561+K14561+K13288+K03264+K03264+K03264+K03264+K03264+K03264+K18532+K18532+K12845+K12845+K13288+K13288+K13288+K13288+K14568+K14568+K14568+K14568+K14568+K14568+K14568+K14569+K11128+K11128+K11128+K07936+K07936+K07936+K07936+K07936+K14560+K14560+K11131+K03538+K03538+K03538+K03538+K03538+K03538+

			K03538+K03538+K03538+K12619+K12619+K12619+K12619+K12619+K12619+K12619+K12619+K12619+K12845+K12845+K12845+K12845+K14568+K14568+K14568+K14568+K14568+K14568+K14556+K14556+K14556+K14556+K14556+K06943+K03537+K03537+K14565+K14565+K14565+K14565+K14565+K14565+K14565+K14565+K14565+K14565+K14565+K14550+K14550+K14569+K14544+K14544+K14544+K14544+K14521+K14521+K14521+K14521+K14521+K14567+K14567+K14567+K14567+K14567+K14567+K14567+K14567+K12619+K12619+K12619+K12619+K12619+K12619+K12619+K12619+K12619+K12619+K12619+K12619+K12619+K14566+K14566+K14566+K14566+K14566+K11131+K11131+K11131+K11131+K14558+K14558+K07562+K07562+K07562+K07562+K07562+K07562+K07562+K07562+K07562+K12619+K12619+K12619+K12619+K12619+K12619+K12619+K12619+K12619+K12619+K14573+K14573+K14571+K14571+K14571+K14571+K14571+K12619+K12619+K12619+K12619+K14546+K14546+K01164+K01164+K01164+K01164+K01164+K01164+K01164+K01164+K01164+K01164+K01164+K03097+K03097+K03097+K03097+K03097+K03097+K14548+K14548+K14548+K14548+K14548+K14554+K14554+K14554+K03115+K03115+K03115+K03115+K11129+K11129+K11129+K11129+K11129+K11129+K11129+K11129+K11129+K11129+K14546+K14546+K14546+K14546+K14546+K14546+K14546+K14546+K14546+K13288+K03097+K03097+K03097+K03097+K03097+K14570+K14570+K14570+K14570+K07178+K07178+K07178+K07178+K07178+K14538+K14538+K14538+K14538+K14538+K14538+K14521+K14521+K14549+K14549+K14549+K14563+K14563+K14563+K14563+K12619+K11108+K11108+K11108+K11108+K11108+K11108+K14557+K14557+K14539+K14539+K14539+K14539+K14539+K14539+K14559+K14559+K14559+K14559+K14559+K03539+K14573+K14552+K06943+K14552+K14552+K07179+K06943+K14553+K12619+K14552+K14552+K06943+K14552+K14572+K14552+K12619+K06943+K14552+K06943+K14552+K06943+K14555+K14555+K14555+K14555+K06943+K14568+K14568+K14568+K14568+K14568+K14568+K14568+K14568+K14568+K14568+K06943+K06943+K14553+K14555+K07179+K07179+K07179+K07179+K07179+K07179+K07179+K07179+K07179+K07179+K07179+K07179+K14536+K12619+K06943+K14555+K14536+K14570+K14570+K11883+K11883+K11883+K14537+K14537+K14537+K14537+K14564+K14564+K14564+K12619
Ribosome	ko03010	946	K02863+K02863+K02863+K02863+K02969+K02969+K02969+K02969+K02903+K02903+K02900+K02900+K02900+K02900+K02900+K02900+K02872+K02991+K02991+K02991+K02943+K02943+K02943+K02943+K02943+K02943+K02872+K02968+K02968+K02968+K02968+K02882+K02882+K02882+K02882+K02939+K02939+K02939+K02939+K02939+K02996+K02996+K02985+K02985+K02985+K02894+K02894+K02894+K02894+K02894+K02894+K02894+K02894+K02895+K02895+K02918+K02918+K02918+K02909+K02909+K02995+K02995+K02995+K02995+K02995+K02868+K02980+K02980+K02980+K02980+K02950+K02929+K02929+K02929+K02929+K02929+K02929+K02929+K02876+K02876+K02876+K02876+K02876+K02876+K02873+K02873+K02873+K02873+K02873+K02873+

K02873+K02873+K02873+K02873+K02993+K02896+K02896+K02896+K02896+K02896+K02948+K02948+K02948+K02940+K02940+
K02875+K02875+K02875+K02884+K02884+K02884+K02884+K02980+K02980+K02980+K02981+K02981+K02926+K02926+K02926+
K02926+K02915+K02915+K02915+K02935+K02935+K02871+K02871+K02871+K02931+K02931+K02931+K02931+K02875+K02875+
K02875+K02921+K02921+K02921+K02921+K02890+K02890+K02891+K02891+K02891+K02891+K02946+K02946+K02897+K02897+
K02891+K02979+K02979+K02979+K02979+K02932+K02932+K02932+K02932+K02932+K02932+K02932+K02932+K02932+K02953+
K02953+K02953+K02953+K02953+K02953+K02893+K02893+K02893+K02898+K02898+K02898+K02898+K02898+K02898+K02898+
K02971+K02993+K02993+K02993+K02993+K02993+K02993+K02993+K02993+K02958+K02958+K02958+K02899+K02899+K02899+
K02899+K02955+K02955+K02955+K02955+K02955+K02973+K02973+K02973+K02973+K02985+K02985+K02892+K02920+K02920+
K02920+K02935+K02935+K02935+K02891+K02891+K02891+K02891+K02891+K02891+K02919+K02919+K02872+K02872+K02977+
K02977+K02977+K02977+K02977+K02977+K02936+K02936+K02936+K02936+K02936+K02936+K02936+K02936+K02941+K02941+
K02941+K02941+K02941+K02941+K02941+K02941+K02941+K02941+K02941+K02941+K02941+K02888+K02888+K02888+K02888+
K02975+K02975+K02975+K02975+K02975+K02975+K02975+K02938+K02938+K02938+K02938+K02938+K02938+K02938+K02938+
K02938+K02938+K02938+K02938+K02974+K02974+K02974+K02962+K02962+K02962+K02962+K02934+K02908+K02908+K02953+
K02953+K02953+K02980+K02980+K02973+K02973+K02973+K02973+K02973+K02973+K02973+K02973+K02905+K02905+K02905+
K02905+K02989+K02989+K02989+K02989+K02989+K02989+K02979+K02925+K02925+K02925+K02925+K02925+K02925+K02882+
K02882+K02882+K02882+K02908+K02908+K02901+K02901+K02901+K02901+K02901+K02901+K02901+K02984+K02984+K02984+
K02984+K02984+K02984+K02984+K02984+K02984+K02984+K02893+K02893+K02893+K02997+K02997+K02951+K02951+K02951+
K02966+K02966+K02940+K02940+K02940+K02940+K02940+K02940+K02942+K02942+K02942+K02935+K02935+K02978+K02927+
K02927+K02915+K02915+K02915+K02915+K02915+K02866+K02866+K02866+K02866+K02866+K02866+K02866+K02866+K02866+
K02866+K02866+K02978+K02978+K02978+K02978+K02874+K02874+K02874+K02874+K02874+K02874+K02947+K02947+K02947+
K02947+K02947+K02947+K02947+K02947+K02947+K02947+K02877+K02877+K02877+K02877+K02877+K02877+K02960+K02960+
K02960+K02960+K02960+K02960+K02960+K02960+K02930+K02930+K02930+K02930+K02930+K02964+K02964+K02964+K02954+
K02954+K02963+K02954+K02954+K02954+K02923+K02923+K02923+K02923+K02910+K02910+K02910+K02910+K02910+K02935+
K02935+K02935+K02971+K02971+K02971+K02971+K02879+K02879+K02951+K02951+K02951+K02942+K02942+K02942+K02942+
K02942+K02942+K02942+K02868+K02868+K02868+K02902+K02902+K02952+K02952+K02921+K02921+K02906+K02906+K02906+
K02906+K02880+K02880+K02880+K02880+K02948+K02948+K02912+K02912+K02996+K02996+K02996+K02875+K02875+K02875+

	K02957+K02957+K02957+K02957+K02957+K02905+K02905+K02905+K02905+K02976+K02976+K02976+K02976+K02976+K02867+K02867+K02946+K02946+K02963+K02904+K02961+K02961+K02961+K02961+K02961+K02934+K02883+K02883+K02883+K02884+K02884+K02933+K02941+K02971+K02971+K02971+K02971+K02870+K02870+K02870+K02870+K02870+K02870+K02870+K02870+K02870+K02961+K02961+K02961+K02961+K02937+K02937+K02937+K02937+K02937+K02937+K02876+K02952+K02952+K02952+K02917+K02917+K02917+K02917+K02917+K02917+K02983+K02983+K02983+K02990+K02990+K02942+K02942+K02942+K02942+K02942+K02942+K02942+K02942+K02922+K02922+K02896+K02896+K02907+K02907+K02974+K02974+K02974+K02974+K02939+K02990+K02990+K02927+K02927+K02927+K02927+K02927+K02882+K02981+K02981+K02981+K02880+K02880+K02880+K02933+K02933+K02933+K02933+K02933+K02933+K02933+K02924+K02924+K02937+K02969+K02969+K02969+K02911+K02911+K02911+K02911+K02887+K02887+K02887+K02887+K02918+K02918+K02918+K02883+K02883+K02916+K02930+K02982+K02982+K02982+K02903+K02903+K02997+K02997+K02997+K02997+K02865+K02865+K02865+K02865+K02865+K02865+K02865+K02954+K02920+K02920+K02896+K02910+K02910+K02910+K02867+K02964+K02964+K02964+K02964+K02881+K02881+K02918+K02918+K02918+K02906+K02906+K02924+K02924+K02924+K02924+K02992+K02992+K02920+K02920+K02983+K02989+K02989+K02989+K02865+K02865+K02881+K02881+K02881+K02881+K02881+K02913+K02913+K02913+K02915+K02915+K02915+K02915+K02883+K02888+K02965+K02965+K02965+K02909+K02872+K02864+K02864+K02922+K02922+K02922+K02922+K02922+K02988+K02988+K02900+K02991+K02991+K02873+K02873+K02995+K02937+K02937+K02937+K02926+K02926+K02945+K02945+K02996+K02997+K02997+K02894+K02992+K02950+K02992+K02877+K02966+K02966+K02868+K02981+K02867+K02889+K02987+K02987+K02987+K02981+K02889+K02987+K02987+K02987+K02889+K02889+K02889+K02889+K02896+K02896+K02896+K02896+K02896+K02896+K02896+K02981+K02889+K02889+K02867+K02871+K02982+K02894+K02894+K02894+K02925+K02925+K02925+K02925+K02925+K02998+K02998+K02998+K02998+K02998+K02998+K02998+K02998+K02998+K02998+K02998+K02998+K02998+K02998+K02895+K02895+K02895+K02971+K02971+K02971+K02971+K02971+K02914+K02914+K02914+K02914+K02914+K02914+K02914+K02914+K02966+K02966+K02966+K02984+K02865+K02906+K02906+K02906+K02906+K02934+K02934+K02934+K02934+K02934+K02934+K02945+K02945+K02945+K02945+K02879+K02941+K02941+K02941+K02941+K02941+K02941+K02941+K02941+K02941+K02941+K02889+K02889+K02889+K02889+K02889+K02889+K02988+K02988+K02988+K02988+K02988+K02879+K02879+K02879+K02879+K02879+K02899+K02899+K02899+K02899+K02899+K02899+K02957+K02957+K02957+K02957+K02957+K02946+K02945+K02987+K02992+K02930+K02987+K02979+K02930+K02950+K02979+K02945+K02993+K02971+K02987+K02993+K02950+K02938+K02938+K02982+K02938+K02911+K02956+K02878+K02963+K02963+K02896+K02896+K02896+K02896+K02896+K02950+K02963+K02954+K02963+

			K02947+K02961+K02963+K02930+K02886+K02992+K02986+K02886+K02930+K02886+K02982+K02886+K02990+K02886+K02930+K02971+K02950+K02986+K02987+K02886+K02983+K02992+K02946+K02961+K02930+K02986+K02986+K02886+K02961+K02992+K02886+K02982+K02886+K02894+K02894+K02894+K02894+K02894+K02912+K02912+K02912+K02959+K02959+K02959+K02959+K02932+K02932+K02932+K02932+K02932+K02932+K02932+K02932+K02949+K02949+K02949+K02949+K02949+K02949+K02949+K02881+K02881+K02914+K02914+K02914+K02885+K02885+K02885+K02885+K02885+K02885+K02885+K02885+K02982+K02878+K02982
RNA transport	ko03013	909	K03259+K03259+K03259+K03259+K03259+K03259+K03259+K03247+K03247+K03231+K03231+K03231+K03231+K03231+K03231+K14290+K14290+K14290+K14290+K14290+K14290+K14290+K03259+K03259+K03259+K12160+K12160+K13137+K13137+K13137+K13137+K14004+K14004+K14004+K14004+K14004+K14004+K14004+K14004+K03241+K03241+K14325+K14325+K14325+K14292+K14292+K14292+K07936+K07936+K07936+K07936+K07936+K07936+K07936+K07936+K07936+K12883+K12883+K12883+K13176+K13176+K13176+K14298+K14298+K14298+K14298+K14298+K03245+K03245+K03245+K13175+K13175+K14324+K12877+K12877+K12877+K12877+K03249+K03249+K03249+K03249+K18213+K14295+K12160+K12160+K12160+K12160+K03248+K03248+K03248+K12881+K00784+K00784+K03231+K03231+K13151+K13151+K13151+K03257+K03257+K03257+K03257+K03257+K03257+K03257+K03257+K03257+K03257+K03257+K03257+K03257+K03257+K03257+K03257+K12160+K12160+K12160+K12160+K03113+K03113+K07936+K07936+K07936+K03239+K03239+K03239+K12812+K12812+K03238+K03238+K03238+K03238+K14299+K14299+K10577+K10577+K10577+K10577+K10577+K10577+K10577+K03243+K12880+K12880+K03238+K03238+K03238+K12881+K14288+K14288+K14288+K14288+K14288+K03113+K03113+K03113+K03113+K03250+K03250+K03250+K03250+K03250+K03243+K14299+K03259+K03259+K14289+K14289+K14289+K14289+K14289+K14289+K07936+K07936+K07936+K07936+K07936+K14324+K03238+K03238+K03238+K03238+K03238+K14297+K14297+K14297+K14297+K14297+K14297+K14297+K14297+K14297+K14297+K12876+K12876+K12876+K12876+K12876+K13114+K13114+K13114+K13114+K13114+K10577+K03259+K03259+K03259+K03538+K03538+K03538+K03538+K03538+K03538+K03538+K03538+K03538+K03246+K03246+K03246+K12881+K14314+K03238+K03238+K03238+K13126+K13025+K13025+K13025+K13025+K13025+K13025+K12879+K12879+K12881+K00784+K00784+K03113+K14328+K03113+K03113+K03537+K03537+K14317+K14317+K14317+K14317+K14317+K14317+K14317+K14305+K14305+K03236+K03236+K03236+K03236+K03231+K03236+K03236+K03236+K03236+K03236+K03236+K03236+K03236+K03236+K03231+K03231+K03231+K03231+K03231+K03231+K03231+K03231+K03231+K03231+K03231+K03231+K03231+K03231+K03231+K03231+K03231+K03254+K03231+K03231+K03231+K03231+K03231+K03231+K03231+K03236+K03231+K03231+K03231+K03231+K03236+K03231+K03231+K03231+

	K03231+K14307+K14307+K12881+K12879+K13171+K13025+K13025+K03260+K03260+K03253+K03253+K03253+K03253+K03253+K03253+K03253+K14309+K14309+K14309+K14309+K14309+K14309+K14309+K14309+K03252+K03252+K03252+K03252+K03252+K03252+K03252+K03252+K18213+K18213+K18213+K18213+K18213+K14320+K14320+K14320+K14320+K14320+K14320+K14320+K14297+K14297+K14297+K14297+K14297+K03260+K12878+K12878+K12878+K12878+K12878+K12878+K12878+K12878+K12878+K12878+K12878+K09291+K13126+K14312+K14312+K14312+K12812+K12812+K12812+K07562+K07562+K07562+K07562+K07562+K07562+K07562+K07562+K07562+K03237+K03237+K03237+K03237+K03237+K03237+K03237+K05019+K05019+K05019+K13174+K13174+K13174+K13174+K13174+K13174+K13174+K13174+K13174+K13174+K18213+K18213+K18213+K14293+K14293+K14293+K14293+K14293+K14293+K14318+K14318+K14318+K14318+K14318+K12160+K12160+K14327+K14327+K14327+K14327+K14327+K14327+K14327+K14327+K14327+K14327+K14327+K14327+K14327+K14327+K14327+K14293+K14293+K14293+K14293+K14293+K14293+K14293+K14293+K14293+K14293+K14293+K14293+K14293+K14293+K00784+K00784+K01164+K01164+K01164+K01164+K01164+K01164+K01164+K01164+K01164+K01164+K01164+K12881+K12881+K12881+K12881+K12881+K14303+K14303+K14303+K14303+K14303+K14303+K14303+K14303+K12875+K12875+K12875+K12875+K12875+K12875+K12875+K12875+K12875+K12875+K12875+K12875+K12875+K12875+K12875+K12875+K14310+K14310+K14310+K14310+K14310+K03257+K03257+K14297+K14297+K14297+K14297+K14297+K03259+K03259+K03259+K03260+K13126+K13126+K13126+K14313+K14313+K14313+K14313+K14313+K14300+K14300+K13126+K12881+K12881+K12881+K12881+K12881+K12881+K13137+K13137+K13137+K13137+K03680+K03680+K03680+K03680+K02516+K02516+K02516+K02516+K02516+K03240+K03240+K03240+K03240+K03240+K03240+K12881+K12881+K14314+K14314+K14314+K14314+K14314+K14314+K14314+K13126+K13126+K13126+K13126+K13126+K03248+K03248+K03248+K03680+K03680+K03680+K03680+K03680+K03680+K03680+K13126+K13126+K14308+K14308+K14308+K14308+K14308+K14308+K03251+K03251+K03251+K03251+K03251+K03251+K03251+K03539+K12881+K12881+K12881+K12881+K12881+K12881+K03262+K03262+K03262+K03262+K14319+K14319+K14319+K14319+K13171+K03231+K14291+K03231+K03231+K03231+K03231+K03231+K03257+K03257+K13126+K03231+K13126+K03262+K13126+K13126+K05749+K03262+K03231+K03231+K13126+K03231+K13126+K03231+K03231+K03262+K03231+K13126+K14292+K14291+K13126+K13126+K14299+K03231+K14291+K05749+K05749+K05749+K05749+K05749+K05749+K05749+K05749+

			K03257+K05749+K03231+K03257+K14292+K12879+K14291+K03231+K13171+K13126+K14292+K13126+K14292+K13171+K03231+K03231+K03238+K03254+K03238+K14292+K14304+K13126+K03262+K03262+K03243+K13171+K14291+K14292+K13126+K14304+K03254+K03262+K03231+K14328+K13114+K03254+K14292+K03262+K03257+K13126+K14292+K13171+K03257+K14292+K03257+K14292+K13171+K03262+K03257+K13126+K13126+K03254+K13171+K13126+K14304+K03231+K13171+K13126+K13171+K14292+K03257+K13171+K03254+K14292+K13171+K10577+K14328+K14328+K14328+K14328+K14328+K14328+K03262+K03262+K03262+K03262+K14307+K14307+K14304+K12882+K14328+K12876+K14304+K12882+K12882+K03254+K14307+K13126+K13126+K13126+K03260+K03250+K14304+K14307+K13171+K03262+K14328+K14304+K13151+K13126+K12879+K13126+K13025+K13126+K03231+K03231+K13126+K13126+K03231+K03243+K12879+K03231+K14299+K13126+K13126+K03243+K03231+K13126+K13126+K03243+K13025+K13126+K13126+K13126+K13025+K13025+K12879+K13025+K12882+K14292+K03259+K03254+K12879+K13151+K13126+K14304+K13126+K13025+K03231+K13126+K14307+K13025+K13126+K13025+K13171+K14299+K14304+K13126+K13126+K13126+K13126+K12879+K03243+K03243+K13126+K12879+K13171+K13126+K13025+K13126+K13025+K13171+K13126+K14299+K14292+K03113+K03113+K03113+K03113+K14306+K14306+K14306+K14306+K09291+K09291+K09291+K09291+K09291+K09291+K09291+K14326+K14326+K14326+K14326+K14326+K14326+K14326+K14326+K14326+K03242+K03242+K03242+K03242+K03242+K03242+K14321+K14321+K14321+K14315+K14315+K03754+K03754+K03754+K03754+K13130+K13130+K13130+K13130+K13130+K13130+K13130+K14319+K14319+K14319+K14301+K14301+K14301+K14301+K03231
mRNA surveillance pathway	ko03015	664	K06269+K06269+K06269+K06269+K06269+K06269+K14411+K14411+K14411+K14411+K14411+K14325+K14325+K14325+K12883+K12883+K12883+K06100+K06100+K06100+K06100+K06100+K14324+K12877+K12877+K12877+K12877+K14376+K14376+K14376+K14376+K14376+K14376+K14376+K14376+K14376+K14376+K14376+K14376+K14376+K14376+K14376+K14376+K14376+K14376+K14376+K14396+K14376+K14376+K14411+K14411+K12881+K14411+K12812+K12812+K14399+K14399+K14411+K14411+K12881+K06269+K06269+K06269+K14411+K14411+K14324+K04382+K04382+K04382+K04382+K04382+K04382+K04382+K06965+K06965+K06965+K06965+K06965+K06965+K06965+K06269+K06269+K06269+K06269+K06269+K12876+K12876+K12876+K12876+K12876+K04382+K04382+K04382+K04382+K04382+K06269+K06269+K06269+K06269+K06269+K13114+K13114+K13114+K13114+K13114+K14396+K14396+K14396+K12881+K14397+K14397+K14397+K14397+K14396+K14396+K13126+K15544+K15544+K13025+K13025+K13025+K13025+K13025+K13025+K14396+K12881+K14397+K14397+K14328+K14409+K14409+K14409+K14409+K14409+K14409+K14409+K14409+K12881+K13171+K15542+K13025+K13025+K13917+K13917+K14400+K14400+K14400+K14400+K14400+K14403+K14403+K14403+K14403+K14403+K14403+K14403+K14403+K14403+K14403+K14403+K14403+K13917+K13917+K13917+K13917+

	K13917+K13917+K13917+K13917+K13917+K13917+K13917+K13917+K13917+K06100+K06100+K06100+K06100+K06100+K11583+K11583+K11583+K11583+K11583+K11583+K11583+K11583+K11583+K11583+K11583+K11583+K11583+K11583+K11583+K13126+K11583+K11583+K11583+K11583+K11583+K11583+K11583+K11583+K11583+K11583+K11583+K11583+K14376+K14376+K14376+K14376+K14376+K14376+K14376+K14376+K14376+K14376+K14376+K14376+K14376+K14376+K14376+K14376+K14376+K12812+K12812+K12812+K04382+K04382+K04382+K04382+K04382+K04382+K14400+K14400+K14400+K06269+K06269+K06269+K06269+K06269+K06269+K06269+K06269+K06269+K06269+K11584+K11584+K11584+K11584+K14376+K14376+K14376+K14411+K14411+K14411+K14411+K14411+K14411+K14411+K14411+K14327+K14327+K14327+K14327+K14327+K14327+K14327+K14327+K14327+K14327+K14327+K14327+K14327+K14327+K03456+K03456+K03456+K03456+K03456+K03456+K03456+K03456+K03456+K03456+K03456+K03456+K03456+K14408+K14408+K14408+K14408+K14408+K14408+K14408+K14408+K14408+K14396+K14411+K08873+K08873+K12881+K12881+K12881+K12881+K12881+K14405+K14405+K14405+K14416+K14416+K14416+K14416+K14416+K14416+K14416+K14416+K14416+K14416+K14416+K14416+K11584+K11584+K11584+K11584+K11584+K11584+K11584+K12875+K12875+K12875+K12875+K12875+K12875+K12875+K12875+K12875+K12875+K12875+K12875+K12875+K12875+K12875+K12875+K03265+K03265+K03265+K03265+K03265+K03265+K03265+K03265+K03265+K03265+K03265+K14398+K14398+K14398+K14398+K14398+K14398+K14398+K14398+K14398+K14396+K14396+K14396+K13126+K13126+K13126+K14376+K13126+K12881+K12881+K12881+K12881+K12881+K12881+K06269+K06269+K06269+K06269+K06269+K06269+K06269+K06269+K06269+K11584+K11584+K11584+K11584+K11584+K11584+K11584+K11584+K11584+K04354+K04354+K04354+K04354+K04354+K04354+K04354+K04354+K04354+K04354+K04354+K14402+K14402+K14402+K14402+K12881+K12881+K14404+K14404+K14404+K14404+K14404+K13126+K13126+K13126+K13126+K13126+K14411+K14411+K14411+K14411+K14411+K14411+K14411+K14411+K14411+K14411+K14411+K11584+K11584+K11584+K11584+K11584+K11584+K11584+K11584+K11584+K11584+K14404+K14404+K13126+K13126+K14397+K14397+K14397+K14397+K14397+K06269+K06269+K11584+K11584+K11584+K11584+K11584+K11584+K12881+K12881+K12881+K12881+K12881+K12881+K13171+K14409+K11583+K11583+K04354+K14409+K13126+K13126+K11583+K14409+K13126+K13126+K11583+K11583+K04354+K13126+K11583+K13126+K11583+K13126+K11583+K14411+K13126+K04354+K13126+K04354+K14411+K14411+K13171+K04354+K13126+K06269+K14407+K13126+K13171+K14409+K13126+K14409+K15542+K11583+K13171+K14409+K14409+K13126+K14328+K13114+K14409+K13126+K13171+K14409+K13171+K14407+K11584+K13126+K14409+K13126+K13171+K13126+K14407+K13171+K13126+K13171+K14411+K14409+K13171

			+K13171+K14411+K14328+K14328+K14328+K14328+K14328+K14328+K14407+K14407+K12882+K14328+K12876+K12882+K12882+K13126+K13126+K13126+K04382+K14962+K13171+K14328+K11584+K13126+K13126+K13025+K13126+K13126+K13126+K06269+K13126+K15542+K04354+K13126+K13126+K04354+K04382+K13126+K13025+K13126+K13126+K13126+K13025+K13025+K13025+K12882+K04354+K04382+K04354+K13126+K04354+K04354+K13126+K13025+K13126+K04354+K13025+K13126+K13025+K13171+K11584+K13126+K13126+K13126+K13126+K04354+K15542+K13126+K04354+K13171+K13126+K13025+K13126+K04354+K11584+K13025+K13171+K13126+K03267+K03267+K03267+K03267+K03267+K03267+K03267+K03267+K03267+K03267+K03267+K11584+K11584+K06269+K06269+K06269+K06269+K06269+K14326+K14326+K14326+K14326+K14326+K14326+K14326+K14326+K14326+K14401+K14401+K14401
RNA degradation	ko03018	553	K04043+K12611+K12611+K12611+K12606+K12606+K11600+K11600+K11600+K11600+K03678+K03678+K03678+K03678+K12620+K12620+K12620+K12620+K12620+K12620+K07573+K07573+K07573+K01689+K12581+K12581+K12581+K12581+K12581+K12581+K12581+K12581+K12581+K12581+K12581+K03681+K03681+K03681+K12623+K12623+K14442+K14442+K14442+K12608+K12608+K12608+K12608+K12608+K12608+K12608+K12608+K12608+K12608+K12625+K12625+K12625+K12625+K12625+K12625+K12581+K12581+K04077+K04077+K04077+K04077+K04077+K04077+K04077+K04077+K04077+K04077+K04077+K04077+K04077+K04077+K04077+K04077+K04077+K12585+K12585+K12585+K12585+K12585+K12585+K12585+K12585+K12585+K12585+K12585+K12585+K12585+K12585+K12585+K12622+K12622+K12622+K12622+K12622+K12621+K12621+K12624+K12624+K12598+K12613+K12613+K12613+K12613+K12613+K12617+K12617+K12617+K04043+K12619+K12619+K12619+K12619+K12619+K12619+K12619+K12619+K12619+K00850+K12608+K12608+K12608+K13126+K12589+K12589+K12589+K12589+K12589+K12589+K12581+K12581+K12581+K12581+K12580+K12627+K12627+K12627+K12627+K12627+K12626+K12626+K12626+K12611+K12611+K03679+K12590+K12590+K01689+K01689+K01689+K01689+K01689+K01689+K01689+K12593+K12593+K12599+K12599+K12604+K01148+K01148+K01148+K01148+K01148+K01148+K01148+K12617+K12617+K12617+K12617+K12617+K12617+K12617+K12617+K12617+K12617+K12617+K10643+K10643+K10643+K10643+K10643+K10643+K10643+K10643+K04077+K04077+K04077+K04077+K12598+K12598+K12598+K12598+K12598+K12598+K12598+K12598+K12598+K12598+K12619+K12619+K12619+K12619+K12619+K12619+K12619+K12619+K12619+K13126+K12619+K12619+K12619+K12619+K10643+K10643+K14442+K14442+K12607+K12607+K12607+K12607+K12607+K12607+K12607+K12619+K12619+K12619+K12619+K12619+K12619+K12619+K12619+K12619+K12619+K00962+K00962+K00962+K00962+K00962+K12619+K12619+K12619+K12619+K12591+K12591+K12591+K12591+K12591+K12616+K12616+K12616+K12616+K12616+K12616+K12616+K12616+K12616+K12616+K12616+K12616+K12616+K12616+K12616+K12616+K03514+K03514+K03514+

			K03514+K03514+K03514+K03514+K12614+K12614+K12614+K12614+K12614+K12614+K12614+K12614+K12613+K03654+K03654+K13126+K13126+K13126+K13126+K01689+K01689+K01689+K01689+K01689+K12606+K12606+K12606+K12606+K12606+K12606+K04077+K04077+K04077+K04077+K04043+K04043+K04043+K12619+K00850+K00850+K00850+K00850+K00850+K00850+K00850+K00850+K00850+K00850+K13126+K13126+K13126+K13126+K13126+K13126+K13126+K04043+K04043+K04043+K04043+K14442+K14442+K00962+K04043+K12580+K12580+K12580+K12580+K12580+K04043+K13126+K12580+K12580+K13126+K12580+K12603+K13126+K13126+K13126+K00850+K12580+K12603+K12580+K12580+K13126+K00850+K12608+K12580+K04043+K12580+K13126+K12580+K04043+K13126+K13126+K12619+K04043+K12580+K12580+K12580+K04077+K12603+K12580+K12580+K04043+K12580+K04077+K00850+K04043+K12580+K13126+K12580+K12619+K04077+K04043+K12580+K04043+K12580+K13126+K12580+K04077+K12603+K13126+K12603+K04077+K13126+K12580+K12600+K12603+K13126+K04077+K00962+K04077+K12580+K12580+K04043+K13126+K04077+K12605+K12605+K12605+K12605+K12605+K13126+K12605+K12605+K12605+K04043+K12605+K12605+K12605+K12605+K12613+K12603+K04077+K13126+K00850+K13126+K12599+K00962+K04077+K00962+K12608+K00962+K00850+K04043+K13126+K13126+K13126+K12608+K12608+K00850+K12608+K04077+K12600+K04077+K12600+K04043+K13126+K04077+K13126+K04043+K12619+K13126+K12627+K04077+K13126+K13126+K12603+K12600+K04077+K13126+K12627+K12581+K13126+K04043+K13126+K12605+K13126+K04043+K13126+K04043+K04043+K04043+K13126+K13126+K04043+K00962+K12603+K12603+K12600+K04077+K12600+K04043+K13126+K13126+K12613+K04043+K13126+K04043+K13126+K12622+K13126+K13126+K13126+K12603+K13126+K12603+K04077+K04043+K12622+K04043+K13126+K13126+K13126+K12603+K13126+K12603+K04077+K04077+K04077+K01689+K01689+K01689+K01689+K00850+K00850+K00850+K00850+K00850+K00850+K00850+K00850+K00850+K00850+K00850+K00850+K01148+K01148+K01148+K00850+K01689+K01689+K01689+K01689+K12581+K12581+K14442+K14442+K14442+K14442+K14442+K14442+K14442+K14442+K14442+K14442+K14442+K14442+K14442+K00850+K00850+K00850+K12619
RNA polymerase	ko03020	127	K03005+K03005+K03005+K03005+K03016+K03016+K03016+K03016+K03008+K03008+K03008+K03015+K03015+K03015+K03009+K03009+K03009+K03009+K03009+K03011+K03011+K03011+K03011+K03024+K03025+K03025+K03040+K03020+K03020+K03013+K03013+K03025+K03025+K03025+K03013+K03014+K03014+K03019+K03007+K03016+K03016+K03013+K03013+K03012+K03012+K03012+K03002+K03002+K03002+K03002+K03013+K03013+K03013+K03000+K03000+K03021+K03021+K03021+K02999+K02999+K02999+K02999+K02999+K03021+K03021+K03021+K03021+K03021+K03021+K03021+K03010+K03010+K03010+K03010+K03010+K03010+K03010+K03010+K03010+K03010+K03010+K14721+K14721+K14721+K14721+K14721+K14721+K14721+K14721+K14721+K14721+K14721+K14721+K03014+K03014+K03014+K03014+K03014+K03014+K03006+K03006+K03006+K03006+K03006+K03043+

			K03043+K03013+K03040+K03040+K03040+K03040+K03040+K03040+K03018+K03018+K03040+K03043+K03046+K03007+K03007+ K03007+K03007+K03012+K03012+K03012+K03012+K03023
Basal transcription factors	ko03022	171	K03125+K03125+K03125+K03125+K03125+K03125+K03125+K03133+K03133+K03133+K03133+K03133+K03133+K03133+K03124+ K03124+K03124+K03124+K03120+K03120+K03120+K03120+K03120+K10845+K10845+K10845+K03124+K06634+K06634+K03137+ K03137+K03137+K03137+K03142+K03142+K03123+K03123+K03123+K03123+K03139+K03139+K03139+K03143+K03143+K10842+ K10842+K03142+K03142+K03142+K03142+K03142+K03142+K03142+K03142+K03142+K03142+K03134+K03134+K03134+K03134+ K03144+K03144+K03144+K03144+K03144+K03144+K03144+K03144+K03144+K03144+K03136+K03136+K03136+K03129+K03129+ K03129+K03129+K03129+K03124+K03124+K03124+K03124+K03124+K03126+K03126+K03126+K03126+K03126+K03141+K03141+ K03141+K10844+K10844+K10844+K10844+K03135+K03135+K03135+K03135+K03135+K03135+K03125+K03138+K03138+K03138+ K03138+K03138+K03138+K03138+K03130+K03130+K03130+K03130+K03127+K03127+K14649+K14649+K03123+K03123+K03123+ K03123+K03122+K03122+K03122+K03122+K03122+K03122+K03122+K10843+K03126+K06634+K06634+K03143+K06634+K03143+ K06634+K06634+K03143+K06634+K03125+K03126+K03143+K03126+K03143+K03125+K03125+K10843+K03126+K03128+K03143+ K03128+K03128+K10843+K03128+K10843+K03128+K10843+K10843+K03128+K03128+K03131+K03131+K14649+K14649+K03131+ K03131+K03131+K03131+K02202+K02202+K02202
DNA replication	ko03030	130	K07466+K07466+K07466+K07466+K10744+K07466+K07466+K07466+K10739+K10739+K10739+K10739+K04799+K04799+K04799+ K04799+K07466+K07466+K07466+K07466+K07466+K07466+K02320+K02320+K02320+K10755+K10755+K10755+K02328+K07466+ K07466+K07466+K03505+K03505+K03505+K10756+K10756+K02685+K02540+K03506+K10756+K10756+K04802+K04802+K02684+ K02684+K02325+K02325+K02326+K07466+K07466+K07466+K02327+K02327+K02327+K02327+K07466+K07466+K07466+K07466+ K07466+K07466+K07466+K07466+K10754+K10754+K10754+K10754+K07466+K07466+K07466+K10755+K03111+K03111+K03111+ K03111+K03111+K02541+K02541+K02541+K10747+K10747+K10747+K10747+K10747+K10747+K10747+K10747+K02210+K02335+ K02335+K02335+K03504+K03504+K03504+K03504+K10747+K10747+K10747+K10747+K10747+K07466+K07466+K07466+K02209+ K02542+K02542+K02327+K02542+K02542+K02542+K07466+K07466+K02540+K02540+K02540+K02540+K02540+K02540+K07466+ K07466+K07466+K02325+K02325+K02325+K02325+K02325+K02325+K04802+K04802
Spliceosome	ko03040	1049	K12826+K12826+K12849+K12849+K12849+K12849+K12849+K12900+K12900+K12900+K12900+K12900+K12900+K12900+K12900+ K11096+K12883+K12883+K12883+K12885+K12885+K11087+K11087+K11087+K12741+K12741+K12741+K12741+K12896+K12896+ K12896+K12844+K12844+K12844+K12741+K12741+K12741+K12741+K12845+K12877+K12877+K12877+K12877+K12874+K12874+

K12874+K11088+K11088+K11088+K12881+K11092+K11092+K11092+K12848+K12848+K12843+K12843+K12873+K12873+K12873+
K12873+K12873+K12873+K12873+K12873+K12834+K12834+K12834+K12834+K12834+K12859+K11091+K11091+K11091+K11091+
K11091+K12812+K12812+K12836+K12836+K12836+K12836+K12836+K12836+K11094+K11094+K11094+K12623+K12623+K11086+
K11086+K12896+K12896+K12741+K12741+K12741+K12890+K12890+K11096+K11096+K12625+K12625+K12625+K12625+K12625+
K12625+K12880+K12880+K03283+K12833+K12833+K12833+K12833+K12833+K12845+K12845+K12861+K12861+K12861+K12868+
K12868+K12868+K12868+K12868+K12868+K12868+K12868+K12828+K12871+K12863+K12863+K12863+K12863+K12863+K12881+
K11093+K11093+K11093+K11093+K11093+K11093+K11093+K11093+K11093+K12891+K12891+K12891+K12891+K12844+K12844+
K12844+K12844+K12844+K12844+K12844+K12844+K12844+K11098+K11098+K12837+K12844+K12844+K12859+K12859+K12859+
K12859+K12859+K12859+K12622+K12622+K12622+K12622+K12622+K12897+K12897+K12897+K12897+K12621+K12621+K12624+
K12624+K12857+K12857+K12857+K11098+K11098+K12741+K12741+K12741+K12741+K12741+K12890+K12890+K12890+K12890+
K12890+K12890+K12890+K12890+K12890+K12896+K12896+K12896+K12896+K12896+K12829+K12829+K11087+K11087+K11097+
K11097+K11097+K11097+K11095+K11095+K11095+K12876+K12876+K12876+K12876+K12876+K11099+K11099+K11099+K11099+
K11099+K11099+K12823+K12823+K12823+K12741+K12741+K12741+K12741+K12741+K12741+K12881+K12831+K12831+K12733+
K12897+K12897+K12741+K12741+K12845+K12845+K12845+K12845+K03283+K12741+K13025+K13025+K13025+K13025+K13025+
K13025+K12879+K12879+K12893+K12893+K12893+K12893+K12881+K12627+K12627+K12627+K12627+K12627+K12626+K12626+
K12626+K12741+K12741+K12830+K12830+K12831+K12896+K12896+K12896+K12896+K12896+K11097+K11097+K12816+K12817+
K12830+K12821+K12821+K09564+K09564+K12822+K12822+K12822+K12822+K12822+K12822+K12822+K12822+K12822+K12822+
K03283+K03283+K03283+K03283+K03283+K12881+K12879+K12818+K12818+K12868+K13025+K13025+K12856+K12856+K12856+
K12823+K12823+K12823+K12818+K12818+K12818+K12818+K12818+K12818+K12818+K12900+K12900+K12900+K12900+K12900+
K12900+K12900+K12900+K12900+K12815+K12815+K12815+K12893+K12893+K12893+K12893+K12893+K12893+K12893+K12893+
K12893+K12893+K12893+K12893+K12893+K12893+K12893+K12893+K12824+K12824+K12824+K12824+K12824+K12824+K12835+
K12835+K12835+K12835+K12835+K12833+K12837+K12837+K12837+K12837+K12837+K12878+K12878+K12878+K12878+K12878+
K12878+K12878+K12878+K12878+K12878+K12878+K12823+K12823+K12823+K12823+K12823+K12823+K12823+K12823+K12823+
K12823+K12867+K12867+K12867+K12867+K12867+K12867+K11984+K11984+K11984+K11984+K12823+K12823+K12823+K12823+
K12823+K12823+K12823+K12823+K12823+K12832+K12832+K12832+K12832+K12832+K12832+K12832+K12812+K12812+K12812+
K12813+K12813+K12813+K12862+K12862+K12855+K12855+K12855+K12855+K12855+K12829+K12829+K12842+K12842+K12852+

	K12852+K12852+K12823+K12823+K12823+K12837+K12837+K12837+K12837+K12837+K12837+K12837+K12837+K12837+K12837+K12837+K12837+K12837+K12885+K12885+K12885+K12885+K12885+K12885+K12885+K12885+K12885+K12741+K12741+K12741+K12741+K12741+K12741+K12741+K12741+K12741+K12741+K12741+K12741+K12741+K12741+K12741+K12741+K12847+K12847+K12847+K12847+K12881+K12881+K12881+K12881+K12881+K12897+K12897+K12897+K12897+K12897+K12897+K12897+K12897+K12897+K12897+K12818+K12818+K12818+K12818+K12818+K12869+K12869+K12875+K12875+K12875+K12875+K12875+K12875+K12875+K12875+K12875+K12875+K12875+K12875+K12875+K12875+K12875+K12875+K12811+K12811+K12811+K12811+K12860+K12860+K12860+K12860+K12860+K12860+K12860+K12860+K12860+K12860+K12897+K12897+K12897+K12897+K12897+K12897+K12897+K12897+K12897+K12897+K12836+K12836+K12836+K12836+K12836+K12836+K12823+K12900+K12900+K12900+K12900+K12900+K12900+K12741+K12741+K12741+K12741+K12741+K12818+K12818+K12818+K12818+K12819+K12819+K12819+K12819+K12819+K12819+K12819+K12819+K12819+K12819+K12819+K12864+K12864+K12864+K12864+K12864+K12890+K12890+K12890+K12890+K12890+K12890+K12890+K12890+K12890+K12890+K12890+K12890+K12846+K12846+K12846+K12846+K11098+K12881+K12881+K12881+K12881+K12881+K12881+K12872+K12872+K12872+K12872+K12850+K12850+K12850+K12850+K12850+K12850+K12850+K12850+K12850+K12850+K12850+K12891+K12891+K12891+K12891+K12891+K12891+K12891+K12869+K12869+K12869+K12869+K12818+K12818+K12818+K12818+K12890+K12890+K12890+K12890+K12890+K12890+K12890+K12890+K12890+K11095+K11095+K11095+K12885+K12885+K12885+K12885+K12885+K12885+K12885+K12885+K12885+K12885+K12885+K12885+K12885+K12881+K12881+K12827+K12827+K12827+K12827+K12827+K12827+K12827+K12827+K12827+K12827+K12843+K12843+K12843+K12843+K12843+K12843+K12843+K12858+K12858+K12858+K12858+K12858+K12858+K12858+K12858+K03283+K03283+K03283+K03283+K06063+K06063+K06063+K06063+K06063+K06063+K06063+K06063+K06063+K06063+K06063+K06063+K06063+K06063+K12893+K12893+K12893+K12893+K12893+K12893+K12893+K12893+K12881+K12881+K12881+K12881+K12881+K12881+K10599+K10599+K10599+K10599+K12830+K12817+K12830+K12830+K12830+K12828+K12825+K12662+K12823+K12823+K12823+K12825+K12816+K03283+K12662+K12820+K12862+K12816+K12896+K03283+K03283+K12825+K12896+K03283+K12862+K03283+K03283+K12829+K12821+K03283+K03283+K03283+K03283+K03283+K12829+K12837+K12820+K12821+K03283+K12823+K12816+K12868+K12829+K12828+K03283+K03283+K12829+K12829+K12821+K03283+K12816+K03283+K12896+K12879+K12821+K12821+K03283+K12829+K03283+K12829+K03283+K03283+K12821+K03283+K12865+K03283+K12823+K12823+K03283+K12821+K12825+K12821+K03283+K03283+K11096+K12825+K03283+K12829+K03283+K03283+K12829+K12825+K12816+K12821+K12825+K12862+K12825+K12825+K03283+K12862+K03283+K12862+K12828+K12821+K12825+K12821+K12828+K12821+K12828+K03283+K12820+K03283+

			K12896+K12896+K12817+K12821+K12821+K12828+K12821+K12825+K12821+K12825+K12825+K12820+K12820+K12828+K12821+K12821+K12741+K12865+K03283+K12820+K12820+K12828+K12820+K12837+K12865+K03283+K12662+K03283+K03283+K03283+K03283+K03283+K03283+K03283+K03283+K12882+K12662+K12662+K12876+K12865+K12865+K12662+K12882+K09564+K09564+K12825+K12882+K12662+K12828+K12865+K12865+K12865+K12865+K12662+K12837+K12837+K12837+K12837+K12837+K12837+K12837+K12837+K12837+K12837+K12837+K12837+K03283+K03283+K12831+K12831+K12868+K12837+K12879+K12862+K13025+K12868+K12627+K12879+K03283+K03283+K12823+K12627+K12868+K12865+K12823+K12820+K12823+K12837+K13025+K12837+K12823+K03283+K12862+K12868+K13025+K12825+K13025+K12879+K12862+K13025+K12882+K12823+K12879+K12868+K12823+K12823+K13025+K12823+K12837+K13025+K13025+K12837+K12823+K12622+K12862+K12622+K12879+K12820+K12823+K03283+K12820+K12879+K03283+K12860+K13025+K12823+K12837+K12837+K13025+K12820+K12821+K12820+K12836+K12836+K12836+K12836+K12836+K12891+K12891+K12891+K12891+K12891+K12891+K12891+K12891+K12900+K12900+K12900+K12900+K12900+K03283+K12741+K12741+K12741+K12741+K12741+K12741+K12741+K12830+K12830+K12830+K12830+K12830+K12830+K12830+K12830+K12830+K12830+K12830+K12830+K12839+K12839+K12839+K12839+K12839+K12854+K12854+K12854+K12854+K12854+K12741+K12741+K12741+K12741+K12741+K12741+K12741+K12741
Proteasome	ko03050	210	K10881+K10881+K10881+K10881+K06699+K06699+K06699+K06699+K06699+K06699+K06699+K03029+K03029+K03029+K03029+K03029+K03029+K03029+K03029+K03029+K02729+K02729+K03063+K03063+K02725+K02725+K02725+K02725+K02725+K03065+K03065+K03065+K03065+K02737+K02737+K02737+K02737+K03062+K03062+K03038+K03038+K03038+K10881+K02732+K02732+K02732+K02732+K02726+K02726+K02726+K02728+K02728+K02728+K02728+K02728+K02728+K02728+K02735+K02735+K02735+K02735+K02735+K02735+K02735+K03039+K03039+K03039+K03039+K03039+K03039+K03030+K03030+K03030+K03030+K03030+K03030+K03030+K03030+K03030+K02731+K02731+K02731+K02731+K02731+K02731+K02727+K02727+K02727+K06700+K06700+K02729+K02729+K02729+K02729+K02736+K02736+K02736+K02738+K02738+K02738+K02738+K02738+K02730+K02730+K02730+K02730+K02730+K02730+K02730+K03064+K03064+K03064+K03064+K03066+K03066+K03066+K03066+K03066+K03066+K03066+K03066+K02739+K03036+K02734+K02734+K02739+K03032+K03032+K03032+K03032+K03032+K03032+K03032+K03031+K03031+K03031+K03031+K03031+K11599+K11599+K11599+K11599+K11599+K11599+K03037+K03037+K03037+K03037+K03037+K03037+K03037+K02739+K02739+K02739+K02739+K02739+K02739+K02739+K02739+K03036+K03036+K03036+K03036+K03036+

			K03036+K03032+K02735+K03062+K03062+K03062+K03062+K03028+K03028+K03028+K03028+K03028+K03028+K03028+K03065+K03065+K03028+K03028+K03028+K03028+K03065+K03028+K03028+K02731+K02737+K03065+K03065+K02737+K03028+K03033+K03033+K03033+K03033+K03033+K03033+K03033+K03061+K03061+K03061+K03061+K03061+K03035+K03035+K03035+K03035
Protein export	ko03060	212	K03217+K03217+K03217+K09648+K07342+K07342+K07342+K07342+K07342+K03100+K09647+K09647+K09647+K09647+K09647+K03116+K03116+K07342+K07342+K07342+K13280+K13280+K13280+K13280+K03104+K03104+K03104+K03104+K12946+K12946+K12946+K03110+K03110+K03110+K03110+K03100+K03100+K12947+K12947+K12947+K09647+K12948+K12948+K12948+K12948+K12272+K12272+K12272+K09540+K03105+K03105+K09481+K09481+K09481+K09481+K09481+K03106+K12947+K03118+K03118+K03118+K03109+K03109+K03109+K03109+K03109+K03109+K03109+K03109+K03109+K09490+K09490+K09490+K03108+K03070+K03070+K03070+K03070+K03070+K03070+K03070+K03070+K03070+K03070+K03070+K10956+K10956+K10956+K10956+K10956+K10956+K10956+K10956+K10956+K10956+K10956+K03070+K03070+K03070+K03070+K03070+K03070+K03070+K03070+K03217+K03217+K03217+K03217+K03217+K03217+K03217+K03217+K03217+K03217+K03106+K03106+K03106+K03106+K03106+K03106+K03106+K03106+K03217+K03217+K03217+K12275+K12275+K12275+K12275+K12275+K09490+K13431+K13431+K13431+K13431+K13431+K13431+K13431+K13431+K13431+K13431+K13431+K09540+K09540+K09540+K09540+K09540+K09540+K09540+K09540+K09540+K09540+K12946+K12946+K12946+K12946+K03106+K03106+K03106+K03106+K03109+K03108+K03217+K03217+K03217+K03217+K09490+K09490+K09490+K09490+K09490+K03217+K09490+K09490+K09490+K09490+K03217+K03217+K09490+K09490+K03109+K09490+K09490+K09490+K09490+K03108+K09490+K09490+K09490+K03108+K03108+K09490+K09490+K09490+K03108+K03108+K03108+K03217+K09490+K10956+K03107+K03107+K03107+K03107+K03107+K03107+K12947+K12947+K12947+K12947+K12947+K10956
Base excision repair	ko03410	111	K10802+K10802+K10802+K10802+K10802+K10802+K10802+K10802+K01246+K01246+K01246+K01246+K03652+K03652+K04799+K04799+K04799+K04799+K01246+K10802+K10802+K10802+K10802+K10802+K10803+K10803+K10802+K10802+K10802+K10802+K02328+K01247+K01247+K01247+K01247+K03505+K03505+K03505+K10772+K10772+K03506+K01246+K04802+K04802+K02325+K02325+K02326+K02327+K02327+K02327+K02327+K10798+K10798+K10798+K10798+K10798+K10747+K10747+K10747+K10747+K10747+K10747+K10747+K10747+K02335+K02335+K02335+K01142+K01142+K01142+K03504+K03504+K03504+K03504+K10747+K10747+K10747+K10747+K10747+K01246+K01246+K01246+K01246+K01246+K01246+K10801+K02327+K10801+K01246+K10801+K10801+K10801+K01246+K01246+K01246+K10563+K10563+K10563+K10563+K10563+K10563+K02325+K02325+K02325+K02325+K02325+K02325+K04802+K04802+K03512+K03512

Nucleotide excision repair	ko03420	237	K10839+K10839+K10839+K07466+K07466+K07466+K07466+K07466+K07466+K07466+K10739+K10739+K10739+K10739+K07466+ K07466+K07466+K10839+K10839+K10839+K10839+K10839+K10839+K07466+K07466+K07466+K10839+K10839+K10839+K10839+ K10839+K10845+K10845+K10845+K06634+K06634+K10755+K10755+K10755+K10848+K10848+K10848+K10848+K02328+K07466+ K07466+K07466+K03505+K03505+K03505+K10839+K03142+K03142+K03868+K03868+K10756+K10756+K03506+K10756+K10756+ K04802+K04802+K03143+K03143+K02325+K02325+K10842+K10842+K02326+K03142+K03142+K03142+K03142+K03142+K03142+ K03142+K03142+K03142+K03142+K07466+K07466+K07466+K10846+K10846+K10846+K02327+K02327+K02327+K02327+K10609+ K10609+K10609+K10609+K10609+K10609+K10609+K10609+K10609+K10609+K10609+K10609+K10609+K10609+K10609+K10609+ K10849+K10849+K10849+K10849+K10849+K10849+K03144+K03144+K03144+K03144+K03144+K03144+K03144+K03144+K03144+ K03144+K07466+K07466+K07466+K07466+K07466+K07466+K07466+K07466+K10754+K10754+K10754+K10754+K10838+K10838+ K10841+K10841+K10841+K10841+K10841+K10841+K07466+K07466+K07466+K10610+K10610+K10610+K10610+K10610+K10610+ K10610+K10610+K10610+K10610+K10610+K10755+K03141+K03141+K03141+K10844+K10844+K10844+K10844+K10747+K10747+ K10747+K10747+K10747+K10747+K10747+K10747+K10570+K10570+K10570+K10570+K10570+K03868+K03868+K03868+K03868+ K03868+K03868+K02335+K02335+K02335+K03504+K03504+K03504+K03504+K10747+K10747+K10747+K10747+K10747+K07466+ K07466+K07466+K10843+K06634+K06634+K03143+K06634+K03143+K06634+K06634+K03143+K06634+K02327+K03143+K03143+ K10839+K07466+K07466+K10843+K03143+K10843+K10843+K10843+K10843+K07466+K07466+K07466+K10140+K10140+K10140+ K10140+K02325+K02325+K02325+K02325+K02325+K02325+K04802+K04802+K02202+K02202+K02202
Mismatch repair	ko03430	114	K07466+K07466+K07466+K07466+K07466+K08737+K07466+K07466+K10739+K10739+K10739+K10739+K07466+K07466+K07466+ K07466+K07466+K07466+K08736+K08736+K10755+K10755+K10755+K02328+K07466+K07466+K07466+K03505+K03505+K03505+ K08739+K08739+K08739+K08739+K08739+K08739+K10756+K10756+K10756+K10756+K04802+K04802+K07466+K07466+K07466+ K02327+K02327+K02327+K02327+K10858+K10858+K10858+K10858+K07466+K07466+K07466+K07466+K07466+K07466+K07466+ K07466+K10754+K10754+K10754+K10754+K08737+K07456+K07456+K07456+K07456+K07456+K08735+K08735+K07466+K07466+ K07466+K08737+K08737+K10755+K03111+K03111+K03111+K03111+K03111+K10747+K10747+K10747+K10747+K10747+K10747+ K10747+K10747+K08734+K08734+K03504+K03504+K03504+K03504+K10747+K10747+K10747+K10747+K10747+K07466+K07466+ K07466+K02327+K07466+K07466+K07466+K07466+K07466+K04802+K04802
Homologous recombination	ko03440	145	K07466+K07466+K07466+K07466+K10881+K10881+K10881+K10881+K07466+K07466+K07466+K08991+K08991+K10739+K10739+ K10739+K10739+K07466+K07466+K07466+K07466+K07466+K07466+K10881+K02328+K07466+K07466+K07466+K03505+K03505+

			K03505+K10882+K10882+K03553+K03553+K03553+K03553+K04482+K04482+K03553+K03553+K03553+K07466+K07466+K07466+K10875+K10875+K10875+K10875+K02327+K02327+K02327+K02327+K07466+K07466+K07466+K07466+K07466+K07466+K07466+K07466+K03165+K03165+K03165+K03165+K03165+K03165+K03165+K10865+K10865+K10865+K10865+K10865+K07466+K07466+K07466+K10875+K10875+K10875+K10875+K10875+K10875+K10875+K03165+K03165+K03165+K03165+K03165+K03165+K03165+K03165+K10866+K10866+K10866+K03111+K03111+K03111+K03111+K03111+K10882+K10882+K10882+K10882+K10882+K10882+K10869+K02335+K02335+K02335+K10901+K10901+K10901+K10901+K10879+K10879+K10879+K10879+K10879+K10879+K03504+K03504+K03504+K03504+K07466+K07466+K07466+K03553+K03553+K02327+K07466+K07466+K07466+K07466+K10867+K10867+K10867+K10867+K03655+K03655+K07466+K03553+K03553+K03553+K03553+K03553
Non-homologous end-joining	ko03450	34	K04799+K04799+K04799+K04799+K10865+K10865+K10865+K10865+K10865+K10866+K10866+K10866+K10777+K10777+K10777+K10777+K10777+K10777+K10777+K10777+K10777+K10777+K10777+K10777+K10777+K10777+K10885+K10885+K10884+K10884+K10884+K10884+K03512+K03512
Phosphatidylinositol signaling system	ko04070	222	K00913+K00913+K00913+K00913+K00913+K00915+K00915+K00915+K00913+K00913+K00921+K00921+K00921+K00921+K00921+K02183+K02183+K02183+K02183+K02183+K02183+K02183+K02183+K02183+K02183+K02183+K02183+K02183+K00921+K00921+K00921+K00921+K00921+K00921+K00921+K10572+K10572+K10572+K10572+K10572+K00921+K00921+K00921+K00921+K00921+K00921+K00921+K00921+K00921+K00921+K00921+K15422+K15422+K02183+K02183+K02183+K01092+K01092+K00999+K00999+K00999+K00999+K00999+K00999+K02183+K00913+K01110+K01110+K01110+K01110+K01110+K01110+K01110+K01110+K01110+K01110+K00901+K01110+K01110+K01110+K00901+K00901+K00889+K00889+K00889+K00889+K00889+K00888+K00888+K01106+K01106+K00889+K00889+K00889+K00889+K02183+K02183+K02183+K02183+K02183+K02183+K02183+K02183+K00914+K00914+K00914+K00914+K00914+K00901+K00901+K00901+K00901+K00889+K00889+K00889+K00889+K00889+K00889+K00889+K00889+K00889+K01110+K01110+K05857+K05857+K05857+K05857+K05857+K05857+K05857+K00888+K00888+K00888+K00888+K00888+K00981+K10572+K10572+K10572+K10572+K00915+K00915+K00915+K00901+K01092+K00981+K00981+K00901+K00901+K00901+K05857+K10572+K00901+K00901+K10572+K00901+K00901+K00901+K01110+K10572+K10572+K00901+K00901+K10572+K00901+K00901+K00901+K10572+K10572+K00901+K10572+K10572+K10572+K10572+K01092+K00901+K00901+K00901+K00901+K05857+K00901+K00901+K00901+K00901+K00901+K00981+K05857+K05857+K05857+K05857+K01110+K05857+K00901+K00981+K00901+K15422+K01110+K05857+K00901+K00901+K00981+K00901+K00901+K00901+K15422+K00901+K00901+K00901+K01110+K00901+K00901+K00901+K05857+K05857+K00901+K00901+K00901+K00913+K00913+K00981+K00981+K00981

Plant hormone signal transduction	ko04075	827	K14484+K14484+K14497+K14497+K14498+K14498+K14498+K14498+K14498+K14498+K14497+K14497+K14497+K14488+K14431+<br>K14431+K14484+K14484+K14484+K13464+K13464+K14496+K14496+K14496+K14492+K14492+K14492+K14492+K14492+K13464+<br>K13464+K13464+K13464+K14431+K14431+K14431+K14432+K14432+K14432+K14432+K14432+K14502+K14502+K14502+K14484+<br>K14484+K14496+K14496+K14496+K14496+K14496+K14496+K14486+K14432+K14432+K14432+K13464+K13464+K13464+K13464+<br>K13946+K13946+K14488+K14484+K14484+K14484+K14484+K14484+K14484+K14484+K14484+K14484+K14484+K14484+K14484+<br>K14484+K14484+K14484+K14498+K14498+K14498+K14498+K14498+K14498+K14498+K14498+K14498+K14498+K14508+K14508+<br>K14489+K14489+K14489+K14489+K14489+K14489+K14489+K14489+K14489+K14489+K14489+K14484+K14484+K14484+K14484+<br>K14484+K14484+K14484+K14484+K14484+K14504+K14504+K14504+K14504+K14504+K14504+K14504+K14504+K14504+K14504+<br>K14504+K14504+K14504+K14504+K14504+K14504+K14504+K14504+K14504+K14504+K14504+K14488+K14515+K14497+K14508+<br>K14508+K14508+K14508+K14508+K14508+K14508+K14508+K14508+K14508+K13449+K13449+K13449+K14488+K14488+K14488+<br>K14488+K14488+K14488+K14488+K14488+K14488+K14488+K14488+K14488+K14488+K14488+K14488+K14488+K14488+K14488+<br>K14488+K13415+K13415+K13415+K13415+K13415+K13415+K13415+K13415+K14502+K14502+K14502+K14502+K14502+K14486+<br>K14484+K14484+K14484+K14484+K14484+K14484+K14484+K14484+K14484+K14484+K14484+K14484+K14484+K14484+K14484+<br>K14484+K14484+K14484+K14484+K14484+K14484+K14484+K14484+K14496+K14496+K14496+K14496+K14503+K14503+K14503+<br>K14503+K14503+K14503+K14432+K14432+K14432+K14484+K14495+K14495+K14484+K14486+K14486+K13946+K13946+K13946+<br>K14505+K14505+K14488+K14488+K14488+K14484+K14484+K14484+K14488+K14488+K14488+K14508+K14508+K14508+K14492+<br>K14484+K14484+K14484+K14489+K14489+K14489+K14489+K14489+K14431+K14492+K14492+K14488+K14492+K14492+K14492+<br>K14488+K14488+K14486+K14486+K14486+K14486+K14486+K14486+K14486+K14486+K14484+K14484+K14496+K14488+K14488+<br>K14488+K14488+K14488+K14488+K14488+K14488+K14498+K14498+K14498+K14431+K14495+K14495+K14488+K14488+K14488+<br>K14488+K14488+K14488+K14488+K14486+K14486+K14486+K14486+K14486+K14486+K14486+K14486+K14505+K14488+K14488+<br>K14488+K14488+K14512+K14512+K14512+K14512+K14512+K14512+K14512+K14512+K14512+K13416+K13422+K13422+K13422+<br>K14499+K14499+K14516+K14516+K14484+K14484+K14484+K14484+K14508+K14497+K14489+K14514+K14514+K13464+K13464+<br>K13464+K13464+K13464+K13464+K13464+K13464+K13464+K14489+K14489+K14489+K14487+K14487+K14487+K14487+K14431+<br>K14431+K14431+K14431+K14431+K14431+K14431+K14431+K14431+K14431+K14485+K14485+K14485+K14485+K14485+K14485+<br>K14485+K14485+K14486+K14486+K14486+K14486+K14486+K14486+K14509+K14509+K14498+K14498+K14498+K14498+K14498+<br>K14498+K14498+K14498+K13946+K13946+K13946+K14514+K14514+K14514+K14514+K14514+K14514+K14514+K14514+K14514+

	K14514+K14514+K14514+K14514+K14514+K14514+K14514+K14514+K14514+K14514+K14491+K14491+K14491+K14497+K14497+K14515+K14515+K14515+K14515+K14515+K14515+K14491+K14432+K14432+K14432+K14432+K14432+K13463+K13463+K13464+K13464+K13464+K13464+K13464+K13464+K14510+K14510+K14510+K14510+K14510+K14510+K14486+K14486+K14486+K14486+K14486+K14486+K14486+K14486+K14484+K14484+K14484+K14484+K14484+K14484+K14484+K14484+K14484+K14497+K14497+K14497+K14497+K14497+K14497+K13463+K13463+K13463+K13463+K13463+K13463+K13463+K13463+K13463+K13463+K13463+K13463+K13463+K14491+K14491+K14491+K14491+K14491+K14491+K14491+K14509+K14509+K14509+K14509+K14500+K14500+K14500+K14500+K14500+K14500+K14500+K14500+K14500+K14500+K14500+K14500+K14500+K14500+K14500+K14500+K14514+K14514+K14514+K14513+K14513+K14513+K14513+K14513+K14513+K14494+K14494+K14494+K14494+K14494+K14494+K14494+K14491+K14491+K14491+K14491+K14491+K14491+K14491+K14491+K14491+K14502+K14502+K14502+K14502+K14502+K14502+K14502+K14502+K14502+K14502+K14502+K14502+K14490+K14490+K14490+K14490+K14490+K13422+K13422+K13422+K13422+K13422+K13422+K14491+K14491+K14491+K14491+K14491+K14432+K14432+K14432+K14432+K14432+K14432+K14498+K14498+K14498+K14498+K14498+K14498+K14498+K14498+K14485+K14485+K14485+K14506+K14506+K14506+K14506+K14506+K14504+K14493+K14487+K14500+K14432+K14487+K14487+K14487+K13416+K14515+K14515+K14515+K14486+K13416+K14497+K14500+K13946+K14500+K12126+K13946+K14500+K14508+K14512+K13416+K14486+K14484+K14508+K14484+K13946+K14497+K14500+K14497+K13946+K14486+K13946+K14484+K14494+K14486+K13946+K14497+K14512+K13946+K14515+K14515+K13946+K13946+K14497+K14486+K14508+K12126+K13416+K13946+K14497+K13946+K14497+K14486+K14515+K14486+K14515+K14508+K14486+K12126+K14486+K14486+K14486+K14497+K14497+K13946+K14486+K14515+K14512+K14486+K14509+K14509+K14509+K14509+K14509+K14509+K14509+K13946+K13946+K14515+K14486+K14497+K14515+K14486+K14515+K14486+K14515+K14512+K14515+K14497+K14508+K14486+K14497+K12126+K12126+K12126+K12126+K12126+K12126+K12126+K12126+K12126+K12126+K12126+K12126+K12126+K13946+K14486+K14486+K14510+K14510+K14510+K14510+K14510+K14512+K14497+K14510+K13946+K13946+K13946+K13946+K14500+K13946+K13416+K14486+K14500+K13946+K13416+K14500+K13946+K14500+K14510+K14486+K13946+K13416+K13946+K14504+K14486+K13416+K14486+K13946+K14497+K13946+K13416+K14497+K14497+K13946+K14486+K12126+K14500+K14508+K13946+K12126+K13416+K13946+K13946+K13946+K14486+K13946+K13416+K14486+K14486+K13946+K14486+K14508+K14491+K14491+K14491+K14491+K14487+K14487+K14487+K14487+K14487+K14486+K14486+K14487+K14487+K13464+K13464+K13464+K13464+K13464+K13464+K13464+K13464+K14431+K14431+K14431+K13422+

			K13422+K13422+K13422+K14432+K14432+K14432+K14432+K14432+K13946+K14431+K14431+K14431+K14484+K14484+K14484+K14484+K14484+K14484+K14484+K14484+K14493+K14493+K14493+K14493+K14493+K14494+K14494+K14494+K14493+K14493+K14484+K14484+K14500+K14500+K14500+K14500+K14500+K14500+K14500+K14500+K14500+K14500+K14500+K14500+K14500+K14500+K14500
Ubiquitin mediated proteolysis	ko04120	616	K06689+K06689+K06689+K06689+K10580+K10580+K10580+K10580+K06689+K06689+K06689+K06689+K10686+K10686+K10686+K10686+K10686+K06689+K06689+K06689+K06689+K06689+K06689+K06689+K06689+K06689+K10144+K10144+K10144+K10144+K10144+K10144+K10576+K10576+K10576+K10576+K10576+K10144+K10144+K10144+K10144+K10144+K10144+K10144+K10144+K10144+K03872+K03872+K03872+K03872+K03872+K03872+K03872+K03358+K03358+K03358+K03358+K10573+K10573+K10573+K04506+K04506+K10581+K10581+K10581+K10581+K10581+K10581+K10581+K10575+K10575+K10575+K10575+K04706+K03094+K10578+K10578+K10578+K10578+K10579+K10579+K10579+K10579+K10579+K10579+K03094+K03094+K10684+K03094+K03094+K03094+K03094+K03094+K03094+K03094+K03094+K06689+K06689+K10577+K10577+K10577+K10577+K10577+K10577+K10577+K10144+K10589+K10589+K10589+K10580+K10580+K10580+K10592+K10592+K10592+K10592+K10575+K10575+K10575+K10688+K10571+K10571+K10571+K10571+K10571+K10571+K10571+K10571+K10571+K10571+K10571+K10571+K03354+K03354+K03354+K04506+K04506+K06689+K06689+K06689+K06689+K06689+K06689+K06689+K06689+K06689+K06689+K06689+K04506+K03347+K04554+K04554+K04554+K04554+K04554+K04554+K04554+K04554+K04554+K10573+K10573+K10573+K10573+K10573+K10573+K10573+K10573+K10573+K06688+K03178+K10144+K03347+K10577+K10583+K10589+K10589+K10688+K10688+K03868+K03868+K04506+K04506+K03875+K03875+K03875+K03875+K03875+K03875+K03875+K12456+K12456+K10581+K10581+K10581+K10581+K10581+K10581+K10581+K10581+K10581+K10581+K10581+K10581+K10581+K10581+K10581+K10581+K03357+K04506+K04506+K04506+K04506+K03094+K03094+K03094+K03178+K03178+K03094+K03094+K04506+K04506+K04506+K09561+K09561+K03347+K03347+K03347+K03347+K03351+K03353+K10580+K10580+K03347+K03347+K10590+K10590+K10590+K10590+K10590+K03347+K03353+K03352+K03348+K03348+K10598+K10598+K10598+K10598+K10143+K10143+K10143+K10143+K10143+K10143+K10143+K06689+K06689+K06689+K06689+K06689+K06689+K06689+K06689+K06689+K06689+K06689+K06689+K06689+K10580+K10581+K10581+K10581+K10581+K10581+K10590+K10590+K10590+K10144+K10601+K10601+K10601+K10601+K10601+K10601+K10601+K10601+K10601+K10601+K10601+K10601+K10601+K10601+K10601+K10601+K10601+K10601+K10609+K10609+K10609+K10609+K10609+K10609+

			K10609+K10609+K10609+K10609+K10609+K10609+K10609+K10609+K10609+K10609+K10590+K10590+K10590+K10590+K10144+K10144+K10144+K03350+K03350+K03350+K03350+K03350+K03350+K03350+K03350+K03350+K03350+K10592+K10592+K10592+K03353+K03353+K03353+K03354+K03354+K03354+K03354+K10610+K10610+K10610+K10610+K10610+K10610+K10610+K10610+K10610+K10610+K10610+K04649+K04649+K04649+K04649+K04649+K04649+K06689+K06689+K06689+K06689+K06689+K06689+K06689+K06689+K06689+K06689+K06689+K06689+K10591+K10591+K10591+K10591+K10591+K10591+K10591+K10591+K10591+K10685+K10685+K03349+K03349+K03349+K03349+K03349+K09561+K09561+K09561+K09561+K09561+K09561+K04506+K10570+K10570+K10570+K10570+K10570+K06689+K06689+K10581+K10581+K10581+K10581+K10581+K10581+K10581+K10581+K10581+K10581+K10576+K03351+K03351+K03351+K03869+K03869+K03869+K03869+K03869+K03869+K03869+K03869+K03869+K03869+K03869+K03869+K03868+K03868+K03868+K03868+K03868+K03868+K03353+K03353+K03353+K03094+K03094+K03094+K03094+K10599+K10599+K10599+K10599+K10588+K10588+K10588+K10588+K10588+K10588+K10588+K03347+K03347+K03178+K03178+K10573+K03347+K03178+K03178+K10143+K10573+K03875+K10579+K03347+K10580+K10573+K03178+K03347+K03347+K03347+K03178+K03178+K03178+K03178+K03178+K03347+K03347+K03352+K03347+K03347+K03347+K03347+K03178+K03347+K10589+K10589+K10589+K10589+K10589+K10589+K10589+K10589+K10589+K10589+K03347+K03178+K03178+K10573+K03347+K03347+K03347+K03178+K03178+K03178+K03347+K03348+K10573+K03178+K10573+K03178+K03347+K03347+K03347+K03347+K03178+K10688+K03178+K03178+K03178+K03347+K10577+K03348+K10143+K10143+K03094+K03178+K03347+K03352+K03347+K03347+K03347+K03347+K03352+K03347+K03347+K03352+K03347+K03347+K03875+K10589+K03352+K03875+K03347+K03347+K03347+K03347+K10143+K03352+K03347+K10143+K03347+K03347+K03347+K03347+K03347+K03352+K03347+K03352+K03347+K03178+K03352+K03347+K03347+K03178+K10597+K10597+K10597+K10597+K03094+K03094+K03094+K03094+K03364+K04706+K04706+K04706+K04706+K04706+K04706+K04706+K04706+K04706+K04706+K04706+K04706+K04706+K04706+K10140+K10140+K10140+K10140
Sulfur relay system	ko04122	41	K04487+K04487+K04487+K14168+K14168+K14168+K14168+K14168+K14168+K12161+K12161+K03635+K03635+K03635+K03635+K03635+K03635+K11996+K11996+K11996+K11996+K11996+K00566+K00566+K00566+K00566+K00566+K00566+K00566+K00566+K00566+K00566+K01011+K01011+K01011+K01011+K01011+K01011+K01011+K01011+K14169
SNARE interactions in vesicular transport	ko04130	105	K08515+K08516+K08516+K08516+K08503+K08503+K08503+K08503+K08490+K08490+K08490+K08490+K08506+K08506+K08506+K08506+K08506+K08496+K08496+K08496+K08496+K08495+K08495+K08495+K08495+K08488+K08488+K08488+K08497+K08497+K08497+K08490+K08486+K08486+K08486+K08515+K08515+K08486+K08486+K08515+K08515+K08515+K08495+K08495+K08495+

			K08495+K08489+K08489+K08489+K08493+K08493+K08493+K08493+K08486+K08486+K08507+K08507+K08507+K08505+K08505+K08506+K08488+K08488+K08488+K08488+K08488+K08488+K08488+K08488+K08488+K08493+K08493+K08493+K08490+K08490+K08490+K08490+K08497+K08497+K08504+K08493+K08493+K08493+K08488+K08488+K08488+K08488+K08488+K08488+K08488+K08488+K08488+K08488+K08488+K08517+K08517+K08517+K08517+K08517+K08517+K08498+K08498+K0849 8+K08498+K08486
Regulation of autophagy	ko04140	154	K08332+K08332+K08332+K08339+K08339+K08339+K08339+K08333+K08333+K08333+K08341+K08341+K08341+K08343+K08343+K08343+K08343+K08341+K08341+K08341+K08341+K08341+K17888+K17888+K17888+K08334+K08334+K08334+K08341+K08341+K08341+K08341+K08341+K08341+K08341+K08341+K08341+K17890+K08341+K08336+K08336+K08269+K07198+K17890+K17890+K17890+K17890+K00914+K00914+K00914+K00914+K00914+K07198+K08334+K08331+K08331+K08331+K08331+K08331+K08331+K08331+K08331+K08331+K08331+K08331+K08331+K08331+K08331+K08331+K08331+K08331+K08343+K08343+K08343+K08334+K08334+K08334+K08334+K08334+K08334+K08334+K07198+K07198+K07198+K07198+K07198+K07198+K07198+K08269+K07198+K07198+K08269+K08269+K08269+K08269+K08269+K08269+K08269+K08341+K08269+K08269+K08269+K08337+K08337+K08269+K08269+K08337+K08337+K08337+K08337+K08337+K08269+K08269+K07198+K08337+K07198+K08269+K07198+K07198+K08337+K07198+K07198+K07198+K07198+K08337+K07198+K07198+K08337+K07198+K08269+K08331+K08331+K07198+K07198+K07198+K07198+K07198+K07198+K07198+K08341+K08341+K08341+K08341+K08341+K08269+K08269+K08269+K08269+K08342+K08342+K08342+K08342+K08342+K08342
Protein processing in endoplasmic reticulum	ko04141	1290	K10839+K10839+K10839+K06689+K06689+K06689+K06689+K09503+K09503+K09503+K09503+K09503+K09503+K09503+K09503+K09503+K09503+K09503+K14012+K14012+K14012+K14012+K14012+K06689+K06689+K06689+K06689+K14015+K14015+K14015+K14015+K14015+K14015+K14004+K14004+K14004+K14004+K14004+K14004+K14004+K14004+K12666+K06689+K06689+K06689+K06689+K06689+K06689+K06689+K06689+K06689+K08057+K08057+K08057+K08057+K08057+K08057+K08057+K13719+K13719+K13719+K13719+K13719+K13719+K12670+K12670+K12670+K12670+K12670+K12670+K09562+K09562+K09562+K10088+K10088+K10088+K12668+K12668+K12668+K12668+K12668+K12668+K12668+K12668+K13984+K13984+K13989+K13989+K07342+K07342+K07342+K07342+K07342+K10575+K10575+K10575+K10575+K09580+K09580+K09580+K09580+K08057+K08057+K08057+K08057+K08057+K08057+K08057+K08057+K03094+K10578+K10578+K10578+K10578+K14012+K14012+K14012+K14012+K14012+K14012+K09518+K09518+K09518+K09584+K09584+K09584+K09584+K09584+K09584+K09584+K10839+K10839+K10839+K10839+K10839+K10839+K13993+

	K13993+K13993+K13993+K13993+K13993+K13993+K13993+K13993+K13993+K11863+K03094+K03094+K09562+K09562+K09562+K12666+K12666+K12666+K12666+K12666+K13993+K13993+K13993+K13993+K13993+K13993+K03094+K03094+K03094+K03094+K03094+K03094+K03094+K03094+K13993+K13993+K13993+K13993+K13993+K13993+K13993+K06689+K06689+K13525+K14016+K14016+K14016+K09580+K09580+K09580+K09580+K09580+K09580+K09580+K04079+K04079+K04079+K04079+K09503+K09503+K09503+K10839+K10839+K10839+K10839+K10839+K07342+K07342+K07342+K13993+K13993+K13993+K13993+K03283+K14009+K14009+K14009+K13250+K13250+K09518+K09518+K09518+K09518+K09518+K09518+K09518+K07953+K07953+K14006+K14006+K10666+K10666+K10575+K10575+K10575+K05546+K08057+K08057+K08057+K08057+K08057+K08057+K08057+K13249+K13249+K13249+K13249+K14016+K14016+K13993+K13993+K13993+K13989+K13989+K07953+K07953+K07953+K07953+K07953+K07953+K07953+K07953+K07953+K07953+K06689+K06689+K06689+K06689+K06689+K06689+K06689+K06689+K06689+K06689+K06689+K03347+K10666+K10666+K09486+K04554+K04554+K04554+K04554+K04554+K04554+K04554+K04554+K04554+K13993+K13993+K13993+K13993+K13993+K13993+K13250+K13250+K13250+K09540+K03347+K09481+K09481+K09481+K09481+K09481+K13993+K10839+K14009+K14009+K14009+K14009+K14009+K14009+K14009+K14009+K09518+K09518+K09518+K09518+K09518+K14003+K14003+K03868+K03868+K13989+K13989+K13989+K13989+K09517+K09517+K09517+K08054+K14003+K14003+K14003+K14003+K08288+K03283+K13993+K13993+K13993+K13993+K13993+K08288+K12668+K10666+K10666+K03094+K03094+K03094+K09487+K03094+K03094+K09490+K09490+K09490+K04079+K04079+K04079+K04079+K09561+K09561+K03347+K03347+K03347+K03347+K14005+K14005+K09503+K14009+K12669+K03283+K03283+K03283+K03283+K03283+K08288+K12669+K03347+K03347+K13993+K08653+K08653+K08653+K08653+K08653+K08653+K08653+K03347+K14007+K14007+K14007+K14007+K14007+K14007+K14007+K14007+K14007+K14007+K14007+K14007+K06689+K06689+K06689+K06689+K06689+K06689+K06689+K06689+K06689+K06689+K06689+K06689+K06689+K16196+K16196+K16196+K12666+K12666+K12666+K12666+K11718+K11718+K11718+K11718+K10601+K10601+K10601+K10601+K10601+K10601+K10601+K10601+K10601+K10601+K10601+K10601+K10601+K10601+K10601+K10601+K10601+K10601+K14009+K14009+K14009+K14009+K14009+K14001+K14001+K14001+K14001+K14001+K10956+K10956+K10956+K10956+K10956+K10956+K10956+K10956+K10956+K10956+K10956+K09580+K10666+K10666+K10666+K10666+K10666+K07151+K07151+K07151+K07151+K07151+K07151+K07151+K07151+K03237+K03237+K03237+K03237+K03237+K03237+K03237+K09584+K09584+K09584+K09584+K09584+K09584+K09584+K10084+K10084+K10084+K10084+K10084+K10084+K10084+K10666+K10666+K10666+K10666+K10666+K10666+K10666+K06689+K06689+K06689+K06689+K06689+K06689+K06689+K06689+K06689+K06689+K06689+K06689+K10950+K10950+K10950+K10950+K10950+K10950+K10950+K10085+K10085+K10085+K10085+K10085+K09518+

	K09518+K09518+K09518+K09518+K05546+K10666+K10666+K10666+K12275+K12275+K12275+K12275+K12275+K13525+K12667+K12667+K12667+K12667+K12667+K12667+K12667+K12667+K12667+K12667+K12667+K12667+K12667+K12667+K12667+K12667+K12667+K12667+K14026+K14026+K14026+K14026+K14026+K14026+K14026+K14026+K14026+K09490+K01230+K01230+K01230+K01230+K01230+K01230+K01230+K01230+K01230+K01230+K08852+K08852+K08852+K08852+K08852+K08852+K08852+K08852+K09561+K09561+K09561+K09561+K09561+K09561+K13525+K13993+K09487+K10950+K10950+K10950+K10950+K10950+K10950+K10950+K10950+K06689+K06689+K07151+K07151+K07151+K07151+K07151+K07151+K08852+K08852+K10636+K10636+K10636+K10636+K10636+K10636+K10636+K10636+K10636+K10636+K10636+K10636+K10636+K10636+K10636+K09580+K09580+K09580+K09580+K09580+K09580+K09580+K09580+K09580+K09580+K10666+K10666+K10666+K10666+K09580+K07765+K07765+K01230+K01230+K01230+K01230+K01230+K14016+K14016+K14016+K14016+K10666+K10666+K10666+K10666+K10666+K03868+K03868+K03868+K03868+K03868+K03868+K09580+K09580+K09580+K09580+K09580+K13989+K13989+K13989+K13989+K13989+K13989+K09540+K09540+K09540+K09540+K09540+K09540+K09540+K09540+K09540+K09540+K03283+K03283+K03283+K03283+K03094+K03094+K03094+K03094+K14011+K14011+K14011+K04523+K04523+K04523+K04523+K04523+K04523+K04523+K04523+K04523+K04523+K08057+K08057+K08057+K08057+K08057+K08057+K08057+K08057+K08057+K08057+K03347+K04079+K01456+K03347+K04079+K09487+K13525+K03347+K13525+K04079+K10661+K09487+K14005+K14005+K05546+K12666+K03283+K08054+K04079+K14012+K03283+K08288+K10661+K14006+K03283+K14003+K04079+K08288+K04079+K08054+K03283+K03283+K08288+K03283+K14018+K04079+K04079+K09487+K04079+K01456+K13525+K09580+K14012+K14003+K09580+K12666+K09580+K03347+K08054+K03283+K03283+K08288+K03283+K03283+K03283+K09487+K04079+K01456+K08288+K03283+K12666+K08288+K12666+K04079+K13993+K04079+K04079+K03283+K03283+K14005+K12666+K08054+K12666+K03283+K10661+K12666+K04079+K04079+K09580+K08288+K03283+K12666+K04079+K04079+K14012+K03347+K03283+K09490+K09490+K09580+K03283+K12666+K09490+K09580+K09580+K08054+K09487+K12666+K04079+K08288+K03283+K03283+K14003+K04079+K03283+K09490+K09490+K01456+K09487+K09487+K03283+K03347+K04079+K03347+K01456+K09580+K03283+K04079+K03283+K01228+K10661+K03283+K03347+K10661+K09487+K03347+K01456+K04079+K10661+K14007+K03347+K03283+K09487+K09490+K09490+K08288+K03283+K04079+K03283+K14006+K09490+K14005+K10661+K03347+K09486+K09490+K13525+K04079+K09487+K14005+K10661+K14005+K03347+K03347+K09487+K04079+K03347+K09487+K10661+K14005+K09580+K08288+K10661+K09487+K01456+K03347+K04079+K09487+K04079+K03283+K04079+K10661+K03283+K04079+K10661+K04079+K01456+K09487+K04079+K04079+K04079+K04079+K10661+K08054+K03347+K04079+K04079+K04079+K03283+K09487+

			K04079+K09490+K04079+K03347+K09487+K09486+K03347+K09487+K03283+K14003+K09487+K09487+K03347+K04079+K01456+K01456+K09487+K09486+K01456+K09487+K04079+K04079+K04079+K01456+K04079+K03347+K01456+K04079+K03347+K04079+K04079+K05546+K03347+K04079+K04079+K01456+K04079+K09487+K04079+K03347+K04079+K10661+K09487+K04079+K01456+K04079+K04079+K04079+K04079+K04079+K04079+K04079+K04079+K09490+K10839+K03347+K03283+K10661+K14003+K04079+K09486+K03283+K03283+K03283+K03283+K03283+K03283+K03283+K03283+K03283+K03283+K03283+K03283+K14012+K14012+K03283+K03283+K03283+K03283+K09490+K03283+K14007+K14007+K03283+K03283+K03283+K03283+K03283+K03283+K14007+K03283+K03283+K03283+K03283+K03283+K03283+K03283+K03283+K03283+K03283+K03283+K03283+K05546+K03283+K03283+K03283+K03283+K03283+K03283+K03283+K03283+K03283+K09490+K03283+K09490+K14007+K09490+K14007+K14018+K14018+K14018+K14018+K14018+K14018+K14007+K14018+K14018+K14018+K14018+K01228+K01228+K01228+K01228+K01228+K01228+K01228+K01228+K13525+K01228+K14007+K14007+K14007+K14007+K14007+K13525+K09490+K09518+K14007+K13525+K09490+K09490+K14007+K14007+K13525+K05546+K14007+K14007+K14007+K03094+K14012+K14006+K14006+K14006+K14006+K14006+K14006+K13525+K14006+K14006+K13525+K09490+K09490+K13525+K09487+K09487+K09490+K09487+K09486+K13525+K14007+K14018+K14006+K03283+K13525+K03283+K03347+K05546+K09486+K09487+K09580+K14005+K03347+K03347+K09487+K14005+K13250+K03347+K03347+K14005+K13525+K09487+K03283+K09580+K03283+K09486+K13525+K04079+K08054+K14005+K03347+K05546+K08054+K03347+K14005+K08054+K13525+K09487+K05546+K08054+K09584+K14006+K08054+K14005+K03347+K03347+K09487+K14006+K09487+K09486+K14005+K04079+K14005+K14005+K08054+K13525+K13525+K09487+K03347+K03283+K09487+K03347+K13525+K09487+K13525+K09580+K14005+K04079+K14005+K09487+K09584+K13525+K09490+K03347+K03347+K14005+K14005+K14005+K13525+K14005+K14005+K08054+K09487+K05546+K03347+K04079+K14005+K14005+K13525+K09486+K08054+K08054+K04079+K03347+K03347+K03347+K04079+K08054+K09487+K09487+K03347+K03347+K09580+K14005+K03347+K09487+K14005+K14005+K04079+K13525+K13525+K13525+K14005+K03347+K09584+K09487+K14005+K03283+K09487+K03283+K01456+K14005+K09487+K09486+K03347+K09486+K03347+K09487+K10956+K10666+K10666+K10666+K08057+K10597+K10597+K10597+K10597+K03283+K03094+K03094+K03094+K03094+K04523+K04523+K04523+K04523+K04523+K09523+K09523+K09580+K09580+K09580+K09580+K09580+K09580+K09580+K09580+K14006+K14006+K14006+K14006+K14006+K14006+K09503+K09503+K10956
Endocytosis	ko04144	568	K12194+K12194+K12194+K12194+K12194+K12194+K12194+K07937+K07937+K07937+K07937+K07937+K07937+K07937+K07937+K07937+K12192+K12192+K12192+K12192+K12192+K12192+K07904+K07904+K07904+K12193+K12193+K12193+K12493+K12493+K12493+K12493+K12493+K12493+K07897+K07897+K07897+K12196+K12196+K12196+K12196+K12196+K12196+K07897+K07897+

	K12198+K12198+K12198+K12198+K12198+K07904+K07904+K07904+K07904+K07937+K12183+K12193+K12193+K12193+K12195+K12195+K12195+K07897+K07897+K07897+K07897+K07897+K12194+K12194+K12194+K12194+K18443+K18443+K12199+K12199+K01115+K07904+K07904+K07904+K12188+K12188+K07904+K07904+K07904+K07937+K07937+K07937+K07937+K07937+K07937+K07937+K07937+K04646+K04646+K07904+K07904+K07904+K07904+K07904+K03283+K07889+K12479+K12479+K07904+K07904+K12185+K07904+K07904+K07904+K07904+K12183+K12183+K12183+K12486+K12486+K12486+K07889+K04646+K04646+K04646+K04646+K04646+K04646+K04646+K04646+K11827+K12194+K12194+K12194+K12194+K12194+K12194+K12194+K12194+K12191+K12191+K12191+K12191+K07937+K07937+K07937+K07904+K01115+K07904+K07904+K07904+K07904+K07897+K07897+K03283+K01115+K01115+K12189+K12189+K12189+K12200+K12471+K07889+K07889+K01528+K11866+K11866+K11866+K11866+K11866+K11866+K12197+K12197+K12197+K12197+K12197+K12197+K12197+K03283+K03283+K03283+K03283+K03283+K12197+K07904+K12197+K12197+K12197+K07889+K07889+K12184+K12184+K01115+K04646+K04646+K18442+K18442+K18442+K18442+K18442+K07904+K07904+K07904+K07904+K07904+K07904+K07904+K01528+K01528+K01528+K01528+K01528+K01528+K01528+K01528+K01115+K01115+K01115+K01115+K01115+K01115+K00889+K00889+K00889+K00889+K00889+K01115+K01115+K01115+K01115+K01115+K01115+K01115+K18443+K18443+K18443+K18443+K18443+K18443+K12471+K12471+K12471+K12471+K12471+K12471+K12471+K12471+K12489+K12489+K12489+K12489+K12489+K12489+K12489+K12489+K12489+K12489+K10591+K10591+K10591+K10591+K10591+K10591+K10591+K10591+K10591+K12489+K12489+K12489+K12489+K12489+K12489+K12489+K12489+K12489+K01115+K01115+K11826+K11826+K11826+K11826+K11826+K07904+K01115+K01115+K01115+K01115+K01115+K01115+K01115+K01115+K01115+K07937+K00889+K00889+K00889+K00889+K01115+K12483+K12483+K12483+K01115+K01115+K01115+K01115+K01115+K01115+K01115+K01115+K01115+K00889+K00889+K00889+K00889+K00889+K00889+K00889+K00889+K00889+K01115+K01115+K01115+K01115+K01115+K01115+K01115+K01115+K11824+K11824+K11824+K11824+K11824+K11824+K11824+K11824+K11824+K11824+K11824+K11824+K01528+K01528+K12198+K12198+K12198+K07889+K07889+K07889+K07889+K07889+K07889+K07889+K12489+K03283+K03283+K03283+K03283+K01528+K07889+K07889+K07889+K07889+K07889+K07889+K07889+K12200+K03283+K07904+K03283+K03283+K12471+K03283+K03283+K04646+K12486+K03283+K07904+K01115+K12486+K03283+K03283+K12471+K03283+K03283+K03283+K01115+K03283+K12471+K12486+K01115+K03283+K03283+K12471+K03283+K03283+K12471+K12471+K03283+K12471+K04646+K03283+K03283+K03283+K03283+K03283+K12471+K12200+K03283+K03283+K03283+K04646+K03283+K03283+K03283+K12200+K01115+K01115+K18442+K03283+K03283+K12

			486+K12200+K01115+K03283+K12200+K04646+K03283+K04646+K12486+K01528+K12200+K01115+K01115+K04646+K01115+K03283+K01115+K18442+K01115+K18442+K04646+K07897+K07897+K07897+K03283+K03283+K03283+K03283+K03283+K03283+K03283+K03283+K03283+K03283+K03283+K03283+K04646+K03283+K01115+K01115+K01115+K01115+K01115+K01115+K01115+K01115+K03283+K03283+K18442+K12486+K03283+K03283+K03283+K12185+K01115+K04646+K07904+K03283+K03283+K04646+K12185+K12185+K12185+K12185+K12185+K03283+K07937+K07937+K07937+K07937+K07937+K07937+K07937+K07937+K07937+K07937+K07937+K12486+K12486+K12486+K12486+K12190+K12492+K12492+K12492+K12492+K12492+K12492+K12492+K12492+K12483+K12483+K12493+K12493+K12493+K12493+K12493+K11866+K11866+K11866
Phagosome	ko04145	399	K07375+K07374+K07374+K07374+K07374+K07374+K07375+K07375+K07374+K07374+K07374+K07374+K07374+K02148+K02148+K02148+K02148+K08057+K08057+K08057+K08057+K08057+K08057+K08057+K08057+K07375+K07375+K07375+K07897+K07897+K07897+K07375+K07375+K07375+K07375+K07375+K07375+K07375+K07375+K07897+K07897+K02155+K02155+K02155+K02153+K02153+K02153+K02153+K02147+K07342+K07342+K07342+K07342+K07342+K07897+K07897+K07897+K07897+K07897+K07374+K07374+K07374+K07374+K07374+K04392+K04392+K04392+K08057+K08057+K08057+K08057+K08057+K08057+K08057+K08057+K02155+K02155+K02146+K02146+K02146+K02146+K02146+K02155+K02155+K02155+K02155+K02155+K02155+K00921+K00921+K00921+K00921+K00921+K07342+K07342+K07342+K00921+K00921+K00921+K00921+K00921+K00921+K00921+K02154+K02154+K02154+K02154+K02154+K02154+K02154+K02154+K02154+K02154+K02154+K04392+K04392+K07889+K07375+K07375+K07375+K07375+K03661+K07374+K04392+K04392+K04392+K04392+K04392+K02152+K02152+K08057+K08057+K08057+K08057+K08057+K08057+K08057+K07889+K00921+K00921+K00921+K00921+K00921+K00921+K00921+K00921+K00921+K00921+K00921+K02151+K02151+K02151+K02151+K02151+K02151+K08488+K08488+K08488+K02152+K02152+K02152+K02152+K02152+K02155+K02155+K09481+K09481+K09481+K09481+K09481+K02150+K02150+K02150+K02150+K02150+K02150+K02150+K07897+K07897+K03661+K03661+K02154+K08054+K02153+K02153+K02153+K02153+K02153+K07375+K07889+K07889+K07375+K02149+K07375+K07375+K07375+K07375+K07375+K07375+K07375+K07375+K07889+K07889+K02154+K04392+K10956+K10956+K10956+K10956+K10956+K10956+K10956+K10956+K10956+K10956+K10956+K02144+K02144+K02144+K02144+K02144+K02144+K08488+K08488+K08488+K08488+K08488+K08488+K08488+K08488+K08488+K02148+K02148+K00914+K00914+

			K00914+K00914+K00914+K02155+K07374+K07374+K07374+K07374+K02145+K02145+K02145+K02145+K02145+K02145+K02145+K02145+K02145+K02145+K02149+K02147+K02147+K02147+K02147+K02147+K02147+K02147+K02147+K02147+K02147+K02147+K02147+K02147+K02147+K07374+K07374+K07374+K07374+K07374+K07374+K07374+K07374+K07374+K07375+K07375+K07889+K07889+K07889+K07889+K07889+K07889+K07889+K07375+K07375+K07375+K07375+K07375+K07375+K07375+K07375+K08488+K08488+K08488+K08488+K08488+K08488+K08488+K08488+K08488+K08488+K08488+K08057+K08057+K08057+K08057+K08057+K08057+K08057+K08057+K08057+K08057+K07889+K07889+K07889+K07889+K07889+K07889+K07889+K08517+K08517+K08517+K08517+K08517+K08517+K08054+K04392+K07375+K02154+K07375+K02154+K08054+K02154+K02154+K02154+K08054+K07375+K02154+K07375+K08054+K02154+K04392+K08054+K02154+K07375+K02154+K07375+K07375+K07374+K08054+K02154+K02154+K07375+K07375+K07897+K07897+K07897+K07375+K07375+K08054+K08054+K08054+K08054+K08054+K08054+K08054+K08054+K08054+K08054+K07375+K07375+K10956+K08057+K04392+K04392+K10956
Basal transcription factors	ko03022	171	K03125+K03125+K03125+K03125+K03125+K03125+K03125+K03133+K03133+K03133+K03133+K03133+K03133+K03133+K03124+K03124+K03124+K03124+K03120+K03120+K03120+K03120+K03120+K10845+K10845+K10845+K03124+K06634+K06634+K03137+K03137+K03137+K03137+K03142+K03142+K03123+K03123+K03123+K03123+K03139+K03139+K03139+K03143+K03143+K10842+K10842+K03142+K03142+K03142+K03142+K03142+K03142+K03142+K03142+K03142+K03142+K03134+K03134+K03134+K03134+K03144+K03144+K03144+K03144+K03144+K03144+K03144+K03144+K03144+K03144+K03136+K03136+K03136+K03129+K03129+K03129+K03129+K03129+K03124+K03124+K03124+K03124+K03124+K03126+K03126+K03126+K03126+K03126+K03141+K03141+K03141+K10844+K10844+K10844+K10844+K03135+K03135+K03135+K03135+K03135+K03135+K03125+K03138+K03138+K03138+K03138+K03138+K03138+K03138+K03130+K03130+K03130+K03130+K03127+K03127+K14649+K14649+K03123+K03123+K03123+K03123+K03122+K03122+K03122+K03122+K03122+K03122+K03122+K10843+K03126+K06634+K06634+K03143+K06634+K03143+K06634+K06634+K03143+K06634+K03125+K03126+K03143+K03126+K03143+K03125+K03125+K10843+K03126+K03128+K03143+K03128+K03128+K10843+K03128+K10843+K03128+K10843+K10843+K03128+K03128+K03131+K03131+K14649+K14649+K03131+K03131+K03131+K03131+K02202+K02202+K02202
Peroxisome	ko04146	420	K01897+K13348+K13348+K13348+K13348+K04565+K04565+K04565+K04565+K04565+K13348+K13348+K13348+K13348+K13354+K13354+K13354+K13335+K13335+K13335+K13335+K13335+K13335+K13348+K13348+K13348+K13348+K13347+K13347+K13347+K00869+K00869+K00869+K00869+K00869+K00869+K11147+K11147+K04565+K04565+K04565+K04565+K04565+K04565+K00031+K00031+K00031+K00031+K00031+K00031+K00031+K00031+K03781+K03781+K03781+K03781+K03781+K03781+K03781+K03781+

			K03781+K03781+K03426+K03426+K13237+K13237+K13237+K11517+K13356+K13356+K13356+K13356+K13356+K13356+K13356+K13356+K13356+K13356+K13356+K13356+K13356+K13356+K13346+K13346+K07513+K07513+K00232+K01640+K01640+K01640+K01640+K13337+K13337+K13337+K04565+K04565+K04565+K04565+K04565+K07513+K06664+K06664+K06664+K06664+K13338+K13338+K13338+K13338+K13338+K04564+K04564+K04564+K13347+K13347+K13347+K12261+K13339+K13339+K13339+K13339+K13339+K13347+K13347+K04565+K04565+K13345+K11517+K13348+K13348+K13341+K13341+K04565+K04565+K12663+K12663+K12663+K13348+K13348+K00232+K13343+K13343+K13343+K13343+K13343+K13343+K13343+K00106+K00106+K00106+K00106+K00106+K00106+K01897+K01897+K01897+K01897+K01897+K01897+K01897+K01897+K01897+K01897+K01897+K01897+K01897+K05677+K13342+K13342+K13342+K13342+K13342+K13342+K13342+K13342+K13342+K00869+K00869+K00869+K00869+K00869+K00869+K13356+K01897+K01897+K01897+K01897+K01897+K01897+K01897+K01897+K00232+K00232+K00232+K00232+K00232+K01897+K00232+K00232+K00232+K11517+K11517+K11517+K11517+K11517+K13336+K13336+K13336+K13336+K13336+K00830+K00830+K00830+K00830+K00830+K00830+K00830+K00830+K01897+K01897+K01897+K01897+K01897+K01897+K01897+K13354+K13354+K13354+K13354+K13356+K01897+K01897+K01897+K01897+K01897+K01897+K01897+K01897+K01897+K01897+K01897+K01897+K07513+K07513+K07513+K07513+K07513+K07513+K07513+K13344+K13344+K13344+K13344+K13344+K13344+K13344+K13346+K01897+K01897+K01897+K00232+K01897+K01897+K01897+K13346+K00031+K01897+K01897+K01897+K01897+K00031+K01897+K01897+K01897+K13346+K01897+K13356+K01897+K01897+K01897+K01897+K01897+K01897+K01897+K01897+K13346+K13346+K13346+K01897+K01897+K12261+K01897+K01897+K13346+K01897+K01897+K01897+K01897+K13346+K01897+K01897+K13346+K13346+K13346+K13346+K07513+K00031+K13356+K12261+K12261+K13356+K13356+K13356+K12261+K12261+K13356+K13356+K12261+K12261+K01897+K13356+K00031+K12261+K13356+K12261+K13356+K13356+K12261+K12261+K13356+K13356+K12261+K13356+K12261+K12261+K13356+K01897+K01897+K00031+K13346+K13346+K01640+K01640+K01640+K01640+K01640+K01640+K01578+K01578+K01578+K01578+K11517+K11517+K11517+K11517+K00031+K00031+K03781+K03781+K03781+K03781+K03781+K03781+K03781+K03781+K00306+K00306
Plant-pathogen interaction	ko04626	701	K13436+K13436+K13436+K13436+K12795+K12795+K12795+K12795+K13458+K13458+K13458+K13458+K12795+K12795+K12795+K12795+K13448+K13448+K13448+K13448+K13448+K04371+K04371+K04371+K04371+K04368+K13436+K13436+K13436+K13436+

	K13436+K13436+K13436+K13436+K13412+K13430+K13457+K13457+K12795+K12795+K12795+K12795+K13457+K13457+K13457+K13457+K13457+K13457+K13457+K13457+K13457+K04079+K04079+K04079+K04079+K02183+K02183+K02183+K02183+K02183+K02183+K02183+K02183+K02183+K02183+K02183+K02183+K02183+K13414+K13414+K13449+K13449+K13449+K13448+K13448+K13448+K04368+K04368+K04368+K13448+K13448+K13448+K13448+K13448+K13448+K13448+K18834+K13448+K13448+K13448+K13448+K13448+K13429+K13448+K13448+K13448+K13448+K13448+K13448+K13448+K13448+K13448+K13448+K13448+K04368+K13448+K13448+K13448+K13412+K13412+K13448+K02183+K02183+K02183+K13436+K13436+K13436+K13448+K13448+K13448+K13448+K13448+K13448+K13448+K13420+K13420+K13456+K13456+K13456+K13448+K13448+K13448+K13448+K13413+K13413+K05391+K05391+K05391+K05391+K05391+K05391+K05391+K05391+K05391+K13413+K13413+K13416+K02183+K09487+K04079+K04079+K04079+K04079+K13448+K13436+K13436+K13436+K13436+K13436+K13448+K13412+K13412+K13412+K13457+K13459+K00864+K00864+K00864+K00864+K00864+K13459+K05391+K13447+K13447+K13447+K18834+K18834+K18834+K18834+K18835+K18835+K18835+K18835+K18835+K18835+K18835+K18835+K18835+K18835+K18835+K13457+K13412+K13412+K05391+K13457+K13457+K13457+K13413+K13448+K13448+K13448+K13448+K13448+K13448+K13448+K02183+K02183+K02183+K02183+K02183+K02183+K02183+K02183+K13412+K13412+K13412+K13412+K13412+K13412+K13412+K13412+K09487+K09487+K09487+K09487+K09487+K09487+K09487+K09487+K09487+K09487+K09487+K09487+K09487+K09487+K09487+K13427+K13427+K13427+K13427+K13427+K13427+K13412+K13457+K13412+K13412+K13412+K13412+K13412+K13412+K13412+K13412+K13412+K13412+K13412+K13412+K13412+K13412+K13412+K05391+K13412+K13412+K13412+K13412+K13412+K13412+K13412+K05391+K13412+K13412+K13412+K13412+K13412+K13412+K13412+K13412+K13412+K13412+K13430+K13430+K13430+K13430+K13430+K13430+K13430+K13430+K13430+K13430+K13430+K05391+K05391+K05391+K05391+K05391+K05391+K05391+K13412+K13412+K05391+K05391+K05391+K05391+K05391+K05391+

	K05391+K04079+K13457+K13429+K04079+K13457+K13424+K09487+K04079+K13416+K13429+K09487+K13459+K13459+K13429+K13459+K13459+K13429+K13459+K13459+K04079+K13416+K13412+K13424+K04079+K13459+K04079+K13457+K04079+K13459+K12795+K04079+K04079+K13429+K13457+K13416+K13424+K13414+K13429+K13429+K13459+K13459+K13429+K13459+K09487+K04079+K13429+K13414+K04079+K13436+K13429+K04079+K04079+K13424+K12795+K04079+K13412+K04079+K13457+K13457+K04079+K04079+K13424+K13459+K13412+K13412+K09487+K04079+K13429+K04079+K09487+K09487+K13424+K13457+K13459+K04079+K04079+K13459+K09487+K13457+K04079+K09487+K13416+K13459+K13459+K04079+K13414+K13414+K04079+K13436+K09487+K13459+K09487+K04079+K13457+K09487+K13457+K13457+K09487+K04079+K13457+K09487+K04079+K13424+K04079+K04079+K13436+K13414+K04079+K09487+K04079+K13429+K04079+K13414+K04079+K13457+K13412+K04079+K04079+K04079+K04079+K09487+K04079+K04079+K13457+K09487+K13457+K09487+K13457+K09487+K09487+K04079+K09487+K13457+K09487+K04079+K13457+K04079+K04079+K04079+K04079+K13414+K04079+K04079+K13436+K13413+K04079+K04079+K04079+K09487+K04079+K13412+K13466+K04079+K09487+K04079+K04079+K04079+K04079+K04079+K04079+K04079+K04079+K13424+K04079+K13414+K13457+K04079+K13457+K13414+K13459+K13424+K13424+K13436+K13436+K09487+K09487+K09487+K13429+K13412+K13412+K13459+K09487+K13429+K13429+K13429+K09487+K13429+K13436+K13429+K13429+K13436+K09487+K13416+K04079+K13459+K13416+K09487+K13429+K13429+K13429+K13429+K09487+K09487+K04079+K13429+K13459+K13457+K09487+K13416+K13459+K09487+K09487+K13459+K13416+K13412+K13429+K04079+K09487+K13459+K13412+K13424+K13459+K13414+K13429+K13416+K13412+K13459+K13429+K04079+K13429+K13459+K13429+K13429+K13459+K13416+K04079+K04079+K09487+K09487+K13459+K13457+K09487+K13429+K13459+K04079+K13429+K09487+K13429+K09487+K13416+K13412+K09487+K13414+K09487+K13424+K13457+K13457+K13414+K18875+K18875+K18875+K18875+K13448+K13448+K13448+K13448+K13448+K13448+K13448+K13448+K13412+K13412+K13412+K13412+K13412+K13412+K13412+K04371+K04371+K04371
