# Supplementary material for: Single-molecule long-read sequencing of the full-length transcriptome of Rhododendron lapponicum L
Source: Sci Rep. 2020 Apr 21;10:6755. doi: 10.1038/s41598-020-63814-x (PMC7174332; doi:10.1038/s41598-020-63814-x)
Supplement: Supplementary file 6 — Supplementary Table S6. [file 41598_2020_63814_MOESM6_ESM.pdf]

Single-molecule long-read sequencing of the full-length transcriptome of *Rhododendron lapponicum* L.

Xinping Jia, Ling Tang, Xueying Mei, Huazhou Liu, Hairong Luo, Yanming Deng, Jiale Su

Institute of Leisure Agriculture, Jiangsu Academy of Agricultural Sciences, Jiangsu Key Laboratory for Horticultural Crop Genetic Improvement, Nanjing 210014, China

Table S6 Summary of the genes in anthocyanin biosynthesis pathway

Gene	Number	Gene ID
Trans-cinnamate 4-monooxygenase (C4H)	7	F01_cb9736_c8/f1p0/2072; F01_cb14089_c2/f1p0/1860; F01_cb14089_c0/f1p3/1777; F01_cb9736_c14/f1p1/1658; F01_cb9736_c11/f1p0/1823; F01_cb9736_c7/f1p1/2054; F01_cb9736_c2/f2p3/1866
Chalcone isomerase (CHI)	12	F01_cb2253_c0/f11p2/991; F01_cb2253_c13/f1p0/860; F01_cb2253_c6/f1p0/2583; F01_cb2253_c4/f1p0/2680; F01_cb2253_c3/f1p0/2434; F01_cb2253_c10/f1p0/982; F01_cb2253_c12/f1p1/962; F01_cb2253_c2/f1p0/2538; F01_cb2253_c9/f1p1/1001; F01_cb2253_c8/f1p1/1139; F01_cb2253_c1/f2p0/3998;F01_cb16358_c0/f3p0/839
Chalcone synthase (CHS)	41	F01_cb8564_c868/f2p9/1907; F01_cb18456_c6019/f1p5/1613; F01_cb18456_c6042/f1p0/1560; F01_cb18456_c6049/f1p1/1546; F01_cb18456_c6515/f1p0/1621; F01_cb8564_c11714/f2p0/2782; F01_cb18456_c6036/f1p0/1694; F01_cb18456_c5973/f1p3/1686; F01_cb18456_c6024/f1p0/1617; F01_cb18456_c6810/f1p0/1586; F01_cb18456_c6500/f1p0/1679; F01_cb8564_c123804/f1p1/2490; F01_cb18456_c6478/f1p2/1469; F01_cb18456_c1168/f22p1/1548; F01_cb18456_c2304/f1p0/1374; F01_cb8564_c126194/f1p0/2132F01_cb18456_c6496/f1p0/1810; F01_cb18456_c6007/f1p1/1591; F01_cb18456_c5940/f1p2/1461; F01_cb18456_c5957/f1p0/965; F01_cb18456_c6490/f1p0/1591; F01_cb18456_c5939/f1p3/1634; F01_cb11599_c10/f5p32/1656; F01_cb18456_c6517/f1p0/994; F01_cb11599_c6/f1p8/1041; F01_cb18456_c5937/f1p0/1763; F01_cb18456_c7798/f2p2/1522; F01_cb18456_c7272/f1p0/1376; F01_cb8564_c124225/f1p0/2875; F01_cb8564_c125928/f1p0/1916; F01_cb18456_c6492/f1p0/1536; F01_cb18456_c6955/f1p0/1531; F01_cb18456_c6048/f1p0/1595; F01_cb18456_c6952/f1p1/1718; F01_cb18456_c6054/f1p0/1241; F01_cb8564_c124293/f1p0/2958; F01_cb18456_c5942/f1p0/1676; F01_cb11599_c11/f1p34/1663;F01_cb18456_c6063/f1p0/1474; F01_cb18456_c5927/f1p0/1603; F01_cb18456_c6812/f1p0/1046
Naringenin 3-dioxygenase (F3H)	9	F01_cb13925_c0/f2p0/1339; F01_cb13925_c1/f1p0/1431; F01_cb13925_c7/f1p0/1449; F01_cb7606_c17/f44p3/1535 F01_cb13925_c4/f1p0/1356; F01_cb7606_c7/f1p1/1457; F01_cb7606_c8/f1p0/1499;

		F01_cb7606_c1/f5p0/2550; F01_cb13925_c3/f1p1/1452
Flavonoid 3′-hydroxylase (F3′H)	8	F01_cb7452_c15/f1p1/1706; F01_cb7452_c16/f1p0/1726; F01_cb7452_c5/f1p1/1888; F01_cb7452_c14/f1p0/1741; F01_cb7452_c3/f2p0/1744; F01_cb7452_c6/f1p1/1917; F01_cb7452_c17/f1p0/1655; F01_cb7452_c20/f1p0/1034
Flavonoid 3′, 5′-hydroxylase (F3′5′H)	8	F01_cb7576_c3/f1p0/2594; F01_cb3444_c2/f1p0/3087; F01_cb7576_c14/f1p1/1754; F01_cb7576_c7/f2p0/1921; F01_cb7576_c17/f34p2/1894; F01_cb7576_c4/f1p0/2124; F01_cb7576_c6/f1p0/2034; F01_cb7576_c5/f1p0/2032
Anthocyanidin synthase (ANS)	5	F01_cb7563_c5/f2p1/2302; F01_cb7563_c31/f1p0/655; F01_cb7563_c21/f1p1/1490; F01_cb7563_c6/f1p2/2589; F01_cb7563_c32/f1p0/597
Dihydroflavonol 4-reductase (DFR)	6	F01_cb10454_c0/f25p3/1319; F01_cb12846_c0/f3p0/1373; F01_cb12234_c0/f2p0/1427; F01_cb12846_c2/f1p0/1720; F01_cb3655_c0/f2p0/2991; F01_cb12846_c1/f1p0/1352
