# Supplementary material for: Single-molecule long-read sequencing of the full-length transcriptome of Rhododendron lapponicum L
Source: Sci Rep. 2020 Apr 21;10:6755. doi: 10.1038/s41598-020-63814-x (PMC7174332; doi:10.1038/s41598-020-63814-x)
Supplement: Supplementary file 7 — Supplementary Table S7. [file 41598_2020_63814_MOESM7_ESM.pdf]

# Single-molecule long-read sequencing of the full-length transcriptome of *Rhododendron lapponicum* L.

Xinping Jia, Ling Tang, Xueying Mei, Huazhou Liu, Hairong Luo, Yanming Deng, Jiale Su

Institute of Leisure Agriculture, Jiangsu Academy of Agricultural Sciences, Jiangsu Key Laboratory for Horticultural Crop Genetic Improvement, Nanjing 210014, China

Table S7 The primer used for qRT-PCR analysis

| Primer ID | Gene ID | Forward Primer | Reverse Primer |
|---|---|---|---|
| C4H | F01_cb9736_c8/f1p0/2072 | GACACAGTCAACCCCTCCAT | TGGTCGAAAGGTGGGTATGT |
| CHS | F01_cb8564_c11714/f2p0/2782 | CCCACCTTGACAGTCTGGTT | GCCATCGCTATCAGGTAGA |
| F3H | F01_cb13925_c0/f2p0/1339 | GCTGGGGGTTTTATCTGAG | CAACAGGGTTATGGTACC |
| F3′5′H | F01_cb7576_c7/f2p0/1921 | GTCTTTCGGTCTTGCTTTGC | AGTTTCAGCCGTTGAGCCTA |
| ANS | F01_cb7563_c31/f1p0/655 | GAGCACAAGTTGTTCAGGA | CCCAAGAAGACCAAAACCAA |
| DFR | F01_cb3655_c0/f2p0/2991 | TGTTAGTGGTCGGTCCCTT | ATCATGGGATGAGCAGATG |
