# Supplementary material for: Single-molecule long-read sequencing of the full-length transcriptome of Rhododendron lapponicum L
Source: Sci Rep. 2020 Apr 21;10:6755. doi: 10.1038/s41598-020-63814-x (PMC7174332; doi:10.1038/s41598-020-63814-x)
Supplement: Supplementary file 2 — Supplementary Table S2. [file 41598_2020_63814_MOESM2_ESM.pdf]

Single-molecule long-read sequencing of the full-length transcriptome of *Rhododendron lapponicum* L.

Xinping Jia, Ling Tang, Xueying Mei, Huazhou Liu, Hairong Luo, Yanming Deng, Jiale Su

Institute of Leisure Agriculture, Jiangsu Academy of Agricultural Sciences, Jiangsu Key Laboratory for Horticultural Crop Genetic Improvement, Nanjing 210014, China

Table S2 Summary of alternative splicing (AS) events predicted

<table>
<tr><th>Query_Name</th><th>Subject_Name</th><th>Qhsp_Start1</th><th>Qhsp_End1</th><th>Qhsp_Start2</th><th>Qhsp_End2</th><th>Shsp_Start1</th><th>Shsp_End1</th><th>Shsp_Start2</th><th>Shsp_End2</th></tr>
<tr><td>cb8564_c21909/f1p2/3933 isoform=cb8564_c21909;full_length_coverage=1;non_full_length_coverage=2;isoform_length=3933;expected_accuracy=0.998</td><td>gnl|BL_ORD_ID|11819 cb8564_c15229/f1p2/4082 isoform=cb8564_c15229;full_length_coverage=1;non_full_length_coverage=2;isoform_length=4082;expected_accuracy=1.000</td><td>131</td><td>3933</td><td>1</td><td>130</td><td>279</td><td>4082</td><td>24</td><td>153</td></tr>
<tr><td>cb8564_c44005/f1p0/3815 isoform=cb8564_c44005;full_length_coverage=1;non_full_length_coverage=0;isoform_length=3815;expected_accuracy=0.993</td><td>gnl|BL_ORD_ID|56019 cb8564_c52510/f6p2/4563</td><td>1</td><td>2046</td><td>2041</td><td>3808</td><td>46</td><td>2094</td><td>2194</td><td>3969</td></tr>
<tr><td>cb5600_c21/f1p0/2784</td><td>gnl|BL_ORD_ID|74133 cb5600_c6/f1p0/2661</td><td>1</td><td>2095</td><td>2098</td><td>2525</td><td>31</td><td>2125</td><td>2237</td><td>2663</td></tr>
<tr><td>cb8564_c147929/f1p0/2042 isoform=cb8564_c147929;full_length_coverage=1;non_full_length_coverage=0;isoform_length=2042;expected_accuracy=0.992</td><td>gnl|BL_ORD_ID|55709 cb8564_c4950/f1p0/2564</td><td>1</td><td>1246</td><td>1247</td><td>2018</td><td>44</td><td>1291</td><td>1656</td><td>2427</td></tr>
<tr><td>cb8564_c81531/f1p0/2743 isoform=cb8564_c81531;full_length_coverage=1;non_full_length_coverage=0;isoform_length=2743;expected_accuracy=0.996</td><td>gnl|BL_ORD_ID|57893 cb8564_c83527/f2p2/3885</td><td>1</td><td>2217</td><td>2214</td><td>2710</td><td>1</td><td>2205</td><td>3349</td><td>3851</td></tr>
<tr><td>cb14724_c0/f4p1/1726 isoform=cb14724_c0;full_length_coverage=4;non_full_length_coverage=1;isoform_length=1726;expected_accuracy=1.000</td><td>gnl|BL_ORD_ID|40277 cb3622_c10/f1p0/3532 isoform=cb3622_c10;full_length_coverage=1;non_full_length_coverage=0;isoform_length=3532;expected_accuracy=0.999</td><td>1</td><td>1352</td><td>1351</td><td>1725</td><td>1</td><td>1351</td><td>2693</td><td>3064</td></tr>
</table>

cb8564_c52723/f2p0/3547 isoform=cb8564_c52723;full_length_coverage=2;non_full_length_coverage=0;isoform_length=3547;expected_accuracy=0.990	gnl|BL_ORD_ID|55991 cb8564_c51339/f1p0/3758	360	3472	4	363	652	3773	3	361
cb4123_c54/f1p3/1372 isoform=cb4123_c54;full_length_coverage=1;non_full_length_coverage=3;isoform_length=1372;expected_accuracy=1.000	gnl|BL_ORD_ID|41188 cb4123_c19/f1p0/2726 isoform=cb4123_c19;full_length_coverage=1;non_full_length_coverage=0;isoform_length=2726;expected_accuracy=1.000	139	1339	2	144	1526	2726	1	143
cb8564_c179457/f1p0/2647 isoform=cb8564_c179457;full_length_coverage=1;non_full_length_coverage=0;isoform_length=2647;expected_accuracy=1.000	gnl|BL_ORD_ID|14058 cb8564_c23936/f1p0/3016 isoform=cb8564_c23936;full_length_coverage=1;non_full_length_coverage=0;isoform_length=3016;expected_accuracy=0.995	1	1692	1690	2563	4	1716	2143	3016
cb8564_c116262/f1p0/2136	gnl|BL_ORD_ID|53028 cb8564_c114755/f1p0/2541	236	2105	3	240	441	2310	79	316
cb12513_c86/f1p1/1373	gnl|BL_ORD_ID|14255 cb8564_c3884/f1p1/2696 isoform=cb8564_c3884;full_length_coverage=1;non_full_length_coverage=1;isoform_length=2696;expected_accuracy=0.993	311	1363	34	312	1542	2590	10	287
cb8564_c844/f2p0/2586 isoform=cb8564_c844;full_length_coverage=2;non_full_length_coverage=0;isoform_length=2586;expected_accuracy=1.000	gnl|BL_ORD_ID|17665 cb8564_c865/f4p0/2761 isoform=cb8564_c865;full_length_coverage=4;non_full_length_coverage=0;isoform_length=2761;expected_accuracy=0.996	1	2343	2341	2528	17	2361	2574	2761
cb1770_c25/f1p0/3573	gnl|BL_ORD_ID|33526 cb1770_c15/f1p0/3678 isoform=cb1770_c15;full_length_coverage=1;non_full_length_coverage=0;isoform_length=3678;expected_accuracy=0.992	1	1748	1747	3373	183	1933	2049	3678

cb1540_c27/f1p0/3741	gnl|BL_ORD_ID|28145 cb1540_c7/f1p0/4274 isoform=cb1540_c7;full_length_coverage=1;non_full_length_coverage=0;isoform_length=4274;expected_accuracy=0.996	304	3535	1	303	993	4246	465	766
cb8456_c128/f1p1/1805 isoform=cb8456_c128;full_length_coverage=1;non_full_length_coverage=1;isoform_length=1805;expected_accuracy=0.994	gnl|BL_ORD_ID|52549 cb8456_c63/f1p0/2043	1	1371	1366	1785	1	1356	1471	1888
cb1494_c17/f1p0/2481	gnl|BL_ORD_ID|29800 cb1494_c10/f1p0/3165 isoform=cb1494_c10;full_length_coverage=1;non_full_length_coverage=0;isoform_length=3165;expected_accuracy=0.994	1	1198	1199	2381	3	1196	1917	3100
cb6730_c14/f1p0/2258	gnl|BL_ORD_ID|5875 cb6730_c2/f5p0/2632 isoform=cb6730_c2;full_length_coverage=5;non_full_length_coverage=0;isoform_length=2632;expected_accuracy=1.000	1	1382	1381	2267	15	1391	1654	2521
cb8564_c128275/f1p1/2721	gnl|BL_ORD_ID|54097 cb8564_c18464/f1p0/3061	430	2708	55	435	578	2857	1	376
cb8564_c35568/f1p0/2997	gnl|BL_ORD_ID|16879 cb8564_c48606/f1p1/3308 isoform=cb8564_c48606;full_length_coverage=1;non_full_length_coverage=1;isoform_length=3308;expected_accuracy=0.998	13	2174	2170	2889	1	2151	2587	3308
cb1822_c3/f1p1/3787 isoform=cb1822_c3;full_length_coverage=1;non_full_length_coverage=1;isoform_length=3787;expected_accuracy=0.998	gnl|BL_ORD_ID|32354 cb1822_c17/f1p0/5987 isoform=cb1822_c17;full_length_coverage=1;non_full_length_coverage=0;isoform_length=5987;expected_accuracy=0.997	1	3205	3206	3786	2033	5241	5410	5987

cb8564_c3632/f1p0/3696 isoform=cb8564_c3632;full_length_coverage=1;non_full_length_coverage=0;isoform_length=3696;expected_accuracy=0.998	gnl|BL_ORD_ID|17598 cb8564_c801/f2p0/4265 isoform=cb8564_c801;full_length_coverage=2;non_full_length_coverage=0;isoform_length=4265;expected_accuracy=0.998	1	2117	2115	3696	1	2121	2683	4264
cb8564_c2692/f2p0/2586 isoform=cb8564_c2692;full_length_coverage=2;non_full_length_coverage=0;isoform_length=2586;expected_accuracy=0.999	gnl|BL_ORD_ID|54793 cb8564_c2694/f1p2/4102	1	1548	1549	2586	467	2018	3068	4105
cb2550_c4/f1p1/2016 isoform=cb2550_c4;full_length_coverage=1;non_full_length_coverage=1;isoform_length=2016;expected_accuracy=0.995	gnl|BL_ORD_ID|37839 cb2550_c3/f1p1/3883 isoform=cb2550_c3;full_length_coverage=1;non_full_length_coverage=1;isoform_length=3883;expected_accuracy=0.992	141	1955	1	143	2065	3879	342	485
cb8564_c5007/f1p0/1941	gnl|BL_ORD_ID|12954 cb8564_c161772/f7p6/3766 isoform=cb8564_c161772;full_length_coverage=7;non_full_length_coverage=6;isoform_length=3766;expected_accuracy=0.997	1	1447	1446	1802	1831	3277	3409	3766
cb2716_c2/f1p0/2568	gnl|BL_ORD_ID|37132 cb2716_c0/f1p0/3829 isoform=cb2716_c0;full_length_coverage=1;non_full_length_coverage=0;isoform_length=3829;expected_accuracy=0.999	1	1563	1560	2341	118	1680	2772	3553
cb8292_c5/f2p0/1876 isoform=cb8292_c5;full_length_coverage=2;non_full_length_coverage=0;isoform_length=1876;expected_accuracy=0.999	gnl|BL_ORD_ID|9293 cb8292_c11/f1p1/2274 isoform=cb8292_c11;full_length_coverage=1;non_full_length_coverage=1;isoform_length=2274;expected_accuracy=0.997	234	1876	75	236	291	1932	13	176
cb8564_c44698/f1p5/2843 isoform=cb8564_c44698;full_length_coverage=1;non_f	gnl|BL_ORD_ID|14399 cb8564_c39189/f1p1/2975 isoform=cb8564_c39189;full_length_coverage=1;non_fu	1	2516	2511	2829	1	2531	2650	2975

ull_length_coverage=5;isoform_length=2843;expected_accuracy=0.997	ll_length_coverage=1;isoform_length=2975;expected_accuracy=0.992								
cb4674_c44/f1p2/2925 isoform=cb4674_c44;full_length_coverage=1;non_full_length_coverage=2;isoform_length=2925;expected_accuracy=0.991	gnl|BL_ORD_ID|72844 cb4674_c33/f1p3/3281	1	2069	2067	2913	165	222 0	2382	322 7
cb3139_c7/f1p0/2420 isoform=cb3139_c7;full_length_coverage=1;non_full_length_coverage=0;isoform_length=2420;expected_accuracy=1.000	gnl|BL_ORD_ID|37016 cb3139_c2/f2p1/3600 isoform=cb3139_c2;full_length_coverage=2;non_full_length_coverage=1;isoform_length=3600;expected_accuracy=0.998	377	2420	40	379	1448	349 0	1	340
cb8564_c129638/f1p0/2479	gnl|BL_ORD_ID|54482 cb8564_c124018/f1p1/3057	1	1627	1623	2474	51	167 7	2082	293 3
cb1935_c14/f1p0/3028	gnl|BL_ORD_ID|35521 cb1935_c10/f2p0/3205 isoform=cb1935_c10;full_length_coverage=2;non_full_length_coverage=0;isoform_length=3205;expected_accuracy=0.995	11	1968	1963	3017	46	200 3	2126	317 6
cb10612_c4/f1p0/1284 isoform=cb10612_c4;full_length_coverage=1;non_full_length_coverage=0;isoform_length=1284;expected_accuracy=1.000	gnl|BL_ORD_ID|2641 cb10612_c5/f4p1/1487 isoform=cb10612_c5;full_length_coverage=4;non_full_length_coverage=1;isoform_length=1487;expected_accuracy=0.999	585	1282	6	589	790	148 7	1	584
cb4123_c103/f1p0/1158	gnl|BL_ORD_ID|70778 cb4123_c26/f1p0/1586	292	1178	9	297	462	134 9	1	288
cb8564_c178999/f1p0/2222 isoform=cb8564_c178999;full_length_coverage=1;non_full_length_coverage=0;isoform_length=2222;expected_accuracy=0.994	gnl|BL_ORD_ID|55912 cb8564_c48681/f1p0/3431	13	1778	1773	2222	6	176 2	2768	322 0

cb1860_c13/f1p0/2150 isoform=cb1860_c13;full_length_coverage=1;non_full_length_coverage=0;isoform_length=2150;expected_accuracy=0.998	gnl|BL_ORD_ID|34591 cb1860_c35/f18p4/2527 isoform=cb1860_c35;full_length_coverage=18;non_full_length_coverage=4;isoform_length=2527;expected_accuracy=1.000	1	1277	1273	2146	115	1392	1645	2527
cb6569_c6/f1p1/2392 isoform=cb6569_c6;full_length_coverage=1;non_full_length_coverage=1;isoform_length=2392;expected_accuracy=0.995	gnl|BL_ORD_ID|3967 cb6569_c2/f2p1/2601 isoform=cb6569_c2;full_length_coverage=2;non_full_length_coverage=1;isoform_length=2601;expected_accuracy=1.000	1	1678	1673	2392	15	1693	1805	2524
cb8564_c173799/f1p3/2289 isoform=cb8564_c173799;full_length_coverage=1;non_full_length_coverage=3;isoform_length=2289;expected_accuracy=1.000	gnl|BL_ORD_ID|11724 cb8564_c124026/f1p1/2665 isoform=cb8564_c124026;full_length_coverage=1;non_full_length_coverage=1;isoform_length=2665;expected_accuracy=1.000	1	1670	1669	2259	1	1676	2068	2661
cb8564_c42654/f1p1/2906 isoform=cb8564_c42654;full_length_coverage=1;non_full_length_coverage=1;isoform_length=2906;expected_accuracy=0.990	gnl|BL_ORD_ID|18852 cb8564_c84488/f1p0/3408 isoform=cb8564_c84488;full_length_coverage=1;non_full_length_coverage=0;isoform_length=3408;expected_accuracy=0.996	221	2824	1	221	790	3388	1	217
cb2770_c2/f3p0/2565 isoform=cb2770_c2;full_length_coverage=3;non_full_length_coverage=0;isoform_length=2565;expected_accuracy=0.999	gnl|BL_ORD_ID|69963 cb2770_c47/f1p0/3578	1	1676	1676	2565	4	1679	2474	3378
cb8564_c10938/f1p1/2321	gnl|BL_ORD_ID|53888 cb8564_c13732/f1p0/4239	1	1315	1310	2292	1326	2640	2759	3741
cb181_c38/f1p0/2538	gnl|BL_ORD_ID|63590 cb181_c41/f1p0/2832	1089	2510	1	1094	1232	2652	1	1104
cb1960_c41/f1p1/2517	gnl|BL_ORD_ID|35970 cb1960_c63/f1p4/2956 isoform=cb1960_c63;full_length_coverage=1;non_full_l	2	2123	2120	2503	15	2136	2533	2899

	ength_coverage=4;isoform_length=2956;expected_accuracy=0.999								
cb7637_c13/f1p1/2121 isoform=cb7637_c13;full_length_coverage=1;non_full_length_coverage=1;isoform_length=2121;expected_accuracy=0.996	gnl|BL_ORD_ID|8865 cb7637_c18/f1p1/2421 isoform=cb7637_c18;full_length_coverage=1;non_full_length_coverage=1;isoform_length=2421;expected_accuracy=0.998	404	2121	5	404	648	2365	1	401
cb7210_c10/f1p0/2452 isoform=cb7210_c10;full_length_coverage=1;non_full_length_coverage=0;isoform_length=2452;expected_accuracy=0.996	gnl|BL_ORD_ID|8857 cb7210_c5/f1p1/3158 isoform=cb7210_c5;full_length_coverage=1;non_full_length_coverage=1;isoform_length=3158;expected_accuracy=0.998	1100	2451	1	1102	1807	3158	1	1102
cb4549_c5/f1p0/1871 isoform=cb4549_c5;full_length_coverage=1;non_full_length_coverage=0;isoform_length=1871;expected_accuracy=0.995	gnl|BL_ORD_ID|41890 cb4549_c0/f2p0/1976 isoform=cb4549_c0;full_length_coverage=2;non_full_length_coverage=0;isoform_length=1976;expected_accuracy=0.998	386	1871	39	385	471	1955	1	364
cb8564_c188097/f1p0/2552 isoform=cb8564_c188097;full_length_coverage=1;non_full_length_coverage=0;isoform_length=2552;expected_accuracy=0.996	gnl|BL_ORD_ID|56010 cb8564_c51971/f1p0/3064	1	1945	1940	2552	10	1954	2057	2669
cb10045_c36/f38p2/1421 isoform=cb10045_c36;full_length_coverage=38;non_full_length_coverage=2;isoform_length=1421;expected_accuracy=1.000	gnl|BL_ORD_ID|1795 cb10045_c18/f1p2/1574 isoform=cb10045_c18;full_length_coverage=1;non_full_length_coverage=2;isoform_length=1574;expected_accuracy=1.000	140	1421	12	140	251	1523	1	129
cb8564_c115486/f1p1/3203	gnl|BL_ORD_ID|52899 cb8564_c111240/f1p1/3895	104	3229	1	102	653	3775	232	334
cb1648_c1/f9p1/2048 isoform=cb1648_c1;full_length_coverage=9;non_full_le	gnl|BL_ORD_ID|66333 cb1648_c27/f1p1/2264	280	2048	49	280	382	2150	1	233

ngth_coverage=1;isoform_length=2048;expected_accuracy=1.000									
cb3388_c6/f1p0/2642 isoform=cb3388_c6;full_length_coverage=1;non_full_length_coverage=0;isoform_length=2642;expected_accuracy=0.994	gnl|BL_ORD_ID|39985 cb3388_c11/f1p0/3143 isoform=cb3388_c11;full_length_coverage=1;non_full_length_coverage=0;isoform_length=3143;expected_accuracy=0.997	1	2525	2526	2642	225	2749	2875	2991
cb2471_c18/f2p0/2036 isoform=cb2471_c18;full_length_coverage=2;non_full_length_coverage=0;isoform_length=2036;expected_accuracy=1.000	gnl|BL_ORD_ID|37463 cb2471_c9/f2p0/2471 isoform=cb2471_c9;full_length_coverage=2;non_full_length_coverage=0;isoform_length=2471;expected_accuracy=0.998	1	1158	1156	1940	47	1203	1688	2471
cb8564_c22390/f1p12/1923 isoform=cb8564_c22390;full_length_coverage=1;non_full_length_coverage=12;isoform_length=1923;expected_accuracy=1.000	gnl|BL_ORD_ID|13722 cb8564_c18567/f1p2/2597 isoform=cb8564_c18567;full_length_coverage=1;non_full_length_coverage=2;isoform_length=2597;expected_accuracy=0.995	1	1078	1079	1902	19	1130	1773	2597
cb6065_c9/f1p0/2661 isoform=cb6065_c9;full_length_coverage=1;non_full_length_coverage=0;isoform_length=2661;expected_accuracy=0.994	gnl|BL_ORD_ID|74850 cb6065_c14/f1p0/3101	1	1434	1429	2643	26	1464	1578	2780
cb188_c9/f1p0/3939 isoform=cb188_c9;full_length_coverage=1;non_full_length_coverage=0;isoform_length=3939;expected_accuracy=0.993	gnl|BL_ORD_ID|36293 cb188_c6/f1p1/4010 isoform=cb188_c6;full_length_coverage=1;non_full_length_coverage=1;isoform_length=4010;expected_accuracy=0.996	124	3581	3579	3939	1	3463	3603	3963
cb8564_c72349/f1p0/3248	gnl|BL_ORD_ID|16445 cb8564_c48521/f3p0/3397 isoform=cb8564_c48521;full_length_coverage=3;non_full_length_coverage=0;isoform_length=3397;expected_accuracy=0.997	1	2662	2659	3068	39	2724	2883	3292

cb750_c40/f1p0/3632 isoform=cb750_c40;full_length_coverage=1;non_full_length_coverage=0;isoform_length=3632;expected_accuracy=0.990	gnl|BL_ORD_ID|7348 cb750_c27/f1p1/4163 isoform=cb750_c27;full_length_coverage=1;non_full_length_coverage=1;isoform_length=4163;expected_accuracy=0.998	11	2164	2163	3611	26	2162	2719	4163
cb560_c8/f1p0/3610 isoform=cb560_c8;full_length_coverage=1;non_full_length_coverage=0;isoform_length=3610;expected_accuracy=0.995	gnl|BL_ORD_ID|73553 cb560_c10/f1p0/3728	2	2430	2431	3610	2	2430	2562	3742
cb8564_c24185/f1p1/2695 isoform=cb8564_c24185;full_length_coverage=1;non_full_length_coverage=1;isoform_length=2695;expected_accuracy=0.996	gnl|BL_ORD_ID|13176 cb8564_c15957/f1p0/2838 isoform=cb8564_c15957;full_length_coverage=1;non_full_length_coverage=0;isoform_length=2838;expected_accuracy=0.994	1	1665	1664	2695	1	1684	1792	2817
cb18773_c9/f1p0/6228	gnl|BL_ORD_ID|67996 cb18773_c6/f1p1/5702	1	3374	3373	5233	114	3487	3600	5460
cb6339_c16/f1p1/2286 isoform=cb6339_c16;full_length_coverage=1;non_full_length_coverage=1;isoform_length=2286;expected_accuracy=0.997	gnl|BL_ORD_ID|4267 cb6339_c88/f27p4/2609 isoform=cb6339_c88;full_length_coverage=27;non_full_length_coverage=4;isoform_length=2609;expected_accuracy=0.995	1	1780	1776	2286	91	1872	2076	2586
cb13753_c0/f1p0/1755	gnl|BL_ORD_ID|70597 cb3759_c2/f1p0/3671	377	1665	7	378	1910	3197	51	420
cb3324_c10/f1p2/5646 isoform=cb3324_c10;full_length_coverage=1;non_full_length_coverage=2;isoform_length=5646;expected_accuracy=0.991	gnl|BL_ORD_ID|71091 cb3324_c7/f1p2/6368	2378	5646	1	2380	3083	6355	1	2356
cb9571_c24/f1p2/1208 isoform=cb9571_c24;full_length_coverage=1;non_full_	gnl|BL_ORD_ID|21300 cb9571_c2/f34p2/1543 isoform=cb9571_c2;full_length_coverage=34;non_full_l	293	1208	35	298	561	1477	20	283

length_coverage=2;isoform_length=1208;expected_accuracy=1.000	ength_coverage=2;isoform_length=1543;expected_accuracy=1.000								
cb8292_c5/f2p0/1876 isoform=cb8292_c5;full_length_coverage=2;non_full_length_coverage=0;isoform_length=1876;expected_accuracy=0.999	gnl|BL_ORD_ID|9292 cb8292_c17/f1p0/1910 isoform=cb8292_c17;full_length_coverage=1;non_full_length_coverage=0;isoform_length=1910;expected_accuracy=0.998	234	1870	54	236	298	1910	1	183
cb7612_c7/f1p0/2393	gnl|BL_ORD_ID|8785 cb7612_c0/f1p1/2574 isoform=cb7612_c0;full_length_coverage=1;non_full_length_coverage=1;isoform_length=2574;expected_accuracy=0.991	1	1724	1722	2142	2	1720	2073	2492
cb8564_c5007/f1p0/1941	gnl|BL_ORD_ID|16235 cb8564_c4984/f1p0/3665 isoform=cb8564_c4984;full_length_coverage=1;non_full_length_coverage=0;isoform_length=3665;expected_accuracy=1.000	1	1447	1446	1713	1819	3265	3397	3665
cb396_c42/f1p0/2343	gnl|BL_ORD_ID|40201 cb396_c30/f1p0/2423 isoform=cb396_c30;full_length_coverage=1;non_full_length_coverage=0;isoform_length=2423;expected_accuracy=0.996	101	1458	1457	2340	3	1369	1539	2423
cb3211_c3/f1p1/3175 isoform=cb3211_c3;full_length_coverage=1;non_full_length_coverage=1;isoform_length=3175;expected_accuracy=0.998	gnl|BL_ORD_ID|70232 cb3211_c4/f1p0/3640	1	2004	2004	3125	134	2142	2502	3623
cb8564_c1229/f1p0/4016	gnl|BL_ORD_ID|12265 cb8564_c1235/f7p1/4220 isoform=cb8564_c1235;full_length_coverage=7;non_full_length_coverage=1;isoform_length=4220;expected_accuracy=0.999	109	2102	2101	3993	1	1994	2164	4056

cb666_c7/f1p0/2038 isoform=cb666_c7;full_length_coverage=1;non_full_length_coverage=0;isoform_length=2038;expected_accuracy=0.996	gnl|BL_ORD_ID|49394 cb666_c14/f1p0/2626	100	2038	1	101	202	2140	2	102
cb862_c7/f1p0/2681	gnl|BL_ORD_ID|17904 cb862_c22/f1p0/3314 isoform=cb862_c22;full_length_coverage=1;non_full_length_coverage=0;isoform_length=3314;expected_accuracy=0.995	1	1294	1294	2671	97	1395	1913	3314
cb2907_c2/f1p1/2369 isoform=cb2907_c2;full_length_coverage=1;non_full_length_coverage=1;isoform_length=2369;expected_accuracy=0.991	gnl|BL_ORD_ID|38599 cb2907_c5/f1p0/3162 isoform=cb2907_c5;full_length_coverage=1;non_full_length_coverage=0;isoform_length=3162;expected_accuracy=0.999	1	1331	1330	2369	18	1337	2121	3161
cb8564_c15653/f1p4/2475	gnl|BL_ORD_ID|56010 cb8564_c51971/f1p0/3064	22	2293	2289	2460	41	2312	2531	2702
cb8564_c12536/f1p0/2333 isoform=cb8564_c12536;full_length_coverage=1;non_full_length_coverage=0;isoform_length=2333;expected_accuracy=0.998	gnl|BL_ORD_ID|54392 cb8564_c23489/f1p0/2469	248	2333	68	247	336	2423	1	180
cb946_c1/f5p0/3149 isoform=cb946_c1;full_length_coverage=5;non_full_length_coverage=0;isoform_length=3149;expected_accuracy=1.000	gnl|BL_ORD_ID|19592 cb946_c2/f3p0/3333 isoform=cb946_c2;full_length_coverage=3;non_full_length_coverage=0;isoform_length=3333;expected_accuracy=1.000	1053	3149	1	1053	1206	3303	46	1098
cb8564_c85646/f1p0/3480	gnl|BL_ORD_ID|55920 cb8564_c48949/f1p0/4357	106	2377	2378	3030	21	2293	2847	3496
cb5672_c16/f1p0/2940 isoform=cb5672_c16;full_length_coverage=1;non_full_	gnl|BL_ORD_ID|44913 cb5672_c6/f1p0/3840 isoform=cb5672_c6;full_length_coverage=1;non_full_le	1	2032	2031	2940	12	2044	2845	3756

length_coverage=0;isoform_length=2940;expected_accuracy=0.999	ngth_coverage=0;isoform_length=3840;expected_accuracy=0.994								
cb5516_c26/f1p0/3029 isoform=cb5516_c26;full_length_coverage=1;non_full_length_coverage=0;isoform_length=3029;expected_accuracy=1.000	gnl|BL_ORD_ID|46454 cb5516_c53/f1p0/3210 isoform=cb5516_c53;full_length_coverage=1;non_full_length_coverage=0;isoform_length=3210;expected_accuracy=0.994	10	2327	2322	2968	114	2435	2561	3210
cb187_c5/f1p0/3727 isoform=cb187_c5;full_length_coverage=1;non_full_length_coverage=0;isoform_length=3727;expected_accuracy=0.996	gnl|BL_ORD_ID|34575 cb187_c6/f9p1/4066 isoform=cb187_c6;full_length_coverage=9;non_full_length_coverage=1;isoform_length=4066;expected_accuracy=1.000	152	3726	1	151	492	4066	98	248
cb8564_c12355/f1p9/2685 isoform=cb8564_c12355;full_length_coverage=1;non_full_length_coverage=9;isoform_length=2685;expected_accuracy=0.999	gnl|BL_ORD_ID|56381 cb8564_c75626/f1p2/3199	1	2141	2139	2685	4	2116	2611	3157
cb8564_c46247/f1p1/3336 isoform=cb8564_c46247;full_length_coverage=1;non_full_length_coverage=1;isoform_length=3336;expected_accuracy=0.992	gnl|BL_ORD_ID|66137 cb18054_c3/f1p0/5284	10	2311	2309	3286	1148	3441	4305	5284
cb8564_c178335/f1p2/3430 isoform=cb8564_c178335;full_length_coverage=1;non_full_length_coverage=2;isoform_length=3430;expected_accuracy=0.998	gnl|BL_ORD_ID|9995 cb8564_c111409/f2p0/3661 isoform=cb8564_c111409;full_length_coverage=2;non_full_length_coverage=0;isoform_length=3661;expected_accuracy=0.999	1	2863	2861	3429	1	2863	3093	3661
cb8564_c36689/f1p1/2368 isoform=cb8564_c36689;full_length_coverage=1;non_full_length_coverage=1;isoform_length=2368;expected_accuracy=0.991	gnl|BL_ORD_ID|61972 cb12931_c30/f1p0/5903	1	1709	1706	2368	1	1701	2200	2864

<table>
<tr><td>cb8564_c37220/f1p2/4071 isoform=cb8564_c37220;full_length_coverage=1;non_full_length_coverage=2;isoform_length=4071;expected_accuracy=0.994</td><td>gnl|BL_ORD_ID|14942 cb8564_c34188/f2p3/4546 isoform=cb8564_c34188;full_length_coverage=2;non_full_length_coverage=3;isoform_length=4546;expected_accuracy=1.000</td><td>104</td><td>3277</td><td>3273</td><td>4071</td><td>207</td><td>3382</td><td>3647</td><td>4446</td></tr>
<tr><td>cb373_c39/f1p0/4415</td><td>gnl|BL_ORD_ID|70839 cb373_c37/f1p0/4690</td><td>16</td><td>3355</td><td>3356</td><td>3763</td><td>11</td><td>3352</td><td>3458</td><td>3865</td></tr>
<tr><td>cb8564_c49821/f1p1/2905</td><td>gnl|BL_ORD_ID|13472 cb8564_c182192/f1p6/3150 isoform=cb8564_c182192;full_length_coverage=1;non_full_length_coverage=6;isoform_length=3150;expected_accuracy=0.995</td><td>1</td><td>1544</td><td>1541</td><td>2881</td><td>19</td><td>1570</td><td>1788</td><td>3130</td></tr>
<tr><td>cb7400_c0/f3p1/2300 isoform=cb7400_c0;full_length_coverage=3;non_full_length_coverage=1;isoform_length=2300;expected_accuracy=1.000</td><td>gnl|BL_ORD_ID|51190 cb7400_c14/f1p0/2608</td><td>1</td><td>1661</td><td>1662</td><td>2300</td><td>1</td><td>1658</td><td>1798</td><td>2425</td></tr>
<tr><td>cb3575_c3/f2p1/3187 isoform=cb3575_c3;full_length_coverage=2;non_full_length_coverage=1;isoform_length=3187;expected_accuracy=1.000</td><td>gnl|BL_ORD_ID|39142 cb3575_c4/f1p2/3595 isoform=cb3575_c4;full_length_coverage=1;non_full_length_coverage=2;isoform_length=3595;expected_accuracy=0.992</td><td>20</td><td>2555</td><td>2555</td><td>3158</td><td>1</td><td>2528</td><td>2993</td><td>3595</td></tr>
<tr><td>cb8564_c18901/f1p1/2269</td><td>gnl|BL_ORD_ID|11254 cb8564_c13282/f1p2/2892 isoform=cb8564_c13282;full_length_coverage=1;non_full_length_coverage=2;isoform_length=2892;expected_accuracy=0.994</td><td>1</td><td>1836</td><td>1835</td><td>2180</td><td>7</td><td>1842</td><td>2548</td><td>2892</td></tr>
<tr><td>cb9472_c3/f1p0/1395 isoform=cb9472_c3;full_length_coverage=1;non_full_length_coverage=0;isoform_length=1395;expected_accuracy=0.998</td><td>gnl|BL_ORD_ID|58745 cb9472_c0/f1p0/2145</td><td>388</td><td>1395</td><td>8</td><td>390</td><td>1066</td><td>2073</td><td>4</td><td>388</td></tr>
</table>

cb8564_c111706/f1p0/2253	gnl|BL_ORD_ID|10765 cb8564_c108620/f6p2/2639 isoform=cb8564_c108620;full_length_coverage=6;non_full_length_coverage=2;isoform_length=2639;expected_accuracy=1.000	1	1681	1678	2175	168	1848	2139	2633
cb10850_c6/f1p0/1579	gnl|BL_ORD_ID|3040 cb10850_c2/f1p0/2696 isoform=cb10850_c2;full_length_coverage=1;non_full_length_coverage=0;isoform_length=2696;expected_accuracy=0.995	1	1137	1138	1415	1114	2250	2366	2644
cb8564_c178999/f1p0/2222 isoform=cb8564_c178999;full_length_coverage=1;non_full_length_coverage=0;isoform_length=2222;expected_accuracy=0.994	gnl|BL_ORD_ID|15401 cb8564_c34720/f1p3/3220 isoform=cb8564_c34720;full_length_coverage=1;non_full_length_coverage=3;isoform_length=3220;expected_accuracy=0.992	10	1778	1773	2221	10	1776	2745	3220
cb8564_c13789/f1p1/3323	gnl|BL_ORD_ID|55761 cb8564_c24976/f1p1/3685	2	2821	2820	3333	55	2874	3187	3701
cb5271_c5/f1p2/2317	gnl|BL_ORD_ID|45450 cb5271_c4/f1p2/3138 isoform=cb5271_c4;full_length_coverage=1;non_full_length_coverage=2;isoform_length=3138;expected_accuracy=0.995	1	1283	1279	2316	1	1283	2101	3138
cb8564_c49821/f1p1/2905	gnl|BL_ORD_ID|15357 cb8564_c44284/f4p1/3133 isoform=cb8564_c44284;full_length_coverage=4;non_full_length_coverage=1;isoform_length=3133;expected_accuracy=1.000	1	1544	1541	2881	9	1575	1793	3133
cb8564_c15653/f1p4/2475	gnl|BL_ORD_ID|13065 cb8564_c157334/f1p21/2777 isoform=cb8564_c157334;full_length_coverage=1;non_full_length_coverage=21;isoform_length=2777;expected_accuracy=0.995	20	2293	2289	2480	42	2316	2535	2727

cb8564_c112971/f1p0/2064 isoform=cb8564_c112971;full_length_coverage=1;non_full_length_coverage=0;isoform_length=2064;expected_accuracy=0.991	gnl|BL_ORD_ID|9281 cb8564_c119279/f1p0/2203 isoform=cb8564_c119279;full_length_coverage=1;non_full_length_coverage=0;isoform_length=2203;expected_accuracy=0.996	1	1422	1422	2062	2	1420	1568	2203
cb2671_c18/f1p0/2982 isoform=cb2671_c18;full_length_coverage=1;non_full_length_coverage=0;isoform_length=2982;expected_accuracy=0.995	gnl|BL_ORD_ID|68701 cb2671_c38/f1p0/3750	1138	2959	102	1141	1929	3755	1	1040
cb8564_c39136/f1p0/3036 isoform=cb8564_c39136;full_length_coverage=1;non_full_length_coverage=0;isoform_length=3036;expected_accuracy=0.998	gnl|BL_ORD_ID|55211 cb8564_c35547/f1p0/3745	1	2089	2085	3036	9	2105	2232	3184
cb18456_c1958/f1p1/1247 isoform=cb18456_c1958;full_length_coverage=1;non_full_length_coverage=1;isoform_length=1247;expected_accuracy=0.998	gnl|BL_ORD_ID|36413 cb18456_c1924/f1p2/1435 isoform=cb18456_c1924;full_length_coverage=1;non_full_length_coverage=2;isoform_length=1435;expected_accuracy=1.000	9	781	779	1217	5	777	878	1314
cb8564_c34553/f1p0/3407	gnl|BL_ORD_ID|14394 cb8564_c39181/f2p0/3796 isoform=cb8564_c39181;full_length_coverage=2;non_full_length_coverage=0;isoform_length=3796;expected_accuracy=0.997	26	2805	2804	3212	20	2793	3369	3796
cb3482_c32/f1p0/1683 isoform=cb3482_c32;full_length_coverage=1;non_full_length_coverage=0;isoform_length=1683;expected_accuracy=1.000	gnl|BL_ORD_ID|41029 cb3482_c38/f13p2/2110 isoform=cb3482_c38;full_length_coverage=13;non_full_length_coverage=2;isoform_length=2110;expected_accuracy=1.000	1	1586	1581	1683	129	1729	2008	2110
cb14220_c21/f1p1/5537	gnl|BL_ORD_ID|25176 cb14220_c10/f1p1/6717 isoform=cb14220_c10;full_length_coverage=1;non_full	1	4570	4569	5549	1002	5554	5675	6650

	_length_coverage=1;isoform_length=6717;expected_accuracy=0.993								
cb1960_c41/f1p1/2517	gnl|BL_ORD_ID|36150 cb1960_c28/f1p0/3094 isoform=cb1960_c28;full_length_coverage=1;non_full_length_coverage=0;isoform_length=3094;expected_accuracy=0.995	1	2123	2120	2503	192	2311	2708	3091
cb7116_c11/f11p3/2162 isoform=cb7116_c11;full_length_coverage=11;non_full_length_coverage=3;isoform_length=2162;expected_accuracy=1.000	gnl|BL_ORD_ID|5769 cb7116_c4/f2p0/2694 isoform=cb7116_c4;full_length_coverage=2;non_full_length_coverage=0;isoform_length=2694;expected_accuracy=0.997	455	2109	8	460	1002	2659	2	454
cb18456_c1748/f1p0/1180	gnl|BL_ORD_ID|69242 cb18456_c1755/f1p0/1422	243	1188	6	248	382	1327	2	244
cb5565_c66/f1p0/2756	gnl|BL_ORD_ID|46929 cb5565_c63/f1p0/3234 isoform=cb5565_c63;full_length_coverage=1;non_full_length_coverage=0;isoform_length=3234;expected_accuracy=0.997	1	2174	2172	2745	7	2176	2661	3234
cb8564_c109730/f3p0/2853 isoform=cb8564_c109730;full_length_coverage=3;non_full_length_coverage=0;isoform_length=2853;expected_accuracy=0.998	gnl|BL_ORD_ID|53093 cb8564_c116144/f1p0/3080	1	2737	2737	2847	64	2796	2907	3018
cb8564_c84130/f1p2/3574 isoform=cb8564_c84130;full_length_coverage=1;non_full_length_coverage=2;isoform_length=3574;expected_accuracy=0.995	gnl|BL_ORD_ID|55537 cb8564_c44585/f1p0/3901	125	3573	1	127	379	3822	81	206
cb1016_c0/f7p1/1298 isoform=cb1016_c0;full_length_coverage=7;non_full_le	gnl|BL_ORD_ID|47223 cb1016_c4/f1p1/2801	1	1042	1041	1289	67	1106	2409	2675

ngth_coverage=1;isoform_length=1298;expected_accuracy=1.000									
cb10054_c49/f1p1/1123 isoform=cb10054_c49;full_length_coverage=1;non_full_length_coverage=1;isoform_length=1123;expected_accuracy=1.000	gnl|BL_ORD_ID|2392 cb10054_c55/f2p0/1317 isoform=cb10054_c55;full_length_coverage=2;non_full_length_coverage=0;isoform_length=1317;expected_accuracy=0.995	258	1120	6	262	458	1317	4	273
cb6277_c19/f1p1/2357 isoform=cb6277_c19;full_length_coverage=1;non_full_length_coverage=1;isoform_length=2357;expected_accuracy=0.995	gnl|BL_ORD_ID|49577 cb6277_c13/f1p0/2679	1	1183	1183	2342	26	1208	1514	2673
cb3362_c27/f1p0/2905 isoform=cb3362_c27;full_length_coverage=1;non_full_length_coverage=0;isoform_length=2905;expected_accuracy=0.995	gnl|BL_ORD_ID|39591 cb3362_c29/f1p0/3618 isoform=cb3362_c29;full_length_coverage=1;non_full_length_coverage=0;isoform_length=3618;expected_accuracy=0.992	1	1996	1997	2905	79	2075	2640	3550
cb7023_c6/f1p0/1630	gnl|BL_ORD_ID|3663 cb7023_c3/f1p1/2449 isoform=cb7023_c3;full_length_coverage=1;non_full_length_coverage=1;isoform_length=2449;expected_accuracy=0.995	129	1564	1	129	1013	2449	19	147
cb8564_c13652/f1p0/2664	gnl|BL_ORD_ID|18458 cb8564_c9882/f2p0/2918 isoform=cb8564_c9882;full_length_coverage=2;non_full_length_coverage=0;isoform_length=2918;expected_accuracy=0.999	1	1476	1475	2664	22	1498	1636	2809
cb3263_c5/f1p0/3305	gnl|BL_ORD_ID|39132 cb3263_c3/f1p0/3378 isoform=cb3263_c3;full_length_coverage=1;non_full_length_coverage=0;isoform_length=3378;expected_accuracy=0.997	1	2752	2748	3144	36	2785	2982	3378

cb7736_c4/f1p0/3571 isoform=cb7736_c4;full_length_coverage=1;non_full_length_coverage=0;isoform_length=3571;expected_accuracy=0.996	gnl|BL_ORD_ID|50678 cb7736_c3/f1p0/4173	1	2535	2536	3568	1	2527	3121	4150
cb9694_c11/f1p0/1693	gnl|BL_ORD_ID|59911 cb9694_c4/f1p0/1868	450	1642	52	454	564	1756	1	403
cb4665_c2/f2p0/2737 isoform=cb4665_c2;full_length_coverage=2;non_full_length_coverage=0;isoform_length=2737;expected_accuracy=1.000	gnl|BL_ORD_ID|72831 cb4665_c3/f1p0/3053	1	1885	1885	2729	21	1905	2155	2999
cb8564_c154694/f2p9/3322 isoform=cb8564_c154694;full_length_coverage=2;non_full_length_coverage=9;isoform_length=3322;expected_accuracy=1.000	gnl|BL_ORD_ID|15223 cb8564_c35037/f1p0/3377 isoform=cb8564_c35037;full_length_coverage=1;non_full_length_coverage=0;isoform_length=3377;expected_accuracy=0.996	1	2757	2755	3264	1	2761	2866	3377
cb14220_c21/f1p1/5537	gnl|BL_ORD_ID|63131 cb14220_c4/f1p1/7240	1	4570	4569	5549	905	5474	5597	6577
cb8564_c72680/f1p0/2685	gnl|BL_ORD_ID|57123 cb8564_c84015/f1p0/3207	12	1735	1730	2636	3	1726	1905	2811
cb8564_c23232/f1p3/2540	gnl|BL_ORD_ID|55935 cb8564_c49443/f1p0/2984	16	2172	2167	2518	39	2192	2294	2645
cb6646_c1/f3p1/2483 isoform=cb6646_c1;full_length_coverage=3;non_full_length_coverage=1;isoform_length=2483;expected_accuracy=1.000	gnl|BL_ORD_ID|4220 cb6646_c6/f1p0/2802 isoform=cb6646_c6;full_length_coverage=1;non_full_length_coverage=0;isoform_length=2802;expected_accuracy=0.994	1	2050	2047	2416	3	2050	2433	2802
cb8564_c40764/f1p0/2563 isoform=cb8564_c40764;full_length_coverage=1;non_f	gnl|BL_ORD_ID|54438 cb8564_c46124/f1p0/3383	1	2167	2162	2563	151	2315	2522	2927

ull_length_coverage=0;isoform_length=2563;expected_accuracy=0.997									
cb1490_c11/f1p1/3876	gnl|BL_ORD_ID|30687 cb1490_c2/f1p1/4303 isoform=cb1490_c2;full_length_coverage=1;non_full_length_coverage=1;isoform_length=4303;expected_accuracy=0.994	20	2559	2556	3884	6	2550	2942	4267
cb8564_c40598/f1p4/3119	gnl|BL_ORD_ID|14471 cb8564_c34518/f1p0/3136 isoform=cb8564_c34518;full_length_coverage=1;non_full_length_coverage=0;isoform_length=3136;expected_accuracy=0.991	1	2732	2729	3053	1	2698	2818	3136
cb8564_c158845/f2p1/2551 isoform=cb8564_c158845;full_length_coverage=2;non_full_length_coverage=1;isoform_length=2551;expected_accuracy=0.995	gnl|BL_ORD_ID|57744 cb8564_c79518/f1p0/2771	1	1901	1897	2546	16	1900	2062	2689
cb1535_c10/f1p0/3272	gnl|BL_ORD_ID|63719 cb1535_c3/f1p0/4275	1250	3241	10	1255	2187	4187	1	1249
cb8564_c121641/f1p0/2089	gnl|BL_ORD_ID|12681 cb8564_c146083/f6p3/2197 isoform=cb8564_c146083;full_length_coverage=6;non_full_length_coverage=3;isoform_length=2197;expected_accuracy=1.000	1	1526	1525	1884	23	1574	1789	2148
cb8564_c43119/f1p4/3194 isoform=cb8564_c43119;full_length_coverage=1;non_full_length_coverage=4;isoform_length=3194;expected_accuracy=0.995	gnl|BL_ORD_ID|55817 cb8564_c40942/f1p0/3608	12	1671	1668	3150	20	1669	1811	3278
cb626_c91/f1p0/2043 isoform=cb626_c91;full_length_coverage=1;non_full_le	gnl|BL_ORD_ID|4060 cb626_c17/f1p0/4935 isoform=cb626_c17;full_length_coverage=1;non_full_le	1	1397	1397	1964	2231	3629	4370	4935

ngth_coverage=0;isoform_length=2043;expected_accuracy=0.997	ngth_coverage=0;isoform_length=4935;expected_accuracy=0.993								
cb4163_c9/f1p0/2277 isoform=cb4163_c9;full_length_coverage=1;non_full_length_coverage=0;isoform_length=2277;expected_accuracy=0.995	gnl|BL_ORD_ID|72300 cb4163_c14/f1p0/3022	1	1952	1948	2205	607	2544	2708	2973
cb2528_c0/f4p1/1435 isoform=cb2528_c0;full_length_coverage=4;non_full_length_coverage=1;isoform_length=1435;expected_accuracy=1.000	gnl|BL_ORD_ID|37491 cb2528_c1/f1p1/3892 isoform=cb2528_c1;full_length_coverage=1;non_full_length_coverage=1;isoform_length=3892;expected_accuracy=1.000	530	1434	64	534	2988	3892	1	471
cb7969_c11/f1p0/1950 isoform=cb7969_c11;full_length_coverage=1;non_full_length_coverage=0;isoform_length=1950;expected_accuracy=0.992	gnl|BL_ORD_ID|50803 cb7969_c22/f1p0/2368	1	1409	1405	1931	101	1511	1756	2283
cb8564_c186356/f1p0/2744 isoform=cb8564_c186356;full_length_coverage=1;non_full_length_coverage=0;isoform_length=2744;expected_accuracy=0.998	gnl|BL_ORD_ID|61972 cb12931_c30/f1p0/5903	1	1396	1393	2744	1	1394	1514	2865
cb8564_c36056/f1p0/2652 isoform=cb8564_c36056;full_length_coverage=1;non_full_length_coverage=0;isoform_length=2652;expected_accuracy=0.999	gnl|BL_ORD_ID|55585 cb8564_c45904/f1p5/3439	248	2652	1	251	456	2861	60	312
cb4528_c38/f2p0/1640 isoform=cb4528_c38;full_length_coverage=2;non_full_length_coverage=0;isoform_length=1640;expected_accuracy=1.000	gnl|BL_ORD_ID|42033 cb4528_c16/f1p0/2037 isoform=cb4528_c16;full_length_coverage=1;non_full_length_coverage=0;isoform_length=2037;expected_accuracy=0.993	1	1497	1495	1616	121	1618	1919	2037

cb8564_c53418/f1p0/2602 isoform=cb8564_c53418;full_length_coverage=1;non_full_length_coverage=0;isoform_length=2602;expected_accuracy=0.991	gnl|BL_ORD_ID|13571 cb8564_c20949/f2p0/2862 isoform=cb8564_c20949;full_length_coverage=2;non_full_length_coverage=0;isoform_length=2862;expected_accuracy=0.997	1163	2602	1	1168	1417	2859	150	1316
cb1119_c16/f1p0/2494	gnl|BL_ORD_ID|2754 cb1119_c18/f1p0/2556 isoform=cb1119_c18;full_length_coverage=1;non_full_length_coverage=0;isoform_length=2556;expected_accuracy=0.997	262	2478	54	261	337	2556	1	208
cb8564_c36731/f1p2/2761	gnl|BL_ORD_ID|15038 cb8564_c39136/f1p0/3036 isoform=cb8564_c39136;full_length_coverage=1;non_full_length_coverage=0;isoform_length=3036;expected_accuracy=0.998	1	1977	1972	2738	1	1973	2271	3036
cb7940_c54/f1p1/1719 isoform=cb7940_c54;full_length_coverage=1;non_full_length_coverage=1;isoform_length=1719;expected_accuracy=0.997	gnl|BL_ORD_ID|7312 cb7940_c49/f1p1/1935 isoform=cb7940_c49;full_length_coverage=1;non_full_length_coverage=1;isoform_length=1935;expected_accuracy=0.995	10	1366	1363	1719	1	1372	1571	1927
cb8564_c37390/f1p0/2683 isoform=cb8564_c37390;full_length_coverage=1;non_full_length_coverage=0;isoform_length=2683;expected_accuracy=1.000	gnl|BL_ORD_ID|15197 cb8564_c43963/f9p0/3230 isoform=cb8564_c43963;full_length_coverage=9;non_full_length_coverage=0;isoform_length=3230;expected_accuracy=0.993	265	2683	1	267	707	3152	1	258
cb2084_c3/f2p0/2121 isoform=cb2084_c3;full_length_coverage=2;non_full_length_coverage=0;isoform_length=2121;expected_accuracy=0.997	gnl|BL_ORD_ID|35414 cb2084_c12/f1p0/3016 isoform=cb2084_c12;full_length_coverage=1;non_full_length_coverage=0;isoform_length=3016;expected_accuracy=0.999	1	1586	1585	2094	3	1588	2506	3016
cb3163_c12/f1p0/2539 isoform=cb3163_c12;full_length_coverage=1;non_full_	gnl|BL_ORD_ID|37284 cb3163_c1/f5p1/2811 isoform=cb3163_c1;full_length_coverage=5;non_full_le	1	1656	1654	2539	76	1731	1854	2739

length_coverage=0;isoform_length=2539;expected_accuracy=0.994	ngth_coverage=1;isoform_length=2811;expected_accuracy=0.998								
cb2770_c36/f1p0/3443	gnl|BL_ORD_ID|71945 cb4543_c1/f1p0/3821	123	2661	2662	3239	3322	767	659	81
cb2707_c24/f1p0/2640	gnl|BL_ORD_ID|36722 cb2707_c55/f1p0/3422 isoform=cb2707_c55;full_length_coverage=1;non_full_length_coverage=0;isoform_length=3422;expected_accuracy=0.995	21	2430	2425	2649	1	2405	2913	3137
cb10736_c0/f1p0/1267 isoform=cb10736_c0;full_length_coverage=1;non_full_length_coverage=0;isoform_length=1267;expected_accuracy=0.992	gnl|BL_ORD_ID|48809 cb10736_c1/f1p0/3095	147	1266	17	147	1931	3051	2	132
cb4452_c2/f3p0/1957 isoform=cb4452_c2;full_length_coverage=3;non_full_length_coverage=0;isoform_length=1957;expected_accuracy=1.000	gnl|BL_ORD_ID|42359 cb4452_c4/f1p0/2965 isoform=cb4452_c4;full_length_coverage=1;non_full_length_coverage=0;isoform_length=2965;expected_accuracy=0.995	1	1509	1507	1931	11	1525	2539	2965
cb5617_c15/f2p0/2303 isoform=cb5617_c15;full_length_coverage=2;non_full_length_coverage=0;isoform_length=2303;expected_accuracy=0.995	gnl|BL_ORD_ID|74165 cb5617_c7/f1p0/3437	1092	2268	1	1093	2212	3388	20	1110
cb261_c15/f1p0/3068 isoform=cb261_c15;full_length_coverage=1;non_full_length_coverage=0;isoform_length=3068;expected_accuracy=0.996	gnl|BL_ORD_ID|69511 cb261_c23/f1p1/3640	1	2524	2522	3068	145	2677	3122	3668
cb5815_c16/f2p0/1366 isoform=cb5815_c16;full_length_coverage=2;non_full_length_coverage=0;isoform_length=1366;expected_accu	gnl|BL_ORD_ID|73448 cb5815_c7/f1p0/2348	235	1340	8	235	1228	2333	21	249

racy=0.993									
cb8564_c36731/f1p2/2761	gnl|BL_ORD_ID|13575 cb8564_c20499/f3p0/3328 isoform=cb8564_c20499;full_length_coverage=3;non_full_length_coverage=0;isoform_length=3328;expected_accuracy=1.000	1	1977	1972	2741	1	1980	2409	3179
cb11952_c19/f1p0/1832	gnl|BL_ORD_ID|14854 cb8564_c3814/f1p0/2566 isoform=cb8564_c3814;full_length_coverage=1;non_full_length_coverage=0;isoform_length=2566;expected_accuracy=0.993	366	1804	5	365	1129	2563	5	362
cb8564_c2516/f2p0/2561 isoform=cb8564_c2516;full_length_coverage=2;non_full_length_coverage=0;isoform_length=2561;expected_accuracy=0.994	gnl|BL_ORD_ID|14698 cb8564_c4069/f2p0/2782 isoform=cb8564_c4069;full_length_coverage=2;non_full_length_coverage=0;isoform_length=2782;expected_accuracy=0.995	1	2112	2110	2499	1	2103	2393	2782
cb7412_c16/f1p0/1476	gnl|BL_ORD_ID|51204 cb7412_c5/f1p2/2647	1	1143	1142	1459	1	1143	2182	2500
cb5516_c26/f1p0/3029 isoform=cb5516_c26;full_length_coverage=1;non_full_length_coverage=0;isoform_length=3029;expected_accuracy=1.000	gnl|BL_ORD_ID|45350 cb5516_c66/f1p0/3072 isoform=cb5516_c66;full_length_coverage=1;non_full_length_coverage=0;isoform_length=3072;expected_accuracy=0.996	1	2327	2322	2964	1	2305	2430	3072
cb3586_c11/f1p0/2822 isoform=cb3586_c11;full_length_coverage=1;non_full_length_coverage=0;isoform_length=2822;expected_accuracy=0.995	gnl|BL_ORD_ID|71356 cb3586_c9/f1p0/3463	278	2822	1	278	914	3471	4	280
cb8564_c4645/f1p1/2620 isoform=cb8564_c4645;full_length_coverage=1;non_full_length_coverage=1;isoform_length=2620;expected_ac	gnl|BL_ORD_ID|12605 cb8564_c147470/f1p2/2765 isoform=cb8564_c147470;full_length_coverage=1;non_full_length_coverage=2;isoform_length=2765;expected_a	1	1828	1829	2557	75	1914	2038	2765

curacy=0.994	ccuracy=0.992								
cb8564_c504/f6p2/3836 isoform=cb8564_c504;full_length_coverage=6;non_full_length_coverage=2;isoform_length=3836;expected_accuracy=1.000	gnl|BL_ORD_ID|16742 cb8564_c628/f3p1/4943 isoform=cb8564_c628;full_length_coverage=3;non_full_length_coverage=1;isoform_length=4943;expected_accuracy=1.000	1	1915	1913	3772	4	1916	3085	4943
cb8564_c85953/f1p0/2986	gnl|BL_ORD_ID|56772 cb8564_c71057/f1p4/4091	175	2717	1	174	1498	4044	12	181
cb8564_c111744/f1p1/2111 isoform=cb8564_c111744;full_length_coverage=1;non_full_length_coverage=1;isoform_length=2111;expected_accuracy=0.998	gnl|BL_ORD_ID|11418 cb8564_c146326/f4p0/2491 isoform=cb8564_c146326;full_length_coverage=4;non_full_length_coverage=0;isoform_length=2491;expected_accuracy=0.997	1	1639	1635	2111	83	1717	1890	2366
cb8564_c118662/f1p0/1941	gnl|BL_ORD_ID|15094 cb8564_c3728/f3p1/2420 isoform=cb8564_c3728;full_length_coverage=3;non_full_length_coverage=1;isoform_length=2420;expected_accuracy=1.000	216	1847	5	219	785	2420	14	240
cb8564_c3432/f1p0/2118	gnl|BL_ORD_ID|13042 cb8564_c161281/f1p7/2816 isoform=cb8564_c161281;full_length_coverage=1;non_full_length_coverage=7;isoform_length=2816;expected_accuracy=0.994	619	2090	7	623	740	2210	10	624
cb5375_c2/f3p0/2503 isoform=cb5375_c2;full_length_coverage=3;non_full_length_coverage=0;isoform_length=2503;expected_accuracy=1.000	gnl|BL_ORD_ID|45225 cb5375_c10/f1p0/2540 isoform=cb5375_c10;full_length_coverage=1;non_full_length_coverage=0;isoform_length=2540;expected_accuracy=0.998	1	1664	1665	2405	1	1665	1800	2540
cb7336_c3/f1p1/2411 isoform=cb7336_c3;full_length_coverage=1;non_full_length_coverage=1;isoform_length=2411;expected_accura	gnl|BL_ORD_ID|6810 cb7336_c2/f1p1/3070 isoform=cb7336_c2;full_length_coverage=1;non_full_length_coverage=1;isoform_length=3070;expected_accura	1	1280	1277	2365	56	1340	1980	3070

cy=0.992	cy=0.996								
cb5608_c33/f1p0/2602	gnl|BL_ORD_ID|46459 cb5608_c24/f1p1/2825 isoform=cb5608_c24;full_length_coverage=1;non_full_length_coverage=1;isoform_length=2825;expected_accuracy=0.998	763	2610	9	762	864	2708	1	755
cb18456_c1776/f1p0/1211	gnl|BL_ORD_ID|12597 cb8564_c123090/f1p10/2452 isoform=cb8564_c123090;full_length_coverage=1;non_full_length_coverage=10;isoform_length=2452;expected_accuracy=0.993	280	1214	9	285	1513	2446	1128	1401
cb8564_c181853/f1p0/2119 isoform=cb8564_c181853;full_length_coverage=1;non_full_length_coverage=0;isoform_length=2119;expected_accuracy=0.997	gnl|BL_ORD_ID|54698 cb8564_c155298/f3p9/3822	1	1339	1334	2118	1	1341	2169	2953
cb1524_c18/f1p0/3747	gnl|BL_ORD_ID|30626 cb1524_c8/f1p0/3674 isoform=cb1524_c8;full_length_coverage=1;non_full_length_coverage=0;isoform_length=3674;expected_accuracy=0.995	153	3581	1	154	267	3674	3	158
cb8564_c38947/f1p0/2650 isoform=cb8564_c38947;full_length_coverage=1;non_full_length_coverage=0;isoform_length=2650;expected_accuracy=0.996	gnl|BL_ORD_ID|56727 cb8564_c66920/f3p3/2894	1	1441	1436	2643	1	1439	1656	2863
cb8564_c38947/f1p0/2650 isoform=cb8564_c38947;full_length_coverage=1;non_full_length_coverage=0;isoform_length=2650;expected_accuracy=0.996	gnl|BL_ORD_ID|55873 cb8564_c47701/f1p0/3144	12	1441	1436	2650	24	1449	1666	2880
cb8564_c40598/f1p4/3119	gnl|BL_ORD_ID|16622 cb8564_c50151/f1p0/3198	1	2732	2729	3051	6	275	2876	319

	isoform=cb8564_c50151;full_length_coverage=1;non_full_length_coverage=0;isoform_length=3198;expected_accuracy=0.995						5		8
cb307_c3/f1p0/4879	gnl|BL_ORD_ID|68186 cb307_c24/f1p0/6707	1	2879	2875	4446	7	2861	4878	6450
cb2097_c50/f1p0/2824	gnl|BL_ORD_ID|34289 cb2097_c20/f10p3/3179 isoform=cb2097_c20;full_length_coverage=10;non_full_length_coverage=3;isoform_length=3179;expected_accuracy=1.000	1	2323	2318	2757	80	2403	2578	3016
cb6273_c2/f2p1/2491 isoform=cb6273_c2;full_length_coverage=2;non_full_length_coverage=1;isoform_length=2491;expected_accuracy=1.000	gnl|BL_ORD_ID|5821 cb6273_c14/f1p1/5047 isoform=cb6273_c14;full_length_coverage=1;non_full_length_coverage=1;isoform_length=5047;expected_accuracy=0.997	1	1332	1330	2491	58	1395	2963	4132
cb8564_c36774/f1p0/2756 isoform=cb8564_c36774;full_length_coverage=1;non_full_length_coverage=0;isoform_length=2756;expected_accuracy=0.990	gnl|BL_ORD_ID|55398 cb8564_c40412/f1p4/3590	145	2756	1	145	854	3471	67	210
cb5491_c9/f1p0/3235	gnl|BL_ORD_ID|45975 cb5491_c6/f1p0/3581 isoform=cb5491_c6;full_length_coverage=1;non_full_length_coverage=0;isoform_length=3581;expected_accuracy=0.999	1	2441	2438	2767	114	2547	3205	3534
cb8564_c36883/f1p0/3072 isoform=cb8564_c36883;full_length_coverage=1;non_full_length_coverage=0;isoform_length=3072;expected_accuracy=0.996	gnl|BL_ORD_ID|55920 cb8564_c48949/f1p0/4357	1	2290	2291	2957	2	2293	2847	3510
cb8564_c118246/f1p0/2200	gnl|BL_ORD_ID|19172 cb8564_c83528/f4p0/3158	1	1819	1819	2196	1	182	2782	315

isoform=cb8564_c118246;full_length_coverage=1;non_full_length_coverage=0;isoform_length=2200;expected_accuracy=0.997	isoform=cb8564_c83528;full_length_coverage=4;non_full_length_coverage=0;isoform_length=3158;expected_accuracy=0.996						6		8
cb5867_c4/f1p1/2853	gnl|BL_ORD_ID|74526 cb5867_c7/f1p0/4489	2	2012	2008	2718	82	2093	3692	4402
cb6125_c1/f1p0/2190 isoform=cb6125_c1;full_length_coverage=1;non_full_length_coverage=0;isoform_length=2190;expected_accuracy=0.994	gnl|BL_ORD_ID|74934 cb6125_c2/f1p0/2571	207	2135	1	207	655	2583	20	227
cb2471_c1/f7p0/2064 isoform=cb2471_c1;full_length_coverage=7;non_full_length_coverage=0;isoform_length=2064;expected_accuracy=1.000	gnl|BL_ORD_ID|37270 cb2471_c8/f3p0/2291 isoform=cb2471_c8;full_length_coverage=3;non_full_length_coverage=0;isoform_length=2291;expected_accuracy=0.996	170	2064	1	172	382	2288	43	224
cb8564_c40598/f1p4/3119	gnl|BL_ORD_ID|13575 cb8564_c20499/f3p0/3328 isoform=cb8564_c20499;full_length_coverage=3;non_full_length_coverage=0;isoform_length=3328;expected_accuracy=1.000	1	2732	2729	3071	1	2731	2852	3195
cb4889_c22/f1p0/2625 isoform=cb4889_c22;full_length_coverage=1;non_full_length_coverage=0;isoform_length=2625;expected_accuracy=0.995	gnl|BL_ORD_ID|73052 cb4889_c29/f1p0/3264	280	2604	7	285	498	2822	52	330
cb1770_c3/f3p0/3565 isoform=cb1770_c3;full_length_coverage=3;non_full_length_coverage=0;isoform_length=3565;expected_accuracy=0.999	gnl|BL_ORD_ID|33844 cb1770_c9/f1p0/3711 isoform=cb1770_c9;full_length_coverage=1;non_full_length_coverage=0;isoform_length=3711;expected_accuracy=0.999	155	3565	1	155	267	3660	8	162
cb8564_c44004/f1p0/2796	gnl|BL_ORD_ID|13089 cb8564_c16897/f1p0/3118	1	1549	1547	2795	33	158	1870	3118

isoform=cb8564_c44004;full_length_coverage=1;non_full_length_coverage=0;isoform_length=2796;expected_accuracy=1.000	isoform=cb8564_c16897;full_length_coverage=1;non_full_length_coverage=0;isoform_length=3118;expected_accuracy=0.998						1		
cb8564_c49540/f1p2/2982	gnl|BL_ORD_ID|11778 cb8564_c150403/f2p9/3190 isoform=cb8564_c150403;full_length_coverage=2;non_full_length_coverage=9;isoform_length=3190;expected_accuracy=0.996	750	2982	77	747	860	3092	1	677
cb8564_c77411/f1p0/2169 isoform=cb8564_c77411;full_length_coverage=1;non_full_length_coverage=0;isoform_length=2169;expected_accuracy=0.993	gnl|BL_ORD_ID|10484 cb8564_c107507/f12p0/2558 isoform=cb8564_c107507;full_length_coverage=12;non_full_length_coverage=0;isoform_length=2558;expected_accuracy=1.000	105	2165	1	106	496	2558	45	151
cb548_c79/f1p2/3153	gnl|BL_ORD_ID|46779 cb548_c109/f1p0/3280 isoform=cb548_c109;full_length_coverage=1;non_full_length_coverage=0;isoform_length=3280;expected_accuracy=0.996	1292	3022	1	1295	1551	3280	45	1340
cb14183_c4/f1p0/1000 isoform=cb14183_c4;full_length_coverage=1;non_full_length_coverage=0;isoform_length=1000;expected_accuracy=0.997	gnl|BL_ORD_ID|63113 cb14183_c3/f1p1/1235	118	1000	3	122	291	1173	1	120
cb14641_c15/f1p0/1682 isoform=cb14641_c15;full_length_coverage=1;non_full_length_coverage=0;isoform_length=1682;expected_accuracy=0.992	gnl|BL_ORD_ID|57976 cb8564_c85640/f1p3/2511	453	1682	71	452	1205	2435	604	987
cb8564_c186356/f1p0/2744 isoform=cb8564_c186356;full_length_coverage=1;non_full_length_coverage=0;isoform_length=2744;expected	gnl|BL_ORD_ID|56601 cb8564_c48153/f1p0/3083	1	1396	1393	2744	13	1405	1525	2876

<table>
<tr><td>_accuracy=0.998</td><td></td><td></td><td></td><td></td><td></td><td></td><td></td><td></td><td></td></tr>
<tr><td>cb3290_c38/f1p0/2345 isoform=cb3290_c38;full_length_coverage=1;non_full_length_coverage=0;isoform_length=2345;expected_accuracy=0.996</td><td>gnl|BL_ORD_ID|70293 cb3290_c160/f1p0/2670</td><td>1150</td><td>2333</td><td>1</td><td>1151</td><td>1340</td><td>2523</td><td>33</td><td>1183</td></tr>
<tr><td>cb8564_c13234/f1p0/3522</td><td>gnl|BL_ORD_ID|54260 cb8564_c21334/f1p0/4233</td><td>23</td><td>2499</td><td>2497</td><td>3238</td><td>26</td><td>2506</td><td>2696</td><td>3437</td></tr>
<tr><td>cb8564_c182028/f1p0/2513 isoform=cb8564_c182028;full_length_coverage=1;non_full_length_coverage=0;isoform_length=2513;expected_accuracy=0.996</td><td>gnl|BL_ORD_ID|14584 cb8564_c24185/f1p1/2695 isoform=cb8564_c24185;full_length_coverage=1;non_full_length_coverage=1;isoform_length=2695;expected_accuracy=0.996</td><td>1</td><td>1971</td><td>1968</td><td>2513</td><td>1</td><td>1942</td><td>2086</td><td>2631</td></tr>
<tr><td>cb8564_c46939/f1p1/2926</td><td>gnl|BL_ORD_ID|12913 cb8564_c182049/f1p0/3195 isoform=cb8564_c182049;full_length_coverage=1;non_full_length_coverage=0;isoform_length=3195;expected_accuracy=0.997</td><td>1</td><td>2134</td><td>2130</td><td>2870</td><td>1</td><td>2157</td><td>2262</td><td>3009</td></tr>
<tr><td>cb2629_c16/f1p0/2938</td><td>gnl|BL_ORD_ID|37707 cb2629_c14/f1p0/3350 isoform=cb2629_c14;full_length_coverage=1;non_full_length_coverage=0;isoform_length=3350;expected_accuracy=0.996</td><td>1</td><td>1728</td><td>1729</td><td>2948</td><td>206</td><td>1930</td><td>2133</td><td>3350</td></tr>
<tr><td>cb8564_c89913/f1p1/3572 isoform=cb8564_c89913;full_length_coverage=1;non_full_length_coverage=1;isoform_length=3572;expected_accuracy=0.998</td><td>gnl|BL_ORD_ID|57143 cb8564_c84495/f1p0/3631</td><td>1</td><td>2463</td><td>2463</td><td>3458</td><td>67</td><td>2535</td><td>2637</td><td>3646</td></tr>
<tr><td>cb8564_c82539/f7p3/2550</td><td>gnl|BL_ORD_ID|13518 cb8564_c20989/f1p1/3206 isoform=cb8564_c20989;full_length_coverage=1;non_full_length_coverage=1;isoform_length=3206;expected_ac</td><td>15</td><td>2040</td><td>2040</td><td>2506</td><td>3</td><td>2023</td><td>2740</td><td>3206</td></tr>
</table>

	curacy=0.990								
cb8564_c185920/f1p0/2777 isoform=cb8564_c185920;full_length_coverage=1;non_full_length_coverage=0;isoform_length=2777;expected_accuracy=0.996	gnl|BL_ORD_ID|13976 cb8564_c43401/f1p2/3025 isoform=cb8564_c43401;full_length_coverage=1;non_full_length_coverage=2;isoform_length=3025;expected_accuracy=0.995	174	2776	1	177	373	3025	18	207
cb13418_c23/f1p0/1284	gnl|BL_ORD_ID|55902 cb8564_c48356/f1p0/3140	528	1212	7	527	2272	2956	1	519
cb10318_c0/f1p0/1601	gnl|BL_ORD_ID|2074 cb10318_c1/f1p0/2040 isoform=cb10318_c1;full_length_coverage=1;non_full_length_coverage=0;isoform_length=2040;expected_accuracy=0.997	694	1554	9	693	819	1680	1	688
cb489_c1/f1p0/2365 isoform=cb489_c1;full_length_coverage=1;non_full_length_coverage=0;isoform_length=2365;expected_accuracy=0.996	gnl|BL_ORD_ID|70931 cb489_c0/f1p0/4793	1	1560	1559	2365	19	1578	3332	4138
cb8564_c50697/f1p3/2172 isoform=cb8564_c50697;full_length_coverage=1;non_full_length_coverage=3;isoform_length=2172;expected_accuracy=0.993	gnl|BL_ORD_ID|57875 cb8564_c83119/f1p1/3046	106	2172	1	108	723	2785	1	106
cb8564_c116163/f1p0/1965 isoform=cb8564_c116163;full_length_coverage=1;non_full_length_coverage=0;isoform_length=1965;expected_accuracy=0.997	gnl|BL_ORD_ID|12400 cb8564_c126704/f1p0/2200 isoform=cb8564_c126704;full_length_coverage=1;non_full_length_coverage=0;isoform_length=2200;expected_accuracy=0.995	1	1686	1685	1961	1	1675	1924	2200
cb446_c16/f1p0/2478 isoform=cb446_c16;full_length_coverage=1;non_full_length_coverage=0;isoform_length=2478;expected_accur	gnl|BL_ORD_ID|72186 cb446_c49/f1p0/2765	758	2478	95	762	823	2543	5	672

acy=1.000									
cb3554_c10/f1p0/3346 isoform=cb3554_c10;full_length_coverage=1;non_full_length_coverage=0;isoform_length=3346;expected_accuracy=0.994	gnl|BL_ORD_ID|41040 cb3554_c8/f1p0/3574 isoform=cb3554_c8;full_length_coverage=1;non_full_length_coverage=0;isoform_length=3574;expected_accuracy=0.996	1	2402	2401	3298	1	2428	2671	3574
cb3011_c11/f1p0/3340 isoform=cb3011_c11;full_length_coverage=1;non_full_length_coverage=0;isoform_length=3340;expected_accuracy=0.999	gnl|BL_ORD_ID|38768 cb3011_c16/f1p0/3695 isoform=cb3011_c16;full_length_coverage=1;non_full_length_coverage=0;isoform_length=3695;expected_accuracy=0.998	1053	3340	1	1053	1329	3614	1	1048
cb1307_c23/f1p0/2549	gnl|BL_ORD_ID|61219 cb1307_c4/f1p0/4042	1	1534	1532	2466	1029	2563	2705	3634
cb4233_c77/f1p1/2209 isoform=cb4233_c77;full_length_coverage=1;non_full_length_coverage=1;isoform_length=2209;expected_accuracy=0.995	gnl|BL_ORD_ID|43581 cb4233_c85/f1p0/2730 isoform=cb4233_c85;full_length_coverage=1;non_full_length_coverage=0;isoform_length=2730;expected_accuracy=0.995	14	1710	1710	2120	454	2147	2318	2730
cb2229_c66/f60p1/2354 isoform=cb2229_c66;full_length_coverage=60;non_full_length_coverage=1;isoform_length=2354;expected_accuracy=0.999	gnl|BL_ORD_ID|38403 cb2229_c71/f3p0/3903 isoform=cb2229_c71;full_length_coverage=3;non_full_length_coverage=0;isoform_length=3903;expected_accuracy=1.000	1	1690	1687	2286	83	1774	3303	3903
cb2882_c2/f1p0/2136 isoform=cb2882_c2;full_length_coverage=1;non_full_length_coverage=0;isoform_length=2136;expected_accuracy=0.994	gnl|BL_ORD_ID|68972 cb2882_c4/f1p0/2356	178	2136	1	177	373	2331	79	255
cb8564_c36689/f1p1/2368 isoform=cb8564_c36689;full_length_coverage=1;non_full_length_coverage=1;isoform_length=2368;expected_	gnl|BL_ORD_ID|14410 cb8564_c44256/f1p1/3267 isoform=cb8564_c44256;full_length_coverage=1;non_full_length_coverage=1;isoform_length=3267;expected_ac	1	1709	1706	2368	1	1669	2605	3267

accuracy=0.991	curacy=0.995								
cb8564_c17422/f1p1/4151	gnl|BL_ORD_ID|13288 cb8564_c20074/f1p2/3928 isoform=cb8564_c20074;full_length_coverage=1;non_full_length_coverage=2;isoform_length=3928;expected_accuracy=0.995	24	3391	3389	3827	1	3368	3481	3919
cb8564_c13505/f1p0/3470	gnl|BL_ORD_ID|18192 cb8564_c9970/f2p1/3736 isoform=cb8564_c9970;full_length_coverage=2;non_full_length_coverage=1;isoform_length=3736;expected_accuracy=0.998	148	3445	1	148	440	3736	3	150
cb8526_c8/f1p0/1787	gnl|BL_ORD_ID|9319 cb8526_c1/f2p0/2143 isoform=cb8526_c1;full_length_coverage=2;non_full_length_coverage=0;isoform_length=2143;expected_accuracy=0.997	252	1782	37	254	610	2141	20	238
cb8564_c16914/f2p0/3868 isoform=cb8564_c16914;full_length_coverage=2;non_full_length_coverage=0;isoform_length=3868;expected_accuracy=1.000	gnl|BL_ORD_ID|34463 cb18696_c0/f1p1/5162 isoform=cb18696_c0;full_length_coverage=1;non_full_length_coverage=1;isoform_length=5162;expected_accuracy=0.999	1	3274	3274	3868	1	3272	3690	4283
cb8564_c111632/f1p0/2604 isoform=cb8564_c111632;full_length_coverage=1;non_full_length_coverage=0;isoform_length=2604;expected_accuracy=0.991	gnl|BL_ORD_ID|16522 cb8564_c74617/f7p2/3311 isoform=cb8564_c74617;full_length_coverage=7;non_full_length_coverage=2;isoform_length=3311;expected_accuracy=1.000	1	1688	1688	2604	188	1866	2403	3311
cb8564_c43546/f1p1/3276	gnl|BL_ORD_ID|16172 cb8564_c53024/f1p0/3159 isoform=cb8564_c53024;full_length_coverage=1;non_full_length_coverage=0;isoform_length=3159;expected_accuracy=0.996	10	1731	1728	2854	1	1699	2017	3142
cb8564_c15230/f1p0/1944	gnl|BL_ORD_ID|55239 cb8564_c36145/f1p0/2868	352	1944	5	357	1063	265	1	353

							3		
cb8564_c87624/f1p0/2450	gnl|BL_ORD_ID|58108 cb8564_c89073/f1p0/3953	1	1650	1649	2405	57	1706	3206	3962
cb8564_c3087/f1p0/2090 isoform=cb8564_c3087;full_length_coverage=1;non_full_length_coverage=0;isoform_length=2090;expected_accuracy=1.000	gnl|BL_ORD_ID|53663 cb8564_c151176/f1p0/2365	13	1914	1909	2090	1	1906	2012	2187
cb8564_c69975/f1p0/2818 isoform=cb8564_c69975;full_length_coverage=1;non_full_length_coverage=0;isoform_length=2818;expected_accuracy=0.999	gnl|BL_ORD_ID|16997 cb8564_c75534/f1p0/3028 isoform=cb8564_c75534;full_length_coverage=1;non_full_length_coverage=0;isoform_length=3028;expected_accuracy=0.994	260	2818	2	258	429	3012	1	267
cb2263_c4/f2p0/2476 isoform=cb2263_c4;full_length_coverage=2;non_full_length_coverage=0;isoform_length=2476;expected_accuracy=0.994	gnl|BL_ORD_ID|36791 cb2263_c5/f1p0/3994 isoform=cb2263_c5;full_length_coverage=1;non_full_length_coverage=0;isoform_length=3994;expected_accuracy=0.998	1	1406	1407	2476	25	1436	2457	3526
cb8564_c148768/f2p6/2332 isoform=cb8564_c148768;full_length_coverage=2;non_full_length_coverage=6;isoform_length=2332;expected_accuracy=0.996	gnl|BL_ORD_ID|56180 cb8564_c71615/f1p1/4167	1	1874	1874	2243	2	1878	3590	3959
cb8564_c42814/f1p0/2151 isoform=cb8564_c42814;full_length_coverage=1;non_full_length_coverage=0;isoform_length=2151;expected_accuracy=0.993	gnl|BL_ORD_ID|56624 cb8564_c49356/f1p0/3133	650	2151	7	652	1329	2850	1	641
cb4758_c4/f1p0/2881 isoform=cb4758_c4;full_length_coverage=1;non_full_length_coverage=0;isoform_length=2881;expected_accur	gnl|BL_ORD_ID|71991 cb4758_c5/f1p0/3624	1	2263	2264	2881	368	2635	2748	3366

acy=0.995									
cb1468_c1/f4p2/2905 isoform=cb1468_c1;full_length_coverage=4;non_full_length_coverage=2;isoform_length=2905;expected_accuracy=1.000	gnl|BL_ORD_ID|63645 cb1468_c3/f1p0/4312	1	2049	2048	2860	94	2140	3043	3855
cb8564_c37147/f1p0/2262 isoform=cb8564_c37147;full_length_coverage=1;non_full_length_coverage=0;isoform_length=2262;expected_accuracy=0.998	gnl|BL_ORD_ID|56693 cb8564_c52693/f2p0/3348	294	2262	5	296	1069	3030	5	294
cb8564_c15653/f1p4/2475	gnl|BL_ORD_ID|11316 cb8564_c12355/f1p9/2685 isoform=cb8564_c12355;full_length_coverage=1;non_full_length_coverage=9;isoform_length=2685;expected_accuracy=0.999	1	2293	2289	2434	1	2321	2540	2685
cb1767_c17/f1p1/2756 isoform=cb1767_c17;full_length_coverage=1;non_full_length_coverage=1;isoform_length=2756;expected_accuracy=0.995	gnl|BL_ORD_ID|32461 cb1767_c16/f1p0/2784 isoform=cb1767_c16;full_length_coverage=1;non_full_length_coverage=0;isoform_length=2784;expected_accuracy=0.995	1	1825	1823	2645	20	1846	1961	2784
cb1545_c26/f1p0/3338	gnl|BL_ORD_ID|63731 cb1545_c18/f1p0/3314	1	2013	2014	3212	115	2127	2011	3210
cb295_c53/f1p0/1719 isoform=cb295_c53;full_length_coverage=1;non_full_length_coverage=0;isoform_length=1719;expected_accuracy=0.994	gnl|BL_ORD_ID|69540 cb295_c48/f1p1/2137	1	1347	1345	1699	249	1598	1722	2075
cb14697_c4/f2p1/1690 isoform=cb14697_c4;full_length_coverage=2;non_full_length_coverage=1;isoform_length=1690;expected_accu	gnl|BL_ORD_ID|9229 cb8564_c120266/f1p0/2493 isoform=cb8564_c120266;full_length_coverage=1;non_full_length_coverage=0;isoform_length=2493;expected_a	641	1687	9	645	1447	2493	46	682

racy=0.995	ccuracy=0.997								
cb5359_c41/f1p0/1175	gnl|BL_ORD_ID|73821 cb5359_c20/f1p0/2586	125	1164	3	126	1432	2471	67	190
cb4493_c8/f1p0/2014 isoform=cb4493_c8;full_length_coverage=1;non_full_length_coverage=0;isoform_length=2014;expected_accuracy=0.991	gnl|BL_ORD_ID|72610 cb4493_c6/f1p0/3333	1	1023	1024	2010	239	1265	2103	3095
cb2818_c54/f1p1/2570	gnl|BL_ORD_ID|38592 cb2818_c9/f2p2/3193 isoform=cb2818_c9;full_length_coverage=2;non_full_length_coverage=2;isoform_length=3193;expected_accuracy=0.997	1	2142	2141	2547	1	2140	2607	3010
cb188_c3/f1p1/3195 isoform=cb188_c3;full_length_coverage=1;non_full_length_coverage=1;isoform_length=3195;expected_accuracy=0.997	gnl|BL_ORD_ID|68083 cb188_c1/f1p1/4971	1369	3161	1	1370	3054	4846	874	2243
cb8564_c20234/f1p0/3982 isoform=cb8564_c20234;full_length_coverage=1;non_full_length_coverage=0;isoform_length=3982;expected_accuracy=0.995	gnl|BL_ORD_ID|54245 cb8564_c21117/f1p0/4721	1215	3982	109	1220	1913	4683	1	1111
cb5643_c66/f1p0/2234 isoform=cb5643_c66;full_length_coverage=1;non_full_length_coverage=0;isoform_length=2234;expected_accuracy=0.996	gnl|BL_ORD_ID|46677 cb5643_c120/f3p1/2448 isoform=cb5643_c120;full_length_coverage=3;non_full_length_coverage=1;isoform_length=2448;expected_accuracy=1.000	201	2234	1	202	392	2425	26	226
cb8564_c54233/f1p1/3194 isoform=cb8564_c54233;full_length_coverage=1;non_full_length_coverage=1;isoform_length=3194;expected_	gnl|BL_ORD_ID|16149 cb8564_c47162/f1p0/3361 isoform=cb8564_c47162;full_length_coverage=1;non_full_length_coverage=0;isoform_length=3361;expected_ac	429	3194	8	430	592	3359	2	402

accuracy=0.995	curacy=0.996								
cb12805_c0/f2p0/1819 isoform=cb12805_c0;full_length_coverage=2;non_full_length_coverage=0;isoform_length=1819;expected_accuracy=0.993	gnl|BL_ORD_ID|54512 cb8564_c124861/f1p0/2013	1	1035	1034	1810	52	1086	1256	2032
cb18649_c3/f1p0/5011	gnl|BL_ORD_ID|69440 cb18649_c7/f1p0/5612	260	4791	1	260	751	5282	9	278
cb3978_c5/f1p0/3304 isoform=cb3978_c5;full_length_coverage=1;non_full_length_coverage=0;isoform_length=3304;expected_accuracy=0.998	gnl|BL_ORD_ID|39556 cb3978_c3/f2p0/5092 isoform=cb3978_c3;full_length_coverage=2;non_full_length_coverage=0;isoform_length=5092;expected_accuracy=1.000	1087	3193	1	1090	2987	5092	31	1128
cb8564_c14344/f1p0/2533 isoform=cb8564_c14344;full_length_coverage=1;non_full_length_coverage=0;isoform_length=2533;expected_accuracy=0.993	gnl|BL_ORD_ID|16004 cb8564_c48473/f1p2/2701 isoform=cb8564_c48473;full_length_coverage=1;non_full_length_coverage=2;isoform_length=2701;expected_accuracy=0.999	148	2525	1	151	322	2701	3	152
cb3733_c6/f1p1/1240	gnl|BL_ORD_ID|70573 cb3733_c4/f1p0/3433	419	1239	56	421	2574	3394	1	366
cb295_c13/f1p1/3878 isoform=cb295_c13;full_length_coverage=1;non_full_length_coverage=1;isoform_length=3878;expected_accuracy=0.994	gnl|BL_ORD_ID|34398 cb18646_c0/f2p2/6586 isoform=cb18646_c0;full_length_coverage=2;non_full_length_coverage=2;isoform_length=6586;expected_accuracy=0.999	1	3634	3633	3827	2503	6158	6391	6586
cb8564_c122994/f1p1/1940 isoform=cb8564_c122994;full_length_coverage=1;non_full_length_coverage=1;isoform_length=1940;expected_accuracy=1.000	gnl|BL_ORD_ID|11859 cb8564_c126680/f3p4/2272 isoform=cb8564_c126680;full_length_coverage=3;non_full_length_coverage=4;isoform_length=2272;expected_accuracy=0.996	1	1748	1748	1940	104	1849	1991	2184
cb6256_c6/f1p1/2316	gnl|BL_ORD_ID|5661 cb6256_c4/f2p2/2901	1137	2284	1	1137	1739	288	1	1140

isoform=cb6256_c6;full_length_coverage=1;non_full_length_coverage=1;isoform_length=2316;expected_accuracy=1.000	isoform=cb6256_c4;full_length_coverage=2;non_full_length_coverage=2;isoform_length=2901;expected_accuracy=0.997						7		
cb4768_c85/f1p0/1983 isoform=cb4768_c85;full_length_coverage=1;non_full_length_coverage=0;isoform_length=1983;expected_accuracy=1.000	gnl|BL_ORD_ID|63765 cb1590_c41/f1p0/3526	103	1966	1	106	1892	29	2312	2206
cb16569_c42/f1p1/1715 isoform=cb16569_c42;full_length_coverage=1;non_full_length_coverage=1;isoform_length=1715;expected_accuracy=1.000	gnl|BL_ORD_ID|19815 cb8916_c26/f1p0/1759 isoform=cb8916_c26;full_length_coverage=1;non_full_length_coverage=0;isoform_length=1759;expected_accuracy=0.999	202	1673	6	204	289	1759	3	187
cb7851_c2/f1p0/2210 isoform=cb7851_c2;full_length_coverage=1;non_full_length_coverage=0;isoform_length=2210;expected_accuracy=0.994	gnl|BL_ORD_ID|8230 cb7851_c3/f1p0/2493 isoform=cb7851_c3;full_length_coverage=1;non_full_length_coverage=0;isoform_length=2493;expected_accuracy=0.991	1	1304	1304	2201	1	1284	1598	2492
cb8564_c14344/f1p0/2533 isoform=cb8564_c14344;full_length_coverage=1;non_full_length_coverage=0;isoform_length=2533;expected_accuracy=0.993	gnl|BL_ORD_ID|55220 cb8564_c35645/f1p0/2804	148	2517	1	151	356	2726	1	151
cb1096_c4/f2p1/2193 isoform=cb1096_c4;full_length_coverage=2;non_full_length_coverage=1;isoform_length=2193;expected_accuracy=0.992	gnl|BL_ORD_ID|47346 cb1096_c13/f1p0/3282	1048	2118	1	1049	2230	3299	13	1064
cb8564_c37748/f1p0/3835 isoform=cb8564_c37748;full_length_coverage=1;non_full_length_coverage=0;isoform_length=3835;expected_	gnl|BL_ORD_ID|15378 cb8564_c37602/f2p1/3926 isoform=cb8564_c37602;full_length_coverage=2;non_full_length_coverage=1;isoform_length=3926;expected_ac	287	3732	1	286	396	3846	6	272

accuracy=0.995	curacy=0.999								
cb606_c5/f1p1/3393 isoform=cb606_c5;full_length_coverage=1;non_full_length_coverage=1;isoform_length=3393;expected_accuracy=0.998	gnl|BL_ORD_ID|73588 cb606_c11/f1p0/4621	1	3229	3228	3393	6	3238	3985	4150
cb5921_c65/f77p8/2262 isoform=cb5921_c65;full_length_coverage=77;non_full_length_coverage=8;isoform_length=2262;expected_accuracy=1.000	gnl|BL_ORD_ID|74654 cb5921_c44/f1p1/2444	1	2068	2069	2220	1	2070	2184	2336
cb8564_c24867/f1p3/2846	gnl|BL_ORD_ID|9350 cb8564_c11826/f1p0/2916 isoform=cb8564_c11826;full_length_coverage=1;non_full_length_coverage=0;isoform_length=2916;expected_accuracy=0.997	1288	2661	12	1289	1543	2916	23	1296
cb8564_c43399/f1p0/2654 isoform=cb8564_c43399;full_length_coverage=1;non_full_length_coverage=0;isoform_length=2654;expected_accuracy=0.999	gnl|BL_ORD_ID|55213 cb8564_c35568/f1p0/2997	1	2367	2363	2654	13	2385	2600	2890
cb6442_c10/f1p1/2623 isoform=cb6442_c10;full_length_coverage=1;non_full_length_coverage=1;isoform_length=2623;expected_accuracy=0.996	gnl|BL_ORD_ID|49727 cb6442_c3/f1p1/2761	1111	2578	1	1112	1235	2704	1	1117
cb2448_c3/f1p0/3231 isoform=cb2448_c3;full_length_coverage=1;non_full_length_coverage=0;isoform_length=3231;expected_accuracy=0.994	gnl|BL_ORD_ID|36831 cb2448_c2/f1p0/3926 isoform=cb2448_c2;full_length_coverage=1;non_full_length_coverage=0;isoform_length=3926;expected_accuracy=0.999	1	2664	2664	3194	17	2684	3397	3926
cb8564_c123093/f1p0/2918	gnl|BL_ORD_ID|10605 cb8564_c114675/f1p2/2937	1214	2924	1	1213	1109	281	5	122

<table>
<tr><td></td><td>isoform=cb8564_c114675;full_length_coverage=1;non_full_length_coverage=2;isoform_length=2937;expected_accuracy=0.998</td><td></td><td></td><td></td><td></td><td></td><td>9</td><td></td><td>5</td></tr>
<tr><td>cb4997_c18/f1p0/2327</td><td>gnl|BL_ORD_ID|73169 cb4997_c12/f1p1/2947</td><td>1</td><td>1547</td><td>1543</td><td>2091</td><td>16</td><td>1561</td><td>2383</td><td>2934</td></tr>
<tr><td>cb8564_c180116/f1p0/2944 isoform=cb8564_c180116;full_length_coverage=1;non_full_length_coverage=0;isoform_length=2944;expected_accuracy=0.996</td><td>gnl|BL_ORD_ID|55182 cb8564_c34825/f1p2/3200</td><td>14</td><td>2522</td><td>2520</td><td>2944</td><td>6</td><td>2542</td><td>2771</td><td>3192</td></tr>
<tr><td>cb8564_c38947/f1p0/2650 isoform=cb8564_c38947;full_length_coverage=1;non_full_length_coverage=0;isoform_length=2650;expected_accuracy=0.996</td><td>gnl|BL_ORD_ID|55213 cb8564_c35568/f1p0/2997</td><td>1</td><td>1441</td><td>1436</td><td>2650</td><td>13</td><td>1460</td><td>1677</td><td>2889</td></tr>
<tr><td>cb868_c17/f1p0/3361</td><td>gnl|BL_ORD_ID|57582 cb868_c5/f1p0/3523</td><td>1</td><td>1833</td><td>1833</td><td>3334</td><td>43</td><td>1875</td><td>1985</td><td>3486</td></tr>
<tr><td>cb5022_c10/f1p0/3124</td><td>gnl|BL_ORD_ID|73204 cb5022_c7/f1p0/3286</td><td>152</td><td>3028</td><td>11</td><td>153</td><td>275</td><td>3152</td><td>3</td><td>144</td></tr>
<tr><td>cb12972_c13/f1p0/1732</td><td>gnl|BL_ORD_ID|14141 cb8564_c2622/f1p1/2613 isoform=cb8564_c2622;full_length_coverage=1;non_full_length_coverage=1;isoform_length=2613;expected_accuracy=0.996</td><td>165</td><td>1725</td><td>1</td><td>166</td><td>1054</td><td>2612</td><td>39</td><td>204</td></tr>
<tr><td>cb13565_c65/f1p4/1614 isoform=cb13565_c65;full_length_coverage=1;non_full_length_coverage=4;isoform_length=1614;expected_accuracy=1.000</td><td>gnl|BL_ORD_ID|4515 cb6452_c4/f1p3/2166 isoform=cb6452_c4;full_length_coverage=1;non_full_length_coverage=3;isoform_length=2166;expected_accuracy=1.000</td><td>435</td><td>1614</td><td>61</td><td>434</td><td>948</td><td>2129</td><td>40</td><td>414</td></tr>
<tr><td>cb9448_c0/f7p1/1767</td><td>gnl|BL_ORD_ID|58727 cb9448_c9/f1p1/1806</td><td>547</td><td>1767</td><td>85</td><td>546</td><td>582</td><td>180</td><td>1</td><td>460</td></tr>
</table>

isoform=cb9448_c0;full_length_coverage=7;non_full_length_coverage=1;isoform_length=1767;expected_accuracy=1.000							2		
cb10045_c38/f10p4/1463 isoform=cb10045_c38;full_length_coverage=10;non_full_length_coverage=4;isoform_length=1463;expected_accuracy=1.000	gnl|BL_ORD_ID|1795 cb10045_c18/f1p2/1574 isoform=cb10045_c18;full_length_coverage=1;non_full_length_coverage=2;isoform_length=1574;expected_accuracy=1.000	141	1463	13	141	251	1573	1	129
cb288_c0/f4p0/4339 isoform=cb288_c0;full_length_coverage=4;non_full_length_coverage=0;isoform_length=4339;expected_accuracy=1.000	gnl|BL_ORD_ID|68165 cb288_c3/f1p0/4898	176	4339	13	175	637	4800	1	162
cb9788_c36/f1p0/1958	gnl|BL_ORD_ID|58889 cb9788_c16/f1p0/3980	13	1340	1339	1901	1	1322	3000	3556
cb7935_c3/f1p0/2325	gnl|BL_ORD_ID|51636 cb7935_c1/f1p0/2562	1	1764	1762	2335	4	1769	1933	2506
cb8564_c15654/f1p1/3152	gnl|BL_ORD_ID|11704 cb8564_c15268/f1p0/3312 isoform=cb8564_c15268;full_length_coverage=1;non_full_length_coverage=0;isoform_length=3312;expected_accuracy=0.998	118	2774	2773	3167	1	2656	2900	3294
cb7209_c6/f1p0/2322 isoform=cb7209_c6;full_length_coverage=1;non_full_length_coverage=0;isoform_length=2322;expected_accuracy=0.999	gnl|BL_ORD_ID|8776 cb7209_c18/f1p1/2480 isoform=cb7209_c18;full_length_coverage=1;non_full_length_coverage=1;isoform_length=2480;expected_accuracy=0.999	1	1583	1580	2322	25	1600	1729	2471
cb8564_c116193/f1p0/2609	gnl|BL_ORD_ID|15835 cb8564_c73664/f5p3/2665 isoform=cb8564_c73664;full_length_coverage=5;non_full_length_coverage=3;isoform_length=2665;expected_ac	2	2262	2258	2658	1	2276	2141	2542

<table>
<tr><td></td><td></td><td></td><td></td><td></td><td></td><td></td><td></td><td></td><td></td></tr>
<tr><td></td><td>curacy=0.994</td><td></td><td></td><td></td><td></td><td></td><td></td><td></td><td></td></tr>
<tr><td>cb8564_c36134/f1p0/3837</td><td>gnl|BL_ORD_ID|14498 cb8564_c35468/f1p0/4536 isoform=cb8564_c35468;full_length_coverage=1;non_full_length_coverage=0;isoform_length=4536;expected_accuracy=0.995</td><td>1416</td><td>3840</td><td>1</td><td>1415</td><td>1929</td><td>4350</td><td>10</td><td>1424</td></tr>
<tr><td>cb8564_c46939/f1p1/2926</td><td>gnl|BL_ORD_ID|16886 cb8564_c50727/f1p0/3201 isoform=cb8564_c50727;full_length_coverage=1;non_full_length_coverage=0;isoform_length=3201;expected_accuracy=0.999</td><td>1</td><td>2134</td><td>2130</td><td>2870</td><td>3</td><td>2169</td><td>2274</td><td>3015</td></tr>
<tr><td>cb1088_c12/f1p0/2593</td><td>gnl|BL_ORD_ID|1950 cb1088_c5/f1p0/3109 isoform=cb1088_c5;full_length_coverage=1;non_full_length_coverage=0;isoform_length=3109;expected_accuracy=0.995</td><td>1</td><td>1648</td><td>1650</td><td>2426</td><td>175</td><td>1820</td><td>2334</td><td>3109</td></tr>
<tr><td>cb1438_c12/f1p0/3268</td><td>gnl|BL_ORD_ID|30573 cb1438_c3/f3p3/4154 isoform=cb1438_c3;full_length_coverage=3;non_full_length_coverage=3;isoform_length=4154;expected_accuracy=1.000</td><td>1435</td><td>3296</td><td>1</td><td>1439</td><td>2221</td><td>4082</td><td>540</td><td>1980</td></tr>
<tr><td>cb965_c9/f2p4/4285 isoform=cb965_c9;full_length_coverage=2;non_full_length_coverage=4;isoform_length=4285;expected_accuracy=0.999</td><td>gnl|BL_ORD_ID|19288 cb965_c14/f1p0/4481 isoform=cb965_c14;full_length_coverage=1;non_full_length_coverage=0;isoform_length=4481;expected_accuracy=0.998</td><td>2086</td><td>4285</td><td>2</td><td>2090</td><td>2225</td><td>4426</td><td>1</td><td>2083</td></tr>
<tr><td>cb8895_c5/f1p0/1953</td><td>gnl|BL_ORD_ID|58442 cb8895_c4/f1p0/2063</td><td>243</td><td>1916</td><td>55</td><td>244</td><td>343</td><td>2014</td><td>25</td><td>214</td></tr>
<tr><td>cb8564_c13084/f1p2/3143 isoform=cb8564_c13084;full_length_coverage=1;non_full_length_coverage=2;isoform_length=3143;expected_</td><td>gnl|BL_ORD_ID|17146 cb8564_c9739/f5p4/3281 isoform=cb8564_c9739;full_length_coverage=5;non_full_length_coverage=4;isoform_length=3281;expected_ac</td><td>1365</td><td>3143</td><td>11</td><td>1365</td><td>1474</td><td>3252</td><td>1</td><td>1354</td></tr>
</table>

accuracy=0.996	curacy=0.996								
cb8564_c46939/f1p1/2926	gnl|BL_ORD_ID|14471 cb8564_c34518/f1p0/3136 isoform=cb8564_c34518;full_length_coverage=1;non_full_length_coverage=0;isoform_length=3136;expected_accuracy=0.991	1	2134	2130	2870	1	2115	2219	2954
cb1508_c6/f1p1/1904	gnl|BL_ORD_ID|63691 cb1508_c5/f1p0/1961	371	1845	42	372	478	1953	5	335
cb6518_c7/f1p0/2783	gnl|BL_ORD_ID|5044 cb6518_c3/f2p0/3835 isoform=cb6518_c3;full_length_coverage=2;non_full_length_coverage=0;isoform_length=3835;expected_accuracy=0.999	1	1809	1807	2706	12	1826	2932	3835
cb8564_c181853/f1p0/2119 isoform=cb8564_c181853;full_length_coverage=1;non_full_length_coverage=0;isoform_length=2119;expected_accuracy=0.997	gnl|BL_ORD_ID|15315 cb8564_c39014/f1p0/3019 isoform=cb8564_c39014;full_length_coverage=1;non_full_length_coverage=0;isoform_length=3019;expected_accuracy=0.992	1	1339	1334	2116	1	1356	2213	3013
cb6691_c13/f1p0/2308	gnl|BL_ORD_ID|3563 cb6691_c6/f2p0/2298 isoform=cb6691_c6;full_length_coverage=2;non_full_length_coverage=0;isoform_length=2298;expected_accuracy=1.000	1010	2135	1	1010	1115	2240	7	1015
cb16430_c1/f1p0/1766 isoform=cb16430_c1;full_length_coverage=1;non_full_length_coverage=0;isoform_length=1766;expected_accuracy=0.996	gnl|BL_ORD_ID|6757 cb7986_c9/f1p0/2279 isoform=cb7986_c9;full_length_coverage=1;non_full_length_coverage=0;isoform_length=2279;expected_accuracy=1.000	414	1766	7	417	935	2276	303	712
cb8564_c23232/f1p3/2540	gnl|BL_ORD_ID|54044 cb8564_c17331/f1p0/3185	16	2172	2167	2517	39	2196	2298	2648
cb8564_c10007/f3p0/2883	gnl|BL_ORD_ID|11423 cb8564_c12647/f1p0/3038	1	2113	2112	2883	48	216	2262	303

isoform=cb8564_c10007;full_length_coverage=3;non_full_length_coverage=0;isoform_length=2883;expected_accuracy=1.000	isoform=cb8564_c12647;full_length_coverage=1;non_full_length_coverage=0;isoform_length=3038;expected_accuracy=1.000						2		3
cb5471_c11/f1p0/2489	gnl|BL_ORD_ID|45003 cb5471_c7/f1p0/3091 isoform=cb5471_c7;full_length_coverage=1;non_full_length_coverage=0;isoform_length=3091;expected_accuracy=0.997	1148	2555	1	1147	1267	2721	1	1146
cb8564_c181853/f1p0/2119 isoform=cb8564_c181853;full_length_coverage=1;non_full_length_coverage=0;isoform_length=2119;expected_accuracy=0.997	gnl|BL_ORD_ID|15496 cb8564_c47853/f1p0/2927 isoform=cb8564_c47853;full_length_coverage=1;non_full_length_coverage=0;isoform_length=2927;expected_accuracy=0.991	1	1339	1334	2116	1	1326	2149	2927
cb4674_c10/f2p0/2688 isoform=cb4674_c10;full_length_coverage=2;non_full_length_coverage=0;isoform_length=2688;expected_accuracy=1.000	gnl|BL_ORD_ID|72846 cb4674_c72/f1p0/2929	2	2046	2047	2688	19	2036	2215	2870
cb7016_c17/f1p0/3309	gnl|BL_ORD_ID|50253 cb7016_c9/f1p0/3305	1	1955	1954	3179	35	1993	2100	3325
cb8564_c43779/f1p0/3021 isoform=cb8564_c43779;full_length_coverage=1;non_full_length_coverage=0;isoform_length=3021;expected_accuracy=0.993	gnl|BL_ORD_ID|55912 cb8564_c48681/f1p0/3431	1	2345	2345	3019	1	2340	2543	3216
cb1138_c15/f1p6/3365 isoform=cb1138_c15;full_length_coverage=1;non_full_length_coverage=6;isoform_length=3365;expected_accuracy=0.998	gnl|BL_ORD_ID|59178 cb1138_c38/f1p0/3611	1	2847	2842	3324	1	2842	2946	3429
cb8564_c89404/f1p1/3577	gnl|BL_ORD_ID|16525 cb8564_c74618/f1p0/3734	1	2495	2490	3571	50	253	2650	373

isoform=cb8564_c89404;full_length_coverage=1;non_full_length_coverage=1;isoform_length=3577;expected_accuracy=0.993	isoform=cb8564_c74618;full_length_coverage=1;non_full_length_coverage=0;isoform_length=3734;expected_accuracy=0.998						6		4
cb8564_c157909/f1p2/2084 isoform=cb8564_c157909;full_length_coverage=1;non_full_length_coverage=2;isoform_length=2084;expected_accuracy=0.999	gnl|BL_ORD_ID|58073 cb8564_c88099/f1p2/2499	180	2084	1	179	428	2323	20	198
cb8617_c4/f1p0/2294 isoform=cb8617_c4;full_length_coverage=1;non_full_length_coverage=0;isoform_length=2294;expected_accuracy=1.000	gnl|BL_ORD_ID|17936 cb8617_c2/f1p0/2817 isoform=cb8617_c2;full_length_coverage=1;non_full_length_coverage=0;isoform_length=2817;expected_accuracy=0.991	1	1463	1462	2197	38	1503	2084	2817
cb2561_c10/f1p0/3960	gnl|BL_ORD_ID|68615 cb2561_c8/f1p0/3657	120	2363	2362	3551	1	2247	2365	3551
cb8564_c2621/f1p1/2401 isoform=cb8564_c2621;full_length_coverage=1;non_full_length_coverage=1;isoform_length=2401;expected_accuracy=1.000	gnl|BL_ORD_ID|14141 cb8564_c2622/f1p1/2613 isoform=cb8564_c2622;full_length_coverage=1;non_full_length_coverage=1;isoform_length=2613;expected_accuracy=0.996	1141	2401	1	1146	1303	2561	6	1150
cb4123_c103/f1p0/1158	gnl|BL_ORD_ID|41009 cb4123_c54/f1p3/1372 isoform=cb4123_c54;full_length_coverage=1;non_full_length_coverage=3;isoform_length=1372;expected_accuracy=1.000	292	1178	9	297	463	1350	1	289
cb5174_c10/f1p1/2391 isoform=cb5174_c10;full_length_coverage=1;non_full_length_coverage=1;isoform_length=2391;expected_accuracy=0.994	gnl|BL_ORD_ID|73354 cb5174_c12/f1p0/2862	133	2391	1	134	605	2862	1	134
cb309_c19/f1p0/1785	gnl|BL_ORD_ID|38927 cb309_c3/f2p2/4443	1	1335	1336	1785	2406	374	3956	440

isoform=cb309_c19;full_length_coverage=1;non_full_length_coverage=0;isoform_length=1785;expected_accuracy=0.996	isoform=cb309_c3;full_length_coverage=2;non_full_length_coverage=2;isoform_length=4443;expected_accuracy=1.000						0		5
cb8564_c21439/f1p0/2587	gnl|BL_ORD_ID|12770 cb8564_c160685/f1p8/2891 isoform=cb8564_c160685;full_length_coverage=1;non_full_length_coverage=8;isoform_length=2891;expected_accuracy=0.993	1226	2572	1	1228	1352	2698	1	1231
cb8118_c4/f2p0/1036 isoform=cb8118_c4;full_length_coverage=2;non_full_length_coverage=0;isoform_length=1036;expected_accuracy=1.000	gnl|BL_ORD_ID|7307 cb8118_c3/f3p0/2457 isoform=cb8118_c3;full_length_coverage=3;non_full_length_coverage=0;isoform_length=2457;expected_accuracy=0.999	105	1030	3	105	1530	2454	20	116
cb8564_c178226/f1p0/2728 isoform=cb8564_c178226;full_length_coverage=1;non_full_length_coverage=0;isoform_length=2728;expected_accuracy=0.998	gnl|BL_ORD_ID|56624 cb8564_c49356/f1p0/3133	1284	2728	1	1288	1387	2850	1	1283
cb8564_c45864/f1p0/2965	gnl|BL_ORD_ID|56381 cb8564_c75626/f1p2/3199	16	2115	2113	2601	16	2116	2611	3099
cb8564_c52246/f1p1/2752	gnl|BL_ORD_ID|12229 cb8564_c12514/f1p0/2626 isoform=cb8564_c12514;full_length_coverage=1;non_full_length_coverage=0;isoform_length=2626;expected_accuracy=0.997	1	1673	1671	2493	14	1679	1779	2626
cb8564_c73853/f1p0/2443 isoform=cb8564_c73853;full_length_coverage=1;non_full_length_coverage=0;isoform_length=2443;expected_accuracy=1.000	gnl|BL_ORD_ID|19163 cb8564_c79821/f1p0/2692 isoform=cb8564_c79821;full_length_coverage=1;non_full_length_coverage=0;isoform_length=2692;expected_accuracy=0.995	1	1281	1276	2352	82	1335	1615	2692
cb8564_c51376/f1p3/3179	gnl|BL_ORD_ID|15193 cb8564_c42308/f1p5/3192	765	3057	8	768	888	318	10	785

	isoform=cb8564_c42308;full_length_coverage=1;non_full_length_coverage=5;isoform_length=3192;expected_accuracy=0.995						7		
cb2929_c8/f1p0/1889 isoform=cb2929_c8;full_length_coverage=1;non_full_length_coverage=0;isoform_length=1889;expected_accuracy=0.998	gnl|BL_ORD_ID|38874 cb2929_c20/f2p4/2085 isoform=cb2929_c20;full_length_coverage=2;non_full_length_coverage=4;isoform_length=2085;expected_accuracy=1.000	1	1361	1357	1889	3	1371	1548	2080
cb2640_c3/f2p1/3206 isoform=cb2640_c3;full_length_coverage=2;non_full_length_coverage=1;isoform_length=3206;expected_accuracy=0.998	gnl|BL_ORD_ID|68659 cb2640_c11/f1p0/3319	334	3205	9	335	459	3331	1	353
cb8564_c147009/f3p4/1975 isoform=cb8564_c147009;full_length_coverage=3;non_full_length_coverage=4;isoform_length=1975;expected_accuracy=1.000	gnl|BL_ORD_ID|55274 cb8564_c37068/f1p0/2402	251	1974	73	250	454	2178	9	187
cb6719_c84/f32p1/2539 isoform=cb6719_c84;full_length_coverage=32;non_full_length_coverage=1;isoform_length=2539;expected_accuracy=1.000	gnl|BL_ORD_ID|4663 cb6719_c20/f1p0/3262 isoform=cb6719_c20;full_length_coverage=1;non_full_length_coverage=0;isoform_length=3262;expected_accuracy=0.996	1	1472	1471	2493	647	2119	2240	3262
cb8564_c110147/f1p0/3399 isoform=cb8564_c110147;full_length_coverage=1;non_full_length_coverage=0;isoform_length=3399;expected_accuracy=0.999	gnl|BL_ORD_ID|11650 cb8564_c152526/f1p6/3802 isoform=cb8564_c152526;full_length_coverage=1;non_full_length_coverage=6;isoform_length=3802;expected_accuracy=0.999	1	2986	2987	3399	9	3015	3282	3694
cb8564_c40598/f1p4/3119	gnl|BL_ORD_ID|57017 cb8564_c80370/f1p0/3493	121	2732	2729	3055	3085	474	353	27
cb8564_c14758/f1p0/4100	gnl|BL_ORD_ID|52768 cb8564_c13351/f1p2/4615	2	3050	3046	3773	4	3047	3910	4637

cb8564_c48153/f1p0/3083	gnl|BL_ORD_ID|16879 cb8564_c48606/f1p1/3308 isoform=cb8564_c48606;full_length_coverage=1;non_full_length_coverage=1;isoform_length=3308;expected_accuracy=0.998	13	2162	2158	2879	1	2151	2587	3308
cb2686_c3/f1p0/2597	gnl|BL_ORD_ID|38040 cb2686_c5/f1p0/3171 isoform=cb2686_c5;full_length_coverage=1;non_full_length_coverage=0;isoform_length=3171;expected_accuracy=0.994	1	1688	1688	2590	1	1687	2284	3170
cb2737_c64/f1p1/2838 isoform=cb2737_c64;full_length_coverage=1;non_full_length_coverage=1;isoform_length=2838;expected_accuracy=0.993	gnl|BL_ORD_ID|38134 cb2737_c22/f1p0/3196 isoform=cb2737_c22;full_length_coverage=1;non_full_length_coverage=0;isoform_length=3196;expected_accuracy=0.995	1	1537	1535	2838	251	1763	1894	3167
cb8564_c44698/f1p5/2843 isoform=cb8564_c44698;full_length_coverage=1;non_full_length_coverage=5;isoform_length=2843;expected_accuracy=0.997	gnl|BL_ORD_ID|15496 cb8564_c47853/f1p0/2927 isoform=cb8564_c47853;full_length_coverage=1;non_full_length_coverage=0;isoform_length=2927;expected_accuracy=0.991	1	2516	2511	2826	1	2494	2614	2927
cb17420_c1/f1p1/1860 isoform=cb17420_c1;full_length_coverage=1;non_full_length_coverage=1;isoform_length=1860;expected_accuracy=1.000	gnl|BL_ORD_ID|55322 cb8564_c38387/f1p0/2341	549	1860	9	552	657	1968	18	555
cb8564_c45787/f1p3/3310	gnl|BL_ORD_ID|15125 cb8564_c45003/f1p0/4015 isoform=cb8564_c45003;full_length_coverage=1;non_full_length_coverage=0;isoform_length=4015;expected_accuracy=0.996	1012	3270	100	1011	1756	4015	4	915
cb8564_c40764/f1p0/2563 isoform=cb8564_c40764;full_length_coverage=1;non_f	gnl|BL_ORD_ID|13976 cb8564_c43401/f1p2/3025 isoform=cb8564_c43401;full_length_coverage=1;non_fu	1	2167	2162	2563	1	2226	2437	2841

ull_length_coverage=0;isoform_length=2563;expected_accuracy=0.997	ll_length_coverage=2;isoform_length=3025;expected_accuracy=0.995								
cb1306_c36/f1p0/2164 isoform=cb1306_c36;full_length_coverage=1;non_full_length_coverage=0;isoform_length=2164;expected_accuracy=1.000	gnl|BL_ORD_ID|25326 cb1306_c10/f1p1/3298 isoform=cb1306_c10;full_length_coverage=1;non_full_length_coverage=1;isoform_length=3298;expected_accuracy=1.000	1	1793	1788	2164	20	1808	2733	3109
cb3747_c11/f1p0/2520 isoform=cb3747_c11;full_length_coverage=1;non_full_length_coverage=0;isoform_length=2520;expected_accuracy=0.994	gnl|BL_ORD_ID|39404 cb3747_c23/f7p0/2837 isoform=cb3747_c23;full_length_coverage=7;non_full_length_coverage=0;isoform_length=2837;expected_accuracy=0.998	1	1743	1742	2520	58	1825	2058	2837
cb12_c40/f1p0/4680	gnl|BL_ORD_ID|61079 cb12_c11/f1p2/4713	470	4507	94	475	659	4704	2	383
cb8564_c50881/f1p2/2125 isoform=cb8564_c50881;full_length_coverage=1;non_full_length_coverage=2;isoform_length=2125;expected_accuracy=0.994	gnl|BL_ORD_ID|55471 cb8564_c42491/f1p0/2564	1	1863	1858	2112	1	1858	2135	2392
cb8564_c107586/f11p1/4059 isoform=cb8564_c107586;full_length_coverage=11;non_full_length_coverage=1;isoform_length=4059;expected_accuracy=1.000	gnl|BL_ORD_ID|3345 cb10480_c36/f1p0/5122 isoform=cb10480_c36;full_length_coverage=1;non_full_length_coverage=0;isoform_length=5122;expected_accuracy=0.997	1	2878	2876	3969	22	2903	4031	5122
cb8564_c40671/f1p0/2871 isoform=cb8564_c40671;full_length_coverage=1;non_full_length_coverage=0;isoform_length=2871;expected_accuracy=0.998	gnl|BL_ORD_ID|55939 cb8564_c49630/f1p4/3487	101	2615	2615	2865	398	2913	3243	3493
cb8564_c14548/f1p0/3030	gnl|BL_ORD_ID|14058 cb8564_c23936/f1p0/3016 isoform=cb8564_c23936;full_length_coverage=1;non_fu	13	1725	1723	2573	4	1716	2143	3016

	ll_length_coverage=0;isoform_length=3016;expected_accuracy=0.995								
cb10695_c2/f1p0/1166 isoform=cb10695_c2;full_length_coverage=1;non_full_length_coverage=0;isoform_length=1166;expected_accuracy=1.000	gnl|BL_ORD_ID|751 cb10695_c1/f1p0/1931 isoform=cb10695_c1;full_length_coverage=1;non_full_length_coverage=0;isoform_length=1931;expected_accuracy=1.000	551	1166	7	556	1251	1866	75	622
cb781_c3/f2p0/4499 isoform=cb781_c3;full_length_coverage=2;non_full_length_coverage=0;isoform_length=4499;expected_accuracy=0.996	gnl|BL_ORD_ID|49464 cb781_c6/f1p1/4637	160	4499	1	162	277	4614	17	177
cb8564_c50128/f1p0/4343	gnl|BL_ORD_ID|55207 cb8564_c35399/f1p0/4553	297	4077	24	301	400	4180	11	281
cb944_c33/f1p1/3119 isoform=cb944_c33;full_length_coverage=1;non_full_length_coverage=1;isoform_length=3119;expected_accuracy=1.000	gnl|BL_ORD_ID|20927 cb944_c35/f1p0/3736 isoform=cb944_c35;full_length_coverage=1;non_full_length_coverage=0;isoform_length=3736;expected_accuracy=0.999	1	2454	2452	3119	27	2462	3060	3715
cb4123_c39/f1p0/1394 isoform=cb4123_c39;full_length_coverage=1;non_full_length_coverage=0;isoform_length=1394;expected_accuracy=1.000	gnl|BL_ORD_ID|41229 cb4123_c16/f2p0/1482 isoform=cb4123_c16;full_length_coverage=2;non_full_length_coverage=0;isoform_length=1482;expected_accuracy=1.000	287	1367	7	286	401	1481	11	286
cb8564_c46939/f1p1/2926	gnl|BL_ORD_ID|53680 cb8564_c153222/f2p39/3454	1	2134	2130	2870	7	2150	2255	2996
cb5659_c78/f1p0/2309	gnl|BL_ORD_ID|47091 cb5659_c59/f1p6/2922 isoform=cb5659_c59;full_length_coverage=1;non_full_length_coverage=6;isoform_length=2922;expected_accuracy=0.997	105	1676	1675	2137	1	1571	2151	2614

cb8564_c117302/f1p0/2004	gnl|BL_ORD_ID|11826 cb8564_c125000/f1p0/2204 isoform=cb8564_c125000;full_length_coverage=1;non_full_length_coverage=0;isoform_length=2204;expected_accuracy=0.997	433	2006	55	433	610	2183	1	379
cb8564_c44698/f1p5/2843 isoform=cb8564_c44698;full_length_coverage=1;non_full_length_coverage=5;isoform_length=2843;expected_accuracy=0.997	gnl|BL_ORD_ID|14529 cb8564_c44074/f1p1/2922 isoform=cb8564_c44074;full_length_coverage=1;non_full_length_coverage=1;isoform_length=2922;expected_accuracy=0.994	1	2515	2511	2830	1	2486	2605	2922
cb3229_c19/f1p0/2319 isoform=cb3229_c19;full_length_coverage=1;non_full_length_coverage=0;isoform_length=2319;expected_accuracy=0.998	gnl|BL_ORD_ID|39753 cb3229_c3/f2p0/2593 isoform=cb3229_c3;full_length_coverage=2;non_full_length_coverage=0;isoform_length=2593;expected_accuracy=0.997	262	2271	7	261	583	2593	1	258
cb2533_c15/f1p0/2210 isoform=cb2533_c15;full_length_coverage=1;non_full_length_coverage=0;isoform_length=2210;expected_accuracy=0.998	gnl|BL_ORD_ID|38794 cb2533_c9/f1p0/3092 isoform=cb2533_c9;full_length_coverage=1;non_full_length_coverage=0;isoform_length=3092;expected_accuracy=1.000	1	1179	1177	2209	6	1186	2060	3092
cb8564_c39136/f1p0/3036 isoform=cb8564_c39136;full_length_coverage=1;non_full_length_coverage=0;isoform_length=3036;expected_accuracy=0.998	gnl|BL_ORD_ID|16681 cb8564_c54233/f1p1/3194 isoform=cb8564_c54233;full_length_coverage=1;non_full_length_coverage=1;isoform_length=3194;expected_accuracy=0.995	2	2089	2085	3032	8	2118	2245	3194
cb3584_c13/f1p0/2550	gnl|BL_ORD_ID|40786 cb3584_c10/f1p1/3385 isoform=cb3584_c10;full_length_coverage=1;non_full_length_coverage=1;isoform_length=3385;expected_accuracy=0.992	1	1613	1612	2533	1	1611	1713	2631
cb3542_c12/f1p0/3330	gnl|BL_ORD_ID|40875 cb3542_c2/f2p3/3510 isoform=cb3542_c2;full_length_coverage=2;non_full_le	1128	3296	1	1133	1345	3510	31	1163

	ngth_coverage=3;isoform_length=3510;expected_accuracy=0.997								
cb8564_c72680/f1p0/2685	gnl|BL_ORD_ID|52842 cb8564_c15468/f1p0/3015	1	1735	1730	2604	32	1766	1945	2819
cb8564_c38035/f1p0/2770	gnl|BL_ORD_ID|55433 cb8564_c41572/f1p1/3265	1	2035	2033	2756	1	2030	2219	2946
cb10912_c3/f1p0/1030 isoform=cb10912_c3;full_length_coverage=1;non_full_length_coverage=0;isoform_length=1030;expected_accuracy=0.997	gnl|BL_ORD_ID|2406 cb10912_c7/f1p0/1564 isoform=cb10912_c7;full_length_coverage=1;non_full_length_coverage=0;isoform_length=1564;expected_accuracy=1.000	220	1025	33	223	759	1564	1	195
cb8564_c148448/f2p13/3821 isoform=cb8564_c148448;full_length_coverage=2;non_full_length_coverage=13;isoform_length=3821;expected_accuracy=0.995	gnl|BL_ORD_ID|64326 cb14859_c10/f1p0/5042	108	1937	1935	3756	1	1830	3004	4825
cb8564_c88853/f1p0/2988	gnl|BL_ORD_ID|14537 cb8564_c36204/f3p0/3065 isoform=cb8564_c36204;full_length_coverage=3;non_full_length_coverage=0;isoform_length=3065;expected_accuracy=0.997	1324	2748	1	1328	1643	3065	50	1377
cb500_c53/f1p0/3672	gnl|BL_ORD_ID|72245 cb500_c44/f1p0/3962	114	1936	1935	3471	66	1888	2351	3887
cb3046_c12/f1p0/2338	gnl|BL_ORD_ID|37103 cb3046_c0/f6p0/2414 isoform=cb3046_c0;full_length_coverage=6;non_full_length_coverage=0;isoform_length=2414;expected_accuracy=1.000	1	2018	2014	2272	36	2053	2155	2412
cb8564_c45596/f1p1/3430	gnl|BL_ORD_ID|55405 cb8564_c40823/f1p2/3537	112	2780	2775	3379	1	2669	2788	3392

cb8564_c35567/f2p1/2669	gnl|BL_ORD_ID|15917 cb8564_c52863/f1p0/2866 isoform=cb8564_c52863;full_length_coverage=1;non_full_length_coverage=0;isoform_length=2866;expected_accuracy=0.997	1	1355	1355	2579	2	1352	1640	2866
cb12714_c29/f1p2/1583	gnl|BL_ORD_ID|13830 cb8564_c188097/f1p0/2552 isoform=cb8564_c188097;full_length_coverage=1;non_full_length_coverage=0;isoform_length=2552;expected_accuracy=0.996	1	1296	1293	1590	1	1296	2255	2552
cb3955_c12/f1p0/1586 isoform=cb3955_c12;full_length_coverage=1;non_full_length_coverage=0;isoform_length=1586;expected_accuracy=1.000	gnl|BL_ORD_ID|39173 cb3955_c11/f1p0/2530 isoform=cb3955_c11;full_length_coverage=1;non_full_length_coverage=0;isoform_length=2530;expected_accuracy=0.998	1	1275	1273	1586	22	1294	2152	2463
cb8564_c9854/f2p0/3197 isoform=cb8564_c9854;full_length_coverage=2;non_full_length_coverage=0;isoform_length=3197;expected_accuracy=1.000	gnl|BL_ORD_ID|13496 cb8564_c18927/f1p0/3935 isoform=cb8564_c18927;full_length_coverage=1;non_full_length_coverage=0;isoform_length=3935;expected_accuracy=0.996	1	2495	2490	3193	154	2636	3234	3935
cb8564_c10736/f3p10/3904 isoform=cb8564_c10736;full_length_coverage=3;non_full_length_coverage=10;isoform_length=3904;expected_accuracy=0.998	gnl|BL_ORD_ID|19170 cb8564_c9757/f2p7/4230 isoform=cb8564_c9757;full_length_coverage=2;non_full_length_coverage=7;isoform_length=4230;expected_accuracy=0.999	101	3264	3265	3904	3	3162	3573	4212
cb8564_c2223/f1p0/2047	gnl|BL_ORD_ID|53847 cb8564_c2458/f1p0/2930	434	2055	8	439	886	2507	12	443
cb8564_c51035/f2p2/4216 isoform=cb8564_c51035;full_length_coverage=2;non_full_length_coverage=2;isoform_length=4216;expected_accuracy=0.999	gnl|BL_ORD_ID|14314 cb8564_c45827/f2p2/4435 isoform=cb8564_c45827;full_length_coverage=2;non_full_length_coverage=2;isoform_length=4435;expected_accuracy=0.999	15	3261	3257	4160	1	3255	3520	4426

cb1333_c101/f1p0/1395 isoform=cb1333_c101;full_length_coverage=1;non_full_length_coverage=0;isoform_length=1395;expected_accuracy=0.997	gnl|BL_ORD_ID|26022 cb1333_c19/f2p1/4599 isoform=cb1333_c19;full_length_coverage=2;non_full_length_coverage=1;isoform_length=4599;expected_accuracy=0.999	146	1395	6	150	3314	4562	1	144
cb8564_c127627/f1p0/2239	gnl|BL_ORD_ID|54524 cb8564_c125248/f1p0/3365	166	2218	1	168	1048	3103	20	187
cb12446_c3/f1p0/1412 isoform=cb12446_c3;full_length_coverage=1;non_full_length_coverage=0;isoform_length=1412;expected_accuracy=1.000	gnl|BL_ORD_ID|54707 cb8564_c157184/f1p3/4810	109	1412	1	111	596	1900	1	111
cb3601_c21/f1p1/1026 isoform=cb3601_c21;full_length_coverage=1;non_full_length_coverage=1;isoform_length=1026;expected_accuracy=1.000	gnl|BL_ORD_ID|70508 cb3601_c11/f1p0/2518	187	991	1	189	1637	2447	96	282
cb5199_c13/f1p2/3718	gnl|BL_ORD_ID|73617 cb5199_c9/f1p0/4034	1	3184	3184	3700	1	3185	3461	3978
cb3143_c17/f1p0/2870 isoform=cb3143_c17;full_length_coverage=1;non_full_length_coverage=0;isoform_length=2870;expected_accuracy=0.993	gnl|BL_ORD_ID|37815 cb3143_c7/f1p1/3040 isoform=cb3143_c7;full_length_coverage=1;non_full_length_coverage=1;isoform_length=3040;expected_accuracy=1.000	162	2870	1	163	321	3021	21	182
cb938_c6/f2p5/3513 isoform=cb938_c6;full_length_coverage=2;non_full_length_coverage=5;isoform_length=3513;expected_accuracy=1.000	gnl|BL_ORD_ID|59101 cb938_c9/f1p0/4170	1	2565	2565	3466	1	2565	3262	4163
cb9549_c8/f1p0/1542 isoform=cb9549_c8;full_length_coverage=1;non_full_le	gnl|BL_ORD_ID|21588 cb9549_c0/f3p0/1849 isoform=cb9549_c0;full_length_coverage=3;non_full_le	114	1542	1	115	345	1777	60	174

ngth_coverage=0;isoform_length=1542;expected_accuracy=1.000	ngth_coverage=0;isoform_length=1849;expected_accuracy=0.999								
cb8564_c11675/f1p0/3333 isoform=cb8564_c11675;full_length_coverage=1;non_full_length_coverage=0;isoform_length=3333;expected_accuracy=0.999	gnl|BL_ORD_ID|54298 cb8564_c22041/f1p0/3578	1	1827	1824	3262	44	1872	2057	3497
cb8564_c90372/f1p0/2157 isoform=cb8564_c90372;full_length_coverage=1;non_full_length_coverage=0;isoform_length=2157;expected_accuracy=0.994	gnl|BL_ORD_ID|19195 cb8564_c90437/f1p0/2888 isoform=cb8564_c90437;full_length_coverage=1;non_full_length_coverage=0;isoform_length=2888;expected_accuracy=0.995	1	1444	1442	2157	91	1533	2133	2845
cb3213_c14/f1p0/2247	gnl|BL_ORD_ID|40431 cb3213_c4/f4p0/2414 isoform=cb3213_c4;full_length_coverage=4;non_full_length_coverage=0;isoform_length=2414;expected_accuracy=0.995	100	2198	1	100	316	2414	92	191
cb4258_c0/f4p0/2993 isoform=cb4258_c0;full_length_coverage=4;non_full_length_coverage=0;isoform_length=2993;expected_accuracy=1.000	gnl|BL_ORD_ID|43235 cb4258_c6/f1p0/3338 isoform=cb4258_c6;full_length_coverage=1;non_full_length_coverage=0;isoform_length=3338;expected_accuracy=0.999	147	2993	1	146	485	3331	18	162
cb5900_c97/f1p0/2618 isoform=cb5900_c97;full_length_coverage=1;non_full_length_coverage=0;isoform_length=2618;expected_accuracy=0.993	gnl|BL_ORD_ID|74605 cb5900_c77/f1p0/3189	1	1833	1830	2615	64	1886	2357	3143
cb13042_c4/f1p0/1356	gnl|BL_ORD_ID|25127 cb13042_c0/f7p0/1302 isoform=cb13042_c0;full_length_coverage=7;non_full_length_coverage=0;isoform_length=1302;expected_accuracy=1.000	321	1300	5	322	164	1146	1	307

cb3213_c12/f1p0/2190 isoform=cb3213_c12;full_length_coverage=1;non_full_length_coverage=0;isoform_length=2190;expected_accuracy=0.995	gnl|BL_ORD_ID|70971 cb3213_c21/f1p0/2529	100	2173	1	100	304	2384	80	179
cb12972_c38/f1p0/1837 isoform=cb12972_c38;full_length_coverage=1;non_full_length_coverage=0;isoform_length=1837;expected_accuracy=0.992	gnl|BL_ORD_ID|14141 cb8564_c2622/f1p1/2613 isoform=cb8564_c2622;full_length_coverage=1;non_full_length_coverage=1;isoform_length=2613;expected_accuracy=0.996	247	1837	45	249	1023	2611	1	205
cb8564_c113778/f1p0/2109 isoform=cb8564_c113778;full_length_coverage=1;non_full_length_coverage=0;isoform_length=2109;expected_accuracy=0.997	gnl|BL_ORD_ID|12337 cb8564_c129133/f1p1/2874 isoform=cb8564_c129133;full_length_coverage=1;non_full_length_coverage=1;isoform_length=2874;expected_accuracy=0.997	1	1144	1141	2109	148	1289	1905	2873
cb8564_c44698/f1p5/2843 isoform=cb8564_c44698;full_length_coverage=1;non_full_length_coverage=5;isoform_length=2843;expected_accuracy=0.997	gnl|BL_ORD_ID|56719 cb8564_c54250/f1p3/3063	1	2516	2511	2827	1	2512	2682	3000
cb4819_c0/f3p0/1276 isoform=cb4819_c0;full_length_coverage=3;non_full_length_coverage=0;isoform_length=1276;expected_accuracy=1.000	gnl|BL_ORD_ID|44405 cb4819_c1/f2p0/3178 isoform=cb4819_c1;full_length_coverage=2;non_full_length_coverage=0;isoform_length=3178;expected_accuracy=1.000	357	1276	57	359	2257	3176	1	303
cb2488_c1/f1p0/3123 isoform=cb2488_c1;full_length_coverage=1;non_full_length_coverage=0;isoform_length=3123;expected_accuracy=0.993	gnl|BL_ORD_ID|38533 cb2533_c16/f1p0/4039 isoform=cb2533_c16;full_length_coverage=1;non_full_length_coverage=0;isoform_length=4039;expected_accuracy=0.994	674	3123	89	675	2836	386	4039	3451
cb297_c7/f1p0/4453 isoform=cb297_c7;full_length_coverage=1;non_full_len	gnl|BL_ORD_ID|36751 cb297_c16/f3p1/4779 isoform=cb297_c16;full_length_coverage=3;non_full_le	2	2873	2872	4453	43	2912	3196	4779

gth_coverage=0;isoform_length=4453;expected_accuracy=0.997	ngth_coverage=1;isoform_length=4779;expected_accuracy=0.998								
cb5382_c3/f1p0/2584 isoform=cb5382_c3;full_length_coverage=1;non_full_length_coverage=0;isoform_length=2584;expected_accuracy=0.995	gnl|BL_ORD_ID|44422 cb5382_c2/f1p0/2750 isoform=cb5382_c2;full_length_coverage=1;non_full_length_coverage=0;isoform_length=2750;expected_accuracy=1.000	1117	2584	1	1118	1236	2707	17	1134
cb7519_c21/f1p0/2280 isoform=cb7519_c21;full_length_coverage=1;non_full_length_coverage=0;isoform_length=2280;expected_accuracy=0.994	gnl|BL_ORD_ID|50569 cb7519_c6/f1p0/2438	11	1593	1593	2280	1	1587	1712	2399
cb454_c24/f1p0/2537	gnl|BL_ORD_ID|42146 cb454_c7/f1p0/2374 isoform=cb454_c7;full_length_coverage=1;non_full_length_coverage=0;isoform_length=2374;expected_accuracy=0.997	225	2264	1	224	331	2374	1	224
cb8564_c41507/f1p0/2100 isoform=cb8564_c41507;full_length_coverage=1;non_full_length_coverage=0;isoform_length=2100;expected_accuracy=0.998	gnl|BL_ORD_ID|14339 cb8564_c36260/f1p0/3626 isoform=cb8564_c36260;full_length_coverage=1;non_full_length_coverage=0;isoform_length=3626;expected_accuracy=0.996	1	1657	1655	2100	1133	2792	3178	3623
cb8980_c2/f4p1/1909 isoform=cb8980_c2;full_length_coverage=4;non_full_length_coverage=1;isoform_length=1909;expected_accuracy=1.000	gnl|BL_ORD_ID|59418 cb8980_c4/f1p1/2063	561	1909	63	565	672	2020	16	518
cb332_c3/f1p1/4544	gnl|BL_ORD_ID|69566 cb332_c6/f1p0/5252	1989	4512	1	1994	2726	5253	9	2014
cb8564_c36145/f1p0/2868	gnl|BL_ORD_ID|14058 cb8564_c23936/f1p0/3016 isoform=cb8564_c23936;full_length_coverage=1;non_fu	1	1707	1705	2578	8	1716	2143	3016

	ll_length_coverage=0;isoform_length=3016;expected_accuracy=0.995								
cb500_c21/f1p0/3434 isoform=cb500_c21;full_length_coverage=1;non_full_length_coverage=0;isoform_length=3434;expected_accuracy=0.996	gnl|BL_ORD_ID|72251 cb500_c68/f1p0/4152	176	3260	1	180	961	4045	124	301
cb8564_c18491/f2p0/4707 isoform=cb8564_c18491;full_length_coverage=2;non_full_length_coverage=0;isoform_length=4707;expected_accuracy=0.996	gnl|BL_ORD_ID|34665 cb18654_c6/f1p0/5389 isoform=cb18654_c6;full_length_coverage=1;non_full_length_coverage=0;isoform_length=5389;expected_accuracy=0.996	1	3997	3998	4699	61	4063	4687	5389
cb8564_c5054/f1p0/2981 isoform=cb8564_c5054;full_length_coverage=1;non_full_length_coverage=0;isoform_length=2981;expected_accuracy=0.996	gnl|BL_ORD_ID|15840 cb8564_c5053/f2p0/3158 isoform=cb8564_c5053;full_length_coverage=2;non_full_length_coverage=0;isoform_length=3158;expected_accuracy=0.996	1	2438	2437	2980	55	2485	2615	3158
cb234_c1/f1p1/4574 isoform=cb234_c1;full_length_coverage=1;non_full_length_coverage=1;isoform_length=4574;expected_accuracy=0.997	gnl|BL_ORD_ID|38043 cb234_c4/f1p1/4715 isoform=cb234_c4;full_length_coverage=1;non_full_length_coverage=1;isoform_length=4715;expected_accuracy=0.994	4	4236	4231	4572	5	4241	4358	4699
cb8564_c71815/f1p0/2753	gnl|BL_ORD_ID|10862 cb8564_c106708/f1p3/2795 isoform=cb8564_c106708;full_length_coverage=1;non_full_length_coverage=3;isoform_length=2795;expected_accuracy=0.991	1	1377	1372	2460	53	1460	1694	2787
cb548_c58/f1p2/3035	gnl|BL_ORD_ID|46779 cb548_c109/f1p0/3280 isoform=cb548_c109;full_length_coverage=1;non_full_length_coverage=0;isoform_length=3280;expected_accuracy=0.996	1326	3053	1	1329	1551	3279	12	1340

cb395_c4/f1p0/3249	gnl|BL_ORD_ID|69601 cb395_c5/f1p0/3523	14	2595	2596	3115	1	2582	2756	3275
cb3427_c3/f1p0/3550	gnl|BL_ORD_ID|39943 cb3427_c4/f1p0/3709 isoform=cb3427_c4;full_length_coverage=1;non_full_length_coverage=0;isoform_length=3709;expected_accuracy=0.993	1	3138	3137	3486	1	3132	3351	3698
cb12714_c29/f1p2/1583	gnl|BL_ORD_ID|17272 cb8564_c91851/f1p1/2644 isoform=cb8564_c91851;full_length_coverage=1;non_full_length_coverage=1;isoform_length=2644;expected_accuracy=0.993	1	1296	1293	1591	1	1296	2345	2644
cb2206_c10/f1p0/2608	gnl|BL_ORD_ID|36911 cb2206_c6/f1p0/3298 isoform=cb2206_c6;full_length_coverage=1;non_full_length_coverage=0;isoform_length=3298;expected_accuracy=0.995	1	2214	2213	2605	1	2206	2905	3297
cb7560_c35/f1p1/2004 isoform=cb7560_c35;full_length_coverage=1;non_full_length_coverage=1;isoform_length=2004;expected_accuracy=0.994	gnl|BL_ORD_ID|50597 cb7560_c32/f1p0/2834	1	1446	1444	1972	234	1681	1928	2456
cb3814_c30/f1p0/2852	gnl|BL_ORD_ID|71529 cb3814_c17/f1p0/3106	15	1737	1735	2486	4	1722	2079	2830
cb2674_c19/f1p0/3539 isoform=cb2674_c19;full_length_coverage=1;non_full_length_coverage=0;isoform_length=3539;expected_accuracy=0.993	gnl|BL_ORD_ID|38975 cb2674_c31/f35p4/3827 isoform=cb2674_c31;full_length_coverage=35;non_full_length_coverage=4;isoform_length=3827;expected_accuracy=1.000	1	2456	2457	3539	141	2607	2743	3827
cb10385_c5/f1p0/1837	gnl|BL_ORD_ID|48457 cb10385_c3/f1p0/3720	235	1663	4	235	2285	3713	3	234

cb8564_c46939/f1p1/2926	gnl|BL_ORD_ID|12440 cb8564_c130435/f1p3/3226 isoform=cb8564_c130435;full_length_coverage=1;non_full_length_coverage=3;isoform_length=3226;expected_accuracy=0.994	1	2134	2130	2870	2	2165	2271	3019
cb4233_c109/f1p3/2092 isoform=cb4233_c109;full_length_coverage=1;non_full_length_coverage=3;isoform_length=2092;expected_accuracy=0.994	gnl|BL_ORD_ID|72374 cb4233_c97/f1p1/2296	1	1522	1517	2086	53	1578	1702	2274
cb8564_c20707/f1p1/3366 isoform=cb8564_c20707;full_length_coverage=1;non_full_length_coverage=1;isoform_length=3366;expected_accuracy=0.993	gnl|BL_ORD_ID|54353 cb8564_c22866/f1p1/4372	1180	3366	112	1180	2108	4300	1	1050
cb8983_c32/f1p0/1686 isoform=cb8983_c32;full_length_coverage=1;non_full_length_coverage=0;isoform_length=1686;expected_accuracy=0.992	gnl|BL_ORD_ID|19399 cb8983_c11/f1p0/2136 isoform=cb8983_c11;full_length_coverage=1;non_full_length_coverage=0;isoform_length=2136;expected_accuracy=0.999	133	1686	1	136	581	2134	1	135
cb8564_c109489/f3p0/2914 isoform=cb8564_c109489;full_length_coverage=3;non_full_length_coverage=0;isoform_length=2914;expected_accuracy=1.000	gnl|BL_ORD_ID|10869 cb8564_c117138/f1p0/4110 isoform=cb8564_c117138;full_length_coverage=1;non_full_length_coverage=0;isoform_length=4110;expected_accuracy=0.995	1	1559	1555	2914	75	1627	2753	4110
cb6238_c4/f1p0/2903 isoform=cb6238_c4;full_length_coverage=1;non_full_length_coverage=0;isoform_length=2903;expected_accuracy=0.997	gnl|BL_ORD_ID|5359 cb6238_c5/f1p0/3446 isoform=cb6238_c5;full_length_coverage=1;non_full_length_coverage=0;isoform_length=3446;expected_accuracy=0.994	13	1936	1937	2890	17	1895	2427	3374
cb7478_c5/f1p0/2384	gnl|BL_ORD_ID|8354 cb7478_c8/f3p0/2393 isoform=cb7478_c8;full_length_coverage=3;non_full_le	12	1877	1874	2250	1	1863	1966	2344

	ngth_coverage=0;isoform_length=2393;expected_accuracy=1.000								
cb2629_c6/f1p0/4836 isoform=cb2629_c6;full_length_coverage=1;non_full_length_coverage=0;isoform_length=4836;expected_accuracy=0.993	gnl|BL_ORD_ID|34935 cb18639_c0/f2p0/5077 isoform=cb18639_c0;full_length_coverage=2;non_full_length_coverage=0;isoform_length=5077;expected_accuracy=0.998	1	2925	2922	4836	1	2950	3115	5035
cb8564_c38947/f1p0/2650 isoform=cb8564_c38947;full_length_coverage=1;non_full_length_coverage=0;isoform_length=2650;expected_accuracy=0.996	gnl|BL_ORD_ID|56602 cb8564_c48192/f1p6/3029	1	1441	1436	2650	1	1441	1658	2871
cb3482_c32/f1p0/1683 isoform=cb3482_c32;full_length_coverage=1;non_full_length_coverage=0;isoform_length=1683;expected_accuracy=1.000	gnl|BL_ORD_ID|40559 cb3482_c26/f1p2/2186 isoform=cb3482_c26;full_length_coverage=1;non_full_length_coverage=2;isoform_length=2186;expected_accuracy=0.992	1	1586	1581	1683	125	1724	2009	2111
cb10027_c13/f4p0/1586 isoform=cb10027_c13;full_length_coverage=4;non_full_length_coverage=0;isoform_length=1586;expected_accuracy=1.000	gnl|BL_ORD_ID|2017 cb10027_c6/f1p0/1792 isoform=cb10027_c6;full_length_coverage=1;non_full_length_coverage=0;isoform_length=1792;expected_accuracy=1.000	227	1586	5	228	439	1792	1	224
cb5472_c0/f1p0/3101	gnl|BL_ORD_ID|45834 cb5472_c6/f1p0/3267 isoform=cb5472_c6;full_length_coverage=1;non_full_length_coverage=0;isoform_length=3267;expected_accuracy=0.991	22	2674	2675	3073	1	2624	2813	3214
cb8564_c45116/f1p0/2875 isoform=cb8564_c45116;full_length_coverage=1;non_full_length_coverage=0;isoform_length=2875;expected_accuracy=1.000	gnl|BL_ORD_ID|15379 cb8564_c37604/f2p0/3150 isoform=cb8564_c37604;full_length_coverage=2;non_full_length_coverage=0;isoform_length=3150;expected_accuracy=0.996	1	1544	1542	2875	85	1604	1744	3081

cb8564_c80412/f1p0/2903 isoform=cb8564_c80412;full_length_coverage=1;non_full_length_coverage=0;isoform_length=2903;expected_accuracy=0.996	gnl|BL_ORD_ID|18170 cb8564_c88512/f1p0/4140 isoform=cb8564_c88512;full_length_coverage=1;non_full_length_coverage=0;isoform_length=4140;expected_accuracy=0.992	1	1774	1771	2903	1053	2824	3011	4140
cb18301_c30/f1p0/1075	gnl|BL_ORD_ID|33896 cb18301_c28/f1p0/1300 isoform=cb18301_c28;full_length_coverage=1;non_full_length_coverage=0;isoform_length=1300;expected_accuracy=0.995	179	1063	19	184	370	1254	46	219
cb3290_c38/f1p0/2345 isoform=cb3290_c38;full_length_coverage=1;non_full_length_coverage=0;isoform_length=2345;expected_accuracy=0.996	gnl|BL_ORD_ID|71045 cb3290_c173/f1p0/3126	1150	2341	1	1151	1632	2831	319	1475
cb7560_c35/f1p1/2004 isoform=cb7560_c35;full_length_coverage=1;non_full_length_coverage=1;isoform_length=2004;expected_accuracy=0.994	gnl|BL_ORD_ID|8568 cb7560_c43/f1p2/2513 isoform=cb7560_c43;full_length_coverage=1;non_full_length_coverage=2;isoform_length=2513;expected_accuracy=0.998	1	1446	1444	2004	223	1668	1915	2475
cb381_c0/f2p0/4859 isoform=cb381_c0;full_length_coverage=2;non_full_length_coverage=0;isoform_length=4859;expected_accuracy=0.997	gnl|BL_ORD_ID|40998 cb381_c6/f1p0/5381 isoform=cb381_c6;full_length_coverage=1;non_full_length_coverage=0;isoform_length=5381;expected_accuracy=0.996	19	3705	3704	4859	1	3681	4229	5380
cb2263_c9/f1p0/2491	gnl|BL_ORD_ID|36791 cb2263_c5/f1p0/3994 isoform=cb2263_c5;full_length_coverage=1;non_full_length_coverage=0;isoform_length=3994;expected_accuracy=0.998	1	1320	1321	2361	117	1436	2457	3497
cb7181_c10/f1p1/1554 isoform=cb7181_c10;full_length_coverage=1;non_full_	gnl|BL_ORD_ID|3706 cb7181_c9/f1p1/2607 isoform=cb7181_c9;full_length_coverage=1;non_full_le	1	1058	1056	1553	1	1050	2110	2607

length_coverage=1;isoform_length=1554;expected_accuracy=0.994	ngth_coverage=1;isoform_length=2607;expected_accuracy=0.999								
cb8564_c36056/f1p0/2652 isoform=cb8564_c36056;full_length_coverage=1;non_full_length_coverage=0;isoform_length=2652;expected_accuracy=0.999	gnl|BL_ORD_ID|56727 cb8564_c66920/f3p3/2894	248	2644	1	251	466	2863	70	322
cb8564_c14548/f1p0/3030	gnl|BL_ORD_ID|52845 cb8564_c15530/f1p0/3247	13	2313	2314	2657	6	2306	2785	3128
cb8564_c123038/f1p0/2682	gnl|BL_ORD_ID|15348 cb8564_c25433/f2p0/2642 isoform=cb8564_c25433;full_length_coverage=2;non_full_length_coverage=0;isoform_length=2642;expected_accuracy=1.000	1245	2531	10	1250	1356	2642	1	1241
cb1380_c9/f3p0/2515 isoform=cb1380_c9;full_length_coverage=3;non_full_length_coverage=0;isoform_length=2515;expected_accuracy=0.994	gnl|BL_ORD_ID|61310 cb1380_c37/f1p0/3199	1	1680	1679	2447	6	1705	2358	3126
cb8564_c24710/f1p0/2430 isoform=cb8564_c24710;full_length_coverage=1;non_full_length_coverage=0;isoform_length=2430;expected_accuracy=0.996	gnl|BL_ORD_ID|56014 cb8564_c52246/f1p1/2752	14	1499	1502	2430	1	1489	1589	2492
cb3143_c8/f1p1/2928 isoform=cb3143_c8;full_length_coverage=1;non_full_length_coverage=1;isoform_length=2928;expected_accuracy=0.992	gnl|BL_ORD_ID|69160 cb3143_c4/f1p0/3643	205	2923	1	206	686	3402	31	236
cb3226_c22/f2p0/2434 isoform=cb3226_c22;full_length_coverage=2;non_full_	gnl|BL_ORD_ID|39676 cb3226_c10/f1p0/3292 isoform=cb3226_c10;full_length_coverage=1;non_full_l	136	2431	1	138	1001	3292	29	165

length_coverage=0;isoform_length=2434;expected_accuracy=1.000	ength_coverage=0;isoform_length=3292;expected_accuracy=0.994								
cb401_c28/f1p0/2163 isoform=cb401_c28;full_length_coverage=1;non_full_length_coverage=0;isoform_length=2163;expected_accuracy=0.992	gnl|BL_ORD_ID|40225 cb401_c88/f3p0/2402 isoform=cb401_c88;full_length_coverage=3;non_full_length_coverage=0;isoform_length=2402;expected_accuracy=0.994	317	2163	4	313	539	2377	21	329
cb3763_c17/f1p0/2834 isoform=cb3763_c17;full_length_coverage=1;non_full_length_coverage=0;isoform_length=2834;expected_accuracy=0.995	gnl|BL_ORD_ID|40108 cb3763_c16/f1p1/3107 isoform=cb3763_c16;full_length_coverage=1;non_full_length_coverage=1;isoform_length=3107;expected_accuracy=0.998	1	1844	1844	2834	1	1850	2015	3005
cb2947_c9/f1p0/3003 isoform=cb2947_c9;full_length_coverage=1;non_full_length_coverage=0;isoform_length=3003;expected_accuracy=1.000	gnl|BL_ORD_ID|38195 cb2947_c15/f1p0/5692 isoform=cb2947_c15;full_length_coverage=1;non_full_length_coverage=0;isoform_length=5692;expected_accuracy=0.995	15	2762	2763	3003	1	2755	2918	3158
cb8564_c44698/f1p5/2843 isoform=cb8564_c44698;full_length_coverage=1;non_full_length_coverage=5;isoform_length=2843;expected_accuracy=0.997	gnl|BL_ORD_ID|9418 cb8564_c119193/f1p2/2975 isoform=cb8564_c119193;full_length_coverage=1;non_full_length_coverage=2;isoform_length=2975;expected_accuracy=0.998	1	2516	2511	2826	1	2532	2655	2975
cb4263_c4/f1p1/2943	gnl|BL_ORD_ID|43863 cb4263_c0/f1p1/3386 isoform=cb4263_c0;full_length_coverage=1;non_full_length_coverage=1;isoform_length=3386;expected_accuracy=0.992	241	2990	1	243	623	3386	108	350
cb2918_c67/f1p0/2483 isoform=cb2918_c67;full_length_coverage=1;non_full_length_coverage=0;isoform_length=2483;expected_accuracy=1.000	gnl|BL_ORD_ID|70088 cb2918_c33/f1p3/2817	1080	2483	1	1085	1299	2702	58	1142

cb4576_c12/f13p3/4007 isoform=cb4576_c12;full_length_coverage=13;non_full_length_coverage=3;isoform_length=4007;expected_accuracy=0.998	gnl|BL_ORD_ID|44415 cb4576_c54/f1p0/4341 isoform=cb4576_c54;full_length_coverage=1;non_full_length_coverage=0;isoform_length=4341;expected_accuracy=0.995	1847	3894	1	1848	2301	4341	1	1837
cb964_c15/f1p0/4325	gnl|BL_ORD_ID|20582 cb964_c26/f1p0/6950 isoform=cb964_c26;full_length_coverage=1;non_full_length_coverage=0;isoform_length=6950;expected_accuracy=0.996	121	2343	2342	4104	1	2222	5212	6950
cb17354_c1/f1p0/1440	gnl|BL_ORD_ID|41507 cb34_c0/f1p0/4992 isoform=cb34_c0;full_length_coverage=1;non_full_length_coverage=0;isoform_length=4992;expected_accuracy=0.999	1	1059	1056	1354	17	1074	1799	2097
cb6491_c6/f1p1/3274 isoform=cb6491_c6;full_length_coverage=1;non_full_length_coverage=1;isoform_length=3274;expected_accuracy=0.999	gnl|BL_ORD_ID|3949 cb6491_c2/f1p0/4290 isoform=cb6491_c2;full_length_coverage=1;non_full_length_coverage=0;isoform_length=4290;expected_accuracy=0.996	15	2950	2948	3274	1	2932	3099	3413
cb8564_c38035/f1p0/2770	gnl|BL_ORD_ID|55327 cb8564_c38462/f1p0/3241	1	2035	2033	2756	1	2028	2218	2945
cb2747_c7/f1p3/3310	gnl|BL_ORD_ID|68804 cb2747_c6/f1p0/3765	1565	3243	1	1566	2019	3697	1	1566
cb8564_c49562/f1p0/2838 isoform=cb8564_c49562;full_length_coverage=1;non_full_length_coverage=0;isoform_length=2838;expected_accuracy=0.995	gnl|BL_ORD_ID|14539 cb8564_c46562/f2p3/3196 isoform=cb8564_c46562;full_length_coverage=2;non_full_length_coverage=3;isoform_length=3196;expected_accuracy=0.995	1	2331	2327	2836	3	2351	2683	3195
cb5659_c32/f1p0/2068 isoform=cb5659_c32;full_length_coverage=1;non_full_	gnl|BL_ORD_ID|45353 cb5659_c43/f1p0/2132 isoform=cb5659_c43;full_length_coverage=1;non_full_l	914	2068	94	919	930	2086	1	829

length_coverage=0;isoform_length=2068;expected_accuracy=0.993	ength_coverage=0;isoform_length=2132;expected_accuracy=0.992								
cb12092_c9/f1p0/1052	gnl|BL_ORD_ID|24711 cb12092_c11/f1p0/1145 isoform=cb12092_c11;full_length_coverage=1;non_full_length_coverage=0;isoform_length=1145;expected_accuracy=0.998	426	983	1	429	547	1107	1	431
cb8564_c25634/f1p0/2542	gnl|BL_ORD_ID|60434 cb11946_c5/f1p1/5186	956	2536	99	957	3619	5200	2229	3087
cb17699_c4/f1p0/1082	gnl|BL_ORD_ID|32243 cb17699_c0/f6p1/1251 isoform=cb17699_c0;full_length_coverage=6;non_full_length_coverage=1;isoform_length=1251;expected_accuracy=1.000	171	1057	4	175	365	1251	1	172
cb8028_c0/f2p0/1473 isoform=cb8028_c0;full_length_coverage=2;non_full_length_coverage=0;isoform_length=1473;expected_accuracy=1.000	gnl|BL_ORD_ID|6436 cb8028_c2/f1p0/2399 isoform=cb8028_c2;full_length_coverage=1;non_full_length_coverage=0;isoform_length=2399;expected_accuracy=0.994	384	1473	7	385	1303	2398	3	390
cb5724_c9/f1p0/2169 isoform=cb5724_c9;full_length_coverage=1;non_full_length_coverage=0;isoform_length=2169;expected_accuracy=0.999	gnl|BL_ORD_ID|46771 cb5724_c8/f1p0/2804 isoform=cb5724_c8;full_length_coverage=1;non_full_length_coverage=0;isoform_length=2804;expected_accuracy=0.996	1	1455	1453	2169	65	1513	2086	2801
cb8564_c24424/f1p0/4115	gnl|BL_ORD_ID|52054 cb8564_c11371/f1p2/3552	12	3217	3216	3380	51	3257	3132	3296
cb8564_c185920/f1p0/2777 isoform=cb8564_c185920;full_length_coverage=1;non_full_length_coverage=0;isoform_length=2777;expected_accuracy=0.996	gnl|BL_ORD_ID|56641 cb8564_c50309/f1p5/3075	174	2770	1	177	350	2947	14	191

cb8564_c148448/f2p13/3821 isoform=cb8564_c148448;full_length_coverage=2;non_full_length_coverage=13;isoform_length=3821;expected_accuracy=0.995	gnl|BL_ORD_ID|16248 cb8564_c5118/f1p1/4889 isoform=cb8564_c5118;full_length_coverage=1;non_full_length_coverage=1;isoform_length=4889;expected_accuracy=0.991	1935	3812	108	1937	2987	4855	1	1822
cb8516_c2/f1p0/2182	gnl|BL_ORD_ID|52576 cb8516_c3/f1p0/2479	1	1854	1854	2139	1	1854	1984	2269
cb8564_c77994/f1p2/2630 isoform=cb8564_c77994;full_length_coverage=1;non_full_length_coverage=2;isoform_length=2630;expected_accuracy=0.998	gnl|BL_ORD_ID|57795 cb8564_c80951/f1p0/2999	1	1819	1815	2630	131	1950	2094	2910
cb8564_c40598/f1p4/3119	gnl|BL_ORD_ID|17429 cb8564_c85447/f1p0/3196 isoform=cb8564_c85447;full_length_coverage=1;non_full_length_coverage=0;isoform_length=3196;expected_accuracy=0.997	1	2732	2729	3054	3	2752	2872	3196
cb8564_c132795/f5p10/3729 isoform=cb8564_c132795;full_length_coverage=5;non_full_length_coverage=10;isoform_length=3729;expected_accuracy=0.999	gnl|BL_ORD_ID|15705 cb8564_c5095/f1p0/3793 isoform=cb8564_c5095;full_length_coverage=1;non_full_length_coverage=0;isoform_length=3793;expected_accuracy=0.995	1521	3640	1	1522	1673	3792	2	1523
cb8564_c120215/f1p1/2248	gnl|BL_ORD_ID|13672 cb8564_c1926/f1p1/2305 isoform=cb8564_c1926;full_length_coverage=1;non_full_length_coverage=1;isoform_length=2305;expected_accuracy=0.995	1002	2133	1	1004	1182	2305	1	998
cb8564_c184250/f1p0/2256 isoform=cb8564_c184250;full_length_coverage=1;non_full_length_coverage=0;isoform_length=2256;expected_accuracy=0.996	gnl|BL_ORD_ID|14601 cb8564_c34940/f1p2/2892 isoform=cb8564_c34940;full_length_coverage=1;non_full_length_coverage=2;isoform_length=2892;expected_accuracy=0.991	1	1642	1638	2256	27	1670	1933	2553

cb8564_c75620/f1p0/2781 isoform=cb8564_c75620;full_length_coverage=1;non_full_length_coverage=0;isoform_length=2781;expected_accuracy=0.996	gnl|BL_ORD_ID|18398 cb8564_c90428/f1p0/2987 isoform=cb8564_c90428;full_length_coverage=1;non_full_length_coverage=0;isoform_length=2987;expected_accuracy=0.996	1	1769	1764	2781	1	173 4	1930	294 7
cb8564_c185920/f1p0/2777 isoform=cb8564_c185920;full_length_coverage=1;non_full_length_coverage=0;isoform_length=2777;expected_accuracy=0.996	gnl|BL_ORD_ID|55433 cb8564_c41572/f1p1/3265	174	2777	1	177	344	294 9	16	185
cb868_c17/f1p0/3361	gnl|BL_ORD_ID|56508 cb868_c12/f1p0/3718	1	1833	1833	3334	24	185 6	1966	346 7
cb1176_c32/f1p0/3993	gnl|BL_ORD_ID|59226 cb1176_c14/f1p0/4251	110	3923	1	112	419	423 0	20	131
cb8564_c46484/f1p0/3151	gnl|BL_ORD_ID|16149 cb8564_c47162/f1p0/3361 isoform=cb8564_c47162;full_length_coverage=1;non_full_length_coverage=0;isoform_length=3361;expected_accuracy=0.996	325	3095	5	326	592	336 1	103	402
cb8564_c68409/f2p0/2141 isoform=cb8564_c68409;full_length_coverage=2;non_full_length_coverage=0;isoform_length=2141;expected_accuracy=0.995	gnl|BL_ORD_ID|15528 cb8564_c72800/f1p0/2322 isoform=cb8564_c72800;full_length_coverage=1;non_full_length_coverage=0;isoform_length=2322;expected_accuracy=0.996	265	2141	52	266	424	230 0	1	213
cb3722_c8/f1p0/3086	gnl|BL_ORD_ID|71469 cb3722_c13/f1p0/3071	114	2336	2335	3011	1	222 3	2334	301 0
cb7735_c2/f2p0/1143 isoform=cb7735_c2;full_length_coverage=2;non_full_length_coverage=0;isoform_length=1143;expected_accuracy=1.000	gnl|BL_ORD_ID|8560 cb7735_c5/f1p0/2560 isoform=cb7735_c5;full_length_coverage=1;non_full_length_coverage=0;isoform_length=2560;expected_accuracy=0.997	434	1141	8	435	1854	256 0	1	428

cb8564_c69139/f2p0/3547	gnl|BL_ORD_ID|56103 cb8564_c69762/f2p0/4058	1	2065	2066	3562	55	2119	2230	3726
cb7463_c5/f1p0/2362 isoform=cb7463_c5;full_length_coverage=1;non_full_length_coverage=0;isoform_length=2362;expected_accuracy=0.995	gnl|BL_ORD_ID|8127 cb7463_c11/f4p1/2609 isoform=cb7463_c11;full_length_coverage=4;non_full_length_coverage=1;isoform_length=2609;expected_accuracy=0.999	177	2362	1	180	422	2608	72	251
cb8345_c2/f1p0/2284 isoform=cb8345_c2;full_length_coverage=1;non_full_length_coverage=0;isoform_length=2284;expected_accuracy=0.994	gnl|BL_ORD_ID|9936 cb8345_c0/f1p0/2394 isoform=cb8345_c0;full_length_coverage=1;non_full_length_coverage=0;isoform_length=2394;expected_accuracy=0.993	1	1512	1512	2284	1	1490	1613	2387
cb8564_c39136/f1p0/3036 isoform=cb8564_c39136;full_length_coverage=1;non_full_length_coverage=0;isoform_length=3036;expected_accuracy=0.998	gnl|BL_ORD_ID|16622 cb8564_c50151/f1p0/3198 isoform=cb8564_c50151;full_length_coverage=1;non_full_length_coverage=0;isoform_length=3198;expected_accuracy=0.995	1	2089	2085	3034	6	2120	2247	3198
cb6405_c6/f1p0/2746 isoform=cb6405_c6;full_length_coverage=1;non_full_length_coverage=0;isoform_length=2746;expected_accuracy=0.997	gnl|BL_ORD_ID|49679 cb6405_c4/f1p0/2864	1	1642	1641	2746	1	1642	1787	2892
cb7181_c10/f1p1/1554 isoform=cb7181_c10;full_length_coverage=1;non_full_length_coverage=1;isoform_length=1554;expected_accuracy=0.994	gnl|BL_ORD_ID|3704 cb7181_c1/f3p1/2633 isoform=cb7181_c1;full_length_coverage=3;non_full_length_coverage=1;isoform_length=2633;expected_accuracy=0.999	1	1058	1056	1519	4	1060	2118	2583
cb8564_c112431/f1p0/2101	gnl|BL_ORD_ID|14374 cb8564_c37670/f2p0/3061 isoform=cb8564_c37670;full_length_coverage=2;non_full_length_coverage=0;isoform_length=3061;expected_accuracy=1.000	164	1864	1	165	1361	3061	50	214

cb18456_c6530/f1p0/1200	gnl|BL_ORD_ID|60328 cb11694_c2/f1p0/2576	260	1166	3	259	947	41	1734	1478
cb4016_c18/f1p0/2605 isoform=cb4016_c18;full_length_coverage=1;non_full_length_coverage=0;isoform_length=2605;expected_accuracy=0.999	gnl|BL_ORD_ID|40529 cb4016_c2/f3p1/2863 isoform=cb4016_c2;full_length_coverage=3;non_full_length_coverage=1;isoform_length=2863;expected_accuracy=0.998	188	2564	1	189	491	2863	2	189
cb8564_c74180/f1p1/2858	gnl|BL_ORD_ID|17040 cb8564_c69197/f5p5/3337 isoform=cb8564_c69197;full_length_coverage=5;non_full_length_coverage=5;isoform_length=3337;expected_accuracy=0.996	1	2587	2585	2854	109	2695	2834	3103
cb2723_c26/f4p0/2737 isoform=cb2723_c26;full_length_coverage=4;non_full_length_coverage=0;isoform_length=2737;expected_accuracy=1.000	gnl|BL_ORD_ID|36753 cb2723_c4/f2p0/4552 isoform=cb2723_c4;full_length_coverage=2;non_full_length_coverage=0;isoform_length=4552;expected_accuracy=1.000	15	2395	2395	2737	1	2388	3276	3618
cb8564_c41692/f1p3/2987 isoform=cb8564_c41692;full_length_coverage=1;non_full_length_coverage=3;isoform_length=2987;expected_accuracy=0.995	gnl|BL_ORD_ID|55920 cb8564_c48949/f1p0/4357	1	2317	2318	2987	2	2293	2847	3504
cb8564_c2241/f1p2/2527 isoform=cb8564_c2241;full_length_coverage=1;non_full_length_coverage=2;isoform_length=2527;expected_accuracy=0.995	gnl|BL_ORD_ID|13611 cb8564_c2184/f1p0/2606 isoform=cb8564_c2184;full_length_coverage=1;non_full_length_coverage=0;isoform_length=2606;expected_accuracy=0.995	294	2527	32	294	384	2605	24	272
cb13748_c51/f1p0/3514	gnl|BL_ORD_ID|26168 cb13748_c41/f1p2/3610 isoform=cb13748_c41;full_length_coverage=1;non_full_length_coverage=2;isoform_length=3610;expected_accuracy=0.999	2	2809	2807	2951	5	2815	3048	3190

cb8564_c185920/f1p0/2777 isoform=cb8564_c185920;full_length_coverage=1;non_full_length_coverage=0;isoform_length=2777;expected_accuracy=0.996	gnl|BL_ORD_ID|15428 cb8564_c42387/f1p4/2973 isoform=cb8564_c42387;full_length_coverage=1;non_full_length_coverage=4;isoform_length=2973;expected_accuracy=0.993	174	2766	1	177	354	2973	15	195
cb8478_c7/f2p0/3290 isoform=cb8478_c7;full_length_coverage=2;non_full_length_coverage=0;isoform_length=3290;expected_accuracy=0.998	gnl|BL_ORD_ID|10224 cb8478_c13/f2p0/3348 isoform=cb8478_c13;full_length_coverage=2;non_full_length_coverage=0;isoform_length=3348;expected_accuracy=1.000	1	3034	3034	3225	1	3031	3157	3348
cb5516_c26/f1p0/3029 isoform=cb5516_c26;full_length_coverage=1;non_full_length_coverage=0;isoform_length=3029;expected_accuracy=1.000	gnl|BL_ORD_ID|46314 cb5516_c2/f40p1/3248 isoform=cb5516_c2;full_length_coverage=40;non_full_length_coverage=1;isoform_length=3248;expected_accuracy=1.000	10	2327	2322	3027	101	2417	2542	3248
cb4461_c3/f1p2/2896	gnl|BL_ORD_ID|44357 cb4461_c2/f1p2/3341 isoform=cb4461_c2;full_length_coverage=1;non_full_length_coverage=2;isoform_length=3341;expected_accuracy=0.996	23	2475	2474	2896	1	2453	2920	3340
cb8564_c181853/f1p0/2119 isoform=cb8564_c181853;full_length_coverage=1;non_full_length_coverage=0;isoform_length=2119;expected_accuracy=0.997	gnl|BL_ORD_ID|15994 cb8564_c54019/f1p1/2940 isoform=cb8564_c54019;full_length_coverage=1;non_full_length_coverage=1;isoform_length=2940;expected_accuracy=0.991	1	1339	1334	2119	7	1333	2158	2935
cb8564_c41162/f1p0/3158	gnl|BL_ORD_ID|55910 cb8564_c48645/f1p1/3287	21	2553	2548	2975	22	2555	2686	3113
cb8564_c4961/f1p0/2082	gnl|BL_ORD_ID|16588 cb8564_c4944/f1p0/2151 isoform=cb8564_c4944;full_length_coverage=1;non_full_length_coverage=0;isoform_length=2151;expected_accuracy=0.997	212	2077	56	212	286	2151	8	162

cb8564_c185945/f1p0/2304 isoform=cb8564_c185945;full_length_coverage=1;non_full_length_coverage=0;isoform_length=2304;expected_accuracy=0.997	gnl|BL_ORD_ID|56102 cb8564_c69761/f1p0/2560	1	1938	1933	2302	1	1935	2160	2527
cb8564_c88915/f1p0/2486 isoform=cb8564_c88915;full_length_coverage=1;non_full_length_coverage=0;isoform_length=2486;expected_accuracy=0.999	gnl|BL_ORD_ID|17082 cb8564_c85207/f1p0/2727 isoform=cb8564_c85207;full_length_coverage=1;non_full_length_coverage=0;isoform_length=2727;expected_accuracy=0.997	1	1537	1535	2486	1	1535	1775	2726
cb9602_c3/f1p0/1779 isoform=cb9602_c3;full_length_coverage=1;non_full_length_coverage=0;isoform_length=1779;expected_accuracy=0.993	gnl|BL_ORD_ID|20056 cb9602_c1/f1p0/2113 isoform=cb9602_c1;full_length_coverage=1;non_full_length_coverage=0;isoform_length=2113;expected_accuracy=0.994	421	1779	72	422	756	2113	1	351
cb8564_c146882/f3p8/4113 isoform=cb8564_c146882;full_length_coverage=3;non_full_length_coverage=8;isoform_length=4113;expected_accuracy=0.998	gnl|BL_ORD_ID|13890 cb8564_c35935/f1p0/4149 isoform=cb8564_c35935;full_length_coverage=1;non_full_length_coverage=0;isoform_length=4149;expected_accuracy=0.993	181	4062	1	185	288	4149	1	183
cb5103_c4/f2p0/1847 isoform=cb5103_c4;full_length_coverage=2;non_full_length_coverage=0;isoform_length=1847;expected_accuracy=0.994	gnl|BL_ORD_ID|73268 cb5103_c10/f1p0/2138	1	1521	1518	1773	4	1530	1846	2102
cb964_c15/f1p0/4325	gnl|BL_ORD_ID|59137 cb964_c2/f1p0/4848	14	2341	2342	4110	185	2513	2718	4486
cb8564_c15653/f1p4/2475	gnl|BL_ORD_ID|12776 cb8564_c163741/f1p20/2690 isoform=cb8564_c163741;full_length_coverage=1;non_full_length_coverage=20;isoform_length=2690;expected_accuracy=1.000	16	2293	2289	2433	48	2327	2546	2690

cb12931_c15/f1p0/1493 isoform=cb12931_c15;full_length_coverage=1;non_full_length_coverage=0;isoform_length=1493;expected_accuracy=1.000	gnl|BL_ORD_ID|55818 cb8564_c41208/f1p0/2901	1	1106	1104	1484	1	1105	2438	2817
cb8564_c40598/f1p4/3119	gnl|BL_ORD_ID|55211 cb8564_c35547/f1p0/3745	1	2732	2729	3055	9	2739	2860	3186
cb8564_c43455/f1p0/2242 isoform=cb8564_c43455;full_length_coverage=1;non_full_length_coverage=0;isoform_length=2242;expected_accuracy=0.995	gnl|BL_ORD_ID|16671 cb8564_c54439/f1p0/2590 isoform=cb8564_c54439;full_length_coverage=1;non_full_length_coverage=0;isoform_length=2590;expected_accuracy=0.994	279	2242	4	283	567	2578	5	290
cb8564_c47011/f1p0/3100	gnl|BL_ORD_ID|55246 cb8564_c36343/f1p0/3448	17	2306	2301	2934	19	2314	2550	3183
cb13417_c4/f1p0/1422 isoform=cb13417_c4;full_length_coverage=1;non_full_length_coverage=0;isoform_length=1422;expected_accuracy=1.000	gnl|BL_ORD_ID|25115 cb13417_c0/f2p0/1787 isoform=cb13417_c0;full_length_coverage=2;non_full_length_coverage=0;isoform_length=1787;expected_accuracy=0.990	1	1170	1170	1421	85	1260	1536	1787
cb8564_c10938/f1p1/2321	gnl|BL_ORD_ID|17270 cb8564_c9070/f5p11/3834 isoform=cb8564_c9070;full_length_coverage=5;non_full_length_coverage=11;isoform_length=3834;expected_accuracy=1.000	1	1315	1310	2292	1313	2627	2746	3728
cb8564_c46212/f3p1/4373 isoform=cb8564_c46212;full_length_coverage=3;non_full_length_coverage=1;isoform_length=4373;expected_accuracy=0.999	gnl|BL_ORD_ID|14314 cb8564_c45827/f2p2/4435 isoform=cb8564_c45827;full_length_coverage=2;non_full_length_coverage=2;isoform_length=4435;expected_accuracy=0.999	184	4334	15	185	279	4434	1	163
cb4016_c34/f1p0/2560 isoform=cb4016_c34;full_length_coverage=1;non_full_	gnl|BL_ORD_ID|39535 cb4016_c27/f1p1/3204 isoform=cb4016_c27;full_length_coverage=1;non_full_l	194	2560	15	195	790	3150	3	181

length_coverage=0;isoform_length=2560;expected_accuracy=0.998	ength_coverage=1;isoform_length=3204;expected_accuracy=0.996								
cb9780_c1/f2p0/1733 isoform=cb9780_c1;full_length_coverage=2;non_full_length_coverage=0;isoform_length=1733;expected_accuracy=0.995	gnl|BL_ORD_ID|21082 cb9780_c12/f1p0/1845 isoform=cb9780_c12;full_length_coverage=1;non_full_length_coverage=0;isoform_length=1845;expected_accuracy=0.995	117	1727	1	120	259	1845	1	121
cb3627_c2/f1p0/1989 isoform=cb3627_c2;full_length_coverage=1;non_full_length_coverage=0;isoform_length=1989;expected_accuracy=0.993	gnl|BL_ORD_ID|41467 cb3627_c1/f1p0/3569 isoform=cb3627_c1;full_length_coverage=1;non_full_length_coverage=0;isoform_length=3569;expected_accuracy=0.991	1	1516	1517	1989	4	1513	2440	2910
cb12_c37/f1p0/4067	gnl|BL_ORD_ID|61090 cb12_c56/f1p0/6478	286	4058	1	288	2515	6295	2098	2385
cb3537_c0/f9p0/3405 isoform=cb3537_c0;full_length_coverage=9;non_full_length_coverage=0;isoform_length=3405;expected_accuracy=1.000	gnl|BL_ORD_ID|71292 cb3537_c10/f1p0/3473	1598	3382	133	1602	1689	3475	1	1468
cb5372_c2/f1p0/2179 isoform=cb5372_c2;full_length_coverage=1;non_full_length_coverage=0;isoform_length=2179;expected_accuracy=0.993	gnl|BL_ORD_ID|73836 cb5372_c0/f1p0/3087	1	1577	1576	2177	131	1708	2288	2889
cb1573_c16/f1p0/3454 isoform=cb1573_c16;full_length_coverage=1;non_full_length_coverage=0;isoform_length=3454;expected_accuracy=0.998	gnl|BL_ORD_ID|30371 cb1573_c7/f1p2/3841 isoform=cb1573_c7;full_length_coverage=1;non_full_length_coverage=2;isoform_length=3841;expected_accuracy=1.000	1249	3454	1	1252	1445	3658	33	1286
cb8564_c4966/f1p0/2245	gnl|BL_ORD_ID|16588 cb8564_c4944/f1p0/2151 isoform=cb8564_c4944;full_length_coverage=1;non_ful	128	1993	1	128	286	2151	36	162

	l_length_coverage=0;isoform_length=2151;expected_accuracy=0.997								
cb6221_c13/f1p0/2080 isoform=cb6221_c13;full_length_coverage=1;non_full_length_coverage=0;isoform_length=2080;expected_accuracy=1.000	gnl|BL_ORD_ID|4880 cb6221_c4/f3p3/2906 isoform=cb6221_c4;full_length_coverage=3;non_full_length_coverage=3;isoform_length=2906;expected_accuracy=1.000	117	2080	1	117	900	2865	73	187
cb8564_c43455/f1p0/2242 isoform=cb8564_c43455;full_length_coverage=1;non_full_length_coverage=0;isoform_length=2242;expected_accuracy=0.995	gnl|BL_ORD_ID|11774 cb8564_c146891/f2p5/2561 isoform=cb8564_c146891;full_length_coverage=2;non_full_length_coverage=5;isoform_length=2561;expected_accuracy=0.999	279	2242	29	283	571	2543	48	302
cb8564_c71742/f1p0/2675	gnl|BL_ORD_ID|54752 cb8564_c167108/f1p0/2919	1	1309	1310	2413	122	1422	1809	2912
cb2044_c6/f1p0/1724 isoform=cb2044_c6;full_length_coverage=1;non_full_length_coverage=0;isoform_length=1724;expected_accuracy=1.000	gnl|BL_ORD_ID|34506 cb2044_c4/f1p0/1805 isoform=cb2044_c4;full_length_coverage=1;non_full_length_coverage=0;isoform_length=1805;expected_accuracy=0.999	140	1674	1	141	285	1805	28	168
cb8564_c43119/f1p4/3194 isoform=cb8564_c43119;full_length_coverage=1;non_full_length_coverage=4;isoform_length=3194;expected_accuracy=0.995	gnl|BL_ORD_ID|56069 cb8564_c54106/f1p2/3479	1	1671	1668	3157	1	1660	1802	3279
cb10471_c5/f1p1/1667 isoform=cb10471_c5;full_length_coverage=1;non_full_length_coverage=1;isoform_length=1667;expected_accuracy=0.999	gnl|BL_ORD_ID|48559 cb10471_c4/f1p1/2111	99	1631	1	101	569	2112	58	158
cb6221_c19/f1p0/1943	gnl|BL_ORD_ID|49542 cb6221_c14/f1p0/2546	247	1873	66	247	756	2385	3	185

cb1913_c44/f1p0/2236 isoform=cb1913_c44;full_length_coverage=1;non_full_length_coverage=0;isoform_length=2236;expected_accuracy=0.998	gnl|BL_ORD_ID|66491 cb1913_c50/f1p1/2689	1	1634	1630	2236	98	172 4	1849	246 7
cb6712_c3/f2p0/2326 isoform=cb6712_c3;full_length_coverage=2;non_full_length_coverage=0;isoform_length=2326;expected_accuracy=1.000	gnl|BL_ORD_ID|4045 cb6712_c5/f1p0/2789 isoform=cb6712_c5;full_length_coverage=1;non_full_length_coverage=0;isoform_length=2789;expected_accuracy=0.999	1	1423	1422	2245	67	148 8	1966	278 9
cb8564_c10819/f1p1/3887 isoform=cb8564_c10819;full_length_coverage=1;non_full_length_coverage=1;isoform_length=3887;expected_accuracy=0.995	gnl|BL_ORD_ID|14495 cb8564_c35464/f1p1/3983 isoform=cb8564_c35464;full_length_coverage=1;non_full_length_coverage=1;isoform_length=3983;expected_accuracy=0.993	1	3553	3549	3854	10	356 0	3677	398 3
cb2279_c0/f10p3/2834 isoform=cb2279_c0;full_length_coverage=10;non_full_length_coverage=3;isoform_length=2834;expected_accuracy=1.000	gnl|BL_ORD_ID|68406 cb2279_c5/f1p1/2918	1	1930	1929	2813	1	193 0	2076	296 0
cb5814_c5/f1p0/2403 isoform=cb5814_c5;full_length_coverage=1;non_full_length_coverage=0;isoform_length=2403;expected_accuracy=0.993	gnl|BL_ORD_ID|45442 cb5814_c10/f1p1/2706 isoform=cb5814_c10;full_length_coverage=1;non_full_length_coverage=1;isoform_length=2706;expected_accuracy=0.993	1	1848	1845	2403	86	193 5	2065	262 2
cb8564_c185920/f1p0/2777 isoform=cb8564_c185920;full_length_coverage=1;non_full_length_coverage=0;isoform_length=2777;expected_accuracy=0.996	gnl|BL_ORD_ID|55839 cb8564_c43677/f1p0/3269	174	2770	1	177	404	299 9	69	245
cb5068_c5/f1p1/2699 isoform=cb5068_c5;full_length_coverage=1;non_full_le	gnl|BL_ORD_ID|73236 cb5068_c3/f1p1/2981	2	2431	2426	2699	147	257 7	2723	299 6

ngth_coverage=1;isoform_length=2699;expected_accuracy=0.995									
cb2629_c15/f1p0/2175 isoform=cb2629_c15;full_length_coverage=1;non_full_length_coverage=0;isoform_length=2175;expected_accuracy=0.991	gnl|BL_ORD_ID|68645 cb2629_c13/f1p0/4067	1	1663	1662	2175	1785	3450	3561	4074
cb13753_c0/f1p0/1755	gnl|BL_ORD_ID|41340 cb3759_c3/f1p0/1980 isoform=cb3759_c3;full_length_coverage=1;non_full_length_coverage=0;isoform_length=1980;expected_accuracy=0.996	377	1707	7	378	635	1964	1	372
cb7576_c14/f1p1/1754	gnl|BL_ORD_ID|50604 cb7576_c6/f1p0/2034	1	1614	1610	1758	1	1608	1716	1868
cb3143_c31/f1p0/2171	gnl|BL_ORD_ID|36686 cb3143_c30/f1p0/2377 isoform=cb3143_c30;full_length_coverage=1;non_full_length_coverage=0;isoform_length=2377;expected_accuracy=1.000	1	1600	1599	2164	1	1605	1749	2315
cb6111_c34/f1p0/1434 isoform=cb6111_c34;full_length_coverage=1;non_full_length_coverage=0;isoform_length=1434;expected_accuracy=0.992	gnl|BL_ORD_ID|74915 cb6111_c17/f1p0/1775	121	1387	12	126	416	1681	14	131
cb4543_c0/f1p0/3310	gnl|BL_ORD_ID|36954 cb2770_c15/f1p1/3337 isoform=cb2770_c15;full_length_coverage=1;non_full_length_coverage=1;isoform_length=3337;expected_accuracy=0.994	652	3117	73	651	2605	139	3294	2715
cb9718_c43/f1p0/1655 isoform=cb9718_c43;full_length_coverage=1;non_full_	gnl|BL_ORD_ID|20841 cb9718_c52/f66p3/1863 isoform=cb9718_c52;full_length_coverage=66;non_full	288	1655	3	290	488	1862	101	388

length_coverage=0;isoform_length=1655;expected_accuracy=0.996	_length_coverage=3;isoform_length=1863;expected_accuracy=1.000								
cb7849_c18/f1p0/2052 isoform=cb7849_c18;full_length_coverage=1;non_full_length_coverage=0;isoform_length=2052;expected_accuracy=0.991	gnl|BL_ORD_ID|51570 cb7849_c31/f1p0/2309	12	1375	1373	2051	55	1418	1551	2234
cb8564_c107494/f8p3/3940 isoform=cb8564_c107494;full_length_coverage=8;non_full_length_coverage=3;isoform_length=3940;expected_accuracy=1.000	gnl|BL_ORD_ID|18179 cb8564_c87968/f1p1/3997 isoform=cb8564_c87968;full_length_coverage=1;non_full_length_coverage=1;isoform_length=3997;expected_accuracy=0.993	505	3914	97	508	580	3997	1	412
cb8564_c90990/f1p3/3338	gnl|BL_ORD_ID|14810 cb8564_c34492/f1p1/3811 isoform=cb8564_c34492;full_length_coverage=1;non_full_length_coverage=1;isoform_length=3811;expected_accuracy=0.991	236	3271	2	237	771	3811	1	239
cb8564_c44290/f1p1/3198	gnl|BL_ORD_ID|14531 cb8564_c41778/f1p9/3482 isoform=cb8564_c41778;full_length_coverage=1;non_full_length_coverage=9;isoform_length=3482;expected_accuracy=0.992	336	2964	7	338	815	3442	5	342
cb4452_c6/f1p0/3034	gnl|BL_ORD_ID|72572 cb4452_c3/f1p0/3340	1293	3023	1	1295	1566	3297	154	1448
cb8564_c51647/f1p0/2214 isoform=cb8564_c51647;full_length_coverage=1;non_full_length_coverage=0;isoform_length=2214;expected_accuracy=0.993	gnl|BL_ORD_ID|55615 cb8564_c46860/f1p0/4047	159	2214	1	158	1582	3637	1209	1366
cb595_c15/f1p0/3791	gnl|BL_ORD_ID|45025 cb595_c8/f1p2/4751 isoform=cb595_c8;full_length_coverage=1;non_full_len	1	2284	2284	3916	9	2292	3065	4696

	gth_coverage=2;isoform_length=4751;expected_accuracy=0.998								
cb9781_c0/f1p0/1976	gnl|BL_ORD_ID|20401 cb9781_c1/f1p0/2121 isoform=cb9781_c1;full_length_coverage=1;non_full_length_coverage=0;isoform_length=2121;expected_accuracy=0.991	117	1976	1	116	220	2076	4	119
cb452_c69/f1p2/3866 isoform=cb452_c69;full_length_coverage=1;non_full_length_coverage=2;isoform_length=3866;expected_accuracy=0.998	gnl|BL_ORD_ID|43247 cb452_c17/f2p0/4042 isoform=cb452_c17;full_length_coverage=2;non_full_length_coverage=0;isoform_length=4042;expected_accuracy=0.997	2	2266	2266	3801	127	2369	2508	4042
cb1404_c30/f1p0/2845 isoform=cb1404_c30;full_length_coverage=1;non_full_length_coverage=0;isoform_length=2845;expected_accuracy=0.999	gnl|BL_ORD_ID|61342 cb1404_c12/f1p0/3355	1	2368	2363	2844	133	2497	2602	3086
cb965_c5/f2p7/4283 isoform=cb965_c5;full_length_coverage=2;non_full_length_coverage=7;isoform_length=4283;expected_accuracy=1.000	gnl|BL_ORD_ID|20577 cb965_c22/f1p1/4708 isoform=cb965_c22;full_length_coverage=1;non_full_length_coverage=1;isoform_length=4708;expected_accuracy=0.994	1	3552	3550	4267	1	3546	3991	4708
cb14897_c7/f1p0/1640 isoform=cb14897_c7;full_length_coverage=1;non_full_length_coverage=0;isoform_length=1640;expected_accuracy=1.000	gnl|BL_ORD_ID|27781 cb14897_c3/f1p1/1884 isoform=cb14897_c3;full_length_coverage=1;non_full_length_coverage=1;isoform_length=1884;expected_accuracy=0.996	12	1245	1240	1639	28	1263	1487	1884
cb7064_c2/f4p0/1297 isoform=cb7064_c2;full_length_coverage=4;non_full_length_coverage=0;isoform_length=1297;expected_accuracy=1.000	gnl|BL_ORD_ID|50308 cb7064_c16/f1p0/3334	155	1297	50	155	1854	2995	24	129

<table>
<tr><td>cb2177_c46/f1p0/2729</td><td>gnl|BL_ORD_ID|35910 cb2177_c22/f1p0/4026 isoform=cb2177_c22;full_length_coverage=1;non_full_length_coverage=0;isoform_length=4026;expected_accuracy=0.997</td><td>1340</td><td>2722</td><td>12</td><td>1341</td><td>2644</td><td>4026</td><td>1</td><td>1330</td></tr>
<tr><td>cb289_c5/f1p0/4379 isoform=cb289_c5;full_length_coverage=1;non_full_length_coverage=0;isoform_length=4379;expected_accuracy=0.997</td><td>gnl|BL_ORD_ID|37736 cb289_c4/f1p0/4413 isoform=cb289_c4;full_length_coverage=1;non_full_length_coverage=0;isoform_length=4413;expected_accuracy=0.994</td><td>1</td><td>4156</td><td>4156</td><td>4305</td><td>6</td><td>4163</td><td>4265</td><td>4413</td></tr>
<tr><td>cb8277_c3/f1p0/1611 isoform=cb8277_c3;full_length_coverage=1;non_full_length_coverage=0;isoform_length=1611;expected_accuracy=0.997</td><td>gnl|BL_ORD_ID|7850 cb8277_c2/f1p0/3699 isoform=cb8277_c2;full_length_coverage=1;non_full_length_coverage=0;isoform_length=3699;expected_accuracy=0.997</td><td>1</td><td>1250</td><td>1251</td><td>1589</td><td>7</td><td>1251</td><td>1777</td><td>2115</td></tr>
<tr><td>cb8564_c17892/f1p0/3277</td><td>gnl|BL_ORD_ID|52815 cb8564_c14758/f1p0/4100</td><td>200</td><td>3271</td><td>1</td><td>201</td><td>699</td><td>3769</td><td>125</td><td>325</td></tr>
<tr><td>cb2072_c47/f1p0/1819</td><td>gnl|BL_ORD_ID|34951 cb2072_c45/f1p0/2282 isoform=cb2072_c45;full_length_coverage=1;non_full_length_coverage=0;isoform_length=2282;expected_accuracy=0.994</td><td>331</td><td>1810</td><td>71</td><td>330</td><td>420</td><td>1899</td><td>2</td><td>261</td></tr>
<tr><td>cb3687_c16/f1p3/2980 isoform=cb3687_c16;full_length_coverage=1;non_full_length_coverage=3;isoform_length=2980;expected_accuracy=0.998</td><td>gnl|BL_ORD_ID|41006 cb3687_c7/f2p0/3068 isoform=cb3687_c7;full_length_coverage=2;non_full_length_coverage=0;isoform_length=3068;expected_accuracy=0.997</td><td>1</td><td>2002</td><td>2001</td><td>2888</td><td>35</td><td>2031</td><td>2181</td><td>3068</td></tr>
<tr><td>cb8564_c44698/f1p5/2843 isoform=cb8564_c44698;full_length_coverage=1;non_full_length_coverage=5;isoform_length=2843;expected_accuracy=0.997</td><td>gnl|BL_ORD_ID|15315 cb8564_c39014/f1p0/3019 isoform=cb8564_c39014;full_length_coverage=1;non_full_length_coverage=0;isoform_length=3019;expected_accuracy=0.992</td><td>1</td><td>2514</td><td>2511</td><td>2826</td><td>1</td><td>2564</td><td>2691</td><td>3013</td></tr>
</table>

cb2033_c16/f1p0/2147 isoform=cb2033_c16;full_length_coverage=1;non_full_length_coverage=0;isoform_length=2147;expected_accuracy=0.994	gnl|BL_ORD_ID|66631 cb2033_c9/f1p0/2609	1	1872	1868	2147	1	1867	2028	2308
cb1905_c32/f1p0/2036 isoform=cb1905_c32;full_length_coverage=1;non_full_length_coverage=0;isoform_length=2036;expected_accuracy=0.996	gnl|BL_ORD_ID|34533 cb1905_c22/f1p0/2509 isoform=cb1905_c22;full_length_coverage=1;non_full_length_coverage=0;isoform_length=2509;expected_accuracy=0.996	553	2036	7	553	786	2269	1	547
cb396_c48/f1p0/2172	gnl|BL_ORD_ID|70866 cb396_c50/f1p0/2950	238	2101	54	238	1103	2966	670	854
cb2156_c24/f1p1/2545 isoform=cb2156_c24;full_length_coverage=1;non_full_length_coverage=1;isoform_length=2545;expected_accuracy=0.991	gnl|BL_ORD_ID|35476 cb2156_c23/f1p1/3009 isoform=cb2156_c23;full_length_coverage=1;non_full_length_coverage=1;isoform_length=3009;expected_accuracy=0.993	1	1422	1420	2545	218	1646	1771	2896
cb9718_c43/f1p0/1655 isoform=cb9718_c43;full_length_coverage=1;non_full_length_coverage=0;isoform_length=1655;expected_accuracy=0.996	gnl|BL_ORD_ID|20739 cb9718_c35/f1p2/1802 isoform=cb9718_c35;full_length_coverage=1;non_full_length_coverage=2;isoform_length=1802;expected_accuracy=1.000	288	1655	56	290	335	1702	1	235
cb8564_c40598/f1p4/3119	gnl|BL_ORD_ID|16681 cb8564_c54233/f1p1/3194 isoform=cb8564_c54233;full_length_coverage=1;non_full_length_coverage=1;isoform_length=3194;expected_accuracy=0.995	2	2732	2729	3049	8	2753	2874	3194
cb5058_c1/f1p0/2699 isoform=cb5058_c1;full_length_coverage=1;non_full_length_coverage=0;isoform_length=2699;expected_accuracy=0.991	gnl|BL_ORD_ID|44019 cb5058_c0/f1p0/3186 isoform=cb5058_c0;full_length_coverage=1;non_full_length_coverage=0;isoform_length=3186;expected_accuracy=0.997	1	2469	2464	2593	481	2953	3057	3186

cb8531_c1/f1p0/2194	gnl|BL_ORD_ID|10091 cb8531_c0/f2p0/2343 isoform=cb8531_c0;full_length_coverage=2;non_full_length_coverage=0;isoform_length=2343;expected_accuracy=0.999	1	1452	1453	2209	29	1480	1580	2336
cb8564_c9728/f7p1/3462 isoform=cb8564_c9728;full_length_coverage=7;non_full_length_coverage=1;isoform_length=3462;expected_accuracy=0.997	gnl|BL_ORD_ID|52659 cb8564_c10244/f1p0/4022	1	2939	2937	3410	41	2965	3383	3869
cb8564_c38035/f1p0/2770	gnl|BL_ORD_ID|56961 cb8564_c78609/f1p0/3194	19	2035	2033	2756	45	2064	2254	2978
cb12931_c15/f1p0/1493 isoform=cb12931_c15;full_length_coverage=1;non_full_length_coverage=0;isoform_length=1493;expected_accuracy=1.000	gnl|BL_ORD_ID|55280 cb8564_c37198/f1p5/2941	1	1106	1104	1493	1	1102	2540	2929
cb2927_c2/f2p0/3116 isoform=cb2927_c2;full_length_coverage=2;non_full_length_coverage=0;isoform_length=3116;expected_accuracy=0.995	gnl|BL_ORD_ID|69003 cb2927_c5/f1p1/3753	12	1680	1676	3096	1	1685	2333	3759
cb8564_c45300/f1p4/3014 isoform=cb8564_c45300;full_length_coverage=1;non_full_length_coverage=4;isoform_length=3014;expected_accuracy=0.996	gnl|BL_ORD_ID|55472 cb8564_c42531/f1p0/3996	243	3014	128	246	1090	3863	816	933
cb4128_c23/f1p0/2488 isoform=cb4128_c23;full_length_coverage=1;non_full_length_coverage=0;isoform_length=2488;expected_accuracy=0.999	gnl|BL_ORD_ID|70785 cb4128_c21/f1p0/3017	1	1757	1756	2439	2	1760	2096	2779

cb8564_c66920/f3p3/2894	gnl|BL_ORD_ID|16879 cb8564_c48606/f1p1/3308 isoform=cb8564_c48606;full_length_coverage=1;non_full_length_coverage=1;isoform_length=3308;expected_accuracy=0.998	1	2153	2149	2863	1	2151	2587	3301
cb781_c2/f3p0/1827 isoform=cb781_c2;full_length_coverage=3;non_full_length_coverage=0;isoform_length=1827;expected_accuracy=0.999	gnl|BL_ORD_ID|6928 cb781_c18/f1p0/2084 isoform=cb781_c18;full_length_coverage=1;non_full_length_coverage=0;isoform_length=2084;expected_accuracy=0.992	1	1243	1243	1827	23	1262	1367	1948
cb649_c40/f1p0/3326	gnl|BL_ORD_ID|49373 cb649_c36/f1p0/4093	1	3135	3133	3349	2	3132	3679	3907
cb964_c10/f1p0/2954 isoform=cb964_c10;full_length_coverage=1;non_full_length_coverage=0;isoform_length=2954;expected_accuracy=0.996	gnl|BL_ORD_ID|59137 cb964_c2/f1p0/4848	147	2954	1	151	1674	4481	213	363
cb9442_c4/f1p0/1965	gnl|BL_ORD_ID|59723 cb9442_c8/f1p1/2068	1	1455	1453	1829	1	1456	1668	2044
cb8564_c2797/f1p0/2268 isoform=cb8564_c2797;full_length_coverage=1;non_full_length_coverage=0;isoform_length=2268;expected_accuracy=0.992	gnl|BL_ORD_ID|56487 cb8564_c77730/f1p0/2866	1	1374	1370	2198	179	1548	1802	2630
cb181_c38/f1p0/2538	gnl|BL_ORD_ID|66303 cb181_c44/f1p0/2945	1089	2510	1	1094	1221	2642	1	1093
cb8564_c122327/f1p0/3916 isoform=cb8564_c122327;full_length_coverage=1;non_full_length_coverage=0;isoform_length=3916;expected_accuracy=0.995	gnl|BL_ORD_ID|54548 cb8564_c125967/f1p0/4340	2	2812	2812	3916	50	2822	3205	4310

cb6508_c15/f1p0/2527	gnl|BL_ORD_ID|47554 cb6508_c8/f1p0/3471	1	1256	1257	2218	2	1257	2543	3504
cb8564_c15844/f1p3/3061 isoform=cb8564_c15844;full_length_coverage=1;non_full_length_coverage=3;isoform_length=3061;expected_accuracy=0.995	gnl|BL_ORD_ID|17091 cb8564_c97736/f2p5/3272 isoform=cb8564_c97736;full_length_coverage=2;non_full_length_coverage=5;isoform_length=3272;expected_accuracy=1.000	1	2692	2688	3060	34	2726	2899	3272
cb4848_c24/f1p0/1860 isoform=cb4848_c24;full_length_coverage=1;non_full_length_coverage=0;isoform_length=1860;expected_accuracy=0.999	gnl|BL_ORD_ID|73029 cb4848_c17/f1p0/2140	206	1860	68	205	274	1926	2	139
cb1626_c14/f1p0/2173 isoform=cb1626_c14;full_length_coverage=1;non_full_length_coverage=0;isoform_length=2173;expected_accuracy=0.999	gnl|BL_ORD_ID|63808 cb1626_c17/f1p0/3006	1001	2172	1	1001	1827	2999	77	1092
cb4503_c12/f1p0/2341 isoform=cb4503_c12;full_length_coverage=1;non_full_length_coverage=0;isoform_length=2341;expected_accuracy=0.999	gnl|BL_ORD_ID|42041 cb4503_c8/f2p0/2921 isoform=cb4503_c8;full_length_coverage=2;non_full_length_coverage=0;isoform_length=2921;expected_accuracy=1.000	1	1488	1486	2329	166	1660	2076	2921
cb8564_c48953/f1p0/2715	gnl|BL_ORD_ID|55975 cb8564_c50843/f1p0/4165	1	2134	2133	2584	1119	3252	3639	4090
cb507_c4/f1p0/3466	gnl|BL_ORD_ID|43270 cb507_c1/f1p0/4600 isoform=cb507_c1;full_length_coverage=1;non_full_length_coverage=0;isoform_length=4600;expected_accuracy=0.997	1	2743	2739	3390	1	2762	3946	4597
cb7774_c5/f1p0/3020	gnl|BL_ORD_ID|51513 cb7774_c3/f1p0/4456	169	3042	1	173	1587	4460	22	194

cb416_c28/f1p0/2988 isoform=cb416_c28;full_length_coverage=1;non_full_length_coverage=0;isoform_length=2988;expected_accuracy=0.996	gnl|BL_ORD_ID|44207 cb416_c73/f3p0/3007 isoform=cb416_c73;full_length_coverage=3;non_full_length_coverage=0;isoform_length=3007;expected_accuracy=1.000	125	2890	1	128	240	3007	1	129
cb7880_c5/f1p0/2230	gnl|BL_ORD_ID|51597 cb7880_c3/f1p0/2527	1	1374	1371	2217	31	1403	1616	2461
cb8564_c36056/f1p0/2652 isoform=cb8564_c36056;full_length_coverage=1;non_full_length_coverage=0;isoform_length=2652;expected_accuracy=0.999	gnl|BL_ORD_ID|55866 cb8564_c47522/f1p0/2954	248	2651	22	251	445	2849	70	301
cb5125_c36/f1p0/1789 isoform=cb5125_c36;full_length_coverage=1;non_full_length_coverage=0;isoform_length=1789;expected_accuracy=0.998	gnl|BL_ORD_ID|42295 cb5125_c41/f11p2/2137 isoform=cb5125_c41;full_length_coverage=11;non_full_length_coverage=2;isoform_length=2137;expected_accuracy=1.000	1	1163	1158	1789	73	1240	1446	2077
cb8564_c17543/f1p10/2689 isoform=cb8564_c17543;full_length_coverage=1;non_full_length_coverage=10;isoform_length=2689;expected_accuracy=0.999	gnl|BL_ORD_ID|14058 cb8564_c23936/f1p0/3016 isoform=cb8564_c23936;full_length_coverage=1;non_full_length_coverage=0;isoform_length=3016;expected_accuracy=0.995	1	1707	1705	2579	1	1716	2143	3016
cb8564_c46939/f1p1/2926	gnl|BL_ORD_ID|15152 cb8564_c38629/f2p1/3246 isoform=cb8564_c38629;full_length_coverage=2;non_full_length_coverage=1;isoform_length=3246;expected_accuracy=0.993	1	2134	2130	2870	1	2173	2278	3020
cb8564_c51661/f1p0/3132	gnl|BL_ORD_ID|14067 cb8564_c33962/f2p1/3330 isoform=cb8564_c33962;full_length_coverage=2;non_full_length_coverage=1;isoform_length=3330;expected_accuracy=1.000	1	2104	2099	3077	1	2105	2262	3240

cb4108_c0/f3p0/2143 isoform=cb4108_c0;full_length_coverage=3;non_full_length_coverage=0;isoform_length=2143;expected_accuracy=1.000	gnl|BL_ORD_ID|40378 cb4108_c1/f1p0/3431 isoform=cb4108_c1;full_length_coverage=1;non_full_length_coverage=0;isoform_length=3431;expected_accuracy=0.998	1	1083	1083	2098	16	1097	2415	3431
cb8418_c0/f2p0/2231 isoform=cb8418_c0;full_length_coverage=2;non_full_length_coverage=0;isoform_length=2231;expected_accuracy=0.997	gnl|BL_ORD_ID|9126 cb8418_c1/f1p0/2365 isoform=cb8418_c1;full_length_coverage=1;non_full_length_coverage=0;isoform_length=2365;expected_accuracy=0.991	595	2231	78	595	717	2361	1	515
cb8564_c180116/f1p0/2944 isoform=cb8564_c180116;full_length_coverage=1;non_full_length_coverage=0;isoform_length=2944;expected_accuracy=0.996	gnl|BL_ORD_ID|55600 cb8564_c46484/f1p0/3151	106	2522	2520	2944	5	2441	2670	3093
cb3941_c92/f1p0/2573	gnl|BL_ORD_ID|39558 cb3941_c41/f1p0/2822 isoform=cb3941_c41;full_length_coverage=1;non_full_length_coverage=0;isoform_length=2822;expected_accuracy=0.991	136	2230	11	137	728	2822	6	131
cb5307_c9/f1p0/2902	gnl|BL_ORD_ID|73771 cb5307_c16/f1p0/3281	1204	2896	11	1204	1295	2988	2	1187
cb8564_c73853/f1p0/2443 isoform=cb8564_c73853;full_length_coverage=1;non_full_length_coverage=0;isoform_length=2443;expected_accuracy=1.000	gnl|BL_ORD_ID|13313 cb8564_c188075/f1p2/2904 isoform=cb8564_c188075;full_length_coverage=1;non_full_length_coverage=2;isoform_length=2904;expected_accuracy=0.996	1	1281	1276	2443	98	1374	1654	2829
cb5819_c17/f2p0/2480 isoform=cb5819_c17;full_length_coverage=2;non_full_length_coverage=0;isoform_length=2480;expected_accuracy=0.996	gnl|BL_ORD_ID|44667 cb5819_c6/f1p0/2565 isoform=cb5819_c6;full_length_coverage=1;non_full_length_coverage=0;isoform_length=2565;expected_accuracy=1.000	1	1752	1748	2456	1	1755	1858	2565

cb626_c60/f1p1/4254	gnl|BL_ORD_ID|4060 cb626_c17/f1p0/4935 isoform=cb626_c17;full_length_coverage=1;non_full_length_coverage=0;isoform_length=4935;expected_accuracy=0.993	32	3661	3659	4223	1	3585	4375	4935
cb500_c17/f1p0/2323 isoform=cb500_c17;full_length_coverage=1;non_full_length_coverage=0;isoform_length=2323;expected_accuracy=0.997	gnl|BL_ORD_ID|70944 cb500_c35/f1p0/3166	1	1293	1288	2216	700	1992	2124	3052
cb6719_c56/f1p0/2578	gnl|BL_ORD_ID|4666 cb6719_c24/f1p0/2613 isoform=cb6719_c24;full_length_coverage=1;non_full_length_coverage=0;isoform_length=2613;expected_accuracy=0.998	1	1366	1365	2419	72	1437	1559	2613
cb8564_c20384/f1p4/2475 isoform=cb8564_c20384;full_length_coverage=1;non_full_length_coverage=4;isoform_length=2475;expected_accuracy=0.999	gnl|BL_ORD_ID|17162 cb8564_c87465/f2p9/3179 isoform=cb8564_c87465;full_length_coverage=2;non_full_length_coverage=9;isoform_length=3179;expected_accuracy=1.000	1	1492	1493	2475	1177	2667	2010	2993
cb398_c5/f1p0/4384	gnl|BL_ORD_ID|39099 cb398_c3/f1p0/4458 isoform=cb398_c3;full_length_coverage=1;non_full_length_coverage=0;isoform_length=4458;expected_accuracy=0.996	168	4235	1	172	396	4458	33	205
cb3848_c1/f4p0/2367 isoform=cb3848_c1;full_length_coverage=4;non_full_length_coverage=0;isoform_length=2367;expected_accuracy=0.995	gnl|BL_ORD_ID|40468 cb3848_c17/f1p0/3076 isoform=cb3848_c17;full_length_coverage=1;non_full_length_coverage=0;isoform_length=3076;expected_accuracy=0.992	2	2023	2021	2315	1	2033	2759	3050
cb9700_c5/f1p0/1823 isoform=cb9700_c5;full_length_coverage=1;non_full_le	gnl|BL_ORD_ID|59913 cb9700_c1/f1p0/2092	1	1520	1519	1823	29	1548	1667	1971

ngth_coverage=0;isoform_length=1823;expected_accuracy=0.999									
cb17756_c15/f1p0/1779 isoform=cb17756_c15;full_length_coverage=1;non_full_length_coverage=0;isoform_length=1779;expected_accuracy=0.996	gnl|BL_ORD_ID|13304 cb8564_c20604/f1p0/4741 isoform=cb8564_c20604;full_length_coverage=1;non_full_length_coverage=0;isoform_length=4741;expected_accuracy=0.996	108	1779	1	110	1322	2989	361	471
cb7836_c3/f1p0/1935	gnl|BL_ORD_ID|6770 cb7836_c0/f1p0/2522 isoform=cb7836_c0;full_length_coverage=1;non_full_length_coverage=0;isoform_length=2522;expected_accuracy=0.993	562	1946	7	563	1138	2519	1	557
cb8564_c1631/f1p0/2766 isoform=cb8564_c1631;full_length_coverage=1;non_full_length_coverage=0;isoform_length=2766;expected_accuracy=0.999	gnl|BL_ORD_ID|56577 cb8564_c7828/f1p0/2920	124	2766	1	126	281	2921	1	124
cb3580_c26/f1p0/2466	gnl|BL_ORD_ID|40393 cb3580_c24/f1p0/3582 isoform=cb3580_c24;full_length_coverage=1;non_full_length_coverage=0;isoform_length=3582;expected_accuracy=0.992	180	2434	1	185	1337	3582	765	949
cb8564_c81580/f1p0/2450 isoform=cb8564_c81580;full_length_coverage=1;non_full_length_coverage=0;isoform_length=2450;expected_accuracy=0.997	gnl|BL_ORD_ID|15502 cb8564_c75170/f2p0/2677 isoform=cb8564_c75170;full_length_coverage=2;non_full_length_coverage=0;isoform_length=2677;expected_accuracy=0.994	1	1786	1781	2450	1	1787	1926	2594
cb1524_c8/f1p0/3674 isoform=cb1524_c8;full_length_coverage=1;non_full_length_coverage=0;isoform_length=3674;expected_accuracy=0.995	gnl|BL_ORD_ID|28439 cb1524_c30/f1p0/3792 isoform=cb1524_c30;full_length_coverage=1;non_full_length_coverage=0;isoform_length=3792;expected_accuracy=0.994	157	3670	1	156	256	3792	2	156

cb993_c8/f2p0/2754 isoform=cb993_c8;full_length_coverage=2;non_full_length_coverage=0;isoform_length=2754;expected_accuracy=0.999	gnl|BL_ORD_ID|19610 cb993_c16/f1p0/4226 isoform=cb993_c16;full_length_coverage=1;non_full_length_coverage=0;isoform_length=4226;expected_accuracy=0.997	1	2282	2280	2754	1	2279	3676	4149
cb938_c15/f1p0/3219	gnl|BL_ORD_ID|19888 cb938_c17/f1p1/3461 isoform=cb938_c17;full_length_coverage=1;non_full_length_coverage=1;isoform_length=3461;expected_accuracy=0.991	1	2546	2541	3185	1	2547	2825	3461
cb792_c8/f3p0/3700 isoform=cb792_c8;full_length_coverage=3;non_full_length_coverage=0;isoform_length=3700;expected_accuracy=1.000	gnl|BL_ORD_ID|50981 cb792_c47/f1p0/3981	186	3673	1	187	411	3901	93	293
cb8658_c2/f1p0/2311	gnl|BL_ORD_ID|18756 cb8658_c6/f1p0/3454 isoform=cb8658_c6;full_length_coverage=1;non_full_length_coverage=0;isoform_length=3454;expected_accuracy=0.996	1	1701	1700	2184	49	1745	2967	3454
cb626_c67/f1p1/4388	gnl|BL_ORD_ID|47167 cb626_c79/f1p0/5202	1831	4309	111	1836	2580	5057	15	1739
cb8564_c4643/f1p1/2554	gnl|BL_ORD_ID|56658 cb8564_c51371/f1p1/2943	1	1705	1700	2540	1	1705	1838	2681
cb1512_c15/f1p1/3322	gnl|BL_ORD_ID|27951 cb1512_c4/f1p1/4292 isoform=cb1512_c4;full_length_coverage=1;non_full_length_coverage=1;isoform_length=4292;expected_accuracy=0.997	1	1681	1681	3271	129	1808	2672	4292
cb8564_c17543/f1p10/2689 isoform=cb8564_c17543;full_length_coverage=1;non_f	gnl|BL_ORD_ID|56381 cb8564_c75626/f1p2/3199	1	2117	2115	2586	1	2116	2611	3081

ull_length_coverage=10;isoform_length=2689;expected_accuracy=0.999									
cb8564_c75626/f1p2/3199	gnl|BL_ORD_ID|52063 cb8564_c11680/f1p1/3676	25	2674	2672	3151	53	2692	2993	3471
cb11867_c3/f1p0/1755 isoform=cb11867_c3;full_length_coverage=1;non_full_length_coverage=0;isoform_length=1755;expected_accuracy=1.000	gnl|BL_ORD_ID|13423 cb8564_c23368/f1p0/2544 isoform=cb8564_c23368;full_length_coverage=1;non_full_length_coverage=0;isoform_length=2544;expected_accuracy=0.998	435	1755	8	440	1225	2544	1	433
cb3800_c3/f3p0/3108 isoform=cb3800_c3;full_length_coverage=3;non_full_length_coverage=0;isoform_length=3108;expected_accuracy=1.000	gnl|BL_ORD_ID|40736 cb3800_c17/f1p0/3180 isoform=cb3800_c17;full_length_coverage=1;non_full_length_coverage=0;isoform_length=3180;expected_accuracy=0.993	1037	3046	1	1038	1171	3180	22	1053
cb5516_c26/f1p0/3029 isoform=cb5516_c26;full_length_coverage=1;non_full_length_coverage=0;isoform_length=3029;expected_accuracy=1.000	gnl|BL_ORD_ID|74009 cb5516_c65/f1p1/3150	1	2327	2322	2971	5	2330	2455	3104
cb6825_c19/f1p0/2295 isoform=cb6825_c19;full_length_coverage=1;non_full_length_coverage=0;isoform_length=2295;expected_accuracy=0.997	gnl|BL_ORD_ID|4067 cb6825_c32/f1p1/2521 isoform=cb6825_c32;full_length_coverage=1;non_full_length_coverage=1;isoform_length=2521;expected_accuracy=0.997	1	1494	1489	2292	4	1497	1670	2472
cb10464_c4/f1p0/1117 isoform=cb10464_c4;full_length_coverage=1;non_full_length_coverage=0;isoform_length=1117;expected_accuracy=0.990	gnl|BL_ORD_ID|1828 cb10464_c0/f4p0/1450 isoform=cb10464_c0;full_length_coverage=4;non_full_length_coverage=0;isoform_length=1450;expected_accuracy=1.000	376	1113	9	380	713	1450	1	373
cb8564_c119678/f2p0/2739 isoform=cb8564_c119678;full_length_coverage=2;non_	gnl|BL_ORD_ID|11469 cb8564_c13203/f2p0/2827 isoform=cb8564_c13203;full_length_coverage=2;non_fu	1	2299	2298	2707	12	2301	2419	2827

full_length_coverage=0;isoform_length=2739;expected_accuracy=1.000	ll_length_coverage=0;isoform_length=2827;expected_accuracy=0.999								
cb8564_c13319/f1p3/2892 isoform=cb8564_c13319;full_length_coverage=1;non_full_length_coverage=3;isoform_length=2892;expected_accuracy=0.996	gnl|BL_ORD_ID|55298 cb8564_c37611/f1p0/3259	155	2786	1	156	323	2956	32	187
cb9780_c1/f2p0/1733 isoform=cb9780_c1;full_length_coverage=2;non_full_length_coverage=0;isoform_length=1733;expected_accuracy=0.995	gnl|BL_ORD_ID|19369 cb9780_c0/f33p0/1865 isoform=cb9780_c0;full_length_coverage=33;non_full_length_coverage=0;isoform_length=1865;expected_accuracy=1.000	117	1727	1	120	259	1865	6	121
cb1956_c5/f2p0/2813 isoform=cb1956_c5;full_length_coverage=2;non_full_length_coverage=0;isoform_length=2813;expected_accuracy=0.997	gnl|BL_ORD_ID|36248 cb1956_c2/f5p0/3073 isoform=cb1956_c2;full_length_coverage=5;non_full_length_coverage=0;isoform_length=3073;expected_accuracy=0.999	1	1453	1451	2812	85	1532	1692	3073
cb8564_c74379/f1p1/2508	gnl|BL_ORD_ID|15835 cb8564_c73664/f5p3/2665 isoform=cb8564_c73664;full_length_coverage=5;non_full_length_coverage=3;isoform_length=2665;expected_accuracy=0.994	1	1645	1642	2352	3	1655	1860	2570
cb9467_c3/f4p0/2200 isoform=cb9467_c3;full_length_coverage=4;non_full_length_coverage=0;isoform_length=2200;expected_accuracy=1.000	gnl|BL_ORD_ID|20200 cb9467_c17/f1p0/3298 isoform=cb9467_c17;full_length_coverage=1;non_full_length_coverage=0;isoform_length=3298;expected_accuracy=1.000	258	2200	96	257	1353	3295	1	162
cb4263_c3/f1p1/3036	gnl|BL_ORD_ID|43863 cb4263_c0/f1p1/3386 isoform=cb4263_c0;full_length_coverage=1;non_full_length_coverage=1;isoform_length=3386;expected_accuracy=0.992	263	3038	1	265	623	3386	86	350

cb8564_c32509/f1p1/2880 isoform=cb8564_c32509;full_length_coverage=1;non_full_length_coverage=1;isoform_length=2880;expected_accuracy=0.991	gnl|BL_ORD_ID|14531 cb8564_c41778/f1p9/3482 isoform=cb8564_c41778;full_length_coverage=1;non_full_length_coverage=9;isoform_length=3482;expected_accuracy=0.992	241	2877	2	245	812	3416	10	247
cb12931_c15/f1p0/1493 isoform=cb12931_c15;full_length_coverage=1;non_full_length_coverage=0;isoform_length=1493;expected_accuracy=1.000	gnl|BL_ORD_ID|13559 cb8564_c183540/f1p0/2769 isoform=cb8564_c183540;full_length_coverage=1;non_full_length_coverage=0;isoform_length=2769;expected_accuracy=0.996	1	1106	1104	1492	1	1102	2382	2769
cb8564_c72680/f1p0/2685	gnl|BL_ORD_ID|12821 cb8564_c156372/f1p3/2909 isoform=cb8564_c156372;full_length_coverage=1;non_full_length_coverage=3;isoform_length=2909;expected_accuracy=1.000	1	1735	1730	2636	45	1782	1961	2867
cb8113_c5/f1p1/2305 isoform=cb8113_c5;full_length_coverage=1;non_full_length_coverage=1;isoform_length=2305;expected_accuracy=0.995	gnl|BL_ORD_ID|8750 cb8113_c2/f1p1/2633 isoform=cb8113_c2;full_length_coverage=1;non_full_length_coverage=1;isoform_length=2633;expected_accuracy=0.997	1139	2305	1	1138	1467	2633	15	1152
cb8564_c148937/f2p0/2793	gnl|BL_ORD_ID|57112 cb8564_c83553/f1p0/3004	306	2796	89	303	349	2840	1	215
cb2692_c3/f1p0/1958	gnl|BL_ORD_ID|37633 cb2692_c2/f1p0/3834 isoform=cb2692_c2;full_length_coverage=1;non_full_length_coverage=0;isoform_length=3834;expected_accuracy=0.997	290	1977	6	291	2142	3823	1	287
cb7037_c5/f1p0/2408	gnl|BL_ORD_ID|47824 cb7037_c1/f1p0/2620	1	2028	2028	2386	103	2130	2245	2610
cb8564_c77710/f1p0/1826	gnl|BL_ORD_ID|13694 cb8564_c19189/f1p1/3379 isoform=cb8564_c19189;full_length_coverage=1;non_fu	479	1645	5	481	2222	3379	1	468

	ll_length_coverage=1;isoform_length=3379;expected_accuracy=0.991								
cb8564_c48153/f1p0/3083	gnl|BL_ORD_ID|14531 cb8564_c41778/f1p9/3482 isoform=cb8564_c41778;full_length_coverage=1;non_full_length_coverage=9;isoform_length=3482;expected_accuracy=0.992	251	2896	13	255	812	3459	1	247
cb8747_c11/f1p0/1868	gnl|BL_ORD_ID|17793 cb8747_c13/f6p0/1942 isoform=cb8747_c13;full_length_coverage=6;non_full_length_coverage=0;isoform_length=1942;expected_accuracy=1.000	526	1805	6	528	663	1942	22	544
cb16245_c3/f1p0/1340	gnl|BL_ORD_ID|48381 cb10317_c4/f1p0/1797	351	1303	9	354	828	1806	3	348
cb944_c103/f2p0/1192 isoform=cb944_c103;full_length_coverage=2;non_full_length_coverage=0;isoform_length=1192;expected_accuracy=0.999	gnl|BL_ORD_ID|21044 cb944_c16/f3p0/1974 isoform=cb944_c16;full_length_coverage=3;non_full_length_coverage=0;isoform_length=1974;expected_accuracy=1.000	391	1192	51	391	1166	1969	149	489
cb8564_c18575/f1p0/1939 isoform=cb8564_c18575;full_length_coverage=1;non_full_length_coverage=0;isoform_length=1939;expected_accuracy=0.990	gnl|BL_ORD_ID|55754 cb8564_c24444/f1p0/4497	1	1427	1422	1939	815	2254	3235	3761
cb9270_c3/f1p0/2645	gnl|BL_ORD_ID|20685 cb9270_c2/f1p0/2756 isoform=cb9270_c2;full_length_coverage=1;non_full_length_coverage=0;isoform_length=2756;expected_accuracy=0.997	632	2534	68	633	857	2756	26	589
cb8564_c50151/f1p0/3198 isoform=cb8564_c50151;full_length_coverage=1;non_f	gnl|BL_ORD_ID|56041 cb8564_c53077/f1p2/3406	341	3198	6	341	452	3287	1	337

ull_length_coverage=0;isoform_length=3198;expected_accuracy=0.995									
cb8564_c53036/f1p0/2917 isoform=cb8564_c53036;full_length_coverage=1;non_full_length_coverage=0;isoform_length=2917;expected_accuracy=0.999	gnl|BL_ORD_ID|15003 cb8564_c43733/f1p0/3701 isoform=cb8564_c43733;full_length_coverage=1;non_full_length_coverage=0;isoform_length=3701;expected_accuracy=0.999	235	2902	1	238	1063	3701	1	238
cb8564_c39136/f1p0/3036 isoform=cb8564_c39136;full_length_coverage=1;non_full_length_coverage=0;isoform_length=3036;expected_accuracy=0.998	gnl|BL_ORD_ID|55910 cb8564_c48645/f1p1/3287	12	2089	2085	2972	17	2098	2225	3113
cb1039_c25/f1p0/3513	gnl|BL_ORD_ID|1556 cb1039_c41/f1p0/4155 isoform=cb1039_c41;full_length_coverage=1;non_full_length_coverage=0;isoform_length=4155;expected_accuracy=0.999	2	2198	2198	3406	415	2626	2732	3940
cb8564_c146299/f10p7/3623 isoform=cb8564_c146299;full_length_coverage=10;non_full_length_coverage=7;isoform_length=3623;expected_accuracy=0.999	gnl|BL_ORD_ID|58077 cb8564_c88175/f1p0/4011	16	2746	2746	3517	12	2742	3254	4020
cb18456_c1419/f5p0/1798 isoform=cb18456_c1419;full_length_coverage=5;non_full_length_coverage=0;isoform_length=1798;expected_accuracy=1.000	gnl|BL_ORD_ID|9181 cb8564_c107375/f7p0/2006 isoform=cb8564_c107375;full_length_coverage=7;non_full_length_coverage=0;isoform_length=2006;expected_accuracy=0.997	247	1798	3	247	451	2002	1	244
cb8564_c42654/f1p1/2906 isoform=cb8564_c42654;full_length_coverage=1;non_full_length_coverage=1;isoform_length=2906;expected_accuracy=0.990	gnl|BL_ORD_ID|14410 cb8564_c44256/f1p1/3267 isoform=cb8564_c44256;full_length_coverage=1;non_full_length_coverage=1;isoform_length=3267;expected_accuracy=0.995	1	2130	2126	2824	1	2119	2553	3247

cb6785_c17/f1p0/2283	gnl|BL_ORD_ID|5434 cb6785_c11/f1p0/2230 isoform=cb6785_c11;full_length_coverage=1;non_full_length_coverage=0;isoform_length=2230;expected_accuracy=0.990	495	2087	84	498	591	2182	1	420
cb14637_c1/f1p0/1701	gnl|BL_ORD_ID|17509 cb8564_c824/f2p0/4292 isoform=cb8564_c824;full_length_coverage=2;non_full_length_coverage=0;isoform_length=4292;expected_accuracy=1.000	331	1706	4	331	2917	4292	1	328
cb2737_c9/f2p0/2910 isoform=cb2737_c9;full_length_coverage=2;non_full_length_coverage=0;isoform_length=2910;expected_accuracy=0.993	gnl|BL_ORD_ID|68778 cb2737_c36/f1p0/3051	210	2886	2	210	396	3079	1	210
cb8564_c21778/f1p0/3229 isoform=cb8564_c21778;full_length_coverage=1;non_full_length_coverage=0;isoform_length=3229;expected_accuracy=0.995	gnl|BL_ORD_ID|52049 cb8564_c11237/f1p0/3521	241	3205	1	242	384	3351	7	248
cb4286_c14/f1p0/2234 isoform=cb4286_c14;full_length_coverage=1;non_full_length_coverage=0;isoform_length=2234;expected_accuracy=0.996	gnl|BL_ORD_ID|42399 cb4286_c2/f1p0/3381 isoform=cb4286_c2;full_length_coverage=1;non_full_length_coverage=0;isoform_length=3381;expected_accuracy=0.998	767	2234	84	769	1908	3376	1	702
cb8564_c180116/f1p0/2944 isoform=cb8564_c180116;full_length_coverage=1;non_full_length_coverage=0;isoform_length=2944;expected_accuracy=0.996	gnl|BL_ORD_ID|55522 cb8564_c44123/f1p1/3230	19	2522	2520	2944	15	2515	2743	3167
cb1549_c11/f1p0/3749 isoform=cb1549_c11;full_length_coverage=1;non_full_l	gnl|BL_ORD_ID|63738 cb1549_c10/f1p1/3944	1	3472	3468	3736	61	3531	3660	3928

ength_coverage=0;isoform_length=3749;expected_accuracy=0.996									
cb2910_c13/f1p0/2551 isoform=cb2910_c13;full_length_coverage=1;non_full_length_coverage=0;isoform_length=2551;expected_accuracy=0.998	gnl|BL_ORD_ID|38136 cb2910_c2/f4p2/2811 isoform=cb2910_c2;full_length_coverage=4;non_full_length_coverage=2;isoform_length=2811;expected_accuracy=1.000	1	1402	1402	2551	99	1502	1626	2774
cb8564_c179291/f1p0/3112 isoform=cb8564_c179291;full_length_coverage=1;non_full_length_coverage=0;isoform_length=3112;expected_accuracy=0.994	gnl|BL_ORD_ID|55162 cb8564_c34284/f1p0/3282	166	3112	1	170	290	3237	9	178
cb8564_c44005/f1p0/3815 isoform=cb8564_c44005;full_length_coverage=1;non_full_length_coverage=0;isoform_length=3815;expected_accuracy=0.993	gnl|BL_ORD_ID|15996 cb8564_c66839/f6p1/4041 isoform=cb8564_c66839;full_length_coverage=6;non_full_length_coverage=1;isoform_length=4041;expected_accuracy=1.000	1	2046	2041	3815	6	2061	2161	3943
cb8564_c123261/f1p0/2282 isoform=cb8564_c123261;full_length_coverage=1;non_full_length_coverage=0;isoform_length=2282;expected_accuracy=1.000	gnl|BL_ORD_ID|17527 cb8564_c84215/f1p0/3183 isoform=cb8564_c84215;full_length_coverage=1;non_full_length_coverage=0;isoform_length=3183;expected_accuracy=0.997	1	1730	1728	2217	83	1810	2697	3183
cb3941_c21/f2p2/3405 isoform=cb3941_c21;full_length_coverage=2;non_full_length_coverage=2;isoform_length=3405;expected_accuracy=0.998	gnl|BL_ORD_ID|40541 cb3941_c38/f1p1/3548 isoform=cb3941_c38;full_length_coverage=1;non_full_length_coverage=1;isoform_length=3548;expected_accuracy=0.997	147	3342	1	147	354	3548	52	198
cb8564_c16897/f1p0/3118 isoform=cb8564_c16897;full_length_coverage=1;non_full_length_coverage=0;isoform_length=3118;expected_accuracy=0.998	gnl|BL_ORD_ID|11821 cb8564_c15227/f1p0/3770 isoform=cb8564_c15227;full_length_coverage=1;non_full_length_coverage=0;isoform_length=3770;expected_accuracy=0.999	1	1584	1579	3118	40	1623	2228	3765

cb1088_c5/f1p0/3109 isoform=cb1088_c5;full_length_coverage=1;non_full_length_coverage=0;isoform_length=3109;expected_accuracy=0.995	gnl|BL_ORD_ID|47337 cb1088_c7/f1p0/3209	1	2686	2685	3109	1	2666	2766	3190
cb18456_c6530/f1p0/1200	gnl|BL_ORD_ID|67485 cb18456_c3994/f1p0/1791	260	1166	3	259	795	1700	8	264
cb2737_c63/f1p2/2799 isoform=cb2737_c63;full_length_coverage=1;non_full_length_coverage=2;isoform_length=2799;expected_accuracy=0.994	gnl|BL_ORD_ID|36735 cb2737_c13/f1p0/3105 isoform=cb2737_c13;full_length_coverage=1;non_full_length_coverage=0;isoform_length=3105;expected_accuracy=0.995	101	1514	1512	2798	265	1676	1808	3103
cb8564_c45609/f1p0/3991	gnl|BL_ORD_ID|15996 cb8564_c66839/f6p1/4041 isoform=cb8564_c66839;full_length_coverage=6;non_full_length_coverage=1;isoform_length=4041;expected_accuracy=1.000	260	3705	1	260	456	3901	1	261
cb748_c24/f1p0/3517	gnl|BL_ORD_ID|6979 cb748_c5/f2p0/3050 isoform=cb748_c5;full_length_coverage=2;non_full_length_coverage=0;isoform_length=3050;expected_accuracy=1.000	12	2626	2626	2936	1	2614	2504	2836
cb8564_c117152/f1p0/2542	gnl|BL_ORD_ID|9561 cb8564_c109796/f2p1/3040 isoform=cb8564_c109796;full_length_coverage=2;non_full_length_coverage=1;isoform_length=3040;expected_accuracy=1.000	1057	2470	1	1058	1627	3040	3	1059
cb1666_c13/f1p0/1804 isoform=cb1666_c13;full_length_coverage=1;non_full_length_coverage=0;isoform_length=1804;expected_accuracy=0.991	gnl|BL_ORD_ID|33315 cb1666_c5/f1p3/2503 isoform=cb1666_c5;full_length_coverage=1;non_full_length_coverage=3;isoform_length=2503;expected_accuracy=0.995	1	1664	1661	1804	560	2225	2337	2481

cb8564_c35314/f1p0/2682 isoform=cb8564_c35314;full_length_coverage=1;non_full_length_coverage=0;isoform_length=2682;expected_accuracy=0.994	gnl|BL_ORD_ID|56381 cb8564_c75626/f1p2/3199	1	2132	2130	2682	1	2116	2611	3158
cb7521_c46/f1p0/2168	gnl|BL_ORD_ID|51306 cb7521_c34/f1p0/2816	1	1630	1626	2041	225	1854	2055	2470
cb8564_c10249/f3p1/3015 isoform=cb8564_c10249;full_length_coverage=3;non_full_length_coverage=1;isoform_length=3015;expected_accuracy=0.995	gnl|BL_ORD_ID|55130 cb8564_c25074/f1p1/3102	1	2261	2260	2935	22	2286	2388	3063
cb4698_c19/f1p1/3082	gnl|BL_ORD_ID|42963 cb4698_c11/f1p0/3141 isoform=cb4698_c11;full_length_coverage=1;non_full_length_coverage=0;isoform_length=3141;expected_accuracy=1.000	1251	2968	1	1251	1427	3141	26	1272
cb8564_c43779/f1p0/3021 isoform=cb8564_c43779;full_length_coverage=1;non_full_length_coverage=0;isoform_length=3021;expected_accuracy=0.993	gnl|BL_ORD_ID|15193 cb8564_c42308/f1p5/3192 isoform=cb8564_c42308;full_length_coverage=1;non_full_length_coverage=5;isoform_length=3192;expected_accuracy=0.995	1	2344	2345	3018	5	2363	2519	3191
cb8564_c46939/f1p1/2926	gnl|BL_ORD_ID|55243 cb8564_c36242/f1p0/3335	1	2134	2130	2870	1	2146	2251	2992
cb14792_c2/f1p0/1864 isoform=cb14792_c2;full_length_coverage=1;non_full_length_coverage=0;isoform_length=1864;expected_accuracy=0.995	gnl|BL_ORD_ID|11547 cb8564_c147115/f6p1/2217 isoform=cb8564_c147115;full_length_coverage=6;non_full_length_coverage=1;isoform_length=2217;expected_accuracy=1.000	1	1524	1519	1864	138	1661	1872	2217
cb3309_c2/f2p0/2095 isoform=cb3309_c2;full_length_coverage=2;non_full_le	gnl|BL_ORD_ID|70307 cb3309_c10/f1p1/3166	162	2056	1	164	1272	3166	34	197

ngth_coverage=0;isoform_length=2095;expected_accuracy=1.000									
cb12714_c29/f1p2/1583	gnl|BL_ORD_ID|56010 cb8564_c51971/f1p0/3064	1	1296	1293	1597	10	1305	2372	2676
cb8564_c51444/f1p0/2956	gnl|BL_ORD_ID|14538 cb8564_c39008/f1p0/3144 isoform=cb8564_c39008;full_length_coverage=1;non_full_length_coverage=0;isoform_length=3144;expected_accuracy=0.995	1	2463	2458	2976	57	2519	2619	3136
cb1224_c20/f1p0/3308 isoform=cb1224_c20;full_length_coverage=1;non_full_length_coverage=0;isoform_length=3308;expected_accuracy=0.997	gnl|BL_ORD_ID|24622 cb1224_c6/f1p0/4422 isoform=cb1224_c6;full_length_coverage=1;non_full_length_coverage=0;isoform_length=4422;expected_accuracy=0.997	1	3180	3175	3291	681	3862	4306	4422
cb2471_c1/f7p0/2064 isoform=cb2471_c1;full_length_coverage=7;non_full_length_coverage=0;isoform_length=2064;expected_accuracy=1.000	gnl|BL_ORD_ID|36817 cb2471_c22/f1p0/2702 isoform=cb2471_c22;full_length_coverage=1;non_full_length_coverage=0;isoform_length=2702;expected_accuracy=0.999	1	1399	1398	2063	6	1403	2034	2700
cb2927_c17/f1p0/2828 isoform=cb2927_c17;full_length_coverage=1;non_full_length_coverage=0;isoform_length=2828;expected_accuracy=0.995	gnl|BL_ORD_ID|38415 cb2927_c2/f2p0/3116 isoform=cb2927_c2;full_length_coverage=2;non_full_length_coverage=0;isoform_length=3116;expected_accuracy=0.995	1	1906	1905	2827	1	1900	2172	3094
cb6390_c5/f1p0/2067 isoform=cb6390_c5;full_length_coverage=1;non_full_length_coverage=0;isoform_length=2067;expected_accuracy=0.994	gnl|BL_ORD_ID|3697 cb6390_c3/f1p0/2298 isoform=cb6390_c3;full_length_coverage=1;non_full_length_coverage=0;isoform_length=2298;expected_accuracy=0.998	359	2067	6	360	540	2242	1	354
cb3309_c2/f2p0/2095 isoform=cb3309_c2;full_length_coverage=2;non_full_le	gnl|BL_ORD_ID|70309 cb3309_c13/f1p0/2443	1	1209	1206	2095	13	1219	1477	2377

ngth_coverage=0;isoform_length=2095;expected_accuracy=1.000									
cb2737_c9/f2p0/2910 isoform=cb2737_c9;full_length_coverage=2;non_full_length_coverage=0;isoform_length=2910;expected_accuracy=0.993	gnl|BL_ORD_ID|38129 cb2737_c25/f1p0/3198 isoform=cb2737_c25;full_length_coverage=1;non_full_length_coverage=0;isoform_length=3198;expected_accuracy=0.997	210	2868	2	210	537	3198	57	266
cb8564_c47011/f1p0/3100	gnl|BL_ORD_ID|57017 cb8564_c80370/f1p0/3493	127	2306	2301	2934	3085	907	669	36
cb3752_c1/f2p0/1494 isoform=cb3752_c1;full_length_coverage=2;non_full_length_coverage=0;isoform_length=1494;expected_accuracy=1.000	gnl|BL_ORD_ID|40664 cb3752_c7/f1p0/3475 isoform=cb3752_c7;full_length_coverage=1;non_full_length_coverage=0;isoform_length=3475;expected_accuracy=0.999	465	1494	5	466	2382	3416	1	464
cb6221_c19/f1p0/1943	gnl|BL_ORD_ID|4878 cb6221_c8/f1p3/4003 isoform=cb6221_c8;full_length_coverage=1;non_full_length_coverage=3;isoform_length=4003;expected_accuracy=0.995	247	1873	75	247	885	2517	1	174
cb2737_c40/f1p0/2830	gnl|BL_ORD_ID|68777 cb2737_c19/f1p0/3583	1	1397	1395	2616	808	2205	2337	3558
cb7495_c2/f2p1/2150 isoform=cb7495_c2;full_length_coverage=2;non_full_length_coverage=1;isoform_length=2150;expected_accuracy=1.000	gnl|BL_ORD_ID|6886 cb7495_c3/f1p5/2591 isoform=cb7495_c3;full_length_coverage=1;non_full_length_coverage=5;isoform_length=2591;expected_accuracy=0.999	133	2150	1	138	267	2284	12	149
cb8564_c120694/f1p0/2801 isoform=cb8564_c120694;full_length_coverage=1;non_full_length_coverage=0;isoform_length=2801;expected_accuracy=0.997	gnl|BL_ORD_ID|55737 cb8564_c5271/f1p0/3205	260	2801	2	260	658	3194	1	259
cb8564_c183526/f1p0/2169	gnl|BL_ORD_ID|12829 cb8564_c161406/f2p1/2330	1	1850	1850	2168	2	188	2003	233

<table>
<tr><td>isoform=cb8564_c183526;full_length_coverage=1;non_full_length_coverage=0;isoform_length=2169;expected_accuracy=0.998</td><td>isoform=cb8564_c161406;full_length_coverage=2;non_full_length_coverage=1;isoform_length=2330;expected_accuracy=0.997</td><td></td><td></td><td></td><td></td><td></td><td>0</td><td></td><td>0</td></tr>
<tr><td>cb8564_c4643/f1p1/2554</td><td>gnl|BL_ORD_ID|14567 cb8564_c4647/f1p2/2774 isoform=cb8564_c4647;full_length_coverage=1;non_full_length_coverage=2;isoform_length=2774;expected_accuracy=0.992</td><td>1</td><td>1705</td><td>1700</td><td>2552</td><td>33</td><td>1756</td><td>1890</td><td>2764</td></tr>
<tr><td>cb8564_c52952/f1p0/3129</td><td>gnl|BL_ORD_ID|18852 cb8564_c84488/f1p0/3408 isoform=cb8564_c84488;full_length_coverage=1;non_full_length_coverage=0;isoform_length=3408;expected_accuracy=0.996</td><td>230</td><td>2848</td><td>22</td><td>230</td><td>790</td><td>3408</td><td>14</td><td>217</td></tr>
<tr><td>cb1487_c5/f1p0/1318 isoform=cb1487_c5;full_length_coverage=1;non_full_length_coverage=0;isoform_length=1318;expected_accuracy=0.991</td><td>gnl|BL_ORD_ID|29668 cb1487_c1/f1p0/3827 isoform=cb1487_c1;full_length_coverage=1;non_full_length_coverage=0;isoform_length=3827;expected_accuracy=0.996</td><td>157</td><td>1318</td><td>50</td><td>159</td><td>2596</td><td>3761</td><td>1</td><td>110</td></tr>
<tr><td>cb322_c15/f1p0/3487 isoform=cb322_c15;full_length_coverage=1;non_full_length_coverage=0;isoform_length=3487;expected_accuracy=0.997</td><td>gnl|BL_ORD_ID|40333 cb322_c18/f1p0/4294 isoform=cb322_c18;full_length_coverage=1;non_full_length_coverage=0;isoform_length=4294;expected_accuracy=0.994</td><td>1</td><td>2729</td><td>2728</td><td>3487</td><td>5</td><td>2750</td><td>2896</td><td>3657</td></tr>
<tr><td>cb8564_c16090/f1p6/2546 isoform=cb8564_c16090;full_length_coverage=1;non_full_length_coverage=6;isoform_length=2546;expected_accuracy=1.000</td><td>gnl|BL_ORD_ID|14058 cb8564_c23936/f1p0/3016 isoform=cb8564_c23936;full_length_coverage=1;non_full_length_coverage=0;isoform_length=3016;expected_accuracy=0.995</td><td>1</td><td>1712</td><td>1710</td><td>2482</td><td>4</td><td>1716</td><td>2143</td><td>2914</td></tr>
<tr><td>cb5057_c18/f1p0/1420 isoform=cb5057_c18;full_length_coverage=1;non_full_length_coverage=0;isoform_length=1420;expected_accu</td><td>gnl|BL_ORD_ID|43289 cb5057_c10/f1p1/1806 isoform=cb5057_c10;full_length_coverage=1;non_full_length_coverage=1;isoform_length=1806;expected_accur</td><td>1</td><td>1020</td><td>1016</td><td>1420</td><td>3</td><td>1028</td><td>1192</td><td>1602</td></tr>
</table>

racy=0.992	acy=1.000								
cb3324_c9/f1p0/5886	gnl|BL_ORD_ID|71091 cb3324_c7/f1p2/6368	2360	5594	1	2362	3083	6317	1	2356
cb2789_c3/f2p0/2507 isoform=cb2789_c3;full_length_coverage=2;non_full_length_coverage=0;isoform_length=2507;expected_accuracy=1.000	gnl|BL_ORD_ID|68853 cb2789_c11/f1p0/2870	284	2507	6	286	442	2666	1	279
cb2825_c1/f4p1/2045 isoform=cb2825_c1;full_length_coverage=4;non_full_length_coverage=1;isoform_length=2045;expected_accuracy=1.000	gnl|BL_ORD_ID|38761 cb2825_c4/f1p1/3802 isoform=cb2825_c4;full_length_coverage=1;non_full_length_coverage=1;isoform_length=3802;expected_accuracy=0.996	137	2045	1	139	1873	3788	20	156
cb402_c23/f1p1/2136 isoform=cb402_c23;full_length_coverage=1;non_full_length_coverage=1;isoform_length=2136;expected_accuracy=1.000	gnl|BL_ORD_ID|39494 cb402_c24/f1p1/3130 isoform=cb402_c24;full_length_coverage=1;non_full_length_coverage=1;isoform_length=3130;expected_accuracy=0.993	116	2080	1	117	1167	3130	9	125
cb8564_c186356/f1p0/2744 isoform=cb8564_c186356;full_length_coverage=1;non_full_length_coverage=0;isoform_length=2744;expected_accuracy=0.998	gnl|BL_ORD_ID|55574 cb8564_c45699/f1p0/3005	1393	2744	1	1396	1498	2845	1	1378
cb8564_c82133/f2p0/2339 isoform=cb8564_c82133;full_length_coverage=2;non_full_length_coverage=0;isoform_length=2339;expected_accuracy=1.000	gnl|BL_ORD_ID|10504 cb8564_c112560/f1p0/2652 isoform=cb8564_c112560;full_length_coverage=1;non_full_length_coverage=0;isoform_length=2652;expected_accuracy=0.996	1	1965	1966	2318	79	2040	2298	2650
cb396_c48/f1p0/2172	gnl|BL_ORD_ID|39335 cb396_c41/f1p0/2356 isoform=cb396_c41;full_length_coverage=1;non_full_length_coverage=0;isoform_length=2356;expected_accura	238	2102	55	238	495	2356	63	246

	cy=0.991								
cb713_c3/f1p2/3872 isoform=cb713_c3;full_length_coverage=1;non_full_length_coverage=2;isoform_length=3872;expected_accuracy=0.999	gnl|BL_ORD_ID|3459 cb713_c6/f1p2/3965 isoform=cb713_c6;full_length_coverage=1;non_full_length_coverage=2;isoform_length=3965;expected_accuracy=0.995	13	2058	2057	3772	34	2084	2249	3965
cb8564_c4705/f1p1/1899	gnl|BL_ORD_ID|55051 cb8564_c4700/f1p0/4682	1	1078	1077	1764	2	1079	3604	4291
cb18788_c0/f1p0/5832	gnl|BL_ORD_ID|67932 cb18657_c2/f1p0/5718	46	5028	5026	5663	9	4991	5099	5737
cb8564_c127660/f1p1/2274 isoform=cb8564_c127660;full_length_coverage=1;non_full_length_coverage=1;isoform_length=2274;expected_accuracy=1.000	gnl|BL_ORD_ID|57869 cb8564_c82906/f1p1/2331	387	2274	98	386	451	2338	1	289
cb8564_c185920/f1p0/2777 isoform=cb8564_c185920;full_length_coverage=1;non_full_length_coverage=0;isoform_length=2777;expected_accuracy=0.996	gnl|BL_ORD_ID|13512 cb8564_c186209/f1p0/2931 isoform=cb8564_c186209;full_length_coverage=1;non_full_length_coverage=0;isoform_length=2931;expected_accuracy=0.996	174	2774	1	177	351	2931	15	192
cb18456_c3841/f1p0/1111	gnl|BL_ORD_ID|35957 cb18456_c3753/f1p2/1269 isoform=cb18456_c3753;full_length_coverage=1;non_full_length_coverage=2;isoform_length=1269;expected_accuracy=0.997	7	588	585	1050	2	605	737	1215
cb8564_c3829/f1p0/2015 isoform=cb8564_c3829;full_length_coverage=1;non_full_length_coverage=0;isoform_length=2015;expected_accuracy=0.997	gnl|BL_ORD_ID|14789 cb8564_c3830/f1p1/2146 isoform=cb8564_c3830;full_length_coverage=1;non_full_length_coverage=1;isoform_length=2146;expected_accuracy=0.997	370	2005	38	372	439	2076	1	330
cb8564_c138545/f4p0/2007	gnl|BL_ORD_ID|9253 cb8564_c121717/f1p0/2419	1	1028	1026	1926	1	102	1519	241

<table>
<tr><td>isoform=cb8564_c138545;full_length_coverage=4;non_full_length_coverage=0;isoform_length=2007;expected_accuracy=1.000</td><td>isoform=cb8564_c121717;full_length_coverage=1;non_full_length_coverage=0;isoform_length=2419;expected_accuracy=0.999</td><td></td><td></td><td></td><td></td><td></td><td>9</td><td></td><td>9</td></tr>
<tr><td>cb8564_c91729/f1p1/2879</td><td>gnl|BL_ORD_ID|52847 cb8564_c101047/f2p0/2937</td><td>362</td><td>2790</td><td>77</td><td>357</td><td>495</td><td>2924</td><td>369</td><td>649</td></tr>
<tr><td>cb8564_c43779/f1p0/3021 isoform=cb8564_c43779;full_length_coverage=1;non_full_length_coverage=0;isoform_length=3021;expected_accuracy=0.993</td><td>gnl|BL_ORD_ID|15169 cb8564_c40218/f1p0/3146 isoform=cb8564_c40218;full_length_coverage=1;non_full_length_coverage=0;isoform_length=3146;expected_accuracy=0.993</td><td>1</td><td>2344</td><td>2343</td><td>3017</td><td>4</td><td>2330</td><td>2482</td><td>3146</td></tr>
<tr><td>cb8564_c127456/f1p1/2149</td><td>gnl|BL_ORD_ID|52413 cb8564_c122074/f1p0/2702</td><td>13</td><td>1731</td><td>1731</td><td>2108</td><td>27</td><td>1745</td><td>1947</td><td>2324</td></tr>
<tr><td>cb7504_c3/f1p0/1972 isoform=cb7504_c3;full_length_coverage=1;non_full_length_coverage=0;isoform_length=1972;expected_accuracy=0.995</td><td>gnl|BL_ORD_ID|7873 cb7504_c11/f15p0/2330 isoform=cb7504_c11;full_length_coverage=15;non_full_length_coverage=0;isoform_length=2330;expected_accuracy=1.000</td><td>1</td><td>1003</td><td>1005</td><td>1972</td><td>63</td><td>1071</td><td>1357</td><td>2326</td></tr>
<tr><td>cb10934_c7/f2p0/1121 isoform=cb10934_c7;full_length_coverage=2;non_full_length_coverage=0;isoform_length=1121;expected_accuracy=1.000</td><td>gnl|BL_ORD_ID|295 cb10934_c3/f1p0/2605 isoform=cb10934_c3;full_length_coverage=1;non_full_length_coverage=0;isoform_length=2605;expected_accuracy=0.996</td><td>130</td><td>1105</td><td>3</td><td>134</td><td>1626</td><td>2605</td><td>2</td><td>131</td></tr>
<tr><td>cb1540_c27/f1p0/3741</td><td>gnl|BL_ORD_ID|28148 cb1540_c3/f2p0/3962 isoform=cb1540_c3;full_length_coverage=2;non_full_length_coverage=0;isoform_length=3962;expected_accuracy=0.996</td><td>304</td><td>3535</td><td>1</td><td>303</td><td>665</td><td>3894</td><td>139</td><td>442</td></tr>
<tr><td>cb8564_c86089/f1p2/3472</td><td>gnl|BL_ORD_ID|16434 cb8564_c69071/f2p1/3650 isoform=cb8564_c69071;full_length_coverage=2;non_full_length_coverage=1;isoform_length=3650;expected_ac</td><td>173</td><td>3436</td><td>1</td><td>177</td><td>391</td><td>3650</td><td>1</td><td>179</td></tr>
</table>

	curacy=0.996								
cb8564_c110398/f1p2/2012	gnl|BL_ORD_ID|16544 cb8564_c75063/f1p1/2293 isoform=cb8564_c75063;full_length_coverage=1;non_full_length_coverage=1;isoform_length=2293;expected_accuracy=0.999	1	1401	1399	1963	1	1401	1703	2265
cb8564_c39136/f1p0/3036 isoform=cb8564_c39136;full_length_coverage=1;non_full_length_coverage=0;isoform_length=3036;expected_accuracy=0.998	gnl|BL_ORD_ID|55437 cb8564_c41690/f1p1/3409	1	2089	2085	3036	1	2100	2227	3180
cb1343_c27/f1p0/3871	gnl|BL_ORD_ID|61275 cb1343_c6/f1p0/4367	2	3229	3228	3816	14	3242	3408	3996
cb14634_c52/f1p1/4305	gnl|BL_ORD_ID|30730 cb14634_c35/f1p0/4383 isoform=cb14634_c35;full_length_coverage=1;non_full_length_coverage=0;isoform_length=4383;expected_accuracy=0.995	1	2658	2653	4282	18	2641	2759	4383
cb8564_c39624/f1p1/3482 isoform=cb8564_c39624;full_length_coverage=1;non_full_length_coverage=1;isoform_length=3482;expected_accuracy=0.996	gnl|BL_ORD_ID|14669 cb8564_c44765/f1p1/4352 isoform=cb8564_c44765;full_length_coverage=1;non_full_length_coverage=1;isoform_length=4352;expected_accuracy=0.995	1532	3482	13	1534	2375	4339	1	1524
cb5421_c9/f1p0/1725	gnl|BL_ORD_ID|44652 cb5421_c4/f1p0/1919 isoform=cb5421_c4;full_length_coverage=1;non_full_length_coverage=0;isoform_length=1919;expected_accuracy=0.997	178	1618	56	179	258	1710	1	124
cb8564_c14767/f1p3/2856	gnl|BL_ORD_ID|17556 cb8564_c9820/f2p0/3032 isoform=cb8564_c9820;full_length_coverage=2;non_full_length_coverage=0;isoform_length=3032;expected_ac	1	1702	1700	2601	1	1704	2131	3032

	curacy=0.995										
cb8564_c145793/f4p0/3585 isoform=cb8564_c145793;full_length_coverage=4;non_full_length_coverage=0;isoform_length=3585;expected_accuracy=0.999	gnl|BL_ORD_ID|16157 cb8564_c77802/f1p0/3651 isoform=cb8564_c77802;full_length_coverage=1;non_full_length_coverage=0;isoform_length=3651;expected_accuracy=0.996	17	2715	2716	3563	1	2693	2810	3651		
cb8564_c38035/f1p0/2770	gnl|BL_ORD_ID|12938 cb8564_c181163/f1p1/2936 isoform=cb8564_c181163;full_length_coverage=1;non_full_length_coverage=1;isoform_length=2936;expected_accuracy=0.993	1	2035	2033	2756	5	2028	2217	2933		
cb6935_c4/f1p0/1785	gnl|BL_ORD_ID|3711 cb6935_c0/f1p0/2736 isoform=cb6935_c0;full_length_coverage=1;non_full_length_coverage=0;isoform_length=2736;expected_accuracy=0.993	1	1017	1015	1797	1	1027	1951	2733		
cb2737_c40/f1p0/2830	gnl|BL_ORD_ID|36733 cb2737_c14/f1p0/2854 isoform=cb2737_c14;full_length_coverage=1;non_full_length_coverage=0;isoform_length=2854;expected_accuracy=0.996	1	1397	1395	2600	120	1518	1650	2854		
cb5304_c17/f1p1/2678 isoform=cb5304_c17;full_length_coverage=1;non_full_length_coverage=1;isoform_length=2678;expected_accuracy=0.998	gnl|BL_ORD_ID|73759 cb5304_c5/f1p0/2736	1	1907	1906	2611	30	1937	2052	2762		
cb8564_c118180/f1p0/2445	gnl|BL_ORD_ID|13236 cb8564_c20610/f2p1/2663 isoform=cb8564_c20610;full_length_coverage=2;non_full_length_coverage=1;isoform_length=2663;expected_accuracy=1.000	1	1664	1669	2263	3	1666	1997	2590		
cb8564_c38035/f1p0/2770	gnl|BL_ORD_ID|55317 cb8564_c38251/f1p0/3243	1	2035	2033	2756	1	205	2247	297		

						7		1	
cb8_c5/f1p1/4485 isoform=cb8_c5;full_length_coverage=1;non_full_length_coverage=1;isoform_length=4485;expected_accuracy=0.997	gnl|BL_ORD_ID|21602 cb8_c2/f3p1/5093 isoform=cb8_c2;full_length_coverage=3;non_full_length_coverage=1;isoform_length=5093;expected_accuracy=0.999	1	3076	3077	4485	18	3097	3685	5093
cb8564_c129474/f1p0/1983	gnl|BL_ORD_ID|52723 cb8564_c12226/f1p0/2278	1	1448	1443	1877	69	1510	1644	2077
cb8564_c41162/f1p0/3158	gnl|BL_ORD_ID|55472 cb8564_c42531/f1p0/3996	130	2553	2548	3038	816	3242	3373	3863
cb6730_c20/f1p0/2001	gnl|BL_ORD_ID|5876 cb6730_c3/f4p0/2261 isoform=cb6730_c3;full_length_coverage=4;non_full_length_coverage=0;isoform_length=2261;expected_accuracy=1.000	1	1181	1180	1993	2	1183	1446	2258
cb8564_c86497/f1p0/2897 isoform=cb8564_c86497;full_length_coverage=1;non_full_length_coverage=0;isoform_length=2897;expected_accuracy=0.996	gnl|BL_ORD_ID|17459 cb8564_c84898/f1p0/3878 isoform=cb8564_c84898;full_length_coverage=1;non_full_length_coverage=0;isoform_length=3878;expected_accuracy=0.997	1	2659	2657	2897	110	2768	3588	3827
cb8564_c53418/f1p0/2602 isoform=cb8564_c53418;full_length_coverage=1;non_full_length_coverage=0;isoform_length=2602;expected_accuracy=0.991	gnl|BL_ORD_ID|15554 cb8564_c47252/f1p0/2795 isoform=cb8564_c47252;full_length_coverage=1;non_full_length_coverage=0;isoform_length=2795;expected_accuracy=0.997	1163	2602	1	1168	1272	2713	4	1171
cb8564_c3985/f1p0/3415	gnl|BL_ORD_ID|65345 cb16578_c3/f1p0/5077	1	2152	2151	3368	1	2152	3822	5039
cb8902_c5/f1p0/2054	gnl|BL_ORD_ID|21805 cb8902_c7/f2p0/2221 isoform=cb8902_c7;full_length_coverage=2;non_full_length_coverage=0;isoform_length=2221;expected_accura	1	1678	1673	2052	56	1733	1841	2221

	cy=0.999								
cb8564_c116509/f1p0/3009	gnl|BL_ORD_ID|12106 cb8564_c146659/f3p0/3534 isoform=cb8564_c146659;full_length_coverage=3;non_full_length_coverage=0;isoform_length=3534;expected_accuracy=0.998	1	2015	2015	2985	75	2095	2551	3529
cb8564_c91149/f1p5/2619 isoform=cb8564_c91149;full_length_coverage=1;non_full_length_coverage=5;isoform_length=2619;expected_accuracy=0.997	gnl|BL_ORD_ID|17556 cb8564_c9820/f2p0/3032 isoform=cb8564_c9820;full_length_coverage=2;non_full_length_coverage=0;isoform_length=3032;expected_accuracy=0.995	1	1710	1708	2614	1	1704	2131	3018
cb12389_c2/f1p0/1783 isoform=cb12389_c2;full_length_coverage=1;non_full_length_coverage=0;isoform_length=1783;expected_accuracy=0.991	gnl|BL_ORD_ID|20474 cb9943_c4/f1p1/1910 isoform=cb9943_c4;full_length_coverage=1;non_full_length_coverage=1;isoform_length=1910;expected_accuracy=1.000	1	1251	1248	1783	10	1265	1370	1904
cb3008_c5/f1p1/1912 isoform=cb3008_c5;full_length_coverage=1;non_full_length_coverage=1;isoform_length=1912;expected_accuracy=0.993	gnl|BL_ORD_ID|36622 cb3008_c1/f2p2/3740 isoform=cb3008_c1;full_length_coverage=2;non_full_length_coverage=2;isoform_length=3740;expected_accuracy=0.998	10	1677	1678	1912	9	1676	2459	2692
cb5637_c11/f7p0/1025 isoform=cb5637_c11;full_length_coverage=7;non_full_length_coverage=0;isoform_length=1025;expected_accuracy=1.000	gnl|BL_ORD_ID|46919 cb5637_c7/f1p1/1131 isoform=cb5637_c7;full_length_coverage=1;non_full_length_coverage=1;isoform_length=1131;expected_accuracy=1.000	2	603	600	994	1	603	737	1131
cb8564_c51169/f1p3/3251 isoform=cb8564_c51169;full_length_coverage=1;non_full_length_coverage=3;isoform_length=3251;expected_accuracy=0.993	gnl|BL_ORD_ID|58096 cb8564_c88702/f1p0/3616	130	3230	1	130	309	3413	27	157
cb8564_c75076/f1p0/3860	gnl|BL_ORD_ID|11480 cb8564_c147875/f1p2/4050	1611	3857	1	1616	1826	404	26	164

isoform=cb8564_c75076;full_length_coverage=1;non_full_length_coverage=0;isoform_length=3860;expected_accuracy=0.996	isoform=cb8564_c147875;full_length_coverage=1;non_full_length_coverage=2;isoform_length=4050;expected_accuracy=0.992						8		2
cb8564_c126557/f1p0/3867 isoform=cb8564_c126557;full_length_coverage=1;non_full_length_coverage=0;isoform_length=3867;expected_accuracy=1.000	gnl|BL_ORD_ID|65215 cb16334_c2/f1p0/5402	1	3427	3427	3867	90	3515	3720	4160
cb6221_c19/f1p0/1943	gnl|BL_ORD_ID|4880 cb6221_c4/f3p3/2906 isoform=cb6221_c4;full_length_coverage=3;non_full_length_coverage=3;isoform_length=2906;expected_accuracy=1.000	247	1873	63	247	900	2530	1	187
cb8564_c181853/f1p0/2119 isoform=cb8564_c181853;full_length_coverage=1;non_full_length_coverage=0;isoform_length=2119;expected_accuracy=0.997	gnl|BL_ORD_ID|15153 cb8564_c36883/f1p0/3072 isoform=cb8564_c36883;full_length_coverage=1;non_full_length_coverage=0;isoform_length=3072;expected_accuracy=0.996	1	1339	1334	2119	1	1341	2169	2954
cb9143_c30/f2p0/2817 isoform=cb9143_c30;full_length_coverage=2;non_full_length_coverage=0;isoform_length=2817;expected_accuracy=0.996	gnl|BL_ORD_ID|20007 cb9143_c59/f2p6/2981 isoform=cb9143_c59;full_length_coverage=2;non_full_length_coverage=6;isoform_length=2981;expected_accuracy=0.996	199	2815	13	203	356	2979	23	222
cb8564_c14882/f1p0/2156	gnl|BL_ORD_ID|52763 cb8564_c13243/f1p0/4432	237	2080	47	238	2440	4283	1	191
cb8564_c17357/f1p1/3002 isoform=cb8564_c17357;full_length_coverage=1;non_full_length_coverage=1;isoform_length=3002;expected_accuracy=0.992	gnl|BL_ORD_ID|13079 cb8564_c18068/f1p2/3340 isoform=cb8564_c18068;full_length_coverage=1;non_full_length_coverage=2;isoform_length=3340;expected_accuracy=0.995	1	1755	1752	3002	6	1765	2100	3335
cb10696_c1/f2p0/1444	gnl|BL_ORD_ID|1517 cb10696_c0/f4p2/1611	132	1444	1	132	264	157	1	132

isoform=cb10696_c1;full_length_coverage=2;non_full_length_coverage=0;isoform_length=1444;expected_accuracy=1.000	isoform=cb10696_c0;full_length_coverage=4;non_full_length_coverage=2;isoform_length=1611;expected_accuracy=0.997						6		
cb7395_c9/f1p0/2016	gnl|BL_ORD_ID|7487 cb7395_c3/f1p0/2630 isoform=cb7395_c3;full_length_coverage=1;non_full_length_coverage=0;isoform_length=2630;expected_accuracy=0.997	1	1363	1360	1814	75	1438	2176	2630
cb8564_c3432/f1p0/2118	gnl|BL_ORD_ID|53641 cb8564_c140752/f1p6/2343	619	2090	7	623	744	2215	12	628
cb965_c21/f1p0/3360	gnl|BL_ORD_ID|20095 cb965_c5/f2p7/4283 isoform=cb965_c5;full_length_coverage=2;non_full_length_coverage=7;isoform_length=4283;expected_accuracy=1.000	1170	3298	1	1171	2067	4186	783	1952
cb4528_c40/f4p0/1916 isoform=cb4528_c40;full_length_coverage=4;non_full_length_coverage=0;isoform_length=1916;expected_accuracy=1.000	gnl|BL_ORD_ID|44099 cb4528_c13/f1p0/3055 isoform=cb4528_c13;full_length_coverage=1;non_full_length_coverage=0;isoform_length=3055;expected_accuracy=0.994	1	1291	1290	1916	4	1295	2347	2972
cb8564_c182592/f1p0/2600 isoform=cb8564_c182592;full_length_coverage=1;non_full_length_coverage=0;isoform_length=2600;expected_accuracy=0.997	gnl|BL_ORD_ID|10296 cb8564_c116976/f1p0/2943 isoform=cb8564_c116976;full_length_coverage=1;non_full_length_coverage=0;isoform_length=2943;expected_accuracy=0.997	1	1582	1583	2597	1	1584	1927	2941
cb7611_c28/f5p0/2201 isoform=cb7611_c28;full_length_coverage=5;non_full_length_coverage=0;isoform_length=2201;expected_accuracy=1.000	gnl|BL_ORD_ID|7566 cb7611_c18/f1p0/3224 isoform=cb7611_c18;full_length_coverage=1;non_full_length_coverage=0;isoform_length=3224;expected_accuracy=1.000	269	2175	29	271	1325	3224	1	243
cb8564_c14344/f1p0/2533	gnl|BL_ORD_ID|57123 cb8564_c84015/f1p0/3207	148	2513	1	151	472	283	3	153

isoform=cb8564_c14344;full_length_coverage=1;non_full_length_coverage=0;isoform_length=2533;expected_accuracy=0.993							8		
cb6307_c26/f1p0/2394 isoform=cb6307_c26;full_length_coverage=1;non_full_length_coverage=0;isoform_length=2394;expected_accuracy=0.996	gnl|BL_ORD_ID|5693 cb6307_c0/f7p1/2712 isoform=cb6307_c0;full_length_coverage=7;non_full_length_coverage=1;isoform_length=2712;expected_accuracy=1.000	11	1856	1852	2394	17	1868	2169	2711
cb7623_c27/f1p0/2082	gnl|BL_ORD_ID|7074 cb7623_c34/f2p0/2410 isoform=cb7623_c34;full_length_coverage=2;non_full_length_coverage=0;isoform_length=2410;expected_accuracy=0.994	1	1243	1240	2074	3	1241	1434	2271
cb3367_c13/f1p0/1942 isoform=cb3367_c13;full_length_coverage=1;non_full_length_coverage=0;isoform_length=1942;expected_accuracy=0.998	gnl|BL_ORD_ID|40686 cb3367_c21/f1p0/3212 isoform=cb3367_c21;full_length_coverage=1;non_full_length_coverage=0;isoform_length=3212;expected_accuracy=0.992	124	1942	1	125	1351	3163	89	213
cb5931_c10/f1p1/2616	gnl|BL_ORD_ID|74667 cb5931_c3/f1p0/2877	2	2034	2030	2613	2	2034	2254	2837
cb162_c1/f1p0/3584 isoform=cb162_c1;full_length_coverage=1;non_full_length_coverage=0;isoform_length=3584;expected_accuracy=0.994	gnl|BL_ORD_ID|63570 cb162_c3/f1p0/4680	1061	3584	1	1064	2217	4743	33	1099
cb8564_c87721/f1p0/2168 isoform=cb8564_c87721;full_length_coverage=1;non_full_length_coverage=0;isoform_length=2168;expected_accuracy=0.997	gnl|BL_ORD_ID|17354 cb8564_c82150/f1p0/2905 isoform=cb8564_c82150;full_length_coverage=1;non_full_length_coverage=0;isoform_length=2905;expected_accuracy=0.994	1	1811	1809	2103	46	1862	2611	2905
cb8564_c122625/f1p0/2416	gnl|BL_ORD_ID|10212 cb8564_c10164/f1p0/2908	262	2190	4	263	978	290	8	260

	isoform=cb8564_c10164;full_length_coverage=1;non_full_length_coverage=0;isoform_length=2908;expected_accuracy=0.996						8		
cb8564_c49562/f1p0/2838 isoform=cb8564_c49562;full_length_coverage=1;non_full_length_coverage=0;isoform_length=2838;expected_accuracy=0.995	gnl|BL_ORD_ID|16681 cb8564_c54233/f1p1/3194 isoform=cb8564_c54233;full_length_coverage=1;non_full_length_coverage=1;isoform_length=3194;expected_accuracy=0.995	1	2331	2327	2836	3	2349	2681	3194
cb2285_c14/f1p0/2217	gnl|BL_ORD_ID|68411 cb2285_c6/f1p0/3984	1	1098	1097	1903	85	1178	3194	3999
cb3485_c9/f1p0/2043 isoform=cb3485_c9;full_length_coverage=1;non_full_length_coverage=0;isoform_length=2043;expected_accuracy=0.993	gnl|BL_ORD_ID|71249 cb3485_c13/f1p0/2609	365	2035	4	365	870	2542	1	362
cb8564_c44063/f1p0/2503 isoform=cb8564_c44063;full_length_coverage=1;non_full_length_coverage=0;isoform_length=2503;expected_accuracy=0.999	gnl|BL_ORD_ID|55860 cb8564_c47209/f1p0/3227	397	2472	63	402	584	2657	100	439
cb8564_c36056/f1p0/2652 isoform=cb8564_c36056;full_length_coverage=1;non_full_length_coverage=0;isoform_length=2652;expected_accuracy=0.999	gnl|BL_ORD_ID|14585 cb8564_c36952/f1p0/3622 isoform=cb8564_c36952;full_length_coverage=1;non_full_length_coverage=0;isoform_length=3622;expected_accuracy=0.994	253	2652	1	251	1229	3622	69	323
cb8564_c43119/f1p4/3194 isoform=cb8564_c43119;full_length_coverage=1;non_full_length_coverage=4;isoform_length=3194;expected_accuracy=0.995	gnl|BL_ORD_ID|56041 cb8564_c53077/f1p2/3406	338	3157	4	338	452	3249	6	337
cb4632_c9/f1p0/2253	gnl|BL_ORD_ID|72781 cb4632_c5/f1p0/2489	1	1837	1834	2216	26	186	2101	248

isoform=cb4632_c9;full_length_coverage=1;non_full_length_coverage=0;isoform_length=2253;expected_accuracy=0.990							5		4
cb9143_c99/f1p0/2956 isoform=cb9143_c99;full_length_coverage=1;non_full_length_coverage=0;isoform_length=2956;expected_accuracy=0.992	gnl|BL_ORD_ID|19764 cb9143_c75/f1p0/4652 isoform=cb9143_c75;full_length_coverage=1;non_full_length_coverage=0;isoform_length=4652;expected_accuracy=0.998	227	2956	1	227	1867	4590	1115	1334
cb3602_c1/f6p3/2221 isoform=cb3602_c1;full_length_coverage=6;non_full_length_coverage=3;isoform_length=2221;expected_accuracy=1.000	gnl|BL_ORD_ID|70514 cb3602_c15/f1p1/2585	1	1453	1451	2221	1	1446	1683	2453
cb8564_c77080/f1p0/2613 isoform=cb8564_c77080;full_length_coverage=1;non_full_length_coverage=0;isoform_length=2613;expected_accuracy=0.995	gnl|BL_ORD_ID|15632 cb8564_c72151/f1p0/2999 isoform=cb8564_c72151;full_length_coverage=1;non_full_length_coverage=0;isoform_length=2999;expected_accuracy=1.000	1	2048	2043	2601	47	2087	2440	2999
cb5608_c19/f1p0/2420 isoform=cb5608_c19;full_length_coverage=1;non_full_length_coverage=0;isoform_length=2420;expected_accuracy=0.999	gnl|BL_ORD_ID|45094 cb5608_c13/f2p0/2807 isoform=cb5608_c13;full_length_coverage=2;non_full_length_coverage=0;isoform_length=2807;expected_accuracy=0.996	1	1699	1694	2420	40	1738	1942	2668
cb8037_c5/f1p0/1371	gnl|BL_ORD_ID|50836 cb8037_c3/f1p0/1939	1	1016	1014	1289	21	1041	1524	1799
cb8564_c38035/f1p0/2770	gnl|BL_ORD_ID|15994 cb8564_c54019/f1p1/2940 isoform=cb8564_c54019;full_length_coverage=1;non_full_length_coverage=1;isoform_length=2940;expected_accuracy=0.991	1	2035	2033	2754	7	2027	2217	2932
cb8813_c1/f2p0/1129	gnl|BL_ORD_ID|18429 cb8813_c2/f1p0/2264	237	1129	35	238	1229	212	1	206

isoform=cb8813_c1;full_length_coverage=2;non_full_length_coverage=0;isoform_length=1129;expected_accuracy=0.999	isoform=cb8813_c2;full_length_coverage=1;non_full_length_coverage=0;isoform_length=2264;expected_accuracy=0.997						0		
cb8564_c85665/f1p0/3087	gnl|BL_ORD_ID|17429 cb8564_c85447/f1p0/3196 isoform=cb8564_c85447;full_length_coverage=1;non_full_length_coverage=0;isoform_length=3196;expected_accuracy=0.997	2	2430	2429	2965	16	2413	2660	3192
cb1891_c12/f1p0/3403 isoform=cb1891_c12;full_length_coverage=1;non_full_length_coverage=0;isoform_length=3403;expected_accuracy=0.999	gnl|BL_ORD_ID|36331 cb1891_c13/f1p1/7475 isoform=cb1891_c13;full_length_coverage=1;non_full_length_coverage=1;isoform_length=7475;expected_accuracy=0.997	1	2744	2740	3403	102	2840	6809	7475
cb8564_c109782/f2p0/1931 isoform=cb8564_c109782;full_length_coverage=2;non_full_length_coverage=0;isoform_length=1931;expected_accuracy=0.999	gnl|BL_ORD_ID|53451 cb8564_c125376/f1p0/2051	1	1551	1551	1878	1	1552	1661	1987
cb8564_c40598/f1p4/3119	gnl|BL_ORD_ID|12440 cb8564_c130435/f1p3/3226 isoform=cb8564_c130435;full_length_coverage=1;non_full_length_coverage=3;isoform_length=3226;expected_accuracy=0.994	1	2732	2729	3055	2	2758	2879	3214
cb454_c33/f1p0/2337 isoform=cb454_c33;full_length_coverage=1;non_full_length_coverage=0;isoform_length=2337;expected_accuracy=0.997	gnl|BL_ORD_ID|42150 cb454_c1/f3p0/2382 isoform=cb454_c1;full_length_coverage=3;non_full_length_coverage=0;isoform_length=2382;expected_accuracy=1.000	252	2335	69	251	296	2379	7	189
cb8564_c387/f2p0/2124 isoform=cb8564_c387;full_length_coverage=2;non_full_length_coverage=0;isoform_length=2124;expected_acc	gnl|BL_ORD_ID|9245 cb8564_c1146/f2p1/2474 isoform=cb8564_c1146;full_length_coverage=2;non_full_length_coverage=1;isoform_length=2474;expected_acc	1	1133	1132	2124	137	1259	1482	2473

uracy=1.000	uracy=0.996								
cb8564_c116509/f1p0/3009	gnl|BL_ORD_ID|12113 cb8564_c147548/f5p1/3243 isoform=cb8564_c147548;full_length_coverage=5;non_full_length_coverage=1;isoform_length=3243;expected_accuracy=0.996	1	2016	2015	2988	24	2040	2264	3241
cb8564_c114742/f2p1/2028 isoform=cb8564_c114742;full_length_coverage=2;non_full_length_coverage=1;isoform_length=2028;expected_accuracy=0.999	gnl|BL_ORD_ID|53307 cb8564_c121613/f1p0/2393	1015	2028	1	1018	1259	2270	2	1019
cb229_c8/f1p1/4650 isoform=cb229_c8;full_length_coverage=1;non_full_length_coverage=1;isoform_length=4650;expected_accuracy=0.996	gnl|BL_ORD_ID|38783 cb229_c3/f1p1/4847 isoform=cb229_c3;full_length_coverage=1;non_full_length_coverage=1;isoform_length=4847;expected_accuracy=0.998	1	2334	2335	4650	27	2362	2479	4794
cb9042_c7/f1p1/1973 isoform=cb9042_c7;full_length_coverage=1;non_full_length_coverage=1;isoform_length=1973;expected_accuracy=0.993	gnl|BL_ORD_ID|20047 cb9042_c1/f3p1/2206 isoform=cb9042_c1;full_length_coverage=3;non_full_length_coverage=1;isoform_length=2206;expected_accuracy=0.997	102	1973	1	101	328	2201	1	100
cb8564_c38035/f1p0/2770	gnl|BL_ORD_ID|15496 cb8564_c47853/f1p0/2927 isoform=cb8564_c47853;full_length_coverage=1;non_full_length_coverage=0;isoform_length=2927;expected_accuracy=0.991	1	2030	2033	2755	1	2016	2208	2927
cb8564_c181853/f1p0/2119 isoform=cb8564_c181853;full_length_coverage=1;non_full_length_coverage=0;isoform_length=2119;expected_accuracy=0.997	gnl|BL_ORD_ID|55839 cb8564_c43677/f1p0/3269	1	1339	1334	2113	55	1394	2222	2999
cb3763_c18/f1p0/2884	gnl|BL_ORD_ID|71497 cb3763_c45/f1p0/3130	1234	2877	1	1237	1361	300	11	124

						4		7	
cb5125_c36/f1p0/1789 isoform=cb5125_c36;full_length_coverage=1;non_full_length_coverage=0;isoform_length=1789;expected_accuracy=0.998	gnl|BL_ORD_ID|43698 cb5125_c6/f8p4/2105 isoform=cb5125_c6;full_length_coverage=8;non_full_length_coverage=4;isoform_length=2105;expected_accuracy=1.000	1	1163	1158	1789	14	1176	1382	2014
cb8564_c13282/f1p2/2892 isoform=cb8564_c13282;full_length_coverage=1;non_full_length_coverage=2;isoform_length=2892;expected_accuracy=0.994	gnl|BL_ORD_ID|13075 cb8564_c17357/f1p1/3002 isoform=cb8564_c17357;full_length_coverage=1;non_full_length_coverage=1;isoform_length=3002;expected_accuracy=0.992	14	1771	1767	2887	1	1755	1888	3002
cb4063_c17/f1p0/2890	gnl|BL_ORD_ID|40735 cb4063_c6/f1p0/2803 isoform=cb4063_c6;full_length_coverage=1;non_full_length_coverage=0;isoform_length=2803;expected_accuracy=0.995	215	2496	103	216	482	2763	1	114
cb567_c15/f1p0/3394	gnl|BL_ORD_ID|45926 cb567_c4/f1p0/3256 isoform=cb567_c4;full_length_coverage=1;non_full_length_coverage=0;isoform_length=3256;expected_accuracy=0.996	2	2687	2685	3112	4	2689	2829	3256
cb2336_c12/f1p1/5436 isoform=cb2336_c12;full_length_coverage=1;non_full_length_coverage=1;isoform_length=5436;expected_accuracy=0.997	gnl|BL_ORD_ID|38632 cb2336_c11/f1p1/6811 isoform=cb2336_c11;full_length_coverage=1;non_full_length_coverage=1;isoform_length=6811;expected_accuracy=0.999	1	3743	3740	5369	1282	5030	5180	6811
cb8564_c4261/f1p0/1862	gnl|BL_ORD_ID|15183 cb8564_c4259/f2p0/2998 isoform=cb8564_c4259;full_length_coverage=2;non_full_length_coverage=0;isoform_length=2998;expected_accuracy=1.000	1	1541	1539	1839	1	1540	2698	2998
cb3618_c25/f1p0/2107	gnl|BL_ORD_ID|71383 cb3618_c11/f1p0/2951	132	2107	1	131	804	277	73	203

<table>
<tr><td></td><td></td><td></td><td></td><td></td><td></td><td></td><td>9</td><td></td><td></td></tr>
<tr><td>cb1433_c8/f1p0/4081</td><td>gnl|BL_ORD_ID|25224 cb1433_c2/f1p0/4326 isoform=cb1433_c2;full_length_coverage=1;non_full_length_coverage=0;isoform_length=4326;expected_accuracy=0.997</td><td>247</td><td>4046</td><td>1</td><td>247</td><td>532</td><td>4326</td><td>3</td><td>249</td></tr>
<tr><td>cb6530_c26/f1p0/2387 isoform=cb6530_c26;full_length_coverage=1;non_full_length_coverage=0;isoform_length=2387;expected_accuracy=0.998</td><td>gnl|BL_ORD_ID|49806 cb6530_c44/f1p0/2497</td><td>1009</td><td>2377</td><td>1</td><td>1010</td><td>1121</td><td>2489</td><td>8</td><td>1017</td></tr>
<tr><td>cb8564_c13319/f1p3/2892 isoform=cb8564_c13319;full_length_coverage=1;non_full_length_coverage=3;isoform_length=2892;expected_accuracy=0.996</td><td>gnl|BL_ORD_ID|57988 cb8564_c86038/f1p0/3338</td><td>155</td><td>2892</td><td>1</td><td>156</td><td>324</td><td>3062</td><td>32</td><td>188</td></tr>
<tr><td>cb10330_c6/f1p0/1244</td><td>gnl|BL_ORD_ID|505 cb10330_c1/f1p0/1746 isoform=cb10330_c1;full_length_coverage=1;non_full_length_coverage=0;isoform_length=1746;expected_accuracy=0.992</td><td>129</td><td>1199</td><td>2</td><td>129</td><td>421</td><td>1489</td><td>66</td><td>194</td></tr>
<tr><td>cb452_c17/f2p0/4042 isoform=cb452_c17;full_length_coverage=2;non_full_length_coverage=0;isoform_length=4042;expected_accuracy=0.997</td><td>gnl|BL_ORD_ID|41873 cb452_c22/f1p0/4230 isoform=cb452_c22;full_length_coverage=1;non_full_length_coverage=0;isoform_length=4230;expected_accuracy=0.997</td><td>130</td><td>3074</td><td>3075</td><td>4017</td><td>1</td><td>2942</td><td>3289</td><td>4230</td></tr>
<tr><td>cb8564_c34047/f2p0/3175 isoform=cb8564_c34047;full_length_coverage=2;non_full_length_coverage=0;isoform_length=3175;expected_accuracy=1.000</td><td>gnl|BL_ORD_ID|15130 cb8564_c33393/f7p2/3346 isoform=cb8564_c33393;full_length_coverage=7;non_full_length_coverage=2;isoform_length=3346;expected_accuracy=0.996</td><td>1</td><td>2174</td><td>2171</td><td>3175</td><td>1</td><td>2173</td><td>2293</td><td>3297</td></tr>
<tr><td>cb388_c34/f1p0/3652</td><td>gnl|BL_ORD_ID|69593 cb388_c51/f1p0/3691</td><td>225</td><td>3487</td><td>21</td><td>224</td><td>364</td><td>362</td><td>21</td><td>222</td></tr>
</table>

<table>
<tr><td></td><td></td><td></td><td></td><td></td><td></td><td></td><td>8</td><td></td><td></td></tr>
<tr><td>cb7189_c8/f1p0/2376</td><td>gnl|BL_ORD_ID|51024 cb7189_c10/f1p0/2311</td><td>1</td><td>1304</td><td>1304</td><td>2063</td><td>242</td><td>1545</td><td>1440</td><td>2199</td></tr>
<tr><td>cb2719_c47/f2p0/2857</td><td>gnl|BL_ORD_ID|37134 cb2719_c32/f1p0/2977 isoform=cb2719_c32;full_length_coverage=1;non_full_length_coverage=0;isoform_length=2977;expected_accuracy=0.997</td><td>234</td><td>2841</td><td>1</td><td>235</td><td>369</td><td>2976</td><td>22</td><td>256</td></tr>
<tr><td>cb4972_c64/f1p0/2792</td><td>gnl|BL_ORD_ID|43215 cb4972_c0/f12p1/2841 isoform=cb4972_c0;full_length_coverage=12;non_full_length_coverage=1;isoform_length=2841;expected_accuracy=1.000</td><td>1</td><td>1345</td><td>1344</td><td>2670</td><td>40</td><td>1383</td><td>1513</td><td>2841</td></tr>
<tr><td>cb10088_c26/f1p0/1030</td><td>gnl|BL_ORD_ID|62560 cb13559_c9/f1p0/1399</td><td>476</td><td>1034</td><td>9</td><td>479</td><td>774</td><td>1332</td><td>1</td><td>471</td></tr>
<tr><td>cb8564_c119657/f1p2/2788</td><td>gnl|BL_ORD_ID|16193 cb8564_c76962/f1p2/3049 isoform=cb8564_c76962;full_length_coverage=1;non_full_length_coverage=2;isoform_length=3049;expected_accuracy=0.997</td><td>15</td><td>1742</td><td>1740</td><td>2676</td><td>7</td><td>1733</td><td>2113</td><td>3049</td></tr>
<tr><td>cb1860_c13/f1p0/2150 isoform=cb1860_c13;full_length_coverage=1;non_full_length_coverage=0;isoform_length=2150;expected_accuracy=0.998</td><td>gnl|BL_ORD_ID|34656 cb1860_c12/f1p3/2509 isoform=cb1860_c12;full_length_coverage=1;non_full_length_coverage=3;isoform_length=2509;expected_accuracy=0.996</td><td>1</td><td>1277</td><td>1273</td><td>2102</td><td>115</td><td>1392</td><td>1645</td><td>2480</td></tr>
<tr><td>cb3557_c22/f1p0/2990</td><td>gnl|BL_ORD_ID|70457 cb3557_c31/f1p0/2861</td><td>1134</td><td>2722</td><td>1</td><td>1135</td><td>1297</td><td>2885</td><td>1</td><td>1135</td></tr>
<tr><td>cb8485_c1/f2p0/2382 isoform=cb8485_c1;full_length_coverage=2;non_full_length_coverage=0;isoform_length=2382;expected_accur</td><td>gnl|BL_ORD_ID|10135 cb8485_c2/f1p0/2791 isoform=cb8485_c2;full_length_coverage=1;non_full_length_coverage=0;isoform_length=2791;expected_accura</td><td>107</td><td>1591</td><td>1588</td><td>2377</td><td>1</td><td>1485</td><td>2004</td><td>2791</td></tr>
</table>

acy=0.996	cy=0.994								
cb3312_c16/f1p0/1452	gnl|BL_ORD_ID|40961 cb3312_c14/f1p1/3482 isoform=cb3312_c14;full_length_coverage=1;non_full_length_coverage=1;isoform_length=3482;expected_accuracy=0.996	1	1094	1094	1456	1	1093	2972	3334
cb8564_c45609/f1p0/3991	gnl|BL_ORD_ID|55964 cb8564_c50435/f1p0/4916	260	3797	23	260	436	3974	4	241
cb4664_c0/f6p1/2463 isoform=cb4664_c0;full_length_coverage=6;non_full_length_coverage=1;isoform_length=2463;expected_accuracy=0.996	gnl|BL_ORD_ID|72828 cb4664_c13/f1p0/2651	232	2463	1	231	383	2613	1	232
cb1333_c96/f1p0/1554 isoform=cb1333_c96;full_length_coverage=1;non_full_length_coverage=0;isoform_length=1554;expected_accuracy=0.991	gnl|BL_ORD_ID|26023 cb1333_c10/f4p0/1937 isoform=cb1333_c10;full_length_coverage=4;non_full_length_coverage=0;isoform_length=1937;expected_accuracy=1.000	143	1554	1	142	503	1916	1	142
cb8564_c118659/f2p0/3437 isoform=cb8564_c118659;full_length_coverage=2;non_full_length_coverage=0;isoform_length=3437;expected_accuracy=0.994	gnl|BL_ORD_ID|10567 cb8564_c118715/f1p0/3592 isoform=cb8564_c118715;full_length_coverage=1;non_full_length_coverage=0;isoform_length=3592;expected_accuracy=0.996	1	2754	2752	3268	83	2848	3078	3592
cb146_c9/f1p0/1387	gnl|BL_ORD_ID|30275 cb146_c5/f1p0/4805 isoform=cb146_c5;full_length_coverage=1;non_full_length_coverage=0;isoform_length=4805;expected_accuracy=0.997	230	1344	29	231	3692	4805	1	203
cb8564_c33962/f2p1/3330 isoform=cb8564_c33962;full_length_coverage=2;non_full_length_coverage=1;isoform_length=3330;expected_	gnl|BL_ORD_ID|57290 cb8564_c89168/f1p0/3652	1	2869	2873	3178	27	2889	3093	3398

accuracy=1.000									
cb18456_c4245/f1p0/1080	gnl|BL_ORD_ID|36303 cb18456_c4072/f1p0/1343 isoform=cb18456_c4072;full_length_coverage=1;non_full_length_coverage=0;isoform_length=1343;expected_accuracy=0.998	7	512	508	1079	1	495	604	1187
cb6111_c25/f1p0/1520 isoform=cb6111_c25;full_length_coverage=1;non_full_length_coverage=0;isoform_length=1520;expected_accuracy=0.993	gnl|BL_ORD_ID|74915 cb6111_c17/f1p0/1775	118	1520	10	123	272	1673	14	128
cb8564_c115923/f1p0/2925	gnl|BL_ORD_ID|14608 cb8564_c23723/f1p0/3118 isoform=cb8564_c23723;full_length_coverage=1;non_full_length_coverage=0;isoform_length=3118;expected_accuracy=0.992	13	1675	1675	2550	2	1659	2242	3118
cb3907_c9/f1p0/2875	gnl|BL_ORD_ID|40128 cb3907_c4/f1p0/3038 isoform=cb3907_c4;full_length_coverage=1;non_full_length_coverage=0;isoform_length=3038;expected_accuracy=0.992	1	1474	1475	2654	16	1502	1872	3037
cb8564_c38035/f1p0/2770	gnl|BL_ORD_ID|16473 cb8564_c51923/f1p1/2966 isoform=cb8564_c51923;full_length_coverage=1;non_full_length_coverage=1;isoform_length=2966;expected_accuracy=0.997	1	2035	2033	2756	1	2049	2239	2963
cb8564_c123093/f1p0/2918	gnl|BL_ORD_ID|11608 cb8564_c146551/f1p1/3093 isoform=cb8564_c146551;full_length_coverage=1;non_full_length_coverage=1;isoform_length=3093;expected_accuracy=1.000	1214	2924	1	1213	1183	2893	87	1299
cb2818_c34/f1p0/3053	gnl|BL_ORD_ID|38982 cb2818_c74/f3p0/3087	114	2904	1	119	297	308	23	141

	isoform=cb2818_c74;full_length_coverage=3;non_full_length_coverage=0;isoform_length=3087;expected_accuracy=1.000						7		
cb2845_c38/f1p0/1808 isoform=cb2845_c38;full_length_coverage=1;non_full_length_coverage=0;isoform_length=1808;expected_accuracy=0.993	gnl|BL_ORD_ID|70029 cb2845_c34/f1p0/3557	1	1403	1403	1788	1580	3008	3131	3516
cb8995_c8/f1p0/2000 isoform=cb8995_c8;full_length_coverage=1;non_full_length_coverage=0;isoform_length=2000;expected_accuracy=1.000	gnl|BL_ORD_ID|20765 cb8995_c10/f1p0/2440 isoform=cb8995_c10;full_length_coverage=1;non_full_length_coverage=0;isoform_length=2440;expected_accuracy=0.999	97	2000	1	100	213	2116	1	101
cb10490_c4/f1p0/1258	gnl|BL_ORD_ID|48584 cb10490_c5/f1p0/2250	427	1072	7	429	1597	2242	36	458
cb18456_c929/f1p1/1404 isoform=cb18456_c929;full_length_coverage=1;non_full_length_coverage=1;isoform_length=1404;expected_accuracy=0.994	gnl|BL_ORD_ID|53691 cb8564_c1249/f1p1/2145	438	1356	8	440	1219	2140	1	430
cb8564_c898/f2p0/2398 isoform=cb8564_c898;full_length_coverage=2;non_full_length_coverage=0;isoform_length=2398;expected_accuracy=0.999	gnl|BL_ORD_ID|15309 cb8564_c4215/f1p0/4987 isoform=cb8564_c4215;full_length_coverage=1;non_full_length_coverage=0;isoform_length=4987;expected_accuracy=0.996	494	2398	63	493	3071	4972	1	430
cb1415_c16/f1p0/3004 isoform=cb1415_c16;full_length_coverage=1;non_full_length_coverage=0;isoform_length=3004;expected_accuracy=0.995	gnl|BL_ORD_ID|27156 cb1415_c7/f1p0/3750 isoform=cb1415_c7;full_length_coverage=1;non_full_length_coverage=0;isoform_length=3750;expected_accuracy=0.992	1464	3004	1	1465	2153	3712	55	1517
cb5714_c11/f1p0/1261	gnl|BL_ORD_ID|74327 cb5714_c3/f1p0/2759	247	1261	43	250	1698	271	65	269

isoform=cb5714_c11;full_length_coverage=1;non_full_length_coverage=0;isoform_length=1261;expected_accuracy=0.990							6		
cb3143_c10/f1p1/2863	gnl|BL_ORD_ID|69160 cb3143_c4/f1p0/3643	163	2870	1	163	687	3393	122	284
cb10539_c0/f13p0/1103	gnl|BL_ORD_ID|466 cb10539_c2/f1p0/4443 isoform=cb10539_c2;full_length_coverage=1;non_full_length_coverage=0;isoform_length=4443;expected_accuracy=0.994	415	1105	52	416	3683	4374	1	371
cb5125_c34/f1p0/1890	gnl|BL_ORD_ID|43698 cb5125_c6/f8p4/2105 isoform=cb5125_c6;full_length_coverage=8;non_full_length_coverage=4;isoform_length=2105;expected_accuracy=1.000	1	1145	1144	1780	7	1148	1340	1975
cb8564_c184250/f1p0/2256 isoform=cb8564_c184250;full_length_coverage=1;non_full_length_coverage=0;isoform_length=2256;expected_accuracy=0.996	gnl|BL_ORD_ID|14274 cb8564_c34179/f3p0/2531 isoform=cb8564_c34179;full_length_coverage=3;non_full_length_coverage=0;isoform_length=2531;expected_accuracy=0.999	1	1642	1638	2256	1	1645	1906	2524
cb18456_c7297/f1p0/1408	gnl|BL_ORD_ID|54892 cb8564_c3430/f1p1/2229	520	1317	8	519	1394	2191	18	530
cb8564_c10938/f1p1/2321	gnl|BL_ORD_ID|11222 cb8564_c13611/f1p0/4658 isoform=cb8564_c13611;full_length_coverage=1;non_full_length_coverage=0;isoform_length=4658;expected_accuracy=0.991	1	1315	1310	2292	2249	3559	3677	4658
cb8941_c4/f1p0/1811 isoform=cb8941_c4;full_length_coverage=1;non_full_length_coverage=0;isoform_length=1811;expected_accura	gnl|BL_ORD_ID|20055 cb8941_c0/f1p0/2230 isoform=cb8941_c0;full_length_coverage=1;non_full_length_coverage=0;isoform_length=2230;expected_accura	1	1183	1183	1811	21	1209	1570	2209

cy=0.999	cy=1.000								
cb4966_c14/f1p0/2442 isoform=cb4966_c14;full_length_coverage=1;non_full_length_coverage=0;isoform_length=2442;expected_accuracy=0.994	gnl|BL_ORD_ID|73135 cb4966_c13/f1p0/3284	1	1524	1522	2430	4	1531	2204	3109
cb15082_c1/f1p0/1736	gnl|BL_ORD_ID|53645 cb8564_c144123/f1p0/2074	120	1736	1	120	406	2022	4	123
cb5422_c6/f1p0/1857	gnl|BL_ORD_ID|45246 cb5422_c11/f1p0/5531 isoform=cb5422_c11;full_length_coverage=1;non_full_length_coverage=0;isoform_length=5531;expected_accuracy=0.998	1	1128	1127	1850	160	1287	4809	5531
cb16877_c1/f1p1/1742	gnl|BL_ORD_ID|53708 cb8564_c1398/f1p0/2214	107	1747	1	108	454	2069	4	111
cb8564_c146028/f1p2/2149 isoform=cb8564_c146028;full_length_coverage=1;non_full_length_coverage=2;isoform_length=2149;expected_accuracy=0.996	gnl|BL_ORD_ID|58073 cb8564_c88099/f1p2/2499	219	2143	27	218	428	2351	7	198
cb7863_c23/f1p0/2057 isoform=cb7863_c23;full_length_coverage=1;non_full_length_coverage=0;isoform_length=2057;expected_accuracy=0.998	gnl|BL_ORD_ID|8929 cb7863_c6/f3p1/2339 isoform=cb7863_c6;full_length_coverage=3;non_full_length_coverage=1;isoform_length=2339;expected_accuracy=0.995	264	2057	64	268	544	2338	56	261
cb8564_c81890/f1p1/2084 isoform=cb8564_c81890;full_length_coverage=1;non_full_length_coverage=1;isoform_length=2084;expected_accuracy=0.994	gnl|BL_ORD_ID|11375 cb8564_c147204/f5p1/2269 isoform=cb8564_c147204;full_length_coverage=5;non_full_length_coverage=1;isoform_length=2269;expected_accuracy=0.996	1	1818	1813	2084	56	1873	1984	2256
cb6802_c12/f1p0/3195	gnl|BL_ORD_ID|50080 cb6802_c20/f1p0/3496	119	3195	1	119	428	351	6	123

isoform=cb6802_c12;full_length_coverage=1;non_full_length_coverage=0;isoform_length=3195;expected_accuracy=0.997							3		
cb3359_c47/f1p0/1712 isoform=cb3359_c47;full_length_coverage=1;non_full_length_coverage=0;isoform_length=1712;expected_accuracy=0.996	gnl|BL_ORD_ID|39778 cb3359_c46/f1p1/1823 isoform=cb3359_c46;full_length_coverage=1;non_full_length_coverage=1;isoform_length=1823;expected_accuracy=0.997	1	1023	1020	1686	5	1021	1157	1823
cb8564_c39136/f1p0/3036 isoform=cb8564_c39136;full_length_coverage=1;non_full_length_coverage=0;isoform_length=3036;expected_accuracy=0.998	gnl|BL_ORD_ID|55472 cb8564_c42531/f1p0/3996	124	2089	2085	3034	816	2785	2912	3863
cb1279_c85/f1p0/1794 isoform=cb1279_c85;full_length_coverage=1;non_full_length_coverage=0;isoform_length=1794;expected_accuracy=0.998	gnl|BL_ORD_ID|25829 cb1279_c10/f2p0/2859 isoform=cb1279_c10;full_length_coverage=2;non_full_length_coverage=0;isoform_length=2859;expected_accuracy=0.999	11	1120	1115	1760	1	1105	2227	2859
cb2683_c0/f3p0/2464 isoform=cb2683_c0;full_length_coverage=3;non_full_length_coverage=0;isoform_length=2464;expected_accuracy=1.000	gnl|BL_ORD_ID|69890 cb2683_c10/f1p0/3502	441	2448	93	441	1384	3389	1	349
cb8564_c77802/f1p0/3651 isoform=cb8564_c77802;full_length_coverage=1;non_full_length_coverage=0;isoform_length=3651;expected_accuracy=0.996	gnl|BL_ORD_ID|15895 cb8564_c75013/f1p0/4362 isoform=cb8564_c75013;full_length_coverage=1;non_full_length_coverage=0;isoform_length=4362;expected_accuracy=0.995	18	2317	2314	3558	1	2306	3117	4362
cb6146_c4/f1p0/2397 isoform=cb6146_c4;full_length_coverage=1;non_full_length_coverage=0;isoform_length=2397;expected_accur	gnl|BL_ORD_ID|45009 cb6146_c5/f1p0/2877 isoform=cb6146_c5;full_length_coverage=1;non_full_length_coverage=0;isoform_length=2877;expected_accura	1	1190	1189	2360	1	1194	1706	2877

acy=0.993	cy=0.997								
cb10432_c12/f1p0/1478	gnl|BL_ORD_ID|1184 cb10432_c5/f1p0/1694 isoform=cb10432_c5;full_length_coverage=1;non_full_length_coverage=0;isoform_length=1694;expected_accuracy=0.995	1	1079	1074	1485	1	1080	1180	1590
cb8101_c9/f1p0/2203 isoform=cb8101_c9;full_length_coverage=1;non_full_length_coverage=0;isoform_length=2203;expected_accuracy=0.997	gnl|BL_ORD_ID|51752 cb8101_c18/f1p0/2567	1	1617	1613	2200	190	1805	1919	2506
cb8564_c32252/f4p1/3260 isoform=cb8564_c32252;full_length_coverage=4;non_full_length_coverage=1;isoform_length=3260;expected_accuracy=1.000	gnl|BL_ORD_ID|55446 cb8564_c41840/f1p0/3409	142	3259	13	141	241	3359	2	130
cb8564_c17357/f1p1/3002 isoform=cb8564_c17357;full_length_coverage=1;non_full_length_coverage=1;isoform_length=3002;expected_accuracy=0.992	gnl|BL_ORD_ID|35901 cb18782_c0/f1p2/5630 isoform=cb18782_c0;full_length_coverage=1;non_full_length_coverage=2;isoform_length=5630;expected_accuracy=0.997	1	1755	1752	3002	8	1769	4303	5538
cb8564_c79364/f1p0/2690 isoform=cb8564_c79364;full_length_coverage=1;non_full_length_coverage=0;isoform_length=2690;expected_accuracy=0.997	gnl|BL_ORD_ID|17061 cb8564_c81806/f2p0/2831 isoform=cb8564_c81806;full_length_coverage=2;non_full_length_coverage=0;isoform_length=2831;expected_accuracy=0.999	1	1379	1377	2638	4	1366	1573	2831
cb8564_c113392/f1p0/2376	gnl|BL_ORD_ID|15266 cb8564_c3333/f1p0/3954 isoform=cb8564_c3333;full_length_coverage=1;non_full_length_coverage=0;isoform_length=3954;expected_accuracy=0.990	407	2308	85	407	2027	3950	1	327
cb1891_c9/f1p0/3776	gnl|BL_ORD_ID|36331 cb1891_c13/f1p1/7475	1	2841	2841	3520	2	284	6770	744

	isoform=cb1891_c13;full_length_coverage=1;non_full_length_coverage=1;isoform_length=7475;expected_accuracy=0.997						1		9
cb12003_c31/f1p0/1799 isoform=cb12003_c31;full_length_coverage=1;non_full_length_coverage=0;isoform_length=1799;expected_accuracy=0.997	gnl|BL_ORD_ID|42024 cb4768_c16/f1p0/1948 isoform=cb4768_c16;full_length_coverage=1;non_full_length_coverage=0;isoform_length=1948;expected_accuracy=0.993	1	1200	1199	1799	5	1210	1315	1915
cb7761_c2/f2p0/1991 isoform=cb7761_c2;full_length_coverage=2;non_full_length_coverage=0;isoform_length=1991;expected_accuracy=0.999	gnl|BL_ORD_ID|7622 cb7761_c6/f1p0/2339 isoform=cb7761_c6;full_length_coverage=1;non_full_length_coverage=0;isoform_length=2339;expected_accuracy=0.994	570	1991	58	571	819	2258	2	512
cb8564_c73216/f1p0/3006	gnl|BL_ORD_ID|55939 cb8564_c49630/f1p4/3487	28	2896	2896	3146	19	2913	3243	3493
cb8564_c15608/f1p3/3110	gnl|BL_ORD_ID|52054 cb8564_c11371/f1p2/3552	1	2790	2785	3102	43	2832	2976	3293
cb8564_c37361/f1p0/3076	gnl|BL_ORD_ID|54425 cb8564_c42906/f1p2/3181	1	2481	2477	3070	1	2460	2561	3154
cb8564_c87721/f1p0/2168 isoform=cb8564_c87721;full_length_coverage=1;non_full_length_coverage=0;isoform_length=2168;expected_accuracy=0.997	gnl|BL_ORD_ID|17522 cb8564_c83241/f1p3/2918 isoform=cb8564_c83241;full_length_coverage=1;non_full_length_coverage=3;isoform_length=2918;expected_accuracy=0.997	1	1811	1809	2164	103	1919	2566	2918
cb8564_c49356/f1p0/3133	gnl|BL_ORD_ID|16172 cb8564_c53024/f1p0/3159 isoform=cb8564_c53024;full_length_coverage=1;non_full_length_coverage=0;isoform_length=3159;expected_accuracy=0.996	1	1696	1693	2851	1	1699	2017	3156
cb8564_c16102/f1p0/2566	gnl|BL_ORD_ID|14608 cb8564_c23723/f1p0/3118	13	1684	1684	2566	15	165	2242	3118

isoform=cb8564_c16102;full_length_coverage=1;non_full_length_coverage=0;isoform_length=2566;expected_accuracy=0.992	isoform=cb8564_c23723;full_length_coverage=1;non_full_length_coverage=0;isoform_length=3118;expected_accuracy=0.992						9		
cb910_c31/f1p1/2978 isoform=cb910_c31;full_length_coverage=1;non_full_length_coverage=1;isoform_length=2978;expected_accuracy=0.997	gnl|BL_ORD_ID|57612 cb910_c84/f1p0/3501	1	2085	2081	2978	27	2111	2243	3140
cb8564_c43779/f1p0/3021 isoform=cb8564_c43779;full_length_coverage=1;non_full_length_coverage=0;isoform_length=3021;expected_accuracy=0.993	gnl|BL_ORD_ID|14471 cb8564_c34518/f1p0/3136 isoform=cb8564_c34518;full_length_coverage=1;non_full_length_coverage=0;isoform_length=3136;expected_accuracy=0.991	1	2341	2345	3021	1	2310	2468	3136
cb8564_c35301/f1p0/2777	gnl|BL_ORD_ID|16879 cb8564_c48606/f1p1/3308 isoform=cb8564_c48606;full_length_coverage=1;non_full_length_coverage=1;isoform_length=3308;expected_accuracy=0.998	125	2013	2009	2729	247	2151	2587	3308
cb8564_c23958/f1p0/2286 isoform=cb8564_c23958;full_length_coverage=1;non_full_length_coverage=0;isoform_length=2286;expected_accuracy=1.000	gnl|BL_ORD_ID|53903 cb8564_c14264/f1p0/3603	1	1235	1232	2286	1132	2365	2527	3579
cb8564_c111744/f1p1/2111 isoform=cb8564_c111744;full_length_coverage=1;non_full_length_coverage=1;isoform_length=2111;expected_accuracy=0.998	gnl|BL_ORD_ID|14439 cb8564_c3345/f1p0/2570 isoform=cb8564_c3345;full_length_coverage=1;non_full_length_coverage=0;isoform_length=2570;expected_accuracy=0.999	13	1639	1635	2111	1	1624	1797	2273
cb8564_c128252/f1p0/2428	gnl|BL_ORD_ID|52094 cb8564_c110646/f1p0/2764	17	1818	1815	2456	1	1810	2110	2751
cb7969_c11/f1p0/1950	gnl|BL_ORD_ID|32342 cb1767_c38/f1p1/2725	1	1409	1405	1906	2181	773	528	27

isoform=cb7969_c11;full_length_coverage=1;non_full_length_coverage=0;isoform_length=1950;expected_accuracy=0.992	isoform=cb1767_c38;full_length_coverage=1;non_full_length_coverage=1;isoform_length=2725;expected_accuracy=0.993								
cb8564_c39136/f1p0/3036 isoform=cb8564_c39136;full_length_coverage=1;non_full_length_coverage=0;isoform_length=3036;expected_accuracy=0.998	gnl|BL_ORD_ID|53680 cb8564_c153222/f2p39/3454	1	2089	2085	3024	7	2102	2229	3170
cb295_c28/f1p1/2235	gnl|BL_ORD_ID|37900 cb295_c23/f1p1/2681 isoform=cb295_c23;full_length_coverage=1;non_full_length_coverage=1;isoform_length=2681;expected_accuracy=0.993	1	1359	1358	2050	1	1349	1987	2681
cb4821_c9/f1p0/1558	gnl|BL_ORD_ID|43064 cb4821_c4/f2p0/3249 isoform=cb4821_c4;full_length_coverage=2;non_full_length_coverage=0;isoform_length=3249;expected_accuracy=0.999	490	1467	7	489	2049	3023	1	484
cb2097_c87/f1p2/2320 isoform=cb2097_c87;full_length_coverage=1;non_full_length_coverage=2;isoform_length=2320;expected_accuracy=0.992	gnl|BL_ORD_ID|66709 cb2097_c98/f1p0/3073	549	2304	57	548	946	2702	196	684
cb12003_c71/f1p0/1043 isoform=cb12003_c71;full_length_coverage=1;non_full_length_coverage=0;isoform_length=1043;expected_accuracy=1.000	gnl|BL_ORD_ID|71997 cb4768_c38/f1p1/2233	336	1043	44	340	789	1496	272	568
cb12470_c9/f1p2/1842 isoform=cb12470_c9;full_length_coverage=1;non_full_length_coverage=2;isoform_length=1842;expected_accu	gnl|BL_ORD_ID|9247 cb8564_c1145/f1p2/1988 isoform=cb8564_c1145;full_length_coverage=1;non_full_length_coverage=2;isoform_length=1988;expected_acc	1	1445	1443	1842	3	1446	1582	1982

racy=0.992	uracy=0.995								
cb2155_c11/f1p0/2668 isoform=cb2155_c11;full_length_coverage=1;non_full_length_coverage=0;isoform_length=2668;expected_accuracy=1.000	gnl|BL_ORD_ID|35419 cb2155_c25/f2p1/2883 isoform=cb2155_c25;full_length_coverage=2;non_full_length_coverage=1;isoform_length=2883;expected_accuracy=0.996	1	2105	2106	2668	91	2194	2312	2873
cb8564_c36731/f1p2/2761	gnl|BL_ORD_ID|55502 cb8564_c43538/f1p0/3563	1	1977	1972	2739	2	1985	2414	3181
cb8564_c45150/f1p0/2940 isoform=cb8564_c45150;full_length_coverage=1;non_full_length_coverage=0;isoform_length=2940;expected_accuracy=0.992	gnl|BL_ORD_ID|55920 cb8564_c48949/f1p0/4357	1	2273	2274	2937	2	2293	2847	3510
cb8564_c675/f4p4/2437 isoform=cb8564_c675;full_length_coverage=4;non_full_length_coverage=4;isoform_length=2437;expected_accuracy=1.000	gnl|BL_ORD_ID|12956 cb8564_c1771/f3p1/3767 isoform=cb8564_c1771;full_length_coverage=3;non_full_length_coverage=1;isoform_length=3767;expected_accuracy=0.996	1	1217	1215	2338	119	1334	2646	3767
cb910_c28/f1p1/2974 isoform=cb910_c28;full_length_coverage=1;non_full_length_coverage=1;isoform_length=2974;expected_accuracy=0.995	gnl|BL_ORD_ID|57612 cb910_c84/f1p0/3501	1	2158	2156	2974	29	2186	2302	3120
cb16925_c1/f1p0/1736 isoform=cb16925_c1;full_length_coverage=1;non_full_length_coverage=0;isoform_length=1736;expected_accuracy=0.999	gnl|BL_ORD_ID|11762 cb8564_c1407/f7p0/2261 isoform=cb8564_c1407;full_length_coverage=7;non_full_length_coverage=0;isoform_length=2261;expected_accuracy=1.000	1	1127	1127	1712	1	1127	1485	2085
cb3265_c60/f1p0/2197	gnl|BL_ORD_ID|41239 cb3265_c50/f1p2/2270 isoform=cb3265_c50;full_length_coverage=1;non_full_length_coverage=2;isoform_length=2270;expected_accur	1	1740	1742	2165	6	1742	1612	2034

	acy=0.993								
cb8564_c87466/f1p1/3006 isoform=cb8564_c87466;full_length_coverage=1;non_full_length_coverage=1;isoform_length=3006;expected_accuracy=0.994	gnl|BL_ORD_ID|14531 cb8564_c41778/f1p9/3482 isoform=cb8564_c41778;full_length_coverage=1;non_full_length_coverage=9;isoform_length=3482;expected_accuracy=0.992	250	2923	1	254	812	3463	1	247
cb12572_c4/f1p1/1439 isoform=cb12572_c4;full_length_coverage=1;non_full_length_coverage=1;isoform_length=1439;expected_accuracy=0.993	gnl|BL_ORD_ID|73926 cb5440_c4/f1p0/3106	1	1106	1104	1429	24	1125	2686	3011
cb8564_c110170/f1p0/2703	gnl|BL_ORD_ID|9197 cb8564_c113014/f1p0/2963 isoform=cb8564_c113014;full_length_coverage=1;non_full_length_coverage=0;isoform_length=2963;expected_accuracy=0.996	1	1606	1607	2610	22	1628	1961	2963
cb8564_c70133/f1p0/2881	gnl|BL_ORD_ID|16704 cb8564_c68755/f6p0/3071 isoform=cb8564_c68755;full_length_coverage=6;non_full_length_coverage=0;isoform_length=3071;expected_accuracy=0.998	13	1940	1941	2860	46	1977	2153	3071
cb14220_c18/f1p1/6379 isoform=cb14220_c18;full_length_coverage=1;non_full_length_coverage=1;isoform_length=6379;expected_accuracy=0.992	gnl|BL_ORD_ID|63134 cb14220_c9/f1p0/6925	2165	6379	106	2167	2708	6921	218	2293
cb10369_c5/f1p0/1703	gnl|BL_ORD_ID|2711 cb10369_c4/f1p0/2035 isoform=cb10369_c4;full_length_coverage=1;non_full_length_coverage=0;isoform_length=2035;expected_accuracy=0.995	184	1605	24	185	628	2035	143	301
cb8564_c77710/f1p0/1826	gnl|BL_ORD_ID|13247 cb8564_c20033/f1p1/3127	479	1645	5	481	1968	312	1	477

	isoform=cb8564_c20033;full_length_coverage=1;non_full_length_coverage=1;isoform_length=3127;expected_accuracy=0.994						6		
cb1648_c46/f32p0/2018 isoform=cb1648_c46;full_length_coverage=32;non_full_length_coverage=0;isoform_length=2018;expected_accuracy=1.000	gnl|BL_ORD_ID|66333 cb1648_c27/f1p1/2264	245	2018	27	245	382	2153	1	233
cb9201_c6/f1p0/2240	gnl|BL_ORD_ID|19588 cb9201_c2/f1p0/3381 isoform=cb9201_c2;full_length_coverage=1;non_full_length_coverage=0;isoform_length=3381;expected_accuracy=0.995	414	2065	6	414	1735	3381	74	479
cb8564_c45440/f1p0/2458 isoform=cb8564_c45440;full_length_coverage=1;non_full_length_coverage=0;isoform_length=2458;expected_accuracy=0.992	gnl|BL_ORD_ID|56073 cb8564_c54187/f1p0/3412	306	2458	49	310	1117	3261	1	261
cb8564_c85288/f1p2/2983 isoform=cb8564_c85288;full_length_coverage=1;non_full_length_coverage=2;isoform_length=2983;expected_accuracy=0.999	gnl|BL_ORD_ID|55821 cb8564_c41766/f1p2/3241	1	2289	2289	2969	40	2334	2575	3255
cb8564_c2150/f1p0/2358	gnl|BL_ORD_ID|13555 cb8564_c2143/f1p1/2668 isoform=cb8564_c2143;full_length_coverage=1;non_full_length_coverage=1;isoform_length=2668;expected_accuracy=0.996	1	2130	2130	2378	1	2127	2255	2503
cb10054_c49/f1p1/1123 isoform=cb10054_c49;full_length_coverage=1;non_full_length_coverage=1;isoform_length=1123;expected_acc	gnl|BL_ORD_ID|574 cb10054_c47/f1p0/1362 isoform=cb10054_c47;full_length_coverage=1;non_full_length_coverage=0;isoform_length=1362;expected_acc	258	1123	3	262	439	1304	1	258

uracy=1.000	uracy=0.994								
cb8564_c87817/f1p0/3148	gnl|BL_ORD_ID|45447 cb5340_c0/f1p0/3122 isoform=cb5340_c0;full_length_coverage=1;non_full_length_coverage=0;isoform_length=3122;expected_accuracy=0.996	103	2301	2300	3027	3069	869	754	27
cb8564_c86614/f1p2/3276	gnl|BL_ORD_ID|18565 cb8564_c92252/f1p1/3402 isoform=cb8564_c92252;full_length_coverage=1;non_full_length_coverage=1;isoform_length=3402;expected_accuracy=0.997	99	3168	1	103	333	3402	11	113
cb8564_c181853/f1p0/2119 isoform=cb8564_c181853;full_length_coverage=1;non_full_length_coverage=0;isoform_length=2119;expected_accuracy=0.997	gnl|BL_ORD_ID|56722 cb8564_c54360/f1p0/3040	1	1339	1334	2106	1	1341	2136	2908
cb8564_c181853/f1p0/2119 isoform=cb8564_c181853;full_length_coverage=1;non_full_length_coverage=0;isoform_length=2119;expected_accuracy=0.997	gnl|BL_ORD_ID|55433 cb8564_c41572/f1p1/3265	1	1339	1334	2119	1	1334	2161	2948
cb1027_c22/f4p1/3590 isoform=cb1027_c22;full_length_coverage=4;non_full_length_coverage=1;isoform_length=3590;expected_accuracy=0.999	gnl|BL_ORD_ID|47239 cb1027_c4/f1p0/4455	126	3069	3068	3590	1	2915	3827	4341
cb1545_c18/f1p0/3314	gnl|BL_ORD_ID|29255 cb1545_c4/f3p0/3538 isoform=cb1545_c4;full_length_coverage=3;non_full_length_coverage=0;isoform_length=3538;expected_accuracy=0.998	1	1983	1982	3300	3	1986	2233	3538
cb17424_c12/f1p1/1401	gnl|BL_ORD_ID|38807 cb2733_c8/f1p1/1938	112	1377	1	113	671	193	1	114

	isoform=cb2733_c8;full_length_coverage=1;non_full_length_coverage=1;isoform_length=1938;expected_accuracy=0.999						6		
cb8564_c46612/f1p1/3367	gnl|BL_ORD_ID|58199 cb8564_c91279/f1p0/3567	509	2965	8	508	749	3200	7	508
cb716_c3/f3p1/1492 isoform=cb716_c3;full_length_coverage=3;non_full_length_coverage=1;isoform_length=1492;expected_accuracy=1.000	gnl|BL_ORD_ID|4081 cb716_c10/f1p1/1798 isoform=cb716_c10;full_length_coverage=1;non_full_length_coverage=1;isoform_length=1798;expected_accuracy=0.995	1	1172	1168	1492	4	1171	1474	1798
cb3586_c7/f1p0/2846	gnl|BL_ORD_ID|71356 cb3586_c9/f1p0/3463	300	2847	73	302	912	3471	1	224
cb8564_c121979/f1p0/2453	gnl|BL_ORD_ID|52094 cb8564_c110646/f1p0/2764	1	1743	1740	2381	83	1810	2110	2751
cb8564_c15996/f1p5/3083 isoform=cb8564_c15996;full_length_coverage=1;non_full_length_coverage=5;isoform_length=3083;expected_accuracy=0.993	gnl|BL_ORD_ID|52057 cb8564_c11435/f1p1/3407	323	3083	4	328	482	3240	18	341
cb4128_c17/f1p0/2338	gnl|BL_ORD_ID|40767 cb4128_c10/f3p1/2897 isoform=cb4128_c10;full_length_coverage=3;non_full_length_coverage=1;isoform_length=2897;expected_accuracy=0.998	484	2272	5	487	1052	2849	3	487
cb8564_c53418/f1p0/2602 isoform=cb8564_c53418;full_length_coverage=1;non_full_length_coverage=0;isoform_length=2602;expected_accuracy=0.991	gnl|BL_ORD_ID|12811 cb8564_c162155/f1p6/2689 isoform=cb8564_c162155;full_length_coverage=1;non_full_length_coverage=6;isoform_length=2689;expected_accuracy=0.997	1163	2479	1	1168	1368	2687	95	1267
cb8564_c38947/f1p0/2650	gnl|BL_ORD_ID|55866 cb8564_c47522/f1p0/2954	1	1441	1436	2650	1	141	1635	284

isoform=cb8564_c38947;full_length_coverage=1;non_full_length_coverage=0;isoform_length=2650;expected_accuracy=0.996							8		9
cb8564_c43399/f1p0/2654 isoform=cb8564_c43399;full_length_coverage=1;non_full_length_coverage=0;isoform_length=2654;expected_accuracy=0.999	gnl|BL_ORD_ID|17161 cb8564_c87466/f1p1/3006 isoform=cb8564_c87466;full_length_coverage=1;non_full_length_coverage=1;isoform_length=3006;expected_accuracy=0.994	1	2367	2363	2654	1	2383	2604	2901
cb8564_c35607/f1p0/2587 isoform=cb8564_c35607;full_length_coverage=1;non_full_length_coverage=0;isoform_length=2587;expected_accuracy=0.993	gnl|BL_ORD_ID|14314 cb8564_c45827/f2p2/4435 isoform=cb8564_c45827;full_length_coverage=2;non_full_length_coverage=2;isoform_length=4435;expected_accuracy=0.999	1	1685	1685	2587	1594	3275	3524	4428
cb2101_c6/f1p0/2473 isoform=cb2101_c6;full_length_coverage=1;non_full_length_coverage=0;isoform_length=2473;expected_accuracy=0.990	gnl|BL_ORD_ID|66714 cb2101_c7/f1p0/2882	1	1937	1934	2473	1	1927	2209	2743
cb12968_c7/f1p0/1320 isoform=cb12968_c7;full_length_coverage=1;non_full_length_coverage=0;isoform_length=1320;expected_accuracy=0.998	gnl|BL_ORD_ID|27058 cb12968_c2/f1p0/1571 isoform=cb12968_c2;full_length_coverage=1;non_full_length_coverage=0;isoform_length=1571;expected_accuracy=0.994	157	1320	29	157	411	1571	1	123
cb8564_c35607/f1p0/2587 isoform=cb8564_c35607;full_length_coverage=1;non_full_length_coverage=0;isoform_length=2587;expected_accuracy=0.993	gnl|BL_ORD_ID|14293 cb8564_c46212/f3p1/4373 isoform=cb8564_c46212;full_length_coverage=3;non_full_length_coverage=1;isoform_length=4373;expected_accuracy=0.999	1	1685	1685	2587	1499	3180	3428	4328
cb5304_c6/f1p0/2203 isoform=cb5304_c6;full_length_coverage=1;non_full_length_coverage=0;isoform_length=2203;expected_accur	gnl|BL_ORD_ID|73763 cb5304_c14/f1p0/2912	1	1509	1508	2203	310	1817	1932	2629

acy=0.997									
cb6090_c0/f5p0/2362 isoform=cb6090_c0;full_length_coverage=5;non_full_length_coverage=0;isoform_length=2362;expected_accuracy=1.000	gnl|BL_ORD_ID|73488 cb6090_c3/f1p0/2843	1	1688	1689	2361	1	1694	2179	2862
cb658_c9/f1p1/2605 isoform=cb658_c9;full_length_coverage=1;non_full_length_coverage=1;isoform_length=2605;expected_accuracy=0.997	gnl|BL_ORD_ID|4084 cb658_c10/f1p1/6427 isoform=cb658_c10;full_length_coverage=1;non_full_length_coverage=1;isoform_length=6427;expected_accuracy=0.993	1	1286	1286	2577	2577	3862	5144	6427
cb454_c1/f3p0/2382 isoform=cb454_c1;full_length_coverage=3;non_full_length_coverage=0;isoform_length=2382;expected_accuracy=1.000	gnl|BL_ORD_ID|72202 cb454_c35/f1p0/2896	1	1422	1426	2348	27	1436	1798	2718
cb8564_c132795/f5p10/3729 isoform=cb8564_c132795;full_length_coverage=5;non_full_length_coverage=10;isoform_length=3729;expected_accuracy=0.999	gnl|BL_ORD_ID|16247 cb8564_c5117/f1p0/4873 isoform=cb8564_c5117;full_length_coverage=1;non_full_length_coverage=0;isoform_length=4873;expected_accuracy=0.999	1	1832	1830	3619	82	1921	3088	4873
cb1406_c5/f1p0/2334 isoform=cb1406_c5;full_length_coverage=1;non_full_length_coverage=0;isoform_length=2334;expected_accuracy=0.996	gnl|BL_ORD_ID|27455 cb1406_c0/f2p1/5413 isoform=cb1406_c0;full_length_coverage=2;non_full_length_coverage=1;isoform_length=5413;expected_accuracy=0.999	214	2334	1	218	3268	5393	13	228
cb3856_c0/f2p1/1619 isoform=cb3856_c0;full_length_coverage=2;non_full_length_coverage=1;isoform_length=1619;expected_accuracy=0.996	gnl|BL_ORD_ID|71563 cb3856_c6/f1p0/1906	130	1619	1	131	363	1853	1	134
cb3999_c22/f1p0/2445	gnl|BL_ORD_ID|41057 cb3999_c13/f1p0/2693	1	1868	1868	2445	1	186	2020	259

isoform=cb3999_c22;full_length_coverage=1;non_full_length_coverage=0;isoform_length=2445;expected_accuracy=0.998	isoform=cb3999_c13;full_length_coverage=1;non_full_length_coverage=0;isoform_length=2693;expected_accuracy=1.000						8		8
cb8564_c18907/f1p2/4138 isoform=cb8564_c18907;full_length_coverage=1;non_full_length_coverage=2;isoform_length=4138;expected_accuracy=0.996	gnl|BL_ORD_ID|64186 cb14630_c30/f1p0/5869	363	4138	1	367	1879	5655	1	367
cb2640_c18/f1p0/3205 isoform=cb2640_c18;full_length_coverage=1;non_full_length_coverage=0;isoform_length=3205;expected_accuracy=0.991	gnl|BL_ORD_ID|68659 cb2640_c11/f1p0/3319	188	3072	1	189	459	3331	37	225
cb8564_c70307/f2p0/3222 isoform=cb8564_c70307;full_length_coverage=2;non_full_length_coverage=0;isoform_length=3222;expected_accuracy=0.999	gnl|BL_ORD_ID|56330 cb8564_c74576/f1p0/4386	1	2253	2253	3187	200	2452	3000	3938
cb8564_c36011/f1p0/3015	gnl|BL_ORD_ID|56381 cb8564_c75626/f1p2/3199	10	2128	2126	2675	12	2116	2611	3160
cb3586_c10/f1p0/2722 isoform=cb3586_c10;full_length_coverage=1;non_full_length_coverage=0;isoform_length=2722;expected_accuracy=0.993	gnl|BL_ORD_ID|71356 cb3586_c9/f1p0/3463	252	2722	1	252	914	3386	25	280
cb8564_c22636/f1p0/2704 isoform=cb8564_c22636;full_length_coverage=1;non_full_length_coverage=0;isoform_length=2704;expected_accuracy=0.994	gnl|BL_ORD_ID|13678 cb8564_c22279/f1p0/2983 isoform=cb8564_c22279;full_length_coverage=1;non_full_length_coverage=0;isoform_length=2983;expected_accuracy=0.997	1	2092	2093	2704	1	2095	2214	2826
cb293_c6/f1p0/3758	gnl|BL_ORD_ID|69536 cb293_c7/f1p0/4528	1	1906	1903	3742	10	191	2645	445

isoform=cb293_c6;full_length_coverage=1;non_full_length_coverage=0;isoform_length=3758;expected_accuracy=0.997							9		6
cb12513_c86/f1p1/1373	gnl|BL_ORD_ID|14254 cb8564_c3885/f1p7/2735 isoform=cb8564_c3885;full_length_coverage=1;non_full_length_coverage=7;isoform_length=2735;expected_accuracy=0.999	311	1363	55	312	1490	2542	17	274
cb12714_c29/f1p2/1583	gnl|BL_ORD_ID|52063 cb8564_c11680/f1p1/3676	1	1296	1293	1591	22	1317	3174	3471
cb141_c1/f3p0/3564 isoform=cb141_c1;full_length_coverage=3;non_full_length_coverage=0;isoform_length=3564;expected_accuracy=1.000	gnl|BL_ORD_ID|61123 cb141_c3/f1p0/4274	1647	3564	1	1649	2388	4305	18	1670
cb8564_c72349/f1p0/3248	gnl|BL_ORD_ID|12934 cb8564_c164095/f2p4/3350 isoform=cb8564_c164095;full_length_coverage=2;non_full_length_coverage=4;isoform_length=3350;expected_accuracy=0.995	1	2662	2659	3068	97	2779	2938	3350
cb1312_c24/f1p0/3018	gnl|BL_ORD_ID|26440 cb1312_c40/f1p1/3867 isoform=cb1312_c40;full_length_coverage=1;non_full_length_coverage=1;isoform_length=3867;expected_accuracy=0.996	1	1532	1531	2888	144	1669	2476	3827
cb8564_c23232/f1p3/2540	gnl|BL_ORD_ID|13302 cb8564_c19160/f1p2/2687 isoform=cb8564_c19160;full_length_coverage=1;non_full_length_coverage=2;isoform_length=2687;expected_accuracy=0.990	16	2172	2167	2518	5	2190	2292	2643
cb1528_c3/f1p0/2290	gnl|BL_ORD_ID|63716 cb1528_c4/f1p0/3332	97	2295	1	100	1084	329	1	100

						6			
cb4528_c38/f2p0/1640 isoform=cb4528_c38;full_length_coverage=2;non_full_length_coverage=0;isoform_length=1640;expected_accuracy=1.000	gnl|BL_ORD_ID|42120 cb4528_c39/f1p0/2046 isoform=cb4528_c39;full_length_coverage=1;non_full_length_coverage=0;isoform_length=2046;expected_accuracy=0.995	1	1497	1495	1639	99	1595	1903	2046
cb12685_c3/f1p2/1466	gnl|BL_ORD_ID|13277 cb8564_c23063/f1p2/2963 isoform=cb8564_c23063;full_length_coverage=1;non_full_length_coverage=2;isoform_length=2963;expected_accuracy=0.996	1	1294	1292	1463	8	1303	2792	2959
cb1178_c21/f1p0/1378	gnl|BL_ORD_ID|23778 cb1178_c16/f1p0/1671 isoform=cb1178_c16;full_length_coverage=1;non_full_length_coverage=0;isoform_length=1671;expected_accuracy=0.993	105	1380	1	104	223	1504	1	103
cb8564_c40764/f1p0/2563 isoform=cb8564_c40764;full_length_coverage=1;non_full_length_coverage=0;isoform_length=2563;expected_accuracy=0.997	gnl|BL_ORD_ID|56623 cb8564_c49310/f1p2/3045	19	2167	2162	2563	65	2211	2418	2819
cb12292_c22/f1p0/1010	gnl|BL_ORD_ID|23626 cb12292_c9/f1p0/1677 isoform=cb12292_c9;full_length_coverage=1;non_full_length_coverage=0;isoform_length=1677;expected_accuracy=0.995	159	1006	8	155	816	1662	1	145
cb8564_c2392/f1p2/2510 isoform=cb8564_c2392;full_length_coverage=1;non_full_length_coverage=2;isoform_length=2510;expected_accuracy=0.992	gnl|BL_ORD_ID|13231 cb8564_c2282/f1p1/3052 isoform=cb8564_c2282;full_length_coverage=1;non_full_length_coverage=1;isoform_length=3052;expected_accuracy=0.995	707	2510	8	709	1259	3046	2	691
cb8564_c71532/f1p0/3010	gnl|BL_ORD_ID|17929 cb8564_c88718/f1p0/4453	18	2515	2514	3010	1	247	2636	3112

isoform=cb8564_c71532;full_length_coverage=1;non_full_length_coverage=0;isoform_length=3010;expected_accuracy=0.995	isoform=cb8564_c88718;full_length_coverage=1;non_full_length_coverage=0;isoform_length=4453;expected_accuracy=0.997						9		
cb8564_c70133/f1p0/2881	gnl|BL_ORD_ID|16978 cb8564_c74124/f1p0/3022 isoform=cb8564_c74124;full_length_coverage=1;non_full_length_coverage=0;isoform_length=3022;expected_accuracy=0.999	1	1940	1941	2807	21	1981	2157	3022
cb8564_c111147/f1p0/3741	gnl|BL_ORD_ID|12399 cb8564_c128675/f2p0/3892 isoform=cb8564_c128675;full_length_coverage=2;non_full_length_coverage=0;isoform_length=3892;expected_accuracy=0.997	694	3722	9	696	858	3890	1	687
cb12_c37/f1p0/4067	gnl|BL_ORD_ID|61087 cb12_c40/f1p0/4680	286	4080	186	288	702	4496	470	572
cb8564_c42409/f1p0/3007	gnl|BL_ORD_ID|16705 cb8564_c53880/f1p0/3484 isoform=cb8564_c53880;full_length_coverage=1;non_full_length_coverage=0;isoform_length=3484;expected_accuracy=0.996	1089	2899	107	1090	1672	3484	75	1056
cb3566_c0/f2p1/1967 isoform=cb3566_c0;full_length_coverage=2;non_full_length_coverage=1;isoform_length=1967;expected_accuracy=0.996	gnl|BL_ORD_ID|40310 cb3566_c1/f1p1/3588 isoform=cb3566_c1;full_length_coverage=1;non_full_length_coverage=1;isoform_length=3588;expected_accuracy=0.993	1	1433	1428	1967	1	1429	2997	3536
cb7100_c0/f1p0/2696 isoform=cb7100_c0;full_length_coverage=1;non_full_length_coverage=0;isoform_length=2696;expected_accuracy=0.993	gnl|BL_ORD_ID|3725 cb7100_c1/f1p0/2838 isoform=cb7100_c1;full_length_coverage=1;non_full_length_coverage=0;isoform_length=2838;expected_accuracy=0.996	1	2188	2186	2693	10	2198	2329	2838
cb8564_c1159/f1p1/2210	gnl|BL_ORD_ID|12031 cb8564_c146119/f9p4/2560	1004	2210	1	1005	1330	253	25	103

<table>
<tr><td>isoform=cb8564_c1159;full_length_coverage=1;non_full_length_coverage=1;isoform_length=2210;expected_accuracy=0.993</td><td>isoform=cb8564_c146119;full_length_coverage=9;non_full_length_coverage=4;isoform_length=2560;expected_accuracy=1.000</td><td></td><td></td><td></td><td></td><td></td><td>5</td><td></td><td>2</td></tr>
<tr><td>cb233_c13/f1p0/2651 isoform=cb233_c13;full_length_coverage=1;non_full_length_coverage=0;isoform_length=2651;expected_accuracy=0.992</td><td>gnl|BL_ORD_ID|38488 cb233_c5/f2p0/2943 isoform=cb233_c5;full_length_coverage=2;non_full_length_coverage=0;isoform_length=2943;expected_accuracy=1.000</td><td>377</td><td>2600</td><td>4</td><td>382</td><td>694</td><td>2943</td><td>36</td><td>418</td></tr>
<tr><td>cb946_c11/f1p0/3138 isoform=cb946_c11;full_length_coverage=1;non_full_length_coverage=0;isoform_length=3138;expected_accuracy=1.000</td><td>gnl|BL_ORD_ID|19593 cb946_c0/f2p0/3227 isoform=cb946_c0;full_length_coverage=2;non_full_length_coverage=0;isoform_length=3227;expected_accuracy=0.992</td><td>1004</td><td>3099</td><td>1</td><td>1004</td><td>1126</td><td>3227</td><td>12</td><td>1015</td></tr>
<tr><td>cb8637_c58/f1p0/1789 isoform=cb8637_c58;full_length_coverage=1;non_full_length_coverage=0;isoform_length=1789;expected_accuracy=0.990</td><td>gnl|BL_ORD_ID|18077 cb8637_c32/f18p0/2009 isoform=cb8637_c32;full_length_coverage=18;non_full_length_coverage=0;isoform_length=2009;expected_accuracy=1.000</td><td>1</td><td>1332</td><td>1332</td><td>1789</td><td>101</td><td>1432</td><td>1535</td><td>1992</td></tr>
<tr><td>cb3838_c13/f1p0/3157 isoform=cb3838_c13;full_length_coverage=1;non_full_length_coverage=0;isoform_length=3157;expected_accuracy=0.993</td><td>gnl|BL_ORD_ID|70647 cb3838_c5/f1p0/4926</td><td>1013</td><td>3140</td><td>1</td><td>1013</td><td>1818</td><td>3949</td><td>3</td><td>1026</td></tr>
<tr><td>cb8564_c150120/f1p3/2376 isoform=cb8564_c150120;full_length_coverage=1;non_full_length_coverage=3;isoform_length=2376;expected_accuracy=1.000</td><td>gnl|BL_ORD_ID|13919 cb8564_c36044/f1p1/2509 isoform=cb8564_c36044;full_length_coverage=1;non_full_length_coverage=1;isoform_length=2509;expected_accuracy=0.995</td><td>1</td><td>2130</td><td>2127</td><td>2376</td><td>7</td><td>2139</td><td>2261</td><td>2509</td></tr>
<tr><td>cb541_c24/f1p0/5418 isoform=cb541_c24;full_length_coverage=1;non_full_length_coverage=0;isoform_length=5418;expected_accur</td><td>gnl|BL_ORD_ID|44620 cb541_c26/f1p1/5713 isoform=cb541_c26;full_length_coverage=1;non_full_length_coverage=1;isoform_length=5713;expected_accura</td><td>2</td><td>4102</td><td>4103</td><td>5418</td><td>1</td><td>4119</td><td>4249</td><td>5571</td></tr>
</table>

acy=0.993	cy=0.997								
cb3898_c0/f8p1/2602 isoform=cb3898_c0;full_length_coverage=8;non_full_length_coverage=1;isoform_length=2602;expected_accuracy=1.000	gnl|BL_ORD_ID|70677 cb3898_c7/f1p0/3325	1288	2602	1	1289	1487	2801	22	1306
cb4016_c34/f1p0/2560 isoform=cb4016_c34;full_length_coverage=1;non_full_length_coverage=0;isoform_length=2560;expected_accuracy=0.998	gnl|BL_ORD_ID|71728 cb4016_c38/f1p0/2815	1264	2560	12	1266	1380	2675	7	1259
cb8564_c116509/f1p0/3009	gnl|BL_ORD_ID|11519 cb8564_c146513/f2p1/3281 isoform=cb8564_c146513;full_length_coverage=2;non_full_length_coverage=1;isoform_length=3281;expected_accuracy=0.997	1	2016	2015	2985	61	2077	2301	3276
cb8564_c844/f2p0/2586 isoform=cb8564_c844;full_length_coverage=2;non_full_length_coverage=0;isoform_length=2586;expected_accuracy=1.000	gnl|BL_ORD_ID|14165 cb8564_c3674/f1p0/3030 isoform=cb8564_c3674;full_length_coverage=1;non_full_length_coverage=0;isoform_length=3030;expected_accuracy=0.996	1	2343	2344	2586	310	2654	2768	3010
cb373_c29/f1p0/4336	gnl|BL_ORD_ID|70842 cb373_c46/f1p0/6511	1	3532	3532	3924	412	3938	4473	4864
cb6802_c57/f1p0/2631	gnl|BL_ORD_ID|4195 cb6802_c5/f2p0/2683 isoform=cb6802_c5;full_length_coverage=2;non_full_length_coverage=0;isoform_length=2683;expected_accuracy=0.998	191	2236	1	192	343	2379	29	218
cb8564_c36689/f1p1/2368 isoform=cb8564_c36689;full_length_coverage=1;non_full_length_coverage=1;isoform_length=2368;expected_	gnl|BL_ORD_ID|56602 cb8564_c48192/f1p6/3029	1	1709	1706	2368	1	1705	2204	2867

accuracy=0.991									
cb280_c71/f1p0/3805 isoform=cb280_c71;full_length_coverage=1;non_full_length_coverage=0;isoform_length=3805;expected_accuracy=0.994	gnl|BL_ORD_ID|36868 cb280_c70/f1p0/4034 isoform=cb280_c70;full_length_coverage=1;non_full_length_coverage=0;isoform_length=4034;expected_accuracy=0.996	1	3085	3085	3805	17	3105	3310	4033
cb1956_c12/f1p0/3120	gnl|BL_ORD_ID|66543 cb1956_c24/f1p0/3428	124	2714	2714	3138	2	2593	2732	3156
cb14060_c0/f1p1/1332	gnl|BL_ORD_ID|39618 cb3307_c26/f1p2/2430 isoform=cb3307_c26;full_length_coverage=1;non_full_length_coverage=2;isoform_length=2430;expected_accuracy=0.997	150	1331	5	153	1248	2429	9	157
cb7650_c9/f2p0/2154 isoform=cb7650_c9;full_length_coverage=2;non_full_length_coverage=0;isoform_length=2154;expected_accuracy=0.998	gnl|BL_ORD_ID|8847 cb7650_c5/f1p0/2571 isoform=cb7650_c5;full_length_coverage=1;non_full_length_coverage=0;isoform_length=2571;expected_accuracy=0.998	603	2154	67	604	997	2547	26	563
cb8564_c14116/f1p0/2968	gnl|BL_ORD_ID|19169 cb8564_c9750/f2p0/3506 isoform=cb8564_c9750;full_length_coverage=2;non_full_length_coverage=0;isoform_length=3506;expected_accuracy=0.994	664	2869	9	667	1296	3506	1	658
cb8564_c4546/f2p0/2151	gnl|BL_ORD_ID|14176 cb8564_c4538/f2p0/2766 isoform=cb8564_c4538;full_length_coverage=2;non_full_length_coverage=0;isoform_length=2766;expected_accuracy=0.994	11	1438	1436	2158	3	1430	2048	2766
cb9604_c7/f1p0/1887	gnl|BL_ORD_ID|21527 cb9604_c2/f2p0/1950 isoform=cb9604_c2;full_length_coverage=2;non_full_length_coverage=0;isoform_length=1950;expected_accura	202	1802	25	200	299	1897	1	157

	cy=0.994								
cb6904_c0/f24p4/1884 isoform=cb6904_c0;full_length_coverage=24;non_full_length_coverage=4;isoform_length=1884;expected_accuracy=1.000	gnl|BL_ORD_ID|47756 cb6904_c9/f1p1/2839	393	1884	75	395	1328	2819	1	318
cb3586_c13/f1p0/3140	gnl|BL_ORD_ID|71356 cb3586_c9/f1p0/3463	314	2935	73	314	765	3386	10	250
cb6204_c6/f1p1/2162 isoform=cb6204_c6;full_length_coverage=1;non_full_length_coverage=1;isoform_length=2162;expected_accuracy=1.000	gnl|BL_ORD_ID|4939 cb6204_c0/f24p2/2501 isoform=cb6204_c0;full_length_coverage=24;non_full_length_coverage=2;isoform_length=2501;expected_accuracy=1.000	1	1249	1244	2162	1	1248	1574	2492
cb8564_c20918/f2p2/2484	gnl|BL_ORD_ID|13115 cb8564_c16916/f7p1/3034 isoform=cb8564_c16916;full_length_coverage=7;non_full_length_coverage=1;isoform_length=3034;expected_accuracy=0.996	1	1497	1495	2453	146	1646	1853	2811
cb5891_c11/f1p0/2563	gnl|BL_ORD_ID|46310 cb5891_c10/f1p0/2609 isoform=cb5891_c10;full_length_coverage=1;non_full_length_coverage=0;isoform_length=2609;expected_accuracy=0.995	201	2520	1	200	308	2604	5	204
cb8564_c111706/f1p0/2253	gnl|BL_ORD_ID|10848 cb8564_c112467/f1p0/3061 isoform=cb8564_c112467;full_length_coverage=1;non_full_length_coverage=0;isoform_length=3061;expected_accuracy=0.994	1	1681	1678	2122	2	1655	2617	3061
cb8564_c38947/f1p0/2650 isoform=cb8564_c38947;full_length_coverage=1;non_full_length_coverage=0;isoform_length=2650;expected_	gnl|BL_ORD_ID|17161 cb8564_c87466/f1p1/3006 isoform=cb8564_c87466;full_length_coverage=1;non_full_length_coverage=1;isoform_length=3006;expected_ac	1	1441	1436	2650	1	1452	1671	2900

accuracy=0.996	curacy=0.994								
cb1525_c7/f1p0/3162 isoform=cb1525_c7;full_length_coverage=1;non_full_length_coverage=0;isoform_length=3162;expected_accuracy=0.995	gnl|BL_ORD_ID|63714 cb1525_c28/f1p1/5628	149	3162	1	149	807	3829	56	204
cb5415_c2/f2p1/1400 isoform=cb5415_c2;full_length_coverage=2;non_full_length_coverage=1;isoform_length=1400;expected_accuracy=0.999	gnl|BL_ORD_ID|46428 cb5415_c1/f2p0/2456 isoform=cb5415_c1;full_length_coverage=2;non_full_length_coverage=0;isoform_length=2456;expected_accuracy=0.995	478	1400	50	480	1529	2455	35	469
cb2981_c2/f1p1/3260 isoform=cb2981_c2;full_length_coverage=1;non_full_length_coverage=1;isoform_length=3260;expected_accuracy=0.997	gnl|BL_ORD_ID|38098 cb2981_c1/f1p0/3757 isoform=cb2981_c1;full_length_coverage=1;non_full_length_coverage=0;isoform_length=3757;expected_accuracy=0.997	1	2519	2516	3162	245	2765	3112	3757
cb5183_c115/f1p0/1016	gnl|BL_ORD_ID|73598 cb5183_c50/f1p0/1765	326	1016	7	330	938	1629	6	329
cb5697_c0/f4p1/2117 isoform=cb5697_c0;full_length_coverage=4;non_full_length_coverage=1;isoform_length=2117;expected_accuracy=1.000	gnl|BL_ORD_ID|46177 cb5697_c2/f3p0/3035 isoform=cb5697_c2;full_length_coverage=3;non_full_length_coverage=0;isoform_length=3035;expected_accuracy=0.998	1	1169	1170	2117	1	1169	2083	3031
cb8564_c182028/f1p0/2513 isoform=cb8564_c182028;full_length_coverage=1;non_full_length_coverage=0;isoform_length=2513;expected_accuracy=0.996	gnl|BL_ORD_ID|16052 cb8564_c69457/f3p14/2821 isoform=cb8564_c69457;full_length_coverage=3;non_full_length_coverage=14;isoform_length=2821;expected_accuracy=0.997	1	1971	1968	2513	1	1970	2114	2659
cb8564_c18491/f2p0/4707 isoform=cb8564_c18491;full_length_coverage=2;non_full_length_coverage=0;isoform_length=4707;expected_	gnl|BL_ORD_ID|67917 cb18654_c10/f1p0/5700	1	4067	4068	4707	815	4886	5111	5750

accuracy=0.996									
cb8564_c3782/f1p0/3164	gnl|BL_ORD_ID|14097 cb8564_c3797/f2p1/2809 isoform=cb8564_c3797;full_length_coverage=2;non_full_length_coverage=1;isoform_length=2809;expected_accuracy=0.996	2	2395	2395	2663	4	2395	2541	2809
cb7257_c2/f1p0/2073 isoform=cb7257_c2;full_length_coverage=1;non_full_length_coverage=0;isoform_length=2073;expected_accuracy=0.993	gnl|BL_ORD_ID|7883 cb7257_c1/f1p0/2322 isoform=cb7257_c1;full_length_coverage=1;non_full_length_coverage=0;isoform_length=2322;expected_accuracy=1.000	1	1564	1562	2073	31	1598	1702	2214
cb1626_c15/f1p0/2204 isoform=cb1626_c15;full_length_coverage=1;non_full_length_coverage=0;isoform_length=2204;expected_accuracy=0.998	gnl|BL_ORD_ID|33068 cb1626_c13/f1p0/4596 isoform=cb1626_c13;full_length_coverage=1;non_full_length_coverage=0;isoform_length=4596;expected_accuracy=1.000	1093	2154	1	1096	3518	4578	1	1078
cb1505_c7/f3p0/2757 isoform=cb1505_c7;full_length_coverage=3;non_full_length_coverage=0;isoform_length=2757;expected_accuracy=0.996	gnl|BL_ORD_ID|63681 cb1505_c18/f1p0/3176	1	2272	2271	2728	1	2272	2434	2891
cb15243_c1/f1p0/1809 isoform=cb15243_c1;full_length_coverage=1;non_full_length_coverage=0;isoform_length=1809;expected_accuracy=0.992	gnl|BL_ORD_ID|10813 cb8564_c121257/f1p0/1906 isoform=cb8564_c121257;full_length_coverage=1;non_full_length_coverage=0;isoform_length=1906;expected_accuracy=0.995	113	1776	1	112	259	1906	1	112
cb295_c13/f1p1/3878 isoform=cb295_c13;full_length_coverage=1;non_full_length_coverage=1;isoform_length=3878;expected_accuracy=0.994	gnl|BL_ORD_ID|34400 cb18646_c6/f1p3/6738 isoform=cb18646_c6;full_length_coverage=1;non_full_length_coverage=3;isoform_length=6738;expected_accuracy=0.995	1	3634	3634	3878	2484	6137	6371	6616
cb3636_c48/f4p1/2561	gnl|BL_ORD_ID|40745 cb3636_c38/f1p1/2658	117	2547	1	117	231	265	4	119

<table>
<tr><td>isoform=cb3636_c48;full_length_coverage=4;non_full_length_coverage=1;isoform_length=2561;expected_accuracy=1.000</td><td>isoform=cb3636_c38;full_length_coverage=1;non_full_length_coverage=1;isoform_length=2658;expected_accuracy=0.999</td><td></td><td></td><td></td><td></td><td></td><td>8</td><td></td><td></td></tr>
<tr><td>cb8564_c51923/f1p1/2966 isoform=cb8564_c51923;full_length_coverage=1;non_full_length_coverage=1;isoform_length=2966;expected_accuracy=0.997</td><td>gnl|BL_ORD_ID|17429 cb8564_c85447/f1p0/3196 isoform=cb8564_c85447;full_length_coverage=1;non_full_length_coverage=0;isoform_length=3196;expected_accuracy=0.997</td><td>1</td><td>2431</td><td>2430</td><td>2966</td><td>3</td><td>2413</td><td>2660</td><td>3196</td></tr>
<tr><td>cb8564_c36731/f1p2/2761</td><td>gnl|BL_ORD_ID|56764 cb8564_c70795/f1p0/3769</td><td>18</td><td>1976</td><td>1972</td><td>2738</td><td>34</td><td>1994</td><td>2424</td><td>3187</td></tr>
<tr><td>cb8564_c42316/f1p0/2699 isoform=cb8564_c42316;full_length_coverage=1;non_full_length_coverage=0;isoform_length=2699;expected_accuracy=0.992</td><td>gnl|BL_ORD_ID|55265 cb8564_c36918/f1p0/3534</td><td>178</td><td>2699</td><td>13</td><td>177</td><td>626</td><td>3147</td><td>1</td><td>168</td></tr>
<tr><td>cb8564_c17694/f1p1/3299</td><td>gnl|BL_ORD_ID|32014 cb16355_c13/f1p3/5303 isoform=cb16355_c13;full_length_coverage=1;non_full_length_coverage=3;isoform_length=5303;expected_accuracy=0.992</td><td>1</td><td>2347</td><td>2347</td><td>3356</td><td>1720</td><td>4066</td><td>4294</td><td>5300</td></tr>
<tr><td>cb8564_c35088/f1p0/3430 isoform=cb8564_c35088;full_length_coverage=1;non_full_length_coverage=0;isoform_length=3430;expected_accuracy=0.991</td><td>gnl|BL_ORD_ID|52067 cb8564_c11914/f1p0/3554</td><td>1</td><td>2060</td><td>2058</td><td>3430</td><td>1</td><td>2048</td><td>2158</td><td>3546</td></tr>
<tr><td>cb7222_c6/f1p0/2441</td><td>gnl|BL_ORD_ID|50431 cb7222_c3/f1p0/2643</td><td>399</td><td>2312</td><td>56</td><td>404</td><td>650</td><td>2563</td><td>1</td><td>348</td></tr>
<tr><td>cb8564_c71802/f1p1/3657 isoform=cb8564_c71802;full_length_coverage=1;non_full_length_coverage=1;isoform_length=3657;expected_</td><td>gnl|BL_ORD_ID|16562 cb8564_c68923/f2p2/3765 isoform=cb8564_c68923;full_length_coverage=2;non_full_length_coverage=2;isoform_length=3765;expected_ac</td><td>188</td><td>3574</td><td>1</td><td>191</td><td>379</td><td>3765</td><td>37</td><td>231</td></tr>
</table>

accuracy=1.000	curacy=0.999								
cb8564_c39014/f1p0/3019 isoform=cb8564_c39014;full_length_coverage=1;non_full_length_coverage=0;isoform_length=3019;expected_accuracy=0.992	gnl|BL_ORD_ID|17429 cb8564_c85447/f1p0/3196 isoform=cb8564_c85447;full_length_coverage=1;non_full_length_coverage=0;isoform_length=3196;expected_accuracy=0.997	1	2467	2469	3013	3	2413	2662	3192
cb8564_c1994/f1p0/2024 isoform=cb8564_c1994;full_length_coverage=1;non_full_length_coverage=0;isoform_length=2024;expected_accuracy=0.995	gnl|BL_ORD_ID|13434 cb8564_c1993/f1p0/2538 isoform=cb8564_c1993;full_length_coverage=1;non_full_length_coverage=0;isoform_length=2538;expected_accuracy=0.993	10	1187	1186	2024	2	1170	1694	2525
cb7611_c26/f1p0/2483	gnl|BL_ORD_ID|7565 cb7611_c19/f1p0/3611 isoform=cb7611_c19;full_length_coverage=1;non_full_length_coverage=0;isoform_length=3611;expected_accuracy=0.991	289	2198	66	290	1674	3589	1	224
cb8564_c42506/f1p0/2909	gnl|BL_ORD_ID|56613 cb8564_c48653/f1p0/4470	1	2200	2199	2856	1059	3258	3131	3796
cb1039_c4/f2p2/4154 isoform=cb1039_c4;full_length_coverage=2;non_full_length_coverage=2;isoform_length=4154;expected_accuracy=1.000	gnl|BL_ORD_ID|1556 cb1039_c41/f1p0/4155 isoform=cb1039_c41;full_length_coverage=1;non_full_length_coverage=0;isoform_length=4155;expected_accuracy=0.999	20	2652	2652	4099	1	2626	2732	4155
cb8564_c16669/f1p0/2894	gnl|BL_ORD_ID|11380 cb8564_c12334/f3p1/4316 isoform=cb8564_c12334;full_length_coverage=3;non_full_length_coverage=1;isoform_length=4316;expected_accuracy=0.998	1	1723	1721	2706	785	2507	3318	4303
cb8564_c36808/f1p0/2756	gnl|BL_ORD_ID|56353 cb8564_c75114/f1p0/2866	19	2050	2050	2555	4	2035	2193	2701
cb6606_c32/f1p0/1651	gnl|BL_ORD_ID|49866 cb6606_c12/f1p0/2893	1	1197	1196	1651	1	1193	1855	230

isoform=cb6606_c32;full_length_coverage=1;non_full_length_coverage=0;isoform_length=1651;expected_accuracy=0.995									8
cb8564_c40810/f1p0/3998 isoform=cb8564_c40810;full_length_coverage=1;non_full_length_coverage=0;isoform_length=3998;expected_accuracy=0.996	gnl|BL_ORD_ID|55207 cb8564_c35399/f1p0/4553	1	2765	2761	3998	73	2836	3039	4276
cb8564_c2326/f1p0/2006 isoform=cb8564_c2326;full_length_coverage=1;non_full_length_coverage=0;isoform_length=2006;expected_accuracy=0.990	gnl|BL_ORD_ID|13231 cb8564_c2282/f1p1/3052 isoform=cb8564_c2282;full_length_coverage=1;non_full_length_coverage=1;isoform_length=3052;expected_accuracy=0.995	230	2006	6	234	1280	3050	1	232
cb2927_c0/f3p1/3122 isoform=cb2927_c0;full_length_coverage=3;non_full_length_coverage=1;isoform_length=3122;expected_accuracy=1.000	gnl|BL_ORD_ID|37704 cb2927_c8/f1p1/3276 isoform=cb2927_c8;full_length_coverage=1;non_full_length_coverage=1;isoform_length=3276;expected_accuracy=1.000	1319	3122	1	1318	1464	3267	1	1318
cb6719_c46/f1p0/2507	gnl|BL_ORD_ID|4666 cb6719_c24/f1p0/2613 isoform=cb6719_c24;full_length_coverage=1;non_full_length_coverage=0;isoform_length=2613;expected_accuracy=0.998	1	1435	1434	2452	1	1437	1559	2576
cb8564_c115640/f1p0/2569 isoform=cb8564_c115640;full_length_coverage=1;non_full_length_coverage=0;isoform_length=2569;expected_accuracy=0.995	gnl|BL_ORD_ID|55680 cb8564_c4257/f1p0/3687	1	1406	1407	2545	1	1406	2525	3664
cb6320_c1/f2p0/1818 isoform=cb6320_c1;full_length_coverage=2;non_full_length_coverage=0;isoform_length=1818;expected_accur	gnl|BL_ORD_ID|5687 cb6320_c2/f1p0/2874 isoform=cb6320_c2;full_length_coverage=1;non_full_length_coverage=0;isoform_length=2874;expected_accura	1	1206	1202	1818	91	1283	2226	2872

acy=0.997	cy=0.995								
cb10132_c18/f1p1/1347	gnl|BL_ORD_ID|57227 cb8564_c87128/f1p2/1996	401	1301	42	401	1078	197 9	129	487
cb8564_c10682/f1p0/1913 isoform=cb8564_c10682;full_length_coverage=1;non_full_length_coverage=0;isoform_length=1913;expected_accuracy=0.994	gnl|BL_ORD_ID|11948 cb8564_c146094/f3p3/2130 isoform=cb8564_c146094;full_length_coverage=3;non_full_length_coverage=3;isoform_length=2130;expected_accuracy=1.000	1	1045	1041	1913	45	108 7	1258	213 0
cb500_c21/f1p0/3434 isoform=cb500_c21;full_length_coverage=1;non_full_length_coverage=0;isoform_length=3434;expected_accuracy=0.996	gnl|BL_ORD_ID|72245 cb500_c44/f1p0/3962	1	1834	1833	3369	57	188 8	2351	388 7
cb8564_c49562/f1p0/2838 isoform=cb8564_c49562;full_length_coverage=1;non_full_length_coverage=0;isoform_length=2838;expected_accuracy=0.995	gnl|BL_ORD_ID|15193 cb8564_c42308/f1p5/3192 isoform=cb8564_c42308;full_length_coverage=1;non_full_length_coverage=5;isoform_length=3192;expected_accuracy=0.995	1	2331	2327	2837	1	234 5	2677	319 1
cb6406_c65/f1p0/1830 isoform=cb6406_c65;full_length_coverage=1;non_full_length_coverage=0;isoform_length=1830;expected_accuracy=0.992	gnl|BL_ORD_ID|49692 cb6406_c59/f1p0/2372	1	1055	1051	1830	142	1197	1328	210 8
cb8564_c68409/f2p0/2141 isoform=cb8564_c68409;full_length_coverage=2;non_full_length_coverage=0;isoform_length=2141;expected_accuracy=0.995	gnl|BL_ORD_ID|12420 cb8564_c125410/f1p0/2472 isoform=cb8564_c125410;full_length_coverage=1;non_full_length_coverage=0;isoform_length=2472;expected_accuracy=0.997	264	2135	57	266	600	247 2	2	211
cb9901_c0/f4p0/1305 isoform=cb9901_c0;full_length_coverage=4;non_full_length_coverage=0;isoform_length=1305;expected_accur	gnl|BL_ORD_ID|19917 cb9901_c2/f2p0/1904 isoform=cb9901_c2;full_length_coverage=2;non_full_length_coverage=0;isoform_length=1904;expected_accura	440	1305	9	441	1014	190 3	1	433

acy=0.997	cy=0.994								
cb16364_c4/f1p5/1679 isoform=cb16364_c4;full_length_coverage=1;non_full_length_coverage=5;isoform_length=1679;expected_accuracy=0.991	gnl|BL_ORD_ID|61352 cb1417_c6/f1p1/4358	438	1619	8	441	3204	4387	10	441
cb8564_c23436/f1p0/3497 isoform=cb8564_c23436;full_length_coverage=1;non_full_length_coverage=0;isoform_length=3497;expected_accuracy=0.994	gnl|BL_ORD_ID|15178 cb8564_c36410/f1p0/4676 isoform=cb8564_c36410;full_length_coverage=1;non_full_length_coverage=0;isoform_length=4676;expected_accuracy=0.991	1	2305	2305	3497	12	2317	3483	4673
cb3290_c194/f3p0/2878 isoform=cb3290_c194;full_length_coverage=3;non_full_length_coverage=0;isoform_length=2878;expected_accuracy=0.997	gnl|BL_ORD_ID|40560 cb3290_c85/f2p1/2883 isoform=cb3290_c85;full_length_coverage=2;non_full_length_coverage=1;isoform_length=2883;expected_accuracy=0.998	1	1497	1498	2767	9	1502	1614	2883
cb6499_c24/f1p0/1697	gnl|BL_ORD_ID|5167 cb6499_c4/f3p1/2065 isoform=cb6499_c4;full_length_coverage=3;non_full_length_coverage=1;isoform_length=2065;expected_accuracy=0.998	1	1336	1333	1708	54	1388	1626	2001
cb1956_c14/f1p0/2988 isoform=cb1956_c14;full_length_coverage=1;non_full_length_coverage=0;isoform_length=2988;expected_accuracy=0.998	gnl|BL_ORD_ID|66543 cb1956_c24/f1p0/3428	1	2571	2571	2988	2	2593	2732	3145
cb8564_c48916/f1p0/3009	gnl|BL_ORD_ID|55176 cb8564_c34738/f1p0/4152	280	2880	1	283	1394	3992	31	311
cb5728_c10/f1p1/1983 isoform=cb5728_c10;full_length_coverage=1;non_full_length_coverage=1;isoform_length=1983;expected_accu	gnl|BL_ORD_ID|74351 cb5728_c13/f1p1/2211	186	1952	1	185	412	2172	39	219

racy=0.992									
cb1279_c21/f1p0/2474	gnl|BL_ORD_ID|61182 cb1279_c29/f1p0/3632	14	1888	1885	2444	1	1875	3100	3659
cb2082_c32/f1p0/2897	gnl|BL_ORD_ID|66682 cb2082_c19/f1p0/2908	1	1434	1433	2635	8	1441	1608	2809
cb8564_c178999/f1p0/2222 isoform=cb8564_c178999;full_length_coverage=1;non_full_length_coverage=0;isoform_length=2222;expected_accuracy=0.994	gnl|BL_ORD_ID|55430 cb8564_c41551/f1p0/3408	14	1778	1773	2222	12	1772	2505	2952
cb7601_c7/f1p0/1884	gnl|BL_ORD_ID|7855 cb7601_c2/f3p0/2000 isoform=cb7601_c2;full_length_coverage=3;non_full_length_coverage=0;isoform_length=2000;expected_accuracy=0.999	14	1492	1497	1805	1	1479	1598	1906
cb7749_c11/f1p1/1246	gnl|BL_ORD_ID|7216 cb7749_c1/f4p1/1314 isoform=cb7749_c1;full_length_coverage=4;non_full_length_coverage=1;isoform_length=1314;expected_accuracy=1.000	169	1151	4	173	325	1303	5	170
cb8564_c146025/f2p1/3347 isoform=cb8564_c146025;full_length_coverage=2;non_full_length_coverage=1;isoform_length=3347;expected_accuracy=0.996	gnl|BL_ORD_ID|13377 cb8564_c22091/f2p0/3351 isoform=cb8564_c22091;full_length_coverage=2;non_full_length_coverage=0;isoform_length=3351;expected_accuracy=0.997	1	1680	1677	3224	1	1682	1805	3351
cb10385_c10/f1p0/1526 isoform=cb10385_c10;full_length_coverage=1;non_full_length_coverage=0;isoform_length=1526;expected_accuracy=0.994	gnl|BL_ORD_ID|48458 cb10385_c5/f1p0/1837	236	1523	3	234	375	1663	4	235
cb2785_c20/f1p2/2883	gnl|BL_ORD_ID|36863 cb2785_c14/f1p0/2954	1	1577	1577	2796	28	160	1737	295

isoform=cb2785_c20;full_length_coverage=1;non_full_length_coverage=2;isoform_length=2883;expected_accuracy=0.997	isoform=cb2785_c14;full_length_coverage=1;non_full_length_coverage=0;isoform_length=2954;expected_accuracy=0.996						9		4
cb15632_c0/f5p0/1384 isoform=cb15632_c0;full_length_coverage=5;non_full_length_coverage=0;isoform_length=1384;expected_accuracy=1.000	gnl|BL_ORD_ID|18694 cb8564_c80201/f2p0/3643 isoform=cb8564_c80201;full_length_coverage=2;non_full_length_coverage=0;isoform_length=3643;expected_accuracy=0.996	577	1384	9	579	2547	3354	1	571
cb8564_c23232/f1p3/2540	gnl|BL_ORD_ID|55328 cb8564_c38464/f1p0/2918	16	2172	2167	2500	5	2161	2263	2596
cb8564_c35865/f2p2/3469 isoform=cb8564_c35865;full_length_coverage=2;non_full_length_coverage=2;isoform_length=3469;expected_accuracy=1.000	gnl|BL_ORD_ID|13863 cb8564_c20625/f1p0/3774 isoform=cb8564_c20625;full_length_coverage=1;non_full_length_coverage=0;isoform_length=3774;expected_accuracy=0.996	1	2925	2924	3467	1	2921	3227	3773
cb3671_c9/f1p1/3273 isoform=cb3671_c9;full_length_coverage=1;non_full_length_coverage=1;isoform_length=3273;expected_accuracy=0.999	gnl|BL_ORD_ID|71434 cb3671_c14/f1p0/3470	1	2910	2910	3262	32	2940	3116	3469
cb6789_c6/f1p0/2385 isoform=cb6789_c6;full_length_coverage=1;non_full_length_coverage=0;isoform_length=2385;expected_accuracy=0.995	gnl|BL_ORD_ID|4622 cb6789_c1/f1p0/2768 isoform=cb6789_c1;full_length_coverage=1;non_full_length_coverage=0;isoform_length=2768;expected_accuracy=0.996	229	2345	1	228	654	2768	206	433
cb7532_c12/f3p1/2769 isoform=cb7532_c12;full_length_coverage=3;non_full_length_coverage=1;isoform_length=2769;expected_accuracy=1.000	gnl|BL_ORD_ID|8323 cb7532_c6/f1p0/2816 isoform=cb7532_c6;full_length_coverage=1;non_full_length_coverage=0;isoform_length=2816;expected_accuracy=1.000	705	2737	77	705	779	2816	1	634
cb4962_c0/f3p0/1119	gnl|BL_ORD_ID|43826 cb4962_c2/f1p0/2493	395	1119	4	397	1770	249	1	388

isoform=cb4962_c0;full_length_coverage=3;non_full_length_coverage=0;isoform_length=1119;expected_accuracy=0.996	isoform=cb4962_c2;full_length_coverage=1;non_full_length_coverage=0;isoform_length=2493;expected_accuracy=0.992						3		
cb499_c41/f1p0/3022	gnl|BL_ORD_ID|72236 cb499_c46/f1p0/3594	15	2092	2091	3012	9	2086	2296	3217
cb8564_c86964/f1p0/2501 isoform=cb8564_c86964;full_length_coverage=1;non_full_length_coverage=0;isoform_length=2501;expected_accuracy=0.993	gnl|BL_ORD_ID|13944 cb8564_c4652/f3p0/3053 isoform=cb8564_c4652;full_length_coverage=3;non_full_length_coverage=0;isoform_length=3053;expected_accuracy=0.996	1	1791	1790	2481	214	1996	2363	3053
cb15591_c1/f1p1/1413 isoform=cb15591_c1;full_length_coverage=1;non_full_length_coverage=1;isoform_length=1413;expected_accuracy=0.999	gnl|BL_ORD_ID|59641 cb9317_c6/f1p0/2118	280	1413	4	284	539	1672	1	278
cb3965_c3/f1p0/2377 isoform=cb3965_c3;full_length_coverage=1;non_full_length_coverage=0;isoform_length=2377;expected_accuracy=0.995	gnl|BL_ORD_ID|71681 cb3965_c1/f1p0/3487	1	1578	1578	2373	937	2514	2641	3435
cb8564_c40598/f1p4/3119	gnl|BL_ORD_ID|55182 cb8564_c34825/f1p2/3200	11	2732	2729	3054	6	2754	2875	3197
cb8564_c16829/f1p3/2089 isoform=cb8564_c16829;full_length_coverage=1;non_full_length_coverage=3;isoform_length=2089;expected_accuracy=0.995	gnl|BL_ORD_ID|52763 cb8564_c13243/f1p0/4432	228	2071	65	229	2440	4283	29	191
cb7735_c2/f2p0/1143 isoform=cb7735_c2;full_length_coverage=2;non_full_length_coverage=0;isoform_length=1143;expected_accura	gnl|BL_ORD_ID|8557 cb7735_c3/f2p0/2531 isoform=cb7735_c3;full_length_coverage=2;non_full_length_coverage=0;isoform_length=2531;expected_accura	434	1143	8	435	1810	2511	1	432

cy=1.000	cy=1.000								
cb2275_c0/f1p0/3981 isoform=cb2275_c0;full_length_coverage=1;non_full_length_coverage=0;isoform_length=3981;expected_accuracy=0.998	gnl|BL_ORD_ID|9389 cb8564_c108071/f9p6/4539 isoform=cb8564_c108071;full_length_coverage=9;non_full_length_coverage=6;isoform_length=4539;expected_accuracy=1.000	15	3840	3835	3981	4494	668	183	23
cb8564_c87721/f1p0/2168 isoform=cb8564_c87721;full_length_coverage=1;non_full_length_coverage=0;isoform_length=2168;expected_accuracy=0.997	gnl|BL_ORD_ID|15882 cb8564_c69162/f3p0/2862 isoform=cb8564_c69162;full_length_coverage=3;non_full_length_coverage=0;isoform_length=2862;expected_accuracy=0.998	1	1811	1809	2074	140	1950	2597	2862
cb5098_c14/f1p0/2880	gnl|BL_ORD_ID|42664 cb5098_c0/f41p2/2921 isoform=cb5098_c0;full_length_coverage=41;non_full_length_coverage=2;isoform_length=2921;expected_accuracy=1.000	1	1987	1985	2698	65	2054	2207	2920
cb8564_c38233/f1p0/2780	gnl|BL_ORD_ID|55373 cb8564_c39785/f1p0/3296	1	1857	1856	2762	346	2202	2327	3233
cb8564_c39720/f1p0/4003	gnl|BL_ORD_ID|54095 cb8564_c18359/f1p0/3917	820	3713	99	821	931	3823	47	769
cb8564_c39546/f1p2/3023	gnl|BL_ORD_ID|18852 cb8564_c84488/f1p0/3408 isoform=cb8564_c84488;full_length_coverage=1;non_full_length_coverage=0;isoform_length=3408;expected_accuracy=0.996	216	2835	1	216	790	3408	1	217
cb3862_c16/f1p2/3488	gnl|BL_ORD_ID|71572 cb3862_c15/f1p0/4329	1665	3479	11	1666	2224	4038	2	1656
cb8564_c54206/f2p0/3081 isoform=cb8564_c54206;full_length_coverage=2;non_full_length_coverage=0;isoform_length=3081;expected_	gnl|BL_ORD_ID|15130 cb8564_c33393/f7p2/3346 isoform=cb8564_c33393;full_length_coverage=7;non_full_length_coverage=2;isoform_length=3346;expected_ac	1	1805	1804	3081	1	1805	1966	3243

accuracy=0.997	curacy=0.996								
cb8564_c181853/f1p0/2119 isoform=cb8564_c181853;full_length_coverage=1;non_full_length_coverage=0;isoform_length=2119;expected_accuracy=0.997	gnl|BL_ORD_ID|55940 cb8564_c49691/f1p0/2905	1	1339	1334	2119	7	1338	2102	2891
cb3665_c20/f1p0/2779	gnl|BL_ORD_ID|39191 cb3665_c2/f1p0/2916 isoform=cb3665_c2;full_length_coverage=1;non_full_length_coverage=0;isoform_length=2916;expected_accuracy=0.993	1	1482	1479	2669	6	1485	1616	2807
cb653_c12/f1p0/3754 isoform=cb653_c12;full_length_coverage=1;non_full_length_coverage=0;isoform_length=3754;expected_accuracy=0.995	gnl|BL_ORD_ID|5129 cb653_c6/f1p0/4151 isoform=cb653_c6;full_length_coverage=1;non_full_length_coverage=0;isoform_length=4151;expected_accuracy=0.996	195	3754	1	195	537	4097	111	309
cb8333_c1/f2p1/2101 isoform=cb8333_c1;full_length_coverage=2;non_full_length_coverage=1;isoform_length=2101;expected_accuracy=1.000	gnl|BL_ORD_ID|52473 cb8333_c6/f1p0/2576	342	2078	4	342	736	2472	19	357
cb388_c28/f1p3/3528 isoform=cb388_c28;full_length_coverage=1;non_full_length_coverage=3;isoform_length=3528;expected_accuracy=1.000	gnl|BL_ORD_ID|69593 cb388_c51/f1p0/3691	222	3474	21	222	377	3630	23	222
cb8564_c42207/f1p0/3158	gnl|BL_ORD_ID|10029 cb8564_c107323/f4p0/3768 isoform=cb8564_c107323;full_length_coverage=4;non_full_length_coverage=0;isoform_length=3768;expected_accuracy=0.999	1	2776	2771	3186	1	2774	3351	3767
cb3366_c7/f1p0/3317	gnl|BL_ORD_ID|71148 cb3366_c6/f1p0/3643	1	2945	2945	3317	1	294	3146	351

isoform=cb3366_c7;full_length_coverage=1;non_full_length_coverage=0;isoform_length=3317;expected_accuracy=0.997							8		8
cb2172_c21/f1p1/2738	gnl|BL_ORD_ID|35668 cb2172_c5/f2p0/3992 isoform=cb2172_c5;full_length_coverage=2;non_full_length_coverage=0;isoform_length=3992;expected_accuracy=1.000	1	1399	1402	2714	46	1472	2678	3992
cb1306_c36/f1p0/2164 isoform=cb1306_c36;full_length_coverage=1;non_full_length_coverage=0;isoform_length=2164;expected_accuracy=1.000	gnl|BL_ORD_ID|61218 cb1306_c35/f1p0/3110	1	1793	1788	2164	53	1845	2701	3077
cb8564_c10872/f1p2/2947 isoform=cb8564_c10872;full_length_coverage=1;non_full_length_coverage=2;isoform_length=2947;expected_accuracy=0.994	gnl|BL_ORD_ID|12601 cb8564_c13656/f1p0/3139 isoform=cb8564_c13656;full_length_coverage=1;non_full_length_coverage=0;isoform_length=3139;expected_accuracy=0.999	22	2667	2666	2922	1	2640	2883	3139
cb8564_c152353/f2p3/2614	gnl|BL_ORD_ID|13115 cb8564_c16916/f7p1/3034 isoform=cb8564_c16916;full_length_coverage=7;non_full_length_coverage=1;isoform_length=3034;expected_accuracy=0.996	1	1504	1502	2500	143	1646	1853	2852
cb17345_c6/f1p1/2375 isoform=cb17345_c6;full_length_coverage=1;non_full_length_coverage=1;isoform_length=2375;expected_accuracy=0.998	gnl|BL_ORD_ID|32580 cb17345_c8/f1p0/3246 isoform=cb17345_c8;full_length_coverage=1;non_full_length_coverage=0;isoform_length=3246;expected_accuracy=0.997	1	1634	1633	2375	2	1635	2499	3240
cb8564_c4775/f1p0/2922 isoform=cb8564_c4775;full_length_coverage=1;non_full_length_coverage=0;isoform_length=2922;expected_ac	gnl|BL_ORD_ID|16021 cb8564_c4813/f1p1/3090 isoform=cb8564_c4813;full_length_coverage=1;non_full_length_coverage=1;isoform_length=3090;expected_ac	1	2387	2385	2872	2	2392	2603	3090

curacy=0.995	curacy=0.999								
cb8564_c110147/f1p0/3399 isoform=cb8564_c110147;full_length_coverage=1;non_full_length_coverage=0;isoform_length=3399;expected_accuracy=0.999	gnl|BL_ORD_ID|9995 cb8564_c111409/f2p0/3661 isoform=cb8564_c111409;full_length_coverage=2;non_full_length_coverage=0;isoform_length=3661;expected_accuracy=0.999	1	2834	2832	3399	9	2863	3093	3661
cb7496_c25/f1p1/1093 isoform=cb7496_c25;full_length_coverage=1;non_full_length_coverage=1;isoform_length=1093;expected_accuracy=0.991	gnl|BL_ORD_ID|7688 cb7496_c9/f2p1/1474 isoform=cb7496_c9;full_length_coverage=2;non_full_length_coverage=1;isoform_length=1474;expected_accuracy=0.995	365	1086	9	367	757	1474	97	457
cb8564_c50128/f1p0/4343	gnl|BL_ORD_ID|14819 cb8564_c40333/f1p2/4446 isoform=cb8564_c40333;full_length_coverage=1;non_full_length_coverage=2;isoform_length=4446;expected_accuracy=0.998	28	3278	3274	4077	1	3280	3545	4349
cb8564_c40598/f1p4/3119	gnl|BL_ORD_ID|53680 cb8564_c153222/f2p39/3454	1	2732	2729	3041	7	2737	2858	3170
cb1656_c19/f1p1/3657 isoform=cb1656_c19;full_length_coverage=1;non_full_length_coverage=1;isoform_length=3657;expected_accuracy=1.000	gnl|BL_ORD_ID|31007 cb1656_c7/f1p1/3974 isoform=cb1656_c7;full_length_coverage=1;non_full_length_coverage=1;isoform_length=3974;expected_accuracy=0.997	2	2977	2976	3629	1	2976	3272	3922
cb8564_c5122/f1p0/3329	gnl|BL_ORD_ID|55727 cb8564_c5115/f1p1/3855	1067	3334	1	1069	1370	3637	9	1078
cb1956_c5/f2p0/2813 isoform=cb1956_c5;full_length_coverage=2;non_full_length_coverage=0;isoform_length=2813;expected_accuracy=0.997	gnl|BL_ORD_ID|35490 cb1956_c14/f1p0/2988 isoform=cb1956_c14;full_length_coverage=1;non_full_length_coverage=0;isoform_length=2988;expected_accuracy=0.998	1451	2811	1	1450	1627	2988	39	1464
cb8564_c41551/f1p0/3408	gnl|BL_ORD_ID|17429 cb8564_c85447/f1p0/3196	12	2419	2418	2952	11	241	2660	319

	isoform=cb8564_c85447;full_length_coverage=1;non_full_length_coverage=0;isoform_length=3196;expected_accuracy=0.997						3		6
cb227_c11/f1p1/3717	gnl|BL_ORD_ID|37093 cb227_c5/f1p0/3888 isoform=cb227_c5;full_length_coverage=1;non_full_length_coverage=0;isoform_length=3888;expected_accuracy=0.998	1	2510	2510	3693	6	2523	2712	3888
cb6657_c4/f1p1/2205 isoform=cb6657_c4;full_length_coverage=1;non_full_length_coverage=1;isoform_length=2205;expected_accuracy=1.000	gnl|BL_ORD_ID|5662 cb6657_c0/f1p1/2805 isoform=cb6657_c0;full_length_coverage=1;non_full_length_coverage=1;isoform_length=2805;expected_accuracy=0.993	1	1818	1817	2205	3	1814	2305	2693
cb4166_c3/f1p0/1896 isoform=cb4166_c3;full_length_coverage=1;non_full_length_coverage=0;isoform_length=1896;expected_accuracy=1.000	gnl|BL_ORD_ID|43549 cb4166_c5/f1p0/2886 isoform=cb4166_c5;full_length_coverage=1;non_full_length_coverage=0;isoform_length=2886;expected_accuracy=0.992	370	1893	47	370	1367	2886	1	327
cb8564_c71815/f1p0/2753	gnl|BL_ORD_ID|9497 cb8564_c107044/f3p8/2832 isoform=cb8564_c107044;full_length_coverage=3;non_full_length_coverage=8;isoform_length=2832;expected_accuracy=0.998	1	1377	1372	2485	104	1478	1712	2829
cb938_c3/f5p1/3495 isoform=cb938_c3;full_length_coverage=5;non_full_length_coverage=1;isoform_length=3495;expected_accuracy=0.999	gnl|BL_ORD_ID|59101 cb938_c9/f1p0/4170	116	2596	2596	3495	85	2565	3262	4161
cb8599_c19/f1p1/2037	gnl|BL_ORD_ID|18446 cb8599_c8/f1p1/2281 isoform=cb8599_c8;full_length_coverage=1;non_full_length_coverage=1;isoform_length=2281;expected_accura	218	2020	45	219	403	2204	4	180

	cy=0.998								
cb3292_c8/f1p0/3523 isoform=cb3292_c8;full_length_coverage=1;non_full_length_coverage=0;isoform_length=3523;expected_accuracy=0.991	gnl|BL_ORD_ID|41083 cb3292_c10/f1p0/3715 isoform=cb3292_c10;full_length_coverage=1;non_full_length_coverage=0;isoform_length=3715;expected_accuracy=0.993	23	2348	2346	3444	1	2332	2618	3715
cb8564_c23645/f1p0/2729	gnl|BL_ORD_ID|54283 cb8564_c21726/f1p0/2757	1	2066	2062	2477	1	2066	2200	2614
cb8564_c50151/f1p0/3198 isoform=cb8564_c50151;full_length_coverage=1;non_full_length_coverage=0;isoform_length=3198;expected_accuracy=0.995	gnl|BL_ORD_ID|55195 cb8564_c35170/f1p0/3386	503	3189	6	502	738	3399	1	497
cb9214_c4/f3p0/1986 isoform=cb9214_c4;full_length_coverage=3;non_full_length_coverage=0;isoform_length=1986;expected_accuracy=0.999	gnl|BL_ORD_ID|21271 cb9214_c11/f1p0/2500 isoform=cb9214_c11;full_length_coverage=1;non_full_length_coverage=0;isoform_length=2500;expected_accuracy=0.999	1	1652	1652	1986	1	1654	2164	2500
cb2842_c11/f1p0/2421 isoform=cb2842_c11;full_length_coverage=1;non_full_length_coverage=0;isoform_length=2421;expected_accuracy=0.994	gnl|BL_ORD_ID|39044 cb2842_c6/f2p1/2719 isoform=cb2842_c6;full_length_coverage=2;non_full_length_coverage=1;isoform_length=2719;expected_accuracy=0.996	147	2421	1	146	269	2549	1	146
cb8564_c4679/f1p1/2608	gnl|BL_ORD_ID|18308 cb8564_c936/f6p4/2901 isoform=cb8564_c936;full_length_coverage=6;non_full_length_coverage=4;isoform_length=2901;expected_accuracy=0.997	251	2604	11	250	349	2702	8	247
cb3309_c9/f1p0/2090 isoform=cb3309_c9;full_length_coverage=1;non_full_length_coverage=0;isoform_length=2090;expected_accur	gnl|BL_ORD_ID|70307 cb3309_c10/f1p1/3166	193	2090	1	195	1272	3160	1	197

acy=0.999									
cb548_c67/f1p3/3075	gnl|BL_ORD_ID|46779 cb548_c109/f1p0/3280 isoform=cb548_c109;full_length_coverage=1;non_full_length_coverage=0;isoform_length=3280;expected_accuracy=0.996	1298	3026	1	1301	1551	3279	45	1340
cb8564_c71585/f1p0/2336	gnl|BL_ORD_ID|12705 cb8564_c15619/f1p0/3068 isoform=cb8564_c15619;full_length_coverage=1;non_full_length_coverage=0;isoform_length=3068;expected_accuracy=0.996	622	2317	80	625	1242	2947	1	546
cb18275_c11/f1p1/1730	gnl|BL_ORD_ID|53227 cb8564_c119414/f1p2/2048	131	1733	1	134	400	2002	78	211
cb4744_c5/f1p0/1551 isoform=cb4744_c5;full_length_coverage=1;non_full_length_coverage=0;isoform_length=1551;expected_accuracy=0.994	gnl|BL_ORD_ID|72925 cb4744_c2/f1p0/3502	1	1151	1150	1551	102	1248	2620	3020
cb8564_c86760/f1p1/2598 isoform=cb8564_c86760;full_length_coverage=1;non_full_length_coverage=1;isoform_length=2598;expected_accuracy=0.998	gnl|BL_ORD_ID|16151 cb8564_c71575/f1p1/3050 isoform=cb8564_c71575;full_length_coverage=1;non_full_length_coverage=1;isoform_length=3050;expected_accuracy=0.993	249	2597	1	250	689	3050	1	249
cb8564_c129638/f1p0/2479	gnl|BL_ORD_ID|12871 cb8564_c167845/f2p2/3087 isoform=cb8564_c167845;full_length_coverage=2;non_full_length_coverage=2;isoform_length=3087;expected_accuracy=0.992	1	1627	1623	2475	173	1798	2202	3054
cb6326_c66/f1p0/1099	gnl|BL_ORD_ID|49616 cb6326_c18/f1p3/1610	216	1105	7	220	668	1555	114	315
cb3061_c4/f2p0/2362	gnl|BL_ORD_ID|38031 cb3061_c7/f1p0/2487	173	2362	17	174	275	246	1	160

isoform=cb3061_c4;full_length_coverage=2;non_full_length_coverage=0;isoform_length=2362;expected_accuracy=0.993	isoform=cb3061_c7;full_length_coverage=1;non_full_length_coverage=0;isoform_length=2487;expected_accuracy=0.993						4		
cb8564_c15653/f1p4/2475	gnl|BL_ORD_ID|55935 cb8564_c49443/f1p0/2984	22	2293	2289	2418	66	2334	2553	2681
cb8564_c45596/f1p1/3430	gnl|BL_ORD_ID|55944 cb8564_c49827/f1p0/4362	2	2780	2775	3379	1	2779	2898	3502
cb3037_c11/f1p1/3242 isoform=cb3037_c11;full_length_coverage=1;non_full_length_coverage=1;isoform_length=3242;expected_accuracy=0.999	gnl|BL_ORD_ID|38293 cb3037_c6/f1p1/3564 isoform=cb3037_c6;full_length_coverage=1;non_full_length_coverage=1;isoform_length=3564;expected_accuracy=1.000	1	2082	2081	3242	13	2095	2223	3384
cb8564_c10938/f1p1/2321	gnl|BL_ORD_ID|52761 cb8564_c13197/f1p0/3718	1	1315	1310	2292	1174	2488	2607	3589
cb4452_c4/f1p0/2965 isoform=cb4452_c4;full_length_coverage=1;non_full_length_coverage=0;isoform_length=2965;expected_accuracy=0.995	gnl|BL_ORD_ID|72572 cb4452_c3/f1p0/3340	1218	2965	1	1220	1566	3297	234	1448
cb8564_c39136/f1p0/3036 isoform=cb8564_c39136;full_length_coverage=1;non_full_length_coverage=0;isoform_length=3036;expected_accuracy=0.998	gnl|BL_ORD_ID|15152 cb8564_c38629/f2p1/3246 isoform=cb8564_c38629;full_length_coverage=2;non_full_length_coverage=1;isoform_length=3246;expected_accuracy=0.993	1	2089	2085	3036	1	2125	2252	3206
cb953_c2/f2p1/3253 isoform=cb953_c2;full_length_coverage=2;non_full_length_coverage=1;isoform_length=3253;expected_accuracy=1.000	gnl|BL_ORD_ID|20812 cb953_c1/f2p0/4567 isoform=cb953_c1;full_length_coverage=2;non_full_length_coverage=0;isoform_length=4567;expected_accuracy=0.999	1	2229	2225	3253	35	2262	3530	4562
cb9058_c14/f1p0/1762	gnl|BL_ORD_ID|21924 cb9058_c9/f1p1/2092	1	1121	1116	1762	32	1153	1283	192

isoform=cb9058_c14;full_length_coverage=1;non_full_length_coverage=0;isoform_length=1762;expected_accuracy=0.995	isoform=cb9058_c9;full_length_coverage=1;non_full_length_coverage=1;isoform_length=2092;expected_accuracy=1.000								9
cb1754_c0/f2p0/1934 isoform=cb1754_c0;full_length_coverage=2;non_full_length_coverage=0;isoform_length=1934;expected_accuracy=1.000	gnl|BL_ORD_ID|63921 cb1754_c1/f1p0/4184	231	1934	65	233	2092	3795	3	171
cb8564_c38815/f1p3/3473 isoform=cb8564_c38815;full_length_coverage=1;non_full_length_coverage=3;isoform_length=3473;expected_accuracy=0.995	gnl|BL_ORD_ID|15059 cb8564_c26915/f3p0/3758 isoform=cb8564_c26915;full_length_coverage=3;non_full_length_coverage=0;isoform_length=3758;expected_accuracy=1.000	1	2729	2727	3368	7	2737	3117	3758
cb8564_c45300/f1p4/3014 isoform=cb8564_c45300;full_length_coverage=1;non_full_length_coverage=4;isoform_length=3014;expected_accuracy=0.996	gnl|BL_ORD_ID|56764 cb8564_c70795/f1p0/3769	243	3014	19	246	415	3185	34	258
cb8564_c38035/f1p0/2770	gnl|BL_ORD_ID|14836 cb8564_c41692/f1p3/2987 isoform=cb8564_c41692;full_length_coverage=1;non_full_length_coverage=3;isoform_length=2987;expected_accuracy=0.995	1	2035	2033	2755	1	2061	2255	2987
cb8564_c74269/f1p0/2347	gnl|BL_ORD_ID|57849 cb8564_c82482/f1p0/3780	1033	2348	1	1034	2350	3668	3	1035
cb7511_c29/f1p1/2416 isoform=cb7511_c29;full_length_coverage=1;non_full_length_coverage=1;isoform_length=2416;expected_accuracy=0.997	gnl|BL_ORD_ID|50563 cb7511_c6/f1p0/3521	107	1950	1950	2416	3	1848	2719	3185
cb8564_c76495/f1p0/3949	gnl|BL_ORD_ID|57678 cb8564_c9837/f2p0/4515	1	2913	2912	3943	95	300	3484	451

							7		5
cb8800_c4/f1p0/1370 isoform=cb8800_c4;full_length_coverage=1;non_full_length_coverage=0;isoform_length=1370;expected_accuracy=0.992	gnl|BL_ORD_ID|58373 cb8800_c1/f1p0/2412	1	1057	1052	1370	667	172 4	1870	218 9
cb8564_c14176/f1p0/4818	gnl|BL_ORD_ID|67919 cb18654_c16/f1p0/5228	2	4085	4086	4566	27	4110	4735	521 4
cb653_c6/f1p0/4151 isoform=cb653_c6;full_length_coverage=1;non_full_length_coverage=0;isoform_length=4151;expected_accuracy=0.996	gnl|BL_ORD_ID|5131 cb653_c8/f1p1/4297 isoform=cb653_c8;full_length_coverage=1;non_full_length_coverage=1;isoform_length=4297;expected_accuracy=0.999	308	4135	1	309	474	429 7	1	307
cb16807_c2/f1p0/1293	gnl|BL_ORD_ID|32276 cb16807_c1/f1p0/1485 isoform=cb16807_c1;full_length_coverage=1;non_full_length_coverage=0;isoform_length=1485;expected_accuracy=0.998	233	1272	56	233	432	147 1	1	178
cb8564_c14280/f1p0/2385	gnl|BL_ORD_ID|13862 cb8564_c20620/f1p1/2688 isoform=cb8564_c20620;full_length_coverage=1;non_full_length_coverage=1;isoform_length=2688;expected_accuracy=0.992	1	2052	2049	2286	247	229 9	2399	263 5
cb1772_c28/f3p2/3809 isoform=cb1772_c28;full_length_coverage=3;non_full_length_coverage=2;isoform_length=3809;expected_accuracy=1.000	gnl|BL_ORD_ID|66372 cb1772_c16/f1p1/4029	309	3809	12	310	431	394 4	12	295
cb8564_c178226/f1p0/2728 isoform=cb8564_c178226;full_length_coverage=1;non_full_length_coverage=0;isoform_length=2728;expected	gnl|BL_ORD_ID|14263 cb8564_c42654/f1p1/2906 isoform=cb8564_c42654;full_length_coverage=1;non_full_length_coverage=1;isoform_length=2906;expected_ac	1284	2706	1	1288	1397	282 4	1	129 2

_accuracy=0.998	curacy=0.990								
cb8564_c87188/f1p1/3426	gnl|BL_ORD_ID|55939 cb8564_c49630/f1p4/3487	127	3021	3021	3271	18	2913	3243	3493
cb1380_c36/f1p0/2344 isoform=cb1380_c36;full_length_coverage=1;non_full_length_coverage=0;isoform_length=2344;expected_accuracy=0.998	gnl|BL_ORD_ID|25713 cb1380_c45/f1p0/3005 isoform=cb1380_c45;full_length_coverage=1;non_full_length_coverage=0;isoform_length=3005;expected_accuracy=0.996	1	1513	1512	2344	1	1514	2168	3005
cb11760_c0/f1p0/1120 isoform=cb11760_c0;full_length_coverage=1;non_full_length_coverage=0;isoform_length=1120;expected_accuracy=1.000	gnl|BL_ORD_ID|60353 cb11760_c1/f1p0/3328	332	1120	43	332	1965	2756	4	293
cb8564_c68610/f6p0/2150 isoform=cb8564_c68610;full_length_coverage=6;non_full_length_coverage=0;isoform_length=2150;expected_accuracy=1.000	gnl|BL_ORD_ID|12420 cb8564_c125410/f1p0/2472 isoform=cb8564_c125410;full_length_coverage=1;non_full_length_coverage=0;isoform_length=2472;expected_accuracy=0.997	212	2083	4	214	600	2472	1	211
cb8564_c123951/f1p1/4450 isoform=cb8564_c123951;full_length_coverage=1;non_full_length_coverage=1;isoform_length=4450;expected_accuracy=0.996	gnl|BL_ORD_ID|52930 cb8564_c112168/f1p0/4870	310	4450	1	311	547	4692	79	390
cb2367_c4/f1p0/1959	gnl|BL_ORD_ID|37426 cb2367_c5/f1p0/2867 isoform=cb2367_c5;full_length_coverage=1;non_full_length_coverage=0;isoform_length=2867;expected_accuracy=0.990	1	1317	1316	1938	5	1329	2163	2796
cb8564_c180116/f1p0/2944 isoform=cb8564_c180116;full_length_coverage=1;non_full_length_coverage=0;isoform_length=2944;expected	gnl|BL_ORD_ID|17429 cb8564_c85447/f1p0/3196 isoform=cb8564_c85447;full_length_coverage=1;non_full_length_coverage=0;isoform_length=3196;expected_ac	2	2522	2520	2944	1	2540	2769	3191

_accuracy=0.996	curacy=0.997								
cb124_c43/f1p0/2759	gnl|BL_ORD_ID|23059 cb124_c7/f2p0/3544 isoform=cb124_c7;full_length_coverage=2;non_full_length_coverage=0;isoform_length=3544;expected_accuracy=1.000	232	2473	13	233	1303	3544	12	232
cb8564_c36689/f1p1/2368 isoform=cb8564_c36689;full_length_coverage=1;non_full_length_coverage=1;isoform_length=2368;expected_accuracy=0.991	gnl|BL_ORD_ID|55391 cb8564_c40278/f3p2/3083	1	1709	1706	2368	1	1703	2202	2866
cb2461_c3/f1p0/3258	gnl|BL_ORD_ID|37876 cb2461_c6/f1p0/5655 isoform=cb2461_c6;full_length_coverage=1;non_full_length_coverage=0;isoform_length=5655;expected_accuracy=0.998	1	2947	2942	3250	1666	4606	4924	5232
cb8564_c16741/f1p0/2997	gnl|BL_ORD_ID|11111 cb8564_c108411/f1p1/3146 isoform=cb8564_c108411;full_length_coverage=1;non_full_length_coverage=1;isoform_length=3146;expected_accuracy=0.991	375	3001	52	376	450	3100	1	330
cb2263_c3/f2p0/2490 isoform=cb2263_c3;full_length_coverage=2;non_full_length_coverage=0;isoform_length=2490;expected_accuracy=0.999	gnl|BL_ORD_ID|36791 cb2263_c5/f1p0/3994 isoform=cb2263_c5;full_length_coverage=1;non_full_length_coverage=0;isoform_length=3994;expected_accuracy=0.998	1	1382	1383	2490	54	1436	2457	3566
cb8564_c45300/f1p4/3014 isoform=cb8564_c45300;full_length_coverage=1;non_full_length_coverage=4;isoform_length=3014;expected_accuracy=0.996	gnl|BL_ORD_ID|12913 cb8564_c182049/f1p0/3195 isoform=cb8564_c182049;full_length_coverage=1;non_full_length_coverage=0;isoform_length=3195;expected_accuracy=0.997	243	3014	1	246	398	3193	1	240
cb8564_c42583/f1p0/1936	gnl|BL_ORD_ID|56037 cb8564_c53029/f1p0/2512	1	1200	1200	1919	1	1199	1795	251

<table>
<tr><td></td><td></td><td></td><td></td><td></td><td></td><td></td><td></td><td></td><td></td></tr>
<tr><td></td><td></td><td></td><td></td><td></td><td></td><td></td><td></td><td></td><td>3</td></tr>
<tr><td>cb1088_c12/f1p0/2593</td><td>gnl|BL_ORD_ID|1951 cb1088_c6/f1p2/3285 isoform=cb1088_c6;full_length_coverage=1;non_full_length_coverage=2;isoform_length=3285;expected_accuracy=0.997</td><td>1</td><td>1648</td><td>1650</td><td>2507</td><td>219</td><td>1867</td><td>2357</td><td>3214</td></tr>
<tr><td>cb8564_c37748/f1p0/3835 isoform=cb8564_c37748;full_length_coverage=1;non_full_length_coverage=0;isoform_length=3835;expected_accuracy=0.995</td><td>gnl|BL_ORD_ID|15300 cb8564_c45205/f10p2/4247 isoform=cb8564_c45205;full_length_coverage=10;non_full_length_coverage=2;isoform_length=4247;expected_accuracy=0.999</td><td>287</td><td>3755</td><td>1</td><td>286</td><td>775</td><td>4247</td><td>366</td><td>651</td></tr>
<tr><td>cb8564_c13348/f1p0/2742 isoform=cb8564_c13348;full_length_coverage=1;non_full_length_coverage=0;isoform_length=2742;expected_accuracy=0.995</td><td>gnl|BL_ORD_ID|56381 cb8564_c75626/f1p2/3199</td><td>1</td><td>2151</td><td>2149</td><td>2731</td><td>4</td><td>2116</td><td>2611</td><td>3184</td></tr>
<tr><td>cb7873_c1/f1p0/2332 isoform=cb7873_c1;full_length_coverage=1;non_full_length_coverage=0;isoform_length=2332;expected_accuracy=0.997</td><td>gnl|BL_ORD_ID|6464 cb7873_c0/f1p0/2514 isoform=cb7873_c0;full_length_coverage=1;non_full_length_coverage=0;isoform_length=2514;expected_accuracy=1.000</td><td>1</td><td>1265</td><td>1266</td><td>2332</td><td>53</td><td>1315</td><td>1417</td><td>2484</td></tr>
<tr><td>cb6889_c17/f1p0/2549</td><td>gnl|BL_ORD_ID|5980 cb6889_c7/f3p1/2671 isoform=cb6889_c7;full_length_coverage=3;non_full_length_coverage=1;isoform_length=2671;expected_accuracy=1.000</td><td>359</td><td>2357</td><td>61</td><td>364</td><td>653</td><td>2652</td><td>1</td><td>295</td></tr>
<tr><td>cb8564_c91068/f1p0/3080</td><td>gnl|BL_ORD_ID|14956 cb8564_c34985/f1p0/3209 isoform=cb8564_c34985;full_length_coverage=1;non_full_length_coverage=0;isoform_length=3209;expected_accuracy=0.991</td><td>2</td><td>2598</td><td>2596</td><td>2821</td><td>485</td><td>3094</td><td>2982</td><td>3208</td></tr>
<tr><td>cb8564_c90349/f1p0/3076</td><td>gnl|BL_ORD_ID|17076 cb8564_c81253/f1p0/4323</td><td>466</td><td>3081</td><td>9</td><td>465</td><td>1695</td><td>432</td><td>1</td><td>457</td></tr>
</table>

	isoform=cb8564_c81253;full_length_coverage=1;non_full_length_coverage=0;isoform_length=4323;expected_accuracy=0.999						1		
cb8564_c79931/f1p3/1874 isoform=cb8564_c79931;full_length_coverage=1;non_full_length_coverage=3;isoform_length=1874;expected_accuracy=0.991	gnl|BL_ORD_ID|53517 cb8564_c127539/f1p0/2402	1	1352	1352	1863	1	1343	1768	2272
cb16052_c1/f2p0/1765 isoform=cb16052_c1;full_length_coverage=2;non_full_length_coverage=0;isoform_length=1765;expected_accuracy=0.993	gnl|BL_ORD_ID|17604 cb8564_c89610/f1p0/1980 isoform=cb8564_c89610;full_length_coverage=1;non_full_length_coverage=0;isoform_length=1980;expected_accuracy=0.999	199	1764	2	202	417	1980	5	202
cb8564_c72680/f1p0/2685	gnl|BL_ORD_ID|56712 cb8564_c53711/f1p0/3201	1	1735	1730	2636	28	1762	1941	2847
cb6712_c1/f5p1/2348 isoform=cb6712_c1;full_length_coverage=5;non_full_length_coverage=1;isoform_length=2348;expected_accuracy=0.997	gnl|BL_ORD_ID|4045 cb6712_c5/f1p0/2789 isoform=cb6712_c5;full_length_coverage=1;non_full_length_coverage=0;isoform_length=2789;expected_accuracy=0.999	1	1500	1499	2323	8	1488	1966	2789
cb124_c2/f4p0/2458 isoform=cb124_c2;full_length_coverage=4;non_full_length_coverage=0;isoform_length=2458;expected_accuracy=0.998	gnl|BL_ORD_ID|24675 cb124_c28/f1p0/2797 isoform=cb124_c28;full_length_coverage=1;non_full_length_coverage=0;isoform_length=2797;expected_accuracy=0.997	217	2419	1	217	595	2797	24	240
cb4016_c33/f1p0/2507	gnl|BL_ORD_ID|71723 cb4016_c11/f1p0/2688	1	1874	1874	2462	32	1908	2016	2604
cb1648_c46/f32p0/2018 isoform=cb1648_c46;full_length_coverage=32;non_full_length_coverage=0;isoform_length=2018;expected_acc	gnl|BL_ORD_ID|32503 cb1648_c15/f1p0/2133 isoform=cb1648_c15;full_length_coverage=1;non_full_length_coverage=0;isoform_length=2133;expected_accur	245	2018	52	245	337	2109	1	195

<table>
<tr><td></td><td></td><td></td><td></td><td></td><td></td><td></td><td></td><td></td><td></td></tr>
<tr><td>uracy=1.000</td><td>acy=1.000</td><td></td><td></td><td></td><td></td><td></td><td></td><td></td><td></td></tr>
<tr><td>cb8564_c31999/f5p0/2360 isoform=cb8564_c31999;full_length_coverage=5;non_full_length_coverage=0;isoform_length=2360;expected_accuracy=1.000</td><td>gnl|BL_ORD_ID|16625 cb8564_c72796/f1p0/2508 isoform=cb8564_c72796;full_length_coverage=1;non_full_length_coverage=0;isoform_length=2508;expected_accuracy=0.997</td><td>164</td><td>2360</td><td>11</td><td>168</td><td>313</td><td>2508</td><td>1</td><td>157</td></tr>
<tr><td>cb2482_c31/f1p1/2865 isoform=cb2482_c31;full_length_coverage=1;non_full_length_coverage=1;isoform_length=2865;expected_accuracy=0.996</td><td>gnl|BL_ORD_ID|38930 cb2482_c30/f13p1/3051 isoform=cb2482_c30;full_length_coverage=13;non_full_length_coverage=1;isoform_length=3051;expected_accuracy=1.000</td><td>26</td><td>2632</td><td>2631</td><td>2862</td><td>93</td><td>2696</td><td>2820</td><td>3051</td></tr>
<tr><td>cb2382_c7/f1p0/5459</td><td>gnl|BL_ORD_ID|69726 cb2382_c6/f1p1/5561</td><td>4</td><td>4592</td><td>4590</td><td>5090</td><td>115</td><td>4712</td><td>4818</td><td>5318</td></tr>
<tr><td>cb2084_c13/f1p0/1992 isoform=cb2084_c13;full_length_coverage=1;non_full_length_coverage=0;isoform_length=1992;expected_accuracy=0.995</td><td>gnl|BL_ORD_ID|35414 cb2084_c12/f1p0/3016 isoform=cb2084_c12;full_length_coverage=1;non_full_length_coverage=0;isoform_length=3016;expected_accuracy=0.999</td><td>1</td><td>1519</td><td>1516</td><td>1992</td><td>3</td><td>1521</td><td>2540</td><td>3016</td></tr>
<tr><td>cb3887_c13/f1p0/2422</td><td>gnl|BL_ORD_ID|39510 cb3887_c4/f1p0/2446 isoform=cb3887_c4;full_length_coverage=1;non_full_length_coverage=0;isoform_length=2446;expected_accuracy=0.994</td><td>260</td><td>2358</td><td>28</td><td>259</td><td>348</td><td>2446</td><td>2</td><td>232</td></tr>
<tr><td>cb5999_c5/f1p0/2393 isoform=cb5999_c5;full_length_coverage=1;non_full_length_coverage=0;isoform_length=2393;expected_accuracy=0.996</td><td>gnl|BL_ORD_ID|74748 cb5999_c6/f1p0/2868</td><td>1</td><td>1285</td><td>1283</td><td>2393</td><td>82</td><td>1369</td><td>1697</td><td>2806</td></tr>
<tr><td>cb8564_c91729/f1p1/2879</td><td>gnl|BL_ORD_ID|56255 cb8564_c72763/f1p0/3108</td><td>362</td><td>2782</td><td>6</td><td>357</td><td>388</td><td>2809</td><td>199</td><td>542</td></tr>
<tr><td>cb3988_c6/f1p0/2823</td><td>gnl|BL_ORD_ID|41321 cb3988_c1/f1p1/3470</td><td>1</td><td>1841</td><td>1837</td><td>2828</td><td>222</td><td>205</td><td>2423</td><td>3411</td></tr>
</table>

	isoform=cb3988_c1;full_length_coverage=1;non_full_length_coverage=1;isoform_length=3470;expected_accuracy=1.000						1		
cb8564_c23232/f1p3/2540	gnl|BL_ORD_ID|13065 cb8564_c157334/f1p21/2777 isoform=cb8564_c157334;full_length_coverage=1;non_full_length_coverage=21;isoform_length=2777;expected_accuracy=0.995	16	2172	2167	2518	11	2174	2276	2627
cb4768_c85/f1p0/1983 isoform=cb4768_c85;full_length_coverage=1;non_full_length_coverage=0;isoform_length=1983;expected_accuracy=1.000	gnl|BL_ORD_ID|71998 cb4768_c50/f1p0/2391	103	1965	1	106	423	2285	3	109
cb8564_c4643/f1p1/2554	gnl|BL_ORD_ID|12102 cb8564_c136340/f3p2/2729 isoform=cb8564_c136340;full_length_coverage=3;non_full_length_coverage=2;isoform_length=2729;expected_accuracy=0.998	1	1705	1700	2521	67	1772	1905	2729
cb10988_c4/f1p0/1116 isoform=cb10988_c4;full_length_coverage=1;non_full_length_coverage=0;isoform_length=1116;expected_accuracy=0.991	gnl|BL_ORD_ID|293 cb10988_c7/f1p3/1227 isoform=cb10988_c7;full_length_coverage=1;non_full_length_coverage=3;isoform_length=1227;expected_accuracy=1.000	6	600	596	1116	1	595	707	1227
cb1178_c27/f1p0/1197 isoform=cb1178_c27;full_length_coverage=1;non_full_length_coverage=0;isoform_length=1197;expected_accuracy=0.992	gnl|BL_ORD_ID|23778 cb1178_c16/f1p0/1671 isoform=cb1178_c16;full_length_coverage=1;non_full_length_coverage=0;isoform_length=1671;expected_accuracy=0.993	111	1197	1	110	223	1308	1	103
cb4802_c34/f1p0/1485 isoform=cb4802_c34;full_length_coverage=1;non_full_length_coverage=0;isoform_length=1485;expected_accu	gnl|BL_ORD_ID|42547 cb4802_c39/f44p4/1860 isoform=cb4802_c39;full_length_coverage=44;non_full_length_coverage=4;isoform_length=1860;expected_acc	156	1485	27	161	508	1839	51	183

racy=0.997	uracy=1.000								
cb2324_c36/f1p1/3463	gnl|BL_ORD_ID|68433 cb2324_c13/f1p0/4526	1	3229	3226	3477	21	3256	4302	4550
cb8564_c39014/f1p0/3019 isoform=cb8564_c39014;full_length_coverage=1;non_full_length_coverage=0;isoform_length=3019;expected_accuracy=0.992	gnl|BL_ORD_ID|55920 cb8564_c48949/f1p0/4357	1	2336	2337	3013	2	2293	2847	3504
cb8564_c124170/f1p0/1999 isoform=cb8564_c124170;full_length_coverage=1;non_full_length_coverage=0;isoform_length=1999;expected_accuracy=0.992	gnl|BL_ORD_ID|54528 cb8564_c125341/f1p0/3014	1	1549	1550	1999	14	1537	2549	2992
cb9473_c9/f1p0/1550 isoform=cb9473_c9;full_length_coverage=1;non_full_length_coverage=0;isoform_length=1550;expected_accuracy=0.994	gnl|BL_ORD_ID|20340 cb9473_c1/f1p1/2077 isoform=cb9473_c1;full_length_coverage=1;non_full_length_coverage=1;isoform_length=2077;expected_accuracy=1.000	1	1225	1224	1550	228	1451	1672	1999
cb8564_c1754/f1p0/4035	gnl|BL_ORD_ID|35566 cb18781_c3/f1p0/7271 isoform=cb18781_c3;full_length_coverage=1;non_full_length_coverage=0;isoform_length=7271;expected_accuracy=0.995	1	3545	3543	4038	2686	6221	6343	6839
cb6499_c4/f3p1/2065 isoform=cb6499_c4;full_length_coverage=3;non_full_length_coverage=1;isoform_length=2065;expected_accuracy=0.998	gnl|BL_ORD_ID|4653 cb6499_c26/f12p0/2255 isoform=cb6499_c26;full_length_coverage=12;non_full_length_coverage=0;isoform_length=2255;expected_accuracy=1.000	170	2063	1	171	357	2250	3	171
cb8564_c46939/f1p1/2926	gnl|BL_ORD_ID|14091 cb8564_c43119/f1p4/3194 isoform=cb8564_c43119;full_length_coverage=1;non_full_length_coverage=4;isoform_length=3194;expected_ac	1	2134	2130	2870	1	2158	2263	3011

	curacy=0.995								
cb8564_c47286/f1p0/3783	gnl|BL_ORD_ID|13491 cb8564_c20651/f3p0/4196 isoform=cb8564_c20651;full_length_coverage=3;non_full_length_coverage=0;isoform_length=4196;expected_accuracy=0.997	1435	3807	1	1435	1721	4093	50	1485
cb8564_c44698/f1p5/2843 isoform=cb8564_c44698;full_length_coverage=1;non_full_length_coverage=5;isoform_length=2843;expected_accuracy=0.997	gnl|BL_ORD_ID|55839 cb8564_c43677/f1p0/3269	1	2516	2511	2823	55	2566	2685	2999
cb12608_c1/f3p1/1172 isoform=cb12608_c1;full_length_coverage=3;non_full_length_coverage=1;isoform_length=1172;expected_accuracy=1.000	gnl|BL_ORD_ID|60962 cb12608_c13/f1p1/1437	100	1169	1	100	352	1420	145	244
cb8564_c118659/f2p0/3437 isoform=cb8564_c118659;full_length_coverage=2;non_full_length_coverage=0;isoform_length=3437;expected_accuracy=0.994	gnl|BL_ORD_ID|52309 cb8564_c117845/f1p1/3666	1	2906	2907	3320	85	2994	3251	3665
cb1333_c101/f1p0/1395 isoform=cb1333_c101;full_length_coverage=1;non_full_length_coverage=0;isoform_length=1395;expected_accuracy=0.997	gnl|BL_ORD_ID|25626 cb1333_c96/f1p0/1554 isoform=cb1333_c96;full_length_coverage=1;non_full_length_coverage=0;isoform_length=1554;expected_accuracy=0.991	149	1395	6	148	254	1497	1	142
cb8564_c146025/f2p1/3347 isoform=cb8564_c146025;full_length_coverage=2;non_full_length_coverage=1;isoform_length=3347;expected_accuracy=0.996	gnl|BL_ORD_ID|12196 cb8564_c12654/f2p0/4122 isoform=cb8564_c12654;full_length_coverage=2;non_full_length_coverage=0;isoform_length=4122;expected_accuracy=1.000	1	1900	1900	3238	1	1901	2784	4122
cb8564_c35568/f1p0/2997	gnl|BL_ORD_ID|14531 cb8564_c41778/f1p9/3482	265	2892	19	269	812	344	5	247

	isoform=cb8564_c41778;full_length_coverage=1;non_full_length_coverage=9;isoform_length=3482;expected_accuracy=0.992						2		
cb8564_c45827/f2p2/4435 isoform=cb8564_c45827;full_length_coverage=2;non_full_length_coverage=2;isoform_length=4435;expected_accuracy=0.999	gnl|BL_ORD_ID|14942 cb8564_c34188/f2p3/4546 isoform=cb8564_c34188;full_length_coverage=2;non_full_length_coverage=3;isoform_length=4546;expected_accuracy=1.000	270	4422	1	274	398	4546	19	293
cb8564_c107877/f2p1/3829 isoform=cb8564_c107877;full_length_coverage=2;non_full_length_coverage=1;isoform_length=3829;expected_accuracy=1.000	gnl|BL_ORD_ID|56428 cb8564_c76764/f1p1/4492	258	3824	1	259	952	4519	147	407
cb1909_c6/f1p0/3557	gnl|BL_ORD_ID|35723 cb1909_c5/f1p0/3837 isoform=cb1909_c5;full_length_coverage=1;non_full_length_coverage=0;isoform_length=3837;expected_accuracy=1.000	2	3344	3342	3520	72	3412	3633	3811
cb2177_c46/f1p0/2729	gnl|BL_ORD_ID|35943 cb2177_c48/f1p0/3555 isoform=cb2177_c48;full_length_coverage=1;non_full_length_coverage=0;isoform_length=3555;expected_accuracy=0.995	15	2187	2185	2713	1	2165	3028	3555
cb5137_c53/f1p0/2672 isoform=cb5137_c53;full_length_coverage=1;non_full_length_coverage=0;isoform_length=2672;expected_accuracy=0.992	gnl|BL_ORD_ID|59209 cb1164_c15/f1p2/3151	12	2565	2562	2664	2803	227	123	22
cb8564_c89179/f1p0/3763 isoform=cb8564_c89179;full_length_coverage=1;non_full_length_coverage=0;isoform_length=3763;expected_	gnl|BL_ORD_ID|16165 cb8564_c75981/f2p0/4046 isoform=cb8564_c75981;full_length_coverage=2;non_full_length_coverage=0;isoform_length=4046;expected_ac	521	3737	9	522	830	4046	1	514

accuracy=0.995	curacy=0.995								
cb8564_c22636/f1p0/2704 isoform=cb8564_c22636;full_length_coverage=1;non_full_length_coverage=0;isoform_length=2704;expected_accuracy=0.994	gnl|BL_ORD_ID|17366 cb8564_c80423/f1p0/3018 isoform=cb8564_c80423;full_length_coverage=1;non_full_length_coverage=0;isoform_length=3018;expected_accuracy=1.000	1	2092	2093	2704	1	2098	2335	2947
cb3201_c33/f1p1/2925	gnl|BL_ORD_ID|70955 cb3201_c29/f1p0/3532	1	1485	1484	2830	2	1486	1862	3208
cb1052_c55/f1p0/2730	gnl|BL_ORD_ID|219 cb1052_c15/f1p0/4466 isoform=cb1052_c15;full_length_coverage=1;non_full_length_coverage=0;isoform_length=4466;expected_accuracy=0.992	599	2743	65	600	2177	4316	1	533
cb8564_c44850/f1p0/3916 isoform=cb8564_c44850;full_length_coverage=1;non_full_length_coverage=0;isoform_length=3916;expected_accuracy=0.993	gnl|BL_ORD_ID|56087 cb8564_c66874/f2p0/4090	1846	3916	1	1846	1967	4037	37	1866
cb1613_c5/f1p0/2054 isoform=cb1613_c5;full_length_coverage=1;non_full_length_coverage=0;isoform_length=2054;expected_accuracy=1.000	gnl|BL_ORD_ID|28653 cb1613_c2/f1p0/2771 isoform=cb1613_c2;full_length_coverage=1;non_full_length_coverage=0;isoform_length=2771;expected_accuracy=1.000	1	1200	1195	1968	34	1215	1999	2771
cb8564_c4742/f1p0/2505 isoform=cb8564_c4742;full_length_coverage=1;non_full_length_coverage=0;isoform_length=2505;expected_accuracy=0.999	gnl|BL_ORD_ID|16010 cb8564_c4743/f1p0/2508 isoform=cb8564_c4743;full_length_coverage=1;non_full_length_coverage=0;isoform_length=2508;expected_accuracy=0.994	1	2089	2084	2398	1	2089	2195	2508
cb581_c4/f1p1/2569 isoform=cb581_c4;full_length_coverage=1;non_full_length_coverage=1;isoform_length=2569;expected_accurac	gnl|BL_ORD_ID|45252 cb581_c3/f1p1/4740 isoform=cb581_c3;full_length_coverage=1;non_full_length_coverage=1;isoform_length=4740;expected_accurac	1007	2569	1	1009	3107	4669	220	1227

y=0.999	y=0.997								
cb8564_c43779/f1p0/3021 isoform=cb8564_c43779;full_length_coverage=1;non_full_length_coverage=0;isoform_length=3021;expected_accuracy=0.993	gnl|BL_ORD_ID|14091 cb8564_c43119/f1p4/3194 isoform=cb8564_c43119;full_length_coverage=1;non_full_length_coverage=4;isoform_length=3194;expected_accuracy=0.995	1	2344	2345	2981	1	2360	2517	3157
cb5585_c8/f1p0/1963 isoform=cb5585_c8;full_length_coverage=1;non_full_length_coverage=0;isoform_length=1963;expected_accuracy=0.995	gnl|BL_ORD_ID|44917 cb5585_c4/f1p0/2892 isoform=cb5585_c4;full_length_coverage=1;non_full_length_coverage=0;isoform_length=2892;expected_accuracy=0.995	1	1097	1095	1963	1	1101	1585	2443
cb2737_c26/f1p0/2992 isoform=cb2737_c26;full_length_coverage=1;non_full_length_coverage=0;isoform_length=2992;expected_accuracy=0.996	gnl|BL_ORD_ID|38132 cb2737_c20/f1p0/3644 isoform=cb2737_c20;full_length_coverage=1;non_full_length_coverage=0;isoform_length=3644;expected_accuracy=0.998	1	1804	1802	2992	475	2278	2410	3602
cb8564_c181853/f1p0/2119 isoform=cb8564_c181853;full_length_coverage=1;non_full_length_coverage=0;isoform_length=2119;expected_accuracy=0.997	gnl|BL_ORD_ID|57846 cb8564_c82432/f1p1/3412	1	1339	1334	2119	1	1341	2221	3010
cb15591_c1/f1p1/1413 isoform=cb15591_c1;full_length_coverage=1;non_full_length_coverage=1;isoform_length=1413;expected_accuracy=0.999	gnl|BL_ORD_ID|59642 cb9317_c8/f1p0/1919	280	1413	4	284	543	1676	2	282
cb8564_c43450/f1p0/2695	gnl|BL_ORD_ID|55480 cb8564_c42677/f1p0/3180	15	1886	1882	2713	21	1896	2011	2842
cb2629_c15/f1p0/2175 isoform=cb2629_c15;full_length_coverage=1;non_full_length_coverage=0;isoform_length=2175;expected_accu	gnl|BL_ORD_ID|69477 cb18844_c1/f1p0/5518	1	1663	1662	2175	2726	4391	4502	5015

racy=0.991									
cb8564_c37361/f1p0/3076	gnl|BL_ORD_ID|14539 cb8564_c46562/f2p3/3196 isoform=cb8564_c46562;full_length_coverage=2;non_full_length_coverage=3;isoform_length=3196;expected_accuracy=0.995	1	2481	2477	3066	7	2506	2607	3195
cb2818_c34/f1p0/3053	gnl|BL_ORD_ID|68900 cb2818_c40/f1p0/3487	114	2923	1	115	587	3395	30	142
cb5516_c26/f1p0/3029 isoform=cb5516_c26;full_length_coverage=1;non_full_length_coverage=0;isoform_length=3029;expected_accuracy=1.000	gnl|BL_ORD_ID|46728 cb5516_c42/f1p2/3202 isoform=cb5516_c42;full_length_coverage=1;non_full_length_coverage=2;isoform_length=3202;expected_accuracy=0.995	1	2327	2322	3025	5	2328	2453	3159
cb8564_c22866/f1p1/4372	gnl|BL_ORD_ID|53911 cb8564_c14704/f1p2/4862	1	2384	2385	4303	289	2672	2825	4744
cb3586_c0/f4p0/3057 isoform=cb3586_c0;full_length_coverage=4;non_full_length_coverage=0;isoform_length=3057;expected_accuracy=1.000	gnl|BL_ORD_ID|71356 cb3586_c9/f1p0/3463	350	3057	73	353	766	3472	1	282
cb8564_c51722/f1p1/3503	gnl|BL_ORD_ID|16149 cb8564_c47162/f1p0/3361 isoform=cb8564_c47162;full_length_coverage=1;non_full_length_coverage=0;isoform_length=3361;expected_accuracy=0.996	443	3214	67	444	592	3361	22	402
cb3941_c50/f1p2/3496 isoform=cb3941_c50;full_length_coverage=1;non_full_length_coverage=2;isoform_length=3496;expected_accuracy=0.997	gnl|BL_ORD_ID|40541 cb3941_c38/f1p1/3548 isoform=cb3941_c38;full_length_coverage=1;non_full_length_coverage=1;isoform_length=3548;expected_accuracy=0.997	310	3496	113	310	354	3543	1	198
cb8564_c40764/f1p0/2563	gnl|BL_ORD_ID|14836 cb8564_c41692/f1p3/2987	1	2167	2164	2563	1	218	2404	280

isoform=cb8564_c40764;full_length_coverage=1;non_full_length_coverage=0;isoform_length=2563;expected_accuracy=0.997	isoform=cb8564_c41692;full_length_coverage=1;non_full_length_coverage=3;isoform_length=2987;expected_accuracy=0.995						8		7
cb1404_c30/f1p0/2845 isoform=cb1404_c30;full_length_coverage=1;non_full_length_coverage=0;isoform_length=2845;expected_accuracy=0.999	gnl|BL_ORD_ID|26083 cb1404_c35/f36p1/3086 isoform=cb1404_c35;full_length_coverage=36;non_full_length_coverage=1;isoform_length=3086;expected_accuracy=1.000	1	2368	2363	2822	151	2520	2625	3086
cb8564_c15653/f1p4/2475	gnl|BL_ORD_ID|12505 cb8564_c13348/f1p0/2742 isoform=cb8564_c13348;full_length_coverage=1;non_full_length_coverage=0;isoform_length=2742;expected_accuracy=0.995	1	2292	2291	2461	1	2333	2558	2731
cb12972_c5/f2p0/1545 isoform=cb12972_c5;full_length_coverage=2;non_full_length_coverage=0;isoform_length=1545;expected_accuracy=1.000	gnl|BL_ORD_ID|53852 cb8564_c2734/f1p0/2131	327	1545	39	332	448	1666	1	295
cb2966_c0/f3p0/2527 isoform=cb2966_c0;full_length_coverage=3;non_full_length_coverage=0;isoform_length=2527;expected_accuracy=1.000	gnl|BL_ORD_ID|38223 cb2966_c4/f1p0/2868 isoform=cb2966_c4;full_length_coverage=1;non_full_length_coverage=0;isoform_length=2868;expected_accuracy=0.997	163	2499	1	166	533	2868	2	163
cb6508_c5/f1p0/2613 isoform=cb6508_c5;full_length_coverage=1;non_full_length_coverage=0;isoform_length=2613;expected_accuracy=0.995	gnl|BL_ORD_ID|3598 cb6508_c4/f1p0/2838 isoform=cb6508_c4;full_length_coverage=1;non_full_length_coverage=0;isoform_length=2838;expected_accuracy=1.000	1	1709	1704	2590	2	1708	1953	2838
cb8564_c2788/f1p0/2682 isoform=cb8564_c2788;full_length_coverage=1;non_full_length_coverage=0;isoform_length=2682;expected_ac	gnl|BL_ORD_ID|14039 cb8564_c2753/f1p0/2971 isoform=cb8564_c2753;full_length_coverage=1;non_full_length_coverage=0;isoform_length=2971;expected_ac	1157	2682	1	1157	1449	2971	1	1158

curacy=0.999	curacy=0.997								
cb8564_c36689/f1p1/2368 isoform=cb8564_c36689;full_length_coverage=1;non_full_length_coverage=1;isoform_length=2368;expected_accuracy=0.991	gnl|BL_ORD_ID|55213 cb8564_c35568/f1p0/2997	1	1709	1706	2368	13	1724	2223	2885
cb8564_c87466/f1p1/3006 isoform=cb8564_c87466;full_length_coverage=1;non_full_length_coverage=1;isoform_length=3006;expected_accuracy=0.994	gnl|BL_ORD_ID|16879 cb8564_c48606/f1p1/3308 isoform=cb8564_c48606;full_length_coverage=1;non_full_length_coverage=1;isoform_length=3308;expected_accuracy=0.998	1	2169	2165	2900	1	2151	2587	3308
cb8564_c53418/f1p0/2602 isoform=cb8564_c53418;full_length_coverage=1;non_full_length_coverage=0;isoform_length=2602;expected_accuracy=0.991	gnl|BL_ORD_ID|55780 cb8564_c36000/f1p16/3134	1163	2499	1	1168	1366	27	2634	1467
cb2737_c63/f1p2/2799 isoform=cb2737_c63;full_length_coverage=1;non_full_length_coverage=2;isoform_length=2799;expected_accuracy=0.994	gnl|BL_ORD_ID|38133 cb2737_c21/f1p2/3479 isoform=cb2737_c21;full_length_coverage=1;non_full_length_coverage=2;isoform_length=3479;expected_accuracy=1.000	109	1514	1512	2798	596	2003	2135	3431
cb8564_c181853/f1p0/2119 isoform=cb8564_c181853;full_length_coverage=1;non_full_length_coverage=0;isoform_length=2119;expected_accuracy=0.997	gnl|BL_ORD_ID|55607 cb8564_c46612/f1p1/3367	1	1339	1334	2119	8	1345	2174	2964
cb322_c9/f2p0/3136 isoform=cb322_c9;full_length_coverage=2;non_full_length_coverage=0;isoform_length=3136;expected_accuracy=1.000	gnl|BL_ORD_ID|69561 cb322_c28/f1p0/3948	1	2718	2718	3136	153	2866	3355	3773
cb8564_c107625/f7p0/2800	gnl|BL_ORD_ID|57052 cb8564_c81772/f1p0/3125	1264	2797	1	1265	1614	314	247	1511

isoform=cb8564_c107625;full_length_coverage=7;non_full_length_coverage=0;isoform_length=2800;expected_accuracy=1.000							8		
cb8564_c85037/f2p2/3207 isoform=cb8564_c85037;full_length_coverage=2;non_full_length_coverage=2;isoform_length=3207;expected_accuracy=0.997	gnl|BL_ORD_ID|15377 cb8564_c35188/f1p4/3470 isoform=cb8564_c35188;full_length_coverage=1;non_full_length_coverage=4;isoform_length=3470;expected_accuracy=0.995	2	2131	2131	3207	1	2129	2293	3368
cb2966_c0/f3p0/2527 isoform=cb2966_c0;full_length_coverage=3;non_full_length_coverage=0;isoform_length=2527;expected_accuracy=1.000	gnl|BL_ORD_ID|70108 cb2966_c2/f1p0/3755	163	2440	1	166	1388	3665	1	163
cb5643_c92/f1p0/2723	gnl|BL_ORD_ID|45379 cb5643_c25/f1p0/3322 isoform=cb5643_c25;full_length_coverage=1;non_full_length_coverage=0;isoform_length=3322;expected_accuracy=0.998	110	2633	1	112	799	3322	1	111
cb3769_c4/f1p0/3111	gnl|BL_ORD_ID|40981 cb3769_c3/f1p0/3241 isoform=cb3769_c3;full_length_coverage=1;non_full_length_coverage=0;isoform_length=3241;expected_accuracy=0.995	12	2376	2375	2961	17	2407	2657	3240
cb8564_c5070/f1p0/2861	gnl|BL_ORD_ID|55743 cb8564_c6593/f1p1/2865	10	1660	1659	2831	55	1703	1596	2768
cb2737_c26/f1p0/2992 isoform=cb2737_c26;full_length_coverage=1;non_full_length_coverage=0;isoform_length=2992;expected_accuracy=0.996	gnl|BL_ORD_ID|68777 cb2737_c19/f1p0/3583	1	1804	1802	2992	402	2205	2337	3550
cb2156_c24/f1p1/2545	gnl|BL_ORD_ID|66761 cb2156_c22/f1p1/3698	1	1422	1420	2545	849	228	2405	353

isoform=cb2156_c24;full_length_coverage=1;non_full_length_coverage=1;isoform_length=2545;expected_accuracy=0.991							0		2
cb17345_c9/f1p0/2238	gnl|BL_ORD_ID|32580 cb17345_c8/f1p0/3246 isoform=cb17345_c8;full_length_coverage=1;non_full_length_coverage=0;isoform_length=3246;expected_accuracy=0.997	1	1617	1614	2223	4	1621	2527	3135
cb9042_c7/f1p1/1973 isoform=cb9042_c7;full_length_coverage=1;non_full_length_coverage=1;isoform_length=1973;expected_accuracy=0.993	gnl|BL_ORD_ID|58515 cb9042_c3/f1p0/2364	102	1970	1	101	336	2187	5	105
cb750_c27/f1p1/4163 isoform=cb750_c27;full_length_coverage=1;non_full_length_coverage=1;isoform_length=4163;expected_accuracy=0.998	gnl|BL_ORD_ID|8746 cb750_c10/f1p0/4646 isoform=cb750_c10;full_length_coverage=1;non_full_length_coverage=0;isoform_length=4646;expected_accuracy=0.997	1814	4163	114	1815	2276	4623	2	1707
cb8564_c161530/f1p7/3594 isoform=cb8564_c161530;full_length_coverage=1;non_full_length_coverage=7;isoform_length=3594;expected_accuracy=0.999	gnl|BL_ORD_ID|57396 cb8564_c91806/f1p1/3620	132	2864	2862	3594	13	2745	2895	3622
cb6063_c19/f1p1/2514	gnl|BL_ORD_ID|45238 cb6063_c0/f15p2/2941 isoform=cb6063_c0;full_length_coverage=15;non_full_length_coverage=2;isoform_length=2941;expected_accuracy=1.000	157	2441	1	156	657	2940	1	154
cb249_c10/f1p0/1963 isoform=cb249_c10;full_length_coverage=1;non_full_length_coverage=0;isoform_length=1963;expected_accur	gnl|BL_ORD_ID|38949 cb249_c1/f2p0/2671 isoform=cb249_c1;full_length_coverage=2;non_full_length_coverage=0;isoform_length=2671;expected_accurac	1	1494	1492	1963	354	1849	2119	2590

acy=0.992	y=1.000								
cb3119_c2/f1p0/2612 isoform=cb3119_c2;full_length_coverage=1;non_full_length_coverage=0;isoform_length=2612;expected_accuracy=0.999	gnl|BL_ORD_ID|69147 cb3119_c7/f1p0/3565	1	1765	1764	2612	5	1788	2033	2882
cb1333_c9/f4p0/1480 isoform=cb1333_c9;full_length_coverage=4;non_full_length_coverage=0;isoform_length=1480;expected_accuracy=1.000	gnl|BL_ORD_ID|61259 cb1333_c109/f1p0/5379	153	1473	29	154	3609	4925	23	148
cb8564_c147327/f4p0/3198 isoform=cb8564_c147327;full_length_coverage=4;non_full_length_coverage=0;isoform_length=3198;expected_accuracy=0.999	gnl|BL_ORD_ID|57195 cb8564_c86311/f1p1/3790	1491	3197	146	1493	1919	3625	415	1762
cb8564_c51035/f2p2/4216 isoform=cb8564_c51035;full_length_coverage=2;non_full_length_coverage=2;isoform_length=4216;expected_accuracy=0.999	gnl|BL_ORD_ID|16628 cb8564_c47737/f7p5/4351 isoform=cb8564_c47737;full_length_coverage=7;non_full_length_coverage=5;isoform_length=4351;expected_accuracy=0.998	286	4215	1	287	404	4339	1	286
cb4211_c2/f2p1/1162 isoform=cb4211_c2;full_length_coverage=2;non_full_length_coverage=1;isoform_length=1162;expected_accuracy=1.000	gnl|BL_ORD_ID|71851 cb4211_c4/f1p2/3385	124	1162	2	126	2198	3236	5	129
cb8564_c84130/f1p2/3574 isoform=cb8564_c84130;full_length_coverage=1;non_full_length_coverage=2;isoform_length=3574;expected_accuracy=0.995	gnl|BL_ORD_ID|55542 cb8564_c44720/f1p0/4093	125	3511	1	127	556	3938	7	132
cb3339_c5/f1p1/3094	gnl|BL_ORD_ID|40811 cb3339_c2/f1p1/3653	1	1878	1878	2998	1	186	2340	346

	isoform=cb3339_c2;full_length_coverage=1;non_full_length_coverage=1;isoform_length=3653;expected_accuracy=0.997						4		0
cb1030_c21/f1p1/3050	gnl|BL_ORD_ID|1808 cb1030_c5/f2p0/3347 isoform=cb1030_c5;full_length_coverage=2;non_full_length_coverage=0;isoform_length=3347;expected_accuracy=0.997	28	2802	2805	2966	1	2774	2900	3060
cb8564_c44698/f1p5/2843 isoform=cb8564_c44698;full_length_coverage=1;non_full_length_coverage=5;isoform_length=2843;expected_accuracy=0.997	gnl|BL_ORD_ID|15153 cb8564_c36883/f1p0/3072 isoform=cb8564_c36883;full_length_coverage=1;non_full_length_coverage=0;isoform_length=3072;expected_accuracy=0.996	1	2516	2511	2843	1	2513	2634	2968
cb5290_c45/f1p0/2555	gnl|BL_ORD_ID|73746 cb5290_c79/f1p0/2804	507	2567	88	512	646	2706	26	450
cb5633_c81/f1p0/2727 isoform=cb5633_c81;full_length_coverage=1;non_full_length_coverage=0;isoform_length=2727;expected_accuracy=0.992	gnl|BL_ORD_ID|45725 cb5633_c20/f1p1/2973 isoform=cb5633_c20;full_length_coverage=1;non_full_length_coverage=1;isoform_length=2973;expected_accuracy=0.994	1	2007	2006	2721	61	1988	2292	2973
cb5550_c11/f1p0/2859 isoform=cb5550_c11;full_length_coverage=1;non_full_length_coverage=0;isoform_length=2859;expected_accuracy=0.994	gnl|BL_ORD_ID|45197 cb5550_c10/f1p0/4832 isoform=cb5550_c10;full_length_coverage=1;non_full_length_coverage=0;isoform_length=4832;expected_accuracy=0.998	1	2505	2500	2787	94	2599	4545	4832
cb3892_c1/f3p0/2858 isoform=cb3892_c1;full_length_coverage=3;non_full_length_coverage=0;isoform_length=2858;expected_accuracy=0.995	gnl|BL_ORD_ID|40678 cb3892_c5/f1p0/3003 isoform=cb3892_c5;full_length_coverage=1;non_full_length_coverage=0;isoform_length=3003;expected_accuracy=0.998	184	2850	1	185	335	3003	1	186
cb8564_c36689/f1p1/2368	gnl|BL_ORD_ID|10510 cb8564_c111713/f6p2/3032	1	1709	1706	2368	1	167	2171	283

isoform=cb8564_c36689;full_length_coverage=1;non_full_length_coverage=1;isoform_length=2368;expected_accuracy=0.991	isoform=cb8564_c111713;full_length_coverage=6;non_full_length_coverage=2;isoform_length=3032;expected_accuracy=0.998						2		4
cb8564_c38035/f1p0/2770	gnl|BL_ORD_ID|56641 cb8564_c50309/f1p5/3075	1	2035	2033	2752	1	2037	2227	2947
cb5960_c4/f1p0/2967	gnl|BL_ORD_ID|74709 cb5960_c13/f1p0/3400	15	1974	1972	2634	26	1984	2733	3395
cb16052_c16/f1p0/1872	gnl|BL_ORD_ID|17604 cb8564_c89610/f1p0/1980 isoform=cb8564_c89610;full_length_coverage=1;non_full_length_coverage=0;isoform_length=1980;expected_accuracy=0.999	157	1720	1	160	417	1980	43	202
cb1666_c5/f1p3/2503 isoform=cb1666_c5;full_length_coverage=1;non_full_length_coverage=3;isoform_length=2503;expected_accuracy=0.995	gnl|BL_ORD_ID|33313 cb1666_c3/f1p1/4226 isoform=cb1666_c3;full_length_coverage=1;non_full_length_coverage=1;isoform_length=4226;expected_accuracy=0.997	533	2399	7	535	2360	4226	1	524
cb8564_c71815/f1p0/2753	gnl|BL_ORD_ID|57875 cb8564_c83119/f1p1/3046	1	1377	1372	2485	29	1405	1639	2752
cb3226_c5/f2p0/2559 isoform=cb3226_c5;full_length_coverage=2;non_full_length_coverage=0;isoform_length=2559;expected_accuracy=1.000	gnl|BL_ORD_ID|39534 cb3226_c8/f1p0/2993 isoform=cb3226_c8;full_length_coverage=1;non_full_length_coverage=0;isoform_length=2993;expected_accuracy=0.990	1	1635	1634	2530	7	1634	2101	2993
cb8564_c38035/f1p0/2770	gnl|BL_ORD_ID|55341 cb8564_c38927/f1p0/3326	1	2035	2033	2756	7	2043	2233	2961
cb8564_c89918/f1p1/2045 isoform=cb8564_c89918;full_length_coverage=1;non_full_length_coverage=1;isoform_length=2045;expected_	gnl|BL_ORD_ID|54984 cb8564_c4106/f1p2/2854	1	1616	1611	2045	297	1884	2079	2512

accuracy=0.991									
cb8564_c48538/f1p0/2599	gnl|BL_ORD_ID|16440 cb8564_c77380/f2p0/3010 isoform=cb8564_c77380;full_length_coverage=2;non_full_length_coverage=0;isoform_length=3010;expected_accuracy=0.998	554	2462	81	556	1082	2993	170	645
cb8564_c119687/f1p1/2659	gnl|BL_ORD_ID|53584 cb8564_c129387/f1p0/3201	16	1941	1941	2650	25	1949	2178	2887
cb295_c13/f1p1/3878 isoform=cb295_c13;full_length_coverage=1;non_full_length_coverage=1;isoform_length=3878;expected_accuracy=0.994	gnl|BL_ORD_ID|34399 cb18646_c1/f2p2/6581 isoform=cb18646_c1;full_length_coverage=2;non_full_length_coverage=2;isoform_length=6581;expected_accuracy=0.999	1	3634	3633	3878	2464	6120	6227	6477
cb8564_c34299/f3p1/3910 isoform=cb8564_c34299;full_length_coverage=3;non_full_length_coverage=1;isoform_length=3910;expected_accuracy=0.998	gnl|BL_ORD_ID|55790 cb8564_c36942/f1p0/4630	25	2794	2793	3832	47	2819	3325	4364
cb12363_c5/f1p0/1088 isoform=cb12363_c5;full_length_coverage=1;non_full_length_coverage=0;isoform_length=1088;expected_accuracy=1.000	gnl|BL_ORD_ID|49952 cb6700_c1/f1p0/2789	158	1036	3	162	1604	2482	1068	1225
cb17234_c4/f1p0/1430 isoform=cb17234_c4;full_length_coverage=1;non_full_length_coverage=0;isoform_length=1430;expected_accuracy=0.995	gnl|BL_ORD_ID|34066 cb17234_c6/f5p1/1693 isoform=cb17234_c6;full_length_coverage=5;non_full_length_coverage=1;isoform_length=1693;expected_accuracy=1.000	148	1429	4	148	410	1691	1	143
cb8564_c3432/f1p0/2118	gnl|BL_ORD_ID|54595 cb8564_c127586/f1p0/2499	619	2090	7	623	745	2216	13	629
cb8102_c19/f1p0/1683	gnl|BL_ORD_ID|8042 cb8102_c20/f21p0/1923	1	1128	1124	1683	33	1160	1339	190

<table>
<tr><td>isoform=cb8102_c19;full_length_coverage=1;non_full_length_coverage=0;isoform_length=1683;expected_accuracy=0.991</td><td>isoform=cb8102_c20;full_length_coverage=21;non_full_length_coverage=0;isoform_length=1923;expected_accuracy=1.000</td><td></td><td></td><td></td><td></td><td></td><td></td><td></td><td>1</td></tr>
<tr><td>cb965_c30/f1p3/4326 isoform=cb965_c30;full_length_coverage=1;non_full_length_coverage=3;isoform_length=4326;expected_accuracy=0.994</td><td>gnl|BL_ORD_ID|22008 cb965_c55/f1p1/5368 isoform=cb965_c55;full_length_coverage=1;non_full_length_coverage=1;isoform_length=5368;expected_accuracy=0.997</td><td>1</td><td>2848</td><td>2847</td><td>4257</td><td>1</td><td>2849</td><td>3960</td><td>5368</td></tr>
<tr><td>cb8564_c44004/f1p0/2796 isoform=cb8564_c44004;full_length_coverage=1;non_full_length_coverage=0;isoform_length=2796;expected_accuracy=1.000</td><td>gnl|BL_ORD_ID|11821 cb8564_c15227/f1p0/3770 isoform=cb8564_c15227;full_length_coverage=1;non_full_length_coverage=0;isoform_length=3770;expected_accuracy=0.999</td><td>1</td><td>1549</td><td>1547</td><td>2796</td><td>72</td><td>1620</td><td>2519</td><td>3766</td></tr>
<tr><td>cb279_c11/f1p1/4423 isoform=cb279_c11;full_length_coverage=1;non_full_length_coverage=1;isoform_length=4423;expected_accuracy=0.999</td><td>gnl|BL_ORD_ID|37181 cb279_c33/f5p1/4837 isoform=cb279_c33;full_length_coverage=5;non_full_length_coverage=1;isoform_length=4837;expected_accuracy=1.000</td><td>147</td><td>4423</td><td>1</td><td>151</td><td>522</td><td>4813</td><td>98</td><td>247</td></tr>
<tr><td>cb8564_c180679/f1p0/2109 isoform=cb8564_c180679;full_length_coverage=1;non_full_length_coverage=0;isoform_length=2109;expected_accuracy=0.999</td><td>gnl|BL_ORD_ID|53846 cb8564_c2435/f1p1/2639</td><td>198</td><td>2109</td><td>1</td><td>202</td><td>605</td><td>2516</td><td>26</td><td>227</td></tr>
<tr><td>cb1875_c0/f5p0/3492 isoform=cb1875_c0;full_length_coverage=5;non_full_length_coverage=0;isoform_length=3492;expected_accuracy=1.000</td><td>gnl|BL_ORD_ID|68211 cb1875_c7/f1p0/4579</td><td>1</td><td>1815</td><td>1814</td><td>3488</td><td>93</td><td>1906</td><td>2751</td><td>4425</td></tr>
<tr><td>cb8564_c77994/f1p2/2630 isoform=cb8564_c77994;full_length_coverage=1;non_full_length_coverage=2;isoform_length=2630;expected_</td><td>gnl|BL_ORD_ID|54709 cb8564_c157281/f2p1/3057</td><td>1</td><td>1819</td><td>1815</td><td>2630</td><td>114</td><td>1935</td><td>2079</td><td>2896</td></tr>
</table>

accuracy=0.998									
cb1238_c0/f3p1/2659 isoform=cb1238_c0;full_length_coverage=3;non_full_length_coverage=1;isoform_length=2659;expected_accuracy=1.000	gnl|BL_ORD_ID|59301 cb1238_c9/f1p0/4165	683	2659	91	687	2121	4097	1	599
cb3744_c16/f1p0/2234	gnl|BL_ORD_ID|39703 cb3744_c0/f27p2/3071 isoform=cb3744_c0;full_length_coverage=27;non_full_length_coverage=2;isoform_length=3071;expected_accuracy=1.000	1	1429	1424	1922	179	1607	2573	3071
cb12714_c29/f1p2/1583	gnl|BL_ORD_ID|55328 cb8564_c38464/f1p0/2918	1	1296	1293	1526	1	1296	2363	2596
cb3485_c2/f3p0/2452 isoform=cb3485_c2;full_length_coverage=3;non_full_length_coverage=0;isoform_length=2452;expected_accuracy=1.000	gnl|BL_ORD_ID|71246 cb3485_c10/f1p0/2515	677	2452	84	678	720	2494	1	597
cb8564_c21138/f1p3/2652	gnl|BL_ORD_ID|9350 cb8564_c11826/f1p0/2916 isoform=cb8564_c11826;full_length_coverage=1;non_full_length_coverage=0;isoform_length=2916;expected_accuracy=0.997	1292	2600	1	1293	1543	2853	4	1296
cb2964_c3/f1p0/1791 isoform=cb2964_c3;full_length_coverage=1;non_full_length_coverage=0;isoform_length=1791;expected_accuracy=0.996	gnl|BL_ORD_ID|37120 cb2964_c6/f1p0/1936 isoform=cb2964_c6;full_length_coverage=1;non_full_length_coverage=0;isoform_length=1936;expected_accuracy=1.000	213	1791	7	215	325	1904	1	206
cb8564_c116509/f1p0/3009	gnl|BL_ORD_ID|9800 cb8564_c109800/f6p1/3270 isoform=cb8564_c109800;full_length_coverage=6;non_full_length_coverage=1;isoform_length=3270;expected_a	1	2016	2015	2985	13	2030	2254	3230

	ccuracy=0.998								
cb7493_c4/f1p0/2146	gnl|BL_ORD_ID|50550 cb7493_c3/f1p0/2634	1	1712	1707	2121	1	1707	2004	2420
cb8564_c40978/f1p0/3073	gnl|BL_ORD_ID|14410 cb8564_c44256/f1p1/3267 isoform=cb8564_c44256;full_length_coverage=1;non_full_length_coverage=1;isoform_length=3267;expected_accuracy=0.995	1	2133	2129	2845	1	2119	2553	3267
cb5921_c35/f1p0/2385	gnl|BL_ORD_ID|74654 cb5921_c44/f1p1/2444	11	2074	2075	2226	9	2070	2184	2336
cb8813_c7/f1p0/1096	gnl|BL_ORD_ID|18429 cb8813_c2/f1p0/2264 isoform=cb8813_c2;full_length_coverage=1;non_full_length_coverage=0;isoform_length=2264;expected_accuracy=0.997	245	1069	39	246	1229	2053	1	206
cb9630_c18/f1p1/2469 isoform=cb9630_c18;full_length_coverage=1;non_full_length_coverage=1;isoform_length=2469;expected_accuracy=0.999	gnl|BL_ORD_ID|59865 cb9630_c26/f1p0/3573	1	1632	1631	2466	1	1632	2720	3555
cb2020_c14/f1p0/2216 isoform=cb2020_c14;full_length_coverage=1;non_full_length_coverage=0;isoform_length=2216;expected_accuracy=0.991	gnl|BL_ORD_ID|66612 cb2020_c17/f1p0/2941	1	1824	1824	2197	1	1822	2554	2929
cb8564_c15573/f1p0/3752 isoform=cb8564_c15573;full_length_coverage=1;non_full_length_coverage=0;isoform_length=3752;expected_accuracy=0.997	gnl|BL_ORD_ID|13527 cb8564_c21762/f3p4/3985 isoform=cb8564_c21762;full_length_coverage=3;non_full_length_coverage=4;isoform_length=3985;expected_accuracy=1.000	1	2900	2896	3752	13	2922	3037	3894
cb4552_c3/f1p0/2538	gnl|BL_ORD_ID|42484 cb4552_c2/f1p0/3315	236	2538	1	237	1008	331	1	236

isoform=cb4552_c3;full_length_coverage=1;non_full_length_coverage=0;isoform_length=2538;expected_accuracy=0.991	isoform=cb4552_c2;full_length_coverage=1;non_full_length_coverage=0;isoform_length=3315;expected_accuracy=0.993						3		
cb8564_c50881/f1p2/2125 isoform=cb8564_c50881;full_length_coverage=1;non_full_length_coverage=2;isoform_length=2125;expected_accuracy=0.994	gnl|BL_ORD_ID|12475 cb8564_c148406/f2p0/2449 isoform=cb8564_c148406;full_length_coverage=2;non_full_length_coverage=0;isoform_length=2449;expected_accuracy=0.990	1	1863	1858	2111	49	1914	2193	2449
cb396_c57/f1p0/2029 isoform=cb396_c57;full_length_coverage=1;non_full_length_coverage=0;isoform_length=2029;expected_accuracy=0.995	gnl|BL_ORD_ID|39504 cb396_c16/f1p0/2356 isoform=cb396_c16;full_length_coverage=1;non_full_length_coverage=0;isoform_length=2356;expected_accuracy=1.000	1	1399	1395	2029	70	1446	1707	2342
cb955_c38/f1p0/2505	gnl|BL_ORD_ID|59129 cb955_c25/f1p0/3018	14	1663	1663	2327	8	1657	2345	3009
cb8564_c36731/f1p2/2761	gnl|BL_ORD_ID|57017 cb8564_c80370/f1p0/3493	119	1977	1972	2740	3085	1225	796	27
cb4862_c13/f1p1/2421 isoform=cb4862_c13;full_length_coverage=1;non_full_length_coverage=1;isoform_length=2421;expected_accuracy=0.999	gnl|BL_ORD_ID|42994 cb4862_c9/f1p1/3086 isoform=cb4862_c9;full_length_coverage=1;non_full_length_coverage=1;isoform_length=3086;expected_accuracy=0.997	1	1384	1380	2418	84	1463	2039	3075
cb8564_c106911/f7p3/3690 isoform=cb8564_c106911;full_length_coverage=7;non_full_length_coverage=3;isoform_length=3690;expected_accuracy=0.998	gnl|BL_ORD_ID|10486 cb8564_c107051/f2p1/3797 isoform=cb8564_c107051;full_length_coverage=2;non_full_length_coverage=1;isoform_length=3797;expected_accuracy=0.997	201	3607	1	203	392	3797	13	215
cb2899_c13/f1p0/1590 isoform=cb2899_c13;full_length_coverage=1;non_full_length_coverage=0;isoform_length=1590;expected_accu	gnl|BL_ORD_ID|37043 cb2899_c1/f2p0/1740 isoform=cb2899_c1;full_length_coverage=2;non_full_length_coverage=0;isoform_length=1740;expected_accura	97	1590	1	101	239	1734	32	132

racy=0.995	cy=0.994								
cb315_c1/f1p0/4753 isoform=cb315_c1;full_length_coverage=1;non_full_length_coverage=0;isoform_length=4753;expected_accuracy=0.993	gnl|BL_ORD_ID|36651 cb315_c0/f1p0/4872 isoform=cb315_c0;full_length_coverage=1;non_full_length_coverage=0;isoform_length=4872;expected_accuracy=0.992	23	3890	3888	4675	1	3817	4102	4872
cb2272_c12/f1p0/5238	gnl|BL_ORD_ID|69666 cb2272_c8/f1p0/6467	16	2695	2691	5202	135	2814	3845	6356
cb8564_c75364/f1p0/2028	gnl|BL_ORD_ID|58065 cb8564_c87948/f1p0/3033	702	2038	88	701	1606	2942	306	919
cb2674_c12/f1p0/3677	gnl|BL_ORD_ID|38975 cb2674_c31/f35p4/3827 isoform=cb2674_c31;full_length_coverage=35;non_full_length_coverage=4;isoform_length=3827;expected_accuracy=1.000	1	2544	2542	3643	66	2612	2727	3827
cb12931_c15/f1p0/1493 isoform=cb12931_c15;full_length_coverage=1;non_full_length_coverage=0;isoform_length=1493;expected_accuracy=1.000	gnl|BL_ORD_ID|55417 cb8564_c41115/f1p3/2751	1	1106	1104	1485	4	1105	2310	2690
cb8564_c2355/f1p4/2493 isoform=cb8564_c2355;full_length_coverage=1;non_full_length_coverage=4;isoform_length=2493;expected_accuracy=0.995	gnl|BL_ORD_ID|13611 cb8564_c2184/f1p0/2606 isoform=cb8564_c2184;full_length_coverage=1;non_full_length_coverage=0;isoform_length=2606;expected_accuracy=0.995	277	2488	4	277	384	2601	4	272
cb8564_c37361/f1p0/3076	gnl|BL_ORD_ID|16622 cb8564_c50151/f1p0/3198 isoform=cb8564_c50151;full_length_coverage=1;non_full_length_coverage=0;isoform_length=3198;expected_accuracy=0.995	1	2481	2477	3068	6	2506	2607	3198
cb3518_c21/f1p0/1435	gnl|BL_ORD_ID|39441 cb3518_c0/f10p1/1768	104	1376	1	107	496	176	54	160

isoform=cb3518_c21;full_length_coverage=1;non_full_length_coverage=0;isoform_length=1435;expected_accuracy=1.000	isoform=cb3518_c0;full_length_coverage=10;non_full_length_coverage=1;isoform_length=1768;expected_accuracy=1.000						8		
cb8564_c43450/f1p0/2695	gnl|BL_ORD_ID|55610 cb8564_c46706/f1p2/2881	133	1886	1882	2714	145	1899	2014	2847
cb8564_c74445/f1p1/2816 isoform=cb8564_c74445;full_length_coverage=1;non_full_length_coverage=1;isoform_length=2816;expected_accuracy=0.992	gnl|BL_ORD_ID|55544 cb8564_c44764/f1p0/3516	1332	2816	13	1334	1807	3291	32	1355
cb4641_c4/f2p1/2180 isoform=cb4641_c4;full_length_coverage=2;non_full_length_coverage=1;isoform_length=2180;expected_accuracy=0.999	gnl|BL_ORD_ID|72794 cb4641_c8/f1p1/2416	1	1173	1172	2178	1	1173	1285	2291
cb2719_c25/f1p0/2916 isoform=cb2719_c25;full_length_coverage=1;non_full_length_coverage=0;isoform_length=2916;expected_accuracy=0.995	gnl|BL_ORD_ID|37134 cb2719_c32/f1p0/2977 isoform=cb2719_c32;full_length_coverage=1;non_full_length_coverage=0;isoform_length=2977;expected_accuracy=0.997	263	2857	1	264	369	2977	1	256
cb8564_c35093/f1p5/2916	gnl|BL_ORD_ID|16879 cb8564_c48606/f1p1/3308 isoform=cb8564_c48606;full_length_coverage=1;non_full_length_coverage=1;isoform_length=3308;expected_accuracy=0.998	20	2169	2165	2885	2	2151	2587	3308
cb6430_c0/f2p1/2342 isoform=cb6430_c0;full_length_coverage=2;non_full_length_coverage=1;isoform_length=2342;expected_accuracy=1.000	gnl|BL_ORD_ID|49714 cb6430_c2/f1p0/2694	1	1727	1728	2308	2	1753	2101	2681
cb8564_c41930/f1p0/3319	gnl|BL_ORD_ID|15281 cb8564_c39484/f1p0/3555	517	3319	57	519	747	355	105	567

isoform=cb8564_c41930;full_length_coverage=1;non_full_length_coverage=0;isoform_length=3319;expected_accuracy=0.996	isoform=cb8564_c39484;full_length_coverage=1;non_full_length_coverage=0;isoform_length=3555;expected_accuracy=0.997						4		
cb13752_c11/f2p0/1820 isoform=cb13752_c11;full_length_coverage=2;non_full_length_coverage=0;isoform_length=1820;expected_accuracy=0.996	gnl|BL_ORD_ID|25257 cb13752_c3/f2p0/1870 isoform=cb13752_c3;full_length_coverage=2;non_full_length_coverage=0;isoform_length=1870;expected_accuracy=0.995	11	1613	1613	1773	5	1608	1708	1870
cb8564_c50309/f1p5/3075	gnl|BL_ORD_ID|17429 cb8564_c85447/f1p0/3196 isoform=cb8564_c85447;full_length_coverage=1;non_full_length_coverage=0;isoform_length=3196;expected_accuracy=0.997	1	2419	2418	2947	3	2413	2660	3189
cb9604_c7/f1p0/1887	gnl|BL_ORD_ID|21528 cb9604_c1/f2p0/1964 isoform=cb9604_c1;full_length_coverage=2;non_full_length_coverage=0;isoform_length=1964;expected_accuracy=0.998	202	1798	6	200	366	1962	27	221
cb12_c37/f1p0/4067	gnl|BL_ORD_ID|61092 cb12_c60/f1p0/5175	286	4080	186	288	931	4733	699	801
cb8564_c2539/f1p1/2062 isoform=cb8564_c2539;full_length_coverage=1;non_full_length_coverage=1;isoform_length=2062;expected_accuracy=0.997	gnl|BL_ORD_ID|54773 cb8564_c2528/f1p0/3357	1	1646	1644	2062	5	1645	2812	3229
cb8564_c45300/f1p4/3014 isoform=cb8564_c45300;full_length_coverage=1;non_full_length_coverage=4;isoform_length=3014;expected_accuracy=0.996	gnl|BL_ORD_ID|15152 cb8564_c38629/f2p1/3246 isoform=cb8564_c38629;full_length_coverage=2;non_full_length_coverage=1;isoform_length=3246;expected_accuracy=0.993	243	3014	1	247	420	3204	1	259
cb17118_c7/f1p0/1440	gnl|BL_ORD_ID|52607 cb8564_c1007/f1p0/1898	1	1141	1137	1429	271	1411	1596	188

									8
cb322_c9/f2p0/3136 isoform=cb322_c9;full_length_coverage=2;non_full_length_coverage=0;isoform_length=3136;expected_accuracy=1.000	gnl|BL_ORD_ID|41025 cb322_c8/f2p0/3977 isoform=cb322_c8;full_length_coverage=2;non_full_length_coverage=0;isoform_length=3977;expected_accuracy=0.999	1	2718	2718	3134	14	2727	3560	3977
cb8564_c844/f2p0/2586 isoform=cb8564_c844;full_length_coverage=2;non_full_length_coverage=0;isoform_length=2586;expected_accuracy=1.000	gnl|BL_ORD_ID|54919 cb8564_c3679/f1p0/2936	1	2343	2344	2586	2	2346	2460	2702
cb3671_c20/f2p1/3263 isoform=cb3671_c20;full_length_coverage=2;non_full_length_coverage=1;isoform_length=3263;expected_accuracy=0.999	gnl|BL_ORD_ID|71434 cb3671_c14/f1p0/3470	1	2858	2858	3211	79	2940	3119	3472
cb6968_c6/f1p0/2273 isoform=cb6968_c6;full_length_coverage=1;non_full_length_coverage=0;isoform_length=2273;expected_accuracy=0.996	gnl|BL_ORD_ID|4815 cb6968_c3/f2p0/2613 isoform=cb6968_c3;full_length_coverage=2;non_full_length_coverage=0;isoform_length=2613;expected_accuracy=0.994	185	2273	1	185	423	2511	79	264
cb7337_c6/f1p0/1841 isoform=cb7337_c6;full_length_coverage=1;non_full_length_coverage=0;isoform_length=1841;expected_accuracy=0.992	gnl|BL_ORD_ID|50485 cb7337_c3/f1p0/2569	1	1684	1682	1841	10	1695	1805	1963
cb4444_c10/f1p0/1986 isoform=cb4444_c10;full_length_coverage=1;non_full_length_coverage=0;isoform_length=1986;expected_accuracy=0.996	gnl|BL_ORD_ID|43661 cb4444_c6/f1p0/3234 isoform=cb4444_c6;full_length_coverage=1;non_full_length_coverage=0;isoform_length=3234;expected_accuracy=0.990	350	1986	5	349	1539	3178	18	359
cb8564_c181853/f1p0/2119	gnl|BL_ORD_ID|13512 cb8564_c186209/f1p0/2931	1	1339	1334	2117	1	134	2148	293

isoform=cb8564_c181853;full_length_coverage=1;non_full_length_coverage=0;isoform_length=2119;expected_accuracy=0.997	isoform=cb8564_c186209;full_length_coverage=1;non_full_length_coverage=0;isoform_length=2931;expected_accuracy=0.996						1		1
cb8564_c4750/f1p1/2456 isoform=cb8564_c4750;full_length_coverage=1;non_full_length_coverage=1;isoform_length=2456;expected_accuracy=0.993	gnl|BL_ORD_ID|55688 cb8564_c4747/f1p0/2838	1	1330	1328	2367	224	1559	1807	2853
cb354_c0/f2p0/4074 isoform=cb354_c0;full_length_coverage=2;non_full_length_coverage=0;isoform_length=4074;expected_accuracy=0.994	gnl|BL_ORD_ID|70822 cb354_c1/f1p0/4589	284	4074	154	287	503	4300	3	136
cb1656_c9/f1p1/3733 isoform=cb1656_c9;full_length_coverage=1;non_full_length_coverage=1;isoform_length=3733;expected_accuracy=0.992	gnl|BL_ORD_ID|31007 cb1656_c7/f1p1/3974 isoform=cb1656_c7;full_length_coverage=1;non_full_length_coverage=1;isoform_length=3974;expected_accuracy=0.997	1	2973	2974	3730	42	2976	3114	3866
cb7484_c4/f1p0/2339	gnl|BL_ORD_ID|7223 cb7484_c2/f1p0/2401 isoform=cb7484_c2;full_length_coverage=1;non_full_length_coverage=0;isoform_length=2401;expected_accuracy=0.996	1	1568	1567	2242	6	1572	1726	2401
cb8160_c2/f1p0/2208 isoform=cb8160_c2;full_length_coverage=1;non_full_length_coverage=0;isoform_length=2208;expected_accuracy=0.998	gnl|BL_ORD_ID|6487 cb8160_c1/f1p1/2577 isoform=cb8160_c1;full_length_coverage=1;non_full_length_coverage=1;isoform_length=2577;expected_accuracy=0.997	99	2208	1	100	379	2492	79	178
cb18456_c4245/f1p0/1080	gnl|BL_ORD_ID|67489 cb18456_c4062/f1p3/1349	7	512	508	1079	1	494	603	1185
cb8564_c72349/f1p0/3248	gnl|BL_ORD_ID|55538 cb8564_c44621/f1p1/3432	1	2662	2659	3068	104	2765	2924	3333

cb2996_c4/f1p0/2559	gnl|BL_ORD_ID|39036 cb2996_c1/f1p0/3750 isoform=cb2996_c1;full_length_coverage=1;non_full_length_coverage=0;isoform_length=3750;expected_accuracy=0.996	1106	2505	1	1107	2353	3750	51	1155
cb8258_c9/f1p0/2131 isoform=cb8258_c9;full_length_coverage=1;non_full_length_coverage=0;isoform_length=2131;expected_accuracy=0.991	gnl|BL_ORD_ID|7647 cb8258_c13/f1p0/2409 isoform=cb8258_c13;full_length_coverage=1;non_full_length_coverage=0;isoform_length=2409;expected_accuracy=0.999	1	1687	1685	2131	161	1858	1960	2408
cb8564_c46484/f1p0/3151	gnl|BL_ORD_ID|55195 cb8564_c35170/f1p0/3386	423	3086	5	422	738	3399	103	497
cb8564_c31961/f6p0/2803 isoform=cb8564_c31961;full_length_coverage=6;non_full_length_coverage=0;isoform_length=2803;expected_accuracy=1.000	gnl|BL_ORD_ID|18405 cb8564_c81089/f1p1/2976 isoform=cb8564_c81089;full_length_coverage=1;non_full_length_coverage=1;isoform_length=2976;expected_accuracy=0.991	102	1525	1526	2803	1	1427	1670	2946
cb8564_c178226/f1p0/2728 isoform=cb8564_c178226;full_length_coverage=1;non_full_length_coverage=0;isoform_length=2728;expected_accuracy=0.998	gnl|BL_ORD_ID|55574 cb8564_c45699/f1p0/3005	1284	2728	1	1288	1404	2846	1	1300
cb2869_c15/f1p1/3426 isoform=cb2869_c15;full_length_coverage=1;non_full_length_coverage=1;isoform_length=3426;expected_accuracy=0.994	gnl|BL_ORD_ID|70052 cb2869_c11/f1p0/3673	1	2078	2077	3382	25	2092	2371	3673
cb373_c29/f1p0/4336	gnl|BL_ORD_ID|40118 cb373_c48/f1p0/5064 isoform=cb373_c48;full_length_coverage=1;non_full_length_coverage=0;isoform_length=5064;expected_accuracy=0.992	16	3532	3532	3924	418	3919	4057	4448

cb8564_c150564/f1p2/1934	gnl|BL_ORD_ID|16400 cb8564_c4928/f2p0/2999 isoform=cb8564_c4928;full_length_coverage=2;non_full_length_coverage=0;isoform_length=2999;expected_accuracy=0.997	259	1944	78	259	1314	2999	1	182
cb12714_c29/f1p2/1583	gnl|BL_ORD_ID|13363 cb8564_c20575/f1p0/3783 isoform=cb8564_c20575;full_length_coverage=1;non_full_length_coverage=0;isoform_length=3783;expected_accuracy=0.997	1	1295	1293	1590	1	1295	3486	3783
cb8564_c16669/f1p0/2894	gnl|BL_ORD_ID|11871 cb8564_c14157/f1p1/4319 isoform=cb8564_c14157;full_length_coverage=1;non_full_length_coverage=1;isoform_length=4319;expected_accuracy=0.996	1	1723	1721	2706	782	2504	3317	4304
cb11941_c14/f1p0/1335	gnl|BL_ORD_ID|24140 cb11941_c6/f2p1/1361 isoform=cb11941_c6;full_length_coverage=2;non_full_length_coverage=1;isoform_length=1361;expected_accuracy=0.999	172	1207	24	173	326	1361	10	159
cb5131_c4/f1p0/1785 isoform=cb5131_c4;full_length_coverage=1;non_full_length_coverage=0;isoform_length=1785;expected_accuracy=0.993	gnl|BL_ORD_ID|44116 cb5131_c0/f1p0/3173 isoform=cb5131_c0;full_length_coverage=1;non_full_length_coverage=0;isoform_length=3173;expected_accuracy=0.995	1	1072	1070	1785	580	1644	2450	3172
cb8564_c90171/f1p0/2007 isoform=cb8564_c90171;full_length_coverage=1;non_full_length_coverage=0;isoform_length=2007;expected_accuracy=1.000	gnl|BL_ORD_ID|17044 cb8564_c77203/f2p0/2820 isoform=cb8564_c77203;full_length_coverage=2;non_full_length_coverage=0;isoform_length=2820;expected_accuracy=0.996	1	1632	1627	2007	1	1631	2233	2613
cb322_c9/f2p0/3136 isoform=cb322_c9;full_length_coverage=2;non_full_len	gnl|BL_ORD_ID|40333 cb322_c18/f1p0/4294 isoform=cb322_c18;full_length_coverage=1;non_full_le	1	2718	2718	3136	21	2749	3239	3658

gth_coverage=0;isoform_length=3136;expected_accuracy=1.000	ngth_coverage=0;isoform_length=4294;expected_accuracy=0.994								
cb8564_c124137/f1p0/2382	gnl|BL_ORD_ID|52866 cb8564_c110370/f1p0/3044	299	2372	4	300	828	2903	9	307
cb6028_c5/f1p0/2368 isoform=cb6028_c5;full_length_coverage=1;non_full_length_coverage=0;isoform_length=2368;expected_accuracy=0.994	gnl|BL_ORD_ID|45124 cb6028_c3/f1p0/2608 isoform=cb6028_c3;full_length_coverage=1;non_full_length_coverage=0;isoform_length=2608;expected_accuracy=0.994	1	1616	1616	2339	1	1617	1887	2608
cb8564_c1403/f1p0/2069	gnl|BL_ORD_ID|11763 cb8564_c1402/f1p0/2306 isoform=cb8564_c1402;full_length_coverage=1;non_full_length_coverage=0;isoform_length=2306;expected_accuracy=0.997	1	1734	1733	2066	56	1799	1949	2284
cb8564_c69018/f2p1/2753 isoform=cb8564_c69018;full_length_coverage=2;non_full_length_coverage=1;isoform_length=2753;expected_accuracy=0.991	gnl|BL_ORD_ID|16392 cb8564_c76010/f1p0/3693 isoform=cb8564_c76010;full_length_coverage=1;non_full_length_coverage=0;isoform_length=3693;expected_accuracy=0.993	489	2753	81	490	1422	3691	1	410
cb12_c30/f1p2/4443 isoform=cb12_c30;full_length_coverage=1;non_full_length_coverage=2;isoform_length=4443;expected_accuracy=0.992	gnl|BL_ORD_ID|25321 cb12_c54/f1p0/8058 isoform=cb12_c54;full_length_coverage=1;non_full_length_coverage=0;isoform_length=8058;expected_accuracy=0.994	369	4371	1	373	4054	8058	1346	1723
cb9933_c3/f1p0/1921	gnl|BL_ORD_ID|20310 cb9933_c4/f1p0/1991 isoform=cb9933_c4;full_length_coverage=1;non_full_length_coverage=0;isoform_length=1991;expected_accuracy=1.000	138	1798	11	138	273	1933	1	122
cb5229_c12/f14p1/1035 isoform=cb5229_c12;full_length_coverage=14;non_full	gnl|BL_ORD_ID|44594 cb5229_c13/f1p1/2074 isoform=cb5229_c13;full_length_coverage=1;non_full_l	369	1035	8	371	1406	2073	1	366

_length_coverage=1;isoform_length=1035;expected_accuracy=1.000	ength_coverage=1;isoform_length=2074;expected_accuracy=0.999								
cb8564_c4961/f1p0/2082	gnl|BL_ORD_ID|53421 cb8564_c124689/f1p0/2316	212	2078	54	212	362	2228	17	174
cb8564_c145840/f9p2/3901 isoform=cb8564_c145840;full_length_coverage=9;non_full_length_coverage=2;isoform_length=3901;expected_accuracy=0.999	gnl|BL_ORD_ID|13983 cb8564_c40988/f2p0/4134 isoform=cb8564_c40988;full_length_coverage=2;non_full_length_coverage=0;isoform_length=4134;expected_accuracy=0.999	228	3884	15	229	477	4132	1	216
cb14323_c0/f7p0/1360 isoform=cb14323_c0;full_length_coverage=7;non_full_length_coverage=0;isoform_length=1360;expected_accuracy=1.000	gnl|BL_ORD_ID|63229 cb14323_c6/f1p0/1451	100	1357	1	101	198	1449	2	98
cb2707_c17/f1p1/3162 isoform=cb2707_c17;full_length_coverage=1;non_full_length_coverage=1;isoform_length=3162;expected_accuracy=0.995	gnl|BL_ORD_ID|36995 cb2707_c28/f1p1/3570 isoform=cb2707_c28;full_length_coverage=1;non_full_length_coverage=1;isoform_length=3570;expected_accuracy=0.996	11	2572	2575	3118	317	2879	3014	3559
cb2316_c8/f1p0/3795	gnl|BL_ORD_ID|69692 cb2316_c1/f1p0/3976	3	3314	3313	3599	1	3311	3698	3998
cb8564_c43779/f1p0/3021 isoform=cb8564_c43779;full_length_coverage=1;non_full_length_coverage=0;isoform_length=3021;expected_accuracy=0.993	gnl|BL_ORD_ID|14539 cb8564_c46562/f2p3/3196 isoform=cb8564_c46562;full_length_coverage=2;non_full_length_coverage=3;isoform_length=3196;expected_accuracy=0.995	1	2344	2345	3017	7	2369	2525	3195
cb8564_c45300/f1p4/3014 isoform=cb8564_c45300;full_length_coverage=1;non_full_length_coverage=4;isoform_length=3014;expected_accuracy=0.996	gnl|BL_ORD_ID|55522 cb8564_c44123/f1p1/3230	243	3014	16	246	398	3169	15	242

cb7189_c8/f1p0/2376	gnl|BL_ORD_ID|9014 cb7189_c16/f9p1/2273 isoform=cb7189_c16;full_length_coverage=9;non_full_length_coverage=1;isoform_length=2273;expected_accuracy=1.000	1	1304	1304	2063	316	1619	1514	2273
cb6904_c3/f4p5/1797 isoform=cb6904_c3;full_length_coverage=4;non_full_length_coverage=5;isoform_length=1797;expected_accuracy=1.000	gnl|BL_ORD_ID|4313 cb6904_c8/f1p1/2657 isoform=cb6904_c8;full_length_coverage=1;non_full_length_coverage=1;isoform_length=2657;expected_accuracy=0.999	395	1797	82	397	1242	2644	1	314
cb8564_c40107/f1p0/4108	gnl|BL_ORD_ID|14247 cb8564_c45575/f3p6/4033 isoform=cb8564_c45575;full_length_coverage=3;non_full_length_coverage=6;isoform_length=4033;expected_accuracy=0.998	1603	3955	1	1599	1558	3910	78	1678
cb8564_c21500/f1p0/4129 isoform=cb8564_c21500;full_length_coverage=1;non_full_length_coverage=0;isoform_length=4129;expected_accuracy=0.999	gnl|BL_ORD_ID|66126 cb18041_c3/f1p0/5098	332	4129	1	331	998	4800	468	800
cb9666_c3/f1p0/1777 isoform=cb9666_c3;full_length_coverage=1;non_full_length_coverage=0;isoform_length=1777;expected_accuracy=0.999	gnl|BL_ORD_ID|58842 cb9666_c1/f1p0/2225	1	1268	1268	1777	77	1356	1690	2201
cb7420_c0/f7p1/2477 isoform=cb7420_c0;full_length_coverage=7;non_full_length_coverage=1;isoform_length=2477;expected_accuracy=1.000	gnl|BL_ORD_ID|51217 cb7420_c9/f1p0/3428	18	1886	1884	2477	2	1865	2849	3442
cb1279_c42/f1p0/3192 isoform=cb1279_c42;full_length_coverage=1;non_full_	gnl|BL_ORD_ID|61184 cb1279_c48/f1p0/3561	1	2738	2735	3192	14	2751	3111	3561

length_coverage=0;isoform_length=3192;expected_accuracy=0.993									
cb8564_c41162/f1p0/3158	gnl|BL_ORD_ID|55600 cb8564_c46484/f1p0/3151	107	2553	2548	3042	5	2475	2606	3099
cb7315_c15/f1p0/1788 isoform=cb7315_c15;full_length_coverage=1;non_full_length_coverage=0;isoform_length=1788;expected_accuracy=0.992	gnl|BL_ORD_ID|50474 cb7315_c7/f1p0/2338	1	1670	1667	1772	42	1715	2200	2305
cb8564_c17122/f1p0/3317	gnl|BL_ORD_ID|12196 cb8564_c12654/f2p0/4122 isoform=cb8564_c12654;full_length_coverage=2;non_full_length_coverage=0;isoform_length=4122;expected_accuracy=1.000	12	1905	1905	3230	9	1901	2784	4109
cb6339_c16/f1p1/2286 isoform=cb6339_c16;full_length_coverage=1;non_full_length_coverage=1;isoform_length=2286;expected_accuracy=0.997	gnl|BL_ORD_ID|5632 cb6339_c91/f6p2/2480 isoform=cb6339_c91;full_length_coverage=6;non_full_length_coverage=2;isoform_length=2480;expected_accuracy=1.000	1	1780	1776	2180	93	1873	2077	2480
cb8564_c47011/f1p0/3100	gnl|BL_ORD_ID|16886 cb8564_c50727/f1p0/3201 isoform=cb8564_c50727;full_length_coverage=1;non_full_length_coverage=0;isoform_length=3201;expected_accuracy=0.999	2	2306	2301	2934	2	2323	2561	3194
cb8564_c118659/f2p0/3437 isoform=cb8564_c118659;full_length_coverage=2;non_full_length_coverage=0;isoform_length=3437;expected_accuracy=0.994	gnl|BL_ORD_ID|54608 cb8564_c127899/f1p0/3880	1	2754	2752	3269	297	3075	3305	3823
cb2737_c39/f1p0/2866	gnl|BL_ORD_ID|36735 cb2737_c13/f1p0/3105 isoform=cb2737_c13;full_length_coverage=1;non_full_l	1	1679	1677	2849	1	1676	1808	2979

	ength_coverage=0;isoform_length=3105;expected_accuracy=0.995								
cb8564_c15230/f1p0/1944	gnl|BL_ORD_ID|16528 cb8564_c54660/f1p21/2689 isoform=cb8564_c54660;full_length_coverage=1;non_full_length_coverage=21;isoform_length=2689;expected_accuracy=0.999	352	1944	38	357	1087	2679	50	377
cb502_c51/f1p1/2358	gnl|BL_ORD_ID|43896 cb502_c9/f2p3/2544 isoform=cb502_c9;full_length_coverage=2;non_full_length_coverage=3;isoform_length=2544;expected_accuracy=0.999	1053	2278	1	1055	1164	2387	7	1061
cb454_c8/f1p0/2277 isoform=cb454_c8;full_length_coverage=1;non_full_length_coverage=0;isoform_length=2277;expected_accuracy=1.000	gnl|BL_ORD_ID|72201 cb454_c19/f1p0/2558	277	2277	94	276	406	2406	118	300
cb2784_c26/f1p0/1619 isoform=cb2784_c26;full_length_coverage=1;non_full_length_coverage=0;isoform_length=1619;expected_accuracy=1.000	gnl|BL_ORD_ID|68844 cb2784_c9/f1p0/3117	595	1619	64	597	1781	2805	5	538
cb3818_c9/f1p0/2837	gnl|BL_ORD_ID|71534 cb3818_c3/f1p0/3470	1	1932	1932	2758	49	1981	2626	3452
cb6981_c20/f1p0/1700 isoform=cb6981_c20;full_length_coverage=1;non_full_length_coverage=0;isoform_length=1700;expected_accuracy=0.999	gnl|BL_ORD_ID|5610 cb6981_c22/f2p0/2125 isoform=cb6981_c22;full_length_coverage=2;non_full_length_coverage=0;isoform_length=2125;expected_accuracy=0.997	270	1700	8	274	531	1966	1	265
cb396_c19/f1p0/2179 isoform=cb396_c19;full_length_coverage=1;non_full_le	gnl|BL_ORD_ID|70865 cb396_c49/f1p0/3077	312	2179	53	312	523	2394	21	282

ngth_coverage=0;isoform_length=2179;expected_accuracy=0.992									
cb1445_c4/f1p0/4026	gnl|BL_ORD_ID|29673 cb1445_c1/f1p0/4325 isoform=cb1445_c1;full_length_coverage=1;non_full_length_coverage=0;isoform_length=4325;expected_accuracy=0.996	1	2730	2729	3592	31	2756	3461	4325
cb18456_c2267/f1p0/1551	gnl|BL_ORD_ID|74175 cb5625_c0/f1p0/3054	178	1563	29	179	1490	2853	2	152
cb8564_c45699/f1p0/3005	gnl|BL_ORD_ID|18852 cb8564_c84488/f1p0/3408 isoform=cb8564_c84488;full_length_coverage=1;non_full_length_coverage=0;isoform_length=3408;expected_accuracy=0.996	233	2844	1	233	790	3408	1	217
cb8564_c40598/f1p4/3119	gnl|BL_ORD_ID|55551 cb8564_c44964/f1p0/3508	1	2732	2729	3053	1	2727	2848	3173
cb5930_c6/f1p0/2076	gnl|BL_ORD_ID|45031 cb5930_c3/f1p0/2974 isoform=cb5930_c3;full_length_coverage=1;non_full_length_coverage=0;isoform_length=2974;expected_accuracy=1.000	159	2013	1	160	1120	2974	5	164
cb5608_c38/f1p0/2614 isoform=cb5608_c38;full_length_coverage=1;non_full_length_coverage=0;isoform_length=2614;expected_accuracy=0.992	gnl|BL_ORD_ID|46459 cb5608_c24/f1p1/2825 isoform=cb5608_c24;full_length_coverage=1;non_full_length_coverage=1;isoform_length=2825;expected_accuracy=0.998	786	2614	80	785	864	2702	52	755
cb8564_c37361/f1p0/3076	gnl|BL_ORD_ID|16886 cb8564_c50727/f1p0/3201 isoform=cb8564_c50727;full_length_coverage=1;non_full_length_coverage=0;isoform_length=3201;expected_accuracy=0.999	1	2481	2477	3070	3	2507	2608	3201

cb8564_c72974/f1p0/4031 isoform=cb8564_c72974;full_length_coverage=1;non_full_length_coverage=0;isoform_length=4031;expected_accuracy=0.996	gnl|BL_ORD_ID|56749 cb8564_c69992/f1p0/4505	409	3972	6	410	971	4542	132	527
cb16366_c0/f1p0/1316	gnl|BL_ORD_ID|43221 cb5153_c7/f1p0/3152 isoform=cb5153_c7;full_length_coverage=1;non_full_length_coverage=0;isoform_length=3152;expected_accuracy=0.993	298	1233	38	298	2210	3148	23	283
cb18456_c4991/f1p0/1426	gnl|BL_ORD_ID|67493 cb18456_c4264/f1p1/1767	252	1214	32	257	803	1765	22	247
cb2021_c6/f1p1/3759	gnl|BL_ORD_ID|34677 cb2021_c4/f1p2/4085 isoform=cb2021_c4;full_length_coverage=1;non_full_length_coverage=2;isoform_length=4085;expected_accuracy=0.996	244	3575	1	249	770	4084	34	277
cb8564_c182028/f1p0/2513 isoform=cb8564_c182028;full_length_coverage=1;non_full_length_coverage=0;isoform_length=2513;expected_accuracy=0.996	gnl|BL_ORD_ID|12770 cb8564_c160685/f1p8/2891 isoform=cb8564_c160685;full_length_coverage=1;non_full_length_coverage=8;isoform_length=2891;expected_accuracy=0.993	1	1971	1968	2513	1	1973	2117	2662
cb5197_c7/f1p0/2919	gnl|BL_ORD_ID|44661 cb5197_c4/f1p0/3085 isoform=cb5197_c4;full_length_coverage=1;non_full_length_coverage=0;isoform_length=3085;expected_accuracy=0.995	1	1528	1527	2910	7	1530	1678	3058
cb8564_c36056/f1p0/2652 isoform=cb8564_c36056;full_length_coverage=1;non_full_length_coverage=0;isoform_length=2652;expected_accuracy=0.999	gnl|BL_ORD_ID|55230 cb8564_c35854/f2p1/3003	248	2639	1	251	480	2870	88	336

cb8564_c39136/f1p0/3036 isoform=cb8564_c39136;full_length_coverage=1;non_full_length_coverage=0;isoform_length=3036;expected_accuracy=0.998	gnl|BL_ORD_ID|13575 cb8564_c20499/f3p0/3328 isoform=cb8564_c20499;full_length_coverage=3;non_full_length_coverage=0;isoform_length=3328;expected_accuracy=1.000	1	2089	2085	3036	1	2096	2223	3176
cb8564_c81778/f1p0/3178	gnl|BL_ORD_ID|11292 cb8564_c153063/f2p1/3553 isoform=cb8564_c153063;full_length_coverage=2;non_full_length_coverage=1;isoform_length=3553;expected_accuracy=0.998	2	2698	2694	2918	8	2700	3302	3526
cb8564_c135684/f4p2/3618 isoform=cb8564_c135684;full_length_coverage=4;non_full_length_coverage=2;isoform_length=3618;expected_accuracy=0.996	gnl|BL_ORD_ID|58077 cb8564_c88175/f1p0/4011	2	2741	2741	3509	1	2742	3254	4022
cb8564_c46939/f1p1/2926	gnl|BL_ORD_ID|15169 cb8564_c40218/f1p0/3146 isoform=cb8564_c40218;full_length_coverage=1;non_full_length_coverage=0;isoform_length=3146;expected_accuracy=0.993	1	2134	2130	2870	4	2130	2235	2968
cb8564_c38233/f1p0/2780	gnl|BL_ORD_ID|11414 cb8564_c147327/f4p0/3198 isoform=cb8564_c147327;full_length_coverage=4;non_full_length_coverage=0;isoform_length=3198;expected_accuracy=0.999	1	1857	1856	2785	289	2145	2270	3198
cb8564_c185920/f1p0/2777 isoform=cb8564_c185920;full_length_coverage=1;non_full_length_coverage=0;isoform_length=2777;expected_accuracy=0.996	gnl|BL_ORD_ID|13138 cb8564_c180315/f1p0/2916 isoform=cb8564_c180315;full_length_coverage=1;non_full_length_coverage=0;isoform_length=2916;expected_accuracy=0.997	174	2777	1	177	313	2916	17	194
cb8564_c16220/f12p0/3116 isoform=cb8564_c16220;full_length_coverage=12;non_	gnl|BL_ORD_ID|14723 cb8564_c38100/f3p0/3175 isoform=cb8564_c38100;full_length_coverage=3;non_fu	13	1764	1762	3034	1	1751	1903	3175

full_length_coverage=0;isoform_length=3116;expected_accuracy=0.996	ll_length_coverage=0;isoform_length=3175;expected_accuracy=0.997								
cb7865_c10/f1p0/2730 isoform=cb7865_c10;full_length_coverage=1;non_full_length_coverage=0;isoform_length=2730;expected_accuracy=0.996	gnl|BL_ORD_ID|8963 cb7865_c7/f1p0/3320 isoform=cb7865_c7;full_length_coverage=1;non_full_length_coverage=0;isoform_length=3320;expected_accuracy=0.996	1127	2730	1	1129	1716	3320	240	1368
cb1494_c8/f1p0/4224	gnl|BL_ORD_ID|66209 cb18199_c3/f1p0/5929	1	2993	2993	4189	1	2993	4740	5935
cb3180_c2/f1p0/3347 isoform=cb3180_c2;full_length_coverage=1;non_full_length_coverage=0;isoform_length=3347;expected_accuracy=0.997	gnl|BL_ORD_ID|38070 cb3180_c0/f1p0/3698 isoform=cb3180_c0;full_length_coverage=1;non_full_length_coverage=0;isoform_length=3698;expected_accuracy=0.996	13	2456	2452	3288	1	2441	2862	3698
cb5999_c8/f1p0/2429 isoform=cb5999_c8;full_length_coverage=1;non_full_length_coverage=0;isoform_length=2429;expected_accuracy=0.996	gnl|BL_ORD_ID|45208 cb5999_c3/f1p0/2890 isoform=cb5999_c3;full_length_coverage=1;non_full_length_coverage=0;isoform_length=2890;expected_accuracy=0.995	1203	2417	1	1207	1670	2890	27	1233
cb9446_c7/f1p0/1576 isoform=cb9446_c7;full_length_coverage=1;non_full_length_coverage=0;isoform_length=1576;expected_accuracy=0.994	gnl|BL_ORD_ID|59725 cb9446_c0/f4p1/1997	189	1576	21	194	357	1744	4	176
cb8564_c14743/f1p0/2123 isoform=cb8564_c14743;full_length_coverage=1;non_full_length_coverage=0;isoform_length=2123;expected_accuracy=1.000	gnl|BL_ORD_ID|12195 cb8564_c12302/f1p1/2294 isoform=cb8564_c12302;full_length_coverage=1;non_full_length_coverage=1;isoform_length=2294;expected_accuracy=0.992	1	1712	1710	2105	47	1758	1901	2294
cb8564_c39756/f1p1/3004	gnl|BL_ORD_ID|18852 cb8564_c84488/f1p0/3408 isoform=cb8564_c84488;full_length_coverage=1;non_fu	219	2847	1	219	790	3406	1	217

	ll_length_coverage=0;isoform_length=3408;expected_accuracy=0.996								
cb4260_c5/f1p1/2120	gnl|BL_ORD_ID|42573 cb4260_c1/f1p0/3391 isoform=cb4260_c1;full_length_coverage=1;non_full_length_coverage=0;isoform_length=3391;expected_accuracy=0.994	1	1332	1329	2119	25	1356	2569	3357
cb8564_c83310/f1p0/2140 isoform=cb8564_c83310;full_length_coverage=1;non_full_length_coverage=0;isoform_length=2140;expected_accuracy=0.993	gnl|BL_ORD_ID|13944 cb8564_c4652/f3p0/3053 isoform=cb8564_c4652;full_length_coverage=3;non_full_length_coverage=0;isoform_length=3053;expected_accuracy=0.996	1	1969	1968	2140	27	1996	2363	2535
cb8564_c45116/f1p0/2875 isoform=cb8564_c45116;full_length_coverage=1;non_full_length_coverage=0;isoform_length=2875;expected_accuracy=1.000	gnl|BL_ORD_ID|56118 cb8564_c70073/f1p0/3142	1	1544	1541	2875	67	1610	1744	3078
cb405_c8/f1p0/1921	gnl|BL_ORD_ID|41390 cb405_c4/f1p0/2728 isoform=cb405_c4;full_length_coverage=1;non_full_length_coverage=0;isoform_length=2728;expected_accuracy=0.998	1	1211	1210	1765	45	1255	2161	2718
cb9852_c11/f1p0/1916	gnl|BL_ORD_ID|19373 cb9852_c8/f1p0/2057 isoform=cb9852_c8;full_length_coverage=1;non_full_length_coverage=0;isoform_length=2057;expected_accuracy=0.993	575	1848	9	580	691	1965	1	570
cb8564_c19831/f2p0/3515 isoform=cb8564_c19831;full_length_coverage=2;non_full_length_coverage=0;isoform_length=3515;expected_accuracy=0.994	gnl|BL_ORD_ID|11871 cb8564_c14157/f1p1/4319 isoform=cb8564_c14157;full_length_coverage=1;non_full_length_coverage=1;isoform_length=4319;expected_accuracy=0.996	1	2517	2515	3515	1	2504	3317	4319

cb3087_c39/f1p0/3065	gnl|BL_ORD_ID|38387 cb3087_c5/f3p1/2943 isoform=cb3087_c5;full_length_coverage=3;non_full_length_coverage=1;isoform_length=2943;expected_accuracy=1.000	20	2059	2054	2496	1	2040	2449	2891
cb2737_c55/f1p1/2936 isoform=cb2737_c55;full_length_coverage=1;non_full_length_coverage=1;isoform_length=2936;expected_accuracy=1.000	gnl|BL_ORD_ID|38132 cb2737_c20/f1p0/3644 isoform=cb2737_c20;full_length_coverage=1;non_full_length_coverage=0;isoform_length=3644;expected_accuracy=0.998	1	1595	1593	2848	687	2278	2410	3644
cb8564_c44698/f1p5/2843 isoform=cb8564_c44698;full_length_coverage=1;non_full_length_coverage=5;isoform_length=2843;expected_accuracy=0.997	gnl|BL_ORD_ID|56961 cb8564_c78609/f1p0/3194	18	2516	2511	2834	45	2539	2660	2985
cb8564_c45596/f1p1/3430	gnl|BL_ORD_ID|53657 cb8564_c149859/f1p1/3614	2	2780	2775	3379	78	2856	2975	3579
cb8564_c37079/f1p0/2659	gnl|BL_ORD_ID|54752 cb8564_c167108/f1p0/2919	1	1322	1321	2423	105	1425	1810	2912
cb8564_c36978/f1p0/2472	gnl|BL_ORD_ID|14359 cb8564_c25630/f2p0/2567 isoform=cb8564_c25630;full_length_coverage=2;non_full_length_coverage=0;isoform_length=2567;expected_accuracy=1.000	1	1385	1381	2274	5	1388	1560	2454
cb8564_c82155/f1p2/3145 isoform=cb8564_c82155;full_length_coverage=1;non_full_length_coverage=2;isoform_length=3145;expected_accuracy=0.995	gnl|BL_ORD_ID|16789 cb8564_c69307/f2p2/3301 isoform=cb8564_c69307;full_length_coverage=2;non_full_length_coverage=2;isoform_length=3301;expected_accuracy=1.000	255	3139	1	254	413	3301	25	279
cb8564_c12805/f1p0/2580 isoform=cb8564_c12805;full_length_coverage=1;non_f	gnl|BL_ORD_ID|56712 cb8564_c53711/f1p0/3201	1	1695	1690	2580	32	1727	1969	2862

<table>
<tr><td></td><td></td><td></td><td></td><td></td><td></td><td></td><td></td><td></td><td></td></tr>
<tr><td>ull_length_coverage=0;isoform_length=2580;expected_accuracy=0.993</td><td></td><td></td><td></td><td></td><td></td><td></td><td></td><td></td><td></td></tr>
<tr><td>cb500_c17/f1p0/2323 isoform=cb500_c17;full_length_coverage=1;non_full_length_coverage=0;isoform_length=2323;expected_accuracy=0.997</td><td>gnl|BL_ORD_ID|72243 cb500_c40/f1p0/3270</td><td>1</td><td>1293</td><td>1288</td><td>2323</td><td>763</td><td>2054</td><td>2186</td><td>3221</td></tr>
<tr><td>cb416_c19/f1p0/2939</td><td>gnl|BL_ORD_ID|42634 cb416_c26/f1p0/3038 isoform=cb416_c26;full_length_coverage=1;non_full_length_coverage=0;isoform_length=3038;expected_accuracy=1.000</td><td>105</td><td>2931</td><td>10</td><td>109</td><td>203</td><td>3029</td><td>4</td><td>103</td></tr>
<tr><td>cb8564_c45300/f1p4/3014 isoform=cb8564_c45300;full_length_coverage=1;non_full_length_coverage=4;isoform_length=3014;expected_accuracy=0.996</td><td>gnl|BL_ORD_ID|57017 cb8564_c80370/f1p0/3493</td><td>243</td><td>3014</td><td>122</td><td>246</td><td>2805</td><td>31</td><td>3085</td><td>2962</td></tr>
<tr><td>cb3022_c6/f1p0/3495 isoform=cb3022_c6;full_length_coverage=1;non_full_length_coverage=0;isoform_length=3495;expected_accuracy=0.998</td><td>gnl|BL_ORD_ID|69057 cb3022_c5/f1p0/3748</td><td>1</td><td>2677</td><td>2673</td><td>3381</td><td>66</td><td>2743</td><td>3049</td><td>3757</td></tr>
<tr><td>cb6471_c23/f1p0/2688</td><td>gnl|BL_ORD_ID|4198 cb6471_c20/f1p0/3013 isoform=cb6471_c20;full_length_coverage=1;non_full_length_coverage=0;isoform_length=3013;expected_accuracy=0.992</td><td>1</td><td>1927</td><td>1928</td><td>2662</td><td>197</td><td>2114</td><td>2275</td><td>3012</td></tr>
<tr><td>cb17651_c0/f1p0/1850 isoform=cb17651_c0;full_length_coverage=1;non_full_length_coverage=0;isoform_length=1850;expected_accuracy=1.000</td><td>gnl|BL_ORD_ID|10159 cb8564_c111800/f1p0/2023 isoform=cb8564_c111800;full_length_coverage=1;non_full_length_coverage=0;isoform_length=2023;expected_accuracy=0.996</td><td>1</td><td>1017</td><td>1017</td><td>1846</td><td>62</td><td>1074</td><td>1194</td><td>2023</td></tr>
</table>

cb8564_c4894/f1p1/2708	gnl|BL_ORD_ID|15477 cb8564_c4890/f2p1/2906 isoform=cb8564_c4890;full_length_coverage=2;non_full_length_coverage=1;isoform_length=2906;expected_accuracy=1.000	1	2353	2352	2680	92	2445	2578	2906
cb8288_c9/f1p0/1125	gnl|BL_ORD_ID|9858 cb8288_c4/f1p0/2081 isoform=cb8288_c4;full_length_coverage=1;non_full_length_coverage=0;isoform_length=2081;expected_accuracy=0.997	165	1015	2	166	1206	2056	3	168
cb2068_c7/f1p1/2788 isoform=cb2068_c7;full_length_coverage=1;non_full_length_coverage=1;isoform_length=2788;expected_accuracy=0.994	gnl|BL_ORD_ID|68270 cb2068_c8/f1p0/3866	1	1500	1501	2782	53	1566	2530	3813
cb8564_c46104/f1p0/3297	gnl|BL_ORD_ID|16877 cb8564_c54048/f1p0/3516 isoform=cb8564_c54048;full_length_coverage=1;non_full_length_coverage=0;isoform_length=3516;expected_accuracy=0.997	1	2256	2256	3216	6	2283	2540	3516
cb8564_c25429/f1p0/2554	gnl|BL_ORD_ID|55231 cb8564_c36011/f1p0/3015	732	2465	9	737	899	2637	10	751
cb8564_c87256/f1p0/2285 isoform=cb8564_c87256;full_length_coverage=1;non_full_length_coverage=0;isoform_length=2285;expected_accuracy=0.992	gnl|BL_ORD_ID|16210 cb8564_c74150/f1p4/3524 isoform=cb8564_c74150;full_length_coverage=1;non_full_length_coverage=4;isoform_length=3524;expected_accuracy=0.998	1	1703	1700	2285	2459	754	620	28
cb8564_c1638/f1p0/2866	gnl|BL_ORD_ID|12808 cb8564_c1599/f1p0/3294 isoform=cb8564_c1599;full_length_coverage=1;non_full_length_coverage=0;isoform_length=3294;expected_accuracy=0.999	285	2855	90	288	547	3116	2	196

cb1574_c3/f1p1/3697	gnl|BL_ORD_ID|63749 cb1574_c1/f1p1/4246	1	2382	2380	3498	5	2386	2965	4082
cb12714_c29/f1p2/1583	gnl|BL_ORD_ID|55935 cb8564_c49443/f1p0/2984	1	1296	1293	1581	35	1330	2394	2681
cb69_c29/f1p0/5122	gnl|BL_ORD_ID|5904 cb69_c5/f1p0/4977 isoform=cb69_c5;full_length_coverage=1;non_full_length_coverage=0;isoform_length=4977;expected_accuracy=0.999	1	2349	2346	4610	101	2450	2630	4893
cb6221_c5/f3p1/2261 isoform=cb6221_c5;full_length_coverage=3;non_full_length_coverage=1;isoform_length=2261;expected_accuracy=0.999	gnl|BL_ORD_ID|4878 cb6221_c8/f1p3/4003 isoform=cb6221_c8;full_length_coverage=1;non_full_length_coverage=3;isoform_length=4003;expected_accuracy=0.995	256	2256	85	256	885	2887	1	174
cb12714_c29/f1p2/1583	gnl|BL_ORD_ID|52715 cb8564_c11997/f1p2/2670	1	1296	1293	1559	1	1296	2363	2629
cb3763_c17/f1p0/2834 isoform=cb3763_c17;full_length_coverage=1;non_full_length_coverage=0;isoform_length=2834;expected_accuracy=0.995	gnl|BL_ORD_ID|71497 cb3763_c45/f1p0/3130	1	1844	1844	2831	3	1852	2017	3004
cb2014_c39/f1p0/2720	gnl|BL_ORD_ID|35262 cb2014_c18/f1p0/3961 isoform=cb2014_c18;full_length_coverage=1;non_full_length_coverage=0;isoform_length=3961;expected_accuracy=0.991	525	2629	56	527	1866	3961	198	666
cb8564_c44063/f1p0/2503 isoform=cb8564_c44063;full_length_coverage=1;non_full_length_coverage=0;isoform_length=2503;expected_accuracy=0.999	gnl|BL_ORD_ID|54119 cb8564_c18913/f1p5/2705	397	2452	49	402	649	2704	150	504

cb3115_c3/f1p0/3418	gnl|BL_ORD_ID|39034 cb3115_c1/f1p0/3824 isoform=cb3115_c1;full_length_coverage=1;non_full_length_coverage=0;isoform_length=3824;expected_accuracy=0.996	1706	3371	1	1709	2159	3824	150	1853
cb8564_c22195/f4p0/2939 isoform=cb8564_c22195;full_length_coverage=4;non_full_length_coverage=0;isoform_length=2939;expected_accuracy=0.996	gnl|BL_ORD_ID|18906 cb8564_c90449/f1p3/3223 isoform=cb8564_c90449;full_length_coverage=1;non_full_length_coverage=3;isoform_length=3223;expected_accuracy=0.996	264	2914	1	264	573	3223	33	295
cb8564_c36689/f1p1/2368 isoform=cb8564_c36689;full_length_coverage=1;non_full_length_coverage=1;isoform_length=2368;expected_accuracy=0.991	gnl|BL_ORD_ID|16879 cb8564_c48606/f1p1/3308 isoform=cb8564_c48606;full_length_coverage=1;non_full_length_coverage=1;isoform_length=3308;expected_accuracy=0.998	1	1709	1706	2368	1	1701	2640	3304
cb8564_c54251/f1p0/2624	gnl|BL_ORD_ID|56693 cb8564_c52693/f2p0/3348	2	2034	2033	2630	3	2035	2676	3273
cb8564_c35645/f1p0/2804	gnl|BL_ORD_ID|57123 cb8564_c84015/f1p0/3207	150	2722	1	151	266	2838	3	153
cb7064_c3/f2p0/1272 isoform=cb7064_c3;full_length_coverage=2;non_full_length_coverage=0;isoform_length=1272;expected_accuracy=0.993	gnl|BL_ORD_ID|3805 cb7064_c24/f6p0/3053 isoform=cb7064_c24;full_length_coverage=6;non_full_length_coverage=0;isoform_length=3053;expected_accuracy=1.000	129	1272	25	129	1899	3052	72	177
cb2206_c4/f1p1/2033 isoform=cb2206_c4;full_length_coverage=1;non_full_length_coverage=1;isoform_length=2033;expected_accuracy=0.994	gnl|BL_ORD_ID|68325 cb2206_c5/f1p0/3375	273	2033	3	275	1600	3361	1	274
cb792_c8/f3p0/3700 isoform=cb792_c8;full_length_coverage=3;non_full_len	gnl|BL_ORD_ID|49470 cb792_c35/f1p1/3973	186	3699	1	187	384	3897	75	266

gth_coverage=0;isoform_length=3700;expected_accuracy=1.000									
cb8564_c16240/f1p3/2671 isoform=cb8564_c16240;full_length_coverage=1;non_full_length_coverage=3;isoform_length=2671;expected_accuracy=0.997	gnl|BL_ORD_ID|54138 cb8564_c19172/f1p1/3470	1	2075	2072	2623	46	2121	2658	3208
cb11999_c13/f1p1/1500 isoform=cb11999_c13;full_length_coverage=1;non_full_length_coverage=1;isoform_length=1500;expected_accuracy=0.993	gnl|BL_ORD_ID|14692 cb8564_c45214/f1p5/1849 isoform=cb8564_c45214;full_length_coverage=1;non_full_length_coverage=5;isoform_length=1849;expected_accuracy=0.992	278	1500	3	280	532	1763	27	306
cb8564_c46500/f1p1/2752	gnl|BL_ORD_ID|9350 cb8564_c11826/f1p0/2916 isoform=cb8564_c11826;full_length_coverage=1;non_full_length_coverage=0;isoform_length=2916;expected_accuracy=0.997	1292	2661	1	1293	1543	2915	3	1296
cb8564_c4558/f1p0/2790 isoform=cb8564_c4558;full_length_coverage=1;non_full_length_coverage=0;isoform_length=2790;expected_accuracy=0.993	gnl|BL_ORD_ID|14126 cb8564_c4556/f1p0/3011 isoform=cb8564_c4556;full_length_coverage=1;non_full_length_coverage=0;isoform_length=3011;expected_accuracy=0.992	1255	2790	1	1258	1416	2974	25	1302
cb5564_c6/f3p0/2246 isoform=cb5564_c6;full_length_coverage=3;non_full_length_coverage=0;isoform_length=2246;expected_accuracy=0.999	gnl|BL_ORD_ID|44673 cb5564_c2/f3p1/2892 isoform=cb5564_c2;full_length_coverage=3;non_full_length_coverage=1;isoform_length=2892;expected_accuracy=0.999	156	2246	1	159	793	2885	104	262
cb8564_c66877/f3p0/2938 isoform=cb8564_c66877;full_length_coverage=3;non_full_length_coverage=0;isoform_length=2938;expected_accuracy=0.996	gnl|BL_ORD_ID|11240 cb8564_c145553/f1p3/3270 isoform=cb8564_c145553;full_length_coverage=1;non_full_length_coverage=3;isoform_length=3270;expected_accuracy=0.996	562	2890	60	564	942	3270	1	505

cb8564_c181853/f1p0/2119 isoform=cb8564_c181853;full_length_coverage=1;non_full_length_coverage=0;isoform_length=2119;expected_accuracy=0.997	gnl|BL_ORD_ID|14529 cb8564_c44074/f1p1/2922 isoform=cb8564_c44074;full_length_coverage=1;non_full_length_coverage=1;isoform_length=2922;expected_accuracy=0.994	1	1339	1334	2119	1	1330	2146	2921
cb5900_c94/f1p0/2004 isoform=cb5900_c94;full_length_coverage=1;non_full_length_coverage=0;isoform_length=2004;expected_accuracy=1.000	gnl|BL_ORD_ID|74605 cb5900_c77/f1p0/3189	1	1222	1219	2004	670	1886	2357	3142
cb16973_c0/f1p1/1620 isoform=cb16973_c0;full_length_coverage=1;non_full_length_coverage=1;isoform_length=1620;expected_accuracy=0.992	gnl|BL_ORD_ID|16537 cb8564_c47909/f1p0/3196 isoform=cb8564_c47909;full_length_coverage=1;non_full_length_coverage=0;isoform_length=3196;expected_accuracy=0.996	166	1618	25	167	1746	3196	1361	1504
cb4016_c32/f1p0/1913	gnl|BL_ORD_ID|71724 cb4016_c31/f1p0/2568	191	1930	25	194	780	2519	181	352
cb14792_c2/f1p0/1864 isoform=cb14792_c2;full_length_coverage=1;non_full_length_coverage=0;isoform_length=1864;expected_accuracy=0.995	gnl|BL_ORD_ID|53171 cb8564_c118027/f1p0/2767	1	1524	1519	1850	167	1690	1946	2277
cb7550_c1/f2p1/1771 isoform=cb7550_c1;full_length_coverage=2;non_full_length_coverage=1;isoform_length=1771;expected_accuracy=0.998	gnl|BL_ORD_ID|50589 cb7550_c4/f1p1/2591	1	1035	1030	1771	90	1130	1729	2481
cb6169_c3/f1p0/1711	gnl|BL_ORD_ID|74997 cb6169_c0/f1p0/2898	1	1352	1350	1729	1	1353	2431	2809
cb16166_c4/f1p0/1739 isoform=cb16166_c4;full_length_coverage=1;non_full_	gnl|BL_ORD_ID|54528 cb8564_c125341/f1p0/3014	1	1297	1296	1739	240	1538	2549	2992

length_coverage=0;isoform_length=1739;expected_accuracy=0.995									
cb1656_c15/f1p1/3875	gnl|BL_ORD_ID|31007 cb1656_c7/f1p1/3974 isoform=cb1656_c7;full_length_coverage=1;non_full_length_coverage=1;isoform_length=3974;expected_accuracy=0.997	2	2978	2979	3840	1	2976	3114	3974
cb8564_c36731/f1p2/2761	gnl|BL_ORD_ID|55377 cb8564_c39822/f1p0/3306	1	1977	1972	2737	1	1976	2405	3169
cb8564_c118486/f1p0/2843 isoform=cb8564_c118486;full_length_coverage=1;non_full_length_coverage=0;isoform_length=2843;expected_accuracy=0.994	gnl|BL_ORD_ID|10969 cb8564_c115641/f1p0/4863 isoform=cb8564_c115641;full_length_coverage=1;non_full_length_coverage=0;isoform_length=4863;expected_accuracy=0.997	16	2311	2311	2843	1839	4136	4331	4863
cb8564_c42959/f1p0/3330	gnl|BL_ORD_ID|13527 cb8564_c21762/f3p4/3985 isoform=cb8564_c21762;full_length_coverage=3;non_full_length_coverage=4;isoform_length=3985;expected_accuracy=1.000	121	3198	1	125	829	3906	14	137
cb8564_c13598/f1p0/3018 isoform=cb8564_c13598;full_length_coverage=1;non_full_length_coverage=0;isoform_length=3018;expected_accuracy=0.997	gnl|BL_ORD_ID|13013 cb8564_c17114/f4p0/3443 isoform=cb8564_c17114;full_length_coverage=4;non_full_length_coverage=0;isoform_length=3443;expected_accuracy=1.000	175	3016	1	180	401	3243	2	181
cb16315_c3/f1p0/1559 isoform=cb16315_c3;full_length_coverage=1;non_full_length_coverage=0;isoform_length=1559;expected_accuracy=0.992	gnl|BL_ORD_ID|43942 cb4391_c2/f1p0/3515 isoform=cb4391_c2;full_length_coverage=1;non_full_length_coverage=0;isoform_length=3515;expected_accuracy=0.997	184	1559	3	187	2063	3429	45	227
cb8564_c77994/f1p2/2630 isoform=cb8564_c77994;full_length_coverage=1;non_f	gnl|BL_ORD_ID|56574 cb8564_c5209/f1p0/3174	1	1819	1815	2630	8	1827	1971	2787

ull_length_coverage=2;isoform_length=2630;expected_accuracy=0.998									
cb416_c58/f1p0/2505 isoform=cb416_c58;full_length_coverage=1;non_full_length_coverage=0;isoform_length=2505;expected_accuracy=0.992	gnl|BL_ORD_ID|72147 cb416_c21/f1p0/3162	286	2505	4	287	743	2943	312	592
cb2735_c7/f1p3/3636 isoform=cb2735_c7;full_length_coverage=1;non_full_length_coverage=3;isoform_length=3636;expected_accuracy=0.997	gnl|BL_ORD_ID|38523 cb2735_c8/f1p3/4461 isoform=cb2735_c8;full_length_coverage=1;non_full_length_coverage=3;isoform_length=4461;expected_accuracy=0.997	1	2976	2974	3539	1	2980	3889	4461
cb8564_c43401/f1p2/3025 isoform=cb8564_c43401;full_length_coverage=1;non_full_length_coverage=2;isoform_length=3025;expected_accuracy=0.995	gnl|BL_ORD_ID|55920 cb8564_c48949/f1p0/4357	1	2352	2353	3025	2	2293	2847	3507
cb8288_c8/f1p0/1153	gnl|BL_ORD_ID|9500 cb8288_c7/f1p0/1418 isoform=cb8288_c7;full_length_coverage=1;non_full_length_coverage=0;isoform_length=1418;expected_accuracy=0.992	170	1062	2	170	526	1418	9	177
cb6456_c1/f2p0/2477 isoform=cb6456_c1;full_length_coverage=2;non_full_length_coverage=0;isoform_length=2477;expected_accuracy=1.000	gnl|BL_ORD_ID|4478 cb6456_c0/f2p0/2553 isoform=cb6456_c0;full_length_coverage=2;non_full_length_coverage=0;isoform_length=2553;expected_accuracy=0.993	1	1963	1962	2454	1	1957	2060	2553
cb8564_c40109/f1p0/2886	gnl|BL_ORD_ID|14291 cb8564_c39261/f1p0/3083 isoform=cb8564_c39261;full_length_coverage=1;non_full_length_coverage=0;isoform_length=3083;expected_accuracy=0.994	1	1598	1596	2862	1	1597	1821	3083

cb8564_c3183/f2p1/2280 isoform=cb8564_c3183;full_length_coverage=2;non_full_length_coverage=1;isoform_length=2280;expected_accuracy=0.994	gnl|BL_ORD_ID|54863 cb8564_c3179/f1p0/3204	355	2280	8	355	1013	2942	1	344
cb6297_c12/f1p0/2314	gnl|BL_ORD_ID|4329 cb6297_c18/f1p0/5398 isoform=cb6297_c18;full_length_coverage=1;non_full_length_coverage=0;isoform_length=5398;expected_accuracy=0.997	423	2174	50	425	3646	5398	2	375
cb6412_c5/f1p0/2729	gnl|BL_ORD_ID|47500 cb6412_c4/f1p0/2858	21	2411	2407	2741	4	2394	2516	2850
cb5900_c142/f1p0/2588 isoform=cb5900_c142;full_length_coverage=1;non_full_length_coverage=0;isoform_length=2588;expected_accuracy=0.993	gnl|BL_ORD_ID|73465 cb5900_c127/f1p0/2926	1	2275	2274	2535	68	2343	2607	2878
cb9143_c30/f2p0/2817 isoform=cb9143_c30;full_length_coverage=2;non_full_length_coverage=0;isoform_length=2817;expected_accuracy=0.996	gnl|BL_ORD_ID|19564 cb9143_c31/f2p0/2946 isoform=cb9143_c31;full_length_coverage=2;non_full_length_coverage=0;isoform_length=2946;expected_accuracy=0.997	199	2779	1	203	331	2912	1	197
cb322_c35/f1p0/3437 isoform=cb322_c35;full_length_coverage=1;non_full_length_coverage=0;isoform_length=3437;expected_accuracy=0.997	gnl|BL_ORD_ID|69560 cb322_c25/f1p0/3845	114	3413	1	119	375	3661	6	123
cb2061_c13/f1p1/1591 isoform=cb2061_c13;full_length_coverage=1;non_full_length_coverage=1;isoform_length=1591;expected_accuracy=0.991	gnl|BL_ORD_ID|35375 cb2061_c7/f1p0/1923 isoform=cb2061_c7;full_length_coverage=1;non_full_length_coverage=0;isoform_length=1923;expected_accuracy=0.993	145	1591	1	144	428	1875	1	145

cb12_c30/f1p2/4443 isoform=cb12_c30;full_length_coverage=1;non_full_length_coverage=2;isoform_length=4443;expected_accuracy=0.992	gnl|BL_ORD_ID|61079 cb12_c11/f1p2/4713	373	4419	2	378	659	4706	2	383
cb8564_c40598/f1p4/3119	gnl|BL_ORD_ID|55521 cb8564_c44014/f1p0/3720	25	2732	2729	3058	40	2745	2866	3195
cb8564_c15348/f1p4/2577 isoform=cb8564_c15348;full_length_coverage=1;non_full_length_coverage=4;isoform_length=2577;expected_accuracy=0.997	gnl|BL_ORD_ID|56381 cb8564_c75626/f1p2/3199	2	2086	2084	2577	4	2116	2611	3104
cb1573_c41/f1p0/1724 isoform=cb1573_c41;full_length_coverage=1;non_full_length_coverage=0;isoform_length=1724;expected_accuracy=1.000	gnl|BL_ORD_ID|30371 cb1573_c7/f1p2/3841 isoform=cb1573_c7;full_length_coverage=1;non_full_length_coverage=2;isoform_length=3841;expected_accuracy=1.000	1	1319	1314	1661	33	1344	3500	3839
cb8564_c186243/f1p0/2948 isoform=cb8564_c186243;full_length_coverage=1;non_full_length_coverage=0;isoform_length=2948;expected_accuracy=0.995	gnl|BL_ORD_ID|12405 cb8564_c146025/f2p1/3347 isoform=cb8564_c146025;full_length_coverage=2;non_full_length_coverage=1;isoform_length=3347;expected_accuracy=0.996	1	1986	1981	2948	1	1986	2150	3118
cb6057_c7/f1p0/1762	gnl|BL_ORD_ID|45370 cb6057_c4/f1p0/1955 isoform=cb6057_c4;full_length_coverage=1;non_full_length_coverage=0;isoform_length=1955;expected_accuracy=0.993	1	1404	1404	1748	31	1432	1608	1952
cb3253_c4/f1p0/2515 isoform=cb3253_c4;full_length_coverage=1;non_full_length_coverage=0;isoform_length=2515;expected_accuracy=0.991	gnl|BL_ORD_ID|41324 cb3253_c1/f1p0/3675 isoform=cb3253_c1;full_length_coverage=1;non_full_length_coverage=0;isoform_length=3675;expected_accuracy=0.997	1183	2514	1	1187	2374	3675	5	1194

cb5408_c1/f1p0/2926 isoform=cb5408_c1;full_length_coverage=1;non_full_length_coverage=0;isoform_length=2926;expected_accuracy=0.993	gnl|BL_ORD_ID|73880 cb5408_c2/f1p0/3049	1	1825	1824	2852	28	1854	1957	2986
cb16925_c1/f1p0/1736 isoform=cb16925_c1;full_length_coverage=1;non_full_length_coverage=0;isoform_length=1736;expected_accuracy=0.999	gnl|BL_ORD_ID|12359 cb8564_c126768/f1p0/2147 isoform=cb8564_c126768;full_length_coverage=1;non_full_length_coverage=0;isoform_length=2147;expected_accuracy=0.993	1	1127	1124	1736	3	1130	1485	2095
cb322_c28/f1p0/3948	gnl|BL_ORD_ID|70802 cb322_c34/f1p0/3998	157	2868	2866	3815	1	2718	3056	4005
cb8564_c39136/f1p0/3036 isoform=cb8564_c39136;full_length_coverage=1;non_full_length_coverage=0;isoform_length=3036;expected_accuracy=0.998	gnl|BL_ORD_ID|12440 cb8564_c130435/f1p3/3226 isoform=cb8564_c130435;full_length_coverage=1;non_full_length_coverage=3;isoform_length=3226;expected_accuracy=0.994	1	2089	2085	3035	2	2117	2245	3210
cb4123_c104/f1p0/1147 isoform=cb4123_c104;full_length_coverage=1;non_full_length_coverage=0;isoform_length=1147;expected_accuracy=0.991	gnl|BL_ORD_ID|39737 cb4123_c69/f2p1/1387 isoform=cb4123_c69;full_length_coverage=2;non_full_length_coverage=1;isoform_length=1387;expected_accuracy=0.996	8	778	773	1124	1	773	986	1340
cb6824_c9/f1p0/2342	gnl|BL_ORD_ID|5935 cb6824_c7/f1p0/2433 isoform=cb6824_c7;full_length_coverage=1;non_full_length_coverage=0;isoform_length=2433;expected_accuracy=0.991	107	2307	1	111	243	2432	1	111
cb8564_c20384/f1p4/2475 isoform=cb8564_c20384;full_length_coverage=1;non_full_length_coverage=4;isoform_length=2475;expected_accuracy=0.999	gnl|BL_ORD_ID|52656 cb8564_c10132/f1p8/3379	1	1492	1493	2363	1	1492	835	1706

cb8564_c53418/f1p0/2602 isoform=cb8564_c53418;full_length_coverage=1;non_full_length_coverage=0;isoform_length=2602;expected_accuracy=0.991	gnl|BL_ORD_ID|55529 cb8564_c44286/f1p0/2747	1171	2548	1	1168	1278	2665	2	1169
cb8564_c45300/f1p4/3014 isoform=cb8564_c45300;full_length_coverage=1;non_full_length_coverage=4;isoform_length=3014;expected_accuracy=0.996	gnl|BL_ORD_ID|16886 cb8564_c50727/f1p0/3201 isoform=cb8564_c50727;full_length_coverage=1;non_full_length_coverage=0;isoform_length=3201;expected_accuracy=0.999	243	3014	1	246	402	3199	3	245
cb8564_c128883/f2p0/2192 isoform=cb8564_c128883;full_length_coverage=2;non_full_length_coverage=0;isoform_length=2192;expected_accuracy=0.999	gnl|BL_ORD_ID|17258 cb8564_c88121/f1p0/3326 isoform=cb8564_c88121;full_length_coverage=1;non_full_length_coverage=0;isoform_length=3326;expected_accuracy=0.998	1	1194	1193	2189	5	1200	2329	3323
cb2304_c1/f2p0/2952 isoform=cb2304_c1;full_length_coverage=2;non_full_length_coverage=0;isoform_length=2952;expected_accuracy=0.997	gnl|BL_ORD_ID|68425 cb2304_c10/f1p0/6051	99	2952	1	101	2726	5587	2019	2119
cb6742_c12/f1p0/2398	gnl|BL_ORD_ID|50019 cb6742_c11/f1p0/2554	1	1343	1342	2280	132	1474	1588	2531
cb1291_c9/f1p1/2168 isoform=cb1291_c9;full_length_coverage=1;non_full_length_coverage=1;isoform_length=2168;expected_accuracy=0.993	gnl|BL_ORD_ID|26065 cb1291_c7/f1p1/2985 isoform=cb1291_c7;full_length_coverage=1;non_full_length_coverage=1;isoform_length=2985;expected_accuracy=0.998	183	2168	1	182	817	2794	271	453
cb8564_c42814/f1p0/2151 isoform=cb8564_c42814;full_length_coverage=1;non_full_length_coverage=0;isoform_length=2151;expected_accuracy=0.993	gnl|BL_ORD_ID|13642 cb8564_c188095/f1p0/3177 isoform=cb8564_c188095;full_length_coverage=1;non_full_length_coverage=0;isoform_length=3177;expected_accuracy=0.998	650	2151	7	652	1653	3155	1	675

cb8564_c188095/f1p0/3177 isoform=cb8564_c188095;full_length_coverage=1;non_full_length_coverage=0;isoform_length=3177;expected_accuracy=0.998	gnl|BL_ORD_ID|14747 cb8564_c44688/f1p1/3602 isoform=cb8564_c44688;full_length_coverage=1;non_full_length_coverage=1;isoform_length=3602;expected_accuracy=0.999	1	2441	2437	3159	1	2444	2880	3602
cb7085_c11/f1p0/1882 isoform=cb7085_c11;full_length_coverage=1;non_full_length_coverage=0;isoform_length=1882;expected_accuracy=0.992	gnl|BL_ORD_ID|4154 cb7085_c8/f1p0/2701 isoform=cb7085_c8;full_length_coverage=1;non_full_length_coverage=0;isoform_length=2701;expected_accuracy=0.996	551	1882	70	553	1349	2677	5	483
cb5896_c10/f2p1/2427 isoform=cb5896_c10;full_length_coverage=2;non_full_length_coverage=1;isoform_length=2427;expected_accuracy=0.998	gnl|BL_ORD_ID|46732 cb5896_c60/f1p2/2803 isoform=cb5896_c60;full_length_coverage=1;non_full_length_coverage=2;isoform_length=2803;expected_accuracy=0.996	1	1304	1303	2427	4	1307	1432	2559
cb8564_c2802/f1p0/3927	gnl|BL_ORD_ID|16376 cb8564_c733/f6p0/4072 isoform=cb8564_c733;full_length_coverage=6;non_full_length_coverage=0;isoform_length=4072;expected_accuracy=1.000	1	2533	2533	3856	1	2535	2750	4072
cb8564_c14743/f1p0/2123 isoform=cb8564_c14743;full_length_coverage=1;non_full_length_coverage=0;isoform_length=2123;expected_accuracy=1.000	gnl|BL_ORD_ID|55196 cb8564_c35205/f1p1/2269	1	1712	1710	2123	2	1713	1850	2263
cb8564_c36689/f1p1/2368 isoform=cb8564_c36689;full_length_coverage=1;non_full_length_coverage=1;isoform_length=2368;expected_accuracy=0.991	gnl|BL_ORD_ID|15104 cb8564_c37866/f1p3/2874 isoform=cb8564_c37866;full_length_coverage=1;non_full_length_coverage=3;isoform_length=2874;expected_accuracy=0.990	1	1709	1706	2368	1	1691	2198	2863
cb8564_c2797/f1p0/2268 isoform=cb8564_c2797;full_length_coverage=1;non_ful	gnl|BL_ORD_ID|57211 cb8564_c86767/f1p0/2702	1	1374	1370	2191	72	1416	1670	2491

l_length_coverage=0;isoform_length=2268;expected_accuracy=0.992									
cb8564_c38219/f2p2/2476 isoform=cb8564_c38219;full_length_coverage=2;non_full_length_coverage=2;isoform_length=2476;expected_accuracy=0.996	gnl|BL_ORD_ID|55688 cb8564_c4747/f1p0/2838	1	1430	1428	2475	142	1559	1807	2853
cb8564_c40764/f1p0/2563 isoform=cb8564_c40764;full_length_coverage=1;non_full_length_coverage=0;isoform_length=2563;expected_accuracy=0.997	gnl|BL_ORD_ID|55839 cb8564_c43677/f1p0/3269	1	2167	2162	2563	55	2218	2425	2824
cb8564_c43399/f1p0/2654 isoform=cb8564_c43399;full_length_coverage=1;non_full_length_coverage=0;isoform_length=2654;expected_accuracy=0.999	gnl|BL_ORD_ID|57289 cb8564_c89122/f1p0/3335	22	2367	2363	2641	81	2421	2637	2915
cb2493_c40/f1p0/3431	gnl|BL_ORD_ID|37222 cb2493_c9/f3p4/3744 isoform=cb2493_c9;full_length_coverage=3;non_full_length_coverage=4;isoform_length=3744;expected_accuracy=0.997	485	3408	56	488	746	3689	5	435
cb912_c15/f1p0/2991 isoform=cb912_c15;full_length_coverage=1;non_full_length_coverage=0;isoform_length=2991;expected_accuracy=0.999	gnl|BL_ORD_ID|59088 cb912_c11/f1p0/3488	1	2472	2469	2991	269	2741	2878	3399
cb9448_c9/f1p1/1806	gnl|BL_ORD_ID|58724 cb9448_c6/f1p1/2077	459	1802	54	460	649	1980	32	438
cb16925_c1/f1p0/1736 isoform=cb16925_c1;full_length_coverage=1;non_full_	gnl|BL_ORD_ID|9596 cb8564_c122271/f1p0/2171 isoform=cb8564_c122271;full_length_coverage=1;non_f	1	1127	1124	1736	1	1128	1482	2092

length_coverage=0;isoform_length=1736;expected_accuracy=0.999	ull_length_coverage=0;isoform_length=2171;expected_accuracy=0.993								
cb8564_c76946/f4p0/2069 isoform=cb8564_c76946;full_length_coverage=4;non_full_length_coverage=0;isoform_length=2069;expected_accuracy=0.998	gnl|BL_ORD_ID|17274 cb8564_c82521/f1p0/2413 isoform=cb8564_c82521;full_length_coverage=1;non_full_length_coverage=0;isoform_length=2413;expected_accuracy=0.999	1	1324	1322	1976	4	1328	1759	2413
cb11915_c7/f1p2/1523 isoform=cb11915_c7;full_length_coverage=1;non_full_length_coverage=2;isoform_length=1523;expected_accuracy=0.992	gnl|BL_ORD_ID|60410 cb11915_c8/f1p3/1864	1	1133	1128	1523	1	1137	1446	1842
cb8564_c12355/f1p9/2685 isoform=cb8564_c12355;full_length_coverage=1;non_full_length_coverage=9;isoform_length=2685;expected_accuracy=0.999	gnl|BL_ORD_ID|9350 cb8564_c11826/f1p0/2916 isoform=cb8564_c11826;full_length_coverage=1;non_full_length_coverage=0;isoform_length=2916;expected_accuracy=0.997	1312	2685	103	1313	1543	2916	86	1296
cb8564_c48551/f1p0/2454 isoform=cb8564_c48551;full_length_coverage=1;non_full_length_coverage=0;isoform_length=2454;expected_accuracy=0.995	gnl|BL_ORD_ID|16169 cb8564_c66877/f3p0/2938 isoform=cb8564_c66877;full_length_coverage=3;non_full_length_coverage=0;isoform_length=2938;expected_accuracy=0.996	1	1438	1438	2454	60	1496	1766	2783
cb8113_c1/f1p1/2789 isoform=cb8113_c1;full_length_coverage=1;non_full_length_coverage=1;isoform_length=2789;expected_accuracy=0.998	gnl|BL_ORD_ID|8749 cb8113_c3/f1p1/3914 isoform=cb8113_c3;full_length_coverage=1;non_full_length_coverage=1;isoform_length=3914;expected_accuracy=1.000	1	1899	1898	2789	21	1915	2994	3886
cb8564_c38947/f1p0/2650 isoform=cb8564_c38947;full_length_coverage=1;non_full_length_coverage=0;isoform_length=2650;expected_accuracy=0.996	gnl|BL_ORD_ID|13273 cb8564_c188193/f1p0/2849 isoform=cb8564_c188193;full_length_coverage=1;non_full_length_coverage=0;isoform_length=2849;expected_accuracy=0.997	1	1441	1436	2650	1	1439	1635	2849

cb8564_c41162/f1p0/3158	gnl|BL_ORD_ID|16681 cb8564_c54233/f1p1/3194 isoform=cb8564_c54233;full_length_coverage=1;non_full_length_coverage=1;isoform_length=3194;expected_accuracy=0.995	2	2553	2548	3036	7	2575	2706	3194
cb8564_c37498/f1p0/3279 isoform=cb8564_c37498;full_length_coverage=1;non_full_length_coverage=0;isoform_length=3279;expected_accuracy=0.999	gnl|BL_ORD_ID|15552 cb8564_c53792/f1p0/4512 isoform=cb8564_c53792;full_length_coverage=1;non_full_length_coverage=0;isoform_length=4512;expected_accuracy=0.994	169	3279	1	170	1412	4512	61	231
cb8564_c69457/f3p14/2821 isoform=cb8564_c69457;full_length_coverage=3;non_full_length_coverage=14;isoform_length=2821;expected_accuracy=0.997	gnl|BL_ORD_ID|13176 cb8564_c15957/f1p0/2838 isoform=cb8564_c15957;full_length_coverage=1;non_full_length_coverage=0;isoform_length=2838;expected_accuracy=0.994	1	1693	1692	2745	1	1684	1792	2838
cb1138_c11/f1p2/3819 isoform=cb1138_c11;full_length_coverage=1;non_full_length_coverage=2;isoform_length=3819;expected_accuracy=0.997	gnl|BL_ORD_ID|59175 cb1138_c19/f1p0/4197	1	1920	1920	3819	47	1967	2251	4153
cb5643_c46/f1p0/3297 isoform=cb5643_c46;full_length_coverage=1;non_full_length_coverage=0;isoform_length=3297;expected_accuracy=0.996	gnl|BL_ORD_ID|45701 cb5643_c45/f1p0/3996 isoform=cb5643_c45;full_length_coverage=1;non_full_length_coverage=0;isoform_length=3996;expected_accuracy=0.999	1	2201	2199	3297	141	2347	2765	3871
cb17137_c7/f1p1/1834	gnl|BL_ORD_ID|11611 cb8564_c146555/f4p2/2300 isoform=cb8564_c146555;full_length_coverage=4;non_full_length_coverage=2;isoform_length=2300;expected_accuracy=0.996	1	1291	1287	1753	97	1387	1758	2224
cb10054_c49/f1p1/1123 isoform=cb10054_c49;full_length_coverage=1;non_full	gnl|BL_ORD_ID|47994 cb10054_c53/f1p2/1445	258	1122	6	262	445	1309	1	264

_length_coverage=1;isoform_length=1123;expected_accuracy=1.000									
cb150_c10/f1p0/1858	gnl|BL_ORD_ID|29110 cb150_c6/f1p0/4821 isoform=cb150_c6;full_length_coverage=1;non_full_length_coverage=0;isoform_length=4821;expected_accuracy=0.997	1	1172	1171	1851	1	1172	4145	4820
cb8564_c34921/f1p4/3083 isoform=cb8564_c34921;full_length_coverage=1;non_full_length_coverage=4;isoform_length=3083;expected_accuracy=0.994	gnl|BL_ORD_ID|14411 cb8564_c44258/f1p0/3202 isoform=cb8564_c44258;full_length_coverage=1;non_full_length_coverage=0;isoform_length=3202;expected_accuracy=0.993	406	3073	8	401	509	3202	5	403
cb8564_c35645/f1p0/2804	gnl|BL_ORD_ID|16331 cb8564_c75969/f1p1/2798 isoform=cb8564_c75969;full_length_coverage=1;non_full_length_coverage=1;isoform_length=2798;expected_accuracy=0.997	150	2677	15	151	250	2798	1	137
cb8564_c35037/f1p0/3377 isoform=cb8564_c35037;full_length_coverage=1;non_full_length_coverage=0;isoform_length=3377;expected_accuracy=0.996	gnl|BL_ORD_ID|14662 cb8564_c42236/f1p0/4544 isoform=cb8564_c42236;full_length_coverage=1;non_full_length_coverage=0;isoform_length=4544;expected_accuracy=0.995	1	2664	2664	3365	6	2670	3838	4542
cb2314_c5/f1p0/3736	gnl|BL_ORD_ID|37916 cb2314_c2/f1p0/3840 isoform=cb2314_c2;full_length_coverage=1;non_full_length_coverage=0;isoform_length=3840;expected_accuracy=0.995	1	3224	3224	3709	1	3220	3335	3819
cb4066_c13/f1p0/2044	gnl|BL_ORD_ID|40793 cb4066_c15/f1p0/3532 isoform=cb4066_c15;full_length_coverage=1;non_full_length_coverage=0;isoform_length=3532;expected_accuracy=0.992	351	2028	43	352	1827	3502	1	306

cb8564_c43399/f1p0/2654 isoform=cb8564_c43399;full_length_coverage=1;non_full_length_coverage=0;isoform_length=2654;expected_accuracy=0.999	gnl|BL_ORD_ID|55574 cb8564_c45699/f1p0/3005	1	2367	2363	2654	1	2344	2560	2849
cb8564_c53418/f1p0/2602 isoform=cb8564_c53418;full_length_coverage=1;non_full_length_coverage=0;isoform_length=2602;expected_accuracy=0.991	gnl|BL_ORD_ID|17747 cb8564_c81734/f1p1/2771 isoform=cb8564_c81734;full_length_coverage=1;non_full_length_coverage=1;isoform_length=2771;expected_accuracy=0.995	1163	2540	1	1168	1391	2771	124	1290
cb1665_c3/f1p0/3650	gnl|BL_ORD_ID|63840 cb1665_c0/f1p0/4001	1	2893	2893	3608	129	3038	3166	3875
cb1333_c101/f1p0/1395 isoform=cb1333_c101;full_length_coverage=1;non_full_length_coverage=0;isoform_length=1395;expected_accuracy=0.997	gnl|BL_ORD_ID|61260 cb1333_c110/f1p0/5352	149	1395	27	150	3730	4974	29	152
cb8564_c109800/f6p1/3270 isoform=cb8564_c109800;full_length_coverage=6;non_full_length_coverage=1;isoform_length=3270;expected_accuracy=0.998	gnl|BL_ORD_ID|12106 cb8564_c146659/f3p0/3534 isoform=cb8564_c146659;full_length_coverage=3;non_full_length_coverage=0;isoform_length=3534;expected_accuracy=0.998	1	2029	2027	3235	63	2095	2324	3534
cb2954_c4/f1p0/3606	gnl|BL_ORD_ID|36660 cb2954_c1/f2p0/3661 isoform=cb2954_c1;full_length_coverage=2;non_full_length_coverage=0;isoform_length=3661;expected_accuracy=0.998	2	2969	2969	3515	20	2985	3090	3635
cb8564_c38035/f1p0/2770	gnl|BL_ORD_ID|15153 cb8564_c36883/f1p0/3072 isoform=cb8564_c36883;full_length_coverage=1;non_full_length_coverage=0;isoform_length=3072;expected_accuracy=0.996	1	2035	2033	2756	1	2038	2228	2952

cb6345_c2/f1p1/2613 isoform=cb6345_c2;full_length_coverage=1;non_full_length_coverage=1;isoform_length=2613;expected_accuracy=0.996	gnl|BL_ORD_ID|5243 cb6345_c0/f2p1/2864 isoform=cb6345_c0;full_length_coverage=2;non_full_length_coverage=1;isoform_length=2864;expected_accuracy=1.000	197	2605	1	197	477	2864	4	200
cb309_c9/f1p2/4191 isoform=cb309_c9;full_length_coverage=1;non_full_length_coverage=2;isoform_length=4191;expected_accuracy=0.998	gnl|BL_ORD_ID|69546 cb309_c5/f1p0/4669	1	3777	3776	4178	206	3975	4184	4585
cb1545_c26/f1p0/3338	gnl|BL_ORD_ID|61383 cb1545_c23/f1p0/3408	1	2013	2014	3189	125	2136	2020	3195
cb12931_c15/f1p0/1493 isoform=cb12931_c15;full_length_coverage=1;non_full_length_coverage=0;isoform_length=1493;expected_accuracy=1.000	gnl|BL_ORD_ID|55414 cb8564_c41002/f1p3/2869	1	1106	1104	1493	6	1107	2426	2813
cb1821_c14/f1p0/2950 isoform=cb1821_c14;full_length_coverage=1;non_full_length_coverage=0;isoform_length=2950;expected_accuracy=0.995	gnl|BL_ORD_ID|63997 cb1821_c16/f1p1/3514	1242	2950	1	1245	1452	3162	74	1322
cb8564_c76764/f1p1/4492	gnl|BL_ORD_ID|56122 cb8564_c70128/f1p0/4834	1	3710	3708	4519	122	3831	3983	4794
cb14110_c13/f14p2/1499 isoform=cb14110_c13;full_length_coverage=14;non_full_length_coverage=2;isoform_length=1499;expected_accuracy=1.000	gnl|BL_ORD_ID|26766 cb14110_c9/f1p0/1874 isoform=cb14110_c9;full_length_coverage=1;non_full_length_coverage=0;isoform_length=1874;expected_accuracy=0.999	1	1024	1022	1498	20	1053	1408	1874
cb8564_c89391/f1p0/3380	gnl|BL_ORD_ID|19115 cb8564_c88760/f1p6/3136 isoform=cb8564_c88760;full_length_coverage=1;non_fu	13	2834	2835	3107	1	2822	2681	2953

	ll_length_coverage=6;isoform_length=3136;expected_accuracy=0.996								
cb8564_c38035/f1p0/2770	gnl|BL_ORD_ID|14556 cb8564_c45150/f1p0/2940 isoform=cb8564_c45150;full_length_coverage=1;non_full_length_coverage=0;isoform_length=2940;expected_accuracy=0.992	1	2035	2033	2756	1	2022	2211	2932
cb51_c26/f1p0/2695	gnl|BL_ORD_ID|46998 cb51_c0/f6p0/2663 isoform=cb51_c0;full_length_coverage=6;non_full_length_coverage=0;isoform_length=2663;expected_accuracy=1.000	1	2090	2090	2432	32	2121	2309	2651
cb8564_c82568/f7p1/2364 isoform=cb8564_c82568;full_length_coverage=7;non_full_length_coverage=1;isoform_length=2364;expected_accuracy=1.000	gnl|BL_ORD_ID|52928 cb8564_c112134/f1p0/2518	479	2364	77	478	565	2450	2	403
cb8564_c81580/f1p0/2450 isoform=cb8564_c81580;full_length_coverage=1;non_full_length_coverage=0;isoform_length=2450;expected_accuracy=0.997	gnl|BL_ORD_ID|11848 cb8564_c146276/f2p0/2672 isoform=cb8564_c146276;full_length_coverage=2;non_full_length_coverage=0;isoform_length=2672;expected_accuracy=0.995	1	1786	1781	2450	42	1818	1957	2625
cb653_c12/f1p0/3754 isoform=cb653_c12;full_length_coverage=1;non_full_length_coverage=0;isoform_length=3754;expected_accuracy=0.995	gnl|BL_ORD_ID|5913 cb653_c14/f1p0/4097 isoform=cb653_c14;full_length_coverage=1;non_full_length_coverage=0;isoform_length=4097;expected_accuracy=0.996	195	3754	1	196	496	4052	76	272
cb16334_c1/f1p0/1310	gnl|BL_ORD_ID|9807 cb8564_c116631/f1p0/2583 isoform=cb8564_c116631;full_length_coverage=1;non_full_length_coverage=0;isoform_length=2583;expected_accuracy=0.999	9	774	775	1327	14	788	2027	2580

cb8564_c121421/f1p1/2345 isoform=cb8564_c121421;full_length_coverage=1;non_full_length_coverage=1;isoform_length=2345;expected_accuracy=0.998	gnl|BL_ORD_ID|52928 cb8564_c112134/f1p0/2518	489	2345	87	488	565	2396	2	403
cb7940_c54/f1p1/1719 isoform=cb7940_c54;full_length_coverage=1;non_full_length_coverage=1;isoform_length=1719;expected_accuracy=0.997	gnl|BL_ORD_ID|51641 cb7940_c15/f1p0/2221	1	1366	1363	1719	73	1438	1634	1991
cb12714_c29/f1p2/1583	gnl|BL_ORD_ID|9350 cb8564_c11826/f1p0/2916 isoform=cb8564_c11826;full_length_coverage=1;non_full_length_coverage=0;isoform_length=2916;expected_accuracy=0.997	1	1295	1293	1597	4	1296	2612	2916
cb3490_c19/f1p0/2989	gnl|BL_ORD_ID|39292 cb3490_c28/f9p1/2949 isoform=cb3490_c28;full_length_coverage=9;non_full_length_coverage=1;isoform_length=2949;expected_accuracy=1.000	483	2827	7	486	591	2935	7	487
cb8564_c17543/f1p10/2689 isoform=cb8564_c17543;full_length_coverage=1;non_full_length_coverage=10;isoform_length=2689;expected_accuracy=0.999	gnl|BL_ORD_ID|9350 cb8564_c11826/f1p0/2916 isoform=cb8564_c11826;full_length_coverage=1;non_full_length_coverage=0;isoform_length=2916;expected_accuracy=0.997	1288	2586	1	1289	1543	2840	1	1296
cb8564_c40764/f1p0/2563 isoform=cb8564_c40764;full_length_coverage=1;non_full_length_coverage=0;isoform_length=2563;expected_accuracy=0.997	gnl|BL_ORD_ID|55341 cb8564_c38927/f1p0/3326	1	2166	2162	2563	7	2169	2377	2777
cb8564_c86938/f8p6/3219 isoform=cb8564_c86938;full_length_coverage=8;non_f	gnl|BL_ORD_ID|58119 cb8564_c89388/f1p0/3860	1	2858	2861	3128	5	2866	3436	3695

ull_length_coverage=6;isoform_length=3219;expected_accuracy=0.994										
cb6798_c16/f1p0/2203	gnl|BL_ORD_ID|50075 cb6798_c12/f1p4/2573	577	2130	8	582	916	2468	105	679
cb4664_c16/f1p0/2007	gnl|BL_ORD_ID|42516 cb4664_c6/f1p0/3256 isoform=cb4664_c6;full_length_coverage=1;non_full_length_coverage=0;isoform_length=3256;expected_accuracy=0.998	280	2004	49	280	1523	3256	1	232
cb1974_c7/f1p1/3734	gnl|BL_ORD_ID|34705 cb1974_c6/f1p0/3999 isoform=cb1974_c6;full_length_coverage=1;non_full_length_coverage=0;isoform_length=3999;expected_accuracy=0.991	1785	3677	1	1786	2021	3906	1	1783
cb8564_c48192/f1p6/3029	gnl|BL_ORD_ID|16879 cb8564_c48606/f1p1/3308 isoform=cb8564_c48606;full_length_coverage=1;non_full_length_coverage=1;isoform_length=3308;expected_accuracy=0.998	1	2155	2151	2871	1	2151	2587	3308
cb8564_c38464/f1p0/2918	gnl|BL_ORD_ID|9350 cb8564_c11826/f1p0/2916 isoform=cb8564_c11826;full_length_coverage=1;non_full_length_coverage=0;isoform_length=2916;expected_accuracy=0.997	1	1295	1294	2596	4	1296	1543	2845
cb3421_c0/f5p1/2836 isoform=cb3421_c0;full_length_coverage=5;non_full_length_coverage=1;isoform_length=2836;expected_accuracy=0.998	gnl|BL_ORD_ID|71191 cb3421_c13/f1p0/4144	319	2825	33	319	1645	4152	64	347
cb8564_c87721/f1p0/2168 isoform=cb8564_c87721;full_length_coverage=1;non_f	gnl|BL_ORD_ID|56177 cb8564_c71533/f1p0/3179	14	1811	1809	2168	1	1804	2451	2808

ull_length_coverage=0;isoform_length=2168;expected_accuracy=0.997									
cb8564_c107573/f11p0/3286 isoform=cb8564_c107573;full_length_coverage=11;non_full_length_coverage=0;isoform_length=3286;expected_accuracy=1.000	gnl|BL_ORD_ID|16806 cb8564_c76074/f1p0/3355 isoform=cb8564_c76074;full_length_coverage=1;non_full_length_coverage=0;isoform_length=3355;expected_accuracy=0.998	1525	3286	141	1526	1594	3355	1	1383
cb8564_c12030/f1p0/2476 isoform=cb8564_c12030;full_length_coverage=1;non_full_length_coverage=0;isoform_length=2476;expected_accuracy=0.996	gnl|BL_ORD_ID|54022 cb8564_c17079/f1p0/3440	1015	2476	1	1016	1801	3260	14	1004
cb8564_c71065/f1p0/2706 isoform=cb8564_c71065;full_length_coverage=1;non_full_length_coverage=0;isoform_length=2706;expected_accuracy=0.995	gnl|BL_ORD_ID|17583 cb8564_c86974/f1p0/2846 isoform=cb8564_c86974;full_length_coverage=1;non_full_length_coverage=0;isoform_length=2846;expected_accuracy=0.995	153	2705	1	155	293	2846	5	159
cb10403_c23/f1p1/1720 isoform=cb10403_c23;full_length_coverage=1;non_full_length_coverage=1;isoform_length=1720;expected_accuracy=0.997	gnl|BL_ORD_ID|48488 cb10403_c27/f1p1/1806	1	1363	1362	1718	218	1579	1445	1801
cb2700_c16/f1p0/2346 isoform=cb2700_c16;full_length_coverage=1;non_full_length_coverage=0;isoform_length=2346;expected_accuracy=0.999	gnl|BL_ORD_ID|68736 cb2700_c13/f1p0/2774	294	2346	9	296	440	2473	4	293
cb10921_c6/f1p0/1495	gnl|BL_ORD_ID|1666 cb10921_c1/f1p0/2459 isoform=cb10921_c1;full_length_coverage=1;non_full_length_coverage=0;isoform_length=2459;expected_accuracy=0.991	532	1417	6	533	1572	2459	22	552

cb8564_c24781/f1p0/2847	gnl|BL_ORD_ID|13098 cb8564_c160798/f1p0/3317 isoform=cb8564_c160798;full_length_coverage=1;non_full_length_coverage=0;isoform_length=3317;expected_accuracy=0.999	13	2521	2518	2833	1	2525	2986	3302
cb3838_c6/f1p0/3274 isoform=cb3838_c6;full_length_coverage=1;non_full_length_coverage=0;isoform_length=3274;expected_accuracy=0.994	gnl|BL_ORD_ID|70647 cb3838_c5/f1p0/4926	1026	3153	1	1026	1818	3947	3	1026
cb8564_c80694/f1p1/4265	gnl|BL_ORD_ID|3345 cb10480_c36/f1p0/5122 isoform=cb10480_c36;full_length_coverage=1;non_full_length_coverage=0;isoform_length=5122;expected_accuracy=0.997	158	3065	3063	4156	1	2903	4031	5122
cb3087_c5/f3p1/2943 isoform=cb3087_c5;full_length_coverage=3;non_full_length_coverage=1;isoform_length=2943;expected_accuracy=1.000	gnl|BL_ORD_ID|37719 cb3087_c29/f1p1/3127 isoform=cb3087_c29;full_length_coverage=1;non_full_length_coverage=1;isoform_length=3127;expected_accuracy=0.993	1	2327	2323	2943	22	2348	2486	3106
cb8564_c66920/f3p3/2894	gnl|BL_ORD_ID|16705 cb8564_c53880/f1p0/3484 isoform=cb8564_c53880;full_length_coverage=1;non_full_length_coverage=0;isoform_length=3484;expected_accuracy=0.996	1056	2863	1	1057	1672	3477	1	1056
cb8564_c15653/f1p4/2475	gnl|BL_ORD_ID|55614 cb8564_c46839/f1p1/2758	1	2293	2289	2430	2	2295	2514	2653
cb1523_c12/f1p0/2619 isoform=cb1523_c12;full_length_coverage=1;non_full_length_coverage=0;isoform_length=2619;expected_accuracy=0.998	gnl|BL_ORD_ID|63706 cb1523_c8/f1p0/4424	1	1748	1746	2619	7	1749	3523	4395

cb6763_c0/f6p1/1948 isoform=cb6763_c0;full_length_coverage=6;non_full_length_coverage=1;isoform_length=1948;expected_accuracy=0.999	gnl|BL_ORD_ID|5331 cb6763_c3/f1p0/2719 isoform=cb6763_c3;full_length_coverage=1;non_full_length_coverage=0;isoform_length=2719;expected_accuracy=0.996	600	1928	64	604	1393	2719	2	542
cb8564_c75620/f1p0/2781 isoform=cb8564_c75620;full_length_coverage=1;non_full_length_coverage=0;isoform_length=2781;expected_accuracy=0.996	gnl|BL_ORD_ID|57373 cb8564_c91193/f1p0/3331	15	1769	1764	2781	58	1812	2008	3025
cb8564_c43779/f1p0/3021 isoform=cb8564_c43779;full_length_coverage=1;non_full_length_coverage=0;isoform_length=3021;expected_accuracy=0.993	gnl|BL_ORD_ID|55894 cb8564_c48160/f1p2/3308	2	2344	2345	3021	2	2364	2520	3195
cb3774_c34/f1p0/3501	gnl|BL_ORD_ID|39195 cb3774_c28/f1p0/4127 isoform=cb3774_c28;full_length_coverage=1;non_full_length_coverage=0;isoform_length=4127;expected_accuracy=0.993	1	2947	2944	3349	1	2940	3722	4127
cb4715_c2/f1p0/2552 isoform=cb4715_c2;full_length_coverage=1;non_full_length_coverage=0;isoform_length=2552;expected_accuracy=0.992	gnl|BL_ORD_ID|42541 cb4715_c0/f1p0/3277 isoform=cb4715_c0;full_length_coverage=1;non_full_length_coverage=0;isoform_length=3277;expected_accuracy=0.995	235	2518	13	238	974	3277	1	227
cb6582_c2/f2p0/2371 isoform=cb6582_c2;full_length_coverage=2;non_full_length_coverage=0;isoform_length=2371;expected_accuracy=0.996	gnl|BL_ORD_ID|4992 cb6582_c18/f1p0/3412 isoform=cb6582_c18;full_length_coverage=1;non_full_length_coverage=0;isoform_length=3412;expected_accuracy=0.992	473	2370	6	475	1454	3403	1	479
cb8564_c45300/f1p4/3014 isoform=cb8564_c45300;full_length_coverage=1;non_f	gnl|BL_ORD_ID|13575 cb8564_c20499/f3p0/3328 isoform=cb8564_c20499;full_length_coverage=3;non_fu	243	3014	1	246	400	3174	1	243

ull_length_coverage=4;isoform_length=3014;expected_accuracy=0.996	ll_length_coverage=0;isoform_length=3328;expected_accuracy=1.000								
cb18456_c1748/f1p0/1180	gnl|BL_ORD_ID|53550 cb8564_c128527/f1p1/2034	243	1188	9	248	976	31	1350	1114
cb8564_c12805/f1p0/2580 isoform=cb8564_c12805;full_length_coverage=1;non_full_length_coverage=0;isoform_length=2580;expected_accuracy=0.993	gnl|BL_ORD_ID|52842 cb8564_c15468/f1p0/3015	1	1695	1690	2533	36	1731	1973	2819
cb8564_c128295/f1p0/2590	gnl|BL_ORD_ID|12383 cb8564_c122520/f1p0/2546 isoform=cb8564_c122520;full_length_coverage=1;non_full_length_coverage=0;isoform_length=2546;expected_accuracy=0.998	1	1703	1705	2509	1	1700	1591	2394
cb2208_c33/f1p0/3174	gnl|BL_ORD_ID|69621 cb2208_c30/f1p0/3607	19	2195	2193	2861	1	2178	2412	3079
cb3406_c2/f2p0/1990 isoform=cb3406_c2;full_length_coverage=2;non_full_length_coverage=0;isoform_length=1990;expected_accuracy=1.000	gnl|BL_ORD_ID|40557 cb3406_c11/f1p0/2573 isoform=cb3406_c11;full_length_coverage=1;non_full_length_coverage=0;isoform_length=2573;expected_accuracy=0.992	177	1923	10	177	832	2573	1	167
cb12703_c1/f1p0/1108	gnl|BL_ORD_ID|22232 cb12703_c2/f1p0/1343 isoform=cb12703_c2;full_length_coverage=1;non_full_length_coverage=0;isoform_length=1343;expected_accuracy=0.997	9	828	826	1103	1	820	1066	1343
cb9170_c5/f1p0/1862 isoform=cb9170_c5;full_length_coverage=1;non_full_length_coverage=0;isoform_length=1862;expected_accuracy=1.000	gnl|BL_ORD_ID|20766 cb9170_c0/f3p0/1973 isoform=cb9170_c0;full_length_coverage=3;non_full_length_coverage=0;isoform_length=1973;expected_accuracy=1.000	246	1861	3	246	358	1973	1	245
cb8564_c40598/f1p4/3119	gnl|BL_ORD_ID|14539 cb8564_c46562/f2p3/3196	10	2732	2729	3049	16	275	2876	319

	isoform=cb8564_c46562;full_length_coverage=2;non_full_length_coverage=3;isoform_length=3196;expected_accuracy=0.995						5		5
cb470_c7/f1p0/2198 isoform=cb470_c7;full_length_coverage=1;non_full_length_coverage=0;isoform_length=2198;expected_accuracy=1.000	gnl|BL_ORD_ID|72212 cb470_c9/f1p0/2469	1	1456	1454	2162	70	1526	1753	2461
cb16300_c1/f1p1/1024	gnl|BL_ORD_ID|3148 cb10555_c9/f1p0/1886 isoform=cb10555_c9;full_length_coverage=1;non_full_length_coverage=0;isoform_length=1886;expected_accuracy=0.997	14	522	522	998	27	534	1410	1886
cb8564_c49348/f15p0/3022 isoform=cb8564_c49348;full_length_coverage=15;non_full_length_coverage=0;isoform_length=3022;expected_accuracy=0.996	gnl|BL_ORD_ID|45447 cb5340_c0/f1p0/3122 isoform=cb5340_c0;full_length_coverage=1;non_full_length_coverage=0;isoform_length=3122;expected_accuracy=0.996	1	2231	2230	2957	3099	869	754	27
cb18456_c1748/f1p0/1180	gnl|BL_ORD_ID|11429 cb8564_c1308/f1p7/2249 isoform=cb8564_c1308;full_length_coverage=1;non_full_length_coverage=7;isoform_length=2249;expected_accuracy=0.998	243	1188	6	248	1299	2243	920	1161
cb5108_c29/f1p0/1830 isoform=cb5108_c29;full_length_coverage=1;non_full_length_coverage=0;isoform_length=1830;expected_accuracy=0.999	gnl|BL_ORD_ID|43672 cb5108_c12/f1p0/2615 isoform=cb5108_c12;full_length_coverage=1;non_full_length_coverage=0;isoform_length=2615;expected_accuracy=0.997	1	1457	1456	1830	1	1482	2109	2482
cb8564_c90797/f1p0/2864	gnl|BL_ORD_ID|16351 cb8564_c70055/f4p2/2889 isoform=cb8564_c70055;full_length_coverage=4;non_full_length_coverage=2;isoform_length=2889;expected_ac	15	2307	2308	2657	82	2378	2505	2851

	curacy=1.000								
cb8564_c25287/f1p0/2199 isoform=cb8564_c25287;full_length_coverage=1;non_full_length_coverage=0;isoform_length=2199;expected_accuracy=0.993	gnl|BL_ORD_ID|16625 cb8564_c72796/f1p0/2508 isoform=cb8564_c72796;full_length_coverage=1;non_full_length_coverage=0;isoform_length=2508;expected_accuracy=0.997	169	2199	1	170	332	2366	30	199
cb5874_c30/f1p0/2210	gnl|BL_ORD_ID|45911 cb5874_c11/f2p1/2975 isoform=cb5874_c11;full_length_coverage=2;non_full_length_coverage=1;isoform_length=2975;expected_accuracy=1.000	145	2190	1	145	832	2877	490	634
cb8564_c4541/f3p0/2161 isoform=cb8564_c4541;full_length_coverage=3;non_full_length_coverage=0;isoform_length=2161;expected_accuracy=0.992	gnl|BL_ORD_ID|55032 cb8564_c4537/f1p0/2998	1	1286	1284	2106	48	1332	2198	3019
cb8564_c37866/f1p3/2874 isoform=cb8564_c37866;full_length_coverage=1;non_full_length_coverage=3;isoform_length=2874;expected_accuracy=0.990	gnl|BL_ORD_ID|14407 cb8564_c44036/f1p0/3258 isoform=cb8564_c44036;full_length_coverage=1;non_full_length_coverage=0;isoform_length=3258;expected_accuracy=0.992	1159	2863	1	1159	1575	3258	1	1147
cb8564_c40203/f1p0/3161	gnl|BL_ORD_ID|16775 cb8564_c57586/f4p1/3438 isoform=cb8564_c57586;full_length_coverage=4;non_full_length_coverage=1;isoform_length=3438;expected_accuracy=1.000	310	3144	1	312	604	3438	1	311
cb2482_c50/f1p0/1645	gnl|BL_ORD_ID|37654 cb2482_c40/f1p0/2979 isoform=cb2482_c40;full_length_coverage=1;non_full_length_coverage=0;isoform_length=2979;expected_accuracy=0.997	489	1649	75	494	1816	2976	1	420
cb8564_c82644/f1p0/3097	gnl|BL_ORD_ID|57755 cb8564_c79781/f1p0/5222	1	2465	2464	3001	2	246	4457	499

							6		3
cb8564_c14636/f1p0/2373	gnl|BL_ORD_ID|58187 cb8564_c91049/f1p0/3717	1	1719	1715	2405	408	2127	2942	3633
cb18301_c37/f1p0/1280	gnl|BL_ORD_ID|12295 cb8564_c123425/f1p0/2833 isoform=cb8564_c123425;full_length_coverage=1;non_full_length_coverage=0;isoform_length=2833;expected_accuracy=0.994	1	1020	1019	1282	63	1083	1204	1467
cb8564_c186356/f1p0/2744 isoform=cb8564_c186356;full_length_coverage=1;non_full_length_coverage=0;isoform_length=2744;expected_accuracy=0.998	gnl|BL_ORD_ID|15154 cb8564_c32509/f1p1/2880 isoform=cb8564_c32509;full_length_coverage=1;non_full_length_coverage=1;isoform_length=2880;expected_accuracy=0.991	11	1396	1393	2722	2	1410	1531	2877
cb8564_c15653/f1p4/2475	gnl|BL_ORD_ID|52809 cb8564_c14548/f1p0/3030	22	2291	2289	2435	44	2313	2511	2657
cb8564_c20840/f1p3/2713 isoform=cb8564_c20840;full_length_coverage=1;non_full_length_coverage=3;isoform_length=2713;expected_accuracy=0.999	gnl|BL_ORD_ID|14290 cb8564_c24078/f1p1/3014 isoform=cb8564_c24078;full_length_coverage=1;non_full_length_coverage=1;isoform_length=3014;expected_accuracy=0.993	315	2683	47	316	629	3014	75	346
cb8564_c84198/f1p0/2418	gnl|BL_ORD_ID|56786 cb8564_c71725/f1p0/3227	1	2008	2006	2428	63	2073	2190	2612
cb2033_c16/f1p0/2147 isoform=cb2033_c16;full_length_coverage=1;non_full_length_coverage=0;isoform_length=2147;expected_accuracy=0.994	gnl|BL_ORD_ID|34871 cb2033_c13/f1p0/2928 isoform=cb2033_c13;full_length_coverage=1;non_full_length_coverage=0;isoform_length=2928;expected_accuracy=0.994	1	1872	1868	2147	694	2541	2648	2924
cb7560_c35/f1p1/2004 isoform=cb7560_c35;full_length_coverage=1;non_full_length_coverage=1;isoform_length=2004;expected_accu	gnl|BL_ORD_ID|8570 cb7560_c40/f1p1/2225 isoform=cb7560_c40;full_length_coverage=1;non_full_length_coverage=1;isoform_length=2225;expected_accur	1	1446	1444	1969	2	1450	1698	2225

racy=0.994	acy=0.995								
cb9718_c43/f1p0/1655 isoform=cb9718_c43;full_length_coverage=1;non_full_length_coverage=0;isoform_length=1655;expected_accuracy=0.996	gnl|BL_ORD_ID|58858 cb9718_c26/f1p0/2005	288	1655	8	290	451	1818	69	351
cb9157_c9/f1p0/1707 isoform=cb9157_c9;full_length_coverage=1;non_full_length_coverage=0;isoform_length=1707;expected_accuracy=0.996	gnl|BL_ORD_ID|21708 cb9157_c5/f1p0/2086 isoform=cb9157_c5;full_length_coverage=1;non_full_length_coverage=0;isoform_length=2086;expected_accuracy=0.993	1	1330	1327	1707	184	1509	1651	2031
cb8564_c70577/f1p0/3111	gnl|BL_ORD_ID|18906 cb8564_c90449/f1p3/3223 isoform=cb8564_c90449;full_length_coverage=1;non_full_length_coverage=3;isoform_length=3223;expected_accuracy=0.996	262	2845	1	263	640	3223	36	296
cb8564_c114729/f1p1/2091 isoform=cb8564_c114729;full_length_coverage=1;non_full_length_coverage=1;isoform_length=2091;expected_accuracy=0.994	gnl|BL_ORD_ID|54773 cb8564_c2528/f1p0/3357	1	1609	1608	2067	32	1644	2766	3229
cb7263_c45/f1p0/1490 isoform=cb7263_c45;full_length_coverage=1;non_full_length_coverage=0;isoform_length=1490;expected_accuracy=0.999	gnl|BL_ORD_ID|8081 cb7263_c14/f3p0/2616 isoform=cb7263_c14;full_length_coverage=3;non_full_length_coverage=0;isoform_length=2616;expected_accuracy=1.000	1	1021	1020	1490	41	1061	2131	2601
cb4649_c2/f1p0/2183 isoform=cb4649_c2;full_length_coverage=1;non_full_length_coverage=0;isoform_length=2183;expected_accuracy=0.997	gnl|BL_ORD_ID|72809 cb4649_c0/f1p0/3217	108	2136	1	108	1155	3185	1	109
cb5999_c8/f1p0/2429	gnl|BL_ORD_ID|74748 cb5999_c6/f1p0/2868	1	1268	1266	2427	108	136	1697	285

isoform=cb5999_c8;full_length_coverage=1;non_full_length_coverage=0;isoform_length=2429;expected_accuracy=0.996							9		3
cb9096_c5/f1p0/2190 isoform=cb9096_c5;full_length_coverage=1;non_full_length_coverage=0;isoform_length=2190;expected_accuracy=0.995	gnl|BL_ORD_ID|20794 cb9096_c2/f1p0/3368 isoform=cb9096_c2;full_length_coverage=1;non_full_length_coverage=0;isoform_length=3368;expected_accuracy=0.996	482	2190	94	481	1659	3364	1	388
cb8564_c37361/f1p0/3076	gnl|BL_ORD_ID|55437 cb8564_c41690/f1p1/3409	1	2481	2477	3070	1	2486	2587	3180
cb8564_c36731/f1p2/2761	gnl|BL_ORD_ID|55910 cb8564_c48645/f1p1/3287	11	1977	1972	2673	17	1982	2411	3113
cb10088_c29/f1p0/1009 isoform=cb10088_c29;full_length_coverage=1;non_full_length_coverage=0;isoform_length=1009;expected_accuracy=1.000	gnl|BL_ORD_ID|25465 cb13559_c0/f2p2/1276 isoform=cb13559_c0;full_length_coverage=2;non_full_length_coverage=2;isoform_length=1276;expected_accuracy=0.997	442	1009	5	447	583	1151	1	444
cb6271_c9/f1p0/1555 isoform=cb6271_c9;full_length_coverage=1;non_full_length_coverage=0;isoform_length=1555;expected_accuracy=1.000	gnl|BL_ORD_ID|49574 cb6271_c5/f1p0/2238	1	1364	1359	1500	19	1383	2037	2179
cb13202_c19/f1p0/1573	gnl|BL_ORD_ID|25400 cb13202_c0/f5p1/1343 isoform=cb13202_c0;full_length_coverage=5;non_full_length_coverage=1;isoform_length=1343;expected_accuracy=0.999	344	1229	53	345	396	1281	1	293
cb14709_c16/f1p0/1225 isoform=cb14709_c16;full_length_coverage=1;non_full_length_coverage=0;isoform_length=1225;expected_acc	gnl|BL_ORD_ID|27948 cb14709_c7/f2p0/1443 isoform=cb14709_c7;full_length_coverage=2;non_full_length_coverage=0;isoform_length=1443;expected_accur	1	1023	1019	1225	19	1045	1235	1443

uracy=0.992	acy=0.999								
cb5091_c4/f1p0/3004	gnl|BL_ORD_ID|73253 cb5091_c2/f1p0/3066	203	2960	2	205	318	3074	1	204
cb8564_c44698/f1p5/2843 isoform=cb8564_c44698;full_length_coverage=1;non_full_length_coverage=5;isoform_length=2843;expected_accuracy=0.997	gnl|BL_ORD_ID|55317 cb8564_c38251/f1p0/3243	1	2516	2511	2829	1	2532	2653	2973
cb8564_c126213/f1p0/1904	gnl|BL_ORD_ID|52103 cb8564_c110975/f1p0/2133	1	1585	1586	1766	58	1642	1833	2013
cb6071_c14/f1p2/5103 isoform=cb6071_c14;full_length_coverage=1;non_full_length_coverage=2;isoform_length=5103;expected_accuracy=0.997	gnl|BL_ORD_ID|45342 cb6071_c10/f1p1/6435 isoform=cb6071_c10;full_length_coverage=1;non_full_length_coverage=1;isoform_length=6435;expected_accuracy=0.997	305	5048	108	304	1692	6435	1329	1525
cb7214_c11/f1p0/1870	gnl|BL_ORD_ID|8880 cb7214_c4/f1p0/2395 isoform=cb7214_c4;full_length_coverage=1;non_full_length_coverage=0;isoform_length=2395;expected_accuracy=0.999	174	1841	1	173	647	2314	28	200
cb2305_c7/f1p0/2918 isoform=cb2305_c7;full_length_coverage=1;non_full_length_coverage=0;isoform_length=2918;expected_accuracy=0.993	gnl|BL_ORD_ID|36688 cb2305_c9/f1p0/3279 isoform=cb2305_c9;full_length_coverage=1;non_full_length_coverage=0;isoform_length=3279;expected_accuracy=0.994	1351	2902	1	1352	1732	3279	198	1543
cb2737_c37/f1p0/2894	gnl|BL_ORD_ID|36735 cb2737_c13/f1p0/3105 isoform=cb2737_c13;full_length_coverage=1;non_full_length_coverage=0;isoform_length=3105;expected_accuracy=0.995	1	1495	1493	2791	186	1676	1808	3105
cb18230_c0/f1p0/1725	gnl|BL_ORD_ID|63865 cb1683_c28/f1p0/3914	547	1725	58	548	2769	394	1727	222

isoform=cb18230_c0;full_length_coverage=1;non_full_length_coverage=0;isoform_length=1725;expected_accuracy=0.992							9		0
cb8564_c1159/f1p1/2210 isoform=cb8564_c1159;full_length_coverage=1;non_full_length_coverage=1;isoform_length=2210;expected_accuracy=0.993	gnl|BL_ORD_ID|52627 cb8564_c1184/f1p0/2806	1004	2170	1	1005	1307	249 2	2	100 9
cb6318_c23/f1p1/2399 isoform=cb6318_c23;full_length_coverage=1;non_full_length_coverage=1;isoform_length=2399;expected_accuracy=0.991	gnl|BL_ORD_ID|4833 cb6318_c33/f7p2/2637 isoform=cb6318_c33;full_length_coverage=7;non_full_length_coverage=2;isoform_length=2637;expected_accuracy=1.000	1	1848	1844	2399	31	187 7	2022	257 6
cb3324_c11/f1p0/5586	gnl|BL_ORD_ID|71091 cb3324_c7/f1p2/6368	2212	5502	1	2214	3083	637 3	143	235 6
cb6471_c23/f1p0/2688	gnl|BL_ORD_ID|5494 cb6471_c7/f2p0/2927 isoform=cb6471_c7;full_length_coverage=2;non_full_length_coverage=0;isoform_length=2927;expected_accuracy=0.997	10	1927	1928	2663	1	191 2	2074	280 9
cb8564_c38947/f1p0/2650 isoform=cb8564_c38947;full_length_coverage=1;non_full_length_coverage=0;isoform_length=2650;expected_accuracy=0.996	gnl|BL_ORD_ID|13054 cb8564_c181182/f1p0/2830 isoform=cb8564_c181182;full_length_coverage=1;non_full_length_coverage=0;isoform_length=2830;expected_accuracy=0.998	1	1441	1436	2650	1	143 7	1617	283 0
cb8564_c74445/f1p1/2816 isoform=cb8564_c74445;full_length_coverage=1;non_full_length_coverage=1;isoform_length=2816;expected_accuracy=0.992	gnl|BL_ORD_ID|54719 cb8564_c159487/f1p6/3403	1332	2816	11	1334	1778	326 2	1	132 6
cb8564_c39136/f1p0/3036	gnl|BL_ORD_ID|55502 cb8564_c43538/f1p0/3563	1	2089	2085	3036	2	210	2228	318

isoform=cb8564_c39136;full_length_coverage=1;non_full_length_coverage=0;isoform_length=3036;expected_accuracy=0.998							1		0
cb4016_c19/f1p0/2644 isoform=cb4016_c19;full_length_coverage=1;non_full_length_coverage=0;isoform_length=2644;expected_accuracy=0.995	gnl|BL_ORD_ID|39535 cb4016_c27/f1p1/3204 isoform=cb4016_c27;full_length_coverage=1;non_full_length_coverage=1;isoform_length=3204;expected_accuracy=0.996	284	2644	5	283	792	3148	3	277
cb8564_c53806/f1p3/3974 isoform=cb8564_c53806;full_length_coverage=1;non_full_length_coverage=3;isoform_length=3974;expected_accuracy=0.990	gnl|BL_ORD_ID|16628 cb8564_c47737/f7p5/4351 isoform=cb8564_c47737;full_length_coverage=7;non_full_length_coverage=5;isoform_length=4351;expected_accuracy=0.998	172	3974	1	173	395	4179	15	178
cb2177_c32/f1p0/2567 isoform=cb2177_c32;full_length_coverage=1;non_full_length_coverage=0;isoform_length=2567;expected_accuracy=0.990	gnl|BL_ORD_ID|34831 cb2177_c34/f1p1/2802 isoform=cb2177_c34;full_length_coverage=1;non_full_length_coverage=1;isoform_length=2802;expected_accuracy=0.994	1	1568	1567	2546	16	1601	1776	2759
cb10563_c2/f1p0/1592	gnl|BL_ORD_ID|2785 cb10563_c1/f1p0/2871 isoform=cb10563_c1;full_length_coverage=1;non_full_length_coverage=0;isoform_length=2871;expected_accuracy=0.995	1	1018	1018	1597	1	1017	2291	2870
cb8564_c50697/f1p3/2172 isoform=cb8564_c50697;full_length_coverage=1;non_full_length_coverage=3;isoform_length=2172;expected_accuracy=0.993	gnl|BL_ORD_ID|58020 cb8564_c86752/f1p1/3346	106	2167	1	108	739	2800	15	122
cb6271_c9/f1p0/1555 isoform=cb6271_c9;full_length_coverage=1;non_full_length_coverage=0;isoform_length=1555;expected_accur	gnl|BL_ORD_ID|4495 cb6271_c7/f1p0/2266 isoform=cb6271_c7;full_length_coverage=1;non_full_length_coverage=0;isoform_length=2266;expected_accura	1	1362	1359	1555	64	1425	2082	2266

acy=1.000	cy=0.996								
cb4260_c2/f2p2/2188 isoform=cb4260_c2;full_length_coverage=2;non_full_length_coverage=2;isoform_length=2188;expected_accuracy=0.997	gnl|BL_ORD_ID|42573 cb4260_c1/f1p0/3391 isoform=cb4260_c1;full_length_coverage=1;non_full_length_coverage=0;isoform_length=3391;expected_accuracy=0.994	1	1295	1292	2115	63	1356	2569	3391
cb15918_c2/f3p0/1168 isoform=cb15918_c2;full_length_coverage=3;non_full_length_coverage=0;isoform_length=1168;expected_accuracy=1.000	gnl|BL_ORD_ID|64973 cb15918_c10/f1p0/1603	499	1164	53	504	1087	1751	7	458
cb1262_c30/f1p0/3820 isoform=cb1262_c30;full_length_coverage=1;non_full_length_coverage=0;isoform_length=3820;expected_accuracy=0.995	gnl|BL_ORD_ID|23602 cb1262_c27/f1p1/4094 isoform=cb1262_c27;full_length_coverage=1;non_full_length_coverage=1;isoform_length=4094;expected_accuracy=0.996	235	3820	20	236	529	4091	1	224
cb8564_c47011/f1p0/3100	gnl|BL_ORD_ID|58037 cb8564_c87188/f1p1/3426	23	2306	2301	2934	127	2403	2641	3274
cb8564_c92358/f1p0/3797 isoform=cb8564_c92358;full_length_coverage=1;non_full_length_coverage=0;isoform_length=3797;expected_accuracy=0.995	gnl|BL_ORD_ID|10455 cb8564_c107877/f2p1/3829 isoform=cb8564_c107877;full_length_coverage=2;non_full_length_coverage=1;isoform_length=3829;expected_accuracy=1.000	155	2855	2854	3797	1	2703	2868	3812
cb500_c17/f1p0/2323 isoform=cb500_c17;full_length_coverage=1;non_full_length_coverage=0;isoform_length=2323;expected_accuracy=0.997	gnl|BL_ORD_ID|72251 cb500_c68/f1p0/4152	1	1293	1288	2323	1577	2869	3001	4036
cb7085_c25/f9p1/1910 isoform=cb7085_c25;full_length_coverage=9;non_full_length_coverage=1;isoform_length=1910;expected_accu	gnl|BL_ORD_ID|4154 cb7085_c8/f1p0/2701 isoform=cb7085_c8;full_length_coverage=1;non_full_length_coverage=0;isoform_length=2701;expected_accura	564	1910	77	566	1349	2695	1	483

racy=1.000	cy=0.996								
cb3888_c8/f1p0/2800 isoform=cb3888_c8;full_length_coverage=1;non_full_length_coverage=0;isoform_length=2800;expected_accuracy=0.996	gnl|BL_ORD_ID|71602 cb3888_c2/f1p0/3488	243	2800	18	244	460	3019	1	228
cb4532_c7/f1p0/1761	gnl|BL_ORD_ID|43745 cb4532_c5/f1p1/2905 isoform=cb4532_c5;full_length_coverage=1;non_full_length_coverage=1;isoform_length=2905;expected_accuracy=0.996	260	1578	40	261	1581	2904	1	227
cb2737_c35/f1p0/2780 isoform=cb2737_c35;full_length_coverage=1;non_full_length_coverage=0;isoform_length=2780;expected_accuracy=0.993	gnl|BL_ORD_ID|36571 cb2737_c54/f1p0/3029 isoform=cb2737_c54;full_length_coverage=1;non_full_length_coverage=0;isoform_length=3029;expected_accuracy=0.995	126	1913	1914	2780	216	1998	2110	2975
cb8564_c186243/f1p0/2948 isoform=cb8564_c186243;full_length_coverage=1;non_full_length_coverage=0;isoform_length=2948;expected_accuracy=0.995	gnl|BL_ORD_ID|57332 cb8564_c90231/f1p1/3635	1	1986	1981	2948	1	1990	2154	3122
cb7007_c6/f1p0/2251 isoform=cb7007_c6;full_length_coverage=1;non_full_length_coverage=0;isoform_length=2251;expected_accuracy=0.997	gnl|BL_ORD_ID|47801 cb7007_c13/f1p0/2402	261	2251	94	264	399	2389	1	171
cb1875_c5/f1p1/3559 isoform=cb1875_c5;full_length_coverage=1;non_full_length_coverage=1;isoform_length=3559;expected_accuracy=0.996	gnl|BL_ORD_ID|68211 cb1875_c7/f1p0/4579	1	1781	1780	3457	120	1906	2751	4425
cb8564_c43399/f1p0/2654	gnl|BL_ORD_ID|55391 cb8564_c40278/f3p2/3083	1	2367	2363	2654	1	236	2580	287

isoform=cb8564_c43399;full_length_coverage=1;non_full_length_coverage=0;isoform_length=2654;expected_accuracy=0.999							4		1
cb8564_c46939/f1p1/2926	gnl|BL_ORD_ID|16622 cb8564_c50151/f1p0/3198 isoform=cb8564_c50151;full_length_coverage=1;non_full_length_coverage=0;isoform_length=3198;expected_accuracy=0.995	1	2134	2130	2870	6	2168	2273	3014
cb2177_c91/f1p0/3417	gnl|BL_ORD_ID|66793 cb2177_c69/f1p0/4458	2	2400	2397	3187	52	2444	3650	4441
cb8564_c17077/f2p1/4019 isoform=cb8564_c17077;full_length_coverage=2;non_full_length_coverage=1;isoform_length=4019;expected_accuracy=0.999	gnl|BL_ORD_ID|56594 cb8564_c47777/f1p0/4391	385	4019	179	386	476	4118	8	215
cb10989_c11/f1p0/3363 isoform=cb10989_c11;full_length_coverage=1;non_full_length_coverage=0;isoform_length=3363;expected_accuracy=0.995	gnl|BL_ORD_ID|1784 cb10989_c8/f1p0/3471 isoform=cb10989_c8;full_length_coverage=1;non_full_length_coverage=0;isoform_length=3471;expected_accuracy=0.998	1472	3305	1	1475	1638	3471	3	1480
cb5357_c1/f3p0/2536 isoform=cb5357_c1;full_length_coverage=3;non_full_length_coverage=0;isoform_length=2536;expected_accuracy=0.996	gnl|BL_ORD_ID|73816 cb5357_c2/f1p0/3122	158	2536	1	161	723	3094	25	190
cb8564_c47011/f1p0/3100	gnl|BL_ORD_ID|17429 cb8564_c85447/f1p0/3196 isoform=cb8564_c85447;full_length_coverage=1;non_full_length_coverage=0;isoform_length=3196;expected_accuracy=0.997	2	2306	2301	2934	2	2297	2557	3188
cb17_c17/f1p0/4756	gnl|BL_ORD_ID|33351 cb17_c14/f1p0/4869	363	4671	207	363	458	476	1	157

	isoform=cb17_c14;full_length_coverage=1;non_full_length_coverage=0;isoform_length=4869;expected_accuracy=0.997						4		
cb8564_c46939/f1p1/2926	gnl|BL_ORD_ID|17429 cb8564_c85447/f1p0/3196 isoform=cb8564_c85447;full_length_coverage=1;non_full_length_coverage=0;isoform_length=3196;expected_accuracy=0.997	1	2134	2130	2870	3	2143	2248	3010
cb8564_c14344/f1p0/2533 isoform=cb8564_c14344;full_length_coverage=1;non_full_length_coverage=0;isoform_length=2533;expected_accuracy=0.993	gnl|BL_ORD_ID|13439 cb8564_c22195/f4p0/2939 isoform=cb8564_c22195;full_length_coverage=4;non_full_length_coverage=0;isoform_length=2939;expected_accuracy=0.996	148	2533	1	151	537	2923	1	151
cb8564_c185920/f1p0/2777 isoform=cb8564_c185920;full_length_coverage=1;non_full_length_coverage=0;isoform_length=2777;expected_accuracy=0.996	gnl|BL_ORD_ID|15315 cb8564_c39014/f1p0/3019 isoform=cb8564_c39014;full_length_coverage=1;non_full_length_coverage=0;isoform_length=3019;expected_accuracy=0.992	176	2773	1	177	348	3013	14	185
cb8564_c19466/f3p0/4669 isoform=cb8564_c19466;full_length_coverage=3;non_full_length_coverage=0;isoform_length=4669;expected_accuracy=0.995	gnl|BL_ORD_ID|36034 cb18797_c1/f1p0/5240 isoform=cb18797_c1;full_length_coverage=1;non_full_length_coverage=0;isoform_length=5240;expected_accuracy=0.997	1	3819	3818	4669	1	3825	4045	4898
cb5643_c112/f1p0/1970 isoform=cb5643_c112;full_length_coverage=1;non_full_length_coverage=0;isoform_length=1970;expected_accuracy=0.992	gnl|BL_ORD_ID|74229 cb5643_c113/f1p0/2786	1	1433	1434	1970	137	1558	1954	2484
cb8564_c54233/f1p1/3194 isoform=cb8564_c54233;full_length_coverage=1;non_full_length_coverage=1;isoform_length=3194;expected_	gnl|BL_ORD_ID|55195 cb8564_c35170/f1p0/3386	527	3187	8	526	738	3399	2	497

<table>
<tr><td>accuracy=0.995</td><td></td><td></td><td></td><td></td><td></td><td></td><td></td><td></td><td></td></tr>
<tr><td>cb8564_c186356/f1p0/2744 isoform=cb8564_c186356;full_length_coverage=1;non_full_length_coverage=0;isoform_length=2744;expected_accuracy=0.998</td><td>gnl|BL_ORD_ID|17161 cb8564_c87466/f1p1/3006 isoform=cb8564_c87466;full_length_coverage=1;non_full_length_coverage=1;isoform_length=3006;expected_accuracy=0.994</td><td>1</td><td>1396</td><td>1393</td><td>2744</td><td>1</td><td>1409</td><td>1530</td><td>2897</td></tr>
<tr><td>cb4674_c10/f2p0/2688 isoform=cb4674_c10;full_length_coverage=2;non_full_length_coverage=0;isoform_length=2688;expected_accuracy=1.000</td><td>gnl|BL_ORD_ID|42124 cb4674_c68/f1p0/2827 isoform=cb4674_c68;full_length_coverage=1;non_full_length_coverage=0;isoform_length=2827;expected_accuracy=0.997</td><td>18</td><td>2047</td><td>2047</td><td>2688</td><td>1</td><td>2025</td><td>2178</td><td>2819</td></tr>
<tr><td>cb8564_c36689/f1p1/2368 isoform=cb8564_c36689;full_length_coverage=1;non_full_length_coverage=1;isoform_length=2368;expected_accuracy=0.991</td><td>gnl|BL_ORD_ID|15154 cb8564_c32509/f1p1/2880 isoform=cb8564_c32509;full_length_coverage=1;non_full_length_coverage=1;isoform_length=2880;expected_accuracy=0.991</td><td>11</td><td>1709</td><td>1706</td><td>2346</td><td>2</td><td>1720</td><td>2230</td><td>2877</td></tr>
<tr><td>cb4702_c18/f1p0/2684</td><td>gnl|BL_ORD_ID|43527 cb4702_c30/f6p2/2783 isoform=cb4702_c30;full_length_coverage=6;non_full_length_coverage=2;isoform_length=2783;expected_accuracy=0.999</td><td>14</td><td>2200</td><td>2195</td><td>2645</td><td>15</td><td>2201</td><td>2309</td><td>2760</td></tr>
<tr><td>cb653_c9/f1p0/4025 isoform=cb653_c9;full_length_coverage=1;non_full_length_coverage=0;isoform_length=4025;expected_accuracy=0.997</td><td>gnl|BL_ORD_ID|5131 cb653_c8/f1p1/4297 isoform=cb653_c8;full_length_coverage=1;non_full_length_coverage=1;isoform_length=4297;expected_accuracy=0.999</td><td>301</td><td>4020</td><td>1</td><td>301</td><td>580</td><td>4297</td><td>6</td><td>306</td></tr>
<tr><td>cb18456_c5612/f1p0/1308</td><td>gnl|BL_ORD_ID|67493 cb18456_c4264/f1p1/1767</td><td>253</td><td>1216</td><td>3</td><td>258</td><td>803</td><td>1765</td><td>4</td><td>247</td></tr>
<tr><td>cb7560_c35/f1p1/2004 isoform=cb7560_c35;full_length_coverage=1;non_full_length_coverage=1;isoform_length=2004;expected_accu</td><td>gnl|BL_ORD_ID|7381 cb7560_c15/f2p1/2269 isoform=cb7560_c15;full_length_coverage=2;non_full_length_coverage=1;isoform_length=2269;expected_accur</td><td>1</td><td>1446</td><td>1444</td><td>1996</td><td>1</td><td>1448</td><td>1695</td><td>2269</td></tr>
</table>

racy=0.994	acy=0.997								
cb8564_c75371/f1p0/1913 isoform=cb8564_c75371;full_length_coverage=1;non_full_length_coverage=0;isoform_length=1913;expected_accuracy=0.998	gnl|BL_ORD_ID|17076 cb8564_c81253/f1p0/4323 isoform=cb8564_c81253;full_length_coverage=1;non_full_length_coverage=0;isoform_length=4323;expected_accuracy=0.999	465	1913	9	464	2855	4305	1	457
cb3699_c5/f1p0/2467 isoform=cb3699_c5;full_length_coverage=1;non_full_length_coverage=0;isoform_length=2467;expected_accuracy=1.000	gnl|BL_ORD_ID|71454 cb3699_c13/f1p0/2720	1161	2467	1	1162	1418	2723	58	1196
cb10090_c7/f1p2/1608 isoform=cb10090_c7;full_length_coverage=1;non_full_length_coverage=2;isoform_length=1608;expected_accuracy=1.000	gnl|BL_ORD_ID|48060 cb10090_c3/f1p1/2009	1	1123	1120	1573	1	1130	1547	2000
cb5874_c27/f1p0/2672	gnl|BL_ORD_ID|74537 cb5874_c33/f1p0/2945	624	2572	7	623	902	2851	96	702
cb2927_c11/f1p2/2856 isoform=cb2927_c11;full_length_coverage=1;non_full_length_coverage=2;isoform_length=2856;expected_accuracy=0.997	gnl|BL_ORD_ID|38415 cb2927_c2/f2p0/3116 isoform=cb2927_c2;full_length_coverage=2;non_full_length_coverage=0;isoform_length=3116;expected_accuracy=0.995	1	1910	1909	2854	5	1900	2172	3116
cb2184_c0/f4p0/2237 isoform=cb2184_c0;full_length_coverage=4;non_full_length_coverage=0;isoform_length=2237;expected_accuracy=1.000	gnl|BL_ORD_ID|56154 cb8564_c70991/f1p13/4645	1114	2237	1	1116	1163	39	3429	2315
cb5304_c17/f1p1/2678 isoform=cb5304_c17;full_length_coverage=1;non_full_length_coverage=1;isoform_length=2678;expected_accu	gnl|BL_ORD_ID|73763 cb5304_c14/f1p0/2912	101	1907	1906	2678	6	1817	1932	2706

racy=0.998									
cb8564_c12600/f1p0/2645	gnl|BL_ORD_ID|11862 cb8564_c13783/f1p0/3760 isoform=cb8564_c13783;full_length_coverage=1;non_full_length_coverage=0;isoform_length=3760;expected_accuracy=0.999	254	2645	1	255	377	2765	21	274
cb8564_c111744/f1p1/2111 isoform=cb8564_c111744;full_length_coverage=1;non_full_length_coverage=1;isoform_length=2111;expected_accuracy=0.998	gnl|BL_ORD_ID|12500 cb8564_c146833/f3p3/2521 isoform=cb8564_c146833;full_length_coverage=3;non_full_length_coverage=3;isoform_length=2521;expected_accuracy=1.000	14	1639	1635	2111	1	1620	1793	2262
cb8564_c48235/f1p1/2252	gnl|BL_ORD_ID|15357 cb8564_c44284/f4p1/3133 isoform=cb8564_c44284;full_length_coverage=4;non_full_length_coverage=1;isoform_length=3133;expected_accuracy=1.000	1	1599	1596	2236	113	1711	2493	3133
cb850_c28/f1p0/3553 isoform=cb850_c28;full_length_coverage=1;non_full_length_coverage=0;isoform_length=3553;expected_accuracy=0.992	gnl|BL_ORD_ID|10568 cb850_c10/f2p0/3928 isoform=cb850_c10;full_length_coverage=2;non_full_length_coverage=0;isoform_length=3928;expected_accuracy=0.998	303	3550	1	307	684	3928	240	545
cb8564_c85953/f1p0/2986	gnl|BL_ORD_ID|16364 cb8564_c72362/f4p1/4130 isoform=cb8564_c72362;full_length_coverage=4;non_full_length_coverage=1;isoform_length=4130;expected_accuracy=0.998	175	2717	1	174	1570	4116	78	250
cb8564_c186967/f1p0/2513 isoform=cb8564_c186967;full_length_coverage=1;non_full_length_coverage=0;isoform_length=2513;expected_accuracy=0.998	gnl|BL_ORD_ID|56983 cb8564_c79327/f1p1/3074	1	1942	1938	2477	43	1984	2275	2815
cb2695_c3/f3p0/3385	gnl|BL_ORD_ID|37071 cb2695_c43/f1p0/3494	1378	3384	11	1383	1491	349	1	137

isoform=cb2695_c3;full_length_coverage=3;non_full_length_coverage=0;isoform_length=3385;expected_accuracy=0.999	isoform=cb2695_c43;full_length_coverage=1;non_full_length_coverage=0;isoform_length=3494;expected_accuracy=0.995						4		4
cb1613_c9/f1p0/2755 isoform=cb1613_c9;full_length_coverage=1;non_full_length_coverage=0;isoform_length=2755;expected_accuracy=0.999	gnl|BL_ORD_ID|28654 cb1613_c1/f1p0/4248 isoform=cb1613_c1;full_length_coverage=1;non_full_length_coverage=0;isoform_length=4248;expected_accuracy=0.996	1	2120	2118	2755	22	2142	3516	4152
cb8564_c81003/f1p0/4196 isoform=cb8564_c81003;full_length_coverage=1;non_full_length_coverage=0;isoform_length=4196;expected_accuracy=0.995	gnl|BL_ORD_ID|56733 cb8564_c69529/f1p0/4528	1	2263	2264	4192	2	2267	2556	4488
cb8564_c48153/f1p0/3083	gnl|BL_ORD_ID|18852 cb8564_c84488/f1p0/3408 isoform=cb8564_c84488;full_length_coverage=1;non_full_length_coverage=0;isoform_length=3408;expected_accuracy=0.996	255	2875	13	255	790	3408	1	217
cb3756_c5/f1p0/3416	gnl|BL_ORD_ID|70591 cb3756_c11/f1p1/4447	1586	3392	134	1589	2673	4479	1	1456
cb8564_c181853/f1p0/2119 isoform=cb8564_c181853;full_length_coverage=1;non_full_length_coverage=0;isoform_length=2119;expected_accuracy=0.997	gnl|BL_ORD_ID|55205 cb8564_c35377/f1p0/2958	1	1339	1334	2119	4	1345	2122	2910
cb18456_c1748/f1p0/1180	gnl|BL_ORD_ID|69245 cb18456_c1770/f1p0/1415	243	1188	3	248	385	1330	2	247
cb10946_c4/f1p0/1694 isoform=cb10946_c4;full_length_coverage=1;non_full_length_coverage=0;isoform_length=1694;expected_accu	gnl|BL_ORD_ID|1142 cb10946_c3/f1p0/3086 isoform=cb10946_c3;full_length_coverage=1;non_full_length_coverage=0;isoform_length=3086;expected_accur	355	1694	58	357	1704	3042	26	325

racy=1.000	acy=0.994								
cb4016_c34/f1p0/2560 isoform=cb4016_c34;full_length_coverage=1;non_full_length_coverage=0;isoform_length=2560;expected_accuracy=0.998	gnl|BL_ORD_ID|40529 cb4016_c2/f3p1/2863 isoform=cb4016_c2;full_length_coverage=3;non_full_length_coverage=1;isoform_length=2863;expected_accuracy=0.998	194	2560	12	195	491	2850	5	189
cb2097_c87/f1p2/2320 isoform=cb2097_c87;full_length_coverage=1;non_full_length_coverage=2;isoform_length=2320;expected_accuracy=0.992	gnl|BL_ORD_ID|36136 cb2097_c106/f1p0/2666 isoform=cb2097_c106;full_length_coverage=1;non_full_length_coverage=0;isoform_length=2666;expected_accuracy=1.000	549	2304	57	548	890	2654	140	628
cb8564_c51723/f1p2/3016	gnl|BL_ORD_ID|14773 cb8564_c31982/f1p3/3384 isoform=cb8564_c31982;full_length_coverage=1;non_full_length_coverage=3;isoform_length=3384;expected_accuracy=0.997	139	3033	1	141	491	3384	11	151
cb4889_c22/f1p0/2625 isoform=cb4889_c22;full_length_coverage=1;non_full_length_coverage=0;isoform_length=2625;expected_accuracy=0.995	gnl|BL_ORD_ID|73055 cb4889_c55/f1p2/2939	280	2622	7	285	483	2824	37	315
cb3339_c12/f13p1/3205 isoform=cb3339_c12;full_length_coverage=13;non_full_length_coverage=1;isoform_length=3205;expected_accuracy=1.000	gnl|BL_ORD_ID|40811 cb3339_c2/f1p1/3653 isoform=cb3339_c2;full_length_coverage=1;non_full_length_coverage=1;isoform_length=3653;expected_accuracy=0.997	1	1875	1875	3191	1	1864	2340	3653
cb2759_c6/f1p0/3412	gnl|BL_ORD_ID|37960 cb2759_c3/f1p0/3834 isoform=cb2759_c3;full_length_coverage=1;non_full_length_coverage=0;isoform_length=3834;expected_accuracy=0.998	194	3085	11	193	944	3833	62	244
cb9093_c2/f1p0/1840	gnl|BL_ORD_ID|58548 cb9093_c0/f1p0/2192	1	1420	1417	1840	151	156	1694	2115

isoform=cb9093_c2;full_length_coverage=1;non_full_length_coverage=0;isoform_length=1840;expected_accuracy=0.996							8		
cb8564_c43401/f1p2/3025 isoform=cb8564_c43401;full_length_coverage=1;non_full_length_coverage=2;isoform_length=3025;expected_accuracy=0.995	gnl|BL_ORD_ID|17429 cb8564_c85447/f1p0/3196 isoform=cb8564_c85447;full_length_coverage=1;non_full_length_coverage=0;isoform_length=3196;expected_accuracy=0.997	1	2485	2484	3025	3	2413	2660	3195
cb8564_c86946/f1p0/3289	gnl|BL_ORD_ID|57846 cb8564_c82432/f1p1/3412	1	1646	1646	2831	58	1703	1818	3007
cb3229_c3/f2p0/2593 isoform=cb3229_c3;full_length_coverage=2;non_full_length_coverage=0;isoform_length=2593;expected_accuracy=0.997	gnl|BL_ORD_ID|40615 cb3229_c17/f1p0/2804 isoform=cb3229_c17;full_length_coverage=1;non_full_length_coverage=0;isoform_length=2804;expected_accuracy=1.000	1	1844	1844	2502	4	1847	2136	2804
cb7241_c2/f1p1/2546 isoform=cb7241_c2;full_length_coverage=1;non_full_length_coverage=1;isoform_length=2546;expected_accuracy=0.999	gnl|BL_ORD_ID|8165 cb7241_c1/f1p1/2663 isoform=cb7241_c1;full_length_coverage=1;non_full_length_coverage=1;isoform_length=2663;expected_accuracy=0.999	1140	2546	1	1141	1264	2662	2	1154
cb8564_c5007/f1p0/1941	gnl|BL_ORD_ID|11479 cb8564_c148217/f3p5/3754 isoform=cb8564_c148217;full_length_coverage=3;non_full_length_coverage=5;isoform_length=3754;expected_accuracy=1.000	1	1447	1446	1802	1820	3265	3397	3754
cb7416_c3/f1p0/2846	gnl|BL_ORD_ID|51208 cb7416_c4/f1p1/3436	13	1342	1337	2587	8	1341	2173	3421
cb1388_c2/f2p0/4190 isoform=cb1388_c2;full_length_coverage=2;non_full_length_coverage=0;isoform_length=4190;expected_accur	gnl|BL_ORD_ID|26097 cb1388_c37/f1p0/5272 isoform=cb1388_c37;full_length_coverage=1;non_full_length_coverage=0;isoform_length=5272;expected_accur	391	4190	1	392	1448	5243	20	415

<table>
<tr><td>acy=0.998</td><td>acy=0.995</td><td></td><td></td><td></td><td></td><td></td><td></td><td></td><td></td></tr>
<tr><td>cb944_c22/f2p0/1418 isoform=cb944_c22;full_length_coverage=2;non_full_length_coverage=0;isoform_length=1418;expected_accuracy=1.000</td><td>gnl|BL_ORD_ID|57627 cb944_c41/f1p0/2631</td><td>299</td><td>1418</td><td>3</td><td>298</td><td>1275</td><td>2394</td><td>4</td><td>300</td></tr>
<tr><td>cb53_c1/f1p1/4723 isoform=cb53_c1;full_length_coverage=1;non_full_length_coverage=1;isoform_length=4723;expected_accuracy=0.996</td><td>gnl|BL_ORD_ID|45270 cb53_c9/f1p3/5277 isoform=cb53_c9;full_length_coverage=1;non_full_length_coverage=3;isoform_length=5277;expected_accuracy=0.997</td><td>1930</td><td>4721</td><td>1</td><td>1933</td><td>2487</td><td>5277</td><td>39</td><td>1974</td></tr>
<tr><td>cb5412_c0/f2p0/2007</td><td>gnl|BL_ORD_ID|45659 cb5412_c2/f1p0/3104 isoform=cb5412_c2;full_length_coverage=1;non_full_length_coverage=0;isoform_length=3104;expected_accuracy=0.996</td><td>275</td><td>2004</td><td>78</td><td>274</td><td>1379</td><td>3103</td><td>1</td><td>196</td></tr>
<tr><td>cb8564_c5070/f1p0/2861</td><td>gnl|BL_ORD_ID|56563 cb8564_c5062/f1p0/3831</td><td>10</td><td>1660</td><td>1659</td><td>2831</td><td>34</td><td>1681</td><td>1574</td><td>2746</td></tr>
<tr><td>cb4042_c8/f1p0/3354</td><td>gnl|BL_ORD_ID|71743 cb4042_c5/f1p1/3481</td><td>213</td><td>3218</td><td>1</td><td>212</td><td>368</td><td>3373</td><td>1</td><td>203</td></tr>
<tr><td>cb7832_c1/f1p0/2374 isoform=cb7832_c1;full_length_coverage=1;non_full_length_coverage=0;isoform_length=2374;expected_accuracy=0.992</td><td>gnl|BL_ORD_ID|50746 cb7832_c2/f1p0/2644</td><td>1</td><td>1274</td><td>1272</td><td>2287</td><td>231</td><td>1510</td><td>1611</td><td>2630</td></tr>
<tr><td>cb8564_c43587/f1p0/2930</td><td>gnl|BL_ORD_ID|55962 cb8564_c50282/f1p0/4402</td><td>15</td><td>1700</td><td>1699</td><td>2936</td><td>1457</td><td>3171</td><td>3065</td><td>4310</td></tr>
<tr><td>cb1973_c16/f1p0/2444</td><td>gnl|BL_ORD_ID|66570 cb1973_c19/f1p0/3182</td><td>1013</td><td>2376</td><td>1</td><td>1013</td><td>1571</td><td>2911</td><td>22</td><td>1034</td></tr>
<tr><td>cb500_c17/f1p0/2323</td><td>gnl|BL_ORD_ID|70945 cb500_c39/f1p0/3153</td><td>1</td><td>1293</td><td>1288</td><td>2323</td><td>702</td><td>199</td><td>2126</td><td>316</td></tr>
</table>

isoform=cb500_c17;full_length_coverage=1;non_full_length_coverage=0;isoform_length=2323;expected_accuracy=0.997							4		1
cb8564_c45242/f1p0/2126 isoform=cb8564_c45242;full_length_coverage=1;non_full_length_coverage=0;isoform_length=2126;expected_accuracy=0.990	gnl|BL_ORD_ID|14407 cb8564_c44036/f1p0/3258 isoform=cb8564_c44036;full_length_coverage=1;non_full_length_coverage=0;isoform_length=3258;expected_accuracy=0.992	443	2121	71	443	1575	3258	775	1147
cb6132_c8/f1p0/1087	gnl|BL_ORD_ID|46172 cb6132_c4/f1p0/2723 isoform=cb6132_c4;full_length_coverage=1;non_full_length_coverage=0;isoform_length=2723;expected_accuracy=0.995	491	1087	9	490	2066	2662	1	481
cb6238_c3/f2p0/2360 isoform=cb6238_c3;full_length_coverage=2;non_full_length_coverage=0;isoform_length=2360;expected_accuracy=1.000	gnl|BL_ORD_ID|5113 cb6238_c2/f2p0/2826 isoform=cb6238_c2;full_length_coverage=2;non_full_length_coverage=0;isoform_length=2826;expected_accuracy=1.000	1	1151	1151	2264	2	1152	1713	2826
cb4653_c0/f1p0/3288 isoform=cb4653_c0;full_length_coverage=1;non_full_length_coverage=0;isoform_length=3288;expected_accuracy=0.997	gnl|BL_ORD_ID|42544 cb4653_c1/f1p0/3426 isoform=cb4653_c1;full_length_coverage=1;non_full_length_coverage=0;isoform_length=3426;expected_accuracy=0.993	245	3288	11	246	416	3423	1	236
cb7334_c4/f1p0/2579 isoform=cb7334_c4;full_length_coverage=1;non_full_length_coverage=0;isoform_length=2579;expected_accuracy=0.999	gnl|BL_ORD_ID|51127 cb7334_c6/f1p0/2961	463	2579	5	464	616	2732	16	478
cb317_c5/f1p0/3451 isoform=cb317_c5;full_length_coverage=1;non_full_length_coverage=0;isoform_length=3451;expected_accurac	gnl|BL_ORD_ID|68195 cb317_c8/f1p1/4711	1	3038	3033	3317	115	3159	3385	3669

y=0.998									
cb8586_c9/f1p3/1411 isoform=cb8586_c9;full_length_coverage=1;non_full_length_coverage=3;isoform_length=1411;expected_accuracy=0.996	gnl|BL_ORD_ID|17412 cb8586_c3/f1p1/2345 isoform=cb8586_c3;full_length_coverage=1;non_full_length_coverage=1;isoform_length=2345;expected_accuracy=0.996	1	1053	1050	1411	1	1047	1948	2309
cb8564_c40107/f1p0/4108	gnl|BL_ORD_ID|15996 cb8564_c66839/f6p1/4041 isoform=cb8564_c66839;full_length_coverage=6;non_full_length_coverage=1;isoform_length=4041;expected_accuracy=1.000	1603	3955	1	1599	1514	3866	35	1634
cb14664_c0/f4p0/1146 isoform=cb14664_c0;full_length_coverage=4;non_full_length_coverage=0;isoform_length=1146;expected_accuracy=0.996	gnl|BL_ORD_ID|64215 cb14664_c5/f1p0/1279	203	1133	5	204	346	1277	1	200
cb8564_c180116/f1p0/2944 isoform=cb8564_c180116;full_length_coverage=1;non_full_length_coverage=0;isoform_length=2944;expected_accuracy=0.996	gnl|BL_ORD_ID|15193 cb8564_c42308/f1p5/3192 isoform=cb8564_c42308;full_length_coverage=1;non_full_length_coverage=5;isoform_length=3192;expected_accuracy=0.995	1	2522	2520	2944	2	2537	2766	3190
cb8564_c46939/f1p1/2926	gnl|BL_ORD_ID|55502 cb8564_c43538/f1p0/3563	1	2134	2130	2870	2	2149	2254	2995
cb13748_c22/f1p0/3447	gnl|BL_ORD_ID|27754 cb13748_c13/f2p0/3522 isoform=cb13748_c13;full_length_coverage=2;non_full_length_coverage=0;isoform_length=3522;expected_accuracy=0.998	2	3102	3098	3404	1	3101	3216	3522
cb548_c65/f1p1/2963	gnl|BL_ORD_ID|46779 cb548_c109/f1p0/3280 isoform=cb548_c109;full_length_coverage=1;non_full_length_coverage=0;isoform_length=3280;expected_accur	1293	2948	1	1296	1551	3204	45	1340

	acy=0.996								
cb398_c5/f1p0/4384	gnl|BL_ORD_ID|39098 cb398_c4/f1p0/4400 isoform=cb398_c4;full_length_coverage=1;non_full_length_coverage=0;isoform_length=4400;expected_accuracy=0.999	177	4179	1	172	400	4400	2	172
cb8564_c73853/f1p0/2443 isoform=cb8564_c73853;full_length_coverage=1;non_full_length_coverage=0;isoform_length=2443;expected_accuracy=1.000	gnl|BL_ORD_ID|53674 cb8564_c152641/f1p6/2794	1	1281	1276	2442	27	1303	1583	2753
cb8564_c139090/f1p2/2322	gnl|BL_ORD_ID|17527 cb8564_c84215/f1p0/3183 isoform=cb8564_c84215;full_length_coverage=1;non_full_length_coverage=0;isoform_length=3183;expected_accuracy=0.997	1	1810	1808	2265	1	1810	2697	3141
cb5307_c22/f2p0/3012 isoform=cb5307_c22;full_length_coverage=2;non_full_length_coverage=0;isoform_length=3012;expected_accuracy=0.995	gnl|BL_ORD_ID|73769 cb5307_c7/f1p0/3894	102	1687	1683	3012	3	1580	2554	3883
cb2324_c2/f3p1/3595 isoform=cb2324_c2;full_length_coverage=3;non_full_length_coverage=1;isoform_length=3595;expected_accuracy=0.996	gnl|BL_ORD_ID|36741 cb2324_c5/f2p1/3722 isoform=cb2324_c5;full_length_coverage=2;non_full_length_coverage=1;isoform_length=3722;expected_accuracy=0.995	274	3595	21	275	404	3722	2	258
cb12714_c29/f1p2/1583	gnl|BL_ORD_ID|11957 cb8564_c135981/f1p0/2840 isoform=cb8564_c135981;full_length_coverage=1;non_full_length_coverage=0;isoform_length=2840;expected_accuracy=0.992	1	1296	1293	1590	189	1480	2545	2840
cb3518_c21/f1p0/1435	gnl|BL_ORD_ID|40086 cb3518_c14/f1p2/1777	104	1420	1	106	465	177	10	117

isoform=cb3518_c21;full_length_coverage=1;non_full_length_coverage=0;isoform_length=1435;expected_accuracy=1.000	isoform=cb3518_c14;full_length_coverage=1;non_full_length_coverage=2;isoform_length=1777;expected_accuracy=0.994						7		
cb2737_c42/f1p0/2881	gnl|BL_ORD_ID|38133 cb2737_c21/f1p2/3479 isoform=cb2737_c21;full_length_coverage=1;non_full_length_coverage=2;isoform_length=3479;expected_accuracy=1.000	1	1427	1425	2632	577	2003	2135	3342
cb8564_c14775/f1p1/2535	gnl|BL_ORD_ID|11111 cb8564_c108411/f1p1/3146 isoform=cb8564_c108411;full_length_coverage=1;non_full_length_coverage=1;isoform_length=3146;expected_accuracy=0.991	1056	2492	1	1058	1695	3145	170	1242
cb316_c44/f1p3/4198 isoform=cb316_c44;full_length_coverage=1;non_full_length_coverage=3;isoform_length=4198;expected_accuracy=0.994	gnl|BL_ORD_ID|69554 cb316_c10/f1p2/4819	24	2660	2656	4198	71	2702	3318	4861
cb16438_c1/f2p0/1049 isoform=cb16438_c1;full_length_coverage=2;non_full_length_coverage=0;isoform_length=1049;expected_accuracy=1.000	gnl|BL_ORD_ID|65268 cb16438_c2/f1p0/1232	336	1049	4	336	512	1226	1	332
cb7642_c139/f1p0/2022 isoform=cb7642_c139;full_length_coverage=1;non_full_length_coverage=0;isoform_length=2022;expected_accuracy=0.993	gnl|BL_ORD_ID|6567 cb7642_c138/f1p0/2345 isoform=cb7642_c138;full_length_coverage=1;non_full_length_coverage=0;isoform_length=2345;expected_accuracy=0.994	1	1475	1476	2020	129	1603	1800	2345
cb124_c26/f1p0/3756	gnl|BL_ORD_ID|24083 cb124_c45/f1p0/3856 isoform=cb124_c45;full_length_coverage=1;non_full_length_coverage=0;isoform_length=3856;expected_accura	1	3076	3073	3720	1	3075	3203	3850

	cy=0.996								
cb737_c27/f1p0/4113	gnl|BL_ORD_ID|7432 cb737_c72/f3p2/4288 isoform=cb737_c72;full_length_coverage=3;non_full_length_coverage=2;isoform_length=4288;expected_accuracy=0.998	1	3348	3347	4115	15	3363	3520	4288
cb5612_c14/f3p0/2416 isoform=cb5612_c14;full_length_coverage=3;non_full_length_coverage=0;isoform_length=2416;expected_accuracy=0.998	gnl|BL_ORD_ID|45308 cb5612_c2/f2p0/2528 isoform=cb5612_c2;full_length_coverage=2;non_full_length_coverage=0;isoform_length=2528;expected_accuracy=0.997	148	2414	1	147	260	2526	9	155
cb8564_c68023/f5p1/3648 isoform=cb8564_c68023;full_length_coverage=5;non_full_length_coverage=1;isoform_length=3648;expected_accuracy=1.000	gnl|BL_ORD_ID|16243 cb8564_c69770/f1p2/3779 isoform=cb8564_c69770;full_length_coverage=1;non_full_length_coverage=2;isoform_length=3779;expected_accuracy=1.000	271	3648	1	274	388	3764	9	278
cb5440_c2/f3p1/1394 isoform=cb5440_c2;full_length_coverage=3;non_full_length_coverage=1;isoform_length=1394;expected_accuracy=0.998	gnl|BL_ORD_ID|46312 cb5440_c8/f1p1/3070 isoform=cb5440_c8;full_length_coverage=1;non_full_length_coverage=1;isoform_length=3070;expected_accuracy=0.991	1	1067	1062	1394	63	1142	2735	3070
cb322_c15/f1p0/3487 isoform=cb322_c15;full_length_coverage=1;non_full_length_coverage=0;isoform_length=3487;expected_accuracy=0.997	gnl|BL_ORD_ID|69560 cb322_c25/f1p0/3845	1	2729	2728	3487	7	2732	2878	3636
cb14792_c2/f1p0/1864 isoform=cb14792_c2;full_length_coverage=1;non_full_length_coverage=0;isoform_length=1864;expected_accuracy=0.995	gnl|BL_ORD_ID|19072 cb8564_c88935/f5p2/2290 isoform=cb8564_c88935;full_length_coverage=5;non_full_length_coverage=2;isoform_length=2290;expected_accuracy=1.000	1	1524	1519	1818	257	1780	1991	2290
cb8564_c52935/f1p0/3498	gnl|BL_ORD_ID|54440 cb8564_c46842/f1p0/3416	1	2027	2025	3322	1	202	2138	343

							8		5
cb2272_c12/f1p0/5238	gnl|BL_ORD_ID|38157 cb2272_c10/f1p0/6304 isoform=cb2272_c10;full_length_coverage=1;non_full_length_coverage=0;isoform_length=6304;expected_accuracy=0.996	128	2695	2691	5193	215	2779	3807	6304
cb8564_c71802/f1p1/3657 isoform=cb8564_c71802;full_length_coverage=1;non_full_length_coverage=1;isoform_length=3657;expected_accuracy=1.000	gnl|BL_ORD_ID|15937 cb8564_c69148/f2p0/3719 isoform=cb8564_c69148;full_length_coverage=2;non_full_length_coverage=0;isoform_length=3719;expected_accuracy=0.997	1	2767	2768	3561	47	2806	2926	3719
cb14322_c5/f1p0/1783	gnl|BL_ORD_ID|53245 cb8564_c119806/f1p0/2166	1	1580	1576	1786	94	1679	1873	2084
cb5460_c7/f1p0/2109 isoform=cb5460_c7;full_length_coverage=1;non_full_length_coverage=0;isoform_length=2109;expected_accuracy=0.997	gnl|BL_ORD_ID|73948 cb5460_c9/f1p1/2896	1	1344	1340	2030	752	2095	2217	2906
cb8564_c36689/f1p1/2368 isoform=cb8564_c36689;full_length_coverage=1;non_full_length_coverage=1;isoform_length=2368;expected_accuracy=0.991	gnl|BL_ORD_ID|55413 cb8564_c40978/f1p0/3073	1	1709	1706	2368	1	1683	2182	2845
cb11885_c4/f1p0/1695 isoform=cb11885_c4;full_length_coverage=1;non_full_length_coverage=0;isoform_length=1695;expected_accuracy=0.998	gnl|BL_ORD_ID|71762 cb4066_c10/f1p1/3649	232	1641	3	232	2227	3636	1	231
cb8564_c21764/f1p0/2599	gnl|BL_ORD_ID|56491 cb8564_c77843/f1p0/2830	1	2030	2030	2607	158	2185	2069	2646
cb4233_c155/f1p1/2407	gnl|BL_ORD_ID|71867 cb4233_c156/f1p1/2451	128	2175	1	132	400	244	7	139

							8		
cb8564_c77203/f2p0/2820 isoform=cb8564_c77203;full_length_coverage=2;non_full_length_coverage=0;isoform_length=2820;expected_accuracy=0.996	gnl|BL_ORD_ID|14537 cb8564_c36204/f3p0/3065 isoform=cb8564_c36204;full_length_coverage=3;non_full_length_coverage=0;isoform_length=3065;expected_accuracy=0.997	1372	2819	1	1374	1618	3065	1	1374
cb8564_c91518/f1p0/3380	gnl|BL_ORD_ID|16911 cb8564_c72734/f1p0/3132 isoform=cb8564_c72734;full_length_coverage=1;non_full_length_coverage=0;isoform_length=3132;expected_accuracy=0.995	1	2710	2709	2852	188	2881	2989	3132
cb8564_c3718/f1p0/2760	gnl|BL_ORD_ID|12841 cb8564_c168338/f2p0/3398 isoform=cb8564_c168338;full_length_coverage=2;non_full_length_coverage=0;isoform_length=3398;expected_accuracy=0.999	1	2149	2148	2740	40	2186	2295	2887
cb8564_c91149/f1p5/2619 isoform=cb8564_c91149;full_length_coverage=1;non_full_length_coverage=5;isoform_length=2619;expected_accuracy=0.997	gnl|BL_ORD_ID|56381 cb8564_c75626/f1p2/3199	1	2125	2123	2614	4	2116	2611	3088
cb8564_c129154/f1p0/2632 isoform=cb8564_c129154;full_length_coverage=1;non_full_length_coverage=0;isoform_length=2632;expected_accuracy=0.997	gnl|BL_ORD_ID|53022 cb8564_c114466/f1p0/3000	1	1586	1587	2590	22	1610	1943	2946
cb13045_c6/f1p0/1293 isoform=cb13045_c6;full_length_coverage=1;non_full_length_coverage=0;isoform_length=1293;expected_accuracy=0.991	gnl|BL_ORD_ID|16485 cb8564_c72854/f1p0/3264 isoform=cb8564_c72854;full_length_coverage=1;non_full_length_coverage=0;isoform_length=3264;expected_accuracy=1.000	1	1011	1008	1293	48	1058	2149	2434
cb693_c7/f1p0/2901	gnl|BL_ORD_ID|4971 cb693_c3/f1p0/3671	1	2459	2460	2828	45	249	3296	366

<table>
<tr><th></th><th></th><th></th><th></th><th></th><th></th><th></th><th></th><th></th><th></th></tr>
<tr><td></td><td>isoform=cb693_c3;full_length_coverage=1;non_full_length_coverage=0;isoform_length=3671;expected_accuracy=0.995</td><td></td><td></td><td></td><td></td><td></td><td>7</td><td></td><td>3</td></tr>
<tr><td>cb9467_c4/f9p0/2081 isoform=cb9467_c4;full_length_coverage=9;non_full_length_coverage=0;isoform_length=2081;expected_accuracy=0.999</td><td>gnl|BL_ORD_ID|20200 cb9467_c17/f1p0/3298 isoform=cb9467_c17;full_length_coverage=1;non_full_length_coverage=0;isoform_length=3298;expected_accuracy=1.000</td><td>163</td><td>2067</td><td>1</td><td>162</td><td>1353</td><td>3251</td><td>1</td><td>162</td></tr>
<tr><td>cb8564_c152519/f2p1/2103 isoform=cb8564_c152519;full_length_coverage=2;non_full_length_coverage=1;isoform_length=2103;expected_accuracy=1.000</td><td>gnl|BL_ORD_ID|11147 cb8564_c120697/f1p0/2276 isoform=cb8564_c120697;full_length_coverage=1;non_full_length_coverage=0;isoform_length=2276;expected_accuracy=0.995</td><td>1</td><td>1562</td><td>1559</td><td>2069</td><td>52</td><td>1614</td><td>1756</td><td>2266</td></tr>
<tr><td>cb7463_c5/f1p0/2362 isoform=cb7463_c5;full_length_coverage=1;non_full_length_coverage=0;isoform_length=2362;expected_accuracy=0.995</td><td>gnl|BL_ORD_ID|7043 cb7463_c6/f1p0/2483 isoform=cb7463_c6;full_length_coverage=1;non_full_length_coverage=0;isoform_length=2483;expected_accuracy=0.997</td><td>177</td><td>2297</td><td>1</td><td>180</td><td>394</td><td>2483</td><td>45</td><td>224</td></tr>
<tr><td>cb8564_c88569/f1p3/3382 isoform=cb8564_c88569;full_length_coverage=1;non_full_length_coverage=3;isoform_length=3382;expected_accuracy=0.998</td><td>gnl|BL_ORD_ID|19263 cb8564_c80691/f1p1/3438 isoform=cb8564_c80691;full_length_coverage=1;non_full_length_coverage=1;isoform_length=3438;expected_accuracy=1.000</td><td>141</td><td>2480</td><td>2479</td><td>3372</td><td>96</td><td>2436</td><td>2546</td><td>3438</td></tr>
<tr><td>cb9813_c128/f1p0/1498</td><td>gnl|BL_ORD_ID|58920 cb9813_c57/f1p0/1921</td><td>175</td><td>1498</td><td>8</td><td>179</td><td>351</td><td>1672</td><td>4</td><td>175</td></tr>
<tr><td>cb12931_c15/f1p0/1493 isoform=cb12931_c15;full_length_coverage=1;non_full_length_coverage=0;isoform_length=1493;expected_accuracy=1.000</td><td>gnl|BL_ORD_ID|14382 cb8564_c38947/f1p0/2650 isoform=cb8564_c38947;full_length_coverage=1;non_full_length_coverage=0;isoform_length=2650;expected_accuracy=0.996</td><td>1</td><td>1106</td><td>1104</td><td>1492</td><td>1</td><td>1104</td><td>2262</td><td>2650</td></tr>
<tr><td>cb6623_c27/f1p1/2886</td><td>gnl|BL_ORD_ID|49890 cb6623_c26/f1p0/3303</td><td>626</td><td>2644</td><td>7</td><td>630</td><td>1234</td><td>325</td><td>50</td><td>673</td></tr>
</table>

							3		
cb8564_c3929/f1p0/2812 isoform=cb8564_c3929;full_length_coverage=1;non_full_length_coverage=0;isoform_length=2812;expected_accuracy=0.998	gnl|BL_ORD_ID|12615 cb8564_c138783/f5p1/3091 isoform=cb8564_c138783;full_length_coverage=5;non_full_length_coverage=1;isoform_length=3091;expected_accuracy=0.999	1	2071	2067	2812	113	2180	2327	3072
cb708_c7/f1p0/3755	gnl|BL_ORD_ID|47202 cb708_c3/f1p0/4948	1	2891	2889	3540	39	2928	4226	4877
cb965_c40/f1p0/4415	gnl|BL_ORD_ID|22111 cb965_c30/f1p3/4326 isoform=cb965_c30;full_length_coverage=1;non_full_length_coverage=3;isoform_length=4326;expected_accuracy=0.994	1	2886	2882	4031	4	2885	3102	4251
cb295_c26/f1p1/1869	gnl|BL_ORD_ID|37900 cb295_c23/f1p1/2681 isoform=cb295_c23;full_length_coverage=1;non_full_length_coverage=1;isoform_length=2681;expected_accuracy=0.993	1	1360	1358	1857	1	1350	2119	2620
cb15801_c5/f1p0/1808	gnl|BL_ORD_ID|40656 cb3306_c16/f1p0/2613 isoform=cb3306_c16;full_length_coverage=1;non_full_length_coverage=0;isoform_length=2613;expected_accuracy=0.998	478	1726	5	480	1256	2503	42	519
cb16679_c2/f1p0/1254	gnl|BL_ORD_ID|33343 cb16679_c3/f2p0/1563 isoform=cb16679_c3;full_length_coverage=2;non_full_length_coverage=0;isoform_length=1563;expected_accuracy=1.000	1	1074	1070	1234	1	1074	1399	1563
cb14192_c1/f2p0/1828 isoform=cb14192_c1;full_length_coverage=2;non_full_length_coverage=0;isoform_length=1828;expected_accu	gnl|BL_ORD_ID|13787 cb8564_c1982/f1p0/2344 isoform=cb8564_c1982;full_length_coverage=1;non_full_length_coverage=0;isoform_length=2344;expected_ac	1	1267	1267	1821	27	1296	1761	2344

racy=0.994	curacy=0.993								
cb7969_c11/f1p0/1950 isoform=cb7969_c11;full_length_coverage=1;non_full_length_coverage=0;isoform_length=1950;expected_accuracy=0.992	gnl|BL_ORD_ID|6552 cb7969_c4/f1p0/2324 isoform=cb7969_c4;full_length_coverage=1;non_full_length_coverage=0;isoform_length=2324;expected_accuracy=0.996	1	1409	1405	1927	122	1532	1777	2321
cb14724_c4/f1p0/1676	gnl|BL_ORD_ID|40277 cb3622_c10/f1p0/3532 isoform=cb3622_c10;full_length_coverage=1;non_full_length_coverage=0;isoform_length=3532;expected_accuracy=0.999	1	1330	1329	1649	25	1351	3211	3531
cb16550_c1/f1p2/1845 isoform=cb16550_c1;full_length_coverage=1;non_full_length_coverage=2;isoform_length=1845;expected_accuracy=0.999	gnl|BL_ORD_ID|11486 cb8564_c127460/f1p2/2151 isoform=cb8564_c127460;full_length_coverage=1;non_full_length_coverage=2;isoform_length=2151;expected_accuracy=0.998	1	1212	1207	1845	73	1287	1510	2143
cb8564_c161530/f1p7/3594 isoform=cb8564_c161530;full_length_coverage=1;non_full_length_coverage=7;isoform_length=3594;expected_accuracy=0.999	gnl|BL_ORD_ID|58077 cb8564_c88175/f1p0/4011	132	2862	2862	3594	12	2742	3254	3981
cb5600_c16/f1p0/2870	gnl|BL_ORD_ID|45243 cb5600_c5/f1p0/2860 isoform=cb5600_c5;full_length_coverage=1;non_full_length_coverage=0;isoform_length=2860;expected_accuracy=0.997	1	2143	2140	2697	33	2173	2304	2860
cb14634_c57/f1p0/2155 isoform=cb14634_c57;full_length_coverage=1;non_full_length_coverage=0;isoform_length=2155;expected_accuracy=0.994	gnl|BL_ORD_ID|64192 cb14634_c60/f1p0/2755	1	1295	1292	2155	39	1338	1910	2775
cb2674_c19/f1p0/3539	gnl|BL_ORD_ID|38772 cb2674_c2/f2p4/3751	1	2456	2457	3511	83	254	2683	375

isoform=cb2674_c19;full_length_coverage=1;non_full_length_coverage=0;isoform_length=3539;expected_accuracy=0.993	isoform=cb2674_c2;full_length_coverage=2;non_full_length_coverage=4;isoform_length=3751;expected_accuracy=0.994						7		0
cb8564_c43399/f1p0/2654 isoform=cb8564_c43399;full_length_coverage=1;non_full_length_coverage=0;isoform_length=2654;expected_accuracy=0.999	gnl|BL_ORD_ID|61972 cb12931_c30/f1p0/5903	1	2367	2363	2654	1	2362	2578	2869
cb3964_c2/f1p0/3477	gnl|BL_ORD_ID|71680 cb3964_c3/f1p0/3409	1555	3301	1	1556	1658	3402	1	1556
cb8564_c38151/f1p3/3560	gnl|BL_ORD_ID|56035 cb8564_c52940/f1p2/3963	13	2308	2307	3454	5	2302	2793	3940
cb4114_c4/f1p0/3095 isoform=cb4114_c4;full_length_coverage=1;non_full_length_coverage=0;isoform_length=3095;expected_accuracy=1.000	gnl|BL_ORD_ID|39263 cb4114_c1/f1p0/3431 isoform=cb4114_c1;full_length_coverage=1;non_full_length_coverage=0;isoform_length=3431;expected_accuracy=0.997	1239	3095	120	1242	1571	3431	32	1153
cb8539_c3/f2p0/2041 isoform=cb8539_c3;full_length_coverage=2;non_full_length_coverage=0;isoform_length=2041;expected_accuracy=1.000	gnl|BL_ORD_ID|10886 cb8539_c5/f1p0/2106 isoform=cb8539_c5;full_length_coverage=1;non_full_length_coverage=0;isoform_length=2106;expected_accuracy=0.996	552	2041	84	553	610	2098	1	471
cb8564_c15230/f1p0/1944	gnl|BL_ORD_ID|55861 cb8564_c47234/f1p0/2932	352	1944	60	357	1104	2695	97	394
cb8564_c115246/f1p0/2687 isoform=cb8564_c115246;full_length_coverage=1;non_full_length_coverage=0;isoform_length=2687;expected_accuracy=0.996	gnl|BL_ORD_ID|11306 cb8564_c14717/f1p0/4274 isoform=cb8564_c14717;full_length_coverage=1;non_full_length_coverage=0;isoform_length=4274;expected_accuracy=0.995	1317	2664	1	1322	2923	4274	761	2078
cb2527_c48/f10p2/3282	gnl|BL_ORD_ID|68590 cb2527_c17/f1p1/3542	1	1906	1902	3203	1	190	2256	355

isoform=cb2527_c48;full_length_coverage=10;non_full_length_coverage=2;isoform_length=3282;expected_accuracy=0.997							6		7
cb8564_c37361/f1p0/3076	gnl|BL_ORD_ID|13575 cb8564_c20499/f3p0/3328 isoform=cb8564_c20499;full_length_coverage=3;non_full_length_coverage=0;isoform_length=3328;expected_accuracy=1.000	1	2481	2477	3070	1	248 2	2583	317 6
cb8564_c42814/f1p0/2151 isoform=cb8564_c42814;full_length_coverage=1;non_full_length_coverage=0;isoform_length=2151;expected_accuracy=0.993	gnl|BL_ORD_ID|55177 cb8564_c34740/f1p0/3512	650	2151	7	652	1691	319 4	1	677
cb3213_c13/f1p0/2292	gnl|BL_ORD_ID|40431 cb3213_c4/f4p0/2414 isoform=cb3213_c4;full_length_coverage=4;non_full_length_coverage=0;isoform_length=2414;expected_accuracy=0.995	104	2196	1	104	316	241 4	88	191
cb8564_c38035/f1p0/2770	gnl|BL_ORD_ID|13512 cb8564_c186209/f1p0/2931 isoform=cb8564_c186209;full_length_coverage=1;non_full_length_coverage=0;isoform_length=2931;expected_accuracy=0.996	1	2035	2033	2756	1	201 7	2207	293 1
cb8564_c113445/f1p1/1964 isoform=cb8564_c113445;full_length_coverage=1;non_full_length_coverage=1;isoform_length=1964;expected_accuracy=0.995	gnl|BL_ORD_ID|17614 cb8564_c92081/f1p1/2901 isoform=cb8564_c92081;full_length_coverage=1;non_full_length_coverage=1;isoform_length=2901;expected_accuracy=0.995	1	1530	1535	1964	1	152 8	2441	286 9
cb8564_c2223/f1p0/2047	gnl|BL_ORD_ID|13707 cb8564_c2273/f1p2/2502 isoform=cb8564_c2273;full_length_coverage=1;non_full_length_coverage=2;isoform_length=2502;expected_ac	434	2040	49	439	888	250 2	56	445

<table>
<tr><td></td><td>curacy=0.995</td><td></td><td></td><td></td><td></td><td></td><td></td><td></td><td></td></tr>
<tr><td>cb8564_c44698/f1p5/2843 isoform=cb8564_c44698;full_length_coverage=1;non_full_length_coverage=5;isoform_length=2843;expected_accuracy=0.997</td><td>gnl|BL_ORD_ID|14556 cb8564_c45150/f1p0/2940 isoform=cb8564_c45150;full_length_coverage=1;non_full_length_coverage=0;isoform_length=2940;expected_accuracy=0.992</td><td>1</td><td>2516</td><td>2511</td><td>2835</td><td>1</td><td>2496</td><td>2616</td><td>2940</td></tr>
<tr><td>cb2471_c2/f7p1/2180 isoform=cb2471_c2;full_length_coverage=7;non_full_length_coverage=1;isoform_length=2180;expected_accuracy=1.000</td><td>gnl|BL_ORD_ID|69758 cb2471_c14/f1p0/2261</td><td>266</td><td>2180</td><td>86</td><td>268</td><td>355</td><td>2256</td><td>1</td><td>183</td></tr>
<tr><td>cb2156_c24/f1p1/2545 isoform=cb2156_c24;full_length_coverage=1;non_full_length_coverage=1;isoform_length=2545;expected_accuracy=0.991</td><td>gnl|BL_ORD_ID|35009 cb2156_c82/f5p1/2836 isoform=cb2156_c82;full_length_coverage=5;non_full_length_coverage=1;isoform_length=2836;expected_accuracy=1.000</td><td>1</td><td>1422</td><td>1420</td><td>2545</td><td>43</td><td>1480</td><td>1605</td><td>2735</td></tr>
<tr><td>cb8564_c90621/f1p2/3030 isoform=cb8564_c90621;full_length_coverage=1;non_full_length_coverage=2;isoform_length=3030;expected_accuracy=0.994</td><td>gnl|BL_ORD_ID|15376 cb8564_c31588/f10p3/3394 isoform=cb8564_c31588;full_length_coverage=10;non_full_length_coverage=3;isoform_length=3394;expected_accuracy=1.000</td><td>206</td><td>3027</td><td>1</td><td>205</td><td>546</td><td>3366</td><td>208</td><td>412</td></tr>
<tr><td>cb3783_c15/f1p2/3106 isoform=cb3783_c15;full_length_coverage=1;non_full_length_coverage=2;isoform_length=3106;expected_accuracy=0.993</td><td>gnl|BL_ORD_ID|40402 cb3783_c2/f1p2/3529 isoform=cb3783_c2;full_length_coverage=1;non_full_length_coverage=2;isoform_length=3529;expected_accuracy=0.998</td><td>1</td><td>1776</td><td>1772</td><td>3106</td><td>7</td><td>1779</td><td>2250</td><td>3521</td></tr>
<tr><td>cb1298_c1/f3p0/2128 isoform=cb1298_c1;full_length_coverage=3;non_full_length_coverage=0;isoform_length=2128;expected_accuracy=0.996</td><td>gnl|BL_ORD_ID|61206 cb1298_c8/f1p0/2338</td><td>1</td><td>1343</td><td>1343</td><td>2074</td><td>38</td><td>1379</td><td>1552</td><td>2283</td></tr>
<tr><td>cb5596_c6/f10p0/2530</td><td>gnl|BL_ORD_ID|74127 cb5596_c16/f1p0/3416</td><td>299</td><td>2528</td><td>88</td><td>302</td><td>1182</td><td>3411</td><td>1</td><td>216</td></tr>
</table>

isoform=cb5596_c6;full_length_coverage=10;non_full_length_coverage=0;isoform_length=2530;expected_accuracy=1.000									
cb12302_c30/f1p0/1673 isoform=cb12302_c30;full_length_coverage=1;non_full_length_coverage=0;isoform_length=1673;expected_accuracy=0.992	gnl|BL_ORD_ID|27346 cb1380_c17/f1p1/1771 isoform=cb1380_c17;full_length_coverage=1;non_full_length_coverage=1;isoform_length=1771;expected_accuracy=0.993	284	1632	3	288	410	1771	3	296
cb8564_c10630/f1p2/2801 isoform=cb8564_c10630;full_length_coverage=1;non_full_length_coverage=2;isoform_length=2801;expected_accuracy=1.000	gnl|BL_ORD_ID|10274 cb8564_c11542/f1p2/2858 isoform=cb8564_c11542;full_length_coverage=1;non_full_length_coverage=2;isoform_length=2858;expected_accuracy=0.997	1	2162	2161	2758	1	2161	2264	2855
cb6835_c30/f1p0/2031	gnl|BL_ORD_ID|4332 cb6835_c11/f1p2/2231 isoform=cb6835_c11;full_length_coverage=1;non_full_length_coverage=2;isoform_length=2231;expected_accuracy=0.998	716	1918	8	719	1029	2231	1	711
cb8564_c41162/f1p0/3158	gnl|BL_ORD_ID|57017 cb8564_c80370/f1p0/3493	124	2553	2548	3042	3085	652	521	27
cb3826_c8/f1p0/3348 isoform=cb3826_c8;full_length_coverage=1;non_full_length_coverage=0;isoform_length=3348;expected_accuracy=0.994	gnl|BL_ORD_ID|71538 cb3826_c3/f1p0/3517	1	2584	2579	3348	1	2587	2693	3461
cb313_c3/f2p0/2434 isoform=cb313_c3;full_length_coverage=2;non_full_length_coverage=0;isoform_length=2434;expected_accuracy=0.995	gnl|BL_ORD_ID|68193 cb313_c25/f1p0/3123	2	2112	2112	2397	8	2116	2681	2966
cb781_c2/f3p0/1827 isoform=cb781_c2;full_length_coverage=3;non_full_len	gnl|BL_ORD_ID|6925 cb781_c19/f1p1/2021 isoform=cb781_c19;full_length_coverage=1;non_full_le	1	1243	1243	1827	5	1246	1351	1935

gth_coverage=0;isoform_length=1827;expected_accuracy=0.999	ngth_coverage=1;isoform_length=2021;expected_accuracy=0.998								
cb5125_c34/f1p0/1890	gnl|BL_ORD_ID|43457 cb5125_c11/f2p1/2545 isoform=cb5125_c11;full_length_coverage=2;non_full_length_coverage=1;isoform_length=2545;expected_accuracy=1.000	1	1145	1144	1780	46	1187	1823	2458
cb15533_c11/f1p0/1222 isoform=cb15533_c11;full_length_coverage=1;non_full_length_coverage=0;isoform_length=1222;expected_accuracy=0.995	gnl|BL_ORD_ID|10970 cb8564_c115643/f1p0/1910 isoform=cb8564_c115643;full_length_coverage=1;non_full_length_coverage=0;isoform_length=1910;expected_accuracy=0.999	9	610	606	1211	1	606	1288	1873
cb322_c15/f1p0/3487 isoform=cb322_c15;full_length_coverage=1;non_full_length_coverage=0;isoform_length=3487;expected_accuracy=0.997	gnl|BL_ORD_ID|41025 cb322_c8/f2p0/3977 isoform=cb322_c8;full_length_coverage=2;non_full_length_coverage=0;isoform_length=3977;expected_accuracy=0.999	1	2729	2728	3486	2	2728	3218	3977
cb2737_c55/f1p1/2936 isoform=cb2737_c55;full_length_coverage=1;non_full_length_coverage=1;isoform_length=2936;expected_accuracy=1.000	gnl|BL_ORD_ID|68786 cb2737_c50/f1p0/3706	1	1595	1593	2936	491	2082	2214	3551
cb8564_c119767/f1p0/1892	gnl|BL_ORD_ID|10350 cb8564_c108942/f5p0/2520 isoform=cb8564_c108942;full_length_coverage=5;non_full_length_coverage=0;isoform_length=2520;expected_accuracy=1.000	186	1885	5	189	826	2490	11	194
cb7085_c12/f1p0/1968	gnl|BL_ORD_ID|4154 cb7085_c8/f1p0/2701 isoform=cb7085_c8;full_length_coverage=1;non_full_length_coverage=0;isoform_length=2701;expected_accuracy=0.996	542	1894	61	544	1349	2701	1	483

cb1333_c101/f1p0/1395 isoform=cb1333_c101;full_length_coverage=1;non_full_length_coverage=0;isoform_length=1395;expected_accuracy=0.997	gnl|BL_ORD_ID|61255 cb1333_c94/f1p0/1764	149	1395	27	147	503	1747	32	152
cb8564_c146882/f3p8/4113 isoform=cb8564_c146882;full_length_coverage=3;non_full_length_coverage=8;isoform_length=4113;expected_accuracy=0.998	gnl|BL_ORD_ID|16628 cb8564_c47737/f7p5/4351 isoform=cb8564_c47737;full_length_coverage=7;non_full_length_coverage=5;isoform_length=4351;expected_accuracy=0.998	184	4063	1	185	395	4274	1	178
cb4016_c42/f1p1/2580 isoform=cb4016_c42;full_length_coverage=1;non_full_length_coverage=1;isoform_length=2580;expected_accuracy=0.993	gnl|BL_ORD_ID|40901 cb4016_c19/f1p0/2644 isoform=cb4016_c19;full_length_coverage=1;non_full_length_coverage=0;isoform_length=2644;expected_accuracy=0.995	139	2494	1	143	284	2644	1	140
cb4685_c6/f1p0/3026 isoform=cb4685_c6;full_length_coverage=1;non_full_length_coverage=0;isoform_length=3026;expected_accuracy=0.999	gnl|BL_ORD_ID|42751 cb4685_c2/f1p0/3259 isoform=cb4685_c2;full_length_coverage=1;non_full_length_coverage=0;isoform_length=3259;expected_accuracy=0.995	1	1918	1917	2998	52	1973	2139	3215
cb8564_c47522/f1p0/2954	gnl|BL_ORD_ID|18852 cb8564_c84488/f1p0/3408 isoform=cb8564_c84488;full_length_coverage=1;non_full_length_coverage=0;isoform_length=3408;expected_accuracy=0.996	226	2845	1	226	790	3408	1	217
cb10141_c13/f24p1/1070 isoform=cb10141_c13;full_length_coverage=24;non_full_length_coverage=1;isoform_length=1070;expected_accuracy=1.000	gnl|BL_ORD_ID|48121 cb10141_c5/f1p0/1325	416	1070	5	419	519	1173	1	415
cb8564_c36056/f1p0/2652 isoform=cb8564_c36056;full_length_coverage=1;non_f	gnl|BL_ORD_ID|15154 cb8564_c32509/f1p1/2880 isoform=cb8564_c32509;full_length_coverage=1;non_fu	248	2626	1	251	465	2877	61	320

ull_length_coverage=0;isoform_length=2652;expected_accuracy=0.999	ll_length_coverage=1;isoform_length=2880;expected_accuracy=0.991								
cb1729_c18/f1p0/3891	gnl|BL_ORD_ID|31731 cb1729_c6/f2p2/3867 isoform=cb1729_c6;full_length_coverage=2;non_full_length_coverage=2;isoform_length=3867;expected_accuracy=0.996	282	3769	2	282	384	386 7	1	281
cb8564_c146124/f7p1/2108 isoform=cb8564_c146124;full_length_coverage=7;non_full_length_coverage=1;isoform_length=2108;expected_accuracy=1.000	gnl|BL_ORD_ID|12361 cb8564_c1392/f1p1/3024 isoform=cb8564_c1392;full_length_coverage=1;non_full_length_coverage=1;isoform_length=3024;expected_accuracy=0.993	1	1515	1515	2106	53	156 2	2433	302 4
cb8564_c41756/f1p0/2738 isoform=cb8564_c41756;full_length_coverage=1;non_full_length_coverage=0;isoform_length=2738;expected_accuracy=0.996	gnl|BL_ORD_ID|55359 cb8564_c39546/f1p2/3023	1	2173	2170	2738	1	214 8	2271	283 9
cb8564_c43399/f1p0/2654 isoform=cb8564_c43399;full_length_coverage=1;non_full_length_coverage=0;isoform_length=2654;expected_accuracy=0.999	gnl|BL_ORD_ID|17164 cb8564_c90856/f1p11/2858 isoform=cb8564_c90856;full_length_coverage=1;non_full_length_coverage=11;isoform_length=2858;expected_accuracy=0.999	23	2367	2363	2651	35	235 5	2572	285 8
cb8564_c36731/f1p2/2761	gnl|BL_ORD_ID|14091 cb8564_c43119/f1p4/3194 isoform=cb8564_c43119;full_length_coverage=1;non_full_length_coverage=4;isoform_length=3194;expected_accuracy=0.995	1	1977	1972	2698	1	199 2	2424	315 7
cb693_c8/f1p0/2842 isoform=cb693_c8;full_length_coverage=1;non_full_length_coverage=0;isoform_length=2842;expected_accuracy=0.993	gnl|BL_ORD_ID|4971 cb693_c3/f1p0/3671 isoform=cb693_c3;full_length_coverage=1;non_full_length_coverage=0;isoform_length=3671;expected_accuracy=0.995	1	2466	2462	2840	3	244 8	3296	367 1

cb2071_c7/f1p0/2109 isoform=cb2071_c7;full_length_coverage=1;non_full_length_coverage=0;isoform_length=2109;expected_accuracy=1.000	gnl|BL_ORD_ID|66664 cb2071_c6/f1p0/4059	1	1150	1145	2051	7	1158	3156	4061
cb5583_c2/f1p0/2107	gnl|BL_ORD_ID|46284 cb5583_c1/f1p0/3059 isoform=cb5583_c1;full_length_coverage=1;non_full_length_coverage=0;isoform_length=3059;expected_accuracy=0.997	132	2060	1	137	1127	3051	1	138
cb4030_c0/f2p0/2589 isoform=cb4030_c0;full_length_coverage=2;non_full_length_coverage=0;isoform_length=2589;expected_accuracy=0.999	gnl|BL_ORD_ID|71738 cb4030_c1/f1p0/3459	1	1555	1556	2589	16	1551	2331	3365
cb3061_c8/f1p0/2430 isoform=cb3061_c8;full_length_coverage=1;non_full_length_coverage=0;isoform_length=2430;expected_accuracy=0.999	gnl|BL_ORD_ID|69090 cb3061_c19/f1p0/3233	197	2429	1	199	871	3105	7	205
cb8564_c38035/f1p0/2770	gnl|BL_ORD_ID|55986 cb8564_c51172/f1p0/3367	2	2035	2033	2756	7	2059	2249	2973
cb6980_c16/f1p0/2284 isoform=cb6980_c16;full_length_coverage=1;non_full_length_coverage=0;isoform_length=2284;expected_accuracy=1.000	gnl|BL_ORD_ID|4579 cb6980_c7/f4p1/2478 isoform=cb6980_c7;full_length_coverage=4;non_full_length_coverage=1;isoform_length=2478;expected_accuracy=0.998	1	1987	1982	2284	18	2006	2130	2432
cb6622_c0/f8p0/2284 isoform=cb6622_c0;full_length_coverage=8;non_full_length_coverage=0;isoform_length=2284;expected_accuracy=1.000	gnl|BL_ORD_ID|3916 cb6622_c4/f1p0/2808 isoform=cb6622_c4;full_length_coverage=1;non_full_length_coverage=0;isoform_length=2808;expected_accuracy=0.998	175	2249	1	177	734	2808	17	195

cb11723_c0/f1p0/1808	gnl|BL_ORD_ID|60342 cb11723_c1/f1p0/2354	1	1303	1302	1751	21	1323	1557	2006
cb8564_c36056/f1p0/2652 isoform=cb8564_c36056;full_length_coverage=1;non_full_length_coverage=0;isoform_length=2652;expected_accuracy=0.999	gnl|BL_ORD_ID|54403 cb8564_c35093/f1p5/2916	248	2651	1	251	482	2885	88	338
cb8564_c43779/f1p0/3021 isoform=cb8564_c43779;full_length_coverage=1;non_full_length_coverage=0;isoform_length=3021;expected_accuracy=0.993	gnl|BL_ORD_ID|57017 cb8564_c80370/f1p0/3493	118	2344	2345	3021	3085	860	704	29
cb4698_c32/f1p0/3247	gnl|BL_ORD_ID|42963 cb4698_c11/f1p0/3141 isoform=cb4698_c11;full_length_coverage=1;non_full_length_coverage=0;isoform_length=3141;expected_accuracy=1.000	1398	3115	124	1398	1427	3141	1	1272
cb6956_c12/f1p0/2437	gnl|BL_ORD_ID|5805 cb6956_c6/f1p0/2394 isoform=cb6956_c6;full_length_coverage=1;non_full_length_coverage=0;isoform_length=2394;expected_accuracy=0.992	410	2255	6	409	555	2394	1	403
cb1404_c8/f1p0/2908 isoform=cb1404_c8;full_length_coverage=1;non_full_length_coverage=0;isoform_length=2908;expected_accuracy=0.996	gnl|BL_ORD_ID|26460 cb1404_c7/f1p0/3636 isoform=cb1404_c7;full_length_coverage=1;non_full_length_coverage=0;isoform_length=3636;expected_accuracy=0.997	1	2543	2539	2907	58	2575	3269	3636
cb9788_c16/f1p0/3980	gnl|BL_ORD_ID|58891 cb9788_c18/f1p0/3674	1253	3583	117	1253	1257	3587	1	1137
cb8564_c71716/f1p0/3226	gnl|BL_ORD_ID|54396 cb8564_c23546/f1p0/4263	1	2398	2398	3133	1	2405	2731	3466

cb16680_c6/f1p0/1706 isoform=cb16680_c6;full_length_coverage=1;non_full_length_coverage=0;isoform_length=1706;expected_accuracy=0.997	gnl|BL_ORD_ID|53383 cb8564_c123815/f1p0/2326	150	1706	1	154	537	209 3	51	204
cb56_c2/f1p0/5001	gnl|BL_ORD_ID|45012 cb56_c5/f1p0/4943 isoform=cb56_c5;full_length_coverage=1;non_full_length_coverage=0;isoform_length=4943;expected_accuracy=0.999	1	3926	3925	4635	167	4110	4233	494 3
cb7254_c5/f1p0/1881	gnl|BL_ORD_ID|7211 cb7254_c7/f1p0/2281 isoform=cb7254_c7;full_length_coverage=1;non_full_length_coverage=0;isoform_length=2281;expected_accuracy=0.994	126	1759	1	125	242	188 0	3	127
cb1279_c10/f2p0/2859 isoform=cb1279_c10;full_length_coverage=2;non_full_length_coverage=0;isoform_length=2859;expected_accuracy=0.999	gnl|BL_ORD_ID|27065 cb1279_c46/f1p3/3121 isoform=cb1279_c46;full_length_coverage=1;non_full_length_coverage=3;isoform_length=3121;expected_accuracy=1.000	202	2859	1	203	420	307 4	47	245
cb8564_c7674/f6p3/2437 isoform=cb8564_c7674;full_length_coverage=6;non_full_length_coverage=3;isoform_length=2437;expected_accuracy=0.995	gnl|BL_ORD_ID|12956 cb8564_c1771/f3p1/3767 isoform=cb8564_c1771;full_length_coverage=3;non_full_length_coverage=1;isoform_length=3767;expected_accuracy=0.996	1	1303	1301	2424	32	133 4	2646	376 7
cb8564_c41756/f1p0/2738 isoform=cb8564_c41756;full_length_coverage=1;non_full_length_coverage=0;isoform_length=2738;expected_accuracy=0.996	gnl|BL_ORD_ID|56036 cb8564_c52952/f1p0/3129	14	2173	2170	2737	22	216 2	2285	285 1
cb8349_c32/f1p0/2256 isoform=cb8349_c32;full_length_coverage=1;non_full_	gnl|BL_ORD_ID|52487 cb8349_c29/f1p0/2734	1	1525	1525	2161	3	152 7	1749	238 4

length_coverage=0;isoform_length=2256;expected_accuracy=0.994									
cb6032_c13/f1p2/1975 isoform=cb6032_c13;full_length_coverage=1;non_full_length_coverage=2;isoform_length=1975;expected_accuracy=0.991	gnl|BL_ORD_ID|46085 cb6032_c14/f1p2/2142 isoform=cb6032_c14;full_length_coverage=1;non_full_length_coverage=2;isoform_length=2142;expected_accuracy=0.996	1	1184	1181	1974	16	1202	1347	2142
cb8564_c38035/f1p0/2770	gnl|BL_ORD_ID|55291 cb8564_c37409/f1p0/3153	1	2035	2033	2754	1	2032	2222	2948
cb2640_c12/f1p0/3046 isoform=cb2640_c12;full_length_coverage=1;non_full_length_coverage=0;isoform_length=3046;expected_accuracy=0.994	gnl|BL_ORD_ID|68659 cb2640_c11/f1p0/3319	192	3046	1	193	459	3310	8	225
cb3143_c17/f1p0/2870 isoform=cb3143_c17;full_length_coverage=1;non_full_length_coverage=0;isoform_length=2870;expected_accuracy=0.993	gnl|BL_ORD_ID|69160 cb3143_c4/f1p0/3643	160	2870	1	163	685	3389	9	171
cb8564_c18383/f1p1/3801 isoform=cb8564_c18383;full_length_coverage=1;non_full_length_coverage=1;isoform_length=3801;expected_accuracy=0.990	gnl|BL_ORD_ID|53917 cb8564_c14852/f1p0/4493	1672	3784	1	1668	1808	3933	16	1671
cb947_c19/f1p1/2164 isoform=cb947_c19;full_length_coverage=1;non_full_length_coverage=1;isoform_length=2164;expected_accuracy=1.000	gnl|BL_ORD_ID|21933 cb947_c14/f1p0/4429 isoform=cb947_c14;full_length_coverage=1;non_full_length_coverage=0;isoform_length=4429;expected_accuracy=0.998	1	1312	1308	2161	7	1309	3575	4423
cb2145_c12/f1p0/2110 isoform=cb2145_c12;full_length_coverage=1;non_full_	gnl|BL_ORD_ID|66745 cb2145_c2/f1p0/4035	698	2110	91	697	2482	3887	1	607

length_coverage=0;isoform_length=2110;expected_accuracy=0.995									
cb8564_c90372/f1p0/2157 isoform=cb8564_c90372;full_length_coverage=1;non_full_length_coverage=0;isoform_length=2157;expected_accuracy=0.994	gnl|BL_ORD_ID|15904 cb8564_c69373/f4p3/2436 isoform=cb8564_c69373;full_length_coverage=4;non_full_length_coverage=3;isoform_length=2436;expected_accuracy=1.000	1	1092	1088	2157	101	1193	1364	2434
cb3678_c20/f11p0/3156 isoform=cb3678_c20;full_length_coverage=11;non_full_length_coverage=0;isoform_length=3156;expected_accuracy=1.000	gnl|BL_ORD_ID|71438 cb3678_c4/f1p0/3559	493	3156	9	496	607	3270	4	478
cb8564_c71725/f1p0/3227	gnl|BL_ORD_ID|15727 cb8564_c69026/f2p1/3011 isoform=cb8564_c69026;full_length_coverage=2;non_full_length_coverage=1;isoform_length=3011;expected_accuracy=0.999	10	1551	1548	2615	1	1534	1936	3002
cb9815_c3/f1p0/1942 isoform=cb9815_c3;full_length_coverage=1;non_full_length_coverage=0;isoform_length=1942;expected_accuracy=0.993	gnl|BL_ORD_ID|21400 cb9815_c2/f1p0/2812 isoform=cb9815_c2;full_length_coverage=1;non_full_length_coverage=0;isoform_length=2812;expected_accuracy=1.000	1	1678	1677	1942	10	1702	1926	2187
cb454_c23/f1p0/2557	gnl|BL_ORD_ID|42150 cb454_c1/f3p0/2382 isoform=cb454_c1;full_length_coverage=3;non_full_length_coverage=0;isoform_length=2382;expected_accuracy=1.000	242	2295	53	241	296	2350	1	189
cb8564_c88311/f1p1/2475 isoform=cb8564_c88311;full_length_coverage=1;non_full_length_coverage=1;isoform_length=2475;expected_accuracy=0.993	gnl|BL_ORD_ID|15376 cb8564_c31588/f10p3/3394 isoform=cb8564_c31588;full_length_coverage=10;non_full_length_coverage=3;isoform_length=3394;expected_accuracy=1.000	1	1814	1812	2473	215	2036	2727	3388

cb1178_c29/f1p0/1155	gnl|BL_ORD_ID|23776 cb1178_c19/f1p0/1460 isoform=cb1178_c19;full_length_coverage=1;non_full_length_coverage=0;isoform_length=1460;expected_accuracy=1.000	117	1167	1	116	297	1347	61	176
cb8564_c36731/f1p2/2761	gnl|BL_ORD_ID|16447 cb8564_c47202/f1p0/3195 isoform=cb8564_c47202;full_length_coverage=1;non_full_length_coverage=0;isoform_length=3195;expected_accuracy=0.996	1	1977	1972	2735	1	1980	2409	3195
cb12586_c4/f1p0/1346	gnl|BL_ORD_ID|23754 cb12586_c0/f2p0/1428 isoform=cb12586_c0;full_length_coverage=2;non_full_length_coverage=0;isoform_length=1428;expected_accuracy=0.996	398	1332	4	398	494	1428	1	390
cb3763_c48/f1p0/3166	gnl|BL_ORD_ID|39926 cb3763_c51/f1p1/2991 isoform=cb3763_c51;full_length_coverage=1;non_full_length_coverage=1;isoform_length=2991;expected_accuracy=0.999	371	2909	41	372	453	2991	6	335
cb5874_c15/f1p0/2254	gnl|BL_ORD_ID|74533 cb5874_c16/f1p0/3263	225	2249	1	224	875	2898	452	675
cb900_c13/f1p0/2723	gnl|BL_ORD_ID|20993 cb900_c14/f1p0/3179 isoform=cb900_c14;full_length_coverage=1;non_full_length_coverage=0;isoform_length=3179;expected_accuracy=0.994	1	1488	1488	2517	102	1598	2130	3161
cb8564_c15996/f1p5/3083 isoform=cb8564_c15996;full_length_coverage=1;non_full_length_coverage=5;isoform_length=3083;expected_accuracy=0.993	gnl|BL_ORD_ID|19212 cb8564_c85033/f2p16/3336 isoform=cb8564_c85033;full_length_coverage=2;non_full_length_coverage=16;isoform_length=3336;expected_accuracy=0.996	323	3083	6	328	469	3227	7	328

cb8564_c88629/f1p0/3306 isoform=cb8564_c88629;full_length_coverage=1;non_full_length_coverage=0;isoform_length=3306;expected_accuracy=0.999	gnl|BL_ORD_ID|11744 cb8564_c151271/f2p2/3622 isoform=cb8564_c151271;full_length_coverage=2;non_full_length_coverage=2;isoform_length=3622;expected_accuracy=0.991	30	3122	3122	3306	1	3099	3281	3465
cb8564_c40764/f1p0/2563 isoform=cb8564_c40764;full_length_coverage=1;non_full_length_coverage=0;isoform_length=2563;expected_accuracy=0.997	gnl|BL_ORD_ID|57175 cb8564_c85646/f1p0/3480	19	2166	2162	2563	106	2251	2459	2860
cb8564_c85288/f1p2/2983 isoform=cb8564_c85288;full_length_coverage=1;non_full_length_coverage=2;isoform_length=2983;expected_accuracy=0.999	gnl|BL_ORD_ID|54703 cb8564_c156696/f1p2/3606	1	2289	2289	2983	3	2297	2911	3605
cb8564_c1947/f1p0/2439	gnl|BL_ORD_ID|14046 cb8564_c3803/f4p0/2310 isoform=cb8564_c3803;full_length_coverage=4;non_full_length_coverage=0;isoform_length=2310;expected_accuracy=0.999	1	1972	1971	2140	57	2026	2137	2304
cb8564_c45300/f1p4/3014 isoform=cb8564_c45300;full_length_coverage=1;non_full_length_coverage=4;isoform_length=3014;expected_accuracy=0.996	gnl|BL_ORD_ID|56331 cb8564_c74623/f1p1/3530	243	3014	12	246	441	3214	18	249
cb415_c43/f1p0/3304 isoform=cb415_c43;full_length_coverage=1;non_full_length_coverage=0;isoform_length=3304;expected_accuracy=0.991	gnl|BL_ORD_ID|72139 cb415_c54/f1p0/3859	24	2924	2922	3304	129	3022	3345	3739
cb8564_c16829/f1p3/2089 isoform=cb8564_c16829;full_length_coverage=1;non_f	gnl|BL_ORD_ID|14463 cb8564_c25154/f1p5/4276 isoform=cb8564_c25154;full_length_coverage=1;non_fu	228	2089	65	229	2415	4276	27	189

ull_length_coverage=3;isoform_length=2089;expected_accuracy=0.995	ll_length_coverage=5;isoform_length=4276;expected_accuracy=0.995								
cb7986_c17/f1p0/2091 isoform=cb7986_c17;full_length_coverage=1;non_full_length_coverage=0;isoform_length=2091;expected_accuracy=0.991	gnl|BL_ORD_ID|6757 cb7986_c9/f1p0/2279 isoform=cb7986_c9;full_length_coverage=1;non_full_length_coverage=0;isoform_length=2279;expected_accuracy=1.000	687	2046	8	690	935	2279	41	712
cb8564_c23898/f1p1/3652	gnl|BL_ORD_ID|14193 cb8564_c31973/f2p1/4589 isoform=cb8564_c31973;full_length_coverage=2;non_full_length_coverage=1;isoform_length=4589;expected_accuracy=0.996	10	3057	3053	3624	5	3055	3965	4540
cb8564_c3832/f1p0/2033 isoform=cb8564_c3832;full_length_coverage=1;non_full_length_coverage=0;isoform_length=2033;expected_accuracy=0.995	gnl|BL_ORD_ID|13992 cb8564_c3828/f5p1/2269 isoform=cb8564_c3828;full_length_coverage=5;non_full_length_coverage=1;isoform_length=2269;expected_accuracy=1.000	1	1611	1610	2022	143	1754	1855	2269
cb8564_c92199/f1p0/2079	gnl|BL_ORD_ID|17306 cb8564_c78972/f1p0/2676 isoform=cb8564_c78972;full_length_coverage=1;non_full_length_coverage=0;isoform_length=2676;expected_accuracy=0.997	1	1795	1794	2063	33	1827	2346	2615
cb8564_c36056/f1p0/2652 isoform=cb8564_c36056;full_length_coverage=1;non_full_length_coverage=0;isoform_length=2652;expected_accuracy=0.999	gnl|BL_ORD_ID|56005 cb8564_c51803/f1p0/3354	248	2524	1	251	480	2758	86	336
cb1667_c7/f1p0/2596 isoform=cb1667_c7;full_length_coverage=1;non_full_length_coverage=0;isoform_length=2596;expected_accuracy=0.995	gnl|BL_ORD_ID|32659 cb1667_c5/f1p0/2649 isoform=cb1667_c5;full_length_coverage=1;non_full_length_coverage=0;isoform_length=2649;expected_accuracy=0.996	1	1719	1718	2511	5	1721	1855	2649

cb8564_c80085/f1p0/3315	gnl|BL_ORD_ID|58199 cb8564_c91279/f1p0/3567	525	2979	81	524	749	3203	64	508
cb4902_c20/f5p1/2187 isoform=cb4902_c20;full_length_coverage=5;non_full_length_coverage=1;isoform_length=2187;expected_accuracy=0.999	gnl|BL_ORD_ID|43250 cb4902_c11/f1p1/2252 isoform=cb4902_c11;full_length_coverage=1;non_full_length_coverage=1;isoform_length=2252;expected_accuracy=0.994	332	2187	45	334	396	2252	1	291
cb8564_c43119/f1p4/3194 isoform=cb8564_c43119;full_length_coverage=1;non_full_length_coverage=4;isoform_length=3194;expected_accuracy=0.995	gnl|BL_ORD_ID|55939 cb8564_c49630/f1p4/3487	2	2937	2937	3157	1	2913	3243	3463
cb4397_c4/f1p0/2292 isoform=cb4397_c4;full_length_coverage=1;non_full_length_coverage=0;isoform_length=2292;expected_accuracy=0.995	gnl|BL_ORD_ID|42920 cb4397_c2/f1p0/3361 isoform=cb4397_c2;full_length_coverage=1;non_full_length_coverage=0;isoform_length=3361;expected_accuracy=0.993	1	1829	1824	2215	988	2815	2969	3361
cb8564_c49356/f1p0/3133	gnl|BL_ORD_ID|14410 cb8564_c44256/f1p1/3267 isoform=cb8564_c44256;full_length_coverage=1;non_full_length_coverage=1;isoform_length=3267;expected_accuracy=0.995	1	2137	2133	2848	1	2119	2553	3267
cb7533_c13/f1p0/2022 isoform=cb7533_c13;full_length_coverage=1;non_full_length_coverage=0;isoform_length=2022;expected_accuracy=0.995	gnl|BL_ORD_ID|51319 cb7533_c5/f1p0/2459	149	2021	1	151	447	2321	63	213
cb8564_c147548/f5p1/3243 isoform=cb8564_c147548;full_length_coverage=5;non_full_length_coverage=1;isoform_length=3243;expected_accuracy=0.996	gnl|BL_ORD_ID|12106 cb8564_c146659/f3p0/3534 isoform=cb8564_c146659;full_length_coverage=3;non_full_length_coverage=0;isoform_length=3534;expected_accuracy=0.998	2	2039	2037	3243	52	2095	2324	3534

cb10299_c1/f3p0/1233 isoform=cb10299_c1;full_length_coverage=3;non_full_length_coverage=0;isoform_length=1233;expected_accuracy=1.000	gnl|BL_ORD_ID|48353 cb10299_c7/f1p0/3268	101	1233	1	104	2035	3167	4	107
cb8564_c91776/f1p2/2099	gnl|BL_ORD_ID|57982 cb8564_c85904/f1p1/2015	165	1834	12	164	301	1970	1	151
cb8564_c73853/f1p0/2443 isoform=cb8564_c73853;full_length_coverage=1;non_full_length_coverage=0;isoform_length=2443;expected_accuracy=1.000	gnl|BL_ORD_ID|57186 cb8564_c85953/f1p0/2986	1	1281	1276	2428	1	1281	1561	2716
cb8564_c40598/f1p4/3119	gnl|BL_ORD_ID|15169 cb8564_c40218/f1p0/3146 isoform=cb8564_c40218;full_length_coverage=1;non_full_length_coverage=0;isoform_length=3146;expected_accuracy=0.993	1	2732	2729	3049	4	2712	2831	3146
cb5137_c91/f1p0/3100	gnl|BL_ORD_ID|43804 cb5137_c27/f1p1/2948 isoform=cb5137_c27;full_length_coverage=1;non_full_length_coverage=1;isoform_length=2948;expected_accuracy=0.991	228	2800	1	231	377	2948	4	234
cb8564_c42814/f1p0/2151 isoform=cb8564_c42814;full_length_coverage=1;non_full_length_coverage=0;isoform_length=2151;expected_accuracy=0.993	gnl|BL_ORD_ID|55413 cb8564_c40978/f1p0/3073	650	2151	7	652	1344	2847	1	656
cb8564_c33962/f2p1/3330 isoform=cb8564_c33962;full_length_coverage=2;non_full_length_coverage=1;isoform_length=3330;expected_accuracy=1.000	gnl|BL_ORD_ID|15223 cb8564_c35037/f1p0/3377 isoform=cb8564_c35037;full_length_coverage=1;non_full_length_coverage=0;isoform_length=3377;expected_accuracy=0.996	1	2746	2744	3255	19	2761	2866	3377

cb8564_c15653/f1p4/2475	gnl|BL_ORD_ID|13063 cb8564_c179457/f1p0/2647 isoform=cb8564_c179457;full_length_coverage=1;non_full_length_coverage=0;isoform_length=2647;expected_accuracy=1.000	22	2293	2289	2435	32	2282	2501	2647
cb3887_c13/f1p0/2422	gnl|BL_ORD_ID|39511 cb3887_c5/f1p0/2830 isoform=cb3887_c5;full_length_coverage=1;non_full_length_coverage=0;isoform_length=2830;expected_accuracy=0.996	1	1827	1823	2410	64	1900	2239	2830
cb8564_c85665/f1p0/3087	gnl|BL_ORD_ID|55920 cb8564_c48949/f1p0/4357	2	2300	2301	2965	16	2293	2847	3504
cb10133_c6/f1p1/1304	gnl|BL_ORD_ID|3296 cb10133_c4/f1p1/1973 isoform=cb10133_c4;full_length_coverage=1;non_full_length_coverage=1;isoform_length=1973;expected_accuracy=0.995	1	1074	1070	1307	441	1518	1642	1879
cb1492_c9/f1p1/2823 isoform=cb1492_c9;full_length_coverage=1;non_full_length_coverage=1;isoform_length=2823;expected_accuracy=1.000	gnl|BL_ORD_ID|29081 cb1492_c1/f1p2/4305 isoform=cb1492_c1;full_length_coverage=1;non_full_length_coverage=2;isoform_length=4305;expected_accuracy=0.998	191	2823	1	196	1671	4302	31	226
cb2717_c10/f1p0/2555	gnl|BL_ORD_ID|38218 cb2717_c3/f1p0/2998 isoform=cb2717_c3;full_length_coverage=1;non_full_length_coverage=0;isoform_length=2998;expected_accuracy=0.993	1	1651	1647	2454	170	1816	2193	2998
cb3101_c20/f1p0/3230	gnl|BL_ORD_ID|69128 cb3101_c14/f1p0/3479	18	1954	1950	2934	3	1939	2235	3219
cb1326_c9/f1p0/3549 isoform=cb1326_c9;full_length_coverage=1;non_full_le	gnl|BL_ORD_ID|25378 cb1326_c34/f5p1/4137 isoform=cb1326_c34;full_length_coverage=5;non_full_l	1	3198	3194	3549	272	3469	3782	4137

ngth_coverage=0;isoform_length=3549;expected_accuracy=0.996	ength_coverage=1;isoform_length=4137;expected_accuracy=1.000								
cb5183_c115/f1p0/1016	gnl|BL_ORD_ID|73602 cb5183_c57/f1p0/1810	326	1016	8	330	940	1630	9	331
cb8564_c17933/f1p0/2278 isoform=cb8564_c17933;full_length_coverage=1;non_full_length_coverage=0;isoform_length=2278;expected_accuracy=1.000	gnl|BL_ORD_ID|11923 cb8564_c15085/f1p0/2489 isoform=cb8564_c15085;full_length_coverage=1;non_full_length_coverage=0;isoform_length=2489;expected_accuracy=0.995	1	1525	1525	2278	1	1541	1677	2430
cb8995_c8/f1p0/2000 isoform=cb8995_c8;full_length_coverage=1;non_full_length_coverage=0;isoform_length=2000;expected_accuracy=1.000	gnl|BL_ORD_ID|58501 cb8995_c5/f1p0/2524	97	2000	1	100	205	2109	2	101
cb4098_c31/f1p2/3666 isoform=cb4098_c31;full_length_coverage=1;non_full_length_coverage=2;isoform_length=3666;expected_accuracy=0.998	gnl|BL_ORD_ID|39575 cb4098_c28/f1p1/4006 isoform=cb4098_c28;full_length_coverage=1;non_full_length_coverage=1;isoform_length=4006;expected_accuracy=0.999	160	2000	1999	3666	17	1866	2277	3961
cb5896_c26/f1p1/3042 isoform=cb5896_c26;full_length_coverage=1;non_full_length_coverage=1;isoform_length=3042;expected_accuracy=0.996	gnl|BL_ORD_ID|74558 cb5896_c23/f1p0/4184	1	1672	1673	2979	7	1678	2860	4167
cb8564_c42506/f1p0/2909	gnl|BL_ORD_ID|55354 cb8564_c39256/f1p1/3847	1	2200	2199	2852	1040	3239	3112	3764
cb6211_c47/f1p0/2820	gnl|BL_ORD_ID|3557 cb6211_c55/f1p0/2825 isoform=cb6211_c55;full_length_coverage=1;non_full_length_coverage=0;isoform_length=2825;expected_accuracy=0.994	395	2595	54	394	550	2776	77	422

cb7076_c17/f11p1/2296 isoform=cb7076_c17;full_length_coverage=11;non_full_length_coverage=1;isoform_length=2296;expected_accuracy=0.997	gnl|BL_ORD_ID|4918 cb7076_c8/f1p1/2468 isoform=cb7076_c8;full_length_coverage=1;non_full_length_coverage=1;isoform_length=2468;expected_accuracy=0.995	104	2254	1	101	317	2468	11	114
cb6063_c19/f1p1/2514	gnl|BL_ORD_ID|46524 cb6063_c4/f2p0/2706 isoform=cb6063_c4;full_length_coverage=2;non_full_length_coverage=0;isoform_length=2706;expected_accuracy=0.999	157	2441	1	156	413	2690	1	155
cb18332_c7/f1p2/1609 isoform=cb18332_c7;full_length_coverage=1;non_full_length_coverage=2;isoform_length=1609;expected_accuracy=0.995	gnl|BL_ORD_ID|67241 cb18332_c6/f1p0/1841	117	1567	1	117	349	1800	87	203
cb13748_c15/f2p0/3413 isoform=cb13748_c15;full_length_coverage=2;non_full_length_coverage=0;isoform_length=3413;expected_accuracy=1.000	gnl|BL_ORD_ID|27754 cb13748_c13/f2p0/3522 isoform=cb13748_c13;full_length_coverage=2;non_full_length_coverage=0;isoform_length=3522;expected_accuracy=0.998	26	3147	3143	3412	1	3101	3216	3490
cb8564_c42654/f1p1/2906 isoform=cb8564_c42654;full_length_coverage=1;non_full_length_coverage=1;isoform_length=2906;expected_accuracy=0.990	gnl|BL_ORD_ID|16172 cb8564_c53024/f1p0/3159 isoform=cb8564_c53024;full_length_coverage=1;non_full_length_coverage=0;isoform_length=3159;expected_accuracy=0.996	1	1706	1703	2824	1	1699	2017	3133
cb8564_c41756/f1p0/2738 isoform=cb8564_c41756;full_length_coverage=1;non_full_length_coverage=0;isoform_length=2738;expected_accuracy=0.996	gnl|BL_ORD_ID|15104 cb8564_c37866/f1p3/2874 isoform=cb8564_c37866;full_length_coverage=1;non_full_length_coverage=3;isoform_length=2874;expected_accuracy=0.990	1	2173	2170	2738	1	2173	2300	2867
cb7940_c54/f1p1/1719 isoform=cb7940_c54;full_length_coverage=1;non_full_	gnl|BL_ORD_ID|8821 cb7940_c58/f68p1/2037 isoform=cb7940_c58;full_length_coverage=68;non_full	1	1366	1363	1719	112	1477	1673	2030

length_coverage=1;isoform_length=1719;expected_accuracy=0.997	_length_coverage=1;isoform_length=2037;expected_accuracy=1.000								
cb8564_c21138/f1p3/2652	gnl|BL_ORD_ID|14058 cb8564_c23936/f1p0/3016 isoform=cb8564_c23936;full_length_coverage=1;non_full_length_coverage=0;isoform_length=3016;expected_accuracy=0.995	1	1710	1708	2580	4	1716	2143	3016
cb12983_c7/f1p2/1542 isoform=cb12983_c7;full_length_coverage=1;non_full_length_coverage=2;isoform_length=1542;expected_accuracy=0.999	gnl|BL_ORD_ID|12784 cb8564_c16498/f2p2/2818 isoform=cb8564_c16498;full_length_coverage=2;non_full_length_coverage=2;isoform_length=2818;expected_accuracy=1.000	239	1542	73	243	1360	2660	411	580
cb8564_c130391/f1p0/2103	gnl|BL_ORD_ID|53375 cb8564_c123599/f1p0/2339	132	1996	1	135	436	2300	1	135
cb9425_c0/f1p0/2087 isoform=cb9425_c0;full_length_coverage=1;non_full_length_coverage=0;isoform_length=2087;expected_accuracy=1.000	gnl|BL_ORD_ID|19736 cb9425_c1/f1p0/2223 isoform=cb9425_c1;full_length_coverage=1;non_full_length_coverage=0;isoform_length=2223;expected_accuracy=0.996	1	1614	1615	2085	1	1615	1754	2223
cb1960_c41/f1p1/2517	gnl|BL_ORD_ID|34968 cb1960_c10/f2p0/3006 isoform=cb1960_c10;full_length_coverage=2;non_full_length_coverage=0;isoform_length=3006;expected_accuracy=1.000	1	2123	2120	2503	30	2175	2573	2956
cb3570_c11/f1p0/3198	gnl|BL_ORD_ID|39906 cb3570_c7/f1p0/3031 isoform=cb3570_c7;full_length_coverage=1;non_full_length_coverage=0;isoform_length=3031;expected_accuracy=0.997	239	2854	20	240	380	2995	15	233
cb2324_c36/f1p1/3463	gnl|BL_ORD_ID|36741 cb2324_c5/f2p1/3722 isoform=cb2324_c5;full_length_coverage=2;non_full_le	100	3477	1	100	329	3701	75	174

	ngth_coverage=1;isoform_length=3722;expected_accuracy=0.995								
cb8564_c107332/f9p1/3308 isoform=cb8564_c107332;full_length_coverage=9;non_full_length_coverage=1;isoform_length=3308;expected_accuracy=0.997	gnl|BL_ORD_ID|16917 cb8564_c48079/f5p1/3512 isoform=cb8564_c48079;full_length_coverage=5;non_full_length_coverage=1;isoform_length=3512;expected_accuracy=0.995	465	3308	78	466	499	336 4	1	358
cb8564_c36145/f1p0/2868	gnl|BL_ORD_ID|9350 cb8564_c11826/f1p0/2916 isoform=cb8564_c11826;full_length_coverage=1;non_full_length_coverage=0;isoform_length=2916;expected_accuracy=0.997	1	1291	1290	2653	8	129 6	1543	290 8
cb3087_c5/f3p1/2943 isoform=cb3087_c5;full_length_coverage=3;non_full_length_coverage=1;isoform_length=2943;expected_accuracy=1.000	gnl|BL_ORD_ID|36601 cb3087_c18/f1p1/3011 isoform=cb3087_c18;full_length_coverage=1;non_full_length_coverage=1;isoform_length=3011;expected_accuracy=1.000	16	2327	2323	2834	1	231 2	2451	296 3
cb8564_c122994/f1p1/1940 isoform=cb8564_c122994;full_length_coverage=1;non_full_length_coverage=1;isoform_length=1940;expected_accuracy=1.000	gnl|BL_ORD_ID|11486 cb8564_c127460/f1p2/2151 isoform=cb8564_c127460;full_length_coverage=1;non_full_length_coverage=2;isoform_length=2151;expected_accuracy=0.998	1	1751	1748	1881	126	187 9	2018	215 1
cb8564_c145342/f2p0/2771 isoform=cb8564_c145342;full_length_coverage=2;non_full_length_coverage=0;isoform_length=2771;expected_accuracy=0.996	gnl|BL_ORD_ID|12841 cb8564_c168338/f2p0/3398 isoform=cb8564_c168338;full_length_coverage=2;non_full_length_coverage=0;isoform_length=3398;expected_accuracy=0.999	441	2771	5	442	564	288 9	2	440
cb7214_c10/f1p0/1871	gnl|BL_ORD_ID|51048 cb7214_c8/f1p0/2224	134	1809	1	136	288	196 3	42	177
cb13386_c19/f1p0/1632	gnl|BL_ORD_ID|21079 cb9143_c23/f4p0/2041 isoform=cb9143_c23;full_length_coverage=4;non_full_l	1	1390	1386	1635	10	139 9	1698	194 7

	ength_coverage=0;isoform_length=2041;expected_accuracy=0.994								
cb3013_c7/f1p0/1974 isoform=cb3013_c7;full_length_coverage=1;non_full_length_coverage=0;isoform_length=1974;expected_accuracy=0.995	gnl|BL_ORD_ID|70137 cb3013_c10/f1p0/3338	1	1405	1404	1972	37	1457	2372	2940
cb8564_c76927/f1p0/2418 isoform=cb8564_c76927;full_length_coverage=1;non_full_length_coverage=0;isoform_length=2418;expected_accuracy=0.998	gnl|BL_ORD_ID|16550 cb8564_c68385/f5p0/2739 isoform=cb8564_c68385;full_length_coverage=5;non_full_length_coverage=0;isoform_length=2739;expected_accuracy=1.000	2	2014	2013	2418	85	2099	2334	2739
cb1163_c15/f1p3/3725 isoform=cb1163_c15;full_length_coverage=1;non_full_length_coverage=3;isoform_length=3725;expected_accuracy=0.999	gnl|BL_ORD_ID|23462 cb1163_c14/f1p1/4695 isoform=cb1163_c14;full_length_coverage=1;non_full_length_coverage=1;isoform_length=4695;expected_accuracy=0.991	1	1951	1950	3616	4	1955	3030	4695
cb8564_c43546/f1p1/3276	gnl|BL_ORD_ID|14407 cb8564_c44036/f1p0/3258 isoform=cb8564_c44036;full_length_coverage=1;non_full_length_coverage=0;isoform_length=3258;expected_accuracy=0.992	1179	2854	10	1179	1575	3247	1	1147
cb9571_c10/f1p1/1876 isoform=cb9571_c10;full_length_coverage=1;non_full_length_coverage=1;isoform_length=1876;expected_accuracy=0.997	gnl|BL_ORD_ID|21299 cb9571_c7/f1p1/2039 isoform=cb9571_c7;full_length_coverage=1;non_full_length_coverage=1;isoform_length=2039;expected_accuracy=0.992	1	1247	1248	1876	1	1252	1411	2038
cb8564_c152641/f1p6/2794	gnl|BL_ORD_ID|16364 cb8564_c72362/f4p1/4130 isoform=cb8564_c72362;full_length_coverage=4;non_full_length_coverage=1;isoform_length=4130;expected_accuracy=0.998	197	2753	12	196	1570	4130	63	250

cb2279_c5/f1p1/2918	gnl|BL_ORD_ID|36529 cb2279_c6/f1p1/3055 isoform=cb2279_c6;full_length_coverage=1;non_full_length_coverage=1;isoform_length=3055;expected_accuracy=0.996	22	2593	2592	2960	16	2580	2692	3054
cb8564_c50881/f1p2/2125 isoform=cb8564_c50881;full_length_coverage=1;non_full_length_coverage=2;isoform_length=2125;expected_accuracy=0.994	gnl|BL_ORD_ID|11977 cb8564_c146395/f9p1/2471 isoform=cb8564_c146395;full_length_coverage=9;non_full_length_coverage=1;isoform_length=2471;expected_accuracy=0.998	1	1863	1858	2112	1	1865	2142	2399
cb6528_c8/f1p0/2123	gnl|BL_ORD_ID|49801 cb6528_c5/f1p0/2611	1	1276	1273	2032	122	1397	1853	2611
cb3586_c10/f1p0/2722 isoform=cb3586_c10;full_length_coverage=1;non_full_length_coverage=0;isoform_length=2722;expected_accuracy=0.993	gnl|BL_ORD_ID|41149 cb3586_c2/f1p0/3545 isoform=cb3586_c2;full_length_coverage=1;non_full_length_coverage=0;isoform_length=3545;expected_accuracy=0.998	252	2722	1	252	950	3422	77	335
cb8564_c42506/f1p0/2909	gnl|BL_ORD_ID|55988 cb8564_c51312/f1p2/3964	1	2203	2199	2857	1057	3259	3129	3787
cb8564_c12355/f1p9/2685 isoform=cb8564_c12355;full_length_coverage=1;non_full_length_coverage=9;isoform_length=2685;expected_accuracy=0.999	gnl|BL_ORD_ID|17556 cb8564_c9820/f2p0/3032 isoform=cb8564_c9820;full_length_coverage=2;non_full_length_coverage=0;isoform_length=3032;expected_accuracy=0.995	1	1731	1729	2630	1	1704	2131	3032
cb4142_c6/f1p0/2339	gnl|BL_ORD_ID|40542 cb4142_c1/f1p0/3435 isoform=cb4142_c1;full_length_coverage=1;non_full_length_coverage=0;isoform_length=3435;expected_accuracy=0.996	550	2034	9	552	1947	3431	30	570
cb8564_c74379/f1p1/2508	gnl|BL_ORD_ID|56412 cb8564_c76337/f1p1/2735	1	1645	1642	2369	2	1647	1852	2579

cb6471_c27/f1p0/2602	gnl|BL_ORD_ID|5494 cb6471_c7/f2p0/2927 isoform=cb6471_c7;full_length_coverage=2;non_full_length_coverage=0;isoform_length=2927;expected_accuracy=0.997	1	1863	1864	2622	52	1914	2074	2832
cb8564_c160490/f1p4/3939	gnl|BL_ORD_ID|9961 cb8564_c12013/f1p0/4420 isoform=cb8564_c12013;full_length_coverage=1;non_full_length_coverage=0;isoform_length=4420;expected_accuracy=0.993	1	3052	3048	3789	35	3077	3654	4397
cb1224_c13/f1p1/3758	gnl|BL_ORD_ID|59284 cb1224_c19/f1p1/4162	1	2859	2863	3647	212	3070	3250	4033
cb8564_c125661/f1p1/2227	gnl|BL_ORD_ID|53602 cb8564_c129771/f1p0/3078	1	1184	1179	2079	1	1183	2152	3052
cb8564_c124328/f1p0/1986 isoform=cb8564_c124328;full_length_coverage=1;non_full_length_coverage=0;isoform_length=1986;expected_accuracy=0.991	gnl|BL_ORD_ID|18994 cb8564_c87879/f1p0/2371 isoform=cb8564_c87879;full_length_coverage=1;non_full_length_coverage=0;isoform_length=2371;expected_accuracy=0.991	1	1479	1475	1986	4	1487	1859	2369
cb535_c2/f1p0/4696	gnl|BL_ORD_ID|45719 cb535_c5/f1p0/4656 isoform=cb535_c5;full_length_coverage=1;non_full_length_coverage=0;isoform_length=4656;expected_accuracy=0.995	1012	4486	1	1012	1192	4656	4	1012
cb8564_c41162/f1p0/3158	gnl|BL_ORD_ID|55578 cb8564_c45787/f1p3/3310	10	2553	2548	3040	104	2647	2778	3270
cb309_c7/f1p2/4109 isoform=cb309_c7;full_length_coverage=1;non_full_length_coverage=2;isoform_length=4109;expected_accuracy=0.997	gnl|BL_ORD_ID|69547 cb309_c21/f1p0/4520	1	3739	3740	4085	2	3720	4055	4400

cb9541_c3/f1p0/2530 isoform=cb9541_c3;full_length_coverage=1;non_full_length_coverage=0;isoform_length=2530;expected_accuracy=0.999	gnl|BL_ORD_ID|19611 cb9541_c2/f1p0/2876 isoform=cb9541_c2;full_length_coverage=1;non_full_length_coverage=0;isoform_length=2876;expected_accuracy=0.999	1	2264	2262	2530	85	2357	2469	2739
cb18456_c1748/f1p0/1180	gnl|BL_ORD_ID|67356 cb18456_c1797/f1p0/1494	243	1188	3	248	420	1365	39	282
cb2640_c23/f1p0/2402	gnl|BL_ORD_ID|37283 cb2640_c31/f3p0/3266 isoform=cb2640_c31;full_length_coverage=3;non_full_length_coverage=0;isoform_length=3266;expected_accuracy=1.000	1	2036	2038	2406	15	2050	2775	3144
cb750_c64/f2p2/3775 isoform=cb750_c64;full_length_coverage=2;non_full_length_coverage=2;isoform_length=3775;expected_accuracy=1.000	gnl|BL_ORD_ID|7348 cb750_c27/f1p1/4163 isoform=cb750_c27;full_length_coverage=1;non_full_length_coverage=1;isoform_length=4163;expected_accuracy=0.998	170	2328	2327	3770	1	2162	2719	4163
cb6794_c9/f2p0/2356 isoform=cb6794_c9;full_length_coverage=2;non_full_length_coverage=0;isoform_length=2356;expected_accuracy=1.000	gnl|BL_ORD_ID|4893 cb6794_c49/f6p1/2503 isoform=cb6794_c49;full_length_coverage=6;non_full_length_coverage=1;isoform_length=2503;expected_accuracy=0.998	1	2119	2117	2354	1	2118	2266	2503
cb8564_c184250/f1p0/2256 isoform=cb8564_c184250;full_length_coverage=1;non_full_length_coverage=0;isoform_length=2256;expected_accuracy=0.996	gnl|BL_ORD_ID|56102 cb8564_c69761/f1p0/2560	1	1642	1638	2255	1	1651	1912	2527
cb8564_c15653/f1p4/2475	gnl|BL_ORD_ID|13571 cb8564_c20949/f2p0/2862 isoform=cb8564_c20949;full_length_coverage=2;non_full_length_coverage=0;isoform_length=2862;expected_accuracy=0.997	1	2293	2289	2482	150	2441	2660	2853

cb8564_c42814/f1p0/2151 isoform=cb8564_c42814;full_length_coverage=1;non_full_length_coverage=0;isoform_length=2151;expected_accuracy=0.993	gnl|BL_ORD_ID|55376 cb8564_c39821/f1p0/3886	650	2148	7	652	2251	3735	1	647
cb3603_c18/f1p1/2591	gnl|BL_ORD_ID|40849 cb3603_c49/f12p4/3214 isoform=cb3603_c49;full_length_coverage=12;non_full_length_coverage=4;isoform_length=3214;expected_accuracy=1.000	201	2583	2	201	802	3184	1	203
cb8564_c89918/f1p1/2045 isoform=cb8564_c89918;full_length_coverage=1;non_full_length_coverage=1;isoform_length=2045;expected_accuracy=0.991	gnl|BL_ORD_ID|52251 cb8564_c115598/f1p0/2466	1	1616	1611	2045	5	1592	1787	2220
cb8564_c128725/f1p1/2771 isoform=cb8564_c128725;full_length_coverage=1;non_full_length_coverage=1;isoform_length=2771;expected_accuracy=1.000	gnl|BL_ORD_ID|56601 cb8564_c48153/f1p0/3083	1	2669	2664	2769	13	2682	2792	2896
cb13770_c0/f5p0/1737 isoform=cb13770_c0;full_length_coverage=5;non_full_length_coverage=0;isoform_length=1737;expected_accuracy=1.000	gnl|BL_ORD_ID|18766 cb8564_c92150/f1p0/4398 isoform=cb8564_c92150;full_length_coverage=1;non_full_length_coverage=0;isoform_length=4398;expected_accuracy=0.997	108	1725	1	110	2784	4398	1	109
cb8564_c69975/f1p0/2818 isoform=cb8564_c69975;full_length_coverage=1;non_full_length_coverage=0;isoform_length=2818;expected_accuracy=0.999	gnl|BL_ORD_ID|12051 cb8564_c147180/f4p0/3043 isoform=cb8564_c147180;full_length_coverage=4;non_full_length_coverage=0;isoform_length=3043;expected_accuracy=0.998	260	2818	1	259	420	2977	1	259
cb18456_c6530/f1p0/1200	gnl|BL_ORD_ID|22961 cb11694_c0/f1p2/1835 isoform=cb11694_c0;full_length_coverage=1;non_full_l	260	1166	3	259	964	59	1751	1495

	ength_coverage=2;isoform_length=1835;expected_accuracy=0.996								
cb5221_c12/f1p1/3269 isoform=cb5221_c12;full_length_coverage=1;non_full_length_coverage=1;isoform_length=3269;expected_accuracy=0.994	gnl|BL_ORD_ID|45742 cb5221_c11/f1p0/3287 isoform=cb5221_c11;full_length_coverage=1;non_full_length_coverage=0;isoform_length=3287;expected_accuracy=0.996	219	3115	1	220	390	3287	1	221
cb8564_c15653/f1p4/2475	gnl|BL_ORD_ID|52715 cb8564_c11997/f1p2/2670	22	2293	2289	2396	32	2303	2522	2629
cb8564_c42814/f1p0/2151 isoform=cb8564_c42814;full_length_coverage=1;non_full_length_coverage=0;isoform_length=2151;expected_accuracy=0.993	gnl|BL_ORD_ID|16805 cb8564_c66657/f4p6/2862 isoform=cb8564_c66657;full_length_coverage=4;non_full_length_coverage=6;isoform_length=2862;expected_accuracy=1.000	650	2149	7	652	1361	2862	29	673
cb8564_c89762/f1p0/2818 isoform=cb8564_c89762;full_length_coverage=1;non_full_length_coverage=0;isoform_length=2818;expected_accuracy=0.994	gnl|BL_ORD_ID|19256 cb8564_c87348/f1p0/3176 isoform=cb8564_c87348;full_length_coverage=1;non_full_length_coverage=0;isoform_length=3176;expected_accuracy=0.997	163	2818	12	168	473	3169	1	162
cb8564_c127715/f2p0/2769 isoform=cb8564_c127715;full_length_coverage=2;non_full_length_coverage=0;isoform_length=2769;expected_accuracy=0.997	gnl|BL_ORD_ID|53688 cb8564_c155311/f1p0/3003	1196	2769	1	1198	1434	3015	94	1289
cb8564_c2737/f1p0/2712	gnl|BL_ORD_ID|14039 cb8564_c2753/f1p0/2971 isoform=cb8564_c2753;full_length_coverage=1;non_full_length_coverage=0;isoform_length=2971;expected_accuracy=0.997	1156	2744	1	1157	1361	2971	1	1157
cb6390_c3/f1p0/2298 isoform=cb6390_c3;full_length_coverage=1;non_full_le	gnl|BL_ORD_ID|3696 cb6390_c2/f1p0/2643 isoform=cb6390_c2;full_length_coverage=1;non_full_le	1	1513	1511	2242	266	1776	1912	2643

ngth_coverage=0;isoform_length=2298;expected_accuracy=0.998	ngth_coverage=0;isoform_length=2643;expected_accuracy=0.998								
cb8564_c15653/f1p4/2475	gnl|BL_ORD_ID|17747 cb8564_c81734/f1p1/2771 isoform=cb8564_c81734;full_length_coverage=1;non_full_length_coverage=1;isoform_length=2771;expected_accuracy=0.995	1	2293	2289	2426	124	2414	2634	2771
cb8564_c15376/f1p0/4060	gnl|BL_ORD_ID|10828 cb8564_c10736/f3p10/3904 isoform=cb8564_c10736;full_length_coverage=3;non_full_length_coverage=10;isoform_length=3904;expected_accuracy=0.998	12	2717	2717	3675	137	2842	2946	3904
cb8564_c78606/f1p0/3454 isoform=cb8564_c78606;full_length_coverage=1;non_full_length_coverage=0;isoform_length=3454;expected_accuracy=0.992	gnl|BL_ORD_ID|56330 cb8564_c74576/f1p0/4386	1	2448	2448	3452	7	2452	3000	4002
cb313_c3/f2p0/2434 isoform=cb313_c3;full_length_coverage=2;non_full_length_coverage=0;isoform_length=2434;expected_accuracy=0.995	gnl|BL_ORD_ID|38276 cb313_c5/f2p0/2828 isoform=cb313_c5;full_length_coverage=2;non_full_length_coverage=0;isoform_length=2828;expected_accuracy=0.997	2	2112	2112	2393	5	2113	2547	2828
cb8564_c41756/f1p0/2738 isoform=cb8564_c41756;full_length_coverage=1;non_full_length_coverage=0;isoform_length=2738;expected_accuracy=0.996	gnl|BL_ORD_ID|55866 cb8564_c47522/f1p0/2954	1	2173	2170	2738	1	2158	2281	2849
cb8564_c5050/f1p0/3151 isoform=cb8564_c5050;full_length_coverage=1;non_full_length_coverage=0;isoform_length=3151;expected_accuracy=0.994	gnl|BL_ORD_ID|16604 cb8564_c5045/f1p0/3665 isoform=cb8564_c5045;full_length_coverage=1;non_full_length_coverage=0;isoform_length=3665;expected_accuracy=0.994	141	3151	1	142	384	3399	54	195

cb8564_c90621/f1p2/3030 isoform=cb8564_c90621;full_length_coverage=1;non_full_length_coverage=2;isoform_length=3030;expected_accuracy=0.994	gnl|BL_ORD_ID|56770 cb8564_c71034/f1p2/3266	206	3030	1	205	361	3184	23	227
cb581_c7/f1p0/2369 isoform=cb581_c7;full_length_coverage=1;non_full_length_coverage=0;isoform_length=2369;expected_accuracy=0.990	gnl|BL_ORD_ID|45251 cb581_c4/f1p1/2569 isoform=cb581_c4;full_length_coverage=1;non_full_length_coverage=1;isoform_length=2569;expected_accuracy=0.999	1	1631	1627	2368	79	1728	1833	2569
cb8564_c20798/f1p1/2960	gnl|BL_ORD_ID|9759 cb8564_c108367/f4p6/3120 isoform=cb8564_c108367;full_length_coverage=4;non_full_length_coverage=6;isoform_length=3120;expected_accuracy=0.999	1314	2932	1	1319	1483	3101	21	1339
cb8564_c38035/f1p0/2770	gnl|BL_ORD_ID|56623 cb8564_c49310/f1p2/3045	19	2035	2033	2756	65	2084	2274	2998
cb6095_c3/f1p0/2445 isoform=cb6095_c3;full_length_coverage=1;non_full_length_coverage=0;isoform_length=2445;expected_accuracy=0.994	gnl|BL_ORD_ID|46748 cb6095_c2/f1p0/2694 isoform=cb6095_c2;full_length_coverage=1;non_full_length_coverage=0;isoform_length=2694;expected_accuracy=0.994	1	1340	1338	2445	12	1349	1516	2624
cb2640_c23/f1p0/2402	gnl|BL_ORD_ID|69857 cb2640_c25/f1p0/3308	1	2036	2038	2400	60	2095	2820	3184
cb8564_c3927/f1p1/2747 isoform=cb8564_c3927;full_length_coverage=1;non_full_length_coverage=1;isoform_length=2747;expected_accuracy=0.995	gnl|BL_ORD_ID|12615 cb8564_c138783/f5p1/3091 isoform=cb8564_c138783;full_length_coverage=5;non_full_length_coverage=1;isoform_length=3091;expected_accuracy=0.999	1	1593	1593	2747	132	1724	1850	3002
cb316_c29/f1p2/4133 isoform=cb316_c29;full_length_coverage=1;non_full_le	gnl|BL_ORD_ID|69554 cb316_c10/f1p2/4819	2	2583	2579	4121	142	2702	3318	4858

ngth_coverage=2;isoform_length=4133;expected_accuracy=0.992									
cb8564_c42814/f1p0/2151 isoform=cb8564_c42814;full_length_coverage=1;non_full_length_coverage=0;isoform_length=2151;expected_accuracy=0.993	gnl|BL_ORD_ID|54403 cb8564_c35093/f1p5/2916	650	2151	8	652	1380	2883	20	692
cb8564_c123192/f1p0/1931 isoform=cb8564_c123192;full_length_coverage=1;non_full_length_coverage=0;isoform_length=1931;expected_accuracy=0.994	gnl|BL_ORD_ID|18186 cb8564_c79521/f2p0/2343 isoform=cb8564_c79521;full_length_coverage=2;non_full_length_coverage=0;isoform_length=2343;expected_accuracy=0.995	1	1173	1172	1931	1	1177	1311	2070
cb18456_c1418/f2p0/1818 isoform=cb18456_c1418;full_length_coverage=2;non_full_length_coverage=0;isoform_length=1818;expected_accuracy=0.992	gnl|BL_ORD_ID|56115 cb8564_c70012/f1p0/2001	1	1002	998	1818	2	998	1098	1913
cb8564_c88409/f1p1/2884	gnl|BL_ORD_ID|67972 cb18738_c0/f1p0/5429	1	1486	1486	2758	13	1504	4055	5327
cb8564_c181853/f1p0/2119 isoform=cb8564_c181853;full_length_coverage=1;non_full_length_coverage=0;isoform_length=2119;expected_accuracy=0.997	gnl|BL_ORD_ID|55383 cb8564_c40109/f1p0/2886	1	1339	1334	2117	1	1340	2085	2866
cb848_c2/f2p2/3920 isoform=cb848_c2;full_length_coverage=2;non_full_length_coverage=2;isoform_length=3920;expected_accuracy=0.993	gnl|BL_ORD_ID|9940 cb848_c9/f1p1/4053 isoform=cb848_c9;full_length_coverage=1;non_full_length_coverage=1;isoform_length=4053;expected_accuracy=0.995	1	3501	3500	3917	4	3521	3632	4048
cb2880_c1/f3p0/2764 isoform=cb2880_c1;full_length_coverage=3;non_full_le	gnl|BL_ORD_ID|38441 cb2880_c3/f1p0/3627 isoform=cb2880_c3;full_length_coverage=1;non_full_le	1	2302	2297	2764	3	2306	3160	3627

ngth_coverage=0;isoform_length=2764;expected_accuracy=1.000	ngth_coverage=0;isoform_length=3627;expected_accuracy=0.991								
cb10963_c2/f1p0/1707 isoform=cb10963_c2;full_length_coverage=1;non_full_length_coverage=0;isoform_length=1707;expected_accuracy=0.996	gnl|BL_ORD_ID|1150 cb10963_c1/f1p0/2063 isoform=cb10963_c1;full_length_coverage=1;non_full_length_coverage=0;isoform_length=2063;expected_accuracy=0.992	560	1698	64	562	925	2063	1	499
cb2733_c21/f1p0/1180 isoform=cb2733_c21;full_length_coverage=1;non_full_length_coverage=0;isoform_length=1180;expected_accuracy=1.000	gnl|BL_ORD_ID|68774 cb2733_c13/f1p0/1472	115	1180	1	115	261	1326	3	118
cb13082_c10/f1p0/1535	gnl|BL_ORD_ID|25743 cb13082_c2/f3p0/1615 isoform=cb13082_c2;full_length_coverage=3;non_full_length_coverage=0;isoform_length=1615;expected_accuracy=1.000	1	1284	1283	1480	2	1285	1418	1615
cb653_c9/f1p0/4025 isoform=cb653_c9;full_length_coverage=1;non_full_length_coverage=0;isoform_length=4025;expected_accuracy=0.997	gnl|BL_ORD_ID|49384 cb653_c26/f1p0/4462	301	4024	3	301	484	4208	7	306
cb2873_c1/f12p3/3025 isoform=cb2873_c1;full_length_coverage=12;non_full_length_coverage=3;isoform_length=3025;expected_accuracy=1.000	gnl|BL_ORD_ID|68966 cb2873_c10/f1p0/3294	191	3025	11	191	321	3153	1	181
cb7764_c6/f1p0/2353 isoform=cb7764_c6;full_length_coverage=1;non_full_length_coverage=0;isoform_length=2353;expected_accuracy=0.999	gnl|BL_ORD_ID|7024 cb7764_c5/f1p0/2511 isoform=cb7764_c5;full_length_coverage=1;non_full_length_coverage=0;isoform_length=2511;expected_accuracy=0.996	245	2320	3	249	439	2510	89	335

cb4674_c86/f89p3/3166 isoform=cb4674_c86;full_length_coverage=89;non_full_length_coverage=3;isoform_length=3166;expected_accuracy=1.000	gnl|BL_ORD_ID|72844 cb4674_c33/f1p3/3281	100	2315	2313	3166	3	2220	2382	3234
cb8564_c185920/f1p0/2777 isoform=cb8564_c185920;full_length_coverage=1;non_full_length_coverage=0;isoform_length=2777;expected_accuracy=0.996	gnl|BL_ORD_ID|14399 cb8564_c39189/f1p1/2975 isoform=cb8564_c39189;full_length_coverage=1;non_full_length_coverage=1;isoform_length=2975;expected_accuracy=0.992	174	2776	1	177	355	2975	15	193
cb6221_c5/f3p1/2261 isoform=cb6221_c5;full_length_coverage=3;non_full_length_coverage=1;isoform_length=2261;expected_accuracy=0.999	gnl|BL_ORD_ID|4880 cb6221_c4/f3p3/2906 isoform=cb6221_c4;full_length_coverage=3;non_full_length_coverage=3;isoform_length=2906;expected_accuracy=1.000	256	2260	73	256	900	2905	1	187
cb5891_c8/f1p0/2721	gnl|BL_ORD_ID|45259 cb5891_c2/f1p0/3660 isoform=cb5891_c2;full_length_coverage=1;non_full_length_coverage=0;isoform_length=3660;expected_accuracy=1.000	1213	2627	1	1212	2247	3660	1	1210
cb8564_c4743/f1p0/2508 isoform=cb8564_c4743;full_length_coverage=1;non_full_length_coverage=0;isoform_length=2508;expected_accuracy=0.994	gnl|BL_ORD_ID|14698 cb8564_c4069/f2p0/2782 isoform=cb8564_c4069;full_length_coverage=2;non_full_length_coverage=0;isoform_length=2782;expected_accuracy=0.995	1	2086	2084	2508	1	2103	2282	2705
cb4508_c10/f1p1/2787	gnl|BL_ORD_ID|42640 cb4508_c4/f1p1/3290 isoform=cb4508_c4;full_length_coverage=1;non_full_length_coverage=1;isoform_length=3290;expected_accuracy=0.996	113	2767	1	109	634	3288	1	109
cb8564_c44074/f1p1/2922 isoform=cb8564_c44074;full_length_coverage=1;non_f	gnl|BL_ORD_ID|17429 cb8564_c85447/f1p0/3196 isoform=cb8564_c85447;full_length_coverage=1;non_fu	1	2397	2396	2922	3	2413	2660	3196

ull_length_coverage=1;isoform_length=2922;expected_accuracy=0.994	ll_length_coverage=0;isoform_length=3196;expected_accuracy=0.997								
cb8785_c2/f1p0/2198 isoform=cb8785_c2;full_length_coverage=1;non_full_length_coverage=0;isoform_length=2198;expected_accuracy=0.998	gnl|BL_ORD_ID|19241 cb8785_c3/f1p0/3260 isoform=cb8785_c3;full_length_coverage=1;non_full_length_coverage=0;isoform_length=3260;expected_accuracy=0.996	1	1641	1639	2123	81	1722	2778	3260
cb317_c13/f1p0/4439	gnl|BL_ORD_ID|37313 cb317_c17/f1p0/4336 isoform=cb317_c17;full_length_coverage=1;non_full_length_coverage=0;isoform_length=4336;expected_accuracy=0.993	109	3147	3142	4219	1	3026	3251	4320
cb750_c21/f1p0/3523	gnl|BL_ORD_ID|8048 cb750_c40/f1p0/3632 isoform=cb750_c40;full_length_coverage=1;non_full_length_coverage=0;isoform_length=3632;expected_accuracy=0.990	673	3466	88	674	827	3632	135	727
cb4674_c89/f1p0/1671 isoform=cb4674_c89;full_length_coverage=1;non_full_length_coverage=0;isoform_length=1671;expected_accuracy=0.995	gnl|BL_ORD_ID|43463 cb4674_c77/f1p2/3189 isoform=cb4674_c77;full_length_coverage=1;non_full_length_coverage=2;isoform_length=3189;expected_accuracy=0.999	1	1114	1110	1671	1269	2380	2547	3106
cb8564_c146679/f2p0/2668 isoform=cb8564_c146679;full_length_coverage=2;non_full_length_coverage=0;isoform_length=2668;expected_accuracy=1.000	gnl|BL_ORD_ID|52101 cb8564_c110923/f1p0/3133	278	2668	74	282	300	2691	2	197
cb2055_c29/f1p0/2360	gnl|BL_ORD_ID|35222 cb2055_c22/f1p0/3295 isoform=cb2055_c22;full_length_coverage=1;non_full_length_coverage=0;isoform_length=3295;expected_accuracy=0.994	1	1859	1857	2198	183	2034	2784	3126

cb8101_c9/f1p0/2203 isoform=cb8101_c9;full_length_coverage=1;non_full_length_coverage=0;isoform_length=2203;expected_accuracy=0.997	gnl|BL_ORD_ID|51750 cb8101_c4/f1p0/2459	1	1617	1613	2105	228	1843	1957	2449
cb8564_c182049/f1p0/3195 isoform=cb8564_c182049;full_length_coverage=1;non_full_length_coverage=0;isoform_length=3195;expected_accuracy=0.997	gnl|BL_ORD_ID|55939 cb8564_c49630/f1p4/3487	2	2935	2935	3185	1	2913	3243	3493
cb8564_c44063/f1p0/2503 isoform=cb8564_c44063;full_length_coverage=1;non_full_length_coverage=0;isoform_length=2503;expected_accuracy=0.999	gnl|BL_ORD_ID|55861 cb8564_c47234/f1p0/2932	397	2503	48	402	597	2702	97	452
cb912_c15/f1p0/2991 isoform=cb912_c15;full_length_coverage=1;non_full_length_coverage=0;isoform_length=2991;expected_accuracy=0.999	gnl|BL_ORD_ID|59087 cb912_c9/f1p1/3267	1	2472	2469	2929	130	2602	2739	3199
cb11851_c28/f1p0/1049 isoform=cb11851_c28;full_length_coverage=1;non_full_length_coverage=0;isoform_length=1049;expected_accuracy=0.994	gnl|BL_ORD_ID|61534 cb11851_c31/f1p1/1766	411	1007	5	416	561	1157	1	413
cb8564_c37361/f1p0/3076	gnl|BL_ORD_ID|14471 cb8564_c34518/f1p0/3136 isoform=cb8564_c34518;full_length_coverage=1;non_full_length_coverage=0;isoform_length=3136;expected_accuracy=0.991	1	2481	2477	3070	1	2449	2550	3136
cb8564_c42285/f1p0/3370	gnl|BL_ORD_ID|17429 cb8564_c85447/f1p0/3196 isoform=cb8564_c85447;full_length_coverage=1;non_fu	1	2418	2417	2953	3	2413	2660	3196

<table>
<tr><td></td><td>ll_length_coverage=0;isoform_length=3196;expected_accuracy=0.997</td><td></td><td></td><td></td><td></td><td></td><td></td><td></td><td></td></tr>
<tr><td>cb8564_c118788/f2p0/2730 isoform=cb8564_c118788;full_length_coverage=2;non_full_length_coverage=0;isoform_length=2730;expected_accuracy=0.999</td><td>gnl|BL_ORD_ID|17627 cb8564_c86161/f2p0/2876 isoform=cb8564_c86161;full_length_coverage=2;non_full_length_coverage=0;isoform_length=2876;expected_accuracy=0.997</td><td>1</td><td>1710</td><td>1709</td><td>2680</td><td>62</td><td>1770</td><td>1904</td><td>2876</td></tr>
<tr><td>cb8564_c87855/f1p1/2779 isoform=cb8564_c87855;full_length_coverage=1;non_full_length_coverage=1;isoform_length=2779;expected_accuracy=0.997</td><td>gnl|BL_ORD_ID|14718 cb8564_c39852/f1p0/3673 isoform=cb8564_c39852;full_length_coverage=1;non_full_length_coverage=0;isoform_length=3673;expected_accuracy=0.995</td><td>134</td><td>1972</td><td>1970</td><td>2779</td><td>1</td><td>1834</td><td>2865</td><td>3673</td></tr>
<tr><td>cb8564_c40598/f1p4/3119</td><td>gnl|BL_ORD_ID|58037 cb8564_c87188/f1p1/3426</td><td>26</td><td>2732</td><td>2729</td><td>3058</td><td>130</td><td>2836</td><td>2957</td><td>3286</td></tr>
<tr><td>cb7884_c5/f1p0/2380 isoform=cb7884_c5;full_length_coverage=1;non_full_length_coverage=0;isoform_length=2380;expected_accuracy=0.994</td><td>gnl|BL_ORD_ID|7114 cb7884_c4/f1p0/2511 isoform=cb7884_c4;full_length_coverage=1;non_full_length_coverage=0;isoform_length=2511;expected_accuracy=0.995</td><td>13</td><td>1565</td><td>1563</td><td>2380</td><td>1</td><td>1554</td><td>1684</td><td>2501</td></tr>
<tr><td>cb435_c6/f1p0/5339</td><td>gnl|BL_ORD_ID|72170 cb435_c7/f1p0/5320</td><td>25</td><td>4329</td><td>4327</td><td>4707</td><td>62</td><td>4368</td><td>4933</td><td>5313</td></tr>
<tr><td>cb18456_c5939/f1p3/1634</td><td>gnl|BL_ORD_ID|17669 cb8564_c868/f2p9/1907 isoform=cb8564_c868;full_length_coverage=2;non_full_length_coverage=9;isoform_length=1907;expected_accuracy=1.000</td><td>283</td><td>1498</td><td>4</td><td>283</td><td>690</td><td>1907</td><td>1</td><td>285</td></tr>
<tr><td>cb8564_c49562/f1p0/2838 isoform=cb8564_c49562;full_length_coverage=1;non_full_length_coverage=0;isoform_length=2838;expected_accuracy=0.995</td><td>gnl|BL_ORD_ID|55341 cb8564_c38927/f1p0/3326</td><td>1</td><td>2331</td><td>2327</td><td>2838</td><td>3</td><td>2337</td><td>2444</td><td>2961</td></tr>
</table>

cb16619_c3/f1p0/1732	gnl|BL_ORD_ID|74658 cb5921_c54/f1p0/2912	119	1569	1	120	1430	288 1	2	122
cb8564_c36663/f1p0/2776 isoform=cb8564_c36663;full_length_coverage=1;non_full_length_coverage=0;isoform_length=2776;expected_accuracy=0.995	gnl|BL_ORD_ID|67977 cb18745_c2/f1p0/5425	1	1636	1636	2776	2387	401 8	4122	526 2
cb12_c37/f1p0/4067	gnl|BL_ORD_ID|61089 cb12_c50/f1p0/4439	286	4080	1	289	503	431 2	21	309
cb11489_c0/f1p0/1094 isoform=cb11489_c0;full_length_coverage=1;non_full_length_coverage=0;isoform_length=1094;expected_accuracy=0.995	gnl|BL_ORD_ID|24432 cb11489_c1/f1p0/3982 isoform=cb11489_c1;full_length_coverage=1;non_full_length_coverage=0;isoform_length=3982;expected_accuracy=0.995	193	1094	21	192	2820	372 6	26	196
cb2388_c9/f1p0/2763	gnl|BL_ORD_ID|38706 cb2388_c8/f1p1/3379 isoform=cb2388_c8;full_length_coverage=1;non_full_length_coverage=1;isoform_length=3379;expected_accuracy=0.998	1203	2534	1	1205	2048	337 9	8	121 7
cb2737_c40/f1p0/2830	gnl|BL_ORD_ID|68786 cb2737_c50/f1p0/3706	1	1397	1395	2651	685	208 2	2214	346 6
cb2458_c10/f1p1/2684 isoform=cb2458_c10;full_length_coverage=1;non_full_length_coverage=1;isoform_length=2684;expected_accuracy=0.992	gnl|BL_ORD_ID|37675 cb2458_c4/f2p1/2839 isoform=cb2458_c4;full_length_coverage=2;non_full_length_coverage=1;isoform_length=2839;expected_accuracy=0.994	379	2684	85	380	516	282 7	1	296
cb13748_c4/f9p0/2089 isoform=cb13748_c4;full_length_coverage=9;non_full_length_coverage=0;isoform_length=2089;expected_accuracy=1.000	gnl|BL_ORD_ID|26479 cb13748_c60/f1p0/3166 isoform=cb13748_c60;full_length_coverage=1;non_full_length_coverage=0;isoform_length=3166;expected_accuracy=0.991	680	2072	76	682	1782	316 6	33	637

cb8564_c182592/f1p0/2600 isoform=cb8564_c182592;full_length_coverage=1;non_full_length_coverage=0;isoform_length=2600;expected_accuracy=0.997	gnl|BL_ORD_ID|53022 cb8564_c114466/f1p0/3000	1	1582	1583	2586	8	1610	1943	2946
cb1146_c14/f5p1/3802 isoform=cb1146_c14;full_length_coverage=5;non_full_length_coverage=1;isoform_length=3802;expected_accuracy=1.000	gnl|BL_ORD_ID|59189 cb1146_c4/f1p0/4749	1463	3802	111	1465	2017	4356	143	1497
cb8564_c21808/f1p0/2228	gnl|BL_ORD_ID|13156 cb8564_c18183/f2p0/2268 isoform=cb8564_c18183;full_length_coverage=2;non_full_length_coverage=0;isoform_length=2268;expected_accuracy=0.999	1	1416	1415	2145	12	1431	1539	2268
cb2926_c7/f2p0/2634 isoform=cb2926_c7;full_length_coverage=2;non_full_length_coverage=0;isoform_length=2634;expected_accuracy=0.993	gnl|BL_ORD_ID|38742 cb2926_c15/f1p0/2713 isoform=cb2926_c15;full_length_coverage=1;non_full_length_coverage=0;isoform_length=2713;expected_accuracy=0.996	11	1791	1791	2610	8	1784	1884	2703
cb7851_c0/f3p0/2191 isoform=cb7851_c0;full_length_coverage=3;non_full_length_coverage=0;isoform_length=2191;expected_accuracy=0.996	gnl|BL_ORD_ID|8228 cb7851_c1/f1p0/2518 isoform=cb7851_c1;full_length_coverage=1;non_full_length_coverage=0;isoform_length=2518;expected_accuracy=0.995	1	1285	1285	2183	3	1306	1620	2518
cb18456_c1988/f1p0/1112	gnl|BL_ORD_ID|67391 cb18456_c2087/f1p0/1448	412	1024	50	416	676	1288	5	371
cb8564_c73853/f1p0/2443 isoform=cb8564_c73853;full_length_coverage=1;non_full_length_coverage=0;isoform_length=2443;expected_accuracy=1.000	gnl|BL_ORD_ID|56983 cb8564_c79327/f1p1/3074	1	1281	1276	2428	99	1375	1655	2814

cb653_c9/f1p0/4025 isoform=cb653_c9;full_length_coverage=1;non_full_length_coverage=0;isoform_length=4025;expected_accuracy=0.997	gnl|BL_ORD_ID|5915 cb653_c13/f1p2/4267 isoform=cb653_c13;full_length_coverage=1;non_full_length_coverage=2;isoform_length=4267;expected_accuracy=0.999	301	4024	1	301	473	4191	6	295
cb8564_c44698/f1p5/2843 isoform=cb8564_c44698;full_length_coverage=1;non_full_length_coverage=5;isoform_length=2843;expected_accuracy=0.997	gnl|BL_ORD_ID|54438 cb8564_c46124/f1p0/3383	1	2516	2511	2836	151	2663	2784	3114
cb12931_c15/f1p0/1493 isoform=cb12931_c15;full_length_coverage=1;non_full_length_coverage=0;isoform_length=1493;expected_accuracy=1.000	gnl|BL_ORD_ID|13544 cb8564_c186356/f1p0/2744 isoform=cb8564_c186356;full_length_coverage=1;non_full_length_coverage=0;isoform_length=2744;expected_accuracy=0.998	1	1106	1104	1489	1	1102	2359	2744
cb12931_c15/f1p0/1493 isoform=cb12931_c15;full_length_coverage=1;non_full_length_coverage=0;isoform_length=1493;expected_accuracy=1.000	gnl|BL_ORD_ID|15154 cb8564_c32509/f1p1/2880 isoform=cb8564_c32509;full_length_coverage=1;non_full_length_coverage=1;isoform_length=2880;expected_accuracy=0.991	11	1106	1104	1467	2	1110	2509	2877
cb8564_c2810/f1p1/2785 isoform=cb8564_c2810;full_length_coverage=1;non_full_length_coverage=1;isoform_length=2785;expected_accuracy=0.996	gnl|BL_ORD_ID|14981 cb8564_c2811/f1p0/3018 isoform=cb8564_c2811;full_length_coverage=1;non_full_length_coverage=0;isoform_length=3018;expected_accuracy=0.997	1	1455	1456	2699	1	1456	1773	3018
cb6106_c23/f14p0/1983 isoform=cb6106_c23;full_length_coverage=14;non_full_length_coverage=0;isoform_length=1983;expected_accuracy=1.000	gnl|BL_ORD_ID|45790 cb6106_c5/f1p0/2598 isoform=cb6106_c5;full_length_coverage=1;non_full_length_coverage=0;isoform_length=2598;expected_accuracy=0.997	1	1322	1319	1979	8	1342	1935	2598
cb1049_c9/f1p0/2974 isoform=cb1049_c9;full_length_coverage=1;non_full_le	gnl|BL_ORD_ID|47282 cb1049_c14/f1p0/3390	1345	2974	10	1346	1500	3134	56	1397

ngth_coverage=0;isoform_length=2974;expected_accuracy=1.000									
cb6816_c3/f1p0/1841 isoform=cb6816_c3;full_length_coverage=1;non_full_length_coverage=0;isoform_length=1841;expected_accuracy=0.997	gnl|BL_ORD_ID|5381 cb6816_c0/f1p0/2763 isoform=cb6816_c0;full_length_coverage=1;non_full_length_coverage=0;isoform_length=2763;expected_accuracy=0.995	1	1015	1010	1841	15	1029	1924	2755
cb13877_c3/f1p0/1270 isoform=cb13877_c3;full_length_coverage=1;non_full_length_coverage=0;isoform_length=1270;expected_accuracy=0.999	gnl|BL_ORD_ID|62848 cb13877_c4/f1p0/1685	247	1270	43	252	449	1472	46	257
cb1178_c19/f1p0/1460 isoform=cb1178_c19;full_length_coverage=1;non_full_length_coverage=0;isoform_length=1460;expected_accuracy=1.000	gnl|BL_ORD_ID|59233 cb1178_c13/f1p0/2397	387	1460	47	388	912	1985	3	343
cb8564_c186356/f1p0/2744 isoform=cb8564_c186356;full_length_coverage=1;non_full_length_coverage=0;isoform_length=2744;expected_accuracy=0.998	gnl|BL_ORD_ID|56602 cb8564_c48192/f1p6/3029	1	1396	1393	2744	1	1398	1518	2868
cb8564_c109682/f3p0/2064 isoform=cb8564_c109682;full_length_coverage=3;non_full_length_coverage=0;isoform_length=2064;expected_accuracy=1.000	gnl|BL_ORD_ID|10872 cb8564_c117686/f1p0/2190 isoform=cb8564_c117686;full_length_coverage=1;non_full_length_coverage=0;isoform_length=2190;expected_accuracy=1.000	530	2064	56	531	655	2189	1	476
cb8564_c72639/f1p3/2409 isoform=cb8564_c72639;full_length_coverage=1;non_full_length_coverage=3;isoform_length=2409;expected_accuracy=0.999	gnl|BL_ORD_ID|17615 cb8564_c91506/f1p0/2592 isoform=cb8564_c91506;full_length_coverage=1;non_full_length_coverage=0;isoform_length=2592;expected_accuracy=0.994	288	2400	8	289	479	2592	1	282

cb8564_c132795/f5p10/3729 isoform=cb8564_c132795;full_length_coverage=5;non_full_length_coverage=10;isoform_length=3729;expected_accuracy=0.999	gnl|BL_ORD_ID|55725 cb8564_c5109/f1p0/3767	1	1832	1831	3618	1	1831	1950	3737
cb8564_c43779/f1p0/3021 isoform=cb8564_c43779;full_length_coverage=1;non_full_length_coverage=0;isoform_length=3021;expected_accuracy=0.993	gnl|BL_ORD_ID|55502 cb8564_c43538/f1p0/3563	1	2344	2345	3021	2	2350	2506	3180
cb13076_c20/f1p0/1742 isoform=cb13076_c20;full_length_coverage=1;non_full_length_coverage=0;isoform_length=1742;expected_accuracy=0.995	gnl|BL_ORD_ID|57982 cb8564_c85904/f1p1/2015	185	1742	70	184	301	1859	37	151
cb8564_c953/f2p0/2607 isoform=cb8564_c953;full_length_coverage=2;non_full_length_coverage=0;isoform_length=2607;expected_accuracy=0.997	gnl|BL_ORD_ID|54954 cb8564_c3903/f1p1/2749	154	2539	1	154	381	2768	102	251
cb8564_c145859/f2p0/2922 isoform=cb8564_c145859;full_length_coverage=2;non_full_length_coverage=0;isoform_length=2922;expected_accuracy=0.999	gnl|BL_ORD_ID|56809 cb8564_c72465/f1p0/4108	1	1672	1669	2922	6	1679	2646	3899
cb8564_c158279/f2p4/3190	gnl|BL_ORD_ID|14233 cb8564_c24054/f1p0/3316 isoform=cb8564_c24054;full_length_coverage=1;non_full_length_coverage=0;isoform_length=3316;expected_accuracy=0.998	1420	3128	1	1422	1608	3316	5	1424
cb8564_c1923/f1p0/2116 isoform=cb8564_c1923;full_length_coverage=1;non_ful	gnl|BL_ORD_ID|53544 cb8564_c128329/f1p0/2686	1	1776	1775	2116	1	1776	1950	2292

l_length_coverage=0;isoform_length=2116;expected_accuracy=0.991									
cb12362_c43/f1p0/1174	gnl|BL_ORD_ID|1903 cb10045_c31/f1p0/1199 isoform=cb10045_c31;full_length_coverage=1;non_full_length_coverage=0;isoform_length=1199;expected_accuracy=0.994	118	1014	4	119	258	1180	1	116
cb8564_c14344/f1p0/2533 isoform=cb8564_c14344;full_length_coverage=1;non_full_length_coverage=0;isoform_length=2533;expected_accuracy=0.993	gnl|BL_ORD_ID|52842 cb8564_c15468/f1p0/3015	148	2454	1	151	512	2819	43	193
cb4016_c33/f1p0/2507	gnl|BL_ORD_ID|40527 cb4016_c6/f1p1/2956 isoform=cb4016_c6;full_length_coverage=1;non_full_length_coverage=1;isoform_length=2956;expected_accuracy=0.996	207	2462	1	208	526	2780	23	227
cb8996_c3/f1p0/1657 isoform=cb8996_c3;full_length_coverage=1;non_full_length_coverage=0;isoform_length=1657;expected_accuracy=0.992	gnl|BL_ORD_ID|22059 cb8996_c1/f1p0/2011 isoform=cb8996_c1;full_length_coverage=1;non_full_length_coverage=0;isoform_length=2011;expected_accuracy=0.996	1	1129	1129	1657	1	1128	1367	1895
cb6161_c7/f1p3/2184 isoform=cb6161_c7;full_length_coverage=1;non_full_length_coverage=3;isoform_length=2184;expected_accuracy=0.990	gnl|BL_ORD_ID|46442 cb6161_c4/f2p0/2920 isoform=cb6161_c4;full_length_coverage=2;non_full_length_coverage=0;isoform_length=2920;expected_accuracy=1.000	162	2161	14	162	920	2920	1	148
cb6657_c5/f1p1/2260 isoform=cb6657_c5;full_length_coverage=1;non_full_length_coverage=1;isoform_length=2260;expected_accuracy=0.999	gnl|BL_ORD_ID|5662 cb6657_c0/f1p1/2805 isoform=cb6657_c0;full_length_coverage=1;non_full_length_coverage=1;isoform_length=2805;expected_accuracy=0.993	1	1808	1808	2258	3	1813	2232	2683

cb7085_c26/f13p0/1893 isoform=cb7085_c26;full_length_coverage=13;non_full_length_coverage=0;isoform_length=1893;expected_accuracy=1.000	gnl|BL_ORD_ID|4154 cb7085_c8/f1p0/2701 isoform=cb7085_c8;full_length_coverage=1;non_full_length_coverage=0;isoform_length=2701;expected_accuracy=0.996	539	1889	70	541	1349	2701	1	483
cb8564_c107237/f5p1/2872 isoform=cb8564_c107237;full_length_coverage=5;non_full_length_coverage=1;isoform_length=2872;expected_accuracy=0.995	gnl|BL_ORD_ID|54964 cb8564_c4015/f1p0/3731	1	2206	2205	2837	34	2239	3116	3748
cb12972_c15/f1p0/1541 isoform=cb12972_c15;full_length_coverage=1;non_full_length_coverage=0;isoform_length=1541;expected_accuracy=0.996	gnl|BL_ORD_ID|62020 cb12972_c13/f1p0/1732	295	1541	40	300	414	1651	1	261
cb7214_c5/f1p0/1888 isoform=cb7214_c5;full_length_coverage=1;non_full_length_coverage=0;isoform_length=1888;expected_accuracy=0.994	gnl|BL_ORD_ID|8880 cb7214_c4/f1p0/2395 isoform=cb7214_c4;full_length_coverage=1;non_full_length_coverage=0;isoform_length=2395;expected_accuracy=0.999	244	1888	3	245	645	2310	21	263
cb18646_c2/f1p5/6412 isoform=cb18646_c2;full_length_coverage=1;non_full_length_coverage=5;isoform_length=6412;expected_accuracy=0.997	gnl|BL_ORD_ID|34398 cb18646_c0/f2p2/6586 isoform=cb18646_c0;full_length_coverage=2;non_full_length_coverage=2;isoform_length=6586;expected_accuracy=0.999	2	6140	6139	6334	3	6158	6391	6586
cb7942_c26/f1p0/1770 isoform=cb7942_c26;full_length_coverage=1;non_full_length_coverage=0;isoform_length=1770;expected_accuracy=0.993	gnl|BL_ORD_ID|6206 cb7942_c31/f5p0/2051 isoform=cb7942_c31;full_length_coverage=5;non_full_length_coverage=0;isoform_length=2051;expected_accuracy=0.993	250	1770	3	252	490	2012	34	283
cb3818_c17/f1p0/2829 isoform=cb3818_c17;full_length_coverage=1;non_full_	gnl|BL_ORD_ID|40376 cb3818_c0/f3p0/2845 isoform=cb3818_c0;full_length_coverage=3;non_full_le	543	2828	98	543	556	2845	1	448

length_coverage=0;isoform_length=2829;expected_accuracy=0.996	ngth_coverage=0;isoform_length=2845;expected_accuracy=1.000								
cb6090_c5/f1p0/2373 isoform=cb6090_c5;full_length_coverage=1;non_full_length_coverage=0;isoform_length=2373;expected_accuracy=0.995	gnl|BL_ORD_ID|44623 cb6090_c1/f1p0/2937 isoform=cb6090_c1;full_length_coverage=1;non_full_length_coverage=0;isoform_length=2937;expected_accuracy=0.997	1	1689	1691	2371	4	1685	2268	2937
cb8564_c35935/f1p0/4149 isoform=cb8564_c35935;full_length_coverage=1;non_full_length_coverage=0;isoform_length=4149;expected_accuracy=0.993	gnl|BL_ORD_ID|16628 cb8564_c47737/f7p5/4351 isoform=cb8564_c47737;full_length_coverage=7;non_full_length_coverage=5;isoform_length=4351;expected_accuracy=0.998	291	4149	1	295	395	4273	1	290
cb3603_c18/f1p1/2591	gnl|BL_ORD_ID|39360 cb3603_c17/f1p3/3266 isoform=cb3603_c17;full_length_coverage=1;non_full_length_coverage=3;isoform_length=3266;expected_accuracy=0.994	201	2586	2	201	795	3180	1	196
cb8564_c163547/f1p1/2420 isoform=cb8564_c163547;full_length_coverage=1;non_full_length_coverage=1;isoform_length=2420;expected_accuracy=0.994	gnl|BL_ORD_ID|16111 cb8564_c75565/f1p1/2477 isoform=cb8564_c75565;full_length_coverage=1;non_full_length_coverage=1;isoform_length=2477;expected_accuracy=0.995	543	2419	66	547	607	2477	7	488
cb8564_c2608/f1p0/2317 isoform=cb8564_c2608;full_length_coverage=1;non_full_length_coverage=0;isoform_length=2317;expected_accuracy=0.998	gnl|BL_ORD_ID|54786 cb8564_c2612/f1p0/3007	1	1346	1345	2317	1	1343	1930	2903
cb12341_c15/f1p0/1821	gnl|BL_ORD_ID|43183 cb5118_c1/f5p8/3138 isoform=cb5118_c1;full_length_coverage=5;non_full_length_coverage=8;isoform_length=3138;expected_accuracy=1.000	99	1595	1	101	1628	3124	1	97

cb8564_c76734/f1p0/3758	gnl|BL_ORD_ID|57198 cb8564_c86412/f1p0/4137	1	3013	3013	3419	169	3181	3496	3901
cb15168_c1/f1p0/1570 isoform=cb15168_c1;full_length_coverage=1;non_full_length_coverage=0;isoform_length=1570;expected_accuracy=0.993	gnl|BL_ORD_ID|64500 cb15168_c4/f1p0/1841	1	1205	1202	1570	51	1255	1355	1723
cb3256_c4/f1p0/2358 isoform=cb3256_c4;full_length_coverage=1;non_full_length_coverage=0;isoform_length=2358;expected_accuracy=0.995	gnl|BL_ORD_ID|40771 cb3256_c0/f1p0/3673 isoform=cb3256_c0;full_length_coverage=1;non_full_length_coverage=0;isoform_length=3673;expected_accuracy=0.998	1142	2289	1	1145	2529	3673	227	1368
cb8564_c129638/f1p0/2479	gnl|BL_ORD_ID|52261 cb8564_c116055/f1p0/3385	1	1627	1623	2475	34	1660	2065	2917
cb8564_c34518/f1p0/3136 isoform=cb8564_c34518;full_length_coverage=1;non_full_length_coverage=0;isoform_length=3136;expected_accuracy=0.991	gnl|BL_ORD_ID|55939 cb8564_c49630/f1p4/3487	2	2882	2882	3126	1	2913	3243	3493
cb6518_c7/f1p0/2783	gnl|BL_ORD_ID|5047 cb6518_c9/f1p1/3889 isoform=cb6518_c9;full_length_coverage=1;non_full_length_coverage=1;isoform_length=3889;expected_accuracy=0.997	1	1809	1807	2725	5	1811	2918	3836
cb14637_c3/f1p0/1759 isoform=cb14637_c3;full_length_coverage=1;non_full_length_coverage=0;isoform_length=1759;expected_accuracy=1.000	gnl|BL_ORD_ID|16851 cb8564_c4800/f2p0/3854 isoform=cb8564_c4800;full_length_coverage=2;non_full_length_coverage=0;isoform_length=3854;expected_accuracy=0.996	1	1385	1384	1713	736	2122	3523	3854
cb6217_c5/f2p1/2238 isoform=cb6217_c5;full_length_coverage=2;non_full_le	gnl|BL_ORD_ID|5726 cb6217_c4/f3p0/2549 isoform=cb6217_c4;full_length_coverage=3;non_full_le	150	2238	1	151	284	2372	5	155

ngth_coverage=1;isoform_length=2238;expected_accuracy=0.999	ngth_coverage=0;isoform_length=2549;expected_accuracy=1.000								
cb8564_c7774/f3p4/1962 isoform=cb8564_c7774;full_length_coverage=3;non_full_length_coverage=4;isoform_length=1962;expected_accuracy=1.000	gnl|BL_ORD_ID|16481 cb8564_c5089/f1p0/2122 isoform=cb8564_c5089;full_length_coverage=1;non_full_length_coverage=0;isoform_length=2122;expected_accuracy=0.995	1	1462	1457	1880	12	1470	1699	2122
cb9467_c9/f6p0/2182 isoform=cb9467_c9;full_length_coverage=6;non_full_length_coverage=0;isoform_length=2182;expected_accuracy=1.000	gnl|BL_ORD_ID|21567 cb9467_c27/f1p0/2294 isoform=cb9467_c27;full_length_coverage=1;non_full_length_coverage=0;isoform_length=2294;expected_accuracy=0.999	165	2105	1	166	352	2294	3	169
cb875_c3/f1p0/4771	gnl|BL_ORD_ID|19102 cb875_c20/f1p3/6635 isoform=cb875_c20;full_length_coverage=1;non_full_length_coverage=3;isoform_length=6635;expected_accuracy=0.997	1	3236	3234	4726	1030	4264	5145	6634
cb3603_c31/f1p0/4927 isoform=cb3603_c31;full_length_coverage=1;non_full_length_coverage=0;isoform_length=4927;expected_accuracy=0.996	gnl|BL_ORD_ID|40851 cb3603_c41/f1p0/5154 isoform=cb3603_c41;full_length_coverage=1;non_full_length_coverage=0;isoform_length=5154;expected_accuracy=0.997	201	4914	1	201	447	5154	1	202
cb8564_c87256/f1p0/2285 isoform=cb8564_c87256;full_length_coverage=1;non_full_length_coverage=0;isoform_length=2285;expected_accuracy=0.992	gnl|BL_ORD_ID|11500 cb8564_c14546/f1p5/3300 isoform=cb8564_c14546;full_length_coverage=1;non_full_length_coverage=5;isoform_length=3300;expected_accuracy=0.996	1	1703	1700	2285	834	2537	2671	3262
cb5057_c18/f1p0/1420 isoform=cb5057_c18;full_length_coverage=1;non_full_length_coverage=0;isoform_length=1420;expected_accuracy=0.992	gnl|BL_ORD_ID|41939 cb5057_c2/f3p0/1613 isoform=cb5057_c2;full_length_coverage=3;non_full_length_coverage=0;isoform_length=1613;expected_accuracy=1.000	1	1020	1016	1420	1	1023	1187	1613

cb4877_c1/f2p1/2369 isoform=cb4877_c1;full_length_coverage=2;non_full_length_coverage=1;isoform_length=2369;expected_accuracy=1.000	gnl|BL_ORD_ID|42790 cb4877_c3/f1p0/2888 isoform=cb4877_c3;full_length_coverage=1;non_full_length_coverage=0;isoform_length=2888;expected_accuracy=0.996	1	1435	1434	2334	4	1438	1989	2888
cb6217_c5/f2p1/2238 isoform=cb6217_c5;full_length_coverage=2;non_full_length_coverage=1;isoform_length=2238;expected_accuracy=0.999	gnl|BL_ORD_ID|49539 cb6217_c35/f1p0/2092	150	2199	1	151	283	2306	4	154
cb964_c1/f1p0/4546	gnl|BL_ORD_ID|59142 cb964_c19/f1p0/4670	1	2394	2393	4166	87	2481	2626	4399
cb5460_c5/f1p0/3237	gnl|BL_ORD_ID|73948 cb5460_c9/f1p1/2896	18	2102	2098	2788	11	2095	2217	2906
cb5487_c6/f1p1/2539	gnl|BL_ORD_ID|73975 cb5487_c11/f1p0/2702	1	1465	1465	2490	13	1477	1646	2671
cb8564_c36689/f1p1/2368 isoform=cb8564_c36689;full_length_coverage=1;non_full_length_coverage=1;isoform_length=2368;expected_accuracy=0.991	gnl|BL_ORD_ID|14368 cb8564_c34638/f1p0/2817 isoform=cb8564_c34638;full_length_coverage=1;non_full_length_coverage=0;isoform_length=2817;expected_accuracy=0.992	1	1709	1706	2368	1	1662	2158	2813
cb7851_c0/f3p0/2191 isoform=cb7851_c0;full_length_coverage=3;non_full_length_coverage=0;isoform_length=2191;expected_accuracy=0.996	gnl|BL_ORD_ID|8230 cb7851_c3/f1p0/2493 isoform=cb7851_c3;full_length_coverage=1;non_full_length_coverage=0;isoform_length=2493;expected_accuracy=0.991	1	1285	1285	2180	5	1284	1598	2492
cb8564_c34060/f2p0/3214 isoform=cb8564_c34060;full_length_coverage=2;non_full_length_coverage=0;isoform_length=3214;expected_accuracy=0.993	gnl|BL_ORD_ID|16149 cb8564_c47162/f1p0/3361 isoform=cb8564_c47162;full_length_coverage=1;non_full_length_coverage=0;isoform_length=3361;expected_accuracy=0.996	433	3214	6	434	592	3361	1	402

cb8564_c69192/f2p0/4443 isoform=cb8564_c69192;full_length_coverage=2;non_full_length_coverage=0;isoform_length=4443;expected_accuracy=0.996	gnl|BL_ORD_ID|67930 cb18656_c10/f1p0/5311	1	3966	3967	4443	123	4102	4852	5325
cb8564_c183213/f1p3/3190 isoform=cb8564_c183213;full_length_coverage=1;non_full_length_coverage=3;isoform_length=3190;expected_accuracy=0.999	gnl|BL_ORD_ID|57083 cb8564_c82622/f1p0/3597	1	2313	2318	3190	3	2315	2596	3468
cb16508_c2/f1p0/1556	gnl|BL_ORD_ID|32526 cb16508_c1/f1p0/1732 isoform=cb16508_c1;full_length_coverage=1;non_full_length_coverage=0;isoform_length=1732;expected_accuracy=1.000	248	1552	33	247	406	1710	17	224
cb18456_c7297/f1p0/1408	gnl|BL_ORD_ID|11779 cb8564_c122762/f1p4/2270 isoform=cb8564_c122762;full_length_coverage=1;non_full_length_coverage=4;isoform_length=2270;expected_accuracy=0.992	520	1342	7	519	1453	2270	6	529
cb8564_c40764/f1p0/2563 isoform=cb8564_c40764;full_length_coverage=1;non_full_length_coverage=0;isoform_length=2563;expected_accuracy=0.997	gnl|BL_ORD_ID|15496 cb8564_c47853/f1p0/2927 isoform=cb8564_c47853;full_length_coverage=1;non_full_length_coverage=0;isoform_length=2927;expected_accuracy=0.991	1	2167	2162	2563	1	2145	2353	2752
cb2155_c26/f5p0/2830 isoform=cb2155_c26;full_length_coverage=5;non_full_length_coverage=0;isoform_length=2830;expected_accuracy=1.000	gnl|BL_ORD_ID|66760 cb2155_c21/f1p0/3343	1	1729	1727	2795	1	1731	2240	3318
cb8564_c15653/f1p4/2475	gnl|BL_ORD_ID|54449 cb8564_c122906/f2p13/2871	22	2293	2289	2482	166	2437	2656	2849

cb8564_c13467/f1p0/3088	gnl|BL_ORD_ID|54145 cb8564_c19339/f1p1/3362	17	2494	2494	3219	27	2501	2612	3337
cb5290_c43/f1p0/2774	gnl|BL_ORD_ID|73390 cb5290_c40/f1p0/2998	235	2691	1	236	379	2835	4	246
cb4798_c33/f4p0/1530 isoform=cb4798_c33;full_length_coverage=4;non_full_length_coverage=0;isoform_length=1530;expected_accuracy=1.000	gnl|BL_ORD_ID|43886 cb4798_c18/f1p0/1786 isoform=cb4798_c18;full_length_coverage=1;non_full_length_coverage=0;isoform_length=1786;expected_accuracy=0.996	515	1530	58	516	770	1785	1	459
cb3192_c10/f1p0/3250 isoform=cb3192_c10;full_length_coverage=1;non_full_length_coverage=0;isoform_length=3250;expected_accuracy=0.998	gnl|BL_ORD_ID|38500 cb3192_c13/f1p1/3411 isoform=cb3192_c13;full_length_coverage=1;non_full_length_coverage=1;isoform_length=3411;expected_accuracy=0.996	1112	3250	105	1114	1242	3379	13	1013
cb3010_c8/f1p0/2925 isoform=cb3010_c8;full_length_coverage=1;non_full_length_coverage=0;isoform_length=2925;expected_accuracy=0.998	gnl|BL_ORD_ID|38462 cb3010_c9/f1p0/3027 isoform=cb3010_c9;full_length_coverage=1;non_full_length_coverage=0;isoform_length=3027;expected_accuracy=0.994	1422	2908	1	1423	1552	3027	4	1422
cb3576_c15/f1p0/3129 isoform=cb3576_c15;full_length_coverage=1;non_full_length_coverage=0;isoform_length=3129;expected_accuracy=0.994	gnl|BL_ORD_ID|41526 cb3576_c2/f1p0/3257 isoform=cb3576_c2;full_length_coverage=1;non_full_length_coverage=0;isoform_length=3257;expected_accuracy=0.997	165	3129	1	165	266	3257	1	165
cb8564_c4679/f1p1/2608	gnl|BL_ORD_ID|12101 cb8564_c126062/f1p2/2804 isoform=cb8564_c126062;full_length_coverage=1;non_full_length_coverage=2;isoform_length=2804;expected_accuracy=0.992	251	2604	11	250	425	2774	8	247
cb8564_c18821/f1p0/2573 isoform=cb8564_c18821;full_length_coverage=1;non_f	gnl|BL_ORD_ID|55760 cb8564_c24919/f1p0/3395	1	1625	1625	2555	537	2161	2299	3229

ull_length_coverage=0;isoform_length=2573;expected_accuracy=0.996									
cb9230_c4/f1p1/1857	gnl|BL_ORD_ID|19941 cb9230_c2/f1p1/2148 isoform=cb9230_c2;full_length_coverage=1;non_full_length_coverage=1;isoform_length=2148;expected_accuracy=0.991	154	1741	1	155	557	2148	13	168
cb8564_c13864/f2p1/3092 isoform=cb8564_c13864;full_length_coverage=2;non_full_length_coverage=1;isoform_length=3092;expected_accuracy=0.996	gnl|BL_ORD_ID|13369 cb8564_c18803/f2p0/3233 isoform=cb8564_c18803;full_length_coverage=2;non_full_length_coverage=0;isoform_length=3233;expected_accuracy=1.000	1101	3090	1	1101	1246	3233	1	1102
cb8564_c37361/f1p0/3076	gnl|BL_ORD_ID|55910 cb8564_c48645/f1p1/3287	11	2481	2477	3005	17	2484	2585	3113
cb8564_c39136/f1p0/3036 isoform=cb8564_c39136;full_length_coverage=1;non_full_length_coverage=0;isoform_length=3036;expected_accuracy=0.998	gnl|BL_ORD_ID|55600 cb8564_c46484/f1p0/3151	101	2089	2085	3036	5	2018	2145	3097
cb8564_c86566/f1p0/2178	gnl|BL_ORD_ID|19138 cb8564_c80714/f1p1/2608 isoform=cb8564_c80714;full_length_coverage=1;non_full_length_coverage=1;isoform_length=2608;expected_accuracy=0.995	384	2194	7	389	571	2379	40	422
cb7837_c0/f3p0/2227 isoform=cb7837_c0;full_length_coverage=3;non_full_length_coverage=0;isoform_length=2227;expected_accuracy=1.000	gnl|BL_ORD_ID|7423 cb7837_c1/f2p0/2384 isoform=cb7837_c1;full_length_coverage=2;non_full_length_coverage=0;isoform_length=2384;expected_accuracy=0.992	112	2226	1	112	272	2384	52	162
cb8564_c4541/f3p0/2161 isoform=cb8564_c4541;full_length_coverage=3;non_ful	gnl|BL_ORD_ID|14171 cb8564_c4536/f1p0/3069 isoform=cb8564_c4536;full_length_coverage=1;non_ful	1	1286	1284	2161	1	1281	2148	3024

l_length_coverage=0;isoform_length=2161;expected_accuracy=0.992	l_length_coverage=0;isoform_length=3069;expected_accuracy=0.993								
cb1524_c18/f1p0/3747	gnl|BL_ORD_ID|28439 cb1524_c30/f1p0/3792 isoform=cb1524_c30;full_length_coverage=1;non_full_length_coverage=0;isoform_length=3792;expected_accuracy=0.994	153	3577	1	152	366	3792	4	156
cb8564_c77080/f1p0/2613 isoform=cb8564_c77080;full_length_coverage=1;non_full_length_coverage=0;isoform_length=2613;expected_accuracy=0.995	gnl|BL_ORD_ID|18501 cb8564_c86501/f1p2/2948 isoform=cb8564_c86501;full_length_coverage=1;non_full_length_coverage=2;isoform_length=2948;expected_accuracy=0.991	1	2048	2043	2613	27	2064	2245	2815
cb8564_c15053/f1p0/3444	gnl|BL_ORD_ID|11890 cb8564_c13642/f1p0/3657 isoform=cb8564_c13642;full_length_coverage=1;non_full_length_coverage=0;isoform_length=3657;expected_accuracy=0.996	1635	3361	1	1636	1810	3535	22	1653
cb8564_c43779/f1p0/3021 isoform=cb8564_c43779;full_length_coverage=1;non_full_length_coverage=0;isoform_length=3021;expected_accuracy=0.993	gnl|BL_ORD_ID|56002 cb8564_c51722/f1p1/3503	21	2344	2345	3021	67	2386	2542	3216
cb693_c5/f1p0/2880	gnl|BL_ORD_ID|4971 cb693_c3/f1p0/3671 isoform=cb693_c3;full_length_coverage=1;non_full_length_coverage=0;isoform_length=3671;expected_accuracy=0.995	1	2434	2435	2810	45	2497	3296	3671
cb8564_c40764/f1p0/2563 isoform=cb8564_c40764;full_length_coverage=1;non_full_length_coverage=0;isoform_length=2563;expected_accuracy=0.997	gnl|BL_ORD_ID|55920 cb8564_c48949/f1p0/4357	1	2166	2162	2563	2	2166	2928	3326

cb5659_c81/f1p0/2132 isoform=cb5659_c81;full_length_coverage=1;non_full_length_coverage=0;isoform_length=2132;expected_accuracy=0.999	gnl|BL_ORD_ID|45302 cb5659_c105/f4p0/2776 isoform=cb5659_c105;full_length_coverage=4;non_full_length_coverage=0;isoform_length=2776;expected_accuracy=1.000	1	1656	1653	2083	136	1787	2346	2776
cb8564_c82549/f1p0/2302	gnl|BL_ORD_ID|53232 cb8564_c119499/f1p2/2534	11	1185	1181	2154	8	1184	1356	2329
cb3132_c0/f2p0/1470 isoform=cb3132_c0;full_length_coverage=2;non_full_length_coverage=0;isoform_length=1470;expected_accuracy=0.998	gnl|BL_ORD_ID|38055 cb3132_c2/f2p0/3713 isoform=cb3132_c2;full_length_coverage=2;non_full_length_coverage=0;isoform_length=3713;expected_accuracy=0.997	1	1075	1075	1470	1	1071	1190	1583
cb8422_c13/f3p0/2035 isoform=cb8422_c13;full_length_coverage=3;non_full_length_coverage=0;isoform_length=2035;expected_accuracy=1.000	gnl|BL_ORD_ID|11122 cb8422_c8/f1p0/2339 isoform=cb8422_c8;full_length_coverage=1;non_full_length_coverage=0;isoform_length=2339;expected_accuracy=0.993	1	1516	1514	1978	3	1514	1874	2339
cb8564_c83100/f1p1/2671 isoform=cb8564_c83100;full_length_coverage=1;non_full_length_coverage=1;isoform_length=2671;expected_accuracy=0.996	gnl|BL_ORD_ID|57891 cb8564_c83487/f1p0/3257	11	1637	1637	2631	12	1639	2005	2993
cb5046_c5/f1p0/1852	gnl|BL_ORD_ID|72091 cb5046_c4/f1p0/2918	1	1248	1245	1842	538	1785	2112	2709
cb8564_c44286/f1p0/2747	gnl|BL_ORD_ID|56381 cb8564_c75626/f1p2/3199	2	2121	2119	2665	4	2116	2611	3157
cb8564_c39824/f1p0/3319 isoform=cb8564_c39824;full_length_coverage=1;non_full_length_coverage=0;isoform_length=3319;expected_accuracy=1.000	gnl|BL_ORD_ID|14377 cb8564_c36097/f1p2/3430 isoform=cb8564_c36097;full_length_coverage=1;non_full_length_coverage=2;isoform_length=3430;expected_accuracy=0.996	1	1737	1737	3176	133	1865	1995	3430

cb8564_c39136/f1p0/3036 isoform=cb8564_c39136;full_length_coverage=1;non_full_length_coverage=0;isoform_length=3036;expected_accuracy=0.998	gnl|BL_ORD_ID|14471 cb8564_c34518/f1p0/3136 isoform=cb8564_c34518;full_length_coverage=1;non_full_length_coverage=0;isoform_length=3136;expected_accuracy=0.991	1	2089	2085	3036	1	2067	2193	3136
cb1505_c40/f1p0/2008	gnl|BL_ORD_ID|29921 cb1505_c16/f1p0/2115 isoform=cb1505_c16;full_length_coverage=1;non_full_length_coverage=0;isoform_length=2115;expected_accuracy=0.997	809	1800	9	808	1101	2093	6	806
cb8564_c112431/f1p0/2101	gnl|BL_ORD_ID|58076 cb8564_c88170/f1p1/2083	163	1864	1	165	333	2034	8	172
cb8564_c38462/f1p0/3241	gnl|BL_ORD_ID|55920 cb8564_c48949/f1p0/4357	1	2280	2281	2946	2	2293	2847	3506
cb6719_c46/f1p0/2507	gnl|BL_ORD_ID|5888 cb6719_c12/f3p0/3100 isoform=cb6719_c12;full_length_coverage=3;non_full_length_coverage=0;isoform_length=3100;expected_accuracy=1.000	998	2452	88	1002	1636	3089	1	917
cb10317_c4/f1p0/1797	gnl|BL_ORD_ID|48379 cb10317_c2/f1p0/3207	1	1216	1215	1806	1	1215	2373	2964
cb8617_c0/f6p0/2265 isoform=cb8617_c0;full_length_coverage=6;non_full_length_coverage=0;isoform_length=2265;expected_accuracy=1.000	gnl|BL_ORD_ID|17936 cb8617_c2/f1p0/2817 isoform=cb8617_c2;full_length_coverage=1;non_full_length_coverage=0;isoform_length=2817;expected_accuracy=0.991	1	1504	1503	2241	1	1503	2084	2817
cb3678_c12/f1p0/2637 isoform=cb3678_c12;full_length_coverage=1;non_full_length_coverage=0;isoform_length=2637;expected_accuracy=0.997	gnl|BL_ORD_ID|41571 cb3678_c5/f1p0/3157 isoform=cb3678_c5;full_length_coverage=1;non_full_length_coverage=0;isoform_length=3157;expected_accuracy=0.994	1	2331	2326	2637	26	2364	2847	3157

cb8564_c185920/f1p0/2777 isoform=cb8564_c185920;full_length_coverage=1;non_full_length_coverage=0;isoform_length=2777;expected_accuracy=0.996	gnl|BL_ORD_ID|55327 cb8564_c38462/f1p0/3241	174	2775	1	177	345	2946	14	186
cb293_c6/f1p0/3758 isoform=cb293_c6;full_length_coverage=1;non_full_length_coverage=0;isoform_length=3758;expected_accuracy=0.997	gnl|BL_ORD_ID|37295 cb293_c1/f1p0/4190 isoform=cb293_c1;full_length_coverage=1;non_full_length_coverage=0;isoform_length=4190;expected_accuracy=0.999	11	1906	1903	3657	1	1899	2444	4190
cb2246_c6/f1p0/3623	gnl|BL_ORD_ID|68375 cb2246_c15/f1p0/3748	23	3187	3188	3630	1	3165	3291	3732
cb9157_c9/f1p0/1707 isoform=cb9157_c9;full_length_coverage=1;non_full_length_coverage=0;isoform_length=1707;expected_accuracy=0.996	gnl|BL_ORD_ID|58595 cb9157_c4/f1p0/2256	1	1330	1327	1661	255	1581	1723	2057
cb8564_c119767/f1p0/1892	gnl|BL_ORD_ID|11659 cb8564_c147719/f1p1/2554 isoform=cb8564_c147719;full_length_coverage=1;non_full_length_coverage=1;isoform_length=2554;expected_accuracy=0.996	186	1882	9	189	818	2514	1	186
cb4350_c8/f1p4/1998 isoform=cb4350_c8;full_length_coverage=1;non_full_length_coverage=4;isoform_length=1998;expected_accuracy=0.999	gnl|BL_ORD_ID|72492 cb4350_c14/f1p1/2136	273	1958	5	268	447	2133	2	263
cb8564_c71904/f1p1/2820 isoform=cb8564_c71904;full_length_coverage=1;non_full_length_coverage=1;isoform_length=2820;expected_accuracy=0.996	gnl|BL_ORD_ID|57330 cb8564_c90173/f1p0/3220	1	1983	1978	2820	186	2162	2351	3193

cb1935_c14/f1p0/3028	gnl|BL_ORD_ID|66507 cb1935_c18/f1p0/3361	15	1968	1963	3017	37	1989	2112	3166
cb8564_c10064/f5p11/3897 isoform=cb8564_c10064;full_length_coverage=5;non_full_length_coverage=11;isoform_length=3897;expected_accuracy=0.996	gnl|BL_ORD_ID|54049 cb8564_c17373/f1p2/4790	159	3134	3135	3897	583	3558	3968	4730
cb8687_c3/f1p0/2296 isoform=cb8687_c3;full_length_coverage=1;non_full_length_coverage=0;isoform_length=2296;expected_accuracy=0.994	gnl|BL_ORD_ID|18632 cb8687_c4/f1p0/3035 isoform=cb8687_c4;full_length_coverage=1;non_full_length_coverage=0;isoform_length=3035;expected_accuracy=0.990	211	2238	1	211	965	3035	412	626
cb8564_c47701/f1p0/3144	gnl|BL_ORD_ID|14531 cb8564_c41778/f1p9/3482 isoform=cb8564_c41778;full_length_coverage=1;non_full_length_coverage=9;isoform_length=3482;expected_accuracy=0.992	253	2880	24	257	812	3439	11	247
cb6169_c4/f1p0/1708 isoform=cb6169_c4;full_length_coverage=1;non_full_length_coverage=0;isoform_length=1708;expected_accuracy=0.994	gnl|BL_ORD_ID|74998 cb6169_c2/f1p0/2943	1	1352	1350	1708	5	1356	2453	2811
cb3290_c38/f1p0/2345 isoform=cb3290_c38;full_length_coverage=1;non_full_length_coverage=0;isoform_length=2345;expected_accuracy=0.996	gnl|BL_ORD_ID|71039 cb3290_c87/f1p11/2956	1150	2345	1	1151	1586	2789	273	1429
cb8564_c78701/f2p2/2927 isoform=cb8564_c78701;full_length_coverage=2;non_full_length_coverage=2;isoform_length=2927;expected_accuracy=1.000	gnl|BL_ORD_ID|17990 cb8564_c81281/f1p1/3440 isoform=cb8564_c81281;full_length_coverage=1;non_full_length_coverage=1;isoform_length=3440;expected_accuracy=1.000	1	2732	2728	2927	104	2810	3194	3391

cb1494_c20/f1p0/3547 isoform=cb1494_c20;full_length_coverage=1;non_full_length_coverage=0;isoform_length=3547;expected_accuracy=0.996	gnl|BL_ORD_ID|61366 cb1494_c21/f1p0/5091	1	2179	2179	3547	111	2293	3746	5114
cb6054_c29/f1p0/1952 isoform=cb6054_c29;full_length_coverage=1;non_full_length_coverage=0;isoform_length=1952;expected_accuracy=0.991	gnl|BL_ORD_ID|74835 cb6054_c23/f1p2/2316	1	1642	1638	1952	25	1667	1796	2112
cb4559_c7/f1p0/1283	gnl|BL_ORD_ID|43630 cb4559_c3/f1p0/3310 isoform=cb4559_c3;full_length_coverage=1;non_full_length_coverage=0;isoform_length=3310;expected_accuracy=0.992	515	1286	60	517	2373	3145	1	458
cb452_c69/f1p2/3866 isoform=cb452_c69;full_length_coverage=1;non_full_length_coverage=2;isoform_length=3866;expected_accuracy=0.998	gnl|BL_ORD_ID|70911 cb452_c24/f1p0/4042	1	2266	2266	3821	95	2363	2502	4059
cb2733_c3/f2p0/1351 isoform=cb2733_c3;full_length_coverage=2;non_full_length_coverage=0;isoform_length=1351;expected_accuracy=1.000	gnl|BL_ORD_ID|37265 cb2733_c5/f2p1/1786 isoform=cb2733_c5;full_length_coverage=2;non_full_length_coverage=1;isoform_length=1786;expected_accuracy=0.995	143	1350	50	142	578	1785	90	190
cb513_c0/f3p0/2985 isoform=cb513_c0;full_length_coverage=3;non_full_length_coverage=0;isoform_length=2985;expected_accuracy=0.995	gnl|BL_ORD_ID|43725 cb513_c4/f1p0/3166 isoform=cb513_c4;full_length_coverage=1;non_full_length_coverage=0;isoform_length=3166;expected_accuracy=0.995	1	2125	2124	2985	24	2149	2304	3165
cb8478_c37/f1p0/3192	gnl|BL_ORD_ID|10548 cb8478_c9/f2p1/3524 isoform=cb8478_c9;full_length_coverage=2;non_full_le	12	2659	2657	3149	1	2648	3005	3497

	ngth_coverage=1;isoform_length=3524;expected_accuracy=0.999								
cb1340_c4/f1p0/1977 isoform=cb1340_c4;full_length_coverage=1;non_full_length_coverage=0;isoform_length=1977;expected_accuracy=0.996	gnl|BL_ORD_ID|25836 cb1340_c2/f1p0/3014 isoform=cb1340_c2;full_length_coverage=1;non_full_length_coverage=0;isoform_length=3014;expected_accuracy=0.993	1	1466	1462	1959	46	1507	2517	3014
cb18456_c4171/f1p0/1189 isoform=cb18456_c4171;full_length_coverage=1;non_full_length_coverage=0;isoform_length=1189;expected_accuracy=0.993	gnl|BL_ORD_ID|67493 cb18456_c4264/f1p1/1767	258	1189	4	263	803	1761	1	247
cb8564_c40218/f1p0/3146 isoform=cb8564_c40218;full_length_coverage=1;non_full_length_coverage=0;isoform_length=3146;expected_accuracy=0.993	gnl|BL_ORD_ID|56041 cb8564_c53077/f1p2/3406	337	3146	4	337	452	3285	1	337
cb3835_c38/f1p0/2346	gnl|BL_ORD_ID|41583 cb3835_c24/f1p1/2989 isoform=cb3835_c24;full_length_coverage=1;non_full_length_coverage=1;isoform_length=2989;expected_accuracy=0.994	326	2254	70	328	942	2871	1	259
cb1468_c4/f1p2/2897 isoform=cb1468_c4;full_length_coverage=1;non_full_length_coverage=2;isoform_length=2897;expected_accuracy=0.997	gnl|BL_ORD_ID|63645 cb1468_c3/f1p0/4312	2	2012	2011	2823	131	2140	3043	3855
cb13565_c28/f1p4/1411 isoform=cb13565_c28;full_length_coverage=1;non_full_length_coverage=4;isoform_length=1411;expected_accuracy=0.998	gnl|BL_ORD_ID|62569 cb13565_c31/f1p9/1644	143	1411	4	147	338	1606	10	153

cb8564_c178999/f1p0/2222 isoform=cb8564_c178999;full_length_coverage=1;non_full_length_coverage=0;isoform_length=2222;expected_accuracy=0.994	gnl|BL_ORD_ID|55894 cb8564_c48160/f1p2/3308	10	1778	1773	2221	4	1787	2745	3195
cb8167_c9/f1p0/2008 isoform=cb8167_c9;full_length_coverage=1;non_full_length_coverage=0;isoform_length=2008;expected_accuracy=0.996	gnl|BL_ORD_ID|7083 cb8167_c6/f1p1/2277 isoform=cb8167_c6;full_length_coverage=1;non_full_length_coverage=1;isoform_length=2277;expected_accuracy=0.991	132	2008	12	130	255	2119	1	116
cb14637_c1/f1p0/1701	gnl|BL_ORD_ID|56533 cb8564_c4774/f1p1/3231	331	1680	6	331	1667	3016	1	326
cb8564_c38233/f1p0/2780	gnl|BL_ORD_ID|55931 cb8564_c49346/f1p3/3240	1	1857	1856	2785	218	2074	2199	3127
cb8564_c44063/f1p0/2503 isoform=cb8564_c44063;full_length_coverage=1;non_full_length_coverage=0;isoform_length=2503;expected_accuracy=0.999	gnl|BL_ORD_ID|16528 cb8564_c54660/f1p21/2689 isoform=cb8564_c54660;full_length_coverage=1;non_full_length_coverage=21;isoform_length=2689;expected_accuracy=0.999	397	2503	49	402	580	2686	80	435
cb1317_c3/f5p1/2427 isoform=cb1317_c3;full_length_coverage=5;non_full_length_coverage=1;isoform_length=2427;expected_accuracy=1.000	gnl|BL_ORD_ID|61229 cb1317_c8/f1p0/4195	182	2406	16	180	2167	4389	74	232
cb8564_c45300/f1p4/3014 isoform=cb8564_c45300;full_length_coverage=1;non_full_length_coverage=4;isoform_length=3014;expected_accuracy=0.996	gnl|BL_ORD_ID|14091 cb8564_c43119/f1p4/3194 isoform=cb8564_c43119;full_length_coverage=1;non_full_length_coverage=4;isoform_length=3194;expected_accuracy=0.995	243	2976	1	246	401	3157	1	244
cb15493_c0/f17p0/1006 isoform=cb15493_c0;full_length_coverage=17;non_full	gnl|BL_ORD_ID|15750 cb8564_c69252/f2p1/3018 isoform=cb8564_c69252;full_length_coverage=2;non_fu	307	1006	4	309	2319	3018	1	306

_length_coverage=0;isoform_length=1006;expected_accuracy=1.000	ll_length_coverage=1;isoform_length=3018;expected_accuracy=0.998								
cb8564_c83720/f1p0/2268	gnl|BL_ORD_ID|9154 cb8564_c11164/f1p0/3234 isoform=cb8564_c11164;full_length_coverage=1;non_full_length_coverage=0;isoform_length=3234;expected_accuracy=0.997	1	1503	1504	2262	4	1501	2474	3234
cb8564_c105574/f4p1/2274 isoform=cb8564_c105574;full_length_coverage=4;non_full_length_coverage=1;isoform_length=2274;expected_accuracy=0.995	gnl|BL_ORD_ID|13351 cb8564_c2055/f1p1/2415 isoform=cb8564_c2055;full_length_coverage=1;non_full_length_coverage=1;isoform_length=2415;expected_accuracy=1.000	110	1522	1521	2274	1	1410	1615	2371
cb2842_c11/f1p0/2421 isoform=cb2842_c11;full_length_coverage=1;non_full_length_coverage=0;isoform_length=2421;expected_accuracy=0.994	gnl|BL_ORD_ID|37105 cb2842_c29/f18p1/2546 isoform=cb2842_c29;full_length_coverage=18;non_full_length_coverage=1;isoform_length=2546;expected_accuracy=1.000	147	2417	1	146	269	2546	1	146
cb5806_c4/f1p2/2764 isoform=cb5806_c4;full_length_coverage=1;non_full_length_coverage=2;isoform_length=2764;expected_accuracy=0.991	gnl|BL_ORD_ID|74450 cb5806_c2/f1p0/2888	106	2069	2068	2764	1	1968	2133	2830
cb5755_c4/f1p0/2811	gnl|BL_ORD_ID|45433 cb5755_c3/f1p0/2933 isoform=cb5755_c3;full_length_coverage=1;non_full_length_coverage=0;isoform_length=2933;expected_accuracy=0.993	1	1504	1502	2803	1	1496	1632	2933
cb18782_c0/f1p2/5630 isoform=cb18782_c0;full_length_coverage=1;non_full_length_coverage=2;isoform_length=5630;expected_accuracy=0.997	gnl|BL_ORD_ID|35902 cb18782_c3/f1p2/5766 isoform=cb18782_c3;full_length_coverage=1;non_full_length_coverage=2;isoform_length=5766;expected_accuracy=0.996	3	4306	4304	5594	1	4321	4455	5766

cb8564_c35301/f1p0/2777	gnl|BL_ORD_ID|55844 cb8564_c44552/f1p0/3216	125	1768	1764	2718	269	1930	2068	3021
cb3143_c8/f1p1/2928 isoform=cb3143_c8;full_length_coverage=1;non_full_length_coverage=1;isoform_length=2928;expected_accuracy=0.992	gnl|BL_ORD_ID|37665 cb3143_c33/f10p3/3441 isoform=cb3143_c33;full_length_coverage=10;non_full_length_coverage=3;isoform_length=3441;expected_accuracy=1.000	205	2927	1	206	722	3441	72	277
cb8564_c36731/f1p2/2761	gnl|BL_ORD_ID|12913 cb8564_c182049/f1p0/3195 isoform=cb8564_c182049;full_length_coverage=1;non_full_length_coverage=0;isoform_length=3195;expected_accuracy=0.997	1	1977	1972	2738	1	1993	2424	3195
cb8564_c24784/f1p1/2695	gnl|BL_ORD_ID|56381 cb8564_c75626/f1p2/3199	2	2114	2112	2599	2	2116	2611	3099
cb2854_c44/f1p0/3207 isoform=cb2854_c44;full_length_coverage=1;non_full_length_coverage=0;isoform_length=3207;expected_accuracy=0.995	gnl|BL_ORD_ID|36848 cb2854_c42/f1p0/3914 isoform=cb2854_c42;full_length_coverage=1;non_full_length_coverage=0;isoform_length=3914;expected_accuracy=0.999	1	2886	2883	3207	4	2890	3590	3913
cb8564_c75620/f1p0/2781 isoform=cb8564_c75620;full_length_coverage=1;non_full_length_coverage=0;isoform_length=2781;expected_accuracy=0.996	gnl|BL_ORD_ID|11006 cb8564_c107982/f2p0/2940 isoform=cb8564_c107982;full_length_coverage=2;non_full_length_coverage=0;isoform_length=2940;expected_accuracy=0.998	1	1769	1764	2781	1	1767	1878	2895
cb5960_c15/f1p0/2801	gnl|BL_ORD_ID|74707 cb5960_c9/f1p0/3444	2	2020	2018	2642	42	2061	2806	3430
cb8564_c69336/f3p0/3731 isoform=cb8564_c69336;full_length_coverage=3;non_full_length_coverage=0;isoform_length=3731;expected_accuracy=0.997	gnl|BL_ORD_ID|57158 cb8564_c85001/f1p0/3853	1575	3728	1	1574	1710	3881	1	1574

cb9549_c8/f1p0/1542 isoform=cb9549_c8;full_length_coverage=1;non_full_length_coverage=0;isoform_length=1542;expected_accuracy=1.000	gnl|BL_ORD_ID|21591 cb9549_c7/f1p0/1719 isoform=cb9549_c7;full_length_coverage=1;non_full_length_coverage=0;isoform_length=1719;expected_accuracy=0.993	114	1542	1	113	279	171 0	25	136
cb8564_c81040/f1p1/2371	gnl|BL_ORD_ID|53111 cb8564_c116552/f1p1/2737	10	1965	1962	2478	1	195 6	2161	267 8
cb18456_c6959/f1p0/1857	gnl|BL_ORD_ID|16450 cb8564_c72442/f2p1/2290 isoform=cb8564_c72442;full_length_coverage=2;non_full_length_coverage=1;isoform_length=2290;expected_accuracy=0.994	653	1782	74	654	1158	228 8	1	583
cb8564_c46939/f1p1/2926	gnl|BL_ORD_ID|56929 cb8564_c77329/f1p0/3886	17	2134	2130	2870	32	215 4	2259	300 0
cb8564_c11853/f1p0/2732	gnl|BL_ORD_ID|17752 cb8564_c80488/f1p0/2745 isoform=cb8564_c80488;full_length_coverage=1;non_full_length_coverage=0;isoform_length=2745;expected_accuracy=0.993	11	2072	2071	2585	11	208 3	2208	273 2
cb14724_c4/f1p0/1676	gnl|BL_ORD_ID|40725 cb3622_c2/f3p1/2136 isoform=cb3622_c2;full_length_coverage=3;non_full_length_coverage=1;isoform_length=2136;expected_accuracy=1.000	1	1332	1330	1668	25	135 6	1765	210 4
cb8564_c23232/f1p3/2540	gnl|BL_ORD_ID|55553 cb8564_c45006/f1p1/2724	16	2172	2173	2518	4	215 7	2265	261 0
cb373_c8/f1p0/3698 isoform=cb373_c8;full_length_coverage=1;non_full_length_coverage=0;isoform_length=3698;expected_accuracy=0.997	gnl|BL_ORD_ID|40699 cb373_c6/f1p0/4615 isoform=cb373_c6;full_length_coverage=1;non_full_length_coverage=0;isoform_length=4615;expected_accuracy=0.997	232	3698	1	232	1118	458 6	56	286

cb4674_c44/f1p2/2925 isoform=cb4674_c44;full_length_coverage=1;non_full_length_coverage=2;isoform_length=2925;expected_accuracy=0.991	gnl|BL_ORD_ID|43463 cb4674_c77/f1p2/3189 isoform=cb4674_c77;full_length_coverage=1;non_full_length_coverage=2;isoform_length=3189;expected_accuracy=0.999	1	2243	2242	2901	164	2392	2533	3189
cb5668_c6/f1p0/2697	gnl|BL_ORD_ID|74261 cb5668_c2/f1p0/3152	1	1663	1660	2711	59	1759	2020	3072
cb8564_c45596/f1p1/3430	gnl|BL_ORD_ID|12503 cb8564_c130989/f3p0/3592 isoform=cb8564_c130989;full_length_coverage=3;non_full_length_coverage=0;isoform_length=3592;expected_accuracy=0.997	2	2780	2775	3378	90	2870	2989	3592
cb5460_c22/f1p1/2849	gnl|BL_ORD_ID|73948 cb5460_c9/f1p1/2896	1	2085	2081	2777	11	2095	2217	2906
cb8564_c176603/f2p2/3262 isoform=cb8564_c176603;full_length_coverage=2;non_full_length_coverage=2;isoform_length=3262;expected_accuracy=0.997	gnl|BL_ORD_ID|18565 cb8564_c92252/f1p1/3402 isoform=cb8564_c92252;full_length_coverage=1;non_full_length_coverage=1;isoform_length=3402;expected_accuracy=0.997	146	3211	1	146	335	3402	6	151
cb8564_c161792/f1p1/2087	gnl|BL_ORD_ID|53698 cb8564_c1307/f1p0/2847	17	1703	1700	2044	104	1790	2349	2693
cb8617_c3/f1p0/2761	gnl|BL_ORD_ID|17936 cb8617_c2/f1p0/2817 isoform=cb8617_c2;full_length_coverage=1;non_full_length_coverage=0;isoform_length=2817;expected_accuracy=0.991	1	1938	1933	2675	3	1932	2080	2817
cb4997_c18/f1p0/2327	gnl|BL_ORD_ID|42221 cb4997_c15/f1p0/2104 isoform=cb4997_c15;full_length_coverage=1;non_full_length_coverage=0;isoform_length=2104;expected_accuracy=0.994	1	1545	1543	1997	1	1547	1647	2103

cb8564_c42814/f1p0/2151 isoform=cb8564_c42814;full_length_coverage=1;non_full_length_coverage=0;isoform_length=2151;expected_accuracy=0.993	gnl|BL_ORD_ID|56833 cb8564_c73203/f1p0/2850	650	2149	7	652	1172	2673	1	646
cb8564_c116328/f1p6/2732	gnl|BL_ORD_ID|9350 cb8564_c11826/f1p0/2916 isoform=cb8564_c11826;full_length_coverage=1;non_full_length_coverage=0;isoform_length=2916;expected_accuracy=0.997	1293	2617	1	1294	1543	2867	5	1296
cb427_c2/f9p1/1897 isoform=cb427_c2;full_length_coverage=9;non_full_length_coverage=1;isoform_length=1897;expected_accuracy=1.000	gnl|BL_ORD_ID|43366 cb427_c9/f1p0/4077 isoform=cb427_c9;full_length_coverage=1;non_full_length_coverage=0;isoform_length=4077;expected_accuracy=0.991	1	1586	1582	1897	2	1586	2370	2686
cb435_c1/f1p0/4816 isoform=cb435_c1;full_length_coverage=1;non_full_length_coverage=0;isoform_length=4816;expected_accuracy=0.999	gnl|BL_ORD_ID|72170 cb435_c7/f1p0/5320	12	4374	4372	4747	6	4368	4933	5313
cb6507_c5/f1p0/2184	gnl|BL_ORD_ID|3952 cb6507_c4/f1p0/2292 isoform=cb6507_c4;full_length_coverage=1;non_full_length_coverage=0;isoform_length=2292;expected_accuracy=0.995	1	1222	1221	2175	10	1229	1338	2292
cb7164_c0/f1p0/2692 isoform=cb7164_c0;full_length_coverage=1;non_full_length_coverage=0;isoform_length=2692;expected_accuracy=0.992	gnl|BL_ORD_ID|47866 cb7164_c1/f1p0/4448	103	2440	2437	2659	4	2349	4041	4262
cb8564_c183213/f1p3/3190 isoform=cb8564_c183213;full_length_coverage=1;non_	gnl|BL_ORD_ID|10365 cb8564_c107731/f4p7/3727 isoform=cb8564_c107731;full_length_coverage=4;non_f	1	2313	2318	3190	106	2418	2745	3617

full_length_coverage=3;isoform_length=3190;expected_accuracy=0.999	ull_length_coverage=7;isoform_length=3727;expected_accuracy=0.998								
cb8564_c15653/f1p4/2475	gnl|BL_ORD_ID|53923 cb8564_c15010/f1p10/2806	1	2293	2289	2446	1	2293	2559	2716
cb466_c13/f1p0/3021	gnl|BL_ORD_ID|41724 cb466_c1/f1p1/3973 isoform=cb466_c1;full_length_coverage=1;non_full_length_coverage=1;isoform_length=3973;expected_accuracy=0.991	213	2955	16	215	1234	3972	13	209
cb1656_c19/f1p1/3657 isoform=cb1656_c19;full_length_coverage=1;non_full_length_coverage=1;isoform_length=3657;expected_accuracy=1.000	gnl|BL_ORD_ID|63828 cb1656_c15/f1p1/3875	1	2977	2976	3629	1	2978	3137	3788
cb8564_c110954/f1p1/2392 isoform=cb8564_c110954;full_length_coverage=1;non_full_length_coverage=1;isoform_length=2392;expected_accuracy=0.994	gnl|BL_ORD_ID|9183 cb8564_c121433/f4p1/2731 isoform=cb8564_c121433;full_length_coverage=4;non_full_length_coverage=1;isoform_length=2731;expected_accuracy=1.000	1	2113	2109	2392	34	2156	2344	2628
cb2284_c10/f1p2/2830 isoform=cb2284_c10;full_length_coverage=1;non_full_length_coverage=2;isoform_length=2830;expected_accuracy=0.997	gnl|BL_ORD_ID|36592 cb2284_c7/f1p0/3988 isoform=cb2284_c7;full_length_coverage=1;non_full_length_coverage=0;isoform_length=3988;expected_accuracy=0.996	268	2789	1	273	1467	3988	1	276
cb8564_c48356/f1p0/3140	gnl|BL_ORD_ID|54429 cb8564_c44507/f1p0/3862	1	2435	2434	2957	53	2487	3025	3548
cb5125_c34/f1p0/1890	gnl|BL_ORD_ID|42295 cb5125_c41/f11p2/2137 isoform=cb5125_c41;full_length_coverage=11;non_full_length_coverage=2;isoform_length=2137;expected_accuracy=1.000	1	1145	1144	1780	66	1212	1404	2038

cb2274_c17/f1p0/2659	gnl|BL_ORD_ID|68398 cb2274_c16/f1p0/2808	270	2427	4	274	473	2626	3	273
cb8564_c23232/f1p3/2540	gnl|BL_ORD_ID|11483 cb8564_c12665/f1p15/2806 isoform=cb8564_c12665;full_length_coverage=1;non_full_length_coverage=15;isoform_length=2806;expected_accuracy=0.995	16	2172	2167	2518	7	2168	2270	2621
cb8564_c181853/f1p0/2119 isoform=cb8564_c181853;full_length_coverage=1;non_full_length_coverage=0;isoform_length=2119;expected_accuracy=0.997	gnl|BL_ORD_ID|12938 cb8564_c181163/f1p1/2936 isoform=cb8564_c181163;full_length_coverage=1;non_full_length_coverage=1;isoform_length=2936;expected_accuracy=0.993	1	1339	1334	2119	5	1339	2158	2935
cb1052_c19/f1p0/2851	gnl|BL_ORD_ID|1189 cb1052_c16/f1p0/2854 isoform=cb1052_c16;full_length_coverage=1;non_full_length_coverage=0;isoform_length=2854;expected_accuracy=0.995	605	2793	76	601	669	2854	1	522
cb8564_c48473/f1p2/2701 isoform=cb8564_c48473;full_length_coverage=1;non_full_length_coverage=2;isoform_length=2701;expected_accuracy=0.999	gnl|BL_ORD_ID|12821 cb8564_c156372/f1p3/2909 isoform=cb8564_c156372;full_length_coverage=1;non_full_length_coverage=3;isoform_length=2909;expected_accuracy=1.000	220	2701	2	225	427	2909	60	283
cb6980_c16/f1p0/2284 isoform=cb6980_c16;full_length_coverage=1;non_full_length_coverage=0;isoform_length=2284;expected_accuracy=1.000	gnl|BL_ORD_ID|50225 cb6980_c9/f1p0/2797	1	1987	1982	2284	67	2057	2181	2483
cb8564_c38035/f1p0/2770	gnl|BL_ORD_ID|9418 cb8564_c119193/f1p2/2975 isoform=cb8564_c119193;full_length_coverage=1;non_full_length_coverage=2;isoform_length=2975;expected_accuracy=0.998	1	2035	2033	2755	1	2055	2246	2975

cb8564_c14344/f1p0/2533 isoform=cb8564_c14344;full_length_coverage=1;non_full_length_coverage=0;isoform_length=2533;expected_accuracy=0.993	gnl|BL_ORD_ID|54405 cb8564_c35890/f1p0/3157	148	2507	1	151	476	2836	7	157
cb8564_c39739/f1p1/2219 isoform=cb8564_c39739;full_length_coverage=1;non_full_length_coverage=1;isoform_length=2219;expected_accuracy=0.993	gnl|BL_ORD_ID|12829 cb8564_c161406/f2p1/2330 isoform=cb8564_c161406;full_length_coverage=2;non_full_length_coverage=1;isoform_length=2330;expected_accuracy=0.997	10	1839	1840	2153	9	1880	2007	2330
cb626_c16/f1p0/3290	gnl|BL_ORD_ID|47162 cb626_c15/f1p0/3353	1279	3049	1	1278	1459	3229	1	1278
cb17446_c1/f1p0/1452 isoform=cb17446_c1;full_length_coverage=1;non_full_length_coverage=0;isoform_length=1452;expected_accuracy=0.991	gnl|BL_ORD_ID|33637 cb17446_c0/f1p0/1723 isoform=cb17446_c0;full_length_coverage=1;non_full_length_coverage=0;isoform_length=1723;expected_accuracy=0.990	1	1208	1206	1452	32	1239	1436	1681
cb8564_c38653/f1p1/2281 isoform=cb8564_c38653;full_length_coverage=1;non_full_length_coverage=1;isoform_length=2281;expected_accuracy=0.991	gnl|BL_ORD_ID|14642 cb8564_c33936/f7p0/2581 isoform=cb8564_c33936;full_length_coverage=7;non_full_length_coverage=0;isoform_length=2581;expected_accuracy=0.998	1	1368	1368	2281	1	1354	1647	2564
cb8564_c38035/f1p0/2770	gnl|BL_ORD_ID|14529 cb8564_c44074/f1p1/2922 isoform=cb8564_c44074;full_length_coverage=1;non_full_length_coverage=1;isoform_length=2922;expected_accuracy=0.994	1	2030	2033	2756	1	2013	2205	2919
cb9589_c0/f3p0/2041 isoform=cb9589_c0;full_length_coverage=3;non_full_length_coverage=0;isoform_length=2041;expected_accuracy=1.000	gnl|BL_ORD_ID|19864 cb9589_c3/f1p0/3277 isoform=cb9589_c3;full_length_coverage=1;non_full_length_coverage=0;isoform_length=3277;expected_accuracy=0.995	1	1224	1226	2041	32	1253	2466	3277

cb8564_c21515/f1p0/3130	gnl|BL_ORD_ID|13377 cb8564_c22091/f2p0/3351 isoform=cb8564_c22091;full_length_coverage=2;non_full_length_coverage=0;isoform_length=3351;expected_accuracy=0.997	1165	2712	1	1168	1805	3351	515	1682
cb4942_c12/f1p1/1339	gnl|BL_ORD_ID|44010 cb4942_c5/f2p1/2283 isoform=cb4942_c5;full_length_coverage=2;non_full_length_coverage=1;isoform_length=2283;expected_accuracy=1.000	1	1243	1241	1341	5	1244	2166	2265
cb4953_c5/f1p1/1763	gnl|BL_ORD_ID|43241 cb4953_c3/f1p0/3107 isoform=cb4953_c3;full_length_coverage=1;non_full_length_coverage=0;isoform_length=3107;expected_accuracy=0.997	1	1084	1083	1688	1	1082	2495	3101
cb8564_c149594/f1p1/2449	gnl|BL_ORD_ID|12956 cb8564_c1771/f3p1/3767 isoform=cb8564_c1771;full_length_coverage=3;non_full_length_coverage=1;isoform_length=3767;expected_accuracy=0.996	1	1242	1240	2366	74	1334	2646	3767
cb8564_c165050/f2p1/2091 isoform=cb8564_c165050;full_length_coverage=2;non_full_length_coverage=1;isoform_length=2091;expected_accuracy=0.997	gnl|BL_ORD_ID|52763 cb8564_c13243/f1p0/4432	271	2091	81	269	2443	4261	2	191
cb3362_c13/f3p2/3516 isoform=cb3362_c13;full_length_coverage=3;non_full_length_coverage=2;isoform_length=3516;expected_accuracy=0.998	gnl|BL_ORD_ID|41033 cb3362_c17/f1p0/3642 isoform=cb3362_c17;full_length_coverage=1;non_full_length_coverage=0;isoform_length=3642;expected_accuracy=0.996	153	3479	1	153	320	3642	1	155
cb8564_c128717/f3p4/2465 isoform=cb8564_c128717;full_length_coverage=3;non_	gnl|BL_ORD_ID|57978 cb8564_c85833/f1p0/2942	1	1711	1709	2355	1	1709	2309	2955

full_length_coverage=4;isoform_length=2465;expected_accuracy=0.994									
cb307_c6/f1p1/4064 isoform=cb307_c6;full_length_coverage=1;non_full_length_coverage=1;isoform_length=4064;expected_accuracy=0.997	gnl|BL_ORD_ID|69545 cb307_c16/f1p1/4761	11	2744	2744	4064	20	2757	3006	4325
cb8564_c39136/f1p0/3036 isoform=cb8564_c39136;full_length_coverage=1;non_full_length_coverage=0;isoform_length=3036;expected_accuracy=0.998	gnl|BL_ORD_ID|14838 cb8564_c34060/f2p0/3214 isoform=cb8564_c34060;full_length_coverage=2;non_full_length_coverage=0;isoform_length=3214;expected_accuracy=0.993	1	2089	2085	3034	6	2137	2264	3214
cb6061_c3/f1p0/2514 isoform=cb6061_c3;full_length_coverage=1;non_full_length_coverage=0;isoform_length=2514;expected_accuracy=0.997	gnl|BL_ORD_ID|45128 cb6061_c2/f1p0/2948 isoform=cb6061_c2;full_length_coverage=1;non_full_length_coverage=0;isoform_length=2948;expected_accuracy=0.995	1056	2513	1	1056	1495	2948	18	1073
cb416_c58/f1p0/2505 isoform=cb416_c58;full_length_coverage=1;non_full_length_coverage=0;isoform_length=2505;expected_accuracy=0.992	gnl|BL_ORD_ID|70897 cb416_c51/f1p0/2774	286	2498	4	287	581	2774	150	430
cb10297_c5/f1p0/1838	gnl|BL_ORD_ID|48347 cb10297_c4/f1p0/2035	1	1207	1207	1674	1	1207	1357	1824
cb8564_c37361/f1p0/3076	gnl|BL_ORD_ID|55182 cb8564_c34825/f1p2/3200	10	2481	2477	3070	6	2505	2606	3196
cb6633_c6/f2p1/2044 isoform=cb6633_c6;full_length_coverage=2;non_full_length_coverage=1;isoform_length=2044;expected_accuracy=0.996	gnl|BL_ORD_ID|3747 cb6633_c9/f1p0/2805 isoform=cb6633_c9;full_length_coverage=1;non_full_length_coverage=0;isoform_length=2805;expected_accuracy=0.997	1	1493	1490	2033	27	1518	2261	2805

cb7486_c6/f1p0/1952 isoform=cb7486_c6;full_length_coverage=1;non_full_length_coverage=0;isoform_length=1952;expected_accuracy=0.990	gnl|BL_ORD_ID|51282 cb7486_c7/f1p0/2565	397	1952	49	398	553	2110	91	442
cb8564_c2273/f1p2/2502 isoform=cb8564_c2273;full_length_coverage=1;non_full_length_coverage=2;isoform_length=2502;expected_accuracy=0.995	gnl|BL_ORD_ID|13611 cb8564_c2184/f1p0/2606 isoform=cb8564_c2184;full_length_coverage=1;non_full_length_coverage=0;isoform_length=2606;expected_accuracy=0.995	280	2502	56	280	384	2606	49	272
cb14792_c2/f1p0/1864 isoform=cb14792_c2;full_length_coverage=1;non_full_length_coverage=0;isoform_length=1864;expected_accuracy=0.995	gnl|BL_ORD_ID|58053 cb8564_c87612/f1p0/2171	1	1524	1519	1864	10	1533	1744	2089
cb1306_c36/f1p0/2164 isoform=cb1306_c36;full_length_coverage=1;non_full_length_coverage=0;isoform_length=2164;expected_accuracy=1.000	gnl|BL_ORD_ID|25571 cb1306_c0/f11p2/3283 isoform=cb1306_c0;full_length_coverage=11;non_full_length_coverage=2;isoform_length=3283;expected_accuracy=1.000	1	1793	1788	2164	190	1982	2907	3283
cb2683_c4/f1p0/3274 isoform=cb2683_c4;full_length_coverage=1;non_full_length_coverage=0;isoform_length=3274;expected_accuracy=0.997	gnl|BL_ORD_ID|37537 cb2683_c3/f1p0/3843 isoform=cb2683_c3;full_length_coverage=1;non_full_length_coverage=0;isoform_length=3843;expected_accuracy=0.995	1	2581	2581	3274	17	2594	2812	3505
cb8564_c138545/f4p0/2007 isoform=cb8564_c138545;full_length_coverage=4;non_full_length_coverage=0;isoform_length=2007;expected_accuracy=1.000	gnl|BL_ORD_ID|10389 cb8564_c110053/f2p0/2311 isoform=cb8564_c110053;full_length_coverage=2;non_full_length_coverage=0;isoform_length=2311;expected_accuracy=0.996	1	1027	1026	1925	1	1032	1413	2311
cb8564_c45300/f1p4/3014 isoform=cb8564_c45300;full_length_coverage=1;non_f	gnl|BL_ORD_ID|55521 cb8564_c44014/f1p0/3720	243	3014	24	246	416	3188	40	259

ull_length_coverage=4;isoform_length=3014;expected_accuracy=0.996									
cb8564_c151935/f1p1/2206	gnl|BL_ORD_ID|11244 cb8564_c13867/f3p0/4487 isoform=cb8564_c13867;full_length_coverage=3;non_full_length_coverage=0;isoform_length=4487;expected_accuracy=0.994	262	2208	5	263	2515	4465	27	284
cb8564_c36689/f1p1/2368 isoform=cb8564_c36689;full_length_coverage=1;non_full_length_coverage=1;isoform_length=2368;expected_accuracy=0.991	gnl|BL_ORD_ID|14726 cb8564_c34020/f2p0/2942 isoform=cb8564_c34020;full_length_coverage=2;non_full_length_coverage=0;isoform_length=2942;expected_accuracy=0.996	1	1709	1706	2368	1	1737	2236	2900
cb1215_c8/f1p0/3148	gnl|BL_ORD_ID|24740 cb1215_c15/f1p0/3339 isoform=cb1215_c15;full_length_coverage=1;non_full_length_coverage=0;isoform_length=3339;expected_accuracy=0.997	1309	3073	1	1308	1576	3339	20	1326
cb9785_c1/f1p0/1855	gnl|BL_ORD_ID|22177 cb9785_c0/f1p0/1978 isoform=cb9785_c0;full_length_coverage=1;non_full_length_coverage=0;isoform_length=1978;expected_accuracy=0.996	191	1844	29	191	321	1975	1	172
cb2640_c12/f1p0/3046 isoform=cb2640_c12;full_length_coverage=1;non_full_length_coverage=0;isoform_length=3046;expected_accuracy=0.994	gnl|BL_ORD_ID|68660 cb2640_c13/f1p0/3176	192	3046	16	193	309	3160	2	204
cb2097_c120/f1p0/1809 isoform=cb2097_c120;full_length_coverage=1;non_full_length_coverage=0;isoform_length=1809;expected_accuracy=0.994	gnl|BL_ORD_ID|66704 cb2097_c75/f1p0/2500	1	1422	1420	1809	509	1930	2040	2428

cb8564_c15653/f1p4/2475	gnl|BL_ORD_ID|10681 cb8564_c10952/f1p1/2673 isoform=cb8564_c10952;full_length_coverage=1;non_full_length_coverage=1;isoform_length=2673;expected_accuracy=0.992	1	2293	2289	2432	2	2303	2525	2671
cb5290_c64/f1p0/3128	gnl|BL_ORD_ID|44595 cb5290_c41/f1p1/3038 isoform=cb5290_c41;full_length_coverage=1;non_full_length_coverage=1;isoform_length=3038;expected_accuracy=0.997	1	1748	1747	2657	177	1924	2050	2960
cb8564_c15653/f1p4/2475	gnl|BL_ORD_ID|14105 cb8564_c35314/f1p0/2682 isoform=cb8564_c35314;full_length_coverage=1;non_full_length_coverage=0;isoform_length=2682;expected_accuracy=0.994	1	2293	2289	2435	4	2314	2536	2682
cb7815_c1/f8p1/2084 isoform=cb7815_c1;full_length_coverage=8;non_full_length_coverage=1;isoform_length=2084;expected_accuracy=1.000	gnl|BL_ORD_ID|51548 cb7815_c8/f1p0/3813	1	1350	1351	2029	84	1435	3031	3709
cb7348_c14/f11p1/1632 isoform=cb7348_c14;full_length_coverage=11;non_full_length_coverage=1;isoform_length=1632;expected_accuracy=1.000	gnl|BL_ORD_ID|8010 cb7348_c6/f1p0/1887 isoform=cb7348_c6;full_length_coverage=1;non_full_length_coverage=0;isoform_length=1887;expected_accuracy=0.998	1	1044	1041	1632	1	1046	1293	1886
cb18456_c6530/f1p0/1200	gnl|BL_ORD_ID|35192 cb18456_c6313/f1p4/1799 isoform=cb18456_c6313;full_length_coverage=1;non_full_length_coverage=4;isoform_length=1799;expected_accuracy=0.990	260	1166	3	259	797	1702	7	266
cb8564_c90990/f1p3/3338	gnl|BL_ORD_ID|16877 cb8564_c54048/f1p0/3516 isoform=cb8564_c54048;full_length_coverage=1;non_fu	1	2272	2272	3249	5	2283	2540	3516

	ll_length_coverage=0;isoform_length=3516;expected_accuracy=0.997								
cb8564_c52191/f1p0/2822 isoform=cb8564_c52191;full_length_coverage=1;non_full_length_coverage=0;isoform_length=2822;expected_accuracy=0.997	gnl|BL_ORD_ID|16172 cb8564_c53024/f1p0/3159 isoform=cb8564_c53024;full_length_coverage=1;non_full_length_coverage=0;isoform_length=3159;expected_accuracy=0.996	114	1683	1680	2822	132	1699	2017	3157
cb8564_c37361/f1p0/3076	gnl|BL_ORD_ID|16447 cb8564_c47202/f1p0/3195 isoform=cb8564_c47202;full_length_coverage=1;non_full_length_coverage=0;isoform_length=3195;expected_accuracy=0.996	1	2481	2477	3067	1	2482	2583	3195
cb6716_c4/f1p0/2291	gnl|BL_ORD_ID|4049 cb6716_c0/f2p2/2584 isoform=cb6716_c0;full_length_coverage=2;non_full_length_coverage=2;isoform_length=2584;expected_accuracy=1.000	1	1816	1815	2239	81	1896	2157	2582
cb18649_c2/f1p0/3942 isoform=cb18649_c2;full_length_coverage=1;non_full_length_coverage=0;isoform_length=3942;expected_accuracy=0.993	gnl|BL_ORD_ID|69440 cb18649_c7/f1p0/5612	1	2588	2587	3940	1111	3700	3930	5282
cb1279_c10/f2p0/2859 isoform=cb1279_c10;full_length_coverage=2;non_full_length_coverage=0;isoform_length=2859;expected_accuracy=0.999	gnl|BL_ORD_ID|27063 cb1279_c42/f1p0/3192 isoform=cb1279_c42;full_length_coverage=1;non_full_length_coverage=0;isoform_length=3192;expected_accuracy=0.993	1	1853	1849	2859	11	1865	2153	3160
cb8564_c3788/f1p1/2424	gnl|BL_ORD_ID|14925 cb8564_c3785/f1p0/2806 isoform=cb8564_c3785;full_length_coverage=1;non_full_length_coverage=0;isoform_length=2806;expected_accuracy=0.990	1	2114	2114	2411	246	2358	2509	2806

cb8564_c150144/f1p7/2784 isoform=cb8564_c150144;full_length_coverage=1;non_full_length_coverage=7;isoform_length=2784;expected_accuracy=0.991	gnl|BL_ORD_ID|55920 cb8564_c48949/f1p0/4357	1	2289	2290	2784	1	2293	2847	3336
cb8478_c39/f1p0/3267 isoform=cb8478_c39;full_length_coverage=1;non_full_length_coverage=0;isoform_length=3267;expected_accuracy=1.000	gnl|BL_ORD_ID|10224 cb8478_c13/f2p0/3348 isoform=cb8478_c13;full_length_coverage=2;non_full_length_coverage=0;isoform_length=3348;expected_accuracy=1.000	2	2959	2959	3149	77	3031	3157	3348
cb17862_c4/f1p0/1707	gnl|BL_ORD_ID|5716 cb6802_c23/f1p0/4851 isoform=cb6802_c23;full_length_coverage=1;non_full_length_coverage=0;isoform_length=4851;expected_accuracy=0.995	517	1682	64	517	3680	4850	718	1173
cb16877_c0/f1p0/1735 isoform=cb16877_c0;full_length_coverage=1;non_full_length_coverage=0;isoform_length=1735;expected_accuracy=0.996	gnl|BL_ORD_ID|53708 cb8564_c1398/f1p0/2214	110	1735	1	111	454	2072	1	111
cb3878_c4/f1p0/2331	gnl|BL_ORD_ID|70667 cb3878_c2/f1p0/3504	1	1163	1162	2197	1	1160	2462	3499
cb4016_c16/f3p2/2553 isoform=cb4016_c16;full_length_coverage=3;non_full_length_coverage=2;isoform_length=2553;expected_accuracy=0.996	gnl|BL_ORD_ID|70718 cb4016_c29/f1p0/2693	1	1400	1401	2464	87	1484	1605	2668
cb762_c17/f1p0/3793	gnl|BL_ORD_ID|8135 cb762_c1/f2p0/4120 isoform=cb762_c1;full_length_coverage=2;non_full_length_coverage=0;isoform_length=4120;expected_accuracy=0.994	1	2720	2718	3630	31	2745	3209	4120

<table>
<tr><th></th><th></th><th></th><th></th><th></th><th></th><th></th><th></th><th></th><th></th></tr>
<tr><td>cb6548_c2/f6p1/2451 isoform=cb6548_c2;full_length_coverage=6;non_full_length_coverage=1;isoform_length=2451;expected_accuracy=0.999</td><td>gnl|BL_ORD_ID|5637 cb6548_c10/f1p1/2515 isoform=cb6548_c10;full_length_coverage=1;non_full_length_coverage=1;isoform_length=2515;expected_accuracy=0.991</td><td>251</td><td>2409</td><td>50</td><td>252</td><td>345</td><td>2515</td><td>1</td><td>209</td></tr>
<tr><td>cb8564_c78476/f1p0/3130</td><td>gnl|BL_ORD_ID|57891 cb8564_c83487/f1p0/3257</td><td>12</td><td>1640</td><td>1640</td><td>2539</td><td>12</td><td>1639</td><td>2005</td><td>2904</td></tr>
<tr><td>cb8564_c123717/f1p0/2918</td><td>gnl|BL_ORD_ID|53318 cb8564_c121948/f1p0/3245</td><td>252</td><td>2909</td><td>14</td><td>253</td><td>370</td><td>3027</td><td>11</td><td>259</td></tr>
<tr><td>cb12989_c1/f1p0/1037</td><td>gnl|BL_ORD_ID|25460 cb12989_c0/f4p0/1107 isoform=cb12989_c0;full_length_coverage=4;non_full_length_coverage=0;isoform_length=1107;expected_accuracy=1.000</td><td>332</td><td>1005</td><td>8</td><td>334</td><td>435</td><td>1107</td><td>1</td><td>324</td></tr>
<tr><td>cb7761_c6/f1p0/2339 isoform=cb7761_c6;full_length_coverage=1;non_full_length_coverage=0;isoform_length=2339;expected_accuracy=0.994</td><td>gnl|BL_ORD_ID|7623 cb7761_c5/f1p0/2537 isoform=cb7761_c5;full_length_coverage=1;non_full_length_coverage=0;isoform_length=2537;expected_accuracy=0.993</td><td>1</td><td>1121</td><td>1121</td><td>2319</td><td>52</td><td>1173</td><td>1358</td><td>2537</td></tr>
<tr><td>cb8564_c75977/f1p0/2165</td><td>gnl|BL_ORD_ID|11679 cb8564_c146299/f10p7/3623 isoform=cb8564_c146299;full_length_coverage=10;non_full_length_coverage=7;isoform_length=3623;expected_accuracy=0.999</td><td>1</td><td>1393</td><td>1394</td><td>2130</td><td>67</td><td>1459</td><td>2747</td><td>3483</td></tr>
<tr><td>cb4487_c20/f1p0/1667</td><td>gnl|BL_ORD_ID|43638 cb4487_c5/f5p0/3334 isoform=cb4487_c5;full_length_coverage=5;non_full_length_coverage=0;isoform_length=3334;expected_accuracy=1.000</td><td>267</td><td>1557</td><td>3</td><td>270</td><td>1984</td><td>3274</td><td>85</td><td>352</td></tr>
<tr><td>cb6816_c2/f1p0/2039</td><td>gnl|BL_ORD_ID|5381 cb6816_c0/f1p0/2763 isoform=cb6816_c0;full_length_coverage=1;non_full_le</td><td>40</td><td>1000</td><td>995</td><td>1834</td><td>51</td><td>1029</td><td>1924</td><td>2763</td></tr>
</table>

	ngth_coverage=0;isoform_length=2763;expected_accuracy=0.995								
cb8205_c18/f1p0/1626 isoform=cb8205_c18;full_length_coverage=1;non_full_length_coverage=0;isoform_length=1626;expected_accuracy=1.000	gnl|BL_ORD_ID|8243 cb8205_c8/f1p0/2259 isoform=cb8205_c8;full_length_coverage=1;non_full_length_coverage=0;isoform_length=2259;expected_accuracy=0.996	185	1626	33	190	818	2258	1	158
cb6682_c6/f1p1/2237 isoform=cb6682_c6;full_length_coverage=1;non_full_length_coverage=1;isoform_length=2237;expected_accuracy=0.996	gnl|BL_ORD_ID|47643 cb6682_c5/f1p0/2779	1	1884	1882	2236	2	1876	2070	2425
cb8564_c40764/f1p0/2563 isoform=cb8564_c40764;full_length_coverage=1;non_full_length_coverage=0;isoform_length=2563;expected_accuracy=0.997	gnl|BL_ORD_ID|14399 cb8564_c39189/f1p1/2975 isoform=cb8564_c39189;full_length_coverage=1;non_full_length_coverage=1;isoform_length=2975;expected_accuracy=0.992	1	2167	2164	2563	1	2177	2391	2791
cb6388_c2/f1p1/2366 isoform=cb6388_c2;full_length_coverage=1;non_full_length_coverage=1;isoform_length=2366;expected_accuracy=0.999	gnl|BL_ORD_ID|4572 cb6388_c3/f1p0/2513 isoform=cb6388_c3;full_length_coverage=1;non_full_length_coverage=0;isoform_length=2513;expected_accuracy=0.996	1	1394	1391	2344	18	1410	1560	2513
cb5125_c36/f1p0/1789 isoform=cb5125_c36;full_length_coverage=1;non_full_length_coverage=0;isoform_length=1789;expected_accuracy=0.998	gnl|BL_ORD_ID|43699 cb5125_c8/f3p3/2148 isoform=cb5125_c8;full_length_coverage=3;non_full_length_coverage=3;isoform_length=2148;expected_accuracy=0.999	1	1163	1158	1789	8	1169	1375	2008
cb8205_c8/f1p0/2259 isoform=cb8205_c8;full_length_coverage=1;non_full_length_coverage=0;isoform_length=2259;expected_accuracy=0.996	gnl|BL_ORD_ID|51822 cb8205_c7/f1p0/2934	11	1607	1607	2234	283	1879	2279	2908

cb8564_c12805/f1p0/2580 isoform=cb8564_c12805;full_length_coverage=1;non_full_length_coverage=0;isoform_length=2580;expected_accuracy=0.993	gnl|BL_ORD_ID|56759 cb8564_c70577/f1p0/3111	11	1695	1690	2580	1	1687	1929	2822
cb5780_c3/f2p0/3092 isoform=cb5780_c3;full_length_coverage=2;non_full_length_coverage=0;isoform_length=3092;expected_accuracy=0.998	gnl|BL_ORD_ID|46054 cb5780_c12/f1p0/3428 isoform=cb5780_c12;full_length_coverage=1;non_full_length_coverage=0;isoform_length=3428;expected_accuracy=0.999	109	2322	2320	3073	1	2214	2675	3428
cb4016_c17/f1p0/2709 isoform=cb4016_c17;full_length_coverage=1;non_full_length_coverage=0;isoform_length=2709;expected_accuracy=0.995	gnl|BL_ORD_ID|40529 cb4016_c2/f3p1/2863 isoform=cb4016_c2;full_length_coverage=3;non_full_length_coverage=1;isoform_length=2863;expected_accuracy=0.998	185	2709	1	187	326	2849	4	189
cb5962_c9/f1p2/2694 isoform=cb5962_c9;full_length_coverage=1;non_full_length_coverage=2;isoform_length=2694;expected_accuracy=0.998	gnl|BL_ORD_ID|45736 cb5962_c4/f3p6/2935 isoform=cb5962_c4;full_length_coverage=3;non_full_length_coverage=6;isoform_length=2935;expected_accuracy=0.998	1	1390	1386	2694	4	1393	1527	2836
cb5610_c20/f1p0/2091	gnl|BL_ORD_ID|45980 cb5610_c17/f1p1/2257 isoform=cb5610_c17;full_length_coverage=1;non_full_length_coverage=1;isoform_length=2257;expected_accuracy=0.990	154	2040	1	155	281	2166	27	181
cb4949_c11/f1p0/2473 isoform=cb4949_c11;full_length_coverage=1;non_full_length_coverage=0;isoform_length=2473;expected_accuracy=0.999	gnl|BL_ORD_ID|42811 cb4949_c3/f1p0/3120 isoform=cb4949_c3;full_length_coverage=1;non_full_length_coverage=0;isoform_length=3120;expected_accuracy=0.991	1	1902	1900	2409	1	1921	2609	3120
cb9199_c4/f4p0/1836 isoform=cb9199_c4;full_length_coverage=4;non_full_le	gnl|BL_ORD_ID|59568 cb9199_c14/f1p0/2004	13	1002	999	1763	1	993	1236	2000

ngth_coverage=0;isoform_length=1836;expected_accuracy=1.000									
cb8564_c51661/f1p0/3132	gnl|BL_ORD_ID|57139 cb8564_c84468/f1p0/3473	121	2104	2099	3076	129	2112	2269	3246
cb8564_c9123/f4p5/3852 isoform=cb8564_c9123;full_length_coverage=4;non_full_length_coverage=5;isoform_length=3852;expected_accuracy=0.997	gnl|BL_ORD_ID|13205 cb8564_c20974/f1p0/4061 isoform=cb8564_c20974;full_length_coverage=1;non_full_length_coverage=0;isoform_length=4061;expected_accuracy=0.995	268	3852	119	272	437	4014	30	182
cb3332_c2/f1p0/1383 isoform=cb3332_c2;full_length_coverage=1;non_full_length_coverage=0;isoform_length=1383;expected_accuracy=0.999	gnl|BL_ORD_ID|40239 cb3332_c1/f1p0/3508 isoform=cb3332_c1;full_length_coverage=1;non_full_length_coverage=0;isoform_length=3508;expected_accuracy=0.996	375	1383	61	376	2471	3479	1	317
cb2284_c9/f1p0/2609	gnl|BL_ORD_ID|37510 cb2284_c29/f1p1/2964 isoform=cb2284_c29;full_length_coverage=1;non_full_length_coverage=1;isoform_length=2964;expected_accuracy=0.995	1	2051	2049	2602	98	2147	2415	2964
cb3359_c22/f1p0/2040	gnl|BL_ORD_ID|40969 cb3359_c15/f1p0/3164 isoform=cb3359_c15;full_length_coverage=1;non_full_length_coverage=0;isoform_length=3164;expected_accuracy=0.993	1	1696	1697	2025	1	1693	1839	2166
cb2802_c6/f1p0/1916 isoform=cb2802_c6;full_length_coverage=1;non_full_length_coverage=0;isoform_length=1916;expected_accuracy=0.993	gnl|BL_ORD_ID|38259 cb2802_c17/f5p0/2060 isoform=cb2802_c17;full_length_coverage=5;non_full_length_coverage=0;isoform_length=2060;expected_accuracy=1.000	1	1173	1171	1916	1	1173	1313	2058
cb18456_c7550/f1p0/1776 isoform=cb18456_c7550;full_length_coverage=1;non_f	gnl|BL_ORD_ID|52366 cb8564_c119851/f1p0/2310	351	1776	64	352	544	1970	2	290

ull_length_coverage=0;isoform_length=1776;expected_accuracy=0.993									
cb8564_c46939/f1p1/2926	gnl|BL_ORD_ID|55211 cb8564_c35547/f1p0/3745	1	2134	2130	2870	9	2153	2258	2998
cb8564_c841/f3p2/2127 isoform=cb8564_c841;full_length_coverage=3;non_full_length_coverage=2;isoform_length=2127;expected_accuracy=0.998	gnl|BL_ORD_ID|12361 cb8564_c1392/f1p1/3024 isoform=cb8564_c1392;full_length_coverage=1;non_full_length_coverage=1;isoform_length=3024;expected_accuracy=0.993	1	1516	1517	2127	51	1561	2412	3022
cb401_c157/f2p0/2398 isoform=cb401_c157;full_length_coverage=2;non_full_length_coverage=0;isoform_length=2398;expected_accuracy=0.995	gnl|BL_ORD_ID|70873 cb401_c42/f1p0/2704	1	1676	1674	2351	3	1679	2018	2698
cb8564_c50835/f1p0/2578	gnl|BL_ORD_ID|55917 cb8564_c48916/f1p0/3009	606	2580	78	609	900	2874	26	556
cb8564_c34638/f1p0/2817 isoform=cb8564_c34638;full_length_coverage=1;non_full_length_coverage=0;isoform_length=2817;expected_accuracy=0.992	gnl|BL_ORD_ID|18852 cb8564_c84488/f1p0/3408 isoform=cb8564_c84488;full_length_coverage=1;non_full_length_coverage=0;isoform_length=3408;expected_accuracy=0.996	219	2813	1	219	790	3408	1	217
cb8564_c87455/f1p0/2321	gnl|BL_ORD_ID|52655 cb8564_c10124/f1p0/3457	1	1494	1492	2223	26	1519	2675	3406
cb8564_c146513/f2p1/3281 isoform=cb8564_c146513;full_length_coverage=2;non_full_length_coverage=1;isoform_length=3281;expected_accuracy=0.997	gnl|BL_ORD_ID|12106 cb8564_c146659/f3p0/3534 isoform=cb8564_c146659;full_length_coverage=3;non_full_length_coverage=0;isoform_length=3534;expected_accuracy=0.998	1	2076	2074	3281	8	2095	2324	3534
cb10132_c18/f1p1/1347	gnl|BL_ORD_ID|18242 cb8564_c83110/f1p1/2442 isoform=cb8564_c83110;full_length_coverage=1;non_fu	281	1302	42	283	1386	2401	122	362

	ll_length_coverage=1;isoform_length=2442;expected_accuracy=0.997								
cb8564_c2600/f1p0/2432 isoform=cb8564_c2600;full_length_coverage=1;non_full_length_coverage=0;isoform_length=2432;expected_accuracy=1.000	gnl|BL_ORD_ID|54001 cb8564_c16726/f1p0/2596	1	1400	1399	2411	4	1391	1508	2520
cb9583_c6/f1p0/1811 isoform=cb9583_c6;full_length_coverage=1;non_full_length_coverage=0;isoform_length=1811;expected_accuracy=0.993	gnl|BL_ORD_ID|19510 cb9583_c3/f1p0/2043 isoform=cb9583_c3;full_length_coverage=1;non_full_length_coverage=0;isoform_length=2043;expected_accuracy=0.999	210	1795	9	209	454	2042	6	203
cb8564_c78626/f1p0/2262 isoform=cb8564_c78626;full_length_coverage=1;non_full_length_coverage=0;isoform_length=2262;expected_accuracy=0.997	gnl|BL_ORD_ID|57135 cb8564_c84378/f1p1/2886	1	1196	1195	2262	1	1196	1717	2784
cb8564_c38035/f1p0/2770	gnl|BL_ORD_ID|55607 cb8564_c46612/f1p1/3367	1	2035	2033	2756	8	2043	2233	2962
cb8564_c46321/f1p2/2987	gnl|BL_ORD_ID|14531 cb8564_c41778/f1p9/3482 isoform=cb8564_c41778;full_length_coverage=1;non_full_length_coverage=9;isoform_length=3482;expected_accuracy=0.992	260	2872	26	264	812	3426	7	247
cb8564_c44004/f1p0/2796 isoform=cb8564_c44004;full_length_coverage=1;non_full_length_coverage=0;isoform_length=2796;expected_accuracy=1.000	gnl|BL_ORD_ID|12978 cb8564_c175946/f10p1/3002 isoform=cb8564_c175946;full_length_coverage=10;non_full_length_coverage=1;isoform_length=3002;expected_accuracy=1.000	1	1549	1546	2795	72	1620	1754	3002
cb8564_c4643/f1p1/2554	gnl|BL_ORD_ID|16733 cb8564_c583/f12p2/2795 isoform=cb8564_c583;full_length_coverage=12;non_ful	1	1705	1700	2552	29	1733	1866	2719

	l_length_coverage=2;isoform_length=2795;expected_accuracy=1.000								
cb6757_c10/f1p0/2627	gnl|BL_ORD_ID|50029 cb6757_c6/f1p0/4618	1	1319	1317	2488	1	1319	3410	4580
cb8564_c10938/f1p1/2321	gnl|BL_ORD_ID|10628 cb8564_c10567/f1p5/3651 isoform=cb8564_c10567;full_length_coverage=1;non_full_length_coverage=5;isoform_length=3651;expected_accuracy=0.996	1	1315	1310	2292	1196	2509	2628	3610
cb3833_c5/f1p0/1849 isoform=cb3833_c5;full_length_coverage=1;non_full_length_coverage=0;isoform_length=1849;expected_accuracy=0.992	gnl|BL_ORD_ID|71542 cb3833_c1/f1p0/3501	312	1849	74	313	1875	3408	1	250
cb897_c39/f1p0/2987 isoform=cb897_c39;full_length_coverage=1;non_full_length_coverage=0;isoform_length=2987;expected_accuracy=0.991	gnl|BL_ORD_ID|59072 cb897_c18/f1p0/3399	1305	2987	126	1308	1509	3195	1	1187
cb2625_c9/f1p0/2161	gnl|BL_ORD_ID|38294 cb2625_c8/f1p0/2457 isoform=cb2625_c8;full_length_coverage=1;non_full_length_coverage=0;isoform_length=2457;expected_accuracy=0.997	1	1470	1468	2136	119	1588	1789	2457
cb8564_c2753/f1p0/2971 isoform=cb8564_c2753;full_length_coverage=1;non_full_length_coverage=0;isoform_length=2971;expected_accuracy=0.997	gnl|BL_ORD_ID|54800 cb8564_c2742/f1p0/3521	1449	2956	1	1449	1639	3145	1	1449
cb8564_c71076/f1p2/2391	gnl|BL_ORD_ID|56072 cb8564_c54171/f1p0/3438	142	2424	1	142	598	2880	297	438

cb8564_c47011/f1p0/3100	gnl|BL_ORD_ID|55472 cb8564_c42531/f1p0/3996	133	2306	2301	2934	816	2987	3225	3858
cb8564_c148479/f1p0/3197 isoform=cb8564_c148479;full_length_coverage=1;non_full_length_coverage=0;isoform_length=3197;expected_accuracy=0.997	gnl|BL_ORD_ID|52737 cb8564_c12549/f1p0/4073	1	2459	2455	3176	24	2482	3341	4062
cb4838_c4/f1p0/1068 isoform=cb4838_c4;full_length_coverage=1;non_full_length_coverage=0;isoform_length=1068;expected_accuracy=1.000	gnl|BL_ORD_ID|41739 cb4838_c3/f1p0/1177 isoform=cb4838_c3;full_length_coverage=1;non_full_length_coverage=0;isoform_length=1177;expected_accuracy=0.999	176	1068	7	177	289	1177	1	170
cb16776_c18/f1p0/6115	gnl|BL_ORD_ID|66941 cb16776_c23/f1p0/5901	2187	5884	199	2188	2099	5797	1	1989
cb1193_c13/f1p1/3683	gnl|BL_ORD_ID|59251 cb1193_c27/f1p0/3958	11	1867	1864	3686	8	1865	2014	3836
cb8564_c110954/f1p1/2392 isoform=cb8564_c110954;full_length_coverage=1;non_full_length_coverage=1;isoform_length=2392;expected_accuracy=0.994	gnl|BL_ORD_ID|54760 cb8564_c174230/f1p1/2759	1	2113	2109	2392	86	2224	2412	2694
cb5057_c2/f3p0/1613 isoform=cb5057_c2;full_length_coverage=3;non_full_length_coverage=0;isoform_length=1613;expected_accuracy=1.000	gnl|BL_ORD_ID|43283 cb5057_c11/f1p0/1761 isoform=cb5057_c11;full_length_coverage=1;non_full_length_coverage=0;isoform_length=1761;expected_accuracy=0.995	1	1264	1263	1613	1	1264	1373	1722
cb12363_c5/f1p0/1088 isoform=cb12363_c5;full_length_coverage=1;non_full_length_coverage=0;isoform_length=1088;expected_accuracy=1.000	gnl|BL_ORD_ID|5094 cb6700_c9/f1p0/2495 isoform=cb6700_c9;full_length_coverage=1;non_full_length_coverage=0;isoform_length=2495;expected_accuracy=0.993	158	1051	3	162	1604	2495	1070	1227

cb7412_c16/f1p0/1476	gnl|BL_ORD_ID|7462 cb7412_c7/f1p2/2560 isoform=cb7412_c7;full_length_coverage=1;non_full_length_coverage=2;isoform_length=2560;expected_accuracy=0.994	1	1143	1142	1448	1	1140	2180	2486
cb8564_c37390/f1p0/2683 isoform=cb8564_c37390;full_length_coverage=1;non_full_length_coverage=0;isoform_length=2683;expected_accuracy=1.000	gnl|BL_ORD_ID|55828 cb8564_c42159/f1p0/3251	265	2683	21	267	704	3123	10	255
cb8564_c2323/f1p0/2212 isoform=cb8564_c2323;full_length_coverage=1;non_full_length_coverage=0;isoform_length=2212;expected_accuracy=0.995	gnl|BL_ORD_ID|53846 cb8564_c2435/f1p1/2639	1	1320	1316	2212	26	1345	1623	2519
cb12685_c3/f1p2/1466	gnl|BL_ORD_ID|53630 cb8564_c134203/f1p0/3255	1	1294	1292	1463	15	1312	2900	3068
cb8564_c120352/f1p2/2487	gnl|BL_ORD_ID|10012 cb8564_c114725/f1p0/2945 isoform=cb8564_c114725;full_length_coverage=1;non_full_length_coverage=0;isoform_length=2945;expected_accuracy=0.991	1	1784	1783	2461	1	1792	2265	2945
cb781_c2/f3p0/1827 isoform=cb781_c2;full_length_coverage=3;non_full_length_coverage=0;isoform_length=1827;expected_accuracy=0.999	gnl|BL_ORD_ID|6782 cb781_c16/f1p0/1990 isoform=cb781_c16;full_length_coverage=1;non_full_length_coverage=0;isoform_length=1990;expected_accuracy=0.998	1	1243	1243	1806	82	1324	1429	1990
cb8564_c182028/f1p0/2513 isoform=cb8564_c182028;full_length_coverage=1;non_full_length_coverage=0;isoform_length=2513;expected_accuracy=0.996	gnl|BL_ORD_ID|19279 cb8564_c8088/f4p4/2776 isoform=cb8564_c8088;full_length_coverage=4;non_full_length_coverage=4;isoform_length=2776;expected_accuracy=0.998	1	1971	1968	2513	32	2001	2145	2690

cb5422_c3/f1p0/2208	gnl|BL_ORD_ID|73898 cb5422_c10/f1p0/5864	1	1312	1311	2019	3	1313	4803	5511
cb8478_c24/f1p0/3383 isoform=cb8478_c24;full_length_coverage=1;non_full_length_coverage=0;isoform_length=3383;expected_accuracy=0.996	gnl|BL_ORD_ID|9933 cb8478_c26/f1p0/3535 isoform=cb8478_c26;full_length_coverage=1;non_full_length_coverage=0;isoform_length=3535;expected_accuracy=0.995	266	3335	1	265	463	3535	1	264
cb3061_c27/f4p0/2371 isoform=cb3061_c27;full_length_coverage=4;non_full_length_coverage=0;isoform_length=2371;expected_accuracy=0.992	gnl|BL_ORD_ID|38031 cb3061_c7/f1p0/2487 isoform=cb3061_c7;full_length_coverage=1;non_full_length_coverage=0;isoform_length=2487;expected_accuracy=0.993	121	2342	1	122	275	2487	39	160
cb8564_c43455/f1p0/2242 isoform=cb8564_c43455;full_length_coverage=1;non_full_length_coverage=0;isoform_length=2242;expected_accuracy=0.995	gnl|BL_ORD_ID|17173 cb8564_c98020/f5p12/4000 isoform=cb8564_c98020;full_length_coverage=5;non_full_length_coverage=12;isoform_length=4000;expected_accuracy=0.995	279	2242	5	282	1979	3944	1	276
cb454_c11/f1p0/2332 isoform=cb454_c11;full_length_coverage=1;non_full_length_coverage=0;isoform_length=2332;expected_accuracy=0.992	gnl|BL_ORD_ID|70924 cb454_c27/f1p0/2557	275	2325	49	274	335	2387	2	229
cb8564_c188097/f1p0/2552 isoform=cb8564_c188097;full_length_coverage=1;non_full_length_coverage=0;isoform_length=2552;expected_accuracy=0.996	gnl|BL_ORD_ID|56670 cb8564_c51772/f1p0/2859	1	1945	1940	2510	1	1946	2094	2664
cb3848_c1/f4p0/2367 isoform=cb3848_c1;full_length_coverage=4;non_full_length_coverage=0;isoform_length=2367;expected_accuracy=0.995	gnl|BL_ORD_ID|39387 cb3848_c22/f1p1/3080 isoform=cb3848_c22;full_length_coverage=1;non_full_length_coverage=1;isoform_length=3080;expected_accuracy=0.998	1	2023	2021	2352	1	2021	2748	3080

<table>
<tr><td>cb2026_c0/f2p0/3902 isoform=cb2026_c0;full_length_coverage=2;non_full_length_coverage=0;isoform_length=3902;expected_accuracy=1.000</td><td>gnl|BL_ORD_ID|35151 cb2026_c1/f1p0/4080 isoform=cb2026_c1;full_length_coverage=1;non_full_length_coverage=0;isoform_length=4080;expected_accuracy=0.994</td><td>1</td><td>3404</td><td>3401</td><td>3869</td><td>1</td><td>3378</td><td>3542</td><td>4006</td></tr>
<tr><td>cb11952_c19/f1p0/1832</td><td>gnl|BL_ORD_ID|14853 cb8564_c3817/f3p0/2841 isoform=cb8564_c3817;full_length_coverage=3;non_full_length_coverage=0;isoform_length=2841;expected_accuracy=0.996</td><td>366</td><td>1804</td><td>5</td><td>365</td><td>1401</td><td>2839</td><td>178</td><td>535</td></tr>
<tr><td>cb12939_c8/f1p0/1310 isoform=cb12939_c8;full_length_coverage=1;non_full_length_coverage=0;isoform_length=1310;expected_accuracy=0.993</td><td>gnl|BL_ORD_ID|55736 cb8564_c5254/f1p0/2278</td><td>109</td><td>1307</td><td>1</td><td>110</td><td>1098</td><td>2293</td><td>25</td><td>136</td></tr>
<tr><td>cb10451_c11/f1p0/1745</td><td>gnl|BL_ORD_ID|48536 cb10451_c7/f1p0/2052</td><td>1</td><td>1312</td><td>1308</td><td>1716</td><td>35</td><td>1346</td><td>1456</td><td>1864</td></tr>
<tr><td>cb1178_c17/f1p0/1559 isoform=cb1178_c17;full_length_coverage=1;non_full_length_coverage=0;isoform_length=1559;expected_accuracy=0.995</td><td>gnl|BL_ORD_ID|59230 cb1178_c9/f1p0/2856</td><td>435</td><td>1528</td><td>48</td><td>435</td><td>1750</td><td>2843</td><td>1</td><td>388</td></tr>
<tr><td>cb5125_c36/f1p0/1789 isoform=cb5125_c36;full_length_coverage=1;non_full_length_coverage=0;isoform_length=1789;expected_accuracy=0.998</td><td>gnl|BL_ORD_ID|42296 cb5125_c42/f1p3/2079 isoform=cb5125_c42;full_length_coverage=1;non_full_length_coverage=3;isoform_length=2079;expected_accuracy=0.992</td><td>1</td><td>1163</td><td>1158</td><td>1789</td><td>8</td><td>1175</td><td>1381</td><td>2014</td></tr>
<tr><td>cb8564_c106740/f7p2/2421 isoform=cb8564_c106740;full_length_coverage=7;non_full_length_coverage=2;isoform_length=2421;expected_accuracy=1.000</td><td>gnl|BL_ORD_ID|52710 cb8564_c11928/f1p0/3418</td><td>242</td><td>2360</td><td>8</td><td>244</td><td>1173</td><td>3291</td><td>1</td><td>239</td></tr>
</table>

cb8564_c51971/f1p0/3064	gnl|BL_ORD_ID|9350 cb8564_c11826/f1p0/2916 isoform=cb8564_c11826;full_length_coverage=1;non_full_length_coverage=0;isoform_length=2916;expected_accuracy=0.997	1303	2676	10	1304	1543	2916	4	1296
cb17388_c6/f1p2/1438 isoform=cb17388_c6;full_length_coverage=1;non_full_length_coverage=2;isoform_length=1438;expected_accuracy=0.992	gnl|BL_ORD_ID|53482 cb8564_c126073/f1p1/2951	1	1088	1086	1438	5	1092	1280	1632
cb2818_c78/f1p0/2043 isoform=cb2818_c78;full_length_coverage=1;non_full_length_coverage=0;isoform_length=2043;expected_accuracy=0.992	gnl|BL_ORD_ID|68908 cb2818_c62/f1p1/3434	1	1936	1935	2039	909	2841	3236	3341
cb7638_c8/f1p0/1378 isoform=cb7638_c8;full_length_coverage=1;non_full_length_coverage=0;isoform_length=1378;expected_accuracy=0.999	gnl|BL_ORD_ID|8195 cb7638_c3/f1p0/1953 isoform=cb7638_c3;full_length_coverage=1;non_full_length_coverage=0;isoform_length=1953;expected_accuracy=0.991	1	1050	1049	1378	46	1093	1239	1569
cb8564_c115486/f1p1/3203	gnl|BL_ORD_ID|53615 cb8564_c130071/f1p0/4218	98	3139	1	102	531	3563	108	210
cb7150_c0/f2p0/2634 isoform=cb7150_c0;full_length_coverage=2;non_full_length_coverage=0;isoform_length=2634;expected_accuracy=1.000	gnl|BL_ORD_ID|5101 cb7150_c3/f1p0/3108 isoform=cb7150_c3;full_length_coverage=1;non_full_length_coverage=0;isoform_length=3108;expected_accuracy=0.993	1	2335	2335	2634	48	2372	2782	3081
cb4848_c24/f1p0/1860 isoform=cb4848_c24;full_length_coverage=1;non_full_length_coverage=0;isoform_length=1860;expected_accuracy=0.999	gnl|BL_ORD_ID|72032 cb4848_c9/f1p1/2843	206	1860	5	205	1010	2662	242	442

cb466_c13/f1p0/3021	gnl|BL_ORD_ID|41725 cb466_c0/f10p1/3978 isoform=cb466_c0;full_length_coverage=10;non_full_length_coverage=1;isoform_length=3978;expected_accuracy=1.000	213	2961	11	215	1227	3975	1	207
cb3653_c3/f1p0/1932 isoform=cb3653_c3;full_length_coverage=1;non_full_length_coverage=0;isoform_length=1932;expected_accuracy=0.994	gnl|BL_ORD_ID|39077 cb3653_c1/f2p0/2318 isoform=cb3653_c1;full_length_coverage=2;non_full_length_coverage=0;isoform_length=2318;expected_accuracy=0.992	457	1910	9	459	869	2318	1	450
cb8564_c53418/f1p0/2602 isoform=cb8564_c53418;full_length_coverage=1;non_full_length_coverage=0;isoform_length=2602;expected_accuracy=0.991	gnl|BL_ORD_ID|12921 cb8564_c17543/f1p10/2689 isoform=cb8564_c17543;full_length_coverage=1;non_full_length_coverage=10;isoform_length=2689;expected_accuracy=0.999	1163	2472	1	1168	1273	2585	4	1172
cb6181_c34/f1p0/2542	gnl|BL_ORD_ID|6168 cb6181_c17/f1p0/2583 isoform=cb6181_c17;full_length_coverage=1;non_full_length_coverage=0;isoform_length=2583;expected_accuracy=0.994	1	1267	1267	2285	1	1269	1487	2499
cb8564_c188097/f1p0/2552 isoform=cb8564_c188097;full_length_coverage=1;non_full_length_coverage=0;isoform_length=2552;expected_accuracy=0.996	gnl|BL_ORD_ID|13302 cb8564_c19160/f1p2/2687 isoform=cb8564_c19160;full_length_coverage=1;non_full_length_coverage=2;isoform_length=2687;expected_accuracy=0.990	1	1945	1940	2551	1	1974	2077	2687
cb2935_c37/f1p0/2974 isoform=cb2935_c37;full_length_coverage=1;non_full_length_coverage=0;isoform_length=2974;expected_accuracy=0.995	gnl|BL_ORD_ID|69017 cb2935_c40/f1p0/3601	1	1742	1737	2974	150	1894	2095	3333
cb8564_c114960/f1p2/3602	gnl|BL_ORD_ID|52943 cb8564_c112587/f1p0/4698	1036	3561	1	1037	1326	3851	176	1213

cb8564_c163331/f2p2/3571 isoform=cb8564_c163331;full_length_coverage=2;non_full_length_coverage=2;isoform_length=3571;expected_accuracy=0.996	gnl|BL_ORD_ID|16525 cb8564_c74618/f1p0/3734 isoform=cb8564_c74618;full_length_coverage=1;non_full_length_coverage=0;isoform_length=3734;expected_accuracy=0.998	1	2488	2483	3566	41	2536	2650	3734
cb8564_c16240/f1p3/2671 isoform=cb8564_c16240;full_length_coverage=1;non_full_length_coverage=3;isoform_length=2671;expected_accuracy=0.997	gnl|BL_ORD_ID|18051 cb8564_c9825/f10p17/3363 isoform=cb8564_c9825;full_length_coverage=10;non_full_length_coverage=17;isoform_length=3363;expected_accuracy=0.999	2	2075	2072	2671	5	2071	2608	3208
cb737_c27/f1p0/4113	gnl|BL_ORD_ID|50933 cb737_c18/f1p0/4382	106	3348	3347	4123	1	3244	3401	4180
cb500_c38/f1p0/2822 isoform=cb500_c38;full_length_coverage=1;non_full_length_coverage=0;isoform_length=2822;expected_accuracy=0.999	gnl|BL_ORD_ID|72247 cb500_c49/f1p0/3666	13	2396	2391	2782	120	2479	2941	3332
cb3941_c93/f1p0/2611	gnl|BL_ORD_ID|71656 cb3941_c90/f1p0/4349	2	2200	2198	2626	29	2226	2466	2894
cb6063_c19/f1p1/2514	gnl|BL_ORD_ID|45237 cb6063_c1/f11p0/2691 isoform=cb6063_c1;full_length_coverage=11;non_full_length_coverage=0;isoform_length=2691;expected_accuracy=1.000	157	2441	1	156	397	2674	74	228
cb500_c6/f4p0/3398 isoform=cb500_c6;full_length_coverage=4;non_full_length_coverage=0;isoform_length=3398;expected_accuracy=1.000	gnl|BL_ORD_ID|72251 cb500_c68/f1p0/4152	272	3356	1	276	961	4045	28	301
cb8564_c123988/f1p0/2683	gnl|BL_ORD_ID|10158 cb8564_c108914/f6p2/2897 isoform=cb8564_c108914;full_length_coverage=6;non_f	1	2343	2344	2638	89	2431	2603	2897

	ull_length_coverage=2;isoform_length=2897;expected_accuracy=0.998								
cb7076_c12/f1p1/2435 isoform=cb7076_c12;full_length_coverage=1;non_full_length_coverage=1;isoform_length=2435;expected_accuracy=0.997	gnl|BL_ORD_ID|50315 cb7076_c3/f1p0/2554	1	1850	1851	2384	10	1863	1995	2529
cb12_c45/f1p0/4319 isoform=cb12_c45;full_length_coverage=1;non_full_length_coverage=0;isoform_length=4319;expected_accuracy=0.993	gnl|BL_ORD_ID|25321 cb12_c54/f1p0/8058 isoform=cb12_c54;full_length_coverage=1;non_full_length_coverage=0;isoform_length=8058;expected_accuracy=0.994	271	4273	1	273	4056	8058	2024	2292
cb3632_c1/f1p0/2963 isoform=cb3632_c1;full_length_coverage=1;non_full_length_coverage=0;isoform_length=2963;expected_accuracy=0.996	gnl|BL_ORD_ID|71392 cb3632_c0/f1p0/3572	1	2629	2629	2961	25	2651	3221	3553
cb1438_c12/f1p0/3268	gnl|BL_ORD_ID|63602 cb1438_c8/f1p0/4349	1435	3296	1	1439	2219	4081	538	1978
cb8564_c71076/f1p2/2391	gnl|BL_ORD_ID|9367 cb8564_c107702/f7p2/2922 isoform=cb8564_c107702;full_length_coverage=7;non_full_length_coverage=2;isoform_length=2922;expected_accuracy=1.000	142	2424	1	142	632	2914	330	472
cb8564_c22331/f1p0/4122 isoform=cb8564_c22331;full_length_coverage=1;non_full_length_coverage=0;isoform_length=4122;expected_accuracy=0.992	gnl|BL_ORD_ID|54049 cb8564_c17373/f1p2/4790	2	3484	3485	4122	93	3558	3968	4607
cb9549_c8/f1p0/1542 isoform=cb9549_c8;full_length_coverage=1;non_full_le	gnl|BL_ORD_ID|21589 cb9549_c3/f1p0/1890 isoform=cb9549_c3;full_length_coverage=1;non_full_le	114	1542	1	115	414	1843	129	243

ngth_coverage=0;isoform_length=1542;expected_accuracy=1.000	ngth_coverage=0;isoform_length=1890;expected_accuracy=1.000								
cb11590_c0/f1p0/1012 isoform=cb11590_c0;full_length_coverage=1;non_full_length_coverage=0;isoform_length=1012;expected_accuracy=0.999	gnl|BL_ORD_ID|24735 cb11590_c1/f1p0/3691 isoform=cb11590_c1;full_length_coverage=1;non_full_length_coverage=0;isoform_length=3691;expected_accuracy=0.996	221	999	2	222	2915	3691	23	243
cb4128_c23/f1p0/2488 isoform=cb4128_c23;full_length_coverage=1;non_full_length_coverage=0;isoform_length=2488;expected_accuracy=0.999	gnl|BL_ORD_ID|71807 cb4128_c11/f1p0/3379	1	1757	1756	2488	70	1830	2166	2906
cb1366_c5/f1p0/1505 isoform=cb1366_c5;full_length_coverage=1;non_full_length_coverage=0;isoform_length=1505;expected_accuracy=0.997	gnl|BL_ORD_ID|25706 cb1366_c4/f1p0/1599 isoform=cb1366_c4;full_length_coverage=1;non_full_length_coverage=0;isoform_length=1599;expected_accuracy=0.995	1	1016	1016	1483	4	1017	1133	1599
cb8564_c52278/f1p1/2746	gnl|BL_ORD_ID|15663 cb8564_c71422/f3p2/3204 isoform=cb8564_c71422;full_length_coverage=3;non_full_length_coverage=2;isoform_length=3204;expected_accuracy=0.998	14	2531	2531	2667	1	2518	3071	3204
cb8564_c48473/f1p2/2701 isoform=cb8564_c48473;full_length_coverage=1;non_full_length_coverage=2;isoform_length=2701;expected_accuracy=0.999	gnl|BL_ORD_ID|52842 cb8564_c15468/f1p0/3015	220	2630	1	225	410	2819	41	266
cb8564_c90372/f1p0/2157 isoform=cb8564_c90372;full_length_coverage=1;non_full_length_coverage=0;isoform_length=2157;expected_accuracy=0.994	gnl|BL_ORD_ID|18439 cb8564_c87900/f1p0/2261 isoform=cb8564_c87900;full_length_coverage=1;non_full_length_coverage=0;isoform_length=2261;expected_accuracy=0.995	1	1092	1088	2081	29	1096	1268	2261

cb4805_c7/f1p0/2117 isoform=cb4805_c7;full_length_coverage=1;non_full_length_coverage=0;isoform_length=2117;expected_accuracy=0.994	gnl|BL_ORD_ID|42029 cb4805_c0/f4p0/2407 isoform=cb4805_c0;full_length_coverage=4;non_full_length_coverage=0;isoform_length=2407;expected_accuracy=1.000	1	1754	1749	2084	22	1775	2069	2407
cb8564_c180116/f1p0/2944 isoform=cb8564_c180116;full_length_coverage=1;non_full_length_coverage=0;isoform_length=2944;expected_accuracy=0.996	gnl|BL_ORD_ID|55472 cb8564_c42531/f1p0/3996	129	2522	2520	2944	816	3208	3437	3861
cb2177_c33/f1p1/2778 isoform=cb2177_c33;full_length_coverage=1;non_full_length_coverage=1;isoform_length=2778;expected_accuracy=0.999	gnl|BL_ORD_ID|35910 cb2177_c22/f1p0/4026 isoform=cb2177_c22;full_length_coverage=1;non_full_length_coverage=0;isoform_length=4026;expected_accuracy=0.997	1279	2661	1	1280	2644	4026	48	1330
cb8564_c938/f1p0/2388	gnl|BL_ORD_ID|18451 cb8564_c956/f2p0/2664 isoform=cb8564_c956;full_length_coverage=2;non_full_length_coverage=0;isoform_length=2664;expected_accuracy=1.000	534	2392	65	536	651	2510	41	518
cb964_c18/f1p0/2779	gnl|BL_ORD_ID|21917 cb964_c11/f1p0/2945 isoform=cb964_c11;full_length_coverage=1;non_full_length_coverage=0;isoform_length=2945;expected_accuracy=0.995	1	1665	1666	2585	257	1914	2026	2945
cb1406_c5/f1p0/2334 isoform=cb1406_c5;full_length_coverage=1;non_full_length_coverage=0;isoform_length=2334;expected_accuracy=0.996	gnl|BL_ORD_ID|25275 cb1406_c14/f1p1/5506 isoform=cb1406_c14;full_length_coverage=1;non_full_length_coverage=1;isoform_length=5506;expected_accuracy=0.998	214	2334	1	218	3315	5439	1	218
cb3341_c13/f1p0/1618 isoform=cb3341_c13;full_length_coverage=1;non_full_	gnl|BL_ORD_ID|39643 cb3341_c4/f3p0/1721 isoform=cb3341_c4;full_length_coverage=3;non_full_le	539	1618	68	544	591	1671	4	488

length_coverage=0;isoform_length=1618;expected_accuracy=0.998	ngth_coverage=0;isoform_length=1721;expected_accuracy=1.000								
cb14968_c6/f1p0/1264	gnl|BL_ORD_ID|64376 cb14968_c10/f1p0/1444	127	1227	8	127	235	1335	6	121
cb8564_c107887/f1p0/2716	gnl|BL_ORD_ID|17627 cb8564_c86161/f2p0/2876 isoform=cb8564_c86161;full_length_coverage=2;non_full_length_coverage=0;isoform_length=2876;expected_accuracy=0.997	1	1773	1772	2734	1	1770	1904	2866
cb292_c26/f1p1/4089	gnl|BL_ORD_ID|38399 cb292_c6/f1p0/4480 isoform=cb292_c6;full_length_coverage=1;non_full_length_coverage=0;isoform_length=4480;expected_accuracy=0.997	1	2048	2044	3906	61	2108	2546	4409
cb4444_c12/f1p0/2629	gnl|BL_ORD_ID|43609 cb4444_c22/f1p0/3073 isoform=cb4444_c22;full_length_coverage=1;non_full_length_coverage=0;isoform_length=3073;expected_accuracy=0.996	363	2613	6	362	823	3070	4	360
cb2274_c9/f1p1/2153 isoform=cb2274_c9;full_length_coverage=1;non_full_length_coverage=1;isoform_length=2153;expected_accuracy=0.994	gnl|BL_ORD_ID|37609 cb2274_c10/f1p0/2302 isoform=cb2274_c10;full_length_coverage=1;non_full_length_coverage=0;isoform_length=2302;expected_accuracy=0.999	1	1152	1148	2107	1	1136	1340	2302
cb8564_c42387/f1p4/2973 isoform=cb8564_c42387;full_length_coverage=1;non_full_length_coverage=4;isoform_length=2973;expected_accuracy=0.993	gnl|BL_ORD_ID|17429 cb8564_c85447/f1p0/3196 isoform=cb8564_c85447;full_length_coverage=1;non_full_length_coverage=0;isoform_length=3196;expected_accuracy=0.997	1	2428	2432	2973	3	2413	2663	3185
cb8564_c87721/f1p0/2168 isoform=cb8564_c87721;full_length_coverage=1;non_f	gnl|BL_ORD_ID|16564 cb8564_c69038/f4p3/2958 isoform=cb8564_c69038;full_length_coverage=4;non_fu	1	1811	1809	2168	14	1827	2474	2833

ull_length_coverage=0;isoform_length=2168;expected_accuracy=0.997	ll_length_coverage=3;isoform_length=2958;expected_accuracy=0.997								
cb16136_c0/f2p0/1338 isoform=cb16136_c0;full_length_coverage=2;non_full_length_coverage=0;isoform_length=1338;expected_accuracy=0.993	gnl|BL_ORD_ID|55829 cb8564_c42176/f1p0/3183	436	1338	50	435	2194	3095	4	389
cb11915_c17/f1p1/1371 isoform=cb11915_c17;full_length_coverage=1;non_full_length_coverage=1;isoform_length=1371;expected_accuracy=0.993	gnl|BL_ORD_ID|53529 cb8564_c127829/f1p1/1946	442	1371	9	446	938	1867	1	439
cb4834_c32/f1p0/2446 isoform=cb4834_c32;full_length_coverage=1;non_full_length_coverage=0;isoform_length=2446;expected_accuracy=0.991	gnl|BL_ORD_ID|73014 cb4834_c36/f1p0/2620	12	1846	1842	2446	3	1826	1934	2527
cb8564_c39136/f1p0/3036 isoform=cb8564_c39136;full_length_coverage=1;non_full_length_coverage=0;isoform_length=3036;expected_accuracy=0.998	gnl|BL_ORD_ID|55522 cb8564_c44123/f1p1/3230	16	2089	2085	3036	15	2092	2219	3171
cb13565_c28/f1p4/1411 isoform=cb13565_c28;full_length_coverage=1;non_full_length_coverage=4;isoform_length=1411;expected_accuracy=0.998	gnl|BL_ORD_ID|62572 cb13565_c52/f1p3/1664	143	1410	23	147	388	1655	80	203
cb1039_c20/f1p0/3998 isoform=cb1039_c20;full_length_coverage=1;non_full_length_coverage=0;isoform_length=3998;expected_accuracy=0.996	gnl|BL_ORD_ID|1556 cb1039_c41/f1p0/4155 isoform=cb1039_c41;full_length_coverage=1;non_full_length_coverage=0;isoform_length=4155;expected_accuracy=0.999	1	2587	2587	3998	26	2626	2732	4123

cb6339_c16/f1p1/2286 isoform=cb6339_c16;full_length_coverage=1;non_full_length_coverage=1;isoform_length=2286;expected_accuracy=0.997	gnl|BL_ORD_ID|49628 cb6339_c40/f1p2/2606	10	1780	1776	2286	15	1787	1991	2501
cb8564_c148933/f2p1/2153	gnl|BL_ORD_ID|12361 cb8564_c1392/f1p1/3024 isoform=cb8564_c1392;full_length_coverage=1;non_full_length_coverage=1;isoform_length=3024;expected_accuracy=0.993	1	1541	1541	2132	22	1562	2433	3024
cb8564_c188097/f1p0/2552 isoform=cb8564_c188097;full_length_coverage=1;non_full_length_coverage=0;isoform_length=2552;expected_accuracy=0.996	gnl|BL_ORD_ID|55583 cb8564_c45873/f1p2/2968	1	1945	1940	2552	1	1943	2046	2658
cb8564_c49748/f1p2/2449	gnl|BL_ORD_ID|11528 cb8564_c146182/f3p2/3172 isoform=cb8564_c146182;full_length_coverage=3;non_full_length_coverage=2;isoform_length=3172;expected_accuracy=1.000	535	2415	7	536	1283	3163	1	530
cb8564_c68409/f2p0/2141 isoform=cb8564_c68409;full_length_coverage=2;non_full_length_coverage=0;isoform_length=2141;expected_accuracy=0.995	gnl|BL_ORD_ID|16835 cb8564_c73948/f1p0/2329 isoform=cb8564_c73948;full_length_coverage=1;non_full_length_coverage=0;isoform_length=2329;expected_accuracy=0.999	416	2110	57	417	634	2329	2	362
cb8564_c110637/f1p2/2491 isoform=cb8564_c110637;full_length_coverage=1;non_full_length_coverage=2;isoform_length=2491;expected_accuracy=0.995	gnl|BL_ORD_ID|13896 cb8564_c4136/f2p1/2697 isoform=cb8564_c4136;full_length_coverage=2;non_full_length_coverage=1;isoform_length=2697;expected_accuracy=1.000	13	2243	2239	2455	1	2246	2472	2694
cb401_c50/f1p0/2173 isoform=cb401_c50;full_length_coverage=1;non_full_le	gnl|BL_ORD_ID|69611 cb401_c144/f1p1/2401	96	2171	1	101	296	2389	55	155

ngth_coverage=0;isoform_length=2173;expected_accuracy=0.998									
cb8564_c132288/f2p0/3421 isoform=cb8564_c132288;full_length_coverage=2;non_full_length_coverage=0;isoform_length=3421;expected_accuracy=0.996	gnl|BL_ORD_ID|14669 cb8564_c44765/f1p1/4352 isoform=cb8564_c44765;full_length_coverage=1;non_full_length_coverage=1;isoform_length=4352;expected_accuracy=0.995	1425	3421	1	1427	2375	4352	111	1524
cb6646_c1/f3p1/2483 isoform=cb6646_c1;full_length_coverage=3;non_full_length_coverage=1;isoform_length=2483;expected_accuracy=1.000	gnl|BL_ORD_ID|3729 cb6646_c22/f1p1/2606 isoform=cb6646_c22;full_length_coverage=1;non_full_length_coverage=1;isoform_length=2606;expected_accuracy=0.996	240	2466	1	241	380	2606	23	263
cb391_c5/f1p0/3775 isoform=cb391_c5;full_length_coverage=1;non_full_length_coverage=0;isoform_length=3775;expected_accuracy=0.994	gnl|BL_ORD_ID|41069 cb391_c0/f1p0/4835 isoform=cb391_c0;full_length_coverage=1;non_full_length_coverage=0;isoform_length=4835;expected_accuracy=0.999	362	3775	1	365	1419	4835	14	378
cb8564_c40598/f1p4/3119	gnl|BL_ORD_ID|56275 cb8564_c73216/f1p0/3006	21	2732	2729	3055	26	2711	2832	3158
cb3618_c22/f1p0/2903	gnl|BL_ORD_ID|71384 cb3618_c17/f1p0/3242	1	2422	2422	2838	21	2443	2801	3217
cb8564_c47011/f1p0/3100	gnl|BL_ORD_ID|14411 cb8564_c44258/f1p0/3202 isoform=cb8564_c44258;full_length_coverage=1;non_full_length_coverage=0;isoform_length=3202;expected_accuracy=0.993	11	2306	2301	2931	8	2328	2572	3202
cb8564_c86900/f1p0/3283	gnl|BL_ORD_ID|10963 cb8564_c106917/f6p1/3331 isoform=cb8564_c106917;full_length_coverage=6;non_full_length_coverage=1;isoform_length=3331;expected_accuracy=1.000	211	3069	2	215	473	3331	3	214

cb5421_c10/f1p0/1589 isoform=cb5421_c10;full_length_coverage=1;non_full_length_coverage=0;isoform_length=1589;expected_accuracy=0.991	gnl|BL_ORD_ID|44652 cb5421_c4/f1p0/1919 isoform=cb5421_c4;full_length_coverage=1;non_full_length_coverage=0;isoform_length=1919;expected_accuracy=0.997	114	1589	1	115	258	1736	10	124
cb5304_c6/f1p0/2203 isoform=cb5304_c6;full_length_coverage=1;non_full_length_coverage=0;isoform_length=2203;expected_accuracy=0.997	gnl|BL_ORD_ID|73758 cb5304_c2/f1p0/3141	1	1509	1508	2203	363	1870	2009	2706
cb8564_c176603/f2p2/3262 isoform=cb8564_c176603;full_length_coverage=2;non_full_length_coverage=2;isoform_length=3262;expected_accuracy=0.997	gnl|BL_ORD_ID|14409 cb8564_c40201/f1p0/3342 isoform=cb8564_c40201;full_length_coverage=1;non_full_length_coverage=0;isoform_length=3342;expected_accuracy=0.993	144	3221	1	146	269	3342	1	147
cb3856_c0/f2p1/1619 isoform=cb3856_c0;full_length_coverage=2;non_full_length_coverage=1;isoform_length=1619;expected_accuracy=0.996	gnl|BL_ORD_ID|40758 cb3856_c1/f2p0/1847 isoform=cb3856_c1;full_length_coverage=2;non_full_length_coverage=0;isoform_length=1847;expected_accuracy=0.996	130	1614	1	131	365	1847	9	136
cb416_c28/f1p0/2988 isoform=cb416_c28;full_length_coverage=1;non_full_length_coverage=0;isoform_length=2988;expected_accuracy=0.996	gnl|BL_ORD_ID|72151 cb416_c41/f1p0/3183	121	2895	1	116	281	3054	53	162
cb4533_c1/f1p0/3594	gnl|BL_ORD_ID|72649 cb4533_c0/f1p0/3317	1523	3175	114	1523	1575	3244	52	1461
cb8564_c90914/f1p0/2521	gnl|BL_ORD_ID|17948 cb8564_c87252/f1p1/2853 isoform=cb8564_c87252;full_length_coverage=1;non_full_length_coverage=1;isoform_length=2853;expected_accuracy=0.993	1	1396	1394	2515	109	1505	1726	2839

cb17442_c0/f2p0/1363 isoform=cb17442_c0;full_length_coverage=2;non_full_length_coverage=0;isoform_length=1363;expected_accuracy=1.000	gnl|BL_ORD_ID|31965 cb17442_c2/f1p1/1710 isoform=cb17442_c2;full_length_coverage=1;non_full_length_coverage=1;isoform_length=1710;expected_accuracy=0.991	212	1363	34	213	356	1507	2	180
cb5772_c10/f1p1/1731	gnl|BL_ORD_ID|46526 cb5772_c11/f1p1/1685 isoform=cb5772_c11;full_length_coverage=1;non_full_length_coverage=1;isoform_length=1685;expected_accuracy=0.992	206	1613	56	208	256	1672	1	153
cb1729_c18/f1p0/3891	gnl|BL_ORD_ID|63907 cb1729_c14/f1p1/3905	282	3740	1	282	430	3879	39	323
cb8564_c36689/f1p1/2368 isoform=cb8564_c36689;full_length_coverage=1;non_full_length_coverage=1;isoform_length=2368;expected_accuracy=0.991	gnl|BL_ORD_ID|56071 cb8564_c54161/f1p4/2912	1	1709	1706	2368	2	1704	2258	2921
cb11723_c2/f1p0/1768	gnl|BL_ORD_ID|60342 cb11723_c1/f1p0/2354	1	1321	1320	1763	12	1323	1557	2000
cb14664_c5/f1p0/1279	gnl|BL_ORD_ID|52959 cb8564_c113186/f1p0/2216	158	1277	47	163	946	2065	105	220
cb8564_c116501/f1p0/3896 isoform=cb8564_c116501;full_length_coverage=1;non_full_length_coverage=0;isoform_length=3896;expected_accuracy=0.994	gnl|BL_ORD_ID|54548 cb8564_c125967/f1p0/4340	1	2762	2762	3893	65	2822	3205	4332
cb1802_c1/f5p0/1943 isoform=cb1802_c1;full_length_coverage=5;non_full_length_coverage=0;isoform_length=1943;expected_accuracy=1.000	gnl|BL_ORD_ID|33202 cb1802_c8/f1p0/3646 isoform=cb1802_c8;full_length_coverage=1;non_full_length_coverage=0;isoform_length=3646;expected_accuracy=0.998	579	1943	61	583	2289	3646	33	554

cb8564_c21439/f1p0/2587	gnl|BL_ORD_ID|19279 cb8564_c8088/f4p4/2776 isoform=cb8564_c8088;full_length_coverage=4;non_full_length_coverage=4;isoform_length=2776;expected_accuracy=0.998	1226	2572	1	1228	1380	2726	32	1259
cb6712_c0/f5p1/2302 isoform=cb6712_c0;full_length_coverage=5;non_full_length_coverage=1;isoform_length=2302;expected_accuracy=1.000	gnl|BL_ORD_ID|4045 cb6712_c5/f1p0/2789 isoform=cb6712_c5;full_length_coverage=1;non_full_length_coverage=0;isoform_length=2789;expected_accuracy=0.999	11	1499	1498	2302	1	1488	1966	2770
cb1065_c17/f1p0/2210	gnl|BL_ORD_ID|47305 cb1065_c14/f1p0/2462	1024	2048	10	1025	1275	2300	2	1012
cb8564_c146690/f2p0/2014 isoform=cb8564_c146690;full_length_coverage=2;non_full_length_coverage=0;isoform_length=2014;expected_accuracy=0.997	gnl|BL_ORD_ID|9761 cb8564_c114521/f1p0/3515 isoform=cb8564_c114521;full_length_coverage=1;non_full_length_coverage=0;isoform_length=3515;expected_accuracy=0.999	449	2014	54	453	1948	3513	78	477
cb8564_c148479/f1p0/3197 isoform=cb8564_c148479;full_length_coverage=1;non_full_length_coverage=0;isoform_length=3197;expected_accuracy=0.997	gnl|BL_ORD_ID|52768 cb8564_c13351/f1p2/4615	1	2459	2455	3182	587	3047	3910	4637
cb7639_c0/f3p0/2226 isoform=cb7639_c0;full_length_coverage=3;non_full_length_coverage=0;isoform_length=2226;expected_accuracy=0.997	gnl|BL_ORD_ID|51427 cb7639_c11/f1p0/2532	1	1800	1799	2226	2	1801	2081	2507
cb5847_c5/f1p0/2404 isoform=cb5847_c5;full_length_coverage=1;non_full_length_coverage=0;isoform_length=2404;expected_accuracy=1.000	gnl|BL_ORD_ID|45644 cb5847_c3/f1p0/2997 isoform=cb5847_c3;full_length_coverage=1;non_full_length_coverage=0;isoform_length=2997;expected_accuracy=0.993	1	1502	1501	2397	155	1653	2106	2997

cb500_c75/f1p0/2105	gnl|BL_ORD_ID|70942 cb500_c16/f1p0/3627	105	1613	1	105	2137	3645	1865	1969
cb8564_c68992/f1p5/3235 isoform=cb8564_c68992;full_length_coverage=1;non_full_length_coverage=5;isoform_length=3235;expected_accuracy=0.995	gnl|BL_ORD_ID|13377 cb8564_c22091/f2p0/3351 isoform=cb8564_c22091;full_length_coverage=2;non_full_length_coverage=0;isoform_length=3351;expected_accuracy=0.997	1	1687	1684	3234	2	1682	1805	3351
cb13081_c9/f1p0/1335	gnl|BL_ORD_ID|10154 cb8564_c11196/f5p0/2303 isoform=cb8564_c11196;full_length_coverage=5;non_full_length_coverage=0;isoform_length=2303;expected_accuracy=1.000	494	1279	8	496	1433	2217	4	492
cb8564_c150144/f1p7/2784 isoform=cb8564_c150144;full_length_coverage=1;non_full_length_coverage=7;isoform_length=2784;expected_accuracy=0.991	gnl|BL_ORD_ID|17429 cb8564_c85447/f1p0/3196 isoform=cb8564_c85447;full_length_coverage=1;non_full_length_coverage=0;isoform_length=3196;expected_accuracy=0.997	2	2419	2418	2784	3	2413	2660	3020
cb8564_c34238/f4p1/4317 isoform=cb8564_c34238;full_length_coverage=4;non_full_length_coverage=1;isoform_length=4317;expected_accuracy=1.000	gnl|BL_ORD_ID|55207 cb8564_c35399/f1p0/4553	285	4299	11	289	396	4411	11	291
cb2854_c50/f1p0/1716	gnl|BL_ORD_ID|36931 cb2854_c26/f1p0/2391 isoform=cb2854_c26;full_length_coverage=1;non_full_length_coverage=0;isoform_length=2391;expected_accuracy=0.997	10	1388	1386	1699	85	1463	2079	2391
cb285_c15/f1p1/2293 isoform=cb285_c15;full_length_coverage=1;non_full_length_coverage=1;isoform_length=2293;expected_accuracy=0.998	gnl|BL_ORD_ID|38370 cb285_c4/f1p0/2341 isoform=cb285_c4;full_length_coverage=1;non_full_length_coverage=0;isoform_length=2341;expected_accuracy=0.993	336	2284	76	337	399	2341	1	262

cb8564_c49194/f2p2/3207 isoform=cb8564_c49194;full_length_coverage=2;non_full_length_coverage=2;isoform_length=3207;expected_accuracy=0.996	gnl|BL_ORD_ID|9472 cb8564_c107869/f7p3/3550 isoform=cb8564_c107869;full_length_coverage=7;non_full_length_coverage=3;isoform_length=3550;expected_accuracy=1.000	218	3207	1	221	552	354 8	91	309
cb3290_c38/f1p0/2345 isoform=cb3290_c38;full_length_coverage=1;non_full_length_coverage=0;isoform_length=2345;expected_accuracy=0.996	gnl|BL_ORD_ID|70294 cb3290_c164/f1p0/3025	1150	2345	1	1151	1592	279 4	281	143 5
cb6794_c24/f1p0/2733	gnl|BL_ORD_ID|50069 cb6794_c18/f1p1/2705	1	1329	1327	2484	60	139 0	1504	266 1
cb3687_c38/f1p0/3245	gnl|BL_ORD_ID|41006 cb3687_c7/f2p0/3068 isoform=cb3687_c7;full_length_coverage=2;non_full_length_coverage=0;isoform_length=3068;expected_accuracy=0.997	1	2031	2030	2917	1	203 1	2181	306 8
cb8564_c85535/f1p0/4529 isoform=cb8564_c85535;full_length_coverage=1;non_full_length_coverage=0;isoform_length=4529;expected_accuracy=0.997	gnl|BL_ORD_ID|64237 cb14704_c11/f1p0/5388	1769	4526	1	1769	2569	532 8	610	237 8
cb313_c8/f1p0/2415 isoform=cb313_c8;full_length_coverage=1;non_full_length_coverage=0;isoform_length=2415;expected_accuracy=1.000	gnl|BL_ORD_ID|36627 cb313_c24/f1p0/3081 isoform=cb313_c24;full_length_coverage=1;non_full_length_coverage=0;isoform_length=3081;expected_accuracy=0.995	1	2171	2171	2415	82	225 5	2820	306 4
cb8995_c8/f1p0/2000 isoform=cb8995_c8;full_length_coverage=1;non_full_length_coverage=0;isoform_length=2000;expected_accuracy=1.000	gnl|BL_ORD_ID|19528 cb8995_c4/f1p0/2502 isoform=cb8995_c4;full_length_coverage=1;non_full_length_coverage=0;isoform_length=2502;expected_accuracy=0.996	97	2000	1	100	277	218 0	65	164

cb2105_c5/f1p0/2500 isoform=cb2105_c5;full_length_coverage=1;non_full_length_coverage=0;isoform_length=2500;expected_accuracy=0.992	gnl|BL_ORD_ID|66723 cb2105_c7/f1p0/2774	143	2460	1	142	327	2640	26	167
cb8564_c77843/f1p0/2830	gnl|BL_ORD_ID|13115 cb8564_c16916/f7p1/3034 isoform=cb8564_c16916;full_length_coverage=7;non_full_length_coverage=1;isoform_length=3034;expected_accuracy=0.996	120	1764	1762	2646	1	1646	1853	2737
cb8564_c38947/f1p0/2650 isoform=cb8564_c38947;full_length_coverage=1;non_full_length_coverage=0;isoform_length=2650;expected_accuracy=0.996	gnl|BL_ORD_ID|16369 cb8564_c52191/f1p0/2822 isoform=cb8564_c52191;full_length_coverage=1;non_full_length_coverage=0;isoform_length=2822;expected_accuracy=0.997	134	1441	1436	2650	84	1391	1608	2822
cb1049_c9/f1p0/2974 isoform=cb1049_c9;full_length_coverage=1;non_full_length_coverage=0;isoform_length=2974;expected_accuracy=1.000	gnl|BL_ORD_ID|1364 cb1049_c18/f10p1/3143 isoform=cb1049_c18;full_length_coverage=10;non_full_length_coverage=1;isoform_length=3143;expected_accuracy=1.000	1345	2974	1	1346	1493	3122	45	1390
cb2870_c18/f2p1/2618 isoform=cb2870_c18;full_length_coverage=2;non_full_length_coverage=1;isoform_length=2618;expected_accuracy=1.000	gnl|BL_ORD_ID|38086 cb2870_c3/f1p1/3794 isoform=cb2870_c3;full_length_coverage=1;non_full_length_coverage=1;isoform_length=3794;expected_accuracy=0.994	1	1908	1904	2598	2	1904	3101	3794
cb3226_c16/f1p0/2457 isoform=cb3226_c16;full_length_coverage=1;non_full_length_coverage=0;isoform_length=2457;expected_accuracy=0.994	gnl|BL_ORD_ID|39676 cb3226_c10/f1p0/3292 isoform=cb3226_c10;full_length_coverage=1;non_full_length_coverage=0;isoform_length=3292;expected_accuracy=0.994	147	2457	1	147	981	3292	22	164
cb6742_c12/f1p0/2398	gnl|BL_ORD_ID|50018 cb6742_c6/f1p0/3431	1	1343	1342	2269	12	1354	2079	3011

cb2959_c2/f1p0/3487 isoform=cb2959_c2;full_length_coverage=1;non_full_length_coverage=0;isoform_length=3487;expected_accuracy=0.995	gnl|BL_ORD_ID|38587 cb2959_c4/f1p0/3532 isoform=cb2959_c4;full_length_coverage=1;non_full_length_coverage=0;isoform_length=3532;expected_accuracy=0.994	1000	3426	1	1000	1138	3532	6	1000
cb8564_c186356/f1p0/2744 isoform=cb8564_c186356;full_length_coverage=1;non_full_length_coverage=0;isoform_length=2744;expected_accuracy=0.998	gnl|BL_ORD_ID|55213 cb8564_c35568/f1p0/2997	1	1396	1393	2744	13	1417	1537	2886
cb3903_c0/f2p0/2622 isoform=cb3903_c0;full_length_coverage=2;non_full_length_coverage=0;isoform_length=2622;expected_accuracy=0.995	gnl|BL_ORD_ID|41371 cb3903_c2/f1p0/3465 isoform=cb3903_c2;full_length_coverage=1;non_full_length_coverage=0;isoform_length=3465;expected_accuracy=0.996	1	2507	2507	2622	882	3385	3265	3380
cb1955_c26/f2p1/2336 isoform=cb1955_c26;full_length_coverage=2;non_full_length_coverage=1;isoform_length=2336;expected_accuracy=1.000	gnl|BL_ORD_ID|35587 cb1955_c12/f1p1/2862 isoform=cb1955_c12;full_length_coverage=1;non_full_length_coverage=1;isoform_length=2862;expected_accuracy=1.000	1117	2336	1	1119	1569	2788	1	1120
cb14220_c15/f1p5/6485 isoform=cb14220_c15;full_length_coverage=1;non_full_length_coverage=5;isoform_length=6485;expected_accuracy=0.999	gnl|BL_ORD_ID|63134 cb14220_c9/f1p0/6925	2180	6426	106	2182	2708	6950	218	2293
cb454_c40/f1p0/2415	gnl|BL_ORD_ID|42150 cb454_c1/f3p0/2382 isoform=cb454_c1;full_length_coverage=3;non_full_length_coverage=0;isoform_length=2382;expected_accuracy=1.000	237	2293	48	236	296	2351	1	189
cb873_c20/f1p0/2479 isoform=cb873_c20;full_length_coverage=1;non_full_le	gnl|BL_ORD_ID|57590 cb873_c16/f1p0/4474	1146	2479	1	1146	2949	4282	17	1140

ngth_coverage=0;isoform_length=2479;expected_accuracy=0.996									
cb1371_c50/f4p1/3964 isoform=cb1371_c50;full_length_coverage=4;non_full_length_coverage=1;isoform_length=3964;expected_accuracy=1.000	gnl|BL_ORD_ID|25357 cb1371_c47/f1p1/5280 isoform=cb1371_c47;full_length_coverage=1;non_full_length_coverage=1;isoform_length=5280;expected_accuracy=0.998	453	3964	66	458	1725	5233	1	393
cb2045_c6/f1p0/2314 isoform=cb2045_c6;full_length_coverage=1;non_full_length_coverage=0;isoform_length=2314;expected_accuracy=0.993	gnl|BL_ORD_ID|35012 cb2045_c4/f1p0/3368 isoform=cb2045_c4;full_length_coverage=1;non_full_length_coverage=0;isoform_length=3368;expected_accuracy=0.996	1	1489	1490	2278	276	1764	2579	3368
cb4007_c11/f3p1/2723 isoform=cb4007_c11;full_length_coverage=3;non_full_length_coverage=1;isoform_length=2723;expected_accuracy=0.997	gnl|BL_ORD_ID|71719 cb4007_c46/f1p0/3154	1	1825	1824	2665	3	1803	2145	2985
cb5659_c81/f1p0/2132 isoform=cb5659_c81;full_length_coverage=1;non_full_length_coverage=0;isoform_length=2132;expected_accuracy=0.999	gnl|BL_ORD_ID|46024 cb5659_c74/f1p3/2885 isoform=cb5659_c74;full_length_coverage=1;non_full_length_coverage=3;isoform_length=2885;expected_accuracy=0.994	1	1656	1653	2132	146	1799	2364	2838
cb8564_c40598/f1p4/3119	gnl|BL_ORD_ID|57028 cb8564_c80926/f1p9/3250	1	2732	2729	3051	1	2728	2946	3268
cb8564_c118011/f1p0/1965	gnl|BL_ORD_ID|54973 cb8564_c4062/f1p0/2826	1	1321	1321	1964	600	1933	2113	2753
cb8564_c145598/f4p6/3779 isoform=cb8564_c145598;full_length_coverage=4;non_full_length_coverage=6;isoform_length=3779;expected_accuracy=1.000	gnl|BL_ORD_ID|48479 cb10398_c17/f1p0/5753	20	2593	2591	3771	31	2604	4060	5240

cb8564_c49562/f1p0/2838 isoform=cb8564_c49562;full_length_coverage=1;non_full_length_coverage=0;isoform_length=2838;expected_accuracy=0.995	gnl|BL_ORD_ID|16622 cb8564_c50151/f1p0/3198 isoform=cb8564_c50151;full_length_coverage=1;non_full_length_coverage=0;isoform_length=3198;expected_accuracy=0.995	2	2331	2327	2838	3	2351	2683	3198
cb99_c12/f1p0/3017 isoform=cb99_c12;full_length_coverage=1;non_full_length_coverage=0;isoform_length=3017;expected_accuracy=0.997	gnl|BL_ORD_ID|68041 cb18819_c2/f1p0/5197	1	2118	2116	2981	1833	3952	4135	5003
cb11915_c7/f1p2/1523 isoform=cb11915_c7;full_length_coverage=1;non_full_length_coverage=2;isoform_length=1523;expected_accuracy=0.992	gnl|BL_ORD_ID|53626 cb8564_c131696/f1p0/1904	1	1133	1128	1485	32	1168	1523	1881
cb7361_c3/f1p0/1750 isoform=cb7361_c3;full_length_coverage=1;non_full_length_coverage=0;isoform_length=1750;expected_accuracy=0.995	gnl|BL_ORD_ID|7513 cb7361_c1/f1p0/2638 isoform=cb7361_c1;full_length_coverage=1;non_full_length_coverage=0;isoform_length=2638;expected_accuracy=0.998	1	1156	1153	1743	44	1197	2010	2600
cb8564_c39136/f1p0/3036 isoform=cb8564_c39136;full_length_coverage=1;non_full_length_coverage=0;isoform_length=3036;expected_accuracy=0.998	gnl|BL_ORD_ID|55623 cb8564_c47027/f1p0/3544	1	2089	2085	3036	1	2120	2247	3200
cb8564_c129967/f2p0/4204 isoform=cb8564_c129967;full_length_coverage=2;non_full_length_coverage=0;isoform_length=4204;expected_accuracy=0.997	gnl|BL_ORD_ID|54583 cb8564_c127138/f1p0/4403	1	3173	3171	4076	42	3214	3444	4349
cb8564_c10716/f1p3/2179	gnl|BL_ORD_ID|54238 cb8564_c21052/f1p2/3145	262	2210	76	263	1109	3057	244	430

cb8564_c178653/f1p0/2779 isoform=cb8564_c178653;full_length_coverage=1;non_full_length_coverage=0;isoform_length=2779;expected_accuracy=0.994	gnl|BL_ORD_ID|55280 cb8564_c37198/f1p5/2941	1	2179	2175	2779	1	2177	2296	2900
cb8564_c15653/f1p4/2475	gnl|BL_ORD_ID|55529 cb8564_c44286/f1p0/2747	1	2293	2289	2434	2	2301	2520	2665
cb1833_c7/f2p4/3738 isoform=cb1833_c7;full_length_coverage=2;non_full_length_coverage=4;isoform_length=3738;expected_accuracy=0.997	gnl|BL_ORD_ID|33409 cb1833_c19/f1p1/3788 isoform=cb1833_c19;full_length_coverage=1;non_full_length_coverage=1;isoform_length=3788;expected_accuracy=0.991	1	2978	2976	3608	1	3019	3139	3788
cb8564_c36635/f1p0/4030 isoform=cb8564_c36635;full_length_coverage=1;non_full_length_coverage=0;isoform_length=4030;expected_accuracy=0.998	gnl|BL_ORD_ID|16592 cb8564_c51035/f2p2/4216 isoform=cb8564_c51035;full_length_coverage=2;non_full_length_coverage=2;isoform_length=4216;expected_accuracy=0.999	184	4026	1	189	318	4160	1	184
cb8564_c34060/f2p0/3214 isoform=cb8564_c34060;full_length_coverage=2;non_full_length_coverage=0;isoform_length=3214;expected_accuracy=0.993	gnl|BL_ORD_ID|55195 cb8564_c35170/f1p0/3386	533	3205	6	530	738	3399	1	497
cb8564_c188097/f1p0/2552 isoform=cb8564_c188097;full_length_coverage=1;non_full_length_coverage=0;isoform_length=2552;expected_accuracy=0.996	gnl|BL_ORD_ID|13063 cb8564_c179457/f1p0/2647 isoform=cb8564_c179457;full_length_coverage=1;non_full_length_coverage=0;isoform_length=2647;expected_accuracy=1.000	1	1945	1940	2552	1	1924	2027	2639
cb6448_c7/f1p0/2778 isoform=cb6448_c7;full_length_coverage=1;non_full_length_coverage=0;isoform_length=2778;expected_accuracy=0.998	gnl|BL_ORD_ID|4720 cb6448_c6/f1p0/3003 isoform=cb6448_c6;full_length_coverage=1;non_full_length_coverage=0;isoform_length=3003;expected_accuracy=0.995	1	2477	2478	2778	2	2472	2630	2931

<table>
<tr><td>cb8564_c78858/f1p0/2927 isoform=cb8564_c78858;full_length_coverage=1;non_full_length_coverage=0;isoform_length=2927;expected_accuracy=0.995</td><td>gnl|BL_ORD_ID|16364 cb8564_c72362/f4p1/4130 isoform=cb8564_c72362;full_length_coverage=4;non_full_length_coverage=1;isoform_length=4130;expected_accuracy=0.998</td><td>175</td><td>2927</td><td>1</td><td>174</td><td>1366</td><td>4125</td><td>78</td><td>250</td></tr>
<tr><td>cb567_c15/f1p0/3394</td><td>gnl|BL_ORD_ID|45929 cb567_c8/f1p0/3388 isoform=cb567_c8;full_length_coverage=1;non_full_length_coverage=0;isoform_length=3388;expected_accuracy=0.993</td><td>424</td><td>3128</td><td>75</td><td>426</td><td>684</td><td>3388</td><td>1</td><td>349</td></tr>
<tr><td>cb8564_c24391/f1p1/2764 isoform=cb8564_c24391;full_length_coverage=1;non_full_length_coverage=1;isoform_length=2764;expected_accuracy=1.000</td><td>gnl|BL_ORD_ID|13437 cb8564_c22776/f2p8/3226 isoform=cb8564_c22776;full_length_coverage=2;non_full_length_coverage=8;isoform_length=3226;expected_accuracy=0.995</td><td>1</td><td>1714</td><td>1713</td><td>2764</td><td>7</td><td>1719</td><td>1976</td><td>3028</td></tr>
<tr><td>cb7863_c23/f1p0/2057 isoform=cb7863_c23;full_length_coverage=1;non_full_length_coverage=0;isoform_length=2057;expected_accuracy=0.998</td><td>gnl|BL_ORD_ID|8931 cb7863_c2/f5p0/2343 isoform=cb7863_c2;full_length_coverage=5;non_full_length_coverage=0;isoform_length=2343;expected_accuracy=1.000</td><td>264</td><td>2057</td><td>37</td><td>268</td><td>517</td><td>2310</td><td>1</td><td>234</td></tr>
<tr><td>cb466_c12/f1p0/3253 isoform=cb466_c12;full_length_coverage=1;non_full_length_coverage=0;isoform_length=3253;expected_accuracy=0.991</td><td>gnl|BL_ORD_ID|41725 cb466_c0/f10p1/3978 isoform=cb466_c0;full_length_coverage=10;non_full_length_coverage=1;isoform_length=3978;expected_accuracy=1.000</td><td>1135</td><td>3251</td><td>1</td><td>1133</td><td>1857</td><td>3978</td><td>35</td><td>1165</td></tr>
<tr><td>cb2279_c20/f1p0/1654</td><td>gnl|BL_ORD_ID|36531 cb2279_c0/f10p3/2834 isoform=cb2279_c0;full_length_coverage=10;non_full_length_coverage=3;isoform_length=2834;expected_accuracy=1.000</td><td>212</td><td>1439</td><td>25</td><td>214</td><td>1580</td><td>2808</td><td>22</td><td>211</td></tr>
<tr><td>cb3899_c2/f1p0/3245</td><td>gnl|BL_ORD_ID|71621 cb3899_c0/f1p0/3500</td><td>1</td><td>1823</td><td>1823</td><td>3262</td><td>153</td><td>1975</td><td>2077</td><td>3517</td></tr>
</table>

cb8564_c43779/f1p0/3021 isoform=cb8564_c43779;full_length_coverage=1;non_full_length_coverage=0;isoform_length=3021;expected_accuracy=0.993	gnl|BL_ORD_ID|56764 cb8564_c70795/f1p0/3769	18	2344	2345	3021	34	2360	2516	3187
cb8564_c44698/f1p5/2843 isoform=cb8564_c44698;full_length_coverage=1;non_full_length_coverage=5;isoform_length=2843;expected_accuracy=0.997	gnl|BL_ORD_ID|55986 cb8564_c51172/f1p0/3367	2	2516	2511	2827	7	2534	2655	2973
cb8564_c146072/f3p3/2176 isoform=cb8564_c146072;full_length_coverage=3;non_full_length_coverage=3;isoform_length=2176;expected_accuracy=1.000	gnl|BL_ORD_ID|11219 cb8564_c14871/f5p3/2424 isoform=cb8564_c14871;full_length_coverage=5;non_full_length_coverage=3;isoform_length=2424;expected_accuracy=0.996	217	2165	1	215	412	2363	28	238
cb8564_c47522/f1p0/2954	gnl|BL_ORD_ID|16172 cb8564_c53024/f1p0/3159 isoform=cb8564_c53024;full_length_coverage=1;non_full_length_coverage=0;isoform_length=3159;expected_accuracy=0.996	1	1710	1707	2849	1	1699	2017	3157
cb151_c6/f1p0/4591 isoform=cb151_c6;full_length_coverage=1;non_full_length_coverage=0;isoform_length=4591;expected_accuracy=0.991	gnl|BL_ORD_ID|30049 cb151_c3/f1p1/4867 isoform=cb151_c3;full_length_coverage=1;non_full_length_coverage=1;isoform_length=4867;expected_accuracy=0.996	454	4588	1	457	743	4867	71	524
cb11213_c0/f1p0/1532	gnl|BL_ORD_ID|3138 cb11213_c1/f1p0/2821 isoform=cb11213_c1;full_length_coverage=1;non_full_length_coverage=0;isoform_length=2821;expected_accuracy=0.991	1	1006	1005	1472	1	992	2308	2783
cb6067_c6/f1p0/2318	gnl|BL_ORD_ID|74858 cb6067_c1/f1p0/2876	12	1201	1201	2269	8	1197	1759	2827

cb7696_c12/f1p1/1985	gnl|BL_ORD_ID|6383 cb7696_c3/f2p1/2434 isoform=cb7696_c3;full_length_coverage=2;non_full_length_coverage=1;isoform_length=2434;expected_accuracy=0.993	1	1746	1744	1980	2	1745	2066	2304
cb4146_c9/f1p0/1658	gnl|BL_ORD_ID|71832 cb4146_c2/f1p0/3419	109	1625	1	109	1658	3172	1	109
cb1333_c101/f1p0/1395 isoform=cb1333_c101;full_length_coverage=1;non_full_length_coverage=0;isoform_length=1395;expected_accuracy=0.997	gnl|BL_ORD_ID|27594 cb1333_c48/f1p0/4759 isoform=cb1333_c48;full_length_coverage=1;non_full_length_coverage=0;isoform_length=4759;expected_accuracy=0.995	149	1395	27	150	3436	4678	31	154
cb3903_c0/f2p0/2622 isoform=cb3903_c0;full_length_coverage=2;non_full_length_coverage=0;isoform_length=2622;expected_accuracy=0.995	gnl|BL_ORD_ID|71627 cb3903_c1/f1p0/3496	1	2505	2507	2621	930	3433	3316	3430
cb8564_c49617/f1p0/3794 isoform=cb8564_c49617;full_length_coverage=1;non_full_length_coverage=0;isoform_length=3794;expected_accuracy=0.999	gnl|BL_ORD_ID|55838 cb8564_c43552/f1p0/3829	124	2019	2019	3794	3	1898	2010	3785
cb8564_c110954/f1p1/2392 isoform=cb8564_c110954;full_length_coverage=1;non_full_length_coverage=1;isoform_length=2392;expected_accuracy=0.994	gnl|BL_ORD_ID|11399 cb8564_c129154/f1p0/2632 isoform=cb8564_c129154;full_length_coverage=1;non_full_length_coverage=0;isoform_length=2632;expected_accuracy=0.997	21	2113	2109	2392	1	2116	2304	2586
cb3061_c27/f4p0/2371 isoform=cb3061_c27;full_length_coverage=4;non_full_length_coverage=0;isoform_length=2371;expected_accuracy=0.992	gnl|BL_ORD_ID|69090 cb3061_c19/f1p0/3233	121	2342	1	126	892	3105	9	133

cb415_c43/f1p0/3304 isoform=cb415_c43;full_length_coverage=1;non_full_length_coverage=0;isoform_length=3304;expected_accuracy=0.991	gnl|BL_ORD_ID|42970 cb415_c31/f1p1/3757 isoform=cb415_c31;full_length_coverage=1;non_full_length_coverage=1;isoform_length=3757;expected_accuracy=0.996	18	2924	2922	3304	119	3010	3335	3712
cb18456_c4245/f1p0/1080	gnl|BL_ORD_ID|67565 cb18456_c5612/f1p0/1308	7	512	508	1079	2	508	616	1199
cb8564_c3316/f1p0/2304	gnl|BL_ORD_ID|55651 cb8564_c3310/f1p1/2761	11	1156	1155	2199	1	1146	1749	2793
cb8564_c42091/f1p0/3606 isoform=cb8564_c42091;full_length_coverage=1;non_full_length_coverage=0;isoform_length=3606;expected_accuracy=0.998	gnl|BL_ORD_ID|9113 cb8564_c107316/f11p3/3897 isoform=cb8564_c107316;full_length_coverage=11;non_full_length_coverage=3;isoform_length=3897;expected_accuracy=1.000	1	2325	2324	3606	40	2363	2598	3884
cb8564_c48661/f1p2/2634 isoform=cb8564_c48661;full_length_coverage=1;non_full_length_coverage=2;isoform_length=2634;expected_accuracy=0.998	gnl|BL_ORD_ID|10862 cb8564_c106708/f1p3/2795 isoform=cb8564_c106708;full_length_coverage=1;non_full_length_coverage=3;isoform_length=2795;expected_accuracy=0.991	396	2630	5	398	539	2787	2	429
cb8564_c109275/f5p1/2435 isoform=cb8564_c109275;full_length_coverage=5;non_full_length_coverage=1;isoform_length=2435;expected_accuracy=1.000	gnl|BL_ORD_ID|11914 cb8564_c129298/f1p0/3269 isoform=cb8564_c129298;full_length_coverage=1;non_full_length_coverage=0;isoform_length=3269;expected_accuracy=0.993	537	2435	89	539	1372	3268	1	451
cb1956_c11/f1p0/2868 isoform=cb1956_c11;full_length_coverage=1;non_full_length_coverage=0;isoform_length=2868;expected_accuracy=0.996	gnl|BL_ORD_ID|66538 cb1956_c12/f1p0/3120	1	1889	1889	2868	166	2050	2152	3137
cb8564_c4940/f1p0/2731 isoform=cb8564_c4940;full_length_coverage=1;non_full_length_coverage=0;isoform_length=2731;expected_ac	gnl|BL_ORD_ID|15618 cb8564_c4939/f1p0/3100 isoform=cb8564_c4939;full_length_coverage=1;non_full_length_coverage=0;isoform_length=3100;expected_ac	1	1756	1755	2731	1	1749	2104	3080

curacy=0.996	curacy=0.995								
cb8564_c120549/f1p0/2685 isoform=cb8564_c120549;full_length_coverage=1;non_full_length_coverage=0;isoform_length=2685;expected_accuracy=0.999	gnl|BL_ORD_ID|54524 cb8564_c125248/f1p0/3365	151	2685	1	153	559	3101	35	187
cb1333_c101/f1p0/1395 isoform=cb1333_c101;full_length_coverage=1;non_full_length_coverage=0;isoform_length=1395;expected_accuracy=0.997	gnl|BL_ORD_ID|61251 cb1333_c76/f1p0/3569	149	1395	27	150	2256	3500	31	154
cb8564_c181853/f1p0/2119 isoform=cb8564_c181853;full_length_coverage=1;non_full_length_coverage=0;isoform_length=2119;expected_accuracy=0.997	gnl|BL_ORD_ID|14399 cb8564_c39189/f1p1/2975 isoform=cb8564_c39189;full_length_coverage=1;non_full_length_coverage=1;isoform_length=2975;expected_accuracy=0.992	1	1339	1334	2119	1	1352	2181	2975
cb5896_c24/f1p0/2963 isoform=cb5896_c24;full_length_coverage=1;non_full_length_coverage=0;isoform_length=2963;expected_accuracy=0.998	gnl|BL_ORD_ID|74558 cb5896_c23/f1p0/4184	1	1692	1693	2963	1	1678	2860	4155
cb8564_c178226/f1p0/2728 isoform=cb8564_c178226;full_length_coverage=1;non_full_length_coverage=0;isoform_length=2728;expected_accuracy=0.998	gnl|BL_ORD_ID|10510 cb8564_c111713/f6p2/3032 isoform=cb8564_c111713;full_length_coverage=6;non_full_length_coverage=2;isoform_length=3032;expected_accuracy=0.998	1284	2728	1	1288	1391	2836	1	1287
cb8564_c36689/f1p1/2368 isoform=cb8564_c36689;full_length_coverage=1;non_full_length_coverage=1;isoform_length=2368;expected_accuracy=0.991	gnl|BL_ORD_ID|55574 cb8564_c45699/f1p0/3005	1	1709	1706	2368	1	1683	2182	2844
cb7786_c15/f7p3/2290	gnl|BL_ORD_ID|6654 cb7786_c5/f1p1/2527	216	2286	1	215	458	252	1	214

isoform=cb7786_c15;full_length_coverage=7;non_full_length_coverage=3;isoform_length=2290;expected_accuracy=1.000	isoform=cb7786_c5;full_length_coverage=1;non_full_length_coverage=1;isoform_length=2527;expected_accuracy=0.999						7		
cb4233_c92/f1p1/2467 isoform=cb4233_c92;full_length_coverage=1;non_full_length_coverage=1;isoform_length=2467;expected_accuracy=0.998	gnl|BL_ORD_ID|72375 cb4233_c106/f1p2/3225	1	1959	1958	2467	32	1986	2666	3169
cb3833_c0/f3p0/1648 isoform=cb3833_c0;full_length_coverage=3;non_full_length_coverage=0;isoform_length=1648;expected_accuracy=1.000	gnl|BL_ORD_ID|40462 cb3833_c2/f1p0/3264 isoform=cb3833_c2;full_length_coverage=1;non_full_length_coverage=0;isoform_length=3264;expected_accuracy=0.994	327	1635	4	328	1956	3264	1	323
cb8564_c40598/f1p4/3119	gnl|BL_ORD_ID|55502 cb8564_c43538/f1p0/3563	1	2732	2729	3054	2	2736	2857	3181
cb3270_c5/f1p0/2990 isoform=cb3270_c5;full_length_coverage=1;non_full_length_coverage=0;isoform_length=2990;expected_accuracy=0.991	gnl|BL_ORD_ID|41534 cb3270_c3/f1p0/3673 isoform=cb3270_c3;full_length_coverage=1;non_full_length_coverage=0;isoform_length=3673;expected_accuracy=0.997	1	1626	1623	2990	51	1698	2302	3673
cb6326_c66/f1p0/1099	gnl|BL_ORD_ID|5375 cb6326_c63/f1p0/1216 isoform=cb6326_c63;full_length_coverage=1;non_full_length_coverage=0;isoform_length=1216;expected_accuracy=0.998	216	1060	7	220	372	1216	1	214
cb5137_c53/f1p0/2672 isoform=cb5137_c53;full_length_coverage=1;non_full_length_coverage=0;isoform_length=2672;expected_accuracy=0.992	gnl|BL_ORD_ID|72120 cb5137_c91/f1p0/3100	19	2565	2562	2672	20	2589	2693	2802
cb1163_c21/f1p5/3705	gnl|BL_ORD_ID|23459 cb1163_c11/f2p1/3742	1	2507	2506	3627	1	250	2616	374

isoform=cb1163_c21;full_length_coverage=1;non_full_length_coverage=5;isoform_length=3705;expected_accuracy=0.994	isoform=cb1163_c11;full_length_coverage=2;non_full_length_coverage=1;isoform_length=3742;expected_accuracy=0.999						4		2
cb14540_c10/f1p0/1846	gnl|BL_ORD_ID|57978 cb8564_c85833/f1p0/2942	1	1104	1102	1721	607	1709	2309	2928
cb18456_c5228/f2p2/1404 isoform=cb18456_c5228;full_length_coverage=2;non_full_length_coverage=2;isoform_length=1404;expected_accuracy=0.999	gnl|BL_ORD_ID|53691 cb8564_c1249/f1p1/2145	439	1360	9	441	1219	2140	1	430
cb8564_c79905/f1p0/2920	gnl|BL_ORD_ID|57797 cb8564_c80971/f1p0/4679	1	1731	1730	2878	731	2461	3283	4431
cb1130_c5/f1p0/2483 isoform=cb1130_c5;full_length_coverage=1;non_full_length_coverage=0;isoform_length=2483;expected_accuracy=0.991	gnl|BL_ORD_ID|22369 cb1130_c6/f1p0/2972 isoform=cb1130_c6;full_length_coverage=1;non_full_length_coverage=0;isoform_length=2972;expected_accuracy=0.996	1	1809	1809	2482	11	1801	2298	2972
cb8564_c41115/f1p3/2751	gnl|BL_ORD_ID|55481 cb8564_c42759/f1p1/2974	10	1542	1539	2690	33	1564	1736	2884
cb6473_c12/f1p0/1867 isoform=cb6473_c12;full_length_coverage=1;non_full_length_coverage=0;isoform_length=1867;expected_accuracy=0.991	gnl|BL_ORD_ID|3723 cb6473_c7/f1p0/1970 isoform=cb6473_c7;full_length_coverage=1;non_full_length_coverage=0;isoform_length=1970;expected_accuracy=1.000	1	1131	1126	1865	1	1110	1231	1970
cb4016_c34/f1p0/2560 isoform=cb4016_c34;full_length_coverage=1;non_full_length_coverage=0;isoform_length=2560;expected_accuracy=0.998	gnl|BL_ORD_ID|40897 cb4016_c17/f1p0/2709 isoform=cb4016_c17;full_length_coverage=1;non_full_length_coverage=0;isoform_length=2709;expected_accuracy=0.995	194	2559	12	195	351	2709	2	187
cb2834_c14/f1p0/3411	gnl|BL_ORD_ID|70018 cb2834_c15/f1p0/3611	263	3407	1	263	373	351	16	270

						7			
cb5814_c5/f1p0/2403 isoform=cb5814_c5;full_length_coverage=1;non_full_length_coverage=0;isoform_length=2403;expected_accuracy=0.993	gnl|BL_ORD_ID|74459 cb5814_c9/f1p0/2718	1	1848	1845	2358	159	200 0	2130	264 3
cb2719_c50/f1p1/2822	gnl|BL_ORD_ID|37134 cb2719_c32/f1p0/2977 isoform=cb2719_c32;full_length_coverage=1;non_full_length_coverage=0;isoform_length=2977;expected_accuracy=0.997	255	2799	1	257	433	297 7	1	257
cb7642_c36/f1p0/2490 isoform=cb7642_c36;full_length_coverage=1;non_full_length_coverage=0;isoform_length=2490;expected_accuracy=0.991	gnl|BL_ORD_ID|8633 cb7642_c35/f1p0/3405 isoform=cb7642_c35;full_length_coverage=1;non_full_length_coverage=0;isoform_length=3405;expected_accuracy=0.991	1	1677	1676	2490	117	180 1	2019	283 7
cb4487_c26/f1p0/1475 isoform=cb4487_c26;full_length_coverage=1;non_full_length_coverage=0;isoform_length=1475;expected_accuracy=0.998	gnl|BL_ORD_ID|72600 cb4487_c15/f1p0/1739	417	1473	6	420	631	168 7	16	431
cb14139_c8/f1p0/1596 isoform=cb14139_c8;full_length_coverage=1;non_full_length_coverage=0;isoform_length=1596;expected_accuracy=0.993	gnl|BL_ORD_ID|25802 cb14139_c4/f1p0/1783 isoform=cb14139_c4;full_length_coverage=1;non_full_length_coverage=0;isoform_length=1783;expected_accuracy=0.994	726	1596	75	726	844	171 5	70	721
cb9283_c8/f3p0/2020 isoform=cb9283_c8;full_length_coverage=3;non_full_length_coverage=0;isoform_length=2020;expected_accuracy=1.000	gnl|BL_ORD_ID|19600 cb9283_c4/f1p0/3059 isoform=cb9283_c4;full_length_coverage=1;non_full_length_coverage=0;isoform_length=3059;expected_accuracy=0.996	1	1156	1154	2020	14	1165	2170	303 5
cb5270_c33/f1p0/2572	gnl|BL_ORD_ID|73716 cb5270_c25/f1p1/2809	1098	2526	1	1097	1294	272	96	1191

							9		
cb3307_c44/f1p0/1073 isoform=cb3307_c44;full_length_coverage=1;non_full_length_coverage=0;isoform_length=1073;expected_accuracy=0.999	gnl|BL_ORD_ID|70305 cb3307_c25/f1p0/2537	157	1073	4	161	1416	2324	5	161
cb8564_c71766/f1p0/2194 isoform=cb8564_c71766;full_length_coverage=1;non_full_length_coverage=0;isoform_length=2194;expected_accuracy=0.996	gnl|BL_ORD_ID|56470 cb8564_c77506/f1p0/3897	296	2194	8	300	2024	3905	1	293
cb5962_c9/f1p2/2694 isoform=cb5962_c9;full_length_coverage=1;non_full_length_coverage=2;isoform_length=2694;expected_accuracy=0.998	gnl|BL_ORD_ID|46987 cb5962_c20/f1p3/2774 isoform=cb5962_c20;full_length_coverage=1;non_full_length_coverage=3;isoform_length=2774;expected_accuracy=0.993	1	1390	1386	2597	21	1419	1552	2774
cb1333_c101/f1p0/1395 isoform=cb1333_c101;full_length_coverage=1;non_full_length_coverage=0;isoform_length=1395;expected_accuracy=0.997	gnl|BL_ORD_ID|61248 cb1333_c59/f1p0/2058	149	1395	27	148	742	1986	31	152
cb8564_c37149/f1p9/3212 isoform=cb8564_c37149;full_length_coverage=1;non_full_length_coverage=9;isoform_length=3212;expected_accuracy=0.991	gnl|BL_ORD_ID|55939 cb8564_c49630/f1p4/3487	114	2957	2957	3205	71	2913	3243	3493
cb8564_c51244/f1p0/2997	gnl|BL_ORD_ID|56025 cb8564_c52735/f1p0/3300	1	2365	2366	2949	17	2383	2700	3283
cb6048_c11/f1p0/2383	gnl|BL_ORD_ID|45391 cb6048_c16/f3p0/2541 isoform=cb6048_c16;full_length_coverage=3;non_full_length_coverage=0;isoform_length=2541;expected_accur	1	1762	1759	2370	64	1826	1930	2541

	acy=1.000								
cb8564_c49540/f1p2/2982	gnl|BL_ORD_ID|15490 cb8564_c54085/f3p6/3186 isoform=cb8564_c54085;full_length_coverage=3;non_full_length_coverage=6;isoform_length=3186;expected_accuracy=0.996	750	2982	81	747	850	3082	1	667
cb7189_c8/f1p0/2376	gnl|BL_ORD_ID|6238 cb7189_c5/f1p1/2266 isoform=cb7189_c5;full_length_coverage=1;non_full_length_coverage=1;isoform_length=2266;expected_accuracy=1.000	1	1304	1304	2063	219	1522	1417	2176
cb8564_c146250/f5p1/2250 isoform=cb8564_c146250;full_length_coverage=5;non_full_length_coverage=1;isoform_length=2250;expected_accuracy=1.000	gnl|BL_ORD_ID|58095 cb8564_c88673/f1p0/3095	1	1476	1474	2181	40	1518	2317	3025
cb965_c21/f1p0/3360	gnl|BL_ORD_ID|20094 cb965_c9/f2p4/4285 isoform=cb965_c9;full_length_coverage=2;non_full_length_coverage=4;isoform_length=4285;expected_accuracy=0.999	1170	3298	1	1171	2094	4221	809	1979
cb6914_c21/f1p1/2085 isoform=cb6914_c21;full_length_coverage=1;non_full_length_coverage=1;isoform_length=2085;expected_accuracy=1.000	gnl|BL_ORD_ID|6059 cb6914_c11/f1p1/2301 isoform=cb6914_c11;full_length_coverage=1;non_full_length_coverage=1;isoform_length=2301;expected_accuracy=0.997	179	2044	1	179	439	2301	54	235
cb8564_c75076/f1p0/3860 isoform=cb8564_c75076;full_length_coverage=1;non_full_length_coverage=0;isoform_length=3860;expected_accuracy=0.996	gnl|BL_ORD_ID|12872 cb8564_c155955/f4p6/4104 isoform=cb8564_c155955;full_length_coverage=4;non_full_length_coverage=6;isoform_length=4104;expected_accuracy=1.000	1611	3860	1	1616	1832	4081	30	1648
cb8564_c45596/f1p1/3430	gnl|BL_ORD_ID|15046 cb8564_c38440/f1p1/3618	2	2780	2775	3379	32	284	2961	356

	isoform=cb8564_c38440;full_length_coverage=1;non_full_length_coverage=1;isoform_length=3618;expected_accuracy=0.993						1		8
cb3290_c38/f1p0/2345 isoform=cb3290_c38;full_length_coverage=1;non_full_length_coverage=0;isoform_length=2345;expected_accuracy=0.996	gnl|BL_ORD_ID|70292 cb3290_c108/f1p0/3138	1150	2343	1	1151	1706	2906	276	1432
cb1581_c11/f1p3/2463 isoform=cb1581_c11;full_length_coverage=1;non_full_length_coverage=3;isoform_length=2463;expected_accuracy=0.991	gnl|BL_ORD_ID|28677 cb1581_c8/f1p0/2559 isoform=cb1581_c8;full_length_coverage=1;non_full_length_coverage=0;isoform_length=2559;expected_accuracy=0.994	253	2414	6	252	402	2559	1	247
cb8564_c84151/f1p0/2425 isoform=cb8564_c84151;full_length_coverage=1;non_full_length_coverage=0;isoform_length=2425;expected_accuracy=0.992	gnl|BL_ORD_ID|15626 cb8564_c74256/f1p0/2961 isoform=cb8564_c74256;full_length_coverage=1;non_full_length_coverage=0;isoform_length=2961;expected_accuracy=0.995	1	1725	1726	2425	58	1799	2205	2909
cb14882_c0/f1p0/1874 isoform=cb14882_c0;full_length_coverage=1;non_full_length_coverage=0;isoform_length=1874;expected_accuracy=1.000	gnl|BL_ORD_ID|12384 cb8564_c123657/f1p0/1999 isoform=cb8564_c123657;full_length_coverage=1;non_full_length_coverage=0;isoform_length=1999;expected_accuracy=0.996	1	1686	1685	1857	1	1688	1826	1999
cb8564_c117596/f1p0/1989 isoform=cb8564_c117596;full_length_coverage=1;non_full_length_coverage=0;isoform_length=1989;expected_accuracy=0.999	gnl|BL_ORD_ID|55034 cb8564_c4546/f2p0/2151	1	1622	1620	1956	10	1631	1825	2160
cb8564_c15996/f1p5/3083 isoform=cb8564_c15996;full_length_coverage=1;non_full_length_coverage=5;isoform_length=3083;expected_	gnl|BL_ORD_ID|56172 cb8564_c71449/f1p0/3507	323	3018	4	328	474	3165	5	333

accuracy=0.993									
cb2739_c7/f1p0/3065	gnl|BL_ORD_ID|38775 cb2739_c0/f2p0/3590 isoform=cb2739_c0;full_length_coverage=2;non_full_length_coverage=0;isoform_length=3590;expected_accuracy=0.997	1	1801	1800	2872	507	2298	2519	3587
cb9583_c8/f1p0/1425	gnl|BL_ORD_ID|19511 cb9583_c6/f1p0/1811 isoform=cb9583_c6;full_length_coverage=1;non_full_length_coverage=0;isoform_length=1811;expected_accuracy=0.993	527	1224	7	529	1115	1811	9	534
cb8564_c16240/f1p3/2671 isoform=cb8564_c16240;full_length_coverage=1;non_full_length_coverage=3;isoform_length=2671;expected_accuracy=0.997	gnl|BL_ORD_ID|52713 cb8564_c11986/f1p1/3355	16	2075	2072	2665	17	2077	2614	3208
cb7611_c26/f1p0/2483	gnl|BL_ORD_ID|7566 cb7611_c18/f1p0/3224 isoform=cb7611_c18;full_length_coverage=1;non_full_length_coverage=0;isoform_length=3224;expected_accuracy=1.000	288	2194	48	290	1325	3224	1	243
cb8564_c89331/f1p0/2344	gnl|BL_ORD_ID|17423 cb8564_c83614/f1p1/3341 isoform=cb8564_c83614;full_length_coverage=1;non_full_length_coverage=1;isoform_length=3341;expected_accuracy=0.995	1	2105	2101	2381	117	2218	3063	3341
cb166_c11/f1p0/4188	gnl|BL_ORD_ID|32848 cb166_c16/f1p0/4452 isoform=cb166_c16;full_length_coverage=1;non_full_length_coverage=0;isoform_length=4452;expected_accuracy=0.991	1392	4118	12	1394	1646	4364	85	1453
cb910_c28/f1p1/2974	gnl|BL_ORD_ID|19839 cb910_c65/f2p1/3148	1	2158	2156	2974	3	216	2280	310

isoform=cb910_c28;full_length_coverage=1;non_full_length_coverage=1;isoform_length=2974;expected_accuracy=0.995	isoform=cb910_c65;full_length_coverage=2;non_full_length_coverage=1;isoform_length=3148;expected_accuracy=0.998						4		4
cb5125_c6/f8p4/2105 isoform=cb5125_c6;full_length_coverage=8;non_full_length_coverage=4;isoform_length=2105;expected_accuracy=1.000	gnl|BL_ORD_ID|43457 cb5125_c11/f2p1/2545 isoform=cb5125_c11;full_length_coverage=2;non_full_length_coverage=1;isoform_length=2545;expected_accuracy=1.000	1	1297	1295	2062	40	1336	1778	2545
cb8564_c51035/f2p2/4216 isoform=cb8564_c51035;full_length_coverage=2;non_full_length_coverage=2;isoform_length=4216;expected_accuracy=0.999	gnl|BL_ORD_ID|55207 cb8564_c35399/f1p0/4553	286	4160	10	287	405	4280	11	287
cb7010_c28/f1p0/2721 isoform=cb7010_c28;full_length_coverage=1;non_full_length_coverage=0;isoform_length=2721;expected_accuracy=0.997	gnl|BL_ORD_ID|47804 cb7010_c20/f1p0/4751	182	2721	1	185	2044	4595	1489	1673
cb9467_c69/f1p0/2737	gnl|BL_ORD_ID|20406 cb9467_c53/f1p0/2583 isoform=cb9467_c53;full_length_coverage=1;non_full_length_coverage=0;isoform_length=2583;expected_accuracy=0.996	153	2026	11	156	696	2564	7	147
cb5564_c17/f1p0/2403	gnl|BL_ORD_ID|44673 cb5564_c2/f3p1/2892 isoform=cb5564_c2;full_length_coverage=3;non_full_length_coverage=1;isoform_length=2892;expected_accuracy=0.999	262	2306	5	265	793	2841	3	262
cb13842_c0/f1p0/1450 isoform=cb13842_c0;full_length_coverage=1;non_full_length_coverage=0;isoform_length=1450;expected_accu	gnl|BL_ORD_ID|18318 cb8564_c86426/f1p0/2857 isoform=cb8564_c86426;full_length_coverage=1;non_full_length_coverage=0;isoform_length=2857;expected_ac	1	1120	1119	1421	5	1122	2491	2793

racy=0.991	curacy=0.996								
cb8564_c47522/f1p0/2954	gnl|BL_ORD_ID|14407 cb8564_c44036/f1p0/3258 isoform=cb8564_c44036;full_length_coverage=1;non_full_length_coverage=0;isoform_length=3258;expected_accuracy=0.992	1158	2845	1	1158	1575	3258	1	1147
cb12766_c11/f1p6/1661	gnl|BL_ORD_ID|72689 cb4564_c15/f1p0/1760	442	1622	7	443	588	1769	10	446
cb8564_c129638/f1p0/2479	gnl|BL_ORD_ID|53319 cb8564_c122043/f1p0/3229	1	1627	1623	2475	28	1654	2059	2911
cb6730_c2/f5p0/2632 isoform=cb6730_c2;full_length_coverage=5;non_full_length_coverage=0;isoform_length=2632;expected_accuracy=1.000	gnl|BL_ORD_ID|49988 cb6730_c7/f1p0/2780	15	1753	1752	2632	1	1739	1896	2776
cb1207_c16/f1p0/3156	gnl|BL_ORD_ID|59265 cb1207_c4/f1p0/4068	222	2975	1	224	1300	4054	961	1184
cb10693_c1/f2p0/1622 isoform=cb10693_c1;full_length_coverage=2;non_full_length_coverage=0;isoform_length=1622;expected_accuracy=1.000	gnl|BL_ORD_ID|48772 cb10693_c4/f1p1/1797	1	1408	1406	1622	3	1410	1577	1793
cb1691_c25/f1p0/2763	gnl|BL_ORD_ID|32447 cb1691_c22/f1p0/3569 isoform=cb1691_c22;full_length_coverage=1;non_full_length_coverage=0;isoform_length=3569;expected_accuracy=0.999	1130	2681	1	1131	2019	3569	128	1257
cb626_c59/f1p0/4159 isoform=cb626_c59;full_length_coverage=1;non_full_length_coverage=0;isoform_length=4159;expected_accur	gnl|BL_ORD_ID|4060 cb626_c17/f1p0/4935 isoform=cb626_c17;full_length_coverage=1;non_full_length_coverage=0;isoform_length=4935;expected_accura	11	3591	3591	4159	110	3629	4370	4934

acy=0.991	cy=0.993								
cb1438_c12/f1p0/3268	gnl|BL_ORD_ID|29145 cb1438_c10/f1p5/4153 isoform=cb1438_c10;full_length_coverage=1;non_full_length_coverage=5;isoform_length=4153;expected_accuracy=0.999	1435	3193	1	1439	2393	4153	712	2152
cb8564_c74180/f1p1/2858	gnl|BL_ORD_ID|56341 cb8564_c74768/f1p0/3399	1	2587	2585	2854	190	2776	2915	3184
cb9448_c18/f2p0/1769 isoform=cb9448_c18;full_length_coverage=2;non_full_length_coverage=0;isoform_length=1769;expected_accuracy=1.000	gnl|BL_ORD_ID|58724 cb9448_c6/f1p1/2077	527	1769	89	526	772	2015	1	438
cb1306_c36/f1p0/2164 isoform=cb1306_c36;full_length_coverage=1;non_full_length_coverage=0;isoform_length=2164;expected_accuracy=1.000	gnl|BL_ORD_ID|25329 cb1306_c16/f1p0/3008 isoform=cb1306_c16;full_length_coverage=1;non_full_length_coverage=0;isoform_length=3008;expected_accuracy=0.996	1	1793	1788	2087	29	1823	2708	3008
cb8564_c38035/f1p0/2770	gnl|BL_ORD_ID|14399 cb8564_c39189/f1p1/2975 isoform=cb8564_c39189;full_length_coverage=1;non_full_length_coverage=1;isoform_length=2975;expected_accuracy=0.992	1	2035	2033	2756	1	2050	2242	2973
cb8564_c87256/f1p0/2285 isoform=cb8564_c87256;full_length_coverage=1;non_full_length_coverage=0;isoform_length=2285;expected_accuracy=0.992	gnl|BL_ORD_ID|11794 cb8564_c146257/f6p6/2574 isoform=cb8564_c146257;full_length_coverage=6;non_full_length_coverage=6;isoform_length=2574;expected_accuracy=1.000	1	1703	1700	2285	135	1839	1972	2562
cb9296_c14/f1p0/1970	gnl|BL_ORD_ID|20303 cb9296_c6/f1p1/1992 isoform=cb9296_c6;full_length_coverage=1;non_full_length_coverage=1;isoform_length=1992;expected_accura	11	1238	1239	1765	13	1240	1461	1992

	cy=1.000								
cb8564_c20760/f2p0/3197 isoform=cb8564_c20760;full_length_coverage=2;non_full_length_coverage=0;isoform_length=3197;expected_accuracy=1.000	gnl|BL_ORD_ID|11305 cb8564_c15446/f1p0/3742 isoform=cb8564_c15446;full_length_coverage=1;non_full_length_coverage=0;isoform_length=3742;expected_accuracy=0.997	1	2862	2860	3197	218	3079	3371	3708
cb14044_c17/f1p0/1065	gnl|BL_ORD_ID|49491 cb6181_c31/f1p1/2199	1	723	719	972	365	1088	1222	1475
cb8658_c3/f1p0/2265	gnl|BL_ORD_ID|18756 cb8658_c6/f1p0/3454 isoform=cb8658_c6;full_length_coverage=1;non_full_length_coverage=0;isoform_length=3454;expected_accuracy=0.996	1	1736	1735	2271	16	1745	2915	3454
cb3759_c6/f1p0/2298	gnl|BL_ORD_ID|40823 cb3759_c16/f1p0/3176 isoform=cb3759_c16;full_length_coverage=1;non_full_length_coverage=0;isoform_length=3176;expected_accuracy=0.997	1	1727	1729	2238	40	1757	2594	3101
cb12893_c39/f1p0/1097 isoform=cb12893_c39;full_length_coverage=1;non_full_length_coverage=0;isoform_length=1097;expected_accuracy=0.996	gnl|BL_ORD_ID|27633 cb12893_c81/f64p5/1418 isoform=cb12893_c81;full_length_coverage=64;non_full_length_coverage=5;isoform_length=1418;expected_accuracy=1.000	234	1095	4	239	489	1363	1	234
cb6340_c14/f1p0/2054 isoform=cb6340_c14;full_length_coverage=1;non_full_length_coverage=0;isoform_length=2054;expected_accuracy=0.993	gnl|BL_ORD_ID|5952 cb6340_c15/f1p0/2223 isoform=cb6340_c15;full_length_coverage=1;non_full_length_coverage=0;isoform_length=2223;expected_accuracy=0.994	219	2054	87	217	376	2211	1	131
cb18654_c5/f1p0/1646 isoform=cb18654_c5;full_length_coverage=1;non_full_length_coverage=0;isoform_length=1646;expected_accu	gnl|BL_ORD_ID|54076 cb8564_c17962/f1p0/3284	1	1007	1007	1646	1251	2257	2646	3283

racy=0.996											
cb8313_c6/f1p0/2290 isoform=cb8313_c6;full_length_coverage=1;non_full_length_coverage=0;isoform_length=2290;expected_accuracy=0.996	gnl|BL_ORD_ID|10395 cb8313_c5/f1p0/2403 isoform=cb8313_c5;full_length_coverage=1;non_full_length_coverage=0;isoform_length=2403;expected_accuracy=0.995	2	2147	2144	2285	3	2161	2261	2403		
cb1905_c8/f1p0/2229 isoform=cb1905_c8;full_length_coverage=1;non_full_length_coverage=0;isoform_length=2229;expected_accuracy=0.994	gnl|BL_ORD_ID|34887 cb1905_c2/f3p0/2454 isoform=cb1905_c2;full_length_coverage=3;non_full_length_coverage=0;isoform_length=2454;expected_accuracy=1.000	1	1719	1719	2215	6	1726	1957	2454		
cb6825_c19/f1p0/2295 isoform=cb6825_c19;full_length_coverage=1;non_full_length_coverage=0;isoform_length=2295;expected_accuracy=0.997	gnl|BL_ORD_ID|4068 cb6825_c37/f3p1/2605 isoform=cb6825_c37;full_length_coverage=3;non_full_length_coverage=1;isoform_length=2605;expected_accuracy=0.995	1	1494	1489	2295	68	1561	1734	2540		
cb1821_c5/f2p1/3329 isoform=cb1821_c5;full_length_coverage=2;non_full_length_coverage=1;isoform_length=3329;expected_accuracy=0.999	gnl|BL_ORD_ID|34014 cb1821_c29/f1p2/4045 isoform=cb1821_c29;full_length_coverage=1;non_full_length_coverage=2;isoform_length=4045;expected_accuracy=0.995	297	3329	1	298	1012	4043	108	404		
cb2677_c13/f1p1/3727 isoform=cb2677_c13;full_length_coverage=1;non_full_length_coverage=1;isoform_length=3727;expected_accuracy=0.998	gnl|BL_ORD_ID|69883 cb2677_c26/f1p0/4011	260	3727	1	262	378	3849	4	267		
cb4745_c12/f1p0/2017 isoform=cb4745_c12;full_length_coverage=1;non_full_length_coverage=0;isoform_length=2017;expected_accuracy=0.996	gnl|BL_ORD_ID|43416 cb4745_c0/f12p0/3046 isoform=cb4745_c0;full_length_coverage=12;non_full_length_coverage=0;isoform_length=3046;expected_accuracy=1.000	156	2016	1	161	1185	3046	14	167		
cb9467_c64/f1p1/2076	gnl|BL_ORD_ID|21843 cb9467_c45/f1p0/2180	672	2033	7	673	816	217	5	672		

	isoform=cb9467_c45;full_length_coverage=1;non_full_length_coverage=0;isoform_length=2180;expected_accuracy=0.999						9		
cb1508_c6/f1p1/1904	gnl|BL_ORD_ID|30077 cb1508_c8/f1p0/1971 isoform=cb1508_c8;full_length_coverage=1;non_full_length_coverage=0;isoform_length=1971;expected_accuracy=1.000	371	1848	56	372	494	1971	1	317
cb2816_c18/f1p0/3156 isoform=cb2816_c18;full_length_coverage=1;non_full_length_coverage=0;isoform_length=3156;expected_accuracy=0.994	gnl|BL_ORD_ID|37556 cb2816_c26/f1p1/3654 isoform=cb2816_c26;full_length_coverage=1;non_full_length_coverage=1;isoform_length=3654;expected_accuracy=0.999	1	1723	1721	3156	78	1803	2085	3536
cb1913_c44/f1p0/2236 isoform=cb1913_c44;full_length_coverage=1;non_full_length_coverage=0;isoform_length=2236;expected_accuracy=0.998	gnl|BL_ORD_ID|66492 cb1913_c51/f1p0/2531	1	1634	1630	2220	54	1687	1821	2411
cb1279_c85/f1p0/1794 isoform=cb1279_c85;full_length_coverage=1;non_full_length_coverage=0;isoform_length=1794;expected_accuracy=0.998	gnl|BL_ORD_ID|27063 cb1279_c42/f1p0/3192 isoform=cb1279_c42;full_length_coverage=1;non_full_length_coverage=0;isoform_length=3192;expected_accuracy=0.993	1	1120	1115	1794	1	1116	2529	3192
cb2097_c120/f1p0/1809 isoform=cb2097_c120;full_length_coverage=1;non_full_length_coverage=0;isoform_length=1809;expected_accuracy=0.994	gnl|BL_ORD_ID|36319 cb2097_c87/f1p2/2320 isoform=cb2097_c87;full_length_coverage=1;non_full_length_coverage=2;isoform_length=2320;expected_accuracy=0.992	1	1422	1420	1799	393	1814	1924	2304
cb8564_c188095/f1p0/3177 isoform=cb8564_c188095;full_length_coverage=1;non_full_length_coverage=0;isoform_length=3177;expected	gnl|BL_ORD_ID|15226 cb8564_c34123/f2p0/3784 isoform=cb8564_c34123;full_length_coverage=2;non_full_length_coverage=0;isoform_length=3784;expected_ac	1345	3158	1	1346	1969	3784	1	1351

<table>
<tr><td>_accuracy=0.998</td><td>curacy=1.000</td><td></td><td></td><td></td><td></td><td></td><td></td><td></td><td></td></tr>
<tr><td>cb4848_c14/f1p0/1912</td><td>gnl|BL_ORD_ID|42205 cb4848_c5/f1p1/3168 isoform=cb4848_c5;full_length_coverage=1;non_full_length_coverage=1;isoform_length=3168;expected_accuracy=0.994</td><td>1</td><td>1551</td><td>1549</td><td>1846</td><td>981</td><td>2530</td><td>2873</td><td>3168</td></tr>
<tr><td>cb4147_c31/f1p0/6049</td><td>gnl|BL_ORD_ID|70796 cb4147_c12/f1p0/6657</td><td>4</td><td>4516</td><td>4517</td><td>5902</td><td>40</td><td>4553</td><td>5192</td><td>6577</td></tr>
<tr><td>cb8564_c12600/f1p0/2645</td><td>gnl|BL_ORD_ID|55766 cb8564_c25127/f1p0/3463</td><td>254</td><td>2645</td><td>1</td><td>255</td><td>370</td><td>2737</td><td>17</td><td>267</td></tr>
<tr><td>cb9170_c5/f1p0/1862 isoform=cb9170_c5;full_length_coverage=1;non_full_length_coverage=0;isoform_length=1862;expected_accuracy=1.000</td><td>gnl|BL_ORD_ID|21012 cb9170_c4/f1p0/1925 isoform=cb9170_c4;full_length_coverage=1;non_full_length_coverage=0;isoform_length=1925;expected_accuracy=0.996</td><td>246</td><td>1862</td><td>74</td><td>246</td><td>287</td><td>1900</td><td>1</td><td>173</td></tr>
<tr><td>cb18456_c5600/f1p0/1216</td><td>gnl|BL_ORD_ID|16844 cb8564_c68630/f3p3/1964 isoform=cb8564_c68630;full_length_coverage=3;non_full_length_coverage=3;isoform_length=1964;expected_accuracy=1.000</td><td>537</td><td>1161</td><td>7</td><td>536</td><td>1288</td><td>1917</td><td>1</td><td>528</td></tr>
<tr><td>cb8564_c68976/f2p0/3533 isoform=cb8564_c68976;full_length_coverage=2;non_full_length_coverage=0;isoform_length=3533;expected_accuracy=1.000</td><td>gnl|BL_ORD_ID|57694 cb8564_c78332/f1p0/4781</td><td>12</td><td>2799</td><td>2797</td><td>3522</td><td>12</td><td>2813</td><td>3635</td><td>4361</td></tr>
<tr><td>cb8564_c36731/f1p2/2761</td><td>gnl|BL_ORD_ID|54425 cb8564_c42906/f1p2/3181</td><td>1</td><td>1977</td><td>1972</td><td>2739</td><td>1</td><td>1958</td><td>2387</td><td>3155</td></tr>
<tr><td>cb8564_c184250/f1p0/2256 isoform=cb8564_c184250;full_length_coverage=1;non_full_length_coverage=0;isoform_length=2256;expected</td><td>gnl|BL_ORD_ID|57213 cb8564_c86813/f1p1/2854</td><td>1</td><td>1642</td><td>1638</td><td>2256</td><td>27</td><td>1668</td><td>1929</td><td>2547</td></tr>
</table>

_accuracy=0.996									
cb8564_c37866/f1p3/2874 isoform=cb8564_c37866;full_length_coverage=1;non_full_length_coverage=3;isoform_length=2874;expected_accuracy=0.990	gnl|BL_ORD_ID|14410 cb8564_c44256/f1p1/3267 isoform=cb8564_c44256;full_length_coverage=1;non_full_length_coverage=1;isoform_length=3267;expected_accuracy=0.995	1	2147	2143	2863	1	2119	2553	3267
cb8564_c21439/f1p0/2587	gnl|BL_ORD_ID|54611 cb8564_c127997/f1p3/2802	1226	2572	1	1228	1351	2697	1	1230
cb2873_c6/f1p1/3087 isoform=cb2873_c6;full_length_coverage=1;non_full_length_coverage=1;isoform_length=3087;expected_accuracy=0.998	gnl|BL_ORD_ID|36904 cb2873_c2/f2p2/3521 isoform=cb2873_c2;full_length_coverage=2;non_full_length_coverage=2;isoform_length=3521;expected_accuracy=0.998	1	2226	2223	3080	1	2220	2644	3521
cb8564_c40598/f1p4/3119	gnl|BL_ORD_ID|55246 cb8564_c36343/f1p0/3448	2	2732	2729	3051	3	2745	2866	3188
cb4641_c2/f2p1/2256 isoform=cb4641_c2;full_length_coverage=2;non_full_length_coverage=1;isoform_length=2256;expected_accuracy=0.999	gnl|BL_ORD_ID|42925 cb4641_c3/f2p1/2387 isoform=cb4641_c3;full_length_coverage=2;non_full_length_coverage=1;isoform_length=2387;expected_accuracy=1.000	1032	2254	1	1033	1164	2387	1	1032
cb8564_c4687/f1p0/2736 isoform=cb8564_c4687;full_length_coverage=1;non_full_length_coverage=0;isoform_length=2736;expected_accuracy=0.995	gnl|BL_ORD_ID|55049 cb8564_c4686/f1p0/3506	1	2436	2435	2727	4	2434	3239	3530
cb8564_c9531/f3p0/2496 isoform=cb8564_c9531;full_length_coverage=3;non_full_length_coverage=0;isoform_length=2496;expected_accuracy=0.999	gnl|BL_ORD_ID|13914 cb8564_c23779/f1p0/3567 isoform=cb8564_c23779;full_length_coverage=1;non_full_length_coverage=0;isoform_length=3567;expected_accuracy=0.996	15	2070	2067	2492	1	2054	3142	3567
cb8564_c53960/f1p0/2993	gnl|BL_ORD_ID|15379 cb8564_c37604/f2p0/3150	1	1604	1602	2993	1	160	1744	314

isoform=cb8564_c53960;full_length_coverage=1;non_full_length_coverage=0;isoform_length=2993;expected_accuracy=0.993	isoform=cb8564_c37604;full_length_coverage=2;non_full_length_coverage=0;isoform_length=3150;expected_accuracy=0.996						4		5
cb5659_c78/f1p0/2309	gnl|BL_ORD_ID|45301 cb5659_c107/f46p4/2736 isoform=cb5659_c107;full_length_coverage=46;non_full_length_coverage=4;isoform_length=2736;expected_accuracy=1.000	1	1676	1675	2137	7	1682	2262	2727
cb7493_c1/f1p0/2337 isoform=cb7493_c1;full_length_coverage=1;non_full_length_coverage=0;isoform_length=2337;expected_accuracy=0.999	gnl|BL_ORD_ID|50550 cb7493_c3/f1p0/2634	1	1866	1864	2336	1	1866	2007	2479
cb416_c58/f1p0/2505 isoform=cb416_c58;full_length_coverage=1;non_full_length_coverage=0;isoform_length=2505;expected_accuracy=0.992	gnl|BL_ORD_ID|42632 cb416_c28/f1p0/2988 isoform=cb416_c28;full_length_coverage=1;non_full_length_coverage=0;isoform_length=2988;expected_accuracy=0.996	286	2505	4	287	696	2896	265	545
cb10172_c11/f1p0/1434 isoform=cb10172_c11;full_length_coverage=1;non_full_length_coverage=0;isoform_length=1434;expected_accuracy=0.993	gnl|BL_ORD_ID|433 cb10172_c1/f8p0/1594 isoform=cb10172_c1;full_length_coverage=8;non_full_length_coverage=0;isoform_length=1594;expected_accuracy=1.000	1	1098	1098	1434	21	1119	1236	1572
cb8564_c46622/f2p0/3338 isoform=cb8564_c46622;full_length_coverage=2;non_full_length_coverage=0;isoform_length=3338;expected_accuracy=0.997	gnl|BL_ORD_ID|15520 cb8564_c52723/f2p0/3547 isoform=cb8564_c52723;full_length_coverage=2;non_full_length_coverage=0;isoform_length=3547;expected_accuracy=0.990	1425	3338	1	1426	1556	3471	1	1421
cb18456_c3695/f1p0/1345	gnl|BL_ORD_ID|17162 cb8564_c87465/f2p9/3179 isoform=cb8564_c87465;full_length_coverage=2;non_full_length_coverage=9;isoform_length=3179;expected_ac	1	1051	1047	1341	1411	2460	2635	2929

	curacy=1.000								
cb862_c22/f1p0/3314 isoform=cb862_c22;full_length_coverage=1;non_full_length_coverage=0;isoform_length=3314;expected_accuracy=0.995	gnl|BL_ORD_ID|57576 cb862_c10/f1p0/3428	182	3255	1	182	305	338 6	7	189
cb4674_c26/f1p1/3006 isoform=cb4674_c26;full_length_coverage=1;non_full_length_coverage=1;isoform_length=3006;expected_accuracy=0.995	gnl|BL_ORD_ID|72844 cb4674_c33/f1p3/3281	1	2157	2155	3006	84	222 0	2382	323 5
cb1540_c27/f1p0/3741	gnl|BL_ORD_ID|61379 cb1540_c20/f1p0/4038	304	3535	1	303	632	386 3	108	410
cb5993_c2/f1p0/2414	gnl|BL_ORD_ID|45179 cb5993_c1/f1p1/2959 isoform=cb5993_c1;full_length_coverage=1;non_full_length_coverage=1;isoform_length=2959;expected_accuracy=1.000	1	1289	1285	2407	196	148 4	1801	292 3
cb8564_c75977/f1p0/2165	gnl|BL_ORD_ID|57396 cb8564_c91806/f1p1/3620	1	1393	1394	2125	64	145 6	2896	362 2
cb4945_c4/f1p0/2080 isoform=cb4945_c4;full_length_coverage=1;non_full_length_coverage=0;isoform_length=2080;expected_accuracy=0.996	gnl|BL_ORD_ID|73110 cb4945_c1/f1p0/3164	1	1222	1219	2047	729	195 2	2295	312 3
cb8564_c51159/f1p0/2634 isoform=cb8564_c51159;full_length_coverage=1;non_full_length_coverage=0;isoform_length=2634;expected_accuracy=0.995	gnl|BL_ORD_ID|56353 cb8564_c75114/f1p0/2866	110	2101	2098	2634	1	199 3	2197	273 1
cb8564_c123147/f1p0/2657	gnl|BL_ORD_ID|16781 cb8564_c76648/f1p0/2870	1	1859	1861	2657	22	188	2075	287

isoform=cb8564_c123147;full_length_coverage=1;non_full_length_coverage=0;isoform_length=2657;expected_accuracy=0.997	isoform=cb8564_c76648;full_length_coverage=1;non_full_length_coverage=0;isoform_length=2870;expected_accuracy=0.999						8		0
cb8456_c128/f1p1/1805 isoform=cb8456_c128;full_length_coverage=1;non_full_length_coverage=1;isoform_length=1805;expected_accuracy=0.994	gnl|BL_ORD_ID|10827 cb8456_c150/f136p3/2032 isoform=cb8456_c150;full_length_coverage=136;non_full_length_coverage=3;isoform_length=2032;expected_accuracy=1.000	1	1371	1366	1805	102	1457	1572	2006
cb5098_c14/f1p0/2880	gnl|BL_ORD_ID|42663 cb5098_c9/f1p4/2945 isoform=cb5098_c9;full_length_coverage=1;non_full_length_coverage=4;isoform_length=2945;expected_accuracy=0.996	1	1987	1985	2698	20	2009	2162	2874
cb416_c41/f1p0/3183	gnl|BL_ORD_ID|42635 cb416_c25/f1p0/3907 isoform=cb416_c25;full_length_coverage=1;non_full_length_coverage=0;isoform_length=3907;expected_accuracy=0.996	1054	3054	101	1053	1912	3907	1	952
cb1648_c14/f1p0/3818 isoform=cb1648_c14;full_length_coverage=1;non_full_length_coverage=0;isoform_length=3818;expected_accuracy=0.995	gnl|BL_ORD_ID|63818 cb1648_c13/f1p0/4230	193	3818	1	193	339	3962	8	196
cb8564_c19160/f1p2/2687 isoform=cb8564_c19160;full_length_coverage=1;non_full_length_coverage=2;isoform_length=2687;expected_accuracy=0.990	gnl|BL_ORD_ID|14058 cb8564_c23936/f1p0/3016 isoform=cb8564_c23936;full_length_coverage=1;non_full_length_coverage=0;isoform_length=3016;expected_accuracy=0.995	1	1743	1741	2613	4	1716	2143	3016
cb322_c34/f1p0/3998	gnl|BL_ORD_ID|40328 cb322_c11/f1p1/4829 isoform=cb322_c11;full_length_coverage=1;non_full_length_coverage=1;isoform_length=4829;expected_accura	1	3649	3646	3960	80	3736	4511	4826

	cy=0.990								
cb8564_c84130/f1p2/3574 isoform=cb8564_c84130;full_length_coverage=1;non_full_length_coverage=2;isoform_length=3574;expected_accuracy=0.995	gnl|BL_ORD_ID|10486 cb8564_c107051/f2p1/3797 isoform=cb8564_c107051;full_length_coverage=2;non_full_length_coverage=1;isoform_length=3797;expected_accuracy=0.997	125	3511	1	127	392	3797	84	215
cb8564_c51172/f1p0/3367	gnl|BL_ORD_ID|58199 cb8564_c91279/f1p0/3567	523	2973	7	522	749	3197	8	508
cb9718_c43/f1p0/1655 isoform=cb9718_c43;full_length_coverage=1;non_full_length_coverage=0;isoform_length=1655;expected_accuracy=0.996	gnl|BL_ORD_ID|59927 cb9718_c15/f1p3/1797	288	1655	59	290	332	1699	1	232
cb8564_c39136/f1p0/3036 isoform=cb8564_c39136;full_length_coverage=1;non_full_length_coverage=0;isoform_length=3036;expected_accuracy=0.998	gnl|BL_ORD_ID|12913 cb8564_c182049/f1p0/3195 isoform=cb8564_c182049;full_length_coverage=1;non_full_length_coverage=0;isoform_length=3195;expected_accuracy=0.997	1	2089	2085	3036	1	2109	2236	3195
cb2854_c43/f1p0/2083 isoform=cb2854_c43;full_length_coverage=1;non_full_length_coverage=0;isoform_length=2083;expected_accuracy=0.994	gnl|BL_ORD_ID|36848 cb2854_c42/f1p0/3914 isoform=cb2854_c42;full_length_coverage=1;non_full_length_coverage=0;isoform_length=3914;expected_accuracy=0.999	1	1705	1702	2027	1186	2890	3590	3914
cb8564_c91851/f1p1/2644 isoform=cb8564_c91851;full_length_coverage=1;non_full_length_coverage=1;isoform_length=2644;expected_accuracy=0.993	gnl|BL_ORD_ID|14058 cb8564_c23936/f1p0/3016 isoform=cb8564_c23936;full_length_coverage=1;non_full_length_coverage=0;isoform_length=3016;expected_accuracy=0.995	1	1713	1711	2567	4	1716	2143	3016
cb7893_c0/f6p1/2246 isoform=cb7893_c0;full_length_coverage=6;non_full_length_coverage=1;isoform_length=2246;expected_accur	gnl|BL_ORD_ID|8161 cb7893_c2/f1p1/2510 isoform=cb7893_c2;full_length_coverage=1;non_full_length_coverage=1;isoform_length=2510;expected_accura	15	1750	1745	2228	2	1736	2028	2510

acy=1.000	cy=0.993								
cb795_c7/f1p0/2330	gnl|BL_ORD_ID|49474 cb795_c15/f1p0/3020	441	2279	45	442	1018	2856	52	450
cb8564_c45699/f1p0/3005	gnl|BL_ORD_ID|14410 cb8564_c44256/f1p1/3267 isoform=cb8564_c44256;full_length_coverage=1;non_full_length_coverage=1;isoform_length=3267;expected_accuracy=0.995	1	2133	2129	2844	1	2119	2553	3267
cb2950_c0/f4p0/2843 isoform=cb2950_c0;full_length_coverage=4;non_full_length_coverage=0;isoform_length=2843;expected_accuracy=1.000	gnl|BL_ORD_ID|36576 cb2950_c14/f1p0/2872 isoform=cb2950_c14;full_length_coverage=1;non_full_length_coverage=0;isoform_length=2872;expected_accuracy=0.999	398	2843	69	399	430	2871	1	329
cb7550_c6/f1p0/1886	gnl|BL_ORD_ID|50589 cb7550_c4/f1p1/2591	1	1051	1046	1754	74	1130	1729	2446
cb8564_c90372/f1p0/2157 isoform=cb8564_c90372;full_length_coverage=1;non_full_length_coverage=0;isoform_length=2157;expected_accuracy=0.994	gnl|BL_ORD_ID|56779 cb8564_c71242/f1p0/2918	1	1092	1090	2157	104	1197	1466	2533
cb8564_c34922/f1p0/2696 isoform=cb8564_c34922;full_length_coverage=1;non_full_length_coverage=0;isoform_length=2696;expected_accuracy=0.993	gnl|BL_ORD_ID|14531 cb8564_c41778/f1p9/3482 isoform=cb8564_c41778;full_length_coverage=1;non_full_length_coverage=9;isoform_length=3482;expected_accuracy=0.992	242	2696	1	246	812	3302	1	247
cb2471_c2/f7p1/2180 isoform=cb2471_c2;full_length_coverage=7;non_full_length_coverage=1;isoform_length=2180;expected_accuracy=1.000	gnl|BL_ORD_ID|37270 cb2471_c8/f3p0/2291 isoform=cb2471_c8;full_length_coverage=3;non_full_length_coverage=0;isoform_length=2291;expected_accuracy=0.996	265	2180	44	268	373	2288	1	225
cb7184_c2/f3p1/1854	gnl|BL_ORD_ID|51021 cb7184_c5/f1p1/2648	371	1854	53	376	1134	262	61	384

isoform=cb7184_c2;full_length_coverage=3;non_full_length_coverage=1;isoform_length=1854;expected_accuracy=1.000							6		
cb15510_c0/f1p0/1378	gnl|BL_ORD_ID|64710 cb15510_c5/f1p1/1580	463	1312	5	459	677	1525	1	457
cb4006_c13/f1p0/2983 isoform=cb4006_c13;full_length_coverage=1;non_full_length_coverage=0;isoform_length=2983;expected_accuracy=0.994	gnl|BL_ORD_ID|39613 cb4006_c2/f4p2/3269 isoform=cb4006_c2;full_length_coverage=4;non_full_length_coverage=2;isoform_length=3269;expected_accuracy=1.000	274	2970	1	276	577	3269	97	373
cb4299_c2/f1p0/2773	gnl|BL_ORD_ID|42791 cb4299_c0/f1p0/3383 isoform=cb4299_c0;full_length_coverage=1;non_full_length_coverage=0;isoform_length=3383;expected_accuracy=0.996	1	2556	2552	2783	133	2682	2939	3169
cb3603_c42/f1p0/5113 isoform=cb3603_c42;full_length_coverage=1;non_full_length_coverage=0;isoform_length=5113;expected_accuracy=0.995	gnl|BL_ORD_ID|40851 cb3603_c41/f1p0/5154 isoform=cb3603_c41;full_length_coverage=1;non_full_length_coverage=0;isoform_length=5154;expected_accuracy=0.997	116	4927	1	112	351	5154	92	202
cb3323_c13/f1p0/3055 isoform=cb3323_c13;full_length_coverage=1;non_full_length_coverage=0;isoform_length=3055;expected_accuracy=0.995	gnl|BL_ORD_ID|40053 cb3323_c11/f1p0/3260 isoform=cb3323_c11;full_length_coverage=1;non_full_length_coverage=0;isoform_length=3260;expected_accuracy=0.998	571	3055	58	575	667	3159	1	521
cb8564_c89391/f1p0/3380	gnl|BL_ORD_ID|53677 cb8564_c152797/f2p3/3371	22	2834	2835	3107	1	2811	2670	2942
cb2737_c40/f1p0/2830	gnl|BL_ORD_ID|38133 cb2737_c21/f1p2/3479 isoform=cb2737_c21;full_length_coverage=1;non_full_length_coverage=2;isoform_length=3479;expected_accur	1	1397	1395	2651	606	2003	2135	3391

	acy=1.000								
cb5244_c15/f1p3/2886	gnl|BL_ORD_ID|45884 cb5244_c7/f1p0/4048 isoform=cb5244_c7;full_length_coverage=1;non_full_length_coverage=0;isoform_length=4048;expected_accuracy=0.997	293	2892	40	294	1155	3759	36	291
cb8564_c181853/f1p0/2119 isoform=cb8564_c181853;full_length_coverage=1;non_full_length_coverage=0;isoform_length=2119;expected_accuracy=0.997	gnl|BL_ORD_ID|54438 cb8564_c46124/f1p0/3383	1	1339	1334	2119	151	1491	2319	3107
cb8564_c69290/f5p0/2257 isoform=cb8564_c69290;full_length_coverage=5;non_full_length_coverage=0;isoform_length=2257;expected_accuracy=1.000	gnl|BL_ORD_ID|11067 cb8564_c111307/f1p0/2509 isoform=cb8564_c111307;full_length_coverage=1;non_full_length_coverage=0;isoform_length=2509;expected_accuracy=0.999	505	2254	99	506	760	2509	1	408
cb8564_c148828/f1p1/3417 isoform=cb8564_c148828;full_length_coverage=1;non_full_length_coverage=1;isoform_length=3417;expected_accuracy=0.993	gnl|BL_ORD_ID|16525 cb8564_c74618/f1p0/3734 isoform=cb8564_c74618;full_length_coverage=1;non_full_length_coverage=0;isoform_length=3734;expected_accuracy=0.998	1	2541	2536	3417	1	2536	2650	3532
cb8564_c70577/f1p0/3111	gnl|BL_ORD_ID|58074 cb8564_c88129/f1p3/3262	1	2109	2106	2866	3	2110	2394	3154
cb18456_c6530/f1p0/1200	gnl|BL_ORD_ID|52890 cb8564_c111083/f1p0/1916	260	1166	3	259	824	1729	37	293
cb8388_c8/f1p0/1792	gnl|BL_ORD_ID|9888 cb8388_c1/f2p0/2359 isoform=cb8388_c1;full_length_coverage=2;non_full_length_coverage=0;isoform_length=2359;expected_accuracy=0.996	415	1655	50	418	1119	2359	1	369
cb8564_c14882/f1p0/2156	gnl|BL_ORD_ID|12527 cb8564_c15023/f2p0/2552	1	1644	1645	2060	30	167	2137	255

	isoform=cb8564_c15023;full_length_coverage=2;non_full_length_coverage=0;isoform_length=2552;expected_accuracy=0.996						4		2
cb9467_c4/f9p0/2081 isoform=cb9467_c4;full_length_coverage=9;non_full_length_coverage=0;isoform_length=2081;expected_accuracy=0.999	gnl|BL_ORD_ID|21567 cb9467_c27/f1p0/2294 isoform=cb9467_c27;full_length_coverage=1;non_full_length_coverage=0;isoform_length=2294;expected_accuracy=0.999	161	2081	1	162	352	2259	3	169
cb8564_c82521/f1p0/2413 isoform=cb8564_c82521;full_length_coverage=1;non_full_length_coverage=0;isoform_length=2413;expected_accuracy=0.999	gnl|BL_ORD_ID|12496 cb8564_c14618/f5p1/3581 isoform=cb8564_c14618;full_length_coverage=5;non_full_length_coverage=1;isoform_length=3581;expected_accuracy=0.997	331	2413	4	333	1410	3494	1	329
cb8564_c70945/f6p3/2821 isoform=cb8564_c70945;full_length_coverage=6;non_full_length_coverage=3;isoform_length=2821;expected_accuracy=1.000	gnl|BL_ORD_ID|57248 cb8564_c88028/f1p1/3776	222	2774	20	224	1248	3800	1	202
cb8564_c20551/f2p0/2429 isoform=cb8564_c20551;full_length_coverage=2;non_full_length_coverage=0;isoform_length=2429;expected_accuracy=0.999	gnl|BL_ORD_ID|16092 cb8564_c72709/f5p1/2784 isoform=cb8564_c72709;full_length_coverage=5;non_full_length_coverage=1;isoform_length=2784;expected_accuracy=0.997	17	2235	2235	2429	1	2218	2507	2701
cb8564_c45300/f1p4/3014 isoform=cb8564_c45300;full_length_coverage=1;non_full_length_coverage=4;isoform_length=3014;expected_accuracy=0.996	gnl|BL_ORD_ID|55182 cb8564_c34825/f1p2/3200	243	3014	11	246	394	3194	6	237
cb8564_c70365/f1p1/3583 isoform=cb8564_c70365;full_length_coverage=1;non_full_length_coverage=1;isoform_length=3583;expected_	gnl|BL_ORD_ID|57853 cb8564_c82558/f1p0/3707	1416	3583	1	1415	1557	3727	7	1421

accuracy=0.997									
cb4745_c12/f1p0/2017 isoform=cb4745_c12;full_length_coverage=1;non_full_length_coverage=0;isoform_length=2017;expected_accuracy=0.996	gnl|BL_ORD_ID|43415 cb4745_c2/f4p1/3063 isoform=cb4745_c2;full_length_coverage=4;non_full_length_coverage=1;isoform_length=3063;expected_accuracy=1.000	156	2017	15	161	1197	3062	14	156
cb5900_c74/f1p0/3322 isoform=cb5900_c74;full_length_coverage=1;non_full_length_coverage=0;isoform_length=3322;expected_accuracy=0.996	gnl|BL_ORD_ID|45474 cb5900_c73/f1p0/3430 isoform=cb5900_c73;full_length_coverage=1;non_full_length_coverage=0;isoform_length=3430;expected_accuracy=0.995	120	3221	1	120	321	3429	12	131
cb8564_c68409/f2p0/2141 isoform=cb8564_c68409;full_length_coverage=2;non_full_length_coverage=0;isoform_length=2141;expected_accuracy=0.995	gnl|BL_ORD_ID|56470 cb8564_c77506/f1p0/3897	264	2141	61	266	2024	3885	1	206
cb6111_c6/f3p1/1599 isoform=cb6111_c6;full_length_coverage=3;non_full_length_coverage=1;isoform_length=1599;expected_accuracy=1.000	gnl|BL_ORD_ID|74915 cb6111_c17/f1p0/1775	165	1598	54	166	251	1681	14	126
cb6039_c51/f1p1/2182 isoform=cb6039_c51;full_length_coverage=1;non_full_length_coverage=1;isoform_length=2182;expected_accuracy=0.991	gnl|BL_ORD_ID|74797 cb6039_c8/f1p0/2609	1	1418	1413	2182	24	1442	1761	2533
cb8564_c46939/f1p1/2926	gnl|BL_ORD_ID|56764 cb8564_c70795/f1p0/3769	19	2134	2130	2870	34	2159	2264	3005
cb8564_c40978/f1p0/3073	gnl|BL_ORD_ID|14407 cb8564_c44036/f1p0/3258 isoform=cb8564_c44036;full_length_coverage=1;non_full_length_coverage=0;isoform_length=3258;expected_ac	1159	2845	1	1159	1575	3258	1	1147

	curacy=0.992								
cb8564_c3239/f1p0/1871	gnl|BL_ORD_ID|54534 cb8564_c125514/f1p0/2141	221	1815	32	222	360	1953	17	204
cb8564_c186243/f1p0/2948 isoform=cb8564_c186243;full_length_coverage=1;non_full_length_coverage=0;isoform_length=2948;expected_accuracy=0.995	gnl|BL_ORD_ID|54377 cb8564_c23287/f1p3/4458	19	1986	1981	2948	1106	3076	3240	4208
cb5013_c16/f1p0/2722	gnl|BL_ORD_ID|42367 cb5013_c15/f1p0/2652 isoform=cb5013_c15;full_length_coverage=1;non_full_length_coverage=0;isoform_length=2652;expected_accuracy=0.999	231	2524	12	231	380	2652	1	221
cb11284_c6/f1p0/1725 isoform=cb11284_c6;full_length_coverage=1;non_full_length_coverage=0;isoform_length=1725;expected_accuracy=0.994	gnl|BL_ORD_ID|60127 cb11284_c3/f1p0/2604	1	1442	1440	1725	27	1468	1858	2143
cb4544_c1/f9p1/2005 isoform=cb4544_c1;full_length_coverage=9;non_full_length_coverage=1;isoform_length=2005;expected_accuracy=1.000	gnl|BL_ORD_ID|72660 cb4544_c8/f1p0/2838	521	1974	9	524	1149	2599	7	518
cb4047_c8/f1p0/2511	gnl|BL_ORD_ID|40224 cb4047_c18/f14p4/2895 isoform=cb4047_c18;full_length_coverage=14;non_full_length_coverage=4;isoform_length=2895;expected_accuracy=1.000	1	1760	1756	2495	31	1799	1922	2661
cb1380_c6/f4p1/2530 isoform=cb1380_c6;full_length_coverage=4;non_full_length_coverage=1;isoform_length=2530;expected_accur	gnl|BL_ORD_ID|61310 cb1380_c37/f1p0/3199	1	1699	1698	2530	7	1705	2358	3190

acy=1.000									
cb6802_c52/f1p0/2612	gnl|BL_ORD_ID|50085 cb6802_c53/f1p0/3720	167	2577	1	168	1251	365 9	3	194
cb8564_c79401/f1p0/4816	gnl|BL_ORD_ID|35084 cb18812_c7/f1p1/5089 isoform=cb18812_c7;full_length_coverage=1;non_full_length_coverage=1;isoform_length=5089;expected_accuracy=0.998	1072	4398	1	1068	1760	508 9	807	187 3
cb8564_c23232/f1p3/2540	gnl|BL_ORD_ID|13063 cb8564_c179457/f1p0/2647 isoform=cb8564_c179457;full_length_coverage=1;non_full_length_coverage=0;isoform_length=2647;expected_accuracy=1.000	16	2172	2167	2518	5	214 0	2242	259 3
cb2520_c14/f1p1/2870 isoform=cb2520_c14;full_length_coverage=1;non_full_length_coverage=1;isoform_length=2870;expected_accuracy=0.996	gnl|BL_ORD_ID|36518 cb2520_c4/f2p0/3911 isoform=cb2520_c4;full_length_coverage=2;non_full_length_coverage=0;isoform_length=3911;expected_accuracy=0.999	103	2840	1	104	1177	3911	571	676
cb4745_c12/f1p0/2017 isoform=cb4745_c12;full_length_coverage=1;non_full_length_coverage=0;isoform_length=2017;expected_accuracy=0.996	gnl|BL_ORD_ID|72928 cb4745_c8/f1p0/2875	156	2016	15	161	1049	291 3	11	156
cb8564_c20081/f1p0/2720 isoform=cb8564_c20081;full_length_coverage=1;non_full_length_coverage=0;isoform_length=2720;expected_accuracy=0.995	gnl|BL_ORD_ID|53927 cb8564_c15139/f1p1/4122	16	1613	1609	2720	225	181 5	2805	391 6
cb8564_c2600/f1p0/2432 isoform=cb8564_c2600;full_length_coverage=1;non_full_length_coverage=0;isoform_length=2432;expected_ac	gnl|BL_ORD_ID|53667 cb8564_c151431/f1p0/2629	1	1400	1399	2432	101	149 0	1607	263 9

curacy=1.000									
cb4576_c161/f5p0/3997 isoform=cb4576_c161;full_length_coverage=5;non_full_length_coverage=0;isoform_length=3997;expected_accuracy=1.000	gnl|BL_ORD_ID|44415 cb4576_c54/f1p0/4341 isoform=cb4576_c54;full_length_coverage=1;non_full_length_coverage=0;isoform_length=4341;expected_accuracy=0.995	1841	3887	1	1842	2301	4341	10	1837
cb4482_c1/f1p0/2752 isoform=cb4482_c1;full_length_coverage=1;non_full_length_coverage=0;isoform_length=2752;expected_accuracy=0.997	gnl|BL_ORD_ID|44277 cb4482_c0/f1p0/3335 isoform=cb4482_c0;full_length_coverage=1;non_full_length_coverage=0;isoform_length=3335;expected_accuracy=0.998	638	2752	90	640	1214	3327	1	550
cb7595_c14/f1p1/2186 isoform=cb7595_c14;full_length_coverage=1;non_full_length_coverage=1;isoform_length=2186;expected_accuracy=0.995	gnl|BL_ORD_ID|7651 cb7595_c13/f1p1/2460 isoform=cb7595_c13;full_length_coverage=1;non_full_length_coverage=1;isoform_length=2460;expected_accuracy=0.994	267	2185	33	266	540	2460	160	398
cb8564_c10682/f1p0/1913 isoform=cb8564_c10682;full_length_coverage=1;non_full_length_coverage=0;isoform_length=1913;expected_accuracy=0.994	gnl|BL_ORD_ID|12478 cb8564_c152519/f2p1/2103 isoform=cb8564_c152519;full_length_coverage=2;non_full_length_coverage=1;isoform_length=2103;expected_accuracy=1.000	10	1045	1041	1913	1	1037	1208	2081
cb18456_c5009/f1p0/1645	gnl|BL_ORD_ID|58142 cb8564_c89861/f1p27/2678	172	1379	4	167	1485	2693	1486	1648
cb1956_c5/f2p0/2813 isoform=cb1956_c5;full_length_coverage=2;non_full_length_coverage=0;isoform_length=2813;expected_accuracy=0.997	gnl|BL_ORD_ID|66542 cb1956_c22/f1p0/3042	14	1450	1451	2813	3	1437	1600	2963
cb8564_c79364/f1p0/2690 isoform=cb8564_c79364;full_length_coverage=1;non_full_length_coverage=0;isoform_length=2690;expected_	gnl|BL_ORD_ID|16128 cb8564_c70982/f4p0/3076 isoform=cb8564_c70982;full_length_coverage=4;non_full_length_coverage=0;isoform_length=3076;expected_ac	1	1379	1377	2690	34	1400	1607	2922

accuracy=0.997	curacy=0.994								
cb454_c33/f1p0/2337 isoform=cb454_c33;full_length_coverage=1;non_full_length_coverage=0;isoform_length=2337;expected_accuracy=0.997	gnl|BL_ORD_ID|72201 cb454_c19/f1p0/2558	252	2302	69	251	406	2453	118	300
cb9549_c8/f1p0/1542 isoform=cb9549_c8;full_length_coverage=1;non_full_length_coverage=0;isoform_length=1542;expected_accuracy=1.000	gnl|BL_ORD_ID|21592 cb9549_c6/f1p0/1758 isoform=cb9549_c6;full_length_coverage=1;non_full_length_coverage=0;isoform_length=1758;expected_accuracy=0.995	114	1542	1	113	256	1688	1	113
cb8564_c42506/f1p0/2909	gnl|BL_ORD_ID|15334 cb8564_c36574/f14p7/3838 isoform=cb8564_c36574;full_length_coverage=14;non_full_length_coverage=7;isoform_length=3838;expected_accuracy=1.000	1	2203	2199	2857	1025	3227	3097	3755
cb8564_c112719/f1p0/2721	gnl|BL_ORD_ID|36102 cb18634_c6/f1p0/5260 isoform=cb18634_c6;full_length_coverage=1;non_full_length_coverage=0;isoform_length=5260;expected_accuracy=0.998	1078	2586	1	1077	3709	5215	2505	3582
cb8564_c18821/f1p0/2573 isoform=cb8564_c18821;full_length_coverage=1;non_full_length_coverage=0;isoform_length=2573;expected_accuracy=0.996	gnl|BL_ORD_ID|12647 cb8564_c146156/f5p1/2754 isoform=cb8564_c146156;full_length_coverage=5;non_full_length_coverage=1;isoform_length=2754;expected_accuracy=1.000	1	1625	1625	2573	41	1665	1803	2752
cb8564_c68180/f8p0/3575 isoform=cb8564_c68180;full_length_coverage=8;non_full_length_coverage=0;isoform_length=3575;expected_accuracy=1.000	gnl|BL_ORD_ID|18350 cb8564_c89194/f2p0/4007 isoform=cb8564_c89194;full_length_coverage=2;non_full_length_coverage=0;isoform_length=4007;expected_accuracy=0.998	1725	3492	1	1729	2239	4007	4	1724
cb8564_c79364/f1p0/2690	gnl|BL_ORD_ID|10197 cb8564_c107583/f8p0/3100	1	1379	1377	2690	200	156	1774	308

isoform=cb8564_c79364;full_length_coverage=1;non_full_length_coverage=0;isoform_length=2690;expected_accuracy=0.997	isoform=cb8564_c107583;full_length_coverage=8;non_full_length_coverage=0;isoform_length=3100;expected_accuracy=1.000						8		9
cb8564_c73244/f1p0/2654 isoform=cb8564_c73244;full_length_coverage=1;non_full_length_coverage=0;isoform_length=2654;expected_accuracy=0.991	gnl|BL_ORD_ID|34790 cb18769_c6/f1p0/7712 isoform=cb18769_c6;full_length_coverage=1;non_full_length_coverage=0;isoform_length=7712;expected_accuracy=0.997	1	1341	1337	2654	2948	4286	6382	7696
cb1915_c3/f7p0/1759 isoform=cb1915_c3;full_length_coverage=7;non_full_length_coverage=0;isoform_length=1759;expected_accuracy=1.000	gnl|BL_ORD_ID|36464 cb1915_c9/f1p0/2392 isoform=cb1915_c9;full_length_coverage=1;non_full_length_coverage=0;isoform_length=2392;expected_accuracy=0.998	496	1759	63	497	1078	2364	2	437
cb6704_c13/f2p1/1820 isoform=cb6704_c13;full_length_coverage=2;non_full_length_coverage=1;isoform_length=1820;expected_accuracy=1.000	gnl|BL_ORD_ID|5106 cb6704_c3/f1p1/2596 isoform=cb6704_c3;full_length_coverage=1;non_full_length_coverage=1;isoform_length=2596;expected_accuracy=1.000	1	1391	1387	1820	1	1386	2151	2584
cb242_c10/f1p2/2788 isoform=cb242_c10;full_length_coverage=1;non_full_length_coverage=2;isoform_length=2788;expected_accuracy=0.995	gnl|BL_ORD_ID|36521 cb242_c4/f1p1/4899 isoform=cb242_c4;full_length_coverage=1;non_full_length_coverage=1;isoform_length=4899;expected_accuracy=0.997	222	2784	2	224	2376	4886	502	719
cb18786_c0/f1p0/5838	gnl|BL_ORD_ID|31380 cb17283_c5/f1p1/5374 isoform=cb17283_c5;full_length_coverage=1;non_full_length_coverage=1;isoform_length=5374;expected_accuracy=0.998	28	4905	4902	5264	1	4878	5012	5374
cb11646_c1/f1p0/1708 isoform=cb11646_c1;full_length_coverage=1;non_full_length_coverage=0;isoform_length=1708;expected_accu	gnl|BL_ORD_ID|23038 cb11646_c2/f1p0/1951 isoform=cb11646_c2;full_length_coverage=1;non_full_length_coverage=0;isoform_length=1951;expected_accur	1	1168	1167	1708	1	1166	1379	1921

racy=0.996	acy=0.993								
cb8564_c123270/f1p0/1927	gnl|BL_ORD_ID|15055 cb8564_c3236/f1p0/1961 isoform=cb8564_c3236;full_length_coverage=1;non_full_length_coverage=0;isoform_length=1961;expected_accuracy=1.000	244	1812	43	245	377	1945	6	208
cb8587_c0/f4p0/1923 isoform=cb8587_c0;full_length_coverage=4;non_full_length_coverage=0;isoform_length=1923;expected_accuracy=1.000	gnl|BL_ORD_ID|19180 cb8587_c3/f1p0/2327 isoform=cb8587_c3;full_length_coverage=1;non_full_length_coverage=0;isoform_length=2327;expected_accuracy=0.995	474	1923	54	474	877	2326	1	420
cb3570_c13/f1p0/3155	gnl|BL_ORD_ID|39906 cb3570_c7/f1p0/3031 isoform=cb3570_c7;full_length_coverage=1;non_full_length_coverage=0;isoform_length=3031;expected_accuracy=0.997	269	2888	16	269	381	2998	27	279
cb8564_c83196/f1p0/2505 isoform=cb8564_c83196;full_length_coverage=1;non_full_length_coverage=0;isoform_length=2505;expected_accuracy=0.995	gnl|BL_ORD_ID|56909 cb8564_c76562/f1p1/4711	1	1642	1642	2503	2023	3646	3759	4620
cb5896_c4/f6p1/3033 isoform=cb5896_c4;full_length_coverage=6;non_full_length_coverage=1;isoform_length=3033;expected_accuracy=1.000	gnl|BL_ORD_ID|45880 cb5896_c35/f1p0/3617 isoform=cb5896_c35;full_length_coverage=1;non_full_length_coverage=0;isoform_length=3617;expected_accuracy=0.997	106	2171	2167	3033	1	2067	2749	3615
cb13565_c52/f1p3/1664	gnl|BL_ORD_ID|49739 cb6452_c8/f1p4/2559	474	1655	64	473	1263	2441	22	431
cb820_c5/f1p0/6444 isoform=cb820_c5;full_length_coverage=1;non_full_length_coverage=0;isoform_length=6444;expected_accurac	gnl|BL_ORD_ID|51001 cb820_c3/f1p0/8069	1577	6348	1	1582	3250	8036	1285	2870

y=0.995									
cb18456_c1776/f1p0/1211	gnl|BL_ORD_ID|69245 cb18456_c1770/f1p0/1415	280	1214	3	285	397	1331	2	285
cb6036_c5/f1p0/2446 isoform=cb6036_c5;full_length_coverage=1;non_full_length_coverage=0;isoform_length=2446;expected_accuracy=0.993	gnl|BL_ORD_ID|74793 cb6036_c12/f1p0/2846	1061	2437	1	1062	1377	2753	5	1066
cb8564_c139315/f1p2/3949 isoform=cb8564_c139315;full_length_coverage=1;non_full_length_coverage=2;isoform_length=3949;expected_accuracy=0.999	gnl|BL_ORD_ID|56594 cb8564_c47777/f1p0/4391	213	3825	1	214	476	4099	2	215
cb8564_c86222/f1p1/3397 isoform=cb8564_c86222;full_length_coverage=1;non_full_length_coverage=1;isoform_length=3397;expected_accuracy=0.999	gnl|BL_ORD_ID|16157 cb8564_c77802/f1p0/3651 isoform=cb8564_c77802;full_length_coverage=1;non_full_length_coverage=0;isoform_length=3651;expected_accuracy=0.996	114	2646	2647	3397	160	2693	2810	3558
cb8564_c145910/f8p2/2344 isoform=cb8564_c145910;full_length_coverage=8;non_full_length_coverage=2;isoform_length=2344;expected_accuracy=0.995	gnl|BL_ORD_ID|13514 cb8564_c21005/f1p1/4445 isoform=cb8564_c21005;full_length_coverage=1;non_full_length_coverage=1;isoform_length=4445;expected_accuracy=0.999	106	1942	1939	2344	1	1837	3560	3964
cb3309_c14/f1p0/2567	gnl|BL_ORD_ID|40140 cb3309_c4/f1p1/3664 isoform=cb3309_c4;full_length_coverage=1;non_full_length_coverage=1;isoform_length=3664;expected_accuracy=0.995	160	2536	1	162	1269	3643	34	200
cb5962_c9/f1p2/2694 isoform=cb5962_c9;full_length_coverage=1;non_full_length_coverage=2;isoform_length=2694;expected_accur	gnl|BL_ORD_ID|74719 cb5962_c18/f1p0/3273	1	1390	1386	2688	1	1390	1524	2827

acy=0.998									
cb6611_c5/f1p0/2660 isoform=cb6611_c5;full_length_coverage=1;non_full_length_coverage=0;isoform_length=2660;expected_accuracy=1.000	gnl|BL_ORD_ID|49870 cb6611_c2/f1p0/2900	1	1663	1662	2660	41	1703	1879	2877
cb5472_c5/f1p0/3130 isoform=cb5472_c5;full_length_coverage=1;non_full_length_coverage=0;isoform_length=3130;expected_accuracy=0.995	gnl|BL_ORD_ID|45834 cb5472_c6/f1p0/3267 isoform=cb5472_c6;full_length_coverage=1;non_full_length_coverage=0;isoform_length=3267;expected_accuracy=0.991	1	2614	2615	3069	22	2624	2813	3267
cb8564_c121763/f1p2/2624 isoform=cb8564_c121763;full_length_coverage=1;non_full_length_coverage=2;isoform_length=2624;expected_accuracy=0.997	gnl|BL_ORD_ID|10158 cb8564_c108914/f6p2/2897 isoform=cb8564_c108914;full_length_coverage=6;non_full_length_coverage=2;isoform_length=2897;expected_accuracy=0.998	1	2416	2417	2624	12	2431	2603	2810
cb3265_c11/f1p0/2135 isoform=cb3265_c11;full_length_coverage=1;non_full_length_coverage=0;isoform_length=2135;expected_accuracy=1.000	gnl|BL_ORD_ID|41416 cb3265_c14/f1p0/2965 isoform=cb3265_c14;full_length_coverage=1;non_full_length_coverage=0;isoform_length=2965;expected_accuracy=0.995	505	2111	66	504	1359	2965	44	482
cb5208_c10/f1p1/2768 isoform=cb5208_c10;full_length_coverage=1;non_full_length_coverage=1;isoform_length=2768;expected_accuracy=0.991	gnl|BL_ORD_ID|44771 cb5208_c4/f1p0/3236 isoform=cb5208_c4;full_length_coverage=1;non_full_length_coverage=0;isoform_length=3236;expected_accuracy=0.997	1	1953	1952	2767	226	2180	2412	3234
cb965_c21/f1p0/3360	gnl|BL_ORD_ID|19288 cb965_c14/f1p0/4481 isoform=cb965_c14;full_length_coverage=1;non_full_length_coverage=0;isoform_length=4481;expected_accuracy=0.998	1170	3298	1	1171	2234	4362	803	1972
cb792_c53/f1p0/3930	gnl|BL_ORD_ID|49470 cb792_c35/f1p1/3973	1	1842	1838	3677	86	191	2047	388

							6		5
cb12698_c17/f1p3/1760 isoform=cb12698_c17;full_length_coverage=1;non_full_length_coverage=3;isoform_length=1760;expected_accuracy=1.000	gnl|BL_ORD_ID|21291 cb9797_c33/f1p1/2260 isoform=cb9797_c33;full_length_coverage=1;non_full_length_coverage=1;isoform_length=2260;expected_accuracy=0.995	1	1222	1219	1715	66	1287	1766	2260
cb8564_c45596/f1p1/3430	gnl|BL_ORD_ID|56080 cb8564_c54387/f1p0/4415	2	2780	2775	3379	2	2783	2902	3506
cb454_c8/f1p0/2277 isoform=cb454_c8;full_length_coverage=1;non_full_length_coverage=0;isoform_length=2277;expected_accuracy=1.000	gnl|BL_ORD_ID|42150 cb454_c1/f3p0/2382 isoform=cb454_c1;full_length_coverage=3;non_full_length_coverage=0;isoform_length=2382;expected_accuracy=1.000	277	2277	94	276	296	2296	7	189
cb14044_c6/f1p0/1650 isoform=cb14044_c6;full_length_coverage=1;non_full_length_coverage=0;isoform_length=1650;expected_accuracy=0.996	gnl|BL_ORD_ID|6014 cb6181_c10/f2p2/1946 isoform=cb6181_c10;full_length_coverage=2;non_full_length_coverage=2;isoform_length=1946;expected_accuracy=0.998	1	1019	1015	1650	16	1045	1179	1814
cb653_c9/f1p0/4025 isoform=cb653_c9;full_length_coverage=1;non_full_length_coverage=0;isoform_length=4025;expected_accuracy=0.997	gnl|BL_ORD_ID|5129 cb653_c6/f1p0/4151 isoform=cb653_c6;full_length_coverage=1;non_full_length_coverage=0;isoform_length=4151;expected_accuracy=0.996	301	4025	1	301	414	4140	6	308
cb15567_c9/f1p1/1733 isoform=cb15567_c9;full_length_coverage=1;non_full_length_coverage=1;isoform_length=1733;expected_accuracy=0.994	gnl|BL_ORD_ID|12512 cb8564_c131767/f1p0/1982 isoform=cb8564_c131767;full_length_coverage=1;non_full_length_coverage=0;isoform_length=1982;expected_accuracy=0.997	1	1626	1622	1733	1	1626	1739	1850
cb18456_c2426/f1p0/1054 isoform=cb18456_c2426;full_length_coverage=1;non_full_length_coverage=0;isoform_length=1054;expected_	gnl|BL_ORD_ID|67421 cb18456_c2446/f1p3/1392	498	1054	5	500	717	1273	13	509

accuracy=0.993									
cb1648_c15/f1p0/2133 isoform=cb1648_c15;full_length_coverage=1;non_full_length_coverage=0;isoform_length=2133;expected_accuracy=1.000	gnl|BL_ORD_ID|63818 cb1648_c13/f1p0/4230	1	1159	1159	2133	3	1162	2988	3962
cb6719_c45/f1p0/1995	gnl|BL_ORD_ID|4665 cb6719_c23/f1p3/2664 isoform=cb6719_c23;full_length_coverage=1;non_full_length_coverage=3;isoform_length=2664;expected_accuracy=1.000	276	2007	7	279	765	2497	169	442
cb8564_c12781/f1p0/3625	gnl|BL_ORD_ID|53989 cb8564_c16516/f1p0/4473	1462	3632	1	1467	1823	3993	1	1468
cb27_c7/f1p0/2206	gnl|BL_ORD_ID|37260 cb27_c6/f1p0/2861 isoform=cb27_c6;full_length_coverage=1;non_full_length_coverage=0;isoform_length=2861;expected_accuracy=0.996	1	1524	1522	2243	130	1652	1966	2688
cb8564_c148330/f2p0/2897 isoform=cb8564_c148330;full_length_coverage=2;non_full_length_coverage=0;isoform_length=2897;expected_accuracy=0.995	gnl|BL_ORD_ID|56345 cb8564_c74914/f1p0/3530	1278	2892	1	1283	1497	3103	2	1284
cb5125_c36/f1p0/1789 isoform=cb5125_c36;full_length_coverage=1;non_full_length_coverage=0;isoform_length=1789;expected_accuracy=0.998	gnl|BL_ORD_ID|43453 cb5125_c17/f1p2/2087 isoform=cb5125_c17;full_length_coverage=1;non_full_length_coverage=2;isoform_length=2087;expected_accuracy=0.998	1	1163	1158	1756	74	1248	1454	2052
cb2758_c6/f1p1/3278 isoform=cb2758_c6;full_length_coverage=1;non_full_length_coverage=1;isoform_length=3278;expected_accur	gnl|BL_ORD_ID|69950 cb2758_c12/f1p0/3518	271	3278	113	272	319	3327	47	206

acy=1.000									
cb1278_c4/f2p0/2603 isoform=cb1278_c4;full_length_coverage=2;non_full_length_coverage=0;isoform_length=2603;expected_accuracy=0.997	gnl|BL_ORD_ID|27260 cb1278_c6/f1p0/3038 isoform=cb1278_c6;full_length_coverage=1;non_full_length_coverage=0;isoform_length=3038;expected_accuracy=0.996	1	2019	2017	2603	9	2026	2416	3002
cb234_c1/f1p1/4574 isoform=cb234_c1;full_length_coverage=1;non_full_length_coverage=1;isoform_length=4574;expected_accuracy=0.997	gnl|BL_ORD_ID|69492 cb234_c6/f1p0/4641	4	4236	4231	4531	4	4252	4369	4669
cb9780_c1/f2p0/1733 isoform=cb9780_c1;full_length_coverage=2;non_full_length_coverage=0;isoform_length=1733;expected_accuracy=0.995	gnl|BL_ORD_ID|58886 cb9780_c3/f1p0/2086	117	1733	1	120	264	1882	6	126
cb8564_c69658/f1p1/2292 isoform=cb8564_c69658;full_length_coverage=1;non_full_length_coverage=1;isoform_length=2292;expected_accuracy=0.996	gnl|BL_ORD_ID|17527 cb8564_c84215/f1p0/3183 isoform=cb8564_c84215;full_length_coverage=1;non_full_length_coverage=0;isoform_length=3183;expected_accuracy=0.997	1	1803	1801	2292	1	1810	2697	3182
cb10513_c3/f1p0/1999	gnl|BL_ORD_ID|55658 cb8564_c3692/f1p0/3493	1	1445	1444	1842	22	1464	2561	2959
cb8564_c39546/f1p2/3023	gnl|BL_ORD_ID|14407 cb8564_c44036/f1p0/3258 isoform=cb8564_c44036;full_length_coverage=1;non_full_length_coverage=0;isoform_length=3258;expected_accuracy=0.992	1148	2835	1	1148	1575	3258	1	1147
cb8564_c2326/f1p0/2006 isoform=cb8564_c2326;full_length_coverage=1;non_full_length_coverage=0;isoform_length=2006;expected_ac	gnl|BL_ORD_ID|13707 cb8564_c2273/f1p2/2502 isoform=cb8564_c2273;full_length_coverage=1;non_full_length_coverage=2;isoform_length=2502;expected_ac	230	2006	48	234	721	2502	56	243

curacy=0.990	curacy=0.995								
cb1279_c77/f1p0/3526 isoform=cb1279_c77;full_length_coverage=1;non_full_length_coverage=0;isoform_length=3526;expected_accuracy=0.994	gnl|BL_ORD_ID|61182 cb1279_c29/f1p0/3632	1	2976	2972	3526	1	2986	3099	3655
cb8564_c53266/f1p2/2976	gnl|BL_ORD_ID|16809 cb8564_c53349/f1p1/4983 isoform=cb8564_c53349;full_length_coverage=1;non_full_length_coverage=1;isoform_length=4983;expected_accuracy=0.997	1	2754	2751	2986	1874	4625	4747	4982
cb1842_c14/f1p0/2978	gnl|BL_ORD_ID|66406 cb1842_c16/f1p0/3507	17	2154	2151	2855	2	2139	2604	3308
cb8564_c42513/f1p2/3770 isoform=cb8564_c42513;full_length_coverage=1;non_full_length_coverage=2;isoform_length=3770;expected_accuracy=0.997	gnl|BL_ORD_ID|55981 cb8564_c51070/f1p0/5238	1	2946	2947	3762	41	2972	3304	4116
cb2838_c1/f1p0/2301 isoform=cb2838_c1;full_length_coverage=1;non_full_length_coverage=0;isoform_length=2301;expected_accuracy=0.996	gnl|BL_ORD_ID|37788 cb2838_c0/f1p0/3795 isoform=cb2838_c0;full_length_coverage=1;non_full_length_coverage=0;isoform_length=3795;expected_accuracy=0.994	1072	2301	1	1072	1184	2412	5	1073
cb6238_c2/f2p0/2826 isoform=cb6238_c2;full_length_coverage=2;non_full_length_coverage=0;isoform_length=2826;expected_accuracy=1.000	gnl|BL_ORD_ID|5359 cb6238_c5/f1p0/3446 isoform=cb6238_c5;full_length_coverage=1;non_full_length_coverage=0;isoform_length=3446;expected_accuracy=0.994	1	1898	1899	2826	3	1895	2427	3348
cb3843_c6/f1p0/2919 isoform=cb3843_c6;full_length_coverage=1;non_full_length_coverage=0;isoform_length=2919;expected_accur	gnl|BL_ORD_ID|39769 cb3843_c4/f1p0/3395 isoform=cb3843_c4;full_length_coverage=1;non_full_length_coverage=0;isoform_length=3395;expected_accura	1	1877	1875	2919	168	2058	2267	3320

acy=0.992	cy=0.992								
cb8564_c39136/f1p0/3036 isoform=cb8564_c39136;full_length_coverage=1;non_full_length_coverage=0;isoform_length=3036;expected_accuracy=0.998	gnl|BL_ORD_ID|55243 cb8564_c36242/f1p0/3335	1	2089	2085	3035	1	2098	2225	3176
cb8564_c3504/f1p0/2587	gnl|BL_ORD_ID|15303 cb8564_c3481/f1p0/2830 isoform=cb8564_c3481;full_length_coverage=1;non_full_length_coverage=0;isoform_length=2830;expected_accuracy=0.995	16	1678	1674	2347	18	1676	2158	2830
cb5306_c6/f1p0/2511 isoform=cb5306_c6;full_length_coverage=1;non_full_length_coverage=0;isoform_length=2511;expected_accuracy=0.996	gnl|BL_ORD_ID|45769 cb5306_c4/f1p0/3133 isoform=cb5306_c4;full_length_coverage=1;non_full_length_coverage=0;isoform_length=3133;expected_accuracy=0.998	1211	2511	1	1214	1800	3102	5	1207
cb7563_c5/f2p1/2302 isoform=cb7563_c5;full_length_coverage=2;non_full_length_coverage=1;isoform_length=2302;expected_accuracy=1.000	gnl|BL_ORD_ID|7631 cb7563_c6/f1p2/2589 isoform=cb7563_c6;full_length_coverage=1;non_full_length_coverage=2;isoform_length=2589;expected_accuracy=1.000	242	2300	5	247	531	2589	1	244
cb146_c1/f3p0/3314 isoform=cb146_c1;full_length_coverage=3;non_full_length_coverage=0;isoform_length=3314;expected_accuracy=0.998	gnl|BL_ORD_ID|63558 cb146_c7/f1p0/3617	1182	3314	1	1177	1068	3209	1	1187
cb3290_c38/f1p0/2345 isoform=cb3290_c38;full_length_coverage=1;non_full_length_coverage=0;isoform_length=2345;expected_accuracy=0.996	gnl|BL_ORD_ID|40352 cb3290_c43/f1p9/2985 isoform=cb3290_c43;full_length_coverage=1;non_full_length_coverage=9;isoform_length=2985;expected_accuracy=0.992	1150	2345	1	1151	1584	2790	276	1426
cb5600_c17/f1p1/2696	gnl|BL_ORD_ID|45243 cb5600_c5/f1p0/2860	1	2172	2169	2696	1	217	2304	282

isoform=cb5600_c17;full_length_coverage=1;non_full_length_coverage=1;isoform_length=2696;expected_accuracy=0.997	isoform=cb5600_c5;full_length_coverage=1;non_full_length_coverage=0;isoform_length=2860;expected_accuracy=0.997						3		7
cb5447_c38/f1p1/3528 isoform=cb5447_c38;full_length_coverage=1;non_full_length_coverage=1;isoform_length=3528;expected_accuracy=0.997	gnl|BL_ORD_ID|45897 cb5447_c33/f1p0/4199 isoform=cb5447_c33;full_length_coverage=1;non_full_length_coverage=0;isoform_length=4199;expected_accuracy=0.997	1	2146	2146	3528	15	2155	2805	4183
cb8564_c178238/f1p1/2052 isoform=cb8564_c178238;full_length_coverage=1;non_full_length_coverage=1;isoform_length=2052;expected_accuracy=0.999	gnl|BL_ORD_ID|18132 cb8564_c89813/f1p0/3303 isoform=cb8564_c89813;full_length_coverage=1;non_full_length_coverage=0;isoform_length=3303;expected_accuracy=0.991	1005	2039	1	1007	2274	3303	35	1029
cb8564_c11653/f1p1/3278 isoform=cb8564_c11653;full_length_coverage=1;non_full_length_coverage=1;isoform_length=3278;expected_accuracy=0.999	gnl|BL_ORD_ID|54175 cb8564_c19847/f1p1/3929	1153	3278	1	1156	1726	3853	5	1162
cb3664_c10/f1p0/2454	gnl|BL_ORD_ID|41173 cb3664_c4/f1p2/3015 isoform=cb3664_c4;full_length_coverage=1;non_full_length_coverage=2;isoform_length=3015;expected_accuracy=0.997	128	2422	1	129	663	2957	97	225
cb1492_c9/f1p1/2823 isoform=cb1492_c9;full_length_coverage=1;non_full_length_coverage=1;isoform_length=2823;expected_accuracy=1.000	gnl|BL_ORD_ID|29399 cb1492_c3/f1p2/4122 isoform=cb1492_c3;full_length_coverage=1;non_full_length_coverage=2;isoform_length=4122;expected_accuracy=0.994	191	2823	1	196	1342	3964	7	201
cb8564_c36689/f1p1/2368 isoform=cb8564_c36689;full_length_coverage=1;non_full_length_coverage=1;isoform_length=2368;expected_	gnl|BL_ORD_ID|56655 cb8564_c51226/f1p0/3111	1	1709	1706	2368	2	1674	2088	2750

accuracy=0.991									
cb18275_c11/f1p1/1730	gnl|BL_ORD_ID|53567 cb8564_c128900/f1p0/3172	131	1733	1	134	1530	3132	1208	1341
cb8564_c44074/f1p1/2922 isoform=cb8564_c44074;full_length_coverage=1;non_full_length_coverage=1;isoform_length=2922;expected_accuracy=0.994	gnl|BL_ORD_ID|55920 cb8564_c48949/f1p0/4357	1	2267	2268	2922	2	2293	2847	3508
cb3061_c16/f1p0/2352 isoform=cb3061_c16;full_length_coverage=1;non_full_length_coverage=0;isoform_length=2352;expected_accuracy=0.990	gnl|BL_ORD_ID|69090 cb3061_c19/f1p0/3233	175	2352	1	180	892	3073	58	237
cb8564_c2602/f1p0/2480 isoform=cb8564_c2602;full_length_coverage=1;non_full_length_coverage=0;isoform_length=2480;expected_accuracy=0.995	gnl|BL_ORD_ID|53667 cb8564_c151431/f1p0/2629	1	1424	1423	2480	50	1482	1586	2639
cb3570_c9/f1p0/2911	gnl|BL_ORD_ID|39903 cb3570_c3/f1p0/3091 isoform=cb3570_c3;full_length_coverage=1;non_full_length_coverage=0;isoform_length=3091;expected_accuracy=0.995	155	2922	1	157	272	3039	1	157
cb8564_c35645/f1p0/2804	gnl|BL_ORD_ID|10800 cb8564_c10037/f1p5/2946 isoform=cb8564_c10037;full_length_coverage=1;non_full_length_coverage=5;isoform_length=2946;expected_accuracy=0.994	149	2726	1	151	365	2938	38	187
cb8564_c3643/f1p0/2518 isoform=cb8564_c3643;full_length_coverage=1;non_full_length_coverage=0;isoform_length=2518;expected_ac	gnl|BL_ORD_ID|54912 cb8564_c3637/f1p0/3675	356	2498	5	358	1459	3607	1	380

curacy=1.000									
cb8564_c112185/f1p1/2186 isoform=cb8564_c112185;full_length_coverage=1;non_full_length_coverage=1;isoform_length=2186;expected_accuracy=0.995	gnl|BL_ORD_ID|53478 cb8564_c126020/f1p1/2281	1	1968	1967	2176	15	1980	2084	2292
cb5270_c33/f1p0/2572	gnl|BL_ORD_ID|45098 cb5270_c38/f6p2/2827 isoform=cb5270_c38;full_length_coverage=6;non_full_length_coverage=2;isoform_length=2827;expected_accuracy=1.000	1098	2566	1	1097	1310	2778	111	1207
cb8564_c41756/f1p0/2738 isoform=cb8564_c41756;full_length_coverage=1;non_full_length_coverage=0;isoform_length=2738;expected_accuracy=0.996	gnl|BL_ORD_ID|13273 cb8564_c188193/f1p0/2849 isoform=cb8564_c188193;full_length_coverage=1;non_full_length_coverage=0;isoform_length=2849;expected_accuracy=0.997	1	2173	2170	2738	1	2158	2281	2849
cb8564_c39822/f1p0/3306	gnl|BL_ORD_ID|55939 cb8564_c49630/f1p4/3487	2	2912	2912	3160	1	2913	3243	3493
cb4098_c31/f1p2/3666 isoform=cb4098_c31;full_length_coverage=1;non_full_length_coverage=2;isoform_length=3666;expected_accuracy=0.998	gnl|BL_ORD_ID|40754 cb4098_c13/f2p1/3772 isoform=cb4098_c13;full_length_coverage=2;non_full_length_coverage=1;isoform_length=3772;expected_accuracy=0.998	160	1999	1999	3666	19	1867	2026	3711
cb8564_c53418/f1p0/2602 isoform=cb8564_c53418;full_length_coverage=1;non_full_length_coverage=0;isoform_length=2602;expected_accuracy=0.991	gnl|BL_ORD_ID|10681 cb8564_c10952/f1p1/2673 isoform=cb8564_c10952;full_length_coverage=1;non_full_length_coverage=1;isoform_length=2673;expected_accuracy=0.992	1163	2546	1	1168	1271	2671	2	1170
cb8564_c39136/f1p0/3036 isoform=cb8564_c39136;full_length_coverage=1;non_full_length_coverage=0;isoform_length=3036;expected_	gnl|BL_ORD_ID|55246 cb8564_c36343/f1p0/3448	2	2089	2085	3034	3	2112	2239	3188

accuracy=0.998									
cb8564_c188097/f1p0/2552 isoform=cb8564_c188097;full_length_coverage=1;non_full_length_coverage=0;isoform_length=2552;expected_accuracy=0.996	gnl|BL_ORD_ID|11957 cb8564_c135981/f1p0/2840 isoform=cb8564_c135981;full_length_coverage=1;non_full_length_coverage=0;isoform_length=2840;expected_accuracy=0.992	1	1945	1940	2552	189	2126	2229	2840
cb8564_c41756/f1p0/2738 isoform=cb8564_c41756;full_length_coverage=1;non_full_length_coverage=0;isoform_length=2738;expected_accuracy=0.996	gnl|BL_ORD_ID|16369 cb8564_c52191/f1p0/2822 isoform=cb8564_c52191;full_length_coverage=1;non_full_length_coverage=0;isoform_length=2822;expected_accuracy=0.997	132	2173	2170	2738	88	2131	2254	2822
cb2033_c17/f1p0/2200	gnl|BL_ORD_ID|66631 cb2033_c9/f1p0/2609	1	1577	1576	2195	2	1577	1689	2308
cb8564_c146670/f2p0/2135 isoform=cb8564_c146670;full_length_coverage=2;non_full_length_coverage=0;isoform_length=2135;expected_accuracy=0.998	gnl|BL_ORD_ID|13735 cb8564_c1944/f1p0/2509 isoform=cb8564_c1944;full_length_coverage=1;non_full_length_coverage=0;isoform_length=2509;expected_accuracy=0.993	129	2135	1	130	486	2492	12	142
cb8564_c181853/f1p0/2119 isoform=cb8564_c181853;full_length_coverage=1;non_full_length_coverage=0;isoform_length=2119;expected_accuracy=0.997	gnl|BL_ORD_ID|55317 cb8564_c38251/f1p0/3243	1	1339	1334	2119	1	1346	2188	2973
cb8564_c180116/f1p0/2944 isoform=cb8564_c180116;full_length_coverage=1;non_full_length_coverage=0;isoform_length=2944;expected_accuracy=0.996	gnl|BL_ORD_ID|16622 cb8564_c50151/f1p0/3198 isoform=cb8564_c50151;full_length_coverage=1;non_full_length_coverage=0;isoform_length=3198;expected_accuracy=0.995	1	2522	2520	2944	3	2543	2772	3196
cb8564_c83476/f1p0/2715 isoform=cb8564_c83476;full_length_coverage=1;non_full_length_coverage=0;isoform_length=2715;expected_	gnl|BL_ORD_ID|16335 cb8564_c72599/f1p2/2911 isoform=cb8564_c72599;full_length_coverage=1;non_full_length_coverage=2;isoform_length=2911;expected_ac	398	2714	4	398	545	2861	31	434

accuracy=0.996	curacy=0.997								
cb5643_c112/f1p0/1970 isoform=cb5643_c112;full_length_coverage=1;non_full_length_coverage=0;isoform_length=1970;expected_accuracy=0.992	gnl|BL_ORD_ID|45701 cb5643_c45/f1p0/3996 isoform=cb5643_c45;full_length_coverage=1;non_full_length_coverage=0;isoform_length=3996;expected_accuracy=0.999	1	1435	1434	1970	50	1473	3375	3905
cb6730_c3/f4p0/2261 isoform=cb6730_c3;full_length_coverage=4;non_full_length_coverage=0;isoform_length=2261;expected_accuracy=1.000	gnl|BL_ORD_ID|4797 cb6730_c26/f1p1/2468 isoform=cb6730_c26;full_length_coverage=1;non_full_length_coverage=1;isoform_length=2468;expected_accuracy=0.998	456	2261	7	455	624	2430	1	448
cb8564_c50881/f1p2/2125 isoform=cb8564_c50881;full_length_coverage=1;non_full_length_coverage=2;isoform_length=2125;expected_accuracy=0.994	gnl|BL_ORD_ID|11756 cb8564_c146467/f10p0/2494 isoform=cb8564_c146467;full_length_coverage=10;non_full_length_coverage=0;isoform_length=2494;expected_accuracy=1.000	1	1863	1858	2112	54	1912	2190	2447
cb8564_c36731/f1p2/2761	gnl|BL_ORD_ID|55243 cb8564_c36242/f1p0/3335	1	1977	1972	2737	1	1982	2411	3176
cb2033_c16/f1p0/2147 isoform=cb2033_c16;full_length_coverage=1;non_full_length_coverage=0;isoform_length=2147;expected_accuracy=0.994	gnl|BL_ORD_ID|36040 cb2033_c22/f1p0/2340 isoform=cb2033_c22;full_length_coverage=1;non_full_length_coverage=0;isoform_length=2340;expected_accuracy=0.991	1	1872	1868	2147	2	1897	2058	2336
cb8564_c75674/f1p0/2870	gnl|BL_ORD_ID|17094 cb8564_c78858/f1p0/2927 isoform=cb8564_c78858;full_length_coverage=1;non_full_length_coverage=0;isoform_length=2927;expected_accuracy=0.995	172	2694	1	173	378	2927	2	176
cb5218_c3/f1p0/2697 isoform=cb5218_c3;full_length_coverage=1;non_full_length_coverage=0;isoform_length=2697;expected_accur	gnl|BL_ORD_ID|46533 cb5218_c2/f1p0/3153 isoform=cb5218_c2;full_length_coverage=1;non_full_length_coverage=0;isoform_length=3153;expected_accura	1	2275	2273	2697	1	2268	2684	3108

acy=0.997	cy=0.996								
cb8564_c13767/f3p3/2285 isoform=cb8564_c13767;full_length_coverage=3;non_full_length_coverage=3;isoform_length=2285;expected_accuracy=0.996	gnl|BL_ORD_ID|55345 cb8564_c39002/f1p2/2677	1	1364	1362	2184	1	1364	1721	2541
cb12_c37/f1p0/4067	gnl|BL_ORD_ID|61079 cb12_c11/f1p2/4713	286	4080	1	288	891	4693	474	761
cb8564_c46839/f1p1/2758	gnl|BL_ORD_ID|56353 cb8564_c75114/f1p0/2866	2	2036	2036	2581	1	2035	2193	2737
cb309_c9/f1p2/4191 isoform=cb309_c9;full_length_coverage=1;non_full_length_coverage=2;isoform_length=4191;expected_accuracy=0.998	gnl|BL_ORD_ID|38927 cb309_c3/f2p2/4443 isoform=cb309_c3;full_length_coverage=2;non_full_length_coverage=2;isoform_length=4443;expected_accuracy=1.000	1	3777	3776	4191	22	3797	4006	4421
cb3229_c12/f1p0/2616	gnl|BL_ORD_ID|39752 cb3229_c2/f2p0/2613 isoform=cb3229_c2;full_length_coverage=2;non_full_length_coverage=0;isoform_length=2613;expected_accuracy=0.996	444	2412	5	448	582	2557	2	418
cb6730_c29/f5p1/2459 isoform=cb6730_c29;full_length_coverage=5;non_full_length_coverage=1;isoform_length=2459;expected_accuracy=1.000	gnl|BL_ORD_ID|3442 cb6730_c30/f4p0/2653 isoform=cb6730_c30;full_length_coverage=4;non_full_length_coverage=0;isoform_length=2653;expected_accuracy=1.000	492	2459	6	492	665	2651	1	494
cb8902_c5/f1p0/2054	gnl|BL_ORD_ID|21806 cb8902_c1/f4p0/2185 isoform=cb8902_c1;full_length_coverage=4;non_full_length_coverage=0;isoform_length=2185;expected_accuracy=1.000	1	1678	1673	2055	5	1678	1786	2168
cb3290_c38/f1p0/2345	gnl|BL_ORD_ID|39267 cb3290_c95/f1p11/2792	1150	2345	1	1151	1589	278	274	143

isoform=cb3290_c38;full_length_coverage=1;non_full_length_coverage=0;isoform_length=2345;expected_accuracy=0.996	isoform=cb3290_c95;full_length_coverage=1;non_full_length_coverage=11;isoform_length=2792;expected_accuracy=0.993						9		2
cb3235_c2/f2p1/3341 isoform=cb3235_c2;full_length_coverage=2;non_full_length_coverage=1;isoform_length=3341;expected_accuracy=0.998	gnl|BL_ORD_ID|40515 cb3235_c6/f1p0/3468 isoform=cb3235_c6;full_length_coverage=1;non_full_length_coverage=0;isoform_length=3468;expected_accuracy=1.000	1	2060	2058	3333	1	2082	2192	3468
cb1049_c13/f1p0/3054	gnl|BL_ORD_ID|1364 cb1049_c18/f10p1/3143 isoform=cb1049_c18;full_length_coverage=10;non_full_length_coverage=1;isoform_length=3143;expected_accuracy=1.000	1	1596	1593	3032	1	1596	1703	3142
cb3438_c26/f1p0/3084	gnl|BL_ORD_ID|41283 cb3438_c34/f1p0/2915 isoform=cb3438_c34;full_length_coverage=1;non_full_length_coverage=0;isoform_length=2915;expected_accuracy=0.994	196	2794	12	197	301	2915	4	187
cb13119_c3/f1p0/1356 isoform=cb13119_c3;full_length_coverage=1;non_full_length_coverage=0;isoform_length=1356;expected_accuracy=0.994	gnl|BL_ORD_ID|71113 cb3343_c7/f1p0/3597	216	1356	31	216	2449	3589	2039	2224
cb1779_c4/f1p0/3691	gnl|BL_ORD_ID|63947 cb1779_c1/f1p0/4179	1	2830	2828	3593	2	2831	3075	3840
cb3580_c76/f1p0/2053 isoform=cb3580_c76;full_length_coverage=1;non_full_length_coverage=0;isoform_length=2053;expected_accuracy=0.992	gnl|BL_ORD_ID|39321 cb3580_c75/f1p0/2886 isoform=cb3580_c75;full_length_coverage=1;non_full_length_coverage=0;isoform_length=2886;expected_accuracy=0.999	517	2053	95	519	1349	2884	1	425
cb11110_c3/f1p0/1566	gnl|BL_ORD_ID|67250 cb18371_c2/f1p0/1680	416	1566	71	419	492	164	1	349

isoform=cb11110_c3;full_length_coverage=1;non_full_length_coverage=0;isoform_length=1566;expected_accuracy=0.991							3		
cb8564_c39136/f1p0/3036 isoform=cb8564_c39136;full_length_coverage=1;non_full_length_coverage=0;isoform_length=3036;expected_accuracy=0.998	gnl|BL_ORD_ID|15169 cb8564_c40218/f1p0/3146 isoform=cb8564_c40218;full_length_coverage=1;non_full_length_coverage=0;isoform_length=3146;expected_accuracy=0.993	1	2089	2085	3032	4	2083	2209	3146
cb14316_c1/f2p0/1416 isoform=cb14316_c1;full_length_coverage=2;non_full_length_coverage=0;isoform_length=1416;expected_accuracy=0.999	gnl|BL_ORD_ID|52951 cb8564_c112776/f1p0/1815	239	1416	43	239	575	1751	2	199
cb8564_c127211/f1p0/2256 isoform=cb8564_c127211;full_length_coverage=1;non_full_length_coverage=0;isoform_length=2256;expected_accuracy=0.998	gnl|BL_ORD_ID|53478 cb8564_c126020/f1p1/2281	1	1951	1950	2157	26	1980	2084	2292
cb8564_c161307/f2p7/2586 isoform=cb8564_c161307;full_length_coverage=2;non_full_length_coverage=7;isoform_length=2586;expected_accuracy=0.993	gnl|BL_ORD_ID|14608 cb8564_c23723/f1p0/3118 isoform=cb8564_c23723;full_length_coverage=1;non_full_length_coverage=0;isoform_length=3118;expected_accuracy=0.992	1	1663	1663	2539	1	1659	2242	3118
cb8564_c20974/f1p0/4061 isoform=cb8564_c20974;full_length_coverage=1;non_full_length_coverage=0;isoform_length=4061;expected_accuracy=0.995	gnl|BL_ORD_ID|65909 cb17646_c3/f1p1/5031	1	3367	3368	4061	47	3455	3865	4554
cb7611_c28/f5p0/2201 isoform=cb7611_c28;full_length_coverage=5;non_full_length_coverage=0;isoform_length=2201;expected_accu	gnl|BL_ORD_ID|7565 cb7611_c19/f1p0/3611 isoform=cb7611_c19;full_length_coverage=1;non_full_length_coverage=0;isoform_length=3611;expected_accur	270	2201	47	271	1674	3611	1	224

racy=1.000	acy=0.991								
cb3584_c18/f3p2/2517 isoform=cb3584_c18;full_length_coverage=3;non_full_length_coverage=2;isoform_length=2517;expected_accuracy=0.996	gnl|BL_ORD_ID|40786 cb3584_c10/f1p1/3385 isoform=cb3584_c10;full_length_coverage=1;non_full_length_coverage=1;isoform_length=3385;expected_accuracy=0.992	1	1598	1597	2517	12	1611	1713	2625
cb5125_c42/f1p3/2079 isoform=cb5125_c42;full_length_coverage=1;non_full_length_coverage=3;isoform_length=2079;expected_accuracy=0.992	gnl|BL_ORD_ID|43457 cb5125_c11/f2p1/2545 isoform=cb5125_c11;full_length_coverage=2;non_full_length_coverage=1;isoform_length=2545;expected_accuracy=1.000	1	1296	1294	2064	46	1336	1778	2544
cb8564_c52191/f1p0/2822 isoform=cb8564_c52191;full_length_coverage=1;non_full_length_coverage=0;isoform_length=2822;expected_accuracy=0.997	gnl|BL_ORD_ID|14585 cb8564_c36952/f1p0/3622 isoform=cb8564_c36952;full_length_coverage=1;non_full_length_coverage=0;isoform_length=3622;expected_accuracy=0.994	423	2822	84	423	1229	3621	133	471
cb10083_c9/f1p0/1254	gnl|BL_ORD_ID|2910 cb10083_c5/f1p0/2795 isoform=cb10083_c5;full_length_coverage=1;non_full_length_coverage=0;isoform_length=2795;expected_accuracy=0.998	382	1169	4	382	1997	2784	27	408
cb8564_c181074/f1p0/2725 isoform=cb8564_c181074;full_length_coverage=1;non_full_length_coverage=0;isoform_length=2725;expected_accuracy=1.000	gnl|BL_ORD_ID|16364 cb8564_c72362/f4p1/4130 isoform=cb8564_c72362;full_length_coverage=4;non_full_length_coverage=1;isoform_length=4130;expected_accuracy=0.998	148	2703	1	147	1570	4130	78	223
cb8564_c178226/f1p0/2728 isoform=cb8564_c178226;full_length_coverage=1;non_full_length_coverage=0;isoform_length=2728;expected_accuracy=0.998	gnl|BL_ORD_ID|55359 cb8564_c39546/f1p2/3023	1284	2728	1	1288	1391	2837	1	1287
cb3011_c28/f1p0/3136	gnl|BL_ORD_ID|38862 cb3011_c30/f1p1/3312	1	2678	2677	3136	1	267	2801	326

isoform=cb3011_c28;full_length_coverage=1;non_full_length_coverage=0;isoform_length=3136;expected_accuracy=0.997	isoform=cb3011_c30;full_length_coverage=1;non_full_length_coverage=1;isoform_length=3312;expected_accuracy=0.999						8		2
cb5137_c53/f1p0/2672 isoform=cb5137_c53;full_length_coverage=1;non_full_length_coverage=0;isoform_length=2672;expected_accuracy=0.992	gnl|BL_ORD_ID|44215 cb5137_c125/f41p0/2869 isoform=cb5137_c125;full_length_coverage=41;non_full_length_coverage=0;isoform_length=2869;expected_accuracy=1.000	19	2565	2562	2672	66	2635	2739	2848
cb3119_c1/f1p0/2995 isoform=cb3119_c1;full_length_coverage=1;non_full_length_coverage=0;isoform_length=2995;expected_accuracy=0.995	gnl|BL_ORD_ID|69147 cb3119_c7/f1p0/3565	1	2761	2762	2994	5	2783	3183	3416
cb1163_c19/f1p0/3148 isoform=cb1163_c19;full_length_coverage=1;non_full_length_coverage=0;isoform_length=3148;expected_accuracy=0.996	gnl|BL_ORD_ID|23462 cb1163_c14/f1p1/4695 isoform=cb1163_c14;full_length_coverage=1;non_full_length_coverage=1;isoform_length=4695;expected_accuracy=0.991	1431	3099	1	1432	3030	4695	524	1955
cb8564_c83545/f1p0/3678	gnl|BL_ORD_ID|16611 cb8564_c71207/f1p0/4053 isoform=cb8564_c71207;full_length_coverage=1;non_full_length_coverage=0;isoform_length=4053;expected_accuracy=0.995	2	2742	2742	3470	61	2811	3326	4053
cb8564_c89114/f1p1/2346 isoform=cb8564_c89114;full_length_coverage=1;non_full_length_coverage=1;isoform_length=2346;expected_accuracy=1.000	gnl|BL_ORD_ID|17527 cb8564_c84215/f1p0/3183 isoform=cb8564_c84215;full_length_coverage=1;non_full_length_coverage=0;isoform_length=3183;expected_accuracy=0.997	1	1800	1798	2290	10	1810	2697	3183
cb173_c16/f1p0/2914	gnl|BL_ORD_ID|63581 cb173_c9/f1p0/4950	227	2727	1	227	1719	4219	29	255
cb8564_c20202/f1p0/2556	gnl|BL_ORD_ID|15221 cb8564_c25162/f1p0/2742	1	2227	2224	2556	17	224	2406	274

isoform=cb8564_c20202;full_length_coverage=1;non_full_length_coverage=0;isoform_length=2556;expected_accuracy=0.999	isoform=cb8564_c25162;full_length_coverage=1;non_full_length_coverage=0;isoform_length=2742;expected_accuracy=0.995						2		0
cb3763_c18/f1p0/2884	gnl|BL_ORD_ID|40108 cb3763_c16/f1p1/3107 isoform=cb3763_c16;full_length_coverage=1;non_full_length_coverage=1;isoform_length=3107;expected_accuracy=0.998	1234	2884	1	1237	1359	3009	9	1245
cb124_c39/f1p0/2659 isoform=cb124_c39;full_length_coverage=1;non_full_length_coverage=0;isoform_length=2659;expected_accuracy=0.993	gnl|BL_ORD_ID|59058 cb124_c18/f1p0/3758	464	2658	79	464	1378	3596	275	661
cb13049_c21/f1p1/1364	gnl|BL_ORD_ID|18258 cb8564_c84027/f1p0/2419 isoform=cb8564_c84027;full_length_coverage=1;non_full_length_coverage=0;isoform_length=2419;expected_accuracy=0.997	346	1350	42	346	1414	2419	1	305
cb8564_c188095/f1p0/3177 isoform=cb8564_c188095;full_length_coverage=1;non_full_length_coverage=0;isoform_length=3177;expected_accuracy=0.998	gnl|BL_ORD_ID|14238 cb8564_c39248/f1p1/3575 isoform=cb8564_c39248;full_length_coverage=1;non_full_length_coverage=1;isoform_length=3575;expected_accuracy=0.993	1	2441	2437	3157	1	2419	2856	3575
cb3367_c13/f1p0/1942 isoform=cb3367_c13;full_length_coverage=1;non_full_length_coverage=0;isoform_length=1942;expected_accuracy=0.998	gnl|BL_ORD_ID|40685 cb3367_c20/f3p0/3095 isoform=cb3367_c20;full_length_coverage=3;non_full_length_coverage=0;isoform_length=3095;expected_accuracy=0.999	124	1942	1	125	1273	3094	8	132
cb1956_c11/f1p0/2868 isoform=cb1956_c11;full_length_coverage=1;non_full_length_coverage=0;isoform_length=2868;expected_accu	gnl|BL_ORD_ID|36248 cb1956_c2/f5p0/3073 isoform=cb1956_c2;full_length_coverage=5;non_full_length_coverage=0;isoform_length=3073;expected_accura	1	1889	1889	2859	89	1972	2074	3073

racy=0.996	cy=0.999								
cb285_c16/f1p1/2281 isoform=cb285_c16;full_length_coverage=1;non_full_length_coverage=1;isoform_length=2281;expected_accuracy=0.999	gnl|BL_ORD_ID|36788 cb285_c0/f3p1/2363 isoform=cb285_c0;full_length_coverage=3;non_full_length_coverage=1;isoform_length=2363;expected_accuracy=0.997	265	2221	5	266	410	2363	1	273
cb8564_c43779/f1p0/3021 isoform=cb8564_c43779;full_length_coverage=1;non_full_length_coverage=0;isoform_length=3021;expected_accuracy=0.993	gnl|BL_ORD_ID|54425 cb8564_c42906/f1p2/3181	1	2344	2345	3021	1	2323	2479	3154
cb3704_c6/f1p1/2658 isoform=cb3704_c6;full_length_coverage=1;non_full_length_coverage=1;isoform_length=2658;expected_accuracy=0.998	gnl|BL_ORD_ID|39409 cb3704_c2/f1p1/2978 isoform=cb3704_c2;full_length_coverage=1;non_full_length_coverage=1;isoform_length=2978;expected_accuracy=1.000	1	2385	2383	2658	132	2516	2677	2953
cb322_c9/f2p0/3136 isoform=cb322_c9;full_length_coverage=2;non_full_length_coverage=0;isoform_length=3136;expected_accuracy=1.000	gnl|BL_ORD_ID|40331 cb322_c15/f1p0/3487 isoform=cb322_c15;full_length_coverage=1;non_full_length_coverage=0;isoform_length=3487;expected_accuracy=0.997	1	2718	2718	3135	13	2728	3070	3487
cb10142_c1/f1p0/1558	gnl|BL_ORD_ID|1637 cb10142_c4/f1p0/1790 isoform=cb10142_c4;full_length_coverage=1;non_full_length_coverage=0;isoform_length=1790;expected_accuracy=0.996	1	1239	1239	1560	1	1240	1439	1760
cb12_c24/f1p0/4291	gnl|BL_ORD_ID|61091 cb12_c58/f1p0/5377	241	4142	1	243	1406	5292	769	1011
cb5256_c5/f1p1/2755 isoform=cb5256_c5;full_length_coverage=1;non_full_length_coverage=1;isoform_length=2755;expected_accur	gnl|BL_ORD_ID|44572 cb5256_c2/f1p1/2847 isoform=cb5256_c2;full_length_coverage=1;non_full_length_coverage=1;isoform_length=2847;expected_accura	200	2744	10	199	320	2847	9	189

acy=0.994	cy=0.997								
cb10903_c12/f1p3/5206 isoform=cb10903_c12;full_length_coverage=1;non_full_length_coverage=3;isoform_length=5206;expected_accuracy=0.996	gnl|BL_ORD_ID|566 cb10903_c8/f1p2/5437 isoform=cb10903_c8;full_length_coverage=1;non_full_length_coverage=2;isoform_length=5437;expected_accuracy=0.998	4	4346	4345	5206	1	4321	4580	5436
cb8564_c15222/f1p0/2320 isoform=cb8564_c15222;full_length_coverage=1;non_full_length_coverage=0;isoform_length=2320;expected_accuracy=0.997	gnl|BL_ORD_ID|56585 cb8564_c47487/f1p0/2550	1	1650	1647	2310	4	1653	1889	2552
cb3309_c8/f1p0/1954 isoform=cb3309_c8;full_length_coverage=1;non_full_length_coverage=0;isoform_length=1954;expected_accuracy=0.995	gnl|BL_ORD_ID|70310 cb3309_c14/f1p0/2567	195	1934	34	196	797	2537	1	161
cb18230_c2/f1p0/2057	gnl|BL_ORD_ID|63865 cb1683_c28/f1p0/3914	420	1645	58	423	2731	3956	1832	2201
cb4624_c0/f2p0/2271 isoform=cb4624_c0;full_length_coverage=2;non_full_length_coverage=0;isoform_length=2271;expected_accuracy=0.996	gnl|BL_ORD_ID|72776 cb4624_c5/f1p0/2432	1	1369	1368	2184	1	1368	1494	2308
cb2156_c24/f1p1/2545 isoform=cb2156_c24;full_length_coverage=1;non_full_length_coverage=1;isoform_length=2545;expected_accuracy=0.991	gnl|BL_ORD_ID|35205 cb2156_c25/f1p0/2891 isoform=cb2156_c25;full_length_coverage=1;non_full_length_coverage=0;isoform_length=2891;expected_accuracy=0.995	1	1422	1420	2545	125	1536	1661	2790
cb8564_c43546/f1p1/3276	gnl|BL_ORD_ID|14410 cb8564_c44256/f1p1/3267 isoform=cb8564_c44256;full_length_coverage=1;non_full_length_coverage=1;isoform_length=3267;expected_ac	10	2153	2149	2854	1	2119	2553	3256

<table>
<tr><td></td><td>curacy=0.995</td><td></td><td></td><td></td><td></td><td></td><td></td><td></td><td></td></tr>
<tr><td>cb1955_c36/f1p0/2749 isoform=cb1955_c36;full_length_coverage=1;non_full_length_coverage=0;isoform_length=2749;expected_accuracy=0.994</td><td>gnl|BL_ORD_ID|66534 cb1955_c27/f1p0/3020</td><td>1</td><td>1902</td><td>1899</td><td>2748</td><td>14</td><td>1916</td><td>2196</td><td>3044</td></tr>
<tr><td>cb8564_c69135/f3p0/3749 isoform=cb8564_c69135;full_length_coverage=3;non_full_length_coverage=0;isoform_length=3749;expected_accuracy=0.998</td><td>gnl|BL_ORD_ID|17407 cb8564_c85285/f1p0/4248 isoform=cb8564_c85285;full_length_coverage=1;non_full_length_coverage=0;isoform_length=4248;expected_accuracy=0.997</td><td>1</td><td>1890</td><td>1889</td><td>3736</td><td>43</td><td>1930</td><td>2402</td><td>4248</td></tr>
<tr><td>cb8564_c48235/f1p1/2252</td><td>gnl|BL_ORD_ID|13472 cb8564_c182192/f1p6/3150 isoform=cb8564_c182192;full_length_coverage=1;non_full_length_coverage=6;isoform_length=3150;expected_accuracy=0.995</td><td>1</td><td>1600</td><td>1596</td><td>2253</td><td>102</td><td>1707</td><td>2488</td><td>3147</td></tr>
<tr><td>cb6277_c19/f1p1/2357 isoform=cb6277_c19;full_length_coverage=1;non_full_length_coverage=1;isoform_length=2357;expected_accuracy=0.995</td><td>gnl|BL_ORD_ID|49578 cb6277_c18/f1p0/2697</td><td>1</td><td>1183</td><td>1183</td><td>2286</td><td>113</td><td>1295</td><td>1601</td><td>2704</td></tr>
<tr><td>cb2229_c66/f60p1/2354 isoform=cb2229_c66;full_length_coverage=60;non_full_length_coverage=1;isoform_length=2354;expected_accuracy=0.999</td><td>gnl|BL_ORD_ID|68360 cb2229_c20/f1p0/3944</td><td>1</td><td>1690</td><td>1687</td><td>2299</td><td>26</td><td>1723</td><td>3265</td><td>3879</td></tr>
<tr><td>cb13761_c2/f1p0/1129 isoform=cb13761_c2;full_length_coverage=1;non_full_length_coverage=0;isoform_length=1129;expected_accuracy=0.997</td><td>gnl|BL_ORD_ID|26686 cb13761_c4/f1p0/1251 isoform=cb13761_c4;full_length_coverage=1;non_full_length_coverage=0;isoform_length=1251;expected_accuracy=0.991</td><td>381</td><td>1105</td><td>6</td><td>380</td><td>528</td><td>1250</td><td>1</td><td>367</td></tr>
<tr><td>cb4016_c18/f1p0/2605</td><td>gnl|BL_ORD_ID|71728 cb4016_c38/f1p0/2815</td><td>1254</td><td>2566</td><td>1</td><td>1256</td><td>1380</td><td>269</td><td>4</td><td>125</td></tr>
</table>

isoform=cb4016_c18;full_length_coverage=1;non_full_length_coverage=0;isoform_length=2605;expected_accuracy=0.999							1		9
cb1956_c11/f1p0/2868 isoform=cb1956_c11;full_length_coverage=1;non_full_length_coverage=0;isoform_length=2868;expected_accuracy=0.996	gnl|BL_ORD_ID|66542 cb1956_c22/f1p0/3042	10	1889	1889	2868	3	1880	1982	2971
cb2954_c4/f1p0/3606	gnl|BL_ORD_ID|37637 cb2954_c5/f1p0/3687 isoform=cb2954_c5;full_length_coverage=1;non_full_length_coverage=0;isoform_length=3687;expected_accuracy=0.998	2	2969	2969	3515	22	3006	3111	3658
cb584_c43/f3p1/3426 isoform=cb584_c43;full_length_coverage=3;non_full_length_coverage=1;isoform_length=3426;expected_accuracy=1.000	gnl|BL_ORD_ID|45633 cb584_c11/f1p0/3581 isoform=cb584_c11;full_length_coverage=1;non_full_length_coverage=0;isoform_length=3581;expected_accuracy=0.997	305	3426	1	306	424	3570	19	312
cb8564_c87534/f1p0/3105	gnl|BL_ORD_ID|14843 cb8564_c44474/f1p1/3915 isoform=cb8564_c44474;full_length_coverage=1;non_full_length_coverage=1;isoform_length=3915;expected_accuracy=0.996	527	2914	79	532	1440	3821	1	454
cb8564_c36643/f1p3/2668	gnl|BL_ORD_ID|17556 cb8564_c9820/f2p0/3032 isoform=cb8564_c9820;full_length_coverage=2;non_full_length_coverage=0;isoform_length=3032;expected_accuracy=0.995	13	1689	1687	2587	7	1704	2131	3032
cb500_c17/f1p0/2323 isoform=cb500_c17;full_length_coverage=1;non_full_length_coverage=0;isoform_length=2323;expected_accur	gnl|BL_ORD_ID|70946 cb500_c74/f1p0/3703	1	1293	1288	2323	846	2138	2270	3304

acy=0.997									
cb1163_c42/f1p0/4122	gnl|BL_ORD_ID|23459 cb1163_c11/f2p1/3742 isoform=cb1163_c11;full_length_coverage=2;non_full_length_coverage=1;isoform_length=3742;expected_accuracy=0.999	120	2535	2534	3651	89	2504	2616	3736
cb862_c22/f1p0/3314 isoform=cb862_c22;full_length_coverage=1;non_full_length_coverage=0;isoform_length=3314;expected_accuracy=0.995	gnl|BL_ORD_ID|17905 cb862_c21/f1p0/3616 isoform=cb862_c21;full_length_coverage=1;non_full_length_coverage=0;isoform_length=3616;expected_accuracy=1.000	182	3314	1	182	474	3614	82	264
cb15591_c1/f1p1/1413 isoform=cb15591_c1;full_length_coverage=1;non_full_length_coverage=1;isoform_length=1413;expected_accuracy=0.999	gnl|BL_ORD_ID|21104 cb9317_c10/f1p1/1720 isoform=cb9317_c10;full_length_coverage=1;non_full_length_coverage=1;isoform_length=1720;expected_accuracy=0.997	280	1413	36	284	510	1646	1	248
cb8564_c4583/f5p0/2597 isoform=cb8564_c4583;full_length_coverage=5;non_full_length_coverage=0;isoform_length=2597;expected_accuracy=0.998	gnl|BL_ORD_ID|15246 cb8564_c4582/f1p0/2713 isoform=cb8564_c4582;full_length_coverage=1;non_full_length_coverage=0;isoform_length=2713;expected_accuracy=0.997	104	2597	1	107	222	2713	1	107
cb8564_c121826/f1p0/2708 isoform=cb8564_c121826;full_length_coverage=1;non_full_length_coverage=0;isoform_length=2708;expected_accuracy=0.998	gnl|BL_ORD_ID|53063 cb8564_c115478/f1p0/2938	1	2046	2045	2708	15	2067	2192	2860
cb8564_c18223/f1p0/3446 isoform=cb8564_c18223;full_length_coverage=1;non_full_length_coverage=0;isoform_length=3446;expected_accuracy=0.998	gnl|BL_ORD_ID|13352 cb8564_c19199/f1p0/3820 isoform=cb8564_c19199;full_length_coverage=1;non_full_length_coverage=0;isoform_length=3820;expected_accuracy=0.998	1153	3445	1	1154	1529	3820	39	1177
cb2831_c22/f1p0/2102	gnl|BL_ORD_ID|70016 cb2831_c50/f1p0/2799	1	1598	1598	2095	1	159	2338	283

							8		4
cb666_c10/f1p0/2176	gnl|BL_ORD_ID|49394 cb666_c14/f1p0/2626	100	2057	1	101	202	2136	2	102
cb8564_c188097/f1p0/2552 isoform=cb8564_c188097;full_length_coverage=1;non_full_length_coverage=0;isoform_length=2552;expected_accuracy=0.996	gnl|BL_ORD_ID|52809 cb8564_c14548/f1p0/3030	1	1945	1940	2552	13	1957	2060	2649
cb1030_c20/f1p1/3218	gnl|BL_ORD_ID|1420 cb1030_c30/f1p0/3343 isoform=cb1030_c30;full_length_coverage=1;non_full_length_coverage=0;isoform_length=3343;expected_accuracy=0.994	2	2775	2778	3173	1	2774	2898	3293
cb16997_c85/f1p0/2068	gnl|BL_ORD_ID|32730 cb16997_c79/f1p0/2350 isoform=cb16997_c79;full_length_coverage=1;non_full_length_coverage=0;isoform_length=2350;expected_accuracy=0.999	1	1557	1556	2072	1	1556	1700	2215
cb500_c17/f1p0/2323 isoform=cb500_c17;full_length_coverage=1;non_full_length_coverage=0;isoform_length=2323;expected_accuracy=0.997	gnl|BL_ORD_ID|42492 cb500_c6/f4p0/3398 isoform=cb500_c6;full_length_coverage=4;non_full_length_coverage=0;isoform_length=3398;expected_accuracy=1.000	1	1293	1288	2323	888	2180	2312	3347
cb6802_c15/f1p0/2731	gnl|BL_ORD_ID|4248 cb6802_c12/f1p0/3195 isoform=cb6802_c12;full_length_coverage=1;non_full_length_coverage=0;isoform_length=3195;expected_accuracy=0.997	231	2553	23	232	745	3088	17	227
cb8564_c110860/f1p0/2539	gnl|BL_ORD_ID|56347 cb8564_c74976/f1p0/3389	1	2108	2106	2414	1	2108	3064	3371
cb8564_c41756/f1p0/2738	gnl|BL_ORD_ID|55391 cb8564_c40278/f3p2/3083	1	2173	2170	2738	1	217	2302	287

isoform=cb8564_c41756;full_length_coverage=1;non_full_length_coverage=0;isoform_length=2738;expected_accuracy=0.996							9		0
cb8564_c163002/f3p0/1920 isoform=cb8564_c163002;full_length_coverage=3;non_full_length_coverage=0;isoform_length=1920;expected_accuracy=1.000	gnl|BL_ORD_ID|14668 cb8564_c3082/f1p0/2032 isoform=cb8564_c3082;full_length_coverage=1;non_full_length_coverage=0;isoform_length=2032;expected_accuracy=0.993	520	1885	9	521	667	2032	45	556
cb8564_c68629/f9p0/2229 isoform=cb8564_c68629;full_length_coverage=9;non_full_length_coverage=0;isoform_length=2229;expected_accuracy=1.000	gnl|BL_ORD_ID|16225 cb8564_c76305/f1p0/2353 isoform=cb8564_c76305;full_length_coverage=1;non_full_length_coverage=0;isoform_length=2353;expected_accuracy=1.000	1	1383	1382	2183	2	1384	1551	2353
cb401_c68/f1p0/2128 isoform=cb401_c68;full_length_coverage=1;non_full_length_coverage=0;isoform_length=2128;expected_accuracy=0.994	gnl|BL_ORD_ID|69611 cb401_c144/f1p1/2401	676	2117	8	674	961	2402	45	711
cb17973_c53/f1p0/1649 isoform=cb17973_c53;full_length_coverage=1;non_full_length_coverage=0;isoform_length=1649;expected_accuracy=0.992	gnl|BL_ORD_ID|56721 cb8564_c54312/f1p0/2789	186	1638	29	186	1270	2717	1	157
cb3573_c5/f1p0/3481	gnl|BL_ORD_ID|39163 cb3573_c0/f1p0/3591 isoform=cb3573_c0;full_length_coverage=1;non_full_length_coverage=0;isoform_length=3591;expected_accuracy=0.997	703	3411	8	707	881	3591	73	760
cb470_c8/f1p0/2206 isoform=cb470_c8;full_length_coverage=1;non_full_length_coverage=0;isoform_length=2206;expected_accurac	gnl|BL_ORD_ID|72212 cb470_c9/f1p0/2469	1	1503	1501	2206	2	1526	1753	2458

y=0.993									
cb5304_c6/f1p0/2203 isoform=cb5304_c6;full_length_coverage=1;non_full_length_coverage=0;isoform_length=2203;expected_accuracy=0.997	gnl|BL_ORD_ID|73759 cb5304_c5/f1p0/2736	1	1509	1508	2203	435	1937	2052	2750
cb2947_c16/f1p0/5590	gnl|BL_ORD_ID|38195 cb2947_c15/f1p0/5692 isoform=cb2947_c15;full_length_coverage=1;non_full_length_coverage=0;isoform_length=5692;expected_accuracy=0.995	2429	5532	24	2428	2589	5692	1	2442
cb3856_c2/f2p0/1678 isoform=cb3856_c2;full_length_coverage=2;non_full_length_coverage=0;isoform_length=1678;expected_accuracy=1.000	gnl|BL_ORD_ID|40756 cb3856_c4/f1p1/1955 isoform=cb3856_c4;full_length_coverage=1;non_full_length_coverage=1;isoform_length=1955;expected_accuracy=0.997	141	1678	12	145	307	1844	1	131
cb8564_c37361/f1p0/3076	gnl|BL_ORD_ID|55377 cb8564_c39822/f1p0/3306	1	2481	2477	3069	1	2478	2579	3169
cb12931_c15/f1p0/1493 isoform=cb12931_c15;full_length_coverage=1;non_full_length_coverage=0;isoform_length=1493;expected_accuracy=1.000	gnl|BL_ORD_ID|14615 cb8564_c41756/f1p0/2738 isoform=cb8564_c41756;full_length_coverage=1;non_full_length_coverage=0;isoform_length=2738;expected_accuracy=0.996	1	1106	1104	1492	1	1098	2350	2738
cb8564_c75707/f1p1/4363 isoform=cb8564_c75707;full_length_coverage=1;non_full_length_coverage=1;isoform_length=4363;expected_accuracy=0.999	gnl|BL_ORD_ID|17119 cb8564_c79662/f1p0/4430 isoform=cb8564_c79662;full_length_coverage=1;non_full_length_coverage=0;isoform_length=4430;expected_accuracy=0.993	3	3347	3347	4363	1	3310	3412	4426
cb8564_c20661/f1p2/3083 isoform=cb8564_c20661;full_length_coverage=1;non_full_length_coverage=2;isoform_length=3083;expected_	gnl|BL_ORD_ID|57416 cb8564_c95210/f1p2/3497	1	2981	2978	3083	31	3009	3233	3327

accuracy=1.000									
cb3119_c2/f1p0/2612 isoform=cb3119_c2;full_length_coverage=1;non_full_length_coverage=0;isoform_length=2612;expected_accuracy=0.999	gnl|BL_ORD_ID|37651 cb3119_c1/f1p0/2995 isoform=cb3119_c1;full_length_coverage=1;non_full_length_coverage=0;isoform_length=2995;expected_accuracy=0.995	1	1763	1764	2513	1	1765	2011	2761
cb322_c28/f1p0/3948	gnl|BL_ORD_ID|41025 cb322_c8/f2p0/3977 isoform=cb322_c8;full_length_coverage=2;non_full_length_coverage=0;isoform_length=3977;expected_accuracy=0.999	140	2868	2866	3771	1	2729	3071	3977
cb8564_c39827/f1p1/2907	gnl|BL_ORD_ID|14585 cb8564_c36952/f1p0/3622 isoform=cb8564_c36952;full_length_coverage=1;non_full_length_coverage=0;isoform_length=3622;expected_accuracy=0.994	486	2879	53	486	1229	3620	34	471
cb5799_c18/f1p0/2629 isoform=cb5799_c18;full_length_coverage=1;non_full_length_coverage=0;isoform_length=2629;expected_accuracy=0.996	gnl|BL_ORD_ID|74443 cb5799_c5/f1p0/2915	1	1913	1912	2629	36	1951	2056	2773
cb6875_c8/f1p0/1799 isoform=cb6875_c8;full_length_coverage=1;non_full_length_coverage=0;isoform_length=1799;expected_accuracy=0.994	gnl|BL_ORD_ID|5625 cb6875_c5/f1p0/2662 isoform=cb6875_c5;full_length_coverage=1;non_full_length_coverage=0;isoform_length=2662;expected_accuracy=0.991	417	1799	54	417	1278	2658	1	365
cb10921_c3/f1p0/1562	gnl|BL_ORD_ID|1666 cb10921_c1/f1p0/2459 isoform=cb10921_c1;full_length_coverage=1;non_full_length_coverage=0;isoform_length=2459;expected_accuracy=0.991	533	1427	7	534	1572	2455	22	552
cb8564_c36643/f1p3/2668	gnl|BL_ORD_ID|56353 cb8564_c75114/f1p0/2866	13	2019	2019	2565	7	203	2193	273

<table>
<tr><td></td><td></td><td></td><td></td><td></td><td></td><td></td><td>5</td><td></td><td>7</td></tr>
<tr><td>cb2707_c10/f3p0/2850 isoform=cb2707_c10;full_length_coverage=3;non_full_length_coverage=0;isoform_length=2850;expected_accuracy=1.000</td><td>gnl|BL_ORD_ID|36722 cb2707_c55/f1p0/3422 isoform=cb2707_c55;full_length_coverage=1;non_full_length_coverage=0;isoform_length=3422;expected_accuracy=0.995</td><td>21</td><td>2379</td><td>2379</td><td>2850</td><td>1</td><td>2351</td><td>2680</td><td>3151</td></tr>
<tr><td>cb8564_c2392/f1p2/2510 isoform=cb8564_c2392;full_length_coverage=1;non_full_length_coverage=2;isoform_length=2510;expected_accuracy=0.992</td><td>gnl|BL_ORD_ID|13611 cb8564_c2184/f1p0/2606 isoform=cb8564_c2184;full_length_coverage=1;non_full_length_coverage=0;isoform_length=2606;expected_accuracy=0.995</td><td>283</td><td>2510</td><td>4</td><td>283</td><td>384</td><td>2602</td><td>4</td><td>272</td></tr>
<tr><td>cb6614_c2/f1p0/2730 isoform=cb6614_c2;full_length_coverage=1;non_full_length_coverage=0;isoform_length=2730;expected_accuracy=0.997</td><td>gnl|BL_ORD_ID|4491 cb6614_c1/f1p0/2809 isoform=cb6614_c1;full_length_coverage=1;non_full_length_coverage=0;isoform_length=2809;expected_accuracy=0.995</td><td>1</td><td>2049</td><td>2050</td><td>2620</td><td>24</td><td>2071</td><td>2239</td><td>2809</td></tr>
<tr><td>cb944_c41/f1p0/2631</td><td>gnl|BL_ORD_ID|59104 cb944_c38/f1p0/3532</td><td>11</td><td>1592</td><td>1592</td><td>2411</td><td>3</td><td>1584</td><td>2261</td><td>3080</td></tr>
<tr><td>cb7748_c1/f3p0/2333 isoform=cb7748_c1;full_length_coverage=3;non_full_length_coverage=0;isoform_length=2333;expected_accuracy=0.998</td><td>gnl|BL_ORD_ID|6409 cb7748_c4/f1p0/4792 isoform=cb7748_c4;full_length_coverage=1;non_full_length_coverage=0;isoform_length=4792;expected_accuracy=0.996</td><td>1</td><td>1797</td><td>1793</td><td>2333</td><td>1</td><td>1828</td><td>2487</td><td>3027</td></tr>
<tr><td>cb8433_c12/f1p0/2395 isoform=cb8433_c12;full_length_coverage=1;non_full_length_coverage=0;isoform_length=2395;expected_accuracy=0.996</td><td>gnl|BL_ORD_ID|10988 cb8433_c22/f1p0/5526 isoform=cb8433_c22;full_length_coverage=1;non_full_length_coverage=0;isoform_length=5526;expected_accuracy=0.996</td><td>1</td><td>1787</td><td>1784</td><td>2395</td><td>12</td><td>1795</td><td>3655</td><td>4268</td></tr>
<tr><td>cb2184_c6/f1p0/2315</td><td>gnl|BL_ORD_ID|56154 cb8564_c70991/f1p13/4645</td><td>1090</td><td>2217</td><td>1</td><td>1092</td><td>1163</td><td>36</td><td>3334</td><td>2243</td></tr>
<tr><td>cb3582_c3/f1p0/3545</td><td>gnl|BL_ORD_ID|71348 cb3582_c2/f1p0/3551</td><td>1312</td><td>3403</td><td>1</td><td>1313</td><td>1444</td><td>353</td><td>2</td><td>131</td></tr>
</table>

							5		4
cb7696_c12/f1p1/1985	gnl|BL_ORD_ID|8427 cb7696_c11/f3p1/2420 isoform=cb7696_c11;full_length_coverage=3;non_full_length_coverage=1;isoform_length=2420;expected_accuracy=0.998	1	1746	1744	1980	60	1804	2126	2364
cb8198_c6/f1p1/1844 isoform=cb8198_c6;full_length_coverage=1;non_full_length_coverage=1;isoform_length=1844;expected_accuracy=0.995	gnl|BL_ORD_ID|9021 cb8198_c2/f1p0/2433 isoform=cb8198_c2;full_length_coverage=1;non_full_length_coverage=0;isoform_length=2433;expected_accuracy=0.993	1	1109	1109	1844	39	1156	1630	2365
cb381_c1/f1p0/3954 isoform=cb381_c1;full_length_coverage=1;non_full_length_coverage=0;isoform_length=3954;expected_accuracy=0.990	gnl|BL_ORD_ID|40998 cb381_c6/f1p0/5381 isoform=cb381_c6;full_length_coverage=1;non_full_length_coverage=0;isoform_length=5381;expected_accuracy=0.996	1	2854	2853	3954	821	3681	4229	5342
cb5771_c23/f15p1/2633 isoform=cb5771_c23;full_length_coverage=15;non_full_length_coverage=1;isoform_length=2633;expected_accuracy=1.000	gnl|BL_ORD_ID|45084 cb5771_c5/f1p1/3484 isoform=cb5771_c5;full_length_coverage=1;non_full_length_coverage=1;isoform_length=3484;expected_accuracy=0.995	1	1983	1981	2617	1	1982	2850	3484
cb12714_c29/f1p2/1583	gnl|BL_ORD_ID|54449 cb8564_c122906/f2p13/2871	1	1296	1293	1597	135	1430	2497	2801
cb8564_c33851/f2p0/3455 isoform=cb8564_c33851;full_length_coverage=2;non_full_length_coverage=0;isoform_length=3455;expected_accuracy=1.000	gnl|BL_ORD_ID|52067 cb8564_c11914/f1p0/3554	1	2078	2076	3455	1	2048	2158	3537
cb573_c9/f1p1/2484 isoform=cb573_c9;full_length_coverage=1;non_full_length_coverage=1;isoform_length=2484;expected_accurac	gnl|BL_ORD_ID|46348 cb573_c10/f1p1/2845 isoform=cb573_c10;full_length_coverage=1;non_full_length_coverage=1;isoform_length=2845;expected_accura	1	1301	1299	2484	59	1381	1656	2842

y=0.998	cy=0.996								
cb7096_c23/f1p0/2569	gnl|BL_ORD_ID|50336 cb7096_c16/f1p0/2638	164	2451	1	165	316	259 5	37	199
cb9813_c128/f1p0/1498	gnl|BL_ORD_ID|58918 cb9813_c53/f1p3/1923	175	1498	3	179	356	167 7	4	180
cb1960_c41/f1p1/2517	gnl|BL_ORD_ID|68238 cb1960_c43/f1p0/3174	1	2123	2120	2503	32	215 4	2552	293 5
cb8564_c48538/f1p0/2599	gnl|BL_ORD_ID|13889 cb8564_c34195/f2p1/2830 isoform=cb8564_c34195;full_length_coverage=2;non_full_length_coverage=1;isoform_length=2830;expected_accuracy=0.997	554	2462	81	556	911	281 9	2	482
cb2818_c26/f1p0/2862 isoform=cb2818_c26;full_length_coverage=1;non_full_length_coverage=0;isoform_length=2862;expected_accuracy=0.993	gnl|BL_ORD_ID|38982 cb2818_c74/f3p0/3087 isoform=cb2818_c74;full_length_coverage=3;non_full_length_coverage=0;isoform_length=3087;expected_accuracy=1.000	1	2471	2466	2841	53	253 1	2709	308 7
cb8564_c91758/f1p1/2726	gnl|BL_ORD_ID|55116 cb8564_c24808/f1p0/3718	286	2672	93	283	961	334 7	1	187
cb1163_c13/f1p1/3869 isoform=cb1163_c13;full_length_coverage=1;non_full_length_coverage=1;isoform_length=3869;expected_accuracy=0.992	gnl|BL_ORD_ID|59200 cb1163_c12/f1p0/4451	1	2493	2492	3868	2	250 3	2617	399 4
cb3290_c38/f1p0/2345 isoform=cb3290_c38;full_length_coverage=1;non_full_length_coverage=0;isoform_length=2345;expected_accuracy=0.996	gnl|BL_ORD_ID|39624 cb3290_c189/f9p0/2939 isoform=cb3290_c189;full_length_coverage=9;non_full_length_coverage=0;isoform_length=2939;expected_accuracy=1.000	1150	2345	1	1151	1667	286 2	360	151 0
cb4771_c5/f1p0/2633	gnl|BL_ORD_ID|72956 cb4771_c3/f1p0/3275	311	2562	34	314	962	321	1	281

							3		
cb2825_c0/f7p1/2163 isoform=cb2825_c0;full_length_coverage=7;non_full_length_coverage=1;isoform_length=2163;expected_accuracy=1.000	gnl|BL_ORD_ID|70008 cb2825_c6/f1p1/2358	242	2162	82	242	445	2365	1	159
cb4367_c10/f1p1/1298 isoform=cb4367_c10;full_length_coverage=1;non_full_length_coverage=1;isoform_length=1298;expected_accuracy=0.995	gnl|BL_ORD_ID|72507 cb4367_c4/f1p0/2078	1	1129	1126	1260	123	1245	1665	1799
cb4380_c4/f1p0/3270	gnl|BL_ORD_ID|41735 cb4380_c0/f1p0/3372 isoform=cb4380_c0;full_length_coverage=1;non_full_length_coverage=0;isoform_length=3372;expected_accuracy=0.998	481	3223	60	480	637	3371	15	431
cb2759_c6/f1p0/3412	gnl|BL_ORD_ID|37958 cb2759_c1/f1p0/3776 isoform=cb2759_c1;full_length_coverage=1;non_full_length_coverage=0;isoform_length=3776;expected_accuracy=0.992	194	3069	11	193	907	3776	27	210
cb3535_c0/f3p1/3398 isoform=cb3535_c0;full_length_coverage=3;non_full_length_coverage=1;isoform_length=3398;expected_accuracy=0.995	gnl|BL_ORD_ID|41049 cb3535_c11/f1p1/3518 isoform=cb3535_c11;full_length_coverage=1;non_full_length_coverage=1;isoform_length=3518;expected_accuracy=1.000	1	2573	2569	3395	1	2579	2682	3518
cb2686_c5/f1p0/3171 isoform=cb2686_c5;full_length_coverage=1;non_full_length_coverage=0;isoform_length=3171;expected_accuracy=0.994	gnl|BL_ORD_ID|69894 cb2686_c2/f1p0/4322	1	2343	2341	3170	26	2368	3515	4343
cb8564_c188097/f1p0/2552	gnl|BL_ORD_ID|52715 cb8564_c11997/f1p2/2670	1	1945	1940	2521	1	194	2048	262

isoform=cb8564_c188097;full_length_coverage=1;non_full_length_coverage=0;isoform_length=2552;expected_accuracy=0.996							5		9
cb12893_c39/f1p0/1097 isoform=cb12893_c39;full_length_coverage=1;non_full_length_coverage=0;isoform_length=1097;expected_accuracy=0.996	gnl|BL_ORD_ID|61928 cb12893_c63/f1p2/1520	234	1095	6	239	494	1367	8	239
cb4380_c7/f1p0/3480	gnl|BL_ORD_ID|41735 cb4380_c0/f1p0/3372 isoform=cb4380_c0;full_length_coverage=1;non_full_length_coverage=0;isoform_length=3372;expected_accuracy=0.998	487	3299	66	490	567	3370	15	435
cb18456_c1419/f5p0/1798 isoform=cb18456_c1419;full_length_coverage=5;non_full_length_coverage=0;isoform_length=1798;expected_accuracy=1.000	gnl|BL_ORD_ID|17050 cb8564_c66818/f5p0/2029 isoform=cb8564_c66818;full_length_coverage=5;non_full_length_coverage=0;isoform_length=2029;expected_accuracy=1.000	247	1798	3	246	474	2025	1	243
cb2737_c63/f1p2/2799 isoform=cb2737_c63;full_length_coverage=1;non_full_length_coverage=2;isoform_length=2799;expected_accuracy=0.994	gnl|BL_ORD_ID|36733 cb2737_c14/f1p0/2854 isoform=cb2737_c14;full_length_coverage=1;non_full_length_coverage=0;isoform_length=2854;expected_accuracy=0.996	1	1514	1512	2711	1	1518	1650	2854
cb8637_c59/f1p0/1766 isoform=cb8637_c59;full_length_coverage=1;non_full_length_coverage=0;isoform_length=1766;expected_accuracy=1.000	gnl|BL_ORD_ID|19166 cb8637_c22/f1p0/1910 isoform=cb8637_c22;full_length_coverage=1;non_full_length_coverage=0;isoform_length=1910;expected_accuracy=0.995	1	1375	1371	1737	56	1429	1544	1910
cb8564_c42814/f1p0/2151 isoform=cb8564_c42814;full_length_coverage=1;non_full_length_coverage=0;isoform_length=2151;expected_	gnl|BL_ORD_ID|55574 cb8564_c45699/f1p0/3005	650	2151	7	652	1346	2846	1	659

accuracy=0.993									
cb16776_c28/f1p0/5224 isoform=cb16776_c28;full_length_coverage=1;non_full_length_coverage=0;isoform_length=5224;expected_accuracy=0.997	gnl|BL_ORD_ID|65466 cb16776_c11/f1p0/6273	16	4960	4959	5224	1	4952	5422	5690
cb8564_c68941/f7p1/2708 isoform=cb8564_c68941;full_length_coverage=7;non_full_length_coverage=1;isoform_length=2708;expected_accuracy=1.000	gnl|BL_ORD_ID|57844 cb8564_c82408/f1p0/2861	583	2708	62	585	690	2814	56	579
cb8564_c42814/f1p0/2151 isoform=cb8564_c42814;full_length_coverage=1;non_full_length_coverage=0;isoform_length=2151;expected_accuracy=0.993	gnl|BL_ORD_ID|55371 cb8564_c39756/f1p1/3004	650	2151	7	652	1336	2852	1	648
cb15300_c0/f1p0/1024	gnl|BL_ORD_ID|28933 cb15300_c1/f1p0/1168 isoform=cb15300_c1;full_length_coverage=1;non_full_length_coverage=0;isoform_length=1168;expected_accuracy=1.000	100	1026	1	102	242	1168	10	113
cb5091_c1/f1p0/2773 isoform=cb5091_c1;full_length_coverage=1;non_full_length_coverage=0;isoform_length=2773;expected_accuracy=0.999	gnl|BL_ORD_ID|73252 cb5091_c0/f1p0/3180	1	2338	2339	2756	271	2608	2736	3152
cb8564_c145840/f9p2/3901 isoform=cb8564_c145840;full_length_coverage=9;non_full_length_coverage=2;isoform_length=3901;expected_accuracy=0.999	gnl|BL_ORD_ID|15430 cb8564_c43033/f1p1/4012 isoform=cb8564_c43033;full_length_coverage=1;non_full_length_coverage=1;isoform_length=4012;expected_accuracy=0.998	1	2123	2122	3867	3	2127	2264	4012
cb8564_c42654/f1p1/2906	gnl|BL_ORD_ID|14407 cb8564_c44036/f1p0/3258	1153	2824	1	1153	1575	323	1	1147

isoform=cb8564_c42654;full_length_coverage=1;non_full_length_coverage=1;isoform_length=2906;expected_accuracy=0.990	isoform=cb8564_c44036;full_length_coverage=1;non_full_length_coverage=0;isoform_length=3258;expected_accuracy=0.992						8		
cb12931_c15/f1p0/1493 isoform=cb12931_c15;full_length_coverage=1;non_full_length_coverage=0;isoform_length=1493;expected_accuracy=1.000	gnl|BL_ORD_ID|56648 cb8564_c50629/f1p3/2919	1	1106	1104	1493	4	1103	2555	2944
cb8564_c45426/f1p0/2266	gnl|BL_ORD_ID|13975 cb8564_c42753/f2p0/2720 isoform=cb8564_c42753;full_length_coverage=2;non_full_length_coverage=0;isoform_length=2720;expected_accuracy=0.992	10	1381	1379	2235	17	1389	1714	2571
cb2836_c25/f1p0/3140 isoform=cb2836_c25;full_length_coverage=1;non_full_length_coverage=0;isoform_length=3140;expected_accuracy=1.000	gnl|BL_ORD_ID|37734 cb2836_c30/f2p1/3305 isoform=cb2836_c30;full_length_coverage=2;non_full_length_coverage=1;isoform_length=3305;expected_accuracy=0.998	1	1919	1916	3138	6	1923	2084	3305
cb5306_c20/f1p0/1640 isoform=cb5306_c20;full_length_coverage=1;non_full_length_coverage=0;isoform_length=1640;expected_accuracy=0.996	gnl|BL_ORD_ID|45775 cb5306_c13/f1p0/2299 isoform=cb5306_c13;full_length_coverage=1;non_full_length_coverage=0;isoform_length=2299;expected_accuracy=0.991	381	1638	9	384	513	1771	3	383
cb8564_c81040/f1p1/2371	gnl|BL_ORD_ID|16951 cb8564_c68678/f7p4/2826 isoform=cb8564_c68678;full_length_coverage=7;non_full_length_coverage=4;isoform_length=2826;expected_accuracy=1.000	1	1965	1962	2481	24	1990	2195	2715
cb8564_c5255/f1p0/2672 isoform=cb8564_c5255;full_length_coverage=1;non_full_length_coverage=0;isoform_length=2672;expected_ac	gnl|BL_ORD_ID|61984 cb12939_c13/f1p0/6119	234	2596	1	235	3349	5722	2440	2682

curacy=0.997									
cb4016_c7/f1p0/2879 isoform=cb4016_c7;full_length_coverage=1;non_full_length_coverage=0;isoform_length=2879;expected_accuracy=0.998	gnl|BL_ORD_ID|41579 cb4016_c30/f1p1/3132 isoform=cb4016_c30;full_length_coverage=1;non_full_length_coverage=1;isoform_length=3132;expected_accuracy=0.994	204	2879	16	205	382	3072	1	194
cb548_c80/f1p0/3220	gnl|BL_ORD_ID|46779 cb548_c109/f1p0/3280 isoform=cb548_c109;full_length_coverage=1;non_full_length_coverage=0;isoform_length=3280;expected_accuracy=0.996	1336	3066	1	1339	1551	3280	2	1340
cb8564_c23232/f1p3/2540	gnl|BL_ORD_ID|10640 cb8564_c100234/f2p5/2744 isoform=cb8564_c100234;full_length_coverage=2;non_full_length_coverage=5;isoform_length=2744;expected_accuracy=0.993	16	2172	2167	2518	10	2176	2278	2629
cb8564_c23232/f1p3/2540	gnl|BL_ORD_ID|13229 cb8564_c19083/f1p6/2707 isoform=cb8564_c19083;full_length_coverage=1;non_full_length_coverage=6;isoform_length=2707;expected_accuracy=0.992	16	2172	2167	2518	8	2201	2303	2654
cb5613_c4/f6p1/2465 isoform=cb5613_c4;full_length_coverage=6;non_full_length_coverage=1;isoform_length=2465;expected_accuracy=1.000	gnl|BL_ORD_ID|44685 cb5613_c26/f1p0/3883 isoform=cb5613_c26;full_length_coverage=1;non_full_length_coverage=0;isoform_length=3883;expected_accuracy=0.994	103	1947	1944	2465	1	1841	3361	3882
cb1119_c6/f1p0/2308 isoform=cb1119_c6;full_length_coverage=1;non_full_length_coverage=0;isoform_length=2308;expected_accuracy=0.994	gnl|BL_ORD_ID|1542 cb1119_c3/f1p1/4475 isoform=cb1119_c3;full_length_coverage=1;non_full_length_coverage=1;isoform_length=4475;expected_accuracy=0.998	1039	2308	1	1040	1572	2844	123	1164
cb4263_c3/f1p1/3036	gnl|BL_ORD_ID|43864 cb4263_c5/f1p1/3378	264	3033	1	265	620	337	78	342

	isoform=cb4263_c5;full_length_coverage=1;non_full_length_coverage=1;isoform_length=3378;expected_accuracy=1.000						8		
cb3163_c7/f2p0/2607 isoform=cb3163_c7;full_length_coverage=2;non_full_length_coverage=0;isoform_length=2607;expected_accuracy=1.000	gnl|BL_ORD_ID|38392 cb3163_c13/f1p0/2685 isoform=cb3163_c13;full_length_coverage=1;non_full_length_coverage=0;isoform_length=2685;expected_accuracy=0.995	337	2604	53	337	422	2685	3	286
cb1333_c101/f1p0/1395 isoform=cb1333_c101;full_length_coverage=1;non_full_length_coverage=0;isoform_length=1395;expected_accuracy=0.997	gnl|BL_ORD_ID|61259 cb1333_c109/f1p0/5379	149	1395	18	150	3609	4853	17	148
cb18275_c11/f1p1/1730	gnl|BL_ORD_ID|11648 cb8564_c124380/f2p2/2924 isoform=cb8564_c124380;full_length_coverage=2;non_full_length_coverage=2;isoform_length=2924;expected_accuracy=1.000	131	1733	1	134	1321	2924	999	1132
cb548_c121/f65p0/2919 isoform=cb548_c121;full_length_coverage=65;non_full_length_coverage=0;isoform_length=2919;expected_accuracy=1.000	gnl|BL_ORD_ID|46779 cb548_c109/f1p0/3280 isoform=cb548_c109;full_length_coverage=1;non_full_length_coverage=0;isoform_length=3280;expected_accuracy=0.996	1334	2919	1	1337	1551	3140	6	1340
cb5063_c0/f2p0/1849	gnl|BL_ORD_ID|42228 cb5063_c9/f1p0/2367 isoform=cb5063_c9;full_length_coverage=1;non_full_length_coverage=0;isoform_length=2367;expected_accuracy=0.994	1	1200	1198	1826	350	1550	1739	2367
cb1956_c5/f2p0/2813 isoform=cb1956_c5;full_length_coverage=2;non_full_length_coverage=0;isoform_length=2813;expected_accur	gnl|BL_ORD_ID|66538 cb1956_c12/f1p0/3120	1	1453	1451	2813	162	1610	1770	3129

acy=0.997									
cb567_c11/f1p1/3120 isoform=cb567_c11;full_length_coverage=1;non_full_length_coverage=1;isoform_length=3120;expected_accuracy=0.993	gnl|BL_ORD_ID|45926 cb567_c4/f1p0/3256 isoform=cb567_c4;full_length_coverage=1;non_full_length_coverage=0;isoform_length=3256;expected_accuracy=0.996	1	2705	2703	3120	20	2689	2829	3240
cb8564_c37361/f1p0/3076	gnl|BL_ORD_ID|15152 cb8564_c38629/f2p1/3246 isoform=cb8564_c38629;full_length_coverage=2;non_full_length_coverage=1;isoform_length=3246;expected_accuracy=0.993	1	2481	2477	3070	1	2511	2612	3206
cb8564_c5240/f1p0/2480	gnl|BL_ORD_ID|55735 cb8564_c5247/f1p0/2805	13	2066	2066	2486	22	2074	2188	2608
cb1540_c27/f1p0/3741	gnl|BL_ORD_ID|28146 cb1540_c4/f2p0/3874 isoform=cb1540_c4;full_length_coverage=2;non_full_length_coverage=0;isoform_length=3874;expected_accuracy=0.998	304	3535	1	303	531	3788	1	302
cb8564_c110147/f1p0/3399 isoform=cb8564_c110147;full_length_coverage=1;non_full_length_coverage=0;isoform_length=3399;expected_accuracy=0.999	gnl|BL_ORD_ID|10567 cb8564_c118715/f1p0/3592 isoform=cb8564_c118715;full_length_coverage=1;non_full_length_coverage=0;isoform_length=3592;expected_accuracy=0.996	1	2834	2832	3347	6	2848	3078	3590
cb1188_c0/f1p0/4430 isoform=cb1188_c0;full_length_coverage=1;non_full_length_coverage=0;isoform_length=4430;expected_accuracy=0.999	gnl|BL_ORD_ID|24353 cb1188_c2/f1p0/5011 isoform=cb1188_c2;full_length_coverage=1;non_full_length_coverage=0;isoform_length=5011;expected_accuracy=0.997	1	3659	3658	4378	54	3736	4160	4853
cb8564_c24235/f1p0/3615	gnl|BL_ORD_ID|11514 cb8564_c12502/f1p0/4194 isoform=cb8564_c12502;full_length_coverage=1;non_full_length_coverage=0;isoform_length=4194;expected_ac	1422	3374	13	1424	2077	4029	72	1480

	curacy=0.998								
cb5158_c1/f2p0/1308 isoform=cb5158_c1;full_length_coverage=2;non_full_length_coverage=0;isoform_length=1308;expected_accuracy=0.998	gnl|BL_ORD_ID|73336 cb5158_c2/f1p0/3008	153	1308	2	156	1790	2949	72	222
cb5600_c17/f1p1/2696 isoform=cb5600_c17;full_length_coverage=1;non_full_length_coverage=1;isoform_length=2696;expected_accuracy=0.997	gnl|BL_ORD_ID|46920 cb5600_c22/f9p0/2763 isoform=cb5600_c22;full_length_coverage=9;non_full_length_coverage=0;isoform_length=2763;expected_accuracy=1.000	1	2172	2169	2615	14	2186	2318	2763
cb8564_c82539/f7p3/2550	gnl|BL_ORD_ID|13431 cb8564_c19588/f1p0/3337 isoform=cb8564_c19588;full_length_coverage=1;non_full_length_coverage=0;isoform_length=3337;expected_accuracy=0.997	1	2040	2040	2475	2	2039	2902	3337
cb8564_c50089/f1p0/2929 isoform=cb8564_c50089;full_length_coverage=1;non_full_length_coverage=0;isoform_length=2929;expected_accuracy=0.993	gnl|BL_ORD_ID|11125 cb8564_c107332/f9p1/3308 isoform=cb8564_c107332;full_length_coverage=9;non_full_length_coverage=1;isoform_length=3308;expected_accuracy=0.997	1	2301	2305	2929	42	2347	2646	3272
cb8564_c36731/f1p2/2761	gnl|BL_ORD_ID|16219 cb8564_c47828/f1p0/3035 isoform=cb8564_c47828;full_length_coverage=1;non_full_length_coverage=0;isoform_length=3035;expected_accuracy=0.991	1	1977	1972	2734	1	1982	2276	3035
cb3344_c6/f1p0/2126 isoform=cb3344_c6;full_length_coverage=1;non_full_length_coverage=0;isoform_length=2126;expected_accuracy=0.997	gnl|BL_ORD_ID|40986 cb3344_c2/f4p1/3653 isoform=cb3344_c2;full_length_coverage=4;non_full_length_coverage=1;isoform_length=3653;expected_accuracy=0.998	1	1431	1430	2125	58	1488	2819	3517
cb8564_c45864/f1p0/2965	gnl|BL_ORD_ID|17556 cb8564_c9820/f2p0/3032	16	1705	1703	2601	13	170	2131	302

	isoform=cb8564_c9820;full_length_coverage=2;non_full_length_coverage=0;isoform_length=3032;expected_accuracy=0.995						4		9
cb3557_c15/f1p0/2674 isoform=cb3557_c15;full_length_coverage=1;non_full_length_coverage=0;isoform_length=2674;expected_accuracy=0.995	gnl|BL_ORD_ID|39538 cb3557_c9/f1p0/3261 isoform=cb3557_c9;full_length_coverage=1;non_full_length_coverage=0;isoform_length=3261;expected_accuracy=0.995	1	1978	1976	2627	83	2056	2608	3253
cb8564_c129638/f1p0/2479	gnl|BL_ORD_ID|13043 cb8564_c170549/f2p3/3030 isoform=cb8564_c170549;full_length_coverage=2;non_full_length_coverage=3;isoform_length=3030;expected_accuracy=0.997	1	1627	1623	2475	79	1705	2110	2962
cb8564_c45300/f1p4/3014 isoform=cb8564_c45300;full_length_coverage=1;non_full_length_coverage=4;isoform_length=3014;expected_accuracy=0.996	gnl|BL_ORD_ID|56002 cb8564_c51722/f1p1/3503	243	3014	22	246	442	3214	67	285
cb1467_c0/f2p0/4330 isoform=cb1467_c0;full_length_coverage=2;non_full_length_coverage=0;isoform_length=4330;expected_accuracy=0.994	gnl|BL_ORD_ID|63644 cb1467_c5/f1p0/5220	382	4205	1	384	1092	4915	31	416
cb8564_c84266/f1p2/3194	gnl|BL_ORD_ID|13766 cb8564_c18520/f2p0/3299 isoform=cb8564_c18520;full_length_coverage=2;non_full_length_coverage=0;isoform_length=3299;expected_accuracy=0.992	1	2064	2064	3165	27	2089	2198	3299
cb6277_c13/f1p0/2679	gnl|BL_ORD_ID|4562 cb6277_c30/f4p0/2856 isoform=cb6277_c30;full_length_coverage=4;non_full_length_coverage=0;isoform_length=2856;expected_accur	202	2669	1	203	392	2856	88	290

	acy=1.000								
cb8564_c11542/f1p2/2858 isoform=cb8564_c11542;full_length_coverage=1;non_full_length_coverage=2;isoform_length=2858;expected_accuracy=0.997	gnl|BL_ORD_ID|13635 cb8564_c21316/f1p2/2982 isoform=cb8564_c21316;full_length_coverage=1;non_full_length_coverage=2;isoform_length=2982;expected_accuracy=0.997	1	1977	1976	2829	1	1978	2146	2982
cb3079_c0/f10p0/1714 isoform=cb3079_c0;full_length_coverage=10;non_full_length_coverage=0;isoform_length=1714;expected_accuracy=1.000	gnl|BL_ORD_ID|38504 cb3079_c4/f1p0/2325 isoform=cb3079_c4;full_length_coverage=1;non_full_length_coverage=0;isoform_length=2325;expected_accuracy=0.992	1	1059	1057	1714	1	1057	1636	2292
cb8564_c124198/f2p8/1922 isoform=cb8564_c124198;full_length_coverage=2;non_full_length_coverage=8;isoform_length=1922;expected_accuracy=0.995	gnl|BL_ORD_ID|13132 cb8564_c16589/f1p7/2477 isoform=cb8564_c16589;full_length_coverage=1;non_full_length_coverage=7;isoform_length=2477;expected_accuracy=0.999	1	1074	1075	1922	1	1074	1632	2477
cb8564_c14344/f1p0/2533 isoform=cb8564_c14344;full_length_coverage=1;non_full_length_coverage=0;isoform_length=2533;expected_accuracy=0.993	gnl|BL_ORD_ID|18906 cb8564_c90449/f1p3/3223 isoform=cb8564_c90449;full_length_coverage=1;non_full_length_coverage=3;isoform_length=3223;expected_accuracy=0.996	148	2524	1	151	846	3223	33	182
cb1279_c85/f1p0/1794 isoform=cb1279_c85;full_length_coverage=1;non_full_length_coverage=0;isoform_length=1794;expected_accuracy=0.998	gnl|BL_ORD_ID|25310 cb1279_c9/f3p1/2959 isoform=cb1279_c9;full_length_coverage=3;non_full_length_coverage=1;isoform_length=2959;expected_accuracy=1.000	1	1120	1115	1794	37	1154	2276	2959
cb8564_c9531/f3p0/2496 isoform=cb8564_c9531;full_length_coverage=3;non_full_length_coverage=0;isoform_length=2496;expected_accuracy=0.999	gnl|BL_ORD_ID|53971 cb8564_c16286/f1p0/3853	1	1996	1993	2496	3	1999	3147	3650
cb8564_c80798/f1p1/3793	gnl|BL_ORD_ID|67953 cb18691_c0/f1p1/5509	425	3603	52	427	2230	540	23	410

							5		
cb373_c39/f1p0/4415	gnl|BL_ORD_ID|41210 cb373_c12/f1p1/3932 isoform=cb373_c12;full_length_coverage=1;non_full_length_coverage=1;isoform_length=3932;expected_accuracy=0.996	16	3355	3354	3759	6	3348	3527	3932
cb8564_c109758/f2p1/2747 isoform=cb8564_c109758;full_length_coverage=2;non_full_length_coverage=1;isoform_length=2747;expected_accuracy=0.997	gnl|BL_ORD_ID|11141 cb8564_c109409/f3p1/2839 isoform=cb8564_c109409;full_length_coverage=3;non_full_length_coverage=1;isoform_length=2839;expected_accuracy=0.995	22	2255	2255	2747	1	2233	2347	2839
cb12931_c15/f1p0/1493 isoform=cb12931_c15;full_length_coverage=1;non_full_length_coverage=0;isoform_length=1493;expected_accuracy=1.000	gnl|BL_ORD_ID|55574 cb8564_c45699/f1p0/3005	1	1106	1104	1493	1	1084	2462	2849
cb8564_c136465/f1p3/3229 isoform=cb8564_c136465;full_length_coverage=1;non_full_length_coverage=3;isoform_length=3229;expected_accuracy=0.992	gnl|BL_ORD_ID|16338 cb8564_c53034/f1p2/3495 isoform=cb8564_c53034;full_length_coverage=1;non_full_length_coverage=2;isoform_length=3495;expected_accuracy=0.998	129	3162	1	133	468	3493	12	145
cb8564_c41756/f1p0/2738 isoform=cb8564_c41756;full_length_coverage=1;non_full_length_coverage=0;isoform_length=2738;expected_accuracy=0.996	gnl|BL_ORD_ID|55213 cb8564_c35568/f1p0/2997	1	2173	2170	2738	13	2200	2323	2889
cb4493_c8/f1p0/2014 isoform=cb4493_c8;full_length_coverage=1;non_full_length_coverage=0;isoform_length=2014;expected_accuracy=0.991	gnl|BL_ORD_ID|42702 cb4493_c7/f1p1/3006 isoform=cb4493_c7;full_length_coverage=1;non_full_length_coverage=1;isoform_length=3006;expected_accuracy=0.995	1	1023	1024	2014	152	1178	1992	3000
cb4674_c38/f1p2/3131	gnl|BL_ORD_ID|43463 cb4674_c77/f1p2/3189	13	2405	2404	3062	1	239	2533	318

<table>
<tr><th></th><th></th><th></th><th></th><th></th><th></th><th></th><th></th><th></th><th></th></tr>
<tr><td></td><td>isoform=cb4674_c77;full_length_coverage=1;non_full_length_coverage=2;isoform_length=3189;expected_accuracy=0.999</td><td></td><td></td><td></td><td></td><td></td><td>2</td><td></td><td>9</td></tr>
<tr><td>cb4469_c6/f1p0/2503</td><td>gnl|BL_ORD_ID|44310 cb4469_c5/f1p0/2979 isoform=cb4469_c5;full_length_coverage=1;non_full_length_coverage=0;isoform_length=2979;expected_accuracy=0.997</td><td>1</td><td>1835</td><td>1834</td><td>2500</td><td>59</td><td>1891</td><td>2257</td><td>2921</td></tr>
<tr><td>cb309_c19/f1p0/1785 isoform=cb309_c19;full_length_coverage=1;non_full_length_coverage=0;isoform_length=1785;expected_accuracy=0.996</td><td>gnl|BL_ORD_ID|69547 cb309_c21/f1p0/4520</td><td>1</td><td>1335</td><td>1336</td><td>1785</td><td>2386</td><td>3720</td><td>3936</td><td>4385</td></tr>
<tr><td>cb8564_c49821/f1p1/2905</td><td>gnl|BL_ORD_ID|14278 cb8564_c36280/f1p0/3133 isoform=cb8564_c36280;full_length_coverage=1;non_full_length_coverage=0;isoform_length=3133;expected_accuracy=1.000</td><td>1</td><td>1544</td><td>1541</td><td>2881</td><td>43</td><td>1586</td><td>1804</td><td>3115</td></tr>
<tr><td>cb8564_c48645/f1p1/3287</td><td>gnl|BL_ORD_ID|55939 cb8564_c49630/f1p4/3487</td><td>17</td><td>2918</td><td>2918</td><td>3113</td><td>10</td><td>2913</td><td>3243</td><td>3438</td></tr>
<tr><td>cb6181_c7/f6p0/2362 isoform=cb6181_c7;full_length_coverage=6;non_full_length_coverage=0;isoform_length=2362;expected_accuracy=1.000</td><td>gnl|BL_ORD_ID|49488 cb6181_c15/f1p0/3250</td><td>1</td><td>1260</td><td>1260</td><td>2362</td><td>11</td><td>1270</td><td>1486</td><td>2590</td></tr>
<tr><td>cb8564_c40598/f1p4/3119</td><td>gnl|BL_ORD_ID|15063 cb8564_c37149/f1p9/3212 isoform=cb8564_c37149;full_length_coverage=1;non_full_length_coverage=9;isoform_length=3212;expected_accuracy=0.991</td><td>19</td><td>2732</td><td>2729</td><td>3050</td><td>61</td><td>2772</td><td>2893</td><td>3212</td></tr>
<tr><td>cb8564_c183213/f1p3/3190</td><td>gnl|BL_ORD_ID|57750 cb8564_c79653/f1p0/3642</td><td>1</td><td>2313</td><td>2318</td><td>3110</td><td>64</td><td>237</td><td>2703</td><td>349</td></tr>
</table>

isoform=cb8564_c183213;full_length_coverage=1;non_full_length_coverage=3;isoform_length=3190;expected_accuracy=0.999							6		5
cb8564_c44698/f1p5/2843 isoform=cb8564_c44698;full_length_coverage=1;non_full_length_coverage=5;isoform_length=2843;expected_accuracy=0.997	gnl|BL_ORD_ID|55327 cb8564_c38462/f1p0/3241	1	2516	2511	2828	1	2503	2624	2946
cb2101_c8/f1p0/1743	gnl|BL_ORD_ID|66713 cb2101_c4/f1p0/3801	13	1455	1454	1736	968	2412	3383	3665
cb8564_c44698/f1p5/2843 isoform=cb8564_c44698;full_length_coverage=1;non_full_length_coverage=5;isoform_length=2843;expected_accuracy=0.997	gnl|BL_ORD_ID|55341 cb8564_c38927/f1p0/3326	1	2516	2511	2832	7	2518	2639	2966
cb965_c18/f1p0/2545 isoform=cb965_c18;full_length_coverage=1;non_full_length_coverage=0;isoform_length=2545;expected_accuracy=1.000	gnl|BL_ORD_ID|20577 cb965_c22/f1p1/4708 isoform=cb965_c22;full_length_coverage=1;non_full_length_coverage=1;isoform_length=4708;expected_accuracy=0.994	1	1828	1826	2543	1497	3320	3990	4708
cb8564_c177939/f1p0/1977 isoform=cb8564_c177939;full_length_coverage=1;non_full_length_coverage=0;isoform_length=1977;expected_accuracy=1.000	gnl|BL_ORD_ID|54765 cb8564_c2442/f1p1/2806	1	1724	1723	1974	2	1723	2241	2492
cb2265_c8/f1p0/3520	gnl|BL_ORD_ID|36890 cb2265_c4/f1p1/3992 isoform=cb2265_c4;full_length_coverage=1;non_full_length_coverage=1;isoform_length=3992;expected_accuracy=0.997	1	2407	2404	3346	20	2427	3049	3990
cb8564_c15653/f1p4/2475	gnl|BL_ORD_ID|58179 cb8564_c90747/f1p0/2795	22	2293	2289	2441	32	227	2495	264

							6		7
cb2097_c58/f1p0/2505 isoform=cb2097_c58;full_length_coverage=1;non_full_length_coverage=0;isoform_length=2505;expected_accuracy=0.998	gnl|BL_ORD_ID|35690 cb2097_c59/f1p0/3019 isoform=cb2097_c59;full_length_coverage=1;non_full_length_coverage=0;isoform_length=3019;expected_accuracy=0.995	1	1583	1581	2505	5	1587	2095	3019
cb8564_c15230/f1p0/1944	gnl|BL_ORD_ID|55860 cb8564_c47209/f1p0/3227	352	1920	8	357	1091	2657	16	381
cb5772_c13/f1p0/1660	gnl|BL_ORD_ID|44721 cb5772_c4/f3p1/1610 isoform=cb5772_c4;full_length_coverage=3;non_full_length_coverage=1;isoform_length=1610;expected_accuracy=1.000	168	1506	9	170	271	1608	4	170
cb8564_c4757/f1p0/2740	gnl|BL_ORD_ID|55688 cb8564_c4747/f1p0/2838	103	1661	1659	2702	1	1559	1807	2853
cb8564_c44063/f1p0/2503 isoform=cb8564_c44063;full_length_coverage=1;non_full_length_coverage=0;isoform_length=2503;expected_accuracy=0.999	gnl|BL_ORD_ID|53915 cb8564_c14767/f1p3/2856	397	2455	4	402	549	2607	5	404
cb17388_c6/f1p2/1438 isoform=cb17388_c6;full_length_coverage=1;non_full_length_coverage=2;isoform_length=1438;expected_accuracy=0.992	gnl|BL_ORD_ID|10293 cb8564_c118743/f1p2/1948 isoform=cb8564_c118743;full_length_coverage=1;non_full_length_coverage=2;isoform_length=1948;expected_accuracy=0.998	1	1088	1086	1438	2	1085	1276	1626
cb8564_c125478/f1p1/2270 isoform=cb8564_c125478;full_length_coverage=1;non_full_length_coverage=1;isoform_length=2270;expected_accuracy=1.000	gnl|BL_ORD_ID|11370 cb8564_c139900/f2p2/3938 isoform=cb8564_c139900;full_length_coverage=2;non_full_length_coverage=2;isoform_length=3938;expected_accuracy=0.998	221	2270	1	222	1861	3912	147	364
cb8564_c68992/f1p5/3235	gnl|BL_ORD_ID|12196 cb8564_c12654/f2p0/4122	1	1908	1908	3235	2	190	2784	410

isoform=cb8564_c68992;full_length_coverage=1;non_full_length_coverage=5;isoform_length=3235;expected_accuracy=0.995	isoform=cb8564_c12654;full_length_coverage=2;non_full_length_coverage=0;isoform_length=4122;expected_accuracy=1.000						1		9
cb8564_c146072/f3p3/2176 isoform=cb8564_c146072;full_length_coverage=3;non_full_length_coverage=3;isoform_length=2176;expected_accuracy=1.000	gnl|BL_ORD_ID|12438 cb8564_c125968/f2p3/2612 isoform=cb8564_c125968;full_length_coverage=2;non_full_length_coverage=3;isoform_length=2612;expected_accuracy=0.997	211	2165	1	215	561	2517	16	231
cb8564_c24286/f1p0/3377	gnl|BL_ORD_ID|13846 cb8564_c22100/f1p0/4833 isoform=cb8564_c22100;full_length_coverage=1;non_full_length_coverage=0;isoform_length=4833;expected_accuracy=0.995	1	2887	2886	3386	1270	4142	4252	4753
cb3665_c20/f1p0/2779	gnl|BL_ORD_ID|40380 cb3665_c0/f7p0/2852 isoform=cb3665_c0;full_length_coverage=7;non_full_length_coverage=0;isoform_length=2852;expected_accuracy=1.000	1	1482	1479	2669	49	1530	1661	2851
cb4098_c13/f2p1/3772 isoform=cb4098_c13;full_length_coverage=2;non_full_length_coverage=1;isoform_length=3772;expected_accuracy=0.998	gnl|BL_ORD_ID|40755 cb4098_c12/f2p1/4674 isoform=cb4098_c12;full_length_coverage=2;non_full_length_coverage=1;isoform_length=4674;expected_accuracy=0.998	1	2629	2627	3772	112	2737	3514	4659
cb938_c15/f1p0/3219	gnl|BL_ORD_ID|19887 cb938_c12/f1p0/3486 isoform=cb938_c12;full_length_coverage=1;non_full_length_coverage=0;isoform_length=3486;expected_accuracy=0.996	1	2546	2541	3181	1	2568	2844	3486
cb8564_c45300/f1p4/3014 isoform=cb8564_c45300;full_length_coverage=1;non_full_length_coverage=4;isoform_length=3014;expected_	gnl|BL_ORD_ID|16681 cb8564_c54233/f1p1/3194 isoform=cb8564_c54233;full_length_coverage=1;non_full_length_coverage=1;isoform_length=3194;expected_ac	243	3012	2	246	428	3194	8	271

<table>
<tr><td>accuracy=0.996</td><td>curacy=0.995</td><td></td><td></td><td></td><td></td><td></td><td></td><td></td><td></td></tr>
<tr><td>cb13434_c67/f1p0/1520</td><td>gnl|BL_ORD_ID|52285 cb8564_c116906/f1p1/2461</td><td>227</td><td>1366</td><td>3</td><td>227</td><td>1360</td><td>2501</td><td>2</td><td>226</td></tr>
<tr><td>cb8564_c39136/f1p0/3036 isoform=cb8564_c39136;full_length_coverage=1;non_full_length_coverage=0;isoform_length=3036;expected_accuracy=0.998</td><td>gnl|BL_ORD_ID|55377 cb8564_c39822/f1p0/3306</td><td>1</td><td>2089</td><td>2085</td><td>3035</td><td>1</td><td>2092</td><td>2219</td><td>3169</td></tr>
<tr><td>cb99_c7/f1p0/3479 isoform=cb99_c7;full_length_coverage=1;non_full_length_coverage=0;isoform_length=3479;expected_accuracy=0.990</td><td>gnl|BL_ORD_ID|59047 cb99_c10/f1p0/4693</td><td>144</td><td>3405</td><td>1</td><td>146</td><td>1335</td><td>4609</td><td>17</td><td>161</td></tr>
<tr><td>cb1417_c11/f1p0/3544 isoform=cb1417_c11;full_length_coverage=1;non_full_length_coverage=0;isoform_length=3544;expected_accuracy=0.991</td><td>gnl|BL_ORD_ID|61352 cb1417_c6/f1p1/4358</td><td>438</td><td>3544</td><td>8</td><td>438</td><td>1279</td><td>4387</td><td>12</td><td>438</td></tr>
<tr><td>cb8564_c36635/f1p0/4030 isoform=cb8564_c36635;full_length_coverage=1;non_full_length_coverage=0;isoform_length=4030;expected_accuracy=0.998</td><td>gnl|BL_ORD_ID|14953 cb8564_c34238/f4p1/4317 isoform=cb8564_c34238;full_length_coverage=4;non_full_length_coverage=1;isoform_length=4317;expected_accuracy=1.000</td><td>184</td><td>4030</td><td>1</td><td>189</td><td>326</td><td>4173</td><td>2</td><td>183</td></tr>
<tr><td>cb8564_c178226/f1p0/2728 isoform=cb8564_c178226;full_length_coverage=1;non_full_length_coverage=0;isoform_length=2728;expected_accuracy=0.998</td><td>gnl|BL_ORD_ID|55504 cb8564_c43546/f1p1/3276</td><td>1284</td><td>2715</td><td>1</td><td>1288</td><td>1422</td><td>2854</td><td>10</td><td>1318</td></tr>
<tr><td>cb5125_c34/f1p0/1890</td><td>gnl|BL_ORD_ID|42296 cb5125_c42/f1p3/2079 isoform=cb5125_c42;full_length_coverage=1;non_full_length_coverage=3;isoform_length=2079;expected_accur</td><td>1</td><td>1145</td><td>1144</td><td>1780</td><td>1</td><td>1147</td><td>1339</td><td>1975</td></tr>
</table>

	acy=0.992								
cb8642_c15/f1p1/2588 isoform=cb8642_c15;full_length_coverage=1;non_full_length_coverage=1;isoform_length=2588;expected_accuracy=0.994	gnl|BL_ORD_ID|18905 cb8642_c7/f1p0/3885 isoform=cb8642_c7;full_length_coverage=1;non_full_length_coverage=0;isoform_length=3885;expected_accuracy=1.000	1	1515	1512	2512	4	1522	2886	3885
cb560_c8/f1p0/3610 isoform=cb560_c8;full_length_coverage=1;non_full_length_coverage=0;isoform_length=3610;expected_accuracy=0.995	gnl|BL_ORD_ID|45710 cb560_c5/f1p0/4128 isoform=cb560_c5;full_length_coverage=1;non_full_length_coverage=0;isoform_length=4128;expected_accuracy=0.998	1	2431	2429	3610	1	2447	2926	4107
cb4449_c1/f2p0/3029 isoform=cb4449_c1;full_length_coverage=2;non_full_length_coverage=0;isoform_length=3029;expected_accuracy=1.000	gnl|BL_ORD_ID|41957 cb4449_c4/f1p0/3414 isoform=cb4449_c4;full_length_coverage=1;non_full_length_coverage=0;isoform_length=3414;expected_accuracy=0.996	1	2505	2504	3029	3	2503	2807	3334
cb10299_c0/f5p0/1323 isoform=cb10299_c0;full_length_coverage=5;non_full_length_coverage=0;isoform_length=1323;expected_accuracy=0.999	gnl|BL_ORD_ID|48353 cb10299_c7/f1p0/3268	99	1323	1	102	2035	3260	10	107
cb8564_c44698/f1p5/2843 isoform=cb8564_c44698;full_length_coverage=1;non_full_length_coverage=5;isoform_length=2843;expected_accuracy=0.997	gnl|BL_ORD_ID|55291 cb8564_c37409/f1p0/3153	1	2516	2511	2825	1	2507	2628	2948
cb2206_c10/f1p0/2608	gnl|BL_ORD_ID|36912 cb2206_c7/f1p0/2717 isoform=cb2206_c7;full_length_coverage=1;non_full_length_coverage=0;isoform_length=2717;expected_accuracy=0.997	1	2059	2059	2605	1	2055	2161	2707
cb12698_c19/f1p4/1796	gnl|BL_ORD_ID|21291 cb9797_c33/f1p1/2260	1	1262	1259	1754	21	128	1766	225

	isoform=cb9797_c33;full_length_coverage=1;non_full_length_coverage=1;isoform_length=2260;expected_accuracy=0.995						7		9
cb8564_c186356/f1p0/2744 isoform=cb8564_c186356;full_length_coverage=1;non_full_length_coverage=0;isoform_length=2744;expected_accuracy=0.998	gnl|BL_ORD_ID|16369 cb8564_c52191/f1p0/2822 isoform=cb8564_c52191;full_length_coverage=1;non_full_length_coverage=0;isoform_length=2822;expected_accuracy=0.997	1393	2744	132	1396	1468	2819	84	1348
cb2737_c57/f1p0/2959	gnl|BL_ORD_ID|36735 cb2737_c13/f1p0/3105 isoform=cb2737_c13;full_length_coverage=1;non_full_length_coverage=0;isoform_length=3105;expected_accuracy=0.995	1	1609	1607	2862	69	1676	1808	3066
cb3366_c9/f1p1/2799 isoform=cb3366_c9;full_length_coverage=1;non_full_length_coverage=1;isoform_length=2799;expected_accuracy=0.996	gnl|BL_ORD_ID|39487 cb3366_c10/f1p0/2875 isoform=cb3366_c10;full_length_coverage=1;non_full_length_coverage=0;isoform_length=2875;expected_accuracy=0.996	1	1619	1618	2730	8	1624	1764	2875
cb322_c45/f1p0/3654	gnl|BL_ORD_ID|40328 cb322_c11/f1p1/4829 isoform=cb322_c11;full_length_coverage=1;non_full_length_coverage=1;isoform_length=4829;expected_accuracy=0.990	1	2715	2715	3645	75	2801	3133	4065
cb1956_c14/f1p0/2988 isoform=cb1956_c14;full_length_coverage=1;non_full_length_coverage=0;isoform_length=2988;expected_accuracy=0.998	gnl|BL_ORD_ID|66540 cb1956_c20/f1p0/3189	1	2566	2571	2988	20	2607	2744	3157
cb6365_c0/f5p2/1956 isoform=cb6365_c0;full_length_coverage=5;non_full_length_coverage=2;isoform_length=1956;expected_accur	gnl|BL_ORD_ID|49656 cb6365_c14/f1p0/2471	115	1956	1	119	247	2087	1	119

acy=0.998											
cb8564_c36242/f1p0/3335	gnl|BL_ORD_ID|55939 cb8564_c49630/f1p4/3487	2	2918	2918	3168	1	2913	3243	3493		
cb4807_c10/f1p0/3240	gnl|BL_ORD_ID|44272 cb4807_c19/f1p0/3259 isoform=cb4807_c19;full_length_coverage=1;non_full_length_coverage=0;isoform_length=3259;expected_accuracy=0.995	403	3196	50	404	462	3259	1	355		
cb14757_c8/f1p0/1464	gnl|BL_ORD_ID|64268 cb14757_c4/f1p0/1874	401	1392	5	403	891	1882	25	423		
cb8564_c44005/f1p0/3815 isoform=cb8564_c44005;full_length_coverage=1;non_full_length_coverage=0;isoform_length=3815;expected_accuracy=0.993	gnl|BL_ORD_ID|14247 cb8564_c45575/f3p6/4033 isoform=cb8564_c45575;full_length_coverage=3;non_full_length_coverage=6;isoform_length=4033;expected_accuracy=0.998	1	2046	2041	3815	48	2105	2205	3987		
cb5659_c78/f1p0/2309	gnl|BL_ORD_ID|45302 cb5659_c105/f4p0/2776 isoform=cb5659_c105;full_length_coverage=4;non_full_length_coverage=0;isoform_length=2776;expected_accuracy=1.000	1	1676	1675	2127	52	1745	2319	2776		
cb4674_c26/f1p1/3006 isoform=cb4674_c26;full_length_coverage=1;non_full_length_coverage=1;isoform_length=3006;expected_accuracy=0.995	gnl|BL_ORD_ID|43463 cb4674_c77/f1p2/3189 isoform=cb4674_c77;full_length_coverage=1;non_full_length_coverage=2;isoform_length=3189;expected_accuracy=0.999	1	2331	2330	2987	83	2392	2533	3189		
cb8564_c38979/f1p0/2930 isoform=cb8564_c38979;full_length_coverage=1;non_full_length_coverage=0;isoform_length=2930;expected_accuracy=0.996	gnl|BL_ORD_ID|56029 cb8564_c52782/f1p2/3327	310	2930	6	309	618	3237	11	289		
cb510_c2/f2p0/3807	gnl|BL_ORD_ID|72263 cb510_c18/f1p0/3980	1	2524	2523	3797	16	253	2638	390		

isoform=cb510_c2;full_length_coverage=2;non_full_length_coverage=0;isoform_length=3807;expected_accuracy=0.993							8		9
cb8564_c119852/f1p0/2378 isoform=cb8564_c119852;full_length_coverage=1;non_full_length_coverage=0;isoform_length=2378;expected_accuracy=1.000	gnl|BL_ORD_ID|52412 cb8564_c122038/f1p0/2819	13	1662	1661	2378	28	1677	1798	2516
cb4674_c38/f1p2/3131	gnl|BL_ORD_ID|72844 cb4674_c33/f1p3/3281	14	2231	2229	3083	3	2220	2382	3237
cb3270_c4/f1p2/3142 isoform=cb3270_c4;full_length_coverage=1;non_full_length_coverage=2;isoform_length=3142;expected_accuracy=0.999	gnl|BL_ORD_ID|41534 cb3270_c3/f1p0/3673 isoform=cb3270_c3;full_length_coverage=1;non_full_length_coverage=0;isoform_length=3673;expected_accuracy=0.997	1	1673	1670	3038	23	1698	2302	3671
cb11186_c0/f2p0/1697 isoform=cb11186_c0;full_length_coverage=2;non_full_length_coverage=0;isoform_length=1697;expected_accuracy=0.999	gnl|BL_ORD_ID|10 cb11186_c1/f1p0/2372 isoform=cb11186_c1;full_length_coverage=1;non_full_length_coverage=0;isoform_length=2372;expected_accuracy=0.992	112	1697	1	113	651	2232	29	147
cb12714_c29/f1p2/1583	gnl|BL_ORD_ID|13063 cb8564_c179457/f1p0/2647 isoform=cb8564_c179457;full_length_coverage=1;non_full_length_coverage=0;isoform_length=2647;expected_accuracy=1.000	1	1296	1293	1597	1	1275	2342	2646
cb8564_c23232/f1p3/2540	gnl|BL_ORD_ID|54246 cb8564_c21138/f1p3/2652	16	2172	2167	2508	5	2157	2259	2600
cb4367_c10/f1p1/1298 isoform=cb4367_c10;full_length_coverage=1;non_full_length_coverage=1;isoform_length=1298;expected_accu	gnl|BL_ORD_ID|42901 cb4367_c3/f1p1/2058 isoform=cb4367_c3;full_length_coverage=1;non_full_length_coverage=1;isoform_length=2058;expected_accura	1	1129	1126	1298	20	1140	1559	1730

racy=0.995	cy=0.991								
cb2629_c15/f1p0/2175 isoform=cb2629_c15;full_length_coverage=1;non_full_length_coverage=0;isoform_length=2175;expected_accuracy=0.991	gnl|BL_ORD_ID|34935 cb18639_c0/f2p0/5077 isoform=cb18639_c0;full_length_coverage=2;non_full_length_coverage=0;isoform_length=5077;expected_accuracy=0.998	1	1663	1662	2175	2572	4240	4351	4864
cb3750_c6/f2p0/2746 isoform=cb3750_c6;full_length_coverage=2;non_full_length_coverage=0;isoform_length=2746;expected_accuracy=0.999	gnl|BL_ORD_ID|40301 cb3750_c20/f1p3/3049 isoform=cb3750_c20;full_length_coverage=1;non_full_length_coverage=3;isoform_length=3049;expected_accuracy=0.992	1	2052	2054	2739	29	2092	2362	3049
cb4016_c32/f1p0/1913	gnl|BL_ORD_ID|41579 cb4016_c30/f1p1/3132 isoform=cb4016_c30;full_length_coverage=1;non_full_length_coverage=1;isoform_length=3132;expected_accuracy=0.994	191	1930	23	194	1297	3037	683	859
cb781_c33/f1p0/1887	gnl|BL_ORD_ID|9056 cb781_c29/f1p0/2108 isoform=cb781_c29;full_length_coverage=1;non_full_length_coverage=0;isoform_length=2108;expected_accuracy=0.993	1	1330	1330	1861	23	1352	1457	1986
cb16550_c1/f1p2/1845 isoform=cb16550_c1;full_length_coverage=1;non_full_length_coverage=2;isoform_length=1845;expected_accuracy=0.999	gnl|BL_ORD_ID|53316 cb8564_c121927/f1p0/2410	1	1212	1207	1833	94	1300	1523	2143
cb5583_c0/f4p0/1845 isoform=cb5583_c0;full_length_coverage=4;non_full_length_coverage=0;isoform_length=1845;expected_accuracy=1.000	gnl|BL_ORD_ID|74103 cb5583_c2/f1p0/2107	132	1772	1	132	422	2060	1	132
cb8564_c41162/f1p0/3158	gnl|BL_ORD_ID|12440 cb8564_c130435/f1p3/3226	11	2553	2548	3042	13	257	2711	321

	isoform=cb8564_c130435;full_length_coverage=1;non_full_length_coverage=3;isoform_length=3226;expected_accuracy=0.994						8		4
cb8564_c15653/f1p4/2475	gnl|BL_ORD_ID|17272 cb8564_c91851/f1p1/2644 isoform=cb8564_c91851;full_length_coverage=1;non_full_length_coverage=1;isoform_length=2644;expected_accuracy=0.993	14	2294	2289	2428	26	2287	2505	2644
cb9313_c5/f1p0/1570	gnl|BL_ORD_ID|20100 cb9313_c0/f2p0/1947 isoform=cb9313_c0;full_length_coverage=2;non_full_length_coverage=0;isoform_length=1947;expected_accuracy=0.999	183	1583	60	184	436	1836	32	156
cb2550_c6/f1p0/1894	gnl|BL_ORD_ID|38963 cb2550_c9/f8p1/2373 isoform=cb2550_c9;full_length_coverage=8;non_full_length_coverage=1;isoform_length=2373;expected_accuracy=1.000	1	1401	1397	1897	57	1460	1824	2321
cb8564_c4178/f1p0/2685	gnl|BL_ORD_ID|18914 cb8564_c972/f3p0/2746 isoform=cb8564_c972;full_length_coverage=3;non_full_length_coverage=0;isoform_length=2746;expected_accuracy=0.999	16	1983	1979	2503	153	2115	2223	2746
cb7849_c36/f57p2/2251 isoform=cb7849_c36;full_length_coverage=57;non_full_length_coverage=2;isoform_length=2251;expected_accuracy=1.000	gnl|BL_ORD_ID|51569 cb7849_c16/f1p1/3573	1	1328	1327	2250	17	1345	2622	3544
cb454_c24/f1p0/2537	gnl|BL_ORD_ID|43127 cb454_c39/f1p0/2534 isoform=cb454_c39;full_length_coverage=1;non_full_length_coverage=0;isoform_length=2534;expected_accura	225	2275	1	224	488	2534	158	381

	cy=1.000								
cb4128_c19/f1p0/2260	gnl|BL_ORD_ID|71807 cb4128_c11/f1p0/3379	1	1761	1760	2263	70	1830	2166	2669
cb8564_c182028/f1p0/2513 isoform=cb8564_c182028;full_length_coverage=1;non_full_length_coverage=0;isoform_length=2513;expected_accuracy=0.996	gnl|BL_ORD_ID|54611 cb8564_c127997/f1p3/2802	1	1971	1968	2513	1	1972	2116	2661
cb3307_c38/f1p0/2373	gnl|BL_ORD_ID|39617 cb3307_c21/f1p0/2162 isoform=cb3307_c21;full_length_coverage=1;non_full_length_coverage=0;isoform_length=2162;expected_accuracy=0.995	135	2020	1	137	278	2162	4	140
cb8422_c7/f1p0/2132	gnl|BL_ORD_ID|11122 cb8422_c8/f1p0/2339 isoform=cb8422_c8;full_length_coverage=1;non_full_length_coverage=0;isoform_length=2339;expected_accuracy=0.993	1	1480	1480	2144	37	1512	1674	2339
cb11067_c13/f1p0/1371 isoform=cb11067_c13;full_length_coverage=1;non_full_length_coverage=0;isoform_length=1371;expected_accuracy=0.993	gnl|BL_ORD_ID|49138 cb11067_c12/f1p0/1497	1	1177	1176	1325	74	1250	1359	1508
cb1334_c12/f1p0/6575	gnl|BL_ORD_ID|61264 cb1334_c10/f1p0/6801	388	6210	12	392	512	6334	1	382
cb1955_c24/f1p0/2186 isoform=cb1955_c24;full_length_coverage=1;non_full_length_coverage=0;isoform_length=2186;expected_accuracy=0.994	gnl|BL_ORD_ID|36339 cb1955_c43/f2p0/2634 isoform=cb1955_c43;full_length_coverage=2;non_full_length_coverage=0;isoform_length=2634;expected_accuracy=1.000	1	1479	1479	2186	101	1579	1902	2610
cb2818_c78/f1p0/2043	gnl|BL_ORD_ID|68900 cb2818_c40/f1p0/3487	1	1936	1935	2041	961	289	3288	339

isoform=cb2818_c78;full_length_coverage=1;non_full_length_coverage=0;isoform_length=2043;expected_accuracy=0.992							3		5
cb13753_c0/f1p0/1755	gnl|BL_ORD_ID|71489 cb3759_c8/f1p0/3290	377	1707	8	378	1864	3192	6	374
cb781_c7/f1p0/2984	gnl|BL_ORD_ID|49464 cb781_c6/f1p1/4637	113	3008	1	115	277	3193	63	177
cb8564_c76041/f1p0/1893	gnl|BL_ORD_ID|18863 cb8564_c82439/f1p4/3856 isoform=cb8564_c82439;full_length_coverage=1;non_full_length_coverage=4;isoform_length=3856;expected_accuracy=0.997	306	1675	31	308	2343	3707	1	276
cb1333_c96/f1p0/1554 isoform=cb1333_c96;full_length_coverage=1;non_full_length_coverage=0;isoform_length=1554;expected_accuracy=0.991	gnl|BL_ORD_ID|61259 cb1333_c109/f1p0/5379	143	1554	13	142	3498	4911	17	146
cb2176_c1/f2p2/3618 isoform=cb2176_c1;full_length_coverage=2;non_full_length_coverage=2;isoform_length=3618;expected_accuracy=1.000	gnl|BL_ORD_ID|34486 cb2176_c4/f1p1/3759 isoform=cb2176_c4;full_length_coverage=1;non_full_length_coverage=1;isoform_length=3759;expected_accuracy=0.994	442	3612	97	439	610	3759	147	488
cb7504_c3/f1p0/1972 isoform=cb7504_c3;full_length_coverage=1;non_full_length_coverage=0;isoform_length=1972;expected_accuracy=0.995	gnl|BL_ORD_ID|7872 cb7504_c10/f4p0/2301 isoform=cb7504_c10;full_length_coverage=4;non_full_length_coverage=0;isoform_length=2301;expected_accuracy=0.993	1	1003	1005	1972	5	1013	1299	2268
cb6730_c26/f1p1/2468 isoform=cb6730_c26;full_length_coverage=1;non_full_length_coverage=1;isoform_length=2468;expected_accu	gnl|BL_ORD_ID|3442 cb6730_c30/f4p0/2653 isoform=cb6730_c30;full_length_coverage=4;non_full_length_coverage=0;isoform_length=2653;expected_accur	1	1841	1842	2431	41	1883	2064	2653

racy=0.998	acy=1.000								
cb8564_c35300/f1p2/3912	gnl|BL_ORD_ID|14953 cb8564_c34238/f4p1/4317 isoform=cb8564_c34238;full_length_coverage=4;non_full_length_coverage=1;isoform_length=4317;expected_accuracy=1.000	185	3922	1	187	427	4165	1	179
cb1931_c1/f1p0/2305 isoform=cb1931_c1;full_length_coverage=1;non_full_length_coverage=0;isoform_length=2305;expected_accuracy=1.000	gnl|BL_ORD_ID|34818 cb1931_c0/f1p0/4121 isoform=cb1931_c0;full_length_coverage=1;non_full_length_coverage=0;isoform_length=4121;expected_accuracy=0.998	1	1914	1909	2305	66	1978	3706	4101
cb8564_c20202/f1p0/2556 isoform=cb8564_c20202;full_length_coverage=1;non_full_length_coverage=0;isoform_length=2556;expected_accuracy=0.999	gnl|BL_ORD_ID|14672 cb8564_c31831/f7p0/2817 isoform=cb8564_c31831;full_length_coverage=7;non_full_length_coverage=0;isoform_length=2817;expected_accuracy=0.997	1	2227	2224	2556	56	2300	2464	2786
cb3290_c38/f1p0/2345 isoform=cb3290_c38;full_length_coverage=1;non_full_length_coverage=0;isoform_length=2345;expected_accuracy=0.996	gnl|BL_ORD_ID|71041 cb3290_c116/f1p0/3359	1150	2331	1	1151	1629	2810	322	1472
cb8564_c40764/f1p0/2563 isoform=cb8564_c40764;full_length_coverage=1;non_full_length_coverage=0;isoform_length=2563;expected_accuracy=0.997	gnl|BL_ORD_ID|55433 cb8564_c41572/f1p1/3265	1	2167	2162	2563	1	2157	2363	2761
cb8564_c39620/f1p0/2913	gnl|BL_ORD_ID|56643 cb8564_c50449/f1p0/3504	2	2112	2111	2852	3	2113	2745	3486
cb1955_c21/f1p1/2842 isoform=cb1955_c21;full_length_coverage=1;non_full_length_coverage=1;isoform_length=2842;expected_accu	gnl|BL_ORD_ID|66532 cb1955_c22/f1p2/3580	1	2568	2569	2840	2	2568	3343	3617

racy=0.995									
cb453_c25/f17p1/2265 isoform=cb453_c25;full_length_coverage=17;non_full_length_coverage=1;isoform_length=2265;expected_accuracy=1.000	gnl|BL_ORD_ID|42376 cb453_c6/f1p0/4805 isoform=cb453_c6;full_length_coverage=1;non_full_length_coverage=0;isoform_length=4805;expected_accuracy=0.997	616	2265	66	618	3155	4805	1	552
cb7404_c15/f1p0/2068	gnl|BL_ORD_ID|51201 cb7404_c23/f1p1/2352	1	1292	1289	2071	6	1297	1441	2223
cb8564_c15653/f1p4/2475	gnl|BL_ORD_ID|54329 cb8564_c22496/f4p7/2826	1	2293	2289	2478	1	2293	2512	2701
cb3939_c29/f1p0/2382	gnl|BL_ORD_ID|39237 cb3939_c13/f1p0/3298 isoform=cb3939_c13;full_length_coverage=1;non_full_length_coverage=0;isoform_length=3298;expected_accuracy=0.997	1208	2324	100	1209	2148	3263	78	1185
cb4576_c12/f13p3/4007 isoform=cb4576_c12;full_length_coverage=13;non_full_length_coverage=3;isoform_length=4007;expected_accuracy=0.998	gnl|BL_ORD_ID|72706 cb4576_c62/f1p0/4079	466	3939	49	464	618	4091	27	443
cb3575_c11/f1p0/3124 isoform=cb3575_c11;full_length_coverage=1;non_full_length_coverage=0;isoform_length=3124;expected_accuracy=0.996	gnl|BL_ORD_ID|40181 cb3575_c12/f1p0/3408 isoform=cb3575_c12;full_length_coverage=1;non_full_length_coverage=0;isoform_length=3408;expected_accuracy=0.993	1	2662	2662	3124	8	2676	2942	3408
cb8564_c37361/f1p0/3076	gnl|BL_ORD_ID|55410 cb8564_c40893/f1p0/3594	1	2481	2477	3070	2	2483	2901	3478
cb8564_c46939/f1p1/2926	gnl|BL_ORD_ID|14539 cb8564_c46562/f2p3/3196 isoform=cb8564_c46562;full_length_coverage=2;non_full_length_coverage=3;isoform_length=3196;expected_ac	10	2134	2130	2870	16	2168	2273	3015

	curacy=0.995								
cb8564_c5023/f2p1/3612 isoform=cb8564_c5023;full_length_coverage=2;non_full_length_coverage=1;isoform_length=3612;expected_accuracy=0.995	gnl|BL_ORD_ID|12193 cb8564_c152656/f1p4/3688 isoform=cb8564_c152656;full_length_coverage=1;non_full_length_coverage=4;isoform_length=3688;expected_accuracy=0.997	1	3278	3277	3518	18	3314	3446	3688
cb8253_c9/f1p0/1760 isoform=cb8253_c9;full_length_coverage=1;non_full_length_coverage=0;isoform_length=1760;expected_accuracy=0.993	gnl|BL_ORD_ID|50912 cb8253_c5/f1p0/2152	1	1594	1594	1760	1	1605	1706	1872
cb8564_c41162/f1p0/3158	gnl|BL_ORD_ID|55182 cb8564_c34825/f1p2/3200	13	2553	2548	3041	6	2576	2707	3197
cb17859_c0/f1p0/1025 isoform=cb17859_c0;full_length_coverage=1;non_full_length_coverage=0;isoform_length=1025;expected_accuracy=0.991	gnl|BL_ORD_ID|12221 cb8564_c13026/f1p0/4872 isoform=cb8564_c13026;full_length_coverage=1;non_full_length_coverage=0;isoform_length=4872;expected_accuracy=0.996	132	1018	7	133	3985	4872	1	128
cb4077_c20/f1p0/1388	gnl|BL_ORD_ID|41226 cb4077_c18/f3p0/3453 isoform=cb4077_c18;full_length_coverage=3;non_full_length_coverage=0;isoform_length=3453;expected_accuracy=0.997	179	1416	30	182	2218	3453	1	152
cb8564_c75977/f1p0/2165	gnl|BL_ORD_ID|16611 cb8564_c71207/f1p0/4053 isoform=cb8564_c71207;full_length_coverage=1;non_full_length_coverage=0;isoform_length=4053;expected_accuracy=0.995	1	1393	1394	2126	122	1519	3327	4053
cb8564_c80368/f1p0/2872 isoform=cb8564_c80368;full_length_coverage=1;non_full_length_coverage=0;isoform_length=2872;expected_	gnl|BL_ORD_ID|53355 cb8564_c123108/f1p0/3035	19	2081	2081	2854	17	2091	2228	3000

accuracy=0.996									
cb8564_c178226/f1p0/2728 isoform=cb8564_c178226;full_length_coverage=1;non_full_length_coverage=0;isoform_length=2728;expected_accuracy=0.998	gnl|BL_ORD_ID|55873 cb8564_c47701/f1p0/3144	1284	2728	12	1288	1432	2878	24	1328
cb8564_c45699/f1p0/3005	gnl|BL_ORD_ID|16879 cb8564_c48606/f1p1/3308 isoform=cb8564_c48606;full_length_coverage=1;non_full_length_coverage=1;isoform_length=3308;expected_accuracy=0.998	1	2133	2129	2848	1	2151	2587	3308
cb1779_c4/f1p0/3691	gnl|BL_ORD_ID|31422 cb1779_c11/f1p0/5053 isoform=cb1779_c11;full_length_coverage=1;non_full_length_coverage=0;isoform_length=5053;expected_accuracy=0.996	1	2830	2828	3673	15	2836	3079	3920
cb1371_c19/f1p3/4073	gnl|BL_ORD_ID|25357 cb1371_c47/f1p1/5280 isoform=cb1371_c47;full_length_coverage=1;non_full_length_coverage=1;isoform_length=5280;expected_accuracy=0.998	491	4058	98	496	1725	5280	7	393
cb8564_c72937/f1p1/3443	gnl|BL_ORD_ID|11744 cb8564_c151271/f2p2/3622 isoform=cb8564_c151271;full_length_coverage=2;non_full_length_coverage=2;isoform_length=3622;expected_accuracy=0.991	1	3086	3086	3421	1	3099	3281	3622
cb8564_c23826/f2p1/3274 isoform=cb8564_c23826;full_length_coverage=2;non_full_length_coverage=1;isoform_length=3274;expected_accuracy=0.996	gnl|BL_ORD_ID|52661 cb8564_c10315/f1p0/4512	432	3239	62	432	1609	4416	1	371
cb8564_c41162/f1p0/3158	gnl|BL_ORD_ID|55522 cb8564_c44123/f1p1/3230	21	2553	2548	3040	16	254	2679	317

						9		1
cb5221_c27/f1p0/3107	gnl|BL_ORD_ID|45742 cb5221_c11/f1p0/3287 isoform=cb5221_c11;full_length_coverage=1;non_full_length_coverage=0;isoform_length=3287;expected_accuracy=0.996	193	3076	1	194	390	3287	28	221
cb8564_c3793/f1p0/2874	gnl|BL_ORD_ID|14097 cb8564_c3797/f2p1/2809 isoform=cb8564_c3797;full_length_coverage=2;non_full_length_coverage=1;isoform_length=2809;expected_accuracy=0.996	1	2399	2399	2667	1	2395	2541	2809
cb8564_c84022/f1p0/2360	gnl|BL_ORD_ID|15835 cb8564_c73664/f5p3/2665 isoform=cb8564_c73664;full_length_coverage=5;non_full_length_coverage=3;isoform_length=2665;expected_accuracy=0.994	1	1561	1559	2334	3	1571	1707	2482
cb12714_c29/f1p2/1583	gnl|BL_ORD_ID|58211 cb8564_c91688/f1p0/2747	1	1296	1293	1542	1	1295	2439	2687
cb8564_c42387/f1p4/2973 isoform=cb8564_c42387;full_length_coverage=1;non_full_length_coverage=4;isoform_length=2973;expected_accuracy=0.993	gnl|BL_ORD_ID|55920 cb8564_c48949/f1p0/4357	1	2295	2296	2973	2	2293	2847	3497
cb3406_c5/f1p0/1947 isoform=cb3406_c5;full_length_coverage=1;non_full_length_coverage=0;isoform_length=1947;expected_accuracy=0.995	gnl|BL_ORD_ID|40557 cb3406_c11/f1p0/2573 isoform=cb3406_c11;full_length_coverage=1;non_full_length_coverage=0;isoform_length=2573;expected_accuracy=0.992	216	1946	3	211	831	2560	41	248
cb8564_c117126/f2p0/2610 isoform=cb8564_c117126;full_length_coverage=2;non_full_length_coverage=0;isoform_length=2610;expected	gnl|BL_ORD_ID|14626 cb8564_c41734/f1p0/2770 isoform=cb8564_c41734;full_length_coverage=1;non_full_length_coverage=0;isoform_length=2770;expected_ac	112	1476	1476	2610	1	1363	1632	2770

_accuracy=0.992	curacy=0.994								
cb8564_c11997/f1p2/2670	gnl|BL_ORD_ID|9350 cb8564_c11826/f1p0/2916 isoform=cb8564_c11826;full_length_coverage=1;non_full_length_coverage=0;isoform_length=2916;expected_accuracy=0.997	1294	2629	1	1295	1543	2878	4	1296
cb2844_c7/f1p0/2064 isoform=cb2844_c7;full_length_coverage=1;non_full_length_coverage=0;isoform_length=2064;expected_accuracy=0.996	gnl|BL_ORD_ID|68936 cb2844_c1/f1p0/2387	298	2062	40	299	423	2186	3	262
cb8564_c180116/f1p0/2944 isoform=cb8564_c180116;full_length_coverage=1;non_full_length_coverage=0;isoform_length=2944;expected_accuracy=0.996	gnl|BL_ORD_ID|15169 cb8564_c40218/f1p0/3146 isoform=cb8564_c40218;full_length_coverage=1;non_full_length_coverage=0;isoform_length=3146;expected_accuracy=0.993	1	2522	2520	2944	1	2502	2729	3146
cb499_c61/f1p0/3335	gnl|BL_ORD_ID|42604 cb499_c4/f4p1/4016 isoform=cb499_c4;full_length_coverage=4;non_full_length_coverage=1;isoform_length=4016;expected_accuracy=1.000	1510	3363	1	1513	2161	4016	112	1627
cb8564_c4096/f1p0/2104 isoform=cb8564_c4096;full_length_coverage=1;non_full_length_coverage=0;isoform_length=2104;expected_accuracy=1.000	gnl|BL_ORD_ID|54979 cb8564_c4095/f1p0/4108	135	2091	1	138	2136	4091	2	139
cb4373_c5/f1p0/3315 isoform=cb4373_c5;full_length_coverage=1;non_full_length_coverage=0;isoform_length=3315;expected_accuracy=0.999	gnl|BL_ORD_ID|42010 cb4373_c11/f7p0/3421 isoform=cb4373_c11;full_length_coverage=7;non_full_length_coverage=0;isoform_length=3421;expected_accuracy=1.000	1	2394	2394	3293	1	2396	2525	3421
cb8564_c129820/f1p0/2313	gnl|BL_ORD_ID|18376 cb8564_c7852/f2p0/2668	217	2313	1	218	443	253	34	251

isoform=cb8564_c129820;full_length_coverage=1;non_full_length_coverage=0;isoform_length=2313;expected_accuracy=0.994	isoform=cb8564_c7852;full_length_coverage=2;non_full_length_coverage=0;isoform_length=2668;expected_accuracy=0.996						2		
cb8564_c42654/f1p1/2906 isoform=cb8564_c42654;full_length_coverage=1;non_full_length_coverage=1;isoform_length=2906;expected_accuracy=0.990	gnl|BL_ORD_ID|16879 cb8564_c48606/f1p1/3308 isoform=cb8564_c48606;full_length_coverage=1;non_full_length_coverage=1;isoform_length=3308;expected_accuracy=0.998	1	2130	2126	2824	1	2151	2587	3284
cb8564_c127627/f1p0/2239	gnl|BL_ORD_ID|11065 cb8564_c120549/f1p0/2685 isoform=cb8564_c120549;full_length_coverage=1;non_full_length_coverage=0;isoform_length=2685;expected_accuracy=0.999	166	2216	16	168	640	2685	1	153
cb1142_c17/f1p1/2208	gnl|BL_ORD_ID|23842 cb1142_c18/f1p2/2810 isoform=cb1142_c18;full_length_coverage=1;non_full_length_coverage=2;isoform_length=2810;expected_accuracy=0.998	1	1127	1123	2211	32	1160	1581	2671
cb6024_c12/f2p1/2451 isoform=cb6024_c12;full_length_coverage=2;non_full_length_coverage=1;isoform_length=2451;expected_accuracy=0.996	gnl|BL_ORD_ID|74781 cb6024_c7/f1p1/2576	1	1247	1244	2451	19	1264	1364	2570
cb8564_c117101/f1p0/2770	gnl|BL_ORD_ID|14951 cb8564_c3667/f1p0/2932 isoform=cb8564_c3667;full_length_coverage=1;non_full_length_coverage=0;isoform_length=2932;expected_accuracy=0.992	1159	2434	10	1160	1659	2932	1	1160
cb8564_c142203/f1p7/2878 isoform=cb8564_c142203;full_length_coverage=1;non_full_length_coverage=7;isoform_length=2878;expected	gnl|BL_ORD_ID|57248 cb8564_c88028/f1p1/3776	210	2760	12	212	1248	3800	1	202

_accuracy=0.994									
cb8564_c79084/f1p0/2498 isoform=cb8564_c79084;full_length_coverage=1;non_full_length_coverage=0;isoform_length=2498;expected_accuracy=0.991	gnl|BL_ORD_ID|12051 cb8564_c147180/f4p0/3043 isoform=cb8564_c147180;full_length_coverage=4;non_full_length_coverage=0;isoform_length=3043;expected_accuracy=0.998	133	2498	1	133	654	3024	1	134
cb16776_c24/f1p0/5826	gnl|BL_ORD_ID|33586 cb16776_c21/f1p0/6046 isoform=cb16776_c21;full_length_coverage=1;non_full_length_coverage=0;isoform_length=6046;expected_accuracy=0.997	2113	5809	1	2114	2354	6046	141	2244
cb14316_c15/f1p0/1874 isoform=cb14316_c15;full_length_coverage=1;non_full_length_coverage=0;isoform_length=1874;expected_accuracy=0.999	gnl|BL_ORD_ID|15569 cb8564_c74232/f3p0/2075 isoform=cb8564_c74232;full_length_coverage=3;non_full_length_coverage=0;isoform_length=2075;expected_accuracy=1.000	1	1228	1225	1874	28	1254	1374	2022
cb8564_c21572/f1p1/3061 isoform=cb8564_c21572;full_length_coverage=1;non_full_length_coverage=1;isoform_length=3061;expected_accuracy=0.991	gnl|BL_ORD_ID|54112 cb8564_c18776/f1p2/3544	1127	3061	1	1125	1338	3253	58	1183
cb12972_c5/f2p0/1545 isoform=cb12972_c5;full_length_coverage=2;non_full_length_coverage=0;isoform_length=1545;expected_accuracy=1.000	gnl|BL_ORD_ID|54792 cb8564_c2623/f1p1/2475	206	1545	9	208	1051	2390	1	200
cb3290_c38/f1p0/2345 isoform=cb3290_c38;full_length_coverage=1;non_full_length_coverage=0;isoform_length=2345;expected_accuracy=0.996	gnl|BL_ORD_ID|71037 cb3290_c60/f1p2/2868	1150	2340	1	1151	1593	2790	280	1436
cb8564_c84198/f1p0/2418	gnl|BL_ORD_ID|53689 cb8564_c155515/f1p4/2826	1	2008	2006	2428	17	202	2144	256

							7		6
cb8564_c43399/f1p0/2654 isoform=cb8564_c43399;full_length_coverage=1;non_full_length_coverage=0;isoform_length=2654;expected_accuracy=0.999	gnl|BL_ORD_ID|56648 cb8564_c50629/f1p3/2919	1	2367	2363	2654	4	2365	2653	2944
cb9215_c0/f4p1/1807 isoform=cb9215_c0;full_length_coverage=4;non_full_length_coverage=1;isoform_length=1807;expected_accuracy=1.000	gnl|BL_ORD_ID|59585 cb9215_c5/f1p0/2151	611	1801	79	612	927	2116	1	533
cb316_c22/f1p0/3262 isoform=cb316_c22;full_length_coverage=1;non_full_length_coverage=0;isoform_length=3262;expected_accuracy=0.998	gnl|BL_ORD_ID|69554 cb316_c10/f1p2/4819	1	1609	1605	3147	1096	2702	3318	4861
cb8564_c129638/f1p0/2479	gnl|BL_ORD_ID|54748 cb8564_c163479/f1p2/3022	1	1627	1623	2475	121	1747	2152	3004
cb12292_c13/f1p0/1000 isoform=cb12292_c13;full_length_coverage=1;non_full_length_coverage=0;isoform_length=1000;expected_accuracy=0.995	gnl|BL_ORD_ID|23626 cb12292_c9/f1p0/1677 isoform=cb12292_c9;full_length_coverage=1;non_full_length_coverage=0;isoform_length=1677;expected_accuracy=0.995	151	997	1	147	816	1674	1	145
cb8564_c90494/f1p0/2950 isoform=cb8564_c90494;full_length_coverage=1;non_full_length_coverage=0;isoform_length=2950;expected_accuracy=0.997	gnl|BL_ORD_ID|18436 cb8564_c88865/f1p0/3123 isoform=cb8564_c88865;full_length_coverage=1;non_full_length_coverage=0;isoform_length=3123;expected_accuracy=0.995	347	2950	4	348	527	3122	1	362
cb452_c42/f1p0/3142	gnl|BL_ORD_ID|43284 cb452_c15/f2p3/3234 isoform=cb452_c15;full_length_coverage=2;non_full_length_coverage=3;isoform_length=3234;expected_accura	1492	2981	1	1492	1633	3123	1	1495

	cy=0.999								
cb8564_c181853/f1p0/2119 isoform=cb8564_c181853;full_length_coverage=1;non_full_length_coverage=0;isoform_length=2119;expected_accuracy=0.997	gnl|BL_ORD_ID|56641 cb8564_c50309/f1p5/3075	1	1339	1334	2113	1	1340	2168	2947
cb9283_c3/f1p0/2129	gnl|BL_ORD_ID|19600 cb9283_c4/f1p0/3059 isoform=cb9283_c4;full_length_coverage=1;non_full_length_coverage=0;isoform_length=3059;expected_accuracy=0.996	1	1122	1120	2010	46	1165	2170	3059
cb8564_c38947/f1p0/2650 isoform=cb8564_c38947;full_length_coverage=1;non_full_length_coverage=0;isoform_length=2650;expected_accuracy=0.996	gnl|BL_ORD_ID|56601 cb8564_c48153/f1p0/3083	1	1441	1436	2650	13	1448	1665	2879
cb6181_c7/f6p0/2362 isoform=cb6181_c7;full_length_coverage=6;non_full_length_coverage=0;isoform_length=2362;expected_accuracy=1.000	gnl|BL_ORD_ID|6168 cb6181_c17/f1p0/2583 isoform=cb6181_c17;full_length_coverage=1;non_full_length_coverage=0;isoform_length=2583;expected_accuracy=0.994	1	1260	1260	2362	7	1269	1487	2583
cb1545_c34/f1p0/3898	gnl|BL_ORD_ID|29257 cb1545_c6/f1p1/3759 isoform=cb1545_c6;full_length_coverage=1;non_full_length_coverage=1;isoform_length=3759;expected_accuracy=0.998	1	1854	1854	3486	1	1855	2013	3645
cb17397_c9/f1p1/1416	gnl|BL_ORD_ID|65805 cb17397_c3/f1p0/2619	268	1419	8	268	1085	2236	3	263
cb2737_c39/f1p0/2866	gnl|BL_ORD_ID|38134 cb2737_c22/f1p0/3196 isoform=cb2737_c22;full_length_coverage=1;non_full_length_coverage=0;isoform_length=3196;expected_accur	1	1679	1677	2849	98	1763	1894	3045

	acy=0.995								
cb6835_c9/f1p3/1996 isoform=cb6835_c9;full_length_coverage=1;non_full_length_coverage=3;isoform_length=1996;expected_accuracy=1.000	gnl|BL_ORD_ID|4332 cb6835_c11/f1p2/2231 isoform=cb6835_c11;full_length_coverage=1;non_full_length_coverage=2;isoform_length=2231;expected_accuracy=0.998	713	1915	8	716	1029	2231	1	711
cb8564_c79084/f1p0/2498 isoform=cb8564_c79084;full_length_coverage=1;non_full_length_coverage=0;isoform_length=2498;expected_accuracy=0.991	gnl|BL_ORD_ID|18398 cb8564_c90428/f1p0/2987 isoform=cb8564_c90428;full_length_coverage=1;non_full_length_coverage=0;isoform_length=2987;expected_accuracy=0.996	133	2493	1	133	656	2987	1	134
cb7462_c9/f1p0/2569	gnl|BL_ORD_ID|7881 cb7462_c3/f3p0/2615 isoform=cb7462_c3;full_length_coverage=3;non_full_length_coverage=0;isoform_length=2615;expected_accuracy=1.000	14	1677	1675	2377	1	1668	1821	2523
cb8564_c44698/f1p5/2843 isoform=cb8564_c44698;full_length_coverage=1;non_full_length_coverage=5;isoform_length=2843;expected_accuracy=0.997	gnl|BL_ORD_ID|14836 cb8564_c41692/f1p3/2987 isoform=cb8564_c41692;full_length_coverage=1;non_full_length_coverage=3;isoform_length=2987;expected_accuracy=0.995	1	2516	2511	2826	1	2544	2667	2987
cb8564_c44698/f1p5/2843 isoform=cb8564_c44698;full_length_coverage=1;non_full_length_coverage=5;isoform_length=2843;expected_accuracy=0.997	gnl|BL_ORD_ID|56641 cb8564_c50309/f1p5/3075	1	2516	2511	2836	1	2512	2633	2958
cb12893_c46/f1p0/1118	gnl|BL_ORD_ID|61928 cb12893_c63/f1p2/1520	275	1108	3	277	521	1367	8	280
cb8564_c45864/f1p0/2965	gnl|BL_ORD_ID|56353 cb8564_c75114/f1p0/2866	16	2036	2036	2582	14	2035	2193	2737
cb8564_c51804/f1p1/3590	gnl|BL_ORD_ID|68032 cb18809_c1/f1p0/5493	253	3494	1	252	2193	545	8	256

isoform=cb8564_c51804;full_length_coverage=1;non_full_length_coverage=1;isoform_length=3590;expected_accuracy=0.993							9		
cb944_c92/f1p0/1128	gnl|BL_ORD_ID|59108 cb944_c59/f1p1/1752	9	693	688	1139	508	1193	1295	1746
cb8564_c178226/f1p0/2728 isoform=cb8564_c178226;full_length_coverage=1;non_full_length_coverage=0;isoform_length=2728;expected_accuracy=0.998	gnl|BL_ORD_ID|15104 cb8564_c37866/f1p3/2874 isoform=cb8564_c37866;full_length_coverage=1;non_full_length_coverage=3;isoform_length=2874;expected_accuracy=0.990	1284	2728	1	1288	1405	2865	1	1299
cb8564_c53806/f1p3/3974 isoform=cb8564_c53806;full_length_coverage=1;non_full_length_coverage=3;isoform_length=3974;expected_accuracy=0.990	gnl|BL_ORD_ID|55207 cb8564_c35399/f1p0/4553	172	3974	1	173	396	4180	16	179
cb12513_c26/f1p0/1379 isoform=cb12513_c26;full_length_coverage=1;non_full_length_coverage=0;isoform_length=1379;expected_accuracy=0.995	gnl|BL_ORD_ID|14254 cb8564_c3885/f1p7/2735 isoform=cb8564_c3885;full_length_coverage=1;non_full_length_coverage=7;isoform_length=2735;expected_accuracy=0.999	254	1379	26	259	1490	2615	17	250
cb1492_c9/f1p1/2823 isoform=cb1492_c9;full_length_coverage=1;non_full_length_coverage=1;isoform_length=2823;expected_accuracy=1.000	gnl|BL_ORD_ID|29080 cb1492_c0/f4p2/4028 isoform=cb1492_c0;full_length_coverage=4;non_full_length_coverage=2;isoform_length=4028;expected_accuracy=1.000	191	2823	1	196	1395	4027	58	253
cb2679_c4/f1p0/2896 isoform=cb2679_c4;full_length_coverage=1;non_full_length_coverage=0;isoform_length=2896;expected_accuracy=0.995	gnl|BL_ORD_ID|68713 cb2679_c3/f1p0/3143	1	2427	2424	2880	4	2436	2660	3116
cb1956_c11/f1p0/2868	gnl|BL_ORD_ID|35490 cb1956_c14/f1p0/2988	1	1889	1889	2858	43	190	2009	298

isoform=cb1956_c11;full_length_coverage=1;non_full_length_coverage=0;isoform_length=2868;expected_accuracy=0.996	isoform=cb1956_c14;full_length_coverage=1;non_full_length_coverage=0;isoform_length=2988;expected_accuracy=0.998						7		8
cb750_c8/f2p0/3775 isoform=cb750_c8;full_length_coverage=2;non_full_length_coverage=0;isoform_length=3775;expected_accuracy=0.996	gnl|BL_ORD_ID|7348 cb750_c27/f1p1/4163 isoform=cb750_c27;full_length_coverage=1;non_full_length_coverage=1;isoform_length=4163;expected_accuracy=0.998	133	2290	2289	3726	1	2162	2719	4163
cb8707_c5/f1p0/2018	gnl|BL_ORD_ID|18165 cb8707_c12/f3p1/2249 isoform=cb8707_c12;full_length_coverage=3;non_full_length_coverage=1;isoform_length=2249;expected_accuracy=1.000	1	1469	1468	1992	3	1442	1722	2246
cb391_c5/f1p0/3775 isoform=cb391_c5;full_length_coverage=1;non_full_length_coverage=0;isoform_length=3775;expected_accuracy=0.994	gnl|BL_ORD_ID|70863 cb391_c4/f1p0/4291	362	3775	148	363	871	4288	33	249
cb8564_c73422/f1p0/3612	gnl|BL_ORD_ID|54095 cb8564_c18359/f1p0/3917	1	3079	3074	3609	30	3108	3246	3781
cb1822_c8/f1p2/4358 isoform=cb1822_c8;full_length_coverage=1;non_full_length_coverage=2;isoform_length=4358;expected_accuracy=0.998	gnl|BL_ORD_ID|63999 cb1822_c16/f1p0/5988	1	4035	4031	4358	7	4043	5440	5765
cb1052_c55/f1p0/2730	gnl|BL_ORD_ID|964 cb1052_c56/f1p1/2849 isoform=cb1052_c56;full_length_coverage=1;non_full_length_coverage=1;isoform_length=2849;expected_accuracy=0.998	599	2732	9	600	719	2849	1	590
cb4491_c1/f3p0/1788	gnl|BL_ORD_ID|41856 cb4491_c3/f1p0/1921	10	1410	1406	1750	1	140	1576	192

isoform=cb4491_c1;full_length_coverage=3;non_full_length_coverage=0;isoform_length=1788;expected_accuracy=1.000	isoform=cb4491_c3;full_length_coverage=1;non_full_length_coverage=0;isoform_length=1921;expected_accuracy=1.000						0		1
cb2737_c63/f1p2/2799 isoform=cb2737_c63;full_length_coverage=1;non_full_length_coverage=2;isoform_length=2799;expected_accuracy=0.994	gnl|BL_ORD_ID|68785 cb2737_c49/f1p0/3341	109	2782	1	109	430	3110	28	137
cb3833_c0/f3p0/1648 isoform=cb3833_c0;full_length_coverage=3;non_full_length_coverage=0;isoform_length=1648;expected_accuracy=1.000	gnl|BL_ORD_ID|71542 cb3833_c1/f1p0/3501	327	1648	79	328	1875	3196	1	250
cb8564_c89331/f1p0/2344	gnl|BL_ORD_ID|12084 cb8564_c146353/f7p2/3353 isoform=cb8564_c146353;full_length_coverage=7;non_full_length_coverage=2;isoform_length=3353;expected_accuracy=0.998	1	2105	2101	2382	114	2218	3046	3329
cb90_c17/f1p1/4529 isoform=cb90_c17;full_length_coverage=1;non_full_length_coverage=1;isoform_length=4529;expected_accuracy=0.992	gnl|BL_ORD_ID|19781 cb90_c9/f1p0/4970 isoform=cb90_c9;full_length_coverage=1;non_full_length_coverage=0;isoform_length=4970;expected_accuracy=0.999	350	4529	21	349	713	4914	150	479
cb12931_c15/f1p0/1493 isoform=cb12931_c15;full_length_coverage=1;non_full_length_coverage=0;isoform_length=1493;expected_accuracy=1.000	gnl|BL_ORD_ID|13749 cb8564_c185495/f1p0/2805 isoform=cb8564_c185495;full_length_coverage=1;non_full_length_coverage=0;isoform_length=2805;expected_accuracy=0.996	1	1106	1104	1488	1	1099	2423	2805
cb8564_c44698/f1p5/2843 isoform=cb8564_c44698;full_length_coverage=1;non_full_length_coverage=5;isoform_length=2843;expected_	gnl|BL_ORD_ID|55607 cb8564_c46612/f1p1/3367	1	2516	2511	2830	8	2518	2639	2965

accuracy=0.997									
cb8564_c12302/f1p1/2294 isoform=cb8564_c12302;full_length_coverage=1;non_full_length_coverage=1;isoform_length=2294;expected_accuracy=0.992	gnl|BL_ORD_ID|12527 cb8564_c15023/f2p0/2552 isoform=cb8564_c15023;full_length_coverage=2;non_full_length_coverage=0;isoform_length=2552;expected_accuracy=0.996	1	1646	1646	2294	30	1675	1879	2529
cb5483_c3/f1p0/2270 isoform=cb5483_c3;full_length_coverage=1;non_full_length_coverage=0;isoform_length=2270;expected_accuracy=0.996	gnl|BL_ORD_ID|73971 cb5483_c7/f1p0/2690	172	2270	1	176	502	2601	26	204
cb8564_c82838/f1p1/3447	gnl|BL_ORD_ID|16338 cb8564_c53034/f1p2/3495 isoform=cb8564_c53034;full_length_coverage=1;non_full_length_coverage=2;isoform_length=3495;expected_accuracy=0.998	137	3172	1	141	468	3495	6	145
cb8564_c42814/f1p0/2151 isoform=cb8564_c42814;full_length_coverage=1;non_full_length_coverage=0;isoform_length=2151;expected_accuracy=0.993	gnl|BL_ORD_ID|10510 cb8564_c111713/f6p2/3032 isoform=cb8564_c111713;full_length_coverage=6;non_full_length_coverage=2;isoform_length=3032;expected_accuracy=0.998	650	2151	7	652	1333	2836	1	645
cb16315_c1/f1p0/1642 isoform=cb16315_c1;full_length_coverage=1;non_full_length_coverage=0;isoform_length=1642;expected_accuracy=0.994	gnl|BL_ORD_ID|72532 cb4391_c5/f1p0/3162	203	1642	47	206	740	2180	1	160
cb9093_c2/f1p0/1840 isoform=cb9093_c2;full_length_coverage=1;non_full_length_coverage=0;isoform_length=1840;expected_accuracy=0.996	gnl|BL_ORD_ID|58549 cb9093_c1/f1p0/2083	1	1420	1417	1840	97	1514	1640	2063
cb8564_c125478/f1p1/2270	gnl|BL_ORD_ID|12289 cb8564_c122903/f5p2/3949	221	2270	1	222	1778	382	53	277

isoform=cb8564_c125478;full_length_coverage=1;non_full_length_coverage=1;isoform_length=2270;expected_accuracy=1.000	isoform=cb8564_c122903;full_length_coverage=5;non_full_length_coverage=2;isoform_length=3949;expected_accuracy=0.998						9		
cb6277_c13/f1p0/2679	gnl|BL_ORD_ID|3491 cb6277_c8/f1p0/2793 isoform=cb6277_c8;full_length_coverage=1;non_full_length_coverage=0;isoform_length=2793;expected_accuracy=0.999	202	2576	1	203	419	279 3	115	317
cb6719_c31/f1p0/2434 isoform=cb6719_c31;full_length_coverage=1;non_full_length_coverage=0;isoform_length=2434;expected_accuracy=0.995	gnl|BL_ORD_ID|4666 cb6719_c24/f1p0/2613 isoform=cb6719_c24;full_length_coverage=1;non_full_length_coverage=0;isoform_length=2613;expected_accuracy=0.998	1	1331	1330	2383	91	143 7	1559	261 3
cb2910_c13/f1p0/2551 isoform=cb2910_c13;full_length_coverage=1;non_full_length_coverage=0;isoform_length=2551;expected_accuracy=0.998	gnl|BL_ORD_ID|70081 cb2910_c26/f1p0/2855	1	1402	1402	2550	99	150 1	1624	277 0
cb8564_c21398/f1p0/3085 isoform=cb8564_c21398;full_length_coverage=1;non_full_length_coverage=0;isoform_length=3085;expected_accuracy=0.997	gnl|BL_ORD_ID|58218 cb8564_c91852/f1p0/3459	13	2362	2357	3085	67	241 2	2606	333 4
cb8564_c38035/f1p0/2770	gnl|BL_ORD_ID|15428 cb8564_c42387/f1p4/2973 isoform=cb8564_c42387;full_length_coverage=1;non_full_length_coverage=4;isoform_length=2973;expected_accuracy=0.993	1	2035	2033	2700	1	204 2	2233	291 3
cb2209_c62/f1p0/2619	gnl|BL_ORD_ID|38726 cb2209_c11/f1p1/2823 isoform=cb2209_c11;full_length_coverage=1;non_full_length_coverage=1;isoform_length=2823;expected_accur	2	2083	2082	2461	223	230 1	2452	282 2

	acy=1.000								
cb5001_c11/f1p0/2067	gnl|BL_ORD_ID|43049 cb5001_c2/f4p3/3059 isoform=cb5001_c2;full_length_coverage=4;non_full_length_coverage=3;isoform_length=3059;expected_accuracy=1.000	1	1585	1580	2073	77	1660	2436	2929
cb8564_c47522/f1p0/2954	gnl|BL_ORD_ID|14410 cb8564_c44256/f1p1/3267 isoform=cb8564_c44256;full_length_coverage=1;non_full_length_coverage=1;isoform_length=3267;expected_accuracy=0.995	1	2132	2128	2845	1	2119	2553	3267
cb8564_c45609/f1p0/3991	gnl|BL_ORD_ID|15822 cb8564_c50372/f4p2/4090 isoform=cb8564_c50372;full_length_coverage=4;non_full_length_coverage=2;isoform_length=4090;expected_accuracy=0.996	260	3701	1	260	649	4090	201	460
cb8564_c87256/f1p0/2285 isoform=cb8564_c87256;full_length_coverage=1;non_full_length_coverage=0;isoform_length=2285;expected_accuracy=0.992	gnl|BL_ORD_ID|16383 cb8564_c75900/f1p3/2677 isoform=cb8564_c75900;full_length_coverage=1;non_full_length_coverage=3;isoform_length=2677;expected_accuracy=0.995	1	1703	1700	2285	239	1941	2075	2664
cb649_c40/f1p0/3326	gnl|BL_ORD_ID|49372 cb649_c33/f1p0/4079	12	3135	3133	3349	1	3124	3671	3887
cb8564_c70216/f1p0/3183 isoform=cb8564_c70216;full_length_coverage=1;non_full_length_coverage=0;isoform_length=3183;expected_accuracy=0.998	gnl|BL_ORD_ID|15737 cb8564_c76570/f1p0/3381 isoform=cb8564_c76570;full_length_coverage=1;non_full_length_coverage=0;isoform_length=3381;expected_accuracy=0.996	1	2594	2592	3183	45	2647	2789	3381
cb938_c17/f1p1/3461 isoform=cb938_c17;full_length_coverage=1;non_full_length_coverage=1;isoform_length=3461;expected_accur	gnl|BL_ORD_ID|21149 cb938_c5/f2p0/4567 isoform=cb938_c5;full_length_coverage=2;non_full_length_coverage=0;isoform_length=4567;expected_accurac	1153	3459	1	1156	2255	4567	1	1155

acy=0.991	y=0.998								
cb3235_c2/f2p1/3341 isoform=cb3235_c2;full_length_coverage=2;non_full_length_coverage=1;isoform_length=3341;expected_accuracy=0.998	gnl|BL_ORD_ID|70995 cb3235_c18/f1p0/3408	1	2409	2407	3261	1	2431	2542	3396
cb8564_c42506/f1p0/2909	gnl|BL_ORD_ID|55361 cb8564_c39566/f1p1/3824	1	2203	2199	2857	914	3116	2986	3652
cb12690_c13/f17p1/1862 isoform=cb12690_c13;full_length_coverage=17;non_full_length_coverage=1;isoform_length=1862;expected_accuracy=1.000	gnl|BL_ORD_ID|18575 cb8564_c90782/f1p0/2732 isoform=cb8564_c90782;full_length_coverage=1;non_full_length_coverage=0;isoform_length=2732;expected_accuracy=0.997	1	1488	1485	1860	5	1489	2357	2732
cb2758_c4/f2p2/3132 isoform=cb2758_c4;full_length_coverage=2;non_full_length_coverage=2;isoform_length=3132;expected_accuracy=1.000	gnl|BL_ORD_ID|69951 cb2758_c13/f1p0/3354	132	3132	1	133	296	3301	4	136
cb1052_c55/f1p0/2730	gnl|BL_ORD_ID|1191 cb1052_c13/f1p0/4367 isoform=cb1052_c13;full_length_coverage=1;non_full_length_coverage=0;isoform_length=4367;expected_accuracy=0.998	600	2743	75	600	2093	4236	1	526
cb12805_c0/f2p0/1819 isoform=cb12805_c0;full_length_coverage=2;non_full_length_coverage=0;isoform_length=1819;expected_accuracy=0.993	gnl|BL_ORD_ID|12334 cb8564_c128068/f1p0/2255 isoform=cb8564_c128068;full_length_coverage=1;non_full_length_coverage=0;isoform_length=2255;expected_accuracy=0.995	1	1035	1034	1819	84	1131	1301	2084
cb8564_c21767/f1p1/2839 isoform=cb8564_c21767;full_length_coverage=1;non_full_length_coverage=1;isoform_length=2839;expected_	gnl|BL_ORD_ID|12866 cb8564_c162614/f1p0/2999 isoform=cb8564_c162614;full_length_coverage=1;non_full_length_coverage=0;isoform_length=2999;expected_a	1040	2788	1	1040	1249	2999	60	1102

accuracy=0.996	ccuracy=0.998								
cb6390_c5/f1p0/2067 isoform=cb6390_c5;full_length_coverage=1;non_full_length_coverage=0;isoform_length=2067;expected_accuracy=0.994	gnl|BL_ORD_ID|3698 cb6390_c0/f5p0/2306 isoform=cb6390_c0;full_length_coverage=5;non_full_length_coverage=0;isoform_length=2306;expected_accuracy=1.000	359	2067	9	360	604	2306	69	418
cb6054_c29/f1p0/1952 isoform=cb6054_c29;full_length_coverage=1;non_full_length_coverage=0;isoform_length=1952;expected_accuracy=0.991	gnl|BL_ORD_ID|74836 cb6054_c31/f1p0/2404	1	1642	1638	1952	109	1751	1880	2194
cb2804_c65/f1p0/3384 isoform=cb2804_c65;full_length_coverage=1;non_full_length_coverage=0;isoform_length=3384;expected_accuracy=0.994	gnl|BL_ORD_ID|38356 cb2804_c93/f1p0/3636 isoform=cb2804_c93;full_length_coverage=1;non_full_length_coverage=0;isoform_length=3636;expected_accuracy=0.991	1	2471	2469	3337	54	2511	2771	3636
cb8564_c43399/f1p0/2654 isoform=cb8564_c43399;full_length_coverage=1;non_full_length_coverage=0;isoform_length=2654;expected_accuracy=0.999	gnl|BL_ORD_ID|15154 cb8564_c32509/f1p1/2880 isoform=cb8564_c32509;full_length_coverage=1;non_full_length_coverage=1;isoform_length=2880;expected_accuracy=0.991	11	2367	2363	2628	2	2392	2610	2877
cb5307_c9/f1p0/2902	gnl|BL_ORD_ID|46362 cb5307_c22/f2p0/3012 isoform=cb5307_c22;full_length_coverage=2;non_full_length_coverage=0;isoform_length=3012;expected_accuracy=0.995	1204	2887	1	1204	1330	3012	1	1222
cb2700_c10/f1p0/2691 isoform=cb2700_c10;full_length_coverage=1;non_full_length_coverage=0;isoform_length=2691;expected_accuracy=0.999	gnl|BL_ORD_ID|38064 cb2700_c1/f1p0/2876 isoform=cb2700_c1;full_length_coverage=1;non_full_length_coverage=0;isoform_length=2876;expected_accuracy=1.000	1086	2691	108	1086	1266	2872	1	980
cb8564_c49090/f1p0/3264	gnl|BL_ORD_ID|55195 cb8564_c35170/f1p0/3386	567	3233	71	566	738	339	3	497

							9		
cb359_c1/f1p0/4935	gnl|BL_ORD_ID|39413 cb359_c5/f1p0/5076 isoform=cb359_c5;full_length_coverage=1;non_full_length_coverage=0;isoform_length=5076;expected_accuracy=0.999	10	3598	3594	4219	45	3652	4402	5033
cb8564_c145672/f9p4/2440 isoform=cb8564_c145672;full_length_coverage=9;non_full_length_coverage=4;isoform_length=2440;expected_accuracy=1.000	gnl|BL_ORD_ID|14369 cb8564_c37858/f1p0/2999 isoform=cb8564_c37858;full_length_coverage=1;non_full_length_coverage=0;isoform_length=2999;expected_accuracy=0.998	468	2420	89	469	1047	2999	1	379
cb2640_c29/f1p0/3441	gnl|BL_ORD_ID|68659 cb2640_c11/f1p0/3319	355	3227	4	356	459	3331	2	353
cb1163_c15/f1p3/3725 isoform=cb1163_c15;full_length_coverage=1;non_full_length_coverage=3;isoform_length=3725;expected_accuracy=0.999	gnl|BL_ORD_ID|23459 cb1163_c11/f2p1/3742 isoform=cb1163_c11;full_length_coverage=2;non_full_length_coverage=1;isoform_length=3742;expected_accuracy=0.999	1	2502	2501	3618	1	2504	2616	3742
cb8564_c11528/f1p1/2758 isoform=cb8564_c11528;full_length_coverage=1;non_full_length_coverage=1;isoform_length=2758;expected_accuracy=0.994	gnl|BL_ORD_ID|14902 cb8564_c42851/f1p1/2963 isoform=cb8564_c42851;full_length_coverage=1;non_full_length_coverage=1;isoform_length=2963;expected_accuracy=0.995	2	2284	2283	2756	108	2383	2492	2963
cb2818_c74/f3p0/3087 isoform=cb2818_c74;full_length_coverage=3;non_full_length_coverage=0;isoform_length=3087;expected_accuracy=1.000	gnl|BL_ORD_ID|68900 cb2818_c40/f1p0/3487	136	3087	1	139	426	3377	8	144
cb8564_c20499/f3p0/3328 isoform=cb8564_c20499;full_length_coverage=3;non_full_length_coverage=0;isoform_length=3328;expected_	gnl|BL_ORD_ID|55939 cb8564_c49630/f1p4/3487	2	2916	2916	3166	1	2913	3243	3493

accuracy=1.000									
cb8564_c90494/f1p0/2950 isoform=cb8564_c90494;full_length_coverage=1;non_full_length_coverage=0;isoform_length=2950;expected_accuracy=0.997	gnl|BL_ORD_ID|16259 cb8564_c68587/f4p0/3092 isoform=cb8564_c68587;full_length_coverage=4;non_full_length_coverage=0;isoform_length=3092;expected_accuracy=1.000	347	2950	60	348	486	3086	1	301
cb2108_c3/f1p1/3950 isoform=cb2108_c3;full_length_coverage=1;non_full_length_coverage=1;isoform_length=3950;expected_accuracy=0.994	gnl|BL_ORD_ID|34468 cb2108_c4/f1p1/4035 isoform=cb2108_c4;full_length_coverage=1;non_full_length_coverage=1;isoform_length=4035;expected_accuracy=0.997	1	3451	3451	3832	50	3501	3652	4035
cb5487_c1/f2p1/2546 isoform=cb5487_c1;full_length_coverage=2;non_full_length_coverage=1;isoform_length=2546;expected_accuracy=0.996	gnl|BL_ORD_ID|73975 cb5487_c11/f1p0/2702	1	1478	1478	2502	4	1477	1646	2671
cb3856_c5/f1p0/2096	gnl|BL_ORD_ID|40760 cb3856_c3/f1p0/3507 isoform=cb3856_c3;full_length_coverage=1;non_full_length_coverage=0;isoform_length=3507;expected_accuracy=0.998	461	1952	7	464	2019	3507	8	465
cb8564_c71747/f1p1/2679 isoform=cb8564_c71747;full_length_coverage=1;non_full_length_coverage=1;isoform_length=2679;expected_accuracy=0.999	gnl|BL_ORD_ID|14459 cb8564_c42140/f1p1/3025 isoform=cb8564_c42140;full_length_coverage=1;non_full_length_coverage=1;isoform_length=3025;expected_accuracy=0.998	1	2002	1998	2673	192	2208	2350	3025
cb8564_c43779/f1p0/3021 isoform=cb8564_c43779;full_length_coverage=1;non_full_length_coverage=0;isoform_length=3021;expected_accuracy=0.993	gnl|BL_ORD_ID|55437 cb8564_c41690/f1p1/3409	1	2344	2345	3021	1	2349	2505	3180
cb500_c17/f1p0/2323	gnl|BL_ORD_ID|41778 cb500_c10/f2p0/3350	1	1293	1288	2323	775	206	2199	323

isoform=cb500_c17;full_length_coverage=1;non_full_length_coverage=0;isoform_length=2323;expected_accuracy=0.997	isoform=cb500_c10;full_length_coverage=2;non_full_length_coverage=0;isoform_length=3350;expected_accuracy=0.998						7		0
cb8564_c39136/f1p0/3036 isoform=cb8564_c39136;full_length_coverage=1;non_full_length_coverage=0;isoform_length=3036;expected_accuracy=0.998	gnl|BL_ORD_ID|17429 cb8564_c85447/f1p0/3196 isoform=cb8564_c85447;full_length_coverage=1;non_full_length_coverage=0;isoform_length=3196;expected_accuracy=0.997	1	2089	2085	3036	3	2095	2222	3195
cb8564_c49562/f1p0/2838 isoform=cb8564_c49562;full_length_coverage=1;non_full_length_coverage=0;isoform_length=2838;expected_accuracy=0.995	gnl|BL_ORD_ID|53680 cb8564_c153222/f2p39/3454	1	2331	2327	2828	3	2333	2665	3170
cb6889_c18/f1p0/2446	gnl|BL_ORD_ID|47747 cb6889_c13/f1p0/2673	317	2410	68	322	588	2680	1	255
cb5046_c5/f1p0/1852	gnl|BL_ORD_ID|72089 cb5046_c1/f1p0/3159	1	1458	1456	1842	438	1898	2733	3126
cb8564_c188097/f1p0/2552 isoform=cb8564_c188097;full_length_coverage=1;non_full_length_coverage=0;isoform_length=2552;expected_accuracy=0.996	gnl|BL_ORD_ID|12970 cb8564_c16702/f2p13/2748 isoform=cb8564_c16702;full_length_coverage=2;non_full_length_coverage=13;isoform_length=2748;expected_accuracy=0.998	1	1945	1940	2495	147	2090	2193	2748
cb309_c7/f1p2/4109 isoform=cb309_c7;full_length_coverage=1;non_full_length_coverage=2;isoform_length=4109;expected_accuracy=0.997	gnl|BL_ORD_ID|38927 cb309_c3/f2p2/4443 isoform=cb309_c3;full_length_coverage=2;non_full_length_coverage=2;isoform_length=4443;expected_accuracy=1.000	1	3739	3740	4108	2	3740	4075	4443
cb8564_c37361/f1p0/3076	gnl|BL_ORD_ID|55472 cb8564_c42531/f1p0/3996	125	2481	2477	3068	816	3171	3272	3863
cb8564_c36635/f1p0/4030	gnl|BL_ORD_ID|16628 cb8564_c47737/f7p5/4351	184	4022	1	189	436	427	1	183

isoform=cb8564_c36635;full_length_coverage=1;non_full_length_coverage=0;isoform_length=4030;expected_accuracy=0.998	isoform=cb8564_c47737;full_length_coverage=7;non_full_length_coverage=5;isoform_length=4351;expected_accuracy=0.998						4		
cb1279_c21/f1p0/2474	gnl|BL_ORD_ID|25800 cb1279_c77/f1p0/3526 isoform=cb1279_c77;full_length_coverage=1;non_full_length_coverage=0;isoform_length=3526;expected_accuracy=0.994	14	1888	1885	2440	1	1872	2973	3526
cb8564_c80845/f1p0/3015 isoform=cb8564_c80845;full_length_coverage=1;non_full_length_coverage=0;isoform_length=3015;expected_accuracy=0.993	gnl|BL_ORD_ID|57039 cb8564_c81313/f1p0/3748	13	1675	1676	2903	56	1723	2133	3362
cb3290_c95/f1p11/2792 isoform=cb3290_c95;full_length_coverage=1;non_full_length_coverage=11;isoform_length=2792;expected_accuracy=0.993	gnl|BL_ORD_ID|40560 cb3290_c85/f2p1/2883 isoform=cb3290_c85;full_length_coverage=2;non_full_length_coverage=1;isoform_length=2883;expected_accuracy=0.998	1	1512	1513	2787	5	1502	1614	2883
cb1770_c5/f2p0/3464 isoform=cb1770_c5;full_length_coverage=2;non_full_length_coverage=0;isoform_length=3464;expected_accuracy=0.991	gnl|BL_ORD_ID|33844 cb1770_c9/f1p0/3711 isoform=cb1770_c9;full_length_coverage=1;non_full_length_coverage=0;isoform_length=3711;expected_accuracy=0.999	1	2033	2031	3464	8	2038	2199	3630
cb6581_c0/f3p2/2004 isoform=cb6581_c0;full_length_coverage=3;non_full_length_coverage=2;isoform_length=2004;expected_accuracy=0.995	gnl|BL_ORD_ID|3444 cb6581_c6/f1p2/2437 isoform=cb6581_c6;full_length_coverage=1;non_full_length_coverage=2;isoform_length=2437;expected_accuracy=0.992	1	1061	1057	1995	2	1076	1497	2437
cb8564_c36643/f1p3/2668	gnl|BL_ORD_ID|56381 cb8564_c75626/f1p2/3199	13	2098	2096	2668	10	2116	2611	3183
cb2698_c6/f1p0/1752	gnl|BL_ORD_ID|68733 cb2698_c4/f1p0/1944	303	1752	65	304	496	194	30	269

isoform=cb2698_c6;full_length_coverage=1;non_full_length_coverage=0;isoform_length=1752;expected_accuracy=0.998							5		
cb6609_c4/f1p0/2108 isoform=cb6609_c4;full_length_coverage=1;non_full_length_coverage=0;isoform_length=2108;expected_accuracy=0.993	gnl|BL_ORD_ID|3768 cb6609_c2/f1p0/2814 isoform=cb6609_c2;full_length_coverage=1;non_full_length_coverage=0;isoform_length=2814;expected_accuracy=0.999	275	2108	61	279	917	2748	1	213
cb5204_c1/f6p1/2806 isoform=cb5204_c1;full_length_coverage=6;non_full_length_coverage=1;isoform_length=2806;expected_accuracy=0.999	gnl|BL_ORD_ID|46181 cb5204_c6/f1p1/2901 isoform=cb5204_c6;full_length_coverage=1;non_full_length_coverage=1;isoform_length=2901;expected_accuracy=0.997	16	1753	1753	2798	1	1739	1855	2901
cb13748_c39/f1p0/3343	gnl|BL_ORD_ID|62722 cb13748_c47/f1p0/3466	508	3204	88	509	572	3268	5	426
cb1665_c3/f1p0/3650	gnl|BL_ORD_ID|31039 cb1665_c2/f1p0/3731 isoform=cb1665_c2;full_length_coverage=1;non_full_length_coverage=0;isoform_length=3731;expected_accuracy=0.996	1	2893	2891	3508	70	2988	3114	3731
cb8564_c53561/f1p0/2359	gnl|BL_ORD_ID|14026 cb8564_c43137/f1p0/2726 isoform=cb8564_c43137;full_length_coverage=1;non_full_length_coverage=0;isoform_length=2726;expected_accuracy=0.996	495	2342	6	499	881	2726	134	627
cb228_c23/f1p0/3387	gnl|BL_ORD_ID|38752 cb228_c14/f1p0/3285 isoform=cb228_c14;full_length_coverage=1;non_full_length_coverage=0;isoform_length=3285;expected_accuracy=1.000	1406	3071	1	1411	1614	3279	27	1436
cb3290_c38/f1p0/2345	gnl|BL_ORD_ID|40424 cb3290_c67/f1p0/2784	1150	2343	1	1151	1592	278	308	143

isoform=cb3290_c38;full_length_coverage=1;non_full_length_coverage=0;isoform_length=2345;expected_accuracy=0.996	isoform=cb3290_c67;full_length_coverage=1;non_full_length_coverage=0;isoform_length=2784;expected_accuracy=0.999						4		5
cb2739_c1/f2p0/3546 isoform=cb2739_c1;full_length_coverage=2;non_full_length_coverage=0;isoform_length=3546;expected_accuracy=0.996	gnl|BL_ORD_ID|69934 cb2739_c3/f1p0/3875	1248	3525	1	1253	1505	3777	152	1401
cb1772_c26/f1p0/3767	gnl|BL_ORD_ID|63938 cb1772_c19/f1p0/4042	232	3695	11	234	440	3891	19	258
cb8564_c1372/f1p0/2553	gnl|BL_ORD_ID|12085 cb8564_c154026/f1p0/2823 isoform=cb8564_c154026;full_length_coverage=1;non_full_length_coverage=0;isoform_length=2823;expected_accuracy=0.992	1	1478	1479	2563	43	1515	1736	2823
cb8564_c90797/f1p0/2864	gnl|BL_ORD_ID|18126 cb8564_c83895/f1p1/2948 isoform=cb8564_c83895;full_length_coverage=1;non_full_length_coverage=1;isoform_length=2948;expected_accuracy=0.995	15	2307	2308	2657	175	2468	2595	2937
cb8564_c51722/f1p1/3503	gnl|BL_ORD_ID|56041 cb8564_c53077/f1p2/3406	379	3215	67	379	452	3288	22	337
cb8564_c39136/f1p0/3036 isoform=cb8564_c39136;full_length_coverage=1;non_full_length_coverage=0;isoform_length=3036;expected_accuracy=0.998	gnl|BL_ORD_ID|15924 cb8564_c52804/f1p5/3221 isoform=cb8564_c52804;full_length_coverage=1;non_full_length_coverage=5;isoform_length=3221;expected_accuracy=0.999	1	2089	2085	3036	1	2094	2221	3175
cb4862_c8/f1p0/2364 isoform=cb4862_c8;full_length_coverage=1;non_full_length_coverage=0;isoform_length=2364;expected_accur	gnl|BL_ORD_ID|42994 cb4862_c9/f1p1/3086 isoform=cb4862_c9;full_length_coverage=1;non_full_length_coverage=1;isoform_length=3086;expected_accura	1	1251	1247	2258	217	1463	2039	3044

acy=0.992	cy=0.997								
cb18409_c22/f2p0/1096	gnl|BL_ORD_ID|48894 cb10833_c4/f1p0/2067	514	1031	7	514	1316	1833	83	600
cb500_c43/f1p0/3109 isoform=cb500_c43;full_length_coverage=1;non_full_length_coverage=0;isoform_length=3109;expected_accuracy=0.992	gnl|BL_ORD_ID|72247 cb500_c49/f1p0/3666	13	2306	2303	3107	118	2395	2527	3332
cb12_c21/f1p0/4378 isoform=cb12_c21;full_length_coverage=1;non_full_length_coverage=0;isoform_length=4378;expected_accuracy=0.999	gnl|BL_ORD_ID|61091 cb12_c58/f1p0/5377	319	4197	1	319	1408	5292	766	1084
cb8564_c5007/f1p0/1941	gnl|BL_ORD_ID|9289 cb8564_c105652/f6p6/3755 isoform=cb8564_c105652;full_length_coverage=6;non_full_length_coverage=6;isoform_length=3755;expected_accuracy=0.999	1	1447	1446	1712	1910	3356	3489	3755
cb8564_c21439/f1p0/2587	gnl|BL_ORD_ID|10074 cb8564_c11613/f1p0/2758 isoform=cb8564_c11613;full_length_coverage=1;non_full_length_coverage=0;isoform_length=2758;expected_accuracy=0.991	1226	2572	1	1228	1383	2758	3	1262
cb1833_c16/f1p2/3675 isoform=cb1833_c16;full_length_coverage=1;non_full_length_coverage=2;isoform_length=3675;expected_accuracy=0.999	gnl|BL_ORD_ID|33409 cb1833_c19/f1p1/3788 isoform=cb1833_c19;full_length_coverage=1;non_full_length_coverage=1;isoform_length=3788;expected_accuracy=0.991	146	3036	3034	3669	91	3019	3139	3788
cb8564_c145558/f4p2/2640 isoform=cb8564_c145558;full_length_coverage=4;non_full_length_coverage=2;isoform_length=2640;expected	gnl|BL_ORD_ID|12099 cb8564_c12612/f1p0/2697 isoform=cb8564_c12612;full_length_coverage=1;non_full_length_coverage=0;isoform_length=2697;expected_ac	1	2474	2477	2610	183	2666	2563	2697

_accuracy=1.000	curacy=0.995								
cb3006_c7/f1p0/1659 isoform=cb3006_c7;full_length_coverage=1;non_full_length_coverage=0;isoform_length=1659;expected_accuracy=0.994	gnl|BL_ORD_ID|37998 cb3006_c4/f1p0/2052 isoform=cb3006_c4;full_length_coverage=1;non_full_length_coverage=0;isoform_length=2052;expected_accuracy=0.997	1	1112	1112	1659	34	1135	1387	1935
cb8564_c91317/f1p0/2833	gnl|BL_ORD_ID|14501 cb8564_c24947/f1p0/3141 isoform=cb8564_c24947;full_length_coverage=1;non_full_length_coverage=0;isoform_length=3141;expected_accuracy=0.997	477	2794	60	482	850	3141	1	423
cb295_c53/f1p0/1719 isoform=cb295_c53;full_length_coverage=1;non_full_length_coverage=0;isoform_length=1719;expected_accuracy=0.994	gnl|BL_ORD_ID|67912 cb18646_c8/f1p2/6225	1	1347	1345	1719	4395	5744	5869	6236
cb5307_c0/f9p0/3058 isoform=cb5307_c0;full_length_coverage=9;non_full_length_coverage=0;isoform_length=3058;expected_accuracy=1.000	gnl|BL_ORD_ID|73769 cb5307_c7/f1p0/3894	137	1714	1710	3047	3	1580	2554	3891
cb8564_c106924/f3p13/3304 isoform=cb8564_c106924;full_length_coverage=3;non_full_length_coverage=13;isoform_length=3304;expected_accuracy=1.000	gnl|BL_ORD_ID|14187 cb8564_c45896/f1p0/3360 isoform=cb8564_c45896;full_length_coverage=1;non_full_length_coverage=0;isoform_length=3360;expected_accuracy=0.996	108	2134	2134	3281	57	2081	2215	3360
cb1658_c15/f1p0/2863 isoform=cb1658_c15;full_length_coverage=1;non_full_length_coverage=0;isoform_length=2863;expected_accuracy=0.994	gnl|BL_ORD_ID|63834 cb1658_c42/f1p0/3937	1153	2863	1	1156	2152	3862	8	1160
cb1326_c9/f1p0/3549	gnl|BL_ORD_ID|61238 cb1326_c5/f1p0/4374	1	3198	3194	3466	135	333	3645	391

isoform=cb1326_c9;full_length_coverage=1;non_full_length_coverage=0;isoform_length=3549;expected_accuracy=0.996							2		7
cb12931_c15/f1p0/1493 isoform=cb12931_c15;full_length_coverage=1;non_full_length_coverage=0;isoform_length=1493;expected_accuracy=1.000	gnl|BL_ORD_ID|16795 cb8564_c73428/f1p0/2966 isoform=cb8564_c73428;full_length_coverage=1;non_full_length_coverage=0;isoform_length=2966;expected_accuracy=0.994	1	1106	1104	1493	7	1107	2579	2966
cb2645_c10/f1p0/3344	gnl|BL_ORD_ID|68675 cb2645_c13/f1p0/3678	1	2820	2817	3371	53	2888	2989	3540
cb8564_c44698/f1p5/2843 isoform=cb8564_c44698;full_length_coverage=1;non_full_length_coverage=5;isoform_length=2843;expected_accuracy=0.997	gnl|BL_ORD_ID|57176 cb8564_c85665/f1p0/3087	13	2516	2511	2826	2	2523	2644	2965
cb4444_c8/f1p0/2558 isoform=cb4444_c8;full_length_coverage=1;non_full_length_coverage=0;isoform_length=2558;expected_accuracy=0.994	gnl|BL_ORD_ID|43662 cb4444_c7/f1p0/2681 isoform=cb4444_c7;full_length_coverage=1;non_full_length_coverage=0;isoform_length=2681;expected_accuracy=0.993	1	1624	1620	2484	3	1633	1792	2681
cb12931_c15/f1p0/1493 isoform=cb12931_c15;full_length_coverage=1;non_full_length_coverage=0;isoform_length=1493;expected_accuracy=1.000	gnl|BL_ORD_ID|13054 cb8564_c181182/f1p0/2830 isoform=cb8564_c181182;full_length_coverage=1;non_full_length_coverage=0;isoform_length=2830;expected_accuracy=0.998	1	1106	1104	1492	1	1100	2442	2830
cb187_c11/f1p1/3861	gnl|BL_ORD_ID|34572 cb187_c9/f1p0/4217 isoform=cb187_c9;full_length_coverage=1;non_full_length_coverage=0;isoform_length=4217;expected_accuracy=1.000	142	3876	1	145	482	4217	21	165
cb1379_c3/f2p0/3748	gnl|BL_ORD_ID|61303 cb1379_c14/f1p0/3762	1	2330	2330	3646	3	233	2434	375

isoform=cb1379_c3;full_length_coverage=2;non_full_length_coverage=0;isoform_length=3748;expected_accuracy=0.996							4		0
cb1768_c14/f1p0/3461	gnl|BL_ORD_ID|32884 cb1768_c0/f20p2/3608 isoform=cb1768_c0;full_length_coverage=20;non_full_length_coverage=2;isoform_length=3608;expected_accuracy=1.000	1	2336	2333	3380	15	2350	2498	3545
cb8564_c83100/f1p1/2671 isoform=cb8564_c83100;full_length_coverage=1;non_full_length_coverage=1;isoform_length=2671;expected_accuracy=0.996	gnl|BL_ORD_ID|9280 cb8564_c107656/f6p3/3035 isoform=cb8564_c107656;full_length_coverage=6;non_full_length_coverage=3;isoform_length=3035;expected_accuracy=0.999	1	1637	1636	2671	1	1633	1998	3021
cb8747_c11/f1p0/1868	gnl|BL_ORD_ID|18977 cb8747_c5/f1p0/2009 isoform=cb8747_c5;full_length_coverage=1;non_full_length_coverage=0;isoform_length=2009;expected_accuracy=0.999	526	1881	57	528	591	1945	1	472
cb8564_c49562/f1p0/2838 isoform=cb8564_c49562;full_length_coverage=1;non_full_length_coverage=0;isoform_length=2838;expected_accuracy=0.995	gnl|BL_ORD_ID|54747 cb8564_c162728/f1p28/3382	2	2331	2327	2838	168	2497	2829	3344
cb910_c28/f1p1/2974 isoform=cb910_c28;full_length_coverage=1;non_full_length_coverage=1;isoform_length=2974;expected_accuracy=0.995	gnl|BL_ORD_ID|21606 cb910_c32/f1p1/3242 isoform=cb910_c32;full_length_coverage=1;non_full_length_coverage=1;isoform_length=3242;expected_accuracy=0.998	1	2158	2156	2974	2	2160	2276	3094
cb6441_c15/f1p0/2019 isoform=cb6441_c15;full_length_coverage=1;non_full_length_coverage=0;isoform_length=2019;expected_accu	gnl|BL_ORD_ID|4984 cb6441_c16/f1p0/2176 isoform=cb6441_c16;full_length_coverage=1;non_full_length_coverage=0;isoform_length=2176;expected_accur	251	2019	56	250	369	2141	1	197

racy=0.993	acy=0.994								
cb15484_c3/f1p0/1383 isoform=cb15484_c3;full_length_coverage=1;non_full_length_coverage=0;isoform_length=1383;expected_accuracy=0.991	gnl|BL_ORD_ID|27887 cb15484_c0/f1p0/1773 isoform=cb15484_c0;full_length_coverage=1;non_full_length_coverage=0;isoform_length=1773;expected_accuracy=0.994	1	1020	1019	1383	275	1294	1413	1773
cb8564_c40333/f1p2/4446 isoform=cb8564_c40333;full_length_coverage=1;non_full_length_coverage=2;isoform_length=4446;expected_accuracy=0.998	gnl|BL_ORD_ID|14942 cb8564_c34188/f2p3/4546 isoform=cb8564_c34188;full_length_coverage=2;non_full_length_coverage=3;isoform_length=4546;expected_accuracy=1.000	271	4446	1	275	398	4546	18	293
cb13119_c5/f1p0/1886	gnl|BL_ORD_ID|71114 cb3343_c9/f1p0/2028	1	1305	1303	1761	22	1326	1467	1926
cb309_c7/f1p2/4109 isoform=cb309_c7;full_length_coverage=1;non_full_length_coverage=2;isoform_length=4109;expected_accuracy=0.997	gnl|BL_ORD_ID|38926 cb309_c9/f1p2/4191 isoform=cb309_c9;full_length_coverage=1;non_full_length_coverage=2;isoform_length=4191;expected_accuracy=0.998	21	3739	3740	4086	1	3720	3845	4191
cb8564_c122886/f1p1/2748 isoform=cb8564_c122886;full_length_coverage=1;non_full_length_coverage=1;isoform_length=2748;expected_accuracy=0.991	gnl|BL_ORD_ID|16505 cb8564_c73731/f2p0/4847 isoform=cb8564_c73731;full_length_coverage=2;non_full_length_coverage=0;isoform_length=4847;expected_accuracy=0.996	1116	2748	1	1118	3074	4704	250	1368
cb11848_c11/f1p0/1203	gnl|BL_ORD_ID|16105 cb8564_c71556/f1p0/1991 isoform=cb8564_c71556;full_length_coverage=1;non_full_length_coverage=0;isoform_length=1991;expected_accuracy=0.997	218	1199	4	217	1006	1991	1	213
cb2927_c2/f2p0/3116 isoform=cb2927_c2;full_length_coverage=2;non_full_length_coverage=0;isoform_length=3116;expected_accura	gnl|BL_ORD_ID|69004 cb2927_c9/f1p1/3279	1327	3109	10	1326	1465	3250	1	1318

cy=0.995									
cb8564_c36011/f1p0/3015	gnl|BL_ORD_ID|56353 cb8564_c75114/f1p0/2866	14	2049	2049	2595	14	2035	2193	2737
cb3010_c18/f1p2/2828 isoform=cb3010_c18;full_length_coverage=1;non_full_length_coverage=2;isoform_length=2828;expected_accuracy=0.999	gnl|BL_ORD_ID|38253 cb3010_c15/f1p0/2932 isoform=cb3010_c15;full_length_coverage=1;non_full_length_coverage=0;isoform_length=2932;expected_accuracy=0.997	1049	2790	1	1050	1192	2932	32	1078
cb1333_c101/f1p0/1395 isoform=cb1333_c101;full_length_coverage=1;non_full_length_coverage=0;isoform_length=1395;expected_accuracy=0.997	gnl|BL_ORD_ID|27473 cb1333_c63/f1p0/4699 isoform=cb1333_c63;full_length_coverage=1;non_full_length_coverage=0;isoform_length=4699;expected_accuracy=0.998	148	1395	6	150	3375	4621	1	144
cb8564_c45300/f1p4/3014 isoform=cb8564_c45300;full_length_coverage=1;non_full_length_coverage=4;isoform_length=3014;expected_accuracy=0.996	gnl|BL_ORD_ID|55910 cb8564_c48645/f1p1/3287	243	2952	12	246	404	3113	17	248
cb8564_c80689/f1p0/2162 isoform=cb8564_c80689;full_length_coverage=1;non_full_length_coverage=0;isoform_length=2162;expected_accuracy=0.996	gnl|BL_ORD_ID|54038 cb8564_c17261/f1p0/3204	1023	2114	1	1024	1999	3094	23	1049
cb5516_c26/f1p0/3029 isoform=cb5516_c26;full_length_coverage=1;non_full_length_coverage=0;isoform_length=3029;expected_accuracy=1.000	gnl|BL_ORD_ID|45043 cb5516_c85/f68p0/3217 isoform=cb5516_c85;full_length_coverage=68;non_full_length_coverage=0;isoform_length=3217;expected_accuracy=1.000	10	2327	2322	2962	136	2452	2577	3217
cb8564_c39136/f1p0/3036 isoform=cb8564_c39136;full_length_coverage=1;non_full_length_coverage=0;isoform_length=3036;expected_	gnl|BL_ORD_ID|56764 cb8564_c70795/f1p0/3769	19	2089	2085	3036	34	2111	2238	3187

accuracy=0.998									
cb3856_c0/f2p1/1619 isoform=cb3856_c0;full_length_coverage=2;non_full_length_coverage=1;isoform_length=1619;expected_accuracy=0.996	gnl|BL_ORD_ID|40760 cb3856_c3/f1p0/3507 isoform=cb3856_c3;full_length_coverage=1;non_full_length_coverage=0;isoform_length=3507;expected_accuracy=0.998	128	1619	1	131	2018	3506	1	132
cb8564_c132795/f5p10/3729 isoform=cb8564_c132795;full_length_coverage=5;non_full_length_coverage=10;isoform_length=3729;expected_accuracy=0.999	gnl|BL_ORD_ID|16248 cb8564_c5118/f1p1/4889 isoform=cb8564_c5118;full_length_coverage=1;non_full_length_coverage=1;isoform_length=4889;expected_accuracy=0.991	1	1832	1830	3640	1	1822	2987	4791
cb944_c33/f1p1/3119 isoform=cb944_c33;full_length_coverage=1;non_full_length_coverage=1;isoform_length=3119;expected_accuracy=1.000	gnl|BL_ORD_ID|19525 cb944_c27/f2p0/3667 isoform=cb944_c27;full_length_coverage=2;non_full_length_coverage=0;isoform_length=3667;expected_accuracy=0.999	1465	3068	1	1467	2068	3667	5	1465
cb8564_c12805/f1p0/2580 isoform=cb8564_c12805;full_length_coverage=1;non_full_length_coverage=0;isoform_length=2580;expected_accuracy=0.993	gnl|BL_ORD_ID|56580 cb8564_c47159/f1p0/3238	16	1695	1690	2580	81	1761	2003	2896
cb8564_c185920/f1p0/2777 isoform=cb8564_c185920;full_length_coverage=1;non_full_length_coverage=0;isoform_length=2777;expected_accuracy=0.996	gnl|BL_ORD_ID|14836 cb8564_c41692/f1p3/2987 isoform=cb8564_c41692;full_length_coverage=1;non_full_length_coverage=3;isoform_length=2987;expected_accuracy=0.995	174	2773	1	177	352	2987	15	193
cb3307_c38/f1p0/2373	gnl|BL_ORD_ID|41080 cb3307_c5/f2p0/3313 isoform=cb3307_c5;full_length_coverage=2;non_full_length_coverage=0;isoform_length=3313;expected_accuracy=0.997	136	2108	1	137	1342	3313	19	155
cb1514_c4/f2p1/3503	gnl|BL_ORD_ID|63697 cb1514_c10/f1p0/3670	1	3277	3277	3439	1	327	3372	353

<table>
<tr><td>isoform=cb1514_c4;full_length_coverage=2;non_full_length_coverage=1;isoform_length=3503;expected_accuracy=0.997</td><td></td><td></td><td></td><td></td><td></td><td></td><td>1</td><td></td><td>5</td></tr>
<tr><td>cb11848_c2/f1p1/1232</td><td>gnl|BL_ORD_ID|15933 cb8564_c68761/f3p4/1935 isoform=cb8564_c68761;full_length_coverage=3;non_full_length_coverage=4;isoform_length=1935;expected_accuracy=1.000</td><td>243</td><td>1178</td><td>39</td><td>242</td><td>1000</td><td>1934</td><td>3</td><td>207</td></tr>
<tr><td>cb4494_c7/f1p0/2870 isoform=cb4494_c7;full_length_coverage=1;non_full_length_coverage=0;isoform_length=2870;expected_accuracy=0.995</td><td>gnl|BL_ORD_ID|72612 cb4494_c1/f1p0/3279</td><td>2</td><td>2637</td><td>2637</td><td>2870</td><td>1</td><td>2646</td><td>2868</td><td>3104</td></tr>
<tr><td>cb850_c20/f1p0/1927</td><td>gnl|BL_ORD_ID|10143 cb850_c28/f1p0/3553 isoform=cb850_c28;full_length_coverage=1;non_full_length_coverage=0;isoform_length=3553;expected_accuracy=0.992</td><td>190</td><td>1904</td><td>2</td><td>192</td><td>1775</td><td>3509</td><td>111</td><td>305</td></tr>
<tr><td>cb8564_c98278/f5p6/2387 isoform=cb8564_c98278;full_length_coverage=5;non_full_length_coverage=6;isoform_length=2387;expected_accuracy=0.995</td><td>gnl|BL_ORD_ID|13975 cb8564_c42753/f2p0/2720 isoform=cb8564_c42753;full_length_coverage=2;non_full_length_coverage=0;isoform_length=2720;expected_accuracy=0.992</td><td>1</td><td>1387</td><td>1385</td><td>2387</td><td>1</td><td>1389</td><td>1714</td><td>2720</td></tr>
<tr><td>cb295_c26/f1p1/1869</td><td>gnl|BL_ORD_ID|68178 cb295_c28/f1p1/2235</td><td>1</td><td>1359</td><td>1358</td><td>1857</td><td>1</td><td>1359</td><td>1490</td><td>1989</td></tr>
<tr><td>cb5221_c27/f1p0/3107</td><td>gnl|BL_ORD_ID|45937 cb5221_c7/f1p0/3686 isoform=cb5221_c7;full_length_coverage=1;non_full_length_coverage=0;isoform_length=3686;expected_accuracy=0.996</td><td>1</td><td>1716</td><td>1713</td><td>3080</td><td>2</td><td>1736</td><td>2170</td><td>3537</td></tr>
<tr><td>cb8564_c11824/f2p0/2433</td><td>gnl|BL_ORD_ID|54126 cb8564_c18992/f1p0/3876</td><td>12</td><td>1397</td><td>1395</td><td>2433</td><td>1</td><td>138</td><td>2624</td><td>366</td></tr>
</table>

isoform=cb8564_c11824;full_length_coverage=2;non_full_length_coverage=0;isoform_length=2433;expected_accuracy=1.000							6		7
cb5125_c34/f1p0/1890	gnl|BL_ORD_ID|43699 cb5125_c8/f3p3/2148 isoform=cb5125_c8;full_length_coverage=3;non_full_length_coverage=3;isoform_length=2148;expected_accuracy=0.999	1	1145	1144	1780	1	1141	1333	1968
cb5851_c6/f1p0/2655	gnl|BL_ORD_ID|45118 cb5851_c9/f1p0/2643 isoform=cb5851_c9;full_length_coverage=1;non_full_length_coverage=0;isoform_length=2643;expected_accuracy=1.000	406	2435	5	409	632	2643	1	393
cb3575_c3/f2p1/3187 isoform=cb3575_c3;full_length_coverage=2;non_full_length_coverage=1;isoform_length=3187;expected_accuracy=1.000	gnl|BL_ORD_ID|70471 cb3575_c6/f1p1/3530	1	2555	2555	3047	1	2561	3027	3519
cb3887_c10/f1p0/2589	gnl|BL_ORD_ID|39511 cb3887_c5/f1p0/2830 isoform=cb3887_c5;full_length_coverage=1;non_full_length_coverage=0;isoform_length=2830;expected_accuracy=0.996	10	1802	1798	2386	107	1900	2239	2830
cb8564_c42814/f1p0/2151 isoform=cb8564_c42814;full_length_coverage=1;non_full_length_coverage=0;isoform_length=2151;expected_accuracy=0.993	gnl|BL_ORD_ID|14263 cb8564_c42654/f1p1/2906 isoform=cb8564_c42654;full_length_coverage=1;non_full_length_coverage=1;isoform_length=2906;expected_accuracy=0.990	650	2129	7	652	1339	2824	1	651
cb9434_c4/f1p0/2082	gnl|BL_ORD_ID|58718 cb9434_c5/f1p0/2667	1	1038	1037	2079	2	1040	1465	2507
cb8564_c36731/f1p2/2761	gnl|BL_ORD_ID|15193 cb8564_c42308/f1p5/3192	1	1977	1972	2735	5	199	2427	319

	isoform=cb8564_c42308;full_length_coverage=1;non_full_length_coverage=5;isoform_length=3192;expected_accuracy=0.995						8		1
cb341_c10/f1p0/3009	gnl|BL_ORD_ID|40575 cb341_c21/f1p0/3212 isoform=cb341_c21;full_length_coverage=1;non_full_length_coverage=0;isoform_length=3212;expected_accuracy=0.991	18	2686	2683	2900	1	2684	2992	3212
cb8564_c53036/f1p0/2917 isoform=cb8564_c53036;full_length_coverage=1;non_full_length_coverage=0;isoform_length=2917;expected_accuracy=0.999	gnl|BL_ORD_ID|15819 cb8564_c51027/f1p0/3064 isoform=cb8564_c51027;full_length_coverage=1;non_full_length_coverage=0;isoform_length=3064;expected_accuracy=0.996	1	2267	2267	2917	1	2268	2376	3024
cb2935_c11/f1p0/3067 isoform=cb2935_c11;full_length_coverage=1;non_full_length_coverage=0;isoform_length=3067;expected_accuracy=0.999	gnl|BL_ORD_ID|70092 cb2935_c30/f1p0/3442	259	3067	2	263	533	3343	8	269
cb4576_c55/f1p2/3996 isoform=cb4576_c55;full_length_coverage=1;non_full_length_coverage=2;isoform_length=3996;expected_accuracy=0.994	gnl|BL_ORD_ID|44415 cb4576_c54/f1p0/4341 isoform=cb4576_c54;full_length_coverage=1;non_full_length_coverage=0;isoform_length=4341;expected_accuracy=0.995	1937	3985	134	1938	2301	4341	46	1837
cb17744_c27/f1p0/1267	gnl|BL_ORD_ID|31103 cb17744_c12/f1p0/5455 isoform=cb17744_c12;full_length_coverage=1;non_full_length_coverage=0;isoform_length=5455;expected_accuracy=0.998	416	1189	9	416	4443	5211	1	407
cb3771_c6/f1p0/3096 isoform=cb3771_c6;full_length_coverage=1;non_full_length_coverage=0;isoform_length=3096;expected_accur	gnl|BL_ORD_ID|40598 cb3771_c5/f1p0/3508 isoform=cb3771_c5;full_length_coverage=1;non_full_length_coverage=0;isoform_length=3508;expected_accura	186	3096	1	187	590	3495	27	213

acy=0.996	cy=0.993								
cb18456_c6530/f1p0/1200	gnl|BL_ORD_ID|56888 cb8564_c75479/f1p1/2639	260	1160	3	259	959	60	1746	1490
cb8564_c14268/f6p2/4833 isoform=cb8564_c14268;full_length_coverage=6;non_full_length_coverage=2;isoform_length=4833;expected_accuracy=0.998	gnl|BL_ORD_ID|50937 cb737_c63/f1p2/5575	1	4212	4212	4832	1	4214	4994	5614
cb8564_c40598/f1p4/3119	gnl|BL_ORD_ID|55243 cb8564_c36242/f1p0/3335	1	2732	2729	3069	1	2733	2854	3192
cb8564_c86614/f1p2/3276	gnl|BL_ORD_ID|14409 cb8564_c40201/f1p0/3342 isoform=cb8564_c40201;full_length_coverage=1;non_full_length_coverage=0;isoform_length=3342;expected_accuracy=0.993	101	3178	1	103	271	3342	6	107
cb1163_c65/f1p1/3715	gnl|BL_ORD_ID|23460 cb1163_c13/f1p1/3869 isoform=cb1163_c13;full_length_coverage=1;non_full_length_coverage=1;isoform_length=3869;expected_accuracy=0.992	1	2651	2651	3707	1	2635	2818	3868
cb8564_c5070/f1p0/2861	gnl|BL_ORD_ID|56565 cb8564_c5064/f1p0/3231	10	1660	1659	2831	30	1679	1572	2744
cb8564_c89748/f3p1/2815 isoform=cb8564_c89748;full_length_coverage=3;non_full_length_coverage=1;isoform_length=2815;expected_accuracy=0.998	gnl|BL_ORD_ID|17430 cb8564_c85912/f1p1/2858 isoform=cb8564_c85912;full_length_coverage=1;non_full_length_coverage=1;isoform_length=2858;expected_accuracy=0.999	1	1737	1736	2737	9	1741	1858	2858
cb2869_c39/f1p0/1988	gnl|BL_ORD_ID|36991 cb2869_c22/f1p0/2751 isoform=cb2869_c22;full_length_coverage=1;non_full_length_coverage=0;isoform_length=2751;expected_accur	12	1262	1262	1796	1	1250	2217	2751

	acy=0.999								
cb1193_c21/f1p1/3672	gnl|BL_ORD_ID|22832 cb1193_c5/f2p1/3708 isoform=cb1193_c5;full_length_coverage=2;non_full_length_coverage=1;isoform_length=3708;expected_accuracy=0.997	1	1867	1864	3554	6	1868	2019	3708
cb5103_c18/f1p0/1858 isoform=cb5103_c18;full_length_coverage=1;non_full_length_coverage=0;isoform_length=1858;expected_accuracy=0.994	gnl|BL_ORD_ID|72111 cb5103_c7/f1p0/2511	1	1522	1519	1857	2	1534	2045	2377
cb4098_c71/f2p1/3728 isoform=cb4098_c71;full_length_coverage=2;non_full_length_coverage=1;isoform_length=3728;expected_accuracy=1.000	gnl|BL_ORD_ID|39575 cb4098_c28/f1p1/4006 isoform=cb4098_c28;full_length_coverage=1;non_full_length_coverage=1;isoform_length=4006;expected_accuracy=0.999	107	1977	1973	3726	1	1869	2252	4006
cb5633_c34/f1p3/2503 isoform=cb5633_c34;full_length_coverage=1;non_full_length_coverage=3;isoform_length=2503;expected_accuracy=0.996	gnl|BL_ORD_ID|45226 cb5633_c81/f1p0/2727 isoform=cb5633_c81;full_length_coverage=1;non_full_length_coverage=0;isoform_length=2727;expected_accuracy=0.992	332	2503	83	335	460	2720	13	286
cb8564_c18662/f1p0/2467 isoform=cb8564_c18662;full_length_coverage=1;non_full_length_coverage=0;isoform_length=2467;expected_accuracy=0.997	gnl|BL_ORD_ID|13115 cb8564_c16916/f7p1/3034 isoform=cb8564_c16916;full_length_coverage=7;non_full_length_coverage=1;isoform_length=3034;expected_accuracy=0.996	1	1823	1824	2464	143	1968	2098	2739
cb12662_c12/f1p0/1497 isoform=cb12662_c12;full_length_coverage=1;non_full_length_coverage=0;isoform_length=1497;expected_accuracy=1.000	gnl|BL_ORD_ID|61792 cb12662_c11/f1p0/1530	125	1423	1	129	234	1532	1	129
cb8831_c9/f1p1/2228	gnl|BL_ORD_ID|17278 cb8831_c5/f1p0/2282	1	1550	1550	2118	2	159	1693	228

isoform=cb8831_c9;full_length_coverage=1;non_full_length_coverage=1;isoform_length=2228;expected_accuracy=0.993	isoform=cb8831_c5;full_length_coverage=1;non_full_length_coverage=0;isoform_length=2282;expected_accuracy=0.997						0		2
cb8253_c9/f1p0/1760 isoform=cb8253_c9;full_length_coverage=1;non_full_length_coverage=0;isoform_length=1760;expected_accuracy=0.993	gnl|BL_ORD_ID|8384 cb8253_c11/f4p0/1869 isoform=cb8253_c11;full_length_coverage=4;non_full_length_coverage=0;isoform_length=1869;expected_accuracy=1.000	1	1594	1594	1760	1	1600	1701	1867
cb1490_c4/f1p3/4064	gnl|BL_ORD_ID|30687 cb1490_c2/f1p1/4303 isoform=cb1490_c2;full_length_coverage=1;non_full_length_coverage=1;isoform_length=4303;expected_accuracy=0.994	1	2550	2547	3910	1	2550	2942	4303
cb8564_c15653/f1p4/2475	gnl|BL_ORD_ID|55583 cb8564_c45873/f1p2/2968	22	2293	2289	2482	32	2301	2520	2713
cb7976_c18/f1p0/2021	gnl|BL_ORD_ID|50806 cb7976_c7/f1p0/2654	735	1990	81	737	1366	2621	1	657
cb8564_c43399/f1p0/2654 isoform=cb8564_c43399;full_length_coverage=1;non_full_length_coverage=0;isoform_length=2654;expected_accuracy=0.999	gnl|BL_ORD_ID|13273 cb8564_c188193/f1p0/2849 isoform=cb8564_c188193;full_length_coverage=1;non_full_length_coverage=0;isoform_length=2849;expected_accuracy=0.997	1	2367	2363	2653	1	2343	2559	2849
cb4233_c62/f1p0/3000	gnl|BL_ORD_ID|72364 cb4233_c56/f1p0/2924	1	1777	1779	2683	1	1782	1992	2876
cb10213_c19/f1p0/1599 isoform=cb10213_c19;full_length_coverage=1;non_full_length_coverage=0;isoform_length=1599;expected_accuracy=0.999	gnl|BL_ORD_ID|48228 cb10213_c5/f1p0/3219	1	1229	1225	1599	8	1233	2789	3162
cb4174_c3/f1p0/3410	gnl|BL_ORD_ID|72313 cb4174_c5/f1p0/3191	131	2403	2404	3149	1	228	2407	315

							4		2
cb5267_c6/f1p0/2120 isoform=cb5267_c6;full_length_coverage=1;non_full_length_coverage=0;isoform_length=2120;expected_accuracy=0.994	gnl|BL_ORD_ID|73708 cb5267_c7/f1p0/2389	262	2084	7	267	435	2261	1	264
cb8564_c46939/f1p1/2926	gnl|BL_ORD_ID|55246 cb8564_c36343/f1p0/3448	2	2134	2130	2870	3	2160	2265	3004
cb8564_c16741/f1p0/2997	gnl|BL_ORD_ID|52661 cb8564_c10315/f1p0/4512	375	3001	5	375	1666	4292	1	371
cb2520_c20/f1p1/2903 isoform=cb2520_c20;full_length_coverage=1;non_full_length_coverage=1;isoform_length=2903;expected_accuracy=0.994	gnl|BL_ORD_ID|36518 cb2520_c4/f2p0/3911 isoform=cb2520_c4;full_length_coverage=2;non_full_length_coverage=0;isoform_length=3911;expected_accuracy=0.999	105	2842	1	106	1177	3911	571	676
cb8564_c112175/f2p0/2623 isoform=cb8564_c112175;full_length_coverage=2;non_full_length_coverage=0;isoform_length=2623;expected_accuracy=0.996	gnl|BL_ORD_ID|53022 cb8564_c114466/f1p0/3000	1	1638	1639	2623	2	1610	1943	2926
cb7257_c2/f1p0/2073 isoform=cb7257_c2;full_length_coverage=1;non_full_length_coverage=0;isoform_length=2073;expected_accuracy=0.993	gnl|BL_ORD_ID|51081 cb7257_c0/f1p0/2663	1	1564	1562	2073	104	1672	1776	2288
cb5271_c8/f1p2/2641 isoform=cb5271_c8;full_length_coverage=1;non_full_length_coverage=2;isoform_length=2641;expected_accuracy=0.994	gnl|BL_ORD_ID|44628 cb5271_c18/f1p2/2761 isoform=cb5271_c18;full_length_coverage=1;non_full_length_coverage=2;isoform_length=2761;expected_accuracy=0.991	1273	2622	1	1275	1419	2761	1	1268
cb548_c122/f43p2/3004	gnl|BL_ORD_ID|46779 cb548_c109/f1p0/3280	1347	3002	13	1350	1551	319	4	134

isoform=cb548_c122;full_length_coverage=43;non_full_length_coverage=2;isoform_length=3004;expected_accuracy=1.000	isoform=cb548_c109;full_length_coverage=1;non_full_length_coverage=0;isoform_length=3280;expected_accuracy=0.996						8		0
cb6499_c15/f1p1/1972 isoform=cb6499_c15;full_length_coverage=1;non_full_length_coverage=1;isoform_length=1972;expected_accuracy=0.994	gnl|BL_ORD_ID|4653 cb6499_c26/f12p0/2255 isoform=cb6499_c26;full_length_coverage=12;non_full_length_coverage=0;isoform_length=2255;expected_accuracy=1.000	125	1906	1	126	357	2138	48	171
cb1270_c1/f1p0/3702 isoform=cb1270_c1;full_length_coverage=1;non_full_length_coverage=0;isoform_length=3702;expected_accuracy=0.998	gnl|BL_ORD_ID|59322 cb1270_c5/f1p0/4305	1	3232	3230	3631	4	3237	3710	4111
cb4759_c15/f1p0/2126	gnl|BL_ORD_ID|43548 cb4759_c13/f1p0/2816 isoform=cb4759_c13;full_length_coverage=1;non_full_length_coverage=0;isoform_length=2816;expected_accuracy=1.000	584	2096	6	585	1306	2816	3	578
cb5538_c7/f1p0/2392 isoform=cb5538_c7;full_length_coverage=1;non_full_length_coverage=0;isoform_length=2392;expected_accuracy=0.993	gnl|BL_ORD_ID|74046 cb5538_c24/f1p0/2729	1	1488	1487	2392	99	1587	1833	2735
cb12047_c103/f1p1/1531	gnl|BL_ORD_ID|12861 cb8564_c15921/f1p11/1869 isoform=cb8564_c15921;full_length_coverage=1;non_full_length_coverage=11;isoform_length=1869;expected_accuracy=0.993	9	870	865	1498	3	876	1171	1836
cb12037_c6/f1p0/1610 isoform=cb12037_c6;full_length_coverage=1;non_full_length_coverage=0;isoform_length=1610;expected_accu	gnl|BL_ORD_ID|11654 cb8564_c124642/f1p0/2345 isoform=cb8564_c124642;full_length_coverage=1;non_full_length_coverage=0;isoform_length=2345;expected_a	1	1435	1434	1610	165	1584	2170	2345

racy=0.993	ccuracy=0.991								
cb4957_c12/f1p0/1876 isoform=cb4957_c12;full_length_coverage=1;non_full_length_coverage=0;isoform_length=1876;expected_accuracy=1.000	gnl|BL_ORD_ID|41849 cb4957_c1/f1p0/3205 isoform=cb4957_c1;full_length_coverage=1;non_full_length_coverage=0;isoform_length=3205;expected_accuracy=0.996	1	1467	1468	1873	74	1548	2800	3205
cb8564_c181074/f1p0/2725 isoform=cb8564_c181074;full_length_coverage=1;non_full_length_coverage=0;isoform_length=2725;expected_accuracy=1.000	gnl|BL_ORD_ID|17094 cb8564_c78858/f1p0/2927 isoform=cb8564_c78858;full_length_coverage=1;non_full_length_coverage=0;isoform_length=2927;expected_accuracy=0.995	148	2698	1	147	378	2927	1	147
cb8564_c118213/f2p0/2393 isoform=cb8564_c118213;full_length_coverage=2;non_full_length_coverage=0;isoform_length=2393;expected_accuracy=0.990	gnl|BL_ORD_ID|14369 cb8564_c37858/f1p0/2999 isoform=cb8564_c37858;full_length_coverage=1;non_full_length_coverage=0;isoform_length=2999;expected_accuracy=0.998	370	2331	9	371	1047	2999	18	379
cb6097_c42/f1p0/1998 isoform=cb6097_c42;full_length_coverage=1;non_full_length_coverage=0;isoform_length=1998;expected_accuracy=0.995	gnl|BL_ORD_ID|45264 cb6097_c35/f1p0/2775 isoform=cb6097_c35;full_length_coverage=1;non_full_length_coverage=0;isoform_length=2775;expected_accuracy=0.999	1	1526	1524	1998	672	2197	2303	2775
cb8564_c40764/f1p0/2563 isoform=cb8564_c40764;full_length_coverage=1;non_full_length_coverage=0;isoform_length=2563;expected_accuracy=0.997	gnl|BL_ORD_ID|16473 cb8564_c51923/f1p1/2966 isoform=cb8564_c51923;full_length_coverage=1;non_full_length_coverage=1;isoform_length=2966;expected_accuracy=0.997	1	2167	2162	2563	1	2176	2383	2784
cb1178_c27/f1p0/1197 isoform=cb1178_c27;full_length_coverage=1;non_full_length_coverage=0;isoform_length=1197;expected_accuracy=0.992	gnl|BL_ORD_ID|61143 cb1178_c5/f2p0/1389	111	1181	1	110	321	1389	97	200
cb5447_c3/f5p2/3155	gnl|BL_ORD_ID|45897 cb5447_c33/f1p0/4199	19	2178	2178	3155	1	215	2805	377

isoform=cb5447_c3;full_length_coverage=5;non_full_length_coverage=2;isoform_length=3155;expected_accuracy=1.000	isoform=cb5447_c33;full_length_coverage=1;non_full_length_coverage=0;isoform_length=4199;expected_accuracy=0.997						5		8
cb762_c6/f1p0/3769 isoform=cb762_c6;full_length_coverage=1;non_full_length_coverage=0;isoform_length=3769;expected_accuracy=0.993	gnl|BL_ORD_ID|8132 cb762_c4/f1p0/4517 isoform=cb762_c4;full_length_coverage=1;non_full_length_coverage=0;isoform_length=4517;expected_accuracy=0.996	1	2843	2841	3769	48	2890	3356	4284
cb5307_c9/f1p0/2902	gnl|BL_ORD_ID|45615 cb5307_c0/f9p0/3058 isoform=cb5307_c0;full_length_coverage=9;non_full_length_coverage=0;isoform_length=3058;expected_accuracy=1.000	1204	2896	1	1204	1356	3048	47	1248
cb8564_c36731/f1p2/2761	gnl|BL_ORD_ID|15169 cb8564_c40218/f1p0/3146 isoform=cb8564_c40218;full_length_coverage=1;non_full_length_coverage=0;isoform_length=3146;expected_accuracy=0.993	1	1977	1972	2734	4	1968	2394	3146
cb3400_c4/f1p0/2972	gnl|BL_ORD_ID|71172 cb3400_c2/f1p0/3309	1	2653	2651	2958	24	2677	3011	3319
cb8564_c75977/f1p0/2165	gnl|BL_ORD_ID|16147 cb8564_c77752/f1p1/3809 isoform=cb8564_c77752;full_length_coverage=1;non_full_length_coverage=1;isoform_length=3809;expected_accuracy=0.991	1	1393	1394	2126	60	1461	2755	3521
cb2637_c16/f1p0/1139 isoform=cb2637_c16;full_length_coverage=1;non_full_length_coverage=0;isoform_length=1139;expected_accuracy=1.000	gnl|BL_ORD_ID|39001 cb2637_c8/f1p0/2971 isoform=cb2637_c8;full_length_coverage=1;non_full_length_coverage=0;isoform_length=2971;expected_accuracy=0.992	382	1139	51	384	1198	1958	1	339
cb3145_c1/f1p0/3709	gnl|BL_ORD_ID|39003 cb3145_c2/f1p0/3679	3	3016	3017	3507	13	302	3147	363

	isoform=cb3145_c2;full_length_coverage=1;non_full_length_coverage=0;isoform_length=3679;expected_accuracy=0.997						5		8
cb8564_c22212/f1p0/2530 isoform=cb8564_c22212;full_length_coverage=1;non_full_length_coverage=0;isoform_length=2530;expected_accuracy=0.992	gnl|BL_ORD_ID|52063 cb8564_c11680/f1p1/3676	1	2118	2113	2530	22	2139	2996	3413
cb18456_c6530/f1p0/1200	gnl|BL_ORD_ID|933 cb10092_c2/f3p19/1736 isoform=cb10092_c2;full_length_coverage=3;non_full_length_coverage=19;isoform_length=1736;expected_accuracy=1.000	260	1166	3	259	803	1708	8	272
cb8564_c90797/f1p0/2864	gnl|BL_ORD_ID|13313 cb8564_c188075/f1p2/2904 isoform=cb8564_c188075;full_length_coverage=1;non_full_length_coverage=2;isoform_length=2904;expected_accuracy=0.996	15	2313	2308	2661	96	2393	2515	2868
cb8564_c129638/f1p0/2479	gnl|BL_ORD_ID|52949 cb8564_c112716/f1p0/3223	1	1627	1623	2475	20	1646	2051	2924
cb7915_c3/f1p0/1964 isoform=cb7915_c3;full_length_coverage=1;non_full_length_coverage=0;isoform_length=1964;expected_accuracy=0.997	gnl|BL_ORD_ID|8620 cb7915_c2/f2p0/2505 isoform=cb7915_c2;full_length_coverage=2;non_full_length_coverage=0;isoform_length=2505;expected_accuracy=1.000	1	1547	1545	1964	46	1581	2065	2485
cb8564_c185920/f1p0/2777 isoform=cb8564_c185920;full_length_coverage=1;non_full_length_coverage=0;isoform_length=2777;expected_accuracy=0.996	gnl|BL_ORD_ID|55341 cb8564_c38927/f1p0/3326	174	2777	1	177	356	2964	20	197
cb4771_c5/f1p0/2633	gnl|BL_ORD_ID|43536 cb4771_c2/f1p0/3261	311	2598	87	314	908	318	1	228

	isoform=cb4771_c2;full_length_coverage=1;non_full_length_coverage=0;isoform_length=3261;expected_accuracy=0.996						7		
cb8564_c43779/f1p0/3021 isoform=cb8564_c43779;full_length_coverage=1;non_full_length_coverage=0;isoform_length=3021;expected_accuracy=0.993	gnl|BL_ORD_ID|17429 cb8564_c85447/f1p0/3196 isoform=cb8564_c85447;full_length_coverage=1;non_full_length_coverage=0;isoform_length=3196;expected_accuracy=0.997	1	2344	2345	3021	3	2344	2522	3195
cb8564_c23645/f1p0/2729	gnl|BL_ORD_ID|52033 cb8564_c10646/f1p1/3031	1	2066	2062	2493	2	2068	2202	2633
cb1770_c13/f1p0/3529 isoform=cb1770_c13;full_length_coverage=1;non_full_length_coverage=0;isoform_length=3529;expected_accuracy=1.000	gnl|BL_ORD_ID|33844 cb1770_c9/f1p0/3711 isoform=cb1770_c9;full_length_coverage=1;non_full_length_coverage=0;isoform_length=3711;expected_accuracy=0.999	1	2029	2027	3529	8	2038	2199	3701
cb8564_c109930/f2p1/2066 isoform=cb8564_c109930;full_length_coverage=2;non_full_length_coverage=1;isoform_length=2066;expected_accuracy=1.000	gnl|BL_ORD_ID|12510 cb8564_c122775/f1p0/2291 isoform=cb8564_c122775;full_length_coverage=1;non_full_length_coverage=0;isoform_length=2291;expected_accuracy=0.995	1	1103	1101	2016	2123	1020	914	1
cb9446_c7/f1p0/1576 isoform=cb9446_c7;full_length_coverage=1;non_full_length_coverage=0;isoform_length=1576;expected_accuracy=0.994	gnl|BL_ORD_ID|21108 cb9446_c1/f1p0/1941 isoform=cb9446_c1;full_length_coverage=1;non_full_length_coverage=0;isoform_length=1941;expected_accuracy=0.996	189	1576	23	194	352	1737	1	171
cb8564_c18575/f1p0/1939 isoform=cb8564_c18575;full_length_coverage=1;non_full_length_coverage=0;isoform_length=1939;expected_accuracy=0.990	gnl|BL_ORD_ID|13618 cb8564_c185222/f1p3/3772 isoform=cb8564_c185222;full_length_coverage=1;non_full_length_coverage=3;isoform_length=3772;expected_accuracy=0.997	1	1427	1422	1939	824	2265	3246	3772
cb3988_c7/f1p1/3083	gnl|BL_ORD_ID|41321 cb3988_c1/f1p1/3470	1	2003	1999	3044	46	205	2423	347

	isoform=cb3988_c1;full_length_coverage=1;non_full_length_coverage=1;isoform_length=3470;expected_accuracy=1.000						1		0
cb8564_c37175/f1p0/3267	gnl|BL_ORD_ID|14738 cb8564_c42029/f1p0/3342 isoform=cb8564_c42029;full_length_coverage=1;non_full_length_coverage=0;isoform_length=3342;expected_accuracy=0.998	116	3184	11	118	270	3341	13	122
cb3449_c13/f1p2/3050 isoform=cb3449_c13;full_length_coverage=1;non_full_length_coverage=2;isoform_length=3050;expected_accuracy=0.993	gnl|BL_ORD_ID|71214 cb3449_c18/f1p2/3268	1	2755	2752	3050	2	2758	2926	3220
cb7969_c11/f1p0/1950 isoform=cb7969_c11;full_length_coverage=1;non_full_length_coverage=0;isoform_length=1950;expected_accuracy=0.992	gnl|BL_ORD_ID|50804 cb7969_c23/f1p0/2373	1	1409	1405	1950	69	1479	1724	2270
cb8564_c73605/f1p0/2967 isoform=cb8564_c73605;full_length_coverage=1;non_full_length_coverage=0;isoform_length=2967;expected_accuracy=0.992	gnl|BL_ORD_ID|56794 cb8564_c71992/f1p0/3077	1	2354	2351	2870	12	2375	2544	3064
cb938_c15/f1p0/3219	gnl|BL_ORD_ID|21551 cb938_c49/f5p1/3515 isoform=cb938_c49;full_length_coverage=5;non_full_length_coverage=1;isoform_length=3515;expected_accuracy=1.000	1	2546	2541	3227	3	2549	2825	3511
cb8564_c35645/f1p0/2804	gnl|BL_ORD_ID|52842 cb8564_c15468/f1p0/3015	150	2663	1	151	306	2819	43	193
cb8564_c50089/f1p0/2929	gnl|BL_ORD_ID|56070 cb8564_c54122/f1p1/3367	1	2301	2301	2907	45	235	2651	325

isoform=cb8564_c50089;full_length_coverage=1;non_full_length_coverage=0;isoform_length=2929;expected_accuracy=0.993							0		8
cb6406_c59/f1p0/2372	gnl|BL_ORD_ID|5551 cb6406_c15/f1p0/2860 isoform=cb6406_c15;full_length_coverage=1;non_full_length_coverage=0;isoform_length=2860;expected_accuracy=0.994	841	2163	9	843	1539	2860	1	836
cb8564_c87256/f1p0/2285 isoform=cb8564_c87256;full_length_coverage=1;non_full_length_coverage=0;isoform_length=2285;expected_accuracy=0.992	gnl|BL_ORD_ID|54699 cb8564_c155769/f1p3/2649	1	1703	1700	2285	205	1910	2044	2636
cb8564_c45609/f1p0/3991	gnl|BL_ORD_ID|14247 cb8564_c45575/f3p6/4033 isoform=cb8564_c45575;full_length_coverage=3;non_full_length_coverage=6;isoform_length=4033;expected_accuracy=0.998	260	3705	1	260	500	3945	43	305
cb912_c15/f1p0/2991 isoform=cb912_c15;full_length_coverage=1;non_full_length_coverage=0;isoform_length=2991;expected_accuracy=0.999	gnl|BL_ORD_ID|59090 cb912_c16/f1p0/3349	1	2472	2469	2859	291	2763	2900	3290
cb18456_c1776/f1p0/1211	gnl|BL_ORD_ID|11429 cb8564_c1308/f1p7/2249 isoform=cb8564_c1308;full_length_coverage=1;non_full_length_coverage=7;isoform_length=2249;expected_accuracy=0.998	280	1214	6	285	1311	2244	921	1199
cb6757_c12/f1p0/2448 isoform=cb6757_c12;full_length_coverage=1;non_full_length_coverage=0;isoform_length=2448;expected_accu	gnl|BL_ORD_ID|50029 cb6757_c6/f1p0/4618	1	1299	1297	2448	1	1319	3410	4583

racy=0.993									
cb7388_c0/f2p0/2378	gnl|BL_ORD_ID|7290 cb7388_c1/f1p0/2631 isoform=cb7388_c1;full_length_coverage=1;non_full_length_coverage=0;isoform_length=2631;expected_accuracy=0.999	1	1892	1891	2345	54	1945	2180	2631
cb7200_c9/f1p0/2478	gnl|BL_ORD_ID|7319 cb7200_c2/f3p1/2679 isoform=cb7200_c2;full_length_coverage=3;non_full_length_coverage=1;isoform_length=2679;expected_accuracy=1.000	526	2269	6	529	734	2473	12	535
cb4020_c0/f2p0/2091 isoform=cb4020_c0;full_length_coverage=2;non_full_length_coverage=0;isoform_length=2091;expected_accuracy=0.998	gnl|BL_ORD_ID|40368 cb4020_c3/f1p0/2582 isoform=cb4020_c3;full_length_coverage=1;non_full_length_coverage=0;isoform_length=2582;expected_accuracy=1.000	1	1217	1215	2091	1	1239	1687	2563
cb6181_c4/f3p0/2235 isoform=cb6181_c4;full_length_coverage=3;non_full_length_coverage=0;isoform_length=2235;expected_accuracy=1.000	gnl|BL_ORD_ID|5503 cb6181_c7/f6p0/2362 isoform=cb6181_c7;full_length_coverage=6;non_full_length_coverage=0;isoform_length=2362;expected_accuracy=1.000	1	1513	1511	2186	1	1513	1687	2362
cb8564_c21222/f1p0/4286	gnl|BL_ORD_ID|54171 cb8564_c19825/f1p0/4566	1949	4288	1	1949	2099	4438	2	1950
cb191_c1/f1p2/4439 isoform=cb191_c1;full_length_coverage=1;non_full_length_coverage=2;isoform_length=4439;expected_accuracy=0.998	gnl|BL_ORD_ID|34772 cb191_c15/f1p1/5569 isoform=cb191_c15;full_length_coverage=1;non_full_length_coverage=1;isoform_length=5569;expected_accuracy=0.996	1044	4439	1	1044	1467	4865	243	1282
cb3285_c19/f1p1/2821	gnl|BL_ORD_ID|41359 cb3285_c14/f1p1/3029 isoform=cb3285_c14;full_length_coverage=1;non_full_length_coverage=1;isoform_length=3029;expected_accur	1	1702	1697	2712	3	1696	2013	3029

	acy=0.997								
cb8564_c12600/f1p0/2645	gnl|BL_ORD_ID|11921 cb8564_c15088/f2p0/3643 isoform=cb8564_c15088;full_length_coverage=2;non_full_length_coverage=0;isoform_length=3643;expected_accuracy=0.994	254	2645	18	255	341	2707	1	238
cb3557_c31/f1p0/2861	gnl|BL_ORD_ID|70455 cb3557_c8/f1p0/3541	21	2233	2231	2885	1	2213	2767	3421
cb15510_c0/f1p0/1378	gnl|BL_ORD_ID|41745 cb5048_c10/f4p1/1631 isoform=cb5048_c10;full_length_coverage=4;non_full_length_coverage=1;isoform_length=1631;expected_accuracy=1.000	463	1314	5	459	677	1528	1	457
cb8564_c85288/f1p2/2983 isoform=cb8564_c85288;full_length_coverage=1;non_full_length_coverage=2;isoform_length=2983;expected_accuracy=0.999	gnl|BL_ORD_ID|11528 cb8564_c146182/f3p2/3172 isoform=cb8564_c146182;full_length_coverage=3;non_full_length_coverage=2;isoform_length=3172;expected_accuracy=1.000	1	2289	2289	2981	41	2335	2479	3172
cb8564_c23232/f1p3/2540	gnl|BL_ORD_ID|11957 cb8564_c135981/f1p0/2840 isoform=cb8564_c135981;full_length_coverage=1;non_full_length_coverage=0;isoform_length=2840;expected_accuracy=0.992	16	2172	2167	2518	193	2342	2445	2794
cb2918_c13/f2p0/2549 isoform=cb2918_c13;full_length_coverage=2;non_full_length_coverage=0;isoform_length=2549;expected_accuracy=1.000	gnl|BL_ORD_ID|68997 cb2918_c66/f1p0/3035	1080	2536	1	1082	1578	3034	60	1141
cb2836_c20/f1p2/3269 isoform=cb2836_c20;full_length_coverage=1;non_full_length_coverage=2;isoform_length=3269;expected_accu	gnl|BL_ORD_ID|68925 cb2836_c11/f1p0/3675	1	2389	2390	3141	170	2559	2666	3418

racy=0.993									
cb8564_c129154/f1p0/2632 isoform=cb8564_c129154;full_length_coverage=1;non_full_length_coverage=0;isoform_length=2632;expected_accuracy=0.997	gnl|BL_ORD_ID|10296 cb8564_c116976/f1p0/2943 isoform=cb8564_c116976;full_length_coverage=1;non_full_length_coverage=0;isoform_length=2943;expected_accuracy=0.997	1	1586	1587	2601	15	1584	1927	2941
cb2818_c78/f1p0/2043 isoform=cb2818_c78;full_length_coverage=1;non_full_length_coverage=0;isoform_length=2043;expected_accuracy=0.992	gnl|BL_ORD_ID|68899 cb2818_c34/f1p0/3053	1	1936	1935	2041	488	2420	2815	2923
cb2985_c4/f1p0/3241 isoform=cb2985_c4;full_length_coverage=1;non_full_length_coverage=0;isoform_length=3241;expected_accuracy=0.994	gnl|BL_ORD_ID|36841 cb2985_c2/f4p2/3365 isoform=cb2985_c2;full_length_coverage=4;non_full_length_coverage=2;isoform_length=3365;expected_accuracy=1.000	10	1966	1967	3175	1	1952	2158	3365
cb8564_c8764/f7p3/3755 isoform=cb8564_c8764;full_length_coverage=7;non_full_length_coverage=3;isoform_length=3755;expected_accuracy=1.000	gnl|BL_ORD_ID|54306 cb8564_c22128/f1p2/3973	1	3056	3056	3755	1	3055	3213	3912
cb1772_c14/f1p0/2582	gnl|BL_ORD_ID|63938 cb1772_c19/f1p0/4042	1	2321	2318	2564	108	2429	3670	3916
cb8564_c5115/f1p1/3855	gnl|BL_ORD_ID|15705 cb8564_c5095/f1p0/3793 isoform=cb8564_c5095;full_length_coverage=1;non_full_length_coverage=0;isoform_length=3793;expected_accuracy=0.995	1523	3639	1	1524	1673	3791	1	1523
cb191_c1/f1p2/4439 isoform=cb191_c1;full_length_coverage=1;non_full_length_coverage=2;isoform_length=4439;expected_accurac	gnl|BL_ORD_ID|66308 cb191_c14/f1p0/5848	1044	4439	1	1044	1520	4918	291	1334

y=0.998									
cb8564_c36731/f1p2/2761	gnl|BL_ORD_ID|53680 cb8564_c153222/f2p39/3454	1	1977	1972	2726	7	198 6	2415	317 0
cb99_c12/f1p0/3017 isoform=cb99_c12;full_length_coverage=1;non_full_length_coverage=0;isoform_length=3017;expected_accuracy=0.997	gnl|BL_ORD_ID|21972 cb99_c4/f1p1/4938 isoform=cb99_c4;full_length_coverage=1;non_full_length_coverage=1;isoform_length=4938;expected_accuracy=0.999	1	2761	2758	2979	1764	452 7	4718	493 8
cb6291_c3/f1p0/2781 isoform=cb6291_c3;full_length_coverage=1;non_full_length_coverage=0;isoform_length=2781;expected_accuracy=0.992	gnl|BL_ORD_ID|4113 cb6291_c0/f2p0/2911 isoform=cb6291_c0;full_length_coverage=2;non_full_length_coverage=0;isoform_length=2911;expected_accuracy=0.997	192	2754	1	191	343	2911	30	224
cb8564_c54518/f1p0/2781	gnl|BL_ORD_ID|12978 cb8564_c175946/f10p1/3002 isoform=cb8564_c175946;full_length_coverage=10;non_full_length_coverage=1;isoform_length=3002;expected_accuracy=1.000	1	1587	1584	2793	34	162 0	1754	296 3
cb8564_c7294/f1p0/2355 isoform=cb8564_c7294;full_length_coverage=1;non_full_length_coverage=0;isoform_length=2355;expected_accuracy=0.997	gnl|BL_ORD_ID|15303 cb8564_c3481/f1p0/2830 isoform=cb8564_c3481;full_length_coverage=1;non_full_length_coverage=0;isoform_length=2830;expected_accuracy=0.995	1	1678	1674	2331	8	167 6	2158	281 4
cb8564_c5245/f7p0/2048 isoform=cb8564_c5245;full_length_coverage=7;non_full_length_coverage=0;isoform_length=2048;expected_accuracy=1.000	gnl|BL_ORD_ID|15463 cb8564_c5246/f1p0/2196 isoform=cb8564_c5246;full_length_coverage=1;non_full_length_coverage=0;isoform_length=2196;expected_accuracy=0.993	16	1641	1641	2047	1	165 5	1787	219 6
cb14_c28/f1p0/3251 isoform=cb14_c28;full_length_coverage=1;non_full_length_coverage=0;isoform_length=3251;expected_accurac	gnl|BL_ORD_ID|27787 cb14_c30/f1p0/3711 isoform=cb14_c30;full_length_coverage=1;non_full_length_coverage=0;isoform_length=3711;expected_accurac	1158	3210	1	1161	1667	3711	3	1156

y=0.991	y=0.996								
cb18848_c1/f1p2/5728 isoform=cb18848_c1;full_length_coverage=1;non_full_length_coverage=2;isoform_length=5728;expected_accuracy=1.000	gnl|BL_ORD_ID|36294 cb18848_c0/f1p0/5897 isoform=cb18848_c0;full_length_coverage=1;non_full_length_coverage=0;isoform_length=5897;expected_accuracy=0.996	1	5222	5223	5727	5	5227	5393	5897
cb8564_c39136/f1p0/3036 isoform=cb8564_c39136;full_length_coverage=1;non_full_length_coverage=0;isoform_length=3036;expected_accuracy=0.998	gnl|BL_ORD_ID|16447 cb8564_c47202/f1p0/3195 isoform=cb8564_c47202;full_length_coverage=1;non_full_length_coverage=0;isoform_length=3195;expected_accuracy=0.996	1	2089	2085	3033	1	2096	2223	3195
cb584_c43/f3p1/3426 isoform=cb584_c43;full_length_coverage=3;non_full_length_coverage=1;isoform_length=3426;expected_accuracy=1.000	gnl|BL_ORD_ID|72281 cb584_c8/f1p0/4733	175	3426	1	180	1496	4749	42	221
cb9773_c9/f1p0/2060	gnl|BL_ORD_ID|58880 cb9773_c6/f1p0/2971	551	2015	60	554	1506	2987	3	501
cb6106_c25/f1p0/2002	gnl|BL_ORD_ID|45790 cb6106_c5/f1p0/2598 isoform=cb6106_c5;full_length_coverage=1;non_full_length_coverage=0;isoform_length=2598;expected_accuracy=0.997	1	1332	1329	1992	12	1342	1935	2598
cb13985_c12/f5p0/1453 isoform=cb13985_c12;full_length_coverage=5;non_full_length_coverage=0;isoform_length=1453;expected_accuracy=1.000	gnl|BL_ORD_ID|62962 cb13985_c10/f1p0/1780	186	1453	23	190	349	1616	1	165
cb8909_c12/f1p0/1836 isoform=cb8909_c12;full_length_coverage=1;non_full_length_coverage=0;isoform_length=1836;expected_accu	gnl|BL_ORD_ID|20551 cb8909_c3/f2p0/1945 isoform=cb8909_c3;full_length_coverage=2;non_full_length_coverage=0;isoform_length=1945;expected_accura	1	1624	1620	1836	1	1625	1726	1945

racy=1.000	cy=1.000								
cb8564_c40598/f1p4/3119	gnl|BL_ORD_ID|14838 cb8564_c34060/f2p0/3214 isoform=cb8564_c34060;full_length_coverage=2;non_full_length_coverage=0;isoform_length=3214;expected_accuracy=0.993	1	2732	2729	3051	6	2772	2893	3214
cb9146_c8/f1p0/1115 isoform=cb9146_c8;full_length_coverage=1;non_full_length_coverage=0;isoform_length=1115;expected_accuracy=0.991	gnl|BL_ORD_ID|20475 cb9146_c4/f1p0/1288 isoform=cb9146_c4;full_length_coverage=1;non_full_length_coverage=0;isoform_length=1288;expected_accuracy=0.996	101	1100	1	101	263	1288	59	159
cb8564_c89014/f1p0/2743	gnl|BL_ORD_ID|15942 cb8564_c77573/f2p2/2771 isoform=cb8564_c77573;full_length_coverage=2;non_full_length_coverage=2;isoform_length=2771;expected_accuracy=1.000	14	1755	1753	2457	1	1747	1880	2584
cb8564_c85953/f1p0/2986	gnl|BL_ORD_ID|17094 cb8564_c78858/f1p0/2927 isoform=cb8564_c78858;full_length_coverage=1;non_full_length_coverage=0;isoform_length=2927;expected_accuracy=0.995	175	2717	1	176	378	2918	1	176
cb18409_c75/f1p0/1035 isoform=cb18409_c75;full_length_coverage=1;non_full_length_coverage=0;isoform_length=1035;expected_accuracy=0.995	gnl|BL_ORD_ID|56968 cb8564_c78887/f1p0/2186	109	1035	2	112	1195	2122	3	116
cb3135_c4/f1p0/3711	gnl|BL_ORD_ID|38272 cb3135_c6/f1p0/3675 isoform=cb3135_c6;full_length_coverage=1;non_full_length_coverage=0;isoform_length=3675;expected_accuracy=0.997	103	3050	3049	3417	1	2938	3300	3666
cb9718_c43/f1p0/1655	gnl|BL_ORD_ID|59924 cb9718_c9/f1p10/1998	288	1655	3	290	389	175	2	289

isoform=cb9718_c43;full_length_coverage=1;non_full_length_coverage=0;isoform_length=1655;expected_accuracy=0.996							6		
cb14183_c4/f1p0/1000 isoform=cb14183_c4;full_length_coverage=1;non_full_length_coverage=0;isoform_length=1000;expected_accuracy=0.997	gnl|BL_ORD_ID|25904 cb14183_c1/f3p1/1153 isoform=cb14183_c1;full_length_coverage=3;non_full_length_coverage=1;isoform_length=1153;expected_accuracy=1.000	118	972	1	122	300	1153	7	129
cb3449_c13/f1p2/3050 isoform=cb3449_c13;full_length_coverage=1;non_full_length_coverage=2;isoform_length=3050;expected_accuracy=0.993	gnl|BL_ORD_ID|71217 cb3449_c38/f1p0/3430	1	2755	2752	3047	100	2855	3023	3319
cb2643_c10/f1p0/2833 isoform=cb2643_c10;full_length_coverage=1;non_full_length_coverage=0;isoform_length=2833;expected_accuracy=0.998	gnl|BL_ORD_ID|68664 cb2643_c13/f1p0/3261	1	2279	2274	2833	11	2293	2651	3210
cb10971_c2/f1p0/1762	gnl|BL_ORD_ID|652 cb10971_c0/f1p1/1867 isoform=cb10971_c0;full_length_coverage=1;non_full_length_coverage=1;isoform_length=1867;expected_accuracy=0.996	328	1710	8	331	490	1867	39	362
cb8564_c18096/f1p0/2378 isoform=cb8564_c18096;full_length_coverage=1;non_full_length_coverage=0;isoform_length=2378;expected_accuracy=1.000	gnl|BL_ORD_ID|56138 cb8564_c70645/f1p0/3759	1	2058	2059	2378	685	2770	3007	3331
cb8564_c46939/f1p1/2926	gnl|BL_ORD_ID|58037 cb8564_c87188/f1p1/3426	21	2134	2130	2870	127	2249	2354	3095
cb8564_c125018/f1p0/3452	gnl|BL_ORD_ID|10275 cb8564_c109614/f3p0/3516	30	3167	3167	3435	3	313	3251	351

isoform=cb8564_c125018;full_length_coverage=1;non_full_length_coverage=0;isoform_length=3452;expected_accuracy=0.993	isoform=cb8564_c109614;full_length_coverage=3;non_full_length_coverage=0;isoform_length=3516;expected_accuracy=0.997						8		6
cb191_c1/f1p2/4439 isoform=cb191_c1;full_length_coverage=1;non_full_length_coverage=2;isoform_length=4439;expected_accuracy=0.998	gnl|BL_ORD_ID|34867 cb191_c7/f1p1/4776 isoform=cb191_c7;full_length_coverage=1;non_full_length_coverage=1;isoform_length=4776;expected_accuracy=0.994	1044	4430	1	1044	1409	4776	188	1223
cb18456_c6478/f1p2/1469 isoform=cb18456_c6478;full_length_coverage=1;non_full_length_coverage=2;isoform_length=1469;expected_accuracy=0.992	gnl|BL_ORD_ID|17669 cb8564_c868/f2p9/1907 isoform=cb8564_c868;full_length_coverage=2;non_full_length_coverage=9;isoform_length=1907;expected_accuracy=1.000	289	1469	4	289	690	1867	1	285
cb6645_c4/f1p0/1775 isoform=cb6645_c4;full_length_coverage=1;non_full_length_coverage=0;isoform_length=1775;expected_accuracy=0.993	gnl|BL_ORD_ID|3658 cb6645_c0/f1p0/2792 isoform=cb6645_c0;full_length_coverage=1;non_full_length_coverage=0;isoform_length=2792;expected_accuracy=0.996	447	1774	64	451	567	1896	7	396
cb8983_c35/f1p0/1566 isoform=cb8983_c35;full_length_coverage=1;non_full_length_coverage=0;isoform_length=1566;expected_accuracy=0.999	gnl|BL_ORD_ID|21824 cb8983_c25/f1p0/1857 isoform=cb8983_c25;full_length_coverage=1;non_full_length_coverage=0;isoform_length=1857;expected_accuracy=0.995	116	1564	1	118	291	1738	42	161
cb8564_c75626/f1p2/3199	gnl|BL_ORD_ID|10307 cb8564_c11483/f8p0/3482 isoform=cb8564_c11483;full_length_coverage=8;non_full_length_coverage=0;isoform_length=3482;expected_accuracy=0.996	1	2674	2672	3182	1	2673	2972	3482
cb1333_c9/f4p0/1480 isoform=cb1333_c9;full_length_coverage=4;non_full_length_coverage=0;isoform_length=1480;expected_accur	gnl|BL_ORD_ID|26228 cb1333_c50/f1p0/4810 isoform=cb1333_c50;full_length_coverage=1;non_full_length_coverage=0;isoform_length=4810;expected_accur	153	1460	8	154	3506	4810	1	146

acy=1.000	acy=0.996								
cb124_c2/f4p0/2458 isoform=cb124_c2;full_length_coverage=4;non_full_length_coverage=0;isoform_length=2458;expected_accuracy=0.998	gnl|BL_ORD_ID|22719 cb124_c29/f1p0/2489 isoform=cb124_c29;full_length_coverage=1;non_full_length_coverage=0;isoform_length=2489;expected_accuracy=0.992	215	2353	1	217	353	2489	5	222
cb4128_c19/f1p0/2260	gnl|BL_ORD_ID|70785 cb4128_c21/f1p0/3017	1	1761	1760	2263	2	1760	2096	2600
cb4077_c12/f1p0/1303 isoform=cb4077_c12;full_length_coverage=1;non_full_length_coverage=0;isoform_length=1303;expected_accuracy=0.991	gnl|BL_ORD_ID|41226 cb4077_c18/f3p0/3453 isoform=cb4077_c18;full_length_coverage=3;non_full_length_coverage=0;isoform_length=3453;expected_accuracy=0.997	115	1303	1	114	2222	3408	9	121
cb8564_c188193/f1p0/2849 isoform=cb8564_c188193;full_length_coverage=1;non_full_length_coverage=0;isoform_length=2849;expected_accuracy=0.997	gnl|BL_ORD_ID|16705 cb8564_c53880/f1p0/3484 isoform=cb8564_c53880;full_length_coverage=1;non_full_length_coverage=0;isoform_length=3484;expected_accuracy=0.996	1056	2849	1	1057	1672	3484	1	1056
cb1203_c25/f1p0/1362	gnl|BL_ORD_ID|59261 cb1203_c4/f1p0/4549	157	1269	3	157	3251	4363	2268	2422
cb5472_c0/f1p0/3101	gnl|BL_ORD_ID|73415 cb5472_c10/f1p0/4117	22	2674	2674	3073	1	2652	3683	4082
cb8564_c34195/f2p1/2830 isoform=cb8564_c34195;full_length_coverage=2;non_full_length_coverage=1;isoform_length=2830;expected_accuracy=0.997	gnl|BL_ORD_ID|11240 cb8564_c145553/f1p3/3270 isoform=cb8564_c145553;full_length_coverage=1;non_full_length_coverage=3;isoform_length=3270;expected_accuracy=0.996	1	1725	1726	2830	29	1757	2144	3248
cb8564_c53806/f1p3/3974 isoform=cb8564_c53806;full_length_coverage=1;non_full_length_coverage=3;isoform_length=3974;expected_	gnl|BL_ORD_ID|14293 cb8564_c46212/f3p1/4373 isoform=cb8564_c46212;full_length_coverage=3;non_full_length_coverage=1;isoform_length=4373;expected_ac	1	3174	3170	3974	15	3160	3424	4227

accuracy=0.990	curacy=0.999								
cb8564_c181853/f1p0/2119 isoform=cb8564_c181853;full_length_coverage=1;non_full_length_coverage=0;isoform_length=2119;expected_accuracy=0.997	gnl|BL_ORD_ID|55327 cb8564_c38462/f1p0/3241	1	1339	1334	2118	1	133 1	2159	294 6
cb4740_c2/f4p0/2097 isoform=cb4740_c2;full_length_coverage=4;non_full_length_coverage=0;isoform_length=2097;expected_accuracy=1.000	gnl|BL_ORD_ID|42535 cb4740_c9/f1p0/3072 isoform=cb4740_c9;full_length_coverage=1;non_full_length_coverage=0;isoform_length=3072;expected_accuracy=0.992	1	1277	1276	2097	72	134 9	2252	307 1
cb4233_c84/f1p1/2344	gnl|BL_ORD_ID|44054 cb4233_c238/f63p1/2736 isoform=cb4233_c238;full_length_coverage=63;non_full_length_coverage=1;isoform_length=2736;expected_accuracy=1.000	227	2370	1	225	593	273 6	569	793
cb8564_c36056/f1p0/2652 isoform=cb8564_c36056;full_length_coverage=1;non_full_length_coverage=0;isoform_length=2652;expected_accuracy=0.999	gnl|BL_ORD_ID|17161 cb8564_c87466/f1p1/3006 isoform=cb8564_c87466;full_length_coverage=1;non_full_length_coverage=1;isoform_length=3006;expected_accuracy=0.994	248	2652	1	251	474	290 1	73	329
cb8564_c24777/f1p3/2493 isoform=cb8564_c24777;full_length_coverage=1;non_full_length_coverage=3;isoform_length=2493;expected_accuracy=0.997	gnl|BL_ORD_ID|54302 cb8564_c22058/f1p5/3725	511	2478	7	511	1750	371 9	1127	163 0
cb8976_c4/f1p0/1816 isoform=cb8976_c4;full_length_coverage=1;non_full_length_coverage=0;isoform_length=1816;expected_accuracy=0.992	gnl|BL_ORD_ID|20719 cb8976_c2/f1p0/1978 isoform=cb8976_c2;full_length_coverage=1;non_full_length_coverage=0;isoform_length=1978;expected_accuracy=0.995	1	1033	1028	1799	42	107 3	1210	197 8
cb8564_c178335/f1p2/3430	gnl|BL_ORD_ID|11650 cb8564_c152526/f1p6/3802	1	3015	3016	3430	1	301	3282	369

isoform=cb8564_c178335;full_length_coverage=1;non_full_length_coverage=2;isoform_length=3430;expected_accuracy=0.998	isoform=cb8564_c152526;full_length_coverage=1;non_full_length_coverage=6;isoform_length=3802;expected_accuracy=0.999						5		5
cb4074_c3/f2p1/2503 isoform=cb4074_c3;full_length_coverage=2;non_full_length_coverage=1;isoform_length=2503;expected_accuracy=1.000	gnl|BL_ORD_ID|39766 cb4074_c7/f1p1/2826 isoform=cb4074_c7;full_length_coverage=1;non_full_length_coverage=1;isoform_length=2826;expected_accuracy=0.994	1	1336	1332	2383	4	1335	1781	2826
cb8564_c49341/f1p0/2817 isoform=cb8564_c49341;full_length_coverage=1;non_full_length_coverage=0;isoform_length=2817;expected_accuracy=0.992	gnl|BL_ORD_ID|13884 cb8564_c37104/f1p0/2985 isoform=cb8564_c37104;full_length_coverage=1;non_full_length_coverage=0;isoform_length=2985;expected_accuracy=0.997	824	2817	97	825	962	2963	129	861
cb8564_c41756/f1p0/2738 isoform=cb8564_c41756;full_length_coverage=1;non_full_length_coverage=0;isoform_length=2738;expected_accuracy=0.996	gnl|BL_ORD_ID|56601 cb8564_c48153/f1p0/3083	1	2173	2170	2738	13	2188	2311	2879
cb2013_c10/f1p0/3941	gnl|BL_ORD_ID|35460 cb2013_c8/f1p0/4696 isoform=cb2013_c8;full_length_coverage=1;non_full_length_coverage=0;isoform_length=4696;expected_accuracy=0.993	1	3096	3091	3774	2	3089	4012	4693
cb7642_c192/f1p0/1882 isoform=cb7642_c192;full_length_coverage=1;non_full_length_coverage=0;isoform_length=1882;expected_accuracy=0.996	gnl|BL_ORD_ID|6565 cb7642_c134/f1p0/2352 isoform=cb7642_c134;full_length_coverage=1;non_full_length_coverage=0;isoform_length=2352;expected_accuracy=0.995	1	1625	1626	1882	5	1629	2016	2272
cb8564_c44681/f1p0/2675	gnl|BL_ORD_ID|58097 cb8564_c88764/f1p0/2888	1105	2685	1	1110	1291	2873	61	1170
cb3981_c8/f1p0/2246	gnl|BL_ORD_ID|71697 cb3981_c10/f1p0/3478	141	2246	1	145	1154	325	4	154

isoform=cb3981_c8;full_length_coverage=1;non_full_length_coverage=0;isoform_length=2246;expected_accuracy=0.995							8		
cb5013_c16/f1p0/2722	gnl|BL_ORD_ID|43895 cb5013_c25/f5p0/2757 isoform=cb5013_c25;full_length_coverage=5;non_full_length_coverage=0;isoform_length=2757;expected_accuracy=1.000	231	2544	12	232	444	2757	1	222
cb4269_c10/f1p0/1931 isoform=cb4269_c10;full_length_coverage=1;non_full_length_coverage=0;isoform_length=1931;expected_accuracy=0.997	gnl|BL_ORD_ID|43569 cb4269_c3/f2p0/2105 isoform=cb4269_c3;full_length_coverage=2;non_full_length_coverage=0;isoform_length=2105;expected_accuracy=0.998	129	1931	1	130	298	2098	1	116
cb8564_c40764/f1p0/2563 isoform=cb8564_c40764;full_length_coverage=1;non_full_length_coverage=0;isoform_length=2563;expected_accuracy=0.997	gnl|BL_ORD_ID|15153 cb8564_c36883/f1p0/3072 isoform=cb8564_c36883;full_length_coverage=1;non_full_length_coverage=0;isoform_length=3072;expected_accuracy=0.996	1	2167	2162	2562	1	2165	2372	2772
cb8564_c48473/f1p2/2701 isoform=cb8564_c48473;full_length_coverage=1;non_full_length_coverage=2;isoform_length=2701;expected_accuracy=0.999	gnl|BL_ORD_ID|18962 cb8564_c79723/f1p6/3025 isoform=cb8564_c79723;full_length_coverage=1;non_full_length_coverage=6;isoform_length=3025;expected_accuracy=0.999	220	2661	1	225	585	3025	216	441
cb2351_c2/f1p0/2558 isoform=cb2351_c2;full_length_coverage=1;non_full_length_coverage=0;isoform_length=2558;expected_accuracy=0.998	gnl|BL_ORD_ID|68453 cb2351_c0/f1p0/3992	1	2093	2092	2558	26	2119	3014	3480
cb3354_c4/f1p0/1508	gnl|BL_ORD_ID|40592 cb3354_c3/f1p0/2584 isoform=cb3354_c3;full_length_coverage=1;non_full_length_coverage=0;isoform_length=2584;expected_accura	1	1116	1116	1476	94	1213	2093	2453

	cy=0.999								
cb7545_c12/f1p0/1529 isoform=cb7545_c12;full_length_coverage=1;non_full_length_coverage=0;isoform_length=1529;expected_accuracy=0.997	gnl|BL_ORD_ID|6787 cb7545_c4/f1p0/2591 isoform=cb7545_c4;full_length_coverage=1;non_full_length_coverage=0;isoform_length=2591;expected_accuracy=0.997	145	1512	1	144	1225	2591	410	553
cb8564_c45596/f1p1/3430	gnl|BL_ORD_ID|13870 cb8564_c39624/f1p1/3482 isoform=cb8564_c39624;full_length_coverage=1;non_full_length_coverage=1;isoform_length=3482;expected_accuracy=0.996	2	2780	2775	3365	13	2773	2892	3482
cb8564_c4138/f5p2/2729 isoform=cb8564_c4138;full_length_coverage=5;non_full_length_coverage=2;isoform_length=2729;expected_accuracy=0.994	gnl|BL_ORD_ID|55672 cb8564_c4135/f1p1/4201	2	2196	2192	2729	7	2201	2427	2965
cb8564_c111805/f1p0/2538 isoform=cb8564_c111805;full_length_coverage=1;non_full_length_coverage=0;isoform_length=2538;expected_accuracy=0.995	gnl|BL_ORD_ID|52389 cb8564_c121284/f1p0/2852	1	1859	1859	2538	1	1861	2186	2865
cb8564_c9477/f9p0/2721 isoform=cb8564_c9477;full_length_coverage=9;non_full_length_coverage=0;isoform_length=2721;expected_accuracy=0.996	gnl|BL_ORD_ID|56999 cb8564_c79815/f1p0/2970	1	2083	2079	2721	16	2098	2287	2929
cb6606_c31/f1p0/1808 isoform=cb6606_c31;full_length_coverage=1;non_full_length_coverage=0;isoform_length=1808;expected_accuracy=0.997	gnl|BL_ORD_ID|3940 cb6606_c21/f1p0/2745 isoform=cb6606_c21;full_length_coverage=1;non_full_length_coverage=0;isoform_length=2745;expected_accuracy=0.996	649	1808	69	649	1587	2745	1	581
cb6246_c7/f1p0/2210	gnl|BL_ORD_ID|49559 cb6246_c5/f1p0/3258	1	1073	1072	2114	41	1113	1893	293

									5
cb8564_c48661/f1p2/2634 isoform=cb8564_c48661;full_length_coverage=1;non_full_length_coverage=2;isoform_length=2634;expected_accuracy=0.998	gnl|BL_ORD_ID|19155 cb8564_c90208/f1p2/2920 isoform=cb8564_c90208;full_length_coverage=1;non_full_length_coverage=2;isoform_length=2920;expected_accuracy=0.990	396	2634	56	399	490	2763	24	385
cb8564_c120048/f1p1/2648	gnl|BL_ORD_ID|52227 cb8564_c115027/f1p0/3027	2	2408	2407	2577	213	2619	2769	2939
cb13187_c8/f1p0/1776	gnl|BL_ORD_ID|58889 cb9788_c16/f1p0/3980	1	1273	1272	1775	50	1322	3000	3485
cb3219_c1/f1p1/3698 isoform=cb3219_c1;full_length_coverage=1;non_full_length_coverage=1;isoform_length=3698;expected_accuracy=0.997	gnl|BL_ORD_ID|40398 cb3219_c2/f1p0/3884 isoform=cb3219_c2;full_length_coverage=1;non_full_length_coverage=0;isoform_length=3884;expected_accuracy=0.996	318	3698	1	319	510	3884	31	345
cb12662_c8/f1p0/1447	gnl|BL_ORD_ID|1517 cb10696_c0/f4p2/1611 isoform=cb10696_c0;full_length_coverage=4;non_full_length_coverage=2;isoform_length=1611;expected_accuracy=0.997	179	1453	56	179	264	1538	1	132
cb6278_c18/f1p0/1671 isoform=cb6278_c18;full_length_coverage=1;non_full_length_coverage=0;isoform_length=1671;expected_accuracy=0.994	gnl|BL_ORD_ID|49582 cb6278_c9/f1p0/2648	144	1671	1	144	1053	2580	75	218
cb8564_c146072/f3p3/2176 isoform=cb8564_c146072;full_length_coverage=3;non_full_length_coverage=3;isoform_length=2176;expected_accuracy=1.000	gnl|BL_ORD_ID|18820 cb8564_c8764/f7p3/3755 isoform=cb8564_c8764;full_length_coverage=7;non_full_length_coverage=3;isoform_length=3755;expected_accuracy=1.000	213	2162	1	215	1805	3752	13	236
cb14044_c6/f1p0/1650	gnl|BL_ORD_ID|6045 cb6181_c37/f1p1/2073	1	1019	1015	1650	206	123	1369	200

isoform=cb14044_c6;full_length_coverage=1;non_full_length_coverage=0;isoform_length=1650;expected_accuracy=0.996	isoform=cb6181_c37;full_length_coverage=1;non_full_length_coverage=1;isoform_length=2073;expected_accuracy=0.996						5		4
cb8564_c36056/f1p0/2652 isoform=cb8564_c36056;full_length_coverage=1;non_full_length_coverage=0;isoform_length=2652;expected_accuracy=0.999	gnl|BL_ORD_ID|17164 cb8564_c90856/f1p11/2858 isoform=cb8564_c90856;full_length_coverage=1;non_full_length_coverage=11;isoform_length=2858;expected_accuracy=0.999	248	2649	1	251	457	2858	86	313
cb1992_c10/f2p2/3165 isoform=cb1992_c10;full_length_coverage=2;non_full_length_coverage=2;isoform_length=3165;expected_accuracy=0.997	gnl|BL_ORD_ID|34846 cb1992_c24/f1p0/3683 isoform=cb1992_c24;full_length_coverage=1;non_full_length_coverage=0;isoform_length=3683;expected_accuracy=0.996	119	2407	2405	3165	2	2288	2911	3671
cb373_c8/f1p0/3698 isoform=cb373_c8;full_length_coverage=1;non_full_length_coverage=0;isoform_length=3698;expected_accuracy=0.997	gnl|BL_ORD_ID|41213 cb373_c11/f1p0/3937 isoform=cb373_c11;full_length_coverage=1;non_full_length_coverage=0;isoform_length=3937;expected_accuracy=0.996	1	3333	3334	3698	62	3395	3501	3865
cb18456_c1419/f5p0/1798 isoform=cb18456_c1419;full_length_coverage=5;non_full_length_coverage=0;isoform_length=1798;expected_accuracy=1.000	gnl|BL_ORD_ID|15336 cb8564_c41228/f1p1/4062 isoform=cb8564_c41228;full_length_coverage=1;non_full_length_coverage=1;isoform_length=4062;expected_accuracy=0.997	245	1791	3	249	2516	4062	1	246
cb8564_c41162/f1p0/3158	gnl|BL_ORD_ID|14411 cb8564_c44258/f1p0/3202 isoform=cb8564_c44258;full_length_coverage=1;non_full_length_coverage=0;isoform_length=3202;expected_accuracy=0.993	21	2553	2548	3030	15	2589	2720	3202
cb8129_c11/f1p0/1889 isoform=cb8129_c11;full_length_coverage=1;non_full_length_coverage=0;isoform_length=1889;expected_accu	gnl|BL_ORD_ID|50878 cb8129_c9/f1p0/3008	1	1104	1105	1889	2	1099	2092	2876

racy=0.994									
cb14630_c37/f1p1/5449 isoform=cb14630_c37;full_length_coverage=1;non_full_length_coverage=1;isoform_length=5449;expected_accuracy=0.996	gnl|BL_ORD_ID|28779 cb14630_c32/f1p2/5767 isoform=cb14630_c32;full_length_coverage=1;non_full_length_coverage=2;isoform_length=5767;expected_accuracy=0.994	1	4273	4268	5449	5	4295	4468	5648
cb873_c19/f1p0/2582 isoform=cb873_c19;full_length_coverage=1;non_full_length_coverage=0;isoform_length=2582;expected_accuracy=0.994	gnl|BL_ORD_ID|57590 cb873_c16/f1p0/4474	1124	2582	1	1124	2949	4412	17	1140
cb6519_c2/f1p0/2447	gnl|BL_ORD_ID|49795 cb6519_c0/f1p0/2728	346	2398	57	348	423	2504	20	309
cb12189_c34/f1p3/1569	gnl|BL_ORD_ID|60618 cb12189_c37/f1p0/1873	559	1499	6	563	788	1728	1	556
cb8564_c5007/f1p0/1941	gnl|BL_ORD_ID|12987 cb8564_c162306/f3p5/3760 isoform=cb8564_c162306;full_length_coverage=3;non_full_length_coverage=5;isoform_length=3760;expected_accuracy=0.998	1	1447	1446	1756	1872	3318	3450	3760
cb8564_c107323/f4p0/3768 isoform=cb8564_c107323;full_length_coverage=4;non_full_length_coverage=0;isoform_length=3768;expected_accuracy=0.999	gnl|BL_ORD_ID|15999 cb8564_c50865/f1p1/3822 isoform=cb8564_c50865;full_length_coverage=1;non_full_length_coverage=1;isoform_length=3822;expected_accuracy=0.994	18	2808	2807	3719	1	2798	2909	3821
cb1333_c9/f4p0/1480 isoform=cb1333_c9;full_length_coverage=4;non_full_length_coverage=0;isoform_length=1480;expected_accuracy=1.000	gnl|BL_ORD_ID|27473 cb1333_c63/f1p0/4699 isoform=cb1333_c63;full_length_coverage=1;non_full_length_coverage=0;isoform_length=4699;expected_accuracy=0.998	152	1473	29	154	3375	4693	19	144
cb5565_c14/f2p1/3093	gnl|BL_ORD_ID|46508 cb5565_c81/f1p2/3263	253	3093	2	252	373	321	5	257

isoform=cb5565_c14;full_length_coverage=2;non_full_length_coverage=1;isoform_length=3093;expected_accuracy=1.000	isoform=cb5565_c81;full_length_coverage=1;non_full_length_coverage=2;isoform_length=3263;expected_accuracy=1.000						2		
cb8564_c77411/f1p0/2169 isoform=cb8564_c77411;full_length_coverage=1;non_full_length_coverage=0;isoform_length=2169;expected_accuracy=0.993	gnl|BL_ORD_ID|57969 cb8564_c85458/f1p0/2732	105	2165	1	106	613	2681	151	256
cb8564_c78147/f1p0/3581	gnl|BL_ORD_ID|56063 cb8564_c53906/f1p0/3730	20	2647	2650	3149	29	2652	2841	3341
cb18456_c7148/f1p0/1595 isoform=cb18456_c7148;full_length_coverage=1;non_full_length_coverage=0;isoform_length=1595;expected_accuracy=0.993	gnl|BL_ORD_ID|9949 cb8564_c118109/f1p0/2145 isoform=cb8564_c118109;full_length_coverage=1;non_full_length_coverage=0;isoform_length=2145;expected_accuracy=1.000	330	1595	8	334	900	2143	43	371
cb4100_c4/f1p0/2480 isoform=cb4100_c4;full_length_coverage=1;non_full_length_coverage=0;isoform_length=2480;expected_accuracy=0.996	gnl|BL_ORD_ID|71787 cb4100_c1/f1p0/3420	1	1792	1792	2480	21	1812	2661	3349
cb1279_c10/f2p0/2859 isoform=cb1279_c10;full_length_coverage=2;non_full_length_coverage=0;isoform_length=2859;expected_accuracy=0.999	gnl|BL_ORD_ID|61185 cb1279_c50/f1p1/3873	202	2859	1	203	1144	3798	4	206
cb5139_c7/f1p1/2751	gnl|BL_ORD_ID|73323 cb5139_c9/f1p0/3187	1	1384	1382	2749	59	1442	1650	3029
cb8564_c42814/f1p0/2151 isoform=cb8564_c42814;full_length_coverage=1;non_full_length_coverage=0;isoform_length=2151;expected_	gnl|BL_ORD_ID|14368 cb8564_c34638/f1p0/2817 isoform=cb8564_c34638;full_length_coverage=1;non_full_length_coverage=0;isoform_length=2817;expected_ac	650	2151	7	652	1330	2815	1	647

accuracy=0.993	curacy=0.992								
cb4997_c15/f1p0/2104 isoform=cb4997_c15;full_length_coverage=1;non_full_length_coverage=0;isoform_length=2104;expected_accuracy=0.994	gnl|BL_ORD_ID|73169 cb4997_c12/f1p1/2947	1	1547	1545	2103	16	1559	2281	2838
cb6622_c9/f1p0/2543	gnl|BL_ORD_ID|3917 cb6622_c7/f1p0/2787 isoform=cb6622_c7;full_length_coverage=1;non_full_length_coverage=0;isoform_length=2787;expected_accuracy=0.997	143	2245	13	139	687	2787	1	127
cb2935_c37/f1p0/2974 isoform=cb2935_c37;full_length_coverage=1;non_full_length_coverage=0;isoform_length=2974;expected_accuracy=0.995	gnl|BL_ORD_ID|70092 cb2935_c30/f1p0/3442	1	1742	1737	2970	126	1876	2077	3311
cb8564_c81944/f1p1/3166 isoform=cb8564_c81944;full_length_coverage=1;non_full_length_coverage=1;isoform_length=3166;expected_accuracy=0.995	gnl|BL_ORD_ID|16500 cb8564_c52758/f1p0/3322 isoform=cb8564_c52758;full_length_coverage=1;non_full_length_coverage=0;isoform_length=3322;expected_accuracy=0.996	1	2661	2660	3166	12	2664	2815	3322
cb6391_c2/f2p0/2236 isoform=cb6391_c2;full_length_coverage=2;non_full_length_coverage=0;isoform_length=2236;expected_accuracy=0.998	gnl|BL_ORD_ID|4281 cb6391_c4/f1p0/2869 isoform=cb6391_c4;full_length_coverage=1;non_full_length_coverage=0;isoform_length=2869;expected_accuracy=1.000	101	1695	1693	2234	1	1614	2328	2869
cb6633_c19/f1p0/1733 isoform=cb6633_c19;full_length_coverage=1;non_full_length_coverage=0;isoform_length=1733;expected_accuracy=1.000	gnl|BL_ORD_ID|3747 cb6633_c9/f1p0/2805 isoform=cb6633_c9;full_length_coverage=1;non_full_length_coverage=0;isoform_length=2805;expected_accuracy=0.997	1	1137	1134	1676	382	1518	2261	2805
cb8564_c122040/f1p0/2864	gnl|BL_ORD_ID|54091 cb8564_c18227/f1p0/4224	1	1886	1886	2767	2	188	2179	306

isoform=cb8564_c122040;full_length_coverage=1;non_full_length_coverage=0;isoform_length=2864;expected_accuracy=1.000							7		0
cb8564_c13291/f1p0/2701	gnl|BL_ORD_ID|54022 cb8564_c17079/f1p0/3440	1003	2637	1	1004	1801	3435	1	1004
cb2965_c3/f1p0/3423	gnl|BL_ORD_ID|70107 cb2965_c4/f1p0/3375	1	2735	2733	3313	1	2735	2845	3414
cb2737_c37/f1p0/2894	gnl|BL_ORD_ID|38134 cb2737_c22/f1p0/3196 isoform=cb2737_c22;full_length_coverage=1;non_full_length_coverage=0;isoform_length=3196;expected_accuracy=0.995	1	1495	1493	2819	282	1763	1894	3196
cb8564_c49562/f1p0/2838 isoform=cb8564_c49562;full_length_coverage=1;non_full_length_coverage=0;isoform_length=2838;expected_accuracy=0.995	gnl|BL_ORD_ID|55334 cb8564_c38684/f1p1/3241	1	2331	2327	2834	1	2328	2736	3247
cb8264_c4/f1p0/1748 isoform=cb8264_c4;full_length_coverage=1;non_full_length_coverage=0;isoform_length=1748;expected_accuracy=0.999	gnl|BL_ORD_ID|51864 cb8264_c2/f1p0/2001	205	1748	28	205	309	1853	5	182
cb6490_c7/f1p0/2432	gnl|BL_ORD_ID|49764 cb6490_c1/f1p0/2838	1	1463	1462	2380	8	1463	1840	2769
cb14724_c6/f1p0/1741	gnl|BL_ORD_ID|40277 cb3622_c10/f1p0/3532 isoform=cb3622_c10;full_length_coverage=1;non_full_length_coverage=0;isoform_length=3532;expected_accuracy=0.999	1	1354	1352	1729	1	1352	1847	2223
cb3226_c7/f1p0/3210	gnl|BL_ORD_ID|39532 cb3226_c6/f1p0/3708	1	2322	2320	3136	100	242	2893	370

isoform=cb3226_c7;full_length_coverage=1;non_full_length_coverage=0;isoform_length=3210;expected_accuracy=0.998	isoform=cb3226_c6;full_length_coverage=1;non_full_length_coverage=0;isoform_length=3708;expected_accuracy=0.999						0		8
cb8564_c38035/f1p0/2770	gnl|BL_ORD_ID|55839 cb8564_c43677/f1p0/3269	1	2035	2033	2752	55	2091	2281	2999
cb8564_c49562/f1p0/2838 isoform=cb8564_c49562;full_length_coverage=1;non_full_length_coverage=0;isoform_length=2838;expected_accuracy=0.995	gnl|BL_ORD_ID|55430 cb8564_c41551/f1p0/3408	13	2331	2327	2838	12	2331	2438	2949
cb6063_c13/f1p1/2429	gnl|BL_ORD_ID|46524 cb6063_c4/f2p0/2706 isoform=cb6063_c4;full_length_coverage=2;non_full_length_coverage=0;isoform_length=2706;expected_accuracy=0.999	119	2436	1	122	376	2687	120	241
cb8564_c81839/f1p0/2332 isoform=cb8564_c81839;full_length_coverage=1;non_full_length_coverage=0;isoform_length=2332;expected_accuracy=0.998	gnl|BL_ORD_ID|18354 cb8564_c88127/f1p0/2488 isoform=cb8564_c88127;full_length_coverage=1;non_full_length_coverage=0;isoform_length=2488;expected_accuracy=0.996	1	1946	1947	2332	3	1948	2077	2457
cb8564_c2326/f1p0/2006 isoform=cb8564_c2326;full_length_coverage=1;non_full_length_coverage=0;isoform_length=2006;expected_accuracy=0.990	gnl|BL_ORD_ID|14743 cb8564_c2392/f1p2/2510 isoform=cb8564_c2392;full_length_coverage=1;non_full_length_coverage=2;isoform_length=2510;expected_accuracy=0.992	230	2002	4	234	728	2510	4	246
cb2737_c14/f1p0/2854 isoform=cb2737_c14;full_length_coverage=1;non_full_length_coverage=0;isoform_length=2854;expected_accuracy=0.996	gnl|BL_ORD_ID|38132 cb2737_c20/f1p0/3644 isoform=cb2737_c20;full_length_coverage=1;non_full_length_coverage=0;isoform_length=3644;expected_accuracy=0.998	110	2854	1	110	871	3594	24	133
cb8564_c68745/f3p0/2856	gnl|BL_ORD_ID|15454 cb8564_c67827/f7p0/3512	292	2856	3	297	948	351	1	295

<table>
<tr><th></th><th></th><th></th><th></th><th></th><th></th><th></th><th></th><th></th><th></th></tr>
<tr><td>isoform=cb8564_c68745;full_length_coverage=3;non_full_length_coverage=0;isoform_length=2856;expected_accuracy=0.996</td><td>isoform=cb8564_c67827;full_length_coverage=7;non_full_length_coverage=0;isoform_length=3512;expected_accuracy=1.000</td><td></td><td></td><td></td><td></td><td></td><td>2</td><td></td><td></td></tr>
<tr><td>cb124_c43/f1p0/2759</td><td>gnl|BL_ORD_ID|22798 cb124_c13/f1p0/3616 isoform=cb124_c13;full_length_coverage=1;non_full_length_coverage=0;isoform_length=3616;expected_accuracy=0.999</td><td>232</td><td>2448</td><td>16</td><td>233</td><td>1400</td><td>3616</td><td>11</td><td>228</td></tr>
<tr><td>cb2461_c12/f1p0/5311</td><td>gnl|BL_ORD_ID|37876 cb2461_c6/f1p0/5655 isoform=cb2461_c6;full_length_coverage=1;non_full_length_coverage=0;isoform_length=5655;expected_accuracy=0.998</td><td>41</td><td>4642</td><td>4637</td><td>4910</td><td>12</td><td>4606</td><td>4924</td><td>5196</td></tr>
<tr><td>cb8564_c14775/f1p1/2535</td><td>gnl|BL_ORD_ID|12253 cb8564_c145424/f8p2/3323 isoform=cb8564_c145424;full_length_coverage=8;non_full_length_coverage=2;isoform_length=3323;expected_accuracy=0.998</td><td>1056</td><td>2492</td><td>1</td><td>1058</td><td>1795</td><td>3231</td><td>287</td><td>1344</td></tr>
<tr><td>cb5603_c0/f4p1/2663 isoform=cb5603_c0;full_length_coverage=4;non_full_length_coverage=1;isoform_length=2663;expected_accuracy=1.000</td><td>gnl|BL_ORD_ID|45848 cb5603_c1/f1p1/3057 isoform=cb5603_c1;full_length_coverage=1;non_full_length_coverage=1;isoform_length=3057;expected_accuracy=0.997</td><td>148</td><td>2650</td><td>1</td><td>149</td><td>540</td><td>3045</td><td>85</td><td>232</td></tr>
<tr><td>cb3941_c69/f1p0/3448</td><td>gnl|BL_ORD_ID|39437 cb3941_c64/f1p2/4241 isoform=cb3941_c64;full_length_coverage=1;non_full_length_coverage=2;isoform_length=4241;expected_accuracy=0.995</td><td>27</td><td>2961</td><td>2958</td><td>3212</td><td>1</td><td>2934</td><td>3933</td><td>4187</td></tr>
<tr><td>cb12714_c29/f1p2/1583</td><td>gnl|BL_ORD_ID|53995 cb8564_c16613/f1p1/4260</td><td>1</td><td>1295</td><td>1293</td><td>1588</td><td>1</td><td>1295</td><td>3915</td><td>4210</td></tr>
<tr><td>cb762_c8/f1p0/3946</td><td>gnl|BL_ORD_ID|8132 cb762_c4/f1p0/4517</td><td>1</td><td>2768</td><td>2766</td><td>3921</td><td>111</td><td>289</td><td>3356</td><td>451</td></tr>
</table>

isoform=cb762_c8;full_length_coverage=1;non_full_length_coverage=0;isoform_length=3946;expected_accuracy=0.990	isoform=cb762_c4;full_length_coverage=1;non_full_length_coverage=0;isoform_length=4517;expected_accuracy=0.996						0		7
cb11547_c1/f1p0/1452 isoform=cb11547_c1;full_length_coverage=1;non_full_length_coverage=0;isoform_length=1452;expected_accuracy=0.996	gnl|BL_ORD_ID|60259 cb11547_c2/f1p0/3211	1	1118	1117	1428	2	1117	2893	320 3
cb4799_c5/f1p0/2439 isoform=cb4799_c5;full_length_coverage=1;non_full_length_coverage=0;isoform_length=2439;expected_accuracy=0.998	gnl|BL_ORD_ID|72007 cb4799_c12/f1p0/2894	113	2439	1	112	252	257 8	32	143
cb2733_c21/f1p0/1180 isoform=cb2733_c21;full_length_coverage=1;non_full_length_coverage=0;isoform_length=1180;expected_accuracy=1.000	gnl|BL_ORD_ID|68775 cb2733_c15/f1p2/1425	115	1180	1	115	260	132 6	3	117
cb8564_c109868/f2p0/2101 isoform=cb8564_c109868;full_length_coverage=2;non_full_length_coverage=0;isoform_length=2101;expected_accuracy=0.999	gnl|BL_ORD_ID|52097 cb8564_c110812/f1p0/2109	1	1005	1005	2001	5	100 8	1111	210 7
cb2737_c42/f1p0/2881	gnl|BL_ORD_ID|68786 cb2737_c50/f1p0/3706	1	1427	1425	2632	656	208 2	2214	341 7
cb8564_c23232/f1p3/2540	gnl|BL_ORD_ID|58179 cb8564_c90747/f1p0/2795	16	2172	2167	2518	5	213 4	2236	258 7
cb7786_c14/f6p3/2319 isoform=cb7786_c14;full_length_coverage=6;non_full_length_coverage=3;isoform_length=2319;expected_accu	gnl|BL_ORD_ID|6654 cb7786_c5/f1p1/2527 isoform=cb7786_c5;full_length_coverage=1;non_full_length_coverage=1;isoform_length=2527;expected_accura	217	2289	1	216	458	252 7	6	214

racy=1.000	cy=0.999								
cb1360_c13/f1p1/7793	gnl|BL_ORD_ID|61281 cb1360_c12/f1p1/7905	1	4804	4803	7369	69	4872	5231	7791
cb452_c36/f1p0/2902 isoform=cb452_c36;full_length_coverage=1;non_full_length_coverage=0;isoform_length=2902;expected_accuracy=0.994	gnl|BL_ORD_ID|43284 cb452_c15/f2p3/3234 isoform=cb452_c15;full_length_coverage=2;non_full_length_coverage=3;isoform_length=3234;expected_accuracy=0.999	1374	2902	1	1374	1633	3153	132	1495
cb2927_c17/f1p0/2828 isoform=cb2927_c17;full_length_coverage=1;non_full_length_coverage=0;isoform_length=2828;expected_accuracy=0.995	gnl|BL_ORD_ID|37697 cb2927_c6/f1p1/3514 isoform=cb2927_c6;full_length_coverage=1;non_full_length_coverage=1;isoform_length=3514;expected_accuracy=0.996	1	1680	1676	2827	17	1711	2359	3506
cb322_c25/f1p0/3845	gnl|BL_ORD_ID|70802 cb322_c34/f1p0/3998	22	2733	2731	3661	1	2718	3056	3986
cb8564_c43450/f1p0/2695	gnl|BL_ORD_ID|55191 cb8564_c35155/f1p0/2975	10	1886	1882	2705	9	1895	2010	2834
cb8564_c180116/f1p0/2944 isoform=cb8564_c180116;full_length_coverage=1;non_full_length_coverage=0;isoform_length=2944;expected_accuracy=0.996	gnl|BL_ORD_ID|55910 cb8564_c48645/f1p1/3287	15	2522	2520	2883	17	2521	2750	3113
cb8564_c66920/f3p3/2894	gnl|BL_ORD_ID|14531 cb8564_c41778/f1p9/3482 isoform=cb8564_c41778;full_length_coverage=1;non_full_length_coverage=9;isoform_length=3482;expected_accuracy=0.992	243	2863	1	247	812	3432	1	247
cb8564_c40810/f1p0/3998 isoform=cb8564_c40810;full_length_coverage=1;non_full_length_coverage=0;isoform_length=3998;expected_	gnl|BL_ORD_ID|16628 cb8564_c47737/f7p5/4351 isoform=cb8564_c47737;full_length_coverage=7;non_full_length_coverage=5;isoform_length=4351;expected_ac	1	2765	2761	3998	72	2835	3038	4274

accuracy=0.996	curacy=0.998								
cb3297_c5/f2p0/2376 isoform=cb3297_c5;full_length_coverage=2;non_full_length_coverage=0;isoform_length=2376;expected_accuracy=1.000	gnl|BL_ORD_ID|71052 cb3297_c9/f1p0/2560	1	1692	1692	2317	46	1741	1951	2577
cb6058_c4/f1p0/2346 isoform=cb6058_c4;full_length_coverage=1;non_full_length_coverage=0;isoform_length=2346;expected_accuracy=0.994	gnl|BL_ORD_ID|74840 cb6058_c8/f1p0/2922	1	1761	1757	2343	393	2154	2282	2869
cb8564_c41076/f2p1/3193	gnl|BL_ORD_ID|55376 cb8564_c39821/f1p0/3886	1316	3124	1	1317	1943	3735	1	1317
cb3756_c5/f1p0/3416	gnl|BL_ORD_ID|39637 cb3756_c10/f1p3/3741 isoform=cb3756_c10;full_length_coverage=1;non_full_length_coverage=3;isoform_length=3741;expected_accuracy=0.997	886	3432	98	888	1058	3605	29	810
cb8564_c52387/f1p5/2863 isoform=cb8564_c52387;full_length_coverage=1;non_full_length_coverage=5;isoform_length=2863;expected_accuracy=0.992	gnl|BL_ORD_ID|56633 cb8564_c49796/f2p5/3077	586	2863	8	582	740	3012	5	555
cb18456_c2426/f1p0/1054 isoform=cb18456_c2426;full_length_coverage=1;non_full_length_coverage=0;isoform_length=1054;expected_accuracy=0.993	gnl|BL_ORD_ID|36016 cb18456_c2454/f1p0/1244 isoform=cb18456_c2454;full_length_coverage=1;non_full_length_coverage=0;isoform_length=1244;expected_accuracy=0.991	498	1054	6	500	627	1185	9	515
cb273_c4/f1p0/1772 isoform=cb273_c4;full_length_coverage=1;non_full_length_coverage=0;isoform_length=1772;expected_accurac	gnl|BL_ORD_ID|68134 cb273_c6/f1p0/5146	1	1084	1084	1745	3099	4182	4322	4984

y=0.999									
cb8564_c37361/f1p0/3076	gnl|BL_ORD_ID|55623 cb8564_c47027/f1p0/3544	1	2481	2477	3070	1	2506	2607	3200
cb7270_c18/f1p0/1814 isoform=cb7270_c18;full_length_coverage=1;non_full_length_coverage=0;isoform_length=1814;expected_accuracy=0.995	gnl|BL_ORD_ID|7000 cb7270_c8/f1p0/2657 isoform=cb7270_c8;full_length_coverage=1;non_full_length_coverage=0;isoform_length=2657;expected_accuracy=0.992	358	1814	7	358	1185	2637	1	348
cb8564_c50881/f1p2/2125 isoform=cb8564_c50881;full_length_coverage=1;non_full_length_coverage=2;isoform_length=2125;expected_accuracy=0.994	gnl|BL_ORD_ID|13455 cb8564_c182847/f1p0/2435 isoform=cb8564_c182847;full_length_coverage=1;non_full_length_coverage=0;isoform_length=2435;expected_accuracy=1.000	1	1863	1858	2107	42	1906	2183	2435
cb8564_c20664/f3p4/3119 isoform=cb8564_c20664;full_length_coverage=3;non_full_length_coverage=4;isoform_length=3119;expected_accuracy=0.996	gnl|BL_ORD_ID|13766 cb8564_c18520/f2p0/3299 isoform=cb8564_c18520;full_length_coverage=2;non_full_length_coverage=0;isoform_length=3299;expected_accuracy=0.992	10	2032	2032	3119	73	2089	2198	3284
cb8564_c157909/f1p2/2084 isoform=cb8564_c157909;full_length_coverage=1;non_full_length_coverage=2;isoform_length=2084;expected_accuracy=0.999	gnl|BL_ORD_ID|13480 cb8564_c19794/f1p0/2446 isoform=cb8564_c19794;full_length_coverage=1;non_full_length_coverage=0;isoform_length=2446;expected_accuracy=0.994	1	1503	1504	2084	10	1502	1864	2445
cb11067_c4/f1p1/1290	gnl|BL_ORD_ID|49137 cb11067_c11/f1p0/1488	324	1288	9	328	502	1443	1	312
cb8564_c37361/f1p0/3076	gnl|BL_ORD_ID|55551 cb8564_c44964/f1p0/3508	1	2481	2477	3070	1	2478	2579	3173
cb5617_c9/f1p0/2638 isoform=cb5617_c9;full_length_coverage=1;non_full_length_coverage=0;isoform_length=2638;expected_accur	gnl|BL_ORD_ID|45940 cb5617_c3/f2p0/2746 isoform=cb5617_c3;full_length_coverage=2;non_full_length_coverage=0;isoform_length=2746;expected_accura	1	1904	1905	2638	1	1895	2014	2746

acy=0.997	cy=0.998								
cb8564_c20620/f1p1/2688 isoform=cb8564_c20620;full_length_coverage=1;non_full_length_coverage=1;isoform_length=2688;expected_accuracy=0.992	gnl|BL_ORD_ID|13176 cb8564_c15957/f1p0/2838 isoform=cb8564_c15957;full_length_coverage=1;non_full_length_coverage=0;isoform_length=2838;expected_accuracy=0.994	1	1695	1694	2635	2	1684	1792	2721
cb2097_c120/f1p0/1809 isoform=cb2097_c120;full_length_coverage=1;non_full_length_coverage=0;isoform_length=1809;expected_accuracy=0.994	gnl|BL_ORD_ID|35231 cb2097_c18/f10p0/2448 isoform=cb2097_c18;full_length_coverage=10;non_full_length_coverage=0;isoform_length=2448;expected_accuracy=1.000	1	1422	1420	1809	515	1945	2055	2446
cb4178_c0/f2p0/2616 isoform=cb4178_c0;full_length_coverage=2;non_full_length_coverage=0;isoform_length=2616;expected_accuracy=1.000	gnl|BL_ORD_ID|43092 cb4178_c2/f1p0/2741 isoform=cb4178_c2;full_length_coverage=1;non_full_length_coverage=0;isoform_length=2741;expected_accuracy=0.994	1272	2616	1	1271	1390	2734	1	1268
cb18837_c1/f1p0/5324 isoform=cb18837_c1;full_length_coverage=1;non_full_length_coverage=0;isoform_length=5324;expected_accuracy=0.998	gnl|BL_ORD_ID|36411 cb18837_c0/f1p0/5358 isoform=cb18837_c0;full_length_coverage=1;non_full_length_coverage=0;isoform_length=5358;expected_accuracy=0.998	1	3292	3290	5192	9	3307	3456	5358
cb6802_c57/f1p0/2631	gnl|BL_ORD_ID|50089 cb6802_c59/f1p0/2826	191	2236	1	192	332	2377	40	207
cb5624_c2/f1p0/2635 isoform=cb5624_c2;full_length_coverage=1;non_full_length_coverage=0;isoform_length=2635;expected_accuracy=0.996	gnl|BL_ORD_ID|74174 cb5624_c5/f1p0/2752	1	1674	1673	2590	1	1675	1844	2761
cb17004_c0/f1p0/1612	gnl|BL_ORD_ID|25706 cb1366_c4/f1p0/1599 isoform=cb1366_c4;full_length_coverage=1;non_full_length_coverage=0;isoform_length=1599;expected_accura	1	1015	1015	1482	5	1017	1133	1599

	cy=0.995								
cb1956_c12/f1p0/3120	gnl|BL_ORD_ID|66540 cb1956_c20/f1p0/3189	106	2709	2714	3133	1	2607	2744	3163
cb2873_c10/f1p0/3294	gnl|BL_ORD_ID|38306 cb2873_c3/f1p0/3789 isoform=cb2873_c3;full_length_coverage=1;non_full_length_coverage=0;isoform_length=3789;expected_accuracy=0.995	320	3125	50	321	989	3789	1	268
cb7226_c3/f1p0/2414	gnl|BL_ORD_ID|6814 cb7226_c0/f1p0/2663 isoform=cb7226_c0;full_length_coverage=1;non_full_length_coverage=0;isoform_length=2663;expected_accuracy=0.996	10	1607	1605	2401	42	1635	1869	2661
cb8564_c111706/f1p0/2253	gnl|BL_ORD_ID|52405 cb8564_c121956/f1p0/2565	1	1681	1678	2175	2	1682	1973	2470
cb9360_c14/f18p2/2034 isoform=cb9360_c14;full_length_coverage=18;non_full_length_coverage=2;isoform_length=2034;expected_accuracy=1.000	gnl|BL_ORD_ID|59676 cb9360_c8/f1p1/2215	1	1126	1123	2034	1	1126	1279	2190
cb11915_c7/f1p2/1523 isoform=cb11915_c7;full_length_coverage=1;non_full_length_coverage=2;isoform_length=1523;expected_accuracy=0.992	gnl|BL_ORD_ID|18218 cb8564_c89018/f4p5/1975 isoform=cb8564_c89018;full_length_coverage=4;non_full_length_coverage=5;isoform_length=1975;expected_accuracy=0.999	1	1133	1128	1523	1	1136	1491	1887
cb8084_c3/f1p1/2128	gnl|BL_ORD_ID|9019 cb8084_c2/f3p1/2279 isoform=cb8084_c2;full_length_coverage=3;non_full_length_coverage=1;isoform_length=2279;expected_accuracy=0.996	1	1853	1853	2100	1	1853	2009	2257
cb8564_c12805/f1p0/2580	gnl|BL_ORD_ID|54405 cb8564_c35890/f1p0/3157	1	1695	1690	2580	1	169	1937	283

isoform=cb8564_c12805;full_length_coverage=1;non_full_length_coverage=0;isoform_length=2580;expected_accuracy=0.993							5		0
cb8564_c127273/f1p0/3035	gnl|BL_ORD_ID|54548 cb8564_c125967/f1p0/4340	1	1629	1629	2760	1204	2822	3205	4332
cb12714_c29/f1p2/1583	gnl|BL_ORD_ID|52654 cb8564_c10111/f1p0/2706	1	1296	1293	1597	1	1296	2335	2639
cb16680_c6/f1p0/1706 isoform=cb16680_c6;full_length_coverage=1;non_full_length_coverage=0;isoform_length=1706;expected_accuracy=0.997	gnl|BL_ORD_ID|17788 cb8564_c83905/f9p1/2112 isoform=cb8564_c83905;full_length_coverage=9;non_full_length_coverage=1;isoform_length=2112;expected_accuracy=0.997	150	1706	1	154	526	2069	42	193
cb8564_c37866/f1p3/2874 isoform=cb8564_c37866;full_length_coverage=1;non_full_length_coverage=3;isoform_length=2874;expected_accuracy=0.990	gnl|BL_ORD_ID|16172 cb8564_c53024/f1p0/3159 isoform=cb8564_c53024;full_length_coverage=1;non_full_length_coverage=0;isoform_length=3159;expected_accuracy=0.996	1	1719	1716	2869	1	1699	2017	3159
cb3759_c13/f1p0/1723 isoform=cb3759_c13;full_length_coverage=1;non_full_length_coverage=0;isoform_length=1723;expected_accuracy=0.994	gnl|BL_ORD_ID|40823 cb3759_c16/f1p0/3176 isoform=cb3759_c16;full_length_coverage=1;non_full_length_coverage=0;isoform_length=3176;expected_accuracy=0.997	372	1686	4	373	1869	3176	20	389
cb8564_c36343/f1p0/3448	gnl|BL_ORD_ID|55939 cb8564_c49630/f1p4/3487	20	2930	2930	3180	13	2913	3243	3493
cb18012_c2/f1p0/1829	gnl|BL_ORD_ID|15055 cb8564_c3236/f1p0/1961 isoform=cb8564_c3236;full_length_coverage=1;non_full_length_coverage=0;isoform_length=1961;expected_accuracy=1.000	175	1755	8	176	377	1959	40	208
cb5469_c10/f1p0/2248	gnl|BL_ORD_ID|73955 cb5469_c13/f1p0/2524	225	2222	70	225	381	238	47	203

isoform=cb5469_c10;full_length_coverage=1;non_full_length_coverage=0;isoform_length=2248;expected_accuracy=0.999							4		
cb8564_c132795/f5p10/3729 isoform=cb8564_c132795;full_length_coverage=5;non_full_length_coverage=10;isoform_length=3729;expected_accuracy=0.999	gnl|BL_ORD_ID|16742 cb8564_c628/f3p1/4943 isoform=cb8564_c628;full_length_coverage=3;non_full_length_coverage=1;isoform_length=4943;expected_accuracy=1.000	1	1832	1830	3640	87	1916	3085	4892
cb3856_c0/f2p1/1619 isoform=cb3856_c0;full_length_coverage=2;non_full_length_coverage=1;isoform_length=1619;expected_accuracy=0.996	gnl|BL_ORD_ID|40756 cb3856_c4/f1p1/1955 isoform=cb3856_c4;full_length_coverage=1;non_full_length_coverage=1;isoform_length=1955;expected_accuracy=0.997	129	1619	1	131	365	1855	1	131
cb237_c14/f1p0/4353	gnl|BL_ORD_ID|38950 cb237_c12/f1p0/4420 isoform=cb237_c12;full_length_coverage=1;non_full_length_coverage=0;isoform_length=4420;expected_accuracy=0.995	192	4179	1	191	455	4420	97	285
cb8564_c15608/f1p3/3110	gnl|BL_ORD_ID|13013 cb8564_c17114/f4p0/3443 isoform=cb8564_c17114;full_length_coverage=4;non_full_length_coverage=0;isoform_length=3443;expected_accuracy=1.000	1	2790	2785	3102	1	2790	2934	3251
cb8564_c24867/f1p3/2846	gnl|BL_ORD_ID|56353 cb8564_c75114/f1p0/2866	10	2038	2038	2584	9	2035	2193	2737
cb6169_c4/f1p0/1708 isoform=cb6169_c4;full_length_coverage=1;non_full_length_coverage=0;isoform_length=1708;expected_accuracy=0.994	gnl|BL_ORD_ID|46016 cb6169_c1/f1p0/2784 isoform=cb6169_c1;full_length_coverage=1;non_full_length_coverage=0;isoform_length=2784;expected_accuracy=0.995	1	1352	1350	1707	1	1351	2428	2784
cb8564_c2788/f1p0/2682	gnl|BL_ORD_ID|54800 cb8564_c2742/f1p0/3521	1155	2667	1	1157	1637	314	1	1158

isoform=cb8564_c2788;full_length_coverage=1;non_full_length_coverage=0;isoform_length=2682;expected_accuracy=0.999							5		
cb12931_c15/f1p0/1493 isoform=cb12931_c15;full_length_coverage=1;non_full_length_coverage=0;isoform_length=1493;expected_accuracy=1.000	gnl|BL_ORD_ID|15602 cb8564_c50271/f1p1/2688 isoform=cb8564_c50271;full_length_coverage=1;non_full_length_coverage=1;isoform_length=2688;expected_accuracy=0.994	1	1106	1104	1488	1	1104	2302	2688
cb8564_c17694/f1p1/3299	gnl|BL_ORD_ID|32013 cb16355_c15/f1p8/5101 isoform=cb16355_c15;full_length_coverage=1;non_full_length_coverage=8;isoform_length=5101;expected_accuracy=1.000	1	2347	2347	3356	1489	3837	4065	5075
cb2242_c3/f2p1/1866 isoform=cb2242_c3;full_length_coverage=2;non_full_length_coverage=1;isoform_length=1866;expected_accuracy=1.000	gnl|BL_ORD_ID|37986 cb2242_c10/f1p1/2221 isoform=cb2242_c10;full_length_coverage=1;non_full_length_coverage=1;isoform_length=2221;expected_accuracy=1.000	352	1823	9	352	751	2221	1	346
cb9290_c0/f2p0/2007 isoform=cb9290_c0;full_length_coverage=2;non_full_length_coverage=0;isoform_length=2007;expected_accuracy=0.996	gnl|BL_ORD_ID|59626 cb9290_c2/f1p0/2119	527	1989	76	527	604	2066	2	454
cb8564_c36689/f1p1/2368 isoform=cb8564_c36689;full_length_coverage=1;non_full_length_coverage=1;isoform_length=2368;expected_accuracy=0.991	gnl|BL_ORD_ID|11085 cb8564_c115872/f1p0/2750 isoform=cb8564_c115872;full_length_coverage=1;non_full_length_coverage=0;isoform_length=2750;expected_accuracy=0.995	1	1709	1706	2368	1	1705	2089	2750
cb3898_c0/f8p1/2602 isoform=cb3898_c0;full_length_coverage=8;non_full_length_coverage=1;isoform_length=2602;expected_accur	gnl|BL_ORD_ID|39329 cb3898_c5/f1p0/2807 isoform=cb3898_c5;full_length_coverage=1;non_full_length_coverage=0;isoform_length=2807;expected_accura	1288	2582	1	1289	1513	2807	47	1333

acy=1.000	cy=0.999								
cb4651_c0/f2p2/1332 isoform=cb4651_c0;full_length_coverage=2;non_full_length_coverage=2;isoform_length=1332;expected_accuracy=0.998	gnl|BL_ORD_ID|72812 cb4651_c3/f1p0/4374	290	1332	9	291	1576	2618	1	283
cb3120_c19/f1p3/2883 isoform=cb3120_c19;full_length_coverage=1;non_full_length_coverage=3;isoform_length=2883;expected_accuracy=0.998	gnl|BL_ORD_ID|37028 cb3120_c15/f1p2/2916 isoform=cb3120_c15;full_length_coverage=1;non_full_length_coverage=2;isoform_length=2916;expected_accuracy=0.994	1	1982	1981	2837	4	1962	2064	2912
cb2263_c2/f3p1/2293 isoform=cb2263_c2;full_length_coverage=3;non_full_length_coverage=1;isoform_length=2293;expected_accuracy=0.996	gnl|BL_ORD_ID|68389 cb2263_c20/f1p0/2614	452	2205	7	455	840	2593	1	450
cb18456_c6478/f1p2/1469 isoform=cb18456_c6478;full_length_coverage=1;non_full_length_coverage=2;isoform_length=1469;expected_accuracy=0.992	gnl|BL_ORD_ID|53487 cb8564_c126194/f1p0/2132	289	1469	4	289	666	1840	1	288
cb8564_c45596/f1p1/3430	gnl|BL_ORD_ID|14990 cb8564_c35477/f1p1/3605 isoform=cb8564_c35477;full_length_coverage=1;non_full_length_coverage=1;isoform_length=3605;expected_accuracy=0.997	2	2780	2775	3379	21	2829	2948	3552
cb8564_c181853/f1p0/2119 isoform=cb8564_c181853;full_length_coverage=1;non_full_length_coverage=0;isoform_length=2119;expected_accuracy=0.997	gnl|BL_ORD_ID|55341 cb8564_c38927/f1p0/3326	1	1339	1334	2119	7	1346	2174	2963
cb8564_c1895/f1p0/2189	gnl|BL_ORD_ID|53781 cb8564_c1882/f1p2/3030	265	1974	5	262	1193	290	9	269

						3			
cb2737_c64/f1p1/2838 isoform=cb2737_c64;full_length_coverage=1;non_full_length_coverage=1;isoform_length=2838;expected_accuracy=0.993	gnl|BL_ORD_ID|36571 cb2737_c54/f1p0/3029 isoform=cb2737_c54;full_length_coverage=1;non_full_length_coverage=0;isoform_length=3029;expected_accuracy=0.995	1	1915	1914	2838	104	1999	2110	3022
cb1535_c1/f2p0/3305 isoform=cb1535_c1;full_length_coverage=2;non_full_length_coverage=0;isoform_length=3305;expected_accuracy=1.000	gnl|BL_ORD_ID|63719 cb1535_c3/f1p0/4275	1245	3305	1	1246	2162	4222	1	1245
cb8564_c71222/f1p0/3190	gnl|BL_ORD_ID|15431 cb8564_c43034/f1p2/3197 isoform=cb8564_c43034;full_length_coverage=1;non_full_length_coverage=2;isoform_length=3197;expected_accuracy=0.996	1	2497	2494	3028	4	2522	2663	3197
cb8564_c71904/f1p1/2820 isoform=cb8564_c71904;full_length_coverage=1;non_full_length_coverage=1;isoform_length=2820;expected_accuracy=0.996	gnl|BL_ORD_ID|19083 cb8564_c80095/f1p2/3116 isoform=cb8564_c80095;full_length_coverage=1;non_full_length_coverage=2;isoform_length=3116;expected_accuracy=0.995	1	1983	1978	2820	75	2060	2249	3090
cb8564_c43450/f1p0/2695	gnl|BL_ORD_ID|55962 cb8564_c50282/f1p0/4402	16	1886	1882	2714	1457	3357	3472	4306
cb8564_c69933/f1p0/3523 isoform=cb8564_c69933;full_length_coverage=1;non_full_length_coverage=0;isoform_length=3523;expected_accuracy=0.994	gnl|BL_ORD_ID|16114 cb8564_c66873/f7p2/3961 isoform=cb8564_c66873;full_length_coverage=7;non_full_length_coverage=2;isoform_length=3961;expected_accuracy=1.000	167	3523	1	169	544	3904	30	195
cb8564_c4910/f1p0/2450 isoform=cb8564_c4910;full_length_coverage=1;non_full_length_coverage=0;isoform_length=2450;expected_ac	gnl|BL_ORD_ID|55705 cb8564_c4909/f1p0/3122	164	2448	15	165	802	3118	1	151

curacy=0.999									
cb8564_c48235/f1p1/2252	gnl|BL_ORD_ID|14278 cb8564_c36280/f1p0/3133 isoform=cb8564_c36280;full_length_coverage=1;non_full_length_coverage=0;isoform_length=3133;expected_accuracy=1.000	1	1600	1596	2253	124	1723	2476	3132
cb8564_c185920/f1p0/2777 isoform=cb8564_c185920;full_length_coverage=1;non_full_length_coverage=0;isoform_length=2777;expected_accuracy=0.996	gnl|BL_ORD_ID|56403 cb8564_c76007/f1p0/2966	174	2774	1	177	354	2946	23	195
cb8564_c77080/f1p0/2613 isoform=cb8564_c77080;full_length_coverage=1;non_full_length_coverage=0;isoform_length=2613;expected_accuracy=0.995	gnl|BL_ORD_ID|12826 cb8564_c168354/f1p1/2790 isoform=cb8564_c168354;full_length_coverage=1;non_full_length_coverage=1;isoform_length=2790;expected_accuracy=0.995	2	2048	2043	2527	86	2127	2308	2790
cb4240_c5/f1p0/2230	gnl|BL_ORD_ID|72395 cb4240_c3/f1p0/3396	1	1667	1666	2163	1	1667	2905	3401
cb10255_c19/f2p2/1507 isoform=cb10255_c19;full_length_coverage=2;non_full_length_coverage=2;isoform_length=1507;expected_accuracy=0.997	gnl|BL_ORD_ID|1898 cb10255_c13/f1p2/1646 isoform=cb10255_c13;full_length_coverage=1;non_full_length_coverage=2;isoform_length=1646;expected_accuracy=0.994	110	1507	1	113	249	1646	9	121
cb14724_c5/f1p0/1750 isoform=cb14724_c5;full_length_coverage=1;non_full_length_coverage=0;isoform_length=1750;expected_accuracy=0.994	gnl|BL_ORD_ID|40725 cb3622_c2/f3p1/2136 isoform=cb3622_c2;full_length_coverage=3;non_full_length_coverage=1;isoform_length=2136;expected_accuracy=1.000	1	1331	1329	1699	25	1356	1765	2136
cb8564_c47011/f1p0/3100	gnl|BL_ORD_ID|15193 cb8564_c42308/f1p5/3192 isoform=cb8564_c42308;full_length_coverage=1;non_full_length_coverage=5;isoform_length=3192;expected_ac	2	2306	2301	2933	4	2316	2554	3187

	curacy=0.995								
cb11989_c4/f1p1/1835	gnl|BL_ORD_ID|32340 cb1767_c37/f1p0/1947 isoform=cb1767_c37;full_length_coverage=1;non_full_length_coverage=0;isoform_length=1947;expected_accuracy=0.995	1	1494	1495	1797	1946	444	327	27
cb16226_c1/f1p0/1638	gnl|BL_ORD_ID|55251 cb8564_c36501/f1p0/2829	466	1595	6	469	1597	2726	35	496
cb8564_c182028/f1p0/2513 isoform=cb8564_c182028;full_length_coverage=1;non_full_length_coverage=0;isoform_length=2513;expected_accuracy=0.996	gnl|BL_ORD_ID|11428 cb8564_c149144/f2p18/3112 isoform=cb8564_c149144;full_length_coverage=2;non_full_length_coverage=18;isoform_length=3112;expected_accuracy=0.994	1	1971	1968	2513	1	1974	2118	2666
cb13042_c4/f1p0/1356	gnl|BL_ORD_ID|25514 cb13042_c1/f1p0/1422 isoform=cb13042_c1;full_length_coverage=1;non_full_length_coverage=0;isoform_length=1422;expected_accuracy=1.000	321	1300	5	322	181	1166	1	327
cb8564_c36731/f1p2/2761	gnl|BL_ORD_ID|55522 cb8564_c44123/f1p1/3230	15	1977	1972	2738	15	1976	2405	3171
cb11067_c13/f1p0/1371 isoform=cb11067_c13;full_length_coverage=1;non_full_length_coverage=0;isoform_length=1371;expected_accuracy=0.993	gnl|BL_ORD_ID|2318 cb11067_c7/f1p0/1522 isoform=cb11067_c7;full_length_coverage=1;non_full_length_coverage=0;isoform_length=1522;expected_accuracy=0.998	1	1177	1176	1371	1	1177	1286	1481
cb8564_c15031/f1p2/3179 isoform=cb8564_c15031;full_length_coverage=1;non_full_length_coverage=2;isoform_length=3179;expected_accuracy=0.995	gnl|BL_ORD_ID|54178 cb8564_c19910/f1p0/4399	2	2486	2487	3179	33	2516	3153	3842
cb313_c30/f3p0/3087	gnl|BL_ORD_ID|38275 cb313_c7/f1p0/4354	1	1940	1938	3062	11	194	3217	435

isoform=cb313_c30;full_length_coverage=3;non_full_length_coverage=0;isoform_length=3087;expected_accuracy=1.000	isoform=cb313_c7;full_length_coverage=1;non_full_length_coverage=0;isoform_length=4354;expected_accuracy=0.994						5		2
cb8564_c115594/f1p0/2839	gnl|BL_ORD_ID|57979 cb8564_c85837/f1p0/3079	17	1722	1720	2501	16	1752	2306	3088
cb6631_c25/f1p0/1946 isoform=cb6631_c25;full_length_coverage=1;non_full_length_coverage=0;isoform_length=1946;expected_accuracy=0.996	gnl|BL_ORD_ID|5050 cb6631_c10/f1p0/2272 isoform=cb6631_c10;full_length_coverage=1;non_full_length_coverage=0;isoform_length=2272;expected_accuracy=0.991	1	1189	1186	1946	17	1207	1355	2126
cb8564_c80434/f1p3/2690 isoform=cb8564_c80434;full_length_coverage=1;non_full_length_coverage=3;isoform_length=2690;expected_accuracy=0.991	gnl|BL_ORD_ID|56852 cb8564_c74048/f1p2/2835	1	1480	1480	2592	43	1523	1725	2842
cb8564_c185920/f1p0/2777 isoform=cb8564_c185920;full_length_coverage=1;non_full_length_coverage=0;isoform_length=2777;expected_accuracy=0.996	gnl|BL_ORD_ID|55607 cb8564_c46612/f1p1/3367	174	2777	1	177	357	2965	22	199
cb8564_c146306/f4p4/2710 isoform=cb8564_c146306;full_length_coverage=4;non_full_length_coverage=4;isoform_length=2710;expected_accuracy=1.000	gnl|BL_ORD_ID|54560 cb8564_c126417/f1p0/2804	1	1702	1700	2666	25	1725	1830	2795
cb7560_c35/f1p1/2004 isoform=cb7560_c35;full_length_coverage=1;non_full_length_coverage=1;isoform_length=2004;expected_accuracy=0.994	gnl|BL_ORD_ID|7066 cb7560_c70/f94p4/2550 isoform=cb7560_c70;full_length_coverage=94;non_full_length_coverage=4;isoform_length=2550;expected_accuracy=1.000	1	1446	1444	2004	285	1732	1979	2539
cb8564_c119623/f1p0/1981	gnl|BL_ORD_ID|12072 cb8564_c140600/f2p3/2228	1	1282	1281	1981	12	128	1438	213

isoform=cb8564_c119623;full_length_coverage=1;non_full_length_coverage=0;isoform_length=1981;expected_accuracy=0.997	isoform=cb8564_c140600;full_length_coverage=2;non_full_length_coverage=3;isoform_length=2228;expected_accuracy=0.993						7		9
cb18456_c1776/f1p0/1211	gnl|BL_ORD_ID|9159 cb8564_c120244/f1p7/1979 isoform=cb8564_c120244;full_length_coverage=1;non_full_length_coverage=7;isoform_length=1979;expected_accuracy=0.993	280	1207	9	285	1052	1979	667	940
cb8564_c156696/f1p2/3606	gnl|BL_ORD_ID|15360 cb8564_c32914/f9p2/4297 isoform=cb8564_c32914;full_length_coverage=9;non_full_length_coverage=2;isoform_length=4297;expected_accuracy=1.000	1756	3589	1	1757	2464	4297	46	1802
cb3294_c3/f1p0/5376	gnl|BL_ORD_ID|71049 cb3294_c2/f1p0/6277	210	3886	3884	5369	281	3957	4601	6086
cb8564_c146250/f5p1/2250 isoform=cb8564_c146250;full_length_coverage=5;non_full_length_coverage=1;isoform_length=2250;expected_accuracy=1.000	gnl|BL_ORD_ID|52655 cb8564_c10124/f1p0/3457	1	1476	1474	2211	41	1519	2675	3413
cb8564_c178335/f1p2/3430 isoform=cb8564_c178335;full_length_coverage=1;non_full_length_coverage=2;isoform_length=3430;expected_accuracy=0.998	gnl|BL_ORD_ID|54608 cb8564_c127899/f1p0/3880	1	2863	2861	3379	213	3075	3305	3823
cb3609_c1/f2p0/2920 isoform=cb3609_c1;full_length_coverage=2;non_full_length_coverage=0;isoform_length=2920;expected_accuracy=0.994	gnl|BL_ORD_ID|41221 cb3609_c4/f1p0/3102 isoform=cb3609_c4;full_length_coverage=1;non_full_length_coverage=0;isoform_length=3102;expected_accuracy=0.997	1	1465	1463	2880	112	1576	1687	3102
cb8564_c89448/f1p0/2859	gnl|BL_ORD_ID|56345 cb8564_c74914/f1p0/3530	1276	2859	1	1281	1497	310	2	128

isoform=cb8564_c89448;full_length_coverage=1;non_full_length_coverage=0;isoform_length=2859;expected_accuracy=0.995							5		4
cb16742_c1/f2p1/1240 isoform=cb16742_c1;full_length_coverage=2;non_full_length_coverage=1;isoform_length=1240;expected_accuracy=0.999	gnl|BL_ORD_ID|33184 cb16742_c2/f1p2/1416 isoform=cb16742_c2;full_length_coverage=1;non_full_length_coverage=2;isoform_length=1416;expected_accuracy=1.000	104	1239	1	106	282	1416	34	140
cb10272_c17/f1p1/1759	gnl|BL_ORD_ID|48324 cb10272_c11/f1p0/2538	11	1157	1157	1632	2	1148	2015	2490
cb17104_c1/f1p0/1292	gnl|BL_ORD_ID|33287 cb17104_c5/f1p0/1481 isoform=cb17104_c5;full_length_coverage=1;non_full_length_coverage=0;isoform_length=1481;expected_accuracy=0.993	232	1296	25	234	416	1480	1	208
cb11421_c0/f2p0/1024 isoform=cb11421_c0;full_length_coverage=2;non_full_length_coverage=0;isoform_length=1024;expected_accuracy=1.000	gnl|BL_ORD_ID|60199 cb11421_c1/f1p0/3136	293	1024	37	295	1856	2585	10	267
cb2737_c40/f1p0/2830	gnl|BL_ORD_ID|38132 cb2737_c20/f1p0/3644 isoform=cb2737_c20;full_length_coverage=1;non_full_length_coverage=0;isoform_length=3644;expected_accuracy=0.998	1	1397	1395	2650	881	2278	2410	3644
cb8564_c146886/f4p5/3319 isoform=cb8564_c146886;full_length_coverage=4;non_full_length_coverage=5;isoform_length=3319;expected_accuracy=0.996	gnl|BL_ORD_ID|54440 cb8564_c46842/f1p0/3416	1	1993	1991	3288	1	2028	2138	3435
cb1178_c29/f1p0/1155	gnl|BL_ORD_ID|61143 cb1178_c5/f2p0/1389	117	1167	1	116	321	137	85	200

							1		
cb2779_c6/f1p0/1723 isoform=cb2779_c6;full_length_coverage=1;non_full_length_coverage=0;isoform_length=1723;expected_accuracy=0.997	gnl|BL_ORD_ID|39065 cb2779_c3/f1p1/3765 isoform=cb2779_c3;full_length_coverage=1;non_full_length_coverage=1;isoform_length=3765;expected_accuracy=0.991	1	1095	1094	1702	1	1098	2990	3603
cb8564_c119015/f1p10/3431	gnl|BL_ORD_ID|16877 cb8564_c54048/f1p0/3516 isoform=cb8564_c54048;full_length_coverage=1;non_full_length_coverage=0;isoform_length=3516;expected_accuracy=0.997	1	2280	2280	3257	1	2283	2540	3516
cb12034_c11/f1p0/1825	gnl|BL_ORD_ID|11594 cb8564_c130303/f1p0/2428 isoform=cb8564_c130303;full_length_coverage=1;non_full_length_coverage=0;isoform_length=2428;expected_accuracy=0.991	282	1827	9	283	859	2428	1	274
cb804_c11/f1p0/3984	gnl|BL_ORD_ID|8129 cb804_c4/f1p0/4404 isoform=cb804_c4;full_length_coverage=1;non_full_length_coverage=0;isoform_length=4404;expected_accuracy=0.995	1	2686	2686	3895	168	2829	3200	4404
cb3482_c32/f1p0/1683 isoform=cb3482_c32;full_length_coverage=1;non_full_length_coverage=0;isoform_length=1683;expected_accuracy=1.000	gnl|BL_ORD_ID|71239 cb3482_c18/f1p1/2089	1	1586	1581	1683	36	1636	1915	2017
cb8916_c4/f9p0/1721 isoform=cb8916_c4;full_length_coverage=9;non_full_length_coverage=0;isoform_length=1721;expected_accuracy=1.000	gnl|BL_ORD_ID|19815 cb8916_c26/f1p0/1759 isoform=cb8916_c26;full_length_coverage=1;non_full_length_coverage=0;isoform_length=1759;expected_accuracy=0.999	255	1721	61	257	289	1758	1	187
cb8564_c40764/f1p0/2563	gnl|BL_ORD_ID|55317 cb8564_c38251/f1p0/3243	1	2166	2162	2563	1	218	2391	279

isoform=cb8564_c40764;full_length_coverage=1;non_full_length_coverage=0;isoform_length=2563;expected_accuracy=0.997							3		2
cb1512_c15/f1p1/3322	gnl|BL_ORD_ID|27953 cb1512_c0/f2p2/4265 isoform=cb1512_c0;full_length_coverage=2;non_full_length_coverage=2;isoform_length=4265;expected_accuracy=0.996	1	1682	1681	3311	118	1797	2563	4221
cb5480_c1/f1p0/3608	gnl|BL_ORD_ID|13864 cb8564_c22844/f1p2/4014 isoform=cb8564_c22844;full_length_coverage=1;non_full_length_coverage=2;isoform_length=4014;expected_accuracy=0.998	106	2688	2687	3160	4014	1433	473	1
cb10054_c49/f1p1/1123 isoform=cb10054_c49;full_length_coverage=1;non_full_length_coverage=1;isoform_length=1123;expected_accuracy=1.000	gnl|BL_ORD_ID|48165 cb10171_c9/f1p0/2516	258	1122	3	262	449	1314	2	268
cb8564_c43450/f1p0/2695	gnl|BL_ORD_ID|56036 cb8564_c52952/f1p0/3129	15	1886	1882	2714	22	1900	2015	2848
cb6719_c31/f1p0/2434 isoform=cb6719_c31;full_length_coverage=1;non_full_length_coverage=0;isoform_length=2434;expected_accuracy=0.995	gnl|BL_ORD_ID|4663 cb6719_c20/f1p0/3262 isoform=cb6719_c20;full_length_coverage=1;non_full_length_coverage=0;isoform_length=3262;expected_accuracy=0.996	1	1331	1330	2351	774	2119	2240	3262
cb5290_c43/f1p0/2774	gnl|BL_ORD_ID|44595 cb5290_c41/f1p1/3038 isoform=cb5290_c41;full_length_coverage=1;non_full_length_coverage=1;isoform_length=3038;expected_accuracy=0.997	235	2771	1	236	381	2917	5	248
cb2707_c10/f3p0/2850	gnl|BL_ORD_ID|38446 cb2707_c18/f1p1/3468	1	2379	2376	2836	304	267	3007	346

isoform=cb2707_c10;full_length_coverage=3;non_full_length_coverage=0;isoform_length=2850;expected_accuracy=1.000	isoform=cb2707_c18;full_length_coverage=1;non_full_length_coverage=1;isoform_length=3468;expected_accuracy=0.997						9		8
cb2825_c0/f7p1/2163 isoform=cb2825_c0;full_length_coverage=7;non_full_length_coverage=1;isoform_length=2163;expected_accuracy=1.000	gnl|BL_ORD_ID|38762 cb2825_c3/f2p1/2885 isoform=cb2825_c3;full_length_coverage=2;non_full_length_coverage=1;isoform_length=2885;expected_accuracy=0.997	242	2163	85	243	966	2885	3	157
cb14125_c138/f1p0/1025	gnl|BL_ORD_ID|57284 cb8564_c88984/f1p0/3187	214	1025	27	217	662	1473	2	192
cb14220_c21/f1p1/5537	gnl|BL_ORD_ID|63136 cb14220_c12/f1p0/6602	1	4570	4569	5549	793	5353	5475	6470
cb6181_c55/f1p1/2098 isoform=cb6181_c55;full_length_coverage=1;non_full_length_coverage=1;isoform_length=2098;expected_accuracy=0.991	gnl|BL_ORD_ID|49492 cb6181_c34/f1p0/2542	1	1528	1526	2098	6	1520	1694	2257
cb8564_c67517/f12p1/3027 isoform=cb8564_c67517;full_length_coverage=12;non_full_length_coverage=1;isoform_length=3027;expected_accuracy=1.000	gnl|BL_ORD_ID|17457 cb8564_c80400/f1p0/3071 isoform=cb8564_c80400;full_length_coverage=1;non_full_length_coverage=0;isoform_length=3071;expected_accuracy=0.998	1329	3026	105	1328	1378	3069	43	1269
cb12714_c29/f1p2/1583	gnl|BL_ORD_ID|52794 cb8564_c14070/f1p1/2667	1	1296	1293	1597	1	1295	2299	2603
cb8564_c42814/f1p0/2151 isoform=cb8564_c42814;full_length_coverage=1;non_full_length_coverage=0;isoform_length=2151;expected_accuracy=0.993	gnl|BL_ORD_ID|55415 cb8564_c41076/f2p1/3193	650	2151	7	652	1624	3127	1	647
cb8564_c112885/f1p1/1941	gnl|BL_ORD_ID|16450 cb8564_c72442/f2p1/2290	585	1713	7	586	1158	228	2	583

	isoform=cb8564_c72442;full_length_coverage=2;non_full_length_coverage=1;isoform_length=2290;expected_accuracy=0.994						8		
cb12714_c29/f1p2/1583	gnl|BL_ORD_ID|52809 cb8564_c14548/f1p0/3030	1	1296	1293	1597	13	1308	2352	2656
cb8_c39/f6p2/3811 isoform=cb8_c39;full_length_coverage=6;non_full_length_coverage=2;isoform_length=3811;expected_accuracy=1.000	gnl|BL_ORD_ID|59029 cb8_c15/f1p0/4140	201	3808	102	201	410	4013	15	115
cb5672_c13/f1p0/3266	gnl|BL_ORD_ID|44913 cb5672_c6/f1p0/3840 isoform=cb5672_c6;full_length_coverage=1;non_full_length_coverage=0;isoform_length=3840;expected_accuracy=0.994	2	2051	2050	3064	5	2044	2764	3770
cb2737_c45/f1p0/2944	gnl|BL_ORD_ID|36735 cb2737_c13/f1p0/3105 isoform=cb2737_c13;full_length_coverage=1;non_full_length_coverage=0;isoform_length=3105;expected_accuracy=0.995	1	1476	1474	2757	206	1676	1808	3097
cb8205_c12/f1p0/2213	gnl|BL_ORD_ID|8245 cb8205_c5/f2p0/2450 isoform=cb8205_c5;full_length_coverage=2;non_full_length_coverage=0;isoform_length=2450;expected_accuracy=0.999	1	1248	1244	2223	20	1250	1471	2448
cb18456_c7627/f1p0/1639 isoform=cb18456_c7627;full_length_coverage=1;non_full_length_coverage=0;isoform_length=1639;expected_accuracy=0.990	gnl|BL_ORD_ID|57276 cb8564_c88788/f1p0/1896	1	1300	1299	1639	11	1304	1553	1893
cb8564_c49562/f1p0/2838	gnl|BL_ORD_ID|15169 cb8564_c40218/f1p0/3146	2	2331	2327	2836	1	231	2641	314

<table>
<tr><td>isoform=cb8564_c49562;full_length_coverage=1;non_full_length_coverage=0;isoform_length=2838;expected_accuracy=0.995</td><td>isoform=cb8564_c40218;full_length_coverage=1;non_full_length_coverage=0;isoform_length=3146;expected_accuracy=0.993</td><td></td><td></td><td></td><td></td><td></td><td>3</td><td></td><td>6</td></tr>
<tr><td>cb8564_c182028/f1p0/2513 isoform=cb8564_c182028;full_length_coverage=1;non_full_length_coverage=0;isoform_length=2513;expected_accuracy=0.996</td><td>gnl|BL_ORD_ID|10074 cb8564_c11613/f1p0/2758 isoform=cb8564_c11613;full_length_coverage=1;non_full_length_coverage=0;isoform_length=2758;expected_accuracy=0.991</td><td>1</td><td>1971</td><td>1968</td><td>2513</td><td>3</td><td>2018</td><td>2165</td><td>2722</td></tr>
<tr><td>cb5037_c0/f6p0/2322 isoform=cb5037_c0;full_length_coverage=6;non_full_length_coverage=0;isoform_length=2322;expected_accuracy=1.000</td><td>gnl|BL_ORD_ID|43673 cb5037_c5/f1p0/2410 isoform=cb5037_c5;full_length_coverage=1;non_full_length_coverage=0;isoform_length=2410;expected_accuracy=1.000</td><td>1080</td><td>2307</td><td>1</td><td>1084</td><td>1183</td><td>2410</td><td>1</td><td>1082</td></tr>
<tr><td>cb15082_c1/f1p0/1736</td><td>gnl|BL_ORD_ID|55711 cb8564_c4953/f1p0/2075</td><td>120</td><td>1736</td><td>1</td><td>120</td><td>347</td><td>1963</td><td>26</td><td>145</td></tr>
<tr><td>cb1770_c20/f1p1/3435</td><td>gnl|BL_ORD_ID|33526 cb1770_c15/f1p0/3678 isoform=cb1770_c15;full_length_coverage=1;non_full_length_coverage=0;isoform_length=3678;expected_accuracy=0.992</td><td>1</td><td>1782</td><td>1781</td><td>3407</td><td>149</td><td>1933</td><td>2049</td><td>3678</td></tr>
<tr><td>cb8564_c85207/f1p0/2727 isoform=cb8564_c85207;full_length_coverage=1;non_full_length_coverage=0;isoform_length=2727;expected_accuracy=0.997</td><td>gnl|BL_ORD_ID|17771 cb8564_c87457/f2p0/2828 isoform=cb8564_c87457;full_length_coverage=2;non_full_length_coverage=0;isoform_length=2828;expected_accuracy=1.000</td><td>430</td><td>2727</td><td>9</td><td>433</td><td>529</td><td>2828</td><td>1</td><td>425</td></tr>
<tr><td>cb8564_c36689/f1p1/2368 isoform=cb8564_c36689;full_length_coverage=1;non_full_length_coverage=1;isoform_length=2368;expected_accuracy=0.991</td><td>gnl|BL_ORD_ID|55371 cb8564_c39756/f1p1/3004</td><td>1</td><td>1709</td><td>1706</td><td>2354</td><td>1</td><td>1675</td><td>2174</td><td>2839</td></tr>
<tr><td>cb8564_c40598/f1p4/3119</td><td>gnl|BL_ORD_ID|55837 cb8564_c43074/f1p0/3464</td><td>26</td><td>2732</td><td>2729</td><td>3053</td><td>37</td><td>275</td><td>2871</td><td>319</td></tr>
</table>

						0		5
cb6221_c19/f1p0/1943	gnl|BL_ORD_ID|49541 cb6221_c7/f1p0/3291	247	1873	75	247	930	2557	6	179
cb8564_c43399/f1p0/2654 isoform=cb8564_c43399;full_length_coverage=1;non_full_length_coverage=0;isoform_length=2654;expected_accuracy=0.999	gnl|BL_ORD_ID|56601 cb8564_c48153/f1p0/3083	1	2367	2363	2653	13	2373	2589	2879
cb8564_c36731/f1p2/2761	gnl|BL_ORD_ID|16622 cb8564_c50151/f1p0/3198 isoform=cb8564_c50151;full_length_coverage=1;non_full_length_coverage=0;isoform_length=3198;expected_accuracy=0.995	1	1977	1972	2736	6	2005	2433	3198
cb8564_c15608/f1p3/3110	gnl|BL_ORD_ID|9831 cb8564_c106778/f15p6/3278 isoform=cb8564_c106778;full_length_coverage=15;non_full_length_coverage=6;isoform_length=3278;expected_accuracy=1.000	1	2790	2785	3102	1	2790	2934	3250
cb1455_c2/f1p1/3990 isoform=cb1455_c2;full_length_coverage=1;non_full_length_coverage=1;isoform_length=3990;expected_accuracy=0.996	gnl|BL_ORD_ID|28490 cb1455_c1/f1p1/4291 isoform=cb1455_c1;full_length_coverage=1;non_full_length_coverage=1;isoform_length=4291;expected_accuracy=0.996	172	3983	1	171	409	4220	1	174
cb500_c17/f1p0/2323 isoform=cb500_c17;full_length_coverage=1;non_full_length_coverage=0;isoform_length=2323;expected_accuracy=0.997	gnl|BL_ORD_ID|43083 cb500_c21/f1p0/3434 isoform=cb500_c21;full_length_coverage=1;non_full_length_coverage=0;isoform_length=3434;expected_accuracy=0.996	1	1293	1288	2323	792	2084	2216	3251
cb8564_c5007/f1p0/1941	gnl|BL_ORD_ID|13110 cb8564_c157825/f2p5/3742 isoform=cb8564_c157825;full_length_coverage=2;non_full_length_coverage=5;isoform_length=3742;expected_a	1	1447	1446	1744	1865	3311	3443	3742

	ccuracy=0.998								
cb6084_c4/f1p0/2878 isoform=cb6084_c4;full_length_coverage=1;non_full_length_coverage=0;isoform_length=2878;expected_accuracy=0.997	gnl|BL_ORD_ID|44988 cb6084_c3/f1p0/4275 isoform=cb6084_c3;full_length_coverage=1;non_full_length_coverage=0;isoform_length=4275;expected_accuracy=0.993	1	1782	1781	2878	79	1858	3139	4239
cb280_c71/f1p0/3805 isoform=cb280_c71;full_length_coverage=1;non_full_length_coverage=0;isoform_length=3805;expected_accuracy=0.994	gnl|BL_ORD_ID|37402 cb280_c74/f1p0/4024 isoform=cb280_c74;full_length_coverage=1;non_full_length_coverage=0;isoform_length=4024;expected_accuracy=0.997	1	3085	3085	3805	17	3100	3304	4022
cb13762_c16/f1p0/1376 isoform=cb13762_c16;full_length_coverage=1;non_full_length_coverage=0;isoform_length=1376;expected_accuracy=0.996	gnl|BL_ORD_ID|25473 cb13762_c8/f1p0/1530 isoform=cb13762_c8;full_length_coverage=1;non_full_length_coverage=0;isoform_length=1530;expected_accuracy=0.991	110	1338	1	111	303	1530	40	150
cb8564_c117686/f1p0/2190 isoform=cb8564_c117686;full_length_coverage=1;non_full_length_coverage=0;isoform_length=2190;expected_accuracy=1.000	gnl|BL_ORD_ID|10151 cb8564_c112138/f1p0/2737 isoform=cb8564_c112138;full_length_coverage=1;non_full_length_coverage=0;isoform_length=2737;expected_accuracy=0.995	1	1607	1606	2189	1	1606	2154	2737
cb8564_c15653/f1p4/2475	gnl|BL_ORD_ID|56021 cb8564_c52557/f1p1/2866	22	2293	2289	2437	86	2354	2573	2720
cb8564_c116421/f1p3/2229 isoform=cb8564_c116421;full_length_coverage=1;non_full_length_coverage=3;isoform_length=2229;expected_accuracy=0.992	gnl|BL_ORD_ID|53584 cb8564_c129387/f1p0/3201	1	1576	1576	2187	375	1949	2178	2796
cb8564_c38947/f1p0/2650 isoform=cb8564_c38947;full_length_coverage=1;non_full_length_coverage=0;isoform_length=2650;expected_	gnl|BL_ORD_ID|55391 cb8564_c40278/f3p2/3083	1	1441	1436	2650	1	1439	1656	2870

accuracy=0.996									
cb1955_c15/f1p0/2361	gnl|BL_ORD_ID|35587 cb1955_c12/f1p1/2862 isoform=cb1955_c12;full_length_coverage=1;non_full_length_coverage=1;isoform_length=2862;expected_accuracy=1.000	1053	2280	1	1055	1569	2789	66	1120
cb912_c7/f1p1/2209 isoform=cb912_c7;full_length_coverage=1;non_full_length_coverage=1;isoform_length=2209;expected_accuracy=1.000	gnl|BL_ORD_ID|59086 cb912_c5/f1p0/3541	1	1426	1424	2169	861	2286	2393	3138
cb8564_c40764/f1p0/2563 isoform=cb8564_c40764;full_length_coverage=1;non_full_length_coverage=0;isoform_length=2563;expected_accuracy=0.997	gnl|BL_ORD_ID|54698 cb8564_c155298/f3p9/3822	1	2167	2162	2563	1	2165	2372	2773
cb11728_c0/f1p0/1316	gnl|BL_ORD_ID|23838 cb11728_c1/f1p0/2287 isoform=cb11728_c1;full_length_coverage=1;non_full_length_coverage=0;isoform_length=2287;expected_accuracy=0.996	616	1316	8	620	1587	2287	3	615
cb6339_c16/f1p1/2286 isoform=cb6339_c16;full_length_coverage=1;non_full_length_coverage=1;isoform_length=2286;expected_accuracy=0.997	gnl|BL_ORD_ID|4268 cb6339_c89/f118p1/2676 isoform=cb6339_c89;full_length_coverage=118;non_full_length_coverage=1;isoform_length=2676;expected_accuracy=1.000	1	1780	1776	2283	151	1932	2136	2643
cb12714_c29/f1p2/1583	gnl|BL_ORD_ID|17008 cb8564_c55271/f1p4/2648 isoform=cb8564_c55271;full_length_coverage=1;non_full_length_coverage=4;isoform_length=2648;expected_accuracy=0.998	1	1296	1293	1537	2	1308	2377	2616
cb560_c8/f1p0/3610	gnl|BL_ORD_ID|45174 cb560_c12/f1p0/3972	1	2430	2431	3459	30	246	2597	362

isoform=cb560_c8;full_length_coverage=1;non_full_length_coverage=0;isoform_length=3610;expected_accuracy=0.995	isoform=cb560_c12;full_length_coverage=1;non_full_length_coverage=0;isoform_length=3972;expected_accuracy=0.996						5		5
cb8564_c1541/f1p0/2233 isoform=cb8564_c1541;full_length_coverage=1;non_full_length_coverage=0;isoform_length=2233;expected_accuracy=0.995	gnl|BL_ORD_ID|13369 cb8564_c18803/f2p0/3233 isoform=cb8564_c18803;full_length_coverage=2;non_full_length_coverage=0;isoform_length=3233;expected_accuracy=1.000	163	2145	1	163	1246	3233	946	1102
cb8564_c36731/f1p2/2761	gnl|BL_ORD_ID|15924 cb8564_c52804/f1p5/3221 isoform=cb8564_c52804;full_length_coverage=1;non_full_length_coverage=5;isoform_length=3221;expected_accuracy=0.999	1	1977	1972	2741	1	1978	2407	3178
cb9823_c2/f1p0/1778	gnl|BL_ORD_ID|58926 cb9823_c1/f1p0/3390	263	1720	76	265	1946	3412	1555	1744
cb1788_c4/f1p0/3161 isoform=cb1788_c4;full_length_coverage=1;non_full_length_coverage=0;isoform_length=3161;expected_accuracy=0.997	gnl|BL_ORD_ID|63962 cb1788_c3/f1p0/3440	2	2706	2704	3097	1	2698	3069	3461
cb8564_c115280/f1p0/2295	gnl|BL_ORD_ID|11667 cb8564_c148356/f4p0/2099 isoform=cb8564_c148356;full_length_coverage=4;non_full_length_coverage=0;isoform_length=2099;expected_accuracy=1.000	1	1025	1025	1760	7	1032	1364	2099
cb3143_c8/f1p1/2928 isoform=cb3143_c8;full_length_coverage=1;non_full_length_coverage=1;isoform_length=2928;expected_accuracy=0.992	gnl|BL_ORD_ID|37814 cb3143_c5/f1p3/3440 isoform=cb3143_c5;full_length_coverage=1;non_full_length_coverage=3;isoform_length=3440;expected_accuracy=0.999	205	2928	1	206	676	3397	22	227
cb8564_c185920/f1p0/2777	gnl|BL_ORD_ID|57176 cb8564_c85665/f1p0/3087	174	2773	1	177	364	296	3	180

isoform=cb8564_c185920;full_length_coverage=1;non_full_length_coverage=0;isoform_length=2777;expected_accuracy=0.996							5		
cb8564_c111805/f1p0/2538 isoform=cb8564_c111805;full_length_coverage=1;non_full_length_coverage=0;isoform_length=2538;expected_accuracy=0.995	gnl|BL_ORD_ID|9773 cb8564_c112780/f1p1/2558 isoform=cb8564_c112780;full_length_coverage=1;non_full_length_coverage=1;isoform_length=2558;expected_accuracy=0.990	1	1859	1859	2436	1	1857	1982	2558
cb8564_c34238/f4p1/4317 isoform=cb8564_c34238;full_length_coverage=4;non_full_length_coverage=1;isoform_length=4317;expected_accuracy=1.000	gnl|BL_ORD_ID|14314 cb8564_c45827/f2p2/4435 isoform=cb8564_c45827;full_length_coverage=2;non_full_length_coverage=2;isoform_length=4435;expected_accuracy=0.999	16	3270	3266	4178	1	3255	3520	4435
cb8564_c23232/f1p3/2540	gnl|BL_ORD_ID|55603 cb8564_c46500/f1p1/2752	16	2172	2167	2518	6	2156	2258	2609
cb2836_c25/f1p0/3140 isoform=cb2836_c25;full_length_coverage=1;non_full_length_coverage=0;isoform_length=3140;expected_accuracy=1.000	gnl|BL_ORD_ID|36507 cb2836_c27/f8p2/3344 isoform=cb2836_c27;full_length_coverage=8;non_full_length_coverage=2;isoform_length=3344;expected_accuracy=1.000	1	1919	1916	3139	42	1960	2121	3344
cb8564_c18812/f1p1/3920 isoform=cb8564_c18812;full_length_coverage=1;non_full_length_coverage=1;isoform_length=3920;expected_accuracy=0.996	gnl|BL_ORD_ID|52818 cb8564_c14781/f1p0/4348	1	3309	3308	3920	12	3320	3444	4056
cb8983_c35/f1p0/1566 isoform=cb8983_c35;full_length_coverage=1;non_full_length_coverage=0;isoform_length=1566;expected_accuracy=0.999	gnl|BL_ORD_ID|58490 cb8983_c15/f1p0/2528	115	1564	1	118	803	2254	314	433
cb8564_c13789/f1p1/3323	gnl|BL_ORD_ID|15789 cb8564_c58034/f2p2/3989	2	2821	2820	3332	4	283	3390	390

	isoform=cb8564_c58034;full_length_coverage=2;non_full_length_coverage=2;isoform_length=3989;expected_accuracy=0.996						0		6
cb8564_c69217/f2p0/2619 isoform=cb8564_c69217;full_length_coverage=2;non_full_length_coverage=0;isoform_length=2619;expected_accuracy=1.000	gnl|BL_ORD_ID|15780 cb8564_c74972/f1p0/2743 isoform=cb8564_c74972;full_length_coverage=1;non_full_length_coverage=0;isoform_length=2743;expected_accuracy=0.997	1	2025	2024	2619	3	2024	2148	2742
cb8564_c47011/f1p0/3100	gnl|BL_ORD_ID|16622 cb8564_c50151/f1p0/3198 isoform=cb8564_c50151;full_length_coverage=1;non_full_length_coverage=0;isoform_length=3198;expected_accuracy=0.995	2	2306	2301	2934	5	2322	2560	3193
cb8564_c43399/f1p0/2654 isoform=cb8564_c43399;full_length_coverage=1;non_full_length_coverage=0;isoform_length=2654;expected_accuracy=0.999	gnl|BL_ORD_ID|16369 cb8564_c52191/f1p0/2822 isoform=cb8564_c52191;full_length_coverage=1;non_full_length_coverage=0;isoform_length=2822;expected_accuracy=0.997	134	2367	2363	2653	84	2316	2532	2822
cb5091_c4/f1p0/3004	gnl|BL_ORD_ID|73252 cb5091_c0/f1p0/3180	1	2580	2581	2975	29	2608	2736	3131
cb8564_c145335/f3p0/1964 isoform=cb8564_c145335;full_length_coverage=3;non_full_length_coverage=0;isoform_length=1964;expected_accuracy=0.999	gnl|BL_ORD_ID|11866 cb8564_c126236/f1p0/2200 isoform=cb8564_c126236;full_length_coverage=1;non_full_length_coverage=0;isoform_length=2200;expected_accuracy=0.999	136	1964	1	133	362	2190	1	133
cb1573_c9/f2p0/3382 isoform=cb1573_c9;full_length_coverage=2;non_full_length_coverage=0;isoform_length=3382;expected_accuracy=0.993	gnl|BL_ORD_ID|30372 cb1573_c3/f4p1/3618 isoform=cb1573_c3;full_length_coverage=4;non_full_length_coverage=1;isoform_length=3618;expected_accuracy=0.996	1274	3382	1	1277	1497	3605	51	1338
cb3073_c2/f1p0/3478	gnl|BL_ORD_ID|69091 cb3073_c3/f1p0/4308	172	3478	1	176	340	367	55	229

isoform=cb3073_c2;full_length_coverage=1;non_full_length_coverage=0;isoform_length=3478;expected_accuracy=0.996							8		
cb8564_c14882/f1p0/2156	gnl|BL_ORD_ID|14463 cb8564_c25154/f1p5/4276 isoform=cb8564_c25154;full_length_coverage=1;non_full_length_coverage=5;isoform_length=4276;expected_accuracy=0.995	237	2098	50	238	2415	4276	1	189
cb8564_c13598/f1p0/3018 isoform=cb8564_c13598;full_length_coverage=1;non_full_length_coverage=0;isoform_length=3018;expected_accuracy=0.997	gnl|BL_ORD_ID|52054 cb8564_c11371/f1p2/3552	175	3016	1	180	443	3285	44	223
cb3482_c32/f1p0/1683 isoform=cb3482_c32;full_length_coverage=1;non_full_length_coverage=0;isoform_length=1683;expected_accuracy=1.000	gnl|BL_ORD_ID|40354 cb3482_c8/f3p1/2169 isoform=cb3482_c8;full_length_coverage=3;non_full_length_coverage=1;isoform_length=2169;expected_accuracy=1.000	1	1586	1581	1683	148	1733	2012	2114
cb8564_c47522/f1p0/2954	gnl|BL_ORD_ID|14531 cb8564_c41778/f1p9/3482 isoform=cb8564_c41778;full_length_coverage=1;non_full_length_coverage=9;isoform_length=3482;expected_accuracy=0.992	222	2849	1	226	812	3439	1	247
cb8564_c45699/f1p0/3005	gnl|BL_ORD_ID|16705 cb8564_c53880/f1p0/3484 isoform=cb8564_c53880;full_length_coverage=1;non_full_length_coverage=0;isoform_length=3484;expected_accuracy=0.996	1039	2848	1	1040	1672	3484	1	1056
cb8101_c9/f1p0/2203 isoform=cb8101_c9;full_length_coverage=1;non_full_length_coverage=0;isoform_length=2203;expected_accur	gnl|BL_ORD_ID|6421 cb8101_c19/f1p0/2535 isoform=cb8101_c19;full_length_coverage=1;non_full_length_coverage=0;isoform_length=2535;expected_accur	1	1617	1613	2203	227	1839	1953	2533

acy=0.997	acy=0.998								
cb8564_c188097/f1p0/2552 isoform=cb8564_c188097;full_length_coverage=1;non_full_length_coverage=0;isoform_length=2552;expected_accuracy=0.996	gnl|BL_ORD_ID|52776 cb8564_c13457/f1p1/2717	1	1945	1940	2551	1	1945	2095	2706
cb965_c18/f1p0/2545 isoform=cb965_c18;full_length_coverage=1;non_full_length_coverage=0;isoform_length=2545;expected_accuracy=1.000	gnl|BL_ORD_ID|20578 cb965_c23/f1p0/4205 isoform=cb965_c23;full_length_coverage=1;non_full_length_coverage=0;isoform_length=4205;expected_accuracy=0.999	1	1828	1831	2496	1480	3304	3531	4205
cb8564_c5122/f1p0/3329	gnl|BL_ORD_ID|12422 cb8564_c132795/f5p10/3729 isoform=cb8564_c132795;full_length_coverage=5;non_full_length_coverage=10;isoform_length=3729;expected_accuracy=0.999	1067	3334	1	1069	1368	3637	6	1076
cb9549_c8/f1p0/1542 isoform=cb9549_c8;full_length_coverage=1;non_full_length_coverage=0;isoform_length=1542;expected_accuracy=1.000	gnl|BL_ORD_ID|59798 cb9549_c2/f1p0/2047	114	1542	1	115	354	1782	69	183
cb8617_c4/f1p0/2294 isoform=cb8617_c4;full_length_coverage=1;non_full_length_coverage=0;isoform_length=2294;expected_accuracy=1.000	gnl|BL_ORD_ID|57451 cb8617_c3/f1p0/2761	1	1463	1461	2220	41	1505	1936	2698
cb6452_c4/f1p3/2166 isoform=cb6452_c4;full_length_coverage=1;non_full_length_coverage=3;isoform_length=2166;expected_accuracy=1.000	gnl|BL_ORD_ID|49738 cb6452_c7/f1p1/2312	1	1320	1316	2130	4	1323	1455	2269
cb5183_c56/f1p0/1814	gnl|BL_ORD_ID|46058 cb5183_c19/f1p0/3158	1	1185	1183	1643	6	1185	2686	314

	isoform=cb5183_c19;full_length_coverage=1;non_full_length_coverage=0;isoform_length=3158;expected_accuracy=0.993								6
cb4063_c27/f2p0/2863 isoform=cb4063_c27;full_length_coverage=2;non_full_length_coverage=0;isoform_length=2863;expected_accuracy=1.000	gnl|BL_ORD_ID|40734 cb4063_c7/f1p0/2935 isoform=cb4063_c7;full_length_coverage=1;non_full_length_coverage=0;isoform_length=2935;expected_accuracy=0.996	113	1512	1512	2861	1	1400	1585	2935
cb8564_c38131/f1p1/2108	gnl|BL_ORD_ID|13750 cb8564_c23112/f1p0/2272 isoform=cb8564_c23112;full_length_coverage=1;non_full_length_coverage=0;isoform_length=2272;expected_accuracy=0.999	10	1587	1585	1955	2	1585	1901	2272
cb3884_c13/f1p0/2172 isoform=cb3884_c13;full_length_coverage=1;non_full_length_coverage=0;isoform_length=2172;expected_accuracy=0.996	gnl|BL_ORD_ID|71594 cb3884_c11/f1p0/2228	655	2172	71	656	708	2226	1	586
cb804_c7/f1p0/3407 isoform=cb804_c7;full_length_coverage=1;non_full_length_coverage=0;isoform_length=3407;expected_accuracy=0.997	gnl|BL_ORD_ID|50990 cb804_c11/f1p0/3984	1521	3407	132	1525	2009	3896	1	1440
cb8564_c178226/f1p0/2728 isoform=cb8564_c178226;full_length_coverage=1;non_full_length_coverage=0;isoform_length=2728;expected_accuracy=0.998	gnl|BL_ORD_ID|16805 cb8564_c66657/f4p6/2862 isoform=cb8564_c66657;full_length_coverage=4;non_full_length_coverage=6;isoform_length=2862;expected_accuracy=1.000	1284	2726	1	1288	1419	2862	29	1315
cb4576_c55/f1p2/3996 isoform=cb4576_c55;full_length_coverage=1;non_full_length_coverage=2;isoform_length=3996;expected_accu	gnl|BL_ORD_ID|72703 cb4576_c56/f1p0/4266	172	3227	3225	3986	1	3051	3497	4259

racy=0.994									
cb7972_c4/f1p0/2284 isoform=cb7972_c4;full_length_coverage=1;non_full_length_coverage=0;isoform_length=2284;expected_accuracy=0.990	gnl|BL_ORD_ID|8321 cb7972_c3/f1p0/2475 isoform=cb7972_c3;full_length_coverage=1;non_full_length_coverage=0;isoform_length=2475;expected_accuracy=0.998	1	1463	1460	2284	3	1461	1641	2468
cb13355_c13/f1p0/1325 isoform=cb13355_c13;full_length_coverage=1;non_full_length_coverage=0;isoform_length=1325;expected_accuracy=0.994	gnl|BL_ORD_ID|25370 cb13355_c3/f2p0/1541 isoform=cb13355_c3;full_length_coverage=2;non_full_length_coverage=0;isoform_length=1541;expected_accuracy=1.000	377	1325	6	378	488	1438	1	372
cb8564_c36731/f1p2/2761	gnl|BL_ORD_ID|55551 cb8564_c44964/f1p0/3508	1	1977	1972	2738	1	1976	2405	3173
cb7774_c9/f1p0/3888 isoform=cb7774_c9;full_length_coverage=1;non_full_length_coverage=0;isoform_length=3888;expected_accuracy=0.999	gnl|BL_ORD_ID|51513 cb7774_c3/f1p0/4456	1	3042	3041	3842	22	3059	3659	4461
cb8564_c49090/f1p0/3264	gnl|BL_ORD_ID|56041 cb8564_c53077/f1p2/3406	405	3242	72	405	452	3287	5	337
cb8564_c21764/f1p0/2599	gnl|BL_ORD_ID|12023 cb8564_c146372/f9p3/2654 isoform=cb8564_c146372;full_length_coverage=9;non_full_length_coverage=3;isoform_length=2654;expected_accuracy=0.999	1	2030	2030	2619	69	2096	1980	2569
cb2804_c229/f1p1/1373 isoform=cb2804_c229;full_length_coverage=1;non_full_length_coverage=1;isoform_length=1373;expected_accuracy=0.991	gnl|BL_ORD_ID|38911 cb2804_c50/f2p0/2193 isoform=cb2804_c50;full_length_coverage=2;non_full_length_coverage=0;isoform_length=2193;expected_accuracy=0.992	137	1371	3	139	619	1850	1	133
cb8564_c927/f7p2/2538	gnl|BL_ORD_ID|13896 cb8564_c4136/f2p1/2697	1	2237	2233	2458	10	224	2472	269

isoform=cb8564_c927;full_length_coverage=7;non_full_length_coverage=2;isoform_length=2538;expected_accuracy=1.000	isoform=cb8564_c4136;full_length_coverage=2;non_full_length_coverage=1;isoform_length=2697;expected_accuracy=1.000						6		7
cb4664_c15/f1p0/2526	gnl|BL_ORD_ID|72828 cb4664_c13/f1p0/2651	229	2260	7	232	588	2612	3	229
cb8564_c106956/f4p6/2390 isoform=cb8564_c106956;full_length_coverage=4;non_full_length_coverage=6;isoform_length=2390;expected_accuracy=0.999	gnl|BL_ORD_ID|14642 cb8564_c33936/f7p0/2581 isoform=cb8564_c33936;full_length_coverage=7;non_full_length_coverage=0;isoform_length=2581;expected_accuracy=0.998	1	1359	1359	2293	1	1354	1647	2581
cb5082_c7/f1p0/2984	gnl|BL_ORD_ID|42252 cb5082_c5/f1p0/3051 isoform=cb5082_c5;full_length_coverage=1;non_full_length_coverage=0;isoform_length=3051;expected_accuracy=0.992	1	1872	1873	2892	25	1894	2019	3051
cb2753_c18/f1p0/2859	gnl|BL_ORD_ID|69948 cb2753_c22/f1p0/3667	1	2193	2191	2568	689	2880	3164	3541
cb12931_c15/f1p0/1493 isoform=cb12931_c15;full_length_coverage=1;non_full_length_coverage=0;isoform_length=1493;expected_accuracy=1.000	gnl|BL_ORD_ID|12863 cb8564_c178653/f1p0/2779 isoform=cb8564_c178653;full_length_coverage=1;non_full_length_coverage=0;isoform_length=2779;expected_accuracy=0.994	1	1106	1104	1464	1	1104	2419	2779
cb8564_c40598/f1p4/3119	gnl|BL_ORD_ID|56764 cb8564_c70795/f1p0/3769	19	2732	2729	3059	34	2746	2867	3192
cb2985_c4/f1p0/3241 isoform=cb2985_c4;full_length_coverage=1;non_full_length_coverage=0;isoform_length=3241;expected_accuracy=0.994	gnl|BL_ORD_ID|37919 cb2985_c15/f1p2/3532 isoform=cb2985_c15;full_length_coverage=1;non_full_length_coverage=2;isoform_length=3532;expected_accuracy=0.998	1	1966	1967	3241	1	1979	2185	3461
cb2155_c25/f2p1/2883	gnl|BL_ORD_ID|35909 cb2155_c6/f2p0/3314	100	1788	1786	2883	1	168	2196	329

isoform=cb2155_c25;full_length_coverage=2;non_full_length_coverage=1;isoform_length=2883;expected_accuracy=0.996	isoform=cb2155_c6;full_length_coverage=2;non_full_length_coverage=0;isoform_length=3314;expected_accuracy=0.997						7		7
cb8564_c39149/f1p0/3375	gnl|BL_ORD_ID|58134 cb8564_c89788/f1p0/3521	136	3211	1	135	294	3369	9	143
cb8564_c43779/f1p0/3021 isoform=cb8564_c43779;full_length_coverage=1;non_full_length_coverage=0;isoform_length=3021;expected_accuracy=0.993	gnl|BL_ORD_ID|53680 cb8564_c153222/f2p39/3454	1	2344	2345	3009	7	2351	2507	3170
cb14968_c11/f1p0/1127	gnl|BL_ORD_ID|64376 cb14968_c10/f1p0/1444	149	1132	30	149	235	1218	6	121
cb8564_c36689/f1p1/2368 isoform=cb8564_c36689;full_length_coverage=1;non_full_length_coverage=1;isoform_length=2368;expected_accuracy=0.991	gnl|BL_ORD_ID|55873 cb8564_c47701/f1p0/3144	12	1709	1706	2368	24	1713	2212	2876
cb8564_c43587/f1p0/2930	gnl|BL_ORD_ID|55362 cb8564_c39584/f2p3/4364	12	1700	1699	2936	15	1705	1599	2842
cb206_c10/f1p0/1588	gnl|BL_ORD_ID|36416 cb206_c9/f1p0/2615 isoform=cb206_c9;full_length_coverage=1;non_full_length_coverage=0;isoform_length=2615;expected_accuracy=1.000	405	1377	43	406	1641	2614	1	364
cb3513_c28/f18p0/2970 isoform=cb3513_c28;full_length_coverage=18;non_full_length_coverage=0;isoform_length=2970;expected_accuracy=1.000	gnl|BL_ORD_ID|40099 cb3513_c7/f2p1/3768 isoform=cb3513_c7;full_length_coverage=2;non_full_length_coverage=1;isoform_length=3768;expected_accuracy=1.000	1	1551	1551	2970	1	1551	1866	3289
cb8564_c40764/f1p0/2563	gnl|BL_ORD_ID|9418 cb8564_c119193/f1p2/2975	1	2167	2162	2563	1	218	2391	279

isoform=cb8564_c40764;full_length_coverage=1;non_full_length_coverage=0;isoform_length=2563;expected_accuracy=0.997	isoform=cb8564_c119193;full_length_coverage=1;non_full_length_coverage=2;isoform_length=2975;expected_accuracy=0.998						2		5
cb1505_c17/f1p1/1887	gnl|BL_ORD_ID|63682 cb1505_c24/f1p0/2552	1	1205	1203	1828	13	1218	1852	2478
cb4016_c32/f1p0/1913	gnl|BL_ORD_ID|40528 cb4016_c3/f2p0/2850 isoform=cb4016_c3;full_length_coverage=2;non_full_length_coverage=0;isoform_length=2850;expected_accuracy=1.000	191	1929	23	194	1111	2850	510	683
cb1573_c41/f1p0/1724 isoform=cb1573_c41;full_length_coverage=1;non_full_length_coverage=0;isoform_length=1724;expected_accuracy=1.000	gnl|BL_ORD_ID|28225 cb1573_c19/f1p1/3913 isoform=cb1573_c19;full_length_coverage=1;non_full_length_coverage=1;isoform_length=3913;expected_accuracy=0.996	1	1319	1314	1721	33	1352	3507	3913
cb8564_c13789/f1p1/3323	gnl|BL_ORD_ID|11922 cb8564_c15087/f1p1/3594 isoform=cb8564_c15087;full_length_coverage=1;non_full_length_coverage=1;isoform_length=3594;expected_accuracy=0.996	2	2821	2820	3311	1	2838	3103	3594
cb7642_c53/f1p0/1947 isoform=cb7642_c53;full_length_coverage=1;non_full_length_coverage=0;isoform_length=1947;expected_accuracy=0.990	gnl|BL_ORD_ID|7246 cb7642_c26/f2p2/2157 isoform=cb7642_c26;full_length_coverage=2;non_full_length_coverage=2;isoform_length=2157;expected_accuracy=1.000	1	1181	1178	1947	1	1183	1290	2060
cb10054_c49/f1p1/1123 isoform=cb10054_c49;full_length_coverage=1;non_full_length_coverage=1;isoform_length=1123;expected_accuracy=1.000	gnl|BL_ORD_ID|1796 cb10054_c3/f1p7/1329 isoform=cb10054_c3;full_length_coverage=1;non_full_length_coverage=7;isoform_length=1329;expected_accuracy=1.000	258	1123	8	262	438	1306	6	257
cb16226_c1/f1p0/1638	gnl|BL_ORD_ID|17167 cb8564_c87931/f1p0/3852	466	1600	6	469	1646	277	78	538

	isoform=cb8564_c87931;full_length_coverage=1;non_full_length_coverage=0;isoform_length=3852;expected_accuracy=0.993						8		
cb18012_c2/f1p0/1829	gnl|BL_ORD_ID|54534 cb8564_c125514/f1p0/2141	175	1819	1	176	360	2004	29	204
cb2873_c11/f1p0/3401	gnl|BL_ORD_ID|36904 cb2873_c2/f2p2/3521 isoform=cb2873_c2;full_length_coverage=2;non_full_length_coverage=2;isoform_length=3521;expected_accuracy=0.998	132	3367	1	132	272	3510	6	137
cb17397_c0/f1p0/1631 isoform=cb17397_c0;full_length_coverage=1;non_full_length_coverage=0;isoform_length=1631;expected_accuracy=0.999	gnl|BL_ORD_ID|31786 cb17397_c2/f1p0/5709 isoform=cb17397_c2;full_length_coverage=1;non_full_length_coverage=0;isoform_length=5709;expected_accuracy=0.995	1	1070	1065	1631	3374	4439	5127	5692
cb8564_c75977/f1p0/2165	gnl|BL_ORD_ID|13003 cb8564_c161530/f1p7/3594 isoform=cb8564_c161530;full_length_coverage=1;non_full_length_coverage=7;isoform_length=3594;expected_accuracy=0.999	1	1393	1394	2125	183	1575	2863	3594
cb8564_c113778/f1p0/2109 isoform=cb8564_c113778;full_length_coverage=1;non_full_length_coverage=0;isoform_length=2109;expected_accuracy=0.997	gnl|BL_ORD_ID|12568 cb8564_c123971/f3p1/2902 isoform=cb8564_c123971;full_length_coverage=3;non_full_length_coverage=1;isoform_length=2902;expected_accuracy=0.996	1	1144	1141	2109	19	1160	1776	2744
cb1333_c60/f1p0/1953 isoform=cb1333_c60;full_length_coverage=1;non_full_length_coverage=0;isoform_length=1953;expected_accuracy=0.999	gnl|BL_ORD_ID|61248 cb1333_c59/f1p0/2058	277	1951	6	280	389	2065	1	275
cb3892_c10/f1p0/2868	gnl|BL_ORD_ID|40678 cb3892_c5/f1p0/3003	183	2832	1	184	335	300	1	186

	isoform=cb3892_c5;full_length_coverage=1;non_full_length_coverage=0;isoform_length=3003;expected_accuracy=0.998						3		
cb541_c24/f1p0/5418 isoform=cb541_c24;full_length_coverage=1;non_full_length_coverage=0;isoform_length=5418;expected_accuracy=0.993	gnl|BL_ORD_ID|44619 cb541_c22/f1p0/5566 isoform=cb541_c22;full_length_coverage=1;non_full_length_coverage=0;isoform_length=5566;expected_accuracy=0.998	1	4102	4103	5416	1	4097	4227	5549
cb8564_c46939/f1p1/2926	gnl|BL_ORD_ID|55600 cb8564_c46484/f1p0/3151	102	2134	2130	2870	5	2066	2171	2911
cb653_c12/f1p0/3754 isoform=cb653_c12;full_length_coverage=1;non_full_length_coverage=0;isoform_length=3754;expected_accuracy=0.995	gnl|BL_ORD_ID|49384 cb653_c26/f1p0/4462	195	3754	1	196	607	4166	109	308
cb3290_c38/f1p0/2345 isoform=cb3290_c38;full_length_coverage=1;non_full_length_coverage=0;isoform_length=2345;expected_accuracy=0.996	gnl|BL_ORD_ID|71043 cb3290_c170/f1p0/3059	1150	2345	1	1151	1624	2828	317	1467
cb11854_c49/f1p0/1279	gnl|BL_ORD_ID|12162 cb8564_c146678/f2p5/2093 isoform=cb8564_c146678;full_length_coverage=2;non_full_length_coverage=5;isoform_length=2093;expected_accuracy=0.997	349	1253	37	350	1144	2048	1	314
cb7642_c53/f1p0/1947 isoform=cb7642_c53;full_length_coverage=1;non_full_length_coverage=0;isoform_length=1947;expected_accuracy=0.990	gnl|BL_ORD_ID|51434 cb7642_c126/f1p0/2123	10	1181	1178	1880	27	1199	1306	2009
cb4469_c6/f1p0/2503	gnl|BL_ORD_ID|44064 cb4469_c10/f1p0/2911	1	1835	1834	2470	70	189	2275	2911

	isoform=cb4469_c10;full_length_coverage=1;non_full_length_coverage=0;isoform_length=2911;expected_accuracy=0.994						7		
cb14996_c0/f1p0/1746	gnl|BL_ORD_ID|74572 cb5896_c102/f1p0/2978	1	1118	1117	1757	26	1143	1714	2353
cb8564_c117926/f1p0/2540	gnl|BL_ORD_ID|10765 cb8564_c108620/f6p2/2639 isoform=cb8564_c108620;full_length_coverage=6;non_full_length_coverage=2;isoform_length=2639;expected_accuracy=1.000	17	1943	1941	2330	143	2068	2254	2639
cb8564_c180116/f1p0/2944 isoform=cb8564_c180116;full_length_coverage=1;non_full_length_coverage=0;isoform_length=2944;expected_accuracy=0.996	gnl|BL_ORD_ID|54747 cb8564_c162728/f1p28/3382	1	2522	2520	2944	168	2689	2918	3342
cb4656_c12/f1p0/1533 isoform=cb4656_c12;full_length_coverage=1;non_full_length_coverage=0;isoform_length=1533;expected_accuracy=0.999	gnl|BL_ORD_ID|41822 cb4656_c6/f1p0/1966 isoform=cb4656_c6;full_length_coverage=1;non_full_length_coverage=0;isoform_length=1966;expected_accuracy=0.990	122	1533	1	121	288	1689	23	143
cb8564_c68629/f9p0/2229 isoform=cb8564_c68629;full_length_coverage=9;non_full_length_coverage=0;isoform_length=2229;expected_accuracy=1.000	gnl|BL_ORD_ID|58043 cb8564_c87344/f1p0/2474	11	1383	1382	2210	17	1389	1567	2394
cb14220_c21/f1p1/5537	gnl|BL_ORD_ID|25173 cb14220_c18/f1p1/6379 isoform=cb14220_c18;full_length_coverage=1;non_full_length_coverage=1;isoform_length=6379;expected_accuracy=0.992	1	4570	4569	5549	708	5251	5373	6366
cb8456_c128/f1p1/1805	gnl|BL_ORD_ID|51952 cb8456_c77/f1p0/2298	12	1371	1366	1805	23	136	1484	192

isoform=cb8456_c128;full_length_coverage=1;non_full_length_coverage=1;isoform_length=1805;expected_accuracy=0.994							9		1
cb8564_c15653/f1p4/2475	gnl|BL_ORD_ID|15068 cb8564_c46958/f1p6/2685 isoform=cb8564_c46958;full_length_coverage=1;non_full_length_coverage=6;isoform_length=2685;expected_accuracy=0.990	22	2293	2289	2446	48	2311	2530	2685
cb8564_c46939/f1p1/2926	gnl|BL_ORD_ID|55623 cb8564_c47027/f1p0/3544	1	2134	2130	2870	1	2168	2273	3014
cb9442_c3/f3p1/1845 isoform=cb9442_c3;full_length_coverage=3;non_full_length_coverage=1;isoform_length=1845;expected_accuracy=1.000	gnl|BL_ORD_ID|59723 cb9442_c8/f1p1/2068	1	1450	1448	1842	7	1456	1668	2044
cb4444_c18/f1p0/2708	gnl|BL_ORD_ID|43662 cb4444_c7/f1p0/2681 isoform=cb4444_c7;full_length_coverage=1;non_full_length_coverage=0;isoform_length=2681;expected_accuracy=0.993	1	1632	1630	2399	1	1632	1779	2573
cb2177_c32/f1p0/2567 isoform=cb2177_c32;full_length_coverage=1;non_full_length_coverage=0;isoform_length=2567;expected_accuracy=0.990	gnl|BL_ORD_ID|34628 cb2177_c160/f4p2/3130 isoform=cb2177_c160;full_length_coverage=4;non_full_length_coverage=2;isoform_length=3130;expected_accuracy=1.000	1	1568	1567	2567	377	1950	2125	3126
cb2834_c14/f1p0/3411	gnl|BL_ORD_ID|68923 cb2834_c8/f1p2/3513	263	3380	1	263	406	3523	41	303
cb9270_c3/f1p0/2645	gnl|BL_ORD_ID|66349 cb1683_c33/f1p0/2889	632	2536	85	633	1956	53	2767	2219
cb3088_c6/f1p0/2002	gnl|BL_ORD_ID|37966 cb3088_c4/f1p0/3723	262	1981	3	266	1949	367	28	294

	isoform=cb3088_c4;full_length_coverage=1;non_full_length_coverage=0;isoform_length=3723;expected_accuracy=0.997						4		
cb8564_c21439/f1p0/2587	gnl|BL_ORD_ID|13686 cb8564_c23425/f1p0/2742 isoform=cb8564_c23425;full_length_coverage=1;non_full_length_coverage=0;isoform_length=2742;expected_accuracy=0.993	1	1228	1226	2572	30	1258	1378	2695
cb6518_c3/f2p0/3835 isoform=cb6518_c3;full_length_coverage=2;non_full_length_coverage=0;isoform_length=3835;expected_accuracy=0.999	gnl|BL_ORD_ID|5046 cb6518_c8/f1p0/3970 isoform=cb6518_c8;full_length_coverage=1;non_full_length_coverage=0;isoform_length=3970;expected_accuracy=0.991	551	3822	8	554	714	3970	1	548
cb3046_c0/f6p0/2414 isoform=cb3046_c0;full_length_coverage=6;non_full_length_coverage=0;isoform_length=2414;expected_accuracy=1.000	gnl|BL_ORD_ID|69072 cb3046_c5/f1p0/3275	15	1900	1898	2414	1	1896	2305	2822
cb8564_c47011/f1p0/3100	gnl|BL_ORD_ID|14539 cb8564_c46562/f2p3/3196 isoform=cb8564_c46562;full_length_coverage=2;non_full_length_coverage=3;isoform_length=3196;expected_accuracy=0.995	2	2306	2301	2934	6	2322	2560	3192
cb8564_c47929/f1p0/3443 isoform=cb8564_c47929;full_length_coverage=1;non_full_length_coverage=0;isoform_length=3443;expected_accuracy=0.992	gnl|BL_ORD_ID|56663 cb8564_c51593/f1p0/4707	1	1893	1889	3442	156	2043	3138	4691
cb8564_c109865/f1p2/1924 isoform=cb8564_c109865;full_length_coverage=1;non_full_length_coverage=2;isoform_length=1924;expected	gnl|BL_ORD_ID|55214 cb8564_c35600/f1p0/2363	1	1802	1798	1907	104	1902	2140	2245

_accuracy=0.994									
cb8564_c36520/f1p0/3415	gnl|BL_ORD_ID|13363 cb8564_c20575/f1p0/3783 isoform=cb8564_c20575;full_length_coverage=1;non_full_length_coverage=0;isoform_length=3783;expected_accuracy=0.997	22	3004	3002	3404	32	3004	3305	3707
cb5183_c115/f1p0/1016	gnl|BL_ORD_ID|73600 cb5183_c53/f1p1/1789	326	1016	4	330	981	1672	46	372
cb8564_c81926/f1p2/3307 isoform=cb8564_c81926;full_length_coverage=1;non_full_length_coverage=2;isoform_length=3307;expected_accuracy=0.994	gnl|BL_ORD_ID|18317 cb8564_c80643/f1p1/3548 isoform=cb8564_c80643;full_length_coverage=1;non_full_length_coverage=1;isoform_length=3548;expected_accuracy=0.991	1	2264	2264	3292	1	2251	2509	3548
cb5183_c62/f1p0/1693	gnl|BL_ORD_ID|46058 cb5183_c19/f1p0/3158 isoform=cb5183_c19;full_length_coverage=1;non_full_length_coverage=0;isoform_length=3158;expected_accuracy=0.993	1	1188	1186	1668	1	1184	2118	2598
cb8564_c40764/f1p0/2563 isoform=cb8564_c40764;full_length_coverage=1;non_full_length_coverage=0;isoform_length=2563;expected_accuracy=0.997	gnl|BL_ORD_ID|15994 cb8564_c54019/f1p1/2940 isoform=cb8564_c54019;full_length_coverage=1;non_full_length_coverage=1;isoform_length=2940;expected_accuracy=0.991	1	2167	2162	2563	7	2154	2358	2757
cb2033_c2/f2p0/3608 isoform=cb2033_c2;full_length_coverage=2;non_full_length_coverage=0;isoform_length=3608;expected_accuracy=1.000	gnl|BL_ORD_ID|66632 cb2033_c12/f1p0/3978	1	1953	1952	3575	73	2024	2299	3921
cb1030_c25/f1p0/3189	gnl|BL_ORD_ID|47255 cb1030_c32/f1p0/3185	11	1795	1793	2836	83	1866	2020	3062
cb8564_c185920/f1p0/2777	gnl|BL_ORD_ID|54438 cb8564_c46124/f1p0/3383	174	2777	1	177	501	310	165	342

isoform=cb8564_c185920;full_length_coverage=1;non_full_length_coverage=0;isoform_length=2777;expected_accuracy=0.996							8		
cb12572_c11/f1p0/1421	gnl|BL_ORD_ID|73926 cb5440_c4/f1p0/3106	1	1109	1113	1405	17	1125	1913	2205
cb8564_c10952/f1p1/2673 isoform=cb8564_c10952;full_length_coverage=1;non_full_length_coverage=1;isoform_length=2673;expected_accuracy=0.992	gnl|BL_ORD_ID|56381 cb8564_c75626/f1p2/3199	2	2122	2120	2671	4	2116	2611	3155
cb8564_c36056/f1p0/2652 isoform=cb8564_c36056;full_length_coverage=1;non_full_length_coverage=0;isoform_length=2652;expected_accuracy=0.999	gnl|BL_ORD_ID|55378 cb8564_c39827/f1p1/2907	248	2650	1	251	481	2879	88	337
cb295_c26/f1p1/1869	gnl|BL_ORD_ID|38640 cb295_c2/f4p5/2058 isoform=cb295_c2;full_length_coverage=4;non_full_length_coverage=5;isoform_length=2058;expected_accuracy=1.000	1	1359	1358	1856	77	1425	1556	2058
cb6040_c11/f1p0/2095	gnl|BL_ORD_ID|74811 cb6040_c7/f1p1/2549	1	1752	1747	1943	66	1816	2039	2238
cb12_c11/f1p2/4713	gnl|BL_ORD_ID|61090 cb12_c56/f1p0/6478	375	4671	2	378	1999	6295	1377	1753
cb8564_c23016/f1p1/2528 isoform=cb8564_c23016;full_length_coverage=1;non_full_length_coverage=1;isoform_length=2528;expected_accuracy=1.000	gnl|BL_ORD_ID|13981 cb8564_c23567/f1p2/3305 isoform=cb8564_c23567;full_length_coverage=1;non_full_length_coverage=2;isoform_length=3305;expected_accuracy=0.993	1	2032	2031	2429	639	2669	2911	3305
cb3170_c4/f1p1/2150	gnl|BL_ORD_ID|38754 cb3170_c5/f1p2/2463	1	1523	1522	2070	191	171	1816	236

	isoform=cb3170_c5;full_length_coverage=1;non_full_length_coverage=2;isoform_length=2463;expected_accuracy=0.997						3		4
cb8295_c1/f6p0/1642 isoform=cb8295_c1;full_length_coverage=6;non_full_length_coverage=0;isoform_length=1642;expected_accuracy=1.000	gnl|BL_ORD_ID|9587 cb8295_c5/f5p0/2401 isoform=cb8295_c5;full_length_coverage=5;non_full_length_coverage=0;isoform_length=2401;expected_accuracy=1.000	1	1207	1204	1642	1	1208	1961	2399
cb8564_c125010/f1p0/1943 isoform=cb8564_c125010;full_length_coverage=1;non_full_length_coverage=0;isoform_length=1943;expected_accuracy=0.992	gnl|BL_ORD_ID|11705 cb8564_c129214/f1p1/2051 isoform=cb8564_c129214;full_length_coverage=1;non_full_length_coverage=1;isoform_length=2051;expected_accuracy=0.994	1	1626	1625	1943	1	1630	1737	2051
cb3426_c24/f1p0/2863	gnl|BL_ORD_ID|39893 cb3426_c18/f1p1/3249 isoform=cb3426_c18;full_length_coverage=1;non_full_length_coverage=1;isoform_length=3249;expected_accuracy=0.999	15	2361	2358	2848	48	2397	2605	3094
cb8564_c86614/f1p2/3276	gnl|BL_ORD_ID|14738 cb8564_c42029/f1p0/3342 isoform=cb8564_c42029;full_length_coverage=1;non_full_length_coverage=0;isoform_length=3342;expected_accuracy=0.998	101	3169	1	103	270	3342	19	122
cb3270_c8/f1p0/2363 isoform=cb3270_c8;full_length_coverage=1;non_full_length_coverage=0;isoform_length=2363;expected_accuracy=0.994	gnl|BL_ORD_ID|71019 cb3270_c22/f1p2/3080	1	1624	1620	2353	4	1628	2325	3058
cb12893_c39/f1p0/1097 isoform=cb12893_c39;full_length_coverage=1;non_full_length_coverage=0;isoform_length=1097;expected_acc	gnl|BL_ORD_ID|26294 cb12893_c37/f1p0/1237 isoform=cb12893_c37;full_length_coverage=1;non_full_length_coverage=0;isoform_length=1237;expected_acc	239	1096	4	239	379	1237	1	236

uracy=0.996	uracy=0.998								
cb15516_c2/f1p0/1333 isoform=cb15516_c2;full_length_coverage=1;non_full_length_coverage=0;isoform_length=1333;expected_accuracy=0.994	gnl|BL_ORD_ID|3778 cb6371_c5/f1p0/2342 isoform=cb6371_c5;full_length_coverage=1;non_full_length_coverage=0;isoform_length=2342;expected_accuracy=0.995	193	1333	36	192	1189	2328	1	157
cb14044_c17/f1p0/1065	gnl|BL_ORD_ID|25840 cb14044_c2/f4p1/1883 isoform=cb14044_c2;full_length_coverage=4;non_full_length_coverage=1;isoform_length=1883;expected_accuracy=1.000	1	723	719	972	307	1030	1164	1417
cb8564_c145947/f8p2/2415 isoform=cb8564_c145947;full_length_coverage=8;non_full_length_coverage=2;isoform_length=2415;expected_accuracy=1.000	gnl|BL_ORD_ID|12956 cb8564_c1771/f3p1/3767 isoform=cb8564_c1771;full_length_coverage=3;non_full_length_coverage=1;isoform_length=3767;expected_accuracy=0.996	1	1291	1289	2415	26	1334	2646	3767
cb5874_c30/f1p0/2210	gnl|BL_ORD_ID|74537 cb5874_c33/f1p0/2945	145	2094	1	145	902	2851	560	704
cb14664_c3/f1p0/1551 isoform=cb14664_c3;full_length_coverage=1;non_full_length_coverage=0;isoform_length=1551;expected_accuracy=1.000	gnl|BL_ORD_ID|13432 cb8564_c1994/f1p0/2024 isoform=cb8564_c1994;full_length_coverage=1;non_full_length_coverage=0;isoform_length=2024;expected_accuracy=0.995	162	1536	2	167	645	2024	1	167
cb285_c15/f1p1/2293 isoform=cb285_c15;full_length_coverage=1;non_full_length_coverage=1;isoform_length=2293;expected_accuracy=0.998	gnl|BL_ORD_ID|36788 cb285_c0/f3p1/2363 isoform=cb285_c0;full_length_coverage=3;non_full_length_coverage=1;isoform_length=2363;expected_accuracy=0.997	336	2292	93	337	410	2363	2	273
cb7521_c46/f1p0/2168	gnl|BL_ORD_ID|8395 cb7521_c70/f1p3/2481 isoform=cb7521_c70;full_length_coverage=1;non_full_length_coverage=3;isoform_length=2481;expected_accur	1	1630	1626	2043	119	1748	1949	2367

<table>
<tr><td></td><td></td><td></td><td></td><td></td><td></td><td></td><td></td><td></td><td></td></tr>
<tr><td></td><td>acy=0.996</td><td></td><td></td><td></td><td></td><td></td><td></td><td></td><td></td></tr>
<tr><td>cb5137_c53/f1p0/2672 isoform=cb5137_c53;full_length_coverage=1;non_full_length_coverage=0;isoform_length=2672;expected_accuracy=0.992</td><td>gnl|BL_ORD_ID|42260 cb5137_c79/f1p1/2853 isoform=cb5137_c79;full_length_coverage=1;non_full_length_coverage=1;isoform_length=2853;expected_accuracy=0.994</td><td>110</td><td>2565</td><td>2562</td><td>2671</td><td>36</td><td>2491</td><td>2596</td><td>2704</td></tr>
<tr><td>cb5204_c24/f11p2/2812 isoform=cb5204_c24;full_length_coverage=11;non_full_length_coverage=2;isoform_length=2812;expected_accuracy=1.000</td><td>gnl|BL_ORD_ID|46181 cb5204_c6/f1p1/2901 isoform=cb5204_c6;full_length_coverage=1;non_full_length_coverage=1;isoform_length=2901;expected_accuracy=0.997</td><td>1</td><td>1725</td><td>1725</td><td>2761</td><td>6</td><td>1739</td><td>1855</td><td>2901</td></tr>
<tr><td>cb1312_c24/f1p0/3018</td><td>gnl|BL_ORD_ID|27492 cb1312_c33/f1p3/3892 isoform=cb1312_c33;full_length_coverage=1;non_full_length_coverage=3;isoform_length=3892;expected_accuracy=0.996</td><td>1</td><td>1532</td><td>1531</td><td>2888</td><td>149</td><td>1675</td><td>2405</td><td>3758</td></tr>
<tr><td>cb3941_c9/f2p0/1926</td><td>gnl|BL_ORD_ID|39242 cb3941_c29/f1p0/3410 isoform=cb3941_c29;full_length_coverage=1;non_full_length_coverage=0;isoform_length=3410;expected_accuracy=0.997</td><td>250</td><td>1897</td><td>48</td><td>250</td><td>1764</td><td>3410</td><td>1</td><td>201</td></tr>
<tr><td>cb10044_c3/f1p0/1568 isoform=cb10044_c3;full_length_coverage=1;non_full_length_coverage=0;isoform_length=1568;expected_accuracy=0.997</td><td>gnl|BL_ORD_ID|1260 cb10044_c4/f1p0/1866 isoform=cb10044_c4;full_length_coverage=1;non_full_length_coverage=0;isoform_length=1866;expected_accuracy=0.998</td><td>502</td><td>1568</td><td>7</td><td>504</td><td>723</td><td>1789</td><td>1</td><td>499</td></tr>
<tr><td>cb4391_c9/f1p0/1712 isoform=cb4391_c9;full_length_coverage=1;non_full_length_coverage=0;isoform_length=1712;expected_accuracy=0.994</td><td>gnl|BL_ORD_ID|72533 cb4391_c6/f1p0/2827</td><td>310</td><td>1712</td><td>6</td><td>315</td><td>1370</td><td>2772</td><td>1</td><td>311</td></tr>
<tr><td>cb5891_c11/f1p0/2563</td><td>gnl|BL_ORD_ID|74547 cb5891_c8/f1p0/2721</td><td>201</td><td>2520</td><td>1</td><td>200</td><td>306</td><td>262</td><td>1</td><td>202</td></tr>
</table>

							7		
cb3593_c2/f1p0/2887 isoform=cb3593_c2;full_length_coverage=1;non_full_length_coverage=0;isoform_length=2887;expected_accuracy=0.997	gnl|BL_ORD_ID|71361 cb3593_c1/f1p0/3600	10	2493	2493	2856	1	2483	3124	3485
cb1770_c11/f1p0/3480 isoform=cb1770_c11;full_length_coverage=1;non_full_length_coverage=0;isoform_length=3480;expected_accuracy=0.997	gnl|BL_ORD_ID|33844 cb1770_c9/f1p0/3711 isoform=cb1770_c9;full_length_coverage=1;non_full_length_coverage=0;isoform_length=3711;expected_accuracy=0.999	1	2313	2308	3480	12	2326	2479	3653
cb8564_c51661/f1p0/3132	gnl|BL_ORD_ID|54719 cb8564_c159487/f1p6/3403	1	2104	2099	3077	19	2122	2279	3257
cb309_c4/f2p2/4112 isoform=cb309_c4;full_length_coverage=2;non_full_length_coverage=2;isoform_length=4112;expected_accuracy=0.996	gnl|BL_ORD_ID|38927 cb309_c3/f2p2/4443 isoform=cb309_c3;full_length_coverage=2;non_full_length_coverage=2;isoform_length=4443;expected_accuracy=1.000	1	3715	3716	4112	22	3739	4027	4424
cb10027_c14/f6p0/1589 isoform=cb10027_c14;full_length_coverage=6;non_full_length_coverage=0;isoform_length=1589;expected_accuracy=1.000	gnl|BL_ORD_ID|2017 cb10027_c6/f1p0/1792 isoform=cb10027_c6;full_length_coverage=1;non_full_length_coverage=0;isoform_length=1792;expected_accuracy=1.000	226	1579	4	227	439	1792	1	224
cb8564_c182028/f1p0/2513 isoform=cb8564_c182028;full_length_coverage=1;non_full_length_coverage=0;isoform_length=2513;expected_accuracy=0.996	gnl|BL_ORD_ID|57311 cb8564_c89547/f1p0/2973	1	1971	1968	2513	91	2062	2206	2751
cb8564_c4845/f1p0/3094 isoform=cb8564_c4845;full_length_coverage=1;non_full_length_coverage=0;isoform_length=3094;expected_ac	gnl|BL_ORD_ID|16120 cb8564_c4844/f1p0/4014 isoform=cb8564_c4844;full_length_coverage=1;non_full_length_coverage=0;isoform_length=4014;expected_ac	1	1933	1932	3088	64	1995	2858	4014

curacy=0.996	curacy=0.995									
cb4016_c32/f1p0/1913	gnl|BL_ORD_ID|40531 cb4016_c8/f1p0/2818 isoform=cb4016_c8;full_length_coverage=1;non_full_length_coverage=0;isoform_length=2818;expected_accuracy=0.993	191	1930	23	194	1047	2783	446	619
cb6143_c0/f2p0/2680 isoform=cb6143_c0;full_length_coverage=2;non_full_length_coverage=0;isoform_length=2680;expected_accuracy=0.997	gnl|BL_ORD_ID|45275 cb6143_c4/f1p1/2929 isoform=cb6143_c4;full_length_coverage=1;non_full_length_coverage=1;isoform_length=2929;expected_accuracy=1.000	1250	2679	1	1251	1501	2929	57	1305
cb822_c6/f2p3/4553 isoform=cb822_c6;full_length_coverage=2;non_full_length_coverage=3;isoform_length=4553;expected_accuracy=1.000	gnl|BL_ORD_ID|6209 cb822_c32/f1p1/5821 isoform=cb822_c32;full_length_coverage=1;non_full_length_coverage=1;isoform_length=5821;expected_accuracy=0.999	1	2792	2792	4552	2	2802	4060	5818
cb8564_c41756/f1p0/2738 isoform=cb8564_c41756;full_length_coverage=1;non_full_length_coverage=0;isoform_length=2738;expected_accuracy=0.996	gnl|BL_ORD_ID|14368 cb8564_c34638/f1p0/2817 isoform=cb8564_c34638;full_length_coverage=1;non_full_length_coverage=0;isoform_length=2817;expected_accuracy=0.992	1	2173	2170	2738	1	2135	2257	2817
cb8564_c73443/f1p0/4382	gnl|BL_ORD_ID|57929 cb8564_c84227/f1p0/4645	2	3293	3292	4343	3	3309	3547	4610
cb6398_c11/f1p0/2579	gnl|BL_ORD_ID|49673 cb6398_c8/f1p0/2747	13	1929	1924	2573	1	1918	2053	2697
cb8564_c14344/f1p0/2533 isoform=cb8564_c14344;full_length_coverage=1;non_full_length_coverage=0;isoform_length=2533;expected_accuracy=0.993	gnl|BL_ORD_ID|18962 cb8564_c79723/f1p6/3025 isoform=cb8564_c79723;full_length_coverage=1;non_full_length_coverage=6;isoform_length=3025;expected_accuracy=0.999	148	2485	1	151	687	3025	218	368
cb5613_c29/f1p3/2436	gnl|BL_ORD_ID|44685 cb5613_c26/f1p0/3883	1	1845	1842	2365	1	184	3361	388

isoform=cb5613_c29;full_length_coverage=1;non_full_length_coverage=3;isoform_length=2436;expected_accuracy=1.000	isoform=cb5613_c26;full_length_coverage=1;non_full_length_coverage=0;isoform_length=3883;expected_accuracy=0.994						1		3
cb3771_c16/f1p0/3239 isoform=cb3771_c16;full_length_coverage=1;non_full_length_coverage=0;isoform_length=3239;expected_accuracy=0.995	gnl|BL_ORD_ID|40598 cb3771_c5/f1p0/3508 isoform=cb3771_c5;full_length_coverage=1;non_full_length_coverage=0;isoform_length=3508;expected_accuracy=0.993	1	2536	2536	3186	192	2723	2859	3508
cb862_c11/f1p0/3220	gnl|BL_ORD_ID|57578 cb862_c12/f1p0/3756	786	3110	83	785	1331	3655	29	731
cb134_c0/f5p0/2267 isoform=cb134_c0;full_length_coverage=5;non_full_length_coverage=0;isoform_length=2267;expected_accuracy=1.000	gnl|BL_ORD_ID|27559 cb134_c11/f1p0/3255 isoform=cb134_c11;full_length_coverage=1;non_full_length_coverage=0;isoform_length=3255;expected_accuracy=0.996	1	1424	1420	2265	3	1426	2412	3255
cb8564_c20359/f1p3/3986 isoform=cb8564_c20359;full_length_coverage=1;non_full_length_coverage=3;isoform_length=3986;expected_accuracy=0.991	gnl|BL_ORD_ID|14242 cb8564_c31910/f18p7/4258 isoform=cb8564_c31910;full_length_coverage=18;non_full_length_coverage=7;isoform_length=4258;expected_accuracy=1.000	1	2550	2551	3981	1	2570	2827	4258
cb8564_c138545/f4p0/2007 isoform=cb8564_c138545;full_length_coverage=4;non_full_length_coverage=0;isoform_length=2007;expected_accuracy=1.000	gnl|BL_ORD_ID|10349 cb8564_c110674/f1p0/2425 isoform=cb8564_c110674;full_length_coverage=1;non_full_length_coverage=0;isoform_length=2425;expected_accuracy=0.996	1	1277	1275	1925	1	1278	1775	2425
cb6063_c19/f1p1/2514	gnl|BL_ORD_ID|45456 cb6063_c27/f3p0/2968 isoform=cb6063_c27;full_length_coverage=3;non_full_length_coverage=0;isoform_length=2968;expected_accuracy=1.000	157	2432	1	156	693	2965	1	153
cb4471_c12/f1p0/2778	gnl|BL_ORD_ID|42093 cb4471_c7/f1p0/2973	21	2300	2300	2777	1	228	2419	289

isoform=cb4471_c12;full_length_coverage=1;non_full_length_coverage=0;isoform_length=2778;expected_accuracy=0.996	isoform=cb4471_c7;full_length_coverage=1;non_full_length_coverage=0;isoform_length=2973;expected_accuracy=0.998						1		4
cb13861_c12/f1p0/1585	gnl|BL_ORD_ID|26339 cb13861_c2/f2p0/1709 isoform=cb13861_c2;full_length_coverage=2;non_full_length_coverage=0;isoform_length=1709;expected_accuracy=0.996	12	1237	1233	1576	4	1229	1366	1709
cb6111_c12/f2p0/1372 isoform=cb6111_c12;full_length_coverage=2;non_full_length_coverage=0;isoform_length=1372;expected_accuracy=0.999	gnl|BL_ORD_ID|74915 cb6111_c17/f1p0/1775	162	1372	54	161	251	1462	14	120
cb8564_c90437/f1p0/2888 isoform=cb8564_c90437;full_length_coverage=1;non_full_length_coverage=0;isoform_length=2888;expected_accuracy=0.995	gnl|BL_ORD_ID|57978 cb8564_c85833/f1p0/2942	1178	2777	1	1180	1354	2955	1	1180
cb713_c5/f1p2/3811 isoform=cb713_c5;full_length_coverage=1;non_full_length_coverage=2;isoform_length=3811;expected_accuracy=0.996	gnl|BL_ORD_ID|5490 cb713_c1/f1p1/4666 isoform=cb713_c1;full_length_coverage=1;non_full_length_coverage=1;isoform_length=4666;expected_accuracy=0.996	128	2817	2816	3811	3	2681	3652	4651
cb9143_c30/f2p0/2817 isoform=cb9143_c30;full_length_coverage=2;non_full_length_coverage=0;isoform_length=2817;expected_accuracy=0.996	gnl|BL_ORD_ID|19566 cb9143_c33/f2p0/3006 isoform=cb9143_c33;full_length_coverage=2;non_full_length_coverage=0;isoform_length=3006;expected_accuracy=0.995	199	2738	1	203	467	3006	128	330
cb354_c5/f1p0/4015	gnl|BL_ORD_ID|70822 cb354_c1/f1p0/4589	244	4039	114	247	503	4298	3	136
cb17052_c0/f2p0/1426	gnl|BL_ORD_ID|31873 cb17052_c2/f1p0/1584	271	1423	4	270	433	157	1	264

isoform=cb17052_c0;full_length_coverage=2;non_full_length_coverage=0;isoform_length=1426;expected_accuracy=0.996	isoform=cb17052_c2;full_length_coverage=1;non_full_length_coverage=0;isoform_length=1584;expected_accuracy=0.995						9		
cb8564_c89842/f1p0/2302 isoform=cb8564_c89842;full_length_coverage=1;non_full_length_coverage=0;isoform_length=2302;expected_accuracy=0.990	gnl|BL_ORD_ID|57182 cb8564_c85892/f1p0/3297	1	1700	1698	2302	160	187 6	2409	302 1
cb1030_c34/f1p0/2904	gnl|BL_ORD_ID|1808 cb1030_c5/f2p0/3347 isoform=cb1030_c5;full_length_coverage=2;non_full_length_coverage=0;isoform_length=3347;expected_accuracy=0.997	1	2740	2743	2873	35	277 4	2900	302 9
cb8564_c188095/f1p0/3177 isoform=cb8564_c188095;full_length_coverage=1;non_full_length_coverage=0;isoform_length=3177;expected_accuracy=0.998	gnl|BL_ORD_ID|14950 cb8564_c44595/f2p0/3305 isoform=cb8564_c44595;full_length_coverage=2;non_full_length_coverage=0;isoform_length=3305;expected_accuracy=0.995	1143	3177	1	1144	1249	328 5	1	1142
cb11012_c4/f1p0/1724 isoform=cb11012_c4;full_length_coverage=1;non_full_length_coverage=0;isoform_length=1724;expected_accuracy=0.997	gnl|BL_ORD_ID|1652 cb11012_c2/f1p0/2546 isoform=cb11012_c2;full_length_coverage=1;non_full_length_coverage=0;isoform_length=2546;expected_accuracy=0.999	1	1356	1356	1724	1	135 1	2146	251 4
cb1913_c44/f1p0/2236 isoform=cb1913_c44;full_length_coverage=1;non_full_length_coverage=0;isoform_length=2236;expected_accuracy=0.998	gnl|BL_ORD_ID|66487 cb1913_c32/f1p0/3800	1	1634	1630	2236	85	171 7	1842	246 1
cb8564_c50697/f1p3/2172 isoform=cb8564_c50697;full_length_coverage=1;non_full_length_coverage=3;isoform_length=2172;expected_	gnl|BL_ORD_ID|56830 cb8564_c73119/f1p0/2955	106	2126	1	108	811	282 7	1	106

accuracy=0.993									
cb17252_c0/f1p0/1799	gnl|BL_ORD_ID|56867 cb8564_c74584/f1p0/3264	257	1788	4	258	1697	3229	833	1087
cb4374_c3/f1p0/2557 isoform=cb4374_c3;full_length_coverage=1;non_full_length_coverage=0;isoform_length=2557;expected_accuracy=0.997	gnl|BL_ORD_ID|72518 cb4374_c8/f1p0/2778	1	1860	1856	2557	4	1809	2047	2751
cb8564_c42130/f1p0/3239	gnl|BL_ORD_ID|55342 cb8564_c38940/f1p0/4032	695	3162	9	697	1133	3580	54	736
cb581_c7/f1p0/2369 isoform=cb581_c7;full_length_coverage=1;non_full_length_coverage=0;isoform_length=2369;expected_accuracy=0.990	gnl|BL_ORD_ID|45250 cb581_c5/f1p1/2742 isoform=cb581_c5;full_length_coverage=1;non_full_length_coverage=1;isoform_length=2742;expected_accuracy=0.996	1	1631	1627	2341	270	1928	2033	2741
cb7064_c20/f1p0/1347	gnl|BL_ORD_ID|50308 cb7064_c16/f1p0/3334	135	1338	7	135	1854	3042	1	129
cb3744_c16/f1p0/2234	gnl|BL_ORD_ID|39704 cb3744_c4/f1p2/3147 isoform=cb3744_c4;full_length_coverage=1;non_full_length_coverage=2;isoform_length=3147;expected_accuracy=1.000	1	1429	1424	1922	172	1600	2566	3064
cb15140_c13/f1p1/5199 isoform=cb15140_c13;full_length_coverage=1;non_full_length_coverage=1;isoform_length=5199;expected_accuracy=0.991	gnl|BL_ORD_ID|64476 cb15140_c10/f1p1/5378	31	5053	5053	5198	57	5093	5204	5349
cb502_c51/f1p1/2358	gnl|BL_ORD_ID|41843 cb502_c10/f2p4/2578 isoform=cb502_c10;full_length_coverage=2;non_full_length_coverage=4;isoform_length=2578;expected_accura	1053	2282	1	1055	1164	2391	7	1061

	cy=1.000								
cb965_c9/f2p4/4285 isoform=cb965_c9;full_length_coverage=2;non_full_length_coverage=4;isoform_length=4285;expected_accuracy=0.999	gnl|BL_ORD_ID|20577 cb965_c22/f1p1/4708 isoform=cb965_c22;full_length_coverage=1;non_full_length_coverage=1;isoform_length=4708;expected_accuracy=0.994	1	3576	3579	4285	1	3545	3995	4691
cb4538_c2/f1p0/2858	gnl|BL_ORD_ID|42377 cb4538_c1/f2p0/3318 isoform=cb4538_c1;full_length_coverage=2;non_full_length_coverage=0;isoform_length=3318;expected_accuracy=0.998	1	1983	1983	2854	141	2123	2424	3294
cb8564_c80798/f1p1/3793	gnl|BL_ORD_ID|35033 cb18691_c2/f1p2/5513 isoform=cb18691_c2;full_length_coverage=1;non_full_length_coverage=2;isoform_length=5513;expected_accuracy=0.994	425	3606	52	427	2331	5513	23	396
cb8564_c36689/f1p1/2368 isoform=cb8564_c36689;full_length_coverage=1;non_full_length_coverage=1;isoform_length=2368;expected_accuracy=0.991	gnl|BL_ORD_ID|55923 cb8564_c49016/f1p3/2947	1	1709	1706	2368	1	1675	2232	2895
cb13661_c3/f1p0/1743 isoform=cb13661_c3;full_length_coverage=1;non_full_length_coverage=0;isoform_length=1743;expected_accuracy=0.993	gnl|BL_ORD_ID|37986 cb2242_c10/f1p1/2221 isoform=cb2242_c10;full_length_coverage=1;non_full_length_coverage=1;isoform_length=2221;expected_accuracy=1.000	411	1743	63	411	751	2077	1	346
cb3534_c14/f1p0/2649 isoform=cb3534_c14;full_length_coverage=1;non_full_length_coverage=0;isoform_length=2649;expected_accuracy=0.998	gnl|BL_ORD_ID|40872 cb3534_c0/f1p0/3598 isoform=cb3534_c0;full_length_coverage=1;non_full_length_coverage=0;isoform_length=3598;expected_accuracy=0.996	1033	2643	1	1033	1967	3598	214	1247
cb4296_c2/f1p0/2951	gnl|BL_ORD_ID|43704 cb4296_c1/f1p0/3294	1	2266	2265	2949	1	226	2613	329

isoform=cb4296_c2;full_length_coverage=1;non_full_length_coverage=0;isoform_length=2951;expected_accuracy=0.998	isoform=cb4296_c1;full_length_coverage=1;non_full_length_coverage=0;isoform_length=3294;expected_accuracy=0.995						5		4
cb6352_c8/f1p0/2668 isoform=cb6352_c8;full_length_coverage=1;non_full_length_coverage=0;isoform_length=2668;expected_accuracy=0.999	gnl|BL_ORD_ID|5177 cb6352_c13/f1p0/5504 isoform=cb6352_c13;full_length_coverage=1;non_full_length_coverage=0;isoform_length=5504;expected_accuracy=0.998	1	2150	2149	2632	15	2159	5021	5504
cb8564_c5070/f1p0/2861	gnl|BL_ORD_ID|10454 cb8564_c112288/f2p7/2851 isoform=cb8564_c112288;full_length_coverage=2;non_full_length_coverage=7;isoform_length=2851;expected_accuracy=0.992	10	1660	1659	2830	78	1730	1623	2794
cb4016_c32/f1p0/1913	gnl|BL_ORD_ID|40896 cb4016_c10/f1p0/2636 isoform=cb4016_c10;full_length_coverage=1;non_full_length_coverage=0;isoform_length=2636;expected_accuracy=0.992	191	1930	25	194	829	2565	232	402
cb16968_c0/f1p0/1336	gnl|BL_ORD_ID|40767 cb4128_c10/f3p1/2897 isoform=cb4128_c10;full_length_coverage=3;non_full_length_coverage=1;isoform_length=2897;expected_accuracy=0.998	488	1258	9	489	2096	2875	3	485
cb2737_c63/f1p2/2799 isoform=cb2737_c63;full_length_coverage=1;non_full_length_coverage=2;isoform_length=2799;expected_accuracy=0.994	gnl|BL_ORD_ID|36571 cb2737_c54/f1p0/3029 isoform=cb2737_c54;full_length_coverage=1;non_full_length_coverage=0;isoform_length=3029;expected_accuracy=0.995	101	1885	1886	2799	216	1998	2110	3023
cb8564_c47011/f1p0/3100	gnl|BL_ORD_ID|55910 cb8564_c48645/f1p1/3287	14	2306	2301	2875	17	2300	2538	3113
cb8564_c181853/f1p0/2119	gnl|BL_ORD_ID|58064 cb8564_c87929/f1p0/3541	1	1339	1334	2112	1	134	2551	332

isoform=cb8564_c181853;full_length_coverage=1;non_full_length_coverage=0;isoform_length=2119;expected_accuracy=0.997							1		9
cb8564_c181853/f1p0/2119 isoform=cb8564_c181853;full_length_coverage=1;non_full_length_coverage=0;isoform_length=2119;expected_accuracy=0.997	gnl|BL_ORD_ID|14291 cb8564_c39261/f1p0/3083 isoform=cb8564_c39261;full_length_coverage=1;non_full_length_coverage=0;isoform_length=3083;expected_accuracy=0.994	1	1339	1334	2113	1	1339	2307	3083
cb8690_c6/f1p0/1987 isoform=cb8690_c6;full_length_coverage=1;non_full_length_coverage=0;isoform_length=1987;expected_accuracy=0.994	gnl|BL_ORD_ID|17410 cb8690_c2/f1p0/2420 isoform=cb8690_c2;full_length_coverage=1;non_full_length_coverage=0;isoform_length=2420;expected_accuracy=0.995	384	1987	56	384	620	2225	1	330
cb3744_c16/f1p0/2234	gnl|BL_ORD_ID|71475 cb3744_c21/f1p2/3189	1	1429	1424	1922	202	1630	2596	3094
cb1795_c9/f1p1/4313	gnl|BL_ORD_ID|66381 cb1795_c5/f1p0/4734	1303	4227	1	1304	1775	4698	10	1312
cb7638_c8/f1p0/1378 isoform=cb7638_c8;full_length_coverage=1;non_full_length_coverage=0;isoform_length=1378;expected_accuracy=0.999	gnl|BL_ORD_ID|51424 cb7638_c4/f1p0/1884	1	1050	1049	1368	45	1090	1236	1556
cb3081_c29/f1p0/3488	gnl|BL_ORD_ID|37158 cb3081_c15/f1p0/3452 isoform=cb3081_c15;full_length_coverage=1;non_full_length_coverage=0;isoform_length=3452;expected_accuracy=0.996	182	3309	16	184	324	3452	1	169
cb5643_c99/f1p2/2746	gnl|BL_ORD_ID|45379 cb5643_c25/f1p0/3322 isoform=cb5643_c25;full_length_coverage=1;non_full_length_coverage=0;isoform_length=3322;expected_accur	109	2632	1	111	799	3322	1	111

	acy=0.998								
cb4016_c32/f1p0/1913	gnl|BL_ORD_ID|40900 cb4016_c14/f1p0/2928 isoform=cb4016_c14;full_length_coverage=1;non_full_length_coverage=0;isoform_length=2928;expected_accuracy=0.994	191	1930	23	194	1148	2890	547	719
cb8564_c16258/f3p3/3048 isoform=cb8564_c16258;full_length_coverage=3;non_full_length_coverage=3;isoform_length=3048;expected_accuracy=1.000	gnl|BL_ORD_ID|13113 cb8564_c16914/f2p0/3868 isoform=cb8564_c16914;full_length_coverage=2;non_full_length_coverage=0;isoform_length=3868;expected_accuracy=1.000	1480	2983	1	1482	1771	3274	6	1484
cb15452_c2/f1p0/1586	gnl|BL_ORD_ID|64677 cb15452_c1/f1p0/1757	305	1555	5	310	436	1685	17	311
cb6829_c3/f3p1/2194 isoform=cb6829_c3;full_length_coverage=3;non_full_length_coverage=1;isoform_length=2194;expected_accuracy=1.000	gnl|BL_ORD_ID|4297 cb6829_c1/f6p2/2641 isoform=cb6829_c1;full_length_coverage=6;non_full_length_coverage=2;isoform_length=2641;expected_accuracy=1.000	1	1832	1832	2194	22	1854	2278	2640
cb173_c34/f1p1/2634 isoform=cb173_c34;full_length_coverage=1;non_full_length_coverage=1;isoform_length=2634;expected_accuracy=0.995	gnl|BL_ORD_ID|63581 cb173_c9/f1p0/4950	254	2634	1	254	1719	4101	1	255
cb8564_c178999/f1p0/2222 isoform=cb8564_c178999;full_length_coverage=1;non_full_length_coverage=0;isoform_length=2222;expected_accuracy=0.994	gnl|BL_ORD_ID|14411 cb8564_c44258/f1p0/3202 isoform=cb8564_c44258;full_length_coverage=1;non_full_length_coverage=0;isoform_length=3202;expected_accuracy=0.993	10	1778	1773	2211	5	1791	2762	3202
cb8279_c0/f5p0/1893 isoform=cb8279_c0;full_length_coverage=5;non_full_length_coverage=0;isoform_length=1893;expected_accur	gnl|BL_ORD_ID|51873 cb8279_c11/f1p0/2005	568	1893	67	569	626	1951	4	506

acy=0.998									
cb8564_c23232/f1p3/2540	gnl|BL_ORD_ID|55239 cb8564_c36145/f1p0/2868	16	2172	2167	2518	1	2155	2257	2608
cb8564_c90797/f1p0/2864	gnl|BL_ORD_ID|53674 cb8564_c152641/f1p6/2794	15	2307	2308	2618	25	2317	2444	2750
cb12446_c3/f1p0/1412 isoform=cb12446_c3;full_length_coverage=1;non_full_length_coverage=0;isoform_length=1412;expected_accuracy=1.000	gnl|BL_ORD_ID|9965 cb8564_c110926/f1p3/2015 isoform=cb8564_c110926;full_length_coverage=1;non_full_length_coverage=3;isoform_length=2015;expected_accuracy=0.994	109	1412	1	111	596	1900	1	111
cb2784_c25/f1p0/1649	gnl|BL_ORD_ID|68847 cb2784_c13/f1p0/3301	600	1727	63	602	2119	3247	1	550
cb8564_c46939/f1p1/2926	gnl|BL_ORD_ID|14838 cb8564_c34060/f2p0/3214 isoform=cb8564_c34060;full_length_coverage=2;non_full_length_coverage=0;isoform_length=3214;expected_accuracy=0.993	1	2134	2130	2870	6	2185	2290	3031
cb8564_c146026/f1p0/2707	gnl|BL_ORD_ID|9991 cb8564_c111546/f1p1/2743 isoform=cb8564_c111546;full_length_coverage=1;non_full_length_coverage=1;isoform_length=2743;expected_accuracy=1.000	316	2679	65	316	380	2743	1	251
cb8564_c91149/f1p5/2619 isoform=cb8564_c91149;full_length_coverage=1;non_full_length_coverage=5;isoform_length=2619;expected_accuracy=0.997	gnl|BL_ORD_ID|56353 cb8564_c75114/f1p0/2866	1	2044	2044	2604	1	2035	2193	2737
cb134_c5/f1p0/2215 isoform=cb134_c5;full_length_coverage=1;non_full_length_coverage=0;isoform_length=2215;expected_accurac	gnl|BL_ORD_ID|27520 cb134_c3/f1p0/2674 isoform=cb134_c3;full_length_coverage=1;non_full_length_coverage=0;isoform_length=2674;expected_accurac	10	1099	1097	2147	1	1089	1626	2674

y=0.999	y=0.995								
cb8564_c41572/f1p1/3265	gnl|BL_ORD_ID|17429 cb8564_c85447/f1p0/3196 isoform=cb8564_c85447;full_length_coverage=1;non_full_length_coverage=0;isoform_length=3196;expected_accuracy=0.997	1	2411	2410	2949	3	241 3	2660	319 6
cb2804_c34/f2p0/2211 isoform=cb2804_c34;full_length_coverage=2;non_full_length_coverage=0;isoform_length=2211;expected_accuracy=1.000	gnl|BL_ORD_ID|38910 cb2804_c55/f2p0/3404 isoform=cb2804_c55;full_length_coverage=2;non_full_length_coverage=0;isoform_length=3404;expected_accuracy=1.000	131	2183	1	133	1353	340 4	1	132
cb8564_c153817/f2p1/2013 isoform=cb8564_c153817;full_length_coverage=2;non_full_length_coverage=1;isoform_length=2013;expected_accuracy=1.000	gnl|BL_ORD_ID|54738 cb8564_c161787/f1p1/2177	684	2010	9	688	814	214 0	6	685
cb8564_c40598/f1p4/3119	gnl|BL_ORD_ID|16447 cb8564_c47202/f1p0/3195 isoform=cb8564_c47202;full_length_coverage=1;non_full_length_coverage=0;isoform_length=3195;expected_accuracy=0.996	1	2732	2729	3050	1	273 1	2852	319 5
cb4374_c2/f1p0/2590 isoform=cb4374_c2;full_length_coverage=1;non_full_length_coverage=0;isoform_length=2590;expected_accuracy=0.994	gnl|BL_ORD_ID|72518 cb4374_c8/f1p0/2778	1	1844	1840	2547	4	180 9	2047	275 8
cb12_c37/f1p0/4067	gnl|BL_ORD_ID|25418 cb12_c30/f1p2/4443 isoform=cb12_c30;full_length_coverage=1;non_full_length_coverage=2;isoform_length=4443;expected_accuracy=0.992	286	4080	186	288	607	440 6	373	476
cb9541_c3/f1p0/2530	gnl|BL_ORD_ID|59795 cb9541_c5/f1p0/2731	14	2264	2262	2530	7	224	2361	262

isoform=cb9541_c3;full_length_coverage=1;non_full_length_coverage=0;isoform_length=2530;expected_accuracy=0.999							9		8
cb1458_c15/f1p0/1854 isoform=cb1458_c15;full_length_coverage=1;non_full_length_coverage=0;isoform_length=1854;expected_accuracy=0.997	gnl|BL_ORD_ID|30590 cb1458_c4/f1p1/2072 isoform=cb1458_c4;full_length_coverage=1;non_full_length_coverage=1;isoform_length=2072;expected_accuracy=0.998	502	1854	63	503	650	2006	83	522
cb8564_c15348/f1p4/2577 isoform=cb8564_c15348;full_length_coverage=1;non_full_length_coverage=4;isoform_length=2577;expected_accuracy=0.997	gnl|BL_ORD_ID|56353 cb8564_c75114/f1p0/2866	2	2007	2007	2553	1	2035	2193	2737
cb2529_c6/f1p0/2581	gnl|BL_ORD_ID|36770 cb2529_c7/f1p0/5178 isoform=cb2529_c7;full_length_coverage=1;non_full_length_coverage=0;isoform_length=5178;expected_accuracy=0.993	1	1714	1714	2542	1576	3289	4341	5165
cb8564_c113064/f1p0/1966 isoform=cb8564_c113064;full_length_coverage=1;non_full_length_coverage=0;isoform_length=1966;expected_accuracy=0.997	gnl|BL_ORD_ID|10268 cb8564_c111650/f1p0/2370 isoform=cb8564_c111650;full_length_coverage=1;non_full_length_coverage=0;isoform_length=2370;expected_accuracy=0.997	113	1966	1	112	485	2337	122	233
cb5204_c26/f1p3/2844	gnl|BL_ORD_ID|46181 cb5204_c6/f1p1/2901 isoform=cb5204_c6;full_length_coverage=1;non_full_length_coverage=1;isoform_length=2901;expected_accuracy=0.997	1	1698	1698	2735	26	1739	1855	2901
cb10133_c6/f1p1/1304	gnl|BL_ORD_ID|48112 cb10133_c5/f1p0/1576	1	1074	1070	1307	70	1147	1271	1508
cb8564_c13234/f1p0/3522	gnl|BL_ORD_ID|54145 cb8564_c19339/f1p1/3362	18	2497	2497	3238	22	250	2612	335

							1		3
cb8564_c182592/f1p0/2600 isoform=cb8564_c182592;full_length_coverage=1;non_full_length_coverage=0;isoform_length=2600;expected_accuracy=0.997	gnl|BL_ORD_ID|52859 cb8564_c110170/f1p0/2703	102	1466	1462	2586	1	1386	1486	2610
cb5183_c115/f1p0/1016	gnl|BL_ORD_ID|45680 cb5183_c82/f1p1/1654 isoform=cb5183_c82;full_length_coverage=1;non_full_length_coverage=1;isoform_length=1654;expected_accuracy=0.994	326	993	1	330	936	1600	1	328
cb8_c39/f6p2/3811 isoform=cb8_c39;full_length_coverage=6;non_full_length_coverage=2;isoform_length=3811;expected_accuracy=1.000	gnl|BL_ORD_ID|21603 cb8_c5/f1p1/4485 isoform=cb8_c5;full_length_coverage=1;non_full_length_coverage=1;isoform_length=4485;expected_accuracy=0.997	102	3323	3322	3807	40	3262	4003	4485
cb9399_c2/f1p0/1800 isoform=cb9399_c2;full_length_coverage=1;non_full_length_coverage=0;isoform_length=1800;expected_accuracy=0.999	gnl|BL_ORD_ID|21508 cb9399_c0/f1p0/2096 isoform=cb9399_c0;full_length_coverage=1;non_full_length_coverage=0;isoform_length=2096;expected_accuracy=0.993	135	1800	1	134	424	2086	8	141
cb8564_c106911/f7p3/3690 isoform=cb8564_c106911;full_length_coverage=7;non_full_length_coverage=3;isoform_length=3690;expected_accuracy=0.998	gnl|BL_ORD_ID|55537 cb8564_c44585/f1p0/3901	201	3690	2	203	379	3844	6	206
cb8564_c14344/f1p0/2533 isoform=cb8564_c14344;full_length_coverage=1;non_full_length_coverage=0;isoform_length=2533;expected_accuracy=0.993	gnl|BL_ORD_ID|10800 cb8564_c10037/f1p5/2946 isoform=cb8564_c10037;full_length_coverage=1;non_full_length_coverage=5;isoform_length=2946;expected_accuracy=0.994	148	2525	1	151	572	2946	38	187
cb8564_c141653/f3p2/3337	gnl|BL_ORD_ID|57694 cb8564_c78332/f1p0/4781	1	2530	2528	3254	9	253	3635	436

							8		1
cb14382_c5/f1p0/1401	gnl|BL_ORD_ID|49473 cb793_c3/f1p0/3227	321	1351	5	326	2127	3163	1	321
cb8564_c180116/f1p0/2944 isoform=cb8564_c180116;full_length_coverage=1;non_full_length_coverage=0;isoform_length=2944;expected_accuracy=0.996	gnl|BL_ORD_ID|58037 cb8564_c87188/f1p1/3426	24	2522	2520	2944	127	2624	2853	3277
cb510_c11/f1p0/3703 isoform=cb510_c11;full_length_coverage=1;non_full_length_coverage=0;isoform_length=3703;expected_accuracy=0.995	gnl|BL_ORD_ID|42975 cb510_c1/f6p1/3896 isoform=cb510_c1;full_length_coverage=6;non_full_length_coverage=1;isoform_length=3896;expected_accuracy=1.000	1	3215	3211	3661	85	3298	3445	3896
cb500_c17/f1p0/2323 isoform=cb500_c17;full_length_coverage=1;non_full_length_coverage=0;isoform_length=2323;expected_accuracy=0.997	gnl|BL_ORD_ID|72244 cb500_c41/f1p0/3311	1	1293	1288	2323	856	2148	2280	3316
cb9201_c7/f1p0/2183	gnl|BL_ORD_ID|19588 cb9201_c2/f1p0/3381 isoform=cb9201_c2;full_length_coverage=1;non_full_length_coverage=0;isoform_length=3381;expected_accuracy=0.995	424	2073	5	424	1735	3381	56	479
cb8564_c181340/f1p0/2326 isoform=cb8564_c181340;full_length_coverage=1;non_full_length_coverage=0;isoform_length=2326;expected_accuracy=0.998	gnl|BL_ORD_ID|54234 cb8564_c20918/f2p2/2484	270	2326	5	270	375	2431	1	262
cb3771_c16/f1p0/3239 isoform=cb3771_c16;full_length_coverage=1;non_full_length_coverage=0;isoform_length=3239;expected_accu	gnl|BL_ORD_ID|71501 cb3771_c10/f1p0/3494	1	2537	2536	3239	88	2629	2765	3467

racy=0.995									
cb8564_c39756/f1p1/3004	gnl|BL_ORD_ID|14410 cb8564_c44256/f1p1/3267 isoform=cb8564_c44256;full_length_coverage=1;non_full_length_coverage=1;isoform_length=3267;expected_accuracy=0.995	1	2125	2121	2847	1	2119	2553	3265
cb8564_c43779/f1p0/3021 isoform=cb8564_c43779;full_length_coverage=1;non_full_length_coverage=0;isoform_length=3021;expected_accuracy=0.993	gnl|BL_ORD_ID|57344 cb8564_c90538/f1p0/3845	1	2344	2345	3021	2	2346	2562	3236
cb9867_c3/f1p0/1688 isoform=cb9867_c3;full_length_coverage=1;non_full_length_coverage=0;isoform_length=1688;expected_accuracy=0.993	gnl|BL_ORD_ID|20641 cb9867_c0/f3p0/1916 isoform=cb9867_c0;full_length_coverage=3;non_full_length_coverage=0;isoform_length=1916;expected_accuracy=1.000	1	1050	1047	1664	100	1156	1292	1916
cb8564_c178226/f1p0/2728 isoform=cb8564_c178226;full_length_coverage=1;non_full_length_coverage=0;isoform_length=2728;expected_accuracy=0.998	gnl|BL_ORD_ID|56601 cb8564_c48153/f1p0/3083	1284	2728	1	1288	1431	2877	13	1327
cb8564_c4731/f1p0/2173	gnl|BL_ORD_ID|18228 cb8564_c912/f2p1/2421 isoform=cb8564_c912;full_length_coverage=2;non_full_length_coverage=1;isoform_length=2421;expected_accuracy=0.999	443	2116	7	444	750	2421	1	440
cb15591_c1/f1p1/1413 isoform=cb15591_c1;full_length_coverage=1;non_full_length_coverage=1;isoform_length=1413;expected_accuracy=0.999	gnl|BL_ORD_ID|21554 cb9317_c5/f2p3/1769 isoform=cb9317_c5;full_length_coverage=2;non_full_length_coverage=3;isoform_length=1769;expected_accuracy=1.000	280	1413	4	284	544	1680	1	281
cb8564_c161476/f1p4/2801	gnl|BL_ORD_ID|17429 cb8564_c85447/f1p0/3196	1	2435	2434	2801	3	241	2660	301

isoform=cb8564_c161476;full_length_coverage=1;non_full_length_coverage=4;isoform_length=2801;expected_accuracy=0.995	isoform=cb8564_c85447;full_length_coverage=1;non_full_length_coverage=0;isoform_length=3196;expected_accuracy=0.997						3		2
cb6719_c45/f1p0/1995	gnl|BL_ORD_ID|49973 cb6719_c29/f1p0/3377	276	2004	9	279	1636	3365	1042	1313
cb8564_c3087/f1p0/2090 isoform=cb8564_c3087;full_length_coverage=1;non_full_length_coverage=0;isoform_length=2090;expected_accuracy=1.000	gnl|BL_ORD_ID|15998 cb8564_c50863/f2p2/2274 isoform=cb8564_c50863;full_length_coverage=2;non_full_length_coverage=2;isoform_length=2274;expected_accuracy=0.995	1	1914	1909	2090	31	1951	2057	2239
cb5538_c31/f1p0/2490	gnl|BL_ORD_ID|45187 cb5538_c6/f1p0/3016 isoform=cb5538_c6;full_length_coverage=1;non_full_length_coverage=0;isoform_length=3016;expected_accuracy=0.994	1	1539	1539	2327	3	1540	2223	3013
cb626_c59/f1p0/4159 isoform=cb626_c59;full_length_coverage=1;non_full_length_coverage=0;isoform_length=4159;expected_accuracy=0.991	gnl|BL_ORD_ID|47167 cb626_c79/f1p0/5202	1642	4159	11	1647	2580	5065	114	1739
cb8564_c73853/f1p0/2443 isoform=cb8564_c73853;full_length_coverage=1;non_full_length_coverage=0;isoform_length=2443;expected_accuracy=1.000	gnl|BL_ORD_ID|16351 cb8564_c70055/f4p2/2889 isoform=cb8564_c70055;full_length_coverage=4;non_full_length_coverage=2;isoform_length=2889;expected_accuracy=1.000	1	1281	1276	2443	84	1364	1644	2816
cb8449_c1/f2p2/2097 isoform=cb8449_c1;full_length_coverage=2;non_full_length_coverage=2;isoform_length=2097;expected_accuracy=0.998	gnl|BL_ORD_ID|9574 cb8449_c3/f1p1/2342 isoform=cb8449_c3;full_length_coverage=1;non_full_length_coverage=1;isoform_length=2342;expected_accuracy=0.997	1	1780	1777	2097	57	1843	1962	2280
cb8564_c16816/f1p2/3599	gnl|BL_ORD_ID|69460 cb18782_c1/f1p2/5627	1761	3599	15	1763	3814	565	1	175

isoform=cb8564_c16816;full_length_coverage=1;non_full_length_coverage=2;isoform_length=3599;expected_accuracy=0.995							4		2
cb2737_c37/f1p0/2894	gnl|BL_ORD_ID|36571 cb2737_c54/f1p0/3029 isoform=cb2737_c54;full_length_coverage=1;non_full_length_coverage=0;isoform_length=3029;expected_accuracy=0.995	1	1867	1868	2796	135	1998	2110	3029
cb8564_c123270/f1p0/1927	gnl|BL_ORD_ID|54534 cb8564_c125514/f1p0/2141	244	1812	55	245	360	1926	17	204
cb12047_c134/f1p0/1727 isoform=cb12047_c134;full_length_coverage=1;non_full_length_coverage=0;isoform_length=1727;expected_accuracy=0.999	gnl|BL_ORD_ID|52056 cb8564_c11430/f1p16/1906	1	1006	1001	1673	51	1056	1162	1834
cb8564_c39355/f1p0/3531	gnl|BL_ORD_ID|16775 cb8564_c57586/f4p1/3438 isoform=cb8564_c57586;full_length_coverage=4;non_full_length_coverage=1;isoform_length=3438;expected_accuracy=1.000	366	3215	4	367	605	3438	75	469
cb551_c11/f15p2/2542 isoform=cb551_c11;full_length_coverage=15;non_full_length_coverage=2;isoform_length=2542;expected_accuracy=1.000	gnl|BL_ORD_ID|73546 cb551_c13/f1p0/2912	100	1433	1431	2512	2	1334	1752	2833
cb8564_c148132/f3p0/2595 isoform=cb8564_c148132;full_length_coverage=3;non_full_length_coverage=0;isoform_length=2595;expected_accuracy=1.000	gnl|BL_ORD_ID|13987 cb8564_c2882/f1p0/2646 isoform=cb8564_c2882;full_length_coverage=1;non_full_length_coverage=0;isoform_length=2646;expected_accuracy=0.996	101	1498	1496	2592	1	1423	1548	2643
cb3147_c7/f1p0/2346	gnl|BL_ORD_ID|38849 cb3147_c3/f1p0/2291	1	1283	1281	2117	31	131	1457	229

	isoform=cb3147_c3;full_length_coverage=1;non_full_length_coverage=0;isoform_length=2291;expected_accuracy=0.993						3		1
cb8564_c53418/f1p0/2602 isoform=cb8564_c53418;full_length_coverage=1;non_full_length_coverage=0;isoform_length=2602;expected_accuracy=0.991	gnl|BL_ORD_ID|54329 cb8564_c22496/f4p7/2826	1163	2602	1	1168	1269	2709	1	1168
cb944_c34/f1p0/3024 isoform=cb944_c34;full_length_coverage=1;non_full_length_coverage=0;isoform_length=3024;expected_accuracy=0.995	gnl|BL_ORD_ID|20927 cb944_c35/f1p0/3736 isoform=cb944_c35;full_length_coverage=1;non_full_length_coverage=0;isoform_length=3736;expected_accuracy=0.999	1	2434	2432	3024	23	2462	3060	3652
cb8564_c45775/f1p0/3138 isoform=cb8564_c45775;full_length_coverage=1;non_full_length_coverage=0;isoform_length=3138;expected_accuracy=0.991	gnl|BL_ORD_ID|14649 cb8564_c45118/f2p0/4438 isoform=cb8564_c45118;full_length_coverage=2;non_full_length_coverage=0;isoform_length=4438;expected_accuracy=1.000	1	2586	2585	3059	98	2702	3959	4438
cb8564_c3239/f1p0/1871	gnl|BL_ORD_ID|54874 cb8564_c3238/f1p0/2188	222	1809	32	222	507	2093	22	209
cb8564_c42409/f1p0/3007	gnl|BL_ORD_ID|16879 cb8564_c48606/f1p1/3308 isoform=cb8564_c48606;full_length_coverage=1;non_full_length_coverage=1;isoform_length=3308;expected_accuracy=0.998	107	2184	2180	2899	75	2151	2587	3308
cb16052_c9/f1p0/1143	gnl|BL_ORD_ID|54642 cb8564_c129201/f1p0/1895	151	1147	34	151	747	1743	44	161
cb8564_c19445/f1p0/3163 isoform=cb8564_c19445;full_length_coverage=1;non_full_length_coverage=0;isoform_length=3163;expected_	gnl|BL_ORD_ID|14208 cb8564_c25268/f1p0/3377 isoform=cb8564_c25268;full_length_coverage=1;non_full_length_coverage=0;isoform_length=3377;expected_ac	1	2299	2298	3163	1	2291	2424	3285

accuracy=0.997	curacy=0.992								
cb6291_c3/f1p0/2781 isoform=cb6291_c3;full_length_coverage=1;non_full_length_coverage=0;isoform_length=2781;expected_accuracy=0.992	gnl|BL_ORD_ID|47420 cb6291_c2/f1p0/3168	192	2775	1	191	466	3055	154	347
cb8564_c53873/f1p0/2658	gnl|BL_ORD_ID|54141 cb8564_c19256/f1p0/3854	736	2503	9	737	1639	3406	1	734
cb8564_c46939/f1p1/2926	gnl|BL_ORD_ID|55437 cb8564_c41690/f1p1/3409	1	2134	2130	2870	1	2148	2253	2994
cb4807_c21/f1p0/3041 isoform=cb4807_c21;full_length_coverage=1;non_full_length_coverage=0;isoform_length=3041;expected_accuracy=0.996	gnl|BL_ORD_ID|43586 cb4807_c0/f4p0/3303 isoform=cb4807_c0;full_length_coverage=4;non_full_length_coverage=0;isoform_length=3303;expected_accuracy=0.998	1146	3041	1	1146	1399	3303	47	1192
cb1312_c24/f1p0/3018	gnl|BL_ORD_ID|61223 cb1312_c8/f1p0/4164	1	1532	1531	2888	154	1685	2417	3774
cb5103_c24/f4p0/1839 isoform=cb5103_c24;full_length_coverage=4;non_full_length_coverage=0;isoform_length=1839;expected_accuracy=1.000	gnl|BL_ORD_ID|72111 cb5103_c7/f1p0/2511	1	1518	1515	1829	19	1534	2045	2359
cb8564_c2326/f1p0/2006 isoform=cb8564_c2326;full_length_coverage=1;non_full_length_coverage=0;isoform_length=2006;expected_accuracy=0.990	gnl|BL_ORD_ID|54764 cb8564_c2424/f1p1/2797	230	2006	4	234	844	2617	133	366
cb1609_c7/f1p0/1655	gnl|BL_ORD_ID|29381 cb1609_c6/f1p0/1785 isoform=cb1609_c6;full_length_coverage=1;non_full_length_coverage=0;isoform_length=1785;expected_accura	1	1078	1077	1658	1	1077	1200	1782

	cy=0.997								
cb8564_c145668/f4p0/2444 isoform=cb8564_c145668;full_length_coverage=4;non_full_length_coverage=0;isoform_length=2444;expected_accuracy=1.000	gnl|BL_ORD_ID|15329 cb8564_c33103/f10p0/2526 isoform=cb8564_c33103;full_length_coverage=10;non_full_length_coverage=0;isoform_length=2526;expected_accuracy=1.000	1	2137	2138	2427	1	2137	2238	2526
cb1936_c2/f1p0/2075	gnl|BL_ORD_ID|36181 cb1936_c1/f1p0/3489 isoform=cb1936_c1;full_length_coverage=1;non_full_length_coverage=0;isoform_length=3489;expected_accuracy=0.993	276	1935	44	275	1804	3461	1	232
cb8564_c51780/f1p2/3067 isoform=cb8564_c51780;full_length_coverage=1;non_full_length_coverage=2;isoform_length=3067;expected_accuracy=0.995	gnl|BL_ORD_ID|55821 cb8564_c41766/f1p2/3241	1	2308	2308	3067	27	2334	2492	3253
cb8564_c3899/f1p0/2507 isoform=cb8564_c3899;full_length_coverage=1;non_full_length_coverage=0;isoform_length=2507;expected_accuracy=0.995	gnl|BL_ORD_ID|54953 cb8564_c3898/f1p0/2998	1151	2429	1	1152	1532	2808	8	1157
cb2918_c79/f1p0/2785 isoform=cb2918_c79;full_length_coverage=1;non_full_length_coverage=0;isoform_length=2785;expected_accuracy=1.000	gnl|BL_ORD_ID|37245 cb2918_c88/f3p0/3004 isoform=cb2918_c88;full_length_coverage=3;non_full_length_coverage=0;isoform_length=3004;expected_accuracy=1.000	1	2540	2536	2782	12	2548	2759	3004
cb2177_c32/f1p0/2567 isoform=cb2177_c32;full_length_coverage=1;non_full_length_coverage=0;isoform_length=2567;expected_accuracy=0.990	gnl|BL_ORD_ID|66791 cb2177_c38/f1p0/3092	1	1568	1567	2546	226	1808	1983	2965
cb7128_c8/f1p0/1081	gnl|BL_ORD_ID|65370 cb16614_c3/f1p0/1379	125	1077	2	126	402	135	3	127

isoform=cb7128_c8;full_length_coverage=1;non_full_length_coverage=0;isoform_length=1081;expected_accuracy=0.992							3		
cb8564_c42814/f1p0/2151 isoform=cb8564_c42814;full_length_coverage=1;non_full_length_coverage=0;isoform_length=2151;expected_accuracy=0.993	gnl|BL_ORD_ID|56601 cb8564_c48153/f1p0/3083	650	2151	7	652	1373	2877	13	684
cb11524_c0/f2p1/1126 isoform=cb11524_c0;full_length_coverage=2;non_full_length_coverage=1;isoform_length=1126;expected_accuracy=0.990	gnl|BL_ORD_ID|23512 cb11524_c1/f1p0/2290 isoform=cb11524_c1;full_length_coverage=1;non_full_length_coverage=0;isoform_length=2290;expected_accuracy=0.998	337	1126	8	337	1498	2289	1	331
cb10558_c10/f1p0/1635 isoform=cb10558_c10;full_length_coverage=1;non_full_length_coverage=0;isoform_length=1635;expected_accuracy=0.991	gnl|BL_ORD_ID|48654 cb10558_c15/f1p0/1780	1	1360	1361	1635	7	1372	1502	1773
cb5199_c11/f1p2/2600	gnl|BL_ORD_ID|73620 cb5199_c19/f1p2/3067	1	1891	1892	2517	1	1902	2461	3086
cb8564_c45300/f1p4/3014 isoform=cb8564_c45300;full_length_coverage=1;non_full_length_coverage=4;isoform_length=3014;expected_accuracy=0.996	gnl|BL_ORD_ID|55211 cb8564_c35547/f1p0/3745	243	3014	1	246	413	3182	9	256
cb3007_c13/f1p0/2385	gnl|BL_ORD_ID|37322 cb3007_c12/f1p0/2890 isoform=cb3007_c12;full_length_coverage=1;non_full_length_coverage=0;isoform_length=2890;expected_accuracy=0.996	117	2345	1	121	666	2890	1	120
cb1368_c2/f1p0/2063	gnl|BL_ORD_ID|61285 cb1368_c0/f1p0/4350	1	1643	1642	2034	1	164	1796	218

						5		8
cb6739_c19/f1p1/2409 isoform=cb6739_c19;full_length_coverage=1;non_full_length_coverage=1;isoform_length=2409;expected_accuracy=0.998	gnl|BL_ORD_ID|50006 cb6739_c17/f1p1/2735	1	2070	2067	2409	1	2079	2202	2546
cb2493_c40/f1p0/3431	gnl|BL_ORD_ID|69776 cb2493_c46/f1p1/3909	485	3408	56	488	775	3697	24	463
cb8564_c10682/f1p0/1913 isoform=cb8564_c10682;full_length_coverage=1;non_full_length_coverage=0;isoform_length=1913;expected_accuracy=0.994	gnl|BL_ORD_ID|55572 cb8564_c45654/f1p0/2343	1	1045	1041	1913	42	1086	1466	2338
cb3143_c17/f1p0/2870 isoform=cb3143_c17;full_length_coverage=1;non_full_length_coverage=0;isoform_length=2870;expected_accuracy=0.993	gnl|BL_ORD_ID|37665 cb3143_c33/f10p3/3441 isoform=cb3143_c33;full_length_coverage=10;non_full_length_coverage=3;isoform_length=3441;expected_accuracy=1.000	160	2870	1	163	721	3424	54	212
cb6202_c7/f1p0/1705 isoform=cb6202_c7;full_length_coverage=1;non_full_length_coverage=0;isoform_length=1705;expected_accuracy=0.998	gnl|BL_ORD_ID|49523 cb6202_c5/f1p0/2275	398	1702	8	403	960	2243	32	424
cb8564_c40764/f1p0/2563 isoform=cb8564_c40764;full_length_coverage=1;non_full_length_coverage=0;isoform_length=2563;expected_accuracy=0.997	gnl|BL_ORD_ID|15428 cb8564_c42387/f1p4/2973 isoform=cb8564_c42387;full_length_coverage=1;non_full_length_coverage=4;isoform_length=2973;expected_accuracy=0.993	1	2167	2162	2563	1	2169	2378	2789
cb3602_c15/f1p1/2585	gnl|BL_ORD_ID|40604 cb3602_c7/f1p2/2820 isoform=cb3602_c7;full_length_coverage=1;non_full_length_coverage=2;isoform_length=2820;expected_accura	1137	2463	1	1139	1482	2812	5	1149

	cy=1.000								
cb6221_c13/f1p0/2080 isoform=cb6221_c13;full_length_coverage=1;non_full_length_coverage=0;isoform_length=2080;expected_accuracy=1.000	gnl|BL_ORD_ID|4878 cb6221_c8/f1p3/4003 isoform=cb6221_c8;full_length_coverage=1;non_full_length_coverage=3;isoform_length=4003;expected_accuracy=0.995	117	2080	1	117	885	2851	60	174
cb5772_c13/f1p0/1660	gnl|BL_ORD_ID|46526 cb5772_c11/f1p1/1685 isoform=cb5772_c11;full_length_coverage=1;non_full_length_coverage=1;isoform_length=1685;expected_accuracy=0.992	168	1509	18	170	256	1606	1	153
cb3513_c10/f1p0/2860	gnl|BL_ORD_ID|40098 cb3513_c6/f2p0/2992 isoform=cb3513_c6;full_length_coverage=2;non_full_length_coverage=0;isoform_length=2992;expected_accuracy=0.997	1074	2808	1	1076	1216	2958	1	1076
cb2677_c13/f1p1/3727 isoform=cb2677_c13;full_length_coverage=1;non_full_length_coverage=1;isoform_length=3727;expected_accuracy=0.998	gnl|BL_ORD_ID|38453 cb2677_c24/f1p0/3934 isoform=cb2677_c24;full_length_coverage=1;non_full_length_coverage=0;isoform_length=3934;expected_accuracy=0.992	260	3727	1	259	426	3907	1	265
cb1626_c17/f1p0/3006	gnl|BL_ORD_ID|33068 cb1626_c13/f1p0/4596 isoform=cb1626_c13;full_length_coverage=1;non_full_length_coverage=0;isoform_length=4596;expected_accuracy=1.000	1092	2996	1	1092	2692	4596	1	1079
cb8564_c14775/f1p1/2535	gnl|BL_ORD_ID|12323 cb8564_c12737/f1p1/3352 isoform=cb8564_c12737;full_length_coverage=1;non_full_length_coverage=1;isoform_length=3352;expected_accuracy=0.997	1056	2492	1	1058	1837	3272	334	1387
cb1240_c4/f1p1/4081	gnl|BL_ORD_ID|67887 cb18607_c0/f1p0/5858	1	2657	2654	4065	4	266	2798	422

isoform=cb1240_c4;full_length_coverage=1;non_full_length_coverage=1;isoform_length=4081;expected_accuracy=0.995							6		4
cb8564_c130024/f1p0/2150	gnl|BL_ORD_ID|12134 cb8564_c129304/f1p0/2288 isoform=cb8564_c129304;full_length_coverage=1;non_full_length_coverage=0;isoform_length=2288;expected_accuracy=0.996	269	2155	52	270	357	2244	1	219
cb11296_c7/f1p1/1597	gnl|BL_ORD_ID|23796 cb11296_c5/f1p1/1722 isoform=cb11296_c5;full_length_coverage=1;non_full_length_coverage=1;isoform_length=1722;expected_accuracy=1.000	593	1532	7	596	767	1707	1	590
cb1524_c31/f1p0/3629 isoform=cb1524_c31;full_length_coverage=1;non_full_length_coverage=0;isoform_length=3629;expected_accuracy=0.997	gnl|BL_ORD_ID|28439 cb1524_c30/f1p0/3792 isoform=cb1524_c30;full_length_coverage=1;non_full_length_coverage=0;isoform_length=3792;expected_accuracy=0.994	107	3628	1	106	256	3777	51	156
cb8564_c44698/f1p5/2843 isoform=cb8564_c44698;full_length_coverage=1;non_full_length_coverage=5;isoform_length=2843;expected_accuracy=0.997	gnl|BL_ORD_ID|12938 cb8564_c181163/f1p1/2936 isoform=cb8564_c181163;full_length_coverage=1;non_full_length_coverage=1;isoform_length=2936;expected_accuracy=0.993	1	2516	2511	2830	5	2499	2619	2936
cb8564_c50697/f1p3/2172 isoform=cb8564_c50697;full_length_coverage=1;non_full_length_coverage=3;isoform_length=2172;expected_accuracy=0.993	gnl|BL_ORD_ID|9497 cb8564_c107044/f3p8/2832 isoform=cb8564_c107044;full_length_coverage=3;non_full_length_coverage=8;isoform_length=2832;expected_accuracy=0.998	106	2142	1	108	796	2832	72	179
cb965_c5/f2p7/4283 isoform=cb965_c5;full_length_coverage=2;non_full_length_coverage=7;isoform_length=4283;expected_accurac	gnl|BL_ORD_ID|19288 cb965_c14/f1p0/4481 isoform=cb965_c14;full_length_coverage=1;non_full_length_coverage=0;isoform_length=4481;expected_accura	2059	4283	2	2063	2225	4459	1	2083

y=1.000	cy=0.998								
cb8564_c47011/f1p0/3100	gnl|BL_ORD_ID|55182 cb8564_c34825/f1p2/3200	13	2306	2301	2934	6	232 1	2559	318 9
cb1333_c10/f4p0/1937 isoform=cb1333_c10;full_length_coverage=4;non_full_length_coverage=0;isoform_length=1937;expected_accuracy=1.000	gnl|BL_ORD_ID|26504 cb1333_c75/f1p0/2099 isoform=cb1333_c75;full_length_coverage=1;non_full_length_coverage=0;isoform_length=2099;expected_accuracy=0.998	1	1095	1096	1903	6	1116	1292	209 9
cb12931_c15/f1p0/1493 isoform=cb12931_c15;full_length_coverage=1;non_full_length_coverage=0;isoform_length=1493;expected_accuracy=1.000	gnl|BL_ORD_ID|17161 cb8564_c87466/f1p1/3006 isoform=cb8564_c87466;full_length_coverage=1;non_full_length_coverage=1;isoform_length=3006;expected_accuracy=0.994	1	1106	1104	1493	1	1115	2502	290 1
cb10142_c11/f1p0/1528 isoform=cb10142_c11;full_length_coverage=1;non_full_length_coverage=0;isoform_length=1528;expected_accuracy=1.000	gnl|BL_ORD_ID|2501 cb10142_c13/f1p0/1836 isoform=cb10142_c13;full_length_coverage=1;non_full_length_coverage=0;isoform_length=1836;expected_accuracy=0.997	1	1360	1358	1464	5	136 7	1730	183 6
cb17118_c7/f1p0/1440	gnl|BL_ORD_ID|33875 cb17118_c4/f1p0/1886 isoform=cb17118_c4;full_length_coverage=1;non_full_length_coverage=0;isoform_length=1886;expected_accuracy=0.995	1	1141	1137	1414	289	142 6	1610	188 6
cb12047_c103/f1p1/1531	gnl|BL_ORD_ID|52769 cb8564_c13372/f1p15/4378	9	870	865	1520	7	868	1154	180 9
cb8564_c133426/f1p2/2797 isoform=cb8564_c133426;full_length_coverage=1;non_full_length_coverage=2;isoform_length=2797;expected_accuracy=0.992	gnl|BL_ORD_ID|55047 cb8564_c4680/f1p0/2928	248	2696	1	248	374	280 1	3	250
cb8564_c21909/f1p2/3933	gnl|BL_ORD_ID|52786 cb8564_c13843/f1p0/4329	131	3933	1	130	384	418	31	160

isoform=cb8564_c21909;full_length_coverage=1;non_full_length_coverage=2;isoform_length=3933;expected_accuracy=0.998							3		
cb765_c15/f1p0/3098	gnl|BL_ORD_ID|7179 cb765_c2/f1p1/4647 isoform=cb765_c2;full_length_coverage=1;non_full_length_coverage=1;isoform_length=4647;expected_accuracy=0.997	1	2374	2372	2838	1522	3891	4032	4498
cb15516_c2/f1p0/1333 isoform=cb15516_c2;full_length_coverage=1;non_full_length_coverage=0;isoform_length=1333;expected_accuracy=0.994	gnl|BL_ORD_ID|47466 cb6371_c4/f1p0/2354	193	1333	41	192	1178	2330	1	152
cb4972_c32/f1p0/3346 isoform=cb4972_c32;full_length_coverage=1;non_full_length_coverage=0;isoform_length=3346;expected_accuracy=0.995	gnl|BL_ORD_ID|41907 cb4972_c52/f1p1/3427 isoform=cb4972_c52;full_length_coverage=1;non_full_length_coverage=1;isoform_length=3427;expected_accuracy=0.991	1647	3346	126	1648	1705	3421	42	1575
cb8564_c47713/f1p0/4233 isoform=cb8564_c47713;full_length_coverage=1;non_full_length_coverage=0;isoform_length=4233;expected_accuracy=0.997	gnl|BL_ORD_ID|52703 cb8564_c11697/f1p0/4709	1	3293	3288	4233	2	3296	3425	4370
cb2737_c55/f1p1/2936 isoform=cb2737_c55;full_length_coverage=1;non_full_length_coverage=1;isoform_length=2936;expected_accuracy=1.000	gnl|BL_ORD_ID|68777 cb2737_c19/f1p0/3583	1	1595	1593	2814	614	2205	2337	3558
cb18287_c3/f1p0/1559	gnl|BL_ORD_ID|32561 cb18287_c2/f1p0/1786 isoform=cb18287_c2;full_length_coverage=1;non_full_length_coverage=0;isoform_length=1786;expected_accur	280	1549	3	276	514	1783	69	342

	acy=0.996								
cb1974_c7/f1p1/3734	gnl|BL_ORD_ID|34704 cb1974_c4/f1p1/4106 isoform=cb1974_c4;full_length_coverage=1;non_full_length_coverage=1;isoform_length=4106;expected_accuracy=0.999	1785	3755	1	1786	2101	4071	79	1862
cb5745_c11/f1p0/1408	gnl|BL_ORD_ID|44881 cb5745_c4/f1p0/2698 isoform=cb5745_c4;full_length_coverage=1;non_full_length_coverage=0;isoform_length=2698;expected_accuracy=0.992	624	1407	7	625	1883	2663	1	622
cb8564_c89361/f1p1/2277 isoform=cb8564_c89361;full_length_coverage=1;non_full_length_coverage=1;isoform_length=2277;expected_accuracy=0.994	gnl|BL_ORD_ID|57088 cb8564_c82765/f1p1/2779	461	2274	96	466	977	2789	480	850
cb6318_c8/f1p1/2518	gnl|BL_ORD_ID|4925 cb6318_c10/f2p0/2888 isoform=cb6318_c10;full_length_coverage=2;non_full_length_coverage=0;isoform_length=2888;expected_accuracy=1.000	101	2469	1	103	386	2755	55	158
cb14897_c14/f1p0/1816	gnl|BL_ORD_ID|15625 cb8564_c73578/f1p1/4243 isoform=cb8564_c73578;full_length_coverage=1;non_full_length_coverage=1;isoform_length=4243;expected_accuracy=0.996	565	1732	60	565	3075	4243	1	504
cb373_c39/f1p0/4415	gnl|BL_ORD_ID|40699 cb373_c6/f1p0/4615 isoform=cb373_c6;full_length_coverage=1;non_full_length_coverage=0;isoform_length=4615;expected_accuracy=0.997	254	3749	16	254	1117	4615	6	243
cb5449_c16/f1p0/2392	gnl|BL_ORD_ID|46752 cb5449_c13/f1p0/2396	1059	2280	1	1059	1177	239	1	105

	isoform=cb5449_c13;full_length_coverage=1;non_full_length_coverage=0;isoform_length=2396;expected_accuracy=0.999						6		7
cb8564_c20384/f1p4/2475 isoform=cb8564_c20384;full_length_coverage=1;non_full_length_coverage=4;isoform_length=2475;expected_accuracy=0.999	gnl|BL_ORD_ID|13743 cb8564_c21615/f1p7/4576 isoform=cb8564_c21615;full_length_coverage=1;non_full_length_coverage=7;isoform_length=4576;expected_accuracy=0.993	1	1492	1493	2363	1	1491	834	1705
cb12_c37/f1p0/4067	gnl|BL_ORD_ID|26827 cb12_c28/f1p0/4906 isoform=cb12_c28;full_length_coverage=1;non_full_length_coverage=0;isoform_length=4906;expected_accuracy=0.998	286	4080	186	288	921	4714	689	791
cb2097_c91/f1p0/2631	gnl|BL_ORD_ID|36136 cb2097_c106/f1p0/2666 isoform=cb2097_c106;full_length_coverage=1;non_full_length_coverage=0;isoform_length=2666;expected_accuracy=1.000	598	2310	72	597	890	2608	103	628
cb8564_c9893/f7p1/3111 isoform=cb8564_c9893;full_length_coverage=7;non_full_length_coverage=1;isoform_length=3111;expected_accuracy=0.998	gnl|BL_ORD_ID|12496 cb8564_c14618/f5p1/3581 isoform=cb8564_c14618;full_length_coverage=5;non_full_length_coverage=1;isoform_length=3581;expected_accuracy=0.997	21	2428	2426	3111	1	2407	2840	3526
cb1770_c25/f1p0/3573	gnl|BL_ORD_ID|33426 cb1770_c13/f1p0/3529 isoform=cb1770_c13;full_length_coverage=1;non_full_length_coverage=0;isoform_length=3529;expected_accuracy=1.000	138	3408	1	139	260	3527	19	157
cb8564_c32509/f1p1/2880 isoform=cb8564_c32509;full_length_coverage=1;non_full_length_coverage=1;isoform_length=2880;expected_	gnl|BL_ORD_ID|16879 cb8564_c48606/f1p1/3308 isoform=cb8564_c48606;full_length_coverage=1;non_full_length_coverage=1;isoform_length=3308;expected_ac	2	2182	2178	2877	11	2151	2587	3283

<table>
<tr><td>accuracy=0.991</td><td>curacy=0.998</td><td></td><td></td><td></td><td></td><td></td><td></td><td></td><td></td></tr>
<tr><td>cb8564_c40764/f1p0/2563 isoform=cb8564_c40764;full_length_coverage=1;non_full_length_coverage=0;isoform_length=2563;expected_accuracy=0.997</td><td>gnl|BL_ORD_ID|13024 cb8564_c161476/f1p4/2801 isoform=cb8564_c161476;full_length_coverage=1;non_full_length_coverage=4;isoform_length=2801;expected_accuracy=0.995</td><td>1</td><td>2167</td><td>2162</td><td>2563</td><td>1</td><td>2176</td><td>2387</td><td>2799</td></tr>
<tr><td>cb8564_c170549/f2p3/3030 isoform=cb8564_c170549;full_length_coverage=2;non_full_length_coverage=3;isoform_length=3030;expected_accuracy=0.997</td><td>gnl|BL_ORD_ID|11649 cb8564_c124383/f1p0/3136 isoform=cb8564_c124383;full_length_coverage=1;non_full_length_coverage=0;isoform_length=3136;expected_accuracy=0.997</td><td>118</td><td>3010</td><td>1</td><td>117</td><td>223</td><td>3114</td><td>10</td><td>120</td></tr>
<tr><td>cb9061_c27/f1p1/2278 isoform=cb9061_c27;full_length_coverage=1;non_full_length_coverage=1;isoform_length=2278;expected_accuracy=0.992</td><td>gnl|BL_ORD_ID|58527 cb9061_c19/f1p0/2376</td><td>1</td><td>1451</td><td>1452</td><td>2201</td><td>22</td><td>1473</td><td>1581</td><td>2331</td></tr>
<tr><td>cb8564_c23232/f1p3/2540</td><td>gnl|BL_ORD_ID|55583 cb8564_c45873/f1p2/2968</td><td>16</td><td>2172</td><td>2167</td><td>2518</td><td>5</td><td>2159</td><td>2261</td><td>2612</td></tr>
<tr><td>cb2804_c159/f1p1/1802</td><td>gnl|BL_ORD_ID|38911 cb2804_c50/f2p0/2193 isoform=cb2804_c50;full_length_coverage=2;non_full_length_coverage=0;isoform_length=2193;expected_accuracy=0.992</td><td>124</td><td>1699</td><td>1</td><td>126</td><td>619</td><td>2193</td><td>8</td><td>133</td></tr>
<tr><td>cb4016_c32/f1p0/1913</td><td>gnl|BL_ORD_ID|40530 cb4016_c9/f1p1/2685 isoform=cb4016_c9;full_length_coverage=1;non_full_length_coverage=1;isoform_length=2685;expected_accuracy=0.996</td><td>191</td><td>1930</td><td>25</td><td>194</td><td>853</td><td>2592</td><td>257</td><td>427</td></tr>
<tr><td>cb3359_c35/f1p0/1632 isoform=cb3359_c35;full_length_coverage=1;non_full_length_coverage=0;isoform_length=1632;expected_accu</td><td>gnl|BL_ORD_ID|40969 cb3359_c15/f1p0/3164 isoform=cb3359_c15;full_length_coverage=1;non_full_length_coverage=0;isoform_length=3164;expected_accur</td><td>1</td><td>1469</td><td>1469</td><td>1632</td><td>52</td><td>1516</td><td>1624</td><td>1784</td></tr>
</table>

racy=1.000	acy=0.993								
cb8564_c17122/f1p0/3317	gnl|BL_ORD_ID|13377 cb8564_c22091/f2p0/3351 isoform=cb8564_c22091;full_length_coverage=2;non_full_length_coverage=0;isoform_length=3351;expected_accuracy=0.997	12	1685	1682	3229	9	1682	1805	3351
cb5224_c19/f1p0/2383 isoform=cb5224_c19;full_length_coverage=1;non_full_length_coverage=0;isoform_length=2383;expected_accuracy=0.995	gnl|BL_ORD_ID|73655 cb5224_c22/f1p0/2626	123	2383	1	124	249	2518	10	128
cb4211_c6/f1p1/2925 isoform=cb4211_c6;full_length_coverage=1;non_full_length_coverage=1;isoform_length=2925;expected_accuracy=0.994	gnl|BL_ORD_ID|71851 cb4211_c4/f1p2/3385	11	1873	1869	2925	5	1872	2197	3256
cb6000_c46/f1p0/1956 isoform=cb6000_c46;full_length_coverage=1;non_full_length_coverage=0;isoform_length=1956;expected_accuracy=0.995	gnl|BL_ORD_ID|45166 cb6000_c2/f10p0/2873 isoform=cb6000_c2;full_length_coverage=10;non_full_length_coverage=0;isoform_length=2873;expected_accuracy=1.000	1	1145	1140	1956	9	1132	2057	2873
cb8564_c42409/f1p0/3007	gnl|BL_ORD_ID|14531 cb8564_c41778/f1p9/3482 isoform=cb8564_c41778;full_length_coverage=1;non_full_length_coverage=9;isoform_length=3482;expected_accuracy=0.992	277	2899	107	281	812	3439	73	247
cb8564_c52978/f1p0/2089 isoform=cb8564_c52978;full_length_coverage=1;non_full_length_coverage=0;isoform_length=2089;expected_accuracy=0.996	gnl|BL_ORD_ID|58087 cb8564_c88385/f1p0/2356	336	2089	66	341	452	2222	2	278
cb8564_c109935/f2p1/2100	gnl|BL_ORD_ID|9761 cb8564_c114521/f1p0/3515	536	2100	70	540	1948	351	1	477

isoform=cb8564_c109935;full_length_coverage=2;non_full_length_coverage=1;isoform_length=2100;expected_accuracy=0.995	isoform=cb8564_c114521;full_length_coverage=1;non_full_length_coverage=0;isoform_length=3515;expected_accuracy=0.999						3		
cb8564_c180116/f1p0/2944 isoform=cb8564_c180116;full_length_coverage=1;non_full_length_coverage=0;isoform_length=2944;expected_accuracy=0.996	gnl|BL_ORD_ID|56002 cb8564_c51722/f1p1/3503	25	2522	2520	2944	67	2560	2789	3212
cb8564_c15404/f1p9/2596 isoform=cb8564_c15404;full_length_coverage=1;non_full_length_coverage=9;isoform_length=2596;expected_accuracy=0.991	gnl|BL_ORD_ID|56644 cb8564_c50505/f1p0/3091	441	2596	7	443	592	2741	8	441
cb18456_c1748/f1p0/1180	gnl|BL_ORD_ID|12159 cb8564_c125300/f1p1/2646 isoform=cb8564_c125300;full_length_coverage=1;non_full_length_coverage=1;isoform_length=2646;expected_accuracy=0.995	243	1188	4	248	976	32	1358	1114
cb6978_c16/f1p1/2475	gnl|BL_ORD_ID|50220 cb6978_c13/f1p0/2528	1	1906	1907	2417	1	1909	2011	2521
cb317_c5/f1p0/3451 isoform=cb317_c5;full_length_coverage=1;non_full_length_coverage=0;isoform_length=3451;expected_accuracy=0.998	gnl|BL_ORD_ID|37313 cb317_c17/f1p0/4336 isoform=cb317_c17;full_length_coverage=1;non_full_length_coverage=0;isoform_length=4336;expected_accuracy=0.993	1	3038	3033	3317	1	3026	3251	3533
cb965_c18/f1p0/2545 isoform=cb965_c18;full_length_coverage=1;non_full_length_coverage=0;isoform_length=2545;expected_accuracy=1.000	gnl|BL_ORD_ID|20097 cb965_c6/f2p0/3258 isoform=cb965_c6;full_length_coverage=2;non_full_length_coverage=0;isoform_length=3258;expected_accuracy=0.996	1	1828	1831	2484	541	2367	2595	3258
cb8564_c956/f2p0/2664	gnl|BL_ORD_ID|14229 cb8564_c4515/f1p0/2986	518	2654	9	517	795	292	1	508

isoform=cb8564_c956;full_length_coverage=2;non_full_length_coverage=0;isoform_length=2664;expected_accuracy=1.000	isoform=cb8564_c4515;full_length_coverage=1;non_full_length_coverage=0;isoform_length=2986;expected_accuracy=1.000						9		
cb2789_c1/f3p0/2541 isoform=cb2789_c1;full_length_coverage=3;non_full_length_coverage=0;isoform_length=2541;expected_accuracy=1.000	gnl|BL_ORD_ID|68853 cb2789_c11/f1p0/2870	317	2541	49	319	442	2666	21	279
cb295_c13/f1p1/3878 isoform=cb295_c13;full_length_coverage=1;non_full_length_coverage=1;isoform_length=3878;expected_accuracy=0.994	gnl|BL_ORD_ID|67912 cb18646_c8/f1p2/6225	1	3634	3633	3878	1980	5636	5870	6117
cb8564_c181853/f1p0/2119 isoform=cb8564_c181853;full_length_coverage=1;non_full_length_coverage=0;isoform_length=2119;expected_accuracy=0.997	gnl|BL_ORD_ID|55464 cb8564_c42285/f1p0/3370	1	1339	1334	2119	1	1339	2167	2952
cb3143_c17/f1p0/2870 isoform=cb3143_c17;full_length_coverage=1;non_full_length_coverage=0;isoform_length=2870;expected_accuracy=0.993	gnl|BL_ORD_ID|70201 cb3143_c20/f1p0/3633	160	2870	1	163	785	3490	25	186
cb8564_c49958/f1p0/3088 isoform=cb8564_c49958;full_length_coverage=1;non_full_length_coverage=0;isoform_length=3088;expected_accuracy=0.995	gnl|BL_ORD_ID|56316 cb8564_c74191/f1p2/3383	1353	3088	1	1355	1550	3284	1	1355
cb3143_c17/f1p0/2870 isoform=cb3143_c17;full_length_coverage=1;non_full_length_coverage=0;isoform_length=2870;expected_accu	gnl|BL_ORD_ID|37814 cb3143_c5/f1p3/3440 isoform=cb3143_c5;full_length_coverage=1;non_full_length_coverage=3;isoform_length=3440;expected_accura	160	2870	10	163	675	3379	11	162

racy=0.993	cy=0.999								
cb8564_c45300/f1p4/3014 isoform=cb8564_c45300;full_length_coverage=1;non_full_length_coverage=4;isoform_length=3014;expected_accuracy=0.996	gnl|BL_ORD_ID|58037 cb8564_c87188/f1p1/3426	243	3014	21	246	505	3279	127	348
cb8564_c48473/f1p2/2701 isoform=cb8564_c48473;full_length_coverage=1;non_full_length_coverage=2;isoform_length=2701;expected_accuracy=0.999	gnl|BL_ORD_ID|16331 cb8564_c75969/f1p1/2798 isoform=cb8564_c75969;full_length_coverage=1;non_full_length_coverage=1;isoform_length=2798;expected_accuracy=0.997	220	2644	17	225	354	2798	1	210
cb6719_c52/f1p0/2293	gnl|BL_ORD_ID|6004 cb6719_c31/f1p0/2434 isoform=cb6719_c31;full_length_coverage=1;non_full_length_coverage=0;isoform_length=2434;expected_accuracy=0.995	641	2223	7	640	779	2344	3	634
cb8564_c90797/f1p0/2864	gnl|BL_ORD_ID|56983 cb8564_c79327/f1p1/3074	15	2313	2308	2607	97	2394	2516	2815
cb1224_c20/f1p0/3308 isoform=cb1224_c20;full_length_coverage=1;non_full_length_coverage=0;isoform_length=3308;expected_accuracy=0.997	gnl|BL_ORD_ID|59284 cb1224_c19/f1p1/4162	1	3180	3175	3308	203	3384	3825	3955
cb4016_c32/f1p0/1913	gnl|BL_ORD_ID|39537 cb4016_c20/f1p1/2959 isoform=cb4016_c20;full_length_coverage=1;non_full_length_coverage=1;isoform_length=2959;expected_accuracy=0.992	191	1930	23	194	1125	2866	526	700
cb18793_c2/f1p2/5878	gnl|BL_ORD_ID|34963 cb18793_c1/f1p1/6347 isoform=cb18793_c1;full_length_coverage=1;non_full_length_coverage=1;isoform_length=6347;expected_accur	1	4389	4387	5867	6	4389	4875	6346

	acy=0.994								
cb5769_c3/f1p0/2310	gnl|BL_ORD_ID|47115 cb5769_c1/f2p0/2914 isoform=cb5769_c1;full_length_coverage=2;non_full_length_coverage=0;isoform_length=2914;expected_accuracy=0.996	201	2277	1	205	839	2914	86	289
cb18456_c1748/f1p0/1180	gnl|BL_ORD_ID|11861 cb8564_c126350/f1p2/2554 isoform=cb8564_c126350;full_length_coverage=1;non_full_length_coverage=2;isoform_length=2554;expected_accuracy=0.996	243	1188	7	248	974	30	1346	1112
cb8564_c40598/f1p4/3119	gnl|BL_ORD_ID|16886 cb8564_c50727/f1p0/3201 isoform=cb8564_c50727;full_length_coverage=1;non_full_length_coverage=0;isoform_length=3201;expected_accuracy=0.999	1	2732	2729	3053	3	2756	2877	3201
cb8564_c35645/f1p0/2804	gnl|BL_ORD_ID|54405 cb8564_c35890/f1p0/3157	150	2716	1	151	270	2836	7	157
cb5204_c7/f1p0/3044	gnl|BL_ORD_ID|46181 cb5204_c6/f1p1/2901 isoform=cb5204_c6;full_length_coverage=1;non_full_length_coverage=1;isoform_length=2901;expected_accuracy=0.997	1	1717	1717	2763	23	1739	1855	2901
cb8564_c132218/f1p5/2319	gnl|BL_ORD_ID|55921 cb8564_c48990/f1p2/2740	1079	2249	1	1075	1549	2719	15	1087
cb5515_c1/f1p0/1999 isoform=cb5515_c1;full_length_coverage=1;non_full_length_coverage=0;isoform_length=1999;expected_accuracy=0.996	gnl|BL_ORD_ID|47062 cb5515_c0/f1p0/3081 isoform=cb5515_c0;full_length_coverage=1;non_full_length_coverage=0;isoform_length=3081;expected_accuracy=0.995	1	1372	1371	1999	5	1379	2244	2876
cb454_c4/f3p0/2268	gnl|BL_ORD_ID|42152 cb454_c9/f1p0/2795	221	2255	1	220	763	279	442	659

isoform=cb454_c4;full_length_coverage=3;non_full_length_coverage=0;isoform_length=2268;expected_accuracy=1.000	isoform=cb454_c9;full_length_coverage=1;non_full_length_coverage=0;isoform_length=2795;expected_accuracy=0.992						5		
cb8564_c15573/f1p0/3752 isoform=cb8564_c15573;full_length_coverage=1;non_full_length_coverage=0;isoform_length=3752;expected_accuracy=0.997	gnl|BL_ORD_ID|14333 cb8564_c39696/f4p3/4068 isoform=cb8564_c39696;full_length_coverage=4;non_full_length_coverage=3;isoform_length=4068;expected_accuracy=0.997	1	2900	2896	3752	168	3079	3194	4050
cb8564_c37361/f1p0/3076	gnl|BL_ORD_ID|16681 cb8564_c54233/f1p1/3194 isoform=cb8564_c54233;full_length_coverage=1;non_full_length_coverage=1;isoform_length=3194;expected_accuracy=0.995	2	2481	2477	3066	8	2504	2605	3194
cb8564_c83113/f1p0/2883 isoform=cb8564_c83113;full_length_coverage=1;non_full_length_coverage=0;isoform_length=2883;expected_accuracy=0.996	gnl|BL_ORD_ID|57807 cb8564_c81227/f1p0/3713	184	2878	1	187	744	3439	11	198
cb4016_c32/f1p0/1913	gnl|BL_ORD_ID|40899 cb4016_c15/f1p1/2488 isoform=cb4016_c15;full_length_coverage=1;non_full_length_coverage=1;isoform_length=2488;expected_accuracy=1.000	191	1930	7	194	749	2488	132	321
cb18456_c3695/f1p0/1345	gnl|BL_ORD_ID|52656 cb8564_c10132/f1p8/3379	1	1051	1047	1293	235	1285	1460	1706
cb8564_c129638/f1p0/2479	gnl|BL_ORD_ID|13105 cb8564_c165846/f1p5/3154 isoform=cb8564_c165846;full_length_coverage=1;non_full_length_coverage=5;isoform_length=3154;expected_accuracy=0.995	1	1627	1623	2475	140	1766	2171	3023
cb8564_c186356/f1p0/2744	gnl|BL_ORD_ID|55391 cb8564_c40278/f3p2/3083	1	1396	1393	2744	1	139	1516	286

isoform=cb8564_c186356;full_length_coverage=1;non_full_length_coverage=0;isoform_length=2744;expected_accuracy=0.998							6		7
cb1030_c20/f1p1/3218	gnl|BL_ORD_ID|1808 cb1030_c5/f2p0/3347 isoform=cb1030_c5;full_length_coverage=2;non_full_length_coverage=0;isoform_length=3347;expected_accuracy=0.997	1	2775	2778	3173	1	2774	2900	3295
cb8564_c31577/f2p6/3328 isoform=cb8564_c31577;full_length_coverage=2;non_full_length_coverage=6;isoform_length=3328;expected_accuracy=0.999	gnl|BL_ORD_ID|13106 cb8564_c16615/f1p2/3838 isoform=cb8564_c16615;full_length_coverage=1;non_full_length_coverage=2;isoform_length=3838;expected_accuracy=1.000	396	3328	85	396	911	3830	1	313
cb4972_c64/f1p0/2792	gnl|BL_ORD_ID|42088 cb4972_c10/f2p1/2837 isoform=cb4972_c10;full_length_coverage=2;non_full_length_coverage=1;isoform_length=2837;expected_accuracy=1.000	1	1345	1344	2678	5	1347	1477	2811
cb1492_c3/f1p2/4122 isoform=cb1492_c3;full_length_coverage=1;non_full_length_coverage=2;isoform_length=4122;expected_accuracy=0.994	gnl|BL_ORD_ID|29081 cb1492_c1/f1p2/4305 isoform=cb1492_c1;full_length_coverage=1;non_full_length_coverage=2;isoform_length=4305;expected_accuracy=0.998	1292	3966	1	1291	1621	4304	25	1316
cb8564_c10713/f1p4/3120 isoform=cb8564_c10713;full_length_coverage=1;non_full_length_coverage=4;isoform_length=3120;expected_accuracy=0.992	gnl|BL_ORD_ID|12601 cb8564_c13656/f1p0/3139 isoform=cb8564_c13656;full_length_coverage=1;non_full_length_coverage=0;isoform_length=3139;expected_accuracy=0.999	24	2667	2666	3062	1	2641	2742	3139
cb4949_c7/f1p0/2266 isoform=cb4949_c7;full_length_coverage=1;non_full_length_coverage=0;isoform_length=2266;expected_accur	gnl|BL_ORD_ID|73116 cb4949_c5/f1p0/2987	10	1758	1755	2266	17	1753	2437	2948

acy=0.999									
cb8564_c83062/f1p1/2429	gnl|BL_ORD_ID|58137 cb8564_c89794/f1p1/2867	1	1638	1637	2423	2	1639	1812	2598
cb8456_c137/f1p0/1507 isoform=cb8456_c137;full_length_coverage=1;non_full_length_coverage=0;isoform_length=1507;expected_accuracy=0.994	gnl|BL_ORD_ID|52551 cb8456_c69/f1p0/2253	650	1507	9	654	1028	1887	9	654
cb4686_c4/f1p0/2845	gnl|BL_ORD_ID|42972 cb4686_c2/f1p0/3164 isoform=cb4686_c2;full_length_coverage=1;non_full_length_coverage=0;isoform_length=3164;expected_accuracy=0.995	106	1851	1850	2807	1	1745	2209	3164
cb868_c29/f1p0/3279	gnl|BL_ORD_ID|57583 cb868_c6/f1p0/3726	1	1514	1512	2901	601	2114	2305	3694
cb12714_c29/f1p2/1583	gnl|BL_ORD_ID|11483 cb8564_c12665/f1p15/2806 isoform=cb8564_c12665;full_length_coverage=1;non_full_length_coverage=15;isoform_length=2806;expected_accuracy=0.995	1	1296	1293	1592	2	1303	2370	2671
cb8564_c44420/f1p0/2853 isoform=cb8564_c44420;full_length_coverage=1;non_full_length_coverage=0;isoform_length=2853;expected_accuracy=0.992	gnl|BL_ORD_ID|13472 cb8564_c182192/f1p6/3150 isoform=cb8564_c182192;full_length_coverage=1;non_full_length_coverage=6;isoform_length=3150;expected_accuracy=0.995	14	1487	1484	2851	98	1570	1788	3150
cb8564_c180679/f1p0/2109 isoform=cb8564_c180679;full_length_coverage=1;non_full_length_coverage=0;isoform_length=2109;expected_accuracy=0.999	gnl|BL_ORD_ID|13611 cb8564_c2184/f1p0/2606 isoform=cb8564_c2184;full_length_coverage=1;non_full_length_coverage=0;isoform_length=2606;expected_accuracy=0.995	198	2109	1	202	692	2603	1	202
cb7337_c6/f1p0/1841	gnl|BL_ORD_ID|50484 cb7337_c1/f1p0/2641	1	1684	1682	1841	13	169	1808	196

isoform=cb7337_c6;full_length_coverage=1;non_full_length_coverage=0;isoform_length=1841;expected_accuracy=0.992							8		6
cb8564_c70954/f1p0/2365	gnl|BL_ORD_ID|17359 cb8564_c89607/f1p0/2600 isoform=cb8564_c89607;full_length_coverage=1;non_full_length_coverage=0;isoform_length=2600;expected_accuracy=1.000	1	1181	1180	2257	1	1183	1441	2516
cb8564_c5070/f1p0/2861	gnl|BL_ORD_ID|56564 cb8564_c5063/f1p0/3368	10	1660	1659	2831	61	1711	1604	2775
cb8564_c4645/f1p1/2620 isoform=cb8564_c4645;full_length_coverage=1;non_full_length_coverage=1;isoform_length=2620;expected_accuracy=0.994	gnl|BL_ORD_ID|14567 cb8564_c4647/f1p2/2774 isoform=cb8564_c4647;full_length_coverage=1;non_full_length_coverage=2;isoform_length=2774;expected_accuracy=0.992	1	1828	1829	2567	32	1890	2017	2774
cb8564_c37361/f1p0/3076	gnl|BL_ORD_ID|15924 cb8564_c52804/f1p5/3221 isoform=cb8564_c52804;full_length_coverage=1;non_full_length_coverage=5;isoform_length=3221;expected_accuracy=0.999	1	2481	2477	3070	1	2480	2581	3175
cb8564_c41756/f1p0/2738 isoform=cb8564_c41756;full_length_coverage=1;non_full_length_coverage=0;isoform_length=2738;expected_accuracy=0.996	gnl|BL_ORD_ID|55574 cb8564_c45699/f1p0/3005	1	2173	2170	2738	1	2159	2282	2848
cb8564_c36011/f1p0/3015	gnl|BL_ORD_ID|17556 cb8564_c9820/f2p0/3032 isoform=cb8564_c9820;full_length_coverage=2;non_full_length_coverage=0;isoform_length=3032;expected_accuracy=0.995	10	1718	1716	2617	9	1704	2131	3032
cb2279_c4/f1p2/2606	gnl|BL_ORD_ID|36531 cb2279_c0/f10p3/2834	1	1359	1357	2593	13	136	1580	281

isoform=cb2279_c4;full_length_coverage=1;non_full_length_coverage=2;isoform_length=2606;expected_accuracy=0.996	isoform=cb2279_c0;full_length_coverage=10;non_full_length_coverage=3;isoform_length=2834;expected_accuracy=1.000						8		6
cb12302_c31/f1p0/1548 isoform=cb12302_c31;full_length_coverage=1;non_full_length_coverage=0;isoform_length=1548;expected_accuracy=1.000	gnl|BL_ORD_ID|27352 cb1380_c18/f1p6/2264 isoform=cb1380_c18;full_length_coverage=1;non_full_length_coverage=6;isoform_length=2264;expected_accuracy=0.997	324	1548	8	329	560	1784	1	316
cb8564_c90797/f1p0/2864	gnl|BL_ORD_ID|12899 cb8564_c181074/f1p0/2725 isoform=cb8564_c181074;full_length_coverage=1;non_full_length_coverage=0;isoform_length=2725;expected_accuracy=1.000	17	2307	2308	2643	1	2268	2395	2725
cb6471_c27/f1p0/2602	gnl|BL_ORD_ID|4198 cb6471_c20/f1p0/3013 isoform=cb6471_c20;full_length_coverage=1;non_full_length_coverage=0;isoform_length=3013;expected_accuracy=0.992	1	1863	1864	2598	257	2116	2275	3012
cb3603_c31/f1p0/4927 isoform=cb3603_c31;full_length_coverage=1;non_full_length_coverage=0;isoform_length=4927;expected_accuracy=0.996	gnl|BL_ORD_ID|39466 cb3603_c36/f1p0/5599 isoform=cb3603_c36;full_length_coverage=1;non_full_length_coverage=0;isoform_length=5599;expected_accuracy=0.992	205	4072	4070	4927	210	4105	4651	5508
cb8564_c46104/f1p0/3297	gnl|BL_ORD_ID|14810 cb8564_c34492/f1p1/3811 isoform=cb8564_c34492;full_length_coverage=1;non_full_length_coverage=1;isoform_length=3811;expected_accuracy=0.991	235	3238	1	236	771	3811	1	239
cb8564_c49562/f1p0/2838 isoform=cb8564_c49562;full_length_coverage=1;non_full_length_coverage=0;isoform_length=2838;expected_	gnl|BL_ORD_ID|56623 cb8564_c49310/f1p2/3045	23	2331	2327	2838	65	2378	2485	2998

<table>
<tr><td>accuracy=0.995</td><td></td><td></td><td></td><td></td><td></td><td></td><td></td><td></td><td></td></tr>
<tr><td>cb8564_c34238/f4p1/4317 isoform=cb8564_c34238;full_length_coverage=4;non_full_length_coverage=1;isoform_length=4317;expected_accuracy=1.000</td><td>gnl|BL_ORD_ID|14819 cb8564_c40333/f1p2/4446 isoform=cb8564_c40333;full_length_coverage=1;non_full_length_coverage=2;isoform_length=4446;expected_accuracy=0.998</td><td>15</td><td>3270</td><td>3266</td><td>4165</td><td>1</td><td>3280</td><td>3545</td><td>4446</td></tr>
<tr><td>cb7023_c0/f2p1/2542 isoform=cb7023_c0;full_length_coverage=2;non_full_length_coverage=1;isoform_length=2542;expected_accuracy=0.996</td><td>gnl|BL_ORD_ID|3664 cb7023_c4/f1p0/2631 isoform=cb7023_c4;full_length_coverage=1;non_full_length_coverage=0;isoform_length=2631;expected_accuracy=0.995</td><td>1</td><td>2041</td><td>2039</td><td>2519</td><td>2</td><td>2043</td><td>2151</td><td>2631</td></tr>
<tr><td>cb8564_c15951/f1p1/3969 isoform=cb8564_c15951;full_length_coverage=1;non_full_length_coverage=1;isoform_length=3969;expected_accuracy=0.997</td><td>gnl|BL_ORD_ID|13700 cb8564_c20054/f1p0/4502 isoform=cb8564_c20054;full_length_coverage=1;non_full_length_coverage=0;isoform_length=4502;expected_accuracy=1.000</td><td>1</td><td>3328</td><td>3329</td><td>3969</td><td>24</td><td>3352</td><td>3762</td><td>4404</td></tr>
<tr><td>cb8564_c77380/f2p0/3010 isoform=cb8564_c77380;full_length_coverage=2;non_full_length_coverage=0;isoform_length=3010;expected_accuracy=0.998</td><td>gnl|BL_ORD_ID|11240 cb8564_c145553/f1p3/3270 isoform=cb8564_c145553;full_length_coverage=1;non_full_length_coverage=3;isoform_length=3270;expected_accuracy=0.996</td><td>141</td><td>1900</td><td>1899</td><td>3010</td><td>1</td><td>1760</td><td>2145</td><td>3253</td></tr>
<tr><td>cb6277_c14/f1p1/2588 isoform=cb6277_c14;full_length_coverage=1;non_full_length_coverage=1;isoform_length=2588;expected_accuracy=0.995</td><td>gnl|BL_ORD_ID|3491 cb6277_c8/f1p0/2793 isoform=cb6277_c8;full_length_coverage=1;non_full_length_coverage=0;isoform_length=2793;expected_accuracy=0.999</td><td>13</td><td>1436</td><td>1436</td><td>2495</td><td>1</td><td>1425</td><td>1731</td><td>2793</td></tr>
<tr><td>cb8564_c39136/f1p0/3036 isoform=cb8564_c39136;full_length_coverage=1;non_full_length_coverage=0;isoform_length=3036;expected_accuracy=0.998</td><td>gnl|BL_ORD_ID|55182 cb8564_c34825/f1p2/3200</td><td>11</td><td>2089</td><td>2085</td><td>3036</td><td>6</td><td>2119</td><td>2246</td><td>3196</td></tr>
<tr><td>cb4016_c32/f1p0/1913</td><td>gnl|BL_ORD_ID|40898 cb4016_c16/f3p2/2553</td><td>191</td><td>1930</td><td>7</td><td>194</td><td>719</td><td>245</td><td>103</td><td>291</td></tr>
</table>

	isoform=cb4016_c16;full_length_coverage=3;non_full_length_coverage=2;isoform_length=2553;expected_accuracy=0.996						8		
cb8564_c134351/f3p2/2684 isoform=cb8564_c134351;full_length_coverage=3;non_full_length_coverage=2;isoform_length=2684;expected_accuracy=0.996	gnl|BL_ORD_ID|11179 cb8564_c11761/f4p0/2940 isoform=cb8564_c11761;full_length_coverage=4;non_full_length_coverage=0;isoform_length=2940;expected_accuracy=0.996	1	1802	1798	2629	1	1807	2043	2876
cb5725_c35/f1p0/2272 isoform=cb5725_c35;full_length_coverage=1;non_full_length_coverage=0;isoform_length=2272;expected_accuracy=1.000	gnl|BL_ORD_ID|74341 cb5725_c19/f1p0/3053	1	1369	1366	2211	51	1418	2201	3046
cb8564_c42677/f1p0/3180	gnl|BL_ORD_ID|14410 cb8564_c44256/f1p1/3267 isoform=cb8564_c44256;full_length_coverage=1;non_full_length_coverage=1;isoform_length=3267;expected_accuracy=0.995	21	2132	2128	2842	14	2119	2553	3266
cb12310_c27/f1p0/1191	gnl|BL_ORD_ID|23378 cb12310_c7/f2p0/1721 isoform=cb12310_c7;full_length_coverage=2;non_full_length_coverage=0;isoform_length=1721;expected_accuracy=1.000	210	991	1	213	940	1721	3	216
cb8564_c15653/f1p4/2475	gnl|BL_ORD_ID|58013 cb8564_c86635/f1p17/2750	1	2293	2289	2482	1	2293	2512	2705
cb4233_c109/f1p3/2092 isoform=cb4233_c109;full_length_coverage=1;non_full_length_coverage=3;isoform_length=2092;expected_accuracy=0.994	gnl|BL_ORD_ID|44053 cb4233_c239/f16p1/2275 isoform=cb4233_c239;full_length_coverage=16;non_full_length_coverage=1;isoform_length=2275;expected_accuracy=1.000	1	1522	1517	2086	50	1580	1704	2275
cb8564_c46081/f1p0/3421	gnl|BL_ORD_ID|16878 cb8564_c48607/f1p0/4177	1299	3321	1	1298	2157	417	22	131

	isoform=cb8564_c48607;full_length_coverage=1;non_full_length_coverage=0;isoform_length=4177;expected_accuracy=0.991						7		5
cb8564_c39546/f1p2/3023	gnl|BL_ORD_ID|14410 cb8564_c44256/f1p1/3267 isoform=cb8564_c44256;full_length_coverage=1;non_full_length_coverage=1;isoform_length=3267;expected_accuracy=0.995	1	2122	2118	2835	1	2119	2553	3267
cb8564_c38251/f1p0/3243	gnl|BL_ORD_ID|17429 cb8564_c85447/f1p0/3196 isoform=cb8564_c85447;full_length_coverage=1;non_full_length_coverage=0;isoform_length=3196;expected_accuracy=0.997	1	2439	2438	2973	3	2413	2660	3195
cb9863_c0/f2p0/1727 isoform=cb9863_c0;full_length_coverage=2;non_full_length_coverage=0;isoform_length=1727;expected_accuracy=1.000	gnl|BL_ORD_ID|60037 cb9863_c3/f1p0/5975	1	1197	1192	1727	6	1202	4862	5398
cb8_c5/f1p1/4485 isoform=cb8_c5;full_length_coverage=1;non_full_length_coverage=1;isoform_length=4485;expected_accuracy=0.997	gnl|BL_ORD_ID|59026 cb8_c6/f1p0/4495	263	4291	1	268	469	4499	6	273
cb8564_c159910/f2p1/2333 isoform=cb8564_c159910;full_length_coverage=2;non_full_length_coverage=1;isoform_length=2333;expected_accuracy=0.991	gnl|BL_ORD_ID|10006 cb8564_c109862/f2p0/3191 isoform=cb8564_c109862;full_length_coverage=2;non_full_length_coverage=0;isoform_length=3191;expected_accuracy=0.999	164	2325	1	166	1032	3191	1	168
cb8564_c10872/f1p2/2947 isoform=cb8564_c10872;full_length_coverage=1;non_full_length_coverage=2;isoform_length=2947;expected_	gnl|BL_ORD_ID|10743 cb8564_c10713/f1p4/3120 isoform=cb8564_c10713;full_length_coverage=1;non_full_length_coverage=4;isoform_length=3120;expected_ac	1	2667	2666	2925	3	2666	2806	3066

accuracy=0.994	curacy=0.992								
cb18287_c2/f1p0/1786 isoform=cb18287_c2;full_length_coverage=1;non_full_length_coverage=0;isoform_length=1786;expected_accuracy=0.996	gnl|BL_ORD_ID|15918 cb8564_c72237/f1p0/2397 isoform=cb8564_c72237;full_length_coverage=1;non_full_length_coverage=0;isoform_length=2397;expected_accuracy=0.995	346	1785	69	345	960	2397	3	278
cb8564_c74375/f1p1/2718 isoform=cb8564_c74375;full_length_coverage=1;non_full_length_coverage=1;isoform_length=2718;expected_accuracy=1.000	gnl|BL_ORD_ID|18074 cb8564_c79813/f1p1/2853 isoform=cb8564_c79813;full_length_coverage=1;non_full_length_coverage=1;isoform_length=2853;expected_accuracy=1.000	1	1609	1608	2717	1	1636	1745	2853
cb781_c2/f3p0/1827 isoform=cb781_c2;full_length_coverage=3;non_full_length_coverage=0;isoform_length=1827;expected_accuracy=0.999	gnl|BL_ORD_ID|7264 cb781_c0/f11p1/1956 isoform=cb781_c0;full_length_coverage=11;non_full_length_coverage=1;isoform_length=1956;expected_accuracy=1.000	1	1243	1243	1827	24	1266	1371	1955
cb8687_c8/f1p0/1570	gnl|BL_ORD_ID|18631 cb8687_c5/f1p0/1932 isoform=cb8687_c5;full_length_coverage=1;non_full_length_coverage=0;isoform_length=1932;expected_accuracy=1.000	581	1549	7	581	929	1897	18	592
cb8564_c7774/f3p4/1962 isoform=cb8564_c7774;full_length_coverage=3;non_full_length_coverage=4;isoform_length=1962;expected_accuracy=1.000	gnl|BL_ORD_ID|56567 cb8564_c5087/f1p0/3205	1	1461	1461	1962	10	1477	2633	3127
cb7143_c5/f1p0/2139	gnl|BL_ORD_ID|6196 cb7143_c4/f1p0/2390 isoform=cb7143_c4;full_length_coverage=1;non_full_length_coverage=0;isoform_length=2390;expected_accuracy=0.993	10	1266	1264	2137	1	1252	1516	2390
cb8564_c5107/f1p2/3747	gnl|BL_ORD_ID|16247 cb8564_c5117/f1p0/4873	15	1936	1934	3722	7	192	3088	487

isoform=cb8564_c5107;full_length_coverage=1;non_full_length_coverage=2;isoform_length=3747;expected_accuracy=0.994	isoform=cb8564_c5117;full_length_coverage=1;non_full_length_coverage=0;isoform_length=4873;expected_accuracy=0.999						1		1
cb5346_c6/f1p0/1741 isoform=cb5346_c6;full_length_coverage=1;non_full_length_coverage=0;isoform_length=1741;expected_accuracy=0.997	gnl|BL_ORD_ID|73808 cb5346_c0/f1p0/3122	185	1740	31	183	1169	2722	4	156
cb8564_c17933/f1p0/2278 isoform=cb8564_c17933;full_length_coverage=1;non_full_length_coverage=0;isoform_length=2278;expected_accuracy=1.000	gnl|BL_ORD_ID|12288 cb8564_c14928/f1p0/2382 isoform=cb8564_c14928;full_length_coverage=1;non_full_length_coverage=0;isoform_length=2382;expected_accuracy=0.995	1	1525	1525	2244	1	1527	1663	2382
cb8564_c42906/f1p2/3181	gnl|BL_ORD_ID|55939 cb8564_c49630/f1p4/3487	2	2894	2894	3144	1	2913	3243	3493
cb8564_c45300/f1p4/3014 isoform=cb8564_c45300;full_length_coverage=1;non_full_length_coverage=4;isoform_length=3014;expected_accuracy=0.996	gnl|BL_ORD_ID|16149 cb8564_c47162/f1p0/3361 isoform=cb8564_c47162;full_length_coverage=1;non_full_length_coverage=0;isoform_length=3361;expected_accuracy=0.996	244	3014	1	246	592	3361	1	243
cb6889_c8/f1p0/2744	gnl|BL_ORD_ID|47747 cb6889_c13/f1p0/2673	330	2363	81	335	588	2620	1	255
cb8564_c36731/f1p2/2761	gnl|BL_ORD_ID|14539 cb8564_c46562/f2p3/3196 isoform=cb8564_c46562;full_length_coverage=2;non_full_length_coverage=3;isoform_length=3196;expected_accuracy=0.995	1	1977	1972	2734	7	2003	2433	3195
cb8564_c178226/f1p0/2728 isoform=cb8564_c178226;full_length_coverage=1;non_full_length_coverage=0;isoform_length=2728;expected	gnl|BL_ORD_ID|55866 cb8564_c47522/f1p0/2954	1284	2728	1	1288	1401	2847	1	1297

<table>
<tr><td>_accuracy=0.998</td><td></td><td></td><td></td><td></td><td></td><td></td><td></td><td></td><td></td></tr>
<tr><td>cb416_c19/f1p0/2939</td><td>gnl|BL_ORD_ID|72149 cb416_c36/f1p0/3277</td><td>11</td><td>1499</td><td>1494</td><td>2849</td><td>9</td><td>1499</td><td>1636</td><td>2997</td></tr>
<tr><td>cb14220_c10/f1p1/6717 isoform=cb14220_c10;full_length_coverage=1;non_full_length_coverage=1;isoform_length=6717;expected_accuracy=0.993</td><td>gnl|BL_ORD_ID|63133 cb14220_c7/f1p0/6933</td><td>2469</td><td>6717</td><td>133</td><td>2471</td><td>2660</td><td>6928</td><td>6</td><td>2351</td></tr>
<tr><td>cb8564_c44123/f1p1/3230</td><td>gnl|BL_ORD_ID|55939 cb8564_c49630/f1p4/3487</td><td>14</td><td>2911</td><td>2911</td><td>3161</td><td>12</td><td>2913</td><td>3243</td><td>3493</td></tr>
<tr><td>cb8564_c178226/f1p0/2728 isoform=cb8564_c178226;full_length_coverage=1;non_full_length_coverage=0;isoform_length=2728;expected_accuracy=0.998</td><td>gnl|BL_ORD_ID|14368 cb8564_c34638/f1p0/2817 isoform=cb8564_c34638;full_length_coverage=1;non_full_length_coverage=0;isoform_length=2817;expected_accuracy=0.992</td><td>1284</td><td>2728</td><td>1</td><td>1288</td><td>1388</td><td>2815</td><td>1</td><td>1284</td></tr>
<tr><td>cb10054_c49/f1p1/1123 isoform=cb10054_c49;full_length_coverage=1;non_full_length_coverage=1;isoform_length=1123;expected_accuracy=1.000</td><td>gnl|BL_ORD_ID|722 cb10054_c29/f1p0/1317 isoform=cb10054_c29;full_length_coverage=1;non_full_length_coverage=0;isoform_length=1317;expected_accuracy=0.994</td><td>258</td><td>1123</td><td>5</td><td>262</td><td>447</td><td>1317</td><td>4</td><td>264</td></tr>
<tr><td>cb8564_c41756/f1p0/2738 isoform=cb8564_c41756;full_length_coverage=1;non_full_length_coverage=0;isoform_length=2738;expected_accuracy=0.996</td><td>gnl|BL_ORD_ID|55962 cb8564_c50282/f1p0/4402</td><td>15</td><td>2173</td><td>2170</td><td>2738</td><td>1457</td><td>3619</td><td>3742</td><td>4310</td></tr>
<tr><td>cb5902_c2/f1p0/2787</td><td>gnl|BL_ORD_ID|74623 cb5902_c0/f1p0/2977</td><td>1</td><td>2521</td><td>2518</td><td>2775</td><td>73</td><td>2598</td><td>2736</td><td>2993</td></tr>
<tr><td>cb8564_c53418/f1p0/2602 isoform=cb8564_c53418;full_length_coverage=1;non_full_length_coverage=0;isoform_length=2602;expected_</td><td>gnl|BL_ORD_ID|53915 cb8564_c14767/f1p3/2856</td><td>1163</td><td>2499</td><td>1</td><td>1168</td><td>1268</td><td>2607</td><td>1</td><td>1167</td></tr>
</table>

accuracy=0.991									
cb3228_c3/f1p0/2752 isoform=cb3228_c3;full_length_coverage=1;non_full_length_coverage=0;isoform_length=2752;expected_accuracy=0.997	gnl|BL_ORD_ID|70986 cb3228_c4/f1p0/4440	1	1663	1663	2648	33	1697	3172	4157
cb12047_c134/f1p0/1727 isoform=cb12047_c134;full_length_coverage=1;non_full_length_coverage=0;isoform_length=1727;expected_accuracy=0.999	gnl|BL_ORD_ID|54487 cb8564_c124166/f1p0/2223	1	1006	1001	1716	22	1027	1133	1848
cb3000_c3/f1p0/1940 isoform=cb3000_c3;full_length_coverage=1;non_full_length_coverage=0;isoform_length=1940;expected_accuracy=0.991	gnl|BL_ORD_ID|36712 cb3000_c1/f1p0/2248 isoform=cb3000_c1;full_length_coverage=1;non_full_length_coverage=0;isoform_length=2248;expected_accuracy=0.997	1	1186	1185	1940	7	1193	1312	2074
cb4174_c11/f2p0/2738 isoform=cb4174_c11;full_length_coverage=2;non_full_length_coverage=0;isoform_length=2738;expected_accuracy=1.000	gnl|BL_ORD_ID|72312 cb4174_c3/f1p0/3410	1	2444	2444	2695	54	2501	2899	3149
cb6391_c2/f2p0/2236 isoform=cb6391_c2;full_length_coverage=2;non_full_length_coverage=0;isoform_length=2236;expected_accuracy=0.998	gnl|BL_ORD_ID|3495 cb6391_c11/f1p0/2658 isoform=cb6391_c11;full_length_coverage=1;non_full_length_coverage=0;isoform_length=2658;expected_accuracy=0.993	225	2236	38	227	521	2527	41	229
cb737_c27/f1p0/4113	gnl|BL_ORD_ID|49447 cb737_c20/f1p0/4370	1	3348	3347	4033	59	3406	3563	4252
cb8564_c16431/f1p2/4911 isoform=cb8564_c16431;full_length_coverage=1;non_full_length_coverage=2;isoform_length=4911;expected_	gnl|BL_ORD_ID|64476 cb15140_c10/f1p1/5378	1	4785	4785	4911	312	5093	5204	5330

accuracy=0.991									
cb8564_c146029/f4p1/2251 isoform=cb8564_c146029;full_length_coverage=4;non_full_length_coverage=1;isoform_length=2251;expected_accuracy=1.000	gnl|BL_ORD_ID|9245 cb8564_c1146/f2p1/2474 isoform=cb8564_c1146;full_length_coverage=2;non_full_length_coverage=1;isoform_length=2474;expected_accuracy=0.996	1	1259	1258	2250	1	1259	1482	2474
cb8564_c36688/f1p0/3356	gnl|BL_ORD_ID|16149 cb8564_c47162/f1p0/3361 isoform=cb8564_c47162;full_length_coverage=1;non_full_length_coverage=0;isoform_length=3361;expected_accuracy=0.996	402	3171	6	403	592	3361	4	402
cb6406_c65/f1p0/1830 isoform=cb6406_c65;full_length_coverage=1;non_full_length_coverage=0;isoform_length=1830;expected_accuracy=0.992	gnl|BL_ORD_ID|49694 cb6406_c61/f1p0/2585	1	1055	1051	1830	255	1310	1441	2221
cb3999_c7/f1p0/2608	gnl|BL_ORD_ID|41059 cb3999_c16/f1p0/2857 isoform=cb3999_c16;full_length_coverage=1;non_full_length_coverage=0;isoform_length=2857;expected_accuracy=0.999	1	2089	2086	2538	10	2098	2401	2857
cb12931_c15/f1p0/1493 isoform=cb12931_c15;full_length_coverage=1;non_full_length_coverage=0;isoform_length=1493;expected_accuracy=1.000	gnl|BL_ORD_ID|55866 cb8564_c47522/f1p0/2954	1	1106	1104	1492	1	1081	2461	2849
cb10088_c29/f1p0/1009 isoform=cb10088_c29;full_length_coverage=1;non_full_length_coverage=0;isoform_length=1009;expected_accuracy=1.000	gnl|BL_ORD_ID|2975 cb10088_c25/f1p3/1453 isoform=cb10088_c25;full_length_coverage=1;non_full_length_coverage=3;isoform_length=1453;expected_accuracy=1.000	442	1009	9	447	578	1145	1	439
cb8227_c9/f1p1/1980	gnl|BL_ORD_ID|7454 cb8227_c3/f1p1/2427	202	1980	8	204	452	222	28	223

isoform=cb8227_c9;full_length_coverage=1;non_full_length_coverage=1;isoform_length=1980;expected_accuracy=0.993	isoform=cb8227_c3;full_length_coverage=1;non_full_length_coverage=1;isoform_length=2427;expected_accuracy=0.994						9		
cb8564_c119193/f1p2/2975 isoform=cb8564_c119193;full_length_coverage=1;non_full_length_coverage=2;isoform_length=2975;expected_accuracy=0.998	gnl|BL_ORD_ID|55920 cb8564_c48949/f1p0/4357	1	2308	2309	2975	2	2293	2847	3504
cb8564_c145668/f4p0/2444 isoform=cb8564_c145668;full_length_coverage=4;non_full_length_coverage=0;isoform_length=2444;expected_accuracy=1.000	gnl|BL_ORD_ID|15060 cb8564_c37490/f1p3/2627 isoform=cb8564_c37490;full_length_coverage=1;non_full_length_coverage=3;isoform_length=2627;expected_accuracy=0.998	1	2137	2137	2428	4	2124	2245	2534
cb5874_c30/f1p0/2210	gnl|BL_ORD_ID|44545 cb5874_c6/f8p0/2864 isoform=cb5874_c6;full_length_coverage=8;non_full_length_coverage=0;isoform_length=2864;expected_accuracy=1.000	145	2094	1	145	915	2864	573	717
cb7345_c8/f1p0/2440 isoform=cb7345_c8;full_length_coverage=1;non_full_length_coverage=0;isoform_length=2440;expected_accuracy=0.996	gnl|BL_ORD_ID|7283 cb7345_c20/f4p2/2585 isoform=cb7345_c20;full_length_coverage=4;non_full_length_coverage=2;isoform_length=2585;expected_accuracy=1.000	1	2257	2255	2440	3	2273	2389	2578
cb8564_c40598/f1p4/3119	gnl|BL_ORD_ID|54425 cb8564_c42906/f1p2/3181	1	2732	2729	3054	1	2709	2830	3155
cb8564_c187111/f1p0/2710 isoform=cb8564_c187111;full_length_coverage=1;non_full_length_coverage=0;isoform_length=2710;expected_accuracy=1.000	gnl|BL_ORD_ID|10605 cb8564_c114675/f1p2/2937 isoform=cb8564_c114675;full_length_coverage=1;non_full_length_coverage=2;isoform_length=2937;expected_accuracy=0.998	404	2710	42	406	524	2831	52	418
cb8564_c37282/f1p0/4051	gnl|BL_ORD_ID|56689 cb8564_c52534/f1p0/4439	1	2281	2279	4003	8	228	2725	445

<table>
<tr><td></td><td></td><td></td><td></td><td></td><td></td><td></td><td>8</td><td></td><td>0</td></tr>
<tr><td>cb8564_c180116/f1p0/2944 isoform=cb8564_c180116;full_length_coverage=1;non_full_length_coverage=0;isoform_length=2944;expected_accuracy=0.996</td><td>gnl|BL_ORD_ID|56275 cb8564_c73216/f1p0/3006</td><td>25</td><td>2522</td><td>2520</td><td>2944</td><td>28</td><td>2499</td><td>2728</td><td>3152</td></tr>
<tr><td>cb8881_c18/f1p0/1628 isoform=cb8881_c18;full_length_coverage=1;non_full_length_coverage=0;isoform_length=1628;expected_accuracy=0.997</td><td>gnl|BL_ORD_ID|59335 cb8881_c7/f1p0/1930</td><td>1</td><td>1226</td><td>1225</td><td>1621</td><td>2</td><td>1236</td><td>1591</td><td>1985</td></tr>
<tr><td>cb8564_c80883/f1p0/3185</td><td>gnl|BL_ORD_ID|12496 cb8564_c14618/f5p1/3581 isoform=cb8564_c14618;full_length_coverage=5;non_full_length_coverage=1;isoform_length=3581;expected_accuracy=0.997</td><td>1</td><td>2169</td><td>2166</td><td>3126</td><td>1</td><td>2168</td><td>2586</td><td>3548</td></tr>
<tr><td>cb8564_c47209/f1p0/3227</td><td>gnl|BL_ORD_ID|14058 cb8564_c23936/f1p0/3016 isoform=cb8564_c23936;full_length_coverage=1;non_full_length_coverage=0;isoform_length=3016;expected_accuracy=0.995</td><td>16</td><td>1738</td><td>1736</td><td>2606</td><td>11</td><td>1716</td><td>2143</td><td>3016</td></tr>
<tr><td>cb1994_c0/f1p0/4099</td><td>gnl|BL_ORD_ID|35307 cb1994_c7/f1p0/5227 isoform=cb1994_c7;full_length_coverage=1;non_full_length_coverage=0;isoform_length=5227;expected_accuracy=0.998</td><td>2</td><td>2568</td><td>2565</td><td>3626</td><td>53</td><td>2622</td><td>4002</td><td>5069</td></tr>
<tr><td>cb9471_c10/f1p0/1522 isoform=cb9471_c10;full_length_coverage=1;non_full_length_coverage=0;isoform_length=1522;expected_accuracy=0.998</td><td>gnl|BL_ORD_ID|58743 cb9471_c3/f1p0/2045</td><td>246</td><td>1522</td><td>3</td><td>251</td><td>488</td><td>1765</td><td>15</td><td>264</td></tr>
<tr><td>cb8564_c146141/f10p6/2566</td><td>gnl|BL_ORD_ID|53954 cb8564_c15976/f1p0/2695</td><td>1051</td><td>2502</td><td>1</td><td>1054</td><td>1158</td><td>260</td><td>2</td><td>105</td></tr>
</table>

isoform=cb8564_c146141;full_length_coverage=10;non_full_length_coverage=6;isoform_length=2566;expected_accuracy=0.998							7		5
cb6788_c3/f1p0/1918 isoform=cb6788_c3;full_length_coverage=1;non_full_length_coverage=0;isoform_length=1918;expected_accuracy=1.000	gnl|BL_ORD_ID|6006 cb6788_c0/f1p0/2764 isoform=cb6788_c0;full_length_coverage=1;non_full_length_coverage=0;isoform_length=2764;expected_accuracy=0.992	130	1902	1	131	985	2756	18	147
cb8564_c23645/f1p0/2729	gnl|BL_ORD_ID|17415 cb8564_c9135/f13p0/2814 isoform=cb8564_c9135;full_length_coverage=13;non_full_length_coverage=0;isoform_length=2814;expected_accuracy=1.000	1	2066	2062	2493	129	2195	2329	2760
cb8564_c43119/f1p4/3194 isoform=cb8564_c43119;full_length_coverage=1;non_full_length_coverage=4;isoform_length=3194;expected_accuracy=0.995	gnl|BL_ORD_ID|55164 cb8564_c34296/f2p2/3371	1	1671	1668	3157	7	1663	1805	3282
cb15567_c9/f1p1/1733 isoform=cb15567_c9;full_length_coverage=1;non_full_length_coverage=1;isoform_length=1733;expected_accuracy=0.994	gnl|BL_ORD_ID|11881 cb8564_c148133/f2p1/1959 isoform=cb8564_c148133;full_length_coverage=2;non_full_length_coverage=1;isoform_length=1959;expected_accuracy=1.000	1	1626	1622	1733	1	1640	1753	1864
cb8564_c45300/f1p4/3014 isoform=cb8564_c45300;full_length_coverage=1;non_full_length_coverage=4;isoform_length=3014;expected_accuracy=0.996	gnl|BL_ORD_ID|56275 cb8564_c73216/f1p0/3006	243	3014	22	246	406	3154	28	249
cb8564_c3679/f1p0/2936	gnl|BL_ORD_ID|14164 cb8564_c3676/f1p0/3441 isoform=cb8564_c3676;full_length_coverage=1;non_full_length_coverage=0;isoform_length=3441;expected_ac	2	2346	2344	2646	68	2412	3139	3441

	curacy=0.997								
cb8564_c22212/f1p0/2530 isoform=cb8564_c22212;full_length_coverage=1;non_full_length_coverage=0;isoform_length=2530;expected_accuracy=0.992	gnl|BL_ORD_ID|55945 cb8564_c49862/f1p0/2807	1	2118	2113	2530	1	2117	2234	2651
cb8564_c79084/f1p0/2498 isoform=cb8564_c79084;full_length_coverage=1;non_full_length_coverage=0;isoform_length=2498;expected_accuracy=0.991	gnl|BL_ORD_ID|15486 cb8564_c69975/f1p0/2818 isoform=cb8564_c69975;full_length_coverage=1;non_full_length_coverage=0;isoform_length=2818;expected_accuracy=0.999	133	2451	1	133	494	2818	1	134
cb12931_c15/f1p0/1493 isoform=cb12931_c15;full_length_coverage=1;non_full_length_coverage=0;isoform_length=1493;expected_accuracy=1.000	gnl|BL_ORD_ID|56727 cb8564_c66920/f3p3/2894	1	1106	1104	1485	1	1102	2482	2863
cb8349_c32/f1p0/2256 isoform=cb8349_c32;full_length_coverage=1;non_full_length_coverage=0;isoform_length=2256;expected_accuracy=0.994	gnl|BL_ORD_ID|9633 cb8349_c50/f2p0/3005 isoform=cb8349_c50;full_length_coverage=2;non_full_length_coverage=0;isoform_length=3005;expected_accuracy=1.000	1	1203	1202	2161	27	1229	1921	2881
cb8564_c89122/f1p0/3335	gnl|BL_ORD_ID|14531 cb8564_c41778/f1p9/3482 isoform=cb8564_c41778;full_length_coverage=1;non_full_length_coverage=9;isoform_length=3482;expected_accuracy=0.992	302	2913	81	306	812	3426	21	247
cb8564_c21439/f1p0/2587	gnl|BL_ORD_ID|18764 cb8564_c84695/f1p1/2710 isoform=cb8564_c84695;full_length_coverage=1;non_full_length_coverage=1;isoform_length=2710;expected_accuracy=0.998	1226	2542	1	1228	1350	2664	1	1229
cb2552_c5/f1p0/1147	gnl|BL_ORD_ID|37671 cb2552_c2/f1p0/2173	284	1147	3	283	1163	202	1	281

isoform=cb2552_c5;full_length_coverage=1;non_full_length_coverage=0;isoform_length=1147;expected_accuracy=0.994	isoform=cb2552_c2;full_length_coverage=1;non_full_length_coverage=0;isoform_length=2173;expected_accuracy=1.000						4		
cb8564_c126213/f1p0/1904	gnl|BL_ORD_ID|11817 cb8564_c146446/f7p1/2026 isoform=cb8564_c146446;full_length_coverage=7;non_full_length_coverage=1;isoform_length=2026;expected_accuracy=1.000	1	1585	1586	1779	59	1642	1833	2026
cb8564_c90372/f1p0/2157 isoform=cb8564_c90372;full_length_coverage=1;non_full_length_coverage=0;isoform_length=2157;expected_accuracy=0.994	gnl|BL_ORD_ID|53232 cb8564_c119499/f1p2/2534	1	1092	1088	2091	92	1184	1356	2359
cb3586_c11/f1p0/2822 isoform=cb3586_c11;full_length_coverage=1;non_full_length_coverage=0;isoform_length=2822;expected_accuracy=0.995	gnl|BL_ORD_ID|41151 cb3586_c0/f4p0/3057 isoform=cb3586_c0;full_length_coverage=4;non_full_length_coverage=0;isoform_length=3057;expected_accuracy=1.000	278	2822	1	277	498	3056	76	349
cb2882_c1/f1p0/3506 isoform=cb2882_c1;full_length_coverage=1;non_full_length_coverage=0;isoform_length=3506;expected_accuracy=0.998	gnl|BL_ORD_ID|37087 cb2882_c0/f1p0/3780 isoform=cb2882_c0;full_length_coverage=1;non_full_length_coverage=0;isoform_length=3780;expected_accuracy=0.999	2	2608	2608	3506	104	2708	2853	3751
cb7076_c17/f11p1/2296 isoform=cb7076_c17;full_length_coverage=11;non_full_length_coverage=1;isoform_length=2296;expected_accuracy=0.997	gnl|BL_ORD_ID|4463 cb7076_c12/f1p1/2435 isoform=cb7076_c12;full_length_coverage=1;non_full_length_coverage=1;isoform_length=2435;expected_accuracy=0.997	1	1637	1636	2296	1	1637	1764	2424
cb12714_c29/f1p2/1583	gnl|BL_ORD_ID|53904 cb8564_c14267/f1p3/2584	1	1296	1293	1588	3	1298	2283	2578
cb8564_c17892/f1p0/3277	gnl|BL_ORD_ID|56666 cb8564_c51678/f1p0/3945	200	3271	2	201	573	364	2	201

							3		
cb8564_c36056/f1p0/2652 isoform=cb8564_c36056;full_length_coverage=1;non_full_length_coverage=0;isoform_length=2652;expected_accuracy=0.999	gnl|BL_ORD_ID|13273 cb8564_c188193/f1p0/2849 isoform=cb8564_c188193;full_length_coverage=1;non_full_length_coverage=0;isoform_length=2849;expected_accuracy=0.997	248	2651	1	251	466	2849	70	322
cb3354_c1/f2p0/1541 isoform=cb3354_c1;full_length_coverage=2;non_full_length_coverage=0;isoform_length=1541;expected_accuracy=1.000	gnl|BL_ORD_ID|40592 cb3354_c3/f1p0/2584 isoform=cb3354_c3;full_length_coverage=1;non_full_length_coverage=0;isoform_length=2584;expected_accuracy=0.999	1	1184	1184	1541	29	1213	2093	2450
cb401_c28/f1p0/2163 isoform=cb401_c28;full_length_coverage=1;non_full_length_coverage=0;isoform_length=2163;expected_accuracy=0.992	gnl|BL_ORD_ID|41314 cb401_c156/f16p0/2365 isoform=cb401_c156;full_length_coverage=16;non_full_length_coverage=0;isoform_length=2365;expected_accuracy=1.000	317	2139	8	313	553	2364	36	342
cb295_c26/f1p1/1869	gnl|BL_ORD_ID|36693 cb295_c32/f1p2/2147 isoform=cb295_c32;full_length_coverage=1;non_full_length_coverage=2;isoform_length=2147;expected_accuracy=0.991	1	1359	1358	1857	3	1380	1512	2016
cb17884_c0/f3p1/1879 isoform=cb17884_c0;full_length_coverage=3;non_full_length_coverage=1;isoform_length=1879;expected_accuracy=1.000	gnl|BL_ORD_ID|11567 cb8564_c125919/f1p0/2530 isoform=cb8564_c125919;full_length_coverage=1;non_full_length_coverage=0;isoform_length=2530;expected_accuracy=1.000	252	1879	68	253	901	2528	52	239
cb11941_c13/f1p0/1210	gnl|BL_ORD_ID|24140 cb11941_c6/f2p1/1361 isoform=cb11941_c6;full_length_coverage=2;non_full_length_coverage=1;isoform_length=1361;expected_accuracy=0.999	166	1177	9	167	326	1361	1	159
cb8564_c86152/f1p2/2957	gnl|BL_ORD_ID|15620 cb8564_c69210/f3p3/3290	115	2955	1	114	450	329	226	339

isoform=cb8564_c86152;full_length_coverage=1;non_full_length_coverage=2;isoform_length=2957;expected_accuracy=0.999	isoform=cb8564_c69210;full_length_coverage=3;non_full_length_coverage=3;isoform_length=3290;expected_accuracy=0.999						0		
cb1458_c18/f1p0/1969 isoform=cb1458_c18;full_length_coverage=1;non_full_length_coverage=0;isoform_length=1969;expected_accuracy=0.992	gnl|BL_ORD_ID|30591 cb1458_c6/f1p0/3084 isoform=cb1458_c6;full_length_coverage=1;non_full_length_coverage=0;isoform_length=3084;expected_accuracy=0.991	1	1036	1036	1966	23	1075	2162	3084
cb8564_c111706/f1p0/2253	gnl|BL_ORD_ID|12795 cb8564_c176017/f8p2/2488 isoform=cb8564_c176017;full_length_coverage=8;non_full_length_coverage=2;isoform_length=2488;expected_accuracy=1.000	1	1681	1678	2181	2	1682	1973	2479
cb5962_c9/f1p2/2694 isoform=cb5962_c9;full_length_coverage=1;non_full_length_coverage=2;isoform_length=2694;expected_accuracy=0.998	gnl|BL_ORD_ID|45735 cb5962_c1/f8p7/2888 isoform=cb5962_c1;full_length_coverage=8;non_full_length_coverage=7;isoform_length=2888;expected_accuracy=1.000	1	1390	1386	2694	19	1409	1543	2852
cb8564_c24901/f1p0/4088 isoform=cb8564_c24901;full_length_coverage=1;non_full_length_coverage=0;isoform_length=4088;expected_accuracy=0.999	gnl|BL_ORD_ID|52811 cb8564_c14584/f2p0/4272	134	4066	1	135	302	4235	65	199
cb8564_c40764/f1p0/2563 isoform=cb8564_c40764;full_length_coverage=1;non_full_length_coverage=0;isoform_length=2563;expected_accuracy=0.997	gnl|BL_ORD_ID|55332 cb8564_c38655/f1p0/3210	1	2167	2162	2563	2	2162	2402	2803
cb5643_c46/f1p0/3297 isoform=cb5643_c46;full_length_coverage=1;non_full_length_coverage=0;isoform_length=3297;expected_accu	gnl|BL_ORD_ID|74220 cb5643_c62/f1p1/3868	10	2201	2199	3297	8	2205	2606	3712

racy=0.996									
cb8564_c40764/f1p0/2563 isoform=cb8564_c40764;full_length_coverage=1;non_full_length_coverage=0;isoform_length=2563;expected_accuracy=0.997	gnl|BL_ORD_ID|55986 cb8564_c51172/f1p0/3367	2	2166	2162	2563	7	2185	2393	2794
cb10846_c1/f1p0/1856	gnl|BL_ORD_ID|48905 cb10846_c4/f1p0/2641	1	1491	1490	1809	41	1531	2094	2413
cb4657_c15/f1p0/4478 isoform=cb4657_c15;full_length_coverage=1;non_full_length_coverage=0;isoform_length=4478;expected_accuracy=0.997	gnl|BL_ORD_ID|42493 cb4657_c10/f1p0/4691 isoform=cb4657_c10;full_length_coverage=1;non_full_length_coverage=0;isoform_length=4691;expected_accuracy=0.994	350	4478	130	351	576	4691	1	222
cb6829_c4/f2p1/2198 isoform=cb6829_c4;full_length_coverage=2;non_full_length_coverage=1;isoform_length=2198;expected_accuracy=0.997	gnl|BL_ORD_ID|4297 cb6829_c1/f6p2/2641 isoform=cb6829_c1;full_length_coverage=6;non_full_length_coverage=2;isoform_length=2641;expected_accuracy=1.000	1	1838	1838	2198	22	1854	2278	2638
cb8564_c188097/f1p0/2552 isoform=cb8564_c188097;full_length_coverage=1;non_full_length_coverage=0;isoform_length=2552;expected_accuracy=0.996	gnl|BL_ORD_ID|17008 cb8564_c55271/f1p4/2648 isoform=cb8564_c55271;full_length_coverage=1;non_full_length_coverage=4;isoform_length=2648;expected_accuracy=0.998	1	1945	1940	2499	2	1958	2061	2616
cb2737_c42/f1p0/2881	gnl|BL_ORD_ID|38132 cb2737_c20/f1p0/3644 isoform=cb2737_c20;full_length_coverage=1;non_full_length_coverage=0;isoform_length=3644;expected_accuracy=0.998	1	1427	1425	2632	852	2278	2410	3596
cb8564_c69975/f1p0/2818 isoform=cb8564_c69975;full_length_coverage=1;non_full_length_coverage=0;isoform_length=2818;expected_	gnl|BL_ORD_ID|57373 cb8564_c91193/f1p0/3331	260	2818	15	259	465	3023	58	303

<table>
<tr><td></td><td></td><td></td><td></td><td></td><td></td><td></td><td></td><td></td><td></td></tr>
<tr><td>accuracy=0.999</td><td></td><td></td><td></td><td></td><td></td><td></td><td></td><td></td><td></td></tr>
<tr><td>cb8564_c4645/f1p1/2620 isoform=cb8564_c4645;full_length_coverage=1;non_full_length_coverage=1;isoform_length=2620;expected_accuracy=0.994</td><td>gnl|BL_ORD_ID|56319 cb8564_c74283/f1p0/3024</td><td>1</td><td>1828</td><td>1829</td><td>2545</td><td>12</td><td>1850</td><td>1974</td><td>2688</td></tr>
<tr><td>cb396_c57/f1p0/2029 isoform=cb396_c57;full_length_coverage=1;non_full_length_coverage=0;isoform_length=2029;expected_accuracy=0.995</td><td>gnl|BL_ORD_ID|39231 cb396_c67/f28p0/2542 isoform=cb396_c67;full_length_coverage=28;non_full_length_coverage=0;isoform_length=2542;expected_accuracy=1.000</td><td>1</td><td>1399</td><td>1395</td><td>2029</td><td>70</td><td>1469</td><td>1730</td><td>2365</td></tr>
<tr><td>cb8564_c14280/f1p0/2385</td><td>gnl|BL_ORD_ID|10074 cb8564_c11613/f1p0/2758 isoform=cb8564_c11613;full_length_coverage=1;non_full_length_coverage=0;isoform_length=2758;expected_accuracy=0.991</td><td>1</td><td>2052</td><td>2050</td><td>2355</td><td>254</td><td>2346</td><td>2447</td><td>2758</td></tr>
<tr><td>cb8564_c50151/f1p0/3198 isoform=cb8564_c50151;full_length_coverage=1;non_full_length_coverage=0;isoform_length=3198;expected_accuracy=0.995</td><td>gnl|BL_ORD_ID|16149 cb8564_c47162/f1p0/3361 isoform=cb8564_c47162;full_length_coverage=1;non_full_length_coverage=0;isoform_length=3361;expected_accuracy=0.996</td><td>405</td><td>3198</td><td>6</td><td>406</td><td>592</td><td>3361</td><td>1</td><td>402</td></tr>
<tr><td>cb5137_c91/f1p0/3100</td><td>gnl|BL_ORD_ID|42185 cb5137_c56/f1p0/3071 isoform=cb5137_c56;full_length_coverage=1;non_full_length_coverage=0;isoform_length=3071;expected_accuracy=0.991</td><td>128</td><td>2800</td><td>1</td><td>132</td><td>398</td><td>3071</td><td>4</td><td>135</td></tr>
<tr><td>cb8564_c118910/f1p0/3194 isoform=cb8564_c118910;full_length_coverage=1;non_full_length_coverage=0;isoform_length=3194;expected_accuracy=0.996</td><td>gnl|BL_ORD_ID|9479 cb8564_c115532/f1p0/3955 isoform=cb8564_c115532;full_length_coverage=1;non_full_length_coverage=0;isoform_length=3955;expected_accuracy=0.998</td><td>129</td><td>2906</td><td>2905</td><td>3194</td><td>1</td><td>2782</td><td>3557</td><td>3847</td></tr>
<tr><td>cb8564_c145318/f11p3/1986</td><td>gnl|BL_ORD_ID|13510 cb8564_c1931/f2p1/2498</td><td>1</td><td>1191</td><td>1190</td><td>1985</td><td>1</td><td>121</td><td>1708</td><td>249</td></tr>
</table>

isoform=cb8564_c145318;full_length_coverage=11;non_full_length_coverage=3;isoform_length=1986;expected_accuracy=1.000	isoform=cb8564_c1931;full_length_coverage=2;non_full_length_coverage=1;isoform_length=2498;expected_accuracy=1.000						0		8
cb8564_c41507/f1p0/2100 isoform=cb8564_c41507;full_length_coverage=1;non_full_length_coverage=0;isoform_length=2100;expected_accuracy=0.998	gnl|BL_ORD_ID|55161 cb8564_c34270/f1p0/4482	1	1657	1655	2100	1853	3512	3900	4346
cb4746_c4/f1p0/2536 isoform=cb4746_c4;full_length_coverage=1;non_full_length_coverage=0;isoform_length=2536;expected_accuracy=0.996	gnl|BL_ORD_ID|72931 cb4746_c3/f1p0/3262	10	1457	1456	2536	1	1454	2180	3264
cb5633_c36/f1p3/2709	gnl|BL_ORD_ID|45725 cb5633_c20/f1p1/2973 isoform=cb5633_c20;full_length_coverage=1;non_full_length_coverage=1;isoform_length=2973;expected_accuracy=0.994	1	1964	1959	2640	29	1991	2292	2973
cb8564_c12805/f1p0/2580 isoform=cb8564_c12805;full_length_coverage=1;non_full_length_coverage=0;isoform_length=2580;expected_accuracy=0.993	gnl|BL_ORD_ID|18962 cb8564_c79723/f1p6/3025 isoform=cb8564_c79723;full_length_coverage=1;non_full_length_coverage=6;isoform_length=3025;expected_accuracy=0.999	1	1695	1690	2564	211	1906	2148	3025
cb2889_c9/f1p0/3389 isoform=cb2889_c9;full_length_coverage=1;non_full_length_coverage=0;isoform_length=3389;expected_accuracy=0.994	gnl|BL_ORD_ID|36509 cb2889_c3/f1p0/3455 isoform=cb2889_c3;full_length_coverage=1;non_full_length_coverage=0;isoform_length=3455;expected_accuracy=0.992	722	3389	93	722	769	3425	1	634
cb8564_c43399/f1p0/2654 isoform=cb8564_c43399;full_length_coverage=1;non_full_length_coverage=0;isoform_length=2654;expected_	gnl|BL_ORD_ID|55395 cb8564_c40401/f1p4/2799	1	2367	2363	2653	1	2360	2500	2790

accuracy=0.999									
cb8564_c36635/f1p0/4030 isoform=cb8564_c36635;full_length_coverage=1;non_full_length_coverage=0;isoform_length=4030;expected_accuracy=0.998	gnl|BL_ORD_ID|13890 cb8564_c35935/f1p0/4149 isoform=cb8564_c35935;full_length_coverage=1;non_full_length_coverage=0;isoform_length=4149;expected_accuracy=0.993	184	4021	1	189	332	414 9	1	188
cb8564_c111706/f1p0/2253	gnl|BL_ORD_ID|54594 cb8564_c127479/f1p0/2956	1	1681	1678	2121	170	185 0	2141	258 4
cb2225_c5/f1p0/3503 isoform=cb2225_c5;full_length_coverage=1;non_full_length_coverage=0;isoform_length=3503;expected_accuracy=0.997	gnl|BL_ORD_ID|38889 cb2225_c11/f2p0/3660 isoform=cb2225_c11;full_length_coverage=2;non_full_1ength_coverage=0;isoform_length=3660;expected_accuracy=1.000	1	3136	3136	3503	19	315 7	3291	365 8
cb541_c24/f1p0/5418 isoform=cb541_c24;full_length_coverage=1;non_full_length_coverage=0;isoform_length=5418;expected_accuracy=0.993	gnl|BL_ORD_ID|44621 cb541_c27/f1p1/5706 isoform=cb541_c27;full_length_coverage=1;non_full_length_coverage=1;isoform_length=5706;expected_accuracy=0.993	3	4102	4103	5418	3	4114	4244	555 9
cb1065_c16/f1p0/2119	gnl|BL_ORD_ID|242 cb1065_c2/f5p0/2384 isoform=cb1065_c2;full_length_coverage=5;non_full_length_coverage=0;isoform_length=2384;expected_accuracy=1.000	1014	2113	1	1015	1286	238 4	4	102 3
cb5897_c2/f1p0/2053 isoform=cb5897_c2;full_length_coverage=1;non_full_length_coverage=0;isoform_length=2053;expected_accuracy=0.998	gnl|BL_ORD_ID|46292 cb5897_c5/f1p0/2583 isoform=cb5897_c5;full_length_coverage=1;non_full_length_coverage=0;isoform_length=2583;expected_accuracy=0.994	1	1430	1429	1978	1	143 4	2034	258 3
cb10297_c2/f1p0/1900	gnl|BL_ORD_ID|48347 cb10297_c4/f1p0/2035	1	1173	1173	1618	35	120 7	1357	180 2
cb8564_c39136/f1p0/3036	gnl|BL_ORD_ID|14091 cb8564_c43119/f1p4/3194	1	2089	2085	2997	1	2110	2237	315

isoform=cb8564_c39136;full_length_coverage=1;non_full_length_coverage=0;isoform_length=3036;expected_accuracy=0.998	isoform=cb8564_c43119;full_length_coverage=1;non_full_length_coverage=4;isoform_length=3194;expected_accuracy=0.995								7
cb8564_c2150/f1p0/2358	gnl|BL_ORD_ID|9520 cb8564_c107219/f7p1/2625 isoform=cb8564_c107219;full_length_coverage=7;non_full_length_coverage=1;isoform_length=2625;expected_accuracy=1.000	1	2130	2130	2378	67	2196	2324	2572
cb2102_c21/f5p3/2180 isoform=cb2102_c21;full_length_coverage=5;non_full_length_coverage=3;isoform_length=2180;expected_accuracy=1.000	gnl|BL_ORD_ID|35266 cb2102_c6/f1p1/3538 isoform=cb2102_c6;full_length_coverage=1;non_full_length_coverage=1;isoform_length=3538;expected_accuracy=0.997	1	1770	1768	2180	4	1778	2523	2935
cb595_c15/f1p0/3791	gnl|BL_ORD_ID|72285 cb595_c22/f1p0/5464	2	2284	2284	3915	3	2285	3145	4776
cb1459_c0/f8p1/1395 isoform=cb1459_c0;full_length_coverage=8;non_full_length_coverage=1;isoform_length=1395;expected_accuracy=1.000	gnl|BL_ORD_ID|63631 cb1459_c2/f1p1/4317	575	1376	6	577	3502	4304	3	576
cb4544_c8/f1p0/2838	gnl|BL_ORD_ID|71947 cb4544_c11/f1p0/2935	16	1633	1634	2599	1	1632	1911	2877
cb7308_c0/f3p1/1955 isoform=cb7308_c0;full_length_coverage=3;non_full_length_coverage=1;isoform_length=1955;expected_accuracy=0.996	gnl|BL_ORD_ID|8226 cb7308_c7/f1p1/2094 isoform=cb7308_c7;full_length_coverage=1;non_full_length_coverage=1;isoform_length=2094;expected_accuracy=0.994	1	1577	1576	1954	5	1578	1716	2094
cb8564_c19992/f1p2/4274	gnl|BL_ORD_ID|13353 cb8564_c22284/f1p2/4673 isoform=cb8564_c22284;full_length_coverage=1;non_full_length_coverage=2;isoform_length=4673;expected_ac	3	3249	3247	4146	13	3256	3775	4673

	curacy=0.996								
cb8564_c1332/f1p0/2677	gnl|BL_ORD_ID|52636 cb8564_c1322/f1p1/4079	300	2717	7	304	1605	4015	2	304
cb1666_c2/f2p0/2331 isoform=cb1666_c2;full_length_coverage=2;non_full_length_coverage=0;isoform_length=2331;expected_accuracy=1.000	gnl|BL_ORD_ID|33315 cb1666_c5/f1p3/2503 isoform=cb1666_c5;full_length_coverage=1;non_full_length_coverage=3;isoform_length=2503;expected_accuracy=0.995	1	2214	2211	2331	18	2225	2337	2456
cb99_c12/f1p0/3017 isoform=cb99_c12;full_length_coverage=1;non_full_length_coverage=0;isoform_length=3017;expected_accuracy=0.997	gnl|BL_ORD_ID|20897 cb99_c11/f1p1/3412 isoform=cb99_c11;full_length_coverage=1;non_full_length_coverage=1;isoform_length=3412;expected_accuracy=0.999	1	2761	2758	3017	198	2961	3152	3408
cb8564_c121616/f1p1/2723	gnl|BL_ORD_ID|11141 cb8564_c109409/f3p1/2839 isoform=cb8564_c109409;full_length_coverage=3;non_full_length_coverage=1;isoform_length=2839;expected_accuracy=0.995	1	2193	2193	2679	40	2233	2347	2832
cb8564_c15230/f1p0/1944	gnl|BL_ORD_ID|16530 cb8564_c54668/f1p8/2639 isoform=cb8564_c54668;full_length_coverage=1;non_full_length_coverage=8;isoform_length=2639;expected_accuracy=0.999	352	1901	50	357	1091	2638	67	381
cb10058_c1/f1p0/1620	gnl|BL_ORD_ID|48005 cb10058_c3/f1p0/1884	1	1049	1048	1486	65	1114	1326	1763
cb8564_c154552/f1p5/2566 isoform=cb8564_c154552;full_length_coverage=1;non_full_length_coverage=5;isoform_length=2566;expected_accuracy=0.995	gnl|BL_ORD_ID|53584 cb8564_c129387/f1p0/3201	1	1941	1941	2566	12	1949	2178	2807
cb7189_c8/f1p0/2376	gnl|BL_ORD_ID|50408 cb7189_c7/f1p1/2301	1	1304	1304	2063	319	162	1517	227

							2		6
cb4487_c11/f1p0/2408	gnl|BL_ORD_ID|72597 cb4487_c10/f1p0/2515	1	1748	1748	2379	38	1785	1889	2520
cb8564_c81040/f1p1/2371	gnl|BL_ORD_ID|58157 cb8564_c90169/f1p0/2658	10	1965	1962	2475	1	1956	2161	2655
cb8564_c51661/f1p0/3132	gnl|BL_ORD_ID|13424 cb8564_c19032/f4p0/3378 isoform=cb8564_c19032;full_length_coverage=4;non_full_length_coverage=0;isoform_length=3378;expected_accuracy=0.997	1	2104	2099	3077	23	2127	2284	3262
cb8564_c23378/f1p1/2211	gnl|BL_ORD_ID|13799 cb8564_c22358/f1p1/4124 isoform=cb8564_c22358;full_length_coverage=1;non_full_length_coverage=1;isoform_length=4124;expected_accuracy=0.994	1	1684	1684	2195	1	1676	1830	2341
cb8564_c118488/f1p0/2066 isoform=cb8564_c118488;full_length_coverage=1;non_full_length_coverage=0;isoform_length=2066;expected_accuracy=0.991	gnl|BL_ORD_ID|11866 cb8564_c126236/f1p0/2200 isoform=cb8564_c126236;full_length_coverage=1;non_full_length_coverage=0;isoform_length=2200;expected_accuracy=0.999	198	2024	11	202	366	2198	1	184
cb8564_c165050/f2p1/2091 isoform=cb8564_c165050;full_length_coverage=2;non_full_length_coverage=1;isoform_length=2091;expected_accuracy=0.997	gnl|BL_ORD_ID|12527 cb8564_c15023/f2p0/2552 isoform=cb8564_c15023;full_length_coverage=2;non_full_length_coverage=0;isoform_length=2552;expected_accuracy=0.996	1	1672	1673	2091	1	1674	2137	2550
cb9943_c0/f2p1/1899 isoform=cb9943_c0;full_length_coverage=2;non_full_length_coverage=1;isoform_length=1899;expected_accuracy=1.000	gnl|BL_ORD_ID|20471 cb9943_c3/f1p0/2123 isoform=cb9943_c3;full_length_coverage=1;non_full_length_coverage=0;isoform_length=2123;expected_accuracy=0.999	1	1498	1495	1820	1	1498	1798	2123
cb8564_c46939/f1p1/2926	gnl|BL_ORD_ID|16447 cb8564_c47202/f1p0/3195	1	2134	2130	2856	1	214	2249	297

	isoform=cb8564_c47202;full_length_coverage=1;non_full_length_coverage=0;isoform_length=3195;expected_accuracy=0.996						4		6
cb8564_c180116/f1p0/2944 isoform=cb8564_c180116;full_length_coverage=1;non_full_length_coverage=0;isoform_length=2944;expected_accuracy=0.996	gnl|BL_ORD_ID|55334 cb8564_c38684/f1p1/3241	1	2522	2520	2942	2	2520	2825	3247
cb6730_c3/f4p0/2261 isoform=cb6730_c3;full_length_coverage=4;non_full_length_coverage=0;isoform_length=2261;expected_accuracy=1.000	gnl|BL_ORD_ID|5878 cb6730_c6/f2p1/2515 isoform=cb6730_c6;full_length_coverage=2;non_full_length_coverage=1;isoform_length=2515;expected_accuracy=0.994	1	1673	1674	2261	24	1696	1877	2465
cb332_c3/f1p1/4544	gnl|BL_ORD_ID|39700 cb332_c11/f1p0/5243 isoform=cb332_c11;full_length_coverage=1;non_full_length_coverage=0;isoform_length=5243;expected_accuracy=0.991	1989	4542	1	1994	2697	5243	7	1990
cb8564_c129018/f3p0/2395 isoform=cb8564_c129018;full_length_coverage=3;non_full_length_coverage=0;isoform_length=2395;expected_accuracy=0.995	gnl|BL_ORD_ID|56347 cb8564_c74976/f1p0/3389	1	2106	2104	2391	1	2108	3064	3349
cb8564_c21572/f1p1/3061 isoform=cb8564_c21572;full_length_coverage=1;non_full_length_coverage=1;isoform_length=3061;expected_accuracy=0.991	gnl|BL_ORD_ID|14977 cb8564_c31966/f8p3/3401 isoform=cb8564_c31966;full_length_coverage=8;non_full_length_coverage=3;isoform_length=3401;expected_accuracy=1.000	1127	3059	1	1125	1488	3401	208	1333
cb4698_c12/f1p2/3047 isoform=cb4698_c12;full_length_coverage=1;non_full_length_coverage=2;isoform_length=3047;expected_accu	gnl|BL_ORD_ID|42963 cb4698_c11/f1p0/3141 isoform=cb4698_c11;full_length_coverage=1;non_full_length_coverage=0;isoform_length=3141;expected_accur	1245	2962	1	1245	1427	3141	30	1272

racy=0.997	acy=1.000								
cb8564_c41756/f1p0/2738 isoform=cb8564_c41756;full_length_coverage=1;non_full_length_coverage=0;isoform_length=2738;expected_accuracy=0.996	gnl|BL_ORD_ID|55413 cb8564_c40978/f1p0/3073	1	2173	2170	2738	1	2159	2282	2849
cb8564_c39136/f1p0/3036 isoform=cb8564_c39136;full_length_coverage=1;non_full_length_coverage=0;isoform_length=3036;expected_accuracy=0.998	gnl|BL_ORD_ID|54425 cb8564_c42906/f1p2/3181	1	2089	2085	3036	1	2074	2201	3154
cb7863_c19/f1p0/2295	gnl|BL_ORD_ID|8930 cb7863_c0/f16p0/2341 isoform=cb7863_c0;full_length_coverage=16;non_full_length_coverage=0;isoform_length=2341;expected_accuracy=1.000	14	1751	1750	2197	1	1736	1894	2341
cb2737_c57/f1p0/2959	gnl|BL_ORD_ID|38134 cb2737_c22/f1p0/3196 isoform=cb2737_c22;full_length_coverage=1;non_full_length_coverage=0;isoform_length=3196;expected_accuracy=0.995	1	1609	1607	2862	166	1763	1894	3131
cb6730_c14/f1p0/2258	gnl|BL_ORD_ID|3442 cb6730_c30/f4p0/2653 isoform=cb6730_c30;full_length_coverage=4;non_full_length_coverage=0;isoform_length=2653;expected_accuracy=1.000	1	1382	1381	2267	10	1393	1656	2542
cb12714_c29/f1p2/1583	gnl|BL_ORD_ID|12970 cb8564_c16702/f2p13/2748 isoform=cb8564_c16702;full_length_coverage=2;non_full_length_coverage=13;isoform_length=2748;expected_accuracy=0.998	1	1296	1293	1533	147	1441	2508	2748
cb8564_c9363/f3p0/2727	gnl|BL_ORD_ID|57310 cb8564_c89545/f1p0/3414	1	1669	1667	2727	7	167	1994	306

isoform=cb8564_c9363;full_length_coverage=3;non_full_length_coverage=0;isoform_length=2727;expected_accuracy=0.999							4		0
cb9146_c8/f1p0/1115 isoform=cb9146_c8;full_length_coverage=1;non_full_length_coverage=0;isoform_length=1115;expected_accuracy=0.991	gnl|BL_ORD_ID|20652 cb9146_c3/f1p0/1900 isoform=cb9146_c3;full_length_coverage=1;non_full_length_coverage=0;isoform_length=1900;expected_accuracy=0.994	101	1080	1	101	245	1224	40	140
cb8564_c72377/f1p0/3026 isoform=cb8564_c72377;full_length_coverage=1;non_full_length_coverage=0;isoform_length=3026;expected_accuracy=0.995	gnl|BL_ORD_ID|16680 cb8564_c68963/f3p0/3243 isoform=cb8564_c68963;full_length_coverage=3;non_full_length_coverage=0;isoform_length=3243;expected_accuracy=1.000	221	3026	1	222	399	3204	2	223
cb1658_c16/f1p1/2736 isoform=cb1658_c16;full_length_coverage=1;non_full_length_coverage=1;isoform_length=2736;expected_accuracy=0.998	gnl|BL_ORD_ID|63830 cb1658_c10/f1p0/3994	1157	2670	1	1160	2370	3883	14	1174
cb6968_c6/f1p0/2273 isoform=cb6968_c6;full_length_coverage=1;non_full_length_coverage=0;isoform_length=2273;expected_accuracy=0.996	gnl|BL_ORD_ID|5944 cb6968_c19/f6p0/2660 isoform=cb6968_c19;full_length_coverage=6;non_full_length_coverage=0;isoform_length=2660;expected_accuracy=0.999	185	2273	1	185	411	2500	64	249
cb8564_c88673/f1p0/3095	gnl|BL_ORD_ID|52655 cb8564_c10124/f1p0/3457	2	2319	2316	3025	3	2321	2674	3383
cb12714_c29/f1p2/1583	gnl|BL_ORD_ID|13065 cb8564_c157334/f1p21/2777 isoform=cb8564_c157334;full_length_coverage=1;non_full_length_coverage=21;isoform_length=2777;expected_accuracy=0.995	1	1296	1293	1597	7	1309	2376	2680
cb8564_c145335/f3p0/1964	gnl|BL_ORD_ID|13736 cb8564_c1945/f1p0/2267	1	1452	1451	1953	7	146	1766	226

isoform=cb8564_c145335;full_length_coverage=3;non_full_length_coverage=0;isoform_length=1964;expected_accuracy=0.999	isoform=cb8564_c1945;full_length_coverage=1;non_full_length_coverage=0;isoform_length=2267;expected_accuracy=0.999						0		7
cb4016_c32/f1p0/1913	gnl|BL_ORD_ID|40527 cb4016_c6/f1p1/2956 isoform=cb4016_c6;full_length_coverage=1;non_full_length_coverage=1;isoform_length=2956;expected_accuracy=0.996	191	1930	23	194	1124	2863	524	697
cb8564_c15653/f1p4/2475	gnl|BL_ORD_ID|11957 cb8564_c135981/f1p0/2840 isoform=cb8564_c135981;full_length_coverage=1;non_full_length_coverage=0;isoform_length=2840;expected_accuracy=0.992	22	2293	2289	2427	220	2484	2703	2840
cb1245_c3/f1p0/3928 isoform=cb1245_c3;full_length_coverage=1;non_full_length_coverage=0;isoform_length=3928;expected_accuracy=0.997	gnl|BL_ORD_ID|24061 cb1245_c0/f6p1/4116 isoform=cb1245_c0;full_length_coverage=6;non_full_length_coverage=1;isoform_length=4116;expected_accuracy=1.000	1	2779	2775	3890	79	2858	3001	4116
cb12034_c11/f1p0/1825	gnl|BL_ORD_ID|16101 cb8564_c52391/f1p0/2080 isoform=cb8564_c52391;full_length_coverage=1;non_full_length_coverage=0;isoform_length=2080;expected_accuracy=0.997	282	1833	50	283	454	2031	1	234
cb8564_c39136/f1p0/3036 isoform=cb8564_c39136;full_length_coverage=1;non_full_length_coverage=0;isoform_length=3036;expected_accuracy=0.998	gnl|BL_ORD_ID|16886 cb8564_c50727/f1p0/3201 isoform=cb8564_c50727;full_length_coverage=1;non_full_length_coverage=0;isoform_length=3201;expected_accuracy=0.999	1	2089	2085	3036	3	2122	2248	3201
cb8564_c45300/f1p4/3014 isoform=cb8564_c45300;full_length_coverage=1;non_full_length_coverage=4;isoform_length=3014;expected_	gnl|BL_ORD_ID|55551 cb8564_c44964/f1p0/3508	243	3014	1	246	400	3171	1	243

accuracy=0.996									
cb6719_c56/f1p0/2578	gnl|BL_ORD_ID|4663 cb6719_c20/f1p0/3262 isoform=cb6719_c20;full_length_coverage=1;non_full_length_coverage=0;isoform_length=3262;expected_accuracy=0.996	1	1366	1365	2387	755	2119	2240	3262
cb16404_c11/f1p0/1451 isoform=cb16404_c11;full_length_coverage=1;non_full_length_coverage=0;isoform_length=1451;expected_accuracy=1.000	gnl|BL_ORD_ID|54733 cb8564_c160742/f1p0/2024	160	1451	17	164	674	1959	1	147
cb1656_c17/f1p0/3666	gnl|BL_ORD_ID|63828 cb1656_c15/f1p1/3875	1	3346	3342	3547	1	3347	3636	3839
cb9472_c4/f1p0/1359 isoform=cb9472_c4;full_length_coverage=1;non_full_length_coverage=0;isoform_length=1359;expected_accuracy=1.000	gnl|BL_ORD_ID|19537 cb9472_c2/f1p0/1675 isoform=cb9472_c2;full_length_coverage=1;non_full_length_coverage=0;isoform_length=1675;expected_accuracy=0.996	387	1359	42	388	659	1631	1	347
cb8564_c83110/f1p1/2442 isoform=cb8564_c83110;full_length_coverage=1;non_full_length_coverage=1;isoform_length=2442;expected_accuracy=0.997	gnl|BL_ORD_ID|56754 cb8564_c70263/f1p2/3393	1	1295	1290	2393	8	1333	2242	3352
cb8564_c22056/f1p0/3563	gnl|BL_ORD_ID|11514 cb8564_c12502/f1p0/4194 isoform=cb8564_c12502;full_length_coverage=1;non_full_length_coverage=0;isoform_length=4194;expected_accuracy=0.998	1465	3466	13	1467	2077	4078	1	1480
cb1935_c14/f1p0/3028	gnl|BL_ORD_ID|34837 cb1935_c4/f2p0/3210 isoform=cb1935_c4;full_length_coverage=2;non_full_length_coverage=0;isoform_length=3210;expected_accura	11	1968	1963	3017	15	2003	2126	3180

	cy=1.000								
cb4203_c2/f1p0/1914	gnl|BL_ORD_ID|72345 cb4203_c0/f1p0/3405	97	1909	2	101	1103	2915	825	925
cb8564_c4645/f1p1/2620 isoform=cb8564_c4645;full_length_coverage=1;non_full_length_coverage=1;isoform_length=2620;expected_accuracy=0.994	gnl|BL_ORD_ID|55045 cb8564_c4649/f1p0/3061	1	1828	1829	2561	56	1894	2018	2749
cb309_c19/f1p0/1785 isoform=cb309_c19;full_length_coverage=1;non_full_length_coverage=0;isoform_length=1785;expected_accuracy=0.996	gnl|BL_ORD_ID|69546 cb309_c5/f1p0/4669	1	1335	1336	1785	2584	3918	4134	4582
cb6211_c9/f5p0/2703 isoform=cb6211_c9;full_length_coverage=5;non_full_length_coverage=0;isoform_length=2703;expected_accuracy=0.996	gnl|BL_ORD_ID|5238 cb6211_c20/f1p0/3258 isoform=cb6211_c20;full_length_coverage=1;non_full_length_coverage=0;isoform_length=3258;expected_accuracy=0.994	1074	2664	1	1076	1669	3258	1	1076
cb8564_c35399/f1p0/4553	gnl|BL_ORD_ID|14942 cb8564_c34188/f2p3/4546 isoform=cb8564_c34188;full_length_coverage=2;non_full_length_coverage=3;isoform_length=4546;expected_accuracy=1.000	11	3381	3377	4276	14	3382	3647	4546
cb8564_c118659/f2p0/3437 isoform=cb8564_c118659;full_length_coverage=2;non_full_length_coverage=0;isoform_length=3437;expected_accuracy=0.994	gnl|BL_ORD_ID|9995 cb8564_c111409/f2p0/3661 isoform=cb8564_c111409;full_length_coverage=2;non_full_length_coverage=0;isoform_length=3661;expected_accuracy=0.999	1	2754	2752	3319	85	2863	3093	3661
cb8564_c21439/f1p0/2587	gnl|BL_ORD_ID|11428 cb8564_c149144/f2p18/3112 isoform=cb8564_c149144;full_length_coverage=2;non_full_length_coverage=18;isoform_length=3112;expected_	1226	2572	1	1228	1353	2702	1	1232

	accuracy=0.994								
cb8564_c132795/f5p10/3729 isoform=cb8564_c132795;full_length_coverage=5;non_full_length_coverage=10;isoform_length=3729;expected_accuracy=0.999	gnl|BL_ORD_ID|64326 cb14859_c10/f1p0/5042	1	1832	1830	3640	1	1830	3004	4814
cb8564_c109867/f2p0/3910 isoform=cb8564_c109867;full_length_coverage=2;non_full_length_coverage=0;isoform_length=3910;expected_accuracy=0.997	gnl|BL_ORD_ID|54548 cb8564_c125967/f1p0/4340	1	2779	2779	3907	44	2822	3205	4332
cb7863_c14/f1p0/2372	gnl|BL_ORD_ID|6733 cb7863_c11/f1p0/2693 isoform=cb7863_c11;full_length_coverage=1;non_full_length_coverage=0;isoform_length=2693;expected_accuracy=0.998	111	1760	1759	2354	47	1697	2086	2681
cb8564_c36731/f1p2/2761	gnl|BL_ORD_ID|55912 cb8564_c48681/f1p0/3431	1	1977	1972	2739	1	1974	2451	3220
cb8564_c79401/f1p0/4816	gnl|BL_ORD_ID|16547 cb8564_c69388/f4p1/4992 isoform=cb8564_c69388;full_length_coverage=4;non_full_length_coverage=1;isoform_length=4992;expected_accuracy=1.000	1072	4321	1	1068	1744	4992	790	1857
cb8564_c75977/f1p0/2165	gnl|BL_ORD_ID|18194 cb8564_c89260/f1p0/3637 isoform=cb8564_c89260;full_length_coverage=1;non_full_length_coverage=0;isoform_length=3637;expected_accuracy=0.995	1	1393	1394	2124	128	1526	2906	3635
cb8564_c71802/f1p1/3657 isoform=cb8564_c71802;full_length_coverage=1;non_full_length_coverage=1;isoform_length=3657;expected_	gnl|BL_ORD_ID|16243 cb8564_c69770/f1p2/3779 isoform=cb8564_c69770;full_length_coverage=1;non_full_length_coverage=2;isoform_length=3779;expected_ac	188	3579	1	191	388	3779	84	278

accuracy=1.000	curacy=1.000								
cb8564_c118840/f1p1/2264 isoform=cb8564_c118840;full_length_coverage=1;non_full_length_coverage=1;isoform_length=2264;expected_accuracy=0.990	gnl|BL_ORD_ID|54891 cb8564_c3340/f1p0/3978	325	2264	4	325	1998	3920	1	320
cb8564_c43399/f1p0/2654 isoform=cb8564_c43399;full_length_coverage=1;non_full_length_coverage=0;isoform_length=2654;expected_accuracy=0.999	gnl|BL_ORD_ID|56727 cb8564_c66920/f3p3/2894	1	2367	2363	2646	1	2364	2580	2863
cb7749_c11/f1p1/1246	gnl|BL_ORD_ID|50687 cb7749_c8/f1p0/1405	169	1151	4	173	327	1308	3	172
cb12714_c29/f1p2/1583	gnl|BL_ORD_ID|13000 cb8564_c179857/f1p0/2640 isoform=cb8564_c179857;full_length_coverage=1;non_full_length_coverage=0;isoform_length=2640;expected_accuracy=0.999	1	1296	1293	1597	4	1299	2334	2638
cb1333_c101/f1p0/1395 isoform=cb1333_c101;full_length_coverage=1;non_full_length_coverage=0;isoform_length=1395;expected_accuracy=0.997	gnl|BL_ORD_ID|26228 cb1333_c50/f1p0/4810 isoform=cb1333_c50;full_length_coverage=1;non_full_length_coverage=0;isoform_length=4810;expected_accuracy=0.996	149	1395	27	150	3506	4745	22	146
cb500_c38/f1p0/2822 isoform=cb500_c38;full_length_coverage=1;non_full_length_coverage=0;isoform_length=2822;expected_accuracy=0.999	gnl|BL_ORD_ID|42492 cb500_c6/f4p0/3398 isoform=cb500_c6;full_length_coverage=4;non_full_length_coverage=0;isoform_length=3398;expected_accuracy=1.000	1	2396	2391	2822	94	2492	2954	3385
cb8564_c24424/f1p0/4115	gnl|BL_ORD_ID|13013 cb8564_c17114/f4p0/3443 isoform=cb8564_c17114;full_length_coverage=4;non_full_length_coverage=0;isoform_length=3443;expected_ac	12	3217	3216	3380	9	3215	3090	3254

	curacy=1.000								
cb8564_c41756/f1p0/2738 isoform=cb8564_c41756;full_length_coverage=1;non_full_length_coverage=0;isoform_length=2738;expected_accuracy=0.996	gnl|BL_ORD_ID|56727 cb8564_c66920/f3p3/2894	1	2173	2170	2731	1	2179	2302	2863
cb10334_c0/f11p0/1199 isoform=cb10334_c0;full_length_coverage=11;non_full_length_coverage=0;isoform_length=1199;expected_accuracy=1.000	gnl|BL_ORD_ID|2340 cb10334_c4/f1p0/2353 isoform=cb10334_c4;full_length_coverage=1;non_full_length_coverage=0;isoform_length=2353;expected_accuracy=0.999	269	1199	8	274	1423	2351	1	267
cb8564_c43399/f1p0/2654 isoform=cb8564_c43399;full_length_coverage=1;non_full_length_coverage=0;isoform_length=2654;expected_accuracy=0.999	gnl|BL_ORD_ID|56602 cb8564_c48192/f1p6/3029	1	2367	2363	2654	1	2366	2582	2872
cb8564_c71815/f1p0/2753	gnl|BL_ORD_ID|58020 cb8564_c86752/f1p1/3346	1	1377	1372	2485	47	1421	1655	2772
cb3543_c6/f1p0/2439	gnl|BL_ORD_ID|39659 cb3543_c3/f2p0/3596 isoform=cb3543_c3;full_length_coverage=2;non_full_length_coverage=0;isoform_length=3596;expected_accuracy=1.000	1	1561	1559	2049	17	1577	3106	3596
cb8564_c41642/f2p0/3998	gnl|BL_ORD_ID|55587 cb8564_c46008/f1p0/4134	1650	3997	1	1652	1752	4099	4	1644
cb500_c17/f1p0/2323 isoform=cb500_c17;full_length_coverage=1;non_full_length_coverage=0;isoform_length=2323;expected_accuracy=0.997	gnl|BL_ORD_ID|72248 cb500_c53/f1p0/3672	1	1293	1288	2323	894	2186	2318	3353
cb750_c23/f1p0/3659	gnl|BL_ORD_ID|7348 cb750_c27/f1p1/4163	120	2134	2133	3575	150	216	2719	416

	isoform=cb750_c27;full_length_coverage=1;non_full_length_coverage=1;isoform_length=4163;expected_accuracy=0.998						2		3
cb8564_c39136/f1p0/3036 isoform=cb8564_c39136;full_length_coverage=1;non_full_length_coverage=0;isoform_length=3036;expected_accuracy=0.998	gnl|BL_ORD_ID|55551 cb8564_c44964/f1p0/3508	1	2089	2085	3036	1	2092	2219	3173
cb8564_c15230/f1p0/1944	gnl|BL_ORD_ID|18416 cb8564_c82572/f1p22/2743 isoform=cb8564_c82572;full_length_coverage=1;non_full_length_coverage=22;isoform_length=2743;expected_accuracy=1.000	352	1944	39	357	1087	2680	55	376
cb8564_c35256/f1p0/3343 isoform=cb8564_c35256;full_length_coverage=1;non_full_length_coverage=0;isoform_length=3343;expected_accuracy=0.995	gnl|BL_ORD_ID|15520 cb8564_c52723/f2p0/3547 isoform=cb8564_c52723;full_length_coverage=2;non_full_length_coverage=0;isoform_length=3547;expected_accuracy=0.990	1069	3343	1	1070	1206	3476	1	1066
cb8564_c47149/f1p1/2601 isoform=cb8564_c47149;full_length_coverage=1;non_full_length_coverage=1;isoform_length=2601;expected_accuracy=0.997	gnl|BL_ORD_ID|55389 cb8564_c40246/f1p1/2822	1	1649	1649	2601	4	1638	1774	2721
cb18456_c1748/f1p0/1180	gnl|BL_ORD_ID|69244 cb18456_c1759/f1p0/1409	243	1188	3	248	423	1368	42	285
cb3297_c15/f1p0/1649	gnl|BL_ORD_ID|39501 cb3297_c10/f1p0/2398 isoform=cb3297_c10;full_length_coverage=1;non_full_length_coverage=0;isoform_length=2398;expected_accuracy=0.999	279	1626	43	279	1051	2398	1	227
cb5274_c1/f5p1/2378	gnl|BL_ORD_ID|46078 cb5274_c3/f1p1/3134	1	1589	1587	2375	38	162	2345	313

isoform=cb5274_c1;full_length_coverage=5;non_full_length_coverage=1;isoform_length=2378;expected_accuracy=1.000	isoform=cb5274_c3;full_length_coverage=1;non_full_length_coverage=1;isoform_length=3134;expected_accuracy=0.994						5		4
cb8564_c74375/f1p1/2718 isoform=cb8564_c74375;full_length_coverage=1;non_full_length_coverage=1;isoform_length=2718;expected_accuracy=1.000	gnl|BL_ORD_ID|11877 cb8564_c154206/f1p1/3098 isoform=cb8564_c154206;full_length_coverage=1;non_full_length_coverage=1;isoform_length=3098;expected_accuracy=0.990	1	1609	1608	2718	284	1882	1990	3098
cb12047_c134/f1p0/1727 isoform=cb12047_c134;full_length_coverage=1;non_full_length_coverage=0;isoform_length=1727;expected_accuracy=0.999	gnl|BL_ORD_ID|13419 cb8564_c22364/f4p28/1901 isoform=cb8564_c22364;full_length_coverage=4;non_full_length_coverage=28;isoform_length=1901;expected_accuracy=0.995	1	1006	1001	1727	1	1002	1108	1834
cb8564_c113392/f1p0/2376	gnl|BL_ORD_ID|54891 cb8564_c3340/f1p0/3978	407	2308	87	407	1998	3899	1	320
cb1573_c35/f1p0/2689	gnl|BL_ORD_ID|28227 cb1573_c15/f1p0/3869 isoform=cb1573_c15;full_length_coverage=1;non_full_length_coverage=0;isoform_length=3869;expected_accuracy=0.997	2	2210	2209	2426	1094	3302	3646	3863
cb8564_c127456/f1p1/2149	gnl|BL_ORD_ID|52085 cb8564_c110321/f1p1/2697	1	1731	1731	2090	282	2012	2214	2573
cb8087_c4/f1p0/2454 isoform=cb8087_c4;full_length_coverage=1;non_full_length_coverage=0;isoform_length=2454;expected_accuracy=0.997	gnl|BL_ORD_ID|8788 cb8087_c5/f1p0/3215 isoform=cb8087_c5;full_length_coverage=1;non_full_length_coverage=0;isoform_length=3215;expected_accuracy=0.996	2	2072	2071	2454	1	2062	2808	3189
cb4023_c5/f1p1/3111 isoform=cb4023_c5;full_length_coverage=1;non_full_length_coverage=1;isoform_length=3111;expected_accura	gnl|BL_ORD_ID|39794 cb4023_c4/f1p1/4182 isoform=cb4023_c4;full_length_coverage=1;non_full_length_coverage=1;isoform_length=4182;expected_accura	1	2249	2247	3089	24	2255	3347	4182

cy=0.994	cy=0.995								
cb8564_c38233/f1p0/2780	gnl|BL_ORD_ID|54713 cb8564_c158279/f2p4/3190	1	1857	1856	2785	218	207 4	2199	312 8
cb5960_c3/f1p0/2695 isoform=cb5960_c3;full_length_coverage=1;non_full_length_coverage=0;isoform_length=2695;expected_accuracy=0.991	gnl|BL_ORD_ID|74709 cb5960_c13/f1p0/3400	1	1927	1928	2594	62	198 2	2733	339 5
cb8564_c35155/f1p0/2975	gnl|BL_ORD_ID|56633 cb8564_c49796/f2p5/3077	571	2834	9	566	740	300 3	8	555
cb8564_c35745/f1p0/3577 isoform=cb8564_c35745;full_length_coverage=1;non_full_length_coverage=0;isoform_length=3577;expected_accuracy=0.996	gnl|BL_ORD_ID|55990 cb8564_c51337/f1p0/4466	1	2773	2772	3576	59	283 2	3667	447 1
cb12931_c15/f1p0/1493 isoform=cb12931_c15;full_length_coverage=1;non_full_length_coverage=0;isoform_length=1493;expected_accuracy=1.000	gnl|BL_ORD_ID|56602 cb8564_c48192/f1p6/3029	1	1106	1104	1493	1	1104	2484	287 2
cb5271_c5/f1p2/2317	gnl|BL_ORD_ID|73721 cb5271_c9/f1p0/2773	1	1284	1279	2322	2	128 5	1586	262 9
cb8564_c119623/f1p0/1981 isoform=cb8564_c119623;full_length_coverage=1;non_full_length_coverage=0;isoform_length=1981;expected_accuracy=0.997	gnl|BL_ORD_ID|52165 cb8564_c113081/f1p1/2205	1	1282	1281	1981	12	129 1	1442	214 3
cb151_c2/f1p0/4262 isoform=cb151_c2;full_length_coverage=1;non_full_length_coverage=0;isoform_length=4262;expected_accurac	gnl|BL_ORD_ID|30050 cb151_c6/f1p0/4591 isoform=cb151_c6;full_length_coverage=1;non_full_length_coverage=0;isoform_length=4591;expected_accurac	307	4260	1	308	621	459 1	163	456

y=0.992	y=0.991								
cb8564_c75076/f1p0/3860 isoform=cb8564_c75076;full_length_coverage=1;non_full_length_coverage=0;isoform_length=3860;expected_accuracy=0.996	gnl|BL_ORD_ID|17579 cb8564_c86687/f1p2/4094 isoform=cb8564_c86687;full_length_coverage=1;non_full_length_coverage=2;isoform_length=4094;expected_accuracy=0.996	1611	3860	1	1616	1823	4094	24	1639
cb3081_c2/f2p1/3441 isoform=cb3081_c2;full_length_coverage=2;non_full_length_coverage=1;isoform_length=3441;expected_accuracy=0.994	gnl|BL_ORD_ID|37158 cb3081_c15/f1p0/3452 isoform=cb3081_c15;full_length_coverage=1;non_full_length_coverage=0;isoform_length=3452;expected_accuracy=0.996	311	3439	145	313	324	3450	1	169
cb3575_c10/f1p0/3008	gnl|BL_ORD_ID|39143 cb3575_c7/f1p1/3444 isoform=cb3575_c7;full_length_coverage=1;non_full_length_coverage=1;isoform_length=3444;expected_accuracy=0.997	1396	2952	1	1398	1869	3444	201	1598
cb8564_c10549/f1p2/3517 isoform=cb8564_c10549;full_length_coverage=1;non_full_length_coverage=2;isoform_length=3517;expected_accuracy=1.000	gnl|BL_ORD_ID|53927 cb8564_c15139/f1p1/4122	187	3517	17	187	584	3915	225	395
cb5189_c1/f3p0/2380 isoform=cb5189_c1;full_length_coverage=3;non_full_length_coverage=0;isoform_length=2380;expected_accuracy=0.995	gnl|BL_ORD_ID|73605 cb5189_c6/f1p0/2847	1047	2380	1	1047	1322	2655	9	1050
cb1301_c5/f1p0/2947 isoform=cb1301_c5;full_length_coverage=1;non_full_length_coverage=0;isoform_length=2947;expected_accuracy=0.995	gnl|BL_ORD_ID|61209 cb1301_c4/f1p0/3013	1	2022	2023	2855	69	2082	2198	3029
cb2907_c2/f1p1/2369	gnl|BL_ORD_ID|38597 cb2907_c3/f1p0/2605	1	1817	1815	2352	7	181	2068	260

isoform=cb2907_c2;full_length_coverage=1;non_full_length_coverage=1;isoform_length=2369;expected_accuracy=0.991	isoform=cb2907_c3;full_length_coverage=1;non_full_length_coverage=0;isoform_length=2605;expected_accuracy=0.998						9		5
cb5898_c8/f1p0/2072	gnl|BL_ORD_ID|45945 cb5898_c6/f1p0/2574 isoform=cb5898_c6;full_length_coverage=1;non_full_length_coverage=0;isoform_length=2574;expected_accuracy=0.994	189	2051	1	190	697	2557	23	212
cb3586_c11/f1p0/2822 isoform=cb3586_c11;full_length_coverage=1;non_full_length_coverage=0;isoform_length=2822;expected_accuracy=0.995	gnl|BL_ORD_ID|41150 cb3586_c3/f1p0/2988 isoform=cb3586_c3;full_length_coverage=1;non_full_length_coverage=0;isoform_length=2988;expected_accuracy=0.993	278	2822	1	278	443	2988	1	274
cb2935_c11/f1p0/3067 isoform=cb2935_c11;full_length_coverage=1;non_full_length_coverage=0;isoform_length=3067;expected_accuracy=0.999	gnl|BL_ORD_ID|37468 cb2935_c23/f1p0/3325 isoform=cb2935_c23;full_length_coverage=1;non_full_length_coverage=0;isoform_length=3325;expected_accuracy=0.994	259	3067	104	263	429	3259	3	164
cb8564_c186209/f1p0/2931 isoform=cb8564_c186209;full_length_coverage=1;non_full_length_coverage=0;isoform_length=2931;expected_accuracy=0.996	gnl|BL_ORD_ID|17429 cb8564_c85447/f1p0/3196 isoform=cb8564_c85447;full_length_coverage=1;non_full_length_coverage=0;isoform_length=3196;expected_accuracy=0.997	1	2399	2398	2931	3	2413	2660	3193
cb8564_c45300/f1p4/3014 isoform=cb8564_c45300;full_length_coverage=1;non_full_length_coverage=4;isoform_length=3014;expected_accuracy=0.996	gnl|BL_ORD_ID|14539 cb8564_c46562/f2p3/3196 isoform=cb8564_c46562;full_length_coverage=2;non_full_length_coverage=3;isoform_length=3196;expected_accuracy=0.995	243	3012	10	246	417	3195	16	260
cb5857_c26/f1p1/2735	gnl|BL_ORD_ID|45453 cb5857_c9/f1p1/2844 isoform=cb5857_c9;full_length_coverage=1;non_full_length_coverage=1;isoform_length=2844;expected_accura	1	1752	1751	2620	59	1809	1967	2835

	cy=0.996								
cb9512_c14/f1p0/1856 isoform=cb9512_c14;full_length_coverage=1;non_full_length_coverage=0;isoform_length=1856;expected_accuracy=0.998	gnl|BL_ORD_ID|22018 cb9512_c6/f1p0/1979 isoform=cb9512_c6;full_length_coverage=1;non_full_length_coverage=0;isoform_length=1979;expected_accuracy=0.993	1	1294	1292	1856	12	1301	1416	1979
cb4233_c84/f1p1/2344	gnl|BL_ORD_ID|42617 cb4233_c37/f2p3/2739 isoform=cb4233_c37;full_length_coverage=2;non_full_length_coverage=3;isoform_length=2739;expected_accuracy=0.992	227	2391	1	225	527	2694	502	727
cb8564_c21052/f1p2/3145	gnl|BL_ORD_ID|18820 cb8564_c8764/f7p3/3755 isoform=cb8564_c8764;full_length_coverage=7;non_full_length_coverage=3;isoform_length=3755;expected_accuracy=1.000	244	3055	16	249	943	3755	1	236
cb8564_c186209/f1p0/2931 isoform=cb8564_c186209;full_length_coverage=1;non_full_length_coverage=0;isoform_length=2931;expected_accuracy=0.996	gnl|BL_ORD_ID|55920 cb8564_c48949/f1p0/4357	1	2269	2270	2931	2	2293	2847	3505
cb2771_c12/f1p1/2633 isoform=cb2771_c12;full_length_coverage=1;non_full_length_coverage=1;isoform_length=2633;expected_accuracy=0.993	gnl|BL_ORD_ID|37782 cb2771_c1/f5p1/3188 isoform=cb2771_c1;full_length_coverage=5;non_full_length_coverage=1;isoform_length=3188;expected_accuracy=1.000	1	1952	1947	2629	51	2004	2496	3188
cb8564_c20798/f1p1/2960	gnl|BL_ORD_ID|53630 cb8564_c134203/f1p0/3255	1314	2932	1	1319	1481	3077	15	1337
cb2097_c120/f1p0/1809 isoform=cb2097_c120;full_length_coverage=1;non_full_length_coverage=0;isoform_length=1809;expected_acc	gnl|BL_ORD_ID|66710 cb2097_c110/f1p0/2370	1	1422	1420	1809	417	1838	1948	2337

uracy=0.994									
cb583_c0/f3p0/1982 isoform=cb583_c0;full_length_coverage=3;non_full_length_coverage=0;isoform_length=1982;expected_accuracy=0.996	gnl|BL_ORD_ID|46617 cb583_c3/f1p0/2797 isoform=cb583_c3;full_length_coverage=1;non_full_length_coverage=0;isoform_length=2797;expected_accuracy=0.995	1	1174	1171	1957	4	1180	1991	2780
cb8564_c2223/f1p0/2047	gnl|BL_ORD_ID|13231 cb8564_c2282/f1p1/3052 isoform=cb8564_c2282;full_length_coverage=1;non_full_length_coverage=1;isoform_length=3052;expected_accuracy=0.995	434	2042	6	439	1446	3052	1	434
cb3613_c4/f1p0/2552	gnl|BL_ORD_ID|41041 cb3613_c3/f1p0/2896 isoform=cb3613_c3;full_length_coverage=1;non_full_length_coverage=0;isoform_length=2896;expected_accuracy=0.995	15	2190	2185	2538	21	2186	2544	2896
cb10190_c4/f1p0/1454 isoform=cb10190_c4;full_length_coverage=1;non_full_length_coverage=0;isoform_length=1454;expected_accuracy=0.992	gnl|BL_ORD_ID|48195 cb10190_c2/f1p0/2194	1	1331	1330	1454	8	1338	1882	2006
cb14139_c2/f1p0/1763 isoform=cb14139_c2;full_length_coverage=1;non_full_length_coverage=0;isoform_length=1763;expected_accuracy=0.991	gnl|BL_ORD_ID|21468 cb9813_c26/f1p0/2710 isoform=cb9813_c26;full_length_coverage=1;non_full_length_coverage=0;isoform_length=2710;expected_accuracy=0.990	10	1035	1035	1763	1	1024	1962	2688
cb1333_c99/f1p0/1469 isoform=cb1333_c99;full_length_coverage=1;non_full_length_coverage=0;isoform_length=1469;expected_accuracy=0.996	gnl|BL_ORD_ID|61257 cb1333_c104/f1p0/5565	145	1468	1	146	3606	4940	1	146
cb1333_c101/f1p0/1395	gnl|BL_ORD_ID|61256 cb1333_c103/f1p0/5663	149	1395	27	150	3805	504	16	139

isoform=cb1333_c101;full_length_coverage=1;non_full_length_coverage=0;isoform_length=1395;expected_accuracy=0.997							9		
cb8564_c44698/f1p5/2843 isoform=cb8564_c44698;full_length_coverage=1;non_full_length_coverage=5;isoform_length=2843;expected_accuracy=0.997	gnl|BL_ORD_ID|13512 cb8564_c186209/f1p0/2931 isoform=cb8564_c186209;full_length_coverage=1;non_full_length_coverage=0;isoform_length=2931;expected_accuracy=0.996	1	2516	2511	2827	1	2492	2613	2931
cb792_c8/f3p0/3700 isoform=cb792_c8;full_length_coverage=3;non_full_length_coverage=0;isoform_length=3700;expected_accuracy=1.000	gnl|BL_ORD_ID|8413 cb792_c27/f1p3/4257 isoform=cb792_c27;full_length_coverage=1;non_full_length_coverage=3;isoform_length=4257;expected_accuracy=0.993	186	3691	1	187	741	4229	431	623
cb3007_c9/f1p0/2400	gnl|BL_ORD_ID|70130 cb3007_c5/f1p0/2390	1134	2300	1	1139	1241	2408	1	1139
cb8564_c43450/f1p0/2695	gnl|BL_ORD_ID|55873 cb8564_c47701/f1p0/3144	13	1886	1882	2714	24	1927	2042	2876
cb233_c19/f1p0/2802 isoform=cb233_c19;full_length_coverage=1;non_full_length_coverage=0;isoform_length=2802;expected_accuracy=0.992	gnl|BL_ORD_ID|38487 cb233_c6/f2p1/3007 isoform=cb233_c6;full_length_coverage=2;non_full_length_coverage=1;isoform_length=3007;expected_accuracy=0.998	1	2203	2202	2802	15	2220	2348	2951
cb8564_c90455/f1p2/3367 isoform=cb8564_c90455;full_length_coverage=1;non_full_length_coverage=2;isoform_length=3367;expected_accuracy=0.993	gnl|BL_ORD_ID|9651 cb8564_c11608/f1p2/3419 isoform=cb8564_c11608;full_length_coverage=1;non_full_length_coverage=2;isoform_length=3419;expected_accuracy=0.993	22	3071	3073	3349	1	3044	3147	3418
cb8564_c51722/f1p1/3503	gnl|BL_ORD_ID|55195 cb8564_c35170/f1p0/3386	541	3206	67	540	738	3399	22	497
cb188_c4/f1p1/2870	gnl|BL_ORD_ID|68083 cb188_c1/f1p1/4971	1026	2818	1	1027	3054	484	1217	224

isoform=cb188_c4;full_length_coverage=1;non_full_length_coverage=1;isoform_length=2870;expected_accuracy=0.998							6		3
cb8564_c76982/f1p0/3523	gnl|BL_ORD_ID|16157 cb8564_c77802/f1p0/3651 isoform=cb8564_c77802;full_length_coverage=1;non_full_length_coverage=0;isoform_length=3651;expected_accuracy=0.996	1	2599	2600	3368	100	2693	2810	3575
cb1138_c38/f1p0/3611	gnl|BL_ORD_ID|23011 cb1138_c11/f1p2/3819 isoform=cb1138_c11;full_length_coverage=1;non_full_length_coverage=2;isoform_length=3819;expected_accuracy=0.997	1	2760	2759	3430	1	2759	3148	3819
cb12302_c31/f1p0/1548 isoform=cb12302_c31;full_length_coverage=1;non_full_length_coverage=0;isoform_length=1548;expected_accuracy=1.000	gnl|BL_ORD_ID|27346 cb1380_c17/f1p1/1771 isoform=cb1380_c17;full_length_coverage=1;non_full_length_coverage=1;isoform_length=1771;expected_accuracy=0.993	324	1502	5	329	586	1771	3	335
cb2966_c1/f2p0/2466 isoform=cb2966_c1;full_length_coverage=2;non_full_length_coverage=0;isoform_length=2466;expected_accuracy=0.999	gnl|BL_ORD_ID|70108 cb2966_c2/f1p0/3755	162	2441	1	165	1388	3665	1	163
cb1926_c7/f1p0/3660 isoform=cb1926_c7;full_length_coverage=1;non_full_length_coverage=0;isoform_length=3660;expected_accuracy=0.994	gnl|BL_ORD_ID|34503 cb1926_c3/f1p1/4309 isoform=cb1926_c3;full_length_coverage=1;non_full_length_coverage=1;isoform_length=4309;expected_accuracy=0.996	1	3453	3452	3660	1	3448	3588	3798
cb6441_c35/f9p0/1985 isoform=cb6441_c35;full_length_coverage=9;non_full_length_coverage=0;isoform_length=1985;expected_accu	gnl|BL_ORD_ID|6112 cb6441_c9/f1p0/2855 isoform=cb6441_c9;full_length_coverage=1;non_full_length_coverage=0;isoform_length=2855;expected_accura	182	1965	1	183	1074	2854	1	180

racy=1.000	cy=0.991								
cb2737_c64/f1p1/2838 isoform=cb2737_c64;full_length_coverage=1;non_full_length_coverage=1;isoform_length=2838;expected_accuracy=0.993	gnl|BL_ORD_ID|68786 cb2737_c50/f1p0/3706	124	1537	1535	2838	673	2082	2214	3503
cb8564_c15376/f1p0/4060	gnl|BL_ORD_ID|11947 cb8564_c146099/f10p9/3818 isoform=cb8564_c146099;full_length_coverage=10;non_full_length_coverage=9;isoform_length=3818;expected_accuracy=1.000	12	2717	2717	3675	39	2742	2846	3804
cb3618_c19/f1p0/3191	gnl|BL_ORD_ID|71384 cb3618_c17/f1p0/3242	1	2434	2434	2868	16	2443	2801	3233
cb8564_c21572/f1p1/3061 isoform=cb8564_c21572;full_length_coverage=1;non_full_length_coverage=1;isoform_length=3061;expected_accuracy=0.991	gnl|BL_ORD_ID|54442 cb8564_c122516/f1p2/3535	1127	3061	1	1125	1461	3376	181	1306
cb8564_c186243/f1p0/2948 isoform=cb8564_c186243;full_length_coverage=1;non_full_length_coverage=0;isoform_length=2948;expected_accuracy=0.995	gnl|BL_ORD_ID|54034 cb8564_c17220/f1p1/3235	1	1986	1981	2948	1	1987	2151	3118
cb8564_c45699/f1p0/3005	gnl|BL_ORD_ID|14531 cb8564_c41778/f1p9/3482 isoform=cb8564_c41778;full_length_coverage=1;non_full_length_coverage=9;isoform_length=3482;expected_accuracy=0.992	229	2850	1	233	812	3441	1	247
cb6121_c28/f7p0/1771 isoform=cb6121_c28;full_length_coverage=7;non_full_length_coverage=0;isoform_length=1771;expected_accu	gnl|BL_ORD_ID|46546 cb6121_c20/f1p0/1804 isoform=cb6121_c20;full_length_coverage=1;non_full_length_coverage=0;isoform_length=1804;expected_accur	657	1771	75	658	688	1801	1	584

racy=1.000	acy=0.995								
cb3381_c21/f1p0/3306	gnl|BL_ORD_ID|71158 cb3381_c7/f1p0/3623	756	3190	77	757	1166	3600	3	683
cb7832_c4/f1p0/2427 isoform=cb7832_c4;full_length_coverage=1;non_full_length_coverage=0;isoform_length=2427;expected_accuracy=0.996	gnl|BL_ORD_ID|50746 cb7832_c2/f1p0/2644	1	1349	1347	2363	161	1510	1611	2630
cb8564_c111493/f3p5/2014 isoform=cb8564_c111493;full_length_coverage=3;non_full_length_coverage=5;isoform_length=2014;expected_accuracy=1.000	gnl|BL_ORD_ID|18502 cb8564_c978/f1p2/2552 isoform=cb8564_c978;full_length_coverage=1;non_full_length_coverage=2;isoform_length=2552;expected_accuracy=0.994	1	1460	1458	1940	1	1456	2074	2552
cb965_c40/f1p0/4415	gnl|BL_ORD_ID|20096 cb965_c4/f20p1/4337 isoform=cb965_c4;full_length_coverage=20;non_full_length_coverage=1;isoform_length=4337;expected_accuracy=1.000	1	2886	2882	4029	89	2973	3190	4337
cb8564_c48153/f1p0/3083	gnl|BL_ORD_ID|16172 cb8564_c53024/f1p0/3159 isoform=cb8564_c53024;full_length_coverage=1;non_full_length_coverage=0;isoform_length=3159;expected_accuracy=0.996	13	1740	1737	2879	1	1699	2017	3157
cb8564_c184250/f1p0/2256 isoform=cb8564_c184250;full_length_coverage=1;non_full_length_coverage=0;isoform_length=2256;expected_accuracy=0.996	gnl|BL_ORD_ID|53144 cb8564_c117565/f1p0/2837	1	1642	1638	2256	1	1650	1911	2527
cb2815_c0/f2p0/3776 isoform=cb2815_c0;full_length_coverage=2;non_full_length_coverage=0;isoform_length=3776;expected_accur	gnl|BL_ORD_ID|37533 cb2815_c3/f1p0/5228 isoform=cb2815_c3;full_length_coverage=1;non_full_length_coverage=0;isoform_length=5228;expected_accura	14	2801	2800	3671	35	2829	4356	5227

acy=1.000	cy=0.995								
cb6631_c25/f1p0/1946 isoform=cb6631_c25;full_length_coverage=1;non_full_length_coverage=0;isoform_length=1946;expected_accuracy=0.996	gnl|BL_ORD_ID|47610 cb6631_c20/f1p1/2421	1	1189	1186	1946	15	1203	1352	2117
cb8564_c21527/f7p0/3631 isoform=cb8564_c21527;full_length_coverage=7;non_full_length_coverage=0;isoform_length=3631;expected_accuracy=1.000	gnl|BL_ORD_ID|11711 cb8564_c14355/f1p0/3726 isoform=cb8564_c14355;full_length_coverage=1;non_full_length_coverage=0;isoform_length=3726;expected_accuracy=0.995	1634	3595	1	1633	1768	3726	1	1632
cb12034_c6/f1p0/1822	gnl|BL_ORD_ID|56457 cb8564_c77357/f1p0/3806	132	1609	1	133	2284	3749	12	141
cb10253_c8/f1p0/1576 isoform=cb10253_c8;full_length_coverage=1;non_full_length_coverage=0;isoform_length=1576;expected_accuracy=0.997	gnl|BL_ORD_ID|48290 cb10253_c5/f1p0/1732	338	1575	8	342	438	1675	2	336
cb8564_c2539/f1p1/2062 isoform=cb8564_c2539;full_length_coverage=1;non_full_length_coverage=1;isoform_length=2062;expected_accuracy=0.997	gnl|BL_ORD_ID|54774 cb8564_c2529/f1p0/3439	1	1646	1644	2062	5	1644	2807	3224
cb233_c19/f1p0/2802 isoform=cb233_c19;full_length_coverage=1;non_full_length_coverage=0;isoform_length=2802;expected_accuracy=0.992	gnl|BL_ORD_ID|38488 cb233_c5/f2p0/2943 isoform=cb233_c5;full_length_coverage=2;non_full_length_coverage=0;isoform_length=2943;expected_accuracy=1.000	1	2203	2202	2767	23	2230	2375	2943
cb8564_c21764/f1p0/2599	gnl|BL_ORD_ID|58004 cb8564_c86464/f1p0/2629	1	2030	2030	2619	85	2112	1996	2585
cb8564_c53418/f1p0/2602	gnl|BL_ORD_ID|9475 cb8564_c108093/f2p11/2745	1163	2593	1	1168	1283	273	11	1182

isoform=cb8564_c53418;full_length_coverage=1;non_full_length_coverage=0;isoform_length=2602;expected_accuracy=0.991	isoform=cb8564_c108093;full_length_coverage=2;non_full_length_coverage=11;isoform_length=2745;expected_accuracy=0.998						8		
cb5422_c6/f1p0/1857	gnl|BL_ORD_ID|73898 cb5422_c10/f1p0/5864	1	1128	1127	1847	186	1313	4803	5521
cb8564_c119193/f1p2/2975 isoform=cb8564_c119193;full_length_coverage=1;non_full_length_coverage=2;isoform_length=2975;expected_accuracy=0.998	gnl|BL_ORD_ID|17429 cb8564_c85447/f1p0/3196 isoform=cb8564_c85447;full_length_coverage=1;non_full_length_coverage=0;isoform_length=3196;expected_accuracy=0.997	1	2439	2438	2975	3	2413	2660	3192
cb17137_c7/f1p1/1834	gnl|BL_ORD_ID|9295 cb8564_c121088/f1p1/2137 isoform=cb8564_c121088;full_length_coverage=1;non_full_length_coverage=1;isoform_length=2137;expected_accuracy=0.998	1	1291	1287	1691	95	1361	1732	2137
cb8564_c37409/f1p0/3153	gnl|BL_ORD_ID|55920 cb8564_c48949/f1p0/4357	1	2284	2285	2948	2	2293	2847	3503
cb8564_c90747/f1p0/2795	gnl|BL_ORD_ID|14058 cb8564_c23936/f1p0/3016 isoform=cb8564_c23936;full_length_coverage=1;non_full_length_coverage=0;isoform_length=3016;expected_accuracy=0.995	1	1686	1684	2557	4	1716	2143	3016
cb8964_c10/f1p0/1945 isoform=cb8964_c10;full_length_coverage=1;non_full_length_coverage=0;isoform_length=1945;expected_accuracy=0.995	gnl|BL_ORD_ID|58475 cb8964_c4/f1p0/2407	1	1503	1503	1902	43	1546	1856	2252
cb18782_c0/f1p2/5630 isoform=cb18782_c0;full_length_coverage=1;non_full_length_coverage=2;isoform_length=5630;expected_accu	gnl|BL_ORD_ID|35903 cb18782_c2/f1p2/5753 isoform=cb18782_c2;full_length_coverage=1;non_full_length_coverage=2;isoform_length=5753;expected_accur	1	4306	4304	5565	35	4339	4474	5753

racy=0.997	acy=0.991								
cb4840_c11/f1p0/1383	gnl|BL_ORD_ID|73021 cb4840_c6/f1p0/2066	215	1341	30	214	567	1693	3	187
cb8564_c77994/f1p2/2630 isoform=cb8564_c77994;full_length_coverage=1;non_full_length_coverage=2;isoform_length=2630;expected_accuracy=0.998	gnl|BL_ORD_ID|56338 cb8564_c74689/f1p0/3037	1	1819	1815	2630	16	1835	1979	2795
cb4798_c12/f1p0/2329 isoform=cb4798_c12;full_length_coverage=1;non_full_length_coverage=0;isoform_length=2329;expected_accuracy=0.996	gnl|BL_ORD_ID|43893 cb4798_c11/f1p0/2939 isoform=cb4798_c11;full_length_coverage=1;non_full_length_coverage=0;isoform_length=2939;expected_accuracy=0.992	1	1240	1239	2329	2	1243	1845	2937
cb8564_c125167/f1p0/2703 isoform=cb8564_c125167;full_length_coverage=1;non_full_length_coverage=0;isoform_length=2703;expected_accuracy=0.996	gnl|BL_ORD_ID|12046 cb8564_c126049/f1p0/3874 isoform=cb8564_c126049;full_length_coverage=1;non_full_length_coverage=0;isoform_length=3874;expected_accuracy=0.997	1	1753	1751	2693	1059	2835	2942	3874
cb8564_c177939/f1p0/1977 isoform=cb8564_c177939;full_length_coverage=1;non_full_length_coverage=0;isoform_length=1977;expected_accuracy=1.000	gnl|BL_ORD_ID|14025 cb8564_c31262/f7p4/2729 isoform=cb8564_c31262;full_length_coverage=7;non_full_length_coverage=4;isoform_length=2729;expected_accuracy=0.994	1	1724	1723	1977	139	1861	2383	2634
cb5819_c9/f1p0/2331	gnl|BL_ORD_ID|45136 cb5819_c17/f2p0/2480 isoform=cb5819_c17;full_length_coverage=2;non_full_length_coverage=0;isoform_length=2480;expected_accuracy=0.996	366	2258	5	369	587	2479	60	424
cb6271_c9/f1p0/1555 isoform=cb6271_c9;full_length_coverage=1;non_full_length_coverage=0;isoform_length=1555;expected_accur	gnl|BL_ORD_ID|49572 cb6271_c2/f1p0/2897	1	1364	1359	1555	93	1457	2112	2314

acy=1.000									
cb8564_c50128/f1p0/4343	gnl|BL_ORD_ID|16628 cb8564_c47737/f7p5/4351 isoform=cb8564_c47737;full_length_coverage=7;non_full_length_coverage=5;isoform_length=4351;expected_accuracy=0.998	297	4077	15	301	399	4179	1	280
cb765_c15/f1p0/3098	gnl|BL_ORD_ID|49457 cb765_c6/f1p0/4514	1	2374	2372	2793	1575	3948	4090	4511
cb2773_c15/f1p0/1745 isoform=cb2773_c15;full_length_coverage=1;non_full_length_coverage=0;isoform_length=1745;expected_accuracy=1.000	gnl|BL_ORD_ID|68835 cb2773_c5/f1p0/2867	1	1395	1395	1745	13	1408	2513	2865
cb2430_c6/f1p0/3031 isoform=cb2430_c6;full_length_coverage=1;non_full_length_coverage=0;isoform_length=3031;expected_accuracy=0.998	gnl|BL_ORD_ID|38548 cb2430_c4/f2p0/3285 isoform=cb2430_c4;full_length_coverage=2;non_full_length_coverage=0;isoform_length=3285;expected_accuracy=0.992	1	2608	2607	3031	1	2616	2857	3285
cb1333_c59/f1p0/2058	gnl|BL_ORD_ID|26654 cb1333_c1/f7p2/5095 isoform=cb1333_c1;full_length_coverage=7;non_full_length_coverage=2;isoform_length=5095;expected_accuracy=0.999	153	2065	1	152	3183	5095	47	198
cb18708_c1/f1p0/1670	gnl|BL_ORD_ID|57291 cb8564_c89181/f1p1/2994	1	1005	1002	1675	715	1719	2317	2990
cb16130_c1/f2p0/1014 isoform=cb16130_c1;full_length_coverage=2;non_full_length_coverage=0;isoform_length=1014;expected_accuracy=0.997	gnl|BL_ORD_ID|12256 cb8564_c129456/f1p0/2332 isoform=cb8564_c129456;full_length_coverage=1;non_full_length_coverage=0;isoform_length=2332;expected_accuracy=0.997	244	1010	8	243	1572	2332	1	235
cb99_c7/f1p0/3479	gnl|BL_ORD_ID|21972 cb99_c4/f1p1/4938	144	3479	1	144	1284	463	32	169

isoform=cb99_c7;full_length_coverage=1;non_full_length_coverage=0;isoform_length=3479;expected_accuracy=0.990	isoform=cb99_c4;full_length_coverage=1;non_full_length_coverage=1;isoform_length=4938;expected_accuracy=0.999						2		
cb1333_c101/f1p0/1395 isoform=cb1333_c101;full_length_coverage=1;non_full_length_coverage=0;isoform_length=1395;expected_accuracy=0.997	gnl|BL_ORD_ID|26023 cb1333_c10/f4p0/1937 isoform=cb1333_c10;full_length_coverage=4;non_full_length_coverage=0;isoform_length=1937;expected_accuracy=1.000	149	1395	6	148	614	1858	1	142
cb8564_c152519/f2p1/2103 isoform=cb8564_c152519;full_length_coverage=2;non_full_length_coverage=1;isoform_length=2103;expected_accuracy=1.000	gnl|BL_ORD_ID|55572 cb8564_c45654/f1p0/2343	1	1153	1152	2082	51	1202	1410	2339
cb8564_c91980/f1p0/1973 isoform=cb8564_c91980;full_length_coverage=1;non_full_length_coverage=0;isoform_length=1973;expected_accuracy=0.999	gnl|BL_ORD_ID|17247 cb8564_c87662/f4p1/3732 isoform=cb8564_c87662;full_length_coverage=4;non_full_length_coverage=1;isoform_length=3732;expected_accuracy=0.999	1	1334	1334	1973	1022	2353	3094	3732
cb10253_c8/f1p0/1576 isoform=cb10253_c8;full_length_coverage=1;non_full_length_coverage=0;isoform_length=1576;expected_accuracy=0.997	gnl|BL_ORD_ID|1693 cb10253_c6/f1p0/1651 isoform=cb10253_c6;full_length_coverage=1;non_full_length_coverage=0;isoform_length=1651;expected_accuracy=1.000	338	1576	78	342	367	1605	1	265
cb4233_c110/f1p1/2847	gnl|BL_ORD_ID|72375 cb4233_c106/f1p2/3225	1	1984	1983	2544	3	1986	2666	3227
cb8564_c13789/f1p1/3323	gnl|BL_ORD_ID|9976 cb8564_c10328/f1p1/3525 isoform=cb8564_c10328;full_length_coverage=1;non_full_length_coverage=1;isoform_length=3525;expected_accuracy=0.995	2	2819	2820	3280	2	2833	3064	3525
cb8564_c119435/f1p0/2633	gnl|BL_ORD_ID|10826 cb8564_c114896/f1p2/3068	500	2389	7	503	1060	294	116	619

	isoform=cb8564_c114896;full_length_coverage=1;non_full_length_coverage=2;isoform_length=3068;expected_accuracy=0.993						3		
cb5487_c2/f1p0/3186	gnl|BL_ORD_ID|73975 cb5487_c11/f1p0/2702	1	1484	1484	2448	1	1477	1646	2608
cb8564_c49562/f1p0/2838 isoform=cb8564_c49562;full_length_coverage=1;non_full_length_coverage=0;isoform_length=2838;expected_accuracy=0.995	gnl|BL_ORD_ID|56961 cb8564_c78609/f1p0/3194	23	2331	2327	2838	45	2358	2465	2978
cb8564_c109969/f3p0/3562 isoform=cb8564_c109969;full_length_coverage=3;non_full_length_coverage=0;isoform_length=3562;expected_accuracy=0.998	gnl|BL_ORD_ID|53494 cb8564_c126420/f1p0/4861	1	3313	3308	3562	18	3328	4495	4747
cb4744_c4/f1p0/1625	gnl|BL_ORD_ID|44018 cb4744_c0/f2p0/2942 isoform=cb4744_c0;full_length_coverage=2;non_full_length_coverage=0;isoform_length=2942;expected_accuracy=0.997	1	1153	1151	1507	1	1152	2567	2923
cb8564_c20112/f1p0/3162 isoform=cb8564_c20112;full_length_coverage=1;non_full_length_coverage=0;isoform_length=3162;expected_accuracy=0.995	gnl|BL_ORD_ID|18821 cb8564_c8768/f7p0/3451 isoform=cb8564_c8768;full_length_coverage=7;non_full_length_coverage=0;isoform_length=3451;expected_accuracy=0.999	1	1640	1638	3162	114	1754	1916	3442
cb2698_c6/f1p0/1752 isoform=cb2698_c6;full_length_coverage=1;non_full_length_coverage=0;isoform_length=1752;expected_accuracy=0.998	gnl|BL_ORD_ID|37643 cb2698_c2/f1p0/1886 isoform=cb2698_c2;full_length_coverage=1;non_full_length_coverage=0;isoform_length=1886;expected_accuracy=0.995	303	1752	65	304	442	1884	26	265
cb8564_c186356/f1p0/2744	gnl|BL_ORD_ID|56727 cb8564_c66920/f3p3/2894	1	1396	1393	2740	1	139	1516	286

isoform=cb8564_c186356;full_length_coverage=1;non_full_length_coverage=0;isoform_length=2744;expected_accuracy=0.998							6		3
cb8564_c20363/f1p3/2558 isoform=cb8564_c20363;full_length_coverage=1;non_full_length_coverage=3;isoform_length=2558;expected_accuracy=0.997	gnl|BL_ORD_ID|14835 cb8564_c24261/f1p1/2563 isoform=cb8564_c24261;full_length_coverage=1;non_full_length_coverage=1;isoform_length=2563;expected_accuracy=0.995	587	2541	84	588	611	2563	1	505
cb3957_c1/f2p1/5488 isoform=cb3957_c1;full_length_coverage=2;non_full_length_coverage=1;isoform_length=5488;expected_accuracy=0.998	gnl|BL_ORD_ID|71673 cb3957_c5/f1p1/7529	2777	5486	119	2778	4833	7540	1	2652
cb1317_c11/f1p1/4136 isoform=cb1317_c11;full_length_coverage=1;non_full_length_coverage=1;isoform_length=4136;expected_accuracy=0.997	gnl|BL_ORD_ID|61229 cb1317_c8/f1p0/4195	227	4136	1	227	461	4354	6	232
cb8564_c43455/f1p0/2242 isoform=cb8564_c43455;full_length_coverage=1;non_full_length_coverage=0;isoform_length=2242;expected_accuracy=0.995	gnl|BL_ORD_ID|53489 cb8564_c126370/f2p7/2635	279	2242	33	283	554	2520	35	285
cb12662_c8/f1p0/1447	gnl|BL_ORD_ID|61792 cb12662_c11/f1p0/1530	179	1453	54	179	258	1532	1	126
cb12422_c8/f1p1/1600 isoform=cb12422_c8;full_length_coverage=1;non_full_length_coverage=1;isoform_length=1600;expected_accuracy=0.996	gnl|BL_ORD_ID|23174 cb12422_c9/f1p2/1757 isoform=cb12422_c9;full_length_coverage=1;non_full_length_coverage=2;isoform_length=1757;expected_accuracy=0.990	281	1600	7	285	392	1711	1	281
cb3644_c22/f1p0/3675	gnl|BL_ORD_ID|39863 cb3644_c15/f1p0/3548	1517	3136	13	1517	1928	354	1	147

	isoform=cb3644_c15;full_length_coverage=1;non_full_length_coverage=0;isoform_length=3548;expected_accuracy=0.995						8		7
cb8564_c86900/f1p0/3283	gnl|BL_ORD_ID|16917 cb8564_c48079/f5p1/3512 isoform=cb8564_c48079;full_length_coverage=5;non_full_length_coverage=1;isoform_length=3512;expected_accuracy=0.995	211	3083	14	213	498	3370	1	200
cb7315_c15/f1p0/1788 isoform=cb7315_c15;full_length_coverage=1;non_full_length_coverage=0;isoform_length=1788;expected_accuracy=0.992	gnl|BL_ORD_ID|6615 cb7315_c1/f3p0/2330 isoform=cb7315_c1;full_length_coverage=3;non_full_length_coverage=0;isoform_length=2330;expected_accuracy=0.995	1	1670	1667	1788	1	1674	2159	2280
cb1581_c17/f1p4/2476 isoform=cb1581_c17;full_length_coverage=1;non_full_length_coverage=4;isoform_length=2476;expected_accuracy=0.997	gnl|BL_ORD_ID|28677 cb1581_c8/f1p0/2559 isoform=cb1581_c8;full_length_coverage=1;non_full_length_coverage=0;isoform_length=2559;expected_accuracy=0.994	229	2386	1	228	402	2559	23	247
cb8564_c116345/f1p1/2198 isoform=cb8564_c116345;full_length_coverage=1;non_full_length_coverage=1;isoform_length=2198;expected_accuracy=0.991	gnl|BL_ORD_ID|11002 cb8564_c119745/f1p1/2751 isoform=cb8564_c119745;full_length_coverage=1;non_full_length_coverage=1;isoform_length=2751;expected_accuracy=0.992	369	2180	4	373	896	2707	1	388
cb9789_c4/f1p0/1770 isoform=cb9789_c4;full_length_coverage=1;non_full_length_coverage=0;isoform_length=1770;expected_accuracy=1.000	gnl|BL_ORD_ID|20884 cb9789_c2/f1p0/1933 isoform=cb9789_c2;full_length_coverage=1;non_full_length_coverage=0;isoform_length=1933;expected_accuracy=0.995	366	1769	5	370	519	1923	14	382
cb17104_c0/f1p0/1194 isoform=cb17104_c0;full_length_coverage=1;non_full_length_coverage=0;isoform_length=1194;expected_accu	gnl|BL_ORD_ID|33287 cb17104_c5/f1p0/1481 isoform=cb17104_c5;full_length_coverage=1;non_full_length_coverage=0;isoform_length=1481;expected_accur	113	1190	1	115	404	1481	95	209

racy=1.000	acy=0.993								
cb13565_c28/f1p4/1411 isoform=cb13565_c28;full_length_coverage=1;non_full_length_coverage=4;isoform_length=1411;expected_accuracy=0.998	gnl|BL_ORD_ID|27684 cb13565_c65/f1p4/1614 isoform=cb13565_c65;full_length_coverage=1;non_full_length_coverage=4;isoform_length=1614;expected_accuracy=1.000	143	1410	44	147	349	1614	61	164
cb8564_c38233/f1p0/2780	gnl|BL_ORD_ID|15649 cb8564_c69003/f5p1/3374 isoform=cb8564_c69003;full_length_coverage=5;non_full_length_coverage=1;isoform_length=3374;expected_accuracy=1.000	1	1857	1856	2785	220	2076	2201	3130
cb8564_c145424/f8p2/3323 isoform=cb8564_c145424;full_length_coverage=8;non_full_length_coverage=2;isoform_length=3323;expected_accuracy=0.998	gnl|BL_ORD_ID|52661 cb8564_c10315/f1p0/4512	443	3310	73	443	1549	4416	1	371
cb2737_c40/f1p0/2830	gnl|BL_ORD_ID|36735 cb2737_c13/f1p0/3105 isoform=cb2737_c13;full_length_coverage=1;non_full_length_coverage=0;isoform_length=3105;expected_accuracy=0.995	1	1397	1395	2651	283	1676	1808	3063
cb4007_c46/f1p0/3154	gnl|BL_ORD_ID|40420 cb4007_c17/f1p0/3468 isoform=cb4007_c17;full_length_coverage=1;non_full_length_coverage=0;isoform_length=3468;expected_accuracy=1.000	1	2403	2399	2985	1	2404	2880	3467
cb8564_c44290/f1p1/3198	gnl|BL_ORD_ID|18852 cb8564_c84488/f1p0/3408 isoform=cb8564_c84488;full_length_coverage=1;non_full_length_coverage=0;isoform_length=3408;expected_accuracy=0.996	339	2957	5	338	791	3408	4	312
cb8889_c1/f2p1/2254	gnl|BL_ORD_ID|58440 cb8889_c5/f1p0/2686	1	1221	1221	2254	1	120	1496	253

isoform=cb8889_c1;full_length_coverage=2;non_full_length_coverage=1;isoform_length=2254;expected_accuracy=0.994							6		7
cb401_c69/f1p0/2279	gnl|BL_ORD_ID|70873 cb401_c42/f1p0/2704	1	1609	1607	2208	71	1679	2018	2619
cb8564_c14699/f1p0/3540 isoform=cb8564_c14699;full_length_coverage=1;non_full_length_coverage=0;isoform_length=3540;expected_accuracy=0.997	gnl|BL_ORD_ID|13803 cb8564_c18538/f1p0/4027 isoform=cb8564_c18538;full_length_coverage=1;non_full_length_coverage=0;isoform_length=4027;expected_accuracy=0.995	108	2874	2871	3540	1	2763	3277	3941
cb9868_c3/f1p0/1466 isoform=cb9868_c3;full_length_coverage=1;non_full_length_coverage=0;isoform_length=1466;expected_accuracy=0.996	gnl|BL_ORD_ID|21338 cb9868_c2/f1p0/1570 isoform=cb9868_c2;full_length_coverage=1;non_full_length_coverage=0;isoform_length=1570;expected_accuracy=0.998	238	1466	66	240	307	1537	1	175
cb8564_c53418/f1p0/2602 isoform=cb8564_c53418;full_length_coverage=1;non_full_length_coverage=0;isoform_length=2602;expected_accuracy=0.991	gnl|BL_ORD_ID|14105 cb8564_c35314/f1p0/2682 isoform=cb8564_c35314;full_length_coverage=1;non_full_length_coverage=0;isoform_length=2682;expected_accuracy=0.994	1163	2549	1	1168	1285	2682	4	1184
cb373_c4/f2p0/3694	gnl|BL_ORD_ID|41213 cb373_c11/f1p0/3937 isoform=cb373_c11;full_length_coverage=1;non_full_length_coverage=0;isoform_length=3937;expected_accuracy=0.996	1	3301	3302	3699	93	3395	3501	3898
cb14220_c12/f1p0/6602	gnl|BL_ORD_ID|63133 cb14220_c7/f1p0/6933	2263	6560	1	2265	2660	6950	90	2351
cb3759_c6/f1p0/2298	gnl|BL_ORD_ID|71489 cb3759_c8/f1p0/3290	1	1727	1729	2238	25	1751	2593	3102
cb5378_c2/f1p0/2616	gnl|BL_ORD_ID|73843 cb5378_c0/f1p0/3125	1	1932	1932	2616	117	205	2342	302

isoform=cb5378_c2;full_length_coverage=1;non_full_length_coverage=0;isoform_length=2616;expected_accuracy=0.999							0		7
cb8564_c36731/f1p2/2761	gnl|BL_ORD_ID|55182 cb8564_c34825/f1p2/3200	10	1977	1972	2739	6	2003	2432	3197
cb3888_c4/f1p0/3311 isoform=cb3888_c4;full_length_coverage=1;non_full_length_coverage=0;isoform_length=3311;expected_accuracy=0.993	gnl|BL_ORD_ID|71602 cb3888_c2/f1p0/3488	226	3229	1	227	460	3473	1	228
cb4016_c32/f1p0/1913	gnl|BL_ORD_ID|40895 cb4016_c12/f1p0/2681 isoform=cb4016_c12;full_length_coverage=1;non_full_length_coverage=0;isoform_length=2681;expected_accuracy=0.992	191	1930	25	194	918	2651	320	490
cb8564_c79528/f2p1/3397 isoform=cb8564_c79528;full_length_coverage=2;non_full_length_coverage=1;isoform_length=3397;expected_accuracy=0.995	gnl|BL_ORD_ID|19049 cb8564_c79989/f1p1/3569 isoform=cb8564_c79989;full_length_coverage=1;non_full_length_coverage=1;isoform_length=3569;expected_accuracy=0.997	1262	3392	108	1261	1441	3569	184	1337
cb8564_c71816/f1p1/3018	gnl|BL_ORD_ID|11730 cb8564_c146015/f9p2/3016 isoform=cb8564_c146015;full_length_coverage=9;non_full_length_coverage=2;isoform_length=3016;expected_accuracy=1.000	1	2019	2020	2916	203	2220	2120	3016
cb5616_c5/f1p0/2496 isoform=cb5616_c5;full_length_coverage=1;non_full_length_coverage=0;isoform_length=2496;expected_accuracy=0.996	gnl|BL_ORD_ID|45329 cb5616_c4/f1p0/3056 isoform=cb5616_c4;full_length_coverage=1;non_full_length_coverage=0;isoform_length=3056;expected_accuracy=1.000	307	2400	4	311	960	3056	1	308
cb12714_c29/f1p2/1583	gnl|BL_ORD_ID|57800 cb8564_c81082/f1p0/3091	1	1296	1293	1596	3	129	2515	281

							8		8
cb3106_c10/f1p1/2435 isoform=cb3106_c10;full_length_coverage=1;non_full_length_coverage=1;isoform_length=2435;expected_accuracy=0.995	gnl|BL_ORD_ID|37403 cb3106_c8/f1p0/3729 isoform=cb3106_c8;full_length_coverage=1;non_full_length_coverage=0;isoform_length=3729;expected_accuracy=0.994	1	1913	1909	2435	1	1906	3143	3666
cb8564_c106810/f5p0/2936 isoform=cb8564_c106810;full_length_coverage=5;non_full_length_coverage=0;isoform_length=2936;expected_accuracy=1.000	gnl|BL_ORD_ID|19026 cb8564_c83568/f1p0/3138 isoform=cb8564_c83568;full_length_coverage=1;non_full_length_coverage=0;isoform_length=3138;expected_accuracy=0.995	1424	2855	1	1424	1711	3138	20	1477
cb5307_c9/f1p0/2902	gnl|BL_ORD_ID|73767 cb5307_c3/f1p0/3130	1204	2896	1	1204	1370	3063	62	1262
cb3887_c12/f2p0/2396 isoform=cb3887_c12;full_length_coverage=2;non_full_length_coverage=0;isoform_length=2396;expected_accuracy=0.994	gnl|BL_ORD_ID|39511 cb3887_c5/f1p0/2830 isoform=cb3887_c5;full_length_coverage=1;non_full_length_coverage=0;isoform_length=2830;expected_accuracy=0.996	1	1834	1830	2396	90	1900	2239	2798
cb416_c19/f1p0/2939	gnl|BL_ORD_ID|44207 cb416_c73/f3p0/3007 isoform=cb416_c73;full_length_coverage=3;non_full_length_coverage=0;isoform_length=3007;expected_accuracy=1.000	105	2849	10	107	259	3006	56	156
cb8564_c109636/f4p0/2195 isoform=cb8564_c109636;full_length_coverage=4;non_full_length_coverage=0;isoform_length=2195;expected_accuracy=0.995	gnl|BL_ORD_ID|53478 cb8564_c126020/f1p1/2281	1	1988	1987	2195	15	1980	2084	2292
cb8564_c114729/f1p1/2091 isoform=cb8564_c114729;full_length_coverage=1;non_full_length_coverage=1;isoform_length=2091;expected	gnl|BL_ORD_ID|54774 cb8564_c2529/f1p0/3439	1	1609	1608	2091	32	1643	2761	3248

_accuracy=0.994									
cb8564_c38233/f1p0/2780	gnl|BL_ORD_ID|12520 cb8564_c146064/f3p0/3212 isoform=cb8564_c146064;full_length_coverage=3;non_full_length_coverage=0;isoform_length=3212;expected_accuracy=1.000	1	1857	1856	2782	306	216 2	2287	321 2
cb8564_c89555/f1p0/3557	gnl|BL_ORD_ID|14775 cb8564_c31987/f2p1/3601 isoform=cb8564_c31987;full_length_coverage=2;non_full_length_coverage=1;isoform_length=3601;expected_accuracy=0.993	2	2714	2715	3462	3	270 6	2854	360 0
cb8564_c20202/f1p0/2556 isoform=cb8564_c20202;full_length_coverage=1;non_full_length_coverage=0;isoform_length=2556;expected_accuracy=0.999	gnl|BL_ORD_ID|54324 cb8564_c22446/f1p0/2866	16	2227	2224	2556	1	221 6	2380	271 2
cb938_c17/f1p1/3461 isoform=cb938_c17;full_length_coverage=1;non_full_length_coverage=1;isoform_length=3461;expected_accuracy=0.991	gnl|BL_ORD_ID|59101 cb938_c9/f1p0/4170	1	2564	2564	3459	1	256 5	3262	416 3
cb7612_c6/f1p0/2279	gnl|BL_ORD_ID|8785 cb7612_c0/f1p1/2574 isoform=cb7612_c0;full_length_coverage=1;non_full_length_coverage=1;isoform_length=2574;expected_accuracy=0.991	1	1684	1682	2183	40	172 0	2073	257 4
cb8564_c7856/f2p2/3151 isoform=cb8564_c7856;full_length_coverage=2;non_full_length_coverage=2;isoform_length=3151;expected_accuracy=0.997	gnl|BL_ORD_ID|55036 cb8564_c4571/f1p0/3389	156	3151	12	153	340	333 4	7	149
cb8564_c36689/f1p1/2368	gnl|BL_ORD_ID|55962 cb8564_c50282/f1p0/4402	15	1709	1706	2368	1457	314	3642	430

isoform=cb8564_c36689;full_length_coverage=1;non_full_length_coverage=1;isoform_length=2368;expected_accuracy=0.991							3		6
cb12422_c8/f1p1/1600 isoform=cb12422_c8;full_length_coverage=1;non_full_length_coverage=1;isoform_length=1600;expected_accuracy=0.996	gnl|BL_ORD_ID|61716 cb12422_c17/f1p1/1730	281	1600	29	285	396	1716	29	285
cb12714_c29/f1p2/1583	gnl|BL_ORD_ID|52845 cb8564_c15530/f1p0/3247	1	1296	1293	1597	6	1301	2823	3127
cb8564_c72963/f1p0/2583	gnl|BL_ORD_ID|58115 cb8564_c89322/f1p0/2588	1	1279	1274	2367	1	1281	1494	2588
cb8564_c52246/f1p1/2752	gnl|BL_ORD_ID|14028 cb8564_c31992/f2p0/4284 isoform=cb8564_c31992;full_length_coverage=2;non_full_length_coverage=0;isoform_length=4284;expected_accuracy=0.997	1	1284	1283	2494	14	1295	3049	4284
cb8564_c47011/f1p0/3100	gnl|BL_ORD_ID|55894 cb8564_c48160/f1p2/3308	11	2306	2301	2934	7	2317	2555	3188
cb6063_c13/f1p1/2429	gnl|BL_ORD_ID|45238 cb6063_c0/f15p2/2941 isoform=cb6063_c0;full_length_coverage=15;non_full_length_coverage=2;isoform_length=2941;expected_accuracy=1.000	119	2436	1	120	620	2937	119	238
cb13515_c9/f1p0/1348	gnl|BL_ORD_ID|26400 cb13515_c10/f2p0/1494 isoform=cb13515_c10;full_length_coverage=2;non_full_length_coverage=0;isoform_length=1494;expected_accuracy=0.997	1	1117	1114	1290	93	1208	1317	1494
cb332_c3/f1p1/4544	gnl|BL_ORD_ID|39701 cb332_c10/f2p0/5265	1989	4553	16	1994	2697	526	1	198

	isoform=cb332_c10;full_length_coverage=2;non_full_length_coverage=0;isoform_length=5265;expected_accuracy=0.999						5		5
cb1788_c6/f1p0/3148	gnl|BL_ORD_ID|31765 cb1788_c0/f1p0/4170 isoform=cb1788_c0;full_length_coverage=1;non_full_length_coverage=0;isoform_length=4170;expected_accuracy=0.996	146	3063	1	148	1159	4077	47	194
cb8564_c148588/f5p0/2542 isoform=cb8564_c148588;full_length_coverage=5;non_full_length_coverage=0;isoform_length=2542;expected_accuracy=1.000	gnl|BL_ORD_ID|55184 cb8564_c34882/f1p0/2687	1	2212	2210	2523	3	2214	2376	2689
cb4047_c8/f1p0/2511	gnl|BL_ORD_ID|40087 cb4047_c4/f3p2/2787 isoform=cb4047_c4;full_length_coverage=3;non_full_length_coverage=2;isoform_length=2787;expected_accuracy=0.999	1	1760	1756	2495	98	1858	1981	2720
cb8564_c182028/f1p0/2513 isoform=cb8564_c182028;full_length_coverage=1;non_full_length_coverage=0;isoform_length=2513;expected_accuracy=0.996	gnl|BL_ORD_ID|18764 cb8564_c84695/f1p1/2710 isoform=cb8564_c84695;full_length_coverage=1;non_full_length_coverage=1;isoform_length=2710;expected_accuracy=0.998	1	1971	1968	2513	1	1967	2111	2658
cb8564_c77371/f1p1/2767	gnl|BL_ORD_ID|13431 cb8564_c19588/f1p0/3337 isoform=cb8564_c19588;full_length_coverage=1;non_full_length_coverage=0;isoform_length=3337;expected_accuracy=0.997	18	2055	2055	2490	1	2039	2902	3337
cb8564_c20519/f1p0/3425 isoform=cb8564_c20519;full_length_coverage=1;non_full_length_coverage=0;isoform_length=3425;expected_	gnl|BL_ORD_ID|13018 cb8564_c17689/f1p0/3643 isoform=cb8564_c17689;full_length_coverage=1;non_full_length_coverage=0;isoform_length=3643;expected_ac	1	1917	1913	3425	8	1933	2038	3551

accuracy=0.994	curacy=0.995								
cb16383_c7/f1p0/1854	gnl|BL_ORD_ID|57305 cb8564_c89458/f2p0/3479	1	1027	1028	1848	1	1027	1158	1978
cb7642_c53/f1p0/1947 isoform=cb7642_c53;full_length_coverage=1;non_full_length_coverage=0;isoform_length=1947;expected_accuracy=0.990	gnl|BL_ORD_ID|8873 cb7642_c75/f1p1/2061 isoform=cb7642_c75;full_length_coverage=1;non_full_length_coverage=1;isoform_length=2061;expected_accuracy=0.997	1	1181	1178	1947	1	1159	1266	2036
cb2770_c58/f1p0/3220 isoform=cb2770_c58;full_length_coverage=1;non_full_length_coverage=0;isoform_length=3220;expected_accuracy=0.995	gnl|BL_ORD_ID|68823 cb2770_c36/f1p0/3443	1	2347	2347	3165	12	2322	2423	3239
cb2533_c10/f1p0/2206 isoform=cb2533_c10;full_length_coverage=1;non_full_length_coverage=0;isoform_length=2206;expected_accuracy=0.995	gnl|BL_ORD_ID|38794 cb2533_c9/f1p0/3092 isoform=cb2533_c9;full_length_coverage=1;non_full_length_coverage=0;isoform_length=3092;expected_accuracy=1.000	1	1146	1144	2200	39	1186	2060	3092
cb3449_c13/f1p2/3050 isoform=cb3449_c13;full_length_coverage=1;non_full_length_coverage=2;isoform_length=3050;expected_accuracy=0.993	gnl|BL_ORD_ID|71216 cb3449_c35/f1p1/3670	1	2755	2752	3050	51	2806	2974	3273
cb90_c9/f1p0/4970 isoform=cb90_c9;full_length_coverage=1;non_full_length_coverage=0;isoform_length=4970;expected_accuracy=0.999	gnl|BL_ORD_ID|20924 cb90_c49/f1p0/5038 isoform=cb90_c49;full_length_coverage=1;non_full_length_coverage=0;isoform_length=5038;expected_accuracy=0.998	1184	4889	1	1183	1333	5038	1	1179
cb5457_c6/f1p0/1981	gnl|BL_ORD_ID|44616 cb5457_c3/f1p0/2089 isoform=cb5457_c3;full_length_coverage=1;non_full_length_coverage=0;isoform_length=2089;expected_accura	1	1670	1670	1873	79	1748	1886	2089

	cy=0.995								
cb8564_c123664/f1p0/3544	gnl|BL_ORD_ID|52107 cb8564_c111147/f1p0/3741	894	3376	93	895	1243	3725	1	780
cb3590_c7/f1p0/2823 isoform=cb3590_c7;full_length_coverage=1;non_full_length_coverage=0;isoform_length=2823;expected_accuracy=0.992	gnl|BL_ORD_ID|71358 cb3590_c4/f1p0/3544	1	2404	2402	2823	31	2433	3111	3531
cb6474_c13/f1p0/1404 isoform=cb6474_c13;full_length_coverage=1;non_full_length_coverage=0;isoform_length=1404;expected_accuracy=0.993	gnl|BL_ORD_ID|4758 cb6474_c12/f1p0/1699 isoform=cb6474_c12;full_length_coverage=1;non_full_length_coverage=0;isoform_length=1699;expected_accuracy=0.994	177	1404	6	180	322	1549	6	171
cb2737_c57/f1p0/2959	gnl|BL_ORD_ID|36571 cb2737_c54/f1p0/3029 isoform=cb2737_c54;full_length_coverage=1;non_full_length_coverage=0;isoform_length=3029;expected_accuracy=0.995	1	1981	1982	2862	25	1998	2110	2988
cb8564_c114347/f1p0/2560	gnl|BL_ORD_ID|14245 cb8564_c33844/f4p0/2678 isoform=cb8564_c33844;full_length_coverage=4;non_full_length_coverage=0;isoform_length=2678;expected_accuracy=0.999	1	1649	1648	2439	1	1648	1876	2667
cb8564_c43779/f1p0/3021 isoform=cb8564_c43779;full_length_coverage=1;non_full_length_coverage=0;isoform_length=3021;expected_accuracy=0.993	gnl|BL_ORD_ID|13575 cb8564_c20499/f3p0/3328 isoform=cb8564_c20499;full_length_coverage=3;non_full_length_coverage=0;isoform_length=3328;expected_accuracy=1.000	1	2344	2345	3021	1	2345	2501	3176
cb4576_c58/f1p1/3963	gnl|BL_ORD_ID|72706 cb4576_c62/f1p0/4079	426	3855	8	424	618	4047	27	443
cb18456_c3695/f1p0/1345	gnl|BL_ORD_ID|13109 cb8564_c16444/f5p10/3053	1	1051	1047	1341	785	183	2010	230

	isoform=cb8564_c16444;full_length_coverage=5;non_full_length_coverage=10;isoform_length=3053;expected_accuracy=0.992						5		4
cb653_c9/f1p0/4025 isoform=cb653_c9;full_length_coverage=1;non_full_length_coverage=0;isoform_length=4025;expected_accuracy=0.997	gnl|BL_ORD_ID|49383 cb653_c20/f1p2/4373	301	4020	28	301	613	4328	70	341
cb6732_c18/f1p0/2510	gnl|BL_ORD_ID|49996 cb6732_c8/f1p1/2366	621	2255	7	624	740	2374	1	625
cb8564_c52278/f1p1/2746	gnl|BL_ORD_ID|16045 cb8564_c75622/f1p0/3602 isoform=cb8564_c75622;full_length_coverage=1;non_full_length_coverage=0;isoform_length=3602;expected_accuracy=0.998	13	2426	2423	2701	1	2414	3327	3602
cb10272_c3/f5p2/1680 isoform=cb10272_c3;full_length_coverage=5;non_full_length_coverage=2;isoform_length=1680;expected_accuracy=1.000	gnl|BL_ORD_ID|48324 cb10272_c11/f1p0/2538	1	1148	1148	1623	1	1148	2015	2490
cb1490_c6/f1p2/3547	gnl|BL_ORD_ID|30687 cb1490_c2/f1p1/4303 isoform=cb1490_c2;full_length_coverage=1;non_full_length_coverage=1;isoform_length=4303;expected_accuracy=0.994	1	2094	2091	3423	457	2550	2942	4272
cb8564_c35607/f1p0/2587 isoform=cb8564_c35607;full_length_coverage=1;non_full_length_coverage=0;isoform_length=2587;expected_accuracy=0.993	gnl|BL_ORD_ID|14942 cb8564_c34188/f2p3/4546 isoform=cb8564_c34188;full_length_coverage=2;non_full_length_coverage=3;isoform_length=4546;expected_accuracy=1.000	1	1685	1685	2581	1721	3402	3651	4546
cb7493_c4/f1p0/2146	gnl|BL_ORD_ID|6865 cb7493_c1/f1p0/2337	1	1712	1710	2121	1	170	1864	227

	isoform=cb7493_c1;full_length_coverage=1;non_full_length_coverage=0;isoform_length=2337;expected_accuracy=0.999						7		7
cb2966_c1/f2p0/2466 isoform=cb2966_c1;full_length_coverage=2;non_full_length_coverage=0;isoform_length=2466;expected_accuracy=0.999	gnl|BL_ORD_ID|38223 cb2966_c4/f1p0/2868 isoform=cb2966_c4;full_length_coverage=1;non_full_length_coverage=0;isoform_length=2868;expected_accuracy=0.997	162	2466	1	165	533	2834	2	163
cb8564_c51533/f1p0/2888 isoform=cb8564_c51533;full_length_coverage=1;non_full_length_coverage=0;isoform_length=2888;expected_accuracy=0.998	gnl|BL_ORD_ID|15949 cb8564_c47887/f1p0/3477 isoform=cb8564_c47887;full_length_coverage=1;non_full_length_coverage=0;isoform_length=3477;expected_accuracy=0.994	240	2884	20	239	858	3477	1	220
cb8564_c177392/f1p0/2423 isoform=cb8564_c177392;full_length_coverage=1;non_full_length_coverage=0;isoform_length=2423;expected_accuracy=0.996	gnl|BL_ORD_ID|55328 cb8564_c38464/f1p0/2918	1	1751	1754	2406	1	1751	1944	2596
cb8564_c23232/f1p3/2540	gnl|BL_ORD_ID|53102 cb8564_c116328/f1p6/2732	16	2172	2167	2518	4	2160	2262	2613
cb8564_c15653/f1p4/2475	gnl|BL_ORD_ID|58171 cb8564_c90592/f1p0/2863	2	2293	2289	2435	3	2272	2416	2562
cb8564_c83196/f1p0/2505 isoform=cb8564_c83196;full_length_coverage=1;non_full_length_coverage=0;isoform_length=2505;expected_accuracy=0.995	gnl|BL_ORD_ID|15583 cb8564_c76490/f3p1/3602 isoform=cb8564_c76490;full_length_coverage=3;non_full_length_coverage=1;isoform_length=3602;expected_accuracy=0.997	1	1642	1642	2505	908	2550	2663	3526
cb7649_c16/f1p0/1932 isoform=cb7649_c16;full_length_coverage=1;non_full_length_coverage=0;isoform_length=1932;expected_accu	gnl|BL_ORD_ID|6350 cb7649_c4/f1p0/2573 isoform=cb7649_c4;full_length_coverage=1;non_full_length_coverage=0;isoform_length=2573;expected_accura	1	1081	1076	1932	1	1083	1694	2551

racy=0.999	cy=0.999								
cb8564_c75977/f1p0/2165	gnl|BL_ORD_ID|19213 cb8564_c85031/f2p0/4106 isoform=cb8564_c85031;full_length_coverage=2;non_full_length_coverage=0;isoform_length=4106;expected_accuracy=0.997	1	1393	1394	2130	1	1393	2681	3417
cb8564_c90372/f1p0/2157 isoform=cb8564_c90372;full_length_coverage=1;non_full_length_coverage=0;isoform_length=2157;expected_accuracy=0.994	gnl|BL_ORD_ID|18990 cb8564_c79575/f2p0/2489 isoform=cb8564_c79575;full_length_coverage=2;non_full_length_coverage=0;isoform_length=2489;expected_accuracy=0.997	1	1092	1088	2157	99	1191	1362	2432
cb151_c2/f1p0/4262 isoform=cb151_c2;full_length_coverage=1;non_full_length_coverage=0;isoform_length=4262;expected_accuracy=0.992	gnl|BL_ORD_ID|30049 cb151_c3/f1p1/4867 isoform=cb151_c3;full_length_coverage=1;non_full_length_coverage=1;isoform_length=4867;expected_accuracy=0.996	307	4257	1	308	911	4867	231	523
cb1065_c12/f1p0/2107 isoform=cb1065_c12;full_length_coverage=1;non_full_length_coverage=0;isoform_length=2107;expected_accuracy=0.998	gnl|BL_ORD_ID|1631 cb1065_c8/f1p0/2427 isoform=cb1065_c8;full_length_coverage=1;non_full_length_coverage=0;isoform_length=2427;expected_accuracy=0.994	1	1093	1091	2106	9	1098	1319	2341
cb8564_c188097/f1p0/2552 isoform=cb8564_c188097;full_length_coverage=1;non_full_length_coverage=0;isoform_length=2552;expected_accuracy=0.996	gnl|BL_ORD_ID|11483 cb8564_c12665/f1p15/2806 isoform=cb8564_c12665;full_length_coverage=1;non_full_length_coverage=15;isoform_length=2806;expected_accuracy=0.995	1	1945	1940	2550	2	1952	2055	2667
cb8564_c3927/f1p1/2747 isoform=cb8564_c3927;full_length_coverage=1;non_full_length_coverage=1;isoform_length=2747;expected_accuracy=0.995	gnl|BL_ORD_ID|14937 cb8564_c3938/f4p2/3036 isoform=cb8564_c3938;full_length_coverage=4;non_full_length_coverage=2;isoform_length=3036;expected_accuracy=0.996	1	1593	1593	2747	36	1628	1754	2908
cb8564_c35063/f1p0/3277	gnl|BL_ORD_ID|56063 cb8564_c53906/f1p0/3730	1	2629	2632	3132	24	265	2841	334

							2		1
cb2893_c2/f1p0/1401 isoform=cb2893_c2;full_length_coverage=1;non_full_length_coverage=0;isoform_length=1401;expected_accuracy=0.996	gnl|BL_ORD_ID|38659 cb2893_c1/f1p0/1726 isoform=cb2893_c1;full_length_coverage=1;non_full_length_coverage=0;isoform_length=1726;expected_accuracy=0.999	1	1239	1239	1401	7	1245	1517	1679
cb8564_c79661/f1p0/2314 isoform=cb8564_c79661;full_length_coverage=1;non_full_length_coverage=0;isoform_length=2314;expected_accuracy=0.994	gnl|BL_ORD_ID|17021 cb8564_c73064/f2p0/2574 isoform=cb8564_c73064;full_length_coverage=2;non_full_length_coverage=0;isoform_length=2574;expected_accuracy=0.996	1	1332	1333	2314	1	1339	1454	2442
cb5896_c135/f1p0/4855 isoform=cb5896_c135;full_length_coverage=1;non_full_length_coverage=0;isoform_length=4855;expected_accuracy=0.995	gnl|BL_ORD_ID|44452 cb5896_c134/f1p0/4910 isoform=cb5896_c134;full_length_coverage=1;non_full_length_coverage=0;isoform_length=4910;expected_accuracy=0.999	224	2796	2796	4855	1	2572	2815	4870
cb8564_c35645/f1p0/2804	gnl|BL_ORD_ID|56712 cb8564_c53711/f1p0/3201	150	2726	1	151	302	2878	39	189
cb6346_c2/f1p0/2403 isoform=cb6346_c2;full_length_coverage=1;non_full_length_coverage=0;isoform_length=2403;expected_accuracy=0.993	gnl|BL_ORD_ID|49634 cb6346_c1/f1p0/2878	1138	2403	10	1142	1247	2512	1	1134
cb8564_c34060/f2p0/3214 isoform=cb8564_c34060;full_length_coverage=2;non_full_length_coverage=0;isoform_length=3214;expected_accuracy=0.993	gnl|BL_ORD_ID|56041 cb8564_c53077/f1p2/3406	368	3214	57	368	452	3287	41	337
cb1913_c44/f1p0/2236 isoform=cb1913_c44;full_length_coverage=1;non_full_length_coverage=0;isoform_length=2236;expected_accu	gnl|BL_ORD_ID|66489 cb1913_c39/f1p0/2723	1	1634	1630	2236	193	1825	1950	2568

racy=0.998									
cb2737_c54/f1p0/3029 isoform=cb2737_c54;full_length_coverage=1;non_full_length_coverage=0;isoform_length=3029;expected_accuracy=0.995	gnl|BL_ORD_ID|36732 cb2737_c15/f1p0/3303 isoform=cb2737_c15;full_length_coverage=1;non_full_length_coverage=0;isoform_length=3303;expected_accuracy=0.995	112	1625	1623	3029	1	1521	1870	3263
cb8569_c9/f1p0/2101	gnl|BL_ORD_ID|57419 cb8569_c7/f1p0/3650	1	1036	1037	2071	164	1199	2616	3650
cb9867_c3/f1p0/1688 isoform=cb9867_c3;full_length_coverage=1;non_full_length_coverage=0;isoform_length=1688;expected_accuracy=0.993	gnl|BL_ORD_ID|60040 cb9867_c2/f1p0/1954	1	1050	1047	1688	40	1095	1231	1879
cb18275_c9/f1p1/1709 isoform=cb18275_c9;full_length_coverage=1;non_full_length_coverage=1;isoform_length=1709;expected_accuracy=0.993	gnl|BL_ORD_ID|11253 cb8564_c151174/f1p2/2106 isoform=cb8564_c151174;full_length_coverage=1;non_full_length_coverage=2;isoform_length=2106;expected_accuracy=0.997	162	1709	1	166	398	1945	133	298
cb4163_c10/f1p0/2735 isoform=cb4163_c10;full_length_coverage=1;non_full_length_coverage=0;isoform_length=2735;expected_accuracy=0.995	gnl|BL_ORD_ID|43708 cb4163_c16/f4p0/2920 isoform=cb4163_c16;full_length_coverage=4;non_full_length_coverage=0;isoform_length=2920;expected_accuracy=1.000	1	1840	1841	2735	2	1841	1988	2885
cb295_c26/f1p1/1869	gnl|BL_ORD_ID|36781 cb295_c59/f1p4/2024 isoform=cb295_c59;full_length_coverage=1;non_full_length_coverage=4;isoform_length=2024;expected_accuracy=0.993	1	1359	1358	1857	1	1368	1499	2003
cb897_c30/f1p0/2403 isoform=cb897_c30;full_length_coverage=1;non_full_length_coverage=0;isoform_length=2403;expected_accur	gnl|BL_ORD_ID|21670 cb897_c17/f1p0/3323 isoform=cb897_c17;full_length_coverage=1;non_full_length_coverage=0;isoform_length=3323;expected_accura	1	2273	2270	2403	42	2327	3052	3184

acy=0.997	cy=0.994								
cb944_c92/f1p0/1128	gnl|BL_ORD_ID|59113 cb944_c85/f1p1/1255	8	693	688	1139	1	687	789	125<br>3
cb8564_c21764/f1p0/2599	gnl|BL_ORD_ID|57107 cb8564_c83478/f1p0/3224	1	2030	2030	2619	3	203<br>0	1914	250<br>3
cb7400_c0/f3p1/2300 isoform=cb7400_c0;full_length_coverage=3;non_full_length_coverage=1;isoform_length=2300;expected_accuracy=1.000	gnl|BL_ORD_ID|50505 cb7400_c8/f1p0/2474	1	1661	1662	2300	48	170<br>5	1845	247<br>6
cb8564_c178999/f1p0/2222 isoform=cb8564_c178999;full_length_coverage=1;non_full_length_coverage=0;isoform_length=2222;expected_accuracy=0.994	gnl|BL_ORD_ID|55182 cb8564_c34825/f1p2/3200	16	1778	1773	2222	6	179<br>1	2749	319<br>7
cb1626_c14/f1p0/2173 isoform=cb1626_c14;full_length_coverage=1;non_full_length_coverage=0;isoform_length=2173;expected_accuracy=0.999	gnl|BL_ORD_ID|32074 cb1626_c4/f2p1/4537 isoform=cb1626_c4;full_length_coverage=2;non_full_length_coverage=1;isoform_length=4537;expected_accuracy=0.998	1001	2168	1	1002	3370	453<br>7	54	107<br>0
cb8564_c47853/f1p0/2927 isoform=cb8564_c47853;full_length_coverage=1;non_full_length_coverage=0;isoform_length=2927;expected_accuracy=0.991	gnl|BL_ORD_ID|55920 cb8564_c48949/f1p0/4357	1	2271	2272	2927	2	229<br>3	2847	350<br>4
cb8564_c186356/f1p0/2744 isoform=cb8564_c186356;full_length_coverage=1;non_full_length_coverage=0;isoform_length=2744;expected_accuracy=0.998	gnl|BL_ORD_ID|55866 cb8564_c47522/f1p0/2954	1393	2744	1	1396	1495	284<br>6	1	137<br>5
cb8564_c54206/f2p0/3081	gnl|BL_ORD_ID|15354 cb8564_c45229/f4p1/3393	1	1805	1804	3081	149	195	2114	339

isoform=cb8564_c54206;full_length_coverage=2;non_full_length_coverage=0;isoform_length=3081;expected_accuracy=0.997	isoform=cb8564_c45229;full_length_coverage=4;non_full_length_coverage=1;isoform_length=3393;expected_accuracy=0.999						3		3
cb14897_c1/f2p3/1721 isoform=cb14897_c1;full_length_coverage=2;non_full_length_coverage=3;isoform_length=1721;expected_accuracy=1.000	gnl|BL_ORD_ID|53163 cb8564_c117857/f1p1/2890	1	1296	1296	1700	28	1327	2470	2876
cb373_c13/f1p0/3706 isoform=cb373_c13;full_length_coverage=1;non_full_length_coverage=0;isoform_length=3706;expected_accuracy=0.995	gnl|BL_ORD_ID|40700 cb373_c7/f1p0/3964 isoform=cb373_c7;full_length_coverage=1;non_full_length_coverage=0;isoform_length=3964;expected_accuracy=0.995	173	3645	1	173	490	3964	6	179
cb8564_c4906/f1p0/2813	gnl|BL_ORD_ID|55701 cb8564_c4898/f1p0/3489	1	2313	2312	2743	37	2349	2843	3274
cb8564_c180116/f1p0/2944 isoform=cb8564_c180116;full_length_coverage=1;non_full_length_coverage=0;isoform_length=2944;expected_accuracy=0.996	gnl|BL_ORD_ID|53680 cb8564_c153222/f2p39/3454	1	2522	2520	2936	4	2525	2754	3170
cb4652_c6/f1p0/1797 isoform=cb4652_c6;full_length_coverage=1;non_full_length_coverage=0;isoform_length=1797;expected_accuracy=0.993	gnl|BL_ORD_ID|43334 cb4652_c3/f1p0/1920 isoform=cb4652_c3;full_length_coverage=1;non_full_length_coverage=0;isoform_length=1920;expected_accuracy=1.000	231	1790	47	233	361	1920	7	193
cb2804_c109/f1p0/1890 isoform=cb2804_c109;full_length_coverage=1;non_full_length_coverage=0;isoform_length=1890;expected_accuracy=0.996	gnl|BL_ORD_ID|68883 cb2804_c151/f1p0/2463	184	1890	1	183	1	1695	1870	2052
cb373_c13/f1p0/3706	gnl|BL_ORD_ID|40699 cb373_c6/f1p0/4615	173	3663	1	173	1118	461	1	173

isoform=cb373_c13;full_length_coverage=1;non_full_length_coverage=0;isoform_length=3706;expected_accuracy=0.995	isoform=cb373_c6;full_length_coverage=1;non_full_length_coverage=0;isoform_length=4615;expected_accuracy=0.997						5		
cb4066_c13/f1p0/2044	gnl|BL_ORD_ID|71763 cb4066_c12/f1p0/2290	12	1242	1242	2028	1	1231	1468	2254
cb8564_c87721/f1p0/2168 isoform=cb8564_c87721;full_length_coverage=1;non_full_length_coverage=0;isoform_length=2168;expected_accuracy=0.997	gnl|BL_ORD_ID|16335 cb8564_c72599/f1p2/2911 isoform=cb8564_c72599;full_length_coverage=1;non_full_length_coverage=2;isoform_length=2911;expected_accuracy=0.997	1	1811	1809	2107	148	1966	2613	2911
cb850_c28/f1p0/3553 isoform=cb850_c28;full_length_coverage=1;non_full_length_coverage=0;isoform_length=3553;expected_accuracy=0.992	gnl|BL_ORD_ID|11099 cb850_c13/f1p1/4040 isoform=cb850_c13;full_length_coverage=1;non_full_length_coverage=1;isoform_length=4040;expected_accuracy=0.994	303	3553	1	307	636	3855	193	496
cb7902_c11/f1p0/1255	gnl|BL_ORD_ID|8205 cb7902_c6/f1p0/2164 isoform=cb7902_c6;full_length_coverage=1;non_full_length_coverage=0;isoform_length=2164;expected_accuracy=0.990	549	1152	8	550	1557	2160	1	543
cb8564_c43979/f1p0/2026	gnl|BL_ORD_ID|18120 cb8564_c92186/f1p0/2894 isoform=cb8564_c92186;full_length_coverage=1;non_full_length_coverage=0;isoform_length=2894;expected_accuracy=0.994	224	2036	6	227	1046	2852	5	232
cb5183_c115/f1p0/1016	gnl|BL_ORD_ID|41757 cb5183_c122/f163p6/1859 isoform=cb5183_c122;full_length_coverage=163;non_full_length_coverage=6;isoform_length=1859;expected_accuracy=1.000	326	1016	4	330	1047	1737	112	438
cb8564_c109931/f3p0/2132	gnl|BL_ORD_ID|11188 cb8564_c114244/f1p0/3314	1031	2124	1	1034	2221	331	12	104

isoform=cb8564_c109931;full_length_coverage=3;non_full_length_coverage=0;isoform_length=2132;expected_accuracy=1.000	isoform=cb8564_c114244;full_length_coverage=1;non_full_length_coverage=0;isoform_length=3314;expected_accuracy=0.998						4		4
cb1956_c18/f1p0/2183	gnl|BL_ORD_ID|36250 cb1956_c4/f3p0/3816 isoform=cb1956_c4;full_length_coverage=3;non_full_length_coverage=0;isoform_length=3816;expected_accuracy=0.997	1	1918	1918	2182	646	2563	2699	2962
cb1415_c16/f1p0/3004 isoform=cb1415_c16;full_length_coverage=1;non_full_length_coverage=0;isoform_length=3004;expected_accuracy=0.995	gnl|BL_ORD_ID|27157 cb1415_c6/f1p0/4270 isoform=cb1415_c6;full_length_coverage=1;non_full_length_coverage=0;isoform_length=4270;expected_accuracy=0.994	1	1469	1464	3004	55	1524	2386	3949
cb8564_c11592/f1p0/4735 isoform=cb8564_c11592;full_length_coverage=1;non_full_length_coverage=0;isoform_length=4735;expected_accuracy=0.997	gnl|BL_ORD_ID|35344 cb18654_c17/f1p0/6253 isoform=cb18654_c17;full_length_coverage=1;non_full_length_coverage=0;isoform_length=6253;expected_accuracy=0.996	1	4169	4169	4564	1215	5405	5858	6253
cb1306_c36/f1p0/2164 isoform=cb1306_c36;full_length_coverage=1;non_full_length_coverage=0;isoform_length=2164;expected_accuracy=1.000	gnl|BL_ORD_ID|61217 cb1306_c28/f1p0/5943	1	1793	1788	2123	2804	4596	5521	5856
cb8564_c15653/f1p4/2475	gnl|BL_ORD_ID|56007 cb8564_c51870/f1p2/2815	22	2293	2289	2432	82	2353	2572	2715
cb8564_c180116/f1p0/2944 isoform=cb8564_c180116;full_length_coverage=1;non_full_length_coverage=0;isoform_length=2944;expected_accuracy=0.996	gnl|BL_ORD_ID|16681 cb8564_c54233/f1p1/3194 isoform=cb8564_c54233;full_length_coverage=1;non_full_length_coverage=1;isoform_length=3194;expected_accuracy=0.995	1	2522	2520	2944	4	2541	2770	3194
cb435_c9/f1p0/4856	gnl|BL_ORD_ID|72171 cb435_c8/f1p0/5270	18	4346	4344	4730	1	433	4896	528

							1		2
cb14630_c37/f1p1/5449 isoform=cb14630_c37;full_length_coverage=1;non_full_length_coverage=1;isoform_length=5449;expected_accuracy=0.996	gnl|BL_ORD_ID|30225 cb14630_c41/f19p4/5749 isoform=cb14630_c41;full_length_coverage=19;non_full_length_coverage=4;isoform_length=5749;expected_accuracy=1.000	1	4273	4268	5449	84	4366	4539	5720
cb8564_c38035/f1p0/2770	gnl|BL_ORD_ID|54438 cb8564_c46124/f1p0/3383	1	2035	2033	2754	151	2188	2378	3104
cb8564_c36731/f1p2/2761	gnl|BL_ORD_ID|55472 cb8564_c42531/f1p0/3996	125	1977	1972	2736	816	2669	3098	3863
cb1545_c23/f1p0/3408	gnl|BL_ORD_ID|63732 cb1545_c19/f1p0/3724	10	1992	1991	3195	7	1988	2235	3439
cb6925_c3/f1p0/2046	gnl|BL_ORD_ID|5120 cb6925_c2/f1p0/2330 isoform=cb6925_c2;full_length_coverage=1;non_full_length_coverage=0;isoform_length=2330;expected_accuracy=0.994	100	1197	1193	2056	31	1130	1428	2292
cb8620_c0/f1p0/2318	gnl|BL_ORD_ID|57454 cb8620_c1/f1p0/2443	1	1434	1429	2184	2	1435	1688	2443
cb8564_c185920/f1p0/2777 isoform=cb8564_c185920;full_length_coverage=1;non_full_length_coverage=0;isoform_length=2777;expected_accuracy=0.996	gnl|BL_ORD_ID|14556 cb8564_c45150/f1p0/2940 isoform=cb8564_c45150;full_length_coverage=1;non_full_length_coverage=0;isoform_length=2940;expected_accuracy=0.992	174	2777	1	178	347	2935	14	192
cb8564_c40107/f1p0/4108	gnl|BL_ORD_ID|55964 cb8564_c50435/f1p0/4916	1603	3955	1	1599	1494	3846	16	1614
cb8564_c52738/f1p0/3595	gnl|BL_ORD_ID|56035 cb8564_c52940/f1p2/3963	14	2316	2315	3445	5	2302	2793	3922
cb8564_c18587/f1p1/2159	gnl|BL_ORD_ID|11450 cb8564_c12878/f1p1/3352	562	2055	9	564	1869	335	1	555

	isoform=cb8564_c12878;full_length_coverage=1;non_full_length_coverage=1;isoform_length=3352;expected_accuracy=0.995						2		
cb9467_c89/f2p0/1589 isoform=cb9467_c89;full_length_coverage=2;non_full_length_coverage=0;isoform_length=1589;expected_accuracy=0.993	gnl|BL_ORD_ID|21842 cb9467_c46/f1p0/2279 isoform=cb9467_c46;full_length_coverage=1;non_full_length_coverage=0;isoform_length=2279;expected_accuracy=0.992	1	1171	1169	1589	436	1609	1855	2275
cb2225_c5/f1p0/3503 isoform=cb2225_c5;full_length_coverage=1;non_full_length_coverage=0;isoform_length=3503;expected_accuracy=0.997	gnl|BL_ORD_ID|38789 cb2225_c1/f2p0/3544 isoform=cb2225_c1;full_length_coverage=2;non_full_length_coverage=0;isoform_length=3544;expected_accuracy=0.998	1	3136	3136	3354	55	3192	3326	3544
cb8564_c148897/f2p2/3139 isoform=cb8564_c148897;full_length_coverage=2;non_full_length_coverage=2;isoform_length=3139;expected_accuracy=0.997	gnl|BL_ORD_ID|13766 cb8564_c18520/f2p0/3299 isoform=cb8564_c18520;full_length_coverage=2;non_full_length_coverage=0;isoform_length=3299;expected_accuracy=0.992	1	2088	2088	3139	2	2089	2198	3245
cb2686_c4/f1p0/2023 isoform=cb2686_c4;full_length_coverage=1;non_full_length_coverage=0;isoform_length=2023;expected_accuracy=0.997	gnl|BL_ORD_ID|38040 cb2686_c5/f1p0/3171 isoform=cb2686_c5;full_length_coverage=1;non_full_length_coverage=0;isoform_length=3171;expected_accuracy=0.994	11	1187	1187	2023	512	1687	2208	3044
cb5132_c1/f2p1/1791 isoform=cb5132_c1;full_length_coverage=2;non_full_length_coverage=1;isoform_length=1791;expected_accuracy=1.000	gnl|BL_ORD_ID|43253 cb5132_c3/f1p0/3552 isoform=cb5132_c3;full_length_coverage=1;non_full_length_coverage=0;isoform_length=3552;expected_accuracy=0.999	1	1068	1067	1791	22	1069	2834	3545
cb4123_c103/f1p0/1158	gnl|BL_ORD_ID|41229 cb4123_c16/f2p0/1482 isoform=cb4123_c16;full_length_coverage=2;non_full_length_coverage=0;isoform_length=1482;expected_accur	292	1178	9	295	576	1463	1	286

	acy=1.000								
cb8564_c36731/f1p2/2761	gnl|BL_ORD_ID|55600 cb8564_c46484/f1p0/3151	102	1977	1972	2741	5	190 2	2331	310 0
cb3842_c0/f2p0/2475 isoform=cb3842_c0;full_length_coverage=2;non_full_length_coverage=0;isoform_length=2475;expected_accuracy=0.997	gnl|BL_ORD_ID|40308 cb3842_c6/f1p0/3329 isoform=cb3842_c6;full_length_coverage=1;non_full_length_coverage=0;isoform_length=3329;expected_accuracy=0.991	1	2194	2195	2455	7	219 9	3071	332 9
cb8564_c46177/f1p0/1967	gnl|BL_ORD_ID|10223 cb8564_c10832/f3p0/2415 isoform=cb8564_c10832;full_length_coverage=3;non_full_length_coverage=0;isoform_length=2415;expected_accuracy=0.998	177	1963	16	180	576	236 3	16	177
cb14139_c0/f5p0/1783 isoform=cb14139_c0;full_length_coverage=5;non_full_length_coverage=0;isoform_length=1783;expected_accuracy=1.000	gnl|BL_ORD_ID|21468 cb9813_c26/f1p0/2710 isoform=cb9813_c26;full_length_coverage=1;non_full_length_coverage=0;isoform_length=2710;expected_accuracy=0.990	1	1005	1005	1756	22	102 4	1962	271 0
cb8564_c35645/f1p0/2804	gnl|BL_ORD_ID|18906 cb8564_c90449/f1p3/3223 isoform=cb8564_c90449;full_length_coverage=1;non_full_length_coverage=3;isoform_length=3223;expected_accuracy=0.996	149	2726	1	151	639	321 6	33	182
cb8564_c13282/f1p2/2892 isoform=cb8564_c13282;full_length_coverage=1;non_full_length_coverage=2;isoform_length=2892;expected_accuracy=0.994	gnl|BL_ORD_ID|35903 cb18782_c2/f1p2/5753 isoform=cb18782_c2;full_length_coverage=1;non_full_length_coverage=2;isoform_length=5753;expected_accuracy=0.991	1	1772	1767	2892	29	179 1	4612	573 1
cb1049_c9/f1p0/2974 isoform=cb1049_c9;full_length_coverage=1;non_full_length_coverage=0;isoform_length=2974;expected_accur	gnl|BL_ORD_ID|1657 cb1049_c5/f2p1/3134 isoform=cb1049_c5;full_length_coverage=2;non_full_length_coverage=1;isoform_length=3134;expected_accura	1345	2974	1	1346	1452	308 1	4	134 9

acy=1.000	cy=1.000								
cb1401_c3/f1p0/3485 isoform=cb1401_c3;full_length_coverage=1;non_full_length_coverage=0;isoform_length=3485;expected_accuracy=0.994	gnl|BL_ORD_ID|25500 cb1401_c2/f1p0/3828 isoform=cb1401_c2;full_length_coverage=1;non_full_length_coverage=0;isoform_length=3828;expected_accuracy=0.992	1149	3485	1	1151	1486	3828	168	1324
cb647_c8/f1p0/1962 isoform=cb647_c8;full_length_coverage=1;non_full_length_coverage=0;isoform_length=1962;expected_accuracy=1.000	gnl|BL_ORD_ID|5577 cb647_c7/f1p0/2791 isoform=cb647_c7;full_length_coverage=1;non_full_length_coverage=0;isoform_length=2791;expected_accuracy=0.993	1	1235	1233	1956	151	1383	2058	2790
cb10594_c0/f6p0/1247 isoform=cb10594_c0;full_length_coverage=6;non_full_length_coverage=0;isoform_length=1247;expected_accuracy=1.000	gnl|BL_ORD_ID|1370 cb10594_c1/f1p0/1672 isoform=cb10594_c1;full_length_coverage=1;non_full_length_coverage=0;isoform_length=1672;expected_accuracy=0.995	340	1247	7	342	769	1672	1	335
cb5183_c100/f1p2/1592 isoform=cb5183_c100;full_length_coverage=1;non_full_length_coverage=2;isoform_length=1592;expected_accuracy=0.998	gnl|BL_ORD_ID|73602 cb5183_c57/f1p0/1810	278	1562	3	282	413	1694	9	288
cb295_c59/f1p4/2024 isoform=cb295_c59;full_length_coverage=1;non_full_length_coverage=4;isoform_length=2024;expected_accuracy=0.993	gnl|BL_ORD_ID|37900 cb295_c23/f1p1/2681 isoform=cb295_c23;full_length_coverage=1;non_full_length_coverage=1;isoform_length=2681;expected_accuracy=0.993	1	1368	1367	2024	1	1349	1987	2641
cb8564_c2505/f1p0/2018	gnl|BL_ORD_ID|55631 cb8564_c2499/f1p0/2257	12	1423	1423	1935	118	1524	1667	2180
cb8564_c51035/f2p2/4216 isoform=cb8564_c51035;full_length_coverage=2;non_full_length_coverage=2;isoform_length=4216;expected_	gnl|BL_ORD_ID|14819 cb8564_c40333/f1p2/4446 isoform=cb8564_c40333;full_length_coverage=1;non_full_length_coverage=2;isoform_length=4446;expected_ac	14	3261	3257	4156	1	3280	3545	4446

accuracy=0.999	curacy=0.998								
cb8564_c36954/f1p1/3314	gnl|BL_ORD_ID|57963 cb8564_c85293/f1p2/3232	2	2145	2144	3191	168	2313	2200	3247
cb8564_c45150/f1p0/2940 isoform=cb8564_c45150;full_length_coverage=1;non_full_length_coverage=0;isoform_length=2940;expected_accuracy=0.992	gnl|BL_ORD_ID|17429 cb8564_c85447/f1p0/3196 isoform=cb8564_c85447;full_length_coverage=1;non_full_length_coverage=0;isoform_length=3196;expected_accuracy=0.997	1	2403	2402	2935	3	2413	2660	3196
cb862_c7/f1p0/2681	gnl|BL_ORD_ID|56507 cb862_c26/f1p0/2964	1	1294	1291	2671	98	1399	1523	2931
cb8564_c2535/f1p0/2190 isoform=cb8564_c2535;full_length_coverage=1;non_full_length_coverage=0;isoform_length=2190;expected_accuracy=1.000	gnl|BL_ORD_ID|15337 cb8564_c2536/f1p0/2410 isoform=cb8564_c2536;full_length_coverage=1;non_full_length_coverage=0;isoform_length=2410;expected_accuracy=1.000	1	1520	1521	2154	66	1610	1777	2410
cb12446_c3/f1p0/1412 isoform=cb12446_c3;full_length_coverage=1;non_full_length_coverage=0;isoform_length=1412;expected_accuracy=1.000	gnl|BL_ORD_ID|9272 cb8564_c101010/f5p2/2025 isoform=cb8564_c101010;full_length_coverage=5;non_full_length_coverage=2;isoform_length=2025;expected_accuracy=0.990	109	1412	1	111	669	1971	83	184
cb5622_c7/f1p0/1600 isoform=cb5622_c7;full_length_coverage=1;non_full_length_coverage=0;isoform_length=1600;expected_accuracy=0.996	gnl|BL_ORD_ID|74169 cb5622_c2/f1p0/2496	12	1227	1227	1600	1	1217	2011	2384
cb7111_c5/f1p0/1972	gnl|BL_ORD_ID|47848 cb7111_c2/f1p0/2690	1	1116	1113	1778	27	1142	2027	2692
cb8564_c117792/f1p0/2284 isoform=cb8564_c117792;full_length_coverage=1;non_full_length_coverage=0;isoform_length=2284;expected	gnl|BL_ORD_ID|13020 cb8564_c16150/f1p0/2824 isoform=cb8564_c16150;full_length_coverage=1;non_full_length_coverage=0;isoform_length=2824;expected_ac	1	1262	1257	2284	1	1263	1798	2824

_accuracy=0.995	curacy=0.997								
cb3769_c1/f2p0/2942 isoform=cb3769_c1;full_length_coverage=2;non_full_length_coverage=0;isoform_length=2942;expected_accuracy=0.996	gnl|BL_ORD_ID|40981 cb3769_c3/f1p0/3241 isoform=cb3769_c3;full_length_coverage=1;non_full_length_coverage=0;isoform_length=3241;expected_accuracy=0.995	1	2358	2357	2942	46	2407	2657	3240
cb18646_c1/f2p2/6581 isoform=cb18646_c1;full_length_coverage=2;non_full_length_coverage=2;isoform_length=6581;expected_accuracy=0.999	gnl|BL_ORD_ID|34400 cb18646_c6/f1p3/6738 isoform=cb18646_c6;full_length_coverage=1;non_full_length_coverage=3;isoform_length=6738;expected_accuracy=0.995	1	6228	6228	6581	23	6245	6371	6720
cb13752_c8/f1p0/1329 isoform=cb13752_c8;full_length_coverage=1;non_full_length_coverage=0;isoform_length=1329;expected_accuracy=0.993	gnl|BL_ORD_ID|25257 cb13752_c3/f2p0/1870 isoform=cb13752_c3;full_length_coverage=2;non_full_length_coverage=0;isoform_length=1870;expected_accuracy=0.995	433	1303	7	434	1000	1870	7	435
cb8564_c78741/f1p0/2473 isoform=cb8564_c78741;full_length_coverage=1;non_full_length_coverage=0;isoform_length=2473;expected_accuracy=1.000	gnl|BL_ORD_ID|19257 cb8564_c83505/f1p0/3415 isoform=cb8564_c83505;full_length_coverage=1;non_full_length_coverage=0;isoform_length=3415;expected_accuracy=1.000	1	1617	1617	2452	415	2031	2579	3415
cb8564_c36056/f1p0/2652 isoform=cb8564_c36056;full_length_coverage=1;non_full_length_coverage=0;isoform_length=2652;expected_accuracy=0.999	gnl|BL_ORD_ID|57134 cb8564_c84358/f1p0/3146	248	2651	1	251	532	2930	72	324
cb8564_c71815/f1p0/2753	gnl|BL_ORD_ID|11343 cb8564_c148508/f1p3/2825 isoform=cb8564_c148508;full_length_coverage=1;non_full_length_coverage=3;isoform_length=2825;expected_accuracy=0.994	1	1377	1372	2484	76	1453	1687	2793
cb8564_c73423/f1p2/2256	gnl|BL_ORD_ID|54984 cb8564_c4106/f1p2/2854	1014	2256	1	1018	1300	254	204	1189

isoform=cb8564_c73423;full_length_coverage=1;non_full_length_coverage=2;isoform_length=2256;expected_accuracy=0.994							1		
cb8564_c110147/f1p0/3399 isoform=cb8564_c110147;full_length_coverage=1;non_full_length_coverage=0;isoform_length=3399;expected_accuracy=0.999	gnl|BL_ORD_ID|52309 cb8564_c117845/f1p1/3666	1	2986	2987	3399	9	2994	3251	3664
cb8564_c69690/f2p0/1977 isoform=cb8564_c69690;full_length_coverage=2;non_full_length_coverage=0;isoform_length=1977;expected_accuracy=1.000	gnl|BL_ORD_ID|18072 cb8564_c85619/f1p0/2084 isoform=cb8564_c85619;full_length_coverage=1;non_full_length_coverage=0;isoform_length=2084;expected_accuracy=0.997	1	1679	1678	1948	2	1680	1814	2084
cb8564_c47737/f7p5/4351 isoform=cb8564_c47737;full_length_coverage=7;non_full_length_coverage=5;isoform_length=4351;expected_accuracy=0.998	gnl|BL_ORD_ID|14942 cb8564_c34188/f2p3/4546 isoform=cb8564_c34188;full_length_coverage=2;non_full_length_coverage=3;isoform_length=4546;expected_accuracy=1.000	1	3380	3376	4274	5	3382	3647	4546
cb8564_c12758/f1p0/2368 isoform=cb8564_c12758;full_length_coverage=1;non_full_length_coverage=0;isoform_length=2368;expected_accuracy=0.995	gnl|BL_ORD_ID|11785 cb8564_c12484/f1p0/2841 isoform=cb8564_c12484;full_length_coverage=1;non_full_length_coverage=0;isoform_length=2841;expected_accuracy=0.992	1	1331	1331	2324	57	1386	1850	2841
cb8564_c5118/f1p1/4889 isoform=cb8564_c5118;full_length_coverage=1;non_full_length_coverage=1;isoform_length=4889;expected_accuracy=0.991	gnl|BL_ORD_ID|30141 cb14859_c7/f1p3/5317 isoform=cb14859_c7;full_length_coverage=1;non_full_length_coverage=3;isoform_length=5317;expected_accuracy=0.995	1398	4787	1	1401	1913	5317	78	1486
cb1514_c4/f2p1/3503 isoform=cb1514_c4;full_length_coverage=2;non_full_length_coverage=1;isoform_length=3503;expected_accur	gnl|BL_ORD_ID|30815 cb1514_c26/f47p2/3605 isoform=cb1514_c26;full_length_coverage=47;non_full_length_coverage=2;isoform_length=3605;expected_acc	1	3277	3277	3500	1	3280	3382	3605

acy=0.997	uracy=1.000								
cb1333_c101/f1p0/1395 isoform=cb1333_c101;full_length_coverage=1;non_full_length_coverage=0;isoform_length=1395;expected_accuracy=0.997	gnl|BL_ORD_ID|61262 cb1333_c125/f1p1/5010	149	1395	27	150	3610	4854	29	152
cb4579_c3/f1p0/2979	gnl|BL_ORD_ID|43920 cb4579_c6/f1p0/3307 isoform=cb4579_c6;full_length_coverage=1;non_full_length_coverage=0;isoform_length=3307;expected_accuracy=0.999	1243	2911	1	1246	1639	3307	33	1278
cb8564_c132934/f1p2/2237 isoform=cb8564_c132934;full_length_coverage=1;non_full_length_coverage=2;isoform_length=2237;expected_accuracy=0.999	gnl|BL_ORD_ID|13514 cb8564_c21005/f1p1/4445 isoform=cb8564_c21005;full_length_coverage=1;non_full_length_coverage=1;isoform_length=4445;expected_accuracy=0.999	1	1837	1834	2182	1	1837	3560	3907
cb8564_c36056/f1p0/2652 isoform=cb8564_c36056;full_length_coverage=1;non_full_length_coverage=0;isoform_length=2652;expected_accuracy=0.999	gnl|BL_ORD_ID|55391 cb8564_c40278/f3p2/3083	248	2652	1	251	466	2871	70	322
cb6530_c80/f1p0/2511 isoform=cb6530_c80;full_length_coverage=1;non_full_length_coverage=0;isoform_length=2511;expected_accuracy=0.992	gnl|BL_ORD_ID|4005 cb6530_c35/f1p0/2668 isoform=cb6530_c35;full_length_coverage=1;non_full_length_coverage=0;isoform_length=2668;expected_accuracy=0.998	1	2002	1997	2511	30	2014	2116	2626
cb8564_c15376/f1p0/4060	gnl|BL_ORD_ID|13816 cb8564_c22331/f1p0/4122 isoform=cb8564_c22331;full_length_coverage=1;non_full_length_coverage=0;isoform_length=4122;expected_accuracy=0.992	147	2717	2717	3675	494	3062	3166	4122
cb8564_c41115/f1p3/2751	gnl|BL_ORD_ID|15154 cb8564_c32509/f1p1/2880	14	1542	1539	2673	2	155	1729	287

	isoform=cb8564_c32509;full_length_coverage=1;non_full_length_coverage=1;isoform_length=2880;expected_accuracy=0.991						4		7
cb7230_c11/f1p0/1539 isoform=cb7230_c11;full_length_coverage=1;non_full_length_coverage=0;isoform_length=1539;expected_accuracy=0.993	gnl|BL_ORD_ID|8684 cb7230_c8/f1p0/1707 isoform=cb7230_c8;full_length_coverage=1;non_full_length_coverage=0;isoform_length=1707;expected_accuracy=1.000	1	1134	1135	1539	31	1169	1299	1705
cb8564_c5122/f1p0/3329	gnl|BL_ORD_ID|16486 cb8564_c504/f6p2/3836 isoform=cb8564_c504;full_length_coverage=6;non_full_length_coverage=2;isoform_length=3836;expected_accuracy=1.000	1067	3334	1	1069	1451	3720	89	1159
cb1030_c34/f1p0/2904	gnl|BL_ORD_ID|1420 cb1030_c30/f1p0/3343 isoform=cb1030_c30;full_length_coverage=1;non_full_length_coverage=0;isoform_length=3343;expected_accuracy=0.994	1	2740	2743	2873	34	2774	2898	3027
cb8564_c45699/f1p0/3005	gnl|BL_ORD_ID|16172 cb8564_c53024/f1p0/3159 isoform=cb8564_c53024;full_length_coverage=1;non_full_length_coverage=0;isoform_length=3159;expected_accuracy=0.996	1	1711	1708	2848	1	1699	2017	3157
cb8428_c5/f1p0/2320	gnl|BL_ORD_ID|10021 cb8428_c4/f1p0/2354 isoform=cb8428_c4;full_length_coverage=1;non_full_length_coverage=0;isoform_length=2354;expected_accuracy=0.993	1	1782	1777	2246	3	1788	1888	2354
cb8205_c18/f1p0/1626 isoform=cb8205_c18;full_length_coverage=1;non_full_length_coverage=0;isoform_length=1626;expected_accu	gnl|BL_ORD_ID|8245 cb8205_c5/f2p0/2450 isoform=cb8205_c5;full_length_coverage=2;non_full_length_coverage=0;isoform_length=2450;expected_accura	185	1605	43	190	1031	2448	241	388

racy=1.000	cy=0.999								
cb8564_c47011/f1p0/3100	gnl|BL_ORD_ID|53680 cb8564_c153222/f2p39/3454	2	2306	2301	2929	6	2304	2542	3170
cb9718_c43/f1p0/1655 isoform=cb9718_c43;full_length_coverage=1;non_full_length_coverage=0;isoform_length=1655;expected_accuracy=0.996	gnl|BL_ORD_ID|59926 cb9718_c14/f1p3/1868	288	1655	4	290	392	1759	6	292
cb8564_c71815/f1p0/2753	gnl|BL_ORD_ID|19155 cb8564_c90208/f1p2/2920 isoform=cb8564_c90208;full_length_coverage=1;non_full_length_coverage=2;isoform_length=2920;expected_accuracy=0.990	11	1377	1372	2485	24	1422	1660	2784
cb7637_c9/f1p1/2355	gnl|BL_ORD_ID|51422 cb7637_c20/f1p0/2581	1	1619	1618	2350	51	1669	1789	2522
cb1585_c28/f1p0/1745 isoform=cb1585_c28;full_length_coverage=1;non_full_length_coverage=0;isoform_length=1745;expected_accuracy=0.996	gnl|BL_ORD_ID|30289 cb1585_c7/f1p0/4254 isoform=cb1585_c7;full_length_coverage=1;non_full_length_coverage=0;isoform_length=4254;expected_accuracy=0.995	672	1742	81	675	3178	4254	1881	2475
cb1312_c24/f1p0/3018	gnl|BL_ORD_ID|27494 cb1312_c37/f6p0/3835 isoform=cb1312_c37;full_length_coverage=6;non_full_length_coverage=0;isoform_length=3835;expected_accuracy=0.999	1	1532	1531	2888	90	1619	2351	3708
cb7036_c15/f1p0/1899	gnl|BL_ORD_ID|5116 cb7036_c19/f1p1/2831 isoform=cb7036_c19;full_length_coverage=1;non_full_length_coverage=1;isoform_length=2831;expected_accuracy=0.996	1	1462	1458	1900	1	1462	2317	2759
cb8564_c152519/f2p1/2103	gnl|BL_ORD_ID|13269 cb8564_c19149/f1p1/2483	114	2102	1	116	458	244	52	168

<table>
<tr><th></th><th></th><th></th><th></th><th></th><th></th><th></th><th></th><th></th><th></th></tr>
<tr><td>isoform=cb8564_c152519;full_length_coverage=2;non_full_length_coverage=1;isoform_length=2103;expected_accuracy=1.000</td><td>isoform=cb8564_c19149;full_length_coverage=1;non_full_length_coverage=1;isoform_length=2483;expected_accuracy=0.998</td><td></td><td></td><td></td><td></td><td></td><td>3</td><td></td><td></td></tr>
<tr><td>cb8564_c86279/f2p1/3248</td><td>gnl|BL_ORD_ID|57409 cb8564_c92262/f1p0/3503</td><td>366</td><td>3248</td><td>82</td><td>368</td><td>456</td><td>3342</td><td>1</td><td>287</td></tr>
<tr><td>cb12047_c103/f1p1/1531</td><td>gnl|BL_ORD_ID|55337 cb8564_c38755/f1p3/1935</td><td>9</td><td>870</td><td>865</td><td>1531</td><td>1</td><td>862</td><td>1148</td><td>1814</td></tr>
<tr><td>cb2101_c6/f1p0/2473 isoform=cb2101_c6;full_length_coverage=1;non_full_length_coverage=0;isoform_length=2473;expected_accuracy=0.990</td><td>gnl|BL_ORD_ID|34649 cb2101_c0/f3p0/2796 isoform=cb2101_c0;full_length_coverage=3;non_full_length_coverage=0;isoform_length=2796;expected_accuracy=0.997</td><td>1</td><td>1937</td><td>1934</td><td>2473</td><td>1</td><td>1913</td><td>2195</td><td>2729</td></tr>
<tr><td>cb8564_c37361/f1p0/3076</td><td>gnl|BL_ORD_ID|55502 cb8564_c43538/f1p0/3563</td><td>1</td><td>2481</td><td>2477</td><td>3070</td><td>2</td><td>2487</td><td>2588</td><td>3180</td></tr>
<tr><td>cb8564_c49593/f1p0/2995 isoform=cb8564_c49593;full_length_coverage=1;non_full_length_coverage=0;isoform_length=2995;expected_accuracy=0.992</td><td>gnl|BL_ORD_ID|15063 cb8564_c37149/f1p9/3212 isoform=cb8564_c37149;full_length_coverage=1;non_full_length_coverage=9;isoform_length=3212;expected_accuracy=0.991</td><td>717</td><td>2972</td><td>74</td><td>721</td><td>950</td><td>3212</td><td>114</td><td>770</td></tr>
<tr><td>cb322_c35/f1p0/3437 isoform=cb322_c35;full_length_coverage=1;non_full_length_coverage=0;isoform_length=3437;expected_accuracy=0.997</td><td>gnl|BL_ORD_ID|40505 cb322_c54/f1p0/3800 isoform=cb322_c54;full_length_coverage=1;non_full_length_coverage=0;isoform_length=3800;expected_accuracy=0.996</td><td>114</td><td>3437</td><td>1</td><td>119</td><td>376</td><td>3689</td><td>8</td><td>125</td></tr>
<tr><td>cb8564_c91845/f1p0/2021 isoform=cb8564_c91845;full_length_coverage=1;non_full_length_coverage=0;isoform_length=2021;expected_accuracy=0.998</td><td>gnl|BL_ORD_ID|16543 cb8564_c75067/f1p1/2650 isoform=cb8564_c75067;full_length_coverage=1;non_full_length_coverage=1;isoform_length=2650;expected_accuracy=0.995</td><td>483</td><td>2021</td><td>91</td><td>481</td><td>1111</td><td>2648</td><td>413</td><td>802</td></tr>
<tr><td>cb6277_c19/f1p1/2357</td><td>gnl|BL_ORD_ID|5386 cb6277_c20/f1p1/2469</td><td>177</td><td>2335</td><td>1</td><td>178</td><td>311</td><td>246</td><td>1</td><td>178</td></tr>
</table>

isoform=cb6277_c19;full_length_coverage=1;non_full_length_coverage=1;isoform_length=2357;expected_accuracy=0.995	isoform=cb6277_c20;full_length_coverage=1;non_full_length_coverage=1;isoform_length=2469;expected_accuracy=0.998						9		
cb1178_c27/f1p0/1197 isoform=cb1178_c27;full_length_coverage=1;non_full_length_coverage=0;isoform_length=1197;expected_accuracy=0.992	gnl|BL_ORD_ID|23776 cb1178_c19/f1p0/1460 isoform=cb1178_c19;full_length_coverage=1;non_full_length_coverage=0;isoform_length=1460;expected_accuracy=1.000	111	1197	1	110	297	1382	73	176
cb8564_c19433/f1p0/3193	gnl|BL_ORD_ID|14052 cb8564_c38815/f1p3/3473 isoform=cb8564_c38815;full_length_coverage=1;non_full_length_coverage=3;isoform_length=3473;expected_accuracy=0.995	623	3126	8	624	814	3315	1	617
cb8276_c1/f2p1/2355 isoform=cb8276_c1;full_length_coverage=2;non_full_length_coverage=1;isoform_length=2355;expected_accuracy=0.996	gnl|BL_ORD_ID|8462 cb8276_c6/f1p1/3242 isoform=cb8276_c6;full_length_coverage=1;non_full_length_coverage=1;isoform_length=3242;expected_accuracy=1.000	1	1379	1377	2323	2	1381	2296	3242
cb5137_c75/f1p0/3221	gnl|BL_ORD_ID|42828 cb5137_c119/f1p0/5065 isoform=cb5137_c119;full_length_coverage=1;non_full_length_coverage=0;isoform_length=5065;expected_accuracy=0.997	317	2874	7	318	2507	5065	65	354
cb8564_c113873/f1p1/4424 isoform=cb8564_c113873;full_length_coverage=1;non_full_length_coverage=1;isoform_length=4424;expected_accuracy=0.997	gnl|BL_ORD_ID|52930 cb8564_c112168/f1p0/4870	205	4363	1	206	547	4707	185	390
cb8564_c83505/f1p0/3415 isoform=cb8564_c83505;full_length_coverage=1;non_full_length_coverage=0;isoform_length=3415;expected_	gnl|BL_ORD_ID|16981 cb8564_c75995/f1p0/3575 isoform=cb8564_c75995;full_length_coverage=1;non_full_length_coverage=0;isoform_length=3575;expected_ac	1489	3415	1	1492	1616	3536	4	1511

accuracy=1.000	curacy=0.993								
cb868_c5/f1p0/3523	gnl|BL_ORD_ID|57583 cb868_c6/f1p0/3726	1	2099	2097	3486	16	2114	2305	369 4
cb5565_c14/f2p1/3093 isoform=cb5565_c14;full_length_coverage=2;non_full_length_coverage=1;isoform_length=3093;expected_accuracy=1.000	gnl|BL_ORD_ID|46322 cb5565_c47/f1p0/3193 isoform=cb5565_c47;full_length_coverage=1;non_full_length_coverage=0;isoform_length=3193;expected_accuracy=0.995	253	3093	1	252	374	319 3	5	258
cb3281_c11/f1p0/1936	gnl|BL_ORD_ID|39579 cb3281_c6/f1p0/2227 isoform=cb3281_c6;full_length_coverage=1;non_full_length_coverage=0;isoform_length=2227;expected_accuracy=0.997	1	1244	1243	1891	5	125 0	1574	222 2
cb7497_c22/f1p0/2606	gnl|BL_ORD_ID|8328 cb7497_c5/f1p1/3232 isoform=cb7497_c5;full_length_coverage=1;non_full_length_coverage=1;isoform_length=3232;expected_accuracy=0.991	998	2581	60	1001	1534	3116	4	967
cb8564_c81040/f1p1/2371	gnl|BL_ORD_ID|16018 cb8564_c74728/f5p3/2841 isoform=cb8564_c74728;full_length_coverage=5;non_full_length_coverage=3;isoform_length=2841;expected_accuracy=1.000	1	1965	1962	2481	114	207 8	2283	280 0
cb9479_c19/f1p0/1725 isoform=cb9479_c19;full_length_coverage=1;non_full_length_coverage=0;isoform_length=1725;expected_accuracy=1.000	gnl|BL_ORD_ID|19570 cb9479_c25/f2p0/2029 isoform=cb9479_c25;full_length_coverage=2;non_full_length_coverage=0;isoform_length=2029;expected_accuracy=1.000	1	1103	1099	1725	3	1105	1400	202 6
cb8564_c22900/f1p1/3151 isoform=cb8564_c22900;full_length_coverage=1;non_full_length_coverage=1;isoform_length=3151;expected_	gnl|BL_ORD_ID|11212 cb8564_c146148/f2p5/3291 isoform=cb8564_c146148;full_length_coverage=2;non_full_length_coverage=5;isoform_length=3291;expected_a	1	1750	1746	3116	15	175 6	1917	328 9

accuracy=0.994	ccuracy=0.998								
cb150_c4/f1p0/3890 isoform=cb150_c4;full_length_coverage=1;non_full_length_coverage=0;isoform_length=3890;expected_accuracy=0.996	gnl|BL_ORD_ID|29111 cb150_c3/f1p0/4197 isoform=cb150_c3;full_length_coverage=1;non_full_length_coverage=0;isoform_length=4197;expected_accuracy=0.995	1	3310	3309	3884	4	3310	3620	4197
cb1278_c11/f10p1/2558 isoform=cb1278_c11;full_length_coverage=10;non_full_length_coverage=1;isoform_length=2558;expected_accuracy=1.000	gnl|BL_ORD_ID|27259 cb1278_c5/f1p0/4398 isoform=cb1278_c5;full_length_coverage=1;non_full_length_coverage=0;isoform_length=4398;expected_accuracy=0.998	1	1951	1949	2536	7	1955	3811	4398
cb1770_c25/f1p0/3573	gnl|BL_ORD_ID|33848 cb1770_c5/f2p0/3464 isoform=cb1770_c5;full_length_coverage=2;non_full_length_coverage=0;isoform_length=3464;expected_accuracy=0.991	138	3339	1	137	264	3464	19	160
cb8564_c75384/f1p1/3644 isoform=cb8564_c75384;full_length_coverage=1;non_full_length_coverage=1;isoform_length=3644;expected_accuracy=0.994	gnl|BL_ORD_ID|57143 cb8564_c84495/f1p0/3631	10	2542	2542	3532	1	2535	2637	3625
cb1016_c3/f1p0/2299 isoform=cb1016_c3;full_length_coverage=1;non_full_length_coverage=0;isoform_length=2299;expected_accuracy=0.996	gnl|BL_ORD_ID|2484 cb1016_c5/f1p0/2657 isoform=cb1016_c5;full_length_coverage=1;non_full_length_coverage=0;isoform_length=2657;expected_accuracy=0.996	365	2299	4	367	740	2641	1	364
cb8564_c84198/f1p0/2418	gnl|BL_ORD_ID|57393 cb8564_c91758/f1p1/2726	1	2008	2006	2428	108	2118	2235	2657
cb6471_c23/f1p0/2688	gnl|BL_ORD_ID|5660 cb6471_c31/f2p2/2905 isoform=cb6471_c31;full_length_coverage=2;non_full_length_coverage=2;isoform_length=2905;expected_accur	1	1927	1928	2663	76	2000	2162	2897

	acy=1.000								
cb2108_c13/f1p0/3439 isoform=cb2108_c13;full_length_coverage=1;non_full_length_coverage=0;isoform_length=3439;expected_accuracy=0.991	gnl|BL_ORD_ID|34468 cb2108_c4/f1p1/4035 isoform=cb2108_c4;full_length_coverage=1;non_full_length_coverage=1;isoform_length=4035;expected_accuracy=0.997	1	2929	2929	3322	603	3501	3652	4035
cb8564_c36808/f1p0/2756	gnl|BL_ORD_ID|56381 cb8564_c75626/f1p2/3199	19	2129	2127	2555	7	2116	2611	3042
cb2275_c0/f1p0/3981 isoform=cb2275_c0;full_length_coverage=1;non_full_length_coverage=0;isoform_length=3981;expected_accuracy=0.998	gnl|BL_ORD_ID|14192 cb8564_c31974/f8p6/4543 isoform=cb8564_c31974;full_length_coverage=8;non_full_length_coverage=6;isoform_length=4543;expected_accuracy=0.997	23	3840	3835	3981	4458	639	154	4
cb13433_c53/f1p0/4827	gnl|BL_ORD_ID|62376 cb13433_c57/f1p0/6389	2413	4789	1	2418	3382	5758	68	2504
cb10133_c6/f1p1/1304	gnl|BL_ORD_ID|3295 cb10133_c7/f1p1/1730 isoform=cb10133_c7;full_length_coverage=1;non_full_length_coverage=1;isoform_length=1730;expected_accuracy=0.994	1	1074	1070	1307	203	1276	1400	1637
cb8564_c66961/f1p1/3021 isoform=cb8564_c66961;full_length_coverage=1;non_full_length_coverage=1;isoform_length=3021;expected_accuracy=0.996	gnl|BL_ORD_ID|55815 cb8564_c40609/f1p0/4032	15	2675	2672	3021	1	2693	3388	3731
cb8564_c41756/f1p0/2738 isoform=cb8564_c41756;full_length_coverage=1;non_full_length_coverage=0;isoform_length=2738;expected_accuracy=0.996	gnl|BL_ORD_ID|56602 cb8564_c48192/f1p6/3029	1	2173	2170	2738	1	2181	2304	2871
cb3491_c0/f3p0/1339	gnl|BL_ORD_ID|70407 cb3491_c4/f1p0/2063	449	1320	7	450	1170	204	1	454

isoform=cb3491_c0;full_length_coverage=3;non_full_length_coverage=0;isoform_length=1339;expected_accuracy=1.000							1		
cb8539_c3/f2p0/2041 isoform=cb8539_c3;full_length_coverage=2;non_full_length_coverage=0;isoform_length=2041;expected_accuracy=1.000	gnl|BL_ORD_ID|10884 cb8539_c1/f2p0/2336 isoform=cb8539_c1;full_length_coverage=2;non_full_length_coverage=0;isoform_length=2336;expected_accuracy=0.997	552	2041	59	553	625	2114	1	486
cb8564_c178335/f1p2/3430 isoform=cb8564_c178335;full_length_coverage=1;non_full_length_coverage=2;isoform_length=3430;expected_accuracy=0.998	gnl|BL_ORD_ID|52309 cb8564_c117845/f1p1/3666	1	3015	3016	3430	1	2994	3251	3665
cb2759_c0/f5p0/3138 isoform=cb2759_c0;full_length_coverage=5;non_full_length_coverage=0;isoform_length=3138;expected_accuracy=1.000	gnl|BL_ORD_ID|37960 cb2759_c3/f1p0/3834 isoform=cb2759_c3;full_length_coverage=1;non_full_length_coverage=0;isoform_length=3834;expected_accuracy=0.998	265	3138	20	264	944	3817	1	244
cb18456_c6530/f1p0/1200	gnl|BL_ORD_ID|34669 cb18456_c6410/f1p1/1766 isoform=cb18456_c6410;full_length_coverage=1;non_full_length_coverage=1;isoform_length=1766;expected_accuracy=1.000	260	1166	45	259	747	1657	1	215
cb8564_c41551/f1p0/3408	gnl|BL_ORD_ID|55920 cb8564_c48949/f1p0/4357	20	2289	2290	2953	18	2293	2847	3509
cb8564_c46939/f1p1/2926	gnl|BL_ORD_ID|57017 cb8564_c80370/f1p0/3493	119	2134	2130	2870	3085	1061	956	215
cb8564_c36559/f1p11/3192 isoform=cb8564_c36559;full_length_coverage=1;non_full_length_coverage=11;isoform_length=3192;expected	gnl|BL_ORD_ID|55939 cb8564_c49630/f1p4/3487	2	2934	2934	3182	3	2913	3243	3493

_accuracy=0.997									
cb8564_c34922/f1p0/2696 isoform=cb8564_c34922;full_length_coverage=1;non_full_length_coverage=0;isoform_length=2696;expected_accuracy=0.993	gnl|BL_ORD_ID|16879 cb8564_c48606/f1p1/3308 isoform=cb8564_c48606;full_length_coverage=1;non_full_length_coverage=1;isoform_length=3308;expected_accuracy=0.998	1	2119	2115	2696	1	2151	2587	3169
cb8564_c43538/f1p0/3563	gnl|BL_ORD_ID|55939 cb8564_c49630/f1p4/3487	10	2921	2921	3170	6	2913	3243	3493
cb4775_c0/f2p1/1973 isoform=cb4775_c0;full_length_coverage=2;non_full_length_coverage=1;isoform_length=1973;expected_accuracy=1.000	gnl|BL_ORD_ID|72004 cb4775_c5/f1p0/2223	1	1660	1658	1973	4	1686	1850	2166
cb1512_c15/f1p1/3322	gnl|BL_ORD_ID|27954 cb1512_c3/f2p2/4035 isoform=cb1512_c3;full_length_coverage=2;non_full_length_coverage=2;isoform_length=4035;expected_accuracy=0.999	1	1682	1681	3271	1	1682	2445	4035
cb18456_c1776/f1p0/1211	gnl|BL_ORD_ID|11861 cb8564_c126350/f1p2/2554 isoform=cb8564_c126350;full_length_coverage=1;non_full_length_coverage=2;isoform_length=2554;expected_accuracy=0.996	280	1214	6	285	962	29	1346	1074
cb3774_c49/f1p0/3335	gnl|BL_ORD_ID|41550 cb3774_c42/f1p0/3959 isoform=cb3774_c42;full_length_coverage=1;non_full_length_coverage=0;isoform_length=3959;expected_accuracy=0.993	1	2620	2617	3145	1	2617	3436	3959
cb8564_c43601/f1p0/2228	gnl|BL_ORD_ID|14729 cb8564_c41849/f1p1/4203 isoform=cb8564_c41849;full_length_coverage=1;non_full_length_coverage=1;isoform_length=4203;expected_ac	1	1814	1814	2083	1712	3524	3889	4159

	curacy=0.996								
cb3061_c14/f1p0/2385	gnl|BL_ORD_ID|69090 cb3061_c19/f1p0/3233	177	2389	1	177	893	3105	27	203
cb8564_c3087/f1p0/2090 isoform=cb8564_c3087;full_length_coverage=1;non_full_length_coverage=0;isoform_length=2090;expected_accuracy=1.000	gnl|BL_ORD_ID|52384 cb8564_c121118/f1p0/2660	10	1914	1909	2090	7	1910	2016	2197
cb18456_c4058/f1p1/1182 isoform=cb18456_c4058;full_length_coverage=1;non_full_length_coverage=1;isoform_length=1182;expected_accuracy=0.997	gnl|BL_ORD_ID|67493 cb18456_c4264/f1p1/1767	251	1182	5	256	803	1728	4	247
cb14165_c2/f1p0/1247	gnl|BL_ORD_ID|39040 cb2856_c4/f1p0/2196 isoform=cb2856_c4;full_length_coverage=1;non_full_length_coverage=0;isoform_length=2196;expected_accuracy=0.996	309	1231	4	312	1254	2196	28	333
cb3270_c8/f1p0/2363 isoform=cb3270_c8;full_length_coverage=1;non_full_length_coverage=0;isoform_length=2363;expected_accuracy=0.994	gnl|BL_ORD_ID|70273 cb3270_c14/f1p0/2972	1	1615	1620	2338	7	1621	2252	2970
cb12043_c1/f2p0/1546 isoform=cb12043_c1;full_length_coverage=2;non_full_length_coverage=0;isoform_length=1546;expected_accuracy=0.998	gnl|BL_ORD_ID|54955 cb8564_c3933/f1p0/4298	122	1508	1	122	2913	4299	1	120
cb5057_c18/f1p0/1420 isoform=cb5057_c18;full_length_coverage=1;non_full_length_coverage=0;isoform_length=1420;expected_accu	gnl|BL_ORD_ID|60447 cb11967_c17/f1p2/1809	1	1020	1016	1420	3	1022	1186	1592

racy=0.992									
cb8564_c3432/f1p0/2118	gnl|BL_ORD_ID|13930 cb8564_c3400/f3p0/2378 isoform=cb8564_c3400;full_length_coverage=3;non_full_length_coverage=0;isoform_length=2378;expected_accuracy=0.992	619	2090	7	623	742	2214	10	626
cb8564_c80524/f1p0/3995 isoform=cb8564_c80524;full_length_coverage=1;non_full_length_coverage=0;isoform_length=3995;expected_accuracy=0.992	gnl|BL_ORD_ID|67930 cb18656_c10/f1p0/5311	1	3658	3657	3991	438	4114	4844	5178
cb4233_c140/f1p1/2272 isoform=cb4233_c140;full_length_coverage=1;non_full_length_coverage=1;isoform_length=2272;expected_accuracy=0.996	gnl|BL_ORD_ID|71865 cb4233_c138/f1p0/3035	1	1586	1581	2267	1	1586	2306	2995
cb8564_c5122/f1p0/3329	gnl|BL_ORD_ID|55731 cb8564_c5169/f1p0/3921	1067	3319	1	1069	1513	3764	87	1155
cb1163_c42/f1p0/4122	gnl|BL_ORD_ID|23462 cb1163_c14/f1p1/4695 isoform=cb1163_c14;full_length_coverage=1;non_full_length_coverage=1;isoform_length=4695;expected_accuracy=0.991	120	1984	1983	3651	92	1955	3030	4692
cb8564_c23232/f1p3/2540	gnl|BL_ORD_ID|12970 cb8564_c16702/f2p13/2748 isoform=cb8564_c16702;full_length_coverage=2;non_full_length_coverage=13;isoform_length=2748;expected_accuracy=0.998	16	2172	2167	2507	151	2306	2408	2748
cb5483_c3/f1p0/2270 isoform=cb5483_c3;full_length_coverage=1;non_full_length_coverage=0;isoform_length=2270;expected_accur	gnl|BL_ORD_ID|46850 cb5483_c4/f1p0/2765 isoform=cb5483_c4;full_length_coverage=1;non_full_length_coverage=0;isoform_length=2765;expected_accura	172	2270	1	176	490	2609	25	207

acy=0.996	cy=0.991								
cb8564_c90372/f1p0/2157 isoform=cb8564_c90372;full_length_coverage=1;non_full_length_coverage=0;isoform_length=2157;expected_accuracy=0.994	gnl|BL_ORD_ID|11880 cb8564_c148138/f2p3/2537 isoform=cb8564_c148138;full_length_coverage=2;non_full_length_coverage=3;isoform_length=2537;expected_accuracy=0.991	1	1092	1091	2154	126	1217	1392	2455
cb8564_c106740/f7p2/2421 isoform=cb8564_c106740;full_length_coverage=7;non_full_length_coverage=2;isoform_length=2421;expected_accuracy=1.000	gnl|BL_ORD_ID|16625 cb8564_c72796/f1p0/2508 isoform=cb8564_c72796;full_length_coverage=1;non_full_length_coverage=0;isoform_length=2508;expected_accuracy=0.997	242	2421	62	244	315	2494	20	200
cb711_c1/f1p0/2532 isoform=cb711_c1;full_length_coverage=1;non_full_length_coverage=0;isoform_length=2532;expected_accuracy=0.992	gnl|BL_ORD_ID|5587 cb711_c4/f1p0/2827 isoform=cb711_c4;full_length_coverage=1;non_full_length_coverage=0;isoform_length=2827;expected_accuracy=0.997	1	1548	1546	2532	1	1557	1697	2690
cb5943_c9/f4p0/1361 isoform=cb5943_c9;full_length_coverage=4;non_full_length_coverage=0;isoform_length=1361;expected_accuracy=1.000	gnl|BL_ORD_ID|46197 cb5943_c4/f1p0/1485 isoform=cb5943_c4;full_length_coverage=1;non_full_length_coverage=0;isoform_length=1485;expected_accuracy=0.992	328	1331	5	329	482	1485	1	323
cb8564_c23232/f1p3/2540	gnl|BL_ORD_ID|55860 cb8564_c47209/f1p0/3227	19	2172	2167	2518	16	2186	2288	2636
cb8564_c75969/f1p1/2798 isoform=cb8564_c75969;full_length_coverage=1;non_full_length_coverage=1;isoform_length=2798;expected_accuracy=0.997	gnl|BL_ORD_ID|58074 cb8564_c88129/f1p3/3262	1	2120	2117	2798	14	2110	2394	3077
cb6271_c9/f1p0/1555 isoform=cb6271_c9;full_length_coverage=1;non_full_length_coverage=0;isoform_length=1555;expected_accur	gnl|BL_ORD_ID|49573 cb6271_c4/f1p0/2714	1	1364	1359	1555	17	1381	2101	2298

acy=1.000									
cb8564_c118509/f1p2/2743	gnl|BL_ORD_ID|56903 cb8564_c76255/f1p0/3009	295	2701	6	294	446	2849	3	291
cb8564_c37079/f1p0/2659	gnl|BL_ORD_ID|16169 cb8564_c66877/f3p0/2938 isoform=cb8564_c66877;full_length_coverage=3;non_full_length_coverage=0;isoform_length=2938;expected_accuracy=0.996	1	1322	1321	2423	60	1380	1765	2867
cb8564_c25287/f1p0/2199 isoform=cb8564_c25287;full_length_coverage=1;non_full_length_coverage=0;isoform_length=2199;expected_accuracy=0.993	gnl|BL_ORD_ID|57291 cb8564_c89181/f1p1/2994	1	1697	1694	2199	3	1719	2317	2823
cb187_c14/f1p0/2733 isoform=cb187_c14;full_length_coverage=1;non_full_length_coverage=0;isoform_length=2733;expected_accuracy=0.996	gnl|BL_ORD_ID|34575 cb187_c6/f9p1/4066 isoform=cb187_c6;full_length_coverage=9;non_full_length_coverage=1;isoform_length=4066;expected_accuracy=1.000	200	2733	1	199	1523	4045	54	248
cb6012_c15/f1p0/1851	gnl|BL_ORD_ID|45694 cb6012_c7/f3p2/2205 isoform=cb6012_c7;full_length_coverage=3;non_full_length_coverage=2;isoform_length=2205;expected_accuracy=0.995	1	1030	1029	1847	23	1052	1177	1995
cb2025_c3/f1p0/3798	gnl|BL_ORD_ID|35814 cb2025_c4/f1p0/3760 isoform=cb2025_c4;full_length_coverage=1;non_full_length_coverage=0;isoform_length=3760;expected_accuracy=0.994	1	2392	2391	3589	71	2458	2560	3760
cb6406_c65/f1p0/1830 isoform=cb6406_c65;full_length_coverage=1;non_full_length_coverage=0;isoform_length=1830;expected_accu	gnl|BL_ORD_ID|47493 cb6406_c21/f1p0/2190	1	1055	1051	1830	77	1132	1348	2128

racy=0.992									
cb8041_c27/f1p2/2321 isoform=cb8041_c27;full_length_coverage=1;non_full_length_coverage=2;isoform_length=2321;expected_accuracy=0.995	gnl|BL_ORD_ID|8355 cb8041_c6/f1p0/2474 isoform=cb8041_c6;full_length_coverage=1;non_full_length_coverage=0;isoform_length=2474;expected_accuracy=0.992	1	1519	1517	2244	25	1530	1743	2467
cb3270_c16/f1p0/3182	gnl|BL_ORD_ID|41534 cb3270_c3/f1p0/3673 isoform=cb3270_c3;full_length_coverage=1;non_full_length_coverage=0;isoform_length=3673;expected_accuracy=0.997	15	1713	1710	3087	1	1698	2302	3673
cb3601_c12/f1p1/1820 isoform=cb3601_c12;full_length_coverage=1;non_full_length_coverage=1;isoform_length=1820;expected_accuracy=0.990	gnl|BL_ORD_ID|70507 cb3601_c9/f1p0/2122	1	1379	1378	1820	55	1432	1534	1975
cb12931_c15/f1p0/1493 isoform=cb12931_c15;full_length_coverage=1;non_full_length_coverage=0;isoform_length=1493;expected_accuracy=1.000	gnl|BL_ORD_ID|56071 cb8564_c54161/f1p4/2912	1	1106	1104	1493	2	1103	2538	2926
cb2195_c26/f1p0/2900 isoform=cb2195_c26;full_length_coverage=1;non_full_length_coverage=0;isoform_length=2900;expected_accuracy=0.994	gnl|BL_ORD_ID|36363 cb2195_c23/f1p0/3248 isoform=cb2195_c23;full_length_coverage=1;non_full_length_coverage=0;isoform_length=3248;expected_accuracy=0.997	1	1678	1675	2900	16	1694	1915	3140
cb246_c3/f1p3/3304 isoform=cb246_c3;full_length_coverage=1;non_full_length_coverage=3;isoform_length=3304;expected_accuracy=0.997	gnl|BL_ORD_ID|36939 cb246_c5/f1p2/3598 isoform=cb246_c5;full_length_coverage=1;non_full_length_coverage=2;isoform_length=3598;expected_accuracy=0.992	1	2715	2712	3304	1	2712	2883	3473
cb8564_c744/f5p1/2823	gnl|BL_ORD_ID|18307 cb8564_c937/f3p0/2970	1	2157	2158	2821	1	215	2307	297

<table>
<tr><td>isoform=cb8564_c744;full_length_coverage=5;non_full_length_coverage=1;isoform_length=2823;expected_accuracy=0.996</td><td>isoform=cb8564_c937;full_length_coverage=3;non_full_length_coverage=0;isoform_length=2970;expected_accuracy=0.999</td><td></td><td></td><td></td><td></td><td></td><td>7</td><td></td><td>0</td></tr>
<tr><td>cb17973_c53/f1p0/1649 isoform=cb17973_c53;full_length_coverage=1;non_full_length_coverage=0;isoform_length=1649;expected_accuracy=0.992</td><td>gnl|BL_ORD_ID|15467 cb8564_c48905/f1p0/2042 isoform=cb8564_c48905;full_length_coverage=1;non_full_length_coverage=0;isoform_length=2042;expected_accuracy=0.995</td><td>186</td><td>1643</td><td>29</td><td>186</td><td>594</td><td>2042</td><td>1</td><td>156</td></tr>
<tr><td>cb1220_c1/f2p1/2616 isoform=cb1220_c1;full_length_coverage=2;non_full_length_coverage=1;isoform_length=2616;expected_accuracy=0.996</td><td>gnl|BL_ORD_ID|59274 cb1220_c8/f1p0/3488</td><td>1</td><td>1352</td><td>1351</td><td>2616</td><td>97</td><td>1449</td><td>1965</td><td>3233</td></tr>
<tr><td>cb8564_c181853/f1p0/2119 isoform=cb8564_c181853;full_length_coverage=1;non_full_length_coverage=0;isoform_length=2119;expected_accuracy=0.997</td><td>gnl|BL_ORD_ID|14556 cb8564_c45150/f1p0/2940 isoform=cb8564_c45150;full_length_coverage=1;non_full_length_coverage=0;isoform_length=2940;expected_accuracy=0.992</td><td>1</td><td>1339</td><td>1334</td><td>2119</td><td>1</td><td>1327</td><td>2152</td><td>2934</td></tr>
<tr><td>cb415_c47/f1p0/3141 isoform=cb415_c47;full_length_coverage=1;non_full_length_coverage=0;isoform_length=3141;expected_accuracy=0.994</td><td>gnl|BL_ORD_ID|72143 cb415_c84/f1p0/3822</td><td>17</td><td>2736</td><td>2734</td><td>3141</td><td>17</td><td>2739</td><td>3304</td><td>3711</td></tr>
<tr><td>cb2527_c30/f1p0/3430</td><td>gnl|BL_ORD_ID|68590 cb2527_c17/f1p1/3542</td><td>1</td><td>1878</td><td>1874</td><td>3175</td><td>29</td><td>1906</td><td>2256</td><td>3557</td></tr>
<tr><td>cb8564_c181853/f1p0/2119 isoform=cb8564_c181853;full_length_coverage=1;non_full_length_coverage=0;isoform_length=2119;expected_accuracy=0.997</td><td>gnl|BL_ORD_ID|14836 cb8564_c41692/f1p3/2987 isoform=cb8564_c41692;full_length_coverage=1;non_full_length_coverage=3;isoform_length=2987;expected_accuracy=0.995</td><td>1</td><td>1339</td><td>1334</td><td>2116</td><td>1</td><td>1355</td><td>2193</td><td>2987</td></tr>
<tr><td>cb8564_c50703/f1p0/3790</td><td>gnl|BL_ORD_ID|16695 cb8564_c53300/f1p1/4218</td><td>168</td><td>3790</td><td>1</td><td>168</td><td>493</td><td>4116</td><td>1</td><td>168</td></tr>
</table>

isoform=cb8564_c50703;full_length_coverage=1;non_full_length_coverage=0;isoform_length=3790;expected_accuracy=1.000	isoform=cb8564_c53300;full_length_coverage=1;non_full_length_coverage=1;isoform_length=4218;expected_accuracy=0.999								
cb1279_c85/f1p0/1794 isoform=cb1279_c85;full_length_coverage=1;non_full_length_coverage=0;isoform_length=1794;expected_accuracy=0.998	gnl|BL_ORD_ID|25719 cb1279_c37/f1p1/2816 isoform=cb1279_c37;full_length_coverage=1;non_full_length_coverage=1;isoform_length=2816;expected_accuracy=0.996	1	1120	1115	1761	38	1154	2180	2816
cb373_c39/f1p0/4415	gnl|BL_ORD_ID|40700 cb373_c7/f1p0/3964 isoform=cb373_c7;full_length_coverage=1;non_full_length_coverage=0;isoform_length=3964;expected_accuracy=0.995	254	3731	16	253	489	3964	11	248
cb8564_c185920/f1p0/2777 isoform=cb8564_c185920;full_length_coverage=1;non_full_length_coverage=0;isoform_length=2777;expected_accuracy=0.996	gnl|BL_ORD_ID|15496 cb8564_c47853/f1p0/2927 isoform=cb8564_c47853;full_length_coverage=1;non_full_length_coverage=0;isoform_length=2927;expected_accuracy=0.991	174	2773	1	177	344	2927	15	188
cb8564_c90417/f1p0/2393 isoform=cb8564_c90417;full_length_coverage=1;non_full_length_coverage=0;isoform_length=2393;expected_accuracy=0.991	gnl|BL_ORD_ID|17649 cb8564_c80259/f2p2/3241 isoform=cb8564_c80259;full_length_coverage=2;non_full_length_coverage=2;isoform_length=3241;expected_accuracy=1.000	1	1727	1724	2393	1	1733	2562	3230
cb11071_c8/f1p0/1469 isoform=cb11071_c8;full_length_coverage=1;non_full_length_coverage=0;isoform_length=1469;expected_accuracy=0.997	gnl|BL_ORD_ID|3378 cb11071_c3/f1p1/1705 isoform=cb11071_c3;full_length_coverage=1;non_full_length_coverage=1;isoform_length=1705;expected_accuracy=0.993	115	1469	1	115	309	1662	1	114
cb3888_c8/f1p0/2800 isoform=cb3888_c8;full_length_coverage=1;non_full_length_coverage=0;isoform_length=2800;expected_accur	gnl|BL_ORD_ID|40212 cb3888_c3/f1p0/3359 isoform=cb3888_c3;full_length_coverage=1;non_full_length_coverage=0;isoform_length=3359;expected_accura	243	2800	18	243	375	2930	1	226

acy=0.996	cy=0.995								
cb1333_c101/f1p0/1395 isoform=cb1333_c101;full_length_coverage=1;non_full_length_coverage=0;isoform_length=1395;expected_accuracy=0.997	gnl|BL_ORD_ID|61249 cb1333_c68/f1p0/4817	149	1395	27	150	3538	4782	29	152
cb7636_c5/f1p0/1712 isoform=cb7636_c5;full_length_coverage=1;non_full_length_coverage=0;isoform_length=1712;expected_accuracy=0.997	gnl|BL_ORD_ID|51419 cb7636_c3/f1p0/2438	382	1712	7	382	1017	2348	1	394
cb8302_c8/f1p0/1717 isoform=cb8302_c8;full_length_coverage=1;non_full_length_coverage=0;isoform_length=1717;expected_accuracy=0.994	gnl|BL_ORD_ID|9284 cb8302_c10/f6p0/2187 isoform=cb8302_c10;full_length_coverage=6;non_full_length_coverage=0;isoform_length=2187;expected_accuracy=1.000	175	1717	5	178	547	2089	1	174
cb8564_c186356/f1p0/2744 isoform=cb8564_c186356;full_length_coverage=1;non_full_length_coverage=0;isoform_length=2744;expected_accuracy=0.998	gnl|BL_ORD_ID|14263 cb8564_c42654/f1p1/2906 isoform=cb8564_c42654;full_length_coverage=1;non_full_length_coverage=1;isoform_length=2906;expected_accuracy=0.990	1393	2723	1	1396	1492	2824	1	1371
cb8564_c40978/f1p0/3073	gnl|BL_ORD_ID|18852 cb8564_c84488/f1p0/3408 isoform=cb8564_c84488;full_length_coverage=1;non_full_length_coverage=0;isoform_length=3408;expected_accuracy=0.996	228	2845	1	228	790	3408	1	217
cb12244_c1/f1p2/1884 isoform=cb12244_c1;full_length_coverage=1;non_full_length_coverage=2;isoform_length=1884;expected_accuracy=0.999	gnl|BL_ORD_ID|11394 cb8564_c150120/f1p3/2376 isoform=cb8564_c150120;full_length_coverage=1;non_full_length_coverage=3;isoform_length=2376;expected_accuracy=1.000	1	1048	1048	1884	29	1072	1539	2375
cb2804_c166/f1p0/1682	gnl|BL_ORD_ID|38911 cb2804_c50/f2p0/2193	161	1448	31	163	619	190	1	133

	isoform=cb2804_c50;full_length_coverage=2;non_full_length_coverage=0;isoform_length=2193;expected_accuracy=0.992						5		
cb7388_c2/f1p0/2288	gnl|BL_ORD_ID|7290 cb7388_c1/f1p0/2631 isoform=cb7388_c1;full_length_coverage=1;non_full_length_coverage=0;isoform_length=2631;expected_accuracy=0.999	1	1860	1857	2312	5	1864	2178	2630
cb5058_c4/f1p0/2291 isoform=cb5058_c4;full_length_coverage=1;non_full_length_coverage=0;isoform_length=2291;expected_accuracy=0.997	gnl|BL_ORD_ID|44021 cb5058_c2/f1p0/2666 isoform=cb5058_c2;full_length_coverage=1;non_full_length_coverage=0;isoform_length=2666;expected_accuracy=0.992	1	2061	2056	2288	276	2328	2431	2666
cb273_c4/f1p0/1772 isoform=cb273_c4;full_length_coverage=1;non_full_length_coverage=0;isoform_length=1772;expected_accuracy=0.999	gnl|BL_ORD_ID|37356 cb273_c3/f1p0/2380 isoform=cb273_c3;full_length_coverage=1;non_full_length_coverage=0;isoform_length=2380;expected_accuracy=0.992	1	1084	1084	1772	484	1559	1699	2380
cb4016_c10/f1p0/2636 isoform=cb4016_c10;full_length_coverage=1;non_full_length_coverage=0;isoform_length=2636;expected_accuracy=0.992	gnl|BL_ORD_ID|71723 cb4016_c11/f1p0/2688	1	1896	1896	2589	8	1908	2016	2711
cb12972_c15/f1p0/1541 isoform=cb12972_c15;full_length_coverage=1;non_full_length_coverage=0;isoform_length=1541;expected_accuracy=0.996	gnl|BL_ORD_ID|62024 cb12972_c37/f1p0/1779	295	1541	7	300	447	1684	1	294
cb4493_c13/f1p1/4300 isoform=cb4493_c13;full_length_coverage=1;non_full_length_coverage=1;isoform_length=4300;expected_accu	gnl|BL_ORD_ID|43098 cb4493_c10/f1p2/4658 isoform=cb4493_c10;full_length_coverage=1;non_full_length_coverage=2;isoform_length=4658;expected_accur	112	2669	2667	4295	1	2558	2961	4590

racy=0.992	acy=0.993								
cb8564_c40598/f1p4/3119	gnl|BL_ORD_ID|55437 cb8564_c41690/f1p1/3409	1	2732	2729	3053	1	2735	2856	3180
cb7521_c46/f1p0/2168	gnl|BL_ORD_ID|50571 cb7521_c26/f1p0/2812	1	1630	1626	2041	252	1880	2081	2496
cb14619_c9/f1p0/1471 isoform=cb14619_c9;full_length_coverage=1;non_full_length_coverage=0;isoform_length=1471;expected_accuracy=1.000	gnl|BL_ORD_ID|54844 cb8564_c3057/f1p0/2651	1	1196	1198	1471	13	1213	2127	2402
cb8564_c146882/f3p8/4113 isoform=cb8564_c146882;full_length_coverage=3;non_full_length_coverage=8;isoform_length=4113;expected_accuracy=0.998	gnl|BL_ORD_ID|14293 cb8564_c46212/f3p1/4373 isoform=cb8564_c46212;full_length_coverage=3;non_full_length_coverage=1;isoform_length=4373;expected_accuracy=0.999	1	3169	3165	4113	1	3160	3424	4372
cb9199_c4/f4p0/1836 isoform=cb9199_c4;full_length_coverage=4;non_full_length_coverage=0;isoform_length=1836;expected_accuracy=1.000	gnl|BL_ORD_ID|20905 cb9199_c13/f1p0/2081 isoform=cb9199_c13;full_length_coverage=1;non_full_length_coverage=0;isoform_length=2081;expected_accuracy=0.995	304	1836	9	303	496	2030	1	293
cb8564_c72680/f1p0/2685	gnl|BL_ORD_ID|18962 cb8564_c79723/f1p6/3025 isoform=cb8564_c79723;full_length_coverage=1;non_full_length_coverage=6;isoform_length=3025;expected_accuracy=0.999	1	1735	1730	2635	206	1941	2120	3025
cb8564_c11802/f1p0/3687 isoform=cb8564_c11802;full_length_coverage=1;non_full_length_coverage=0;isoform_length=3687;expected_accuracy=1.000	gnl|BL_ORD_ID|55146 cb8564_c25395/f1p0/4267	12	2152	2151	3683	5	2151	2619	4151
cb8564_c34965/f1p0/2700	gnl|BL_ORD_ID|57063 cb8564_c82099/f1p2/3297	1	1485	1481	2687	40	152	1934	314

							4		0
cb4233_c84/f1p1/2344	gnl|BL_ORD_ID|71861 cb4233_c130/f1p0/2863	227	2381	1	225	570	2724	546	770
cb8564_c82332/f1p0/2714 isoform=cb8564_c82332;full_length_coverage=1;non_full_length_coverage=0;isoform_length=2714;expected_accuracy=0.994	gnl|BL_ORD_ID|56896 cb8564_c75866/f1p0/3458	374	2714	50	374	852	3196	1	332
cb8564_c69975/f1p0/2818 isoform=cb8564_c69975;full_length_coverage=1;non_full_length_coverage=0;isoform_length=2818;expected_accuracy=0.999	gnl|BL_ORD_ID|58178 cb8564_c90729/f1p0/2969	260	2818	16	259	408	2966	29	246
cb8564_c41115/f1p3/2751	gnl|BL_ORD_ID|55873 cb8564_c47701/f1p0/3144	15	1542	1539	2690	24	1549	1721	2873
cb8564_c37361/f1p0/3076	gnl|BL_ORD_ID|56275 cb8564_c73216/f1p0/3006	21	2481	2477	3070	28	2462	2563	3156
cb8564_c45300/f1p4/3014 isoform=cb8564_c45300;full_length_coverage=1;non_full_length_coverage=4;isoform_length=3014;expected_accuracy=0.996	gnl|BL_ORD_ID|53680 cb8564_c153222/f2p39/3454	243	3005	1	246	406	3170	7	249
cb8564_c185920/f1p0/2777 isoform=cb8564_c185920;full_length_coverage=1;non_full_length_coverage=0;isoform_length=2777;expected_accuracy=0.996	gnl|BL_ORD_ID|16473 cb8564_c51923/f1p1/2966 isoform=cb8564_c51923;full_length_coverage=1;non_full_length_coverage=1;isoform_length=2966;expected_accuracy=0.997	174	2777	1	177	361	2966	18	203
cb8564_c24391/f1p1/2764 isoform=cb8564_c24391;full_length_coverage=1;non_full_length_coverage=1;isoform_length=2764;expected_	gnl|BL_ORD_ID|58202 cb8564_c91398/f1p0/3444	1	1714	1713	2764	37	1766	2023	3077

accuracy=1.000									
cb13495_c1/f1p1/1287 isoform=cb13495_c1;full_length_coverage=1;non_full_length_coverage=1;isoform_length=1287;expected_accuracy=1.000	gnl|BL_ORD_ID|16241 cb8564_c76946/f4p0/2069 isoform=cb8564_c76946;full_length_coverage=4;non_full_length_coverage=0;isoform_length=2069;expected_accuracy=0.998	332	1287	6	334	1047	2002	1	329
cb1333_c101/f1p0/1395 isoform=cb1333_c101;full_length_coverage=1;non_full_length_coverage=0;isoform_length=1395;expected_accuracy=0.997	gnl|BL_ORD_ID|61250 cb1333_c73/f1p0/4863	149	1395	27	150	3442	4686	29	152
cb5469_c6/f1p0/2317	gnl|BL_ORD_ID|44581 cb5469_c3/f1p0/2770 isoform=cb5469_c3;full_length_coverage=1;non_full_length_coverage=0;isoform_length=2770;expected_accuracy=0.993	268	2229	32	269	809	2770	90	327
cb3007_c18/f1p0/3150	gnl|BL_ORD_ID|37278 cb3007_c3/f1p0/3424 isoform=cb3007_c3;full_length_coverage=1;non_full_length_coverage=0;isoform_length=3424;expected_accuracy=0.991	880	2940	9	881	1346	3424	1	876
cb2737_c40/f1p0/2830	gnl|BL_ORD_ID|38134 cb2737_c22/f1p0/3196 isoform=cb2737_c22;full_length_coverage=1;non_full_length_coverage=0;isoform_length=3196;expected_accuracy=0.995	1	1397	1395	2651	377	1763	1894	3128
cb9436_c13/f1p0/2211	gnl|BL_ORD_ID|21678 cb9436_c4/f1p0/2516 isoform=cb9436_c4;full_length_coverage=1;non_full_length_coverage=0;isoform_length=2516;expected_accuracy=0.998	696	2125	72	697	1088	2516	1	627
cb16997_c22/f1p0/1844	gnl|BL_ORD_ID|31858 cb16997_c84/f1p0/2527	12	1451	1446	1775	1	143	2199	252

isoform=cb16997_c22;full_length_coverage=1;non_full_length_coverage=0;isoform_length=1844;expected_accuracy=0.994	isoform=cb16997_c84;full_length_coverage=1;non_full_length_coverage=0;isoform_length=2527;expected_accuracy=0.996						7		7
cb7214_c10/f1p0/1871	gnl|BL_ORD_ID|51049 cb7214_c9/f1p0/2049	134	1886	1	136	268	2020	22	157
cb6247_c2/f1p0/2904 isoform=cb6247_c2;full_length_coverage=1;non_full_length_coverage=0;isoform_length=2904;expected_accuracy=0.996	gnl|BL_ORD_ID|47407 cb6247_c3/f1p0/3766	18	2056	2054	2780	1	2045	2994	3720
cb8564_c905/f7p9/2409 isoform=cb8564_c905;full_length_coverage=7;non_full_length_coverage=9;isoform_length=2409;expected_accuracy=1.000	gnl|BL_ORD_ID|15303 cb8564_c3481/f1p0/2830 isoform=cb8564_c3481;full_length_coverage=1;non_full_length_coverage=0;isoform_length=2830;expected_accuracy=0.995	1	1668	1664	2337	3	1676	2158	2830
cb5963_c1/f1p0/2966 isoform=cb5963_c1;full_length_coverage=1;non_full_length_coverage=0;isoform_length=2966;expected_accuracy=0.994	gnl|BL_ORD_ID|46501 cb5963_c2/f1p0/3029 isoform=cb5963_c2;full_length_coverage=1;non_full_length_coverage=0;isoform_length=3029;expected_accuracy=0.993	1	1809	1807	2907	1	1814	1926	3029
cb1259_c21/f1p0/3481	gnl|BL_ORD_ID|23434 cb1259_c4/f1p0/4412 isoform=cb1259_c4;full_length_coverage=1;non_full_length_coverage=0;isoform_length=4412;expected_accuracy=0.995	29	3003	3003	3258	13	2985	4158	4411
cb2779_c5/f1p0/1790 isoform=cb2779_c5;full_length_coverage=1;non_full_length_coverage=0;isoform_length=1790;expected_accuracy=0.993	gnl|BL_ORD_ID|69969 cb2779_c1/f1p1/3812	1	1078	1077	1788	14	1093	2971	3679
cb8564_c122989/f1p0/4055	gnl|BL_ORD_ID|9374 cb8564_c109035/f10p1/3794	10	2587	2588	3750	144	275	2629	379

	isoform=cb8564_c109035;full_length_coverage=10;non_full_length_coverage=1;isoform_length=3794;expected_accuracy=1.000						4		1
cb7771_c6/f1p0/2053 isoform=cb7771_c6;full_length_coverage=1;non_full_length_coverage=0;isoform_length=2053;expected_accuracy=0.998	gnl|BL_ORD_ID|7436 cb7771_c3/f1p0/2176 isoform=cb7771_c3;full_length_coverage=1;non_full_length_coverage=0;isoform_length=2176;expected_accuracy=0.994	1	1327	1325	2053	1	1329	1432	2157
cb6161_c3/f2p1/1827 isoform=cb6161_c3;full_length_coverage=2;non_full_length_coverage=1;isoform_length=1827;expected_accuracy=0.991	gnl|BL_ORD_ID|46442 cb6161_c4/f2p0/2920 isoform=cb6161_c4;full_length_coverage=2;non_full_length_coverage=0;isoform_length=2920;expected_accuracy=1.000	148	1827	1	148	920	2599	5	148
cb8564_c45300/f1p4/3014 isoform=cb8564_c45300;full_length_coverage=1;non_full_length_coverage=4;isoform_length=3014;expected_accuracy=0.996	gnl|BL_ORD_ID|17429 cb8564_c85447/f1p0/3196 isoform=cb8564_c85447;full_length_coverage=1;non_full_length_coverage=0;isoform_length=3196;expected_accuracy=0.997	243	3014	1	246	399	3193	3	242
cb3941_c43/f1p0/4055	gnl|BL_ORD_ID|40540 cb3941_c39/f1p0/4473 isoform=cb3941_c39;full_length_coverage=1;non_full_length_coverage=0;isoform_length=4473;expected_accuracy=0.994	318	4096	1	318	484	4255	11	326
cb8564_c69975/f1p0/2818 isoform=cb8564_c69975;full_length_coverage=1;non_full_length_coverage=0;isoform_length=2818;expected_accuracy=0.999	gnl|BL_ORD_ID|18398 cb8564_c90428/f1p0/2987 isoform=cb8564_c90428;full_length_coverage=1;non_full_length_coverage=0;isoform_length=2987;expected_accuracy=0.996	260	2818	1	259	422	2945	1	260
cb8564_c110210/f1p0/2628 isoform=cb8564_c110210;full_length_coverage=1;non_full_length_coverage=0;isoform_length=2628;expected	gnl|BL_ORD_ID|9685 cb8564_c115488/f1p1/3915 isoform=cb8564_c115488;full_length_coverage=1;non_full_length_coverage=1;isoform_length=3915;expected_a	1	1869	1868	2627	17	1890	3155	3915

_accuracy=0.995	ccuracy=0.993								
cb5808_c3/f1p0/2793	gnl|BL_ORD_ID|46403 cb5808_c2/f1p0/3006 isoform=cb5808_c2;full_length_coverage=1;non_full_length_coverage=0;isoform_length=3006;expected_accuracy=0.996	1	2080	2079	2587	39	2119	2471	2979
cb3039_c11/f1p0/3305	gnl|BL_ORD_ID|69064 cb3039_c7/f1p0/3656	131	3353	1	135	279	3501	19	153
cb280_c75/f1p0/3730	gnl|BL_ORD_ID|68157 cb280_c88/f1p0/5515	307	3609	13	306	2181	5483	3	295
cb8564_c128290/f1p0/2765 isoform=cb8564_c128290;full_length_coverage=1;non_full_length_coverage=0;isoform_length=2765;expected_accuracy=0.996	gnl|BL_ORD_ID|11608 cb8564_c146551/f1p1/3093 isoform=cb8564_c146551;full_length_coverage=1;non_full_length_coverage=1;isoform_length=3093;expected_accuracy=1.000	1	1820	1820	2765	84	1903	2032	2978
cb1406_c5/f1p0/2334 isoform=cb1406_c5;full_length_coverage=1;non_full_length_coverage=0;isoform_length=2334;expected_accuracy=0.996	gnl|BL_ORD_ID|25273 cb1406_c13/f1p1/5633 isoform=cb1406_c13;full_length_coverage=1;non_full_length_coverage=1;isoform_length=5633;expected_accuracy=0.997	214	2334	11	218	3505	5629	1	207
cb8564_c179457/f1p0/2647 isoform=cb8564_c179457;full_length_coverage=1;non_full_length_coverage=0;isoform_length=2647;expected_accuracy=1.000	gnl|BL_ORD_ID|9350 cb8564_c11826/f1p0/2916 isoform=cb8564_c11826;full_length_coverage=1;non_full_length_coverage=0;isoform_length=2916;expected_accuracy=0.997	1273	2646	1	1274	1543	2916	4	1296
cb8564_c44258/f1p0/3202 isoform=cb8564_c44258;full_length_coverage=1;non_full_length_coverage=0;isoform_length=3202;expected_accuracy=0.993	gnl|BL_ORD_ID|16149 cb8564_c47162/f1p0/3361 isoform=cb8564_c47162;full_length_coverage=1;non_full_length_coverage=0;isoform_length=3361;expected_accuracy=0.996	408	3202	5	409	592	3353	5	402
cb2645_c20/f3p0/3491	gnl|BL_ORD_ID|37179 cb2645_c5/f1p0/3610	1	2875	2872	3491	24	289	2994	361

isoform=cb2645_c20;full_length_coverage=3;non_full_length_coverage=0;isoform_length=3491;expected_accuracy=1.000	isoform=cb2645_c5;full_length_coverage=1;non_full_length_coverage=0;isoform_length=3610;expected_accuracy=0.996						3		0
cb2430_c18/f1p0/3122	gnl|BL_ORD_ID|38548 cb2430_c4/f2p0/3285 isoform=cb2430_c4;full_length_coverage=2;non_full_length_coverage=0;isoform_length=3285;expected_accuracy=0.992	1	1838	1836	2427	4	1837	2245	2833
cb5896_c154/f1p0/2291 isoform=cb5896_c154;full_length_coverage=1;non_full_length_coverage=0;isoform_length=2291;expected_accuracy=0.998	gnl|BL_ORD_ID|44479 cb5896_c55/f1p0/2592 isoform=cb5896_c55;full_length_coverage=1;non_full_length_coverage=0;isoform_length=2592;expected_accuracy=0.993	1	1681	1680	2291	51	1727	1937	2548
cb8637_c58/f1p0/1789 isoform=cb8637_c58;full_length_coverage=1;non_full_length_coverage=0;isoform_length=1789;expected_accuracy=0.990	gnl|BL_ORD_ID|17073 cb8637_c67/f7p0/1947 isoform=cb8637_c67;full_length_coverage=7;non_full_length_coverage=0;isoform_length=1947;expected_accuracy=1.000	1	1332	1332	1773	73	1404	1507	1947
cb8564_c40598/f1p4/3119	gnl|BL_ORD_ID|12913 cb8564_c182049/f1p0/3195 isoform=cb8564_c182049;full_length_coverage=1;non_full_length_coverage=0;isoform_length=3195;expected_accuracy=0.997	1	2732	2729	3053	1	2748	2871	3195
cb8564_c49562/f1p0/2838 isoform=cb8564_c49562;full_length_coverage=1;non_full_length_coverage=0;isoform_length=2838;expected_accuracy=0.995	gnl|BL_ORD_ID|57175 cb8564_c85646/f1p0/3480	23	2331	2327	2829	106	2419	2526	3030
cb8564_c10736/f3p10/3904 isoform=cb8564_c10736;full_length_coverage=3;non_full_length_coverage=10;isoform_length=3904;expected	gnl|BL_ORD_ID|11693 cb8564_c13094/f1p5/4181 isoform=cb8564_c13094;full_length_coverage=1;non_full_length_coverage=5;isoform_length=4181;expected_ac	131	3264	3265	3904	1	3132	3542	4181

_accuracy=0.998	curacy=0.999								
cb606_c5/f1p1/3393 isoform=cb606_c5;full_length_coverage=1;non_full_length_coverage=1;isoform_length=3393;expected_accuracy=0.998	gnl|BL_ORD_ID|46196 cb606_c14/f1p4/4386 isoform=cb606_c14;full_length_coverage=1;non_full_length_coverage=4;isoform_length=4386;expected_accuracy=0.997	1	3229	3228	3393	5	3236	3986	4151
cb396_c48/f1p0/2172	gnl|BL_ORD_ID|70867 cb396_c51/f1p1/2465	238	2054	3	238	496	2312	12	247
cb2707_c10/f3p0/2850 isoform=cb2707_c10;full_length_coverage=3;non_full_length_coverage=0;isoform_length=2850;expected_accuracy=1.000	gnl|BL_ORD_ID|36994 cb2707_c29/f1p1/3207 isoform=cb2707_c29;full_length_coverage=1;non_full_length_coverage=1;isoform_length=3207;expected_accuracy=0.991	2	2379	2379	2850	1	2382	2707	3178
cb8564_c54518/f1p0/2781	gnl|BL_ORD_ID|11821 cb8564_c15227/f1p0/3770 isoform=cb8564_c15227;full_length_coverage=1;non_full_length_coverage=0;isoform_length=3770;expected_accuracy=0.999	1	1587	1585	2793	34	1620	2519	3727
cb8564_c15771/f1p1/3660	gnl|BL_ORD_ID|14775 cb8564_c31987/f2p1/3601 isoform=cb8564_c31987;full_length_coverage=2;non_full_length_coverage=1;isoform_length=3601;expected_accuracy=0.993	1	2711	2712	3436	5	2706	2854	3576
cb8564_c42019/f1p0/3526	gnl|BL_ORD_ID|55939 cb8564_c49630/f1p4/3487	130	3008	3008	3257	35	2913	3243	3492
cb626_c67/f1p1/4388	gnl|BL_ORD_ID|4060 cb626_c17/f1p0/4935 isoform=cb626_c17;full_length_coverage=1;non_full_length_coverage=0;isoform_length=4935;expected_accuracy=0.993	111	3749	3749	4309	12	3629	4370	4926
cb8564_c40598/f1p4/3119	gnl|BL_ORD_ID|56002 cb8564_c51722/f1p1/3503	23	2732	2729	3056	67	277	2893	321

							2		9
cb7642_c201/f1p0/1831 isoform=cb7642_c201;full_length_coverage=1;non_full_length_coverage=0;isoform_length=1831;expected_accuracy=0.992	gnl|BL_ORD_ID|51433 cb7642_c125/f1p0/2713	1	1029	1028	1831	108	1145	1364	2171
cb5125_c8/f3p3/2148 isoform=cb5125_c8;full_length_coverage=3;non_full_length_coverage=3;isoform_length=2148;expected_accuracy=0.999	gnl|BL_ORD_ID|43457 cb5125_c11/f2p1/2545 isoform=cb5125_c11;full_length_coverage=2;non_full_length_coverage=1;isoform_length=2545;expected_accuracy=1.000	1	1290	1288	2056	46	1336	1778	2545
cb15243_c1/f1p0/1809 isoform=cb15243_c1;full_length_coverage=1;non_full_length_coverage=0;isoform_length=1809;expected_accuracy=0.992	gnl|BL_ORD_ID|10268 cb8564_c111650/f1p0/2370 isoform=cb8564_c111650;full_length_coverage=1;non_full_length_coverage=0;isoform_length=2370;expected_accuracy=0.997	113	1808	1	112	485	2163	122	233
cb8564_c28079/f2p1/2021 isoform=cb8564_c28079;full_length_coverage=2;non_full_length_coverage=1;isoform_length=2021;expected_accuracy=0.998	gnl|BL_ORD_ID|13165 cb8564_c17237/f1p1/2698 isoform=cb8564_c17237;full_length_coverage=1;non_full_length_coverage=1;isoform_length=2698;expected_accuracy=0.999	398	2017	44	395	1079	2697	1	338
cb13938_c10/f1p0/1187	gnl|BL_ORD_ID|72022 cb4825_c13/f1p0/4647	151	1197	4	150	3608	4649	2595	2741
cb8564_c188097/f1p0/2552 isoform=cb8564_c188097;full_length_coverage=1;non_full_length_coverage=0;isoform_length=2552;expected_accuracy=0.996	gnl|BL_ORD_ID|13065 cb8564_c157334/f1p21/2777 isoform=cb8564_c157334;full_length_coverage=1;non_full_length_coverage=21;isoform_length=2777;expected_accuracy=0.995	1	1945	1940	2552	7	1958	2061	2673
cb1772_c26/f1p0/3767	gnl|BL_ORD_ID|66372 cb1772_c16/f1p1/4029	232	3695	13	234	430	3894	25	246
cb124_c2/f4p0/2458	gnl|BL_ORD_ID|23061 cb124_c8/f2p0/3745	216	2420	1	217	1540	374	3	217

isoform=cb124_c2;full_length_coverage=4;non_full_length_coverage=0;isoform_length=2458;expected_accuracy=0.998	isoform=cb124_c8;full_length_coverage=2;non_full_length_coverage=0;isoform_length=3745;expected_accuracy=0.999						5		
cb3557_c15/f1p0/2674 isoform=cb3557_c15;full_length_coverage=1;non_full_length_coverage=0;isoform_length=2674;expected_accuracy=0.995	gnl|BL_ORD_ID|70457 cb3557_c31/f1p0/2861	1037	2627	1	1038	1297	2877	98	1135
cb8564_c90797/f1p0/2864	gnl|BL_ORD_ID|57186 cb8564_c85953/f1p0/2986	17	2307	2308	2607	1	2295	2422	2717
cb8564_c19160/f1p2/2687 isoform=cb8564_c19160;full_length_coverage=1;non_full_length_coverage=2;isoform_length=2687;expected_accuracy=0.990	gnl|BL_ORD_ID|9350 cb8564_c11826/f1p0/2916 isoform=cb8564_c11826;full_length_coverage=1;non_full_length_coverage=0;isoform_length=2916;expected_accuracy=0.997	1325	2687	1	1326	1543	2908	4	1296
cb2818_c54/f1p1/2570	gnl|BL_ORD_ID|68906 cb2818_c57/f1p0/3403	2	2144	2141	2547	3	2144	2611	3017
cb1913_c44/f1p0/2236 isoform=cb1913_c44;full_length_coverage=1;non_full_length_coverage=0;isoform_length=2236;expected_accuracy=0.998	gnl|BL_ORD_ID|66490 cb1913_c46/f1p1/2592	1	1634	1630	2236	43	1675	1809	2415
cb5583_c0/f4p0/1845 isoform=cb5583_c0;full_length_coverage=4;non_full_length_coverage=0;isoform_length=1845;expected_accuracy=1.000	gnl|BL_ORD_ID|74104 cb5583_c3/f1p0/2163	132	1845	1	132	422	2137	1	132
cb8564_c37802/f1p0/2973	gnl|BL_ORD_ID|14978 cb8564_c39294/f1p0/3287 isoform=cb8564_c39294;full_length_coverage=1;non_full_length_coverage=0;isoform_length=3287;expected_ac	1	2292	2287	2777	1	2286	2782	3270

	curacy=0.999								
cb965_c18/f1p0/2545 isoform=cb965_c18;full_length_coverage=1;non_full_length_coverage=0;isoform_length=2545;expected_accuracy=1.000	gnl|BL_ORD_ID|20479 cb965_c42/f1p0/3993 isoform=cb965_c42;full_length_coverage=1;non_full_length_coverage=0;isoform_length=3993;expected_accuracy=0.998	1	1828	1831	2487	1274	3100	3328	3993
cb2707_c24/f1p0/2640	gnl|BL_ORD_ID|36994 cb2707_c29/f1p1/3207 isoform=cb2707_c29;full_length_coverage=1;non_full_length_coverage=1;isoform_length=3207;expected_accuracy=0.991	2	2430	2425	2649	1	2436	2940	3164
cb8564_c17092/f1p0/3523	gnl|BL_ORD_ID|13106 cb8564_c16615/f1p2/3838 isoform=cb8564_c16615;full_length_coverage=1;non_full_length_coverage=2;isoform_length=3838;expected_accuracy=1.000	314	3252	1	314	911	3837	1	313
cb1685_c9/f1p1/3226 isoform=cb1685_c9;full_length_coverage=1;non_full_length_coverage=1;isoform_length=3226;expected_accuracy=0.994	gnl|BL_ORD_ID|31292 cb1685_c11/f2p1/3604 isoform=cb1685_c11;full_length_coverage=2;non_full_length_coverage=1;isoform_length=3604;expected_accuracy=0.995	1	2295	2294	3226	84	2385	2644	3573
cb8637_c58/f1p0/1789 isoform=cb8637_c58;full_length_coverage=1;non_full_length_coverage=0;isoform_length=1789;expected_accuracy=0.990	gnl|BL_ORD_ID|57461 cb8637_c31/f1p0/2012	1	1332	1332	1762	126	1457	1560	1989
cb8564_c91083/f1p0/2192 isoform=cb8564_c91083;full_length_coverage=1;non_full_length_coverage=0;isoform_length=2192;expected_accuracy=0.997	gnl|BL_ORD_ID|18222 cb8564_c84564/f1p0/2312 isoform=cb8564_c84564;full_length_coverage=1;non_full_length_coverage=0;isoform_length=2312;expected_accuracy=0.991	1	1532	1530	2184	3	1529	1629	2312
cb7128_c3/f1p0/1951	gnl|BL_ORD_ID|4728 cb7128_c4/f1p0/2477	427	1920	62	428	931	242	21	374

	isoform=cb7128_c4;full_length_coverage=1;non_full_length_coverage=0;isoform_length=2477;expected_accuracy=0.999						3		
cb658_c18/f1p1/5197	gnl|BL_ORD_ID|4084 cb658_c10/f1p1/6427 isoform=cb658_c10;full_length_coverage=1;non_full_length_coverage=1;isoform_length=6427;expected_accuracy=0.993	28	3899	3899	5188	2	3862	5144	6422
cb8564_c35806/f1p0/2697	gnl|BL_ORD_ID|55939 cb8564_c49630/f1p4/3487	126	2454	2454	2703	580	2913	3243	3493
cb8564_c182028/f1p0/2513 isoform=cb8564_c182028;full_length_coverage=1;non_full_length_coverage=0;isoform_length=2513;expected_accuracy=0.996	gnl|BL_ORD_ID|13686 cb8564_c23425/f1p0/2742 isoform=cb8564_c23425;full_length_coverage=1;non_full_length_coverage=0;isoform_length=2742;expected_accuracy=0.993	1	1971	1968	2513	30	1997	2140	2660
cb8564_c161476/f1p4/2801 isoform=cb8564_c161476;full_length_coverage=1;non_full_length_coverage=4;isoform_length=2801;expected_accuracy=0.995	gnl|BL_ORD_ID|55920 cb8564_c48949/f1p0/4357	16	2302	2303	2801	16	2293	2847	3328
cb8564_c94261/f1p0/3116 isoform=cb8564_c94261;full_length_coverage=1;non_full_length_coverage=0;isoform_length=3116;expected_accuracy=0.994	gnl|BL_ORD_ID|13864 cb8564_c22844/f1p2/4014 isoform=cb8564_c22844;full_length_coverage=1;non_full_length_coverage=2;isoform_length=4014;expected_accuracy=0.998	521	3095	60	522	1433	4012	12	473
cb8564_c16741/f1p0/2997	gnl|BL_ORD_ID|12323 cb8564_c12737/f1p1/3352 isoform=cb8564_c12737;full_length_coverage=1;non_full_length_coverage=1;isoform_length=3352;expected_accuracy=0.997	375	3001	71	376	606	3227	186	490
cb4233_c84/f1p1/2344	gnl|BL_ORD_ID|72376 cb4233_c129/f1p1/2970	227	2372	1	225	520	266	496	720

						5			
cb8564_c49090/f1p0/3264	gnl|BL_ORD_ID|16149 cb8564_c47162/f1p0/3361 isoform=cb8564_c47162;full_length_coverage=1;non_full_length_coverage=0;isoform_length=3361;expected_accuracy=0.996	469	3242	71	470	592	336 1	3	402
cb8564_c37802/f1p0/2973	gnl|BL_ORD_ID|15374 cb8564_c43905/f1p0/3073 isoform=cb8564_c43905;full_length_coverage=1;non_full_length_coverage=0;isoform_length=3073;expected_accuracy=0.994	1	2292	2287	2754	1	228 4	2608	307 3
cb8533_c1/f2p0/1399 isoform=cb8533_c1;full_length_coverage=2;non_full_length_coverage=0;isoform_length=1399;expected_accuracy=1.000	gnl|BL_ORD_ID|10098 cb8533_c8/f1p0/1587 isoform=cb8533_c8;full_length_coverage=1;non_full_length_coverage=0;isoform_length=1587;expected_accuracy=0.994	11	1112	1107	1399	1	1103	1251	154 3
cb3031_c0/f1p0/3742 isoform=cb3031_c0;full_length_coverage=1;non_full_length_coverage=0;isoform_length=3742;expected_accuracy=0.999	gnl|BL_ORD_ID|36999 cb3031_c1/f1p0/3822 isoform=cb3031_c1;full_length_coverage=1;non_full_length_coverage=0;isoform_length=3822;expected_accuracy=0.996	165	3427	3423	3742	1	327 5	3503	382 2
cb2960_c2/f1p0/2155 isoform=cb2960_c2;full_length_coverage=1;non_full_length_coverage=0;isoform_length=2155;expected_accuracy=0.995	gnl|BL_ORD_ID|36641 cb2960_c3/f1p0/2973 isoform=cb2960_c3;full_length_coverage=1;non_full_length_coverage=0;isoform_length=2973;expected_accuracy=0.996	1	1832	1831	2144	41	187 4	2660	297 3
cb7198_c2/f1p0/2361	gnl|BL_ORD_ID|50415 cb7198_c3/f1p0/2567	1049	2334	1	1049	1266	255 0	2	105 0
cb12893_c39/f1p0/1097 isoform=cb12893_c39;full_length_coverage=1;non_full_length_coverage=0;isoform_length=1097;expected_acc	gnl|BL_ORD_ID|61932 cb12893_c72/f1p0/1628	234	1079	4	239	490	134 7	1	235

uracy=0.996									
cb8564_c41572/f1p1/3265	gnl|BL_ORD_ID|55920 cb8564_c48949/f1p0/4357	1	2281	2282	2951	2	2293	2847	3510
cb388_c19/f1p0/3492 isoform=cb388_c19;full_length_coverage=1;non_full_length_coverage=0;isoform_length=3492;expected_accuracy=0.997	gnl|BL_ORD_ID|40929 cb388_c25/f1p0/4272 isoform=cb388_c25;full_length_coverage=1;non_full_length_coverage=0;isoform_length=4272;expected_accuracy=0.994	1	3046	3045	3492	1	3094	3800	4246
cb7016_c13/f1p0/3328	gnl|BL_ORD_ID|50254 cb7016_c11/f1p0/3457	16	1957	1956	3303	1	1933	2040	3389
cb1791_c1/f1p0/2228 isoform=cb1791_c1;full_length_coverage=1;non_full_length_coverage=0;isoform_length=2228;expected_accuracy=0.995	gnl|BL_ORD_ID|66379 cb1791_c0/f1p0/4169	1	1558	1559	2228	1231	2791	3505	4174
cb2737_c40/f1p0/2830	gnl|BL_ORD_ID|36571 cb2737_c54/f1p0/3029 isoform=cb2737_c54;full_length_coverage=1;non_full_length_coverage=0;isoform_length=3029;expected_accuracy=0.995	1	1769	1770	2651	234	1998	2110	2985
cb8564_c37361/f1p0/3076	gnl|BL_ORD_ID|55246 cb8564_c36343/f1p0/3448	2	2481	2477	3068	3	2496	2597	3188
cb944_c10/f5p1/1315 isoform=cb944_c10;full_length_coverage=5;non_full_length_coverage=1;isoform_length=1315;expected_accuracy=1.000	gnl|BL_ORD_ID|21044 cb944_c16/f3p0/1974 isoform=cb944_c16;full_length_coverage=3;non_full_length_coverage=0;isoform_length=1974;expected_accuracy=1.000	466	1274	8	466	1166	1974	12	489
cb8564_c165050/f2p1/2091 isoform=cb8564_c165050;full_length_coverage=2;non_full_length_coverage=1;isoform_length=2091;expected	gnl|BL_ORD_ID|54351 cb8564_c22815/f1p0/4618	271	2059	5	269	2511	4303	3	266

_accuracy=0.997									
cb12446_c3/f1p0/1412 isoform=cb12446_c3;full_length_coverage=1;non_full_length_coverage=0;isoform_length=1412;expected_accuracy=1.000	gnl|BL_ORD_ID|12144 cb8564_c146050/f14p3/2042 isoform=cb8564_c146050;full_length_coverage=14;non_full_length_coverage=3;isoform_length=2042;expected_accuracy=1.000	109	1411	1	111	738	2042	144	253
cb8564_c3432/f1p0/2118	gnl|BL_ORD_ID|13290 cb8564_c1937/f1p5/2510 isoform=cb8564_c1937;full_length_coverage=1;non_full_length_coverage=5;isoform_length=2510;expected_accuracy=0.991	619	2090	8	623	786	2257	18	670
cb7749_c11/f1p1/1246	gnl|BL_ORD_ID|50686 cb7749_c7/f1p0/1366	169	1103	4	173	390	1323	66	235
cb6569_c6/f1p1/2392 isoform=cb6569_c6;full_length_coverage=1;non_full_length_coverage=1;isoform_length=2392;expected_accuracy=0.995	gnl|BL_ORD_ID|47579 cb6569_c3/f1p1/2683	1	1678	1673	2324	74	1753	1865	2516
cb8564_c12355/f1p9/2685 isoform=cb8564_c12355;full_length_coverage=1;non_full_length_coverage=9;isoform_length=2685;expected_accuracy=0.999	gnl|BL_ORD_ID|56353 cb8564_c75114/f1p0/2866	1	2062	2062	2608	1	2035	2193	2737
cb8564_c43779/f1p0/3021 isoform=cb8564_c43779;full_length_coverage=1;non_full_length_coverage=0;isoform_length=3021;expected_accuracy=0.993	gnl|BL_ORD_ID|55551 cb8564_c44964/f1p0/3508	1	2344	2345	3021	1	2341	2497	3173
cb7253_c3/f1p0/2607	gnl|BL_ORD_ID|6517 cb7253_c9/f1p0/2565 isoform=cb7253_c9;full_length_coverage=1;non_full_length_coverage=0;isoform_length=2565;expected_accura	114	1801	1801	2542	1	1690	1827	2565

	cy=0.992								
cb8564_c38629/f2p1/3246 isoform=cb8564_c38629;full_length_coverage=2;non_full_length_coverage=1;isoform_length=3246;expected_accuracy=0.993	gnl|BL_ORD_ID|55939 cb8564_c49630/f1p4/3487	10	2946	2946	3196	6	2913	3243	3493
cb6159_c9/f1p0/2712 isoform=cb6159_c9;full_length_coverage=1;non_full_length_coverage=0;isoform_length=2712;expected_accuracy=0.994	gnl|BL_ORD_ID|46029 cb6159_c13/f4p1/2851 isoform=cb6159_c13;full_length_coverage=4;non_full_length_coverage=1;isoform_length=2851;expected_accuracy=0.997	1037	2641	1	1037	1246	2851	27	1066
cb11848_c1/f2p0/1107 isoform=cb11848_c1;full_length_coverage=2;non_full_length_coverage=0;isoform_length=1107;expected_accuracy=0.996	gnl|BL_ORD_ID|15459 cb8564_c76939/f1p3/2221 isoform=cb8564_c76939;full_length_coverage=1;non_full_length_coverage=3;isoform_length=2221;expected_accuracy=0.993	474	1107	5	473	1288	1929	1	465
cb541_c26/f1p1/5713 isoform=cb541_c26;full_length_coverage=1;non_full_length_coverage=1;isoform_length=5713;expected_accuracy=0.997	gnl|BL_ORD_ID|73517 cb541_c17/f1p0/6031	114	5713	1	111	224	5814	1	110
cb8564_c128725/f1p1/2771 isoform=cb8564_c128725;full_length_coverage=1;non_full_length_coverage=1;isoform_length=2771;expected_accuracy=1.000	gnl|BL_ORD_ID|55391 cb8564_c40278/f3p2/3083	1	2669	2664	2771	1	2673	2783	2891
cb8564_c47252/f1p0/2795 isoform=cb8564_c47252;full_length_coverage=1;non_full_length_coverage=0;isoform_length=2795;expected_accuracy=0.997	gnl|BL_ORD_ID|56381 cb8564_c75626/f1p2/3199	1	2116	2114	2701	1	2116	2611	3199
cb4674_c44/f1p2/2925	gnl|BL_ORD_ID|72841 cb4674_c25/f1p0/3544	1	2069	2070	2906	86	214	2657	349

isoform=cb4674_c44;full_length_coverage=1;non_full_length_coverage=2;isoform_length=2925;expected_accuracy=0.991							1		2
cb8564_c42959/f1p0/3330	gnl|BL_ORD_ID|14333 cb8564_c39696/f4p3/4068 isoform=cb8564_c39696;full_length_coverage=4;non_full_length_coverage=3;isoform_length=4068;expected_accuracy=0.997	121	3198	1	125	986	4062	169	294
cb8564_c185920/f1p0/2777 isoform=cb8564_c185920;full_length_coverage=1;non_full_length_coverage=0;isoform_length=2777;expected_accuracy=0.996	gnl|BL_ORD_ID|55464 cb8564_c42285/f1p0/3370	174	2777	1	177	351	2953	15	192
cb8564_c49310/f1p2/3045	gnl|BL_ORD_ID|58199 cb8564_c91279/f1p0/3567	550	3004	65	549	749	3203	24	508
cb8564_c14344/f1p0/2533 isoform=cb8564_c14344;full_length_coverage=1;non_full_length_coverage=0;isoform_length=2533;expected_accuracy=0.993	gnl|BL_ORD_ID|56712 cb8564_c53711/f1p0/3201	148	2533	1	151	508	2894	39	189
cb8794_c14/f1p0/1326	gnl|BL_ORD_ID|17971 cb8794_c9/f1p0/1826 isoform=cb8794_c9;full_length_coverage=1;non_full_length_coverage=0;isoform_length=1826;expected_accuracy=0.998	495	1177	6	496	1008	1691	2	492
cb14220_c10/f1p1/6717 isoform=cb14220_c10;full_length_coverage=1;non_full_length_coverage=1;isoform_length=6717;expected_accuracy=0.993	gnl|BL_ORD_ID|63134 cb14220_c9/f1p0/6925	2469	6691	185	2471	2708	6950	1	2293
cb99_c7/f1p0/3479	gnl|BL_ORD_ID|21971 cb99_c3/f1p1/4977	144	3479	1	146	1407	475	156	300

isoform=cb99_c7;full_length_coverage=1;non_full_length_coverage=0;isoform_length=3479;expected_accuracy=0.990	isoform=cb99_c3;full_length_coverage=1;non_full_length_coverage=1;isoform_length=4977;expected_accuracy=1.000						6		
cb8564_c36689/f1p1/2368 isoform=cb8564_c36689;full_length_coverage=1;non_full_length_coverage=1;isoform_length=2368;expected_accuracy=0.991	gnl|BL_ORD_ID|14263 cb8564_c42654/f1p1/2906 isoform=cb8564_c42654;full_length_coverage=1;non_full_length_coverage=1;isoform_length=2906;expected_accuracy=0.990	1	1709	1706	2347	1	1678	2178	2824
cb7815_c11/f1p1/2093 isoform=cb7815_c11;full_length_coverage=1;non_full_length_coverage=1;isoform_length=2093;expected_accuracy=0.993	gnl|BL_ORD_ID|51548 cb7815_c8/f1p0/3813	1	1318	1319	1999	117	1435	3031	3709
cb8564_c68610/f6p0/2150 isoform=cb8564_c68610;full_length_coverage=6;non_full_length_coverage=0;isoform_length=2150;expected_accuracy=1.000	gnl|BL_ORD_ID|16835 cb8564_c73948/f1p0/2329 isoform=cb8564_c73948;full_length_coverage=1;non_full_length_coverage=0;isoform_length=2329;expected_accuracy=0.999	364	2058	4	365	634	2329	1	362
cb438_c5/f1p0/1932 isoform=cb438_c5;full_length_coverage=1;non_full_length_coverage=0;isoform_length=1932;expected_accuracy=0.993	gnl|BL_ORD_ID|42326 cb438_c2/f1p0/4812 isoform=cb438_c2;full_length_coverage=1;non_full_length_coverage=0;isoform_length=4812;expected_accuracy=0.997	331	1932	9	335	1416	3024	1	321
cb3670_c5/f1p0/2095 isoform=cb3670_c5;full_length_coverage=1;non_full_length_coverage=0;isoform_length=2095;expected_accuracy=1.000	gnl|BL_ORD_ID|71428 cb3670_c3/f1p0/2218	1	1232	1231	2033	22	1261	1386	2188
cb4798_c10/f1p0/2855 isoform=cb4798_c10;full_length_coverage=1;non_full_length_coverage=0;isoform_length=2855;expected_accu	gnl|BL_ORD_ID|42780 cb4798_c7/f1p0/3256 isoform=cb4798_c7;full_length_coverage=1;non_full_length_coverage=0;isoform_length=3256;expected_accura	1012	2855	1	1013	1355	3189	163	1171

racy=0.994	cy=0.997								
cb8564_c71747/f1p1/2679 isoform=cb8564_c71747;full_length_coverage=1;non_full_length_coverage=1;isoform_length=2679;expected_accuracy=0.999	gnl|BL_ORD_ID|15322 cb8564_c34141/f2p2/3036 isoform=cb8564_c34141;full_length_coverage=2;non_full_length_coverage=2;isoform_length=3036;expected_accuracy=0.993	1	1999	1998	2679	88	2099	2242	2921
cb2737_c1/f4p0/2862 isoform=cb2737_c1;full_length_coverage=4;non_full_length_coverage=0;isoform_length=2862;expected_accuracy=0.999	gnl|BL_ORD_ID|36571 cb2737_c54/f1p0/3029 isoform=cb2737_c54;full_length_coverage=1;non_full_length_coverage=0;isoform_length=3029;expected_accuracy=0.995	1	1942	1943	2862	63	1998	2110	3025
cb666_c2/f1p0/2897 isoform=cb666_c2;full_length_coverage=1;non_full_length_coverage=0;isoform_length=2897;expected_accuracy=0.996	gnl|BL_ORD_ID|47188 cb666_c0/f1p0/4630	1128	2897	1	1131	2803	4574	449	1582
cb7209_c6/f1p0/2322 isoform=cb7209_c6;full_length_coverage=1;non_full_length_coverage=0;isoform_length=2322;expected_accuracy=0.999	gnl|BL_ORD_ID|7147 cb7209_c20/f1p1/2581 isoform=cb7209_c20;full_length_coverage=1;non_full_length_coverage=1;isoform_length=2581;expected_accuracy=0.996	1	1583	1580	2322	119	1702	1832	2575
cb8564_c11435/f1p1/3407	gnl|BL_ORD_ID|12196 cb8564_c12654/f2p0/4122 isoform=cb8564_c12654;full_length_coverage=2;non_full_length_coverage=0;isoform_length=4122;expected_accuracy=1.000	18	1916	1916	3254	4	1901	2784	4122
cb18409_c104/f1p0/1443	gnl|BL_ORD_ID|12988 cb8564_c16263/f1p0/2381 isoform=cb8564_c16263;full_length_coverage=1;non_full_length_coverage=0;isoform_length=2381;expected_accuracy=0.995	110	1438	1	113	1051	2381	24	136
cb8564_c43779/f1p0/3021	gnl|BL_ORD_ID|55600 cb8564_c46484/f1p0/3151	101	2344	2345	3021	5	226	2423	309

isoform=cb8564_c43779;full_length_coverage=1;non_full_length_coverage=0;isoform_length=3021;expected_accuracy=0.993							7		7
cb2770_c52/f1p0/2648	gnl|BL_ORD_ID|69963 cb2770_c47/f1p0/3578	1	1608	1608	2517	72	1679	2474	3398
cb1462_c20/f1p0/3600 isoform=cb1462_c20;full_length_coverage=1;non_full_length_coverage=0;isoform_length=3600;expected_accuracy=0.995	gnl|BL_ORD_ID|28514 cb1462_c5/f2p1/3927 isoform=cb1462_c5;full_length_coverage=2;non_full_length_coverage=1;isoform_length=3927;expected_accuracy=0.998	167	3524	1	167	566	3927	78	244
cb5183_c100/f1p2/1592 isoform=cb5183_c100;full_length_coverage=1;non_full_length_coverage=2;isoform_length=1592;expected_accuracy=0.998	gnl|BL_ORD_ID|73601 cb5183_c56/f1p0/1814	278	1519	7	282	402	1643	1	277
cb8564_c123951/f1p1/4450 isoform=cb8564_c123951;full_length_coverage=1;non_full_length_coverage=1;isoform_length=4450;expected_accuracy=0.996	gnl|BL_ORD_ID|52889 cb8564_c110962/f2p1/4818	310	4450	1	311	531	4676	43	374
cb10304_c5/f1p0/1455 isoform=cb10304_c5;full_length_coverage=1;non_full_length_coverage=0;isoform_length=1455;expected_accuracy=1.000	gnl|BL_ORD_ID|2574 cb10304_c2/f2p1/1614 isoform=cb10304_c2;full_length_coverage=2;non_full_length_coverage=1;isoform_length=1614;expected_accuracy=1.000	11	1333	1332	1455	56	1377	1493	1614
cb8971_c24/f1p1/3712	gnl|BL_ORD_ID|19877 cb8971_c6/f1p2/4360 isoform=cb8971_c6;full_length_coverage=1;non_full_length_coverage=2;isoform_length=4360;expected_accuracy=0.997	1	2344	2341	3594	1	2344	3052	4303
cb11749_c2/f1p0/1220	gnl|BL_ORD_ID|60349 cb11749_c1/f1p0/3050	385	1180	8	390	2236	303	14	396

							1		
cb8564_c118011/f1p0/1965	gnl|BL_ORD_ID|9341 cb8564_c107041/f1p2/2670 isoform=cb8564_c107041;full_length_coverage=1;non_full_length_coverage=2;isoform_length=2670;expected_accuracy=0.994	1	1321	1317	1964	508	1824	2003	2648
cb1415_c16/f1p0/3004 isoform=cb1415_c16;full_length_coverage=1;non_full_length_coverage=0;isoform_length=3004;expected_accuracy=0.995	gnl|BL_ORD_ID|61345 cb1415_c11/f1p0/4135	1	1467	1464	3004	56	1525	2186	3749
cb6528_c10/f1p0/2365	gnl|BL_ORD_ID|4353 cb6528_c4/f1p0/2794 isoform=cb6528_c4;full_length_coverage=1;non_full_length_coverage=0;isoform_length=2794;expected_accuracy=0.995	1	1468	1465	2245	9	1486	1945	2729
cb5999_c5/f1p0/2393 isoform=cb5999_c5;full_length_coverage=1;non_full_length_coverage=0;isoform_length=2393;expected_accuracy=0.996	gnl|BL_ORD_ID|45208 cb5999_c3/f1p0/2890 isoform=cb5999_c3;full_length_coverage=1;non_full_length_coverage=0;isoform_length=2890;expected_accuracy=0.995	1	1224	1220	2393	1	1233	1670	2853
cb5001_c11/f1p0/2067	gnl|BL_ORD_ID|73186 cb5001_c6/f1p0/3148	1	1585	1580	2073	1	1584	2360	2853
cb8564_c80867/f1p8/2842 isoform=cb8564_c80867;full_length_coverage=1;non_full_length_coverage=8;isoform_length=2842;expected_accuracy=0.995	gnl|BL_ORD_ID|57868 cb8564_c82903/f1p0/3274	1	1853	1855	2761	36	1887	1994	2903
cb6719_c46/f1p0/2507	gnl|BL_ORD_ID|4663 cb6719_c20/f1p0/3262 isoform=cb6719_c20;full_length_coverage=1;non_full_length_coverage=0;isoform_length=3262;expected_accur	1	1435	1434	2452	684	2119	2240	3257

	acy=0.996								
cb8564_c3679/f1p0/2936	gnl|BL_ORD_ID|17665 cb8564_c865/f4p0/2761 isoform=cb8564_c865;full_length_coverage=4;non_full_length_coverage=0;isoform_length=2761;expected_accuracy=0.996	2	2461	2460	2644	17	2476	2577	2761
cb8564_c40764/f1p0/2563 isoform=cb8564_c40764;full_length_coverage=1;non_full_length_coverage=0;isoform_length=2563;expected_accuracy=0.997	gnl|BL_ORD_ID|56641 cb8564_c50309/f1p5/3075	1	2167	2162	2563	1	2164	2371	2772
cb1406_c5/f1p0/2334 isoform=cb1406_c5;full_length_coverage=1;non_full_length_coverage=0;isoform_length=2334;expected_accuracy=0.996	gnl|BL_ORD_ID|25274 cb1406_c12/f1p1/5814 isoform=cb1406_c12;full_length_coverage=1;non_full_length_coverage=1;isoform_length=5814;expected_accuracy=0.996	214	2334	1	218	3680	5804	1	218
cb246_c3/f1p3/3304 isoform=cb246_c3;full_length_coverage=1;non_full_length_coverage=3;isoform_length=3304;expected_accuracy=0.997	gnl|BL_ORD_ID|38903 cb246_c16/f30p3/3550 isoform=cb246_c16;full_length_coverage=30;non_full_length_coverage=3;isoform_length=3550;expected_accuracy=1.000	1	2715	2712	3303	70	2786	2957	3550
cb293_c1/f1p0/4190 isoform=cb293_c1;full_length_coverage=1;non_full_length_coverage=0;isoform_length=4190;expected_accuracy=0.999	gnl|BL_ORD_ID|69536 cb293_c7/f1p0/4528	1	2211	2208	4190	20	2232	2409	4370
cb581_c7/f1p0/2369 isoform=cb581_c7;full_length_coverage=1;non_full_length_coverage=0;isoform_length=2369;expected_accuracy=0.990	gnl|BL_ORD_ID|45254 cb581_c1/f2p1/2803 isoform=cb581_c1;full_length_coverage=2;non_full_length_coverage=1;isoform_length=2803;expected_accuracy=1.000	1	1631	1627	2369	326	1956	2061	2799
cb8564_c5117/f1p0/4873	gnl|BL_ORD_ID|29247 cb14859_c9/f1p3/5221	722	4873	82	723	998	515	1	641

isoform=cb8564_c5117;full_length_coverage=1;non_full_length_coverage=0;isoform_length=4873;expected_accuracy=0.999	isoform=cb14859_c9;full_length_coverage=1;non_full_length_coverage=3;isoform_length=5221;expected_accuracy=0.997						0		
cb8564_c2150/f1p0/2358	gnl|BL_ORD_ID|11337 cb8564_c123109/f2p0/3168 isoform=cb8564_c123109;full_length_coverage=2;non_full_length_coverage=0;isoform_length=3168;expected_accuracy=0.998	12	2130	2130	2332	685	2806	2934	3136
cb8564_c34638/f1p0/2817 isoform=cb8564_c34638;full_length_coverage=1;non_full_length_coverage=0;isoform_length=2817;expected_accuracy=0.992	gnl|BL_ORD_ID|14407 cb8564_c44036/f1p0/3258 isoform=cb8564_c44036;full_length_coverage=1;non_full_length_coverage=0;isoform_length=3258;expected_accuracy=0.992	1146	2813	1	1146	1575	3258	1	1147
cb8564_c41756/f1p0/2738 isoform=cb8564_c41756;full_length_coverage=1;non_full_length_coverage=0;isoform_length=2738;expected_accuracy=0.996	gnl|BL_ORD_ID|10510 cb8564_c111713/f6p2/3032 isoform=cb8564_c111713;full_length_coverage=6;non_full_length_coverage=2;isoform_length=3032;expected_accuracy=0.998	1	2173	2170	2738	1	2148	2271	2838
cb9199_c16/f1p0/1711 isoform=cb9199_c16;full_length_coverage=1;non_full_length_coverage=0;isoform_length=1711;expected_accuracy=0.998	gnl|BL_ORD_ID|20905 cb9199_c13/f1p0/2081 isoform=cb9199_c13;full_length_coverage=1;non_full_length_coverage=0;isoform_length=2081;expected_accuracy=0.995	157	1711	1	156	496	2051	138	293
cb15493_c2/f1p0/1044	gnl|BL_ORD_ID|15750 cb8564_c69252/f2p1/3018 isoform=cb8564_c69252;full_length_coverage=2;non_full_length_coverage=1;isoform_length=3018;expected_accuracy=0.998	305	1003	6	307	2319	3018	1	306
cb2785_c20/f1p2/2883 isoform=cb2785_c20;full_length_coverage=1;non_full_length_coverage=2;isoform_length=2883;expected_accu	gnl|BL_ORD_ID|36581 cb2785_c9/f1p1/3811 isoform=cb2785_c9;full_length_coverage=1;non_full_length_coverage=1;isoform_length=3811;expected_accura	1	2058	2060	2803	101	2156	3065	3806

racy=0.997	cy=0.995								
cb9604_c7/f1p0/1887	gnl|BL_ORD_ID|20879 cb9604_c0/f3p0/1972 isoform=cb9604_c0;full_length_coverage=3;non_full_length_coverage=0;isoform_length=1972;expected_accuracy=0.997	202	1802	27	200	321	1920	1	176
cb8564_c19794/f1p0/2446 isoform=cb8564_c19794;full_length_coverage=1;non_full_length_coverage=0;isoform_length=2446;expected_accuracy=0.994	gnl|BL_ORD_ID|57737 cb8564_c79357/f1p0/2727	189	2444	1	188	434	2688	15	202
cb3618_c26/f1p0/2964	gnl|BL_ORD_ID|71384 cb3618_c17/f1p0/3242	2	2434	2434	2849	17	2443	2801	3217
cb8564_c44698/f1p5/2843 isoform=cb8564_c44698;full_length_coverage=1;non_full_length_coverage=5;isoform_length=2843;expected_accuracy=0.997	gnl|BL_ORD_ID|56623 cb8564_c49310/f1p2/3045	18	2516	2511	2834	65	2559	2680	3005
cb4651_c1/f1p0/3282	gnl|BL_ORD_ID|72812 cb4651_c3/f1p0/4374	21	2348	2347	3292	15	2342	3089	4034
cb3471_c3/f1p1/1814 isoform=cb3471_c3;full_length_coverage=1;non_full_length_coverage=1;isoform_length=1814;expected_accuracy=0.996	gnl|BL_ORD_ID|70396 cb3471_c0/f1p0/3611	1	1009	1007	1783	1	1044	2775	3563
cb944_c92/f1p0/1128	gnl|BL_ORD_ID|59112 cb944_c83/f1p0/1294	8	693	688	1139	11	697	799	1250
cb1821_c16/f1p1/3514	gnl|BL_ORD_ID|63996 cb1821_c15/f1p0/3876	655	3314	77	655	1142	3792	2	579
cb7725_c2/f1p1/2358	gnl|BL_ORD_ID|8968 cb7725_c0/f1p1/2549	216	2358	1	217	368	250	51	267

isoform=cb7725_c2;full_length_coverage=1;non_full_length_coverage=1;isoform_length=2358;expected_accuracy=0.992	isoform=cb7725_c0;full_length_coverage=1;non_full_length_coverage=1;isoform_length=2549;expected_accuracy=0.996						8		
cb8564_c69489/f1p0/1983 isoform=cb8564_c69489;full_length_coverage=1;non_full_length_coverage=0;isoform_length=1983;expected_accuracy=0.994	gnl|BL_ORD_ID|19270 cb8564_c79478/f1p0/2124 isoform=cb8564_c79478;full_length_coverage=1;non_full_length_coverage=0;isoform_length=2124;expected_accuracy=0.994	173	1983	11	175	314	2124	1	165
cb3485_c10/f1p0/2515	gnl|BL_ORD_ID|40922 cb3485_c4/f1p0/3162 isoform=cb3485_c4;full_length_coverage=1;non_full_length_coverage=0;isoform_length=3162;expected_accuracy=0.996	1	1299	1298	2523	77	1375	1809	3033
cb8564_c38947/f1p0/2650 isoform=cb8564_c38947;full_length_coverage=1;non_full_length_coverage=0;isoform_length=2650;expected_accuracy=0.996	gnl|BL_ORD_ID|61972 cb12931_c30/f1p0/5903	1	1441	1436	2650	1	1437	1654	2868
cb5699_c30/f4p1/2374 isoform=cb5699_c30;full_length_coverage=4;non_full_length_coverage=1;isoform_length=2374;expected_accuracy=1.000	gnl|BL_ORD_ID|46122 cb5699_c5/f1p0/3030 isoform=cb5699_c5;full_length_coverage=1;non_full_length_coverage=0;isoform_length=3030;expected_accuracy=0.995	225	2365	1	226	889	3030	1	229
cb3981_c17/f1p0/2982	gnl|BL_ORD_ID|71698 cb3981_c11/f1p0/3288	495	2937	5	496	759	3201	1	495
cb8564_c90231/f1p1/3635	gnl|BL_ORD_ID|12196 cb8564_c12654/f2p0/4122 isoform=cb8564_c12654;full_length_coverage=2;non_full_length_coverage=0;isoform_length=4122;expected_accuracy=1.000	1	1904	1904	3242	1	1901	2784	4122
cb8564_c44420/f1p0/2853	gnl|BL_ORD_ID|15357 cb8564_c44284/f4p1/3133	1	1487	1484	2831	97	157	1793	313

isoform=cb8564_c44420;full_length_coverage=1;non_full_length_coverage=0;isoform_length=2853;expected_accuracy=0.992	isoform=cb8564_c44284;full_length_coverage=4;non_full_length_coverage=1;isoform_length=3133;expected_accuracy=1.000						5		3
cb4042_c14/f1p0/3138 isoform=cb4042_c14;full_length_coverage=1;non_full_length_coverage=0;isoform_length=3138;expected_accuracy=0.993	gnl|BL_ORD_ID|39856 cb4042_c10/f1p0/3274 isoform=cb4042_c10;full_length_coverage=1;non_full_length_coverage=0;isoform_length=3274;expected_accuracy=0.992	1	2786	2786	3076	14	2795	2986	3274
cb964_c15/f1p0/4325	gnl|BL_ORD_ID|59142 cb964_c19/f1p0/4670	1	2341	2342	4110	81	2421	2626	4394
cb8564_c21052/f1p2/3145	gnl|BL_ORD_ID|13276 cb8564_c23062/f1p3/3805 isoform=cb8564_c23062;full_length_coverage=1;non_full_length_coverage=3;isoform_length=3805;expected_accuracy=0.995	244	3057	13	249	925	3742	1	229
cb8564_c84198/f1p0/2418	gnl|BL_ORD_ID|12430 cb8564_c147762/f1p5/2779 isoform=cb8564_c147762;full_length_coverage=1;non_full_length_coverage=5;isoform_length=2779;expected_accuracy=0.995	1	2008	2006	2428	83	2094	2211	2632
cb8564_c41076/f2p1/3193	gnl|BL_ORD_ID|14238 cb8564_c39248/f1p1/3575 isoform=cb8564_c39248;full_length_coverage=1;non_full_length_coverage=1;isoform_length=3575;expected_accuracy=0.993	1	2413	2409	3128	1	2419	2856	3574
cb8564_c75534/f1p0/3028 isoform=cb8564_c75534;full_length_coverage=1;non_full_length_coverage=0;isoform_length=3028;expected_accuracy=0.994	gnl|BL_ORD_ID|56483 cb8564_c77669/f1p0/3318	1	1798	1797	2897	35	1825	2128	3225
cb7642_c36/f1p0/2490	gnl|BL_ORD_ID|51431 cb7642_c72/f1p0/3432	1	1677	1676	2490	137	181	2029	284

isoform=cb7642_c36;full_length_coverage=1;non_full_length_coverage=0;isoform_length=2490;expected_accuracy=0.991							3		5
cb12047_c134/f1p0/1727 isoform=cb12047_c134;full_length_coverage=1;non_full_length_coverage=0;isoform_length=1727;expected_accuracy=0.999	gnl|BL_ORD_ID|60511 cb12047_c47/f1p7/1851	1	1006	1001	1711	1	1006	1112	1822
cb1262_c13/f1p0/3920 isoform=cb1262_c13;full_length_coverage=1;non_full_length_coverage=0;isoform_length=3920;expected_accuracy=0.996	gnl|BL_ORD_ID|23602 cb1262_c27/f1p1/4094 isoform=cb1262_c27;full_length_coverage=1;non_full_length_coverage=1;isoform_length=4094;expected_accuracy=0.996	352	3920	133	353	529	4094	1	224
cb6802_c57/f1p0/2631	gnl|BL_ORD_ID|50084 cb6802_c52/f1p0/2612	191	2236	1	192	293	2338	1	168
cb6346_c2/f1p0/2403 isoform=cb6346_c2;full_length_coverage=1;non_full_length_coverage=0;isoform_length=2403;expected_accuracy=0.993	gnl|BL_ORD_ID|4866 cb6346_c3/f1p0/2550 isoform=cb6346_c3;full_length_coverage=1;non_full_length_coverage=0;isoform_length=2550;expected_accuracy=0.996	1138	2403	10	1142	1243	2507	1	1130
cb452_c42/f1p0/3142	gnl|BL_ORD_ID|70914 cb452_c29/f1p1/3123	1296	2981	1	1297	1405	3091	1	1302
cb8564_c71747/f1p1/2679 isoform=cb8564_c71747;full_length_coverage=1;non_full_length_coverage=1;isoform_length=2679;expected_accuracy=0.999	gnl|BL_ORD_ID|16786 cb8564_c52175/f1p3/3008 isoform=cb8564_c52175;full_length_coverage=1;non_full_length_coverage=3;isoform_length=3008;expected_accuracy=0.999	1	2002	1998	2679	25	2094	2236	2917
cb8564_c38947/f1p0/2650 isoform=cb8564_c38947;full_length_coverage=1;non_full_length_coverage=0;isoform_length=2650;expected_	gnl|BL_ORD_ID|14263 cb8564_c42654/f1p1/2906 isoform=cb8564_c42654;full_length_coverage=1;non_full_length_coverage=1;isoform_length=2906;expected_ac	1	1441	1436	2626	1	1414	1631	2824

accuracy=0.996	curacy=0.990								
cb8564_c113599/f1p0/2144	gnl|BL_ORD_ID|16225 cb8564_c76305/f1p0/2353 isoform=cb8564_c76305;full_length_coverage=1;non_full_length_coverage=0;isoform_length=2353;expected_accuracy=1.000	1	1347	1346	2125	39	1384	1551	2331
cb3143_c11/f1p1/3005 isoform=cb3143_c11;full_length_coverage=1;non_full_length_coverage=1;isoform_length=3005;expected_accuracy=0.997	gnl|BL_ORD_ID|69160 cb3143_c4/f1p0/3643	294	3005	14	294	687	3402	1	284
cb8097_c6/f1p0/2610 isoform=cb8097_c6;full_length_coverage=1;non_full_length_coverage=0;isoform_length=2610;expected_accuracy=0.998	gnl|BL_ORD_ID|50861 cb8097_c4/f1p0/3641	1	2175	2175	2608	100	2278	3227	3662
cb8564_c38251/f1p0/3243	gnl|BL_ORD_ID|55920 cb8564_c48949/f1p0/4357	1	2309	2310	2973	2	2293	2847	3507
cb6606_c20/f1p0/2268	gnl|BL_ORD_ID|49866 cb6606_c12/f1p0/2893	1	1174	1173	2229	24	1193	1855	2900
cb6548_c16/f1p0/2550	gnl|BL_ORD_ID|5637 cb6548_c10/f1p1/2515 isoform=cb6548_c10;full_length_coverage=1;non_full_length_coverage=1;isoform_length=2515;expected_accuracy=0.991	272	2430	73	273	345	2515	1	209
cb9061_c56/f10p1/2114 isoform=cb9061_c56;full_length_coverage=10;non_full_length_coverage=1;isoform_length=2114;expected_accuracy=1.000	gnl|BL_ORD_ID|58527 cb9061_c19/f1p0/2376	1	1472	1473	2114	2	1473	1581	2226
cb1001_c9/f1p0/3094	gnl|BL_ORD_ID|2951 cb1001_c2/f1p0/3812	257	3023	1	257	1036	381	639	893

	isoform=cb1001_c2;full_length_coverage=1;non_full_length_coverage=0;isoform_length=3812;expected_accuracy=0.995						2		
cb1953_c8/f1p0/2581 isoform=cb1953_c8;full_length_coverage=1;non_full_length_coverage=0;isoform_length=2581;expected_accuracy=0.993	gnl|BL_ORD_ID|35584 cb1953_c6/f1p0/3949 isoform=cb1953_c6;full_length_coverage=1;non_full_length_coverage=0;isoform_length=3949;expected_accuracy=0.999	1	1516	1515	2558	612	2127	2903	3949
cb8564_c21439/f1p0/2587	gnl|BL_ORD_ID|16052 cb8564_c69457/f3p14/2821 isoform=cb8564_c69457;full_length_coverage=3;non_full_length_coverage=14;isoform_length=2821;expected_accuracy=0.997	1226	2572	1	1228	1349	2695	1	1228
cb18456_c7297/f1p0/1408	gnl|BL_ORD_ID|54626 cb8564_c128456/f1p0/2321	520	1324	7	519	1434	2238	1	514
cb3087_c39/f1p0/3065	gnl|BL_ORD_ID|37719 cb3087_c29/f1p1/3127 isoform=cb3087_c29;full_length_coverage=1;non_full_length_coverage=1;isoform_length=3127;expected_accuracy=0.993	1	2059	2054	2496	1	2061	2613	3054
cb8564_c17237/f1p1/2698 isoform=cb8564_c17237;full_length_coverage=1;non_full_length_coverage=1;isoform_length=2698;expected_accuracy=0.999	gnl|BL_ORD_ID|54208 cb8564_c20515/f1p0/3776	2	2449	2445	2698	1	2445	3325	3578
cb8564_c11511/f2p0/2718 isoform=cb8564_c11511;full_length_coverage=2;non_full_length_coverage=0;isoform_length=2718;expected_accuracy=0.992	gnl|BL_ORD_ID|57148 cb8564_c84620/f1p0/3289	195	2715	1	194	528	3054	10	197
cb8564_c132288/f2p0/3421	gnl|BL_ORD_ID|16467 cb8564_c49872/f2p1/3689	1	1918	1918	3408	137	203	2209	368

isoform=cb8564_c132288;full_length_coverage=2;non_full_length_coverage=0;isoform_length=3421;expected_accuracy=0.996	isoform=cb8564_c49872;full_length_coverage=2;non_full_length_coverage=1;isoform_length=3689;expected_accuracy=1.000						1		9
cb7007_c7/f1p0/2328	gnl|BL_ORD_ID|47801 cb7007_c13/f1p0/2402	656	2330	93	657	713	2387	1	565
cb341_c7/f1p0/2819 isoform=cb341_c7;full_length_coverage=1;non_full_length_coverage=0;isoform_length=2819;expected_accuracy=1.000	gnl|BL_ORD_ID|40575 cb341_c21/f1p0/3212 isoform=cb341_c21;full_length_coverage=1;non_full_length_coverage=0;isoform_length=3212;expected_accuracy=0.991	16	2507	2507	2723	1	2506	2993	3212
cb8564_c4697/f8p0/2707 isoform=cb8564_c4697;full_length_coverage=8;non_full_length_coverage=0;isoform_length=2707;expected_accuracy=1.000	gnl|BL_ORD_ID|11448 cb8564_c124301/f1p0/2778 isoform=cb8564_c124301;full_length_coverage=1;non_full_length_coverage=0;isoform_length=2778;expected_accuracy=0.997	313	2644	75	317	449	2778	1	244
cb1468_c0/f7p1/2932 isoform=cb1468_c0;full_length_coverage=7;non_full_length_coverage=1;isoform_length=2932;expected_accuracy=1.000	gnl|BL_ORD_ID|63645 cb1468_c3/f1p0/4312	1	2062	2061	2864	94	2140	3043	3855
cb1279_c62/f1p0/2083	gnl|BL_ORD_ID|61188 cb1279_c66/f1p1/3600	177	2116	1	177	1485	3425	4	180
cb8564_c44063/f1p0/2503 isoform=cb8564_c44063;full_length_coverage=1;non_full_length_coverage=0;isoform_length=2503;expected_accuracy=0.999	gnl|BL_ORD_ID|55785 cb8564_c36643/f1p3/2668	397	2503	5	402	536	2641	13	411
cb1317_c16/f1p1/2428	gnl|BL_ORD_ID|27409 cb1317_c11/f1p1/4136 isoform=cb1317_c11;full_length_coverage=1;non_full_length_coverage=1;isoform_length=4136;expected_accur	229	2361	1	228	1953	4080	4	228

	acy=0.997								
cb16828_c0/f2p0/1022 isoform=cb16828_c0;full_length_coverage=2;non_full_length_coverage=0;isoform_length=1022;expected_accuracy=0.999	gnl|BL_ORD_ID|32793 cb16828_c1/f1p0/1216 isoform=cb16828_c1;full_length_coverage=1;non_full_length_coverage=0;isoform_length=1216;expected_accuracy=0.995	134	1022	30	136	337	1216	2	111
cb6036_c7/f1p0/2226	gnl|BL_ORD_ID|74791 cb6036_c9/f1p0/2767	1	1671	1671	2172	74	1744	2180	2681
cb8564_c42506/f1p0/2909	gnl|BL_ORD_ID|56038 cb8564_c53031/f1p4/4126	1	2200	2199	2857	1316	3515	3388	4054
cb12714_c29/f1p2/1583	gnl|BL_ORD_ID|54246 cb8564_c21138/f1p3/2652	1	1296	1293	1534	1	1294	2359	2600
cb1581_c2/f2p4/2471 isoform=cb1581_c2;full_length_coverage=2;non_full_length_coverage=4;isoform_length=2471;expected_accuracy=1.000	gnl|BL_ORD_ID|28677 cb1581_c8/f1p0/2559 isoform=cb1581_c8;full_length_coverage=1;non_full_length_coverage=0;isoform_length=2559;expected_accuracy=0.994	225	2383	1	224	402	2559	23	247
cb8564_c21926/f1p0/3009	gnl|BL_ORD_ID|52043 cb8564_c11099/f1p1/4643	1246	2638	1	1246	3249	4641	1861	3106
cb5931_c19/f2p1/1950 isoform=cb5931_c19;full_length_coverage=2;non_full_length_coverage=1;isoform_length=1950;expected_accuracy=1.000	gnl|BL_ORD_ID|74667 cb5931_c3/f1p0/2877	196	1950	2	198	502	2252	2	198
cb4007_c27/f1p0/2752	gnl|BL_ORD_ID|70713 cb4007_c23/f1p0/3263	11	1810	1809	2705	3	1828	2373	3269
cb4646_c27/f1p0/2961	gnl|BL_ORD_ID|43219 cb4646_c7/f1p1/3035 isoform=cb4646_c7;full_length_coverage=1;non_full_length_coverage=1;isoform_length=3035;expected_accura	841	2589	9	845	1284	3034	13	851

	cy=0.999								
cb9467_c49/f1p1/2123 isoform=cb9467_c49;full_length_coverage=1;non_full_length_coverage=1;isoform_length=2123;expected_accuracy=0.993	gnl|BL_ORD_ID|21842 cb9467_c46/f1p0/2279 isoform=cb9467_c46;full_length_coverage=1;non_full_length_coverage=0;isoform_length=2279;expected_accuracy=0.992	1	1610	1608	2034	4	1609	1855	2279
cb8564_c36689/f1p1/2368 isoform=cb8564_c36689;full_length_coverage=1;non_full_length_coverage=1;isoform_length=2368;expected_accuracy=0.991	gnl|BL_ORD_ID|55866 cb8564_c47522/f1p0/2954	1	1709	1706	2368	1	1682	2181	2845
cb8102_c19/f1p0/1683 isoform=cb8102_c19;full_length_coverage=1;non_full_length_coverage=0;isoform_length=1683;expected_accuracy=0.991	gnl|BL_ORD_ID|7932 cb8102_c8/f1p3/1987 isoform=cb8102_c8;full_length_coverage=1;non_full_length_coverage=3;isoform_length=1987;expected_accuracy=1.000	1	1128	1124	1683	23	1150	1329	1889
cb8564_c10952/f1p1/2673 isoform=cb8564_c10952;full_length_coverage=1;non_full_length_coverage=1;isoform_length=2673;expected_accuracy=0.992	gnl|BL_ORD_ID|56353 cb8564_c75114/f1p0/2866	2	2041	2039	2594	1	2035	2193	2737
cb8564_c81734/f1p1/2771 isoform=cb8564_c81734;full_length_coverage=1;non_full_length_coverage=1;isoform_length=2771;expected_accuracy=0.995	gnl|BL_ORD_ID|56381 cb8564_c75626/f1p2/3199	121	2234	2232	2771	1	2116	2611	3149
cb17137_c7/f1p1/1834	gnl|BL_ORD_ID|12667 cb8564_c127556/f1p2/2131 isoform=cb8564_c127556;full_length_coverage=1;non_full_length_coverage=2;isoform_length=2131;expected_accuracy=0.999	1	1291	1287	1691	1	1292	1663	2067
cb10931_c3/f1p0/1364	gnl|BL_ORD_ID|1042 cb10931_c5/f1p0/1833	532	1364	6	531	975	180	1	516

isoform=cb10931_c3;full_length_coverage=1;non_full_length_coverage=0;isoform_length=1364;expected_accuracy=0.997	isoform=cb10931_c5;full_length_coverage=1;non_full_length_coverage=0;isoform_length=1833;expected_accuracy=0.996						7		
cb3664_c1/f3p0/2420 isoform=cb3664_c1;full_length_coverage=3;non_full_length_coverage=0;isoform_length=2420;expected_accuracy=1.000	gnl|BL_ORD_ID|41173 cb3664_c4/f1p2/3015 isoform=cb3664_c4;full_length_coverage=1;non_full_length_coverage=2;isoform_length=3015;expected_accuracy=0.997	200	2420	1	201	663	2883	18	225
cb11173_c5/f1p0/1147	gnl|BL_ORD_ID|1689 cb11173_c3/f1p0/3143 isoform=cb11173_c3;full_length_coverage=1;non_full_length_coverage=0;isoform_length=3143;expected_accuracy=0.994	6	581	580	1082	81	649	2644	3143
cb8760_c4/f1p0/1054	gnl|BL_ORD_ID|58346 cb8760_c2/f1p0/2371	160	1018	2	161	1199	2057	1	160
cb8564_c125304/f1p3/1973	gnl|BL_ORD_ID|53517 cb8564_c127539/f1p0/2402	1	1343	1343	1823	1	1343	1768	2248
cb15584_c0/f3p0/1259 isoform=cb15584_c0;full_length_coverage=3;non_full_length_coverage=0;isoform_length=1259;expected_accuracy=0.992	gnl|BL_ORD_ID|64763 cb15584_c4/f1p0/1780	371	1248	8	371	851	1727	15	377
cb8564_c36689/f1p1/2368 isoform=cb8564_c36689;full_length_coverage=1;non_full_length_coverage=1;isoform_length=2368;expected_accuracy=0.991	gnl|BL_ORD_ID|13273 cb8564_c188193/f1p0/2849 isoform=cb8564_c188193;full_length_coverage=1;non_full_length_coverage=0;isoform_length=2849;expected_accuracy=0.997	1	1709	1706	2368	1	1682	2181	2845
cb18132_c26/f1p0/1670	gnl|BL_ORD_ID|56415 cb8564_c76420/f1p0/2783	490	1654	9	492	1513	2676	11	494
cb8564_c42906/f1p2/3181	gnl|BL_ORD_ID|56041 cb8564_c53077/f1p2/3406	336	3153	4	336	452	328	6	337

						8			
cb8564_c48661/f1p2/2634 isoform=cb8564_c48661;full_length_coverage=1;non_full_length_coverage=2;isoform_length=2634;expected_accuracy=0.998	gnl|BL_ORD_ID|11343 cb8564_c148508/f1p3/2825 isoform=cb8564_c148508;full_length_coverage=1;non_full_length_coverage=3;isoform_length=2825;expected_accuracy=0.994	396	2631	8	399	539	2774	40	433
cb8564_c54387/f1p0/4415	gnl|BL_ORD_ID|16467 cb8564_c49872/f2p1/3689 isoform=cb8564_c49872;full_length_coverage=2;non_full_length_coverage=1;isoform_length=3689;expected_accuracy=1.000	2	2011	2011	3492	27	2031	2209	3689
cb8564_c19221/f2p0/2424 isoform=cb8564_c19221;full_length_coverage=2;non_full_length_coverage=0;isoform_length=2424;expected_accuracy=0.995	gnl|BL_ORD_ID|54080 cb8564_c18027/f1p0/3948	390	2423	50	389	1755	3788	4	343
cb8564_c76252/f1p1/3205 isoform=cb8564_c76252;full_length_coverage=1;non_full_length_coverage=1;isoform_length=3205;expected_accuracy=1.000	gnl|BL_ORD_ID|57993 cb8564_c86203/f1p0/3428	1052	3205	1	1053	1267	3400	60	1112
cb9358_c9/f1p0/1857 isoform=cb9358_c9;full_length_coverage=1;non_full_length_coverage=0;isoform_length=1857;expected_accuracy=1.000	gnl|BL_ORD_ID|19548 cb9358_c4/f1p0/2124 isoform=cb9358_c4;full_length_coverage=1;non_full_length_coverage=0;isoform_length=2124;expected_accuracy=1.000	155	1857	1	156	420	2124	37	191
cb1163_c21/f1p5/3705 isoform=cb1163_c21;full_length_coverage=1;non_full_length_coverage=5;isoform_length=3705;expected_accuracy=0.994	gnl|BL_ORD_ID|23462 cb1163_c14/f1p1/4695 isoform=cb1163_c14;full_length_coverage=1;non_full_length_coverage=1;isoform_length=4695;expected_accuracy=0.991	1	1957	1956	3625	2	1955	3030	4695
cb8564_c80845/f1p0/3015	gnl|BL_ORD_ID|11236 cb8564_c145778/f9p1/3503	1	1675	1676	3015	13	169	2143	348

isoform=cb8564_c80845;full_length_coverage=1;non_full_length_coverage=0;isoform_length=3015;expected_accuracy=0.993	isoform=cb8564_c145778;full_length_coverage=9;non_full_length_coverage=1;isoform_length=3503;expected_accuracy=1.000						2		5
cb8564_c35645/f1p0/2804	gnl|BL_ORD_ID|18962 cb8564_c79723/f1p6/3025 isoform=cb8564_c79723;full_length_coverage=1;non_full_length_coverage=6;isoform_length=3025;expected_accuracy=0.999	150	2694	1	151	481	3025	218	368
cb5048_c7/f1p1/1621 isoform=cb5048_c7;full_length_coverage=1;non_full_length_coverage=1;isoform_length=1621;expected_accuracy=0.990	gnl|BL_ORD_ID|72095 cb5048_c6/f1p0/2038	1	1263	1263	1541	1	1265	1706	1984
cb8564_c4092/f1p0/3913	gnl|BL_ORD_ID|54976 cb8564_c4091/f1p0/3908	251	3517	1	253	461	3727	2	255
cb5633_c34/f1p3/2503 isoform=cb5633_c34;full_length_coverage=1;non_full_length_coverage=3;isoform_length=2503;expected_accuracy=0.996	gnl|BL_ORD_ID|74193 cb5633_c36/f1p3/2709	332	2503	38	335	464	2639	1	295
cb8564_c180679/f1p0/2109 isoform=cb8564_c180679;full_length_coverage=1;non_full_length_coverage=0;isoform_length=2109;expected_accuracy=0.999	gnl|BL_ORD_ID|14025 cb8564_c31262/f7p4/2729 isoform=cb8564_c31262;full_length_coverage=7;non_full_length_coverage=4;isoform_length=2729;expected_accuracy=0.994	198	2109	1	202	718	2631	139	340
cb2527_c19/f1p0/2292 isoform=cb2527_c19;full_length_coverage=1;non_full_length_coverage=0;isoform_length=2292;expected_accuracy=0.997	gnl|BL_ORD_ID|38908 cb2527_c48/f10p2/3282 isoform=cb2527_c48;full_length_coverage=10;non_full_length_coverage=2;isoform_length=3282;expected_accuracy=0.997	132	2291	1	136	1088	3248	26	154
cb8564_c181853/f1p0/2119	gnl|BL_ORD_ID|15428 cb8564_c42387/f1p4/2973	1	1339	1334	2109	1	134	2173	297

isoform=cb8564_c181853;full_length_coverage=1;non_full_length_coverage=0;isoform_length=2119;expected_accuracy=0.997	isoform=cb8564_c42387;full_length_coverage=1;non_full_length_coverage=4;isoform_length=2973;expected_accuracy=0.993						4		3
cb8564_c111594/f1p0/3002	gnl|BL_ORD_ID|53251 cb8564_c119935/f1p0/3487	108	2897	1	109	726	3515	26	134
cb8564_c35300/f1p2/3912	gnl|BL_ORD_ID|16776 cb8564_c53806/f1p3/3974 isoform=cb8564_c53806;full_length_coverage=1;non_full_length_coverage=3;isoform_length=3974;expected_accuracy=0.990	185	3827	16	186	313	3974	1	173
cb124_c43/f1p0/2759	gnl|BL_ORD_ID|23058 cb124_c6/f3p0/2872 isoform=cb124_c6;full_length_coverage=3;non_full_length_coverage=0;isoform_length=2872;expected_accuracy=1.000	233	2491	13	233	614	2872	39	259
cb12714_c29/f1p2/1583	gnl|BL_ORD_ID|54044 cb8564_c17331/f1p0/3185	1	1296	1293	1543	35	1331	2398	2648
cb3229_c12/f1p0/2616	gnl|BL_ORD_ID|39750 cb3229_c0/f3p0/2667 isoform=cb3229_c0;full_length_coverage=3;non_full_length_coverage=0;isoform_length=2667;expected_accuracy=0.998	444	2412	45	448	572	2549	1	409
cb8881_c18/f1p0/1628 isoform=cb8881_c18;full_length_coverage=1;non_full_length_coverage=0;isoform_length=1628;expected_accuracy=0.997	gnl|BL_ORD_ID|20988 cb8881_c0/f8p0/2063 isoform=cb8881_c0;full_length_coverage=8;non_full_length_coverage=0;isoform_length=2063;expected_accuracy=1.000	1	1226	1225	1628	13	1239	1646	2047
cb8564_c12792/f1p0/3267 isoform=cb8564_c12792;full_length_coverage=1;non_full_length_coverage=0;isoform_length=3267;expected_	gnl|BL_ORD_ID|12663 cb8564_c14856/f1p0/3558 isoform=cb8564_c14856;full_length_coverage=1;non_full_length_coverage=0;isoform_length=3558;expected_ac	2	2932	2930	3265	1	2932	3224	3558

accuracy=0.996	curacy=0.991								
cb8564_c185920/f1p0/2777 isoform=cb8564_c185920;full_length_coverage=1;non_full_length_coverage=0;isoform_length=2777;expected_accuracy=0.996	gnl|BL_ORD_ID|14529 cb8564_c44074/f1p1/2922 isoform=cb8564_c44074;full_length_coverage=1;non_full_length_coverage=1;isoform_length=2922;expected_accuracy=0.994	174	2777	1	177	346	2922	14	190
cb3170_c3/f1p1/2075 isoform=cb3170_c3;full_length_coverage=1;non_full_length_coverage=1;isoform_length=2075;expected_accuracy=0.996	gnl|BL_ORD_ID|38754 cb3170_c5/f1p2/2463 isoform=cb3170_c5;full_length_coverage=1;non_full_length_coverage=2;isoform_length=2463;expected_accuracy=0.997	1	1519	1518	2075	191	1713	1816	2373
cb8564_c123988/f1p0/2683	gnl|BL_ORD_ID|53109 cb8564_c116513/f1p1/2958	1	2343	2344	2556	142	2484	2656	2868
cb500_c49/f1p0/3666	gnl|BL_ORD_ID|72251 cb500_c68/f1p0/4152	284	3332	11	288	961	4034	34	301
cb4190_c0/f2p1/2052 isoform=cb4190_c0;full_length_coverage=2;non_full_length_coverage=1;isoform_length=2052;expected_accuracy=0.998	gnl|BL_ORD_ID|43320 cb4190_c1/f1p0/3409 isoform=cb4190_c1;full_length_coverage=1;non_full_length_coverage=0;isoform_length=3409;expected_accuracy=0.999	1	1338	1335	2052	42	1383	2688	3406
cb7639_c15/f1p0/2203 isoform=cb7639_c15;full_length_coverage=1;non_full_length_coverage=0;isoform_length=2203;expected_accuracy=0.998	gnl|BL_ORD_ID|51428 cb7639_c14/f1p0/2787	1	1804	1803	2203	37	1834	2114	2541
cb4269_c10/f1p0/1931 isoform=cb4269_c10;full_length_coverage=1;non_full_length_coverage=0;isoform_length=1931;expected_accuracy=0.997	gnl|BL_ORD_ID|43058 cb4269_c11/f2p0/2147 isoform=cb4269_c11;full_length_coverage=2;non_full_length_coverage=0;isoform_length=2147;expected_accuracy=0.997	129	1931	1	130	340	2142	105	225
cb8564_c146667/f2p0/2543	gnl|BL_ORD_ID|10739 cb8564_c120058/f5p2/2713	1	2247	2243	2498	2	225	2458	271

isoform=cb8564_c146667;full_length_coverage=2;non_full_length_coverage=0;isoform_length=2543;expected_accuracy=1.000	isoform=cb8564_c120058;full_length_coverage=5;non_full_length_coverage=2;isoform_length=2713;expected_accuracy=0.996						3		3
cb2601_c2/f2p0/2981 isoform=cb2601_c2;full_length_coverage=2;non_full_length_coverage=0;isoform_length=2981;expected_accuracy=0.999	gnl|BL_ORD_ID|68634 cb2601_c3/f1p0/3863	1	1831	1832	2981	1	1833	2369	3520
cb6636_c4/f1p0/1118 isoform=cb6636_c4;full_length_coverage=1;non_full_length_coverage=0;isoform_length=1118;expected_accuracy=0.994	gnl|BL_ORD_ID|49909 cb6636_c3/f1p0/1512	126	1118	2	129	453	1445	27	153
cb8564_c86536/f1p0/2772 isoform=cb8564_c86536;full_length_coverage=1;non_full_length_coverage=0;isoform_length=2772;expected_accuracy=0.997	gnl|BL_ORD_ID|10590 cb8564_c107836/f2p0/3713 isoform=cb8564_c107836;full_length_coverage=2;non_full_length_coverage=0;isoform_length=3713;expected_accuracy=0.996	109	2772	1	112	227	2899	1	108
cb8564_c75566/f1p0/2337 isoform=cb8564_c75566;full_length_coverage=1;non_full_length_coverage=0;isoform_length=2337;expected_accuracy=0.994	gnl|BL_ORD_ID|18567 cb8564_c88386/f1p0/3550 isoform=cb8564_c88386;full_length_coverage=1;non_full_length_coverage=0;isoform_length=3550;expected_accuracy=1.000	1	1176	1171	2337	7	1182	2382	3545
cb12513_c19/f1p3/1447	gnl|BL_ORD_ID|14254 cb8564_c3885/f1p7/2735 isoform=cb8564_c3885;full_length_coverage=1;non_full_length_coverage=7;isoform_length=2735;expected_accuracy=0.999	269	1414	3	270	1490	2630	7	274
cb6633_c10/f1p0/1980 isoform=cb6633_c10;full_length_coverage=1;non_full_length_coverage=0;isoform_length=1980;expected_accu	gnl|BL_ORD_ID|3747 cb6633_c9/f1p0/2805 isoform=cb6633_c9;full_length_coverage=1;non_full_length_coverage=0;isoform_length=2805;expected_accura	1	1409	1406	1948	110	1518	2261	2805

racy=0.999	cy=0.997								
cb7088_c9/f1p0/2325	gnl|BL_ORD_ID|4800 cb7088_c10/f1p0/2704 isoform=cb7088_c10;full_length_coverage=1;non_full_length_coverage=0;isoform_length=2704;expected_accuracy=0.994	180	2342	1	179	470	2634	81	265
cb8564_c78476/f1p0/3130	gnl|BL_ORD_ID|9280 cb8564_c107656/f6p3/3035 isoform=cb8564_c107656;full_length_coverage=6;non_full_length_coverage=3;isoform_length=3035;expected_accuracy=0.999	11	1640	1639	2539	12	1633	1998	2893
cb2238_c51/f18p0/2624 isoform=cb2238_c51;full_length_coverage=18;non_full_length_coverage=0;isoform_length=2624;expected_accuracy=1.000	gnl|BL_ORD_ID|68369 cb2238_c21/f1p0/3677	138	2604	1	139	797	3263	5	145
cb1333_c10/f4p0/1937 isoform=cb1333_c10;full_length_coverage=4;non_full_length_coverage=0;isoform_length=1937;expected_accuracy=1.000	gnl|BL_ORD_ID|61259 cb1333_c109/f1p0/5379	143	1936	13	142	3138	4931	17	146
cb309_c4/f2p2/4112 isoform=cb309_c4;full_length_coverage=2;non_full_length_coverage=2;isoform_length=4112;expected_accuracy=0.996	gnl|BL_ORD_ID|69547 cb309_c21/f1p0/4520	1	3715	3716	4108	22	3719	4007	4400
cb4487_c26/f1p0/1475 isoform=cb4487_c26;full_length_coverage=1;non_full_length_coverage=0;isoform_length=1475;expected_accuracy=0.998	gnl|BL_ORD_ID|72601 cb4487_c19/f1p1/1740	417	1475	46	420	576	1634	1	376
cb454_c24/f1p0/2537	gnl|BL_ORD_ID|70924 cb454_c27/f1p0/2557	225	2275	1	224	335	238	6	229

							6		
cb9664_c0/f1p0/2005 isoform=cb9664_c0;full_length_coverage=1;non_full_length_coverage=0;isoform_length=2005;expected_accuracy=0.996	gnl|BL_ORD_ID|58841 cb9664_c2/f1p0/2566	1	1087	1086	2004	3	1092	1642	2559
cb2927_c1/f2p1/2815 isoform=cb2927_c1;full_length_coverage=2;non_full_length_coverage=1;isoform_length=2815;expected_accuracy=0.994	gnl|BL_ORD_ID|38415 cb2927_c2/f2p0/3116 isoform=cb2927_c2;full_length_coverage=2;non_full_length_coverage=0;isoform_length=3116;expected_accuracy=0.995	1	1879	1878	2815	16	1900	2172	3109
cb309_c4/f2p2/4112 isoform=cb309_c4;full_length_coverage=2;non_full_length_coverage=2;isoform_length=4112;expected_accuracy=0.996	gnl|BL_ORD_ID|69546 cb309_c5/f1p0/4669	1	3715	3716	4096	206	3917	4205	4585
cb8564_c90610/f1p0/2901	gnl|BL_ORD_ID|56373 cb8564_c75497/f1p0/3111	1087	2722	1	1087	1185	2821	226	1312
cb8564_c40598/f1p4/3119	gnl|BL_ORD_ID|15152 cb8564_c38629/f2p1/3246 isoform=cb8564_c38629;full_length_coverage=2;non_full_length_coverage=1;isoform_length=3246;expected_accuracy=0.993	1	2732	2729	3055	1	2761	2882	3208
cb7940_c54/f1p1/1719 isoform=cb7940_c54;full_length_coverage=1;non_full_length_coverage=1;isoform_length=1719;expected_accuracy=0.997	gnl|BL_ORD_ID|51643 cb7940_c42/f1p0/2040	1	1366	1363	1704	58	1423	1619	1962
cb1291_c9/f1p1/2168 isoform=cb1291_c9;full_length_coverage=1;non_full_length_coverage=1;isoform_length=2168;expected_accur	gnl|BL_ORD_ID|61199 cb1291_c14/f1p0/2892	183	2168	1	182	545	2531	2	184

acy=0.993										
cb8564_c38462/f1p0/3241	gnl|BL_ORD_ID|17429 cb8564_c85447/f1p0/3196 isoform=cb8564_c85447;full_length_coverage=1;non_full_length_coverage=0;isoform_length=3196;expected_accuracy=0.997	1	2410	2409	2946	3	2413	2660	3194
cb12931_c15/f1p0/1493 isoform=cb12931_c15;full_length_coverage=1;non_full_length_coverage=0;isoform_length=1493;expected_accuracy=1.000	gnl|BL_ORD_ID|55213 cb8564_c35568/f1p0/2997	1	1106	1104	1493	13	1123	2503	2890
cb1326_c9/f1p0/3549 isoform=cb1326_c9;full_length_coverage=1;non_full_length_coverage=0;isoform_length=3549;expected_accuracy=0.996	gnl|BL_ORD_ID|25268 cb1326_c13/f1p1/4140 isoform=cb1326_c13;full_length_coverage=1;non_full_length_coverage=1;isoform_length=4140;expected_accuracy=0.991	1	3198	3194	3508	298	3493	3805	4137
cb8564_c81778/f1p0/3178	gnl|BL_ORD_ID|55135 cb8564_c25151/f1p2/3689	2	2698	2694	2918	1	2692	3295	3519
cb8564_c87490/f1p0/2768	gnl|BL_ORD_ID|18365 cb8564_c81851/f2p0/3845 isoform=cb8564_c81851;full_length_coverage=2;non_full_length_coverage=0;isoform_length=3845;expected_accuracy=0.997	1	1736	1735	2772	4	1742	2688	3725
cb16925_c1/f1p0/1736 isoform=cb16925_c1;full_length_coverage=1;non_full_length_coverage=0;isoform_length=1736;expected_accuracy=0.999	gnl|BL_ORD_ID|53594 cb8564_c129578/f1p0/2063	1	1127	1124	1712	1	1135	1490	2078
cb8564_c36689/f1p1/2368 isoform=cb8564_c36689;full_length_coverage=1;non_full_length_coverage=1;isoform_length=2368;expected_	gnl|BL_ORD_ID|15602 cb8564_c50271/f1p1/2688 isoform=cb8564_c50271;full_length_coverage=1;non_full_length_coverage=1;isoform_length=2688;expected_ac	1	1709	1706	2368	1	1704	2022	2688

accuracy=0.991	curacy=0.994								
cb17349_c3/f2p0/1249 isoform=cb17349_c3;full_length_coverage=2;non_full_length_coverage=0;isoform_length=1249;expected_accuracy=0.993	gnl|BL_ORD_ID|32156 cb17349_c2/f2p0/1517 isoform=cb17349_c2;full_length_coverage=2;non_full_length_coverage=0;isoform_length=1517;expected_accuracy=1.000	9	681	677	1239	1	671	899	1459
cb8564_c109747/f3p1/2083 isoform=cb8564_c109747;full_length_coverage=3;non_full_length_coverage=1;isoform_length=2083;expected_accuracy=0.998	gnl|BL_ORD_ID|15976 cb8564_c53159/f1p0/2602 isoform=cb8564_c53159;full_length_coverage=1;non_full_length_coverage=0;isoform_length=2602;expected_accuracy=0.994	1	1051	1050	2023	1	1053	1471	2442
cb4370_c5/f1p0/2518 isoform=cb4370_c5;full_length_coverage=1;non_full_length_coverage=0;isoform_length=2518;expected_accuracy=0.998	gnl|BL_ORD_ID|72511 cb4370_c3/f1p0/3357	329	2482	33	330	1186	3339	1	298
cb12362_c44/f1p1/1106	gnl|BL_ORD_ID|1903 cb10045_c31/f1p0/1199 isoform=cb10045_c31;full_length_coverage=1;non_full_length_coverage=0;isoform_length=1199;expected_accuracy=0.994	117	1058	2	118	258	1199	1	116
cb8564_c35356/f1p0/2738 isoform=cb8564_c35356;full_length_coverage=1;non_full_length_coverage=0;isoform_length=2738;expected_accuracy=0.994	gnl|BL_ORD_ID|15169 cb8564_c40218/f1p0/3146 isoform=cb8564_c40218;full_length_coverage=1;non_full_length_coverage=0;isoform_length=3146;expected_accuracy=0.993	1	2071	2070	2737	4	2065	2486	3146
cb5359_c41/f1p0/1175	gnl|BL_ORD_ID|44627 cb5359_c37/f13p1/2486 isoform=cb5359_c37;full_length_coverage=13;non_full_length_coverage=1;isoform_length=2486;expected_accuracy=0.996	124	1164	3	126	1432	2471	73	194
cb8564_c20575/f1p0/3783	gnl|BL_ORD_ID|53995 cb8564_c16613/f1p1/4260	1	2046	2044	3781	1	204	2473	421

isoform=cb8564_c20575;full_length_coverage=1;non_full_length_coverage=0;isoform_length=3783;expected_accuracy=0.997							6		0
cb8564_c185920/f1p0/2777 isoform=cb8564_c185920;full_length_coverage=1;non_full_length_coverage=0;isoform_length=2777;expected_accuracy=0.996	gnl|BL_ORD_ID|12938 cb8564_c181163/f1p1/2936 isoform=cb8564_c181163;full_length_coverage=1;non_full_length_coverage=1;isoform_length=2936;expected_accuracy=0.993	174	2777	1	177	352	293 6	19	195
cb2513_c28/f1p0/2811 isoform=cb2513_c28;full_length_coverage=1;non_full_length_coverage=0;isoform_length=2811;expected_accuracy=0.995	gnl|BL_ORD_ID|36412 cb18837_c1/f1p0/5324 isoform=cb18837_c1;full_length_coverage=1;non_full_length_coverage=0;isoform_length=5324;expected_accuracy=0.998	679	2811	97	681	3137	527 7	22	601
cb8564_c48192/f1p6/3029	gnl|BL_ORD_ID|14531 cb8564_c41778/f1p9/3482 isoform=cb8564_c41778;full_length_coverage=1;non_full_length_coverage=9;isoform_length=3482;expected_accuracy=0.992	245	2874	1	249	812	344 2	1	247
cb388_c33/f1p0/3488	gnl|BL_ORD_ID|69589 cb388_c34/f1p0/3652	20	2859	2859	3293	43	289 6	3039	347 0
cb8564_c76575/f2p0/3760 isoform=cb8564_c76575;full_length_coverage=2;non_full_length_coverage=0;isoform_length=3760;expected_accuracy=1.000	gnl|BL_ORD_ID|15880 cb8564_c69166/f2p0/4243 isoform=cb8564_c69166;full_length_coverage=2;non_full_length_coverage=0;isoform_length=4243;expected_accuracy=0.997	1462	3722	1	1463	1986	424 3	12	147 1
cb900_c13/f1p0/2723	gnl|BL_ORD_ID|57606 cb900_c8/f1p0/3263	1	1986	1981	2445	35	202 0	2665	313 0
cb18456_c6530/f1p0/1200	gnl|BL_ORD_ID|34789 cb18456_c3977/f1p2/1752 isoform=cb18456_c3977;full_length_coverage=1;non_full_length_coverage=2;isoform_length=1752;expected_ac	260	1156	4	259	794	168 2	9	262

	curacy=0.992								
cb3644_c20/f1p0/3151	gnl|BL_ORD_ID|71404 cb3644_c9/f1p0/3316	1	1893	1894	3105	5	1897	2030	3241
cb7969_c11/f1p0/1950 isoform=cb7969_c11;full_length_coverage=1;non_full_length_coverage=0;isoform_length=1950;expected_accuracy=0.992	gnl|BL_ORD_ID|8012 cb7969_c33/f4p1/2302 isoform=cb7969_c33;full_length_coverage=4;non_full_length_coverage=1;isoform_length=2302;expected_accuracy=0.997	1	1409	1405	1950	73	1483	1728	2295
cb8564_c70156/f1p0/2250 isoform=cb8564_c70156;full_length_coverage=1;non_full_length_coverage=0;isoform_length=2250;expected_accuracy=0.996	gnl|BL_ORD_ID|12057 cb8564_c145727/f5p1/2610 isoform=cb8564_c145727;full_length_coverage=5;non_full_length_coverage=1;isoform_length=2610;expected_accuracy=1.000	108	2247	1	110	471	2607	51	160
cb8564_c15653/f1p4/2475	gnl|BL_ORD_ID|52845 cb8564_c15530/f1p0/3247	22	2293	2289	2481	37	2308	2982	3174
cb8564_c4676/f1p1/2570 isoform=cb8564_c4676;full_length_coverage=1;non_full_length_coverage=1;isoform_length=2570;expected_accuracy=0.994	gnl|BL_ORD_ID|10588 cb8564_c117037/f1p1/2690 isoform=cb8564_c117037;full_length_coverage=1;non_full_length_coverage=1;isoform_length=2690;expected_accuracy=0.995	250	2529	2	249	409	2690	1	250
cb9146_c8/f1p0/1115 isoform=cb9146_c8;full_length_coverage=1;non_full_length_coverage=0;isoform_length=1115;expected_accuracy=0.991	gnl|BL_ORD_ID|21134 cb9146_c11/f5p0/1276 isoform=cb9146_c11;full_length_coverage=5;non_full_length_coverage=0;isoform_length=1276;expected_accuracy=1.000	101	1113	1	101	265	1276	61	161
cb500_c17/f1p0/2323 isoform=cb500_c17;full_length_coverage=1;non_full_length_coverage=0;isoform_length=2323;expected_accuracy=0.997	gnl|BL_ORD_ID|43084 cb500_c26/f1p0/3434 isoform=cb500_c26;full_length_coverage=1;non_full_length_coverage=0;isoform_length=3434;expected_accuracy=0.998	1	1293	1288	2323	725	2017	2149	3182
cb8564_c47011/f1p0/3100	gnl|BL_ORD_ID|56764 cb8564_c70795/f1p0/3769	21	2306	2301	2934	34	231	2551	318

<table>
<tr><td></td><td></td><td></td><td></td><td></td><td></td><td></td><td>3</td><td></td><td>0</td></tr>
<tr><td>cb5690_c8/f11p0/1087 isoform=cb5690_c8;full_length_coverage=11;non_full_length_coverage=0;isoform_length=1087;expected_accuracy=1.000</td><td>gnl|BL_ORD_ID|46833 cb5690_c4/f1p0/3237 isoform=cb5690_c4;full_length_coverage=1;non_full_length_coverage=0;isoform_length=3237;expected_accuracy=0.996</td><td>348</td><td>1084</td><td>49</td><td>352</td><td>2502</td><td>3237</td><td>1</td><td>304</td></tr>
<tr><td>cb8564_c90630/f1p0/2172 isoform=cb8564_c90630;full_length_coverage=1;non_full_length_coverage=0;isoform_length=2172;expected_accuracy=0.995</td><td>gnl|BL_ORD_ID|18665 cb8564_c78920/f1p1/3989 isoform=cb8564_c78920;full_length_coverage=1;non_full_length_coverage=1;isoform_length=3989;expected_accuracy=0.996</td><td>1</td><td>1185</td><td>1181</td><td>2110</td><td>1407</td><td>2588</td><td>3073</td><td>3989</td></tr>
<tr><td>cb8564_c43779/f1p0/3021 isoform=cb8564_c43779;full_length_coverage=1;non_full_length_coverage=0;isoform_length=3021;expected_accuracy=0.993</td><td>gnl|BL_ORD_ID|55910 cb8564_c48645/f1p1/3287</td><td>11</td><td>2344</td><td>2345</td><td>2956</td><td>17</td><td>2347</td><td>2503</td><td>3113</td></tr>
<tr><td>cb8564_c72349/f1p0/3248</td><td>gnl|BL_ORD_ID|56713 cb8564_c53754/f1p0/3446</td><td>105</td><td>2662</td><td>2659</td><td>3068</td><td>181</td><td>2738</td><td>2897</td><td>3306</td></tr>
<tr><td>cb8596_c0/f2p0/1748 isoform=cb8596_c0;full_length_coverage=2;non_full_length_coverage=0;isoform_length=1748;expected_accuracy=0.999</td><td>gnl|BL_ORD_ID|18634 cb8596_c1/f1p0/2325 isoform=cb8596_c1;full_length_coverage=1;non_full_length_coverage=0;isoform_length=2325;expected_accuracy=0.994</td><td>1</td><td>1202</td><td>1202</td><td>1748</td><td>21</td><td>1233</td><td>1760</td><td>2306</td></tr>
<tr><td>cb12972_c15/f1p0/1541 isoform=cb12972_c15;full_length_coverage=1;non_full_length_coverage=0;isoform_length=1541;expected_accuracy=0.996</td><td>gnl|BL_ORD_ID|15126 cb8564_c2621/f1p1/2401 isoform=cb8564_c2621;full_length_coverage=1;non_full_length_coverage=1;isoform_length=2401;expected_accuracy=1.000</td><td>205</td><td>1541</td><td>7</td><td>206</td><td>1051</td><td>2378</td><td>1</td><td>199</td></tr>
<tr><td>cb998_c14/f1p0/3632 isoform=cb998_c14;full_length_coverage=1;non_full_length_coverage=0;isoform_length=3632;expected_accur</td><td>gnl|BL_ORD_ID|67910 cb18641_c0/f1p0/4940</td><td>1774</td><td>3632</td><td>16</td><td>1773</td><td>2925</td><td>4789</td><td>1</td><td>1761</td></tr>
</table>

acy=0.995									
cb5447_c34/f1p0/3194 isoform=cb5447_c34;full_length_coverage=1;non_full_length_coverage=0;isoform_length=3194;expected_accuracy=0.996	gnl|BL_ORD_ID|45897 cb5447_c33/f1p0/4199 isoform=cb5447_c33;full_length_coverage=1;non_full_length_coverage=0;isoform_length=4199;expected_accuracy=0.997	1	2170	2170	3194	4	2155	2805	3824
cb3290_c38/f1p0/2345 isoform=cb3290_c38;full_length_coverage=1;non_full_length_coverage=0;isoform_length=2345;expected_accuracy=0.996	gnl|BL_ORD_ID|40880 cb3290_c196/f17p2/2965 isoform=cb3290_c196;full_length_coverage=17;non_full_length_coverage=2;isoform_length=2965;expected_accuracy=1.000	1150	2345	1	1151	1692	2894	379	1535
cb626_c44/f1p1/4252 isoform=cb626_c44;full_length_coverage=1;non_full_length_coverage=1;isoform_length=4252;expected_accuracy=0.990	gnl|BL_ORD_ID|4060 cb626_c17/f1p0/4935 isoform=cb626_c17;full_length_coverage=1;non_full_length_coverage=0;isoform_length=4935;expected_accuracy=0.993	21	3677	3677	4245	48	3629	4370	4935
cb2960_c2/f1p0/2155 isoform=cb2960_c2;full_length_coverage=1;non_full_length_coverage=0;isoform_length=2155;expected_accuracy=0.995	gnl|BL_ORD_ID|36645 cb2960_c4/f1p0/2302 isoform=cb2960_c4;full_length_coverage=1;non_full_length_coverage=0;isoform_length=2302;expected_accuracy=0.995	1	1707	1702	2144	16	1722	1859	2302
cb8564_c2726/f1p0/4270	gnl|BL_ORD_ID|13887 cb8564_c2723/f1p0/4402 isoform=cb8564_c2723;full_length_coverage=1;non_full_length_coverage=0;isoform_length=4402;expected_accuracy=0.996	1	3089	3088	4209	1	3084	3238	4359
cb2831_c57/f3p1/2669 isoform=cb2831_c57;full_length_coverage=3;non_full_length_coverage=1;isoform_length=2669;expected_accuracy=0.997	gnl|BL_ORD_ID|38321 cb2831_c8/f2p0/3387 isoform=cb2831_c8;full_length_coverage=2;non_full_length_coverage=0;isoform_length=3387;expected_accuracy=1.000	121	2360	2361	2668	1	2238	3072	3380
cb8564_c11802/f1p0/3687	gnl|BL_ORD_ID|15343 cb8564_c25122/f1p0/3884	1	2152	2151	3687	1	215	2346	388

isoform=cb8564_c11802;full_length_coverage=1;non_full_length_coverage=0;isoform_length=3687;expected_accuracy=1.000	isoform=cb8564_c25122;full_length_coverage=1;non_full_length_coverage=0;isoform_length=3884;expected_accuracy=0.999						2		4
cb8564_c88176/f1p7/3178 isoform=cb8564_c88176;full_length_coverage=1;non_full_length_coverage=7;isoform_length=3178;expected_accuracy=0.998	gnl|BL_ORD_ID|17532 cb8564_c96234/f5p13/3443 isoform=cb8564_c96234;full_length_coverage=5;non_full_length_coverage=13;isoform_length=3443;expected_accuracy=0.994	611	3178	63	615	716	3288	1	550
cb8564_c110158/f1p0/1948 isoform=cb8564_c110158;full_length_coverage=1;non_full_length_coverage=0;isoform_length=1948;expected_accuracy=0.999	gnl|BL_ORD_ID|12332 cb8564_c146529/f7p0/2311 isoform=cb8564_c146529;full_length_coverage=7;non_full_length_coverage=0;isoform_length=2311;expected_accuracy=1.000	129	1948	10	130	415	2233	1	121
cb6530_c26/f1p0/2387 isoform=cb6530_c26;full_length_coverage=1;non_full_length_coverage=0;isoform_length=2387;expected_accuracy=0.998	gnl|BL_ORD_ID|5366 cb6530_c23/f1p0/2764 isoform=cb6530_c23;full_length_coverage=1;non_full_length_coverage=0;isoform_length=2764;expected_accuracy=0.996	1009	2351	1	1010	1422	2764	311	1319
cb12227_c15/f1p2/1760	gnl|BL_ORD_ID|54244 cb8564_c21108/f1p1/1969	10	1643	1641	1742	106	1736	1840	1941
cb4433_c10/f11p1/2900 isoform=cb4433_c10;full_length_coverage=11;non_full_length_coverage=1;isoform_length=2900;expected_accuracy=1.000	gnl|BL_ORD_ID|71906 cb4433_c22/f1p0/3300	212	2900	100	212	466	3154	1	113
cb8564_c110003/f2p0/3361 isoform=cb8564_c110003;full_length_coverage=2;non_full_length_coverage=0;isoform_length=3361;expected_accuracy=0.997	gnl|BL_ORD_ID|12046 cb8564_c126049/f1p0/3874 isoform=cb8564_c126049;full_length_coverage=1;non_full_length_coverage=0;isoform_length=3874;expected_accuracy=0.997	1	2830	2828	3316	6	2835	2942	3431
cb8564_c44005/f1p0/3815	gnl|BL_ORD_ID|15822 cb8564_c50372/f4p2/4090	1	2046	2041	3769	206	225	2354	409

isoform=cb8564_c44005;full_length_coverage=1;non_full_length_coverage=0;isoform_length=3815;expected_accuracy=0.993	isoform=cb8564_c50372;full_length_coverage=4;non_full_length_coverage=2;isoform_length=4090;expected_accuracy=0.996						4		0
cb8564_c51169/f1p3/3251 isoform=cb8564_c51169;full_length_coverage=1;non_full_length_coverage=3;isoform_length=3251;expected_accuracy=0.993	gnl|BL_ORD_ID|55830 cb8564_c42264/f1p0/3734	130	3205	1	130	288	3365	6	136
cb8564_c4900/f4p1/3332 isoform=cb8564_c4900;full_length_coverage=4;non_full_length_coverage=1;isoform_length=3332;expected_accuracy=1.000	gnl|BL_ORD_ID|15781 cb8564_c4899/f1p0/3376 isoform=cb8564_c4899;full_length_coverage=1;non_full_length_coverage=0;isoform_length=3376;expected_accuracy=0.996	143	2255	2254	3332	1	2110	2292	3367
cb3824_c7/f1p1/2719 isoform=cb3824_c7;full_length_coverage=1;non_full_length_coverage=1;isoform_length=2719;expected_accuracy=0.996	gnl|BL_ORD_ID|70639 cb3824_c3/f1p2/3052	252	2716	2	252	501	2964	1	243
cb9530_c4/f1p1/1777	gnl|BL_ORD_ID|19496 cb9530_c1/f1p0/2055 isoform=cb9530_c1;full_length_coverage=1;non_full_length_coverage=0;isoform_length=2055;expected_accuracy=1.000	1	1494	1493	1686	6	1499	1866	2055
cb5726_c3/f1p0/2930	gnl|BL_ORD_ID|46682 cb5726_c0/f1p0/3016 isoform=cb5726_c0;full_length_coverage=1;non_full_length_coverage=0;isoform_length=3016;expected_accuracy=0.994	598	2785	8	599	818	3008	1	595
cb3290_c38/f1p0/2345 isoform=cb3290_c38;full_length_coverage=1;non_full_length_coverage=0;isoform_length=2345;expected_accu	gnl|BL_ORD_ID|40560 cb3290_c85/f2p1/2883 isoform=cb3290_c85;full_length_coverage=2;non_full_length_coverage=1;isoform_length=2883;expected_accur	1150	2343	1	1151	1690	2883	273	1422

racy=0.996	acy=0.998								
cb8564_c113232/f1p0/3087	gnl|BL_ORD_ID|54608 cb8564_c127899/f1p0/3880	2	2841	2839	2997	215	3075	3305	3463
cb8564_c51780/f1p2/3067 isoform=cb8564_c51780;full_length_coverage=1;non_full_length_coverage=2;isoform_length=3067;expected_accuracy=0.995	gnl|BL_ORD_ID|54703 cb8564_c156696/f1p2/3606	12	2308	2308	3067	1	2297	2828	3589
cb4163_c10/f1p0/2735 isoform=cb4163_c10;full_length_coverage=1;non_full_length_coverage=0;isoform_length=2735;expected_accuracy=0.995	gnl|BL_ORD_ID|43332 cb4163_c19/f2p1/2896 isoform=cb4163_c19;full_length_coverage=2;non_full_length_coverage=1;isoform_length=2896;expected_accuracy=0.997	1	1840	1841	2715	42	1878	2025	2896
cb7757_c12/f1p1/1610 isoform=cb7757_c12;full_length_coverage=1;non_full_length_coverage=1;isoform_length=1610;expected_accuracy=0.997	gnl|BL_ORD_ID|6689 cb7757_c10/f1p1/1977 isoform=cb7757_c10;full_length_coverage=1;non_full_length_coverage=1;isoform_length=1977;expected_accuracy=0.998	1	1399	1397	1608	1	1399	1764	1977
cb5472_c5/f1p0/3130 isoform=cb5472_c5;full_length_coverage=1;non_full_length_coverage=0;isoform_length=3130;expected_accuracy=0.995	gnl|BL_ORD_ID|73415 cb5472_c10/f1p0/4117	1	2614	2614	3015	21	2652	3683	4084
cb359_c1/f1p0/4935	gnl|BL_ORD_ID|40519 cb359_c0/f1p1/4862 isoform=cb359_c0;full_length_coverage=1;non_full_length_coverage=1;isoform_length=4862;expected_accuracy=0.996	10	3598	3593	4027	97	3683	4427	4862
cb8564_c129284/f1p0/2118 isoform=cb8564_c129284;full_length_coverage=1;non_full_length_coverage=0;isoform_length=2118;expected_	gnl|BL_ORD_ID|11756 cb8564_c146467/f10p0/2494 isoform=cb8564_c146467;full_length_coverage=10;non_full_length_coverage=0;isoform_length=2494;expected	1	1970	1965	2118	54	2029	2213	2367

accuracy=0.991	_accuracy=1.000								
cb2770_c49/f1p0/2592	gnl|BL_ORD_ID|69963 cb2770_c47/f1p0/3578	10	1688	1688	2592	1	1679	2474	3378
cb2737_c49/f1p0/3341	gnl|BL_ORD_ID|38129 cb2737_c25/f1p0/3198 isoform=cb2737_c25;full_length_coverage=1;non_full_length_coverage=0;isoform_length=3198;expected_accuracy=0.997	429	3092	91	430	536	3198	1	340
cb5137_c92/f1p0/2632 isoform=cb5137_c92;full_length_coverage=1;non_full_length_coverage=0;isoform_length=2632;expected_accuracy=1.000	gnl|BL_ORD_ID|41994 cb5137_c96/f1p2/2956 isoform=cb5137_c96;full_length_coverage=1;non_full_length_coverage=2;isoform_length=2956;expected_accuracy=0.997	377	2632	52	381	661	2917	155	470
cb4916_c5/f1p0/1981	gnl|BL_ORD_ID|42009 cb4916_c0/f6p1/2108 isoform=cb4916_c0;full_length_coverage=6;non_full_length_coverage=1;isoform_length=2108;expected_accuracy=0.995	1	1417	1412	2006	1	1412	1512	2107
cb401_c50/f1p0/2173 isoform=cb401_c50;full_length_coverage=1;non_full_length_coverage=0;isoform_length=2173;expected_accuracy=0.998	gnl|BL_ORD_ID|70874 cb401_c49/f1p4/2600	96	2171	1	101	259	2352	18	118
cb16315_c0/f1p0/1511	gnl|BL_ORD_ID|43942 cb4391_c2/f1p0/3515 isoform=cb4391_c2;full_length_coverage=1;non_full_length_coverage=0;isoform_length=3515;expected_accuracy=0.997	113	1512	1	114	1978	3377	812	924
cb510_c11/f1p0/3703 isoform=cb510_c11;full_length_coverage=1;non_full_length_coverage=0;isoform_length=3703;expected_accur	gnl|BL_ORD_ID|42977 cb510_c2/f2p0/3807 isoform=cb510_c2;full_length_coverage=2;non_full_length_coverage=0;isoform_length=3807;expected_accurac	15	3215	3211	3653	1	3201	3347	3797

acy=0.995	y=0.993								
cb8564_c11123/f1p2/2599	gnl|BL_ORD_ID|11179 cb8564_c11761/f4p0/2940 isoform=cb8564_c11761;full_length_coverage=4;non_full_length_coverage=0;isoform_length=2940;expected_accuracy=0.996	1	1693	1689	2491	115	1807	2043	2845
cb8564_c36689/f1p1/2368 isoform=cb8564_c36689;full_length_coverage=1;non_full_length_coverage=1;isoform_length=2368;expected_accuracy=0.991	gnl|BL_ORD_ID|55359 cb8564_c39546/f1p2/3023	1	1709	1706	2368	1	1672	2171	2835
cb2218_c8/f1p1/2956 isoform=cb2218_c8;full_length_coverage=1;non_full_length_coverage=1;isoform_length=2956;expected_accuracy=0.996	gnl|BL_ORD_ID|68349 cb2218_c5/f1p0/4008	109	2956	1	112	313	3162	30	141
cb8374_c4/f1p0/2094 isoform=cb8374_c4;full_length_coverage=1;non_full_length_coverage=0;isoform_length=2094;expected_accuracy=0.992	gnl|BL_ORD_ID|9986 cb8374_c2/f1p0/2333 isoform=cb8374_c2;full_length_coverage=1;non_full_length_coverage=0;isoform_length=2333;expected_accuracy=0.996	14	1890	1887	2091	1	1875	2061	2273
cb3907_c1/f4p0/2661 isoform=cb3907_c1;full_length_coverage=4;non_full_length_coverage=0;isoform_length=2661;expected_accuracy=0.997	gnl|BL_ORD_ID|40128 cb3907_c4/f1p0/3038 isoform=cb3907_c4;full_length_coverage=1;non_full_length_coverage=0;isoform_length=3038;expected_accuracy=0.992	1	1494	1495	2661	14	1502	1872	3038
cb6111_c34/f1p0/1434 isoform=cb6111_c34;full_length_coverage=1;non_full_length_coverage=0;isoform_length=1434;expected_accuracy=0.992	gnl|BL_ORD_ID|46715 cb6111_c23/f1p0/1599 isoform=cb6111_c23;full_length_coverage=1;non_full_length_coverage=0;isoform_length=1599;expected_accuracy=0.993	121	1434	1	122	269	1579	3	126
cb3451_c8/f1p0/1800	gnl|BL_ORD_ID|41079 cb3451_c7/f2p0/2047	103	1800	1	106	345	204	78	183

isoform=cb3451_c8;full_length_coverage=1;non_full_length_coverage=0;isoform_length=1800;expected_accuracy=0.997	isoform=cb3451_c7;full_length_coverage=2;non_full_length_coverage=0;isoform_length=2047;expected_accuracy=1.000						1		
cb8564_c37361/f1p0/3076	gnl|BL_ORD_ID|56002 cb8564_c51722/f1p1/3503	21	2481	2477	3070	67	2523	2624	3216
cb8564_c16240/f1p3/2671 isoform=cb8564_c16240;full_length_coverage=1;non_full_length_coverage=3;isoform_length=2671;expected_accuracy=0.997	gnl|BL_ORD_ID|11985 cb8564_c146172/f11p0/3320 isoform=cb8564_c146172;full_length_coverage=11;non_full_length_coverage=0;isoform_length=3320;expected_accuracy=1.000	1	2075	2072	2671	47	2122	2659	3259
cb12422_c8/f1p1/1600 isoform=cb12422_c8;full_length_coverage=1;non_full_length_coverage=1;isoform_length=1600;expected_accuracy=0.996	gnl|BL_ORD_ID|23066 cb12422_c22/f35p1/1776 isoform=cb12422_c22;full_length_coverage=35;non_full_length_coverage=1;isoform_length=1776;expected_accuracy=1.000	281	1600	4	285	456	1776	60	345
cb1569_c4/f1p0/2788 isoform=cb1569_c4;full_length_coverage=1;non_full_length_coverage=0;isoform_length=2788;expected_accuracy=0.993	gnl|BL_ORD_ID|29609 cb1569_c3/f1p0/4264 isoform=cb1569_c3;full_length_coverage=1;non_full_length_coverage=0;isoform_length=4264;expected_accuracy=0.994	1	2367	2363	2788	1	2362	3771	4195
cb2803_c6/f1p0/2449 isoform=cb2803_c6;full_length_coverage=1;non_full_length_coverage=0;isoform_length=2449;expected_accuracy=0.990	gnl|BL_ORD_ID|38212 cb2803_c5/f1p0/2932 isoform=cb2803_c5;full_length_coverage=1;non_full_length_coverage=0;isoform_length=2932;expected_accuracy=0.998	1	2024	2023	2449	18	2036	2505	2932
cb2087_c5/f1p0/3142 isoform=cb2087_c5;full_length_coverage=1;non_full_length_coverage=0;isoform_length=3142;expected_accuracy=0.991	gnl|BL_ORD_ID|36296 cb2087_c11/f1p1/3204 isoform=cb2087_c11;full_length_coverage=1;non_full_length_coverage=1;isoform_length=3204;expected_accuracy=0.994	325	3131	34	328	398	3204	1	297
cb289_c5/f1p0/4379	gnl|BL_ORD_ID|37169 cb289_c16/f1p0/4532	1	4156	4156	4379	1	415	4253	447

isoform=cb289_c5;full_length_coverage=1;non_full_length_coverage=0;isoform_length=4379;expected_accuracy=0.997	isoform=cb289_c16;full_length_coverage=1;non_full_length_coverage=0;isoform_length=4532;expected_accuracy=0.992						2		4
cb8564_c10872/f1p2/2947 isoform=cb8564_c10872;full_length_coverage=1;non_full_length_coverage=2;isoform_length=2947;expected_accuracy=0.994	gnl|BL_ORD_ID|53939 cb8564_c15654/f1p1/3152	1	2667	2666	2917	114	2774	2915	3167
cb938_c15/f1p0/3219	gnl|BL_ORD_ID|59101 cb938_c9/f1p0/4170	1	2546	2541	3183	1	2548	3521	4163
cb1333_c101/f1p0/1395 isoform=cb1333_c101;full_length_coverage=1;non_full_length_coverage=0;isoform_length=1395;expected_accuracy=0.997	gnl|BL_ORD_ID|26655 cb1333_c7/f6p1/4935 isoform=cb1333_c7;full_length_coverage=6;non_full_length_coverage=1;isoform_length=4935;expected_accuracy=1.000	149	1395	27	150	3610	4854	31	154
cb8564_c38035/f1p0/2770	gnl|BL_ORD_ID|57175 cb8564_c85646/f1p0/3480	19	2035	2033	2747	106	2125	2315	3030
cb8564_c41756/f1p0/2738 isoform=cb8564_c41756;full_length_coverage=1;non_full_length_coverage=0;isoform_length=2738;expected_accuracy=0.996	gnl|BL_ORD_ID|16805 cb8564_c66657/f4p6/2862 isoform=cb8564_c66657;full_length_coverage=4;non_full_length_coverage=6;isoform_length=2862;expected_accuracy=1.000	1	2173	2170	2734	29	2176	2299	2862
cb3290_c35/f1p0/2163 isoform=cb3290_c35;full_length_coverage=1;non_full_length_coverage=0;isoform_length=2163;expected_accuracy=0.995	gnl|BL_ORD_ID|41313 cb3290_c33/f1p0/3704 isoform=cb3290_c33;full_length_coverage=1;non_full_length_coverage=0;isoform_length=3704;expected_accuracy=0.998	14	1507	1508	2163	6	1501	1619	2275
cb1052_c55/f1p0/2730	gnl|BL_ORD_ID|1189 cb1052_c16/f1p0/2854 isoform=cb1052_c16;full_length_coverage=1;non_full_length_coverage=0;isoform_length=2854;expected_accur	599	2726	75	604	730	2854	1	525

	acy=0.995								
cb1812_c29/f1p0/2465 isoform=cb1812_c29;full_length_coverage=1;non_full_length_coverage=0;isoform_length=2465;expected_accuracy=0.992	gnl|BL_ORD_ID|34232 cb1812_c24/f1p0/4164 isoform=cb1812_c24;full_length_coverage=1;non_full_length_coverage=0;isoform_length=4164;expected_accuracy=0.996	280	2465	3	282	1981	4164	1	281
cb15510_c5/f1p1/1580	gnl|BL_ORD_ID|72095 cb5048_c6/f1p0/2038	1	1243	1243	1518	23	1265	1706	1981
cb8564_c41756/f1p0/2738 isoform=cb8564_c41756;full_length_coverage=1;non_full_length_coverage=0;isoform_length=2738;expected_accuracy=0.996	gnl|BL_ORD_ID|55873 cb8564_c47701/f1p0/3144	12	2173	2170	2738	24	2189	2312	2880
cb8564_c36689/f1p1/2368 isoform=cb8564_c36689;full_length_coverage=1;non_full_length_coverage=1;isoform_length=2368;expected_accuracy=0.991	gnl|BL_ORD_ID|55280 cb8564_c37198/f1p5/2941	1	1709	1706	2368	1	1703	2260	2924
cb4367_c10/f1p1/1298 isoform=cb4367_c10;full_length_coverage=1;non_full_length_coverage=1;isoform_length=1298;expected_accuracy=0.995	gnl|BL_ORD_ID|42900 cb4367_c2/f1p1/2510 isoform=cb4367_c2;full_length_coverage=1;non_full_length_coverage=1;isoform_length=2510;expected_accuracy=0.991	1	1129	1126	1298	20	1143	2289	2457
cb8564_c49562/f1p0/2838 isoform=cb8564_c49562;full_length_coverage=1;non_full_length_coverage=0;isoform_length=2838;expected_accuracy=0.995	gnl|BL_ORD_ID|54438 cb8564_c46124/f1p0/3383	1	2331	2327	2836	147	2482	2589	3104
cb8564_c39136/f1p0/3036 isoform=cb8564_c39136;full_length_coverage=1;non_full_length_coverage=0;isoform_length=3036;expected_	gnl|BL_ORD_ID|57017 cb8564_c80370/f1p0/3493	118	2089	2085	3036	3085	1109	982	29

accuracy=0.998									
cb3147_c6/f1p0/2136 isoform=cb3147_c6;full_length_coverage=1;non_full_length_coverage=0;isoform_length=2136;expected_accuracy=1.000	gnl|BL_ORD_ID|38849 cb3147_c3/f1p0/2291 isoform=cb3147_c3;full_length_coverage=1;non_full_length_coverage=0;isoform_length=2291;expected_accuracy=0.993	1	1270	1268	2130	32	1306	1457	2291
cb1279_c10/f2p0/2859 isoform=cb1279_c10;full_length_coverage=2;non_full_length_coverage=0;isoform_length=2859;expected_accuracy=0.999	gnl|BL_ORD_ID|27064 cb1279_c45/f1p3/3848 isoform=cb1279_c45;full_length_coverage=1;non_full_length_coverage=3;isoform_length=3848;expected_accuracy=0.999	202	2859	13	203	1146	3800	3	195
cb8564_c40764/f1p0/2563 isoform=cb8564_c40764;full_length_coverage=1;non_full_length_coverage=0;isoform_length=2563;expected_accuracy=0.997	gnl|BL_ORD_ID|13512 cb8564_c186209/f1p0/2931 isoform=cb8564_c186209;full_length_coverage=1;non_full_length_coverage=0;isoform_length=2931;expected_accuracy=0.996	1	2167	2162	2563	1	2144	2351	2752
cb1684_c4/f1p0/3388 isoform=cb1684_c4;full_length_coverage=1;non_full_length_coverage=0;isoform_length=3388;expected_accuracy=0.999	gnl|BL_ORD_ID|31193 cb1684_c0/f1p0/4194 isoform=cb1684_c0;full_length_coverage=1;non_full_length_coverage=0;isoform_length=4194;expected_accuracy=0.995	1	1873	1872	3388	38	1909	2479	4001
cb6084_c10/f1p0/2883 isoform=cb6084_c10;full_length_coverage=1;non_full_length_coverage=0;isoform_length=2883;expected_accuracy=0.992	gnl|BL_ORD_ID|44988 cb6084_c3/f1p0/4275 isoform=cb6084_c3;full_length_coverage=1;non_full_length_coverage=0;isoform_length=4275;expected_accuracy=0.993	1	1850	1849	2883	1	1858	3139	4179
cb8564_c15653/f1p4/2475	gnl|BL_ORD_ID|15554 cb8564_c47252/f1p0/2795 isoform=cb8564_c47252;full_length_coverage=1;non_full_length_coverage=0;isoform_length=2795;expected_accuracy=0.997	1	2293	2289	2482	4	2296	2514	2707
cb8564_c41162/f1p0/3158	gnl|BL_ORD_ID|58037 cb8564_c87188/f1p1/3426	25	2553	2548	3042	127	265	2789	328

							8		3
cb8564_c51172/f1p0/3367	gnl|BL_ORD_ID|55920 cb8564_c48949/f1p0/4357	21	2311	2312	2973	18	2293	2847	3505
cb8564_c43779/f1p0/3021 isoform=cb8564_c43779;full_length_coverage=1;non_full_length_coverage=0;isoform_length=3021;expected_accuracy=0.993	gnl|BL_ORD_ID|58037 cb8564_c87188/f1p1/3426	20	2344	2345	3021	127	2450	2606	3281
cb8564_c5094/f3p0/3851 isoform=cb8564_c5094;full_length_coverage=3;non_full_length_coverage=0;isoform_length=3851;expected_accuracy=1.000	gnl|BL_ORD_ID|16742 cb8564_c628/f3p1/4943 isoform=cb8564_c628;full_length_coverage=3;non_full_length_coverage=1;isoform_length=4943;expected_accuracy=1.000	1	1913	1911	3767	4	1916	3085	4943
cb18456_c6530/f1p0/1200	gnl|BL_ORD_ID|35900 cb18456_c3965/f1p2/1734 isoform=cb18456_c3965;full_length_coverage=1;non_full_length_coverage=2;isoform_length=1734;expected_accuracy=0.995	260	1166	3	259	759	1663	8	264
cb7639_c15/f1p0/2203 isoform=cb7639_c15;full_length_coverage=1;non_full_length_coverage=0;isoform_length=2203;expected_accuracy=0.998	gnl|BL_ORD_ID|51427 cb7639_c11/f1p0/2532	1	1804	1803	2203	2	1801	2081	2508
cb8564_c2323/f1p0/2212 isoform=cb8564_c2323;full_length_coverage=1;non_full_length_coverage=0;isoform_length=2212;expected_accuracy=0.995	gnl|BL_ORD_ID|13199 cb8564_c182089/f1p0/2445 isoform=cb8564_c182089;full_length_coverage=1;non_full_length_coverage=0;isoform_length=2445;expected_accuracy=1.000	1	1320	1316	2208	1	1320	1553	2445
cb2662_c6/f1p0/2449 isoform=cb2662_c6;full_length_coverage=1;non_full_length_coverage=0;isoform_length=2449;expected_accur	gnl|BL_ORD_ID|38855 cb2662_c2/f1p0/3909 isoform=cb2662_c2;full_length_coverage=1;non_full_length_coverage=0;isoform_length=3909;expected_accura	1	2243	2238	2449	680	2920	3635	3847

acy=0.993	cy=0.995								
cb8564_c185920/f1p0/2777 isoform=cb8564_c185920;full_length_coverage=1;non_full_length_coverage=0;isoform_length=2777;expected_accuracy=0.996	gnl|BL_ORD_ID|15153 cb8564_c36883/f1p0/3072 isoform=cb8564_c36883;full_length_coverage=1;non_full_length_coverage=0;isoform_length=3072;expected_accuracy=0.996	174	2777	1	177	351	295 5	15	192
cb3678_c20/f11p0/3156 isoform=cb3678_c20;full_length_coverage=11;non_full_length_coverage=0;isoform_length=3156;expected_accuracy=1.000	gnl|BL_ORD_ID|41572 cb3678_c2/f2p0/3245 isoform=cb3678_c2;full_length_coverage=2;non_full_length_coverage=0;isoform_length=3245;expected_accuracy=1.000	493	3154	64	496	583	324 5	22	454
cb3586_c10/f1p0/2722 isoform=cb3586_c10;full_length_coverage=1;non_full_length_coverage=0;isoform_length=2722;expected_accuracy=0.993	gnl|BL_ORD_ID|41150 cb3586_c3/f1p0/2988 isoform=cb3586_c3;full_length_coverage=1;non_full_length_coverage=0;isoform_length=2988;expected_accuracy=0.993	252	2722	1	252	443	290 3	22	274
cb4016_c33/f1p0/2507	gnl|BL_ORD_ID|40900 cb4016_c14/f1p0/2928 isoform=cb4016_c14;full_length_coverage=1;non_full_length_coverage=0;isoform_length=2928;expected_accuracy=0.994	207	2462	1	208	549	280 7	25	249
cb8564_c20551/f2p0/2429 isoform=cb8564_c20551;full_length_coverage=2;non_full_length_coverage=0;isoform_length=2429;expected_accuracy=0.999	gnl|BL_ORD_ID|57228 cb8564_c87142/f1p0/3290	1	2235	2235	2429	75	230 9	2598	279 2
cb7642_c139/f1p0/2022 isoform=cb7642_c139;full_length_coverage=1;non_full_length_coverage=0;isoform_length=2022;expected_accuracy=0.993	gnl|BL_ORD_ID|50637 cb7642_c158/f1p0/2441	1	1475	1476	1994	200	167 4	1871	239 0
cb8564_c45699/f1p0/3005	gnl|BL_ORD_ID|55844 cb8564_c44552/f1p0/3216	101	1888	1884	2842	129	193	2068	302

							0		8
cb8995_c8/f1p0/2000 isoform=cb8995_c8;full_length_coverage=1;non_full_length_coverage=0;isoform_length=2000;expected_accuracy=1.000	gnl|BL_ORD_ID|59425 cb8995_c11/f1p0/2278	97	2000	1	100	277	2181	66	165
cb6199_c22/f1p0/2552 isoform=cb6199_c22;full_length_coverage=1;non_full_length_coverage=0;isoform_length=2552;expected_accuracy=0.998	gnl|BL_ORD_ID|4354 cb6199_c20/f1p0/3192 isoform=cb6199_c20;full_length_coverage=1;non_full_length_coverage=0;isoform_length=3192;expected_accuracy=0.995	259	2552	3	261	749	3041	3	246
cb8564_c45300/f1p4/3014 isoform=cb8564_c45300;full_length_coverage=1;non_full_length_coverage=4;isoform_length=3014;expected_accuracy=0.996	gnl|BL_ORD_ID|14838 cb8564_c34060/f2p0/3214 isoform=cb8564_c34060;full_length_coverage=2;non_full_length_coverage=0;isoform_length=3214;expected_accuracy=0.993	243	3014	1	246	432	3214	6	273
cb964_c10/f1p0/2954 isoform=cb964_c10;full_length_coverage=1;non_full_length_coverage=0;isoform_length=2954;expected_accuracy=0.996	gnl|BL_ORD_ID|59142 cb964_c19/f1p0/4670	147	2954	1	151	1582	4389	122	272
cb8564_c43917/f1p0/2346 isoform=cb8564_c43917;full_length_coverage=1;non_full_length_coverage=0;isoform_length=2346;expected_accuracy=1.000	gnl|BL_ORD_ID|55201 cb8564_c35323/f1p0/3479	1	1728	1729	2242	93	1819	2851	3364
cb1956_c25/f1p0/3810	gnl|BL_ORD_ID|36250 cb1956_c4/f3p0/3816 isoform=cb1956_c4;full_length_coverage=3;non_full_length_coverage=0;isoform_length=3816;expected_accuracy=0.997	11	2553	2553	2990	1	2541	3392	3816
cb8564_c68314/f3p1/1908	gnl|BL_ORD_ID|11097 cb8564_c111419/f2p1/2850	1	1318	1317	1908	8	133	2255	283

isoform=cb8564_c68314;full_length_coverage=3;non_full_length_coverage=1;isoform_length=1908;expected_accuracy=1.000	isoform=cb8564_c111419;full_length_coverage=2;non_full_length_coverage=1;isoform_length=2850;expected_accuracy=1.000						1		0
cb12685_c3/f1p2/1466	gnl|BL_ORD_ID|14860 cb8564_c24783/f1p1/3102 isoform=cb8564_c24783;full_length_coverage=1;non_full_length_coverage=1;isoform_length=3102;expected_accuracy=0.992	1	1294	1292	1463	16	1340	2929	3100
cb90_c17/f1p1/4529 isoform=cb90_c17;full_length_coverage=1;non_full_length_coverage=1;isoform_length=4529;expected_accuracy=0.992	gnl|BL_ORD_ID|59036 cb90_c11/f1p0/4874	350	4529	21	349	712	4887	148	477
cb8564_c3183/f2p1/2280 isoform=cb8564_c3183;full_length_coverage=2;non_full_length_coverage=1;isoform_length=2280;expected_accuracy=0.994	gnl|BL_ORD_ID|14565 cb8564_c3180/f1p0/3132 isoform=cb8564_c3180;full_length_coverage=1;non_full_length_coverage=0;isoform_length=3132;expected_accuracy=0.993	355	2280	63	352	961	2886	1	290
cb3988_c15/f1p0/2853 isoform=cb3988_c15;full_length_coverage=1;non_full_length_coverage=0;isoform_length=2853;expected_accuracy=0.997	gnl|BL_ORD_ID|41321 cb3988_c1/f1p1/3470 isoform=cb3988_c1;full_length_coverage=1;non_full_length_coverage=1;isoform_length=3470;expected_accuracy=1.000	1	1802	1798	2846	261	2051	2423	3470
cb1988_c2/f4p2/3928 isoform=cb1988_c2;full_length_coverage=4;non_full_length_coverage=2;isoform_length=3928;expected_accuracy=1.000	gnl|BL_ORD_ID|35813 cb1988_c27/f1p0/5263 isoform=cb1988_c27;full_length_coverage=1;non_full_length_coverage=0;isoform_length=5263;expected_accuracy=0.997	1	2672	2670	3892	1	2675	4043	5263
cb8564_c51223/f1p4/1975	gnl|BL_ORD_ID|52763 cb8564_c13243/f1p0/4432	207	1937	21	208	2440	4170	4	191
cb4945_c3/f1p0/2690	gnl|BL_ORD_ID|73110 cb4945_c1/f1p0/3164	1	1933	1930	2690	14	195	2295	305

isoform=cb4945_c3;full_length_coverage=1;non_full_length_coverage=0;isoform_length=2690;expected_accuracy=0.999							2		5
cb8564_c44063/f1p0/2503 isoform=cb8564_c44063;full_length_coverage=1;non_full_length_coverage=0;isoform_length=2503;expected_accuracy=0.999	gnl|BL_ORD_ID|55231 cb8564_c36011/f1p0/3015	397	2503	7	402	553	2671	10	408
cb8564_c87466/f1p1/3006 isoform=cb8564_c87466;full_length_coverage=1;non_full_length_coverage=1;isoform_length=3006;expected_accuracy=0.994	gnl|BL_ORD_ID|16705 cb8564_c53880/f1p0/3484 isoform=cb8564_c53880;full_length_coverage=1;non_full_length_coverage=0;isoform_length=3484;expected_accuracy=0.996	1069	2900	1	1070	1672	3484	1	1056
cb8564_c22279/f1p0/2983 isoform=cb8564_c22279;full_length_coverage=1;non_full_length_coverage=0;isoform_length=2983;expected_accuracy=0.997	gnl|BL_ORD_ID|17366 cb8564_c80423/f1p0/3018 isoform=cb8564_c80423;full_length_coverage=1;non_full_length_coverage=0;isoform_length=3018;expected_accuracy=1.000	1	2095	2096	2897	1	2098	2219	3018
cb8564_c86566/f1p0/2178	gnl|BL_ORD_ID|57978 cb8564_c85833/f1p0/2942	10	1709	1707	2194	12	1709	2309	2795
cb8564_c43399/f1p0/2654 isoform=cb8564_c43399;full_length_coverage=1;non_full_length_coverage=0;isoform_length=2654;expected_accuracy=0.999	gnl|BL_ORD_ID|55962 cb8564_c50282/f1p0/4402	15	2367	2363	2653	1457	3804	4020	4310
cb8564_c50076/f1p1/2461 isoform=cb8564_c50076;full_length_coverage=1;non_full_length_coverage=1;isoform_length=2461;expected_accuracy=0.993	gnl|BL_ORD_ID|12455 cb8564_c12908/f1p0/2790 isoform=cb8564_c12908;full_length_coverage=1;non_full_length_coverage=0;isoform_length=2790;expected_accuracy=0.996	1	1679	1675	2452	5	1686	2009	2790
cb8564_c120694/f1p0/2801	gnl|BL_ORD_ID|17728 cb8564_c87595/f1p0/4323	260	2800	2	260	1793	432	1	257

isoform=cb8564_c120694;full_length_coverage=1;non_full_length_coverage=0;isoform_length=2801;expected_accuracy=0.997	isoform=cb8564_c87595;full_length_coverage=1;non_full_length_coverage=0;isoform_length=4323;expected_accuracy=0.997						3		
cb5125_c34/f1p0/1890	gnl|BL_ORD_ID|43453 cb5125_c17/f1p2/2087 isoform=cb5125_c17;full_length_coverage=1;non_full_length_coverage=2;isoform_length=2087;expected_accuracy=0.998	1	1145	1144	1780	67	1220	1412	2046
cb8564_c84381/f1p0/2854 isoform=cb8564_c84381;full_length_coverage=1;non_full_length_coverage=0;isoform_length=2854;expected_accuracy=0.992	gnl|BL_ORD_ID|56971 cb8564_c78998/f1p0/3377	1	1944	1943	2854	1	1939	2473	3386
cb1467_c1/f1p1/4278	gnl|BL_ORD_ID|63644 cb1467_c5/f1p0/5220	489	4286	78	491	1092	4889	3	416
cb7795_c1/f2p1/2486 isoform=cb7795_c1;full_length_coverage=2;non_full_length_coverage=1;isoform_length=2486;expected_accuracy=0.996	gnl|BL_ORD_ID|6861 cb7795_c2/f1p0/3536 isoform=cb7795_c2;full_length_coverage=1;non_full_length_coverage=0;isoform_length=3536;expected_accuracy=0.996	1	2059	2057	2449	23	2083	3143	3534
cb2687_c4/f1p0/2406	gnl|BL_ORD_ID|38801 cb2687_c1/f1p0/3839 isoform=cb2687_c1;full_length_coverage=1;non_full_length_coverage=0;isoform_length=3839;expected_accuracy=1.000	117	1989	1989	2414	1	1872	3333	3758
cb18456_c1776/f1p0/1211	gnl|BL_ORD_ID|34562 cb18456_c1614/f2p5/1371 isoform=cb18456_c1614;full_length_coverage=2;non_full_length_coverage=5;isoform_length=1371;expected_accuracy=1.000	280	1214	9	285	384	1318	2	272
cb8564_c180116/f1p0/2944	gnl|BL_ORD_ID|56764 cb8564_c70795/f1p0/3769	22	2522	2520	2944	34	253	2763	318

isoform=cb8564_c180116;full_length_coverage=1;non_full_length_coverage=0;isoform_length=2944;expected_accuracy=0.996							4		3
cb8564_c145335/f3p0/1964 isoform=cb8564_c145335;full_length_coverage=3;non_full_length_coverage=0;isoform_length=1964;expected_accuracy=0.999	gnl|BL_ORD_ID|53375 cb8564_c123599/f1p0/2339	136	1964	1	133	431	2259	1	133
cb8564_c77701/f1p0/2478 isoform=cb8564_c77701;full_length_coverage=1;non_full_length_coverage=0;isoform_length=2478;expected_accuracy=0.997	gnl|BL_ORD_ID|55735 cb8564_c5247/f1p0/2805	15	2077	2074	2478	22	2077	2319	2724
cb8564_c47522/f1p0/2954	gnl|BL_ORD_ID|16879 cb8564_c48606/f1p1/3308 isoform=cb8564_c48606;full_length_coverage=1;non_full_length_coverage=1;isoform_length=3308;expected_accuracy=0.998	1	2132	2128	2849	1	2151	2587	3308
cb8564_c107877/f2p1/3829 isoform=cb8564_c107877;full_length_coverage=2;non_full_length_coverage=1;isoform_length=3829;expected_accuracy=1.000	gnl|BL_ORD_ID|16294 cb8564_c70697/f1p0/4245 isoform=cb8564_c70697;full_length_coverage=1;non_full_length_coverage=0;isoform_length=4245;expected_accuracy=0.993	1	2732	2730	3829	213	2954	3120	4220
cb8564_c128609/f1p0/3058 isoform=cb8564_c128609;full_length_coverage=1;non_full_length_coverage=0;isoform_length=3058;expected_accuracy=0.996	gnl|BL_ORD_ID|54634 cb8564_c128804/f1p0/3259	1	2766	2767	3018	36	2798	2970	3222
cb7837_c4/f1p0/2386	gnl|BL_ORD_ID|7423 cb7837_c1/f2p0/2384 isoform=cb7837_c1;full_length_coverage=2;non_full_length_coverage=0;isoform_length=2384;expected_accura	158	2270	1	158	272	2382	5	162

	cy=0.992								
cb12714_c29/f1p2/1583	gnl|BL_ORD_ID|13093 cb8564_c177392/f1p0/2423 isoform=cb8564_c177392;full_length_coverage=1;non_full_length_coverage=0;isoform_length=2423;expected_accuracy=0.996	1	1296	1293	1543	1	1296	2173	2423
cb8564_c108344/f8p1/2362 isoform=cb8564_c108344;full_length_coverage=8;non_full_length_coverage=1;isoform_length=2362;expected_accuracy=1.000	gnl|BL_ORD_ID|55987 cb8564_c51257/f1p0/4656	12	1613	1612	2325	1	1613	3621	4334
cb8564_c188193/f1p0/2849 isoform=cb8564_c188193;full_length_coverage=1;non_full_length_coverage=0;isoform_length=2849;expected_accuracy=0.997	gnl|BL_ORD_ID|16879 cb8564_c48606/f1p1/3308 isoform=cb8564_c48606;full_length_coverage=1;non_full_length_coverage=1;isoform_length=3308;expected_accuracy=0.998	1	2132	2128	2849	1	2151	2587	3308
cb8564_c43779/f1p0/3021 isoform=cb8564_c43779;full_length_coverage=1;non_full_length_coverage=0;isoform_length=3021;expected_accuracy=0.993	gnl|BL_ORD_ID|15924 cb8564_c52804/f1p5/3221 isoform=cb8564_c52804;full_length_coverage=1;non_full_length_coverage=5;isoform_length=3221;expected_accuracy=0.999	1	2344	2345	3021	1	2343	2499	3175
cb649_c18/f1p1/3876	gnl|BL_ORD_ID|4016 cb649_c21/f1p0/4098 isoform=cb649_c21;full_length_coverage=1;non_full_length_coverage=0;isoform_length=4098;expected_accuracy=0.990	265	3847	1	265	445	4035	57	322
cb6277_c7/f1p0/2614 isoform=cb6277_c7;full_length_coverage=1;non_full_length_coverage=0;isoform_length=2614;expected_accuracy=0.996	gnl|BL_ORD_ID|4562 cb6277_c30/f4p0/2856 isoform=cb6277_c30;full_length_coverage=4;non_full_length_coverage=0;isoform_length=2856;expected_accuracy=1.000	1	1457	1459	2614	113	1569	1703	2855
cb8564_c123270/f1p0/1927	gnl|BL_ORD_ID|54874 cb8564_c3238/f1p0/2188	245	1812	55	245	507	207	22	209

							2		
cb8564_c9136/f9p5/2606 isoform=cb8564_c9136;full_length_coverage=9;non_full_length_coverage=5;isoform_length=2606;expected_accuracy=1.000	gnl|BL_ORD_ID|53954 cb8564_c15976/f1p0/2695	1052	2524	1	1055	1158	2631	2	1055
cb6204_c6/f1p1/2162 isoform=cb6204_c6;full_length_coverage=1;non_full_length_coverage=1;isoform_length=2162;expected_accuracy=1.000	gnl|BL_ORD_ID|4437 cb6204_c9/f1p3/2742 isoform=cb6204_c9;full_length_coverage=1;non_full_length_coverage=3;isoform_length=2742;expected_accuracy=0.991	11	1249	1244	2162	1	1231	1556	2475
cb15584_c0/f3p0/1259 isoform=cb15584_c0;full_length_coverage=3;non_full_length_coverage=0;isoform_length=1259;expected_accuracy=0.992	gnl|BL_ORD_ID|64762 cb15584_c2/f1p0/1708	371	1226	7	371	856	1712	20	375
cb1489_c2/f1p0/3842	gnl|BL_ORD_ID|68232 cb1945_c14/f1p2/3923	106	3607	3607	3869	3904	403	295	33
cb8564_c91920/f1p0/2089	gnl|BL_ORD_ID|57938 cb8564_c84460/f1p0/2544	581	2080	64	581	911	2410	1	517
cb12972_c5/f2p0/1545 isoform=cb12972_c5;full_length_coverage=2;non_full_length_coverage=0;isoform_length=1545;expected_accuracy=1.000	gnl|BL_ORD_ID|15126 cb8564_c2621/f1p1/2401 isoform=cb8564_c2621;full_length_coverage=1;non_full_length_coverage=1;isoform_length=2401;expected_accuracy=1.000	206	1545	9	208	1020	2359	1	200
cb8564_c21767/f1p1/2839 isoform=cb8564_c21767;full_length_coverage=1;non_full_length_coverage=1;isoform_length=2839;expected_accuracy=0.996	gnl|BL_ORD_ID|12828 cb8564_c171649/f2p1/3023 isoform=cb8564_c171649;full_length_coverage=2;non_full_length_coverage=1;isoform_length=3023;expected_accuracy=0.994	1040	2839	1	1040	1217	3020	32	1070
cb4528_c30/f1p0/1646	gnl|BL_ORD_ID|42120 cb4528_c39/f1p0/2046 isoform=cb4528_c39;full_length_coverage=1;non_full_l	1	1425	1423	1566	171	1595	1903	2046

	ength_coverage=0;isoform_length=2046;expected_accuracy=0.995								
cb6237_c2/f1p0/1699 isoform=cb6237_c2;full_length_coverage=1;non_full_length_coverage=0;isoform_length=1699;expected_accuracy=0.993	gnl|BL_ORD_ID|5892 cb6237_c0/f1p0/2907 isoform=cb6237_c0;full_length_coverage=1;non_full_length_coverage=0;isoform_length=2907;expected_accuracy=1.000	1	1112	1110	1699	10	1129	2250	2839
cb9305_c15/f1p0/1454 isoform=cb9305_c15;full_length_coverage=1;non_full_length_coverage=0;isoform_length=1454;expected_accuracy=0.993	gnl|BL_ORD_ID|20900 cb9305_c3/f1p1/2433 isoform=cb9305_c3;full_length_coverage=1;non_full_length_coverage=1;isoform_length=2433;expected_accuracy=0.992	1	1009	1008	1401	220	1231	2037	2433
cb3537_c13/f1p0/2488 isoform=cb3537_c13;full_length_coverage=1;non_full_length_coverage=0;isoform_length=2488;expected_accuracy=1.000	gnl|BL_ORD_ID|39687 cb3537_c0/f9p0/3405 isoform=cb3537_c0;full_length_coverage=9;non_full_length_coverage=0;isoform_length=3405;expected_accuracy=1.000	2	2302	2297	2488	1	2304	3158	3344
cb90_c17/f1p1/4529 isoform=cb90_c17;full_length_coverage=1;non_full_length_coverage=1;isoform_length=4529;expected_accuracy=0.992	gnl|BL_ORD_ID|20807 cb90_c26/f1p2/4751 isoform=cb90_c26;full_length_coverage=1;non_full_length_coverage=2;isoform_length=4751;expected_accuracy=0.996	350	4494	2	349	570	4713	29	380
cb8564_c23232/f1p3/2540	gnl|BL_ORD_ID|56010 cb8564_c51971/f1p0/3064	16	2172	2167	2518	14	2170	2272	2623
cb15958_c9/f1p0/1815 isoform=cb15958_c9;full_length_coverage=1;non_full_length_coverage=0;isoform_length=1815;expected_accuracy=0.998	gnl|BL_ORD_ID|18457 cb8564_c9889/f2p0/4040 isoform=cb8564_c9889;full_length_coverage=2;non_full_length_coverage=0;isoform_length=4040;expected_accuracy=0.999	441	1812	8	442	2661	4040	1	434
cb7642_c158/f1p0/2441	gnl|BL_ORD_ID|51433 cb7642_c125/f1p0/2713	1030	2348	1	1031	1364	2682	115	1145

cb8564_c186967/f1p0/2513 isoform=cb8564_c186967;full_length_coverage=1;non_full_length_coverage=0;isoform_length=2513;expected_accuracy=0.998	gnl|BL_ORD_ID|13313 cb8564_c188075/f1p2/2904 isoform=cb8564_c188075;full_length_coverage=1;non_full_length_coverage=2;isoform_length=2904;expected_accuracy=0.996	1	1942	1938	2513	40	1983	2274	2850
cb5755_c9/f1p0/2841	gnl|BL_ORD_ID|45433 cb5755_c3/f1p0/2933 isoform=cb5755_c3;full_length_coverage=1;non_full_length_coverage=0;isoform_length=2933;expected_accuracy=0.993	1	1495	1493	2769	5	1496	1632	2933
cb8564_c46562/f2p3/3196 isoform=cb8564_c46562;full_length_coverage=2;non_full_length_coverage=3;isoform_length=3196;expected_accuracy=0.995	gnl|BL_ORD_ID|55939 cb8564_c49630/f1p4/3487	16	2940	2940	3189	8	2913	3243	3493
cb2138_c7/f2p0/1767 isoform=cb2138_c7;full_length_coverage=2;non_full_length_coverage=0;isoform_length=1767;expected_accuracy=1.000	gnl|BL_ORD_ID|34570 cb2138_c3/f1p0/2395 isoform=cb2138_c3;full_length_coverage=1;non_full_length_coverage=0;isoform_length=2395;expected_accuracy=0.997	216	1767	5	220	818	2369	3	219
cb2527_c19/f1p0/2292 isoform=cb2527_c19;full_length_coverage=1;non_full_length_coverage=0;isoform_length=2292;expected_accuracy=0.997	gnl|BL_ORD_ID|69796 cb2527_c31/f1p1/3358	132	2248	1	136	1222	3339	160	288
cb5909_c2/f3p0/1152 isoform=cb5909_c2;full_length_coverage=3;non_full_length_coverage=0;isoform_length=1152;expected_accuracy=0.997	gnl|BL_ORD_ID|46383 cb5909_c4/f2p1/3043 isoform=cb5909_c4;full_length_coverage=2;non_full_length_coverage=1;isoform_length=3043;expected_accuracy=0.996	532	1152	7	536	2418	3038	1	530
cb18456_c1988/f1p0/1112	gnl|BL_ORD_ID|69255 cb18456_c2252/f1p0/1653	412	1026	5	416	728	1340	9	423

cb2733_c21/f1p0/1180 isoform=cb2733_c21;full_length_coverage=1;non_full_length_coverage=0;isoform_length=1180;expected_accuracy=1.000	gnl|BL_ORD_ID|39010 cb2733_c26/f22p0/1425 isoform=cb2733_c26;full_length_coverage=22;non_full_length_coverage=0;isoform_length=1425;expected_accuracy=1.000	115	1180	1	115	281	1346	21	138
cb2831_c26/f1p0/2685	gnl|BL_ORD_ID|68914 cb2831_c15/f1p0/3921	1	2172	2173	2480	70	2230	3098	3411
cb18649_c2/f1p0/3942 isoform=cb18649_c2;full_length_coverage=1;non_full_length_coverage=0;isoform_length=3942;expected_accuracy=0.993	gnl|BL_ORD_ID|67913 cb18649_c3/f1p0/5011	1	2588	2587	3942	620	3209	3439	4793
cb8564_c40764/f1p0/2563 isoform=cb8564_c40764;full_length_coverage=1;non_full_length_coverage=0;isoform_length=2563;expected_accuracy=0.997	gnl|BL_ORD_ID|56961 cb8564_c78609/f1p0/3194	19	2167	2162	2563	45	2191	2398	2799
cb322_c35/f1p0/3437 isoform=cb322_c35;full_length_coverage=1;non_full_length_coverage=0;isoform_length=3437;expected_accuracy=0.997	gnl|BL_ORD_ID|69561 cb322_c28/f1p0/3948	114	3432	1	119	510	3815	140	258
cb8564_c72639/f1p3/2409 isoform=cb8564_c72639;full_length_coverage=1;non_full_length_coverage=3;isoform_length=2409;expected_accuracy=0.999	gnl|BL_ORD_ID|58004 cb8564_c86464/f1p0/2629	288	2409	35	288	456	2579	83	336
cb3753_c4/f1p0/2624 isoform=cb3753_c4;full_length_coverage=1;non_full_length_coverage=0;isoform_length=2624;expected_accuracy=0.992	gnl|BL_ORD_ID|39849 cb3753_c0/f1p0/3525 isoform=cb3753_c0;full_length_coverage=1;non_full_length_coverage=0;isoform_length=3525;expected_accuracy=0.991	13	1659	1657	2610	17	1657	2219	3182

cb8564_c153580/f2p1/2306 isoform=cb8564_c153580;full_length_coverage=2;non_full_length_coverage=1;isoform_length=2306;expected_accuracy=1.000	gnl|BL_ORD_ID|10006 cb8564_c109862/f2p0/3191 isoform=cb8564_c109862;full_length_coverage=2;non_full_length_coverage=0;isoform_length=3191;expected_accuracy=0.999	155	2305	15	157	1032	3176	35	168
cb3449_c13/f1p2/3050 isoform=cb3449_c13;full_length_coverage=1;non_full_length_coverage=2;isoform_length=3050;expected_accuracy=0.993	gnl|BL_ORD_ID|40995 cb3449_c50/f83p4/3327 isoform=cb3449_c50;full_length_coverage=83;non_full_length_coverage=4;isoform_length=3327;expected_accuracy=1.000	1	2755	2752	3050	95	2850	3018	3311
cb8564_c176017/f8p2/2488 isoform=cb8564_c176017;full_length_coverage=8;non_full_length_coverage=2;isoform_length=2488;expected_accuracy=1.000	gnl|BL_ORD_ID|10058 cb8564_c119283/f1p1/2611 isoform=cb8564_c119283;full_length_coverage=1;non_full_length_coverage=1;isoform_length=2611;expected_accuracy=0.999	1048	2474	1	1048	1190	2610	1	1046
cb18456_c3761/f1p0/1206	gnl|BL_ORD_ID|69281 cb18456_c3828/f1p4/1353	9	806	809	1120	4	804	907	1217
cb4935_c5/f1p0/2681 isoform=cb4935_c5;full_length_coverage=1;non_full_length_coverage=0;isoform_length=2681;expected_accuracy=0.998	gnl|BL_ORD_ID|72053 cb4935_c9/f1p1/3034	482	2681	49	482	612	2811	1	434
cb6277_c7/f1p0/2614 isoform=cb6277_c7;full_length_coverage=1;non_full_length_coverage=0;isoform_length=2614;expected_accuracy=0.996	gnl|BL_ORD_ID|3491 cb6277_c8/f1p0/2793 isoform=cb6277_c8;full_length_coverage=1;non_full_length_coverage=0;isoform_length=2793;expected_accuracy=0.999	1	1457	1459	2522	140	1596	1730	2793
cb2873_c7/f1p4/3001 isoform=cb2873_c7;full_length_coverage=1;non_full_length_coverage=4;isoform_length=3001;expected_accuracy=0.999	gnl|BL_ORD_ID|68966 cb2873_c10/f1p0/3294	143	2976	1	143	321	3155	44	181

cb8564_c42677/f1p0/3180	gnl|BL_ORD_ID|18852 cb8564_c84488/f1p0/3408 isoform=cb8564_c84488;full_length_coverage=1;non_full_length_coverage=0;isoform_length=3408;expected_accuracy=0.996	231	2842	21	231	790	3407	14	217
cb12685_c3/f1p2/1466	gnl|BL_ORD_ID|9759 cb8564_c108367/f4p6/3120 isoform=cb8564_c108367;full_length_coverage=4;non_full_length_coverage=6;isoform_length=3120;expected_accuracy=0.999	1	1295	1292	1463	21	1315	2921	3092
cb4496_c3/f1p0/3329	gnl|BL_ORD_ID|72614 cb4496_c4/f1p0/4050	1352	3332	1	1354	1871	3851	70	1423
cb8564_c75977/f1p0/2165	gnl|BL_ORD_ID|58077 cb8564_c88175/f1p0/4011	1	1393	1394	2130	63	1455	3255	3986
cb653_c12/f1p0/3754 isoform=cb653_c12;full_length_coverage=1;non_full_length_coverage=0;isoform_length=3754;expected_accuracy=0.995	gnl|BL_ORD_ID|5130 cb653_c9/f1p0/4025 isoform=cb653_c9;full_length_coverage=1;non_full_length_coverage=0;isoform_length=4025;expected_accuracy=0.997	195	3754	1	194	424	3982	104	301
cb7341_c3/f1p0/1290 isoform=cb7341_c3;full_length_coverage=1;non_full_length_coverage=0;isoform_length=1290;expected_accuracy=1.000	gnl|BL_ORD_ID|8731 cb7341_c1/f2p0/2288 isoform=cb7341_c1;full_length_coverage=2;non_full_length_coverage=0;isoform_length=2288;expected_accuracy=0.996	220	1286	41	220	1222	2288	1	181
cb8564_c91136/f1p0/3087	gnl|BL_ORD_ID|12830 cb8564_c158006/f2p1/3461 isoform=cb8564_c158006;full_length_coverage=2;non_full_length_coverage=1;isoform_length=3461;expected_accuracy=0.990	383	3072	9	384	509	3197	4	376
cb8564_c124042/f1p0/1795	gnl|BL_ORD_ID|27781 cb14897_c3/f1p1/1884 isoform=cb14897_c3;full_length_coverage=1;non_full_l	11	1293	1293	1677	1	1285	1492	1875

	ength_coverage=1;isoform_length=1884;expected_accuracy=0.996								
cb4211_c2/f2p1/1162 isoform=cb4211_c2;full_length_coverage=2;non_full_length_coverage=1;isoform_length=1162;expected_accuracy=1.000	gnl|BL_ORD_ID|41903 cb4211_c6/f1p1/2925 isoform=cb4211_c6;full_length_coverage=1;non_full_length_coverage=1;isoform_length=2925;expected_accuracy=0.994	124	1162	2	126	1870	2905	11	135
cb7601_c7/f1p0/1884	gnl|BL_ORD_ID|8183 cb7601_c9/f1p0/2160 isoform=cb7601_c9;full_length_coverage=1;non_full_length_coverage=0;isoform_length=2160;expected_accuracy=0.994	1	1492	1497	1805	34	1545	1745	2053
cb2177_c80/f1p0/3411	gnl|BL_ORD_ID|66793 cb2177_c69/f1p0/4458	126	2569	2566	3357	1	2444	3650	4441
cb2818_c78/f1p0/2043 isoform=cb2818_c78;full_length_coverage=1;non_full_length_coverage=0;isoform_length=2043;expected_accuracy=0.992	gnl|BL_ORD_ID|68897 cb2818_c28/f1p1/3460	1	1936	1935	2043	934	2866	3361	3470
cb9123_c0/f1p0/2192	gnl|BL_ORD_ID|21774 cb9123_c1/f1p0/2566 isoform=cb9123_c1;full_length_coverage=1;non_full_length_coverage=0;isoform_length=2566;expected_accuracy=0.994	1	1758	1757	2168	140	1897	2148	2566
cb8564_c10064/f5p11/3897 isoform=cb8564_c10064;full_length_coverage=5;non_full_length_coverage=11;isoform_length=3897;expected_accuracy=0.996	gnl|BL_ORD_ID|65909 cb17646_c3/f1p1/5031	191	3134	3135	3897	512	3455	3865	4627
cb3656_c9/f1p0/2680 isoform=cb3656_c9;full_length_coverage=1;non_full_le	gnl|BL_ORD_ID|40790 cb3656_c5/f1p0/3464 isoform=cb3656_c5;full_length_coverage=1;non_full_le	1	2058	2057	2680	4	2060	2839	3461

ngth_coverage=0;isoform_length=2680;expected_accuracy=0.995	ngth_coverage=0;isoform_length=3464;expected_accuracy=0.999								
cb8564_c37361/f1p0/3076	gnl|BL_ORD_ID|58037 cb8564_c87188/f1p1/3426	20	2481	2477	3070	127	2587	2688	3281
cb3037_c20/f1p0/2362 isoform=cb3037_c20;full_length_coverage=1;non_full_length_coverage=0;isoform_length=2362;expected_accuracy=0.998	gnl|BL_ORD_ID|38434 cb3037_c15/f1p0/3566 isoform=cb3037_c15;full_length_coverage=1;non_full_length_coverage=0;isoform_length=3566;expected_accuracy=0.998	1	2130	2126	2362	63	2190	3248	3483
cb12_c30/f1p2/4443 isoform=cb12_c30;full_length_coverage=1;non_full_length_coverage=2;isoform_length=4443;expected_accuracy=0.992	gnl|BL_ORD_ID|61090 cb12_c56/f1p0/6478	373	4384	1	373	2283	6295	1376	1753
cb8564_c119362/f1p0/2391	gnl|BL_ORD_ID|11724 cb8564_c124026/f1p1/2665 isoform=cb8564_c124026;full_length_coverage=1;non_full_length_coverage=1;isoform_length=2665;expected_accuracy=1.000	15	1662	1663	2240	29	1677	2071	2665
cb5307_c9/f1p0/2902	gnl|BL_ORD_ID|44817 cb5307_c14/f1p0/3080 isoform=cb5307_c14;full_length_coverage=1;non_full_length_coverage=0;isoform_length=3080;expected_accuracy=0.993	1204	2896	1	1204	1307	2998	1	1199
cb18456_c1523/f3p0/1472 isoform=cb18456_c1523;full_length_coverage=3;non_full_length_coverage=0;isoform_length=1472;expected_accuracy=0.994	gnl|BL_ORD_ID|19097 cb8564_c86387/f1p0/3075 isoform=cb8564_c86387;full_length_coverage=1;non_full_length_coverage=0;isoform_length=3075;expected_accuracy=0.995	224	1472	49	225	1617	2869	1	177
cb9823_c2/f1p0/1778	gnl|BL_ORD_ID|19699 cb9823_c0/f1p0/1963 isoform=cb9823_c0;full_length_coverage=1;non_full_le	1	1304	1303	1720	107	1413	1517	1933

	ngth_coverage=0;isoform_length=1963;expected_accuracy=0.992								
cb8564_c5122/f1p0/3329	gnl|BL_ORD_ID|12581 cb8564_c148448/f2p13/3821 isoform=cb8564_c148448;full_length_coverage=2;non_full_length_coverage=13;isoform_length=3821;expected_accuracy=0.995	1067	3334	1	1069	1473	3742	113	1181
cb2493_c40/f1p0/3431	gnl|BL_ORD_ID|36784 cb2493_c80/f1p4/3679 isoform=cb2493_c80;full_length_coverage=1;non_full_length_coverage=4;isoform_length=3679;expected_accuracy=0.995	485	3408	76	488	754	3679	10	438
cb8564_c25105/f3p1/4062 isoform=cb8564_c25105;full_length_coverage=3;non_full_length_coverage=1;isoform_length=4062;expected_accuracy=0.995	gnl|BL_ORD_ID|53968 cb8564_c16162/f1p1/4558	1	3253	3252	4041	77	3329	3593	4382
cb3907_c5/f1p0/2446 isoform=cb3907_c5;full_length_coverage=1;non_full_length_coverage=0;isoform_length=2446;expected_accuracy=0.994	gnl|BL_ORD_ID|71630 cb3907_c9/f1p0/2875	225	2446	1	225	470	2691	26	254
cb1960_c41/f1p1/2517	gnl|BL_ORD_ID|66556 cb1960_c58/f1p0/2977	1	2123	2120	2503	30	2122	2520	2903
cb7209_c12/f1p0/2362	gnl|BL_ORD_ID|7147 cb7209_c20/f1p1/2581 isoform=cb7209_c20;full_length_coverage=1;non_full_length_coverage=1;isoform_length=2581;expected_accuracy=0.996	212	2366	1	211	422	2575	93	313
cb17884_c1/f1p0/1875	gnl|BL_ORD_ID|17831 cb8564_c84290/f1p0/3355 isoform=cb8564_c84290;full_length_coverage=1;non_fu	391	1881	75	394	1872	3355	86	407

	ll_length_coverage=0;isoform_length=3355;expected_accuracy=0.998								
cb7798_c5/f1p0/1815	gnl|BL_ORD_ID|8084 cb7798_c1/f1p0/2530 isoform=cb7798_c1;full_length_coverage=1;non_full_length_coverage=0;isoform_length=2530;expected_accuracy=0.996	1	1452	1450	1809	151	1602	2166	2525
cb8564_c42654/f1p1/2906 isoform=cb8564_c42654;full_length_coverage=1;non_full_length_coverage=1;isoform_length=2906;expected_accuracy=0.990	gnl|BL_ORD_ID|16705 cb8564_c53880/f1p0/3484 isoform=cb8564_c53880;full_length_coverage=1;non_full_length_coverage=0;isoform_length=3484;expected_accuracy=0.996	1031	2824	1	1032	1672	3460	1	1056
cb8564_c43779/f1p0/3021 isoform=cb8564_c43779;full_length_coverage=1;non_full_length_coverage=0;isoform_length=3021;expected_accuracy=0.993	gnl|BL_ORD_ID|16447 cb8564_c47202/f1p0/3195 isoform=cb8564_c47202;full_length_coverage=1;non_full_length_coverage=0;isoform_length=3195;expected_accuracy=0.996	1	2344	2345	3018	1	2345	2501	3195
cb500_c19/f1p1/3637 isoform=cb500_c19;full_length_coverage=1;non_full_length_coverage=1;isoform_length=3637;expected_accuracy=0.996	gnl|BL_ORD_ID|72252 cb500_c85/f1p1/5346	176	3613	1	177	1883	5322	764	941
cb18646_c2/f1p5/6412 isoform=cb18646_c2;full_length_coverage=1;non_full_length_coverage=5;isoform_length=6412;expected_accuracy=0.997	gnl|BL_ORD_ID|34399 cb18646_c1/f2p2/6581 isoform=cb18646_c1;full_length_coverage=2;non_full_length_coverage=2;isoform_length=6581;expected_accuracy=0.999	23	6140	6139	6412	3	6120	6227	6504
cb10263_c1/f1p0/1823	gnl|BL_ORD_ID|19715 cb8960_c0/f3p0/1872 isoform=cb8960_c0;full_length_coverage=3;non_full_length_coverage=0;isoform_length=1872;expected_accuracy=1.000	100	1621	1	103	330	1852	114	218

cb8564_c5070/f1p0/2861	gnl|BL_ORD_ID|11412 cb8564_c147227/f33p12/2882 isoform=cb8564_c147227;full_length_coverage=33;non_full_length_coverage=12;isoform_length=2882;expected_accuracy=1.000	10	1660	1659	2831	58	1708	1601	2772
cb8564_c49540/f1p2/2982	gnl|BL_ORD_ID|56386 cb8564_c75670/f1p2/3240	750	2973	76	747	855	3077	1	672
cb2825_c1/f4p1/2045 isoform=cb2825_c1;full_length_coverage=4;non_full_length_coverage=1;isoform_length=2045;expected_accuracy=1.000	gnl|BL_ORD_ID|38762 cb2825_c3/f2p1/2885 isoform=cb2825_c3;full_length_coverage=2;non_full_length_coverage=1;isoform_length=2885;expected_accuracy=0.997	138	2045	1	139	966	2881	22	157
cb8564_c130435/f1p3/3226 isoform=cb8564_c130435;full_length_coverage=1;non_full_length_coverage=3;isoform_length=3226;expected_accuracy=0.994	gnl|BL_ORD_ID|55939 cb8564_c49630/f1p4/3487	13	2943	2943	3201	7	2913	3243	3493
cb8564_c180116/f1p0/2944 isoform=cb8564_c180116;full_length_coverage=1;non_full_length_coverage=0;isoform_length=2944;expected_accuracy=0.996	gnl|BL_ORD_ID|16886 cb8564_c50727/f1p0/3201 isoform=cb8564_c50727;full_length_coverage=1;non_full_length_coverage=0;isoform_length=3201;expected_accuracy=0.999	2	2522	2520	2944	1	2544	2773	3197
cb8564_c49562/f1p0/2838 isoform=cb8564_c49562;full_length_coverage=1;non_full_length_coverage=0;isoform_length=2838;expected_accuracy=0.995	gnl|BL_ORD_ID|56764 cb8564_c70795/f1p0/3769	23	2331	2327	2838	34	2342	2674	3185
cb10514_c1/f2p0/1767 isoform=cb10514_c1;full_length_coverage=2;non_full_length_coverage=0;isoform_length=1767;expected_accuracy=0.999	gnl|BL_ORD_ID|48607 cb10514_c2/f1p0/1908	1	1142	1141	1767	15	1150	1258	1884

cb18058_c1/f1p0/1872 isoform=cb18058_c1;full_length_coverage=1;non_full_length_coverage=0;isoform_length=1872;expected_accuracy=0.999	gnl|BL_ORD_ID|16779 cb8564_c77829/f1p1/2227 isoform=cb8564_c77829;full_length_coverage=1;non_full_length_coverage=1;isoform_length=2227;expected_accuracy=0.999	205	1844	3	209	590	2227	1	206
cb12714_c29/f1p2/1583	gnl|BL_ORD_ID|52776 cb8564_c13457/f1p1/2717	1	1296	1293	1589	1	1296	2410	2706
cb12893_c39/f1p0/1097 isoform=cb12893_c39;full_length_coverage=1;non_full_length_coverage=0;isoform_length=1097;expected_accuracy=0.996	gnl|BL_ORD_ID|26178 cb12893_c16/f1p5/1413 isoform=cb12893_c16;full_length_coverage=1;non_full_length_coverage=5;isoform_length=1413;expected_accuracy=0.998	234	1095	4	239	490	1365	1	235
cb8564_c125603/f1p0/2366	gnl|BL_ORD_ID|12485 cb8564_c122733/f1p0/3383 isoform=cb8564_c122733;full_length_coverage=1;non_full_length_coverage=0;isoform_length=3383;expected_accuracy=0.993	274	2372	30	276	1289	3383	26	270
cb8564_c127997/f1p3/2802	gnl|BL_ORD_ID|13176 cb8564_c15957/f1p0/2838 isoform=cb8564_c15957;full_length_coverage=1;non_full_length_coverage=0;isoform_length=2838;expected_accuracy=0.994	1	1695	1694	2746	1	1684	1792	2838
cb2356_c20/f1p2/3214 isoform=cb2356_c20;full_length_coverage=1;non_full_length_coverage=2;isoform_length=3214;expected_accuracy=1.000	gnl|BL_ORD_ID|38122 cb2356_c8/f1p0/3478 isoform=cb2356_c8;full_length_coverage=1;non_full_length_coverage=0;isoform_length=3478;expected_accuracy=0.995	1	2372	2369	3147	254	2596	2700	3478
cb1992_c44/f5p1/2983 isoform=cb1992_c44;full_length_coverage=5;non_full_length_coverage=1;isoform_length=2983;expected_accuracy=1.000	gnl|BL_ORD_ID|36459 cb1992_c8/f3p0/3590 isoform=cb1992_c8;full_length_coverage=3;non_full_length_coverage=0;isoform_length=3590;expected_accuracy=0.999	23	2452	2450	2983	1	2430	3055	3589

cb8800_c4/f1p0/1370 isoform=cb8800_c4;full_length_coverage=1;non_full_length_coverage=0;isoform_length=1370;expected_accuracy=0.992	gnl|BL_ORD_ID|19216 cb8800_c3/f1p0/2213 isoform=cb8800_c3;full_length_coverage=1;non_full_length_coverage=0;isoform_length=2213;expected_accuracy=0.993	1	1057	1052	1370	689	174 4	1890	2211
cb5679_c4/f1p0/1908 isoform=cb5679_c4;full_length_coverage=1;non_full_length_coverage=0;isoform_length=1908;expected_accuracy=0.995	gnl|BL_ORD_ID|47066 cb5679_c1/f1p0/2272 isoform=cb5679_c1;full_length_coverage=1;non_full_length_coverage=0;isoform_length=2272;expected_accuracy=1.000	1	1286	1287	1902	43	133 7	1660	227 2
cb8564_c3634/f2p0/3601 isoform=cb8564_c3634;full_length_coverage=2;non_full_length_coverage=0;isoform_length=3601;expected_accuracy=0.999	gnl|BL_ORD_ID|15111 cb8564_c46812/f3p0/4253 isoform=cb8564_c46812;full_length_coverage=3;non_full_length_coverage=0;isoform_length=4253;expected_accuracy=0.993	1	2017	2015	3586	103	212 0	2682	425 0
cb6221_c23/f2p1/1839 isoform=cb6221_c23;full_length_coverage=2;non_full_length_coverage=1;isoform_length=1839;expected_accuracy=0.999	gnl|BL_ORD_ID|49542 cb6221_c14/f1p0/2546	186	1839	4	186	756	2411	3	185
cb4861_c2/f1p0/2642	gnl|BL_ORD_ID|72035 cb4861_c0/f1p0/3233	1	2057	2054	2538	104	213 9	2733	321 8
cb8564_c4742/f1p0/2505 isoform=cb8564_c4742;full_length_coverage=1;non_full_length_coverage=0;isoform_length=2505;expected_accuracy=0.999	gnl|BL_ORD_ID|14698 cb8564_c4069/f2p0/2782 isoform=cb8564_c4069;full_length_coverage=2;non_full_length_coverage=0;isoform_length=2782;expected_accuracy=0.995	1	2086	2084	2473	1	210 3	2393	278 2
cb11147_c1/f1p0/1304 isoform=cb11147_c1;full_length_coverage=1;non_full_length_coverage=0;isoform_length=1304;expected_accuracy=1.000	gnl|BL_ORD_ID|49209 cb11147_c2/f1p0/2053	128	1304	13	132	715	189 7	2	121

cb10255_c12/f1p0/1559 isoform=cb10255_c12;full_length_coverage=1;non_full_length_coverage=0;isoform_length=1559;expected_accuracy=0.992	gnl|BL_ORD_ID|48295 cb10255_c18/f1p0/1844	144	1559	1	147	380	1779	96	242
cb10784_c5/f4p0/1248 isoform=cb10784_c5;full_length_coverage=4;non_full_length_coverage=0;isoform_length=1248;expected_accuracy=0.999	gnl|BL_ORD_ID|324 cb10784_c2/f1p0/2941 isoform=cb10784_c2;full_length_coverage=1;non_full_length_coverage=0;isoform_length=2941;expected_accuracy=0.992	127	1248	8	125	1800	2920	1	118
cb6221_c19/f1p0/1943	gnl|BL_ORD_ID|49543 cb6221_c15/f1p0/2712	247	1873	60	247	900	2529	1	189
cb124_c43/f1p0/2759	gnl|BL_ORD_ID|59063 cb124_c36/f1p0/2750	234	2490	13	233	478	2734	12	232
cb3483_c14/f1p0/2857	gnl|BL_ORD_ID|41441 cb3483_c26/f7p1/2926 isoform=cb3483_c26;full_length_coverage=7;non_full_length_coverage=1;isoform_length=2926;expected_accuracy=1.000	161	2772	10	163	313	2923	2	157
cb8564_c10064/f5p11/3897 isoform=cb8564_c10064;full_length_coverage=5;non_full_length_coverage=11;isoform_length=3897;expected_accuracy=0.996	gnl|BL_ORD_ID|19170 cb8564_c9757/f2p7/4230 isoform=cb8564_c9757;full_length_coverage=2;non_full_length_coverage=7;isoform_length=4230;expected_accuracy=0.999	1	3134	3135	3792	30	3162	3573	4230
cb14664_c3/f1p0/1551 isoform=cb14664_c3;full_length_coverage=1;non_full_length_coverage=0;isoform_length=1551;expected_accuracy=1.000	gnl|BL_ORD_ID|52959 cb8564_c113186/f1p0/2216	162	1551	55	167	692	2081	107	219
cb2737_c42/f1p0/2881	gnl|BL_ORD_ID|68777 cb2737_c19/f1p0/3583	1	1427	1425	2632	779	2205	2337	3544

cb293_c6/f1p0/3758 isoform=cb293_c6;full_length_coverage=1;non_full_length_coverage=0;isoform_length=3758;expected_accuracy=0.997	gnl|BL_ORD_ID|37297 cb293_c2/f1p1/4354 isoform=cb293_c2;full_length_coverage=1;non_full_length_coverage=1;isoform_length=4354;expected_accuracy=0.994	1	1906	1903	3718	32	1936	2554	4354
cb8564_c53418/f1p0/2602 isoform=cb8564_c53418;full_length_coverage=1;non_full_length_coverage=0;isoform_length=2602;expected_accuracy=0.991	gnl|BL_ORD_ID|55614 cb8564_c46839/f1p1/2758	1171	2544	1	1168	1279	2653	2	1170
cb14634_c52/f1p1/4305	gnl|BL_ORD_ID|63292 cb14634_c71/f1p0/5060	1	2658	2653	4320	59	2717	2835	4502
cb8564_c38947/f1p0/2650 isoform=cb8564_c38947;full_length_coverage=1;non_full_length_coverage=0;isoform_length=2650;expected_accuracy=0.996	gnl|BL_ORD_ID|15154 cb8564_c32509/f1p1/2880 isoform=cb8564_c32509;full_length_coverage=1;non_full_length_coverage=1;isoform_length=2880;expected_accuracy=0.991	11	1441	1436	2625	2	1454	1673	2877
cb8417_c0/f14p1/2119 isoform=cb8417_c0;full_length_coverage=14;non_full_length_coverage=1;isoform_length=2119;expected_accuracy=1.000	gnl|BL_ORD_ID|51937 cb8417_c8/f1p1/2695	542	2119	74	543	999	2576	3	472
cb8564_c45787/f1p3/3310	gnl|BL_ORD_ID|55939 cb8564_c49630/f1p4/3487	101	3010	3010	3260	3	2913	3243	3493
cb3309_c10/f1p1/3166	gnl|BL_ORD_ID|40140 cb3309_c4/f1p1/3664 isoform=cb3309_c4;full_length_coverage=1;non_full_length_coverage=1;isoform_length=3664;expected_accuracy=0.995	1467	3166	1	1470	1906	3605	1	1466
cb8564_c36056/f1p0/2652 isoform=cb8564_c36056;full_length_coverage=1;non_f	gnl|BL_ORD_ID|57289 cb8564_c89122/f1p0/3335	248	2639	1	251	523	2915	129	381

ull_length_coverage=0;isoform_length=2652;expected_accuracy=0.999									
cb5583_c0/f4p0/1845 isoform=cb5583_c0;full_length_coverage=4;non_full_length_coverage=0;isoform_length=1845;expected_accuracy=1.000	gnl|BL_ORD_ID|46284 cb5583_c1/f1p0/3059 isoform=cb5583_c1;full_length_coverage=1;non_full_length_coverage=0;isoform_length=3059;expected_accuracy=0.997	132	1780	1	132	1417	3059	1	132
cb14044_c6/f1p0/1650 isoform=cb14044_c6;full_length_coverage=1;non_full_length_coverage=0;isoform_length=1650;expected_accuracy=0.996	gnl|BL_ORD_ID|25840 cb14044_c2/f4p1/1883 isoform=cb14044_c2;full_length_coverage=4;non_full_length_coverage=1;isoform_length=1883;expected_accuracy=1.000	1	1019	1015	1650	1	1030	1164	1800
cb6596_c4/f1p0/1084	gnl|BL_ORD_ID|4473 cb6596_c0/f2p0/2822 isoform=cb6596_c0;full_length_coverage=2;non_full_length_coverage=0;isoform_length=2822;expected_accuracy=0.997	105	1096	1	105	1831	2822	5	110
cb2737_c45/f1p0/2944	gnl|BL_ORD_ID|38134 cb2737_c22/f1p0/3196 isoform=cb2737_c22;full_length_coverage=1;non_full_length_coverage=0;isoform_length=3196;expected_accuracy=0.995	1	1476	1474	2756	301	1763	1894	3161
cb1648_c4/f10p3/2031 isoform=cb1648_c4;full_length_coverage=10;non_full_length_coverage=3;isoform_length=2031;expected_accuracy=1.000	gnl|BL_ORD_ID|32503 cb1648_c15/f1p0/2133 isoform=cb1648_c15;full_length_coverage=1;non_full_length_coverage=0;isoform_length=2133;expected_accuracy=1.000	234	2030	26	234	337	2133	1	195
cb1772_c6/f2p1/3674 isoform=cb1772_c6;full_length_coverage=2;non_full_length_coverage=1;isoform_length=3674;expected_accuracy=1.000	gnl|BL_ORD_ID|66372 cb1772_c16/f1p1/4029	216	3674	1	218	430	3901	44	246

cb8564_c46939/f1p1/2926	gnl|BL_ORD_ID|13575 cb8564_c20499/f3p0/3328 isoform=cb8564_c20499;full_length_coverage=3;non_full_length_coverage=0;isoform_length=3328;expected_accuracy=1.000	1	2134	2130	2870	1	2144	2249	2990
cb8564_c4971/f2p0/2890 isoform=cb8564_c4971;full_length_coverage=2;non_full_length_coverage=0;isoform_length=2890;expected_accuracy=1.000	gnl|BL_ORD_ID|15755 cb8564_c4970/f1p0/3133 isoform=cb8564_c4970;full_length_coverage=1;non_full_length_coverage=0;isoform_length=3133;expected_accuracy=0.996	105	2890	1	106	292	3051	5	111
cb8564_c36689/f1p1/2368 isoform=cb8564_c36689;full_length_coverage=1;non_full_length_coverage=1;isoform_length=2368;expected_accuracy=0.991	gnl|BL_ORD_ID|55170 cb8564_c34431/f1p0/3665	1	1709	1706	2368	4	1712	2511	3175
cb2728_c6/f1p0/2834 isoform=cb2728_c6;full_length_coverage=1;non_full_length_coverage=0;isoform_length=2834;expected_accuracy=0.999	gnl|BL_ORD_ID|38993 cb2728_c3/f1p0/3818 isoform=cb2728_c3;full_length_coverage=1;non_full_length_coverage=0;isoform_length=3818;expected_accuracy=0.991	589	2834	7	593	1546	3786	146	735
cb8564_c42091/f1p0/3606 isoform=cb8564_c42091;full_length_coverage=1;non_full_length_coverage=0;isoform_length=3606;expected_accuracy=0.998	gnl|BL_ORD_ID|16529 cb8564_c48301/f1p2/3934 isoform=cb8564_c48301;full_length_coverage=1;non_full_length_coverage=2;isoform_length=3934;expected_accuracy=0.990	1	2325	2324	3606	1	2325	2618	3905
cb5125_c34/f1p0/1890	gnl|BL_ORD_ID|72116 cb5125_c21/f1p3/2083	1	1145	1144	1780	34	1178	1370	2005
cb2071_c1/f5p0/2098 isoform=cb2071_c1;full_length_coverage=5;non_full_length_coverage=0;isoform_length=2098;expected_accuracy=1.000	gnl|BL_ORD_ID|66664 cb2071_c6/f1p0/4059	1	1118	1113	2019	35	1158	3156	4061

<table>
<tr><td>cb8909_c12/f1p0/1836 isoform=cb8909_c12;full_length_coverage=1;non_full_length_coverage=0;isoform_length=1836;expected_accuracy=1.000</td><td>gnl|BL_ORD_ID|59367 cb8909_c4/f1p0/2185</td><td>1</td><td>1624</td><td>1620</td><td>1836</td><td>51</td><td>1667</td><td>1768</td><td>1981</td></tr>
<tr><td>cb3010_c5/f2p0/2793 isoform=cb3010_c5;full_length_coverage=2;non_full_length_coverage=0;isoform_length=2793;expected_accuracy=1.000</td><td>gnl|BL_ORD_ID|70131 cb3010_c17/f1p1/3095</td><td>1307</td><td>2793</td><td>1</td><td>1308</td><td>1442</td><td>2928</td><td>4</td><td>1311</td></tr>
<tr><td>cb1585_c21/f1p1/1983</td><td>gnl|BL_ORD_ID|27809 cb1585_c10/f1p0/2444 isoform=cb1585_c10;full_length_coverage=1;non_full_length_coverage=0;isoform_length=2444;expected_accuracy=1.000</td><td>1</td><td>1456</td><td>1455</td><td>1953</td><td>17</td><td>1473</td><td>1946</td><td>2444</td></tr>
<tr><td>cb792_c31/f1p0/3278 isoform=cb792_c31;full_length_coverage=1;non_full_length_coverage=0;isoform_length=3278;expected_accuracy=0.993</td><td>gnl|BL_ORD_ID|49470 cb792_c35/f1p1/3973</td><td>566</td><td>3278</td><td>7</td><td>567</td><td>1168</td><td>3887</td><td>6</td><td>569</td></tr>
<tr><td>cb5270_c33/f1p0/2572</td><td>gnl|BL_ORD_ID|73713 cb5270_c16/f1p0/2707</td><td>1098</td><td>2566</td><td>1</td><td>1097</td><td>1199</td><td>2670</td><td>1</td><td>1096</td></tr>
<tr><td>cb5422_c6/f1p0/1857</td><td>gnl|BL_ORD_ID|45247 cb5422_c12/f1p0/5493 isoform=cb5422_c12;full_length_coverage=1;non_full_length_coverage=0;isoform_length=5493;expected_accuracy=0.993</td><td>1</td><td>1128</td><td>1127</td><td>1788</td><td>234</td><td>1357</td><td>4836</td><td>5493</td></tr>
<tr><td>cb8564_c5271/f1p0/3205</td><td>gnl|BL_ORD_ID|17728 cb8564_c87595/f1p0/4323 isoform=cb8564_c87595;full_length_coverage=1;non_full_length_coverage=0;isoform_length=4323;expected_accuracy=0.997</td><td>259</td><td>3193</td><td>1</td><td>261</td><td>1395</td><td>4323</td><td>1</td><td>259</td></tr>
</table>

cb317_c5/f1p0/3451 isoform=cb317_c5;full_length_coverage=1;non_full_length_coverage=0;isoform_length=3451;expected_accuracy=0.998	gnl|BL_ORD_ID|37312 cb317_c14/f1p0/4548 isoform=cb317_c14;full_length_coverage=1;non_full_length_coverage=0;isoform_length=4548;expected_accuracy=0.992	1	3038	3033	3317	28	3053	3278	3562
cb313_c22/f1p0/3103	gnl|BL_ORD_ID|38275 cb313_c7/f1p0/4354 isoform=cb313_c7;full_length_coverage=1;non_full_length_coverage=0;isoform_length=4354;expected_accuracy=0.994	1	1950	1948	2985	1	1945	3217	4253
cb8564_c84473/f1p0/2481	gnl|BL_ORD_ID|57836 cb8564_c82188/f1p1/2590	1109	2426	1	1113	1279	2596	30	1145
cb8564_c35093/f1p5/2916	gnl|BL_ORD_ID|14410 cb8564_c44256/f1p1/3267 isoform=cb8564_c44256;full_length_coverage=1;non_full_length_coverage=1;isoform_length=3267;expected_accuracy=0.995	20	2169	2165	2881	2	2119	2553	3267
cb6646_c13/f1p0/2790	gnl|BL_ORD_ID|3729 cb6646_c22/f1p1/2606 isoform=cb6646_c22;full_length_coverage=1;non_full_length_coverage=1;isoform_length=2606;expected_accuracy=0.996	314	2386	53	315	380	2452	1	263
cb1822_c15/f1p0/1826 isoform=cb1822_c15;full_length_coverage=1;non_full_length_coverage=0;isoform_length=1826;expected_accuracy=0.998	gnl|BL_ORD_ID|32354 cb1822_c17/f1p0/5987 isoform=cb1822_c17;full_length_coverage=1;non_full_length_coverage=0;isoform_length=5987;expected_accuracy=0.997	1	1676	1671	1826	769	2449	5178	5333
cb2831_c4/f3p1/2824 isoform=cb2831_c4;full_length_coverage=3;non_full_length_coverage=1;isoform_length=2824;expected_accuracy=0.998	gnl|BL_ORD_ID|68914 cb2831_c15/f1p0/3921	124	2352	2353	2808	2	2230	3098	3553

<table>
<tr><td>cb8564_c107982/f2p0/2940 isoform=cb8564_c107982;full_length_coverage=2;non_full_length_coverage=0;isoform_length=2940;expected_accuracy=0.998</td><td>gnl|BL_ORD_ID|16997 cb8564_c75534/f1p0/3028 isoform=cb8564_c75534;full_length_coverage=1;non_full_length_coverage=0;isoform_length=3028;expected_accuracy=0.994</td><td>17</td><td>1784</td><td>1785</td><td>2909</td><td>21</td><td>1796</td><td>1904</td><td>3028</td></tr>
<tr><td>cb5290_c64/f1p0/3128</td><td>gnl|BL_ORD_ID|73746 cb5290_c79/f1p0/2804</td><td>15</td><td>1748</td><td>1747</td><td>2651</td><td>26</td><td>1759</td><td>1885</td><td>2790</td></tr>
<tr><td>cb8564_c41162/f1p0/3158</td><td>gnl|BL_ORD_ID|56764 cb8564_c70795/f1p0/3769</td><td>23</td><td>2553</td><td>2548</td><td>3040</td><td>34</td><td>2568</td><td>2699</td><td>3187</td></tr>
<tr><td>cb7771_c4/f1p0/2059 isoform=cb7771_c4;full_length_coverage=1;non_full_length_coverage=0;isoform_length=2059;expected_accuracy=1.000</td><td>gnl|BL_ORD_ID|7436 cb7771_c3/f1p0/2176 isoform=cb7771_c3;full_length_coverage=1;non_full_length_coverage=0;isoform_length=2176;expected_accuracy=0.994</td><td>1</td><td>1351</td><td>1349</td><td>2059</td><td>16</td><td>1329</td><td>1432</td><td>2139</td></tr>
<tr><td>cb8564_c45699/f1p0/3005</td><td>gnl|BL_ORD_ID|14407 cb8564_c44036/f1p0/3258 isoform=cb8564_c44036;full_length_coverage=1;non_full_length_coverage=0;isoform_length=3258;expected_accuracy=0.992</td><td>1161</td><td>2844</td><td>1</td><td>1161</td><td>1575</td><td>3258</td><td>1</td><td>1147</td></tr>
<tr><td>cb8564_c48235/f1p1/2252</td><td>gnl|BL_ORD_ID|15623 cb8564_c51515/f1p1/2999 isoform=cb8564_c51515;full_length_coverage=1;non_full_length_coverage=1;isoform_length=2999;expected_accuracy=0.992</td><td>1</td><td>1600</td><td>1596</td><td>2234</td><td>4</td><td>1588</td><td>2367</td><td>2999</td></tr>
<tr><td>cb12931_c15/f1p0/1493 isoform=cb12931_c15;full_length_coverage=1;non_full_length_coverage=0;isoform_length=1493;expected_accuracy=1.000</td><td>gnl|BL_ORD_ID|56601 cb8564_c48153/f1p0/3083</td><td>1</td><td>1106</td><td>1104</td><td>1492</td><td>13</td><td>1109</td><td>2491</td><td>2879</td></tr>
<tr><td>cb666_c12/f1p0/3434 isoform=cb666_c12;full_length_coverage=1;non_full_le</td><td>gnl|BL_ORD_ID|47188 cb666_c0/f1p0/4630</td><td>334</td><td>3434</td><td>1</td><td>334</td><td>1444</td><td>4578</td><td>133</td><td>467</td></tr>
</table>

ngth_coverage=0;isoform_length=3434;expected_accuracy=0.991									
cb2252_c0/f2p0/1094 isoform=cb2252_c0;full_length_coverage=2;non_full_length_coverage=0;isoform_length=1094;expected_accuracy=1.000	gnl|BL_ORD_ID|69656 cb2252_c1/f1p0/4037	367	1094	7	367	3052	3779	4	363
cb6875_c10/f1p0/1769	gnl|BL_ORD_ID|5625 cb6875_c5/f1p0/2662 isoform=cb6875_c5;full_length_coverage=1;non_full_length_coverage=0;isoform_length=2662;expected_accuracy=0.991	417	1743	55	417	1278	2600	1	365
cb8564_c24281/f1p0/3435	gnl|BL_ORD_ID|15789 cb8564_c58034/f2p2/3989 isoform=cb8564_c58034;full_length_coverage=2;non_full_length_coverage=2;isoform_length=3989;expected_accuracy=0.996	15	3170	3166	3284	27	3169	3288	3404
cb8564_c15376/f1p0/4060	gnl|BL_ORD_ID|9876 cb8564_c10064/f5p11/3897 isoform=cb8564_c10064;full_length_coverage=5;non_full_length_coverage=11;isoform_length=3897;expected_accuracy=0.996	12	2717	2717	3675	10	2712	2816	3774
cb8564_c44063/f1p0/2503 isoform=cb8564_c44063;full_length_coverage=1;non_full_length_coverage=0;isoform_length=2503;expected_accuracy=0.999	gnl|BL_ORD_ID|55114 cb8564_c24784/f1p1/2695	398	2446	4	402	551	2599	9	408
cb2747_c15/f1p2/3376 isoform=cb2747_c15;full_length_coverage=1;non_full_length_coverage=2;isoform_length=3376;expected_accuracy=0.994	gnl|BL_ORD_ID|68803 cb2747_c3/f1p0/3786	13	2335	2331	3286	4	2320	2741	3714

cb8564_c51661/f1p0/3132	gnl|BL_ORD_ID|55544 cb8564_c44764/f1p0/3516	1	2104	2099	3077	47	2151	2308	3286
cb3383_c3/f1p0/3067 isoform=cb3383_c3;full_length_coverage=1;non_full_length_coverage=0;isoform_length=3067;expected_accuracy=0.996	gnl|BL_ORD_ID|71165 cb3383_c2/f1p0/3641	1	2046	2045	3065	44	2077	2217	3235
cb4452_c2/f3p0/1957 isoform=cb4452_c2;full_length_coverage=3;non_full_length_coverage=0;isoform_length=1957;expected_accuracy=1.000	gnl|BL_ORD_ID|72573 cb4452_c6/f1p0/3034	1	1509	1507	1931	91	1600	2600	3023
cb292_c11/f1p1/4167	gnl|BL_ORD_ID|38399 cb292_c6/f1p0/4480 isoform=cb292_c6;full_length_coverage=1;non_full_length_coverage=0;isoform_length=4480;expected_accuracy=0.997	104	2211	2207	4142	1	2108	2546	4480
cb8564_c146072/f3p3/2176 isoform=cb8564_c146072;full_length_coverage=3;non_full_length_coverage=3;isoform_length=2176;expected_accuracy=1.000	gnl|BL_ORD_ID|54238 cb8564_c21052/f1p2/3145	213	2165	1	215	1105	3056	28	249
cb8564_c40598/f1p4/3119	gnl|BL_ORD_ID|55910 cb8564_c48645/f1p1/3287	12	2732	2729	2988	17	2733	2854	3113
cb8564_c188097/f1p0/2552 isoform=cb8564_c188097;full_length_coverage=1;non_full_length_coverage=0;isoform_length=2552;expected_accuracy=0.996	gnl|BL_ORD_ID|58179 cb8564_c90747/f1p0/2795	1	1945	1940	2552	1	1918	2021	2633
cb5271_c13/f1p0/2572 isoform=cb5271_c13;full_length_coverage=1;non_full_	gnl|BL_ORD_ID|73722 cb5271_c15/f1p0/2907	1042	2553	1	1044	1245	2758	100	1142

length_coverage=0;isoform_length=2572;expected_accuracy=0.993									
cb8564_c87022/f1p0/2331 isoform=cb8564_c87022;full_length_coverage=1;non_full_length_coverage=0;isoform_length=2331;expected_accuracy=0.994	gnl|BL_ORD_ID|17359 cb8564_c89607/f1p0/2600 isoform=cb8564_c89607;full_length_coverage=1;non_full_length_coverage=0;isoform_length=2600;expected_accuracy=1.000	1	1183	1182	2330	1	1183	1441	2600
cb5874_c15/f1p0/2254	gnl|BL_ORD_ID|45911 cb5874_c11/f2p1/2975 isoform=cb5874_c11;full_length_coverage=2;non_full_length_coverage=1;isoform_length=2975;expected_accuracy=1.000	225	2249	1	224	832	2856	409	632
cb8564_c180116/f1p0/2944 isoform=cb8564_c180116;full_length_coverage=1;non_full_length_coverage=0;isoform_length=2944;expected_accuracy=0.996	gnl|BL_ORD_ID|57017 cb8564_c80370/f1p0/3493	123	2522	2520	2944	3085	686	457	33
cb8564_c36056/f1p0/2652 isoform=cb8564_c36056;full_length_coverage=1;non_full_length_coverage=0;isoform_length=2652;expected_accuracy=0.999	gnl|BL_ORD_ID|56601 cb8564_c48153/f1p0/3083	248	2651	1	251	474	2879	82	330
cb4795_c3/f1p0/1980 isoform=cb4795_c3;full_length_coverage=1;non_full_length_coverage=0;isoform_length=1980;expected_accuracy=0.999	gnl|BL_ORD_ID|43899 cb4795_c2/f1p0/3264 isoform=cb4795_c2;full_length_coverage=1;non_full_length_coverage=0;isoform_length=3264;expected_accuracy=0.998	262	1980	45	262	1546	3264	112	329
cb3747_c11/f1p0/2520 isoform=cb3747_c11;full_length_coverage=1;non_full_length_coverage=0;isoform_length=2520;expected_accuracy=0.994	gnl|BL_ORD_ID|70583 cb3747_c16/f1p1/3060	1	1743	1742	2520	71	1813	2046	2824

cb500_c43/f1p0/3109 isoform=cb500_c43;full_length_coverage=1;non_full_length_coverage=0;isoform_length=3109;expected_accuracy=0.992	gnl|BL_ORD_ID|72248 cb500_c53/f1p0/3672	13	2306	2303	3109	112	2414	2546	3353
cb8564_c37936/f3p0/3317 isoform=cb8564_c37936;full_length_coverage=3;non_full_length_coverage=0;isoform_length=3317;expected_accuracy=0.998	gnl|BL_ORD_ID|55806 cb8564_c38998/f1p0/4043	25	2554	2553	3205	1	2527	3096	3748
cb8564_c4643/f1p1/2554	gnl|BL_ORD_ID|56319 cb8564_c74283/f1p0/3024	1	1705	1700	2542	13	1717	1850	2692
cb9667_c4/f1p0/1542 isoform=cb9667_c4;full_length_coverage=1;non_full_length_coverage=0;isoform_length=1542;expected_accuracy=1.000	gnl|BL_ORD_ID|22169 cb9667_c5/f7p0/1958 isoform=cb9667_c5;full_length_coverage=7;non_full_length_coverage=0;isoform_length=1958;expected_accuracy=0.999	1	1231	1231	1542	1	1232	1646	1958
cb8564_c121435/f1p0/2218 isoform=cb8564_c121435;full_length_coverage=1;non_full_length_coverage=0;isoform_length=2218;expected_accuracy=0.997	gnl|BL_ORD_ID|55611 cb8564_c46736/f1p1/2681	12	1691	1693	2150	30	1705	2092	2550
cb1317_c16/f1p1/2428	gnl|BL_ORD_ID|61229 cb1317_c8/f1p0/4195	229	2361	1	227	2167	4297	9	232
cb841_c30/f1p1/3368 isoform=cb841_c30;full_length_coverage=1;non_full_length_coverage=1;isoform_length=3368;expected_accuracy=0.997	gnl|BL_ORD_ID|9504 cb841_c6/f1p1/4702 isoform=cb841_c6;full_length_coverage=1;non_full_length_coverage=1;isoform_length=4702;expected_accuracy=0.993	1	3072	3073	3368	1	3064	3200	3495
cb8564_c34638/f1p0/2817 isoform=cb8564_c34638;full_length_coverage=1;non_f	gnl|BL_ORD_ID|16172 cb8564_c53024/f1p0/3159 isoform=cb8564_c53024;full_length_coverage=1;non_fu	1	1690	1687	2817	1	1699	2017	3157

ull_length_coverage=0;isoform_length=2817;expected_accuracy=0.992	ll_length_coverage=0;isoform_length=3159;expected_accuracy=0.996								
cb8971_c13/f1p6/3627 isoform=cb8971_c13;full_length_coverage=1;non_full_length_coverage=6;isoform_length=3627;expected_accuracy=0.996	gnl|BL_ORD_ID|19877 cb8971_c6/f1p2/4360 isoform=cb8971_c6;full_length_coverage=1;non_full_length_coverage=2;isoform_length=4360;expected_accuracy=0.997	1	2329	2326	3627	23	2344	3052	4350
cb9696_c0/f1p0/1994 isoform=cb9696_c0;full_length_coverage=1;non_full_length_coverage=0;isoform_length=1994;expected_accuracy=1.000	gnl|BL_ORD_ID|58853 cb9696_c1/f1p0/2146	1	1363	1360	1993	39	1401	1524	2154
cb4273_c3/f1p0/3269 isoform=cb4273_c3;full_length_coverage=1;non_full_length_coverage=0;isoform_length=3269;expected_accuracy=1.000	gnl|BL_ORD_ID|72419 cb4273_c1/f2p0/3358	107	3183	1	106	278	3361	1	104
cb4889_c22/f1p0/2625 isoform=cb4889_c22;full_length_coverage=1;non_full_length_coverage=0;isoform_length=2625;expected_accuracy=0.995	gnl|BL_ORD_ID|73050 cb4889_c18/f1p4/2830	280	2622	29	285	425	2767	1	257
cb8564_c75453/f1p2/2270 isoform=cb8564_c75453;full_length_coverage=1;non_full_length_coverage=2;isoform_length=2270;expected_accuracy=0.996	gnl|BL_ORD_ID|57038 cb8564_c81286/f1p2/3636	265	2270	84	267	1340	3344	859	1042
cb7696_c12/f1p1/1985	gnl|BL_ORD_ID|50658 cb7696_c6/f1p0/2680	1	1746	1748	1980	4	1749	2076	2308
cb3482_c32/f1p0/1683 isoform=cb3482_c32;full_length_coverage=1;non_full_	gnl|BL_ORD_ID|40350 cb3482_c6/f7p2/2166 isoform=cb3482_c6;full_length_coverage=7;non_full_le	1	1586	1581	1683	110	1710	1989	2091

length_coverage=0;isoform_length=1683;expected_accuracy=1.000	ngth_coverage=2;isoform_length=2166;expected_accuracy=0.997								
cb5643_c42/f1p0/3067	gnl|BL_ORD_ID|45379 cb5643_c25/f1p0/3322 isoform=cb5643_c25;full_length_coverage=1;non_full_length_coverage=0;isoform_length=3322;expected_accuracy=0.998	109	2631	1	111	799	3322	1	111
cb8564_c21572/f1p1/3061 isoform=cb8564_c21572;full_length_coverage=1;non_full_length_coverage=1;isoform_length=3061;expected_accuracy=0.991	gnl|BL_ORD_ID|17336 cb8564_c9188/f4p3/3321 isoform=cb8564_c9188;full_length_coverage=4;non_full_length_coverage=3;isoform_length=3321;expected_accuracy=0.998	1127	3061	1	1125	1365	3305	85	1210
cb8564_c46939/f1p1/2926	gnl|BL_ORD_ID|15924 cb8564_c52804/f1p5/3221 isoform=cb8564_c52804;full_length_coverage=1;non_full_length_coverage=5;isoform_length=3221;expected_accuracy=0.999	1	2134	2130	2870	1	2142	2247	2988
cb8564_c13598/f1p0/3018 isoform=cb8564_c13598;full_length_coverage=1;non_full_length_coverage=0;isoform_length=3018;expected_accuracy=0.997	gnl|BL_ORD_ID|9831 cb8564_c106778/f15p6/3278 isoform=cb8564_c106778;full_length_coverage=15;non_full_length_coverage=6;isoform_length=3278;expected_accuracy=1.000	175	3016	1	180	401	3242	2	181
cb1142_c17/f1p1/2208	gnl|BL_ORD_ID|22503 cb1142_c24/f9p2/2781 isoform=cb1142_c24;full_length_coverage=9;non_full_length_coverage=2;isoform_length=2781;expected_accuracy=1.000	1	1127	1123	2211	52	1178	1600	2688
cb8564_c37409/f1p0/3153	gnl|BL_ORD_ID|17429 cb8564_c85447/f1p0/3196 isoform=cb8564_c85447;full_length_coverage=1;non_full_length_coverage=0;isoform_length=3196;expected_accuracy=0.997	1	2414	2413	2948	3	2413	2660	3191

cb8564_c40107/f1p0/4108	gnl|BL_ORD_ID|15822 cb8564_c50372/f4p2/4090 isoform=cb8564_c50372;full_length_coverage=4;non_full_length_coverage=2;isoform_length=4090;expected_accuracy=0.996	1603	3955	1	1599	1707	4059	235	1827
cb396_c18/f1p0/2281 isoform=cb396_c18;full_length_coverage=1;non_full_length_coverage=0;isoform_length=2281;expected_accuracy=0.999	gnl|BL_ORD_ID|39231 cb396_c67/f28p0/2542 isoform=cb396_c67;full_length_coverage=28;non_full_length_coverage=0;isoform_length=2542;expected_accuracy=1.000	250	2275	58	251	523	2542	1	191
cb8564_c111744/f1p1/2111 isoform=cb8564_c111744;full_length_coverage=1;non_full_length_coverage=1;isoform_length=2111;expected_accuracy=0.998	gnl|BL_ORD_ID|12654 cb8564_c126593/f1p2/2518 isoform=cb8564_c126593;full_length_coverage=1;non_full_length_coverage=2;isoform_length=2518;expected_accuracy=0.992	1	1639	1635	2111	49	1683	1856	2331
cb8564_c15653/f1p4/2475	gnl|BL_ORD_ID|9475 cb8564_c108093/f2p11/2745 isoform=cb8564_c108093;full_length_coverage=2;non_full_length_coverage=11;isoform_length=2745;expected_accuracy=0.998	1	2293	2289	2478	11	2307	2526	2738
cb3712_c4/f1p1/2242 isoform=cb3712_c4;full_length_coverage=1;non_full_length_coverage=1;isoform_length=2242;expected_accuracy=0.995	gnl|BL_ORD_ID|71460 cb3712_c5/f1p1/2972	272	2235	30	274	861	2821	1	243
cb6696_c3/f1p0/1462	gnl|BL_ORD_ID|47655 cb6696_c0/f1p0/2790	383	1461	6	384	1683	2761	1	380
cb7550_c8/f1p0/1702	gnl|BL_ORD_ID|6825 cb7550_c0/f3p1/1956 isoform=cb7550_c0;full_length_coverage=3;non_full_length_coverage=1;isoform_length=1956;expected_accuracy=1.000	1	1177	1174	1652	45	1223	1432	1910

cb8564_c106924/f3p13/3304 isoform=cb8564_c106924;full_length_coverage=3;non_full_length_coverage=13;isoform_length=3304;expected_accuracy=1.000	gnl|BL_ORD_ID|14377 cb8564_c36097/f1p2/3430 isoform=cb8564_c36097;full_length_coverage=1;non_full_length_coverage=2;isoform_length=3430;expected_accuracy=0.996	156	1895	1895	3304	130	1865	1995	3400
cb3941_c92/f1p0/2573	gnl|BL_ORD_ID|70694 cb3941_c42/f1p0/2695	136	2167	10	141	669	2700	46	177
cb3436_c2/f1p0/2155 isoform=cb3436_c2;full_length_coverage=1;non_full_length_coverage=0;isoform_length=2155;expected_accuracy=0.997	gnl|BL_ORD_ID|41176 cb3436_c1/f1p0/3048 isoform=cb3436_c1;full_length_coverage=1;non_full_length_coverage=0;isoform_length=3048;expected_accuracy=1.000	1	1429	1426	2151	12	1438	2321	3046
cb8564_c40764/f1p0/2563 isoform=cb8564_c40764;full_length_coverage=1;non_full_length_coverage=0;isoform_length=2563;expected_accuracy=0.997	gnl|BL_ORD_ID|13312 cb8564_c188074/f1p0/2869 isoform=cb8564_c188074;full_length_coverage=1;non_full_length_coverage=0;isoform_length=2869;expected_accuracy=0.996	1	2161	2162	2563	1	2158	2287	2688
cb8564_c68409/f2p0/2141 isoform=cb8564_c68409;full_length_coverage=2;non_full_length_coverage=0;isoform_length=2141;expected_accuracy=0.995	gnl|BL_ORD_ID|58113 cb8564_c89263/f1p0/2300	264	2124	53	266	352	2213	1	214
cb12972_c35/f1p0/1854	gnl|BL_ORD_ID|14141 cb8564_c2622/f1p1/2613 isoform=cb8564_c2622;full_length_coverage=1;non_full_length_coverage=1;isoform_length=2613;expected_accuracy=0.996	247	1729	45	248	1054	2523	1	204
cb8564_c48551/f1p0/2454 isoform=cb8564_c48551;full_length_coverage=1;non_full_length_coverage=0;isoform_length=2454;expected_accuracy=0.995	gnl|BL_ORD_ID|54752 cb8564_c167108/f1p0/2919	1	1438	1438	2454	105	1541	1811	2828

cb14420_c0/f4p0/1483 isoform=cb14420_c0;full_length_coverage=4;non_full_length_coverage=0;isoform_length=1483;expected_accuracy=1.000	gnl|BL_ORD_ID|11669 cb8564_c124605/f1p0/2372 isoform=cb8564_c124605;full_length_coverage=1;non_full_length_coverage=0;isoform_length=2372;expected_accuracy=0.999	595	1483	6	596	1484	2372	1	592
cb1913_c44/f1p0/2236 isoform=cb1913_c44;full_length_coverage=1;non_full_length_coverage=0;isoform_length=2236;expected_accuracy=0.998	gnl|BL_ORD_ID|66488 cb1913_c37/f1p0/2644	1	1634	1630	2236	115	1747	1881	2487
cb8564_c178226/f1p0/2728 isoform=cb8564_c178226;full_length_coverage=1;non_full_length_coverage=0;isoform_length=2728;expected_accuracy=0.998	gnl|BL_ORD_ID|55413 cb8564_c40978/f1p0/3073	1284	2728	1	1288	1402	2847	1	1298
cb8564_c34047/f2p0/3175 isoform=cb8564_c34047;full_length_coverage=2;non_full_length_coverage=0;isoform_length=3175;expected_accuracy=1.000	gnl|BL_ORD_ID|15354 cb8564_c45229/f4p1/3393 isoform=cb8564_c45229;full_length_coverage=4;non_full_length_coverage=1;isoform_length=3393;expected_accuracy=0.999	1	2174	2171	3121	149	2321	2441	3393
cb16375_c1/f1p0/1380 isoform=cb16375_c1;full_length_coverage=1;non_full_length_coverage=0;isoform_length=1380;expected_accuracy=0.999	gnl|BL_ORD_ID|16832 cb8564_c48460/f1p0/2462 isoform=cb8564_c48460;full_length_coverage=1;non_full_length_coverage=0;isoform_length=2462;expected_accuracy=0.996	1	1051	1050	1380	93	1143	2048	2375
cb8564_c21767/f1p1/2839 isoform=cb8564_c21767;full_length_coverage=1;non_full_length_coverage=1;isoform_length=2839;expected_accuracy=0.996	gnl|BL_ORD_ID|11214 cb8564_c146144/f12p2/3048 isoform=cb8564_c146144;full_length_coverage=12;non_full_length_coverage=2;isoform_length=3048;expected_accuracy=0.999	1040	2839	1	1040	1244	3047	57	1097
cb3586_c3/f1p0/2988 isoform=cb3586_c3;full_length_coverage=1;non_full_le	gnl|BL_ORD_ID|71356 cb3586_c9/f1p0/3463	292	2988	1	295	763	3471	4	301

ngth_coverage=0;isoform_length=2988;expected_accuracy=0.993									
cb9674_c0/f4p2/2017 isoform=cb9674_c0;full_length_coverage=4;non_full_length_coverage=2;isoform_length=2017;expected_accuracy=1.000	gnl|BL_ORD_ID|20444 cb9674_c9/f1p0/2070 isoform=cb9674_c9;full_length_coverage=1;non_full_length_coverage=0;isoform_length=2070;expected_accuracy=0.996	1	1134	1135	1974	1	1131	1231	2070
cb90_c17/f1p1/4529 isoform=cb90_c17;full_length_coverage=1;non_full_length_coverage=1;isoform_length=4529;expected_accuracy=0.992	gnl|BL_ORD_ID|59041 cb90_c48/f1p0/5124	350	4494	2	349	602	4743	17	367
cb8564_c34212/f2p0/3637 isoform=cb8564_c34212;full_length_coverage=2;non_full_length_coverage=0;isoform_length=3637;expected_accuracy=1.000	gnl|BL_ORD_ID|55975 cb8564_c50843/f1p0/4165	2	3244	3243	3637	8	3252	3639	4034
cb8564_c4758/f1p0/2319	gnl|BL_ORD_ID|14468 cb8564_c38219/f2p2/2476 isoform=cb8564_c38219;full_length_coverage=2;non_full_length_coverage=2;isoform_length=2476;expected_accuracy=0.996	14	1703	1700	2224	1	1702	1960	2476
cb8564_c35935/f1p0/4149 isoform=cb8564_c35935;full_length_coverage=1;non_full_length_coverage=0;isoform_length=4149;expected_accuracy=0.993	gnl|BL_ORD_ID|14314 cb8564_c45827/f2p2/4435 isoform=cb8564_c45827;full_length_coverage=2;non_full_length_coverage=2;isoform_length=4435;expected_accuracy=0.999	15	3258	3254	4149	1	3255	3520	4421
cb313_c5/f2p0/2828 isoform=cb313_c5;full_length_coverage=2;non_full_length_coverage=0;isoform_length=2828;expected_accuracy=0.997	gnl|BL_ORD_ID|36627 cb313_c24/f1p0/3081 isoform=cb313_c24;full_length_coverage=1;non_full_length_coverage=0;isoform_length=3081;expected_accuracy=0.995	1	2547	2545	2808	143	2688	2818	3081

cb12_c37/f1p0/4067	gnl|BL_ORD_ID|61080 cb12_c15/f1p1/4774	286	4080	186	289	747	4549	450	553
cb8564_c36635/f1p0/4030 isoform=cb8564_c36635;full_length_coverage=1;non_full_length_coverage=0;isoform_length=4030;expected_accuracy=0.998	gnl|BL_ORD_ID|55207 cb8564_c35399/f1p0/4553	184	4030	10	189	437	4284	11	184
cb2753_c18/f1p0/2859	gnl|BL_ORD_ID|37926 cb2753_c3/f3p1/3689 isoform=cb2753_c3;full_length_coverage=3;non_full_length_coverage=1;isoform_length=3689;expected_accuracy=0.997	1	2193	2191	2700	687	2885	3169	3679
cb7320_c3/f3p2/2470 isoform=cb7320_c3;full_length_coverage=3;non_full_length_coverage=2;isoform_length=2470;expected_accuracy=0.999	gnl|BL_ORD_ID|7016 cb7320_c7/f1p1/2801 isoform=cb7320_c7;full_length_coverage=1;non_full_length_coverage=1;isoform_length=2801;expected_accuracy=0.995	1241	2469	116	1241	1573	2801	13	1137
cb8564_c3788/f1p1/2424	gnl|BL_ORD_ID|14097 cb8564_c3797/f2p1/2809 isoform=cb8564_c3797;full_length_coverage=2;non_full_length_coverage=1;isoform_length=2809;expected_accuracy=0.996	1	2114	2114	2382	281	2395	2541	2809
cb8564_c44734/f1p1/3476 isoform=cb8564_c44734;full_length_coverage=1;non_full_length_coverage=1;isoform_length=3476;expected_accuracy=0.990	gnl|BL_ORD_ID|68032 cb18809_c1/f1p0/5493	276	3476	17	275	2193	5413	1	256
cb8564_c35935/f1p0/4149 isoform=cb8564_c35935;full_length_coverage=1;non_full_length_coverage=0;isoform_length=4149;expected_accuracy=0.993	gnl|BL_ORD_ID|14819 cb8564_c40333/f1p2/4446 isoform=cb8564_c40333;full_length_coverage=1;non_full_length_coverage=2;isoform_length=4446;expected_accuracy=0.998	14	3258	3254	4149	1	3280	3545	4445

cb8564_c1933/f4p3/2042 isoform=cb8564_c1933;full_length_coverage=4;non_full_length_coverage=3;isoform_length=2042;expected_accuracy=0.992	gnl|BL_ORD_ID|13510 cb8564_c1931/f2p1/2498 isoform=cb8564_c1931;full_length_coverage=2;non_full_length_coverage=1;isoform_length=2498;expected_accuracy=1.000	1	1210	1209	2000	1	1210	1708	2498
cb4881_c4/f1p0/2342 isoform=cb4881_c4;full_length_coverage=1;non_full_length_coverage=0;isoform_length=2342;expected_accuracy=0.996	gnl|BL_ORD_ID|43200 cb4881_c1/f2p1/2532 isoform=cb4881_c1;full_length_coverage=2;non_full_length_coverage=1;isoform_length=2532;expected_accuracy=0.999	136	2313	1	137	357	2532	99	236
cb3366_c9/f1p1/2799 isoform=cb3366_c9;full_length_coverage=1;non_full_length_coverage=1;isoform_length=2799;expected_accuracy=0.996	gnl|BL_ORD_ID|41548 cb3366_c7/f1p0/3317 isoform=cb3366_c7;full_length_coverage=1;non_full_length_coverage=0;isoform_length=3317;expected_accuracy=0.997	1	2217	2215	2669	8	2221	2863	3317
cb8564_c108411/f1p1/3146 isoform=cb8564_c108411;full_length_coverage=1;non_full_length_coverage=1;isoform_length=3146;expected_accuracy=0.991	gnl|BL_ORD_ID|9449 cb8564_c12123/f1p1/3473 isoform=cb8564_c12123;full_length_coverage=1;non_full_length_coverage=1;isoform_length=3473;expected_accuracy=1.000	1	2424	2422	3053	106	2501	2847	3473
cb3229_c6/f1p0/2608 isoform=cb3229_c6;full_length_coverage=1;non_full_length_coverage=0;isoform_length=2608;expected_accuracy=1.000	gnl|BL_ORD_ID|40614 cb3229_c16/f1p0/2853 isoform=cb3229_c16;full_length_coverage=1;non_full_length_coverage=0;isoform_length=2853;expected_accuracy=0.997	1	2322	2321	2608	16	2311	2451	2738
cb2834_c17/f1p0/3060	gnl|BL_ORD_ID|68923 cb2834_c8/f1p2/3513	1355	2943	1	1360	1937	3523	1	1360
cb8564_c36689/f1p1/2368 isoform=cb8564_c36689;full_length_coverage=1;non_full_length_coverage=1;isoform_length=2368;expected_accuracy=0.991	gnl|BL_ORD_ID|16172 cb8564_c53024/f1p0/3159 isoform=cb8564_c53024;full_length_coverage=1;non_full_length_coverage=0;isoform_length=3159;expected_accuracy=0.996	1	1709	1706	2368	1	1671	2490	3153

cb5900_c97/f1p0/2618 isoform=cb5900_c97;full_length_coverage=1;non_full_length_coverage=0;isoform_length=2618;expected_accuracy=0.993	gnl|BL_ORD_ID|44981 cb5900_c96/f1p0/2763 isoform=cb5900_c96;full_length_coverage=1;non_full_length_coverage=0;isoform_length=2763;expected_accuracy=0.996	1039	2614	1	1041	1194	2763	1	1032
cb5842_c5/f1p0/1317	gnl|BL_ORD_ID|47117 cb5842_c4/f1p0/1486 isoform=cb5842_c4;full_length_coverage=1;non_full_length_coverage=0;isoform_length=1486;expected_accuracy=0.998	112	1329	1	114	268	1486	42	155
cb2779_c4/f1p0/1936 isoform=cb2779_c4;full_length_coverage=1;non_full_length_coverage=0;isoform_length=1936;expected_accuracy=1.000	gnl|BL_ORD_ID|39065 cb2779_c3/f1p1/3765 isoform=cb2779_c3;full_length_coverage=1;non_full_length_coverage=1;isoform_length=3765;expected_accuracy=0.991	1	1093	1092	1859	1	1098	2990	3765
cb18456_c6530/f1p0/1200	gnl|BL_ORD_ID|35899 cb18456_c3967/f1p4/1871 isoform=cb18456_c3967;full_length_coverage=1;non_full_length_coverage=4;isoform_length=1871;expected_accuracy=0.997	260	1166	3	259	926	1832	139	395
cb12047_c103/f1p1/1531	gnl|BL_ORD_ID|10309 cb8564_c106087/f1p13/1857 isoform=cb8564_c106087;full_length_coverage=1;non_full_length_coverage=13;isoform_length=1857;expected_accuracy=1.000	9	870	865	1546	4	874	1160	1842
cb6904_c3/f4p5/1797 isoform=cb6904_c3;full_length_coverage=4;non_full_length_coverage=5;isoform_length=1797;expected_accuracy=1.000	gnl|BL_ORD_ID|47756 cb6904_c9/f1p1/2839	395	1797	78	397	1328	2731	1	318
cb13081_c10/f1p0/1495	gnl|BL_ORD_ID|10154 cb8564_c11196/f5p0/2303 isoform=cb8564_c11196;full_length_coverage=5;non_fu	486	1284	7	488	1433	2230	11	492

	ll_length_coverage=0;isoform_length=2303;expected_accuracy=1.000								
cb3309_c8/f1p0/1954 isoform=cb3309_c8;full_length_coverage=1;non_full_length_coverage=0;isoform_length=1954;expected_accuracy=0.995	gnl|BL_ORD_ID|40139 cb3309_c2/f2p0/2095 isoform=cb3309_c2;full_length_coverage=2;non_full_length_coverage=0;isoform_length=2095;expected_accuracy=1.000	197	1934	34	196	357	2095	1	163
cb3010_c4/f2p0/2751 isoform=cb3010_c4;full_length_coverage=2;non_full_length_coverage=0;isoform_length=2751;expected_accuracy=1.000	gnl|BL_ORD_ID|38254 cb3010_c18/f1p2/2828 isoform=cb3010_c18;full_length_coverage=1;non_full_length_coverage=2;isoform_length=2828;expected_accuracy=0.999	1163	2657	1	1164	1299	2793	3	1168
cb1684_c6/f1p0/2386	gnl|BL_ORD_ID|32264 cb1684_c4/f1p0/3388 isoform=cb1684_c4;full_length_coverage=1;non_full_length_coverage=0;isoform_length=3388;expected_accuracy=0.999	15	1888	1886	2342	1	1873	2923	3378
cb8564_c3432/f1p0/2118	gnl|BL_ORD_ID|11779 cb8564_c122762/f1p4/2270 isoform=cb8564_c122762;full_length_coverage=1;non_full_length_coverage=4;isoform_length=2270;expected_accuracy=0.992	619	2090	7	623	756	2226	12	638
cb8564_c86741/f1p0/3612	gnl|BL_ORD_ID|19017 cb8564_c81704/f1p0/3492 isoform=cb8564_c81704;full_length_coverage=1;non_full_length_coverage=0;isoform_length=3492;expected_accuracy=0.994	171	3343	1	170	322	3492	2	171
cb3471_c3/f1p1/1814 isoform=cb3471_c3;full_length_coverage=1;non_full_length_coverage=1;isoform_length=1814;expected_accuracy=0.996	gnl|BL_ORD_ID|39528 cb3471_c1/f1p0/3483 isoform=cb3471_c1;full_length_coverage=1;non_full_length_coverage=0;isoform_length=3483;expected_accuracy=0.998	1	1009	1007	1754	1	1009	2738	3483

cb8564_c34238/f4p1/4317 isoform=cb8564_c34238;full_length_coverage=4;non_full_length_coverage=1;isoform_length=4317;expected_accuracy=1.000	gnl|BL_ORD_ID|16628 cb8564_c47737/f7p5/4351 isoform=cb8564_c47737;full_length_coverage=7;non_full_length_coverage=5;isoform_length=4351;expected_accuracy=0.998	285	4165	2	289	395	4274	1	290
cb8564_c75620/f1p0/2781 isoform=cb8564_c75620;full_length_coverage=1;non_full_length_coverage=0;isoform_length=2781;expected_accuracy=0.996	gnl|BL_ORD_ID|12051 cb8564_c147180/f4p0/3043 isoform=cb8564_c147180;full_length_coverage=4;non_full_length_coverage=0;isoform_length=3043;expected_accuracy=0.998	1	1769	1764	2781	1	1767	1963	2979
cb1573_c17/f1p1/3566	gnl|BL_ORD_ID|28225 cb1573_c19/f1p1/3913 isoform=cb1573_c19;full_length_coverage=1;non_full_length_coverage=1;isoform_length=3913;expected_accuracy=0.996	1217	3594	1	1220	1453	3837	69	1294
cb8564_c183213/f1p3/3190 isoform=cb8564_c183213;full_length_coverage=1;non_full_length_coverage=3;isoform_length=3190;expected_accuracy=0.999	gnl|BL_ORD_ID|15728 cb8564_c69021/f5p5/3899 isoform=cb8564_c69021;full_length_coverage=5;non_full_length_coverage=5;isoform_length=3899;expected_accuracy=1.000	1	2313	2318	3190	347	2660	2987	3848
cb16752_c0/f4p2/1249 isoform=cb16752_c0;full_length_coverage=4;non_full_length_coverage=2;isoform_length=1249;expected_accuracy=1.000	gnl|BL_ORD_ID|43429 cb423_c5/f1p0/2036 isoform=cb423_c5;full_length_coverage=1;non_full_length_coverage=0;isoform_length=2036;expected_accuracy=0.999	189	1249	3	191	975	2036	15	204
cb292_c24/f1p0/3303 isoform=cb292_c24;full_length_coverage=1;non_full_length_coverage=0;isoform_length=3303;expected_accuracy=0.991	gnl|BL_ORD_ID|38399 cb292_c6/f1p0/4480 isoform=cb292_c6;full_length_coverage=1;non_full_length_coverage=0;isoform_length=4480;expected_accuracy=0.997	1484	3303	1	1488	2546	4358	625	2108
cb12714_c29/f1p2/1583	gnl|BL_ORD_ID|14796 cb8564_c24108/f1p0/2227 isoform=cb8564_c24108;full_length_coverage=1;non_fu	1	1296	1293	1597	2	1295	1867	2171

	ll_length_coverage=0;isoform_length=2227;expected_accuracy=0.993								
cb8564_c121956/f1p0/2565	gnl|BL_ORD_ID|10058 cb8564_c119283/f1p1/2611 isoform=cb8564_c119283;full_length_coverage=1;non_full_length_coverage=1;isoform_length=2611;expected_accuracy=0.999	1048	2471	1	1048	1190	2611	1	1046
cb8564_c70069/f1p0/2273	gnl|BL_ORD_ID|19157 cb8564_c90750/f1p0/2475 isoform=cb8564_c90750;full_length_coverage=1;non_full_length_coverage=0;isoform_length=2475;expected_accuracy=0.998	1	1762	1760	2275	31	1795	1898	2413
cb8564_c90372/f1p0/2157 isoform=cb8564_c90372;full_length_coverage=1;non_full_length_coverage=0;isoform_length=2157;expected_accuracy=0.994	gnl|BL_ORD_ID|11600 cb8564_c128717/f3p4/2465 isoform=cb8564_c128717;full_length_coverage=3;non_full_length_coverage=4;isoform_length=2465;expected_accuracy=0.994	1	1092	1088	2157	91	1183	1355	2425
cb8564_c126213/f1p0/1904	gnl|BL_ORD_ID|12129 cb8564_c146480/f11p0/2032 isoform=cb8564_c146480;full_length_coverage=11;non_full_length_coverage=0;isoform_length=2032;expected_accuracy=1.000	1	1585	1586	1805	35	1619	1801	2020
cb8564_c41756/f1p0/2738 isoform=cb8564_c41756;full_length_coverage=1;non_full_length_coverage=0;isoform_length=2738;expected_accuracy=0.996	gnl|BL_ORD_ID|61972 cb12931_c30/f1p0/5903	1	2173	2170	2738	1	2177	2300	2868
cb6719_c45/f1p0/1995	gnl|BL_ORD_ID|47668 cb6719_c58/f1p2/2659	276	2007	7	279	671	2404	75	348
cb742_c31/f10p0/2606 isoform=cb742_c31;full_length_coverage=10;non_full_	gnl|BL_ORD_ID|50944 cb742_c26/f1p0/5447	631	2606	81	633	3057	5032	4	555

length_coverage=0;isoform_length=2606;expected_accuracy=1.000									
cb3450_c6/f1p0/2269 isoform=cb3450_c6;full_length_coverage=1;non_full_length_coverage=0;isoform_length=2269;expected_accuracy=0.997	gnl|BL_ORD_ID|41482 cb3450_c1/f3p0/3084 isoform=cb3450_c1;full_length_coverage=3;non_full_length_coverage=0;isoform_length=3084;expected_accuracy=1.000	12	1623	1621	2216	97	1702	2468	3064
cb2707_c24/f1p0/2640	gnl|BL_ORD_ID|38446 cb2707_c18/f1p1/3468 isoform=cb2707_c18;full_length_coverage=1;non_full_length_coverage=1;isoform_length=3468;expected_accuracy=0.997	1	2430	2425	2649	304	2733	3244	3468
cb5432_c4/f1p1/1364 isoform=cb5432_c4;full_length_coverage=1;non_full_length_coverage=1;isoform_length=1364;expected_accuracy=0.996	gnl|BL_ORD_ID|45887 cb5432_c1/f2p1/3036 isoform=cb5432_c1;full_length_coverage=2;non_full_length_coverage=1;isoform_length=3036;expected_accuracy=1.000	285	1362	46	286	1961	3036	1	240
cb716_c1/f3p1/1472 isoform=cb716_c1;full_length_coverage=3;non_full_length_coverage=1;isoform_length=1472;expected_accuracy=1.000	gnl|BL_ORD_ID|4081 cb716_c10/f1p1/1798 isoform=cb716_c10;full_length_coverage=1;non_full_length_coverage=1;isoform_length=1798;expected_accuracy=0.995	11	1182	1178	1472	1	1171	1474	1761
cb8564_c86152/f1p2/2957 isoform=cb8564_c86152;full_length_coverage=1;non_full_length_coverage=2;isoform_length=2957;expected_accuracy=0.999	gnl|BL_ORD_ID|57169 cb8564_c85269/f1p0/3455	115	2957	1	114	225	3067	1	114
cb6889_c6/f4p3/2381 isoform=cb6889_c6;full_length_coverage=4;non_full_length_coverage=3;isoform_length=2381;expected_accuracy=1.000	gnl|BL_ORD_ID|47747 cb6889_c13/f1p0/2673	308	2375	59	313	588	2655	1	255

cb8564_c40764/f1p0/2563 isoform=cb8564_c40764;full_length_coverage=1;non_full_length_coverage=0;isoform_length=2563;expected_accuracy=0.997	gnl|BL_ORD_ID|55607 cb8564_c46612/f1p1/3367	1	2166	2162	2563	8	2169	2377	2778
cb6608_c5/f1p1/2467 isoform=cb6608_c5;full_length_coverage=1;non_full_length_coverage=1;isoform_length=2467;expected_accuracy=0.993	gnl|BL_ORD_ID|5962 cb6608_c2/f2p0/3012 isoform=cb6608_c2;full_length_coverage=2;non_full_length_coverage=0;isoform_length=3012;expected_accuracy=0.997	233	2429	10	234	813	3012	3	227
cb8564_c45596/f1p1/3430	gnl|BL_ORD_ID|12535 cb8564_c132288/f2p0/3421 isoform=cb8564_c132288;full_length_coverage=2;non_full_length_coverage=0;isoform_length=3421;expected_accuracy=0.996	112	2780	2775	3378	1	2696	2816	3421
cb14968_c1/f5p0/1289 isoform=cb14968_c1;full_length_coverage=5;non_full_length_coverage=0;isoform_length=1289;expected_accuracy=1.000	gnl|BL_ORD_ID|64376 cb14968_c10/f1p0/1444	117	1287	1	117	235	1405	6	121
cb3747_c11/f1p0/2520 isoform=cb3747_c11;full_length_coverage=1;non_full_length_coverage=0;isoform_length=2520;expected_accuracy=0.994	gnl|BL_ORD_ID|39693 cb3747_c15/f1p1/2985 isoform=cb3747_c15;full_length_coverage=1;non_full_length_coverage=1;isoform_length=2985;expected_accuracy=0.990	1	1743	1742	2520	10	1750	1985	2764
cb7096_c28/f1p0/2545	gnl|BL_ORD_ID|50336 cb7096_c16/f1p0/2638	283	2570	86	284	316	2595	1	199
cb8564_c46939/f1p1/2926	gnl|BL_ORD_ID|55910 cb8564_c48645/f1p1/3287	12	2134	2130	2870	17	2146	2251	2992
cb8564_c118242/f1p2/3724 isoform=cb8564_c118242;full_length_coverage=1;non_	gnl|BL_ORD_ID|52933 cb8564_c112272/f1p0/3791	121	2554	2550	3724	1	2428	2624	3798

full_length_coverage=2;isoform_length=3724;expected_accuracy=0.990									
cb3939_c16/f1p0/2356 isoform=cb3939_c16;full_length_coverage=1;non_full_length_coverage=0;isoform_length=2356;expected_accuracy=0.992	gnl|BL_ORD_ID|39237 cb3939_c13/f1p0/3298 isoform=cb3939_c13;full_length_coverage=1;non_full_length_coverage=0;isoform_length=3298;expected_accuracy=0.997	1	1186	1185	2315	6	1185	2148	3266
cb18456_c1776/f1p0/1211	gnl|BL_ORD_ID|36325 cb18456_c1779/f1p1/1333 isoform=cb18456_c1779;full_length_coverage=1;non_full_length_coverage=1;isoform_length=1333;expected_accuracy=0.996	280	1209	9	285	384	1333	2	271
cb5274_c6/f1p1/2237 isoform=cb5274_c6;full_length_coverage=1;non_full_length_coverage=1;isoform_length=2237;expected_accuracy=0.994	gnl|BL_ORD_ID|46078 cb5274_c3/f1p1/3134 isoform=cb5274_c3;full_length_coverage=1;non_full_length_coverage=1;isoform_length=3134;expected_accuracy=0.994	1	1587	1585	2235	53	1625	2345	3006
cb8564_c146097/f5p0/2205 isoform=cb8564_c146097;full_length_coverage=5;non_full_length_coverage=0;isoform_length=2205;expected_accuracy=1.000	gnl|BL_ORD_ID|13817 cb8564_c22336/f1p0/2652 isoform=cb8564_c22336;full_length_coverage=1;non_full_length_coverage=0;isoform_length=2652;expected_accuracy=0.996	1	1749	1750	2178	30	1764	2219	2652
cb2947_c0/f1p0/3761	gnl|BL_ORD_ID|70099 cb2947_c16/f1p0/5590	341	3557	1	342	2369	5585	54	395
cb2662_c3/f1p0/3620 isoform=cb2662_c3;full_length_coverage=1;non_full_length_coverage=0;isoform_length=3620;expected_accuracy=0.999	gnl|BL_ORD_ID|38855 cb2662_c2/f1p0/3909 isoform=cb2662_c2;full_length_coverage=1;non_full_length_coverage=0;isoform_length=3909;expected_accuracy=0.995	1	2214	2211	3620	12	2227	2383	3792
cb8564_c45440/f1p0/2458 isoform=cb8564_c45440;full_length_coverage=1;non_f	gnl|BL_ORD_ID|11125 cb8564_c107332/f9p1/3308 isoform=cb8564_c107332;full_length_coverage=9;non_f	306	2458	4	310	1162	3300	1	306

ull_length_coverage=0;isoform_length=2458;expected_accuracy=0.992	ull_length_coverage=1;isoform_length=3308;expected_accuracy=0.997								
cb8564_c23232/f1p3/2540	gnl|BL_ORD_ID|11988 cb8564_c13468/f1p0/3695 isoform=cb8564_c13468;full_length_coverage=1;non_full_length_coverage=0;isoform_length=3695;expected_accuracy=0.996	16	2172	2167	2402	10	2166	2268	2503
cb8564_c24812/f1p0/2023 isoform=cb8564_c24812;full_length_coverage=1;non_full_length_coverage=0;isoform_length=2023;expected_accuracy=0.997	gnl|BL_ORD_ID|56609 cb8564_c48466/f1p0/2603	615	2004	81	619	1236	2626	1	540
cb5667_c2/f3p2/2650 isoform=cb5667_c2;full_length_coverage=3;non_full_length_coverage=2;isoform_length=2650;expected_accuracy=0.995	gnl|BL_ORD_ID|74259 cb5667_c10/f1p0/2842	1135	2607	1	1135	1356	2826	6	1148
cb8564_c37361/f1p0/3076	gnl|BL_ORD_ID|55521 cb8564_c44014/f1p0/3720	23	2481	2477	3070	40	2496	2597	3190
cb8909_c12/f1p0/1836 isoform=cb8909_c12;full_length_coverage=1;non_full_length_coverage=0;isoform_length=1836;expected_accuracy=1.000	gnl|BL_ORD_ID|20550 cb8909_c2/f3p0/1988 isoform=cb8909_c2;full_length_coverage=3;non_full_length_coverage=0;isoform_length=1988;expected_accuracy=0.996	1	1624	1620	1805	78	1702	1803	1988
cb4016_c9/f1p1/2685 isoform=cb4016_c9;full_length_coverage=1;non_full_length_coverage=1;isoform_length=2685;expected_accuracy=0.996	gnl|BL_ORD_ID|71723 cb4016_c11/f1p0/2688	1	1921	1921	2616	4	1908	2016	2711
cb5633_c78/f1p0/2529	gnl|BL_ORD_ID|74205 cb5633_c80/f1p0/2818	16	1917	1912	2422	27	1922	2151	2656

cb8564_c38035/f1p0/2770	gnl|BL_ORD_ID|15315 cb8564_c39014/f1p0/3019 isoform=cb8564_c39014;full_length_coverage=1;non_full_length_coverage=0;isoform_length=3019;expected_accuracy=0.992	1	2035	2033	2755	1	2077	2273	3013
cb8564_c1526/f1p0/1956 isoform=cb8564_c1526;full_length_coverage=1;non_full_length_coverage=0;isoform_length=1956;expected_accuracy=0.993	gnl|BL_ORD_ID|12063 cb8564_c1525/f1p0/2356 isoform=cb8564_c1525;full_length_coverage=1;non_full_length_coverage=0;isoform_length=2356;expected_accuracy=0.996	317	1956	71	317	708	2353	1	248
cb8564_c15573/f1p0/3752 isoform=cb8564_c15573;full_length_coverage=1;non_full_length_coverage=0;isoform_length=3752;expected_accuracy=0.997	gnl|BL_ORD_ID|11490 cb8564_c145650/f1p1/3791 isoform=cb8564_c145650;full_length_coverage=1;non_full_length_coverage=1;isoform_length=3791;expected_accuracy=0.994	1	2900	2896	3593	40	2970	3086	3791
cb8564_c41756/f1p0/2738 isoform=cb8564_c41756;full_length_coverage=1;non_full_length_coverage=0;isoform_length=2738;expected_accuracy=0.996	gnl|BL_ORD_ID|54403 cb8564_c35093/f1p5/2916	2	2173	2170	2738	20	2195	2318	2885
cb8564_c44850/f1p0/3916 isoform=cb8564_c44850;full_length_coverage=1;non_full_length_coverage=0;isoform_length=3916;expected_accuracy=0.993	gnl|BL_ORD_ID|55445 cb8564_c41831/f1p0/4874	1846	3842	1	1846	1991	3987	61	1890
cb51_c21/f1p0/2442 isoform=cb51_c21;full_length_coverage=1;non_full_length_coverage=0;isoform_length=2442;expected_accuracy=0.995	gnl|BL_ORD_ID|46998 cb51_c0/f6p0/2663 isoform=cb51_c0;full_length_coverage=6;non_full_length_coverage=0;isoform_length=2663;expected_accuracy=1.000	1	2129	2129	2442	46	2171	2310	2624
cb8564_c178999/f1p0/2222 isoform=cb8564_c178999;full_length_coverage=1;non_	gnl|BL_ORD_ID|16681 cb8564_c54233/f1p1/3194 isoform=cb8564_c54233;full_length_coverage=1;non_fu	1	1778	1773	2217	3	1791	2748	3194

full_length_coverage=0;isoform_length=2222;expected_accuracy=0.994	ll_length_coverage=1;isoform_length=3194;expected_accuracy=0.995								
cb12714_c29/f1p2/1583	gnl|BL_ORD_ID|13595 cb8564_c22212/f1p0/2530 isoform=cb8564_c22212;full_length_coverage=1;non_full_length_coverage=0;isoform_length=2530;expected_accuracy=0.992	1	1296	1293	1532	1	1296	2291	2530
cb5137_c53/f1p0/2672 isoform=cb5137_c53;full_length_coverage=1;non_full_length_coverage=0;isoform_length=2672;expected_accuracy=0.992	gnl|BL_ORD_ID|41994 cb5137_c96/f1p2/2956 isoform=cb5137_c96;full_length_coverage=1;non_full_length_coverage=2;isoform_length=2956;expected_accuracy=0.997	14	2565	2562	2670	155	2715	2849	2956
cb12714_c29/f1p2/1583	gnl|BL_ORD_ID|13302 cb8564_c19160/f1p2/2687 isoform=cb8564_c19160;full_length_coverage=1;non_full_length_coverage=2;isoform_length=2687;expected_accuracy=0.990	1	1296	1293	1589	1	1327	2392	2687
cb1030_c0/f14p0/2912 isoform=cb1030_c0;full_length_coverage=14;non_full_length_coverage=0;isoform_length=2912;expected_accuracy=1.000	gnl|BL_ORD_ID|47255 cb1030_c32/f1p0/3185	1	1855	1853	2912	4	1866	2020	3073
cb105_c15/f1p0/2861	gnl|BL_ORD_ID|368 cb105_c10/f1p0/4473 isoform=cb105_c10;full_length_coverage=1;non_full_length_coverage=0;isoform_length=4473;expected_accuracy=0.992	1	1911	1910	2789	1615	3518	3618	4473
cb12714_c31/f1p3/1578 isoform=cb12714_c31;full_length_coverage=1;non_full_length_coverage=3;isoform_length=1578;expected_accuracy=0.991	gnl|BL_ORD_ID|9350 cb8564_c11826/f1p0/2916 isoform=cb8564_c11826;full_length_coverage=1;non_full_length_coverage=0;isoform_length=2916;expected_accuracy=0.997	282	1578	3	283	1543	2833	1022	1296

cb2527_c40/f1p0/3341	gnl|BL_ORD_ID|38907 cb2527_c49/f15p2/3322 isoform=cb2527_c49;full_length_coverage=15;non_full_length_coverage=2;isoform_length=3322;expected_accuracy=1.000	24	2505	2505	3050	135	2627	2777	3322
cb12714_c29/f1p2/1583	gnl|BL_ORD_ID|56670 cb8564_c51772/f1p0/2859	1	1296	1293	1548	1	1297	2409	2664
cb5258_c4/f2p0/1009 isoform=cb5258_c4;full_length_coverage=2;non_full_length_coverage=0;isoform_length=1009;expected_accuracy=1.000	gnl|BL_ORD_ID|46339 cb5258_c2/f1p0/3657 isoform=cb5258_c2;full_length_coverage=1;non_full_length_coverage=0;isoform_length=3657;expected_accuracy=0.994	391	1009	8	393	2542	3167	1	386
cb8662_c5/f1p0/2097 isoform=cb8662_c5;full_length_coverage=1;non_full_length_coverage=0;isoform_length=2097;expected_accuracy=1.000	gnl|BL_ORD_ID|17389 cb8662_c2/f1p0/2305 isoform=cb8662_c2;full_length_coverage=1;non_full_length_coverage=0;isoform_length=2305;expected_accuracy=0.997	1	1527	1528	2096	18	1572	1737	2305
cb12931_c15/f1p0/1493 isoform=cb12931_c15;full_length_coverage=1;non_full_length_coverage=0;isoform_length=1493;expected_accuracy=1.000	gnl|BL_ORD_ID|14844 cb8564_c36689/f1p1/2368 isoform=cb8564_c36689;full_length_coverage=1;non_full_length_coverage=1;isoform_length=2368;expected_accuracy=0.991	1	1106	1104	1488	1	1105	1984	2368
cb246_c3/f1p3/3304 isoform=cb246_c3;full_length_coverage=1;non_full_length_coverage=3;isoform_length=3304;expected_accuracy=0.997	gnl|BL_ORD_ID|68112 cb246_c8/f1p2/3603	10	2715	2712	3291	7	2712	2883	3462
cb500_c17/f1p0/2323 isoform=cb500_c17;full_length_coverage=1;non_full_length_coverage=0;isoform_length=2323;expected_accuracy=0.997	gnl|BL_ORD_ID|72247 cb500_c49/f1p0/3666	1	1293	1288	2321	900	2167	2299	3332

cb5896_c51/f1p0/2173 isoform=cb5896_c51;full_length_coverage=1;non_full_length_coverage=0;isoform_length=2173;expected_accuracy=1.000	gnl|BL_ORD_ID|44481 cb5896_c50/f1p0/2391 isoform=cb5896_c50;full_length_coverage=1;non_full_length_coverage=0;isoform_length=2391;expected_accuracy=0.997	1	1371	1370	2173	7	1378	1591	2380
cb6063_c13/f1p1/2429	gnl|BL_ORD_ID|74844 cb6063_c11/f1p1/3128	119	2325	1	120	662	2866	161	280
cb8564_c45300/f1p4/3014 isoform=cb8564_c45300;full_length_coverage=1;non_full_length_coverage=4;isoform_length=3014;expected_accuracy=0.996	gnl|BL_ORD_ID|55377 cb8564_c39822/f1p0/3306	243	3014	1	246	399	3168	1	242
cb2674_c18/f1p2/3873 isoform=cb2674_c18;full_length_coverage=1;non_full_length_coverage=2;isoform_length=3873;expected_accuracy=0.998	gnl|BL_ORD_ID|38116 cb2674_c9/f1p2/4073 isoform=cb2674_c9;full_length_coverage=1;non_full_length_coverage=2;isoform_length=4073;expected_accuracy=0.993	1	2622	2623	3873	58	2679	2807	4063
cb6716_c4/f1p0/2291	gnl|BL_ORD_ID|47664 cb6716_c3/f1p0/2637	1	1816	1815	2239	3	1818	2079	2503
cb2533_c28/f1p0/3624	gnl|BL_ORD_ID|38533 cb2533_c16/f1p0/4039 isoform=cb2533_c16;full_length_coverage=1;non_full_length_coverage=0;isoform_length=4039;expected_accuracy=0.994	1	2640	2639	3230	201	2836	3451	4039
cb2804_c101/f1p0/2548 isoform=cb2804_c101;full_length_coverage=1;non_full_length_coverage=0;isoform_length=2548;expected_accuracy=0.995	gnl|BL_ORD_ID|38356 cb2804_c93/f1p0/3636 isoform=cb2804_c93;full_length_coverage=1;non_full_length_coverage=0;isoform_length=3636;expected_accuracy=0.991	132	2548	1	135	858	3262	1	131
cb7506_c3/f1p0/2275	gnl|BL_ORD_ID|7930 cb7506_c0/f1p0/2600 isoform=cb7506_c0;full_length_coverage=1;non_full_le	1	1972	1975	2263	430	2398	2290	2583

	ngth_coverage=0;isoform_length=2600;expected_accuracy=0.999								
cb7442_c32/f1p0/1813	gnl|BL_ORD_ID|8272 cb7442_c31/f1p0/1802 isoform=cb7442_c31;full_length_coverage=1;non_full_length_coverage=0;isoform_length=1802;expected_accuracy=0.993	1	1401	1399	1669	20	1420	1532	1802
cb8564_c91851/f1p1/2644 isoform=cb8564_c91851;full_length_coverage=1;non_full_length_coverage=1;isoform_length=2644;expected_accuracy=0.993	gnl|BL_ORD_ID|9350 cb8564_c11826/f1p0/2916 isoform=cb8564_c11826;full_length_coverage=1;non_full_length_coverage=0;isoform_length=2916;expected_accuracy=0.997	1	1295	1294	2644	4	1296	1543	2910
cb3999_c3/f4p0/1978 isoform=cb3999_c3;full_length_coverage=4;non_full_length_coverage=0;isoform_length=1978;expected_accuracy=1.000	gnl|BL_ORD_ID|39622 cb3999_c22/f1p0/2445 isoform=cb3999_c22;full_length_coverage=1;non_full_length_coverage=0;isoform_length=2445;expected_accuracy=0.998	1	1305	1305	1882	1	1305	1868	2445
cb16997_c99/f1p0/3209 isoform=cb16997_c99;full_length_coverage=1;non_full_length_coverage=0;isoform_length=3209;expected_accuracy=0.993	gnl|BL_ORD_ID|31274 cb16997_c23/f1p0/6285 isoform=cb16997_c23;full_length_coverage=1;non_full_length_coverage=0;isoform_length=6285;expected_accuracy=0.998	1518	3209	1	1521	4347	6035	21	1541
cb8564_c9882/f2p0/2918 isoform=cb8564_c9882;full_length_coverage=2;non_full_length_coverage=0;isoform_length=2918;expected_accuracy=0.999	gnl|BL_ORD_ID|13661 cb8564_c21127/f1p0/3234 isoform=cb8564_c21127;full_length_coverage=1;non_full_length_coverage=0;isoform_length=3234;expected_accuracy=0.997	1	1742	1737	2887	4	1742	2084	3234
cb8564_c608/f6p2/2608 isoform=cb8564_c608;full_length_coverage=6;non_full_length_coverage=2;isoform_length=2608;expected_accuracy=1.000	gnl|BL_ORD_ID|9903 cb8564_c1073/f1p1/3199 isoform=cb8564_c1073;full_length_coverage=1;non_full_length_coverage=1;isoform_length=3199;expected_accuracy=1.000	12	1433	1430	2608	1	1424	2019	3197

cb8564_c39546/f1p2/3023	gnl|BL_ORD_ID|16172 cb8564_c53024/f1p0/3159 isoform=cb8564_c53024;full_length_coverage=1;non_full_length_coverage=0;isoform_length=3159;expected_accuracy=0.996	1	1700	1697	2841	1	1699	2017	3159
cb5960_c4/f1p0/2967	gnl|BL_ORD_ID|74707 cb5960_c9/f1p0/3444	12	1974	1972	2596	99	2061	2806	3430
cb1462_c20/f1p0/3600 isoform=cb1462_c20;full_length_coverage=1;non_full_length_coverage=0;isoform_length=3600;expected_accuracy=0.995	gnl|BL_ORD_ID|28515 cb1462_c7/f1p0/3911 isoform=cb1462_c7;full_length_coverage=1;non_full_length_coverage=0;isoform_length=3911;expected_accuracy=0.993	167	3518	1	167	579	3909	141	319
cb8564_c44698/f1p5/2843 isoform=cb8564_c44698;full_length_coverage=1;non_full_length_coverage=5;isoform_length=2843;expected_accuracy=0.997	gnl|BL_ORD_ID|55433 cb8564_c41572/f1p1/3265	1	2516	2511	2833	1	2504	2623	2952
cb8992_c2/f1p0/1469 isoform=cb8992_c2;full_length_coverage=1;non_full_length_coverage=0;isoform_length=1469;expected_accuracy=0.996	gnl|BL_ORD_ID|21307 cb8992_c1/f1p0/2472 isoform=cb8992_c1;full_length_coverage=1;non_full_length_coverage=0;isoform_length=2472;expected_accuracy=0.996	1	1016	1015	1469	1	1015	1920	2373
cb8564_c40598/f1p4/3119	gnl|BL_ORD_ID|55472 cb8564_c42531/f1p0/3996	127	2732	2729	3051	816	3420	3541	3863
cb8564_c91948/f1p0/3509	gnl|BL_ORD_ID|17316 cb8564_c81235/f1p0/3279 isoform=cb8564_c81235;full_length_coverage=1;non_full_length_coverage=0;isoform_length=3279;expected_accuracy=0.999	13	2139	2142	3059	11	2140	2362	3279
cb6277_c19/f1p1/2357 isoform=cb6277_c19;full_length_coverage=1;non_full_	gnl|BL_ORD_ID|4096 cb6277_c14/f1p1/2588 isoform=cb6277_c14;full_length_coverage=1;non_full_l	177	2338	1	178	430	2588	152	328

length_coverage=1;isoform_length=2357;expected_accuracy=0.995	ength_coverage=1;isoform_length=2588;expected_accuracy=0.995								
cb2242_c14/f1p0/1775 isoform=cb2242_c14;full_length_coverage=1;non_full_length_coverage=0;isoform_length=1775;expected_accuracy=0.992	gnl|BL_ORD_ID|36765 cb2242_c1/f4p0/1990 isoform=cb2242_c1;full_length_coverage=4;non_full_length_coverage=0;isoform_length=1990;expected_accuracy=1.000	1	1489	1484	1764	56	1546	1710	1990
cb8450_c0/f1p0/2366 isoform=cb8450_c0;full_length_coverage=1;non_full_length_coverage=0;isoform_length=2366;expected_accuracy=0.993	gnl|BL_ORD_ID|11010 cb8450_c1/f1p0/2835 isoform=cb8450_c1;full_length_coverage=1;non_full_length_coverage=0;isoform_length=2835;expected_accuracy=0.991	1	1957	1955	2293	7	1961	2499	2835
cb8637_c55/f1p0/1852 isoform=cb8637_c55;full_length_coverage=1;non_full_length_coverage=0;isoform_length=1852;expected_accuracy=0.991	gnl|BL_ORD_ID|18514 cb8637_c28/f1p0/1911 isoform=cb8637_c28;full_length_coverage=1;non_full_length_coverage=0;isoform_length=1911;expected_accuracy=0.994	678	1840	74	681	716	1878	1	608
cb9230_c7/f1p0/1014	gnl|BL_ORD_ID|19942 cb9230_c3/f1p0/2239 isoform=cb9230_c3;full_length_coverage=1;non_full_length_coverage=0;isoform_length=2239;expected_accuracy=0.993	187	991	1	189	1064	1868	24	210
cb8564_c17220/f1p1/3235	gnl|BL_ORD_ID|12196 cb8564_c12654/f2p0/4122 isoform=cb8564_c12654;full_length_coverage=2;non_full_length_coverage=0;isoform_length=4122;expected_accuracy=1.000	1	1901	1901	3157	1	1901	2784	4041
cb15992_c0/f1p0/1875	gnl|BL_ORD_ID|36216 cb1854_c8/f1p0/2451 isoform=cb1854_c8;full_length_coverage=1;non_full_length_coverage=0;isoform_length=2451;expected_accuracy=0.994	435	1889	5	436	943	2419	1	432

cb8564_c37361/f1p0/3076	gnl|BL_ORD_ID|55522 cb8564_c44123/f1p1/3230	15	2481	2477	3070	15	247 8	2579	317 1
cb8916_c33/f12p1/1738 isoform=cb8916_c33;full_length_coverage=12;non_full_length_coverage=1;isoform_length=1738;expected_accuracy=1.000	gnl|BL_ORD_ID|19815 cb8916_c26/f1p0/1759 isoform=cb8916_c26;full_length_coverage=1;non_full_length_coverage=0;isoform_length=1759;expected_accuracy=0.999	229	1698	33	231	289	175 9	1	187
cb8564_c129474/f1p0/1983	gnl|BL_ORD_ID|53245 cb8564_c119806/f1p0/2166	1	1448	1443	1881	69	151 6	1650	208 8
cb8925_c12/f1p0/1705 isoform=cb8925_c12;full_length_coverage=1;non_full_length_coverage=0;isoform_length=1705;expected_accuracy=0.995	gnl|BL_ORD_ID|19880 cb8925_c6/f1p2/1925 isoform=cb8925_c6;full_length_coverage=1;non_full_length_coverage=2;isoform_length=1925;expected_accuracy=1.000	1	1312	1307	1705	1	129 0	1392	179 0
cb8564_c36056/f1p0/2652 isoform=cb8564_c36056;full_length_coverage=1;non_full_length_coverage=0;isoform_length=2652;expected_accuracy=0.999	gnl|BL_ORD_ID|55596 cb8564_c46321/f1p2/2987	248	2638	1	251	483	287 3	87	339
cb8564_c44698/f1p5/2843 isoform=cb8564_c44698;full_length_coverage=1;non_full_length_coverage=5;isoform_length=2843;expected_accuracy=0.997	gnl|BL_ORD_ID|55464 cb8564_c42285/f1p0/3370	1	2516	2511	2830	1	2511	2632	295 3
cb11915_c17/f1p1/1371 isoform=cb11915_c17;full_length_coverage=1;non_full_length_coverage=1;isoform_length=1371;expected_accuracy=0.993	gnl|BL_ORD_ID|52316 cb8564_c117940/f1p0/1903	442	1348	7	446	940	184 6	1	441
cb1945_c12/f1p2/4118 isoform=cb1945_c12;full_length_coverage=1;non_full_	gnl|BL_ORD_ID|34797 cb1945_c5/f1p2/4276 isoform=cb1945_c5;full_length_coverage=1;non_full_le	229	4118	20	231	399	427 4	73	286

length_coverage=2;isoform_length=4118;expected_accuracy=0.999	ngth_coverage=2;isoform_length=4276;expected_accuracy=0.999								
cb2389_c15/f1p0/2930 isoform=cb2389_c15;full_length_coverage=1;non_full_length_coverage=0;isoform_length=2930;expected_accuracy=0.994	gnl|BL_ORD_ID|68486 cb2389_c9/f1p0/3504	1	2471	2471	2930	53	2519	2699	3158
cb7495_c10/f3p2/2201 isoform=cb7495_c10;full_length_coverage=3;non_full_length_coverage=2;isoform_length=2201;expected_accuracy=0.998	gnl|BL_ORD_ID|6886 cb7495_c3/f1p5/2591 isoform=cb7495_c3;full_length_coverage=1;non_full_length_coverage=5;isoform_length=2591;expected_accuracy=0.999	99	2201	2	103	268	2367	1	102
cb8564_c35890/f1p0/3157	gnl|BL_ORD_ID|18906 cb8564_c90449/f1p3/3223 isoform=cb8564_c90449;full_length_coverage=1;non_full_length_coverage=3;isoform_length=3223;expected_accuracy=0.996	270	2836	1	271	640	3206	26	296
cb10222_c5/f1p0/1560	gnl|BL_ORD_ID|48246 cb10222_c2/f1p0/3592	269	1489	38	272	2382	3602	1	235
cb4163_c10/f1p0/2735 isoform=cb4163_c10;full_length_coverage=1;non_full_length_coverage=0;isoform_length=2735;expected_accuracy=0.995	gnl|BL_ORD_ID|71845 cb4163_c5/f1p0/3401	1	1840	1841	2730	46	1885	2032	2924
cb8564_c177939/f1p0/1977 isoform=cb8564_c177939;full_length_coverage=1;non_full_length_coverage=0;isoform_length=1977;expected_accuracy=1.000	gnl|BL_ORD_ID|54766 cb8564_c2448/f1p0/2648	1	1724	1723	1975	1	1722	2314	2566
cb9864_c0/f1p0/1948 isoform=cb9864_c0;full_length_coverage=1;non_full_le	gnl|BL_ORD_ID|21236 cb9864_c1/f1p0/2873 isoform=cb9864_c1;full_length_coverage=1;non_full_le	1	1338	1336	1948	1	1338	1839	2451

ngth_coverage=0;isoform_length=1948;expected_accuracy=0.995	ngth_coverage=0;isoform_length=2873;expected_accuracy=0.995								
cb3586_c3/f1p0/2988 isoform=cb3586_c3;full_length_coverage=1;non_full_length_coverage=0;isoform_length=2988;expected_accuracy=0.993	gnl|BL_ORD_ID|41149 cb3586_c2/f1p0/3545 isoform=cb3586_c2;full_length_coverage=1;non_full_length_coverage=0;isoform_length=3545;expected_accuracy=0.998	293	2988	2	295	820	3495	54	356
cb8564_c22900/f1p1/3151 isoform=cb8564_c22900;full_length_coverage=1;non_full_length_coverage=1;isoform_length=3151;expected_accuracy=0.994	gnl|BL_ORD_ID|17251 cb8564_c93761/f8p4/3293 isoform=cb8564_c93761;full_length_coverage=8;non_full_length_coverage=4;isoform_length=3293;expected_accuracy=0.998	15	1750	1746	3146	1	1731	1892	3293
cb4077_c12/f1p0/1303 isoform=cb4077_c12;full_length_coverage=1;non_full_length_coverage=0;isoform_length=1303;expected_accuracy=0.991	gnl|BL_ORD_ID|39141 cb4077_c6/f1p0/3369 isoform=cb4077_c6;full_length_coverage=1;non_full_length_coverage=0;isoform_length=3369;expected_accuracy=0.997	115	1275	10	114	2210	3369	1	108
cb8564_c157909/f1p2/2084 isoform=cb8564_c157909;full_length_coverage=1;non_full_length_coverage=2;isoform_length=2084;expected_accuracy=0.999	gnl|BL_ORD_ID|13447 cb8564_c22344/f1p0/2596 isoform=cb8564_c22344;full_length_coverage=1;non_full_length_coverage=0;isoform_length=2596;expected_accuracy=0.996	11	1503	1502	2083	1	1492	2015	2596
cb3341_c8/f1p0/1832	gnl|BL_ORD_ID|41612 cb3341_c7/f1p0/2281 isoform=cb3341_c7;full_length_coverage=1;non_full_length_coverage=0;isoform_length=2281;expected_accuracy=0.996	1	1048	1046	1819	1	1045	1512	2281
cb8564_c53036/f1p0/2917 isoform=cb8564_c53036;full_length_coverage=1;non_full_length_coverage=0;isoform_length=2917;expected_accuracy=0.999	gnl|BL_ORD_ID|14064 cb8564_c44301/f1p0/3723 isoform=cb8564_c44301;full_length_coverage=1;non_full_length_coverage=0;isoform_length=3723;expected_accuracy=0.995	235	2900	1	238	1060	3723	1	238

<table>
<tr><td>cb5772_c4/f3p1/1610 isoform=cb5772_c4;full_length_coverage=3;non_full_length_coverage=1;isoform_length=1610;expected_accuracy=1.000</td><td>gnl|BL_ORD_ID|74412 cb5772_c6/f1p0/2531</td><td>370</td><td>1606</td><td>43</td><td>371</td><td>1304</td><td>2541</td><td>1</td><td>327</td></tr>
<tr><td>cb8564_c45882/f1p0/3354</td><td>gnl|BL_ORD_ID|48479 cb10398_c17/f1p0/5753</td><td>1</td><td>2021</td><td>2019</td><td>3199</td><td>584</td><td>2604</td><td>4060</td><td>5240</td></tr>
<tr><td>cb12003_c142/f1p2/1168 isoform=cb12003_c142;full_length_coverage=1;non_full_length_coverage=2;isoform_length=1168;expected_accuracy=0.996</td><td>gnl|BL_ORD_ID|22350 cb12003_c33/f1p0/1360 isoform=cb12003_c33;full_length_coverage=1;non_full_length_coverage=0;isoform_length=1360;expected_accuracy=0.993</td><td>254</td><td>1163</td><td>3</td><td>254</td><td>407</td><td>1317</td><td>2</td><td>250</td></tr>
<tr><td>cb8564_c70588/f1p0/2189 isoform=cb8564_c70588;full_length_coverage=1;non_full_length_coverage=0;isoform_length=2189;expected_accuracy=0.994</td><td>gnl|BL_ORD_ID|16435 cb8564_c69070/f2p0/2710 isoform=cb8564_c69070;full_length_coverage=2;non_full_length_coverage=0;isoform_length=2710;expected_accuracy=0.997</td><td>1</td><td>1196</td><td>1195</td><td>2189</td><td>2</td><td>1195</td><td>1716</td><td>2710</td></tr>
<tr><td>cb7863_c23/f1p0/2057 isoform=cb7863_c23;full_length_coverage=1;non_full_length_coverage=0;isoform_length=2057;expected_accuracy=0.998</td><td>gnl|BL_ORD_ID|50766 cb7863_c14/f1p0/2372</td><td>264</td><td>2044</td><td>88</td><td>268</td><td>574</td><td>2354</td><td>111</td><td>291</td></tr>
<tr><td>cb3744_c16/f1p0/2234</td><td>gnl|BL_ORD_ID|39623 cb3744_c17/f1p2/3148 isoform=cb3744_c17;full_length_coverage=1;non_full_length_coverage=2;isoform_length=3148;expected_accuracy=0.999</td><td>1</td><td>1429</td><td>1424</td><td>1922</td><td>172</td><td>1599</td><td>2565</td><td>3064</td></tr>
<tr><td>cb953_c5/f1p0/2793</td><td>gnl|BL_ORD_ID|20814 cb953_c3/f1p1/3159 isoform=cb953_c3;full_length_coverage=1;non_full_length_coverage=1;isoform_length=3159;expected_accuracy=0.994</td><td>1098</td><td>2645</td><td>1</td><td>1102</td><td>1611</td><td>3159</td><td>18</td><td>1116</td></tr>
</table>

cb8564_c31910/f18p7/4258 isoform=cb8564_c31910;full_length_coverage=18;non_full_length_coverage=7;isoform_length=4258;expected_accuracy=1.000	gnl|BL_ORD_ID|13238 cb8564_c19346/f1p3/4270 isoform=cb8564_c19346;full_length_coverage=1;non_full_length_coverage=3;isoform_length=4270;expected_accuracy=0.995	2097	4257	121	2097	2112	4270	1	1970
cb8760_c0/f3p0/1017 isoform=cb8760_c0;full_length_coverage=3;non_full_length_coverage=0;isoform_length=1017;expected_accuracy=1.000	gnl|BL_ORD_ID|58346 cb8760_c2/f1p0/2371	157	994	1	158	1199	2031	1	160
cb938_c12/f1p0/3486 isoform=cb938_c12;full_length_coverage=1;non_full_length_coverage=0;isoform_length=3486;expected_accuracy=0.996	gnl|BL_ORD_ID|59101 cb938_c9/f1p0/4170	1	2585	2585	3486	1	2565	3262	4161
cb8564_c43779/f1p0/3021 isoform=cb8564_c43779;full_length_coverage=1;non_full_length_coverage=0;isoform_length=3021;expected_accuracy=0.993	gnl|BL_ORD_ID|14411 cb8564_c44258/f1p0/3202 isoform=cb8564_c44258;full_length_coverage=1;non_full_length_coverage=0;isoform_length=3202;expected_accuracy=0.993	2	2344	2345	3011	3	2376	2535	3202
cb1955_c26/f2p1/2336 isoform=cb1955_c26;full_length_coverage=2;non_full_length_coverage=1;isoform_length=2336;expected_accuracy=1.000	gnl|BL_ORD_ID|36339 cb1955_c43/f2p0/2634 isoform=cb1955_c43;full_length_coverage=2;non_full_length_coverage=0;isoform_length=2634;expected_accuracy=1.000	1	1569	1567	2335	1	1579	1858	2634
cb1306_c36/f1p0/2164 isoform=cb1306_c36;full_length_coverage=1;non_full_length_coverage=0;isoform_length=2164;expected_accuracy=1.000	gnl|BL_ORD_ID|25572 cb1306_c1/f7p1/3196 isoform=cb1306_c1;full_length_coverage=7;non_full_length_coverage=1;isoform_length=3196;expected_accuracy=0.997	1	1793	1788	2164	106	1895	2820	3196
cb1333_c76/f1p0/3569	gnl|BL_ORD_ID|26655 cb1333_c7/f6p1/4935 isoform=cb1333_c7;full_length_coverage=6;non_full_le	1	1784	1782	3554	1	1790	3135	4910

	ngth_coverage=1;isoform_length=4935;expected_accuracy=1.000								
cb5022_c0/f4p0/3002 isoform=cb5022_c0;full_length_coverage=4;non_full_length_coverage=0;isoform_length=3002;expected_accuracy=1.000	gnl|BL_ORD_ID|73204 cb5022_c7/f1p0/3286	154	3002	11	155	257	3107	1	144
cb8564_c84130/f1p2/3574 isoform=cb8564_c84130;full_length_coverage=1;non_full_length_coverage=2;isoform_length=3574;expected_accuracy=0.995	gnl|BL_ORD_ID|14727 cb8564_c37594/f3p1/3734 isoform=cb8564_c37594;full_length_coverage=3;non_full_length_coverage=1;isoform_length=3734;expected_accuracy=0.999	125	3514	1	127	317	3704	18	145
cb8564_c47839/f1p0/4186	gnl|BL_ORD_ID|14377 cb8564_c36097/f1p2/3430 isoform=cb8564_c36097;full_length_coverage=1;non_full_length_coverage=2;isoform_length=3430;expected_accuracy=0.996	131	1870	1870	3263	130	1865	1995	3384
cb2747_c7/f1p3/3310	gnl|BL_ORD_ID|68803 cb2747_c3/f1p0/3786	1	2315	2311	3243	6	2320	2741	3673
cb965_c18/f1p0/2545 isoform=cb965_c18;full_length_coverage=1;non_full_length_coverage=0;isoform_length=2545;expected_accuracy=1.000	gnl|BL_ORD_ID|20095 cb965_c5/f2p7/4283 isoform=cb965_c5;full_length_coverage=2;non_full_length_coverage=7;isoform_length=4283;expected_accuracy=1.000	1	1828	1827	2544	1499	3325	3550	4268
cb8564_c115454/f1p0/3174 isoform=cb8564_c115454;full_length_coverage=1;non_full_length_coverage=0;isoform_length=3174;expected_accuracy=0.995	gnl|BL_ORD_ID|54602 cb8564_c127814/f1p0/3705	216	3174	10	220	624	3582	1	207
cb2831_c26/f1p0/2685	gnl|BL_ORD_ID|38321 cb2831_c8/f2p0/3387 isoform=cb2831_c8;full_length_coverage=2;non_full_le	1	2172	2173	2480	67	2238	3072	3380

	ngth_coverage=0;isoform_length=3387;expected_accuracy=1.000								
cb18456_c7557/f1p1/1605 isoform=cb18456_c7557;full_length_coverage=1;non_full_length_coverage=1;isoform_length=1605;expected_accuracy=0.995	gnl|BL_ORD_ID|18147 cb8564_c89748/f3p1/2815 isoform=cb8564_c89748;full_length_coverage=3;non_full_length_coverage=1;isoform_length=2815;expected_accuracy=0.998	582	1605	9	584	1677	2700	1	575
cb8564_c107079/f4p7/4804 isoform=cb8564_c107079;full_length_coverage=4;non_full_length_coverage=7;isoform_length=4804;expected_accuracy=1.000	gnl|BL_ORD_ID|32013 cb16355_c15/f1p8/5101 isoform=cb16355_c15;full_length_coverage=1;non_full_length_coverage=8;isoform_length=5101;expected_accuracy=1.000	3	4575	4575	4782	203	4774	4891	5101
cb8564_c73853/f1p0/2443 isoform=cb8564_c73853;full_length_coverage=1;non_full_length_coverage=0;isoform_length=2443;expected_accuracy=1.000	gnl|BL_ORD_ID|18126 cb8564_c83895/f1p1/2948 isoform=cb8564_c83895;full_length_coverage=1;non_full_length_coverage=1;isoform_length=2948;expected_accuracy=0.995	1	1281	1276	2443	177	1457	1737	2902
cb8564_c22496/f4p7/2826	gnl|BL_ORD_ID|56381 cb8564_c75626/f1p2/3199	1	2113	2111	2699	4	2116	2611	3199
cb13433_c53/f1p0/4827	gnl|BL_ORD_ID|62375 cb13433_c54/f1p0/6911	1	2418	2413	4789	79	2496	3374	5750
cb5057_c18/f1p0/1420 isoform=cb5057_c18;full_length_coverage=1;non_full_length_coverage=0;isoform_length=1420;expected_accuracy=0.992	gnl|BL_ORD_ID|60445 cb11967_c8/f1p1/1821	1	1020	1016	1420	5	1023	1187	1596
cb8564_c70156/f1p0/2250 isoform=cb8564_c70156;full_length_coverage=1;non_full_length_coverage=0;isoform_length=2250;expected_accuracy=0.996	gnl|BL_ORD_ID|15726 cb8564_c69028/f2p0/2446 isoform=cb8564_c69028;full_length_coverage=2;non_full_length_coverage=0;isoform_length=2446;expected_accuracy=1.000	110	2240	1	110	314	2446	5	122

cb5773_c1/f5p0/2852 isoform=cb5773_c1;full_length_coverage=5;non_full_length_coverage=0;isoform_length=2852;expected_accuracy=1.000	gnl|BL_ORD_ID|46835 cb5773_c5/f1p0/3003 isoform=cb5773_c5;full_length_coverage=1;non_full_length_coverage=0;isoform_length=3003;expected_accuracy=0.999	151	2852	1	151	307	3002	1	145
cb8564_c4930/f4p1/3125 isoform=cb8564_c4930;full_length_coverage=4;non_full_length_coverage=1;isoform_length=3125;expected_accuracy=0.999	gnl|BL_ORD_ID|57405 cb8564_c92124/f1p0/3563	469	2973	6	471	1043	3547	9	474
cb3888_c4/f1p0/3311 isoform=cb3888_c4;full_length_coverage=1;non_full_length_coverage=0;isoform_length=3311;expected_accuracy=0.993	gnl|BL_ORD_ID|40212 cb3888_c3/f1p0/3359 isoform=cb3888_c3;full_length_coverage=1;non_full_length_coverage=0;isoform_length=3359;expected_accuracy=0.995	226	3206	1	226	375	3359	1	226
cb2889_c6/f1p0/3520	gnl|BL_ORD_ID|36512 cb2889_c4/f1p0/3702 isoform=cb2889_c4;full_length_coverage=1;non_full_length_coverage=0;isoform_length=3702;expected_accuracy=0.998	1363	3377	1	1368	1689	3702	69	1436
cb8564_c23645/f1p0/2729	gnl|BL_ORD_ID|12425 cb8564_c15273/f1p0/2696 isoform=cb8564_c15273;full_length_coverage=1;non_full_length_coverage=0;isoform_length=2696;expected_accuracy=0.991	1	2066	2062	2493	2	2086	2219	2643
cb9533_c10/f1p0/1798 isoform=cb9533_c10;full_length_coverage=1;non_full_length_coverage=0;isoform_length=1798;expected_accuracy=0.994	gnl|BL_ORD_ID|21716 cb9533_c6/f1p0/3583 isoform=cb9533_c6;full_length_coverage=1;non_full_length_coverage=0;isoform_length=3583;expected_accuracy=0.998	309	1798	74	313	1998	3487	83	322
cb2599_c18/f1p0/3075	gnl|BL_ORD_ID|37160 cb2599_c7/f1p0/3524 isoform=cb2599_c7;full_length_coverage=1;non_full_le	282	3047	125	284	454	3217	1	157

	ngth_coverage=0;isoform_length=3524;expected_accuracy=0.996								
cb3485_c9/f1p0/2043 isoform=cb3485_c9;full_length_coverage=1;non_full_length_coverage=0;isoform_length=2043;expected_accuracy=0.993	gnl|BL_ORD_ID|40920 cb3485_c8/f1p0/2724 isoform=cb3485_c8;full_length_coverage=1;non_full_length_coverage=0;isoform_length=2724;expected_accuracy=0.991	365	2043	4	365	895	2589	2	365
cb7263_c20/f1p0/2377 isoform=cb7263_c20;full_length_coverage=1;non_full_length_coverage=0;isoform_length=2377;expected_accuracy=1.000	gnl|BL_ORD_ID|8081 cb7263_c14/f3p0/2616 isoform=cb7263_c14;full_length_coverage=3;non_full_length_coverage=0;isoform_length=2616;expected_accuracy=1.000	1	2167	2162	2366	43	2211	2412	2616
cb6391_c3/f2p0/2237 isoform=cb6391_c3;full_length_coverage=2;non_full_length_coverage=0;isoform_length=2237;expected_accuracy=0.994	gnl|BL_ORD_ID|3495 cb6391_c11/f1p0/2658 isoform=cb6391_c11;full_length_coverage=1;non_full_length_coverage=0;isoform_length=2658;expected_accuracy=0.993	125	2237	1	127	520	2627	122	246
cb8564_c40598/f1p4/3119	gnl|BL_ORD_ID|15924 cb8564_c52804/f1p5/3221 isoform=cb8564_c52804;full_length_coverage=1;non_full_length_coverage=5;isoform_length=3221;expected_accuracy=0.999	1	2732	2729	3071	1	2729	2850	3192
cb6298_c2/f1p0/2038 isoform=cb6298_c2;full_length_coverage=1;non_full_length_coverage=0;isoform_length=2038;expected_accuracy=0.991	gnl|BL_ORD_ID|3607 cb6298_c1/f1p0/2892 isoform=cb6298_c1;full_length_coverage=1;non_full_length_coverage=0;isoform_length=2892;expected_accuracy=0.995	1	1564	1562	2038	74	1638	2286	2765
cb3248_c3/f1p0/2838	gnl|BL_ORD_ID|70262 cb3248_c1/f1p0/3306	1	1884	1883	2822	2	1885	2355	3294
cb8564_c15653/f1p4/2475	gnl|BL_ORD_ID|55553 cb8564_c45006/f1p1/2724	22	2293	2289	2433	31	2299	2518	2662

cb8564_c44474/f1p1/3915 isoform=cb8564_c44474;full_length_coverage=1;non_full_length_coverage=1;isoform_length=3915;expected_accuracy=0.996	gnl|BL_ORD_ID|15868 cb8564_c47725/f1p0/4368 isoform=cb8564_c47725;full_length_coverage=1;non_full_length_coverage=0;isoform_length=4368;expected_accuracy=0.995	1	2090	2089	3821	3	2080	2608	4346
cb8564_c128725/f1p1/2771 isoform=cb8564_c128725;full_length_coverage=1;non_full_length_coverage=1;isoform_length=2771;expected_accuracy=1.000	gnl|BL_ORD_ID|17161 cb8564_c87466/f1p1/3006 isoform=cb8564_c87466;full_length_coverage=1;non_full_length_coverage=1;isoform_length=3006;expected_accuracy=0.994	1	2669	2664	2771	1	2697	2811	2921
cb309_c7/f1p2/4109 isoform=cb309_c7;full_length_coverage=1;non_full_length_coverage=2;isoform_length=4109;expected_accuracy=0.997	gnl|BL_ORD_ID|69546 cb309_c5/f1p0/4669	1	3739	3740	4073	186	3918	4253	4585
cb3543_c6/f1p0/2439	gnl|BL_ORD_ID|70446 cb3543_c4/f1p0/3845	1	1561	1559	2051	11	1570	3099	3591
cb3570_c10/f1p0/2945 isoform=cb3570_c10;full_length_coverage=1;non_full_length_coverage=0;isoform_length=2945;expected_accuracy=0.991	gnl|BL_ORD_ID|39906 cb3570_c7/f1p0/3031 isoform=cb3570_c7;full_length_coverage=1;non_full_length_coverage=0;isoform_length=3031;expected_accuracy=0.997	285	2943	1	282	381	3029	1	274
cb626_c52/f1p0/4764	gnl|BL_ORD_ID|47167 cb626_c79/f1p0/5202	1921	4416	190	1926	2580	5074	1	1739
cb8564_c659/f1p3/2696 isoform=cb8564_c659;full_length_coverage=1;non_full_length_coverage=3;isoform_length=2696;expected_accuracy=0.990	gnl|BL_ORD_ID|55047 cb8564_c4680/f1p0/2928	249	2682	1	253	385	2801	1	250
cb1613_c5/f1p0/2054 isoform=cb1613_c5;full_length_coverage=1;non_full_le	gnl|BL_ORD_ID|30939 cb1613_c11/f1p0/2425 isoform=cb1613_c11;full_length_coverage=1;non_full_l	1	1200	1198	2052	12	1191	1569	2421

ngth_coverage=0;isoform_length=2054;expected_accuracy=1.000	ength_coverage=0;isoform_length=2425;expected_accuracy=0.996								
cb8564_c36056/f1p0/2652 isoform=cb8564_c36056;full_length_coverage=1;non_full_length_coverage=0;isoform_length=2652;expected_accuracy=0.999	gnl|BL_ORD_ID|56602 cb8564_c48192/f1p6/3029	248	2652	1	251	468	2872	70	324
cb8564_c11997/f1p2/2670	gnl|BL_ORD_ID|14058 cb8564_c23936/f1p0/3016 isoform=cb8564_c23936;full_length_coverage=1;non_full_length_coverage=0;isoform_length=3016;expected_accuracy=0.995	1	1713	1711	2584	4	1716	2143	3016
cb15973_c0/f4p0/1082 isoform=cb15973_c0;full_length_coverage=4;non_full_length_coverage=0;isoform_length=1082;expected_accuracy=0.992	gnl|BL_ORD_ID|29028 cb15973_c1/f1p0/1135 isoform=cb15973_c1;full_length_coverage=1;non_full_length_coverage=0;isoform_length=1135;expected_accuracy=0.999	430	1082	55	430	476	1128	1	376
cb6597_c35/f1p1/2276	gnl|BL_ORD_ID|49853 cb6597_c19/f1p0/3033	1019	2147	1	1019	1938	3066	3	1020
cb6540_c5/f1p1/1737 isoform=cb6540_c5;full_length_coverage=1;non_full_length_coverage=1;isoform_length=1737;expected_accuracy=0.998	gnl|BL_ORD_ID|5261 cb6540_c3/f1p0/2887 isoform=cb6540_c3;full_length_coverage=1;non_full_length_coverage=0;isoform_length=2887;expected_accuracy=0.998	1	1001	1002	1657	1	1002	2231	2887
cb4016_c32/f1p0/1913	gnl|BL_ORD_ID|39428 cb4016_c42/f1p1/2580 isoform=cb4016_c42;full_length_coverage=1;non_full_length_coverage=1;isoform_length=2580;expected_accuracy=0.993	191	1930	25	194	734	2471	137	308
cb8564_c44698/f1p5/2843 isoform=cb8564_c44698;full_length_coverage=1;non_f	gnl|BL_ORD_ID|57175 cb8564_c85646/f1p0/3480	18	2516	2511	2818	106	2600	2721	3030

ull_length_coverage=5;isoform_length=2843;expected_accuracy=0.997									
cb8564_c181853/f1p0/2119 isoform=cb8564_c181853;full_length_coverage=1;non_full_length_coverage=0;isoform_length=2119;expected_accuracy=0.997	gnl|BL_ORD_ID|9418 cb8564_c119193/f1p2/2975 isoform=cb8564_c119193;full_length_coverage=1;non_full_length_coverage=2;isoform_length=2975;expected_accuracy=0.998	1	1339	1334	2116	1	135 2	2186	297 5
cb8564_c2326/f1p0/2006 isoform=cb8564_c2326;full_length_coverage=1;non_full_length_coverage=0;isoform_length=2006;expected_accuracy=0.990	gnl|BL_ORD_ID|53847 cb8564_c2458/f1p0/2930	230	2006	8	234	719	249 2	12	241
cb6111_c18/f1p0/1808	gnl|BL_ORD_ID|45375 cb6111_c11/f2p0/2025 isoform=cb6111_c11;full_length_coverage=2;non_full_length_coverage=0;isoform_length=2025;expected_accuracy=1.000	290	1600	7	292	715	202 5	120	404
cb8564_c17220/f1p1/3235	gnl|BL_ORD_ID|13377 cb8564_c22091/f2p0/3351 isoform=cb8564_c22091;full_length_coverage=2;non_full_length_coverage=0;isoform_length=3351;expected_accuracy=0.997	1	1681	1678	3157	1	168 2	1805	328 4
cb6517_c67/f1p2/2665	gnl|BL_ORD_ID|49791 cb6517_c27/f1p0/2894	222	2649	1	219	346	276 8	1	220
cb3057_c0/f4p0/1599 isoform=cb3057_c0;full_length_coverage=4;non_full_length_coverage=0;isoform_length=1599;expected_accuracy=0.999	gnl|BL_ORD_ID|37411 cb3057_c1/f2p0/2029 isoform=cb3057_c1;full_length_coverage=2;non_full_length_coverage=0;isoform_length=2029;expected_accuracy=0.999	346	1585	63	348	791	202 9	1	284
cb2838_c1/f1p0/2301 isoform=cb2838_c1;full_length_coverage=1;non_full_le	gnl|BL_ORD_ID|37790 cb2838_c2/f1p0/2336 isoform=cb2838_c2;full_length_coverage=1;non_full_le	100	1544	1543	2301	1	144 6	1576	233 6

<table>
<tr><td>ngth_coverage=0;isoform_length=2301;expected_accuracy=0.996</td><td>ngth_coverage=0;isoform_length=2336;expected_accuracy=1.000</td><td></td><td></td><td></td><td></td><td></td><td></td><td></td><td></td></tr>
<tr><td>cb8564_c44698/f1p5/2843 isoform=cb8564_c44698;full_length_coverage=1;non_full_length_coverage=5;isoform_length=2843;expected_accuracy=0.997</td><td>gnl|BL_ORD_ID|54698 cb8564_c155298/f3p9/3822</td><td>1</td><td>2516</td><td>2511</td><td>2828</td><td>1</td><td>2513</td><td>2634</td><td>2953</td></tr>
<tr><td>cb7560_c35/f1p1/2004 isoform=cb7560_c35;full_length_coverage=1;non_full_length_coverage=1;isoform_length=2004;expected_accuracy=0.994</td><td>gnl|BL_ORD_ID|51343 cb7560_c37/f1p0/2673</td><td>1</td><td>1446</td><td>1444</td><td>1996</td><td>230</td><td>1677</td><td>1924</td><td>2476</td></tr>
<tr><td>cb8564_c86230/f1p0/2481 isoform=cb8564_c86230;full_length_coverage=1;non_full_length_coverage=0;isoform_length=2481;expected_accuracy=0.993</td><td>gnl|BL_ORD_ID|16238 cb8564_c75535/f2p0/3461 isoform=cb8564_c75535;full_length_coverage=2;non_full_length_coverage=0;isoform_length=3461;expected_accuracy=0.990</td><td>455</td><td>2480</td><td>59</td><td>456</td><td>1051</td><td>3051</td><td>1</td><td>388</td></tr>
<tr><td>cb8564_c41363/f1p0/2861 isoform=cb8564_c41363;full_length_coverage=1;non_full_length_coverage=0;isoform_length=2861;expected_accuracy=0.997</td><td>gnl|BL_ORD_ID|17053 cb8564_c74966/f1p0/3265 isoform=cb8564_c74966;full_length_coverage=1;non_full_length_coverage=0;isoform_length=3265;expected_accuracy=0.992</td><td>1</td><td>2122</td><td>2123</td><td>2859</td><td>1</td><td>2129</td><td>2529</td><td>3265</td></tr>
<tr><td>cb8564_c78992/f1p0/2856</td><td>gnl|BL_ORD_ID|12600 cb8564_c137667/f2p2/2686 isoform=cb8564_c137667;full_length_coverage=2;non_full_length_coverage=2;isoform_length=2686;expected_accuracy=0.996</td><td>196</td><td>2645</td><td>1</td><td>196</td><td>82</td><td>2533</td><td>20</td><td>214</td></tr>
<tr><td>cb8564_c108411/f1p1/3146 isoform=cb8564_c108411;full_length_coverage=1;non_full_length_coverage=1;isoform_length=3146;expected_accuracy=0.991</td><td>gnl|BL_ORD_ID|10077 cb8564_c11751/f1p1/3395 isoform=cb8564_c11751;full_length_coverage=1;non_full_length_coverage=1;isoform_length=3395;expected_accuracy=0.995</td><td>1</td><td>2012</td><td>2012</td><td>3146</td><td>52</td><td>2038</td><td>2190</td><td>3316</td></tr>
</table>

cb8564_c3432/f1p0/2118	gnl|BL_ORD_ID|54626 cb8564_c128456/f1p0/2321	619	2090	7	623	739	2210	7	623
cb3362_c27/f1p0/2905 isoform=cb3362_c27;full_length_coverage=1;non_full_length_coverage=0;isoform_length=2905;expected_accuracy=0.995	gnl|BL_ORD_ID|39772 cb3362_c35/f1p3/3531 isoform=cb3362_c35;full_length_coverage=1;non_full_length_coverage=3;isoform_length=3531;expected_accuracy=0.996	1	1996	1997	2905	15	2022	2587	3497
cb2927_c11/f1p2/2856 isoform=cb2927_c11;full_length_coverage=1;non_full_length_coverage=2;isoform_length=2856;expected_accuracy=0.997	gnl|BL_ORD_ID|37697 cb2927_c6/f1p1/3514 isoform=cb2927_c6;full_length_coverage=1;non_full_length_coverage=1;isoform_length=3514;expected_accuracy=0.996	1	1689	1685	2840	21	1711	2359	3514
cb8564_c53551/f3p3/2373 isoform=cb8564_c53551;full_length_coverage=3;non_full_length_coverage=3;isoform_length=2373;expected_accuracy=1.000	gnl|BL_ORD_ID|14582 cb8564_c41536/f1p0/3183 isoform=cb8564_c41536;full_length_coverage=1;non_full_length_coverage=0;isoform_length=3183;expected_accuracy=0.996	1	1304	1302	2314	3	1305	2171	3183
cb8564_c48473/f1p2/2701 isoform=cb8564_c48473;full_length_coverage=1;non_full_length_coverage=2;isoform_length=2701;expected_accuracy=0.999	gnl|BL_ORD_ID|56712 cb8564_c53711/f1p0/3201	220	2701	1	225	406	2886	37	262
cb9351_c2/f1p0/1990	gnl|BL_ORD_ID|15293 cb8564_c25416/f2p0/1980 isoform=cb8564_c25416;full_length_coverage=2;non_full_length_coverage=0;isoform_length=1980;expected_accuracy=0.994	744	1878	83	745	1167	33	1980	1317
cb313_c26/f1p0/3095 isoform=cb313_c26;full_length_coverage=1;non_full_length_coverage=0;isoform_length=3095;expected_accuracy=0.996	gnl|BL_ORD_ID|38275 cb313_c7/f1p0/4354 isoform=cb313_c7;full_length_coverage=1;non_full_length_coverage=0;isoform_length=4354;expected_accuracy=0.994	126	2072	2070	3095	1	1945	3217	4244

cb6111_c34/f1p0/1434 isoform=cb6111_c34;full_length_coverage=1;non_full_length_coverage=0;isoform_length=1434;expected_accuracy=0.992	gnl|BL_ORD_ID|44806 cb6111_c6/f3p1/1599 isoform=cb6111_c6;full_length_coverage=3;non_full_length_coverage=1;isoform_length=1599;expected_accuracy=1.000	121	1388	1	122	330	1599	43	166
cb8564_c78968/f1p1/3043 isoform=cb8564_c78968;full_length_coverage=1;non_full_length_coverage=1;isoform_length=3043;expected_accuracy=0.996	gnl|BL_ORD_ID|11491 cb8564_c147498/f6p0/3283 isoform=cb8564_c147498;full_length_coverage=6;non_full_length_coverage=0;isoform_length=3283;expected_accuracy=0.997	244	3043	1	246	482	3281	40	284
cb2737_c42/f1p0/2881	gnl|BL_ORD_ID|36571 cb2737_c54/f1p0/3029 isoform=cb2737_c54;full_length_coverage=1;non_full_length_coverage=0;isoform_length=3029;expected_accuracy=0.995	18	1799	1800	2632	222	1998	2110	2936
cb8564_c88543/f1p0/3439 isoform=cb8564_c88543;full_length_coverage=1;non_full_length_coverage=0;isoform_length=3439;expected_accuracy=0.996	gnl|BL_ORD_ID|13600 cb8564_c21527/f7p0/3631 isoform=cb8564_c21527;full_length_coverage=7;non_full_length_coverage=0;isoform_length=3631;expected_accuracy=1.000	235	3439	2	240	397	3588	34	273
cb3253_c0/f2p0/2494 isoform=cb3253_c0;full_length_coverage=2;non_full_length_coverage=0;isoform_length=2494;expected_accuracy=1.000	gnl|BL_ORD_ID|41324 cb3253_c1/f1p0/3675 isoform=cb3253_c1;full_length_coverage=1;non_full_length_coverage=0;isoform_length=3675;expected_accuracy=0.997	1190	2491	1	1194	2374	3675	1	1194
cb8564_c41756/f1p0/2738 isoform=cb8564_c41756;full_length_coverage=1;non_full_length_coverage=0;isoform_length=2738;expected_accuracy=0.996	gnl|BL_ORD_ID|16795 cb8564_c73428/f1p0/2966 isoform=cb8564_c73428;full_length_coverage=1;non_full_length_coverage=0;isoform_length=2966;expected_accuracy=0.994	1	2173	2170	2738	7	2182	2400	2965
cb6047_c0/f2p0/2861 isoform=cb6047_c0;full_length_coverage=2;non_full_le	gnl|BL_ORD_ID|44894 cb6047_c1/f1p0/2949 isoform=cb6047_c1;full_length_coverage=1;non_full_le	242	2861	100	247	325	2945	1	148

ngth_coverage=0;isoform_length=2861;expected_accuracy=0.995	ngth_coverage=0;isoform_length=2949;expected_accuracy=0.996								
cb8564_c38710/f1p0/1997	gnl|BL_ORD_ID|56700 cb8564_c52920/f1p0/1915	244	1801	42	246	357	1913	1	205
cb8564_c128290/f1p0/2765 isoform=cb8564_c128290;full_length_coverage=1;non_full_length_coverage=0;isoform_length=2765;expected_accuracy=0.996	gnl|BL_ORD_ID|13152 cb8564_c179082/f1p1/2856 isoform=cb8564_c179082;full_length_coverage=1;non_full_length_coverage=1;isoform_length=2856;expected_accuracy=0.998	1	1820	1820	2679	4	1822	1951	2809
cb6785_c11/f1p0/2230 isoform=cb6785_c11;full_length_coverage=1;non_full_length_coverage=0;isoform_length=2230;expected_accuracy=0.990	gnl|BL_ORD_ID|4712 cb6785_c4/f1p0/2768 isoform=cb6785_c4;full_length_coverage=1;non_full_length_coverage=0;isoform_length=2768;expected_accuracy=0.995	1	1562	1561	2154	84	1642	2176	2768
cb7639_c0/f3p0/2226 isoform=cb7639_c0;full_length_coverage=3;non_full_length_coverage=0;isoform_length=2226;expected_accuracy=0.997	gnl|BL_ORD_ID|51428 cb7639_c14/f1p0/2787	1	1800	1799	2226	37	1834	2114	2540
cb8_c22/f1p1/3941 isoform=cb8_c22;full_length_coverage=1;non_full_length_coverage=1;isoform_length=3941;expected_accuracy=0.997	gnl|BL_ORD_ID|59029 cb8_c15/f1p0/4140	375	3938	22	376	456	4010	1	355
cb1974_c7/f1p1/3734	gnl|BL_ORD_ID|66571 cb1974_c2/f1p0/4104	1785	3677	1	1786	2040	3931	25	1801
cb8564_c40598/f1p4/3119	gnl|BL_ORD_ID|14091 cb8564_c43119/f1p4/3194 isoform=cb8564_c43119;full_length_coverage=1;non_full_length_coverage=4;isoform_length=3194;expected_accuracy=0.995	1	2732	2729	3013	1	2749	2871	3157

cb8564_c77994/f1p2/2630 isoform=cb8564_c77994;full_length_coverage=1;non_full_length_coverage=2;isoform_length=2630;expected_accuracy=0.998	gnl|BL_ORD_ID|18842 cb8564_c85964/f7p5/2973 isoform=cb8564_c85964;full_length_coverage=7;non_full_length_coverage=5;isoform_length=2973;expected_accuracy=0.996	1	1819	1815	2630	52	1873	2017	2834
cb8564_c107741/f4p0/3601 isoform=cb8564_c107741;full_length_coverage=4;non_full_length_coverage=0;isoform_length=3601;expected_accuracy=1.000	gnl|BL_ORD_ID|57760 cb8564_c79901/f1p0/4124	137	2748	2744	3601	3	2614	2807	3666
cb1955_c6/f2p0/2436 isoform=cb1955_c6;full_length_coverage=2;non_full_length_coverage=0;isoform_length=2436;expected_accuracy=0.993	gnl|BL_ORD_ID|35587 cb1955_c12/f1p1/2862 isoform=cb1955_c12;full_length_coverage=1;non_full_length_coverage=1;isoform_length=2862;expected_accuracy=1.000	1027	2327	1	1029	1569	2862	92	1120
cb8564_c3812/f2p0/2257	gnl|BL_ORD_ID|14851 cb8564_c3811/f1p0/2840 isoform=cb8564_c3811;full_length_coverage=1;non_full_length_coverage=0;isoform_length=2840;expected_accuracy=0.996	1	1554	1553	2205	7	1567	2188	2840
cb8564_c40598/f1p4/3119	gnl|BL_ORD_ID|55600 cb8564_c46484/f1p0/3151	104	2732	2729	3067	5	2653	2773	3110
cb8564_c66201/f2p1/2384 isoform=cb8564_c66201;full_length_coverage=2;non_full_length_coverage=1;isoform_length=2384;expected_accuracy=0.994	gnl|BL_ORD_ID|55080 cb8564_c24180/f1p0/4190	499	2384	5	499	2245	4103	7	501
cb8564_c184250/f1p0/2256 isoform=cb8564_c184250;full_length_coverage=1;non_full_length_coverage=0;isoform_length=2256;expected_accuracy=0.996	gnl|BL_ORD_ID|56378 cb8564_c75558/f1p0/2635	1	1642	1638	2256	3	1644	1905	2523

cb3584_c15/f1p2/2442 isoform=cb3584_c15;full_length_coverage=1;non_full_length_coverage=2;isoform_length=2442;expected_accuracy=0.994	gnl|BL_ORD_ID|40786 cb3584_c10/f1p1/3385 isoform=cb3584_c10;full_length_coverage=1;non_full_length_coverage=1;isoform_length=3385;expected_accuracy=0.992	14	1617	1616	2442	1	1611	1713	2535
cb8564_c37361/f1p0/3076	gnl|BL_ORD_ID|12913 cb8564_c182049/f1p0/3195 isoform=cb8564_c182049;full_length_coverage=1;non_full_length_coverage=0;isoform_length=3195;expected_accuracy=0.997	1	2481	2477	3070	1	2497	2599	3195
cb6730_c30/f4p0/2653 isoform=cb6730_c30;full_length_coverage=4;non_full_length_coverage=0;isoform_length=2653;expected_accuracy=1.000	gnl|BL_ORD_ID|49988 cb6730_c7/f1p0/2780	10	1755	1754	2653	1	1739	1896	2776
cb8564_c53418/f1p0/2602 isoform=cb8564_c53418;full_length_coverage=1;non_full_length_coverage=0;isoform_length=2602;expected_accuracy=0.991	gnl|BL_ORD_ID|58013 cb8564_c86635/f1p17/2750	1163	2598	1	1168	1269	2707	1	1168
cb1279_c85/f1p0/1794 isoform=cb1279_c85;full_length_coverage=1;non_full_length_coverage=0;isoform_length=1794;expected_accuracy=0.998	gnl|BL_ORD_ID|26844 cb1279_c82/f1p1/2771 isoform=cb1279_c82;full_length_coverage=1;non_full_length_coverage=1;isoform_length=2771;expected_accuracy=0.996	1	1120	1115	1794	24	1141	2088	2771
cb8564_c39756/f1p1/3004	gnl|BL_ORD_ID|14407 cb8564_c44036/f1p0/3258 isoform=cb8564_c44036;full_length_coverage=1;non_full_length_coverage=0;isoform_length=3258;expected_accuracy=0.992	1151	2847	1	1151	1575	3256	1	1147
cb8564_c16421/f1p0/2249	gnl|BL_ORD_ID|54126 cb8564_c18992/f1p0/3876	1	1694	1692	2259	113	1806	2624	3191

cb653_c12/f1p0/3754 isoform=cb653_c12;full_length_coverage=1;non_full_length_coverage=0;isoform_length=3754;expected_accuracy=0.995	gnl|BL_ORD_ID|49383 cb653_c20/f1p2/4373	195	3754	1	196	736	4292	147	343
cb2735_c14/f1p3/3537 isoform=cb2735_c14;full_length_coverage=1;non_full_length_coverage=3;isoform_length=3537;expected_accuracy=0.992	gnl|BL_ORD_ID|38523 cb2735_c8/f1p3/4461 isoform=cb2735_c8;full_length_coverage=1;non_full_length_coverage=3;isoform_length=4461;expected_accuracy=0.997	1	2978	2976	3537	1	2980	3889	4461
cb3216_c5/f1p0/2919	gnl|BL_ORD_ID|39686 cb3216_c3/f1p0/3686 isoform=cb3216_c3;full_length_coverage=1;non_full_length_coverage=0;isoform_length=3686;expected_accuracy=0.998	1	1869	1867	2683	56	1922	2871	3686
cb10162_c5/f1p1/1783 isoform=cb10162_c5;full_length_coverage=1;non_full_length_coverage=1;isoform_length=1783;expected_accuracy=0.991	gnl|BL_ORD_ID|48152 cb10162_c4/f1p0/1934	1	1292	1293	1718	50	1339	1506	1933
cb10896_c0/f8p0/1202 isoform=cb10896_c0;full_length_coverage=8;non_full_length_coverage=0;isoform_length=1202;expected_accuracy=1.000	gnl|BL_ORD_ID|48959 cb10896_c2/f1p0/1971	256	1202	37	256	1002	1947	1	223
cb4805_c7/f1p0/2117 isoform=cb4805_c7;full_length_coverage=1;non_full_length_coverage=0;isoform_length=2117;expected_accuracy=0.994	gnl|BL_ORD_ID|72016 cb4805_c19/f1p0/2494	1	1754	1749	2077	2	1755	2049	2379
cb12529_c35/f1p0/1800 isoform=cb12529_c35;full_length_coverage=1;non_full	gnl|BL_ORD_ID|52013 cb8564_c1207/f1p0/2117	1	1157	1157	1780	22	1185	1497	2119

_length_coverage=0;isoform_length=1800;expected_accuracy=0.997									
cb8564_c43601/f1p0/2228	gnl|BL_ORD_ID|55229 cb8564_c35853/f1p0/4871	1	1815	1814	2076	2011	3826	4179	4444
cb8564_c125377/f1p0/2751	gnl|BL_ORD_ID|17343 cb8564_c84317/f1p0/2567 isoform=cb8564_c84317;full_length_coverage=1;non_full_length_coverage=0;isoform_length=2567;expected_accuracy=0.995	321	2477	61	321	412	2567	1	261
cb8564_c53561/f1p0/2359	gnl|BL_ORD_ID|16440 cb8564_c77380/f2p0/3010 isoform=cb8564_c77380;full_length_coverage=2;non_full_length_coverage=0;isoform_length=3010;expected_accuracy=0.998	495	2342	6	499	1155	3004	144	637
cb6739_c19/f1p1/2409 isoform=cb6739_c19;full_length_coverage=1;non_full_length_coverage=1;isoform_length=2409;expected_accuracy=0.998	gnl|BL_ORD_ID|47681 cb6739_c22/f1p1/2639	10	2070	2067	2402	11	2081	2204	2541
cb8564_c20202/f1p0/2556 isoform=cb8564_c20202;full_length_coverage=1;non_full_length_coverage=0;isoform_length=2556;expected_accuracy=0.999	gnl|BL_ORD_ID|54270 cb8564_c21511/f1p0/2859	1	2227	2224	2532	81	2329	2493	2791
cb8564_c4058/f1p0/2228 isoform=cb8564_c4058;full_length_coverage=1;non_full_length_coverage=0;isoform_length=2228;expected_accuracy=0.999	gnl|BL_ORD_ID|15435 cb8564_c4057/f1p0/2872 isoform=cb8564_c4057;full_length_coverage=1;non_full_length_coverage=0;isoform_length=2872;expected_accuracy=1.000	1046	2228	1	1051	1681	2865	22	1068
cb8564_c75565/f1p1/2477 isoform=cb8564_c75565;full_length_coverage=1;non_f	gnl|BL_ORD_ID|15922 cb8564_c75221/f1p1/2651 isoform=cb8564_c75221;full_length_coverage=1;non_fu	485	2477	7	485	632	2630	51	530

ull_length_coverage=1;isoform_length=2477;expected_accuracy=0.995	ll_length_coverage=1;isoform_length=2651;expected_accuracy=0.997								
cb1665_c5/f1p0/2596 isoform=cb1665_c5;full_length_coverage=1;non_full_length_coverage=0;isoform_length=2596;expected_accuracy=0.997	gnl|BL_ORD_ID|31040 cb1665_c6/f1p0/2770 isoform=cb1665_c6;full_length_coverage=1;non_full_length_coverage=0;isoform_length=2770;expected_accuracy=0.996	1	2258	2258	2592	55	2291	2435	2767
cb1573_c17/f1p1/3566	gnl|BL_ORD_ID|30371 cb1573_c7/f1p2/3841 isoform=cb1573_c7;full_length_coverage=1;non_full_length_coverage=2;isoform_length=3841;expected_accuracy=1.000	1217	3594	1	1220	1445	3822	69	1286
cb5564_c21/f1p0/1889	gnl|BL_ORD_ID|45841 cb5564_c6/f3p0/2246 isoform=cb5564_c6;full_length_coverage=3;non_full_length_coverage=0;isoform_length=2246;expected_accuracy=0.999	355	1872	5	359	710	2233	113	471
cb5108_c3/f2p0/2026 isoform=cb5108_c3;full_length_coverage=2;non_full_length_coverage=0;isoform_length=2026;expected_accuracy=0.998	gnl|BL_ORD_ID|43672 cb5108_c12/f1p0/2615 isoform=cb5108_c12;full_length_coverage=1;non_full_length_coverage=0;isoform_length=2615;expected_accuracy=0.997	1	1480	1479	1986	1	1482	2109	2615
cb8564_c47202/f1p0/3195 isoform=cb8564_c47202;full_length_coverage=1;non_full_length_coverage=0;isoform_length=3195;expected_accuracy=0.996	gnl|BL_ORD_ID|55939 cb8564_c49630/f1p4/3487	2	2916	2916	3188	1	2913	3243	3493
cb2959_c6/f1p0/3470 isoform=cb2959_c6;full_length_coverage=1;non_full_length_coverage=0;isoform_length=3470;expected_accuracy=0.995	gnl|BL_ORD_ID|38587 cb2959_c4/f1p0/3532 isoform=cb2959_c4;full_length_coverage=1;non_full_length_coverage=0;isoform_length=3532;expected_accuracy=0.994	1008	3409	1	1008	1138	3532	1	1000

cb2242_c12/f1p0/1844	gnl|BL_ORD_ID|37986 cb2242_c10/f1p1/2221 isoform=cb2242_c10;full_length_coverage=1;non_full_length_coverage=1;isoform_length=2221;expected_accuracy=1.000	390	1778	45	390	751	2139	1	346
cb2133_c24/f1p0/3763 isoform=cb2133_c24;full_length_coverage=1;non_full_length_coverage=0;isoform_length=3763;expected_accuracy=0.995	gnl|BL_ORD_ID|68291 cb2133_c31/f1p0/3897	1	1952	1950	3763	21	1966	2076	3884
cb357_c7/f1p0/1386 isoform=cb357_c7;full_length_coverage=1;non_full_length_coverage=0;isoform_length=1386;expected_accuracy=1.000	gnl|BL_ORD_ID|70825 cb357_c2/f1p0/1897	266	1386	53	268	652	1772	92	307
cb8564_c4966/f1p0/2245	gnl|BL_ORD_ID|53421 cb8564_c124689/f1p0/2316	128	1994	1	128	362	2228	47	174
cb2507_c1/f3p1/3829 isoform=cb2507_c1;full_length_coverage=3;non_full_length_coverage=1;isoform_length=3829;expected_accuracy=1.000	gnl|BL_ORD_ID|37052 cb2507_c18/f1p0/5572 isoform=cb2507_c18;full_length_coverage=1;non_full_length_coverage=0;isoform_length=5572;expected_accuracy=0.994	1	2536	2535	3774	15	2537	4325	5572
cb5472_c6/f1p0/3267 isoform=cb5472_c6;full_length_coverage=1;non_full_length_coverage=0;isoform_length=3267;expected_accuracy=0.991	gnl|BL_ORD_ID|73415 cb5472_c10/f1p0/4117	1	2812	2813	3216	1	2839	3684	4084
cb8564_c87900/f1p0/2261 isoform=cb8564_c87900;full_length_coverage=1;non_full_length_coverage=0;isoform_length=2261;expected_accuracy=0.995	gnl|BL_ORD_ID|57978 cb8564_c85833/f1p0/2942	1	1624	1622	2261	61	1709	2309	2948

cb3697_c0/f2p0/2080 isoform=cb3697_c0;full_length_coverage=2;non_full_length_coverage=0;isoform_length=2080;expected_accuracy=1.000	gnl|BL_ORD_ID|71450 cb3697_c1/f1p0/3564	1	1084	1084	2073	1	1079	2504	3498
cb507_c4/f1p0/3466	gnl|BL_ORD_ID|72257 cb507_c0/f1p0/4657	1	2743	2739	3390	1	2753	3944	4596
cb1119_c6/f1p0/2308 isoform=cb1119_c6;full_length_coverage=1;non_full_length_coverage=0;isoform_length=2308;expected_accuracy=0.994	gnl|BL_ORD_ID|1538 cb1119_c4/f1p1/4438 isoform=cb1119_c4;full_length_coverage=1;non_full_length_coverage=1;isoform_length=4438;expected_accuracy=0.999	1039	2308	1	1040	1468	2738	22	1060
cb8564_c41162/f1p0/3158	gnl|BL_ORD_ID|14539 cb8564_c46562/f2p3/3196 isoform=cb8564_c46562;full_length_coverage=2;non_full_length_coverage=3;isoform_length=3196;expected_accuracy=0.995	12	2553	2548	3036	16	2577	2708	3195
cb8564_c16090/f1p6/2546 isoform=cb8564_c16090;full_length_coverage=1;non_full_length_coverage=6;isoform_length=2546;expected_accuracy=1.000	gnl|BL_ORD_ID|9350 cb8564_c11826/f1p0/2916 isoform=cb8564_c11826;full_length_coverage=1;non_full_length_coverage=0;isoform_length=2916;expected_accuracy=0.997	1	1294	1293	2482	4	1296	1543	2731
cb5220_c14/f1p2/2375 isoform=cb5220_c14;full_length_coverage=1;non_full_length_coverage=2;isoform_length=2375;expected_accuracy=0.994	gnl|BL_ORD_ID|46711 cb5220_c9/f1p0/2464 isoform=cb5220_c9;full_length_coverage=1;non_full_length_coverage=0;isoform_length=2464;expected_accuracy=0.991	188	2319	1	188	349	2464	2	190
cb14382_c6/f1p0/1278	gnl|BL_ORD_ID|49473 cb793_c3/f1p0/3227	296	1248	6	297	2162	3114	1	292
cb8564_c185920/f1p0/2777 isoform=cb8564_c185920;full_length_coverage=1;non_full_length_coverage=0;isoform_length=2777;expected	gnl|BL_ORD_ID|54698 cb8564_c155298/f3p9/3822	174	2775	1	177	351	2953	15	192

_accuracy=0.996									
cb8564_c15230/f1p0/1944	gnl|BL_ORD_ID|11316 cb8564_c12355/f1p9/2685 isoform=cb8564_c12355;full_length_coverage=1;non_full_length_coverage=9;isoform_length=2685;expected_accuracy=0.999	352	1944	85	357	1085	2677	103	375
cb8564_c71195/f1p0/3415	gnl|BL_ORD_ID|57409 cb8564_c92262/f1p0/3503	1	1755	1756	3373	47	1801	1681	3298
cb7564_c36/f1p0/1940 isoform=cb7564_c36;full_length_coverage=1;non_full_length_coverage=0;isoform_length=1940;expected_accuracy=0.998	gnl|BL_ORD_ID|51351 cb7564_c35/f1p0/2473	231	1939	5	232	767	2474	12	240
cb7456_c0/f2p0/2350 isoform=cb7456_c0;full_length_coverage=2;non_full_length_coverage=0;isoform_length=2350;expected_accuracy=1.000	gnl|BL_ORD_ID|6358 cb7456_c2/f1p0/2610 isoform=cb7456_c2;full_length_coverage=1;non_full_length_coverage=0;isoform_length=2610;expected_accuracy=0.998	1	1613	1613	2350	3	1615	1841	2578
cb1259_c13/f1p1/3391	gnl|BL_ORD_ID|23434 cb1259_c4/f1p0/4412 isoform=cb1259_c4;full_length_coverage=1;non_full_length_coverage=0;isoform_length=4412;expected_accuracy=0.995	19	3002	3002	3258	10	2985	4158	4412
cb8564_c115486/f1p1/3203	gnl|BL_ORD_ID|10287 cb8564_c118242/f1p2/3724 isoform=cb8564_c118242;full_length_coverage=1;non_full_length_coverage=2;isoform_length=3724;expected_accuracy=0.990	98	3222	1	102	600	3724	177	279
cb6063_c13/f1p1/2429	gnl|BL_ORD_ID|45456 cb6063_c27/f3p0/2968 isoform=cb6063_c27;full_length_coverage=3;non_full_length_coverage=0;isoform_length=2968;expected_accur	119	2430	1	120	656	2965	118	237

	acy=1.000								
cb5931_c13/f1p0/2426 isoform=cb5931_c13;full_length_coverage=1;non_full_length_coverage=0;isoform_length=2426;expected_accuracy=0.996	gnl|BL_ORD_ID|74667 cb5931_c3/f1p0/2877	183	2371	1	185	502	2690	16	198
cb13355_c8/f1p0/1342 isoform=cb13355_c8;full_length_coverage=1;non_full_length_coverage=0;isoform_length=1342;expected_accuracy=0.998	gnl|BL_ORD_ID|26684 cb13355_c19/f36p0/1483 isoform=cb13355_c19;full_length_coverage=36;non_full_length_coverage=0;isoform_length=1483;expected_accuracy=1.000	1	1017	1016	1335	39	1051	1164	1483
cb8564_c181853/f1p0/2119 isoform=cb8564_c181853;full_length_coverage=1;non_full_length_coverage=0;isoform_length=2119;expected_accuracy=0.997	gnl|BL_ORD_ID|55291 cb8564_c37409/f1p0/3153	1	1339	1334	2115	1	1335	2163	2948
cb8564_c49562/f1p0/2838 isoform=cb8564_c49562;full_length_coverage=1;non_full_length_coverage=0;isoform_length=2838;expected_accuracy=0.995	gnl|BL_ORD_ID|57176 cb8564_c85665/f1p0/3087	18	2331	2327	2837	2	2342	2449	2965
cb1279_c77/f1p0/3526 isoform=cb1279_c77;full_length_coverage=1;non_full_length_coverage=0;isoform_length=3526;expected_accuracy=0.994	gnl|BL_ORD_ID|61178 cb1279_c17/f1p0/4392	1	2976	2974	3398	1	2986	3860	4285
cb1279_c29/f1p0/3632	gnl|BL_ORD_ID|61178 cb1279_c17/f1p0/4392	1	3104	3101	3526	1	3104	3860	4285
cb6360_c3/f2p0/2156 isoform=cb6360_c3;full_length_coverage=2;non_full_length_coverage=0;isoform_length=2156;expected_accur	gnl|BL_ORD_ID|47452 cb6360_c5/f1p1/2881	1	1546	1542	2156	1	1536	2202	2812

acy=0.996									
cb4016_c2/f3p1/2863 isoform=cb4016_c2;full_length_coverage=3;non_full_length_coverage=1;isoform_length=2863;expected_accuracy=0.998	gnl|BL_ORD_ID|39535 cb4016_c27/f1p1/3204 isoform=cb4016_c27;full_length_coverage=1;non_full_length_coverage=1;isoform_length=3204;expected_accuracy=0.996	489	2863	8	493	788	3163	3	485
cb938_c6/f2p5/3513 isoform=cb938_c6;full_length_coverage=2;non_full_length_coverage=5;isoform_length=3513;expected_accuracy=1.000	gnl|BL_ORD_ID|21149 cb938_c5/f2p0/4567 isoform=cb938_c5;full_length_coverage=2;non_full_length_coverage=0;isoform_length=4567;expected_accuracy=0.998	1153	3466	1	1156	2255	4567	1	1155
cb7842_c1/f2p0/2139 isoform=cb7842_c1;full_length_coverage=2;non_full_length_coverage=0;isoform_length=2139;expected_accuracy=1.000	gnl|BL_ORD_ID|6874 cb7842_c3/f1p0/2515 isoform=cb7842_c3;full_length_coverage=1;non_full_length_coverage=0;isoform_length=2515;expected_accuracy=0.996	1	1215	1213	2056	57	1272	1672	2515
cb416_c58/f1p0/2505 isoform=cb416_c58;full_length_coverage=1;non_full_length_coverage=0;isoform_length=2505;expected_accuracy=0.992	gnl|BL_ORD_ID|44315 cb416_c53/f1p0/2998 isoform=cb416_c53;full_length_coverage=1;non_full_length_coverage=0;isoform_length=2998;expected_accuracy=0.999	286	2505	4	287	680	2880	247	529
cb8564_c36689/f1p1/2368 isoform=cb8564_c36689;full_length_coverage=1;non_full_length_coverage=1;isoform_length=2368;expected_accuracy=0.991	gnl|BL_ORD_ID|55414 cb8564_c41002/f1p3/2869	1	1709	1706	2368	6	1708	2146	2809
cb1380_c36/f1p0/2344 isoform=cb1380_c36;full_length_coverage=1;non_full_length_coverage=0;isoform_length=2344;expected_accuracy=0.998	gnl|BL_ORD_ID|25511 cb1380_c6/f4p1/2530 isoform=cb1380_c6;full_length_coverage=4;non_full_length_coverage=1;isoform_length=2530;expected_accuracy=1.000	259	2344	38	262	436	2530	1	223
cb416_c19/f1p0/2939	gnl|BL_ORD_ID|72151 cb416_c41/f1p0/3183	105	2855	10	109	304	305	104	203

						4			
cb8564_c42814/f1p0/2151 isoform=cb8564_c42814;full_length_coverage=1;non_full_length_coverage=0;isoform_length=2151;expected_accuracy=0.993	gnl|BL_ORD_ID|15104 cb8564_c37866/f1p3/2874 isoform=cb8564_c37866;full_length_coverage=1;non_full_length_coverage=3;isoform_length=2874;expected_accuracy=0.990	650	2151	7	652	1345	2865	1	648
cb293_c8/f1p0/4316	gnl|BL_ORD_ID|69536 cb293_c7/f1p0/4528	1	2223	2220	4295	10	2232	2409	4456
cb2208_c27/f1p0/2987	gnl|BL_ORD_ID|38174 cb2208_c2/f4p0/3275 isoform=cb2208_c2;full_length_coverage=4;non_full_length_coverage=0;isoform_length=3275;expected_accuracy=0.999	2	2173	2171	2961	75	2251	2485	3274
cb2735_c15/f1p0/2847 isoform=cb2735_c15;full_length_coverage=1;non_full_length_coverage=0;isoform_length=2847;expected_accuracy=0.995	gnl|BL_ORD_ID|38523 cb2735_c8/f1p3/4461 isoform=cb2735_c8;full_length_coverage=1;non_full_length_coverage=3;isoform_length=4461;expected_accuracy=0.997	1	2286	2284	2847	690	2980	3889	4459
cb8564_c72190/f1p0/2570	gnl|BL_ORD_ID|56976 cb8564_c79194/f1p0/3436	16	1899	1898	2524	59	1938	2125	2755
cb1821_c16/f1p1/3514	gnl|BL_ORD_ID|33210 cb1821_c9/f1p0/3939 isoform=cb1821_c9;full_length_coverage=1;non_full_length_coverage=0;isoform_length=3939;expected_accuracy=0.997	655	3312	76	655	1283	3939	1	579
cb3671_c15/f1p1/3196 isoform=cb3671_c15;full_length_coverage=1;non_full_length_coverage=1;isoform_length=3196;expected_accuracy=0.996	gnl|BL_ORD_ID|71434 cb3671_c14/f1p0/3470	1	2731	2731	3083	209	2940	3116	3469
cb7296_c2/f2p1/2070	gnl|BL_ORD_ID|6916 cb7296_c3/f1p1/2653	1	1752	1751	2057	12	176	2347	265

isoform=cb7296_c2;full_length_coverage=2;non_full_length_coverage=1;isoform_length=2070;expected_accuracy=1.000	isoform=cb7296_c3;full_length_coverage=1;non_full_length_coverage=1;isoform_length=2653;expected_accuracy=0.998						2		3
cb18456_c4812/f1p2/1223 isoform=cb18456_c4812;full_length_coverage=1;non_full_length_coverage=2;isoform_length=1223;expected_accuracy=0.996	gnl|BL_ORD_ID|67493 cb18456_c4264/f1p1/1767	256	1223	7	256	805	1765	4	245
cb8564_c122963/f1p0/3278	gnl|BL_ORD_ID|52933 cb8564_c112272/f1p0/3791	142	2569	2565	3259	1	2428	2624	3318
cb3470_c2/f1p0/2829 isoform=cb3470_c2;full_length_coverage=1;non_full_length_coverage=0;isoform_length=2829;expected_accuracy=0.998	gnl|BL_ORD_ID|71231 cb3470_c3/f1p0/3191	1	2208	2205	2823	218	2426	2531	3148
cb4123_c103/f1p0/1158	gnl|BL_ORD_ID|70776 cb4123_c24/f1p0/1575	292	1146	3	297	498	1352	30	324
cb8564_c72116/f1p1/3397	gnl|BL_ORD_ID|66137 cb18054_c3/f1p0/5284	1	2317	2315	3294	1125	3441	4305	5284
cb8564_c15573/f1p0/3752 isoform=cb8564_c15573;full_length_coverage=1;non_full_length_coverage=0;isoform_length=3752;expected_accuracy=0.997	gnl|BL_ORD_ID|53955 cb8564_c15989/f1p0/4352	26	2900	2896	3752	2	2877	2992	3849
cb5199_c3/f8p5/2525 isoform=cb5199_c3;full_length_coverage=8;non_full_length_coverage=5;isoform_length=2525;expected_accuracy=0.995	gnl|BL_ORD_ID|73620 cb5199_c19/f1p2/3067	1	1899	1900	2525	1	1902	2461	3086
cb6605_c12/f1p0/1976	gnl|BL_ORD_ID|4293 cb6605_c11/f1p1/2320	1	1295	1291	1976	1	129	1452	213

isoform=cb6605_c12;full_length_coverage=1;non_full_length_coverage=0;isoform_length=1976;expected_accuracy=0.995	isoform=cb6605_c11;full_length_coverage=1;non_full_length_coverage=1;isoform_length=2320;expected_accuracy=0.993						1		7
cb18456_c4960/f1p0/1460	gnl|BL_ORD_ID|67493 cb18456_c4264/f1p1/1767	246	1213	5	251	803	1765	1	247
cb8564_c68409/f2p0/2141 isoform=cb8564_c68409;full_length_coverage=2;non_full_length_coverage=0;isoform_length=2141;expected_accuracy=0.995	gnl|BL_ORD_ID|57867 cb8564_c82898/f1p0/2307	265	2141	5	266	390	2266	1	264
cb8564_c36731/f1p2/2761	gnl|BL_ORD_ID|55437 cb8564_c41690/f1p1/3409	1	1977	1972	2738	1	1984	2413	3180
cb322_c15/f1p0/3487 isoform=cb322_c15;full_length_coverage=1;non_full_length_coverage=0;isoform_length=3487;expected_accuracy=0.997	gnl|BL_ORD_ID|69561 cb322_c28/f1p0/3948	1	2729	2728	3487	141	2867	3013	3772
cb8564_c15996/f1p5/3083 isoform=cb8564_c15996;full_length_coverage=1;non_full_length_coverage=5;isoform_length=3083;expected_accuracy=0.993	gnl|BL_ORD_ID|14057 cb8564_c25576/f1p5/3310 isoform=cb8564_c25576;full_length_coverage=1;non_full_length_coverage=5;isoform_length=3310;expected_accuracy=0.990	323	3083	61	328	511	3280	77	363
cb18848_c1/f1p2/5728 isoform=cb18848_c1;full_length_coverage=1;non_full_length_coverage=2;isoform_length=5728;expected_accuracy=1.000	gnl|BL_ORD_ID|32354 cb1822_c17/f1p0/5987 isoform=cb1822_c17;full_length_coverage=1;non_full_length_coverage=0;isoform_length=5987;expected_accuracy=0.997	1	5222	5223	5728	19	5241	5410	5915
cb10846_c9/f1p0/1820 isoform=cb10846_c9;full_length_coverage=1;non_full_length_coverage=0;isoform_length=1820;expected_accu	gnl|BL_ORD_ID|48905 cb10846_c4/f1p0/2641	11	1518	1517	1820	25	1531	2094	2398

racy=0.993									
cb17002_c1/f1p0/1846 isoform=cb17002_c1;full_length_coverage=1;non_full_length_coverage=0;isoform_length=1846;expected_accuracy=0.998	gnl|BL_ORD_ID|74699 cb5951_c1/f1p0/2950	160	1846	1	161	1248	2932	3	164
cb6217_c5/f2p1/2238 isoform=cb6217_c5;full_length_coverage=2;non_full_length_coverage=1;isoform_length=2238;expected_accuracy=0.999	gnl|BL_ORD_ID|3520 cb6217_c16/f1p0/2367 isoform=cb6217_c16;full_length_coverage=1;non_full_length_coverage=0;isoform_length=2367;expected_accuracy=0.991	150	2189	1	151	281	2325	1	151
cb357_c5/f1p0/1448 isoform=cb357_c5;full_length_coverage=1;non_full_length_coverage=0;isoform_length=1448;expected_accuracy=1.000	gnl|BL_ORD_ID|70825 cb357_c2/f1p0/1897	262	1448	43	264	652	1839	84	307
cb8564_c48473/f1p2/2701 isoform=cb8564_c48473;full_length_coverage=1;non_full_length_coverage=2;isoform_length=2701;expected_accuracy=0.999	gnl|BL_ORD_ID|18906 cb8564_c90449/f1p3/3223 isoform=cb8564_c90449;full_length_coverage=1;non_full_length_coverage=3;isoform_length=3223;expected_accuracy=0.996	220	2700	1	225	744	3223	31	255
cb3087_c5/f3p1/2943 isoform=cb3087_c5;full_length_coverage=3;non_full_length_coverage=1;isoform_length=2943;expected_accuracy=1.000	gnl|BL_ORD_ID|37309 cb3087_c41/f8p0/3089 isoform=cb3087_c41;full_length_coverage=8;non_full_length_coverage=0;isoform_length=3089;expected_accuracy=1.000	1	2327	2323	2908	28	2364	2503	3089
cb1824_c1/f1p0/3448 isoform=cb1824_c1;full_length_coverage=1;non_full_length_coverage=0;isoform_length=3448;expected_accuracy=0.996	gnl|BL_ORD_ID|64001 cb1824_c4/f1p0/3840	13	3224	3229	3448	5	3202	3405	3624
cb8564_c39189/f1p1/2975	gnl|BL_ORD_ID|17429 cb8564_c85447/f1p0/3196	1	2436	2435	2975	3	241	2660	319

isoform=cb8564_c39189;full_length_coverage=1;non_full_length_coverage=1;isoform_length=2975;expected_accuracy=0.992	isoform=cb8564_c85447;full_length_coverage=1;non_full_length_coverage=0;isoform_length=3196;expected_accuracy=0.997						3		5
cb5058_c4/f1p0/2291 isoform=cb5058_c4;full_length_coverage=1;non_full_length_coverage=0;isoform_length=2291;expected_accuracy=0.997	gnl|BL_ORD_ID|44019 cb5058_c0/f1p0/3186 isoform=cb5058_c0;full_length_coverage=1;non_full_length_coverage=0;isoform_length=3186;expected_accuracy=0.997	1	2061	2056	2185	894	2953	3057	3186
cb8205_c5/f2p0/2450 isoform=cb8205_c5;full_length_coverage=2;non_full_length_coverage=0;isoform_length=2450;expected_accuracy=0.999	gnl|BL_ORD_ID|51822 cb8205_c7/f1p0/2934	1	1820	1820	2445	43	1879	2279	2908
cb7736_c7/f1p0/3106	gnl|BL_ORD_ID|6316 cb7736_c4/f1p0/3571 isoform=cb7736_c4;full_length_coverage=1;non_full_length_coverage=0;isoform_length=3571;expected_accuracy=0.996	1	1592	1587	2841	20	1615	2305	3565
cb7965_c0/f10p0/2364 isoform=cb7965_c0;full_length_coverage=10;non_full_length_coverage=0;isoform_length=2364;expected_accuracy=1.000	gnl|BL_ORD_ID|51667 cb7965_c3/f1p0/3001	1	1783	1783	2360	95	1878	2197	2774
cb8564_c177939/f1p0/1977 isoform=cb8564_c177939;full_length_coverage=1;non_full_length_coverage=0;isoform_length=1977;expected_accuracy=1.000	gnl|BL_ORD_ID|53846 cb8564_c2435/f1p1/2639	1	1724	1723	1977	26	1747	2265	2519
cb12685_c3/f1p2/1466	gnl|BL_ORD_ID|54220 cb8564_c20798/f1p1/2960	1	1295	1292	1463	1	1295	2752	2923
cb5900_c24/f1p0/2855	gnl|BL_ORD_ID|45290 cb5900_c36/f1p1/3423	1	2184	2183	2855	7	219	2606	327

isoform=cb5900_c24;full_length_coverage=1;non_full_length_coverage=0;isoform_length=2855;expected_accuracy=0.996	isoform=cb5900_c36;full_length_coverage=1;non_full_length_coverage=1;isoform_length=3423;expected_accuracy=0.996						0		8
cb6473_c12/f1p0/1867 isoform=cb6473_c12;full_length_coverage=1;non_full_length_coverage=0;isoform_length=1867;expected_accuracy=0.991	gnl|BL_ORD_ID|47525 cb6473_c6/f1p0/2233	1	1131	1126	1867	95	1226	1347	2088
cb8564_c43779/f1p0/3021 isoform=cb8564_c43779;full_length_coverage=1;non_full_length_coverage=0;isoform_length=3021;expected_accuracy=0.993	gnl|BL_ORD_ID|56275 cb8564_c73216/f1p0/3006	21	2344	2345	3021	28	2325	2481	3156
cb8564_c15376/f1p0/4060	gnl|BL_ORD_ID|54224 cb8564_c20826/f1p0/4119	147	2717	2717	3673	383	2953	3057	4013
cb2529_c5/f1p0/3561 isoform=cb2529_c5;full_length_coverage=1;non_full_length_coverage=0;isoform_length=3561;expected_accuracy=0.997	gnl|BL_ORD_ID|36768 cb2529_c0/f2p0/3703 isoform=cb2529_c0;full_length_coverage=2;non_full_length_coverage=0;isoform_length=3703;expected_accuracy=0.996	1	2868	2868	3561	1	2863	2992	3686
cb10696_c5/f1p0/1470 isoform=cb10696_c5;full_length_coverage=1;non_full_length_coverage=0;isoform_length=1470;expected_accuracy=0.990	gnl|BL_ORD_ID|61792 cb12662_c11/f1p0/1530	134	1431	1	138	234	1532	1	129
cb8564_c46939/f1p1/2926	gnl|BL_ORD_ID|54425 cb8564_c42906/f1p2/3181	1	2134	2130	2870	1	2122	2227	2968
cb822_c9/f1p3/4612 isoform=cb822_c9;full_length_coverage=1;non_full_length_coverage=3;isoform_length=4612;expected_accurac	gnl|BL_ORD_ID|6209 cb822_c32/f1p1/5821 isoform=cb822_c32;full_length_coverage=1;non_full_length_coverage=1;isoform_length=5821;expected_accura	1	2798	2798	4557	4	2802	4060	5821

y=0.998	cy=0.999								
cb3895_c21/f1p0/3228	gnl|BL_ORD_ID|41273 cb3895_c5/f3p0/3228 isoform=cb3895_c5;full_length_coverage=3;non_full_length_coverage=0;isoform_length=3228;expected_accuracy=0.998	1	2861	2857	3027	75	2935	3058	3228
cb8564_c138545/f4p0/2007 isoform=cb8564_c138545;full_length_coverage=4;non_full_length_coverage=0;isoform_length=2007;expected_accuracy=1.000	gnl|BL_ORD_ID|53270 cb8564_c120391/f1p0/2526	1	1016	1012	1927	1	1020	1504	2419
cb1291_c9/f1p1/2168 isoform=cb1291_c9;full_length_coverage=1;non_full_length_coverage=1;isoform_length=2168;expected_accuracy=0.993	gnl|BL_ORD_ID|26063 cb1291_c2/f6p1/2729 isoform=cb1291_c2;full_length_coverage=6;non_full_length_coverage=1;isoform_length=2729;expected_accuracy=1.000	183	2168	1	182	630	2616	87	269
cb396_c48/f1p0/2172	gnl|BL_ORD_ID|70865 cb396_c49/f1p0/3077	238	2104	5	238	553	2419	72	304
cb3875_c6/f1p0/2825 isoform=cb3875_c6;full_length_coverage=1;non_full_length_coverage=0;isoform_length=2825;expected_accuracy=0.991	gnl|BL_ORD_ID|40472 cb3875_c0/f5p0/2930 isoform=cb3875_c0;full_length_coverage=5;non_full_length_coverage=0;isoform_length=2930;expected_accuracy=1.000	1	2616	2611	2813	1	2628	2728	2930
cb8564_c89331/f1p0/2344	gnl|BL_ORD_ID|56303 cb8564_c73808/f1p0/3527	1	2105	2101	2382	90	2193	3007	3290
cb8564_c91771/f1p0/2716 isoform=cb8564_c91771;full_length_coverage=1;non_full_length_coverage=0;isoform_length=2716;expected_accuracy=0.991	gnl|BL_ORD_ID|15663 cb8564_c71422/f3p2/3204 isoform=cb8564_c71422;full_length_coverage=3;non_full_length_coverage=2;isoform_length=3204;expected_accuracy=0.998	328	2716	48	329	504	2893	1	281
cb8564_c4679/f1p1/2608	gnl|BL_ORD_ID|55047 cb8564_c4680/f1p0/2928	251	2577	1	250	476	280	1	250

							1		
cb8564_c146882/f3p8/4113 isoform=cb8564_c146882;full_length_coverage=3;non_full_length_coverage=8;isoform_length=4113;expected_accuracy=0.998	gnl|BL_ORD_ID|14953 cb8564_c34238/f4p1/4317 isoform=cb8564_c34238;full_length_coverage=4;non_full_length_coverage=1;isoform_length=4317;expected_accuracy=1.000	181	4111	1	185	282	4213	2	178
cb4767_c5/f2p0/1630 isoform=cb4767_c5;full_length_coverage=2;non_full_length_coverage=0;isoform_length=1630;expected_accuracy=1.000	gnl|BL_ORD_ID|71993 cb4767_c2/f1p0/2894	378	1624	7	381	1491	2737	2	376
cb653_c12/f1p0/3754 isoform=cb653_c12;full_length_coverage=1;non_full_length_coverage=0;isoform_length=3754;expected_accuracy=0.995	gnl|BL_ORD_ID|5915 cb653_c13/f1p2/4267 isoform=cb653_c13;full_length_coverage=1;non_full_length_coverage=2;isoform_length=4267;expected_accuracy=0.999	195	3754	1	196	596	4152	101	297
cb426_c5/f1p0/2309 isoform=cb426_c5;full_length_coverage=1;non_full_length_coverage=0;isoform_length=2309;expected_accuracy=0.998	gnl|BL_ORD_ID|42759 cb426_c0/f1p0/4818 isoform=cb426_c0;full_length_coverage=1;non_full_length_coverage=0;isoform_length=4818;expected_accuracy=0.996	1053	2307	1	1056	3567	4818	36	1096
cb3599_c8/f1p1/2994 isoform=cb3599_c8;full_length_coverage=1;non_full_length_coverage=1;isoform_length=2994;expected_accuracy=0.992	gnl|BL_ORD_ID|71369 cb3599_c12/f1p0/3100	1	1992	1993	2952	5	1994	2115	3071
cb6633_c1/f5p1/1966 isoform=cb6633_c1;full_length_coverage=5;non_full_length_coverage=1;isoform_length=1966;expected_accuracy=1.000	gnl|BL_ORD_ID|3747 cb6633_c9/f1p0/2805 isoform=cb6633_c9;full_length_coverage=1;non_full_length_coverage=0;isoform_length=2805;expected_accuracy=0.997	1	1519	1516	1966	1	1518	2261	2713
cb3485_c9/f1p0/2043	gnl|BL_ORD_ID|71246 cb3485_c10/f1p0/2515	365	2043	76	365	815	249	1	290

isoform=cb3485_c9;full_length_coverage=1;non_full_length_coverage=0;isoform_length=2043;expected_accuracy=0.993							4		
cb8564_c39624/f1p1/3482 isoform=cb8564_c39624;full_length_coverage=1;non_full_length_coverage=1;isoform_length=3482;expected_accuracy=0.996	gnl|BL_ORD_ID|16467 cb8564_c49872/f2p1/3689 isoform=cb8564_c49872;full_length_coverage=2;non_full_length_coverage=1;isoform_length=3689;expected_accuracy=1.000	1	2003	2003	3482	15	2031	2209	3689
cb8564_c43779/f1p0/3021 isoform=cb8564_c43779;full_length_coverage=1;non_full_length_coverage=0;isoform_length=3021;expected_accuracy=0.993	gnl|BL_ORD_ID|55182 cb8564_c34825/f1p2/3200	10	2344	2345	3021	6	2368	2524	3196
cb6580_c2/f1p0/1593	gnl|BL_ORD_ID|4017 cb6580_c1/f1p0/2538 isoform=cb6580_c1;full_length_coverage=1;non_full_length_coverage=0;isoform_length=2538;expected_accuracy=0.996	351	1603	53	353	1250	2502	26	326
cb1822_c15/f1p0/1826 isoform=cb1822_c15;full_length_coverage=1;non_full_length_coverage=0;isoform_length=1826;expected_accuracy=0.998	gnl|BL_ORD_ID|36294 cb18848_c0/f1p0/5897 isoform=cb18848_c0;full_length_coverage=1;non_full_length_coverage=0;isoform_length=5897;expected_accuracy=0.996	1	1676	1671	1826	753	2433	5164	5316
cb16550_c1/f1p2/1845 isoform=cb16550_c1;full_length_coverage=1;non_full_length_coverage=2;isoform_length=1845;expected_accuracy=0.999	gnl|BL_ORD_ID|12468 cb8564_c129985/f1p2/2126 isoform=cb8564_c129985;full_length_coverage=1;non_full_length_coverage=2;isoform_length=2126;expected_accuracy=0.996	1	1212	1207	1845	13	1220	1443	2076
cb416_c58/f1p0/2505 isoform=cb416_c58;full_length_coverage=1;non_full_length_coverage=0;isoform_length=2505;expected_accur	gnl|BL_ORD_ID|44207 cb416_c73/f3p0/3007 isoform=cb416_c73;full_length_coverage=3;non_full_length_coverage=0;isoform_length=3007;expected_accura	286	2498	4	287	813	3006	380	662

acy=0.992	cy=1.000								
cb5613_c56/f1p1/2284 isoform=cb5613_c56;full_length_coverage=1;non_full_length_coverage=1;isoform_length=2284;expected_accuracy=0.991	gnl|BL_ORD_ID|44685 cb5613_c26/f1p0/3883 isoform=cb5613_c26;full_length_coverage=1;non_full_length_coverage=0;isoform_length=3883;expected_accuracy=0.994	1	1740	1737	2262	157	1841	3361	3883
cb8564_c34825/f1p2/3200	gnl|BL_ORD_ID|55195 cb8564_c35170/f1p0/3386	493	3185	6	492	738	3399	11	497
cb13565_c30/f1p12/1678 isoform=cb13565_c30;full_length_coverage=1;non_full_length_coverage=12;isoform_length=1678;expected_accuracy=0.999	gnl|BL_ORD_ID|4515 cb6452_c4/f1p3/2166 isoform=cb6452_c4;full_length_coverage=1;non_full_length_coverage=3;isoform_length=2166;expected_accuracy=1.000	429	1624	5	428	948	2146	2	414
cb7023_c7/f1p0/1690	gnl|BL_ORD_ID|3662 cb7023_c0/f2p1/2542 isoform=cb7023_c0;full_length_coverage=2;non_full_length_coverage=1;isoform_length=2542;expected_accuracy=0.996	256	1693	27	258	975	2412	1	231
cb2238_c51/f18p0/2624 isoform=cb2238_c51;full_length_coverage=18;non_full_length_coverage=0;isoform_length=2624;expected_accuracy=1.000	gnl|BL_ORD_ID|69645 cb2238_c16/f1p0/3348	138	2624	1	139	815	3301	1	139
cb51_c21/f1p0/2442 isoform=cb51_c21;full_length_coverage=1;non_full_length_coverage=0;isoform_length=2442;expected_accuracy=0.995	gnl|BL_ORD_ID|73496 cb51_c27/f1p0/3119	13	2129	2129	2442	1	2114	2253	2567
cb13752_c0/f4p0/1812 isoform=cb13752_c0;full_length_coverage=4;non_full_length_coverage=0;isoform_length=1812;expected_accu	gnl|BL_ORD_ID|11857 cb8564_c1417/f1p0/3518 isoform=cb8564_c1417;full_length_coverage=1;non_full_length_coverage=0;isoform_length=3518;expected_ac	1	1376	1374	1812	80	1453	3081	3518

racy=0.999	curacy=0.997								
cb1333_c9/f4p0/1480 isoform=cb1333_c9;full_length_coverage=4;non_full_length_coverage=0;isoform_length=1480;expected_accuracy=1.000	gnl|BL_ORD_ID|25626 cb1333_c96/f1p0/1554 isoform=cb1333_c96;full_length_coverage=1;non_full_length_coverage=0;isoform_length=1554;expected_accuracy=0.991	153	1459	29	152	254	1554	19	142
cb5643_c66/f1p0/2234 isoform=cb5643_c66;full_length_coverage=1;non_full_length_coverage=0;isoform_length=2234;expected_accuracy=0.996	gnl|BL_ORD_ID|74222 cb5643_c69/f1p0/2630	201	2234	18	203	557	2605	156	352
cb8564_c185920/f1p0/2777 isoform=cb8564_c185920;full_length_coverage=1;non_full_length_coverage=0;isoform_length=2777;expected_accuracy=0.996	gnl|BL_ORD_ID|55317 cb8564_c38251/f1p0/3243	174	2776	1	177	358	2973	15	189
cb18456_c6530/f1p0/1200	gnl|BL_ORD_ID|35801 cb18456_c7725/f178p4/1767 isoform=cb18456_c7725;full_length_coverage=178;non_full_length_coverage=4;isoform_length=1767;expected_accuracy=1.000	260	1166	6	259	785	1691	1	254
cb2018_c2/f1p0/1820 isoform=cb2018_c2;full_length_coverage=1;non_full_length_coverage=0;isoform_length=1820;expected_accuracy=0.990	gnl|BL_ORD_ID|68251 cb2018_c0/f1p0/4091	199	1820	91	202	2102	3725	3	116
cb7320_c16/f1p1/1794 isoform=cb7320_c16;full_length_coverage=1;non_full_length_coverage=1;isoform_length=1794;expected_accuracy=0.995	gnl|BL_ORD_ID|7017 cb7320_c2/f3p2/2394 isoform=cb7320_c2;full_length_coverage=3;non_full_length_coverage=2;isoform_length=2394;expected_accuracy=1.000	1	1400	1399	1794	1	1403	1985	2381
cb8564_c17694/f1p1/3299	gnl|BL_ORD_ID|65228 cb16355_c11/f1p4/5263	1	2347	2347	3251	1757	410	4333	523

						5		7
cb12302_c31/f1p0/1548 isoform=cb12302_c31;full_length_coverage=1;non_full_length_coverage=0;isoform_length=1548;expected_accuracy=1.000	gnl|BL_ORD_ID|27348 cb1380_c15/f1p6/2382 isoform=cb1380_c15;full_length_coverage=1;non_full_length_coverage=6;isoform_length=2382;expected_accuracy=0.995	324	1548	4	329	565	1781	1	321
cb8564_c125661/f1p1/2227	gnl|BL_ORD_ID|11919 cb8564_c124232/f1p0/2536 isoform=cb8564_c124232;full_length_coverage=1;non_full_length_coverage=0;isoform_length=2536;expected_accuracy=0.997	1	1180	1179	2059	175	1355	1656	2536
cb8564_c2743/f3p0/2883 isoform=cb8564_c2743;full_length_coverage=3;non_full_length_coverage=0;isoform_length=2883;expected_accuracy=0.997	gnl|BL_ORD_ID|14039 cb8564_c2753/f1p0/2971 isoform=cb8564_c2753;full_length_coverage=1;non_full_length_coverage=0;isoform_length=2971;expected_accuracy=0.997	1273	2882	118	1274	1361	2971	1	1157
cb8564_c23232/f1p3/2540	gnl|BL_ORD_ID|52715 cb8564_c11997/f1p2/2670	16	2172	2167	2518	5	2161	2263	2614
cb8564_c182028/f1p0/2513 isoform=cb8564_c182028;full_length_coverage=1;non_full_length_coverage=0;isoform_length=2513;expected_accuracy=0.996	gnl|BL_ORD_ID|19105 cb8564_c98209/f1p10/2805 isoform=cb8564_c98209;full_length_coverage=1;non_full_length_coverage=10;isoform_length=2805;expected_accuracy=1.000	1	1971	1968	2513	3	1972	2116	2661
cb1203_c25/f1p0/1362	gnl|BL_ORD_ID|59262 cb1203_c12/f1p2/4460	157	1269	3	157	3269	4380	2286	2440
cb1301_c3/f1p0/2903	gnl|BL_ORD_ID|61209 cb1301_c4/f1p0/3013	1	2061	2062	2886	22	2082	2198	3022
cb3586_c10/f1p0/2722 isoform=cb3586_c10;full_length_coverage=1;non_full_length_coverage=0;isoform_length=2722;expected_accu	gnl|BL_ORD_ID|41151 cb3586_c0/f4p0/3057 isoform=cb3586_c0;full_length_coverage=4;non_full_length_coverage=0;isoform_length=3057;expected_accura	252	2722	1	251	498	2970	97	349

racy=0.993	cy=1.000								
cb8564_c146516/f7p0/2412 isoform=cb8564_c146516;full_length_coverage=7;non_full_length_coverage=0;isoform_length=2412;expected_accuracy=1.000	gnl|BL_ORD_ID|52094 cb8564_c110646/f1p0/2764	1	1773	1770	2411	59	1810	2110	2751
cb9434_c3/f2p0/1464 isoform=cb9434_c3;full_length_coverage=2;non_full_length_coverage=0;isoform_length=1464;expected_accuracy=1.000	gnl|BL_ORD_ID|58717 cb9434_c4/f1p0/2082	1	1192	1190	1448	5	1197	1821	2079
cb7972_c4/f1p0/2284 isoform=cb7972_c4;full_length_coverage=1;non_full_length_coverage=0;isoform_length=2284;expected_accuracy=0.990	gnl|BL_ORD_ID|8320 cb7972_c0/f3p0/2450 isoform=cb7972_c0;full_length_coverage=3;non_full_length_coverage=0;isoform_length=2450;expected_accuracy=0.998	1	1463	1460	2224	40	1504	1682	2448
cb1279_c11/f2p3/3084 isoform=cb1279_c11;full_length_coverage=2;non_full_length_coverage=3;isoform_length=3084;expected_accuracy=1.000	gnl|BL_ORD_ID|27065 cb1279_c46/f1p3/3121 isoform=cb1279_c46;full_length_coverage=1;non_full_length_coverage=3;isoform_length=3121;expected_accuracy=1.000	236	2937	1	237	420	3121	2	245
cb8564_c188097/f1p0/2552 isoform=cb8564_c188097;full_length_coverage=1;non_full_length_coverage=0;isoform_length=2552;expected_accuracy=0.996	gnl|BL_ORD_ID|54449 cb8564_c122906/f2p13/2871	1	1945	1940	2552	135	2079	2182	2794
cb5221_c7/f1p0/3686 isoform=cb5221_c7;full_length_coverage=1;non_full_length_coverage=0;isoform_length=3686;expected_accuracy=0.996	gnl|BL_ORD_ID|45936 cb5221_c6/f1p0/4147 isoform=cb5221_c6;full_length_coverage=1;non_full_length_coverage=0;isoform_length=4147;expected_accuracy=0.998	1106	3529	1	1105	1726	4147	1	1105
cb3163_c12/f1p0/2539	gnl|BL_ORD_ID|37035 cb3163_c23/f9p0/2749	1	1656	1654	2539	76	173	1861	274

isoform=cb3163_c12;full_length_coverage=1;non_full_length_coverage=0;isoform_length=2539;expected_accuracy=0.994	isoform=cb3163_c23;full_length_coverage=9;non_full_length_coverage=0;isoform_length=2749;expected_accuracy=1.000						8		7
cb8564_c49562/f1p0/2838 isoform=cb8564_c49562;full_length_coverage=1;non_full_length_coverage=0;isoform_length=2838;expected_accuracy=0.995	gnl|BL_ORD_ID|55912 cb8564_c48681/f1p0/3431	12	2331	2327	2838	6	2321	2701	3216
cb8564_c10938/f1p1/2321	gnl|BL_ORD_ID|53631 cb8564_c135683/f2p4/3808	1	1315	1310	2292	1169	2483	2602	3584
cb8564_c34825/f1p2/3200	gnl|BL_ORD_ID|16149 cb8564_c47162/f1p0/3361 isoform=cb8564_c47162;full_length_coverage=1;non_full_length_coverage=0;isoform_length=3361;expected_accuracy=0.996	395	3194	6	396	592	3361	11	402
cb12714_c29/f1p2/1583	gnl|BL_ORD_ID|55945 cb8564_c49862/f1p0/2807	1	1296	1293	1594	1	1295	2412	2713
cb4744_c6/f1p0/1496 isoform=cb4744_c6;full_length_coverage=1;non_full_length_coverage=0;isoform_length=1496;expected_accuracy=0.997	gnl|BL_ORD_ID|44018 cb4744_c0/f2p0/2942 isoform=cb4744_c0;full_length_coverage=2;non_full_length_coverage=0;isoform_length=2942;expected_accuracy=0.997	1	1159	1160	1496	1	1151	2565	2901
cb4957_c12/f1p0/1876 isoform=cb4957_c12;full_length_coverage=1;non_full_length_coverage=0;isoform_length=1876;expected_accuracy=1.000	gnl|BL_ORD_ID|41850 cb4957_c0/f1p0/3218 isoform=cb4957_c0;full_length_coverage=1;non_full_length_coverage=0;isoform_length=3218;expected_accuracy=0.996	1	1467	1468	1869	131	1574	2818	3218
cb8564_c48473/f1p2/2701 isoform=cb8564_c48473;full_length_coverage=1;non_full_length_coverage=2;isoform_length=2701;expected_	gnl|BL_ORD_ID|10800 cb8564_c10037/f1p5/2946 isoform=cb8564_c10037;full_length_coverage=1;non_full_length_coverage=5;isoform_length=2946;expected_ac	220	2701	1	225	470	2946	36	260

<table>
<tr><td>accuracy=0.999</td><td>curacy=0.994</td><td></td><td></td><td></td><td></td><td></td><td></td><td></td><td></td></tr>
<tr><td>cb8564_c5070/f1p0/2861</td><td>gnl|BL_ORD_ID|56566 cb8564_c5065/f1p0/3147</td><td>10</td><td>1660</td><td>1659</td><td>2831</td><td>35</td><td>1685</td><td>1578</td><td>2749</td></tr>
<tr><td>cb17884_c0/f3p1/1879 isoform=cb17884_c0;full_length_coverage=3;non_full_length_coverage=1;isoform_length=1879;expected_accuracy=1.000</td><td>gnl|BL_ORD_ID|17831 cb8564_c84290/f1p0/3355 isoform=cb8564_c84290;full_length_coverage=1;non_full_length_coverage=0;isoform_length=3355;expected_accuracy=0.998</td><td>391</td><td>1879</td><td>4</td><td>394</td><td>1872</td><td>3353</td><td>14</td><td>407</td></tr>
<tr><td>cb6390_c0/f5p0/2306 isoform=cb6390_c0;full_length_coverage=5;non_full_length_coverage=0;isoform_length=2306;expected_accuracy=1.000</td><td>gnl|BL_ORD_ID|3696 cb6390_c2/f1p0/2643 isoform=cb6390_c2;full_length_coverage=1;non_full_length_coverage=0;isoform_length=2643;expected_accuracy=0.998</td><td>1</td><td>1577</td><td>1575</td><td>2306</td><td>203</td><td>1776</td><td>1912</td><td>2643</td></tr>
<tr><td>cb3485_c11/f1p0/2449</td><td>gnl|BL_ORD_ID|40922 cb3485_c4/f1p0/3162 isoform=cb3485_c4;full_length_coverage=1;non_full_length_coverage=0;isoform_length=3162;expected_accuracy=0.996</td><td>1</td><td>1248</td><td>1246</td><td>2416</td><td>129</td><td>1375</td><td>1834</td><td>3003</td></tr>
<tr><td>cb8564_c36827/f1p1/3524 isoform=cb8564_c36827;full_length_coverage=1;non_full_length_coverage=1;isoform_length=3524;expected_accuracy=0.997</td><td>gnl|BL_ORD_ID|13964 cb8564_c46693/f1p0/4471 isoform=cb8564_c46693;full_length_coverage=1;non_full_length_coverage=0;isoform_length=4471;expected_accuracy=0.994</td><td>2</td><td>3014</td><td>3012</td><td>3524</td><td>1</td><td>3044</td><td>3299</td><td>3823</td></tr>
<tr><td>cb8564_c23232/f1p3/2540</td><td>gnl|BL_ORD_ID|55450 cb8564_c41908/f1p0/2743</td><td>16</td><td>2172</td><td>2167</td><td>2495</td><td>3</td><td>2158</td><td>2260</td><td>2586</td></tr>
<tr><td>cb8564_c44005/f1p0/3815 isoform=cb8564_c44005;full_length_coverage=1;non_full_length_coverage=0;isoform_length=3815;expected_accuracy=0.993</td><td>gnl|BL_ORD_ID|55964 cb8564_c50435/f1p0/4916</td><td>18</td><td>2046</td><td>2041</td><td>3773</td><td>4</td><td>2041</td><td>2141</td><td>3881</td></tr>
<tr><td>cb4007_c19/f1p0/2782</td><td>gnl|BL_ORD_ID|71719 cb4007_c46/f1p0/3154</td><td>136</td><td>1938</td><td>1937</td><td>2778</td><td>1</td><td>180</td><td>2145</td><td>298</td></tr>
</table>

isoform=cb4007_c19;full_length_coverage=1;non_full_length_coverage=0;isoform_length=2782;expected_accuracy=0.995							3		5
cb5550_c20/f1p0/3027	gnl|BL_ORD_ID|45197 cb5550_c10/f1p0/4832 isoform=cb5550_c10;full_length_coverage=1;non_full_length_coverage=0;isoform_length=4832;expected_accuracy=0.998	10	2608	2603	2890	1	2599	4545	4832
cb14634_c52/f1p1/4305	gnl|BL_ORD_ID|27757 cb14634_c75/f2p3/4631 isoform=cb14634_c75;full_length_coverage=2;non_full_length_coverage=3;isoform_length=4631;expected_accuracy=0.996	23	2658	2653	4320	1	2636	2754	4418
cb8564_c23232/f1p3/2540	gnl|BL_ORD_ID|17008 cb8564_c55271/f1p4/2648 isoform=cb8564_c55271;full_length_coverage=1;non_full_length_coverage=4;isoform_length=2648;expected_accuracy=0.998	16	2172	2167	2511	6	2175	2277	2616
cb12931_c15/f1p0/1493 isoform=cb12931_c15;full_length_coverage=1;non_full_length_coverage=0;isoform_length=1493;expected_accuracy=1.000	gnl|BL_ORD_ID|61972 cb12931_c30/f1p0/5903	1	1106	1104	1493	1	1100	2480	2869
cb2804_c78/f1p1/3184 isoform=cb2804_c78;full_length_coverage=1;non_full_length_coverage=1;isoform_length=3184;expected_accuracy=0.994	gnl|BL_ORD_ID|38356 cb2804_c93/f1p0/3636 isoform=cb2804_c93;full_length_coverage=1;non_full_length_coverage=0;isoform_length=3636;expected_accuracy=0.991	2	2528	2526	3148	1	2511	2771	3390
cb2707_c10/f3p0/2850 isoform=cb2707_c10;full_length_coverage=3;non_full_length_coverage=0;isoform_length=2850;expected_accu	gnl|BL_ORD_ID|36995 cb2707_c28/f1p1/3570 isoform=cb2707_c28;full_length_coverage=1;non_full_length_coverage=1;isoform_length=3570;expected_accur	1	2379	2379	2850	309	2684	3012	3483

racy=1.000	acy=0.996								
cb8564_c40764/f1p0/2563 isoform=cb8564_c40764;full_length_coverage=1;non_full_length_coverage=0;isoform_length=2563;expected_accuracy=0.997	gnl|BL_ORD_ID|55464 cb8564_c42285/f1p0/3370	1	2166	2162	2563	1	2162	2370	2771
cb2779_c5/f1p0/1790 isoform=cb2779_c5;full_length_coverage=1;non_full_length_coverage=0;isoform_length=1790;expected_accuracy=0.993	gnl|BL_ORD_ID|39065 cb2779_c3/f1p1/3765 isoform=cb2779_c3;full_length_coverage=1;non_full_length_coverage=1;isoform_length=3765;expected_accuracy=0.991	1	1078	1077	1788	17	1098	2990	3697
cb8564_c71816/f1p1/3018	gnl|BL_ORD_ID|17471 cb8564_c8798/f12p2/2958 isoform=cb8564_c8798;full_length_coverage=12;non_full_length_coverage=2;isoform_length=2958;expected_accuracy=1.000	1	2019	2020	2923	138	2155	2055	2958
cb18456_c1776/f1p0/1211	gnl|BL_ORD_ID|53550 cb8564_c128527/f1p1/2034	280	1214	8	285	964	30	1350	1076
cb792_c53/f1p0/3930	gnl|BL_ORD_ID|50981 cb792_c47/f1p0/3981	1	1842	1838	3663	104	1945	2076	3901
cb2804_c187/f1p0/1491	gnl|BL_ORD_ID|38911 cb2804_c50/f2p0/2193 isoform=cb2804_c50;full_length_coverage=2;non_full_length_coverage=0;isoform_length=2193;expected_accuracy=0.992	133	1425	1	135	619	1909	1	133
cb6889_c8/f1p0/2744	gnl|BL_ORD_ID|5980 cb6889_c7/f3p1/2671 isoform=cb6889_c7;full_length_coverage=3;non_full_length_coverage=1;isoform_length=2671;expected_accuracy=1.000	330	2348	41	335	653	2671	1	295
cb426_c6/f1p0/2161	gnl|BL_ORD_ID|42757 cb426_c2/f1p0/2463	1	1852	1847	2110	47	189	2201	246

	isoform=cb426_c2;full_length_coverage=1;non_full_length_coverage=0;isoform_length=2463;expected_accuracy=0.998						7		3
cb6448_c5/f2p0/2744 isoform=cb6448_c5;full_length_coverage=2;non_full_length_coverage=0;isoform_length=2744;expected_accuracy=1.000	gnl|BL_ORD_ID|4720 cb6448_c6/f1p0/3003 isoform=cb6448_c6;full_length_coverage=1;non_full_length_coverage=0;isoform_length=3003;expected_accuracy=0.995	1	2401	2402	2744	84	247 2	2630	297 3
cb8564_c36056/f1p0/2652 isoform=cb8564_c36056;full_length_coverage=1;non_full_length_coverage=0;isoform_length=2652;expected_accuracy=0.999	gnl|BL_ORD_ID|55160 cb8564_c34262/f1p0/3120	248	2652	131	251	419	282 4	155	275
cb8564_c86635/f1p17/2750	gnl|BL_ORD_ID|56381 cb8564_c75626/f1p2/3199	1	2113	2111	2699	4	2116	2611	319 9
cb1626_c14/f1p0/2173 isoform=cb1626_c14;full_length_coverage=1;non_full_length_coverage=0;isoform_length=2173;expected_accuracy=0.999	gnl|BL_ORD_ID|33068 cb1626_c13/f1p0/4596 isoform=cb1626_c13;full_length_coverage=1;non_full_length_coverage=0;isoform_length=4596;expected_accuracy=1.000	1001	2169	1	1002	3426	459 6	77	108 0
cb6621_c2/f1p0/2658	gnl|BL_ORD_ID|6173 cb6621_c0/f1p0/2807 isoform=cb6621_c0;full_length_coverage=1;non_full_length_coverage=0;isoform_length=2807;expected_accuracy=0.996	214	2557	17	217	463	280 6	1	201
cb5431_c1/f1p0/2992 isoform=cb5431_c1;full_length_coverage=1;non_full_length_coverage=0;isoform_length=2992;expected_accuracy=0.994	gnl|BL_ORD_ID|73915 cb5431_c0/f1p0/3094	14	2811	2808	2992	1	279 5	2895	307 8
cb8564_c89122/f1p0/3335	gnl|BL_ORD_ID|14585 cb8564_c36952/f1p0/3622	528	2915	81	528	1229	360	22	471

	isoform=cb8564_c36952;full_length_coverage=1;non_full_length_coverage=0;isoform_length=3622;expected_accuracy=0.994						9		
cb8564_c49356/f1p0/3133	gnl|BL_ORD_ID|14407 cb8564_c44036/f1p0/3258 isoform=cb8564_c44036;full_length_coverage=1;non_full_length_coverage=0;isoform_length=3258;expected_accuracy=0.992	1144	2848	1	1144	1575	3258	1	1147
cb8564_c79401/f1p0/4816	gnl|BL_ORD_ID|35088 cb18812_c9/f1p1/5153 isoform=cb18812_c9;full_length_coverage=1;non_full_length_coverage=1;isoform_length=5153;expected_accuracy=0.993	1072	4399	1	1068	1823	5153	866	1936
cb396_c48/f1p0/2172	gnl|BL_ORD_ID|39524 cb396_c4/f9p0/2621 isoform=cb396_c4;full_length_coverage=9;non_full_length_coverage=0;isoform_length=2621;expected_accuracy=1.000	238	2104	4	238	644	2509	161	395
cb8564_c183213/f1p3/3190 isoform=cb8564_c183213;full_length_coverage=1;non_full_length_coverage=3;isoform_length=3190;expected_accuracy=0.999	gnl|BL_ORD_ID|18665 cb8564_c78920/f1p1/3989 isoform=cb8564_c78920;full_length_coverage=1;non_full_length_coverage=1;isoform_length=3989;expected_accuracy=0.996	1	2313	2318	3190	1	2312	3128	3989
cb3898_c1/f3p1/2663 isoform=cb3898_c1;full_length_coverage=3;non_full_length_coverage=1;isoform_length=2663;expected_accuracy=0.999	gnl|BL_ORD_ID|39329 cb3898_c5/f1p0/2807 isoform=cb3898_c5;full_length_coverage=1;non_full_length_coverage=0;isoform_length=2807;expected_accuracy=0.999	1	1316	1315	2609	14	1333	1513	2807
cb8564_c129284/f1p0/2118 isoform=cb8564_c129284;full_length_coverage=1;non_full_length_coverage=0;isoform_length=2118;expected_	gnl|BL_ORD_ID|13455 cb8564_c182847/f1p0/2435 isoform=cb8564_c182847;full_length_coverage=1;non_full_length_coverage=0;isoform_length=2435;expected_a	1	1970	1965	2118	42	2023	2206	2360

accuracy=0.991	ccuracy=1.000								
cb8564_c45242/f1p0/2126 isoform=cb8564_c45242;full_length_coverage=1;non_full_length_coverage=0;isoform_length=2126;expected_accuracy=0.990	gnl|BL_ORD_ID|16705 cb8564_c53880/f1p0/3484 isoform=cb8564_c53880;full_length_coverage=1;non_full_length_coverage=0;isoform_length=3484;expected_accuracy=0.996	320	2125	71	321	1672	3484	807	1056
cb3039_c11/f1p0/3305	gnl|BL_ORD_ID|69065 cb3039_c9/f1p0/3676	131	3353	1	133	362	3584	53	185
cb8564_c38947/f1p0/2650 isoform=cb8564_c38947;full_length_coverage=1;non_full_length_coverage=0;isoform_length=2650;expected_accuracy=0.996	gnl|BL_ORD_ID|55574 cb8564_c45699/f1p0/3005	1	1441	1436	2650	1	1421	1636	2848
cb14630_c37/f1p1/5449 isoform=cb14630_c37;full_length_coverage=1;non_full_length_coverage=1;isoform_length=5449;expected_accuracy=0.996	gnl|BL_ORD_ID|64186 cb14630_c30/f1p0/5869	1	4273	4268	5449	31	4301	4474	5657
cb8637_c62/f1p0/1631	gnl|BL_ORD_ID|19203 cb8637_c50/f9p0/1981 isoform=cb8637_c50;full_length_coverage=9;non_full_length_coverage=0;isoform_length=1981;expected_accuracy=1.000	673	1595	75	678	1030	1952	111	714
cb10162_c7/f1p0/1683 isoform=cb10162_c7;full_length_coverage=1;non_full_length_coverage=0;isoform_length=1683;expected_accuracy=0.995	gnl|BL_ORD_ID|48152 cb10162_c4/f1p0/1934	1	1235	1238	1679	105	1338	1506	1933
cb18456_c6049/f1p1/1546 isoform=cb18456_c6049;full_length_coverage=1;non_full_length_coverage=1;isoform_length=1546;expected_	gnl|BL_ORD_ID|17669 cb8564_c868/f2p9/1907 isoform=cb8564_c868;full_length_coverage=2;non_full_length_coverage=9;isoform_length=1907;expected_acc	294	1512	5	294	690	1907	1	285

accuracy=0.991	uracy=1.000								
cb7642_c201/f1p0/1831 isoform=cb7642_c201;full_length_coverage=1;non_full_length_coverage=0;isoform_length=1831;expected_accuracy=0.992	gnl|BL_ORD_ID|6564 cb7642_c135/f1p0/2317 isoform=cb7642_c135;full_length_coverage=1;non_full_length_coverage=0;isoform_length=2317;expected_accuracy=0.992	1	1029	1028	1831	263	1296	1513	2315
cb8564_c41692/f1p3/2987 isoform=cb8564_c41692;full_length_coverage=1;non_full_length_coverage=3;isoform_length=2987;expected_accuracy=0.995	gnl|BL_ORD_ID|17429 cb8564_c85447/f1p0/3196 isoform=cb8564_c85447;full_length_coverage=1;non_full_length_coverage=0;isoform_length=3196;expected_accuracy=0.997	1	2450	2449	2987	3	2413	2660	3192
cb6968_c6/f1p0/2273 isoform=cb6968_c6;full_length_coverage=1;non_full_length_coverage=0;isoform_length=2273;expected_accuracy=0.996	gnl|BL_ORD_ID|6148 cb6968_c18/f2p0/2681 isoform=cb6968_c18;full_length_coverage=2;non_full_length_coverage=0;isoform_length=2681;expected_accuracy=1.000	185	2273	1	185	434	2523	89	274
cb4932_c8/f1p1/1956 isoform=cb4932_c8;full_length_coverage=1;non_full_length_coverage=1;isoform_length=1956;expected_accuracy=0.992	gnl|BL_ORD_ID|42883 cb4932_c5/f1p0/2219 isoform=cb4932_c5;full_length_coverage=1;non_full_length_coverage=0;isoform_length=2219;expected_accuracy=1.000	1	1130	1129	1871	1	1132	1475	2219
cb2156_c24/f1p1/2545 isoform=cb2156_c24;full_length_coverage=1;non_full_length_coverage=1;isoform_length=2545;expected_accuracy=0.991	gnl|BL_ORD_ID|35008 cb2156_c83/f2p1/2873 isoform=cb2156_c83;full_length_coverage=2;non_full_length_coverage=1;isoform_length=2873;expected_accuracy=1.000	1	1422	1420	2545	164	1605	1730	2860
cb8564_c86591/f1p1/3640 isoform=cb8564_c86591;full_length_coverage=1;non_full_length_coverage=1;isoform_length=3640;expected_accuracy=0.997	gnl|BL_ORD_ID|57819 cb8564_c81742/f1p0/3716	1791	3515	1	1792	1981	3704	1	1778
cb8564_c40953/f1p3/3141	gnl|BL_ORD_ID|16338 cb8564_c53034/f1p2/3495	113	3119	1	117	468	349	29	145

isoform=cb8564_c40953;full_length_coverage=1;non_full_length_coverage=3;isoform_length=3141;expected_accuracy=0.997	isoform=cb8564_c53034;full_length_coverage=1;non_full_length_coverage=2;isoform_length=3495;expected_accuracy=0.998						5		
cb1279_c85/f1p0/1794 isoform=cb1279_c85;full_length_coverage=1;non_full_length_coverage=0;isoform_length=1794;expected_accuracy=0.998	gnl|BL_ORD_ID|27062 cb1279_c41/f1p1/2811 isoform=cb1279_c41;full_length_coverage=1;non_full_length_coverage=1;isoform_length=2811;expected_accuracy=0.995	1	1120	1115	1794	41	1155	2143	2811
cb8564_c42814/f1p0/2151 isoform=cb8564_c42814;full_length_coverage=1;non_full_length_coverage=0;isoform_length=2151;expected_accuracy=0.993	gnl|BL_ORD_ID|61972 cb12931_c30/f1p0/5903	650	2151	7	652	1362	2866	1	674
cb4016_c32/f1p0/1913	gnl|BL_ORD_ID|40526 cb4016_c7/f1p0/2879 isoform=cb4016_c7;full_length_coverage=1;non_full_length_coverage=0;isoform_length=2879;expected_accuracy=0.998	191	1930	23	194	1105	2844	504	677
cb2640_c23/f1p0/2402	gnl|BL_ORD_ID|37200 cb2640_c10/f1p0/3278 isoform=cb2640_c10;full_length_coverage=1;non_full_length_coverage=0;isoform_length=3278;expected_accuracy=0.993	1	2036	2038	2406	1	2061	2788	3157
cb7168_c7/f1p1/2326	gnl|BL_ORD_ID|4849 cb7168_c13/f1p1/2387 isoform=cb7168_c13;full_length_coverage=1;non_full_length_coverage=1;isoform_length=2387;expected_accuracy=0.993	1	1935	1934	2218	1	1932	2103	2387
cb8564_c87089/f1p0/4524	gnl|BL_ORD_ID|67930 cb18656_c10/f1p0/5311	1	3980	3981	4466	123	4102	4844	5328
cb15911_c2/f1p0/1696	gnl|BL_ORD_ID|16165 cb8564_c75981/f2p0/4046	1	1122	1120	1696	9	1129	3470	404

isoform=cb15911_c2;full_length_coverage=1;non_full_length_coverage=0;isoform_length=1696;expected_accuracy=0.993	isoform=cb8564_c75981;full_length_coverage=2;non_full_length_coverage=0;isoform_length=4046;expected_accuracy=0.995								5
cb17744_c21/f1p1/1357 isoform=cb17744_c21;full_length_coverage=1;non_full_length_coverage=1;isoform_length=1357;expected_accuracy=0.997	gnl|BL_ORD_ID|31103 cb17744_c12/f1p0/5455 isoform=cb17744_c12;full_length_coverage=1;non_full_length_coverage=0;isoform_length=5455;expected_accuracy=0.998	470	1357	63	470	4443	5326	1	407
cb8564_c129284/f1p0/2118 isoform=cb8564_c129284;full_length_coverage=1;non_full_length_coverage=0;isoform_length=2118;expected_accuracy=0.991	gnl|BL_ORD_ID|11977 cb8564_c146395/f9p1/2471 isoform=cb8564_c146395;full_length_coverage=9;non_full_length_coverage=1;isoform_length=2471;expected_accuracy=0.998	1	1970	1965	2118	1	1982	2165	2319
cb1038_c3/f1p0/3023	gnl|BL_ORD_ID|53 cb1038_c2/f1p0/3151 isoform=cb1038_c2;full_length_coverage=1;non_full_length_coverage=0;isoform_length=3151;expected_accuracy=0.995	1	2444	2440	2839	54	2495	2733	3130
cb8564_c34922/f1p0/2696 isoform=cb8564_c34922;full_length_coverage=1;non_full_length_coverage=0;isoform_length=2696;expected_accuracy=0.993	gnl|BL_ORD_ID|16705 cb8564_c53880/f1p0/3484 isoform=cb8564_c53880;full_length_coverage=1;non_full_length_coverage=0;isoform_length=3484;expected_accuracy=0.996	1053	2696	1	1054	1672	3345	1	1056
cb513_c3/f1p0/2903 isoform=cb513_c3;full_length_coverage=1;non_full_length_coverage=0;isoform_length=2903;expected_accuracy=1.000	gnl|BL_ORD_ID|43725 cb513_c4/f1p0/3166 isoform=cb513_c4;full_length_coverage=1;non_full_length_coverage=0;isoform_length=3166;expected_accuracy=0.995	1	2041	2040	2902	85	2149	2304	3166
cb8123_c3/f1p0/2320 isoform=cb8123_c3;full_length_coverage=1;non_full_length_coverage=0;isoform_length=2320;expected_accur	gnl|BL_ORD_ID|8831 cb8123_c4/f1p0/3382 isoform=cb8123_c4;full_length_coverage=1;non_full_length_coverage=0;isoform_length=3382;expected_accura	251	2306	45	250	1270	3322	1	208

acy=0.992	cy=0.996								
cb13938_c10/f1p0/1187	gnl|BL_ORD_ID|72021 cb4825_c12/f1p0/4476	151	1146	4	150	3496	4491	2473	2619
cb8564_c89918/f1p1/2045 isoform=cb8564_c89918;full_length_coverage=1;non_full_length_coverage=1;isoform_length=2045;expected_accuracy=0.991	gnl|BL_ORD_ID|12415 cb8564_c147167/f5p5/2396 isoform=cb8564_c147167;full_length_coverage=5;non_full_length_coverage=5;isoform_length=2396;expected_accuracy=0.996	1	1616	1611	2045	92	1713	1908	2342
cb4363_c5/f1p0/1115 isoform=cb4363_c5;full_length_coverage=1;non_full_length_coverage=0;isoform_length=1115;expected_accuracy=1.000	gnl|BL_ORD_ID|41671 cb4363_c4/f1p0/1267 isoform=cb4363_c4;full_length_coverage=1;non_full_length_coverage=0;isoform_length=1267;expected_accuracy=1.000	556	1115	56	558	691	1250	38	540
cb18646_c1/f2p2/6581 isoform=cb18646_c1;full_length_coverage=2;non_full_length_coverage=2;isoform_length=6581;expected_accuracy=0.999	gnl|BL_ORD_ID|34398 cb18646_c0/f2p2/6586 isoform=cb18646_c0;full_length_coverage=2;non_full_length_coverage=2;isoform_length=6586;expected_accuracy=0.999	1	6228	6226	6425	23	6265	6390	6586
cb8564_c4687/f1p0/2736 isoform=cb8564_c4687;full_length_coverage=1;non_full_length_coverage=0;isoform_length=2736;expected_accuracy=0.995	gnl|BL_ORD_ID|15232 cb8564_c4689/f1p0/4442 isoform=cb8564_c4689;full_length_coverage=1;non_full_length_coverage=0;isoform_length=4442;expected_accuracy=0.997	247	2736	1	248	1870	4363	1	245
cb8564_c21515/f1p0/3130	gnl|BL_ORD_ID|12196 cb8564_c12654/f2p0/4122 isoform=cb8564_c12654;full_length_coverage=2;non_full_length_coverage=0;isoform_length=4122;expected_accuracy=1.000	1	1388	1388	2726	514	1901	2784	4122
cb415_c43/f1p0/3304 isoform=cb415_c43;full_length_coverage=1;non_full_length_coverage=0;isoform_length=3304;expected_accur	gnl|BL_ORD_ID|72143 cb415_c84/f1p0/3822	24	2924	2922	3304	88	2981	3304	3698

acy=0.991									
cb10206_c17/f1p0/1410 isoform=cb10206_c17;full_length_coverage=1;non_full_length_coverage=0;isoform_length=1410;expected_accuracy=0.995	gnl|BL_ORD_ID|52307 cb8564_c117746/f1p12/3020	1	1001	1001	1410	1365	2367	2475	2887
cb16544_c4/f1p0/1718 isoform=cb16544_c4;full_length_coverage=1;non_full_length_coverage=0;isoform_length=1718;expected_accuracy=0.995	gnl|BL_ORD_ID|43096 cb4493_c17/f1p0/3073 isoform=cb4493_c17;full_length_coverage=1;non_full_length_coverage=0;isoform_length=3073;expected_accuracy=0.999	1	1329	1325	1718	92	1414	2643	3037
cb15567_c9/f1p1/1733 isoform=cb15567_c9;full_length_coverage=1;non_full_length_coverage=1;isoform_length=1733;expected_accuracy=0.994	gnl|BL_ORD_ID|9735 cb8564_c108152/f2p1/1972 isoform=cb8564_c108152;full_length_coverage=2;non_full_length_coverage=1;isoform_length=1972;expected_accuracy=1.000	1	1626	1622	1733	2	1626	1739	1850
cb8564_c71195/f1p0/3415	gnl|BL_ORD_ID|15376 cb8564_c31588/f10p3/3394 isoform=cb8564_c31588;full_length_coverage=10;non_full_length_coverage=3;isoform_length=3394;expected_accuracy=1.000	1	1755	1756	3373	134	1888	1768	3384
cb8516_c0/f3p1/2129 isoform=cb8516_c0;full_length_coverage=3;non_full_length_coverage=1;isoform_length=2129;expected_accuracy=0.999	gnl|BL_ORD_ID|52576 cb8516_c3/f1p0/2479	1	1817	1817	2129	20	1854	1984	2312
cb8564_c129787/f1p0/2756	gnl|BL_ORD_ID|12485 cb8564_c122733/f1p0/3383 isoform=cb8564_c122733;full_length_coverage=1;non_full_length_coverage=0;isoform_length=3383;expected_accuracy=0.993	154	2252	1	156	1289	3383	120	270
cb2229_c69/f3p1/2289	gnl|BL_ORD_ID|38403 cb2229_c71/f3p0/3903	1	1771	1768	2289	5	177	3303	382

isoform=cb2229_c69;full_length_coverage=3;non_full_length_coverage=1;isoform_length=2289;expected_accuracy=1.000	isoform=cb2229_c71;full_length_coverage=3;non_full_length_coverage=0;isoform_length=3903;expected_accuracy=1.000						4		4
cb8564_c71076/f1p2/2391	gnl|BL_ORD_ID|56913 cb8564_c76756/f1p1/3429	142	2363	1	142	1206	3427	905	1046
cb8564_c47011/f1p0/3100	gnl|BL_ORD_ID|15169 cb8564_c40218/f1p0/3146 isoform=cb8564_c40218;full_length_coverage=1;non_full_length_coverage=0;isoform_length=3146;expected_accuracy=0.993	2	2306	2301	2934	3	2284	2519	3143
cb1462_c18/f1p0/3693	gnl|BL_ORD_ID|28515 cb1462_c7/f1p0/3911 isoform=cb1462_c7;full_length_coverage=1;non_full_length_coverage=0;isoform_length=3911;expected_accuracy=0.993	250	3603	1	250	579	3907	53	319
cb8564_c51444/f1p0/2956	gnl|BL_ORD_ID|57736 cb8564_c79306/f1p0/3361	2	2463	2458	2975	1	2462	2562	3078
cb8564_c129284/f1p0/2118 isoform=cb8564_c129284;full_length_coverage=1;non_full_length_coverage=0;isoform_length=2118;expected_accuracy=0.991	gnl|BL_ORD_ID|55471 cb8564_c42491/f1p0/2564	1	1970	1965	2118	1	1975	2158	2312
cb12003_c138/f1p0/1068 isoform=cb12003_c138;full_length_coverage=1;non_full_length_coverage=0;isoform_length=1068;expected_accuracy=1.000	gnl|BL_ORD_ID|42020 cb4768_c19/f1p4/2022 isoform=cb4768_c19;full_length_coverage=1;non_full_length_coverage=4;isoform_length=2022;expected_accuracy=0.997	420	1068	9	424	636	1285	1	415
cb18456_c2265/f1p0/1523 isoform=cb18456_c2265;full_length_coverage=1;non_full_length_coverage=0;isoform_length=1523;expected_	gnl|BL_ORD_ID|74175 cb5625_c0/f1p0/3054	164	1523	9	164	1517	2855	2	151

accuracy=0.997									
cb7584_c15/f1p0/1293	gnl|BL_ORD_ID|6937 cb7584_c7/f1p0/2579 isoform=cb7584_c7;full_length_coverage=1;non_full_length_coverage=0;isoform_length=2579;expected_accuracy=0.997	221	1310	37	225	1482	2579	1	190
cb5688_c3/f2p0/1460 isoform=cb5688_c3;full_length_coverage=2;non_full_length_coverage=0;isoform_length=1460;expected_accuracy=1.000	gnl|BL_ORD_ID|44795 cb5688_c9/f3p1/2411 isoform=cb5688_c9;full_length_coverage=3;non_full_length_coverage=1;isoform_length=2411;expected_accuracy=1.000	124	1423	1	126	1112	2411	113	237
cb8564_c40764/f1p0/2563 isoform=cb8564_c40764;full_length_coverage=1;non_full_length_coverage=0;isoform_length=2563;expected_accuracy=0.997	gnl|BL_ORD_ID|12938 cb8564_c181163/f1p1/2936 isoform=cb8564_c181163;full_length_coverage=1;non_full_length_coverage=1;isoform_length=2936;expected_accuracy=0.993	1	2166	2165	2563	5	2154	2363	2757
cb8564_c176017/f8p2/2488 isoform=cb8564_c176017;full_length_coverage=8;non_full_length_coverage=2;isoform_length=2488;expected_accuracy=1.000	gnl|BL_ORD_ID|54500 cb8564_c124528/f1p0/4445	1	1225	1225	2395	1	1225	3079	4249
cb454_c4/f3p0/2268 isoform=cb454_c4;full_length_coverage=3;non_full_length_coverage=0;isoform_length=2268;expected_accuracy=1.000	gnl|BL_ORD_ID|72201 cb454_c19/f1p0/2558	221	2264	1	220	406	2453	81	300
cb9079_c4/f1p0/1895	gnl|BL_ORD_ID|59491 cb9079_c2/f1p0/2193	292	1711	5	295	461	1881	15	305
cb8564_c51223/f1p4/1975	gnl|BL_ORD_ID|14463 cb8564_c25154/f1p5/4276 isoform=cb8564_c25154;full_length_coverage=1;non_full_length_coverage=5;isoform_length=4276;expected_ac	207	1937	21	208	2415	4145	2	189

	curacy=0.995								
cb8564_c118011/f1p0/1965	gnl|BL_ORD_ID|13895 cb8564_c23584/f1p4/2247 isoform=cb8564_c23584;full_length_coverage=1;non_full_length_coverage=4;isoform_length=2247;expected_accuracy=0.990	1	1321	1317	1964	60	1377	1555	2202
cb862_c26/f1p0/2964	gnl|BL_ORD_ID|17904 cb862_c22/f1p0/3314 isoform=cb862_c22;full_length_coverage=1;non_full_length_coverage=0;isoform_length=3314;expected_accuracy=0.995	1	1527	1526	2931	1	1523	1914	3314
cb8564_c40764/f1p0/2563 isoform=cb8564_c40764;full_length_coverage=1;non_full_length_coverage=0;isoform_length=2563;expected_accuracy=0.997	gnl|BL_ORD_ID|14529 cb8564_c44074/f1p1/2922 isoform=cb8564_c44074;full_length_coverage=1;non_full_length_coverage=1;isoform_length=2922;expected_accuracy=0.994	1	2167	2162	2563	1	2142	2349	2743
cb8072_c0/f18p1/2410 isoform=cb8072_c0;full_length_coverage=18;non_full_length_coverage=1;isoform_length=2410;expected_accuracy=1.000	gnl|BL_ORD_ID|6594 cb8072_c3/f1p0/2585 isoform=cb8072_c3;full_length_coverage=1;non_full_length_coverage=0;isoform_length=2585;expected_accuracy=0.996	1	2130	2129	2407	1	2128	2308	2585
cb3665_c20/f1p0/2779	gnl|BL_ORD_ID|71424 cb3665_c5/f1p0/2924	12	1482	1479	2669	1	1471	1602	2792
cb8564_c76007/f1p0/2966	gnl|BL_ORD_ID|17429 cb8564_c85447/f1p0/3196 isoform=cb8564_c85447;full_length_coverage=1;non_full_length_coverage=0;isoform_length=3196;expected_accuracy=0.997	12	2421	2420	2946	6	2413	2660	3193
cb8564_c84695/f1p1/2710 isoform=cb8564_c84695;full_length_coverage=1;non_full_length_coverage=1;isoform_length=2710;expected_	gnl|BL_ORD_ID|13176 cb8564_c15957/f1p0/2838 isoform=cb8564_c15957;full_length_coverage=1;non_full_length_coverage=0;isoform_length=2838;expected_ac	1	1692	1691	2660	1	1684	1792	2756

accuracy=0.998	curacy=0.994								
cb5421_c1/f2p0/1551 isoform=cb5421_c1;full_length_coverage=2;non_full_length_coverage=0;isoform_length=1551;expected_accuracy=0.996	gnl|BL_ORD_ID|73402 cb5421_c7/f1p0/2594	127	1523	1	127	1207	2604	1	127
cb5714_c13/f1p0/1198 isoform=cb5714_c13;full_length_coverage=1;non_full_length_coverage=0;isoform_length=1198;expected_accuracy=0.996	gnl|BL_ORD_ID|74326 cb5714_c1/f1p0/3032	127	1198	16	129	1572	2645	1	113
cb4641_c4/f2p1/2180 isoform=cb4641_c4;full_length_coverage=2;non_full_length_coverage=1;isoform_length=2180;expected_accuracy=0.999	gnl|BL_ORD_ID|42928 cb4641_c7/f1p1/2278 isoform=cb4641_c7;full_length_coverage=1;non_full_length_coverage=1;isoform_length=2278;expected_accuracy=0.995	10	1173	1172	2178	4	1155	1267	2275
cb4997_c16/f1p0/2058	gnl|BL_ORD_ID|73169 cb4997_c12/f1p1/2947	1	1406	1404	1998	154	1559	2281	2876
cb8564_c188097/f1p0/2552 isoform=cb8564_c188097;full_length_coverage=1;non_full_length_coverage=0;isoform_length=2552;expected_accuracy=0.996	gnl|BL_ORD_ID|55603 cb8564_c46500/f1p1/2752	1	1945	1940	2552	2	1942	2043	2655
cb8564_c18838/f2p0/2444 isoform=cb8564_c18838;full_length_coverage=2;non_full_length_coverage=0;isoform_length=2444;expected_accuracy=0.994	gnl|BL_ORD_ID|55156 cb8564_c25661/f1p0/2727	154	2444	1	154	437	2727	57	210
cb8564_c18662/f1p0/2467 isoform=cb8564_c18662;full_length_coverage=1;non_full_length_coverage=0;isoform_length=2467;expected_	gnl|BL_ORD_ID|10489 cb8564_c11876/f2p2/3220 isoform=cb8564_c11876;full_length_coverage=2;non_full_length_coverage=2;isoform_length=3220;expected_ac	1	1823	1824	2464	176	2001	2469	3110

accuracy=0.997	curacy=0.998								
cb18012_c2/f1p0/1829	gnl|BL_ORD_ID|54874 cb8564_c3238/f1p0/2188	176	1762	1	176	507	2093	34	209
cb3229_c12/f1p0/2616	gnl|BL_ORD_ID|39328 cb3229_c23/f1p0/2590 isoform=cb3229_c23;full_length_coverage=1;non_full_length_coverage=0;isoform_length=2590;expected_accuracy=0.996	444	2405	5	448	630	2590	12	466
cb5896_c44/f1p0/2069 isoform=cb5896_c44;full_length_coverage=1;non_full_length_coverage=0;isoform_length=2069;expected_accuracy=0.991	gnl|BL_ORD_ID|74562 cb5896_c45/f1p0/2541	1	1372	1371	2069	16	1387	1599	2297
cb7345_c17/f1p0/1467 isoform=cb7345_c17;full_length_coverage=1;non_full_length_coverage=0;isoform_length=1467;expected_accuracy=0.990	gnl|BL_ORD_ID|51135 cb7345_c10/f1p2/1653	1	1237	1235	1467	59	1295	1411	1643
cb8564_c90427/f2p1/2823 isoform=cb8564_c90427;full_length_coverage=2;non_full_length_coverage=1;isoform_length=2823;expected_accuracy=0.994	gnl|BL_ORD_ID|57868 cb8564_c82903/f1p0/3274	1	1912	1914	2823	1	1887	1994	2902
cb307_c6/f1p1/4064 isoform=cb307_c6;full_length_coverage=1;non_full_length_coverage=1;isoform_length=4064;expected_accuracy=0.997	gnl|BL_ORD_ID|37483 cb307_c5/f1p1/4350 isoform=cb307_c5;full_length_coverage=1;non_full_length_coverage=1;isoform_length=4350;expected_accuracy=0.997	1	2744	2744	4064	4	2729	2978	4294
cb8564_c181853/f1p0/2119 isoform=cb8564_c181853;full_length_coverage=1;non_full_length_coverage=0;isoform_length=2119;expected_	gnl|BL_ORD_ID|17100 cb8564_c78503/f1p0/2935 isoform=cb8564_c78503;full_length_coverage=1;non_full_length_coverage=0;isoform_length=2935;expected_ac	1	1339	1334	2119	1	1339	2137	2921

accuracy=0.997	curacy=0.997								
cb2208_c33/f1p0/3174	gnl|BL_ORD_ID|69620 cb2208_c18/f1p0/3335	1	2195	2193	2861	58	2252	2486	3154
cb4016_c33/f1p0/2507	gnl|BL_ORD_ID|71727 cb4016_c35/f1p0/2920	207	2462	1	209	468	2722	22	210
cb1193_c21/f1p1/3672	gnl|BL_ORD_ID|59249 cb1193_c13/f1p1/3683	1	2033	2031	3559	1	2033	2158	3686
cb5013_c16/f1p0/2722	gnl|BL_ORD_ID|42368 cb5013_c14/f1p0/2747 isoform=cb5013_c14;full_length_coverage=1;non_full_length_coverage=0;isoform_length=2747;expected_accuracy=0.993	231	2594	12	231	374	2735	1	215
cb4674_c83/f1p0/2467	gnl|BL_ORD_ID|43546 cb4674_c13/f1p0/2776 isoform=cb4674_c13;full_length_coverage=1;non_full_length_coverage=0;isoform_length=2776;expected_accuracy=0.996	1	1913	1912	2438	12	1948	2057	2583
cb8564_c90478/f1p0/2233 isoform=cb8564_c90478;full_length_coverage=1;non_full_length_coverage=0;isoform_length=2233;expected_accuracy=1.000	gnl|BL_ORD_ID|13061 cb8564_c163547/f1p1/2420 isoform=cb8564_c163547;full_length_coverage=1;non_full_length_coverage=1;isoform_length=2420;expected_accuracy=0.994	1064	2233	1	1066	1251	2419	16	1085
cb8564_c39136/f1p0/3036 isoform=cb8564_c39136;full_length_coverage=1;non_full_length_coverage=0;isoform_length=3036;expected_accuracy=0.998	gnl|BL_ORD_ID|14539 cb8564_c46562/f2p3/3196 isoform=cb8564_c46562;full_length_coverage=2;non_full_length_coverage=3;isoform_length=3196;expected_accuracy=0.995	10	2089	2085	3032	16	2120	2247	3195
cb5643_c124/f5p1/2491 isoform=cb5643_c124;full_length_coverage=5;non_full_length_coverage=1;isoform_length=2491;expected_acc	gnl|BL_ORD_ID|74221 cb5643_c68/f1p0/3247	1131	2472	100	1134	1897	3238	1	1035

uracy=0.997									
cb8564_c48473/f1p2/2701 isoform=cb8564_c48473;full_length_coverage=1;non_full_length_coverage=2;isoform_length=2701;expected_accuracy=0.999	gnl|BL_ORD_ID|56580 cb8564_c47159/f1p0/3238	220	2701	11	225	440	2920	81	296
cb7531_c5/f2p1/2362 isoform=cb7531_c5;full_length_coverage=2;non_full_length_coverage=1;isoform_length=2362;expected_accuracy=1.000	gnl|BL_ORD_ID|51316 cb7531_c18/f1p0/2730	573	2325	6	574	744	2496	22	590
cb4233_c84/f1p1/2344	gnl|BL_ORD_ID|43592 cb4233_c82/f1p1/2791 isoform=cb4233_c82;full_length_coverage=1;non_full_length_coverage=1;isoform_length=2791;expected_accuracy=0.993	227	2389	1	225	527	2687	503	726
cb8564_c110954/f1p1/2392 isoform=cb8564_c110954;full_length_coverage=1;non_full_length_coverage=1;isoform_length=2392;expected_accuracy=0.994	gnl|BL_ORD_ID|11994 cb8564_c130264/f1p0/2624 isoform=cb8564_c130264;full_length_coverage=1;non_full_length_coverage=0;isoform_length=2624;expected_accuracy=0.995	1	2113	2109	2392	18	2155	2323	2605
cb4286_c9/f1p1/2064 isoform=cb4286_c9;full_length_coverage=1;non_full_length_coverage=1;isoform_length=2064;expected_accuracy=0.993	gnl|BL_ORD_ID|43994 cb4286_c20/f6p0/2191 isoform=cb4286_c20;full_length_coverage=6;non_full_length_coverage=0;isoform_length=2191;expected_accuracy=1.000	13	1563	1562	2048	1	1562	1705	2191
cb8564_c4643/f1p1/2554	gnl|BL_ORD_ID|55045 cb8564_c4649/f1p0/3061	1	1705	1700	2551	57	1761	1894	2744
cb8564_c21439/f1p0/2587	gnl|BL_ORD_ID|52675 cb8564_c10772/f1p3/2796	1226	2572	1	1228	1383	2729	32	1262
cb415_c43/f1p0/3304	gnl|BL_ORD_ID|43085 cb415_c15/f3p0/3787	17	2924	2922	3304	86	298	3310	369

isoform=cb415_c43;full_length_coverage=1;non_full_length_coverage=0;isoform_length=3304;expected_accuracy=0.991	isoform=cb415_c15;full_length_coverage=3;non_full_length_coverage=0;isoform_length=3787;expected_accuracy=0.998						5		2
cb13752_c8/f1p0/1329 isoform=cb13752_c8;full_length_coverage=1;non_full_length_coverage=0;isoform_length=1329;expected_accuracy=0.993	gnl|BL_ORD_ID|15482 cb8564_c488/f6p0/1932 isoform=cb8564_c488;full_length_coverage=6;non_full_length_coverage=0;isoform_length=1932;expected_accuracy=1.000	433	1329	7	434	1039	1932	44	473
cb8564_c36689/f1p1/2368 isoform=cb8564_c36689;full_length_coverage=1;non_full_length_coverage=1;isoform_length=2368;expected_accuracy=0.991	gnl|BL_ORD_ID|56601 cb8564_c48153/f1p0/3083	1	1709	1706	2368	13	1712	2211	2875
cb1379_c0/f10p0/3693 isoform=cb1379_c0;full_length_coverage=10;non_full_length_coverage=0;isoform_length=3693;expected_accuracy=1.000	gnl|BL_ORD_ID|27262 cb1379_c4/f1p0/4351 isoform=cb1379_c4;full_length_coverage=1;non_full_length_coverage=0;isoform_length=4351;expected_accuracy=0.999	1209	3693	1	1211	1805	4291	28	1240
cb5564_c13/f1p0/1988 isoform=cb5564_c13;full_length_coverage=1;non_full_length_coverage=0;isoform_length=1988;expected_accuracy=0.995	gnl|BL_ORD_ID|45842 cb5564_c9/f1p0/2154 isoform=cb5564_c9;full_length_coverage=1;non_full_length_coverage=0;isoform_length=2154;expected_accuracy=0.992	1	1082	1083	1981	21	1102	1256	2154
cb8564_c42776/f1p0/2186	gnl|BL_ORD_ID|55276 cb8564_c37079/f1p0/2659	508	2204	6	508	725	2421	1	504
cb3535_c16/f1p0/3449	gnl|BL_ORD_ID|41049 cb3535_c11/f1p1/3518 isoform=cb3535_c11;full_length_coverage=1;non_full_length_coverage=1;isoform_length=3518;expected_accuracy=1.000	11	2589	2585	3416	1	2579	2682	3512
cb8564_c45300/f1p4/3014	gnl|BL_ORD_ID|55243 cb8564_c36242/f1p0/3335	243	3014	1	246	401	317	1	244

isoform=cb8564_c45300;full_length_coverage=1;non_full_length_coverage=4;isoform_length=3014;expected_accuracy=0.996							5		
cb373_c4/f2p0/3694	gnl|BL_ORD_ID|40699 cb373_c6/f1p0/4615 isoform=cb373_c6;full_length_coverage=1;non_full_length_coverage=0;isoform_length=4615;expected_accuracy=0.997	201	3695	1	201	1118	4615	87	286
cb16130_c1/f2p0/1014 isoform=cb16130_c1;full_length_coverage=2;non_full_length_coverage=0;isoform_length=1014;expected_accuracy=0.997	gnl|BL_ORD_ID|29529 cb16130_c3/f1p0/1347 isoform=cb16130_c3;full_length_coverage=1;non_full_length_coverage=0;isoform_length=1347;expected_accuracy=0.993	244	1012	8	243	394	1157	58	290
cb4456_c4/f1p0/1832 isoform=cb4456_c4;full_length_coverage=1;non_full_length_coverage=0;isoform_length=1832;expected_accuracy=0.997	gnl|BL_ORD_ID|43644 cb4456_c0/f1p0/3343 isoform=cb4456_c0;full_length_coverage=1;non_full_length_coverage=0;isoform_length=3343;expected_accuracy=0.996	418	1830	50	423	1929	3341	1	360
cb17535_c10/f1p0/1710 isoform=cb17535_c10;full_length_coverage=1;non_full_length_coverage=0;isoform_length=1710;expected_accuracy=0.994	gnl|BL_ORD_ID|4407 cb7016_c6/f1p0/1894 isoform=cb7016_c6;full_length_coverage=1;non_full_length_coverage=0;isoform_length=1894;expected_accuracy=1.000	1	1127	1128	1688	1	1127	1337	1894
cb2739_c7/f1p0/3065	gnl|BL_ORD_ID|38774 cb2739_c1/f2p0/3546 isoform=cb2739_c1;full_length_coverage=2;non_full_length_coverage=0;isoform_length=3546;expected_accuracy=0.996	1	1801	1800	2872	430	2228	2453	3526
cb8564_c113599/f1p0/2144	gnl|BL_ORD_ID|58043 cb8564_c87344/f1p0/2474	1	1347	1346	2125	44	1389	1567	2345
cb8455_c9/f1p0/2404	gnl|BL_ORD_ID|9508 cb8455_c0/f5p0/2372	655	2220	89	654	775	234	66	630

	isoform=cb8455_c0;full_length_coverage=5;non_full_length_coverage=0;isoform_length=2372;expected_accuracy=1.000						0		
cb8564_c180679/f1p0/2109 isoform=cb8564_c180679;full_length_coverage=1;non_full_length_coverage=0;isoform_length=2109;expected_accuracy=0.999	gnl|BL_ORD_ID|54765 cb8564_c2442/f1p1/2806	198	2109	1	202	581	2492	2	203
cb12336_c11/f1p0/1558	gnl|BL_ORD_ID|58847 cb9677_c8/f1p0/2271	368	1460	8	371	1126	2221	21	384
cb6143_c2/f2p0/2755 isoform=cb6143_c2;full_length_coverage=2;non_full_length_coverage=0;isoform_length=2755;expected_accuracy=0.998	gnl|BL_ORD_ID|45275 cb6143_c4/f1p1/2929 isoform=cb6143_c4;full_length_coverage=1;non_full_length_coverage=1;isoform_length=2929;expected_accuracy=1.000	1249	2752	1	1250	1425	2929	57	1305
cb2215_c11/f1p1/2465 isoform=cb2215_c11;full_length_coverage=1;non_full_length_coverage=1;isoform_length=2465;expected_accuracy=0.997	gnl|BL_ORD_ID|38573 cb2215_c5/f1p0/4352 isoform=cb2215_c5;full_length_coverage=1;non_full_length_coverage=0;isoform_length=4352;expected_accuracy=0.993	1	1442	1442	2447	1303	2744	3336	4352
cb1317_c3/f5p1/2427 isoform=cb1317_c3;full_length_coverage=5;non_full_length_coverage=1;isoform_length=2427;expected_accuracy=1.000	gnl|BL_ORD_ID|27409 cb1317_c11/f1p1/4136 isoform=cb1317_c11;full_length_coverage=1;non_full_length_coverage=1;isoform_length=4136;expected_accuracy=0.997	182	2371	16	181	1953	4136	69	228
cb8564_c186356/f1p0/2744 isoform=cb8564_c186356;full_length_coverage=1;non_full_length_coverage=0;isoform_length=2744;expected_accuracy=0.998	gnl|BL_ORD_ID|55873 cb8564_c47701/f1p0/3144	12	1396	1393	2744	24	1406	1526	2877
cb8564_c50128/f1p0/4343	gnl|BL_ORD_ID|14314 cb8564_c45827/f2p2/4435	29	3278	3274	4077	1	325	3520	432

	isoform=cb8564_c45827;full_length_coverage=2;non_full_length_coverage=2;isoform_length=4435;expected_accuracy=0.999						5		3
cb6802_c46/f1p0/2539 isoform=cb6802_c46;full_length_coverage=1;non_full_length_coverage=0;isoform_length=2539;expected_accuracy=0.991	gnl|BL_ORD_ID|4195 cb6802_c5/f2p0/2683 isoform=cb6802_c5;full_length_coverage=2;non_full_length_coverage=0;isoform_length=2683;expected_accuracy=0.998	205	2539	10	206	343	2670	1	218
cb8564_c89788/f1p0/3521	gnl|BL_ORD_ID|56953 cb8564_c78479/f1p0/3692	12	2063	2061	3367	2	2052	2162	3468
cb5523_c3/f1p0/2036 isoform=cb5523_c3;full_length_coverage=1;non_full_length_coverage=0;isoform_length=2036;expected_accuracy=0.997	gnl|BL_ORD_ID|73421 cb5523_c1/f1p0/3096	16	1720	1719	2036	8	1712	2237	2561
cb8564_c162907/f1p0/2069 isoform=cb8564_c162907;full_length_coverage=1;non_full_length_coverage=0;isoform_length=2069;expected_accuracy=0.993	gnl|BL_ORD_ID|52763 cb8564_c13243/f1p0/4432	261	2057	68	259	2443	4235	2	191
cb16569_c16/f1p0/1481 isoform=cb16569_c16;full_length_coverage=1;non_full_length_coverage=0;isoform_length=1481;expected_accuracy=0.999	gnl|BL_ORD_ID|59373 cb8916_c10/f1p0/2236	612	1481	9	608	833	1702	17	617
cb910_c31/f1p1/2978 isoform=cb910_c31;full_length_coverage=1;non_full_length_coverage=1;isoform_length=2978;expected_accuracy=0.997	gnl|BL_ORD_ID|21606 cb910_c32/f1p1/3242 isoform=cb910_c32;full_length_coverage=1;non_full_length_coverage=1;isoform_length=3242;expected_accuracy=0.998	2	2085	2081	2978	1	2085	2217	3114
cb4006_c13/f1p0/2983	gnl|BL_ORD_ID|39614 cb4006_c6/f2p3/3293	274	2983	1	276	508	321	29	304

isoform=cb4006_c13;full_length_coverage=1;non_full_length_coverage=0;isoform_length=2983;expected_accuracy=0.994	isoform=cb4006_c6;full_length_coverage=2;non_full_length_coverage=3;isoform_length=3293;expected_accuracy=1.000						2		
cb4403_c2/f1p0/1938	gnl|BL_ORD_ID|44241 cb4403_c0/f4p0/1959 isoform=cb4403_c0;full_length_coverage=4;non_full_length_coverage=0;isoform_length=1959;expected_accuracy=1.000	1	1185	1186	1829	2	1183	1316	1959
cb947_c27/f1p1/2208	gnl|BL_ORD_ID|21933 cb947_c14/f1p0/4429 isoform=cb947_c14;full_length_coverage=1;non_full_length_coverage=0;isoform_length=4429;expected_accuracy=0.998	1	1300	1296	2091	10	1309	3575	4370
cb8564_c38233/f1p0/2780	gnl|BL_ORD_ID|58067 cb8564_c87983/f1p0/3569	1	1857	1856	2785	335	2191	2316	3244
cb8564_c44621/f1p1/3432	gnl|BL_ORD_ID|14377 cb8564_c36097/f1p2/3430 isoform=cb8564_c36097;full_length_coverage=1;non_full_length_coverage=2;isoform_length=3430;expected_accuracy=0.996	105	1947	1947	3335	3	1865	1995	3379
cb7521_c46/f1p0/2168	gnl|BL_ORD_ID|51305 cb7521_c32/f1p0/2623	1	1630	1626	2041	231	1860	2061	2476
cb3848_c4/f2p0/2376 isoform=cb3848_c4;full_length_coverage=2;non_full_length_coverage=0;isoform_length=2376;expected_accuracy=0.995	gnl|BL_ORD_ID|40471 cb3848_c18/f1p1/3165 isoform=cb3848_c18;full_length_coverage=1;non_full_length_coverage=1;isoform_length=3165;expected_accuracy=0.996	1	2051	2049	2376	2	2035	2763	3093
cb13565_c43/f1p0/1770	gnl|BL_ORD_ID|49739 cb6452_c8/f1p4/2559	446	1629	51	445	1263	2443	38	431
cb8564_c115640/f1p0/2569	gnl|BL_ORD_ID|15184 cb8564_c4258/f1p0/2766	1	1409	1407	2569	11	141	1519	268

isoform=cb8564_c115640;full_length_coverage=1;non_full_length_coverage=0;isoform_length=2569;expected_accuracy=0.995	isoform=cb8564_c4258;full_length_coverage=1;non_full_length_coverage=0;isoform_length=2766;expected_accuracy=0.994						9		0
cb8564_c81238/f1p0/2739	gnl|BL_ORD_ID|12122 cb8564_c152265/f2p1/2512 isoform=cb8564_c152265;full_length_coverage=2;non_full_length_coverage=1;isoform_length=2512;expected_accuracy=0.996	309	2510	7	307	223	2440	43	343
cb8564_c35300/f1p2/3912	gnl|BL_ORD_ID|55207 cb8564_c35399/f1p0/4553	185	3922	11	187	538	4276	11	180
cb12038_c26/f2p3/1762 isoform=cb12038_c26;full_length_coverage=2;non_full_length_coverage=3;isoform_length=1762;expected_accuracy=1.000	gnl|BL_ORD_ID|18863 cb8564_c82439/f1p4/3856 isoform=cb8564_c82439;full_length_coverage=1;non_full_length_coverage=4;isoform_length=3856;expected_accuracy=0.997	268	1762	57	270	2343	3834	43	276
cb8564_c48192/f1p6/3029	gnl|BL_ORD_ID|16705 cb8564_c53880/f1p0/3484 isoform=cb8564_c53880;full_length_coverage=1;non_full_length_coverage=0;isoform_length=3484;expected_accuracy=0.996	1058	2871	1	1059	1672	3484	1	1056
cb8564_c6125/f1p3/2690 isoform=cb8564_c6125;full_length_coverage=1;non_full_length_coverage=3;isoform_length=2690;expected_accuracy=0.996	gnl|BL_ORD_ID|14097 cb8564_c3797/f2p1/2809 isoform=cb8564_c3797;full_length_coverage=2;non_full_length_coverage=1;isoform_length=2809;expected_accuracy=0.996	1	2394	2394	2662	5	2395	2541	2809
cb12189_c14/f1p1/1379 isoform=cb12189_c14;full_length_coverage=1;non_full_length_coverage=1;isoform_length=1379;expected_accuracy=1.000	gnl|BL_ORD_ID|71985 cb4719_c15/f1p4/2908	291	1361	31	291	1856	2925	1455	1714
cb15591_c1/f1p1/1413	gnl|BL_ORD_ID|59643 cb9317_c9/f1p1/1734	280	1413	4	284	542	167	1	281

isoform=cb15591_c1;full_length_coverage=1;non_full_length_coverage=1;isoform_length=1413;expected_accuracy=0.999							5		
cb1245_c3/f1p0/3928 isoform=cb1245_c3;full_length_coverage=1;non_full_length_coverage=0;isoform_length=3928;expected_accuracy=0.997	gnl|BL_ORD_ID|59306 cb1245_c4/f1p0/4425	1	2779	2775	3928	20	2798	2941	4094
cb13131_c34/f1p1/1713 isoform=cb13131_c34;full_length_coverage=1;non_full_length_coverage=1;isoform_length=1713;expected_accuracy=0.997	gnl|BL_ORD_ID|59771 cb9513_c46/f1p2/3668	1	1292	1294	1713	5	1297	1401	1837
cb750_c29/f1p0/3123 isoform=cb750_c29;full_length_coverage=1;non_full_length_coverage=0;isoform_length=3123;expected_accuracy=0.995	gnl|BL_ORD_ID|50949 cb750_c23/f1p0/3659	1	2339	2338	3123	57	2399	2767	3551
cb8478_c39/f1p0/3267 isoform=cb8478_c39;full_length_coverage=1;non_full_length_coverage=0;isoform_length=3267;expected_accuracy=1.000	gnl|BL_ORD_ID|9996 cb8478_c24/f1p0/3383 isoform=cb8478_c24;full_length_coverage=1;non_full_length_coverage=0;isoform_length=3383;expected_accuracy=0.996	2	2959	2959	3219	44	2999	3125	3383
cb6406_c37/f1p0/3004 isoform=cb6406_c37;full_length_coverage=1;non_full_length_coverage=0;isoform_length=3004;expected_accuracy=0.992	gnl|BL_ORD_ID|5639 cb6406_c38/f1p0/3157 isoform=cb6406_c38;full_length_coverage=1;non_full_length_coverage=0;isoform_length=3157;expected_accuracy=0.998	297	2997	1	298	450	3155	10	308
cb313_c5/f2p0/2828 isoform=cb313_c5;full_length_coverage=2;non_full_length_coverage=0;isoform_length=2828;expected_accurac	gnl|BL_ORD_ID|68193 cb313_c25/f1p0/3123	1	2547	2545	2828	4	2550	2679	2962

y=0.997									
cb8586_c12/f11p3/1403 isoform=cb8586_c12;full_length_coverage=11;non_full_length_coverage=3;isoform_length=1403;expected_accuracy=1.000	gnl|BL_ORD_ID|17411 cb8586_c5/f1p1/2288 isoform=cb8586_c5;full_length_coverage=1;non_full_length_coverage=1;isoform_length=2288;expected_accuracy=0.994	1	1112	1109	1364	1	1118	2033	2288
cb8564_c185920/f1p0/2777 isoform=cb8564_c185920;full_length_coverage=1;non_full_length_coverage=0;isoform_length=2777;expected_accuracy=0.996	gnl|BL_ORD_ID|9418 cb8564_c119193/f1p2/2975 isoform=cb8564_c119193;full_length_coverage=1;non_full_length_coverage=2;isoform_length=2975;expected_accuracy=0.998	174	2773	1	177	353	2975	15	193
cb8564_c37361/f1p0/3076	gnl|BL_ORD_ID|15169 cb8564_c40218/f1p0/3146 isoform=cb8564_c40218;full_length_coverage=1;non_full_length_coverage=0;isoform_length=3146;expected_accuracy=0.993	1	2481	2477	3066	4	2466	2566	3146
cb8564_c71904/f1p1/2820 isoform=cb8564_c71904;full_length_coverage=1;non_full_length_coverage=1;isoform_length=2820;expected_accuracy=0.996	gnl|BL_ORD_ID|16025 cb8564_c69594/f2p2/3247 isoform=cb8564_c69594;full_length_coverage=2;non_full_length_coverage=2;isoform_length=3247;expected_accuracy=1.000	1	1983	1978	2768	292	2267	2456	3247
cb4374_c2/f1p0/2590 isoform=cb4374_c2;full_length_coverage=1;non_full_length_coverage=0;isoform_length=2590;expected_accuracy=0.994	gnl|BL_ORD_ID|72517 cb4374_c5/f1p0/2755	1	2014	2011	2539	15	1990	2230	2762
cb6835_c19/f1p0/2028	gnl|BL_ORD_ID|4332 cb6835_c11/f1p2/2231 isoform=cb6835_c11;full_length_coverage=1;non_full_length_coverage=2;isoform_length=2231;expected_accuracy=0.998	673	1860	72	676	1029	2216	106	711
cb6511_c0/f1p0/2835	gnl|BL_ORD_ID|49789 cb6511_c1/f1p0/3675	109	2835	1	114	817	354	1	111

isoform=cb6511_c0;full_length_coverage=1;non_full_length_coverage=0;isoform_length=2835;expected_accuracy=0.994							1		
cb2097_c120/f1p0/1809 isoform=cb2097_c120;full_length_coverage=1;non_full_length_coverage=0;isoform_length=1809;expected_accuracy=0.994	gnl|BL_ORD_ID|36227 cb2097_c125/f48p3/2452 isoform=cb2097_c125;full_length_coverage=48;non_full_length_coverage=3;isoform_length=2452;expected_accuracy=1.000	1	1422	1420	1809	530	1951	2061	2452
cb8564_c81580/f1p0/2450 isoform=cb8564_c81580;full_length_coverage=1;non_full_length_coverage=0;isoform_length=2450;expected_accuracy=0.997	gnl|BL_ORD_ID|11400 cb8564_c12818/f1p0/2687 isoform=cb8564_c12818;full_length_coverage=1;non_full_length_coverage=0;isoform_length=2687;expected_accuracy=0.993	1	1786	1781	2450	28	1834	1973	2641
cb8564_c85607/f1p0/2557	gnl|BL_ORD_ID|58169 cb8564_c90564/f1p0/2758	1	2134	2131	2568	8	2141	2362	2798
cb1821_c18/f1p0/3660	gnl|BL_ORD_ID|32457 cb1821_c11/f1p0/3799 isoform=cb1821_c11;full_length_coverage=1;non_full_length_coverage=0;isoform_length=3799;expected_accuracy=0.998	1410	3473	1	1414	1595	3659	2	1413
cb8564_c80900/f1p1/2361 isoform=cb8564_c80900;full_length_coverage=1;non_full_length_coverage=1;isoform_length=2361;expected_accuracy=0.999	gnl|BL_ORD_ID|56423 cb8564_c76611/f1p0/2650	1	1541	1540	2361	10	1549	1673	2494
cb8564_c81107/f1p0/2975	gnl|BL_ORD_ID|56624 cb8564_c49356/f1p0/3133	156	2714	1	159	271	2848	1	154
cb8564_c43779/f1p0/3021 isoform=cb8564_c43779;full_length_coverage=1;non_full_length_coverage=0;isoform_length=3021;expected_	gnl|BL_ORD_ID|55243 cb8564_c36242/f1p0/3335	1	2344	2345	3020	1	2347	2503	3176

accuracy=0.993									
cb8564_c36688/f1p0/3356	gnl|BL_ORD_ID|55195 cb8564_c35170/f1p0/3386	500	3162	6	499	738	3399	4	497
cb8564_c80488/f1p0/2745 isoform=cb8564_c80488;full_length_coverage=1;non_full_length_coverage=0;isoform_length=2745;expected_accuracy=0.993	gnl|BL_ORD_ID|11179 cb8564_c11761/f4p0/2940 isoform=cb8564_c11761;full_length_coverage=4;non_full_length_coverage=0;isoform_length=2940;expected_accuracy=0.996	11	1821	1817	2687	8	1807	2043	2903
cb6802_c57/f1p0/2631	gnl|BL_ORD_ID|50085 cb6802_c53/f1p0/3720	191	2236	1	193	1377	3422	3	195
cb8564_c88629/f1p0/3306 isoform=cb8564_c88629;full_length_coverage=1;non_full_length_coverage=0;isoform_length=3306;expected_accuracy=0.999	gnl|BL_ORD_ID|11814 cb8564_c155627/f1p2/3483 isoform=cb8564_c155627;full_length_coverage=1;non_full_length_coverage=2;isoform_length=3483;expected_accuracy=0.998	1	3122	3122	3306	8	3119	3300	3483
cb8942_c20/f1p0/2709	gnl|BL_ORD_ID|19916 cb8942_c15/f1p2/2976 isoform=cb8942_c15;full_length_coverage=1;non_full_length_coverage=2;isoform_length=2976;expected_accuracy=0.999	219	2678	1	224	383	2842	1	225
cb8564_c113232/f1p0/3087	gnl|BL_ORD_ID|9995 cb8564_c111409/f2p0/3661 isoform=cb8564_c111409;full_length_coverage=2;non_full_length_coverage=0;isoform_length=3661;expected_accuracy=0.999	2	2841	2839	2997	3	2863	3093	3251
cb4023_c5/f1p1/3111 isoform=cb4023_c5;full_length_coverage=1;non_full_length_coverage=1;isoform_length=3111;expected_accuracy=0.994	gnl|BL_ORD_ID|39792 cb4023_c6/f1p1/3725 isoform=cb4023_c6;full_length_coverage=1;non_full_length_coverage=1;isoform_length=3725;expected_accuracy=0.993	207	3111	1	207	839	3724	175	380
cb6063_c19/f1p1/2514	gnl|BL_ORD_ID|74844 cb6063_c11/f1p1/3128	157	2327	1	156	699	286	42	196

							6		
cb8564_c89122/f1p0/3335	gnl|BL_ORD_ID|55844 cb8564_c44552/f1p0/3216	110	1965	1961	2915	68	1930	2068	3022
cb322_c35/f1p0/3437 isoform=cb322_c35;full_length_coverage=1;non_full_length_coverage=0;isoform_length=3437;expected_accuracy=0.997	gnl|BL_ORD_ID|40333 cb322_c18/f1p0/4294 isoform=cb322_c18;full_length_coverage=1;non_full_length_coverage=0;isoform_length=4294;expected_accuracy=0.994	114	3437	1	119	389	3707	4	129
cb8564_c36056/f1p0/2652 isoform=cb8564_c36056;full_length_coverage=1;non_full_length_coverage=0;isoform_length=2652;expected_accuracy=0.999	gnl|BL_ORD_ID|61972 cb12931_c30/f1p0/5903	248	2652	1	251	464	2869	70	320
cb8564_c15844/f1p3/3061 isoform=cb8564_c15844;full_length_coverage=1;non_full_length_coverage=3;isoform_length=3061;expected_accuracy=0.995	gnl|BL_ORD_ID|53642 cb8564_c141128/f1p1/3401	1	2692	2688	3061	32	2720	2894	3263
cb1371_c15/f1p2/3990 isoform=cb1371_c15;full_length_coverage=1;non_full_length_coverage=2;isoform_length=3990;expected_accuracy=0.993	gnl|BL_ORD_ID|25357 cb1371_c47/f1p1/5280 isoform=cb1371_c47;full_length_coverage=1;non_full_length_coverage=1;isoform_length=5280;expected_accuracy=0.998	380	3924	1	385	1725	5280	9	393
cb2324_c35/f1p1/3632 isoform=cb2324_c35;full_length_coverage=1;non_full_length_coverage=1;isoform_length=3632;expected_accuracy=0.996	gnl|BL_ORD_ID|68434 cb2324_c22/f1p0/3794	205	3607	1	210	354	3751	1	206
cb1512_c15/f1p1/3322	gnl|BL_ORD_ID|28156 cb1512_c10/f1p1/4211 isoform=cb1512_c10;full_length_coverage=1;non_full_length_coverage=1;isoform_length=4211;expected_accur	1	1681	1681	3228	138	1809	2668	4211

	acy=0.991								
cb4007_c32/f1p2/2576	gnl|BL_ORD_ID|70713 cb4007_c23/f1p0/3263	1	1627	1626	2466	203	182 8	2373	321 4
cb6391_c2/f2p0/2236 isoform=cb6391_c2;full_length_coverage=2;non_full_length_coverage=0;isoform_length=2236;expected_accuracy=0.998	gnl|BL_ORD_ID|3496 cb6391_c13/f1p0/2480 isoform=cb6391_c13;full_length_coverage=1;non_full_length_coverage=0;isoform_length=2480;expected_accuracy=0.999	225	2236	93	227	467	247 8	4	138
cb8393_c4/f1p0/1595 isoform=cb8393_c4;full_length_coverage=1;non_full_length_coverage=0;isoform_length=1595;expected_accuracy=0.999	gnl|BL_ORD_ID|9724 cb8393_c0/f1p0/2381 isoform=cb8393_c0;full_length_coverage=1;non_full_length_coverage=0;isoform_length=2381;expected_accuracy=1.000	205	1590	21	206	996	238 1	15	194
cb6000_c52/f40p0/2861 isoform=cb6000_c52;full_length_coverage=40;non_full_length_coverage=0;isoform_length=2861;expected_accuracy=1.000	gnl|BL_ORD_ID|74753 cb6000_c20/f1p0/3241	541	2846	91	543	933	323 7	1	447
cb8564_c5118/f1p1/4889 isoform=cb8564_c5118;full_length_coverage=1;non_full_length_coverage=1;isoform_length=4889;expected_accuracy=0.991	gnl|BL_ORD_ID|30140 cb14859_c6/f1p4/5345 isoform=cb14859_c6;full_length_coverage=1;non_full_length_coverage=4;isoform_length=5345;expected_accuracy=0.999	1398	4854	1	1401	1837	531 5	1	140 9
cb13415_c1/f2p0/1813 isoform=cb13415_c1;full_length_coverage=2;non_full_length_coverage=0;isoform_length=1813;expected_accuracy=0.998	gnl|BL_ORD_ID|44844 cb5659_c28/f2p0/1973 isoform=cb5659_c28;full_length_coverage=2;non_full_length_coverage=0;isoform_length=1973;expected_accuracy=0.996	235	1813	67	237	388	196 4	1	171
cb8564_c181853/f1p0/2119 isoform=cb8564_c181853;full_length_coverage=1;non_full_length_coverage=0;isoform_length=2119;expected_	gnl|BL_ORD_ID|13976 cb8564_c43401/f1p2/3025 isoform=cb8564_c43401;full_length_coverage=1;non_full_length_coverage=2;isoform_length=3025;expected_ac	1	1339	1334	2119	1	139 1	2230	302 5

accuracy=0.997	curacy=0.995								
cb4684_c3/f1p0/3284	gnl|BL_ORD_ID|41678 cb4684_c5/f1p0/3247 isoform=cb4684_c5;full_length_coverage=1;non_full_length_coverage=0;isoform_length=3247;expected_accuracy=0.997	1443	2997	1	1445	1694	3247	90	1534
cb18064_c0/f1p0/1618 isoform=cb18064_c0;full_length_coverage=1;non_full_length_coverage=0;isoform_length=1618;expected_accuracy=0.993	gnl|BL_ORD_ID|9171 cb8564_c119467/f1p0/2716 isoform=cb8564_c119467;full_length_coverage=1;non_full_length_coverage=0;isoform_length=2716;expected_accuracy=0.997	1	1034	1033	1618	23	1057	1900	2485
cb1049_c13/f1p0/3054	gnl|BL_ORD_ID|47283 cb1049_c16/f1p0/3709	10	1596	1593	3032	28	1611	1718	3162
cb649_c18/f1p1/3876	gnl|BL_ORD_ID|5275 cb649_c14/f1p2/4095 isoform=cb649_c14;full_length_coverage=1;non_full_length_coverage=2;isoform_length=4095;expected_accuracy=0.995	265	3847	1	265	404	4000	26	289
cb1770_c20/f1p1/3435	gnl|BL_ORD_ID|33845 cb1770_c3/f3p0/3565 isoform=cb1770_c3;full_length_coverage=3;non_full_length_coverage=0;isoform_length=3565;expected_accuracy=0.999	18	1944	1942	3403	1	1931	2096	3565
cb8564_c51244/f1p0/2997	gnl|BL_ORD_ID|55929 cb8564_c49294/f1p0/4356	1	2130	2130	2967	16	2145	2758	3595
cb8564_c53418/f1p0/2602 isoform=cb8564_c53418;full_length_coverage=1;non_full_length_coverage=0;isoform_length=2602;expected_accuracy=0.991	gnl|BL_ORD_ID|11316 cb8564_c12355/f1p9/2685 isoform=cb8564_c12355;full_length_coverage=1;non_full_length_coverage=9;isoform_length=2685;expected_accuracy=0.999	1163	2548	1	1168	1297	2685	1	1196
cb8564_c113842/f1p0/1982	gnl|BL_ORD_ID|9467 cb8564_c109054/f5p0/2034	216	1960	3	215	314	203	1	206

isoform=cb8564_c113842;full_length_coverage=1;non_full_length_coverage=0;isoform_length=1982;expected_accuracy=0.996	isoform=cb8564_c109054;full_length_coverage=5;non_full_length_coverage=0;isoform_length=2034;expected_accuracy=1.000						4		
cb4674_c27/f1p1/2865 isoform=cb4674_c27;full_length_coverage=1;non_full_length_coverage=1;isoform_length=2865;expected_accuracy=0.993	gnl|BL_ORD_ID|72844 cb4674_c33/f1p3/3281	1	2046	2041	2865	84	2136	2379	3207
cb8564_c4541/f3p0/2161 isoform=cb8564_c4541;full_length_coverage=3;non_full_length_coverage=0;isoform_length=2161;expected_accuracy=0.992	gnl|BL_ORD_ID|14176 cb8564_c4538/f2p0/2766 isoform=cb8564_c4538;full_length_coverage=2;non_full_length_coverage=0;isoform_length=2766;expected_accuracy=0.994	1	1395	1393	2112	40	1430	2048	2766
cb8564_c41115/f1p3/2751	gnl|BL_ORD_ID|55596 cb8564_c46321/f1p2/2987	11	1542	1539	2685	26	1556	1728	2873
cb8564_c162907/f1p0/2069 isoform=cb8564_c162907;full_length_coverage=1;non_full_length_coverage=0;isoform_length=2069;expected_accuracy=0.993	gnl|BL_ORD_ID|14463 cb8564_c25154/f1p5/4276 isoform=cb8564_c25154;full_length_coverage=1;non_full_length_coverage=5;isoform_length=4276;expected_accuracy=0.995	261	2057	72	259	2418	4210	4	189
cb9436_c10/f1p0/2077 isoform=cb9436_c10;full_length_coverage=1;non_full_length_coverage=0;isoform_length=2077;expected_accuracy=0.994	gnl|BL_ORD_ID|21678 cb9436_c4/f1p0/2516 isoform=cb9436_c4;full_length_coverage=1;non_full_length_coverage=0;isoform_length=2516;expected_accuracy=0.998	688	2077	72	689	1088	2480	1	627
cb2482_c31/f1p1/2865 isoform=cb2482_c31;full_length_coverage=1;non_full_length_coverage=1;isoform_length=2865;expected_accuracy=0.996	gnl|BL_ORD_ID|39015 cb2482_c57/f13p2/3062 isoform=cb2482_c57;full_length_coverage=13;non_full_length_coverage=2;isoform_length=3062;expected_accuracy=1.000	1	2632	2631	2865	14	2636	2760	2994
cb12495_c2/f1p0/1842	gnl|BL_ORD_ID|72653 cb4536_c4/f1p0/2190	12	1407	1406	1840	1	136	1641	206

isoform=cb12495_c2;full_length_coverage=1;non_full_length_coverage=0;isoform_length=1842;expected_accuracy=0.992							9		0
cb8564_c117152/f1p0/2542	gnl|BL_ORD_ID|52915 cb8564_c111606/f1p0/3227	1057	2487	1	1058	1618	3048	3	1062
cb5583_c3/f1p0/2163	gnl|BL_ORD_ID|46284 cb5583_c1/f1p0/3059 isoform=cb5583_c1;full_length_coverage=1;non_full_length_coverage=0;isoform_length=3059;expected_accuracy=0.997	132	2071	1	137	1127	3059	1	138
cb4128_c23/f1p0/2488 isoform=cb4128_c23;full_length_coverage=1;non_full_length_coverage=0;isoform_length=2488;expected_accuracy=0.999	gnl|BL_ORD_ID|70784 cb4128_c20/f1p0/3149	1	1757	1756	2373	28	1785	2121	2738
cb8564_c45300/f1p4/3014 isoform=cb8564_c45300;full_length_coverage=1;non_full_length_coverage=4;isoform_length=3014;expected_accuracy=0.996	gnl|BL_ORD_ID|55246 cb8564_c36343/f1p0/3448	243	3014	2	246	410	3188	3	247
cb6742_c12/f1p0/2398	gnl|BL_ORD_ID|5508 cb6742_c3/f1p0/2514 isoform=cb6742_c3;full_length_coverage=1;non_full_length_coverage=0;isoform_length=2514;expected_accuracy=0.998	1	1343	1342	2304	55	1394	1508	2475
cb2739_c7/f1p0/3065	gnl|BL_ORD_ID|69937 cb2739_c8/f1p0/3454	1	1801	1800	2810	428	2227	2450	3463
cb8564_c42814/f1p0/2151 isoform=cb8564_c42814;full_length_coverage=1;non_full_length_coverage=0;isoform_length=2151;expected_	gnl|BL_ORD_ID|13871 cb8564_c33702/f1p6/2850 isoform=cb8564_c33702;full_length_coverage=1;non_full_length_coverage=6;isoform_length=2850;expected_ac	650	2150	9	652	1350	2848	5	663

accuracy=0.993	curacy=0.993								
cb5440_c11/f2p0/1312 isoform=cb5440_c11;full_length_coverage=2;non_full_length_coverage=0;isoform_length=1312;expected_accuracy=0.995	gnl|BL_ORD_ID|46312 cb5440_c8/f1p1/3070 isoform=cb5440_c8;full_length_coverage=1;non_full_length_coverage=1;isoform_length=3070;expected_accuracy=0.991	1	1019	1014	1312	126	1142	2735	3037
cb8564_c72680/f1p0/2685	gnl|BL_ORD_ID|54696 cb8564_c154234/f1p3/3007	1	1735	1730	2636	13	1747	1926	2832
cb2389_c15/f1p0/2930 isoform=cb2389_c15;full_length_coverage=1;non_full_length_coverage=0;isoform_length=2930;expected_accuracy=0.994	gnl|BL_ORD_ID|68485 cb2389_c8/f1p0/3551	1	2472	2471	2930	102	2571	2750	3209
cb8564_c49356/f1p0/3133	gnl|BL_ORD_ID|18852 cb8564_c84488/f1p0/3408 isoform=cb8564_c84488;full_length_coverage=1;non_full_length_coverage=0;isoform_length=3408;expected_accuracy=0.996	212	2848	1	212	790	3408	1	217
cb6283_c1/f1p0/2894 isoform=cb6283_c1;full_length_coverage=1;non_full_length_coverage=0;isoform_length=2894;expected_accuracy=1.000	gnl|BL_ORD_ID|5266 cb6283_c2/f1p0/3571 isoform=cb6283_c2;full_length_coverage=1;non_full_length_coverage=0;isoform_length=3571;expected_accuracy=0.996	1	2231	2228	2817	1	2229	2982	3571
cb3537_c9/f1p0/3437	gnl|BL_ORD_ID|39687 cb3537_c0/f9p0/3405 isoform=cb3537_c0;full_length_coverage=9;non_full_length_coverage=0;isoform_length=3405;expected_accuracy=1.000	369	3101	5	372	594	3328	38	405
cb1462_c0/f7p0/3659 isoform=cb1462_c0;full_length_coverage=7;non_full_length_coverage=0;isoform_length=3659;expected_accur	gnl|BL_ORD_ID|28515 cb1462_c7/f1p0/3911 isoform=cb1462_c7;full_length_coverage=1;non_full_length_coverage=0;isoform_length=3911;expected_accura	302	3655	1	302	579	3911	20	319

acy=1.000	cy=0.993								
cb8564_c47149/f1p1/2601 isoform=cb8564_c47149;full_length_coverage=1;non_full_length_coverage=1;isoform_length=2601;expected_accuracy=0.997	gnl|BL_ORD_ID|19224 cb8564_c87855/f1p1/2779 isoform=cb8564_c87855;full_length_coverage=1;non_full_length_coverage=1;isoform_length=2779;expected_accuracy=0.997	1	1649	1649	2587	58	1705	1841	2779
cb3449_c13/f1p2/3050 isoform=cb3449_c13;full_length_coverage=1;non_full_length_coverage=2;isoform_length=3050;expected_accuracy=0.993	gnl|BL_ORD_ID|39721 cb3449_c7/f3p0/3278 isoform=cb3449_c7;full_length_coverage=3;non_full_length_coverage=0;isoform_length=3278;expected_accuracy=1.000	1	2755	2752	3045	21	2776	2944	3234
cb8564_c181954/f1p0/3278 isoform=cb8564_c181954;full_length_coverage=1;non_full_length_coverage=0;isoform_length=3278;expected_accuracy=0.994	gnl|BL_ORD_ID|55939 cb8564_c49630/f1p4/3487	118	3024	3024	3272	3	2913	3243	3493
cb7521_c46/f1p0/2168	gnl|BL_ORD_ID|8990 cb7521_c33/f14p4/2475 isoform=cb7521_c33;full_length_coverage=14;non_full_length_coverage=4;isoform_length=2475;expected_accuracy=1.000	1	1630	1626	2043	89	1718	1919	2337
cb8564_c89509/f1p4/2644 isoform=cb8564_c89509;full_length_coverage=1;non_full_length_coverage=4;isoform_length=2644;expected_accuracy=1.000	gnl|BL_ORD_ID|15727 cb8564_c69026/f2p1/3011 isoform=cb8564_c69026;full_length_coverage=2;non_full_length_coverage=1;isoform_length=3011;expected_accuracy=0.999	13	1512	1509	2585	48	1534	1936	3011
cb8564_c110973/f1p0/1916 isoform=cb8564_c110973;full_length_coverage=1;non_full_length_coverage=0;isoform_length=1916;expected_accuracy=0.997	gnl|BL_ORD_ID|52629 cb8564_c1216/f1p0/2122	10	1667	1667	1916	1	1654	1868	2117
cb3354_c0/f6p0/1572	gnl|BL_ORD_ID|40592 cb3354_c3/f1p0/2584	1	1213	1213	1572	2	121	2093	245

isoform=cb3354_c0;full_length_coverage=6;non_full_length_coverage=0;isoform_length=1572;expected_accuracy=1.000	isoform=cb3354_c3;full_length_coverage=1;non_full_length_coverage=0;isoform_length=2584;expected_accuracy=0.999						3		2
cb13617_c12/f1p0/1738	gnl|BL_ORD_ID|25065 cb13617_c5/f1p0/1777 isoform=cb13617_c5;full_length_coverage=1;non_full_length_coverage=0;isoform_length=1777;expected_accuracy=0.997	235	1663	5	236	348	1776	4	235
cb17137_c7/f1p1/1834	gnl|BL_ORD_ID|10118 cb8564_c109435/f1p1/2094 isoform=cb8564_c109435;full_length_coverage=1;non_full_length_coverage=1;isoform_length=2094;expected_accuracy=0.991	1	1291	1287	1692	1	1317	1688	2094
cb3599_c13/f1p0/2987 isoform=cb3599_c13;full_length_coverage=1;non_full_length_coverage=0;isoform_length=2987;expected_accuracy=0.998	gnl|BL_ORD_ID|39122 cb3599_c8/f1p1/2994 isoform=cb3599_c8;full_length_coverage=1;non_full_length_coverage=1;isoform_length=2994;expected_accuracy=0.992	1	2339	2334	2863	1	2341	2460	2991
cb10092_c12/f1p5/1646 isoform=cb10092_c12;full_length_coverage=1;non_full_length_coverage=5;isoform_length=1646;expected_accuracy=0.992	gnl|BL_ORD_ID|16450 cb8564_c72442/f2p1/2290 isoform=cb8564_c72442;full_length_coverage=2;non_full_length_coverage=1;isoform_length=2290;expected_accuracy=0.994	569	1643	6	570	1158	2234	14	583
cb16968_c0/f1p0/1336	gnl|BL_ORD_ID|71810 cb4128_c17/f1p0/2338	488	1232	6	489	1528	2272	2	485
cb2661_c7/f1p1/2908	gnl|BL_ORD_ID|38208 cb2661_c14/f6p2/3147 isoform=cb2661_c14;full_length_coverage=6;non_full_length_coverage=2;isoform_length=3147;expected_accuracy=1.000	1	2336	2332	2912	46	2381	2545	3125
cb8564_c14767/f1p3/2856	gnl|BL_ORD_ID|56381 cb8564_c75626/f1p2/3199	1	2112	2110	2607	4	2116	2611	310

									8
cb8564_c90231/f1p1/3635	gnl|BL_ORD_ID|13377 cb8564_c22091/f2p0/3351 isoform=cb8564_c22091;full_length_coverage=2;non_full_length_coverage=0;isoform_length=3351;expected_accuracy=0.997	1	1684	1681	3228	1	1682	1805	3351
cb6622_c3/f2p0/2264 isoform=cb6622_c3;full_length_coverage=2;non_full_length_coverage=0;isoform_length=2264;expected_accuracy=0.999	gnl|BL_ORD_ID|3916 cb6622_c4/f1p0/2808 isoform=cb6622_c4;full_length_coverage=1;non_full_length_coverage=0;isoform_length=2808;expected_accuracy=0.998	165	2229	1	162	743	2808	28	189
cb5224_c19/f1p0/2383 isoform=cb5224_c19;full_length_coverage=1;non_full_length_coverage=0;isoform_length=2383;expected_accuracy=0.995	gnl|BL_ORD_ID|73654 cb5224_c18/f1p0/2731	123	2383	1	124	300	2569	1	124
cb8167_c9/f1p0/2008 isoform=cb8167_c9;full_length_coverage=1;non_full_length_coverage=0;isoform_length=2008;expected_accuracy=0.996	gnl|BL_ORD_ID|8220 cb8167_c17/f9p1/2166 isoform=cb8167_c17;full_length_coverage=9;non_full_length_coverage=1;isoform_length=2166;expected_accuracy=1.000	132	2006	1	130	292	2166	24	152
cb8564_c10808/f1p0/2532	gnl|BL_ORD_ID|54302 cb8564_c22058/f1p5/3725	426	2359	7	426	1786	3719	1127	1546
cb8564_c1737/f1p0/3386	gnl|BL_ORD_ID|53750 cb8564_c1734/f1p0/3808	1636	3399	1	1638	2011	3774	1	1638
cb8564_c41756/f1p0/2738 isoform=cb8564_c41756;full_length_coverage=1;non_full_length_coverage=0;isoform_length=2738;expected_accuracy=0.996	gnl|BL_ORD_ID|55371 cb8564_c39756/f1p1/3004	1	2173	2170	2738	1	2151	2274	2854
cb8564_c129284/f1p0/2118	gnl|BL_ORD_ID|12475 cb8564_c148406/f2p0/2449	1	1970	1965	2118	49	203	2216	237

isoform=cb8564_c129284;full_length_coverage=1;non_full_length_coverage=0;isoform_length=2118;expected_accuracy=0.991	isoform=cb8564_c148406;full_length_coverage=2;non_full_length_coverage=0;isoform_length=2449;expected_accuracy=0.990						1		0
cb17137_c7/f1p1/1834	gnl|BL_ORD_ID|9597 cb8564_c122279/f1p2/2123 isoform=cb8564_c122279;full_length_coverage=1;non_full_length_coverage=2;isoform_length=2123;expected_accuracy=0.992	1	1291	1287	1694	1	1289	1660	2067
cb8564_c145335/f3p0/1964 isoform=cb8564_c145335;full_length_coverage=3;non_full_length_coverage=0;isoform_length=1964;expected_accuracy=0.999	gnl|BL_ORD_ID|12164 cb8564_c146670/f2p0/2135 isoform=cb8564_c146670;full_length_coverage=2;non_full_length_coverage=0;isoform_length=2135;expected_accuracy=0.998	136	1960	1	132	312	2135	1	130
cb8564_c188097/f1p0/2552 isoform=cb8564_c188097;full_length_coverage=1;non_full_length_coverage=0;isoform_length=2552;expected_accuracy=0.996	gnl|BL_ORD_ID|10640 cb8564_c100234/f2p5/2744 isoform=cb8564_c100234;full_length_coverage=2;non_full_length_coverage=5;isoform_length=2744;expected_accuracy=0.993	1	1945	1940	2552	6	1960	2063	2675
cb548_c39/f1p3/2934 isoform=cb548_c39;full_length_coverage=1;non_full_length_coverage=3;isoform_length=2934;expected_accuracy=0.996	gnl|BL_ORD_ID|46779 cb548_c109/f1p0/3280 isoform=cb548_c109;full_length_coverage=1;non_full_length_coverage=0;isoform_length=3280;expected_accuracy=0.996	1313	2934	1	1316	1551	3165	45	1340
cb6798_c22/f1p0/2208	gnl|BL_ORD_ID|50075 cb6798_c12/f1p4/2573	619	2195	8	624	916	2492	76	679
cb1573_c16/f1p0/3454 isoform=cb1573_c16;full_length_coverage=1;non_full_length_coverage=0;isoform_length=3454;expected_accuracy=0.998	gnl|BL_ORD_ID|30864 cb1573_c21/f1p2/3728 isoform=cb1573_c21;full_length_coverage=1;non_full_length_coverage=2;isoform_length=3728;expected_accuracy=0.991	1249	3454	1	1252	1446	3669	27	1287
cb5904_c10/f1p1/1410	gnl|BL_ORD_ID|46482 cb5904_c12/f1p0/1469	437	1353	6	436	554	146	7	435

	isoform=cb5904_c12;full_length_coverage=1;non_full_length_coverage=0;isoform_length=1469;expected_accuracy=0.997						9		
cb2785_c14/f1p0/2954 isoform=cb2785_c14;full_length_coverage=1;non_full_length_coverage=0;isoform_length=2954;expected_accuracy=0.996	gnl|BL_ORD_ID|36869 cb2785_c10/f1p0/3807 isoform=cb2785_c10;full_length_coverage=1;non_full_length_coverage=0;isoform_length=3807;expected_accuracy=0.996	1	2218	2220	2909	1	2210	3119	3807
cb8234_c42/f3p0/2289 isoform=cb8234_c42;full_length_coverage=3;non_full_length_coverage=0;isoform_length=2289;expected_accuracy=0.995	gnl|BL_ORD_ID|8908 cb8234_c5/f4p2/3010 isoform=cb8234_c5;full_length_coverage=4;non_full_length_coverage=2;isoform_length=3010;expected_accuracy=1.000	176	2244	1	179	949	3010	55	232
cb9813_c144/f1p0/1034 isoform=cb9813_c144;full_length_coverage=1;non_full_length_coverage=0;isoform_length=1034;expected_accuracy=1.000	gnl|BL_ORD_ID|58920 cb9813_c57/f1p0/1921	8	766	762	1034	4	762	1487	1758
cb7642_c53/f1p0/1947 isoform=cb7642_c53;full_length_coverage=1;non_full_length_coverage=0;isoform_length=1947;expected_accuracy=0.990	gnl|BL_ORD_ID|6848 cb7642_c9/f1p2/2076 isoform=cb7642_c9;full_length_coverage=1;non_full_length_coverage=2;isoform_length=2076;expected_accuracy=0.999	1	1181	1178	1946	6	1194	1302	2073
cb8564_c836/f4p0/2537 isoform=cb8564_c836;full_length_coverage=4;non_full_length_coverage=0;isoform_length=2537;expected_accuracy=1.000	gnl|BL_ORD_ID|17399 cb8564_c984/f3p0/2649 isoform=cb8564_c984;full_length_coverage=3;non_full_length_coverage=0;isoform_length=2649;expected_accuracy=1.000	148	2511	1	151	285	2649	2	154
cb653_c12/f1p0/3754 isoform=cb653_c12;full_length_coverage=1;non_full_length_coverage=0;isoform_length=3754;expected_accur	gnl|BL_ORD_ID|5131 cb653_c8/f1p1/4297 isoform=cb653_c8;full_length_coverage=1;non_full_length_coverage=1;isoform_length=4297;expected_accurac	195	3754	1	196	703	4259	110	308

acy=0.995	y=0.999								
cb401_c94/f1p0/2488	gnl|BL_ORD_ID|70873 cb401_c42/f1p0/2704	1	1605	1603	2280	90	1679	2018	2698
cb8564_c44290/f1p1/3198	gnl|BL_ORD_ID|56029 cb8564_c52782/f1p2/3327	339	2983	7	338	618	3261	11	320
cb8564_c89918/f1p1/2045 isoform=cb8564_c89918;full_length_coverage=1;non_full_length_coverage=1;isoform_length=2045;expected_accuracy=0.991	gnl|BL_ORD_ID|53524 cb8564_c127653/f1p0/2575	1	1616	1611	2045	29	1616	1811	2244
cb18199_c0/f1p0/1356 isoform=cb18199_c0;full_length_coverage=1;non_full_length_coverage=0;isoform_length=1356;expected_accuracy=0.992	gnl|BL_ORD_ID|29800 cb1494_c10/f1p0/3165 isoform=cb1494_c10;full_length_coverage=1;non_full_length_coverage=0;isoform_length=3165;expected_accuracy=0.994	101	1344	1	102	1854	3100	666	767
cb6491_c8/f1p1/3298 isoform=cb6491_c8;full_length_coverage=1;non_full_length_coverage=1;isoform_length=3298;expected_accuracy=0.991	gnl|BL_ORD_ID|3949 cb6491_c2/f1p0/4290 isoform=cb6491_c2;full_length_coverage=1;non_full_length_coverage=0;isoform_length=4290;expected_accuracy=0.996	1	2917	2915	3295	11	2932	3099	3462
cb5600_c21/f1p0/2784	gnl|BL_ORD_ID|46920 cb5600_c22/f9p0/2763 isoform=cb5600_c22;full_length_coverage=9;non_full_length_coverage=0;isoform_length=2763;expected_accuracy=1.000	1	2095	2098	2525	44	2138	2322	2748
cb1312_c24/f1p0/3018	gnl|BL_ORD_ID|27493 cb1312_c35/f1p0/3895 isoform=cb1312_c35;full_length_coverage=1;non_full_length_coverage=0;isoform_length=3895;expected_accuracy=0.990	1	1532	1531	2888	146	1674	2478	3828
cb8564_c127097/f1p0/2380	gnl|BL_ORD_ID|14828 cb8564_c4561/f2p0/3092	1	1714	1713	2380	23	173	2383	304

isoform=cb8564_c127097;full_length_coverage=1;non_full_length_coverage=0;isoform_length=2380;expected_accuracy=0.994	isoform=cb8564_c4561;full_length_coverage=2;non_full_length_coverage=0;isoform_length=3092;expected_accuracy=0.997						6		9
cb4684_c0/f6p1/3038 isoform=cb4684_c0;full_length_coverage=6;non_full_length_coverage=1;isoform_length=3038;expected_accuracy=1.000	gnl|BL_ORD_ID|41678 cb4684_c5/f1p0/3247 isoform=cb4684_c5;full_length_coverage=1;non_full_length_coverage=0;isoform_length=3247;expected_accuracy=0.997	1	1525	1523	3038	7	1534	1694	3211
cb8564_c38570/f2p2/2760 isoform=cb8564_c38570;full_length_coverage=2;non_full_length_coverage=2;isoform_length=2760;expected_accuracy=0.996	gnl|BL_ORD_ID|16477 cb8564_c53604/f2p1/4117 isoform=cb8564_c53604;full_length_coverage=2;non_full_length_coverage=1;isoform_length=4117;expected_accuracy=0.997	198	2732	1	198	1581	4117	1260	1465
cb16651_c20/f1p1/3052 isoform=cb16651_c20;full_length_coverage=1;non_full_length_coverage=1;isoform_length=3052;expected_accuracy=0.991	gnl|BL_ORD_ID|31471 cb16651_c8/f1p3/3966 isoform=cb16651_c8;full_length_coverage=1;non_full_length_coverage=3;isoform_length=3966;expected_accuracy=0.999	440	3052	5	441	1295	3905	1	420
cb8564_c50697/f1p3/2172 isoform=cb8564_c50697;full_length_coverage=1;non_full_length_coverage=3;isoform_length=2172;expected_accuracy=0.993	gnl|BL_ORD_ID|15568 cb8564_c48661/f1p2/2634 isoform=cb8564_c48661;full_length_coverage=1;non_full_length_coverage=2;isoform_length=2634;expected_accuracy=0.998	106	2118	1	108	627	2634	15	122
cb8564_c9734/f3p3/2491 isoform=cb8564_c9734;full_length_coverage=3;non_full_length_coverage=3;isoform_length=2491;expected_accuracy=1.000	gnl|BL_ORD_ID|55287 cb8564_c37310/f1p0/2716	13	1753	1751	2438	1	1754	1860	2547
cb8564_c35865/f2p2/3469 isoform=cb8564_c35865;full_length_coverage=2;non_full_length_coverage=2;isoform_length=3469;expected_	gnl|BL_ORD_ID|55825 cb8564_c41971/f1p0/3567	1	2819	2816	3452	1	2814	2943	3582

accuracy=1.000									
cb8564_c47522/f1p0/2954	gnl|BL_ORD_ID|16705 cb8564_c53880/f1p0/3484 isoform=cb8564_c53880;full_length_coverage=1;non_full_length_coverage=0;isoform_length=3484;expected_accuracy=0.996	1035	2849	1	1036	1672	3484	1	1056
cb8564_c54537/f1p0/3163	gnl|BL_ORD_ID|56029 cb8564_c52782/f1p2/3327	313	2933	6	312	618	3238	11	320
cb8564_c14548/f1p0/3030	gnl|BL_ORD_ID|9350 cb8564_c11826/f1p0/2916 isoform=cb8564_c11826;full_length_coverage=1;non_full_length_coverage=0;isoform_length=2916;expected_accuracy=0.997	1306	2656	13	1307	1543	2916	4	1296
cb8564_c43601/f1p0/2228	gnl|BL_ORD_ID|55236 cb8564_c36133/f1p0/4635	1	1814	1814	2058	1817	3630	3984	4231
cb2640_c12/f1p0/3046 isoform=cb2640_c12;full_length_coverage=1;non_full_length_coverage=0;isoform_length=3046;expected_accuracy=0.994	gnl|BL_ORD_ID|69859 cb2640_c29/f1p0/3441	192	3046	1	193	355	3206	10	228
cb13985_c3/f1p0/1395 isoform=cb13985_c3;full_length_coverage=1;non_full_length_coverage=0;isoform_length=1395;expected_accuracy=1.000	gnl|BL_ORD_ID|62962 cb13985_c10/f1p0/1780	107	1389	1	111	347	1629	42	149
cb8437_c0/f1p0/2371	gnl|BL_ORD_ID|51940 cb8437_c1/f1p0/2316	1	1824	1823	2201	1	1823	1947	2325
cb6090_c5/f1p0/2373 isoform=cb6090_c5;full_length_coverage=1;non_full_length_coverage=0;isoform_length=2373;expected_accur	gnl|BL_ORD_ID|73488 cb6090_c3/f1p0/2843	1	1689	1688	2372	4	1693	2176	2862

acy=0.995									
cb1065_c16/f1p0/2119	gnl|BL_ORD_ID|1631 cb1065_c8/f1p0/2427 isoform=cb1065_c8;full_length_coverage=1;non_full_length_coverage=0;isoform_length=2427;expected_accuracy=0.994	1014	2114	1	1016	1319	2427	81	1098
cb2737_c35/f1p0/2780 isoform=cb2737_c35;full_length_coverage=1;non_full_length_coverage=0;isoform_length=2780;expected_accuracy=0.993	gnl|BL_ORD_ID|68786 cb2737_c50/f1p0/3706	134	1541	1539	2780	675	2082	2214	3456
cb5183_c100/f1p2/1592 isoform=cb5183_c100;full_length_coverage=1;non_full_length_coverage=2;isoform_length=1592;expected_accuracy=0.998	gnl|BL_ORD_ID|73597 cb5183_c43/f1p0/2049	278	1577	66	282	436	1735	95	311
cb8564_c36689/f1p1/2368 isoform=cb8564_c36689;full_length_coverage=1;non_full_length_coverage=1;isoform_length=2368;expected_accuracy=0.991	gnl|BL_ORD_ID|16805 cb8564_c66657/f4p6/2862 isoform=cb8564_c66657;full_length_coverage=4;non_full_length_coverage=6;isoform_length=2862;expected_accuracy=1.000	1	1709	1706	2368	29	1700	2199	2862
cb3783_c10/f1p0/3098	gnl|BL_ORD_ID|40402 cb3783_c2/f1p2/3529 isoform=cb3783_c2;full_length_coverage=1;non_full_length_coverage=2;isoform_length=3529;expected_accuracy=0.998	1	1765	1761	3029	15	1779	2250	3518
cb8564_c46939/f1p1/2926	gnl|BL_ORD_ID|55522 cb8564_c44123/f1p1/3230	16	2134	2130	2870	15	2140	2245	2985
cb8564_c38035/f1p0/2770	gnl|BL_ORD_ID|13976 cb8564_c43401/f1p2/3025 isoform=cb8564_c43401;full_length_coverage=1;non_full_length_coverage=2;isoform_length=3025;expected_ac	1	2035	2033	2756	1	2099	2289	3023

	curacy=0.995								
cb8564_c43399/f1p0/2654 isoform=cb8564_c43399;full_length_coverage=1;non_full_length_coverage=0;isoform_length=2654;expected_accuracy=0.999	gnl|BL_ORD_ID|14263 cb8564_c42654/f1p1/2906 isoform=cb8564_c42654;full_length_coverage=1;non_full_length_coverage=1;isoform_length=2906;expected_accuracy=0.990	1	2367	2363	2629	1	2340	2557	2824
cb8564_c37361/f1p0/3076	gnl|BL_ORD_ID|56764 cb8564_c70795/f1p0/3769	18	2481	2477	3070	34	2497	2598	3187
cb3759_c13/f1p0/1723 isoform=cb3759_c13;full_length_coverage=1;non_full_length_coverage=0;isoform_length=1723;expected_accuracy=0.994	gnl|BL_ORD_ID|41340 cb3759_c3/f1p0/1980 isoform=cb3759_c3;full_length_coverage=1;non_full_length_coverage=0;isoform_length=1980;expected_accuracy=0.996	372	1716	4	373	635	1980	1	372
cb8564_c88311/f1p1/2475 isoform=cb8564_c88311;full_length_coverage=1;non_full_length_coverage=1;isoform_length=2475;expected_accuracy=0.993	gnl|BL_ORD_ID|56770 cb8564_c71034/f1p2/3266	1	1814	1812	2473	30	1851	2542	3204
cb2033_c17/f1p0/2200	gnl|BL_ORD_ID|36040 cb2033_c22/f1p0/2340 isoform=cb2033_c22;full_length_coverage=1;non_full_length_coverage=0;isoform_length=2340;expected_accuracy=0.991	1	1577	1576	2195	3	1606	1719	2336
cb8564_c43399/f1p0/2654 isoform=cb8564_c43399;full_length_coverage=1;non_full_length_coverage=0;isoform_length=2654;expected_accuracy=0.999	gnl|BL_ORD_ID|55873 cb8564_c47701/f1p0/3144	12	2367	2363	2653	24	2374	2590	2880
cb8160_c2/f1p0/2208 isoform=cb8160_c2;full_length_coverage=1;non_full_length_coverage=0;isoform_length=2208;expected_accur	gnl|BL_ORD_ID|6488 cb8160_c0/f1p1/2444 isoform=cb8160_c0;full_length_coverage=1;non_full_length_coverage=1;isoform_length=2444;expected_accura	99	2206	1	100	315	2439	13	114

acy=0.998	cy=0.993								
cb8564_c118011/f1p0/1965	gnl|BL_ORD_ID|54731 cb8564_c160518/f1p2/2775	1	1321	1321	1964	530	1863	2043	2683
cb8564_c43399/f1p0/2654 isoform=cb8564_c43399;full_length_coverage=1;non_full_length_coverage=0;isoform_length=2654;expected_accuracy=0.999	gnl|BL_ORD_ID|54403 cb8564_c35093/f1p5/2916	2	2367	2363	2653	20	2380	2596	2885
cb3856_c1/f2p0/1847 isoform=cb3856_c1;full_length_coverage=2;non_full_length_coverage=0;isoform_length=1847;expected_accuracy=0.996	gnl|BL_ORD_ID|40760 cb3856_c3/f1p0/3507 isoform=cb3856_c3;full_length_coverage=1;non_full_length_coverage=0;isoform_length=3507;expected_accuracy=0.998	365	1847	9	367	2020	3501	1	363
cb8564_c44698/f1p5/2843 isoform=cb8564_c44698;full_length_coverage=1;non_full_length_coverage=5;isoform_length=2843;expected_accuracy=0.997	gnl|BL_ORD_ID|13976 cb8564_c43401/f1p2/3025 isoform=cb8564_c43401;full_length_coverage=1;non_full_length_coverage=2;isoform_length=3025;expected_accuracy=0.995	1	2516	2511	2829	1	2578	2701	3025
cb7584_c3/f2p2/1694 isoform=cb7584_c3;full_length_coverage=2;non_full_length_coverage=2;isoform_length=1694;expected_accuracy=0.999	gnl|BL_ORD_ID|50612 cb7584_c12/f1p0/1881	1	1346	1345	1694	1	1345	1495	1844
cb6342_c4/f1p0/2662 isoform=cb6342_c4;full_length_coverage=1;non_full_length_coverage=0;isoform_length=2662;expected_accuracy=0.998	gnl|BL_ORD_ID|5291 cb6342_c3/f1p0/2889 isoform=cb6342_c3;full_length_coverage=1;non_full_length_coverage=0;isoform_length=2889;expected_accuracy=0.999	1259	2593	1	1259	1555	2889	196	1453
cb513_c6/f1p0/2997 isoform=cb513_c6;full_length_coverage=1;non_full_length_coverage=0;isoform_length=2997;expected_accurac	gnl|BL_ORD_ID|43725 cb513_c4/f1p0/3166 isoform=cb513_c4;full_length_coverage=1;non_full_length_coverage=0;isoform_length=3166;expected_accurac	1	2137	2136	2997	35	2149	2304	3165

y=0.997	y=0.995								
cb8564_c15996/f1p5/3083 isoform=cb8564_c15996;full_length_coverage=1;non_full_length_coverage=5;isoform_length=3083;expected_accuracy=0.993	gnl|BL_ORD_ID|54027 cb8564_c17122/f1p0/3317	323	3083	9	328	471	3229	12	330
cb5921_c27/f1p2/2226 isoform=cb5921_c27;full_length_coverage=1;non_full_length_coverage=2;isoform_length=2226;expected_accuracy=0.995	gnl|BL_ORD_ID|74654 cb5921_c44/f1p1/2444	1	2024	2025	2176	63	2070	2184	2336
cb13842_c5/f1p0/1399 isoform=cb13842_c5;full_length_coverage=1;non_full_length_coverage=0;isoform_length=1399;expected_accuracy=0.992	gnl|BL_ORD_ID|16206 cb8564_c74486/f1p0/4128 isoform=cb8564_c74486;full_length_coverage=1;non_full_length_coverage=0;isoform_length=4128;expected_accuracy=0.993	1	1043	1038	1398	89	1137	3757	4128
cb8564_c41385/f1p1/3331 isoform=cb8564_c41385;full_length_coverage=1;non_full_length_coverage=1;isoform_length=3331;expected_accuracy=0.996	gnl|BL_ORD_ID|18317 cb8564_c80643/f1p1/3548 isoform=cb8564_c80643;full_length_coverage=1;non_full_length_coverage=1;isoform_length=3548;expected_accuracy=0.991	2	2276	2276	3316	1	2251	2509	3548
cb14752_c10/f1p1/6425	gnl|BL_ORD_ID|64264 cb14752_c8/f1p0/6702	3	5073	5073	6196	3	5073	5181	6304
cb8564_c46857/f1p0/2753 isoform=cb8564_c46857;full_length_coverage=1;non_full_length_coverage=0;isoform_length=2753;expected_accuracy=0.997	gnl|BL_ORD_ID|16567 cb8564_c50175/f1p0/2779 isoform=cb8564_c50175;full_length_coverage=1;non_full_length_coverage=0;isoform_length=2779;expected_accuracy=1.000	1	1659	1658	2692	4	1646	1747	2779
cb8564_c146219/f5p0/2824 isoform=cb8564_c146219;full_length_coverage=5;non_full_length_coverage=0;isoform_length=2824;expected	gnl|BL_ORD_ID|11423 cb8564_c12647/f1p0/3038 isoform=cb8564_c12647;full_length_coverage=1;non_full_length_coverage=0;isoform_length=3038;expected_ac	1	2012	2011	2788	150	2162	2262	3038

_accuracy=0.997	curacy=1.000								
cb15484_c3/f1p0/1383 isoform=cb15484_c3;full_length_coverage=1;non_full_length_coverage=0;isoform_length=1383;expected_accuracy=0.991	gnl|BL_ORD_ID|63438 cb15484_c1/f1p0/1723	1	1020	1019	1383	194	1213	1332	1692
cb862_c7/f1p0/2681	gnl|BL_ORD_ID|17512 cb862_c1/f2p0/3252 isoform=cb862_c1;full_length_coverage=2;non_full_length_coverage=0;isoform_length=3252;expected_accuracy=0.998	1294	2697	1	1294	1810	3213	1	1295
cb3143_c10/f1p1/2863	gnl|BL_ORD_ID|37665 cb3143_c33/f10p3/3441 isoform=cb3143_c33;full_length_coverage=10;non_full_length_coverage=3;isoform_length=3441;expected_accuracy=1.000	163	2870	1	163	723	3428	163	325
cb341_c7/f1p0/2819 isoform=cb341_c7;full_length_coverage=1;non_full_length_coverage=0;isoform_length=2819;expected_accuracy=1.000	gnl|BL_ORD_ID|69575 cb341_c16/f1p0/3691	1	2507	2507	2819	13	2520	3002	3314
cb8564_c42285/f1p0/3370	gnl|BL_ORD_ID|55920 cb8564_c48949/f1p0/4357	1	2288	2289	2953	2	2293	2847	3508
cb3143_c7/f1p1/3040 isoform=cb3143_c7;full_length_coverage=1;non_full_length_coverage=1;isoform_length=3040;expected_accuracy=1.000	gnl|BL_ORD_ID|37665 cb3143_c33/f10p3/3441 isoform=cb3143_c33;full_length_coverage=10;non_full_length_coverage=3;isoform_length=3441;expected_accuracy=1.000	321	3038	6	321	723	3441	39	349
cb1291_c2/f6p1/2729 isoform=cb1291_c2;full_length_coverage=6;non_full_length_coverage=1;isoform_length=2729;expected_accur	gnl|BL_ORD_ID|26064 cb1291_c4/f1p1/4391 isoform=cb1291_c4;full_length_coverage=1;non_full_length_coverage=1;isoform_length=4391;expected_accura	1	2082	2081	2709	13	2092	3764	4391

acy=1.000	cy=1.000								
cb6071_c14/f1p2/5103 isoform=cb6071_c14;full_length_coverage=1;non_full_length_coverage=2;isoform_length=5103;expected_accuracy=0.997	gnl|BL_ORD_ID|45341 cb6071_c15/f2p2/5278 isoform=cb6071_c15;full_length_coverage=2;non_full_length_coverage=2;isoform_length=5278;expected_accuracy=0.997	305	5103	1	304	483	5278	10	316
cb8564_c188097/f1p0/2552 isoform=cb8564_c188097;full_length_coverage=1;non_full_length_coverage=0;isoform_length=2552;expected_accuracy=0.996	gnl|BL_ORD_ID|17272 cb8564_c91851/f1p1/2644 isoform=cb8564_c91851;full_length_coverage=1;non_full_length_coverage=1;isoform_length=2644;expected_accuracy=0.993	1	1945	1940	2552	1	1944	2047	2643
cb8564_c112420/f1p0/2041	gnl|BL_ORD_ID|16302 cb8564_c5251/f1p0/3184 isoform=cb8564_c5251;full_length_coverage=1;non_full_length_coverage=0;isoform_length=3184;expected_accuracy=0.992	627	1728	8	628	2073	3171	43	665
cb8564_c123294/f1p0/1926	gnl|BL_ORD_ID|11640 cb8564_c127145/f1p0/2046 isoform=cb8564_c127145;full_length_coverage=1;non_full_length_coverage=0;isoform_length=2046;expected_accuracy=0.992	1	1473	1472	1827	35	1505	1691	2046
cb14220_c21/f1p1/5537	gnl|BL_ORD_ID|25175 cb14220_c15/f1p5/6485 isoform=cb14220_c15;full_length_coverage=1;non_full_length_coverage=5;isoform_length=6485;expected_accuracy=0.999	1	4570	4569	5549	712	5268	5390	6384
cb8564_c43455/f1p0/2242 isoform=cb8564_c43455;full_length_coverage=1;non_full_length_coverage=0;isoform_length=2242;expected_accuracy=0.995	gnl|BL_ORD_ID|55431 cb8564_c41557/f1p0/3003	279	2242	4	283	550	2520	2	281
cb11044_c2/f1p0/1717	gnl|BL_ORD_ID|2336 cb11044_c1/f1p1/2587	331	1691	58	331	1221	258	1	276

isoform=cb11044_c2;full_length_coverage=1;non_full_length_coverage=0;isoform_length=1717;expected_accuracy=0.998	isoform=cb11044_c1;full_length_coverage=1;non_full_length_coverage=1;isoform_length=2587;expected_accuracy=0.997						7		
cb12349_c8/f1p0/1434 isoform=cb12349_c8;full_length_coverage=1;non_full_length_coverage=0;isoform_length=1434;expected_accuracy=0.995	gnl|BL_ORD_ID|61692 cb12349_c11/f1p0/1764	1	1193	1191	1434	7	1192	1293	1535
cb8564_c43779/f1p0/3021 isoform=cb8564_c43779;full_length_coverage=1;non_full_length_coverage=0;isoform_length=3021;expected_accuracy=0.993	gnl|BL_ORD_ID|12913 cb8564_c182049/f1p0/3195 isoform=cb8564_c182049;full_length_coverage=1;non_full_length_coverage=0;isoform_length=3195;expected_accuracy=0.997	1	2344	2345	3021	1	2360	2516	3195
cb1837_c7/f1p2/3835	gnl|BL_ORD_ID|66399 cb1837_c9/f1p1/3972	170	3792	1	169	298	3923	21	189
cb8564_c74541/f1p1/3268 isoform=cb8564_c74541;full_length_coverage=1;non_full_length_coverage=1;isoform_length=3268;expected_accuracy=0.992	gnl|BL_ORD_ID|57975 cb8564_c85634/f1p0/3815	601	3268	8	597	813	3488	6	597
cb8564_c72349/f1p0/3248	gnl|BL_ORD_ID|14019 cb8564_c39824/f1p0/3319 isoform=cb8564_c39824;full_length_coverage=1;non_full_length_coverage=0;isoform_length=3319;expected_accuracy=1.000	108	2662	2659	3068	1	2555	2714	3123
cb8564_c41690/f1p1/3409	gnl|BL_ORD_ID|55939 cb8564_c49630/f1p4/3487	2	2920	2920	3170	1	2913	3243	3493
cb3101_c12/f1p3/3007 isoform=cb3101_c12;full_length_coverage=1;non_full_length_coverage=3;isoform_length=3007;expected_accu	gnl|BL_ORD_ID|69128 cb3101_c14/f1p0/3479	13	1950	1946	2932	3	1939	2235	3219

racy=0.993									
cb850_c28/f1p0/3553 isoform=cb850_c28;full_length_coverage=1;non_full_length_coverage=0;isoform_length=3553;expected_accuracy=0.992	gnl|BL_ORD_ID|52431 cb850_c22/f1p0/4086	303	3520	1	307	709	3905	263	568
cb2369_c2/f1p0/3530 isoform=cb2369_c2;full_length_coverage=1;non_full_length_coverage=0;isoform_length=3530;expected_accuracy=0.994	gnl|BL_ORD_ID|38850 cb2369_c1/f1p0/3700 isoform=cb2369_c1;full_length_coverage=1;non_full_length_coverage=0;isoform_length=3700;expected_accuracy=0.995	109	2853	2851	3530	1	2745	2874	3551
cb8564_c35300/f1p2/3912	gnl|BL_ORD_ID|55865 cb8564_c47461/f1p0/4325	185	3827	1	186	318	3960	1	186
cb1581_c3/f2p4/2456 isoform=cb1581_c3;full_length_coverage=2;non_full_length_coverage=4;isoform_length=2456;expected_accuracy=0.999	gnl|BL_ORD_ID|28676 cb1581_c9/f1p2/2552 isoform=cb1581_c9;full_length_coverage=1;non_full_length_coverage=2;isoform_length=2552;expected_accuracy=0.999	250	2450	47	249	353	2552	1	204
cb10054_c49/f1p1/1123 isoform=cb10054_c49;full_length_coverage=1;non_full_length_coverage=1;isoform_length=1123;expected_accuracy=1.000	gnl|BL_ORD_ID|2389 cb10054_c52/f1p2/1369 isoform=cb10054_c52;full_length_coverage=1;non_full_length_coverage=2;isoform_length=1369;expected_accuracy=0.997	258	1123	48	262	498	1363	101	317
cb8564_c4945/f1p0/1950 isoform=cb8564_c4945;full_length_coverage=1;non_full_length_coverage=0;isoform_length=1950;expected_accuracy=1.000	gnl|BL_ORD_ID|16586 cb8564_c4940/f1p0/2731 isoform=cb8564_c4940;full_length_coverage=1;non_full_length_coverage=0;isoform_length=2731;expected_accuracy=0.996	969	1950	99	973	1751	2731	89	962
cb3183_c8/f1p3/3030 isoform=cb3183_c8;full_length_coverage=1;non_full_length_coverage=3;isoform_length=3030;expected_accur	gnl|BL_ORD_ID|37887 cb3183_c3/f2p4/3655 isoform=cb3183_c3;full_length_coverage=2;non_full_length_coverage=4;isoform_length=3655;expected_accura	147	3030	1	148	774	3655	202	349

acy=0.999	cy=1.000								
cb8564_c42308/f1p5/3192 isoform=cb8564_c42308;full_length_coverage=1;non_full_length_coverage=5;isoform_length=3192;expected_accuracy=0.995	gnl|BL_ORD_ID|55939 cb8564_c49630/f1p4/3487	11	2934	2934	3190	6	2913	3243	3497
cb2629_c6/f1p0/4836 isoform=cb2629_c6;full_length_coverage=1;non_full_length_coverage=0;isoform_length=4836;expected_accuracy=0.993	gnl|BL_ORD_ID|69477 cb18844_c1/f1p0/5518	1	2925	2922	4812	167	3101	3266	5162
cb5667_c26/f1p1/2495 isoform=cb5667_c26;full_length_coverage=1;non_full_length_coverage=1;isoform_length=2495;expected_accuracy=0.995	gnl|BL_ORD_ID|74259 cb5667_c10/f1p0/2842	1099	2487	1	1099	1356	2741	50	1148
cb6532_c2/f1p0/2075	gnl|BL_ORD_ID|4405 cb6532_c1/f1p0/2254 isoform=cb6532_c1;full_length_coverage=1;non_full_length_coverage=0;isoform_length=2254;expected_accuracy=0.992	1	1306	1304	2064	34	1337	1478	2237
cb16655_c3/f1p0/1854 isoform=cb16655_c3;full_length_coverage=1;non_full_length_coverage=0;isoform_length=1854;expected_accuracy=0.997	gnl|BL_ORD_ID|10633 cb8564_c1210/f1p0/2120 isoform=cb8564_c1210;full_length_coverage=1;non_full_length_coverage=0;isoform_length=2120;expected_accuracy=0.999	148	1786	11	148	479	2120	1	137
cb4835_c1/f3p1/2340 isoform=cb4835_c1;full_length_coverage=3;non_full_length_coverage=1;isoform_length=2340;expected_accuracy=1.000	gnl|BL_ORD_ID|72029 cb4835_c11/f1p0/2717	1	1418	1413	2338	9	1423	1631	2556
cb227_c14/f1p0/2789	gnl|BL_ORD_ID|37093 cb227_c5/f1p0/3888	1	2438	2438	2745	82	252	2712	301

	isoform=cb227_c5;full_length_coverage=1;non_full_length_coverage=0;isoform_length=3888;expected_accuracy=0.998						3		9
cb8564_c165050/f2p1/2091 isoform=cb8564_c165050;full_length_coverage=2;non_full_length_coverage=1;isoform_length=2091;expected_accuracy=0.997	gnl|BL_ORD_ID|14463 cb8564_c25154/f1p5/4276 isoform=cb8564_c25154;full_length_coverage=1;non_full_length_coverage=5;isoform_length=4276;expected_accuracy=0.995	271	2091	85	269	2418	4236	4	189
cb3010_c5/f2p0/2793 isoform=cb3010_c5;full_length_coverage=2;non_full_length_coverage=0;isoform_length=2793;expected_accuracy=1.000	gnl|BL_ORD_ID|38461 cb3010_c8/f1p0/2925 isoform=cb3010_c8;full_length_coverage=1;non_full_length_coverage=0;isoform_length=2925;expected_accuracy=0.998	1189	2793	1	1190	1304	2908	1	1190
cb8564_c42814/f1p0/2151 isoform=cb8564_c42814;full_length_coverage=1;non_full_length_coverage=0;isoform_length=2151;expected_accuracy=0.993	gnl|BL_ORD_ID|15229 cb8564_c40059/f1p0/3005 isoform=cb8564_c40059;full_length_coverage=1;non_full_length_coverage=0;isoform_length=3005;expected_accuracy=0.991	650	2151	7	652	1495	3003	1	678
cb2527_c31/f1p1/3358	gnl|BL_ORD_ID|68590 cb2527_c17/f1p1/3542	135	2040	2036	3337	1	1906	2256	3557
cb6548_c8/f1p0/2532	gnl|BL_ORD_ID|5637 cb6548_c10/f1p1/2515 isoform=cb6548_c10;full_length_coverage=1;non_full_length_coverage=1;isoform_length=2515;expected_accuracy=0.991	265	2327	58	267	451	2515	1	210
cb18339_c0/f1p1/1860 isoform=cb18339_c0;full_length_coverage=1;non_full_length_coverage=1;isoform_length=1860;expected_accuracy=0.999	gnl|BL_ORD_ID|47594 cb6602_c12/f1p0/2867	291	1838	8	295	485	2034	1	280
cb8564_c90410/f1p0/3234	gnl|BL_ORD_ID|15394 cb8564_c24855/f2p0/3424	1	2091	2090	3200	104	219	2315	342

	isoform=cb8564_c24855;full_length_coverage=2;non_full_length_coverage=0;isoform_length=3424;expected_accuracy=0.999						0		4
cb8564_c122951/f1p0/3293	gnl|BL_ORD_ID|12327 cb8564_c123675/f1p1/3399 isoform=cb8564_c123675;full_length_coverage=1;non_full_length_coverage=1;isoform_length=3399;expected_accuracy=0.995	2	2591	2589	3041	157	2744	2931	3381
cb4128_c14/f1p1/2357	gnl|BL_ORD_ID|40767 cb4128_c10/f3p1/2897 isoform=cb4128_c10;full_length_coverage=3;non_full_length_coverage=1;isoform_length=2897;expected_accuracy=0.998	546	2341	64	549	1052	2847	3	487
cb1626_c15/f1p0/2204 isoform=cb1626_c15;full_length_coverage=1;non_full_length_coverage=0;isoform_length=2204;expected_accuracy=0.998	gnl|BL_ORD_ID|63808 cb1626_c17/f1p0/3006	1	1096	1093	2154	1	1091	1919	2976
cb8564_c181074/f1p0/2725 isoform=cb8564_c181074;full_length_coverage=1;non_full_length_coverage=0;isoform_length=2725;expected_accuracy=1.000	gnl|BL_ORD_ID|56772 cb8564_c71057/f1p4/4091	148	2725	1	147	1498	4080	12	154
cb6609_c8/f1p0/2192	gnl|BL_ORD_ID|3768 cb6609_c2/f1p0/2814 isoform=cb6609_c2;full_length_coverage=1;non_full_length_coverage=0;isoform_length=2814;expected_accuracy=0.999	239	1956	29	243	917	2633	1	213
cb5773_c11/f1p0/2633 isoform=cb5773_c11;full_length_coverage=1;non_full_length_coverage=0;isoform_length=2633;expected_accu	gnl|BL_ORD_ID|46836 cb5773_c0/f8p0/2821 isoform=cb5773_c0;full_length_coverage=8;non_full_length_coverage=0;isoform_length=2821;expected_accura	1	1756	1754	2631	1	1759	1944	2821

racy=0.995	cy=1.000								
cb808_c3/f1p0/3813	gnl|BL_ORD_ID|50993 cb808_c1/f1p0/4621	1	2545	2543	3568	89	2633	3609	4633
cb18456_c6530/f1p0/1200	gnl|BL_ORD_ID|53228 cb8564_c119430/f1p0/1961	260	1166	3	259	799	1705	12	268
cb8564_c40978/f1p0/3073	gnl|BL_ORD_ID|16172 cb8564_c53024/f1p0/3159 isoform=cb8564_c53024;full_length_coverage=1;non_full_length_coverage=0;isoform_length=3159;expected_accuracy=0.996	1	1711	1708	2850	1	1699	2017	3158
cb11589_c0/f1p0/1162	gnl|BL_ORD_ID|60276 cb11589_c1/f1p0/2770	131	1138	3	134	1724	2727	734	865
cb1176_c32/f1p0/3993	gnl|BL_ORD_ID|22295 cb1176_c4/f1p0/4212 isoform=cb1176_c4;full_length_coverage=1;non_full_length_coverage=0;isoform_length=4212;expected_accuracy=0.997	111	3923	1	112	374	4184	24	135
cb8564_c83489/f1p1/2514 isoform=cb8564_c83489;full_length_coverage=1;non_full_length_coverage=1;isoform_length=2514;expected_accuracy=0.995	gnl|BL_ORD_ID|18587 cb8564_c84653/f6p1/2944 isoform=cb8564_c84653;full_length_coverage=6;non_full_length_coverage=1;isoform_length=2944;expected_accuracy=0.994	342	2514	5	347	553	2729	1	343
cb8564_c53654/f2p0/3797 isoform=cb8564_c53654;full_length_coverage=2;non_full_length_coverage=0;isoform_length=3797;expected_accuracy=0.996	gnl|BL_ORD_ID|55975 cb8564_c50843/f1p0/4165	2	3272	3271	3761	8	3252	3639	4130
cb6548_c12/f1p1/2464	gnl|BL_ORD_ID|5637 cb6548_c10/f1p1/2515 isoform=cb6548_c10;full_length_coverage=1;non_full_length_coverage=1;isoform_length=2515;expected_accur	259	2427	52	260	345	2515	1	209

	acy=0.991								
cb938_c15/f1p0/3219	gnl|BL_ORD_ID|21148 cb938_c6/f2p5/3513 isoform=cb938_c6;full_length_coverage=2;non_full_length_coverage=5;isoform_length=3513;expected_accuracy=1.000	1	2546	2541	3188	1	2548	2824	3471
cb4706_c4/f1p0/1315 isoform=cb4706_c4;full_length_coverage=1;non_full_length_coverage=0;isoform_length=1315;expected_accuracy=0.995	gnl|BL_ORD_ID|71982 cb4706_c2/f1p0/1537	1	1019	1018	1315	4	1023	1156	1455
cb9427_c6/f1p0/2067 isoform=cb9427_c6;full_length_coverage=1;non_full_length_coverage=0;isoform_length=2067;expected_accuracy=0.994	gnl|BL_ORD_ID|20412 cb9427_c3/f1p0/2379 isoform=cb9427_c3;full_length_coverage=1;non_full_length_coverage=0;isoform_length=2379;expected_accuracy=0.991	881	2067	97	882	1148	2336	1	783
cb7023_c6/f1p0/1630	gnl|BL_ORD_ID|47818 cb7023_c7/f1p0/1690	129	1564	1	129	258	1693	10	138
cb1952_c4/f5p0/2247 isoform=cb1952_c4;full_length_coverage=5;non_full_length_coverage=0;isoform_length=2247;expected_accuracy=1.000	gnl|BL_ORD_ID|66522 cb1952_c18/f1p0/2527	1	2025	2022	2194	21	2057	2196	2366
cb4579_c3/f1p0/2979	gnl|BL_ORD_ID|42018 cb4579_c0/f2p0/3304 isoform=cb4579_c0;full_length_coverage=2;non_full_length_coverage=0;isoform_length=3304;expected_accuracy=1.000	1243	2872	1	1246	1677	3304	71	1316
cb8564_c125514/f1p0/2141	gnl|BL_ORD_ID|54874 cb8564_c3238/f1p0/2188	361	1947	4	361	507	2093	9	366
cb3939_c29/f1p0/2382	gnl|BL_ORD_ID|39238 cb3939_c10/f3p0/3353	1208	2324	101	1209	2181	329	108	121

	isoform=cb3939_c10;full_length_coverage=3;non_full_length_coverage=0;isoform_length=3353;expected_accuracy=0.998						7		6
cb3580_c20/f2p2/2486 isoform=cb3580_c20;full_length_coverage=2;non_full_length_coverage=2;isoform_length=2486;expected_accuracy=0.997	gnl|BL_ORD_ID|40393 cb3580_c24/f1p0/3582 isoform=cb3580_c24;full_length_coverage=1;non_full_length_coverage=0;isoform_length=3582;expected_accuracy=0.992	179	2414	1	183	1337	3567	765	948
cb12424_c19/f1p0/1100	gnl|BL_ORD_ID|61720 cb12424_c18/f1p0/1829	203	1009	28	207	770	1576	33	212
cb8564_c14964/f3p2/2488 isoform=cb8564_c14964;full_length_coverage=3;non_full_length_coverage=2;isoform_length=2488;expected_accuracy=0.995	gnl|BL_ORD_ID|52423 cb8564_c122458/f1p0/2664	1	2156	2155	2487	1	2157	2310	2642
cb8564_c10938/f1p1/2321	gnl|BL_ORD_ID|54156 cb8564_c19563/f1p0/4354	1	1315	1310	2292	1318	2632	2751	3733
cb8564_c87256/f1p0/2285 isoform=cb8564_c87256;full_length_coverage=1;non_full_length_coverage=0;isoform_length=2285;expected_accuracy=0.992	gnl|BL_ORD_ID|16094 cb8564_c72701/f1p0/2931 isoform=cb8564_c72701;full_length_coverage=1;non_full_length_coverage=0;isoform_length=2931;expected_accuracy=0.996	1	1703	1700	2270	515	2221	2355	2931
cb8564_c176017/f8p2/2488 isoform=cb8564_c176017;full_length_coverage=8;non_full_length_coverage=2;isoform_length=2488;expected_accuracy=1.000	gnl|BL_ORD_ID|10848 cb8564_c112467/f1p0/3061 isoform=cb8564_c112467;full_length_coverage=1;non_full_length_coverage=0;isoform_length=3061;expected_accuracy=0.994	1	1875	1874	2417	1	1846	2518	3061
cb6348_c12/f4p0/1764 isoform=cb6348_c12;full_length_coverage=4;non_full_length_coverage=0;isoform_length=1764;expected_accu	gnl|BL_ORD_ID|4828 cb6348_c4/f1p0/2643 isoform=cb6348_c4;full_length_coverage=1;non_full_length_coverage=0;isoform_length=2643;expected_accura	1	1103	1099	1764	15	1121	1952	2618

racy=0.998	cy=0.999								
cb8564_c178653/f1p0/2779 isoform=cb8564_c178653;full_length_coverage=1;non_full_length_coverage=0;isoform_length=2779;expected_accuracy=0.994	gnl|BL_ORD_ID|16795 cb8564_c73428/f1p0/2966 isoform=cb8564_c73428;full_length_coverage=1;non_full_length_coverage=0;isoform_length=2966;expected_accuracy=0.994	1	2179	2177	2779	7	2180	2338	2937
cb2155_c11/f1p0/2668 isoform=cb2155_c11;full_length_coverage=1;non_full_length_coverage=0;isoform_length=2668;expected_accuracy=1.000	gnl|BL_ORD_ID|35418 cb2155_c26/f5p0/2830 isoform=cb2155_c26;full_length_coverage=5;non_full_length_coverage=0;isoform_length=2830;expected_accuracy=1.000	1	2105	2106	2668	38	2135	2253	2805
cb3576_c2/f1p0/3257 isoform=cb3576_c2;full_length_coverage=1;non_full_length_coverage=0;isoform_length=3257;expected_accuracy=0.997	gnl|BL_ORD_ID|41240 cb3576_c1/f1p0/3587 isoform=cb3576_c1;full_length_coverage=1;non_full_length_coverage=0;isoform_length=3587;expected_accuracy=0.995	1	1704	1703	3257	15	1719	1898	3451
cb8564_c49958/f1p0/3088 isoform=cb8564_c49958;full_length_coverage=1;non_full_length_coverage=0;isoform_length=3088;expected_accuracy=0.995	gnl|BL_ORD_ID|55804 cb8564_c38892/f1p1/3313	1353	3080	1	1355	1481	3206	1	1356
cb3387_c4/f1p0/2366 isoform=cb3387_c4;full_length_coverage=1;non_full_length_coverage=0;isoform_length=2366;expected_accuracy=0.997	gnl|BL_ORD_ID|70355 cb3387_c7/f1p0/3232	1	1821	1818	2341	74	1895	2683	3205
cb8564_c33702/f1p6/2850 isoform=cb8564_c33702;full_length_coverage=1;non_full_length_coverage=6;isoform_length=2850;expected_accuracy=0.993	gnl|BL_ORD_ID|56029 cb8564_c52782/f1p2/3327	236	2848	1	236	617	3236	1	225
cb16807_c2/f1p0/1293	gnl|BL_ORD_ID|9869 cb8564_c109825/f2p0/2137	233	1272	5	233	1098	213	10	238

	isoform=cb8564_c109825;full_length_coverage=2;non_full_length_coverage=0;isoform_length=2137;expected_accuracy=0.995						7		
cb6090_c0/f5p0/2362 isoform=cb6090_c0;full_length_coverage=5;non_full_length_coverage=0;isoform_length=2362;expected_accuracy=1.000	gnl|BL_ORD_ID|44623 cb6090_c1/f1p0/2937 isoform=cb6090_c1;full_length_coverage=1;non_full_length_coverage=0;isoform_length=2937;expected_accuracy=0.997	1	1688	1686	2360	1	1686	2265	2937
cb8564_c131385/f1p1/2665 isoform=cb8564_c131385;full_length_coverage=1;non_full_length_coverage=1;isoform_length=2665;expected_accuracy=0.996	gnl|BL_ORD_ID|54560 cb8564_c126417/f1p0/2804	1	1724	1722	2665	2	1725	1830	2773
cb8564_c74375/f1p1/2718 isoform=cb8564_c74375;full_length_coverage=1;non_full_length_coverage=1;isoform_length=2718;expected_accuracy=1.000	gnl|BL_ORD_ID|57029 cb8564_c81055/f1p0/3110	1	1609	1608	2718	132	1744	1853	2962
cb396_c42/f1p0/2343	gnl|BL_ORD_ID|39231 cb396_c67/f28p0/2542 isoform=cb396_c67;full_length_coverage=28;non_full_length_coverage=0;isoform_length=2542;expected_accuracy=1.000	1	1458	1457	2346	7	1465	1635	2524
cb8564_c41756/f1p0/2738 isoform=cb8564_c41756;full_length_coverage=1;non_full_length_coverage=0;isoform_length=2738;expected_accuracy=0.996	gnl|BL_ORD_ID|17161 cb8564_c87466/f1p1/3006 isoform=cb8564_c87466;full_length_coverage=1;non_full_length_coverage=1;isoform_length=3006;expected_accuracy=0.994	1	2173	2170	2738	1	2196	2321	2900
cb4444_c24/f1p1/1945	gnl|BL_ORD_ID|43663 cb4444_c8/f1p0/2558 isoform=cb4444_c8;full_length_coverage=1;non_full_length_coverage=0;isoform_length=2558;expected_accura	592	1943	6	592	1068	2405	1	583

	cy=0.994								
cb8564_c15653/f1p4/2475	gnl|BL_ORD_ID|10640 cb8564_c100234/f2p5/2744 isoform=cb8564_c100234;full_length_coverage=2;non_full_length_coverage=5;isoform_length=2744;expected_accuracy=0.993	15	2293	2289	2482	38	2318	2537	2734
cb8564_c21439/f1p0/2587	gnl|BL_ORD_ID|57311 cb8564_c89547/f1p0/2973	1226	2572	1	1228	1441	2787	91	1320
cb5564_c21/f1p0/1889	gnl|BL_ORD_ID|45842 cb5564_c9/f1p0/2154 isoform=cb5564_c9;full_length_coverage=1;non_full_length_coverage=0;isoform_length=2154;expected_accuracy=0.992	355	1872	46	359	619	2137	67	380
cb2430_c25/f5p0/2633 isoform=cb2430_c25;full_length_coverage=5;non_full_length_coverage=0;isoform_length=2633;expected_accuracy=1.000	gnl|BL_ORD_ID|38549 cb2430_c6/f1p0/3031 isoform=cb2430_c6;full_length_coverage=1;non_full_length_coverage=0;isoform_length=3031;expected_accuracy=0.998	1	1837	1835	2632	1	1832	2239	3031
cb2747_c0/f11p2/3395 isoform=cb2747_c0;full_length_coverage=11;non_full_length_coverage=2;isoform_length=3395;expected_accuracy=1.000	gnl|BL_ORD_ID|68803 cb2747_c3/f1p0/3786	19	2335	2331	3304	4	2320	2741	3714
cb8564_c83196/f1p0/2505 isoform=cb8564_c83196;full_length_coverage=1;non_full_length_coverage=0;isoform_length=2505;expected_accuracy=0.995	gnl|BL_ORD_ID|57277 cb8564_c88819/f1p0/3550	1	1642	1642	2485	945	2587	2700	3543
cb12389_c6/f9p1/1860 isoform=cb12389_c6;full_length_coverage=9;non_full_length_coverage=1;isoform_length=1860;expected_accu	gnl|BL_ORD_ID|20471 cb9943_c3/f1p0/2123 isoform=cb9943_c3;full_length_coverage=1;non_full_length_coverage=0;isoform_length=2123;expected_accura	1	1490	1487	1812	8	1498	1798	2123

racy=0.995	cy=0.999								
cb2640_c12/f1p0/3046 isoform=cb2640_c12;full_length_coverage=1;non_full_length_coverage=0;isoform_length=3046;expected_accuracy=0.994	gnl|BL_ORD_ID|39029 cb2640_c3/f2p1/3206 isoform=cb2640_c3;full_length_coverage=2;non_full_length_coverage=1;isoform_length=3206;expected_accuracy=0.998	192	3046	1	193	334	3184	16	207
cb9100_c7/f1p0/2182	gnl|BL_ORD_ID|20675 cb9100_c8/f1p0/2665 isoform=cb9100_c8;full_length_coverage=1;non_full_length_coverage=0;isoform_length=2665;expected_accuracy=0.998	19	1934	1931	2182	20	1934	2413	2664
cb8564_c81333/f1p0/2975	gnl|BL_ORD_ID|13635 cb8564_c21316/f1p2/2982 isoform=cb8564_c21316;full_length_coverage=1;non_full_length_coverage=2;isoform_length=2982;expected_accuracy=0.997	1	1864	1863	2700	115	1978	2146	2982
cb10304_c5/f1p0/1455 isoform=cb10304_c5;full_length_coverage=1;non_full_length_coverage=0;isoform_length=1455;expected_accuracy=1.000	gnl|BL_ORD_ID|2572 cb10304_c8/f3p0/1648 isoform=cb10304_c8;full_length_coverage=3;non_full_length_coverage=0;isoform_length=1648;expected_accuracy=0.994	1	1333	1328	1455	5	1337	1522	1648
cb1772_c11/f1p0/3842 isoform=cb1772_c11;full_length_coverage=1;non_full_length_coverage=0;isoform_length=3842;expected_accuracy=0.994	gnl|BL_ORD_ID|63938 cb1772_c19/f1p0/4042	285	3814	2	286	441	3970	4	307
cb1573_c16/f1p0/3454 isoform=cb1573_c16;full_length_coverage=1;non_full_length_coverage=0;isoform_length=3454;expected_accuracy=0.998	gnl|BL_ORD_ID|30372 cb1573_c3/f4p1/3618 isoform=cb1573_c3;full_length_coverage=4;non_full_length_coverage=1;isoform_length=3618;expected_accuracy=0.996	1249	3363	1	1252	1497	3618	85	1338
cb7214_c10/f1p0/1871	gnl|BL_ORD_ID|8880 cb7214_c4/f1p0/2395	134	1878	1	136	650	239	21	156

	isoform=cb7214_c4;full_length_coverage=1;non_full_length_coverage=0;isoform_length=2395;expected_accuracy=0.999						5		
cb3411_c39/f1p2/3337	gnl|BL_ORD_ID|40175 cb3411_c12/f1p0/3570 isoform=cb3411_c12;full_length_coverage=1;non_full_length_coverage=0;isoform_length=3570;expected_accuracy=0.995	273	3239	1	274	608	3570	42	316
cb9157_c9/f1p0/1707 isoform=cb9157_c9;full_length_coverage=1;non_full_length_coverage=0;isoform_length=1707;expected_accuracy=0.996	gnl|BL_ORD_ID|19853 cb9157_c6/f1p0/2119 isoform=cb9157_c6;full_length_coverage=1;non_full_length_coverage=0;isoform_length=2119;expected_accuracy=0.998	1	1330	1327	1707	102	1429	1571	1951
cb1016_c1/f2p1/1266 isoform=cb1016_c1;full_length_coverage=2;non_full_length_coverage=1;isoform_length=1266;expected_accuracy=1.000	gnl|BL_ORD_ID|47223 cb1016_c4/f1p1/2801	1	1008	1007	1266	99	1106	2409	2668
cb90_c4/f2p0/4575 isoform=cb90_c4;full_length_coverage=2;non_full_length_coverage=0;isoform_length=4575;expected_accuracy=0.999	gnl|BL_ORD_ID|57571 cb90_c32/f1p0/4642	1615	4574	114	1618	1731	4690	102	1605
cb12513_c62/f1p0/1798	gnl|BL_ORD_ID|14254 cb8564_c3885/f1p7/2735 isoform=cb8564_c3885;full_length_coverage=1;non_full_length_coverage=7;isoform_length=2735;expected_accuracy=0.999	294	1539	38	295	1490	2735	17	274
cb8564_c135684/f4p2/3618 isoform=cb8564_c135684;full_length_coverage=4;non_full_length_coverage=2;isoform_length=3618;expected	gnl|BL_ORD_ID|57396 cb8564_c91806/f1p1/3620	1	2743	2741	3468	1	2745	2895	3622

_accuracy=0.996									
cb13515_c9/f1p0/1348	gnl|BL_ORD_ID|26579 cb13515_c1/f2p1/1462 isoform=cb13515_c1;full_length_coverage=2;non_full_length_coverage=1;isoform_length=1462;expected_accuracy=1.000	1	1117	1114	1295	62	1173	1282	1462
cb6430_c3/f1p1/2522	gnl|BL_ORD_ID|49714 cb6430_c2/f1p0/2694	2	2071	2068	2463	2	2098	2286	2681
cb322_c15/f1p0/3487 isoform=cb322_c15;full_length_coverage=1;non_full_length_coverage=0;isoform_length=3487;expected_accuracy=0.997	gnl|BL_ORD_ID|70802 cb322_c34/f1p0/3998	17	2729	2728	3487	1	2717	3203	3962
cb998_c6/f1p0/3781 isoform=cb998_c6;full_length_coverage=1;non_full_length_coverage=0;isoform_length=3781;expected_accuracy=0.995	gnl|BL_ORD_ID|67910 cb18641_c0/f1p0/4940	1682	3781	16	1687	2393	4496	1	1677
cb8564_c2323/f1p0/2212 isoform=cb8564_c2323;full_length_coverage=1;non_full_length_coverage=0;isoform_length=2212;expected_accuracy=0.995	gnl|BL_ORD_ID|14025 cb8564_c31262/f7p4/2729 isoform=cb8564_c31262;full_length_coverage=7;non_full_length_coverage=4;isoform_length=2729;expected_accuracy=0.994	1	1320	1316	2212	139	1458	1737	2634
cb12047_c134/f1p0/1727 isoform=cb12047_c134;full_length_coverage=1;non_full_length_coverage=0;isoform_length=1727;expected_accuracy=0.999	gnl|BL_ORD_ID|54402 cb8564_c34765/f1p1/1931	1	1006	1001	1727	1	1006	1112	1838
cb12189_c18/f1p5/1510 isoform=cb12189_c18;full_length_coverage=1;non_full_length_coverage=5;isoform_length=1510;expected_acc	gnl|BL_ORD_ID|60618 cb12189_c37/f1p0/1873	556	1505	6	560	788	1732	1	556

uracy=0.991									
cb5622_c4/f1p0/1701	gnl|BL_ORD_ID|74169 cb5622_c2/f1p0/2496	1	1188	1188	1567	31	1217	2011	2390
cb8564_c40764/f1p0/2563 isoform=cb8564_c40764;full_length_coverage=1;non_full_length_coverage=0;isoform_length=2563;expected_accuracy=0.997	gnl|BL_ORD_ID|11225 cb8564_c150144/f1p7/2784 isoform=cb8564_c150144;full_length_coverage=1;non_full_length_coverage=7;isoform_length=2784;expected_accuracy=0.991	1	2166	2162	2563	2	2164	2371	2773
cb13515_c9/f1p0/1348	gnl|BL_ORD_ID|26398 cb13515_c13/f4p0/1446 isoform=cb13515_c13;full_length_coverage=4;non_full_length_coverage=0;isoform_length=1446;expected_accuracy=0.993	1	1117	1114	1318	18	1133	1242	1446
cb8564_c36774/f1p0/2756 isoform=cb8564_c36774;full_length_coverage=1;non_full_length_coverage=0;isoform_length=2756;expected_accuracy=0.990	gnl|BL_ORD_ID|55187 cb8564_c34994/f1p0/3482	145	2756	1	145	881	3500	152	295
cb12_c30/f1p2/4443 isoform=cb12_c30;full_length_coverage=1;non_full_length_coverage=2;isoform_length=4443;expected_accuracy=0.992	gnl|BL_ORD_ID|61091 cb12_c58/f1p0/5377	369	4256	1	373	1404	5292	73	450
cb6360_c2/f2p1/2193 isoform=cb6360_c2;full_length_coverage=2;non_full_length_coverage=1;isoform_length=2193;expected_accuracy=0.995	gnl|BL_ORD_ID|47452 cb6360_c5/f1p1/2881	1	1542	1538	2192	1	1536	2202	2858
cb8564_c88594/f2p0/3794 isoform=cb8564_c88594;full_length_coverage=2;non_full_length_coverage=0;isoform_length=3794;expected_	gnl|BL_ORD_ID|17407 cb8564_c85285/f1p0/4248 isoform=cb8564_c85285;full_length_coverage=1;non_full_length_coverage=0;isoform_length=4248;expected_ac	1	1942	1941	3786	1	1930	2402	4248

accuracy=0.997	curacy=0.997								
cb3690_c7/f1p1/3164 isoform=cb3690_c7;full_length_coverage=1;non_full_length_coverage=1;isoform_length=3164;expected_accuracy=1.000	gnl|BL_ORD_ID|39399 cb3690_c1/f1p0/3553 isoform=cb3690_c1;full_length_coverage=1;non_full_length_coverage=0;isoform_length=3553;expected_accuracy=0.996	147	1796	1794	3164	1	1648	2183	3553
cb14241_c0/f3p0/1412 isoform=cb14241_c0;full_length_coverage=3;non_full_length_coverage=0;isoform_length=1412;expected_accuracy=1.000	gnl|BL_ORD_ID|25524 cb14241_c1/f2p0/1587 isoform=cb14241_c1;full_length_coverage=2;non_full_length_coverage=0;isoform_length=1587;expected_accuracy=1.000	169	1412	55	174	343	1585	1	120
cb2640_c13/f1p0/3176	gnl|BL_ORD_ID|68659 cb2640_c11/f1p0/3319	203	3176	2	206	352	3326	23	227
cb12972_c15/f1p0/1541 isoform=cb12972_c15;full_length_coverage=1;non_full_length_coverage=0;isoform_length=1541;expected_accuracy=0.996	gnl|BL_ORD_ID|54792 cb8564_c2623/f1p1/2475	205	1541	7	206	1082	2409	1	199
cb9467_c19/f1p0/3033 isoform=cb9467_c19;full_length_coverage=1;non_full_length_coverage=0;isoform_length=3033;expected_accuracy=0.991	gnl|BL_ORD_ID|20200 cb9467_c17/f1p0/3298 isoform=cb9467_c17;full_length_coverage=1;non_full_length_coverage=0;isoform_length=3298;expected_accuracy=1.000	1477	3033	1	1477	1614	3173	7	1504
cb500_c17/f1p0/2323 isoform=cb500_c17;full_length_coverage=1;non_full_length_coverage=0;isoform_length=2323;expected_accuracy=0.997	gnl|BL_ORD_ID|41883 cb500_c72/f1p0/4040 isoform=cb500_c72;full_length_coverage=1;non_full_length_coverage=0;isoform_length=4040;expected_accuracy=0.996	1	1293	1288	2323	1378	2670	2802	3836
cb3813_c6/f1p0/1430 isoform=cb3813_c6;full_length_coverage=1;non_full_length_coverage=0;isoform_length=1430;expected_accur	gnl|BL_ORD_ID|39713 cb3813_c1/f1p0/3520 isoform=cb3813_c1;full_length_coverage=1;non_full_length_coverage=0;isoform_length=3520;expected_accura	144	1430	31	147	2236	3516	1937	2051

acy=1.000	cy=0.997								
cb5564_c6/f3p0/2246 isoform=cb5564_c6;full_length_coverage=3;non_full_length_coverage=0;isoform_length=2246;expected_accuracy=0.999	gnl|BL_ORD_ID|44963 cb5564_c14/f14p1/2858 isoform=cb5564_c14;full_length_coverage=14;non_full_length_coverage=1;isoform_length=2858;expected_accuracy=1.000	156	2203	1	159	810	285 8	121	279
cb1514_c4/f2p1/3503 isoform=cb1514_c4;full_length_coverage=2;non_full_length_coverage=1;isoform_length=3503;expected_accuracy=0.997	gnl|BL_ORD_ID|29253 cb1514_c14/f1p1/3711 isoform=cb1514_c14;full_length_coverage=1;non_full_length_coverage=1;isoform_length=3711;expected_accuracy=0.997	1	3277	3277	3480	131	340 8	3508	3711
cb8564_c78480/f1p0/2950	gnl|BL_ORD_ID|16743 cb8564_c68940/f2p0/3028 isoform=cb8564_c68940;full_length_coverage=2;non_full_length_coverage=0;isoform_length=3028;expected_accuracy=0.998	1	1506	1506	2889	22	152 9	1645	302 8
cb17862_c4/f1p0/1707	gnl|BL_ORD_ID|5715 cb6802_c24/f1p0/4072 isoform=cb6802_c24;full_length_coverage=1;non_full_length_coverage=0;isoform_length=4072;expected_accuracy=0.995	358	1682	64	360	2752	407 2	224	520
cb8564_c52869/f3p2/3055 isoform=cb8564_c52869;full_length_coverage=3;non_full_length_coverage=2;isoform_length=3055;expected_accuracy=1.000	gnl|BL_ORD_ID|11261 cb8564_c146388/f5p4/3129 isoform=cb8564_c146388;full_length_coverage=5;non_full_length_coverage=4;isoform_length=3129;expected_accuracy=0.999	130	3004	1	129	255	312 9	1	129
cb8564_c46939/f1p1/2926	gnl|BL_ORD_ID|55182 cb8564_c34825/f1p2/3200	11	2134	2130	2870	6	216 7	2272	301 3
cb8564_c41756/f1p0/2738 isoform=cb8564_c41756;full_length_coverage=1;non_full_length_coverage=0;isoform_length=2738;expected_	gnl|BL_ORD_ID|55280 cb8564_c37198/f1p5/2941	1	2173	2170	2738	1	217 9	2360	292 8

accuracy=0.996									
cb289_c5/f1p0/4379 isoform=cb289_c5;full_length_coverage=1;non_full_length_coverage=0;isoform_length=4379;expected_accuracy=0.997	gnl|BL_ORD_ID|38647 cb289_c27/f5p1/4551 isoform=cb289_c27;full_length_coverage=5;non_full_length_coverage=1;isoform_length=4551;expected_accuracy=0.999	1	4156	4156	4378	69	4226	4328	4551
cb7680_c12/f1p1/2361 isoform=cb7680_c12;full_length_coverage=1;non_full_length_coverage=1;isoform_length=2361;expected_accuracy=0.997	gnl|BL_ORD_ID|51460 cb7680_c7/f1p3/2568	1	1797	1794	2361	7	1805	1955	2523
cb3450_c6/f1p0/2269 isoform=cb3450_c6;full_length_coverage=1;non_full_length_coverage=0;isoform_length=2269;expected_accuracy=0.997	gnl|BL_ORD_ID|41480 cb3450_c4/f1p1/3102 isoform=cb3450_c4;full_length_coverage=1;non_full_length_coverage=1;isoform_length=3102;expected_accuracy=0.997	1	1625	1621	2269	30	1646	2409	3059
cb293_c2/f1p1/4354 isoform=cb293_c2;full_length_coverage=1;non_full_length_coverage=1;isoform_length=4354;expected_accuracy=0.994	gnl|BL_ORD_ID|69536 cb293_c7/f1p0/4528	23	2319	2318	4354	1	2302	2411	4432
cb8564_c154696/f1p1/2190 isoform=cb8564_c154696;full_length_coverage=1;non_full_length_coverage=1;isoform_length=2190;expected_accuracy=0.993	gnl|BL_ORD_ID|9841 cb8564_c115378/f1p0/2599 isoform=cb8564_c115378;full_length_coverage=1;non_full_length_coverage=0;isoform_length=2599;expected_accuracy=0.991	224	2190	48	225	604	2563	1	180
cb8564_c38570/f2p2/2760 isoform=cb8564_c38570;full_length_coverage=2;non_full_length_coverage=2;isoform_length=2760;expected_accuracy=0.996	gnl|BL_ORD_ID|56650 cb8564_c50769/f1p0/3074	198	2732	1	198	333	2855	22	219
cb4563_c37/f5p0/2841	gnl|BL_ORD_ID|42501 cb4563_c10/f1p1/4024	1	2337	2332	2748	1	233	3608	402

isoform=cb4563_c37;full_length_coverage=5;non_full_length_coverage=0;isoform_length=2841;expected_accuracy=0.997	isoform=cb4563_c10;full_length_coverage=1;non_full_length_coverage=1;isoform_length=4024;expected_accuracy=1.000						6		4
cb8564_c44698/f1p5/2843 isoform=cb8564_c44698;full_length_coverage=1;non_full_length_coverage=5;isoform_length=2843;expected_accuracy=0.997	gnl|BL_ORD_ID|15428 cb8564_c42387/f1p4/2973 isoform=cb8564_c42387;full_length_coverage=1;non_full_length_coverage=4;isoform_length=2973;expected_accuracy=0.993	1	2516	2511	2819	1	252 6	2648	297 3
cb4674_c22/f1p0/2988 isoform=cb4674_c22;full_length_coverage=1;non_full_length_coverage=0;isoform_length=2988;expected_accuracy=0.997	gnl|BL_ORD_ID|72844 cb4674_c33/f1p3/3281	1	2095	2096	2988	126	222 0	2324	321 5
cb8564_c40107/f1p0/4108	gnl|BL_ORD_ID|56019 cb8564_c52510/f6p2/4563	1603	3955	1	1599	1547	389 9	75	166 7
cb1616_c1/f4p1/3029 isoform=cb1616_c1;full_length_coverage=4;non_full_length_coverage=1;isoform_length=3029;expected_accuracy=1.000	gnl|BL_ORD_ID|63795 cb1616_c6/f1p1/3449	15	2097	2098	2994	55	214 0	2558	345 5
cb8564_c116345/f1p1/2198 isoform=cb8564_c116345;full_length_coverage=1;non_full_length_coverage=1;isoform_length=2198;expected_accuracy=0.991	gnl|BL_ORD_ID|52360 cb8564_c119657/f1p2/2788	369	2198	9	373	888	271 8	15	380
cb8564_c40598/f1p4/3119	gnl|BL_ORD_ID|55377 cb8564_c39822/f1p0/3306	1	2732	2729	3052	1	272 7	2848	316 9
cb8564_c17237/f1p1/2698 isoform=cb8564_c17237;full_length_coverage=1;non_full_length_coverage=1;isoform_length=2698;expected_	gnl|BL_ORD_ID|10873 cb8564_c11906/f1p0/3462 isoform=cb8564_c11906;full_length_coverage=1;non_full_length_coverage=0;isoform_length=3462;expected_ac	1	2449	2445	2610	73	251 8	3297	346 2

accuracy=0.999	curacy=0.998								
cb8564_c77080/f1p0/2613 isoform=cb8564_c77080;full_length_coverage=1;non_full_length_coverage=0;isoform_length=2613;expected_accuracy=0.995	gnl|BL_ORD_ID|57804 cb8564_c81209/f1p0/3275	1	2048	2043	2613	117	2159	2340	2911
cb946_c19/f1p0/3156 isoform=cb946_c19;full_length_coverage=1;non_full_length_coverage=0;isoform_length=3156;expected_accuracy=0.997	gnl|BL_ORD_ID|19592 cb946_c2/f3p0/3333 isoform=cb946_c2;full_length_coverage=3;non_full_length_coverage=0;isoform_length=3333;expected_accuracy=1.000	1096	3156	1	1096	1206	3267	3	1098
cb4286_c9/f1p1/2064 isoform=cb4286_c9;full_length_coverage=1;non_full_length_coverage=1;isoform_length=2064;expected_accuracy=0.993	gnl|BL_ORD_ID|42550 cb4286_c14/f1p0/2234 isoform=cb4286_c14;full_length_coverage=1;non_full_length_coverage=0;isoform_length=2234;expected_accuracy=0.996	1	1563	1562	2042	56	1612	1755	2234
cb18456_c3695/f1p0/1345	gnl|BL_ORD_ID|13743 cb8564_c21615/f1p7/4576 isoform=cb8564_c21615;full_length_coverage=1;non_full_length_coverage=7;isoform_length=4576;expected_accuracy=0.993	1	1051	1047	1293	235	1284	1459	1705
cb8564_c39756/f1p1/3004	gnl|BL_ORD_ID|16172 cb8564_c53024/f1p0/3159 isoform=cb8564_c53024;full_length_coverage=1;non_full_length_coverage=0;isoform_length=3159;expected_accuracy=0.996	1	1703	1700	2854	1	1699	2017	3157
cb233_c23/f1p0/2935	gnl|BL_ORD_ID|37995 cb233_c12/f1p0/3006 isoform=cb233_c12;full_length_coverage=1;non_full_length_coverage=0;isoform_length=3006;expected_accuracy=0.997	1	1912	1912	2911	2	1907	2008	3006
cb8564_c4543/f1p0/2457	gnl|BL_ORD_ID|14176 cb8564_c4538/f2p0/2766	1	2225	2223	2457	39	224	2436	266

isoform=cb8564_c4543;full_length_coverage=1;non_full_length_coverage=0;isoform_length=2457;expected_accuracy=0.995	isoform=cb8564_c4538;full_length_coverage=2;non_full_length_coverage=0;isoform_length=2766;expected_accuracy=0.994						3		4
cb8564_c118011/f1p0/1965	gnl|BL_ORD_ID|55292 cb8564_c37449/f1p0/2947	1	1321	1317	1964	482	1802	1981	2628
cb2981_c7/f1p2/3345 isoform=cb2981_c7;full_length_coverage=1;non_full_length_coverage=2;isoform_length=3345;expected_accuracy=0.992	gnl|BL_ORD_ID|38098 cb2981_c1/f1p0/3757 isoform=cb2981_c1;full_length_coverage=1;non_full_length_coverage=0;isoform_length=3757;expected_accuracy=0.997	1	2656	2653	3296	118	2765	3112	3757
cb388_c28/f1p3/3528 isoform=cb388_c28;full_length_coverage=1;non_full_length_coverage=3;isoform_length=3528;expected_accuracy=1.000	gnl|BL_ORD_ID|39449 cb388_c17/f1p0/3583 isoform=cb388_c17;full_length_coverage=1;non_full_length_coverage=0;isoform_length=3583;expected_accuracy=0.996	223	3466	21	222	339	3583	22	221
cb6947_c8/f2p0/1909	gnl|BL_ORD_ID|4835 cb6947_c6/f2p0/2118 isoform=cb6947_c6;full_length_coverage=2;non_full_length_coverage=0;isoform_length=2118;expected_accuracy=0.998	604	1898	9	604	807	2101	1	592
cb10480_c25/f1p0/2414	gnl|BL_ORD_ID|48572 cb10480_c27/f1p0/3699	1	2076	2074	2429	712	2789	3173	3529
cb8564_c178226/f1p0/2728 isoform=cb8564_c178226;full_length_coverage=1;non_full_length_coverage=0;isoform_length=2728;expected_accuracy=0.998	gnl|BL_ORD_ID|61972 cb12931_c30/f1p0/5903	1284	2728	1	1288	1420	2866	1	1316
cb8564_c111744/f1p1/2111 isoform=cb8564_c111744;full_length_coverage=1;non_full_length_coverage=1;isoform_length=2111;expected_	gnl|BL_ORD_ID|52178 cb8564_c113392/f1p0/2376	1	1639	1635	2111	22	1657	1830	2306

accuracy=0.998									
cb8564_c37390/f1p0/2683 isoform=cb8564_c37390;full_length_coverage=1;non_full_length_coverage=0;isoform_length=2683;expected_accuracy=1.000	gnl|BL_ORD_ID|57026 cb8564_c80880/f1p0/3252	265	2683	1	267	837	3256	122	388
cb7296_c1/f4p1/2068 isoform=cb7296_c1;full_length_coverage=4;non_full_length_coverage=1;isoform_length=2068;expected_accuracy=0.997	gnl|BL_ORD_ID|6916 cb7296_c3/f1p1/2653 isoform=cb7296_c3;full_length_coverage=1;non_full_length_coverage=1;isoform_length=2653;expected_accuracy=0.998	1	1704	1703	1978	39	1741	2347	2622
cb9143_c30/f2p0/2817 isoform=cb9143_c30;full_length_coverage=2;non_full_length_coverage=0;isoform_length=2817;expected_accuracy=0.996	gnl|BL_ORD_ID|20976 cb9143_c192/f178p0/3055 isoform=cb9143_c192;full_length_coverage=178;non_full_length_coverage=0;isoform_length=3055;expected_accuracy=1.000	199	2817	1	203	437	3055	101	303
cb2739_c8/f1p0/3454	gnl|BL_ORD_ID|69934 cb2739_c3/f1p0/3875	1247	3463	1	1252	1505	3715	157	1401
cb7984_c7/f1p0/1936	gnl|BL_ORD_ID|7814 cb7984_c6/f1p0/2519 isoform=cb7984_c6;full_length_coverage=1;non_full_length_coverage=0;isoform_length=2519;expected_accuracy=0.994	304	1937	35	306	754	2392	1	270
cb3334_c2/f1p0/2904	gnl|BL_ORD_ID|41020 cb3334_c1/f1p0/3655 isoform=cb3334_c1;full_length_coverage=1;non_full_length_coverage=0;isoform_length=3655;expected_accuracy=0.996	552	2916	71	555	1291	3655	1	482
cb8564_c116509/f1p0/3009	gnl|BL_ORD_ID|52900 cb8564_c111266/f1p0/3561	10	2016	2015	2985	37	2043	2267	3237
cb3290_c38/f1p0/2345	gnl|BL_ORD_ID|40879 cb3290_c194/f3p0/2878	1150	2345	1	1151	1574	276	267	141

isoform=cb3290_c38;full_length_coverage=1;non_full_length_coverage=0;isoform_length=2345;expected_accuracy=0.996	isoform=cb3290_c194;full_length_coverage=3;non_full_length_coverage=0;isoform_length=2878;expected_accuracy=0.997						9		7
cb8564_c24710/f1p0/2430 isoform=cb8564_c24710;full_length_coverage=1;non_full_length_coverage=0;isoform_length=2430;expected_accuracy=0.996	gnl|BL_ORD_ID|10372 cb8564_c10402/f1p1/2617 isoform=cb8564_c10402;full_length_coverage=1;non_full_length_coverage=1;isoform_length=2617;expected_accuracy=0.994	1	1499	1502	2430	2	1505	1605	2539
cb8564_c68610/f6p0/2150 isoform=cb8564_c68610;full_length_coverage=6;non_full_length_coverage=0;isoform_length=2150;expected_accuracy=1.000	gnl|BL_ORD_ID|58113 cb8564_c89263/f1p0/2300	212	2072	4	214	352	2213	4	214
cb8564_c45300/f1p4/3014 isoform=cb8564_c45300;full_length_coverage=1;non_full_length_coverage=4;isoform_length=3014;expected_accuracy=0.996	gnl|BL_ORD_ID|16622 cb8564_c50151/f1p0/3198 isoform=cb8564_c50151;full_length_coverage=1;non_full_length_coverage=0;isoform_length=3198;expected_accuracy=0.995	243	3014	1	246	404	3198	6	247
cb5943_c6/f1p0/1364	gnl|BL_ORD_ID|46197 cb5943_c4/f1p0/1485 isoform=cb5943_c4;full_length_coverage=1;non_full_length_coverage=0;isoform_length=1485;expected_accuracy=0.992	326	1329	5	327	482	1485	1	323
cb14220_c12/f1p0/6602	gnl|BL_ORD_ID|63134 cb14220_c9/f1p0/6925	2263	6512	1	2265	2708	6950	32	2293
cb8564_c182028/f1p0/2513 isoform=cb8564_c182028;full_length_coverage=1;non_full_length_coverage=0;isoform_length=2513;expected_accuracy=0.996	gnl|BL_ORD_ID|52675 cb8564_c10772/f1p3/2796	1	1971	1968	2513	32	2004	2148	2693
cb8790_c5/f1p0/1874	gnl|BL_ORD_ID|58367 cb8790_c3/f1p0/2841	1	1375	1375	1696	17	139	2511	283

							3		2
cb12714_c29/f1p2/1583	gnl|BL_ORD_ID|10640 cb8564_c100234/f2p5/2744 isoform=cb8564_c100234;full_length_coverage=2;non_full_length_coverage=5;isoform_length=2744;expected_accuracy=0.993	1	1296	1293	1597	6	1311	2378	2682
cb5440_c11/f2p0/1312 isoform=cb5440_c11;full_length_coverage=2;non_full_length_coverage=0;isoform_length=1312;expected_accuracy=0.995	gnl|BL_ORD_ID|73927 cb5440_c7/f1p1/3066	1	1019	1014	1312	61	1073	2668	2964
cb1240_c4/f1p1/4081 isoform=cb1240_c4;full_length_coverage=1;non_full_length_coverage=1;isoform_length=4081;expected_accuracy=0.995	gnl|BL_ORD_ID|61170 cb1240_c6/f1p0/4543	1	2657	2654	4071	84	2746	2878	4301
cb8564_c46212/f3p1/4373 isoform=cb8564_c46212;full_length_coverage=3;non_full_length_coverage=1;isoform_length=4373;expected_accuracy=0.999	gnl|BL_ORD_ID|14942 cb8564_c34188/f2p3/4546 isoform=cb8564_c34188;full_length_coverage=2;non_full_length_coverage=3;isoform_length=4546;expected_accuracy=1.000	184	4322	1	185	407	4546	5	181
cb8564_c42814/f1p0/2151 isoform=cb8564_c42814;full_length_coverage=1;non_full_length_coverage=0;isoform_length=2151;expected_accuracy=0.993	gnl|BL_ORD_ID|55359 cb8564_c39546/f1p2/3023	650	2151	7	652	1333	2837	1	645
cb2460_c3/f1p0/2273 isoform=cb2460_c3;full_length_coverage=1;non_full_length_coverage=0;isoform_length=2273;expected_accuracy=0.994	gnl|BL_ORD_ID|68527 cb2460_c4/f1p0/3681	294	2246	47	293	1737	3689	55	301
cb955_c17/f1p0/2026	gnl|BL_ORD_ID|59133 cb955_c46/f1p0/2377	229	2026	4	228	403	219	6	230

isoform=cb955_c17;full_length_coverage=1;non_full_length_coverage=0;isoform_length=2026;expected_accuracy=0.990							7		
cb8564_c1140/f1p3/1962 isoform=cb8564_c1140;full_length_coverage=1;non_full_length_coverage=3;isoform_length=1962;expected_accuracy=0.997	gnl|BL_ORD_ID|12027 cb8564_c146113/f15p5/2092 isoform=cb8564_c146113;full_length_coverage=15;non_full_length_coverage=5;isoform_length=2092;expected_accuracy=1.000	112	1962	1	112	235	2084	14	123
cb8564_c45300/f1p4/3014 isoform=cb8564_c45300;full_length_coverage=1;non_full_length_coverage=4;isoform_length=3014;expected_accuracy=0.996	gnl|BL_ORD_ID|12440 cb8564_c130435/f1p3/3226 isoform=cb8564_c130435;full_length_coverage=1;non_full_length_coverage=3;isoform_length=3226;expected_accuracy=0.994	243	3014	1	246	417	3209	2	260
cb3493_c11/f1p0/3332	gnl|BL_ORD_ID|40022 cb3493_c4/f1p0/3469 isoform=cb3493_c4;full_length_coverage=1;non_full_length_coverage=0;isoform_length=3469;expected_accuracy=1.000	1	2347	2342	3014	22	2368	2477	3148
cb8564_c76007/f1p0/2966	gnl|BL_ORD_ID|55920 cb8564_c48949/f1p0/4357	13	2291	2292	2946	5	2293	2847	3505
cb8564_c126419/f1p0/1910 isoform=cb8564_c126419;full_length_coverage=1;non_full_length_coverage=0;isoform_length=1910;expected_accuracy=0.995	gnl|BL_ORD_ID|12781 cb8564_c159074/f1p3/2275 isoform=cb8564_c159074;full_length_coverage=1;non_full_length_coverage=3;isoform_length=2275;expected_accuracy=0.997	11	1731	1728	1899	1	1718	2102	2275
cb8564_c36689/f1p1/2368 isoform=cb8564_c36689;full_length_coverage=1;non_full_length_coverage=1;isoform_length=2368;expected_accuracy=0.991	gnl|BL_ORD_ID|17161 cb8564_c87466/f1p1/3006 isoform=cb8564_c87466;full_length_coverage=1;non_full_length_coverage=1;isoform_length=3006;expected_accuracy=0.994	1	1709	1706	2368	1	1718	2219	2896
cb18009_c1/f1p0/1857	gnl|BL_ORD_ID|42205 cb4848_c5/f1p1/3168	1	1504	1502	1799	1028	253	2873	316

	isoform=cb4848_c5;full_length_coverage=1;non_full_length_coverage=1;isoform_length=3168;expected_accuracy=0.994						0		8
cb8564_c9734/f3p3/2491 isoform=cb8564_c9734;full_length_coverage=3;non_full_length_coverage=3;isoform_length=2491;expected_accuracy=1.000	gnl|BL_ORD_ID|13265 cb8564_c21898/f1p1/2652 isoform=cb8564_c21898;full_length_coverage=1;non_full_length_coverage=1;isoform_length=2652;expected_accuracy=0.999	1214	2440	1	1219	1425	2652	1	1222
cb10352_c15/f1p1/1494 isoform=cb10352_c15;full_length_coverage=1;non_full_length_coverage=1;isoform_length=1494;expected_accuracy=0.991	gnl|BL_ORD_ID|2478 cb10352_c4/f1p0/2586 isoform=cb10352_c4;full_length_coverage=1;non_full_length_coverage=0;isoform_length=2586;expected_accuracy=0.996	1	1159	1159	1494	79	1213	2244	2579
cb8564_c43779/f1p0/3021 isoform=cb8564_c43779;full_length_coverage=1;non_full_length_coverage=0;isoform_length=3021;expected_accuracy=0.993	gnl|BL_ORD_ID|55377 cb8564_c39822/f1p0/3306	1	2344	2345	3020	1	2341	2497	3169
cb5001_c11/f1p0/2067	gnl|BL_ORD_ID|73185 cb5001_c5/f1p0/3084	1	1585	1580	2073	7	1591	2367	2860
cb8564_c188193/f1p0/2849 isoform=cb8564_c188193;full_length_coverage=1;non_full_length_coverage=0;isoform_length=2849;expected_accuracy=0.997	gnl|BL_ORD_ID|14531 cb8564_c41778/f1p9/3482 isoform=cb8564_c41778;full_length_coverage=1;non_full_length_coverage=9;isoform_length=3482;expected_accuracy=0.992	243	2849	1	247	812	3439	1	247
cb8564_c122963/f1p0/3278	gnl|BL_ORD_ID|53405 cb8564_c124255/f1p0/3874	1	2240	2237	3259	3	2221	2351	3379
cb7429_c17/f6p1/1677 isoform=cb7429_c17;full_length_coverage=6;non_full_length_coverage=1;isoform_length=1677;expected_accu	gnl|BL_ORD_ID|8417 cb7429_c2/f1p0/2599 isoform=cb7429_c2;full_length_coverage=1;non_full_length_coverage=0;isoform_length=2599;expected_accura	251	1677	48	252	1156	2581	43	248

racy=1.000	cy=0.997								
cb6028_c3/f1p0/2608 isoform=cb6028_c3;full_length_coverage=1;non_full_length_coverage=0;isoform_length=2608;expected_accuracy=0.994	gnl|BL_ORD_ID|45690 cb6028_c4/f1p0/3558 isoform=cb6028_c4;full_length_coverage=1;non_full_length_coverage=0;isoform_length=3558;expected_accuracy=0.998	1142	2583	1	1144	2114	3558	1	1144
cb8564_c35356/f1p0/2738 isoform=cb8564_c35356;full_length_coverage=1;non_full_length_coverage=0;isoform_length=2738;expected_accuracy=0.994	gnl|BL_ORD_ID|14539 cb8564_c46562/f2p3/3196 isoform=cb8564_c46562;full_length_coverage=2;non_full_length_coverage=3;isoform_length=3196;expected_accuracy=0.995	10	2072	2068	2737	16	2101	2525	3195
cb3699_c5/f1p0/2467 isoform=cb3699_c5;full_length_coverage=1;non_full_length_coverage=0;isoform_length=2467;expected_accuracy=1.000	gnl|BL_ORD_ID|40092 cb3699_c9/f1p0/2741 isoform=cb3699_c9;full_length_coverage=1;non_full_length_coverage=0;isoform_length=2741;expected_accuracy=0.996	1161	2445	1	1162	1458	2741	1	1126
cb7639_c0/f3p0/2226 isoform=cb7639_c0;full_length_coverage=3;non_full_length_coverage=0;isoform_length=2226;expected_accuracy=0.997	gnl|BL_ORD_ID|51425 cb7639_c7/f1p0/2500	1	1800	1799	2226	1	1800	2079	2530
cb1279_c9/f3p1/2959 isoform=cb1279_c9;full_length_coverage=3;non_full_length_coverage=1;isoform_length=2959;expected_accuracy=1.000	gnl|BL_ORD_ID|27065 cb1279_c46/f1p3/3121 isoform=cb1279_c46;full_length_coverage=1;non_full_length_coverage=3;isoform_length=3121;expected_accuracy=1.000	248	2959	1	249	420	3106	1	245
cb8564_c89263/f1p0/2300	gnl|BL_ORD_ID|12420 cb8564_c125410/f1p0/2472 isoform=cb8564_c125410;full_length_coverage=1;non_full_length_coverage=0;isoform_length=2472;expected_accuracy=0.997	304	2213	4	300	552	2461	1	297
cb8564_c146112/f14p3/2729	gnl|BL_ORD_ID|11493 cb8564_c1323/f1p1/2810	1	1508	1508	2699	5	151	1618	280

isoform=cb8564_c146112;full_length_coverage=14;non_full_length_coverage=3;isoform_length=2729;expected_accuracy=1.000	isoform=cb8564_c1323;full_length_coverage=1;non_full_length_coverage=1;isoform_length=2810;expected_accuracy=1.000						2		9
cb2710_c2/f1p0/2584 isoform=cb2710_c2;full_length_coverage=1;non_full_length_coverage=0;isoform_length=2584;expected_accuracy=0.995	gnl|BL_ORD_ID|37908 cb2710_c1/f1p0/3833 isoform=cb2710_c1;full_length_coverage=1;non_full_length_coverage=0;isoform_length=3833;expected_accuracy=0.997	363	2584	77	362	402	2629	1	289
cb317_c13/f1p0/4439	gnl|BL_ORD_ID|68195 cb317_c8/f1p1/4711	2	3147	3142	4217	8	3159	3385	4460
cb9533_c10/f1p0/1798 isoform=cb9533_c10;full_length_coverage=1;non_full_length_coverage=0;isoform_length=1798;expected_accuracy=0.994	gnl|BL_ORD_ID|21714 cb9533_c2/f1p0/1999 isoform=cb9533_c2;full_length_coverage=1;non_full_length_coverage=0;isoform_length=1999;expected_accuracy=0.996	313	1798	74	313	419	1905	2	241
cb8564_c68409/f2p0/2141 isoform=cb8564_c68409;full_length_coverage=2;non_full_length_coverage=0;isoform_length=2141;expected_accuracy=0.995	gnl|BL_ORD_ID|57089 cb8564_c82833/f1p0/2593	265	2110	53	265	590	2435	1	212
cb1494_c16/f1p0/2427	gnl|BL_ORD_ID|29800 cb1494_c10/f1p0/3165 isoform=cb1494_c10;full_length_coverage=1;non_full_length_coverage=0;isoform_length=3165;expected_accuracy=0.994	1199	2433	1	1199	1864	3100	3	1197
cb4981_c3/f1p0/3014 isoform=cb4981_c3;full_length_coverage=1;non_full_length_coverage=0;isoform_length=3014;expected_accuracy=0.994	gnl|BL_ORD_ID|42532 cb4981_c2/f1p0/3208 isoform=cb4981_c2;full_length_coverage=1;non_full_length_coverage=0;isoform_length=3208;expected_accuracy=0.994	1	2434	2432	3013	19	2454	2626	3208
cb16404_c11/f1p0/1451	gnl|BL_ORD_ID|13367 cb8564_c18807/f5p0/1966	160	1451	32	164	660	195	1	133

isoform=cb16404_c11;full_length_coverage=1;non_full_length_coverage=0;isoform_length=1451;expected_accuracy=1.000	isoform=cb8564_c18807;full_length_coverage=5;non_full_length_coverage=0;isoform_length=1966;expected_accuracy=0.997						0		
cb8564_c91716/f1p2/1902	gnl|BL_ORD_ID|18575 cb8564_c90782/f1p0/2732 isoform=cb8564_c90782;full_length_coverage=1;non_full_length_coverage=0;isoform_length=2732;expected_accuracy=0.997	1	1445	1442	1817	48	1489	2357	2732
cb8564_c2326/f1p0/2006 isoform=cb8564_c2326;full_length_coverage=1;non_full_length_coverage=0;isoform_length=2006;expected_accuracy=0.990	gnl|BL_ORD_ID|14440 cb8564_c2355/f1p4/2493 isoform=cb8564_c2355;full_length_coverage=1;non_full_length_coverage=4;isoform_length=2493;expected_accuracy=0.995	230	2001	4	234	720	2488	4	240
cb8564_c45884/f1p0/3837	gnl|BL_ORD_ID|55587 cb8564_c46008/f1p0/4134	1523	3846	1	1525	1752	4100	120	1644
cb2415_c18/f1p5/5570 isoform=cb2415_c18;full_length_coverage=1;non_full_length_coverage=5;isoform_length=5570;expected_accuracy=0.998	gnl|BL_ORD_ID|37646 cb2415_c15/f1p3/5862 isoform=cb2415_c15;full_length_coverage=1;non_full_length_coverage=3;isoform_length=5862;expected_accuracy=0.993	1	4609	4609	5513	5	4597	4960	5862
cb1142_c17/f1p1/2208	gnl|BL_ORD_ID|59186 cb1142_c19/f1p0/2913	1123	2211	1	1127	1597	2685	22	1175
cb4932_c18/f1p0/1876	gnl|BL_ORD_ID|73087 cb4932_c9/f1p0/2989	173	1852	14	173	1225	2904	7	165
cb1545_c34/f1p0/3898	gnl|BL_ORD_ID|63730 cb1545_c15/f1p0/3646	1	1854	1854	3462	10	1862	2020	3627
cb18456_c7053/f1p3/1132 isoform=cb18456_c7053;full_length_coverage=1;non_full_length_coverage=3;isoform_length=1132;expected_	gnl|BL_ORD_ID|20952 cb9521_c5/f1p0/2804 isoform=cb9521_c5;full_length_coverage=1;non_full_length_coverage=0;isoform_length=2804;expected_accura	395	1132	5	399	1999	2735	3	393

accuracy=0.997	cy=0.991								
cb8564_c81775/f1p0/2506 isoform=cb8564_c81775;full_length_coverage=1;non_full_length_coverage=0;isoform_length=2506;expected_accuracy=0.990	gnl|BL_ORD_ID|58115 cb8564_c89322/f1p0/2588	1	1376	1371	2457	1	1379	1498	2588
cb8564_c44063/f1p0/2503 isoform=cb8564_c44063;full_length_coverage=1;non_full_length_coverage=0;isoform_length=2503;expected_accuracy=0.999	gnl|BL_ORD_ID|55407 cb8564_c40851/f1p0/2747	397	2421	4	402	553	2572	9	408
cb8564_c112962/f1p0/2169 isoform=cb8564_c112962;full_length_coverage=1;non_full_length_coverage=0;isoform_length=2169;expected_accuracy=0.990	gnl|BL_ORD_ID|15991 cb8564_c69437/f1p0/2197 isoform=cb8564_c69437;full_length_coverage=1;non_full_length_coverage=0;isoform_length=2197;expected_accuracy=0.996	1	1194	1194	2108	1	1185	1294	2197
cb8564_c126213/f1p0/1904	gnl|BL_ORD_ID|52157 cb8564_c112829/f1p1/2052	1	1585	1586	1773	27	1611	1802	1989
cb1540_c17/f1p0/3387 isoform=cb1540_c17;full_length_coverage=1;non_full_length_coverage=0;isoform_length=3387;expected_accuracy=0.996	gnl|BL_ORD_ID|28145 cb1540_c7/f1p0/4274 isoform=cb1540_c7;full_length_coverage=1;non_full_length_coverage=0;isoform_length=4274;expected_accuracy=0.996	136	3387	1	137	991	4265	315	462
cb953_c5/f1p0/2793	gnl|BL_ORD_ID|20813 cb953_c2/f2p1/3253 isoform=cb953_c2;full_length_coverage=2;non_full_length_coverage=1;isoform_length=3253;expected_accuracy=1.000	1098	2641	1	1103	1711	3253	18	1123
cb2737_c26/f1p0/2992 isoform=cb2737_c26;full_length_coverage=1;non_full_length_coverage=0;isoform_length=2992;expected_accu	gnl|BL_ORD_ID|38133 cb2737_c21/f1p2/3479 isoform=cb2737_c21;full_length_coverage=1;non_full_length_coverage=2;isoform_length=3479;expected_accur	1	1804	1802	2992	196	2003	2135	3348

racy=0.996	acy=1.000								
cb8564_c51533/f1p0/2888 isoform=cb8564_c51533;full_length_coverage=1;non_full_length_coverage=0;isoform_length=2888;expected_accuracy=0.998	gnl|BL_ORD_ID|17053 cb8564_c74966/f1p0/3265 isoform=cb8564_c74966;full_length_coverage=1;non_full_length_coverage=0;isoform_length=3265;expected_accuracy=0.992	1	2144	2145	2881	15	2129	2529	3265
cb3694_c3/f1p0/2055 isoform=cb3694_c3;full_length_coverage=1;non_full_length_coverage=0;isoform_length=2055;expected_accuracy=0.997	gnl|BL_ORD_ID|41488 cb3694_c1/f1p0/2763 isoform=cb3694_c1;full_length_coverage=1;non_full_length_coverage=0;isoform_length=2763;expected_accuracy=0.996	1	1427	1426	2028	621	2057	2158	2763
cb16334_c1/f1p0/1310	gnl|BL_ORD_ID|12345 cb8564_c126557/f1p0/3867 isoform=cb8564_c126557;full_length_coverage=1;non_full_length_coverage=0;isoform_length=3867;expected_accuracy=1.000	9	774	773	1317	19	795	3323	3867
cb8564_c2073/f1p0/2565 isoform=cb8564_c2073;full_length_coverage=1;non_full_length_coverage=0;isoform_length=2565;expected_accuracy=0.994	gnl|BL_ORD_ID|53822 cb8564_c2075/f2p0/3362	1003	2565	1	1003	1796	3358	149	1152
cb8564_c71904/f1p1/2820 isoform=cb8564_c71904;full_length_coverage=1;non_full_length_coverage=1;isoform_length=2820;expected_accuracy=0.996	gnl|BL_ORD_ID|9611 cb8564_c107820/f9p3/3289 isoform=cb8564_c107820;full_length_coverage=9;non_full_length_coverage=3;isoform_length=3289;expected_accuracy=1.000	1	1983	1978	2768	324	2309	2498	3289
cb5741_c21/f7p1/1853 isoform=cb5741_c21;full_length_coverage=7;non_full_length_coverage=1;isoform_length=1853;expected_accuracy=1.000	gnl|BL_ORD_ID|44912 cb5741_c6/f2p0/2553 isoform=cb5741_c6;full_length_coverage=2;non_full_length_coverage=0;isoform_length=2553;expected_accuracy=0.996	12	1607	1605	1795	2	1584	2365	2553
cb7442_c43/f1p0/1720	gnl|BL_ORD_ID|8272 cb7442_c31/f1p0/1802	1	1391	1389	1659	28	142	1532	180

	isoform=cb7442_c31;full_length_coverage=1;non_full_length_coverage=0;isoform_length=1802;expected_accuracy=0.993						0		2
cb6646_c26/f1p0/2555	gnl|BL_ORD_ID|3729 cb6646_c22/f1p1/2606 isoform=cb6646_c22;full_length_coverage=1;non_full_length_coverage=1;isoform_length=2606;expected_accuracy=0.996	277	2504	93	278	380	2606	78	263
cb395_c4/f1p0/3249	gnl|BL_ORD_ID|40012 cb395_c1/f1p0/4857 isoform=cb395_c1;full_length_coverage=1;non_full_length_coverage=0;isoform_length=4857;expected_accuracy=0.999	13	2127	2123	3014	1	2109	3967	4857
cb8564_c40598/f1p4/3119	gnl|BL_ORD_ID|55522 cb8564_c44123/f1p1/3230	16	2732	2729	3053	15	2726	2847	3171
cb4746_c2/f2p1/2607 isoform=cb4746_c2;full_length_coverage=2;non_full_length_coverage=1;isoform_length=2607;expected_accuracy=1.000	gnl|BL_ORD_ID|72931 cb4746_c3/f1p0/3262	113	1543	1542	2607	25	1454	2180	3245
cb293_c6/f1p0/3758 isoform=cb293_c6;full_length_coverage=1;non_full_length_coverage=0;isoform_length=3758;expected_accuracy=0.997	gnl|BL_ORD_ID|68176 cb293_c8/f1p0/4316	1	1906	1903	3758	1	1910	2456	4311
cb500_c47/f1p0/3506	gnl|BL_ORD_ID|72251 cb500_c68/f1p0/4152	302	3375	23	302	962	4035	133	424
cb5013_c16/f1p0/2722	gnl|BL_ORD_ID|44297 cb5013_c1/f4p0/2651 isoform=cb5013_c1;full_length_coverage=4;non_full_length_coverage=0;isoform_length=2651;expected_accura	231	2499	12	231	383	2651	7	224

	cy=1.000								
cb13433_c61/f1p1/3251 isoform=cb13433_c61;full_length_coverage=1;non_full_length_coverage=1;isoform_length=3251;expected_accuracy=0.995	gnl|BL_ORD_ID|25932 cb13433_c33/f2p1/4835 isoform=cb13433_c33;full_length_coverage=2;non_full_length_coverage=1;isoform_length=4835;expected_accuracy=1.000	105	2302	2301	3221	1	2196	3915	4835
cb4016_c34/f1p0/2560 isoform=cb4016_c34;full_length_coverage=1;non_full_length_coverage=0;isoform_length=2560;expected_accuracy=0.998	gnl|BL_ORD_ID|39429 cb4016_c43/f1p0/2750 isoform=cb4016_c43;full_length_coverage=1;non_full_length_coverage=0;isoform_length=2750;expected_accuracy=0.994	194	2560	12	195	360	2721	2	186
cb13565_c12/f1p6/1695 isoform=cb13565_c12;full_length_coverage=1;non_full_length_coverage=6;isoform_length=1695;expected_accuracy=0.993	gnl|BL_ORD_ID|4515 cb6452_c4/f1p3/2166 isoform=cb6452_c4;full_length_coverage=1;non_full_length_coverage=3;isoform_length=2166;expected_accuracy=1.000	428	1641	5	427	948	2163	2	414
cb3580_c45/f1p0/2434 isoform=cb3580_c45;full_length_coverage=1;non_full_length_coverage=0;isoform_length=2434;expected_accuracy=0.994	gnl|BL_ORD_ID|70487 cb3580_c58/f1p0/3103	220	2434	1	219	795	2988	91	309
cb8564_c121763/f1p2/2624 isoform=cb8564_c121763;full_length_coverage=1;non_full_length_coverage=2;isoform_length=2624;expected_accuracy=0.997	gnl|BL_ORD_ID|53109 cb8564_c116513/f1p1/2958	1	2416	2417	2624	65	2484	2656	2863
cb6065_c10/f1p0/2720 isoform=cb6065_c10;full_length_coverage=1;non_full_length_coverage=0;isoform_length=2720;expected_accuracy=0.998	gnl|BL_ORD_ID|46672 cb6065_c8/f1p0/3176 isoform=cb6065_c8;full_length_coverage=1;non_full_length_coverage=0;isoform_length=3176;expected_accuracy=0.995	1	2044	2042	2705	5	2050	2525	3176
cb8564_c179140/f1p0/2477	gnl|BL_ORD_ID|17272 cb8564_c91851/f1p1/2644	14	2041	2037	2471	26	205	2210	264

<table>
<tr><th></th><th></th><th></th><th></th><th></th><th></th><th></th><th></th><th></th><th></th></tr>
<tr><td>isoform=cb8564_c179140;full_length_coverage=1;non_full_length_coverage=0;isoform_length=2477;expected_accuracy=0.998</td><td>isoform=cb8564_c91851;full_length_coverage=1;non_full_length_coverage=1;isoform_length=2644;expected_accuracy=0.993</td><td></td><td></td><td></td><td></td><td></td><td>0</td><td></td><td>4</td></tr>
<tr><td>cb9571_c24/f1p2/1208 isoform=cb9571_c24;full_length_coverage=1;non_full_length_coverage=2;isoform_length=1208;expected_accuracy=1.000</td><td>gnl|BL_ORD_ID|20599 cb9571_c21/f1p2/1540 isoform=cb9571_c21;full_length_coverage=1;non_full_length_coverage=2;isoform_length=1540;expected_accuracy=0.999</td><td>293</td><td>1208</td><td>35</td><td>298</td><td>561</td><td>1477</td><td>20</td><td>283</td></tr>
<tr><td>cb8564_c4645/f1p1/2620 isoform=cb8564_c4645;full_length_coverage=1;non_full_length_coverage=1;isoform_length=2620;expected_accuracy=0.994</td><td>gnl|BL_ORD_ID|16733 cb8564_c583/f12p2/2795 isoform=cb8564_c583;full_length_coverage=12;non_full_length_coverage=2;isoform_length=2795;expected_accuracy=1.000</td><td>1</td><td>1828</td><td>1829</td><td>2620</td><td>28</td><td>1866</td><td>1990</td><td>2780</td></tr>
<tr><td>cb8564_c42091/f1p0/3606 isoform=cb8564_c42091;full_length_coverage=1;non_full_length_coverage=0;isoform_length=3606;expected_accuracy=0.998</td><td>gnl|BL_ORD_ID|15834 cb8564_c66814/f4p0/3927 isoform=cb8564_c66814;full_length_coverage=4;non_full_length_coverage=0;isoform_length=3927;expected_accuracy=1.000</td><td>1</td><td>2325</td><td>2324</td><td>3606</td><td>4</td><td>2330</td><td>2640</td><td>3926</td></tr>
<tr><td>cb6730_c26/f1p1/2468 isoform=cb6730_c26;full_length_coverage=1;non_full_length_coverage=1;isoform_length=2468;expected_accuracy=0.998</td><td>gnl|BL_ORD_ID|5875 cb6730_c2/f5p0/2632 isoform=cb6730_c2;full_length_coverage=5;non_full_length_coverage=0;isoform_length=2632;expected_accuracy=1.000</td><td>1</td><td>1841</td><td>1842</td><td>2431</td><td>46</td><td>1881</td><td>2062</td><td>2632</td></tr>
<tr><td>cb396_c48/f1p0/2172</td><td>gnl|BL_ORD_ID|39504 cb396_c16/f1p0/2356 isoform=cb396_c16;full_length_coverage=1;non_full_length_coverage=0;isoform_length=2356;expected_accuracy=1.000</td><td>238</td><td>2063</td><td>3</td><td>238</td><td>531</td><td>2356</td><td>70</td><td>282</td></tr>
<tr><td>cb322_c54/f1p0/3800 isoform=cb322_c54;full_length_coverage=1;non_full_length_coverage=0;isoform_length=3800;expected_accur</td><td>gnl|BL_ORD_ID|70802 cb322_c34/f1p0/3998</td><td>25</td><td>2736</td><td>2734</td><td>3691</td><td>1</td><td>2718</td><td>3056</td><td>4012</td></tr>
</table>

<table>
<tr><td>acy=0.996</td><td></td><td></td><td></td><td></td><td></td><td></td><td></td><td></td><td></td></tr>
<tr><td>cb2723_c2/f5p0/2798 isoform=cb2723_c2;full_length_coverage=5;non_full_length_coverage=0;isoform_length=2798;expected_accuracy=0.999</td><td>gnl|BL_ORD_ID|36753 cb2723_c4/f2p0/4552 isoform=cb2723_c4;full_length_coverage=2;non_full_length_coverage=0;isoform_length=4552;expected_accuracy=1.000</td><td>22</td><td>2410</td><td>2410</td><td>2798</td><td>1</td><td>2388</td><td>3276</td><td>3664</td></tr>
<tr><td>cb4226_c3/f1p0/2436 isoform=cb4226_c3;full_length_coverage=1;non_full_length_coverage=0;isoform_length=2436;expected_accuracy=1.000</td><td>gnl|BL_ORD_ID|42622 cb4226_c4/f1p0/2756 isoform=cb4226_c4;full_length_coverage=1;non_full_length_coverage=0;isoform_length=2756;expected_accuracy=0.995</td><td>1</td><td>1768</td><td>1766</td><td>2334</td><td>23</td><td>1788</td><td>1969</td><td>2535</td></tr>
<tr><td>cb12972_c26/f1p0/1766</td><td>gnl|BL_ORD_ID|53852 cb8564_c2734/f1p0/2131</td><td>203</td><td>1675</td><td>35</td><td>206</td><td>289</td><td>1760</td><td>1</td><td>172</td></tr>
<tr><td>cb5017_c3/f1p0/2006 isoform=cb5017_c3;full_length_coverage=1;non_full_length_coverage=0;isoform_length=2006;expected_accuracy=0.997</td><td>gnl|BL_ORD_ID|42855 cb5017_c2/f1p0/2301 isoform=cb5017_c2;full_length_coverage=1;non_full_length_coverage=0;isoform_length=2301;expected_accuracy=1.000</td><td>1</td><td>1365</td><td>1363</td><td>2006</td><td>91</td><td>1455</td><td>1658</td><td>2301</td></tr>
<tr><td>cb16315_c3/f1p0/1559 isoform=cb16315_c3;full_length_coverage=1;non_full_length_coverage=0;isoform_length=1559;expected_accuracy=0.992</td><td>gnl|BL_ORD_ID|72532 cb4391_c5/f1p0/3162</td><td>184</td><td>1559</td><td>25</td><td>187</td><td>827</td><td>2193</td><td>1</td><td>161</td></tr>
<tr><td>cb8564_c48153/f1p0/3083</td><td>gnl|BL_ORD_ID|14410 cb8564_c44256/f1p1/3267 isoform=cb8564_c44256;full_length_coverage=1;non_full_length_coverage=1;isoform_length=3267;expected_accuracy=0.995</td><td>13</td><td>2162</td><td>2158</td><td>2875</td><td>1</td><td>2119</td><td>2553</td><td>3267</td></tr>
<tr><td>cb8564_c139315/f1p2/3949 isoform=cb8564_c139315;full_length_coverage=1;non_full_length_coverage=2;isoform_length=3949;expected</td><td>gnl|BL_ORD_ID|13983 cb8564_c40988/f2p0/4134 isoform=cb8564_c40988;full_length_coverage=2;non_full_length_coverage=0;isoform_length=4134;expected_ac</td><td>213</td><td>3825</td><td>1</td><td>214</td><td>477</td><td>4095</td><td>2</td><td>216</td></tr>
</table>

_accuracy=0.999	curacy=0.999								
cb8564_c87455/f1p0/2321	gnl|BL_ORD_ID|58095 cb8564_c88673/f1p0/3095	1	1494	1492	2200	25	1518	2317	3025
cb6491_c8/f1p1/3298 isoform=cb6491_c8;full_length_coverage=1;non_full_length_coverage=1;isoform_length=3298;expected_accuracy=0.991	gnl|BL_ORD_ID|3950 cb6491_c3/f1p0/3487 isoform=cb6491_c3;full_length_coverage=1;non_full_length_coverage=0;isoform_length=3487;expected_accuracy=0.996	1	2917	2915	3181	135	3056	3224	3487
cb8564_c36056/f1p0/2652 isoform=cb8564_c36056;full_length_coverage=1;non_full_length_coverage=0;isoform_length=2652;expected_accuracy=0.999	gnl|BL_ORD_ID|55481 cb8564_c42759/f1p1/2974	248	2652	1	251	491	2892	96	347
cb8564_c87900/f1p0/2261 isoform=cb8564_c87900;full_length_coverage=1;non_full_length_coverage=0;isoform_length=2261;expected_accuracy=0.995	gnl|BL_ORD_ID|57363 cb8564_c90914/f1p0/2521	322	2261	47	327	424	2384	1	283
cb8564_c71659/f1p0/1905	gnl|BL_ORD_ID|15614 cb8564_c4930/f4p1/3125 isoform=cb8564_c4930;full_length_coverage=4;non_full_length_coverage=1;isoform_length=3125;expected_accuracy=0.999	749	1692	9	748	2020	2963	1	740
cb5125_c36/f1p0/1789 isoform=cb5125_c36;full_length_coverage=1;non_full_length_coverage=0;isoform_length=1789;expected_accuracy=0.998	gnl|BL_ORD_ID|72116 cb5125_c21/f1p3/2083	1	1163	1158	1782	41	1206	1412	2037
cb2084_c13/f1p0/1992 isoform=cb2084_c13;full_length_coverage=1;non_full_length_coverage=0;isoform_length=1992;expected_accu	gnl|BL_ORD_ID|34453 cb2084_c3/f2p0/2121 isoform=cb2084_c3;full_length_coverage=2;non_full_length_coverage=0;isoform_length=2121;expected_accura	1	1519	1516	1992	1	1519	1619	2094

racy=0.995	cy=0.997								
cb1508_c6/f1p1/1904	gnl|BL_ORD_ID|30078 cb1508_c2/f2p0/1941 isoform=cb1508_c2;full_length_coverage=2;non_full_length_coverage=0;isoform_length=1941;expected_accuracy=1.000	372	1847	65	373	464	1941	1	309
cb12931_c15/f1p0/1493 isoform=cb12931_c15;full_length_coverage=1;non_full_length_coverage=0;isoform_length=1493;expected_accuracy=1.000	gnl|BL_ORD_ID|16879 cb8564_c48606/f1p1/3308 isoform=cb8564_c48606;full_length_coverage=1;non_full_length_coverage=1;isoform_length=3308;expected_accuracy=0.998	1	1106	1104	1492	1	1101	2920	3308
cb2097_c120/f1p0/1809 isoform=cb2097_c120;full_length_coverage=1;non_full_length_coverage=0;isoform_length=1809;expected_accuracy=0.994	gnl|BL_ORD_ID|66707 cb2097_c91/f1p0/2631	1	1422	1420	1753	442	1867	1977	2310
cb8564_c45300/f1p4/3014 isoform=cb8564_c45300;full_length_coverage=1;non_full_length_coverage=4;isoform_length=3014;expected_accuracy=0.996	gnl|BL_ORD_ID|15169 cb8564_c40218/f1p0/3146 isoform=cb8564_c40218;full_length_coverage=1;non_full_length_coverage=0;isoform_length=3146;expected_accuracy=0.993	243	3012	1	246	399	3146	4	243
cb1648_c40/f1p0/1455 isoform=cb1648_c40;full_length_coverage=1;non_full_length_coverage=0;isoform_length=1455;expected_accuracy=0.992	gnl|BL_ORD_ID|32504 cb1648_c14/f1p0/3818 isoform=cb1648_c14;full_length_coverage=1;non_full_length_coverage=0;isoform_length=3818;expected_accuracy=0.995	579	1455	6	583	2897	3775	1	585
cb8564_c23528/f1p0/4715	gnl|BL_ORD_ID|13659 cb8564_c20252/f1p0/4765 isoform=cb8564_c20252;full_length_coverage=1;non_full_length_coverage=0;isoform_length=4765;expected_accuracy=0.997	138	3161	3161	4485	1	3024	3408	4730
cb8564_c88543/f1p0/3439	gnl|BL_ORD_ID|11721 cb8564_c145906/f6p0/3616	235	3439	2	240	403	3611	40	279

isoform=cb8564_c88543;full_length_coverage=1;non_full_length_coverage=0;isoform_length=3439;expected_accuracy=0.996	isoform=cb8564_c145906;full_length_coverage=6;non_full_length_coverage=0;isoform_length=3616;expected_accuracy=1.000								
cb3088_c2/f1p0/3439 isoform=cb3088_c2;full_length_coverage=1;non_full_length_coverage=0;isoform_length=3439;expected_accuracy=0.994	gnl|BL_ORD_ID|37821 cb3088_c1/f1p0/3724 isoform=cb3088_c1;full_length_coverage=1;non_full_length_coverage=0;isoform_length=3724;expected_accuracy=0.998	17	3121	3121	3439	1	3103	3392	3709
cb581_c7/f1p0/2369 isoform=cb581_c7;full_length_coverage=1;non_full_length_coverage=0;isoform_length=2369;expected_accuracy=0.990	gnl|BL_ORD_ID|45253 cb581_c2/f2p1/2578 isoform=cb581_c2;full_length_coverage=2;non_full_length_coverage=1;isoform_length=2578;expected_accuracy=0.998	1	1631	1627	2369	106	1735	1840	2578
cb4768_c85/f1p0/1983 isoform=cb4768_c85;full_length_coverage=1;non_full_length_coverage=0;isoform_length=1983;expected_accuracy=1.000	gnl|BL_ORD_ID|43790 cb4768_c6/f5p3/2655 isoform=cb4768_c6;full_length_coverage=5;non_full_length_coverage=3;isoform_length=2655;expected_accuracy=1.000	103	1983	1	106	773	2654	353	459
cb273_c4/f1p0/1772 isoform=cb273_c4;full_length_coverage=1;non_full_length_coverage=0;isoform_length=1772;expected_accuracy=0.999	gnl|BL_ORD_ID|68135 cb273_c7/f1p0/5028	1	1084	1084	1771	3098	4181	4321	4980
cb2097_c50/f1p0/2824	gnl|BL_ORD_ID|66695 cb2097_c41/f1p3/3227	1	2323	2318	2754	76	2399	2574	3011
cb8564_c15230/f1p0/1944	gnl|BL_ORD_ID|12686 cb8564_c15404/f1p9/2596 isoform=cb8564_c15404;full_length_coverage=1;non_full_length_coverage=9;isoform_length=2596;expected_accuracy=0.991	352	1944	6	357	1002	2596	7	360
cb6067_c6/f1p0/2318	gnl|BL_ORD_ID|74859 cb6067_c3/f1p0/2859	12	1201	1201	2269	29	121	1780	286

<table>
<tr><td></td><td></td><td></td><td></td><td></td><td></td><td></td><td>8</td><td></td><td>2</td></tr>
<tr><td>cb8564_c15653/f1p4/2475</td><td>gnl|BL_ORD_ID|55119 cb8564_c24867/f1p3/2846</td><td>22</td><td>2293</td><td>2289</td><td>2436</td><td>24</td><td>2297</td><td>2516</td><td>2663</td></tr>
<tr><td>cb18456_c1776/f1p0/1211</td><td>gnl|BL_ORD_ID|18624 cb8564_c83199/f1p1/2355 isoform=cb8564_c83199;full_length_coverage=1;non_full_length_coverage=1;isoform_length=2355;expected_accuracy=0.994</td><td>280</td><td>1214</td><td>9</td><td>285</td><td>977</td><td>43</td><td>1361</td><td>1088</td></tr>
<tr><td>cb3143_c10/f1p1/2863</td><td>gnl|BL_ORD_ID|37814 cb3143_c5/f1p3/3440 isoform=cb3143_c5;full_length_coverage=1;non_full_length_coverage=3;isoform_length=3440;expected_accuracy=0.999</td><td>163</td><td>2870</td><td>1</td><td>163</td><td>677</td><td>3383</td><td>113</td><td>275</td></tr>
<tr><td>cb8564_c112645/f1p0/3358 isoform=cb8564_c112645;full_length_coverage=1;non_full_length_coverage=0;isoform_length=3358;expected_accuracy=0.999</td><td>gnl|BL_ORD_ID|9642 cb8564_c117476/f1p1/3616 isoform=cb8564_c117476;full_length_coverage=1;non_full_length_coverage=1;isoform_length=3616;expected_accuracy=0.997</td><td>1</td><td>2943</td><td>2944</td><td>3357</td><td>4</td><td>2937</td><td>3205</td><td>3616</td></tr>
<tr><td>cb8564_c37361/f1p0/3076</td><td>gnl|BL_ORD_ID|55600 cb8564_c46484/f1p0/3151</td><td>102</td><td>2481</td><td>2477</td><td>3070</td><td>5</td><td>2404</td><td>2505</td><td>3097</td></tr>
<tr><td>cb8564_c13735/f1p1/2121 isoform=cb8564_c13735;full_length_coverage=1;non_full_length_coverage=1;isoform_length=2121;expected_accuracy=0.994</td><td>gnl|BL_ORD_ID|18458 cb8564_c9882/f2p0/2918 isoform=cb8564_c9882;full_length_coverage=2;non_full_length_coverage=0;isoform_length=2918;expected_accuracy=0.999</td><td>264</td><td>2121</td><td>4</td><td>265</td><td>1056</td><td>2918</td><td>1</td><td>263</td></tr>
<tr><td>cb8564_c16237/f1p1/3663 isoform=cb8564_c16237;full_length_coverage=1;non_full_length_coverage=1;isoform_length=3663;expected_accuracy=0.997</td><td>gnl|BL_ORD_ID|10513 cb8564_c11019/f3p1/4099 isoform=cb8564_c11019;full_length_coverage=3;non_full_length_coverage=1;isoform_length=4099;expected_accuracy=0.997</td><td>190</td><td>3663</td><td>1</td><td>190</td><td>615</td><td>4092</td><td>4</td><td>193</td></tr>
<tr><td>cb4889_c22/f1p0/2625</td><td>gnl|BL_ORD_ID|73053 cb4889_c51/f1p5/2911</td><td>280</td><td>2625</td><td>5</td><td>285</td><td>451</td><td>279</td><td>3</td><td>283</td></tr>
</table>

isoform=cb4889_c22;full_length_coverage=1;non_full_length_coverage=0;isoform_length=2625;expected_accuracy=0.995							6		
cb8564_c37866/f1p3/2874 isoform=cb8564_c37866;full_length_coverage=1;non_full_length_coverage=3;isoform_length=2874;expected_accuracy=0.990	gnl|BL_ORD_ID|18852 cb8564_c84488/f1p0/3408 isoform=cb8564_c84488;full_length_coverage=1;non_full_length_coverage=0;isoform_length=3408;expected_accuracy=0.996	220	2863	1	220	790	3408	1	217
cb8564_c83196/f1p0/2505 isoform=cb8564_c83196;full_length_coverage=1;non_full_length_coverage=0;isoform_length=2505;expected_accuracy=0.995	gnl|BL_ORD_ID|16320 cb8564_c68304/f3p0/3488 isoform=cb8564_c68304;full_length_coverage=3;non_full_length_coverage=0;isoform_length=3488;expected_accuracy=1.000	1	1642	1642	2479	897	2539	2652	3488
cb234_c1/f1p1/4574 isoform=cb234_c1;full_length_coverage=1;non_full_length_coverage=1;isoform_length=4574;expected_accuracy=0.997	gnl|BL_ORD_ID|38041 cb234_c2/f1p2/4728 isoform=cb234_c2;full_length_coverage=1;non_full_length_coverage=2;isoform_length=4728;expected_accuracy=0.997	1	4236	4231	4574	4	4240	4357	4699
cb8564_c51923/f1p1/2966 isoform=cb8564_c51923;full_length_coverage=1;non_full_length_coverage=1;isoform_length=2966;expected_accuracy=0.997	gnl|BL_ORD_ID|55920 cb8564_c48949/f1p0/4357	17	2301	2302	2966	16	2293	2847	3508
cb2831_c22/f1p0/2102	gnl|BL_ORD_ID|68917 cb2831_c35/f1p0/2886	1	1598	1598	2104	7	1604	2344	2850
cb8564_c35093/f1p5/2916	gnl|BL_ORD_ID|14531 cb8564_c41778/f1p9/3482 isoform=cb8564_c41778;full_length_coverage=1;non_full_length_coverage=9;isoform_length=3482;expected_accuracy=0.992	259	2885	20	263	812	3439	2	247
cb8564_c47159/f1p0/3238	gnl|BL_ORD_ID|18906 cb8564_c90449/f1p3/3223	336	2919	81	337	640	322	41	296

	isoform=cb8564_c90449;full_length_coverage=1;non_full_length_coverage=3;isoform_length=3223;expected_accuracy=0.996						3		
cb12972_c26/f1p0/1766	gnl|BL_ORD_ID|14141 cb8564_c2622/f1p1/2613 isoform=cb8564_c2622;full_length_coverage=1;non_full_length_coverage=1;isoform_length=2613;expected_accuracy=0.996	203	1675	6	204	1144	2613	7	205
cb4489_c12/f1p0/1870 isoform=cb4489_c12;full_length_coverage=1;non_full_length_coverage=0;isoform_length=1870;expected_accuracy=0.997	gnl|BL_ORD_ID|43268 cb4489_c6/f2p1/2678 isoform=cb4489_c6;full_length_coverage=2;non_full_length_coverage=1;isoform_length=2678;expected_accuracy=1.000	1	1118	1115	1870	1	1111	1862	2622
cb1573_c16/f1p0/3454 isoform=cb1573_c16;full_length_coverage=1;non_full_length_coverage=0;isoform_length=3454;expected_accuracy=0.998	gnl|BL_ORD_ID|28225 cb1573_c19/f1p1/3913 isoform=cb1573_c19;full_length_coverage=1;non_full_length_coverage=1;isoform_length=3913;expected_accuracy=0.996	1249	3454	1	1252	1453	3665	33	1294
cb1913_c44/f1p0/2236 isoform=cb1913_c44;full_length_coverage=1;non_full_length_coverage=0;isoform_length=2236;expected_accuracy=0.998	gnl|BL_ORD_ID|34517 cb1913_c65/f5p2/2595 isoform=cb1913_c65;full_length_coverage=5;non_full_length_coverage=2;isoform_length=2595;expected_accuracy=1.000	1	1634	1630	2236	111	1743	1868	2485
cb8564_c41863/f2p3/2080	gnl|BL_ORD_ID|56001 cb8564_c51701/f1p0/2495	127	1982	1	132	516	2371	1	132
cb581_c5/f1p1/2742 isoform=cb581_c5;full_length_coverage=1;non_full_length_coverage=1;isoform_length=2742;expected_accuracy=0.996	gnl|BL_ORD_ID|45252 cb581_c3/f1p1/4740 isoform=cb581_c3;full_length_coverage=1;non_full_length_coverage=1;isoform_length=4740;expected_accuracy=0.997	1207	2742	1	1209	3107	4642	32	1227
cb3037_c32/f1p0/2518	gnl|BL_ORD_ID|38434 cb3037_c15/f1p0/3566	1	2228	2225	2473	30	224	3318	356

	isoform=cb3037_c15;full_length_coverage=1;non_full_length_coverage=0;isoform_length=3566;expected_accuracy=0.998						9		4
cb4543_c0/f1p0/3310	gnl|BL_ORD_ID|71945 cb4543_c1/f1p0/3821	652	3159	73	651	767	3286	81	659
cb8564_c38233/f1p0/2780	gnl|BL_ORD_ID|54732 cb8564_c160631/f1p4/3629	1	1857	1856	2785	716	2572	2697	3625
cb4263_c4/f1p1/2943	gnl|BL_ORD_ID|43864 cb4263_c5/f1p1/3378 isoform=cb4263_c5;full_length_coverage=1;non_full_length_coverage=1;isoform_length=3378;expected_accuracy=1.000	242	2985	1	243	620	3378	100	342
cb7637_c9/f1p1/2355	gnl|BL_ORD_ID|7361 cb7637_c21/f8p0/2509 isoform=cb7637_c21;full_length_coverage=8;non_full_length_coverage=0;isoform_length=2509;expected_accuracy=1.000	14	1619	1618	2347	55	1660	1780	2507
cb2520_c14/f1p1/2870 isoform=cb2520_c14;full_length_coverage=1;non_full_length_coverage=1;isoform_length=2870;expected_accuracy=0.996	gnl|BL_ORD_ID|69793 cb2520_c6/f1p0/4295	103	2740	1	104	1637	4274	1031	1136
cb1909_c6/f1p0/3557	gnl|BL_ORD_ID|36390 cb1909_c3/f1p0/3823 isoform=cb1909_c3;full_length_coverage=1;non_full_length_coverage=0;isoform_length=3823;expected_accuracy=0.997	1	3344	3342	3484	117	3459	3681	3823
cb2482_c31/f1p1/2865 isoform=cb2482_c31;full_length_coverage=1;non_full_length_coverage=1;isoform_length=2865;expected_accu	gnl|BL_ORD_ID|68553 cb2482_c45/f1p0/3357	26	2632	2631	2865	61	2664	2788	3022

racy=0.996									
cb3307_c0/f16p2/2144 isoform=cb3307_c0;full_length_coverage=16;non_full_length_coverage=2;isoform_length=2144;expected_accuracy=1.000	gnl|BL_ORD_ID|41080 cb3307_c5/f2p0/3313 isoform=cb3307_c5;full_length_coverage=2;non_full_length_coverage=0;isoform_length=3313;expected_accuracy=0.997	168	2144	14	169	1342	3313	1	155
cb5748_c6/f1p0/1754 isoform=cb5748_c6;full_length_coverage=1;non_full_length_coverage=0;isoform_length=1754;expected_accuracy=0.997	gnl|BL_ORD_ID|74381 cb5748_c4/f1p0/2096	1	1063	1063	1754	1	1063	1359	2050
cb8564_c2818/f1p0/2964 isoform=cb8564_c2818;full_length_coverage=1;non_full_length_coverage=0;isoform_length=2964;expected_accuracy=0.993	gnl|BL_ORD_ID|11783 cb8564_c149467/f1p0/3280 isoform=cb8564_c149467;full_length_coverage=1;non_full_length_coverage=0;isoform_length=3280;expected_accuracy=0.990	1127	2942	1	1126	1461	3279	1	1123
cb8564_c40764/f1p0/2563 isoform=cb8564_c40764;full_length_coverage=1;non_full_length_coverage=0;isoform_length=2563;expected_accuracy=0.997	gnl|BL_ORD_ID|57176 cb8564_c85665/f1p0/3087	14	2166	2162	2563	2	2174	2382	2787
cb5641_c4/f1p1/1901 isoform=cb5641_c4;full_length_coverage=1;non_full_length_coverage=1;isoform_length=1901;expected_accuracy=0.996	gnl|BL_ORD_ID|74212 cb5641_c5/f1p0/2136	1	1251	1250	1899	55	1305	1491	2140
cb5743_c23/f1p0/2696	gnl|BL_ORD_ID|74377 cb5743_c16/f1p0/2959	12	1581	1577	2602	4	1573	1679	2704
cb1176_c32/f1p0/3993	gnl|BL_ORD_ID|23232 cb1176_c18/f1p2/4249 isoform=cb1176_c18;full_length_coverage=1;non_full_length_coverage=2;isoform_length=4249;expected_accur	110	3923	1	112	410	4199	10	122

	acy=0.994								
cb416_c19/f1p0/2939	gnl|BL_ORD_ID|72153 cb416_c49/f1p0/3164	1042	2856	10	1043	1254	3070	7	1042
cb4674_c26/f1p1/3006 isoform=cb4674_c26;full_length_coverage=1;non_full_length_coverage=1;isoform_length=3006;expected_accuracy=0.995	gnl|BL_ORD_ID|72841 cb4674_c25/f1p0/3544	1	2157	2158	3004	5	2141	2657	3503
cb11067_c13/f1p0/1371 isoform=cb11067_c13;full_length_coverage=1;non_full_length_coverage=0;isoform_length=1371;expected_accuracy=0.993	gnl|BL_ORD_ID|49133 cb11067_c3/f1p0/1547	1	1177	1176	1368	27	1203	1312	1504
cb8041_c10/f1p2/2193 isoform=cb8041_c10;full_length_coverage=1;non_full_length_coverage=2;isoform_length=2193;expected_accuracy=1.000	gnl|BL_ORD_ID|8273 cb8041_c27/f1p2/2321 isoform=cb8041_c27;full_length_coverage=1;non_full_length_coverage=2;isoform_length=2321;expected_accuracy=0.995	1	1495	1494	2133	25	1519	1682	2321
cb7736_c8/f1p0/3317 isoform=cb7736_c8;full_length_coverage=1;non_full_length_coverage=0;isoform_length=3317;expected_accuracy=0.994	gnl|BL_ORD_ID|6316 cb7736_c4/f1p0/3571 isoform=cb7736_c4;full_length_coverage=1;non_full_length_coverage=0;isoform_length=3571;expected_accuracy=0.996	1192	3317	1	1192	1439	3565	17	1216
cb5874_c30/f1p0/2210	gnl|BL_ORD_ID|74533 cb5874_c16/f1p0/3263	145	2190	1	145	875	2921	533	677
cb373_c6/f1p0/4615 isoform=cb373_c6;full_length_coverage=1;non_full_length_coverage=0;isoform_length=4615;expected_accuracy=0.997	gnl|BL_ORD_ID|40118 cb373_c48/f1p0/5064 isoform=cb373_c48;full_length_coverage=1;non_full_length_coverage=0;isoform_length=5064;expected_accuracy=0.992	1	4223	4221	4474	6	4200	4810	5064
cb8564_c53266/f1p2/2976	gnl|BL_ORD_ID|60642 cb12244_c4/f1p1/4997	1	2754	2751	2985	1670	442	4545	477

							3		9
cb3106_c11/f1p0/2728	gnl|BL_ORD_ID|37403 cb3106_c8/f1p0/3729 isoform=cb3106_c8;full_length_coverage=1;non_full_length_coverage=0;isoform_length=3729;expected_accuracy=0.994	1	1889	1885	2475	21	1906	3143	3727
cb8564_c2323/f1p0/2212 isoform=cb8564_c2323;full_length_coverage=1;non_full_length_coverage=0;isoform_length=2212;expected_accuracy=0.995	gnl|BL_ORD_ID|54765 cb8564_c2442/f1p1/2806	1	1320	1316	2209	2	1321	1599	2492
cb341_c7/f1p0/2819 isoform=cb341_c7;full_length_coverage=1;non_full_length_coverage=0;isoform_length=2819;expected_accuracy=1.000	gnl|BL_ORD_ID|69573 cb341_c10/f1p0/3009	1	2507	2507	2819	4	2509	2684	2996
cb8564_c46939/f1p1/2926	gnl|BL_ORD_ID|55377 cb8564_c39822/f1p0/3306	1	2134	2130	2870	1	2140	2245	2986
cb16651_c18/f1p1/3071 isoform=cb16651_c18;full_length_coverage=1;non_full_length_coverage=1;isoform_length=3071;expected_accuracy=0.998	gnl|BL_ORD_ID|31471 cb16651_c8/f1p3/3966 isoform=cb16651_c8;full_length_coverage=1;non_full_length_coverage=3;isoform_length=3966;expected_accuracy=0.999	426	3071	5	428	1261	3910	1	420
cb8564_c4940/f1p0/2731 isoform=cb8564_c4940;full_length_coverage=1;non_full_length_coverage=0;isoform_length=2731;expected_accuracy=0.996	gnl|BL_ORD_ID|16554 cb8564_c701/f3p0/3014 isoform=cb8564_c701;full_length_coverage=3;non_full_length_coverage=0;isoform_length=3014;expected_accuracy=0.997	1	1756	1755	2731	8	1770	2014	2992
cb12972_c26/f1p0/1766	gnl|BL_ORD_ID|26048 cb12972_c38/f1p0/1837 isoform=cb12972_c38;full_length_coverage=1;non_full_length_coverage=0;isoform_length=1837;expected_acc	203	1673	6	206	368	1837	51	251

	uracy=0.992								
cb8564_c44063/f1p0/2503 isoform=cb8564_c44063;full_length_coverage=1;non_full_length_coverage=0;isoform_length=2503;expected_accuracy=0.999	gnl|BL_ORD_ID|13302 cb8564_c19160/f1p2/2687 isoform=cb8564_c19160;full_length_coverage=1;non_full_length_coverage=2;isoform_length=2687;expected_accuracy=0.990	397	2496	72	402	591	2687	105	446
cb3665_c20/f1p0/2779	gnl|BL_ORD_ID|39192 cb3665_c4/f1p0/2857 isoform=cb3665_c4;full_length_coverage=1;non_full_length_coverage=0;isoform_length=2857;expected_accuracy=1.000	1	1482	1479	2669	11	1492	1623	2813
cb9214_c10/f1p0/1968	gnl|BL_ORD_ID|59579 cb9214_c8/f1p0/2150	1	1370	1368	1973	1	1370	1516	2121
cb8889_c1/f2p1/2254 isoform=cb8889_c1;full_length_coverage=2;non_full_length_coverage=1;isoform_length=2254;expected_accuracy=0.994	gnl|BL_ORD_ID|20509 cb8889_c9/f1p1/2462 isoform=cb8889_c9;full_length_coverage=1;non_full_length_coverage=1;isoform_length=2462;expected_accuracy=0.995	1	1221	1221	2252	1	1205	1423	2462
cb2658_c0/f3p1/7250 isoform=cb2658_c0;full_length_coverage=3;non_full_length_coverage=1;isoform_length=7250;expected_accuracy=0.998	gnl|BL_ORD_ID|68687 cb2658_c3/f1p0/8266	3612	7248	12	3617	4576	8215	5	3612
cb5121_c4/f1p0/2356 isoform=cb5121_c4;full_length_coverage=1;non_full_length_coverage=0;isoform_length=2356;expected_accuracy=0.993	gnl|BL_ORD_ID|43684 cb5121_c3/f1p0/3174 isoform=cb5121_c3;full_length_coverage=1;non_full_length_coverage=0;isoform_length=3174;expected_accuracy=0.997	1	1949	1946	2356	31	1984	2763	3173
cb16776_c28/f1p0/5224 isoform=cb16776_c28;full_length_coverage=1;non_full_length_coverage=0;isoform_length=5224;expected_acc	gnl|BL_ORD_ID|31806 cb16776_c17/f1p1/6143 isoform=cb16776_c17;full_length_coverage=1;non_full_length_coverage=1;isoform_length=6143;expected_acc	2	4960	4959	5224	120	5077	5545	5812

uracy=0.997	uracy=0.995								
cb3421_c16/f1p0/2946	gnl|BL_ORD_ID|71191 cb3421_c13/f1p0/4144	389	2896	43	389	1645	415 2	1	347
cb1616_c20/f1p1/2895 isoform=cb1616_c20;full_length_coverage=1;non_full_length_coverage=1;isoform_length=2895;expected_accuracy=0.997	gnl|BL_ORD_ID|63795 cb1616_c6/f1p1/3449	1	1968	1966	2864	79	204 3	2556	345 5
cb2125_c25/f1p0/3582 isoform=cb2125_c25;full_length_coverage=1;non_full_length_coverage=0;isoform_length=3582;expected_accuracy=0.995	gnl|BL_ORD_ID|35696 cb2125_c8/f2p0/3728 isoform=cb2125_c8;full_length_coverage=2;non_full_length_coverage=0;isoform_length=3728;expected_accuracy=0.995	236	3582	1	235	384	372 4	4	237
cb8564_c48153/f1p0/3083	gnl|BL_ORD_ID|56029 cb8564_c52782/f1p2/3327	255	2878	18	255	617	323 8	11	225
cb8564_c4683/f1p0/2938	gnl|BL_ORD_ID|55047 cb8564_c4680/f1p0/2928	243	2660	1	247	385	280 1	3	250
cb10829_c0/f5p0/1551 isoform=cb10829_c0;full_length_coverage=5;non_full_length_coverage=0;isoform_length=1551;expected_accuracy=1.000	gnl|BL_ORD_ID|450 cb10829_c1/f1p0/2092 isoform=cb10829_c1;full_length_coverage=1;non_full_length_coverage=0;isoform_length=2092;expected_accuracy=0.995	272	1550	57	277	814	209 2	1	221
cb2875_c0/f7p0/3313 isoform=cb2875_c0;full_length_coverage=7;non_full_length_coverage=0;isoform_length=3313;expected_accuracy=0.997	gnl|BL_ORD_ID|38285 cb2875_c11/f1p0/3661 isoform=cb2875_c11;full_length_coverage=1;non_full_length_coverage=0;isoform_length=3661;expected_accuracy=0.997	1590	3313	1	1591	1942	366 0	1	158 6
cb7114_c7/f1p0/2312	gnl|BL_ORD_ID|5846 cb7114_c6/f1p0/2697 isoform=cb7114_c6;full_length_coverage=1;non_full_length_coverage=0;isoform_length=2697;expected_accura	1	1515	1513	2306	7	152 4	1890	268 3

	cy=0.995								
cb8564_c628/f3p1/4943 isoform=cb8564_c628;full_length_coverage=3;non_full_length_coverage=1;isoform_length=4943;expected_accuracy=1.000	gnl|BL_ORD_ID|29247 cb14859_c9/f1p3/5221 isoform=cb14859_c9;full_length_coverage=1;non_full_length_coverage=3;isoform_length=5221;expected_accuracy=0.997	725	4943	87	726	998	5220	1	641
cb8564_c37361/f1p0/3076	gnl|BL_ORD_ID|17429 cb8564_c85447/f1p0/3196 isoform=cb8564_c85447;full_length_coverage=1;non_full_length_coverage=0;isoform_length=3196;expected_accuracy=0.997	1	2481	2477	3070	3	2503	2604	3195
cb8564_c37361/f1p0/3076	gnl|BL_ORD_ID|53680 cb8564_c153222/f2p39/3454	1	2481	2477	3058	7	2488	2589	3170
cb8564_c77994/f1p2/2630 isoform=cb8564_c77994;full_length_coverage=1;non_full_length_coverage=2;isoform_length=2630;expected_accuracy=0.998	gnl|BL_ORD_ID|17478 cb8564_c85115/f1p6/2975 isoform=cb8564_c85115;full_length_coverage=1;non_full_length_coverage=6;isoform_length=2975;expected_accuracy=0.994	1	1819	1815	2630	13	1832	1976	2791
cb2155_c6/f2p0/3314 isoform=cb2155_c6;full_length_coverage=2;non_full_length_coverage=0;isoform_length=3314;expected_accuracy=0.997	gnl|BL_ORD_ID|35904 cb2155_c9/f1p0/3407 isoform=cb2155_c9;full_length_coverage=1;non_full_length_coverage=0;isoform_length=3407;expected_accuracy=0.997	1	2846	2846	3275	8	2858	2978	3407
cb8564_c38940/f1p0/4032	gnl|BL_ORD_ID|10486 cb8564_c107051/f2p1/3797 isoform=cb8564_c107051;full_length_coverage=2;non_full_length_coverage=1;isoform_length=3797;expected_accuracy=0.997	202	3586	2	204	392	3797	13	215
cb5702_c2/f1p0/2693 isoform=cb5702_c2;full_length_coverage=1;non_full_length_coverage=0;isoform_length=2693;expected_accur	gnl|BL_ORD_ID|45978 cb5702_c0/f1p0/3031 isoform=cb5702_c0;full_length_coverage=1;non_full_length_coverage=0;isoform_length=3031;expected_accura	1	2078	2075	2568	1	2079	2538	3031

acy=0.995	cy=0.998								
cb8564_c43779/f1p0/3021 isoform=cb8564_c43779;full_length_coverage=1;non_full_length_coverage=0;isoform_length=3021;expected_accuracy=0.993	gnl|BL_ORD_ID|55472 cb8564_c42531/f1p0/3996	124	2344	2345	3019	816	3034	3190	3863
cb8564_c91159/f1p0/3159 isoform=cb8564_c91159;full_length_coverage=1;non_full_length_coverage=0;isoform_length=3159;expected_accuracy=0.998	gnl|BL_ORD_ID|15570 cb8564_c75349/f1p0/3566 isoform=cb8564_c75349;full_length_coverage=1;non_full_length_coverage=0;isoform_length=3566;expected_accuracy=0.995	1	1630	1628	3107	48	1678	2088	3566
cb8564_c42814/f1p0/2151 isoform=cb8564_c42814;full_length_coverage=1;non_full_length_coverage=0;isoform_length=2151;expected_accuracy=0.993	gnl|BL_ORD_ID|14407 cb8564_c44036/f1p0/3258 isoform=cb8564_c44036;full_length_coverage=1;non_full_length_coverage=0;isoform_length=3258;expected_accuracy=0.992	650	2149	7	652	1757	3258	1	645
cb7010_c28/f1p0/2721 isoform=cb7010_c28;full_length_coverage=1;non_full_length_coverage=0;isoform_length=2721;expected_accuracy=0.997	gnl|BL_ORD_ID|4517 cb7010_c8/f3p3/3171 isoform=cb7010_c8;full_length_coverage=3;non_full_length_coverage=3;isoform_length=3171;expected_accuracy=1.000	182	2721	1	185	559	3097	5	189
cb7096_c21/f1p0/2561	gnl|BL_ORD_ID|50336 cb7096_c16/f1p0/2638	184	2463	1	185	316	2595	15	199
cb9445_c2/f1p0/1972 isoform=cb9445_c2;full_length_coverage=1;non_full_length_coverage=0;isoform_length=1972;expected_accuracy=0.991	gnl|BL_ORD_ID|34648 cb2101_c3/f1p0/4051 isoform=cb2101_c3;full_length_coverage=1;non_full_length_coverage=0;isoform_length=4051;expected_accuracy=0.996	262	1972	36	263	2782	1080	3971	3752
cb8564_c54537/f1p0/3163	gnl|BL_ORD_ID|18852 cb8564_c84488/f1p0/3408 isoform=cb8564_c84488;full_length_coverage=1;non_full_length_coverage=0;isoform_length=3408;expected_ac	313	2930	4	312	791	3408	4	312

	curacy=0.996								
cb8120_c4/f1p0/2175	gnl|BL_ORD_ID|51763 cb8120_c3/f1p0/2751	945	2171	98	946	1477	2703	1	849
cb8564_c44014/f1p0/3720	gnl|BL_ORD_ID|56041 cb8564_c53077/f1p2/3406	353	3215	40	353	452	3314	24	337
cb8564_c43546/f1p1/3276	gnl|BL_ORD_ID|18852 cb8564_c84488/f1p0/3408 isoform=cb8564_c84488;full_length_coverage=1;non_full_length_coverage=0;isoform_length=3408;expected_accuracy=0.996	247	2854	10	247	790	3397	1	217
cb8564_c4643/f1p1/2554	gnl|BL_ORD_ID|12605 cb8564_c147470/f1p2/2765 isoform=cb8564_c147470;full_length_coverage=1;non_full_length_coverage=2;isoform_length=2765;expected_accuracy=0.992	1	1705	1700	2551	76	1781	1914	2765
cb8564_c73853/f1p0/2443 isoform=cb8564_c73853;full_length_coverage=1;non_full_length_coverage=0;isoform_length=2443;expected_accuracy=1.000	gnl|BL_ORD_ID|12899 cb8564_c181074/f1p0/2725 isoform=cb8564_c181074;full_length_coverage=1;non_full_length_coverage=0;isoform_length=2725;expected_accuracy=1.000	1	1281	1276	2443	1	1254	1534	2704
cb18646_c2/f1p5/6412 isoform=cb18646_c2;full_length_coverage=1;non_full_length_coverage=5;isoform_length=6412;expected_accuracy=0.997	gnl|BL_ORD_ID|34400 cb18646_c6/f1p3/6738 isoform=cb18646_c6;full_length_coverage=1;non_full_length_coverage=3;isoform_length=6738;expected_accuracy=0.995	2	6140	6140	6412	3	6137	6371	6644
cb2324_c36/f1p1/3463	gnl|BL_ORD_ID|68434 cb2324_c22/f1p0/3794	100	3473	1	100	382	3751	20	118
cb1163_c19/f1p0/3148 isoform=cb1163_c19;full_length_coverage=1;non_full_length_coverage=0;isoform_length=3148;expected_accu	gnl|BL_ORD_ID|23459 cb1163_c11/f2p1/3742 isoform=cb1163_c11;full_length_coverage=2;non_full_length_coverage=1;isoform_length=3742;expected_accur	1	1982	1981	3101	522	2504	2616	3742

racy=0.996	acy=0.999								
cb415_c102/f1p0/3685	gnl|BL_ORD_ID|72143 cb415_c84/f1p0/3822	15	2090	2085	3634	17	2105	2225	3785
cb1088_c12/f1p0/2593	gnl|BL_ORD_ID|2693 cb1088_c21/f6p2/3255 isoform=cb1088_c21;full_length_coverage=6;non_full_length_coverage=2;isoform_length=3255;expected_accuracy=1.000	1	1648	1650	2496	271	1919	2409	3255
cb8564_c25160/f1p0/3126 isoform=cb8564_c25160;full_length_coverage=1;non_full_length_coverage=0;isoform_length=3126;expected_accuracy=0.996	gnl|BL_ORD_ID|13835 cb8564_c22647/f1p0/3442 isoform=cb8564_c22647;full_length_coverage=1;non_full_length_coverage=0;isoform_length=3442;expected_accuracy=0.995	1199	3081	1	1200	1570	3442	26	1222
cb18275_c11/f1p1/1730	gnl|BL_ORD_ID|11253 cb8564_c151174/f1p2/2106 isoform=cb8564_c151174;full_length_coverage=1;non_full_length_coverage=2;isoform_length=2106;expected_accuracy=0.997	131	1733	1	134	392	1994	68	202
cb309_c9/f1p2/4191 isoform=cb309_c9;full_length_coverage=1;non_full_length_coverage=2;isoform_length=4191;expected_accuracy=0.998	gnl|BL_ORD_ID|69547 cb309_c21/f1p0/4520	1	3777	3776	4190	22	3777	3986	4400
cb8564_c2579/f1p0/2358	gnl|BL_ORD_ID|15217 cb8564_c2575/f1p0/3633 isoform=cb8564_c2575;full_length_coverage=1;non_full_length_coverage=0;isoform_length=3633;expected_accuracy=0.995	1050	2369	1	1050	2174	3493	6	1060
cb2211_c8/f1p0/2631 isoform=cb2211_c8;full_length_coverage=1;non_full_length_coverage=0;isoform_length=2631;expected_accur	gnl|BL_ORD_ID|38588 cb2211_c5/f1p0/2638 isoform=cb2211_c5;full_length_coverage=1;non_full_length_coverage=0;isoform_length=2638;expected_accura	105	2535	1	105	207	2638	1	107

acy=0.991	cy=1.000								
cb3971_c4/f1p0/2436 isoform=cb3971_c4;full_length_coverage=1;non_full_length_coverage=0;isoform_length=2436;expected_accuracy=0.995	gnl|BL_ORD_ID|71682 cb3971_c1/f1p0/3443	1	1594	1592	2436	241	1837	2317	3162
cb8564_c14624/f1p0/1901 isoform=cb8564_c14624;full_length_coverage=1;non_full_length_coverage=0;isoform_length=1901;expected_accuracy=0.994	gnl|BL_ORD_ID|11748 cb8564_c12743/f1p0/2150 isoform=cb8564_c12743;full_length_coverage=1;non_full_length_coverage=0;isoform_length=2150;expected_accuracy=0.995	880	1901	9	881	1129	2150	1	892
cb8564_c73422/f1p0/3612	gnl|BL_ORD_ID|14394 cb8564_c39181/f2p0/3796 isoform=cb8564_c39181;full_length_coverage=2;non_full_length_coverage=0;isoform_length=3796;expected_accuracy=0.997	1	3079	3074	3608	1	3077	3215	3768
cb955_c24/f1p2/2194	gnl|BL_ORD_ID|59129 cb955_c25/f1p0/3018	1	1538	1538	2190	120	1657	2345	2997
cb8564_c70156/f1p0/2250 isoform=cb8564_c70156;full_length_coverage=1;non_full_length_coverage=0;isoform_length=2250;expected_accuracy=0.996	gnl|BL_ORD_ID|17866 cb8564_c81520/f1p0/2464 isoform=cb8564_c81520;full_length_coverage=1;non_full_length_coverage=0;isoform_length=2464;expected_accuracy=1.000	110	2239	1	110	334	2464	52	162
cb8564_c157572/f1p0/3807 isoform=cb8564_c157572;full_length_coverage=1;non_full_length_coverage=0;isoform_length=3807;expected_accuracy=0.994	gnl|BL_ORD_ID|54591 cb8564_c127438/f1p0/3910	316	3807	118	318	445	3936	33	232
cb500_c17/f1p0/2323 isoform=cb500_c17;full_length_coverage=1;non_full_length_coverage=0;isoform_length=2323;expected_accur	gnl|BL_ORD_ID|72249 cb500_c64/f1p0/4276	1	1293	1288	2323	1520	2812	2944	3979

acy=0.997									
cb8564_c145840/f9p2/3901 isoform=cb8564_c145840;full_length_coverage=9;non_full_length_coverage=2;isoform_length=3901;expected_accuracy=0.999	gnl|BL_ORD_ID|56594 cb8564_c47777/f1p0/4391	228	3882	15	229	476	4133	1	215
cb8564_c1783/f1p0/2556	gnl|BL_ORD_ID|12956 cb8564_c1771/f3p1/3767 isoform=cb8564_c1771;full_length_coverage=3;non_full_length_coverage=1;isoform_length=3767;expected_accuracy=0.996	1	1222	1220	2340	115	1334	2646	3764
cb16416_c3/f1p0/1533 isoform=cb16416_c3;full_length_coverage=1;non_full_length_coverage=0;isoform_length=1533;expected_accuracy=0.998	gnl|BL_ORD_ID|31689 cb16416_c0/f2p0/1780 isoform=cb16416_c0;full_length_coverage=2;non_full_length_coverage=0;isoform_length=1780;expected_accuracy=0.999	1	1011	1006	1533	4	1015	1241	1770
cb6879_c5/f1p0/2258 isoform=cb6879_c5;full_length_coverage=1;non_full_length_coverage=0;isoform_length=2258;expected_accuracy=0.991	gnl|BL_ORD_ID|47740 cb6879_c1/f1p0/2571	401	2258	5	401	723	2577	29	441
cb2918_c67/f1p0/2483 isoform=cb2918_c67;full_length_coverage=1;non_full_length_coverage=0;isoform_length=2483;expected_accuracy=1.000	gnl|BL_ORD_ID|37248 cb2918_c86/f74p0/2712 isoform=cb2918_c86;full_length_coverage=74;non_full_length_coverage=0;isoform_length=2712;expected_accuracy=1.000	1080	2483	1	1085	1309	2712	69	1152
cb8564_c158845/f2p1/2551 isoform=cb8564_c158845;full_length_coverage=2;non_full_length_coverage=1;isoform_length=2551;expected_accuracy=0.995	gnl|BL_ORD_ID|19058 cb8564_c9477/f9p0/2721 isoform=cb8564_c9477;full_length_coverage=9;non_full_length_coverage=0;isoform_length=2721;expected_accuracy=0.996	1	2202	2200	2546	55	2239	2392	2718
cb8564_c43779/f1p0/3021	gnl|BL_ORD_ID|55521 cb8564_c44014/f1p0/3720	23	2344	2345	3021	40	235	2515	319

isoform=cb8564_c43779;full_length_coverage=1;non_full_length_coverage=0;isoform_length=3021;expected_accuracy=0.993							9		0
cb2661_c14/f6p2/3147 isoform=cb2661_c14;full_length_coverage=6;non_full_length_coverage=2;isoform_length=3147;expected_accuracy=1.000	gnl|BL_ORD_ID|68689 cb2661_c5/f1p0/3850	1	2275	2276	3147	37	2311	2804	3675
cb7601_c7/f1p0/1884	gnl|BL_ORD_ID|7860 cb7601_c1/f3p0/1952 isoform=cb7601_c1;full_length_coverage=3;non_full_length_coverage=0;isoform_length=1952;expected_accuracy=1.000	1	1492	1497	1805	34	1525	1644	1952
cb295_c53/f1p0/1719 isoform=cb295_c53;full_length_coverage=1;non_full_length_coverage=0;isoform_length=1719;expected_accuracy=0.994	gnl|BL_ORD_ID|34400 cb18646_c6/f1p3/6738 isoform=cb18646_c6;full_length_coverage=1;non_full_length_coverage=3;isoform_length=6738;expected_accuracy=0.995	1	1347	1347	1719	4898	6245	6371	6737
cb3143_c17/f1p0/2870 isoform=cb3143_c17;full_length_coverage=1;non_full_length_coverage=0;isoform_length=2870;expected_accuracy=0.993	gnl|BL_ORD_ID|36612 cb3143_c11/f1p1/3005 isoform=cb3143_c11;full_length_coverage=1;non_full_length_coverage=1;isoform_length=3005;expected_accuracy=0.997	162	2870	1	163	294	2992	21	181
cb1935_c14/f1p0/3028	gnl|BL_ORD_ID|66508 cb1935_c19/f1p0/3130	11	1968	1963	2978	12	1968	2091	3106
cb4128_c19/f1p0/2260	gnl|BL_ORD_ID|70784 cb4128_c20/f1p0/3149	1	1761	1760	2263	28	1785	2121	2625
cb8564_c15608/f1p3/3110	gnl|BL_ORD_ID|54322 cb8564_c22408/f1p2/3368	10	2790	2785	3095	1	2781	2925	3235
cb8564_c40764/f1p0/2563	gnl|BL_ORD_ID|15315 cb8564_c39014/f1p0/3019	1	2167	2164	2563	1	220	2421	283

isoform=cb8564_c40764;full_length_coverage=1;non_full_length_coverage=0;isoform_length=2563;expected_accuracy=0.997	isoform=cb8564_c39014;full_length_coverage=1;non_full_length_coverage=0;isoform_length=3019;expected_accuracy=0.992						9		1
cb9467_c3/f4p0/2200 isoform=cb9467_c3;full_length_coverage=4;non_full_length_coverage=0;isoform_length=2200;expected_accuracy=1.000	gnl|BL_ORD_ID|21565 cb9467_c22/f1p0/2220 isoform=cb9467_c22;full_length_coverage=1;non_full_length_coverage=0;isoform_length=2220;expected_accuracy=0.995	258	2197	96	257	281	2220	1	161
cb7774_c10/f1p0/2476	gnl|BL_ORD_ID|51514 cb7774_c5/f1p0/3020	1	1551	1550	2351	91	1641	2241	3042
cb1262_c28/f1p1/3930 isoform=cb1262_c28;full_length_coverage=1;non_full_length_coverage=1;isoform_length=3930;expected_accuracy=0.994	gnl|BL_ORD_ID|23602 cb1262_c27/f1p1/4094 isoform=cb1262_c27;full_length_coverage=1;non_full_length_coverage=1;isoform_length=4094;expected_accuracy=0.996	189	3744	1	190	529	4094	32	224
cb8564_c43779/f1p0/3021 isoform=cb8564_c43779;full_length_coverage=1;non_full_length_coverage=0;isoform_length=3021;expected_accuracy=0.993	gnl|BL_ORD_ID|55522 cb8564_c44123/f1p1/3230	15	2344	2345	3021	15	2341	2497	3171
cb8564_c41756/f1p0/2738 isoform=cb8564_c41756;full_length_coverage=1;non_full_length_coverage=0;isoform_length=2738;expected_accuracy=0.996	gnl|BL_ORD_ID|15154 cb8564_c32509/f1p1/2880 isoform=cb8564_c32509;full_length_coverage=1;non_full_length_coverage=1;isoform_length=2880;expected_accuracy=0.991	11	2173	2170	2713	2	2207	2330	2877
cb8942_c20/f1p0/2709	gnl|BL_ORD_ID|21372 cb8942_c67/f49p2/2933 isoform=cb8942_c67;full_length_coverage=49;non_full_length_coverage=2;isoform_length=2933;expected_accuracy=1.000	219	2678	1	224	473	2932	90	315
cb4016_c16/f3p2/2553	gnl|BL_ORD_ID|71722 cb4016_c5/f1p0/3485	121	1787	1787	2553	1008	267	2783	354

isoform=cb4016_c16;full_length_coverage=3;non_full_length_coverage=2;isoform_length=2553;expected_accuracy=0.996							5		9
cb8564_c1780/f1p1/3143 isoform=cb8564_c1780;full_length_coverage=1;non_full_length_coverage=1;isoform_length=3143;expected_accuracy=0.995	gnl|BL_ORD_ID|12956 cb8564_c1771/f3p1/3767 isoform=cb8564_c1771;full_length_coverage=3;non_full_length_coverage=1;isoform_length=3767;expected_accuracy=0.996	192	3110	1	194	849	3767	119	312
cb8564_c40764/f1p0/2563 isoform=cb8564_c40764;full_length_coverage=1;non_full_length_coverage=0;isoform_length=2563;expected_accuracy=0.997	gnl|BL_ORD_ID|55327 cb8564_c38462/f1p0/3241	1	2166	2162	2563	1	2154	2362	2762
cb4067_c11/f1p0/2801	gnl|BL_ORD_ID|71765 cb4067_c2/f1p1/3451	231	2802	1	228	820	3391	77	304
cb8564_c188097/f1p0/2552 isoform=cb8564_c188097;full_length_coverage=1;non_full_length_coverage=0;isoform_length=2552;expected_accuracy=0.996	gnl|BL_ORD_ID|54246 cb8564_c21138/f1p3/2652	1	1945	1940	2496	1	1942	2045	2600
cb848_c2/f2p2/3920 isoform=cb848_c2;full_length_coverage=2;non_full_length_coverage=2;isoform_length=3920;expected_accuracy=0.993	gnl|BL_ORD_ID|51017 cb848_c11/f1p1/4011	1	3501	3500	3899	23	3516	3627	4025
cb5714_c6/f1p0/1426 isoform=cb5714_c6;full_length_coverage=1;non_full_length_coverage=0;isoform_length=1426;expected_accuracy=0.996	gnl|BL_ORD_ID|45534 cb5714_c2/f1p0/2670 isoform=cb5714_c2;full_length_coverage=1;non_full_length_coverage=0;isoform_length=2670;expected_accuracy=0.998	263	1426	32	265	1506	2669	1	234
cb2744_c3/f1p0/1362	gnl|BL_ORD_ID|38153 cb2744_c0/f1p1/3821	1	1042	1042	1362	196	123	1877	219

isoform=cb2744_c3;full_length_coverage=1;non_full_length_coverage=0;isoform_length=1362;expected_accuracy=0.991	isoform=cb2744_c0;full_length_coverage=1;non_full_length_coverage=1;isoform_length=3821;expected_accuracy=0.997						9		7
cb1115_c11/f6p4/3674 isoform=cb1115_c11;full_length_coverage=6;non_full_length_coverage=4;isoform_length=3674;expected_accuracy=1.000	gnl|BL_ORD_ID|3245 cb1115_c1/f1p1/4461 isoform=cb1115_c1;full_length_coverage=1;non_full_length_coverage=1;isoform_length=4461;expected_accuracy=0.994	1647	3666	119	1649	2445	4461	1	1529
cb8564_c4691/f1p0/3446 isoform=cb8564_c4691;full_length_coverage=1;non_full_length_coverage=0;isoform_length=3446;expected_accuracy=0.992	gnl|BL_ORD_ID|15232 cb8564_c4689/f1p0/4442 isoform=cb8564_c4689;full_length_coverage=1;non_full_length_coverage=0;isoform_length=4442;expected_accuracy=0.997	1	3048	3045	3443	1	3042	4012	4394
cb8564_c82539/f7p3/2550	gnl|BL_ORD_ID|16732 cb8564_c71389/f1p1/2659 isoform=cb8564_c71389;full_length_coverage=1;non_full_length_coverage=1;isoform_length=2659;expected_accuracy=0.994	1	1356	1352	2471	2	1361	1538	2656
cb8564_c72349/f1p0/3248	gnl|BL_ORD_ID|11241 cb8564_c145555/f2p1/3366 isoform=cb8564_c145555;full_length_coverage=2;non_full_length_coverage=1;isoform_length=3366;expected_accuracy=0.997	1	2662	2659	3068	62	2723	2882	3291
cb3281_c0/f4p0/1900 isoform=cb3281_c0;full_length_coverage=4;non_full_length_coverage=0;isoform_length=1900;expected_accuracy=1.000	gnl|BL_ORD_ID|39579 cb3281_c6/f1p0/2227 isoform=cb3281_c6;full_length_coverage=1;non_full_length_coverage=0;isoform_length=2227;expected_accuracy=0.997	1	1183	1180	1895	68	1251	1513	2227
cb18456_c1776/f1p0/1211	gnl|BL_ORD_ID|69242 cb18456_c1755/f1p0/1422	280	1214	6	285	394	1328	3	282
cb233_c13/f1p0/2651	gnl|BL_ORD_ID|38487 cb233_c6/f2p1/3007	377	2651	4	382	684	296	28	408

isoform=cb233_c13;full_length_coverage=1;non_full_length_coverage=0;isoform_length=2651;expected_accuracy=0.992	isoform=cb233_c6;full_length_coverage=2;non_full_length_coverage=1;isoform_length=3007;expected_accuracy=0.998						6		
cb1691_c9/f1p0/2851	gnl|BL_ORD_ID|32447 cb1691_c22/f1p0/3569 isoform=cb1691_c22;full_length_coverage=1;non_full_length_coverage=0;isoform_length=3569;expected_accuracy=0.999	1256	2807	1	1257	2019	3569	1	1257
cb14859_c10/f1p0/5042	gnl|BL_ORD_ID|30141 cb14859_c7/f1p3/5317 isoform=cb14859_c7;full_length_coverage=1;non_full_length_coverage=3;isoform_length=5317;expected_accuracy=0.995	1404	4810	1	1407	1913	5317	78	1486
cb5643_c112/f1p0/1970 isoform=cb5643_c112;full_length_coverage=1;non_full_length_coverage=0;isoform_length=1970;expected_accuracy=0.992	gnl|BL_ORD_ID|46678 cb5643_c124/f5p1/2491 isoform=cb5643_c124;full_length_coverage=5;non_full_length_coverage=1;isoform_length=2491;expected_accuracy=0.997	1	1433	1434	1970	53	1475	1871	2401
cb500_c43/f1p0/3109 isoform=cb500_c43;full_length_coverage=1;non_full_length_coverage=0;isoform_length=3109;expected_accuracy=0.992	gnl|BL_ORD_ID|42492 cb500_c6/f4p0/3398 isoform=cb500_c6;full_length_coverage=4;non_full_length_coverage=0;isoform_length=3398;expected_accuracy=1.000	1	2306	2303	3109	94	2408	2540	3347
cb8564_c51223/f1p4/1975	gnl|BL_ORD_ID|54351 cb8564_c22815/f1p0/4618	207	1937	21	208	2508	4238	79	266
cb6320_c0/f6p0/1902 isoform=cb6320_c0;full_length_coverage=6;non_full_length_coverage=0;isoform_length=1902;expected_accuracy=1.000	gnl|BL_ORD_ID|5687 cb6320_c2/f1p0/2874 isoform=cb6320_c2;full_length_coverage=1;non_full_length_coverage=0;isoform_length=2874;expected_accuracy=0.995	1	1257	1253	1901	31	1283	2226	2874
cb427_c10/f1p0/1930	gnl|BL_ORD_ID|43369 cb427_c7/f2p0/4808	1	1561	1560	1928	18	157	3184	355

isoform=cb427_c10;full_length_coverage=1;non_full_length_coverage=0;isoform_length=1930;expected_accuracy=0.996	isoform=cb427_c7;full_length_coverage=2;non_full_length_coverage=0;isoform_length=4808;expected_accuracy=0.998						0		0
cb1193_c34/f1p0/4247	gnl|BL_ORD_ID|59249 cb1193_c13/f1p1/3683	2	2038	2036	3564	1	2033	2158	3686
cb8564_c40598/f1p4/3119	gnl|BL_ORD_ID|55623 cb8564_c47027/f1p0/3544	1	2732	2729	3054	1	2755	2876	3201
cb8564_c38035/f1p0/2770	gnl|BL_ORD_ID|57176 cb8564_c85665/f1p0/3087	14	2035	2033	2755	2	2048	2238	2965
cb3773_c9/f1p0/2032 isoform=cb3773_c9;full_length_coverage=1;non_full_length_coverage=0;isoform_length=2032;expected_accuracy=0.995	gnl|BL_ORD_ID|40493 cb3773_c4/f1p1/2259 isoform=cb3773_c4;full_length_coverage=1;non_full_length_coverage=1;isoform_length=2259;expected_accuracy=0.995	1	1355	1355	2032	1	1352	1454	2131
cb135_c26/f1p0/3634	gnl|BL_ORD_ID|27202 cb135_c17/f1p0/4316 isoform=cb135_c17;full_length_coverage=1;non_full_length_coverage=0;isoform_length=4316;expected_accuracy=0.995	1	2610	2609	3662	1	2598	3236	4286
cb3406_c10/f1p0/2005	gnl|BL_ORD_ID|40557 cb3406_c11/f1p0/2573 isoform=cb3406_c11;full_length_coverage=1;non_full_length_coverage=0;isoform_length=2573;expected_accuracy=0.992	179	1922	12	179	832	2573	1	167
cb6518_c6/f1p0/2920 isoform=cb6518_c6;full_length_coverage=1;non_full_length_coverage=0;isoform_length=2920;expected_accuracy=0.997	gnl|BL_ORD_ID|5046 cb6518_c8/f1p0/3970 isoform=cb6518_c8;full_length_coverage=1;non_full_length_coverage=0;isoform_length=3970;expected_accuracy=0.991	1	1982	1980	2866	1	1973	3080	3970
cb3747_c11/f1p0/2520	gnl|BL_ORD_ID|39414 cb3747_c3/f6p1/2970	1	1743	1742	2520	11	177	2010	278

isoform=cb3747_c11;full_length_coverage=1;non_full_length_coverage=0;isoform_length=2520;expected_accuracy=0.994	isoform=cb3747_c3;full_length_coverage=6;non_full_length_coverage=1;isoform_length=2970;expected_accuracy=1.000						7		8
cb7377_c9/f1p0/2341	gnl|BL_ORD_ID|51155 cb7377_c0/f1p0/2631	162	2233	1	162	511	2582	127	289
cb7359_c4/f1p0/2420	gnl|BL_ORD_ID|51144 cb7359_c2/f1p0/2609	318	2269	4	320	589	2540	150	466
cb3281_c8/f1p0/2225 isoform=cb3281_c8;full_length_coverage=1;non_full_length_coverage=0;isoform_length=2225;expected_accuracy=0.996	gnl|BL_ORD_ID|39582 cb3281_c5/f1p0/2482 isoform=cb3281_c5;full_length_coverage=1;non_full_length_coverage=0;isoform_length=2482;expected_accuracy=0.995	1087	2201	1	1090	1357	2482	5	1095
cb2927_c1/f2p1/2815 isoform=cb2927_c1;full_length_coverage=2;non_full_length_coverage=1;isoform_length=2815;expected_accuracy=0.994	gnl|BL_ORD_ID|37697 cb2927_c6/f1p1/3514 isoform=cb2927_c6;full_length_coverage=1;non_full_length_coverage=1;isoform_length=3514;expected_accuracy=0.996	1	1658	1654	2808	32	1711	2359	3514
cb6365_c8/f1p1/2052 isoform=cb6365_c8;full_length_coverage=1;non_full_length_coverage=1;isoform_length=2052;expected_accuracy=0.997	gnl|BL_ORD_ID|49655 cb6365_c12/f1p0/2331	10	1762	1762	2050	10	1763	2044	2332
cb3716_c27/f1p2/1719 isoform=cb3716_c27;full_length_coverage=1;non_full_length_coverage=2;isoform_length=1719;expected_accuracy=0.997	gnl|BL_ORD_ID|40690 cb3716_c31/f11p2/1866 isoform=cb3716_c31;full_length_coverage=11;non_full_length_coverage=2;isoform_length=1866;expected_accuracy=1.000	1	1492	1492	1719	31	1526	1635	1863
cb8564_c119745/f1p1/2751 isoform=cb8564_c119745;full_length_coverage=1;non_full_length_coverage=1;isoform_length=2751;expected	gnl|BL_ORD_ID|16193 cb8564_c76962/f1p2/3049 isoform=cb8564_c76962;full_length_coverage=1;non_full_length_coverage=2;isoform_length=3049;expected_ac	1	1745	1745	2684	2	1730	2113	3049

_accuracy=0.992	curacy=0.997								
cb9143_c30/f2p0/2817 isoform=cb9143_c30;full_length_coverage=2;non_full_length_coverage=0;isoform_length=2817;expected_accuracy=0.996	gnl|BL_ORD_ID|19844 cb9143_c57/f1p0/3069 isoform=cb9143_c57;full_length_coverage=1;non_full_length_coverage=0;isoform_length=3069;expected_accuracy=1.000	199	2817	1	203	441	3054	105	307
cb13938_c20/f1p0/1134 isoform=cb13938_c20;full_length_coverage=1;non_full_length_coverage=0;isoform_length=1134;expected_accuracy=1.000	gnl|BL_ORD_ID|72020 cb4825_c11/f1p0/4859	272	1134	7	272	3903	4765	6	271
cb9612_c1/f1p0/2025	gnl|BL_ORD_ID|59850 cb9612_c2/f1p0/2534	1	1606	1601	1980	15	1611	1894	2272
cb7786_c9/f1p1/2310 isoform=cb7786_c9;full_length_coverage=1;non_full_length_coverage=1;isoform_length=2310;expected_accuracy=0.995	gnl|BL_ORD_ID|6654 cb7786_c5/f1p1/2527 isoform=cb7786_c5;full_length_coverage=1;non_full_length_coverage=1;isoform_length=2527;expected_accuracy=0.999	228	2310	16	227	458	2527	14	214
cb8564_c122989/f1p0/4055	gnl|BL_ORD_ID|10539 cb8564_c109091/f5p0/3765 isoform=cb8564_c109091;full_length_coverage=5;non_full_length_coverage=0;isoform_length=3765;expected_accuracy=1.000	1	2587	2589	3750	139	2725	2601	3762
cb8564_c42814/f1p0/2151 isoform=cb8564_c42814;full_length_coverage=1;non_full_length_coverage=0;isoform_length=2151;expected_accuracy=0.993	gnl|BL_ORD_ID|55866 cb8564_c47522/f1p0/2954	650	2151	7	652	1343	2847	1	655
cb6629_c5/f1p2/1294 isoform=cb6629_c5;full_length_coverage=1;non_full_length_coverage=2;isoform_length=1294;expected_accur	gnl|BL_ORD_ID|4820 cb6629_c2/f1p1/2552 isoform=cb6629_c2;full_length_coverage=1;non_full_length_coverage=1;isoform_length=2552;expected_accura	9	781	778	1292	15	788	2040	2550

acy=1.000	cy=0.998								
cb8564_c46212/f3p1/4373 isoform=cb8564_c46212;full_length_coverage=3;non_full_length_coverage=1;isoform_length=4373;expected_accuracy=0.999	gnl|BL_ORD_ID|14819 cb8564_c40333/f1p2/4446 isoform=cb8564_c40333;full_length_coverage=1;non_full_length_coverage=2;isoform_length=4446;expected_accuracy=0.998	184	4322	14	185	280	4446	1	165
cb8564_c45300/f1p4/3014 isoform=cb8564_c45300;full_length_coverage=1;non_full_length_coverage=4;isoform_length=3014;expected_accuracy=0.996	gnl|BL_ORD_ID|14471 cb8564_c34518/f1p0/3136 isoform=cb8564_c34518;full_length_coverage=1;non_full_length_coverage=0;isoform_length=3136;expected_accuracy=0.991	243	3014	1	246	393	3134	1	238
cb8564_c44698/f1p5/2843 isoform=cb8564_c44698;full_length_coverage=1;non_full_length_coverage=5;isoform_length=2843;expected_accuracy=0.997	gnl|BL_ORD_ID|15994 cb8564_c54019/f1p1/2940 isoform=cb8564_c54019;full_length_coverage=1;non_full_length_coverage=1;isoform_length=2940;expected_accuracy=0.991	1	2516	2511	2834	7	2498	2619	2940
cb18456_c6530/f1p0/1200	gnl|BL_ORD_ID|67487 cb18456_c4043/f1p0/1830	260	1161	3	259	794	1694	8	264
cb5896_c76/f1p0/4056	gnl|BL_ORD_ID|44038 cb4253_c10/f1p0/5750 isoform=cb4253_c10;full_length_coverage=1;non_full_length_coverage=0;isoform_length=5750;expected_accuracy=0.995	336	3654	19	338	2428	5750	1	320
cb7521_c46/f1p0/2168	gnl|BL_ORD_ID|7810 cb7521_c1/f5p2/2475 isoform=cb7521_c1;full_length_coverage=5;non_full_length_coverage=2;isoform_length=2475;expected_accuracy=1.000	1	1630	1626	2043	210	1839	2040	2458
cb8564_c36056/f1p0/2652 isoform=cb8564_c36056;full_length_coverage=1;non_full_length_coverage=0;isoform_length=2652;expected_	gnl|BL_ORD_ID|55873 cb8564_c47701/f1p0/3144	248	2651	1	251	476	2880	82	332

accuracy=0.999									
cb18301_c34/f1p0/1551	gnl|BL_ORD_ID|12295 cb8564_c123425/f1p0/2833 isoform=cb8564_c123425;full_length_coverage=1;non_full_length_coverage=0;isoform_length=2833;expected_accuracy=0.994	1	1075	1072	1468	14	1085	2437	2833
cb8564_c188097/f1p0/2552 isoform=cb8564_c188097;full_length_coverage=1;non_full_length_coverage=0;isoform_length=2552;expected_accuracy=0.996	gnl|BL_ORD_ID|55935 cb8564_c49443/f1p0/2984	1	1945	1940	2543	35	1979	2080	2681
cb6033_c2/f1p0/1294 isoform=cb6033_c2;full_length_coverage=1;non_full_length_coverage=0;isoform_length=1294;expected_accuracy=0.998	gnl|BL_ORD_ID|46393 cb6033_c1/f1p0/2556 isoform=cb6033_c1;full_length_coverage=1;non_full_length_coverage=0;isoform_length=2556;expected_accuracy=0.996	1	1065	1063	1294	968	2038	2323	2553
cb8564_c74699/f1p0/3492	gnl|BL_ORD_ID|57694 cb8564_c78332/f1p0/4781	20	2738	2736	3461	12	2742	3636	4361
cb401_c50/f1p0/2173 isoform=cb401_c50;full_length_coverage=1;non_full_length_coverage=0;isoform_length=2173;expected_accuracy=0.998	gnl|BL_ORD_ID|70875 cb401_c74/f1p1/2481	96	2171	1	101	290	2383	49	149
cb8564_c75977/f1p0/2165	gnl|BL_ORD_ID|17175 cb8564_c90057/f1p0/3709 isoform=cb8564_c90057;full_length_coverage=1;non_full_length_coverage=0;isoform_length=3709;expected_accuracy=0.997	1	1393	1394	2124	60	1451	2981	3709
cb1788_c6/f1p0/3148	gnl|BL_ORD_ID|31766 cb1788_c1/f1p0/3299 isoform=cb1788_c1;full_length_coverage=1;non_full_length_coverage=0;isoform_length=3299;expected_accura	146	3063	1	145	295	3208	1	145

	cy=0.992								
cb466_c12/f1p0/3253 isoform=cb466_c12;full_length_coverage=1;non_full_length_coverage=0;isoform_length=3253;expected_accuracy=0.991	gnl|BL_ORD_ID|72211 cb466_c17/f1p0/4598	1135	3123	1	1133	1870	3865	48	1178
cb1626_c16/f1p0/2872	gnl|BL_ORD_ID|33068 cb1626_c13/f1p0/4596 isoform=cb1626_c13;full_length_coverage=1;non_full_length_coverage=0;isoform_length=4596;expected_accuracy=1.000	1003	2842	1	1003	2692	4530	78	1079
cb1468_c12/f1p1/3132	gnl|BL_ORD_ID|63645 cb1468_c3/f1p0/4312	1	2095	2094	2905	46	2140	3043	3855
cb7126_c3/f1p0/2121 isoform=cb7126_c3;full_length_coverage=1;non_full_length_coverage=0;isoform_length=2121;expected_accuracy=0.998	gnl|BL_ORD_ID|47851 cb7126_c4/f1p0/2520	122	2121	1	125	272	2275	1	134
cb1626_c16/f1p0/2872	gnl|BL_ORD_ID|32074 cb1626_c4/f2p1/4537 isoform=cb1626_c4;full_length_coverage=2;non_full_length_coverage=1;isoform_length=4537;expected_accuracy=0.998	1003	2842	1	1003	2635	4469	55	1069
cb9009_c2/f8p1/1614 isoform=cb9009_c2;full_length_coverage=8;non_full_length_coverage=1;isoform_length=1614;expected_accuracy=0.996	gnl|BL_ORD_ID|20136 cb9009_c6/f1p0/4294 isoform=cb9009_c6;full_length_coverage=1;non_full_length_coverage=0;isoform_length=4294;expected_accuracy=0.992	1	1190	1188	1614	1	1179	3472	3898
cb8564_c4159/f1p2/3687 isoform=cb8564_c4159;full_length_coverage=1;non_full_length_coverage=2;isoform_length=3687;expected_ac	gnl|BL_ORD_ID|17221 cb8564_c945/f5p5/4929 isoform=cb8564_c945;full_length_coverage=5;non_full_length_coverage=5;isoform_length=4929;expected_acc	1	2927	2923	3687	57	2982	4164	4927

curacy=0.999	uracy=1.000								
cb6499_c24/f1p0/1697	gnl|BL_ORD_ID|5990 cb6499_c15/f1p1/1972 isoform=cb6499_c15;full_length_coverage=1;non_full_length_coverage=1;isoform_length=1972;expected_accuracy=0.994	1	1336	1333	1658	8	1342	1581	1906
cb6340_c37/f1p2/2383 isoform=cb6340_c37;full_length_coverage=1;non_full_length_coverage=2;isoform_length=2383;expected_accuracy=0.994	gnl|BL_ORD_ID|47442 cb6340_c10/f1p0/2833	223	2314	1	228	497	2590	98	325
cb8564_c18575/f1p0/1939 isoform=cb8564_c18575;full_length_coverage=1;non_full_length_coverage=0;isoform_length=1939;expected_accuracy=0.990	gnl|BL_ORD_ID|13824 cb8564_c20404/f1p3/3974 isoform=cb8564_c20404;full_length_coverage=1;non_full_length_coverage=3;isoform_length=3974;expected_accuracy=0.999	1	1427	1422	1939	774	2213	3194	3718
cb13025_c13/f1p0/1286	gnl|BL_ORD_ID|27037 cb13025_c3/f2p1/1451 isoform=cb13025_c3;full_length_coverage=2;non_full_length_coverage=1;isoform_length=1451;expected_accuracy=1.000	244	1285	41	243	395	1451	1	203
cb8564_c42409/f1p0/3007	gnl|BL_ORD_ID|55844 cb8564_c44552/f1p0/3216	108	1939	1936	2893	94	1929	2069	3028
cb12038_c22/f5p6/1857 isoform=cb12038_c22;full_length_coverage=5;non_full_length_coverage=6;isoform_length=1857;expected_accuracy=1.000	gnl|BL_ORD_ID|18863 cb8564_c82439/f1p4/3856 isoform=cb8564_c82439;full_length_coverage=1;non_full_length_coverage=4;isoform_length=3856;expected_accuracy=0.997	342	1857	76	344	2343	3855	5	276
cb8564_c40996/f1p0/1966 isoform=cb8564_c40996;full_length_coverage=1;non_full_length_coverage=0;isoform_length=1966;expected_	gnl|BL_ORD_ID|55939 cb8564_c49630/f1p4/3487	1	1700	1700	1955	1230	2913	3243	3493

accuracy=0.991									
cb8564_c110053/f2p0/2311 isoform=cb8564_c110053;full_length_coverage=2;non_full_length_coverage=0;isoform_length=2311;expected_accuracy=0.996	gnl|BL_ORD_ID|9253 cb8564_c121717/f1p0/2419 isoform=cb8564_c121717;full_length_coverage=1;non_full_length_coverage=0;isoform_length=2419;expected_accuracy=0.999	1027	2311	1	1032	1132	2418	1	1028
cb396_c57/f1p0/2029 isoform=cb396_c57;full_length_coverage=1;non_full_length_coverage=0;isoform_length=2029;expected_accuracy=0.995	gnl|BL_ORD_ID|70867 cb396_c51/f1p1/2465	1	1399	1395	2029	12	1411	1672	2307
cb8564_c90747/f1p0/2795	gnl|BL_ORD_ID|9350 cb8564_c11826/f1p0/2916 isoform=cb8564_c11826;full_length_coverage=1;non_full_length_coverage=0;isoform_length=2916;expected_accuracy=0.997	1	1295	1294	2640	4	1296	1543	2916
cb8564_c45242/f1p0/2126 isoform=cb8564_c45242;full_length_coverage=1;non_full_length_coverage=0;isoform_length=2126;expected_accuracy=0.990	gnl|BL_ORD_ID|15226 cb8564_c34123/f2p0/3784 isoform=cb8564_c34123;full_length_coverage=2;non_full_length_coverage=0;isoform_length=3784;expected_accuracy=1.000	320	2126	71	321	1969	3784	1101	1351
cb1224_c13/f1p1/3758	gnl|BL_ORD_ID|24622 cb1224_c6/f1p0/4422 isoform=cb1224_c6;full_length_coverage=1;non_full_length_coverage=0;isoform_length=4422;expected_accuracy=0.997	1	2859	2863	3552	690	3548	3728	4422
cb3907_c5/f1p0/2446 isoform=cb3907_c5;full_length_coverage=1;non_full_length_coverage=0;isoform_length=2446;expected_accuracy=0.994	gnl|BL_ORD_ID|71631 cb3907_c10/f1p0/2898	225	2420	1	225	450	2642	6	234
cb1333_c9/f4p0/1480	gnl|BL_ORD_ID|61260 cb1333_c110/f1p0/5352	153	1473	3	154	3730	504	1	152

isoform=cb1333_c9;full_length_coverage=4;non_full_length_coverage=0;isoform_length=1480;expected_accuracy=1.000							6		
cb8564_c179418/f1p0/2282 isoform=cb8564_c179418;full_length_coverage=1;non_full_length_coverage=0;isoform_length=2282;expected_accuracy=1.000	gnl|BL_ORD_ID|55013 cb8564_c4374/f2p4/2560	631	2282	90	629	884	2535	137	671
cb18456_c1748/f1p0/1180	gnl|BL_ORD_ID|12316 cb8564_c129924/f1p10/2394 isoform=cb8564_c129924;full_length_coverage=1;non_full_length_coverage=10;isoform_length=2394;expected_accuracy=1.000	243	1168	4	248	1469	2394	1087	1331
cb15516_c3/f1p0/1355 isoform=cb15516_c3;full_length_coverage=1;non_full_length_coverage=0;isoform_length=1355;expected_accuracy=0.992	gnl|BL_ORD_ID|47466 cb6371_c4/f1p0/2354	127	1308	1	126	1178	2370	27	152
cb3119_c8/f1p2/3090 isoform=cb3119_c8;full_length_coverage=1;non_full_length_coverage=2;isoform_length=3090;expected_accuracy=1.000	gnl|BL_ORD_ID|69147 cb3119_c7/f1p0/3565	1	1707	1706	3089	64	1788	2033	3416
cb8564_c953/f2p0/2607 isoform=cb8564_c953;full_length_coverage=2;non_full_length_coverage=0;isoform_length=2607;expected_accuracy=0.997	gnl|BL_ORD_ID|53635 cb8564_c137444/f2p1/2869	154	2607	1	154	314	2768	35	184
cb3492_c2/f1p0/3445 isoform=cb3492_c2;full_length_coverage=1;non_full_length_coverage=0;isoform_length=3445;expected_accur	gnl|BL_ORD_ID|70408 cb3492_c0/f1p0/3604	1	3103	3104	3444	32	3143	3264	3605

<table>
<tr><td>acy=0.995</td><td></td><td></td><td></td><td></td><td></td><td></td><td></td><td></td><td></td></tr>
<tr><td>cb396_c48/f1p0/2172</td><td>gnl|BL_ORD_ID|39231 cb396_c67/f28p0/2542 isoform=cb396_c67;full_length_coverage=28;non_full_length_coverage=0;isoform_length=2542;expected_accuracy=1.000</td><td>238</td><td>2104</td><td>3</td><td>238</td><td>554</td><td>2420</td><td>70</td><td>305</td></tr>
<tr><td>cb8564_c4546/f2p0/2151</td><td>gnl|BL_ORD_ID|55032 cb8564_c4537/f1p0/2998</td><td>1</td><td>1329</td><td>1327</td><td>2152</td><td>1</td><td>1332</td><td>2198</td><td>3019</td></tr>
<tr><td>cb8564_c69012/f2p0/2088 isoform=cb8564_c69012;full_length_coverage=2;non_full_length_coverage=0;isoform_length=2088;expected_accuracy=0.995</td><td>gnl|BL_ORD_ID|16545 cb8564_c71990/f1p0/2407 isoform=cb8564_c71990;full_length_coverage=1;non_full_length_coverage=0;isoform_length=2407;expected_accuracy=0.998</td><td>569</td><td>2088</td><td>74</td><td>569</td><td>889</td><td>2406</td><td>1</td><td>496</td></tr>
<tr><td>cb6063_c4/f2p0/2706 isoform=cb6063_c4;full_length_coverage=2;non_full_length_coverage=0;isoform_length=2706;expected_accuracy=0.999</td><td>gnl|BL_ORD_ID|45238 cb6063_c0/f15p2/2941 isoform=cb6063_c0;full_length_coverage=15;non_full_length_coverage=2;isoform_length=2941;expected_accuracy=1.000</td><td>241</td><td>2691</td><td>1</td><td>239</td><td>485</td><td>2941</td><td>1</td><td>238</td></tr>
<tr><td>cb8564_c20798/f1p1/2960</td><td>gnl|BL_ORD_ID|14860 cb8564_c24783/f1p1/3102 isoform=cb8564_c24783;full_length_coverage=1;non_full_length_coverage=1;isoform_length=3102;expected_accuracy=0.992</td><td>1314</td><td>2925</td><td>1</td><td>1319</td><td>1509</td><td>3102</td><td>16</td><td>1365</td></tr>
<tr><td>cb8564_c49562/f1p0/2838 isoform=cb8564_c49562;full_length_coverage=1;non_full_length_coverage=0;isoform_length=2838;expected_accuracy=0.995</td><td>gnl|BL_ORD_ID|55182 cb8564_c34825/f1p2/3200</td><td>15</td><td>2331</td><td>2327</td><td>2838</td><td>6</td><td>2350</td><td>2682</td><td>3194</td></tr>
<tr><td>cb8564_c88311/f1p1/2475 isoform=cb8564_c88311;full_length_coverage=1;non_full_length_coverage=1;isoform_length=2475;expected_</td><td>gnl|BL_ORD_ID|57409 cb8564_c92262/f1p0/3503</td><td>1</td><td>1814</td><td>1812</td><td>2473</td><td>128</td><td>1949</td><td>2640</td><td>3302</td></tr>
</table>

accuracy=0.993									
cb8564_c35645/f1p0/2804	gnl|BL_ORD_ID|13439 cb8564_c22195/f4p0/2939 isoform=cb8564_c22195;full_length_coverage=4;non_full_length_coverage=0;isoform_length=2939;expected_accuracy=0.996	149	2726	1	151	330	2907	1	151
cb4142_c2/f1p0/1937 isoform=cb4142_c2;full_length_coverage=1;non_full_length_coverage=0;isoform_length=1937;expected_accuracy=1.000	gnl|BL_ORD_ID|40544 cb4142_c3/f1p0/2089 isoform=cb4142_c3;full_length_coverage=1;non_full_length_coverage=0;isoform_length=2089;expected_accuracy=0.991	1	1594	1592	1927	1	1594	1706	2041
cb1972_c0/f2p0/3980 isoform=cb1972_c0;full_length_coverage=2;non_full_length_coverage=0;isoform_length=3980;expected_accuracy=1.000	gnl|BL_ORD_ID|35212 cb1972_c8/f1p1/3986 isoform=cb1972_c8;full_length_coverage=1;non_full_length_coverage=1;isoform_length=3986;expected_accuracy=0.998	1346	3876	11	1347	1456	3986	1	1334
cb2707_c17/f1p1/3162 isoform=cb2707_c17;full_length_coverage=1;non_full_length_coverage=1;isoform_length=3162;expected_accuracy=0.995	gnl|BL_ORD_ID|38446 cb2707_c18/f1p1/3468 isoform=cb2707_c18;full_length_coverage=1;non_full_length_coverage=1;isoform_length=3468;expected_accuracy=0.997	11	2572	2571	3030	312	2877	3009	3468
cb8564_c72680/f1p0/2685	gnl|BL_ORD_ID|56759 cb8564_c70577/f1p0/3111	15	1735	1730	2636	1	1722	1901	2807
cb8564_c12637/f1p2/3697	gnl|BL_ORD_ID|9961 cb8564_c12013/f1p0/4420 isoform=cb8564_c12013;full_length_coverage=1;non_full_length_coverage=0;isoform_length=4420;expected_accuracy=0.993	1	2998	2994	3575	86	3077	3654	4233
cb7497_c22/f1p0/2606	gnl|BL_ORD_ID|6379 cb7497_c10/f1p1/3177 isoform=cb7497_c10;full_length_coverage=1;non_full_length_coverage=1;isoform_length=3177;expected_accur	998	2581	1	1001	1572	3156	1	1004

	acy=0.995								
cb5068_c5/f1p1/2699 isoform=cb5068_c5;full_length_coverage=1;non_full_length_coverage=1;isoform_length=2699;expected_accuracy=0.995	gnl|BL_ORD_ID|73235 cb5068_c2/f1p0/3358	2	2431	2426	2699	10	2440	2586	2859
cb3082_c6/f1p1/2400 isoform=cb3082_c6;full_length_coverage=1;non_full_length_coverage=1;isoform_length=2400;expected_accuracy=0.995	gnl|BL_ORD_ID|69100 cb3082_c8/f1p0/2797	110	2367	1	110	437	2692	3	108
cb8564_c41756/f1p0/2738 isoform=cb8564_c41756;full_length_coverage=1;non_full_length_coverage=0;isoform_length=2738;expected_accuracy=0.996	gnl|BL_ORD_ID|14263 cb8564_c42654/f1p1/2906 isoform=cb8564_c42654;full_length_coverage=1;non_full_length_coverage=1;isoform_length=2906;expected_accuracy=0.990	1	2173	2170	2714	1	2155	2278	2824
cb595_c0/f3p1/4752 isoform=cb595_c0;full_length_coverage=3;non_full_length_coverage=1;isoform_length=4752;expected_accuracy=0.999	gnl|BL_ORD_ID|72286 cb595_c23/f1p1/5099	3	4402	4399	4665	1	4412	4576	4842
cb12446_c3/f1p0/1412 isoform=cb12446_c3;full_length_coverage=1;non_full_length_coverage=0;isoform_length=1412;expected_accuracy=1.000	gnl|BL_ORD_ID|11282 cb8564_c150160/f1p3/2042 isoform=cb8564_c150160;full_length_coverage=1;non_full_length_coverage=3;isoform_length=2042;expected_accuracy=0.991	109	1412	1	111	712	2013	117	227
cb3557_c22/f1p0/2990	gnl|BL_ORD_ID|39538 cb3557_c9/f1p0/3261 isoform=cb3557_c9;full_length_coverage=1;non_full_length_coverage=0;isoform_length=3261;expected_accuracy=0.995	16	2070	2068	2722	1	2056	2608	3261
cb8564_c72849/f1p0/2326	gnl|BL_ORD_ID|12630 cb8564_c123147/f1p0/2657	1	1520	1520	2332	37	155	1757	257

	isoform=cb8564_c123147;full_length_coverage=1;non_full_length_coverage=0;isoform_length=2657;expected_accuracy=0.997						4		6
cb3771_c11/f1p0/3160 isoform=cb3771_c11;full_length_coverage=1;non_full_length_coverage=0;isoform_length=3160;expected_accuracy=0.992	gnl|BL_ORD_ID|40598 cb3771_c5/f1p0/3508 isoform=cb3771_c5;full_length_coverage=1;non_full_length_coverage=0;isoform_length=3508;expected_accuracy=0.993	166	3084	1	167	590	3508	43	213
cb8564_c39739/f1p1/2219 isoform=cb8564_c39739;full_length_coverage=1;non_full_length_coverage=1;isoform_length=2219;expected_accuracy=0.993	gnl|BL_ORD_ID|16312 cb8564_c53551/f3p3/2373 isoform=cb8564_c53551;full_length_coverage=3;non_full_length_coverage=3;isoform_length=2373;expected_accuracy=1.000	10	1840	1840	2195	7	1850	1969	2325
cb8564_c35607/f1p0/2587 isoform=cb8564_c35607;full_length_coverage=1;non_full_length_coverage=0;isoform_length=2587;expected_accuracy=0.993	gnl|BL_ORD_ID|14819 cb8564_c40333/f1p2/4446 isoform=cb8564_c40333;full_length_coverage=1;non_full_length_coverage=2;isoform_length=4446;expected_accuracy=0.998	1	1685	1685	2581	1619	3300	3549	4446
cb8564_c118488/f1p0/2066 isoform=cb8564_c118488;full_length_coverage=1;non_full_length_coverage=0;isoform_length=2066;expected_accuracy=0.991	gnl|BL_ORD_ID|53375 cb8564_c123599/f1p0/2339	198	2055	11	200	435	2300	1	181
cb4564_c26/f74p3/1694 isoform=cb4564_c26;full_length_coverage=74;non_full_length_coverage=3;isoform_length=1694;expected_accuracy=0.999	gnl|BL_ORD_ID|72689 cb4564_c15/f1p0/1760	447	1635	6	448	588	1771	1	446
cb5725_c17/f1p0/2282 isoform=cb5725_c17;full_length_coverage=1;non_full_length_coverage=0;isoform_length=2282;expected_accu	gnl|BL_ORD_ID|74341 cb5725_c19/f1p0/3053	1	1367	1364	2202	55	1418	2201	3040

racy=0.991									
cb8564_c50697/f1p3/2172 isoform=cb8564_c50697;full_length_coverage=1;non_full_length_coverage=3;isoform_length=2172;expected_accuracy=0.993	gnl|BL_ORD_ID|11343 cb8564_c148508/f1p3/2825 isoform=cb8564_c148508;full_length_coverage=1;non_full_length_coverage=3;isoform_length=2825;expected_accuracy=0.994	106	2171	1	108	771	2825	47	153
cb762_c11/f1p0/4026	gnl|BL_ORD_ID|8132 cb762_c4/f1p0/4517 isoform=cb762_c4;full_length_coverage=1;non_full_length_coverage=0;isoform_length=4517;expected_accuracy=0.996	1	2803	2801	3954	88	2890	3356	4508
cb8564_c81975/f1p2/2386 isoform=cb8564_c81975;full_length_coverage=1;non_full_length_coverage=2;isoform_length=2386;expected_accuracy=0.996	gnl|BL_ORD_ID|17162 cb8564_c87465/f2p9/3179 isoform=cb8564_c87465;full_length_coverage=2;non_full_length_coverage=9;isoform_length=3179;expected_accuracy=1.000	1	1315	1310	2386	625	1939	2083	3160
cb7872_c9/f1p0/2221	gnl|BL_ORD_ID|50770 cb7872_c10/f1p0/2367	199	2041	11	197	353	2196	25	211
cb7296_c10/f1p1/1975 isoform=cb7296_c10;full_length_coverage=1;non_full_length_coverage=1;isoform_length=1975;expected_accuracy=0.999	gnl|BL_ORD_ID|6916 cb7296_c3/f1p1/2653 isoform=cb7296_c3;full_length_coverage=1;non_full_length_coverage=1;isoform_length=2653;expected_accuracy=0.998	1	1658	1657	1963	62	1720	2347	2653
cb8564_c38464/f1p0/2918	gnl|BL_ORD_ID|14058 cb8564_c23936/f1p0/3016 isoform=cb8564_c23936;full_length_coverage=1;non_full_length_coverage=0;isoform_length=3016;expected_accuracy=0.995	1	1713	1711	2584	4	1716	2143	3016
cb8564_c36635/f1p0/4030 isoform=cb8564_c36635;full_length_coverage=1;non_full_length_coverage=0;isoform_length=4030;expected_	gnl|BL_ORD_ID|55955 cb8564_c50128/f1p0/4343	184	3927	1	189	334	4077	15	204

accuracy=0.998									
cb6519_c1/f1p0/2399 isoform=cb6519_c1;full_length_coverage=1;non_full_length_coverage=0;isoform_length=2399;expected_accuracy=0.996	gnl|BL_ORD_ID|49795 cb6519_c0/f1p0/2728	305	2385	5	307	423	2532	6	309
cb1658_c23/f1p0/2662 isoform=cb1658_c23;full_length_coverage=1;non_full_length_coverage=0;isoform_length=2662;expected_accuracy=0.996	gnl|BL_ORD_ID|63834 cb1658_c42/f1p0/3937	1132	2620	1	1135	2400	3893	18	1161
cb499_c21/f1p0/3092	gnl|BL_ORD_ID|72233 cb499_c34/f1p0/3463	1420	3084	1	1420	1763	3427	29	1448
cb8564_c49562/f1p0/2838 isoform=cb8564_c49562;full_length_coverage=1;non_full_length_coverage=0;isoform_length=2838;expected_accuracy=0.995	gnl|BL_ORD_ID|55910 cb8564_c48645/f1p1/3287	16	2331	2327	2776	17	2329	2661	3113
cb12003_c138/f1p0/1068 isoform=cb12003_c138;full_length_coverage=1;non_full_length_coverage=0;isoform_length=1068;expected_accuracy=1.000	gnl|BL_ORD_ID|60477 cb12003_c35/f1p0/1418	420	1068	5	421	729	1378	1	417
cb500_c38/f1p0/2822 isoform=cb500_c38;full_length_coverage=1;non_full_length_coverage=0;isoform_length=2822;expected_accuracy=0.999	gnl|BL_ORD_ID|72248 cb500_c53/f1p0/3672	13	2396	2391	2822	114	2498	2960	3391
cb13515_c9/f1p0/1348	gnl|BL_ORD_ID|62505 cb13515_c5/f1p0/1553	1	1117	1114	1291	53	1168	1277	1454
cb8564_c117803/f1p0/3246	gnl|BL_ORD_ID|9642 cb8564_c117476/f1p1/3616	1	2822	2823	3236	99	293	3205	361

isoform=cb8564_c117803;full_length_coverage=1;non_full_length_coverage=0;isoform_length=3246;expected_accuracy=0.995	isoform=cb8564_c117476;full_length_coverage=1;non_full_length_coverage=1;isoform_length=3616;expected_accuracy=0.997						7		6
cb4674_c27/f1p1/2865 isoform=cb4674_c27;full_length_coverage=1;non_full_length_coverage=1;isoform_length=2865;expected_accuracy=0.993	gnl|BL_ORD_ID|72841 cb4674_c25/f1p0/3544	1	2046	2046	2865	5	2057	2656	3478
cb8564_c46939/f1p1/2926	gnl|BL_ORD_ID|55472 cb8564_c42531/f1p0/3996	125	2134	2130	2870	816	2833	2938	3679
cb4128_c10/f3p1/2897 isoform=cb4128_c10;full_length_coverage=3;non_full_length_coverage=1;isoform_length=2897;expected_accuracy=0.998	gnl|BL_ORD_ID|71809 cb4128_c15/f1p0/3851	1	2152	2151	2777	1	2154	3114	3740
cb3451_c8/f1p0/1800 isoform=cb3451_c8;full_length_coverage=1;non_full_length_coverage=0;isoform_length=1800;expected_accuracy=0.997	gnl|BL_ORD_ID|39654 cb3451_c0/f1p0/2080 isoform=cb3451_c0;full_length_coverage=1;non_full_length_coverage=0;isoform_length=2080;expected_accuracy=0.992	103	1776	1	106	410	2080	143	248
cb8564_c36689/f1p1/2368 isoform=cb8564_c36689;full_length_coverage=1;non_full_length_coverage=1;isoform_length=2368;expected_accuracy=0.991	gnl|BL_ORD_ID|56727 cb8564_c66920/f3p3/2894	1	1709	1706	2365	1	1703	2202	2863
cb416_c58/f1p0/2505 isoform=cb416_c58;full_length_coverage=1;non_full_length_coverage=0;isoform_length=2505;expected_accuracy=0.992	gnl|BL_ORD_ID|42631 cb416_c29/f1p0/2757 isoform=cb416_c29;full_length_coverage=1;non_full_length_coverage=0;isoform_length=2757;expected_accuracy=0.993	286	2505	4	287	550	2747	121	400
cb8564_c109958/f2p0/2267	gnl|BL_ORD_ID|53549 cb8564_c128508/f1p0/4775	1	1291	1289	2266	1	129	3791	476

isoform=cb8564_c109958;full_length_coverage=2;non_full_length_coverage=0;isoform_length=2267;expected_accuracy=1.000							1		8
cb1926_c7/f1p0/3660 isoform=cb1926_c7;full_length_coverage=1;non_full_length_coverage=0;isoform_length=3660;expected_accuracy=0.994	gnl|BL_ORD_ID|34504 cb1926_c1/f2p1/3871 isoform=cb1926_c1;full_length_coverage=2;non_full_length_coverage=1;isoform_length=3871;expected_accuracy=1.000	18	3453	3452	3660	51	3486	3626	3833
cb8564_c75527/f1p1/1992 isoform=cb8564_c75527;full_length_coverage=1;non_full_length_coverage=1;isoform_length=1992;expected_accuracy=0.993	gnl|BL_ORD_ID|13172 cb8564_c159486/f2p1/2104 isoform=cb8564_c159486;full_length_coverage=2;non_full_length_coverage=1;isoform_length=2104;expected_accuracy=1.000	1	1063	1059	1954	1	1063	1209	2104
cb8564_c86643/f1p0/2138	gnl|BL_ORD_ID|57913 cb8564_c83955/f1p0/2930	1	1776	1776	2024	20	1793	2505	2753
cb8564_c79401/f1p0/4816	gnl|BL_ORD_ID|36231 cb18812_c11/f1p2/5013 isoform=cb18812_c11;full_length_coverage=1;non_full_length_coverage=2;isoform_length=5013;expected_accuracy=0.997	1072	4317	1	1068	1763	5009	809	1876
cb17354_c1/f1p0/1440	gnl|BL_ORD_ID|65774 cb17354_c3/f1p0/1686	1	1059	1056	1321	45	1103	1408	1673
cb4074_c19/f3p1/2467 isoform=cb4074_c19;full_length_coverage=3;non_full_length_coverage=1;isoform_length=2467;expected_accuracy=1.000	gnl|BL_ORD_ID|39766 cb4074_c7/f1p1/2826 isoform=cb4074_c7;full_length_coverage=1;non_full_length_coverage=1;isoform_length=2826;expected_accuracy=0.994	1	1361	1357	2408	2	1335	1781	2826
cb2430_c24/f16p1/2568 isoform=cb2430_c24;full_length_coverage=16;non_full_length_coverage=1;isoform_length=2568;expected_acc	gnl|BL_ORD_ID|36773 cb2430_c19/f1p0/2700 isoform=cb2430_c19;full_length_coverage=1;non_full_length_coverage=0;isoform_length=2700;expected_accur	2	2130	2125	2564	8	2125	2263	2700

uracy=1.000	acy=0.993								
cb1955_c12/f1p1/2862 isoform=cb1955_c12;full_length_coverage=1;non_full_length_coverage=1;isoform_length=2862;expected_accuracy=1.000	gnl|BL_ORD_ID|66534 cb1955_c27/f1p0/3020	107	2021	2019	2861	1	1915	2196	3044
cb15911_c1/f1p0/1420	gnl|BL_ORD_ID|30166 cb15911_c0/f1p0/1558 isoform=cb15911_c0;full_length_coverage=1;non_full_length_coverage=0;isoform_length=1558;expected_accuracy=0.990	292	1291	32	291	559	1558	1	259
cb2533_c26/f1p1/3568 isoform=cb2533_c26;full_length_coverage=1;non_full_length_coverage=1;isoform_length=3568;expected_accuracy=0.997	gnl|BL_ORD_ID|38533 cb2533_c16/f1p0/4039 isoform=cb2533_c16;full_length_coverage=1;non_full_length_coverage=0;isoform_length=4039;expected_accuracy=0.994	133	2983	2982	3565	1	2836	3451	4031
cb7804_c1/f1p0/1940	gnl|BL_ORD_ID|8602 cb7804_c0/f1p0/2529 isoform=cb7804_c0;full_length_coverage=1;non_full_length_coverage=0;isoform_length=2529;expected_accuracy=0.998	1	1128	1126	1952	84	1211	1704	2529
cb12931_c15/f1p0/1493 isoform=cb12931_c15;full_length_coverage=1;non_full_length_coverage=0;isoform_length=1493;expected_accuracy=1.000	gnl|BL_ORD_ID|55391 cb8564_c40278/f3p2/3083	1	1106	1104	1493	1	1102	2482	2871
cb4664_c15/f1p0/2526	gnl|BL_ORD_ID|42518 cb4664_c0/f6p1/2463 isoform=cb4664_c0;full_length_coverage=6;non_full_length_coverage=1;isoform_length=2463;expected_accuracy=0.996	229	2260	7	232	437	2463	3	228
cb8564_c953/f2p0/2607	gnl|BL_ORD_ID|14419 cb8564_c3905/f3p1/2854	154	2603	1	154	380	282	96	248

isoform=cb8564_c953;full_length_coverage=2;non_full_length_coverage=0;isoform_length=2607;expected_accuracy=0.997	isoform=cb8564_c3905;full_length_coverage=3;non_full_length_coverage=1;isoform_length=2854;expected_accuracy=0.999						9		
cb4370_c6/f1p0/2015 isoform=cb4370_c6;full_length_coverage=1;non_full_length_coverage=0;isoform_length=2015;expected_accuracy=0.993	gnl|BL_ORD_ID|44190 cb4370_c7/f1p0/2660 isoform=cb4370_c7;full_length_coverage=1;non_full_length_coverage=0;isoform_length=2660;expected_accuracy=0.996	1	1310	1310	1981	135	1444	1987	2660
cb48_c3/f1p2/4932 isoform=cb48_c3;full_length_coverage=1;non_full_length_coverage=2;isoform_length=4932;expected_accuracy=0.996	gnl|BL_ORD_ID|42658 cb48_c8/f1p2/5695 isoform=cb48_c8;full_length_coverage=1;non_full_length_coverage=2;isoform_length=5695;expected_accuracy=0.998	1	4362	4362	4932	1	4364	5032	5600
cb7432_c6/f2p0/2148 isoform=cb7432_c6;full_length_coverage=2;non_full_length_coverage=0;isoform_length=2148;expected_accuracy=1.000	gnl|BL_ORD_ID|6353 cb7432_c13/f1p0/2615 isoform=cb7432_c13;full_length_coverage=1;non_full_length_coverage=0;isoform_length=2615;expected_accuracy=0.995	154	2148	1	158	622	2615	41	196
cb8564_c122994/f1p1/1940 isoform=cb8564_c122994;full_length_coverage=1;non_full_length_coverage=1;isoform_length=1940;expected_accuracy=1.000	gnl|BL_ORD_ID|54581 cb8564_c127099/f1p0/2249	13	1748	1748	1940	104	1839	1981	2173
cb8564_c43779/f1p0/3021 isoform=cb8564_c43779;full_length_coverage=1;non_full_length_coverage=0;isoform_length=3021;expected_accuracy=0.993	gnl|BL_ORD_ID|16622 cb8564_c50151/f1p0/3198 isoform=cb8564_c50151;full_length_coverage=1;non_full_length_coverage=0;isoform_length=3198;expected_accuracy=0.995	1	2344	2345	3019	6	2369	2525	3198
cb8564_c25160/f1p0/3126 isoform=cb8564_c25160;full_length_coverage=1;non_full_length_coverage=0;isoform_length=3126;expected_	gnl|BL_ORD_ID|54223 cb8564_c20818/f1p0/3419	1	2710	2706	2995	2	2712	3070	3359

accuracy=0.996									
cb8564_c24921/f3p0/3854 isoform=cb8564_c24921;full_length_coverage=3;non_full_length_coverage=0;isoform_length=3854;expected_accuracy=0.996	gnl|BL_ORD_ID|52730 cb8564_c12303/f1p0/3960	1	2016	2016	3852	4	2020	2135	3972
cb8564_c4544/f1p1/2154	gnl|BL_ORD_ID|14176 cb8564_c4538/f2p0/2766 isoform=cb8564_c4538;full_length_coverage=2;non_full_length_coverage=0;isoform_length=2766;expected_accuracy=0.994	1	1433	1431	2073	1	1430	2048	2690
cb8564_c90610/f1p0/2901	gnl|BL_ORD_ID|12722 cb8564_c164043/f3p3/2848 isoform=cb8564_c164043;full_length_coverage=3;non_full_length_coverage=3;isoform_length=2848;expected_accuracy=1.000	1087	2722	1	1087	1169	2803	210	1296
cb8564_c180116/f1p0/2944 isoform=cb8564_c180116;full_length_coverage=1;non_full_length_coverage=0;isoform_length=2944;expected_accuracy=0.996	gnl|BL_ORD_ID|14539 cb8564_c46562/f2p3/3196 isoform=cb8564_c46562;full_length_coverage=2;non_full_length_coverage=3;isoform_length=3196;expected_accuracy=0.995	1	2522	2520	2944	4	2543	2772	3195
cb4269_c10/f1p0/1931 isoform=cb4269_c10;full_length_coverage=1;non_full_length_coverage=0;isoform_length=1931;expected_accuracy=0.997	gnl|BL_ORD_ID|72418 cb4269_c7/f1p0/2973	129	1931	1	130	1121	2922	2	122
cb500_c16/f1p0/3627	gnl|BL_ORD_ID|41882 cb500_c76/f1p0/3763 isoform=cb500_c76;full_length_coverage=1;non_full_length_coverage=0;isoform_length=3763;expected_accuracy=0.997	1	1882	1880	3554	1	1884	2090	3763
cb8564_c33702/f1p6/2850	gnl|BL_ORD_ID|56633 cb8564_c49796/f2p5/3077	580	2847	6	575	740	301	3	555

isoform=cb8564_c33702;full_length_coverage=1;non_full_length_coverage=6;isoform_length=2850;expected_accuracy=0.993							2		
cb16802_c3/f1p0/1096 isoform=cb16802_c3;full_length_coverage=1;non_full_length_coverage=0;isoform_length=1096;expected_accuracy=0.994	gnl|BL_ORD_ID|33032 cb16802_c8/f1p1/1398 isoform=cb16802_c8;full_length_coverage=1;non_full_length_coverage=1;isoform_length=1398;expected_accuracy=0.995	265	1096	7	270	489	1331	4	265
cb2825_c1/f4p1/2045 isoform=cb2825_c1;full_length_coverage=4;non_full_length_coverage=1;isoform_length=2045;expected_accuracy=1.000	gnl|BL_ORD_ID|70008 cb2825_c6/f1p1/2358	138	2045	1	135	445	2362	24	156
cb8564_c35935/f1p0/4149 isoform=cb8564_c35935;full_length_coverage=1;non_full_length_coverage=0;isoform_length=4149;expected_accuracy=0.993	gnl|BL_ORD_ID|55207 cb8564_c35399/f1p0/4553	291	4149	10	295	396	4275	11	291
cb8449_c4/f1p1/2010 isoform=cb8449_c4;full_length_coverage=1;non_full_length_coverage=1;isoform_length=2010;expected_accuracy=0.998	gnl|BL_ORD_ID|9574 cb8449_c3/f1p1/2342 isoform=cb8449_c3;full_length_coverage=1;non_full_length_coverage=1;isoform_length=2342;expected_accuracy=0.997	1	1830	1827	2010	1	1843	1962	2144
cb6742_c12/f1p0/2398	gnl|BL_ORD_ID|5502 cb6742_c9/f1p0/2591 isoform=cb6742_c9;full_length_coverage=1;non_full_length_coverage=0;isoform_length=2591;expected_accuracy=0.998	1	1343	1342	2304	156	1500	1614	2581
cb4042_c14/f1p0/3138 isoform=cb4042_c14;full_length_coverage=1;non_full_length_coverage=0;isoform_length=3138;expected_accu	gnl|BL_ORD_ID|71744 cb4042_c8/f1p0/3354	1	2785	2786	3137	13	2798	2916	3268

racy=0.993									
cb8564_c148448/f2p13/3821 isoform=cb8564_c148448;full_length_coverage=2;non_full_length_coverage=13;isoform_length=3821;expected_accuracy=0.995	gnl|BL_ORD_ID|16742 cb8564_c628/f3p1/4943 isoform=cb8564_c628;full_length_coverage=3;non_full_length_coverage=1;isoform_length=4943;expected_accuracy=1.000	17	1937	1935	3787	1	1916	3085	4937
cb8564_c10484/f1p0/2516	gnl|BL_ORD_ID|9857 cb8564_c10041/f6p0/3368 isoform=cb8564_c10041;full_length_coverage=6;non_full_length_coverage=0;isoform_length=3368;expected_accuracy=0.998	2	2227	2227	2464	3	2243	3131	3368
cb6318_c23/f1p1/2399 isoform=cb6318_c23;full_length_coverage=1;non_full_length_coverage=1;isoform_length=2399;expected_accuracy=0.991	gnl|BL_ORD_ID|4834 cb6318_c36/f11p0/2697 isoform=cb6318_c36;full_length_coverage=11;non_full_length_coverage=0;isoform_length=2697;expected_accuracy=1.000	1	1848	1844	2399	55	1892	2037	2592
cb416_c19/f1p0/2939	gnl|BL_ORD_ID|72150 cb416_c40/f1p0/3116	105	2873	10	109	224	2970	24	123
cb8564_c178226/f1p0/2728 isoform=cb8564_c178226;full_length_coverage=1;non_full_length_coverage=0;isoform_length=2728;expected_accuracy=0.998	gnl|BL_ORD_ID|55371 cb8564_c39756/f1p1/3004	1284	2728	1	1288	1394	2852	1	1290
cb16508_c2/f1p0/1556	gnl|BL_ORD_ID|65309 cb16508_c0/f1p0/1682	248	1552	34	247	358	1662	4	209
cb8564_c91425/f1p1/3198	gnl|BL_ORD_ID|15431 cb8564_c43034/f1p2/3197 isoform=cb8564_c43034;full_length_coverage=1;non_full_length_coverage=2;isoform_length=3197;expected_accuracy=0.996	1	2525	2522	3025	4	2522	2663	3165
cb5631_c16/f1p0/2621	gnl|BL_ORD_ID|74185 cb5631_c19/f1p0/3377	15	1949	1945	2621	1	193	2234	291

isoform=cb5631_c16;full_length_coverage=1;non_full_length_coverage=0;isoform_length=2621;expected_accuracy=0.991							3		5
cb322_c9/f2p0/3136 isoform=cb322_c9;full_length_coverage=2;non_full_length_coverage=0;isoform_length=3136;expected_accuracy=1.000	gnl|BL_ORD_ID|69560 cb322_c25/f1p0/3845	1	2718	2718	3135	18	2731	3220	3636
cb187_c5/f1p0/3727 isoform=cb187_c5;full_length_coverage=1;non_full_length_coverage=0;isoform_length=3727;expected_accuracy=0.996	gnl|BL_ORD_ID|68081 cb187_c18/f1p0/4376	152	3727	1	151	737	4312	20	174
cb5125_c36/f1p0/1789 isoform=cb5125_c36;full_length_coverage=1;non_full_length_coverage=0;isoform_length=1789;expected_accuracy=0.998	gnl|BL_ORD_ID|43457 cb5125_c11/f2p1/2545 isoform=cb5125_c11;full_length_coverage=2;non_full_length_coverage=1;isoform_length=2545;expected_accuracy=1.000	1	1163	1158	1789	53	1215	1865	2497
cb8564_c118011/f1p0/1965	gnl|BL_ORD_ID|52770 cb8564_c13377/f1p0/4350	1	1321	1321	1964	1986	3319	3499	4139
cb2208_c33/f1p0/3174	gnl|BL_ORD_ID|38174 cb2208_c2/f4p0/3275 isoform=cb2208_c2;full_length_coverage=4;non_full_length_coverage=0;isoform_length=3275;expected_accuracy=0.999	1	2195	2193	2861	60	2251	2485	3153
cb8564_c180776/f1p0/2895 isoform=cb8564_c180776;full_length_coverage=1;non_full_length_coverage=0;isoform_length=2895;expected_accuracy=0.995	gnl|BL_ORD_ID|18852 cb8564_c84488/f1p0/3408 isoform=cb8564_c84488;full_length_coverage=1;non_full_length_coverage=0;isoform_length=3408;expected_accuracy=0.996	281	2895	1	280	791	3407	1	281
cb166_c11/f1p0/4188	gnl|BL_ORD_ID|32008 cb166_c21/f4p1/4388	1392	4118	1	1394	1662	438	81	146

	isoform=cb166_c21;full_length_coverage=4;non_full_length_coverage=1;isoform_length=4388;expected_accuracy=1.000						8		9
cb313_c3/f2p0/2434 isoform=cb313_c3;full_length_coverage=2;non_full_length_coverage=0;isoform_length=2434;expected_accuracy=0.995	gnl|BL_ORD_ID|36627 cb313_c24/f1p0/3081 isoform=cb313_c24;full_length_coverage=1;non_full_length_coverage=0;isoform_length=3081;expected_accuracy=0.995	1	2112	2112	2373	146	225 5	2820	308 1
cb8564_c4645/f1p1/2620 isoform=cb8564_c4645;full_length_coverage=1;non_full_length_coverage=1;isoform_length=2620;expected_accuracy=0.994	gnl|BL_ORD_ID|12102 cb8564_c136340/f3p2/2729 isoform=cb8564_c136340;full_length_coverage=3;non_full_length_coverage=2;isoform_length=2729;expected_accuracy=0.998	1	1828	1829	2528	66	190 5	2029	272 9
cb227_c12/f1p1/2303 isoform=cb227_c12;full_length_coverage=1;non_full_length_coverage=1;isoform_length=2303;expected_accuracy=0.990	gnl|BL_ORD_ID|37093 cb227_c5/f1p0/3888 isoform=cb227_c5;full_length_coverage=1;non_full_length_coverage=0;isoform_length=3888;expected_accuracy=0.998	1	2073	2073	2303	428	252 3	2712	294 8
cb3290_c199/f1p0/1897 isoform=cb3290_c199;full_length_coverage=1;non_full_length_coverage=0;isoform_length=1897;expected_accuracy=0.993	gnl|BL_ORD_ID|40424 cb3290_c67/f1p0/2784 isoform=cb3290_c67;full_length_coverage=1;non_full_length_coverage=0;isoform_length=2784;expected_accuracy=0.999	255	1896	4	258	1145	278 4	37	293
cb8564_c23232/f1p3/2540	gnl|BL_ORD_ID|55416 cb8564_c41107/f1p0/2832	16	2172	2167	2518	6	216 3	2265	261 5
cb8564_c129801/f1p0/2269 isoform=cb8564_c129801;full_length_coverage=1;non_full_length_coverage=0;isoform_length=2269;expected_accuracy=0.993	gnl|BL_ORD_ID|54524 cb8564_c125248/f1p0/3365	196	2197	1	197	1114	310 6	1	186
cb8564_c81944/f1p1/3166	gnl|BL_ORD_ID|56083 cb8564_c54532/f1p0/3806	2	2661	2666	3166	2	265	2794	329

isoform=cb8564_c81944;full_length_coverage=1;non_full_length_coverage=1;isoform_length=3166;expected_accuracy=0.995							3		8
cb1658_c18/f1p0/2729 isoform=cb1658_c18;full_length_coverage=1;non_full_length_coverage=0;isoform_length=2729;expected_accuracy=0.999	gnl|BL_ORD_ID|31084 cb1658_c11/f1p0/3913 isoform=cb1658_c11;full_length_coverage=1;non_full_length_coverage=0;isoform_length=3913;expected_accuracy=0.994	1147	2661	1	1150	2404	3913	29	1174
cb12587_c9/f1p0/1679	gnl|BL_ORD_ID|53017 cb8564_c114342/f1p0/2738	160	1616	1	160	1321	2777	30	189
cb2737_c35/f1p0/2780 isoform=cb2737_c35;full_length_coverage=1;non_full_length_coverage=0;isoform_length=2780;expected_accuracy=0.993	gnl|BL_ORD_ID|68785 cb2737_c49/f1p0/3341	134	2780	1	134	430	3081	4	137
cb3456_c3/f1p0/2774	gnl|BL_ORD_ID|41300 cb3456_c0/f4p1/3471 isoform=cb3456_c0;full_length_coverage=4;non_full_length_coverage=1;isoform_length=3471;expected_accuracy=0.997	1221	2781	1	1223	1452	3011	53	1275
cb7845_c1/f1p0/2303 isoform=cb7845_c1;full_length_coverage=1;non_full_length_coverage=0;isoform_length=2303;expected_accuracy=0.991	gnl|BL_ORD_ID|8537 cb7845_c4/f1p0/2414 isoform=cb7845_c4;full_length_coverage=1;non_full_length_coverage=0;isoform_length=2414;expected_accuracy=0.997	182	2225	1	183	365	2414	1	178
cb8564_c146099/f10p9/3818 isoform=cb8564_c146099;full_length_coverage=10;non_full_length_coverage=9;isoform_length=3818;expected_accuracy=1.000	gnl|BL_ORD_ID|19170 cb8564_c9757/f2p7/4230 isoform=cb8564_c9757;full_length_coverage=2;non_full_length_coverage=7;isoform_length=4230;expected_accuracy=0.999	1	3164	3165	3818	1	3162	3573	4226
cb4437_c10/f1p0/2497	gnl|BL_ORD_ID|72558 cb4437_c8/f1p0/2629	269	2494	9	270	402	262	2	263

isoform=cb4437_c10;full_length_coverage=1;non_full_length_coverage=0;isoform_length=2497;expected_accuracy=0.996							8		
cb1235_c4/f2p0/2309 isoform=cb1235_c4;full_length_coverage=2;non_full_length_coverage=0;isoform_length=2309;expected_accuracy=0.997	gnl|BL_ORD_ID|59297 cb1235_c21/f1p0/3452	1	1981	1980	2309	128	2108	3041	3370
cb1772_c6/f2p1/3674 isoform=cb1772_c6;full_length_coverage=2;non_full_length_coverage=1;isoform_length=3674;expected_accuracy=1.000	gnl|BL_ORD_ID|63938 cb1772_c19/f1p0/4042	216	3674	1	218	440	3898	40	258
cb8564_c111233/f1p0/3006 isoform=cb8564_c111233;full_length_coverage=1;non_full_length_coverage=0;isoform_length=3006;expected_accuracy=0.998	gnl|BL_ORD_ID|53359 cb8564_c123159/f1p0/3209	1214	2955	1	1215	1441	3182	71	1265
cb8564_c53806/f1p3/3974 isoform=cb8564_c53806;full_length_coverage=1;non_full_length_coverage=3;isoform_length=3974;expected_accuracy=0.990	gnl|BL_ORD_ID|13890 cb8564_c35935/f1p0/4149 isoform=cb8564_c35935;full_length_coverage=1;non_full_length_coverage=0;isoform_length=4149;expected_accuracy=0.993	169	3974	1	173	288	4055	15	183
cb288_c0/f4p0/4339 isoform=cb288_c0;full_length_coverage=4;non_full_length_coverage=0;isoform_length=4339;expected_accuracy=1.000	gnl|BL_ORD_ID|68166 cb288_c5/f1p0/4834	176	4339	1	175	638	4818	4	191
cb1658_c23/f1p0/2662 isoform=cb1658_c23;full_length_coverage=1;non_full_length_coverage=0;isoform_length=2662;expected_accu	gnl|BL_ORD_ID|31082 cb1658_c15/f1p0/2863 isoform=cb1658_c15;full_length_coverage=1;non_full_length_coverage=0;isoform_length=2863;expected_accur	1132	2590	1	1134	1401	2863	10	1156

racy=0.996	acy=0.994								
cb8564_c71631/f1p0/1923	gnl|BL_ORD_ID|13387 cb8564_c22413/f1p0/3916 isoform=cb8564_c22413;full_length_coverage=1;non_full_length_coverage=0;isoform_length=3916;expected_accuracy=0.991	198	1902	8	200	2213	3916	1	197
cb7771_c5/f1p0/2063	gnl|BL_ORD_ID|7436 cb7771_c3/f1p0/2176 isoform=cb7771_c3;full_length_coverage=1;non_full_length_coverage=0;isoform_length=2176;expected_accuracy=0.994	1	1339	1337	1982	1	1329	1432	2076
cb4487_c20/f1p0/1667	gnl|BL_ORD_ID|43657 cb4487_c12/f1p0/3251 isoform=cb4487_c12;full_length_coverage=1;non_full_length_coverage=0;isoform_length=3251;expected_accuracy=0.997	267	1557	3	270	1931	3221	73	340
cb3493_c11/f1p0/3332	gnl|BL_ORD_ID|70409 cb3493_c9/f1p0/3311	1	2347	2342	3014	123	2469	2578	3249
cb2218_c8/f1p1/2956 isoform=cb2218_c8;full_length_coverage=1;non_full_length_coverage=1;isoform_length=2956;expected_accuracy=0.996	gnl|BL_ORD_ID|68350 cb2218_c14/f1p0/3547	109	2907	1	112	357	3151	74	185
cb2324_c36/f1p1/3463	gnl|BL_ORD_ID|37505 cb2324_c35/f1p1/3632 isoform=cb2324_c35;full_length_coverage=1;non_full_length_coverage=1;isoform_length=3632;expected_accuracy=0.996	100	3477	1	100	234	3611	21	120
cb8564_c84468/f1p0/3473	gnl|BL_ORD_ID|15223 cb8564_c35037/f1p0/3377 isoform=cb8564_c35037;full_length_coverage=1;non_full_length_coverage=0;isoform_length=3377;expected_ac	129	2753	2751	3246	138	2761	2866	3361

	curacy=0.996								
cb3534_c9/f1p0/2857	gnl|BL_ORD_ID|71286 cb3534_c10/f1p0/3148	1310	2716	1	1313	1678	3084	1	1312
cb8564_c146072/f3p3/2176 isoform=cb8564_c146072;full_length_coverage=3;non_full_length_coverage=3;isoform_length=2176;expected_accuracy=1.000	gnl|BL_ORD_ID|55345 cb8564_c39002/f1p2/2677	1	1351	1349	2165	13	1364	1721	2539
cb13049_c13/f1p1/1357 isoform=cb13049_c13;full_length_coverage=1;non_full_length_coverage=1;isoform_length=1357;expected_accuracy=0.993	gnl|BL_ORD_ID|18258 cb8564_c84027/f1p0/2419 isoform=cb8564_c84027;full_length_coverage=1;non_full_length_coverage=0;isoform_length=2419;expected_accuracy=0.997	314	1332	5	314	1414	2419	4	305
cb8564_c188097/f1p0/2552 isoform=cb8564_c188097;full_length_coverage=1;non_full_length_coverage=0;isoform_length=2552;expected_accuracy=0.996	gnl|BL_ORD_ID|54044 cb8564_c17331/f1p0/3185	1	1945	1940	2505	35	1980	2083	2648
cb2737_c55/f1p1/2936 isoform=cb2737_c55;full_length_coverage=1;non_full_length_coverage=1;isoform_length=2936;expected_accuracy=1.000	gnl|BL_ORD_ID|38133 cb2737_c21/f1p2/3479 isoform=cb2737_c21;full_length_coverage=1;non_full_length_coverage=2;isoform_length=3479;expected_accuracy=1.000	1	1595	1593	2936	413	2003	2135	3479
cb4536_c2/f1p0/1954 isoform=cb4536_c2;full_length_coverage=1;non_full_length_coverage=0;isoform_length=1954;expected_accuracy=0.990	gnl|BL_ORD_ID|72653 cb4536_c4/f1p0/2190	11	1377	1376	1943	1	1369	1641	2198
cb3763_c32/f1p1/2854 isoform=cb3763_c32;full_length_coverage=1;non_full_length_coverage=1;isoform_length=2854;expected_accu	gnl|BL_ORD_ID|39926 cb3763_c51/f1p1/2991 isoform=cb3763_c51;full_length_coverage=1;non_full_length_coverage=1;isoform_length=2991;expected_accur	334	2825	6	335	497	2991	1	335

racy=0.996	acy=0.999								
cb3975_c6/f1p0/1965	gnl|BL_ORD_ID|41591 cb3975_c7/f1p1/2550 isoform=cb3975_c7;full_length_coverage=1;non_full_length_coverage=1;isoform_length=2550;expected_accuracy=0.990	1	1559	1558	1964	3	1564	2014	2414
cb1166_c1/f2p0/2173 isoform=cb1166_c1;full_length_coverage=2;non_full_length_coverage=0;isoform_length=2173;expected_accuracy=0.995	gnl|BL_ORD_ID|23338 cb1166_c2/f4p1/2715 isoform=cb1166_c2;full_length_coverage=4;non_full_length_coverage=1;isoform_length=2715;expected_accuracy=1.000	435	2168	9	440	984	2715	40	461
cb8564_c40823/f1p2/3537	gnl|BL_ORD_ID|14669 cb8564_c44765/f1p1/4352 isoform=cb8564_c44765;full_length_coverage=1;non_full_length_coverage=1;isoform_length=4352;expected_accuracy=0.995	1412	3391	1	1414	2375	4352	111	1524
cb8881_c18/f1p0/1628 isoform=cb8881_c18;full_length_coverage=1;non_full_length_coverage=0;isoform_length=1628;expected_accuracy=0.997	gnl|BL_ORD_ID|20989 cb8881_c4/f1p0/2063 isoform=cb8881_c4;full_length_coverage=1;non_full_length_coverage=0;isoform_length=2063;expected_accuracy=0.993	1	1226	1225	1628	3	1231	1636	2036
cb8564_c47011/f1p0/3100	gnl|BL_ORD_ID|12440 cb8564_c130435/f1p3/3226 isoform=cb8564_c130435;full_length_coverage=1;non_full_length_coverage=3;isoform_length=3226;expected_accuracy=0.994	12	2306	2301	2934	14	2320	2561	3204
cb8564_c89271/f1p0/2023	gnl|BL_ORD_ID|18863 cb8564_c82439/f1p4/3856 isoform=cb8564_c82439;full_length_coverage=1;non_full_length_coverage=4;isoform_length=3856;expected_accuracy=0.997	309	1808	55	311	2343	3837	5	276
cb8564_c50309/f1p5/3075	gnl|BL_ORD_ID|55920 cb8564_c48949/f1p0/4357	1	2289	2290	2954	2	229	2847	351

							3		0
cb8564_c71742/f1p0/2675	gnl|BL_ORD_ID|16169 cb8564_c66877/f3p0/2938 isoform=cb8564_c66877;full_length_coverage=3;non_full_length_coverage=0;isoform_length=2938;expected_accuracy=0.996	1	1309	1310	2484	77	1377	1764	2938
cb8564_c1521/f1p0/2226	gnl|BL_ORD_ID|52647 cb8564_c1520/f1p0/2355	1105	2186	105	1108	1221	2302	1	1004
cb8824_c3/f1p0/1749 isoform=cb8824_c3;full_length_coverage=1;non_full_length_coverage=0;isoform_length=1749;expected_accuracy=0.994	gnl|BL_ORD_ID|18297 cb8824_c1/f1p0/2259 isoform=cb8824_c1;full_length_coverage=1;non_full_length_coverage=0;isoform_length=2259;expected_accuracy=0.994	1	1315	1310	1749	1	1312	1687	2125
cb500_c17/f1p0/2323 isoform=cb500_c17;full_length_coverage=1;non_full_length_coverage=0;isoform_length=2323;expected_accuracy=0.997	gnl|BL_ORD_ID|72246 cb500_c47/f1p0/3506	1	1293	1288	2322	917	2209	2341	3375
cb295_c26/f1p1/1869	gnl|BL_ORD_ID|69537 cb295_c9/f1p0/4883	1	1359	1358	1857	2	1352	1483	1986
cb8564_c2326/f1p0/2006 isoform=cb8564_c2326;full_length_coverage=1;non_full_length_coverage=0;isoform_length=2006;expected_accuracy=0.990	gnl|BL_ORD_ID|53844 cb8564_c2432/f1p2/2768	230	2006	4	234	784	2557	73	306
cb8564_c75969/f1p1/2798 isoform=cb8564_c75969;full_length_coverage=1;non_full_length_coverage=1;isoform_length=2798;expected_accuracy=0.997	gnl|BL_ORD_ID|18906 cb8564_c90449/f1p3/3223 isoform=cb8564_c90449;full_length_coverage=1;non_full_length_coverage=3;isoform_length=3223;expected_accuracy=0.996	250	2798	1	251	640	3167	47	296
cb8564_c87358/f1p0/2586	gnl|BL_ORD_ID|57228 cb8564_c87142/f1p0/3290	2	2283	2281	2477	69	235	2596	279

isoform=cb8564_c87358;full_length_coverage=1;non_full_length_coverage=0;isoform_length=2586;expected_accuracy=0.994							0		3
cb3618_c23/f1p0/2587 isoform=cb3618_c23;full_length_coverage=1;non_full_length_coverage=0;isoform_length=2587;expected_accuracy=0.999	gnl|BL_ORD_ID|71383 cb3618_c11/f1p0/2951	1	2348	2346	2585	76	2426	2555	2794
cb7432_c15/f1p0/2249 isoform=cb7432_c15;full_length_coverage=1;non_full_length_coverage=0;isoform_length=2249;expected_accuracy=0.995	gnl|BL_ORD_ID|51232 cb7432_c24/f1p0/2569	156	2249	12	156	331	2425	13	159
cb12714_c29/f1p2/1583	gnl|BL_ORD_ID|12810 cb8564_c18099/f1p1/2597 isoform=cb8564_c18099;full_length_coverage=1;non_full_length_coverage=1;isoform_length=2597;expected_accuracy=0.992	1	1296	1293	1590	1	1296	2299	2597
cb2815_c2/f1p0/1100 isoform=cb2815_c2;full_length_coverage=1;non_full_length_coverage=0;isoform_length=1100;expected_accuracy=0.994	gnl|BL_ORD_ID|37531 cb2815_c0/f2p0/3776 isoform=cb2815_c0;full_length_coverage=2;non_full_length_coverage=0;isoform_length=3776;expected_accuracy=1.000	165	1100	41	166	2800	3735	14	142
cb5999_c2/f1p0/2957	gnl|BL_ORD_ID|45208 cb5999_c3/f1p0/2890 isoform=cb5999_c3;full_length_coverage=1;non_full_length_coverage=0;isoform_length=2890;expected_accuracy=0.995	635	2645	87	638	889	2890	1	552
cb15326_c7/f1p0/1319 isoform=cb15326_c7;full_length_coverage=1;non_full_length_coverage=0;isoform_length=1319;expected_accu	gnl|BL_ORD_ID|57950 cb8564_c84847/f1p0/2279	1	1151	1150	1319	396	1549	1976	2145

racy=0.998									
cb6941_c2/f1p0/1794	gnl|BL_ORD_ID|3443 cb6941_c1/f1p0/2504 isoform=cb6941_c1;full_length_coverage=1;non_full_length_coverage=0;isoform_length=2504;expected_accuracy=0.997	1	1097	1096	1774	27	1116	1828	2502
cb7498_c3/f1p0/1859	gnl|BL_ORD_ID|51292 cb7498_c1/f1p0/2180	547	1650	6	551	1103	2207	50	595
cb8564_c10716/f1p3/2179	gnl|BL_ORD_ID|12438 cb8564_c125968/f2p3/2612 isoform=cb8564_c125968;full_length_coverage=2;non_full_length_coverage=3;isoform_length=2612;expected_accuracy=0.997	258	2210	76	263	564	2518	226	405
cb5659_c78/f1p0/2309	gnl|BL_ORD_ID|46024 cb5659_c74/f1p3/2885 isoform=cb5659_c74;full_length_coverage=1;non_full_length_coverage=3;isoform_length=2885;expected_accuracy=0.994	10	1676	1675	2137	72	1757	2337	2799
cb8564_c78858/f1p0/2927 isoform=cb8564_c78858;full_length_coverage=1;non_full_length_coverage=0;isoform_length=2927;expected_accuracy=0.995	gnl|BL_ORD_ID|56772 cb8564_c71057/f1p4/4091	175	2927	1	174	1294	4053	12	181
cb3292_c11/f1p0/2790 isoform=cb3292_c11;full_length_coverage=1;non_full_length_coverage=0;isoform_length=2790;expected_accuracy=0.996	gnl|BL_ORD_ID|41083 cb3292_c10/f1p0/3715 isoform=cb3292_c10;full_length_coverage=1;non_full_length_coverage=0;isoform_length=3715;expected_accuracy=0.993	1298	2788	1	1300	2235	3715	817	2112
cb7845_c2/f1p0/2265 isoform=cb7845_c2;full_length_coverage=1;non_full_length_coverage=0;isoform_length=2265;expected_accur	gnl|BL_ORD_ID|8537 cb7845_c4/f1p0/2414 isoform=cb7845_c4;full_length_coverage=1;non_full_length_coverage=0;isoform_length=2414;expected_accura	183	2233	1	184	365	2414	1	178

acy=1.000	cy=0.997								
cb2097_c120/f1p0/1809 isoform=cb2097_c120;full_length_coverage=1;non_full_length_coverage=0;isoform_length=1809;expected_accuracy=0.994	gnl|BL_ORD_ID|66706 cb2097_c89/f1p2/2414	1	1422	1420	1809	413	1834	1944	2333
cb8564_c42513/f1p2/3770 isoform=cb8564_c42513;full_length_coverage=1;non_full_length_coverage=2;isoform_length=3770;expected_accuracy=0.997	gnl|BL_ORD_ID|15360 cb8564_c32914/f9p2/4297 isoform=cb8564_c32914;full_length_coverage=9;non_full_length_coverage=2;isoform_length=4297;expected_accuracy=1.000	1	2951	2947	3767	72	3008	3480	4297
cb1489_c4/f1p0/3013 isoform=cb1489_c4;full_length_coverage=1;non_full_length_coverage=0;isoform_length=3013;expected_accuracy=0.993	gnl|BL_ORD_ID|63666 cb1489_c2/f1p0/3842	99	3013	1	103	959	3869	61	168
cb965_c18/f1p0/2545 isoform=cb965_c18;full_length_coverage=1;non_full_length_coverage=0;isoform_length=2545;expected_accuracy=1.000	gnl|BL_ORD_ID|20094 cb965_c9/f2p4/4285 isoform=cb965_c9;full_length_coverage=2;non_full_length_coverage=4;isoform_length=4285;expected_accuracy=0.999	1	1828	1831	2527	1526	3351	3579	4285
cb8564_c70954/f1p0/2365	gnl|BL_ORD_ID|57978 cb8564_c85833/f1p0/2942	1	1708	1707	2257	1	1708	2376	2926
cb1772_c20/f1p0/3984	gnl|BL_ORD_ID|63938 cb1772_c19/f1p0/4042	193	3722	1	194	441	3970	114	307
cb5137_c53/f1p0/2672 isoform=cb5137_c53;full_length_coverage=1;non_full_length_coverage=0;isoform_length=2672;expected_accuracy=0.992	gnl|BL_ORD_ID|73312 cb5137_c30/f1p2/3623	19	2565	2562	2672	847	3397	3501	3610
cb8564_c38035/f1p0/2770	gnl|BL_ORD_ID|55464 cb8564_c42285/f1p0/3370	1	2035	2033	2756	1	203	2226	295

							6		0
cb8564_c18641/f1p0/2364	gnl|BL_ORD_ID|54398 cb8564_c24870/f1p0/2605	15	1650	1645	2321	38	1673	1883	2559
cb8564_c92076/f1p0/3037	gnl|BL_ORD_ID|15755 cb8564_c4970/f1p0/3133 isoform=cb8564_c4970;full_length_coverage=1;non_full_length_coverage=0;isoform_length=3133;expected_accuracy=0.996	111	2954	1	112	292	3133	1	111
cb7126_c8/f1p0/1757	gnl|BL_ORD_ID|4216 cb7126_c5/f1p0/1933 isoform=cb7126_c5;full_length_coverage=1;non_full_length_coverage=0;isoform_length=1933;expected_accuracy=0.996	122	1749	1	125	295	1921	33	157
cb8564_c4766/f1p0/2565	gnl|BL_ORD_ID|55688 cb8564_c4747/f1p0/2838	1	1336	1334	2377	225	1559	1807	2853
cb8564_c34205/f6p1/2161 isoform=cb8564_c34205;full_length_coverage=6;non_full_length_coverage=1;isoform_length=2161;expected_accuracy=0.996	gnl|BL_ORD_ID|14555 cb8564_c40580/f3p1/2616 isoform=cb8564_c40580;full_length_coverage=3;non_full_length_coverage=1;isoform_length=2616;expected_accuracy=0.992	1	1993	1991	2161	1	1958	2204	2373
cb7377_c9/f1p0/2341	gnl|BL_ORD_ID|8470 cb7377_c2/f1p0/2583 isoform=cb7377_c2;full_length_coverage=1;non_full_length_coverage=0;isoform_length=2583;expected_accuracy=0.997	162	2233	1	162	418	2488	128	290
cb6388_c7/f1p1/2217 isoform=cb6388_c7;full_length_coverage=1;non_full_length_coverage=1;isoform_length=2217;expected_accuracy=0.999	gnl|BL_ORD_ID|4572 cb6388_c3/f1p0/2513 isoform=cb6388_c3;full_length_coverage=1;non_full_length_coverage=0;isoform_length=2513;expected_accuracy=0.996	1	1379	1376	2206	31	1410	1560	2391
cb2177_c32/f1p0/2567	gnl|BL_ORD_ID|36019 cb2177_c26/f1p2/3500	1	1568	1567	2534	768	235	2526	350

isoform=cb2177_c32;full_length_coverage=1;non_full_length_coverage=0;isoform_length=2567;expected_accuracy=0.990	isoform=cb2177_c26;full_length_coverage=1;non_full_length_coverage=2;isoform_length=3500;expected_accuracy=0.999						1		0
cb8564_c43399/f1p0/2654 isoform=cb8564_c43399;full_length_coverage=1;non_full_length_coverage=0;isoform_length=2654;expected_accuracy=0.999	gnl|BL_ORD_ID|55866 cb8564_c47522/f1p0/2954	1	2367	2363	2653	1	2343	2559	2849
cb955_c24/f1p2/2194	gnl|BL_ORD_ID|22154 cb955_c13/f2p0/3026 isoform=cb955_c13;full_length_coverage=2;non_full_length_coverage=0;isoform_length=3026;expected_accuracy=0.998	1	1850	1849	2190	4	1853	2641	2982
cb2122_c5/f1p0/3100 isoform=cb2122_c5;full_length_coverage=1;non_full_length_coverage=0;isoform_length=3100;expected_accuracy=0.999	gnl|BL_ORD_ID|34252 cb2122_c9/f1p0/3797 isoform=cb2122_c9;full_length_coverage=1;non_full_length_coverage=0;isoform_length=3797;expected_accuracy=0.997	160	3097	1	165	862	3797	1	165
cb6066_c7/f1p0/1915	gnl|BL_ORD_ID|45957 cb6066_c2/f1p0/2806 isoform=cb6066_c2;full_length_coverage=1;non_full_length_coverage=0;isoform_length=2806;expected_accuracy=0.999	1	1074	1070	1899	3	1075	1978	2805
cb12931_c15/f1p0/1493 isoform=cb12931_c15;full_length_coverage=1;non_full_length_coverage=0;isoform_length=1493;expected_accuracy=1.000	gnl|BL_ORD_ID|55170 cb8564_c34431/f1p0/3665	1	1106	1104	1492	4	1111	2791	3179
cb4233_c84/f1p1/2344	gnl|BL_ORD_ID|71860 cb4233_c118/f1p0/3085	227	2391	1	225	584	2748	560	784
cb8564_c47853/f1p0/2927	gnl|BL_ORD_ID|17429 cb8564_c85447/f1p0/3196	1	2401	2400	2927	3	241	2660	319

isoform=cb8564_c47853;full_length_coverage=1;non_full_length_coverage=0;isoform_length=2927;expected_accuracy=0.991	isoform=cb8564_c85447;full_length_coverage=1;non_full_length_coverage=0;isoform_length=3196;expected_accuracy=0.997						3		2
cb8564_c88629/f1p0/3306 isoform=cb8564_c88629;full_length_coverage=1;non_full_length_coverage=0;isoform_length=3306;expected_accuracy=0.999	gnl|BL_ORD_ID|11236 cb8564_c145778/f9p1/3503 isoform=cb8564_c145778;full_length_coverage=9;non_full_length_coverage=1;isoform_length=3503;expected_accuracy=1.000	1	3122	3122	3306	1	3124	3306	3490
cb8478_c37/f1p0/3192	gnl|BL_ORD_ID|51961 cb8478_c22/f1p0/3564	16	2659	2657	3154	1	2644	2998	3495
cb2229_c66/f60p1/2354 isoform=cb2229_c66;full_length_coverage=60;non_full_length_coverage=1;isoform_length=2354;expected_accuracy=0.999	gnl|BL_ORD_ID|37324 cb2229_c24/f1p0/2736 isoform=cb2229_c24;full_length_coverage=1;non_full_length_coverage=0;isoform_length=2736;expected_accuracy=0.994	1	1758	1759	2317	61	1821	2181	2736
cb1648_c4/f10p3/2031 isoform=cb1648_c4;full_length_coverage=10;non_full_length_coverage=3;isoform_length=2031;expected_accuracy=1.000	gnl|BL_ORD_ID|66333 cb1648_c27/f1p1/2264	234	2031	7	234	382	2178	9	233
cb8564_c116345/f1p1/2198 isoform=cb8564_c116345;full_length_coverage=1;non_full_length_coverage=1;isoform_length=2198;expected_accuracy=0.991	gnl|BL_ORD_ID|11911 cb8564_c132760/f5p6/2734 isoform=cb8564_c132760;full_length_coverage=5;non_full_length_coverage=6;isoform_length=2734;expected_accuracy=0.999	369	2142	5	373	876	2653	4	369
cb8564_c47011/f1p0/3100	gnl|BL_ORD_ID|55522 cb8564_c44123/f1p1/3230	18	2306	2301	2934	15	2294	2532	3164
cb4098_c71/f2p1/3728 isoform=cb4098_c71;full_length_coverage=2;non_full_length_coverage=1;isoform_length=3728;expected_accu	gnl|BL_ORD_ID|40754 cb4098_c13/f2p1/3772 isoform=cb4098_c13;full_length_coverage=2;non_full_length_coverage=1;isoform_length=3772;expected_accur	108	1973	1974	3728	1	1867	2002	3758

racy=1.000	acy=0.998								
cb8564_c127097/f1p0/2380 isoform=cb8564_c127097;full_length_coverage=1;non_full_length_coverage=0;isoform_length=2380;expected_accuracy=0.994	gnl|BL_ORD_ID|9453 cb8564_c113395/f1p0/2950 isoform=cb8564_c113395;full_length_coverage=1;non_full_length_coverage=0;isoform_length=2950;expected_accuracy=0.995	1	1714	1713	2379	26	1738	2283	2950
cb8564_c20384/f1p4/2475 isoform=cb8564_c20384;full_length_coverage=1;non_full_length_coverage=4;isoform_length=2475;expected_accuracy=0.999	gnl|BL_ORD_ID|13109 cb8564_c16444/f5p10/3053 isoform=cb8564_c16444;full_length_coverage=5;non_full_length_coverage=10;isoform_length=3053;expected_accuracy=0.992	1	1492	1493	2475	551	2042	1385	2368
cb8564_c41162/f1p0/3158	gnl|BL_ORD_ID|55246 cb8564_c36343/f1p0/3448	21	2553	2548	3038	21	2567	2698	3188
cb8564_c72639/f1p3/2409 isoform=cb8564_c72639;full_length_coverage=1;non_full_length_coverage=3;isoform_length=2409;expected_accuracy=0.999	gnl|BL_ORD_ID|57107 cb8564_c83478/f1p0/3224	288	2409	36	288	374	2497	2	254
cb3183_c8/f1p3/3030 isoform=cb3183_c8;full_length_coverage=1;non_full_length_coverage=3;isoform_length=3030;expected_accuracy=0.999	gnl|BL_ORD_ID|37888 cb3183_c6/f2p4/3666 isoform=cb3183_c6;full_length_coverage=2;non_full_length_coverage=4;isoform_length=3666;expected_accuracy=1.000	147	3029	1	148	785	3666	211	358
cb8564_c188095/f1p0/3177 isoform=cb8564_c188095;full_length_coverage=1;non_full_length_coverage=0;isoform_length=3177;expected_accuracy=0.998	gnl|BL_ORD_ID|55376 cb8564_c39821/f1p0/3886	1345	3152	1	1346	1943	3735	1	1317
cb5896_c134/f1p0/4910 isoform=cb5896_c134;full_length_coverage=1;non_full_length_coverage=0;isoform_length=4910;expected_acc	gnl|BL_ORD_ID|67969 cb18734_c0/f1p0/5102	1	4256	4255	4870	62	4328	4439	5056

uracy=0.999									
cb8564_c37802/f1p0/2973	gnl|BL_ORD_ID|15464 cb8564_c48625/f1p1/3232 isoform=cb8564_c48625;full_length_coverage=1;non_full_length_coverage=1;isoform_length=3232;expected_accuracy=0.991	1	2292	2287	2755	1	2269	2764	3232
cb8564_c46939/f1p1/2926	gnl|BL_ORD_ID|55551 cb8564_c44964/f1p0/3508	1	2134	2130	2870	1	2140	2245	2988
cb6407_c4/f3p0/2587 isoform=cb6407_c4;full_length_coverage=3;non_full_length_coverage=0;isoform_length=2587;expected_accuracy=1.000	gnl|BL_ORD_ID|6061 cb6407_c18/f1p1/2768 isoform=cb6407_c18;full_length_coverage=1;non_full_length_coverage=1;isoform_length=2768;expected_accuracy=0.996	1	1340	1337	2587	1	1338	1503	2753
cb8564_c73607/f1p0/2892 isoform=cb8564_c73607;full_length_coverage=1;non_full_length_coverage=0;isoform_length=2892;expected_accuracy=0.995	gnl|BL_ORD_ID|56103 cb8564_c69762/f2p0/4058	1	2114	2115	2892	6	2119	2230	3009
cb8564_c74556/f2p1/3463 isoform=cb8564_c74556;full_length_coverage=2;non_full_length_coverage=1;isoform_length=3463;expected_accuracy=0.997	gnl|BL_ORD_ID|52659 cb8564_c10244/f1p0/4022	1	2906	2904	3391	58	2965	3383	3870
cb8564_c37361/f1p0/3076	gnl|BL_ORD_ID|57017 cb8564_c80370/f1p0/3493	119	2481	2477	3070	3085	723	622	29
cb4211_c3/f2p5/1253 isoform=cb4211_c3;full_length_coverage=2;non_full_length_coverage=5;isoform_length=1253;expected_accuracy=1.000	gnl|BL_ORD_ID|71851 cb4211_c4/f1p2/3385	155	1253	8	156	2200	3298	1	148
cb8564_c146882/f3p8/4113 isoform=cb8564_c146882;full_length_coverage=3;non_	gnl|BL_ORD_ID|55207 cb8564_c35399/f1p0/4553	184	4111	10	185	396	4324	11	179

full_length_coverage=8;isoform_length=4113;expected_accuracy=0.998									
cb3301_c3/f1p0/2960 isoform=cb3301_c3;full_length_coverage=1;non_full_length_coverage=0;isoform_length=2960;expected_accuracy=0.996	gnl|BL_ORD_ID|41447 cb3301_c0/f1p0/3662 isoform=cb3301_c0;full_length_coverage=1;non_full_length_coverage=0;isoform_length=3662;expected_accuracy=0.994	1368	2946	1	1369	2063	3662	90	1459
cb8564_c68941/f7p1/2708 isoform=cb8564_c68941;full_length_coverage=7;non_full_length_coverage=1;isoform_length=2708;expected_accuracy=1.000	gnl|BL_ORD_ID|10231 cb8564_c107778/f4p4/2753 isoform=cb8564_c107778;full_length_coverage=4;non_full_length_coverage=4;isoform_length=2753;expected_accuracy=0.996	583	2708	62	585	633	2753	10	525
cb293_c5/f1p0/4488	gnl|BL_ORD_ID|69536 cb293_c7/f1p0/4528	2	2289	2288	4337	15	2302	2411	4432
cb8564_c36731/f1p2/2761	gnl|BL_ORD_ID|55531 cb8564_c44297/f1p0/3146	1	1977	1972	2737	1	1977	2362	3127
cb8564_c102411/f1p1/2832 isoform=cb8564_c102411;full_length_coverage=1;non_full_length_coverage=1;isoform_length=2832;expected_accuracy=0.994	gnl|BL_ORD_ID|14785 cb8564_c36335/f1p0/3172 isoform=cb8564_c36335;full_length_coverage=1;non_full_length_coverage=0;isoform_length=3172;expected_accuracy=0.997	231	2770	2	234	631	3172	3	235
cb8052_c0/f2p0/1908 isoform=cb8052_c0;full_length_coverage=2;non_full_length_coverage=0;isoform_length=1908;expected_accuracy=0.998	gnl|BL_ORD_ID|6981 cb8052_c1/f1p0/2472 isoform=cb8052_c1;full_length_coverage=1;non_full_length_coverage=0;isoform_length=2472;expected_accuracy=0.996	1	1081	1080	1908	112	1191	1644	2472
cb6376_c0/f2p0/2298 isoform=cb6376_c0;full_length_coverage=2;non_full_length_coverage=0;isoform_length=2298;expected_accuracy=0.996	gnl|BL_ORD_ID|3753 cb6376_c2/f1p0/2859 isoform=cb6376_c2;full_length_coverage=1;non_full_length_coverage=0;isoform_length=2859;expected_accuracy=0.997	1	1438	1437	2294	33	1459	2004	2859

cb8564_c47011/f1p0/3100	gnl|BL_ORD_ID|55600 cb8564_c46484/f1p0/3151	110	2306	2301	2934	5	2220	2458	3090
cb8564_c146191/f1p0/2060 isoform=cb8564_c146191;full_length_coverage=1;non_full_length_coverage=0;isoform_length=2060;expected_accuracy=0.999	gnl|BL_ORD_ID|17345 cb8564_c9893/f7p1/3111 isoform=cb8564_c9893;full_length_coverage=7;non_full_length_coverage=1;isoform_length=3111;expected_accuracy=0.998	407	2060	61	409	1431	3085	1	350
cb8564_c21541/f1p0/2864 isoform=cb8564_c21541;full_length_coverage=1;non_full_length_coverage=0;isoform_length=2864;expected_accuracy=0.997	gnl|BL_ORD_ID|54386 cb8564_c23407/f1p0/2923	1133	2798	1	1132	1256	2921	6	1138
cb4838_c4/f1p0/1068 isoform=cb4838_c4;full_length_coverage=1;non_full_length_coverage=0;isoform_length=1068;expected_accuracy=1.000	gnl|BL_ORD_ID|72030 cb4838_c1/f1p0/3640	296	1057	3	297	2488	3249	3	298
cb1178_c11/f1p0/2043	gnl|BL_ORD_ID|59231 cb1178_c10/f1p0/2304	1	1000	996	1921	2	989	1378	2302
cb9813_c128/f1p0/1498	gnl|BL_ORD_ID|65622 cb17070_c2/f1p0/1878	175	1498	3	179	353	1674	1	177
cb820_c4/f1p0/7677 isoform=cb820_c4;full_length_coverage=1;non_full_length_coverage=0;isoform_length=7677;expected_accuracy=0.992	gnl|BL_ORD_ID|51001 cb820_c3/f1p0/8069	2856	7636	1	2861	3250	8036	1	2870
cb8564_c23232/f1p3/2540	gnl|BL_ORD_ID|54449 cb8564_c122906/f2p13/2871	16	2172	2167	2518	139	2295	2397	2748
cb8564_c34638/f1p0/2817 isoform=cb8564_c34638;full_length_coverage=1;non_f	gnl|BL_ORD_ID|14410 cb8564_c44256/f1p1/3267 isoform=cb8564_c44256;full_length_coverage=1;non_fu	1	2109	2105	2813	1	2119	2553	3267

ull_length_coverage=0;isoform_length=2817;expected_accuracy=0.992	ll_length_coverage=1;isoform_length=3267;expected_accuracy=0.995								
cb8564_c45300/f1p4/3014 isoform=cb8564_c45300;full_length_coverage=1;non_full_length_coverage=4;isoform_length=3014;expected_accuracy=0.996	gnl|BL_ORD_ID|56041 cb8564_c53077/f1p2/3406	243	3014	1	246	515	3287	1	243
cb3599_c13/f1p0/2987 isoform=cb3599_c13;full_length_coverage=1;non_full_length_coverage=0;isoform_length=2987;expected_accuracy=0.998	gnl|BL_ORD_ID|41082 cb3599_c17/f12p0/3112 isoform=cb3599_c17;full_length_coverage=12;non_full_length_coverage=0;isoform_length=3112;expected_accuracy=1.000	1	2339	2334	2987	1	2339	2458	3111
cb3586_c11/f1p0/2822 isoform=cb3586_c11;full_length_coverage=1;non_full_length_coverage=0;isoform_length=2822;expected_accuracy=0.995	gnl|BL_ORD_ID|41149 cb3586_c2/f1p0/3545 isoform=cb3586_c2;full_length_coverage=1;non_full_length_coverage=0;isoform_length=3545;expected_accuracy=0.998	278	2822	2	278	950	3495	54	335
cb1770_c25/f1p0/3573	gnl|BL_ORD_ID|33845 cb1770_c3/f3p0/3565 isoform=cb1770_c3;full_length_coverage=3;non_full_length_coverage=0;isoform_length=3565;expected_accuracy=0.999	1	1910	1908	3369	19	1931	2096	3565
cb2831_c22/f1p0/2102	gnl|BL_ORD_ID|38322 cb2831_c7/f3p0/2914 isoform=cb2831_c7;full_length_coverage=3;non_full_length_coverage=0;isoform_length=2914;expected_accuracy=1.000	1	1598	1598	2097	65	1661	2401	2914
cb1138_c21/f1p4/3386 isoform=cb1138_c21;full_length_coverage=1;non_full_length_coverage=4;isoform_length=3386;expected_accuracy=0.995	gnl|BL_ORD_ID|59178 cb1138_c38/f1p0/3611	1	2841	2836	3321	1	2842	2946	3431

cb8564_c77604/f1p0/3355	gnl|BL_ORD_ID|17154 cb8564_c82503/f1p0/4455 isoform=cb8564_c82503;full_length_coverage=1;non_full_length_coverage=0;isoform_length=4455;expected_accuracy=0.995	12	2619	2617	3351	47	2632	3722	4453
cb8564_c116701/f1p0/2471 isoform=cb8564_c116701;full_length_coverage=1;non_full_length_coverage=0;isoform_length=2471;expected_accuracy=0.992	gnl|BL_ORD_ID|9882 cb8564_c116615/f1p0/3195 isoform=cb8564_c116615;full_length_coverage=1;non_full_length_coverage=0;isoform_length=3195;expected_accuracy=0.996	1	1929	1927	2471	603	2527	2631	3175
cb4977_c0/f1p0/3210 isoform=cb4977_c0;full_length_coverage=1;non_full_length_coverage=0;isoform_length=3210;expected_accuracy=0.994	gnl|BL_ORD_ID|44251 cb4977_c1/f1p0/3648 isoform=cb4977_c1;full_length_coverage=1;non_full_length_coverage=0;isoform_length=3648;expected_accuracy=0.999	23	2832	2830	3210	1	2825	2998	3381
cb2707_c24/f1p0/2640	gnl|BL_ORD_ID|36995 cb2707_c28/f1p1/3570 isoform=cb2707_c28;full_length_coverage=1;non_full_length_coverage=1;isoform_length=3570;expected_accuracy=0.996	1	2430	2425	2649	309	2738	3245	3469
cb3411_c18/f1p0/3101 isoform=cb3411_c18;full_length_coverage=1;non_full_length_coverage=0;isoform_length=3101;expected_accuracy=0.998	gnl|BL_ORD_ID|39887 cb3411_c9/f1p0/3617 isoform=cb3411_c9;full_length_coverage=1;non_full_length_coverage=0;isoform_length=3617;expected_accuracy=0.997	1238	3100	104	1241	1756	3617	258	1393
cb1496_c3/f1p2/1807 isoform=cb1496_c3;full_length_coverage=1;non_full_length_coverage=2;isoform_length=1807;expected_accuracy=0.994	gnl|BL_ORD_ID|30710 cb1496_c9/f1p1/3684 isoform=cb1496_c9;full_length_coverage=1;non_full_length_coverage=1;isoform_length=3684;expected_accuracy=0.994	440	1800	58	444	2304	3664	6	390
cb4528_c29/f1p0/1646	gnl|BL_ORD_ID|43229 cb4528_c40/f4p0/1916 isoform=cb4528_c40;full_length_coverage=4;non_full_l	1	1441	1439	1580	30	1470	1775	1916

	ength_coverage=0;isoform_length=1916;expected_accuracy=1.000								
cb2918_c29/f1p0/2588	gnl|BL_ORD_ID|70088 cb2918_c33/f1p3/2817	18	2300	2297	2554	52	2335	2445	2702
cb6716_c4/f1p0/2291	gnl|BL_ORD_ID|47663 cb6716_c1/f1p1/2898	1	1816	1817	2239	70	1886	2167	2584
cb8564_c72800/f1p0/2322 isoform=cb8564_c72800;full_length_coverage=1;non_full_length_coverage=0;isoform_length=2322;expected_accuracy=0.996	gnl|BL_ORD_ID|57089 cb8564_c82833/f1p0/2593	210	2269	2	212	376	2435	1	212
cb13434_c28/f1p1/1476 isoform=cb13434_c28;full_length_coverage=1;non_full_length_coverage=1;isoform_length=1476;expected_accuracy=0.997	gnl|BL_ORD_ID|53863 cb8564_c4654/f1p2/3045	8	728	727	1418	8	730	2131	2817
cb14517_c18/f1p1/2779 isoform=cb14517_c18;full_length_coverage=1;non_full_length_coverage=1;isoform_length=2779;expected_accuracy=0.990	gnl|BL_ORD_ID|30661 cb14517_c1/f4p1/5912 isoform=cb14517_c1;full_length_coverage=4;non_full_length_coverage=1;isoform_length=5912;expected_accuracy=0.999	1339	2779	1	1343	4387	5830	64	1409
cb8564_c78992/f1p0/2856	gnl|BL_ORD_ID|16701 cb8564_c69366/f5p4/2938 isoform=cb8564_c69366;full_length_coverage=5;non_full_length_coverage=4;isoform_length=2938;expected_accuracy=1.000	196	2645	1	196	72	2523	10	205
cb8564_c89322/f1p0/2588	gnl|BL_ORD_ID|17419 cb8564_c87724/f1p0/2766 isoform=cb8564_c87724;full_length_coverage=1;non_full_length_coverage=0;isoform_length=2766;expected_accuracy=0.993	397	2588	5	402	534	2718	1	395

cb910_c16/f1p2/3352	gnl|BL_ORD_ID|21892 cb910_c15/f1p2/4577 isoform=cb910_c15;full_length_coverage=1;non_full_length_coverage=2;isoform_length=4577;expected_accuracy=0.997	1500	3367	1	1501	2127	3988	208	1697
cb2177_c32/f1p0/2567 isoform=cb2177_c32;full_length_coverage=1;non_full_length_coverage=0;isoform_length=2567;expected_accuracy=0.990	gnl|BL_ORD_ID|35944 cb2177_c49/f1p2/3375 isoform=cb2177_c49;full_length_coverage=1;non_full_length_coverage=2;isoform_length=3375;expected_accuracy=0.993	1	1568	1567	2543	650	2224	2399	3375
cb8564_c40764/f1p0/2563 isoform=cb8564_c40764;full_length_coverage=1;non_full_length_coverage=0;isoform_length=2563;expected_accuracy=0.997	gnl|BL_ORD_ID|14556 cb8564_c45150/f1p0/2940 isoform=cb8564_c45150;full_length_coverage=1;non_full_length_coverage=0;isoform_length=2940;expected_accuracy=0.992	1	2166	2162	2563	1	2148	2355	2755
cb8564_c118659/f2p0/3437 isoform=cb8564_c118659;full_length_coverage=2;non_full_length_coverage=0;isoform_length=3437;expected_accuracy=0.994	gnl|BL_ORD_ID|11650 cb8564_c152526/f1p6/3802 isoform=cb8564_c152526;full_length_coverage=1;non_full_length_coverage=6;isoform_length=3802;expected_accuracy=0.999	1	2906	2907	3427	85	3015	3282	3802
cb3427_c5/f1p0/3487 isoform=cb3427_c5;full_length_coverage=1;non_full_length_coverage=0;isoform_length=3487;expected_accuracy=0.997	gnl|BL_ORD_ID|39943 cb3427_c4/f1p0/3709 isoform=cb3427_c4;full_length_coverage=1;non_full_length_coverage=0;isoform_length=3709;expected_accuracy=0.993	1	3084	3086	3447	43	3129	3351	3709
cb12714_c29/f1p2/1583	gnl|BL_ORD_ID|58179 cb8564_c90747/f1p0/2795	1	1296	1293	1597	1	1296	2336	2640
cb2208_c27/f1p0/2987	gnl|BL_ORD_ID|69620 cb2208_c18/f1p0/3335	2	2173	2171	2949	73	2252	2486	3264
cb8564_c53401/f1p1/3828	gnl|BL_ORD_ID|14193 cb8564_c31973/f2p1/4589 isoform=cb8564_c31973;full_length_coverage=2;non_fu	2	2988	2984	3601	69	3055	3965	4586

	ll_length_coverage=1;isoform_length=4589;expected_accuracy=0.996								
cb567_c14/f1p0/3377	gnl|BL_ORD_ID|45927 cb567_c3/f1p0/3308 isoform=cb567_c3;full_length_coverage=1;non_full_length_coverage=0;isoform_length=3308;expected_accuracy=1.000	381	3058	94	383	631	3308	1	293
cb8564_c43601/f1p0/2228	gnl|BL_ORD_ID|16946 cb8564_c49998/f1p1/4102 isoform=cb8564_c49998;full_length_coverage=1;non_full_length_coverage=1;isoform_length=4102;expected_accuracy=0.998	1	1814	1814	2071	1667	3479	3844	4102
cb8564_c128725/f1p1/2771 isoform=cb8564_c128725;full_length_coverage=1;non_full_length_coverage=1;isoform_length=2771;expected_accuracy=1.000	gnl|BL_ORD_ID|10510 cb8564_c111713/f6p2/3032 isoform=cb8564_c111713;full_length_coverage=6;non_full_length_coverage=2;isoform_length=3032;expected_accuracy=0.998	1	2669	2664	2771	1	2642	2751	2859
cb12931_c15/f1p0/1493 isoform=cb12931_c15;full_length_coverage=1;non_full_length_coverage=0;isoform_length=1493;expected_accuracy=1.000	gnl|BL_ORD_ID|13273 cb8564_c188193/f1p0/2849 isoform=cb8564_c188193;full_length_coverage=1;non_full_length_coverage=0;isoform_length=2849;expected_accuracy=0.997	1	1106	1104	1492	1	1102	2461	2849
cb396_c42/f1p0/2343	gnl|BL_ORD_ID|39524 cb396_c4/f9p0/2621 isoform=cb396_c4;full_length_coverage=9;non_full_length_coverage=0;isoform_length=2621;expected_accuracy=1.000	1	1458	1457	2346	99	1555	1725	2618
cb8564_c75977/f1p0/2165	gnl|BL_ORD_ID|57894 cb8564_c83545/f1p0/3678	1	1393	1394	2135	63	1455	2743	3480
cb401_c50/f1p0/2173 isoform=cb401_c50;full_length_coverage=1;non_full_le	gnl|BL_ORD_ID|70878 cb401_c109/f1p1/2463	96	2171	1	101	266	2338	25	125

ngth_coverage=0;isoform_length=2173;expected_accuracy=0.998									
cb2279_c20/f1p0/1654	gnl|BL_ORD_ID|36528 cb2279_c4/f1p2/2606 isoform=cb2279_c4;full_length_coverage=1;non_full_length_coverage=2;isoform_length=2606;expected_accuracy=0.996	212	1455	24	214	1357	2602	11	201
cb12931_c15/f1p0/1493 isoform=cb12931_c15;full_length_coverage=1;non_full_length_coverage=0;isoform_length=1493;expected_accuracy=1.000	gnl|BL_ORD_ID|11085 cb8564_c115872/f1p0/2750 isoform=cb8564_c115872;full_length_coverage=1;non_full_length_coverage=0;isoform_length=2750;expected_accuracy=0.995	1	1106	1104	1488	1	1104	2368	2750
cb18537_c0/f1p0/1839 isoform=cb18537_c0;full_length_coverage=1;non_full_length_coverage=0;isoform_length=1839;expected_accuracy=0.993	gnl|BL_ORD_ID|54218 cb8564_c20754/f1p0/2472	1	1495	1492	1789	26	1522	2166	2463
cb7849_c18/f1p0/2052 isoform=cb7849_c18;full_length_coverage=1;non_full_length_coverage=0;isoform_length=2052;expected_accuracy=0.991	gnl|BL_ORD_ID|50752 cb7849_c20/f1p0/2279	12	1375	1373	2048	22	1385	1518	2198
cb4509_c18/f1p0/2940 isoform=cb4509_c18;full_length_coverage=1;non_full_length_coverage=0;isoform_length=2940;expected_accuracy=0.997	gnl|BL_ORD_ID|72624 cb4509_c10/f1p1/3560	1	1499	1499	2940	243	1741	2113	3554
cb1955_c15/f1p0/2361	gnl|BL_ORD_ID|36339 cb1955_c43/f2p0/2634 isoform=cb1955_c43;full_length_coverage=2;non_full_length_coverage=0;isoform_length=2634;expected_accuracy=1.000	1	1505	1503	2278	74	1579	1858	2634

cb8564_c54461/f1p0/2024 isoform=cb8564_c54461;full_length_coverage=1;non_full_length_coverage=0;isoform_length=2024;expected_accuracy=0.992	gnl|BL_ORD_ID|10792 cb8564_c117151/f1p0/2256 isoform=cb8564_c117151;full_length_coverage=1;non_full_length_coverage=0;isoform_length=2256;expected_accuracy=0.993	671	2024	7	670	868	2233	1	670
cb9061_c16/f1p1/2248 isoform=cb9061_c16;full_length_coverage=1;non_full_length_coverage=1;isoform_length=2248;expected_accuracy=0.999	gnl|BL_ORD_ID|58527 cb9061_c19/f1p0/2376	1	1438	1439	2189	36	1473	1581	2331
cb3759_c6/f1p0/2298	gnl|BL_ORD_ID|70597 cb3759_c2/f1p0/3671	1	1727	1729	2238	71	1797	2639	3148
cb792_c53/f1p0/3930	gnl|BL_ORD_ID|8413 cb792_c27/f1p3/4257 isoform=cb792_c27;full_length_coverage=1;non_full_length_coverage=3;isoform_length=4257;expected_accuracy=0.993	1	1842	1838	3677	442	2268	2399	4225
cb4374_c3/f1p0/2557 isoform=cb4374_c3;full_length_coverage=1;non_full_length_coverage=0;isoform_length=2557;expected_accuracy=0.997	gnl|BL_ORD_ID|72517 cb4374_c5/f1p0/2755	1	2030	2027	2556	15	1990	2230	2762
cb5466_c1/f1p0/2860	gnl|BL_ORD_ID|47101 cb5466_c0/f1p0/3093 isoform=cb5466_c0;full_length_coverage=1;non_full_length_coverage=0;isoform_length=3093;expected_accuracy=0.995	121	2744	1	120	470	3093	44	162
cb822_c36/f4p1/2420 isoform=cb822_c36;full_length_coverage=4;non_full_length_coverage=1;isoform_length=2420;expected_accuracy=1.000	gnl|BL_ORD_ID|51002 cb822_c19/f1p2/4163	655	2417	96	656	2386	4147	2	562

cb8564_c40823/f1p2/3537	gnl|BL_ORD_ID|16467 cb8564_c49872/f2p1/3689 isoform=cb8564_c49872;full_length_coverage=2;non_full_length_coverage=1;isoform_length=3689;expected_accuracy=1.000	1	1897	1897	3378	137	2031	2209	3689
cb6376_c0/f2p0/2298 isoform=cb6376_c0;full_length_coverage=2;non_full_length_coverage=0;isoform_length=2298;expected_accuracy=0.996	gnl|BL_ORD_ID|4338 cb6376_c1/f1p0/2877 isoform=cb6376_c1;full_length_coverage=1;non_full_length_coverage=0;isoform_length=2877;expected_accuracy=0.996	1	1438	1437	2292	23	1477	2022	2877
cb8564_c45300/f1p4/3014 isoform=cb8564_c45300;full_length_coverage=1;non_full_length_coverage=4;isoform_length=3014;expected_accuracy=0.996	gnl|BL_ORD_ID|55623 cb8564_c47027/f1p0/3544	243	3014	1	246	401	3198	1	244
cb5896_c10/f2p1/2427 isoform=cb5896_c10;full_length_coverage=2;non_full_length_coverage=1;isoform_length=2427;expected_accuracy=0.998	gnl|BL_ORD_ID|44558 cb5896_c2/f6p2/2670 isoform=cb5896_c2;full_length_coverage=6;non_full_length_coverage=2;isoform_length=2670;expected_accuracy=1.000	1	1304	1303	2427	54	1357	1482	2609
cb8564_c38035/f1p0/2770	gnl|BL_ORD_ID|56722 cb8564_c54360/f1p0/3040	1	2035	2033	2745	1	2038	2195	2908
cb7781_c0/f2p0/1925 isoform=cb7781_c0;full_length_coverage=2;non_full_length_coverage=0;isoform_length=1925;expected_accuracy=0.999	gnl|BL_ORD_ID|7844 cb7781_c1/f1p0/2534 isoform=cb7781_c1;full_length_coverage=1;non_full_length_coverage=0;isoform_length=2534;expected_accuracy=0.996	500	1853	6	504	1181	2534	1	496
cb2737_c63/f1p2/2799 isoform=cb2737_c63;full_length_coverage=1;non_full_length_coverage=2;isoform_length=2799;expected_accuracy=0.994	gnl|BL_ORD_ID|68786 cb2737_c50/f1p0/3706	109	1514	1512	2799	675	2082	2214	3504

cb8564_c48538/f1p0/2599	gnl|BL_ORD_ID|14026 cb8564_c43137/f1p0/2726 isoform=cb8564_c43137;full_length_coverage=1;non_full_length_coverage=0;isoform_length=2726;expected_accuracy=0.996	553	2462	81	558	807	2715	160	637
cb8757_c7/f1p1/1789	gnl|BL_ORD_ID|17194 cb8757_c2/f1p1/2156 isoform=cb8757_c2;full_length_coverage=1;non_full_length_coverage=1;isoform_length=2156;expected_accuracy=0.993	221	1756	6	223	623	2155	3	220
cb2304_c1/f2p0/2952 isoform=cb2304_c1;full_length_coverage=2;non_full_length_coverage=0;isoform_length=2952;expected_accuracy=0.997	gnl|BL_ORD_ID|68424 cb2304_c9/f1p0/6807	99	2914	1	101	4069	6892	3362	3462
cb12939_c8/f1p0/1310 isoform=cb12939_c8;full_length_coverage=1;non_full_length_coverage=0;isoform_length=1310;expected_accuracy=0.993	gnl|BL_ORD_ID|61984 cb12939_c13/f1p0/6119	109	1283	1	110	4551	5722	5	116
cb584_c19/f1p0/3377 isoform=cb584_c19;full_length_coverage=1;non_full_length_coverage=0;isoform_length=3377;expected_accuracy=0.995	gnl|BL_ORD_ID|46932 cb584_c20/f1p0/4710 isoform=cb584_c20;full_length_coverage=1;non_full_length_coverage=0;isoform_length=4710;expected_accuracy=0.995	629	3377	66	631	1035	3772	1	568
cb8564_c43779/f1p0/3021 isoform=cb8564_c43779;full_length_coverage=1;non_full_length_coverage=0;isoform_length=3021;expected_accuracy=0.993	gnl|BL_ORD_ID|16886 cb8564_c50727/f1p0/3201 isoform=cb8564_c50727;full_length_coverage=1;non_full_length_coverage=0;isoform_length=3201;expected_accuracy=0.999	1	2344	2345	3021	3	2370	2526	3201
cb6063_c4/f2p0/2706 isoform=cb6063_c4;full_length_coverage=2;non_full_le	gnl|BL_ORD_ID|45456 cb6063_c27/f3p0/2968 isoform=cb6063_c27;full_length_coverage=3;non_full_l	238	2681	1	239	518	2965	1	237

ngth_coverage=0;isoform_length=2706;expected_accuracy=0.999	ength_coverage=0;isoform_length=2968;expected_accuracy=1.000								
cb8564_c5122/f1p0/3329	gnl|BL_ORD_ID|15704 cb8564_c5094/f3p0/3851 isoform=cb8564_c5094;full_length_coverage=3;non_full_length_coverage=0;isoform_length=3851;expected_accuracy=1.000	1067	3334	1	1069	1449	3716	89	1157
cb581_c2/f2p1/2578 isoform=cb581_c2;full_length_coverage=2;non_full_length_coverage=1;isoform_length=2578;expected_accuracy=0.998	gnl|BL_ORD_ID|45252 cb581_c3/f1p1/4740 isoform=cb581_c3;full_length_coverage=1;non_full_length_coverage=1;isoform_length=4740;expected_accuracy=0.997	1014	2578	1	1016	3107	4671	192	1227
cb5909_c2/f3p0/1152 isoform=cb5909_c2;full_length_coverage=3;non_full_length_coverage=0;isoform_length=1152;expected_accuracy=0.997	gnl|BL_ORD_ID|74631 cb5909_c6/f1p1/3343	532	1141	6	536	2511	3120	1	530
cb396_c48/f1p0/2172	gnl|BL_ORD_ID|40201 cb396_c30/f1p0/2423 isoform=cb396_c30;full_length_coverage=1;non_full_length_coverage=0;isoform_length=2423;expected_accuracy=0.996	238	2104	40	238	458	2325	3	208
cb8564_c49562/f1p0/2838 isoform=cb8564_c49562;full_length_coverage=1;non_full_length_coverage=0;isoform_length=2838;expected_accuracy=0.995	gnl|BL_ORD_ID|12938 cb8564_c181163/f1p1/2936 isoform=cb8564_c181163;full_length_coverage=1;non_full_length_coverage=1;isoform_length=2936;expected_accuracy=0.993	1	2331	2327	2838	1	2321	2427	2933
cb5281_c9/f1p0/3211 isoform=cb5281_c9;full_length_coverage=1;non_full_length_coverage=0;isoform_length=3211;expected_accuracy=0.995	gnl|BL_ORD_ID|73727 cb5281_c5/f1p0/3493	1309	3211	1	1313	1471	3375	1	1302

cb5487_c5/f1p0/2472	gnl|BL_ORD_ID|73975 cb5487_c11/f1p0/2702	1	1481	1481	2482	4	147 7	1646	267 1
cb8564_c52804/f1p5/3221 isoform=cb8564_c52804;full_length_coverage=1;non_full_length_coverage=5;isoform_length=3221;expected_accuracy=0.999	gnl|BL_ORD_ID|55939 cb8564_c49630/f1p4/3487	2	2914	2914	3164	1	291 3	3243	349 2
cb8564_c46939/f1p1/2926	gnl|BL_ORD_ID|16681 cb8564_c54233/f1p1/3194 isoform=cb8564_c54233;full_length_coverage=1;non_full_length_coverage=1;isoform_length=3194;expected_accuracy=0.995	2	2134	2130	2870	8	216 6	2271	301 2
cb2055_c16/f1p0/2452	gnl|BL_ORD_ID|35222 cb2055_c22/f1p0/3295 isoform=cb2055_c22;full_length_coverage=1;non_full_length_coverage=0;isoform_length=3295;expected_accuracy=0.994	1	1898	1896	2406	145	203 4	2784	329 5
cb396_c18/f1p0/2281 isoform=cb396_c18;full_length_coverage=1;non_full_length_coverage=0;isoform_length=2281;expected_accuracy=0.999	gnl|BL_ORD_ID|70865 cb396_c49/f1p0/3077	249	2265	84	251	521	253 5	21	190
cb2875_c0/f7p0/3313 isoform=cb2875_c0;full_length_coverage=7;non_full_length_coverage=0;isoform_length=3313;expected_accuracy=0.997	gnl|BL_ORD_ID|70060 cb2875_c14/f1p1/3543	1592	3298	1	1591	1816	352 3	1	158 7
cb8564_c45300/f1p4/3014 isoform=cb8564_c45300;full_length_coverage=1;non_full_length_coverage=4;isoform_length=3014;expected_accuracy=0.996	gnl|BL_ORD_ID|16447 cb8564_c47202/f1p0/3195 isoform=cb8564_c47202;full_length_coverage=1;non_full_length_coverage=0;isoform_length=3195;expected_accuracy=0.996	243	3013	1	246	400	319 5	1	243

cb12302_c31/f1p0/1548 isoform=cb12302_c31;full_length_coverage=1;non_full_length_coverage=0;isoform_length=1548;expected_accuracy=1.000	gnl|BL_ORD_ID|61307 cb1380_c29/f1p2/1918	324	1548	8	329	579	180 3	14	335
cb8564_c45300/f1p4/3014 isoform=cb8564_c45300;full_length_coverage=1;non_full_length_coverage=4;isoform_length=3014;expected_accuracy=0.996	gnl|BL_ORD_ID|15924 cb8564_c52804/f1p5/3221 isoform=cb8564_c52804;full_length_coverage=1;non_full_length_coverage=5;isoform_length=3221;expected_accuracy=0.999	243	3014	1	246	398	317 3	1	241
cb13355_c8/f1p0/1342 isoform=cb13355_c8;full_length_coverage=1;non_full_length_coverage=0;isoform_length=1342;expected_accuracy=0.998	gnl|BL_ORD_ID|25368 cb13355_c6/f5p0/1516 isoform=cb13355_c6;full_length_coverage=5;non_full_length_coverage=0;isoform_length=1516;expected_accuracy=1.000	1	1017	1016	1342	11	102 1	1134	146 0
cb395_c4/f1p0/3249	gnl|BL_ORD_ID|40013 cb395_c2/f1p0/3218 isoform=cb395_c2;full_length_coverage=1;non_full_length_coverage=0;isoform_length=3218;expected_accuracy=0.992	15	2595	2598	3082	1	256 3	2738	321 8
cb8564_c36731/f1p2/2761	gnl|BL_ORD_ID|16886 cb8564_c50727/f1p0/3201 isoform=cb8564_c50727;full_length_coverage=1;non_full_length_coverage=0;isoform_length=3201;expected_accuracy=0.999	1	1977	1972	2738	3	198 2	2434	320 1
cb3427_c11/f1p1/1420 isoform=cb3427_c11;full_length_coverage=1;non_full_length_coverage=1;isoform_length=1420;expected_accuracy=1.000	gnl|BL_ORD_ID|39944 cb3427_c5/f1p0/3487 isoform=cb3427_c5;full_length_coverage=1;non_full_length_coverage=0;isoform_length=3487;expected_accuracy=0.997	478	1375	8	483	2590	348 7	1	465
cb8564_c110147/f1p0/3399 isoform=cb8564_c110147;full_length_coverage=1;non_	gnl|BL_ORD_ID|54608 cb8564_c127899/f1p0/3880	1	2834	2832	3347	221	307 5	3305	382 0

full_length_coverage=0;isoform_length=3399;expected_accuracy=0.999									
cb12934_c0/f2p0/1538 isoform=cb12934_c0;full_length_coverage=2;non_full_length_coverage=0;isoform_length=1538;expected_accuracy=1.000	gnl|BL_ORD_ID|64566 cb15252_c15/f1p0/2529	1	1201	1199	1512	1	1187	2147	2463
cb4233_c169/f1p0/2382	gnl|BL_ORD_ID|71865 cb4233_c138/f1p0/3035	119	1697	1692	2378	7	1586	2306	2993
cb8564_c36056/f1p0/2652 isoform=cb8564_c36056;full_length_coverage=1;non_full_length_coverage=0;isoform_length=2652;expected_accuracy=0.999	gnl|BL_ORD_ID|55962 cb8564_c50282/f1p0/4402	248	2651	1	251	1906	4310	1512	1762
cb1928_c2/f3p3/3017 isoform=cb1928_c2;full_length_coverage=3;non_full_length_coverage=3;isoform_length=3017;expected_accuracy=0.998	gnl|BL_ORD_ID|66501 cb1928_c9/f1p0/3652	193	3017	1	195	359	3185	3	200
cb8564_c43074/f1p0/3464	gnl|BL_ORD_ID|56041 cb8564_c53077/f1p2/3406	357	3198	37	357	452	3292	25	337
cb2804_c108/f1p0/3215 isoform=cb2804_c108;full_length_coverage=1;non_full_length_coverage=0;isoform_length=3215;expected_accuracy=0.999	gnl|BL_ORD_ID|37800 cb2804_c106/f1p0/3501 isoform=cb2804_c106;full_length_coverage=1;non_full_length_coverage=0;isoform_length=3501;expected_accuracy=0.996	1	1879	1876	3215	3	1884	2062	3396
cb1203_c25/f1p0/1362	gnl|BL_ORD_ID|22718 cb1203_c2/f1p2/4432 isoform=cb1203_c2;full_length_coverage=1;non_full_length_coverage=2;isoform_length=4432;expected_accuracy=0.997	157	1269	4	157	3204	4316	2222	2375

cb8564_c38035/f1p0/2770	gnl|BL_ORD_ID|54698 cb8564_c155298/f3p9/3822	1	2035	2033	2756	1	2038	2228	2952
cb4563_c33/f1p0/2825	gnl|BL_ORD_ID|72683 cb4563_c12/f1p0/3843	229	2605	2	225	1426	3791	636	860
cb567_c14/f1p0/3377	gnl|BL_ORD_ID|45926 cb567_c4/f1p0/3256 isoform=cb567_c4;full_length_coverage=1;non_full_length_coverage=0;isoform_length=3256;expected_accuracy=0.996	1	2644	2642	3073	54	2689	2829	3256
cb2640_c18/f1p0/3205 isoform=cb2640_c18;full_length_coverage=1;non_full_length_coverage=0;isoform_length=3205;expected_accuracy=0.991	gnl|BL_ORD_ID|69859 cb2640_c29/f1p0/3441	188	3094	1	189	355	3249	40	228
cb8564_c74180/f1p1/2858	gnl|BL_ORD_ID|42781 cb4681_c0/f1p1/3285 isoform=cb4681_c0;full_length_coverage=1;non_full_length_coverage=1;isoform_length=3285;expected_accuracy=0.995	1	2587	2585	2854	3161	575	436	167
cb14859_c10/f1p0/5042	gnl|BL_ORD_ID|30140 cb14859_c6/f1p4/5345 isoform=cb14859_c6;full_length_coverage=1;non_full_length_coverage=4;isoform_length=5345;expected_accuracy=0.999	1404	4825	1	1407	1837	5261	1	1409
cb6691_c13/f1p0/2308	gnl|BL_ORD_ID|5097 cb6691_c22/f13p0/2328 isoform=cb6691_c22;full_length_coverage=13;non_full_length_coverage=0;isoform_length=2328;expected_accuracy=1.000	1010	2135	1	1010	1192	2317	83	1092
cb8288_c8/f1p0/1153	gnl|BL_ORD_ID|9352 cb8288_c2/f2p0/2253 isoform=cb8288_c2;full_length_coverage=2;non_full_le	170	1071	2	171	1344	2245	11	181

	ngth_coverage=0;isoform_length=2253;expected_accuracy=1.000								
cb12714_c29/f1p2/1583	gnl|BL_ORD_ID|55603 cb8564_c46500/f1p1/2752	1	1296	1293	1596	2	1294	2358	2661
cb8564_c21439/f1p0/2587	gnl|BL_ORD_ID|19105 cb8564_c98209/f1p10/2805 isoform=cb8564_c98209;full_length_coverage=1;non_full_length_coverage=10;isoform_length=2805;expected_accuracy=1.000	1226	2572	1	1228	1351	2697	3	1230
cb8564_c5239/f2p0/2655 isoform=cb8564_c5239;full_length_coverage=2;non_full_length_coverage=0;isoform_length=2655;expected_accuracy=0.996	gnl|BL_ORD_ID|16140 cb8564_c5238/f1p0/2896 isoform=cb8564_c5238;full_length_coverage=1;non_full_length_coverage=0;isoform_length=2896;expected_accuracy=0.996	1	2253	2250	2654	13	2248	2490	2896
cb7214_c2/f1p0/2219 isoform=cb7214_c2;full_length_coverage=1;non_full_length_coverage=0;isoform_length=2219;expected_accuracy=0.992	gnl|BL_ORD_ID|7790 cb7214_c1/f1p0/2667 isoform=cb7214_c1;full_length_coverage=1;non_full_length_coverage=0;isoform_length=2667;expected_accuracy=0.997	1	1470	1468	2217	2	1479	1909	2663
cb1822_c3/f1p1/3787 isoform=cb1822_c3;full_length_coverage=1;non_full_length_coverage=1;isoform_length=3787;expected_accuracy=0.998	gnl|BL_ORD_ID|36294 cb18848_c0/f1p0/5897 isoform=cb18848_c0;full_length_coverage=1;non_full_length_coverage=0;isoform_length=5897;expected_accuracy=0.996	1	3205	3206	3711	2017	5227	5393	5897
cb8564_c40764/f1p0/2563 isoform=cb8564_c40764;full_length_coverage=1;non_full_length_coverage=0;isoform_length=2563;expected_accuracy=0.997	gnl|BL_ORD_ID|55291 cb8564_c37409/f1p0/3153	1	2167	2162	2563	1	2159	2366	2766
cb8564_c42506/f1p0/2909	gnl|BL_ORD_ID|14992 cb8564_c45033/f1p5/3781 isoform=cb8564_c45033;full_length_coverage=1;non_fu	1	2203	2199	2850	1057	3258	3128	3781

<table>
<tr><th></th><th></th><th></th><th></th><th></th><th></th><th></th><th></th><th></th><th></th></tr>
<tr><td></td><td>ll_length_coverage=5;isoform_length=3781;expected_accuracy=1.000</td><td></td><td></td><td></td><td></td><td></td><td></td><td></td><td></td></tr>
<tr><td>cb4744_c3/f1p0/2848</td><td>gnl|BL_ORD_ID|44018 cb4744_c0/f2p0/2942 isoform=cb4744_c0;full_length_coverage=2;non_full_length_coverage=0;isoform_length=2942;expected_accuracy=0.997</td><td>1</td><td>2456</td><td>2452</td><td>2829</td><td>8</td><td>2454</td><td>2565</td><td>2942</td></tr>
<tr><td>cb1253_c8/f1p0/3618</td><td>gnl|BL_ORD_ID|23170 cb1253_c2/f2p0/3323 isoform=cb1253_c2;full_length_coverage=2;non_full_length_coverage=0;isoform_length=3323;expected_accuracy=0.999</td><td>197</td><td>3164</td><td>101</td><td>199</td><td>313</td><td>3278</td><td>3</td><td>99</td></tr>
<tr><td>cb944_c34/f1p0/3024 isoform=cb944_c34;full_length_coverage=1;non_full_length_coverage=0;isoform_length=3024;expected_accuracy=0.995</td><td>gnl|BL_ORD_ID|19525 cb944_c27/f2p0/3667 isoform=cb944_c27;full_length_coverage=2;non_full_length_coverage=0;isoform_length=3667;expected_accuracy=0.999</td><td>1449</td><td>3024</td><td>1</td><td>1451</td><td>2068</td><td>3656</td><td>1</td><td>1465</td></tr>
<tr><td>cb4342_c4/f1p0/2299 isoform=cb4342_c4;full_length_coverage=1;non_full_length_coverage=0;isoform_length=2299;expected_accuracy=1.000</td><td>gnl|BL_ORD_ID|43927 cb4342_c1/f2p0/3323 isoform=cb4342_c1;full_length_coverage=2;non_full_length_coverage=0;isoform_length=3323;expected_accuracy=0.996</td><td>1</td><td>1739</td><td>1737</td><td>2299</td><td>12</td><td>1758</td><td>2561</td><td>3115</td></tr>
<tr><td>cb8645_c5/f1p0/1752 isoform=cb8645_c5;full_length_coverage=1;non_full_length_coverage=0;isoform_length=1752;expected_accuracy=0.997</td><td>gnl|BL_ORD_ID|18589 cb8645_c4/f1p0/2467 isoform=cb8645_c4;full_length_coverage=1;non_full_length_coverage=0;isoform_length=2467;expected_accuracy=0.999</td><td>486</td><td>1752</td><td>82</td><td>488</td><td>1099</td><td>2365</td><td>3</td><td>410</td></tr>
<tr><td>cb1467_c0/f2p0/4330 isoform=cb1467_c0;full_length_coverage=2;non_full_length_coverage=0;isoform_length=4330;expected_accuracy=0.994</td><td>gnl|BL_ORD_ID|30647 cb1467_c6/f1p0/5175 isoform=cb1467_c6;full_length_coverage=1;non_full_length_coverage=0;isoform_length=5175;expected_accuracy=0.994</td><td>382</td><td>4330</td><td>1</td><td>384</td><td>1187</td><td>5156</td><td>127</td><td>512</td></tr>
</table>

cb9718_c43/f1p0/1655 isoform=cb9718_c43;full_length_coverage=1;non_full_length_coverage=0;isoform_length=1655;expected_accuracy=0.996	gnl|BL_ORD_ID|59925 cb9718_c13/f1p0/1961	288	1655	3	290	391	1758	4	291
cb5345_c7/f1p0/1521 isoform=cb5345_c7;full_length_coverage=1;non_full_length_coverage=0;isoform_length=1521;expected_accuracy=0.998	gnl|BL_ORD_ID|73806 cb5345_c0/f1p1/3127	1	1141	1141	1521	28	1168	2440	2820
cb4761_c1/f8p0/2802 isoform=cb4761_c1;full_length_coverage=8;non_full_length_coverage=0;isoform_length=2802;expected_accuracy=0.999	gnl|BL_ORD_ID|43068 cb4761_c2/f1p0/3263 isoform=cb4761_c2;full_length_coverage=1;non_full_length_coverage=0;isoform_length=3263;expected_accuracy=0.996	285	2793	41	285	756	3263	1	245
cb8564_c37361/f1p0/3076	gnl|BL_ORD_ID|55243 cb8564_c36242/f1p0/3335	1	2481	2477	3069	1	2484	2585	3176
cb8564_c45609/f1p0/3991	gnl|BL_ORD_ID|56019 cb8564_c52510/f6p2/4563	260	3797	1	260	489	4040	41	300
cb9467_c4/f9p0/2081 isoform=cb9467_c4;full_length_coverage=9;non_full_length_coverage=0;isoform_length=2081;expected_accuracy=0.999	gnl|BL_ORD_ID|21565 cb9467_c22/f1p0/2220 isoform=cb9467_c22;full_length_coverage=1;non_full_length_coverage=0;isoform_length=2220;expected_accuracy=0.995	163	2067	1	162	281	2179	1	161
cb10088_c26/f1p0/1030	gnl|BL_ORD_ID|2975 cb10088_c25/f1p3/1453 isoform=cb10088_c25;full_length_coverage=1;non_full_length_coverage=3;isoform_length=1453;expected_accuracy=1.000	476	1034	7	479	716	1274	1	473
cb10486_c1/f13p1/1234 isoform=cb10486_c1;full_length_coverage=13;non_full	gnl|BL_ORD_ID|1068 cb10486_c2/f1p0/2550 isoform=cb10486_c2;full_length_coverage=1;non_full_l	525	1234	6	526	1841	2550	3	523

_length_coverage=1;isoform_length=1234;expected_accuracy=1.000	ength_coverage=0;isoform_length=2550;expected_accuracy=0.997								
cb8564_c36731/f1p2/2761	gnl|BL_ORD_ID|14471 cb8564_c34518/f1p0/3136 isoform=cb8564_c34518;full_length_coverage=1;non_full_length_coverage=0;isoform_length=3136;expected_accuracy=0.991	1	1977	1972	2738	1	195<br>2	2376	313<br>6
cb1913_c44/f1p0/2236 isoform=cb1913_c44;full_length_coverage=1;non_full_length_coverage=0;isoform_length=2236;expected_accuracy=0.998	gnl|BL_ORD_ID|35835 cb1913_c34/f1p1/2444 isoform=cb1913_c34;full_length_coverage=1;non_full_length_coverage=1;isoform_length=2444;expected_accuracy=0.993	1	1634	1630	2234	42	169<br>2	1817	243<br>1
cb5425_c29/f1p0/1623	gnl|BL_ORD_ID|45676 cb5425_c6/f4p0/1999 isoform=cb5425_c6;full_length_coverage=4;non_full_length_coverage=0;isoform_length=1999;expected_accuracy=1.000	1	1218	1216	1628	5	123<br>1	1558	197<br>0
cb2737_c64/f1p1/2838 isoform=cb2737_c64;full_length_coverage=1;non_full_length_coverage=1;isoform_length=2838;expected_accuracy=0.993	gnl|BL_ORD_ID|36735 cb2737_c13/f1p0/3105 isoform=cb2737_c13;full_length_coverage=1;non_full_length_coverage=0;isoform_length=3105;expected_accuracy=0.995	1	1537	1535	2838	154	167<br>6	1808	310<br>3
cb6391_c14/f1p1/2258 isoform=cb6391_c14;full_length_coverage=1;non_full_length_coverage=1;isoform_length=2258;expected_accuracy=0.994	gnl|BL_ORD_ID|3495 cb6391_c11/f1p0/2658 isoform=cb6391_c11;full_length_coverage=1;non_full_length_coverage=0;isoform_length=2658;expected_accuracy=0.993	197	2258	1	199	520	257<br>8	49	246
cb4848_c10/f1p0/1930 isoform=cb4848_c10;full_length_coverage=1;non_full_length_coverage=0;isoform_length=1930;expected_accuracy=0.993	gnl|BL_ORD_ID|72032 cb4848_c9/f1p1/2843	138	1930	1	137	1010	280<br>1	304	442

cb8564_c45873/f1p2/2968	gnl|BL_ORD_ID|9350 cb8564_c11826/f1p0/2916 isoform=cb8564_c11826;full_length_coverage=1;non_full_length_coverage=0;isoform_length=2916;expected_accuracy=0.997	1293	2665	1	1294	1543	2916	4	1296
cb11024_c1/f1p0/1252	gnl|BL_ORD_ID|49094 cb11024_c2/f1p0/2609	431	1251	8	433	1811	2631	6	431
cb6258_c22/f1p0/2295 isoform=cb6258_c22;full_length_coverage=1;non_full_length_coverage=0;isoform_length=2295;expected_accuracy=0.997	gnl|BL_ORD_ID|6049 cb6258_c8/f2p1/2472 isoform=cb6258_c8;full_length_coverage=2;non_full_length_coverage=1;isoform_length=2472;expected_accuracy=0.995	449	2295	53	451	535	2382	1	399
cb7635_c12/f1p0/1743 isoform=cb7635_c12;full_length_coverage=1;non_full_length_coverage=0;isoform_length=1743;expected_accuracy=0.995	gnl|BL_ORD_ID|7418 cb7635_c1/f5p2/2333 isoform=cb7635_c1;full_length_coverage=5;non_full_length_coverage=2;isoform_length=2333;expected_accuracy=1.000	1	1347	1344	1743	1	1335	1868	2272
cb14663_c3/f1p0/1061 isoform=cb14663_c3;full_length_coverage=1;non_full_length_coverage=0;isoform_length=1061;expected_accuracy=0.998	gnl|BL_ORD_ID|902 cb10034_c28/f1p5/2081 isoform=cb10034_c28;full_length_coverage=1;non_full_length_coverage=5;isoform_length=2081;expected_accuracy=1.000	6	599	599	1039	1557	966	464	23
cb8564_c23436/f1p0/3497 isoform=cb8564_c23436;full_length_coverage=1;non_full_length_coverage=0;isoform_length=3497;expected_accuracy=0.994	gnl|BL_ORD_ID|11711 cb8564_c14355/f1p0/3726 isoform=cb8564_c14355;full_length_coverage=1;non_full_length_coverage=0;isoform_length=3726;expected_accuracy=0.995	179	3497	1	182	397	3715	1	182
cb8564_c4904/f1p0/3104 isoform=cb8564_c4904;full_length_coverage=1;non_full_length_coverage=0;isoform_length=3104;expected_accuracy=0.998	gnl|BL_ORD_ID|55701 cb8564_c4898/f1p0/3489	237	3082	1	236	454	3303	31	266

cb2533_c30/f1p0/3420 isoform=cb2533_c30;full_length_coverage=1;non_full_length_coverage=0;isoform_length=3420;expected_accuracy=0.996	gnl|BL_ORD_ID|69802 cb2533_c17/f1p0/3689	17	2837	2834	3420	16	2834	3018	3605
cb8564_c90621/f1p2/3030 isoform=cb8564_c90621;full_length_coverage=1;non_full_length_coverage=2;isoform_length=3030;expected_accuracy=0.994	gnl|BL_ORD_ID|57409 cb8564_c92262/f1p0/3503	206	3030	1	205	459	3282	121	325
cb8564_c49562/f1p0/2838 isoform=cb8564_c49562;full_length_coverage=1;non_full_length_coverage=0;isoform_length=2838;expected_accuracy=0.995	gnl|BL_ORD_ID|15994 cb8564_c54019/f1p1/2940 isoform=cb8564_c54019;full_length_coverage=1;non_full_length_coverage=1;isoform_length=2940;expected_accuracy=0.991	1	2331	2327	2836	3	2320	2425	2932
cb5564_c21/f1p0/1889	gnl|BL_ORD_ID|74068 cb5564_c17/f1p0/2403	355	1844	5	359	816	2306	219	577
cb5896_c25/f1p1/3090 isoform=cb5896_c25;full_length_coverage=1;non_full_length_coverage=1;isoform_length=3090;expected_accuracy=1.000	gnl|BL_ORD_ID|74558 cb5896_c23/f1p0/4184	114	1791	1792	3090	1	1678	2860	4157
cb8564_c108739/f7p0/2789 isoform=cb8564_c108739;full_length_coverage=7;non_full_length_coverage=0;isoform_length=2789;expected_accuracy=1.000	gnl|BL_ORD_ID|11086 cb8564_c118096/f1p0/2820 isoform=cb8564_c118096;full_length_coverage=1;non_full_length_coverage=0;isoform_length=2820;expected_accuracy=0.991	1	1546	1543	2720	7	1544	1653	2820
cb6490_c6/f1p0/2575	gnl|BL_ORD_ID|49764 cb6490_c1/f1p0/2838	1	1469	1468	2465	2	1463	1840	2837
cb8564_c123436/f1p0/3559	gnl|BL_ORD_ID|10971 cb8564_c115642/f1p0/3794 isoform=cb8564_c115642;full_length_coverage=1;non_f	1011	3262	1	1012	1550	3794	42	1048

	ull_length_coverage=0;isoform_length=3794;expected_accuracy=0.992								
cb581_c6/f1p0/2672 isoform=cb581_c6;full_length_coverage=1;non_full_length_coverage=0;isoform_length=2672;expected_accuracy=0.998	gnl|BL_ORD_ID|45252 cb581_c3/f1p1/4740 isoform=cb581_c3;full_length_coverage=1;non_full_length_coverage=1;isoform_length=4740;expected_accuracy=0.997	1229	2672	1	1231	3107	4550	1	1227
cb8564_c185920/f1p0/2777 isoform=cb8564_c185920;full_length_coverage=1;non_full_length_coverage=0;isoform_length=2777;expected_accuracy=0.996	gnl|BL_ORD_ID|55291 cb8564_c37409/f1p0/3153	174	2772	1	177	351	2948	15	192
cb1333_c9/f4p0/1480 isoform=cb1333_c9;full_length_coverage=4;non_full_length_coverage=0;isoform_length=1480;expected_accuracy=1.000	gnl|BL_ORD_ID|26023 cb1333_c10/f4p0/1937 isoform=cb1333_c10;full_length_coverage=4;non_full_length_coverage=0;isoform_length=1937;expected_accuracy=1.000	153	1473	29	152	614	1930	19	142
cb8564_c84530/f1p0/2483 isoform=cb8564_c84530;full_length_coverage=1;non_full_length_coverage=0;isoform_length=2483;expected_accuracy=1.000	gnl|BL_ORD_ID|15869 cb8564_c77158/f1p0/4524 isoform=cb8564_c77158;full_length_coverage=1;non_full_length_coverage=0;isoform_length=4524;expected_accuracy=0.995	236	2483	1	235	2135	4376	30	265
cb2471_c1/f7p0/2064 isoform=cb2471_c1;full_length_coverage=7;non_full_length_coverage=0;isoform_length=2064;expected_accuracy=1.000	gnl|BL_ORD_ID|69758 cb2471_c14/f1p0/2261	170	2064	1	172	363	2256	1	182
cb10696_c1/f2p0/1444 isoform=cb10696_c1;full_length_coverage=2;non_full_length_coverage=0;isoform_length=1444;expected_accuracy=1.000	gnl|BL_ORD_ID|61792 cb12662_c11/f1p0/1530	132	1406	1	132	258	1532	3	126

cb816_c20/f5p2/2306 isoform=cb816_c20;full_length_coverage=5;non_full_length_coverage=2;isoform_length=2306;expected_accuracy=1.000	gnl|BL_ORD_ID|49480 cb816_c12/f1p0/3158	1	1340	1340	2228	1	1335	2121	3006
cb8564_c84198/f1p0/2418	gnl|BL_ORD_ID|17748 cb8564_c89509/f1p4/2644 isoform=cb8564_c89509;full_length_coverage=1;non_full_length_coverage=4;isoform_length=2644;expected_accuracy=1.000	1	2008	2006	2428	12	2034	2151	2573
cb341_c10/f1p0/3009	gnl|BL_ORD_ID|69575 cb341_c16/f1p0/3691	1	2686	2683	3015	10	2697	3001	3333
cb584_c29/f1p0/4045	gnl|BL_ORD_ID|45629 cb584_c18/f1p0/3716 isoform=cb584_c18;full_length_coverage=1;non_full_length_coverage=0;isoform_length=3716;expected_accuracy=0.996	227	3444	18	228	481	3715	1	218
cb4016_c32/f1p0/1913	gnl|BL_ORD_ID|71727 cb4016_c35/f1p0/2920	191	1930	23	194	1066	2805	466	638
cb18456_c1988/f1p0/1112	gnl|BL_ORD_ID|67384 cb18456_c1991/f1p0/1481	412	1017	8	416	716	1320	1	411
cb8564_c74541/f1p1/3268 isoform=cb8564_c74541;full_length_coverage=1;non_full_length_coverage=1;isoform_length=3268;expected_accuracy=0.992	gnl|BL_ORD_ID|15223 cb8564_c35037/f1p0/3377 isoform=cb8564_c35037;full_length_coverage=1;non_full_length_coverage=0;isoform_length=3377;expected_accuracy=0.996	14	2774	2772	3268	2	2761	2866	3367
cb2918_c65/f1p0/3088	gnl|BL_ORD_ID|70088 cb2918_c33/f1p3/2817	1	1818	1818	2770	118	1934	1755	2708
cb8687_c5/f1p0/1932 isoform=cb8687_c5;full_length_coverage=1;non_full_le	gnl|BL_ORD_ID|18632 cb8687_c4/f1p0/3035 isoform=cb8687_c4;full_length_coverage=1;non_full_le	1	1322	1321	1897	31	1360	2459	3035

ngth_coverage=0;isoform_length=1932;expected_accuracy=1.000	ngth_coverage=0;isoform_length=3035;expected_accuracy=0.990								
cb8564_c45300/f1p4/3014 isoform=cb8564_c45300;full_length_coverage=1;non_full_length_coverage=4;isoform_length=3014;expected_accuracy=0.996	gnl|BL_ORD_ID|54425 cb8564_c42906/f1p2/3181	243	3014	1	246	399	3152	1	242
cb8564_c84022/f1p0/2360	gnl|BL_ORD_ID|56412 cb8564_c76337/f1p1/2735	1	1561	1559	2334	2	1563	1699	2474
