# Supplementary material for: Single-molecule long-read sequencing of the full-length transcriptome of Rhododendron lapponicum L
Source: Sci Rep. 2020 Apr 21;10:6755. doi: 10.1038/s41598-020-63814-x (PMC7174332; doi:10.1038/s41598-020-63814-x)
Supplement: Supplementary file 8 — Supplementary Table S8. [file 41598_2020_63814_MOESM8_ESM.pdf]

Single-molecule long-read sequencing of the full-length transcriptome of *Rhododendron lapponicum* L.

Xinping Jia, Ling Tang, Xueying Mei, Huazhou Liu, Hairong Luo, Yanming Deng, Jiale Su

Institute of Leisure Agriculture, Jiangsu Academy of Agricultural Sciences, Jiangsu Key Laboratory for Horticultural Crop Genetic Improvement, Nanjing 210014, China

Table S8 The characterizations of EST-SSR markers

Primer ID	Gene ID	Forward Primer (5'- 3')	Reverse Primer (5'- 3')	Product Size (bp)	Repeat motif	SSR Length	Amplification result
RL-1	R01_cb8564_c84685/f1p0/3092	CCCATTTCTGTGCTCCTTGT	CACGTCTTCTTCGCCTTTCT	212	(TC)7	14	S
RL-2	R01_cb7730_c11/f1p0/2324	AGCAAGCAAGGCATTCTGTT	CAAACGTCTCCCATTGTGTG	123	(CAG)5	15	S
RL-3	R01_cb14480_c7/f1p0/1514	CTTTGGGGAGTGCAATCAAT	CCACCATCTCATCTCCACCT	249	(T)13	13	S
RL-4	R01_cb8564_c3089/f1p0/2326	TAGCAGTCCAAAACCCATCC	GTATTTCATGTCCTGCGCCT	227	(CT)16	32	S
RL-5	R01_cb2533_c20/f1p0/3689	TCCTCCTTCCCTCTACCACA	AACGGGCAATTTCGTATCTG	172	(AG)12	24	F
RL-6	R01_cb7418_c22/f1p0/2362	GATAGTGGTTCAGGCTCCGA	CCATGAGCAAGCAAAACAAA	108	(TTTTG)5	25	S
RL-7	R01_cb8564_c125704/f1p0/3725	ACTACGTGTGATTTTCGGGC	CTTTGTCCCCTACGAAACCA	196	(A)18	18	S
RL-8	R01_cb8564_c81078/f1p0/3804	CCCTAGGGTTTATGTGGTGTG	CTGAAGCAAACGGAAATGGT	230	(TC)8	16	S
RL-9	R01_cb11851_c41/f1p1/1532	CATAAGGAGCCACCAAAGGA	CAAAACAAGCAAAATTGCGA	249	(TGA)5	15	S
RL-10	R01_cb7067_c8/f1p0/2552	GCACAAGACCAGCATCTCAA	CGCCGAGAGAAAGAGAGAAA	159	(CT)10	20	S
RL-11	R01_cb15596_c6/f1p0/1526	CCTGCCTACTGTCCCCTCTA	GGTTCAATACGTCCACACCC	243	(TTTTCT)7	42	S
RL-12	R01_cb5595_c24/f1p0/2545	ACCAAAACACTCTCATCGGG	GCAGAACTTGAGGGTCTGGA	196	(TCC)5	15	S
RL-13	R01_cb3103_c1/f1p0/2830	GCCTGGACGCTTGAGTTATC	AATGTAATCCCGGATCATGC	280	(A)13	13	S
RL-14	R01_cb5517_c0/f1p0/3079	GTCCCACATCCAGGACACTT	TGCCGAGTTGAGTTCTGAAA	267	(C)49	49	S
RL-15	R01_cb9002_c3/f1p0/1775	AACCGATCACTCCGTCACTC	GAAGGTAATCGGTTGAGGCA	122	(ACC)5	15	F
RL-16	R01_cb1196_c7/f1p0/2899	GATAGGCGGATGGGTATTCA	GATTGCTGCCACCAAATTCT	258	(G)12	12	S
RL-17	R01_cb8564_c23779/f1p0/3567	TTATGGTTCCCTGTTCCCAA	CAAGGGTCAGTTTGCTCTCC	206	(AC)6	12	S
RL-18	R01_cb2095_c14/f1p0/4037	GTGGACTTCGGACAGGTGTT	CGAAGCTTGGCAAAGAAATC	242	(TTTTCT)5	30	S
RL-19	R01_cb8564_c13026/f1p0/4872	TTGTCCACTCACGCATTAGC	CAGTAGAGTTGCCGGAGGAG	247	(CA)13	26	S
RL-20	R01_cb8564_c9501/f5p0/2413	GTACATGTCCATGTCCGTGC	ATGGTGCTCAAAACCCAGAG	272	(CCGCCC)6	36	S
RL-21	R01_cb10015_c288/f1p0/705	CTGGGTCCTTTCCTCATCAA	ACCATGAAACCATGGCAACT	141	(TC)10	20	S
RL-22	R01_cb12646_c21/f1p0/5253	TTGATTGGGAATGGGTGAAT	CTGTGGGTGAAGGAGGAGAG	181	(C)14	14	S
RL-23	R01_cb10249_c3/f1p0/2012	AACAAGGCATTGAAACTCGG	TTTAGGAGCTTCCTCCCTCC	206	(TCCCAC)5	30	F
RL-24	R01_cb12608_c6/f1p1/1493	GACAATCCCTGTGGACCTGT	CGAATTTGGATTGAATCGCT	218	(T)24	24	S

RL-25	R01_cb8564_c146119/f9p4/2560	GGTCCGAATTTCTGATTGGA	CTTGCGAGATGTGGACGTAA	263	(GA)30	60	F
RL-26	R01_cb6640_c1/f1p0/2800	GATGGACAATCGACAATCCC	TCTCTCCAAACCCTCCATTG	267	(TCG)5	15	S
RL-27	R01_cb12691_c3/f3p0/823	AGCTCAAAGGTTTCCCCAGT	GTCGCCAACCTCTTCTTGTC	260	(TCC)6	18	S
RL-28	R01_cb17042_c0/f1p1/1015	ATTCCTCCCTCTGACGGTCT	CGGTGATTGAGAAGCAGACA	131	(GATA)5	20	S
RL-29	R01_cb12916_c4/f2p4/756	TCGGAGCTCGAAACCTACTC	GAAATGGCGGTAGAGGAACA	240	(AAC)5	15	S
RL-30	R01_cb1969_c1/f1p0/4106	GAGGGATGGGAAACTGGACT	TCCTAACATCGAATGGGCTC	265	(TA)6	12	S
RL-31	R01_cb5494_c3/f1p0/2060	GTCGACAGGGAGGAACTGAA	TGAGGCCCCACAGATTTAAG	266	(CCG)7	21	S
RL-32	R01_cb8564_c37057/f1p2/3976	TATGTCAGGAAATCTGCCCC	GAAGGGATTCACGGACAAAA	228	(A)29	29	S
RL-33	R01_cb5290_c33/f1p0/3137	GACAACGCTCCATTCTCCAT	AAAGTATGCATGGAGCCGAC	270	(CT)14	28	F
RL-34	R01_cb6802_c83/f1p0/2222	GCAGAGCAACCATATCGTCA	AACTGCCATTGTACCTTCCG	257	(GAA)7	21	S
RL-35	R01_cb11118_c1/f1p0/2955	GATTCCCATGGATGATGGAC	CTGCTATTGTGTTTTCCGCA	181	(AT)10	20	S
RL-36	R01_cb2930_c10/f1p2/2925	ATAGGTGGAAGAACCAGGCA	CTACCAAACACTACGCCGGT	234	(TCA)5	15	S
RL-37	R01_cb538_c3/f1p0/4762	TCACCACCACCTTTCACAAA	CCGTAAAAATGATGGTCGCT	224	(G)36	36	S
RL-38	R01_cb5119_c0/f1p0/3133	CGGACACTCTCCTATCATACACC	TGTGAGAACAATCAGCTCCG	260	(CT)12	24	S
RL-39	R01_cb629_c5/f1p1/4671	GCCTCCCACTGCAAGTAAAG	TGGCAACACCAGCATATTGT	224	(CCG)6	18	S
RL-40	R01_cb1283_c5/f1p0/3409	CATGAGTGACGGGTTGATTG	TTTGTGGTCTGTGGTGGAAA	174	(CTG)7	21	F
RL-41	R01_cb6638_c6/f1p1/2651	CTTCTGTTTCGCGTAGGGAG	CGCATCGCAAAAGTTATGAA	172	(T)16	16	S
RL-42	R01_cb6290_c1/f1p0/2815	TTTTATTTCGCACCCTCGTC	AAACCGCTTGCTTTTGTGTT	142	(GA)13	26	S
RL-43	R01_cb3471_c0/f1p0/3611	TGCTCATCTTGTGTCCTGCT	GTTGGGGTGTTGTCTGGTTT	200	(AGT)5	15	S
RL-44	R01_cb6239_c11/f1p0/2522	TTACCCAGTGGCACAATTCA	GCGGGTTAAAATGAGCAGAG	255	(CTG)6	18	S
RL-45	R01_cb13274_c4/f1p1/936	ATAACCACAACGAACCCCAA	ACTGCTAATCCAGTCCACCG	113	(TC)22	44	S
RL-46	R01_cb6692_c15/f1p0/2891	CTACCGCAATTCACAGCAGA	TTCGGAGAGAGCGAGAGAAG	133	(CCTC)6	24	S
RL-47	R01_cb427_c22/f1p0/2136	GCAACGGAGGAGAACAAGAG	AAACAGCCTCCTTGACGAGA	195	(GAG)10	30	S
RL-48	R01_cb18521_c1/f1p0/1653	CATCCCCCTTCTTCTAAGCC	GGAGTCCATGGAAGAGGTGA	186	(CT)6	12	S
RL-49	R01_cb8564_c41535/f1p0/3083	GTGTCCTTTATTCTCCGCCA	CAAAGGATTCACGGGATCATA	216	(A)40	40	F
RL-50	R01_cb8564_c77584/f1p0/4530	TGACTTTGGAAGCCATCTCC	CTTGAAGGGGTCCCTGATCT	188	(C)13	13	S

RL-51	R01_cb3984_c14/f6p0/3196	GGTGTTACTTCGCCCGATTA	TGGCGTGAATTCTACTGCTG	149	(AGT)5	15	S
RL-52	R01_cb8564_c125253/f1p0/2315	ACCTACTCATTCCACTGCGG	CAAGTAGCGGAGCTGGTTTC	269	(TCGA)5	20	S
RL-53	R01_cb10048_c1/f6p0/1604	GGCTTATTAACGACACGGGA	ATCATCGTGACCTTGACCGT	263	(GGTCAC)11	66	S
RL-54	R01_cb7182_c11/f1p0/2681	AACACTCTTGTCCCCTGCAC	AGTGTCCTCCTTTCCTGGGT	272	(AGA)13	39	S
RL-55	R01_cb7863_c17/f1p0/2552	ACTCATCAGTTTTCCCCCAA	CTTTCGGATCTCGACCATGT	208	(TC)11	22	S
RL-56	R01_cb8564_c84227/f1p0/4645	CACCTGGAAGTTTTTGGGAA	AGGAGCAACATCGAGCCTAA	174	(GAAAAG)5	30	S
RL-57	R01_cb7494_c0/f2p0/2606	CCATTGACCTCTCTCCTCCA	GGGGTTTTCGAAATGGAGAT	158	(CTC)5	15	S
RL-58	R01_cb10800_c1/f1p0/1522	CGATTTTGCTCCTCCTCATC	GGCACCTTCCATCTTCCATA	187	(GA)10	20	S
RL-59	R01_cb9994_c53/f1p0/1698	TAGCAAAGCTGGACATGCAC	CCAACATTGTTACGAGCCAA	257	(TTG)5	15	S
RL-60	R01_cb15452_c0/f2p0/1734	TGGGTACTTCACTTCCGGTT	AAAGGCACGGAGAGAACAAA	241	(CTT)22	66	S
RL-61	R01_cb3318_c0/f1p0/3663	CAGTGGGGGAAGATGAAGAA	CAACCTCAATTTTCCTCCGA	189	(GAG)5	15	S
RL-62	R01_cb2407_c3/f1p0/2341	TGCATATGATGCTTTCAGGG	AGCCCAACAAACACAACACA	136	(CT)18	36	F
RL-63	R01_cb3010_c17/f1p1/3095	GTCGTCCTCTCTATCTGGCG	CCCACCTGACTGTGAAGGAT	127	(CAG)7	21	S
RL-64	R01_cb8564_c46715/f1p0/3637	CGTTAGCTCTTGCGTTTTCC	ATTCCCAAGAAAGCTCCGAT	227	(CT)38	76	S
RL-65	R01_cb12624_c2/f1p0/1580	AAGATAGATGAGGCGGGGTT	TGCACTCTTCTCACCCTCCT	246	(GAA)18	54	S
RL-66	R01_cb7969_c11/f1p0/1950	CAACCACACTTGATTGCACC	ACACCATCCCCACATAGGAA	184	(TGC)5	15	S
RL-67	R01_cb3229_c12/f1p0/2616	GAAGCACCGGTGAAACAAGT	GGTCCATAATCTCCAAGGCA	274	(GA)19	38	S
RL-68	R01_cb7249_c7/f1p0/2600	GGGTTGAGTTCCCCTCTTTC	CCGAACCTGTGAATGAATCC	212	(AT)11	22	S
RL-69	R01_cb3862_c58/f1p0/1466	GCTCTTTCTCTCTCCACCGA	CTGCTGCTGCTGTTGTTGTT	171	(GA)8	16	S
RL-70	R01_cb322_c45/f1p0/3654	GCGGAGGAGTCATCATCATT	AACCTGGTCTTTCCGAAGGT	277	(TAA)5	15	F
RL-71	R01_cb8785_c1/f3p0/2272	TCTAGCCCTGCCAATACTGC	GCACCTTGTGAACCATGTTG	260	(CAA)5	15	S
RL-72	R01_cb2571_c24/f1p0/3923	AAAGGGCTGCAGTTCAGAGA	TTGGTTCATTCTTGCAGCAG	266	(GCT)5	15	S
RL-73	R01_cb8564_c929/f3p7/2427	GAAGTCATGTGGAGGAGGGA	TCATGTAAAGTGGGAGCCCT	208	(TTG)6	18	S
RL-74	R01_cb8564_c71665/f3p0/2489	TGTTTTCTGTGTTGTGCCGT	CACCACATACAAATTCGCCA	159	(AGT)11	33	S
RL-75	R01_cb8564_c127920/f1p0/2676	AAGACGAAGAAGCTGAACACG	CTGTCTCTCGAAACCCTTGC	189	(GA)13	26	S
RL-76	R01_cb9840_c0/f1p0/1950	GATTGACTGTTGAGGGGGAA	CCCAGTCAATTATCAAAGGTC	266	(TTTGTT)15	90	F

RL-77	R01_cb7337_c3/f1p0/2569	TGGTTGAGGCCTTGGATAAC	ATCGGTACGGTGAAGGTGAG	271	(CTCTCC)5	30	S
RL-78	R01_cb8564_c22336/f1p0/2652	CATCCACCCGTAACGACTCT	TTCTGCATGCCATTCGTTTA	225	(TC)10	20	S
RL-79	R01_cb5421_c4/f1p0/1919	TACGCATAACGGAATCCTCC	CTCAGGAGGATGAGACCCAG	233	(GAA)7	21	S
RL-80	R01_cb17467_c0/f1p0/802	CGTCGGATTAGACTTTTGGC	TGAGGACACCACCGTAAACA	263	(T)16	16	S
RL-81	R01_cb1881_c0/f2p0/1523	CGTCCCTCTTTTCACCCATA	ACTACGTTCGCTATGCACCC	205	(AG)13	26	F
RL-82	R01_cb8564_c69187/f6p2/3623	AGCTCCAGAGCAGCGACTAC	AAGAGTGGTGACTGCGGACT	167	(CT)6	12	S
RL-83	R01_cb8564_c36175/f1p0/2801	CAGTGAACATCTTCGGGTCA	GGATCTCATAACGAGGCCAA	212	(G)19	19	S
RL-84	R01_cb8954_c26/f14p1/1054	ATGGTTGCTAGCTTGATGGG	TCTCGCCAACAACAAAATCA	220	(TC)27	54	S
RL-85	R01_cb5159_c1/f2p0/1967	TAATCGTGCCCTAAACGGAC	CTGCTGTCGGAATCATCGTA	245	(CCTT)5	20	S
RL-86	R01_cb5725_c15/f1p0/3169	TTCATAGGCTTCTTTCCCCA	GGCACAAGCTTCTCCAAATC	211	(T)20	20	S
RL-87	R01_cb8463_c0/f1p0/2363	GTTTGCTGCGTGGACTGAT	AATTCTGAAACCCCCAATCC	109	(GA)8	16	F
RL-88	R01_cb17473_c1/f1p0/1511	CAGCAAGCAATTCAAGGACA	AGTCAAATCAGAAGCCGGTG	268	(CCA)11	33	S
RL-89	R01_cb7496_c19/f1p0/1899	GAAAAACACGACGAGGAGGA	CCGGAGGATGTCGTGATAGT	248	(GAC)5	15	S
RL-90	R01_cb2223_c1/f1p0/2395	TGTCGAGGGAAAGACACTCC	CTGCGTTTGGATCAAGGATT	143	(TTTTTA)7	42	S
RL-91	R01_cb6479_c3/f1p0/1511	CCAGTCAACCTCCACTCTCC	TCCACTACCAACAACCGTGA	184	(CAC)5	15	S
RL-92	R01_cb16396_c1/f1p0/1781	CTTTTTCTCCTTTCCCCAGG	AACAAAAATCGACCGCCTC	267	(TTTA)5	20	S
RL-93	R01_cb12114_c17/f1p0/944	TGCTAGAGATCTGCCCCAAT	ACGTAAATTGGCGAACTTGG	263	(CACCAA)7	42	S
RL-94	R01_cb6039_c33/f1p0/2697	TTCCCCAAACCCTTCTCTTT	CAAGGAAAACAAAACCTGGG	234	(CT)9	18	S
RL-95	R01_cb4708_c2/f1p0/3281	GGCCATTGTTTGAAGGCTTA	GATCTGGATCTGGACCTGGA	216	(AGGTCA)6	36	S
RL-96	R01_cb18409_c47/f1p0/1378	CGATCGGGAGAGAACTGAAG	GAGAAATCCCCTTCTCCCAC	270	(GAG)5	15	F
RL-97	R01_cb5630_c2/f1p0/2741	TCATCGTTGTCACTGGGGTA	GGCGTAGGCAAAAGCAAATA	197	(TTTG)5	20	S
RL-98	R01_cb6951_c7/f1p0/2994	GCGAGAACTGAGAGCAGGAG	TGTGAACCACCTTAGTCGCA	115	(GAGTGG)5	30	S
RL-99	R01_cb3499_c6/f1p1/3214	TTCAGCTTATCCGATGGTCC	GAAAATGGGAATGAGAGGCA	117	(TC)19	38	S
RL-100	R01_cb8564_c109799/f2p0/2167	CCGAAAGTTGGTATCCCTCA	CGCAAGCAATTTTAAGAGCC	234	(C)12	12	S
RL-101	R01_cb3447_c11/f1p0/3760	GATTTGGATCAAGTTGCCGT	GACTTCCGTTTCCTTTTCCC	221	(GAT)6	18	S
RL-102	R01_cb1071_c11/f1p0/3671	TGGACTGGAAGAGGATTTGG	GTTCCAATCGAAGAGCTGGA	196	(CGT)5	15	F

RL-103	R01_cb8564_c107524/f9p1/2447	TCTCTTCTCCTCGCATCCAT	GACTCGTCGTTTTCGAGTCC	226	(TA)7	14	S
RL-104	R01_cb8430_c13/f1p0/2161	CACAGTTCTCAACCCCCAGT	TAATTCGGAGCACCAACTCC	279	(AGG)7	21	S
RL-105	R01_cb8081_c3/f1p0/2053	GTCCGATCTTTCCAATTCCA	AGATGTTGGCGAAGGTCTTG	265	(CCTCTC)6	36	S
RL-106	R01_cb7094_c1/f2p0/2178	GGCTACATCCCCATCTCTGA	CGCTATGAGGAGTCTTGCGT	238	(ATTGT)10	50	S
RL-107	R01_cb2374_c14/f1p1/1832	CCACGAAAAACCACGAATCT	GTCACTGCACCCTTGGATTT	207	(GAA)5	15	F
RL-108	R01_cb10932_c5/f1p0/1663	GGTTTCCGACTCCCTTATCC	CTCGCTCTTTTGTTGCAGTG	107	(CTCC)5	20	S
RL-109	R01_cb4149_c17/f1p0/3287	GCTCTTACCACATCTATCCAAAG	GGTTCTTCATCAACCGGAGA	217	(T)25	25	S
RL-110	R01_cb9216_c23/f1p0/2136	CGCACTCGTCCTTTCAATTT	TCTTCGTCTGAGAATTGGGG	174	(CGA)5	15	F
RL-111	R01_cb8564_c3944/f1p1/2003	ATGACATAACCTTGGCCTCG	TGCATACAGGGGCAGACATA	168	(T)13	13	S
RL-112	R01_cb9460_c1/f1p1/2232	GGCATTAAACCAACCACCTG	ACTACTTGCAGTCACGCCCT	134	(GA)12	24	S
RL-113	R01_cb4273_c8/f1p1/2969	ACCCACAGCTGCTGCTACTT	GCAGTTTTCCTTCTGCCAAG	135	(CTC)6	18	S
RL-114	R01_cb1049_c14/f1p0/3390	CTTCTCACACTCCTCCCTCC	TGCTTATGCGATCAAGCAAC	267	(CT)13	26	S
RL-115	R01_cb4083_c1/f1p0/3439	GCCCAAAAGAGAAATCAGCA	AATTAGCCCCTCAAATCCGT	172	(TTTTC)5	25	F
RL-116	R01_cb3007_c4/f1p0/3121	TTGTTCAATTCAAGGGAGGG	CTGACCAGAGTTCCACCCAT	203	(AGA)17	51	S
RL-117	R01_cb12642_c3/f1p1/1037	ACCCATCCAAAAACAACCAA	TCCAACTTCCAACTTTTCCG	269	(CCCAT)5	25	S
RL-118	R01_cb8564_c38965/f1p0/4733	ATATCACAAGGTTCGGCTCG	TGGTTAAACCGAGCTTGGAG	280	(GA)78	156	S
RL-119	R01_cb9355_c3/f1p0/2134	TAATCGAGGATCTGGATCGG	CTTGTGGTCCTTGAGCCCTA	273	(TTC)5	15	S
RL-120	R01_cb8154_c9/f1p0/2424	AGCTTGATTACCAACCCACG	AGGGTTCTTATCAGGGTGCC	247	(AGGAG)6	30	S
RL-121	R01_cb2209_c60/f1p0/2964	CAAGATGACGAAAACAGCCA	GTACCATTGTTGGTGGGAGG	178	(TCC)5	15	S
RL-122	R01_cb10210_c2/f1p0/963	GACAGCACTTCTAGGGCACC	CTTCGACGGAGGAGAAAGTG	214	(CGA)6	18	S
RL-123	R01_cb8564_c41144/f1p0/3785	GATTGAGGAGGCGATTTTGA	ATGTTGCTTCGCACTTGTTG	255	(CT)9	18	F
RL-124	R01_cb6844_c9/f1p0/2576	GATCTTCATCACTCCAGTCTCC	GACGGATGTAGTGATCCGGT	173	(GA)21	42	S
RL-125	R01_cb6445_c2/f3p0/1815	GCATGCAGCTTGGCATACTA	CCAGGGGAACAATTAGGGTT	258	(TCA)5	15	S
RL-126	R01_cb8910_c5/f1p1/1988	AAAAGACAACCGTTTCCACG	TTCAAAACCCTGACCACCTC	226	(CGG)6	18	S
RL-127	R01_cb5219_c6/f1p0/2921	ATTCTCAACCTCCGTCATCG	ACAATGCATCCGCATACTCA	249	(TC)12	24	S
RL-128	R01_cb8564_c71334/f1p2/4671	GGTCGGGAATTACGTCAAGA	CAAACCCTCCCTTCCTTTTC	168	(GGA)6	18	S

RL-129	R01_cb5373_c4/f1p0/2557	TTGAAGCCTTACAGGATGGG	AACACCGAATTTGGGTCAAG	187	(AAGGGA)9	54	S
RL-130	R01_cb8055_c3/f1p0/2885	GCATGGCGAAGTTTTGAGTT	GACCCTGTTGTTCTCCGTGT	160	(TC)15	30	F
RL-131	R01_cb8564_c89073/f1p0/3953	CATCGTTGAAGAAGCTTTGG	CCCAAAGGATAGGGGGTAAA	111	(G)16	16	F
RL-132	R01_cb3464_c2/f1p0/4818	AAAAGACCCCCTCAGAGGAC	ATTTGAATTCCCCAGTTCCC	246	(AAAGAC)8	48	S
RL-133	R01_cb123_c6/f1p0/5119	GTCAAAGCTGTGCAAAACCA	AAACATATTCGTCCCCCACA	228	(TGG)8	24	S
RL-134	R01_cb1626_c21/f5p1/2846	CTGCAATCGGTTTTGGTTTT	AGGGTTATTGGGATTCAGGG	260	(GA)9	18	S
RL-135	R01_cb4629_c1/f1p0/3400	TGAGGACGGAGGAGAGGTTA	CAACATCCTCGCCTTCAACT	266	(GGGAGA)5	30	S
RL-136	R01_cb12167_c8/f1p1/1686	TCTATTTTTGCAGGCAAGGG	GGTGTGCATCAGCAGCTAAA	198	(AG)6	12	S
RL-137	R01_cb816_c19/f7p1/2220	AGGGAAGTCGGAGAGGAGAG	AGAATCACCACCGGCAATAG	190	(AGG)5	15	S
RL-138	R01_cb9894_c3/f1p0/2048	TTCTTTTCGGCCCATACTTG	TTCCCTCCTAATTGATTGGC	158	(TTA)7	21	S
RL-139	R01_cb7910_c1/f1p0/2504	AACGTGGTACCAGGGAGTTG	TGTTTCACAAACCACATGATT	256	(ATAAA)5	25	S
RL-140	R01_cb3756_c11/f1p1/4447	TGGGGAGATTGAGGGTACAG	AGTTTGGCTGCGTGCTTACT	194	(CGT)7	21	F
RL-141	R01_cb6517_c51/f1p0/2780	CCTTCCCAAGTCCTGAACAA	AGCTTCGCTTTCCTCCATTT	136	(TC)9	18	S
RL-142	R01_cb12923_c5/f1p0/841	AATTGACCGCGTCTGGTAAG	CCCCTCATTTCCTCTTCCTC	216	(CGAAGC)5	30	S
RL-143	R01_cb8564_c84790/f1p0/2014	TCAAGAAATGGCCCTGAAAC	GGCACGGCTCTAATGATCTC	200	(T)21	21	S
RL-144	R01_cb3448_c1/f1p1/3690	CTGATCACAATGCCACCTTG	CTTTCTCGGCAGTTCAAACC	273	(AGA)6	18	S
RL-145	R01_cb3077_c6/f1p0/2976	GAACATGCTTCTCCCTCAGC	TGGAGGTGGGTGGAGTAGAG	162	(CCA)5	15	F
RL-146	R01_cb13264_c4/f1p0/876	AGATGGATTGGGTGTGGAAA	AAGCAAAGGGGTTCCAAGAT	112	(AT)6	12	S
RL-147	R01_cb9355_c7/f1p0/1785	TCAAAGATAGCTCCCCTCCA	TGAACACAGCAATTTGACAAC	222	(TTC)15	45	S
RL-148	R01_cb9788_c14/f1p0/4165	GGAAAGCAGAAAATCACCCA	CCCCATCTAGGCTGGAAAAT	199	(TTCTT)5	25	S
RL-149	R01_cb10624_c1/f1p0/1176	GGCTTTGAAGCATTCTCCTG	ACGTTTTACACAGCCTCGCT	177	(GA)11	22	F
RL-150	R01_cb3500_c30/f4p2/3424	GCTCTAGAATTGGCGACGAC	CCACCTATCCTCCTCGATCA	279	(GCC)6	18	S

S and F separately represent amplification success and amplification failure.
